# From Proteomics to Personalized Medicine: The Importance of Isoflavone Dose and Estrogen Receptor Status in Breast Cancer Cells

**DOI:** 10.3390/jpm10040292

**Published:** 2020-12-19

**Authors:** Maria Ilieș, Alina Uifălean, Sergiu Pașca, Vishnu Mukund Dhople, Michael Lalk, Cristina Adela Iuga, Elke Hammer

**Affiliations:** 1 MedFuture Research Center for Advanced Medicine, Department of Proteomics and Metabolomics, “Iuliu Hațieganu” University of Medicine and Pharmacy, no. 4–6 Louis Pasteur st., 400349 Cluj-Napoca, Romania; ilies.maria@umfcluj.ro (M.I.); pasca.sergiu123@gmail.com (S.P.); iugac@umfcluj.ro (C.A.I.); 2 Department of Pharmaceutical Analysis, Faculty of Pharmacy, “Iuliu Hațieganu” University of Medicine and Pharmacy, Louis Pasteur Street 6, 400349 Cluj-Napoca, Romania; 3 Department of Hematology, “Iuliu Hațieganu” University of Medicine and Pharmacy, 400012 Cluj-Napoca, Romania; 4 Interfaculty Institute for Genetics and Functional Genomics, University Medicine Greifswald, Felix-Hausdorff-Straße 8, 17475 Greifswald, Germany; dhoplevm@uni-greifswald.de (V.M.D.); hammer@uni-greifswald.de (E.H.); 5 Institute of Biochemistry, University of Greifswald, Felix-Hausdorff-Straße 4, 17489 Greifswald, Germany; lalk@uni-greifswald.de; 6 DZHK (German Center for Cardiovascular Research), Partner Site Greifswald, 17475 Greifswald, Germany

**Keywords:** soy isoflavones, breast cancer, proteomics, genistein, daidzein, estrogen receptor

## Abstract

Continuing efforts are directed towards finding alternative breast cancer chemotherapeutics, with improved safety and efficacy profiles. Soy isoflavones represent promising agents but, despite extensive research, limited information exists regarding their impact on the breast cancer cell proteome. The purpose of this study was to compare the proteomic profiles of MCF-7 (estrogen responsive) and MDA-MB-231 (estrogen non-responsive) breast cancer cells exposed to different concentrations of genistein, daidzein, and a soy seed extract, using a high throughput LC–UDMS^E^ protein profiling approach. The 3-(4,5-dimethylthiazol-2-yl)-2,5-diphenyltetrazolium bromide (MTT) assay confirmed the dual activity of soy isoflavones on MCF-7 cells and the inhibitory effect on MDA-MB-231 cells. Proteome profiling of paramagnetic beads prepared peptides by nano-LC UDMS^E^ and pathway enrichment analysis revealed that isoflavones affected distinct molecular pathways in MCF-7 and MDA-MB-231 cells, such as tyrosine kinases signaling pathway, cytoskeleton organization, lipid and phospholipid catabolism, extracellular matrix degradation and mRNA splicing. Also, in MCF-7 cells, low and high isoflavone doses induced different changes of the proteome, including cell cycle alterations. Therefore, the expression of estrogen receptors and the isoflavone dose are determinant factors for the molecular impact of isoflavones and must be taken into account when considering adjuvant breast cancer therapy towards personalized medicine.

## 1. Introduction

Breast cancer is the most frequent malignancy among women and the second most common cancer amongst both sexes [1,2]. Perhaps one of the most characteristic feature of breast cancer is its molecular, phenotypic, and functional diversity, also known as tumor heterogeneity, which challenges both prognosis and therapeutic strategies. The molecular features typically used for breast tumor subtyping include expression of the estrogen receptor (ER) and the progesterone receptor (PR), activation of human epidermal growth factor receptor 2 (HER2/neu, encoded by ERBB2), and/or the presence of BRCA1/2 mutations [3,4]. Although chemotherapy has brought major improvements in controlling cancer’s progression, the treatment of breast cancer by means of chemotherapy is often accompanied by toxic side effects on the nontumoral tissues. Added to this, drug resistance can also be developed by the malignant clone as a response to different therapeutic compounds. Thus, continuing efforts are directed towards finding alternative anticancer agents, with improved safety and efficacy profiles [5].

Soy isoflavones have sparked increased interest as promising anticancer agents due to their capacity to modulate multiple molecular targets or to interfere with various cell signaling pathways [6]. Their structural resemblance to endogenous estrogens allows them to bind to the intranuclear ER, either mimicking or blocking the effects of estrogen in different tissues. However, soy isoflavones, especially genistein (Gen) and daidzein (Dai), can also induce non-ER-mediated effects, such as regulation of apoptosis, cell proliferation and survival, inhibition of angiogenesis and metastasis or antioxidant properties [7]. Most of these mechanisms have been investigated mainly by using traditional biochemical methods, with limited throughput and thoroughness. Nowadays, advanced omics technologies can offer in-depth information, supporting the precise understanding of isoflavone mechanisms in breast cancer.

Nanoproteomics has become a robust analytical platform for highly sensitive, selective, and cost-effective detection of low-abundance proteins by enabling high-throughput analysis of various samples [8]. So far, only few studies have explored the protein profile of breast cancer cells after exposure to soy isoflavones. A proteomic analysis of MCF-7 breast cancer cells using surface-enhanced laser desorption/ionization time of flight (SELDI-TOF) mass spectrometry showed that mixtures of 17β-estradiol, Gen, bisphenol A, and endosulfan exhibit simple similar action, respecting the principles of dose–response and dose additivity [9]. A more recent study has used quantitative phosphoproteomics to show that Gen can modulate cell cycle and DNA damage response pathways in MDA-MB-231 triple-negative breast cancer cells [10].

Both studies investigated the impact of Gen on the proteome using a single breast cancer cell line. However, the protein profile of breast cancer cells exposed to isoflavones depends greatly on the expression level of cell receptors, especially the ER status. Moreover, the isoflavone concentration is crucial, as soy isoflavones exert dose-dependent effects in ER-positive cells, stimulating the proliferation at low concentrations and inhibiting tumor growth at higher doses [7]. Starting from these observations, our group performed a global metabolomic profile of two breast cancer cell lines with different ER expression, in response to various doses of soy isoflavones. Results revealed that, depending on the dose, isoflavones can restrict glucose and glutamine uptake to limit cell proliferation or may uphold the pentose phosphate pathway in order sustain the cell growth [11].

These metabolic changes are caused, at least partially, by alterations occurred in the protein profiles of breast cancer cells exposed to isoflavones. Therefore, to gain a comprehensive understanding of isoflavones impact on breast cancer cells, we performed herein a high throughput protein profiling study. Using a similar design, we compared the proteome of MCF-7 (ER-positive) and MDA-MB-231 (ER-negative), in response to Gen, Dai, and a soy seed extract (SSE). In order to obtain comparable effects, MCF-7 cells were exposed to test concentrations that led to a 20% inhibition of cell growth compared to control (IC_20_) and to a 20% stimulation of cell growth compared to control (SC_20_), respectively. For MDA-MB-231 cells, only the IC_20_ concentrations of test compounds were selected.

Thus, the purpose of our study was: (i) to compare the protein profiles of two breast cancer cell lines with different ER expression when exposed to equivalent concentrations of isoflavones; (ii) to identify which proteomic modulations are specific to breast cancer cell inhibition or proliferation, by comparing the proteomic profile of MCF-7 cells exposed to IC_20_ and SC_20_ of test compounds.

Results indicated that isoflavones exert distinct molecular pathways, targeting tyrosine kinases signaling pathway and cytoskeleton organization in MCF-7 cells and lipid and phospholipid catabolism, extracellular matrix degradation and mRNA splicing in MDA-MB-231 cells. Furthermore, in MCF-7 cells, low and high isoflavone doses induced different changes of the proteome, including cell cycle alterations. The present observations come to complete our previous metabolomic results, emphasizing once again the importance of estrogen receptor status and isoflavone dose when considering soy isoflavones as adjuvant breast cancer therapy towards personalized medicine.

## 2. Materials and Methods

The SSE was received from Hunan Goldliloo Pharmaceutical Co., Ltd. (Changsha, China) and contained 26.87% daidzin, 10.95% glycitin, 3.55% genistin, 1.50% Dai, 0.12% glycitein, and 0.02% Gen. This isoflavone distribution was confirmed by a validated HPLC-UV method [12]. Stock solutions of standard Gen (>98% purity, Sigma-Aldrich, Taufkirchen, Germany), Dai (>98% purity, Sigma-Aldrich, Taufkirchen, Germany), or SSE were prepared in dimethyl sulfoxide and stored at −20 °C. All chemicals were of analytical grade.

### 2.1. Cell Culture

The MCF-7 and MDA-MB-231 breast adenocarcinoma cell lines were obtained from CLS Cell Lines Service (Eppelheim, Germany) and cultured as previously described [11].

### 2.2. MTT Assay

The cytotoxic potential of each test compounds was evaluated 72 h after treatment using the 3-(4,5-dimethylthiazol-2-yl)-2,5-diphenyltetrazolium bromide (MTT) assay, as previously described [11].

### 2.3. Sample Preparation for Proteomics Analysis

#### 2.3.1. Protein Extraction

After 72 h of exposure to test compounds, MCF-7 and MDA-MB-231 cells were lysed in order to extract the protein content. Briefly, the medium was discarded, and the cell layer was washed three times with a total of 40 mL cold phosphate buffer saline (PBS). Cell lysis buffer (Cell Signaling Technology, Leiden, The Netherlands) supplemented with 1% protease inhibitor cocktail (Sigma-Aldrich, Taufkirchen, Germany) was added and the plate was incubated on ice for 5 min. Next, the cells were rapidly scraped and transferred into a reaction tube. After a brief sonication, the cell lysates were centrifuged for 10 min at 14,000× *g* in a cold microfuge. At the end, the supernatant was aliquoted and stored at −80 °C.

#### 2.3.2. Protein Concentration Determination

Protein concentration was determined by Bicinchoninic Assay (BCA) (Thermo Fisher Scientific, Waltham, MA, USA) according to the manufacturer’s protocol and using bovine serum albumin to generate the standard curve.

#### 2.3.3. Trypsin Digestion on Paramagnetic Beads

Four µg of each protein sample were subjected to reduction (dithiothreitol 2.5 mM, 30 min at 37 °C) and alkylation (iodoacetamide 10 mM, 15 min at 37 °C). The reaction was quenched with dithiothreitol (4.5 mM). Hydrophobic and hydrophilic magnetic beads (Speedbeads™ magnetic carboxylated modified particles GE45152105050250 and GE65152105050250) (Cytiva, former GE Healthcare) were prepared shortly before usage as described by Hughes et al. [13] and mixed with the protein suspension in a 2:1 ration (2 µL beads: 1 µg protein).

Further, protein to bead binding and tryptic on-bead digestion were performed following Blankenburg et al. [14] protocol adapted to our cell line samples. To ensure protein to bead binding, acetonitrile (ACN) was added to the mixture (end concentration 70% (*v*/*v*)) and samples were incubated at room temperature for 18 min at 1400 rpm on a ThermoMixer^®^ (Eppendorf, Hamburg, Germany). Beads were separated by placing samples on a magnetic separation rack (DynaMag™-2 Magnet, Invitrogen, Thermo Fisher Scientific) for 2 min and the supernatant was discarded. Next, the beads were washed twice with 180 µL ethanol 70% (*v*/*v*) and twice with 180 µL ACN with intermittent bead magnetic sedimentation for 2 min and supernatant discharge.

On-bead protein digestion was performed by trypsin (Promega, Madison, WI, USA) at 1:25 trypsin to protein ratio (overnight at 37 °C) in 20 mM ammonium bicarbonate buffer. Digestion was stopped by adding ACN (end concentration 95% (*v*/*v*), 18 min, 1400 rpm on a ThermoMixer (Eppendorf). After bead separation, again supernatant was discarded. Beads were washed with 180 µL ACN and air-dried. To release the peptides, beads were resuspended in 10 µL 2% (*m*/*v*) DMSO (3 min sonication). The peptide solution was transferred into a LC vial avoiding any bead transfer and mixed with 10 µL two-fold concentrated buffer A (4% [*v*/*v*] ACN and 0.2% [*v*/*v*] acetic acid) to obtain the required concentration for nano-liquid chromatography-ultra definition mass spectrometry (nanoLC-UDMS^E^) analysis (0.1 µg/µL).

### 2.4. Protein Identification and Quantification by Nano-LC-UDMS^E^

Peptides (300 ng) were separated on an ACQUITY UPLC^®^ M-Class HSS T3 column within 110 min with a non-linear gradient of 3% to 35% acetonitrile 0.1% acetic acid at a flow rate of 300 nL/min. As previously reported [15], an on-line coupled travelling wave ion-mobility-enabled hybrid quadrupole orthogonal acceleration time-of-flight mass spectrometer (SYNAPT G2-Si HDMS, Waters Corporation, Milford, MA, USA) was used to detect eluting peptides. For data acquisition, independent acquisition mode was employed (a programmed feature for parent- and product ions measurement by switching between low energy (MS) and elevated energy (MS^E^)) and collision voltage ramping was set according to Distler et al. [16]. Samples were measured in three technical replicates. Raw data were acquired using MassLynx™ Software Version 1.53.1398 (Waters Corporation). Detailed settings can be found in the Supplementary methods.

### 2.5. Database Search

LC–UDMS^E^ data were processed as previously reported [15,17]. In brief, Progenesis QI v2.0 (Waters Corporation) was used for automated peak picking and alignment. Spectra were searched using the built-in search engine against a Uniprot/Swissprot database (03/2019) limited to human entries (20,404) and the following parameters were set: enzyme specificity: trypsin allowing a maximum of 1 missed cleavage; carbamidomethylation of cysteine as fixed and oxidation of methionine as a variable modification. Search tolerance parameters were set for a false discovery rate of <4% and proteins considered as significantly identified when the following ion matching requirements were passed: fragments/peptide ≥2, fragments/protein ≥5 and peptides/protein ≥1. Further, peptide identification was restricted to absolute mass error <10 ppm, sequence length >5, score >5. Protein relative quantification was performed on the summed peptide abundance by using only peptides which have no conflicting protein identification.

### 2.6. Statistical Analysis

In our analysis we considered only proteins that presented abundances in all three replicates of an experimental condition in either cell line. In the table containing the raw protein abundances, missing values (MCF-7 cell line: 0.4–1.3%; MDA-MB-231: 0.3–0.6%) were replaced by the half-minimal abundance of that sample. Data were log10 scaled and median normalized. Welch’s *t* test was used to identify differences between two groups.

### 2.7. Bioinformatics Analysis

The pathways and protein interactions of proteins with changed abundance were identified with the help of the expression analysis tool of Metascape [18]. This bioinformatics tool ensures pathway and enrichment analysis with the following ontology sources: KEGG Pathway, GO Biological Processes, Reactome Gene Sets, Canonical Pathways, CORUM, TRRUST, DisGeNET and PaGenBase. Enriched pathways and processes are presented as bar charts.

**Figure 1:**
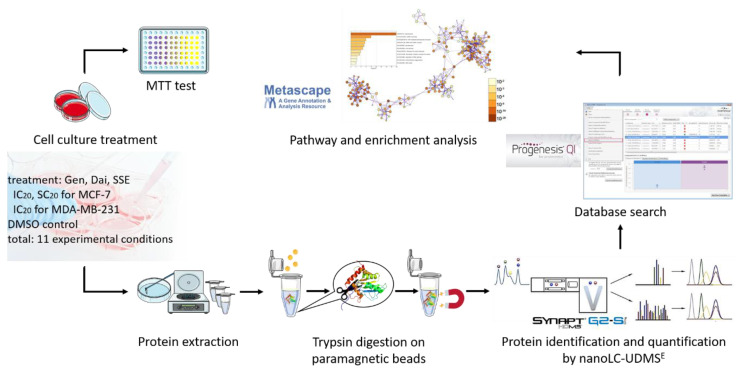
Overview of the complete workflow of the study. First, we performed 3-(4,5-dimethylthiazol-2-yl)-2,5-diphenyltetrazolium bromide (MTT) assay to assess the cytotoxic or stimulatory potential of genistein (Gen), daidzein (Dai), and the soy seed extract (SSE). Second, we subjected MCF-7 and MDA-MB-231 breast cancer cells exposed to different concentrations of isoflavones to protein extraction, trypsin digestion of proteins using paramagnetic beads and nano-liquid chromatography-ultra definition mass spectrometry (nanoLC-UDMS^E^) analysis for protein identification and quantification. Database search combined with statistical and bioinformatical analysis allowed us the identification of most affected signaling pathways and processes. SC_20_, IC_20_: concentrations of Gen, Dai, and SSE that induced a 20% growth stimulation or inhibition, respectively, compared to control.

The complete workflow is presented in Figure 1.

## 3. Results and Discussion

MCF-7 and MDA-MB-231 breast cancer cells are representative models for estrogen-dependent and estrogen-independent human breast adenocarcinoma, respectively. MCF-7 cells, belonging to the luminal A molecular subtype, also express progesterone receptors, but are negative for HER2/neu overexpression. MDA-MB-231 cells are often referred as “triple negative”, as they do not express any estrogen, progesterone or HER-2/neu receptor. Still, they express receptors for epidermal growth factor (EGF) and transforming growth factor alpha (TGF alpha) [19].

Both cell lines were exposed to Gen, Dai and the SSE individually. Gen and Dai are the two main aglycones found in soy seeds, which present structural and functional similarities to 17-estradiol. Gen’s binding affinity for estrogen receptors alpha (ERα) and beta (ERβ) has been estimated to one to three orders of magnitude lower than that of 17-estradiol [20]. The SSE used in this experiment represents a well characterized mixture of Gen, Dai and glycitein, along with their glycosidic conjugates [12]. According to the producer’s certificate of analysis, the extract meets the proper characteristics to be added into various health products, including food supplements.

In order to evaluate the cytotoxic or the stimulatory potential of each test compound, an MTT assay was conducted. As previously described [11], when exposed to relatively low concentrations of isoflavones (1.56–13.06 μM Gen, 1.56–34.28 μM Dai and 6.25–67.61 μg/mL SSE), MCF-7 cells not only survived, but proliferated more compared to control, while higher concentrations inhibited the cell growth. It is well known that isoflavones, especially Gen, can act in a dose-dependent manner, to both positively and negatively regulate tumorigenesis [21,22]. For MDA-MB-231 cells, only a dose-dependent inhibitory effect was observed.

As Gen is a more potent inhibitor than Dai, having a 40 times higher affinity for ERα compared to Dai [23], and as the SSE contains a mixture of isoflavones with various effects, we refrained from selecting equal concentrations for all test compounds. Instead, we selected equi-effective concentrations, which triggered the same level of cell inhibition or cell proliferation.

Therefore, for MCF-7 cells, we selected the test concentrations that resulted in a 20% higher proliferation compared to control (SC_20_), and test concentrations that inhibited cell growth by 20% compared to control (IC_20_). For MDA-MB-231 cells, only the IC_20_ concentrations were selected (Table 1). For both cell lines, we used DMSO as solvent control. In all samples, the final concentration of DMSO in medium was below 0.01% [11].

By selecting the IC_20_ and SC_20_, we attained a range of concentrations which can be physiologically achievable in the human serum. Despite the large variability of serum isoflavone concentrations found in the literature, in the case of Gen, 10 μM can be considered the maximal dietary concentration, while 25 μM represents the pharmacological concentration which has been found in human plasma [24].

The selected concentrations were used for the following proteomic experiments (Table 1).

### 3.1. Proteome Profiling by Data-Independent Nano-LC UDMS^E^ Analysis 

Our proteome profiling study was based on a state-of-the-art sample preparation method using paramagnetic beads towards improving the proteome coverage of complex samples, such as breast cancer cell lines [13,14]. Even more, the selected method was compatible with a wide range of detergents, chaotropes, salts and solvents present in a higher concentration as compared to common in solution digestion methods [13]. The compatibility of the paramagnetic bead method with highly concentrated detergent solutions was an essential aspect of our sample preparation protocol, as the cell lysis buffer used for protein extraction contained 1% Triton X-100, a nonionic surfactant. It is known that standard protein assays, such as Bradford or Lowry, are only suitable with low concentrations of detergents, therefore BCA assay was employed for the protein concentration determination in our study. More importantly, detergents suppress the electrospray ionization of peptides, which would have hampered our protein profiling study by mass spectrometry.

Mass spectrometry data were acquired using a data independent acquisition (DIA) method, known as a powerful tool, developed towards enhancing both protein identification and quantification. This technique was reviewed with respect to protein biomarker studies [25] and it is in line with current investigations on breast cancer.

The breast cancer proteome profiling research was based on the analysis of MCF-7 (ER-positive) and MDA-MB-231 (ER-negative) breast cancer cells, exposed to different isoflavone concentrations. Each sample was analyzed by nano-LC UDMS^E^ in triplicate. As such, we were able to identify 55,240 unique peptides for the MCF-7 control sample and 40,939 unique peptides for the MDA-MB-231 control sample. Only the proteins identified in all the three injections were considered for further analysis. Furthermore, we considered for further analysis the unique proteins identified for each cell line, as well as the common proteins.

In the MCF-7 cell line, 4203 proteins were identified, (1555 unique proteins), whereas in the MDA-MB-231 cell line 3625 proteins were identified (976 unique proteins). A number of 2649 were common proteins. Thus, a total of 5180 proteins were subjected to comprehensive analysis. A complete view on the individual abundance of these proteins can be found in the Supplementary Table S1.

**Figure 2:**
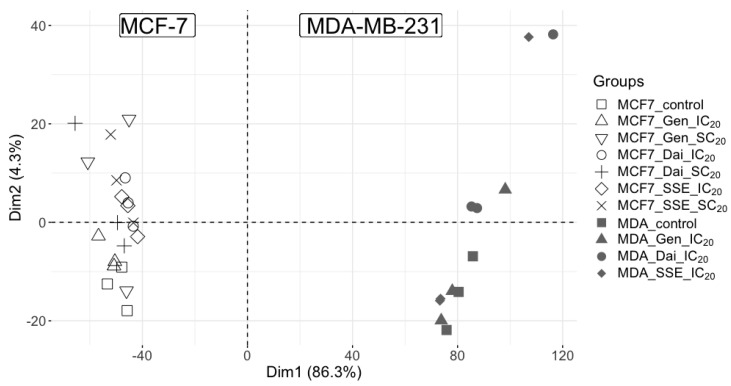
Principal component analysis displaying the sources of variance among all samples. The major variance (86.3%) is attributed to the cell type.

Our first attempt in characterizing the two-breast cell line proteomes was to evaluate the general protein distribution following different treatment conditions. Thus, principal component analysis (PCA) was performed using protein data of all samples (Figure 2). Distinct protein patterns between the two cell lines were disclosed, confirming that the major variance source (PC1 86.3%) was the cell type, as the two cell lines belong to different genotypes. This protein pattern of breast cancer cells exposed to isoflavones is in line with our metabolomic data, as previously published [11].

Second, each cell line data was individually subjected to PCA. For MCF-7 cells, the different isoflavone treatments along with the DMSO control, displayed a clear overlap of the treatments, whereas the control clustered separately. A similar pattern could be also noticed for the MDA-MB-231 cell line. These results suggest that, in each cell line, all isoflavone treatments affect the same protein pattern (Supplementary Figure S1). Again, these observations are in line with our previous metabolomic data, which showed that most of the altered molecular pathways are common to all test compounds [11].

**Figure 3:**
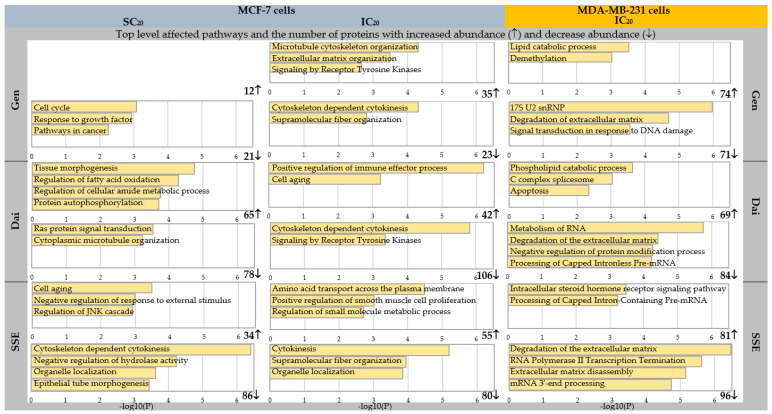
Comparative view on affected pathways as indicated by enrichment pathway analysis and the number of proteins with significantly increased (↑) or reduced (↓) abundance identified in MCF-7 and MDA-MB-231 breast cancer cells following exposure to Gen, Dai, and SSE at stimulatory (SC_20_) and inhibitory (IC_20_) concentrations, respectively.

We further interrogated the different treatment effects on the two breast cancer cell lines and performed statistical analysis on the normalized data. As expected, all test compounds induced statistically significant changes in a portion of the identified proteins. Only proteins with a fold change (FC) greater than |2| (*p* < 0.05) were subjected to pathway and enrichment analysis by Metascape. A complete view on the statistical analysis results can be found in the Supplementary Table S2. Figure 3 presents a complete overview on the number of affected proteins and the top corresponding enriched pathways and in Table 2, we listed the proteins involved in these pathways, along with their *p* value and log2FC.

### 3.2. The Impact of Estrogen Receptor Status on Isoflavone Altered Pathways

The ER is expressed in most invasive breast cancers and is considered an important predictive and prognostic marker. Herein, we exposed both MCF-7 and MDA-MB-231 to equivalent inhibitory doses (IC_20_) of Gen, Dai, and SSE, therefore the same level of inhibition was obtained. Based on the protein profiles revealed after exposure, we searched for the most likely affected pathways and tracked the specific protein alterations which may be associated to the status of ER.

As presented in Figure 3, the same test substances induced significant abundance changes in a different number of proteins in both cell lines. Enrichment analysis revealed that isoflavones affected different molecular pathways in the two cell lines studied. At least partially, these differences may be attributed to the interaction of isoflavones with the ER, in the case of MCF-7 cells.

In MCF-7 cells, inhibitory concentrations of our tested compounds significantly modified (increase or decrease) the abundance of the proteins involved in microtubule cytoskeleton organization (Gen), cytoskeleton-dependent cytokinesis (Gen, Dai), and supramolecular fiber organization (Gen, SSE). The proteins involved in these processes, Rho-related GTP-binding protein RhoB, Formin-2, Tubulin gamma-1 chain were mostly common to all test compounds, as presented in Table 2. Almost all these processes are part of cellular component organization (GO:0016043), a process that results in the assembly, arrangement of constituent parts, or disassembly of a cellular component [26]. A previous quantitative proteomics and transcriptomics study confirmed that Gen can trigger cellular assembly and organization through cytoskeletal rearrangement in breast cancer cells depending on the ERα expression of the cells [27]. However, limited data exist so far about the potential of isoflavones to modulate the cellular cytoskeleton. A native flax root extract containing Gen, Dai, and other phytoestrogens were shown to decrease the proliferation of MCF-7 cells by inhibiting cell impedance, initial cell adhesion, and cell migration ability as a result of increased formation of actin stress fibers [28]. It would be of great interest to further investigate the exact mechanism by which high isoflavone doses act on cell cytoskeleton architecture to impede proliferation and cell growth.

Furthermore, top level enriched pathways indicated Receptor Tyrosine Kinases signaling pathway as significantly affected by inhibitory concentrations of Gen and Dai. These results correlate with the current knowledge about soy isoflavones, particularly Gen, a recognized inhibitor of tyrosine kinase activity of growth factor receptors, such as epidermal growth factor receptor (EGFR) [29,30]. Membrane receptor tyrosine kinases can activate downstream signaling pathways such as mitogen-activated protein kinase (MAPK/ERK) pathway and phosphoinositide 3-kinase (Pl3K/AKT) pathway, resulting in activation of nuclear ER. Gen is able to block tyrosine kinase receptors by competing with adenosine triphosphate (ATP) at its binding site, thereby blocking the downstream cascade and the activation of ER [30]. Gen can also act on a more downstream level in the PI3K/Akt pathway, inducing the expression of phosphatase and tensin homolog deleted on chromosome 10 (PTEN), a natural inhibitor of PI3K/Akt signaling pathway [31]. This growth-inhibitory mechanism is highly dependent by the presence of ER.

Particularly, in the case of SSE, specific molecular processes were identified, such as amino acid transport across the plasma membrane and cytokinesis. These alterations might be caused by other bioactive compounds found in SSE or by the synergic action of all isoflavones present in the SSE.

In MDA-MB-231 cells, the test compounds induced significant abundance changes in the proteins involved in lipid and phospholipid catabolic processes (for Gen and Dai), extracellular matrix degradation, and RNA splicing.

Fatty acid synthesis and oxidation are important parts of the metabolic phenotype of triple negative breast cancer cells, such as MDA-MB-231 cells. In order to produce building blocks for cell development, various oncogenic genes are upregulated to promote oxidation of fatty acids. As presented in Figure 3, Gen and Dai have significantly increased the abundance of proteins involved in lipid and phospholipid catabolic processes, respectively. This suggests that isoflavones can hinder cell growth by favoring lipid and phospholipid degradation, as also observed in the study of Liang et al. [32]. The study revealed that treatment with Gen or Dai inhibited the accumulation of lipid droplets in colon cancer cells by downregulating the expression of Perilipin-1, adipose differentiation-related protein and Tip-47 family proteins and vimentin levels. Furthermore, these compounds significantly induced the mRNA expression of peroxisome proliferator-activated receptor gamma (PPAR-γ), Fas, fatty acid binding protein (FABP), glycerol-3-phosphate acyltransferase (GPAT3), and microsomal TG transfer protein (MTTP), and reduced the mRNA level of mitochondrial uncoupling protein 2 (UCP2) [32]. By regulating these lipid droplet-related factors, isoflavones inhibited the proliferation of colon cancer cells by induction of apoptosis. More specific studies are required to confirm if Gen and Dai elicit the same biochemical behavior also in breast cancer cells.

Degradation of extracellular matrices is required for breast cancer progression, invasion, and metastasis. This process can be accomplished by matrix-degrading proteinases such as metalloproteinases (MMPs) and their key regulators, tissue inhibitors of metalloproteinases (TIMPs). Gen was shown to suppress the invasive potential of MDA-MB-231 breast cancer cells through down-regulation of the transcription of MMP genes and by increasing the expression of TIMP-1 and -2 genes [33]. As MDA-MB-231 cells notably express more MMPs compared to the low-invasive MCF-7 cells, Gen had a greater inhibitory and anti-invasive effect on these cells. In our study, Gen induced the same inhibitory effect at a much lower concentration in MDA-MB-231 cells (IC_20_ = 11.04 μM) compared to MCF-7 cells (IC_20_ = 22.44 μM), confirming that MDA-MB-231 cells are more sensitive to Gen’s action. Similar antiangiogenic effects were observed also for Dai and its metabolites, which significantly reduced the invasive capacity of MDA-MB-231 cells by down-regulation of MMP-2 [34]. In the present study, SSE determined a significant reduction of MMP-10, while Gen affected disintegrin and metalloproteinase domain-containing protein 9, confirming the anti-invasive properties of isoflavones. The precise molecular mechanisms by which isoflavones interfere with the activity of MMPs is, most likely, independent of ER expression and rely on other signaling pathways. For instance, a study performed on hepatocellular carcinoma cells concluded that Gen suppressed MMP-9 transcription by inhibiting nuclear factor-κ B (NF-κB) activity [35,36].

While the antiangiogenic properties of soy isoflavones are well-documented, less information is available about their potential to alter mRNA splicing. The mRNA splicing takes place in nucleosomes and involves removal of intervening sequences (introns) from pre-mRNA to generate a fully functional, mature, mRNA. In our study, both Gen and SSE induced significantly changes in the abundance of Small nuclear ribonucleoprotein-associated proteins B and B’ and Small nuclear ribonucleoprotein Sm D3, two core components of spliceosomal small nuclear ribonucleoproteins (snRNP), with important roles in pre-mRNA splicing (Table 2). To our knowledge, only a gene expression study, performed on PC3 prostate cancer cells, has revealed that Gen down-regulated genes responsible for RNA binding, transcription factors, protein kinases including NF-κB-inducing kinase, and MAP kinase [37]. Further studies are necessary to evaluate the impact of soy isoflavones on DNA transcription and mRNA splicing in breast cancer and to identify the exact mechanisms.

**Figure 4:**
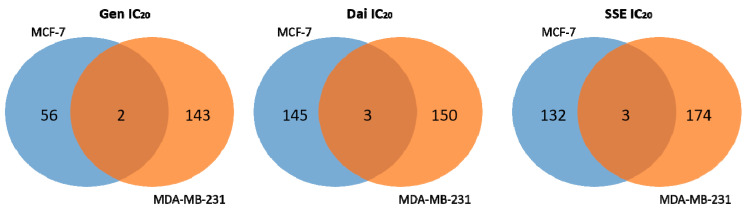
Venn diagram presenting the number of common and unique proteins with significantly changed abundance (*p* < 0.05, FC > |2|) identified following exposure of MCF-7 and MDA-MB-231 breast cancer cells to Gen IC_20_, Dai IC_20,_ and SSE IC_20_, respectively.

The distinct molecular mechanisms observed in ER-positive and ER-negative breast cancer cells are confirmed by the fact that only few proteins with significantly changed abundance were found common for both cell lines (Figure 4).

Therefore, the same test substance, given in the equivalent inhibitory concentrations, induced different molecular responses on protein level in MCF-7 cells (ER-positive) and MDA-MB-231 cells (ER-negative), suggesting that the expression of ER is a determinant factor for isoflavone mechanisms of action.

### 3.3. The Impact of Isoflavone Dose on Protein Profile in MCF-7 Cells

The matter of isoflavone dose has been long debated in the past few years. In vitro studies have shown that relatively low doses of isoflavones (<10 μM for Gen) can trigger cell proliferation, while higher doses inhibit the cell growth. In ER-negative cells, such as MDA-MB-231 cells, this biphasic effect was not observed, all phytoestrogens exhibiting an anti-proliferative effect only. Thus, the proliferative effect of isoflavones, as observed at low doses, can be attributed to the interaction with ER (mainly the ERα receptor). More specifically, Gen was shown to induce *ASAH1* gene expression by activating a GPR30-dependent pathway that culminates in ERK1/2 phosphorylation. The ERK1/2 activation will induce phosphorylation and activation of ERα [22]. However, isoflavones can trigger cell growth through several other mechanisms, less characterized. To investigate these mechanisms, we evaluated the protein profiles obtained after exposing MCF-7 cells to inhibitory and stimulatory concentrations of Gen, Dai, and SSE, respectively, and we searched for the most likely affected pathways.

As presented in Figure 3 and Table 2, low concentrations of Gen stimulate MCF-7 cells growth by reducing the abundance of proteins involved mainly in the cell cycle. In line with a similar study, which exposed MCF-7 cells to 1 μM Gen for 48 h, Gen stimulated the cell growth by increasing the cell proliferative phase (S) and decreasing the resting phase (G0/G1). The proposed underlying mechanism was that Gen induced insulin-like growth factor receptor (IGF-IR) and insulin receptor substrate (IRS-1) expression, enhancing the Insulin-Like Growth Factor signaling pathway. As Gen, in low doses, acts as an ER agonist and estrogen-mediated expansion of breast cancer can be prompted via IGF-I signaling, it is possible that Gen proliferative effects are a consequence of enhanced cross-talks between ER and INF-IR signaling pathways [38].

## 4. Conclusions and Further Perspectives

In this study, we explored the proteomic profiles of two different breast cancer cells, MCF-7 and MDA-MB-231, exposed to different concentrations of Gen, Dai, and an SSE. All test compounds affected distinct molecular pathways in ER-positive and ER-negative breast cancer cells, suggesting that the expression of ER and the isoflavone dose are determinant factors for isoflavone mechanisms of action. Our study revealed a possible effect of isoflavones on cytoskeleton organization and, for the first time, a possible effect on mRNA splicing in breast cancer cells, mechanisms which are worth further investigation through more specific, targeted studies.

Several studies have underlined that after breast cancer diagnosis, many patients prompt an interest in alternative dietary habits and report significant changes in their dietary intake and supplement use [39,40,41]. A recent study pointed that the percentage of women using estrogenic botanical supplements was more than double after diagnosis [41]. Giving the large interest in using soy dietary supplements, several clinical studies have tried to assess the association of soy consumption with the risk of breast cancer. The results of several major clinical trials uphold our preclinical results regarding the importance of the ER status and the isoflavones dose. A large prospective study, which aimed to evaluate the role of hormone receptor on the association between soy food intake and breast cancer risk, concluded that adult soy intake was associated with significantly decreased risk of ER-positive/PR-positive breast cancer in postmenopausal women and decreased risk of ER-negative/PR-negative breast cancer in premenopausal women. The soy association did not vary by HER2/neu status [42]. More recent studies have reported opposing associations of soy supplementation with breast cancer risk depending on the ER status [43] and that each 10 mg/day increment in soy isoflavone intake was associated with a 3% reduced risk of breast cancer, admitting that a higher soy intake might provide a reasonable benefit in breast cancer chemoprevention [44].

In this light, precise dietary recommendations for each individual disease situation become essential, especially when proliferative effects might occur, as it is the case for soy isoflavones. Therefore, in the matter of personalized soy adjuvant therapy in breast cancer, the status of ER and the selection of proper isoflavone dose must be seen as decisive elements for achieving therapeutic safety and efficacy.

## Figures and Tables

**Table 1:** The concentrations of Gen, Dai, and SSE used in the proteomic experiments. The concentrations were selected based on the MTT assay, as described in our previous study [11].

Test Compound	MCF-7		MDA-MB-231
	SC_20_	IC_20_	IC_20_
**Gen (μM)**	5.62	22.44	11.04
**Dai (μM)**	19.01	52.24	36.39
**SSE (μg/mL)**	22.59	166.34	26.36

SC_20_, IC_20_: concentrations of Gen, Dai, and SSE that induced a 20% growth stimulation or inhibition, respectively, compared to control.

**Table 2:** Comprehensive view of the most altered molecular pathways, as indicated by the enrichment pathway analysis, along with the identified associated proteins following exposure of MCF-7 and MDA-MB-231 breast cancer cells to Gen, Dai and SSE.

**MCF-7 Cells**					**MDA-MB-231 Cells**				
	**UniProt**	**Protein Name**	***p* Value**	**log2FC**		**UniProt**	**Protein Name**	***p* Value**	**log2FC**
**Cellular Component Organization**					**Lipid Catabolism**				
***Microtubule Cytoskeleton Organization***					***Lipid Catabolic Process***				
Gen IC_20_	Q96N67	Dedicator of cytokinesis protein 7	0.0342	5.06	Gen IC_20_	Q9NTX5	Ethylmalonyl-CoA decarboxylase	0.0015	7.97
	P16591	Tyrosine-protein kinase Fer	0.0007	4.98		Q7Z5M8	Abhydrolase domain containing 12B	0.0000	4.85
	O43663	Protein regulator of cytokinesis 1	0.0000	1.28		Q9NY59	Sphingomyelin phosphodiesterase 3	0.0067	1.67
***Cytoskeleton Dependent Cytokinesis***					***Phospholipid catabolic process***				
Gen IC_20_	P62745	Rho-related GTP-binding protein RhoB	0.0051	−9.20	Dai IC_20_	Q13093	Platelet-activating factor acetylhydrolase	0.0022	9.76
	Q9NZ56	Formin-2	0.0057	−9.11		Q7Z5M8	Abhydrolase domain containing 12B	0.0004	3.61
	O95630	STAM-binding protein	0.0150	−1.21		Q9NY59	Sphingomyelin phosphodiesterase 3	0.0238	1.25
Dai IC_20_	P62745	Rho-related GTP-binding protein RhoB	0.0201	−6.44	**Degradation of Extracellular Matrix**				
	Q9NZ56	Formin-2	0.0013	−6.34	Gen IC_20_	P42574	Caspase-3	0.0002	−2.95
	Q13464	Rho-associated protein kinase 1	0.0049	−3.44		P07858	Cathepsin B	0.0001	−2.71
***Supramolecular fiber organization***						Q13443	Disintegrin and metalloproteinase domain-containing protein 9	0.0001	−1.05
Gen IC_20_	P62745	Rho-related GTP-binding protein RhoB	0.0051	−9.20	Dai IC_20_	P42574	Caspase-3	0.0034	−2.43
	Q9NZ56	Formin-2	0.0057	−9.11		O15230	Laminin subunit alpha-5	0.0014	−1.67
	P23258	Tubulin gamma-1 chain	0.0473	−2.80		P07711	Cathepsin L1	0.0123	−1.66
SSE IC_20_	P62745	Rho-related GTP-binding protein RhoB	0.0168	−7.24	SSE IC_20_	P42574	Caspase-3	0.0141	−3.28
	Q9NZ56	Formin-2	0.0075	−7.14		P07858	Cathepsin B	0.0049	−2.53
	P23258	Tubulin gamma-1 chain	0.0207	−6.03		P09238	Stromelysin-2 (Matrix metalloproteinase-10)	0.0011	−1.00
**Signaling by Receptor Tyrosine Kinases**					**mRNA Splicing**				
Gen IC_20_	P13942	Collagen alpha-2(XI) chain	0.0209	7.06	***17S U2 snRNP***				
	Q96N67 P16591	Dedicator of cytokinesis protein 7 Tyrosine-protein kinase Fer	0.0342 0.0007	5.06 4.98	Gen IC_20_	P14678	Small nuclear ribonucleoprotein-associated proteins B and B’	0.0000	−2.44
						P62318	Small nuclear ribonucleoprotein Sm D3	0.0171	−1.20
Dai IC_20_	P19388	DNA-directed RNA polymerases I_ II_ and III subunit RPABC1	0.0031	−8.48		Q7L014	Probable ATP-dependent RNA helicase DDX46	0.0004	−1.16
	Q9H6T0	Epithelial splicing regulatory protein 2	0.0003	−3.74	***RNA Polymerase II Transcription Termination***				
	Q13464	Rho-associated protein kinase 1	0.0049	−3.44	SSE IC_20_	P14678	Small nuclear ribonucleoprotein-associated proteins B and B’	0.0034	−2.60
**Cell Cycle**						P62318	Small nuclear ribonucleoprotein Sm D3	0.0028	−1.92
Gen SC_20_	P23258	Tubulin gamma-1 chain	0.0396	−11.85		P26368	Splicing factor U2AF 65 kDa subunit	0.0003	−1.54
	P50402	Emerin	0.0437	−11.60					
	P19388	DNA-directed RNA polymerases I_ II_ and III subunit RPABC1	0.0472	−11.12					

## Supplementary Materials

The following are available online at https://www.mdpi.com/2075-4426/10/4/292/s1, Supplementary methods LC-MS^E^ and search parameters, Figure S1: Principal component analysis displaying the sources of variance for each cell line, Table S1: Overview on the abundance of all relatively quantified 5180 proteins, Table S2: Overview on all relatively quantified 5180 proteins statistical analysis.
