# Supplementary material for: From Proteomics to Personalized Medicine: The Importance of Isoflavone Dose and Estrogen Receptor Status in Breast Cancer Cells
Source: J Pers Med. 2020 Dec 19;10(4):292. doi: 10.3390/jpm10040292 (PMC7766658; doi:10.3390/jpm10040292)
Supplement: Supplementary file 1 [file jpm-10-00292-s001.zip › Supplementary methods_LC-MSE and search parameters.pdf]

Supplementary methods. LC-MS^E and search parameters

Description of experiment settings for LC-MS^E analysis and identification of proteins

**LC-Parameters**	
LC gradient<br><br>A: 0.1 % acetic acid in water<br><br>B: 0.1 % acetic acid in acetonitrile	0min-1% B-2m-3%-100m-25%-110m-35%-112m-99%-114m-1%-130m-1%
Flow rate	0.300µL/min
Column temperature	45°C
**MS^E-Parameters**	
Lock spray	GluFib precursor 785.8426 m/z
Acquisition times	10-110min
Acquisition mode	positive, resolution
Mass range	50-2000Da
Capillary voltage	2kV
Sampling cone	40V
Source offset	80V
Source temperature	80°C
Nebuliser gas flow	7 bar
Trap collision energy	4V
Transfer collision energy	2V

Protein search parameter	Settings
Name of peak list-generating software and release version (number or date)	Progenesis QI v2.0 (Waters Corporation)
Name of the search engine and release version (number or date)	MS^E built in search engine of Progenesis QI v2.0 (Waters Corporation)
Name of database searched and release version/date	Swissprot database release 2019/03 limited to human entries
Enzyme specificity considered	Fully tryptic
# of missed cleavages permitted	1
Fixed modification(s) (including residue specificity)	carbamidomethylation of cysteine
Variable modification(s) (including residue specificity)	oxidation of methionine
Fragment ions per protein	5
Fragment ions per peptide	2
Threshold score for accepting protein identification	≥1 significant peptides
Threshold score/E-value for accepting *individual* MS/MS Spectra	False discovery rate at peptide level <4%
software/method used to evaluate site assignment	No PTM reported

Presentation of Protein Identification Results

Information requested	Reported
Accession number	UniProtKB number and Entry name
Number of *unique* (in terms of amino acid sequence) peptides identified	Only *non-conflicting* peptides have been considered (Progenesis QI v2.0)
