# Supplementary material for: From Proteomics to Personalized Medicine: The Importance of Isoflavone Dose and Estrogen Receptor Status in Breast Cancer Cells
Source: J Pers Med. 2020 Dec 19;10(4):292. doi: 10.3390/jpm10040292 (PMC7766658; doi:10.3390/jpm10040292)
Supplement: Supplementary file 1 [file jpm-10-00292-s001.zip › Supplementary Table S1. Overview on the abundance of all relatively quantified 5180 proteins.pdf]

Supplementary Table S1. Overview on the abundance of all relatively quantified 5180 proteins

			MCF-7																	
	Protein name	UniProt	control 01	control 02	control 03	Dai IC20 01	Dai IC20 02	Dai IC20 03	Dai SC20 01	Dai SC20 02	Dai SC20 03	Gen IC20 01	Gen IC20 02	Gen IC20 03	Gen SC20 01	Gen SC20 02	Gen SC20 03	SSE IC20 01	SSE IC20 02	SSE IC20 03
1	(E2-independent) E3 ubiquitin-conjugating enzyme FATS	Q96M02	117.06	137.88	152.50	130.26	189.60	156.27	155.05	164.63	159.90	321.93	239.85	344.45	233.33	159.88	118.81	121.89	169.08	153.55
2	(E3-independent) E2 ubiquitin-conjugating enzyme	Q9C0C9	13019.97	13280.76	13113.12	9851.63	9362.35	9020.85	10350.59	10367.64	10366.16	11514.97	10834.53	10619.25	11016.42	10439.49	9310.26	8275.94	8223.79	8107.43
3	[F-actin]-monooxygenase MICAL1	Q8TDZ2	0.14	2.53	0.81	1.07	2.77	0.68	0.96	0.36	0.63	0.35	0.75	0.38	2.13	0.97	0.60	4.64	1.07	0.47
4	[F-actin]-monooxygenase MICAL3	Q7RTP6	103949.32	92637.50	86013.44	79015.05	75250.62	66776.60	83995.62	83599.45	76082.57	94012.87	90113.78	81133.59	87875.79	82451.44	57775.84	78692.75	74820.27	71794.76
5	[Protein ADP-ribosylarginine] hydrolase-like protein 1	Q8NDY3	33128.18	31755.91	31180.59	25500.43	24864.88	23601.00	27427.46	27127.28	25595.54	28111.64	27508.88	25909.27	28497.21	24748.58	21502.98	24526.92	23496.16	23816.56
6	[Pyruvate dehydrogenase [acetyl-transferring]]-phosphatase 1_ mitochondrial	Q9P0J1	0.14	2.53	0.81	3.49	3095.74	3618.59	790.06	506.88	420.66	0.35	0.75	0.38	2.13	0.97	0.60	4065.97	350.37	3025.25
7	[Pyruvate dehydrogenase [acetyl-transferring]]-phosphatase 2_ mitochondrial	Q9P2J9	0.14	2.53	0.81	1.07	2.77	0.68	0.96	0.36	0.63	0.35	0.75	0.38	2.13	0.97	0.60	4.64	1.07	0.47
8	1_25-dihydroxyvitamin D(3) 24-hydroxylase_ mitochondrial	Q07973	9283.21	8665.61	8511.87	6320.38	6514.54	6072.82	6804.36	6807.75	6567.66	7411.26	6926.97	6724.18	7703.36	6726.21	6445.46	5441.11	5968.06	5596.40
9	1_2-dihydroxy-3-keto-5-methylthiopentene dioxygenase	Q9BV57	149972.30	148931.94	130601.96	105231.12	98612.10	93124.75	123273.84	119128.87	118833.92	126163.02	118480.94	114100.76	128179.00	124867.47	100158.33	107951.19	98532.29	96051.80
10	1_4-alpha-glucan-branching enzyme	Q04446	159639.31	154552.77	145684.41	100831.45	86747.14	89231.34	112781.47	109332.43	105426.19	123309.46	121053.39	112128.42	142827.05	111120.79	57869.63	88073.49	80336.67	82112.57
11	10 kDa heat shock protein_ mitochondrial	P61604	594427.45	578141.78	536022.11	388746.69	380174.92	354353.57	504463.60	480174.52	471955.83	480285.34	455572.43	426608.68	466287.95	441034.66	362637.16	404370.84	373999.85	369024.87
12	116 kDa U5 small nuclear ribonucleoprotein component	Q15029	138349.61	139871.58	123450.14	90435.30	87747.90	83276.54	102816.58	97984.73	92909.51	108161.66	105872.59	98335.75	102083.82	105857.12	89280.87	84169.16	81084.86	77389.88
13	11-cis retinol dehydrogenase	Q92781	14282.88	15470.58	14703.98	10789.08	9983.69	9274.94	11889.26	11361.07	11804.02	12913.64	12069.13	11893.18	12779.07	13097.91	10393.10	10661.75	9889.78	9880.43
14	14 kDa phosphohistidine phosphatase	Q9NRX4	67637.50	63084.98	57852.50	53485.20	52200.92	45379.42	58086.85	51483.85	47967.67	67020.42	63202.03	61537.81	57178.45	57167.53	43167.28	54909.34	57843.81	49066.82
15	14-3-3 protein beta/alpha	P31946	1133233.06	1083410.73	1054919.12	820051.25	797725.24	731160.77	974192.23	910806.47	895986.73	964925.75	928336.69	891577.57	994912.10	924617.34	827705.81	835517.93	796344.04	750542.80
16	14-3-3 protein epsilon	P62258	1107312.58	1083394.30	1013850.53	827462.30	806228.48	766374.78	869651.58	835682.79	819242.07	962659.17	893955.60	861366.61	901695.86	905206.76	778400.41	775413.99	729557.99	717461.79
17	14-3-3 protein eta	Q04917	251646.45	244951.59	230734.71	184002.97	179952.04	172814.30	207998.64	200248.99	196965.93	219040.47	207320.76	197407.19	218051.46	209317.55	185750.32	178697.59	165401.39	163045.18
18	14-3-3 protein gamma	P61981	372025.88	369044.62	356615.06	289713.33	285198.67	274756.37	300874.50	292094.11	278068.10	351025.05	334766.21	332296.20	326470.44	311153.31	258790.56	249980.87	240739.60	246134.84
19	14-3-3 protein sigma	P31947	169366.83	153994.53	143469.31	115975.22	107467.15	103086.87	127134.98	121543.49	116823.61	147310.22	138203.31	127995.12	139396.20	145112.69	104439.86	105389.39	102093.17	96789.98
20	14-3-3 protein theta	P27348	1006078.45	980263.25	912238.27	690219.34	658454.94	642122.14	855922.96	836568.93	815200.67	817758.87	781363.92	740555.65	874208.66	782750.86	724011.62	752724.34	695647.97	685825.52
21	14-3-3 protein zeta/delta	P63104	1253188.46	1222300.15	1112990.54	947874.63	920188.70	849852.49	1092742.50	1040552.52	976128.46	1160508.08	1112093.78	1048369.17	1086746.00	1044242.42	882305.52	959912.30	902043.45	852829.26
22	182 kDa tankyrase-1-binding protein	Q9C0C2	303918.09	291051.40	266359.62	214846.98	207886.39	189265.40	234880.01	234893.75	214320.48	273147.50	254998.97	245531.25	258236.44	242543.75	183038.57	198432.89	195521.34	184241.51
23	1-aminocyclopropane-1-carboxylate synthase-like protein 1	Q96QU6	453.51	316.83	120.78	356.60	147.45	269.14	260.06	271.98	306.55	673.14	570.12	740.94	590.64	542.88	193.92	298.80	306.24	210.33
24	1-phosphatidylinositol 3-phosphate 5-kinase	Q9Y2I7	0.14	2.53	0.81	1.07	2.77	0.68	0.96	0.36	0.63	0.35	0.75	0.38	2.13	0.97	0.60	4.64	1.07	0.47
25	1-phosphatidylinositol 4_5-bisphosphate phosphodiesterase beta-2	Q00722	9947.77	8846.18	7400.64	7970.03	8629.17	7069.49	6802.61	5990.57	5159.05	17111.87	15490.52	15271.49	11670.69	10232.61	7929.36	7728.40	6853.83	6734.17
26	1-phosphatidylinositol 4_5-bisphosphate phosphodiesterase beta-3	Q01970	18634.89	17208.75	15686.58	8884.53	8895.95	7906.06	8646.32	8195.68	8038.53	12271.61	11860.72	10699.74	12626.83	9770.53	7371.15	9572.38	9302.00	8907.14
27	1-phosphatidylinositol 4_5-bisphosphate phosphodiesterase beta-4	Q15147	14335.69	12911.17	12568.52	9536.59	9142.72	8687.69	12227.14	11947.89	12812.83	11807.41	10948.68	11337.93	11130.24	11089.00	9019.01	9977.42	9904.81	9610.60
28	1-phosphatidylinositol 4_5-bisphosphate phosphodiesterase gamma-1	P19174	202056.02	198693.39	180035.40	134455.78	129449.99	121453.17	161970.28	157999.59	152265.56	170315.37	163477.87	150511.89	166831.80	155664.57	126836.61	135031.93	124899.20	124272.82
29	2'_3'-cyclic-nucleotide 3'-phosphodiesterase	P09543	86205.83	83832.43	69608.95	62077.67	64041.46	56397.71	61350.72	63144.60	58786.57	101699.16	106461.76	105720.24	78539.12	73526.91	57121.30	53561.74	53121.73	50321.69
30	2_4-dienoyl-CoA reductase_ mitochondrial	Q16698	73420.55	69932.13	67316.82	41282.27	39450.59	36477.90	49855.10	45952.31	44834.49	57168.92	55020.89	51328.55	53146.91	39575.53	29798.11	46648.24	40980.90	40557.59
31	26S proteasome non-ATPase regulatory subunit 1	Q99460	658755.99	630521.20	586949.45	453306.60	442818.54	400783.03	547473.72	513793.59	479515.28	551175.27	518910.37	483535.08	554831.09	503640.67	410076.06	473532.00	454520.81	426041.61
32	26S proteasome non-ATPase regulatory subunit 10	O75832	51441.32	47923.64	43925.94	33527.69	33476.96	30106.52	39015.50	36095.22	34603.98	44210.49	42308.31	40599.63	39937.80	35038.72	27888.10	35823.99	32647.66	30503.75
33	26S proteasome non-ATPase regulatory subunit 11	O00231	194187.54	183598.79	163218.32	133065.65	122421.57	119947.02	148535.68	138484.54	131940.81	147206.76	145099.92	134854.55	150087.56	150467.76	122801.22	127218.04	119927.09	121518.40
34	26S proteasome non-ATPase regulatory subunit 12	O00232	225811.06	212833.63	197829.05	149327.48	147762.83	136361.92	183338.02	176197.59	166117.39	178659.19	169770.02	159580.63	184206.65	175469.84	151268.21	155752.43	145524.64	137433.51
35	26S proteasome non-ATPase regulatory subunit 13	Q9UNM6	266039.46	258302.85	230099.47	163876.06	158465.82	146151.91	206608.87	198416.71	185411.01	204332.55	190901.99	175470.50	210516.41	190826.51	149173.06	172907.58	162903.27	152473.91
36	26S proteasome non-ATPase regulatory subunit 14	O00487	41214.33	38353.87	37352.46	24678.07	27167.66	24023.82	38112.01	37297.62	32701.01	29648.14	27721.05	27341.12	30141.54	30099.77	22582.95	32206.32	32258.96	28023.35
37	26S proteasome non-ATPase regulatory subunit 2	Q13200	363131.55	332826.23	304585.79	223842.73	217593.64	196653.94	268751.85	252432.27	242908.56	266874.05	252786.17	228916.54	269892.77	250579.82	207021.53	228689.21	229467.22	206551.70
38	26S proteasome non-ATPase regulatory subunit 3	O43242	462534.37	454297.02	429989.09	329305.91	319816.85	303685.08	384507.71	362759.74	350771.52	387030.40	370734.58	349439.19	383621.69	378318.37	326474.67	328933.32	308923.41	299256.66
39	26S proteasome non-ATPase regulatory subunit 4	P55036	197581.97	192982.59	178211.93	130824.18	129419.58	121035.26	153859.81	149738.79	146377.42	151528.26	143606.80	139580.65	152442.62	143166.47	124125.65	133628.56	128143.55	121305.59
40	26S proteasome non-ATPase regulatory subunit 5	Q16401	61747.02	55379.09	51683.89	31216.07	31001.93	27787.77	41319.83	39607.92	36255.61	45377.10	43062.59	39283.43	44800.86	35577.37	27900.71	33255.62	31346.39	30021.49
41	26S proteasome non-ATPase regulatory subunit 6	Q15008	253240.53	246673.07	243499.98	174865.50	168945.57	159523.73	194361.86	187556.80	183219.56	216125.98	207647.96	194899.78	193924.11	189202.91	157483.60	172691.15	165510.87	159942.34
42	26S proteasome non-ATPase regulatory subunit 7	P51665	30559.40	23525.06	22353.14	21176.69	19219.50	15187.80	21599.15	20107.91	17912.45	24699.75	22775.74	17835.96	24292.69	23118.16	10169.41	19155.06	18600.54	15773.14
43	26S proteasome non-ATPase regulatory subunit 8	P48556	34570.94	36635.33	31713.41	25165.66	24877.36	23472.09	23111.89	23454.46	21181.60	31894.50	32105.63	30612.04	31536.89	24358.12	26344.25	23559.41	24034.57	21878.04
44	26S proteasome non-ATPase regulatory subunit 9	O00233	100030.01	99530.38	92382.00	72435.90	69413.60	67799.29	81807.34	75890.78	70443.68	83397.20	79301.80	75972.84	79785.05	74144.83	65155.75	67529.93	69024.00	66382.27
45	26S proteasome regulatory subunit 10B	P62333	337348.56	299901.22	278322.31	224435.22	217648.06	194254.52	261816.70	248775.50	240282.29	269221.17	253608.90	233229.78	268516.33	245379.17	190959.47	220323.54	214236.60	204540.83
46	26S proteasome regulatory subunit 4	P62191	345270.28	330927.90	306290.22	240502.07	228332.84	211070.06	276974.93	247008.70	244459.10	287161.47	267815.73	248454.20	270852.13	253635.87	197449.86	238384.07	227262.71	215076.25
47	26S proteasome regulatory subunit 6A	P17980	596168.91	575397.55	544729.51	392059.76	395633.99	356113.87	494984.33	440985.20	421209.88	481667.81	461092.92	431614.70	485161.15	463097.74	376401.59	404394.97	381323.01	368158.25
48	26S proteasome regulatory subunit 6B	P43686	178480.79	165346.76	143203.98	127968.41	124687.05	109080.98	141478.80	132265.09	123201.67	145616.34	139032.29	126535.79	141744.03	140281.04	96916.19	124470.54	121130.06	111346.95
49	26S proteasome regulatory subunit 7	P35998	407688.86	396926.41	368121.58	297480.58	284550.41	255259.70	308702.37	294271.13	277234.60	346634.61	324781.02	308624.98	341377.25	313163.54	255522.24	288063.79	259337.37	256922.10
50	26S proteasome regulatory subunit 8	P62195	265951.93	250488.38	222273.05	177116.78	169479.17	151319.84	210315.33	193751.21	184846.20	215170.32	205878.88	191370.69	220496.93	186058.40	139959.67	182046.64	172944.47	158929.06
51	28 kDa heat- and acid-stable phosphoprotein	Q13442	145208.17	142235.82	126871.41	95934.21	92464.83	85275.65	113226.55	106972.87	100808.99	114226.73	106118.04	99424.06	122997.45	116656.24	91087.18	91963.40	86389.12	82413.43
52	28S ribosomal protein S14_ mitochondrial	O60783	1688.58	1462.93	1360.91	1446.54	1255.94	1237.67	1499.75	1447.53	1342.95	1631.86	1567.76	1344.54	1650.08	1626.19	1331.25	1131.11	1102.32	975.57
53	28S ribosomal protein S16_ mitochondrial	Q9Y3D3	28053.37	27298.31	24796.97	17485.65	17426.78	15284.75	22016.24	20318.06	19402.98	21760.71	19612.47	20154.65	20753.80	19742.57	17125.76	17023.87	16712.21	15029.48
54	28S ribosomal protein S17_ mitochondrial	Q9Y2R5	13819.09	11700.47	8618.05	5835.11	5623.75	4112.09	8056.83	7700.59	6524.67	10061.69	9290.93	7817.24	10055.02	6428.15	3043.58	4986.52	4877.58	4545.04
55	28S ribosomal protein S18b_ mitochondrial	Q9Y676	25678.10	24000.08	21914.79	16786.18	15950.90	14997.56	17274.57	17922.97	15985.86	21979.76	20579.69	19216.52	20909.66	20411.55	16534.31	15885.25	15528.20	14746.74
56	28S ribosomal protein S2_ mitochondrial	Q9Y399	8657.94	8455.48	8722.34	6537.40	6531.24	6471.55	7029.75	6810.00	6032.83	9359.79	8569.92	8408.94	7532.79	8003.29	6824.60	6010.83	5470.60	5798.23
57	28S ribosomal protein S21_ mitochondrial	P82921	0.14	2.53	0.81	1.07	2.77	0.68	0.96	0.36	0.63	0.35	0.75	0.38	2.13	0.97	0.60	4.64	1.07	0.47
58	28S ribosomal protein S22_ mitochondrial	P82650	59715.25	58454.43	55586.39	40617.30	39657.06	38342.96	41253.68	39568.57	40739.86	52224.75	50114.33	45548.45	46190.87	44661.95	34511.00	45086.30	37751.45	37020.35
59	28S ribosomal protein S23_ mitochondrial	Q9Y3D9	88162.51	84479.02	79834.18	55781.82	52711.57	48373.15	67687.85	67828.90	57704.47	69561.00	68502.84	66096.58	68743.25	62859.35	49212.95	53742.18	52305.89	54011.13
60	28S ribosomal protein S25_ mitochondrial	P82663	8280.59	11538.29	11564.04	8708.52	6340.92	4468.51	6172.62	9064.48	5688.47	10807.66	9946.50	11159.22	9790.08	11056.62	10164.32	6827.67	4076.25	6214.87
61	28S ribosomal protein S26_ mitochondrial	Q9BYN8	7752.95	6532.00	5979.85	5217.23	5222.21	5231.04	5875.55	6507.65	5727.11	6261.39	6300.82	5920.07	6588.15	6125.60	4396.84	5084.69	4766.47	4371.89
62	28S ribosomal protein S27_ mitochondrial	Q92552	13817.51	12828.94	12743.34	10424.23	10224.47	9716.51	12247.96	12067.07	11835.88	11451.38	11045.92	9886.63	12593.51	11503.73	8509.08	11064.28	10706.75	9626.75
63	28S ribosomal protein S29_ mitochondrial	P51398	32499.13	30197.44	27026.23	18317.92	18412.82	15797.29	23809.30	21790.74	20474.17	24594.73	23298.61	20562.31	22565.04	21239.96	14497.93	20538.28	19758.19	19135.05
64	28S ribosomal protein S31_ mitochondrial	Q92665	1060.78	980.03	1282.37	672.23	555.10	581.52	1018.54	1075.63	1036.15	652.81	708.78	680.00	927.82	698.01	687.17	885.33	711.97	694.28
65	28S ribosomal protein S33_ mitochondrial	Q9Y291	21734.86	19704.16	17552.51	18503.15	17106.53	15574.11	16612.07	14388.62	14931.86	19742.01	19011.49	16009.66	19470.73	18395.46	16797.07	13662.07	12505.31	13129.53
66	28S ribosomal protein S34_ mitochondrial	P82930	41710.75	38172.71	34600.52	25151.13	25294.93	22971.40	28238.15	28841.81	27199.54	34830.35	33341.66	31127.39	32522.28	31026.81	28112.16	25419.27	24442.09	23554.45
67	28S ribosomal protein S35_ mitochondrial	P82673	57846.25	55986.23	54834.26	42852.41	42606.66	40551.05	47914.07	47028.30	46877.94	49719.08	46212.99	46505.43	53574.69	51059.74	42422.23	38477.10	38544.40	36312.99
68	28S ribosomal protein S36_ mitochondrial	P82909	1593.66	1438.38	1404.10	970.20	895.22	786.64	1742.26	1654.54	1088.08	1352.20	1380.13	1145.47	1275.39	1394.02	723.93	1382.54	1412.37	1313.72
69	28S ribosomal protein S5_ mitochondrial	P82675	0.14	2.53	0.81	1.07	2.77	0.68	0.96	0.36	0.63	0.35	0.75	0.38	2.13	0.97	0.60	4.64	1.07	0.47
70	28S ribosomal protein S6_ mitochondrial	P82932	9587.34	9553.10	9273.69	7018.54	6627.73	6045.63	8502.35	8549.31	8420.79	9434.57	9389.47	8858.08	8913.51	8110.82	6568.00	7088.27	6197.80	5866.18
71	28S ribosomal protein S7_ mitochondrial	Q9Y2R9	82107.96	78004.07	76235.93	47963.51	44041.56	44090.63	60852.18	58564.77	52689.70	61682.40	60268.91	54906.06	59623.50	53955.90	44948.07	59569.68	54029.62	52694.67
72	28S ribosomal protein S9_ mitochondrial	P82933	85560.61	80557.05	75592.67	59609.14	59713.39	54753.52	67712.43	66918.41	60339.88	77013.29	71867.84	65290.53	65197.34	65971.90	49688.28	61750.48	61311.52	55969.39
73	2'-deoxynucleoside 5'-phosphate N-hydrolase 1	O43598	89986.00	85302.36	82192.82	56994.92	51500.01	50245.05	67472.93	65138.83	60190.47	70617.25	67840.12	66478.32	70154.88	60966.42	38699.97	44549.97	52030.36	52788.55
74	2-iminobutanoate/2-iminopropanoate deaminase	P52758	0.14	2.53	0.81	1.07	2.77	0.68	0.96	0.36	0.63	0.35	0.75	0.38	2.13	0.97	0.60	4.64	1.07	0.47
75	2-methoxy-6-polyprenyl-1_4-benzoquinol methylase_ mitochondrial	Q5HYK3	0.14	2.53	0.81	2.13	259.02	145.25	1.92	2.05	1.25	0.35	0.75	0.38	2.13	0.97	0.60	866.25	2.47	258.56
76	2-oxoglutarate dehydrogenase_ mitochondrial	Q02218	131665.09	125413.54	118796.76	92421.23	87758.54	84156.86	104853.57	101652.20	98628.24	110821.21	108678.06	100744.56	111968.74	97456.32	97521.70	92712.71	90836.87	89432.34
77	2-oxoisovalerate dehydrogenase subunit alpha_ mitochondrial	P12694	1936.05	2038.26	2030.32	1031.41	825.17	674.67	1389.73	1285.69	1386.37	1658.97	1753.16	2123.92	1697.07	1321.19	1003.23	881.30	1029.03	1174.25
78	3'-5' RNA helicase YTHDC2	Q9H6S0	107357.74	85231.19	64399.92	75017.89	71231.18	62028.06	80672.85	86674.23	77397.79	85744.96	81773.35	73967.35	90703.40	86943.50	50121.47	62575.82	60666.53	54365.08
79	39S ribosomal protein L1_ mitochondrial	Q9BYD6	21821.44	21255.71	21802.34	12687.20	13022.99	10477.10	16639.57	16218.98	15228.35	16437.16	15729.42	14679.03	15803.67	16147.10	11834.59	14344.21	13986.88	12810.54
80	39S ribosomal protein L10_ mitochondrial	Q7Z7H8	686.42	351.67	468.65	847.81	819.85	627.31	296.70	507.60	452.88	756.06	863.34	755.01	754.87	540.12	651.90	413.32	544.69	518.31
81	39S ribosomal protein L11_ mitochondrial	Q9Y3B7	21266.84	20070.02	19948.97	13864.38	14244.55	11541.20	17890.87	17429.33	16625.40	18022.96	17754.48	15313.90	17120.60	16865.95	14474.12	15765.91	15234.57	14250.63
82	39S ribosomal protein L12_ mitochondrial	P52815	77051.73	75675.73	67905.29	48862.17	47648.82	41750.15	58048.89	57169.95	54861.63	55764.61	52063.13	49621.30	54147.90	53658.95	37238.36	46802.19	44607.69	42592.70
83	39S ribosomal protein L13_ mitochondrial	Q9BYD1	35478.19	36872.77	31765.74	24243.99	21530.45	20681.19	26425.32	25416.24	24217.27	28201.96	26686.66	25237.81	29252.11	26311.99	18970.17	23152.15	21208.73	21243.11
84	39S ribosomal protein L14_ mitochondrial	Q6P1L8	336.24	419.09	446.73	328.41	328.60	245.13	373.65	374.67	277.88	490.09	612.51	421.71	475.89	426.11	225.31	323.67	370.88	322.42
85	39S ribosomal protein L15_ mitochondrial	Q9P015	4995.81	5099.01	4609.32	3267.51	3465.60	3033.40	4592.97	3462.72	3767.71	4699.81	4645.91	4063.41	4685.66	3406.91	2976.57	3796.80	3110.17	3379.43
86	39S ribosomal protein L16_ mitochondrial	Q9NX20	4290.78	3878.57	3548.57	2304.81	1997.13	2320.24	2969.91	2844.74	2710.36	2810.18	2424.20	2359.02	2623.94	2367.60	1740.24	3123.62	2839.74	2925.77
87	39S ribosomal protein L17_ mitochondrial	Q9NRX2	65879.11	59376.58	59275.05	45863.57	48610.27	51518.28	48930.32	54128.45	50180.42	56332.94	53879.47	53055.26	54644.02	51336.68	43002.07	42051.81	53023.07	50813.50
88	39S ribosomal protein L19_ mitochondrial	P49406	47359.35	48807.30	47844.65	37716.81	36623.70	34997.69	33975.43	30939.29	28762.28	41820.76	41171.92	37728.48	39520.08	35691.15	32177.87	34667.67	31558.52	31786.61
89	39S ribosomal protein L2_ mitochondrial	Q5T653	13028.21	11370.39	10066.49	8539.77	8906.68	8248.17	8301.10	7768.58	7529.01	10635.64	10571.78	9514.28	10264.66	9953.29	6813.87	7902.09	7395.46	8167.06
90	39S ribosomal protein L21_ mitochondrial	Q7Z2W9	6713.59	6892.62	5707.59	4875.77	4543.08	4093.44	4525.16	4621.71	4259.24	5606.25	5462.82	5705.46	5692.09	5282.81	4764.98	3846.14	4112.11	3773.55
91	39S ribosomal protein L22_ mitochondrial	Q9NWU5	6311.24	5577.80	4717.25	3767.25	3820.07	3516.95	4081.50	3865.09	3538.72	5231.55	5418.76	4855.58	4902.35	4284.76	2905.18	4327.10	3784.20	3991.02
92	39S ribosomal protein L24_ mitochondrial	Q96A35	16431.35	18950.37	17836.43	12774.42	12193.56	11223.69	12226.51	11583.91	11893.81	14173.39	14060.85	14482.91	13652.35	11368.52	11068.11	12683.46	12069.79	11390.63
93	39S ribosomal protein L28_ mitochondrial	Q13084	21099.06	20489.40	19689.57	13927.40	14223.44	13277.10	17251.84	16193.45	16307.38	16715.98	15172.09	15439.59	17462.18	15546.92	13058.19	13618.73	13215.86	12539.42
94	39S ribosomal protein L3_ mitochondrial	P09001	7148.94	6820.11	5608.22	4311.24	3769.48	2627.83	5358.53	5439.94	3846.83	6036.40	5235.04	4515.61	4868.07	5910.80	2115.57	4798.74	4681.15	3548.19
95	39S ribosomal protein L30_ mitochondrial	Q8TCC3	1915.04	1595.24	1508.31	1491.69	1261.12	1021.42	1416.45	1453.13	1201.96	1706.71	1752.56	1698.49	1359.82	1589.26	990.85	1566.32	1361.11	1183.35
96	39S ribosomal protein L32_ mitochondrial	Q9BYC8	1963.18	1505.54	1731.80	1269.97	1413.82	1286.96	815.69	828.53	889.66	1166.49	1378.42	1371.05	1404.23	1485.44	1422.00	905.06	928.35	972.82
97	39S ribosomal protein L37_ mitochondrial	Q9BZE1	78642.55	71239.25	71602.86	60219.33	56498.16	57503.76	62490.50	64865.79	58566.17	73870.21	67278.11	63487.61	65814.69	63972.27	58821.94	54602.35	53194.40	53699.86
98	39S ribosomal protein L38_ mitochondrial	Q96DV4	0.14	2.53	0.81	1.07	2.77	0.68	0.96	0.36	0.63	0.35	0.75	0.38	2.13	0.97	0.60	4.64	1.07	0.47
99	39S ribosomal protein L39_ mitochondrial	Q9NYK5	22872.32	22968.27	21968.62	19212.85	18440.93	18047.84	19264.45	19338.92	18704.81	20849.47	19885.46	19063.26	21232.52	19853.70	13960.71	18387.99	16984.61	16001.17
100	39S ribosomal protein L4_ mitochondrial	Q9BYD3	2184.51	2385.37	2237.32	1446.10	1239.74	1273.73	1944.64	1900.85	1719.09	1805.31	1617.23	1446.58	2000.45	1788.28	1264.09	1396.33	1183.14	1279.11
101	39S ribosomal protein L40_ mitochondrial	Q9NQ50	57583.59	52918.52	53318.17	35444.64	35429.54	32220.89	46823.07	44496.19	40285.39	44597.97	44054.34	43227.59	48168.71	46762.88	37330.98	38065.44	38363.30	33378.50
102	39S ribosomal protein L41_ mitochondrial	Q8IXM3	1090.90	692.45	402.87	1.07	2.77	0.68	0.96	0.36	0.63	325.84	217.03	208.75	2.13	0.97	0.60	4.64	1.07	0.47
103	39S ribosomal protein L43_ mitochondrial	Q8N983	3693.26	3776.95	3637.73	2565.71	2500.19	2185.08	2347.54	2201.31	2100.27	2999.26	2822.50	2602.35	2621.37	2679.03	2419.77	2236.27	2369.52	2142.65
104	39S ribosomal protein L44_ mitochondrial	Q9H9J2	15134.31	14688.63	13023.63	9529.56	9678.32	9947.36	12047.06	12601.06	12312.56	15793.91	15124.83	14138.60	11296.07	10289.45	8837.63	11141.83	10467.36	10531.70
105	39S ribosomal protein L45_ mitochondrial	Q9BRJ2	11646.24	10551.18	8454.61	3209.11	3179.28	3114.58	3693.41	3931.58	3772.15	9930.23	7545.79	6442.45	8922.10	4758.51	3229.33	3150.74	3133.47	3036.68
106	39S ribosomal protein L46_ mitochondrial	Q9H2W6	0.14	2.53	0.81	1.07	2.77	0.68	0.96	0.36	0.63	0.35	0.75	0.38	2.13	0.97	0.60	4.64	1.07	0.47
107	39S ribosomal protein L47_ mitochondrial	Q9HD33	13060.08	11289.05	10288.29	7641.79	6792.76	5194.13	8182.21	12419.94	7207.29	10273.13	8946.78	7222.07	10647.14	11951.51	7044.80	6179.20	9115.86	4417.38
108	39S ribosomal protein L48_ mitochondrial	Q96GC5	15104.41	15003.11	14886.27	10949.94	10566.11	11027.68	13318.60	12435.10	12074.87	13595.30	13326.84	12875.21	13636.09	12123.27	10540.84	11483.65	10471.94	10480.23
109	39S ribosomal protein L49_ mitochondrial	Q13405	21980.13	21607.73	18338.09	15556.20	14910.34	13302.43	17101.28	17849.64	16737.09	18177.43	17168.12	16027.35	17680.78	16979.28	13188.61	17807.50	15751.19	15691.86
110	39S ribosomal protein L50_ mitochondrial	Q8N5N7	40442.06	39725.57	35406.70	25178.26	24075.03	23050.92	29428.46	28956.70	27135.04	32293.90	32340.74	30288.41	31460.40	31284.49	23213.11	25263.73	24576.35	23785.57
111	39S ribosomal protein L52_ mitochondrial	Q86TS9	6584.38	6308.15	6089.72	4829.98	4501.54	4042.01	4357.61	4132.59	4836.44	6159.75	5750.35	5696.87	6006.96	5533.91	4212.44	4272.98	4433.25	4267.25
112	39S ribosomal protein L53_ mitochondrial	Q96EL3	31802.05	31840.98	30783.97	28713.38	25087.13	24597.21	32475.08	28991.97	28352.87	31770.24	30294.13	29870.80	27444.29	25282.72	22542.23	26204.95	24032.52	25550.70
113	39S ribosomal protein L54_ mitochondrial	Q6P161	21693.49	20496.64	19729.40	14138.94	14801.77	14159.41	19427.53	18527.43	18261.00	19286.81	18775.52	17533.26	17934.88	18295.97	14303.58	15231.29	13187.43	13827.63
114	39S ribosomal protein L9_ mitochondrial	Q9BYD2	17367.61	15781.43	13048.90	10139.84	9875.92	9068.45	11029.26	10146.66	9406.76	13147.75	13137.93	12832.94	11972.68	12396.86	7484.14	11187.78	8297.58	7062.20
115	39S ribosomal protein S30_ mitochondrial	Q9NP92	9769.59	7455.72	6111.18	2316.40	2438.84	1708.72	3536.22	3063.80	2713.93	5406.73	4974.95	3790.33	8452.55	3234.97	1587.56	2787.00	2447.19	2087.55
116	3-hydroxy-3-methylglutaryl-coenzyme A reductase	P04035	13925.81	15653.53	12694.66	8880.96	8013.84	6505.03	10581.36	10260.25	9582.17	10368.37	9686.35	9158.04	11231.98	9578.17	7683.08	10074.63	8963.82	8402.53

				MDA-MB-231													
	SSE SC20 01	SSE SC20 02	SSE SC20 03	control 01	control 02	control 03	Dai IC20 01	Dai IC20 02	Dai IC20 03	Gen IC20 01	Gen IC20 02	Gen IC20 03	SSE IC20 01	SSE IC20 02	SSE IC20 03	unique to MCF-7	unique to MDA-MB-231
1	130.20	192.21	166.97	2.27	1.49	0.65	0.84	1.31	0.69	4.16	2.47	0.11	4.90	4.98	0.12	x	
2	9439.78	8601.68	8769.68	297.81	10.06	223.28	206.53	261.52	56.36	313.86	334.68	354.85	288.50	216.63	20.04		
3	2.22	0.19	0.06	46581.79	44123.66	38020.70	25732.39	25615.22	21916.18	38866.22	38955.17	35026.82	36908.09	36599.26	24738.72		x
4	81009.22	72634.84	66972.30	23245.06	19431.22	19446.23	16102.07	16217.02	14418.50	18165.83	17550.41	16033.79	18072.15	17826.21	23702.50		
5	24397.00	21919.52	20408.34	39028.78	38270.84	36682.71	26035.56	26939.87	26525.17	31269.45	30138.44	30244.25	35067.12	33099.81	31070.86		
6	2.22	0.19	0.06	40320.81	38905.70	37021.75	29028.76	28493.24	23883.64	33704.09	33698.78	31538.16	32272.61	32669.19	21063.54		
7	2.22	0.19	0.06	11012.78	11991.24	10107.88	6837.92	6144.03	4728.93	9395.32	9041.86	8175.67	8527.75	7977.33	4659.73		x
8	6781.64	6618.57	6411.94	7491.76	7660.28	7300.79	6178.34	5976.19	5415.23	6129.95	6514.49	6580.22	5781.92	5661.74	3991.12		
9	115352.21	103970.75	102204.55	7199.96	7230.79	6582.07	5399.75	4936.08	4318.70	5603.36	5410.69	5052.73	5740.00	5711.35	3708.73		
10	108042.11	100132.27	97297.47	83800.52	74139.52	69913.94	55963.71	53821.19	48199.57	64515.42	60612.66	53182.82	66616.47	64972.68	46601.48		
11	413992.42	399024.76	367839.50	437658.80	397629.33	396018.64	267871.22	263247.75	234840.87	297732.00	279605.78	271309.12	301811.27	302703.53	264309.68		
12	92700.18	88511.22	84307.65	60616.54	53199.08	47103.16	38204.79	34667.71	30299.20	46260.88	43558.85	42019.56	43225.08	40808.85	27204.64		
13	9838.79	9157.39	9312.53	2.27	1.49	0.65	0.84	1.31	0.69	4.16	2.47	0.11	4.90	4.98	0.12	x	
14	48563.54	48297.30	44244.74	22511.90	20396.39	19540.78	10426.11	9267.46	7608.64	12919.06	12164.96	11385.13	11675.58	11763.71	7189.74		
15	854163.06	800594.93	757148.43	856532.22	797758.29	740116.10	634075.61	620240.94	578106.19	678008.22	650566.48	640748.24	700345.71	653509.29	374486.61		
16	799887.98	770975.59	739612.53	1643267.07	1527877.07	1453184.40	1098132.96	1085124.31	1002589.45	1295178.63	1255611.35	1208358.46	1277772.14	1224332.89	900173.06		
17	194739.54	185931.75	178839.87	241146.65	220632.42	205505.04	155974.01	154914.64	136897.64	175730.21	166704.09	160235.45	181501.05	180541.52	120411.65		
18	278431.23	273983.80	258999.75	117626.67	118176.70	109302.04	89778.54	88162.68	85645.48	92772.52	89113.64	87950.51	99131.05	95051.58	76638.40		
19	112525.35	107881.58	101005.64	219741.08	204142.92	186821.02	150327.50	144513.36	129098.42	159814.50	150560.75	143457.16	170933.51	169879.14	104903.33		
20	757046.73	690766.33	680104.87	338994.60	306087.83	301264.45	211795.03	205329.49	189600.12	257822.77	240743.51	230429.60	254837.78	242520.47	174573.87		
21	903281.81	867528.39	820842.04	1027029.35	980567.32	878509.52	548178.76	521103.07	496215.43	579512.58	563406.67	533097.18	638723.25	608643.69	393408.95		
22	228534.83	201970.71	188744.36	73500.58	64131.21	62862.75	32567.75	32831.76	28473.40	62818.68	60604.18	60017.46	44385.79	42057.38	31835.84		
23	236.34	234.46	206.26	2.27	1.49	0.65	0.84	1.31	0.69	4.16	2.47	0.11	4.90	4.98	0.12	x	
24	2.22	0.19	0.06	96.12	77.79	16.15	0.84	1.31	0.69	94.50	116.29	41.68	98.45	111.42	25.88		x
25	6249.53	6717.35	6986.88	269.64	278.68	285.45	268.46	272.44	244.49	283.76	278.95	301.90	388.14	310.09	215.30		
26	10981.21	10725.52	9293.50	6196.47	5836.79	5408.36	3300.35	3507.36	3028.04	4765.83	4715.01	4455.54	5212.34	4805.08	3104.12		
27	10113.74	9529.35	8695.32	2130.83	1796.92	1832.23	246.50	214.50	131.14	387.00	358.20	324.91	134.13	168.70	30.81		
28	140157.74	131392.10	123604.53	10206.31	9474.92	8327.98	6121.01	5741.83	4470.33	7379.38	7209.50	6552.37	7371.64	7044.16	4487.95		
29	61728.53	61011.42	60613.77	197674.99	181063.77	158900.68	135434.60	124721.27	113349.41	152907.48	149355.88	143180.05	151490.57	143885.95	99854.57		
30	45531.41	43755.81	40471.95	14483.19	13338.63	12667.71	8679.05	8663.69	7359.09	10782.62	10388.97	10032.27	12314.48	11188.31	5267.17		
31	489522.74	483450.92	446286.55	228730.14	210064.76	192375.79	155012.71	148195.51	134346.71	177842.42	162635.75	155419.12	186892.24	176270.72	110052.57		
32	32709.57	32018.31	28808.55	2190.82	2032.54	1909.41	200.44	254.13	250.32	292.60	270.28	244.68	190.49	147.39	93.28		
33	123961.54	121541.55	113545.01	115507.43	111851.38	105199.96	67909.87	67619.13	55442.12	92551.47	88870.56	85656.33	87924.52	82986.55	53683.82		
34	149627.41	141176.38	138214.28	190365.29	174915.47	160235.38	126242.97	122692.58	102942.13	149396.77	143417.25	132751.87	156862.11	149803.86	113620.17		
35	167454.11	159154.80	147159.08	237603.52	226810.88	199947.58	144119.57	141214.15	125116.17	169470.88	162912.61	153623.13	168557.32	160735.97	115849.88		
36	26608.39	24933.08	24058.32	52511.82	49136.28	44740.21	32984.37	31678.89	26230.46	40276.81	40516.04	39955.68	40286.77	40487.15	27197.14		
37	231327.83	209691.53	186040.57	269365.32	256895.10	233675.73	177796.31	171550.04	159509.39	220318.80	209110.49	200566.82	215290.60	206502.33	157909.12		
38	337830.88	321433.78	310972.39	220803.14	202162.10	190322.45	145851.10	143979.92	121508.07	182343.19	172496.82	165653.40	177845.98	169168.35	113399.09		
39	130862.19	123352.69	118790.31	125378.45	113433.60	107020.40	80868.83	79030.02	66921.39	88243.51	86748.74	82851.92	96387.67	94165.71	60414.52		
40	31145.34	30484.04	28039.67	53797.42	44538.09	39990.12	24137.94	24672.86	19593.21	34067.48	34119.93	31872.33	35438.43	32541.42	21554.43		
41	160966.81	157276.39	149509.72	221537.14	214033.55	192790.18	136122.67	134223.07	126297.52	170998.17	166468.53	156721.24	168062.95	167630.47	117911.13		
42	20178.65	19543.91	14650.68	20036.63	17752.59	16197.01	13141.92	11641.41	9052.30	15549.63	13519.72	12226.78	18984.91	17414.37	16292.82		
43	26097.65	23853.24	24185.16	86511.66	80176.79	76037.53	55002.20	55866.70	52303.18	63330.35	63601.65	60816.94	63358.98	61567.91	48888.40		
44	71291.22	67172.28	66134.82	52042.19	48625.58	46059.04	35902.01	35141.99	33172.69	42437.95	41403.58	39655.81	41830.96	40779.51	27902.90		
45	224542.09	212666.28	198688.07	254750.08	245924.30	206017.23	161302.84	160325.31	135397.15	196668.12	186368.54	169652.13	212335.65	198777.41	129600.80		
46	238102.41	234673.97	209825.94	139172.64	128928.77	117907.34	97485.07	91532.01	76396.15	126168.10	116047.68	112411.63	122916.71	118249.26	76715.02		
47	388346.00	382221.36	360741.67	191827.10	168509.40	149899.48	116219.40	111680.43	95299.86	160650.58	151877.13	140466.64	154066.98	145750.82	93543.88		
48	132303.85	125252.16	109710.73	137426.46	131315.18	120301.51	91099.38	89037.73	74516.57	116851.40	103953.22	97492.26	118972.29	116082.15	75595.48		
49	296225.07	284709.36	263226.89	246435.92	227651.57	215555.20	162645.52	154113.96	133813.64	188002.81	173584.32	167874.90	183459.23	174681.27	113857.93		
50	176660.78	167485.00	152370.05	327390.79	310133.27	281650.97	191625.86	183711.10	152281.91	228509.32	216393.06	205804.03	220160.77	208349.33	143676.97		
51	103837.43	98006.85	90442.33	11517.47	11031.01	10514.83	7647.32	7414.39	6698.83	10324.98	9223.58	8310.97	10497.40	10262.37	6317.24		
52	1279.42	1111.69	1238.78	138.38	111.87	101.79	83.54	90.35	9.46	89.07	58.12	34.09	129.97	126.48	21.91		
53	17723.20	16515.61	15154.74	6843.40	6944.50	7035.25	5029.85	4843.60	4085.30	5832.28	5866.02	5320.41	5961.45	5720.30	4119.44		
54	7645.96	5565.05	5296.43	4952.19	4360.06	4039.90	1290.73	1328.14	895.31	703.02	688.61	657.07	761.18	751.65	331.38		
55	18767.36	16899.55	15759.72	2002.94	1710.63	1442.59	1576.41	1522.66	1276.31	1630.58	1506.61	1226.88	1999.50	1975.85	1275.04		
56	7051.13	6586.88	5304.07	35494.65	34248.79	30341.96	22989.48	23333.35	21102.24	24955.99	24209.52	23965.75	30575.33	29291.13	22485.10		
57	2.22	0.19	0.06	2904.49	2634.24	2477.66	1300.37	1316.17	891.35	1572.88	1353.17	1315.76	1299.32	1207.52	536.85		x
58	44731.68	42914.80	41565.72	42808.94	40800.82	37781.05	27960.52	26433.31	22622.29	30307.42	29283.25	26896.88	35636.43	33086.45	22570.43		
59	58895.31	53343.89	51221.81	6055.24	8944.56	9439.65	6874.65	6231.48	5889.10	8278.93	8354.94	8358.07	7620.08	7033.82	5188.95		
60	10019.38	7649.18	7184.81	2.27	1.49	0.65	0.84	1.31	0.69	4.16	2.47	0.11	4.90	4.98	0.12	x	
61	4900.58	4676.48	4581.51	2.27	1.49	0.65	0.84	1.31	0.69	4.16	2.47	0.11	4.90	4.98	0.12	x	
62	9449.96	8928.31	8392.28	41163.56	40276.92	39535.46	29730.15	30337.07	26953.37	33348.42	31897.39	30012.45	35952.84	34425.08	25650.25		
63	19515.80	18154.20	17475.83	14872.85	14196.60	11936.57	8624.46	7935.27	6107.12	10802.40	10176.04	9249.20	11330.81	10477.64	6140.84		
64	715.40	581.29	593.41	2.27	1.49	0.65	0.84	1.31	0.69	4.16	2.47	0.11	4.90	4.98	0.12	x	
65	15353.61	14698.17	14963.84	2.27	1.49	0.65	0.84	1.31	0.69	4.16	2.47	0.11	4.90	4.98	0.12	x	
66	27482.73	25577.96	23810.23	8338.50	7940.77	6621.89	4153.70	4101.77	3448.02	5623.96	5489.06	5388.35	5756.51	5156.62	2964.55		
67	39352.14	36708.23	37676.16	39630.25	39853.04	37300.92	27773.81	26556.57	24760.15	31810.92	29936.46	29460.25	28873.66	26703.25	17713.70		
68	1081.52	1099.24	988.45	2.27	1.49	0.65	0.84	1.31	0.69	4.16	2.47	0.11	4.90	4.98	0.12	x	
69	2.22	0.19	0.06	6542.01	6148.01	4980.00	4372.41	3962.52	3622.78	5467.49	4490.93	5164.75	5644.91	4736.48	3495.15		x
70	6539.92	6696.24	5809.16	4316.93	4614.54	3543.62	3413.30	3205.59	3423.67	3954.64	3162.66	3657.02	4710.01	4021.96	1449.25		
71	51569.62	48190.00	45691.97	37138.81	33499.47	32706.18	21632.20	21912.49	18833.10	36043.61	33930.59	32326.05	25388.80	25317.20	16993.36		
72	64952.71	61081.80	55264.55	77048.72	66740.78	61933.95	54538.51	51460.61	39855.15	59913.33	57470.95	49685.53	68918.31	66728.76	41646.68		
73	57753.69	52121.16	47468.78	2.27	1.49	0.65	0.84	1.31	0.69	4.16	2.47	0.11	4.90	4.98	0.12	x	
74	2.22	0.19	0.06	1590.73	1517.61	1472.99	1136.73	778.14	784.07	1407.66	1292.66	1379.41	1516.36	1402.13	724.74		
75	2.22	0.19	0.06	2.27	1.49	0.65	0.84	1.31	0.69	4.16	2.47	0.11	4.90	4.98	0.12	x	
76	103248.63	93780.57	83806.93	29191.33	26470.17	25079.93	19855.59	19598.12	16414.72	22308.81	20739.63	20326.12	23122.02	22909.64	16644.71		
77	1211.95	1091.56	1128.99	2.27	1.49	0.65	0.84	1.31	0.69	4.16	2.47	0.11	4.90	4.98	0.12	x	
78	82686.18	74691.28	61678.20	9282.26	9130.12	8138.02	5453.11	5383.65	3467.38	8017.22	6792.34	5901.69	7853.58	7465.26	3842.15		
79	14568.59	14324.06	12010.55	2.27	1.49	0.65	0.84	1.31	0.69	4.16	2.47	0.11	4.90	4.98	0.12	x	
80	534.74	536.70	664.31	2.27	1.49	0.65	0.84	1.31	0.69	4.16	2.47	0.11	4.90	4.98	0.12	x	
81	14511.56	13240.22	13035.22	5287.71	5624.81	6039.19	3831.94	3464.60	3197.58	4249.25	4184.86	4199.48	4747.47	4843.06	2559.72		
82	49792.32	46297.91	40830.65	19019.23	16212.05	14429.40	10143.26	9422.54	8200.68	13119.24	12295.68	10919.76	12509.53	12158.77	8255.94		
83	24258.37	23054.89	20599.29	2.27	1.49	0.65	0.84	1.31	0.69	4.16	2.47	0.11	4.90	4.98	0.12	x	
84	704.25	538.70	471.87	9802.88	8386.18	8099.76	5836.09	5564.83	5530.68	6388.97	5777.81	5857.47	6596.36	6718.47	4872.90		
85	3841.88	3328.91	3107.79	12455.14	12377.94	12142.06	7988.92	7671.92	6798.85	11030.84	10037.26	9858.69	10855.65	9657.35	6907.89		
86	2823.04	2506.99	2309.17	2.27	1.49	0.65	0.84	1.31	0.69	4.16	2.47	0.11	4.90	4.98	0.12	x	
87	51181.85	45005.15	44235.00	19113.63	18042.83	18590.29	14820.93	14667.74	13285.50	16157.30	15651.14	15073.95	17432.58	17399.54	6822.76		
88	31831.96	30689.22	27007.85	2.27	1.49	0.65	0.84	1.31	0.69	4.16	2.47	0.11	4.90	4.98	0.12	x	
89	8856.14	8033.49	8091.34	4067.61	3985.99	3740.88	2871.05	2895.75	2076.73	3250.96	3093.80	2940.55	4800.03	4600.41	2259.51		
90	4594.50	4071.59	3794.09	2499.49	2133.19	1946.33	1440.45	1348.29	1061.79	1949.59	1742.85	1734.58	1716.05	1663.68	1222.67		
91	3630.95	3414.05	3298.81	38859.34	36909.85	39139.44	27631.16	25174.47	24691.93	32585.79	31537.38	31406.21	32269.85	31397.87	14262.90		
92	13057.89	13097.27	10516.79	79712.58	74230.51	64866.17	49131.65	48154.04	44355.17	64643.53	63012.89	59130.19	71075.31	68380.70	42433.90		
93	14573.69	14881.88	13895.72	14644.47	12571.44	11267.04	8361.33	8171.23	7442.86	9745.62	8876.62	9512.03	10312.93	9523.47	7412.42		
94	4810.14	4254.77	3421.70	2.27	1.49	0.65	0.84	1.31	0.69	4.16	2.47	0.11	4.90	4.98	0.12	x	
95	1301.91	1293.50	1056.96	2.27	1.49	0.65	0.84	1.31	0.69	4.16	2.47	0.11	4.90	4.98	0.12	x	
96	1075.27	1024.92	1211.49	2.27	1.49	0.65	0.84	1.31	0.69	4.16	2.47	0.11	4.90	4.98	0.12	x	
97	58191.41	53924.17	54015.44	38668.12	32385.24	30267.31	24387.89	25137.11	21344.19	28913.98	29964.71	28579.71	31691.39	32420.53	23733.98		
98	2.22	0.19	0.06	8.74	38.34	5.74	0.84	1.31	0.69	4.16	2.47	0.11	4.90	4.98	0.12		x
99	17490.59	17115.83	16201.68	2.27	1.49	0.65	0.84	1.31	0.69	4.16	2.47	0.11	4.90	4.98	0.12	x	
100	1593.06	1569.27	1562.13	721.05	676.89	679.65	575.95	541.50	389.38	524.14	474.58	478.68	701.07	585.26	349.77		
101	34961.83	33206.30	30711.65	2.27	1.49	0.65	312.53	354.02	144.37	361.73	344.63	24.58	321.16	260.08	175.12		
102	2.22	0.19	0.06	2.27	1.49	0.65	0.84	1.31	0.69	4.16	2.47	0.11	4.90	4.98	0.12	x	
103	2327.31	2493.35	2566.74	5209.77	6788.90	5209.67	4193.15	4293.30	3703.92	4687.19	4238.45	4568.32	4350.14	4676.61	3260.61		
104	9162.01	8806.10	8455.69	2841.90	2141.14	2340.87	1797.50	1648.15	1242.53	2100.77	1964.95	1723.75	2114.71	1927.34	1264.06		
105	3218.82	3089.16	2903.15	2.27	1.49	0.65	0.84	1.31	0.69	4.16	2.47	0.11	4.90	4.98	0.12	x	
106	2.22	0.19	0.06	11503.57	10413.49	9444.35	7887.08	8066.11	6626.48	8574.74	8815.82	8252.10	9291.12	8607.10	6394.26		x
107	7436.18	7344.57	6486.56	5243.39	5137.18	4813.06	2995.95	3029.88	2922.36	2854.10	2822.53	3121.66	2911.85	3187.06	2362.97		
108	11420.22	10959.11	10391.83	6999.57	7787.00	6468.53	6258.77	5470.81	5075.65	5461.38	6000.92	5843.94	6538.54	5720.04	4183.35		
109	16602.41	16595.48	14003.80	43770.26	43718.32	38004.12	31044.97	30254.70	28409.27	34418.62	31779.10	31651.82	40661.11	41441.71	24242.67		
110	26473.59	23528.46	23588.93	5740.60	5486.19	5458.48	4366.97	4589.11	4126.39	4454.85	4406.40	3858.87	5346.65	5133.08	3944.80		
111	4551.24	4018.48	4223.33	2.27	1.49	0.65	0.84	1.31	0.69	4.16	2.47	0.11	4.90	4.98	0.12	x	
112	24735.86	21939.13	20643.27	195.29	559.18	300.89	1565.63	1303.19	1471.65	380.24	1391.21	1053.96	1854.82	1576.96	765.04		
113	14432.99	15278.72	14468.94	2.27	1.49	0.65	0.84	1.31	0.69	4.16	2.47	0.11	4.90	4.98	0.12	x	
114	8252.48	9526.68	7948.43	927.48	819.88	759.60	509.62	594.25	253.30	1114.85	1106.88	865.31	1110.42	1088.74	456.47		
115	2914.97	3122.31	2009.25	4344.80	4306.80	4321.96	2796.95	2700.75	2171.40	2796.05	2603.45	2438.42	3385.90	3186.61	1855.10		
116	9516.06	9072.49	8113.78	2.27	1.49	0.65	0.84	1.31	0.69	4.16	2.47	0.11	4.90	4.98	0.12	x	

Supplementary Table S1. Overview on the abundance of all relatively quantified 5180 proteins

			MCF-7																								MDA-MB-231													
	Protein name	UniProt	control 01	control 02	control 03	Dai IC20 01	Dai IC20 02	Dai IC20 03	Dai SC20 01	Dai SC20 02	Dai SC20 03	Gen IC20 01	Gen IC20 02	Gen IC20 03	Gen SC20 01	Gen SC20 02	Gen SC20 03	SSE IC20 01	SSE IC20 02	SSE IC20 03	SSE SC20 01	SSE SC20 02	SSE SC20 03	control 01	control 02	control 03	Dai IC20 01	Dai IC20 02	Dai IC20 03	Gen IC20 01	Gen IC20 02	Gen IC20 03	SSE IC20 01	SSE IC20 02	SSE IC20 03	unique to MCF-7	unique to MDA-MB-231
117	3-hydroxyacyl-CoA dehydrogenase type-2	Q99714	216869.99	208218.16	195006.60	118071.53	120046.56	108588.73	150886.81	143315.39	139649.12	161393.41	151717.10	144284.78	159557.63	149195.91	120755.63	137193.26	132826.44	127635.33	127639.10	121392.58	117182.04	94493.18	93460.88	88312.35	54213.36	52750.97	46762.90	81007.72	73441.75	73077.73	71621.72	68563.92	49997.86		
118	3-hydroxybutyrate dehydrogenase type 2	Q9BUT1	46073.76	43881.03	42617.46	30587.30	30610.55	29408.07	33283.99	32469.30	30439.69	36603.43	35796.26	35638.71	37448.35	36179.86	30972.12	27975.51	26686.30	24998.62	28787.00	28803.20	28552.70	24276.36	23177.07	22483.10	26379.14	26900.18	22157.79	49839.31	48976.76	43654.05	28225.32	27232.37	18776.47		
119	3-hydroxyisobutyrate dehydrogenase_ mitochondrial	P31937	51908.11	47160.09	39645.18	32217.05	31875.71	28354.28	36789.49	34756.71	33682.93	45627.23	43903.24	40742.95	39203.36	36214.50	26213.05	35541.29	31821.84	30395.49	34127.55	32877.01	28129.88	24711.67	20681.29	15772.40	13585.59	13374.86	12938.59	16180.33	15287.27	13955.79	14909.61	14113.67	9893.21		
120	3-hydroxyisobutyryl-CoA hydrolase_ mitochondrial	Q6NVY1	0.14	2.53	0.81	1.07	2.77	0.68	0.96	0.36	0.63	0.35	0.75	0.38	2.13	0.97	0.60	4.64	1.07	0.47	2.22	0.19	0.06	15653.67	12809.38	9339.28	9124.46	7524.87	7638.40	11177.29	9185.57	8588.83	11238.43	8661.34	4699.97		x
121	3-ketoacyl-CoA thiolase_ mitochondrial	P42765	79786.96	78299.57	70946.36	58213.24	57962.39	52932.83	59018.67	53742.92	56726.77	71740.03	67783.00	61766.58	70017.97	61627.43	41697.84	51824.48	50656.29	49456.74	59476.63	54890.37	52115.32	122495.99	107901.42	108823.92	73419.40	67685.28	60302.50	93609.84	83036.50	82982.30	93757.81	89826.82	59256.46		
122	3-ketoacyl-CoA thiolase_ peroxisomal	P09110	43866.29	43534.51	40886.77	31181.21	28557.83	27859.56	30367.12	29809.06	28320.43	37354.44	34807.22	33231.77	32864.32	31253.76	28388.35	33009.67	31200.68	28681.40	28519.50	25506.47	24944.00	9902.12	7663.19	7200.95	5447.33	5343.94	4179.41	6525.37	6840.89	5970.30	8153.46	7783.29	4595.54		
123	3-mercaptopyruvate sulfurtransferase	P25325	13557.30	11477.31	9497.83	6573.88	6373.00	5757.90	7157.10	8095.52	8041.48	12698.93	11514.48	10478.52	10377.01	7266.30	5444.17	5380.11	5539.88	4637.84	6051.85	6136.51	5716.78	1468.67	332.59	281.59	1190.61	1186.95	693.76	1663.70	1521.40	437.74	2038.63	1966.40	863.86		
124	3-phosphoinositide-dependent protein kinase 1	O15530	4692.50	4722.70	4354.22	3370.12	3087.96	2885.17	3685.03	4109.43	3948.72	4789.65	4434.11	4366.15	3810.75	4038.10	3253.26	3336.00	2722.35	3053.79	3378.43	3221.26	3466.97	2.27	1.49	0.65	0.84	1.31	0.69	4.16	2.47	0.11	4.90	4.98	0.12	x	
125	40S ribosomal protein S10	P46783	351231.28	346014.88	356454.30	231096.07	221978.68	213215.08	239028.66	238420.69	224882.69	280257.40	281206.64	276099.03	253895.53	232798.94	192072.93	209186.85	209376.92	210809.59	248097.08	241141.79	223292.70	202728.34	194081.82	181322.81	133013.91	130444.00	122715.13	155942.82	153483.21	148234.65	153933.43	149473.37	115784.03		
126	40S ribosomal protein S11	P62280	437987.72	431516.48	411702.38	308839.54	305131.69	302637.86	361960.37	327817.17	318081.07	359968.79	344589.86	344883.94	353434.03	348922.43	315879.10	303134.90	296376.02	300690.08	331189.95	343294.39	290291.10	313702.47	300452.61	281009.02	228117.71	217423.55	226311.63	228332.87	228332.07	214946.23	280954.39	263815.20	172589.33		
127	40S ribosomal protein S12	P25398	279801.35	271423.09	249969.58	172666.86	165118.29	155441.91	242144.83	234079.79	225436.10	205124.03	196650.41	181559.57	209657.91	193266.89	161851.46	181755.02	173290.13	167297.11	184676.09	173178.75	157691.05	171509.66	155009.90	142607.55	92151.13	89201.45	74358.22	113494.87	108272.81	101620.61	107086.08	105826.29	70908.20		
128	40S ribosomal protein S13	P62277	573803.15	540000.10	521106.94	381700.16	368916.65	358599.28	416949.52	416858.26	404736.48	451546.09	434840.17	413828.90	431303.87	418658.52	347659.49	353620.66	341645.82	336605.00	394033.83	381894.14	360743.36	304662.09	287692.81	269214.56	186983.31	184953.00	167128.58	207093.06	201321.22	194314.18	215017.43	213008.71	156686.00		
129	40S ribosomal protein S14	P62263	657932.47	643367.68	620034.22	487867.84	476898.35	460590.02	544151.65	539032.26	530254.06	545134.88	532966.71	504595.01	553628.32	538608.86	489088.99	485993.66	475232.62	449991.71	537379.05	514123.03	492570.96	309717.02	291743.76	280355.49	224226.33	221248.56	201427.58	239751.23	226579.74	224097.06	269054.77	265593.82	193202.12		
130	40S ribosomal protein S15	P62841	212668.59	210032.73	193874.87	158954.60	157389.75	148076.47	164073.16	156059.83	154941.47	176940.80	169662.09	163471.12	175545.54	164700.41	153299.05	137971.50	137211.20	131168.18	160117.75	149397.05	149060.78	97860.83	86839.76	84594.28	70554.10	71174.28	69440.88	67084.14	67273.24	63464.36	80758.83	79973.55	58504.40		
131	40S ribosomal protein S15a	P62244	376394.15	350464.48	324915.77	248945.22	227653.15	212191.33	287736.87	280399.68	256585.66	282100.44	282144.31	241294.57	262408.42	246446.81	205034.48	247295.24	238110.81	225647.28	264829.95	233026.03	234069.85	196883.33	191857.59	170997.46	114747.42	116739.75	109370.15	131511.58	129939.82	119666.89	143761.77	141053.49	96357.29		
132	40S ribosomal protein S16	P62249	661321.59	656476.18	622975.34	419029.70	397834.10	386975.64	498130.55	471273.85	454263.06	470909.20	466083.24	430112.43	470091.27	451341.17	410735.58	437793.37	417124.84	410816.71	446777.55	421217.29	407867.60	321321.23	291908.73	274901.46	177266.62	173938.06	162148.10	245518.88	227305.09	208583.80	226641.69	217886.87	161674.45		
133	40S ribosomal protein S17	P08708	35120.09	30854.70	31421.57	23013.45	21980.04	20861.88	28115.99	28201.47	25567.29	26973.16	24118.28	24154.78	27502.97	29435.60	21642.34	23542.85	23042.67	21359.59	21563.32	21454.63	20412.06	115665.04	105500.57	105173.41	48441.17	44763.95	40206.38	48642.54	46977.94	43095.18	56694.41	50696.65	33555.33		
134	40S ribosomal protein S18	P62269	657797.58	645791.84	620581.42	464836.79	459340.58	442077.15	548478.48	528934.77	504155.33	513925.77	495514.81	478471.15	509257.14	485090.83	437728.92	454842.90	434614.84	423957.50	475174.22	454879.16	431553.80	475208.76	444138.29	415652.69	293176.51	293585.70	272760.93	327804.49	322484.36	307417.59	340200.30	330261.52	214361.91		
135	40S ribosomal protein S19	P39019	470749.44	467452.86	437726.96	280862.50	272432.38	254620.67	415472.55	400871.45	372255.06	350356.74	333819.96	315381.58	346408.98	333503.11	267754.01	311718.82	297762.25	289474.34	300905.32	290894.65	275203.43	103798.90	96596.23	96514.27	66156.83	65369.31	56232.64	71192.74	69324.78	64314.49	76253.53	76808.43	55799.62		
136	40S ribosomal protein S2	P15880	682710.10	666578.45	630596.24	437747.62	420855.38	401521.29	510765.23	485437.36	456278.35	528816.58	500743.72	485648.07	496888.85	493013.42	407069.27	433929.38	408151.57	393319.23	465318.47	444720.36	420539.71	624087.04	596783.98	539928.54	394231.22	379313.91	353617.56	444067.07	431273.62	412534.24	462803.03	449879.92	324155.81		
137	40S ribosomal protein S20	P60866	511264.33	500423.31	484743.87	372664.32	353977.04	349221.37	489840.60	468717.10	467763.87	426576.79	399812.46	379047.08	391709.28	398851.54	358089.12	379140.66	360315.93	346507.82	389585.09	373875.62	354118.59	223737.89	209737.13	191623.52	117497.51	119079.96	110712.57	129749.41	126087.05	123949.58	122573.41	119470.78	90581.49		
138	40S ribosomal protein S21	P63220	346886.44	342257.34	329122.05	230539.36	222452.33	214878.84	270698.51	269150.71	265243.72	266598.02	252586.37	245035.78	245471.87	249636.36	211250.76	234669.79	214548.34	218911.32	219410.39	212963.96	206384.00	197211.07	183497.64	177633.24	124934.81	127092.78	119510.73	162077.74	156155.83	148299.52	153167.51	148046.47	105128.56		
139	40S ribosomal protein S23	P62266	19115.16	16603.62	13190.55	6408.61	6940.78	5800.15	15001.45	15304.02	13455.16	9347.97	7760.19	5913.20	14441.72	12627.73	7494.43	13644.95	11131.71	11099.20	7464.80	6568.75	5324.51	11028.03	8676.91	8259.42	3226.57	2808.21	1888.15	11643.90	10827.56	9505.96	7515.93	6741.73	3790.29		
140	40S ribosomal protein S24	P62847	113509.10	110771.09	97542.25	69704.43	63702.38	60875.33	96917.56	94246.44	89266.94	85445.96	82742.64	76086.89	83007.31	76747.89	58128.28	82029.13	74522.27	73718.30	79520.99	74296.21	69481.44	34638.97	33160.42	28961.29	19056.54	18337.80	12077.55	21255.13	18346.95	16314.79	20363.34	19510.03	10819.97		
141	40S ribosomal protein S25	P62851	634333.98	627085.57	607861.27	441330.63	416442.33	410381.84	524305.74	508018.12	489634.64	488940.78	478493.12	448941.12	511983.34	457102.41	421574.03	441122.28	416505.00	409435.91	476995.09	457794.30	429211.36	307973.23	300285.94	276539.68	189628.09	188926.52	180756.88	227698.87	210574.97	206107.00	221367.89	216098.88	132534.03		
142	40S ribosomal protein S26	P62854	134688.79	124045.76	112270.60	95413.75	89742.02	80614.58	121374.82	116142.82	102190.33	104908.97	104816.97	94620.75	108708.62	102830.74	75739.76	86468.90	84283.40	74478.07	96010.03	92715.91	84140.34	96475.05	88532.44	77342.20	58208.17	58724.41	50822.23	56940.37	52105.91	47037.56	58684.55	58869.21	40258.37		
143	40S ribosomal protein S27	P42677	63039.19	61562.55	59047.34	40220.37	40518.01	39219.58	49488.97	46473.84	44755.30	41922.72	40353.08	39104.26	42200.39	42437.66	38825.11	35646.87	34158.85	32455.19	41518.98	41206.68	40282.61	190178.98	178567.76	168596.18	110071.89	110642.96	105333.15	126613.77	128481.39	125748.90	124184.65	121324.03	57665.97		
144	40S ribosomal protein S27-like	Q71UM5	188907.54	179587.52	166962.98	111898.93	111427.52	98814.93	162224.77	154586.69	145932.18	149952.95	144868.15	134980.96	145296.77	130918.48	115086.96	119137.10	114296.82	107995.53	120254.05	117337.15	111852.57	2.27	1.49	0.65	0.84	1.31	0.69	4.16	2.47	0.11	4.90	4.98	0.12	x	
145	40S ribosomal protein S28	P62857	198932.51	192879.94	183697.30	155852.46	149254.20	141401.91	173526.64	171580.14	163025.81	173141.72	163458.85	156900.46	153664.13	161633.21	139840.02	147443.11	142486.80	142128.21	155230.89	149415.43	147874.52	159605.68	152365.11	141944.82	108842.05	105626.80	95945.26	111812.83	105015.44	103222.24	119544.35	115206.63	84941.09		
146	40S ribosomal protein S29	P62273	145004.28	147573.17	143770.85	99939.72	101452.58	97079.90	114725.02	114533.30	113582.01	121125.61	116783.39	110485.75	115129.72	109272.82	76141.43	91226.12	90066.11	88353.39	103145.98	100179.62	96856.70	69663.73	66130.40	64244.32	38614.64	37289.32	33572.75	41779.99	38809.06	38117.08	45492.17	42883.95	32624.51		
147	40S ribosomal protein S3	P23396	1542842.05	1504829.61	1407034.22	1030298.45	1011677.76	949776.76	1241623.77	1174042.43	1134078.98	1199969.49	1132643.70	1082824.28	1198848.00	1129866.55	951064.21	1002117.77	979588.17	930712.83	1058901.67	1029785.74	965050.55	896765.60	830447.55	781825.69	546005.12	515342.65	464010.27	627858.40	592641.67	582853.21	604627.61	573863.61	438146.85		
148	40S ribosomal protein S30	P62861	6113.73	5531.05	5111.58	2920.75	2789.11	2907.62	4089.06	4388.17	4271.67	3954.17	3937.98	3205.72	4126.68	3965.20	3522.04	3706.49	3733.89	3625.19	3802.68	3547.59	3351.20	3299.68	3233.62	3104.39	1382.53	1283.11	1206.03	1939.10	1949.53	1777.53	1970.18	2033.69	1366.80		
149	40S ribosomal protein S3a	P61247	1219767.86	1201296.20	1131860.49	856345.24	820663.34	779888.29	998613.44	954716.09	908854.67	1006280.64	979206.01	914094.65	972774.38	903521.54	693862.17	801782.87	790000.61	766388.96	900720.97	843094.60	787548.04	710866.74	682791.16	640779.75	431606.54	429401.14	391211.68	443341.48	428790.20	415741.68	477797.16	465275.13	356781.00		
150	40S ribosomal protein S4_ X isoform	P62701	783027.84	754196.81	697774.46	525402.15	503067.09	473132.90	622016.69	592325.68	563724.36	616836.93	581347.55	551466.85	617219.67	566603.62	487081.35	496895.65	467330.80	459130.24	518357.41	499231.20	482548.32	404840.19	367398.02	345410.68	259098.78	252021.36	225262.70	275482.05	256779.29	242695.72	271850.37	265466.95	190112.86		
151	40S ribosomal protein S5	P46782	372927.82	348494.64	301824.39	252240.30	248269.70	224447.84	304719.88	297562.74	276527.06	285839.84	276062.18	234583.95	294966.56	275422.64	214356.39	271057.96	242038.53	245643.59	294801.00	267120.07	252667.80	308179.97	296858.72	269118.31	188659.18	197540.92	178738.74	217612.27	202159.27	196400.98	234083.71	225464.89	115174.25		
152	40S ribosomal protein S6	P62753	473576.29	461654.37	429193.41	327019.19	316434.03	293098.80	363960.94	350168.04	337155.70	376814.37	347503.97	331385.68	375856.53	350864.54	284289.91	321353.05	303511.25	294618.88	339844.25	320859.89	299515.40	131277.08	121917.62	117884.85	82728.71	80070.07	69293.30	88706.25	80539.36	80865.64	103610.28	98024.17	71044.55		
153	40S ribosomal protein S7	P62081	613051.68	561506.61	537055.47	413231.76	391524.10	363634.35	509334.99	482763.38	466737.51	469991.64	454114.75	432660.69	487989.71	471051.22	396468.01	458444.44	441257.54	424134.89	438920.23	424833.86	401939.91	370364.34	357083.01	318808.20	307231.08	297723.80	292716.05	382136.25	359321.95	345508.60	309570.51	303151.26	286367.66		
154	40S ribosomal protein S8	P62241	1001842.87	973848.58	911894.75	680380.22	660650.35	613143.59	824844.90	778115.12	776345.89	808900.66	759873.68	709179.19	800334.90	751124.79	633437.08	665278.12	631653.97	621190.88	710918.43	687897.13	659221.59	531061.86	497051.16	480614.49	345672.11	324766.18	298589.37	369842.96	347657.80	336326.82	391618.36	368386.62	251088.12		
155	40S ribosomal protein S9	P46781	619625.80	631398.27	592839.79	433923.55	429386.17	418627.54	534528.04	519820.61	499103.33	484855.43	470120.86	458749.58	483257.85	469234.88	407070.17	418386.18	420875.35	403128.42	450763.10	431193.96	402140.95	492745.66	445770.57	433248.23	316523.00	303836.16	287363.04	335405.66	314582.51	313404.71	330984.25	333285.30	249539.64		
156	40S ribosomal protein SA	P08865	708908.77	670608.84	617005.65	443326.26	430447.26	413542.43	552603.19	541306.09	516076.98	533442.82	503671.93	468433.64	523404.58	492985.15	397696.11	452530.97	430425.09	419203.30	466757.99	452940.05	426184.47	311358.46	300459.12	279308.59	180535.14	187112.41	155483.92	256387.51	245107.72	228650.25	236582.25	234387.10	157369.20		
157	45 kDa calcium-binding protein	Q9BRK5	23728.28	23829.77	20523.32	14641.29	13447.43	13121.30	14841.66	13888.59	13853.42	21714.99	19804.33	17950.32	19859.22	16995.81	17098.87	14373.48	13629.50	12974.12	15661.92	13652.16	13505.23	33616.25	33259.20	30048.43	18752.38	18493.22	15346.37	24592.46	25399.05	24272.39	22937.72	23351.87	14658.63		
158	4-aminobutyrate aminotransferase_ mitochondrial	P80404	179676.08	169143.35	159164.01	124456.60	118706.98	106292.40	144623.48	139747.26	132901.86	176542.91	170907.78	157946.29	151427.65	138784.50	105976.51	120651.95	116821.69	109735.16	129349.29	121096.34	110524.52	3960.11	3245.97	4004.95	2629.20	2678.12	2477.16	3747.63	3173.38	3249.61	2769.26	2768.23	1948.63		
159	4F2 cell-surface antigen heavy chain	P08195	2085118.63	1988217.29	1862551.83	2090836.10	2027526.99	1862514.70	2330662.91	2231187.71	2127255.18	2417139.17	2323711.06	2184689.33	1782817.31	1669437.81	1419279.65	2323969.34	2216793.51	2130541.33	2096436.18	2007569.61	1911521.88	163500.27	152284.56	137250.21	131011.26	126954.86	110069.15	144479.54	135123.59	134130.53	151809.09	146762.71	99790.42		
160	4-trimethylaminobutyraldehyde dehydrogenase	P49189	107054.27	101994.86	90312.55	78173.65	73338.02	65884.17	94193.92	91479.54	84946.75	94102.01	89542.01	80185.86	92572.22	85775.58	58394.07	77489.94	71270.58	65830.09	88675.22	77256.69	69131.45	100925.71	92145.49	86740.99	62809.19	59670.26	49386.82	78068.61	72004.68	68043.43	78519.98	74708.56	48770.43		
161	5'-3' exoribonuclease 1	Q8IZH2	50649.04	53058.15	44400.19	32429.62	31222.65	28834.26	38019.14	35230.72	33505.05	39412.14	37290.34	35127.98	40365.25	35196.58	33846.44	32314.29	30398.94	27069.50	34207.52	32552.68	29657.72	2.27	1.49	0.65	0.84	1.31	0.69	4.16	2.47	0.11	4.90	4.98	0.12	x	
162	5'-3' exoribonuclease 2	Q9H0D6	145595.74	144031.67	136022.30	91759.95	91274.88	81372.15	101370.79	95352.50	93249.02	120969.84	117507.01	107868.18	110108.22	109305.83	94119.87	98658.39	101466.23	93024.29	116479.48	112807.17	107892.28	28247.73	27232.14	24042.82	16814.12	16761.67	14102.50	20001.24	19567.12	18790.43	20583.85	19906.25	10049.30		
163	5'-AMP-activated protein kinase catalytic subunit alpha-1	Q13131	0.14	2.53	0.81	1.07	2.77	0.68	0.96	0.36	0.63	0.35	0.75	0.38	2.13	0.97	0.60	4.64	1.07	0.47	2.22	0.19	0.06	2192.58	2847.93	2939.86	2259.05	2272.03	2036.91	2881.08	2968.40	2871.92	3149.31	2716.60	868.60		x
164	5'-AMP-activated protein kinase subunit beta-1	Q9Y478	36882.04	35085.77	29150.44	25357.89	25934.55	20583.08	24669.05	22768.50	22202.54	30013.47	29413.93	26075.45	28927.58	27069.95	23888.95	20957.36	18181.67	17289.11	28389.62	27534.08	23746.20	2.27	1.49	0.65	0.84	1.31	0.69	4.16	2.47	0.11	4.90	4.98	0.12	x	
165	5'-AMP-activated protein kinase subunit beta-2	O43741	0.14	2.53	0.81	1.07	2.77	0.68	0.96	0.36	0.63	0.35	0.75	0.38	2.13	0.97	0.60	4.64	1.07	0.47	2.22	0.19	0.06	46239.20	44127.27	40227.09	38206.57	37835.17	26409.29	43968.10	40176.32	36524.06	50128.12	48718.56	25155.02		x
166	5'-AMP-activated protein kinase subunit gamma-1	P54619	0.14	2.53	0.81	1.07	2.77	0.68	0.96	0.36	0.63	0.35	0.75	0.38	2.13	0.97	0.60	4.64	1.07	0.47	2.22	0.19	0.06	3621.31	3518.05	4301.48	3028.39	2882.95	2561.64	3272.76	2747.12	2553.09	2564.72	2287.32	1689.32		x
167	5-methylcytosine rRNA methyltransferase NSUN4	Q96CB9	9858.92	9939.21	8464.01	6460.28	6048.51	5684.48	8080.74	7532.17	7746.40	8040.86	7257.97	6639.17	8293.95	7276.88	6825.12	7040.90	6306.87	6511.97	6568.92	6117.95	6628.91	2.27	1.49	0.65	0.84	1.31	0.69	4.16	2.47	0.11	4.90	4.98	0.12	x	
168	5'-nucleotidase domain-containing protein 1	Q5TFE4	123999.94	121331.23	114529.52	79693.11	74184.61	69454.76	90709.47	77726.30	70816.45	112374.40	106161.08	96067.04	104798.52	77759.33	63988.27	101605.32	83987.41	86692.81	90964.12	81898.44	74922.63	73200.77	63062.08	51775.34	48869.25	45557.90	32796.26	59090.05	53063.50	44337.44	64258.55	61392.66	39175.60		
169	5'-nucleotidase domain-containing protein 3	Q86UY8	4173.03	3279.22	2817.19	1956.49	1640.00	1553.37	2408.14	2281.56	2162.03	3271.20	3626.61	3397.42	3406.98	2620.06	1827.05	1805.56	1630.77	1887.28	2014.92	1884.39	1998.11	2.27	1.49	0.65	0.84	1.31	0.69	4.16	2.47	0.11	4.90	4.98	0.12	x	
170	60 kDa heat shock protein_ mitochondrial	P10809	3361332.10	3145252.82	2838155.28	2411583.32	2342188.94	2103068.38	2909944.20	2777422.67	2624777.96	2874940.44	2701431.66	2494932.53	2856972.40	2622700.73	2006836.84	2499185.36	2332717.47	2219958.04	2627837.31	2474080.50	2261145.67	2109431.97	1943776.49	1791047.15	1387557.54	1360051.32	1117734.24	1588531.36	1501655.89	1378762.76	1663418.89	1639345.65	1176968.55		
171	60 kDa SS-A/Ro ribonucleoprotein	P10155	22200.90	21611.47	22395.09	15233.04	15478.81	14506.47	21346.42	19888.56	20084.78	19204.55	18474.34	17014.77	19922.12	18475.58	14145.58	18832.75	15525.46	16147.70	15490.98	14214.19	13799.27	44886.32	40127.43	39378.57	31171.94	29755.56	26320.24	39245.53	34430.00	34560.96	36359.88	35329.79	25978.18		
172	60S acidic ribosomal protein P0	P05388	427159.78	409949.63	377103.36	290407.76	278766.88	275935.87	398209.14	385867.65	364358.15	327648.82	314662.16	292241.12	348016.66	325778.01	277014.93	305550.97	281118.28	268589.18	308879.45	291901.44	279755.82	292880.73	271058.18	244522.15	136299.77	124204.15	114942.43	147167.39	132377.52	130029.06	135820.04	132488.87	60463.43		
173	60S acidic ribosomal protein P0-like	Q8NHW5	14579.41	12127.21	10027.64	9683.78	8511.87	7769.53	8716.09	6838.81	8066.21	10943.56	10726.95	10024.17	11653.96	7229.97	5684.86	8670.21	7910.78	8510.74	8433.16	7366.74	6363.44	2.27	1.49	0.65	0.84	1.31	0.69	4.16	2.47	0.11	4.90	4.98	0.12	x	
174	60S acidic ribosomal protein P1	P05386	44439.82	40103.24	38038.85	22853.65	22958.26	21148.70	36893.98	20419.59	19947.89	29121.34	26926.46	25675.19	36289.56	35664.07	22490.76	28800.24	17036.99	28621.88	23747.02	22894.78	21025.84	24073.15	24665.15	12405.68	6066.15	6003.74	3845.96	18322.51	16105.25	14806.76	10690.78	10348.29	5065.95		
175	60S acidic ribosomal protein P2	P05387	646367.14	635364.63	607393.18	441835.06	440843.47	405503.01	561025.48	548580.83	522653.37	532971.01	513320.88	479660.25	536052.37	497226.80	415654.12	463166.96	446444.71	423837.45	488198.87	478935.03	442801.46	336498.19	301734.71	281176.82	204313.86	194524.01	158510.90	226305.82	212434.09	206399.73	243565.59	230518.89	156093.40		
176	60S ribosomal export protein NMD3	Q96D46	13772.64	13501.64	12575.16	8470.60	8308.59	8440.68	10018.16	10150.76	9480.05	10896.68	10599.06	9818.58	10132.39	8987.75	8805.30	9113.61	8585.23	7489.01	7951.09	7530.38	7141.32	1251.14	1196.80	1220.22	748.10	728.48	607.95	920.10	913.50	882.06	776.79	790.09	563.08		
177	60S ribosomal protein L10	P27635	296798.73	274886.90	232477.75	199853.76	196520.39	170675.36	229527.74	208277.59	193307.12	252993.80	232115.02	203603.39	242490.82	218560.56	154651.30	194527.36	182854.51	174488.98	221574.52	201567.34	175109.40	267538.76	246724.28	204559.38	194234.73	180628.06	153783.90	184463.52	174996.63	159368.24	234187.71	221235.79	132089.26		
178	60s ribosomal protein L10a	P62906	582645.62	559784.24	547483.88	365506.34	350223.91	339586.97	470384.13	458394.01	441777.77	426457.75	402477.75	387279.27	426878.75	404946.76	365946.70	371970.50	352181.74	341722.42	389902.24	366185.47	349456.36	358931.02	345175.36	312700.42	215636.60	207038.61	186606.76	236779.37	231471.46	223371.77	244434.60	232003.94	174620.22		
179	60S ribosomal protein L10-like	Q96L21	17349.13	14926.21	13452.78	5755.71	9058.07	6994.67	11671.09	7857.96	5242.22	9981.95	9288.74	8557.54	12551.67	7426.85	5013.42	9770.63	10577.64	9515.94	9862.39	9429.36	7419.09	26432.65	17937.37	16549.80	14251.26	13883.26	11828.83	18261.95	17194.73	15005.17	17218.10	16834.28	10882.46		
180	60S ribosomal protein L11	P62913	486576.34	469331.01	458172.12	325402.02	310928.13	301971.08	383096.07	374038.49	396217.18	377462.09	365347.88	350095.40	413337.99	362002.72	311630.63	365354.63	330846.40	329480.71	382492.12	341884.65	339770.07	236337.93	216888.08	214988.03	153358.88	154065.95	141289.69	171370.57	163974.91	160047.71	163108.40	173447.68	128257.56		
181	60S ribosomal protein L12	P30050	234017.87	218956.29	186323.56	122889.93	119046.24	103312.83	213396.86	198325.27	189300.78	159356.94	152839.85	133354.46	155299.66	145881.91	98712.00	136818.73	126802.48	119855.24	135429.54	127375.46	115396.29	224730.35	210584.33	196238.66	96015.73	93494.66	82018.76	115355.48	111127.17	104055.91	104099.07	98557.15	63578.74		
182	60S ribosomal protein L13	P26373	769421.26	745811.36	734266.14	499916.98	484656.80	458000.71	584287.10	568886.96	548821.35	599957.28	572914.25	564844.81	614539.82	566373.83	497915.64	480987.75	452915.48	463462.50	554590.27	504579.91	485124.66	375085.17	361870.64	338082.03	263021.45	258293.40	244822.65	259329.50	263430.34	259107.39	285200.23	275300.93	205219.27		
183	60S ribosomal protein L13a	P40429	419935.40	402225.08	382368.96	293586.92	276692.17	276631.09	317526.60	317717.70	308486.11	337376.64	316746.13	311540.16	313566.37	328173.04	281626.12	273894.04	250571.54	249957.40	289629.61	288885.54	280402.59	231485.23	216373.74	207947.70	156825.87	155825.53	142229.89	176059.91	165170.32	158546.88	182697.45	180369.21	133335.38		
184	60S ribosomal protein L14	P50914	380113.44	370932.90	350763.00	258663.84	256478.17	243660.72	322775.10	313107.75	304556.46	311128.32	296254.21	275379.18	290876.23	292705.75	244837.43	248975.04	244457.53	237843.83	265841.36	257942.00	237592.88	311769.48	299490.93	279554.34	189288.56	191711.36	176354.20	209276.26	203046.09	196269.30	206046.42	199856.32	149847.63		
185	60S ribosomal protein L15	P61313	549302.72	530672.95	507363.51	365107.68	361707.14	333748.26	434792.88	416199.70	390764.17	447900.19	427159.31	408541.40	441460.03	417399.91	371367.79	361192.43	334064.39	322614.51	392933.37	373146.25	342877.11	323836.41	302238.75	276866.25	203258.38	196717.86	187480.65	211005.68	202921.35	197885.19	228587.50	215065.57	163278.13		
186	60S ribosomal protein L17	P18621	509526.46	484554.09	458248.98	349945.90	334132.11	320881.30	386385.71	385191.15	367341.99	386049.33	368540.72	351450.13	386403.26	390330.22	325781.95	322338.63	304213.23	296560.49	356062.59	339016.25	326837.01	321479.97	297444.20	277411.78	221373.71	214529.43	196625.67	227455.50	216060.21	210055.92	237959.67	228402.96	171849.10		
187	60S ribosomal protein L18	Q07020	422131.36	417021.23	402413.88	270604.53	264018.11	254955.70	356503.21	333223.88	337097.53	325334.98	312405.19	296591.04	319935.85	312966.61	269595.99	272429.24	257823.29	255594.58	299682.60	281458.23	274371.71	196223.47	185716.83	175460.83	114390.28	112426.19	100409.34	110161.65	104672.51	103657.35	121652.88	118849.24	90795.55		
188	60S ribosomal protein L18a	Q02543	278280.32	274381.63	266175.27	192839.98	179895.84	175666.16	259983.06	249343.92	240468.69	232721.18	223261.02	210410.37	243643.29	228043.43	197107.82	218254.93	202314.86	199815.71	207605.41	197677.53	187330.15	225020.73	232220.87	206366.12	140702.86	136667.47	125884.54	142852.61	145267.46	142114.65	150856.32	145149.88	88648.53		
189	60S ribosomal protein L19	P84098	389606.39	386878.84	363669.79	271554.85	259897.43	246810.17	318706.29	299180.41	290475.72	313613.13	293757.46	283250.64	338986.80	308485.83	276222.60	256471.54	240413.39	247871.22	283445.57	271537.75	258897.57	208338.17	190394.58	181743.10	145076.54	141598.44	135050.60	142017.07	138558.85	133334.14	155119.38	153449.57	108653.43		
190	60S ribosomal protein L21	P46778	273661.76	262694.36	245251.72	163997.13	161833.84	151697.29	198130.22	187774.62	188723.24	207737.37	201433.45	190046.71	212893.62	201555.69	170282.10	169650.16	161573.74	155829.08	178576.69	169204.13	164718.01	72263.01	66783.09	58292.09	40139.19	38997.50	34085.19	51723.43	49740.90	47643.90	52636.83	50860.96	33493.44		
191	60S ribosomal protein L22	P35268	293118.94	289006.99	258582.52	200716.25	191430.23	183905.63	232215.40	227611.85	226120.22	232123.23	224726.25	210660.50	237187.92	228456.37	192382.28	194118.05	189042.27	185057.83	217984.79	200036.03	188299.36	103565.87	92661.95	82429.91	56740.24	56274.73	49697.70	68764.75	63841.00	57973.38	68469.93	70981.91	54173.80		
192	60S ribosomal protein L22-like 1	Q6P5R6	11964.29	11740.06	11403.71	11469.20	10866.92	10320.54	11190.93	10473.42	10585.25	9094.73	8256.44	7710.52	10516.28	9542.52	8966.51	7183.01	6181.55	6799.70	10197.38	10553.13	10411.47	2.27	1.49	0.65	0.84	1.31	0.69	4.16	2.47	0.11	4.90	4.98	0.12	x	
193	60S ribosomal protein L23	P62829	312315.77	307794.00	296297.93	209897.59	204253.61	198193.38	269385.99	255362.55	247702.06	243771.90	234094.66	225954.46	260321.21	245432.79	205216.94	201435.67	196420.20	188637.36	226534.22	220517.53	211103.00	215214.90	199370.52	187637.96	132253.57	129532.41	112199.21	152083.68	146446.72	141864.41	142106.03	136319.59	83022.77		
194	60S ribosomal protein L23a	P62750	444267.49	448470.25	425591.67	326192.88	306917.76	303622.53	390088.51	388573.48	396058.75	372938.67	355398.16	350434.12	382965.94	352596.84	313892.08	318362.60	299879.34	304284.06	344368.55	333692.68	316611.25	301761.33	268057.21	271535.66	183539.40	183470.30	166760.90	181538.98	175959.30	167997.76	187221.12	189818.68	125161.05		
195	60S ribosomal protein L24	P83731	255181.24	245391.55	226242.37	131900.11	128727.52	119781.61	207646.73	197176.59	185654.30	164710.25	154570.91	145038.73	173531.46	154795.55	143168.46	142440.16	137182.04	129487.79	147834.31	141229.59	130809.55	199316.09	176268.98	171980.72	100898.93	97397.90	84867.88	123359.10	117454.31	107342.19	109457.20	107211.42	72706.18		
196	60S ribosomal protein L26	P61254	382247.44	375352.69	359275.36	289783.48	283889.87	273260.08	320470.55	307630.33	306340.42	322609.27	305521.33	301837.13	309508.62	299393.41	271146.93	276213.71	256963.76	250446.90	276549.64	270195.68	254387.24	74511.64	71259.49	67707.52	59482.64	60189.39	70660.69	65733.27	62186.64	62144.38	76462.75	62741.14	52650.86		
197	60S ribosomal protein L27	P61353	568382.04	546263.31	512236.32	359168.77	336002.17	319085.14	441419.83	428499.80	414414.40	435430.30	403774.40	385712.00	432641.82	404944.86	345995.81	368339.02	340695.63	342608.98	390394.64	370509.07	364055.06	250899.63	230820.52	216260.38	168868.87	158373.91	138522.59	194143.13	180422.86	167175.90	197040.01	196269.03	133720.89		
198	60S ribosomal protein L27a	P46776	343372.76	339397.91	328243.72	230223.12	227454.77	217874.51	292876.74	276223.85	274955.29	266090.76	254521.06	248335.37	272406.40	253596.25	220216.93	212618.86	211173.94	208985.86	238760.08	232496.64	225030.84	143883.60	135348.83	133802.43	98233.02	91309.27	83349.26	99588.40	94052.60	95288.73	100133.05	94203.23	66291.68		
199	60S ribosomal protein L28	P46779	374384.98	367904.83	353961.53	275697.86	269433.40	264700.62	320294.37	306667.88	321698.76	310634.16	298152.72	285656.38	337742.10	296916.96	286273.17	274283.55	253213.23	253053.02	290541.23	290986.80	277979.69	253328.27	253803.40	236287.20	180025.06	178841.82	168896.84	175150.34	173463.51	171072.25	186023.78	185927.92	142481.01		
200	60S ribosomal protein L29	P47914	171505.19	171250.25	162046.19	105776.60	106863.14	104043.28	124839.42	122328.29	112861.85	130801.71	127822.86	126054.59	138123.48	119336.84	113414.24	113181.06	102155.27	101138.06	135430.10	126792.29	117168.59	109783.19	101745.79	97302.86	72499.53	73534.05	71807.02	68200.68	68982.44	68085.69	87277.22	82392.29	64410.55		
201	60S ribosomal protein L3	P39023	821681.20	780496.59	732477.66	573213.08	541368.16	521054.49	656504.87	636181.25	610567.93	640069.06	615615.32	569337.71	633917.40	619443.61	539188.13	517237.26	508194.46	487920.71	630202.25	587975.82	556185.83	358936.38	326789.06	319201.60	228758.19	224147.88	200118.07	227277.35	221700.98	213316.06	255139.24	244339.01	168002.23		
202	60S ribosomal protein L30	P62888	214251.61	196161.51	197914.50	144162.93	137092.14	130839.82	209902.01	194722.67	194272.11	160467.48	156288.35	146063.26	182786.27	172941.62	142925.61	143422.29	131276.83	135095.72	158190.40	148031.07	143004.53	144587.29	134551.21	125789.36	61539.39	59465.20	52620.23	77025.59	74210.78	68604.47	73423.39	76146.41	30095.10		
203	60S ribosomal protein L31	P62899	305796.74	292702.66	277711.85	220559.49	210790.80	199713.75	250971.37	239018.70	233152.43	251606.65	240310.30	229261.30	253248.19	243324.35	217771.32	199733.80	191547.90	184694.47	214451.26	200841.37	197020.33	124481.72	113588.20	108136.91	84989.52	86858.49	82181.93	79660.07	80786.03	76316.87	89486.95	90057.16	73092.08		
204	60S ribosomal protein L32	P62910	257837.41	241274.60	220181.01	152111.34	148314.60	139665.63	181246.86	172920.08	164843.59	180042.39	172929.12	162264.83	186916.96	172473.42	141186.77	148642.75	140937.34	140528.44	171232.80	160385.09	145924.94	123038.31	122360.61	115992.28	71164.09	70298.39	62618.24	81022.13	76997.95	72697.31	92952.26	89816.86	60211.05		
205	60S ribosomal protein L34	P49207	55073.85	64519.43	74708.62	30893.66	25889.18	21695.26	22663.82	24477.40	15114.82	61860.62	53332.72	61933.87	57043.16	20968.40	20556.14	19453.67	27350.70	36358.83	66086.85	45722.07	37673.55	167465.48	162133.47	155681.06	121428.77	117784.16	109137.71	116553.11	112832.49	118537.19	126350.42	119155.64	69148.62		
206	60S ribosomal protein L35	P42766	268652.45	244138.31	232223.77	173147.75	167959.20	157487.64	179243.47	168534.83	171841.52	186462.50	180414.38	172932.77	196123.79	180448.05	142377.47	149233.23	150639.96	152960.23	172620.81	162773.73	156995.08	117551.70	116281.86	110780.87	75285.68	79762.62	69052.47	89784.19	85926.22	81016.79	114382.60	108356.04	79235.75		
207	60S ribosomal protein L35a	P18077	227771.14	225546.55	221383.63	168450.96	159457.68	151535.23	192930.12	188246.64	193939.88	183739.67	174302.15	166424.90	192469.32	186013.94	161906.55	162454.45	150574.28	147712.87	177521.11	162342.60	159179.79	77446.81	72147.63	71578.18	54222.42	56064.42	52425.72	56402.32	54846.31	52182.77	60641.05	63164.85	65506.34		
208	60S ribosomal protein L36	Q9Y3U8	172167.90	168307.48	155418.07	109013.95	108250.57	102806.39	181266.81	169322.96	156021.07	130079.74	123371.18	111594.39	129621.85	121963.39	108305.79	108724.89	104652.63	106570.45	112528.41	119898.49	112868.81	65042.37	57686.34	52355.12	44271.52	38048.44	30720.95	52829.58	44568.86	41310.97	64422.66	57322.38	36212.04		
209	60S ribosomal protein L36a-like	Q969Q0	189884.46	186062.33	174483.64	143354.12	135702.87	131835.52	158236.24	155249.28	152665.30	158495.11	154884.30	146560.15	157638.96	154941.31	148961.96	141425.76	132636.55	130817.77	164140.25	140035.59	137506.35	119853.29	114588.67	99982.00	71206.47	71979.47	67812.81	71737.48	64731.88	63777.24	75173.47	72735.86	65649.38		
210	60S ribosomal protein L37	P61927	42041.99	39251.10	39046.51	28264.33	25625.32	26062.78	30664.49	32264.79	33574.04	33139.79	32814.78	31721.26	36573.27	32026.31	28670.96	30130.92	26458.34	27809.75	35779.35	32936.14	32308.97	21950.50	22336.05	20868.65	14792.05	13860.85	14673.56	15025.67	14520.49	14052.22	17598.78	19115.94	3616.26		
211	60S ribosomal protein L37a	P61513	85553.46	83639.32	76981.38	52587.88	50509.04	48829.68	71244.05	68797.45	71824.92	63546.28	60230.62	61070.35	69432.41	67315.74	53727.59	54180.82	52925.11	51901.48	68710.77	56687.01	51540.27	47382.57	42811.54	43331.19	19549.31	18283.15	18719.10	18953.48	16850.42	17530.27	19284.34	19103.26	1775.69		
212	60S ribosomal protein L38	P63173	14786.72	14449.07	13854.86	7865.19	7832.45	7237.19	13028.18	12395.74	12024.32	9448.88	9148.88	7943.78	10049.82	9927.37	8207.44	8638.19	7963.39	7596.07	10334.33	10004.90	9038.71	48462.49	44106.75	44358.44	23887.02	23570.16	20772.27	31682.49	29732.27	31041.10	33330.36	31102.42	23767.86		
213	60S ribosomal protein L39	P62891	11529.16	11557.26	10202.28	5501.46	5978.94	5015.09	8214.67	7576.99	7247.00	6291.40	6366.76	7134.16	6809.10	7536.01	4757.83	6868.43	7273.67	6592.63	8023.62	7131.39	6712.44	2.27	1.49	0.65	0.84	1.31	0.69	4.16	2.47	0.11	4.90	4.98	0.12	x	
214	60S ribosomal protein L3-like	Q92901	14127.66	14244.86	13257.45	10282.70	11435.81	11090.79	9369.12	9109.25	8896.51	10346.13	9838.54	9467.09	13386.66	13453.61	12329.12	8440.96	7966.92	7881.92	10892.45	10672.01	10176.52	2.27	1.49	0.65	0.84	1.31	0.69	4.16	2.47	0.11	4.90	4.98	0.12	x	
215	60S ribosomal protein L4	P36578	1056472.55	1009089.73	976082.38	637494.69	614962.43	577111.03	795783.50	767268.28	747203.35	756848.84	718402.25	683896.08	793269.41	731073.12	597623.21	616581.43	609095.73	591625.32	698104.03	676591.37	647378.89	507345.39	476463.73	436899.16	318505.87	311202.65	282533.23	349193.28	331614.14	319924.64	366430.48	357751.92	236214.83		
216	60S ribosomal protein L5	P46777	756644.64	742025.66	718414.33	555596.07	515083.29	502603.57	608676.28	600579.34	567615.29	637292.06	625944.13	589424.24	620450.79	612418.39	477360.18	493395.03	483261.21	480345.77	574252.94	542793.54	522549.40	515420.80	495011.30	447870.83	322834.12	322006.07	291740.86	376086.88	363401.17	344640.33	380688.67	383475.35	255144.54		
217	60S ribosomal protein L6	Q02878	638765.01	638389.52	591948.57	429307.75	423674.30	406379.85	515930.55	495708.69	484791.54	495933.53	474482.92	448283.45	478702.60	476198.69	449814.54	420429.14	402778.96	391741.10	480885.24	437476.04	424119.76	303125.60	287142.42	267119.45	199382.42	200433.15	191805.23	191605.30	190750.69	183904.39	204870.20	198502.08	151084.77		
218	60S ribosomal protein L7	P18124	805512.45	791524.56	758486.83	571213.84	546970.70	520642.81	668329.77	654933.29	599148.15	640112.09	618212.19	598742.89	646571.53	641322.33	557535.67	555248.68	530207.74	511775.33	602414.62	565629.00	531363.60	615516.18	564015.54	526535.00	403431.97	388480.11	363393.00	456616.58	438222.45	420408.26	483671.67	446475.40	326798.23		
219	60S ribosomal protein L7a	P62424	848359.01	814414.36	773678.44	566161.63	539442.49	521845.13	666649.42	636206.67	631364.80	632096.78	613064.11	577403.49	657371.36	627651.26	574514.66	539065.16	511221.55	488782.45	596134.59	578255.64	550271.80	386611.15	361646.81	329091.12	243112.92	239955.09	214953.21	259537.63	250528.22	241505.66	270678.17	266274.69	210520.62		
220	60S ribosomal protein L8	P62917	384629.10	377014.14	365728.81	279410.66	266420.76	259111.21	286515.59	288402.21	286400.08	313040.21	290760.55	276408.68	317507.40	305356.03	282098.96	250086.97	232422.83	239633.42	271117.95	261414.75	267847.87	242196.17	225103.90	225071.50	175511.68	173345.80	165983.99	190870.93	181336.50	179785.07	201185.01	191012.83	159663.88		
221	60S ribosomal protein L9	P32969	178172.23	170446.19	155357.37	102741.82	102518.93	89607.90	145808.43	144132.88	134562.54	127956.19	119650.91	107080.46	138398.03	127834.08	96696.13	107920.25	106784.10	98054.70	107529.47	101247.07	95473.28	122303.17	116808.29	106668.90	64133.50	59086.75	52801.26	70443.32	66930.03	60602.52	65982.73	64219.84	39941.18		
222	6-phosphofructo-2-kinase/fructose-2_6-bisphosphatase 1	P16118	0.14	2.53	0.81	1.07	2.77	0.68	0.96	0.36	0.63	0.35	0.75	0.38	2.13	0.97	0.60	4.64	1.07	0.47	2.22	0.19	0.06	13288.67	10824.21	9494.29	7115.61	6869.09	6289.99	8139.36	7296.28	6783.49	9154.82	9262.63	5704.44		x
223	6-phosphofructo-2-kinase/fructose-2_6-bisphosphatase 2	O60825	867.92	1115.79	911.62	1042.16	702.30	913.61	1318.94	1390.21	1237.15	1274.00	1230.22	1117.56	1091.58	1067.86	898.37	1069.85	1006.77	820.97	928.86	981.46	875.09	29088.40	25256.08	22914.91	14994.74	14651.88	13738.41	21183.54	18941.13	18509.27	17442.33	16790.40	11204.97		
224	6-phosphofructo-2-kinase/fructose-2_6-bisphosphatase 3	Q16875	13024.94	12252.47	11784.34	9246.80	8964.24	9233.06	10842.57	9556.75	9477.31	10498.48	10098.97	9508.87	9607.76	10721.32	8224.47	7869.62	7744.57	7576.46	8665.67	8353.52	8042.90	1556.66	492.16	534.30	783.73	547.58	290.94	1239.94	917.00	286.74	1384.23	1133.02	481.74		
225	6-phosphofructo-2-kinase/fructose-2_6-bisphosphatase 4	Q16877	5965.46	5564.98	4659.60	6910.13	6447.02	4949.26	5689.77	7541.11	4846.04	9736.19	9401.95	8383.91	5893.78	7753.52	1971.61	6005.24	7524.94	5075.64	5284.28	4822.71	3821.38	16974.49	15010.28	12541.57	10613.88	10775.65	9810.72	13328.32	12918.17	12079.07	13383.29	12479.17	10519.17		
226	6-phosphogluconate dehydrogenase_ decarboxylating	P52209	591395.84	570039.16	553650.07	520557.86	509112.87	484803.01	634476.75	595318.16	567946.09	567810.99	541331.24	512225.41	501501.18	473815.15	413626.09	508307.10	487268.78	475324.27	440211.79	411916.16	393582.16	364402.10	342029.92	319016.28	204310.74	200274.61	177402.13	251494.97	236580.25	226285.26	249947.07	238090.72	208762.60		
227	6-phosphogluconolactonase	O95336	120786.61	113157.91	102285.70	70718.19	76571.68	68737.40	89651.16	85624.11	81381.39	95865.16	89957.37	84185.60	92290.86	83778.48	64744.26	76722.60	74147.20	71393.08	63602.30	63680.04	59743.48	7673.07	7419.17	8851.61	4546.35	4212.65	3552.21	5123.73	5856.42	5582.59	6411.13	6022.28	4229.27		
228	7_8-dihydro-8-oxoguanine triphosphatase	P36639	22147.51	21511.06	20083.09	15491.91	15856.83	15084.04	17381.81	16392.11	15256.87	17667.15	17260.02	16654.68	18319.98	16680.86	15248.88	14927.91	14590.90	15172.38	15886.83	14590.29	13681.56	55802.47	49611.28	42179.53	31888.36	31311.54	25842.24	46757.64	44396.58	40406.52	41871.55	39705.17	26336.20		
229	7-dehydrocholesterol reductase	Q9UBM7	157864.09	148250.05	143731.04	107993.45	102507.36	98573.58	128440.22	122665.89	118649.86	127133.58	124908.01	117019.20	119721.96	113160.04	98934.97	108227.80	105020.34	102552.04	101401.02	106063.53	92430.63	18397.85	17784.50	16324.92	12213.44	11556.32	10636.06	12098.58	10966.89	11379.15	11951.50	12443.77	8963.20		
230	7SK snRNA methylphosphate capping enzyme	Q7L2J0	39452.50	40724.63	34334.30	26992.67	26749.45	23489.79	32502.07	30182.37	29682.20	33627.96	32702.57	30653.56	36056.05	27970.04	23939.29	29292.82	27304.37	26556.00	30940.55	28253.70	26468.08	2.27	1.49	0.65	0.84	1.31	0.69	4.16	2.47	0.11	4.90	4.98	0.12	x	
231	A disintegrin and metalloproteinase with thrombospondin motifs 12	P58397	0.14	2.53	0.81	1.07	2.77	0.68	0.96	0.36	0.63	0.35	0.75	0.38	2.13	0.97	0.60	4.64	1.07	0.47	2.22	0.19	0.06	1983.80	3377.60	1648.29	1263.37	1249.38	986.28	1984.46	1992.52	1765.05	1782.08	1637.81	947.48		x
232	A disintegrin and metalloproteinase with thrombospondin motifs 17	Q8TE56	4960.43	4790.85	4661.16	3308.42	3108.05	3067.21	3632.56	3553.66	3476.46	4279.27	3667.48	3792.00	3681.98	3686.38	3245.10	3013.41	2832.69	2719.25	3347.83	2975.81	2911.26	2.27	1.49	0.65	0.84	1.31	0.69	4.16	2.47	0.11	4.90	4.98	0.12	x	
233	A disintegrin and metalloproteinase with thrombospondin motifs 19	Q8TE59	69162.97	68583.76	65040.95	51290.21	49458.16	48475.53	70776.36	67774.51	65262.63	61728.18	57699.80	56140.77	60643.17	59974.47	52608.33	52130.96	51241.67	50323.94	54199.13	53382.00	49327.04	1657.09	1682.07	1633.57	849.87	775.57	732.50	1573.20	1550.58	1472.66	1364.29	1147.18	388.45		
234	ABI gene family member 3	Q9P2A4	2304.98	1729.83	1476.64	1184.44	1112.53	967.72	1567.87	1428.79	1263.03	1642.17	1705.12	1401.90	1576.03	1344.60	648.84	1364.45	1323.05	1075.04	1506.80	1302.46	1181.76	2.27	1.49	0.65	0.84	1.31	0.69	4.16	2.47	0.11	4.90	4.98	0.12	x	
235	Abl interactor 1	Q8IZP0	10419.87	11672.69	11310.65	7920.81	7072.35	7526.89	9563.38	8719.08	8476.61	9387.44	8713.70	9011.35	8681.43	9616.00	7832.90	7416.79	6594.50	6897.24	9986.94	7399.97	6816.87	11375.66	10691.66	10063.89	7952.05	7294.39	6874.83	9944.70	9203.53	9325.69	9077.55	8667.78	6346.73		
236	Abl interactor 2	Q9NYB9	0.14	2.53	0.81	1.07	2.77	0.68	0.96	0.36	0.63	0.35	0.75	0.38	2.13	0.97	0.60	4.64	1.07	0.47	2.22	0.19	0.06	695.15	697.58	737.02	423.49	363.08	338.81	608.51	647.00	585.65	730.37	643.56	362.39		x
237	Abnormal spindle-like microcephaly-associated protein	Q8IZT6	203382.82	200153.30	189935.60	155356.33	150400.48	147237.51	166553.85	162106.35	157624.08	184769.21	179707.41	175385.38	194125.00	185289.25	145416.59	141370.59	139459.48	144476.31	157993.11	153411.65	138406.08	107566.18	97942.46	82838.38	59737.07	58817.38	49521.63	69983.77	68033.30	61644.49	74289.34	73840.08	50223.04		
238	Acetoacetyl-CoA synthetase	Q86V21	70771.97	64367.95	62326.78	49528.36	47729.60	46965.68	48804.13	47761.11	47005.73	60599.13	58603.51	57912.15	54118.08	52053.77	48064.87	47807.99	43558.87	44344.78	44866.88	45008.66	43800.61	2.27	1.49	0.65	0.84	1.31	0.69	4.16	2.47	0.11	4.90	4.98	0.12	x	
239	Acetolactate synthase-like protein	A1L0T0	13132.39	12758.56	11636.15	6367.68	6137.08	6010.33	9140.93	7448.12	8431.14	9460.14	8569.93	7158.30	12181.00	9375.91	7228.76	7546.73	5852.15	6743.84	7475.80	6278.49	5766.06	2.27	1.49	0.65	0.84	1.31	0.69	4.16	2.47	0.11	4.90	4.98	0.12	x	
240	Acetyl-CoA acetyltransferase_ cytosolic	Q9BWD1	16488.59	13410.29	11588.15	6253.72	5721.02	4544.54	7918.94	10737.43	9776.41	11046.39	9921.04	8614.82	11775.34	11754.27	4441.08	8418.95	7150.52	5452.69	7668.81	6071.78	5001.94	47245.67	35190.32	33233.90	21762.05	21767.24	16371.11	38646.72	35982.20	34273.99	34974.89	32763.67	18841.65		
241	Acetyl-CoA acetyltransferase_ mitochondrial	P24752	40764.97	39370.94	36071.07	29760.21	30571.91	29244.15	30370.10	30993.84	28229.73	36507.15	35621.89	34852.23	36095.48	29635.51	20078.29	31765.88	30789.69	29724.32	31463.90	29930.40	28049.23	85507.63	80195.92	81699.70	57787.73	58125.00	52751.91	68147.17	65441.47	65122.98	60234.56	56399.70	40510.65		
242	Acetyl-CoA carboxylase 1	Q13085	425654.17	401830.79	359709.97	315262.50	300940.61	274414.84	307331.58	301703.47	278699.22	395486.48	378488.09	324132.33	359356.66	330472.82	245789.34	295816.39	283886.38	269964.73	316493.79	287104.17	275643.81	105308.98	91578.03	82100.72	61910.91	60546.68	48279.48	74275.92	70539.91	63705.01	90289.26	85067.59	50675.69		
243	Acetyl-CoA carboxylase 2	O00763	6339.25	5753.95	5415.68	3742.22	3965.89	3724.58	3815.65	4750.15	3623.57	5064.04	5027.98	4275.02	5252.57	3578.93	2651.69	3321.63	3297.90	3628.21	3595.19	3958.14	4423.71	2.27	1.49	0.65	0.84	1.31	0.69	4.16	2.47	0.11	4.90	4.98	0.12	x	
244	Acidic fibroblast growth factor intracellular-binding protein	O43427	13271.90	13732.57	11898.79	9800.38	9995.70	9989.86	10197.48	10221.24	9634.22	12318.35	12234.87	11984.78	11022.56	9967.70	8463.97	10075.24	9936.40	10175.59	9295.33	8546.69	8205.90	10234.98	11112.30	10206.08	7918.81	6641.83	5837.99	6565.99	5870.03	6198.51	9225.56	8293.08	1880.76		

Supplementary Table S1. Overview on the abundance of all relatively quantified 5180 proteins

			MCF-7																					MDA-MB-231													
	Protein name	UniProt	control 01	control 02	control 03	Dai IC20 01	Dai IC20 02	Dai IC20 03	Dai SC20 01	Dai SC20 02	Dai SC20 03	Gen IC20 01	Gen IC20 02	Gen IC20 03	Gen SC20 01	Gen SC20 02	Gen SC20 03	SSE IC20 01	SSE IC20 02	SSE IC20 03	SSE SC20 01	SSE SC20 02	SSE SC20 03	control 01	control 02	control 03	Dai IC20 01	Dai IC20 02	Dai IC20 03	Gen IC20 01	Gen IC20 02	Gen IC20 03	SSE IC20 01	SSE IC20 02	SSE IC20 03	unique to MCF-7	unique to MDA-MB-231
245	Acidic leucine-rich nuclear phosphoprotein 32 family member A	P39687	298466.77	296717.92	278618.56	217232.73	204588.69	205120.90	269274.28	269665.89	260834.15	241534.33	233864.53	223477.14	265264.11	264746.79	208100.21	209632.23	198725.30	196385.13	231822.32	213253.74	203625.28	54254.75	51529.14	38952.50	27431.74	27446.16	26954.75	31335.83	29503.29	28812.87	34618.62	31895.17	20844.70		
246	Acidic leucine-rich nuclear phosphoprotein 32 family member B	Q92688	62347.59	69570.21	55330.06	55287.79	53016.20	54345.45	51563.85	61453.43	53673.54	46095.76	42647.55	43500.88	57968.30	53576.18	20511.24	48866.25	47369.87	45589.21	66254.42	61816.90	52972.91	204334.04	184673.01	174340.57	86501.01	85855.62	74154.25	110994.59	103431.60	97814.12	109046.61	103672.19	63793.32		
247	Acidic leucine-rich nuclear phosphoprotein 32 family member C	O43423	467.94	259.25	405.78	546.80	518.58	373.89	453.50	417.29	486.58	886.77	885.14	758.91	604.14	499.00	378.58	430.83	408.44	326.70	502.62	354.64	415.73	273.52	204.81	143.05	66.07	79.91	27.35	147.18	115.17	112.08	296.72	254.58	43.22		
248	Acidic leucine-rich nuclear phosphoprotein 32 family member D	O95626	5753.37	9137.62	8231.57	7051.58	6486.06	6650.70	5859.75	5121.10	4932.64	6303.56	6387.41	6444.55	7513.44	6578.63	2585.12	4050.28	6342.37	6547.39	7390.67	6142.97	6104.61	3660.89	6342.60	4214.92	3215.64	2600.42	2682.82	3817.59	3224.24	3755.58	4318.05	3685.43	2333.53		
249	Acidic leucine-rich nuclear phosphoprotein 32 family member E	Q9BTT0	65926.02	64135.71	59481.39	23723.13	22932.72	21186.32	43419.17	38823.54	38848.16	32606.91	29201.92	28376.27	41935.48	35761.13	29143.73	26853.15	24654.72	24149.50	29367.65	30023.33	28418.65	30100.00	30170.85	25116.88	16430.25	16034.67	13328.24	19688.60	18776.58	18681.77	17837.81	16673.02	10781.87		
250	Acid-sensing ion channel 1	P78348	38636.62	31034.92	23030.99	25040.93	20799.44	16028.88	25418.78	22240.80	21484.84	33865.30	31370.32	23269.61	30146.27	21599.15	6578.37	22173.14	19165.58	16770.70	21596.48	20007.64	13452.21	2.27	1.49	0.65	0.84	1.31	0.69	4.16	2.47	0.11	4.90	4.98	0.12	x	
251	Aconitate hydratase_ mitochondrial	Q99798	516852.21	495122.94	473301.75	358707.63	341958.23	327686.83	397165.27	386199.83	365773.32	438631.60	425608.21	392271.02	412508.63	396116.92	335530.26	363467.62	347480.22	335356.16	343915.11	330488.39	313300.50	93702.42	85005.16	72972.87	55231.74	54815.35	47751.94	74289.90	69894.35	65717.14	71536.91	66946.61	44292.05		
252	Acrosin-binding protein	Q8NEB7	4559.09	4057.59	3568.96	2811.51	2576.78	2345.24	3273.97	3187.91	3063.45	3547.34	3557.35	3027.46	3249.07	3117.04	2475.38	3049.57	2848.91	2619.89	2914.63	2748.42	2501.40	2.27	1.49	0.65	0.84	1.31	0.69	4.16	2.47	0.11	4.90	4.98	0.12	x	
253	Actin_ alpha skeletal muscle	P68133	1457689.77	1427304.97	1261351.04	892714.34	887428.84	761919.87	1141235.95	1090277.03	961183.94	1202595.29	1097399.17	1006715.96	1028313.62	920018.77	680354.10	1121178.08	1058951.33	965687.37	1039290.83	1012657.49	902189.05	114881.53	104493.23	98106.24	85805.47	78870.36	74077.28	105303.45	96267.91	89623.56	103629.93	103460.53	67585.46		
254	Actin_ aortic smooth muscle	P62736	835.99	615.23	609.69	367.65	481.06	305.57	466.55	406.04	277.49	594.90	669.94	507.12	714.86	621.74	415.88	613.96	578.30	377.56	285.76	185.66	200.84	2.27	1.49	0.65	0.84	1.31	0.69	4.16	2.47	0.11	4.90	4.98	0.12	x	
255	Actin_ cytoplasmic 1	P60709	5494800.80	5415441.59	5048153.93	4070088.92	3952660.27	3692655.30	4680935.44	4555891.60	4315244.86	4589149.46	4395618.67	4169052.14	4334570.70	4133372.22	3315251.56	4611482.15	4339804.89	4248173.05	4333118.87	4119622.35	3802672.90	3795683.80	3532346.05	3191393.16	2305582.55	2249962.98	1933830.48	2906571.13	2702432.38	2488030.13	3275352.94	3124620.03	2115774.73		
256	Actin_ cytoplasmic 2	P63261	6296.98	5837.30	5205.83	4295.32	4127.28	3616.69	5793.17	5271.88	5433.00	5543.93	5570.78	4842.24	5222.91	4429.55	2538.62	5040.08	4461.38	4473.63	4505.18	4113.56	3673.27	6294.70	5470.84	5035.47	3591.11	3232.55	2312.32	4739.12	4083.11	3753.69	4621.28	4324.84	2519.85		
257	Actin_ gamma-enteric smooth muscle	P63267	1399.46	1419.73	1803.37	519.35	600.78	526.31	1180.54	905.99	942.06	882.83	828.86	859.73	1411.94	1398.70	1224.55	2245.50	1556.79	2210.84	658.77	655.73	593.77	2.27	1.49	0.65	0.84	1.31	0.69	4.16	2.47	0.11	4.90	4.98	0.12	x	
258	Actin-binding LIM protein 3	O94929	0.14	2.53	0.81	1.07	2.77	0.68	0.96	0.36	0.63	0.35	0.75	0.38	2.13	0.97	0.60	4.64	1.07	0.47	2.22	0.19	0.06	1665.97	1839.41	1250.54	652.54	689.59	755.72	489.90	467.54	320.72	792.93	796.21	26.73		x
259	Actin-histidine N-methyltransferase	Q86TU7	53055.19	50939.61	50456.55	39758.84	37530.88	34855.41	45521.74	43396.53	40851.74	50587.99	48988.07	47200.48	49542.45	46868.20	37975.22	35547.45	34170.80	33989.71	41534.32	38841.53	37755.16	2.27	1.49	0.65	0.84	1.31	0.69	4.16	2.47	0.11	4.90	4.98	0.12	x	
260	Actin-like protein 6A	O96019	48243.83	50594.60	42179.41	26891.92	27369.59	24538.31	36787.89	34824.30	34604.85	34281.80	33079.15	29865.66	32107.01	28669.34	21310.12	31014.11	29013.77	27352.85	28250.43	27418.98	23488.19	26924.81	26630.09	22599.42	15333.24	14942.96	11086.37	26716.57	25257.87	22541.85	23440.35	22696.71	13389.33		
261	Actin-like protein 7A	Q9Y615	682.68	556.10	899.31	573.51	569.00	563.52	796.06	701.30	459.37	759.33	653.08	597.58	707.87	619.51	571.43	515.22	492.37	514.14	577.66	557.83	603.08	2.27	1.49	0.65	0.84	1.31	0.69	4.16	2.47	0.11	4.90	4.98	0.12	x	
262	Actin-like protein 7B	Q9Y614	0.14	2.53	0.81	94.06	36.92	8.30	1544.39	1231.90	813.89	0.35	0.75	0.38	38.12	21.69	4.97	71.57	1142.47	9.27	2.22	0.19	0.06	4825.58	4632.84	3764.05	3577.94	3767.50	2191.35	5377.23	4063.43	3575.10	5826.77	5478.15	0.12		
263	Actin-related protein 2	P61160	130420.11	124096.77	109486.05	79188.33	76818.85	68011.99	105888.40	102631.94	96519.83	106465.35	101749.93	93191.24	98967.05	90505.38	71023.14	82170.04	79965.33	72505.67	77697.46	75017.88	71680.86	167533.94	162406.56	146278.57	89569.35	89989.12	80932.49	103305.84	97427.55	92613.51	108529.46	106495.45	72834.83		
264	Actin-related protein 2/3 complex subunit 1A	Q92747	0.14	2.53	0.81	1.07	2.77	0.68	0.96	0.36	0.63	0.35	0.75	0.38	2.13	0.97	0.60	4.64	1.07	0.47	2.22	0.19	0.06	100749.83	95910.42	94547.40	68948.19	71067.80	62657.41	86457.11	80998.40	79340.93	82487.23	79682.62	61449.19		x
265	Actin-related protein 2/3 complex subunit 1B	O15143	242758.58	223763.00	219050.91	143261.36	139225.72	117503.04	161333.30	160512.68	149234.67	186869.11	180092.82	158900.39	183086.47	158220.16	143559.35	147429.97	140293.70	130000.20	150979.27	139612.06	132884.24	117193.97	105791.06	99200.52	79672.21	78579.30	77007.92	82645.16	84203.61	78401.40	102712.20	97568.91	68704.47		
266	Actin-related protein 2/3 complex subunit 2	O15144	159913.39	155018.34	145648.19	120905.80	116423.55	114246.63	120016.50	118098.15	112034.97	154859.12	140999.22	138273.60	135285.30	134508.53	117706.77	115562.37	107895.50	105753.00	113088.98	107258.74	102564.84	107304.97	101022.61	99065.51	80153.49	77664.45	68539.39	89413.34	84534.22	81376.78	97333.95	91911.56	70049.77		
267	Actin-related protein 2/3 complex subunit 3	O15145	148677.18	136357.76	124264.38	97980.60	90453.37	83380.24	101641.48	87795.73	87784.81	116724.55	114658.47	104953.83	110495.36	98108.70	78165.92	91034.53	85198.34	76919.22	92210.06	86029.74	79871.13	99674.81	96953.38	91915.32	62468.58	63482.27	52902.13	76350.82	72291.37	71508.26	85570.73	85981.55	61779.03		
268	Actin-related protein 2/3 complex subunit 4	P59998	120407.69	115231.80	115309.40	61954.47	59775.61	58760.80	92466.21	91964.57	89575.03	80116.35	77444.51	74078.93	84699.72	75500.47	67644.15	69402.13	66593.21	64396.87	63917.09	59837.44	56413.84	89057.33	85264.60	80719.63	44787.16	43772.09	38012.10	59129.91	58332.40	54993.39	51496.22	50989.94	36012.83		
269	Actin-related protein 2/3 complex subunit 5	O15511	130019.25	127111.77	103754.40	77154.28	78245.00	65236.77	113381.72	108075.18	100058.63	99615.54	91580.15	84026.29	94946.93	94919.89	62261.61	109584.87	104878.29	96669.42	89332.38	83077.06	76714.53	36950.55	30566.32	29751.61	20973.80	18669.32	14360.99	25768.32	24297.51	22607.45	29688.97	28077.66	18393.00		
270	Actin-related protein 2/3 complex subunit 5-like protein	Q9BPX5	39281.24	36765.66	32870.11	22356.71	20914.33	18932.75	25470.94	24729.47	23452.51	30420.96	31146.49	27374.43	31728.67	26957.00	21472.23	22825.85	22114.94	21392.47	25095.12	23541.18	22238.87	2.27	1.49	0.65	0.84	1.31	0.69	4.16	2.47	0.11	4.90	4.98	0.12	x	
271	Actin-related protein 3	P61158	193904.74	173348.74	158600.90	128692.34	121240.20	105064.58	138243.22	132758.61	122289.90	164624.29	155680.95	135603.58	161506.03	147576.59	101305.22	123386.96	115325.86	108470.60	135731.44	124886.25	110804.30	277412.70	264403.18	236222.07	171107.30	164358.26	135298.49	192537.96	179239.63	163720.15	220290.10	213706.69	128101.37		
272	Actin-related protein 3B	Q9P1U1	7215.00	7156.73	5977.19	3058.94	3278.21	2171.88	5317.10	4695.08	4803.97	4351.25	3753.13	3698.56	5029.86	4714.98	2813.57	3471.73	3430.79	2721.91	3725.60	3245.43	3208.85	36895.33	36136.73	31601.76	22395.18	21965.27	18181.96	27031.78	24823.51	23098.76	27437.95	26690.78	18579.13		
273	Actin-related protein 8	Q9H981	0.14	2.53	0.81	1.07	2.77	0.68	0.96	0.36	0.63	0.35	0.75	0.38	2.13	0.97	0.60	4.64	1.07	0.47	2.22	0.19	0.06	56599.16	53968.34	52377.65	40091.43	40723.85	38266.42	46183.84	44483.84	43218.72	51038.30	48176.17	37837.21		x
274	Activated RNA polymerase II transcriptional coactivator p15	P53999	259012.46	249791.59	233691.86	161147.19	157878.95	146985.70	196838.76	181238.80	171537.03	214125.00	204217.36	193876.69	196813.21	181868.20	157652.12	164620.05	153792.20	149902.93	167507.92	162293.45	145693.10	283468.68	260524.91	245809.70	200505.70	190849.33	174661.07	241226.18	234329.73	220449.30	230999.69	221534.23	152724.18		
275	Activating signal cointegrator 1 complex subunit 3	Q8N3C0	0.14	2.53	0.81	1.07	2.77	0.68	0.96	0.36	0.63	0.35	0.75	0.38	2.13	0.97	0.60	4.64	1.07	0.47	2.22	0.19	0.06	19150.07	18524.95	15158.34	13346.96	13386.12	11009.53	13692.33	13172.61	12472.26	14606.14	14524.96	9435.15		x
276	Activating signal cointegrator 1	Q15650	0.14	2.53	0.81	1.07	2.77	0.68	0.96	0.36	0.63	0.35	0.75	0.38	2.13	0.97	0.60	4.64	1.07	0.47	2.22	0.19	0.06	432.30	467.33	509.81	516.75	685.36	627.00	691.41	562.32	443.09	1208.25	1237.52	645.01		x
277	Activator of 90 kDa heat shock protein ATPase homolog 1	O95433	799230.79	770463.39	709849.17	530437.90	508163.48	474590.64	649535.60	596958.91	574351.32	669524.92	634313.43	603266.96	676614.19	637755.63	507368.27	522375.05	477844.52	474489.77	567325.22	527198.32	506930.98	192970.17	179920.26	166649.21	114812.13	112172.23	95200.35	149313.59	138534.92	134752.29	144684.94	137631.27	98997.00		
278	Active breakpoint cluster region-related protein	Q12979	0.14	2.53	0.81	1.07	2.77	0.68	0.96	0.36	0.63	0.35	0.75	0.38	2.13	0.97	0.60	4.64	1.07	0.47	2.22	0.19	0.06	1551.97	1264.22	1124.98	596.24	517.72	145.36	730.24	563.02	451.82	961.63	779.74	279.53		x
279	Activin receptor type-2B	Q13705	0.14	2.53	0.81	1.07	2.77	0.68	0.96	0.36	0.63	0.35	0.75	0.38	2.13	0.97	0.60	4.64	1.07	0.47	2.22	0.19	0.06	1620.56	1806.92	1558.84	1235.24	1262.43	1083.65	1255.53	1405.50	1351.63	1416.99	1182.62	867.55		x
280	Activity-dependent neuroprotector homeobox protein 2	Q6IQ32	4581.50	3265.32	3985.83	2992.96	2522.25	2513.06	3557.98	3031.50	3550.12	2418.86	2797.01	2680.50	3064.28	2114.57	1894.77	2753.11	2902.35	2709.84	3120.86	2438.56	2043.93	2.27	1.49	0.65	0.84	1.31	0.69	4.16	2.47	0.11	4.90	4.98	0.12	x	
281	Acyl carrier protein_ mitochondrial	O14561	124198.16	115474.98	107124.51	72483.14	69433.12	64796.96	84902.90	74179.42	79772.58	90567.82	84614.32	78857.23	93185.18	78746.22	62833.95	72651.92	64422.84	67140.44	79853.48	74062.94	71214.11	2.27	1.49	0.65	0.84	1.31	0.69	4.16	2.47	0.11	4.90	4.98	0.12	x	
282	Acylamino-acid-releasing enzyme	P13798	77266.92	74374.05	71993.34	55105.12	53393.24	49736.46	67060.63	65734.36	64350.38	65077.37	62222.96	60367.85	70895.38	63883.07	50750.77	52831.73	51466.44	48510.54	60223.46	56355.26	54580.27	94323.90	87080.95	77226.02	60675.46	59251.10	49642.75	67464.66	62668.73	60016.76	74175.07	71006.40	43417.26		
283	Acyl-CoA dehydrogenase family member 9_ mitochondrial	Q9H845	383549.79	380544.49	360219.78	173617.39	165120.30	160423.35	292714.47	286683.85	276229.08	300190.72	281550.67	251538.61	251946.91	222266.94	203785.72	285694.88	265357.90	259415.05	223102.96	206518.00	200534.13	6097.46	5963.28	5222.81	3849.32	3641.66	3115.93	4722.95	4405.46	4335.20	5773.84	5472.30	3300.60		
284	Acyl-CoA desaturase	O00767	153264.14	143547.66	133995.73	145435.45	135804.87	127071.99	135287.70	133551.75	123403.07	115110.40	110137.92	100318.66	121789.47	114923.74	83141.40	184001.14	174593.21	167915.05	101158.73	97079.47	88688.64	8524.27	8088.02	6485.80	7002.58	6576.28	6331.83	7945.18	7272.48	6838.04	8436.37	7881.86	5044.03		
285	Acyl-CoA synthetase family member 2_ mitochondrial	Q96CM8	110705.83	103138.33	95994.11	75480.10	72040.56	67746.26	82262.38	75632.70	74157.99	94412.34	87956.92	84085.76	86250.38	83724.70	69655.31	79037.37	71620.38	71577.54	75095.52	71737.65	66740.30	1176.07	1179.51	1163.19	974.55	1076.97	1092.51	947.28	966.80	893.48	1149.08	1139.86	774.68		
286	Acyl-CoA-binding protein	P07108	368883.42	351257.62	342597.18	252267.15	252360.90	228419.76	302692.81	286525.47	269582.63	309504.12	287325.36	265948.87	282583.93	272597.44	220465.22	287896.17	275231.53	255216.08	245902.51	238416.75	214249.94	182777.46	165785.41	151903.81	108210.02	100678.08	90814.09	128619.21	122077.48	123938.82	146618.07	134061.41	91143.82		
287	Acyl-coenzyme A synthetase ACSM5_ mitochondrial	Q6NUN0	21834.46	21072.11	20694.75	15639.89	14543.24	14454.12	17938.48	17984.11	18104.00	21552.47	21615.66	20056.28	19748.78	18366.46	15115.65	14753.18	15038.92	13732.51	16615.64	16902.80	16363.14	228.68	259.87	149.19	371.05	409.68	429.80	515.62	545.94	469.29	394.01	461.92	44.30		
288	Acyl-coenzyme A thioesterase 2_ mitochondrial	P49753	0.14	2.53	0.81	1.07	2.77	0.68	0.96	0.36	0.63	0.35	0.75	0.38	2.13	0.97	0.60	4.64	1.07	0.47	2.22	0.19	0.06	31582.91	32940.93	34394.48	25332.84	25415.76	24190.19	30748.17	32218.20	30758.33	32222.43	32399.50	17827.87		x
289	Acyl-coenzyme A thioesterase 8	O14734	46097.43	43347.14	39086.16	32984.12	30712.39	28403.81	30848.61	28169.12	29252.19	39508.11	35930.47	35039.29	36795.69	36131.60	28616.02	29543.77	27939.72	26599.85	30088.74	30235.46	27340.31	14949.13	14099.51	14621.30	10531.25	9448.90	8612.24	11109.66	10605.78	10109.89	13581.87	13264.61	9414.18		
290	Acyl-coenzyme A thioesterase 9_ mitochondrial	Q9Y305	0.14	2.53	0.81	1.07	2.77	0.68	0.96	0.36	0.63	0.35	0.75	0.38	2.13	0.97	0.60	4.64	1.07	0.47	2.22	0.19	0.06	66724.00	66681.24	67864.72	47355.59	47635.21	42330.84	55223.74	52094.43	48023.76	52611.69	51889.99	37302.86		x
291	Acylglycerol kinase_ mitochondrial	Q53H12	60409.52	58012.40	52204.50	42071.43	38017.19	38175.73	43330.80	42873.99	42272.80	53155.55	53500.92	49697.54	54125.99	48192.25	39306.90	41099.78	39651.83	39485.77	39202.66	37416.29	37397.62	7121.17	7121.10	6625.14	4735.15	4043.17	3306.37	9755.84	9205.68	7849.44	6276.20	5728.09	3487.97		
292	Acylphosphatase-1	P07311	0.14	2.53	0.81	1.07	2.77	0.68	0.96	0.36	0.63	0.35	0.75	0.38	2.13	0.97	0.60	4.64	1.07	0.47	2.22	0.19	0.06	18638.28	17067.01	11478.88	5861.69	6435.40	8292.63	12760.14	12524.02	10755.37	15347.86	14410.49	7526.07		x
293	Acyl-protein thioesterase 1	O75608	29664.84	30073.85	26726.37	20001.06	20119.69	17985.11	24063.90	22589.22	20862.80	27545.30	25865.79	25402.06	23419.85	23009.16	20372.11	18810.49	18542.57	17878.43	21092.78	19785.31	18798.04	9223.67	8191.41	7117.95	3872.10	3957.40	3135.88	5266.36	4842.28	4551.88	5028.89	4634.19	3048.03		
294	Acyl-protein thioesterase 2	O95372	13088.39	12274.69	13468.00	7773.46	6909.10	6356.66	8589.08	8161.57	7699.41	11936.16	10386.94	9353.72	9952.24	9858.34	8329.15	7624.86	6188.77	6916.19	6412.86	6481.50	5911.96	7121.10	6549.53	6444.18	4134.79	3457.04	2918.44	6476.35	5830.35	4350.92	6061.20	5452.28	3681.67		
295	Acylpyruvase FAHD1_ mitochondrial	Q6P587	9871.83	9646.19	8322.71	5427.30	5318.81	5085.05	7676.48	8256.57	8112.90	7011.22	6669.87	5989.82	7719.48	6601.37	4231.38	5090.41	5229.48	5073.47	5891.67	4860.42	4423.91	2792.81	2678.18	2242.09	1506.61	1255.88	993.97	1900.58	1780.67	1492.41	1792.95	1659.12	522.56		
296	Adapter molecule crk	P46108	85072.73	82782.34	78602.94	59042.77	56497.46	54568.23	66141.51	66506.05	61841.11	72530.01	68009.12	63779.16	66550.96	69981.34	53867.37	58802.09	53198.19	51781.26	58704.71	56884.36	53588.42	210653.81	182545.41	178017.25	142564.56	135670.84	123050.72	160565.40	151561.98	146884.47	155909.47	146077.19	108133.06		
297	Adaptin ear-binding coat-associated protein 2	Q9NVZ3	7516.37	6239.46	5197.22	3455.45	3171.42	2333.11	4800.59	4828.96	3930.25	4475.89	4007.57	3512.36	4724.73	4125.61	1559.94	3695.78	3153.41	2787.89	4095.62	3733.59	2779.36	10183.06	9367.37	8577.38	8914.36	7663.60	6208.65	8017.33	9214.09	8323.85	7809.34	7896.96	6033.59		
298	Adenine phosphoribosyltransferase	P07741	405403.66	391307.21	372640.46	283988.83	273339.69	248808.28	363200.69	352105.54	328952.98	359406.39	345922.48	321931.21	329424.03	312167.13	280065.95	293299.10	275624.81	256072.48	275295.35	262332.93	246460.30	138014.87	129199.93	120090.44	84153.88	80629.55	74063.13	93949.31	92583.83	89460.17	93221.75	91781.10	62237.99		
299	Adenomatous polyposis coli protein 2	O95996	0.14	2.53	0.81	1.07	2.77	0.68	0.96	0.36	0.63	0.35	0.75	0.38	2.13	0.97	0.60	4.64	1.07	0.47	2.22	0.19	0.06	1821.35	1383.12	1425.58	855.69	796.59	695.30	1116.13	1178.15	1046.50	1284.29	1341.48	827.56		x
300	Adenomatous polyposis coli protein	P25054	54352.51	48449.00	42135.43	39062.87	37713.03	32243.01	49538.55	47308.22	44520.88	49346.01	47461.07	42125.18	44291.34	42437.67	24231.13	41041.83	41670.45	37915.58	34406.08	32614.45	30279.17	175037.37	169941.20	152775.99	107644.86	107814.48	99039.13	115948.75	109978.29	105080.78	116384.61	111043.04	102379.00		
301	Adenosine 3'-phospho 5'-phosphosulfate transporter 1	Q8TB61	32079.58	28327.36	25057.15	19767.68	18575.40	17251.11	20134.54	19681.36	17707.33	26991.02	25415.53	23681.50	26423.08	19107.13	12073.84	15269.48	15409.34	14325.60	21989.29	18724.74	14535.27	2.27	1.49	0.65	0.84	1.31	0.69	4.16	2.47	0.11	4.90	4.98	0.12	x	
302	Adenosine deaminase domain-containing protein 2	Q8NCV1	0.14	2.53	0.81	1.07	2.77	0.68	0.96	0.36	0.63	0.35	0.75	0.38	2.13	0.97	0.60	4.64	1.07	0.47	2.22	0.19	0.06	2580.67	2581.06	2306.74	1281.03	1244.98	1174.88	1148.31	1021.45	1089.53	985.05	909.21	208.71		x
303	Adenosine kinase	P55263	31688.08	30419.21	29118.67	22248.07	21184.48	19616.46	22518.86	21497.24	20889.93	27143.06	26135.51	25001.07	26584.38	23148.69	23076.37	20968.02	19364.53	19034.18	20112.61	20041.66	19085.49	33901.55	34706.93	33077.18	25833.55	24840.29	21276.47	31575.73	30023.44	27878.20	30694.08	29239.83	17104.92		
304	Adenosylhomocysteinase 3	Q96HN2	1766.91	1532.61	1222.01	912.16	741.63	589.50	1055.11	958.16	958.95	1483.45	1578.27	1180.28	1221.33	733.02	407.79	1115.86	860.47	895.29	934.92	903.42	695.64	2.27	1.49	0.65	0.84	1.31	0.69	4.16	2.47	0.11	4.90	4.98	0.12	x	
305	Adenosylhomocysteinase	P23526	773934.27	732987.61	675039.94	505194.24	516083.99	484850.10	636512.69	609486.11	589564.46	668782.31	651259.52	606604.30	593799.99	530310.34	452050.07	554490.28	529206.29	504416.55	495438.75	467929.14	450652.14	801953.59	747199.57	686167.76	516946.67	504580.91	460544.01	639124.10	612501.94	576878.70	606386.10	604339.06	438036.24		
306	Adenylate cyclase type 6	O43306	4033.00	3731.42	3854.67	2447.33	2590.48	2516.77	2613.21	2759.35	2948.34	3676.34	3654.00	3373.89	3362.48	2920.05	2249.96	2398.76	2395.57	2628.21	3220.40	3160.12	3108.77	2.27	1.49	0.65	0.84	1.31	0.69	4.16	2.47	0.11	4.90	4.98	0.12	x	
307	Adenylate cyclase type 8	P40145	0.14	2.53	0.81	1.07	2.77	0.68	0.96	0.36	0.63	0.35	0.75	0.38	2.13	0.97	0.60	4.64	1.07	0.47	2.22	0.19	0.06	7343.13	3740.25	2418.22	4268.31	3918.64	2005.74	6572.99	5539.77	3966.62	7413.87	6850.52	3024.06		x
308	Adenylate kinase 2_ mitochondrial	P54819	473351.51	452811.88	426313.91	443257.97	410442.39	393627.15	482043.66	462484.37	451434.88	546661.15	523769.92	487345.68	435357.79	411171.23	349535.29	451944.02	432525.61	418266.19	399666.93	380229.24	368503.39	102072.88	89074.02	81953.54	53898.31	55276.28	47830.50	69997.60	67919.80	64220.98	68599.05	66523.21	41959.40		
309	Adenylate kinase 4_ mitochondrial	P27144	54090.74	52671.52	49744.04	21627.32	22231.78	21413.95	35300.14	34211.45	32302.44	33352.31	32394.76	31402.59	31841.34	30592.57	28046.75	29298.42	29276.79	29000.94	33550.53	32532.19	29787.69	40731.14	37167.47	35203.15	23647.31	22882.71	20008.78	31613.88	31775.96	30492.63	33654.60	34031.36	21858.55		
310	Adenylate kinase isoenzyme 1	P00568	18108.22	19261.48	16356.24	12467.98	13027.19	11112.30	13288.15	12290.54	12977.28	18402.59	17520.54	16803.59	16305.50	12493.53	8531.21	12041.11	12656.31	13433.26	12451.83	12204.02	11538.52	9748.07	8267.16	7435.76	4912.15	5400.20	4934.44	7998.03	7024.57	6710.78	5728.14	7346.03	6407.91		
311	Adenylosuccinate lyase	P30566	68492.22	63605.44	58721.64	46942.59	45859.29	41818.20	53385.09	49406.48	44633.90	65323.73	63535.24	57141.14	59454.11	51890.57	38741.98	44262.50	42407.23	43033.14	45143.61	45900.75	45464.26	33239.62	26468.41	26163.70	21945.19	21808.51	18583.61	29759.85	26969.87	25116.29	29189.35	28154.43	18455.58		
312	Adenylosuccinate synthetase isozyme 2	P30520	85443.53	85113.37	78589.93	65029.98	64056.33	57718.27	73670.60	72515.16	65437.55	79999.16	78132.84	70337.17	80165.10	71135.36	54176.13	64847.97	61793.23	61254.25	72091.16	68864.17	61632.71	99877.61	95144.47	80950.46	49929.27	51548.32	39612.07	60693.97	57984.54	53083.50	65994.26	65284.50	40326.12		
313	Adenylyl cyclase-associated protein 1	Q01518	281961.72	270643.55	260097.76	180046.53	179990.16	171487.06	215353.87	205003.50	197869.92	228138.37	215155.40	204659.37	231588.91	218043.78	182296.64	194250.62	178313.82	173687.73	181393.02	177029.94	163798.43	760458.17	725765.79	679046.90	446336.99	435749.31	394256.75	478818.69	470418.14	448400.01	504247.52	495669.38	350588.67		
314	Adenylyl cyclase-associated protein 2	P40123	2083.72	1720.12	1311.90	519.47	450.79	336.42	615.56	23.10	563.77	1602.91	1739.88	1477.46	1557.24	0.97	165.97	535.76	452.43	531.06	768.44	684.43	468.18	3288.09	3384.13	1304.49	2213.16	1977.31	1216.85	2989.13	2555.46	2013.47	3703.94	3501.93	1561.92		
315	Adhesion G protein-coupled receptor B3	O60242	8562.19	7528.44	6789.75	6278.54	5933.30	5283.94	6833.22	6916.14	6285.91	8494.00	8604.20	7891.02	7934.01	7279.00	5475.71	5715.03	5929.55	5727.72	7517.68	6717.80	6322.48	2.27	1.49	0.65	0.84	1.31	0.69	4.16	2.47	0.11	4.90	4.98	0.12	x	
316	Adhesion G-protein coupled receptor G6	Q86SQ4	4021.53	4090.75	3925.48	2641.67	2761.00	2515.34	2726.87	2317.19	2286.77	3394.37	3222.67	3051.50	3225.45	3225.61	2876.56	2538.53	2399.12	2602.94	1969.57	2040.04	1886.31	2.27	1.49	0.65	0.84	1.31	0.69	4.16	2.47	0.11	4.90	4.98	0.12	x	
317	Adhesion G-protein coupled receptor G7	Q96K78	16212.94	14686.85	13874.64	11314.73	11043.43	11116.09	13566.13	12476.55	11911.76	14631.92	13853.48	13049.32	12865.86	12376.11	10926.93	11596.06	11775.07	10292.57	11330.84	11042.65	10035.07	2.27	1.49	0.65	0.84	1.31	0.69	4.16	2.47	0.11	4.90	4.98	0.12	x	
318	Adipocyte plasma membrane-associated protein	Q9HDC9	152014.83	141092.02	136585.40	101674.72	98573.75	92808.93	114214.27	107884.14	101239.43	127029.42	121398.34	115116.70	121449.57	117697.32	91267.48	106061.95	94344.36	95260.76	90042.85	92313.28	81836.67	81666.90	84233.47	80615.08	47478.91	45426.71	39640.83	66005.99	61266.94	56253.45	48046.92	49075.72	31072.12		
319	ADP/ATP translocase 1	P12235	15284.81	17683.75	14780.30	9425.73	10626.01	8042.46	16373.40	13037.37	12606.41	13148.70	13022.15	12115.53	11465.35	10826.25	7323.60	14293.31	10528.72	9988.84	9597.40	9023.83	9009.69	2.27	1.49	0.65	0.84	1.31	0.69	4.16	2.47	0.11	4.90	4.98	0.12	x	
320	ADP/ATP translocase 2	P05141	2107875.71	2050413.19	1961569.88	1332517.40	1262471.23	1197872.45	1664557.53	1635640.65	1608469.57	1589078.71	1524942.41	1435753.59	1557217.48	1466372.74	1249504.56	1480199.46	1387159.39	1359964.36	1421719.62	1343414.02	1287795.30	575439.82	530470.51	510226.00	333062.62	323056.42	306150.99	454791.13	423678.17	404798.16	422389.37	411985.86	326065.70		
321	ADP/ATP translocase 3	P12236	406304.60	387135.79	367214.86	225382.32	222777.05	200990.70	301209.35	303848.78	273537.54	310066.68	293374.14	272683.70	315330.87	291624.30	215761.30	252927.82	246325.08	227417.70	268947.96	258859.83	240496.97	20517.98	18260.90	17627.05	8759.44	7768.19	5753.16	17886.42	17198.65	14731.99	13922.38	12426.51	7675.66		
322	ADP/ATP translocase 4	Q9H0C2	50009.27	49926.31	45678.36	36485.17	33521.57	32282.70	40123.08	38102.71	34982.73	46306.73	44081.79	40549.70	45032.11	41815.24	33155.65	36633.43	33318.85	31897.64	34476.60	32876.32	29690.33	7276.68	7555.57	8032.81	5784.88	5909.68	5641.73	6229.81	6405.42	6280.44	6568.38	6310.08	4516.28		
323	ADP-ribose glycohydrolase ARH3	Q9NX46	50358.13	53396.13	50374.80	35144.77	33780.40	30402.84	46404.61	45375.79	46624.96	44316.06	42069.37	36584.82	46416.89	40638.88	33758.71	42031.71	37731.07	38992.19	43485.38	37617.99	36064.42	20050.89	18571.55	15414.10	11348.95	11538.79	10775.62	12489.82	12287.49	11450.89	13978.01	13173.33	10342.15		
324	ADP-ribose glycohydrolase MACROD1	Q9BQ69	21434.16	20004.11	17547.68	8997.48	7925.99	7340.29	11804.80	11666.08	10743.69	8898.12	8818.08	8092.89	14366.02	12389.84	9428.32	10367.70	8949.74	9362.51	10063.36	9363.61	8522.39	2.27	1.49	0.65	0.84	1.31	0.69	4.16	2.47	0.11	4.90	4.98	0.12	x	
325	ADP-ribose glycohydrolase OARD1	Q9Y530	22568.50	29349.75	29673.90	14102.21	17852.97	16365.78	18719.27	17794.87	12435.80	22123.45	22685.99	16946.75	16846.58	23242.59	20726.60	22881.23	12759.26	14685.99	22162.29	15748.83	16319.85	15555.74	13788.60	9405.39	10045.62	10291.78	9594.65	12960.67	12226.44	10718.39	12922.03	12212.73	9362.22		
326	ADP-ribose pyrophosphatase_ mitochondrial	Q9BW91	66850.13	65658.90	72773.66	55111.49	51356.64	49310.52	59851.33	62774.04	63701.24	72672.73	68486.11	63548.51	65947.52	63692.17	46167.46	50381.19	48861.00	54770.33	47196.26	44856.55	42574.40	2.27	1.49	0.65	0.84	1.31	0.69	4.16	2.47	0.11	4.90	4.98	0.12	x	
327	ADP-ribosyl cyclase/cyclic ADP-ribose hydrolase 2	Q10588	4872.81	5187.09	5138.11	3960.65	4101.31	3915.25	4797.97	4374.98	4178.80	4979.88	4467.27	4247.72	4955.93	4714.14	4055.46	3857.23	3660.10	3647.72	4184.40	4108.33	4169.88	4738.21	4003.99	3828.62	2985.06	2751.89	2472.37	5319.86	4821.52	4643.35	4166.36	3817.33	2506.61		
328	ADP-ribosylation factor 1	P84077	525144.91	489752.64	449131.88	356743.23	336234.44	299329.00	382698.24	391315.34	358658.08	438806.67	418124.27	384985.82	439945.13	393082.64	296017.50	351699.66	322401.88	305603.75	335661.44	315116.02	292559.32	2.27	1.49	0.65	0.84	1.31	0.69	4.16	2.47	0.11	4.90	4.98	0.12	x	
329	ADP-ribosylation factor 3	P61204	0.14	2.53	0.81	1.07	2.77	0.68	0.96	0.36	0.63	0.35	0.75	0.38	2.13	0.97	0.60	4.64	1.07	0.47	2.22	0.19	0.06	317794.92	287024.42	275499.32	222016.67	208367.31	179477.93	284633.17	261680.28	241283.77	270332.11	258474.83	163212.16		x
330	ADP-ribosylation factor 4	P18085	62676.71	62090.02	60142.31	44985.22	43086.04	38714.75	50130.60	47884.66	46615.88	57529.03	53774.92	48526.13	48722.59	46455.03	39691.01	46023.70	43471.36	42103.14	39731.60	38686.26	36669.80	110123.21	106068.81	100280.14	79006.96	77546.08	68278.43	91759.19	89425.94	85181.00	91714.75	88169.78	64315.03		
331	ADP-ribosylation factor 5	P84085	116741.10	106446.56	97289.22	77977.68	74832.20	75575.37	95653.47	95379.28	89673.43	95954.33	90172.37	81455.85	106046.06	96784.00	89257.04	81531.92	75295.78	72924.99	85972.87	85064.96	76603.33	42065.57	40002.44	37715.58	27990.56	28593.31	26459.80	33798.92	33379.55	32169.99	33957.59	34173.30	31949.73		
332	ADP-ribosylation factor 6	P62330	103589.60	103477.88	100618.13	65617.68	66534.52	63704.39	69837.62	69121.60	69256.53	72717.11	66471.40	68047.18	81479.20	80195.17	71980.33	58575.98	56653.23	53808.53	63346.05	63252.96	57373.01	94480.60	88045.50	80549.58	59846.44	59574.73	52506.46	75396.35	74199.16	71196.69	75333.79	73466.70	53429.11		
333	ADP-ribosylation factor GTPase-activating protein 1	Q8N6T3	132391.40	126713.71	125309.42	98716.21	97026.10	91267.47	124245.58	125465.04	120536.88	126598.56	122793.75	116537.43	117007.46	107018.32	67211.87	89800.46	96401.74	93818.64	100454.94	97361.81	93029.75	88313.41	77609.66	72935.34	63001.27	59319.93	49972.83	77515.34	72901.02	69761.76	73880.19	73866.13	37371.89		
334	ADP-ribosylation factor GTPase-activating protein 2	Q8N6H7	97519.60	94414.26	85528.28	55995.37	53587.52	45704.31	71044.85	66548.75	63065.39	75487.14	71885.59	64703.48	77576.22	64098.83	45553.33	54631.12	50498.97	48251.35	59644.89	50708.70	48466.14	11724.31	10850.87	10228.89	7720.36	7976.53	5873.33	9355.56	8738.23	7583.67	11135.94	10211.06	5919.75		
335	ADP-ribosylation factor GTPase-activating protein 3	Q9NP61	41047.42	40064.22	32106.40	26613.68	24965.92	21974.64	32279.85	30273.81	28164.29	34386.73	33422.93	28578.47	31861.63	30593.38	22208.24	27842.07	27628.27	24693.02	29221.81	27685.37	24771.70	2.27	1.49	0.65	0.84	1.31	0.69	4.16	2.47	0.11	4.90	4.98	0.12	x	
336	ADP-ribosylation factor-like protein 1	P40616	49230.21	43777.51	38011.23	39271.21	36969.43	33809.09	36122.78	33807.49	33933.20	44926.79	44428.82	39239.26	45817.53	40300.11	26336.86	40303.12	36332.50	37219.34	41125.17	38059.89	33005.39	17276.70	8439.19	7971.85	8772.76	8202.60	4200.48	13281.64	11238.03	7105.00	16424.56	15377.07	7192.65		
337	ADP-ribosylation factor-like protein 2	P36404	0.14	2.53	0.81	1.07	2.77	0.68	0.96	0.36	0.63	0.35	0.75	0.38	2.13	0.97	0.60	4.64	1.07	0.47	2.22	0.19	0.06	14250.94	14482.23	11333.10	7345.02	6898.02	7533.04	5891.09	6430.34	5642.74	8833.16	5167.97	3067.86		x
338	ADP-ribosylation factor-like protein 3	P36405	107392.52	103668.02	100063.74	72941.18	66359.35	62034.59	81302.89	78823.82	80558.83	88347.41	83812.99	79737.42	87678.91	78081.47	74905.94	73329.56	71143.60	68058.42	86863.83	72444.60	70774.41	9979.98	9060.01	9069.49	6486.15	5613.09	4562.95	7537.20	7340.45	6771.54	8567.85	7977.77	5210.14		
339	ADP-ribosylation factor-like protein 6-interacting protein 1	Q15041	63172.15	61186.77	58248.67	41592.70	40927.10	40436.09	39542.61	39508.44	40252.57	52588.08	50887.51	48386.49	49208.43	35045.75	8225.99	37943.03	42847.68	38020.95	40962.28	37616.39	34306.17	82211.08	80727.66	72274.26	55629.74	53913.73	55204.27	54350.60	53871.81	53699.37	61553.59	57164.51	41895.11		
340	ADP-ribosylation factor-like protein 6-interacting protein 4	Q66PJ3	17521.77	17873.40	16856.63	11567.73	10981.23	10051.86	13429.78	13328.75	10598.48	12445.52	11611.52	10585.37	14792.32	12625.43	10507.00	10033.64	9754.55	9449.33	10661.85	10179.89	9499.65	2.27	1.49	0.65	0.84	1.31	0.69	4.16	2.47	0.11	4.90	4.98	0.12	x	
341	ADP-ribosylation factor-like protein 8A	Q96BM9	0.14	2.53	0.81	1.07	2.77	0.68	0.96	0.36	0.63	0.35	0.75	0.38	2.13	0.97	0.60	4.64	1.07	0.47	2.22	0.19	0.06	2454.93	2156.40	1881.47	1788.72	1599.04	1423.83	2111.27	1861.59	1720.23	2232.04	1920.07	1199.04		x
342	ADP-ribosylation factor-like protein 8B	Q9NVJ2	122410.36	123939.11	116039.48	86529.27	86854.42	81619.01	102651.93	102601.47	96970.75	108204.03	102602.33	96110.48	100349.81	100273.84	81618.78	89167.30	81917.84	80868.78	80139.73	79116.88	75761.73	739.43	736.67	703.89	355.84	329.93	271.37	518.04	512.99	475.51	495.34	462.09	223.63		
343	ADP-sugar pyrophosphatase	Q9UKK9	46684.74	44097.55	38816.71	32412.10	31634.97	27079.42	37357.72	34444.69	32840.07	41240.27	36942.62	34951.29	41702.81	37565.93	25834.87	31715.42	30087.25	27965.00	32820.00	32003.07	28073.00	36576.09	34353.83	28225.35	32393.43	30615.80	23324.72	50606.61	48588.81	46380.68	39461.33	40517.47	19462.75		
344	Adrenocortical dysplasia protein homolog	Q96AP0	193.66	170.21	70.81	38.50	419.96	518.30	2666.53	1483.36	1058.70	80.51	41.00	29.42	138.97	97.45	5.83	533.60	2457.11	774.90	108.31	354.15	239.11	2.27	1.49	0.65	0.84	1.31	0.69	4.16	2.47	0.11	4.90	4.98	0.12	x	
345	Advillin	O75366	58425.28	56444.49	48808.66	35996.93	35711.82	31864.23	44251.53	42669.79	34218.28	51973.78	47462.73	43945.32	44532.23	41545.80	36012.64	38354.09	35086.31	34281.55	37233.66	35108.94	32444.90	2.27	1.49	0.65	0.84	1.31	0.69	4.16	2.47	0.11	4.90	4.98	0.12	x	
346	AF4/FMR2 family member 3	P51826	6655.06	6336.14	5537.08	4356.39	4184.98	4224.12	4449.49	4648.26	4120.17	5359.76	4844.11	4903.71	5705.21	5215.99	5370.64	4590.84	3809.45	4737.45	4801.87	3876.22	3696.08	2.27	1.49	0.65	0.84	1.31	0.69	4.16	2.47	0.11	4.90	4.98	0.12	x	
347	Afadin	P55196	1076.49	1116.24	1031.72	870.24	948.75	864.42	834.03	826.15	895.23	1608.81	1502.22	1465.18	1292.37	1145.05	960.47	822.90	722.49	766.58	857.69	665.70	689.96	28269.49	21016.40	15106.99	15734.66	11630.36	5839.08	26885.44	21930.12	14506.86	22158.73	18768.99	9093.63		
348	Afamin	P43652	33829.26	31645.93	28543.91	19388.19	19698.90	18770.33	25083.36	25808.93	22705.00	26366.27	25431.19	22925.89	28877.74	25765.08	19294.33	23515.11	21697.65	20383.32	21859.40	20655.39	18291.98	6071.34	4591.52	5163.15	5433.61	6219.59	4813.75	6833.62	6838.80	7038.40	8096.75	7467.87	3083.51		
349	AFG3-like protein 2	Q9Y4W6	127184.28	121124.49	111492.94	79236.10	77732.91	70129.24	93755.86	88931.38	87156.81	97412.72	93727.29	87385.87	102306.07	92845.88	71234.53	84319.67	78951.93	75421.18	84731.06	80936.94	71931.62	68992.30	61460.70	58154.14	52191.93	51244.15	47806.82	55467.71	54909.48	50572.57	57299.06	54403.56	39583.32		
350	Aflatoxin B1 aldehyde reductase member 2	O43488	10272.91	9767.30	8602.25	5589.43	5345.00	4900.27	7013.41	6549.53	6006.90	8341.62	7969.20	8270.61	7830.02	7236.07	6120.73	6239.41	6427.02	5939.59	5088.04	4925.04	5075.13	18136.71	16965.68	15131.61	10631.73	9430.83	8545.59	13954.98	14110.80	13545.44	11939.46	11762.56	7919.58		
351	Aflatoxin B1 aldehyde reductase member 4	Q8NHP1	10441.73	8481.19	7600.93	5148.72	4656.05	3449.47	5993.89	6216.39	5660.53	6203.96	5921.25	4697.58	6490.55	6084.04	3747.84	5269.62	5308.74	4874.99	5609.97	4801.44	4526.16	37785.44	35006.86	29886.13	19496.86	18308.40	11923.22	32453.65	28883.49	24634.87	30256.74	28586.30	15151.14		
352	Agrin	O00468	2920.48	2535.56	2092.45	2074.06	1980.67	2180.05	2564.18	2648.22	2329.09	3345.31	3136.52	3077.20	2127.36	1939.14	2239.87	3291.23	3081.19	3130.50	1965.86	1915.45	2092.32	2.27	1.49	0.65	0.84	1.31	0.69	4.16	2.47	0.11	4.90	4.98	0.12	x	
353	AH receptor-interacting protein	O00170	46131.79	43041.16	38577.63	33545.29	31443.36	30334.40	38312.48	33672.62	34816.92	39653.67	38574.14	36471.16	40596.77	36428.72	26019.34	31111.07	29322.01	27907.87	32121.47	29463.87	26696.41	11543.51	9887.53	7927.65	6057.99	5997.89	4477.16	6833.73	6767.15	5976.90	7611.96	7455.12	4572.80		
354	A-kinase anchor protein 12	Q02952	3282.83	2768.12	2910.02	2082.49	2060.52	2018.38	2819.74	2021.18	2156.82	2780.06	2966.90	2396.75	2390.28	2576.27	1999.16	2001.72	1977.27	1888.68	2048.10	2024.50	1871.95	262023.49	244572.72	223637.61	184625.69	177261.00	158764.68	227170.90	212251.36	205038.68	237742.15	225025.76	148329.53		
355	A-kinase anchor protein 3	O75969	4184.57	3923.75	4056.80	2430.53	2427.29	2399.58	1973.93	2128.70	1885.79	3782.56	3822.58	4537.90	3377.96	3462.09	289.23	827.16	1666.27	2013.40	3802.80	2854.29	2692.34	2.27	1.49	0.65	0.84	1.31	0.69	4.16	2.47	0.11	4.90	4.98	0.12	x	
356	A-kinase anchor protein 8-like	Q9ULX6	24008.79	23431.57	21991.91	17003.00	16425.03	15478.25	20006.42	19150.10	18512.87	20539.50	20431.96	18303.34	21026.44	19291.12	15821.14	16467.27	15642.87	14710.10	17414.80	17203.77	16594.87	2.27	1.49	0.65	0.84	1.31	0.69	4.16	2.47	0.11	4.90	4.98	0.12	x	
357	A-kinase anchor protein 9	Q99996	68477.99	63622.32	61073.99	48072.12	45579.48	42326.43	54811.46	55706.83	51693.34	56845.38	54726.54	51947.33	59332.30	53764.04	35410.44	43034.05	43904.56	44210.05	50038.49	46611.55	45128.54	32120.17	37432.20	31707.74	34056.47	33250.85	31441.44	27096.20	31463.92	32143.52	31816.62	38598.14	58983.21		
358	A-kinase anchor protein SPHKAP	Q2M3C7	34693.13	32527.49	35321.78	17870.79	16566.77	15682.44	19591.64	20999.17	18072.40	30568.42	29618.80	34543.56	29938.26	26091.76	15486.47	17391.09	19512.91	20933.55	40657.55	27457.88	21284.49	2.27	1.49	0.65	0.84	1.31	0.69	4.16	2.47	0.11	4.90	4.98	0.12	x	
359	Alanine aminotransferase 2	Q8TD30	7072.82	6651.78	6556.59	4764.39	4305.61	4215.80	5027.77	5027.46	4831.99	5556.19	5284.95	5150.29	5876.18	5409.31	4037.08	4353.30	3998.43	4155.21	5317.61	5020.16	4890.16	2.27	1.49	0.65	0.84	1.31	0.69	4.16	2.47	0.11	4.90	4.98	0.12	x	
360	Alanine--tRNA ligase_ cytoplasmic	P49588	1483814.31	1425919.31	1311371.41	1026630.58	982030.04	909461.17	1218755.35	1161479.26	1107473.55	1109332.38	1040494.23	978791.42	1179927.92	1081709.17	874295.16	1058987.04	993332.81	949209.91	1001057.01	948238.22	890283.81	505436.51	460324.66	410401.33	346080.71	332526.27	287041.45	393919.68	369293.33	336783.71	399347.00	385119.70	260432.64		
361	Alanine--tRNA ligase_ mitochondrial	Q5JTZ9	1386.02	1715.31	1628.96	1244.86	1147.34	1053.18	1582.79	1291.46	1318.38	1203.02	1170.20	1228.74	1666.52	1294.68	1170.80	1099.24	959.83	1007.10	1128.74	1047.89	1004.21	2.27	1.49	0.65	0.84	1.31	0.69	4.16	2.47	0.11	4.90	4.98	0.12	x	
362	Alanyl-tRNA editing protein Aarsd1	Q9BTE6	9299.19	9388.79	8282.35	7025.27	7629.40	6940.02	6720.69	6770.19	7008.53	9734.76	9163.96	8182.55	7369.74	8326.79	6805.14	6538.19	6703.52	6435.39	8082.46	7198.60	7205.65	2104.50	1914.27	1618.12	663.80	624.94	432.22	910.81	906.40	848.51	992.61	985.30	474.07		
363	Alcohol dehydrogenase class-3	P11766	234216.07	225121.82	207655.13	149781.75	145487.34	135989.77	177436.28	171004.67	163578.17	198771.15	186568.09	177871.24	189733.91	178424.37	149237.46	157875.97	150735.15	145039.56	160074.98	153213.37	142008.12	69642.16	65044.66	58023.51	48022.81	47070.41	41171.85	58186.20	57311.58	54889.39	60323.72	58369.48	40205.65		
364	Aldehyde dehydrogenase family 1 member A3	P47895	0.14	2.53	0.81	1.07	2.77	0.68	0.96	0.36	0.63	0.35	0.75	0.38	2.13	0.97	0.60	4.64	1.07	0.47	2.22	0.19	0.06	1404.36	745.64	1364.50	787.05	763.92	616.68	947.60	844.21	760.84	896.13	919.53	711.44		x
365	Aldehyde dehydrogenase family 16 member A1	Q8IZ83	7960.20	9316.69	9409.60	9162.76	8529.58	9001.46	13722.79	13383.15	10111.91	10536.29	8750.26	7872.81	9548.21	9361.06	9007.17	8207.82	7104.62	6214.57	8263.31	7818.17	7642.36	10635.06	10337.54	10366.34	7669.69	7588.37	7165.42	8312.58	7551.20	7754.15	8429.22	7592.15	6095.87		
366	Aldehyde dehydrogenase X_ mitochondrial	P30837	16454.66	16036.82	16508.13	10482.28	10443.34	9566.93	12467.82	12090.57	11275.71	14994.63	13623.17	14324.70	14656.47	13527.11	12080.15	9967.23	9771.45	9415.79	11507.78	10598.32	10290.14	7756.89	7097.70	6632.51	4884.78	4459.14	3723.77	6554.28	6000.66	5489.55	7159.98	6808.15	4025.60		
367	Aldo-keto reductase family 1 member A1	P14550	7360.81	6337.47	4890.51	4969.41	668.70	500.57	1053.63	651.60	670.98	8134.89	7357.93	6855.56	8141.32	5359.30	961.19	972.73	925.69	593.68	5350.89	5177.20	3549.65	45581.12	34019.70	36161.77	28795.65	27808.24	21832.77	42411.59	39343.87	31488.06	43015.28	41203.95	25576.16		
368	Aldose reductase	P15121	0.14	2.53	0.81	1.07	2.77	0.68	0.96	0.36	0.63	0.35	0.75	0.38	2.13	0.97	0.60	4.64	1.07	0.47	2.22	0.19	0.06	184918.83	163504.38	159228.84	86028.40	87418.59	84405.32	103538.95	101909.49	95724.91	103442.39	98841.95	104418.94		

Supplementary Table S1. Overview on the abundance of all relatively quantified 5180 proteins

	Protein name	UniProt	MCF-7 control_01	control_02	control_03	Dai_IC20_01	Dai_IC20_02	Dai_IC20_03	Dai_SC20_01	Dai_SC20_02	Dai_SC20_03	Gen_IC20_01	Gen_IC20_02	Gen_IC20_03	Gen_SC20_01	Gen_SC20_02	Gen_SC20_03	SSE_IC20_01
369	Alkyldihydroxyacetonephosphate synthase_ peroxisomal	O00116	0.14	2.53	0.81	1.07	2.77	0.68	0.96	0.36	0.63	0.35	0.75	0.38	2.13	0.97	0.60	4.64
370	Allograft inflammatory factor 1-like	Q9BQI0	26360.99	20905.99	19814.58	14697.82	13588.52	13458.02	21884.02	19121.89	18726.20	20115.88	18890.66	17044.17	20954.01	19614.91	16320.63	14688.49
371	Alpha N-terminal protein methyltransferase 1B	Q5VVY1	2107.15	2127.49	2037.86	934.67	963.77	914.29	1407.94	1270.19	1396.04	1041.31	1025.42	944.03	1117.03	1249.10	1126.96	1031.08
372	Alpha/beta hydrolase domain-containing protein 17A	Q96GS6	22165.00	22893.21	14996.95	14121.49	14256.19	12208.28	13537.37	12705.29	12346.05	18086.18	16557.43	16220.96	17310.00	15139.50	14660.65	15158.24
373	Alpha-1_3/1_6-mannosyltransferase ALG2	Q9H553	23058.73	21945.34	20916.88	15280.86	15551.66	14926.33	18165.24	17707.99	17064.74	20642.99	20540.20	20234.46	20266.26	17468.38	13972.59	15365.73
374	Alpha-2-HS-glycoprotein	P02765	178181.75	167257.30	160536.67	110155.51	105270.26	101524.80	151512.99	145355.88	141512.16	154197.35	146590.59	139789.66	139047.52	129269.57	115164.60	143533.15
375	Alpha-2-macroglobulin	P01023	12999.03	12316.13	11114.54	8556.80	7817.81	7720.24	9288.43	9539.10	8499.50	13034.10	12415.89	11264.39	11260.51	10438.51	8538.54	7586.30
376	Alpha-2-macroglobulin receptor-associated protein	P30533	19045.25	18882.99	17819.91	13248.69	12846.28	12034.23	13476.18	12970.46	12331.59	21517.55	19314.05	18957.97	17454.92	16046.74	13889.54	13803.15
377	Alpha-actinin-1	P12814	777905.31	750841.37	691286.38	524933.74	507013.46	468424.15	615457.75	605337.41	568853.40	659469.84	621359.43	578774.07	644955.10	612960.73	514453.23	503160.80
378	Alpha-actinin-2	P35609	71227.43	71173.79	63513.36	50878.38	50594.92	47757.24	56219.63	52729.03	53697.11	61682.11	57952.00	55120.93	58537.86	58363.17	50008.08	46960.53
379	Alpha-actinin-3	Q08043	309848.80	292181.69	260995.75	255376.91	244514.14	222794.74	269954.76	226704.30	212237.98	274504.39	264353.04	245423.84	254435.91	264787.13	199075.00	264877.97
380	Alpha-actinin-4	O43707	2103516.59	2064789.71	1885304.57	1252471.09	1220941.45	1100742.16	1576357.23	1498020.92	1420327.05	1562415.40	1478563.93	1401811.03	1554473.00	1454083.42	1157483.83	1342050.64
381	Alpha-aminoadipic semialdehyde dehydrogenase	P49419	125357.06	125866.19	112637.35	88684.54	85628.08	81179.91	95915.58	92373.71	89154.60	112747.96	104298.85	101933.93	100516.48	98269.94	81074.82	93899.37
382	Alpha-centractin	P61163	157289.59	151875.08	147972.28	122022.65	117135.67	108243.92	123805.77	123845.99	110953.40	157372.78	149093.59	144508.63	139394.94	130063.92	104255.34	107704.22
383	Alpha-endosulfine	O43768	250595.76	252375.21	239592.47	153417.16	144595.12	135358.98	202155.33	187750.04	184816.77	204986.17	198138.54	177439.37	189794.69	183667.68	166455.62	153161.48
384	Alpha-enolase	P06733	4509288.20	4254142.02	3875709.08	3380720.75	3253919.76	2981015.89	4030546.19	3838269.76	3658111.17	3894940.75	3730849.96	3425098.65	4067818.75	3795909.48	2963913.05	3474705.34
385	Alpha-galactosidase A	P06280	12326.51	11558.19	9964.64	6892.29	6186.64	5168.92	9492.65	9027.40	8030.06	10246.83	9491.90	8247.24	9169.98	8305.63	3876.35	7517.72
386	Alpha-internexin	Q16352	0.14	2.53	0.81	1.07	2.77	0.68	0.96	0.36	0.63	0.35	0.75	0.38	2.13	0.97	0.60	4.64
387	Alpha-ketoglutarate-dependent dioxygenase alkB homolog 3	Q96Q83	11291.43	11891.01	11350.86	7497.48	7381.17	6640.35	10254.97	10348.81	9338.01	8569.86	8605.30	7499.71	9745.96	8799.45	7386.40	7897.64
388	Alpha-ketoglutarate-dependent dioxygenase alkB homolog 4	Q9NXW9	5010.57	3955.33	3704.21	422.76	324.99	188.59	627.05	572.17	350.62	3545.71	3450.04	2631.17	5591.86	770.35	83.46	372.94
389	Alpha-ketoglutarate-dependent dioxygenase FTO	Q9C0B1	0.14	2.53	0.81	1.07	2.77	0.68	0.96	0.36	0.63	0.35	0.75	0.38	2.13	0.97	0.60	4.64
390	Alpha-N-acetylgalactosaminidase	P17050	18184.54	19023.92	16996.71	9738.27	9171.06	8090.35	18084.61	17137.55	15729.67	15388.46	15229.22	13778.48	18334.05	17282.62	13345.43	11371.27
391	Alpha-N-acetylglucosaminidase	P54802	14703.01	12403.01	12182.92	9148.25	8467.52	7614.53	13123.55	12671.77	11886.15	11389.47	11597.18	9593.90	10599.25	9578.14	8187.79	9619.61
392	Alpha-parvin	Q9NVD7	2664.51	2764.35	2279.36	1683.74	1745.77	1626.16	2197.98	2082.61	1996.16	2465.24	2221.62	2128.45	2682.99	2329.51	1727.25	1638.49
393	Alpha-protein kinase 2	Q86TB3	0.14	2.53	0.81	1.07	2.77	0.68	0.96	0.36	0.63	0.35	0.75	0.38	2.13	0.97	0.60	4.64
394	Alpha-S1-casein	P47710	8842.51	8783.08	8377.96	6158.23	5547.59	5506.20	6867.18	7483.07	6793.19	7519.21	7628.90	7983.49	8659.11	6884.83	1624.56	3890.32
395	Alpha-soluble NSF attachment protein	P54920	71943.51	63263.34	56124.32	41173.42	37424.60	34458.88	45494.93	44274.75	40892.01	55637.44	52736.74	48042.04	55127.86	47249.26	36108.23	39379.37
396	Alpha-taxilin	P40222	37026.54	35527.61	36112.78	30503.34	28886.87	27710.88	28551.27	28642.79	27587.81	44048.22	41000.77	43042.16	38232.63	37030.10	25208.01	20609.15
397	Amidophosphoribosyltransferase	Q06203	53217.79	50377.83	48301.46	32412.94	29877.94	29690.31	40746.72	40508.41	38535.50	42039.51	41335.17	39603.19	41331.74	37156.82	31591.06	31435.49
398	Amine oxidase [flavin-containing] A	P21397	53631.95	50606.59	52133.20	33458.94	33481.87	33058.88	42222.60	37549.18	38179.25	41760.94	38021.74	40315.79	37659.70	39815.71	34574.22	35272.55
399	Aminoacyl tRNA synthase complex-interacting multifunctional protein 1	Q12904	157274.16	155671.86	142322.00	114749.50	110424.20	100825.61	133074.39	122090.32	118723.51	146838.25	138294.49	125352.68	129495.98	120178.79	100322.91	118490.69
400	Aminoacyl tRNA synthase complex-interacting multifunctional protein 2	Q13155	98095.85	95451.12	88149.42	67797.54	65562.56	61072.15	88831.84	85405.95	81627.87	82074.17	79607.32	77485.71	82795.67	78980.30	59408.99	66523.82
401	Aminopeptidase B	Q9H4A4	184930.23	179925.26	166621.68	129949.25	122304.40	116203.75	128662.30	125113.09	122642.18	162164.88	156353.19	151491.65	146322.18	144310.70	111288.71	119314.82
402	Amphiregulin	P15514	1426.54	1609.61	1288.36	1543.56	1380.39	1278.66	2416.92	1986.10	2194.49	2177.28	2209.09	2086.22	1403.62	1039.00	712.47	1203.80
403	Amyloid-beta A4 precursor protein-binding family B member 2	Q92870	0.14	2.53	0.81	1.07	2.77	0.68	0.96	0.36	0.63	0.35	0.75	0.38	2.13	0.97	0.60	4.64
404	Amyloid-beta A4 precursor protein-binding family B member 3	O95704	0.14	2.53	0.81	1.07	2.77	0.68	0.96	0.36	0.63	0.35	0.75	0.38	2.13	0.97	0.60	4.64
405	Amyloid-beta precursor protein	P05067	1289.56	1155.85	1102.67	811.12	1024.02	912.69	943.32	995.49	947.54	1424.03	1306.29	1564.65	876.89	808.75	531.76	938.86
406	Amyotrophic lateral sclerosis 2 chromosomal region candidate gene 12 protein	Q96Q35	0.14	2.53	0.81	1.07	2.77	0.68	0.96	0.36	0.63	0.35	0.75	0.38	2.13	0.97	0.60	4.64
407	AN1-type zinc finger protein 1	Q8TCF1	7468.99	8579.09	7285.06	4695.12	4150.03	5142.89	5270.93	4399.13	4516.64	5612.77	5738.71	4970.31	5807.01	5315.82	5587.94	4347.92
408	AN1-type zinc finger protein 6	Q6FIF0	4595.11	4687.22	4420.52	3061.22	3016.28	2912.73	3337.22	3441.47	3509.01	4438.70	3722.33	3587.16	3778.42	3039.90	4241.59	4599.37
409	Anamorsin	Q6FI81	81916.30	76216.39	70450.51	55169.08	54113.98	48959.96	63574.67	61017.08	54013.10	69461.58	65795.97	63858.15	62207.72	58378.78	51225.83	56596.95
410	Androglobin	Q8N7X0	0.14	2.53	0.81	1.07	2.77	0.68	0.96	0.36	0.63	0.35	0.75	0.38	2.13	0.97	0.60	4.64
411	Angio-associated migratory cell protein	Q13685	7685.21	6952.62	5258.54	2310.14	2308.99	1797.77	4525.89	4518.37	4034.69	5165.31	4926.87	4280.15	4325.29	3795.58	1691.04	6346.82
412	Angiopoietin-1	Q15389	2363.88	2477.18	2496.47	1810.78	1562.59	1609.52	1595.58	1695.91	1566.15	2266.85	1914.55	2227.88	1919.66	2284.28	1757.14	1645.44
413	Angiopoietin-2	O15123	0.14	2.53	0.81	1.07	2.77	0.68	0.96	0.36	0.63	0.35	0.75	0.38	2.13	0.97	0.60	4.64
414	Anillin	Q9NQW6	450.66	440.56	355.48	281.77	243.14	202.74	216.86	170.51	183.56	190.96	251.51	149.83	183.49	234.02	252.40	118.38
415	Anion exchange protein 3	P48751	38416.53	43872.70	44334.96	30725.88	26489.09	21282.41	31864.63	26983.31	22182.70	37845.52	37135.20	41132.26	35118.27	22903.52	19807.12	26187.11
416	Ankycorbin	Q9P0K7	0.14	2.53	0.81	1.07	2.77	0.68	0.96	0.36	0.63	0.35	0.75	0.38	2.13	0.97	0.60	4.64
417	Ankyrin and armadillo repeat-containing protein	Q7Z5J8	612.67	363.54	453.15	428.59	337.07	372.11	477.08	410.59	360.83	550.70	539.87	540.85	461.44	442.16	192.10	394.77
418	Ankyrin repeat and KH domain-containing protein 1	Q8IWZ3	18307.09	18356.16	17632.88	13529.47	12330.38	11734.75	14757.67	14318.13	13599.40	18101.54	16249.67	16350.88	15373.37	15274.28	11886.39	12592.41
419	Ankyrin repeat and LEM domain-containing protein 1	Q8NAG6	2580.44	3191.68	3706.49	1459.94	1152.77	1129.43	1146.95	1011.97	874.62	3053.92	3174.98	6667.25	2575.17	321.34	1242.13	818.15
420	Ankyrin repeat and MYND domain-containing protein 2	Q8IV38	0.14	2.53	0.81	1.07	2.77	0.68	0.96	0.36	0.63	0.35	0.75	0.38	2.13	0.97	0.60	4.64
421	Ankyrin repeat domain-containing protein 11	Q6UB99	0.14	2.53	0.81	1.07	2.77	0.68	0.96	0.36	0.63	0.35	0.75	0.38	2.13	0.97	0.60	4.64
422	Ankyrin repeat domain-containing protein 12	Q6UB98	0.14	2.53	0.81	1.07	2.77	0.68	0.96	0.36	0.63	0.35	0.75	0.38	2.13	0.97	0.60	4.64
423	Ankyrin repeat domain-containing protein 16	Q6P6B7	1495.81	1424.60	1495.49	1107.60	916.73	987.64	739.50	999.52	822.68	1228.41	1264.61	1101.47	996.65	1166.54	902.61	822.53
424	Ankyrin repeat domain-containing protein 17	O75179	0.14	2.53	0.81	1.07	2.77	0.68	0.96	0.36	0.63	0.35	0.75	0.38	2.13	0.97	0.60	4.64
425	Ankyrin repeat domain-containing protein 18A	Q8IVF6	3838.51	3374.93	2452.16	1965.00	2257.05	1858.06	2124.61	2144.61	1929.40	3078.83	2732.95	2778.49	3626.83	2980.68	2081.45	2423.59
426	Ankyrin repeat domain-containing protein 20A3	Q5VUR7	127602.92	122153.21	111151.79	82063.04	73759.54	71022.35	93400.10	91432.43	86017.87	110432.50	105539.34	97925.22	96664.68	93882.62	69145.52	87187.97
427	Ankyrin repeat domain-containing protein 20A4	Q4UJ75	0.14	2.53	0.81	1.07	2.77	0.68	0.96	0.36	0.63	0.35	0.75	0.38	2.13	0.97	0.60	4.64
428	Ankyrin repeat domain-containing protein 20B	Q5CZ79	37295.90	37465.20	34574.42	32185.75	30320.64	28968.62	32889.95	31042.41	29840.92	36260.79	33982.05	31682.87	35960.85	33834.36	28419.88	26887.99
429	Ankyrin repeat domain-containing protein 26	Q9UPS8	16389.99	16523.70	16191.70	12150.85	11901.65	11248.35	12536.60	12147.38	11987.19	14663.06	14223.70	13502.83	12834.24	13453.99	11165.88	11608.77
430	Ankyrin repeat domain-containing protein 35	Q8N283	25584.68	25422.91	23444.70	15737.69	16065.63	14777.56	21672.41	17950.78	18108.26	19830.71	18540.48	18020.50	21399.00	20072.46	15578.50	17182.65
431	Ankyrin repeat domain-containing protein 36C	Q5JPF3	176.80	171.03	127.31	99.91	65.13	46.75	193.20	151.09	151.43	91.12	126.08	39.62	121.32	124.66	33.33	183.10
432	Ankyrin repeat domain-containing protein 50	Q9ULJ7	7189.38	7352.33	6695.34	6476.14	6060.96	5284.46	5708.60	5691.65	5671.21	6769.86	6261.81	6606.80	7153.20	7067.96	7964.91	5843.76
433	Ankyrin repeat domain-containing protein 54	Q6NXT1	2198.88	2049.23	1987.89	1715.01	1745.60	1637.53	2363.45	2311.20	2242.64	2717.07	2602.21	2766.19	2527.10	2139.09	1768.21	2032.85
434	Ankyrin repeat domain-containing protein 62	A6NC57	31120.10	27992.99	24853.67	20924.78	21099.07	19976.90	22130.62	22657.73	20625.51	29835.46	28857.23	27370.19	32326.72	29187.85	23296.65	25368.80
435	Ankyrin-2	Q01484	236166.51	222980.02	209393.19	162365.32	156993.95	143298.20	176001.09	180382.06	171350.64	218548.07	211191.61	200136.30	203334.26	197965.82	152287.56	152707.77
436	Annexin A1	P04083	0.14	2.53	0.81	1.07	2.77	0.68	0.96	0.36	0.63	0.35	0.75	0.38	2.13	0.97	0.60	4.64
437	Annexin A11	P50995	210233.42	200271.95	189508.87	126943.22	125417.86	115481.74	148985.50	146721.57	141909.22	159867.98	155757.88	152437.01	156848.46	143791.14	120601.85	140116.05
438	Annexin A13	P27216	23981.55	23788.70	24279.94	15017.07	14078.90	13646.22	16427.64	16348.76	16575.47	17674.82	17803.68	15838.08	18452.80	17608.34	14774.07	15017.40
439	Annexin A2	P07355	1028094.30	966432.24	883579.91	764936.69	722275.70	665790.19	822477.32	785267.73	736861.67	1045529.37	983113.07	907975.13	830292.76	794941.37	643261.54	778102.53
440	Annexin A3	P12429	0.14	2.53	0.81	1.07	2.77	0.68	0.96	0.36	0.63	0.35	0.75	0.38	2.13	0.97	0.60	4.64
441	Annexin A4	P09525	331252.89	298416.93	276212.33	209730.78	192721.26	178001.68	219034.95	206348.12	195860.12	291341.44	277007.75	247479.91	261199.31	250208.97	186332.61	203920.85
442	Annexin A5	P08758	690853.19	660060.75	597858.60	438206.61	429557.85	393688.04	532379.09	502458.63	485572.05	531726.14	496751.10	463995.58	553098.93	523079.47	401365.26	469939.46
443	Annexin A6	P08133	1070593.66	1016450.94	936744.53	671873.07	651187.42	593020.37	759922.85	744668.98	708329.84	885535.46	855502.94	783409.22	907128.47	804704.56	655964.78	671743.98
444	Annexin A7	P20073	192897.74	188032.09	185624.26	148603.84	142310.76	141088.81	169526.35	163239.70	159920.18	176040.91	164897.93	158215.66	166801.24	155874.35	133587.15	148796.40
445	Annexin A8	P13928	92.81	69.02	58.54	4478.97	184.18	177.01	3564.71	2924.16	2615.78	10.13	25.51	16.61	168.79	2841.89	269.90	491.65
446	Annexin A9	O76027	2682.30	2992.87	2932.71	1234.71	1339.02	968.22	1661.86	1712.41	1440.16	1663.28	1700.27	1725.96	1835.90	1790.48	1420.51	1195.61
447	Anosmin-1	P23352	0.14	2.53	0.81	1.07	2.77	0.68	0.96	0.36	0.63	0.35	0.75	0.38	2.13	0.97	0.60	4.64
448	Anterior gradient protein 2 homolog	O95994	280101.82	260510.72	249762.61	215206.82	208034.96	201039.98	211228.19	202915.79	196140.85	269568.29	259916.82	240646.72	245920.01	218430.20	190580.09	223328.11
449	AP-1 complex subunit beta-1	Q10567	154060.94	145815.06	154533.81	101261.91	93974.86	86278.22	119533.37	120054.39	105468.14	151633.38	145772.21	138504.76	131028.01	132291.87	95116.53	100191.55
450	AP-1 complex subunit gamma-1	O43747	166604.07	161237.04	154955.16	112759.26	113472.31	100981.72	139549.54	125980.91	125482.47	138313.38	131614.85	125306.24	141557.82	127492.97	105312.58	112193.95
451	AP-1 complex subunit gamma-like 2	O75843	5774.07	5576.64	5567.66	3151.02	3445.61	3239.17	4040.06	4239.22	3612.26	4050.83	4052.77	4508.21	4731.28	3914.08	1165.02	2417.97
452	AP-1 complex subunit mu-1	Q9BXS5	169408.44	158645.49	142533.87	100444.12	99966.38	89314.56	126174.93	110544.14	108024.86	141113.87	131343.75	127732.19	129225.95	128178.69	90970.10	104143.64
453	AP-1 complex subunit mu-2	Q9Y6Q5	90686.31	88026.46	82883.34	78325.75	76731.41	74708.39	77469.24	75260.77	74113.40	93917.47	88852.59	89463.71	80428.40	80966.02	73855.66	71904.75
454	AP-1 complex subunit sigma-1A	P61966	38720.68	40324.73	37747.28	24149.16	27210.40	22078.82	35035.22	33195.95	28640.18	29730.84	30012.07	28502.67	29676.67	28433.14	23170.87	27026.33
455	AP-2 complex subunit alpha-1	O95782	54413.98	50993.60	43788.85	32163.70	32062.25	27319.66	38136.64	37283.12	33821.00	44815.66	43228.80	38672.81	42472.43	37759.06	28261.42	32453.80
456	AP-2 complex subunit alpha-2	O94973	0.14	2.53	0.81	1.07	2.77	0.68	0.96	0.36	0.63	0.35	0.75	0.38	2.13	0.97	0.60	4.64
457	AP-2 complex subunit beta	P63010	62599.97	56349.33	50855.14	42811.72	40251.84	37457.16	47865.64	43936.01	42539.13	57894.00	54186.43	51454.29	52628.54	46671.65	36460.43	44969.26
458	AP-2 complex subunit mu	Q96CW1	31723.47	29046.30	30925.28	22799.85	23589.18	21264.88	24266.66	22933.50	23289.01	27605.66	26667.59	26314.44	26779.05	25812.20	22641.02	22726.84
459	AP2-associated protein kinase 1	Q2M2I8	1427.44	1366.23	1682.68	1169.49	945.46	990.68	985.46	776.84	808.03	1234.55	1065.61	1115.25	1255.33	1122.47	1070.97	1128.90
460	AP-3 complex subunit beta-1	O00203	61292.47	58071.70	56580.65	44391.13	41296.37	42703.63	47581.77	46524.79	43310.85	49121.04	47365.65	45445.66	50467.18	50300.97	47235.08	43005.34
461	AP-3 complex subunit delta-1	O14617	37145.80	38127.18	34507.49	29881.19	27657.73	28055.03	28352.23	29241.79	28512.88	32591.07	31963.54	29651.52	35603.83	30879.01	25575.64	28754.96
462	AP-3 complex subunit mu-1	Q9Y2T2	15023.66	14019.55	11843.71	8684.65	8942.04	7756.44	12753.43	11907.83	10988.81	12758.46	11309.30	10466.21	11231.62	10882.45	7837.92	10413.24
463	AP-4 complex subunit beta-1	Q9Y6B7	13058.72	12735.09	11883.52	8803.59	8840.00	8382.44	11367.39	11340.78	10522.41	10643.61	10399.97	10503.32	9695.59	10506.27	9196.69	10252.19
464	Apolipoprotein A-I	P02647	17390.17	15775.95	14220.67	7246.73	7718.16	6571.23	13078.84	12927.79	11260.39	15490.90	14961.06	13026.49	14681.62	11168.39	7294.18	9046.01
465	Apolipoprotein A-V	Q6Q788	84424.23	80949.25	80675.08	61596.94	59420.34	56904.08	69440.76	67344.90	65995.60	67190.38	64556.68	61137.00	70044.58	68593.31	62477.91	55413.59
466	Apolipoprotein B receptor	Q0VD83	5276.89	4903.26	4858.45	5611.35	5928.07	5395.02	3811.62	3662.27	3445.02	8282.42	8099.51	8077.35	4803.77	3884.09	3524.15	4804.08
467	Apolipoprotein B-100	P04114	195431.90	189652.37	166963.71	133845.99	128837.94	118009.82	147995.42	139954.08	137645.21	170479.14	159056.39	151307.85	159389.64	144255.58	117496.19	136013.17
468	Apoptogenic protein 1_ mitochondrial	Q96IL0	3010.74	2944.62	2784.31	2188.79	2128.38	2084.76	2814.16	2769.61	2743.91	3814.17	3554.77	3400.54	3213.37	2679.37	2339.64	2305.92
469	Apoptosis inhibitor 5	Q9BZZ5	120908.31	121456.15	100957.52	89303.46	94352.72	87719.64	97499.19	96766.99	85823.65	96368.32	94810.27	86629.41	92278.11	94325.59	83496.13	93916.43
470	Apoptosis regulator BAX	Q07812	61706.83	57919.83	54723.79	44281.92	42542.31	42527.71	51474.91	52295.87	47859.92	69226.37	64441.71	61177.60	54232.19	50183.35	43210.65	40771.56
471	Apoptosis-associated speck-like protein containing a CARD	Q9ULZ3	62988.51	55409.59	52822.12	43647.13	46448.82	41206.34	68834.96	54340.09	46225.80	63004.97	57043.82	50765.26	55888.36	50870.34	29223.67	44944.74
472	Apoptosis-inducing factor 1_ mitochondrial	O95831	258239.36	243799.85	223939.91	158507.67	153547.93	138565.03	182608.93	178068.02	166625.84	212702.26	203393.36	187831.62	196319.38	180058.52	145120.93	165234.93
473	Arachidonate 15-lipoxygenase	P16050	3884.92	3615.87	2776.08	2247.91	2291.25	2042.39	2854.38	3343.56	2472.52	3183.87	2798.52	2938.76	3680.95	2735.19	2107.54	2365.87
474	Arfaptin-1	P53367	72383.22	66601.58	65243.81	50485.79	45604.01	44545.88	52208.16	49951.84	48233.65	58227.67	56105.36	52569.75	57596.29	53244.25	45908.81	46552.32
475	Arfaptin-2	P53365	36334.64	36692.67	35400.22	23961.51	22792.96	22720.58	26560.74	25006.38	24441.29	27365.70	26065.60	24451.08	28629.57	28401.71	22389.78	21650.95
476	Arf-GAP domain and FG repeat-containing protein 1	P52594	22529.74	24084.61	21703.70	16562.01	15913.49	15221.00	19434.53	17716.47	17223.79	19073.83	18119.44	16472.00	18926.33	18196.33	14911.34	14380.80
477	Arf-GAP with coiled-coil_ ANK repeat and PH domain-containing protein 1	Q15027	0.14	2.53	0.81	1.07	2.77	0.68	0.96	0.36	0.63	0.35	0.75	0.38	2.13	0.97	0.60	4.64
478	Arf-GAP with coiled-coil_ ANK repeat and PH domain-containing protein 2	Q15057	14123.32	13932.41	12886.44	9248.75	9870.65	8664.93	10560.90	9919.66	9271.38	12721.22	12582.29	12371.70	13678.07	12148.43	10635.20	9021.10
479	Arf-GAP with dual PH domain-containing protein 1	O75689	3090.81	3341.61	3449.30	2478.35	2380.90	2217.42	2701.96	2655.19	2414.66	3062.49	3112.51	2993.26	2788.78	2746.00	2443.52	1543.41
480	Arf-GAP with GTPase_ ANK repeat and PH domain-containing protein 11	Q8TF27	800.05	858.51	777.52	372.15	330.04	274.63	478.13	577.54	547.92	382.92	491.46	715.37	632.03	610.19	309.19	446.33
481	Arf-GAP with GTPase_ ANK repeat and PH domain-containing protein 5	A6NIR3	1639.67	1595.15	1445.65	1157.88	1037.81	759.05	1706.99	1456.91	1298.33	1383.67	1401.86	1179.37	1842.38	1563.63	482.97	958.13
482	Arf-GAP with GTPase_ ANK repeat and PH domain-containing protein 9	Q5VTM2	0.14	2.53	0.81	1.07	2.77	0.68	0.96	0.36	0.63	0.35	0.75	0.38	2.13	0.97	0.60	4.64
483	Arginase-1	P05089	688.54	469.03	351.64	258.47	200.86	137.70	280.93	145.36	222.27	449.29	470.66	127.60	467.93	121.38	23.94	220.69
484	Arginine and glutamate-rich protein 1	Q9NWB6	1650.19	1159.76	919.95	1159.25	1156.15	870.32	2923.00	2731.64	2604.54	1113.17	1229.70	1208.24	1551.50	1670.97	849.59	1021.77
485	Arginine--tRNA ligase_ cytoplasmic	P54136	329163.96	327753.85	319173.35	224359.84	218702.80	206086.72	259016.24	250886.49	240229.08	281934.81	267031.44	250636.47	263213.74	256729.40	214458.38	227148.76
486	Argininosuccinate lyase	P04424	10699.40	9518.92	8925.10	4730.17	4511.12	4240.12	6700.36	6869.07	6445.30	6595.63	6383.31	5935.14	6326.29	6043.10	5085.33	6677.60
487	Argininosuccinate synthase	P00966	868001.54	809663.59	755547.26	769186.26	740997.78	690212.43	937285.72	904098.23	871408.81	824703.91	768705.39	713907.60	820141.10	812000.86	606614.24	710489.00
488	Arginyl-tRNA--protein transferase 1	O95260	5211.93	5315.24	4292.61	4890.65	3912.53	2942.15	3305.92	2620.37	2386.91	4039.95	4111.11	3582.41	3975.56	4856.46	2030.37	3990.35
489	ARL14 effector protein	Q8N8R7	328.08	309.88	443.85	77.11	87.17	84.25	105.43	138.82	141.23	340.85	324.42	358.53	237.66	212.12	51.93	69.13
490	Armadillo repeat protein deleted in velo-cardio-facial syndrome	O00192	0.14	2.53	0.81	1.07	2.77	0.68	0.96	0.36	0.63	0.35	0.75	0.38	2.13	0.97	0.60	4.64

	SSE_IC20_02	SSE_IC20_03	SSE_SC20_01	SSE_SC20_02	SSE_SC20_03	MDA-MB-231 control_01	control_02	control_03	Dai_IC20_01	Dai_IC20_02	Dai_IC20_03	Gen_IC20_01	Gen_IC20_02	Gen_IC20_03	SSE_IC20_01	SSE_IC20_02	SSE_IC20_03	unique to MCF-7	unique to MDA-MB-231
369	1.07	0.47	2.22	0.19	0.06	56837.83	54498.74	49528.70	35992.57	34178.19	28181.31	46423.53	43742.46	40005.53	48519.55	46426.20	29098.57		x
370	14217.23	13654.19	13936.14	14171.34	7495.01	2.27	1.49	0.65	0.84	1.31	0.69	4.16	2.47	0.11	4.90	4.98	0.12	x	
371	1067.59	1094.41	959.13	831.21	836.07	2.27	1.49	0.65	0.84	1.31	0.69	4.16	2.47	0.11	4.90	4.98	0.12	x	
372	14352.22	12630.15	14501.34	13761.66	14627.07	2.27	1.49	0.65	0.84	1.31	0.69	4.16	2.47	0.11	4.90	4.98	0.12	x	
373	15319.52	15052.68	18402.90	17755.16	17294.68	8866.98	8555.88	8089.24	5740.66	5617.39	5593.48	6700.41	6641.68	6418.54	6615.06	5920.60	4425.34		
374	127471.86	130257.67	119934.41	118775.93	116185.77	152625.18	147656.72	138357.86	123092.35	121211.66	114908.29	142149.70	134217.79	130923.19	93218.83	89439.84	72033.94		
375	8574.53	7761.25	7686.82	7543.66	7724.26	46095.52	35465.97	31690.23	40754.12	39508.37	35234.76	56872.47	52672.44	49367.60	47164.63	46254.05	28393.30		
376	12839.82	12380.93	13377.05	12208.07	11907.87	2.27	1.49	0.65	0.84	1.31	0.69	4.16	2.47	0.11	4.90	4.98	0.12	x	
377	476195.65	462175.82	600213.27	565833.52	528585.05	976674.13	914085.04	854949.91	649804.84	643208.36	561797.38	828288.25	776462.72	725938.27	859563.25	840834.26	572602.36		
378	45669.22	43448.41	49200.67	46225.04	43286.89	27500.83	25683.21	24366.01	21964.93	21477.82	17957.45	25275.71	24595.97	23921.54	25405.65	25246.94	15281.90		
379	239468.37	237024.06	215790.46	223170.26	200467.36	15310.51	14711.51	14378.14	10198.04	9965.29	7830.79	12708.00	12167.59	11752.36	17474.07	15837.57	9302.08		
380	1287844.48	1224959.86	1411922.89	1351860.02	1229947.33	2011698.93	1877276.58	1697302.83	1308834.34	1259993.08	1117663.38	1530477.20	1469504.66	1385125.53	1585314.24	1536301.48	1030581.21		
381	86021.79	82421.44	85487.95	81473.51	77719.05	118663.61	109493.79	99658.62	74312.45	72607.88	64981.46	99888.43	93642.37	90515.03	84952.22	83448.26	58019.86		
382	111105.20	109254.84	116530.23	110302.54	104508.90	84358.96	77546.76	67488.64	55290.96	52218.29	45535.01	68506.70	63271.33	59014.22	67914.11	63978.03	41239.95		
383	156694.45	136138.10	144602.24	138596.69	135670.45	31606.86	29202.24	29085.67	21654.09	20962.19	19663.22	24220.66	23594.94	22076.36	26153.21	24443.25	16553.48		
384	3291784.64	3090519.35	3352755.36	3147474.53	2918652.95	4370884.90	4048793.30	3725345.11	2355015.74	2278905.23	1926417.32	3133736.92	2921755.83	2652433.96	2973455.47	2864686.25	1912361.26		
385	7880.71	6774.03	7073.05	6362.63	5464.78	2.27	1.49	0.65	0.84	1.31	0.69	4.16	2.47	0.11	4.90	4.98	0.12	x	
386	1.07	0.47	2.22	0.19	0.06	856.32	981.10	810.67	702.65	789.96	667.87	1048.11	1031.16	905.21	1007.33	810.57	656.23		x
387	6801.66	6629.41	7707.01	7438.25	7348.96	2.27	1.49	0.65	0.84	1.31	0.69	4.16	2.47	0.11	4.90	4.98	0.12	x	
388	306.91	243.66	1324.06	1334.50	750.69	2.27	1.49	0.65	0.84	1.31	0.69	4.16	2.47	0.11	4.90	4.98	0.12	x	
389	1.07	0.47	2.22	0.19	0.06	8923.93	8588.31	7767.99	5807.54	5935.25	5601.21	8119.42	7946.87	7654.46	6961.34	7245.77	4723.41		x
390	9750.29	9364.74	10784.42	9680.47	8738.67	222941.50	195464.82	160355.26	111028.15	101938.11	73269.81	135103.28	119852.19	93688.89	141035.66	138242.35	80634.40		
391	8949.61	8530.95	9550.14	8280.40	7994.85	2.27	1.49	0.65	0.84	1.31	0.69	4.16	2.47	0.11	4.90	4.98	0.12	x	
392	1659.25	1678.48	1588.17	1633.23	1426.62	7999.41	7613.03	7553.85	4949.04	4813.71	3773.80	6471.98	6390.50	5476.66	6064.51	5640.50	3909.37		
393	1.07	0.47	2.22	0.19	0.06	8972.67	8041.48	7670.24	12369.81	11356.05	7506.53	15099.94	13963.52	12033.14	11334.43	11155.14	5326.39		x
394	5489.36	5704.06	8255.12	7597.96	7025.09	2.27	1.49	0.65	0.84	1.31	0.69	4.16	2.47	0.11	4.90	4.98	0.12	x	
395	34683.35	33792.20	38429.76	37785.71	34106.28	18823.27	17754.97	15854.98	11044.96	10945.67	8807.68	13993.35	13047.41	12532.21	13981.50	13520.49	8280.47		
396	21774.56	22697.01	31507.14	27872.24	28989.97	81801.19	76828.62	70290.88	58765.11	57228.93	54941.85	60755.94	60608.75	60400.89	69379.06	64128.67	37878.82		
397	28862.77	27860.60	37596.76	33620.50	32123.58	15741.11	14099.10	12543.24	7945.04	7452.75	6614.94	10471.16	9717.49	9391.13	9562.86	9767.31	5834.60		
398	30898.73	32257.58	35877.27	37883.56	33355.22	2.27	1.49	0.65	0.84	1.31	0.69	4.16	2.47	0.11	4.90	4.98	0.12	x	
399	107304.93	103290.90	118958.05	113061.76	102387.19	72501.38	67781.90	57971.63	44443.83	41291.84	35446.10	50297.40	47136.30	44775.16	53688.37	51169.66	34058.62		
400	63274.70	63794.15	74722.43	67637.20	62540.18	40686.94	37823.61	34242.05	15136.33	14312.60	12328.27	18354.99	17104.41	15419.26	19702.54	18811.48	10416.22		
401	118592.30	113454.39	133285.37	124254.83	121238.96	224687.81	217355.47	194581.73	76482.13	77039.76	68758.57	84266.44	82702.10	79443.56	83068.82	80058.97	50963.05		
402	1114.23	1127.34	1778.03	1724.34	1656.38	2.27	1.49	0.65	0.84	1.31	0.69	4.16	2.47	0.11	4.90	4.98	0.12	x	
403	1.07	0.47	2.22	0.19	0.06	652.12	640.37	536.09	467.34	367.07	262.26	803.51	752.63	612.77	858.10	761.83	364.50		x
404	1.07	0.47	2.22	0.19	0.06	34625.48	32691.32	31339.59	26637.43	26847.08	20382.45	30664.63	28596.88	26781.61	33423.11	32507.01	20975.70		x
405	960.22	980.35	744.59	530.05	580.40	2.27	1.49	0.65	0.84	1.31	0.69	4.16	2.47	0.11	4.90	4.98	0.12	x	
406	1.07	0.47	2.22	0.19	0.06	111640.64	107646.42	96590.63	75545.14	73784.77	64430.38	77182.72	70274.85	73987.93	83230.07	85987.13	57089.11		x
407	3843.93	4882.05	5686.75	5718.82	4820.94	2.27	1.49	0.65	0.84	1.31	0.69	4.16	2.47	0.11	4.90	4.98	0.12	x	
408	3488.22	3484.87	4102.65	3140.77	2906.53	2.27	1.49	0.65	0.84	1.31	0.69	4.16	2.47	0.11	4.90	4.98	0.12	x	
409	55738.82	50358.36	56643.29	52828.47	49839.06	63481.68	53514.82	54241.29	47852.33	42485.75	40619.55	52653.13	50654.87	45929.08	53147.20	49559.35	37491.70		
410	1.07	0.47	2.22	0.19	0.06	4211.77	3571.74	4051.92	3104.59	3128.13	2860.19	3343.12	3475.78	3284.35	3642.04	3258.92	2544.11		x
411	5944.35	5037.64	4507.13	4126.44	3390.37	2.27	1.49	0.65	0.84	1.31	0.69	4.16	2.47	0.11	4.90	4.98	0.12	x	
412	1720.24	1870.60	1832.76	1696.85	1580.80	2.27	1.49	0.65	0.84	1.31	0.69	4.16	2.47	0.11	4.90	4.98	0.12	x	
413	1.07	0.47	2.22	0.19	0.06	6908.95	6329.79	6445.15	4788.34	4636.94	4519.06	5057.79	5086.21	4881.52	5312.61	5484.75	7660.57		x
414	115.60	104.84	182.46	213.61	156.67	184358.01	174286.60	169188.64	131783.39	137600.90	122138.14	149046.50	147428.17	146831.64	177061.45	172221.56	107174.83		
415	26948.22	26811.95	36150.30	27283.35	26245.11	10363.55	10081.19	16034.50	7142.37	7233.95	6094.72	10194.50	9591.23	8522.01	9244.80	9109.28	5965.27		
416	1.07	0.47	2.22	0.19	0.06	18782.64	17870.50	15889.12	8939.69	8939.46	7457.69	13689.72	12592.74	11575.94	11966.52	11279.51	7138.21		x
417	367.38	337.84	456.93	469.97	388.80	2.27	1.49	0.65	0.84	1.31	0.69	4.16	2.47	0.11	4.90	4.98	0.12	x	
418	12740.62	12458.22	13150.36	12037.83	12268.87	37261.11	35901.23	32903.59	28575.57	27910.92	26530.79	32969.11	31480.97	29510.78	31121.88	30257.48	22145.51		
419	706.78	982.41	2475.54	1234.89	1218.46	2.27	1.49	0.65	0.84	1.31	0.69	4.16	2.47	0.11	4.90	4.98	0.12	x	
420	1.07	0.47	2.22	0.19	0.06	4519.80	4436.60	4216.97	2907.36	2840.32	2305.89	3107.39	3064.36	3067.93	3401.88	3148.17	2230.01		x
421	1.07	0.47	2.22	0.19	0.06	11539.82	10386.35	9699.64	8363.86	7913.64	7179.52	8045.93	8590.53	8358.03	7942.07	8562.47	2677.77		x
422	1.07	0.47	2.22	0.19	0.06	10675.36	10228.67	9280.76	7007.47	6535.06	6017.45	9439.55	8833.40	8697.81	8561.09	9055.63	5879.09		x
423	932.79	878.12	845.25	861.34	551.37	2.27	1.49	0.65	0.84	1.31	0.69	4.16	2.47	0.11	4.90	4.98	0.12	x	
424	1.07	0.47	2.22	0.19	0.06	2132.35	1980.58	1652.65	1573.59	1414.73	1331.63	1770.99	1610.97	1621.09	1382.97	1404.11	1123.60		x
425	2128.44	2203.57	1581.02	1645.48	1942.63	4205.28	4762.06	4998.29	3398.66	3450.62	3288.33	4787.66	3986.35	4055.00	4390.56	3542.00	2641.73		
426	81547.55	77743.36	82359.83	78376.13	72655.28	2.27	1.49	0.65	0.84	1.31	0.69	4.16	2.47	0.11	4.90	4.98	0.12	x	
427	1.07	0.47	2.22	0.19	0.06	8196.17	7835.70	7556.16	6086.07	5982.96	5339.22	6632.14	6208.18	5927.32	6733.35	6531.56	5199.64		x
428	26792.50	25603.14	32299.01	30627.32	29036.51	6747.04	6441.42	6465.44	7514.40	7359.68	6990.16	9347.37	8849.78	8941.08	5793.00	5375.68	3757.29		
429	10745.88	10535.56	11492.13	10779.27	10314.74	10397.97	10254.87	8640.69	6326.54	6380.40	5732.86	8510.87	8030.41	7684.44	8354.76	7587.29	6006.28		
430	15330.40	15575.83	16272.44	16064.37	15073.55	19419.10	18602.53	17286.46	12534.13	13602.59	12613.70	15942.60	15446.53	14052.92	13482.02	13828.71	11242.74		
431	218.81	246.76	116.29	71.81	54.82	2.27	1.49	0.65	0.84	1.31	0.69	4.16	2.47	0.11	4.90	4.98	0.12	x	
432	5985.26	5146.71	7500.66	6387.76	5290.94	2.27	1.49	0.65	0.84	1.31	0.69	4.16	2.47	0.11	4.90	4.98	0.12	x	
433	1838.23	1721.41	1519.90	1570.30	1670.71	7645.20	9462.92	7618.50	5866.01	6077.18	6076.29	6306.37	6350.00	6349.03	6665.41	6315.23	4146.59		
434	24885.46	24091.38	19593.22	18233.28	17493.15	22625.58	21996.76	21060.76	16818.57	16895.80	15672.18	17666.03	17362.88	17086.00	18661.40	17809.56	18135.87		
435	153033.23	143296.45	162542.90	159588.69	150575.71	8749.92	8008.12	7673.22	4768.32	4494.55	3988.22	7768.92	7835.63	7963.74	6811.75	6576.45	4183.03		
436	1.07	0.47	2.22	0.19	0.06	782610.87	712878.73	673821.90	429912.07	416317.32	358025.60	546281.43	498701.68	474826.34	550842.64	526997.51	369294.18		x
437	129676.77	128461.71	116138.83	111812.34	103939.97	139274.72	129648.58	123493.70	86871.25	86744.42	77635.33	107342.35	105359.50	104479.01	102926.42	98688.30	74008.81		
438	14833.43	13506.68	16181.97	14297.47	13648.91	2.27	1.49	0.65	0.84	1.31	0.69	4.16	2.47	0.11	4.90	4.98	0.12	x	
439	729648.35	700836.17	718080.33	660985.58	628446.41	3485589.45	3259593.39	3042751.15	2314176.82	2269724.84	1973808.53	2573357.96	2425999.91	2294764.22	2628068.87	2553921.95	1856076.59		
440	1.07	0.47	2.22	0.19	0.06	134036.13	131123.06	113954.47	69986.96	68581.77	59455.13	90984.03	84130.93	76848.42	80259.24	79251.55	52949.16		x
441	190164.07	188181.54	200389.25	194900.78	172969.76	145792.88	132076.56	113552.63	82324.97	81896.00	72529.87	95570.68	94821.07	87365.44	98699.26	93935.82	64633.15		
442	438448.05	427844.66	454853.51	412799.09	371809.30	467242.68	451546.84	404468.17	295401.02	284930.00	262004.19	319698.22	307115.54	286363.41	336642.06	311920.63	199804.65		
443	634464.68	610616.61	700979.15	648676.96	606684.84	492007.70	470624.11	431045.24	303288.61	297132.50	254494.19	378403.42	359787.39	340702.31	390251.54	369531.30	243578.12		
444	139869.11	135996.31	142059.33	129419.60	128146.76	209443.28	215001.61	200863.14	144264.10	141740.87	128298.45	163655.08	160002.68	148665.30	170338.75	161574.10	103336.09		
445	3187.53	126.32	1632.95	1319.46	735.32	2418.49	1970.97	2134.22	1595.73	1517.40	1288.40	2627.71	1338.87	1586.80	1941.10	2249.17	1307.09		
446	1181.26	1113.73	1215.73	1125.05	936.21	2.27	1.49	0.65	0.84	1.31	0.69	4.16	2.47	0.11	4.90	4.98	0.12	x	
447	1.07	0.47	2.22	0.19	0.06	22971.73	28674.90	17381.92	15695.81	14366.85	12488.66	18486.07	21244.93	19014.51	19975.84	17412.79	11951.28		x
448	217908.33	218218.04	209845.58	194503.29	196069.69	2.27	1.49	0.65	0.84	1.31	0.69	4.16	2.47	0.11	4.90	4.98	0.12	x	
449	94104.31	91494.67	100939.68	95690.40	89163.30	44768.34	41630.20	36846.59	25281.27	24849.96	19924.76	30038.37	27542.79	24762.98	30477.84	29009.72	19085.86		
450	106419.44	107677.46	108948.54	110034.53	103359.43	136085.00	133168.91	118193.47	71772.92	67621.16	57729.32	97465.70	89891.54	89343.77	89563.22	84256.02	53911.49		
451	3920.31	3469.73	5579.43	4679.56	3844.00	2.27	1.49	0.65	0.84	1.31	0.69	4.16	2.47	0.11	4.90	4.98	0.12	x	
452	97882.80	93574.16	97199.50	100968.48	93477.25	64595.91	63589.04	58560.68	45642.70	45466.76	40030.30	54955.87	55049.98	52073.92	53607.18	50763.14	35797.72		
453	69137.20	68757.06	67707.74	67748.57	69341.57	2.27	1.49	0.65	0.84	1.31	0.69	4.16	2.47	0.11	4.90	4.98	0.12	x	
454	26056.26	24325.10	24119.22	24753.58	21780.10	11444.50	10250.82	9832.31	5765.38	5901.75	5439.05	6586.82	6140.86	6258.17	7234.59	6510.80	5324.30		
455	31225.36	30208.25	34480.54	33088.92	30969.91	71635.96	65517.78	60464.34	38380.12	37818.72	33904.39	43745.79	43585.11	41227.10	48625.20	44113.23	26405.48		
456	1.07	0.47	2.22	0.19	0.06	17720.09	16439.97	15778.47	14596.99	14461.54	12422.26	17019.31	16710.71	16410.57	16209.46	15334.67	10264.69		x
457	40489.97	38597.38	46417.57	43824.96	41082.83	98348.21	90412.42	79843.04	58210.77	55879.15	50212.26	76781.03	73888.22	70560.34	71828.99	69867.23	45818.03		
458	20215.46	21141.86	19606.90	20071.21	19460.84	68900.08	65551.93	64833.52	33289.50	32029.12	29267.51	34128.60	30935.97	29888.99	29004.56	28193.45	22565.72		
459	925.77	1039.49	881.09	852.34	793.06	2666.53	1876.31	1936.54	1136.41	1061.32	904.58	1475.16	1112.33	1087.97	885.26	858.98	482.97		
460	39745.26	38086.72	37511.97	41179.04	38995.24	36510.19	34797.24	32830.83	22593.96	21740.72	19499.46	30441.80	29392.06	28374.87	29254.21	27126.03	19686.50		
461	24011.37	26192.73	27766.57	26456.55	25755.20	19806.27	18250.35	18649.71	13530.83	13435.77	11643.24	15793.32	14145.79	12798.26	17529.34	17205.88	11385.18		
462	10405.56	10130.66	10359.35	10226.14	8682.05	5838.78	4649.97	4809.35	2771.48	2635.88	1925.50	2817.50	2450.19	2205.51	3878.10	3541.36	2313.10		
463	8890.70	8623.36	8592.96	7987.28	7499.49	2.27	1.49	0.65	0.84	1.31	0.69	4.16	2.47	0.11	4.90	4.98	0.12	x	
464	9308.67	8964.76	8854.81	7436.03	6726.52	2.27	1.49	0.65	0.84	1.31	0.69	4.16	2.47	0.11	4.90	4.98	0.12	x	
465	51803.00	50880.12	57135.03	54971.48	50221.67	2.27	1.49	0.65	0.84	1.31	0.69	4.16	2.47	0.11	4.90	4.98	0.12	x	
466	4234.00	4030.26	4522.92	4141.98	4285.23	2.27	1.49	0.65	0.84	1.31	0.69	4.16	2.47	0.11	4.90	4.98	0.12	x	
467	122650.85	123085.58	140179.90	131152.10	124287.65	42013.78	39515.82	35264.18	18477.24	18596.59	15531.82	27074.85	26946.66	24832.68	25236.58	24288.33	15466.50		
468	1987.33	2186.71	2295.41	2246.37	2135.64	2.27	1.49	0.65	0.84	1.31	0.69	4.16	2.47	0.11	4.90	4.98	0.12	x	
469	86620.06	82595.53	82554.94	82759.99	79988.29	95875.19	91099.91	79787.74	64975.08	63468.94	55455.45	82528.28	76646.34	71080.93	79812.33	77091.32	53251.05		
470	39889.58	41333.65	42968.82	39460.00	38517.60	72548.31	69831.30	64245.74	50518.31	50177.42	42179.11	58753.25	54567.77	51214.48	59146.54	58142.98	43146.11		
471	40949.60	44521.93	45723.45	40565.90	32323.62	2.27	1.49	0.65	0.84	1.31	0.69	4.16	2.47	0.11	4.90	4.98	0.12	x	
472	155639.93	151393.66	165047.52	154376.37	145684.99	66197.45	63180.41	60097.60	47674.43	47587.75	43440.33	52379.18	52024.80	50033.17	57882.74	55619.47	41181.50		
473	2346.35	2139.49	2222.43	2378.47	2186.43	2.27	1.49	0.65	0.84	1.31	0.69	4.16	2.47	0.11	4.90	4.98	0.12	x	
474	43407.90	44033.14	48177.90	43658.75	41893.00	719.20	674.67	739.55	373.09	413.52	351.65	684.20	522.68	593.63	745.01	641.31	314.56		
475	20640.88	19864.29	24776.40	24064.96	22672.26	2.27	1.49	0.65	0.84	1.31	0.69	4.16	2.47	0.11	4.90	4.98	0.12	x	
476	13756.69	14515.50	16357.48	14485.41	14780.57	4887.99	4709.26	4540.56	3172.03	2903.80	2648.08	4564.58	4326.76	4189.69	5067.57	5003.56	3567.52		
477	1.07	0.47	2.22	0.19	0.06	134.31	196.32	2759.39	0.84	1.31	0.69	193.31	140.53	29.25	159.16	101.54	13.70		x
478	8435.24	7895.18	10846.25	10296.99	10218.39	2.27	1.49	0.65	0.84	1.31	0.69	4.16	2.47	0.11	4.90	4.98	0.12	x	
479	2208.72	1479.32	2274.87	2422.16	2262.92	25504.18	23633.71	20293.62	10192.50	10426.16	7512.46	16278.00	14990.84	12667.84	15406.43	15192.81	8207.63		
480	383.59	403.48	440.04	452.68	514.73	2.27	1.49	0.65	0.84	1.31	0.69	4.16	2.47	0.11	4.90	4.98	0.12	x	
481	941.82	832.05	1135.84	1149.29	893.80	2.27	1.49	0.65	0.84	1.31	0.69	4.16	2.47	0.11	4.90	4.98	0.12	x	
482	1.07	0.47	2.22	0.19	0.06	12423.56	12564.74	11143.01	7126.74	7192.79	6243.44	10820.93	10505.05	10780.78	9295.80	9454.50	6723.45		x
483	136.90	181.33	302.97	226.53	115.99	2.27	1.49	0.65	0.84	1.31	0.69	4.16	2.47	0.11	4.90	4.98	0.12	x	
484	741.79	761.07	820.99	677.86	738.58	10179.84	9936.31	9688.86	6843.26	6424.90	5747.39	6894.21	6891.80	6995.86	7782.64	7745.43	471.68		
485	222107.56	210679.70	220100.52	216593.98	198667.34	237703.98	223105.46	206279.25	158125.82	154846.65	137950.76	184388.27	177478.82	173328.84	183849.85	179472.92	127005.35		
486	6369.82	6160.32	5278.71	4838.77	4697.46	8895.81	8498.11	8377.03	7916.22	7862.97	6805.71	8669.09	8201.02	7785.75	9661.43	10292.37	4607.21		
487	685128.55	653726.53	748432.72	699275.24	656409.15	2.27	1.49	0.65	0.84	1.31	0.69	4.16	2.47	0.11	4.90	4.98	0.12	x	
488	3362.14	3149.97	3977.89	3429.10	3353.48	2.27	1.49	0.65	0.84	1.31	0.69	4.16	2.47	0.11	4.90	4.98	0.12	x	
489	106.76	74.97	218.46	109.24	62.07	2.27	1.49	0.65	0.84	1.31	0.69	4.16	2.47	0.11	4.90	4.98	0.12	x	
490	1.07	0.47	2.22	0.19	0.06	141277.84	140542.37	128434.36	160679.12	160091.39	156232.22	218973.05	204309.87	205102.60	137313.46	138334.78	104778.26		x

Supplementary Table S1. Overview on the abundance of all relatively quantified 5180 proteins

	Protein name	UniProt	MCF-7																								MDA-MB-231													
			control 01	control 02	control 03	Dai IC20 01	Dai IC20 02	Dai IC20 03	Dai SC20 01	Dai SC20 02	Dai SC20 03	Gen IC20 01	Gen IC20 02	Gen IC20 03	Gen SC20 01	Gen SC20 02	Gen SC20 03	SSE IC20 01	SSE IC20 02	SSE IC20 03	SSE SC20 01	SSE SC20 02	SSE SC20 03	control 01	control 02	control 03	Dai IC20 01	Dai IC20 02	Dai IC20 03	Gen IC20 01	Gen IC20 02	Gen IC20 03	SSE IC20 01	SSE IC20 02	SSE IC20 03	unique to MCF-7	unique to MDA-MB-231
491	Armadillo repeat-containing protein 10	Q8N2F6	15599.99	15481.79	14005.10	12197.04	11545.77	12017.02	21242.49	15076.14	20570.70	13259.12	11672.13	12465.74	13466.33	11735.45	9584.43	11332.87	10969.03	10031.32	11386.28	10667.18	9013.60	231.44	153.48	110.57	65.75	62.35	8.82	133.69	76.00	37.42	234.42	155.01	24.03		
492	Armadillo repeat-containing protein 2	Q8NEN0	16024.97	14545.36	13839.09	12930.15	13298.03	12795.72	16060.30	15404.78	14791.69	17297.03	16457.54	16131.96	14741.48	14209.46	12977.63	13010.65	13005.51	12332.53	13369.49	13193.57	12770.99	2.27	1.49	0.65	0.84	1.31	0.69	4.16	2.47	0.11	4.90	4.98	0.12	x	
493	Armadillo repeat-containing protein 6	Q6NXE6	1604.28	1124.45	780.39	693.63	796.12	525.27	1121.72	812.34	760.81	1302.27	1283.80	980.79	1062.42	996.15	392.65	850.95	851.74	731.52	1192.45	972.27	814.59	2.27	1.49	0.65	0.84	1.31	0.69	4.16	2.47	0.11	4.90	4.98	0.12	x	
494	Armadillo repeat-containing X-linked protein 3	Q9UH62	4155.02	4247.60	4418.04	4086.24	4149.90	4231.19	4649.33	4530.58	4710.86	6024.48	5403.50	5255.96	4230.76	4178.83	3318.03	4032.08	3715.86	3893.43	4265.35	4535.73	4254.69	2277.39	1733.49	1945.74	1658.95	1557.03	1315.29	1695.39	1626.44	1691.96	1431.89	1408.70	1153.87		
495	Arrestin domain-containing protein 5	A6NEK1	0.14	2.53	0.81	1.07	2.77	0.68	0.96	0.36	0.63	0.35	0.75	0.38	2.13	0.97	0.60	4.64	1.07	0.47	2.22	0.19	0.06	160.64	69.78	52.07	56.69	67.88	49.74	499.08	373.40	275.74	935.24	875.79	516.50		x
496	Asialoglycoprotein receptor 1	P07306	0.14	2.53	0.81	1.07	2.77	0.68	0.96	0.36	0.63	0.35	0.75	0.38	2.13	0.97	0.60	4.64	1.07	0.47	2.22	0.19	0.06	4800.61	4965.46	4762.08	2416.20	2165.48	2003.93	3315.21	2688.26	3099.49	3227.02	3159.81	1719.85		x
497	ASNSD1 upstream open reading frame protein	L0R819	0.14	2.53	0.81	1.07	2.77	0.68	0.96	0.36	0.63	0.35	0.75	0.38	2.13	0.97	0.60	4.64	1.07	0.47	2.22	0.19	0.06	64679.42	60914.61	56465.64	42207.33	43203.22	33224.40	51598.02	46661.86	44123.22	54893.82	52564.68	33535.11		x
498	Asparagine synthetase [glutamine-hydrolyzing]	P08243	1096503.39	1054637.82	983751.76	1080976.19	1045872.65	971709.35	1041226.60	990968.11	961401.29	1033946.67	985273.91	936397.09	813778.93	772656.86	627256.11	1348065.10	1251724.88	1222085.66	864798.33	835342.62	772772.31	74865.46	69640.22	61797.88	51627.06	50474.69	39564.40	57317.49	52424.64	49866.86	59510.20	56791.34	36643.77		
499	Asparagine--tRNA ligase_ cytoplasmic	O43776	168259.60	166531.94	150372.10	112280.43	107986.28	97816.49	139915.24	130242.19	122417.61	131794.94	126554.45	122031.07	130515.19	123752.22	100694.72	119800.52	115841.11	111239.44	117344.95	114611.46	106881.96	165526.63	157423.84	144586.98	104671.92	100838.48	80942.67	118573.24	112342.62	101793.22	131767.59	126001.39	80350.19		
500	Aspartate aminotransferase_ cytoplasmic	P17174	211870.34	209486.31	191137.73	141233.32	143523.36	134706.80	176773.29	175703.35	168089.34	171444.72	157553.21	148926.33	167508.98	164420.14	129588.74	150954.86	132463.42	143820.39	138742.62	133927.72	127276.97	73442.65	68602.26	65196.02	42334.73	40759.52	38785.83	48554.39	45622.45	42778.89	49807.25	49751.84	100531.46		
501	Aspartate aminotransferase_ mitochondrial	P00505	191245.64	181292.63	171477.17	132139.41	131532.36	123319.64	155988.16	152784.59	150831.71	161479.89	153791.55	150045.19	156832.14	153797.98	121774.01	133839.84	128442.09	125285.25	127174.95	122395.75	114470.30	327630.18	307187.27	285418.74	173256.97	170522.86	149018.96	180689.04	175076.91	171346.64	179135.97	176220.32	134576.01		
502	Aspartate-rich protein 1	Q6PGQ1	0.14	2.53	0.81	1.07	2.77	0.68	0.96	0.36	0.63	0.35	0.75	0.38	2.13	0.97	0.60	4.64	1.07	0.47	2.22	0.19	0.06	52565.31	49428.74	53505.56	45927.26	42312.81	37541.76	52319.43	47707.28	50239.12	54433.67	49509.82	37290.36		x
503	Aspartate--tRNA ligase_ cytoplasmic	P14868	387815.35	373628.59	350706.58	271586.24	264331.57	243335.41	305232.48	303130.07	304565.50	321388.28	306964.13	289130.55	324297.63	306515.43	269426.48	279163.16	267144.40	255711.15	293500.58	264931.97	250619.20	627688.60	597738.84	560993.45	414297.92	389721.10	361939.34	472873.42	461668.77	441796.28	469630.49	465804.69	319582.28		
504	Aspartate--tRNA ligase_ mitochondrial	Q6PI48	23097.99	22609.46	23461.75	16010.13	13432.81	12796.44	15001.67	16259.08	14431.54	18854.00	18864.61	17801.17	21372.09	13250.40	10522.04	17073.93	11373.02	13395.40	16436.04	16209.41	14816.56	1410.45	1262.39	1344.74	895.04	825.66	794.04	1105.31	890.96	773.03	976.08	899.30	736.07		
505	Aspartyl aminopeptidase	Q9ULA0	131583.20	128533.39	122864.09	82081.90	79641.24	75753.51	101617.85	97494.90	95466.55	103544.52	102294.93	96416.87	111645.51	103653.19	88119.73	79121.14	75428.50	73617.73	83728.36	78461.24	74763.37	16712.37	16137.53	14512.57	7572.13	7582.50	5821.85	13946.80	12661.13	11730.59	12402.00	11330.88	6756.26		
506	Aspartyl/asparaginyl beta-hydroxylase	Q12797	56806.58	54807.28	51489.35	40295.89	38201.05	37235.15	41260.80	42644.04	41042.89	49439.97	47347.62	44209.26	48121.45	43479.93	34650.27	35908.55	34437.32	33125.61	41803.34	36923.97	30627.34	2.27	1.49	0.65	0.84	1.31	0.69	4.16	2.47	0.11	4.90	4.98	0.12	x	
507	Astrocytic phosphoprotein PEA-15	Q15121	23926.09	24024.11	23031.99	20501.01	19165.12	18103.15	19270.70	17739.86	17361.04	21957.83	21817.39	20596.58	21909.53	21101.48	18432.67	18140.60	17947.62	16806.02	17382.20	17088.33	16313.60	42384.01	40504.45	35984.30	31340.79	30915.12	29995.74	34003.82	32219.23	31772.39	35092.27	33684.66	26997.37		
508	Astrotactin-1	O14525	30884.11	28494.50	26620.21	20367.36	19452.34	20125.57	22464.36	20706.91	20012.29	23974.34	23183.58	23528.11	25957.84	24510.41	24948.12	15971.28	16543.49	18273.21	21601.08	20603.16	20541.75	303.09	172.08	150.57	365.21	223.68	109.08	570.46	362.42	172.17	546.47	461.16	130.90		
509	Ataxin-10	Q9UBB4	93770.92	85860.91	79032.51	58215.75	54801.02	50546.22	62294.77	61518.54	59243.33	73824.06	73342.14	67638.34	72720.65	66498.71	54323.83	57970.33	53772.70	52224.08	58062.56	55313.42	52001.40	37704.86	34357.01	31325.78	22556.30	21793.86	17354.84	28597.46	27495.19	26197.25	31519.18	29738.01	18470.16		
510	Ataxin-2-like protein	Q8WWM	64436.06	59879.66	56497.34	38283.26	39612.34	35440.85	45391.31	44510.21	41256.57	50094.07	47512.89	45668.23	49627.36	46116.12	34194.16	36769.25	35701.20	34146.42	42775.97	40594.09	38955.93	36058.93	36035.20	33023.87	26806.33	26677.91	25132.77	26881.09	27916.34	27402.34	33892.19	29824.13	17819.46		
511	Ataxin-3	P54252	16896.08	15615.13	14808.74	10364.94	10959.12	9762.27	12088.79	12086.65	11843.64	14195.29	13359.72	13440.66	14420.03	13376.10	10258.69	9943.34	9609.09	9267.27	14184.89	12888.46	11447.81	2.27	1.49	0.65	0.84	1.31	0.69	4.16	2.47	0.11	4.90	4.98	0.12	x	
512	Atlastin-2	Q8NHH9	4571.18	4296.85	3039.43	2862.15	2651.73	2393.64	3527.46	3547.72	2897.95	3190.71	3198.48	2881.70	4182.79	3432.44	3036.34	2230.90	2441.30	2202.21	2647.93	2539.03	2400.94	2.27	1.49	0.65	0.84	1.31	0.69	4.16	2.47	0.11	4.90	4.98	0.12	x	
513	Atlastin-3	Q6DD88	169491.77	164817.09	159355.27	106526.88	104542.82	100188.02	138979.22	132626.07	124020.25	141095.32	133371.27	126980.39	146189.04	143521.51	116488.68	107230.85	96897.75	96392.08	116099.28	113613.97	100628.84	27327.04	29864.62	23253.94	18983.36	18300.93	15036.12	24046.63	24329.34	22801.94	25707.71	23816.99	15674.73		
514	ATP synthase F(0) complex subunit B1_ mitochondrial	P24539	318290.54	310182.15	279469.94	222238.59	221649.35	209305.88	246268.61	237574.57	224735.31	281147.56	266847.53	248207.31	255955.25	246243.23	218829.03	217707.93	205046.43	199680.71	221763.59	214275.98	198373.64	150550.98	139837.74	128843.44	98098.53	94890.87	80868.87	113667.39	109676.05	106375.17	123850.84	117819.87	82728.79		
515	ATP synthase mitochondrial F1 complex assembly factor 2	Q8N5M1	4717.24	4098.29	3463.04	2308.44	2412.84	1629.57	3303.56	2728.87	2528.88	3872.30	3333.13	2807.29	3294.95	2242.61	1255.80	2943.03	2141.22	2269.78	2833.89	2343.21	2051.59	2.27	1.49	0.65	0.84	1.31	0.69	4.16	2.47	0.11	4.90	4.98	0.12	x	
516	ATP synthase subunit a	P00846	33978.20	32260.70	30215.64	23943.10	22841.48	21680.18	25125.80	24314.56	22279.23	28893.21	28443.15	26621.73	29218.80	26094.23	24737.41	21931.01	21208.51	22345.65	25111.72	22187.24	20489.08	9934.13	9193.32	7977.32	7917.78	7626.87	7179.41	8990.75	8322.20	7833.78	8722.48	8549.69	4274.06		
517	ATP synthase subunit alpha_ mitochondrial	P25705	1667795.34	1613343.53	1495378.84	1196817.67	1188231.80	1114218.56	1385684.98	1336310.22	1264349.77	1528102.71	1441223.78	1366743.59	1395410.92	1334098.16	1131402.02	1244505.96	1192426.96	1136831.12	1163999.33	1118396.04	1042860.65	808244.01	751714.34	696439.38	470743.67	455213.46	409790.70	566919.15	534116.33	515091.83	549220.94	526270.29	362621.30		
518	ATP synthase subunit beta_ mitochondrial	P06576	1072471.88	1008433.32	899857.49	629298.52	601152.99	548788.17	833582.35	798239.55	749946.29	834989.54	794014.19	715322.07	811226.70	730647.11	573835.67	727065.66	677521.76	646360.51	646949.36	621775.88	576998.25	629304.27	585761.67	525863.64	347591.23	339216.58	287796.91	481956.62	454044.13	411740.17	455732.88	438333.77	273362.63		
519	ATP synthase subunit d_ mitochondrial	O75947	224717.54	205268.86	178267.56	149638.16	132147.45	117560.85	167366.49	150434.65	148642.96	178553.20	165149.08	152683.69	174996.00	165435.38	107154.88	136458.40	132547.66	126628.65	154394.43	137835.10	120772.33	126655.48	112682.15	102037.27	90107.77	87811.33	72092.38	104449.14	97648.90	87987.93	116191.32	107418.63	76557.06		
520	ATP synthase subunit delta_ mitochondrial	P30049	63850.60	54851.14	58840.44	40182.05	39784.91	40569.10	49788.23	47145.69	48978.30	54057.57	50835.12	51675.39	47070.78	47466.60	42418.67	40252.52	38536.27	39789.79	40701.20	43375.99	37620.24	2.27	1.49	0.65	0.84	1.31	0.69	4.16	2.47	0.11	4.90	4.98	0.12	x	
521	ATP synthase subunit e_ mitochondrial	P56385	66791.74	78182.35	93167.47	68530.01	63962.37	69869.35	72845.96	66920.11	70373.90	70759.10	69658.32	79441.27	72859.44	69575.46	70871.06	68447.58	91048.73	65003.29	61165.68	58534.56	56502.24	2.27	1.49	0.65	0.84	1.31	0.69	4.16	2.47	0.11	4.90	4.98	0.12	x	
522	ATP synthase subunit epsilon_ mitochondrial	P56381	1858.36	1823.64	2370.27	1697.61	1528.13	1276.63	1492.84	1419.67	1492.60	2356.09	1907.01	1902.60	2013.33	1987.10	1296.05	1470.78	1409.65	1254.00	857.31	966.92	1209.22	5840.29	5701.33	5481.30	3351.07	3239.18	2581.26	4843.31	3805.42	3527.40	4313.66	4339.27	3381.91		
523	ATP synthase subunit f_ mitochondrial	P56134	19135.87	16759.36	12977.48	2171.06	1960.57	661.38	2428.22	2092.13	1087.50	12013.06	10585.99	9904.66	12718.70	3799.15	623.82	3266.91	2109.53	1403.78	8104.63	890.20	1747.78	11761.49	10112.99	4753.93	5166.75	5272.13	3131.81	10749.70	9398.14	7927.50	8375.39	7702.02	0.12		
524	ATP synthase subunit g_ mitochondrial	O75964	8809.91	7541.69	6735.97	4040.10	4126.73	4179.23	5105.70	4857.92	4954.73	6445.26	6112.69	6631.76	5778.06	4488.70	4243.65	4468.98	4316.77	3852.60	5178.77	4632.62	4926.82	52890.55	43371.59	46493.23	24115.70	25940.48	24311.79	27339.37	28073.22	27365.12	27354.64	24444.24	22145.51		
525	ATP synthase subunit gamma_ mitochondrial	P36542	71848.51	65708.12	63666.00	41751.60	35714.93	34307.11	49806.46	45039.97	43917.96	54157.31	55654.26	55675.97	56069.86	48439.37	29535.82	40616.60	40234.59	39266.68	49461.72	41379.96	37819.88	66456.90	62549.05	59536.02	45354.97	43852.83	41044.57	44557.08	46475.17	46140.62	48026.45	46386.36	19613.21		
526	ATP synthase subunit O_ mitochondrial	P48047	172298.68	170075.20	171387.49	117990.51	116583.84	110109.77	131279.23	121716.24	124206.25	151721.61	145443.77	139921.91	134994.88	131717.46	117989.46	120490.15	117894.18	112711.20	115967.08	112778.30	106136.20	90920.14	84259.24	79999.43	60644.77	57184.78	52229.90	72358.65	69163.16	65167.01	69900.79	68886.62	50038.47		
527	ATP synthase-coupling factor 6_ mitochondrial	P18859	153178.75	151103.61	138978.88	100631.50	95120.39	85675.52	113935.01	104296.59	101548.12	128637.63	117565.84	112407.99	107495.55	97464.39	84267.54	111412.12	104522.35	95061.71	94317.95	92242.14	83325.37	86396.11	77054.56	73123.25	56132.51	54542.44	47229.34	65130.13	61462.20	59254.55	68103.90	68229.63	46935.91		
528	ATPase ASNA1	O43681	110463.11	97620.93	83445.06	56059.10	53640.26	43702.33	69777.63	67342.52	59539.70	77562.08	72579.32	61910.34	80593.69	71406.85	36387.70	59479.78	56809.60	50932.52	59853.19	53449.32	45999.87	5093.92	3051.14	1677.85	2774.00	2538.75	1311.23	3555.18	3526.33	2251.73	4259.90	3836.82	1860.31		
529	ATPase family AAA domain-containing protein 1	Q8NBU5	9967.20	8968.37	8842.08	7555.67	7663.46	7419.98	8397.57	7774.53	7093.11	9219.34	9057.80	7688.95	7828.96	7227.77	5930.49	9096.87	8313.43	8374.57	7512.06	7827.58	7508.92	2505.07	2293.95	2075.62	2780.12	2509.35	1720.28	3556.54	3087.37	2706.10	3745.42	4039.26	1967.03		
530	ATPase family AAA domain-containing protein 2	Q6PL18	10462.13	5418.24	4046.34	85.34	285.56	67.17	803.52	512.87	280.29	4400.14	3849.80	1632.33	2.13	0.97	0.60	516.88	421.83	184.24	66.89	56.86	19.59	84.08	171.62	169.26	119.54	186.53	69.74	156.02	118.61	177.95	57.80	340.95	0.12		
531	ATPase family AAA domain-containing protein 2B	Q9ULI0	3047.67	2916.61	2732.76	2023.44	1953.01	1846.07	1826.17	2101.12	1815.95	2779.80	2430.81	2242.02	2461.58	2522.00	2253.80	2200.54	2087.17	1949.81	2057.87	1837.11	1731.97	2.27	1.49	0.65	0.84	1.31	0.69	4.16	2.47	0.11	4.90	4.98	0.12	x	
532	ATPase family AAA domain-containing protein 3A	Q9NVI7	37812.80	33624.00	30280.54	27236.28	26754.39	25188.66	27452.96	27302.10	25642.37	38276.23	37060.63	35653.16	27856.02	28239.32	24886.31	25176.33	23643.29	23425.36	25070.54	24289.48	23962.83	91947.05	92780.23	79154.20	77983.38	75627.02	75169.96	77614.66	76676.84	74072.93	92749.87	83363.07	60737.92		
533	ATPase family AAA domain-containing protein 3B	Q5T9A4	7584.07	6383.86	6025.62	5160.87	4643.92	4432.57	5215.13	4804.80	4457.54	6035.93	5793.81	5463.39	5784.45	5265.95	4503.04	4825.35	4323.30	3946.71	4613.89	4292.57	3911.68	7053.71	7194.89	6573.90	5027.35	5457.70	4391.15	7190.25	7291.65	6789.66	10586.87	10204.51	6233.68		
534	ATPase family protein 2 homolog	Q8NB90	22686.61	22773.23	20881.39	17542.82	18018.46	16494.25	18988.91	17600.74	16792.32	21019.36	19847.47	19396.44	19910.25	18452.12	15449.24	18703.51	18037.58	17079.15	15959.23	15787.83	15182.85	16886.99	14893.33	12289.05	9623.03	9232.43	5832.66	12461.51	10830.34	8409.68	14383.95	13165.40	6584.12		
535	ATPase inhibitor_ mitochondrial	Q9UII2	0.14	2.53	0.81	1.07	2.77	0.68	0.96	0.36	0.63	0.35	0.75	0.38	2.13	0.97	0.60	4.64	1.07	0.47	2.22	0.19	0.06	213.32	222.39	170.51	155.20	146.02	122.60	164.21	150.14	144.65	146.39	123.07	2312.72		x
536	ATPase MORC2	Q9Y6X9	921.12	705.74	624.43	1000.48	458.96	411.38	695.23	694.53	735.30	739.52	708.09	485.49	566.95	916.14	357.95	442.42	551.84	472.79	712.67	653.68	503.31	2.27	1.49	0.65	0.84	1.31	0.69	4.16	2.47	0.11	4.90	4.98	0.12	x	
537	ATP-binding cassette sub-family A member 12	Q86UK0	77168.87	76213.36	71103.04	53155.51	50247.86	49335.24	65258.27	63644.53	67811.63	64741.60	63186.22	59134.22	68039.46	61085.90	53878.51	60240.43	54620.44	55765.30	62131.41	53226.87	51703.26	42750.92	38976.96	40295.06	34750.17	32865.85	32623.92	36352.64	35880.91	35024.13	41554.17	40418.42	21692.35		
538	ATP-binding cassette sub-family B member 6_ mitochondrial	Q9NP58	1144.94	939.64	694.69	878.77	753.90	561.12	1074.53	875.21	926.58	1110.39	1342.70	982.74	851.51	824.76	149.48	923.29	1008.54	911.39	1077.20	1005.63	789.78	2.27	1.49	0.65	0.84	1.31	0.69	4.16	2.47	0.11	4.90	4.98	0.12	x	
539	ATP-binding cassette sub-family C member 11	Q96J66	3697.53	3642.02	3861.91	2358.38	2199.70	2160.16	2924.60	2790.60	2838.96	3507.65	2935.92	3006.27	3714.27	3102.98	2306.01	2623.35	2383.13	2265.21	2653.50	2539.68	2352.56	2.27	1.49	0.65	0.84	1.31	0.69	4.16	2.47	0.11	4.90	4.98	0.12	x	
540	ATP-binding cassette sub-family D member 3	P28288	27528.16	25617.02	21445.42	15766.01	15731.79	12636.33	18912.16	18415.68	17220.36	21241.98	20803.49	17980.35	19207.30	17511.22	11047.12	17047.25	16575.97	13875.64	16119.78	14788.83	13435.58	28849.79	26646.79	24709.08	20502.01	19898.98	17234.01	23344.04	22963.07	21597.11	23048.14	22645.59	13479.13		
541	ATP-binding cassette sub-family E member 1	P61221	286690.16	278765.63	269174.86	201504.55	196095.71	184461.20	230845.37	225800.45	239926.10	254465.64	238439.31	225701.00	239793.62	217245.34	169276.54	211158.07	190135.79	185041.31	208827.61	194455.09	193255.26	81559.78	76704.09	70959.07	55720.02	54749.21	47347.17	68467.19	66467.59	65650.97	71818.79	63253.76	49044.45		
542	ATP-binding cassette sub-family F member 1	Q8NE71	381969.23	366643.76	344603.53	273660.21	260923.75	244098.39	329200.72	320302.22	307109.34	338643.85	335513.70	303587.51	313248.44	288059.59	241145.84	287553.97	274588.30	266194.65	305836.21	282521.31	257195.44	70318.98	66423.58	58506.48	50656.68	47763.99	40785.12	63369.78	61253.22	57208.73	63784.93	60783.00	40444.83		
543	ATP-binding cassette sub-family F member 2	Q9UG63	132914.34	133279.12	120017.23	85171.02	82056.27	76676.27	95469.41	93622.36	86378.50	107231.35	102445.54	100362.86	99951.14	97948.68	82411.89	79141.65	75688.77	72055.93	87867.95	82083.16	77352.30	47317.88	48533.85	37214.54	31408.58	32546.30	28858.46	35340.93	34269.31	31765.67	42827.44	37555.13	31706.24		
544	ATP-binding cassette sub-family F member 3	Q9NUQ8	12543.21	12404.76	11766.08	8623.86	7913.80	8591.32	9879.57	9965.26	9493.80	9622.84	9152.92	8839.82	10893.66	10450.46	9601.30	8752.58	7811.73	8319.77	8691.15	8360.89	8230.12	2.27	1.49	0.65	0.84	1.31	0.69	4.16	2.47	0.11	4.90	4.98	0.12	x	
545	ATP-binding cassette sub-family G member 1	P45844	1803.16	1795.98	1736.81	2415.11	2006.96	2249.86	2594.86	2544.81	2492.35	2161.86	1963.18	1799.51	2170.18	2646.05	2757.34	2460.03	2490.26	2312.93	2773.63	2386.52	2090.11	2.27	1.49	0.65	0.84	1.31	0.69	4.16	2.47	0.11	4.90	4.98	0.12	x	
546	ATP-citrate synthase	P53396	815777.81	791601.76	758874.59	474084.37	454704.61	419790.24	573707.17	551418.54	525761.88	675951.70	640289.35	599651.54	586084.23	561128.21	421660.66	574069.68	538942.75	524037.05	503353.93	475777.14	443646.99	451737.70	425932.77	401951.48	275626.27	265904.60	224032.14	390283.95	359471.22	338272.86	332366.50	332599.81	213881.37		
547	ATP-dependent 6-phosphofructokinase_ liver type	P17858	525382.42	505500.52	456387.41	311943.05	294008.22	277106.67	383272.36	381441.36	359128.13	386499.54	365419.17	344066.56	390452.97	359616.09	287787.45	331153.90	317121.97	309659.94	327335.07	305903.22	281705.96	95426.30	85155.05	74537.23	52444.56	48426.68	44012.75	63966.90	59865.57	53752.09	61196.01	59479.94	35211.92		
548	ATP-dependent 6-phosphofructokinase_ muscle type	P08237	87614.98	81629.47	72191.07	48791.44	49073.73	42165.03	59582.62	57402.84	54074.52	66320.34	61851.99	55211.79	58755.20	53976.40	38038.22	53710.21	49159.17	49289.04	51012.10	50330.11	45800.45	13912.55	13625.04	11538.79	9688.06	9335.13	7915.96	10889.74	10384.41	9819.68	12458.68	11185.98	6133.39		
549	ATP-dependent 6-phosphofructokinase_ platelet type	Q01813	1072050.13	1030131.30	987488.85	696510.72	682743.66	642749.33	908653.54	885411.47	848021.00	869929.58	832126.33	777623.54	892359.31	828453.27	707005.29	696910.03	655658.29	636635.57	758324.49	707086.23	668757.64	915817.47	872828.90	795033.84	551490.02	537940.33	471281.08	690559.98	674439.27	646139.22	666413.54	636372.40	457035.96		
550	ATP-dependent Clp protease ATP-binding subunit clpX-like_ mitochondrial	O76031	15475.47	13083.81	12638.19	8019.91	7070.46	6881.52	9527.17	9240.02	9201.31	8683.69	9159.57	7972.29	10101.59	8855.16	7918.44	7614.11	7664.38	7350.39	9387.36	7409.24	6533.17	10414.72	9563.02	7687.98	7618.37	7120.51	6525.00	10844.31	11098.32	8915.38	12092.00	11444.84	6458.75		
551	ATP-dependent Clp protease proteolytic subunit_ mitochondrial	Q16740	106818.79	98051.93	89818.62	67012.06	63057.08	59928.08	85750.91	81064.98	75830.60	84574.16	80458.47	74866.46	89264.86	83311.89	69167.41	64781.34	62098.83	57230.79	73908.59	67302.96	63152.84	3533.18	3206.26	3110.41	2178.96	2019.02	1311.72	2361.79	2092.02	1901.05	2481.61	2602.30	1157.72		
552	ATP-dependent DNA helicase DDX11	Q96FC9	150326.01	143866.06	145460.05	98019.59	98713.29	91052.22	125223.01	123107.51	121125.48	120513.22	117717.70	105777.48	123227.14	113675.06	102501.32	101498.72	99367.92	94189.97	116171.08	107355.66	102443.55	2.27	1.49	0.65	0.84	1.31	0.69	4.16	2.47	0.11	4.90	4.98	0.12	x	
553	ATP-dependent DNA helicase Q1	P46063	25690.89	25290.75	23115.49	17885.05	24568.40	20660.03	21403.00	20277.26	19317.52	20519.94	19768.29	19871.46	21389.30	20889.06	20429.40	22301.99	17382.08	19305.68	20837.54	18674.80	17886.66	200572.58	198550.71	177998.92	129824.19	130243.25	114532.06	142671.09	143920.97	139345.06	149587.11	145127.94	132780.99		
554	ATP-dependent RNA helicase A	Q08211	298700.49	282690.37	265969.07	193603.98	187658.96	176248.87	231344.72	224336.39	216659.42	227054.11	223845.53	204326.06	245768.12	230210.66	173049.88	183935.16	185634.37	181490.03	233993.99	210786.47	200313.43	163633.08	148048.25	134795.42	119597.74	118398.64	105367.23	125274.52	119321.94	114856.96	148488.86	140421.33	98859.98		
555	ATP-dependent RNA helicase DDX1	Q92499	220792.23	191363.32	197665.83	128609.65	131239.33	114172.60	157858.76	154105.42	145829.48	163516.06	155058.48	151620.09	158500.05	147051.37	116404.45	130268.65	119037.88	123354.67	136418.15	128916.96	118803.63	161188.26	146162.56	131666.03	98099.19	95218.99	80932.41	125920.89	123834.88	123523.52	115804.01	112874.96	80728.34		
556	ATP-dependent RNA helicase DDX18	Q9NVP1	0.14	2.53	0.81	1.07	2.77	0.68	0.96	0.36	0.63	0.35	0.75	0.38	2.13	0.97	0.60	4.64	1.07	0.47	2.22	0.19	0.06	70013.12	66423.06	64176.72	42660.08	43465.21	36172.90	45120.82	43789.91	41508.80	45575.88	45835.01	31168.00		x
557	ATP-dependent RNA helicase DDX19A	Q9NUU7	60191.32	55518.48	51314.08	36934.41	36869.98	33428.70	45088.97	42181.09	36623.98	50075.42	45359.19	41441.76	46179.51	44980.27	31571.59	35695.97	32769.40	31806.13	35575.88	35589.53	31087.18	78028.23	72762.98	68399.68	48453.84	45111.73	40371.30	58926.85	56936.95	51705.89	61473.25	58975.93	35430.28		
558	ATP-dependent RNA helicase DDX19B	Q9UMR2	1257.94	1352.38	968.53	456.90	80.96	343.85	0.96	0.36	0.63	798.05	801.03	763.22	2.13	0.97	0.60	513.44	22.00	245.81	497.28	512.85	62.12	1574.74	1463.41	128.24	852.19	801.08	586.49	1175.87	1084.48	826.84	1354.89	1285.63	594.86		
559	ATP-dependent RNA helicase DDX39A	O00148	117124.28	115182.07	109991.11	77037.56	75322.58	68506.59	93197.30	88311.33	84243.16	92023.18	88771.62	82823.57	91272.21	86064.63	72936.51	75624.79	75306.75	69320.65	77042.69	72810.48	69371.19	40796.82	39756.60	33645.42	30137.90	27468.97	20947.36	32869.89	29976.49	27061.79	37000.95	36806.60	22137.32		
560	ATP-dependent RNA helicase DDX3X	O00571	217007.56	222298.39	203940.60	140374.75	135343.00	127833.95	162236.92	146695.17	144689.58	167640.29	165090.73	159231.21	171599.64	126413.03	98277.39	131648.30	119802.63	120096.14	143534.56	144283.15	131284.60	294322.47	275369.14	255161.98	191730.08	185398.65	164708.32	217364.24	214851.16	204644.98	221742.70	211829.91	152585.31		
561	ATP-dependent RNA helicase DDX3Y	O15523	232999.11	232661.93	205379.15	152470.04	148498.81	123872.28	196956.97	185861.58	166333.33	213241.71	201963.09	186139.54	178244.40	165326.92	137460.77	158882.13	154193.24	134868.11	152392.22	143187.52	133807.04	51238.78	48340.76	46100.64	37429.04	35079.19	31474.85	44580.99	39516.30	38372.32	42538.60	39640.42	30413.95		
562	ATP-dependent RNA helicase DDX42	Q86XP3	31589.37	29471.98	27028.91	17208.08	16456.85	13941.55	21204.51	20045.59	18220.87	22120.75	21468.16	19403.16	21443.33	19537.73	14970.00	15350.83	15934.86	14662.45	17402.08	17265.02	15216.49	7757.01	5423.02	4877.20	3496.46	3635.21	2628.23	6807.83	6493.62	4346.49	6364.49	6038.15	3357.57		
563	ATP-dependent RNA helicase DDX50	Q9BQ39	5116.70	6220.46	5364.90	4407.60	4119.54	4166.62	4543.02	5089.33	5102.59	5910.55	5278.31	4981.20	5453.97	5551.48	4276.15	3697.25	3289.79	3427.52	3863.60	4235.90	3868.68	13629.02	13484.21	11278.39	9552.34	10302.03	8274.52	11637.57	12487.54	10747.69	12756.22	13722.69	8678.37		
564	ATP-dependent RNA helicase DDX54	Q8TDD1	0.14	2.53	0.81	1.07	2.77	0.68	0.96	0.36	0.63	0.35	0.75	0.38	2.13	0.97	0.60	4.64	1.07	0.47	2.22	0.19	0.06	2.27	1.49	0.65	0.84	1.31	0.69	4.16	2.47	0.11	397.96	348.27	116.51		x
565	ATP-dependent zinc metalloprotease YME1L1	Q96TA2	3473.58	3807.37	3911.26	3557.64	3199.98	3074.13	3425.20	3538.99	3435.10	4694.32	4005.22	3886.61	3302.66	3972.48	3352.32	3123.21	2472.34	2899.87	3498.19	3352.65	4252.31	2.27	1.49	0.65	0.84	1.31	0.69	4.16	2.47	0.11	4.90	4.98	0.12	x	
566	Atrial natriuretic peptide-converting enzyme	Q9Y5Q5	15962.16	14751.44	14110.13	56233.08	53747.63	58639.97	41449.75	44688.75	45630.84	15333.08	16732.77	14561.88	34806.63	29510.73	37237.41	57056.82	45748.95	47427.16	44890.90	47813.72	40341.30	2.27	1.49	0.65	0.84	1.31	0.69	4.16	2.47	0.11	4.90	4.98	0.12	x	
567	AT-rich interactive domain-containing protein 3B	Q8IVW6	2830.36	2906.19	1917.40	1515.91	1419.53	1404.59	1764.38	1788.10	1600.52	1792.70	1781.11	1501.56	1770.83	1843.51	1453.26	1633.05	1382.86	1207.79	1576.27	1390.72	1370.76	2.27	1.49	0.65	0.84	1.31	0.69	4.16	2.47	0.11	4.90	4.98	0.12	x	
568	AT-rich interactive domain-containing protein 4A	P29374	52304.85	40708.17	42311.03	30117.38	28772.23	24744.35	49019.31	47068.59	45437.47	41784.05	42135.47	36223.98	41936.29	36910.27	27370.41	33002.04	29233.03	28303.24	31108.96	30116.49	27532.57	2.27	1.49	0.65	0.84	1.31	0.69	4.16	2.47	0.11	4.90	4.98	0.12	x	
569	AT-rich interactive domain-containing protein 4B	Q4LE39	15902.08	16144.38	15087.02	11806.77	11968.71	10860.72	19513.00	18403.75	16980.34	14002.23	13552.39	13511.32	12945.55	14600.74	12328.04	11583.25	11080.05	10736.14	11752.41	10496.71	10091.22	49567.03	45766.28	42234.32	34260.99	36124.44	30078.76	46160.95	43979.74	39860.87	44872.72	41217.13	38173.67		
570	Aurora kinase B	Q96GD4	0.14	2.53	0.81	1.07	2.77	0.68	0.96	0.36	0.63	0.35	0.75	0.38	2.13	0.97	0.60	4.64	1.07	0.47	2.22	0.19	0.06	727.83	653.32	611.05	438.90	411.90	378.81	546.69	572.41	624.12	532.41	584.92	102.95		x
571	Autophagy protein 5	Q9H1Y0	0.14	2.53	0.81	1.07	2.77	0.68	0.96	0.36	0.63	0.35	0.75	0.38	2.13	0.97	0.60	4.64	1.07	0.47	2.22	0.19	0.06	6003.61	4268.82	2983.71	4010.69	3568.64	2568.70	5084.02	4824.63	3866.19	5023.49	4563.99	2623.83		x
572	Autophagy-related protein 101	Q9BSB4	8756.22	10458.63	8765.70	9268.82	9215.64	8449.27	8863.85	7403.40	8202.72	13662.07	13005.47	13354.63	9739.63	9534.66	7959.98	7251.85	7646.69	7153.88	7552.76	6387.78	8051.92	2.27	1.49	0.65	0.84	1.31	0.69	4.16	2.47	0.11	4.90	4.98	0.12	x	
573	Axin interactor_ dorsalization-associated protein	Q96BJ3	0.14	2.53	0.81	1.07	2.77	0.68	0.96	0.36	0.63	0.35	0.75	0.38	2.13	0.97	0.60	4.64	1.07	0.47	2.22	0.19	0.06	1708.89	1705.71	1553.15	708.73	672.11	452.40	978.65	979.20	777.35	785.98	794.27	272.07		x
574	B box and SPRY domain-containing protein	Q5W0U4	14350.27	14970.20	14190.53	10111.77	10020.92	9412.16	12406.85	11974.59	10809.75	14113.70	13854.37	13530.39	11715.64	5738.43	3622.17	6450.14	8526.27	8202.90	9984.88	10012.57	10624.76	2.27	1.49	0.65	0.84	1.31	0.69	4.16	2.47	0.11	4.90	4.98	0.12	x	
575	Baculoviral IAP repeat-containing protein 6	Q9NR09	219845.05	216442.15	206058.76	146113.96	145818.02	129508.89	158377.06	159898.08	161140.14	176310.18	178546.04	167575.92	162965.49	154212.40	135846.85	171804.12	170972.95	170005.31	179183.43	147203.79	138622.12	225400.88	210329.82	194851.62	125930.20	123968.80	111973.36	171597.95	170924.84	160272.20	182042.85	179696.02	139821.90		
576	BAG family molecular chaperone regulator 1	Q99933	16728.39	14907.58	14529.57	14717.06	14231.47	12352.12	14391.61	14110.13	13527.34	18033.87	17012.97	17306.89	16856.81	6823.93	2264.24	12458.05	12984.30	11902.08	13372.83	14488.08	14761.41	2.27	1.49	0.65	0.84	1.31	0.69	4.16	2.47	0.11	4.90	4.98	0.12	x	
577	BAG family molecular chaperone regulator 2	O95816	0.14	2.53	0.81	1.07	2.77	0.68	0.96	0.36	0.63	0.35	0.75	0.38	2.13	0.97	0.60	4.64	1.07	0.47	2.22	0.19	0.06	46537.57	46217.67	39440.23	29785.60	29287.89	25203.51	32683.89	31368.53	29381.80	37765.51	35851.00	18637.86		x
578	BAG family molecular chaperone regulator 3	O95817	365947.37	344995.67	320038.51	191495.78	184626.20	170843.92	227118.03	213891.68	205353.55	266301.09	252015.81	236618.65	272548.19	256383.04	214399.73	191270.70	172923.53	168506.90	204468.97	195730.98	187131.99	2.27	1.49	0.65	0.84	1.31	0.69	4.16	2.47	0.11	4.90	4.98	0.12	x	
579	BAI1-associated protein 3	O94812	0.14	2.53	0.81	1.07	2.77	0.68	0.96	0.36	0.63	0.35	0.75	0.38	2.13	0.97	0.60	4.64	1.07	0.47	2.22	0.19	0.06	252.89	315.15	140.24	72.48	135.29	99.27	297.96	278.88	171.95	324.87	265.64	129.62		x
580	Band 3 anion transport protein	P02730	1331.84	1016.06	1124.21	186.42	99.22	21.91	95.06	4.49	45.32	414.96	219.76	261.09	797.07	217.18	32.80	81.79	55.36	107.31	46.27	267.16	171.15	48.27	51.90	25.01	0.84	1.31	0.69	4.16	2.47	0.11	4.90	4.98	0.12		
581	Band 4.1-like protein 2	O43491	4761.56	4747.74	4202.59	3532.49	3688.62	3383.55	3870.59	3639.34	3353.37	4965.01	5104.69	5025.11	4643.53	4232.39	3036.19	3747.20	3186.36	3423.13	3222.41	3238.65	2770.31	167392.63	153170.14	140192.87	96294.29	93285.86	81129.65	126050.47	115995.36	109976.86	127228.16	118253.88	71431.01		
582	Band 4.1-like protein 4A	Q9HCS5	0.14	2.53	0.81	1.07	2.77	0.68	0.96	0.36	0.63	0.35	0.75	0.38	2.13	0.97	0.60	4.64	1.07	0.47	2.22	0.19	0.06	15456.20	14725.72	13548.38	10277.29	10415.17	7395.45	11324.32	7853.42	9680.95	15566.90	15259.90	8931.54		x
583	Band 4.1-like protein 5	Q9HCM4	72821.06	70270.39	64419.73	42040.30	39834.66	37137.07	45856.00	42497.96	42255.50	52816.61	50215.28	47801.55	56295.60	51988.91	43558.73	42007.08	39203.13	36761.10	38789.60	37371.64	34749.73	8047.33	7823.63	7305.11	3975.91	3687.81	2880.27	4516.32	4067.99	3637.30	3742.02	3456.33	2312.66		
584	Bardet-Biedl syndrome 10 protein	Q8TAM1	3027.78	2071.97	2573.34	875.62	814.40	724.34	801.24	746.92	665.22	1156.34	752.30	918.54	1161.43	1051.90	836.81	1016.06	1077.96	857.52	1062.90	1280.33	966.17	2.27	1.49	0.65	0.84	1.31	0.69	4.16	2.47	0.11	4.90	4.98	0.12	x	
585	Bargin	Q6ZT62	251.12	347.47	237.01	130.74	122.57	125.25	230.56	249.00	267.94	61.91	67.71	78.70	215.49	261.50	193.50	131.93	125.39	136.08	175.07	174.18	123.85	2.27	1.49	0.65	0.84	1.31	0.69	4.16	2.47	0.11	4.90	4.98	0.12	x	
586	Barrier-to-autointegration factor	O75531	59416.88	51710.03	47195.86	38566.33	38642.78	34590.55	44280.67	44546.11	42102.38	44058.25	41915.82	39285.19	48433.47	45518.13	32741.65	40359.91	39255.69	40834.74	37408.46	34023.75	32036.04	70856.85	68464.48	62799.23	45140.05	46006.59	40768.05	51196.57	48435.83	45870.55	53991.13	50721.97	36967.96		
587	Basal body-orientation factor 1	Q8ND07	0.14	2.53	0.81	1.07	2.77	0.68	0.96	0.36	0.63	0.35	0.75	0.38	2.13	0.97	0.60	4.64	1.07	0.47	2.22	0.19	0.06	6405.72	6056.54	6395.41	4771.39	4944.39	4601.45	4840.46	4445.44	4465.68	5046.19	4952.25	3706.69		x
588	Basal cell adhesion molecule	P50895	0.14	2.53	0.81	1.07	2.77	0.68	0.96	0.36	0.63	0.35	0.75	0.38	2.13	0.97	0.60	4.64	1.07	0.47	2.22	0.19	0.06	6727.04	6345.76	5069.57	3290.92	3047.53	3050.63	3942.98	4118.81	4402.45	4241.83	4224.44	2424.72		x
589	Basic leucine zipper and W2 domain-containing protein 1	Q7L1Q6	380773.72	376437.69	362138.83	240700.29	229466.90	216007.15	258366.98	247916.85	213467.52	298945.68	285702.15	270602.45	313861.70	255544.51	202262.30	232146.12	222873.28	220142.49	251496.19	239232.15	228004.57	76514.43	69353.34	62740.04	43515.57	40562.66	35196.81	52552.69	49025.66	44499.04	50982.17	49618.93	33798.48		
590	Basic leucine zipper and W2 domain-containing protein 2	Q9Y6E2	99463.26	103848.75	97881.79	80652.58	77533.66	72648.69	86739.57	82189.23	70375.13	96086.55	91804.66	74802.28	96401.44	98346.93	76830.40	65912.47	59988.58	65290.03	78844.00	74067.47	70938.12	1248.64	1045.69	1165.57	743.92	954.01	325.90	1115.89	981.85	736.85	1488.89	1373.92	471.84		
591	Basic leucine zipper transcriptional factor ATF-like	Q16520	220.04	147.20	190.73	168.51	167.98	159.63	1330.69	1158.55	1112.01	227.87	291.55	131.07	780.68	83.80	303.76	1046.02	913.69	931.65	1127.58	1335.73	1160.72	2.27	1.49	0.65	0.84	1.31	0.69	4.16	2.47	0.11	4.90	4.98	0.12	x	
592	Basigin	P35613	142414.10	137512.88	124358.60	108991.41	105979.76	96903.35	120998.02	114988.61	114539.57	136864.19	130297.00	122758.31	112074.73	101498.82	80598.83	122532.04	111668.07	107265.07	106503.09	96063.74	88453.72	283979.96	262639.38	232361.74	185749.53	179298.97	149873.73	208057.57	203115.23	188135.59	222745.48	213753.65	144814.01		
593	B-cell lymphoma/leukemia 10	O95999	0.14	2.53	0.81	1.07	2.77	0.68	0.96	0.36	0.63	0.35	0.75	0.38	2.13	0.97	0.60	4.64	1.07	0.47	2.22	0.19	0.06	5493.67	5934.42	5812.27	3400.14	3275.02	3528.16	3641.52	3799.73	3280.56	3022.34	3393.54	2145.63		x
594	B-cell receptor-associated protein 31	P51572	257685.67	252933.31	241128.84	198178.29	200551.77	182105.00	200250.59	191533.47	190582.82	240872.11	216853.85	211589.24	206222.99	198324.11	171236.57	201035.82	189922.17	184505.81	183702.10	172379.58	171559.50	115902.70	109913.02	106188.07	77509.13	77076.34	70534.37	90792.57	88319.42	85120.81	99385.62	95669.56	74803.11		
595	Bcl-2 homologous antagonist/killer	Q16611	543.12	525.10	500.57	697.40	556.89	609.78	695.26	707.34	672.64	652.05	563.20	694.88	702.41	540.87	412.76	851.04	637.61	724.37	540.51	590.45	537.32	2.27	1.49	0.65	0.84	1.31	0.69	4.16	2.47	0.11	4.90	4.98	0.12	x	
596	BCL2/adenovirus E1B 19 kDa protein-interacting protein 3	Q12983	0.14	2.53	0.81	1.07	2.77	0.68	0.96	0.36	0.63	0.35	0.75	0.38	2.13	0.97	0.60	4.64	1.07	0.47	2.22	0.19	0.06	2173.65	2302.20	2251.74	1677.63	1597.62	1716.52	2381.65	2333.73	2355.40	2117.25	692.88	513.14		x
597	Bcl2-associated agonist of cell death	Q92934	0.14	2.53	0.81	1.07	2.77	0.68	0.96	0.36	0.63	0.35	0.75	0.38	2.13	0.97	0.60	4.64	1.07	0.47	2.22	0.19	0.06	7152.72	6909.19	6593.86	7748.37	7426.34	6689.94	8474.99	8122.08	7492.58	8662.47	8517.16	5002.46		x
598	Bcl-2-associated transcription factor 1	Q9NYF8	11848.09	11744.60	11040.50	6875.51	6354.24	7096.58	8257.54	8108.29	8370.18	8503.48	8244.17	8022.86	9386.81	7669.64	7614.01	6363.87	6968.83	6218.57	8983.65	7999.71	8040.78	16934.41	15560.74	15206.02	15799.34	13858.91	13882.86	15126.54	14824.82	14929.19	16477.07	14845.76	6599.06		
599	Bcl-2-like protein 15	Q5TBC7	9137.75	9056.18	9464.76	6857.03	7422.92	6334.62	14522.45	13647.93	13487.43	7812.34	7688.31	6878.56	8984.31	8350.63	6423.03	8606.02	9894.09	7643.25	9150.91	8027.08	7311.33	2.27	1.49	0.65	0.84	1.31	0.69	4.16	2.47	0.11	4.90	4.98	0.12	x	
600	BCL-6 corepressor	Q6W2J9	158560.59	149403.62	139760.39	126888.02	124586.98	117728.35	131992.99	123060.62	124185.92	150708.74	142419.94	134715.14	143045.77	129127.06	113632.00	111200.30	106573.98	109298.97	118891.90	115288.63	113706.75	208193.48	200261.06	192984.27	153578.82	152024.37	134371.31	165580.30	160964.43	154929.71	181608.28	174054.14	120700.55		
601	BEN domain-containing protein 5	Q7L4P6	0.14	2.53	0.81	1.07	2.77	0.68	0.96	0.36	0.63	0.35	0.75	0.38	2.13	0.97	0.60	4.64	1.07	0.47	2.22	0.19	0.06	2040.48	1948.80	1813.07	1377.71	1238.58	1206.62	1409.79	1382.41	1352.46	4.90	4.98	0.12		x
602	Beta-1_3-galactosyl-O-glycosyl-glycoprotein beta-1_6-N-acetylglucosaminyltransferase 4	Q9P109	45310.73	41419.63	38189.48	37081.43	37507.03	35426.18	40132.88	39003.40	38007.23	51166.95	48935.57	47106.62	37104.23	34241.48	32549.39	39986.67	37639.50	36574.32	36940.81	34432.40	33955.59	13107.09	12500.62	11050.86	9019.40	9162.70	8337.69	8374.12	8204.09	7578.67	7709.04	8329.53	2944.58		
603	Beta-1_3-galactosyl-O-glycosyl-glycoprotein beta-1_6-N-acetylglucosaminyltransferase	Q02742	4159.41	3478.02	3248.24	1991.07	2172.30	2072.31	2575.93	2672.87	2362.17	2969.56	2967.47	3172.96	3087.52	2852.82	2600.26	2069.37	1942.75	1869.99	2253.40	2054.55	1845.75	2.27	1.49	0.65	0.84	1.31	0.69	4.16	2.47	0.11	4.90	4.98	0.12	x	
604	Beta-1_3-galactosyltransferase 2	O43825	16537.15	15446.21	15191.06	10604.47	10385.49	9126.96	11207.23	11111.01	11200.86	13592.74	13132.78	12931.37	11112.24	11929.15	10010.66	12843.64	12490.84	12668.19	12185.75	10223.18	10551.44	16505.51	15582.76	14434.96	9292.58	9292.57	8667.72	11980.56	11887.26	12011.94	13957.50	13523.74	10166.24		
605	Beta-actin-like protein 2	Q562R1	149078.59	134937.99	122584.96	79142.23	75902.87	71222.28	115220.00	112502.83	102896.82	143857.54	136892.78	128358.96	98090.81	98662.46	62109.14	138910.16	137736.24	130888.26	107861.57	103211.63	93729.57	27519.88	26050.67	25616.18	20947.15	20991.06	19780.65	21988.41	21243.60	18865.74	23065.89	22582.99	16935.60		
606	Beta-adrenergic receptor kinase 2	P35626	0.14	2.53	0.81	1.07	2.77	0.68	0.96	0.36	0.63	0.35	0.75	0.38	2.13	0.97	0.60	4.64	1.07	0.47	2.22	0.19	0.06	4647.06	3051.51	1666.16	3200.20	3017.52	1694.75	3841.81	3300.65	2696.12	5484.54	4929.88	2312.07		x
607	Beta-arrestin-1	P49407	2371.29	1931.41	2092.33	1090.59	996.24	1028.25	1171.85	1147.70	1009.14	1536.33	1401.53	1148.64	1582.76	1644.82	1551.29	1187.77	1020.75	998.12	1052.56	1158.30	992.49	2.27	1.49	0.65	0.84	1.31	0.69	4.16	2.47	0.11	4.90	4.98	0.12	x	
608	Beta-catenin-like protein 1	Q8WYA6	0.14	2.53	0.81	1.07	2.77	0.68	0.96	0.36	0.63	0.35	0.75	0.38	2.13	0.97	0.60	4.64	1.07	0.47	2.22	0.19	0.06	4862.16	4261.06	4011.61	3389.76	3456.74	3455.02	3971.61	3960.63	3822.98	4456.59	4544.57	3470.06		x
609	Beta-centractin	P42025	8076.86	7874.16	7293.19	8573.72	7231.56	7572.45	7143.08	6522.84	6269.66	9536.92	9621.22	8430.44	7433.77	6878.84	5959.93	7975.95	7582.76	7345.15	6537.70	6759.56	6418.34	1731.34	1501.05	1350.55	1814.87	1666.60	1373.22	1389.13	1280.06	1147.65	2295.43	2278.29	1222.63		
610	Beta-crystallin A3	P05813	6352.08	5218.75	3338.84	2191.43	1271.18	1419.13	2505.74	441.53	2099.74	3678.79	3448.33	2569.53	3333.59	717.71	171.44	2656.39	1285.43	1401.29	2480.62	2127.98	810.04	2.27	1.49	0.65	0.84	1.31	0.69	4.16	2.47	0.11	4.90	4.98	0.12	x	
611	Beta-defensin 116	Q30KQ4	4600.03	5190.03	5724.80	4181.87	3976.26	3910.88	5540.04	5006.90	4787.91	5061.04	4829.77	4598.84	6026.22	4986.75	4207.02	3933.38	3907.95	3775.64	5548.63	5225.02	4583.69	2.27	1.49	0.65	0.84	1.31	0.69	4.16	2.47	0.11	4.90	4.98	0.12	x	
612	Beta-defensin 119	Q8N690	11124.92	10482.40	10290.67	6202.65	5869.92	5871.02	8132.58	7944.69	8284.41	7445.58	6347.28	6457.14	8194.17	7316.04	7080.82	6847.87	6483.32	6201.70	7797.87	7284.74	7492.72	2.27	1.49	0.65	0.84	1.31	0.69	4.16	2.47	0.11	4.90	4.98	0.12	x	
613	Beta-defensin 132	Q7Z7B7	0.14	2.53	0.81	1.07	2.77	0.68	0.96	0.36	0.63	0.35	0.75	0.38	2.13	0.97	0.60	4.64	1.07	0.47	2.22	0.19	0.06	834.00	751.82	926.81	711.64	695.50	633.80	1012.49	912.95	710.81	854.43	797.36	465.83		x
614	Beta-enolase	P13929	101559.85	95081.14	90978.26	83731.33	80082.80	79148.72	85488.07	84424.75	84846.13	96699.05	94452.06	88279.89	94223.07	83178.72	66831.21	82516.97	76982.48	79149.18	73001.03	72032.50	71075.40	24488.77	22987.44	23293.97	14502.77	14722.02	12936.28	16455.68	15342.71	15757.31	21528.80	20746.81	6435.65		
615	Beta-hexosaminidase subunit alpha	P06865	53513.25	50699.36	46658.19	34002.29	32214.36	27441.46	40976.27	37499.58	35996.86	46447.24	45453.20	39602.30	41624.01	39072.91	26680.34	34250.16	34941.38	31654.35	31902.01	31198.26	28756.08	14719.37	13259.55	11627.02	10618.15	10017.13	6708.17	12432.28	11915.14	10228.63	13778.22	13356.18	6739.84		
616	Beta-hexosaminidase subunit beta	P07686	108340.89	95618.29	85414.79	54016.50	60729.20	51284.30	67330.08	62725.02	58944.83	91409.91	84922.28	76658.99	82531.70	70642.14	49969.31	70606.31	64358.84	62410.54	60787.79	58017.84	50541.85	71929.98	66489.12	57595.31	63314.41	60153.83	48664.06	114383.93	108553.99	100609.94	74403.53	72652.42	44586.53		

Supplementary Table S1. Overview on the abundance of all relatively quantified 5180 proteins

			MCF-7																					MDA-MB-231													
	Protein name	UniProt	control_01	control_02	control_03	Dai_IC20_01	Dai_IC20_02	Dai_IC20_03	Dai_SC20_01	Dai_SC20_02	Dai_SC20_03	Gen_IC20_01	Gen_IC20_02	Gen_IC20_03	Gen_SC20_01	Gen_SC20_02	Gen_SC20_03	SSE_IC20_01	SSE_IC20_02	SSE_IC20_03	SSE_SC20_01	SSE_SC20_02	SSE_SC20_03	control_01	control_02	control_03	Dai_IC20_01	Dai_IC20_02	Dai_IC20_03	Gen_IC20_01	Gen_IC20_02	Gen_IC20_03	SSE_IC20_01	SSE_IC20_02	SSE_IC20_03	unique to MCF-7	unique to MDA-MB-231
617	Beta-klotho	Q86Z14	394395.51	336621.38	296626.64	201264.31	189527.15	161784.90	241310.13	228885.86	210834.41	275009.54	259549.28	229784.78	243816.07	212899.63	119187.59	208075.61	205529.66	202980.64	231524.69	212883.51	185877.73	200296.96	189925.35	165938.85	107521.99	105923.33	66404.14	165547.06	145693.26	131542.26	165641.69	162745.44	83079.10		
618	Beta-parvin	Q9HBI1	1297.01	1246.99	1082.73	711.47	600.06	586.55	885.47	823.07	757.74	893.20	778.00	871.88	849.65	949.86	737.72	764.31	700.43	751.77	780.48	730.83	775.75	2.27	1.49	0.65	0.84	1.31	0.69	4.16	2.47	0.11	4.90	4.98	0.12	x	
619	Beta-soluble NSF attachment protein	Q9H115	2650.71	2759.12	2648.98	2388.05	2663.02	2084.59	2217.71	2021.88	1675.57	3168.70	3010.10	2567.66	3177.90	3059.80	1973.40	2366.22	2283.91	1933.94	2403.09	2176.72	2126.90	7060.94	6202.64	6550.98	5613.40	4798.73	4463.03	6278.81	5875.03	4282.24	4717.44	4660.02	3834.62		
620	Beta-ureidopropionase	Q9UBR1	161.32	135.15	75.36	870.97	1770.65	1142.69	2654.34	302.72	22.32	127.67	39.40	48.81	176.03	854.88	224.98	1848.56	1389.58	1734.19	188.23	270.82	46.69	2.27	1.49	0.65	0.84	1.31	0.69	4.16	2.47	0.11	4.90	4.98	689.67	x	
621	BH3-interacting domain death agonist	P55957	65824.87	57712.16	46190.32	43633.92	37400.77	31636.51	41830.44	41343.78	36426.56	53537.67	47857.79	42992.42	51989.71	49944.28	31265.94	34531.40	37028.78	31305.41	40333.24	37752.32	30531.14	31436.01	30453.69	25358.65	21994.06	21242.11	19676.66	25208.17	24643.64	23307.47	26671.53	27568.21	17297.86		
622	Bifunctional 3'-phosphoadenosine 5'-phosphosulfate synthase 1	O43252	148730.48	139898.19	121621.69	84413.75	77590.73	72653.22	90429.52	87672.39	81948.33	145673.90	136868.49	120805.99	132440.34	89193.66	71594.22	84322.11	80014.90	76497.39	84557.10	81314.31	71714.86	62110.64	54741.61	48025.93	40619.26	38205.18	34637.97	44640.38	44554.17	40662.06	54128.34	52624.52	35944.13		
623	Bifunctional 3'-phosphoadenosine 5'-phosphosulfate synthase 2	O95340	377413.01	339570.89	333933.71	171936.39	167058.80	156075.66	215836.67	201388.29	191631.46	261352.40	251479.60	239994.29	253599.63	247070.23	206676.68	169537.94	163990.11	154706.42	168138.08	162876.44	152246.79	21218.28	18400.48	20019.53	16270.73	15226.96	14252.25	19341.77	18929.62	19393.76	17940.29	18024.24	10431.09		
624	Bifunctional arginine demethylase and lysyl-hydroxylase JMJD6	Q6NYC1	5833.99	5655.34	4948.90	4592.17	4465.16	4093.18	4764.50	4598.83	5485.50	5953.08	5705.72	5578.02	5953.36	5824.03	4724.56	6045.48	5292.45	4799.96	6284.91	5100.15	4846.47	2.27	1.49	0.65	0.84	1.31	0.69	4.16	2.47	0.11	4.90	4.98	0.12	x	
625	Bifunctional coenzyme A synthase	Q13057	9929.64	9554.91	7668.01	7273.73	6112.35	5842.66	7316.29	7117.14	6487.94	7708.70	7655.61	7368.20	8391.32	8087.00	5954.19	6690.30	6204.84	5465.98	8146.90	6449.06	5195.77	2.27	1.49	0.65	0.84	1.31	0.69	4.16	2.47	0.11	4.90	4.98	0.12	x	
626	Bifunctional glutamate/proline--tRNA ligase	P07814	492629.78	474001.92	437769.92	321764.13	309640.30	288043.42	379544.16	363951.77	344334.73	413863.22	396966.70	364873.57	373766.61	353387.18	286019.36	354568.44	327175.09	321558.13	333659.43	321999.03	299541.44	734371.88	688959.92	633195.72	525780.10	510090.24	468117.99	610487.74	583484.26	556488.33	655017.47	637022.61	443013.84		
627	Bifunctional methylenetetrahydrofolate dehydrogenase/cyclohydrolase_ mitochondrial	P13995	27460.73	25434.17	23025.32	26339.57	28132.60	24527.79	28016.46	24267.56	25369.89	25927.81	24452.43	23724.42	22068.99	27899.49	15298.02	27244.06	26590.87	27242.53	21018.94	24503.39	21891.43	32626.85	29615.11	27928.18	26590.74	26165.00	22785.37	31033.83	27881.09	26832.41	35466.65	34254.72	22234.09		
628	Bifunctional purine biosynthesis protein PURH	P31939	599629.50	544307.10	509226.73	384169.82	371445.24	345905.07	437349.40	407856.09	389234.83	500388.36	469266.73	434138.47	484534.90	452561.03	344622.29	381786.57	355303.56	343853.21	377925.19	363574.71	334892.98	211820.76	193718.22	174311.55	121135.50	120417.05	94669.18	161773.22	146746.17	130445.58	162501.06	160982.74	94498.18		
629	Biliverdin reductase A	P53004	76568.81	76144.46	73286.71	63569.43	62437.38	57824.11	68409.63	65661.49	61447.86	78935.30	75414.72	69432.54	66722.35	65428.08	56425.92	60971.02	60245.53	56379.45	57166.97	55735.08	53871.62	44475.21	41063.02	40354.21	30538.60	28938.11	25611.28	33733.06	33665.69	32645.90	34954.77	34325.89	25935.24		
630	Biogenesis of lysosome-related organelles complex 1 subunit 1	P78537	459.04	490.92	83.27	1.07	2.77	0.68	9.51	25.64	4.00	532.77	352.10	146.00	2.13	0.97	0.60	4.64	1.07	0.47	2.22	0.19	0.06	2.27	1.49	0.65	0.84	1.31	0.69	4.16	2.47	0.11	4.90	4.98	0.12	x	
631	Biogenesis of lysosome-related organelles complex 1 subunit 2	Q6QNY1	2563.59	2217.46	1651.39	1678.71	1252.81	1203.91	2074.63	1970.19	1401.76	2380.52	2226.99	2057.15	1994.61	1575.97	1241.13	2185.42	1991.81	1697.11	2047.10	1839.02	1655.99	2.27	1.49	0.65	0.84	1.31	0.69	4.16	2.47	0.11	4.90	4.98	0.12	x	
632	Biorientation of chromosomes in cell division protein 1-like 1	Q8NFC6	9457.34	9283.05	8397.70	6402.41	7046.89	7140.81	6382.56	6107.61	5866.46	8487.47	7398.81	7193.36	6701.63	7164.95	6837.18	6361.24	6009.89	5568.96	8256.73	5376.02	8773.92	138143.67	140446.32	128451.45	91529.55	86489.33	70632.73	115493.78	108095.12	99678.15	130595.51	126191.45	76990.85		
633	Bleomycin hydrolase	Q13867	157.05	185.30	157.58	39.52	19.45	20.74	46.61	30.99	8.40	38.24	19.40	45.63	2.13	0.97	0.60	45.65	6.35	27.43	100.16	35.47	54.43	252.74	6.85	28.49	264.52	206.30	80.02	262.02	157.94	10.08	242.76	298.13	1579.90		
634	Blood vessel epicardial substance	Q8NE79	1652.86	1739.07	1566.80	1708.81	1732.41	1677.24	1314.02	1216.17	1242.54	2217.81	2325.34	2098.90	1416.02	1571.08	576.40	1613.22	1547.41	1416.93	1877.16	1648.61	1564.08	2.27	1.49	0.65	0.84	1.31	0.69	4.16	2.47	0.11	4.90	4.98	0.12	x	
635	Bloom syndrome protein	P54132	2150.76	2151.67	1925.74	1594.06	1763.23	1756.91	1584.62	1677.09	1365.18	1539.10	1549.97	1622.23	1853.14	1763.27	1575.91	1434.11	1264.85	1734.09	2158.47	1839.39	1437.81	22143.31	20123.98	18523.47	12545.32	11344.65	9486.89	13547.41	12550.10	12247.83	14682.07	14203.13	3891.85		
636	BMP-binding endothelial regulator protein	Q8N8U9	31636.03	31143.93	29404.41	21663.98	20115.77	20075.53	27450.63	26481.29	24791.02	26247.80	24708.75	23521.59	25466.51	25522.16	21546.95	21540.42	20808.67	21140.78	21254.63	20082.62	19682.11	2.27	1.49	0.65	0.84	1.31	0.69	4.16	2.47	0.11	4.90	4.98	0.12	x	
637	BolA-like protein 1	Q9Y3E2	10630.71	10853.03	12097.09	6816.83	6062.74	6064.31	7011.34	5904.55	6816.32	7758.46	7785.75	6309.89	6318.60	7639.77	5983.77	5908.93	5658.44	4171.09	7768.88	6812.54	7076.36	2.27	1.49	0.65	0.84	1.31	0.69	4.16	2.47	0.11	4.90	4.98	0.12	x	
638	BolA-like protein 2	Q9H3K6	176008.11	170246.80	152862.19	116430.21	107308.19	102086.51	139558.89	127919.99	128466.82	148430.27	138612.35	127877.94	152284.35	140004.59	103315.42	124407.59	110663.85	105687.88	122914.53	115306.59	107708.52	13713.28	12583.46	11354.94	10683.58	10853.94	8699.93	13939.27	13007.64	11894.26	13387.44	13247.60	9617.63		
639	Bone marrow proteoglycan	P13727	516.36	453.68	453.69	922.24	583.19	379.79	905.43	820.81	124.24	438.96	473.30	413.74	770.64	1017.77	230.06	624.63	380.35	459.52	2683.66	2768.19	1852.57	2.27	1.49	0.65	0.84	1.31	0.69	4.16	2.47	0.11	4.90	4.98	0.12	x	
640	Bone morphogenetic protein 5	P22003	0.14	2.53	0.81	1.07	2.77	0.68	0.96	0.36	0.63	0.35	0.75	0.38	2.13	0.97	0.60	4.64	1.07	0.47	2.22	0.19	0.06	3300.74	2855.15	2736.97	1740.27	1645.30	1449.96	2637.63	2577.58	2335.24	2382.60	2358.83	1673.53		x
641	Bone morphogenetic protein receptor type-1A	P36894	9706.28	14158.26	10494.65	22803.30	18614.29	20605.35	18429.62	23835.34	21842.06	12338.74	19574.45	10128.19	18479.74	10012.96	7944.42	21072.28	14936.59	16713.05	20497.97	19514.23	16895.63	2.27	1.49	0.65	0.84	1.31	0.69	4.16	2.47	0.11	4.90	4.98	0.12	x	
642	Box C/D snoRNA protein 1	Q9NWK9	0.14	2.53	0.81	1.07	2.77	0.68	0.96	0.36	0.63	0.35	0.75	0.38	2.13	0.97	0.60	4.64	1.07	0.47	2.22	0.19	0.06	21673.72	20571.86	19310.94	14714.44	13979.09	13731.97	15563.70	14718.11	14374.41	16449.70	16047.39	13374.28		x
643	Brain acid soluble protein 1	P80723	128685.03	124897.49	122735.63	86609.45	83865.11	77034.85	68995.16	65450.67	60260.47	127904.20	119777.69	115765.73	105035.55	106354.95	99949.13	78663.89	70565.67	68946.33	64815.81	66196.95	64896.02	163712.74	162095.60	146004.37	172175.99	170410.55	166040.37	166168.64	161336.28	158684.17	213023.66	196389.18	146060.40		
644	Brain-specific angiogenesis inhibitor 1-associated protein 2	Q9UQB8	114683.80	103187.89	91568.05	79288.53	76103.75	65112.99	86869.22	83663.02	77957.12	101149.53	95756.84	83672.00	94404.23	81587.47	52999.36	79577.62	75462.46	70223.89	82206.31	76596.85	67730.28	20237.18	21032.44	17809.46	10606.24	10305.31	8609.83	19425.44	19163.66	16648.94	15471.10	16640.82	10900.34		
645	Brain-specific angiogenesis inhibitor 1-associated protein 2-like protein 1	Q9UHR4	113257.99	108526.49	104284.48	87933.81	83344.20	78321.82	92277.15	90465.09	86276.27	94047.96	91098.09	86881.30	95318.30	88948.84	66480.83	76197.33	79664.74	77182.35	91827.72	80974.65	80714.66	3961.54	3645.51	2498.79	2292.97	2549.44	2441.23	2289.04	2199.79	2081.54	2934.40	2745.85	2487.71		
646	Branched-chain-amino-acid aminotransferase_ mitochondrial	O15382	5332.65	4529.65	4128.98	3142.50	3073.92	2671.10	4468.57	4299.33	4004.73	3653.20	3303.39	3028.03	4032.75	3823.82	2056.43	3292.44	2910.74	2615.18	3565.03	3159.61	2690.77	2.27	1.49	0.65	0.84	1.31	0.69	4.16	2.47	0.11	4.90	4.98	0.12	x	
647	BRCA1-A complex subunit RAP80	Q96RL1	2601.08	2077.24	2245.96	1335.94	1342.11	1381.36	2027.77	1781.59	1753.42	1927.72	1574.52	1359.59	2070.14	2039.40	1925.97	1496.94	1000.63	1401.14	1414.85	1597.77	1556.92	2.27	1.49	0.65	0.84	1.31	0.69	4.16	2.47	0.11	4.90	4.98	0.12	x	
648	BRCA1-associated ATM activator 1	Q6PJG6	1603.06	1555.37	1439.46	1187.14	1067.75	1282.53	968.42	1170.60	1168.40	1476.77	1724.67	1603.44	1852.37	1532.51	1362.31	941.78	962.93	969.60	1110.66	883.63	1115.88	2.27	1.49	0.65	0.84	1.31	0.69	4.16	2.47	0.11	4.90	4.98	0.12	x	
649	BRCA1-associated protein	Q7Z569	0.14	2.53	0.81	1.07	2.77	0.68	0.96	0.36	0.63	0.35	0.75	0.38	2.13	0.97	0.60	4.64	1.07	0.47	2.22	0.19	0.06	4517.32	4035.71	3476.26	3557.64	3146.23	1839.74	3990.52	3641.82	2949.95	4998.48	4977.04	2455.65		x
650	BRCA2 and CDKN1A-interacting protein	Q9P287	15143.60	11949.53	9376.49	7002.58	6763.25	4914.98	10241.38	9892.15	8929.15	10835.36	9796.33	7907.38	11458.03	10339.77	3922.68	7101.38	7216.67	6207.95	8864.89	7873.57	5919.81	9291.97	8245.58	7404.22	4293.29	4325.35	2650.00	7419.70	7041.95	5852.03	6993.93	7256.23	2818.45		
651	Breast cancer anti-estrogen resistance protein 1	P56945	10811.67	10418.53	10337.28	7294.78	6802.93	6425.76	8624.02	8542.57	7959.13	9275.51	8790.65	8417.59	8853.41	8684.98	7010.86	7036.01	7080.56	6572.96	6606.49	6108.83	5753.66	21859.85	19532.78	17780.18	14178.79	13560.24	12294.55	18132.33	17810.14	16541.27	15687.31	14800.44	11277.93		
652	Breast cancer anti-estrogen resistance protein 3	O75815	67475.56	65065.33	62689.53	48531.15	47014.41	42886.95	50876.73	47274.46	45456.70	52409.89	53827.17	52249.70	50709.41	49436.81	43448.54	49371.34	46517.54	44619.98	50882.65	48829.81	48671.48	5911.59	5708.92	5105.32	3719.83	3779.88	2914.21	5411.66	4735.89	4065.82	5093.52	5131.26	2589.14		
653	Breast carcinoma-amplified sequence 1	O75363	39924.31	40488.42	37693.34	28387.33	26550.75	26125.99	27391.41	27026.97	26541.67	40420.33	38229.69	38394.06	37932.32	36219.02	32434.42	25787.92	23419.99	24238.55	25631.95	24062.70	24497.43	2.27	1.49	0.65	0.84	1.31	0.69	4.16	2.47	0.11	4.90	4.98	0.12	x	
654	Brefeldin A-inhibited guanine nucleotide-exchange protein 1	Q9Y6D6	5104.38	4806.73	4734.78	2811.35	2946.02	2549.06	2014.63	2030.94	1797.48	4028.19	3941.45	3947.94	3493.50	3366.90	2881.81	3534.39	3554.51	3309.11	3600.09	3494.72	3106.57	2.27	1.49	0.65	0.84	1.31	0.69	4.16	2.47	0.11	4.90	4.98	0.12	x	
655	Brefeldin A-inhibited guanine nucleotide-exchange protein 2	Q9Y6D5	56916.03	64281.46	67692.73	31600.23	27342.19	22212.00	19188.58	22265.72	15022.18	63137.72	69341.30	86908.45	62155.04	56078.57	3814.44	14636.57	24514.99	31802.48	66598.44	54573.86	45402.87	333.59	355.48	256.73	247.75	246.47	88.52	280.90	312.15	240.61	260.31	312.73	67.54		
656	BRI3-binding protein	Q8WY22	25406.98	24551.48	21464.97	15124.97	15626.79	12830.32	17900.60	16383.21	16233.85	20834.50	18806.74	16533.07	19672.99	17629.06	11978.30	17073.95	17500.42	15689.18	17366.72	15899.29	14464.05	2.27	1.49	0.65	0.84	1.31	0.69	4.16	2.47	0.11	4.90	4.98	0.12	x	
657	BRISC and BRCA1-A complex member 2	Q9NXR7	588.44	575.49	436.18	266.25	289.91	246.10	750.18	775.68	703.73	519.83	535.03	575.47	553.23	641.68	432.32	349.40	298.30	226.68	420.64	392.93	332.58	857.02	791.59	763.38	141.39	171.49	154.76	120.78	102.33	132.29	135.20	101.61	46.90		
658	BRISC complex subunit Abraxas 2	Q15018	23060.24	22803.97	20864.68	16868.38	16825.28	13455.69	17634.80	16884.97	16368.17	24069.74	22798.93	20718.87	20772.85	19734.89	15418.87	16657.07	17006.62	15487.82	17683.57	17154.01	14953.30	2473.66	2477.23	2519.42	1796.09	1768.09	1642.92	1875.63	1839.85	1904.52	2067.37	1601.26	1285.08		
659	BRO1 domain-containing protein BROX	Q5VW32	24520.94	21970.88	22531.43	16382.65	15338.09	14363.74	20236.64	18544.82	20610.93	20042.42	19066.04	18954.20	20696.88	20617.03	16962.92	18290.41	19786.63	18719.45	19931.62	18248.51	16615.66	9154.30	8856.30	8517.25	5457.73	5615.02	4821.38	5828.90	5210.95	5908.04	5627.97	5339.26	3435.57		
660	Bromodomain adjacent to zinc finger domain protein 2B	Q9UIF8	0.14	2.53	0.81	1.07	2.77	0.68	0.96	0.36	0.63	0.35	0.75	0.38	2.13	0.97	0.60	4.64	1.07	0.47	2.22	0.19	0.06	15273.23	14032.14	13492.02	12137.25	11410.72	9983.97	13705.01	13172.16	12861.61	12973.01	13176.06	8761.24		x
661	Bromodomain-containing protein 2	P25440	0.14	2.53	0.81	1.07	2.77	0.68	0.96	0.36	0.63	0.35	0.75	0.38	2.13	0.97	0.60	4.64	1.07	0.47	2.22	0.19	0.06	62.68	98.88	37.63	36.87	29.62	38.76	37.89	23.77	22.88	4.90	4.98	0.12		x
662	Bromodomain-containing protein 3	Q15059	0.14	2.53	0.81	1.07	2.77	0.68	0.96	0.36	0.63	0.35	0.75	0.38	2.13	0.97	0.60	4.64	1.07	0.47	2.22	0.19	0.06	13573.07	12519.08	12397.20	8513.67	9220.43	8371.37	10602.59	9792.29	9524.03	10946.67	10513.92	7580.26		x
663	Bromodomain-containing protein 4	O60885	0.14	2.53	0.81	1.07	2.77	0.68	0.96	0.36	0.63	0.35	0.75	0.38	2.13	0.97	0.60	4.64	1.07	0.47	2.22	0.19	0.06	2981.68	2609.33	2349.58	1304.02	1234.73	1404.81	1808.93	2096.08	1956.71	1655.60	1867.29	1024.91		x
664	Bromodomain-containing protein 7	Q9NPI1	0.14	2.53	0.81	1.07	2.77	0.68	0.96	0.36	0.63	0.35	0.75	0.38	2.13	0.97	0.60	4.64	1.07	0.47	2.22	0.19	0.06	19874.04	18525.11	18102.45	11494.06	10875.61	12856.18	13247.24	11313.45	11509.56	13919.98	11956.69	4189.72		x
665	BTB/POZ domain-containing adapter for CUL3-mediated RhoA degradation protein 1	Q8WZ19	0.14	2.53	0.81	1.07	2.77	0.68	0.96	0.36	0.63	0.35	0.75	0.38	2.13	0.97	0.60	4.64	1.07	0.47	2.22	0.19	0.06	30050.20	27805.86	25392.34	16941.29	16090.84	15619.70	28431.67	26664.49	25506.87	19636.52	18444.00	12916.41		x
666	BTB/POZ domain-containing protein 9	Q96Q07	0.14	2.53	0.81	1.07	2.77	0.68	0.96	0.36	0.63	0.35	0.75	0.38	2.13	0.97	0.60	4.64	1.07	0.47	2.22	0.19	0.06	4955.64	4523.69	3818.12	2489.10	2406.69	1646.55	2943.33	2878.48	2775.19	3356.82	2914.01	1716.94		x
667	BTB/POZ domain-containing protein KCTD15	Q96SI1	51456.53	62504.07	59344.87	38038.51	32122.71	40888.30	49187.39	56444.77	46823.60	57838.04	50432.42	38818.30	50429.17	50526.54	35599.86	31757.77	31017.43	34192.60	43908.71	41104.76	39217.48	2.27	1.49	0.65	0.84	1.31	0.69	4.16	2.47	0.11	4.90	4.98	0.12	x	
668	BTB/POZ domain-containing protein KCTD2	Q14681	918.02	706.94	591.16	586.05	531.83	564.84	710.22	671.13	556.16	774.58	675.03	651.23	705.25	647.41	289.72	600.88	512.44	625.61	520.51	596.61	513.64	2.27	1.49	0.65	0.84	1.31	0.69	4.16	2.47	0.11	4.90	4.98	0.12	x	
669	BTB/POZ domain-containing protein KCTD3	Q9Y597	0.14	2.53	0.81	1.07	2.77	0.68	0.96	0.36	0.63	0.35	0.75	0.38	2.13	0.97	0.60	762.05	6.96	1000.24	2.22	0.19	0.06	2.27	1.49	0.65	0.84	1.31	0.69	4.16	2.47	0.11	4.90	4.98	0.12	x	
670	BTB/POZ domain-containing protein KCTD9	Q7L273	33287.67	29140.12	26341.64	19773.54	19778.22	17692.64	21341.37	20475.80	19780.81	25601.40	23586.51	24592.66	24588.06	21886.48	20299.26	19908.28	19267.55	17832.24	18370.39	17636.10	18338.89	11717.00	8633.57	8958.47	5985.33	5296.18	2405.92	11969.74	10397.15	7258.94	10294.99	9775.07	4027.72		
671	BUB3-interacting and GLEBS motif-containing protein ZNF207	O43670	47144.67	46969.20	44864.50	31829.50	31516.96	30871.62	36538.87	34553.01	33105.87	37161.77	36616.35	33901.05	39673.00	38643.63	30418.07	31855.19	29534.47	28616.64	29905.56	30224.19	27842.75	19252.28	17978.35	16346.27	11680.18	11234.30	9787.30	17102.63	15194.61	15062.84	15541.97	13858.52	10421.03		
672	Butyrophilin subfamily 3 member A3	O00478	0.14	2.53	0.81	1.07	2.77	0.68	0.96	0.36	0.63	0.35	0.75	0.38	2.13	0.97	0.60	4.64	1.07	0.47	2.22	0.19	0.06	1063.35	976.20	804.83	560.51	438.99	358.23	672.75	738.97	652.62	603.69	575.37	302.66		x
673	Bystin	Q13895	3482.42	3409.27	3217.98	1575.85	1500.39	1233.35	3141.42	3229.38	2530.70	1868.44	1815.95	1715.12	2726.10	2476.39	1571.02	2250.82	2255.73	1942.44	1885.12	1885.57	1579.84	9709.49	9153.82	8972.26	4626.69	4508.19	3935.44	6670.33	6198.77	5851.72	5527.88	5017.52	2408.63		
674	C-1-tetrahydrofolate synthase_ cytoplasmic	P11586	1796089.41	1755870.84	1646533.02	1154561.70	1116293.80	1028585.57	1428072.29	1365902.69	1313464.94	1354878.02	1287627.95	1237417.99	1418027.31	1327951.07	1100059.92	1142869.54	1081923.73	1034601.90	1169022.11	1119376.74	1051740.97	736753.75	696407.50	640621.47	461881.13	451834.03	411144.92	554175.91	536428.85	515596.74	517828.65	508828.48	338707.56		
675	CAAX box protein 1	O15255	249.77	194.03	181.06	166.69	142.91	124.43	127.24	60.61	48.25	455.26	386.15	405.39	126.02	92.10	30.29	203.34	311.52	177.58	188.01	197.49	174.65	2.27	1.49	0.65	0.84	1.31	0.69	4.16	2.47	0.11	4.90	4.98	0.12	x	
676	CAAX prenyl protease 1 homolog	O75844	4463.11	4058.17	3457.14	2524.93	2429.85	2207.59	3026.28	3167.89	2785.15	3230.39	3248.65	2776.20	3378.47	3049.82	2233.40	3274.53	2786.29	2623.33	2698.00	2737.61	2396.68	2.27	1.49	0.65	0.84	1.31	0.69	4.16	2.47	0.11	4.90	4.98	0.12	x	
677	CAD protein	P27708	467681.03	438022.71	411254.02	318017.62	311429.49	298486.36	370297.54	346760.91	333440.88	395307.80	371525.08	352884.43	390532.01	352918.92	289860.84	311051.45	291482.12	297631.12	334115.56	315009.75	292640.24	159738.50	150320.42	143589.26	107106.47	105126.61	96566.09	126069.75	119934.65	115247.87	127463.21	122170.76	86360.64		
678	Cadherin EGF LAG seven-pass G-type receptor 1	Q9NYQ6	25750.38	21307.09	14718.28	33033.43	29301.20	20743.91	26835.33	19786.50	19105.07	26138.66	22926.37	19304.74	26386.07	27622.50	7510.88	24893.40	24096.22	20651.77	17569.26	27673.65	18762.66	33940.95	34169.42	31836.48	24834.59	25190.13	25325.27	28216.83	27557.97	26880.91	28834.39	27692.25	23417.72		
679	Cadherin EGF LAG seven-pass G-type receptor 3	Q9NYQ7	0.14	2.53	0.81	1.07	2.77	0.68	0.96	0.36	0.63	0.35	0.75	0.38	2.13	0.97	0.60	4.64	1.07	0.47	2.22	0.19	0.06	10181.23	9834.01	8558.16	6261.34	5950.02	4774.66	8618.49	8252.71	7721.05	9578.37	9358.60	5730.18		x
680	Cadherin-1	P12830	78395.86	78148.83	68993.06	49861.71	47718.32	48382.55	61242.92	58706.94	56174.90	65215.80	64016.11	58651.17	64782.11	54343.59	42156.73	52094.44	49996.57	50679.08	55243.26	53962.65	41657.02	2.27	1.49	0.65	0.84	1.31	0.69	4.16	2.47	0.11	4.90	4.98	0.12	x	
681	Cadherin-18	Q13634	2043.63	1408.04	1846.56	1.07	2.77	0.68	56.05	47.32	14.03	1930.40	1496.79	965.01	2.13	0.97	0.60	159.50	82.20	81.87	2.22	0.19	0.06	3108.89	417.77	487.79	2592.61	2627.62	1280.15	3248.33	2658.99	1849.21	4271.77	3395.31	2508.09		
682	Cadherin-23	Q9H251	2856.93	2575.76	2602.65	1369.09	1325.19	1176.43	1773.20	1809.68	1747.19	1994.42	1744.61	1842.98	2313.74	2125.13	1593.84	1499.00	1433.23	1450.27	1218.60	1187.19	1202.83	2.27	1.49	0.65	0.84	1.31	0.69	4.16	2.47	0.11	4.90	4.98	0.12	x	
683	Cadherin-5	P33151	5804.32	5334.31	4662.86	4029.60	3915.80	3375.18	4318.97	3880.03	3670.91	5096.85	4789.49	4518.87	4929.29	4530.42	3353.82	3876.84	3712.61	3468.74	4432.96	4310.43	4244.65	2.27	1.49	0.65	0.84	1.31	0.69	4.16	2.47	0.11	4.90	4.98	0.12	x	
684	Cadherin-9	Q9ULB4	5021.85	4753.21	3658.87	2834.16	2409.29	2103.09	4033.69	3881.03	3718.26	4410.07	3919.42	3510.96	3457.00	3069.26	1281.73	4184.06	3974.31	3719.98	2126.22	2137.10	1598.68	2.27	1.49	0.65	0.84	1.31	0.69	4.16	2.47	0.11	4.90	4.98	0.12	x	
685	Calcineurin B homologous protein 1	Q99653	810.14	535.60	274.45	278.59	299.15	173.70	293.42	250.90	242.28	674.18	570.00	338.62	645.95	324.52	65.98	253.62	193.47	189.93	348.97	333.41	192.85	2.27	1.49	0.65	0.84	1.31	0.69	4.16	2.47	0.11	4.90	4.98	0.12	x	
686	Calcineurin B homologous protein 2	O43745	0.14	2.53	0.81	1.07	2.77	0.68	0.96	0.36	0.63	0.35	0.75	0.38	2.13	0.97	0.60	4.64	1.07	0.47	2.22	0.19	0.06	11811.06	12204.69	11307.43	8057.87	7774.56	7275.39	9631.22	9223.06	9237.81	9881.20	8796.61	602.78		x
687	Calcineurin subunit B type 1	P63098	25751.12	24006.54	21433.60	14650.88	12848.28	13377.12	16626.76	15393.00	16290.12	18719.60	17891.71	15367.35	19386.43	16888.45	14936.94	13715.39	13840.28	13771.51	17440.00	15438.60	14511.02	2.27	1.49	0.65	0.84	1.31	0.69	4.16	2.47	0.11	4.90	4.98	0.12	x	
688	Calcineurin-binding protein cabin-1	Q9Y6J0	3047.82	3402.34	2946.81	2480.89	2337.91	2159.39	2466.59	2315.21	1904.73	2868.56	2921.03	2933.07	3122.89	2978.61	2621.63	2768.55	2315.44	2262.38	2616.79	2567.65	2632.97	2.27	1.49	0.65	0.84	1.31	0.69	4.16	2.47	0.11	4.90	4.98	0.12	x	
689	Calcium and integrin-binding protein 1	Q99828	1402.18	1663.22	1282.75	1723.60	1753.68	1521.56	1691.71	1687.55	1581.05	1930.02	1975.34	2038.37	2153.42	1792.28	1561.07	1849.25	1884.21	1954.21	1836.86	1765.28	1712.44	2.27	1.49	0.65	0.84	1.31	0.69	4.16	2.47	0.11	4.90	4.98	0.12	x	
690	Calcium load-activated calcium channel	Q9UM00	0.14	2.53	0.81	1.07	2.77	0.68	0.96	0.36	0.63	0.35	0.75	0.38	2.13	0.97	0.60	4.64	1.07	0.47	2.22	0.19	0.06	20765.72	19245.02	18505.27	13012.57	12737.21	11578.06	15858.86	15267.63	13591.73	16096.58	15396.82	9884.00		x
691	Calcium uniporter regulatory subunit MCUb_ mitochondrial	Q9NWR8	0.14	2.53	0.81	1.07	2.77	0.68	0.96	0.36	0.63	0.35	0.75	0.38	2.13	0.97	0.60	4.64	1.07	0.47	2.22	0.19	0.06	23712.91	23226.64	20770.86	25291.85	24912.80	23629.48	27544.56	27093.62	26035.98	33834.40	32685.57	25273.42		x
692	Calcium uptake protein 2_ mitochondrial	Q8IYU8	7341.96	5744.84	4485.46	3927.57	3863.33	3311.22	5852.23	5425.09	5004.75	5698.68	4795.25	4807.04	5599.67	5186.76	3249.07	4505.59	3996.87	4041.39	4280.89	4555.10	3743.06	2.27	1.49	0.65	0.84	1.31	0.69	4.16	2.47	0.11	4.90	4.98	0.12	x	
693	Calcium/calmodulin-dependent 3'_5'-cyclic nucleotide phosphodiesterase 1B	Q01064	0.14	2.53	0.81	1.07	2.77	0.68	0.96	0.36	0.63	0.35	0.75	0.38	2.13	0.97	0.60	4.64	1.07	0.47	2.22	0.19	0.06	8799.24	8893.83	8096.10	5335.96	5673.66	4451.67	6351.75	6928.77	5912.18	7093.38	6729.47	4605.52		x
694	Calcium/calmodulin-dependent protein kinase type 1	Q14012	0.14	2.53	0.81	1.07	2.77	0.68	0.96	0.36	0.63	0.35	0.75	0.38	2.13	0.97	0.60	4.64	1.07	0.47	2.22	0.19	0.06	776.56	599.05	730.45	478.83	435.78	171.67	635.73	464.65	396.00	884.93	794.16	313.76		x
695	Calcium/calmodulin-dependent protein kinase type II subunit beta	Q13554	0.14	2.53	0.81	1.07	2.77	0.68	0.96	0.36	0.63	0.35	0.75	0.38	2.13	0.97	0.60	4.64	1.07	0.47	2.22	0.19	0.06	12867.92	11450.73	11187.27	8920.04	8018.99	7491.23	10723.19	10088.25	9480.48	11110.63	10799.88	6090.88		x
696	Calcium/calmodulin-dependent protein kinase type II subunit delta	Q13557	28413.54	27509.94	25900.65	16862.79	17026.30	15574.06	21586.51	21873.23	18395.89	23169.14	21571.48	19559.76	23108.55	21249.95	15713.58	19245.07	18550.89	16445.06	17537.64	15659.47	14224.78	5765.46	4859.43	4642.92	3984.77	4198.61	3012.86	5878.85	5279.16	4726.10	5124.13	4882.83	3052.01		
697	Calcium/calmodulin-dependent protein kinase type II subunit gamma	Q13555	26777.70	22318.85	17709.60	13533.51	12314.83	10449.94	18832.09	16692.81	16549.29	18797.46	17991.02	15073.57	18858.94	14265.99	8988.24	15508.99	13390.60	11723.06	14690.60	13230.47	9124.10	2.27	1.49	0.65	0.84	1.31	0.69	4.16	2.47	0.11	4.90	4.98	0.12	x	
698	Calcium-binding mitochondrial carrier protein Aralar1	O75746	0.14	2.53	0.81	1.07	2.77	0.68	0.96	0.36	0.63	0.35	0.75	0.38	2.13	0.97	0.60	4.64	1.07	0.47	2.22	0.19	0.06	10105.25	10462.56	9772.89	6784.91	6986.17	7225.43	8244.11	8316.89	7491.08	8440.09	7707.68	5386.38		x
699	Calcium-binding mitochondrial carrier protein Aralar2	Q9UJS0	144079.27	137556.15	130324.31	101187.54	100354.25	93405.59	111034.82	107802.23	102650.37	141369.17	137846.92	132097.55	114178.16	110403.52	86290.65	107173.02	99693.42	96329.32	95163.55	94324.66	87899.41	5288.23	5034.12	4486.56	2690.81	2618.47	1955.72	3983.39	3710.73	3266.62	3843.47	3458.00	1880.45		
700	Calcium-binding mitochondrial carrier protein SCaMC-1	Q6NUK1	530046.87	533395.98	491856.80	356387.86	337267.57	320689.31	399655.82	388568.53	363834.48	457365.38	441039.15	412903.27	422542.65	402019.81	314304.00	331378.35	323023.78	312278.49	362044.05	339800.76	314160.77	44327.68	44161.33	41096.73	26639.47	26611.61	22572.99	35168.51	33529.59	31093.16	35088.59	33826.23	22218.18		
701	Calcium-binding protein 1	Q9NZU7	395.58	186.20	173.82	135.91	84.86	82.44	128.04	8.27	115.57	394.95	409.25	275.66	2.13	0.97	0.60	56.85	54.46	61.38	169.09	117.77	76.18	6507.24	5615.32	6182.76	3614.25	3225.68	2990.25	5767.12	5885.14	5299.49	4284.17	4102.61	2763.61		
702	Calcium-binding protein 2	Q9NPB3	532.10	656.03	613.30	183.11	169.32	150.41	483.45	405.40	365.24	215.69	291.74	328.88	215.71	253.12	179.44	346.90	346.34	349.14	283.06	247.22	252.21	2.27	1.49	0.65	0.84	1.31	0.69	4.16	2.47	0.11	4.90	4.98	0.12	x	
703	Calcium-binding protein 39	Q9Y376	0.14	2.53	0.81	1.07	2.77	0.68	0.96	0.36	0.63	0.35	0.75	0.38	2.13	0.97	0.60	4.64	1.07	0.47	2.22	0.19	0.06	2174.85	2462.51	2188.73	2151.32	2245.72	1904.03	2207.32	2261.40	2158.08	2031.74	2246.23	1895.07		x
704	Calcium-binding protein 39-like	Q9H9S4	8088.43	8461.32	7443.40	6128.10	5835.03	5200.44	8120.71	7287.05	6417.20	6839.20	6637.25	5741.72	7949.58	5908.78	5541.22	6807.44	5934.84	5908.49	6123.35	6948.64	7636.63	2.27	1.49	0.65	0.84	1.31	0.69	4.16	2.47	0.11	4.90	4.98	0.12	x	
705	Calcium-binding protein 5	Q9NP86	40074.45	40405.94	33190.34	24450.21	24419.03	21879.36	27319.94	26335.29	25368.87	33666.43	34465.32	28861.65	27082.77	26688.83	17494.41	27786.36	25810.47	27840.93	24876.72	22623.36	22273.34	53615.32	53069.02	46854.40	32363.85	31820.44	26166.05	44762.46	41993.24	39886.90	44485.81	42827.81	26188.14		
706	Calcium-dependent secretion activator 1	Q9ULU8	49253.76	46551.98	44053.38	34095.69	31229.44	29350.10	37391.29	37871.09	35121.42	40694.94	40327.11	36833.52	43009.34	40319.95	30424.10	30560.79	30121.92	28236.38	33776.46	31564.24	28660.58	24626.57	21897.59	20346.40	15484.86	15909.23	13483.71	16916.31	16878.60	15747.35	17943.92	16468.37	9484.25		
707	Calcium-regulated heat-stable protein 1	Q9Y2V2	15605.74	15834.40	13275.26	10967.64	13078.24	12178.36	18507.49	15101.47	12985.90	13611.89	12867.40	13117.62	12417.82	11161.65	11914.80	12415.01	12210.73	12032.22	11837.30	11533.94	10583.95	2.27	1.49	0.65	0.84	1.31	0.69	4.16	2.47	0.11	4.90	4.98	0.12	x	
708	Calcyclin-binding protein	Q9HB71	480071.59	454532.59	420352.39	303153.42	296181.65	275072.04	376847.31	357474.87	343991.59	372179.01	348158.59	331683.25	371125.47	361611.38	304160.31	316477.68	295695.95	281399.37	329317.07	312141.69	300636.60	287884.64	261826.37	234024.91	182636.85	176095.43	161289.61	218480.34	212126.22	195682.02	222740.78	213384.79	137059.50		
709	Calcyphosin-2	Q9BXY5	3818.05	3468.88	3417.94	2780.73	2768.57	2685.97	3012.93	3075.94	2821.35	4270.62	4237.73	4271.11	3714.68	3170.59	2769.52	2876.23	2533.77	2771.41	3002.23	2875.17	2626.88	2.27	1.49	0.65	0.84	1.31	0.69	4.16	2.47	0.11	4.90	4.98	0.12	x	
710	Calcyphosin-like protein	Q8WWF8	3787.97	2787.27	1652.68	219.10	88.35	5.39	108.03	107.26	62.35	2023.88	1649.24	1551.63	2055.08	455.57	10.51	101.81	53.43	49.84	818.78	339.36	189.33	2.27	1.49	0.65	0.84	1.31	0.69	4.16	2.47	0.11	4.90	4.98	0.12	x	
711	Caldesmon	Q05682	5298.05	5102.83	5182.94	3348.68	3064.18	2866.71	4502.17	4295.54	3779.52	4656.49	4451.09	3963.68	4781.00	4449.59	3220.32	3373.07	3566.03	3064.14	4135.74	4125.49	3651.38	40009.69	30504.67	28639.12	25044.83	26028.72	18098.48	33302.75	30164.71	26119.87	37066.71	34417.98	23085.15		
712	Calicin	Q13939	22316.61	23215.79	20048.03	13261.29	13738.21	12463.84	16255.86	14852.72	14388.32	21969.64	20007.79	19310.02	22239.68	6318.30	3908.58	13783.90	12051.27	12135.40	15227.09	14804.26	14705.85	2.27	1.49	0.65	0.84	1.31	0.69	4.16	2.47	0.11	4.90	4.98	0.12	x	
713	Calmodulin-1	P0DP23	1418003.32	1354626.90	1289704.48	947096.70	897935.18	839067.07	1080301.00	1055737.93	1001629.35	1141904.81	1112006.28	1002704.52	1175596.84	1084781.30	898600.49	1018920.00	957840.97	930548.24	975087.86	911586.14	859055.30	1033337.31	971000.38	875457.69	636821.94	633757.45	555331.12	861001.11	809349.12	764245.55	864052.20	838891.45	572592.93		
714	Calmodulin-like protein 3	P27482	57544.64	55109.44	50091.51	34889.14	35484.89	32603.97	49237.16	43675.03	46164.54	46632.48	45650.35	37791.79	44684.86	41934.19	32694.68	38629.85	36124.97	34867.10	35330.42	33912.08	30975.15	7022.21	7164.39	6074.38	6020.52	5927.94	4090.40	6411.64	5589.28	5034.01	11419.88	10789.46	6668.42		
715	Calmodulin-like protein 5	Q9NZT1	5278.41	5189.02	4575.40	3144.91	2800.40	2775.52	4939.35	4842.12	4388.16	4449.54	3991.34	4008.15	4432.34	3817.58	3293.37	3179.13	3134.74	2699.18	3340.43	3110.12	3103.63	2.27	1.49	0.65	0.84	1.31	0.69	4.16	2.47	0.11	4.90	4.98	0.12	x	
716	Calmodulin-like protein 6	Q8TD86	0.14	2.53	0.81	1.07	2.77	0.68	0.96	0.36	0.63	0.35	0.75	0.38	2.13	0.97	0.60	4.64	1.07	0.47	2.22	0.19	0.06	3779.85	2166.27	1955.98	2766.81	1928.00	734.09	5342.55	4130.08	1586.64	5936.26	5106.83	2129.25		x
717	Calmodulin-regulated spectrin-associated protein 2	Q08AD1	0.14	2.53	0.81	1.07	2.77	0.68	0.96	0.36	0.63	0.35	0.75	0.38	2.13	0.97	0.60	4.64	1.07	0.47	2.22	0.19	0.06	273222.03	256931.63	233554.45	192276.85	191424.36	180237.99	197168.90	197719.26	191795.44	215195.47	209728.50	148420.32		x
718	Calmodulin-regulated spectrin-associated protein 3	Q9P1Y5	0.14	2.53	0.81	1.07	2.77	0.68	0.96	0.36	0.63	0.35	0.75	0.38	2.13	0.97	0.60	4.64	1.07	0.47	2.22	0.19	0.06	1009.07	1066.97	840.64	660.73	730.30	644.21	870.72	930.26	814.02	791.09	897.69	650.72		x
719	Calnexin	P27824	1048097.99	951007.57	857693.08	707919.46	676085.55	585505.78	865728.46	832855.94	757840.82	929477.64	892419.28	787072.33	845869.15	773105.28	584668.88	790727.47	750146.31	704440.51	746935.65	688909.68	610173.34	447372.41	425417.99	373905.67	275059.44	263174.43	217657.80	364290.59	342933.14	317782.03	348795.90	339426.42	186259.75		
720	Calpain small subunit 1	P04632	165962.67	160594.66	147594.35	107248.05	111428.37	100055.09	119813.57	112243.37	105945.34	141691.03	138376.02	130232.82	140234.77	135026.43	106549.10	111974.01	106746.89	98755.36	97901.21	95490.96	86454.48	191209.17	183826.95	174953.39	122903.85	118906.29	109320.93	128990.45	128482.04	125295.40	130181.16	124625.20	91105.06		
721	Calpain small subunit 2	Q96L46	16931.74	14723.10	14716.29	11193.58	10526.15	10394.75	14033.87	13462.57	12465.03	15271.05	14835.49	13526.52	14379.08	13913.01	11207.81	10560.18	9525.61	9432.35	10519.40	10287.42	10015.10	44896.51	40328.03	41065.41	17343.62	16507.53	13462.52	18960.68	18172.14	17172.69	16670.27	17088.23	11918.35		
722	Calpain-1 catalytic subunit	P07384	681606.37	652248.46	577476.43	366940.29	354000.21	319893.98	476738.54	446417.82	422013.28	507317.33	477776.38	438715.54	503402.64	474341.19	360713.62	398835.76	372946.15	349572.32	384133.06	364375.84	339666.99	254290.54	239968.01	218982.83	134485.15	133250.44	107929.01	181331.40	166912.91	152717.07	168425.65	167146.39	100389.62		
723	Calpain-2 catalytic subunit	P17655	0.14	2.53	0.81	1.07	2.77	0.68	0.96	0.36	0.63	0.35	0.75	0.38	2.13	0.97	0.60	4.64	1.07	0.47	2.22	0.19	0.06	370928.69	326492.43	304654.60	272861.25	262306.46	238602.94	342760.23	319929.01	305889.68	297617.99	288050.55	222636.90		x
724	Calpain-7	Q9Y6W3	4218.59	4119.81	4183.01	3680.13	3440.02	2884.76	2486.87	2155.96	2482.79	4395.26	4528.03	4287.77	3445.87	2350.40	1504.04	2403.24	2251.86	2366.33	2341.31	3008.47	3143.03	1417.45	789.69	1285.61	1426.71	1686.39	1413.15	1065.92	1257.04	1301.39	1485.42	1596.00	392.40		
725	Calpain-8	A6NHC0	19243.33	19101.57	16407.29	12548.24	12311.65	11320.58	14173.26	13592.72	12726.10	14791.54	13038.79	12688.90	14017.08	12390.13	10384.42	12247.55	11361.64	11167.88	12601.07	10786.01	10590.83	2.27	1.49	0.65	0.84	1.31	0.69	4.16	2.47	0.11	4.90	4.98	0.12	x	
726	Calpain-9	O14815	0.14	2.53	0.81	1.07	2.77	0.68	0.96	0.36	0.63	0.35	0.75	0.38	2.13	0.97	0.60	4.64	1.07	0.47	2.22	0.19	0.06	2667.08	3078.47	2573.85	2682.47	2383.68	1776.52	2794.35	2600.55	2220.88	2901.83	2624.05	2001.21		x
727	Calpastatin	P20810	205856.25	199207.44	190563.50	155884.83	146370.71	148638.33	174755.44	176297.24	165650.33	161891.71	157279.73	150158.45	176726.87	166087.90	152167.56	172968.47	153347.49	156968.94	157801.28	157549.10	140004.56	127063.28	121754.19	109453.61	90007.70	84110.45	78176.51	106987.55	102617.08	94371.79	106215.39	105798.63	72246.08		
728	Calponin-2	Q99439	45699.91	39078.34	28935.63	31509.93	31372.79	26519.74	32872.44	28913.23	28040.52	41146.92	39617.59	32942.79	29828.73	23589.93	15641.63	35696.88	34947.60	35633.89	36908.67	33751.53	31652.32	237109.24	225640.14	214010.37	127321.61	126846.27	115585.85	164956.92	156405.77	150630.77	163345.29	153527.74	99620.83		
729	Calponin-3	Q15417	0.14	2.53	0.81	1.07	2.77	0.68	0.96	0.36	0.63	0.35	0.75	0.38	2.13	0.97	0.60	4.64	1.07	0.47	2.22	0.19	0.06	160123.05	152835.47	143097.93	108189.03	107599.28	101053.27	122130.87	114709.87	115616.51	119006.50	115519.02	82852.10		x
730	Calreticulin	P27797	1814187.47	1735858.09	1499631.46	1255501.90	1190755.44	1059209.39	1335530.74	1243271.53	1164360.44	1719931.15	1573868.70	1420776.50	1532900.16	1441391.14	1041594.56	1339234.28	1234677.08	1154432.26	1266053.46	1178919.44	1062943.50	472620.75	429560.70	395362.64	317648.22	300615.58	261150.94	368295.99	342611.99	318554.92	390590.45	381313.95	232737.27		
731	Calretinin	P22676	24693.31	23030.08	19931.79	20431.65	20926.12	16757.65	29971.17	28954.54	25625.92	31828.01	29708.69	26573.94	30594.81	29463.68	15059.02	18323.51	17972.35	15892.75	22331.21	20960.36	17017.06	100244.06	97541.08	92663.65	82980.64	85020.90	83957.24	84282.18	86577.80	84874.67	97602.56	88548.37	5233.11		
732	Calumenin	O43852	347069.93	325726.57	306336.09	272184.47	251459.23	232363.86	262873.27	260319.10	236759.99	334446.93	306704.36	287311.06	289362.18	276116.80	209091.50	254276.16	257494.80	241649.48	260700.07	249170.16	227687.61	188151.92	169895.15	159725.83	126127.68	121688.01	105572.48	150952.45	143961.94	133516.61	150427.75	148197.53	102876.13		
733	CaM kinase-like vesicle-associated protein	Q8NCB2	0.14	2.53	0.81	1.07	2.77	0.68	0.96	0.36	0.63	0.35	0.75	0.38	2.13	0.97	0.60	4.64	1.07	0.47	2.22	0.19	0.06	2161.94	2550.97	1914.04	1529.00	1489.66	1442.13	1648.08	1869.24	1647.32	1630.47	1584.08	1196.61		x

Supplementary Table S1. Overview on the abundance of all relatively quantified 5180 proteins

			MCF-7																					MDA-MB-231													
	Protein name	UniProt	control 01	control 02	control 03	Dai IC20 01	Dai IC20 02	Dai IC20 03	Dai SC20 01	Dai SC20 02	Dai SC20 03	Gen IC20 01	Gen IC20 02	Gen IC20 03	Gen SC20 01	Gen SC20 02	Gen SC20 03	SSE IC20 01	SSE IC20 02	SSE IC20 03	SSE SC20 01	SSE SC20 02	SSE SC20 03	control 01	control 02	control 03	Dai IC20 01	Dai IC20 02	Dai IC20 03	Gen IC20 01	Gen IC20 02	Gen IC20 03	SSE IC20 01	SSE IC20 02	SSE IC20 03	unique to MCF-7	unique to MDA-MB-231
734	cAMP-dependent protein kinase catalytic subunit alpha	P17612	44578.91	44232.19	38478.88	29093.57	28812.98	25810.36	33411.61	31780.31	30369.76	38831.26	36152.00	34067.45	36334.01	34635.08	28308.00	24594.08	27303.57	25872.63	28153.81	27634.97	24821.36	2.27	1.49	0.65	0.84	1.31	0.69	4.16	2.47	0.11	4.90	4.98	0.12	x	
735	cAMP-dependent protein kinase catalytic subunit beta	P22694	5115.71	5045.43	4982.13	3841.90	3432.30	3103.08	4665.15	4204.01	4057.51	4867.55	4541.73	4081.57	4793.52	4587.90	3720.26	3613.91	3124.10	3329.32	3718.51	3688.00	3444.99	2.27	1.49	0.65	0.84	1.31	0.69	4.16	2.47	0.11	4.90	4.98	0.12	x	
736	cAMP-dependent protein kinase inhibitor beta	Q9C010	3626.24	2550.14	1851.01	3007.49	2608.89	2176.70	4152.89	3934.73	3406.97	4123.98	3829.43	3395.74	3043.05	2615.77	812.78	3108.53	2944.50	2453.69	3371.48	2998.16	2518.37	2.27	1.49	0.65	0.84	1.31	0.69	4.16	2.47	0.11	4.90	4.98	0.12	x	
737	cAMP-dependent protein kinase type I-alpha regulatory subunit	P10644	70421.21	66174.77	63887.04	54058.99	51929.57	48223.24	58806.50	55537.10	54532.94	62610.88	62452.95	58134.69	64017.02	59464.47	53260.72	49237.01	46551.00	45463.04	50586.59	50045.09	45566.64	2.27	1.49	0.65	0.84	1.31	0.69	4.16	2.47	0.11	4.90	4.98	0.12	x	
738	cAMP-dependent protein kinase type II-alpha regulatory subunit	P13861	5167.27	3959.27	3649.45	4268.01	2509.41	2087.53	4384.17	4758.36	3721.58	2896.59	3073.37	2213.06	5686.21	7402.32	3158.24	6572.45	6514.79	5431.54	2091.48	1875.89	1843.19	28846.86	28438.54	25391.93	22664.97	21322.34	18572.88	26452.97	26159.80	23167.50	25632.65	24341.17	15865.25		
739	cAMP-dependent protein kinase type II-beta regulatory subunit	P31323	30769.20	29540.95	24955.76	38425.28	36900.42	33622.36	37014.17	34638.21	33290.01	48901.51	49096.77	48352.52	38760.67	38146.53	31405.21	32286.79	30963.31	27371.79	30850.36	30665.17	31414.18	27614.64	27276.46	23639.55	25502.68	25461.05	23943.85	23002.65	23694.46	23154.87	27004.78	26408.95	20216.91		
740	cAMP-regulated phosphoprotein 19	P56211	93033.22	83371.32	84296.69	53120.53	47593.80	46415.93	63925.84	62681.77	57683.15	67120.70	64215.11	56537.27	64396.73	56307.14	48790.62	56139.55	52914.89	49687.95	55404.57	51731.91	50440.49	2.27	1.49	0.65	0.84	1.31	0.69	4.16	2.47	0.11	4.90	4.98	0.12	x	
741	cAMP-specific 3'_5'-cyclic phosphodiesterase 4A	P27815	273.94	372.52	219.96	228.06	675.91	120.40	53.35	68.04	9.01	162.47	134.52	200.19	313.91	2054.44	55.56	91.46	261.51	229.04	295.39	139.66	171.08	1476.66	1669.57	1056.50	1201.20	1079.37	1127.18	1083.70	1085.09	1062.29	1126.74	1113.20	881.76		
742	Cannabinoid receptor 2	P34972	0.14	2.53	0.81	1.07	2.77	0.68	0.96	0.36	0.63	0.35	0.75	0.38	2.13	0.97	0.60	4.64	1.07	0.47	2.22	0.19	0.06	42732.07	40303.37	35223.19	32659.61	33859.87	24332.78	41461.15	40707.36	35005.33	40898.88	38364.74	24127.86		x
743	CAP-Gly domain-containing linker protein 1	P30622	105239.23	104487.97	97631.96	89721.76	85785.77	81884.06	90588.04	85084.78	82999.85	104461.27	100201.75	93384.56	94124.43	92152.39	74122.33	89417.06	82765.58	80086.61	80044.94	75841.35	71514.00	93636.16	80470.26	76596.87	55484.88	57890.69	48179.67	69435.95	66184.77	61909.85	76466.29	71141.57	50939.21		
744	CAP-Gly domain-containing linker protein 2	Q9UDT6	63813.06	61912.36	59714.97	42729.38	39353.80	37174.26	46401.93	46379.14	47002.83	50087.61	49561.86	45234.88	48287.92	50362.28	43061.71	41972.99	40839.14	37711.51	41155.30	41851.58	41182.21	52324.95	49241.70	44700.71	25564.87	25776.75	18782.74	29478.67	29457.68	25420.77	32693.83	29382.04	17798.05		
745	Caprin-1	Q14444	228707.20	220717.88	208528.25	157731.89	150220.57	146552.69	173932.97	167178.28	165083.88	180761.38	173634.85	163898.95	175310.80	174276.49	148459.26	145415.61	132003.52	132151.17	162552.71	151795.73	145234.26	221593.58	206744.31	193510.56	145347.82	142773.91	128698.51	169549.65	162432.62	156382.69	157783.62	154612.34	116055.74		
746	Caprin-2	Q6IMN6	13760.11	12888.88	12120.34	9778.51	10616.25	9774.62	11538.15	11004.41	10250.49	13644.36	12948.19	12848.77	14133.09	12876.03	10371.77	9394.65	8815.08	8900.94	10501.58	10091.34	9196.48	26545.17	25268.23	23712.49	20651.25	20273.86	19002.15	28853.86	29094.52	29773.88	25481.66	24650.60	18332.33		
747	Carbamoyl-phosphate synthase [ammonia]_ mitochondrial	P31327	3609.32	2991.43	2771.25	1705.46	1495.54	1486.77	1473.93	1458.69	1455.27	2388.59	2405.28	2332.81	2469.15	2448.31	2108.98	1636.60	1472.68	1837.43	2082.21	1573.16	1465.58	2.27	1.49	0.65	0.84	1.31	0.69	4.16	2.47	0.11	4.90	4.98	0.12	x	
748	Carbohydrate sulfotransferase 1	O43916	869.97	1989.44	1609.90	1326.22	1368.65	1211.99	1257.20	1066.41	893.40	2170.52	1783.21	1725.64	1046.71	594.38	1001.03	1188.66	1143.91	1020.73	1516.64	1469.24	1448.27	15510.07	15085.91	15593.08	10191.50	9938.75	9400.68	10826.54	10214.63	10524.71	13094.42	12080.74	8932.55		
749	Carbohydrate sulfotransferase 12	Q9NRB3	30733.01	29437.59	29244.27	16284.30	15646.17	15295.19	21524.37	20892.15	20431.67	19787.13	19309.96	17151.85	20858.92	19997.94	15204.28	18708.24	17161.82	17044.66	18664.49	17373.04	17050.37	4106.96	3920.27	3400.68	2817.53	2545.70	1916.45	3355.94	3135.67	2851.80	3930.47	3668.05	1903.82		
750	Carbonic anhydrase 12	O43570	7760.02	7686.88	7393.19	6102.41	5852.25	4891.71	7441.40	7451.77	6457.72	8500.10	8105.90	6694.73	6934.52	8627.61	4645.27	5351.25	4844.68	4472.11	8163.11	7761.41	7175.02	2.27	1.49	0.65	0.84	1.31	0.69	4.16	2.47	0.11	4.90	4.98	0.12	x	
751	Carbonyl reductase [NADPH] 1	P16152	369796.88	367517.20	336300.81	275828.48	273763.08	248748.20	309844.02	301253.44	274190.78	341535.01	324744.10	316428.67	312445.53	293732.16	237492.16	266192.49	255205.13	251465.44	278225.89	266898.69	252552.30	51112.73	47840.70	44632.19	32606.61	31069.61	25756.05	37991.15	36882.48	33333.41	40495.89	37410.89	25117.47		
752	Carbonyl reductase [NADPH] 3	O75828	20429.00	19707.93	17224.89	16885.56	16092.63	15588.78	20112.44	19141.57	18049.51	19780.76	19339.19	17707.65	21512.02	18442.24	12987.63	17447.62	16202.17	16076.63	18377.23	17439.47	15621.59	74857.74	67434.55	64418.92	44879.19	44349.78	39867.81	53519.72	53323.54	49570.81	57008.97	55190.14	38809.14		
753	Carbonyl reductase family member 4	Q8N4T8	0.14	2.53	0.81	1.07	2.77	0.68	0.96	0.36	0.63	0.35	0.75	0.38	2.13	0.97	0.60	4.64	1.07	0.47	2.22	0.19	0.06	2334.97	2242.14	1953.36	1344.89	1369.74	1210.80	1463.25	1601.54	1474.41	1310.46	1199.29	896.87		x
754	Carboxylesterase 3	Q6UWW	790.98	890.08	565.23	617.69	593.35	561.68	545.49	487.70	339.30	880.56	1115.58	996.80	702.79	644.19	535.97	499.23	424.51	510.31	645.02	626.40	565.57	2.27	1.49	0.65	0.84	1.31	0.69	4.16	2.47	0.11	4.90	4.98	0.12	x	
755	Carboxymethylenebutenolidase homolog	Q96DG6	0.14	2.53	0.81	1.07	2.77	0.68	0.96	0.36	0.63	0.35	0.75	0.38	2.13	0.97	0.60	4.64	1.07	0.47	2.22	0.19	0.06	16312.26	15723.88	16872.53	10840.04	11135.82	9803.00	11008.42	11153.64	10669.61	9990.61	11148.77	9216.19		x
756	Carboxy-terminal domain RNA polymerase II polypeptide A small phosphatase 1	Q9GZU7	41987.99	39808.56	37668.51	30436.98	29338.79	28448.90	31412.17	29874.57	30514.30	36786.59	35756.04	34591.34	36684.07	34694.41	29123.20	31939.67	32265.94	30206.18	34274.89	30364.27	29160.10	2.27	1.49	0.65	0.84	1.31	0.69	4.16	2.47	0.11	4.90	4.98	0.12	x	
757	Carnitine O-palmitoyltransferase 1 liver isoform	P50416	267038.63	266858.73	254453.45	174296.52	166604.01	159423.77	185889.60	180990.18	176836.88	202393.60	193128.57	178388.76	206854.43	201752.33	171148.28	162438.62	153069.13	153182.61	167020.62	156690.39	153309.58	117685.27	110911.52	107913.23	88340.41	83569.17	78980.71	90372.02	87068.09	85968.76	93568.23	91562.01	71058.65		
758	Carnitine O-palmitoyltransferase 2_ mitochondrial	P23786	51394.23	53434.71	50627.47	34461.61	33309.02	32412.41	44819.03	42376.48	42753.65	41881.80	38433.51	37396.62	40447.88	38988.45	33655.54	35554.71	34162.41	34450.17	43197.17	35582.59	34131.46	2.27	1.49	0.65	0.84	1.31	0.69	4.16	2.47	0.11	4.90	4.98	0.12	x	
759	Cartilage oligomeric matrix protein	P49747	90390.88	90327.00	80752.92	64217.41	59265.24	52059.90	81452.13	76676.43	70305.07	88756.55	87494.08	80621.88	71529.58	72537.46	52915.93	67185.90	63900.24	60582.80	58032.07	56034.50	51441.60	383439.98	354662.02	308747.97	227018.05	212407.36	185968.55	249208.83	236837.64	217001.10	273049.45	267584.31	170183.90		
760	Cartilage-associated protein	O75718	9160.38	8601.41	8036.12	7006.32	6371.80	6329.81	8676.81	8071.12	7670.68	9604.41	8817.82	8221.32	8491.14	8145.11	6807.02	6599.49	6334.70	6848.67	6894.37	6694.54	6493.20	35780.30	32871.08	31635.31	19282.09	17294.43	15528.47	28160.21	25659.66	25280.39	26320.65	24943.04	16042.23		
761	Casein kinase I isoform alpha	P48729	4165.25	4209.37	4200.90	2026.78	2122.63	1634.46	3068.24	3233.53	2703.95	2508.93	2479.75	2391.43	2806.16	2876.77	1867.80	2429.14	2258.54	2141.55	2433.58	2376.52	2052.46	810.25	849.47	672.62	602.27	426.77	326.15	579.45	613.62	559.37	439.92	374.77	269.95		
762	Casein kinase II subunit alpha 3	Q8NEV1	348867.51	412590.50	379641.73	291660.58	275406.67	252962.29	287647.91	281797.80	259381.62	382100.19	357125.52	330070.32	369551.67	289515.70	234380.85	261950.63	240104.71	237656.82	288106.23	278729.01	276474.48	2.27	1.49	0.65	0.84	1.31	0.69	4.16	2.47	0.11	4.90	4.98	0.12	x	
763	Casein kinase II subunit alpha	P68400	0.14	2.53	0.81	1.07	2.77	0.68	0.96	0.36	0.63	0.35	0.75	0.38	2.13	0.97	0.60	4.64	1.07	0.47	2.22	0.19	0.06	48060.92	41383.30	35652.81	27477.27	25787.34	20497.21	41462.84	39346.76	34742.40	37749.75	35463.34	22871.65		x
764	Casein kinase II subunit alpha'	P19784	13183.79	11266.08	10730.36	7666.57	7326.52	6260.43	9266.10	10295.90	8768.14	10582.92	9417.78	8218.89	10527.65	9967.15	6438.96	8219.27	7237.72	6792.53	8645.68	7061.53	6403.94	52300.77	48770.03	45534.45	31673.84	29125.63	25843.25	37939.11	35095.56	34557.27	37280.98	34527.68	24132.58		
765	Casein kinase II subunit beta	P67870	0.14	2.53	0.81	1.07	2.77	0.68	0.96	0.36	0.63	0.35	0.75	0.38	2.13	0.97	0.60	4.64	1.07	0.47	2.22	0.19	0.06	24851.85	23757.23	22547.01	10837.04	10527.03	9954.66	10177.50	9547.50	9854.06	9958.00	9977.24	4416.44		x
766	Caseinolytic peptidase B protein homolog	Q9H078	98053.35	93173.93	90914.89	71060.10	68131.13	65474.13	80022.51	76433.90	74266.98	88516.10	84716.28	79295.09	81083.50	78077.06	65244.34	69786.15	66424.97	63813.96	70940.01	67074.52	64304.23	2.27	1.49	0.65	0.84	1.31	0.69	4.16	2.47	0.11	4.90	4.98	0.12	x	
767	CASP8 and FADD-like apoptosis regulator	O15519	0.14	2.53	0.81	1.07	2.77	0.68	0.96	0.36	0.63	0.35	0.75	0.38	2.13	0.97	0.60	4.64	1.07	0.47	2.22	0.19	0.06	28128.50	30088.99	28851.97	17603.73	16887.61	13645.80	22449.81	22514.78	19578.04	21911.28	19602.91	12564.90		x
768	CASP8-associated protein 2	Q9UKL3	156544.63	147580.79	137514.57	116268.83	113764.45	107753.98	137184.70	125762.21	119159.91	143578.32	132498.21	122953.92	133285.56	136957.62	101946.57	117486.36	112854.01	111240.63	116053.59	110908.81	100843.95	53276.07	50610.50	45179.53	34153.82	33830.57	25205.14	37837.07	36726.50	32984.10	37733.39	35186.72	21625.97		
769	Caspase recruitment domain-containing protein 11	Q9BXL7	30594.16	24041.80	20242.92	9090.88	10695.42	8677.35	10310.64	10825.22	8817.44	20810.62	19482.12	16156.66	20037.47	11153.93	7892.42	9983.49	8620.20	7576.27	10122.66	9446.55	8201.48	2.27	1.49	0.65	0.84	1.31	0.69	4.16	2.47	0.11	4.90	4.98	0.12	x	
770	Caspase-3	P42574	0.14	2.53	0.81	1.07	2.77	0.68	0.96	0.36	0.63	0.35	0.75	0.38	2.13	0.97	0.60	4.64	1.07	0.47	2.22	0.19	0.06	2615.74	2285.92	2395.67	447.30	387.69	257.24	344.09	259.56	301.75	277.06	224.24	69.09		x
771	Caspase-6	P55212	32187.98	27629.19	31882.05	3708.09	2796.97	2341.63	4619.93	3833.83	3979.51	29759.77	25327.57	18981.59	13826.60	5783.26	2599.07	3236.16	3116.81	3237.93	3406.94	3292.60	2898.78	2.27	1.49	0.65	0.84	1.31	0.69	4.16	2.47	0.11	4.90	4.98	8964.74	x	
772	Caspase-7	P55210	5439.15	5652.36	4882.70	3317.16	3241.26	3077.71	3526.64	3394.41	2893.97	4112.59	3728.46	4041.02	4217.96	3811.01	3339.88	3206.42	3458.64	3310.29	3768.39	3218.56	3016.05	2.27	1.49	0.65	0.84	1.31	0.69	4.16	2.47	0.11	4.90	4.98	0.12	x	
773	Caspase-8	Q14790	1941.19	3550.81	3344.14	2451.40	2937.67	2317.28	2469.51	2677.06	2328.50	3149.07	3213.44	3524.15	2498.45	3277.60	3099.26	2485.94	2238.46	2356.49	2733.25	2727.99	1930.85	2.27	1.49	0.65	0.84	1.31	0.69	4.16	2.47	0.11	4.90	4.98	0.12	x	
774	Catalase	P04040	68725.34	65539.52	58141.36	52390.44	50368.48	46166.06	57291.32	56022.53	52567.83	59916.96	58935.21	56639.78	53706.23	59579.01	40564.74	58513.84	55707.00	51458.13	50389.93	41440.60	42772.11	39739.79	36461.00	31108.38	24498.93	23601.42	20805.89	26146.06	25028.14	23174.98	31316.16	29027.05	19260.17		
775	Catechol O-methyltransferase	P21964	223327.09	208785.01	185053.99	105541.40	97967.66	89894.82	122904.58	114399.61	107712.88	151884.02	144382.86	127042.62	170423.65	142125.62	106161.67	111823.18	103456.85	102976.44	102269.40	92517.05	85282.94	10204.13	9297.81	9694.56	4208.59	3945.11	2863.11	4684.21	5917.46	3830.48	5558.47	7039.63	3057.01		
776	Catenin alpha-1	P35221	327211.09	309811.08	287658.81	203284.71	196815.35	184039.43	236256.16	230788.79	217449.77	259424.31	246297.70	234860.59	256315.04	235944.06	188832.58	209617.36	197872.31	192123.02	203964.68	199971.09	185519.62	126032.03	113590.04	107418.74	86855.09	83517.29	71286.08	106354.14	100858.43	99670.36	105275.90	103374.02	71417.85		
777	Catenin alpha-2	P26232	128595.60	122441.63	112145.58	86899.38	83736.76	81121.92	96448.17	94970.58	91778.86	101827.31	99720.70	94118.97	99892.56	97376.25	82682.57	82680.28	80766.25	78205.73	88808.28	89795.21	81864.18	59399.80	59181.97	57992.31	33818.37	35672.80	34249.42	36306.66	37505.70	37042.16	36771.16	35921.94	26819.53		
778	Catenin beta-1	P35222	81568.41	81639.16	71179.92	57926.28	52878.39	53970.90	60758.34	63336.17	60968.57	72218.82	69713.82	63429.51	60731.34	60830.66	49227.98	59392.08	55964.44	56537.58	59595.13	56107.22	53983.07	5113.20	5063.60	4857.92	3115.69	3414.66	3017.88	3894.37	3857.17	3384.21	4342.35	4336.86	4858.37		
779	Catenin delta-1	O60716	259482.78	252549.77	239449.06	192490.64	184138.08	171489.44	198260.60	183631.74	178653.87	222167.42	214509.62	204745.75	207204.98	204068.81	173111.49	187202.84	179082.32	173197.95	181559.96	166824.94	154491.19	75741.38	71260.90	71443.50	59865.25	56721.41	49453.11	60200.81	54476.12	56733.28	62999.20	60484.78	44567.85		
780	Catenin delta-2	Q9UQB3	29030.68	25100.81	23964.05	20192.93	19447.61	17222.47	19686.18	19809.97	18378.26	22033.83	21577.44	19326.48	24936.66	21906.51	16600.09	18515.80	18030.74	18489.06	22509.00	20396.77	19206.71	2.27	1.49	0.65	0.84	1.31	0.69	4.16	2.47	0.11	4.90	4.98	0.12	x	
781	Cathepsin B	P07858	0.14	2.53	0.81	1.07	2.77	0.68	0.96	0.36	0.63	0.35	0.75	0.38	2.13	0.97	0.60	4.64	1.07	0.47	2.22	0.19	0.06	5015.16	3778.62	4442.53	1456.85	1456.75	1181.37	624.79	677.44	572.94	813.56	649.87	324.74		x
782	Cathepsin D	P07339	507230.49	497485.73	477813.29	281606.90	285981.90	269117.30	459161.05	445432.63	422946.29	505258.31	479864.91	460013.99	409216.61	385704.30	337710.55	280002.45	271036.20	260059.54	346087.66	331852.70	326664.30	39649.69	36801.45	32462.61	16886.75	16166.82	13847.82	20557.06	20383.59	19267.66	22467.08	22213.02	15632.23		
783	Cathepsin L1	P07711	0.14	2.53	0.81	1.07	2.77	0.68	0.96	0.36	0.63	0.35	0.75	0.38	2.13	0.97	0.60	4.64	1.07	0.47	2.22	0.19	0.06	10836.35	9643.98	8178.63	2571.40	2560.29	1434.91	3262.50	3018.87	2350.03	4233.86	3916.76	1882.18		x
784	Cathepsin L2	O60911	399.14	405.01	270.47	191.10	178.93	176.30	447.51	393.31	300.42	242.23	242.00	225.44	309.73	441.19	183.39	252.42	325.15	174.96	270.58	180.20	156.92	546.78	734.33	606.03	982.13	1295.48	872.42	1193.28	1303.22	954.47	1814.12	1701.85	975.60		
785	Cathepsin Z	Q9UBR2	0.14	2.53	0.81	1.07	2.77	0.68	0.96	0.36	0.63	0.35	0.75	0.38	2.13	0.97	0.60	4.64	1.07	0.47	2.22	0.19	0.06	62549.72	58541.66	58489.59	43252.29	44373.58	38319.96	53112.67	49169.73	46799.83	51200.06	48326.99	36633.77		x
786	Cation-dependent mannose-6-phosphate receptor	P20645	10410.37	8187.43	10572.19	6313.27	6124.82	5896.68	7558.75	6506.46	6837.83	8449.46	8009.36	8276.94	8597.39	8180.83	6674.75	5996.27	5707.38	5606.28	5777.72	6043.15	5818.18	9976.16	9842.85	9332.32	5729.55	5863.18	5054.59	6479.44	6730.21	6377.46	6383.46	6037.97	4028.42		
787	Cationic amino acid transporter 2	P52569	5538.32	5823.33	5365.87	6986.46	6896.94	6658.47	6413.78	6309.17	5886.70	7772.05	8165.94	7794.63	6801.72	5417.07	4511.93	6891.87	5407.00	5990.80	6934.70	5895.26	6000.92	2.27	1.49	0.65	0.84	1.31	0.69	4.16	2.47	0.11	4.90	4.98	0.12	x	
788	Cation-independent mannose-6-phosphate receptor	P11717	0.14	2.53	0.81	1.07	2.77	0.68	0.96	0.36	0.63	0.35	0.75	0.38	2.13	0.97	0.60	4.64	1.07	0.47	2.22	0.19	0.06	16951.23	15760.96	14978.93	18919.97	17111.63	15602.57	29507.68	25081.88	26471.32	18603.71	16780.28	14835.27		x
789	Caveolae-associated protein 1	Q6NZI2	0.14	2.53	0.81	1.07	2.77	0.68	0.96	0.36	0.63	0.35	0.75	0.38	2.13	0.97	0.60	4.64	1.07	0.47	2.22	0.19	0.06	94844.53	87673.48	86722.73	59325.62	57607.18	47869.28	70908.60	64689.95	64385.00	78273.08	76040.32	48133.85		x
790	Caveolin-1	Q03135	0.14	2.53	0.81	1.07	2.77	0.68	0.96	0.36	0.63	0.35	0.75	0.38	2.13	0.97	0.60	4.64	1.07	0.47	2.22	0.19	0.06	99000.01	92088.17	79002.27	66875.09	61371.06	54070.14	79132.12	74371.61	64117.34	88634.77	76225.93	50384.97		x
791	Caveolin-2	P51636	0.14	2.53	0.81	1.07	2.77	0.68	0.96	0.36	0.63	0.35	0.75	0.38	2.13	0.97	0.60	4.64	1.07	0.47	2.22	0.19	0.06	413.11	319.43	359.88	932.11	893.40	827.19	919.36	1012.71	720.32	1240.09	648.56	143.41		x
792	Caveolin-3	P56539	0.14	2.53	0.81	1.07	2.77	0.68	0.96	0.36	0.63	0.35	0.75	0.38	2.13	0.97	0.60	4.64	1.07	0.47	2.22	0.19	0.06	6468.95	6356.27	5699.62	3702.79	3459.45	2617.43	5042.98	4596.24	4325.58	4864.49	4524.57	2868.39		x
793	CCA tRNA nucleotidyltransferase 1_ mitochondrial	Q96Q11	34470.75	33520.03	31759.22	27674.79	27001.64	26491.28	31158.56	27639.29	27352.58	26492.82	24753.17	24275.83	29938.13	29607.79	29242.70	24092.14	24878.60	26706.71	24601.51	25872.46	24030.96	2.27	1.49	0.65	0.84	1.31	0.69	4.16	2.47	0.11	4.90	4.98	0.12	x	
794	CCR4-NOT transcription complex subunit 1	A5YKK6	22146.62	17707.80	12501.16	5128.10	4757.08	4623.75	7049.73	6483.15	5609.56	12652.20	11001.89	8626.22	14172.00	6179.55	5027.51	4954.58	4726.11	4561.50	5814.28	5423.63	3897.15	11604.91	11683.25	10458.15	8000.19	8032.80	7062.85	9587.55	9109.73	9672.54	9309.61	8978.97	6398.89		
795	CCR4-NOT transcription complex subunit 10	Q9H9A5	0.14	2.53	0.81	1.07	2.77	0.68	0.96	0.36	0.63	0.35	0.75	0.38	2.13	0.97	0.60	4.64	1.07	0.47	2.22	0.19	0.06	1457.52	1427.63	1419.20	845.19	739.81	522.80	992.79	921.23	928.06	1185.70	1145.09	512.94		x
796	CCR4-NOT transcription complex subunit 2	Q9NZN8	10553.39	10034.55	9748.87	6264.57	6704.45	5818.12	7131.04	7112.24	6843.38	7459.03	7222.07	6864.02	8614.81	7829.17	7067.01	6077.99	5778.60	5397.48	7037.62	6834.69	5791.73	2.27	1.49	0.65	0.84	1.31	0.69	4.16	2.47	0.11	4.90	4.98	0.12	x	
797	CCR4-NOT transcription complex subunit 3	O75175	41491.89	41118.97	35461.75	24751.88	24456.37	21726.49	28238.05	26135.52	25867.21	35790.67	34077.89	31742.82	32358.02	28201.65	18493.03	24020.02	22052.36	21779.73	27138.73	24594.91	22695.21	581.99	1106.84	614.07	767.13	706.20	404.13	861.80	830.90	799.55	650.31	708.22	557.50		
798	CD151 antigen	P48509	0.14	2.53	0.81	1.07	2.77	0.68	0.96	0.36	0.63	0.35	0.75	0.38	2.13	0.97	0.60	4.64	1.07	0.47	2.22	0.19	0.06	11582.65	11283.01	11707.70	7122.63	6877.11	5996.05	8413.41	8738.46	8554.23	7638.74	7225.24	5812.79		x
799	CD164 sialomucin-like 2 protein	Q6UWJ8	27770.30	27563.83	24847.44	21932.57	21036.09	19769.93	22052.63	22123.65	19358.64	23124.57	22463.98	21607.52	24840.28	25259.85	22105.30	20583.26	19476.33	18296.80	22042.98	20516.37	20708.43	2.27	1.49	0.65	0.84	1.31	0.69	4.16	2.47	0.11	4.90	4.98	0.12	x	
800	CD166 antigen	Q13740	0.14	2.53	0.81	1.07	2.77	0.68	0.96	0.36	0.63	0.35	0.75	0.38	2.13	0.97	0.60	4.64	1.07	0.47	2.22	0.19	0.06	2282.08	2161.75	1928.82	1447.63	1657.66	1627.26	1570.42	1433.58	1511.81	4270.01	4138.02	913.62		x
801	CD2 antigen cytoplasmic tail-binding protein 2	O95400	93938.46	89486.70	80355.02	66285.44	65319.86	61474.31	70851.87	69552.64	63913.85	81278.91	76975.05	73749.44	74166.04	72508.71	65616.53	62704.82	57579.49	56803.94	72160.21	66584.34	64202.39	8543.03	7680.62	7070.93	3718.55	3492.65	2911.33	4702.87	5217.30	4019.35	4826.88	5441.38	2860.63		
802	CD2-associated protein	Q9Y5K6	32923.40	29616.92	28997.22	20712.35	20322.47	17140.81	22862.89	19779.12	20281.97	24445.42	24086.32	22410.57	24077.74	22772.33	18626.75	21323.89	19231.55	19203.93	20331.83	19921.98	16762.36	18666.61	15774.39	14868.66	9426.92	10853.18	8529.32	12731.73	12791.69	13419.62	15020.09	13675.54	13873.51		
803	CD44 antigen	P16070	0.14	2.53	0.81	1.07	2.77	0.68	0.96	0.36	0.63	0.35	0.75	0.38	2.13	0.97	0.60	4.64	1.07	0.47	2.22	0.19	0.06	1022280.64	941646.30	888454.70	726497.55	723448.34	605980.15	799136.11	754573.87	711646.09	872445.03	861547.53	641479.93		x
804	CD5 antigen-like	O43866	0.14	2.53	0.81	1.07	2.77	0.68	0.96	0.36	0.63	0.35	0.75	0.38	2.13	0.97	0.60	4.64	1.07	0.47	2.22	0.19	0.06	71069.73	74589.56	60794.48	25649.65	25527.91	18559.81	27004.33	26545.47	26588.16	25614.93	25146.40	15998.13		x
805	CD59 glycoprotein	P13987	0.14	2.53	0.81	1.07	2.77	0.68	0.96	0.36	0.63	0.35	0.75	0.38	2.13	0.97	0.60	4.64	1.07	0.47	2.22	0.19	0.06	10014.11	9642.63	9006.56	11545.21	11227.65	10379.37	11732.12	10970.36	10818.96	14476.03	14800.05	9111.25		x
806	CD63 antigen	P08962	21733.33	22451.90	19843.13	16803.41	16015.49	15243.26	18330.22	17824.40	16428.00	22111.40	20627.40	20089.17	18611.17	18107.04	15965.09	15123.27	14822.91	13842.54	14727.92	14139.57	13751.38	2.27	1.49	0.65	0.84	1.31	0.69	4.16	2.47	0.11	4.90	4.98	0.12	x	
807	CD9 antigen	P21926	179232.49	174796.54	157595.78	111891.03	114557.02	109470.16	120302.22	113938.83	111527.02	137868.46	131369.65	128390.82	139107.55	138399.67	125250.05	112282.30	104687.56	104363.18	106172.34	111627.76	100444.43	46378.94	39909.03	39291.96	31286.62	31720.32	27040.17	36326.50	36602.83	35057.88	40036.50	37311.30	25615.32		
808	CD97 antigen	P48960	2975.42	2870.75	2814.70	2701.03	2941.59	2694.13	2296.74	2246.64	2126.07	3483.93	3292.61	3242.70	3000.49	2722.09	2677.77	2674.22	2429.83	2430.47	2227.18	2162.94	2073.74	2706.70	2486.65	2605.69	1665.70	1493.96	1427.20	2058.81	2003.52	1914.47	2473.45	2390.54	327.95		
809	CD99 antigen	P14209	23866.49	23432.59	22457.65	19883.75	19102.65	17473.34	18484.51	16927.74	18714.37	23815.91	23104.36	24894.53	23559.65	20331.42	17749.13	14386.88	16820.42	12888.93	16558.45	15862.15	16485.58	3940.55	7553.22	3637.56	5112.63	5274.02	5973.88	2439.45	3129.68	3022.71	5630.88	4656.30	7579.37		
810	CD99 antigen-like protein 2	Q8TCZ2	1624.38	1362.12	1132.75	1058.63	956.06	840.21	1622.35	1470.95	1365.16	1277.05	1269.25	1294.01	1532.75	1434.36	1009.92	1165.14	1045.37	1075.76	1296.80	1348.38	790.59	2.27	1.49	0.65	0.84	1.31	0.69	4.16	2.47	0.11	4.90	4.98	0.12	x	
811	Cdc42 effector protein 4	Q9H3Q1	22086.63	21205.13	19880.26	9644.09	10125.25	8981.21	12293.05	12338.10	11444.47	12099.65	11260.71	10625.53	13401.71	12815.00	10137.02	10659.72	9587.52	9270.84	13282.28	12340.67	11173.76	321.17	305.05	84.10	95.26	83.39	51.85	252.11	360.36	93.39	149.84	212.64	128.92		
812	Cdc42-interacting protein 4	Q15642	0.14	2.53	0.81	1.07	2.77	0.68	0.96	0.36	0.63	0.35	0.75	0.38	2.13	0.97	0.60	4.64	1.07	0.47	2.22	0.19	0.06	7825.25	7324.30	6418.99	5135.67	4909.38	3832.39	6290.90	5763.96	4954.11	6851.19	6838.97	4595.78		x
813	CDGSH iron-sulfur domain-containing protein 1	Q9NZ45	11827.56	12277.68	11842.64	6654.21	6652.56	6190.45	9458.24	9315.02	8350.58	8614.59	8753.82	7760.52	8999.82	9087.36	7433.05	7811.93	7757.47	6818.52	7429.21	7207.78	6760.50	2.27	1.49	0.65	0.84	1.31	0.69	4.16	2.47	0.11	4.90	4.98	0.12	x	
814	CDGSH iron-sulfur domain-containing protein 2	Q8N5K1	68153.82	70593.71	66487.88	45175.80	45464.06	41260.60	54968.21	52094.73	51446.75	55565.31	54311.72	49920.49	53601.36	49551.24	38580.47	46703.00	44074.99	43420.85	42865.51	41276.45	41370.08	8506.77	7813.56	6360.20	5070.57	4437.00	4114.46	5686.67	6552.99	5378.29	6163.80	6370.39	3978.49		
815	CDK5 and ABL1 enzyme substrate 1	Q8TDN4	472.36	205.40	207.83	107.55	114.27	124.38	246.64	27.51	109.32	173.35	206.54	264.95	274.24	37.75	15.45	193.50	134.61	134.65	61.79	59.76	97.41	725.62	693.75	1075.90	326.83	301.49	68.19	710.94	495.30	255.73	525.75	461.99	85.02		
816	CDK5 regulatory subunit-associated protein 3	Q96JB5	47864.52	43873.74	42786.10	35019.78	33794.31	32633.74	35624.92	33673.60	33153.05	44064.42	41739.15	38795.14	39381.74	38654.93	32064.51	34390.08	31534.25	30656.03	29474.27	28871.65	27025.19	5758.57	5180.19	1695.89	3180.19	3380.43	2093.23	3951.10	3312.76	3009.52	3611.01	3389.52	1885.73		
817	CDK-activating kinase assembly factor MAT1	P51948	561.82	559.29	545.02	442.39	468.59	392.63	683.45	750.42	697.96	532.40	495.38	677.32	561.61	572.54	304.71	537.87	485.09	421.52	552.83	532.09	514.04	2.27	1.49	0.65	0.84	1.31	0.69	4.16	2.47	0.11	4.90	4.98	0.12	x	
818	CDKN2A-interacting protein	Q9NXV6	1536.14	1569.82	1434.46	1417.07	1438.35	1496.37	1300.22	1294.92	1116.60	2149.09	2268.29	2101.92	1653.39	1330.49	1077.50	1617.06	1478.62	1487.68	1784.55	1462.99	1533.66	1512.82	1319.83	1205.91	976.32	1046.81	958.21	1441.52	1185.89	1234.36	1436.24	1386.46	665.78		
819	Cell cycle and apoptosis regulator protein 2	Q8N163	81192.07	81331.07	74325.58	57308.99	50759.76	43817.60	59246.48	57486.31	53451.81	58995.05	58773.29	53418.54	61287.08	53299.76	47929.74	55201.65	47228.93	46246.35	48166.99	48139.52	46964.33	25253.08	24895.81	23983.47	18464.17	17459.85	16558.41	18443.94	17591.52	18712.65	21173.68	20136.72	16665.30		
820	Cell cycle checkpoint control protein RAD9A	Q99638	2902.29	2479.34	2439.14	1644.29	1986.15	1876.12	1851.42	2098.18	1825.85	2449.48	2165.59	2316.10	2445.58	1989.19	1788.22	1573.57	1512.70	1532.63	1686.53	1838.61	1544.68	2.27	1.49	0.65	0.84	1.31	0.69	4.16	2.47	0.11	4.90	4.98	0.12	x	
821	Cell cycle progression protein 1	Q9ULG6	3504.87	3780.00	3296.40	2425.96	2473.51	2263.50	2966.74	2574.36	2912.58	4207.39	4136.36	3240.77	2947.10	3614.84	2499.75	2886.58	2425.90	2073.89	2264.22	2657.08	2811.00	9346.73	8970.23	8266.69	5394.45	5261.35	4167.16	6725.11	6801.92	6335.20	5274.16	5363.31	3279.34		
822	Cell death activator CIDE-B	Q9UHD4	2038.58	1838.61	1609.02	2422.01	2369.25	2100.75	2173.37	2167.15	2101.71	3195.02	3026.01	3100.99	2565.81	2725.56	2138.22	2085.84	1902.32	1956.77	2105.21	2284.08	2231.95	2.27	1.49	0.65	0.84	1.31	0.69	4.16	2.47	0.11	4.90	4.98	0.12	x	
823	Cell division control protein 42 homolog	P60953	62923.96	62798.38	58662.75	42111.15	57837.35	48973.12	78117.48	71889.95	66532.58	53284.35	50303.33	50915.82	53290.76	50650.98	40876.02	54447.90	47468.41	46968.83	37841.28	37190.31	36577.04	146923.89	145708.09	126308.41	80770.89	82441.80	82889.90	71110.24	73091.77	66690.05	100610.16	89326.93	67447.96		
824	Cell division cycle 5-like protein	Q99459	76511.41	73727.18	63313.96	44984.85	44937.52	42239.84	52039.46	54168.59	51519.78	64216.99	59245.64	56024.22	50466.12	53313.53	55988.69	56433.34	57601.01	51826.30	45549.45	46103.33	44881.86	42635.00	37787.11	37521.46	32306.69	29635.17	24203.90	34744.32	33583.27	31547.97	42048.59	37305.01	24418.89		
825	Cell division cycle and apoptosis regulator protein 1	Q8IX12	28770.27	32582.44	27937.56	26303.09	24859.37	24436.65	26425.30	26698.32	26138.41	27032.10	28643.18	24056.20	32386.68	12162.76	9298.00	24995.56	20565.41	22260.84	30114.61	26010.86	23479.98	2.27	1.49	0.65	0.84	1.31	0.69	4.16	2.47	0.11	4.90	4.98	0.12	x	
826	Cell division cycle protein 123 homolog	O75794	31795.40	31264.40	28038.87	24860.78	23669.59	23212.96	24571.88	23797.36	22460.98	33670.65	32140.27	32759.37	26485.66	24325.85	21392.03	28383.41	26583.26	24362.26	25952.68	25500.66	25500.74	29437.81	26139.47	21166.14	20148.60	18216.48	16350.39	26291.52	22872.97	22985.02	23953.07	24096.13	16992.18		
827	Cell division cycle protein 20 homolog B	Q86Y33	25877.33	24082.60	24473.05	16955.48	16414.15	16474.37	18968.89	17919.23	17659.52	21169.16	20951.80	18860.02	20773.65	19109.61	18371.86	16727.30	15228.46	14891.65	17472.24	16581.75	14954.81	2.27	1.49	0.65	0.84	1.31	0.69	4.16	2.47	0.11	4.90	4.98	0.12	x	
828	Cell division cycle protein 23 homolog	Q9UJX2	0.14	2.53	0.81	1.07	2.77	0.68	0.96	0.36	0.63	0.35	0.75	0.38	2.13	0.97	0.60	4.64	1.07	0.47	2.22	0.19	0.06	9523.43	8397.23	8720.17	7090.72	7223.62	6576.55	8823.94	8046.78	7489.91	7514.02	8445.37	6594.20		x
829	Cell division cycle protein 27 homolog	P30260	0.14	2.53	0.81	1.07	2.77	0.68	0.96	0.36	0.63	0.35	0.75	0.38	2.13	0.97	0.60	4.64	1.07	0.47	2.22	0.19	0.06	911.72	883.82	885.21	838.88	819.18	736.64	885.19	848.37	717.37	1019.64	889.00	417.54		x
830	Cell division cycle-associated 7-like protein	Q96GN5	0.14	2.53	0.81	1.07	2.77	0.68	0.96	0.36	0.63	0.35	0.75	0.38	2.13	0.97	0.60	4.64	1.07	0.47	2.22	0.19	0.06	4448.86	4512.62	3824.61	2210.69	2383.16	2432.07	2380.69	2467.48	2278.62	2442.33	2243.97	1872.93		x
831	Cell division cycle-associated protein 2	Q69YH5	81020.23	78655.10	73839.33	55009.02	53023.83	47559.29	58598.47	56016.19	54351.66	70593.17	68447.26	60452.45	65869.63	64611.10	44098.25	47728.64	54710.42	49349.60	55618.36	52798.01	52967.92	48216.40	45956.95	43827.93	33597.12	33047.10	24998.33	40477.97	39886.11	34737.98	45711.69	41895.03	30724.83		
832	Cell division cycle-associated protein 7	Q9BWT1	1690.34	1429.16	1496.80	1600.85	1301.73	1248.41	1840.04	1717.51	1672.48	1821.38	1729.29	1480.34	1893.37	1461.65	1046.69	1113.77	1192.64	1146.54	1344.50	1425.15	1323.67	2.27	1.49	0.65	0.84	1.31	0.69	4.16	2.47	0.11	4.90	4.98	0.12	x	
833	Cell growth regulator with EF hand domain protein 1	Q99674	250.00	336.58	300.51	111.77	162.91	119.81	144.03	182.77	173.87	234.36	174.69	214.85	188.67	260.37	155.20	97.94	67.99	105.68	148.86	146.88	167.58	2.27	1.49	0.65	0.84	1.31	0.69	4.16	2.47	0.11	4.90	4.98	0.12	x	
834	Cell growth-regulating nucleolar protein	Q9NX58	0.14	2.53	0.81	1.07	2.77	0.68	0.96	0.36	0.63	0.35	0.75	0.38	2.13	0.97	0.60	4.64	1.07	0.47	2.22	0.19	0.06	2733.14	2348.44	2134.10	1192.79	1156.42	921.87	1353.53	2026.38	1692.41	2822.41	2611.65	1470.74		x
835	Cell surface glycoprotein MUC18	P43121	0.14	2.53	0.81	1.07	2.77	0.68	0.96	0.36	0.63	0.35	0.75	0.38	2.13	0.97	0.60	4.64	1.07	0.47	2.22	0.19	0.06	134350.96	131078.04	123133.24	93194.97	94709.59	84678.77	105714.46	109880.56	109615.85	110151.13	107366.29	74221.90		x
836	Cellular nucleic acid-binding protein	P62633	257751.00	249000.85	238444.18	167346.03	167174.20	155266.94	178645.02	173174.98	169302.74	184908.59	179885.74	170573.05	205082.04	193925.95	129634.80	123839.95	142168.19	141044.12	177583.98	167365.60	159916.58	189737.00	180950.45	172499.94	111949.60	112195.03	104442.75	120985.92	118797.83	116778.56	134066.75	130514.74	74816.79		
837	Cellular retinoic acid-binding protein 1	P29762	63905.19	64177.57	63143.11	37354.60	35470.15	34100.56	45859.80	45913.51	43906.16	48832.21	47245.86	44394.47	50123.58	46493.58	39629.42	43215.24	41942.46	40142.43	41254.45	38678.67	37318.52	2.27	1.49	0.65	0.84	1.31	0.69	4.16	2.47	0.11	4.90	4.98	0.12	x	
838	Cellular retinoic acid-binding protein 2	P29373	805738.10	792461.12	756272.16	478440.01	459397.35	447837.01	578912.37	552677.30	540366.95	589180.75	563590.47	544027.05	616390.77	587768.58	517251.16	469650.06	443728.95	440988.73	451228.91	433925.85	408903.90	18763.28	16703.20	16476.45	12691.09	12329.35	12478.30	13766.18	12684.02	12470.01	13296.45	13414.54	11409.11		
839	Centrin-1	Q12798	233.74	202.47	145.17	19.08	12.15	1.34	15.57	55.80	26.12	157.30	214.62	161.06	269.58	75.95	1.87	19.09	8.35	0.94	139.07	16.47	0.12	89801.23	78079.66	66780.39	28104.01	26679.45	20254.52	27013.45	24567.65	21337.34	26930.91	25401.13	11165.58		
840	Centrin-2	P41208	0.14	2.53	0.81	1.07	2.77	0.68	0.96	0.36	0.63	0.35	0.75	0.38	2.13	0.97	0.60	4.64	1.07	0.47	2.22	0.19	0.06	4557.64	4326.76	4346.20	3659.75	3493.83	3337.85	3483.17	3355.19	3394.69	4288.91	3960.49	3280.71		x
841	Centriolin	Q7Z7A1	34059.84	34392.41	30177.47	29605.31	27480.79	25423.28	29189.05	29631.22	28200.23	41780.99	40591.40	38541.46	31459.87	30375.95	25566.27	26595.29	26110.07	24284.57	26764.88	26468.65	24844.48	68180.67	64015.47	57912.86	58916.69	59198.20	51658.78	103580.54	109932.21	103558.61	64548.82	66439.71	49585.79		
842	Centromere protein F	P49454	0.14	2.53	0.81	1.07	2.77	0.68	0.96	0.36	0.63	0.35	0.75	0.38	2.13	0.97	0.60	4.64	1.07	0.47	2.22	0.19	0.06	330031.44	312548.27	308286.94	246863.63	236053.27	222033.46	262521.96	244022.38	235986.26	285959.94	275183.50	187702.91		x
843	Centromere protein J	Q9HC77	0.14	2.53	0.81	1.07	2.77	0.68	0.96	0.36	0.63	0.35	0.75	0.38	2.13	0.97	0.60	4.64	1.07	0.47	2.22	0.19	0.06	11370.97	10141.09	8401.34	4471.67	4724.66	4594.98	7586.03	6502.67	6810.36	7164.10	6321.71	4481.37		x
844	Centromere protein R	Q13352	152473.44	153592.12	130623.16	113017.62	107164.19	93495.18	145302.00	139428.76	127911.37	130103.92	120771.28	109624.91	137024.03	132385.82	97714.52	128352.88	121045.09	110263.88	118280.48	115744.90	103172.11	2.27	1.49	0.65	0.84	1.31	0.69	4.16	2.47	0.11	4.90	4.98	0.12	x	
845	Centromere protein V	Q7Z7K6	0.14	2.53	0.81	1.07	2.77	0.68	0.96	0.36	0.63	0.35	0.75	0.38	2.13	0.97	0.60	4.64	1.07	0.47	2.22	0.19	0.06	12345.52	11734.47	9921.88	9742.42	8934.02	6841.46	9213.80	8742.78	8231.65	11124.66	10259.57	6544.34		x
846	Centromere protein X	A8MT69	35387.22	35109.91	27794.53	24633.43	26446.00	21760.20	29806.46	27584.80	25494.65	41209.49	39136.85	37910.07	35632.63	33168.26	24262.15	24614.62	23747.41	21280.51	25298.90	26460.38	22814.51	2.27	1.49	0.65	0.84	1.31	0.69	4.16	2.47	0.11	4.90	4.98	0.12	x	
847	Centromere/kinetochore protein zw10 homolog	O43264	10435.20	9993.79	8585.77	6411.38	6216.46	5252.97	7244.48	6310.36	5994.73	9108.23	8993.58	7690.12	8302.11	6620.61	3828.34	6415.38	6252.82	5959.10	6255.38	5387.76	4849.59	4081.84	2284.42	2771.54	2168.60	2207.47	1461.36	3247.03	3074.33	2191.34	3238.46	3115.20	1567.77		
848	Centromere-associated protein E	Q02224	192591.68	189872.18	172773.56	142880.30	133183.61	121099.87	168509.18	163509.95	153662.05	166298.85	161707.81	146895.50	160764.06	150620.87	121230.52	146317.83	138279.72	130876.42	136556.57	135075.00	123575.05	147731.86	146036.17	129282.03	93811.42	92920.75	85497.88	123118.36	117470.26	110502.31	126996.31	123392.67	86421.95		
849	Centrosomal protein of 128 kDa	Q6ZU80	0.14	2.53	0.81	1.07	2.77	0.68	0.96	0.36	0.63	0.35	0.75	0.38	2.13	0.97	0.60	4.64	1.07	0.47	2.22	0.19	0.06	11522.76	9671.51	8316.34	6463.35	6935.18	6148.18	7799.03	6866.78	6682.78	8409.72	8135.34	4651.51		x
850	Centrosomal protein of 135 kDa	Q66GS9	42818.05	42412.56	39146.37	33996.68	32628.24	32321.80	34198.82	33595.16	33328.42	41774.46	38649.42	36044.40	38673.23	36414.34	29221.48	29518.53	27726.63	28276.26	29498.25	27259.03	28191.27	131415.64	126993.27	121532.87	59926.21	61442.16	55689.76	87188.09	86870.79	88387.77	86336.12	85035.71	64050.37		
851	Centrosomal protein of 152 kDa	O94986	0.14	2.53	0.81	1.07	2.77	0.68	0.96	0.36	0.63	0.35	0.75	0.38	2.13	0.97	0.60	4.64	1.07	0.47	2.22	0.19	0.06	928.76	742.27	346.31	400.28	418.99	307.40	557.21	620.53	572.27	705.79	547.01	327.47		x
852	Centrosomal protein of 162 kDa	Q5TB80	4108.01	4029.05	3593.11	2687.59	2589.98	2403.12	3064.58	2654.45	2903.22	4218.78	4035.59	3533.91	3286.98	3259.12	2014.38	2906.06	2716.49	2663.42	3024.43	2990.59	2508.03	2.27	1.49	0.65	0.84	1.31	0.69	4.16	2.47	0.11	4.90	4.98	0.12	x	
853	Centrosomal protein of 170 kDa	Q5SW79	16507.74	15547.90	14535.14	10481.54	10966.12	10414.00	15018.78	14608.76	13656.21	13830.19	13793.73	13156.15	16006.20	14508.46	12818.63	11045.84	10642.97	9989.17	11263.54	11014.02	10875.86	37923.03	35833.37	31361.05	27500.26	27135.52	21522.73	31672.15	30414.55	28430.71	37930.62	35277.55	21329.84		
854	Centrosomal protein of 192 kDa	Q8TEP8	48226.07	40486.35	34346.81	29667.65	28960.26	23812.50	34680.72	34318.50	31443.05	37159.26	33732.17	31727.49	33549.72	33905.90	26930.80	28918.91	32239.99	30177.19	33725.72	29443.06	27244.78	2.27	1.49	0.65	0.84	1.31	0.69	4.16	2.47	0.11	4.90	4.98	0.12	x	
855	Centrosomal protein of 290 kDa	O15078	486829.85	473241.19	454221.59	345436.01	342583.88	322702.74	435166.58	427914.27	396477.32	456242.65	430805.68	407770.41	421519.01	366805.52	308011.92	380462.95	348065.85	337466.09	376504.41	351742.98	332993.78	149726.00	136530.46	129210.29	110096.34	104053.90	86044.92	129704.85	123038.28	116752.48	121307.81	114054.87	91086.91		
856	Centrosomal protein of 295 kDa	Q9C0D2	27449.17	22503.34	17686.77	17081.34	15654.34	12738.96	18832.62	17010.23	16222.49	22558.68	21958.63	16887.46	21055.37	20202.90	11832.42	17779.16	15553.46	13333.91	18780.86	15785.18	13937.29	2.27	1.49	0.65	0.84	1.31	0.69	4.16	2.47	0.11	4.90	4.98	0.12	x	
857	Centrosomal protein of 41 kDa	Q9BYV8	78306.94	78980.03	74662.14	53237.21	54599.20	48865.74	63624.29	60830.75	58924.86	69178.26	66616.54	63643.72	63694.89	63161.73	55321.53	56884.88	53499.79	51023.45	53709.96	52905.88	48868.97	5783.29	5130.75	5132.79	3875.28	4020.14	3321.98	4284.30	4319.62	4239.12	4346.55	4390.07	3145.56		
858	Centrosomal protein of 83 kDa	Q9Y592	59445.59	55472.51	52692.72	42705.09	40294.74	38321.60	44582.42	45069.73	43700.43	50312.37	50069.19	48291.62	52815.22	48309.49	39355.36	40250.83	36128.99	38073.64	46380.23	42894.67	39186.02	14062.82	13129.88	12722.69	10946.91	10466.75	10076.09	11564.39	11671.79	11507.90	12922.60	11222.79	5765.57		
859	Centrosomal protein of 85 kDa-like	Q5SZL2	0.14	2.53	0.81	1.07	2.77	0.68	0.96	0.36	0.63	0.35	0.75	0.38	2.13	0.97	0.60	4.64	1.07	0.47	2.22	0.19	0.06	2771.92	2866.61	2517.61	2056.36	1932.12	1055.75	3229.91	2769.10	2321.94	4188.39	4113.40	1465.62		x
860	Centrosomal protein of 95 kDa	Q96GE4	28833.95	30455.44	58946.47	21273.72	21014.70	18021.40	25173.63	23376.94	22389.15	61285.89	59592.99	56830.52	25363.48	35820.89	16122.91	21574.47	21405.46	19744.54	22208.13	20910.96	20120.33	2.27	1.49	0.65	0.84	1.31	0.69	4.16	2.47	0.11	4.90	4.98	0.12	x	
861	Centrosome and spindle pole-associated protein 1	Q1MSJ5	5336.54	4895.46	2779.20	1534.09	1080.22	1591.58	1881.48	1423.97	2349.26	4523.82	4231.99	2938.63	5429.83	1280.62	1074.88	1467.36	993.49	1041.08	1101.10	1746.82	1545.46	2.27	1.49	0.65	0.84	1.31	0.69	4.16	2.47	0.11	4.90	4.98	0.12	x	
862	Centrosome-associated protein 350	Q5VT06	66063.19	59966.12	49097.20	42978.53	41668.84	32033.76	49064.18	48399.74	42174.02	50652.39	46445.90	38755.63	54176.03	49231.86	24659.96	39968.14	38164.51	32844.95	56270.01	52483.65	42024.41	47190.65	48452.86	41543.35	35598.10	35845.97	35231.02	37053.63	35162.14	35917.27	45124.15	46333.70	29492.24		
863	Centrosome-associated protein CEP250	Q9BV73	0.14	2.53	0.81	1.07	2.77	0.68	0.96	0.36	0.63	0.35	0.75	0.38	2.13	0.97	0.60	4.64	1.07	0.47	2.22	0.19	0.06	38.48	29.78	41.49	40.41	41.54	22.12	69.49	52.87	28.17	4.90	4.98	0.12		x
864	Ceramide synthase 2	Q96G23	100435.63	95242.48	90193.54	53804.42	52260.61	47192.19	71064.29	69909.64	64135.35	70066.25	68189.39	64763.57	71188.24	64251.00	54382.92	61280.46	59573.64	57385.33	54459.49	49046.01	46950.21	17432.15	14434.17	15238.08	19939.23	18609.57	16303.59	28518.33	27403.03	25156.72	12688.75	12730.26	8776.27		
865	cGMP-gated cation channel alpha-1	P29973	0.14	2.53	0.81	1.07	2.77	0.68	0.96	0.36	0.63	0.35	0.75	0.38	2.13	0.97	0.60	4.64	1.07	0.47	2.22	0.19	0.06	22468.55	21078.49	18073.71	10001.60	9579.41	8810.10	11207.71	10918.56	10531.68	11900.51	11434.15	6888.55		x

Supplementary Table S1. Overview on the abundance of all relatively quantified 5180 proteins

			MCF-7																				
	Protein name	UniProt	control_01	control_02	control_03	Dai_IC20_01	Dai_IC20_02	Dai_IC20_03	Dai_SC20_01	Dai_SC20_02	Dai_SC20_03	Gen_IC20_01	Gen_IC20_02	Gen_IC20_03	Gen_SC20_01	Gen_SC20_02	Gen_SC20_03	SSE_IC20_01	SSE_IC20_02	SSE_IC20_03	SSE_SC20_01	SSE_SC20_02	SSE_SC20_03
866	Charged multivesicular body protein 1a	Q9HD42	17217.31	16020.91	15024.06	10478.43	10219.24	9847.78	12594.96	12539.55	11273.08	13394.38	13361.33	12104.21	15224.04	12698.70	11235.99	9878.92	9318.87	8968.89	10024.34	9647.37	8750.43
867	Charged multivesicular body protein 2b	Q9UQN3	15248.48	15129.71	13259.73	12681.96	12205.25	11389.15	9920.60	10973.77	10571.46	14950.40	13964.02	12903.70	13960.57	12464.67	10957.03	10549.79	11244.12	9765.57	10789.49	9802.02	8922.95
868	Charged multivesicular body protein 3	Q9Y3E7	4897.38	4721.13	4319.93	3067.25	2751.28	2541.95	2988.93	3210.82	2446.13	3599.36	3430.36	3311.38	3397.77	3175.43	2770.24	2940.59	2592.76	2953.56	2723.15	2644.34	2487.96
869	Charged multivesicular body protein 4a	Q9BY43	85754.71	79602.81	70300.98	65801.93	59996.97	59818.60	66460.90	64460.64	61196.41	75263.65	71629.57	66798.13	72262.85	60593.56	49196.73	61072.51	58671.02	56729.96	66134.47	61976.14	55858.20
870	Charged multivesicular body protein 4b	Q9H444	158278.08	156559.20	142274.54	106947.61	105776.53	99473.69	131213.70	128496.81	118631.66	140356.15	133528.86	128325.53	135870.53	130571.13	114446.07	104017.46	99324.30	97367.11	109526.26	109832.37	100792.59
871	Charged multivesicular body protein 4c	Q96CF2	4078.21	3743.72	3143.20	5286.08	4998.33	4784.88	4575.47	4678.32	4396.49	7176.02	7410.16	7214.68	4571.12	4008.51	3265.30	5303.99	5137.29	5151.42	3665.07	3502.36	3514.47
872	Charged multivesicular body protein 5	Q9NZZ3	68382.03	63536.06	62401.86	44695.81	44035.09	41738.86	49166.24	46882.50	44993.55	55062.50	53194.54	49579.40	59693.15	53878.37	47528.81	43433.14	41674.15	42018.83	47038.15	44061.48	41926.72
873	Charged multivesicular body protein 6	Q96FZ7	1899.77	2483.33	1870.40	1678.90	1730.49	1455.47	1921.92	1752.99	2021.23	2063.94	2182.81	1689.29	2180.75	2084.21	1718.11	1295.17	1371.63	1303.11	1438.23	1311.87	1800.08
874	Chitinase domain-containing protein 1	Q9BWS9	1603.43	2057.60	1547.49	1911.85	1679.28	1675.73	1629.53	1660.10	1421.64	1900.63	2072.70	1884.22	2505.37	1923.87	1784.61	2059.81	1994.77	1842.27	1603.11	1820.36	1207.63
875	Chloride channel CLIC-like protein 1	Q96S66	15053.33	12870.36	13178.95	13032.38	12073.41	11840.57	12975.13	12609.52	11858.46	13327.93	12466.92	13763.25	13434.79	12208.38	9566.72	11315.27	11181.66	10724.11	10748.76	10210.49	10043.20
876	Chloride intracellular channel protein 1	O00299	590170.20	559701.68	520928.91	367882.14	358951.69	341411.02	418878.97	409857.79	392396.74	452388.52	436631.26	416117.71	476364.02	452166.90	403936.84	349861.29	324188.06	331727.21	373759.37	354987.70	335123.34
877	Chloride intracellular channel protein 2	O15247	0.14	2.53	0.81	1.07	2.77	0.68	0.96	0.36	0.63	0.35	0.75	0.38	2.13	0.97	0.60	4.64	1.07	0.47	2.22	0.19	0.06
878	Chloride intracellular channel protein 3	O95833	83628.60	80944.10	72976.12	38749.51	38597.29	33807.15	45088.49	42239.12	39745.61	77222.62	71911.73	66213.09	80357.07	70442.44	52302.73	36650.92	34423.45	31849.30	33036.14	32843.90	29649.30
879	Chloride intracellular channel protein 4	Q9Y696	86261.21	81044.00	79906.92	72869.88	67910.85	61689.91	69153.68	66991.90	62134.67	71421.86	68837.76	64572.64	78655.62	79772.76	70713.79	67323.80	58500.77	60353.23	68057.20	61028.11	60412.97
880	Chloride intracellular channel protein 5	Q9NZA1	1012.53	691.82	422.93	540.21	431.10	285.97	861.18	111.18	713.63	628.19	653.83	442.12	844.83	96.50	73.08	673.24	199.49	367.21	514.50	385.63	227.20
881	Choline O-acetyltransferase	P28329	0.14	2.53	0.81	1.07	2.77	0.68	0.96	0.36	0.63	0.35	0.75	0.38	2.13	0.97	0.60	4.64	1.07	0.47	2.22	0.19	0.06
882	Choline transporter-like protein 1	Q8WWI5	0.14	2.53	0.81	1.07	2.77	0.68	0.96	0.36	0.63	0.35	0.75	0.38	2.13	0.97	0.60	4.64	1.07	0.47	2.22	0.19	0.06
883	Choline-phosphate cytidylyltransferase A	P49585	81935.09	77782.45	71524.60	52480.19	46048.08	42969.23	56338.87	53418.95	51250.36	65929.23	62900.39	56419.83	61844.73	59811.65	42831.45	51123.09	49922.32	46285.95	58033.37	55765.45	52695.78
884	Choline-phosphate cytidylyltransferase B	Q9Y5K3	270683.38	269596.82	247959.94	177032.14	171300.87	155113.98	187048.99	180122.13	168872.33	219156.94	213575.70	199528.89	206636.93	201138.63	167500.82	173539.65	163341.71	152020.14	190246.71	181276.82	170159.44
885	Chondrolectin	Q9H9P2	35697.72	30694.02	28172.26	24765.90	23951.37	22268.13	24244.03	22251.24	24875.52	29141.70	27280.13	26927.73	28569.19	27677.47	21183.14	25673.21	22762.19	21176.32	26304.78	25231.46	24340.33
886	Chromatin accessibility complex protein 1	Q9NRG0	0.14	2.53	0.81	1.07	2.77	0.68	0.96	0.36	0.63	0.35	0.75	0.38	2.13	0.97	0.60	4.64	1.07	0.47	2.22	0.19	0.06
887	Chromatin complexes subunit BAP18	Q8IXM2	0.14	2.53	0.81	1.07	2.77	0.68	0.96	0.36	0.63	0.35	0.75	0.38	2.13	0.97	0.60	4.64	1.07	0.47	2.22	0.19	0.06
888	Chromatin target of PRMT1 protein	Q9Y3Y2	0.14	2.53	0.81	1.07	2.77	0.68	0.96	0.36	0.63	0.35	0.75	0.38	2.13	0.97	0.60	4.64	1.07	0.47	2.22	0.19	0.06
889	Chromobox protein homolog 1	P83916	10711.62	10455.07	10313.26	6879.24	6808.66	6116.68	7943.04	7486.32	7086.61	12008.49	11064.54	9895.01	6835.67	7300.40	4779.65	7923.98	8106.79	8102.64	7911.77	7185.27	6183.37
890	Chromobox protein homolog 3	Q13185	348671.91	340864.90	313018.30	243144.96	236869.43	225297.61	271501.43	253571.58	249461.90	271895.20	257945.80	242785.75	274559.41	265844.03	224442.60	216762.17	213677.88	202078.91	248883.69	235392.29	224159.14
891	Chromobox protein homolog 5	P45973	51211.88	49500.05	46005.31	33429.25	33737.36	28705.22	40285.37	37439.66	36255.15	36263.29	34808.06	32882.71	39203.31	35854.16	27600.43	32584.83	33113.81	30969.40	33238.48	32070.46	29088.45
892	Chromodomain-helicase-DNA-binding protein 1	O14646	7291.00	7641.92	6718.52	5083.47	4947.88	4872.31	4910.56	5153.78	4711.08	7143.70	6423.18	6519.24	5415.24	5349.38	3327.31	5842.63	5297.84	5132.04	5647.38	6514.60	6127.49
893	Chromodomain-helicase-DNA-binding protein 1-like	Q86WJ1	68990.06	64172.71	60511.03	44029.31	42895.58	41936.19	48876.04	48139.28	45430.38	56458.02	56007.97	51592.17	55328.72	48164.70	28965.69	39365.86	44044.69	44040.54	43811.80	43492.93	40621.51
894	Chromodomain-helicase-DNA-binding protein 3	Q12873	0.14	2.53	0.81	1.07	2.77	0.68	0.96	0.36	0.63	0.35	0.75	0.38	2.13	0.97	0.60	4.64	1.07	0.47	2.22	0.19	0.06
895	Chromodomain-helicase-DNA-binding protein 4	Q14839	69752.35	70287.66	64795.56	45087.59	44053.17	43040.61	54938.46	54207.48	54302.84	54628.74	52969.39	49937.61	58020.12	54263.15	46745.25	43751.04	43168.58	43570.52	51052.27	48725.41	46950.64
896	Chromosome-associated kinesin KIF4A	O95239	0.14	2.53	0.81	1.07	2.77	0.68	0.96	0.36	0.63	0.35	0.75	0.38	2.13	0.97	0.60	4.64	1.07	0.47	2.22	0.19	0.06
897	Chromosome-associated kinesin KIF4B	Q2VIQ3	46074.72	42562.24	39251.68	34191.32	33588.07	31852.20	38014.46	36864.15	35051.55	38577.12	36598.01	36104.50	39541.20	39028.97	30733.84	30849.16	27902.51	29074.77	29715.60	29347.69	27188.36
898	Chymotrypsin-C	Q99895	0.14	2.53	0.81	1.07	2.77	0.68	0.96	0.36	0.63	0.35	0.75	0.38	2.13	0.97	0.60	4.64	1.07	0.47	2.22	0.19	0.06
899	Chymotrypsin-like elastase family member 2A	P08217	8798.23	7798.38	7289.88	3828.16	3222.47	2948.86	6482.29	5955.89	5566.95	5361.58	5168.04	4656.95	5920.69	5325.52	3682.42	5036.59	4781.12	4374.72	4362.71	3978.62	3279.10
900	Cilia- and flagella-associated protein 161	Q6P656	0.14	2.53	0.81	1.07	2.77	0.68	0.96	0.36	0.63	0.35	0.75	0.38	2.13	0.97	0.60	4.64	1.07	0.47	2.22	0.19	0.06
901	Cilia- and flagella-associated protein 20	Q9Y6A4	0.14	2.53	0.81	1.07	2.77	0.68	0.96	0.36	0.63	0.35	0.75	0.38	2.13	0.97	0.60	4.64	1.07	0.47	2.22	0.19	0.06
902	Cilia- and flagella-associated protein 298	P57076	12179.44	10245.63	7732.68	6491.69	6197.58	4701.36	7165.80	6913.09	6756.28	10009.07	9604.95	7180.69	8726.22	7245.29	4427.58	7176.57	6155.94	4742.58	7469.96	6260.74	5364.36
903	Cilia- and flagella-associated protein 43	Q8NDM7	0.14	2.53	0.81	1.07	2.77	0.68	0.96	0.36	0.63	0.35	0.75	0.38	2.13	0.97	0.60	4.64	1.07	0.47	2.22	0.19	0.06
904	Cilia- and flagella-associated protein 52	Q8N1V2	6951.13	5931.92	6912.77	4307.49	3771.90	3150.81	6112.16	5395.39	5562.56	5031.18	5291.86	4884.71	5116.09	4641.17	4102.64	4568.38	4413.71	4370.68	4094.37	3700.25	3794.66
905	Cilia- and flagella-associated protein 54	Q96N23	0.14	2.53	0.81	1.07	2.77	0.68	0.96	0.36	0.63	0.35	0.75	0.38	2.13	0.97	0.60	4.64	1.07	0.47	2.22	0.19	0.06
906	Cilia- and flagella-associated protein 58	Q5T655	11148.41	10802.54	10959.79	7172.06	6217.86	6627.42	5648.76	5959.94	6766.65	8291.51	7831.40	7608.00	8868.56	8288.75	6511.05	6244.30	6118.97	5745.08	7334.77	7052.69	6536.89
907	Cilia- and flagella-associated protein 69	A5D8W1	20537.58	19159.74	18423.08	13330.02	12930.87	12270.93	17203.89	16496.93	16712.03	18519.21	18286.47	17237.49	19175.20	16618.45	15567.26	15371.11	14224.46	14279.99	15775.77	14667.30	14968.79
908	Cilia- and flagella-associated protein 74	Q9C0B2	215577.20	199104.28	179349.84	116458.81	115569.71	103266.54	162659.16	155548.43	146790.25	178248.52	162942.42	151206.94	159613.85	146980.40	115366.64	166739.99	162541.24	147658.47	153663.05	145106.46	129584.71
909	Cilia- and flagella-associated protein 91	Q7Z4T9	53831.94	55018.39	51554.11	35176.88	30285.61	34415.36	47018.47	46033.82	45836.86	46542.08	45209.80	41969.75	47913.22	45976.90	36884.11	39167.86	36373.71	35802.79	35918.66	36923.75	33479.86
910	Cilia- and flagella-associated protein 97	Q9P2B7	6258.45	4835.48	2978.61	16808.88	16495.87	11350.75	13314.92	1853.63	1226.18	5302.90	4480.68	3225.12	5383.68	18506.00	3908.89	11729.47	4725.26	9569.47	12683.45	12247.70	9120.09
911	Cingulin	Q9P2M7	136076.41	134512.02	124585.69	95966.75	94054.14	88298.22	106648.26	105128.08	101070.46	121345.11	115021.58	108414.04	111938.75	109706.37	87507.35	96970.57	90684.54	89539.89	95267.52	88092.03	87078.92
912	Citrate synthase_ mitochondrial	O75390	363487.96	344382.30	304296.25	246054.39	242202.95	220443.03	281410.27	286720.88	266237.44	308520.93	298013.98	267207.29	300663.26	286707.47	210777.09	236163.51	234120.21	216446.72	270698.56	250779.93	230770.41
913	C-Jun-amino-terminal kinase-interacting protein 4	O60271	103269.98	102830.64	97152.43	76005.59	75345.01	70005.13	91502.03	85867.14	83990.23	91055.49	86694.24	83053.73	84758.97	83494.88	69791.04	74466.03	72694.03	71935.59	74965.03	71319.31	69116.12
914	Clathrin heavy chain 1	Q00610	3097082.59	2943689.90	2785784.92	1900068.62	1853729.42	1703752.71	2295386.91	2197604.10	2066896.11	2501959.15	2391594.98	2220978.60	2409889.11	2273300.39	1910265.07	2016498.86	1901067.47	1822913.63	1959402.72	1874831.96	1732758.78
915	Clathrin heavy chain 2	P53675	254113.70	250590.71	231944.14	192034.40	179646.01	167798.78	200348.28	195916.32	183515.97	217912.93	212245.13	191614.47	207966.86	183815.10	165972.61	183481.92	161394.63	159640.05	193635.72	174113.55	159810.38
916	Clathrin interactor 1	Q14677	90991.12	86918.56	83245.07	60397.65	56942.43	53197.33	65843.68	62921.13	64167.37	71788.70	70023.60	64345.66	74003.80	65176.29	53688.28	62321.59	54422.08	55271.78	64323.16	61730.53	53793.32
917	Clathrin light chain A	P09496	30569.10	21953.86	27323.59	20766.34	17452.76	17057.99	20509.46	20309.07	24228.35	24469.96	28220.15	28306.83	24511.30	24619.09	20206.24	22573.19	18161.15	17178.38	24579.60	21359.78	16518.44
918	Clathrin light chain B	P09497	106510.95	101627.92	97334.31	85044.79	80276.34	78074.46	81098.67	75902.32	77378.84	92352.65	87656.49	86199.58	96413.94	85859.37	88074.44	80156.03	75718.61	71441.09	78376.30	78546.59	73911.09
919	Claudin-1	O95832	0.14	2.53	0.81	1.07	2.77	0.68	0.96	0.36	0.63	0.35	0.75	0.38	2.13	0.97	0.60	4.64	1.07	0.47	2.22	0.19	0.06
920	Claudin-3	O15551	29008.25	27895.05	25809.37	22796.71	22823.48	21902.18	25012.83	22931.20	22353.74	29170.48	27530.17	27509.34	23642.00	23627.76	21689.30	19555.87	19349.47	18671.95	21903.97	20993.63	19634.37
921	Cleavage and polyadenylation specificity factor subunit 5	O43809	59878.12	57369.48	50055.05	35507.36	33606.78	29748.14	40286.80	37615.81	34492.05	44191.50	40628.22	38126.06	48616.09	43858.89	35309.09	34124.65	31257.21	29241.24	36775.66	33711.42	32607.03
922	Cleavage and polyadenylation specificity factor subunit 6	Q16630	37738.61	35745.54	30808.85	22338.23	21788.52	18977.89	26643.46	27226.53	24052.03	28769.71	27389.26	24218.40	29358.10	26088.75	18578.12	23260.59	22329.14	20615.74	25643.80	24124.44	20444.17
923	Cleavage and polyadenylation specificity factor subunit 7	Q8N684	11094.33	12744.46	11003.20	9724.83	9294.75	8036.61	9043.53	8977.69	7732.01	10734.08	10611.14	9526.76	10122.36	8819.98	7498.87	8642.19	7803.61	7587.59	9368.65	9301.34	8250.80
924	Cleavage stimulation factor subunit 1	Q05048	57076.28	56505.47	51389.91	38272.80	38970.84	34817.28	48925.45	45518.71	42834.95	45121.27	43511.66	40257.06	51493.07	43070.73	42586.85	39353.82	35508.44	35199.50	42329.47	41894.44	42206.82
925	Cleavage stimulation factor subunit 2	P33240	13936.64	11070.36	12071.55	9125.00	9108.50	8294.14	10486.96	10924.67	9693.34	10147.76	10119.04	10246.09	10363.51	9886.55	8577.45	9807.68	8920.80	8973.16	9527.55	8959.94	8613.71
926	Cleft lip and palate transmembrane protein 1	O96005	54411.60	50474.02	44812.39	34649.54	32788.02	31008.66	33957.38	32507.41	31300.51	47908.61	45061.25	42709.65	42149.57	42409.97	34982.49	32113.46	31446.43	31109.78	38791.14	35517.28	32432.37
927	Cleft lip and palate transmembrane protein 1-like protein	Q96KA5	0.14	2.53	0.81	1.07	2.77	0.68	0.96	0.36	0.63	0.35	0.75	0.38	2.13	0.97	0.60	4.64	1.07	0.47	2.22	0.19	0.06
928	CLIP-associating protein 1	Q7Z460	46661.46	47277.68	43792.38	34795.66	35442.13	31831.53	39609.49	38362.02	34139.01	38984.12	36442.04	34971.65	37038.10	36460.24	30591.95	35216.03	32647.26	31386.27	28169.25	30122.33	27937.63
929	CLIP-associating protein 2	O75122	0.14	2.53	0.81	1.07	2.77	0.68	0.96	0.36	0.63	0.35	0.75	0.38	2.13	0.97	0.60	4.64	1.07	0.47	2.22	0.19	0.06
930	Clustered mitochondria protein homolog	O75153	64087.55	63195.39	62666.05	40523.61	39506.81	39119.18	50037.12	45387.48	48060.21	49266.77	47258.71	46194.88	52031.26	50195.32	41943.47	38281.19	35866.61	35637.09	40390.63	38996.69	36923.94
931	Clusterin	P10909	2828.97	2818.97	2657.84	2320.45	2076.99	2177.69	2087.77	2059.69	1954.06	2469.85	2646.60	2377.37	3217.46	2589.81	2296.02	2152.62	2139.93	2369.95	1887.25	1865.63	1699.65
932	Clusterin-associated protein 1	Q96AJ1	2046.72	1824.81	1588.42	1284.29	1219.07	1012.44	1697.98	1675.31	1612.48	1181.65	1305.76	1204.12	1294.41	1055.22	963.59	1430.30	1240.29	1158.57	1090.40	1020.03	970.65
933	CMP-sialic acid transporter	P78382	3255.22	3358.13	3173.09	2173.31	2206.81	2053.05	2156.66	1997.18	1995.62	3135.90	2989.30	2882.21	3219.93	3036.52	2942.77	1809.01	1682.36	1621.43	2243.88	2289.69	1820.26
934	C-myc promoter-binding protein	Q7Z401	25542.97	34810.38	32582.22	8408.49	7594.18	6361.23	6802.72	6963.77	6667.87	21963.48	30606.81	28580.09	21651.81	11759.76	3101.05	4827.63	5767.91	6303.11	19257.63	14036.42	8650.54
935	c-Myc-binding protein	Q99417	8850.26	8650.27	8285.52	6536.92	6147.28	5991.00	5897.62	5478.16	5970.04	8308.48	7678.96	7761.31	7660.86	7403.22	5510.83	5794.78	5218.84	5295.84	6859.71	6132.08	6151.16
936	CNK3/IPCEF1 fusion protein	G9CGD6	0.14	2.53	0.81	1.07	2.77	0.68	0.96	0.36	0.63	0.35	0.75	0.38	10.46	8.53	3.46	4.64	1.07	0.47	2.22	0.19	0.06
937	Coactosin-like protein	Q14019	64607.74	62361.17	64476.46	48726.84	47154.45	45483.58	63062.51	58227.54	53757.24	57053.71	57582.90	51866.26	55302.88	49810.94	43652.55	54587.13	49993.65	48942.22	49718.50	47323.08	43099.67
938	Coagulation factor X	P00742	212801.85	204982.77	200932.49	201557.80	188894.46	184539.24	180346.61	174186.17	176643.42	207820.30	207067.11	198365.12	212801.32	173279.36	130917.87	171109.15	173846.61	169743.73	190001.65	187801.00	188543.36
939	Coatomer subunit alpha	P53621	638750.72	610880.10	592496.69	422214.24	408445.68	394933.76	492308.97	476283.36	464431.39	537603.80	513069.44	492601.47	502523.49	472952.34	402883.80	431569.97	411472.22	398881.07	417942.24	394080.14	373313.99
940	Coatomer subunit beta	P53618	451796.65	455225.67	428065.75	305156.59	295121.54	276353.31	348724.51	341563.87	324980.63	376146.54	358022.24	336761.69	360709.00	336587.72	273355.15	310064.19	294744.08	277170.92	299784.98	286201.40	267325.69
941	Coatomer subunit beta'	P35606	646960.51	622699.08	580625.30	442323.03	419781.70	391368.70	502725.94	475141.01	461699.86	562425.22	532062.00	500931.55	513963.37	503761.13	393818.91	441650.29	416016.82	397474.44	425260.83	398926.25	373242.78
942	Coatomer subunit delta	P48444	688709.68	668791.20	660792.06	525478.22	516514.76	485822.97	571088.50	550678.61	528027.00	647184.79	628562.98	590475.59	588271.11	539505.38	434233.34	519801.42	494953.07	488374.62	517610.10	494876.86	481846.97
943	Coatomer subunit epsilon	O14579	124353.07	115583.83	106806.10	82705.02	79660.41	72425.94	87997.23	85789.49	79266.48	107407.81	102924.09	95790.85	96654.79	92097.63	68139.13	84937.37	82053.06	79034.86	84460.08	80491.72	72624.37
944	Coatomer subunit gamma-1	Q9Y678	408231.36	398457.49	375784.62	274418.03	264549.21	241022.01	324882.48	314938.34	293085.30	344587.29	330271.02	310908.51	329213.94	305884.72	260682.57	272517.97	265481.43	253534.61	284943.43	268186.49	250769.41
945	Coatomer subunit gamma-2	Q9UBF2	250735.23	239028.86	225266.45	187229.08	176556.84	169132.62	210673.76	199947.04	190842.86	220908.92	210492.75	195328.62	209812.19	196637.35	160552.32	195558.40	178168.61	172123.52	178979.23	170659.82	161093.86
946	Coatomer subunit zeta-1	P61923	73761.31	67264.63	58617.13	46352.58	44830.48	41041.67	53459.73	51639.68	46162.95	58246.31	57064.69	50748.63	54459.22	51338.81	37639.06	48195.25	44468.42	43741.74	44808.42	43693.45	41366.43
947	Cofilin-1	P23528	1243040.80	1173189.67	1075669.83	857783.03	828108.72	756245.71	1097469.48	1022738.71	974348.05	1062172.63	1006431.82	941943.78	1066742.67	985424.97	767863.34	837773.24	784902.43	749153.06	823737.55	791084.34	738557.80
948	Cofilin-2	Q9Y281	39981.18	34604.92	28798.87	27270.33	30053.37	24377.13	31086.00	28294.61	26849.23	31205.21	29516.40	26125.76	31466.13	28717.48	22072.97	23541.16	26533.07	26098.24	26168.10	26565.24	27168.31
949	Cohesin subunit SA-2	Q8N3U4	69422.64	83737.89	54477.91	71555.87	73591.10	51097.70	70401.45	67957.49	54944.50	54421.98	53676.28	36338.89	59233.76	101509.50	25027.89	60109.29	57327.44	47849.80	48434.75	48429.78	38565.45
950	Coiled-coil alpha-helical rod protein 1	Q8TD31	0.14	2.53	0.81	1.07	2.77	0.68	0.96	0.36	0.63	0.35	0.75	0.38	2.13	0.97	0.60	4.64	1.07	0.47	2.22	0.19	0.06
951	Coiled-coil and C2 domain-containing protein 1A	Q6P1N0	52822.70	50763.34	51026.13	40499.98	38491.27	36638.00	43690.94	42923.93	41082.34	44020.40	43691.04	41082.82	44189.69	43979.87	38564.57	36361.43	35364.32	32912.41	40422.70	38798.13	37558.09
952	Coiled-coil domain-containing glutamate-rich protein 2	I3L3R5	0.14	2.53	0.81	1.07	2.77	0.68	0.96	0.36	0.63	0.35	0.75	0.38	2.13	0.97	0.60	4.64	1.07	0.47	2.22	0.19	0.06
953	Coiled-coil domain-containing protein 102A	Q96A19	2513.85	2089.99	2478.56	2477.02	2204.88	1810.96	1311.71	677.28	1392.23	4713.28	4740.86	5237.16	2853.77	969.70	591.60	999.47	1647.68	2017.47	3040.59	2601.70	2634.55
954	Coiled-coil domain-containing protein 121	Q6ZUS5	4696.58	4324.76	4776.47	3012.65	2686.97	2287.07	1803.13	1917.82	1731.01	4104.55	4289.71	4763.87	4112.63	3121.84	208.02	831.88	1721.07	2208.71	4797.94	3975.37	3351.30
955	Coiled-coil domain-containing protein 122	Q5T0U0	0.14	2.53	0.81	1.07	2.77	0.68	0.96	0.36	0.63	0.35	0.75	0.38	2.13	0.97	0.60	4.64	1.07	0.47	2.22	0.19	0.06
956	Coiled-coil domain-containing protein 124	Q96CT7	32052.08	30336.19	28628.39	22591.31	21655.63	20430.20	25986.28	24615.46	23378.99	29955.10	28960.70	26863.06	27683.95	24356.51	19943.91	23646.29	20911.27	20375.19	22646.16	21295.72	19524.79
957	Coiled-coil domain-containing protein 136	Q96JN2	192236.00	183416.27	171655.04	130047.65	118072.26	112504.52	149550.41	144020.98	134310.87	151113.02	147435.50	130506.94	156686.11	145849.31	115454.67	119308.53	111130.14	100392.77	137881.83	122127.49	119386.38
958	Coiled-coil domain-containing protein 14	Q49A88	220.34	596.21	537.22	1.07	2.77	0.68	0.96	0.36	0.63	188.41	182.88	21.32	2.13	0.97	0.60	4.64	1.07	0.47	2.22	0.19	0.06
959	Coiled-coil domain-containing protein 141	Q6ZP82	14107.62	14082.49	13008.32	10385.56	9731.61	9039.50	10798.06	10612.26	9106.72	13820.31	12953.20	13030.72	12776.80	12179.57	8431.25	9026.58	9100.77	8798.17	9487.89	9594.94	8993.56
960	Coiled-coil domain-containing protein 144A	A2RUR9	159704.09	153080.25	157715.27	120123.97	113533.87	112991.18	120097.64	116736.28	121180.05	126418.85	116900.51	118820.83	127941.03	124334.87	107479.54	123432.66	118638.74	117811.41	112466.81	103142.57	107778.98
961	Coiled-coil domain-containing protein 144B	Q3MJ40	30665.39	31756.34	29567.18	16937.82	15658.28	14185.30	21688.58	21333.50	19219.12	21455.60	20291.01	18972.02	23323.33	21127.71	17062.37	17931.72	17260.94	16609.47	17349.37	15731.09	15579.42
962	Coiled-coil domain-containing protein 146	Q8IYE0	1938.92	2042.67	1535.48	706.81	917.86	815.72	1200.98	1198.05	1357.17	1957.33	1925.36	1613.88	824.77	944.88	638.25	1533.79	1609.08	1475.51	1380.06	1387.19	1174.65
963	Coiled-coil domain-containing protein 150	Q8NCX0	273802.94	261720.92	260283.94	265882.50	244905.85	245900.09	274704.34	269085.31	258421.35	259260.49	249123.97	239708.79	282217.54	285905.62	240410.48	233345.67	219263.26	219285.45	249291.14	236066.51	229855.41
964	Coiled-coil domain-containing protein 153	Q494R4	11757.15	12753.70	10342.96	18851.47	19454.42	20147.87	16063.45	15434.57	13159.33	15407.11	15109.91	15576.63	13556.47	11874.00	14106.02	20385.98	18374.19	15294.53	11989.41	10463.05	11413.37
965	Coiled-coil domain-containing protein 168	Q8NDH2	0.14	2.53	0.81	1.07	2.77	0.68	0.96	0.36	0.63	0.35	0.75	0.38	2.13	0.97	0.60	4.64	1.07	0.47	2.22	0.19	0.06
966	Coiled-coil domain-containing protein 170	Q8IYT3	3590.13	3073.48	2925.28	2663.82	2712.64	2139.97	2360.36	2454.09	2145.74	3008.64	3192.46	2910.16	3124.66	2856.18	1963.96	2320.74	2095.17	2160.04	2965.37	2705.45	2510.88
967	Coiled-coil domain-containing protein 171	Q6TFL3	11382.82	11132.75	10617.01	8252.12	8360.66	7922.30	9661.96	8881.22	9392.00	10700.32	9847.67	9843.47	10790.64	10450.37	9176.45	8420.82	7945.29	8119.42	8658.06	8559.83	8696.25
968	Coiled-coil domain-containing protein 175	P0C221	1154.25	1005.87	1230.53	773.20	664.97	562.10	953.64	1046.65	1206.70	745.31	901.16	751.20	1274.11	927.98	880.41	1124.22	819.96	994.00	1118.11	856.62	934.80
969	Coiled-coil domain-containing protein 180	Q9P1Z9	0.14	2.53	0.81	1.07	2.77	0.68	0.96	0.36	0.63	0.35	0.75	0.38	2.13	0.97	0.60	4.64	1.07	0.47	2.22	0.19	0.06
970	Coiled-coil domain-containing protein 181	Q5TID7	8129.71	8257.51	7922.45	5968.32	6009.08	5174.24	5781.12	5504.46	5802.03	6518.37	6260.69	6973.43	6070.06	6663.01	6330.72	5392.06	4326.62	4504.17	4658.29	5125.04	4546.24
971	Coiled-coil domain-containing protein 186	Q7Z3E2	0.14	2.53	0.81	1.07	2.77	0.68	0.96	0.36	0.63	0.35	0.75	0.38	2.13	0.97	0.60	4.64	1.07	0.47	2.22	0.19	0.06
972	Coiled-coil domain-containing protein 22	O60826	12176.59	12268.06	12295.55	8597.82	8256.44	7314.78	8742.30	7801.84	8068.38	11973.71	11888.96	10423.00	10913.08	10855.41	8080.50	7313.73	7186.57	7179.33	9049.91	8281.99	8047.44
973	Coiled-coil domain-containing protein 25	Q86WR0	16782.34	16474.41	15272.37	13753.25	12514.68	12089.71	18862.81	18768.01	18594.30	18759.04	17334.13	16624.70	15766.49	15733.10	12671.04	13597.41	12640.49	13017.88	12865.17	12260.31	12023.52
974	Coiled-coil domain-containing protein 30	Q5VVM6	0.14	2.53	0.81	1.07	2.77	0.68	0.96	0.36	0.63	0.35	0.75	0.38	2.13	0.97	0.60	4.64	1.07	0.47	2.22	0.19	0.06
975	Coiled-coil domain-containing protein 39	Q9UFE4	64.06	9.76	25.10	505.77	16.12	10.95	27.45	10.60	0.63	28.21	45.93	7.23	2.13	0.97	0.60	50.41	6.97	30.64	2.22	0.19	0.06
976	Coiled-coil domain-containing protein 43	Q96MW[illegible]	22111.86	21768.34	21433.63	16265.18	15442.93	13809.28	16162.72	17494.49	15989.23	18944.19	18608.50	17291.43	19828.65	18568.79	15962.48	15006.89	14477.02	14635.79	15772.63	14518.49	13525.29
977	Coiled-coil domain-containing protein 47	Q96A33	218788.84	203902.01	179400.21	142647.15	133804.13	124452.31	155589.63	148793.16	139801.00	162249.17	151052.10	141295.20	152757.58	150743.42	115242.57	150913.16	134534.59	127474.67	137652.93	121992.02	115076.77
978	Coiled-coil domain-containing protein 50	Q8IVM0	0.14	2.53	0.81	1.07	2.77	0.68	0.96	0.36	0.63	0.35	0.75	0.38	2.13	0.97	0.60	4.64	1.07	0.47	2.22	0.19	0.06
979	Coiled-coil domain-containing protein 51	Q96ER9	2446.34	3112.20	3093.31	2032.28	2188.12	1988.96	2097.48	1781.95	1924.91	3056.28	2804.64	2261.63	2400.78	2474.96	2161.47	1848.55	1758.31	1567.11	1942.28	2041.99	2003.24
980	Coiled-coil domain-containing protein 58	Q4VC31	9397.15	8730.72	9306.06	4849.34	4787.91	4555.39	7047.18	6635.24	6817.61	7065.22	6287.36	6332.12	7370.48	5677.65	5097.64	6720.05	5084.68	5016.85	4790.09	4832.17	4477.00
981	Coiled-coil domain-containing protein 6	Q16204	175528.16	169710.71	161345.25	116579.30	111120.83	108305.02	130214.26	131842.85	140498.46	136570.24	132028.14	127899.05	149618.63	136181.60	116815.50	126996.56	118581.66	118686.45	139418.81	118921.42	115181.29
982	Coiled-coil domain-containing protein 60	Q8IWA6	0.14	2.53	0.81	1.07	2.77	0.68	1273.79	0.72	31.49	0.35	0.75	0.38	180.69	8.36	90.39	4.64	1.07	0.47	2.22	0.19	0.06
983	Coiled-coil domain-containing protein 62	Q6P9F0	37917.78	35104.00	33937.48	26561.23	26476.64	25529.74	26991.06	25595.98	24813.29	33898.94	32703.75	31215.32	32085.73	31090.70	27156.82	24109.67	23843.49	23007.05	27075.48	26813.26	25728.28
984	Coiled-coil domain-containing protein 66	A2RUB6	10592.50	10124.97	9395.93	5294.88	4640.11	4788.72	7641.92	7514.76	6893.00	7367.16	7383.65	6357.97	7110.69	6147.08	5129.55	5251.37	5490.85	5192.53	6965.77	6206.87	5807.99
985	Coiled-coil domain-containing protein 68	Q9H2F9	0.14	2.53	0.81	1.07	2.77	0.68	0.96	0.36	0.63	0.35	0.75	0.38	2.13	0.97	0.60	4.64	1.07	0.47	2.22	0.19	0.06
986	Coiled-coil domain-containing protein 7	Q96M83	249813.48	246122.18	234039.84	123888.65	113830.48	113357.71	153812.66	153991.51	153824.78	179342.51	182434.07	168905.41	170172.61	163961.77	135309.54	190602.62	182410.72	182248.00	159710.42	144672.68	146159.74
987	Coiled-coil domain-containing protein 83	Q8IWF9	78451.11	69254.80	70927.00	49581.79	50735.18	50767.82	64825.60	63224.93	61627.76	60585.65	57986.12	57559.43	66729.53	43829.54	26325.53	56488.72	53304.59	49185.63	60570.32	55155.15	50112.86
988	Coiled-coil domain-containing protein 85B	Q15834	7467.09	7275.23	5602.44	5930.04	4858.57	4769.25	6136.44	5783.43	5656.73	7461.80	6674.36	5913.27	7013.97	6254.33	4777.69	5315.98	5125.06	4643.23	5488.92	4918.78	4512.53
989	Coiled-coil domain-containing protein 91	Q7Z6B0	0.14	2.53	0.81	1.07	2.77	0.68	0.96	0.36	0.63	0.35	0.75	0.38	2.13	0.97	0.60	4.64	1.07	0.47	2.22	0.19	0.06
990	Coiled-coil domain-containing protein 93	Q567U6	0.14	2.53	0.81	1.07	2.77	0.68	0.96	0.36	0.63	0.35	0.75	0.38	2.13	0.97	0.60	4.64	1.07	0.47	2.22	0.19	0.06
991	Coiled-coil domain-containing protein 97	Q96F63	8663.29	7632.97	5917.40	6672.50	6108.54	5161.68	6527.59	6620.43	6000.79	7774.59	7432.53	6818.19	7476.20	6950.21	3871.26	6202.06	6262.23	5598.01	6089.35	6300.83	4909.20
992	Coiled-coil-helix-coiled-coil-helix domain-containing protein 5	Q9BSY4	826.19	894.15	917.89	1004.35	888.50	946.67	1040.70	994.00	884.52	2078.02	1755.49	1985.09	1340.30	1283.90	800.12	911.00	755.40	731.93	1187.55	1046.42	1220.85
993	Cold shock domain-containing protein E1	O75534	372719.90	371319.91	350687.55	300371.47	290611.49	274644.30	349887.59	339043.06	317187.03	322926.83	302642.44	289254.92	288681.74	261933.92	200317.87	284170.27	270179.19	257417.57	271145.83	256920.88	249231.47
994	Cold-inducible RNA-binding protein	Q14011	76722.87	69268.11	67617.50	45935.05	43920.72	41488.62	45754.97	44023.89	41438.50	50738.22	47513.11	47014.48	50838.13	48835.14	39342.35	41864.18	40768.95	39622.55	44938.32	39830.31	37721.00
995	Collagen alpha-1(V) chain	P20908	141220.00	131709.22	119160.86	101117.58	103442.47	96234.40	113147.51	109326.24	112886.39	137721.39	130815.27	124688.70	117694.28	109199.26	87824.36	120599.64	119249.04	113809.60	116609.30	108159.77	102844.59
996	Collagen alpha-1(XVIII) chain	P39060	17615.15	15904.73	15274.72	12218.19	10640.44	10818.30	11821.41	11523.73	9963.08	14247.45	13694.03	12897.47	14397.84	13316.72	9159.09	9681.88	9928.58	10059.46	11758.07	10837.66	10351.24
997	Collagen alpha-1(XXIII) chain	Q86Y22	73477.94	69834.69	68069.59	48516.53	47227.20	43770.47	52686.94	51772.63	49351.06	62450.47	57785.91	56841.53	59728.77	59539.64	49698.17	45222.55	43309.27	41606.76	49546.63	47350.65	46895.50
998	Collagen alpha-1(XXVIII) chain	Q2UY09	8573.52	8049.04	6546.68	7182.42	6493.58	6874.24	6335.34	7536.97	6676.86	10676.96	10255.96	9086.31	8435.54	7715.78	6159.43	6458.50	6108.53	6505.15	5708.23	6968.49	6422.84
999	Collagen alpha-2(XI) chain	P13942	0.14	2.53	0.81	41.01	100.54	57.61	50.19	38.51	20.82	115.35	81.26	150.34	33.21	19.28	54.26	104.32	78.88	94.83	67.28	49.78	21.43
1000	Collagen alpha-3(VI) chain	P12111	3143.21	2674.32	2862.12	2466.54	2146.77	2458.46	3180.24	2096.23	1412.96	2782.20	2570.88	2619.35	2122.28	3211.35	1968.56	2514.85	1945.50	2991.85	1072.79	2510.43	2000.36
1001	Collagen alpha-5(VI) chain	A8TX70	0.14	2.53	0.81	1.07	2.77	0.68	0.96	0.36	0.63	0.35	0.75	0.38	2.13	0.97	0.60	4.64	1.07	0.47	2.22	0.19	0.06
1002	Collagen alpha-6(VI) chain	A6NMZ7	0.14	2.53	0.81	1.07	2.77	0.68	0.96	0.36	0.63	0.35	0.75	0.38	2.13	0.97	0.60	4.64	1.07	0.47	2.22	0.19	0.06

	MDA-MB-231													
	control_01	control_02	control_03	Dai_IC20_01	Dai_IC20_02	Dai_IC20_03	Gen_IC20_01	Gen_IC20_02	Gen_IC20_03	SSE_IC20_01	SSE_IC20_02	SSE_IC20_03	unique to MCF-7	unique to MDA-MB-231
866	14560.86	15207.10	14383.50	9622.20	9149.66	7712.87	12059.45	11329.55	10606.09	12907.55	10446.15	8016.32		
867	2.27	1.49	0.65	0.84	1.31	0.69	4.16	2.47	0.11	4.90	4.98	0.12	x	
868	2.27	1.49	0.65	0.84	1.31	0.69	4.16	2.47	0.11	4.90	4.98	0.12	x	
869	23657.97	22129.26	19900.80	16204.90	17139.30	13847.13	19074.98	19110.45	16831.42	20948.80	21012.72	13900.34		
870	146875.60	138925.69	134970.77	93596.09	91796.53	77352.57	102985.54	99524.30	96781.10	99930.68	98801.24	69371.02		
871	2.27	1.49	0.65	0.84	1.31	0.69	4.16	2.47	0.11	4.90	4.98	0.12	x	
872	6334.62	5679.23	5059.24	2823.82	2879.96	1961.05	3009.36	2763.27	2284.25	2835.07	2939.23	1192.89		
873	2.27	1.49	0.65	0.84	1.31	0.69	4.16	2.47	0.11	4.90	4.98	0.12	x	
874	2.27	1.49	0.65	0.84	1.31	0.69	4.16	2.47	0.11	4.90	4.98	0.12	x	
875	1545.96	1564.92	1502.27	1281.39	1296.54	1209.80	1456.46	1227.76	1311.84	1630.37	1599.91	1109.71		
876	551603.88	513114.50	489419.65	326257.27	321033.56	293526.27	368269.12	363921.38	343169.91	387971.63	379026.01	280411.13		
877	140.06	155.45	145.77	193.88	126.37	109.12	231.19	174.75	132.74	248.17	342.81	151.73		x
878	2.27	1.49	0.65	0.84	1.31	0.69	4.16	2.47	0.11	4.90	4.98	0.12	x	
879	156978.17	144762.96	131684.78	99728.09	96492.67	83574.75	121208.53	110400.02	108412.19	121084.76	117194.08	78462.34		
880	683.70	439.40	361.97	231.21	175.86	61.08	434.06	366.89	183.18	417.54	414.19	101.73		
881	9834.23	9264.24	7101.74	7795.81	6934.29	7259.34	5385.14	5973.87	6115.91	10087.46	7454.48	1177.74		x
882	950.17	899.74	859.12	1119.76	1097.76	988.60	1091.36	1174.96	1105.16	1213.54	966.73	760.35		x
883	13374.60	12353.20	11679.70	9435.67	9052.07	7927.44	10434.61	10158.95	9428.79	12114.79	11311.04	7736.90		
884	2.27	1.49	0.65	0.84	1.31	0.69	4.16	2.47	0.11	4.90	4.98	0.12	x	
885	2.27	1.49	0.65	0.84	1.31	0.69	4.16	2.47	0.11	4.90	4.98	0.12	x	
886	1408.10	1285.91	1153.41	847.93	712.77	556.73	1261.06	1355.74	1245.27	1070.27	1070.31	637.66		x
887	17649.30	18636.61	16943.16	10737.44	9937.26	8238.08	12431.32	11797.33	11466.61	11838.80	11474.22	6844.31		x
888	7993.94	10444.09	11049.84	7697.69	7642.70	7459.85	9102.22	9095.15	8682.16	19494.17	19164.84	9882.68		x
889	1137.07	1200.09	1057.03	921.32	858.32	845.11	927.56	931.71	970.79	1300.44	1326.06	914.31		
890	117229.01	110092.44	103693.02	77671.86	72611.92	63103.03	98444.29	87517.34	86736.02	87074.04	83729.98	61454.28		
891	7937.71	7797.13	6755.42	5124.77	4783.13	3361.88	6295.71	6127.60	5453.69	6611.73	6744.98	3678.40		
892	2.27	1.49	0.65	0.84	1.31	0.69	4.16	2.47	0.11	4.90	4.98	0.12	x	
893	40664.90	47052.75	38871.06	39101.78	38537.71	41992.06	28399.03	33567.60	32760.07	49390.01	41033.28	33625.73		
894	25836.95	24741.73	19966.38	15762.48	15844.89	15832.86	19505.60	18072.83	17384.59	19622.43	20195.51	15216.49		x
895	187228.31	170014.52	155250.61	127810.82	126853.57	105179.77	131064.91	119627.06	119568.01	144001.13	138720.20	99139.00		
896	21237.84	19590.86	18707.95	12903.58	12273.80	10336.47	23430.31	20179.89	20732.14	19545.13	19074.25	8169.97		x
897	2.27	1.49	0.65	0.84	1.31	0.69	4.16	2.47	0.11	4.90	4.98	0.12	x	
898	1672.76	1593.79	1780.87	1152.72	1070.31	923.21	1583.79	1513.16	1580.79	1467.08	1272.86	858.32		x
899	2.27	1.49	0.65	0.84	1.31	0.69	4.16	2.47	0.11	4.90	4.98	0.12	x	
900	11596.67	11016.76	10720.65	5030.14	4490.14	3601.17	7455.55	6930.91	6177.89	7930.54	7235.91	4746.60		x
901	10582.76	11007.82	9997.56	7679.21	7291.50	7312.52	8712.91	9568.22	7897.51	8140.91	8216.24	5513.80		x
902	1078.18	980.94	1042.23	971.05	1010.32	970.71	1096.81	1149.69	1172.44	1480.13	1531.49	1159.79		
903	1907.65	2369.59	2029.76	1637.35	1507.26	1457.50	2317.82	2131.92	1963.80	1934.31	1861.58	1248.24		x
904	4191.18	3769.75	3664.60	2392.49	2251.17	1645.39	3173.26	3045.67	2593.39	2789.68	2788.95	1507.64		
905	56952.18	55560.17	47621.32	35027.48	34018.50	27712.43	40846.26	40384.53	37178.28	41976.64	40656.14	26951.81		x
906	33147.03	31402.79	28067.55	23171.85	22838.71	22122.22	22167.42	20447.07	20567.35	22679.53	21538.56	5575.93		
907	2.27	1.49	0.65	0.84	1.31	0.69	4.16	2.47	0.11	4.90	4.98	0.12	x	
908	130046.25	119087.00	108273.25	107384.74	104573.10	79593.80	164552.69	155577.75	143693.02	105361.80	108004.54	72293.56		
909	28143.69	34304.28	23988.91	19989.36	18240.68	16135.52	26456.01	24410.46	23363.22	26509.95	23921.95	16047.21		
910	3510.06	3322.49	3193.30	2122.78	2081.17	1450.12	2784.54	2655.26	2364.24	2833.75	2628.14	0.12		
911	13314.01	13090.71	11969.40	8801.60	8552.16	7921.71	8711.87	9196.87	9206.44	9086.95	9538.71	9145.14		
912	165017.26	156016.44	143823.06	98602.89	98726.47	83962.12	123962.02	114620.14	105630.18	121752.62	119087.11	87925.92		
913	60170.70	56502.06	50032.63	42512.82	41217.59	39061.46	46849.85	46273.36	43926.34	52425.49	47297.84	30543.62		
914	1700769.05	1600348.59	1468071.58	1010292.57	987559.46	882998.73	1269957.58	1230982.44	1168854.77	1272226.65	1239331.70	857212.32		
915	129748.78	124602.59	114332.71	88047.02	84577.08	79326.25	104962.87	105172.42	93288.59	111061.55	106129.12	77442.05		
916	29007.36	26140.35	25390.29	18503.69	17395.40	15500.94	22280.80	20137.23	19564.91	22115.28	20615.48	11109.85		
917	285017.08	262967.32	254140.48	187714.00	188838.01	180563.93	204003.20	194722.46	189251.66	208409.67	212315.47	178791.77		
918	23718.66	20468.17	21915.03	13550.21	15162.21	15190.55	16352.19	15762.96	15346.75	18592.50	15038.34	15658.57		
919	5703.81	5393.15	4665.26	3454.40	3064.81	2161.27	4409.14	4386.06	3861.90	5251.73	4613.85	2510.45		x
920	2.27	1.49	0.65	0.84	1.31	0.69	4.16	2.47	0.11	4.90	4.98	0.12	x	
921	75199.40	74187.93	64985.46	47513.72	45476.68	41337.15	50471.37	50383.31	48445.94	56183.25	52790.28	42105.81		
922	5822.25	5304.67	4929.52	3737.88	3365.87	2958.17	4416.55	4589.09	4143.72	4629.52	4376.19	2647.39		
923	15223.79	13922.44	12347.33	12356.97	11054.78	9937.63	12597.28	13484.71	11476.80	10916.52	11009.46	9414.40		
924	2.27	1.49	0.65	0.84	1.31	0.69	4.16	2.47	0.11	4.90	4.98	0.12	x	
925	7228.75	6638.52	6676.21	6175.99	5873.71	5331.36	6363.84	5976.13	5442.09	7424.37	7682.96	3916.84		
926	5906.23	5332.21	4457.14	2874.81	2565.86	2141.34	3562.51	3380.74	3158.79	3786.28	3189.57	1420.94		
927	19465.44	18354.50	16854.49	11411.92	11136.25	9675.37	16434.19	15652.11	14786.41	15619.86	14647.36	9562.85		x
928	33916.91	29749.58	26635.94	23586.90	21816.06	18688.50	28738.31	27666.05	25698.82	28617.29	26877.51	17267.32		
929	4234.93	4567.36	3670.32	3232.57	3026.59	2834.30	3909.19	3729.76	3487.60	3600.76	3594.38	3192.28		x
930	156851.47	159126.71	146411.71	109234.43	107597.18	93571.47	126958.04	123048.13	123197.55	127250.78	124451.97	82151.91		
931	2.27	1.49	0.65	0.84	1.31	0.69	4.16	2.47	0.11	4.90	4.98	0.12	x	
932	3674.36	3294.71	3192.37	2326.91	2234.51	1923.43	3155.07	3570.69	2922.67	2701.26	2917.98	2074.87		
933	2.27	1.49	0.65	0.84	1.31	0.69	4.16	2.47	0.11	4.90	4.98	0.12	x	
934	79082.48	73931.74	69290.62	83411.28	77049.86	76493.08	105730.06	97590.06	96949.62	99409.80	97612.43	70337.18		
935	23237.74	21138.86	20872.01	15570.82	14863.69	13995.09	19156.24	17456.30	17440.22	17816.26	17278.56	12449.29		
936	2.27	1.49	0.65	0.84	1.31	0.69	4.16	2.47	0.11	4.90	4.98	0.12	x	
937	227305.29	213394.81	202403.28	156621.58	159436.09	147703.59	186279.51	180003.11	173207.74	184541.31	175996.96	124176.60		
938	2.27	1.49	0.65	0.84	1.31	0.69	4.16	2.47	0.11	4.90	4.98	0.12	x	
939	310316.16	287433.34	265867.11	203785.29	196801.08	175301.83	231385.90	214127.32	211799.34	238894.81	231709.61	163841.25		
940	117081.53	95548.30	90557.43	66134.02	63635.28	56585.62	100709.34	94986.96	90318.28	89614.71	83672.59	55624.36		
941	172921.48	162860.92	150942.80	115031.34	110594.93	96055.30	133968.96	129732.97	120927.04	135028.66	130314.67	91326.57		
942	282813.46	265741.67	245499.98	208983.62	204929.13	187539.51	218450.51	214964.52	204126.00	233759.87	224720.89	134986.99		
943	56052.64	53087.41	46658.01	37298.66	36752.00	26917.60	40167.34	39375.62	35148.20	50309.20	47136.11	26993.30		
944	140393.36	132166.34	121572.79	97564.76	92969.35	83565.30	114589.18	112968.43	105405.05	118927.15	114435.37	73242.58		
945	21120.96	20133.44	20346.26	15173.35	14874.46	13961.64	17881.53	17223.05	17480.91	18915.86	18466.16	14386.80		
946	50346.35	47930.89	44668.27	39927.84	39601.60	34350.34	50055.92	47313.35	44844.36	48387.47	47387.62	32127.69		
947	1188458.18	1106162.54	1014067.12	650669.95	633791.77	554431.60	748512.46	722492.65	680543.34	703718.10	684190.31	458606.59		
948	24345.11	24615.51	20706.52	12098.99	12241.94	9515.66	13574.13	14236.47	12998.96	13851.06	13095.21	7403.89		
949	2.27	1.49	0.65	0.84	1.31	0.69	4.16	2.47	0.11	4.90	4.98	0.12	x	
950	4191.68	6761.18	3929.05	5083.18	5347.45	5972.05	2208.64	4139.72	3978.34	6717.76	5438.67	7307.04		x
951	2.27	1.49	0.65	0.84	1.31	0.69	4.16	2.47	0.11	4.90	4.98	0.12	x	
952	2509.29	2155.35	1831.14	1224.92	1225.25	645.26	1725.05	1494.93	1194.75	2159.07	1956.53	833.37		x
953	2.27	1.49	0.65	0.84	1.31	0.69	4.16	2.47	0.11	4.90	4.98	0.12	x	
954	12619.28	12255.19	10761.72	10284.51	10212.79	9984.03	7735.96	8479.00	8666.72	12135.16	11911.20	4928.83		
955	3739.63	3574.11	3225.28	2314.46	2181.98	2294.56	2745.78	2655.61	2487.76	2928.83	3116.41	1567.43		x
956	17331.34	35123.97	15767.47	21574.26	19727.43	30313.37	13408.88	17424.86	15027.12	31251.47	21630.42	8263.41		
957	309748.04	286551.36	270019.49	103564.80	97612.02	84020.76	119863.88	113524.84	111894.60	115154.68	110989.57	71574.87		
958	2.27	1.49	0.65	0.84	1.31	0.69	4.16	2.47	0.11	4.90	4.98	9467.10	x	
959	2.27	1.49	0.65	0.84	1.31	0.69	4.16	2.47	0.11	4.90	4.98	0.12	x	
960	140494.12	142554.04	133128.42	103271.74	100378.67	91971.64	123907.93	115938.68	117415.90	128125.96	120233.34	90224.87		
961	18260.97	17164.50	16371.88	17929.06	18573.45	16680.42	22315.59	21594.71	20035.69	28532.20	28363.90	22025.89		
962	2.27	1.49	0.65	0.84	1.31	0.69	4.16	2.47	0.11	4.90	4.98	0.12	x	
963	40270.11	38339.25	35106.84	25939.27	25051.27	22663.25	26101.91	24990.60	24023.00	27328.74	27230.61	18863.30		
964	211.75	123.57	211.42	270.27	174.01	147.67	234.55	237.79	183.20	400.92	378.97	189.64		
965	2227.93	626.90	525.07	1410.61	1226.57	728.38	1832.71	1545.61	970.99	2477.06	2243.18	814.19		x
966	71341.51	65189.51	61870.67	42271.38	43222.14	41570.31	49309.48	46146.27	45571.45	52282.84	46201.40	33199.00		
967	60626.03	65447.36	54053.08	43973.79	42227.33	44939.62	42387.28	42546.19	42014.68	50892.40	44566.23	31873.54		
968	2.27	1.49	0.65	0.84	1.31	0.69	4.16	2.47	0.11	4.90	4.98	0.12	x	
969	396.88	459.20	382.05	343.43	406.63	342.38	354.56	419.31	412.26	433.01	444.39	208.44		x
970	2.27	1.49	0.65	0.84	1.31	0.69	4.16	2.47	0.11	4.90	4.98	0.12	x	
971	43792.10	38584.41	34698.23	28280.43	27288.96	23849.36	31518.35	29991.81	27278.02	33214.07	30790.85	30921.06		x
972	10278.30	9194.74	8230.99	5944.83	5932.92	5095.01	7013.59	6658.85	6363.29	6936.22	6407.55	3857.48		
973	2.27	1.49	0.65	0.84	1.31	0.69	4.16	2.47	0.11	4.90	4.98	0.12	x	
974	26489.24	24715.06	23893.62	20450.14	20466.68	19060.13	22433.82	21566.47	20791.87	25308.81	23572.84	17454.73		x
975	2.27	1.49	0.65	0.84	1.31	0.69	4.16	2.47	0.11	4.90	4.98	0.12	x	
976	2.27	1.49	0.65	0.84	1.31	0.69	4.16	2.47	0.11	4.90	4.98	0.12	x	
977	48609.11	43285.73	38914.24	28351.64	26576.49	23123.67	34388.47	32499.02	28636.80	33111.08	29915.79	22199.84		
978	2597.24	2833.36	1221.08	2021.94	1922.04	2060.46	2220.48	2028.70	1717.74	2519.44	2545.55	406.08		x
979	2.27	1.49	0.65	0.84	1.31	0.69	4.16	2.47	0.11	4.90	4.98	0.12	x	
980	2.27	1.49	0.65	0.84	1.31	0.69	4.16	2.47	0.11	4.90	4.98	0.12	x	
981	28651.62	27038.58	23420.32	19102.87	18555.53	16589.84	24177.97	22440.06	20367.24	24548.45	22706.09	15055.21		
982	2.27	1.49	0.65	0.84	1.31	0.69	4.16	2.47	0.11	4.90	4.98	0.12	x	
983	2.27	1.49	0.65	0.84	1.31	0.69	4.16	2.47	0.11	4.90	4.98	0.12	x	
984	2.27	1.49	0.65	0.84	1.31	0.69	4.16	2.47	0.11	4.90	4.98	0.12	x	
985	28191.40	26892.18	25480.83	24218.46	24993.87	23739.79	27436.29	28241.21	27573.49	26744.73	25422.91	19071.34		x
986	13236.66	11547.08	9516.55	11042.17	10452.24	8327.29	13392.89	12606.30	10664.02	14303.51	13396.05	8392.88		
987	2800.52	2238.66	1695.82	1342.79	1250.79	729.03	2226.17	1883.90	1454.07	2781.91	2111.76	694.63		
988	998.55	919.81	897.13	905.84	829.60	583.23	1169.42	961.52	890.95	1135.11	1046.81	581.10		
989	5486.58	4991.32	4368.01	4160.81	4316.79	4092.38	4542.75	4258.05	3184.07	6055.17	5651.77	3808.76		x
990	6905.87	5904.56	6432.57	6196.98	5456.06	5117.76	5950.68	5687.67	4582.23	5935.69	5181.09	4281.07		x
991	110.32	89.43	126.98	194.46	180.43	151.35	158.07	154.93	144.26	245.91	237.64	126.96		
992	2.27	1.49	0.65	0.84	1.31	0.69	4.16	2.47	0.11	4.90	4.98	0.12	x	
993	55333.72	54888.88	50619.32	34654.03	35289.00	32604.85	42627.11	42736.93	40561.92	41233.79	40339.73	28866.13		
994	6977.08	5390.68	5649.78	5429.46	5417.61	4589.62	6074.34	5568.58	4724.26	6003.85	6255.81	4425.72		
995	2.27	1.49	0.65	0.84	1.31	0.69	4.16	2.47	0.11	4.90	4.98	0.12	x	
996	8076.50	8967.58	7428.39	2661.98	2532.45	2195.89	3656.08	3847.25	3594.81	3398.82	3291.43	2097.56		
997	2.27	1.49	0.65	0.84	1.31	0.69	4.16	2.47	0.11	4.90	4.98	0.12	x	
998	2.27	1.49	0.65	0.84	1.31	0.69	4.16	2.47	0.11	4.90	4.98	0.12	x	
999	2.27	1.49	0.65	0.84	1.31	0.69	4.16	2.47	0.11	4.90	4.98	24499.87	x	
1000	2.27	1.49	0.65	0.84	1.31	0.69	4.16	2.47	0.11	4.90	4.98	0.12	x	
1001	107100.35	98141.36	93352.78	50019.03	47106.50	41307.53	78515.72	73653.92	67582.93	53193.58	49895.72	33910.70		x
1002	1734.73	788.42	1322.80	1531.81	1328.18	982.59	1499.28	1981.90	1250.41	2353.34	2547.58	1058.12		x

Supplementary Table S1. Overview on the abundance of all relatively quantified 5180 proteins

			MCF-7																					MDA-MB-231													
	Protein name	UniProt	control_01	control_02	control_03	Dai_IC20_01	Dai_IC20_02	Dai_IC20_03	Dai_SC20_01	Dai_SC20_02	Dai_SC20_03	Gen_IC20_01	Gen_IC20_02	Gen_IC20_03	Gen_SC20_01	Gen_SC20_02	Gen_SC20_03	SSE_IC20_01	SSE_IC20_02	SSE_IC20_03	SSE_SC20_01	SSE_SC20_02	SSE_SC20_03	control_01	control_02	control_03	Dai_IC20_01	Dai_IC20_02	Dai_IC20_03	Gen_IC20_01	Gen_IC20_02	Gen_IC20_03	SSE_IC20_01	SSE_IC20_02	SSE_IC20_03	unique to MCF-7	unique to MDA-MB-231
1003	Collagen triple helix repeat-containing protein 1	Q96CG8	2451.17	2070.55	2162.18	1654.38	1784.99	1757.45	2771.87	2645.31	2959.37	2528.19	2224.61	2296.49	2593.31	2366.64	1976.23	1824.82	1477.39	1738.18	1749.60	1596.16	1620.22	2.27	1.49	0.65	0.84	1.31	0.69	4.16	2.47	0.11	4.90	4.98	0.12	x	
1004	Collagen type IV alpha-3-binding protein	Q9Y5P4	626.20	290.42	139.96	1.07	2.77	0.68	0.96	0.36	0.63	333.44	207.10	62.44	2.13	0.97	0.60	4.64	1.07	0.47	2.22	0.19	0.06	2.27	1.49	0.65	0.84	1.31	0.69	4.16	2.47	0.11	4.90	4.98	1439.89	x	
1005	Collectrin	Q9HBJ8	71108.44	67699.30	61121.88	55346.12	54140.90	50813.04	53941.46	52738.98	50125.43	72637.89	70415.99	70481.36	57327.76	53118.71	47063.51	62837.85	59546.27	54728.78	58194.26	56706.46	57814.87	2.27	1.49	0.65	0.84	1.31	0.69	4.16	2.47	0.11	4.90	4.98	0.12	x	
1006	COMM domain-containing protein 1	Q8N668	0.14	2.53	0.81	1.07	2.77	0.68	0.96	0.36	0.63	0.35	0.75	0.38	2.13	0.97	0.60	4.64	1.07	0.47	2.22	0.19	0.06	5341.18	5152.28	4875.61	3995.71	4001.64	3568.92	4718.00	4402.45	4197.92	4904.63	4866.25	3508.71		x
1007	COMM domain-containing protein 3	Q9UBI1	13001.96	12652.80	10235.37	9861.25	9760.93	7977.77	10441.72	8423.67	9259.55	12715.83	11358.29	10309.54	11327.72	9441.86	4660.68	9599.83	8387.24	8286.02	9947.15	9267.95	7939.10	2.27	1.49	0.65	0.84	1.31	0.69	4.16	2.47	0.11	4.90	4.98	0.12	x	
1008	Complement C1q tumor necrosis factor-related protein 6	Q9BXI9	168.49	167.49	164.86	232.81	223.59	273.23	2423.05	2114.98	2012.37	339.99	247.02	308.98	548.10	429.80	482.51	287.49	308.92	193.42	267.56	323.74	453.42	9544.72	9929.22	7735.69	6904.41	7140.33	5904.10	7288.50	6302.35	7700.69	9332.44	9238.78	1243.20		
1009	Complement C2	P06681	73355.22	71758.66	63729.20	55524.94	57567.82	53304.01	53043.94	51852.00	48792.65	68679.40	64998.82	61530.06	53834.91	54948.95	48694.43	52256.97	49774.63	47674.33	49012.82	46592.05	44210.08	2.27	1.49	0.65	0.84	1.31	0.69	4.16	2.47	0.11	4.90	4.98	0.12	x	
1010	Complement C3	P01024	0.14	2.53	0.81	1.07	2.77	0.68	0.96	0.36	0.63	0.35	0.75	0.38	2.13	0.97	0.60	4.64	1.07	0.47	2.22	0.19	0.06	6691.08	5103.48	4499.27	4867.59	4861.69	3698.67	7143.78	6331.59	5531.45	6416.89	5964.72	3132.30		x
1011	Complement C5	P01031	0.14	2.53	0.81	1.07	2.77	0.68	0.96	0.36	0.63	0.35	0.75	0.38	2.13	0.97	0.60	4.64	1.07	0.47	2.22	0.19	0.06	114.68	66.59	72.36	17.24	12.70	28.71	49.42	48.11	48.86	51.98	38.57	39.20		x
1012	Complement component 1 Q subcomponent-binding protein mitochondrial	Q07021	78342.88	71581.38	64837.75	56958.82	57551.29	48842.94	59693.16	56002.67	55538.78	69371.14	66149.48	59627.77	70440.78	65390.18	43104.86	54323.66	53803.67	50636.88	63783.81	58862.71	51638.46	65443.29	59700.85	53164.18	60705.91	60680.41	43355.99	66790.83	66471.50	61133.92	76616.64	72451.92	43121.17		
1013	Complement component C7	P10643	0.14	2.53	0.81	1.07	2.77	0.68	0.96	0.36	0.63	0.35	0.75	0.38	2.13	0.97	0.60	4.64	1.07	0.47	2.22	0.19	0.06	6983.94	6736.42	7208.93	5153.28	4326.92	3670.69	5906.98	5137.45	5280.07	7584.14	7509.45	4933.34		x
1014	Complement component receptor 1-like protein	Q2VPA4	29021.16	27282.85	27036.48	21491.46	21244.42	19886.51	22137.65	22409.93	21758.89	32480.13	32803.53	35992.51	29804.49	24025.74	13922.79	18449.18	19411.91	19369.79	28698.59	24084.19	21627.00	2.27	1.49	0.65	0.84	1.31	0.69	4.16	2.47	0.11	4.90	4.98	0.12	x	
1015	Complement factor H	P08603	1118.37	848.07	932.54	734.39	680.78	612.83	1072.76	878.21	1006.95	680.10	1047.97	816.61	909.30	714.90	607.07	900.84	620.52	835.87	896.36	740.41	662.90	31881.65	29662.07	24925.11	23936.31	21804.75	18727.83	27017.27	24744.05	24107.07	21460.23	21026.55	13867.56		
1016	Complex I assembly factor TIMMDC1_ mitochondrial	Q9NPL8	22767.49	22910.32	20284.65	16318.70	15829.90	14643.33	15855.72	14563.31	14476.35	20258.15	18198.92	17158.28	16304.72	16514.06	12996.29	14938.38	14592.82	14011.59	14990.06	15024.31	14177.96	2.27	1.49	0.65	0.84	1.31	0.69	4.16	2.47	0.11	4.90	4.98	0.12	x	
1017	Complex I intermediate-associated protein 30_ mitochondrial	Q9Y375	6601.69	6022.73	6319.18	4867.85	3900.33	4167.94	5644.83	5640.08	5724.35	6080.11	6194.05	5606.44	5434.25	5275.39	3804.82	4422.26	4089.87	4018.27	4674.43	4479.74	4034.77	2.27	1.49	0.65	0.84	1.31	0.69	4.16	2.47	0.11	4.90	4.98	0.12	x	
1018	Complex III assembly factor LYRM7	Q5U5X0	29287.00	27598.92	26827.79	20805.28	19637.95	19439.06	21927.49	21560.66	20467.13	20157.57	20033.81	19682.76	22964.45	21877.63	18498.98	21632.88	18349.72	28785.76	29464.16	19699.06	20632.22	2.27	1.49	0.65	0.84	1.31	0.69	4.16	2.47	0.11	4.90	4.98	0.12	x	
1019	Complexin-1	O14810	0.14	2.53	0.81	1.07	2.77	0.68	0.96	0.36	0.63	0.35	0.75	0.38	2.13	0.97	0.60	4.64	1.07	0.47	2.22	0.19	0.06	88.19	93.38	69.64	33.84	34.26	37.92	47.47	32.75	42.93	199.84	169.05	69.47		x
1020	Complexin-4	Q7Z7G2	85705.20	84485.26	72070.20	57612.97	56061.55	49185.56	81400.03	76503.51	69601.99	85228.51	74760.77	69101.03	66995.81	64957.56	52909.38	64984.46	60844.89	53755.75	64577.50	62450.31	57233.01	2.27	1.49	0.65	0.84	1.31	0.69	4.16	2.47	0.11	4.90	4.98	0.12	x	
1021	Condensin complex subunit 1	Q15021	80358.76	73569.50	65424.60	46744.76	46699.64	42149.27	62065.95	55075.37	53497.54	57617.65	54390.62	50673.56	58714.99	53612.73	40774.66	59243.75	52560.63	52507.33	49005.73	46166.31	42036.37	149286.94	139813.00	133835.06	105022.79	100099.12	82030.50	141641.21	129316.67	120409.37	143223.51	135776.48	86012.17		
1022	Condensin complex subunit 2	Q15003	0.14	2.53	0.81	1.07	2.77	0.68	0.96	0.36	0.63	0.35	0.75	0.38	2.13	0.97	0.60	4.64	1.07	0.47	2.22	0.19	0.06	55928.77	50521.01	46013.48	41334.35	39414.21	35398.13	53077.43	51486.38	48723.85	51760.72	50289.33	33420.65		x
1023	Condensin complex subunit 3	Q9BPX3	38633.45	35244.65	31685.69	20826.02	19850.75	17224.07	24826.46	24188.56	22855.54	27895.86	26910.61	23631.90	28046.02	26276.60	15389.87	22151.41	20926.69	20793.85	24845.62	22870.70	19424.64	47092.67	44674.31	38612.50	26129.90	24827.64	17809.78	35174.79	33042.79	30743.22	34718.53	32716.89	19117.43		
1024	Condensin-2 complex subunit H2	Q6IBW4	0.14	2.53	0.81	1.07	2.77	0.68	0.96	0.36	0.63	0.35	0.75	0.38	2.13	0.97	0.60	4.64	1.07	0.47	2.22	0.19	0.06	739.28	1516.24	725.43	1314.00	1336.01	1482.75	474.85	773.07	721.19	4.90	4.98	0.12		x
1025	Connector enhancer of kinase suppressor of ras 1	Q969H4	22117.34	22368.26	20270.29	19359.29	16780.86	14600.33	18077.38	16980.58	16820.92	20952.19	19795.27	19075.26	19741.47	23522.64	17352.39	14948.00	14546.33	14489.15	14897.51	15527.08	14888.19	2775.66	2581.93	2017.06	1480.29	1602.35	1291.13	3475.54	3364.50	2751.73	2643.45	2605.28	1531.09		
1026	Connector enhancer of kinase suppressor of ras 2	Q8WXI2	277.19	311.09	328.07	428.39	320.52	345.39	373.35	330.61	380.92	278.62	261.85	301.54	445.13	395.96	368.33	288.62	255.02	321.38	242.54	219.93	194.47	2.27	1.49	0.65	0.84	1.31	0.69	4.16	2.47	0.11	4.90	4.98	0.12	x	
1027	Conserved oligomeric Golgi complex subunit 1	Q8WTW3	7182.78	7463.13	7738.11	4179.41	3904.08	4036.78	5523.20	5694.00	5007.44	5825.42	5251.55	4713.29	5686.91	4983.37	4607.52	5056.22	5019.94	4214.78	4507.07	4249.76	4124.93	2.27	1.49	0.65	0.84	1.31	0.69	4.16	2.47	0.11	4.90	4.98	0.12	x	
1028	Conserved oligomeric Golgi complex subunit 2	Q14746	6904.53	6162.85	6356.60	4971.59	4711.65	4247.09	5309.94	5034.19	4517.61	5664.73	6008.07	5534.43	6637.16	6690.06	5498.95	3871.04	3432.38	3408.61	5590.09	5573.81	4939.77	2.27	1.49	0.65	0.84	1.31	0.69	4.16	2.47	0.11	4.90	4.98	0.12	x	
1029	Conserved oligomeric Golgi complex subunit 4	Q9H9E3	33223.22	27372.09	24817.41	19643.49	18731.31	18764.38	23565.32	20158.69	21265.69	24931.10	24098.61	23591.78	23112.34	22699.68	18419.92	21265.04	20009.88	19154.25	19119.82	19655.95	18025.16	2.27	1.49	0.65	0.84	1.31	0.69	4.16	2.47	0.11	4.90	4.98	0.12	x	
1030	Conserved oligomeric Golgi complex subunit 5	Q9UP83	8086.89	8666.55	7592.27	4230.66	4387.15	4287.61	5675.05	5462.48	4980.17	6018.85	5731.54	5407.29	5714.27	5537.53	4332.62	5719.72	5427.12	4749.46	5239.85	4901.24	4021.23	2.27	1.49	0.65	0.84	1.31	0.69	4.16	2.47	0.11	4.90	4.98	0.12	x	
1031	Constitutive coactivator of peroxisome proliferator-activated receptor gamma	Q96EK7	8168.39	7595.97	6966.88	5760.39	5879.09	5550.26	6497.87	6141.71	6387.53	7956.79	7687.02	7660.08	6984.96	7103.62	5990.96	5944.22	5850.34	5679.70	6465.01	5683.80	5369.55	2.27	1.49	0.65	0.84	1.31	0.69	4.16	2.47	0.11	4.90	4.98	0.12	x	
1032	Constitutive coactivator of PPAR-gamma-like protein 1	Q9NZB2	426872.20	421303.41	404611.62	308632.51	293067.32	282810.01	336196.13	323434.05	314617.98	379992.52	364401.29	329885.51	365684.59	357073.12	297027.02	295018.19	272745.13	269817.27	312103.70	301802.48	283582.05	110537.05	103860.83	93699.17	71487.63	70032.30	60979.42	90638.71	84259.81	78962.28	88658.79	83566.56	57258.18		
1033	Contactin-associated protein-like 2	Q9UHC6	0.14	2.53	0.81	1.07	2.77	0.68	0.96	0.36	0.63	0.35	0.75	0.38	2.13	0.97	0.60	4.64	1.07	0.47	2.22	0.19	0.06	2436.04	2539.99	2444.54	1575.55	1784.26	1796.92	2081.32	1970.22	1997.08	2631.93	2428.36	1854.26		x
1034	Contactin-associated protein-like 3	Q9BZ76	34592.46	34549.09	26928.98	26748.25	27468.11	26733.69	15595.04	13289.34	12578.57	31392.02	30385.70	28481.81	23885.10	23309.90	23488.76	21992.94	21907.14	20342.07	32490.78	32920.95	28625.84	2.27	1.49	0.65	0.84	1.31	0.69	4.16	2.47	0.11	4.90	4.98	0.12	x	
1035	Contactin-associated protein-like 4	Q9C0A0	81752.55	70629.10	72216.03	66629.28	61313.84	58975.96	70941.04	73359.70	64613.88	78236.31	72192.50	71787.92	66918.19	67762.53	57495.10	64866.28	59845.88	54494.77	54534.36	53813.89	48675.99	12628.73	13065.50	10850.59	7523.76	6926.09	5183.45	10332.29	9408.35	8608.62	10654.64	10191.11	5882.10		
1036	COP9 signalosome complex subunit 1	Q13098	60465.55	58635.72	56998.29	43334.53	42481.27	39210.30	50233.70	49702.32	47686.59	53314.33	50542.84	48364.70	51343.19	48684.85	39402.38	40580.19	37750.04	36680.86	42247.80	39608.96	37929.50	19367.76	18478.02	17935.30	15968.41	15568.33	15044.97	17030.11	16091.52	16084.97	17868.33	17795.28	14775.58		
1037	COP9 signalosome complex subunit 2	P61201	121216.89	116545.53	112151.65	76318.25	72923.63	68680.70	97460.39	91670.01	86935.26	97137.03	90370.41	86024.93	98235.74	89991.96	73494.84	79811.56	74073.26	71864.45	79124.58	78537.02	73082.51	22389.65	24126.29	22359.54	15455.20	13890.77	11445.24	17958.40	17930.44	16719.13	18296.10	16377.86	12062.38		
1038	COP9 signalosome complex subunit 3	Q9UNS2	30804.80	25846.54	27602.52	18354.42	16817.33	17525.28	24252.39	23839.68	22543.21	25177.29	24659.73	22777.03	24140.10	22748.40	18794.90	20062.24	19743.84	19139.86	20865.94	20383.34	19289.24	15799.56	14070.98	14986.09	13042.74	12919.26	9546.11	16257.80	15619.69	14447.74	14282.21	14401.64	13598.29		
1039	COP9 signalosome complex subunit 4	Q9BT78	82448.99	79339.71	77483.76	54842.02	50458.84	48053.17	58310.81	62368.80	58964.79	69059.93	65784.27	59616.67	63771.05	59120.88	47224.83	49718.79	49721.05	48579.87	54819.39	51930.75	51103.93	55212.81	49528.92	48224.47	33827.70	31547.07	27807.72	45313.60	43881.89	41665.65	41689.16	40192.95	26597.31		
1040	COP9 signalosome complex subunit 5	Q92905	36208.81	31952.81	30314.55	22873.74	21407.77	18688.64	24895.60	24084.90	22529.63	30291.32	29853.51	24687.39	31145.79	26967.81	20776.06	23979.63	21035.94	20014.05	22760.76	20679.36	19176.00	2.27	1.49	0.65	0.84	1.31	0.69	4.16	2.47	0.11	4.90	4.98	0.12	x	
1041	COP9 signalosome complex subunit 6	Q7L5N1	53299.89	47442.36	42324.41	26052.91	24090.75	21574.46	34362.55	32629.40	29628.32	38467.54	36640.07	30237.76	34301.28	30037.38	22467.77	29354.59	27015.32	23589.01	26584.76	25075.52	21548.53	29214.22	27521.16	25314.03	15101.90	16225.89	14784.69	22491.94	20752.93	19544.89	19501.74	19941.33	13407.40		
1042	COP9 signalosome complex subunit 7a	Q9UBW8	39523.29	37588.45	38210.61	25698.17	23348.54	22797.48	30108.02	29078.59	26794.25	33680.39	32211.24	31050.93	31562.81	29430.88	24601.47	25715.32	24678.65	24723.93	25124.87	24779.60	20703.59	3084.57	2957.61	2603.41	1403.89	1376.07	1300.39	2362.53	2398.99	2117.09	2076.64	2015.57	1238.02		
1043	COP9 signalosome complex subunit 8	Q99627	37390.37	35829.89	33069.72	21544.72	19688.60	18214.98	28170.00	27041.60	25377.67	31631.94	30997.14	26924.96	27284.21	25509.98	21848.59	22822.61	22386.01	20373.53	25438.59	21922.20	21560.44	24559.93	22695.12	22864.84	19651.12	19215.89	16632.23	25195.39	23626.22	22664.60	27781.76	24978.04	16957.94		
1044	Copine-1	Q99829	0.14	2.53	0.81	1.07	2.77	0.68	0.96	0.36	0.63	0.35	0.75	0.38	2.13	0.97	0.60	4.64	1.07	0.47	2.22	0.19	0.06	103736.95	87932.64	81097.10	60665.90	57799.90	49603.77	80391.15	73037.83	68355.86	67389.65	62585.88	42397.49		x
1045	Copine-2	Q96FN4	0.14	2.53	0.81	1.07	2.77	0.68	0.96	0.36	0.63	0.35	0.75	0.38	2.13	0.97	0.60	4.64	1.07	0.47	2.22	0.19	0.06	42208.74	38485.54	37202.83	27917.74	27876.69	24570.71	32666.13	31396.28	28924.97	33613.03	32514.54	23992.22		x
1046	Copine-3	O75131	90337.28	86232.18	83178.67	66937.52	64466.09	59077.36	79278.90	77161.45	74163.63	79168.54	75306.66	70085.39	71987.47	67964.80	57400.91	67949.57	65052.53	64478.48	56833.10	54837.39	53741.65	40195.42	37255.88	37629.38	29711.47	27519.74	26205.85	33209.06	30197.00	30552.56	30851.91	29227.93	22519.30		
1047	Copine-5	Q9HCH3	10004.16	7821.51	6943.32	3077.74	4834.22	3018.26	5264.99	9801.55	6706.72	6709.98	5266.62	3862.55	6931.39	3608.36	1711.98	4928.18	3638.79	3196.05	2338.34	2140.68	1922.22	1312.47	1147.43	1199.27	952.93	846.64	732.20	996.05	1006.88	866.64	1015.24	945.83	356.83		
1048	Copine-8	Q86YQ8	0.14	2.53	0.81	1.07	2.77	0.68	0.96	0.36	0.63	0.35	0.75	0.38	2.13	0.97	0.60	4.64	1.07	0.47	2.22	0.19	0.06	11670.44	10833.52	9795.08	6014.06	6012.03	4718.03	6722.58	6966.78	6669.27	7314.89	6914.20	4428.43		x
1049	Copper transport protein ATOX1	O00244	90109.57	84700.87	75402.98	58772.54	54236.50	48514.41	69819.65	64848.67	61930.45	79659.37	75054.22	68410.14	67589.41	65420.88	48485.94	58343.65	55743.94	50341.43	57154.21	54143.82	50988.53	38691.84	34624.35	32914.73	21673.86	21313.11	18446.02	25173.02	22216.08	21609.59	25483.02	25861.46	16702.06		
1050	Cordon-bleu protein-like 1	Q53SF7	15690.38	20226.76	20482.92	17116.23	16038.89	13459.05	16184.28	14439.31	12886.94	21557.28	20265.08	20811.47	19787.97	13404.15	9717.05	12488.72	14643.24	14417.45	17426.30	16076.08	15614.80	2.27	1.49	0.65	0.84	1.31	0.69	4.16	2.47	0.11	4.90	4.98	0.12	x	
1051	Coronin-1A	P31146	14074.52	14070.44	13780.61	8481.89	10922.07	9172.74	13585.66	13209.30	12461.96	11143.11	10341.17	9230.17	10114.31	11352.36	8818.86	10564.14	9033.30	9316.28	12495.38	10404.31	10105.01	559.14	473.28	465.84	343.43	281.03	168.68	366.77	373.89	383.15	397.90	393.94	134.32		
1052	Coronin-1B	Q9BR76	177443.80	164334.97	159066.10	103001.11	96679.77	91914.98	103302.00	103613.98	97067.41	122370.75	116524.17	105758.08	133283.24	110029.27	90327.27	102610.60	99660.87	96657.41	105836.44	94167.52	88279.54	387012.85	360308.05	333718.36	253933.67	243355.86	209083.75	305237.19	300667.82	283135.62	285658.74	298893.64	202997.43		
1053	Coronin-1C	Q9ULV4	0.14	2.53	0.81	1.07	2.77	0.68	0.96	0.36	0.63	0.35	0.75	0.38	2.13	0.97	0.60	4.64	1.07	0.47	2.22	0.19	0.06	9307.77	8145.92	7622.45	7764.30	7837.36	7235.50	7616.15	7427.37	7197.34	13081.71	12460.09	8370.46		x
1054	Coronin-2A	Q92828	0.14	2.53	0.81	1.07	2.77	0.68	0.96	0.36	0.63	0.35	0.75	0.38	2.13	0.97	0.60	4.64	1.07	0.47	2.22	0.19	0.06	3569.98	3250.84	2939.25	2322.76	2274.65	1533.77	2801.82	2609.13	2122.11	3794.68	3472.10	1667.25		x
1055	Coronin-7	P57737	74783.40	67637.44	63089.71	46287.32	48601.65	42172.27	56848.22	55561.71	50091.48	66773.43	64762.17	57750.18	65840.38	61212.76	38534.83	44031.17	44374.44	46227.58	47742.62	45945.38	46335.82	2852.08	2807.35	2800.10	1671.35	1649.38	1412.09	2219.71	2362.07	2152.66	2046.64	1903.94	1140.07		
1056	Corrinoid adenosyltransferase	Q96EY8	4523.40	3500.45	4309.08	2634.31	2765.03	2443.07	3857.59	2837.28	2734.53	3240.65	3064.24	3196.60	3212.04	3113.75	2629.23	2853.36	2603.73	2402.42	2827.88	2567.36	2469.20	2.27	1.49	0.65	0.84	1.31	0.69	4.16	2.47	0.11	4.90	4.98	0.12	x	
1057	Costars family protein ABRACL	Q9P1F3	10362.22	10175.83	10868.51	7173.40	7028.45	6872.65	9077.40	8868.37	8614.18	9926.96	9379.37	9280.39	8237.26	8151.82	7097.18	8103.56	7885.01	8059.35	7250.67	7252.82	6775.91	2.27	1.49	0.65	0.84	1.31	0.69	4.16	2.47	0.11	4.90	4.98	0.12	x	
1058	COX assembly mitochondrial protein 2 homolog	Q9NRP2	3053.08	3310.60	3361.60	1920.15	1629.84	1588.65	860.94	615.44	229.21	2611.04	2591.63	3517.44	2491.61	2263.60	858.73	610.76	1197.64	1446.41	3738.52	2280.89	2056.05	2.27	1.49	0.65	0.84	1.31	0.69	4.16	2.47	0.11	4.90	4.98	0.12	x	
1059	Coxsackievirus and adenovirus receptor	P78310	0.14	2.53	0.81	1.07	2.77	0.68	0.96	0.36	0.63	0.35	0.75	0.38	2.13	0.97	0.60	4.64	1.07	0.47	2.22	0.19	0.06	7370.32	5829.73	4961.88	3652.88	3396.90	2604.01	6156.23	5224.43	4668.87	5089.46	5031.57	3069.64		x
1060	Craniofacial development protein 1	Q9UEE9	17409.76	16301.08	14787.95	8023.57	7747.08	7042.12	11681.85	10623.55	10085.02	10273.69	9180.27	9386.25	10781.44	9617.18	8309.96	9318.65	8022.55	8136.23	8833.68	9022.55	8605.67	21410.40	17621.70	16995.46	12792.65	12884.97	11231.29	15301.21	15247.74	14028.36	13973.20	13545.21	9204.06		
1061	Creatine kinase B-type	P12277	0.14	2.53	0.81	1.07	2.77	0.68	0.96	0.36	0.63	0.35	0.75	0.38	2.13	0.97	0.60	4.64	1.07	0.47	2.22	0.19	0.06	60941.81	41032.48	36666.09	32196.38	30910.27	23822.50	43608.40	41036.73	33426.47	47968.62	43026.01	23616.80		x
1062	Creatine kinase U-type mitochondrial	P12532	30384.98	29435.00	29406.22	22802.89	22263.83	21097.74	23671.90	23989.98	22355.47	26528.59	26672.24	25177.84	27568.13	26347.98	14407.52	25645.77	21557.57	22268.15	22908.41	21676.71	21351.86	2.27	1.49	0.65	0.84	1.31	0.69	4.16	2.47	0.11	4.90	4.98	0.12	x	
1063	Crk-like protein	P46109	47091.51	42954.39	38909.94	25602.71	25379.39	23229.60	33664.91	29113.85	27828.78	37110.49	34910.90	32174.63	37015.73	30201.44	23529.30	26215.11	22851.59	24019.05	26359.17	25989.51	24448.71	26275.37	22912.38	21187.30	17302.06	16509.86	13457.37	19474.64	19098.58	15708.57	20161.67	19607.85	12758.52		
1064	Crooked neck-like protein 1	Q9BZJ0	0.14	2.53	0.81	1.07	2.77	0.68	0.96	0.36	0.63	0.35	0.75	0.38	2.13	0.97	0.60	4.64	1.07	0.47	2.22	0.19	0.06	5726.62	5273.58	5054.67	4488.81	4368.72	3770.08	5402.49	4863.31	4620.05	4842.85	4799.02	4011.45		x
1065	Cryptochrome-2	Q49AN0	0.14	2.53	0.81	1.07	2.77	0.68	0.96	0.36	0.63	0.35	0.75	0.38	2.13	0.97	0.60	4.64	1.07	0.47	2.22	0.19	0.06	33652.41	32645.56	31983.10	22504.96	22703.38	21411.94	22685.82	20460.21	20668.58	21755.63	20242.29	16234.07		x
1066	CST complex subunit STN1	Q9H668	11275.61	11536.54	10086.80	7157.72	7170.42	5847.24	8659.28	7765.70	8341.34	10035.93	10017.69	8403.32	9390.72	7712.58	6474.78	7721.59	7808.97	7246.11	7584.81	7306.76	6651.93	2.27	1.49	0.65	0.84	1.31	0.69	4.16	2.47	0.11	4.90	4.98	0.12	x	
1067	cTAGE family member 2	Q96RT6	367.87	104.51	85.53	143.22	41.70	86.98	101.46	57.92	42.92	178.24	225.91	142.35	26.26	91.49	113.75	89.78	171.30	132.42	224.47	130.34	81.66	2.27	1.49	0.65	0.84	1.31	0.69	4.16	2.47	0.11	4.90	4.98	0.12	x	
1068	C-terminal-binding protein 1	Q13363	1574.72	1313.50	1039.79	1037.04	853.09	669.86	864.45	1035.37	634.43	1045.33	955.55	805.29	1154.46	1032.68	426.85	888.08	881.52	668.52	817.91	702.93	535.56	2.27	1.49	0.65	0.84	1.31	0.69	4.16	2.47	0.11	4.90	4.98	0.12	x	
1069	C-terminal-binding protein 2	P56545	45668.47	40808.76	38186.37	26473.70	26489.08	25175.93	31019.65	30150.30	31393.56	34414.55	33826.50	30503.56	40128.79	28370.25	24527.21	30632.18	26324.19	26518.15	32248.88	28448.70	24860.78	2.27	1.49	0.65	0.84	1.31	0.69	4.16	2.47	0.11	4.90	4.98	0.12	x	
1070	CTP synthase 1	P17812	115233.70	115209.36	115187.38	78265.10	75352.54	71416.98	100362.03	98168.52	93699.11	98825.29	94157.87	90895.20	94745.34	98919.48	85464.84	77538.74	74019.97	72090.76	81555.38	77981.37	75887.08	228276.35	210868.37	200173.43	143900.70	141622.71	128878.91	191109.66	178392.86	170106.00	174061.25	173311.51	124209.84		
1071	CTP synthase 2	Q9NRF8	30387.87	28441.83	24594.82	17084.83	15359.45	13423.30	20606.07	21359.16	18407.73	22139.83	20891.14	17631.48	21571.85	21926.81	14739.23	17487.91	15674.11	14872.55	17216.18	16420.67	14889.86	2.27	1.49	0.65	0.84	1.31	0.69	4.16	2.47	0.11	4.90	4.98	0.12	x	
1072	CTTNBP2 N-terminal-like protein	Q9P2B4	0.14	2.53	0.81	1.07	2.77	0.68	0.96	0.36	0.63	0.35	0.75	0.38	2.13	0.97	0.60	4.64	1.07	0.47	2.22	0.19	0.06	6608.69	6084.61	6227.36	4131.17	4212.21	3548.74	4751.23	4429.94	4297.78	5459.01	4575.78	2851.36		x
1073	C-type lectin domain family 12 member A	Q5QGZ9	0.14	2.53	0.81	1.07	2.77	0.68	0.96	0.36	0.63	0.35	0.75	0.38	2.13	0.97	0.60	4.64	1.07	0.47	2.22	0.19	0.06	24898.18	22658.35	19038.48	9610.99	9738.86	5677.71	25217.88	21454.19	16463.29	20369.41	20791.45	8345.54		x
1074	C-type lectin domain family 2 member B	Q92478	0.14	2.53	0.81	1.07	2.77	0.68	0.96	0.36	0.63	0.35	0.75	0.38	2.13	0.97	0.60	4.64	1.07	0.47	2.22	0.19	0.06	158.27	120.49	118.07	163.74	129.79	54.89	89.78	89.86	71.20	213.26	255.43	96.60		x
1075	C-type lectin domain family 2 member D	Q9UHP7	550.06	472.26	438.35	255.15	272.01	190.03	373.83	333.08	299.69	321.69	254.31	282.01	384.33	198.40	104.52	348.98	125.22	329.21	267.91	203.71	180.40	2.27	1.49	0.65	0.84	1.31	0.69	4.16	2.47	0.11	4.90	4.98	0.12	x	
1076	C-type lectin domain family 4 member A	Q9UMR7	9425.85	8958.52	7599.80	8575.05	7702.33	7042.13	7791.60	7538.32	6746.28	9331.74	8832.59	7806.06	8579.99	7327.06	5090.96	9315.16	9464.46	8785.36	7835.66	7470.89	6160.70	2.27	1.49	0.65	0.84	1.31	0.69	4.16	2.47	0.11	4.90	4.98	0.12	x	
1077	C-type lectin domain family 4 member D	Q8WXI8	58136.56	54279.06	53291.83	36337.43	34916.07	31197.91	44782.23	42934.88	38893.60	46755.10	42610.77	37530.56	40446.49	36492.75	29311.67	37495.56	37111.67	34935.92	37796.12	34732.58	25997.67	2.27	1.49	0.65	0.84	1.31	0.69	4.16	2.47	0.11	4.90	4.98	0.12	x	
1078	C-type lectin domain family 4 member G	Q6UXB4	5297.59	3853.70	3296.81	2282.06	2491.78	1533.19	2970.76	2792.96	2666.31	3016.47	2919.44	2189.93	4135.10	2899.91	911.99	2732.17	2689.03	2012.00	2684.91	2331.07	1893.76	2.27	1.49	0.65	0.84	1.31	0.69	4.16	2.47	0.11	4.90	4.98	0.12	x	
1079	C-type mannose receptor 2	Q9UBG0	0.14	2.53	0.81	1.07	2.77	0.68	0.96	0.36	0.63	0.35	0.75	0.38	2.13	0.97	0.60	4.64	1.07	0.47	2.22	0.19	0.06	3736.40	3626.71	3317.16	1298.07	1280.47	1110.91	962.24	1133.04	1021.33	1056.43	1034.17	531.54		x
1080	CUB domain-containing protein 1	Q9H5V8	0.14	2.53	0.81	1.07	2.77	0.68	0.96	0.36	0.63	0.35	0.75	0.38	2.13	0.97	0.60	4.64	1.07	0.47	2.22	0.19	0.06	79748.69	79455.71	73569.24	46031.95	45238.22	41429.61	57237.06	53874.22	53291.77	56852.48	55393.90	38039.02		x
1081	CUE domain-containing protein 1	Q9NWM	7168.43	6153.19	5619.56	2317.63	1985.72	1750.64	5284.31	6531.63	5037.32	7882.99	7695.10	7241.44	3940.67	4632.62	2290.44	7817.24	8732.30	7673.43	5512.53	5167.64	4270.75	2.27	1.49	0.65	0.84	1.31	0.69	4.16	2.47	0.11	4.90	4.98	0.12	x	
1082	CUGBP Elav-like family member 1	Q92879	2848.09	3109.86	3049.70	2385.17	2330.47	2265.16	5647.01	5740.38	5383.19	2459.97	2413.04	2074.29	2921.68	3020.50	2390.38	2144.84	1995.03	2234.38	2609.73	2012.61	2515.58	15450.81	14120.96	15403.42	6773.48	6939.29	6075.68	5127.73	5232.72	4899.86	4960.02	4683.26	3360.42		
1083	Cullin-1	Q13616	50877.48	44974.68	42352.34	42990.53	31731.33	27553.09	33412.83	31604.73	31876.31	43303.47	39577.71	36721.37	43442.59	48796.92	29111.73	26603.55	25513.59	24590.73	41151.74	37546.53	35393.41	35380.96	31963.87	31814.44	24121.14	23974.42	20460.59	28162.54	26936.89	24992.36	29118.68	28054.37	22476.97		
1084	Cullin-2	Q13617	237827.26	213230.63	197949.14	136283.99	137505.85	120646.63	186747.27	180402.31	157554.80	171936.68	164466.56	149604.22	161752.63	157754.45	118360.06	155865.98	145141.62	135528.29	138772.70	129703.13	124333.80	115830.04	108355.94	90007.33	68559.24	63326.71	51429.95	89256.12	80281.03	69859.87	78121.22	76939.75	52023.93		
1085	Cullin-3	Q13618	179520.62	167868.13	151626.81	105150.97	98887.59	88960.38	137799.34	132517.28	131107.39	139750.32	136812.88	125179.00	137850.24	131759.73	97030.31	119125.72	124087.82	112074.78	118226.42	109747.26	104864.46	64668.89	62463.58	58109.34	58757.03	58628.21	52583.69	87087.19	83983.94	80749.10	50284.20	50405.55	34757.75		
1086	Cullin-4A	Q13619	10431.43	9840.75	9026.97	6778.90	17711.38	14104.19	24069.91	19826.86	17859.26	8840.92	8761.04	7940.44	8373.45	8351.39	7467.71	16952.63	15925.55	14721.26	6924.80	6624.20	6199.12	31492.61	37245.43	32663.01	23381.59	23228.88	17955.04	27175.75	26094.26	23918.20	30427.81	29279.80	18298.45		
1087	Cullin-4B	Q13620	65292.77	57869.47	47777.81	43911.57	42233.23	38001.99	46178.35	43792.01	40163.85	57956.27	54331.65	47866.04	57980.85	50494.33	33454.60	38972.34	40234.60	37338.79	46219.76	43062.81	35690.19	33657.49	34831.24	29069.79	21915.99	21438.63	19661.55	26510.43	25226.49	23419.68	28061.66	25401.73	18325.32		
1088	Cullin-5	Q93034	0.14	2.53	0.81	1.07	2.77	0.68	0.96	0.36	0.63	0.35	0.75	0.38	2.13	0.97	0.60	4.64	1.07	0.47	2.22	0.19	0.06	1898.36	1905.51	1553.73	1103.34	1029.79	797.13	1333.61	1314.70	1398.25	1291.65	1258.50	858.31		x
1089	Cullin-associated NEDD8-dissociated protein 1	Q86VP6	627381.78	616520.52	575366.30	415847.50	393737.62	374322.76	513674.61	498590.94	472862.59	499555.39	483871.35	445658.37	516394.39	468721.40	409291.55	432492.78	404482.95	401860.92	426800.88	409295.87	383851.75	175902.45	168263.42	151921.71	117195.65	115357.35	101605.22	142807.78	135949.97	126729.59	156229.70	148733.37	102645.62		
1090	Cullin-associated NEDD8-dissociated protein 2	O75155	6731.06	6041.53	6213.36	2597.01	2412.76	2343.39	4863.74	5699.76	5324.50	6721.17	6195.96	6263.51	3587.55	3529.01	3284.58	2222.08	2095.96	1997.06	3723.20	4063.74	3549.56	2.27	1.49	0.65	0.84	1.31	0.69	4.16	2.47	0.11	4.90	4.98	0.12	x	
1091	CWF19-like protein 2	Q2TBE0	479.18	497.61	432.38	216.74	255.82	259.43	597.10	485.11	477.31	252.29	235.35	292.35	369.76	325.87	222.16	293.28	300.00	280.41	280.64	350.77	472.96	2.27	1.49	0.65	0.84	1.31	0.69	4.16	2.47	0.11	4.90	4.98	0.12	x	
1092	CXXC motif containing zinc binding protein	Q9NWV4	43501.04	43538.44	37530.23	28685.86	28432.76	26346.32	32389.69	29062.57	29161.74	35219.93	33625.49	29607.95	33080.15	29527.50	22885.18	31814.55	28812.40	29173.80	28508.87	26928.80	23673.25	8305.35	9478.78	8272.88	8104.67	7947.44	6198.87	8692.72	7921.46	7079.03	10777.86	10367.09	6586.63		
1093	CXXC-type zinc finger protein 1	Q9P0U4	21763.18	20495.43	18217.64	13803.44	12868.46	12282.93	16868.38	14601.42	14926.11	17493.81	16820.52	14748.85	17948.66	15954.81	15634.76	14532.92	13344.01	13133.20	13704.74	13581.95	12557.46	2.27	1.49	0.65	0.84	1.31	0.69	4.16	2.47	0.11	4.90	4.98	0.12	x	
1094	Cyclic AMP-responsive element-binding protein 3-like protein 4	Q8TEY5	0.14	2.53	0.81	1.07	2.77	0.68	0.96	0.36	0.63	0.35	0.75	0.38	2.13	0.97	0.60	4.64	1.07	0.47	2.22	0.19	0.06	14808.93	12002.39	14239.31	10536.14	10985.34	10732.35	13080.39	13360.79	12899.47	14261.51	13990.79	10248.55		x
1095	Cyclic nucleotide-gated cation channel alpha-3	Q16281	16047.40	15433.23	15555.37	11866.81	12497.30	12064.24	14549.45	13893.31	14982.59	16212.19	15582.70	15060.55	15208.35	13609.34	11254.85	13724.13	10854.76	12210.88	9452.75	11451.03	11917.93	23771.43	22036.15	21199.83	12752.18	12879.38	11982.10	17067.46	16094.44	16228.74	17660.73	16707.20	12060.69		
1096	Cyclic nucleotide-gated cation channel alpha-4	Q8IV77	945.92	914.69	619.72	511.74	405.24	367.20	1067.80	1082.58	1331.85	554.76	557.85	485.41	740.11	603.22	286.72	656.29	666.03	589.02	690.25	468.30	381.33	2.27	1.49	0.65	0.84	1.31	0.69	4.16	2.47	0.11	4.90	4.98	0.12	x	
1097	Cyclin-dependent kinase 1	P06493	131062.12	125150.22	120925.52	84567.47	78891.57	76360.01	100775.06	99595.76	97473.99	87656.11	82949.69	81559.15	93538.24	85811.04	70391.19	78781.55	74025.01	74440.30	88747.04	86016.92	79996.70	210195.79	197438.24	185270.96	120894.51	117105.22	104563.09	151514.91	145393.64	138176.96	143940.38	139249.98	97737.79		
1098	Cyclin-dependent kinase 12	Q9NYV4	0.14	2.53	0.81	1.07	2.77	0.68	0.96	0.36	0.63	0.35	0.75	0.38	2.13	0.97	0.60	4.64	1.07	0.47	2.22	0.19	0.06	14003.96	12506.43	11385.71	9556.80	9798.23	9764.50	9443.16	9186.29	9209.12	11254.71	10724.64	9646.27		x
1099	Cyclin-dependent kinase 13	Q14004	8379.64	7769.19	7260.91	8699.12	8509.37	7788.37	8748.07	8288.03	8063.45	10772.72	10669.57	10712.67	7061.39	7612.73	6418.74	9691.97	8805.67	8216.91	8674.81	8723.86	8546.53	2681.28	2579.86	2514.21	1273.42	1363.48	1072.33	3451.52	3203.55	3005.49	2404.63	2307.20	979.74		
1100	Cyclin-dependent kinase 15	Q96Q40	16863.50	14903.44	13973.02	10064.45	8941.24	8288.66	14754.24	14675.82	13908.07	12594.41	14005.20	11433.58	14302.63	12235.43	8683.99	10293.41	9684.48	9175.14	10859.31	10575.14	9277.87	2.27	1.49	0.65	0.84	1.31	0.69	4.16	2.47	0.11	4.90	4.98	0.12	x	
1101	Cyclin-dependent kinase 16	Q00536	2391.88	2053.45	1792.38	1445.87	1469.51	957.62	2019.08	1807.81	1599.24	2052.05	2046.66	1746.75	1734.91	1615.20	1003.02	1520.76	1329.68	1376.37	1746.53	1730.67	1238.26	2.27	1.49	0.65	0.84	1.31	0.69	4.16	2.47	0.11	4.90	4.98	0.12	x	
1102	Cyclin-dependent kinase 17	Q00537	0.14	2.53	0.81	1.07	2.77	0.68	0.96	0.36	0.63	0.35	0.75	0.38	2.13	0.97	0.60	4.64	1.07	0.47	2.22	0.19	0.06	1886.68	2023.37	1815.02	1644.65	1793.28	1546.10	2246.37	2114.16	2108.33	2162.98	2427.83	1381.22		x
1103	Cyclin-dependent kinase 18	Q07002	168.32	164.97	71.11	137.18	156.22	80.81	49.10	98.99	36.17	121.68	154.14	20.12	203.09	106.40	165.05	85.91	112.16	95.99	166.20	156.50	166.02	2.27	1.49	0.65	0.84	1.31	0.69	4.16	2.47	0.11	4.90	4.98	0.12	x	
1104	Cyclin-dependent kinase 2	P24941	1386.26	1546.81	1158.75	874.69	961.45	809.44	1228.65	1114.54	979.73	1254.80	1233.95	1178.34	1309.51	1474.96	1044.87	1052.67	916.04	771.83	942.38	873.46	980.14	4186.25	4117.53	3561.45	2221.12	2375.92	1809.44	2855.37	3245.78	3033.07	2942.56	2882.13	1758.97		
1105	Cyclin-dependent kinase 3	Q00526	161112.99	159832.45	146270.94	103222.58	101781.12	92202.54	119203.99	119344.95	111577.99	123279.17	117095.52	108834.95	126121.68	117127.22	91525.28	117750.35	109578.63	106917.22	96420.90	91689.76	84395.08	80945.19	78151.32	72703.28	43684.79	43786.77	37951.56	78013.39	74960.91	70433.24	64512.89	59734.20	40659.43		
1106	Cyclin-dependent kinase 4	P11802	597.03	684.73	472.17	284.37	221.84	209.79	313.15	258.15	275.17	546.51	583.51	518.33	336.17	291.71	249.58	325.40	264.54	207.99	232.47	274.40	215.13	1253.48	954.19	751.32	532.86	500.60	217.36	1085.33	932.02	538.87	966.63	902.76	388.05		
1107	Cyclin-dependent kinase 6	Q00534	206.99	181.39	297.31	58.55	85.96	73.99	52.27	65.77	99.85	86.07	28.37	68.22	276.49	179.74	173.14	247.19	196.26	124.40	113.47	94.59	60.86	2861.85	1842.28	1258.16	1754.45	1773.96	1391.94	3764.91	3466.52	3290.46	2019.70	1702.71	946.74		
1108	Cyclin-dependent kinase 9	P50750	13507.16	13095.89	13436.50	7388.94	6967.19	6682.81	9455.19	9469.56	8225.45	10327.26	9926.84	9441.09	10017.15	8663.55	7418.07	7225.78	7176.25	7106.24	9639.29	8816.20	8413.62	5337.19	3098.94	3129.61	3825.75	3697.87	3135.91	3792.30	3705.74	3063.39	4838.90	4558.95	2948.74		
1109	Cyclin-dependent kinase inhibitor 1	P38936	129431.47	110196.85	86167.01	84159.95	77135.82	62623.86	35900.81	35803.15	28131.48	84040.55	83670.06	69562.75	76701.50	69617.79	35299.70	59593.01	56658.63	49775.83	73317.47	73077.07	61576.43	902.68	924.66	852.16	197.57	167.77	152.20	184.41	111.45	128.38	147.75	164.62	71.05		
1110	Cyclin-dependent kinase inhibitor 3	Q16667	31886.59	30627.50	28285.53	24236.83	23538.78	23098.48	23110.07	23485.33	22296.12	31363.16	29484.20	28010.81	30125.42	29811.72	24709.57	22599.29	19630.32	20399.68	22163.34	21196.73	21546.25	27747.42	26083.66	25626.02	23384.29	21998.95	21634.96	26340.26	25602.82	25524.30	29383.47	27107.51	21500.12		
1111	Cyclin-dependent kinase-like 5	O76039	38139.38	36126.51	36546.71	23688.01	22456.02	21700.59	24258.25	22571.22	23438.73	34820.45	32980.77	38766.81	33520.29	29735.47	22275.77	18773.20	18401.14	19102.11	25180.59	21982.40	20984.28	27325.27	26839.32	24909.52	19398.37	19729.78	18654.54	22150.17	21142.09	21147.06	22814.74	21748.56	13913.20		
1112	Cyclin-dependent kinases regulatory subunit 1	P61024	3616.24	4557.45	3757.39	4424.34	4249.72	3577.55	4025.00	3696.25	3565.60	4070.45	3838.32	3728.67	5516.97	4445.45	2568.21	3326.84	3175.82	2999.42	3914.21	4102.41	3304.49	2.27	1.49	0.65	0.84	1.31	0.69	4.16	2.47	0.11	4.90	4.98	0.12	x	
1113	Cyclin-dependent-like kinase 5	Q00535	27808.02	28759.79	23570.58	14611.47	14492.41	12603.92	23674.44	20698.93	20240.90	19181.58	17966.47	16565.82	20014.61	18024.78	15661.88	15493.02	14755.64	13142.10	17351.50	16052.07	15840.26	2178.74	2334.17	2073.04	827.54	794.60	561.15	1558.08	1390.93	1385.60	1269.89	947.69	479.35		
1114	Cyclin-K	O75909	1822.58	1674.15	1563.63	814.73	816.57	750.99	1078.28	1048.78	1073.31	1168.98	1204.86	1080.68	1280.57	1096.64	815.80	900.43	745.96	723.45	1185.59	998.53	899.82	2.27	1.49	0.65	0.84	1.31	0.69	4.16	2.47	0.11	4.90	4.98	0.12	x	
1115	Cystathionine beta-synthase	P35520	0.14	2.53	0.81	1.07	2.77	0.68	0.96	0.36	0.63	0.35	0.75	0.38	2.13	0.97	0.60	4.64	1.07	0.47	2.22	0.19	0.06	127528.90	121117.30	118182.76	94283.02	91649.55	85594.66	97026.43	96935.01	94523.75	104300.22	94232.01	64710.23		x
1116	Cystathionine beta-synthase-like protein	P0DN79	200073.19	199905.08	189686.80	141758.15	151003.74	132344.00	192284.33	185965.92	173024.70	176265.67	166060.92	164507.88	176260.75	187846.42	119972.78	167968.31	162779.36	156856.60	147272.80	125410.53	129112.81	2.27	1.49	0.65	0.84	1.31	0.69	4.16	2.47	0.11	4.90	4.98	0.12	x	
1117	Cystatin-B	P04080	130338.58	124172.27	112355.29	77681.70	71690.39	68875.38	94939.19	90286.67	87492.36	100634.68	97291.29	92695.69	86717.44	81715.75	69802.84	93298.67	88886.74	86196.95	80508.96	71928.39	68622.62	63508.38	58642.66	56432.50	33590.39	32979.97	29743.62	52482.84	50057.05	48054.89	41044.02	44618.44	29488.30		
1118	Cystatin-C	P01034	6452.86	6733.25	5455.78	5104.22	4380.55	3988.23	3826.07	3655.46	3569.76	5190.89	5299.22	4745.05	6374.63	6489.23	5059.32	4302.00	4413.45	3789.26	3874.43	3336.50	3227.85	2.27	1.49	0.65	0.84	1.31	0.69	4.16	2.47	0.11	4.90	4.98	0.12	x	
1119	Cysteine and glycine-rich protein 1	P21291	346332.89	348494.77	331113.18	233111.08	224687.96	202977.94	251427.86	234122.00	227929.56	308188.27	285995.01	281019.04	283932.31	258245.62	198903.07	234981.43	229656.87	213795.62	211419.07	202908.43	187271.89	209058.42	202639.30	187579.53	122166.48	114587.91	100773.49	148086.80	140072.95	136949.06	148193.91	136458.81	82719.03		
1120	Cysteine and glycine-rich protein 2	Q16527	6555.52	6721.02	6664.52	4841.26	4424.70	3876.40	5712.99	5678.18	5217.46	5489.00	5896.91	5283.70	5562.75	6007.68	4847.26	4534.42	4450.10	4359.84	4240.45	4471.92	4310.37	30232.15	28453.88	26756.02	24673.45	23597.04	20411.36	25478.42	25495.68	25226.96	26230.54	25403.71	17617.39		
1121	Cysteine and histidine-rich domain-containing protein 1	Q9UHD1	108532.37	103118.47	87292.95	66011.28	62098.19	54356.37	81741.32	78041.20	70309.46	84411.58	81460.98	71879.16	82063.74	74763.92	49880.06	69772.61	64659.31	60298.29	68098.07	63637.84	57023.30	158308.93	152103.80	136986.59	89577.79	86377.23	73117.94	118893.68	112716.76	104993.22	125447.37	120253.05	73986.48		
1122	Cysteine and histidine-rich protein 1	Q6ZMK1	3339.34	4156.87	2518.26	1858.02	1996.23	1228.14	2744.09	2763.55	2435.29	2224.75	2020.79	2090.27	2632.68	1772.84	1603.40	1976.51	2140.88	1794.60	2179.95	1706.65	1908.74	2.27	1.49	0.65	0.84	1.31	0.69	4.16	2.47	0.11	4.90	4.98	0.12	x	
1123	Cysteine dioxygenase type 1	Q16878	0.14	2.53	0.81	1.07	2.77	0.68	0.96	0.36	0.63	0.35	0.75	0.38	2.13	0.97	0.60	4.64	1.07	0.47	2.22	0.19	0.06	176.05	164.13	146.45	129.53	104.24	108.94	168.66	162.42	155.43	145.51	141.93	101.30		x
1124	Cysteine protease ATG4B	Q9Y4P1	11146.64	11190.03	9936.20	7892.83	6674.47	6378.69	9115.20	8875.68	8619.67	9505.14	8906.71	8273.45	10565.34	10410.45	7239.96	7155.37	6703.70	6672.75	7607.69	7749.28	6838.74	2293.77	1845.55	1526.43	1229.80	1086.69	551.33	1832.17	1722.99	1043.49	2268.21	2045.92	796.03		
1125	Cysteine protease ATG4C	Q96DT6	0.14	2.53	0.81	1.07	2.77	0.68	0.96	0.36	0.63	0.35	0.75	0.38	2.13	0.97	0.60	4.64	1.07	0.47	2.22	0.19	0.06	15895.55	14998.52	13890.09	13553.08	14213.33	14369.26	16618.62	15277.71	14826.51	16443.35	15015.47	12501.99		x
1126	Cysteine protease ATG4D	Q86TL0	9152.03	7771.07	6541.37	6079.72	6081.00	4514.01	6894.24	6370.72	5203.46	7231.30	6551.68	5524.40	7907.53	7865.68	3427.93	5590.37	5072.03	4240.94	5616.43	5027.90	4555.02	13388.16	10742.67	9979.19	11819.09	11454.47	8278.85	14848.68	13767.47	10806.15	15548.45	14534.78	8151.76		
1127	Cysteine/serine-rich nuclear protein 3	Q8WYN3	0.14	2.53	0.81	1.07	2.77	0.68	0.96	0.36	0.63	0.35	0.75	0.38	2.13	0.97	0.60	4.64	1.07	0.47	2.22	0.19	0.06	869.36	828.03	845.77	687.37	578.83	610.63	1072.49	890.92	924.72	724.33	747.71	596.04		x
1128	Cysteine-rich protein 1	P50238	0.14	2.53	0.81	1.07	2.77	0.68	0.96	0.36	0.63	0.35	0.75	0.38	2.13	0.97	0.60	4.64	1.07	0.47	2.22	0.19	0.06	33935.30	31371.06	30458.85	23300.21	21756.97	21193.01	22636.35	20397.53	20601.54	24733.32	22659.31	18589.82		x
1129	Cysteine-rich protein 2	P52943	291280.89	277428.31	265121.31	191123.46	183692.01	171793.88	199043.44	193424.88	196933.91	254333.85	238894.80	236393.08	243065.94	230937.87	209669.47	175638.94	171064.79	161536.66	181540.76	176525.75	172219.61	4485.44	4389.30	3840.62	2636.93	2580.89	2212.87	3097.40	2993.44	2716.47	3129.83	3068.92	1966.47		
1130	Cysteine-rich with EGF-like domain protein 2	Q6UXH1	60363.99	60199.10	56060.86	41892.50	39724.44	38240.27	51322.74	49995.28	46440.46	58347.42	54868.99	54223.37	53465.58	49003.74	39635.46	41779.47	39957.46	38183.28	45446.75	41836.17	40214.21	2.27	1.49	0.65	0.84	1.31	0.69	4.16	2.47	0.11	4.90	4.98	0.12	x	
1131	Cysteine--tRNA ligase cytoplasmic	P49589	135021.05	122780.99	113589.07	103300.31	104724.87	89853.26	128683.56	104446.18	95810.99	117311.68	119194.74	105677.32	111664.06	123197.09	74038.14	125713.31	102563.75	107596.11	104367.18	97350.62	83961.09	22211.84	21959.21	19894.20	17975.94	16772.71	13274.54	20255.34	19315.02	18557.78	20165.51	19143.84	13549.10		
1132	Cytochrome b-245 light chain	P13498	15548.00	15043.48	13344.48	15692.81	14744.55	13839.89	13389.76	11751.25	11293.29	18366.98	17573.74	16386.87	10352.26	9859.85	8234.60	25094.05	22542.87	22105.22	10999.12	10802.28	9952.46	3548.55	3194.75	2814.08	4169.85	3688.89	2968.88	3648.59	3551.80	3033.33	4969.59	4965.43	2821.74		
1133	Cytochrome b5	P00167	28667.10	27686.32	29506.00	23711.84	23554.19	20682.25	24732.87	23532.83	22797.91	30957.18	29803.99	27616.32	27509.00	26275.13	23674.08	23238.01	20529.49	21053.89	20583.50	19626.05	18878.50	10139.84	10090.71	9582.60	6409.54	6164.87	5417.63	9981.94	9668.24	9258.62	9271.99	9435.97	6145.70		
1134	Cytochrome b5 type B	O43169	122295.43	115808.52	111397.91	76692.91	69916.72	66480.02	85222.94	84576.34	79354.97	92060.27	87316.08	77619.95	94303.33	87187.16	72008.45	74562.01	74048.98	72884.42	77773.16	72942.00	68739.89	66453.24	65523.63	62027.84	46464.90	43124.56	36868.51	63896.27	60084.77	55691.30	65623.52	66594.97	38513.20		
1135	Cytochrome b561	P49447	8133.37	7630.10	6601.08	4610.28	4374.71	3731.58	4569.50	4020.36	4138.03	5663.91	5811.70	5054.75	6232.05	6040.37	4716.74	3864.38	3761.35	3306.49	4315.80	3984.74	3747.81	2.27	1.49	0.65	0.84	1.31	0.69	4.16	2.47	0.11	4.90	4.98	0.12	x	
1136	Cytochrome b-c1 complex subunit 1_ mitochondrial	P31930	308695.02	298914.57	281932.33	204854.69	194859.68	182630.48	244287.11	239721.48	222783.70	254902.75	248214.92	226537.68	243947.96	239845.98	188900.41	206036.22	197646.93	194291.84	219840.46	205082.78	195571.91	249911.39	230528.24	217408.86	162874.47	158890.99	141492.95	193831.90	190526.96	179119.95	217437.99	205831.69	146525.39		

Supplementary Table S1. Overview on the abundance of all relatively quantified 5180 proteins

			MCF-7																					MDA-MB-231													
	Protein name	UniProt	control 01	control 02	control 03	Dai IC20 01	Dai IC20 02	Dai IC20 03	Dai SC20 01	Dai SC20 02	Dai SC20 03	Gen IC20 01	Gen IC20 02	Gen IC20 03	Gen SC20 01	Gen SC20 02	Gen SC20 03	SSE IC20 01	SSE IC20 02	SSE IC20 03	SSE SC20 01	SSE SC20 02	SSE SC20 03	control 01	control 02	control 03	Dai IC20 01	Dai IC20 02	Dai IC20 03	Gen IC20 01	Gen IC20 02	Gen IC20 03	SSE IC20 01	SSE IC20 02	SSE IC20 03	unique to MCF-7	unique to MDA-MB-231
1137	Cytochrome b-c1 complex subunit 2_ mitochondrial	P22695	340303.01	330221.47	309752.89	219229.85	206903.43	195679.87	270558.08	254250.26	248278.19	286948.52	271988.19	254284.50	266824.41	247499.87	209056.83	217045.03	210161.78	203393.09	224263.80	214393.31	198062.61	183969.22	166856.00	154604.43	114734.87	107287.32	95230.81	143629.43	130819.48	128487.57	147204.68	137221.95	94797.29		
1138	Cytochrome b-c1 complex subunit 6_ mitochondrial	P07919	42584.66	41345.65	38052.65	31210.14	29448.11	24933.77	26592.96	25199.31	24937.46	42972.25	43385.67	39440.19	34667.65	28684.07	11074.58	23208.52	26623.50	27697.58	36801.46	34057.14	30393.45	28411.68	27040.16	23922.75	17816.91	17468.52	15148.14	18508.03	17408.24	17179.76	20042.34	17217.82	5357.91		
1139	Cytochrome b-c1 complex subunit 6-like_ mitochondrial	A0A096L	13164.28	13533.03	12644.25	10759.08	10117.37	9687.89	11320.80	10962.87	10587.73	13159.46	12141.88	12026.68	11143.87	11800.37	10161.52	10214.70	9630.26	9442.94	11088.90	10628.96	10411.83	2.27	1.49	0.65	0.84	1.31	0.69	4.16	2.47	0.11	4.90	4.98	0.12	x	
1140	Cytochrome b-c1 complex subunit 7	P14927	199859.65	193466.62	178777.28	136835.19	124059.10	112856.88	155987.19	144910.46	137703.57	170823.35	162091.78	144782.62	159242.62	145221.35	120219.68	130475.75	125796.09	119787.99	144457.55	135813.17	124545.27	75507.61	69897.38	65738.47	58901.57	57877.99	53085.16	57530.05	58024.68	54666.92	70256.91	62775.41	11540.82		
1141	Cytochrome b-c1 complex subunit 9	Q9UDW1	104961.80	105714.02	94229.39	76218.98	74713.94	63867.31	80087.61	77094.56	71870.80	94270.39	89161.58	80213.16	84113.89	74141.51	65231.14	76946.74	71559.69	63533.31	79705.60	74358.05	68196.16	50786.02	48360.87	41859.33	37751.22	36470.44	32332.54	43617.31	43355.96	40472.24	49025.59	45235.65	30201.52		
1142	Cytochrome b-c1 complex subunit Rieske_ mitochondrial	P47985	31767.23	28837.20	24945.75	20109.11	19530.18	16990.79	23220.16	21567.51	20792.87	26624.95	25769.11	23403.04	24914.83	22896.83	14964.37	20968.85	19762.14	18692.69	20932.57	20440.90	18329.50	15584.43	14640.92	14435.65	11224.21	10947.21	10120.03	15648.20	14479.68	14776.32	13958.94	13263.37	9776.53		
1143	Cytochrome c	P99999	128334.44	116806.22	103378.53	99140.49	91249.10	83690.48	114464.83	100044.98	95206.25	125065.62	112910.26	104949.69	98253.29	92873.55	62968.21	104416.00	99804.03	100884.21	93912.53	83994.68	80970.78	103956.19	90263.52	91713.70	73098.97	71169.96	61296.00	99171.99	88682.71	87132.04	85531.05	82564.15	57576.52		
1144	Cytochrome c oxidase assembly factor 6 homolog	Q5JTJ3	1837.20	1613.37	1444.32	1274.79	1302.29	1201.32	1444.94	1059.07	1063.86	1341.92	1262.56	1194.54	1452.17	1557.12	1175.24	1312.22	1187.56	1155.73	1261.83	1219.00	921.35	3682.93	3395.65	2859.64	2495.73	2240.53	2360.83	3001.48	3129.16	2831.07	2731.80	2573.16	1907.52		
1145	Cytochrome c oxidase assembly factor 7	Q96BR5	4637.91	4680.81	5274.39	2587.04	2576.13	2467.01	3308.76	3385.67	3087.97	3530.47	3352.86	2944.15	3283.71	3061.27	2523.86	2585.67	2595.12	2648.04	2658.69	2631.76	2354.00	12982.22	12580.44	11711.37	8201.68	8361.82	8012.56	7929.45	7714.65	7238.90	7698.49	7137.16	5625.03		
1146	Cytochrome c oxidase assembly protein COX16 homolog_ mitochondrial	Q9P0S2	32464.87	33439.21	27050.50	22863.39	21728.92	21841.79	28441.46	27967.31	25060.30	29124.37	25950.62	25425.04	24642.75	24428.66	20452.63	24217.88	24030.09	20702.75	21510.56	21378.03	20144.69	2.27	1.49	0.65	0.84	1.31	0.69	4.16	2.47	0.11	4.90	4.98	0.12	x	
1147	Cytochrome c oxidase assembly protein COX20_ mitochondrial	Q5RI15	23431.62	23089.80	23599.17	16047.85	15067.98	13886.36	16071.21	15455.14	14497.36	22163.26	22011.48	23597.69	20887.97	21075.09	14187.90	12560.15	13195.48	14028.81	18550.81	17014.80	15817.06	10204.78	9260.66	9003.54	6660.17	6643.58	5750.12	8749.89	8342.16	8259.43	7822.51	7135.38	5506.59		
1148	Cytochrome c oxidase copper chaperone	Q14061	42320.59	43486.18	46759.86	31726.34	29971.15	30594.52	36774.85	33002.24	33787.18	40189.51	36729.40	35325.76	33602.90	32293.64	28648.47	32405.51	33013.44	32240.73	33605.79	31468.40	31390.83	31906.93	30905.22	30427.00	23371.67	22943.24	22825.87	26729.91	25298.51	25325.83	28294.66	27251.26	21680.00		
1149	Cytochrome c oxidase subunit 2	P00403	21947.25	21704.75	23982.30	11937.57	10632.87	10787.79	16798.20	17774.68	18234.33	16655.86	16785.85	16513.58	17155.20	16430.60	14500.49	11535.76	11621.10	11015.95	14593.71	12657.60	11909.88	21026.49	19579.77	17811.97	11127.80	10266.44	8344.40	10147.09	10199.33	9043.29	9353.82	8910.89	5262.69		
1150	Cytochrome c oxidase subunit 4 isoform 1_ mitochondrial	P13073	521258.16	508353.10	469911.30	342921.93	330180.40	310261.84	384555.50	360867.39	342589.54	402011.15	384835.64	359987.49	367354.68	348736.57	316627.18	350910.23	334155.74	315374.97	353365.09	335245.95	314969.88	215075.06	198854.62	174106.92	125201.17	115253.78	104412.89	147449.12	140648.86	136816.74	147363.63	146121.27	97310.36		
1151	Cytochrome c oxidase subunit 4 isoform 2_ mitochondrial	Q96KJ9	0.14	2.53	0.81	1.07	2.77	0.68	0.96	0.36	0.63	0.35	0.75	0.38	2.13	0.97	0.60	4.64	1.07	0.47	2.22	0.19	0.06	42438.24	39249.04	34650.61	15377.23	13968.49	8490.82	13541.53	11321.16	8602.12	22492.31	22415.19	11540.31		x
1152	Cytochrome c oxidase subunit 5A_ mitochondrial	P20674	81277.63	74118.76	65817.66	37144.82	37897.52	33640.74	54939.41	50620.12	45275.70	51309.81	48571.04	42964.94	49647.61	45898.62	31267.98	53398.33	45997.96	45533.73	40277.76	38032.01	36107.21	20644.37	20165.37	19474.17	12185.51	11465.07	9990.32	17462.88	15001.20	14659.78	14990.58	14211.04	9498.87		
1153	Cytochrome c oxidase subunit 5B_ mitochondrial	P10606	130335.73	131070.82	127830.69	84348.90	85485.68	81017.24	88419.98	83767.37	84515.83	98576.27	93393.18	88297.76	89432.28	90598.23	78551.40	81599.09	78266.50	77482.38	84750.10	79929.89	78706.99	32853.82	31117.95	29346.36	27824.82	26830.31	24989.68	32681.73	31240.91	30921.14	33341.99	32109.06	24619.69		
1154	Cytochrome c oxidase subunit 6B1	P14854	88476.97	84604.41	86704.38	44892.46	40816.08	38888.27	52635.67	49665.34	44016.51	71728.77	71618.96	87333.00	68855.90	59995.73	30304.86	37425.08	40096.48	39530.42	73976.15	51700.00	48031.68	58439.83	67761.98	55456.73	46958.38	46427.16	46676.26	37983.92	43353.86	42907.01	56600.90	53490.55	38651.83		
1155	Cytochrome c oxidase subunit 6C	P09669	50662.59	49452.95	44398.31	32460.38	31181.89	27750.19	36660.98	36064.72	33657.27	39704.90	35999.19	34800.08	38316.42	32868.58	25688.25	33462.98	30717.40	28291.71	33441.28	31676.34	27956.02	28557.02	29150.15	27677.86	21604.03	19729.74	18621.01	26900.50	22866.21	21106.04	24196.16	22387.32	17683.35		
1156	Cytochrome c oxidase subunit 7A2_ mitochondrial	P14406	44761.77	41525.24	36414.24	30164.64	29412.25	25079.54	35170.43	34861.47	31363.35	35888.17	33892.50	31549.70	37780.80	35592.34	25018.38	30700.78	29026.17	27602.83	36308.74	34373.88	32049.06	2.27	1.49	0.65	0.84	1.31	0.69	4.16	2.47	0.11	4.90	4.98	0.12	x	
1157	Cytochrome c oxidase subunit NDUFA4	O00483	29066.87	27241.03	24450.72	14394.30	13912.92	11871.89	19710.06	19375.37	18065.54	18764.90	18283.31	16698.98	19783.88	17859.50	13093.74	16794.24	15157.67	15041.17	15950.37	15223.80	13443.24	39734.16	36595.02	34487.70	25113.20	25230.84	22701.78	27917.90	26765.89	25726.79	27901.93	26762.70	17786.81		
1158	Cytochrome c1_ heme protein_ mitochondrial	P08574	118651.37	117441.02	109679.40	68822.92	63863.60	58288.62	75748.86	75163.76	72049.09	90789.21	87211.68	83126.08	76348.98	69381.92	48244.34	70159.36	70434.67	70871.67	80083.27	74890.45	68181.23	75619.18	68370.10	59471.49	44401.86	43360.63	35038.24	61799.13	55837.66	50082.16	59334.13	55118.53	31299.25		
1159	Cytochrome c-type heme lyase	P53701	71056.12	69280.04	63101.71	43311.24	41554.53	39639.03	56241.49	55056.86	48852.29	55523.76	52943.54	48489.88	56593.70	51251.35	42314.66	44220.77	41444.82	39658.15	45036.45	43389.24	40965.12	10927.57	10238.90	9165.53	8911.60	8341.82	8966.84	9982.26	8664.84	8481.44	10920.90	10109.81	2172.09		
1160	Cytochrome P450 1A1	P04798	0.14	2.53	0.81	1.07	2.77	0.68	0.96	0.36	0.63	0.35	0.75	0.38	2.13	0.97	0.60	4.64	1.07	0.47	2.22	0.19	0.06	5093.07	5066.60	4563.19	1909.29	1924.91	1511.14	4473.47	4444.73	3662.70	3260.36	2942.56	1720.23		x
1161	Cytochrome P450 20A1	Q6UW02	21783.25	22296.33	21414.05	15794.07	14803.58	14204.16	19848.75	19570.46	18736.68	20549.08	20400.39	19188.00	18596.32	15448.33	12496.33	16655.02	15232.46	14984.97	15143.47	15050.62	14073.53	4810.47	4478.00	4224.43	4198.03	4325.98	4194.05	4153.23	4267.77	4050.96	4755.43	4930.89	1471.71		
1162	Cytochrome P450 26C1	Q6V0L0	1278.54	1026.52	1209.24	896.84	812.76	740.87	726.39	838.17	856.69	819.97	815.15	975.12	856.07	954.45	763.49	704.74	711.74	674.05	815.70	669.48	753.55	2.27	1.49	0.65	0.84	1.31	0.69	4.16	2.47	0.11	4.90	4.98	0.12	x	
1163	Cytochrome P450 2C9	P11712	1729.84	1555.23	1449.22	1369.36	1298.89	1357.11	1064.07	1181.84	1188.61	1773.33	1797.75	1522.99	1290.06	1348.12	1152.15	1253.95	1207.23	1176.34	997.07	969.20	749.81	2.27	1.49	0.65	0.84	1.31	0.69	4.16	2.47	0.11	4.90	4.98	0.12	x	
1164	Cytochrome P450 4X1	Q8N118	22640.42	21618.15	22714.50	16193.84	15986.31	15900.34	23135.13	22845.02	21834.72	20813.03	19134.91	18696.46	18576.14	20211.37	16428.94	17390.83	15477.58	16123.40	15582.09	15764.24	14712.45	2.27	1.49	0.65	0.84	1.31	0.69	4.16	2.47	0.11	4.90	4.98	0.12	x	
1165	Cytochrome P450 4Z1	Q86W10	102637.09	102852.16	100305.49	87099.29	86159.12	82765.59	103607.90	89868.78	96773.15	115355.27	108434.57	104600.15	97476.48	91689.94	74583.02	84421.57	85756.10	82713.18	77473.08	70989.96	66786.62	2.27	1.49	0.65	0.84	1.31	0.69	4.16	2.47	0.11	4.90	4.98	0.12	x	
1166	Cytohesin-2	Q99418	22524.42	20533.81	18736.05	16053.90	15183.23	13613.10	19461.13	29424.27	22877.80	23876.86	23122.95	19351.68	19459.80	18573.07	12565.79	17005.99	18097.16	14878.85	16956.75	15470.76	14124.12	2.27	1.49	0.65	0.84	1.31	0.69	4.16	2.47	0.11	4.90	4.98	0.12	x	
1167	Cytohesin-3	O43739	21134.16	20370.44	20294.60	12551.27	12275.79	11745.26	15873.31	16051.26	14987.69	14353.00	14234.28	13264.38	16270.75	14898.45	11371.18	13316.61	13250.87	12389.16	13034.61	12313.10	12181.35	2.27	1.49	0.65	0.84	1.31	0.69	4.16	2.47	0.11	4.90	4.98	0.12	x	
1168	Cytohesin-4	Q9UIA0	146.85	152.04	216.36	56.23	1929.47	1247.74	2206.73	2492.79	1591.43	108.00	126.55	71.84	500.21	1048.11	9.36	1997.15	2040.60	1271.20	769.78	684.23	627.14	2.27	1.49	0.65	0.84	1.31	0.69	4.16	2.47	0.11	4.90	4.98	0.12	x	
1169	Cytokine receptor-like factor 3	Q8IUI8	0.14	2.53	0.81	1.07	2.77	0.68	0.96	0.36	0.63	0.35	0.75	0.38	2.13	0.97	0.60	4.64	1.07	0.47	2.22	0.19	0.06	38228.44	34334.08	33994.44	26304.42	25684.17	20652.95	30123.69	27787.23	27095.46	31919.37	31645.62	20033.18		x
1170	Cytoplasmic aconitate hydratase	P21399	5924.90	4568.67	3382.08	2739.09	2854.92	2148.92	3282.18	2627.77	2581.17	4746.36	4152.68	3459.68	3793.64	2122.12	852.57	3703.35	2834.97	2657.22	2618.39	2210.52	1397.36	104252.91	99585.12	91299.09	77036.70	81017.20	77194.77	95145.04	96859.94	93045.66	97035.45	91195.18	65574.31		
1171	Cytoplasmic dynein 1 heavy chain 1	Q14204	1414673.58	1330150.27	1238837.05	918691.56	927515.92	849899.70	1104528.53	1040544.55	997205.34	1225302.73	1159277.53	1080492.99	1121166.06	1064163.71	825407.99	1030637.74	985791.73	958552.45	936096.82	911963.66	851450.81	1098860.09	1022657.16	968616.93	751490.96	735262.75	637513.86	925499.22	886514.43	844934.87	912414.30	865363.25	584422.41		
1172	Cytoplasmic dynein 1 intermediate chain 2	Q13409	35739.46	33141.88	31158.20	27669.10	25414.25	25291.57	26283.74	28872.33	26828.36	37165.22	36778.21	36450.09	29912.03	30049.42	22520.69	30448.52	29268.47	28516.55	26685.31	26990.50	25609.51	31911.26	29730.76	29075.82	28495.70	28597.61	25805.70	30792.85	29554.13	29117.07	33591.15	32306.35	24669.25		
1173	Cytoplasmic dynein 1 light intermediate chain 1	Q9Y6G9	68345.27	60603.37	59502.61	38104.80	36189.75	32405.71	48196.09	47332.30	45145.34	53675.77	48306.06	46936.54	50848.94	43277.48	36755.03	40551.44	37100.09	37237.35	40121.42	39993.84	37112.59	53176.40	50084.31	45280.15	35313.26	33590.07	29260.96	42385.12	37972.65	38984.20	42950.66	42345.75	23618.71		
1174	Cytoplasmic dynein 1 light intermediate chain 2	O43237	38539.92	36726.65	34282.04	25787.88	24407.83	20662.90	29037.20	26962.70	28544.96	31714.31	29713.07	28199.93	30056.62	28206.04	23591.28	23426.39	23266.48	20950.07	23484.69	21910.06	20461.17	25986.73	27247.03	26898.62	20472.84	20167.65	17706.88	24689.67	22757.37	21847.31	23728.85	23826.04	15122.66		
1175	Cytoplasmic dynein 2 heavy chain 1	Q8NCM8	464698.26	450746.67	436062.52	325309.23	318832.75	297010.19	385815.34	363207.51	353736.31	399037.51	387713.77	363207.10	381657.85	368675.27	307912.45	317864.62	314091.53	297307.50	324623.06	309683.53	306357.98	45336.28	20641.31	19554.11	21397.36	19168.08	14929.95	29866.55	25658.15	17502.33	25969.02	24155.88	14987.14		
1176	Cytoplasmic FMR1-interacting protein 1	Q7L576	72879.50	64266.22	58309.34	39934.75	40719.10	34315.86	46325.09	50738.96	41509.84	57410.58	54973.71	50921.62	58451.61	54244.85	34416.04	39054.15	38126.43	33802.77	38280.60	37333.59	34792.22	88963.19	81175.94	76674.42	54629.74	52743.01	45525.28	69021.06	65727.13	61259.69	59672.41	58963.82	43085.13		
1177	Cytoplasmic protein NCK1	P16333	527.58	383.23	356.72	174.00	150.04	88.51	241.56	259.54	237.71	221.29	275.95	167.08	224.74	325.35	98.57	233.45	183.61	140.44	185.31	212.17	168.83	2.27	1.49	0.65	0.84	1.31	0.69	4.16	2.47	0.11	4.90	4.98	0.12	x	
1178	Cytoplasmic tRNA 2-thiolation protein 2	Q2VPK5	2126.15	1773.60	1984.82	1141.02	1369.96	1134.77	1914.26	1513.04	1545.90	1367.21	1575.42	1630.54	1598.83	1614.72	1433.49	1115.95	1103.95	993.54	1457.23	1411.02	1224.19	2.27	1.49	0.65	0.84	1.31	0.69	4.16	2.47	0.11	4.90	4.98	0.12	x	
1179	Cytoskeleton-associated protein 4	Q07065	127979.73	125523.97	117363.02	87925.61	85344.51	79713.45	98169.59	93408.92	91101.77	119160.58	110384.30	105109.54	107523.05	100699.46	84173.64	88000.94	82189.17	80643.32	87459.65	81246.62	80746.39	5278.93	4913.97	4776.91	3613.96	3450.35	3069.80	3731.65	3685.09	3670.01	4070.81	3955.47	5347.90		
1180	Cytoskeleton-associated protein 5	Q14008	202133.56	197486.19	180844.85	123714.19	119027.20	111598.62	148525.40	143263.64	139139.75	160053.81	153005.61	147581.57	150208.11	135816.31	125609.57	126786.96	120886.01	120529.32	135505.20	125885.43	119158.89	167597.79	169985.10	149665.35	107454.17	102488.58	89973.92	125868.10	121664.45	113857.66	134105.83	125653.70	80474.38		
1181	Cytosol aminopeptidase	P28838	122497.64	126812.62	111232.96	88213.49	84378.68	78439.12	97784.69	95656.57	89488.09	110976.50	104873.68	100597.91	103686.29	99314.95	83936.66	82535.31	78236.78	75888.16	82895.14	80935.70	78023.29	105992.33	100168.13	92855.63	68828.62	68078.58	61546.70	91679.24	90738.80	86770.79	85978.88	82723.02	55742.84		
1182	Cytosolic 5'-nucleotidase 1A	Q9BXI3	68963.04	66986.09	65797.21	49267.56	46524.75	44601.55	52973.81	51385.64	53227.11	55726.96	51933.89	51643.75	54495.61	54713.24	46442.73	44760.06	42037.23	41181.66	46075.66	45242.40	44120.81	2.27	1.49	0.65	0.84	1.31	0.69	4.16	2.47	0.11	4.90	4.98	0.12	x	
1183	Cytosolic acyl coenzyme A thioester hydrolase	O00154	57536.23	56182.00	52206.82	31661.81	30104.23	29919.66	43370.40	41440.12	33315.39	45050.27	43622.38	41107.20	48186.09	39191.87	27325.63	36198.97	31809.95	31297.08	33638.75	35017.55	30660.08	60306.63	60535.90	55468.97	35809.48	33763.43	30585.36	57060.21	52869.82	54048.96	50131.00	46248.94	27643.45		
1184	Cytosolic beta-glucosidase	Q9H227	505.11	455.31	299.10	317.44	319.75	274.39	273.41	159.19	340.30	803.82	612.91	630.11	479.88	450.10	284.76	289.55	201.81	311.36	325.07	403.61	337.80	2.27	1.49	0.65	0.84	1.31	0.69	4.16	2.47	0.11	4.90	4.98	0.12	x	
1185	Cytosolic carboxypeptidase 2	Q5U5Z8	155454.71	147596.70	136797.68	111393.21	107546.85	100207.32	144103.65	137962.99	131550.38	161153.33	151665.27	143015.62	126474.73	120295.44	88514.32	113603.31	114383.72	112701.19	114950.80	105332.38	98035.35	190570.35	175291.53	165831.61	112317.05	104014.50	85863.43	133874.48	125338.17	118848.23	111649.73	106518.11	70846.85		
1186	Cytosolic carboxypeptidase 3	Q8NEM8	0.14	2.53	0.81	1.07	2.77	0.68	0.96	0.36	0.63	0.35	0.75	0.38	2.13	0.97	0.60	4.64	1.07	0.47	2.22	0.19	0.06	5772.79	5235.82	4565.48	4580.34	4533.57	3476.64	5145.23	4773.01	4169.26	6021.79	6084.68	3611.14		x
1187	Cytosolic Fe-S cluster assembly factor NUBP1	P53384	4307.79	4545.50	3632.89	2845.95	2978.04	2556.12	3499.48	3162.59	2825.57	4127.68	3424.74	3266.42	3276.76	3047.21	1738.70	3721.28	3432.75	3294.68	3098.66	3278.60	3106.87	2.27	1.49	0.65	0.84	1.31	0.69	4.16	2.47	0.11	4.90	4.98	0.12	x	
1188	Cytosolic Fe-S cluster assembly factor NUBP2	Q9Y5Y2	94881.61	86171.50	79972.37	50494.09	51694.36	48434.85	70876.75	70265.69	64333.39	69856.65	63337.54	59308.02	66330.32	62237.11	49718.53	60492.89	52638.12	52709.48	57324.06	56270.17	52124.97	2.27	1.49	0.65	0.84	1.31	0.69	4.16	2.47	0.11	4.90	4.98	0.12	x	
1189	Cytosolic iron-sulfur assembly component 2A	Q9H5X1	7083.49	6566.25	6211.62	5852.30	5657.47	5002.56	6902.48	6295.24	6671.14	7134.88	6849.32	6110.71	6175.41	6196.69	5353.95	6217.65	5399.32	5906.70	5479.25	5448.66	4814.47	2.27	1.49	0.65	0.84	1.31	0.69	4.16	2.47	0.11	4.90	4.98	0.12	x	
1190	Cytosolic iron-sulfur assembly component 2B	Q9Y3D0	8847.14	6793.97	5406.30	4852.03	5101.10	4396.58	6562.50	6355.00	5249.58	7501.96	6837.40	5669.87	7023.66	6212.00	2764.22	5809.11	5333.69	4894.78	6255.93	4958.12	4400.76	5976.10	5601.82	5480.48	4971.67	5252.27	4054.97	6950.65	6436.17	5822.14	8458.52	7908.89	5080.14		
1191	Cytosolic non-specific dipeptidase	Q96KP4	148944.69	143541.05	131121.21	102440.09	96305.78	81911.49	100566.86	101009.63	93301.93	124830.03	115791.53	107508.09	123178.63	98484.40	64952.19	94964.70	87702.43	86236.54	101530.73	88455.72	79964.15	116136.80	106941.33	91866.94	77217.63	75126.36	65572.43	92779.59	89322.34	82293.35	95029.46	90364.41	60506.91		
1192	Cytosolic phospholipase A2	P47712	5134.46	3760.28	2691.70	1162.31	1521.83	1020.06	1262.40	887.98	677.34	4014.82	3611.69	2746.26	3461.07	2132.28	315.93	1283.76	694.65	984.93	428.68	894.90	479.07	2.27	1.49	0.65	0.84	1.31	0.69	4.16	2.47	0.11	4.90	4.98	0.12	x	
1193	Cytosolic purine 5'-nucleotidase	P49902	491270.27	485667.80	449576.88	328147.05	312280.18	291508.84	378007.38	368128.83	344428.39	386123.02	378228.96	344279.63	413619.82	383645.67	323083.23	329075.55	306718.16	294806.56	334314.61	318877.79	294364.66	20296.58	16077.95	17828.62	11585.37	12072.13	11529.49	16148.01	15251.26	13871.40	15750.12	13254.82	10737.32		
1194	Cytospin-B	Q5M775	0.14	2.53	0.81	1.07	2.77	0.68	0.96	0.36	0.63	0.35	0.75	0.38	2.13	0.97	0.60	4.64	1.07	0.47	2.22	0.19	0.06	27116.79	26554.31	23739.22	21434.20	21567.78	16608.86	24069.41	22752.71	20972.53	28445.76	27177.33	18583.87		x
1195	D-3-phosphoglycerate dehydrogenase	O43175	1240121.18	1166183.50	1136902.92	1003245.02	959824.43	928899.18	1176031.43	1150102.41	1113075.68	977262.74	936082.64	893802.78	1016941.10	989675.46	853710.86	1036164.59	956073.22	947843.82	897323.35	854256.07	821630.95	265571.68	248292.34	234880.94	187383.43	184711.45	172341.14	189235.30	182166.76	176525.45	194547.62	195662.85	163269.39		
1196	D-aminoacyl-tRNA deacylase 1	Q8TEA8	65082.68	63089.51	56550.10	41339.73	40896.22	39971.07	44930.67	47161.19	43140.71	53301.34	52090.58	47124.68	52377.89	48455.99	42336.87	43335.68	40917.99	39710.99	46956.87	42436.98	39051.15	27220.70	23900.68	22516.12	16158.37	16123.91	12859.07	22018.09	20648.65	18989.33	21651.52	22124.41	14138.22		
1197	DAZ-associated protein 1	Q96EP5	11152.81	10123.66	8913.05	7526.22	6028.62	5394.96	7323.34	6564.38	6119.99	8566.01	8140.17	7274.07	8158.29	7143.65	4907.97	6275.63	5650.89	5494.75	14754.08	14876.62	5970.08	9795.14	9287.58	8723.10	6008.93	6698.05	5645.08	7458.57	7126.93	6727.92	8544.41	8412.83	5160.55		
1198	D-beta-hydroxybutyrate dehydrogenase_ mitochondrial	Q02338	90642.09	84019.14	78323.90	63486.70	64101.91	57333.25	91949.50	86358.92	84271.99	84016.01	79567.53	75445.11	83126.55	77001.66	63729.50	64154.35	64853.31	58189.17	80609.71	72757.90	73587.17	69049.54	65396.58	58128.06	42452.35	45518.14	36934.54	48187.82	42480.19	50479.26	57927.58	55886.42	3713.94		
1199	DCC-interacting protein 13-alpha	Q9UKG1	5395.82	4504.44	4112.91	3760.04	3637.07	2786.39	2540.75	2695.55	1895.93	4047.41	3900.93	3621.78	3809.66	4225.18	2045.56	2767.90	3086.05	2501.05	3444.09	3113.11	2560.14	49611.82	46865.78	41734.01	30034.58	29188.67	27003.83	42074.84	40258.60	38095.53	40753.78	39439.71	25819.99		
1200	DCC-interacting protein 13-beta	Q8NEU8	0.14	2.53	0.81	42.86	270.20	105.06	420.63	80.83	29.16	4.88	1.92	3.17	2.13	0.97	0.60	317.32	76.34	142.73	161.87	14.19	164.29	2.27	1.49	0.65	0.84	1.31	0.69	4.16	2.47	0.11	4.90	4.98	16046.16	x	
1201	DCN1-like protein 1	Q96GG9	54962.06	50321.07	49552.33	40218.58	37259.32	34774.74	42157.26	43144.58	44289.71	47782.21	45309.22	43094.67	48163.44	43415.99	35359.22	44177.32	41432.75	38785.50	41176.82	34217.07	34082.56	2.27	1.49	0.65	0.84	1.31	0.69	4.16	2.47	0.11	4.90	4.98	0.12	x	
1202	DCN1-like protein 2	Q6PH85	3298.94	2904.72	2023.98	3454.11	3411.78	2370.75	3238.06	3451.23	3343.04	2890.35	3458.36	3947.59	3803.51	3526.83	2885.87	3358.79	3026.48	2475.99	3025.58	3157.49	2546.83	2.27	1.49	0.65	0.84	1.31	0.69	4.16	2.47	0.11	4.90	4.98	0.12	x	
1203	DCN1-like protein 5	Q9BTE7	7353.94	6578.06	6563.64	5336.57	5359.65	5136.75	5483.97	5412.39	4958.61	6791.10	6524.55	6492.87	6254.14	6077.37	5702.53	5036.70	4935.20	4681.35	5465.04	5111.27	4515.70	22248.65	21574.26	19623.27	13591.59	12805.47	10342.88	16650.24	16059.21	15057.26	18530.42	16989.91	11300.09		
1204	dCTP pyrophosphatase 1	Q9H773	194404.18	187836.99	174705.66	111605.88	108938.54	99279.61	142519.86	132027.29	121994.89	136432.79	127452.01	119464.64	136795.58	128748.39	98319.93	114756.06	106118.51	101687.53	128356.81	122299.60	114818.98	28881.89	26506.07	22512.88	17783.94	16069.62	12971.93	21716.58	19732.45	18551.59	25523.18	23430.98	15408.07		
1205	DDB1- and CUL4-associated factor 11	Q8TEB1	17149.49	16357.56	14325.76	9690.58	8863.03	8938.42	11712.04	11408.95	11576.18	14473.41	14680.57	13741.21	14840.21	12535.23	10786.22	9859.20	9131.31	9519.33	10170.65	9451.11	9378.72	277.62	216.45	227.88	159.25	140.90	156.32	257.01	284.30	251.33	196.58	271.40	183.45		
1206	DDB1- and CUL4-associated factor 12	Q5T6F0	21708.45	19975.06	16630.74	14930.50	15238.99	15293.11	15740.74	15467.68	14452.89	18489.77	17669.71	17990.03	19061.11	17483.69	15141.32	14323.88	12626.67	11788.35	13603.95	14142.58	13251.99	2.27	1.49	0.65	0.84	1.31	0.69	4.16	2.47	0.11	4.90	4.98	0.12	x	
1207	DDB1- and CUL4-associated factor 17	Q5H9S7	0.14	2.53	0.81	1.07	2.77	0.68	0.96	0.36	0.63	0.35	0.75	0.38	2.13	0.97	0.60	4.64	1.07	0.47	2.22	0.19	0.06	201.41	194.37	126.15	137.08	66.99	81.76	362.03	363.97	252.62	285.80	258.27	71.92		x
1208	DDB1- and CUL4-associated factor 5	Q96JK2	11836.02	10347.13	9213.76	5865.40	6172.96	5480.65	7604.89	7186.21	6855.71	8177.20	7909.94	7109.51	8539.59	7472.43	4675.19	7101.25	6326.01	5669.11	6989.95	6386.35	5341.96	2.27	1.49	0.65	0.84	1.31	0.69	4.16	2.47	0.11	4.90	4.98	0.12	x	
1209	DDB1- and CUL4-associated factor 7	P61962	1610.34	1605.65	1533.10	1082.67	1066.80	970.39	1359.67	1347.56	1229.67	1483.36	1456.50	1267.22	1548.34	1315.68	1022.72	1278.84	1199.34	1089.96	1526.47	1241.22	1262.46	2.27	1.49	0.65	0.84	1.31	0.69	4.16	2.47	0.11	4.90	4.98	0.12	x	
1210	DDB1- and CUL4-associated factor 8	Q5TAQ9	7879.98	8341.11	7530.52	5954.74	5595.69	5496.52	6160.89	6485.62	6726.37	6957.64	6840.29	6861.58	7250.08	5855.37	3201.72	4314.92	4487.89	4805.73	6309.07	5842.88	5096.09	1596.99	1272.61	1313.25	916.57	981.05	796.67	1068.26	985.82	907.66	897.63	1145.03	1024.98		
1211	D-dopachrome decarboxylase	P30046	5992.97	5502.59	5692.84	2842.84	2875.38	2195.81	4251.97	4059.78	4161.33	4487.85	4256.39	3607.60	3092.26	4403.04	2798.41	3358.94	3572.78	3366.03	3391.82	3026.69	3212.09	2.27	1.49	0.65	0.84	1.31	0.69	4.16	2.47	0.11	4.90	4.98	0.12	x	
1212	D-dopachrome decarboxylase-like protein	A6NHG4	0.14	2.53	0.81	1.07	2.77	0.68	0.96	0.36	0.63	0.35	0.75	0.38	2.13	0.97	0.60	4.64	1.07	0.47	2.22	0.19	0.06	309.51	290.89	228.18	0.84	1.31	0.69	4.16	2.47	0.11	4.90	4.98	0.12		x
1213	DDRGK domain-containing protein 1	Q96HY6	17775.42	17620.16	16300.52	13173.68	13121.19	12692.38	12752.96	12535.68	11442.00	17506.66	16305.62	14718.94	13911.63	13675.04	12772.29	11923.28	12028.96	11772.50	12035.07	11584.96	11372.26	5390.89	5045.72	5114.95	4276.74	4168.58	4089.60	4236.60	4046.96	4077.19	4972.54	4826.18	3949.41		
1214	Death domain-containing protein 1	Q6ZMT9	0.14	2.53	0.81	1.07	2.77	0.68	0.96	0.36	0.63	0.35	0.75	0.38	2.13	0.97	0.60	4.64	1.07	0.47	2.22	0.19	0.06	11003.14	8154.35	6799.20	7035.81	6231.75	4904.45	9601.99	9269.41	7133.68	9539.11	9040.14	5859.18		x
1215	Death-associated protein kinase 1	P53355	34372.70	33873.64	32072.14	28135.90	28772.48	28471.53	31916.55	30862.43	28878.01	37108.03	35347.31	33363.63	29430.33	30140.21	25298.69	26529.50	27687.67	27768.57	24465.83	24083.02	21550.47	3875.90	3913.75	3424.98	2228.50	1929.63	1294.34	2917.24	2624.47	2132.72	2460.35	2192.25	884.16		
1216	Death-inducer obliterator 1	Q9BTC0	20567.87	19740.24	17950.57	12740.41	12419.85	11658.63	13646.63	12907.85	12830.51	18832.04	16766.80	16790.78	17500.53	16236.92	14907.07	12145.96	11565.95	11212.34	13392.35	13173.88	13582.33	1982.00	1771.98	1572.44	1268.84	1220.78	1018.99	1765.45	1638.75	1440.17	2170.82	2262.47	1102.09		
1217	Dedicator of cytokinesis protein 11	Q5JSL3	138892.76	138062.46	123677.26	115707.10	110370.42	104615.87	107211.29	98778.14	95148.18	129431.50	121262.50	115860.46	110192.61	101502.22	87433.06	117670.20	108457.71	107223.74	98544.36	94827.57	93459.11	2.27	1.49	0.65	0.84	1.31	0.69	4.16	2.47	0.11	4.90	4.98	0.12	x	
1218	Dedicator of cytokinesis protein 6	Q96HP0	0.14	2.53	0.81	1.07	2.77	0.68	0.96	0.36	0.63	0.35	0.75	0.38	2.13	0.97	0.60	4.64	1.07	0.47	2.22	0.19	0.06	4442.44	4334.56	4121.80	2871.40	2850.39	2722.06	2508.24	2289.73	2314.97	2308.33	2110.60	1611.97		x
1219	Dedicator of cytokinesis protein 7	Q96N67	0.14	2.53	0.81	1.07	2.77	0.68	0.96	0.36	0.63	14.06	36.15	35.44	2.13	0.97	0.60	18.48	4.22	9.08	2.22	0.19	0.06	1966.64	1686.57	1595.30	1301.30	1464.65	1192.01	2055.40	1918.83	1593.35	1868.50	2075.59	1228.24		
1220	Dedicator of cytokinesis protein 8	Q8NF50	0.14	2.53	0.81	1.07	2.77	0.68	0.96	0.36	0.63	0.35	0.75	0.38	2.13	0.97	0.60	4.64	1.07	0.47	2.22	0.19	0.06	22056.23	20519.06	19230.37	11819.15	11757.62	10950.20	14877.70	13840.81	13344.88	12635.76	12208.72	8800.47		x
1221	Dehydrogenase/reductase SDR family member 1	Q96LJ7	23490.43	23359.04	22783.92	24357.88	21762.02	21289.85	22270.94	24481.78	21412.50	26810.26	28117.77	23224.97	24899.34	18802.46	9995.47	26626.70	19067.85	22192.43	25314.94	21148.54	19926.39	11467.57	11000.26	10580.50	9413.19	9197.26	8498.36	12137.23	11648.63	10400.46	10889.81	10843.81	8051.78		
1222	Dehydrogenase/reductase SDR family member 7	Q9Y394	152794.46	137666.83	137237.27	112154.81	101109.48	100321.34	132651.57	136430.40	128214.57	126277.88	121824.76	113736.51	130513.69	125890.05	121563.90	107736.92	107174.10	98646.69	118087.44	108028.87	106786.43	34107.92	30597.61	27976.69	22419.23	21897.09	19253.34	26499.33	24722.52	22818.31	28264.30	25920.94	17716.13		
1223	Dehydrogenase/reductase SDR family member 7B	Q6IAN0	32370.97	32595.92	30219.39	24388.37	24599.93	23229.07	25318.74	24936.98	23309.31	28591.36	27543.48	26740.47	28139.74	27749.10	23257.07	22598.86	22017.03	20459.83	23285.36	22441.26	20943.28	2.27	1.49	0.65	0.84	1.31	0.69	4.16	2.47	0.11	4.90	4.98	0.12	x	
1224	Delphilin	A4D2P6	54553.39	54811.30	55887.80	49650.53	46286.57	38761.49	40039.80	42164.43	35913.88	58874.22	57339.53	58935.51	51162.79	26218.81	15119.05	35409.64	44899.65	45330.88	65703.48	55065.98	52032.76	17948.25	16273.87	13972.88	10108.28	10223.31	7226.65	11295.13	10281.34	8805.00	11920.69	11546.23	6817.33		
1225	Delta(14)-sterol reductase	Q14739	0.14	2.53	0.81	1.07	2.77	0.68	0.96	0.36	0.63	0.35	0.75	0.38	2.13	0.97	0.60	4.64	1.07	0.47	2.22	0.19	0.06	18902.09	17868.66	16025.75	11660.09	11432.15	10556.61	13651.67	12749.02	12463.60	11683.38	11526.66	8367.15		x
1226	Delta(24)-sterol reductase	Q15392	1332.74	1295.08	867.64	342.71	2350.28	1451.26	3763.26	760.22	537.43	605.12	571.85	398.32	516.42	520.64	144.01	3192.26	2270.69	2128.65	302.31	176.72	204.24	2.27	1.49	0.65	0.84	1.31	0.69	4.16	2.47	0.11	4.90	4.98	0.12	x	
1227	Delta(3_5)-Delta(2_4)-dienoyl-CoA isomerase_ mitochondrial	Q13011	110954.57	107970.29	98313.89	69773.38	68721.23	62944.09	84204.66	80849.24	76029.95	89977.55	89480.57	80679.71	88702.23	82109.48	66920.42	79990.54	76117.64	67373.94	64921.27	62875.94	58480.41	80505.14	76004.92	64815.82	44600.30	43784.80	37827.29	55529.55	55086.88	52046.36	53399.44	50264.81	46570.11		
1228	Delta-1-pyrroline-5-carboxylate dehydrogenase_ mitochondrial	P30038	15787.98	15058.22	12663.51	7191.80	6552.09	6220.03	8513.18	8175.71	7362.42	9309.75	8855.01	8226.43	10166.68	9303.52	7462.68	8329.64	7756.29	7453.55	6642.20	6246.20	5923.63	38032.52	37131.68	31202.37	25932.75	26866.09	26381.83	28940.40	29101.88	26786.57	30971.09	29534.39	33131.17		
1229	Delta-1-pyrroline-5-carboxylate synthase	P54886	84748.32	82475.12	82295.83	56385.04	52246.04	47802.12	57590.84	56630.94	54126.36	67367.48	67287.25	69332.52	73714.34	62858.98	48430.55	46062.65	47242.45	49642.58	64493.58	58093.72	56900.24	9175.84	8756.39	9284.49	6626.62	6494.56	5897.14	7389.97	7992.20	7325.54	10641.82	10407.25	6672.18		
1230	Delta-aminolevulinic acid dehydratase	P13716	55322.73	50926.32	51235.60	34130.98	33157.64	29576.19	37744.28	37436.11	34486.18	42914.50	41085.75	36729.35	44559.04	42843.32	33663.21	32843.19	32421.62	31256.89	33114.06	31400.21	28525.49	6320.75	6868.57	5522.15	5238.04	4589.42	3532.12	5293.61	5682.39	4919.60	6300.53	6124.78	3630.70		
1231	DENN domain-containing protein 2A	Q9ULE3	80179.43	76114.77	68420.98	63944.46	62704.17	56980.61	71184.57	106678.72	97806.84	68494.48	65676.89	64743.68	64449.68	63553.34	48787.46	68008.83	65963.29	63137.21	56228.89	52427.85	49565.29	85832.82	80902.91	78216.10	64077.50	62923.01	59764.80	61010.16	57804.96	58074.50	60529.71	59976.73	44736.85		
1232	DENN domain-containing protein 2C	Q68D51	977.10	1069.80	734.31	742.01	758.92	664.30	703.95	649.93	518.69	1011.42	1127.00	990.22	937.92	918.76	681.94	744.11	798.43	623.37	989.13	854.14	890.66	2.27	1.49	0.65	0.84	1.31	0.69	4.16	2.47	0.11	4.90	4.98	0.12	x	
1233	DENN domain-containing protein 2D	Q9H6A0	2568.09	2259.06	2227.95	1424.22	1509.06	1385.04	1430.51	1406.95	1445.91	2272.91	2104.85	2184.40	2491.75	2227.22	2064.34	1444.77	1429.05	1225.76	1356.16	1474.17	1534.87	2.27	1.49	0.65	0.84	1.31	0.69	4.16	2.47	0.11	4.90	4.98	0.12	x	
1234	DENN domain-containing protein 4C	Q5VZ89	2144.47	2168.30	2167.33	1391.15	1360.20	1207.29	2020.08	1891.15	1795.41	1717.57	1609.92	1476.78	2080.48	1955.24	1449.91	1532.07	1578.21	1200.71	1743.00	1795.90	1403.26	2.27	1.49	0.65	0.84	1.31	0.69	4.16	2.47	0.11	4.90	4.98	0.12	x	
1235	Density-regulated protein	O43583	1182.74	1116.23	810.59	656.00	581.41	282.65	1129.47	923.90	909.30	876.41	847.27	769.45	1054.08	859.82	316.40	951.83	756.50	705.58	585.69	495.37	311.34	1024.89	921.17	835.50	25.45	36.21	18.50	289.45	202.58	198.17	120.04	132.67	20.97		
1236	Deoxycytidine kinase	P27707	2802.06	2863.64	2763.82	1489.35	1277.41	1232.07	1739.64	1378.95	1488.48	1712.87	1627.62	1469.56	2050.06	1920.71	1638.22	1337.24	1457.70	1449.66	1828.22	1800.12	1612.04	2605.41	2481.09	1952.35	1124.44	974.13	604.96	1271.67	1178.92	916.83	1275.10	1314.95	574.95		
1237	Deoxycytidylate deaminase	P32321	4637.60	4442.03	4560.58	2326.13	1942.13	2232.94	2906.11	2630.88	2341.79	2597.18	2717.24	2268.04	2915.73	2601.94	2416.04	2571.14	2242.34	2215.74	2543.04	2199.86	1900.15	8739.72	8841.00	7786.65	5356.59	5056.70	4554.19	5607.13	5205.37	5579.27	6357.96	6324.60	4087.85		
1238	Deoxyhypusine synthase	P49366	38652.55	37590.62	37150.64	22333.51	22160.23	19504.66	22690.96	22409.13	21670.27	27070.27	25836.23	23974.25	27867.73	27335.74	22746.29	24372.96	23702.36	22812.23	18589.49	17418.28	16683.97	2.27	1.49	0.65	0.84	1.31	0.69	4.16	2.47	0.11	4.90	4.98	0.12	x	
1239	Deoxynucleoside triphosphate triphosphohydrolase SAMHD1	Q9Y3Z3	123445.73	114511.70	105597.81	67789.63	65407.85	58192.77	86810.71	81899.49	76004.01	96003.29	93156.22	82859.01	93635.30	79542.38	60139.53	73348.21	64792.59	65528.42	76563.66	70973.11	64615.96	128210.35	117012.06	104794.68	76513.14	73692.99	62886.46	97516.94	91450.24	84587.92	94185.18	90713.74	60340.17		
1240	Deoxyribonuclease-2-alpha	O00115	2047.18	1725.21	1470.77	1362.63	1270.72	1204.79	1518.35	1416.90	1436.77	1485.73	1262.40	1255.31	1513.99	1602.20	569.69	1448.71	1273.97	1333.94	1443.99	1399.48	1248.76	29752.98	26226.15	24706.82	17971.22	17844.87	15597.53	20471.79	19535.42	18722.40	23995.70	24359.75	15867.00		
1241	Deoxyribose-phosphate aldolase	Q9Y315	0.14	2.53	0.81	1.07	2.77	0.68	0.96	0.36	0.63	0.35	0.75	0.38	2.13	0.97	0.60	4.64	1.07	0.47	2.22	0.19	0.06	916.53	847.96	702.77	941.89	597.97	342.54	884.93	966.14	581.56	987.45	939.64	490.05		x
1242	Deoxyuridine 5'-triphosphate nucleotidohydrolase_ mitochondrial	P33316	158832.87	151774.10	147188.54	106346.66	102333.47	94541.20	123699.99	120439.05	115069.97	119151.79	113986.07	110388.37	126493.93	116519.87	92212.42	91977.21	88968.24	88348.10	112219.88	106651.96	99606.61	148522.80	146631.02	144448.50	97474.74	95477.74	85877.88	97838.04	99918.97	97676.16	114773.96	113190.09	77208.64		
1243	Derlin-1	Q9BUN8	75836.57	74817.10	66372.01	47257.76	46630.42	39680.13	55614.30	53897.44	50154.16	56958.04	53996.44	53210.00	59681.89	54580.87	44657.33	50404.61	48091.08	45632.12	44878.39	41409.87	40348.27	2.27	1.49	0.65	0.84	1.31	0.69	4.16	2.47	0.11	4.90	4.98	0.12	x	
1244	Desmin	P17661	17477.42	15236.36	14960.19	10179.07	11011.88	10040.27	12286.10	12085.27	11694.20	14743.31	13108.33	13472.55	14178.82	13339.56	11622.00	11204.72	10105.49	9870.50	10582.62	10884.42	11117.77	307.65	1603.79	306.26	224.95	190.04	137.43	342.75	248.97	261.21	385.75	392.01	124.53		
1245	Desmoglein-1	Q02413	33883.38	32524.78	29320.83	24892.01	24074.78	22577.63	28534.20	25583.86	26710.68	28751.92	28446.62	26452.55	28031.57	23261.21	17865.11	21864.49	22115.75	21382.70	26219.03	22584.32	21036.18	13608.33	13165.35	11521.83	2785.09	2620.53	1865.95	2230.67	1840.94	1696.19	2505.17	2122.39	920.65		
1246	Desmoglein-2	Q14126	8287.96	10718.14	10229.28	4893.82	3571.05	2265.62	5908.16	4962.39	1684.98	10277.40	9819.24	10013.49	7301.27	7803.84	3220.23	9072.74	7034.10	6975.07	7862.64	7918.00	7443.20	1415.60	1538.03	1606.11	923.82	1016.66	551.87	1281.74	1148.54	904.75	2045.81	1970.69	1042.43		
1247	Desmoplakin	P15924	237867.23	223246.75	209473.52	168843.43	166146.06	154059.73	195794.91	184550.01	178825.60	210121.12	198899.22	192492.49	208134.09	174737.03	128876.61	164598.68	158164.68	151089.85	170345.88	167017.87	165074.65	2.27	1.49	0.65	0.84	1.31	0.69	4.16	2.47	0.11	4.90	4.98	0.12	x	
1248	Destrin	P60981	302267.49	302738.19	282384.03	205343.18	197126.71	173771.01	261514.74	248802.53	234328.33	259236.54	251172.77	233380.14	254393.12	211952.20	192529.58	200839.29	182133.46	184627.66	185016.15	188993.22	179758.87	175343.90	164228.98	148562.21	104778.78	98970.86	93061.65	118388.93	109925.90	109031.76	106851.59	104403.81	63114.83		
1249	Deuterosome assembly protein 1	Q05D60	20807.19	20340.43	18678.08	13490.38	13448.38	12353.06	14997.70	15073.50	14470.80	18394.66	18102.86	17856.00	20055.41	19536.11	16337.91	13426.15	12672.00	12312.54	14484.88	13341.86	12506.80	24930.61	22801.47	19580.89	17804.92	17421.14	15795.26	19789.05	19135.52	18280.77	24232.67	22791.79	4808.38		
1250	Developmentally-regulated GTP-binding protein 1	Q9Y295	18521.69	15454.63	12428.82	7601.61	7835.03	6804.53	11741.93	11740.32	10625.46	13449.50	12022.99	10010.58	13488.27	9521.48	7581.31	9365.37	7960.34	7709.91	8565.70	7481.71	7114.93	39391.91	33920.68	31176.65	22417.21	21469.32	19030.48	25813.66	25032.25	21737.53	26772.74	24729.64	15973.08		
1251	Developmentally-regulated GTP-binding protein 2	P55039	0.14	2.53	0.81	1.07	2.77	0.68	0.96	0.36	0.63	0.35	0.75	0.38	2.13	0.97	0.60	4.64	1.07	0.47	2.22	0.19	0.06	1031.19	857.83	784.32	506.23	542.43	344.72	838.75	702.62	637.66	852.84	757.82	402.41		x
1252	Diablo homolog_ mitochondrial	Q9NR28	84861.94	79464.82	71661.42	52475.48	51660.35	48091.01	61297.29	63453.95	56702.46	76261.99	70736.85	68043.06	66898.54	60200.12	50621.43	52221.85	45486.94	43881.55	54669.00	53483.36	50351.10	70123.77	67023.44	61351.49	44797.35	44856.85	42889.97	43114.35	41555.64	41652.16	42021.25	40229.66	34952.17		
1253	Diacylglycerol kinase theta	P52824	0.14	2.53	0.81	1.07	2.77	0.68	0.96	0.36	0.63	0.35	0.75	0.38	2.13	0.97	0.60	4.64	1.07	0.47	2.22	0.19	0.06	38714.58	36773.61	33390.25	25567.39	21643.57	19813.02	25555.50	23306.52	24160.36	28634.30	25858.61	17908.57		x
1254	Diacylglycerol O-acyltransferase 2	Q96PD7	2085.36	1967.48	1234.04	3359.51	4581.30	2726.16	5736.18	3747.76	3075.36	1905.41	1660.51	980.84	2579.45	3612.31	289.75	5316.19	5072.43	2896.39	2494.90	2797.13	1784.78	2.27	1.49	0.65	0.84	1.31	0.69	4.16	2.47	0.11	4.90	4.98	0.12	x	
1255	Diamine acetyltransferase 1	P21673	1149.79	759.58	482.83	287.56	264.99	192.98	418.65	372.17	394.83	708.07	660.21	431.91	615.90	526.69	48.94	320.40	305.55	210.92	434.37	253.88	192.46	2.27	1.49	0.65	0.84	1.31	0.69	4.16	2.47	0.11	4.90	4.98	0.12	x	
1256	Differentially expressed in FDCP 6 homolog	Q9H4E7	139262.21	133698.19	126648.27	102313.91	102737.60	99344.45	106822.67	101662.90	102018.46	123188.12	118121.47	117351.75	117305.75	110256.99	96903.73	86916.76	82317.31	83401.96	103357.59	101477.77	98008.39	212867.41	197810.16	188628.03	173483.67	163441.46	138261.90	199232.84	186839.16	178071.45	195695.08	191031.02	128569.48		
1257	Digestive organ expansion factor homolog	Q68CQ4	0.14	2.53	0.81	1.07	2.77	0.68	0.96	0.36	0.63	0.35	0.75	0.38	2.13	0.97	0.60	4.64	1.07	0.47	2.22	0.19	0.06	3682.60	3300.03	2915.54	1878.31	1972.03	856.64	2896.27	2522.63	1706.35	3605.72	3179.46	1384.76		x
1258	Dihydrofolate reductase	P00374	19612.63	20036.54	20886.55	14201.71	13332.59	12489.92	12838.57	18568.56	10340.19	14737.51	15465.63	17759.84	15935.49	20470.66	7879.87	6437.23	8895.13	9641.78	15056.68	14464.65	13826.79	72130.07	73600.90	60912.39	43142.26	43249.65	38367.87	59197.45	56451.59	56016.49	57184.94	59224.40	35466.67		
1259	Dihydrolipoyl dehydrogenase_ mitochondrial	P09622	365828.38	347521.40	355065.58	280951.06	267837.24	254901.67	305030.22	288821.07	283758.73	373069.13	353280.11	326327.24	323995.32	329132.77	254978.31	267112.67	262792.34	255669.93	266036.93	253372.94	240378.39	38728.66	33920.14	29697.67	22412.58	22102.10	20627.44	25451.16	24807.11	21701.49	27429.88	23820.35	18976.71		

Supplementary Table S1. Overview on the abundance of all relatively quantified 5180 proteins

			MCF-7																					MDA-MB-231													
	Protein name	UniProt	control 01	control 02	control 03	Dai IC20 01	Dai IC20 02	Dai IC20 03	Dai SC20 01	Dai SC20 02	Dai SC20 03	Gen IC20 01	Gen IC20 02	Gen IC20 03	Gen SC20 01	Gen SC20 02	Gen SC20 03	SSE IC20 01	SSE IC20 02	SSE IC20 03	SSE SC20 01	SSE SC20 02	SSE SC20 03	control 01	control 02	control 03	Dai IC20 01	Dai IC20 02	Dai IC20 03	Gen IC20 01	Gen IC20 02	Gen IC20 03	SSE IC20 01	SSE IC20 02	SSE IC20 03	unique to MCF-7	unique to MDA-MB-231
1260	Dihydrolipoyllysine-residue acetyltransferase component of pyruvate dehydrogenase complex_ mitochondrial	P10515	0.14	2.53	0.81	1.07	2.77	0.68	0.96	0.36	0.63	0.35	0.75	0.38	2.13	0.97	0.60	4.64	1.07	0.47	2.22	0.19	0.06	10588.85	9837.91	9481.83	5826.82	5726.53	5445.90	8415.93	7818.79	7919.64	7040.88	7287.72	4716.74		x
1261	Dihydrolipoyllysine-residue succinyltransferase component of 2-oxoglutarate dehydrogenase complex_ mitochondrial	P36957	218461.85	215752.70	199534.57	142003.08	137756.27	124873.28	164121.51	162975.72	152032.81	180681.61	174819.82	159023.74	175265.11	167078.88	122884.31	143808.05	136193.19	135168.45	150150.05	142362.00	131279.51	98769.28	90639.66	76251.22	52438.75	53733.69	44571.59	65852.30	68591.87	64179.16	57875.51	58333.11	45291.66		
1262	Dihydroorotate dehydrogenase (quinone)_ mitochondrial	Q02127	11369.31	11540.95	11244.58	8418.37	7589.29	7386.97	8712.92	8243.56	8672.40	9232.10	8664.68	8214.13	8646.60	8698.95	6643.69	7409.38	7225.56	7082.08	6122.73	5916.09	6050.83	1442.06	1490.05	1345.46	1000.92	898.48	794.61	1312.61	1161.03	1258.24	1156.70	1103.19	645.47		
1263	Dihydropteridine reductase	P09417	0.14	2.53	0.81	1.07	2.77	0.68	0.96	0.36	0.63	0.35	0.75	0.38	2.13	0.97	0.60	4.64	1.07	0.47	2.22	0.19	0.06	978.82	872.15	735.21	838.92	741.22	678.15	725.65	749.09	653.18	1014.65	947.98	420.33		x
1264	Dihydropyrimidinase-related protein 1	Q14194	17286.12	14644.98	11960.55	5361.99	5652.98	2762.69	8231.93	7511.93	4771.59	13221.81	13678.48	10261.15	9712.89	13923.60	338.62	7298.57	6386.30	3503.42	8501.84	6826.28	6065.57	5539.44	15.91	24.73	4637.27	3593.89	2185.17	7105.41	6633.59	396.27	8179.65	9425.55	4148.66		
1265	Dihydropyrimidinase-related protein 2	Q16555	37934.24	33243.38	27233.54	26173.32	24440.10	20787.02	29536.35	28061.51	24774.05	32190.70	30960.19	26389.63	30065.40	26836.76	15995.67	27556.57	23196.48	23768.64	27475.56	25885.02	21462.76	234981.84	215565.17	194955.48	128123.80	124488.82	101434.75	158951.27	146827.60	135324.07	164837.81	161099.01	114592.96		
1266	Dihydropyrimidinase-related protein 3	Q14195	0.14	2.53	0.81	1.07	2.77	0.68	0.96	0.36	0.63	0.35	0.75	0.38	2.13	0.97	0.60	4.64	1.07	0.47	2.22	0.19	0.06	39461.17	36584.93	30077.26	16811.55	16153.62	12492.66	23020.64	20432.17	18376.70	22867.21	22491.67	12823.36		x
1267	Dihydropyrimidinase-related protein 4	O14531	4330.35	3965.79	3156.59	3530.82	3388.32	3123.44	3204.09	3108.75	2799.86	5968.81	5387.70	5793.59	4677.42	5036.93	2720.24	3280.37	2965.28	3374.08	2902.19	2991.09	3134.26	2.27	1.49	0.65	0.84	1.31	0.69	4.16	2.47	0.11	4.90	4.98	0.12	x	
1268	Dihydropyrimidinase-related protein 5	Q9BPU6	18173.26	17384.17	17023.39	10619.35	10418.59	9700.47	13348.97	12769.74	12680.01	12028.18	11889.33	11375.06	13081.16	12279.50	11613.85	10924.16	10513.43	10099.37	11053.92	10619.35	10567.21	2.27	1.49	0.65	0.84	1.31	0.69	4.16	2.47	0.11	4.90	4.98	0.12	x	
1269	Dipeptidase 2	Q9H4A9	2144.66	1756.77	1161.76	1273.13	1216.69	855.78	1188.32	1260.04	1120.16	1398.18	1349.78	1071.09	1585.31	1444.03	412.29	1032.97	1136.33	870.34	1783.28	1414.59	1096.15	2.27	1.49	0.65	0.84	1.31	0.69	4.16	2.47	0.11	4.90	4.98	0.12	x	
1270	Dipeptidyl peptidase 1	P53634	27942.04	28805.22	27791.83	19325.27	19055.77	19058.82	21359.27	20322.48	21320.63	26910.65	25304.14	23157.36	23996.78	22884.19	18613.38	20242.04	19356.20	19923.04	19134.68	19623.75	19130.73	2.27	1.49	0.65	0.84	1.31	0.69	4.16	2.47	0.11	4.90	4.98	0.12	x	
1271	Dipeptidyl peptidase 2	Q9UHL4	8424.32	7642.50	7065.62	6232.26	5770.56	5116.40	7113.39	6798.50	7212.97	7514.19	7850.73	6745.99	7723.22	7417.99	5648.07	5265.85	5493.98	5405.96	5714.18	5413.68	5774.83	2.27	1.49	0.65	0.84	1.31	0.69	4.16	2.47	0.11	4.90	4.98	0.12	x	
1272	Dipeptidyl peptidase 3	Q9NY33	556411.01	527775.58	494884.34	385807.26	378636.67	352565.57	427700.19	411376.28	397533.23	500280.42	477354.55	444299.00	460848.92	423778.36	346562.43	369735.72	368151.33	346243.47	373451.43	354811.86	333817.18	88672.87	84832.18	79621.54	56881.90	54635.13	47184.17	67304.45	63308.90	59034.04	67791.27	65426.74	42467.00		
1273	Diphosphomevalonate decarboxylase	P53602	12235.63	11336.22	9710.14	7690.26	7206.66	6394.32	9590.65	9485.52	8031.19	10746.26	9399.18	9842.59	10584.26	9208.28	5493.95	8587.27	7998.99	7218.40	8175.65	7600.15	6950.62	12311.74	11215.46	10751.23	7982.12	7844.96	6845.17	10008.26	9547.07	9034.55	10225.48	9837.80	6958.16		
1274	DIS3-like exonuclease 1	Q8TF46	4165.92	4216.78	4128.37	2851.20	2678.71	2432.58	3874.37	3970.44	3813.38	3882.04	3705.65	3718.87	3362.90	3229.63	2743.75	2873.04	2718.13	2726.24	3119.31	3196.91	3204.93	26270.33	22248.47	18135.89	22763.79	22647.82	18660.06	28877.04	27660.28	24095.15	26881.44	25413.97	17645.33		
1275	DIS3-like exonuclease 2	Q8IYB7	2836.83	2820.00	2865.72	2192.95	2307.61	2145.39	2128.48	2039.59	2336.23	2585.74	2203.29	2608.99	2608.78	2553.75	2104.52	2083.15	1918.15	1895.29	2597.71	2045.51	2763.05	4076.86	2980.73	3542.68	2570.68	2389.65	1935.42	3462.10	3654.39	3359.96	3753.58	3688.21	2172.05		
1276	Discoidin_ CUB and LCCL domain-containing protein 2	Q96PD2	0.14	2.53	0.81	1.07	2.77	0.68	0.96	0.36	0.63	0.35	0.75	0.38	2.13	0.97	0.60	4.64	1.07	0.47	2.22	0.19	0.06	29856.30	29097.42	25284.53	17657.46	16696.30	12341.24	19441.29	19284.44	16559.07	19908.28	19290.05	10749.44		x
1277	Disco-interacting protein 2 homolog B	Q9P265	16162.27	15626.23	14681.76	9595.64	9215.06	8066.85	12474.68	11364.11	11303.79	12655.19	12373.23	9745.76	11603.23	11935.72	9466.56	10864.92	10328.97	9589.56	11204.28	9981.48	9607.55	2.27	1.49	0.65	0.84	1.31	0.69	4.16	2.47	0.11	4.90	4.98	0.12	x	
1278	Disheveled-associated activator of morphogenesis 1	Q9Y4D1	1777.11	1621.81	1448.52	657.29	755.24	620.52	1460.61	1475.76	1402.23	1010.77	903.43	950.40	1110.51	1136.86	568.15	996.07	994.79	791.59	728.48	766.14	646.24	2.27	1.49	0.65	0.84	1.31	0.69	4.16	2.47	0.11	4.90	4.98	0.12	x	
1279	Disheveled-associated activator of morphogenesis 2	Q86T65	42338.59	40784.85	37542.40	34913.05	31592.50	34306.96	40210.57	39251.32	38700.46	44436.14	44915.95	49053.43	45097.36	43693.40	37460.07	32438.05	29515.07	33818.75	42133.50	36894.22	34403.78	2.27	1.49	0.65	0.84	1.31	0.69	4.16	2.47	0.11	4.90	4.98	0.12	x	
1280	Disintegrin and metalloproteinase domain-containing protein 10	O14672	0.14	2.53	0.81	1.07	2.77	0.68	0.96	0.36	0.63	0.35	0.75	0.38	2.13	0.97	0.60	4.64	1.07	0.47	2.22	0.19	0.06	39509.58	35927.42	30790.91	21663.39	21857.97	14387.21	24132.50	21649.49	18673.30	27063.04	26766.79	14411.52		x
1281	Disintegrin and metalloproteinase domain-containing protein 17	P78536	27.84	61.31	93.01	281.75	198.50	68.98	0.96	0.36	0.63	46.79	70.46	14.67	158.49	118.67	7.47	203.73	314.46	31.42	2.22	0.19	0.06	2.27	1.49	0.65	0.84	1.31	0.69	4.16	2.47	0.11	4.90	4.98	0.12	x	
1282	Disintegrin and metalloproteinase domain-containing protein 22	Q9P0K1	19350.33	18606.82	18651.99	18522.06	16584.33	16531.67	15770.74	14351.14	15106.14	9611.71	9917.00	8402.30	18379.76	7570.96	7369.03	8748.16	13274.14	12913.37	17014.72	14582.34	15155.05	11740.55	12287.05	11531.37	10971.15	11158.08	9475.00	14963.27	14389.14	13433.01	15680.66	13281.16	8266.50		
1283	Disintegrin and metalloproteinase domain-containing protein 7	Q9H2U9	452.97	318.06	442.35	296.62	223.75	265.94	217.63	104.79	133.65	498.90	459.56	584.50	210.31	563.10	433.42	263.66	177.21	123.05	182.71	163.04	162.05	2.27	1.49	0.65	0.84	1.31	0.69	4.16	2.47	0.11	4.90	4.98	0.12	x	
1284	Disintegrin and metalloproteinase domain-containing protein 9	Q13443	0.14	2.53	0.81	1.07	2.77	0.68	0.96	0.36	0.63	0.35	0.75	0.38	2.13	0.97	0.60	4.64	1.07	0.47	2.22	0.19	0.06	4100.91	3873.17	3256.54	1733.32	1840.17	1389.60	1745.91	1476.41	1469.71	1669.61	1697.34	999.46		x
1285	Disks large homolog 1	Q12959	31718.27	29835.98	30294.46	22071.65	20108.99	19585.73	25302.22	25267.81	25560.29	27917.18	26722.41	25316.31	27284.03	26689.07	22489.14	20311.67	19292.18	18087.58	21249.49	18890.39	19537.09	28434.54	21528.06	20267.22	20125.34	19791.14	14322.29	25388.00	22991.37	20172.80	26601.76	25647.32	15904.08		x
1286	Disks large homolog 2	Q15700	183.55	115.11	180.42	171.78	160.97	139.91	160.03	201.90	150.41	207.72	206.50	164.97	216.43	218.56	95.24	95.70	136.17	120.25	112.68	104.67	103.67	2.27	1.49	0.65	0.84	1.31	0.69	4.16	2.47	0.11	4.90	4.98	0.12	x	
1287	Disks large homolog 5	Q8TDM6	11311.54	10663.06	9434.53	7184.51	7171.51	6662.00	9679.31	9851.25	9857.35	9626.70	9750.48	8830.35	10819.42	9156.70	7266.59	8681.06	7749.94	7616.64	9536.51	8584.29	8312.22	2.27	1.49	0.65	0.84	1.31	0.69	4.16	2.47	0.11	4.90	4.98	0.12	x	
1288	Disks large-associated protein 3	O95886	44838.24	43204.64	41644.97	17270.61	15971.14	15740.74	23542.82	23266.29	23433.66	29471.51	30280.29	28623.10	22656.06	22546.45	19718.81	36622.87	35876.28	34341.40	23814.91	24287.33	23626.00	2.27	1.49	0.65	0.84	1.31	0.69	4.16	2.47	0.11	4.90	4.98	0.12	x	
1289	Disks large-associated protein 5	Q15398	0.14	2.53	0.81	1.07	2.77	0.68	0.96	0.36	0.63	0.35	0.75	0.38	2.13	0.97	0.60	4.64	1.07	0.47	2.22	0.19	0.06	6148.12	5739.23	5612.69	3598.35	3504.37	3125.96	5921.07	5755.79	5565.11	5126.11	5072.45	2954.41		x
1290	Dixin	Q155Q3	16566.21	15538.91	14866.20	10004.28	9493.22	9807.84	15104.16	15082.30	12938.44	12753.64	12329.87	11872.33	13941.69	12236.31	9897.63	10519.63	10989.64	10112.41	10659.46	10059.11	8617.39	2.27	1.49	0.65	0.84	1.31	0.69	4.16	2.47	0.11	4.90	4.98	0.12	x	
1291	DNA (cytosine-5)-methyltransferase 1	P26358	275674.37	258061.77	226083.67	182170.17	174750.40	162163.72	217582.28	209453.76	199581.76	216113.63	205809.47	188236.98	219720.55	209935.20	151113.15	195642.58	183307.36	181911.95	179396.22	166055.61	153121.64	301384.58	299853.72	263774.39	234377.43	222408.73	200552.70	339980.45	309895.64	297386.46	287108.96	276198.22	60146.92		
1292	DNA (cytosine-5)-methyltransferase 3-like	Q9UJW3	0.14	2.53	0.81	1.07	2.77	0.68	0.96	0.36	0.63	0.35	0.75	0.38	2.13	0.97	0.60	4.64	1.07	0.47	2.22	0.19	0.06	722.36	172.04	129.35	756.96	384.14	106.89	1003.30	716.03	73.67	959.38	922.74	243.92		x
1293	DNA damage-binding protein 1	Q16531	248286.95	242503.85	224406.79	175019.42	168867.05	162605.24	187059.17	182017.58	175725.90	205031.94	194194.62	186619.36	208554.82	202210.00	160167.60	163949.91	162136.67	149918.34	166264.04	157124.78	143639.75	122131.96	109663.83	101680.56	78420.46	73949.83	61668.02	105826.87	98308.79	91030.44	98613.63	94756.14	62897.60		
1294	DNA damage-induced apoptosis suppressor protein	Q8IXT1	16229.60	15605.61	14805.39	9096.57	8283.81	7592.38	8323.99	7912.06	6962.07	10737.44	10640.15	10198.08	11010.54	10778.00	8430.90	6668.99	6725.75	6379.20	9810.37	9942.01	8313.95	2.27	1.49	0.65	0.84	1.31	0.69	4.16	2.47	0.11	4.90	4.98	0.12	x	
1295	DNA dC->dU-editing enzyme APOBEC-3B	Q9UH17	0.14	2.53	0.81	1.07	2.77	0.68	0.96	0.36	0.63	0.35	0.75	0.38	2.13	0.97	0.60	4.64	1.07	0.47	2.22	0.19	0.06	3555.77	3556.34	3931.46	2998.94	2976.78	2555.92	3663.09	3368.55	3536.83	3615.53	3570.82	2523.43		x
1296	DNA dC->dU-editing enzyme APOBEC-3C	Q9NRW3	2507.32	2173.46	1629.53	7019.23	6259.61	3805.87	10396.56	9957.61	6666.46	2101.50	1968.21	1753.19	2488.24	9073.31	1328.67	6313.96	6184.13	3800.94	1809.48	7132.14	4305.91	8493.69	6089.52	4951.22	5440.08	5438.38	3537.33	5389.34	6411.15	5180.97	8212.97	7427.66	2655.62		
1297	DNA dC->dU-editing enzyme APOBEC-3G	Q9HC16	0.14	2.53	0.81	1.07	2.77	0.68	0.96	0.36	0.63	0.35	0.75	0.38	2.13	0.97	0.60	4.64	1.07	0.47	2.22	0.19	0.06	56724.27	46517.93	46746.32	23549.29	22659.78	18545.93	49556.71	46380.53	42749.63	30926.67	33464.55	22169.33		x
1298	DNA endonuclease RBBP8	Q99708	52.10	72.89	79.63	124.70	91.99	85.50	206.10	108.22	79.17	112.35	114.47	153.29	169.06	92.55	143.67	53.29	78.71	47.08	153.31	154.59	102.43	2.27	1.49	0.65	0.84	1.31	0.69	4.16	2.47	0.11	4.90	4.98	0.12	x	
1299	DNA excision repair protein ERCC-6	Q03468	173086.11	176199.10	165151.42	132464.51	131346.62	121966.30	143328.83	142020.07	136483.43	150275.37	148345.03	134210.05	150249.77	135522.53	124981.78	130259.90	123220.07	117753.79	126389.90	118841.16	112025.46	47428.83	47711.28	42882.26	37967.32	36978.22	33885.57	36659.00	37416.66	36063.98	42828.39	40356.24	33646.36		
1300	DNA excision repair protein ERCC-6-like	Q2NKX8	0.14	2.53	0.81	1.07	2.77	0.68	0.96	0.36	0.63	0.35	0.75	0.38	2.13	0.97	0.60	4.64	1.07	0.47	2.22	0.19	0.06	32436.50	36314.91	28730.99	22305.54	22082.53	21570.26	19294.25	20940.12	20962.99	24250.27	25562.32	10036.48		x
1301	DNA fragmentation factor subunit alpha	O00273	138172.68	136207.81	126165.61	85307.88	84407.78	78291.12	111258.20	104944.69	98465.81	108105.49	104473.08	97024.22	106633.79	100693.67	85960.75	87915.77	82749.12	79768.63	86562.99	85970.89	84352.89	84196.67	67436.93	63406.93	49897.81	48810.94	43200.78	64711.82	62620.12	57963.63	63625.21	59373.62	39350.23		
1302	DNA helicase MCM8	Q9UJA3	29242.02	31103.00	26945.60	19194.20	18338.37	18998.24	25317.84	23006.97	23472.18	21884.51	21213.09	21108.83	21968.69	20909.07	16552.29	22395.43	19526.32	19047.18	18709.41	20786.70	15810.87	20375.71	20160.01	17530.08	17842.64	18764.25	16293.08	23090.68	21458.32	19562.30	12273.73	11302.75	8353.39		
1303	DNA ligase 1	P18858	0.14	2.53	0.81	1.07	2.77	0.68	0.96	0.36	0.63	0.35	0.75	0.38	2.13	0.97	0.60	4.64	1.07	0.47	2.22	0.19	0.06	40575.08	42427.70	41180.40	23511.78	24344.07	20503.36	30908.06	28696.79	25522.91	28282.04	26397.03	19031.21		x
1304	DNA methyltransferase 1-associated protein 1	Q9NPF5	18039.60	18375.15	15920.39	13300.34	12599.95	12183.74	14035.57	13867.15	15005.77	16514.84	16245.25	15843.00	17326.52	15899.54	14492.86	13808.43	12872.75	12457.21	18082.43	15931.87	16105.05	20171.23	19144.34	17982.76	15428.74	15103.12	14403.42	14935.49	14561.06	14298.71	18222.06	18609.34	22982.68		
1305	DNA mismatch repair protein Msh2	P43246	153736.68	143347.69	141136.57	108631.93	104394.58	99629.75	121660.93	126431.75	124138.22	147182.84	134375.08	132036.76	138328.50	127727.41	98547.03	113824.14	109570.62	106969.43	116917.76	106319.15	111786.17	136333.73	118960.59	122642.88	82374.07	81541.72	74189.56	106851.73	99699.38	94824.29	103636.26	106084.38	54205.21		
1306	DNA mismatch repair protein Msh3	P20585	0.14	2.53	0.81	1.07	2.77	0.68	0.96	0.36	0.63	0.35	0.75	0.38	2.13	0.97	0.60	4.64	1.07	0.47	2.22	0.19	0.06	3167.02	1994.82	1726.10	2234.40	2205.02	1487.41	2788.49	2489.17	2059.01	3152.39	2875.24	950.43		x
1307	DNA mismatch repair protein Msh6	P52701	153079.05	142964.43	135059.81	100985.65	97609.19	87823.71	115500.04	115911.05	103911.05	128361.39	124528.89	115271.43	118950.27	123198.24	97955.07	92815.54	91525.61	88998.70	98505.16	95103.89	93512.92	82226.86	78316.40	74992.18	52945.83	52685.43	46283.92	66039.48	62780.24	60241.74	63025.06	62339.30	43294.70		
1308	DNA polymerase alpha catalytic subunit	P09884	6726.94	7793.77	5538.55	5065.26	5505.35	4571.18	5668.40	5498.72	4557.47	5701.46	5392.00	4789.89	5219.96	5334.53	4931.99	5133.55	4561.85	4594.66	5673.04	4582.00	4339.08	2.27	1.49	0.65	0.84	1.31	0.69	4.16	2.47	0.11	4.90	4.98	0.12	x	
1309	DNA polymerase beta	P06746	0.14	2.53	0.81	1.07	2.77	0.68	0.96	0.36	0.63	0.35	0.75	0.38	2.13	0.97	0.60	4.64	1.07	0.47	2.22	0.19	0.06	17223.53	17270.30	15045.12	10652.47	10128.46	9249.23	10258.17	10433.72	10508.43	10712.68	9714.00	6228.61		x
1310	DNA polymerase delta catalytic subunit	P28340	0.14	2.53	0.81	1.07	2.77	0.68	0.96	0.36	0.63	0.35	0.75	0.38	2.13	0.97	0.60	4.64	1.07	0.47	2.22	0.19	0.06	17030.03	16475.51	13638.30	7541.85	8151.23	7115.02	9973.33	10001.10	9894.84	11114.80	10266.23	6806.78		x
1311	DNA polymerase delta subunit 2	P49005	6054.60	6358.86	5596.15	3814.10	3965.32	3948.31	4461.54	4510.47	4367.50	5159.44	4441.12	4261.40	5117.86	4584.03	4257.11	3555.11	3290.97	3367.38	4550.80	4249.67	4377.79	3859.17	3031.82	3606.86	2588.85	2395.60	1965.48	3963.82	3942.32	3530.01	3683.43	3068.04	2351.89		
1312	DNA polymerase delta subunit 3	Q15054	0.14	2.53	0.81	1.07	2.77	0.68	0.96	0.36	0.63	0.35	0.75	0.38	2.13	0.97	0.60	4.64	1.07	0.47	2.22	0.19	0.06	8461.62	8480.10	7559.94	6354.81	5791.17	4865.05	7180.61	6525.14	6240.78	6787.91	5855.31	4701.44		x
1313	DNA polymerase epsilon catalytic subunit A	Q07864	165262.80	155685.03	138253.87	125102.73	120178.66	102738.82	131431.62	128513.21	113952.26	154121.84	152715.27	135126.19	147582.57	134040.07	85376.19	124629.82	118449.59	110006.62	132218.67	122360.56	109381.49	2.27	1.49	0.65	0.84	1.31	0.69	4.16	2.47	0.11	4.90	4.98	0.12	x	
1314	DNA polymerase epsilon subunit 3	Q9NRF9	14567.30	14274.49	14148.89	13720.88	12979.24	11783.47	16613.20	17072.37	16533.54	14655.35	15091.87	13561.89	14453.17	13385.72	12416.39	18912.03	15334.06	17760.54	16528.10	11485.04	10558.69	4428.43	3213.13	2678.78	2628.90	2375.75	1970.23	3190.48	2610.41	2568.95	3033.76	2788.46	1820.28		
1315	DNA polymerase epsilon subunit 4	Q9NR33	11002.70	8614.21	7090.00	4953.55	5207.41	4678.43	5474.24	4272.93	4104.46	8476.57	7543.59	6084.17	5088.77	2994.03	5207.06	4128.89	4581.21	3963.22	7549.88	6219.71	7138.30	2.27	1.49	0.65	0.84	1.31	0.69	4.16	2.47	0.11	4.90	4.98	0.12	x	
1316	DNA polymerase kappa	Q9UBT6	22788.39	22249.19	19204.63	15019.76	14252.28	13353.97	16577.61	15928.65	15521.19	23060.17	22364.26	22168.94	24295.87	22053.75	16195.16	14084.83	13022.55	13380.87	15217.07	15226.93	14310.94	62415.38	68387.35	56145.98	48738.91	46587.71	46459.20	49499.94	52664.11	50078.61	59815.03	57920.83	46133.69		
1317	DNA polymerase zeta catalytic subunit	O60673	0.14	2.53	0.81	1.07	2.77	0.68	0.96	0.36	0.63	0.35	0.75	0.38	2.13	0.97	0.60	4.64	1.07	0.47	2.22	0.19	0.06	26806.57	24549.01	24417.49	19549.02	18970.07	17474.86	23793.71	22881.26	21627.32	23047.64	22783.28	15905.63		x
1318	DNA primase small subunit	P49642	3706.28	4169.49	3757.78	2770.82	2691.30	2693.66	2882.67	2902.08	2692.67	3047.41	2828.47	2788.09	3571.24	2984.75	2863.71	2251.44	2255.23	2133.57	2553.12	2340.89	2060.89	221.30	259.69	160.03	139.02	177.98	158.29	278.60	248.91	159.68	154.45	264.21	170.16		
1319	DNA repair and recombination protein RAD54-like	Q92698	1880.91	1499.53	1576.86	805.58	840.80	714.43	1268.24	1290.17	1179.59	1724.72	1556.04	1557.97	1256.97	1154.59	1150.40	1701.74	1640.54	1675.26	1070.55	1089.19	1206.05	7409.71	7784.45	6452.25	6365.47	6549.23	5634.47	8672.45	7812.44	7529.24	6903.64	6457.58	4510.24		
1320	DNA repair protein complementing XP-G cells	P28715	12911.14	12695.80	11077.26	5878.14	5417.11	5056.21	8272.44	8224.52	8589.25	8266.36	7867.18	7184.79	9083.37	7802.43	6361.48	7333.12	6989.27	6472.49	6499.93	6441.33	5982.35	2.27	1.49	0.65	0.84	1.31	0.69	4.16	2.47	0.11	4.90	4.98	0.12	x	
1321	DNA repair protein RAD50	Q92878	8133.43	6613.22	5391.29	4408.90	4105.54	3164.32	5911.80	5176.36	4678.17	6323.16	6048.57	4980.76	6211.96	5547.60	2204.69	4226.16	4233.74	4121.13	4523.82	3891.90	3406.27	188621.39	180462.03	177632.46	145400.67	144993.99	131422.09	167207.37	160519.98	153829.56	171043.59	162302.86	119653.10		
1322	DNA repair protein RAD51 homolog 1	Q06609	22350.78	19842.14	15028.15	10695.85	22114.61	15326.75	29980.34	21080.35	17624.83	12310.27	11283.41	9251.02	14566.13	15942.17	8210.37	27734.69	22798.27	17129.99	11418.07	11373.08	9321.49	2.27	1.49	0.65	0.84	1.31	0.69	4.16	2.47	0.11	4.90	4.98	0.12	x	
1323	DNA repair protein REV1	Q9UBZ9	40487.73	36912.86	35085.98	39160.97	37847.56	34657.60	34468.63	33429.59	33162.25	50080.18	45066.16	44946.27	37341.87	34473.85	29456.60	37983.57	36684.91	35371.26	31072.27	29259.99	29815.47	9941.89	9274.51	7611.13	6756.56	6853.44	6341.21	8916.57	8778.94	8492.60	6008.99	5199.53	3863.60		
1324	DNA repair protein XRCC1	P18887	19033.02	18235.09	16174.61	13127.97	11764.87	11440.32	11772.49	11197.93	10704.46	16545.99	16397.37	14698.16	15188.33	13339.52	10649.44	11615.01	10756.89	10436.03	12597.61	11809.04	11045.47	2.27	1.49	0.65	0.84	1.31	0.69	4.16	2.47	0.11	4.90	4.98	0.12	x	
1325	DNA repair protein XRCC2	O43543	0.14	2.53	0.81	1.07	2.77	0.68	0.96	0.36	0.63	0.35	0.75	0.38	2.13	0.97	0.60	4.64	1.07	0.47	2.22	0.19	0.06	32941.46	32112.54	27453.96	26442.25	25071.76	22832.09	35454.65	34563.24	32827.31	28528.44	28292.54	19335.93		x
1326	DNA repair protein XRCC4	Q13426	0.14	2.53	0.81	1.07	2.77	0.68	0.96	0.36	0.63	0.35	0.75	0.38	2.13	0.97	0.60	4.64	1.07	0.47	2.22	0.19	0.06	8847.07	8476.28	7724.29	5249.14	5264.13	4741.82	4887.39	4759.09	4619.80	4376.78	4117.08	2803.39		x
1327	DNA replication ATP-dependent helicase/nuclease DNA2	P51530	7488.43	7583.09	6694.47	7323.97	6780.51	6244.60	6698.86	6271.70	6164.07	10123.01	9574.55	9582.86	8426.32	8080.85	8137.15	5739.36	5859.09	5650.53	5116.56	4958.15	4906.86	3121.72	2926.26	2576.21	1520.84	1431.41	1461.81	1525.70	1496.34	1316.18	1620.02	1387.04	750.33		
1328	DNA replication complex GINS protein PSF1	Q14691	24259.95	26312.68	25038.30	22835.70	22129.14	19820.59	26556.38	25946.41	23345.80	29452.81	29431.39	27454.86	27770.54	26039.44	18695.81	23248.96	21246.08	19820.45	23267.23	22343.96	19950.30	89310.29	81979.10	72947.54	87882.89	84140.31	63576.91	83353.44	76074.56	68824.32	106455.16	101152.54	63696.39		
1329	DNA replication complex GINS protein PSF2	Q9Y248	15792.41	17118.72	12732.63	8099.23	8626.50	7277.80	11939.85	11490.67	9763.69	10039.06	7764.81	8693.82	11049.65	9400.15	7430.71	10582.26	9188.90	9371.62	9712.15	9989.68	7754.57	4191.08	4103.82	3314.76	2860.34	2832.46	1988.87	3153.63	2889.51	2621.22	3968.57	3852.97	2169.74		
1330	DNA replication complex GINS protein PSF3	Q9BRX5	1311.59	1186.68	1085.28	834.41	781.57	794.34	880.91	742.10	861.79	618.87	650.30	645.79	932.51	209.70	8.16	642.44	616.42	602.02	1018.44	845.44	904.38	808.24	756.08	661.29	721.23	731.65	660.68	845.44	773.56	740.66	4.90	4.98	0.12		
1331	DNA replication complex GINS protein SLD5	Q9BRT9	0.14	2.53	0.81	1.07	2.77	0.68	0.96	0.36	0.63	0.35	0.75	0.38	2.13	0.97	0.60	4.64	1.07	0.47	2.22	0.19	0.06	14058.26	11084.72	10025.64	7954.22	7055.45	6440.28	9236.69	9131.77	8601.88	10396.63	9089.18	5949.71		x
1332	DNA replication licensing factor MCM2	P49736	377142.32	352648.96	329219.41	229956.67	219293.07	195451.67	283435.17	266555.23	246120.88	270700.55	253341.31	221963.32	302150.14	275124.04	209399.15	205751.13	188507.18	181998.94	247201.58	237761.53	213545.65	146736.48	128994.94	116525.29	97265.58	91954.42	73944.97	122918.86	120980.38	109776.63	129510.98	123946.38	78284.57		
1333	DNA replication licensing factor MCM3	P25205	90527.30	80016.48	77838.62	50481.87	46745.18	45784.43	60857.39	57569.98	54279.01	58667.29	56097.92	50959.51	65321.04	64076.19	46657.19	47867.17	44148.71	45387.48	57528.99	52693.08	47533.74	95806.74	89922.90	84067.99	61580.59	58533.39	49146.32	71531.85	69871.53	66327.35	69225.93	67927.46	43662.16		
1334	DNA replication licensing factor MCM4	P33991	381877.46	363218.43	334167.83	260222.10	255020.10	234820.41	268839.17	258078.49	247717.22	316256.69	305398.48	280448.54	310989.36	276725.95	224963.97	222301.21	204091.19	196556.21	272861.09	249341.58	233407.49	116264.21	105206.07	98987.58	74012.38	69964.79	60327.16	94139.06	91480.13	84241.89	88160.41	82168.65	55008.96		
1335	DNA replication licensing factor MCM5	P33992	334739.19	318693.74	284719.29	213765.17	204847.29	189409.38	237383.76	225485.09	214686.02	237346.01	223013.01	212510.90	277241.43	239104.28	193428.48	199569.12	193959.69	189902.12	229499.10	209645.66	196796.76	193482.41	168758.72	159230.04	115691.35	114481.88	82740.99	151277.59	141568.44	128845.89	150715.61	140645.85	84659.31		
1336	DNA replication licensing factor MCM6	Q14566	527568.00	507283.25	473583.77	413187.40	397296.95	382335.98	387178.35	382980.06	360715.57	480486.00	460756.11	450753.53	462346.54	432368.63	349643.54	376986.02	381464.12	375512.60	406934.33	389228.79	376359.19	336392.93	318424.89	299402.90	234030.58	227026.78	204989.57	281061.08	268723.85	260515.20	287427.27	274929.70	177000.70		
1337	DNA replication licensing factor MCM7	P33993	416331.38	380191.80	372514.04	241549.22	237718.80	223066.22	306141.15	299741.18	286991.20	330616.91	316871.26	311354.54	320542.43	297122.95	242054.70	304562.06	297540.76	292613.14	298517.99	283772.77	262982.79	176736.78	167069.57	147246.35	120232.04	115000.42	105493.22	143721.30	140135.86	131799.97	142304.07	134855.02	101690.00		
1338	DNA topoisomerase 1	P11387	481290.52	470293.11	428588.08	291411.45	280527.19	258393.59	344522.40	332966.03	321405.42	349930.35	332790.67	312923.16	368482.07	342945.03	290719.05	301762.26	285279.67	272922.22	331312.56	313386.56	284437.61	140796.93	135197.95	123865.65	96755.16	92474.97	79775.88	106191.38	99497.08	96698.99	106240.22	104452.80	65885.06		
1339	DNA topoisomerase 2-alpha	P11388	0.14	2.53	0.81	1.07	2.77	0.68	0.96	0.36	0.63	0.35	0.75	0.38	2.13	0.97	0.60	4.64	1.07	0.47	2.22	0.19	0.06	9278.59	8256.93	8182.48	6362.29	6055.13	5336.15	7863.77	7918.27	6776.10	6728.02	6094.81	4641.30		x
1340	DNA topoisomerase 2-beta	Q02880	0.14	2.53	0.81	1.07	2.77	0.68	0.96	0.36	0.63	0.35	0.75	0.38	2.13	0.97	0.60	4.64	1.07	0.47	2.22	0.19	0.06	41898.78	38578.86	36891.29	23948.33	24115.27	24733.66	26127.02	24980.96	23666.52	26792.16	25761.41	18456.88		x
1341	DNA topoisomerase 3-alpha	Q13472	2340.50	2484.54	2226.56	1814.34	1654.75	1656.20	2489.85	1859.11	1985.77	2180.12	1841.74	1915.91	2053.37	2302.99	1398.84	1656.37	1818.77	1659.21	1466.12	1600.79	1531.75	2.27	1.49	0.65	0.84	1.31	0.69	4.16	2.47	0.11	4.90	4.98	0.12	x	
1342	DNA topoisomerase I_ mitochondrial	Q969P6	84210.48	81827.86	78830.83	61545.36	57466.19	54009.13	68205.94	63669.33	61525.44	69103.65	64020.01	61947.34	66636.11	61922.43	55549.08	61853.03	58661.60	55301.23	59688.88	56902.01	54573.27	814.66	604.50	509.90	281.20	308.71	159.17	696.47	731.27	485.35	739.58	692.42	266.72		
1343	DNA-(apurinic or apyrimidinic site) lyase	P27695	117794.09	110835.61	102827.50	85685.55	81820.52	77161.06	106064.28	96954.25	95295.06	101824.87	96132.25	89290.05	107174.82	91878.95	91032.30	89873.25	84518.01	79678.77	95947.66	88083.64	85333.23	113688.12	107472.32	104632.67	98655.35	97209.98	91003.12	106738.11	102977.59	100621.13	114217.65	108699.51	74900.61		
1344	DNA-3-methyladenine glycosylase	P29372	3116.54	2879.55	2660.29	2466.44	2430.77	2388.19	2006.82	1734.96	1897.35	3847.62	3592.03	3652.62	3382.66	3416.45	2505.92	1936.84	2023.96	1789.76	2038.44	2098.57	1960.04	2.27	1.49	0.65	0.84	1.31	0.69	4.16	2.47	0.11	4.90	4.98	0.12	x	
1345	DNA-binding protein SATB2	Q9UPW6	6386.36	5029.15	4976.78	9529.87	8758.90	7983.09	10381.79	8929.21	7081.05	14998.04	12914.71	12393.43	5080.49	5720.00	2668.76	8359.71	10699.35	11395.83	4048.73	4413.92	3138.03	6651.78	5894.25	5462.91	5913.46	5207.73	4803.67	5925.00	5815.62	5726.23	4803.90	5114.15	4165.56		
1346	DNA-dependent protein kinase catalytic subunit	P78527	1282581.52	1196414.22	1093816.67	828886.32	813441.11	739462.41	988769.61	915027.36	870221.37	1031886.47	973598.76	894856.45	1034324.71	945631.43	721733.54	813286.84	771487.71	742956.10	840199.06	782489.71	727381.33	699924.77	651323.09	568209.98	459160.14	436197.23	369851.16	559563.84	528571.11	470723.15	587035.97	558122.88	344848.09		
1347	DNA-directed RNA polymerase II subunit RPB2	P30876	18939.94	18343.78	16297.92	15038.19	14899.80	16139.53	21271.03	22542.41	19414.11	19124.57	18878.51	16187.08	15525.03	15021.23	12332.44	16759.85	18227.46	14333.68	16540.58	13764.40	15004.10	9042.70	8622.56	8385.60	6295.54	5804.48	5319.98	7131.12	7013.08	6857.12	6941.43	7116.19	5270.69		
1348	DNA-directed RNA polymerase III subunit RPC5	Q9NVU0	0.14	2.53	0.81	1.07	2.77	0.68	0.96	0.36	0.63	0.35	0.75	0.38	2.13	0.97	0.60	4.64	1.07	0.47	2.22	0.19	0.06	24571.23	23730.42	18984.09	12714.62	13191.52	9994.45	16014.68	15271.51	13721.08	16711.01	17210.24	9261.92		x
1349	DNA-directed RNA polymerase III subunit RPC9	O75575	4608.45	4249.35	3786.81	1971.23	2371.44	2096.67	2635.78	2449.64	2489.35	2934.78	2884.43	2758.28	2796.69	3171.78	2738.94	2484.29	1925.27	1895.69	2896.75	2689.91	2324.25	2.27	1.49	0.65	0.84	1.31	0.69	4.16	2.47	0.11	4.90	4.98	0.12	x	
1350	DNA-directed RNA polymerases I and III subunit RPAC1	O15160	39694.36	39014.95	51093.06	25975.97	24578.36	23262.81	30213.64	27061.58	26660.66	46256.22	43572.56	38797.91	33731.67	31212.23	23340.09	23377.10	24288.19	23004.93	29896.06	28831.05	25837.58	10647.35	10015.81	9530.34	7448.74	7410.94	7054.85	8105.91	8130.14	7944.81	9051.02	8833.15	6272.07		
1351	DNA-directed RNA polymerases I_ II_ and III subunit RPABC1	P19388	796.00	740.01	517.88	1.07	2.77	0.68	0.96	0.36	0.63	744.41	619.30	498.66	2.13	0.97	0.60	4.64	1.07	0.47	2.22	0.19	0.06	2.27	1.49	0.65	246.30	293.35	41.99	433.03	377.59	224.03	603.33	522.69	0.12		
1352	DNA-directed RNA polymerases I_ II_ and III subunit RPABC3	P52434	48853.99	48735.27	43702.24	39932.23	36998.53	36225.34	42228.10	39249.87	37039.90	42236.95	39614.54	38168.15	39884.86	41711.83	38795.56	29599.42	31798.73	26384.94	39433.35	35182.41	33351.82	13838.84	11174.67	11831.39	10017.92	9576.20	8640.49	11097.75	10754.22	11014.07	11806.06	11424.63	8712.64		
1353	DnaJ homolog subfamily A member 1	P31689	66780.09	62458.37	61808.69	44997.75	42931.99	38426.19	54110.99	50356.09	48425.02	59245.51	56249.15	52297.66	57176.20	45859.73	40389.00	42257.18	39727.32	37995.24	46297.50	44203.25	39707.02	108573.97	98493.02	89509.18	57112.34	53068.83	44233.75	73832.94	70474.98	66614.75	71776.91	70212.35	46263.80		
1354	DnaJ homolog subfamily A member 2	O60884	77635.29	73127.40	67500.39	45823.06	43842.95	42363.86	55067.43	53888.26	53261.91	59535.40	55075.72	51043.29	61407.12	53946.28	43025.48	48332.98	44891.72	41781.09	45603.69	42275.24	40378.63	120962.18	113727.02	103308.92	73873.84	71611.57	67000.73	88159.72	83902.78	77151.85	89523.97	89493.36	66809.29		
1355	DnaJ homolog subfamily A member 3_ mitochondrial	Q96EY1	0.14	2.53	0.81	1.07	2.77	0.68	0.96	0.36	0.63	0.35	0.75	0.38	2.13	0.97	0.60	4.64	1.07	0.47	2.22	0.19	0.06	13903.53	12635.59	11478.27	5462.62	5493.70	4663.21	7691.31	7997.35	6579.41	6987.99	6825.15	3921.12		x
1356	DnaJ homolog subfamily A member 4	Q8WW2[illegible]	191.87	204.32	137.13	130.92	103.19	110.99	136.38	132.38	117.02	174.61	144.06	184.82	192.96	7.45	15.66	102.91	107.58	114.13	148.05	135.87	130.09	2.27	1.49	0.65	0.84	1.31	0.69	4.16	2.47	0.11	4.90	4.98	0.12	x	
1357	DnaJ homolog subfamily B member 1	P25685	107758.04	101868.46	91014.50	66627.53	64016.20	57482.32	78788.58	75811.81	69159.73	79714.95	76744.87	66912.44	82749.68	75644.18	57247.83	66952.12	64279.46	61089.44	71225.89	66469.90	54478.69	762.62	587.00	30.41	333.85	259.70	123.79	552.86	431.52	287.87	774.96	630.24	211.57		
1358	DnaJ homolog subfamily B member 11	Q9UBS4	130721.83	126138.63	120715.31	105969.95	108610.58	98983.12	112546.44	106059.75	101481.39	134167.24	128555.10	118831.90	119431.88	111978.21	90567.07	112229.25	103752.36	100134.17	104902.25	99222.11	91158.35	52739.39	48048.89	43154.16	38969.17	35682.10	28926.67	42325.22	41088.32	39865.93	46159.19	43700.96	31169.42		
1359	DnaJ homolog subfamily B member 12	Q9NXW2	11041.94	10653.86	9469.46	7055.56	6955.47	6953.40	8489.27	7741.02	6740.95	7893.65	8069.68	7204.46	7359.67	7935.24	6302.63	6818.26	7174.76	6649.96	7779.10	7341.20	5739.04	2.27	1.49	0.65	0.84	1.31	0.69	4.16	2.47	0.11	4.90	4.98	0.12	x	
1360	DnaJ homolog subfamily B member 3	Q8WWF[illegible]	0.14	2.53	0.81	1.07	2.77	0.68	0.96	0.36	0.63	0.35	0.75	0.38	2.13	0.97	0.60	4.64	1.07	0.47	2.22	0.19	0.06	6172.67	5202.99	5249.82	2472.10	2505.22	1852.04	1650.92	771.92	1625.18	1851.49	1760.91	909.94		x
1361	DnaJ homolog subfamily B member 4	Q9UDY4	5538.89	5614.14	5931.68	3994.53	4109.55	4007.97	4639.67	5041.83	5037.80	5555.23	5189.78	5269.66	5649.39	5456.96	1728.27	2536.21	3716.01	3598.77	5482.28	4206.53	3922.63	3533.31	3318.08	2675.61	1519.97	1378.96	916.92	2344.95	2429.74	2225.35	1483.92	2215.41	1003.27		
1362	DnaJ homolog subfamily B member 6	O75190	0.14	2.53	0.81	1.07	2.77	0.68	0.96	0.36	0.63	0.35	0.75	0.38	2.13	0.97	0.60	4.64	1.07	0.47	2.22	0.19	0.06	8002.30	7300.72	7238.46	7447.80	7115.14	5961.07	8369.05	7865.07	7429.53	7325.25	7630.19	4738.82		x
1363	DnaJ homolog subfamily B member 9	Q9UBS3	0.14	2.53	0.81	1.07	2.77	0.68	0.96	0.36	0.63	0.35	0.75	0.38	2.13	0.97	0.60	4.64	1.07	0.47	2.22	0.19	0.06	18007.57	13026.86	13495.51	10994.93	11369.84	8780.06	13579.60	12473.60	11263.37	13422.41	15547.19	9335.81		x
1364	DnaJ homolog subfamily C member 10	Q8IXB1	14851.98	15313.13	14482.86	10226.09	10446.32	10419.01	11165.63	10496.21	10985.88	13621.76	13068.11	13022.35	12869.04	11804.13	7912.46	9944.55	8201.03	9062.68	11171.22	10612.68	9845.52	4210.15	4119.07	3712.08	3260.80	3108.66	2847.19	3668.69	3619.11	3406.39	3434.38	3348.70	2499.92		
1365	DnaJ homolog subfamily C member 13	O75165	188032.55	188862.77	185739.11	122465.84	118590.65	113863.32	155995.76	144098.51	147064.88	160995.61	151170.76	140219.96	151685.65	148096.45	120509.33	123094.56	117667.90	115148.81	128082.59	121981.67	121958.50	2.27	1.49	0.65	0.84	1.31	0.69	4.16	2.47	0.11	4.90	4.98	0.12	x	
1366	DnaJ homolog subfamily C member 2	Q99543	380442.53	367142.89	343845.72	280668.30	266075.69	257979.50	321187.32	315578.42	302973.93	377693.73	359925.19	362331.28	333951.41	302991.68	240686.70	287632.00	284696.99	283278.85	276320.96	267823.13	264469.38	24633.74	24269.36	23599.39	18196.95	18410.41	15569.88	22087.69	22445.26	20619.65	20567.28	20279.11	14037.80		
1367	DnaJ homolog subfamily C member 21	Q5F1R6	39395.85	33975.74	31928.55	27159.62	24786.69	23801.99	29178.61	26429.30	24192.22	31191.10	30481.49	29024.35	30106.53	29151.39	26448.09	23622.10	22106.69	22701.83	23769.95	25242.48	24042.87	486.68	508.75	417.06	475.11	447.88	456.61	426.08	389.92	360.64	569.92	587.50	454.36		
1368	DnaJ homolog subfamily C member 3	Q13217	0.14	2.53	0.81	1.07	2.77	0.68	0.96	0.36	0.63	0.35	0.75	0.38	2.13	0.97	0.60	4.64	1.07	0.47	2.22	0.19	0.06	925.52	777.78	782.43	539.49	455.24	306.68	521.56	1018.32	493.26	750.33	466.04	329.91		x
1369	DnaJ homolog subfamily C member 7	Q99615	59268.96	57167.49	52766.70	44609.14	43432.29	38920.03	46752.86	43735.49	42916.48	52872.03	51007.90	48539.41	52919.62	50276.37	41339.79	40045.80	37948.46	37076.13	44584.83	43046.19	40465.32	62026.56	53256.95	55952.15	42971.29	40022.94	35637.75	49550.96	47747.87	45225.72	46773.71	45305.45	33191.13		
1370	DnaJ homolog subfamily C member 8	O75937	35143.32	33579.01	33620.33	23911.73	23630.42	21206.98	30817.26	28927.40	27131.50	30153.73	30494.44	29292.89	30882.29	28661.40	24770.01	22750.77	19896.90	21333.29	25136.91	23350.16	21823.75	32810.07	32662.78	27949.79	21286.05	20882.40	20185.83	25048.67	24957.45	24948.05	28555.53	26500.89	16335.54		
1371	DnaJ homolog subfamily C member 9	Q8WXX5	313282.49	306989.73	284462.22	297800.57	280975.84	265522.11	303189.73	296553.09	284995.24	314082.31	299523.93	298348.78	252053.09	269696.65	227604.52	294054.06	278569.11	263540.30	240752.40	241718.43	232269.85	115965.62	106320.93	99715.44	77136.18	74249.41	62150.41	89941.99	85377.38	78410.74	91139.03	87854.35	58133.54		
1372	DNL-type zinc finger protein	Q5SXM8	9054.34	8707.94	8263.48	7175.08	7414.93	6884.40	7485.39	6986.18	6765.67	10006.58	9973.40	9610.52	7690.08	7264.76	6287.38	6145.01	5981.72	5821.70	6534.94	6159.80	6082.91	2.27	1.49	0.65	0.84	1.31	0.69	4.16	2.47	0.11	4.90	4.98	0.12	x	
1373	Docking protein 5	Q9P104	0.14	2.53	0.81	1.07	2.77	0.68	0.96	0.36	0.63	0.35	0.75	0.38	2.13	0.97	0.60	4.64	1.07	0.47	2.22	0.19	0.06	17228.81	16779.83	14574.88	12968.42	13258.50	9777.49	14903.26	13819.15	12799.85	16288.90	16243.81	9931.20		x
1374	Dolichol-phosphate mannosyltransferase subunit 1	O60762	73995.39	72702.27	67101.25	54366.89	54120.96	49617.67	63946.43	61525.31	59593.81	61840.88	59660.72	55379.35	56566.05	62124.67	49914.77	55277.44	56779.48	53214.70	45481.85	50379.37	44389.71	1464.65	1315.04	1216.45	672.81	638.63	424.06	874.73	752.73	694.70	827.61	752.50	281.10		
1375	Dolichol-phosphate mannosyltransferase subunit 3	Q9P2X0	66078.52	71074.94	68262.74	54708.85	51149.63	50991.57	53970.17	50547.38	55041.12	60253.25	58526.84	53159.25	63393.38	57844.84	50487.44	54405.63	48869.28	52175.03	59248.16	51790.45	47199.14	3451.12	2703.00	3173.87	2895.24	2481.85	2329.46	2902.13	2783.46	2729.46	3114.81	3031.85	353.70		
1376	Dolichyl-diphosphooligosaccharide--protein glycosyltransferase 48 kDa subunit	P39656	80756.62	72072.98	70930.43	44419.15	44940.66	42122.00	69644.55	63523.75	60186.87	62351.73	56321.90	53815.46	59287.29	50627.66	41467.11	53041.48	48575.80	50241.23	42649.49	41955.58	37783.70	22424.52	20016.54	17558.02	10946.39	10198.35	7126.64	20023.98	17283.53	15143.85	15836.31	14462.70	8407.14		
1377	Dolichyl-diphosphooligosaccharide--protein glycosyltransferase subunit 1	P04843	506017.79	489794.91	450642.90	387145.01	371263.98	350801.10	413845.66	400541.78	391683.80	449461.06	422601.16	403139.63	418987.25	397234.95	348238.69	377725.02	357763.30	349905.00	367607.80	353044.52	330179.12	337122.95	310685.40	288489.38	198027.47	189578.31	173794.99	230285.66	215003.79	203092.77	224371.21	212347.72	157310.49		
1378	Dolichyl-diphosphooligosaccharide--protein glycosyltransferase subunit 2	P04844	301204.85	277664.44	250136.69	199291.34	194197.32	183393.59	231067.22	221919.06	207720.56	256848.93	250793.52	225515.49	245533.25	213791.82	158278.84	216491.33	205046.89	193164.53	210989.85	194150.36	179471.84	132937.72	123888.24	108925.69	89575.85	85030.77	71710.27	122083.38	111454.71	99957.93	115472.82	110616.51	75099.53		
1379	Dolichyl-diphosphooligosaccharide--protein glycosyltransferase subunit DAD1	P61803	11054.76	9603.65	8340.88	5274.95	5335.83	4406.10	8139.69	7990.56	6918.16	8573.91	8066.06	6911.14	7242.07	6757.36	4728.44	7053.40	6627.07	6799.46	6636.59	6045.32	5861.74	8482.93	8179.30	7616.15	5193.85	4944.18	4027.24	6771.23	6692.71	6275.28	6285.19	5940.93	3856.42		

Supplementary Table S1. Overview on the abundance of all relatively quantified 5180 proteins

			MCF-7																					MDA-MB-231													
	Protein name	UniProt	control 01	control 02	control 03	Dai IC20 01	Dai IC20 02	Dai IC20 03	Dai SC20 01	Dai SC20 02	Dai SC20 03	Gen IC20 01	Gen IC20 02	Gen IC20 03	Gen SC20 01	Gen SC20 02	Gen SC20 03	SSE IC20 01	SSE IC20 02	SSE IC20 03	SSE SC20 01	SSE SC20 02	SSE SC20 03	control 01	control 02	control 03	Dai IC20 01	Dai IC20 02	Dai IC20 03	Gen IC20 01	Gen IC20 02	Gen IC20 03	SSE IC20 01	SSE IC20 02	SSE IC20 03	unique to MCF-7	unique to MDA-MB-231
1380	Dolichyl-diphosphooligosaccharide--protein glycosyltransferase subunit STT3A	P46977	383335.37	363399.50	346384.69	264896.70	263587.46	259136.52	313799.14	302258.47	293338.79	318201.67	309080.75	296346.79	328286.57	283398.27	246050.66	259198.65	249020.40	264146.23	282719.34	251736.35	237954.40	124611.10	114014.71	104434.22	76374.31	75002.73	67760.63	97262.44	89962.44	87612.83	94417.14	88144.06	60873.62		
1381	Dolichyl-diphosphooligosaccharide--protein glycosyltransferase subunit STT3B	Q8TCJ2	0.14	2.53	0.81	1.07	2.77	0.68	0.96	0.36	0.63	0.35	0.75	0.38	2.13	0.97	0.60	4.64	1.07	0.47	2.22	0.19	0.06	24368.33	24009.38	21005.58	17327.87	16827.76	15041.40	19372.35	19132.95	18140.37	18725.02	18507.61	9697.10		x
1382	Dolichyl-phosphate beta-glucosyltransferase	Q9Y673	5373.15	4926.04	4066.73	3855.65	3542.18	3695.85	3669.08	3623.23	3010.50	4849.59	4582.77	4070.82	3773.55	4214.02	3563.18	3562.45	3904.89	3657.70	3988.69	4138.66	3658.53	2.27	1.49	0.65	0.84	1.31	0.69	4.16	2.47	0.11	4.90	4.98	0.12	x	
1383	Dol-P-Man:Man(5)GlcNAc(2)-PP-Dol alpha-1_3-mannosyltransferase	Q92685	28182.28	24737.11	21287.09	27564.17	21699.08	16343.28	23080.79	22132.98	16670.99	18077.81	17092.05	14657.02	25801.47	18775.64	15982.85	14014.00	27319.81	27859.59	41421.19	29821.19	24115.82	2.27	1.49	0.65	0.84	1.31	0.69	4.16	2.47	0.11	4.90	4.98	0.12	x	
1384	Doublecortin domain-containing protein 1	M0R2J8	3094.43	3037.30	2761.03	2469.49	2426.65	2362.88	2361.21	2273.82	2581.08	3206.36	3175.30	3117.56	3182.91	2944.20	2222.81	2588.58	2385.36	2434.56	2593.49	2676.29	2548.26	2.27	1.49	0.65	0.84	1.31	0.69	4.16	2.47	0.11	4.90	4.98	0.12	x	
1385	Doublecortin domain-containing protein 2B	A2VCK2	0.14	2.53	0.81	1.07	2.77	0.68	0.96	0.36	0.63	0.35	0.75	0.38	2.13	0.97	0.60	4.64	1.07	0.47	2.22	0.19	0.06	11476.48	1094.81	339.23	9696.76	8780.85	4917.38	12364.72	9285.29	4124.70	14459.81	13504.57	5662.89		x
1386	Doublecortin domain-containing protein 2C	A8MYV0	0.14	2.53	0.81	1.07	2.77	0.68	0.96	0.36	0.63	0.35	0.75	0.38	2.13	0.97	0.60	4.64	1.07	0.47	2.22	0.19	0.06	2.27	1.49	0.65	334.69	299.62	216.87	332.21	404.05	383.82	326.70	350.34	165.13		x
1387	Double-strand break repair protein MRE11	P49959	0.14	2.53	0.81	1.07	2.77	0.68	0.96	0.36	0.63	0.35	0.75	0.38	2.13	0.97	0.60	4.64	1.07	0.47	2.22	0.19	0.06	19152.20	18624.01	13180.51	13336.82	13216.88	9662.50	15657.85	15383.08	14502.27	17451.09	18549.25	7254.39		x
1388	Double-stranded RNA-binding protein Staufen homolog 1	O95793	23049.60	21139.41	20502.39	15620.37	14773.66	13343.27	16477.62	16525.34	15259.18	20922.73	19058.93	18582.88	19345.28	17035.51	13209.82	14803.02	13477.81	13081.93	15420.89	14684.34	14205.11	98437.39	89111.65	82459.30	57584.57	56266.90	48444.11	70958.38	67390.50	63960.86	69865.21	65667.28	45624.19		
1389	Double-stranded RNA-binding protein Staufen homolog 2	Q9NUL3	9242.36	9560.08	7930.87	6631.49	6262.90	5664.11	7290.10	6950.69	6953.41	7808.16	7114.98	6741.29	7571.17	7208.48	5469.10	6315.86	5858.63	5556.03	6395.81	5667.63	6142.30	115.47	64.03	84.83	93.78	71.11	84.26	54.63	70.66	58.88	63.73	107.72	68.37		
1390	Double-stranded RNA-specific adenosine deaminase	P55265	4652.27	4047.32	3581.65	2756.79	3133.22	2663.04	3044.52	3266.08	3443.89	3430.30	3029.65	3283.94	4049.30	3444.96	3027.23	2573.72	2245.28	1800.70	2780.69	2724.57	3018.03	2.27	1.49	0.65	0.84	1.31	0.69	4.16	2.47	0.11	4.90	4.98	0.12	x	
1391	Drebrin	Q16643	40400.58	37907.55	37991.82	29669.85	30755.75	27696.05	31723.97	29205.50	29624.29	38144.55	35780.46	36200.44	31421.20	32195.99	27660.56	29892.09	28571.09	25345.53	27690.04	27099.79	26906.63	67611.02	62275.98	56632.13	48863.43	47569.44	43911.94	58350.87	55545.54	54341.32	74758.91	69559.43	45450.25		
1392	Drebrin-like protein	Q9UJU6	132359.57	128543.05	121923.51	98457.62	93685.53	88276.38	109696.93	106619.46	102834.74	117962.78	114881.48	107013.11	121068.75	103747.20	86783.46	95682.83	89535.90	86845.54	91512.82	86610.71	84565.49	130884.24	127337.31	111610.63	103754.27	98017.80	86613.09	113192.90	111928.25	103679.93	115549.60	111383.02	72252.82		
1393	DTW domain-containing protein 2	Q8NBA8	25107.62	24355.45	23745.51	11933.82	11462.48	11410.57	16164.65	16551.48	16172.29	17908.05	17591.05	16507.26	17505.65	15449.71	15038.57	18805.16	17409.04	17905.55	16170.02	14912.91	14661.02	2.27	1.49	0.65	0.84	1.31	0.69	4.16	2.47	0.11	4.90	4.98	0.12	x	
1394	Dual oxidase 2	Q9NRD8	0.14	2.53	0.81	1.07	2.77	0.68	0.96	0.36	0.63	0.35	0.75	0.38	2.13	0.97	0.60	4.64	1.07	0.47	2.22	0.19	0.06	886.00	752.78	597.45	647.62	366.48	128.01	557.68	488.15	408.25	606.75	502.95	122.34		x
1395	Dual serine/threonine and tyrosine protein kinase	Q6XUX3	30435.87	29352.94	29014.54	23632.25	22851.91	22837.67	25363.18	20304.69	19541.19	28658.28	27941.33	23632.85	25290.86	25878.01	21524.58	22862.20	20156.99	22962.43	23073.78	22142.52	20452.08	2.27	1.49	0.65	0.84	1.31	0.69	4.16	2.47	0.11	4.90	4.98	0.12	x	
1396	Dual specificity mitogen-activated protein kinase kinase 1	Q02750	42395.97	37300.83	37606.58	26363.02	24911.20	23219.93	29255.92	28358.26	26013.13	34359.60	32147.75	30513.40	33044.52	29057.74	23606.23	26615.73	24276.14	24410.74	28972.45	24707.82	23142.59	2.27	1.49	0.65	0.84	1.31	0.69	4.16	2.47	0.11	4.90	4.98	0.12	x	
1397	Dual specificity mitogen-activated protein kinase kinase 2	P36507	52655.38	52360.94	50896.31	30871.29	28614.79	26598.63	29823.26	30064.62	29637.71	46661.85	43434.45	38378.61	43354.12	36609.10	30043.62	28340.02	26766.91	26746.09	32897.81	28509.87	29842.86	19680.62	18208.54	17134.93	15540.61	15110.30	13103.12	20577.22	19089.35	18015.44	22056.48	21263.24	14350.86		
1398	Dual specificity mitogen-activated protein kinase kinase 3	P46734	3518.51	3417.43	3026.75	2539.28	2501.37	2378.13	2521.40	2457.58	2381.13	3441.55	3364.55	3004.32	3459.66	3228.22	2852.68	2597.37	2305.92	2381.66	2537.24	2443.98	2356.06	1843.72	1920.95	1523.62	1721.16	1743.26	1555.73	1994.81	2028.66	1926.79	2043.27	2006.92	1384.20		
1399	Dual specificity mitogen-activated protein kinase kinase 4	P45985	38334.98	37309.16	34901.97	24879.05	23764.05	22324.58	29891.43	29164.01	28179.31	30286.31	28726.86	26957.90	28129.95	28002.35	24173.02	25986.27	23748.02	22874.93	24889.34	24512.19	22099.69	2.27	1.49	0.65	0.84	1.31	0.69	4.16	2.47	0.11	4.90	4.98	0.12	x	
1400	Dual specificity protein kinase TTK	P33981	0.14	2.53	0.81	1.07	2.77	0.68	0.96	0.36	0.63	0.35	0.75	0.38	2.13	0.97	0.60	4.64	1.07	0.47	2.22	0.19	0.06	1791.86	1914.67	1552.18	1513.69	1586.79	1290.17	1742.11	1689.33	1680.47	1866.51	1805.93	1246.48		x
1401	Dual specificity protein phosphatase 3	P51452	53.65	147.15	46.54	39.61	363.30	204.40	561.40	824.25	528.92	0.35	0.75	0.38	2.13	0.97	0.60	384.82	284.48	260.41	797.04	711.20	371.47	20050.38	17926.41	16078.08	12923.09	13028.60	11375.56	16814.62	15256.72	14684.00	15114.41	14574.27	10036.11		
1402	Dynactin subunit 1	Q14203	196835.80	183561.64	171112.57	150119.67	143825.40	137730.84	157929.42	156986.52	151434.68	186676.49	179193.32	172536.87	175224.23	162503.16	142014.45	132060.66	132327.95	126473.46	140651.55	134272.42	129483.30	224808.76	210999.23	197562.34	156286.45	151656.23	131495.13	179724.38	175702.83	166556.90	188748.62	188596.36	121520.45		
1403	Dynactin subunit 2	Q13561	45063.90	40040.91	38367.99	32704.98	31532.51	30110.39	38857.19	36604.23	32091.80	40994.86	38777.62	36027.46	40049.48	36995.44	26066.18	31504.43	30818.58	30303.50	30888.37	30177.18	27039.49	58917.63	52989.88	47589.77	28493.10	26622.11	18189.78	32323.97	28920.89	24613.36	33733.98	31641.10	16280.28		
1404	Dynactin subunit 4	Q9UJW0	68018.10	63978.71	61982.31	40457.03	38190.98	36332.74	45541.23	44180.79	42540.16	49846.37	46748.02	45169.62	47328.54	46300.58	36937.42	40711.77	37289.56	35947.38	40698.13	37662.98	34369.53	18617.24	18195.95	17526.08	14151.96	13643.27	13299.41	15920.00	15582.46	14969.07	15166.47	15091.39	11149.39		
1405	Dynactin subunit 5	Q9BTE1	1116.55	1188.04	995.85	528.19	466.06	529.77	1068.35	973.46	1154.75	815.19	795.77	800.38	965.89	1032.62	770.67	509.52	435.04	467.55	404.51	399.16	496.63	6388.95	5540.94	8457.68	5969.14	6297.17	5936.80	6567.05	7102.22	6637.70	6454.81	6430.30	16974.82		
1406	Dynamin-1	Q05193	88428.83	88836.19	83681.36	53180.42	51322.55	47712.11	84266.67	82776.69	77449.56	68796.16	65727.26	60702.78	61285.77	59129.26	52670.83	56130.82	57358.97	51515.96	52466.17	48984.95	48512.37	5227.88	5536.31	29098.11	3035.09	2760.82	1453.71	4885.47	4068.69	2787.59	5514.31	5123.25	2477.81		
1407	Dynamin-1-like protein	O00429	201864.58	192430.17	186246.20	150381.53	143137.82	135508.68	177182.72	173972.34	168108.72	174022.06	173053.59	155292.37	165319.91	144119.58	128548.22	162906.48	154844.67	148540.85	151359.33	140736.89	140753.08	103364.31	96697.03	85364.69	62979.04	61153.60	54775.52	71792.22	70188.92	63480.98	82242.32	76979.14	51113.38		
1408	Dynamin-2	P50570	99561.76	97496.82	91730.25	67010.72	64077.04	60398.60	76833.46	72598.90	71032.29	81926.18	79097.85	73879.40	86395.67	81177.17	69146.46	66453.34	63437.95	61792.67	69167.92	64966.72	62444.37	46779.72	43126.28	38039.33	32107.49	30759.64	28394.64	35524.89	33182.41	32034.87	39665.17	37870.13	21780.92		
1409	Dynamin-3	Q9UQ16	0.14	2.53	0.81	1.07	2.77	0.68	0.96	0.36	0.63	0.35	0.75	0.38	2.13	0.97	0.60	4.64	1.07	0.47	2.22	0.19	0.06	2.27	1.49	0.65	0.84	1.31	0.69	4.16	2.47	0.11	4.90	4.98	0.12	x	
1410	Dynamin-like 120 kDa protein_ mitochondrial	O60313	248781.25	232358.11	219957.80	178329.45	171861.70	161894.22	197066.02	191006.49	182764.21	215442.17	201961.50	189472.40	203187.31	201582.80	163785.26	186896.70	168273.54	167705.77	180478.48	167565.36	160073.65	96172.79	87950.27	81995.04	66448.90	66806.70	61635.92	76151.10	73114.65	69274.94	77801.66	81948.97	63257.11		
1411	Dynein assembly factor 1_ axonemal	Q8NEP3	487.96	394.56	414.76	284.60	298.27	237.24	294.20	271.02	345.97	271.55	317.73	316.24	279.63	325.92	97.27	385.53	357.07	341.93	359.98	317.21	237.94	519.47	521.17	422.31	311.34	351.24	490.78	256.65	284.74	355.11	410.41	454.75	23.52		
1412	Dynein assembly factor 5_ axonemal	Q86Y56	23310.32	20059.44	20478.25	19147.26	17709.60	15079.12	23872.07	23447.68	20076.75	17230.99	17355.70	15584.91	19738.68	27634.38	15117.73	19055.73	17414.46	14336.64	20045.52	18638.35	16229.98	11474.51	11242.68	10785.59	5214.58	5420.07	4691.69	8120.36	7606.79	6974.53	7064.28	6954.01	4240.61		
1413	Dynein heavy chain 1_ axonemal	Q9P2D7	0.14	2.53	0.81	1.07	2.77	0.68	0.96	0.36	0.63	0.35	0.75	0.38	2.13	0.97	0.60	4.64	1.07	0.47	2.22	0.19	0.06	10493.44	11310.10	9027.94	6476.61	6497.22	5316.54	8603.05	7825.18	7525.91	7304.86	7835.37	4481.75		x
1414	Dynein heavy chain 10_ axonemal	Q8IVF4	10873.00	11491.25	9761.61	8767.29	7708.31	8734.43	10080.77	8732.23	8660.19	9757.58	9455.82	9434.85	9430.82	9114.35	7938.53	6986.65	6935.29	7071.53	9131.91	8663.58	8449.01	55911.77	53922.49	47605.33	38520.82	38893.77	36870.34	41266.21	40161.73	37667.69	42704.86	42520.62	34576.04		
1415	Dynein heavy chain 12_ axonemal	Q6ZR08	63861.06	59375.04	54028.23	37463.17	35564.53	30183.89	49250.55	49037.12	42975.03	43656.52	41980.50	37831.80	46609.43	44751.08	29548.42	37321.98	35165.84	32240.09	35299.37	34995.89	31246.27	2.27	1.49	0.65	0.84	1.31	0.69	4.16	2.47	0.11	4.90	4.98	0.12	x	
1416	Dynein heavy chain 14_ axonemal	Q0VDD8	284984.60	259007.92	217638.72	183561.23	176113.11	161878.17	205576.05	218065.76	204601.26	250490.55	234426.61	210627.62	230630.85	209191.88	129876.42	165098.25	164843.37	146279.89	217631.26	196784.41	173239.32	112907.30	104670.79	97008.11	77634.17	75723.70	64218.93	102420.97	96739.16	90020.71	106705.01	101773.57	63458.47		
1417	Dynein heavy chain 17_ axonemal	Q9UFH2	88844.25	73243.74	60638.89	35183.46	32836.00	28509.62	41887.46	38556.93	34277.10	66715.49	59893.02	48974.51	65471.77	42721.43	23930.19	34679.84	31752.03	32088.85	34162.34	33796.25	28018.47	41012.60	34319.12	30419.71	24027.48	22577.08	17080.96	34255.20	31287.53	24206.26	31762.61	29786.98	17028.26		
1418	Dynein heavy chain 2_ axonemal	Q9P225	138408.29	131866.74	126493.83	97094.83	98598.09	90163.75	119232.10	111179.36	109093.19	121945.15	118664.72	110703.28	116752.75	101737.97	85154.82	102252.76	96670.69	96044.69	95513.12	88997.41	82221.81	2.27	1.49	0.65	0.84	1.31	0.69	4.16	2.47	0.11	4.90	4.98	0.12	x	
1419	Dynein heavy chain 3_ axonemal	Q8TD57	0.14	2.53	0.81	1.07	2.77	0.68	0.96	0.36	0.63	0.35	0.75	0.38	2.13	0.97	0.60	4.64	1.07	0.47	2.22	0.19	0.06	24776.45	22220.85	19600.35	20976.64	20003.39	16656.25	22482.45	21005.60	19733.90	23388.06	22537.85	14350.75		x
1420	Dynein heavy chain 5_ axonemal	Q8TE73	2809.97	2673.69	2338.75	1866.28	1662.05	1504.39	2595.75	2137.71	2235.57	2790.03	2656.25	2875.09	2649.73	2235.53	1833.27	1698.87	1642.18	1542.28	1597.67	1779.80	1528.25	45770.85	44146.26	43219.39	27763.22	26911.87	24786.72	27748.77	26987.82	26884.63	26301.85	24726.26	17093.49		
1421	Dynein heavy chain 6_ axonemal	Q9C0G6	36692.75	33160.84	29763.88	25799.29	25122.12	22179.87	26841.57	24186.65	23330.21	32999.16	32597.78	29883.80	30274.74	29029.54	17473.72	27640.41	26837.38	25367.70	24275.62	22931.24	20998.38	2.27	1.49	0.65	0.84	1.31	0.69	4.16	2.47	0.11	4.90	4.98	0.12	x	
1422	Dynein heavy chain 7_ axonemal	Q8WXX0	576.25	386.14	352.88	188.67	117.54	105.42	223.90	44.37	210.63	211.36	212.49	255.13	391.69	231.45	109.51	222.82	32.55	166.76	151.94	171.18	165.32	2.27	1.49	0.65	0.84	1.31	0.69	4.16	2.47	0.11	4.90	4.98	0.12	x	
1423	Dynein heavy chain 8_ axonemal	Q96JB1	15051.03	13761.31	13048.22	4960.34	5243.98	4783.65	9158.42	8669.53	7965.71	12438.95	11603.02	10500.97	8751.55	8259.46	6222.08	12172.66	12633.37	12306.38	9598.80	9025.03	8646.71	68377.20	65699.97	59969.64	45056.57	46844.50	39723.67	63562.00	62043.76	58552.48	56234.33	57163.51	34905.96		
1424	Dynein heavy chain 9_ axonemal	Q9NYC9	0.14	2.53	0.81	1.07	2.77	0.68	0.96	0.36	0.63	0.35	0.75	0.38	2.13	0.97	0.60	4.64	1.07	0.47	2.22	0.19	0.06	51708.10	55203.49	46209.27	42029.08	42085.36	45105.61	37974.40	40445.16	39875.96	55391.17	49481.94	23594.40		x
1425	Dynein heavy chain domain-containing protein 1	Q96M86	26510.60	25118.94	27343.95	17312.37	17533.80	17473.97	23078.54	20336.64	21614.35	22009.91	21197.72	20871.92	24039.95	22556.70	18679.32	17979.17	16108.62	16147.66	16668.78	16358.53	15914.90	2.27	1.49	0.65	0.84	1.31	0.69	4.16	2.47	0.11	4.90	4.98	0.12	x	
1426	Dynein light chain 1_ cytoplasmic	P63167	34072.98	33980.24	30997.88	28084.89	26871.10	26306.41	30182.08	29147.85	26891.76	32585.40	31032.26	29093.39	35091.57	33083.66	27892.43	27474.41	24899.60	24639.26	26014.01	24870.96	23388.03	5551.55	5305.39	4998.80	5895.71	5197.91	4368.07	5518.51	5145.51	4550.71	6763.29	6928.30	4265.27		
1427	Dynein light chain 2_ cytoplasmic	Q96FJ2	15651.98	14957.08	13956.04	7959.90	8078.05	7116.98	12399.08	12541.29	11484.49	11667.30	11070.03	9280.09	10451.87	9437.87	6680.04	12177.87	10736.59	10546.78	8593.31	7981.14	7653.56	2.27	1.49	0.65	0.84	1.31	0.69	4.16	2.47	0.11	4.90	4.98	0.12	x	
1428	Dynein light chain roadblock-type 1	Q9NP97	0.14	2.53	0.81	1.07	2.77	0.68	0.96	0.36	0.63	0.35	0.75	0.38	2.13	0.97	0.60	4.64	1.07	0.47	2.22	0.19	0.06	25337.85	24120.83	22159.85	12910.37	11723.15	8634.24	16797.78	15382.00	14255.92	13612.20	12993.40	7059.70		x
1429	Dynein light chain roadblock-type 2	Q8TF09	15657.10	14806.31	12671.07	11379.17	10928.34	9489.11	15359.38	14536.78	13751.04	15089.65	14228.03	13066.59	13597.99	12971.22	9140.33	12297.97	11769.85	11074.59	11616.77	10281.52	9747.67	2.27	1.49	0.65	0.84	1.31	0.69	4.16	2.47	0.11	4.90	4.98	0.12	x	
1430	Dynein light chain Tctex-type 1	P63172	425.83	418.72	353.30	366.42	436.10	433.74	184.39	261.00	240.81	288.14	269.52	306.74	351.97	315.83	359.26	446.05	287.23	342.01	311.86	283.23	235.49	233.20	212.56	154.82	121.94	145.42	151.12	217.89	172.82	242.34	169.11	207.43	118.59		
1431	Dynein light chain Tctex-type 3	P51808	0.14	2.53	0.81	1.07	2.77	0.68	0.96	0.36	0.63	0.35	0.75	0.38	2.13	0.97	0.60	4.64	1.07	0.47	2.22	0.19	0.06	2378.26	2437.32	2156.23	1434.84	1360.65	1265.95	1833.95	1779.73	1693.00	1753.73	1992.81	1207.62		x
1432	Dynein regulatory complex protein 8	Q5VUJ9	15486.52	14278.21	12292.82	6727.03	6556.15	5589.65	11492.66	12018.92	10634.26	9403.61	9097.65	7286.73	12100.01	11319.03	7920.98	8635.38	8905.74	8549.03	7704.36	6939.83	6204.11	2.27	1.49	0.65	0.84	1.31	0.69	4.16	2.47	0.11	4.90	4.98	0.12	x	
1433	Dynein regulatory complex protein 9	Q9H095	14783.29	14462.58	11946.21	8421.35	8587.18	7269.70	8864.78	8725.66	9096.19	10964.15	11128.32	11040.17	11470.45	10635.51	10489.90	6214.43	6759.22	6685.69	6821.27	7786.41	8443.74	2.27	1.49	0.65	0.84	1.31	0.69	4.16	2.47	0.11	4.90	4.98	0.12	x	
1434	Dystonin	Q03001	0.14	2.53	0.81	1.07	2.77	0.68	0.96	0.36	0.63	0.35	0.75	0.38	2.13	0.97	0.60	4.64	1.07	0.47	2.22	0.19	0.06	212936.71	179407.63	134731.63	149113.83	134694.31	111593.60	187553.08	172527.43	147804.14	205553.48	193063.68	113941.64		x
1435	Dystrobrevin alpha	Q9Y4J8	347.91	164.99	194.00	133.85	59.19	30.00	84.27	33.54	96.43	143.68	126.11	80.06	216.22	41.31	44.62	72.00	45.12	55.84	157.65	103.64	107.32	2.27	1.49	0.65	0.84	1.31	0.69	4.16	2.47	0.11	4.90	4.98	0.12	x	
1436	Dystrobrevin beta	O60941	0.14	2.53	0.81	1.07	2.77	0.68	0.96	0.36	0.63	0.35	0.75	0.38	2.13	0.97	0.60	4.64	1.07	0.47	2.22	0.19	0.06	10446.04	9905.33	8847.51	6960.71	7056.85	4887.18	8055.10	7230.35	6836.44	11018.51	11123.53	5421.00		x
1437	Dystroglycan	Q14118	13954.69	13207.28	11977.58	7763.85	8224.28	6548.45	11244.16	9434.44	9089.59	10686.74	10198.65	8536.06	8691.15	10691.67	6131.45	9515.94	8626.39	9132.69	11929.00	12387.01	12077.65	20635.47	19216.99	18068.02	17249.00	15652.87	12371.09	21118.68	19960.10	19387.06	19614.49	19815.83	11272.84		
1438	Dystrophin	P11532	267631.65	249811.07	234159.79	180039.01	179203.52	156988.64	188060.38	182355.96	172763.26	215316.01	206725.48	195170.23	207045.00	192564.18	155684.37	183116.28	171540.94	163371.93	173108.98	169068.10	155077.16	3828.41	3150.94	3378.29	2205.97	2016.81	1576.11	3276.37	3254.72	2793.45	2849.93	2569.83	1800.49		
1439	E1A-binding protein p400	Q96L91	4138.23	3459.24	3733.78	2978.57	2680.70	3063.45	2791.51	2649.54	2971.05	3920.43	3533.14	3936.71	3470.79	3241.39	3185.36	3157.02	2602.04	2956.76	3610.93	3135.63	2959.61	2.27	1.49	0.65	0.84	1.31	0.69	4.16	2.47	0.11	4.90	4.98	0.12	x	
1440	E2F-associated phosphoprotein	Q56P03	99279.43	96546.16	97378.07	77875.08	73660.46	72291.98	94960.06	92928.04	90342.81	87616.82	82209.94	78519.47	91064.16	90966.23	79417.10	71339.09	70687.12	66169.46	77106.48	75411.08	68752.61	2.27	1.49	0.65	0.84	1.31	0.69	4.16	2.47	0.11	4.90	4.98	0.12	x	
1441	E3 SUMO-protein ligase PIAS3	Q9Y6X2	23042.28	22096.61	18028.26	19512.84	18840.30	18161.94	21015.48	22685.50	20521.33	21885.92	20957.38	20850.05	22507.08	19682.10	16923.88	16814.94	16603.16	16006.02	14115.02	13265.33	13321.06	2.27	1.49	0.65	0.84	1.31	0.69	4.16	2.47	0.11	4.90	4.98	0.12	x	
1442	E3 SUMO-protein ligase RanBP2	P49792	536117.34	514341.12	471353.34	337547.65	336861.27	301459.47	387572.94	372577.56	345130.80	449714.31	428898.41	399338.49	422787.49	387635.92	306918.30	358340.36	339437.36	320983.91	368460.25	350855.26	332223.81	234932.30	213902.62	187583.99	151600.22	149594.18	131948.52	170001.62	159703.04	149666.61	181308.33	168448.14	109440.13		
1443	E3 ubiquitin/ISG15 ligase TRIM25	Q14258	181092.07	175853.21	166563.45	130452.52	124834.13	114809.08	147078.74	149077.29	144427.29	151339.87	139966.06	133015.16	143014.13	138059.87	111829.18	124983.77	122808.85	115390.40	120578.24	114064.61	108882.65	36502.18	35316.17	33478.47	24872.84	25006.48	22828.48	26384.10	24528.39	25220.01	30210.40	27017.26	18826.48		
1444	E3 ubiquitin-protein ligase ARIH1	Q9Y4X5	5926.97	6622.25	6513.72	4564.04	4250.58	3764.44	5201.64	5250.76	4028.09	5403.68	4995.93	4727.98	3968.17	4017.52	3226.62	5217.16	5503.24	4484.74	3734.37	3539.27	3444.36	2.27	1.49	0.65	0.84	1.31	0.69	4.16	2.47	0.11	4.90	4.98	0.12	x	
1445	E3 ubiquitin-protein ligase ARIH2	O95376	6288.48	5942.11	6101.74	3875.94	3181.61	3600.85	4172.31	4296.03	4049.64	5890.57	5398.58	3520.29	3840.07	4502.25	3358.52	3737.49	3005.45	3379.60	3118.45	3122.97	2643.60	2.27	1.49	0.65	0.84	1.31	0.69	4.16	2.47	0.11	4.90	4.98	0.12	x	
1446	E3 ubiquitin-protein ligase BRE1A	Q5VTR2	197799.05	184702.37	173785.33	143392.81	133867.34	128351.39	161508.00	156503.08	151409.62	197289.81	190589.87	171520.48	162106.86	151802.90	126392.37	172768.66	165288.85	159914.57	176587.74	154233.64	143631.69	29183.28	28635.36	27124.61	20177.16	19402.49	18687.51	25045.42	23926.82	23774.36	23383.86	21700.91	17426.99		
1447	E3 ubiquitin-protein ligase BRE1B	O75150	4559.16	4010.09	3801.50	3910.77	3549.06	3678.59	3856.03	3600.85	3661.22	5627.46	5449.84	5517.30	4298.01	3577.30	3182.76	3672.95	3204.70	3038.64	3722.78	3492.12	3340.29	7124.34	7914.86	6415.51	5465.55	5880.98	5708.04	5890.07	5516.95	5219.39	5507.96	6011.14	4158.32		
1448	E3 ubiquitin-protein ligase CBL	P22681	0.14	2.53	0.81	1.07	2.77	0.68	0.96	0.36	0.63	0.35	0.75	0.38	2.13	0.97	0.60	4.64	1.07	0.47	2.22	0.19	0.06	846.51	851.18	892.04	840.25	801.47	623.73	1277.00	1335.17	1172.79	797.66	728.39	500.56		x
1449	E3 ubiquitin-protein ligase CHIP	Q9UNE7	190625.22	183168.43	170262.63	132945.25	121475.97	106587.43	144397.04	140652.79	128252.19	174345.63	164581.91	149061.58	147580.09	127677.65	95205.07	130767.31	129495.37	120005.44	130957.40	122444.43	114290.26	958.57	191.17	143.81	1878.21	1954.29	1122.71	2109.61	2114.79	149.00	3763.12	3497.90	1870.27		
1450	E3 ubiquitin-protein ligase DCST1	Q5T197	33807.47	29641.07	29231.82	22152.52	21207.75	20839.35	26385.17	23039.25	21242.01	26325.74	24747.77	24480.94	24799.24	25823.63	22875.20	21524.40	20324.10	18476.16	23317.42	21161.00	19731.06	2.27	1.49	0.65	0.84	1.31	0.69	4.16	2.47	0.11	4.90	4.98	0.12	x	
1451	E3 ubiquitin-protein ligase HECTD1	Q9ULT8	21245.69	21127.49	20486.05	11259.87	12008.16	10815.05	16027.67	15459.60	15311.18	15317.81	14905.95	13580.25	14461.18	13064.74	12285.75	12485.63	11549.76	11241.93	11188.76	10980.35	10466.53	2.27	1.49	0.65	0.84	1.31	0.69	4.16	2.47	0.11	4.90	4.98	0.12	x	
1452	E3 ubiquitin-protein ligase HECTD3	Q5T447	2105.86	1344.72	1061.09	7055.00	697.00	451.47	1307.47	1917.66	1582.32	936.99	945.17	706.00	1746.96	11687.94	485.99	822.48	1121.17	522.20	4722.65	4334.09	3126.31	2.27	1.49	0.65	0.84	1.31	0.69	4.16	2.47	0.11	4.90	4.98	0.12	x	
1453	E3 ubiquitin-protein ligase HERC2	O95714	95785.43	89917.48	79862.04	89011.36	84203.88	78715.40	95971.87	95434.86	88999.04	113158.18	111008.11	100480.78	84459.53	58901.81	42701.69	78869.21	74290.49	68914.69	67537.66	62207.38	59802.78	27180.39	26263.35	23873.51	20360.75	19449.91	15753.01	23706.06	22513.65	20390.39	26334.24	24389.67	9430.01		
1454	E3 ubiquitin-protein ligase HUWE1	Q7Z6Z7	199085.27	198499.59	185779.18	145936.77	141737.95	137121.65	165078.01	154645.62	149445.59	186468.52	181367.35	171526.79	173632.66	168157.92	137519.37	136989.04	137790.48	132187.50	144061.76	140744.33	133720.09	71786.45	66105.45	63477.22	50306.51	48122.73	43436.81	60160.77	60802.16	56326.24	75725.32	72460.62	54915.22		
1455	E3 ubiquitin-protein ligase Itchy homolog	Q96J02	0.14	2.53	0.81	1.07	2.77	0.68	0.96	0.36	0.63	0.35	0.75	0.38	2.13	0.97	0.60	4.64	1.07	0.47	2.22	0.19	0.06	23887.70	19038.86	17990.39	11863.88	10993.81	8768.62	15271.94	13177.59	11598.18	14868.20	14389.82	8521.93		x
1456	E3 ubiquitin-protein ligase LRSAM1	Q6UWE0	50070.42	52552.07	49670.54	44261.44	40772.38	38085.83	46291.57	44020.75	40575.26	52217.85	48756.28	46372.73	46304.32	40137.03	30317.01	36053.46	36233.96	35228.11	41279.28	36810.61	34237.98	1361.25	1409.54	1374.23	1363.22	1335.18	1222.51	1373.59	1291.55	1123.23	1482.62	1344.02	1113.55		
1457	E3 ubiquitin-protein ligase MARCH11	A6NNE9	12995.88	12145.28	11628.51	9785.59	9200.43	8992.17	11541.15	11063.11	10339.85	13186.31	13162.26	12682.77	12043.99	10313.17	9324.27	8798.79	8927.86	9159.81	9021.05	8943.15	9082.36	2.27	1.49	0.65	0.84	1.31	0.69	4.16	2.47	0.11	4.90	4.98	0.12	x	
1458	E3 ubiquitin-protein ligase MARCH5	Q9NX47	26645.19	26286.47	24625.43	18641.37	18186.38	16940.67	20716.96	19750.85	19721.68	24916.44	23920.35	22878.07	23622.58	22863.27	18795.95	17827.55	17263.12	17299.49	17380.93	17768.29	16466.52	2.27	1.49	0.65	0.84	1.31	0.69	4.16	2.47	0.11	4.90	4.98	0.12	x	
1459	E3 ubiquitin-protein ligase MARCH7	Q9H992	4976.70	5139.83	4455.68	4282.78	4092.48	4062.22	4150.42	4496.31	4283.99	5657.42	5336.99	5199.29	4742.57	4819.87	4264.42	4370.11	3546.01	3612.15	4464.62	4135.33	4154.64	2.27	1.49	0.65	0.84	1.31	0.69	4.16	2.47	0.11	4.90	4.98	0.12	x	
1460	E3 ubiquitin-protein ligase Mdm2	Q00987	19086.07	20359.57	17939.67	12664.17	12731.23	12103.04	16806.16	15213.11	14766.37	15526.78	14845.60	14420.91	16212.15	14861.26	13768.78	13344.56	13139.47	12378.01	14049.63	15303.02	15459.11	2.27	1.49	0.65	0.84	1.31	0.69	4.16	2.47	0.11	4.90	4.98	0.12	x	
1461	E3 ubiquitin-protein ligase MGRN1	O60291	0.14	2.53	0.81	1.07	2.77	0.68	0.96	0.36	0.63	0.35	0.75	0.38	2.13	0.97	0.60	4.64	1.07	0.47	2.22	0.19	0.06	5078.64	4784.26	4405.68	1154.80	1100.78	1120.91	7662.96	7051.13	7119.93	3350.69	3003.12	2118.78		x
1462	E3 ubiquitin-protein ligase MIB1	Q86YT6	7865.22	7547.29	8548.84	5518.06	5199.00	5281.31	7120.64	7278.78	7238.87	7037.09	6435.85	6787.42	6577.92	6764.80	5251.76	5690.65	5226.36	5055.00	5707.18	5549.52	5323.16	2.27	1.49	0.65	0.84	1.31	0.69	4.16	2.47	0.11	4.90	4.98	0.12	x	
1463	E3 ubiquitin-protein ligase NEDD4	P46934	686.77	709.06	650.73	307.63	380.41	299.95	441.31	358.70	397.64	421.73	375.69	459.54	526.76	471.52	387.72	459.29	458.40	471.97	441.85	396.03	403.64	2.27	1.49	0.65	0.84	1.31	0.69	4.16	2.47	0.11	4.90	4.98	0.12	x	
1464	E3 ubiquitin-protein ligase NEDD4-like	Q96PU5	0.14	2.53	0.81	1.07	2.77	0.68	0.96	0.36	0.63	0.35	0.75	0.38	2.13	0.97	0.60	4.64	1.07	0.47	2.22	0.19	0.06	17064.12	16265.03	14994.33	12352.25	12180.37	10968.43	14287.66	13860.04	13742.55	14186.90	13384.89	11133.02		x
1465	E3 ubiquitin-protein ligase PDZRN3	Q9UPQ7	0.14	2.53	0.81	1.07	2.77	0.68	0.96	0.36	0.63	0.35	0.75	0.38	2.13	0.97	0.60	4.64	1.07	0.47	2.22	0.19	0.06	51142.37	41275.50	33350.45	30623.15	29486.04	17779.42	37285.88	34736.86	23227.22	55841.04	54429.80	28792.06		x
1466	E3 ubiquitin-protein ligase pellino homolog 3	Q8N2H9	0.14	2.53	0.81	1.07	2.77	0.68	0.96	0.36	0.63	0.35	0.75	0.38	2.13	0.97	0.60	4.64	1.07	0.47	2.22	0.19	0.06	13690.75	14867.37	13102.54	9492.04	9276.92	8976.74	13512.96	13497.45	12652.13	11712.77	12636.00	9831.26		x
1467	E3 ubiquitin-protein ligase PPP1R11	O60927	9304.65	10492.77	9165.71	8997.70	7442.08	10523.42	10194.80	11691.79	10637.88	9502.01	9560.82	8150.45	10611.10	3269.97	4505.91	9134.85	7177.57	9702.74	9727.92	9992.36	7706.23	2.27	1.49	0.65	0.84	1.31	0.69	4.16	2.47	0.11	4.90	4.98	0.12	x	
1468	E3 ubiquitin-protein ligase RAD18	Q9NS91	0.14	2.53	0.81	1.07	2.77	0.68	0.96	0.36	0.63	0.35	0.75	0.38	2.13	0.97	0.60	4.64	1.07	0.47	2.22	0.19	0.06	62317.98	59166.32	51798.55	49867.73	46488.05	32823.70	56593.00	50935.10	48069.25	63943.20	62701.37	32972.48		x
1469	E3 ubiquitin-protein ligase RBBP6	Q7Z6E9	98654.55	99376.27	87652.76	74269.44	75890.99	71746.34	79212.41	79909.61	72023.03	81528.95	79353.24	77390.96	73723.84	79780.00	72947.94	71607.25	69864.52	67292.09	74733.22	75066.02	73811.28	2.27	1.49	0.65	0.84	1.31	0.69	4.16	2.47	0.11	4.90	4.98	0.12	x	
1470	E3 ubiquitin-protein ligase RBX1	P62877	1175.53	1043.93	1241.77	914.92	828.93	668.55	617.50	461.04	381.21	993.81	904.56	827.05	886.63	965.11	804.61	591.09	648.20	575.10	741.21	648.15	748.49	2.27	1.49	0.65	0.84	1.31	0.69	4.16	2.47	0.11	4.90	4.98	0.12	x	
1471	E3 ubiquitin-protein ligase RNF113A	O15541	10617.57	11530.11	11145.25	8456.25	7810.89	7114.89	8669.93	8213.32	7844.56	8413.56	8630.13	8084.80	10397.51	8661.83	7373.79	7354.18	6790.31	6925.44	7235.94	7995.58	5565.13	247.78	231.37	252.19	138.39	148.80	127.01	160.45	116.03	122.88	75.03	121.03	119.11		
1472	E3 ubiquitin-protein ligase RNF114	Q9Y508	242497.03	239466.90	226513.58	181307.47	174981.58	168275.99	197833.24	190754.61	185650.11	206793.08	197857.28	191398.38	199157.62	193096.98	161309.46	168014.18	161764.10	159147.04	190742.30	177515.55	170504.13	110417.30	102072.68	93139.29	78513.53	76811.81	65706.02	88296.67	83518.90	80402.95	88727.87	88710.35	54021.91		
1473	E3 ubiquitin-protein ligase RNF123	Q5XPI4	825.40	881.61	286.17	370.78	5982.11	4579.79	5280.90	1458.92	797.66	542.16	312.03	118.58	565.75	677.76	47.63	7043.88	1351.70	6459.96	371.54	566.24	121.13	2.27	1.49	0.65	0.84	1.31	0.69	4.16	2.47	0.11	4.90	4.98	0.12	x	
1474	E3 ubiquitin-protein ligase RNF126	Q9BV68	6090.70	6176.80	4596.59	4537.44	4246.36	3714.71	4478.37	4344.00	3970.48	5938.57	5585.56	4716.26	5895.42	5156.10	2997.84	3996.26	3801.77	3832.35	5289.99	4810.57	4562.04	2283.09	2410.08	1965.49	1907.52	1904.03	1443.10	2210.57	2043.14	1883.03	3276.70	3306.39	1906.83		
1475	E3 ubiquitin-protein ligase RNF14	Q9UBS8	0.14	2.53	0.81	1.07	2.77	0.68	0.96	0.36	0.63	0.35	0.75	0.38	2.13	0.97	0.60	4.64	1.07	0.47	2.22	0.19	0.06	21586.61	20009.04	18092.64	26310.63	26303.85	18628.67	28786.18	26554.94	22939.24	38761.60	37468.18	21638.26		x
1476	E3 ubiquitin-protein ligase RNF187	Q5TA31	41677.11	45660.39	42585.93	33232.06	31391.60	30171.16	37310.75	35425.86	34249.67	37325.04	34605.54	35043.41	35067.92	37262.90	31840.51	30837.93	27368.15	25529.64	33133.41	31053.52	30309.30	2.27	1.49	0.65	0.84	1.31	0.69	4.16	2.47	0.11	4.90	4.98	0.12	x	
1477	E3 ubiquitin-protein ligase RNF213	Q63HN8	0.14	2.53	0.81	1.07	2.77	0.68	0.96	0.36	0.63	0.35	0.75	0.38	2.13	0.97	0.60	4.64	1.07	0.47	2.22	0.19	0.06	3285.32	7902.92	4608.65	2982.99	3285.49	3758.42	3739.32	3625.23	3137.29	2907.80	4392.22	705.57		x
1478	E3 ubiquitin-protein ligase RNF216	Q9NWF9	149.64	28.96	155.62	73.23	116.11	89.76	78.06	39.05	87.48	114.53	136.36	206.52	76.78	115.33	85.36	85.01	52.14	115.55	94.02	79.46	96.57	2.27	1.49	0.65	0.84	1.31	0.69	4.16	2.47	0.11	4.90	4.98	0.12	x	
1479	E3 ubiquitin-protein ligase RNF220	Q5VTB9	1005.10	1312.58	1032.40	12.42	492.82	300.87	394.78	182.08	79.56	717.73	453.81	402.32	1000.91	70.53	6.59	445.07	212.04	243.59	2.22	0.19	0.06	9850.03	8387.40	7316.11	6690.85	6465.59	6049.30	7692.43	7118.58	5293.42	7212.65	6715.10	4186.75		
1480	E3 ubiquitin-protein ligase RNF34	Q969K3	4205.06	4563.46	3611.31	2226.32	2473.43	2039.79	3211.41	3196.79	2611.74	2985.27	3241.55	2594.80	2910.61	2839.15	2000.79	2661.26	2473.85	2429.12	2624.92	2656.50	2185.78	2.27	1.49	0.65	0.84	1.31	0.69	4.16	2.47	0.11	4.90	4.98	0.12	x	
1481	E3 ubiquitin-protein ligase RNF6	Q9Y252	64028.70	61708.21	61403.42	46222.81	44574.57	43806.09	57096.83	54167.72	54124.01	56990.79	51324.79	49354.16	51004.86	50097.03	41345.19	47676.70	45843.00	44521.07	44595.23	41754.82	39922.69	2.27	1.49	0.65	0.84	1.31	0.69	4.16	2.47	0.11	4.90	4.98	0.12	x	
1482	E3 ubiquitin-protein ligase TRIM13	O60858	80830.53	75465.16	72796.93	64161.78	61719.18	57420.82	68312.14	72234.05	67720.50	73354.23	70267.29	68101.55	71784.46	69191.02	56012.19	56790.30	55167.47	52141.78	74364.32	71608.14	69501.52	2.27	1.49	0.65	0.84	1.31	0.69	4.16	2.47	0.11	4.90	4.98	0.12	x	
1483	E3 ubiquitin-protein ligase TRIM17	Q9Y577	0.14	2.53	0.81	1.07	2.77	0.68	0.96	0.36	0.63	0.35	0.75	0.38	2.13	0.97	0.60	4.64	1.07	0.47	2.22	0.19	0.06	394.91	374.28	356.87	148.90	178.40	34.30	298.02	238.12	169.85	396.40	305.62	113.56		x
1484	E3 ubiquitin-protein ligase TRIM33	Q9UPN9	336186.27	325803.03	292286.60	209369.96	201608.35	184458.55	258967.90	242282.90	224275.94	283375.78	265181.93	238376.74	262737.96	246058.16	181654.19	215756.24	202656.26	195637.67	227880.14	219615.15	199722.44	2.27	1.49	0.65	0.84	1.31	0.69	4.16	2.47	0.11	4.90	4.98	0.12	x	
1485	E3 ubiquitin-protein ligase TRIM41	Q8WV44	0.14	2.53	0.81	1.07	2.77	0.68	0.96	0.36	0.63	0.35	0.75	0.38	2.13	0.97	0.60	4.64	1.07	0.47	2.22	0.19	0.06	7288.26	7655.14	7629.87	4831.73	4662.82	3913.05	5883.64	5049.52	5380.63	6662.27	6599.05	3817.09		x
1486	E3 ubiquitin-protein ligase TRIM56	Q9BRZ2	0.14	2.53	0.81	1.07	2.77	0.68	0.96	0.36	0.63	0.35	0.75	0.38	2.13	0.97	0.60	4.64	1.07	0.47	2.22	0.19	0.06	18702.79	18963.04	17614.25	14829.07	13961.30	14449.08	16600.60	15142.10	15091.98	21735.44	20412.33	5945.65		x
1487	E3 ubiquitin-protein ligase TRIM58	Q8NG06	5337.18	5324.96	5056.67	6206.44	5804.26	4488.02	5150.95	5392.65	6751.63	5751.16	5672.74	5419.94	5400.43	2526.08	645.49	3733.42	4928.88	4454.15	5686.30	5079.67	5117.16	32221.71	28847.97	26366.52	18323.06	17345.42	14660.85	23702.39	22007.15	19375.17	24220.07	22985.60	12588.38		
1488	E3 ubiquitin-protein ligase TRIM63	Q969Q1	2396.57	2565.54	2091.06	2216.96	2069.38	2218.63	2367.46	2125.99	2112.77	3194.05	2944.32	2601.94	1979.98	2077.86	1755.96	2031.38	1926.05	2008.62	2393.84	2063.40	2033.53	2.27	1.49	0.65	0.84	1.31	0.69	4.16	2.47	0.11	4.90	4.98	0.12	x	
1489	E3 ubiquitin-protein ligase TRIM69	Q86WT6	1240.24	962.81	820.81	486.99	534.76	385.91	656.23	725.99	656.86	683.92	624.76	573.20	936.75	824.51	274.06	599.27	577.89	572.32	658.17	621.68	519.13	2594.24	1991.60	1281.11	2220.40	2064.64	1338.11	2414.38	2782.85	1112.56	3503.53	3905.45	2067.64		
1490	E3 ubiquitin-protein ligase TRIM7	Q9C029	17183.18	16271.68	14336.90	9994.88	9524.16	8982.84	12322.78	11795.18	11100.79	13944.91	14117.49	12760.14	14026.97	11847.22	11096.74	10715.81	9562.39	8541.61	10169.47	10030.51	9661.42	7016.24	6450.16	7316.24	6454.97	5858.14	5183.90	7138.84	6418.00	6743.62	5978.53	6619.27	4981.81		
1491	E3 ubiquitin-protein ligase TRIP12	Q14669	20061.88	18291.40	15618.77	13224.57	13985.13	13102.72	16463.06	15422.87	13877.84	17465.56	17468.69	15690.93	14991.96	14045.73	11988.03	13861.62	15485.79	11988.34	11950.77	11210.71	10525.45	3763.41	3408.35	3693.49	3230.34	3167.75	2750.69	3344.55	3026.99	3314.79	4555.29	3752.43	2385.20		
1492	E3 ubiquitin-protein ligase UBR4	Q5T4S7	159335.53	153792.13	143008.44	108778.57	105006.16	100742.12	116386.76	112336.17	109001.50	136863.21	131247.93	121184.61	149337.78	137612.65	118061.23	120732.80	115465.87	110783.86	121192.67	108475.20	106895.66	169561.39	162771.54	152175.34	115693.16	112921.58	101787.35	147563.97	142263.71	135758.93	140755.20	139606.87	83752.05		
1493	E3 ubiquitin-protein ligase UHRF1	Q96T88	2088.51	1940.00	2037.59	1764.15	1546.27	1333.47	2044.73	996.64	1637.49	1620.07	1775.90	1734.26	1585.75	1648.95	858.58	838.52	1256.75	1236.44	2295.80	1460.61	1977.22	1163.43	1089.40	1021.14	639.17	643.16	497.80	884.60	942.07	788.12	862.55	1054.78	590.19		
1494	E3 ubiquitin-protein ligase ZFP91	Q96JP5	0.14	2.53	0.81	1.07	2.77	0.68	0.96	0.36	0.63	0.35	0.75	0.38	2.13	0.97	0.60	4.64	1.07	0.47	2.22	0.19	0.06	144447.40	139383.10	133158.00	110930.25	106504.49	104369.81	117358.00	119157.73	116026.17	128262.69	128293.53	84697.37		x
1495	E3 ubiquitin-protein ligase ZNF598	Q86UK7	3744.83	3275.08	3045.01	3963.60	3936.66	3774.62	7160.70	6723.22	6341.89	4360.10	4240.73	4066.21	3029.75	2947.07	2226.22	2137.03	2207.36	2205.23	2125.68	2140.72	1925.70	26721.21	25303.99	22441.49	18914.67	18468.18	16121.97	19156.77	18067.47	17299.18	19759.70	17796.97	13928.35		
1496	E3 ubiquitin-protein transferase MAEA	Q7L5Y9	13323.23	12601.62	12512.60	11190.24	10633.30	10612.66	11339.80	11227.89	10526.03	14349.02	14172.65	13623.83	12043.25	10820.71	11510.81	11356.43	10376.62	10446.41	12156.82	11552.50	10859.60	2.27	1.49	0.65	0.84	1.31	0.69	4.16	2.47	0.11	4.90	4.98	0.12	x	
1497	E3 UFM1-protein ligase 1	O94874	87898.43	94754.30	86375.08	66199.89	66424.04	58293.66	68237.70	66575.24	68042.13	84261.55	82113.11	76903.02	86704.27	70784.09	59400.21	59242.41	55596.47	56357.54	82522.84	68084.07	64730.25	2.27	1.49	0.65	0.84	1.31	0.69	4.16	2.47	0.11	4.90	4.98	0.12	x	
1498	Early endosome antigen 1	Q15075	102868.70	102185.03	95724.95	78365.63	75030.30	71012.93	72897.83	70893.55	69367.48	86422.81	81426.39	80757.65	77430.88	78334.44	68033.10	73917.19	71070.41	69403.68	71312.53	68479.05	68031.34	107.15	47.56	123.23	75.41	64.84	80.38	87.52	110.10	69.34	116.86	117.84	42.26		
1499	EARP and GARP complex-interacting protein 1	Q53HC9	209494.91	188324.23	169443.11	131671.59	128352.72	123337.78	135344.45	142650.23	140059.93	183194.74	180866.42	176600.02	183203.19	162231.67	137556.67	109086.94	117277.08	104399.20	131102.22	118522.65	129624.51	2.27	1.49	0.65	0.84	1.31	0.69	4.16	2.47	0.11	4.90	4.98	0.12	x	
1500	Echinoderm microtubule-associated protein-like 2	O95834	63350.01	62815.81	60369.04	47186.56	46617.46	41718.93	52982.27	46958.64	45868.82	53422.64	51906.00	52266.55	52058.52	58194.34	49176.12	44335.15	43539.68	42017.62	32394.06	40879.26	40389.37	2.27	1.49	0.65	0.84	1.31	0.69	4.16	2.47	0.11	4.90	4.98	0.12	x	
1501	Echinoderm microtubule-associated protein-like 3	Q32P44	6777.61	7265.93	6681.47	4037.74	4324.49	3837.63	5625.53	5330.62	5055.73	5600.62	4892.49	4343.75	5657.85	4825.38	3775.23	4219.26	5315.04	4442.23	4403.47	4017.90	3705.80	2.27	1.49	0.65	0.84	1.31	0.69	4.16	2.47	0.11	4.90	4.98	0.12	x	
1502	Echinoderm microtubule-associated protein-like 4	Q9HC35	15192.39	15001.19	14471.47	10224.50	10386.31	9129.55	12289.66	12632.22	11830.94	13580.44	13439.69	11556.73	12119.07	12034.05	8407.91	11557.77	10637.51	10643.48	9989.38	9523.40	9422.13	21445.95	20928.36	18323.40	13695.57	13650.95	11418.98	21215.48	20253.30	18718.18	19239.69	18606.44	11791.93		
1503	Echinoderm microtubule-associated protein-like 6	Q6ZMW3	51103.98	49354.59	46634.97	35474.20	35730.08	31949.73	42618.77	39418.26	40034.07	42468.79	41930.38	38312.21	40759.22	42370.94	31862.80	35502.27	33062.97	34267.23	34064.50	33965.06	35059.40	224.60	199.06	158.99	123.24	147.75	90.97	240.24	217.31	166.93	426.67	294.79	110.12		
1504	Ecto-NOX disulfide-thiol exchanger 1	Q8TC92	70282.37	65096.20	64800.55	54150.64	51402.94	45192.99	49558.57	45907.29	43740.94	66824.48	64227.37	58524.78	56600.69	54721.35	41593.82	50651.77	49506.82	50918.48	47659.94	45891.74	42419.07	2.27	1.49	0.65	0.84	1.31	0.69	4.16	2.47	0.11	4.90	4.98	0.12	x	
1505	eEF1A lysine and N-terminal methyltransferase	Q8N6R0	1474.02	1304.39	999.78	615.42	306.19	288.54	873.31	312.51	816.83	1198.53	1319.74	886.01	1132.54	183.91	136.41	948.98	346.68	461.01	1076.01	618.19	249.85	2.27	1.49	0.65	0.84	1.31	0.69	4.16	2.47	0.11	4.90	4.98	0.12	x	
1506	EEF1A lysine methyltransferase 1	Q8WVE0	2593.69	2322.81	2179.85	1456.75	1344.55	869.48	1632.72	1455.79	1369.72	2023.24	2077.46	1353.37	2003.96	1446.35	510.24	1206.60	978.78	999.70	1544.22	1381.16	1031.67	46981.14	40036.82	29727.73	47061.07	46472.16	27673.77	89974.22	88780.86	66620.68	53159.90	48142.33	24583.74		
1507	EEF1A lysine methyltransferase 2	Q5JPI9	0.14	2.53	0.81	1.07	2.77	0.68	0.96	0.36	0.63	0.35	0.75	0.38	2.13	0.97	0.60	4.64	1.07	0.47	2.22	0.19	0.06	828.72	694.61	590.76	0.84	1.31	0.69	4.16	2.47	0.11	4.90	4.98	0.12		x
1508	EEF1AKMT4-ECE2 readthrough transcript protein	P0DPD8	11550.67	10232.35	10763.50	7589.53	7171.37	7099.90	8343.31	8519.52	8162.06	9306.97	9143.55	8583.36	9915.68	9143.27	7431.67	6831.66	6410.33	6000.20	6745.73	6113.01	6973.30	2.27	1.49	0.65	0.84	1.31	0.69	4.16	2.47	0.11	4.90	4.98	0.12	x	
1509	EF-hand domain-containing protein 1	Q5JVL4	2791.16	2467.36	2220.12	2380.57	2305.42	1982.30	2455.55	2583.42	2313.99	2504.49	2526.35	2128.75	2810.69	2465.94	1223.24	2192.00	2484.62	1974.47	2518.78	2524.78	1992.44	2.27	1.49	0.65	0.84	1.31	0.69	4.16	2.47	0.11	4.90	4.98	0.12	x	
1510	EF-hand domain-containing protein D1	Q9BUP0	0.14	2.53	0.81	1.07	2.77	0.68	0.96	0.36	0.63	0.35	0.75	0.38	2.13	0.97	0.60	4.64	1.07	0.47	2.22	0.19	0.06	22123.19	21023.16	19481.59	12719.81	12226.56	10126.18	18006.61	17552.67	16236.40	16446.93	14914.21	9880.07		x
1511	EF-hand domain-containing protein D2	Q96C19	3504.00	3063.28	2960.04	1837.83	1419.64	1273.00	3975.69	3816.18	3278.77	2005.99	2199.45	2350.67	2504.08	2348.95	1878.44	1785.47	1570.64	1641.93	1613.20	1941.50	1339.63	54050.18	55095.74	50434.14	35150.62	34875.42	31067.38	36982.49	35440.44	34314.07	40749.32	38242.37	27685.68		

Supplementary Table S1. Overview on the abundance of all relatively quantified 5180 proteins

			MCF-7																					MDA-MB-231													
	Protein name	UniProt	control 01	control 02	control 03	Dai IC20 01	Dai IC20 02	Dai IC20 03	Dai SC20 01	Dai SC20 02	Dai SC20 03	Gen IC20 01	Gen IC20 02	Gen IC20 03	Gen SC20 01	Gen SC20 02	Gen SC20 03	SSE IC20 01	SSE IC20 02	SSE IC20 03	SSE SC20 01	SSE SC20 02	SSE SC20 03	control 01	control 02	control 03	Dai IC20 01	Dai IC20 02	Dai IC20 03	Gen IC20 01	Gen IC20 02	Gen IC20 03	SSE IC20 01	SSE IC20 02	SSE IC20 03	unique to MCF-7	unique to MDA-MB-231
1512	EH domain-binding protein 1-like protein 1	Q8N3D4	0.14	2.53	0.81	1.07	2.77	0.68	0.96	0.36	0.63	0.35	0.75	0.38	2.13	0.97	0.60	4.64	1.07	0.47	2.22	0.19	0.06	7137.62	7384.61	5920.36	6108.18	6023.92	4592.28	8106.33	7329.23	6666.96	7949.33	8665.97	4502.75		x
1513	EH domain-containing protein 1	Q9H4M9	118401.59	107452.03	98623.94	75143.71	76357.28	67923.60	88883.03	81747.05	79726.57	104198.06	99713.03	94857.72	89306.22	85222.68	63555.74	81455.17	78043.28	74080.43	77174.51	74640.54	65195.02	216179.99	200544.01	184387.45	141502.93	137524.15	112100.31	178879.68	166458.69	155205.31	180074.55	171686.98	109943.36		
1514	EH domain-containing protein 2	Q9NZN4	53024.56	55667.04	48009.58	37657.69	35031.42	30439.89	39288.52	37400.54	33989.10	44079.27	41592.97	35713.20	45339.28	41996.19	33972.41	36061.63	33743.72	31386.33	41458.28	38284.65	33991.06	59212.39	51764.93	46611.23	39298.26	37972.82	27780.38	46403.99	43766.03	36418.14	48862.88	46772.83	26838.01		
1515	EH domain-containing protein 3	Q9NZN3	0.14	2.53	0.81	1.07	2.77	0.68	0.96	0.36	0.63	0.35	0.75	0.38	2.13	0.97	0.60	4.64	1.07	0.47	2.22	0.19	0.06	556.40	438.19	421.10	324.81	369.03	305.14	328.27	421.47	374.49	432.02	407.31	285.06		x
1516	EH domain-containing protein 4	Q9H223	31524.50	28872.24	27136.57	20270.28	18055.06	18280.00	23102.61	21099.05	20684.18	26952.82	26559.18	25011.82	27836.73	24115.34	19602.22	19172.23	16990.67	17037.10	18669.93	18670.54	16455.24	26281.77	23296.42	19839.29	16359.43	15586.74	10847.12	20702.32	19518.53	16109.14	24081.64	22238.99	11248.93		
1517	eIF-2-alpha kinase activator GCN1	Q92616	354967.37	341209.69	312512.90	240232.79	228069.32	215346.98	271336.02	263565.95	253070.46	291912.09	282227.49	261083.70	294771.56	266768.11	227089.35	230235.68	215573.22	211851.57	232084.16	222101.96	211985.30	85887.00	72249.61	70977.39	55055.72	52869.72	47088.28	82913.25	75731.50	72394.69	65364.70	62847.04	46168.37		
1518	eIF-2-alpha kinase GCN2	Q9P2K8	3654.02	3636.00	3671.51	3415.69	2781.81	3126.24	3544.13	3262.09	3494.30	3635.64	3761.49	3468.07	3540.65	3471.91	2808.29	3497.94	3051.88	2981.08	2755.10	3033.73	2795.34	2.27	1.49	0.65	0.84	1.31	0.69	4.16	2.47	0.11	4.90	4.98	0.12	x	
1519	EKC/KEOPS complex subunit GON7	Q9BXV9	27733.26	22849.81	17534.70	5751.48	10970.03	7794.23	29731.40	11493.97	9007.12	23574.89	20489.95	17861.62	20721.51	7525.56	1824.00	10164.75	16469.41	10157.90	7612.78	7089.01	4715.81	2.27	1.49	0.65	0.84	1.31	0.69	4.16	2.47	0.11	4.90	4.98	0.12	x	
1520	EKC/KEOPS complex subunit LAGE3	Q14657	10100.16	11174.01	11435.74	8765.31	9424.78	8526.21	8853.82	8713.86	8194.02	10097.38	8687.79	8135.58	9687.35	10268.69	8382.83	8867.92	8131.22	7930.50	8064.55	7379.44	7871.20	2882.26	1632.69	2264.31	1659.41	1734.22	1505.98	1849.19	2061.24	1663.74	2165.01	1599.35	988.87		
1521	EKC/KEOPS complex subunit TP53RK	Q96S44	0.14	2.53	0.81	1.07	2.77	0.68	0.96	0.36	0.63	0.35	0.75	0.38	2.13	0.97	0.60	4.64	1.07	0.47	2.22	0.19	0.06	337.22	276.14	274.26	293.02	291.57	263.86	318.17	309.59	205.84	348.48	327.63	144.05		x
1522	EKC/KEOPS complex subunit TPRKB	Q9Y3C4	15181.06	14554.45	13590.05	8692.00	9230.45	8120.81	10145.22	10500.94	10471.31	14397.18	13831.19	13707.01	13618.93	11143.74	5905.51	10072.77	10181.09	9934.31	11668.85	9752.22	9659.30	2.27	1.49	0.65	0.84	1.31	0.69	4.16	2.47	0.11	4.90	4.98	0.12	x	
1523	ELAV-like protein 1	Q15717	40140.41	36832.71	34804.20	24947.05	24431.54	22028.76	31964.39	31350.53	27680.18	30384.57	29398.33	25334.65	32543.07	29352.38	20837.53	26595.67	25200.83	22920.75	29934.61	25812.33	24449.59	13655.78	13100.75	10910.63	9965.70	9620.28	7337.41	9763.83	8328.76	8581.19	12494.12	11794.48	7322.83		
1524	Electrogenic sodium bicarbonate cotransporter 1	Q9Y6R1	0.14	2.53	0.81	1.07	2.77	0.68	0.96	0.36	0.63	0.35	0.75	0.38	2.13	0.97	0.60	4.64	1.07	0.47	2.22	0.19	0.06	338.20	313.48	381.77	255.02	224.45	279.87	247.21	300.86	320.30	376.13	298.85	172.69		x
1525	Electron transfer flavoprotein subunit alpha_ mitochondrial	P13804	170235.82	161202.88	158765.25	112382.63	109081.90	100725.43	124746.05	123726.05	117963.27	145914.65	139536.23	129041.45	144770.77	131185.98	103584.11	110944.39	106598.48	107587.47	109755.13	104286.72	98592.20	83190.17	80734.32	78479.95	54076.35	52206.59	48133.27	60104.97	57322.59	56358.23	58026.11	54243.69	36294.34		
1526	Electron transfer flavoprotein subunit beta	P38117	160769.14	134183.59	122875.95	98985.19	90881.69	81053.69	105599.88	94762.55	95667.76	125981.10	121839.97	104289.43	122319.77	107356.62	71571.16	100270.93	86199.10	88757.85	88461.70	84415.39	75747.56	41796.06	39760.11	34690.39	26794.34	27241.15	22560.23	33385.93	32332.36	29225.78	37804.03	33153.39	21671.24		
1527	ELKS/Rab6-interacting/CAST family member 1	Q8IUD2	0.14	2.53	0.81	1.07	2.77	0.68	0.96	0.36	0.63	0.35	0.75	0.38	2.13	0.97	0.60	4.64	1.07	0.47	2.22	0.19	0.06	66072.09	62306.64	55601.82	42229.52	41267.15	35571.80	40874.92	41642.03	41378.32	39923.01	39670.19	25860.61		x
1528	Ellis-van Creveld syndrome protein	P57679	25025.31	23626.82	23268.74	13052.07	12696.89	10431.97	17279.28	16271.59	14745.46	16699.13	16140.63	13590.20	20711.42	20584.29	9343.00	16927.12	17136.32	15481.00	12291.76	11580.76	9742.74	2.27	1.49	0.65	0.84	1.31	0.69	4.16	2.47	0.11	4.90	4.98	0.12	x	
1529	Elongation factor 1-alpha 1	P68104	0.14	2.53	0.81	1.07	2.77	0.68	0.96	0.36	0.63	0.35	0.75	0.38	2.13	0.97	0.60	4.64	1.07	0.47	2.22	0.19	0.06	2922502.55	2719038.84	2589265.82	1683773.58	1667089.85	1495000.46	2596554.77	2410986.63	2303718.48	2234570.82	2175846.86	1445506.80		x
1530	Elongation factor 1-alpha 2	Q05639	365650.91	339299.60	318886.05	203866.81	191944.26	184665.35	265932.00	253701.79	254079.56	265509.64	255722.96	243276.09	292832.26	273077.93	208900.59	217712.05	201798.55	198420.34	196458.13	175714.52	170044.35	166670.39	159120.49	148020.15	82019.75	79336.22	76186.03	118857.79	110784.84	102424.75	94083.99	94361.46	53894.08		
1531	Elongation factor 1-beta	P24534	588848.53	559132.34	501192.34	362982.00	340859.92	310694.03	443394.98	424453.16	395330.17	467793.35	445561.21	399862.00	448017.61	396620.55	302470.97	367484.08	352281.12	340621.75	376461.60	346535.53	320128.51	300556.06	283368.36	249610.19	199359.92	196060.33	167406.49	258313.47	241072.35	219214.49	263677.98	241431.57	155125.12		
1532	Elongation factor 1-delta	P29692	940740.15	918464.97	888488.85	666998.76	662212.36	628398.49	768119.33	726723.16	724644.32	841593.53	814958.37	770152.83	739180.66	695818.62	594442.33	707672.94	678651.43	667390.08	674741.61	638153.63	613369.14	531214.04	493862.52	473498.00	341266.64	331576.84	301231.07	443355.38	411166.84	402443.47	431583.16	407089.39	239784.88		
1533	Elongation factor 1-gamma	P26641	1496376.35	1448036.67	1357149.68	1037259.61	990226.92	945719.21	1211433.46	1190567.14	1135103.52	1354917.86	1292193.27	1189621.81	1205812.24	1150071.55	970800.30	1103447.67	1045078.05	1000038.79	1120845.58	1068656.26	998588.33	761241.57	729702.96	675435.66	507269.16	493541.20	454274.56	665915.18	638219.95	609391.75	611750.98	578871.84	408804.58		
1534	Elongation factor 2	P13639	3971309.96	3856377.01	3584921.07	2680488.09	2631316.65	2474173.83	3372411.63	3193334.14	3045408.61	3359925.36	3181910.66	3008190.01	3167061.80	2886599.13	2458079.86	2716984.47	2558173.41	2460380.64	2940370.77	2794208.90	2550751.14	1964175.99	1844435.24	1725712.21	1187097.91	1150470.97	1055021.26	1486397.06	1417250.49	1371724.33	1447061.36	1379780.48	904665.05		
1535	Elongation factor G mitochondrial	Q96RP9	90135.75	88818.11	91329.83	60804.44	57371.11	53178.96	62477.78	65887.03	61199.80	76116.82	73141.09	64965.27	73489.90	68074.37	53773.90	55352.75	54586.21	50692.73	58753.58	55004.60	53658.41	33017.58	32661.90	30724.79	19883.03	19061.47	16614.46	26130.96	24559.81	23532.93	25668.73	26371.59	26787.79		
1536	Elongation factor Ts mitochondrial	P43897	30353.36	26955.46	25016.42	21175.64	18916.09	18666.07	22131.25	21591.73	21126.53	22851.56	24199.83	22328.79	26152.49	23797.08	19230.65	19370.31	18465.53	19270.27	21239.06	19866.33	18365.83	132510.18	123872.49	100983.56	66872.69	69430.14	60008.54	79830.33	85353.65	77588.28	85585.70	87017.94	60644.64		
1537	Elongation factor Tu_ mitochondrial	P49411	796286.02	762467.21	728020.21	592971.91	573800.06	538438.13	667905.33	637139.42	631617.29	716450.37	681360.47	626401.88	669716.30	618449.11	528262.45	608244.91	567440.12	560126.69	587390.66	570080.48	563790.41	305881.19	284695.79	276736.45	198526.74	189681.81	177055.84	235756.23	224548.40	215313.03	233637.45	223716.42	151836.24		
1538	Elongation of very long chain fatty acids protein 1	Q9BW60	0.14	2.53	0.81	1.07	2.77	0.68	0.96	0.36	0.63	0.35	0.75	0.38	2.13	0.97	0.60	4.64	1.07	0.47	2.22	0.19	0.06	3392.90	2631.02	2517.65	2022.49	2071.98	1846.47	2644.76	2534.02	2224.14	2492.50	2555.63	1166.60		x
1539	Elongator complex protein 1	O95163	5688.46	3723.09	2816.91	2646.30	2723.50	1505.11	3337.67	3381.52	2083.37	4433.22	3912.31	3250.78	4660.31	3767.98	1268.90	2791.81	2809.08	1994.00	2542.41	1996.78	1654.94	13592.58	15287.44	12703.08	8843.44	8501.43	8705.61	6845.53	7046.82	7096.63	8607.10	7722.83	7711.33		
1540	Elongator complex protein 5	Q8TE02	0.14	2.53	0.81	1.07	2.77	0.68	0.96	0.36	0.63	0.35	0.75	0.38	2.13	0.97	0.60	4.64	1.07	0.47	2.22	0.19	0.06	7180.98	5571.67	4445.90	2838.85	2562.57	1802.17	3868.92	4105.97	3467.31	3723.13	3620.24	2029.81		x
1541	Elongin-B	Q15370	52126.95	50449.60	52834.60	39046.47	38578.02	35220.43	40017.76	38128.86	37850.72	48224.03	44836.51	43136.62	43356.58	40909.87	39291.40	36959.66	36234.13	34442.79	35465.24	32892.11	32891.68	15589.16	16143.12	14661.90	10129.17	9399.95	9664.08	11942.17	10264.73	11255.31	11806.87	10415.83	9656.36		
1542	Elongin-C	Q15369	56956.12	56119.83	51136.51	52513.26	47433.93	46434.26	48416.80	50401.48	44745.79	48220.93	46498.87	44748.01	55098.74	53226.32	33131.18	45682.23	44683.07	47607.61	56919.27	53815.67	46051.65	100061.16	96941.42	88387.17	63471.29	62300.23	54376.10	71634.78	68870.97	65114.24	69491.51	70757.41	43595.24		
1543	Embryonic stem cell-specific 5-hydroxymethylcytosine-binding protein	Q96FZ2	48667.49	47713.05	47615.26	33718.67	32000.21	29048.11	37530.48	39966.89	37284.72	41007.09	40546.15	37680.78	40459.43	37850.47	28430.32	35831.96	32287.61	31205.37	31242.17	31011.27	30572.09	2.27	1.49	0.65	0.84	1.31	0.69	4.16	2.47	0.11	4.90	4.98	0.12	x	
1544	Embryonic testis differentiation protein homolog B	P0DPP9	0.14	2.53	0.81	1.07	2.77	0.68	0.96	0.36	0.63	0.35	0.75	0.38	2.13	0.97	0.60	4.64	1.07	0.47	2.22	0.19	0.06	2291.06	1877.62	2021.72	2101.96	1940.46	1649.14	3339.88	3267.11	3200.00	2378.17	2394.46	1695.24		x
1545	Emerin	P50402	1087.54	798.21	1004.63	514.68	662.04	524.01	647.29	508.52	569.07	873.80	743.20	774.65	2.13	0.97	0.60	553.55	517.02	516.10	652.58	633.87	642.18	51209.24	48604.12	44132.41	32281.26	31730.96	28509.96	35061.63	32765.08	33322.82	38598.68	39616.07	42523.02		
1546	Ena/VASP-like protein	Q9UI08	1884.32	1611.20	1429.42	959.81	965.82	789.51	918.05	932.47	1082.62	1225.56	1241.22	1353.21	1566.49	1102.56	891.11	1119.93	1075.89	1073.18	1093.77	1159.94	1010.50	2.27	1.49	0.65	0.84	1.31	0.69	4.16	2.47	0.11	4.90	4.98	0.12	x	
1547	Endogenous Bornavirus-like nucleoprotein 2	Q6P2I7	0.14	2.53	0.81	1.07	2.77	0.68	0.96	0.36	0.63	0.35	0.75	0.38	2.13	0.97	0.60	4.64	1.07	0.47	2.22	0.19	0.06	25805.67	25749.62	24094.50	15496.58	15340.81	14173.00	13998.77	13453.26	12822.34	14058.22	12282.22	9317.88		x
1548	Endogenous retrovirus group 3 member 1 Env polyprotein	Q14264	0.14	2.53	0.81	1.07	2.77	0.68	0.96	0.36	0.63	0.35	0.75	0.38	2.13	0.97	0.60	4.64	1.07	0.47	2.22	0.19	0.06	75930.89	71403.70	59125.20	60076.57	64218.09	47428.04	80397.79	77520.53	64218.70	85657.66	79732.23	48405.93		x
1549	Endogenous retrovirus group K member 104 Rec protein	P61576	5308.46	4097.86	3300.28	2566.11	2369.06	1992.63	2494.28	2195.19	2145.21	3439.77	3101.35	2666.80	3397.01	2973.91	1329.83	2754.60	2662.62	2485.26	1940.04	1711.01	1313.49	2.27	1.49	0.65	0.84	1.31	0.69	4.16	2.47	0.11	4.90	4.98	0.12	x	
1550	Endonuclease 8-like 2	Q969S2	2202.80	2481.95	1797.73	1617.93	1755.75	1527.65	1903.49	2030.74	1664.71	2296.79	2228.01	2071.02	2453.44	1186.99	1289.76	1747.40	1128.35	1608.02	1193.22	1605.50	1880.94	2.27	1.49	0.65	0.84	1.31	0.69	4.16	2.47	0.11	4.90	4.98	0.12	x	
1551	Endonuclease 8-like 3	Q8TAT5	3586.65	2861.09	2286.07	1326.50	1363.88	1011.36	2309.99	1615.06	2010.26	1987.29	1994.80	1566.72	2428.69	1212.29	571.74	1758.79	1532.51	1788.48	1693.54	1482.17	1160.51	2.27	1.49	0.65	0.84	1.31	0.69	4.16	2.47	0.11	4.90	4.98	0.12	x	
1552	Endonuclease/exonuclease/phosphatase family domain-containing protein 1	Q7L9B9	0.14	2.53	0.81	1.07	2.77	0.68	0.96	0.36	0.63	0.35	0.75	0.38	2.13	0.97	0.60	4.64	1.07	0.47	2.22	0.19	0.06	6656.14	1265.44	959.55	3057.22	3224.46	1581.58	4955.33	4301.11	3652.58	4512.34	3945.70	2037.63		x
1553	Endophilin-A1	Q99962	0.14	2.53	0.81	1.07	2.77	0.68	0.96	0.36	0.63	0.35	0.75	0.38	2.13	0.97	0.60	4.64	1.07	0.47	2.22	0.19	0.06	4540.52	5318.48	3043.20	4424.67	3941.78	3480.78	5514.68	5420.05	4194.45	4951.44	4975.74	3635.78		x
1554	Endophilin-A2	Q99961	29888.39	26671.25	24215.77	16409.92	15771.76	14802.69	18745.95	15602.01	17378.87	20686.75	19119.04	17140.43	23137.94	18819.64	14454.40	16073.36	13582.79	14004.92	16705.75	16301.83	14065.74	26470.75	26224.15	23533.69	17377.31	16735.32	14036.21	22697.88	21472.04	20684.43	22560.77	21830.34	14484.45		
1555	Endophilin-B2	Q9NR46	222496.53	216177.68	207387.84	159341.03	146427.55	144372.78	171773.33	166836.63	172837.17	180285.50	169629.87	161494.91	196048.55	178997.33	163573.13	161743.78	147056.32	147825.58	164915.03	153412.85	147411.02	4567.90	5139.39	4837.79	3685.66	3836.18	3230.89	3552.24	3673.92	3397.15	3556.31	3355.11	2300.34		
1556	Endoplasmic reticulum aminopeptidase 1	Q9NZ08	0.14	2.53	0.81	1.07	2.77	0.68	0.96	0.36	0.63	0.35	0.75	0.38	2.13	0.97	0.60	4.64	1.07	0.47	2.22	0.19	0.06	29083.35	24137.17	22775.59	15483.91	17676.31	13451.81	27167.69	23238.10	18032.39	23970.44	21403.28	15234.77		x
1557	Endoplasmic reticulum chaperone BiP	P11021	2846061.90	2741926.32	2590973.24	2347677.48	2243462.62	2085228.53	2602981.64	2509557.59	2403831.04	2838699.59	2763786.15	2551033.30	2377688.17	2170298.53	1839742.21	2585849.28	2463477.71	2366512.28	2248557.42	2125864.34	2011954.27	2093094.09	1987363.79	1819980.95	1679566.82	1649604.74	1485079.64	1718535.28	1649579.36	1577468.14	1904566.78	1850035.04	1233019.49		
1558	Endoplasmic reticulum junction formation protein lunapark	Q9C0E8	9098.73	9736.15	9285.87	7121.42	7065.91	6789.21	6789.84	6554.45	6207.35	9645.56	8665.35	8847.39	6529.73	7138.99	6335.49	7143.43	7402.17	6971.90	5493.29	5894.90	5663.86	2423.40	2174.45	1918.41	1534.93	1502.26	1420.41	1869.62	1844.45	1738.40	2046.26	1897.80	1211.89		
1559	Endoplasmic reticulum metallopeptidase 1	Q7Z2K6	36187.00	36680.21	34553.60	21095.87	20431.08	19317.03	24853.58	21741.92	23226.37	26333.74	24280.85	24024.35	28932.53	26702.51	24795.38	22350.01	20267.54	18585.37	19836.55	19771.81	20284.41	2.27	1.49	0.65	0.84	1.31	0.69	4.16	2.47	0.11	4.90	4.98	0.12	x	
1560	Endoplasmic reticulum protein SC65	Q92791	6977.77	6146.13	3865.19	604.58	426.87	112.51	494.20	2088.73	310.67	5422.14	4620.92	3855.36	5599.48	1586.38	93.56	328.48	299.18	187.41	1645.22	587.23	480.13	2.27	1.49	0.65	0.84	1.31	0.69	4.16	2.47	0.11	4.90	4.98	8698.49	x	
1561	Endoplasmic reticulum resident protein 29	P30040	432730.23	417093.09	394809.18	329692.88	312636.35	302459.74	359323.44	357985.11	327923.12	395063.02	382075.59	355118.98	359865.71	308178.47	256768.59	348777.16	332244.32	315296.62	312511.25	291282.53	271625.75	90451.59	85247.72	79006.97	56734.41	53747.26	48957.21	65703.37	63944.29	60248.87	65484.91	61509.82	27963.34		
1562	Endoplasmic reticulum resident protein 44	Q9BS26	117471.65	114686.63	109573.82	92174.07	88779.76	82802.74	112198.55	105817.38	104460.02	107870.03	101893.89	97074.82	104805.14	108326.05	99597.37	90734.89	87243.57	86602.58	85067.24	80525.17	77701.77	29909.37	28104.59	26957.11	20631.41	19673.50	19107.12	24697.57	23805.54	22607.49	24826.50	23526.98	18563.69		
1563	Endoplasmic reticulum-Golgi intermediate compartment protein 1	Q969X5	93047.31	83702.61	75865.01	54801.08	50070.26	47236.02	62220.63	56191.43	56305.71	70712.58	67148.18	62037.39	72304.56	67601.31	50313.57	52822.79	50798.69	49772.28	50712.08	49100.59	45838.68	2.27	1.49	0.65	0.84	1.31	0.69	4.16	2.47	0.11	4.90	4.98	0.12	x	
1564	Endoplasmic reticulum-Golgi intermediate compartment protein 2	Q96RQ1	0.14	2.53	0.81	1.07	2.77	0.68	0.96	0.36	0.63	0.35	0.75	0.38	2.13	0.97	0.60	4.64	1.07	0.47	2.22	0.19	0.06	10987.49	10878.85	9632.94	2368.24	2712.83	2443.12	3687.89	3525.83	2537.31	2901.62	2805.36	1660.60		x
1565	Endoplasmic reticulum-Golgi intermediate compartment protein 3	Q9Y282	10919.45	10271.86	9546.37	7399.49	6971.68	6723.00	7633.99	7278.87	6551.38	9719.41	9506.03	8955.49	9163.64	8578.46	7722.48	6741.69	6688.44	6962.19	6993.30	6535.43	6124.16	2.27	1.49	0.65	0.84	1.31	0.69	4.16	2.47	0.11	4.90	4.98	0.12	x	
1566	Endoplasmin	P14625	2075038.04	2002701.34	1914264.29	1615988.39	1510627.59	1428783.79	1856835.92	1797438.80	1741070.16	1957927.11	1876698.55	1752461.21	1746367.86	1632391.72	1403761.59	1691737.59	1556948.90	1516544.84	1629661.69	1549895.94	1470105.35	1339476.09	1261997.39	1174951.06	872000.34	853744.25	772358.21	1019502.56	966148.47	928661.21	1009287.35	983001.42	673629.94		
1567	Endoribonuclease LACTB2	Q53H82	1280.57	1331.42	1127.84	953.90	870.28	797.71	735.90	982.17	749.41	1277.33	1030.74	949.24	1246.56	1209.10	731.11	1014.02	1005.18	1013.38	920.23	921.81	859.75	2.27	1.49	0.65	0.84	1.31	0.69	4.16	2.47	0.11	4.90	4.98	0.12	x	
1568	Endothelial differentiation-related factor 1	O60869	155163.33	147866.23	143676.07	102430.66	99102.20	96346.70	115456.06	115850.78	113299.78	122097.84	115349.31	113384.45	123939.39	121430.98	104041.77	93371.87	90302.37	88629.51	107051.87	100106.29	93894.77	266983.78	245507.90	246796.80	176869.94	170750.17	163035.75	219581.80	213597.71	205357.67	178525.12	166637.22	121385.51		
1569	Endothelin-converting enzyme 1	P42892	0.14	2.53	0.81	1.07	2.77	0.68	0.96	0.36	0.63	0.35	0.75	0.38	2.13	0.97	0.60	4.64	1.07	0.47	2.22	0.19	0.06	5336.54	5551.74	5341.02	4253.45	4091.92	4012.85	6200.20	5745.44	5462.23	5369.95	4595.57	3598.36		x
1570	Enhancer of filamentation 1	Q14511	0.14	2.53	0.81	1.07	2.77	0.68	0.96	0.36	0.63	0.35	0.75	0.38	2.13	0.97	0.60	4.64	1.07	0.47	2.22	0.19	0.06	21138.49	21697.90	18875.82	14304.22	14166.03	13762.67	14714.49	14651.13	14615.75	15027.72	14300.62	7774.08		x
1571	Enhancer of mRNA-decapping protein 4	Q6P2E9	87756.84	84689.89	74503.82	52571.74	49854.25	41887.02	65905.25	60957.19	59748.52	68373.68	63340.21	58647.06	61812.65	59234.62	45230.11	57709.57	56193.63	50851.17	56664.01	52601.01	48381.17	20432.75	18230.56	16715.09	10930.64	10990.54	9196.01	17518.39	14151.44	13849.02	15342.22	16139.94	7238.82		
1572	Enhancer of rudimentary homolog	P84090	41739.33	39390.12	42281.72	27466.84	26149.43	25179.95	26461.54	23538.92	21350.78	35681.01	32818.13	30370.51	30001.33	30078.21	28702.95	21781.36	25370.28	22893.03	26818.85	25576.20	27041.73	12753.40	12041.58	11051.73	11898.67	11552.25	10203.43	11570.87	11426.63	10894.27	12758.50	12823.78	11310.54		
1573	Enolase-phosphatase E1	Q9UHY7	11650.68	10096.24	7714.24	4943.50	4658.48	3702.44	6524.88	6344.87	6042.20	8468.81	7751.07	6551.57	7672.52	5766.52	3956.70	5066.31	4927.86	4231.90	5359.38	5213.63	4393.16	2.27	1.49	0.65	0.84	1.31	0.69	4.16	2.47	0.11	4.90	4.98	0.12	x	
1574	Enoyl-CoA delta isomerase 1 mitochondrial	P42126	114426.78	100533.09	103629.27	75808.99	71917.49	66861.73	100536.67	95519.79	92104.04	87218.37	84251.10	77139.25	86549.89	81232.71	64378.85	76123.50	71771.92	69273.50	73020.41	68316.63	66284.94	38614.55	31822.06	34025.86	24035.07	24624.53	21428.43	26825.73	24813.69	24165.45	36137.18	36778.44	19642.05		
1575	Enoyl-CoA delta isomerase 2 mitochondrial	O75521	43073.39	41857.73	41455.18	31141.05	29580.90	25988.61	35002.44	35512.26	33755.21	36840.45	35978.44	34745.59	36162.38	34820.82	24956.69	28880.04	26047.77	26623.94	30375.56	28743.68	29638.53	8219.58	7821.08	7751.57	6224.92	6030.40	5560.05	6526.04	6473.77	6319.56	7107.32	6188.90	4878.25		
1576	Enoyl-CoA hydratase domain-containing protein 2_ mitochondrial	Q86YB7	96049.09	90387.52	88912.33	71272.61	66448.15	66245.83	75309.17	76514.70	74332.72	78138.31	74003.68	71053.47	77111.86	77807.29	71271.55	63922.27	58621.54	58715.02	70080.63	66729.50	65921.34	2.27	1.49	0.65	0.84	1.31	0.69	4.16	2.47	0.11	4.90	4.98	0.12	x	
1577	Enoyl-CoA hydratase mitochondrial	P30084	249127.60	232978.38	216777.53	150188.16	141430.56	126230.10	198852.94	187199.48	179758.76	185660.94	177269.53	161511.50	216832.26	205223.15	149990.10	153823.52	141726.05	139633.14	146696.95	135321.24	127811.38	132868.24	120693.38	108155.36	66196.29	63219.14	52446.94	90237.70	84489.16	75124.87	80336.76	76588.85	50634.34		
1578	Ensconsin	Q14244	509763.33	492040.24	481394.92	380192.38	371769.27	361847.02	459194.27	430296.77	423492.94	436155.50	422839.09	401693.85	470703.80	391971.30	376277.45	385873.68	372487.02	365463.07	442811.73	439122.07	408403.51	2.27	1.49	0.65	0.84	1.31	0.69	4.16	2.47	0.11	4.90	4.98	0.12	x	
1579	Envoplakin	Q92817	39112.48	39410.85	36826.49	25137.43	23782.22	24410.90	28941.96	29506.93	27559.13	35132.02	32993.02	32993.28	34303.08	31716.05	28144.71	23716.21	21488.27	22230.80	25521.86	23304.17	22344.47	40863.86	39704.44	36841.06	25400.55	25305.26	22178.55	35321.68	32962.36	30774.63	33755.87	32211.20	23211.71		
1580	Envoplakin-like protein	A8MZ36	0.14	2.53	0.81	1.07	2.77	0.68	0.96	0.36	0.63	0.35	0.75	0.38	2.13	0.97	0.60	4.64	1.07	0.47	2.22	0.19	0.06	11493.44	12788.96	11148.09	8784.73	8054.97	6162.08	9793.66	9685.95	9618.83	10490.99	10065.79	5669.14		x
1581	Ephrin type-A receptor 2	P29317	0.14	2.53	0.81	1.07	2.77	0.68	0.96	0.36	0.63	0.35	0.75	0.38	2.13	0.97	0.60	4.64	1.07	0.47	2.22	0.19	0.06	46122.20	41971.71	41378.54	32766.05	31854.54	27271.69	39034.93	36945.33	36637.55	35735.55	34958.56	24427.74		x
1582	Ephrin type-A receptor 4	P54764	72.54	122.21	75.45	173.56	121.05	135.46	191.19	230.56	223.43	232.22	241.59	265.56	165.30	199.72	192.70	269.22	218.65	226.71	199.66	218.34	228.24	2.27	1.49	0.65	0.84	1.31	0.69	4.16	2.47	0.11	4.90	4.98	0.12	x	
1583	Ephrin type-A receptor 6	Q9UF33	0.14	2.53	0.81	1.07	2.77	0.68	0.96	0.36	0.63	0.35	0.75	0.38	2.13	0.97	0.60	4.64	1.07	0.47	2.22	0.19	0.06	3300.96	3089.40	2682.83	3206.81	3171.12	1969.92	3997.83	3641.46	3079.41	4566.36	4294.14	2244.04		x
1584	Ephrin type-A receptor 7	Q15375	0.14	2.53	0.81	1.07	2.77	0.68	0.96	0.36	0.63	0.35	0.75	0.38	2.13	0.97	0.60	4.64	1.07	0.47	2.22	0.19	0.06	42.76	33.35	17.32	106.62	43.03	34.71	386.79	304.72	276.43	362.16	340.93	67.80		x
1585	Ephrin type-B receptor 3	P54753	36627.68	36769.72	35955.91	28175.27	29041.22	26879.69	30874.59	28328.74	28259.38	31147.83	31550.59	29372.46	32104.65	31260.28	27932.30	27472.83	26009.81	25164.15	29647.09	28229.32	26239.23	2.27	1.49	0.65	0.84	1.31	0.69	4.16	2.47	0.11	4.90	4.98	0.12	x	
1586	Ephrin type-B receptor 4	P54760	3056.11	3959.39	3013.57	2515.72	2228.66	2005.35	4189.92	3370.50	2944.87	3533.76	3278.16	3158.85	3422.17	3824.79	2541.31	2741.34	2659.99	2356.82	2293.49	2620.28	2533.14	2.27	1.49	0.65	0.84	1.31	0.69	4.16	2.47	0.11	4.90	4.98	0.12	x	
1587	Epidermal growth factor receptor kinase substrate 8	Q12929	0.14	2.53	0.81	1.07	2.77	0.68	0.96	0.36	0.63	0.35	0.75	0.38	2.13	0.97	0.60	4.64	1.07	0.47	2.22	0.19	0.06	505.62	861.38	654.18	547.37	548.94	397.55	448.05	527.97	365.22	581.91	643.19	251.57		x
1588	Epidermal growth factor receptor kinase substrate 8-like protein 1	Q8TE68	0.27	102.80	86.10	1.07	2.77	0.68	0.96	0.36	0.63	0.35	0.75	0.38	2.13	0.97	0.60	15.52	4.45	8.38	2.22	0.19	0.06	2.27	1.49	0.65	0.84	1.31	0.69	4.16	2.47	0.11	4.90	4.98	0.12	x	
1589	Epidermal growth factor receptor kinase substrate 8-like protein 2	Q9H6S3	22048.40	21167.11	19028.75	11810.72	11682.95	10818.64	18209.69	16328.50	16000.10	19160.28	17650.27	14498.39	17666.06	15277.75	11115.98	12561.06	12022.73	11514.68	10916.16	10459.38	10321.08	3029.60	2510.66	2291.32	1205.13	1325.24	958.74	1819.55	1691.03	1332.04	2376.18	2057.16	1039.08		
1590	Epidermal growth factor receptor	P00533	0.14	2.53	0.81	1.07	2.77	0.68	0.96	0.36	0.63	0.35	0.75	0.38	2.13	0.97	0.60	4.64	1.07	0.47	2.22	0.19	0.06	225849.31	217360.95	204800.06	150084.30	147554.35	137837.97	182521.85	179808.89	170148.68	175764.72	169664.55	119597.91		x
1591	Epidermal growth factor receptor substrate 15	P42566	4332.89	3739.83	3174.78	3308.83	3135.16	2945.02	3676.30	2727.12	3179.97	4220.31	4652.22	3767.80	4196.66	4032.40	3108.59	4120.51	3413.36	3055.10	3668.87	3148.80	2207.57	2.27	1.49	0.65	0.84	1.31	0.69	4.16	2.47	0.11	4.90	4.98	0.12	x	
1592	Epidermal growth factor receptor substrate 15-like 1	Q9UBC2	5962.82	5830.50	4645.10	2998.17	3137.32	2441.91	3209.17	3305.80	3121.29	4262.76	3824.23	3972.69	4428.54	4119.53	2512.02	2733.53	2370.72	2275.72	3755.34	3590.57	3367.89	2.27	1.49	0.65	0.84	1.31	0.69	4.16	2.47	0.11	4.90	4.98	0.12	x	
1593	Epididymal sperm-binding protein 1	Q96BH3	747.80	539.06	300.05	113.54	14.37	42.36	67.91	9.33	25.02	839.76	690.14	489.81	778.87	22.80	1.19	76.94	3.82	16.76	514.58	9.82	29.80	1396.03	931.13	42.47	997.13	954.18	470.97	1376.59	1097.06	646.74	2097.76	1961.53	1137.08		
1594	Epiplakin	P58107	1500856.84	1480677.66	1356323.77	982301.08	956958.60	893104.01	1064857.57	1022176.78	987889.82	1190150.70	1120957.45	1060307.55	1245691.21	1149240.91	883100.79	962349.00	906823.93	870309.24	952887.30	912658.42	854756.56	2677.80	2647.40	2490.13	2273.97	2487.00	2218.87	2564.51	2963.24	2625.69	3219.83	2950.07	2164.01		
1595	Epithelial cell adhesion molecule	P16422	93385.20	84999.10	74117.16	68235.92	67721.55	57670.49	73600.85	72488.24	66141.00	91877.84	88982.94	75404.82	82968.12	72535.97	41473.39	69746.90	67935.27	63432.18	73393.96	68487.18	59792.26	2.27	1.49	0.65	0.84	1.31	0.69	4.16	2.47	0.11	4.90	4.98	0.12	x	
1596	Epithelial splicing regulatory protein 1	Q6NXG1	0.14	2.53	0.81	1.07	2.77	0.68	0.96	0.36	0.63	0.35	0.75	0.38	2.13	0.97	0.60	4.64	1.07	0.47	2.22	0.19	0.06	3152.93	2535.06	2304.12	1527.56	1367.49	1034.79	3560.59	3609.41	3134.33	2414.14	2211.70	1357.88		x
1597	Epithelial splicing regulatory protein 2	Q9H6T0	1040.67	1066.88	988.88	49.15	61.54	52.82	266.92	296.06	285.21	590.68	541.10	578.78	304.42	259.95	238.55	932.44	826.88	890.63	293.09	279.65	343.59	2.27	1.49	0.65	0.84	1.31	0.69	4.16	2.47	0.11	4.90	4.98	0.12	x	
1598	Epithelial-stromal interaction protein 1	Q96J88	8334.49	8129.53	8400.64	4454.73	4391.74	4286.63	6864.21	6136.22	6281.59	6302.17	5394.99	4802.16	6928.23	6775.26	5721.74	5879.67	5780.44	5615.50	5262.34	4367.35	4217.97	2.27	1.49	0.65	0.84	1.31	0.69	4.16	2.47	0.11	4.90	4.98	0.12	x	
1599	Epoxide hydrolase 1	P07099	6407.22	5179.10	5394.98	5474.08	3979.89	4032.26	5694.85	5486.43	4706.39	6628.30	7050.30	6399.97	4874.03	4539.20	3101.97	6745.63	5806.03	5930.08	4625.17	4242.65	4224.32	18199.63	18356.88	16707.77	14844.97	13754.23	11228.77	17679.16	16252.52	16019.53	16823.53	15479.27	10040.16		
1600	Eppin	O95925	8682.91	8380.11	8118.87	7012.16	6820.26	6504.66	8090.82	8444.68	9348.36	8512.78	8091.16	8297.58	8734.73	7568.33	6421.99	8335.29	7913.26	8111.65	7620.17	7563.95	7916.48	2.27	1.49	0.65	0.84	1.31	0.69	4.16	2.47	0.11	4.90	4.98	0.12	x	
1601	Epsilon-sarcoglycan	O43556	136.69	54.91	39.83	471.25	57.84	56.38	251.81	5120.31	4154.33	67.80	71.35	13.51	71.53	821.03	26.54	216.99	120.09	110.65	147.91	335.71	106.96	2.27	1.49	0.65	0.84	1.31	0.69	4.16	2.47	0.11	4.90	4.98	0.12	x	
1602	Epsin-1	Q9Y6I3	0.14	2.53	0.81	1.07	2.77	0.68	0.96	0.36	0.63	0.35	0.75	0.38	2.13	0.97	0.60	4.64	1.07	0.47	2.22	0.19	0.06	12268.46	12022.02	12005.26	6877.85	6704.38	6596.57	8781.98	9504.02	8787.22	9224.45	8368.80	5338.42		x
1603	Epsin-2	O95208	0.14	2.53	0.81	1.07	2.77	0.68	0.96	0.36	0.63	0.35	0.75	0.38	2.13	0.97	0.60	4.64	1.07	0.47	2.22	0.19	0.06	7078.23	7880.26	6855.56	3927.33	3623.71	2637.63	4584.31	4045.08	3823.11	4783.20	3819.94	2682.33		x
1604	Equilibrative nucleoside transporter 1	Q99808	17505.42	16914.18	16500.50	14337.89	13552.20	13084.70	14285.63	14141.88	14116.84	16200.13	15131.55	14637.05	14248.89	15193.94	13646.10	12445.26	12205.54	11827.27	13199.41	12812.79	12921.48	45006.59	42591.51	38240.41	33038.11	32959.46	33396.48	38071.34	36532.69	33027.76	40112.79	40257.13	43041.68		
1605	ER lumen protein-retaining receptor 1	P24390	3842.04	3704.80	3810.09	2181.84	2046.08	2068.68	2298.98	2160.62	2329.29	2580.88	2459.92	2391.66	2531.74	2525.35	2289.97	2138.59	1994.05	2191.65	2088.14	2048.85	2095.19	2.27	1.49	0.65	0.84	1.31	0.69	4.16	2.47	0.11	4.90	4.98	0.12	x	
1606	ER membrane protein complex subunit 1	Q8N766	33437.53	32505.07	26048.93	19574.30	21949.54	20484.68	24748.03	23283.33	22438.54	27482.27	25307.04	22070.37	26550.07	21870.70	17253.34	20500.97	19481.81	18510.77	19995.14	19411.37	17640.08	34753.94	32746.92	29094.39	19672.43	18265.39	14294.40	25361.02	23021.04	21486.20	26881.42	25769.72	14571.42		
1607	ER membrane protein complex subunit 2	Q15006	40994.47	35661.81	30547.02	27996.05	26799.30	20790.36	30676.01	27668.93	25622.37	35817.82	33753.63	28622.30	34188.07	28495.53	16245.78	26450.18	24664.10	21496.10	30559.02	28442.41	24648.98	5391.19	3779.87	3657.66	3150.22	3344.31	2113.64	3794.37	3909.84	2717.56	4125.69	4359.16	2394.00		
1608	ER membrane protein complex subunit 3	Q9P0I2	1684.50	1531.06	1588.03	1016.95	934.69	1128.41	1673.41	1600.59	1520.65	1202.81	1275.02	1277.65	1162.38	1114.01	924.52	1377.99	1221.45	1315.19	1159.70	1165.08	1091.32	1926.51	1816.39	1551.07	1003.76	993.05	638.86	1691.77	1649.73	1525.62	1671.92	1502.72	616.81		
1609	ER membrane protein complex subunit 4	Q5J8M3	26540.44	25311.25	24125.61	18017.52	16919.08	15954.01	18978.48	18751.83	17419.99	20688.29	20003.27	18948.24	23168.90	20863.70	17190.66	17148.49	16799.71	16736.41	17929.36	17132.62	15821.48	13679.11	13360.04	12110.11	5578.10	6004.85	5266.67	6734.51	6659.27	6315.71	5843.89	5747.93	4437.50		
1610	ER membrane protein complex subunit 7	Q9NPA0	82857.39	75023.82	73723.59	43673.18	41266.77	38478.24	38501.34	30638.81	35367.53	56282.54	51220.48	46626.22	56941.68	42940.51	36800.20	38541.35	37501.30	36273.04	42988.95	40432.47	38346.15	2.27	1.49	0.65	0.84	1.31	0.69	4.16	2.47	0.11	4.90	4.98	0.12	x	
1611	ER membrane protein complex subunit 8	O43402	41601.25	38360.85	37795.01	27529.95	25900.25	24819.05	31748.58	30622.28	30185.31	31098.01	28676.94	29028.10	30633.63	29553.25	25614.70	25576.61	26131.97	23593.61	26608.00	25340.05	21853.77	2.27	1.49	0.65	0.84	1.31	0.69	4.16	2.47	0.11	4.90	4.98	0.12	x	
1612	Erbin	Q96RT1	0.14	2.53	0.81	1.07	2.77	0.68	0.96	0.36	0.63	0.35	0.75	0.38	2.13	0.97	0.60	4.64	1.07	0.47	2.22	0.19	0.06	57304.34	51623.89	50595.31	51407.21	48920.23	44441.55	68047.28	62878.10	61197.16	64226.44	59401.86	43619.89		x
1613	ERC protein 2	O15083	0.14	2.53	0.81	1.07	2.77	0.68	0.96	0.36	0.63	0.35	0.75	0.38	2.13	0.97	0.60	4.64	1.07	0.47	2.22	0.19	0.06	10268.56	9615.26	8968.24	6593.76	6335.69	5193.97	7966.88	7716.85	6054.91	8735.81	8377.14	5153.77		x
1614	Erlin-1	O75477	103095.19	97990.27	95945.03	74402.36	71887.10	66517.62	83817.18	85491.04	79119.14	93817.73	91641.83	89313.89	85750.30	79385.73	67412.61	79362.62	68069.67	68804.01	76227.58	73706.96	68161.21	281.27	382.48	331.83	156.99	83.22	60.42	94.91	67.20	65.57	185.20	225.64	66.50		
1615	Erlin-2	O94905	39817.76	39599.19	37186.65	26596.33	25132.25	22928.85	34533.53	33685.78	31494.90	35868.38	34719.43	32466.61	33381.71	33190.88	27341.50	27258.21	25105.16	25284.22	30093.41	27038.34	25633.54	24721.24	23883.57	21384.92	17567.39	16604.23	13253.85	20450.71	19167.68	17406.15	20556.54	19006.43	11209.11		
1616	ERO1-like protein alpha	Q96HE7	167086.88	162182.45	158018.95	120069.97	118174.70	105982.55	132752.08	128843.39	126338.36	150251.87	145888.10	139022.72	143010.49	132216.03	102816.43	113507.96	110254.69	108056.28	118369.45	117210.04	108998.65	89524.74	82006.91	76412.92	53371.13	51487.41	43260.98	67397.79	62635.46	56593.57	68711.75	64676.29	46824.82		
1617	ERO1-like protein beta	Q86YB8	48771.94	49661.33	44319.29	36341.79	32326.97	31614.14	42204.26	37971.14	40031.70	42253.20	39491.75	37216.52	42400.61	38979.90	32655.08	34828.78	29968.35	31456.22	36107.44	34368.51	32103.47	42539.91	40062.00	37249.47	31521.47	31437.70	27984.12	35875.12	34738.51	33308.56	38214.21	36884.71	27675.81		
1618	Erythrocyte band 7 integral membrane protein	P27105	5762.46	5909.09	5248.74	8110.13	8277.87	8097.53	41636.03	40019.26	41694.20	7439.45	7234.44	6142.08	4581.03	4328.30	3964.21	4638.34	4654.28	4447.76	3942.41	3761.46	3784.74	3668.83	3467.65	3056.40	2744.53	2792.67	2688.48	3317.76	3231.55	3032.88	4504.10	4322.81	3243.91		
1619	Erythroid differentiation-related factor 1	Q3B7T1	0.14	2.53	0.81	1.07	2.77	0.68	0.96	0.36	0.63	0.35	0.75	0.38	2.13	0.97	0.60	4.64	1.07	0.47	2.22	0.19	0.06	14257.34	12330.36	11523.94	11302.69	11716.22	7393.76	14431.80	13758.87	11209.79	10644.86	10402.62	4695.65		x
1620	ESF1 homolog	Q9H501	62004.89	58202.26	56694.60	44862.55	41651.15	41773.51	56290.46	59936.23	63663.78	55178.09	52754.10	51323.50	58892.17	53182.93	46487.80	47988.79	45578.28	45900.19	55558.31	51978.35	50822.52	2.27	1.49	0.65	0.84	1.31	0.69	4.16	2.47	0.11	4.90	4.98	0.12	x	
1621	Ester hydrolase C11orf54	Q9H0W9	0.14	2.53	0.81	1.07	2.77	0.68	0.96	0.36	0.63	0.35	0.75	0.38	2.13	0.97	0.60	4.64	1.07	0.47	2.22	0.19	0.06	39205.84	40001.79	36970.67	20985.60	20149.86	20127.69	19903.68	18990.11	19377.47	19401.12	21301.07	777.16		x
1622	Esterase OVCA2	Q8WZ82	661.14	707.53	830.30	505.15	608.57	701.46	857.15	807.09	761.22	1034.96	722.98	781.16	999.17	1092.36	829.86	453.10	521.90	441.31	601.84	544.72	664.85	2.27	1.49	0.65	0.84	1.31	0.69	4.16	2.47	0.11	4.90	4.98	0.12	x	
1623	Estrogen receptor beta	Q92731	77983.26	69997.90	64081.52	66556.73	60616.97	56489.10	57019.38	52705.23	58406.18	72387.14	65619.20	65772.40	62436.15	67118.47	54175.96	60928.05	58987.02	52049.29	61115.62	60465.56	60173.20	2.27	1.49	0.65	0.84	1.31	0.69	4.16	2.47	0.11	4.90	4.98	0.12	x	
1624	Ethanolamine-phosphate cytidylyltransferase	Q99447	9126.35	8654.38	8719.15	6623.44	5586.13	4818.91	6916.67	6580.61	5602.85	7236.71	6526.16	6239.79	7848.08	6288.39	4535.33	6699.40	5564.90	5610.33	6996.42	6344.88	5999.79	1849.15	742.50	677.49	832.62	712.01	528.66	1542.96	1509.75	348.46	1384.85	1114.91	474.04		
1625	Ethylmalonyl-CoA decarboxylase	Q9NTX5	0.14	2.53	0.81	1.07	2.77	0.68	0.96	0.36	0.63	0.35	0.75	0.38	2.13	0.97	0.60	4.64	1.07	0.47	2.22	0.19	0.06	2.27	1.49	0.65	151.94	153.48	81.11	305.70	235.24	163.94	438.69	325.67	107.54		x
1626	ETS domain-containing protein Elk-4	P28324	600.14	520.71	671.95	467.38	396.83	434.09	380.92	424.70	450.09	589.09	594.58	665.57	451.16	466.74	418.79	413.45	431.00	445.78	760.47	576.56	415.72	2.27	1.49	0.65	0.84	1.31	0.69	4.16	2.47	0.11	4.90	4.98	0.12	x	
1627	ETS translocation variant 3	P41162	7399.74	7732.76	6857.75	3923.58	3678.49	3374.63	5388.67	5498.19	3797.69	5097.57	4999.85	4672.21	5515.43	5096.81	3649.61	4776.75	4455.23	4114.14	5415.79	4729.52	3897.85	2.27	1.49	0.65	0.84	1.31	0.69	4.16	2.47	0.11	4.90	4.98	0.12	x	
1628	ETS translocation variant 5	P41161	21573.49	21299.99	20721.32	14310.68	14186.19	14247.10	17656.44	17036.71	16445.81	20348.82	18854.45	17892.42	18283.67	17430.06	14783.03	13880.93	13462.77	13437.60	15647.64	15267.11	14176.93	14167.66	14711.92	13894.73	9119.15	9415.61	8592.89	11047.47	12789.81	12748.59	12338.48	11986.16	9054.27		
1629	ETS-related transcription factor Elf-4	Q99607	5254.19	4813.42	5106.58	4104.46	3984.18	4695.76	3504.77	3240.09	3420.06	5171.33	5552.11	4236.35	4930.91	5527.86	4857.78	3300.41	3094.21	3179.24	3567.47	3599.58	3558.51	212.88	231.33	235.76	73.42	153.93	98.52	234.11	180.92	141.13	198.39	204.97	110.23		
1630	Eukaryotic initiation factor 4A-I	P60842	1675030.73	1614019.69	1526367.51	1094923.88	1065449.15	995080.14	1338302.96	1291110.92	1238211.69	1318361.79	1248874.42	1176079.84	1332068.45	1240701.04	1057649.79	1092729.97	1045845.89	991910.18	1164731.07	1108055.49	1032080.66	1210613.11	1139199.31	1059013.84	747249.65	732417.53	637446.08	1015560.99	969934.79	936296.21	940120.40	911797.67	651915.36		
1631	Eukaryotic initiation factor 4A-II	Q14240	53218.27	56017.75	57864.23	31331.91	29966.66	27934.04	41461.97	39419.71	40817.55	44510.18	42397.94	39258.73	39185.96	36273.56	29366.11	37440.39	32586.88	33131.01	30027.15	29134.21	27801.90	20477.64	18260.91	17978.04	12228.27	11895.84	10815.76	18096.39	16602.77	15194.06	16813.37	16355.62	10952.62		
1632	Eukaryotic initiation factor 4A-III	P38919	221489.66	212246.20	203129.47	155025.31	147883.93	142380.56	174207.24	168139.48	159722.07	172825.13	167588.74	159181.13	175142.97	170391.40	143349.27	139733.65	136805.97	130434.99	149338.47	147758.35	141201.64	37166.26	33294.46	31076.55	27557.49	27460.01	20525.47	40756.09	37152.39	32619.14	39767.90	38004.74	22692.65		
1633	Eukaryotic peptide chain release factor GTP-binding subunit ERF3A	P15170	250253.13	244459.39	235881.93	162870.24	157280.67	148972.25	187002.75	179920.58	178138.56	189575.18	179849.36	173818.14	191512.03	191202.62	160881.43	153197.54	148773.74	149732.00	166560.08	157594.86	152843.20	79124.52	83078.74	72946.47	56678.88	54500.44	58431.80	58214.02	57607.20	58083.02	78228.26	58887.93	35233.10		
1634	Eukaryotic peptide chain release factor GTP-binding subunit ERF3B	Q8IYD1	3132.70	2242.27	1355.34	1252.76	1090.64	832.00	2067.44	2184.59	2032.01	1649.21	1728.40	1312.11	2481.12	2864.27	681.39	1917.57	1830.60	1765.51	1008.01	865.89	524.16	119529.05	115274.61	104521.04	71062.37	71582.18	57631.71	86032.93	79649.66	75522.47	92600.81	89851.53	55991.39		
1635	Eukaryotic peptide chain release factor subunit 1	P62495	103713.70	100897.85	89972.23	71681.26	67470.07	63676.22	84131.26	82006.66	79415.98	79902.09	75939.14	73100.65	87476.56	79422.86	69656.59	69289.28	65692.97	65076.71	74459.98	71296.12	68464.08	68643.09	71044.05	66153.88	45697.81	44251.20	38106.90	55780.33	51927.91	53578.60	51287.72	50658.85	34270.13		
1636	Eukaryotic translation elongation factor 1 epsilon-1	O43324	23759.06	21181.10	21195.26	18314.36	18299.20	16228.55	17977.82	16748.84	17726.31	19920.86	19238.98	19066.39	19463.76	18831.56	15676.11	17229.35	14735.96	14594.66	18278.98	16839.04	15749.41	42729.86	38258.59	37026.22	30233.26	30057.08	28529.82	34592.37	34060.18	31820.52	37744.91	35773.18	26256.96		
1637	Eukaryotic translation initiation factor 1A_ X-chromosomal	P47813	207961.10	199405.08	191575.22	165792.21	157710.38	149684.49	170988.55	163467.57	162993.27	181230.06	173677.31	163970.85	176670.52	168222.04	141112.76	144047.95	139948.39	135960.64	166455.33	157238.46	142709.50	62256.03	53987.48	50721.46	41835.46	41085.26	35539.35	48956.92	46927.44	45826.08	46792.35	50706.31	37063.65		
1638	Eukaryotic translation initiation factor 1b	O60739	40528.67	39897.58	33811.03	27031.57	25988.14	23398.16	37033.58	36884.94	33235.31	35426.06	34483.37	30996.17	32291.34	31001.56	20448.14	30150.21	29988.67	26068.84	27369.68	26309.28	24266.39	6503.25	6597.96	5773.92	5008.12	4828.31	3666.65	5625.06	5497.49	6238.12	6175.34	5686.42	3617.43		

Supplementary Table S1. Overview on the abundance of all relatively quantified 5180 proteins

			MCF-7																					MDA-MB-231													
	Protein name	UniProt	control 01	control 02	control 03	Dai IC20 01	Dai IC20 02	Dai IC20 03	Dai SC20 01	Dai SC20 02	Dai SC20 03	Gen IC20 01	Gen IC20 02	Gen IC20 03	Gen SC20 01	Gen SC20 02	Gen SC20 03	SSE IC20 01	SSE IC20 02	SSE IC20 03	SSE SC20 01	SSE SC20 02	SSE SC20 03	control 01	control 02	control 03	Dai IC20 01	Dai IC20 02	Dai IC20 03	Gen IC20 01	Gen IC20 02	Gen IC20 03	SSE IC20 01	SSE IC20 02	SSE IC20 03	unique to MCF-7	unique to MDA-MB-231
1639	Eukaryotic translation initiation factor 2 subunit 1	P05198	344726.40	326145.99	303272.38	230625.86	220592.31	213938.80	279423.55	275912.90	261723.96	288549.59	272127.86	263798.81	253916.50	258333.67	211089.09	230083.95	218795.47	214043.06	224552.38	228973.14	215240.39	152711.42	137176.38	128637.05	84488.85	83150.43	73052.26	97522.77	88820.93	85884.76	95657.40	92297.41	62402.76		
1640	Eukaryotic translation initiation factor 2 subunit 2	P20042	444786.15	439431.70	414529.91	299410.16	291140.43	267071.93	366085.36	341861.39	332024.52	378256.73	367150.24	343399.02	387664.83	318628.78	263283.72	301362.26	284724.15	277419.81	326703.69	314389.12	292664.83	152606.62	128260.22	101380.11	79660.26	83131.49	66109.98	83949.82	90125.17	81656.20	105486.82	102811.47	64074.20		
1641	Eukaryotic translation initiation factor 2 subunit 3	P41091	249542.02	249250.66	229174.90	183691.39	174413.95	173299.88	221136.08	214500.48	211355.69	217075.71	206432.49	198540.58	211796.08	210057.23	180819.82	178947.67	170553.87	171713.20	179715.87	180078.36	170368.47	102458.03	100150.63	94400.90	48764.82	48641.41	44316.67	52439.90	53144.18	50659.54	50806.42	50463.57	40621.48		
1642	Eukaryotic translation initiation factor 2 subunit 3B	Q2VIR3	0.14	2.53	0.81	1.07	2.77	0.68	0.96	0.36	0.63	0.35	0.75	0.38	2.13	0.97	0.60	4.64	1.07	0.47	2.22	0.19	0.06	4025.21	3830.91	3876.79	4294.97	4173.31	3928.20	4158.91	3798.54	3422.11	4100.18	4963.86	3027.03		x
1643	Eukaryotic translation initiation factor 2A	Q9BY44	170862.45	165080.44	154501.82	118285.99	115602.17	109640.44	138107.22	139098.23	130866.45	150732.67	143543.75	138365.30	137735.86	131285.67	105741.19	127181.83	114101.42	112319.80	122922.01	119576.13	112578.23	27364.36	22963.72	22657.84	17983.84	17744.59	17159.63	19359.92	19637.33	17659.28	21483.68	21836.87	13460.88		
1644	Eukaryotic translation initiation factor 2-alpha kinase 3	Q9NZJ5	6060.24	4554.94	2995.84	903.47	870.51	469.18	1601.71	628.09	584.27	3551.70	3193.91	2427.84	3546.85	584.47	472.39	1867.81	1069.45	1411.88	3055.26	660.30	715.40	2.27	1.49	0.65	0.84	1.31	0.69	4.16	2.47	0.11	4.90	4.98	0.12	x	
1645	Eukaryotic translation initiation factor 2D	P41214	19110.73	18490.30	19277.05	14349.95	13122.25	12737.90	14390.13	15270.38	14829.76	17498.55	16561.49	15166.86	14783.85	14889.55	12768.92	13891.06	12792.29	12910.21	14554.32	13431.03	13255.59	2.27	1.49	0.65	0.84	1.31	0.69	4.16	2.47	0.11	4.90	4.98	0.12	x	
1646	Eukaryotic translation initiation factor 3 subunit A	Q14152	509076.28	515893.88	471107.37	353536.50	341906.91	333590.87	393163.76	389623.24	368977.73	430505.02	414655.04	389994.57	417739.16	407542.85	357670.28	337104.72	327082.47	311699.08	368736.76	352520.52	323409.09	215122.63	215004.56	201698.76	142827.35	138851.55	123109.70	176062.88	167209.42	162315.65	167481.44	161575.54	108411.22		
1647	Eukaryotic translation initiation factor 3 subunit B	P55884	492325.97	476318.15	455565.86	338174.13	320619.75	303897.99	395649.50	379887.48	358083.13	405023.65	390459.31	370076.21	394341.62	380767.10	293048.92	317714.70	284469.52	277544.34	345011.99	327034.79	309227.13	274481.08	257550.90	237348.82	150580.42	147249.52	127789.74	189348.85	179074.87	170011.57	185280.66	178897.20	122456.04		
1648	Eukaryotic translation initiation factor 3 subunit C	Q99613	0.14	2.53	0.81	1.07	2.77	0.68	0.96	0.36	0.63	0.35	0.75	0.38	2.13	0.97	0.60	4.64	1.07	0.47	2.22	0.19	0.06	153957.92	142174.86	134477.45	104059.33	100274.65	92032.47	124960.86	120002.39	116396.89	122476.87	115643.34	98319.39		x
1649	Eukaryotic translation initiation factor 3 subunit C-like protein	B5ME19	346583.83	344879.46	316939.24	239158.10	233872.37	225929.27	276127.51	260264.95	254066.90	275567.63	264972.73	250828.61	270550.09	252723.18	220164.82	219969.21	210362.86	200375.52	228995.11	221480.50	209181.09	2.27	1.49	0.65	0.84	1.31	0.69	4.16	2.47	0.11	4.90	4.98	0.12	x	
1650	Eukaryotic translation initiation factor 3 subunit D	O15371	131865.59	123604.82	117544.31	85609.76	82613.79	74692.35	101990.89	96719.79	92544.05	104489.74	102348.54	95424.08	102758.86	98794.99	76463.97	86275.19	78202.83	75627.65	88519.18	85702.75	78637.79	84977.84	79029.23	70201.06	49077.56	48165.45	42544.62	61892.45	57445.42	53224.64	60757.20	59550.55	41987.50		
1651	Eukaryotic translation initiation factor 3 subunit E	P60228	181047.33	174769.23	172047.64	104813.04	100347.43	94378.78	137997.87	129928.78	126273.51	135368.98	127430.14	119848.88	124639.42	122527.50	100960.48	102831.91	101005.86	97202.49	107553.50	105469.47	97590.79	84777.61	79738.46	73584.69	51759.95	50099.38	42744.22	73173.32	68577.76	64068.75	69658.61	64408.27	42528.59		
1652	Eukaryotic translation initiation factor 3 subunit F	O00303	91715.78	88326.65	84627.64	60864.44	61336.96	59970.97	60697.04	60192.66	58909.49	72358.25	73095.91	68550.66	69564.52	62896.40	42483.69	52994.02	49207.34	52922.43	59748.74	62088.98	58067.67	47774.68	46245.67	38827.58	31463.42	30540.72	28469.70	40363.40	39395.29	38724.98	40090.03	37584.15	20115.99		
1653	Eukaryotic translation initiation factor 3 subunit G	O75821	170057.54	161455.22	156166.97	123758.94	122250.08	117200.71	139808.61	135653.61	131340.31	148677.28	142911.31	132241.89	141901.54	130183.47	111609.50	118637.73	114365.58	108690.19	134497.48	127731.37	118987.90	80085.31	80081.94	71984.62	59880.49	60008.23	57823.59	70956.21	69667.26	67102.70	72838.65	70757.29	51416.03		
1654	Eukaryotic translation initiation factor 3 subunit H	O15372	75066.15	72356.06	67664.88	48220.00	46848.31	42835.28	53684.60	50036.94	48855.89	63240.32	59765.62	59069.10	61434.31	57759.04	46787.39	45715.41	44570.32	43254.47	47145.25	47038.68	46441.10	48366.80	37302.18	35639.40	26930.72	26059.29	21962.67	37849.52	33107.56	29545.96	34566.04	32496.70	21182.69		
1655	Eukaryotic translation initiation factor 3 subunit I	Q13347	365719.11	362274.81	349199.37	233621.91	232217.24	214029.31	301399.61	284291.55	272509.03	301951.40	286572.37	279130.82	282523.47	275273.06	221598.02	235556.71	226306.99	212210.21	238604.01	233392.23	218690.50	160865.60	148560.16	139223.21	99243.62	97322.39	88111.23	121821.80	115937.75	110718.02	116432.76	111100.31	77540.33		
1656	Eukaryotic translation initiation factor 3 subunit J	O75822	40424.11	42458.75	38566.32	32229.25	30273.74	27920.16	35235.06	33371.93	34284.94	35032.24	32107.30	31017.70	40366.87	36613.83	32799.49	28706.69	28717.05	28897.85	34615.13	26225.29	28134.06	22139.57	18256.06	18820.70	14819.68	14313.32	13764.51	20830.53	19425.99	17715.24	17847.64	16440.37	12383.87		
1657	Eukaryotic translation initiation factor 3 subunit K	Q9UBQ5	39587.55	38301.98	33755.27	27536.70	26935.33	22775.92	33441.67	33222.68	32576.65	32222.53	30332.19	28392.38	34337.73	32544.26	22780.36	26268.67	24880.28	23545.31	25694.64	24331.90	22675.98	54962.79	56862.28	47532.51	35058.51	34771.11	29758.89	42772.20	42289.45	40107.39	41828.04	38861.84	26571.02		
1658	Eukaryotic translation initiation factor 3 subunit L	Q9Y262	151759.43	149847.85	139696.76	105447.25	100856.65	92112.03	121257.12	111976.06	109677.32	133138.21	127145.86	118322.26	133157.11	111939.78	92966.07	97584.22	97293.87	92783.66	123480.63	115248.41	105231.29	136358.95	125432.97	119713.00	80305.28	82649.14	68612.79	101354.10	95399.18	92046.92	106718.23	103125.25	60471.45		
1659	Eukaryotic translation initiation factor 3 subunit M	Q7L2H7	110650.02	111263.55	95714.05	78320.36	78182.86	67139.25	93499.06	95800.17	90055.50	95063.99	90336.70	79713.08	88926.29	97138.56	66052.83	73164.97	76526.15	71070.49	70525.40	74195.92	65930.92	124662.29	117547.32	108152.90	91016.91	89880.16	81063.25	103700.02	99842.51	95512.86	108486.10	107045.37	93517.55		
1660	Eukaryotic translation initiation factor 4 gamma 1	Q04637	762912.11	734977.97	688671.60	510313.05	493296.56	452224.14	567108.05	555309.35	528706.86	638227.06	605937.07	576548.55	603883.21	549597.83	474060.99	502962.54	481432.49	460956.87	530735.95	510516.88	490232.53	251068.24	234411.85	220386.30	162833.40	161383.40	139115.09	194174.95	187021.47	179756.28	207583.62	199922.00	142834.94		
1661	Eukaryotic translation initiation factor 4 gamma 2	P78344	262938.35	248004.51	232284.92	176141.05	169980.02	158550.06	194741.28	190797.94	193792.69	214859.20	206308.21	198498.86	202286.96	195404.94	172232.04	197338.52	188944.12	182906.47	204244.33	177528.65	177492.27	130786.01	121840.87	112176.24	77898.51	77024.77	70343.64	103574.09	99330.71	98637.39	106887.97	107482.53	84068.79		
1662	Eukaryotic translation initiation factor 4 gamma 3	O43432	344694.97	334215.36	315986.74	248566.84	241766.48	229139.12	264529.93	259219.04	257550.50	280790.17	275784.03	266484.28	274579.85	269738.78	225614.61	241846.12	234749.54	224841.44	262778.70	256196.18	242364.19	5752.90	5598.14	4668.69	3834.01	3717.03	3106.72	4684.75	4680.28	4090.06	4754.71	4598.47	2844.35		
1663	Eukaryotic translation initiation factor 4B	P23588	331164.64	320165.89	306734.85	228386.42	223347.81	210532.97	244264.13	238473.33	233488.20	282546.30	265523.78	257106.78	269234.07	255254.95	198300.50	199875.06	189938.80	192948.50	245420.27	232948.94	229188.98	79390.09	73585.24	74763.22	52691.94	51873.94	49199.19	66583.64	62220.39	60668.09	60837.74	61320.14	39387.04		
1664	Eukaryotic translation initiation factor 4E	P06730	21333.83	21073.88	19558.54	12414.48	11886.36	9560.08	16364.55	13444.97	14119.64	15258.29	15429.19	13808.96	14088.31	14194.11	9914.54	15134.84	13710.20	13436.62	11956.61	11907.85	10490.68	992.26	974.18	1000.14	536.26	450.05	448.34	887.36	707.01	783.97	740.14	677.85	336.99		
1665	Eukaryotic translation initiation factor 4E type 2	O60573	0.14	2.53	0.81	1.07	2.77	0.68	0.96	0.36	0.63	0.35	0.75	0.38	2.13	0.97	0.60	4.64	1.07	0.47	2.22	0.19	0.06	852.72	247.95	854.29	472.64	528.74	402.14	678.39	598.72	598.75	1049.04	811.50	194.17		x
1666	Eukaryotic translation initiation factor 4E-binding protein 1	Q13541	5050.58	4750.25	2929.25	3263.58	3254.49	2648.51	2548.20	2542.02	2560.13	3963.84	3609.72	3359.68	4503.03	3225.21	3377.42	2738.65	2500.50	2720.08	2627.89	2532.68	2848.90	2.27	1.49	0.65	0.84	1.31	0.69	4.16	2.47	0.11	4.90	4.98	0.12	x	
1667	Eukaryotic translation initiation factor 4H	Q15056	300647.78	279515.35	272492.88	205214.56	193070.62	180459.35	232598.39	224307.13	224109.44	253023.10	240910.67	235229.15	236518.81	252723.10	204308.99	193401.18	190502.15	177328.60	204643.95	195045.41	193028.23	103600.87	103253.78	87885.29	81543.09	80551.04	73117.48	92512.10	91318.52	85023.80	103999.78	103934.82	73549.44		
1668	Eukaryotic translation initiation factor 5	P55010	92206.02	90326.33	88457.28	65792.33	59813.14	57886.62	75064.00	76894.53	72986.20	55809.10	54312.09	52027.38	76150.47	63775.45	53470.57	54530.80	53471.16	52778.01	68642.29	67477.04	63058.79	38494.56	36417.08	32879.72	22451.33	22352.99	20832.39	27446.27	25725.17	24161.99	29055.10	28280.85	17810.59		
1669	Eukaryotic translation initiation factor 5A-1	P63241	645470.99	631481.06	580400.52	400186.93	389110.26	370432.41	542689.12	514093.36	478914.36	502642.19	471829.22	438617.26	526789.07	509844.41	428167.89	395472.96	381234.97	361838.86	426374.46	412795.51	370424.53	793769.02	726824.18	673277.85	476345.85	456475.13	391918.22	635333.16	580216.77	574519.66	555355.31	532453.29	339776.81		
1670	Eukaryotic translation initiation factor 5A-2	Q9GZV4	8613.59	7607.83	6099.62	5609.86	5703.12	4620.98	5119.95	4501.75	4510.86	6869.13	6656.43	5814.38	7319.93	6058.89	2506.75	5465.50	5330.62	5099.17	6054.25	6598.76	7310.12	2374.07	1190.28	284.85	1731.20	1572.62	828.06	2417.60	2170.91	1394.48	3686.89	3068.41	1259.04		
1671	Eukaryotic translation initiation factor 5B	O60841	264941.91	252673.24	241748.11	189344.45	174248.40	160734.87	192196.59	187919.51	181231.28	225588.58	215144.80	205476.15	222507.75	210860.51	164287.35	182409.55	171596.52	170953.58	203513.92	185459.67	173553.67	348792.78	321990.73	300277.77	252864.83	242297.20	211179.67	322704.35	302130.33	290748.82	290106.20	281465.10	151116.73		
1672	Eukaryotic translation initiation factor 6	P56537	84033.64	83503.93	76491.40	61694.97	63530.67	58668.69	72403.49	73326.54	67354.39	76483.74	71729.32	69524.33	72738.65	71291.85	61651.44	60105.89	57013.73	54719.99	60847.29	57941.86	54830.39	240500.65	222393.34	205359.33	120437.20	118452.66	103305.05	149274.90	143320.94	134373.66	153553.51	143960.59	101693.57		
1673	EVI5-like protein	Q96CN4	66712.68	67613.59	63493.51	57722.68	54929.86	51772.69	62239.24	57040.85	61453.20	71133.11	67408.08	67509.24	67503.29	62894.69	53609.65	49339.61	46016.88	47379.66	57248.77	55656.51	54814.29	2.27	1.49	0.65	0.84	1.31	0.69	4.16	2.47	0.11	4.90	4.98	0.12	x	
1674	Exocyst complex component 1	Q9NV70	17255.58	16466.28	14746.83	10825.69	11045.58	9684.53	13177.27	11191.18	11321.00	15207.78	14080.14	12505.59	14282.50	11777.00	7062.05	11545.37	10682.54	10518.16	11710.84	11218.22	9078.25	2.27	1.49	0.65	0.84	1.31	0.69	4.16	2.47	0.11	4.90	4.98	0.12	x	
1675	Exocyst complex component 4	Q96A65	84064.17	79714.90	74531.82	57030.93	55924.52	53398.76	67785.74	66313.41	63292.15	69681.01	67297.64	63299.54	74123.60	62004.51	60735.88	63430.00	57549.59	58612.90	65906.05	63498.49	59449.67	10758.21	11164.72	9911.25	8717.84	8197.60	7822.14	9147.75	8266.89	8355.16	9279.88	9392.72	5577.93		
1676	Exocyst complex component 5	O00471	4029.30	4134.61	3837.60	2872.62	2935.51	2787.56	3459.81	3343.72	3299.28	3414.50	3254.22	3362.38	3605.76	3231.77	2766.79	2640.01	2678.86	2592.33	2856.00	2561.31	2135.32	2.27	1.49	0.65	0.84	1.31	0.69	4.16	2.47	0.11	4.90	4.98	0.12	x	
1677	Exocyst complex component 6B	Q9Y2D4	0.14	2.53	0.81	1.07	2.77	0.68	0.96	0.36	0.63	0.35	0.75	0.38	2.13	0.97	0.60	4.64	1.07	0.47	2.22	0.19	0.06	1315.85	1186.93	1038.43	1186.49	1325.17	896.85	2150.38	1827.92	1484.45	1360.93	1258.70	664.71		x
1678	Exocyst complex component 7	Q9UPT5	9329.43	8756.82	8666.00	6807.53	6287.77	6634.27	7207.90	6826.11	7004.12	6612.28	6431.28	6663.35	6964.15	6875.47	6023.94	7262.96	6778.92	6674.35	5492.87	6310.39	5518.88	2.27	1.49	0.65	0.84	1.31	0.69	4.16	2.47	0.11	4.90	4.98	0.12	x	
1679	Exocyst complex component 8	Q8IYI6	168976.56	161923.98	149209.70	80422.26	77518.46	75665.23	104938.66	99941.10	100104.87	121106.98	121186.58	115049.98	102002.61	97199.83	89789.07	131399.01	123659.90	129080.87	116486.66	114096.38	104933.89	5742.56	5672.68	5082.70	5121.28	5099.98	5020.30	4843.19	5041.78	5143.08	5623.72	5636.82	3323.43		
1680	Exonuclease 3'-5' domain-containing protein 2	Q9NVH0	2773.31	3122.91	2821.56	1962.12	2068.58	1940.57	2125.03	1904.78	1843.90	2424.30	2194.31	2059.21	1980.02	1952.75	1672.29	2124.34	1973.05	1952.92	1940.51	1898.96	1745.16	2.27	1.49	0.65	0.84	1.31	0.69	4.16	2.47	0.11	4.90	4.98	0.12	x	
1681	Exosome complex component CSL4	Q9Y3B2	1072.40	1052.18	948.23	814.60	772.21	738.49	1284.96	1208.66	1011.23	708.52	725.85	644.77	999.18	789.66	803.12	818.75	644.02	691.33	910.72	892.48	879.66	3276.08	2763.03	3132.29	1904.17	1943.71	1653.21	2442.23	2618.92	2332.88	2139.29	2111.81	1491.33		
1682	Exosome complex component MTR3	Q5RKV6	0.14	2.53	0.81	1.07	2.77	0.68	0.96	0.36	0.63	0.35	0.75	0.38	2.13	0.97	0.60	4.64	1.07	0.47	2.22	0.19	0.06	13007.81	12141.73	10833.53	9278.96	8972.25	8098.67	7854.57	8624.00	8442.16	11612.51	9989.10	431.78		x
1683	Exosome complex component RRP42	Q15024	47488.29	43796.85	37022.72	30699.15	28797.21	24668.77	38185.45	36234.82	32678.97	38938.87	37465.63	31406.41	39385.82	33207.35	19358.49	30669.16	29791.60	26258.39	33724.89	32508.39	27605.48	6098.57	4426.98	2450.90	3647.56	3426.41	2326.92	5342.48	4756.31	3784.63	5580.84	5171.69	2632.02		
1684	Exosome complex component RRP45	Q06265	0.14	2.53	0.81	1.07	2.77	0.68	0.96	0.36	0.63	0.35	0.75	0.38	2.13	0.97	0.60	4.64	1.07	0.47	2.22	0.19	0.06	2961.58	3080.39	2828.61	2097.58	2013.30	1858.60	2735.15	2424.58	2836.64	2550.46	2578.25	1774.13		x
1685	Exosome complex exonuclease RRP44	Q9Y2L1	10803.38	10699.63	9995.07	8240.98	7780.90	7474.18	8140.60	7581.84	7666.26	10990.82	11077.50	11226.46	10942.42	9538.11	7300.99	8344.94	7697.13	7632.09	9641.11	8617.12	8418.78	6343.50	5768.46	5344.62	4084.73	4123.05	3437.21	5164.36	5278.42	4676.30	5141.80	4965.21	2477.96		x
1686	Exosome component 10	Q01780	42792.06	38232.99	36494.41	29623.51	28489.07	22913.01	36311.16	37267.38	32631.21	38662.54	37057.29	32497.30	34302.41	31254.49	17906.81	33373.02	29892.98	27866.69	30061.84	28873.66	24354.75	54757.90	51462.22	45396.73	33633.98	31568.39	25023.57	41631.72	37262.88	33745.34	34839.12	31281.86	17294.04		
1687	Exosome RNA helicase MTR4	P42285	73156.28	68828.37	65300.44	46336.66	43896.28	41802.72	50434.61	49587.59	48492.88	57629.19	54046.55	49773.46	52581.38	50808.28	40904.78	45567.28	40484.86	42205.53	48776.11	43097.02	42876.32	195100.59	186706.49	164926.39	148196.36	144943.98	137030.56	154534.71	151159.92	148801.07	190164.28	191170.85	128626.98		
1688	Exostosin-like 3	O43909	6686.50	6506.82	6225.15	4510.96	4492.83	4175.54	4796.98	4550.84	4496.46	5384.24	5733.93	5587.62	6510.05	5927.08	5594.87	3484.99	3775.80	3646.38	4819.22	5218.83	5234.91	2.27	1.49	0.65	0.84	1.31	0.69	4.16	2.47	0.11	4.90	4.98	0.12	x	
1689	Exportin-1	O14980	242240.26	232659.31	213972.91	152858.46	151719.73	139049.31	183180.67	174023.87	165125.14	191678.81	177969.00	165722.27	200307.92	188098.03	148349.65	148508.67	136117.37	135661.91	156275.12	153927.05	136121.76	284874.81	266374.63	244162.94	180810.73	173482.25	144417.54	235375.92	224366.47	207330.78	221510.99	214310.20	141332.10		
1690	Exportin-2	P55060	236767.19	226024.78	210681.20	173752.97	163258.22	158972.85	185683.40	180981.92	173927.09	199812.86	187904.21	180329.42	192361.07	185206.30	156157.50	162148.51	146161.94	150916.86	161863.12	153320.13	146947.56	574972.28	553857.63	512367.61	377024.08	367120.95	337297.13	439102.27	428539.21	415307.74	432301.63	419008.64	304212.47		
1691	Exportin-5	Q9HAV4	108056.66	105896.63	99229.62	82775.37	79670.25	69336.27	93431.33	92410.24	86017.62	93017.30	88273.70	81893.92	99205.86	85119.85	81721.02	70089.44	70468.71	67418.82	87066.13	84635.42	78724.57	5157.11	5034.41	4491.07	3887.15	3226.66	2543.83	4465.00	4033.51	3730.96	5896.06	5675.28	3521.51		
1692	Exportin-7	Q9UIA9	82868.64	80003.02	75349.28	53819.14	53199.47	49730.60	72322.84	63529.74	57986.37	73293.37	67186.55	61365.90	71619.70	66431.00	51016.66	49847.58	48454.05	46344.75	52543.82	52031.20	48660.72	2.27	1.49	0.65	0.84	1.31	0.69	4.16	2.47	0.11	4.90	4.98	0.12	x	
1693	Exportin-T	O43592	90263.32	90552.90	81095.07	62366.10	60335.23	55656.58	73093.19	73521.56	68751.56	71392.20	69506.47	63490.06	76613.99	70860.89	55562.81	54619.46	54710.36	52185.59	62210.82	60483.88	52764.29	967.00	967.67	770.17	243.18	240.80	168.55	591.00	501.05	491.04	416.10	312.51	134.63		
1694	Extended synaptotagmin-1	Q9BSJ8	202259.17	194828.16	182076.87	131186.23	128560.25	115015.57	163165.21	162119.53	147249.17	177815.07	167861.52	154016.98	164395.25	164074.64	127622.12	142944.09	135024.47	123366.35	139810.75	132222.76	121568.52	124744.46	119601.31	107540.72	77487.90	73156.80	61408.21	98361.89	93369.88	87071.07	101506.19	97187.47	61223.72		
1695	Extended synaptotagmin-2	A0FGR8	92865.49	90343.65	73983.07	55821.20	51914.10	46275.29	63123.52	60729.44	55363.18	72922.04	69151.00	62606.38	69526.56	65103.81	44908.97	57223.05	54063.07	48527.87	54766.55	50163.87	46508.90	7498.14	6774.01	5612.99	4079.56	4307.36	3100.73	6135.85	6076.62	5643.21	6297.13	6450.67	3161.94		
1696	Ezrin	P15311	510866.01	486432.73	471490.80	318327.50	301373.40	276948.21	350059.91	337308.23	305280.86	385827.65	366260.04	346398.48	414513.71	378233.35	311710.44	309884.07	299704.49	279276.63	320564.46	306902.72	287977.24	1451226.77	1371350.66	1268827.35	984914.75	953442.69	881003.70	1146537.34	1092767.32	1069062.49	1111097.12	1079075.62	784151.14		
1697	FACT complex subunit SPT16	Q9Y5B9	464462.14	449710.52	413249.32	326338.50	315518.88	298156.39	413569.68	387600.61	383064.97	403567.16	383118.16	367408.47	401091.65	374041.82	307294.21	316397.34	299726.66	286652.91	326385.95	313731.02	299844.68	389303.37	370628.37	341071.83	264897.93	256811.87	224229.64	330373.73	310990.81	294911.71	300491.93	291876.03	198371.55		
1698	FACT complex subunit SSRP1	Q08945	308112.71	290498.49	266790.17	190882.25	184780.00	166440.24	232068.53	217466.15	204206.79	239302.55	229852.10	210364.20	253194.06	230748.44	179019.96	184846.80	172492.04	170655.35	206669.48	190620.40	177350.42	181076.14	163531.86	159203.67	110120.22	106926.77	93506.90	133261.66	128554.58	122705.69	117409.83	112638.74	73416.62		
1699	F-actin-capping protein subunit alpha-1	P52907	173115.62	160245.66	156407.41	100431.32	95839.22	88508.46	126735.70	117533.97	115913.11	134067.54	127114.46	118467.10	133795.78	123274.12	96373.00	108064.01	101022.47	99788.39	104522.39	102894.24	95709.28	145568.50	136967.81	122597.25	91174.02	88408.63	75894.88	103994.04	96517.45	92762.96	109592.86	108592.76	72519.52		
1700	F-actin-capping protein subunit alpha-2	P47755	10916.86	10858.09	67637.52	10789.95	11599.81	10187.03	18755.79	18314.78	17408.65	63038.55	63017.48	67853.11	9827.57	32199.60	8991.80	10678.71	10868.96	9667.92	8881.57	8276.54	8320.39	24097.45	22881.79	18865.58	12020.20	10689.62	9783.19	14497.32	13202.64	12064.45	15031.39	13491.22	8868.85		
1701	F-actin-capping protein subunit beta	P47756	271491.82	260206.47	239847.15	177036.03	172583.76	159860.71	187880.81	178532.45	169387.36	228175.46	218134.06	208082.97	221084.03	214571.11	172418.98	171978.38	164835.08	156020.47	175232.01	168907.48	154616.85	345354.32	314422.59	293135.17	216192.05	209294.36	189384.59	281051.81	260688.17	252934.22	306068.38	289204.05	198169.46		
1702	FAD synthase	Q8NFF5	74224.79	75826.17	67917.30	62755.39	62050.56	58133.85	66181.82	62715.45	59945.99	73923.39	70178.43	68684.38	63267.11	59964.56	52541.48	67997.90	63995.60	60399.88	51099.65	50926.02	46578.50	2.27	1.49	0.65	0.84	1.31	0.69	4.16	2.47	0.11	4.90	4.98	0.12	x	
1703	FAD-linked sulfhydryl oxidase ALR	P55789	30231.06	28050.06	25754.83	25591.82	23783.55	23841.34	18113.52	17945.88	17836.43	28774.22	25156.23	23866.12	26404.77	25807.68	23748.10	20958.10	16778.40	18820.03	23164.33	21744.98	22715.99	2.27	1.49	0.65	0.84	1.31	0.69	4.16	2.47	0.11	4.90	4.98	0.12	x	
1704	Fanconi anemia group A protein	O15360	0.14	2.53	0.81	1.07	2.77	0.68	0.96	0.36	0.63	0.35	0.75	0.38	2.13	0.97	0.60	4.64	1.07	0.47	2.22	0.19	0.06	2696.28	2081.12	1957.59	1464.45	1475.88	961.08	2797.06	2701.63	1717.55	2834.35	2556.06	1154.57		x
1705	Fanconi anemia group C protein	Q00597	430568.91	387618.35	346722.86	247016.93	236501.45	206549.44	295192.58	281069.00	255911.68	333063.35	310812.74	270518.66	301381.15	268120.24	186044.55	296234.99	272579.67	254520.05	239997.23	225055.37	196782.43	1596.60	1535.64	1252.77	1324.70	1462.64	1318.69	1436.11	1266.48	1607.82	1888.47	2536.72	1960.05		
1706	Fanconi anemia group I protein	Q9NVI1	50890.64	46673.39	43851.15	43653.73	40060.04	39555.34	39545.71	37852.18	35059.34	49934.85	48328.24	46956.33	45358.20	44109.22	40177.64	40325.07	35527.82	38290.47	42169.89	35825.93	35712.89	172.76	203.28	199.72	191.81	179.65	197.02	158.34	152.51	147.14	154.74	151.20	15.55		
1707	Fanconi anemia group M protein	Q8IYD8	103676.75	99267.44	90886.40	68582.72	67801.46	62749.83	75574.12	74419.86	70207.32	85232.13	83168.57	78198.99	78237.58	75208.71	63835.47	67502.02	65423.28	63158.86	72323.99	70431.74	64070.24	2098.51	2007.53	1956.59	1200.58	1165.22	947.17	1691.55	1485.64	1845.65	1758.59	1703.98	1220.61		
1708	Far upstream element-binding protein 1	Q96AE4	439547.82	420776.54	397629.39	289220.79	277335.12	264427.49	331982.40	328202.30	307024.48	354113.63	332366.29	320477.81	343424.90	324631.75	282949.67	283136.69	264619.02	254643.77	302983.80	293961.48	280996.27	440082.52	411572.43	393190.56	280761.56	276319.50	260015.30	318581.15	304596.73	290965.28	329088.59	308675.72	215890.36		
1709	Far upstream element-binding protein 2	Q92945	249818.73	239206.78	229831.13	170871.10	162469.90	155959.68	185519.45	181236.45	171797.60	199699.11	194154.13	185775.80	199266.35	192364.17	168658.13	160191.71	152440.51	149599.41	172640.82	162576.95	156710.64	82686.93	77268.21	73139.09	52614.29	51132.19	47505.87	72504.72	70065.03	65256.00	69509.41	70048.12	54404.61		
1710	Far upstream element-binding protein 3	Q96I24	163731.73	158635.74	147670.49	116435.84	110079.61	103252.78	123250.36	120525.98	116056.76	136614.71	126417.68	125978.49	139729.07	131784.70	111202.20	102440.22	102748.28	99326.36	113765.70	106092.17	101874.82	57124.25	52487.77	47000.99	40970.62	38888.99	36930.74	42694.53	43894.09	39790.76	44621.64	44073.16	32805.43		
1711	Farnesyl pyrophosphate synthase	P14324	491173.02	478125.71	459710.05	391796.73	384604.55	361328.11	449320.53	433430.99	413206.39	435047.59	421918.77	398705.66	424191.32	398963.38	373096.15	376423.77	361526.18	336721.44	381969.37	369356.93	347977.22	327784.29	317088.46	291989.10	221036.45	212808.48	192672.74	248795.15	238579.71	232998.53	241482.54	241142.91	163784.84		
1712	FAS-associated death domain protein	Q13158	12804.76	11380.84	7015.91	340.46	198.45	24.14	217.49	242.28	129.60	6797.91	5712.58	4024.92	2.13	0.97	0.60	269.53	160.25	79.13	402.24	687.81	300.31	34143.59	19253.03	14921.54	13487.64	12183.16	9593.24	28767.18	26699.89	18543.47	24337.15	21899.41	10434.01		
1713	FAS-associated factor 1	Q9UNN5	0.14	2.53	0.81	1.07	2.77	0.68	0.96	0.36	0.63	0.35	0.75	0.38	2.13	0.97	0.60	4.64	1.07	0.47	2.22	0.19	0.06	50800.95	46601.83	36874.21	29332.24	29148.11	23202.31	37776.57	33837.03	30387.13	41105.36	39161.81	21883.83		x
1714	FAS-associated factor 2	Q96CS3	160810.02	145579.89	127523.58	87794.38	88354.02	75175.06	106059.27	101097.69	91988.45	119152.04	112933.56	102451.80	113511.20	101832.14	72572.91	99309.31	88335.53	84193.71	91435.52	90555.75	84314.65	79196.01	70431.45	64312.50	46754.45	43323.13	40050.85	63179.19	60606.43	55426.15	62862.74	58611.90	35545.26		
1715	Fascin	Q16658	36376.22	33048.08	30398.38	23206.08	22294.69	20266.72	28998.91	27133.34	25260.19	31697.97	29681.02	25951.03	27569.16	22243.09	25844.72	21972.15	22109.54	20553.24	27380.84	23101.32	26494.09	239986.06	218834.07	198250.13	108945.97	107873.40	100382.27	121829.65	119892.63	112074.79	134375.63	128602.24	103805.91		
1716	Fat storage-inducing transmembrane protein 2	Q8N6M3	26965.50	25077.00	25201.20	16600.86	15147.42	15523.15	18008.42	17899.14	17361.17	21946.00	21254.47	19552.48	20136.56	19162.77	15628.26	18557.12	16409.11	16938.51	16119.67	15806.09	14698.90	2.27	1.49	0.65	0.84	1.31	0.69	4.16	2.47	0.11	4.90	4.98	0.12	x	
1717	Fatty acid synthase	P49327	4927479.10	4768773.49	4430224.36	3390948.96	3276376.43	3048910.24	3709596.87	3538427.39	3426536.50	3561134.90	3387606.67	3185045.71	3738808.94	3505115.21	2815425.09	3750610.09	3584432.34	3465081.55	3062445.31	2918537.66	2719882.40	1060333.98	1018105.33	935595.38	686992.09	673377.47	581507.45	807375.26	769650.09	727777.75	883392.26	842564.23	577239.96		
1718	Fatty acid-binding protein 5	Q01469	39134.34	38271.87	37326.65	35683.84	34198.23	32491.78	47224.49	44079.51	42559.00	40491.86	38099.09	38608.84	37780.69	37987.61	36735.95	31695.69	32307.12	33377.66	38765.51	36648.55	34341.94	429985.30	393614.64	379318.06	190354.37	180775.52	165883.17	186273.36	176605.22	178029.82	182220.30	176285.82	114338.11		
1719	Fatty acyl-CoA reductase 1	Q8WVX9	25390.02	24342.84	23657.09	17175.21	16752.95	16406.72	18409.66	19297.72	17024.49	23621.05	21890.23	21631.48	19064.66	18361.76	15163.01	18881.01	18523.14	18468.31	15453.94	15723.59	14815.88	2439.22	2775.51	2199.57	3593.76	3584.75	3354.14	3281.56	2935.94	2678.72	3201.23	3375.59	2564.14		
1720	Fatty aldehyde dehydrogenase	P51648	156166.76	147142.54	138970.81	111770.85	111198.05	105048.56	105521.73	101698.45	97188.90	135324.18	128444.26	124485.09	127680.80	121216.28	98777.77	106749.56	100531.98	102836.61	105004.61	97108.46	91268.62	49742.83	48056.61	42710.76	28660.24	28036.24	25271.07	30883.28	30385.27	29256.33	32301.02	30921.95	22236.59		
1721	Fatty-acid amide hydrolase 1	O00519	2615.27	2015.25	2564.41	2176.44	2169.77	2076.13	2069.02	2933.27	2641.27	2211.07	2005.37	1843.88	2517.59	1610.12	1285.66	1761.79	2641.50	2152.82	2032.88	1905.18	2647.13	2.27	1.49	0.65	0.84	1.31	0.69	4.16	2.47	0.11	4.90	4.98	0.12	x	
1722	F-box only protein 15	Q8NCQ5	0.14	2.53	0.81	1.07	2.77	0.68	0.96	0.36	0.63	0.35	0.75	0.38	2.13	0.97	0.60	4.64	1.07	0.47	2.22	0.19	0.06	2438.60	3128.11	1174.17	1246.83	1250.14	1825.15	1099.73	1037.63	1324.21	1621.82	1086.14	2269.56		x
1723	F-box only protein 22	Q8NEZ5	20445.24	20939.31	17947.39	13466.24	12875.64	11309.91	16197.47	15881.33	15145.74	19107.89	19274.89	17433.38	16397.84	15632.99	10157.96	15773.16	15121.00	14183.88	14322.29	15055.18	14688.12	2.27	1.49	0.65	0.84	1.31	0.69	4.16	2.47	0.11	4.90	4.98	0.12	x	
1724	F-box only protein 43	Q4G163	28332.43	27879.58	27965.95	18110.32	17635.32	18082.97	20664.23	18614.47	17750.80	25381.91	25924.36	23798.99	22555.10	20640.91	17315.02	18375.21	18359.94	16524.69	15503.08	17474.76	15814.84	7631.61	6624.35	5771.47	5928.08	6327.34	4549.25	5637.09	5619.59	5274.67	9795.36	8231.03	7752.77		
1725	F-box only protein 48	Q5FWF7	7073.93	6927.05	6427.16	4896.29	4882.85	4418.58	5830.74	5901.83	5685.83	6532.44	6568.00	5967.86	6730.05	6335.83	4762.76	4788.53	4678.46	4218.81	5196.86	4993.35	4380.01	2.27	1.49	0.65	0.84	1.31	0.69	4.16	2.47	0.11	4.90	4.98	0.12	x	
1726	F-box/WD repeat-containing protein 7	Q969H0	22044.65	22335.02	21898.72	15365.67	14114.17	14504.38	16942.38	16487.32	16260.22	16596.93	17015.73	16341.21	17478.51	17356.71	15459.20	15439.80	14626.42	13599.08	14357.13	14277.50	14532.91	2.27	1.49	0.65	0.84	1.31	0.69	4.16	2.47	0.11	4.90	4.98	0.12	x	
1727	F-box-like/WD repeat-containing protein TBL1XR1	Q9BZK7	0.14	2.53	0.81	1.07	2.77	0.68	0.96	0.36	0.63	0.35	0.75	0.38	2.13	0.97	0.60	4.64	1.07	0.47	2.22	0.19	0.06	7.47	6.74	15.81	62.75	42.59	15.61	601.22	508.88	473.02	318.90	314.85	115.52		x
1728	Fer-1-like protein 5	A0AVI2	0.14	2.53	0.81	1.07	2.77	0.68	0.96	0.36	0.63	0.35	0.75	0.38	2.13	0.97	0.60	4.64	1.07	0.47	2.22	0.19	0.06	10453.62	10272.37	9883.58	7393.94	8274.16	7747.44	8555.12	8630.36	8332.61	8596.90	8127.96	7310.97		x
1729	Fer-1-like protein 6	Q2WGJ9	4030.04	3662.98	3596.89	2998.97	2756.40	2278.05	3817.51	3813.72	3689.56	3536.66	3328.69	3016.91	3319.14	3087.87	1705.68	2669.22	2608.31	1751.79	3762.53	2743.46	2490.29	821.54	36.80	9.72	497.09	449.65	202.55	535.53	524.99	188.77	696.11	537.99	222.68		
1730	FERM and PDZ domain-containing protein 2	Q68DX3	31520.96	31554.87	30003.91	20370.61	19361.04	18216.91	23169.81	22016.90	20492.74	23680.14	23823.44	22190.42	24781.66	23492.58	20699.06	20976.33	19336.71	19760.64	19845.63	18786.67	18727.36	7887.40	8355.21	6890.35	6252.26	6392.51	5659.27	6402.62	6442.49	5889.40	7782.70	7346.59	7245.88		
1731	FERM domain-containing protein 4B	Q9Y2L6	0.14	2.53	0.81	1.07	2.77	0.68	0.96	0.36	0.63	0.35	0.75	0.38	2.13	0.97	0.60	4.64	1.07	0.47	2.22	0.19	0.06	12922.96	12361.54	12265.54	9711.59	8802.54	6833.37	13117.45	11287.86	10270.93	13556.63	12819.08	8254.88		x
1732	FERM domain-containing protein 8	Q9BZ67	3237.69	3384.55	3501.88	2987.68	2728.88	2710.93	2572.98	2413.81	2427.50	4165.70	3761.97	3235.34	2876.50	3147.10	2745.46	2834.02	2657.91	2754.75	2326.15	2293.16	2283.62	2.27	1.49	0.65	0.84	1.31	0.69	4.16	2.47	0.11	4.90	4.98	0.12	x	
1733	FERM_ ARHGEF and pleckstrin domain-containing protein 1	Q9Y4F1	654.65	667.42	570.88	541.58	712.31	548.17	536.16	522.13	460.13	1265.69	1236.44	1286.34	674.26	361.78	60.76	497.71	581.07	486.19	55.20	558.95	610.31	3627.42	3403.51	3525.91	2670.22	2379.89	1904.31	2986.55	2883.73	2438.95	3033.54	2628.08	2147.15		
1734	FERM_ ARHGEF and pleckstrin domain-containing protein 2	O94887	11549.12	9852.68	9400.27	7010.44	6585.46	5911.61	7323.76	7043.77	6548.79	9691.31	9243.08	8401.28	8295.26	8499.66	5846.55	7998.90	7206.18	7114.67	7415.22	7313.71	6971.12	27035.35	22690.55	21020.34	15091.83	14570.22	13072.77	18127.27	17409.55	14815.09	16126.08	17192.17	17883.38		
1735	Fermitin family homolog 2	Q96AC1	24069.00	21850.65	18047.83	15557.89	13548.57	11228.59	17220.78	15523.10	13421.34	16233.05	14457.88	15433.90	18075.13	13762.37	8718.62	14012.54	12283.58	13007.03	14315.77	13472.26	13582.04	48187.34	41253.89	31860.12	26538.41	24893.96	13631.70	33938.17	27617.28	18648.71	42551.24	38265.53	18251.70		
1736	Fermitin family homolog 3	Q86UX7	18545.59	19519.22	16658.05	24395.18	25619.22	15284.44	17286.67	24898.38	24134.47	16493.81	15324.40	15461.94	24096.98	6955.64	7386.98	23594.07	22791.07	19041.10	27638.31	24660.89	17834.84	19719.47	17904.95	15358.54	11142.44	10338.32	9767.32	11096.57	10687.05	10744.54	13873.58	11038.48	8369.63		
1737	Ferritin heavy chain	P02794	60435.30	61408.69	57873.95	54308.95	50125.06	46993.65	49746.75	45377.86	38068.11	54271.11	53464.18	50632.77	52990.90	47505.91	32576.16	44293.58	42934.03	42751.82	43811.85	46216.95	43276.87	10037.55	8276.03	8954.76	11424.57	9886.22	8328.64	11596.50	9737.30	8709.38	11136.74	10299.17	6754.39		
1738	Ferrochelatase mitochondrial	P22830	0.14	2.53	0.81	1.07	2.77	0.68	0.96	0.36	0.63	0.35	0.75	0.38	2.13	0.97	0.60	4.64	1.07	0.47	2.22	0.19	0.06	4572.16	3908.55	4197.93	4068.44	3786.06	2895.38	4959.11	4786.29	4348.68	6304.59	5814.39	3514.21		x
1739	FGFR1 oncogene partner	O95684	0.14	2.53	0.81	1.07	2.77	0.68	0.96	0.36	0.63	0.35	0.75	0.38	2.13	0.97	0.60	4.64	1.07	0.47	2.22	0.19	0.06	974.77	1016.16	706.24	791.42	697.25	479.16	921.22	783.25	652.20	1242.56	1266.65	588.00		x
1740	Fibrinogen beta chain	P02675	50076.73	48721.19	41802.85	35644.28	33220.69	27991.73	36777.11	34735.49	32032.26	47687.84	45611.02	38948.90	39448.50	33105.40	19795.14	37121.53	36178.21	32902.05	35584.94	33108.23	27458.11	35186.98	33502.29	31850.94	24535.10	23432.63	21400.23	25935.41	25781.44	24965.96	26551.80	25780.12	16659.71		
1741	Fibrinogen C domain-containing protein 1	Q8N539	69695.04	67049.46	65823.66	61505.93	58851.13	56495.48	66981.41	66793.86	66207.55	72443.18	66116.15	65286.11	59255.67	61932.04	54124.15	58623.91	54740.67	54622.61	50622.90	47531.55	48414.10	68350.12	65903.82	64310.63	42241.24	40722.36	37944.07	43858.40	40856.40	40200.34	34074.20	32398.75	26313.11		
1742	Fibrinogen gamma chain	P02679	0.14	2.53	0.81	1.07	2.77	0.68	0.96	0.36	0.63	0.35	0.75	0.38	2.13	0.97	0.60	4.64	1.07	0.47	2.22	0.19	0.06	7608.71	6765.76	6393.37	4601.57	3772.91	2054.56	7054.81	6177.76	4989.43	6584.42	6960.19	2955.20		x
1743	Fibroblast growth factor receptor 2	P21802	0.14	2.53	0.81	1.07	2.77	0.68	0.96	0.36	0.63	0.35	0.75	0.38	2.13	0.97	0.60	4.64	1.07	0.47	2.22	0.19	0.06	5929.08	5318.31	5062.91	4456.52	4034.74	3640.10	4856.73	4301.67	4328.28	5177.70	5110.92	3871.42		x
1744	Fibroblast growth factor receptor 4	P22455	0.14	2.53	0.81	1.07	2.77	0.68	0.96	0.36	0.63	0.35	0.75	0.38	2.13	0.97	0.60	4.64	1.07	0.47	2.22	0.19	0.06	2171.51	1915.63	1954.03	1447.43	1390.67	914.66	1810.66	1705.96	1710.43	2257.98	2035.36	1114.19		x
1745	Fibrocystin	P08F94	0.14	2.53	0.81	1.07	2.77	0.68	0.96	0.36	0.63	0.35	0.75	0.38	2.13	0.97	0.60	4.64	1.07	0.47	2.22	0.19	0.06	1044.02	977.15	857.73	654.51	639.74	407.31	1037.78	951.46	722.68	916.80	966.89	493.88		x
1746	Fibrocystin-L	Q86WI1	25809.87	24644.58	21604.97	18408.51	17672.22	15762.84	19552.77	19167.85	18837.79	23462.80	22799.78	22548.12	22106.37	20687.08	15254.52	19115.55	18692.29	17777.88	18453.28	18440.82	18430.64	2.27	1.49	0.65	0.84	1.31	0.69	4.16	2.47	0.11	4.90	4.98	0.12	x	
1747	Fibronectin type III and SPRY domain-containing protein 1	Q9BTV5	1288.73	1385.86	1175.07	1113.27	1121.43	1062.05	1315.93	1319.21	1353.58	1731.03	1559.24	1651.13	1600.00	1611.88	1195.96	1021.19	892.73	857.52	1036.20	972.13	882.18	2.27	1.49	0.65	0.84	1.31	0.69	4.16	2.47	0.11	4.90	4.98	0.12	x	
1748	Fibronectin type III and SPRY domain-containing protein 2	A1L4K1	15622.40	15657.13	14383.29	9612.90	8487.01	8484.40	10915.79	10180.38	9355.33	12681.33	12073.76	11534.66	12414.51	11162.15	9122.96	8680.45	8924.86	8077.77	10206.12	11074.54	9954.82	10313.78	11760.25	14223.81	15438.73	14325.80	11198.61	17992.12	16734.91	15146.94	22472.07	20211.77	13856.22		
1749	Fibronectin type III domain-containing protein 5	Q8NAU1	222.19	250.99	221.53	337.62	347.18	298.30	205.26	244.23	154.77	518.67	461.71	506.72	395.08	413.09	315.28	287.05	256.20	198.71	288.11	303.48	283.88	2.27	1.49	0.65	0.84	1.31	0.69	4.16	2.47	0.11	4.90	4.98	0.12	x	
1750	Fibrosin-1-like protein	Q9HCM7	21871.04	23302.68	21638.26	16736.67	16496.22	16191.39	16787.49	17457.08	16346.21	20020.02	19659.37	17957.64	19787.52	19167.81	16179.97	14539.32	14310.65	14725.04	15439.60	15931.88	16181.53	4857.29	4665.52	4016.74	2464.02	2595.91	2268.16	3629.66	3386.70	3141.49	3599.75	3569.28	2638.91		
1751	Filaggrin	P20930	0.14	2.53	0.81	1.07	2.77	0.68	0.96	0.36	0.63	0.35	0.75	0.38	2.13	0.97	0.60	4.64	1.07	0.47	2.22	0.19	0.06	74.27	74.00	72.74	120.50	85.55	84.19	69.72	53.77	82.32	42.83	46.51	17.96		x
1752	Filamin A-interacting protein 1-like	Q4L180	0.14	2.53	0.81	1.07	2.77	0.68	0.96	0.36	0.63	0.35	0.75	0.38	2.13	0.97	0.60	4.64	1.07	0.47	2.22	0.19	0.06	13264.75	12842.58	12064.43	8001.04	7483.27	6980.96	8079.32	7270.50	7079.30	7321.67	7108.25	4957.67		x
1753	Filamin-A	P21333	2277561.76	2192682.20	2038206.75	1898777.82	1795888.74	1696774.76	2052089.02	2002645.43	1895959.50	2379729.19	2301990.96	2169137.93	1956562.69	1841474.91	1563065.34	1879546.80	1733645.60	1673457.10	1761296.13	1696215.70	1589750.71	2463921.31	2346749.49	2170195.79	1486263.95	1463067.11	1314428.09	1811155.27	1733383.77	1651411.18	1828042.13	1782191.05	1252422.61		
1754	Filamin-A-interacting protein 1	Q7Z7B0	6379.26	5720.86	4188.37	2979.23	3040.53	2322.02	4003.84	3825.38	3363.31	5059.64	4863.51	4376.77	4458.10	4464.47	1379.91	4590.74	4310.88	3497.57	3012.51	2547.03	2126.38	22558.34	18595.54	17556.04	15039.58	14861.14	13188.28	19003.77	18159.59	17369.91	17279.63	16517.57	12270.50		
1755	Filamin-B	O75369	496438.49	483468.47	461419.15	367352.34	354161.44	341241.46	424344.07	415467.37	393523.72	481428.75	461502.14	443279.65	423274.64	407916.54	340325.87	370206.30	350432.12	344363.75	367069.99	359913.86	336699.74	1520425.05	1434850.26	1321307.85	989822.81	970442.48	872943.31	1167912.04	1112119.11	1074525.91	1158077.41	1128983.30	793362.24		
1756	Filamin-binding LIM protein 1	Q8WUP2	0.14	2.53	0.81	1.07	2.77	0.68	0.96	0.36	0.63	0.35	0.75	0.38	2.13	0.97	0.60	4.64	1.07	0.47	2.22	0.19	0.06	81714.22	79482.83	75577.56	60380.37	58920.11	56386.07	67271.59	66077.18	64193.35	71469.74	67160.67	49740.01		x

Supplementary Table S1. Overview on the abundance of all relatively quantified 5180 proteins

			MCF-7																					MDA-MB-231													
	Protein name	UniProt	control 01	control 02	control 03	Dai IC20 01	Dai IC20 02	Dai IC20 03	Dai SC20 01	Dai SC20 02	Dai SC20 03	Gen IC20 01	Gen IC20 02	Gen IC20 03	Gen SC20 01	Gen SC20 02	Gen SC20 03	SSE IC20 01	SSE IC20 02	SSE IC20 03	SSE SC20 01	SSE SC20 02	SSE SC20 03	control 01	control 02	control 03	Dai IC20 01	Dai IC20 02	Dai IC20 03	Gen IC20 01	Gen IC20 02	Gen IC20 03	SSE IC20 01	SSE IC20 02	SSE IC20 03	unique to MCF-7	unique to MDA-MB-231
1757	Filamin-C	Q14315	0.14	2.53	0.81	1.07	2.77	0.68	0.96	0.36	0.63	0.35	0.75	0.38	2.13	0.97	0.60	4.64	1.07	0.47	2.22	0.19	0.06	58410.55	54890.83	47153.20	43543.54	41468.84	34660.62	53528.70	50726.67	48078.14	53426.00	49992.69	27911.57		x
1758	FK506-binding protein 15	Q5T1M5	85283.78	80227.32	84640.87	58784.36	57676.75	55310.00	70542.80	63897.65	68192.40	75799.22	70797.13	69615.38	78961.09	73501.41	57939.05	60215.42	52934.10	52592.43	62291.12	61916.24	58736.87	2.27	1.49	0.65	0.84	1.31	0.69	4.16	2.47	0.11	4.90	4.98	0.12	x	
1759	Flap endonuclease 1	P39748	346475.40	309740.67	288651.00	219564.80	199760.21	190051.18	246999.67	224044.99	220877.96	237556.37	226650.35	209321.17	267653.51	242627.66	181085.43	197751.65	180546.98	180934.87	228156.27	215287.58	195959.34	126391.86	120808.93	108384.26	78802.44	81133.57	63607.97	95366.00	92092.04	83295.74	106953.06	104877.14	64946.53		
1760	Flavin reductase (NADPH)	P30043	184715.13	175911.56	161690.31	124829.66	130525.98	122448.11	181408.40	165991.52	158456.54	152862.29	141627.77	136622.08	163034.59	152634.95	126781.33	135430.17	132396.52	123096.72	112587.88	112717.04	108161.57	77882.41	72972.26	70131.28	40611.40	37921.74	34582.30	44054.67	41309.74	41462.63	40818.55	38442.56	27217.00		
1761	Flotillin-1	O75955	0.14	2.53	0.81	1.07	2.77	0.68	0.96	0.36	0.63	0.35	0.75	0.38	2.13	0.97	0.60	4.64	1.07	0.47	2.22	0.19	0.06	63729.11	60045.60	57484.26	19197.68	18744.87	14131.11	20412.91	18858.60	16817.14	18912.57	18346.18	10680.31		x
1762	FLYWCH family member 2	Q96CP2	55738.02	54688.65	51210.67	33984.11	33753.22	32357.93	37959.59	36221.51	34157.90	39968.14	38767.20	37845.49	43449.64	42649.17	38363.55	31957.75	30777.56	29691.75	35929.54	35155.22	33528.56	2973.32	2929.60	2566.09	2211.16	2246.53	2106.52	2760.10	2450.90	2409.60	2695.52	2510.76	1673.79		
1763	FLYWCH-type zinc finger-containing protein 1	Q4VC44	2987.96	3064.97	2563.40	2785.24	2751.03	2548.48	1626.57	1452.48	1620.20	2854.96	2657.86	2452.39	3114.09	2842.41	2298.36	2031.83	1706.98	1823.97	2767.16	2491.09	2405.10	2.27	1.49	0.65	0.84	1.31	0.69	4.16	2.47	0.11	4.90	4.98	0.12	x	
1764	Folate receptor beta	P14207	1070.02	894.41	789.48	700.84	796.13	594.98	935.17	915.55	890.49	825.97	1153.99	1276.96	1412.11	1081.37	788.88	791.77	600.22	574.60	923.00	933.94	840.36	2.27	1.49	0.65	0.84	1.31	0.69	4.16	2.47	0.11	4.90	4.98	0.12	x	
1765	Folliculin-interacting protein 1	Q8TF40	5665.66	5489.63	5504.78	4095.03	4284.13	4131.12	4867.32	4998.00	4998.56	5477.66	5292.14	5113.60	5290.06	5462.14	4376.37	3793.38	3839.31	3905.20	4604.55	4242.00	4264.16	2.27	1.49	0.65	0.84	1.31	0.69	4.16	2.47	0.11	4.90	4.98	0.12	x	
1766	Folliculin-interacting protein 2	Q9P278	286.51	178.98	310.07	211.15	201.53	301.38	249.83	259.78	293.84	510.95	460.56	348.88	298.68	327.64	254.31	251.88	203.99	230.06	349.56	275.52	321.57	2.27	1.49	0.65	0.84	1.31	0.69	4.16	2.47	0.11	4.90	4.98	0.12	x	
1767	Follistatin	P19883	22819.90	22151.54	20461.92	14997.56	14621.05	14911.72	15319.54	14655.38	14479.94	17664.18	16929.64	15773.53	15112.88	14514.79	13805.45	15660.50	13850.55	12667.49	13850.14	11953.50	12217.97	420.99	431.33	342.67	268.19	265.32	247.24	287.52	306.53	262.66	4.90	4.98	0.12		
1768	Follistatin-related protein 1	Q12841	0.14	2.53	0.81	1.07	2.77	0.68	0.96	0.36	0.63	0.35	0.75	0.38	2.13	0.97	0.60	4.64	1.07	0.47	2.22	0.19	0.06	6915.59	6510.79	6118.68	3482.43	2946.72	2764.07	3578.78	3806.43	3739.24	3393.93	3326.42	1739.19		x
1769	Follitropin subunit beta	P01225	34677.48	29567.60	29234.06	22530.11	21337.24	21074.79	25095.94	26967.76	22780.29	26401.58	26306.18	21579.46	26211.43	28497.01	23428.60	21379.34	19913.26	20044.39	18813.24	21064.56	19478.21	88.30	1617.35	1176.90	10.69	2.62	2.49	77.04	66.54	7.55	55.64	9.95	2.75		
1770	Folylpolyglutamate synthase_ mitochondrial	Q05932	0.14	2.53	0.81	1.07	2.77	0.68	0.96	0.36	0.63	0.35	0.75	0.38	2.13	0.97	0.60	4.64	1.07	0.47	2.22	0.19	0.06	23168.56	22363.93	20191.65	14846.04	14121.81	13618.18	20007.29	19164.27	18480.39	18131.24	17945.83	12591.91		x
1771	Forkhead box protein D4-like 1	Q9NU39	725.37	702.79	714.94	587.83	521.43	677.73	679.27	748.88	717.10	597.11	505.24	569.80	633.26	691.70	472.14	623.68	543.14	590.75	769.21	777.43	694.64	2.27	1.49	0.65	0.84	1.31	0.69	4.16	2.47	0.11	4.90	4.98	0.12	x	
1772	Forkhead box protein M1	Q08050	0.14	2.53	0.81	1.07	2.77	0.68	0.96	0.36	0.63	0.35	0.75	0.38	2.13	0.97	0.60	4.64	1.07	0.47	2.22	0.19	0.06	31932.85	29136.21	30657.14	16837.69	16203.92	15492.30	16894.77	15674.36	15915.04	14388.68	13615.85	11479.85		x
1773	Formin-1	Q68DA7	32878.04	30265.54	28050.45	26245.37	27086.26	23522.45	27174.09	25063.28	25945.66	34853.88	34975.33	30443.96	28599.35	21789.35	16748.18	23530.02	22292.53	22578.26	32340.99	30844.83	25604.27	22145.13	20307.46	17903.45	12052.54	11556.66	8324.01	15497.87	14117.43	12533.21	18532.62	18104.01	9616.18		
1774	Formin-2	Q9NZ56	236.93	159.25	69.49	1.07	2.77	0.68	0.96	0.36	0.63	0.35	0.75	0.38	2.13	0.97	0.60	4.64	1.07	0.47	2.22	0.19	0.06	2.27	1.49	0.65	353.84	281.54	306.31	511.11	560.49	423.67	695.31	726.94	0.12		
1775	Four and a half LIM domains protein 1	Q13642	0.14	2.53	0.81	1.07	2.77	0.68	0.96	0.36	0.63	0.35	0.75	0.38	2.13	0.97	0.60	4.64	1.07	0.47	2.22	0.19	0.06	67695.56	63880.35	57764.50	43962.68	42333.22	37464.45	50865.53	48156.03	46494.75	50127.22	48322.27	39486.55		x
1776	Four and a half LIM domains protein 2	Q14192	0.14	2.53	0.81	1.07	2.77	0.68	0.96	0.36	0.63	0.35	0.75	0.38	2.13	0.97	0.60	4.64	1.07	0.47	2.22	0.19	0.06	123684.89	101843.63	83554.51	88826.01	86032.43	63546.25	101721.00	95091.00	74536.22	135618.07	125053.96	66102.23		x
1777	Four and a half LIM domains protein 5	Q5TD97	231183.11	231210.66	210464.02	186758.83	181471.82	172142.80	180497.00	174935.85	174468.23	246971.51	242885.65	245459.50	219527.22	205242.36	173392.99	166828.24	160050.47	150622.76	184748.75	177729.28	180260.23	2.27	1.49	0.65	0.84	1.31	0.69	4.16	2.47	0.11	4.90	4.98	0.12	x	
1778	Fragile X mental retardation syndrome-related protein 1	P51114	191360.61	177479.80	166832.57	124738.07	121946.37	112587.40	150561.87	149126.69	142436.05	157298.70	150487.13	140653.69	158965.42	147816.38	119213.48	132327.32	127867.28	119640.16	141929.35	136508.47	128005.96	105203.00	97621.20	91901.40	73005.39	70891.59	62330.73	82236.27	75766.71	73578.24	84279.10	79975.61	58712.26		
1779	Fragile X mental retardation syndrome-related protein 2	P51116	26826.43	25598.87	22736.56	15922.49	16793.61	15614.03	19556.87	19116.92	16180.10	26863.29	26594.63	23838.29	24784.27	25644.66	13309.74	12179.89	15579.09	15035.49	18976.09	18558.13	17693.44	17397.11	18451.11	14458.43	13795.36	11867.52	9187.14	16749.63	16640.12	13858.51	17753.58	17135.27	10107.73		
1780	Free fatty acid receptor 2	O15552	36081.12	36751.49	34863.09	25427.16	24951.88	22324.65	27592.78	26081.71	24666.84	27851.19	28151.48	26251.88	26138.02	26314.47	22792.88	25934.37	24501.53	23403.04	26646.79	25010.97	25682.96	2.27	1.49	0.65	0.84	1.31	0.69	4.16	2.47	0.11	4.90	4.98	0.12	x	
1781	Fructose-1 6-bisphosphatase 1	P09467	435505.06	418958.12	399316.42	240661.85	236567.23	214568.86	309913.45	297931.83	277332.76	317051.96	297755.50	275540.74	360659.45	342838.71	301485.73	242627.60	228385.96	216409.25	242325.73	235629.22	221566.16	2.27	1.49	0.65	0.84	1.31	0.69	4.16	2.47	0.11	4.90	4.98	0.12	x	
1782	Fructose-1_6-bisphosphatase isozyme 2	O00757	95359.25	89202.58	85385.32	58394.03	57161.11	53007.60	67238.92	64938.05	62748.56	66175.41	65771.98	65709.95	77024.91	77888.43	66849.54	55672.04	52423.73	47951.75	56655.91	59002.46	55394.08	2.27	1.49	0.65	0.84	1.31	0.69	4.16	2.47	0.11	4.90	4.98	0.12	x	
1783	Fructose-2 6-bisphosphatase TIGAR	Q9NQ88	341448.76	322533.99	306648.41	285775.36	278189.14	262901.36	298761.31	292252.29	288507.59	289920.02	291771.72	276229.05	322711.78	297833.47	284473.68	275015.13	257667.31	251376.37	311359.14	301220.95	293879.56	112669.97	109476.70	99056.52	79298.45	77597.51	74208.65	85808.80	83403.32	80635.30	94320.94	92597.30	71237.30		
1784	Fructose-bisphosphate aldolase A	P04075	5707163.16	5554438.25	5253273.96	4397790.47	4250634.51	3995164.28	4943954.87	4789277.67	4532981.50	5239417.58	4998176.23	4796554.98	4809187.79	4369111.24	3425297.18	4782928.39	4605258.49	4509215.80	4052084.70	3861651.08	3567750.55	1839832.80	1727831.14	1618249.56	1173249.69	1168102.17	1058993.97	1300581.66	1244301.00	1208882.89	1359854.83	1317838.59	886752.56		
1785	Fructose-bisphosphate aldolase B	P05062	87205.80	82667.51	72567.23	61777.44	55264.75	47171.79	61915.23	58913.74	57169.61	77864.05	74560.39	63501.52	73254.38	65400.83	37783.57	59016.56	54625.79	49646.53	67495.39	59325.26	52152.62	12449.38	12034.18	11077.94	8026.40	8064.53	7649.70	8975.06	8906.61	8646.45	8463.67	7321.03	4941.98		
1786	Fructose-bisphosphate aldolase C	P09972	774451.41	738236.17	698234.62	667052.98	639712.24	609620.81	634060.10	609431.63	587638.62	713912.04	709385.74	651124.46	658595.44	608482.90	538902.06	681295.56	646851.68	643294.27	574502.14	543151.41	510405.30	140976.95	134463.27	123053.00	102436.38	96859.49	79911.19	113075.27	108962.62	103794.18	133036.05	128562.94	87861.68		
1787	Fumarate hydratase_ mitochondrial	P07954	154700.52	135127.88	118604.45	64888.37	62959.97	58352.27	78131.32	75034.22	73545.09	112179.83	101131.82	87377.45	118732.51	79219.97	58890.98	62922.09	60276.61	57702.86	62217.32	60545.58	54766.19	63064.39	62022.63	58350.72	44818.23	43909.53	37046.82	53589.38	50381.66	48378.60	55618.11	53316.16	35970.79		
1788	Fumarylacetoacetate hydrolase domain-containing protein 2A	Q96GK7	512.68	595.13	429.58	554.46	389.58	516.12	490.86	427.69	559.07	674.01	634.23	643.18	322.61	312.27	364.97	690.71	520.64	733.17	529.41	511.46	466.61	2.27	1.49	0.65	0.84	1.31	0.69	4.16	2.47	0.11	4.90	4.98	0.12	x	
1789	Fumarylacetoacetate hydrolase domain-containing protein 2B	Q6P2I3	0.14	2.53	0.81	1.07	2.77	0.68	0.96	0.36	0.63	0.35	0.75	0.38	2.13	0.97	0.60	4.64	1.07	0.47	2.22	0.19	0.06	6732.46	3922.70	4595.45	3733.37	4466.43	3205.58	4249.83	4118.93	4167.08	5756.59	4971.15	3760.83		x
1790	FXYD domain-containing ion transport regulator 3	Q14802	32797.63	29106.34	28818.72	21552.25	21010.11	19902.02	20631.34	20357.82	20088.46	34261.70	33332.52	31796.91	26662.92	27204.86	22013.47	21121.11	21280.00	19985.17	17300.05	16224.79	14696.32	2.27	1.49	0.65	0.84	1.31	0.69	4.16	2.47	0.11	4.90	4.98	0.12	x	
1791	FYVE and coiled-coil domain-containing protein 1	Q9BQS8	83692.43	79034.21	78109.53	56937.82	51507.02	45997.25	51281.36	50271.20	46664.37	90149.22	88288.52	96738.89	76822.35	55799.90	51930.18	44215.05	47761.13	51007.09	76951.20	65856.61	58626.39	53319.45	41032.49	35425.95	35450.00	35203.53	31143.75	44898.99	41499.51	39892.63	44100.34	43370.11	29140.21		
1792	FYVE_ RhoGEF and PH domain-containing protein 5	Q6ZNL6	4282.94	3852.17	3708.84	2856.95	2957.83	2618.16	3440.33	3285.10	3312.78	5126.53	4803.91	4693.82	4029.04	3121.30	3019.38	2501.25	2507.73	2550.83	3131.28	2775.74	2769.30	2.27	1.49	0.65	0.84	1.31	0.69	4.16	2.47	0.11	4.90	4.98	0.12	x	
1793	G protein-activated inward rectifier potassium channel 3	Q92806	0.14	2.53	0.81	1.07	2.77	0.68	0.96	0.36	0.63	0.35	0.75	0.38	2.13	0.97	0.60	4.64	1.07	0.47	2.22	0.19	0.06	2781.49	2404.11	2372.48	1371.86	1229.78	973.26	1417.55	1606.01	1237.02	1289.74	1298.60	615.75		x
1794	G protein-coupled receptor kinase 4	P32298	3647.89	2929.26	2529.62	1916.48	1849.63	1243.85	2448.08	2185.71	1906.58	2853.09	2748.03	2638.43	2830.32	2620.68	932.17	2250.89	1856.56	1922.68	2350.34	1602.53	1628.80	2.27	1.49	0.65	0.84	1.31	0.69	4.16	2.47	0.11	4.90	4.98	0.12	x	
1795	G2/M phase-specific E3 ubiquitin-protein ligase	Q7L622	16572.81	14295.16	11821.42	5302.38	5420.68	4610.60	7124.41	6811.81	6160.64	13980.83	12418.66	11509.85	16223.18	6705.92	4540.43	6576.32	6147.96	5925.81	5586.37	5511.55	5013.53	2.27	1.49	0.65	0.84	1.31	0.69	4.16	2.47	0.11	4.90	4.98	0.12	x	
1796	G2/mitotic-specific cyclin-B1	P14635	12974.35	11709.93	11071.05	10219.35	9812.68	9471.04	11707.08	11635.96	11858.36	9902.67	10269.96	9982.95	10638.70	9446.13	8644.75	10299.17	9683.04	9411.17	10038.91	9939.70	9865.56	9054.33	8979.08	8631.94	5630.61	5453.40	5405.50	7490.53	7319.18	6849.65	7846.30	8701.45	3506.36		
1797	Galactokinase	P51570	139408.05	131622.28	123685.18	88846.24	88725.77	79892.65	102423.43	95275.72	91157.12	118333.35	109218.47	106504.26	107179.06	101443.09	85667.65	89166.77	84483.54	79965.78	86036.33	86389.29	79550.42	13752.28	15931.52	10970.66	9219.12	8542.87	7160.58	10338.28	8667.93	8828.41	11014.51	10130.37	5877.00		
1798	Galactosylgalactosylxylosylprotein 3-beta-glucuronosyltransferase 3	O94766	2128.99	1950.08	1927.79	1259.06	1236.01	976.42	2886.75	2498.96	2632.94	1652.08	1830.20	1425.59	1406.17	1281.09	1047.97	1375.56	1194.66	1284.72	1485.85	1184.60	1126.06	2.27	1.49	0.65	0.84	1.31	0.69	4.16	2.47	0.11	4.90	4.98	0.12	x	
1799	Galectin-1	P09382	321504.54	305699.10	288977.29	264971.45	254971.45	241564.58	299022.07	291687.75	284607.36	345983.31	324423.82	306901.64	252226.03	248919.97	203024.01	291645.41	275016.00	265456.47	220158.43	213987.09	200153.91	717981.67	674474.74	653295.79	371828.51	364515.17	327324.52	418997.50	397499.19	389408.13	382732.07	373231.59	274573.78		
1800	Galectin-3	P17931	162634.27	161534.36	155750.36	105076.99	98217.00	95870.26	117791.97	116025.36	111818.36	137406.79	131839.26	125903.31	105132.08	105938.15	89825.99	128212.25	118181.37	117462.12	92628.93	90420.29	85996.47	2.27	1.49	0.65	0.84	1.31	0.69	4.16	2.47	0.11	4.90	4.98	0.12	x	
1801	Galectin-3-binding protein	Q08380	11751.88	12860.37	10515.40	9177.32	8193.11	7677.37	8618.84	7737.90	8230.91	9791.91	9363.90	8775.94	10809.77	9746.60	8061.67	7765.35	7248.68	7024.87	7364.44	5450.12	5556.82	2.27	1.49	0.65	0.84	1.31	0.69	4.16	2.47	0.11	4.90	4.98	0.12	x	
1802	Gametocyte-specific factor 1	Q8WW3	34601.10	34201.06	34992.53	25477.20	24645.45	23534.15	28030.95	26091.54	26924.29	27581.21	26683.49	25903.13	29726.41	29737.97	24898.49	22786.95	23459.50	22515.32	25087.11	24096.29	22043.31	2.27	1.49	0.65	0.84	1.31	0.69	4.16	2.47	0.11	4.90	4.98	0.12	x	
1803	Gamma-aminobutyric acid receptor-associated protein-like 2	P60520	166890.25	161137.09	147466.77	92905.82	92855.90	86020.03	121061.13	120820.91	118458.92	107142.03	104792.34	98667.69	117402.38	111944.23	92315.89	92744.61	86437.68	88796.99	106059.29	102144.66	99026.65	4421.46	4403.75	3627.10	2987.54	3194.32	2999.35	2851.42	3010.39	2931.21	3118.28	2961.50	2427.25		
1804	Gamma-crystallin A	P11844	25153.42	22361.97	19078.05	23358.20	22351.36	18853.85	18660.62	19151.57	17092.30	23076.97	22625.14	18971.95	26776.69	24849.37	14208.06	18377.97	17239.17	16522.05	23327.30	21027.00	19058.22	2.27	1.49	0.65	0.84	1.31	0.69	4.16	2.47	0.11	4.90	4.98	0.12	x	
1805	Gamma-crystallin S	P22914	2461.00	2375.07	2399.04	1465.57	1491.16	1420.64	1724.46	1554.35	1580.22	2147.99	2053.80	1967.22	1770.25	1720.40	1590.34	1541.37	1594.96	1416.13	1533.80	1333.07	1332.27	2.27	1.49	0.65	0.84	1.31	0.69	4.16	2.47	0.11	4.90	4.98	0.12	x	
1806	Gamma-enolase	P09104	18715.48	17461.91	15547.58	18807.42	18386.73	15242.05	18145.18	14472.88	12987.35	19010.33	17382.82	16376.38	18411.08	24955.36	12917.11	15939.97	14889.84	14157.10	15278.53	14057.84	12167.16	85289.02	85361.00	76378.97	44613.03	42906.06	35568.36	71109.34	64295.40	53196.37	64738.69	61003.67	39227.15		
1807	Gamma-glutamyl hydrolase	Q92820	12106.70	10799.55	10213.80	7950.75	7732.30	6020.88	6618.59	7494.63	7325.75	9347.61	8921.16	7867.31	9074.59	7888.04	3985.92	7380.62	7467.01	7459.98	7580.84	7738.07	6294.38	2.27	1.49	0.65	0.84	1.31	0.69	4.16	2.47	0.11	4.90	4.98	0.12	x	
1808	Gamma-glutamylcyclotransferase	O75223	151695.74	151426.73	138778.11	118917.32	112203.86	107636.40	134249.72	126042.37	121967.20	139730.28	130621.72	123171.72	137403.93	128701.15	105667.12	106308.18	101529.56	99085.60	112810.18	110394.78	101855.59	16944.30	16697.35	16151.16	10404.01	10713.06	9723.31	13735.22	13066.28	12989.13	13172.96	12099.03	8388.86		
1809	Gamma-soluble NSF attachment protein	Q99747	39178.30	39806.54	37228.67	33159.01	36342.06	31996.45	36522.88	37238.15	36183.47	35648.60	34034.55	34669.85	35341.79	27439.67	21181.82	36048.60	32280.24	32342.58	33206.82	29006.23	28004.06	55239.90	54601.41	50532.26	37463.55	37132.04	28855.72	49063.27	45708.41	41913.44	44826.71	45138.35	29466.58		
1810	Gamma-synuclein	O76070	1233.80	1848.71	2626.31	847.30	935.28	1144.47	1569.65	2005.26	2073.05	1254.48	1049.20	854.38	1135.22	1374.26	860.47	1611.18	1217.48	1309.66	1297.65	1475.25	951.18	2.27	1.49	0.65	0.84	1.31	0.69	4.16	2.47	0.11	4.90	4.98	0.12	x	
1811	Gamma-taxilin	Q9NUQ3	9167.29	8967.08	8342.14	6402.73	6496.97	6217.53	6868.80	6805.49	6611.94	9402.87	8569.50	8258.80	8094.17	7140.81	4968.92	5830.71	5675.79	5805.56	6834.79	7102.42	6941.45	2.27	1.49	0.65	0.84	1.31	0.69	4.16	2.47	0.11	4.90	4.98	0.12	x	
1812	Gamma-tubulin complex component 5	Q96RT8	0.14	2.53	0.81	1.07	2.77	0.68	0.96	0.36	0.63	0.35	0.75	0.38	2.13	0.97	0.60	4.64	1.07	0.47	2.22	0.19	0.06	4854.92	5306.48	6135.96	2935.21	2932.21	2225.27	3802.04	3759.85	3687.83	3147.62	2542.66	1937.74		x
1813	GAS2-like protein 2	Q8NHY3	3223.47	3139.94	3482.75	2295.75	2267.40	2107.18	3866.27	4132.21	4300.17	3124.32	2959.00	2619.16	2543.53	2566.44	1897.49	2272.50	2183.11	2264.46	1956.49	1720.97	2027.08	2.27	1.49	0.65	0.84	1.31	0.69	4.16	2.47	0.11	4.90	4.98	0.12	x	
1814	Gastrin/cholecystokinin type B receptor	P32239	2482.75	2642.24	2513.51	1996.84	2138.70	1944.82	1902.48	2405.82	1993.57	3033.73	2615.20	2628.24	2418.94	2253.88	2324.81	2046.90	2045.60	1925.95	1726.14	1854.69	1904.97	2.27	1.49	0.65	0.84	1.31	0.69	4.16	2.47	0.11	4.90	4.98	0.12	x	
1815	GATA zinc finger domain-containing protein 1	Q8WUU	0.14	2.53	0.81	1.07	2.77	0.68	0.96	0.36	0.63	0.35	0.75	0.38	2.13	0.97	0.60	4.64	1.07	0.47	2.22	0.19	0.06	7632.78	6900.48	6179.90	7833.35	7635.07	4854.84	7641.42	6751.65	6213.29	7761.98	7406.35	4435.87		x
1816	GATOR complex protein DEPDC5	O75140	15098.19	15118.55	13992.71	9825.58	9993.84	9549.13	12063.69	11522.78	11075.50	11395.71	11087.06	10996.16	13133.33	11955.69	10390.81	9377.06	8627.13	8597.39	9921.93	10077.11	9261.45	2.27	1.49	0.65	0.84	1.31	0.69	4.16	2.47	0.11	4.90	4.98	0.12	x	
1817	GATOR complex protein WDR24	Q96S15	0.14	2.53	0.81	1.07	2.77	0.68	0.96	0.36	0.63	0.35	0.75	0.38	2.13	0.97	0.60	4.64	1.07	0.47	2.22	0.19	0.06	4011.75	4292.05	3839.86	1895.33	2175.95	1832.69	3306.88	3283.00	3063.53	2921.95	3245.74	1863.11		x
1818	GC-rich sequence DNA-binding factor 2	P16383	6342.04	6090.90	5528.93	4582.47	4980.99	4751.41	4676.60	4451.73	4916.83	7462.36	7185.12	7355.20	6254.57	5654.32	4619.53	4581.66	4170.29	4002.55	5103.79	4830.45	4741.91	298.87	241.65	175.31	230.67	185.51	225.25	278.87	234.51	231.61	349.31	288.71	221.22		
1819	GDNF family receptor alpha-1	P56159	186823.98	187379.77	189196.36	143896.17	136789.30	135914.97	147781.13	149908.93	150640.40	169857.99	156039.38	147503.00	147364.04	148934.91	131330.67	157955.44	147884.92	146849.55	138139.51	134847.39	126502.99	2.27	1.49	0.65	0.84	1.31	0.69	4.16	2.47	0.11	4.90	4.98	0.12	x	
1820	GDNF family receptor alpha-2	O00451	0.14	2.53	0.81	1.07	2.77	0.68	0.96	0.36	0.63	0.35	0.75	0.38	2.13	0.97	0.60	4.64	1.07	0.47	2.22	0.19	0.06	5681.85	6024.07	5542.28	4576.36	4209.67	4183.64	5269.67	4970.23	4450.22	5112.86	4922.22	2158.90		x
1821	GDNF-inducible zinc finger protein 1	Q9H116	155344.84	152590.90	137289.57	153009.21	149512.05	117776.46	158546.96	160575.67	135260.05	143685.98	118521.26	128638.70	145132.23	166644.62	97289.89	151150.64	153704.57	136437.11	124545.15	149997.59	119343.59	2.27	1.49	0.65	0.84	1.31	0.69	4.16	2.47	0.11	4.90	4.98	0.12	x	
1822	GDP-fucose protein O-fucosyltransferase 1	Q9H488	100822.79	88145.41	74211.74	59517.31	58164.83	47997.81	73221.70	72081.41	64090.70	73654.00	70066.29	61387.35	71842.33	64910.08	39931.67	60999.28	56875.46	52855.57	63829.76	57447.64	49854.56	37798.60	34963.32	32326.34	18019.35	17147.36	14256.96	21374.12	20558.19	18910.55	18752.11	17986.11	11205.81		
1823	GDP-fucose protein O-fucosyltransferase 2	Q9Y2G5	5979.93	5775.06	5420.36	4249.40	4083.11	3614.85	4449.07	4082.73	3934.06	5231.05	5217.58	4926.33	4998.57	4780.02	4108.27	4061.99	3610.68	3395.66	3976.38	3669.71	3746.80	2.27	1.49	0.65	0.84	1.31	0.69	4.16	2.47	0.11	4.90	4.98	0.12	x	
1824	GDP-L-fucose synthase	Q13630	16119.81	16531.41	12871.34	9133.96	8607.63	8606.99	13203.13	12335.66	11598.82	13154.69	13225.60	12377.67	13971.43	12639.42	9202.96	9794.94	9364.66	8543.43	10542.48	9753.33	9012.86	57978.58	52830.87	48363.76	35668.42	36061.18	28549.32	42128.39	38820.00	35149.54	45786.14	43197.66	27412.00		
1825	GDP-mannose 4_6 dehydratase	O60547	0.14	2.53	0.81	1.07	2.77	0.68	0.96	0.36	0.63	0.35	0.75	0.38	2.13	0.97	0.60	4.64	1.07	0.47	2.22	0.19	0.06	4479.19	3087.45	3353.47	3112.46	2771.72	1996.87	4531.45	3458.76	3242.38	3565.50	3563.98	2335.13		x
1826	Gelsolin	P06396	200071.92	192479.04	173724.51	122707.37	115622.62	106937.57	141639.09	132288.76	126865.65	253606.79	243794.78	222692.40	162758.74	147613.87	113281.46	150272.79	143511.83	138153.85	120782.12	112964.28	104002.94	139858.27	126818.31	116385.96	84891.72	83636.07	76737.54	93226.16	91114.96	86668.45	113188.85	107945.16	73440.50		
1827	Gem-associated protein 4	P57678	45171.94	42034.26	36581.12	29316.02	27828.22	25122.05	34476.17	33567.04	31145.00	37657.11	35257.82	33359.10	39700.71	35784.46	31020.30	27357.48	25471.83	25059.52	28611.19	27691.13	27043.24	4231.13	4142.32	3635.94	2714.62	2400.63	2017.09	3566.16	2461.15	2949.21	3377.25	3677.02	2167.08		
1828	Gem-associated protein 5	Q8TEQ6	155677.44	157572.00	143884.55	110459.20	109223.69	103572.07	128512.95	125743.38	117412.58	135408.70	126049.37	121802.27	134940.49	127134.22	104618.93	103344.43	100934.44	101083.63	112761.09	108667.72	105704.76	31825.18	32631.26	29732.23	21883.29	20505.24	17394.49	29937.35	29481.65	27502.23	25206.38	22683.80	15509.70		
1829	Gem-associated protein 6	Q8WXD5	2479.78	3072.99	3056.85	2149.53	2339.97	2185.19	1942.99	2172.71	2010.08	2777.25	2512.62	2508.35	2697.92	2736.20	2566.83	2024.55	1488.21	1790.22	1799.43	1811.38	1648.96	2.27	1.49	0.65	0.84	1.31	0.69	4.16	2.47	0.11	4.90	4.98	0.12	x	
1830	General transcription factor 3C polypeptide 4	Q9UKN8	37756.03	34569.04	30800.62	28986.56	27192.19	25452.45	33794.11	32658.56	32182.48	39455.78	40143.03	36685.11	33602.72	29363.32	25708.70	28976.97	26742.01	26240.13	29604.92	27175.13	25513.15	2.27	1.49	0.65	0.84	1.31	0.69	4.16	2.47	0.11	4.90	4.98	0.12	x	
1831	General transcription factor IIF subunit 1	P35269	4394.04	4246.00	3914.36	3143.67	2996.74	2795.65	3615.47	3632.89	3133.05	4005.29	4045.25	3663.18	4274.80	4037.32	3286.16	2975.86	2805.73	2584.08	3862.41	3626.10	3515.36	7947.21	6823.95	5668.04	5677.43	5317.57	4879.58	7913.97	8085.14	6432.25	8932.80	8644.13	5170.90		
1832	General transcription factor IIF subunit 2	P13984	36642.53	34740.55	34911.07	22114.36	22707.88	20177.61	26770.74	25419.61	26115.54	27957.39	27037.21	28081.42	29160.71	29109.23	23111.79	26115.71	23166.39	21358.48	21155.15	20355.77	19932.05	1053.03	900.84	627.25	907.04	953.73	647.37	865.75	699.98	523.86	994.50	1068.41	483.02		
1833	General transcription factor II-I	P78347	486754.07	480342.37	461258.36	290134.22	290258.05	261906.03	355967.77	338301.27	325116.33	370129.05	340650.74	327091.45	345845.26	331544.28	272999.88	298721.15	282763.63	266287.69	258401.56	254233.85	235984.44	23594.31	20126.41	19541.86	12937.65	13075.45	12163.92	17217.33	16725.18	15720.05	20384.71	17705.83	12570.24		
1834	General transcription factor II-I repeat domain-containing protein 2B	Q6EKJ0	12188.45	11172.52	11073.15	9490.86	9284.18	8664.93	11445.49	10707.51	11075.00	11436.47	10510.29	10543.08	10290.97	10107.44	8097.94	9393.57	8284.92	8827.65	8853.92	8509.58	8498.91	2.27	1.49	0.65	0.84	1.31	0.69	4.16	2.47	0.11	4.90	4.98	0.12	x	
1835	General vesicular transport factor p115	O60763	175989.95	167573.61	160444.37	137567.66	135597.35	127388.21	155430.48	147367.10	143382.65	193151.78	183172.26	174384.77	150418.19	144228.93	118471.13	142020.32	137234.52	132325.12	139901.12	132863.61	125320.57	72259.63	69336.43	66014.04	51262.52	49701.64	45536.60	53607.72	51975.57	50699.32	58095.97	56791.87	34975.59		
1836	Geranylgeranyl transferase type-2 subunit alpha	Q92696	117314.96	113511.83	103487.11	83935.06	79913.68	75548.09	87835.40	89011.58	85443.79	92213.09	93130.60	89619.63	87242.42	85242.74	74040.59	75122.04	73893.90	69187.29	79260.05	76051.42	74435.44	2.27	1.49	0.65	0.84	1.31	0.69	4.16	2.47	0.11	4.90	4.98	0.12	x	
1837	Geranylgeranyl transferase type-2 subunit beta	P53611	13052.66	12631.21	10697.32	9257.58	9542.25	8954.83	8496.17	9240.76	8386.44	12590.63	13205.65	11099.66	9924.78	10341.22	8907.66	9014.08	7923.80	8643.79	9493.77	9361.45	8338.10	2.27	1.49	0.65	0.84	1.31	0.69	4.16	2.47	0.11	4.90	4.98	0.12	x	
1838	Germ cell-less protein-like 2	Q8NEA9	0.14	2.53	0.81	1.07	2.77	0.68	0.96	0.36	0.63	0.35	0.75	0.38	2.13	0.97	0.60	4.64	1.07	0.47	2.22	0.19	0.06	401.85	380.19	343.82	574.33	449.42	449.03	405.49	326.01	369.21	444.89	384.01	245.95		x
1839	Germinal-center associated nuclear protein	O60318	1495.29	1036.70	511.55	359.71	260.17	284.31	308.74	290.71	294.47	834.13	658.67	384.78	1103.86	409.17	375.77	311.63	193.68	265.62	319.78	300.73	247.46	3282.07	3489.57	3100.28	1939.37	1850.16	1508.03	2610.68	2419.91	2263.75	2330.45	2275.51	1355.32		
1840	Gigaxonin	Q9H2C0	103301.23	79912.10	45698.32	1727.30	1174.99	482.72	1492.85	940.59	715.64	77361.38	65372.85	43033.62	60587.04	2656.18	132.20	1065.44	736.36	480.02	3850.21	3368.83	973.66	2.27	1.49	0.65	0.84	1.31	0.69	4.16	2.47	0.11	4.90	4.98	0.12	x	
1841	Girdin	Q3V6T2	235550.58	223551.57	215723.26	166281.23	162742.31	155579.03	183910.46	181950.65	174980.65	208782.64	199768.04	190486.65	203579.66	183726.93	143153.14	153519.32	147019.60	149005.23	168040.67	156796.85	149277.71	78814.00	80068.00	72327.02	55940.24	56002.30	51180.62	64497.86	62330.52	63958.01	65453.60	62034.23	39362.68		
1842	Glia maturation factor beta	P60983	55435.66	52023.87	44915.82	39323.03	37100.45	32138.44	40857.32	39235.74	35657.29	50936.76	48374.72	42372.76	47161.17	42510.42	27862.82	40304.02	37891.00	34562.44	40505.60	39005.94	34769.82	55479.25	49590.32	45975.66	41852.50	40934.76	35280.30	47566.08	43382.77	40566.89	53393.85	51692.44	33963.77		
1843	Glial fibrillary acidic protein	P14136	162919.90	140494.68	115412.30	118738.78	119800.73	99561.70	139633.08	139266.61	122041.78	134994.88	127759.27	101610.48	119657.24	120265.50	59984.23	123035.54	128230.06	107939.74	105647.27	98370.68	79852.22	209688.20	181114.80	138846.03	149967.74	143115.52	83658.28	209152.22	187757.14	137009.85	232853.53	216630.12	106869.44		
1844	GLIPR1-like protein 2	Q4G1C9	309.51	362.51	106.38	460.90	240.49	284.03	519.23	326.77	389.84	368.05	426.88	286.66	296.14	250.94	411.27	305.34	414.20	324.72	324.14	430.58	359.46	2.27	1.49	0.65	0.84	1.31	0.69	4.16	2.47	0.11	4.90	4.98	0.12	x	
1845	Glucocorticoid modulatory element-binding protein 2	Q9UKD1	182.28	79.11	141.89	94.73	136.64	75.35	90.29	126.04	92.44	160.15	224.27	165.38	91.13	162.54	89.59	140.99	113.23	162.64	149.11	152.53	89.78	2.27	1.49	0.65	0.84	1.31	0.69	4.16	2.47	0.11	4.90	4.98	0.12	x	
1846	Glucosamine 6-phosphate N-acetyltransferase	Q96EK6	38940.68	35124.81	31239.76	24729.36	23302.01	21782.94	30509.69	29386.60	28584.66	32407.83	31003.22	28787.03	30745.33	29419.07	21119.94	27329.27	25880.50	25809.40	25152.17	23122.84	20083.11	7221.57	6493.93	6024.36	2972.00	2823.13	1792.98	5295.95	4892.71	4098.49	4785.27	4578.76	2281.82		
1847	Glucosamine-6-phosphate isomerase 1	P46926	5377.57	5166.46	4404.50	2598.17	2499.28	2102.90	5109.95	4908.48	4259.44	3472.71	3168.97	3107.67	3763.86	3392.28	2530.00	3392.55	3390.13	3185.41	3126.09	2799.66	2485.08	36505.22	33452.89	29653.53	26136.95	24384.71	22381.85	26113.13	26907.01	24918.95	23538.13	22686.98	18735.77		
1848	Glucose-6-phosphate 1-dehydrogenase	P11413	2244256.84	2182201.77	2058762.96	1924417.86	1845284.54	1757993.55	2030972.35	1948276.37	1904401.04	2264142.46	2183790.11	2062139.47	1813143.38	1693308.11	1416682.20	2154134.61	2037083.88	2023930.54	1728113.93	1589173.83	1512503.31	171938.85	166515.90	152789.09	135307.96	127474.81	117403.61	148055.16	142836.55	139894.45	149094.52	146130.88	106146.65		
1849	Glucose-6-phosphate isomerase	P06744	1667155.31	1637878.53	1537269.43	1114472.66	1064293.22	1016108.89	1321242.96	1247759.27	1211149.94	1376391.36	1335815.97	1257229.53	1430770.50	1316185.50	1099080.74	1092136.11	1047467.53	1021050.40	1145955.22	1083323.98	995993.85	826151.27	775422.64	711575.50	475902.10	463985.01	444041.98	626473.23	598833.21	574087.11	579802.13	553978.15	383028.19		
1850	Glucose-fructose oxidoreductase domain-containing protein 2	Q3B7J2	0.14	2.53	0.81	1.07	2.77	0.68	0.96	0.36	0.63	0.35	0.75	0.38	2.13	0.97	0.60	4.64	1.07	0.47	2.22	0.19	0.06	6317.01	11554.34	9406.57	4942.99	4439.19	2255.13	8641.05	6341.15	5755.39	9130.59	8313.16	3743.07		x
1851	Glucose-induced degradation protein 8 homolog	Q9NWU	1941.89	1923.91	1822.40	1524.62	1542.90	1168.99	1584.27	1530.36	1445.08	1788.55	1719.75	1487.75	2077.35	1955.81	887.03	1374.12	1400.78	1091.55	1541.63	1642.12	1218.23	2532.61	2275.03	1938.49	839.82	828.87	375.00	2628.83	2389.90	1785.01	2177.63	1864.58	819.04		
1852	Glucosidase 2 subunit beta	P14314	512997.94	503925.35	482694.75	370081.09	357359.90	344224.55	440089.53	429534.42	417551.40	423612.58	412250.34	393005.14	424191.04	401801.85	360240.05	375041.80	353860.94	351623.55	346021.75	330732.67	316550.93	301369.67	288015.65	267575.54	179063.78	174143.82	152782.81	224477.77	208639.76	195653.37	205131.71	199097.96	144692.58		
1853	Glutamate dehydrogenase 1_ mitochondrial	P00367	406456.36	395928.90	372237.75	262093.36	253293.18	233927.95	310519.15	287410.43	278202.72	325721.00	310322.46	289850.74	325643.31	313183.90	254733.87	258190.24	241989.71	237527.09	265201.73	246073.23	237301.85	297652.97	274439.32	250106.91	179337.47	173661.23	153070.17	210351.05	197832.53	194362.63	203552.20	198461.20	140466.62		
1854	Glutamate dehydrogenase 2_ mitochondrial	P49448	262105.11	251567.26	231069.68	158409.34	152994.22	143913.41	185787.04	185080.75	176662.47	208349.48	205208.52	187258.48	205437.27	193054.65	148853.00	166548.28	161454.33	158314.71	183821.85	162940.13	155531.59	17482.06	15514.51	14348.90	9512.19	9626.57	7385.51	7983.55	7970.48	7057.65	7040.54	6950.54	3899.34		
1855	Glutamate receptor ionotropic_ NMDA 2A	Q12879	7938.09	6957.20	7458.46	4539.04	4254.80	4017.19	6333.19	6324.24	6101.53	5260.62	5055.00	4824.45	6011.12	5639.27	4476.81	4921.73	4937.79	4510.96	4324.30	4265.02	4199.77	31709.53	29477.10	26102.02	17322.46	14458.81	11994.80	16988.15	11575.09	12915.01	12036.07	12451.44	8142.64		
1856	Glutamate--cysteine ligase regulatory subunit	P48507	0.14	2.53	0.81	1.07	2.77	0.68	0.96	0.36	0.63	0.35	0.75	0.38	2.13	0.97	0.60	4.64	1.07	0.47	2.22	0.19	0.06	54619.04	49427.71	45275.72	42468.73	41008.77	36235.30	41585.01	37964.25	37017.16	44180.90	42629.33	25905.61		x
1857	Glutamate-rich protein 4	A6NGS2	524910.26	475674.25	414377.83	284354.81	268617.13	222609.01	343200.59	335342.46	306344.19	384497.91	372423.80	341233.32	356124.03	305552.21	174306.18	296671.70	293330.62	290685.94	330125.07	305664.51	266463.24	290779.80	279654.38	245048.19	162585.80	160439.98	105584.80	246646.30	218047.13	201558.01	247063.39	241687.33	132432.53		
1858	Glutamate-rich protein 6B	Q5W0A0	796.31	431.65	443.75	404.69	325.57	142.43	375.16	488.86	304.17	739.15	566.98	487.40	593.51	415.84	117.20	310.97	222.97	291.28	360.93	396.95	243.27	2.27	1.49	0.65	0.84	1.31	0.69	4.16	2.47	0.11	4.90	4.98	0.12	x	
1859	Glutamate-rich WD repeat-containing protein 1	Q9BQ67	31740.20	27669.90	23613.22	16893.44	16508.42	13768.38	23575.18	23434.81	22027.24	29536.49	29089.35	25517.57	20872.39	19909.10	11894.89	28495.09	27605.99	26492.55	24188.24	21691.26	18948.91	9423.45	9393.58	8966.83	7177.60	6779.40	5850.17	8343.52	7071.00	6988.53	8366.44	7614.71	5731.06		
1860	Glutaminase kidney isoform_ mitochondrial	O94925	25184.26	23775.41	24337.17	15621.12	15323.51	13940.36	20707.47	19434.22	17872.17	20452.83	18577.87	16916.97	18945.97	18574.54	15010.87	17388.05	16672.06	16023.77	15664.18	15938.75	16052.20	26689.65	25955.04	22569.23	18261.20	17152.60	15526.50	23459.12	22540.93	21910.73	24647.79	21910.39	14820.63		
1861	Glutamine amidotransferase-like class 1 domain-containing protein 3B_ mitochondrial	A0A0B4J	22770.67	23180.06	18502.81	13237.88	13361.27	12160.64	15361.55	15079.82	13762.28	15907.87	15160.41	13482.49	14479.89	14167.51	10614.14	14296.91	14908.83	13937.26	14326.96	13493.96	12171.33	2.27	1.49	0.65	0.84	1.31	0.69	4.16	2.47	0.11	4.90	4.98	0.12	x	
1862	Glutamine--fructose-6-phosphate aminotransferase [isomerizing] 1	Q06210	382531.10	358924.84	343532.25	257585.99	248536.92	231420.49	297347.07	295577.10	277125.37	323478.55	306402.36	286205.43	311766.77	298336.31	241109.43	252308.87	243784.43	237428.35	260877.64	247907.49	230652.58	140501.16	139808.25	129358.84	84290.93	84113.87	70878.56	119922.44	115906.39	110766.04	106598.35	103086.44	70786.97		
1863	Glutamine--fructose-6-phosphate aminotransferase [isomerizing] 2	O94808	50771.65	48714.75	45380.23	38285.74	36042.24	34494.28	37954.35	37198.06	36216.31	43309.38	39279.68	41190.35	41286.53	41736.97	36196.04	34969.48	30612.86	30883.21	36606.56	33983.75	35452.09	36441.70	33468.45	28416.68	20488.31	18941.65	13582.81	24039.54	22140.66	19186.20	28177.88	26848.03	14350.71		
1864	Glutamine-rich protein 1	Q2TAL8	0.14	2.53	0.81	1.07	2.77	0.68	0.96	0.36	0.63	0.35	0.75	0.38	2.13	0.97	0.60	4.64	1.07	0.47	2.22	0.19	0.06	2941.59	2765.02	2915.98	2649.04	2432.31	2654.55	2648.52	2755.92	2698.53	3487.80	878.76	1742.92		x
1865	Glutamine--tRNA ligase	P47897	220287.20	213997.77	201022.97	145191.95	139596.02	132191.40	163791.98	159830.42	155417.93	186530.36	177678.35	167337.14	169891.66	165015.06	134650.03	148742.28	137307.11	134021.06	138920.75	133330.78	128214.55	264435.91	259407.24	235457.59	201715.85	196842.28	180642.21	215094.02	215896.80	214508.41	246905.49	243009.93	183227.22		
1866	Glutamyl-tRNA(Gln) amidotransferase subunit A_ mitochondrial	Q9H0R6	0.14	2.53	0.81	1.07	2.77	0.68	0.96	0.36	0.63	0.35	0.75	0.38	2.13	0.97	0.60	4.64	1.07	0.47	2.22	0.19	0.06	552.86	605.81	613.39	337.19	389.68	274.27	391.06	493.65	517.86	443.48	606.06	281.98		x
1867	Glutamyl-tRNA(Gln) amidotransferase subunit C_ mitochondrial	O43716	0.14	2.53	0.81	1.07	2.77	0.68	0.96	0.36	0.63	0.35	0.75	0.38	2.13	0.97	0.60	4.64	1.07	0.47	2.22	0.19	0.06	799.66	787.49	587.51	494.50	414.55	389.30	601.79	603.37	608.80	647.69	633.65	422.74		x
1868	Glutaredoxin-3	O76003	168115.84	157828.71	141233.20	105281.27	102832.67	93572.95	135176.83	128908.90	122445.93	133409.58	123174.21	112281.66	128205.61	125280.32	93093.94	108097.85	103683.77	96787.12	109424.04	106572.22	95978.29	127975.08	123323.81	115776.43	117457.77	112477.99	99004.68	172955.28	166902.32	162733.01	154698.53	150451.09	100614.64		
1869	Glutaredoxin-related protein 5_ mitochondrial	Q86SX6	5798.25	5863.27	6488.46	4017.70	3738.69	4020.06	4829.42	5047.93	4546.52	4525.67	4852.38	4344.07	4760.58	4140.88	4239.07	4126.38	4864.73	4121.38	3755.84	3517.77	3045.22	123625.82	112341.89	100875.11	73661.33	78003.47	64945.75	79331.53	78991.78	72157.22	96475.08	91727.38	63090.19		
1870	Glutathione peroxidase 2	P18283	6158.00	5698.53	6447.61	4398.23	4420.63	3727.19	5624.05	5086.63	5220.70	5947.16	5436.60	5053.10	5538.43	5428.00	4417.57	4339.28	4806.56	4305.79	4284.21	4245.31	3968.06	2.27	1.49	0.65	0.84	1.31	0.69	4.16	2.47	0.11	4.90	4.98	0.12	x	
1871	Glutathione peroxidase 6	P59796	179.81	120.86	179.81	1130.00	1092.55	1129.77	18487.70	16953.38	16457.15	1062.40	941.57	1031.13	245.79	161.15	49.61	47.30	46.79	95.88	56.54	104.48	58.52	2.27	1.49	0.65	0.84	1.31	0.69	4.16	2.47	0.11	4.90	4.98	0.12	x	
1872	Glutathione reductase_ mitochondrial	P00390	133387.85	131845.11	125205.02	95480.59	91112.93	85435.62	114876.74	110120.55	107129.98	115497.87	111961.29	107862.01	112006.24	109464.34	92657.57	93323.90	87505.84	85103.71	92645.92	92211.50	87270.21	6356.02	4976.44	5411.12	3785.12	3582.07	3428.92	4857.27	4665.58	4333.21	4700.99	5219.81	5217.31		
1873	Glutathione S-transferase kappa 1	Q9Y2Q3	92251.14	89434.45	87071.06	55797.78	53347.20	52148.87	66495.84	63404.46	60985.33	74819.49	69256.91	68387.82	67666.07	67543.14	55528.31	60681.02	57071.30	55811.55	54804.58	54576.86	51372.73	46346.02	43905.22	39661.38	28580.58	28749.59	23699.28	35981.98	34368.86	32470.27	38006.22	36940.67	25402.44		
1874	Glutathione S-transferase LANCL1	O43813	26530.97	24488.26	22417.11	18620.17	17553.41	17202.31	21047.18	20061.99	21238.49	23126.19	20965.49	21169.60	23420.26	20979.98	17748.48	17357.18	15890.95	15529.61	17087.38	15982.66	15950.17	9330.09	4715.48	6618.89	3412.29	3468.86	2520.64	4514.24	4571.23	3085.06	4095.47	3287.80	1997.02		
1875	Glutathione S-transferase Mu 1	P09488	461.61	473.28	541.40	281.32	325.72	249.44	644.12	602.58	484.50	404.47	471.96	497.99	430.69	356.84	308.03	292.72	256.00	241.23	385.64	313.69	318.36	2.27	1.49	0.65	0.84	1.31	0.69	4.16	2.47	0.11	4.90	4.98	0.12	x	
1876	Glutathione S-transferase Mu 2	P28161	6641.87	6071.63	5978.34	4429.23	4401.57	4047.63	4286.95	4325.53	4455.17	5820.26	5696.18	5481.74	5945.83	5709.17	5188.75	4220.21	3622.49	3354.71	4254.92	4112.50	4392.97	2.27	1.49	0.65	0.84	1.31	0.69	4.16	2.47	0.11	4.90	4.98	0.12	x	
1877	Glutathione S-transferase Mu 3	P21266	125252.63	115128.58	100932.72	98476.30	96203.23	79973.02	138781.38	127658.24	121057.46	97071.11	91137.21	82016.74	94961.85	121589.25	71888.07	104228.63	96935.56	89008.64	75282.64	98281.93	83882.97	3638.75	3375.36	3019.87	2635.69	2577.49	2427.42	2733.55	2690.23	2454.64	3097.64	2741.56	1874.95		
1878	Glutathione S-transferase Mu 5	P46439	7076.13	7498.62	7587.13	5735.84	5189.41	5207.10	7782.22	7483.19	7479.12	5876.65	5422.47	5448.34	6151.90	6345.29	5982.61	5833.45	5669.74	5569.74	4844.37	4879.68	4542.09	2.27	1.49	0.65	0.84	1.31	0.69	4.16	2.47	0.11	4.90	4.98	0.12	x	
1879	Glutathione S-transferase omega-1	P78417	247399.36	238451.37	226959.22	172967.41	164846.36	161785.68	200966.92	201337.43	199261.78	211655.99	201943.07	190181.95	213000.02	199806.52	170685.60	164959.27	157468.17	155894.01	182116.24	167827.74	159408.57	119070.30	109057.80	106623.04	72191.40	71125.42	65475.15	85122.49	77136.58	76264.88	74284.27	71284.19	51971.79		
1880	Glutathione S-transferase P	P09211	0.14	2.53	0.81	1.07	2.77	0.68	0.96	0.36	0.63	0.35	0.75	0.38	2.13	0.97	0.60	4.64	1.07	0.47	2.22	0.19	0.06	352434.77	322429.90	288851.58	207329.40	201429.17	156629.66	321119.72	295423.52	266346.55	307650.75	292412.82	190767.96		x
1881	Glutathione S-transferase theta-4	A0A1W2	1183.23	1209.24	1071.34	776.75	683.10	596.73	660.80	1041.07	637.15	940.93	697.51	767.17	879.03	1055.01	494.29	663.07	761.51	628.17	638.68	789.95	617.14	2.27	1.49	0.65	0.84	1.31	0.69	4.16	2.47	0.11	4.90	4.98	0.12	x	
1882	Glutathione synthetase	P48637	110630.88	113808.06	106593.97	81243.76	79078.96	75562.10	95844.34	92264.77	86716.45	91848.30	90120.67	86016.47	89670.84	88526.00	77411.21	73485.96	70770.07	69191.02	73477.47	67546.28	68093.74	169491.63	167776.59	152417.80	114451.33	111158.61	97922.15	133810.20	125184.26	116991.93	130065.53	128144.56	87692.81		

Supplementary Table S1. Overview on the abundance of all relatively quantified 5180 proteins

			MCF-7																					MDA-MB-231													
	Protein name	UniProt	control_01	control_02	control_03	Dai_IC20_01	Dai_IC20_02	Dai_IC20_03	Dai_SC20_01	Dai_SC20_02	Dai_SC20_03	Gen_IC20_01	Gen_IC20_02	Gen_IC20_03	Gen_SC20_01	Gen_SC20_02	Gen_SC20_03	SSE_IC20_01	SSE_IC20_02	SSE_IC20_03	SSE_SC20_01	SSE_SC20_02	SSE_SC20_03	control_01	control_02	control_03	Dai_IC20_01	Dai_IC20_02	Dai_IC20_03	Gen_IC20_01	Gen_IC20_02	Gen_IC20_03	SSE_IC20_01	SSE_IC20_02	SSE_IC20_03	unique to MCF-7	unique to MDA-MB-231
1883	Glyceraldehyde-3-phosphate dehydrogenase	P04406	4517103.99	4272751.55	3713055.54	3195250.13	3085156.08	2664231.81	4315191.00	4150225.38	3765715.72	3923037.87	3780968.61	3315156.98	3458584.43	3264540.68	2182724.51	3663851.63	3454651.95	3226441.91	2960256.62	2833101.19	2477423.72	3781775.73	3509092.64	3231116.14	1602590.39	1558179.67	1282348.55	2228233.11	2101271.83	1875486.71	1900712.22	1834182.08	1168415.12		
1884	Glyceraldehyde-3-phosphate dehydrogenase_ testis-specific	O14556	106.08	36.59	20.39	1.07	2.77	0.68	0.96	0.36	0.63	0.35	0.75	0.38	2.13	0.97	0.60	4.64	1.07	0.47	2.22	0.19	0.06	3985.41	3986.25	4172.28	1858.39	1974.80	1437.76	2131.70	1801.70	1669.30	2244.29	1794.28	0.12		
1885	Glycerol-3-phosphate dehydrogenase [NAD(+)]_ cytoplasmic	P21695	0.14	2.53	0.81	1.07	2.77	0.68	0.96	0.36	0.63	0.35	0.75	0.38	2.13	0.97	0.60	4.64	1.07	0.47	2.22	0.19	0.06	25091.01	24737.98	22168.38	17464.85	16499.09	13724.20	17065.74	17639.12	17651.15	17458.67	17712.59	11325.19		x
1886	Glycerol-3-phosphate dehydrogenase_ mitochondrial	P43304	346990.08	341544.97	323199.21	176415.64	172178.10	165329.54	244665.41	234778.23	227494.71	242264.14	226123.91	211188.76	241054.99	227334.09	190886.20	205699.18	196407.07	192711.19	201179.47	193627.33	182311.06	95313.15	90565.51	80664.27	48638.63	44127.66	37346.17	82704.81	78569.57	75716.45	68766.31	64926.72	42989.53		
1887	Glycerol-3-phosphate phosphatase	A6NDG6	62733.16	58064.86	55724.44	44946.59	43742.06	41859.09	49811.41	46234.73	45282.59	57326.21	53694.69	48147.61	47547.16	41961.57	41755.27	41851.32	41069.99	41435.92	47741.74	44633.28	45648.69	2.27	1.49	0.65	0.84	1.31	0.69	4.16	2.47	0.11	4.90	4.98	0.12	x	
1888	Glycine cleavage system H protein_ mitochondrial	P23434	14908.82	13340.75	12142.74	14801.00	13904.33	11926.32	12202.93	11147.43	11095.77	16940.30	16067.55	13800.52	14301.46	12830.15	5276.41	13591.34	12743.38	12305.53	14872.81	13230.29	10371.66	9397.36	8524.53	6630.28	2880.99	2802.20	3003.87	3591.97	2489.84	3459.04	2794.35	2209.28	1996.82		
1889	Glycine receptor subunit alpha-2	P23416	0.14	2.53	0.81	1.07	2.77	0.68	0.96	0.36	0.63	0.35	0.75	0.38	2.13	0.97	0.60	4.64	1.07	0.47	2.22	0.19	0.06	5721.54	6436.84	5069.88	4430.47	4295.87	5195.74	2164.69	2876.51	2944.49	5011.80	3564.75	706.38		x
1890	Glycine receptor subunit alpha-3	O75311	13383.38	12735.70	11863.44	8179.37	7860.08	7262.49	11191.06	11031.59	10079.03	10752.50	9849.40	9411.28	10272.53	9953.83	8376.71	9143.70	8546.30	8101.49	8250.45	7865.15	7349.61	2.27	1.49	0.65	0.84	1.31	0.69	4.16	2.47	0.11	4.90	4.98	0.12	x	
1891	Glycine--tRNA ligase	P41250	573529.96	566113.59	527818.46	456006.54	436930.48	408574.75	488682.59	483622.47	463434.75	501074.59	481918.49	458601.31	480006.17	432915.60	349797.57	443273.70	414555.30	397337.82	449581.26	418300.38	401090.80	287873.02	252008.33	229411.78	215014.50	200995.14	179489.53	235950.54	225340.48	200596.39	255235.81	239284.65	155725.46		
1892	Glycogen [starch] synthase_ muscle	P13807	135843.47	129642.16	125058.21	89596.89	86610.72	83634.34	105661.54	102425.71	98654.13	107040.92	101372.06	95590.21	117431.41	109009.83	95512.93	81708.98	75507.15	76173.54	93859.04	89870.82	86656.82	2.27	1.49	0.65	0.84	1.31	0.69	4.16	2.47	0.11	4.90	4.98	0.12	x	
1893	Glycogen debranching enzyme	P35573	0.14	2.53	0.81	1.07	2.77	0.68	0.96	0.36	0.63	0.35	0.75	0.38	2.13	0.97	0.60	4.64	1.07	0.47	2.22	0.19	0.06	2261.61	2549.94	2831.56	2407.26	2220.17	1984.38	3023.66	2669.77	3027.99	2889.22	2697.56	1877.02		x
1894	Glycogen phosphorylase_ brain form	P11216	313550.26	301431.98	290685.40	218942.73	208666.49	195542.92	259473.10	247657.80	242824.06	279950.01	265879.82	255733.53	257992.12	239822.09	197278.08	221437.50	214788.33	209350.92	209703.11	194861.73	184795.52	220450.45	208767.89	196747.36	149807.76	143957.96	126264.52	163380.65	155973.97	146412.70	164575.92	153170.65	110472.08		
1895	Glycogen phosphorylase_ liver form	P06737	1014114.13	992467.03	948302.48	722310.95	691138.91	647141.53	832910.00	813402.74	774830.34	884189.58	843661.72	790564.56	824821.84	776180.30	661313.34	718175.75	664749.51	657030.58	712512.04	665047.28	635128.38	714690.50	676513.73	621822.94	470510.02	449651.54	393993.62	552110.53	526388.23	507275.22	521708.83	502326.31	342147.24		
1896	Glycogen phosphorylase_ muscle form	P11217	214545.78	216129.43	208455.52	137527.32	134396.88	127401.49	162352.58	152471.49	154733.15	160196.17	154147.26	146838.30	158371.95	148551.06	129991.42	148360.41	143632.06	136947.71	145146.18	139574.54	131561.25	57114.20	51054.24	46114.29	29784.52	29418.82	23913.77	44411.60	39864.55	37918.28	50515.38	49587.47	30991.83		
1897	Glycogen synthase kinase-3 alpha	P49840	19286.33	18881.55	18033.78	20022.54	19150.97	17725.62	22344.21	22621.33	22012.12	21727.91	20834.34	18989.98	17007.62	15851.96	15208.76	21385.55	20590.31	19063.97	20108.61	18542.34	17621.61	3878.47	3863.04	3215.52	2692.59	2423.22	2083.24	3250.85	3203.61	3047.31	2844.17	2699.83	1708.72		
1898	Glycogen synthase kinase-3 beta	P49841	70665.09	68878.64	64316.64	48950.88	46927.11	44369.17	58398.06	55254.90	54350.42	58439.86	55080.46	52908.78	52989.75	51110.73	44152.82	47872.78	45529.38	43871.09	43943.59	44003.75	43110.02	27457.70	27469.27	23107.07	14722.89	14899.92	13773.39	19472.40	19084.74	19353.59	17561.09	17866.47	12933.30		
1899	Glycogenin-1	P46976	68159.53	61871.16	55951.10	25679.72	25490.28	21139.32	30985.80	28709.93	25588.19	40627.85	39129.15	32535.20	46248.00	42919.02	27767.40	26300.80	25408.73	24588.39	31600.49	30111.03	26686.93	11485.94	8787.29	6645.86	5937.60	5444.74	4463.77	8336.00	7637.89	6176.72	8872.38	8130.20	4820.14		
1900	Glycophorin-A	P02724	0.14	2.53	0.81	1.07	2.77	0.68	0.96	0.36	0.63	0.35	0.75	0.38	2.13	0.97	0.60	4.64	1.07	0.47	2.22	0.19	0.06	4702.52	5221.99	3950.83	1702.71	1854.81	1422.93	2323.56	2023.82	2256.80	1528.28	1821.29	1199.95		x
1901	Glycosaminoglycan xylosylkinase	O75063	15505.68	18707.36	17110.26	12890.51	11746.46	9751.11	14274.65	13106.62	13474.78	15941.96	15350.22	13507.90	15076.26	15222.34	12439.29	13793.56	11655.82	10837.19	11544.22	10551.60	10780.26	2.27	1.49	0.65	0.84	1.31	0.69	4.16	2.47	0.11	4.90	4.98	0.12	x	
1902	Glycylpeptide N-tetradecanoyltransferase 1	P30419	8871.57	7120.86	6392.29	6179.54	5804.63	4167.50	6372.96	5599.30	4592.67	6719.84	6439.83	6023.39	6973.18	6922.91	6844.30	6424.97	3896.27	4885.09	6463.56	5614.62	5521.04	64457.62	61862.29	55260.10	52292.95	51178.60	42531.80	53483.58	57076.55	50388.73	58125.39	61194.92	44153.56		
1903	Glycylpeptide N-tetradecanoyltransferase 2	O60551	3390.94	2875.26	2980.08	2600.88	2506.53	2402.34	2740.90	2615.65	2738.62	3116.79	3117.40	2913.73	3203.64	2932.46	2364.25	2628.97	2351.05	2378.83	2033.71	2229.11	2199.62	6392.92	5729.08	5754.49	3628.11	3624.71	3223.13	4184.67	3778.34	3871.99	4188.70	3806.97	4380.00		
1904	Glyoxalase domain-containing protein 4	Q9HC38	51324.40	48735.89	46342.51	34348.35	33561.87	30258.30	43296.58	45057.00	40504.86	41201.90	39223.98	34758.87	41137.77	39353.65	31444.22	34411.90	33655.32	31636.13	34518.92	32421.31	30386.27	62727.30	59985.82	52056.04	41743.67	38016.45	29532.09	46662.10	42623.67	38812.57	51184.66	50062.54	30871.89		
1905	Glyoxylate reductase/hydroxypyruvate reductase	Q9UBQ7	0.14	2.53	0.81	1.07	2.77	0.68	0.96	0.36	0.63	0.35	0.75	0.38	2.13	0.97	0.60	4.64	1.07	0.47	2.22	0.19	0.06	27748.85	25195.77	22395.96	18255.29	16805.64	14816.87	25934.32	20432.05	21770.89	21186.06	24493.60	14748.58		x
1906	GMP reductase 2	Q9P2T1	0.14	2.53	0.81	1.07	2.77	0.68	0.96	0.36	0.63	0.35	0.75	0.38	2.13	0.97	0.60	4.64	1.07	0.47	2.22	0.19	0.06	9235.55	8242.31	8391.53	3896.24	3610.57	3211.53	5098.56	4879.86	4496.99	5330.79	4648.47	3126.68		x
1907	GMP synthase [glutamine-hydrolyzing]	P49915	487398.82	456455.35	427268.30	315851.19	306665.67	282083.13	372382.56	354128.24	332428.32	395244.28	382638.13	369467.60	410162.91	379006.42	294169.76	296014.99	284677.14	279210.27	348181.07	330438.42	306861.59	123852.01	127921.92	112384.67	82714.37	79613.59	63464.69	98743.29	90800.88	83728.24	110424.25	106006.65	69712.29		
1908	Golgi apparatus protein 1	Q92896	311584.84	292373.39	261930.30	227166.84	224918.03	203547.21	229126.76	238138.63	218708.64	271253.67	262981.76	243324.26	261766.11	259309.06	280534.00	209672.05	203536.23	199038.15	220817.27	210121.80	202543.46	3776.58	3082.53	3082.44	1583.71	1526.77	1156.38	2800.29	2504.71	2049.99	2145.22	1976.20	1239.64		
1909	Golgi membrane protein 1	Q8NBJ4	1145.30	840.98	458.63	130.99	281.74	186.12	176.63	281.33	213.80	827.87	748.31	362.93	953.46	104.31	20.37	265.38	142.32	185.27	13.07	30.70	10.35	2.27	1.49	0.65	0.84	1.31	0.69	4.16	2.47	0.11	4.90	4.98	0.12	x	
1910	Golgi phosphoprotein 3	Q9H4A6	7993.67	6918.72	19500.06	5321.35	4976.62	4627.86	5810.13	5330.25	5327.86	20015.66	21706.11	18545.98	6830.48	11737.75	4557.28	4937.92	4595.29	4574.27	5804.28	5283.67	4679.55	23646.96	22504.29	19061.95	19646.54	17814.75	13783.10	18155.56	17548.71	15892.15	20555.54	21063.77	11423.60		
1911	Golgi reassembly-stacking protein 2	Q9H8Y8	123640.32	118776.10	116768.23	76365.78	74011.04	71147.17	90895.36	89678.79	87230.23	87546.24	86605.21	80169.39	95849.57	86713.42	77148.40	78692.35	75045.67	72286.86	73687.94	69408.33	67965.05	1585.29	1306.64	1386.99	1014.51	883.28	867.89	1663.81	1426.77	1484.96	1419.62	1537.92	519.57		
1912	Golgi resident protein GCP60	Q9H3P7	77995.83	76205.56	71462.17	57104.28	55994.55	54083.74	64788.72	61844.74	63948.25	71792.52	67907.03	66317.86	69393.41	64561.56	54997.46	63257.59	58904.51	57610.13	59331.68	53459.51	51388.09	33066.37	30597.16	27560.11	21512.62	19307.31	18959.38	22692.90	22061.31	22295.62	27451.06	24932.83	16937.50		
1913	Golgi SNAP receptor complex member 2	O14653	428.99	203.45	123.27	574.10	475.97	303.92	410.19	320.32	249.01	513.40	445.05	342.30	473.18	503.67	30.87	387.07	317.26	247.01	508.42	331.68	200.26	2.27	1.49	0.65	0.84	1.31	0.69	4.16	2.47	0.11	4.90	4.98	0.12	x	
1914	Golgi to ER traffic protein 4 homolog	Q7L5D6	13342.10	12258.44	10978.05	6566.62	6558.69	6067.04	10501.70	9720.47	9571.24	9064.11	8229.11	7990.58	10118.74	8388.73	7241.45	7676.69	6775.64	7152.94	7125.04	6494.68	6146.74	12.27	269.98	10.99	475.74	410.35	748.68	8.31	140.79	69.18	824.73	521.16	1.04		
1915	Golgin subfamily A member 1	Q92805	14568.38	15066.68	13847.08	10671.28	10727.10	9846.49	10756.30	11281.77	11193.66	12211.63	11667.80	11793.24	12079.98	11582.48	11175.25	10498.10	9445.99	9268.54	10963.72	10060.50	9622.08	2.27	1.49	0.65	0.84	1.31	0.69	4.16	2.47	0.11	4.90	4.98	0.12	x	
1916	Golgin subfamily A member 2	Q08379	80672.85	78851.40	73058.94	61457.41	58007.49	54324.23	69670.50	68093.79	66087.85	73283.07	69005.98	63791.01	69092.64	62841.44	52335.59	60618.04	56609.86	54383.34	59805.00	53835.72	53684.49	650.07	834.21	728.25	523.66	451.67	466.63	424.52	403.82	474.41	4.90	4.98	0.12		
1917	Golgin subfamily A member 3	Q08378	22216.10	22081.43	20455.20	15683.47	14746.98	13489.89	18206.02	17836.49	16875.94	21102.98	20004.35	18655.12	17131.95	15914.31	12796.79	17078.28	16842.34	15426.55	17085.75	16741.04	14287.64	28617.29	25932.90	22010.58	20564.76	18267.28	13901.43	25950.35	25545.49	21639.26	31238.64	30729.85	16494.57		
1918	Golgin subfamily A member 4	Q13439	24273.00	23326.96	21679.48	17351.77	17070.31	16040.50	19109.49	18457.84	17726.38	21574.33	20423.47	19970.34	19981.59	20572.08	16442.76	15081.11	15222.92	14976.72	17572.71	16120.82	16063.69	42124.34	43550.51	34444.30	24989.58	24824.44	20645.35	30202.49	28749.87	26492.39	33208.47	31443.44	17927.08		
1919	Golgin subfamily A member 5	Q8TBA6	66107.80	63280.61	59396.65	47866.80	42607.24	38720.69	50092.12	48684.33	46291.56	59134.49	54910.47	51863.87	56474.30	59829.72	42420.62	42634.79	41162.42	39378.25	46514.64	43196.43	41902.50	2.27	1.49	0.65	0.84	1.31	0.69	4.16	2.47	0.11	4.90	4.98	0.12	x	
1920	Golgin subfamily A member 8C	A6NN73	0.14	2.53	0.81	1.07	2.77	0.68	0.96	0.36	0.63	0.35	0.75	0.38	2.13	0.97	0.60	4.64	1.07	0.47	2.22	0.19	0.06	260.53	147.24	117.58	275.50	228.81	275.13	227.10	262.90	314.77	278.35	306.53	289.76		x
1921	Golgin subfamily A member 8R	I6L899	29522.62	28940.11	26417.77	20619.70	19619.67	19038.37	21243.69	21203.88	21266.03	23522.25	21510.82	21398.28	24452.32	25450.74	21371.37	18544.65	17785.02	19040.08	19343.30	18035.93	18242.23	22.46	43.34	6.35	50.63	60.45	57.05	76.70	78.86	59.85	64.12	144.17	89.51		
1922	Golgin subfamily B member 1	Q14789	423740.34	405903.21	369734.35	309449.95	300405.89	275712.41	342802.18	340094.36	316488.95	384677.54	373516.01	343037.68	369489.94	339954.65	258684.01	300572.49	285042.89	280071.51	320838.37	300606.44	278669.85	39276.22	37106.18	35290.31	26418.78	25639.52	22200.42	32648.41	31190.92	30272.97	33206.43	30717.38	20237.54		
1923	Golgi-specific brefeldin A-resistance guanine nucleotide exchange factor 1	Q92538	25490.95	24991.72	21709.21	14810.75	13958.87	12834.36	17537.67	16747.59	16086.80	20995.57	20044.04	17888.80	20046.56	19074.52	12199.87	15974.51	16630.86	14730.22	16869.32	15738.03	13942.61	16237.81	17447.83	14849.84	11388.04	11102.29	10810.71	12679.61	12444.36	11737.31	12816.49	12969.49	10860.08		
1924	GON-4-like protein	Q3T8J9	3448.62	3348.62	3623.26	2525.06	2377.88	2341.73	3249.58	3435.00	3258.14	2580.12	2635.98	2569.46	3210.19	3025.90	2408.23	3125.88	2744.94	2411.39	2870.37	2773.07	2592.17	2.27	1.49	0.65	0.84	1.31	0.69	4.16	2.47	0.11	4.90	4.98	0.12	x	
1925	G-patch domain and KOW motifs-containing protein	Q92917	32794.30	29115.67	25473.47	15321.49	14424.84	13198.54	18083.45	16379.21	15900.85	21906.13	21149.55	19003.86	22682.63	15378.33	10817.21	15142.45	12841.58	13088.10	18257.58	14628.25	12873.99	29111.78	26203.11	23444.29	17478.53	17081.61	15241.22	23835.91	22829.93	21787.03	26266.22	22347.47	10750.73		
1926	GPI transamidase component PIG-T	Q969N2	17054.34	14318.34	11878.88	12447.73	10666.36	11504.66	11130.58	10219.73	9090.62	13191.71	12855.62	13483.02	13489.85	12513.26	9487.29	9931.13	9051.10	9685.45	8287.46	9534.55	8332.47	9508.67	9170.02	7927.87	5461.98	5088.96	4989.05	6476.64	6075.94	5876.24	5250.65	5171.64	3776.87		
1927	GPN-loop GTPase 1	Q9HCN4	1957.36	1852.89	2051.66	1333.00	1344.67	1201.82	1047.93	34.33	986.77	1649.19	1745.94	1932.40	1599.89	59.29	539.84	465.24	875.47	936.65	1633.63	1489.53	1375.63	1510.79	1499.09	1327.21	1100.03	979.97	1097.54	1185.22	1060.21	1149.47	1281.29	86.41	10.41		
1928	GPN-loop GTPase 3	Q9UHW5	2030.91	1807.50	1245.45	1253.56	1468.45	1522.18	1203.97	480.94	1678.93	1959.90	1850.83	1634.98	1675.10	474.42	126.77	1141.36	487.28	700.21	1476.38	1776.57	1324.37	2500.25	2390.66	2194.77	1063.34	1081.43	986.65	1236.82	1256.42	1069.39	898.74	876.26	623.72		
1929	G-protein-signaling modulator 1	Q86YR5	0.14	2.53	0.81	1.07	2.77	0.68	0.96	0.36	0.63	0.35	0.75	0.38	2.13	0.97	0.60	4.64	1.07	0.47	2.22	0.19	0.06	13083.90	13032.18	10830.33	6286.50	6357.22	5802.80	8872.14	8785.39	8794.72	6207.25	5730.98	4253.34		x
1930	G-protein-signaling modulator 2	P81274	1946.60	1568.11	1347.03	832.93	919.65	716.81	917.09	963.93	1117.80	1367.82	1369.72	1144.11	1183.62	1198.86	389.76	1044.19	1309.84	1412.89	1646.99	1675.04	1711.02	2.27	1.49	0.65	0.84	1.31	0.69	4.16	2.47	0.11	4.90	4.98	0.12	x	
1931	Grancalcin	P28676	74905.19	76827.53	68621.68	42079.03	38145.13	35704.63	48252.75	44995.01	43987.93	65213.36	61467.08	58690.53	56308.09	50789.68	44891.66	43657.20	40861.66	38262.61	41186.93	37439.26	35938.72	2.27	1.49	0.65	0.84	1.31	0.69	4.16	2.47	0.11	4.90	4.98	0.12	x	
1932	GRB10-interacting GYF protein 2	Q6Y7W6	35508.77	36748.91	34611.23	25568.49	25612.82	23908.09	30019.47	27261.99	25816.66	31947.81	31105.17	29096.96	30186.42	28829.07	24763.03	23757.08	23642.87	22215.38	22086.69	23354.97	22077.54	2.27	1.49	0.65	0.84	1.31	0.69	4.16	2.47	0.11	4.90	4.98	0.12	x	
1933	GRB2-related adapter protein	Q13588	22570.36	18981.58	14304.20	4109.09	2469.73	1394.79	2123.50	829.59	1263.54	16803.71	14569.15	12646.21	15439.33	2239.25	608.39	2877.57	1568.39	1758.85	7715.92	3617.98	3063.45	2.27	1.49	0.65	0.84	1.31	0.69	4.16	2.47	0.11	4.90	4.98	49785.83	x	
1934	G-rich sequence factor 1	Q12849	35400.00	32583.47	29674.14	24583.88	22928.96	21087.85	53343.71	50699.70	49053.30	31465.55	28902.75	26653.74	31215.70	28636.27	22284.35	23739.55	21865.86	20752.79	24956.48	23148.32	20680.66	2.27	1.49	0.65	0.84	1.31	0.69	4.16	2.47	0.11	4.90	4.98	0.12	x	
1935	GRIP1-associated protein 1	Q4V328	52566.52	51490.36	49089.57	36057.67	34092.67	32150.72	38853.08	35928.48	35688.91	47861.75	42881.25	42814.86	42402.43	39121.27	30632.27	39376.85	35753.34	34880.07	32351.66	32391.46	28393.46	48184.49	44703.59	41235.25	38771.61	36429.83	34632.93	60954.91	58378.97	55566.62	46310.30	46507.31	29638.05		
1936	Growth arrest and DNA damage-inducible proteins-interacting protein 1	Q8TAE8	84899.47	84437.12	80596.68	57809.10	57015.34	51342.59	65714.00	66779.40	61380.52	76914.95	74592.98	71199.90	69538.29	67821.89	43248.65	54307.24	52279.61	53002.56	61537.99	56519.90	52698.92	16838.44	16163.52	14427.06	11945.00	10729.22	8660.83	14505.09	13501.94	11948.80	14191.29	13656.56	6435.62		
1937	Growth factor receptor-bound protein 2	P62993	162903.66	154876.36	148514.73	122302.31	113268.39	107532.93	126872.22	125102.28	124785.26	147188.81	138941.49	131682.72	137013.18	128868.51	106104.99	116934.71	110500.78	106942.38	120770.75	109376.50	106161.16	41276.62	38061.82	36081.38	33917.77	34375.54	29847.25	50177.14	50627.55	47349.03	36426.64	35752.99	25621.81		
1938	Growth hormone-inducible transmembrane protein	Q9H3K2	0.14	2.53	0.81	1.07	2.77	0.68	0.96	0.36	0.63	0.35	0.75	0.38	2.13	0.97	0.60	4.64	1.07	0.47	2.22	0.19	0.06	9734.31	8834.92	7842.94	6238.61	6132.04	5892.95	6993.41	6778.33	6627.57	7036.81	6926.36	5010.68		x
1939	Growth/differentiation factor 5	P43026	16696.31	13704.93	12342.47	8464.40	8014.02	6490.44	11624.68	10237.31	9430.35	13023.20	12053.95	8630.89	11916.50	11658.48	6815.79	9719.83	9087.92	7979.03	12119.35	10044.74	9016.51	2.27	1.49	0.65	0.84	1.31	0.69	4.16	2.47	0.11	4.90	4.98	0.12	x	
1940	GrpE protein homolog 1_ mitochondrial	Q9HAV7	52844.87	50563.38	44234.18	34628.65	32323.94	27922.82	41345.09	35480.56	35302.67	39846.34	37446.49	33519.57	41507.85	31364.77	28626.13	34269.47	32238.63	27927.37	35066.15	33748.16	30982.24	17758.10	16645.88	14076.78	12141.59	12539.77	10747.08	14420.83	13391.47	12964.86	15908.59	14720.21	9701.36		
1941	GTP:AMP phosphotransferase AK3_ mitochondrial	Q9UIJ7	16228.49	14154.48	13765.28	8899.41	9909.33	9165.70	10984.02	10961.90	10573.53	12539.91	11859.18	12164.96	10518.94	11785.43	11792.93	12094.00	9137.91	8857.15	10169.99	10291.49	9258.23	2.27	1.49	0.65	0.84	1.31	0.69	4.16	2.47	0.11	4.90	4.98	0.12	x	
1942	GTPase HRas	P01112	356454.51	323579.16	266125.98	217626.92	211621.60	173479.74	263886.29	265473.93	232468.65	277462.22	262152.83	220125.78	274293.81	251824.87	153583.36	235117.49	226114.28	198490.86	209053.90	197532.17	165649.96	157611.06	151625.49	135474.80	64442.62	63649.47	53631.61	72170.24	70881.66	66114.83	54494.58	52930.11	34265.94		
1943	GTPase IMAP family member 4	Q9NUV9	5175.37	5526.31	5284.34	3871.74	3688.68	2990.89	2725.79	2905.31	4189.91	4667.47	5201.35	5455.37	4045.30	3392.66	4032.26	2139.29	1737.10	1958.89	3358.96	3085.33	2853.40	2.27	1.49	0.65	0.84	1.31	0.69	4.16	2.47	0.11	4.90	4.98	0.12	x	
1944	GTPase IMAP family member 5	Q96F15	0.14	2.53	0.81	1.07	2.77	0.68	0.96	0.36	0.63	0.35	0.75	0.38	2.13	0.97	0.60	4.64	1.07	0.47	2.22	0.19	0.06	10150.32	9457.89	7980.66	4388.04	4398.43	2160.59	4518.12	4419.72	3632.01	7372.11	7049.30	3289.07		x
1945	GTPase KRas	P01116	20835.12	22716.90	20141.62	12949.28	12826.07	12663.56	16224.15	17606.24	16394.73	16289.28	15605.74	15543.44	14578.67	16227.65	13006.43	14756.50	14755.44	13513.60	15120.79	14794.18	14154.98	2.27	1.49	0.65	0.84	1.31	0.69	4.16	2.47	0.11	4.90	4.98	0.12	x	
1946	GTPase NRas	P01111	81117.62	80251.47	74273.17	45201.30	41978.08	40632.48	67301.86	63339.85	60074.73	61545.83	59282.12	55341.40	64667.96	57099.63	49750.19	50725.58	47270.31	47285.40	49376.93	47899.76	44294.74	519.10	535.78	451.29	378.97	384.70	161.59	395.90	393.64	310.43	502.58	439.57	220.94		
1947	GTPase-activating protein and VPS9 domain-containing protein 1	Q14C86	142570.39	134500.20	129968.44	110259.57	103661.53	101796.91	119551.17	114560.91	111710.87	121087.62	117330.60	111859.79	123378.22	112128.93	104838.59	102780.70	98920.91	102180.20	124137.09	111054.80	103420.81	2.27	1.49	0.65	0.84	1.31	0.69	4.16	2.47	0.11	4.90	4.98	0.12	x	
1948	GTP-binding nuclear protein Ran	P62826	747559.05	719962.81	699075.36	490495.79	480449.56	469244.81	664932.78	655443.76	625799.91	575529.12	550178.85	518611.68	615214.77	583021.46	540553.97	505245.87	473091.72	467610.58	525834.19	507290.13	483868.15	682889.29	637844.46	601175.11	373607.52	363120.56	329714.38	458363.13	434202.05	419432.37	406973.83	397928.75	262929.30		
1949	GTP-binding protein Rheb	Q15382	57975.63	56777.07	54121.05	40367.97	40999.69	37297.37	49915.51	48318.46	44853.81	50909.27	47276.81	44008.01	47639.69	45914.45	35527.04	40734.29	39760.69	37254.83	41563.88	40595.36	37429.97	32993.00	30930.81	28522.65	22186.41	21908.71	18118.96	26319.66	25976.13	24201.14	26991.43	25242.90	17428.31		
1950	GTP-binding protein Rhes	Q96D21	0.14	2.53	0.81	1.07	2.77	0.68	0.96	0.36	0.63	0.35	0.75	0.38	2.13	0.97	0.60	4.64	1.07	0.47	2.22	0.19	0.06	5354.33	4170.87	4579.07	4316.80	4145.60	3890.61	3530.01	4113.13	3687.14	4600.13	4752.65	3494.53		x
1951	GTP-binding protein Rit1	Q92963	0.14	2.53	0.81	1.07	2.77	0.68	0.96	0.36	0.63	0.35	0.75	0.38	2.13	0.97	0.60	4.64	1.07	0.47	2.22	0.19	0.06	2.27	1.49	0.65	137.72	211.23	100.80	53.32	65.91	3.42	483.62	417.52	130.10		x
1952	GTP-binding protein SAR1a	Q9NR31	57289.80	55357.71	47150.14	29105.09	28275.46	25580.10	40971.36	39972.74	35462.28	43584.03	40954.95	35311.47	36926.94	35916.92	30924.55	35679.41	32994.15	32061.90	36706.90	34000.69	34958.50	7785.99	6574.12	8253.40	4666.56	4624.18	4427.42	6486.09	6417.64	4949.52	5903.31	6183.96	2838.76		
1953	GTP-binding protein SAR1b	Q9Y6B6	47380.14	43832.05	40568.65	31322.19	30190.60	27192.64	37593.35	34472.95	32589.08	41950.32	40127.02	39055.78	40622.47	36491.63	27398.35	32134.44	30256.01	29704.84	30718.92	30263.24	27322.50	22193.17	20918.72	18943.18	13515.80	12694.17	10665.60	15933.53	15760.58	14230.48	16467.02	15421.14	9412.55		
1954	Guanidinoacetate N-methyltransferase	Q14353	0.14	2.53	0.81	1.07	2.77	0.68	0.96	0.36	0.63	0.35	0.75	0.38	2.13	0.97	0.60	4.64	1.07	0.47	2.22	0.19	0.06	2765.65	2442.45	2135.98	2314.44	2213.28	1232.37	2455.91	2131.96	1724.41	3471.71	3376.01	1631.40		x
1955	Guanine nucleotide exchange factor DBS	O15068	11980.70	11519.41	11490.52	7396.94	7227.23	6858.67	8845.84	9008.72	7955.87	9533.54	8904.19	8492.22	9856.26	9322.33	7367.91	7893.38	7605.58	7053.57	7374.96	7411.02	6722.19	1085.04	848.08	746.59	689.02	651.82	333.79	781.18	680.15	482.68	1010.89	1046.48	347.52		
1956	Guanine nucleotide exchange factor VAV2	P52735	35564.06	32712.04	28819.85	31960.41	31289.10	26642.16	35104.62	27729.02	25124.04	32383.99	31723.74	28451.72	33325.21	36942.80	25642.88	30137.34	25263.00	27488.38	34522.32	32037.40	28355.76	74325.25	69894.63	60259.20	41714.43	40192.88	39029.16	61625.52	58784.37	55614.24	50612.76	47548.88	34264.21		
1957	Guanine nucleotide-binding protein G(i) subunit alpha-1	P63096	1615.82	1526.12	1137.16	531.50	507.69	628.97	723.16	703.03	846.81	1115.48	1135.44	1045.58	884.84	1018.41	481.53	942.37	652.39	705.00	736.92	684.84	551.16	9483.43	6997.16	8413.26	7233.27	7816.25	6886.22	7143.83	7128.30	6872.81	7630.42	7910.39	4716.39		
1958	Guanine nucleotide-binding protein G(i) subunit alpha-2	P04899	87378.99	74681.11	67097.81	58915.97	56488.30	48489.75	68312.15	62989.82	59313.66	78356.09	74250.44	67192.11	71491.92	66795.87	41925.69	57539.16	53708.43	47096.44	65454.27	63553.05	55377.24	50789.83	47392.22	40593.03	35660.89	35004.90	32161.79	41373.50	39772.04	36786.84	47681.41	45003.05	32329.01		
1959	Guanine nucleotide-binding protein G(I)/G(S)/G(O) subunit gamma-12	Q9UBI6	0.14	2.53	0.81	1.07	2.77	0.68	0.96	0.36	0.63	0.35	0.75	0.38	2.13	0.97	0.60	4.64	1.07	0.47	2.22	0.19	0.06	75506.12	71533.31	65426.07	54718.93	53333.79	49703.42	52537.31	52368.45	51861.73	65539.23	68202.87	49456.24		x
1960	Guanine nucleotide-binding protein G(I)/G(S)/G(T) subunit beta-1	P62873	71157.74	70930.93	64776.68	46361.50	46397.59	43323.35	57362.12	55012.98	53279.94	57676.10	56995.81	54866.75	58189.27	58588.90	49471.73	44902.77	42757.72	42565.28	46759.39	45054.27	43759.79	114588.46	102724.27	99636.91	60720.90	57922.48	54335.08	71077.10	66085.84	62953.91	73865.68	71505.52	56278.94		
1961	Guanine nucleotide-binding protein G(I)/G(S)/G(T) subunit beta-2	P62879	175634.85	172019.82	158633.51	111659.74	106947.26	100874.61	141257.84	136876.65	133704.09	136104.74	128327.76	120067.03	139139.66	137447.75	118489.68	110568.05	101029.13	102488.17	112587.18	106744.98	106246.07	11887.73	10992.26	10118.03	5905.11	5728.07	5258.68	6250.09	6100.90	5324.00	6526.31	5703.06	4574.10		
1962	Guanine nucleotide-binding protein G(I)/G(S)/G(T) subunit beta-3	P16520	19659.69	15637.82	18099.46	14831.14	12435.45	12650.73	13099.51	13448.70	13264.60	18016.52	16374.52	15853.65	17198.01	17607.95	14114.28	11058.08	10826.68	10566.79	13020.54	12311.44	14006.43	14871.78	13698.87	11617.57	11501.54	10742.31	11276.89	11207.51	11020.33	10568.01	13684.69	11649.37	6886.92		
1963	Guanine nucleotide-binding protein G(k) subunit alpha	P08754	20490.04	19829.33	18160.81	11451.98	11869.92	9643.69	13890.49	13672.22	11080.23	15293.45	14110.46	12556.29	15007.00	13968.39	10439.74	14685.17	12023.78	13643.02	14449.69	13368.28	11805.03	37284.11	36996.17	33219.41	34829.34	31991.50	23283.12	41065.44	38566.21	34444.83	45851.66	43680.07	26599.71		
1964	Guanine nucleotide-binding protein G(o) subunit alpha	P09471	21249.16	20188.81	20473.71	15015.00	15116.86	11574.96	18277.94	19129.21	17236.77	19446.92	17868.90	16069.12	19843.67	17741.39	10481.75	15028.39	14972.24	13778.51	16039.73	15137.38	14789.77	729.66	1016.75	821.18	620.87	593.16	372.57	768.86	599.37	484.60	883.02	788.03	497.52		
1965	Guanine nucleotide-binding protein G(olf) subunit alpha	P38405	12220.03	11075.55	10420.15	8634.14	8206.54	7588.27	11189.17	9898.89	10387.30	12704.19	11784.15	11155.85	11171.39	9891.55	7983.30	7866.81	7298.84	7579.10	8484.71	7811.42	7599.89	18857.60	16838.99	15324.15	10795.28	11252.32	10301.02	11829.23	11739.02	10590.46	13132.48	11646.63	14630.28		
1966	Guanine nucleotide-binding protein G(s) subunit alpha isoforms XLas	Q5JWF2	141619.27	133048.51	127460.36	93738.79	91650.05	77475.83	108051.49	103807.01	96419.91	120003.98	111590.60	106835.21	112941.24	109428.68	100485.52	92204.21	89921.20	84885.97	96013.21	88216.01	85385.23	55436.08	49660.51	46106.37	36685.36	35199.65	31666.09	44085.24	44074.71	40543.45	49172.78	49587.55	34128.33		
1967	Guanine nucleotide-binding protein G(t) subunit alpha-1	P11488	36297.24	34709.56	31333.57	24405.26	23717.51	22169.43	26436.30	25988.71	23643.35	36230.50	35738.68	34727.76	32021.21	28181.07	25264.70	22982.88	22684.40	22224.35	25689.14	24357.26	24033.84	2.27	1.49	0.65	0.84	1.31	0.69	4.16	2.47	0.11	4.90	4.98	0.12	x	
1968	Guanine nucleotide-binding protein G(t) subunit alpha-2	P19087	9925.17	7853.77	5414.90	4552.01	4627.53	3424.65	5576.52	5178.24	4874.18	7688.91	7038.32	5179.84	6314.12	6118.67	1784.99	5181.28	4802.04	4668.56	5098.61	4235.30	3677.41	12696.89	10970.89	10369.71	6797.05	6500.55	5454.96	7217.83	7402.68	6579.97	7513.75	7922.35	4752.23		
1969	Guanine nucleotide-binding protein G(t) subunit alpha-3	A8MTJ3	694.95	699.81	670.10	616.74	712.51	671.51	1000.46	924.67	860.20	1018.86	1025.50	1059.14	791.45	859.77	718.55	714.52	694.31	690.08	556.31	565.37	628.07	8012.05	7714.21	6585.81	5419.95	4927.19	4021.79	6279.41	5839.86	5346.07	6582.89	6185.42	7281.73		
1970	Guanine nucleotide-binding protein subunit alpha-11	P29992	1497.64	1128.96	1106.82	1199.72	1065.87	1086.64	1243.67	1404.93	1249.36	1241.20	1396.76	1473.20	1465.76	1648.97	1404.23	1243.29	1169.72	1189.67	1325.32	1381.17	1416.73	60175.70	55391.42	53238.09	69278.24	68823.59	66661.14	90146.88	83731.73	83985.85	61342.94	59067.40	46910.36		
1971	Guanine nucleotide-binding protein subunit alpha-12	Q03113	0.14	2.53	0.81	1.07	2.77	0.68	0.96	0.36	0.63	0.35	0.75	0.38	2.13	0.97	0.60	4.64	1.07	0.47	2.22	0.19	0.06	3779.98	3373.85	2942.00	2220.33	2010.99	1648.46	3744.80	3303.18	2649.13	2855.83	2648.79	1518.86		x
1972	Guanine nucleotide-binding protein subunit alpha-13	Q14344	514471.34	505739.63	474298.07	349652.08	327502.50	306770.14	404152.62	397795.75	374104.52	410313.72	394707.96	356422.29	426495.12	376118.82	316157.47	345043.04	323010.30	306851.41	379699.98	349068.91	330627.11	84015.52	75546.51	70618.57	50292.03	49452.78	41266.70	67535.37	65710.35	59888.14	60371.25	61286.42	40884.36		
1973	Guanine nucleotide-binding protein subunit alpha-14	O95837	0.14	2.53	0.81	1.07	2.77	0.68	0.96	0.36	0.63	0.35	0.75	0.38	2.13	0.97	0.60	4.64	1.07	0.47	2.22	0.19	0.06	5201.66	4467.38	4148.88	3005.69	2801.58	2307.06	4123.88	3982.43	3766.27	3540.12	3179.73	2325.21		x
1974	Guanine nucleotide-binding protein subunit beta-4	Q9HAV0	32244.31	30309.15	22730.68	20496.45	20156.82	16114.03	19700.16	17639.55	16950.21	28902.49	27103.48	23442.04	24944.02	23610.45	13462.42	22520.01	19093.92	17807.78	23164.97	22051.76	20297.95	4033.01	3730.04	3376.88	1936.56	1905.27	1605.43	2907.26	2835.63	2613.47	2717.92	2539.75	1814.74		
1975	Guanine nucleotide-binding protein subunit beta-5	O14775	3369.13	3206.78	2993.59	2209.59	2254.35	2193.94	2543.06	2301.24	2426.11	2881.07	2816.56	2612.29	3367.26	2790.57	2141.89	2254.05	2032.23	1955.40	2509.01	2074.03	2635.75	2.27	1.49	0.65	0.84	1.31	0.69	4.16	2.47	0.11	4.90	4.98	0.12	x	
1976	Guanine nucleotide-binding protein-like 1	P36915	99219.58	94398.73	91427.36	71000.73	71966.64	66500.61	84543.69	83278.07	80694.01	89391.36	86773.15	83805.41	89774.43	79226.59	75175.87	77009.22	70877.86	70431.63	84185.53	81824.20	78523.44	3957.73	3719.66	4087.86	3127.87	2639.49	2253.68	3473.92	3172.47	3221.01	4158.62	3555.50	2547.57		
1977	Guanylate kinase	Q16774	9171.57	9550.06	7835.64	6875.59	7045.97	6591.44	8008.45	7872.68	7525.47	8488.51	7995.05	8518.89	7750.21	6487.60	6596.08	7400.45	6991.38	6498.76	7495.82	7143.30	6247.87	2.27	1.49	0.65	0.84	1.31	0.69	4.16	2.47	0.11	4.90	4.98	0.12	x	
1978	Guanylate-binding protein 2	P32456	1440.48	1388.97	1155.64	1179.73	1010.58	907.50	1475.28	1322.44	1181.99	1258.58	1269.87	1001.15	1478.40	1553.20	515.07	1184.88	1138.73	1040.94	1733.35	1413.30	1234.85	28.56	16.18	1.29	93.20	103.03	39.33	137.74	80.26	91.66	74.22	93.28	70.42		
1979	Guanylate-binding protein 7	Q8N8V2	3588.52	2851.50	3219.11	1514.54	1719.30	1368.74	2233.82	2157.33	2459.91	2161.78	2207.27	2064.22	2749.56	2268.44	2025.21	1892.75	1818.49	2325.22	1783.79	1597.92	1553.62	2.27	1.49	0.65	0.84	1.31	0.69	4.16	2.47	0.11	4.90	4.98	0.12	x	
1980	Guanylyl cyclase-activating protein 2	Q9UMX6	4260.53	3622.84	3735.37	1889.72	2223.74	2148.67	3455.14	3056.97	2191.84	3068.97	2577.23	2683.99	2824.54	3321.78	2077.36	2590.46	2493.25	2218.88	2401.86	2367.95	2030.66	2488.34	3087.43	2211.49	1839.73	1892.40	1249.61	2066.83	2685.45	2365.01	3059.11	2787.63	1658.84		
1981	Haloacid dehalogenase-like hydrolase domain-containing 5	Q9BXW7	36262.13	32914.52	32126.23	28977.77	25240.64	28312.91	34818.24	32254.02	30865.09	30428.06	29264.92	27285.18	30481.69	27060.66	23953.04	28707.56	23704.69	27840.61	30282.55	28584.60	25385.95	17083.20	15639.60	13821.68	10319.26	9387.03	8625.80	15800.39	12903.07	10597.44	12039.21	12062.72	7760.95		
1982	Hamartin	Q92574	236.53	376.90	259.55	302.30	280.04	117.27	244.94	274.99	224.93	392.34	225.91	281.51	355.42	307.57	131.23	255.89	254.86	248.67	314.26	273.38	188.77	2.27	1.49	0.65	0.84	1.31	0.69	4.16	2.47	0.11	4.90	4.98	0.12	x	
1983	HAUS augmin-like complex subunit 5	O94927	4768.39	4073.54	3076.84	1630.89	1694.35	1512.90	2986.29	3224.52	2587.66	1945.37	1902.83	1679.48	3469.72	3378.73	1670.95	3169.35	2581.31	2566.86	1733.55	1672.59	1258.39	2535.72	2288.53	1782.46	1545.21	1573.95	1360.97	4760.03	4969.55	4312.57	2782.29	2519.27	1585.71		
1984	HBS1-like protein	Q9Y450	26190.64	27055.82	25778.65	17654.87	17877.90	17049.94	22034.73	22973.21	21578.21	24123.11	23725.48	22592.42	21748.09	21781.05	17707.16	17807.97	17003.42	15764.32	19094.87	16660.65	17378.64	5589.21	5125.71	4454.39	3813.14	3663.29	3453.80	3894.73	3983.75	3694.73	4071.77	3979.42	3182.87		
1985	HCLS1-associated protein X-1	O00165	11506.59	11099.51	10693.13	10296.90	9121.23	8769.34	8459.35	9492.45	8822.69	10839.63	9613.45	9243.24	10236.99	10871.06	8385.33	8952.30	7831.70	8173.34	8431.55	7802.82	7926.61	2.27	1.49	0.65	0.84	1.31	0.69	4.16	2.47	0.11	4.90	4.98	0.12	x	
1986	HD domain-containing protein 2	Q7Z4H3	44951.59	42499.93	42368.02	30061.59	29735.32	27886.25	35238.08	33840.95	33989.51	37693.18	37967.72	35698.29	37843.62	35913.98	31468.85	29945.65	27647.59	28557.22	30976.14	29345.26	28209.97	4998.75	5596.64	3009.38	2725.97	2614.75	2667.52	2967.82	2570.20	2151.74	3626.71	3098.41	1025.35		
1987	HEAT repeat-containing protein 3	Q7Z4Q2	0.14	2.53	0.81	1.07	2.77	0.68	0.96	0.36	0.63	0.35	0.75	0.38	2.13	0.97	0.60	4.64	1.07	0.47	2.22	0.19	0.06	54514.56	51987.31	46102.62	39407.85	38544.38	28821.82	43983.19	41926.64	37287.84	45470.03	44748.15	26010.89		x
1988	HEAT repeat-containing protein 6	Q6AI08	195085.22	185335.22	169117.13	134159.92	131835.13	119430.13	139687.95	129802.29	123360.07	179284.56	168193.96	157341.99	156479.03	152273.60	105128.56	130314.99	120593.58	119363.17	133971.15	128979.26	118123.70	2.27	1.49	0.65	0.84	1.31	0.69	4.16	2.47	0.11	4.90	4.98	0.12	x	
1989	Heat shock 70 kDa protein 14	Q0VDF9	10191.79	9273.12	9802.32	6064.61	6037.24	5474.92	7383.12	6829.26	6338.61	7395.84	7145.39	6432.98	6769.11	6725.22	5210.64	6576.12	6918.56	6033.78	5557.36	5881.89	5275.97	2.27	1.49	0.65	0.84	1.31	0.69	4.16	2.47	0.11	4.90	4.98	0.12	x	
1990	Heat shock 70 kDa protein 1A	P0DMV8	2174191.81	2147049.99	2070137.47	1536318.93	1464730.17	1409437.29	1962339.17	1883131.69	1852582.45	1752680.35	1662509.45	1582331.71	1888650.79	1788597.98	1519303.52	1550957.35	1459381.97	1486043.80	1669383.58	1584749.30	1466210.73	1031699.05	963159.48	920071.31	750861.02	724826.83	641642.09	777842.40	732251.50	721053.90	791662.92	763820.22	581108.27		
1991	Heat shock 70 kDa protein 1-like	P34931	59006.45	57383.39	51534.83	80338.51	73263.76	84461.84	72379.39	76218.86	71254.89	48678.65	47751.02	45199.23	63481.76	72051.71	90912.25	71670.00	64384.21	67448.11	67047.66	65857.84	55689.03	11328.29	10668.67	8831.58	9644.27	8980.60	9227.23	8451.57	9083.46	8241.58	12128.63	10209.26	5727.88		
1992	Heat shock 70 kDa protein 4	P34932	912618.76	885418.52	806432.80	681101.34	657399.83	622858.85	760177.07	743545.84	723462.12	871664.13	843396.23	804513.03	822892.67	762679.34	634102.93	663409.11	617829.10	619967.94	724358.09	682790.49	637921.39	1194150.63	1110067.12	992251.68	788797.78	773114.92	650923.16	899874.85	834362.59	793634.15	1035602.40	1024710.69	667141.92		
1993	Heat shock 70 kDa protein 4L	O95757	283792.65	277450.03	260800.68	212561.44	209392.57	194651.17	231450.39	222380.90	214829.29	260820.60	248751.31	238983.43	243712.21	233296.70	190808.84	196969.52	191700.21	188769.40	200680.71	194000.91	185725.70	63703.21	57403.19	53995.04	47159.24	46354.37	42809.68	55095.05	51279.42	49119.66	55331.45	53867.07	34108.73		
1994	Heat shock 70 kDa protein 6	P17066	59457.50	56557.67	57146.50	34518.54	33787.99	31436.59	44369.72	41902.76	40501.83	47154.55	43896.01	42280.94	37605.14	35235.02	25861.61	39569.91	38955.30	38012.03	34569.05	33363.83	31323.43	10538.86	8864.96	8383.32	6419.42	5860.16	5339.67	7728.19	7541.54	6527.50	7019.65	6202.64	3575.59		
1995	Heat shock cognate 71 kDa protein	P11142	5947700.70	5839754.00	5494788.61	4059680.90	3956367.99	3728860.05	5012489.14	4820934.26	4606667.42	5006659.10	4783220.35	4583290.73	4636258.41	4381889.85	3654641.25	4191498.01	4014867.92	3871147.96	4220747.46	4099267.40	3835066.67	3022337.28	2876242.87	2668247.60	1948584.80	1926683.35	1719226.95	2341523.20	2258077.51	2168847.92	2281125.59	2228408.31	1605423.26		
1996	Heat shock protein 105 kDa	Q92598	1045716.32	1001428.47	960759.10	722506.60	693888.58	658191.80	855890.71	817986.95	784910.49	882313.31	837434.04	772063.08	852402.90	786263.62	656842.55	707937.82	664393.48	647566.44	774143.85	730876.70	689409.19	464108.56	441504.77	405579.11	291835.35	277536.02	239273.05	342132.63	321984.13	308598.51	337566.18	326062.01	214418.97		
1997	Heat shock protein 75 kDa_ mitochondrial	Q12931	704802.45	666697.83	623062.78	470846.22	450294.84	423020.86	572662.77	537083.63	516252.49	554304.92	524475.78	493814.95	549235.58	519168.52	416058.52	458973.80	441637.43	426589.65	493454.46	459744.45	428533.96	86979.45	78491.89	71673.39	53811.94	52568.86	44046.52	69946.70	66335.51	60633.42	69142.81	68244.29	44465.11		
1998	Heat shock protein beta-1	P04792	3209933.55	3100814.38	2863755.15	1852950.12	1801986.61	1688617.30	2455304.18	2357161.19	2218364.41	2535761.55	2438025.54	2300654.64	2756414.36	2533854.67	1877124.24	1930045.28	1859265.36	1810926.36	2142390.89	2069091.71	1902214.02	101027.61	92877.79	91746.63	45327.36	45437.69	39033.43	52277.54	55926.88	52326.53	60517.79	54916.01	32948.63		
1999	Heat shock protein HSP 90-alpha A2	Q14568	5365.22	5546.50	5557.52	3678.13	3761.53	3642.25	4594.02	4522.55	4391.75	4587.61	4617.71	4416.75	4989.22	4426.94	3845.46	3673.64	3609.96	3368.34	3239.49	3608.04	3293.67	5242.47	4559.35	4470.72	2531.07	2319.20	1752.42	3674.91	3582.02	3194.53	3500.81	3085.59	1595.32		

Supplementary Table S1. Overview on the abundance of all relatively quantified 5180 proteins

			MCF-7																					MDA-MB-231													
	Protein name	UniProt	control 01	control 02	control 03	Dai IC20 01	Dai IC20 02	Dai IC20 03	Dai SC20 01	Dai SC20 02	Dai SC20 03	Gen IC20 01	Gen IC20 02	Gen IC20 03	Gen SC20 01	Gen SC20 02	Gen SC20 03	SSE IC20 01	SSE IC20 02	SSE IC20 03	SSE SC20 01	SSE SC20 02	SSE SC20 03	control 01	control 02	control 03	Dai IC20 01	Dai IC20 02	Dai IC20 03	Gen IC20 01	Gen IC20 02	Gen IC20 03	SSE IC20 01	SSE IC20 02	SSE IC20 03	unique to MCF-7	unique to MDA-MB-231
2000	Heat shock protein HSP 90-alpha	P07900	3815103.97	3710227.43	3502988.41	2678345.49	2575018.19	2436665.47	3295118.75	3194159.32	3006024.10	3300372.54	3178995.82	2959342.69	3129933.39	2952916.95	2444388.95	2837579.85	2680966.40	2591355.13	2801641.00	2668195.14	2467070.52	2305476.19	2128837.47	1983679.48	1604969.79	1583175.00	1458487.16	1736713.26	1699733.62	1616564.26	1734910.33	1723514.26	1266402.31		
2001	Heat shock protein HSP 90-beta	P08238	7932286.85	7636111.76	7153657.32	5514394.49	5331284.75	4990132.35	6685153.68	6384124.55	6106547.00	6595431.26	6311896.05	5932156.71	6547204.15	6224018.51	5298274.90	5323838.89	5074357.15	4894881.76	5728794.31	5525499.90	5226355.39	5613261.69	5270662.32	4941629.63	3718635.28	3675518.58	3353697.71	4361979.91	4233651.30	4067608.20	4385375.82	4276395.95	3080470.98		
2002	Heat shock-related 70 kDa protein 2	P54652	22170.77	20744.91	16599.76	16441.66	15897.79	13867.29	16403.79	15279.63	13969.90	21047.35	19706.64	18088.90	17785.30	17778.81	12129.26	15954.54	15074.63	14299.62	15759.90	15638.63	13682.45	59567.23	54138.89	48823.52	53623.24	52555.55	47783.21	62771.95	60769.29	56795.49	74799.70	71230.55	47458.69		
2003	Helicase MOV-10	Q9HCE1	72756.99	65901.21	62936.96	54009.83	42004.32	39069.53	48893.26	50570.64	47734.85	57464.64	56498.31	51923.77	57724.98	63214.18	44598.85	42544.67	42483.45	41894.78	46760.54	41917.06	40111.88	54612.46	37052.65	36561.70	34168.75	34338.61	27408.94	46359.61	42675.36	37003.10	53603.23	49475.50	25728.72		
2004	Helicase SKI2W	Q15477	19001.23	17586.02	14805.44	11322.89	11602.96	9558.33	13379.89	12441.52	11268.40	16778.07	15934.48	15533.49	14933.75	12296.68	8816.43	13951.28	12740.07	11282.74	11783.33	10771.31	10350.87	9687.93	10382.85	9374.49	4451.45	4723.94	3899.66	6104.19	5876.34	5577.31	6429.13	6330.92	4226.68		
2005	Helicase SRCAP	Q6ZRS2	0.14	2.53	0.81	1.07	2.77	0.68	0.96	0.36	0.63	0.35	0.75	0.38	2.13	0.97	0.60	4.64	1.07	0.47	2.22	0.19	0.06	889.24	852.07	897.37	887.01	931.24	843.76	996.36	922.74	829.42	902.14	852.29	540.78		x
2006	Helicase with zinc finger domain 2	Q9BYK8	155868.95	167313.38	158087.03	153071.98	147294.37	139974.97	158364.54	150099.33	135829.16	152413.97	162999.04	133625.42	161270.21	133783.74	115286.86	134380.18	121706.49	123193.25	151513.89	138389.69	135769.82	42506.35	40071.54	39859.92	32719.02	29631.11	27622.75	34239.52	32126.70	30805.57	36442.96	32868.75	26913.55		
2007	Helicase-like transcription factor	Q14527	34808.23	29967.37	27696.93	14638.67	19217.21	12393.96	25608.30	26205.24	21278.00	22028.95	21121.39	20060.71	23530.89	18131.21	15111.57	18092.13	17555.14	17819.50	20323.28	15378.65	16059.90	2.27	1.49	0.65	0.84	1.31	0.69	4.16	2.47	0.11	4.90	4.98	0.12	x	
2008	Helix-loop-helix protein 2	Q02577	0.14	2.53	0.81	1.07	2.77	0.68	0.96	0.36	0.63	0.35	0.75	0.38	2.13	0.97	0.60	4.64	1.07	0.47	2.22	0.19	0.06	738.96	667.33	675.29	429.33	396.11	401.99	368.45	351.07	310.41	317.57	327.44	262.05		x
2009	Hematopoietically-expressed homeobox protein HHEX	Q03014	9007.26	8724.59	8089.53	6858.16	6795.28	6566.25	11196.59	10663.95	10420.31	9805.92	9029.62	8541.36	9059.44	7480.44	7697.97	7866.98	7438.01	7244.77	7900.95	7882.72	7910.16	104265.53	98984.66	104740.66	56922.50	57166.00	57160.74	54979.43	56240.71	55745.21	54149.90	61581.88	41606.70		
2010	Heme oxygenase 1	P09601	0.14	2.53	0.81	1.07	2.77	0.68	0.96	0.36	0.63	0.35	0.75	0.38	2.13	0.97	0.60	4.64	1.07	0.47	2.22	0.19	0.06	2959.14	2599.79	2087.14	1413.03	1355.88	769.82	2100.57	1690.73	1306.77	2436.66	2271.53	1070.21		x
2011	Heme oxygenase 2	P30519	169004.19	165908.61	158387.18	117763.56	112978.98	108938.61	140856.53	131598.35	128323.79	134380.86	130604.13	123960.04	145265.59	143726.37	131760.06	114930.46	104463.86	102569.81	117813.54	112487.37	107016.87	17609.79	15103.81	15749.00	11106.22	10485.18	9924.94	13804.96	13103.78	12251.64	13906.76	13308.14	6233.05		
2012	Heme-binding protein 1	Q9NRV9	56466.12	49636.96	40329.92	25620.21	23913.22	24130.83	31118.89	31922.50	27638.27	35324.96	34327.28	29070.44	40161.10	30726.01	16847.37	25214.73	29058.60	27962.89	27672.21	24807.26	23909.84	23577.88	22067.55	21361.16	19803.74	18932.68	18842.40	19679.48	18454.01	18745.75	21158.29	19868.78	18729.98		
2013	Heme-binding protein 2	Q9Y5Z4	79815.89	70334.46	62623.50	42389.44	39828.47	37664.12	61251.36	59386.52	53637.36	56396.24	54038.49	50316.50	69832.44	65993.77	47126.86	49373.38	48003.56	44720.03	44601.65	39224.46	37148.94	17062.02	14933.72	14401.35	13177.87	13846.84	10476.85	16509.30	16084.67	15669.94	17646.28	16382.30	12250.88		
2014	Hemicentin-2	Q8NDA2	22749.63	22307.72	21596.83	18678.14	17797.36	15075.67	19571.43	18445.41	18477.32	23737.53	23238.01	22395.38	21601.59	17901.46	12626.32	17933.34	18751.74	17336.17	20012.28	19848.37	21236.96	2.27	1.49	0.65	0.84	1.31	0.69	4.16	2.47	0.11	4.90	4.98	0.12	x	
2015	Hemogen	Q9BXL5	17278.45	17369.03	17031.21	11534.66	10963.96	10681.71	13516.34	13424.61	13541.26	14495.87	14014.48	13342.01	14266.75	13562.99	11139.67	12009.48	10258.21	10660.43	10876.51	10770.02	10461.18	23658.49	24619.08	20740.93	11690.54	11809.59	10493.57	16444.00	15470.93	14685.06	15628.72	15233.37	10900.36		
2016	Hemoglobin subunit alpha	P69905	37985.23	33562.81	37257.06	28987.65	25863.66	27765.33	43341.83	37806.59	42659.30	34256.79	32441.10	35781.77	38376.87	42995.12	40849.06	34703.20	35695.34	31818.06	25164.58	38639.22	28428.81	44284.53	39477.34	39299.55	36391.63	35993.81	34661.02	43638.71	42620.62	41916.02	38941.39	34725.41	25903.70		
2017	Hemoglobin subunit delta	P02042	37.80	27.38	102.41	50.54	20.37	27.87	90.82	80.81	133.60	68.13	61.09	51.47	82.23	72.19	51.80	64.45	50.12	68.96	41.94	47.69	36.66	2.27	1.49	0.65	0.84	1.31	0.69	4.16	2.47	0.11	4.90	4.98	0.12	x	
2018	Heparan-sulfate 6-O-sulfotransferase 1	O60243	0.14	2.53	0.81	1.07	2.77	0.68	0.96	0.36	0.63	0.35	0.75	0.38	2.13	0.97	0.60	4.64	1.07	0.47	2.22	0.19	0.06	91020.15	86630.33	81419.65	51867.22	51372.72	49033.37	58317.00	55061.66	52949.02	51573.85	50816.73	40805.68		x
2019	Heparan-sulfate 6-O-sulfotransferase 2	Q96MM7	20674.13	21631.86	21319.55	16308.27	16109.12	15814.32	18227.96	17632.50	18468.76	20064.18	18954.43	17943.00	18248.65	18620.60	14083.95	17460.04	15509.94	16436.87	12811.48	14130.50	13546.95	2.27	1.49	0.65	0.84	1.31	0.69	4.16	2.47	0.11	4.90	4.98	0.12	x	
2020	Hepatocyte growth factor-regulated tyrosine kinase substrate	O14964	53112.09	55262.29	50739.95	43188.75	38835.55	37618.42	74119.68	72756.93	72246.79	54080.44	53827.29	49496.09	57855.45	46986.37	35563.72	44930.42	40065.59	39211.72	62410.49	59353.15	48307.20	33829.21	31362.99	28418.32	24510.49	23887.05	21986.99	25837.53	25191.10	23845.14	25888.19	24802.56	18812.89		
2021	Hepatocyte nuclear factor 4-gamma	Q14541	0.14	2.53	0.81	1.07	2.77	0.68	0.96	0.36	0.63	0.35	0.75	0.38	2.13	0.97	0.60	4.64	1.07	0.47	2.22	0.19	0.06	21600.16	18712.42	19140.40	13740.52	13820.89	13162.11	15549.10	14867.07	14205.63	17479.63	16991.31	13556.53		x
2022	Hepatoma-derived growth factor	P51858	357033.43	341034.51	328736.54	218485.76	204382.65	198957.69	264445.02	256847.14	244034.66	270944.74	259616.35	239751.98	290796.26	268027.32	221357.34	214973.09	204772.87	198690.11	238931.76	224086.97	212786.99	124631.51	116284.55	107057.26	72645.64	71553.49	63607.20	91191.04	86329.07	79918.62	94841.43	86741.82	65318.74		
2023	Hepatoma-derived growth factor-like protein 1	Q5TGJ6	11788.24	11096.37	11139.27	7901.88	8131.32	7095.86	8709.90	9157.43	8869.58	9883.63	9719.98	9163.50	9369.72	7578.23	5005.26	5460.92	5740.76	5950.85	8048.06	7497.06	7873.83	12203.26	11243.40	10866.53	8106.73	8294.89	7401.64	7697.57	7631.46	7290.74	8249.62	7847.44	6576.11		
2024	Hepatoma-derived growth factor-related protein 2	Q7Z4V5	17895.42	17426.07	30542.91	12906.45	12930.56	12640.97	13554.28	13036.11	12565.71	28447.40	27981.11	27754.34	15313.19	19380.12	12998.71	10612.84	10683.37	11747.45	15637.97	14089.08	13098.30	12641.50	12160.66	11227.42	12624.45	9562.10	8810.47	11739.86	12538.38	11842.39	11287.09	10210.23	4669.19		
2025	Hermansky-Pudlak syndrome 3 protein	Q969F9	2243.64	2014.69	1748.80	1486.68	1353.76	1382.39	1752.55	1233.61	1242.86	1532.23	1632.15	1395.95	1714.90	1540.06	1155.96	1407.04	1178.13	1252.04	1419.66	1238.96	1130.26	2.27	1.49	0.65	0.84	1.31	0.69	4.16	2.47	0.11	4.90	4.98	0.12	x	
2026	Heterogeneous nuclear ribonucleoprotein A/B	Q99729	94623.07	82996.93	68600.43	49992.91	45062.86	38895.86	55283.42	55592.52	49622.90	52633.61	47974.36	40752.86	65541.00	59307.97	37785.47	43209.39	36129.70	32739.61	55995.56	53036.23	45271.71	26349.75	23695.20	19847.46	22284.42	21098.66	17491.02	22985.60	21033.74	18177.03	29545.20	27492.20	17537.58		
2027	Heterogeneous nuclear ribonucleoprotein A0	Q13151	51095.85	50816.39	48154.11	33094.30	30372.97	29532.41	35290.01	33709.12	34100.22	37142.07	36568.64	34589.69	39776.18	38702.18	34268.32	30627.51	28480.83	27776.69	35135.40	34169.79	33610.38	12449.22	11850.19	10434.06	10248.00	9932.37	9228.11	13731.60	13013.84	12912.17	12204.25	11630.48	7108.47		
2028	Heterogeneous nuclear ribonucleoprotein A1	P09651	456109.76	424889.42	392080.19	291862.09	289056.72	267425.42	333358.06	328012.08	307060.32	320549.67	314475.33	285686.88	357354.39	319315.17	249977.55	314314.05	296310.38	275958.87	364980.73	341845.13	302610.85	218260.49	208126.49	190327.54	221481.10	219680.91	186520.66	236483.19	223098.59	209706.46	293568.41	280018.09	169719.59		
2029	Heterogeneous nuclear ribonucleoprotein A1-like 2	Q32P51	4057.58	4122.40	2680.89	3395.03	3324.62	2708.11	3316.58	3036.36	2815.36	4196.92	3604.43	3332.80	3794.39	3650.62	2482.44	3546.28	3174.29	2768.76	3447.54	3288.82	2950.60	2735.53	1980.87	2343.69	1659.52	1689.84	1517.28	1690.13	1819.13	1793.33	1884.71	1994.08	1168.37		
2030	Heterogeneous nuclear ribonucleoprotein A3	P51991	192629.38	185114.85	178695.68	116216.68	113519.23	104225.57	137603.25	128995.73	124093.51	137370.97	131255.10	125713.85	150714.47	136094.74	111334.62	121274.48	113191.45	110423.69	130886.12	121198.45	113753.18	99493.06	100737.90	87808.63	97338.68	92778.71	79927.90	103005.67	98554.86	90311.00	124783.85	124584.06	82524.43		
2031	Heterogeneous nuclear ribonucleoprotein C-like 2	B2RXH8	2247.32	2282.42	2157.97	1245.63	1100.85	1213.63	1469.44	1417.32	1376.36	1469.13	1251.34	1067.60	1609.04	1525.83	1393.97	1371.47	1359.93	1405.40	1772.21	1442.56	1356.12	20937.51	21151.49	17204.08	8917.55	8595.50	6635.68	8686.51	8335.52	8103.73	8968.37	9462.32	5129.25		
2032	Heterogeneous nuclear ribonucleoprotein C-like 3	B7ZW38	0.14	2.53	0.81	1.07	2.77	0.68	0.96	0.36	0.63	0.35	0.75	0.38	2.13	0.97	0.60	4.64	1.07	0.47	2.22	0.19	0.06	1323.53	1470.67	1791.60	1234.80	1405.84	1369.51	1607.53	1335.67	1419.74	2248.77	1956.44	1056.98		x
2033	Heterogeneous nuclear ribonucleoprotein D0	Q14103	197980.82	182239.93	174690.99	116085.71	107915.71	104674.87	131984.42	130338.80	125789.28	143195.94	137323.39	123625.36	156736.63	126790.31	111744.35	108018.73	100891.88	101038.30	129120.53	119856.27	118122.47	201993.80	192731.79	178597.47	127563.93	124366.14	111226.82	135994.70	128824.80	124615.22	150167.62	144964.56	97067.96		
2034	Heterogeneous nuclear ribonucleoprotein D-like	O14979	1540.68	1405.06	1389.63	1020.87	891.40	837.23	1402.41	1163.10	1135.13	1234.14	1182.63	1193.51	1356.52	1144.38	1136.28	1133.42	1136.84	994.93	1196.48	1101.28	1016.89	15735.07	14899.87	14037.78	14580.68	13053.03	12493.27	15082.95	14363.19	14441.31	16294.96	14956.36	10500.61		
2035	Heterogeneous nuclear ribonucleoprotein F	P52597	227348.76	215692.14	192176.98	112650.45	108235.30	93244.38	141616.82	140175.82	132294.24	129315.89	123768.49	108627.41	158718.66	150787.19	111779.09	109483.27	103934.68	95407.88	124171.87	108638.79	98889.20	155905.92	146973.38	141067.88	101103.70	97538.46	81819.86	112571.42	106183.05	98899.97	113660.75	109809.65	68453.22		
2036	Heterogeneous nuclear ribonucleoprotein H	P31943	304986.46	289177.21	268502.26	192861.83	191649.97	170135.73	231761.14	219412.58	209381.93	246739.78	225154.09	216120.06	225965.29	208992.31	165535.15	211852.81	194970.78	184270.82	225919.09	213330.12	189570.58	296814.71	274035.11	247851.05	205066.90	201369.20	175008.84	233510.07	228053.69	212878.45	247859.99	240443.75	168116.38		
2037	Heterogeneous nuclear ribonucleoprotein H2	P55795	84905.23	79077.96	66928.55	48397.35	45637.58	39090.80	60632.09	57502.75	54584.07	68939.93	63486.01	57413.73	61997.06	56225.73	37247.57	57130.67	54187.65	48228.63	53113.88	48959.87	44654.27	12455.15	10351.54	9004.03	6518.27	6086.05	4348.29	8112.06	7478.56	6391.88	8986.72	8309.35	5261.94		
2038	Heterogeneous nuclear ribonucleoprotein H3	P31942	80740.04	76124.07	70707.32	45609.51	43659.28	39368.64	52413.00	51394.43	47210.34	62088.60	55592.95	52155.05	54484.91	49187.27	40248.86	46765.34	44541.99	42999.25	44130.81	41581.24	38009.21	40994.34	39414.82	36139.06	29761.98	29228.85	26750.26	36629.17	34279.44	33057.62	35965.73	34810.79	26314.59		
2039	Heterogeneous nuclear ribonucleoprotein K	P61978	790394.01	763039.36	695764.25	509520.46	494341.32	465040.39	605740.08	578879.04	564136.69	582006.55	549006.69	530430.89	600895.62	580852.42	473565.20	480963.69	455550.93	440743.45	521839.66	503293.54	471593.89	729718.94	678174.34	630488.95	482003.93	469785.84	420738.69	553901.12	537960.52	512394.21	553785.52	539903.71	394067.12		
2040	Heterogeneous nuclear ribonucleoprotein L	P14866	272145.92	254534.13	244460.52	163934.01	155118.17	145866.75	202923.66	196218.38	166330.63	204123.47	196382.78	183824.34	197423.57	175337.76	152915.82	160748.97	155258.45	155406.74	182506.55	179010.81	154708.89	78841.96	75346.36	68424.46	78260.60	73162.50	67609.06	73467.18	72560.73	69907.08	112535.93	105528.81	60271.09		
2041	Heterogeneous nuclear ribonucleoprotein L-like	Q8WVV9	24443.71	21488.71	19417.12	12997.11	12174.04	11410.25	17537.86	16019.84	13983.89	16603.89	16571.61	14454.12	18450.81	16363.76	13834.36	13031.59	13989.63	12083.65	14439.91	13410.20	12169.70	9870.62	8710.43	8025.43	4933.85	4308.63	3428.07	6957.36	6438.79	5772.98	6494.40	6796.06	3636.45		
2042	Heterogeneous nuclear ribonucleoprotein M	P52272	315144.32	302188.16	277943.52	203821.93	196078.71	180259.23	268635.06	255193.82	246275.63	250777.37	235393.49	222267.07	264283.23	251844.46	210276.36	203189.68	191778.89	187826.63	207385.53	199462.11	188092.05	228381.07	222045.57	201683.41	151710.16	144585.57	129892.78	167671.10	159534.49	151370.80	185740.25	177466.22	115717.29		
2043	Heterogeneous nuclear ribonucleoprotein Q	O60506	469401.81	450006.96	401642.62	302816.66	291497.81	260932.32	339162.51	327409.31	302362.83	351494.96	337536.55	307280.23	361326.74	333165.35	234822.46	296527.33	288620.88	283451.77	340485.43	312052.27	285259.35	406267.37	389210.51	350722.53	300302.15	291687.38	254886.76	363304.29	349617.63	332888.03	359164.52	341294.28	222266.47		
2044	Heterogeneous nuclear ribonucleoprotein R	O43390	172180.36	169321.18	159850.94	123361.49	122810.43	116475.38	137044.25	134739.64	135891.61	164642.56	151193.09	142694.31	141540.42	147872.57	125362.17	122487.23	108411.34	111142.85	129472.30	119207.77	120948.50	23639.26	23272.55	19434.11	18728.34	18435.32	14777.35	20506.37	19276.55	17141.54	25883.07	23785.82	12413.57		
2045	Heterogeneous nuclear ribonucleoprotein U	Q00839	239913.86	217930.71	194873.16	114124.70	108561.04	94434.09	154822.32	151111.77	136636.88	153268.56	143590.88	128472.89	170668.65	158774.20	101705.13	124472.90	119388.92	113501.77	159834.40	141585.35	133038.91	178548.18	167371.42	141102.72	142286.36	136140.56	122905.61	169751.06	162488.79	151536.14	193824.75	180136.53	112315.89		
2046	Heterogeneous nuclear ribonucleoprotein U-like protein 1	Q9BUJ2	94359.55	88462.61	85430.51	74388.63	69906.95	64381.43	69923.07	70169.88	69543.44	87451.81	87115.99	79628.26	80157.94	71119.08	62394.09	71577.28	68782.04	63760.80	81697.94	70229.84	68381.97	273248.84	262359.88	235201.39	188754.30	185882.74	167093.37	223107.42	213331.96	196281.74	235523.55	228339.63	157546.95		
2047	Heterogeneous nuclear ribonucleoprotein U-like protein 2	Q1KMD3	2296.27	2487.04	1998.79	1265.97	1365.38	1267.36	2183.51	1746.49	1374.60	1560.82	1265.46	1250.55	1548.70	1536.46	1186.10	1668.37	1578.84	1454.81	1520.96	1805.43	1228.27	2.27	1.49	0.65	0.84	1.31	0.69	4.16	2.47	0.11	4.90	4.98	0.12	x	
2048	Heterogeneous nuclear ribonucleoproteins A2/B1	P22626	343466.86	342326.56	318381.11	198947.74	193427.19	180290.84	242312.22	233482.44	225462.45	207129.51	202037.07	191120.17	255407.80	231868.92	189517.05	195453.06	187489.63	183252.13	254519.20	244168.55	231732.22	269379.34	258065.56	242291.42	274442.50	267762.31	241974.54	251986.95	245656.92	235949.37	353023.38	341287.08	251200.18		
2049	Heterogeneous nuclear ribonucleoproteins C1/C2	P07910	72620.76	69326.88	62119.14	41514.46	40038.61	37013.79	51151.19	49115.04	49698.10	51048.50	47415.51	45002.93	55181.55	46618.71	39253.71	43569.70	40384.54	38960.49	43403.99	41525.10	38315.07	71071.46	68902.63	60535.92	44695.41	44199.06	38931.17	58234.71	54475.57	53380.48	68221.41	66144.52	43161.22		
2050	Hexokinase-1	P19367	444443.74	422244.88	395058.17	270953.90	260934.48	245436.98	399848.76	390432.73	378053.45	368177.37	349675.91	326369.48	348055.35	324224.53	260089.19	304456.12	278991.75	274534.55	288676.29	272649.35	249841.63	56611.03	54387.34	51371.79	32631.96	32761.23	27791.42	41732.21	40250.66	35483.20	42843.15	40927.69	25356.53		
2051	Hexokinase-2	P52789	536799.91	437220.17	342252.50	339843.95	328625.15	248446.79	360351.51	349689.04	294703.94	453179.84	412865.46	322536.84	400474.90	349121.40	135956.36	317502.14	299488.30	259358.35	314524.55	294284.58	229423.56	75051.51	71277.39	62324.00	41685.36	40047.48	29153.89	48714.92	44752.06	41255.71	53845.54	50242.88	28107.54		
2052	Hexokinase-3	P52790	11385.36	10500.77	10819.20	4069.97	4116.92	4049.13	6892.65	6779.01	6821.17	6264.88	6314.29	6108.88	5949.73	5283.39	4663.61	8092.68	8185.36	7428.77	4958.57	4779.78	4388.95	2.27	1.49	0.65	0.84	1.31	0.69	4.16	2.47	0.11	4.90	4.98	0.12	x	
2053	HHIP-like protein 1	Q96JK4	3521.19	3079.44	2401.73	2174.94	2023.74	1589.55	679.82	580.12	464.47	3319.46	3115.08	3551.63	2855.80	2824.08	720.75	1660.94	1425.57	1264.34	2079.75	1785.93	1398.18	2286.40	2054.28	1967.18	1499.64	1382.95	1295.67	1708.60	1547.05	1383.24	1811.43	1781.70	1254.26		
2054	High affinity cAMP-specific 3'_5'-cyclic phosphodiesterase 7A	Q13946	5870.81	5512.31	5159.14	3645.66	3624.47	3691.72	3738.49	3594.51	3041.45	4246.74	4461.98	3993.64	5204.82	4699.96	3822.52	3426.48	4120.80	4239.70	3888.37	2911.75	2452.91	2.27	1.49	0.65	0.84	1.31	0.69	4.16	2.47	0.11	4.90	4.98	0.12	x	
2055	High affinity cAMP-specific and IBMX-insensitive 3'_5'-cyclic phosphodiesterase 8A	O60658	2330.61	2299.64	1462.42	1343.82	1457.85	1128.98	1016.00	885.19	1400.96	1823.68	1769.97	1877.68	1569.46	1601.98	1403.87	2189.92	1137.62	1508.71	1325.40	1447.89	860.07	2.27	1.49	0.65	0.84	1.31	0.69	4.16	2.47	0.11	4.90	4.98	0.12	x	
2056	High mobility group nucleosome-binding domain-containing protein 5	P82970	24665.78	21764.15	19423.75	23565.87	22838.99	22343.19	23675.85	23249.16	23493.99	46865.35	43759.77	42154.58	32931.01	29487.67	26471.80	21004.10	19788.30	19808.09	29020.51	27769.27	27415.39	9332.65	8151.82	7334.27	7486.43	6685.76	6172.59	7555.16	7485.79	7381.07	6383.28	6594.02	5769.40		
2057	High mobility group protein B1	P09429	697045.34	672254.65	604915.87	389436.31	373885.92	351020.71	475527.57	465654.70	442882.32	411089.09	404825.80	386989.59	478930.74	490506.37	433351.08	372608.42	353150.32	339035.01	419818.17	416362.69	401156.29	330237.40	314977.94	282286.75	193712.06	194632.39	178713.50	248745.09	232122.47	230095.52	244200.55	237211.33	179861.68		
2058	High mobility group protein B2	P26583	452663.55	444819.30	412879.86	317265.78	310520.59	296772.21	359002.17	349963.83	333985.00	340893.71	325539.42	317901.28	381537.89	356620.89	330773.78	293225.78	276279.77	275475.81	321971.27	311174.07	304400.23	100570.99	90491.55	82795.03	72048.20	68994.51	66321.50	84721.66	77479.96	75679.28	80136.60	79810.20	58841.81		
2059	High mobility group protein B3	O15347	249475.97	248296.74	236598.17	169782.18	159704.86	159683.54	202206.73	198748.10	190740.32	187954.18	174857.65	170082.75	209595.14	202092.40	191875.90	168493.74	150444.44	150841.88	179132.95	178933.63	166505.59	102471.93	93290.44	89328.24	59664.17	58349.92	54025.43	70029.72	69377.45	68911.71	75457.26	68657.19	57397.65		
2060	High mobility group protein HMG-I/HMG-Y	P17096	0.14	2.53	0.81	1.07	2.77	0.68	0.96	0.36	0.63	0.35	0.75	0.38	2.13	0.97	0.60	4.64	1.07	0.47	2.22	0.19	0.06	27605.44	26620.77	25391.70	20371.84	19886.21	20906.93	20460.33	19166.94	20404.29	21289.13	21118.38	11121.96		x
2061	Hippocalcin-like protein 1	P37235	27210.02	25309.20	23176.38	18853.67	17975.46	16351.09	28451.02	25855.19	23503.69	22286.03	22261.00	19459.53	21799.81	20810.60	15350.02	19803.80	26306.27	17045.40	18769.37	17396.87	15744.99	8512.93	7018.16	5956.69	4676.03	4552.72	2221.35	9731.10	8352.27	5582.77	8848.55	8126.35	3677.48		
2062	Hippocalcin-like protein 4	Q9UM19	69.21	171.88	120.10	394.85	81.05	45.16	126.42	46.43	83.09	125.39	146.80	172.08	81.54	50.34	174.49	167.64	89.54	70.35	14.31	4.83	13.80	2.27	1.49	0.65	0.84	1.31	0.69	4.16	2.47	0.11	4.90	4.98	0.12	x	
2063	Histidine triad nucleotide-binding protein 1	P49773	151171.71	146658.84	134431.52	110743.85	107271.51	103416.37	119200.33	114315.74	110354.03	125678.54	119426.38	117850.30	117353.11	122385.12	108471.02	104223.97	100881.93	95727.79	106855.07	104872.62	100642.35	129823.98	124642.23	113775.42	89483.93	88370.98	81518.93	116762.66	114443.22	111985.13	109683.52	107749.76	81796.39		
2064	Histidine triad nucleotide-binding protein 2_ mitochondrial	Q9BX68	32264.50	29349.39	28197.33	19020.84	17679.82	16309.75	22716.29	20997.58	20459.98	25636.13	25602.12	23783.54	24613.83	20798.63	13704.96	20755.98	18904.29	19070.48	20543.64	19470.59	16182.15	3497.75	3397.42	3032.91	2790.59	2396.11	1962.18	3450.39	3294.65	3326.03	2897.12	2465.10	1613.44		
2065	Histidine--tRNA ligase cytoplasmic	P12081	185957.05	170724.85	169219.47	128180.66	123351.13	113930.95	153489.89	137037.36	135170.21	159792.92	153858.87	137897.13	151574.94	142043.49	114796.96	120910.66	111286.77	112198.16	130843.07	124326.66	115989.22	116776.55	109217.33	101557.26	75453.15	72385.22	65892.31	87536.63	82441.25	77979.85	81640.33	79688.83	55684.49		
2066	Histone acetyltransferase KAT8	Q9H7Z6	23107.99	20230.97	19701.64	14492.62	13015.81	12525.30	15605.63	15823.44	15356.02	16177.36	14636.38	15232.38	15912.04	15855.90	12152.04	14385.56	13752.37	14385.68	18469.04	16684.13	16276.32	12786.72	12216.25	11415.62	9018.92	9105.61	8135.86	9553.27	9217.67	8959.19	10081.02	9629.84	7115.21		
2067	Histone acetyltransferase p300	Q09472	0.14	2.53	0.81	1.07	2.77	0.68	0.96	0.36	0.63	0.35	0.75	0.38	2.13	0.97	0.60	4.64	1.07	0.47	2.22	0.19	0.06	58084.85	54535.91	50886.57	39352.06	39554.33	37434.10	42065.55	41990.19	40454.44	40885.05	40076.21	31011.00		x
2068	Histone acetyltransferase type B catalytic subunit	O14929	15493.65	12993.20	9817.09	11786.39	21595.71	14157.03	17582.66	15346.12	12448.27	11276.07	10500.27	7907.72	11643.03	14201.74	4189.44	19219.10	15655.83	13617.49	8337.05	7222.46	5772.33	51218.04	47577.51	43920.24	31510.40	30815.46	25331.45	38097.53	34477.10	32252.55	39048.06	38899.23	22051.90		
2069	Histone chaperone ASF1A	Q9Y294	5184.39	4874.59	3833.27	2480.91	2646.96	1843.16	4209.31	3377.50	3448.52	3689.22	3658.96	2877.81	3328.47	3135.58	1262.69	3737.92	3192.77	2952.32	3361.53	2608.19	2352.37	2.27	1.49	0.65	0.84	1.31	0.69	4.16	2.47	0.11	4.90	4.98	0.12	x	
2070	Histone chaperone ASF1B	Q9NVP2	2129.67	2115.32	1974.29	1043.77	826.36	860.88	1304.92	1096.28	959.38	778.22	838.66	838.07	1477.93	1145.18	783.86	646.03	576.46	646.04	1091.53	1057.59	973.89	2.27	1.49	0.65	0.84	1.31	0.69	4.16	2.47	0.11	4.90	4.98	0.12	x	
2071	Histone deacetylase 1	Q13547	56531.05	54351.69	48816.23	31577.36	32701.08	29430.45	37360.08	36309.00	34336.39	41023.90	39212.06	36525.03	41864.65	38588.08	32346.08	34611.93	33947.55	32495.38	35375.69	33970.33	29725.48	50923.81	48376.80	44499.44	43996.53	44048.87	40386.86	51324.93	48618.54	47241.69	51196.05	51037.17	38797.23		
2072	Histone deacetylase 11	Q96DB2	3423.65	3275.22	3014.56	2283.05	2404.57	2071.47	2550.60	2421.87	2400.89	2983.41	3054.43	3073.56	2565.75	2455.10	2187.85	2011.59	2230.51	1921.18	2320.20	2381.74	2382.57	2.27	1.49	0.65	0.84	1.31	0.69	4.16	2.47	0.11	4.90	4.98	0.12	x	
2073	Histone deacetylase 2	Q92769	58043.66	52737.90	45183.41	29750.50	27341.92	24014.03	37703.57	38289.34	33934.01	35360.93	35256.05	30324.63	39544.29	35685.73	24930.62	31610.95	29600.76	27021.22	33918.17	32889.26	30228.49	69020.75	65237.20	59902.49	42142.74	41371.51	36905.64	56217.56	53772.79	50226.16	54238.36	52539.63	34382.80		
2074	Histone deacetylase 7	Q8WUI4	799.09	823.07	855.81	139.67	121.52	179.59	434.17	415.95	544.56	646.16	514.35	642.44	472.46	365.96	355.37	837.05	760.94	856.37	407.79	389.97	378.99	2.27	1.49	0.65	0.84	1.31	0.69	4.16	2.47	0.11	4.90	4.98	0.12	x	
2075	Histone H1.0	P07305	30695.79	26309.99	28337.29	23309.49	21285.90	18411.79	21278.77	27760.42	27221.87	20728.21	24198.45	20168.12	27972.02	12416.40	11139.98	24980.41	19554.44	16586.97	28847.85	25248.88	23327.36	8084.86	6256.86	6924.18	9359.86	8780.86	8644.29	5645.01	6283.57	7005.46	10385.96	6035.61	4159.37		
2076	Histone H1.2	P16403	1240.09	1317.69	1363.35	641.90	612.02	552.86	870.06	826.54	651.24	650.37	655.59	586.61	1032.55	922.58	754.55	554.29	448.37	555.40	779.26	694.71	718.37	240498.24	225583.75	215429.68	185455.20	179241.59	171525.49	175793.83	173008.92	162294.31	235550.17	231494.77	183183.94		
2077	Histone H1.4	P10412	533452.33	526378.71	506316.36	312452.96	299048.71	295007.50	361524.56	356446.75	338978.17	286568.36	274269.48	268670.00	374737.75	380736.16	339738.58	294532.71	278975.63	269313.04	352417.74	339991.65	327178.75	2.27	1.49	0.65	0.84	1.31	0.69	4.16	2.47	0.11	4.90	4.98	0.12	x	
2078	Histone H1.5	P16401	361103.68	350513.23	346013.51	249961.58	246571.30	234250.92	298898.04	278565.83	267834.89	245729.40	241663.67	229428.97	273257.90	273017.81	242669.10	235441.22	222246.55	207989.51	261065.77	248330.36	250485.11	123362.79	114538.77	104807.65	88773.38	89213.85	84800.15	90358.15	89025.86	85690.09	128703.60	127895.42	97353.40		
2079	Histone H1t	P22492	45249.46	44837.56	44548.29	30636.17	27109.50	28746.26	31703.11	32484.17	32435.26	37282.33	34566.23	32455.00	34433.40	35559.20	29090.55	30138.07	26814.19	28329.66	29397.54	29473.03	27733.04	2.27	1.49	0.65	0.84	1.31	0.69	4.16	2.47	0.11	4.90	4.98	0.12	x	
2080	Histone H1x	Q92522	163545.00	159471.77	145169.72	79790.64	76552.21	71863.79	111036.60	101066.97	97270.46	82458.27	80190.62	73736.30	110675.07	101413.27	77836.35	79927.31	78442.58	71827.63	89365.97	89569.59	80009.08	24301.06	20746.65	20458.86	14835.52	14958.26	12771.06	19276.28	18147.38	17177.02	19287.43	18906.35	11590.39		
2081	Histone H2A type 1	P0C0S8	152407.22	154700.26	143591.78	102230.83	103431.41	99872.43	113431.35	104000.10	105935.32	85288.80	83654.03	77806.03	98914.97	98964.05	83404.50	104599.19	99514.60	96628.19	88809.79	91629.29	84015.47	54910.13	53388.58	51550.42	48833.15	48164.07	43433.73	58793.72	54873.39	57405.25	149906.35	153160.35	115830.82		
2082	Histone H2A.Z	P0C0S5	0.14	2.53	0.81	1.07	2.77	0.68	0.96	0.36	0.63	0.35	0.75	0.38	2.13	0.97	0.60	4.64	1.07	0.47	2.22	0.19	0.06	17760.15	17447.81	16341.96	9834.28	9763.00	9290.05	12620.38	12297.95	12725.86	15254.08	14914.61	12168.02		x
2083	Histone H2B type 1-K	O60814	230879.35	261313.02	255634.49	191465.43	183060.89	163583.70	178437.50	177675.93	135412.42	187212.80	189770.09	183510.30	211391.21	123912.98	63436.65	147661.43	161720.24	159356.83	199735.04	178711.28	155863.64	2.27	1.49	0.65	0.84	1.31	0.69	4.16	2.47	0.11	4.90	4.98	0.12	x	
2084	Histone H2B type 1-L	Q99880	0.14	2.53	0.81	1.07	2.77	0.68	0.96	0.36	0.63	0.35	0.75	0.38	2.13	0.97	0.60	4.64	1.07	0.47	2.22	0.19	0.06	109650.79	106975.65	92912.55	84345.35	85191.71	77544.62	86533.43	87414.24	86912.53	224606.97	217214.26	89418.53		x
2085	Histone H3.1	P68431	82168.24	79221.87	77408.86	59224.88	57018.84	53459.24	66338.61	62047.23	60267.25	59887.14	57846.20	55711.34	65459.13	63022.77	54878.27	50461.90	47464.54	46971.06	49120.80	47738.99	49562.49	82126.31	78136.71	79223.30	65621.24	64497.18	60346.89	63948.62	61360.37	61670.96	156208.60	146824.89	117552.84		
2086	Histone H4	P62805	161290.82	166456.02	147009.95	126639.99	123425.52	118529.79	121178.89	115569.12	110354.50	73275.23	71653.70	68416.18	120886.46	120224.82	107311.94	96567.61	91566.26	90432.23	95583.67	94417.32	88260.27	173283.53	160553.56	150881.12	139025.23	138367.67	129184.04	160940.07	155007.77	150293.05	453060.98	423551.47	272406.10		
2087	Histone PARylation factor 1	Q9NWY4	2279.17	2433.99	2135.69	1579.43	1166.82	1398.22	1830.68	1783.82	1750.28	1743.36	1820.67	1520.00	2080.95	1693.56	1574.20	1659.66	1537.39	1357.38	1619.88	1613.84	1424.67	2.27	1.49	0.65	0.84	1.31	0.69	4.16	2.47	0.11	4.90	4.98	0.12	x	
2088	Histone-arginine methyltransferase CARM1	Q86X55	23438.98	23697.62	23307.65	15980.66	14919.21	15240.95	20542.14	19555.33	20118.38	20140.61	18625.60	18705.64	20338.95	20167.18	17741.65	16084.44	14389.27	15566.82	15765.96	16259.56	14602.85	2.27	1.49	0.65	0.84	1.31	0.69	4.16	2.47	0.11	4.90	4.98	0.12	x	
2089	Histone-binding protein RBBP4	Q09028	9898.51	9224.59	8439.19	7955.32	7364.46	6971.95	8744.97	8567.06	8030.17	10267.06	9989.28	8623.88	9298.75	8302.68	6088.77	9392.81	8272.11	7928.67	8732.53	8250.26	7604.15	2153.48	1322.56	1361.94	1346.44	1417.56	951.57	1629.43	1456.79	823.50	2193.82	1920.63	811.06		
2090	Histone-binding protein RBBP7	Q16576	161427.93	156230.24	137279.97	107748.59	105710.86	97456.34	134082.77	124168.94	121157.84	127072.27	117044.79	107421.19	125477.77	113128.18	92991.40	118910.20	110572.02	104350.29	114940.02	112006.18	103278.10	277291.75	267344.24	234892.03	155412.66	141998.31	122985.12	197523.64	196419.69	190878.49	193343.06	178603.12	131613.15		
2091	Histone-lysine N-methyltransferase 2D	O14686	0.14	2.53	0.81	1.07	2.77	0.68	0.96	0.36	0.63	0.35	0.75	0.38	2.13	0.97	0.60	4.64	1.07	0.47	2.22	0.19	0.06	2477.36	2646.89	2679.81	2283.55	1896.30	1633.04	3008.68	2646.75	1694.60	3054.10	2487.14	1510.84		x
2092	Histone-lysine N-methyltransferase ASH1L	Q9NR48	70081.57	66678.31	59354.84	53060.32	51522.49	49067.04	54171.75	51630.48	53482.75	65435.41	61897.21	58788.15	60462.55	52692.83	42202.01	49842.40	48837.83	48181.32	53150.44	49524.59	47080.45	2.27	1.49	0.65	0.84	1.31	0.69	4.16	2.47	0.11	4.90	4.98	0.12	x	
2093	Histone-lysine N-methyltransferase EZH2	Q15910	123.70	133.16	142.59	88.86	74.77	57.32	101.98	102.45	145.03	160.01	70.58	65.32	134.67	55.16	174.13	127.11	116.03	108.19	221.14	90.03	154.54	2.27	1.49	0.65	0.84	1.31	0.69	4.16	2.47	0.11	4.90	4.98	0.12	x	
2094	Histone-lysine N-methyltransferase KMT5B	Q4FZB7	3416.50	3084.24	3050.56	1815.07	1843.35	1577.73	3044.38	2961.03	2728.35	2868.78	2919.46	2763.28	2746.70	2488.48	1411.71	2197.09	2051.29	1920.09	2370.95	2285.70	2099.26	2.27	1.49	0.65	0.84	1.31	0.69	4.16	2.47	0.11	4.90	4.98	0.12	x	
2095	Histone-lysine N-methyltransferase SETD7	Q8WTS6	9389.26	9038.16	8058.10	6610.31	6511.78	5813.93	7125.64	6640.10	6668.36	8650.74	8523.03	6931.19	7396.85	6790.45	5337.27	6928.15	6211.83	5881.92	7275.36	6619.33	5586.48	2.27	1.49	0.65	0.84	1.31	0.69	4.16	2.47	0.11	4.90	4.98	0.12	x	
2096	Histone-lysine N-methyltransferase SUV39H1	O43463	12986.73	11496.84	13282.52	12592.02	11530.20	11847.88	13500.91	12836.97	11952.29	15283.58	13896.18	13260.57	12966.09	13607.70	11804.23	10985.23	10821.75	10568.58	12822.18	10706.11	10298.17	5918.67	5666.75	5208.16	5466.75	5444.15	4749.00	6266.16	6203.94	5404.80	7355.13	7556.26	5584.40		
2097	HIV Tat-specific factor 1	O43719	190994.10	182792.52	170878.71	145967.68	139960.06	124120.08	168744.63	156479.80	147751.07	164560.80	154400.66	143534.18	169232.52	172590.33	121028.35	139108.63	133095.47	123658.17	144466.36	137028.85	124927.62	50222.07	43869.06	30378.74	33477.22	32307.41	20789.52	45631.82	41896.72	32223.34	50504.04	48475.33	26098.77		
2098	HLA class I histocompatibility antigen_ A-1 alpha chain	P30443	0.14	2.53	0.81	1.07	2.77	0.68	0.96	0.36	0.63	0.35	0.75	0.38	2.13	0.97	0.60	4.64	1.07	0.47	2.22	0.19	0.06	106681.80	93360.68	87605.59	70371.89	69257.34	56292.06	81138.79	77088.66	71117.18	82945.41	80822.58	52062.67		x
2099	HLA class I histocompatibility antigen_ A-2 alpha chain	P01892	554.48	515.80	458.02	325.15	282.62	216.29	314.92	319.95	222.16	522.35	474.78	291.35	298.05	334.72	135.02	347.17	311.36	294.73	411.02	383.89	303.28	2.27	1.49	0.65	0.84	1.31	0.69	4.16	2.47	0.11	4.90	4.98	0.12	x	
2100	HLA class I histocompatibility antigen_ A-23 alpha chain	P30447	8613.02	8799.15	8557.11	7538.05	5123.25	5836.93	6088.97	7890.46	7097.81	8112.65	7388.24	7109.96	8132.09	9086.13	4979.25	5637.71	5940.42	5707.00	9632.53	6285.24	6397.18	2.27	1.49	0.65	0.84	1.31	0.69	4.16	2.47	0.11	4.90	4.98	0.12	x	
2101	HLA class I histocompatibility antigen_ A-24 alpha chain	P05534	6812.52	7117.79	6431.28	4695.95	4599.07	4521.95	5062.84	4553.05	4814.47	5323.44	4675.98	4705.71	5061.57	5348.73	4725.50	4048.60	4128.28	3652.93	4737.49	5235.09	5359.89	2.27	1.49	0.65	0.84	1.31	0.69	4.16	2.47	0.11	4.90	4.98	0.12	x	
2102	HLA class I histocompatibility antigen_ A-3 alpha chain	P04439	4632.43	3890.45	2837.28	5952.74	5967.11	4725.44	3279.17	3316.91	2716.88	6476.91	6235.07	4952.16	4700.55	4727.47	1839.92	5743.21	5829.11	4731.29	4563.32	4254.65	3308.87	14259.55	13622.61	11958.00	9212.74	8567.60	6325.44	10362.50	9832.25	8517.83	10622.53	10793.88	6343.52		
2103	HLA class I histocompatibility antigen_ A-30 alpha chain	P16188	0.14	2.53	0.81	1.07	2.77	0.68	0.96	0.36	0.63	0.35	0.75	0.38	2.13	0.97	0.60	4.64	1.07	0.47	2.22	0.19	0.06	42668.41	35659.58	34145.82	28147.79	28972.87	26580.17	30476.48	29500.70	28606.70	33593.98	30299.62	26311.37		x
2104	HLA class I histocompatibility antigen_ A-32 alpha chain	P10314	0.14	2.53	0.81	1.07	2.77	0.68	0.96	0.36	0.63	0.35	0.75	0.38	2.13	0.97	0.60	4.64	1.07	0.47	2.22	0.19	0.06	1355.01	1847.86	2003.16	672.51	692.29	702.40	846.46	860.90	703.62	839.54	713.58	464.80		x
2105	HLA class I histocompatibility antigen_ A-68 alpha chain	P01891	0.14	2.53	0.81	1.07	2.77	0.68	0.96	0.36	0.63	0.35	0.75	0.38	2.13	0.97	0.60	4.64	1.07	0.47	2.22	0.19	0.06	40484.44	37114.37	32505.06	27692.81	25675.95	21222.39	31420.27	29410.66	28537.61	28060.87	29829.03	20632.51		x
2106	HLA class I histocompatibility antigen_ alpha chain E	P13747	99153.65	83888.05	63009.39	20532.23	24478.89	17171.53	25688.74	25467.95	20718.68	85726.00	77120.34	66013.11	69990.42	21818.48	9065.44	24696.67	21887.21	17826.65	16725.46	13386.50	13525.05	67864.75	58980.52	48634.54	43600.24	40383.97	26950.62	57227.16	52149.09	41535.40	60686.82	55875.58	31172.74		
2107	HLA class I histocompatibility antigen_ alpha chain F	P30511	0.14	2.53	0.81	1.07	2.77	0.68	0.96	0.36	0.63	0.35	0.75	0.38	2.13	0.97	0.60	4.64	1.07	0.47	2.22	0.19	0.06	4.53	14.28	9.21	143.43	134.18	37.76	273.19	226.76	195.54	364.22	292.66	87.16		x
2108	HLA class I histocompatibility antigen_ alpha chain G	P17693	61369.99	60705.51	53907.17	45507.33	45536.64	43697.82	41896.91	39181.96	39802.31	60504.76	54126.01	53529.34	53846.63	46102.97	41756.36	40952.18	35460.69	39324.76	45143.23	43275.79	43447.71	28665.85	28172.21	28261.43	19372.89	18562.37	17483.73	18032.67	17666.11	16405.39	22798.14	21733.20	16861.15		
2109	HLA class I histocompatibility antigen_ B-13 alpha chain	P30461	7930.77	7215.36	7523.14	6232.61	5888.87	4736.36	4776.65	4170.03	4271.91	6757.84	6997.34	7879.50	7558.24	8148.13	2009.87	2427.75	4062.96	4437.00	6396.98	6847.83	6066.46	18174.01	16732.44	14680.09	12124.24	10684.30	8988.25	15358.10	14221.38	12395.63	14613.83	14286.00	9621.46		
2110	HLA class I histocompatibility antigen_ B-27 alpha chain	P03989	0.14	2.53	0.81	1.07	2.77	0.68	0.96	0.36	0.63	0.35	0.75	0.38	2.13	0.97	0.60	4.64	1.07	0.47	2.22	0.19	0.06	9435.03	8924.75	7788.91	5552.85	5107.20	3482.24	8470.74	7730.74	6795.72	8924.12	7888.82	4432.57		
2111	HLA class I histocompatibility antigen_ B-45 alpha chain	P30483	0.14	2.53	0.81	1.07	2.77	0.68	0.96	0.36	0.63	0.35	0.75	0.38	2.13	0.97	0.60	4.64	1.07	0.47	2.22	0.19	0.06	22271.33	23531.79	20837.70	15446.45	15928.22	14745.34	19792.20	19347.23	18784.13	19198.54	19847.80	14914.77		x
2112	HLA class I histocompatibility antigen_ B-49 alpha chain	P30487	16758.90	15606.48	15041.37	10964.06	11287.91	10950.87	11881.30	12458.23	11502.08	15958.04	15928.47	15005.03	14438.17	14170.51	13673.36	11302.93	9945.05	10238.48	10644.00	10474.40	9944.80	2.27	1.49	0.65	0.84	1.31	0.69	4.16	2.47	0.11	4.90	4.98	0.12	x	
2113	HLA class I histocompatibility antigen_ B-53 alpha chain	P30491	0.14	2.53	0.81	1.07	2.77	0.68	0.96	0.36	0.63	0.35	0.75	0.38	2.13	0.97	0.60	4.64	1.07	0.47	2.22	0.19	0.06	3795.25	3733.72	3010.06	2882.85	2958.96	2271.95	3120.96	3034.17	2859.16	3929.41	3698.91	2097.02		x

Supplementary Table S1. Overview on the abundance of all relatively quantified 5180 proteins

			MCF-7																					MDA-MB-231													
	Protein name	UniProt	control 01	control 02	control 03	Dai IC20 01	Dai IC20 02	Dai IC20 03	Dai SC20 01	Dai SC20 02	Dai SC20 03	Gen IC20 01	Gen IC20 02	Gen IC20 03	Gen SC20 01	Gen SC20 02	Gen SC20 03	SSE IC20 01	SSE IC20 02	SSE IC20 03	SSE SC20 01	SSE SC20 02	SSE SC20 03	control 01	control 02	control 03	Dai IC20 01	Dai IC20 02	Dai IC20 03	Gen IC20 01	Gen IC20 02	Gen IC20 03	SSE IC20 01	SSE IC20 02	SSE IC20 03	unique to MCF-7	unique to MDA-MB-231
2114	HLA class I histocompatibility antigen_ B-7 alpha chain	P01889	31897.91	28894.73	28373.48	19133.19	19555.67	17742.21	25112.64	26276.84	22765.23	25528.56	23367.62	21438.77	24453.36	22543.53	19900.47	19793.29	18221.29	18208.01	18023.16	16747.66	18098.10	2.27	1.49	0.65	0.84	1.31	0.69	4.16	2.47	0.11	4.90	4.98	0.12	x	
2115	HLA class I histocompatibility antigen_ B-8 alpha chain	P30460	7073.25	5511.01	5275.24	5148.90	4581.76	4171.40	4643.57	4628.94	3885.64	5286.08	4805.60	5557.72	6937.40	4885.31	4102.34	3504.04	3652.89	4165.15	4749.44	3979.62	3830.17	2.27	1.49	0.65	0.84	1.31	0.69	4.16	2.47	0.11	4.90	4.98	0.12	x	
2116	HLA class I histocompatibility antigen_ Cw-12 alpha chain	P30508	0.14	2.53	0.81	1.07	2.77	0.68	0.96	0.36	0.63	0.35	0.75	0.38	2.13	0.97	0.60	4.64	1.07	0.47	2.22	0.19	0.06	12047.75	11503.97	10258.62	8256.63	7839.33	5836.67	10750.65	9711.01	8620.88	10827.85	9488.17	6694.39		x
2117	HLA class I histocompatibility antigen_ Cw-16 alpha chain	Q29960	0.14	2.53	0.81	1.07	2.77	0.68	0.96	0.36	0.63	0.35	0.75	0.38	2.13	0.97	0.60	4.64	1.07	0.47	2.22	0.19	0.06	485.19	539.50	514.09	408.93	413.89	375.21	461.47	487.62	474.87	570.64	480.96	287.20		x
2118	HLA class I histocompatibility antigen_ Cw-17 alpha chain	Q95604	0.14	2.53	0.81	1.07	2.77	0.68	0.96	0.36	0.63	0.35	0.75	0.38	2.13	0.97	0.60	4.64	1.07	0.47	2.22	0.19	0.06	533.18	461.72	452.94	296.80	241.10	229.28	397.69	332.66	272.13	230.29	236.88	45.04		x
2119	HLA class I histocompatibility antigen_ Cw-2 alpha chain	P30501	23570.80	23414.55	23095.26	14005.53	13780.83	14541.21	16737.59	16099.50	15276.53	17650.73	16965.73	15480.95	18078.86	15340.33	14528.58	14778.62	14620.91	13930.49	16039.68	15644.78	14468.47	2.27	1.49	0.65	0.84	1.31	0.69	4.16	2.47	0.11	4.90	4.98	0.12	x	
2120	HLA class I histocompatibility antigen_ Cw-4 alpha chain	P30504	0.14	2.53	0.81	1.07	2.77	0.68	0.96	0.36	0.63	0.35	0.75	0.38	2.13	0.97	0.60	4.64	1.07	0.47	2.22	0.19	0.06	5270.86	4318.52	3848.82	3506.58	3313.78	2570.43	3914.08	3792.31	3239.62	4507.40	4217.07	2562.58		x
2121	HLA class I histocompatibility antigen_ Cw-7 alpha chain	P10321	5281.15	5830.30	4489.21	4113.03	4296.39	4437.85	4291.09	4119.71	4011.48	5375.49	5214.43	4743.46	3967.15	4085.80	3757.29	3697.99	3744.53	3813.50	4396.55	4322.69	3706.12	2.27	1.49	0.65	0.84	1.31	0.69	4.16	2.47	0.11	4.90	4.98	0.12	x	
2122	HLA class II histocompatibility antigen_ DRB1-10 beta chain	Q30167	0.14	2.53	0.81	1.07	2.77	0.68	0.96	0.36	0.63	0.35	0.75	0.38	2.13	0.97	0.60	4.64	1.07	0.47	2.22	0.19	0.06	6271.61	6129.24	5077.37	3199.95	3018.56	2289.25	3538.12	3418.91	3101.44	3921.70	3391.21	1954.75		x
2123	Holliday junction recognition protein	Q8NCD3	0.14	2.53	0.81	1.07	2.77	0.68	0.96	0.36	0.63	0.35	0.75	0.38	2.13	0.97	0.60	4.64	1.07	0.47	2.22	0.19	0.06	5068.21	5149.25	4477.65	3432.73	3002.42	2320.00	3999.95	3781.19	3520.03	4282.25	4225.89	2654.56		x
2124	Homeobox protein aristaless-like 4	Q9H161	76500.30	76274.73	72352.24	66988.24	60699.35	62403.46	62646.98	62384.16	61677.97	73057.90	72996.76	68397.78	72547.61	49408.00	20523.20	55567.65	54002.64	54179.14	68258.96	65870.01	62587.65	2.27	1.49	0.65	0.84	1.31	0.69	4.16	2.47	0.11	4.90	4.98	0.12	x	
2125	Homeobox protein cut-like 1	P39880	12802.16	12121.97	10207.66	7613.19	7486.49	6605.43	9537.27	8743.59	8707.52	9497.45	8822.37	8053.44	9645.63	9219.41	7891.29	7623.69	7459.36	7221.70	9012.13	9001.16	8471.97	2.27	1.49	0.65	0.84	1.31	0.69	4.16	2.47	0.11	4.90	4.98	0.12	x	
2126	Homeobox protein cut-like 2	O14529	8852.44	9600.27	9130.67	7858.25	8347.37	7396.69	8239.67	7969.05	9088.61	9189.10	9010.09	9621.61	8583.10	9358.01	6163.73	6940.36	9282.91	7372.19	10093.20	6665.71	6600.10	2214.56	2275.23	2002.31	2227.22	2123.41	2131.58	2262.06	2457.37	2009.16	2077.61	2093.75	1263.50		
2127	Homeobox protein engrailed-2	P19622	12756.01	12349.58	11466.03	11195.31	11164.85	10507.86	11322.55	10078.92	9749.04	11321.31	10342.85	10151.95	11600.91	11120.60	9075.33	9911.35	9905.05	9561.74	8483.60	8478.09	7669.84	2.27	1.49	0.65	0.84	1.31	0.69	4.16	2.47	0.11	4.90	4.98	0.12	x	
2128	Homeobox protein Hox-A13	P31271	932.42	761.69	390.62	68.36	345.08	157.62	228.11	284.48	123.36	601.43	499.08	169.98	855.18	231.82	13.70	322.44	316.11	183.50	65.33	336.07	7.68	2.27	1.49	0.65	0.84	1.31	0.69	4.16	2.47	0.11	4.90	4.98	0.12	x	
2129	Homeobox protein Hox-B5	P09067	6304.12	5658.58	6129.99	4257.49	4133.18	4196.93	5583.87	5129.56	4783.94	5156.28	4929.11	5005.04	4874.60	4835.18	3625.87	4476.94	4653.75	4735.11	4081.71	4075.52	4016.63	2.27	1.49	0.65	0.84	1.31	0.69	4.16	2.47	0.11	4.90	4.98	0.12	x	
2130	Homeobox protein Hox-C11	O43248	284.37	214.33	314.78	265.83	193.42	207.26	261.82	209.08	196.60	155.55	290.26	226.49	243.26	304.12	179.77	221.18	186.97	177.22	189.29	233.63	207.50	2.27	1.49	0.65	0.84	1.31	0.69	4.16	2.47	0.11	4.90	4.98	0.12	x	
2131	Homeobox protein Hox-C5	Q00444	23674.19	21299.64	20325.70	12593.37	12036.28	11557.48	14875.48	14082.72	13944.29	16890.21	17866.51	17451.86	18828.63	16853.06	15707.01	12031.77	11525.10	10843.79	14344.45	13528.53	13683.85	2.27	1.49	0.65	0.84	1.31	0.69	4.16	2.47	0.11	4.90	4.98	0.12	x	
2132	Homeobox protein Hox-C6	P09630	5826.81	6313.24	5466.33	3505.63	3669.77	3163.37	3718.98	3671.45	3558.42	4839.93	4577.16	4292.61	4908.88	4370.96	4685.09	3277.34	2994.42	3306.76	3182.34	3168.33	2928.91	8834.92	7977.56	6416.25	5610.64	5354.82	3714.49	7302.83	7149.80	5768.83	8678.88	8307.15	4983.28		
2133	Homeobox protein Nkx-2.8	O15522	2370.45	2234.39	2261.94	1897.89	1762.31	1739.12	1905.01	1915.69	1773.10	2548.92	2280.79	2130.22	2318.17	2222.47	1645.85	1662.20	1471.08	1695.87	1717.52	1480.34	1856.37	2.27	1.49	0.65	0.84	1.31	0.69	4.16	2.47	0.11	4.90	4.98	0.12	x	
2134	Homeobox protein Nkx-6.1	P78426	187.37	264.93	163.04	90.92	140.10	109.47	164.29	359.14	269.90	219.99	210.56	183.65	221.51	302.51	181.76	224.52	157.82	263.46	213.12	224.66	297.60	2.27	1.49	0.65	0.84	1.31	0.69	4.16	2.47	0.11	4.90	4.98	0.12	x	
2135	Homeobox protein SIX1	Q15475	241.32	291.41	431.73	502.37	493.36	371.20	606.84	739.97	826.06	365.21	322.44	426.34	621.77	279.52	456.76	1006.07	558.79	606.13	475.11	551.57	402.74	2.27	1.49	0.65	0.84	1.31	0.69	4.16	2.47	0.11	4.90	4.98	0.12	x	
2136	Host cell factor 1	P51610	0.14	2.53	0.81	1.07	2.77	0.68	0.96	0.36	0.63	0.35	0.75	0.38	2.13	0.97	0.60	4.64	1.07	0.47	2.22	0.19	0.06	12229.13	12063.86	10384.05	7959.62	7536.41	6021.21	10899.83	10697.13	10185.97	10977.18	10504.63	6783.68		x
2137	Hsc70-interacting protein	P50502	314130.15	303207.15	279050.11	219501.21	218449.13	199479.02	258362.82	251157.96	229404.16	288299.05	270032.81	244123.46	266324.76	254646.04	200943.94	215459.94	205377.66	188846.75	223711.95	217486.36	191043.34	2.27	1.49	0.65	0.84	1.31	0.69	4.16	2.47	0.11	4.90	4.98	0.12	x	
2138	Hsp70-binding protein 1	Q9NZL4	3628.92	3220.11	3614.40	2306.57	2378.80	2077.32	4578.53	4083.46	3485.28	3140.97	2983.17	2763.24	2962.25	3663.01	2142.80	2790.27	5310.00	3050.44	2264.84	2354.58	2112.77	36126.89	27534.06	24653.89	16931.29	16252.75	11405.56	22457.04	21739.74	18792.19	22905.30	20931.70	11811.29		
2139	Hsp90 co-chaperone Cdc37	Q16543	383675.03	363983.64	333958.22	235994.73	211453.96	189803.40	268079.27	272668.77	244792.58	317220.71	298667.82	282112.22	317054.62	287787.65	200592.54	213125.65	206743.92	195938.26	252045.71	233993.22	218873.69	342636.67	319398.51	284739.79	203319.39	199689.12	176757.67	236054.80	231564.88	221393.39	265278.91	253621.16	164959.39		
2140	Huntingtin-associated protein 1	P54257	0.14	2.53	0.81	1.07	2.77	0.68	0.96	0.36	0.63	0.35	0.75	0.38	2.13	0.97	0.60	4.64	1.07	0.47	2.22	0.19	0.06	23972.89	22802.14	20745.43	16733.55	16779.46	14919.85	17716.67	17578.96	15721.49	17417.17	18319.50	14546.25		x
2141	Huntingtin-interacting protein 1-related protein	O75146	278964.54	265869.86	236035.18	179299.94	184770.21	155164.11	208078.79	192116.97	174210.91	240133.73	209525.42	201529.71	199626.23	187991.95	134715.13	222765.17	205730.99	183667.14	193809.82	192269.92	173611.64	1003.81	1027.93	763.38	880.45	667.21	555.49	946.11	837.87	798.48	1333.57	1174.99	632.94		
2142	Hydroxyacyl-coenzyme A dehydrogenase_ mitochondrial	Q16836	98708.20	99599.17	91447.17	65138.61	56201.16	55590.24	74217.73	68011.13	60737.54	77785.79	75813.41	66661.42	72987.99	73009.73	52495.65	64865.41	59183.72	60665.65	60544.25	60873.50	56477.46	2.27	1.49	0.65	0.84	1.31	0.69	4.16	2.47	0.11	4.90	4.98	0.12	x	
2143	Hydroxyacylglutathione hydrolase_ mitochondrial	Q16775	26863.74	19280.22	20091.74	18551.07	16445.59	15041.54	14903.57	18379.27	19472.40	23038.92	23410.40	19015.15	19094.12	20149.07	17048.43	15401.86	16275.33	15179.48	19275.45	15767.00	16445.13	10786.87	10262.59	8606.79	7372.12	6955.42	7535.38	9751.87	7126.25	8194.71	10297.59	7577.19	4821.12		
2144	Hydroxymethylglutaryl-CoA lyase mitochondrial	P35914	547.97	553.66	437.74	391.90	314.44	378.91	404.99	441.67	307.23	404.56	437.60	338.29	473.27	389.20	436.33	432.55	457.30	397.73	459.77	369.96	413.08	3837.03	3103.76	2965.78	2477.43	2616.53	1894.30	3136.33	2991.09	2693.99	2768.10	2633.79	1649.06		
2145	Hydroxymethylglutaryl-CoA synthase_ cytoplasmic	Q01581	95634.37	91863.09	82768.21	65865.35	59464.08	56935.51	75677.94	70605.64	65518.03	74543.52	72141.40	69567.41	79121.54	73236.73	64096.32	63602.72	59248.87	58271.57	67433.56	65973.97	60587.04	74313.41	73001.44	63146.71	48033.64	44453.95	40416.01	65689.37	63544.96	61466.81	64343.06	61482.36	42107.70		
2146	Hydroxyproline dehydrogenase	Q9UF12	6134.48	5694.25	5421.75	3996.97	4616.78	4438.47	4381.40	5212.48	4370.73	5874.52	6005.50	5959.82	5761.48	4713.02	3121.56	4442.81	4355.24	3950.31	5524.48	5127.62	5231.79	35218.42	33472.19	29674.18	19231.78	18628.76	13822.32	23255.94	21509.33	19657.92	18217.22	17117.78	9176.11		
2147	Hydroxysteroid dehydrogenase-like protein 2	Q6YN16	13627.45	13831.44	12561.78	11082.13	11271.16	9647.43	11570.70	11265.77	10356.20	12702.41	12419.24	10826.00	12889.33	11614.63	7643.68	10324.20	9282.96	9341.41	8822.51	8459.30	7214.50	6537.59	5903.06	5065.87	3508.03	3284.78	2497.10	5585.77	4494.91	3715.76	5083.13	4483.53	2439.23		
2148	Hypoxanthine-guanine phosphoribosyltransferase	P00492	186973.26	172234.35	158180.02	132306.99	126843.33	109578.51	185191.21	173990.55	167098.43	154985.35	145730.22	131374.33	158728.13	142839.25	112809.65	125461.56	121523.68	108018.45	136256.84	124868.69	114637.33	169305.33	158255.90	143702.63	95551.94	92400.41	78428.96	98907.04	92185.17	85365.29	101963.41	96460.11	53443.10		
2149	Hypoxia up-regulated protein 1	Q9Y4L1	1085576.47	1018109.46	943220.08	836281.05	816701.93	761058.91	938741.17	885148.60	855805.13	1009501.73	946897.87	892738.58	878144.93	839363.39	679277.73	858166.90	807582.19	769519.51	853157.80	780331.63	735664.81	269431.91	250052.33	227794.37	185080.78	180846.21	163313.27	203174.47	190414.43	185042.29	219411.93	213565.59	119187.26		
2150	Hypoxia-inducible factor 1-alpha inhibitor	Q9NWT6	223.64	226.06	389.80	573.19	516.32	484.67	515.81	701.06	512.95	646.80	654.45	662.66	692.18	579.60	460.35	506.00	506.15	501.81	488.18	402.87	460.59	2.27	1.49	0.65	0.84	1.31	0.69	4.16	2.47	0.11	4.90	4.98	0.12	x	
2151	Iduronate 2-sulfatase	P22304	9371.90	8967.76	7907.24	7372.70	7105.82	6396.67	7447.51	7195.68	6490.86	9071.77	8148.77	8088.69	8358.84	6873.94	6152.20	6866.95	7284.51	6872.91	7744.69	7187.46	6285.96	2.27	1.49	0.65	0.84	1.31	0.69	4.16	2.47	0.11	4.90	4.98	0.12	x	
2152	Immunity-related GTPase family Q protein	Q8WZA9	0.14	2.53	0.81	1.07	2.77	0.68	0.96	0.36	0.63	0.35	0.75	0.38	2.13	0.97	0.60	4.64	1.07	0.47	2.22	0.19	0.06	991.55	859.76	1130.67	655.14	569.57	693.36	862.10	780.89	765.29	1091.79	943.92	808.31		x
2153	Immunoglobulin heavy constant alpha 1	P01876	267.43	162.47	247.25	151.48	140.19	159.11	277.37	182.99	286.81	280.93	244.24	300.38	229.40	209.96	117.43	192.22	124.11	133.06	161.93	116.67	129.81	2.27	1.49	0.65	0.84	1.31	0.69	4.16	2.47	0.11	4.90	4.98	0.12	x	
2154	Immunoglobulin heavy variable 1-69D	A0A0B4J	56.31	126.41	66.28	281.94	717.91	531.30	1019.37	210.33	208.91	75.59	60.46	44.63	218.93	840.08	187.05	651.99	248.35	833.33	116.83	144.78	81.12	2.27	1.49	0.65	0.84	1.31	0.69	4.16	2.47	0.11	4.90	4.98	0.12	x	
2155	Immunoglobulin heavy variable 3-43	A0A0B4J	802.86	672.44	365.69	209.19	228.58	86.89	331.16	32.20	32.87	854.40	769.96	416.85	2.13	0.97	0.60	195.23	573.07	237.05	60.17	17.13	241.64	2.27	1.49	0.65	0.84	1.31	0.69	4.16	2.47	0.11	4.90	4.98	0.12	x	
2156	Immunoglobulin superfamily member 3	O75054	20151.34	19180.05	18502.69	12903.50	12253.78	12229.31	12747.97	12298.30	12438.08	16632.02	16127.15	15468.68	19175.29	16843.94	13648.08	12053.43	11574.21	11518.61	11261.37	10657.57	11125.97	2.27	1.49	0.65	0.84	1.31	0.69	4.16	2.47	0.11	4.90	4.98	0.12	x	
2157	Importin subunit alpha-1	P52292	93243.59	91296.83	86341.28	56089.98	53353.41	49816.99	65720.37	59431.35	56042.41	70250.51	66876.71	63285.54	60631.84	56907.71	45774.92	50517.78	46340.05	45970.56	54279.41	51519.97	46679.36	71841.66	68106.26	67804.43	50579.31	49447.34	44860.70	71214.38	69041.41	68270.21	66707.02	63411.26	45651.73		
2158	Importin subunit alpha-3	O00629	1400.72	1266.81	1077.31	993.72	1029.02	819.98	889.00	925.19	920.09	1638.02	1646.60	1316.59	1363.50	314.51	228.69	617.13	855.35	833.87	1026.91	1035.29	1146.52	143.04	157.26	127.77	160.42	162.72	167.71	152.52	215.34	172.69	206.83	201.26	41.32		
2159	Importin subunit alpha-5	P52294	140129.95	118923.51	100351.85	80497.43	74338.47	65128.02	95589.66	89018.64	85386.47	120711.11	113531.61	100013.46	104340.56	96454.48	63365.46	90660.05	83522.77	77796.33	88597.24	80782.76	74929.48	33465.15	30485.35	29956.04	25104.68	24747.21	22712.45	27955.91	26382.37	26762.46	29914.40	29470.08	30806.26		
2160	Importin subunit alpha-6	O15131	1796.16	1899.63	1717.32	954.53	996.12	945.72	1373.81	1414.41	1345.93	1437.08	1442.19	1333.65	1890.14	1585.87	1210.22	1139.84	985.94	1037.10	1184.81	1198.89	1174.26	2.27	1.49	0.65	0.84	1.31	0.69	4.16	2.47	0.11	4.90	4.98	0.12	x	
2161	Importin subunit alpha-7	O60684	4749.15	4467.21	4641.37	4083.63	4125.73	3792.74	4116.10	3657.34	3437.03	5334.46	5014.71	4778.62	4490.74	4057.63	3825.54	3926.38	3869.73	3725.76	3910.34	3827.18	3771.69	9541.14	8876.72	7809.98	6040.39	5447.26	4978.40	7472.88	7367.37	6872.53	6887.53	6498.39	3850.18		
2162	Importin subunit beta-1	Q14974	679022.88	654504.53	638840.93	479508.36	457353.04	440493.28	540492.01	520480.77	512008.52	535604.89	496189.75	477638.13	582486.24	544428.40	461451.61	455504.07	412496.46	422517.06	508372.30	466102.65	452932.97	341309.40	313824.15	302506.33	231401.41	223714.56	205739.26	296220.27	273054.10	272350.87	294807.97	284394.86	187168.47		
2163	Importin-4	Q8TEX9	122611.35	119965.76	109816.97	72149.00	70467.29	65270.89	118969.52	113669.89	109295.88	97324.05	95212.52	85997.99	95810.26	88210.94	73109.11	70814.50	66809.63	66494.06	76206.22	73196.27	69873.37	86082.41	78326.61	75459.78	49068.37	47664.73	44160.47	64955.06	62975.01	59299.51	58835.82	55429.61	38003.86		
2164	Importin-5	O00410	137578.81	122986.19	106989.23	73213.42	73701.91	64588.46	91502.51	88749.41	79126.94	106784.03	99001.47	87858.55	106921.74	97453.04	64440.39	72969.45	73027.91	67261.34	71579.98	71574.78	61980.39	264712.08	254567.61	222416.48	149687.56	144142.69	117738.81	206370.06	192695.97	186167.89	196723.54	183999.23	117533.61		
2165	Importin-7	O95373	178654.54	165394.96	139608.47	115513.14	111308.16	94400.91	127803.17	124417.79	110596.21	145165.65	134521.47	123202.72	148036.25	133262.53	96826.81	110451.11	103163.44	99459.71	118114.45	111215.19	104988.11	124092.72	119566.77	111257.48	87255.96	87139.06	74655.88	117455.56	109549.56	104292.78	102776.26	98846.64	64498.81		
2166	Importin-9	Q96P70	19083.76	18137.55	17373.38	10605.12	9948.80	9439.87	13088.75	12505.05	12565.39	16012.63	14416.19	13507.55	15914.86	13437.52	10647.51	10599.68	9814.04	9890.55	10365.00	9483.16	9259.39	47777.88	44812.90	41579.41	31332.35	31222.98	25323.55	43982.24	40632.53	35515.15	44868.80	41687.98	25325.35		
2167	Inactive C-alpha-formylglycine-generating enzyme 2	Q8NBJ7	86196.53	85680.23	83769.71	72404.45	72423.53	68994.77	87462.46	87025.18	86210.77	83169.51	77715.65	74194.67	78152.39	77477.37	63318.84	70763.27	68957.40	69406.37	68490.92	62439.45	64336.51	28233.72	25503.74	23988.39	18315.23	16989.79	15664.50	23213.39	21081.25	21283.05	22650.23	21466.02	14108.07		
2168	Inactive peptidyl-prolyl cis-trans isomerase FKBP6	O75344	3938.06	3730.05	2880.91	3139.18	2879.59	2098.64	2863.12	2748.25	2441.85	4048.79	3731.24	3410.33	4232.55	3256.27	1639.29	2842.23	2931.90	2297.44	3150.38	2949.78	2479.16	2.27	1.49	0.65	0.84	1.31	0.69	4.16	2.47	0.11	4.90	4.98	0.12	x	
2169	Inactive polypeptide N-acetylgalactosaminyltransferase-like protein 5	Q7Z4T8	395.08	270.36	571.38	168.01	88.29	203.60	285.02	184.13	269.56	327.20	416.04	413.80	261.54	257.05	96.78	300.60	154.95	254.33	145.81	200.52	182.00	4380.12	3398.75	2294.64	2325.16	2300.18	940.22	3564.00	2832.51	2020.62	4142.23	3992.21	1532.91		
2170	Inactive rhomboid protein 2	Q6PJF5	0.14	2.53	0.81	1.07	2.77	0.68	0.96	0.36	0.63	0.35	0.75	0.38	2.13	0.97	0.60	4.64	1.07	0.47	2.22	0.19	0.06	17265.12	19564.52	17263.48	13804.03	13179.27	11353.43	16517.15	16045.44	13173.20	16750.35	16177.25	10932.83		x
2171	Inactive tyrosine-protein kinase 7	Q13308	0.14	2.53	0.81	1.07	2.77	0.68	0.96	0.36	0.63	0.35	0.75	0.38	2.13	0.97	0.60	4.64	1.07	0.47	2.22	0.19	0.06	11371.57	10827.44	9877.63	5706.38	5619.14	4554.50	7829.68	7243.87	6863.72	8542.58	7347.32	4632.19		x
2172	Inactive tyrosine-protein kinase PEAK1	Q9H792	0.14	2.53	0.81	1.07	2.77	0.68	0.96	0.36	0.63	0.35	0.75	0.38	2.13	0.97	0.60	4.64	1.07	0.47	2.22	0.19	0.06	4604.21	3281.65	3090.41	2691.50	2704.55	2597.98	3481.38	3230.20	3055.78	3731.77	3034.67	2282.19		x
2173	Inactive ubiquitin thioesterase OTULINL	Q9NUU6	10588.14	11157.14	9888.18	12199.64	11907.50	11681.46	11317.25	10495.02	10835.09	18990.88	17525.01	16938.87	11837.22	10973.48	8687.65	11136.66	11652.65	11246.31	10097.44	9696.80	9960.78	2.27	1.49	0.65	0.84	1.31	0.69	4.16	2.47	0.11	4.90	4.98	0.12	x	
2174	Influenza virus NS1A-binding protein	Q9Y6Y0	13819.15	13573.57	13064.62	8081.59	7549.39	6660.14	14835.55	13006.38	13362.47	11280.85	10376.42	9698.66	14244.84	13087.17	11207.04	12155.60	11303.57	10230.29	8658.99	8204.53	7370.12	1270.63	1435.64	1539.57	1022.96	967.29	939.66	1053.00	1142.92	968.73	1060.16	1086.15	658.70		
2175	Inhibitor of Bruton tyrosine kinase	Q9P2D0	23750.10	20210.34	18933.50	13840.67	12852.88	12154.22	15522.78	14081.46	14490.73	20986.55	19911.69	17040.85	16747.30	15899.19	12125.58	15449.99	13509.43	13408.45	14330.66	13328.40	12551.64	33349.63	31500.73	29352.71	24973.52	23337.23	19693.85	27960.94	25595.07	25180.88	30573.72	31633.42	21435.30		
2176	Inhibitor of nuclear factor kappa-B kinase subunit beta	O14920	10508.82	10106.56	9743.30	31722.74	5180.44	4179.98	8262.24	8532.12	7318.47	6641.98	6060.08	5823.46	7911.46	35555.40	8204.63	7198.48	6883.27	6251.53	5708.97	5509.97	5515.65	13766.35	15269.70	12665.35	9130.86	9384.19	7294.34	11356.36	11735.23	10810.55	11930.81	11789.10	7716.33		
2177	Inhibitor of nuclear factor kappa-B kinase subunit epsilon	Q14164	23842.74	20409.81	20167.81	15463.75	13408.87	13150.05	17580.67	16699.96	15758.64	19367.12	18166.79	16539.44	18045.95	16379.84	13705.52	13492.95	13379.91	12553.45	14752.79	13961.41	13884.04	2.27	1.49	0.65	0.84	1.31	0.69	4.16	2.47	0.11	4.90	4.98	0.12	x	
2178	Inhibitor of nuclear factor kappa-B kinase-interacting protein	Q70UQ0	0.14	2.53	0.81	1.07	2.77	0.68	0.96	0.36	0.63	0.35	0.75	0.38	2.13	0.97	0.60	4.64	1.07	0.47	2.22	0.19	0.06	32056.85	25912.78	19024.28	22350.42	21496.33	21819.77	26310.14	30183.01	15267.48	24478.02	22085.44	11646.66		x
2179	Inner nuclear membrane protein Man1	Q9Y2U8	4254.31	3858.49	3677.53	3093.33	2856.38	3079.64	3153.07	3362.34	3363.97	3294.34	3090.88	2935.48	3669.19	3006.72	2839.74	2997.08	2681.54	3011.86	3900.19	3257.96	3239.85	2.27	1.49	0.65	0.84	1.31	0.69	4.16	2.47	0.11	4.90	4.98	0.12	x	
2180	Inorganic pyrophosphatase 2 mitochondrial	Q9H2U2	204631.16	189721.71	178682.51	128693.45	123310.58	119323.89	147726.79	146279.89	133780.19	159299.16	150577.40	135656.47	146252.79	145939.60	122033.89	131256.01	121235.42	118191.26	145781.20	134740.93	118696.69	106307.32	99177.77	90288.91	65203.35	64206.55	57391.20	93754.90	89402.58	80015.10	96365.43	91693.15	64728.30		
2181	Inorganic pyrophosphatase	Q15181	277790.12	262255.52	240282.43	183589.35	172963.47	159958.72	229254.43	221507.85	205662.55	238491.54	223179.81	210836.61	231888.45	214185.72	165331.80	189727.71	179543.49	169480.08	185296.81	176705.32	142880.32	182794.51	164744.51	155982.85	109489.33	103930.59	89321.06	145011.17	135058.63	130493.28	142987.88	138138.40	89318.38		
2182	Inosine triphosphate pyrophosphatase	Q9BY32	0.14	2.53	0.81	1.07	2.77	0.68	0.96	0.36	0.63	0.35	0.75	0.38	2.13	0.97	0.60	4.64	1.07	0.47	2.22	0.19	0.06	1397.66	913.85	1221.53	251.95	202.36	42.28	247.33	147.75	98.15	400.33	179.06	54.26		x
2183	Inosine-5'-monophosphate dehydrogenase 1	P20839	326.65	318.36	246.76	161.98	224.47	156.68	179.50	87.06	147.40	542.86	551.30	547.71	193.81	328.85	138.24	191.02	280.26	172.82	178.82	195.34	216.48	25830.17	25012.75	24587.25	16894.35	16752.36	14609.61	20033.07	20639.98	20998.23	18212.59	17976.59	13878.42		
2184	Inosine-5'-monophosphate dehydrogenase 2	P12268	332335.42	319020.39	296445.51	233748.45	221366.59	209816.79	264377.38	256592.27	238566.52	286455.09	274794.17	259226.28	260009.90	243966.62	218070.67	234014.84	220325.53	212666.66	244165.61	226485.65	213952.78	236205.45	212032.70	196278.64	156104.05	153621.76	140130.97	188764.29	176486.99	161952.75	183445.73	176480.93	107356.83		
2185	Inositol 1 4 5-trisphosphate receptor type 1	Q14643	1027.29	710.27	696.99	937.77	788.66	893.63	843.06	577.23	762.56	983.09	1008.06	866.17	675.13	454.21	318.13	1181.43	914.92	990.15	722.32	636.01	508.32	601.13	726.36	893.87	362.30	340.61	225.91	467.98	491.33	579.61	530.63	644.59	251.13		
2186	Inositol 1_4_5-trisphosphate receptor type 2	Q14571	0.14	2.53	0.81	1.07	2.77	0.68	0.96	0.36	0.63	0.35	0.75	0.38	2.13	0.97	0.60	4.64	1.07	0.47	2.22	0.19	0.06	30562.02	29565.46	28485.81	17341.80	16908.95	15027.79	21170.59	18887.99	18421.57	24724.17	22488.90	16779.41		x
2187	Inositol monophosphatase 1	P29218	0.14	2.53	0.81	1.07	2.77	0.68	0.96	0.36	0.63	0.35	0.75	0.38	2.13	0.97	0.60	4.64	1.07	0.47	2.22	0.19	0.06	12664.62	10697.79	9999.35	8033.34	7529.78	7159.46	10802.81	9847.89	8444.35	8529.46	8639.29	5914.45		x
2188	Inositol monophosphatase 3	Q9NX62	18803.09	19299.66	18110.17	12724.07	11441.45	11691.88	15025.24	15725.53	11895.18	14091.80	13881.51	13121.53	14029.69	13116.64	11013.01	11218.10	11422.20	10284.91	11818.17	9794.00	10486.30	2.27	1.49	0.65	0.84	1.31	0.69	4.16	2.47	0.11	4.90	4.98	0.12	x	
2189	Inositol polyphosphate 1-phosphatase	P49441	15377.74	14447.73	13630.69	11491.40	10986.14	9721.50	11162.71	11483.77	9468.52	16010.57	15076.80	14648.32	13592.78	12431.37	6246.76	8460.93	9908.29	9785.16	14064.94	12327.68	11226.23	2.27	1.49	0.65	0.84	1.31	0.69	4.16	2.47	0.11	4.90	4.98	0.12	x	
2190	Inositol polyphosphate 5-phosphatase OCRL-1	Q01968	92048.79	89704.21	83871.12	54419.56	54532.09	48627.36	65565.92	66840.26	66290.05	76126.04	72351.49	70482.32	72769.18	67264.35	56492.26	62860.89	60371.13	58807.59	59081.72	56798.60	55595.97	17788.88	15871.25	14308.63	12304.04	11617.05	9263.65	16514.97	15568.22	14590.75	15956.83	15142.04	9243.77		
2191	Inositol-3-phosphate synthase 1	Q9NPH2	33046.68	30903.24	30832.26	21013.47	19958.18	19195.92	24500.81	23019.34	23349.68	27546.58	26116.64	25500.82	25404.80	23676.69	19591.07	20984.50	20991.02	20061.23	18904.95	19329.35	17645.37	104699.68	96344.26	89117.31	67471.06	66184.25	63313.80	75229.58	73183.65	69345.37	74389.53	73313.02	57220.45		
2192	Inositol-tetrakisphosphate 1-kinase	Q13572	3163.18	2557.11	1950.35	2017.93	1864.73	1364.72	3475.59	2914.75	2671.63	3027.09	2860.77	2306.33	2833.08	2328.45	920.07	1919.17	1721.63	1240.65	2366.48	2377.82	1837.73	2.27	1.49	0.65	0.84	1.31	0.69	4.16	2.47	0.11	4.90	4.98	0.12	x	
2193	Insulin	P01308	0.14	2.53	0.81	1.07	2.77	0.68	0.96	0.36	0.63	0.35	0.75	0.38	2.13	0.97	0.60	4.64	1.07	0.47	2.22	0.19	0.06	5716.92	6018.83	4658.05	4451.35	4373.54	2539.99	4377.96	3573.37	2635.92	6113.58	5612.76	2809.10		x
2194	Insulin receptor substrate 1	P35568	22210.96	22332.41	23023.73	15744.41	14623.97	14675.79	23832.30	24144.69	23182.31	17759.79	17054.01	16224.91	20524.52	19353.91	16589.27	16473.76	16610.34	16358.71	16097.29	15573.41	13526.46	2.27	1.49	0.65	0.84	1.31	0.69	4.16	2.47	0.11	4.90	4.98	0.12	x	
2195	Insulin-degrading enzyme	P14735	94864.48	89410.16	88002.98	61702.86	59119.95	57506.50	74847.51	71358.35	66379.29	78823.32	73698.05	69316.71	73775.58	70513.13	59344.53	64169.59	59833.00	58719.18	63582.54	63292.07	57865.11	15529.41	12374.47	10192.32	10405.10	9556.12	7348.74	14919.19	13858.80	9162.15	14978.31	14664.03	8557.81		
2196	Insulin-like growth factor 1 receptor	P08069	166722.90	166152.00	151461.90	76960.18	71659.01	69275.27	95774.26	94784.52	92078.99	123077.91	123517.05	117269.04	97501.62	93287.24	81197.65	139184.35	130376.69	125420.28	99014.21	98458.95	94481.42	2.27	1.49	0.65	0.84	1.31	0.69	4.16	2.47	0.11	4.90	4.98	0.12	x	
2197	Insulin-like growth factor 2 mRNA-binding protein 1	Q9NZI8	0.14	2.53	0.81	1.07	2.77	0.68	0.96	0.36	0.63	0.35	0.75	0.38	2.13	0.97	0.60	4.64	1.07	0.47	2.22	0.19	0.06	6399.68	6436.16	6399.44	4960.97	4726.65	3989.20	5964.89	5540.89	4774.01	6634.68	6422.24	4225.59		x
2198	Insulin-like growth factor 2 mRNA-binding protein 2	Q9Y6M1	0.14	2.53	0.81	1.07	2.77	0.68	0.96	0.36	0.63	0.35	0.75	0.38	2.13	0.97	0.60	4.64	1.07	0.47	2.22	0.19	0.06	60086.29	56412.47	52501.16	43576.92	41656.88	38981.09	43103.14	42925.90	41787.27	46762.54	45164.19	38188.86		x
2199	Insulin-like growth factor 2 mRNA-binding protein 3	O00425	1742.35	1583.16	1482.94	1983.84	1069.50	1038.04	1381.98	1510.56	1645.67	1355.11	1329.76	1283.02	1576.10	1367.73	1135.01	1605.42	1523.13	1362.78	1941.86	1282.67	1265.12	17180.85	15486.97	15245.74	11455.38	11024.18	10442.42	11198.36	10359.25	11116.52	12737.40	11895.68	7425.79		
2200	Insulin-like growth factor-binding protein 7	Q16270	0.14	2.53	0.81	1.07	2.77	0.68	0.96	0.36	0.63	0.35	0.75	0.38	2.13	0.97	0.60	4.64	1.07	0.47	2.22	0.19	0.06	9131.24	8882.88	7879.53	4505.17	4445.05	3963.93	5724.39	5837.42	5446.01	5838.25	5612.45	4107.57		x
2201	Integrator complex subunit 1	Q8N201	33258.09	33112.98	29136.48	26897.45	25845.53	23997.74	28312.77	28173.60	26628.98	32235.15	30751.21	29145.27	34374.85	33374.48	29302.31	23298.60	23458.79	24785.33	28092.56	24691.09	23779.36	42360.33	42086.85	41502.09	28070.03	29953.96	29023.14	36300.27	37047.56	36741.60	39313.89	36803.68	28540.49		
2202	Integrator complex subunit 13	Q9NVM9	58188.88	58237.08	55830.62	39716.42	36722.75	34138.71	42440.73	40054.16	37664.20	42955.79	39389.24	39320.69	48799.54	49530.68	36036.21	36686.05	35525.38	32925.42	44163.49	40687.41	36494.02	2.27	1.49	0.65	0.84	1.31	0.69	4.16	2.47	0.11	4.90	4.98	0.12	x	
2203	Integrator complex subunit 3	Q68E01	93836.46	99404.44	87678.00	74678.76	74395.64	65328.18	73841.93	75230.10	61451.33	83248.06	77812.97	73291.40	80716.72	71303.36	52589.09	91571.36	84092.93	76695.31	84929.75	82202.13	78770.57	2.27	1.49	0.65	0.84	1.31	0.69	4.16	2.47	0.11	4.90	4.98	0.12	x	
2204	Integrator complex subunit 5	Q6P9B9	0.14	2.53	0.81	1.07	2.77	0.68	0.96	0.36	0.63	0.35	0.75	0.38	2.13	0.97	0.60	4.64	1.07	0.47	2.22	0.19	0.06	82.94	71.62	200.52	179.65	158.67	162.81	177.16	186.52	214.16	240.07	262.24	206.64		x
2205	Integrin alpha-2	P17301	25903.09	24696.50	22677.14	21204.63	19777.61	19557.95	24466.65	23283.54	23518.85	25686.37	25219.17	22739.11	23681.45	21240.75	18266.07	20790.30	18947.88	18315.55	20490.09	19226.95	18060.25	2.27	1.49	0.65	0.84	1.31	0.69	4.16	2.47	0.11	4.90	4.98	0.12	x	
2206	Integrin alpha-3	P26006	6205.79	5797.63	5625.13	5494.49	5487.11	5100.01	4854.84	4839.27	4193.70	7808.07	6938.98	7082.99	5215.87	4975.87	4322.79	6056.51	5766.03	5624.69	4683.99	4335.45	3982.82	158196.37	146269.36	120850.36	118403.11	119475.08	103221.26	128638.82	124769.36	117533.09	139596.02	137243.62	90343.77		
2207	Integrin alpha-E	P38570	3820.45	3325.70	2721.24	760.48	695.12	737.06	1622.80	1724.55	1565.82	2758.28	2335.48	2218.69	3150.65	1560.96	1302.05	1595.13	1775.19	1417.72	1012.97	982.83	960.15	7706.60	7192.80	6317.08	4988.67	4989.97	4942.41	7681.40	7354.19	7243.75	6141.85	6660.69	4067.81		
2208	Integrin alpha-V	P06756	0.14	2.53	0.81	1.07	2.77	0.68	0.96	0.36	0.63	0.35	0.75	0.38	2.13	0.97	0.60	4.64	1.07	0.47	2.22	0.19	0.06	59105.30	52336.81	47791.99	26888.01	25531.15	21662.74	31575.84	28257.22	27744.57	24265.26	22263.35	14760.18		x
2209	Integrin alpha-X	P20702	0.14	2.53	0.81	1.07	2.77	0.68	0.96	0.36	0.63	0.35	0.75	0.38	2.13	0.97	0.60	4.64	1.07	0.47	2.22	0.19	0.06	24.63	2685.76	24.20	1.67	10.87	3.21	16.06	16.84	43.49	29.72	19.28	10.40		x
2210	Integrin beta-1	P05556	102212.89	93604.71	94368.00	68509.06	65332.70	59942.43	73556.99	70255.63	66585.20	89655.67	86383.41	83129.26	77655.41	73858.35	76748.34	75340.70	70943.86	70591.61	69305.54	65228.83	63145.27	276448.26	254248.15	236793.55	192192.60	186434.27	164835.15	240585.70	227176.89	216433.19	216587.76	208335.98	145884.94		
2211	Integrin beta-5	P18084	24497.16	25618.69	20632.73	14310.42	15756.23	13730.39	18809.06	17816.60	17353.16	24562.19	22531.35	24097.88	19700.46	16898.54	13142.53	14179.13	13371.73	13207.38	15301.80	13766.16	13840.86	318535.53	298635.42	275921.50	239980.59	236221.11	235017.23	236112.08	234374.30	226342.28	250396.65	254283.20	206461.25		
2212	Integrin beta-like protein 1	O95965	77196.84	73987.44	65464.65	58797.26	59109.63	60413.22	58882.28	56577.33	50913.23	68307.10	64965.02	62904.83	59028.81	61244.05	54107.72	51446.86	51504.30	48281.87	55423.18	55525.10	52881.65	2.27	1.49	0.65	0.84	1.31	0.69	4.16	2.47	0.11	4.90	4.98	0.12	x	
2213	Integrin-linked kinase-associated serine/threonine phosphatase 2C	Q9H0C8	0.14	2.53	0.81	1.07	2.77	0.68	0.96	0.36	0.63	0.35	0.75	0.38	2.13	0.97	0.60	4.64	1.07	0.47	2.22	0.19	0.06	4102.82	6281.36	4126.84	3132.89	3222.48	2814.28	3163.04	3088.32	3008.71	3227.77	3243.90	1373.57		x
2214	Integrin-linked protein kinase	Q13418	582.33	502.66	485.68	808.31	630.93	805.04	607.37	653.42	614.72	1041.88	1047.43	1222.35	638.71	786.93	825.61	569.70	557.02	601.87	787.49	737.24	628.92	38421.51	35704.59	31050.82	22701.88	22635.08	18821.13	29350.34	26952.59	25822.02	30832.33	29380.90	19676.52		
2215	Interferon alpha-5	P01569	0.14	2.53	0.81	1.07	2.77	0.68	0.96	0.36	0.63	0.35	0.75	0.38	2.13	0.97	0.60	4.64	1.07	0.47	2.22	0.19	0.06	7149.18	5491.66	5713.61	5160.37	5140.44	4115.88	6456.96	6380.59	5349.31	6014.92	6332.77	3926.14		x
2216	Interferon regulatory factor 2-binding protein 2	Q7Z5L9	2279.70	1875.20	1965.34	1437.27	1546.45	1480.69	1811.41	1737.68	1800.93	2275.07	2187.03	2106.24	1992.20	1740.23	1692.19	1294.02	1215.95	1219.29	1790.19	1739.27	1632.67	2.27	1.49	0.65	0.84	1.31	0.69	4.16	2.47	0.11	4.90	4.98	0.12	x	
2217	Interferon regulatory factor 2-binding protein-like	Q9H1B7	59908.81	60525.90	74585.23	41611.11	39994.70	38049.77	43352.61	42177.06	39736.63	68534.31	64357.68	63316.77	47896.91	52294.58	42099.89	37222.34	36646.80	35097.81	42008.95	42014.71	40818.86	12179.03	12269.90	10542.70	7504.76	6438.07	4997.99	8639.37	7447.75	6862.99	8952.77	8852.68	4833.37		
2218	Interferon regulatory factor 4	Q15306	10720.39	8606.78	7809.11	5620.31	5173.27	4492.00	8884.78	8752.66	8306.83	7192.50	7420.36	5696.14	8470.64	7557.52	4790.09	6294.93	5723.68	5011.93	7137.93	6294.18	5797.53	2.27	1.49	0.65	0.84	1.31	0.69	4.16	2.47	0.11	4.90	4.98	0.12	x	
2219	Interferon-induced helicase C domain-containing protein 1	Q9BYX4	81438.57	83446.70	76371.52	67276.15	59442.71	63217.07	66404.54	68702.12	66301.28	71230.84	67645.79	65863.72	72176.41	68774.35	57452.45	63015.47	57517.00	57709.30	58552.29	59495.84	55524.33	7344.92	6201.81	5846.72	3938.22	3973.39	3468.23	4050.52	3668.55	3747.18	4043.77	3922.96	2500.46		
2220	Interferon-induced protein with tetratricopeptide repeats 3	O14879	48724.70	49989.34	51335.29	36567.53	34704.68	34182.87	40164.34	37385.65	39760.84	41055.43	39841.47	38719.47	38908.94	40501.02	33666.42	34452.47	33140.53	34135.17	36266.27	36544.22	35761.45	2.27	1.49	0.65	0.84	1.31	0.69	4.16	2.47	0.11	4.90	4.98	0.12	x	
2221	Interferon-induced protein with tetratricopeptide repeats 5	Q13325	0.14	2.53	0.81	1.07	2.77	0.68	0.96	0.36	0.63	0.35	0.75	0.38	2.13	0.97	0.60	4.64	1.07	0.47	2.22	0.19	0.06	1759.08	1482.98	1064.82	534.79	490.89	283.17	1044.87	851.46	610.88	1111.93	1016.81	477.56		x
2222	Interferon-induced transmembrane protein 3	Q01628	5862.98	4985.32	13508.21	3792.47	3859.23	3373.75	5512.84	4162.37	4539.20	13602.03	13461.27	12623.97	4647.70	6722.30	2467.94	3851.33	3767.68	3880.36	3903.52	3938.52	3224.14	2.27	1.49	0.65	0.84	1.31	0.69	4.16	2.47	0.11	4.90	4.98	0.12	x	
2223	Interferon-induced_ double-stranded RNA-activated protein kinase	P19525	41952.66	40145.43	37029.67	24238.46	22993.52	19926.06	33288.75	32676.60	30287.50	32361.71	31503.35	29240.04	32177.11	28955.64	21913.43	24401.33	23329.75	22571.31	24008.22	24191.57	21904.77	10734.30	10384.95	9114.29	6286.73	6130.68	5026.55	7920.74	7841.65	7271.84	7928.26	8084.21	5353.97		
2224	Interferon-inducible double-stranded RNA-dependent protein kinase activator A	O75569	1643.77	1678.35	1661.30	1305.15	1379.91	1336.43	2419.14	2618.45	2519.22	2183.02	1803.25	1705.98	1753.58	1748.39	1437.41	1586.41	1368.56	1318.46	1265.13	1281.40	1174.64	2.27	1.49	0.65	0.84	1.31	0.69	4.16	2.47	0.11	4.90	4.98	0.12	x	
2225	Interferon-stimulated gene 20 kDa protein	Q96AZ6	2753.83	3034.64	2939.09	2650.95	2352.23	2440.78	3894.01	3376.04	2934.12	3376.91	3334.62	3061.79	2317.83	2478.38	1947.58	2826.84	2597.79	2557.61	1988.31	2027.76	1815.93	2.27	1.49	0.65	0.84	1.31	0.69	4.16	2.47	0.11	4.90	4.98	0.12	x	
2226	Interleukin enhancer-binding factor 2	Q12905	182609.82	163945.34	150470.60	133513.45	127646.78	118218.65	149503.29	149609.67	140673.00	147976.73	142690.44	131180.26	151413.24	141413.79	108120.42	130670.08	126825.34	121606.53	145805.03	127194.78	120074.70	31583.19	27329.01	24691.56	19867.50	18912.03	16562.37	21677.59	20049.55	20190.67	26217.90	24817.17	16517.19		
2227	Interleukin enhancer-binding factor 3	Q12906	258462.46	251994.87	237889.99	161837.24	155724.09	151372.97	195806.86	189709.36	185056.19	194437.45	181932.25	171795.93	203646.73	197640.83	169115.70	153370.39	145085.48	142597.19	172241.09	165674.92	157172.33	124678.74	114987.67	112941.58	81008.73	75673.67	69428.74	86226.69	79664.26	76003.70	95599.63	90069.01	63349.21		
2228	Interleukin-1 receptor accessory protein-like 1	Q9NZN1	1068.08	684.46	439.78	588.45	293.50	204.98	708.97	320.56	465.16	586.50	609.51	229.05	714.07	407.07	5.40	659.18	274.16	259.18	598.80	477.68	225.98	2.27	1.49	0.65	0.84	1.31	0.69	4.16	2.47	0.11	4.90	4.98	0.12	x	
2229	Interleukin-1 receptor-associated kinase 3	Q9Y616	25422.45	24760.45	22350.93	18005.94	18557.80	18021.39	18485.84	18631.71	17335.59	24407.62	22891.21	21552.81	20537.57	21192.68	18207.53	17238.78	16208.16	16638.66	18287.64	17559.16	17467.59	2.27	1.49	0.65	0.84	1.31	0.69	4.16	2.47	0.11	4.90	4.98	0.12	x	
2230	Interleukin-1 receptor-like 2	Q9HB29	0.14	2.53	0.81	1.07	2.77	0.68	0.96	0.36	0.63	0.35	0.75	0.38	2.13	0.97	0.60	4.64	1.07	0.47	2.22	0.19	0.06	10219.24	8807.47	7862.11	6819.89	6092.13	4804.48	7561.72	7380.55	5823.47	10306.81	10290.74	5956.08		x

Supplementary Table S1. Overview on the abundance of all relatively quantified 5180 proteins

			MCF-7																					MDA-MB-231													
	Protein name	UniProt	control 01	control 02	control 03	Dai IC20 01	Dai IC20 02	Dai IC20 03	Dai SC20 01	Dai SC20 02	Dai SC20 03	Gen IC20 01	Gen IC20 02	Gen IC20 03	Gen SC20 01	Gen SC20 02	Gen SC20 03	SSE IC20 01	SSE IC20 02	SSE IC20 03	SSE SC20 01	SSE SC20 02	SSE SC20 03	control 01	control 02	control 03	Dai IC20 01	Dai IC20 02	Dai IC20 03	Gen IC20 01	Gen IC20 02	Gen IC20 03	SSE IC20 01	SSE IC20 02	SSE IC20 03	unique to MCF-7	unique to MDA-MB-231
2231	Interleukin-12 receptor subunit beta-1	P42701	0.14	2.53	0.81	15.40	2248.78	1325.62	0.96	0.36	0.63	0.35	0.75	0.38	2.13	0.97	0.60	4.64	1.07	0.47	13.99	19.00	1.59	2.27	1.49	0.65	0.84	1.31	0.69	4.16	2.47	0.11	4.90	4.98	0.12	x	
2232	Interleukin-13 receptor subunit alpha-1	P78552	0.14	2.53	0.81	1.07	2.77	0.68	0.96	0.36	0.63	0.35	0.75	0.38	2.13	0.97	0.60	4.64	1.07	0.47	2.22	0.19	0.06	22491.56	24185.82	22199.09	21021.29	20161.12	14169.02	55496.99	47288.11	43515.79	23315.95	24993.79	15094.77		x
2233	Interleukin-17 receptor B	Q9NRM6	534.19	482.27	551.65	662.22	474.71	498.57	405.56	472.41	354.12	544.18	621.52	498.50	617.49	477.48	479.39	469.76	326.97	405.98	455.89	392.69	365.61	2.27	1.49	0.65	0.84	1.31	0.69	4.16	2.47	0.11	4.90	4.98	0.12	x	
2234	Interleukin-17B	Q9UHF5	0.14	2.53	0.81	1.07	2.77	0.68	0.96	0.36	0.63	0.35	0.75	0.38	2.13	0.97	0.60	4.64	1.07	0.47	2.22	0.19	0.06	1713.29	1622.09	1348.84	813.39	794.55	761.90	1403.00	1170.70	1182.20	1325.73	1201.65	788.71		x
2235	Interleukin-6 receptor subunit beta	P40189	0.14	2.53	0.81	1.07	2.77	0.68	0.96	0.36	0.63	0.35	0.75	0.38	2.13	0.97	0.60	4.64	1.07	0.47	2.22	0.19	0.06	17159.96	15782.97	14935.72	11955.30	11230.90	10354.46	13842.37	12762.47	11809.03	16263.91	15518.04	9383.67		x
2236	Intermediate filament family orphan 1	Q0D2I5	0.14	2.53	0.81	1.07	2.77	0.68	0.96	0.36	0.63	0.35	0.75	0.38	2.13	0.97	0.60	4.64	1.07	0.47	2.22	0.19	0.06	1560.51	1472.32	1070.87	747.77	690.26	460.76	1033.63	977.14	823.11	1132.96	1001.64	496.17		x
2237	Intersectin-1	Q15811	159226.70	155383.36	147169.46	118211.37	113954.23	110733.83	128125.93	122687.22	115420.22	143306.30	138876.70	128300.70	142214.96	124669.82	108741.14	119776.44	115485.32	112069.47	109465.20	104100.86	94010.40	13269.35	12931.61	12683.85	8088.82	8378.31	6940.87	9303.66	8603.83	8988.06	11233.26	11631.89	2747.16		
2238	Intracellular hyaluronan-binding protein 4	Q5JVS0	568.54	517.66	289.60	588.76	493.98	317.81	497.28	533.85	449.02	652.78	634.50	504.36	598.35	470.71	294.29	708.89	553.18	626.44	677.81	605.68	474.21	2.27	1.49	0.65	0.84	1.31	0.69	4.16	2.47	0.11	4.90	4.98	0.12	x	
2239	Intraflagellar transport protein 122 homolog	Q9HBG6	37745.44	35135.01	32397.69	22852.74	23196.30	19646.14	31295.72	29419.18	28275.13	30147.42	28750.11	26672.11	29550.59	29478.58	20471.38	26870.07	24585.42	24444.16	23819.26	22898.97	21015.67	408.27	522.07	371.89	296.64	189.48	170.20	377.84	368.93	350.46	403.63	396.82	204.30		
2240	Intraflagellar transport protein 172 homolog	Q9UG01	6965.52	4653.86	2404.26	2830.28	282.63	192.83	435.88	506.71	411.83	4231.14	3433.33	2307.43	4394.42	2754.03	349.13	1032.96	243.88	232.45	492.70	397.36	460.01	2.27	1.49	0.65	0.84	1.31	0.69	4.16	2.47	0.11	4.90	4.98	0.12	x	
2241	Intraflagellar transport protein 74 homolog	Q96LB3	38837.03	37184.62	33162.38	20237.27	20098.45	17390.88	26181.58	26478.81	25542.72	23850.88	23640.19	21617.27	26241.52	24054.71	21069.86	27877.39	26580.39	27262.07	19032.78	17122.64	15492.11	1535.02	1493.75	1098.43	1644.41	1306.69	1138.48	2022.10	2029.25	1749.14	1804.64	1303.03	1305.44		
2242	Inverted formin-2	Q27J81	59580.56	55527.91	49523.62	32676.62	31311.30	27578.25	39123.29	36897.76	34961.66	49533.78	46070.17	41529.73	49854.67	50837.23	41811.49	30470.28	28689.93	26805.22	40503.20	37352.91	34093.38	2.27	1.49	0.65	0.84	1.31	0.69	4.16	2.47	0.11	4.90	4.98	0.12	x	
2243	Iporin	Q8N2Y8	30399.35	31012.95	29027.58	22598.04	21755.75	21114.34	24811.54	24971.54	20610.35	30859.30	29351.35	25394.33	26066.02	23691.00	17834.20	25361.35	25961.05	23974.44	23731.68	23182.57	21446.47	2429.53	2235.34	2651.84	1782.45	1583.85	1479.76	3195.66	2645.01	2665.79	2628.13	2565.86	1378.25		
2244	IQ domain-containing protein E	Q6IPM2	0.14	2.53	0.81	1.07	2.77	0.68	0.96	0.36	0.63	0.35	0.75	0.38	2.13	0.97	0.60	4.64	1.07	0.47	2.22	0.19	0.06	1682.63	1477.72	1356.40	1761.78	1654.09	1700.94	1434.73	1541.17	1473.76	3191.26	3214.17	197.75		x
2245	IQ motif and SEC7 domain-containing protein 1	Q6DN90	12306.14	13060.95	12151.57	11687.77	11622.85	11070.29	10446.76	10018.00	10170.33	12378.85	11242.82	11479.86	12631.97	12657.96	9558.77	9865.33	9471.11	9730.25	8365.18	7750.80	8687.14	5904.02	5682.95	5068.16	3760.99	3667.77	3214.71	4483.60	4056.34	4041.03	4777.99	4607.91	1275.57		
2246	Iron-sulfur cluster assembly enzyme ISCU_ mitochondrial	Q9H1K1	2735.07	5288.16	4967.71	4103.36	3038.29	1830.47	2981.01	4511.54	2819.62	5462.31	4777.94	4503.52	4095.74	2900.95	1661.88	4225.34	1719.01	2887.82	3637.13	3626.56	3746.11	2.27	1.49	0.65	0.84	1.31	0.69	4.16	2.47	0.11	4.90	4.98	0.12	x	
2247	Islet cell autoantigen 1	Q05084	20873.64	19811.26	16866.95	11185.67	10554.17	9018.06	14155.78	12673.46	12407.92	15105.33	14307.87	12737.57	14547.04	12420.82	10815.01	11949.12	11015.71	10605.55	12973.07	11713.67	10654.47	2.27	1.49	0.65	0.84	1.31	0.69	4.16	2.47	0.11	4.90	4.98	0.12	x	
2248	Isobutyryl-CoA dehydrogenase mitochondrial	Q9UKU7	0.14	2.53	0.81	1.07	2.77	0.68	0.96	0.36	0.63	0.35	0.75	0.38	2.13	0.97	0.60	4.64	1.07	0.47	2.22	0.19	0.06	13916.00	14218.35	13848.25	12788.67	12022.70	12749.50	14317.36	13470.88	13408.56	18035.36	14644.38	10329.72		x
2249	Isochorismatase domain-containing protein 1	Q96CN7	37960.70	35605.17	33263.32	22235.05	21847.17	21876.80	23350.76	22095.55	23919.85	26868.18	26377.81	27606.43	30995.49	30154.83	23434.20	22815.89	19116.11	20199.73	21695.35	22166.67	17283.64	2.27	1.49	0.65	0.84	1.31	0.69	4.16	2.47	0.11	4.90	4.98	0.12	x	
2250	Isochorismatase domain-containing protein 2	Q96AB3	18781.76	17864.50	15282.36	10276.61	9388.75	8708.01	13303.45	13134.63	12035.24	12768.61	11902.08	11206.37	15007.60	12252.84	9276.27	10639.85	10059.75	9917.29	9943.11	9158.79	8963.03	2.27	1.49	0.65	0.84	1.31	0.69	4.16	2.47	0.11	4.90	4.98	0.12	x	
2251	Isocitrate dehydrogenase [NAD] subunit alpha_ mitochondrial	P50213	123394.27	120540.64	108226.91	92736.43	87517.29	82887.33	95268.60	91320.61	91193.65	108801.08	99977.39	98436.15	98871.39	96730.76	76433.66	87770.92	77241.47	77928.41	85287.23	80238.41	78617.00	16248.41	14697.11	14737.02	12786.06	11840.17	10377.46	12943.10	12117.20	11908.23	11753.72	11781.81	9998.02		
2252	Isocitrate dehydrogenase [NAD] subunit beta_ mitochondrial	O43837	43038.60	43193.51	38717.68	29340.99	27515.98	25917.77	35026.46	34514.61	31412.85	36435.88	34786.26	33761.00	34398.45	33207.15	27501.68	29891.33	28571.25	26547.78	28815.57	26380.25	24386.85	2.27	1.49	0.65	0.84	1.31	0.69	4.16	2.47	0.11	4.90	4.98	0.12	x	
2253	Isocitrate dehydrogenase [NADP] cytoplasmic	O75874	171462.83	175185.80	155833.60	150807.06	144879.84	134374.81	154255.69	145983.15	136744.89	163819.92	154135.49	148077.54	143314.22	127695.86	86724.12	175782.61	163446.60	158818.64	131615.41	122314.39	118634.57	254599.75	239765.03	217661.08	186847.26	185861.21	158114.01	195542.78	188127.76	184224.30	206631.40	197603.60	150772.41		
2254	Isocitrate dehydrogenase [NADP]_ mitochondrial	P48735	404250.33	387801.77	360000.27	221591.49	214479.82	191319.34	249569.51	237459.99	228416.09	268561.83	258415.28	245333.34	293623.00	274141.89	210093.89	223668.84	219147.14	203413.89	210998.67	204336.46	188140.77	32810.25	32856.74	29905.11	26982.93	27319.82	26145.77	27755.27	28957.92	28176.68	31991.23	30699.82	19100.69		
2255	Isoleucine--tRNA ligase cytoplasmic	P41252	554272.99	515602.59	477311.80	380393.06	367728.33	331357.88	421664.59	405919.87	394566.25	457035.00	431086.46	394294.10	438430.84	419367.53	318531.49	381090.27	362150.69	347353.06	380873.17	360796.75	329141.40	155802.53	138589.64	117591.87	91115.63	87231.19	67598.19	116935.42	107992.05	91060.74	117854.66	113711.47	65583.68		
2256	Isoleucine--tRNA ligase mitochondrial	Q9NSE4	175437.94	171830.17	183026.30	121071.00	117266.00	107165.10	140366.22	135486.74	126244.01	186543.13	177533.03	173426.15	156814.92	140550.46	107679.21	114074.00	108046.09	107984.64	120186.95	113874.27	109809.64	164240.78	169815.25	154191.94	81935.06	78336.29	67134.49	147517.94	135472.16	136362.45	109665.65	108997.28	75468.56		
2257	Isopentenyl-diphosphate Delta-isomerase 1	Q13907	38474.40	35180.12	31425.34	32861.68	29571.96	26882.90	32070.61	33705.12	33886.07	35854.43	33453.10	31260.66	33841.69	28741.38	22073.54	37687.73	34930.72	32602.66	36098.76	31394.83	26777.26	2909.71	3130.82	2689.69	2132.57	1955.04	1795.99	2470.79	2376.67	2397.88	2214.58	2141.69	1579.00		
2258	Isopentenyl-diphosphate delta-isomerase 2	Q9BXS1	37420.81	33696.18	29558.16	25262.92	22566.12	21276.98	27982.29	27843.47	27031.41	28225.79	27849.66	26385.97	27515.81	26238.64	21369.12	25337.73	22271.80	21676.28	31650.52	21849.24	21136.35	2.27	1.49	0.65	0.84	1.31	0.69	4.16	2.47	0.11	4.90	4.98	0.12	x	
2259	IST1 homolog	P53990	124028.64	119694.10	113942.36	89796.12	86398.01	86282.01	89376.49	90763.03	90190.17	104350.79	100334.13	93241.57	107559.39	83377.40	70311.30	84729.17	76847.71	74898.04	90587.64	79517.28	75048.90	52056.98	49384.35	47618.84	36149.60	36895.93	34936.02	39843.69	39103.21	37907.03	41937.06	38027.36	26425.96		
2260	Izumo sperm-egg fusion protein 2	Q6UXV1	35130.44	36176.47	32250.65	25857.88	25816.93	23737.76	24524.89	24476.44	24345.71	33726.56	30896.81	28632.51	27943.66	27426.05	21452.87	24396.39	22851.04	23215.21	23093.77	20108.77	21960.55	2.27	1.49	0.65	0.84	1.31	0.69	4.16	2.47	0.11	4.90	4.98	0.12	x	
2261	Izumo sperm-egg fusion protein 3	Q5VZ72	0.14	2.53	0.81	1.07	2.77	0.68	0.96	0.36	0.63	0.35	0.75	0.38	2.13	0.97	0.60	4.64	1.07	0.47	2.22	0.19	0.06	4533.98	4309.47	2686.46	2462.01	2488.83	1981.34	2919.85	2720.17	3049.15	3184.65	3116.25	2077.16		x
2262	Jerky protein homolog	O75564	1580.31	1479.49	1617.90	1155.68	1168.93	1071.73	1343.99	1424.92	1379.75	1390.82	1283.33	1261.45	1353.59	1350.68	1143.19	1132.10	1144.20	1045.80	1292.17	1108.60	1002.78	2.27	1.49	0.65	0.84	1.31	0.69	4.16	2.47	0.11	4.90	4.98	0.12	x	
2263	Junction plakoglobin	P14923	120741.31	118656.52	110080.92	65538.15	64043.02	60996.83	86306.45	79209.26	77655.09	83294.46	77398.59	73817.64	87441.69	85877.64	74550.45	73203.73	67512.82	69995.11	65570.46	66106.53	65813.91	3830.27	4019.80	3904.34	2684.96	2502.97	1819.05	3381.40	2906.29	3086.17	3880.76	4106.13	2309.02		
2264	Junctional protein associated with coronary artery disease	Q9P266	1381.16	1300.01	1269.89	1036.52	1118.16	973.36	1208.24	1123.62	1086.78	953.52	982.86	1116.60	1462.41	1406.17	1103.18	1303.16	1307.86	1351.44	1610.86	1867.21	1495.99	2.27	1.49	0.65	0.84	1.31	0.69	4.16	2.47	0.11	4.90	4.98	0.12	x	
2265	Junctophilin-3	Q8WXH2	607.75	544.94	334.87	655.09	4598.26	2386.16	4971.88	5020.42	4693.10	855.01	773.85	860.38	748.41	5908.46	821.97	790.72	4248.49	3877.73	542.42	914.62	578.83	1061.65	1129.18	1064.25	1089.15	1006.90	805.44	1283.44	1222.77	1132.16	1284.78	1316.77	805.77		
2266	Jupiter microtubule associated homolog 1	Q9UK76	221623.76	209598.32	189815.53	141800.00	142756.84	132379.22	160325.63	150627.66	143245.84	190367.16	184357.33	175102.16	181509.59	160435.00	135990.81	143053.57	140189.56	130736.86	147391.25	137509.87	124220.73	252188.30	231967.41	213165.31	123777.76	123262.04	113627.67	149122.55	150115.86	141683.49	149989.64	144918.24	101450.16		
2267	Jupiter microtubule associated homolog 2	Q9H910	232827.61	223341.49	214222.84	139429.55	136769.01	131618.70	159152.34	142623.01	144389.84	171314.97	165676.16	157290.64	179270.22	171252.58	148812.18	140786.72	129920.25	128868.10	143076.22	133731.04	127557.14	92274.24	87541.51	81314.98	57940.35	54703.16	47498.78	75297.50	73622.04	70064.13	77094.16	74856.75	49387.57		
2268	Kalirin	O60229	0.14	2.53	0.81	1.07	2.77	0.68	0.96	0.36	0.63	0.35	0.75	0.38	2.13	0.97	0.60	4.64	1.07	0.47	2.22	0.19	0.06	3956.47	2933.30	2635.10	2665.11	2501.94	1770.16	3009.19	2823.29	2430.69	2968.45	2894.41	2084.80		x
2269	Kallikrein-14	Q9P0G3	947.30	1005.02	930.85	633.92	617.67	580.99	4424.88	4466.70	4363.85	884.62	921.90	930.00	1316.77	1057.80	940.73	850.89	874.24	896.05	877.57	675.37	862.70	2.27	1.49	0.65	0.84	1.31	0.69	4.16	2.47	0.11	4.90	4.98	0.12	x	
2270	Kanadaptin	Q9BWU0	40007.36	37451.15	35913.10	25852.20	25335.30	22425.82	28103.41	25733.53	24282.48	33322.25	31272.75	30334.46	32013.32	27349.84	22779.89	22642.03	22752.91	21815.62	27334.87	28372.92	25192.11	2437.45	2414.68	2303.47	1450.69	1478.97	1126.78	2205.58	2007.40	1936.23	2464.60	2186.91	1282.24		
2271	Katanin p60 ATPase-containing subunit A1	O75449	0.14	2.53	0.81	1.07	2.77	0.68	0.96	0.36	0.63	0.35	0.75	0.38	2.13	0.97	0.60	4.64	1.07	0.47	2.22	0.19	0.06	679.52	732.94	687.40	568.91	481.51	293.15	531.18	487.22	419.45	817.64	941.44	401.39		x
2272	Katanin p60 ATPase-containing subunit A-like 1	Q9BW62	0.14	2.53	0.81	1.07	2.77	0.68	0.96	0.36	0.63	0.35	0.75	0.38	2.13	0.97	0.60	4.64	1.07	0.47	2.22	0.19	0.06	4322.82	2472.90	2043.44	1903.48	2023.90	1699.18	3133.50	2902.36	2038.75	3071.62	2628.09	2267.44		x
2273	Katanin p60 ATPase-containing subunit A-like 2	Q8IYT4	9836.72	9369.74	8413.06	7114.55	7318.54	6860.49	5319.64	5550.23	5248.79	7401.22	7024.88	6649.56	8277.55	6000.23	4846.52	6522.74	6730.30	6581.30	6090.77	6081.76	5179.88	2.27	1.49	0.65	0.84	1.31	0.69	4.16	2.47	0.11	4.90	4.98	0.12	x	
2274	Kazrin	Q674X7	0.14	2.53	0.81	1.07	2.77	0.68	0.96	0.36	0.63	0.35	0.75	0.38	2.13	0.97	0.60	4.64	1.07	0.47	2.22	0.19	0.06	3931.54	3715.70	3019.16	2236.56	2197.16	1929.55	2317.27	2099.50	2118.34	2580.69	2439.79	1305.38		x
2275	Kelch domain-containing protein 4	Q8TBB5	4964.97	6516.11	6093.24	4079.91	3861.91	3542.60	3281.44	2924.63	3802.34	4300.46	4598.07	4904.92	4234.54	4606.18	3551.05	3030.60	3339.39	2952.46	4373.81	3492.73	2741.65	2.27	1.49	0.65	0.84	1.31	0.69	4.16	2.47	0.11	4.90	4.98	0.12	x	
2276	Kelch domain-containing protein 8A	Q8IYD2	0.14	2.53	0.81	1.07	2.77	0.68	0.96	0.36	0.63	0.35	0.75	0.38	2.13	0.97	0.60	4.64	1.07	0.47	2.22	0.19	0.06	8348.79	7472.60	7898.10	6109.41	5255.34	5097.36	7648.17	7275.74	7243.97	7303.96	7114.16	5525.64		x
2277	Kelch domain-containing protein 9	Q8NEP7	16348.91	14950.59	11718.05	7024.69	7180.16	6572.33	8318.91	8312.68	7492.00	9437.19	8407.73	7578.36	9905.83	9147.90	6755.92	7094.26	6630.50	5841.71	7246.33	7048.66	6572.50	2.27	1.49	0.65	0.84	1.31	0.69	4.16	2.47	0.11	4.90	4.98	0.12	x	
2278	Kelch repeat and BTB domain-containing protein 11	O94819	0.14	2.53	0.81	1.07	2.77	0.68	0.96	0.36	0.63	0.35	0.75	0.38	2.13	0.97	0.60	4.64	1.07	0.47	2.22	0.19	0.06	3885.08	3657.09	3590.60	2798.40	2634.02	2490.59	3135.60	2785.95	2745.53	3237.71	2742.58	2270.65		x
2279	Kelch-like protein 13	Q9P2N7	355.91	246.20	238.14	251.59	240.84	195.77	303.98	138.44	267.38	343.78	406.55	274.40	204.14	197.29	12.93	337.85	294.76	313.91	323.02	200.80	136.93	2.27	1.49	0.65	0.84	1.31	0.69	4.16	2.47	0.11	4.90	4.98	0.12	x	
2280	Kelch-like protein 15	Q96M94	467.83	166.45	428.75	3320.44	253.42	338.63	140.74	312.99	153.67	437.99	486.22	636.39	395.03	370.90	1130.88	367.94	332.20	343.14	391.70	418.11	224.46	2.27	1.49	0.65	0.84	1.31	0.69	4.16	2.47	0.11	4.90	4.98	0.12	x	
2281	Kelch-like protein 20	Q9Y2M5	102617.00	93644.61	87172.62	65650.40	63643.84	56901.23	102847.38	101452.27	92873.87	81330.49	79512.24	73441.99	81349.76	73384.10	57655.33	68008.21	64019.22	61815.28	68630.04	65003.40	60386.23	13215.46	12450.37	9507.08	8694.37	8517.44	6617.74	10463.62	9902.01	8642.28	11208.80	10804.66	5558.67		
2282	Kelch-like protein 24	Q6TFL4	9754.39	8224.90	7203.47	4501.33	4033.18	3424.39	5584.97	6103.39	5534.95	7200.78	6912.60	6237.08	6787.21	6314.67	3303.69	5209.04	5259.88	5074.01	5393.41	5078.72	4371.72	2.27	1.49	0.65	0.84	1.31	0.69	4.16	2.47	0.11	4.90	4.98	0.12	x	
2283	Kelch-like protein 25	Q9H0H3	10559.14	8922.12	9951.64	7843.27	7627.04	8071.40	9294.16	8946.63	8358.33	9081.33	9012.65	8517.07	9013.15	9027.98	7883.68	8367.45	7315.13	6661.80	9332.71	7691.40	6813.31	2.27	1.49	0.65	0.84	1.31	0.69	4.16	2.47	0.11	4.90	4.98	0.12	x	
2284	Kelch-like protein 26	Q53HC5	0.14	2.53	0.81	1.07	2.77	0.68	0.96	0.36	0.63	0.35	0.75	0.38	2.13	0.97	0.60	4.64	1.07	0.47	2.22	0.19	0.06	19020.91	17509.15	15627.20	13187.77	11406.10	10689.41	14523.11	13454.80	12739.21	13274.15	13555.45	9562.38		x
2285	Kelch-like protein 31	Q9H511	0.14	2.53	0.81	1.07	2.77	0.68	0.96	0.36	0.63	0.35	0.75	0.38	2.13	0.97	0.60	4.64	1.07	0.47	2.22	0.19	0.06	3617.31	3147.57	2687.19	970.62	961.54	471.15	2870.03	2850.41	2106.63	2207.55	2146.54	831.51		x
2286	Kelch-like protein 5	Q96PQ7	3805.75	3760.30	3765.00	2457.21	2553.14	2315.46	2832.23	2954.52	2856.88	4544.40	3868.91	3623.99	3689.69	3286.38	2903.25	2653.79	2422.98	2407.05	2564.42	2268.48	2534.90	2.27	1.49	0.65	0.84	1.31	0.69	4.16	2.47	0.11	4.90	4.98	0.12	x	
2287	Keratin_ type I cuticular Ha1	Q15323	744.22	639.20	753.79	88.97	105.29	30.08	327.71	364.48	296.32	450.55	524.60	428.87	275.41	197.89	68.75	1331.34	1340.54	1084.75	264.73	277.05	207.87	4883.97	4402.02	3428.98	3981.56	3743.33	3717.57	4430.08	4253.95	3771.98	4796.25	3762.56	0.12		
2288	Keratin_ type I cuticular Ha2	Q14532	15846.41	15883.34	15597.02	12357.13	12097.57	11613.35	13577.80	13347.34	12936.48	15572.82	14483.60	14212.63	14979.76	14205.51	11861.31	11577.41	11402.02	11770.51	11436.77	11156.64	10183.75	2.27	1.49	0.65	0.84	1.31	0.69	4.16	2.47	0.11	4.90	4.98	0.12	x	
2289	Keratin_ type I cuticular Ha3-II	Q14525	5770.64	6272.62	4946.16	4500.42	4452.16	3615.49	5783.04	5157.46	5064.47	5449.55	5982.76	5538.60	5551.75	4894.01	3638.80	3902.29	4191.28	3930.52	3779.24	4022.62	2829.15	318.84	271.44	244.04	214.81	247.53	250.46	237.63	203.61	223.34	322.62	306.79	339.33		
2290	Keratin_ type I cuticular Ha5	Q92764	154910.26	155287.98	145303.48	109136.22	103307.26	99739.33	115330.46	113648.63	108448.83	131097.22	123249.66	121997.82	118215.97	123251.45	107681.44	102798.61	96378.86	95710.39	103108.57	98186.63	96121.55	6898.10	6864.77	6261.35	4457.48	4242.83	4121.21	5032.41	4470.42	4194.45	5517.89	4618.36	1074.88		
2291	Keratin_ type I cuticular Ha6	O76013	4860.10	4408.55	4373.74	3606.84	3348.45	3326.97	4775.36	4898.53	4880.60	4930.73	4726.61	4691.02	4944.69	4157.64	3571.78	3215.13	3023.43	3186.51	3489.26	3232.68	3156.85	7511.31	7109.42	7032.47	1018.57	1023.89	932.11	766.51	712.00	731.65	602.89	632.89	469.16		
2292	Keratin_ type I cuticular Ha7	O76014	1319.05	1269.99	1223.84	798.12	651.36	645.38	913.82	848.27	838.02	1141.24	1125.46	1015.62	1015.06	904.70	487.65	816.27	1064.88	684.57	914.93	1289.94	1763.58	2.27	1.49	0.65	0.84	1.31	0.69	4.16	2.47	0.11	4.90	4.98	0.12	x	
2293	Keratin_ type I cuticular Ha8	O76015	24397.66	20701.96	18111.52	14674.93	12432.12	10664.87	12096.86	10564.14	10540.61	17906.52	16930.18	13987.27	18326.50	13776.01	6155.04	13802.63	12358.46	11214.12	13809.98	12644.37	10576.83	14371.81	12017.80	11177.19	7033.07	6872.39	6679.57	8975.13	8067.86	7962.84	8746.90	8602.77	6148.75		
2294	Keratin_ type I cytoskeletal 12	Q99456	110114.26	104621.18	98743.85	68279.88	65787.36	60713.66	61695.63	60855.31	56428.51	85728.54	82043.39	76995.89	80743.43	76658.07	63333.28	59170.11	56612.04	53298.71	72385.52	70704.23	69748.60	5753.48	5619.26	6047.07	15641.67	15544.12	14476.44	28486.88	27706.14	27715.63	25928.54	25215.94	20442.04		
2295	Keratin_ type I cytoskeletal 13	P13646	76442.68	73280.52	59866.62	44168.35	46262.59	38454.15	56817.45	50047.70	51942.58	56530.12	53184.67	56370.26	59349.67	51662.20	47104.73	44995.03	50024.98	45420.24	48554.13	39154.59	39658.83	803.40	743.59	636.28	622.91	650.38	588.06	692.18	640.32	641.21	739.00	718.14	519.65		
2296	Keratin_ type I cytoskeletal 14	P02533	26064.89	27431.03	25104.22	20165.40	18186.90	16643.89	23157.17	24278.95	22330.22	25314.72	24565.31	23322.53	27608.79	19891.73	17020.21	20154.70	20437.49	20400.41	25701.39	25383.40	21559.63	45829.11	46254.38	42707.40	29485.38	30128.43	26982.27	44935.16	43710.09	41209.88	38113.73	35821.68	26516.74		
2297	Keratin_ type I cytoskeletal 15	P19012	248310.31	241710.97	227522.10	179453.73	173591.19	158165.79	202374.59	191112.69	183422.65	237840.19	222413.43	210329.04	206612.61	195484.28	146679.82	187298.85	179809.90	167263.85	180229.20	174164.41	161123.36	102657.82	96748.14	89609.79	57677.24	57814.09	50990.22	81557.71	74139.83	73814.86	85745.08	81272.47	54661.61		
2298	Keratin_ type I cytoskeletal 16	P08779	175453.87	154723.64	152943.01	117842.84	115819.75	111692.55	130322.18	130042.49	129578.37	149460.97	144553.03	148812.31	139151.83	140464.54	118920.63	118086.18	104516.89	104754.21	118896.17	116199.17	117575.16	10029.09	10344.61	9526.26	11280.14	10972.67	9336.33	19103.84	18268.03	17315.06	10507.17	10736.60	7699.38		
2299	Keratin_ type I cytoskeletal 17	Q04695	25880.64	24870.41	24119.30	20996.84	21165.80	19129.46	41985.35	39839.73	39132.00	26508.63	26846.49	25425.51	25686.66	25285.29	18952.97	19811.04	18382.27	17254.55	19417.51	18847.73	17699.11	3333.19	3207.46	3486.58	2431.55	2369.09	2141.54	2615.38	2553.97	2499.97	2452.23	2310.54	1578.00		
2300	Keratin_ type I cytoskeletal 18	P05783	1756432.47	1634783.24	1508587.93	1476760.87	1408107.56	1291220.71	1441315.77	1399814.45	1375431.42	1513218.25	1410158.44	1318349.65	1474421.61	1374259.26	1065467.46	1301324.44	1218207.29	1183184.54	1121893.08	1066980.06	1008508.83	547683.24	511912.73	488837.80	323891.75	317388.84	278419.08	357563.26	338063.57	316951.20	421245.33	400268.54	274908.23		
2301	Keratin_ type I cytoskeletal 19	P08727	1186377.91	1156573.64	1107535.65	948339.55	932263.70	889624.71	1092689.18	1046067.43	1015214.40	1100002.61	1054522.65	999072.14	1007121.98	962108.52	829839.36	881376.31	848045.45	810602.20	749659.96	719358.88	682324.16	46963.00	43422.82	42336.43	21417.96	21219.68	18949.43	25092.21	23713.10	23059.38	24669.94	23122.46	16531.19		
2302	Keratin_ type I cytoskeletal 20	P35900	2928.54	2886.68	2708.52	2085.36	2202.95	2076.78	2006.97	2075.75	2460.65	3082.97	2884.18	2680.80	2590.67	2560.73	2210.19	2404.89	2202.26	2078.94	1808.38	1861.83	2093.98	2.27	1.49	0.65	0.84	1.31	0.69	4.16	2.47	0.11	4.90	4.98	0.12	x	
2303	Keratin_ type I cytoskeletal 24	Q2M2I5	25279.71	24060.50	21867.81	17810.07	17204.10	15638.93	19003.13	18464.58	18376.33	20035.38	20170.47	18311.30	20940.21	19037.87	17077.09	15987.10	14689.11	14878.86	16947.81	17007.30	16152.17	16336.06	15376.88	12533.03	9337.85	8988.62	7661.68	13029.88	11246.38	10239.75	12484.26	12608.24	7399.18		
2304	Keratin_ type I cytoskeletal 25	Q7Z3Z0	4037.88	3013.86	2472.40	1306.61	1013.43	708.49	1797.98	1320.42	1598.67	2418.41	2160.20	1638.37	3020.04	1339.42	507.25	1961.32	1247.95	1135.93	1656.58	1181.79	787.54	1042.36	969.06	853.75	684.38	564.82	259.13	764.85	636.87	510.57	873.99	981.15	343.97		
2305	Keratin_ type I cytoskeletal 26	Q7Z3Y9	9364.95	9678.15	9447.44	6896.63	7454.36	6774.46	7983.37	7901.43	6908.59	8672.83	8466.71	8139.96	8391.66	8833.31	7037.87	6645.50	6557.06	6479.87	6634.82	6380.44	6709.38	251.61	117.77	187.51	275.99	303.47	140.94	279.01	243.30	262.89	260.61	170.09	130.77		
2306	Keratin_ type I cytoskeletal 28	Q7Z3Y7	12743.73	12140.33	11504.85	9436.75	9109.09	8556.45	10694.93	9911.71	10255.70	10817.85	9937.16	9284.81	11528.37	10364.52	7022.65	9285.95	8899.35	8381.80	8737.49	8699.06	8187.30	6865.22	6373.74	5291.73	1329.39	1372.62	1167.27	1299.57	1060.44	1099.50	2069.15	1826.49	1079.63		
2307	Keratin_ type I cytoskeletal 39	Q6A163	0.14	2.53	0.81	1.07	2.77	0.68	0.96	0.36	0.63	0.35	0.75	0.38	2.13	0.97	0.60	4.64	1.07	0.47	2.22	0.19	0.06	17770.70	17029.03	15800.43	9922.56	10053.51	8887.76	11039.15	10533.57	9862.52	10025.79	9443.00	6614.62		x
2308	Keratin_ type II cuticular Hb2	Q9NSB4	17604.50	16834.71	14919.27	15250.23	15461.24	13926.97	15258.58	15176.64	14189.85	17058.60	16523.27	16132.81	16048.26	14100.71	13691.21	15381.13	14241.34	14426.25	14691.45	13926.98	12528.54	2.27	1.49	0.65	0.84	1.31	0.69	4.16	2.47	0.11	4.90	4.98	0.12	x	
2309	Keratin_ type II cuticular Hb4	Q9NSB2	92491.29	93464.48	87555.94	69364.97	69222.28	63629.92	78693.83	73980.44	73473.15	86136.78	80876.33	75715.19	77985.27	77579.13	66548.86	67029.62	66262.53	65242.06	69756.53	64653.86	62121.62	25496.10	22753.55	21667.42	16956.37	17027.77	14847.87	20714.53	19815.01	18815.98	21561.15	20842.51	13912.90		
2310	Keratin_ type II cuticular Hb5	P78386	12497.31	11634.18	10787.21	7522.21	7523.16	6598.31	9726.05	9081.32	8049.77	9638.25	9552.92	8737.52	10749.27	9354.12	8113.72	7740.25	6992.53	6855.38	7959.90	7292.15	7050.81	542.55	372.96	179.11	392.36	516.18	336.60	500.36	523.97	344.69	618.05	668.66	241.92		
2311	Keratin_ type II cuticular Hb6	O43790	621209.24	587474.99	564707.78	412050.60	395434.92	394715.99	402804.95	388281.93	398957.64	471512.77	459940.88	443912.86	489520.32	459699.95	413671.84	455673.27	423953.20	422463.19	342249.71	340513.81	330689.17	786.12	792.82	738.09	545.04	496.75	423.82	667.79	687.74	554.48	836.68	842.74	464.92		
2312	Keratin_ type II cytoskeletal 3	P12035	35222.64	34353.00	32211.81	23581.83	22701.32	21097.75	28948.30	33414.50	28957.91	29038.04	28303.59	25806.56	28461.10	25221.28	22709.85	24137.74	23291.27	22204.65	24180.63	23641.30	21005.50	6573.87	6959.45	7458.82	5349.11	5010.92	4753.11	6343.62	5876.82	5582.09	5971.46	6140.89	3399.22		
2313	Keratin_ type II cytoskeletal 4	P19013	20849.25	19043.55	17180.95	13841.01	14457.97	12373.53	15865.69	15993.83	15323.15	16374.80	15601.20	13868.48	16213.88	14858.08	11108.10	12999.31	13248.44	12488.86	14466.08	13512.58	12094.54	12736.77	11950.64	12710.14	8281.41	7810.60	7392.61	10527.02	10585.77	10263.88	9632.83	9619.98	6104.41		
2314	Keratin_ type II cytoskeletal 5	P13647	36690.39	34719.18	32079.89	30537.94	29452.59	26883.60	79726.71	73294.69	75475.80	38293.51	38561.80	39552.63	30927.12	29963.16	24448.75	24013.10	21469.20	22108.74	23546.86	23377.27	20396.97	66741.05	62145.07	55660.39	53195.44	50963.09	41218.29	63600.67	60222.16	55273.14	75827.38	74655.90	47037.37		
2315	Keratin_ type II cytoskeletal 6A	P02538	30259.65	28322.10	25742.16	24394.68	22631.32	22941.44	25090.40	24944.58	24843.05	29053.12	26939.13	26354.91	25067.55	26014.31	22102.64	21795.32	23275.61	21346.90	23235.91	22251.41	20087.55	5698.06	5670.72	5157.80	3452.92	3328.98	2823.41	5100.33	5171.61	4462.32	4524.68	4586.17	2872.25		
2316	Keratin_ type II cytoskeletal 6B	P04259	110379.91	110637.42	100362.89	74626.86	70943.60	67489.65	83753.84	82823.13	78675.82	88944.01	86297.79	83712.78	81462.46	76327.13	65549.41	71687.73	69647.09	68942.49	81601.29	77597.65	76811.31	77969.05	72798.91	68240.92	48631.46	48931.11	45548.18	66966.71	66059.80	63738.69	61193.71	60608.25	42958.63		
2317	Keratin_ type II cytoskeletal 7	P08729	77624.18	71064.87	64940.09	48381.64	47835.78	44151.47	55356.84	52939.49	49184.35	60185.10	59285.49	57111.94	59205.52	53575.34	44652.72	49103.68	44943.48	43343.75	49426.48	47828.62	42397.82	249064.99	230232.65	216736.58	140834.55	138161.55	126618.05	161490.49	154355.01	150740.36	175713.76	164642.16	119793.92		
2318	Keratin_ type II cytoskeletal 71	Q3SY84	562600.68	569744.97	548321.08	404539.52	384358.31	369745.55	500109.24	484284.02	473701.34	478160.75	462024.11	442344.17	471342.12	451336.41	397759.35	415114.99	403526.57	393495.80	432433.59	413748.64	388541.79	80413.09	93877.86	76685.81	56798.89	58293.00	55194.61	64176.34	62763.38	60205.29	56412.49	65675.69	57346.75		
2319	Keratin_ type II cytoskeletal 72	Q14CN4	29092.19	27726.42	23423.79	20386.40	20749.04	17862.53	23934.45	23350.38	20900.57	26028.52	25006.02	22182.69	23935.34	22448.86	15153.58	22485.28	21054.56	19751.80	22832.28	20887.53	18283.27	13375.00	12315.71	11307.11	7327.48	7212.61	6173.79	10438.25	9471.66	8622.19	10747.71	10154.72	6090.55		
2320	Keratin_ type II cytoskeletal 73	Q86Y46	78817.85	73670.42	70039.61	58368.82	54641.91	51230.72	69154.54	66976.77	63630.49	66853.62	59448.98	59315.35	64554.51	67847.90	53842.28	59230.45	52749.70	50236.09	57057.47	51571.07	49717.84	2163.05	2035.57	1932.94	1136.66	1257.48	754.20	1845.22	1620.61	1436.81	2449.03	2285.27	1096.24		
2321	Keratin_ type II cytoskeletal 74	Q7RTS7	33387.55	32256.44	29090.71	24709.16	24061.16	22490.69	34423.85	33238.41	32259.97	30994.48	29468.59	28264.51	34514.29	27869.25	22771.94	28623.24	28309.71	26935.66	30802.51	31236.86	29754.55	1114.83	4831.21	1097.79	3495.97	3184.14	6859.56	1191.57	1933.99	1832.69	8086.60	4469.57	708.15		
2322	Keratin_ type II cytoskeletal 75	O95678	45088.20	43832.64	43993.96	40366.37	38036.64	38394.35	41862.25	43716.49	42684.28	38854.99	38750.25	36333.80	39479.52	39306.32	33401.07	37376.60	34626.96	36060.05	30456.32	28953.62	28355.23	12627.65	11830.90	11270.91	7520.07	7116.99	6022.44	8766.30	8741.06	8314.45	9409.77	9194.11	5524.64		
2323	Keratin_ type II cytoskeletal 78	Q8N1N4	19591.78	19658.67	18695.71	13221.07	12433.10	12341.84	15544.29	14943.99	14706.42	16906.88	16022.50	15377.95	17976.37	16441.32	14113.11	11838.47	11721.55	11163.11	14856.29	14160.18	14872.50	4629.37	4414.53	3630.54	3187.68	3120.17	2448.70	3979.68	3712.90	3750.64	6810.39	6727.34	4699.76		
2324	Keratin_ type II cytoskeletal 79	Q5XKE5	42010.41	40287.60	36723.27	29462.80	28624.62	26493.31	33060.98	31920.58	29484.62	39074.46	37344.55	36093.96	37462.01	35865.40	26729.97	28447.24	26183.51	24944.94	28741.31	27171.29	25312.54	6315.30	6112.98	5756.38	4028.60	3897.35	3122.60	5624.08	5094.18	4848.15	5000.63	4859.78	4169.28		
2325	Keratin_ type II cytoskeletal 8	P05787	5344351.72	5192997.18	4976248.19	4688156.69	4521586.53	4300699.63	5004636.04	4853923.65	4627293.36	4694586.74	4537909.01	4287113.33	4642073.59	4396616.44	3728888.87	4358852.42	4146507.02	4048052.78	3765409.47	3632861.69	3443801.76	1590883.84	1519005.58	1377576.45	977914.12	982898.82	896890.36	1047743.35	1003093.96	980565.60	1254974.63	1201074.06	948097.78		
2326	Keratin_ type II cytoskeletal 80	Q6KB66	85916.96	84424.23	76823.13	61346.50	58063.96	55248.65	69506.66	67784.00	64688.38	74873.27	70036.74	69084.50	70640.18	69651.20	57404.86	58147.82	55299.58	53641.04	55354.96	56866.51	53331.85	2.27	1.49	0.65	0.84	1.31	0.69	4.16	2.47	0.11	4.90	4.98	0.12	x	
2327	Keratin-like protein KRT222	Q8N1A0	25155.17	24035.21	21955.01	17200.35	17236.10	16919.81	20613.32	21080.36	20532.32	21852.40	21192.13	21140.81	22814.20	21186.91	12646.63	15546.62	15537.04	17399.65	24936.97	19911.37	17339.70	2.27	1.49	0.65	0.84	1.31	0.69	4.16	2.47	0.11	4.90	4.98	0.12	x	
2328	Keratinocyte-associated protein 2	Q8N6L1	3199.29	2785.66	2930.40	1588.27	1581.06	1663.91	2257.90	2241.78	2169.97	2287.72	2126.67	2368.14	1833.53	1525.46	1613.33	2413.62	2261.02	2198.08	1741.56	1640.50	1476.12	2.27	1.49	0.65	0.84	1.31	0.69	4.16	2.47	0.11	4.90	4.98	0.12	x	
2329	KH domain-containing_ RNA-binding_ signal transduction-associated protein 1	Q07666	6006.80	5851.37	5182.17	4361.97	4253.07	3504.42	4602.77	3958.17	4552.84	4202.33	4419.85	4111.62	4568.25	4338.30	2537.69	4972.38	3466.22	4242.62	3477.98	3481.96	3107.55	37815.88	34036.22	31722.69	29491.39	29148.18	26917.78	31248.41	30114.74	28726.83	33326.29	33423.74	15776.39		
2330	KH domain-containing_ RNA-binding_ signal transduction-associated protein 2	Q5VWX1	0.14	2.53	0.81	1.07	2.77	0.68	0.96	0.36	0.63	0.35	0.75	0.38	2.13	0.97	0.60	4.64	1.07	0.47	2.22	0.19	0.06	963.82	1062.23	1109.80	718.51	801.99	777.85	976.03	936.98	920.74	913.71	906.23	704.57		x
2331	KICSTOR complex protein SZT2	Q5T011	0.14	2.53	0.81	1.07	2.77	0.68	0.96	0.36	0.63	0.35	0.75	0.38	2.13	0.97	0.60	4.64	1.07	0.47	2.22	0.19	0.06	3186.62	3143.63	2915.56	2121.38	1923.57	1776.87	2732.05	2736.22	2630.78	2568.19	2649.88	1922.88		x
2332	KIF1-binding protein	Q96EK5	135161.98	132868.83	134469.89	115863.03	108166.56	105323.96	108148.29	110369.71	107233.10	130700.15	125855.13	116151.11	120191.08	116398.13	102889.07	104364.27	98894.39	96842.01	113948.92	106688.04	103970.15	38836.19	37451.85	35198.56	27761.45	27446.44	24225.07	31962.58	31286.89	29866.59	29061.78	28106.85	20299.28		
2333	Killer cell immunoglobulin-like receptor 2DL3	P43628	2186.66	2293.98	2285.23	1047.02	917.22	961.71	1169.30	1441.74	1452.72	1811.88	1736.50	1693.76	1429.17	1344.69	1084.44	1805.98	2123.95	2172.88	1316.86	1463.37	1354.53	2.27	1.49	0.65	0.84	1.31	0.69	4.16	2.47	0.11	4.90	4.98	0.12	x	
2334	Killer cell lectin-like receptor subfamily B member 1	Q12918	11578.99	11314.35	9722.13	18529.90	16735.58	11351.09	19103.18	11998.85	8047.04	8331.64	7396.46	6235.30	9788.18	24421.24	5892.00	18752.82	16017.03	14498.24	10663.68	9261.30	7752.98	2.27	1.49	0.65	0.84	1.31	0.69	4.16	2.47	0.11	4.90	4.98	0.12	x	
2335	Kinase D-interacting substrate of 220 kDa	Q9ULH0	4556.23	5648.78	5293.00	3686.64	3491.82	3009.96	3838.80	3342.16	3514.25	4612.72	4021.58	3375.54	4267.34	4300.45	4095.32	3062.29	3034.19	2909.17	3133.69	3355.02	4051.49	2.27	1.49	0.65	0.84	1.31	0.69	4.16	2.47	0.11	4.90	4.98	0.12	x	
2336	Kinectin	Q86UP2	510911.19	476261.53	441034.77	327215.68	318428.64	297953.93	377939.60	366654.16	340935.21	413530.37	397631.33	363415.54	400548.71	369189.79	291786.52	321951.82	306430.98	298652.19	326775.87	328724.62	294002.39	419216.64	390735.37	361611.29	277629.63	266708.31	236835.92	342360.55	322770.74	305547.26	351170.27	334205.85	222170.45		
2337	Kinesin heavy chain isoform 5A	Q12840	50497.18	46836.13	42818.50	32278.21	30914.27	28789.87	36140.68	37189.29	35787.47	42059.75	40880.09	37979.98	40958.46	38407.15	30123.78	31578.51	31781.53	29580.57	33078.07	33588.08	31431.06	108364.30	101034.71	90806.45	66227.29	62593.48	51068.62	77515.04	72309.04	69023.72	96872.19	93797.95	60246.90		
2338	Kinesin heavy chain isoform 5C	O60282	52977.09	52042.81	47097.09	45430.71	43493.81	44793.50	50377.42	49230.15	46786.57	46800.26	45292.47	40988.75	50216.49	49727.14	43578.48	42259.15	38121.92	39906.95	44445.60	40885.64	39311.31	151769.78	146599.83	136502.83	120718.20	118763.69	106085.17	138056.56	134357.01	130693.85	142392.86	139683.09	103632.05		
2339	Kinesin light chain 1	Q07866	99819.42	95866.47	83856.09	70584.87	70409.44	63022.81	70926.78	67641.87	62659.56	88158.93	84699.15	79742.51	78318.76	76063.97	68903.76	70359.70	66095.02	63838.48	72471.80	64931.11	64535.36	34356.13	33364.06	28399.75	24745.43	24086.96	21597.10	34118.48	32662.36	29996.31	30381.26	28253.32	20641.85		
2340	Kinesin light chain 2	Q9H0B6	10513.43	10498.71	9694.12	6656.00	7001.01	7028.96	7692.90	7225.84	6500.25	8641.00	8317.50	8024.57	7521.47	8655.72	6837.70	7191.53	6039.87	6624.47	6990.01	7500.99	8032.18	19725.18	18239.67	16950.37	18957.01	19577.54	17785.91	16560.97	16262.64	15609.20	25235.31	24684.40	17690.14		
2341	Kinesin light chain 4	Q9NSK0	7833.17	8174.49	7195.38	7284.64	6651.81	6198.14	6674.35	6496.99	6092.65	8949.99	8092.54	7687.13	7285.75	7120.12	5935.78	7614.96	7237.60	6870.86	6583.69	6370.90	6082.35	2249.99	1952.20	1343.10	1196.34	1117.56	656.64	1355.38	1078.34	900.03	1408.46	1309.35	621.00		
2342	Kinesin-1 heavy chain	P33176	520812.58	502407.90	499303.10	342335.64	337066.33	296237.53	401814.67	386560.52	365561.36	472899.52	450752.89	428743.21	442496.07	404972.19	326665.27	332382.07	322229.38	303618.09	339798.77	339758.34	328035.13	366967.80	346152.58	304041.12	251155.36	242778.45	215676.36	296025.89	282739.89	265041.53	312919.79	294945.41	188114.33		
2343	Kinesin-like protein KIF11	P52732	0.14	2.53	0.81	1.07	2.77	0.68	0.96	0.36	0.63	0.35	0.75	0.38	2.13	0.97	0.60	4.64	1.07	0.47	2.22	0.19	0.06	5295.53	5545.29	5651.48	3682.77	3613.14	3362.59	5070.26	4216.32	4125.45	4865.92	4446.38	3128.60		x
2344	Kinesin-like protein KIF13B	Q9NQT8	0.14	2.53	0.81	1.07	2.77	0.68	0.96	0.36	0.63	0.35	0.75	0.38	2.13	0.97	0.60	4.64	1.07	0.47	2.22	0.19	0.06	42057.33	40092.83	33985.02	30606.50	30751.44	29766.07	33035.32	32251.51	31325.06	32809.22	32131.95	23103.19		x
2345	Kinesin-like protein KIF15	Q9NS87	0.14	2.53	0.81	1.07	2.77	0.68	0.96	0.36	0.63	0.35	0.75	0.38	2.13	0.97	0.60	4.64	1.07	0.47	2.22	0.19	0.06	8552.24	8344.91	7623.63	5681.33	5891.53	5381.48	6778.86	6396.58	6249.75	7456.12	7116.69	5146.96		x
2346	Kinesin-like protein KIF16B	Q96L93	96214.67	93163.63	90070.25	68131.54	56282.06	60026.60	69211.57	72854.23	66123.81	77351.85	76627.92	71790.96	80185.96	98347.54	50447.12	70733.58	59747.02	60489.35	78889.65	63440.91	56937.85	4944.71	3837.01	4973.47	3306.45	3595.31	3479.03	4408.20	4136.67	4281.55	4214.01	3634.47	3771.00		
2347	Kinesin-like protein KIF18A	Q8NI77	15963.90	13183.84	13570.10	8313.88	7662.02	8294.98	11385.62	10471.19	9842.55	10388.72	9926.25	9563.01	11604.58	10167.56	8566.69	9032.14	8417.97	8362.55	10160.17	10486.01	9121.00	2.27	1.49	0.65	0.84	1.31	0.69	4.16	2.47	0.11	4.90	4.98	0.12	x	
2348	Kinesin-like protein KIF1A	Q12756	0.14	2.53	0.81	1.07	2.77	0.68	0.96	0.36	0.63	0.35	0.75	0.38	2.13	0.97	0.60	4.64	1.07	0.47	2.22	0.19	0.06	337088.75	304538.20	260428.81	187649.90	180040.48	107787.56	307272.86	269237.44	214345.37	317692.60	313004.08	157175.53		x
2349	Kinesin-like protein KIF1B	O60333	34206.74	32382.10	30320.89	25880.78	24356.22	23360.29	27764.42	24401.85	25884.61	29274.44	29422.64	27800.92	29136.15	27568.40	22777.76	25940.30	24939.19	27078.52	29217.42	23220.77	20401.52	3350.37	2892.72	2711.44	2253.59	2128.88	1941.75	2648.66	2420.81	2248.33	2304.55	2161.10	1516.96		
2350	Kinesin-like protein KIF27	Q86VH2	25950.19	24249.45	22496.10	22159.79	22218.26	21264.24	25020.03	23247.37	22956.84	28850.37	28237.08	27609.69	24762.88	21287.51	18317.97	22636.41	20799.62	20840.60	22000.65	20647.46	20545.34	9184.87	9114.40	8585.41	8878.89	9066.29	8529.78	8626.64	8240.15	7777.75	10055.99	9220.47	2107.47		
2351	Kinesin-like protein KIF28P	B7ZC32	30545.33	29982.84	26630.22	21937.00	20642.16	19019.53	24955.88	22646.90	22338.72	27659.89	26376.61	25919.43	26486.62	25112.10	19740.17	21971.03	21066.67	19059.39	22098.45	21241.07	19662.36	64181.66	59505.26	58557.96	43912.28	45337.60	42148.86	40583.53	40034.02	39979.09	44518.94	42410.65	31098.51		
2352	Kinesin-like protein KIF2A	O00139	72152.11	69331.43	67360.96	49606.83	46403.58	47356.33	56287.96	59191.37	55775.92	60599.41	57699.39	54770.18	61610.61	53760.84	44743.52	50405.09	44588.95	42435.14	58128.12	53603.27	48171.51	140411.13	135272.46	132182.81	104993.92	101312.50	94214.50	116162.18	111498.47	108157.72	104687.72	101209.89	78099.14		
2353	Kinesin-like protein KIF2C	Q99661	244945.70	248082.01	209456.36	180147.80	183110.93	163508.55	206431.68	183681.37	162863.46	203415.59	190000.95	168214.29	172002.46	156274.55	147882.99	214734.52	206817.51	197730.58	206029.08	209298.43	191560.64	48340.61	47235.48	40460.68	31037.42	29803.91	26605.80	36339.02	35225.50	33969.21	37421.67	36662.19	23906.33		
2354	Kinesin-like protein KIF3B	O15066	0.14	2.53	0.81	1.07	2.77	0.68	0.96	0.36	0.63	0.35	0.75	0.38	2.13	0.97	0.60	4.64	1.07	0.47	2.22	0.19	0.06	42343.77	41340.21	39139.77	30985.20	33088.71	29811.42	34229.91	32345.35	32976.41	38300.69	37090.36	36156.39		x
2355	Kinesin-like protein KIF3C	O14782	82182.96	71187.31	57708.26	119871.20	118826.50	92328.90	125876.39	117200.16	102314.04	69025.03	67374.17	54960.74	70853.97	135929.86	51407.98	102338.97	98081.98	86102.23	93279.93	88332.80	75605.20	96921.22	94490.12	93616.36	60799.57	64021.28	59081.27	59601.36	58645.46	56751.79	51795.61	49559.69	39193.10		
2356	Kinesin-like protein KIF7	Q2M1P5	0.14	2.53	0.81	1.07	2.77	0.68	0.96	0.36	0.63	0.35	0.75	0.38	2.13	0.97	0.60	4.64	1.07	0.47	2.22	0.19	0.06	831.77	833.14	663.71	522.65	493.95	502.89	476.92	472.76	535.44	551.77	507.67	378.10		x
2357	Kinesin-like protein KIF9	Q9HAQ2	14343.54	13645.47	13322.31	11622.32	11415.05	10517.59	12146.17	12842.95	11067.75	15902.35	14824.74	13523.69	12160.30	11070.85	9087.15	11965.09	10845.50	10490.98	12355.03	11785.60	10690.30	2.27	1.49	0.65	0.84	1.31	0.69	4.16	2.47	0.11	4.90	4.98	0.12	x	
2358	Kinesin-like protein KIFC1	Q9BW19	43473.81	37834.58	37543.44	28962.83	26938.26	27530.90	35551.17	33225.77	34288.06	33629.86	32792.67	31290.43	35986.14	36384.87	28496.91	31498.49	30968.72	30425.23	40506.51	35030.24	33010.54	89081.40	84151.42	73431.48	49533.97	51652.45	41492.29	59411.13	54901.74	52292.49	67127.50	63436.87	40881.17		
2359	Kinetochore protein NDC80 homolog	O14777	0.14	2.53	0.81	1.07	2.77	0.68	0.96	0.36	0.63	0.35	0.75	0.38	2.13	0.97	0.60	4.64	1.07	0.47	2.22	0.19	0.06	46994.59	41708.98	39197.51	29006.45	29987.32	23055.64	30088.30	29082.75	25383.56	38012.70	37377.33	21675.83		x
2360	Kinetochore protein Spc24	Q8NBT2	14323.73	12724.23	11041.25	11619.34	10639.69	9188.77	39402.65	37318.50	35370.38	14464.77	13224.08	12232.19	15522.49	14421.86	8555.14	15007.85	14163.88	13464.76	9772.16	9450.33	7946.78	2.27	1.49	0.65	0.84	1.31	0.69	4.16	2.47	0.11	4.90	4.98	0.12	x	
2361	Kinetochore protein Spc25	Q9HBM1	1336.21	1064.79	644.99	796.64	659.38	381.36	897.29	938.82	704.12	928.68	935.35	588.61	1296.35	1135.02	142.95	534.71	582.17	511.44	771.08	653.46	387.56	2.27	1.49	0.65	0.84	1.31	0.69	4.16	2.47	0.11	4.90	4.98	0.12	x	
2362	Klotho	Q9UEF7	1409.40	1312.37	869.47	1577.51	1593.04	1428.61	1514.72	1445.33	1248.78	2711.87	2403.54	2680.26	2166.51	1607.31	1485.74	1552.64	1311.98	1275.97	1529.30	1604.55	1673.86	2.27	1.49	0.65	0.84	1.31	0.69	4.16	2.47	0.11	4.90	4.98	0.12	x	
2363	Krueppel-like factor 11	O14901	22632.29	21066.08	20771.71	16642.80	16068.82	14639.26	19382.96	19094.02	18129.73	19713.47	18599.15	17912.38	19822.39	18695.67	17012.08	14237.22	15017.14	15007.87	17187.58	16328.24	16762.09	2.27	1.49	0.65	0.84	1.31	0.69	4.16	2.47	0.11	4.90	4.98	0.12	x	
2364	Kunitz-type protease inhibitor 1	O43278	22372.12	22229.62	22223.69	17504.34	16721.96	16626.97	18373.53	18121.19	17602.99	19721.29	18193.71	17916.93	19832.63	19178.43	16316.00	17065.79	16364.85	16210.61	16840.62	15332.22	14177.99	2.27	1.49	0.65	0.84	1.31	0.69	4.16	2.47	0.11	4.90	4.98	0.12	x	
2365	Kunitz-type protease inhibitor 2	O43291	10068.48	10480.06	9585.79	6309.78	6207.45	6140.11	8984.70	8684.76	7797.88	8599.69	8460.76	7741.83	8011.42	7464.32	5498.66	7409.32	7188.97	5717.36	5666.43	5811.47	5004.46	2.27	1.49	0.65	0.84	1.31	0.69	4.16	2.47	0.11	4.90	4.98	0.12	x	
2366	Kunitz-type protease inhibitor 4	Q6UDR6	0.14	2.53	0.81	1.07	2.77	0.68	0.96	0.36	0.63	0.35	0.75	0.38	2.13	0.97	0.60	4.64	1.07	0.47	2.22	0.19	0.06	1431.67	1346.27	1353.07	1151.49	1022.60	861.70	1244.01	1268.71	1159.80	1037.28	983.06	526.13		x
2367	Kv channel-interacting protein 1	Q9NZI2	76932.03	73027.88	63627.32	56081.14	54118.93	46538.57	58511.13	56380.32	49819.96	64056.77	61533.17	55000.92	61668.95	56887.16	42684.98	57709.53	53278.80	50330.15	59143.89	57912.60	53245.73	2.27	1.49	0.65	0.84	1.31	0.69	4.16	2.47	0.11	4.90	4.98	0.12	x	
2368	Kv channel-interacting protein 2	Q9NS61	4077.17	4138.20	3234.60	2088.99	1827.71	2021.88	3100.34	3104.18	2765.58	3196.42	2644.48	2688.09	2862.08	2834.40	2430.62	2457.78	2304.78	2011.77	2262.32	2413.51	2105.85	30547.01	29863.08	27417.33	22840.04	22398.86	19963.53	25716.73	24835.22	24221.21	24065.27	22161.12	16856.80		

Supplementary Table S1. Overview on the abundance of all relatively quantified 5180 proteins

			MCF-7																					MDA-MB-231													
	Protein name	UniProt	control 01	control 02	control 03	Dai IC20 01	Dai IC20 02	Dai IC20 03	Dai SC20 01	Dai SC20 02	Dai SC20 03	Gen IC20 01	Gen IC20 02	Gen IC20 03	Gen SC20 01	Gen SC20 02	Gen SC20 03	SSE IC20 01	SSE IC20 02	SSE IC20 03	SSE SC20 01	SSE SC20 02	SSE SC20 03	control 01	control 02	control 03	Dai IC20 01	Dai IC20 02	Dai IC20 03	Gen IC20 01	Gen IC20 02	Gen IC20 03	SSE IC20 01	SSE IC20 02	SSE IC20 03	unique to MCF-7	unique to MDA-MB-231
2369	Kynureninase	Q16719	41337.90	38510.81	34508.21	16617.64	16881.30	13927.92	19994.34	21176.11	18094.10	23280.11	22257.94	17794.72	28043.61	26502.30	16586.01	16147.58	17490.43	14447.71	18177.01	16843.45	15789.02	2.27	1.49	0.65	0.84	1.31	0.69	4.16	2.47	0.11	4.90	4.98	0.12	x	
2370	L-2-hydroxyglutarate dehydrogenase_ mitochondrial	Q9H9P8	165283.18	156427.59	127786.94	78362.27	80414.39	69817.92	103001.62	101557.17	89408.82	120551.01	112350.21	97060.42	125298.39	119716.18	79795.85	86765.44	85289.62	76994.74	94933.49	95863.92	85516.67	2.27	1.49	0.65	0.84	1.31	0.69	4.16	2.47	0.11	4.90	4.98	0.12	x	
2371	Laccase domain-containing protein 1	Q8IV20	972.50	645.87	451.47	420.20	419.40	490.57	492.46	397.85	398.27	683.86	534.95	565.91	588.46	609.87	390.63	472.15	417.82	518.54	557.00	581.85	494.01	2.27	1.49	0.65	0.84	1.31	0.69	4.16	2.47	0.11	4.90	4.98	0.12	x	
2372	Lactotransferrin	P02788	13853.22	13819.58	11309.29	8628.78	9791.00	6923.49	10885.76	15265.81	11967.38	12071.17	10744.24	9584.48	11705.71	10638.95	4861.84	10491.98	9486.46	9240.03	11296.22	10297.02	7777.62	36897.18	32617.85	28892.77	31036.22	29353.57	22963.38	58578.19	51589.48	47415.64	44738.85	41756.77	25903.07		
2373	Lactoylglutathione lyase	Q04760	99116.86	97212.37	95299.15	63394.32	59793.49	58439.07	88545.38	82279.75	79835.93	80206.28	73532.40	69971.62	77042.87	75565.92	60095.51	65418.69	66608.00	63074.63	65615.80	63778.98	59986.53	140616.64	133027.67	130674.80	74791.24	75318.22	70726.32	94140.39	88056.42	88092.10	83602.34	82235.96	66028.98		
2374	Ladinin-1	O00515	11597.45	11306.78	10727.87	6999.68	6484.05	6252.04	5645.50	5916.60	5320.12	7766.63	7194.11	7072.67	8370.82	6944.26	5823.79	6725.31	6754.06	6124.62	6976.63	6647.70	6241.31	2.27	1.49	0.65	0.84	1.31	0.69	4.16	2.47	0.11	4.90	4.98	0.12	x	
2375	Lambda-crystallin homolog	Q9Y2S2	19128.09	15013.44	16737.39	9673.61	8536.65	7938.10	5852.50	5141.05	5956.85	11600.83	10958.54	10838.73	11156.59	10597.67	9166.23	7486.23	7890.69	7056.91	10009.02	9641.81	8433.04	2.27	1.49	0.65	0.84	1.31	0.69	4.16	2.47	0.11	4.90	4.98	0.12	x	
2376	Lamina-associated polypeptide 2_ isoform alpha	P42166	197104.46	187318.67	175366.99	122763.69	120115.77	112606.99	151699.18	145151.54	138340.37	128947.73	126115.51	117706.30	155713.87	151107.35	125848.78	116315.36	112812.65	107389.83	128180.72	125728.92	116785.21	7103.86	7304.89	6230.97	4915.40	4807.22	3945.60	6537.42	6147.60	6207.86	6298.49	6732.37	4318.20		
2377	Lamina-associated polypeptide 2_ isoforms beta/gamma	P42167	12199.74	12656.10	12454.00	6690.36	6292.08	5962.94	8837.79	8326.66	8770.11	8624.18	8200.67	7988.89	9659.01	7519.15	6107.69	5885.05	6251.82	6223.90	7264.21	7317.44	6750.08	115765.68	109115.81	105902.91	74058.03	71849.41	62506.50	88201.09	84849.03	82005.47	97554.51	94225.06	66864.99		
2378	Lamin-B1	P20700	210766.53	199607.14	197766.08	143846.30	139485.02	134236.78	160345.44	157716.69	152036.37	176679.98	168468.86	162986.72	169973.49	161447.44	138053.93	140120.58	134466.11	132195.35	153219.06	141921.04	139208.97	50662.93	48919.97	46445.50	38065.39	37124.27	32234.05	41336.50	39106.32	38341.93	56331.26	53631.45	36691.65		
2379	Lamin-B2	Q03252	0.14	2.53	0.81	1.07	2.77	0.68	0.96	0.36	0.63	0.35	0.75	0.38	2.13	0.97	0.60	4.64	1.07	0.47	2.22	0.19	0.06	70357.99	66140.96	73453.59	64916.79	64262.58	61552.17	68809.47	66012.96	64983.13	71222.19	68090.80	52738.25		x
2380	Laminin subunit alpha-5	O15230	207596.85	192655.11	182686.82	143148.54	138524.37	128729.77	150092.68	147307.87	141826.03	171688.02	162849.37	151868.92	170654.95	154634.42	124336.15	135776.68	126921.27	126332.33	141063.75	129071.61	124149.92	315.43	229.33	220.56	69.62	80.90	52.06	133.48	105.85	81.11	99.09	126.69	93.42		
2381	Laminin subunit beta-1	P07942	6880.76	6623.73	5912.98	4693.17	4521.19	4049.91	5949.42	5814.94	5543.19	7165.58	6886.83	6450.46	6761.20	6137.45	5015.48	4250.00	3733.75	4025.36	4775.27	4488.31	4525.59	609.18	464.44	411.23	739.07	588.03	328.49	860.08	718.02	623.41	759.55	857.05	292.56		
2382	L-aminoadipate-semialdehyde dehydrogenase-phosphopantetheinyl transferase	Q9NRN7	48173.73	45661.68	45399.66	31278.21	28774.24	27591.85	26885.77	24461.68	24859.23	41460.08	42199.90	37225.13	37772.69	31856.96	24927.20	29484.85	26764.49	27173.88	33001.36	33230.61	30053.33	219772.65	197613.35	194409.52	155867.87	153274.51	151003.24	204878.32	183234.98	181328.26	188390.26	177254.73	55094.16		
2383	LanC-like protein 2	Q9NS86	0.14	2.53	0.81	1.07	2.77	0.68	0.96	0.36	0.63	0.35	0.75	0.38	2.13	0.97	0.60	4.64	1.07	0.47	2.22	0.19	0.06	25897.63	23256.00	22579.52	15214.79	14479.72	12477.52	20970.13	18824.83	16768.89	21050.97	19494.38	12201.67		x
2384	Lanosterol 14-alpha demethylase	Q16850	25269.16	25249.30	22685.22	16871.12	15839.01	13775.57	18123.43	17412.16	15853.50	23230.01	22382.34	21990.19	20361.66	18548.54	11721.17	15130.31	15797.17	15561.13	19669.31	17845.68	16088.59	15105.83	14894.77	12991.36	11722.13	11693.96	9268.09	14467.41	13279.37	12256.71	15291.12	15075.83	11754.22		
2385	Lanosterol synthase	P48449	0.14	2.53	0.81	1.07	2.77	0.68	0.96	0.36	0.63	0.35	0.75	0.38	2.13	0.97	0.60	4.64	1.07	0.47	2.22	0.19	0.06	21265.25	19170.47	17134.83	15606.81	15434.11	13807.34	18501.21	17624.72	17917.04	17787.87	16624.44	14568.44		x
2386	La-related protein 1	Q6PKG0	185043.42	179701.68	158438.57	130718.13	120791.75	107613.42	145233.53	138017.31	133840.49	160699.46	156067.16	140428.52	162259.90	159699.95	101725.42	125973.88	119626.81	114713.07	135179.36	130680.19	120690.77	11263.46	9490.22	9969.21	7213.63	7203.52	6405.84	9194.17	8744.48	7943.90	7994.76	7706.00	6767.73		
2387	La-related protein 1B	Q659C4	81793.70	77758.43	79733.42	76497.02	67449.71	69584.25	73454.54	73589.31	68165.38	77991.46	76475.91	74301.14	77952.26	80550.07	61240.67	59892.29	70165.83	70366.84	81084.47	71947.35	70346.56	2.27	1.49	0.65	0.84	1.31	0.69	4.16	2.47	0.11	4.90	4.98	0.12	x	
2388	La-related protein 4	Q71RC2	40932.32	39769.71	41457.79	30293.26	29144.84	28477.69	34918.59	32480.82	32958.72	34621.90	33519.89	31389.65	34055.73	31270.59	25598.46	29607.78	26536.27	26823.98	29890.41	27451.87	25174.77	1328.08	400.56	340.90	740.15	784.96	538.18	1005.71	978.99	309.81	758.56	757.89	467.80		
2389	La-related protein 4B	Q92615	1824.92	1498.72	1170.97	2043.37	1126.66	1016.85	1178.92	1063.76	1086.29	1045.53	1053.40	1038.15	1479.95	2646.57	1165.25	1272.36	847.97	959.21	1414.47	1138.20	1229.32	15174.07	13134.20	14499.55	10726.54	10665.24	9182.93	14226.01	13698.36	13765.11	14101.20	13506.14	8766.56		
2390	La-related protein 7	Q4G0J3	2999.64	3052.35	2593.03	2735.50	3151.79	3127.71	1311.41	815.96	470.65	1827.85	1364.80	1499.47	2509.19	367.68	387.51	1256.48	1808.63	1574.32	2710.57	2463.35	2059.96	5826.70	5319.69	5187.21	3647.21	3573.83	3239.76	3136.68	3082.72	2979.21	2901.40	2721.72	2115.78		
2391	Large neutral amino acids transporter small subunit 1	Q01650	259480.83	255125.67	237556.55	305882.71	307396.65	292870.44	314544.32	299709.18	283582.24	367999.56	354728.88	337380.54	265962.86	247588.50	218438.82	322418.14	306037.73	305218.76	311406.63	302785.81	281353.91	27323.38	24547.30	22738.47	25811.13	26097.13	22878.59	27596.57	26951.29	25753.58	26863.59	31775.60	13254.16		
2392	Large proline-rich protein BAG6	P46379	89140.23	84674.25	79141.03	60689.59	57117.53	53097.11	70421.60	68301.30	66105.42	75664.23	73933.75	70152.12	78111.47	71966.03	65300.91	60513.56	55392.91	57714.29	59056.03	52700.34	53968.23	1595.74	1899.08	1870.25	1111.94	1277.28	1083.82	1525.21	1566.47	1468.74	1982.15	1643.30	1232.02		
2393	Large subunit GTPase 1 homolog	Q9H089	3453.71	3432.90	3097.14	1842.60	1952.00	1822.64	3312.77	3075.15	2483.06	2685.06	2727.74	2346.44	2796.34	2278.62	1774.22	2177.57	2082.08	2031.56	2482.50	2536.49	2364.30	2.27	1.49	0.65	0.84	1.31	0.69	4.16	2.47	0.11	4.90	4.98	0.12	x	
2394	Lariat debranching enzyme	Q9UK59	139123.59	114149.43	87521.94	86499.64	80093.74	65770.36	92270.45	89616.08	82911.54	109133.65	101775.46	83521.65	112289.41	90346.61	46038.85	77009.94	72479.70	64263.01	96745.95	86051.49	67344.55	1546.86	919.52	1737.01	1588.27	1731.02	1765.59	468.79	1884.86	1472.53	2771.66	1573.88	376.37		
2395	Lateral signaling target protein 2 homolog	Q9HCC9	1303.40	1076.03	1135.50	1061.27	988.82	868.84	1027.57	1001.67	992.38	1559.91	1521.97	1497.71	1305.13	1299.55	1036.16	811.61	921.04	794.49	778.06	836.11	831.41	697.05	650.11	832.95	294.54	252.55	251.60	515.87	443.57	432.35	639.29	622.09	190.64		
2396	Latexin	Q9BS40	75783.53	74387.23	70652.67	21421.81	21242.14	19602.99	34861.68	32820.37	31665.80	35358.54	34249.71	32911.66	47138.57	43583.39	39289.35	27108.76	25716.02	24035.24	22148.53	21756.96	19454.88	2.27	1.49	0.65	0.84	1.31	0.69	4.16	2.47	0.11	4.90	4.98	0.12	x	
2397	Legumain	Q99538	67215.48	61092.10	56278.03	42749.60	41964.52	37362.85	49296.69	46885.78	44290.80	52578.67	50108.90	46053.13	52792.88	49361.73	33435.66	44697.26	40138.88	41015.07	44763.62	40950.56	36350.59	2.27	1.49	0.65	0.84	1.31	0.69	4.16	2.47	0.11	4.90	4.98	0.12	x	
2398	Leiomodin-2	Q6P5Q4	0.14	2.53	0.81	1.07	2.77	0.68	0.96	0.36	0.63	0.35	0.75	0.38	2.13	0.97	0.60	4.64	1.07	0.47	2.22	0.19	0.06	6356.51	6424.96	5332.90	3742.74	3766.07	3157.02	4448.01	4456.88	4611.09	4258.75	4439.16	3050.46		x
2399	Leptin receptor	P48357	91227.73	66517.65	42044.30	25778.46	17934.92	10817.94	19197.23	11830.41	6701.73	55994.00	44504.67	27360.21	59274.01	28014.39	4546.93	17023.82	15618.46	11906.28	16794.06	15841.45	10671.42	2.27	1.49	0.65	0.84	1.31	0.69	4.16	2.47	0.11	4.90	4.98	0.12	x	
2400	Lethal(2) giant larvae protein homolog 1	Q15334	0.14	2.53	0.81	1.07	2.77	0.68	0.96	0.36	0.63	0.35	0.75	0.38	2.13	0.97	0.60	4.64	1.07	0.47	2.22	0.19	0.06	8425.71	7764.91	7451.28	6010.68	5471.20	4535.63	6404.38	5981.98	5756.03	6372.66	6316.55	4709.35		x
2401	Lethal(3)malignant brain tumor-like protein 4	Q8NA19	0.14	2.53	0.81	1.07	2.77	0.68	0.96	0.36	0.63	0.35	0.75	0.38	2.13	0.97	0.60	4.64	1.07	0.47	2.22	0.19	0.06	24643.47	3036.72	3228.03	6387.41	5043.13	2550.51	20760.29	16410.21	6771.03	13409.24	11638.39	4765.15		x
2402	Leucine rich adaptor protein 1	Q96LR2	0.14	2.53	0.81	1.07	2.77	0.68	0.96	0.36	0.63	0.35	0.75	0.38	2.13	0.97	0.60	4.64	1.07	0.47	2.22	0.19	0.06	6184.72	5960.94	5751.15	4203.30	3788.11	3364.97	4483.15	4270.45	4388.22	4924.21	4960.38	3399.45		x
2403	Leucine zipper protein 1	Q86V48	17193.74	17172.05	15581.43	10937.69	11496.49	10595.53	12384.92	11617.03	10970.27	14902.89	14116.57	13499.06	14494.35	13474.70	12249.40	9996.01	9400.00	9837.05	11122.99	10849.96	10572.44	33581.70	33581.79	29681.60	28655.96	27973.75	26173.32	25476.57	26153.43	25214.88	34895.14	31790.25	23817.56		
2404	Leucine zipper protein 4	Q9P127	1353.56	1124.91	1157.83	916.50	784.15	751.46	761.04	777.37	908.48	1364.71	1380.17	1036.57	1248.03	1039.32	746.78	700.44	646.43	731.79	734.17	726.81	866.28	2.27	1.49	0.65	0.84	1.31	0.69	4.16	2.47	0.11	4.90	4.98	0.12	x	
2405	Leucine zipper putative tumor suppressor 1	Q9Y250	0.14	2.53	0.81	1.07	2.77	0.68	0.96	0.36	0.63	0.35	0.75	0.38	2.13	0.97	0.60	4.64	1.07	0.47	2.22	0.19	0.06	6303.75	5159.13	4468.26	4592.73	4070.16	4242.21	5234.13	4718.45	4802.96	5963.65	5471.56	4094.90		x
2406	Leucine zipper putative tumor suppressor 2	Q9BRK4	0.14	2.53	0.81	1.07	2.77	0.68	0.96	0.36	0.63	0.35	0.75	0.38	2.13	0.97	0.60	4.64	1.07	0.47	2.22	0.19	0.06	24021.55	22415.07	20178.30	23711.49	22870.23	21611.74	30464.81	31403.08	30175.27	23658.55	23253.80	17200.94		x
2407	Leucine zipper transcription factor-like protein 1	Q9NQ48	0.14	2.53	0.81	1.07	2.77	0.68	0.96	0.36	0.63	0.35	0.75	0.38	2.13	0.97	0.60	4.64	1.07	0.47	2.22	0.19	0.06	5641.39	6422.92	6458.45	5441.63	5173.40	5370.34	5238.15	5714.97	5509.06	5933.41	5602.19	4677.51		x
2408	Leucine-rich PPR motif-containing protein_ mitochondrial	P42704	895523.97	824197.53	743716.25	534699.30	514408.15	481554.70	656593.70	623425.80	596075.83	699727.68	660799.98	603582.26	700402.39	636088.80	497765.40	525862.34	498199.31	478705.21	554525.26	523075.87	479346.68	491254.55	466006.51	420465.63	320494.07	316075.76	289881.50	406903.63	391241.81	366268.89	396983.06	379957.92	275745.92		
2409	Leucine-rich repeat flightless-interacting protein 1	Q32MZ4	77744.99	74580.86	69843.12	56468.18	54431.88	51703.25	64758.49	61595.88	58526.16	67446.06	66111.38	62227.42	70368.72	66315.71	60363.45	54019.49	52924.46	53741.82	61857.19	57985.08	56501.82	48636.44	42233.95	41827.13	35080.35	34122.27	31321.80	38621.27	36295.00	35580.77	38851.75	38182.04	28057.06		
2410	Leucine-rich repeat flightless-interacting protein 2	Q9Y608	58684.07	44098.47	34957.18	26028.52	24216.33	20422.67	33369.66	31484.84	31669.28	35642.38	33573.79	28482.31	37380.04	27324.51	8945.56	26590.17	25935.38	22982.33	30276.08	27067.71	20754.07	29260.00	27335.66	22144.35	14357.51	13661.18	10033.66	22077.77	18892.46	15514.17	21515.11	20865.01	12581.64		
2411	Leucine-rich repeat serine/threonine-protein kinase 1	Q38SD2	11224.31	9458.79	7799.76	7121.75	6312.79	5453.73	10099.19	9295.19	8226.32	8172.71	8036.69	6983.89	10263.61	8997.97	4526.93	5756.92	5731.55	5018.84	5976.56	4998.76	4296.70	17256.48	16636.50	15893.55	12967.97	13349.35	11959.24	13725.29	12865.56	12851.01	15462.05	14609.48	11325.45		
2412	Leucine-rich repeat transmembrane protein FLRT1	Q9NZU1	0.14	2.53	0.81	1.07	2.77	0.68	0.96	0.36	0.63	0.35	0.75	0.38	2.13	0.97	0.60	4.64	1.07	0.47	2.22	0.19	0.06	2115.89	1810.98	1521.78	1445.88	1371.31	1009.01	2257.57	1958.61	1593.45	2111.84	2224.91	961.12		x
2413	Leucine-rich repeat-containing G-protein coupled receptor 5	O75473	0.14	2.53	0.81	1.07	2.77	0.68	0.96	0.36	0.63	0.35	0.75	0.38	2.13	0.97	0.60	4.64	1.07	0.47	2.22	0.19	0.06	19648.90	18688.18	18504.41	15188.15	14556.46	12846.91	16293.13	15675.74	14860.90	14901.41	14036.94	10830.93		x
2414	Leucine-rich repeat-containing protein 1	Q9BTT6	40632.33	33777.42	28545.88	23419.63	23815.89	19739.28	22167.93	21237.57	19524.05	30625.22	29374.26	27787.67	30207.01	26533.14	15838.22	20843.95	16065.82	19199.18	30191.32	28578.99	22102.25	240.51	156.54	115.81	172.66	143.56	151.44	200.42	187.00	156.23	262.02	246.70	102.24		
2415	Leucine-rich repeat-containing protein 14	Q15048	10517.10	10037.89	9716.14	7737.86	7641.09	7273.43	6799.09	6977.87	6866.25	9664.20	9186.10	9217.55	8290.97	8516.19	7350.97	8514.30	8091.69	7853.20	6837.19	6577.04	6425.70	2.27	1.49	0.65	0.84	1.31	0.69	4.16	2.47	0.11	4.90	4.98	0.12	x	
2416	Leucine-rich repeat-containing protein 23	Q53EV4	28850.47	29836.07	28345.59	21300.47	21576.34	22216.55	26136.95	24733.01	24288.98	25043.28	23859.35	22774.90	27817.09	26184.21	22031.26	19540.76	19008.20	19690.40	20127.64	20890.35	18574.58	2.27	1.49	0.65	0.84	1.31	0.69	4.16	2.47	0.11	4.90	4.98	0.12	x	
2417	Leucine-rich repeat-containing protein 40	Q9H9A6	0.14	2.53	0.81	1.07	2.77	0.68	0.96	0.36	0.63	0.35	0.75	0.38	2.13	0.97	0.60	4.64	1.07	0.47	2.22	0.19	0.06	199.17	3.80	53.17	0.84	1.31	0.69	4.16	2.47	0.11	167.38	106.28	23.61		x
2418	Leucine-rich repeat-containing protein 42	Q9Y546	0.14	2.53	0.81	1.07	2.77	0.68	0.96	0.36	0.63	0.35	0.75	0.38	2.13	0.97	0.60	4.64	1.07	0.47	2.22	0.19	0.06	4282.56	4053.66	3938.76	3211.82	3036.59	2518.29	8096.45	7868.14	7785.09	5024.18	4775.49	2924.19		x
2419	Leucine-rich repeat-containing protein 47	Q8N1G4	34523.29	34296.74	32125.23	22089.39	21431.36	18424.39	26607.77	26518.53	26266.89	29767.75	29608.41	26422.41	31218.02	28183.39	21099.10	22316.51	20520.99	21492.05	23073.46	21293.68	20339.31	7311.67	6517.59	6231.88	4769.30	4769.13	4018.90	5513.75	5110.31	4667.34	5328.10	5753.02	3408.54		
2420	Leucine-rich repeat-containing protein 59	Q96AG4	203758.65	188084.44	174718.06	144668.13	142093.03	119851.78	173754.79	160204.85	151212.81	178669.22	173477.61	147497.22	173230.68	154317.88	122959.03	141537.47	137127.10	128927.90	142766.91	136435.91	124313.75	47104.85	43698.34	42470.28	17280.17	18630.97	15763.47	22351.01	21976.23	20984.41	20247.07	19773.88	13376.22		
2421	Leucine--tRNA ligase cytoplasmic	Q9P2J5	203846.69	200191.86	189259.68	138434.71	138671.17	123768.21	157327.18	154984.06	141868.88	177201.58	169379.02	158705.94	169574.21	149202.93	120961.20	142621.89	133673.96	124543.12	143208.18	137812.62	124440.74	141275.01	128416.49	118247.46	95069.26	90551.61	79308.96	119210.00	112073.20	108243.15	117985.17	109590.32	73449.96		
2422	Leucine-zipper-like transcriptional regulator 1	Q8N653	2481.55	1738.50	2174.88	1798.01	1832.60	1746.54	3055.57	2340.69	2190.06	2187.10	2264.46	1960.30	2275.20	1983.92	2356.19	1822.31	1712.77	1711.95	2160.34	2162.55	2000.94	2.27	1.49	0.65	0.84	1.31	0.69	4.16	2.47	0.11	4.90	4.98	0.12	x	
2423	Leucyl-cystinyl aminopeptidase	Q9UIQ6	106839.67	87920.74	73582.56	59931.59	57385.36	48742.67	66234.42	64649.55	63416.06	73222.19	75013.85	62144.80	77606.55	69518.15	44541.98	56976.30	58503.95	50838.12	62895.70	56675.86	47673.87	178.34	189.63	167.22	150.13	162.15	153.81	226.77	228.32	243.60	232.99	250.47	187.48		
2424	Leukocyte elastase inhibitor	P30740	29517.53	29107.57	28016.19	19038.67	19054.65	17429.98	21120.90	21713.91	20007.29	22739.85	21936.03	21941.31	24684.50	21910.60	18335.26	20505.98	19346.68	19053.10	17830.16	16523.67	15357.97	2.27	1.49	0.65	0.84	1.31	0.69	4.16	2.47	0.11	4.90	4.98	0.12	x	
2425	Leukocyte immunoglobulin-like receptor subfamily B member 1	Q8NHL6	6056.80	6151.11	5666.86	4029.10	3761.50	3878.47	4286.97	4021.46	4175.16	5459.22	5243.94	5097.03	5403.42	5465.92	4378.11	3874.07	3385.82	3690.46	4041.38	4149.91	4110.98	341.68	247.28	206.47	307.23	261.47	257.92	285.95	253.53	254.97	280.35	276.63	233.90		
2426	Leukotriene A-4 hydrolase	P09960	238318.56	230547.61	206260.63	168665.76	163502.27	142259.32	198124.29	185905.21	172151.26	206478.55	195951.80	177952.30	195482.95	173691.90	140224.95	184662.26	172882.93	155544.23	171005.29	162401.60	155620.92	139414.32	132077.50	117166.37	109507.82	103620.67	91621.21	124817.91	125013.95	117087.37	148917.84	139627.49	97472.22		
2427	LIM and cysteine-rich domains protein 1	Q9NZU5	0.14	2.53	0.81	1.07	2.77	0.68	0.96	0.36	0.63	0.35	0.75	0.38	2.13	0.97	0.60	4.64	1.07	0.47	2.22	0.19	0.06	26617.42	26316.93	21813.46	13822.71	13416.91	12316.20	14962.39	14567.14	14119.21	14868.84	14131.87	10687.02		x
2428	LIM and senescent cell antigen-like-containing domain protein 1	P48059	40349.27	38669.97	35138.53	26235.92	25433.34	23952.18	28162.34	29377.94	28453.94	33175.76	30054.37	30429.98	32496.07	31674.49	26821.47	24792.63	24025.44	24964.55	26329.96	26595.90	24356.16	44453.55	41930.97	36951.89	24854.97	25265.90	20816.50	29043.62	28830.92	27012.46	31915.59	29829.38	20008.45		
2429	LIM and senescent cell antigen-like-containing domain protein 2	Q7Z4I7	0.14	2.53	0.81	1.07	2.77	0.68	0.96	0.36	0.63	0.35	0.75	0.38	2.13	0.97	0.60	4.64	1.07	0.47	2.22	0.19	0.06	1525.92	1364.74	1979.36	1331.73	1240.60	939.15	1701.07	1638.42	1335.17	1949.59	1521.96	944.01		x
2430	LIM and SH3 domain protein 1	Q14847	248214.36	241702.63	232790.78	211089.95	203555.23	194268.20	231734.97	227762.86	216249.71	257630.48	248278.28	236454.99	233205.54	211743.39	175395.51	228074.94	219308.13	214194.05	227213.59	210594.50	199633.56	236091.15	221571.32	206203.08	153534.17	154266.22	138820.66	177112.23	165156.82	165424.07	186778.89	181668.91	105022.73		
2431	LIM domain and actin-binding protein 1	Q9UHB6	4312.39	4207.80	3803.93	3156.65	3035.25	2583.52	2758.17	2746.52	2525.49	3620.34	3632.09	3616.06	3026.59	2828.00	2636.13	3252.23	3248.60	3134.02	2471.92	2257.16	2227.37	2.27	1.49	0.65	0.84	1.31	0.69	4.16	2.47	0.11	4.90	4.98	0.12	x	
2432	LIM domain kinase 1	P53667	0.14	2.53	0.81	1.07	2.77	0.68	0.96	0.36	0.63	0.35	0.75	0.38	2.13	0.97	0.60	4.64	1.07	0.47	2.22	0.19	0.06	34340.89	33432.60	29066.71	18521.60	18332.38	14164.72	29026.68	25988.88	24464.68	28177.71	26506.18	14999.40		x
2433	LIM domain kinase 2	P53671	3300.13	3312.61	3009.92	2134.42	2305.58	2198.04	2186.30	2126.42	1838.95	2714.92	2548.03	2621.67	2038.44	2848.08	1992.92	1946.58	1754.36	1998.76	1962.27	2071.95	2060.32	2.27	1.49	0.65	0.84	1.31	0.69	4.16	2.47	0.11	4.90	4.98	0.12	x	
2434	LIM domain only protein 7	Q8WWI1	78781.39	73998.66	66780.27	53336.88	50845.76	46568.12	56292.27	55134.23	50982.62	59641.29	57411.13	50913.78	62866.72	60416.37	48027.89	54831.05	51520.02	52213.80	54361.48	50804.24	46974.26	3090.21	2976.47	2673.47	1770.07	1941.74	1836.47	2052.97	2077.22	1936.34	2442.50	2420.31	1480.72		
2435	LIM/homeobox protein Lhx4	Q969G2	24051.73	22122.13	18528.73	14641.62	14222.00	12660.43	15194.10	14669.70	12011.46	18636.93	17331.64	16852.22	17793.13	15614.84	12299.79	13997.21	13236.26	12409.40	17964.77	17441.07	15375.58	2.27	1.49	0.65	0.84	1.31	0.69	4.16	2.47	0.11	4.90	4.98	0.12	x	
2436	Limbin	Q86UK5	0.14	2.53	0.81	1.07	2.77	0.68	0.96	0.36	0.63	0.35	0.75	0.38	2.13	0.97	0.60	4.64	1.07	0.47	2.22	0.19	0.06	18472.17	17501.65	15560.36	18905.86	19009.35	15974.08	25365.13	23678.99	23376.25	27119.24	27516.84	18678.52		x
2437	LINE-1 retrotransposable element ORF1 protein	Q9UN81	159424.79	148266.89	143109.83	99424.60	96540.49	94118.50	111942.64	107630.73	107098.82	127766.61	123247.83	114822.29	126767.28	118505.29	107543.38	95395.07	89289.15	85685.83	108532.89	100992.57	99381.25	2640.35	2593.28	2722.08	1712.51	1790.59	1671.34	2344.37	2169.91	2225.83	2271.92	2339.86	1713.03		
2438	LINE-1 type transposase domain-containing protein 1	Q5T7N2	88669.15	86344.79	82074.77	63898.26	60076.78	59093.04	70407.92	71887.56	67248.08	83505.77	80169.89	76000.95	74406.17	66304.89	52596.84	61275.23	63769.69	60959.73	69054.48	62714.25	63287.65	22436.76	20174.04	20167.45	13801.07	13940.05	12550.12	15661.71	15682.51	14294.68	17637.75	16478.12	11512.57		
2439	Lipid droplet-associated hydrolase	Q9H6V9	1968.73	1746.90	1700.54	1227.65	1611.89	1317.42	1570.93	1490.05	1428.95	1848.84	1633.15	1651.13	1505.26	1804.34	1525.79	1385.87	1168.32	1170.41	1493.66	1415.55	1551.50	2.27	1.49	0.65	0.84	1.31	0.69	4.16	2.47	0.11	4.90	4.98	0.12	x	
2440	Lipocalin-15	Q6UWW	0.14	2.53	0.81	1.07	2.77	0.68	0.96	0.36	0.63	0.35	0.75	0.38	2.13	0.97	0.60	4.64	1.07	0.47	2.22	0.19	0.06	6777.43	6303.78	5800.77	4030.75	4423.13	2315.78	5567.97	5007.38	4065.37	6173.99	6260.82	3233.36		x
2441	Lipolysis-stimulated lipoprotein receptor	Q86X29	249762.24	252839.15	243231.27	196841.58	190125.57	188557.27	206406.30	189224.68	190465.83	220298.42	210199.69	196013.29	198952.33	192769.83	159851.11	190176.55	180771.58	177415.09	185320.36	174260.99	167353.16	46598.51	43809.41	38560.10	28191.71	27272.25	23831.80	34705.89	32338.95	29896.87	36093.99	34271.38	23615.60		
2442	Lipoma-preferred partner	Q93052	22966.87	22351.66	20267.91	15948.30	15564.89	14342.85	13761.91	13799.44	12926.34	21273.27	20563.71	19572.49	17065.94	17923.47	15077.23	15655.93	14889.46	14270.25	13878.64	13766.16	12650.45	21246.30	19990.71	17579.50	11750.03	10951.29	9427.70	14876.30	14288.70	13655.82	15957.75	14963.70	10326.19		
2443	Lipopolysaccharide-responsive and beige-like anchor protein	P50851	42844.63	41891.78	39836.41	34669.27	32029.64	31011.37	34127.73	34538.88	32595.32	45290.09	42411.17	40499.65	38786.37	36558.08	30586.26	33089.41	30455.73	29183.30	30285.39	29970.54	28637.71	2.27	1.49	0.65	0.84	1.31	0.69	4.16	2.47	0.11	4.90	4.98	0.12	x	
2444	Liprin-alpha-1	Q13136	54324.37	52122.25	47817.51	40878.87	40035.71	37388.82	47693.15	44349.37	44133.55	50618.32	47828.23	43750.16	49138.79	44997.82	36509.74	42141.45	37929.12	36680.19	37511.79	38292.87	37014.53	1310.09	1364.54	1395.47	1648.95	1437.61	1361.83	1778.41	1621.66	1628.90	2268.87	1918.62	1065.04		
2445	Liprin-alpha-3	O75145	495664.28	478000.20	457805.92	368153.46	351589.54	328804.35	385209.72	369335.08	357234.52	448139.60	423260.05	400703.08	418518.60	380155.88	328378.93	372980.81	355182.85	350020.74	359761.45	344650.56	326240.19	89028.86	82659.19	74335.66	62698.44	60631.76	51377.76	82842.20	74352.77	64690.97	88668.03	80126.20	50683.08		
2446	Liprin-beta-1	Q86W92	54557.83	50821.79	47124.76	27728.32	28026.46	23775.15	32211.74	30092.34	28526.61	45616.30	42671.69	37858.79	43760.94	32510.75	21378.28	28221.84	27603.55	26673.17	30832.84	30303.35	26765.65	2690.09	2204.75	2265.28	1431.78	1469.61	1319.76	1607.46	1619.30	1420.39	1987.70	1712.00	1511.38		
2447	L-lactate dehydrogenase A chain	P00338	2667757.02	2633162.56	2504801.74	2142821.67	2094875.26	2005997.04	2499472.42	2486848.26	2407533.90	2512366.06	2461839.40	2301806.62	2478749.01	2457210.75	2107570.13	2004020.88	1911315.55	1861072.03	2140974.61	2056565.72	1980870.38	1733592.56	1569043.75	1541855.44	1056386.38	1046745.47	934629.31	1345173.93	1314663.38	1235605.17	1260143.49	1256008.38	1016849.20		
2448	L-lactate dehydrogenase B chain	P07195	12119.05	11667.39	10517.82	7197.12	6731.58	6604.56	7723.42	7871.75	7353.45	7985.76	7643.12	7255.45	7992.58	7864.63	6754.85	7204.04	6852.53	6652.33	6110.66	6542.65	6382.80	2252769.16	2059569.67	1977401.14	1364786.09	1334189.34	1240068.07	1617808.26	1582906.57	1527498.02	1542126.22	1473089.20	1080374.45		
2449	L-lactate dehydrogenase C chain	P07864	14797.39	15293.10	16369.20	9163.83	8759.89	8638.92	10701.67	10309.96	9852.72	13467.63	12843.32	11412.95	10713.09	10690.86	7361.22	9638.88	8666.71	10299.07	8997.00	8946.90	8472.02	17932.12	15345.54	13111.76	10544.72	9552.33	6911.58	15186.67	13505.25	12495.58	14205.31	12370.59	7746.73		
2450	LON peptidase N-terminal domain and RING finger protein 2	Q1L5Z9	33142.59	33116.23	30844.28	21164.50	19649.27	18663.68	26687.22	25515.26	24199.15	26860.59	25481.75	23212.54	25989.03	24705.17	17769.69	24438.07	23769.36	22439.65	24031.52	23473.66	21351.22	2.27	1.49	0.65	0.84	1.31	0.69	4.16	2.47	0.11	4.90	4.98	0.12	x	
2451	Lon protease homolog mitochondrial	P36776	414812.74	397764.44	380424.39	319137.52	292831.93	298457.91	358920.02	350017.91	338589.47	357703.56	347312.35	320321.99	382731.06	345646.35	319336.58	319867.39	287740.71	285458.26	328390.94	312905.67	307054.65	2.27	1.49	0.65	0.84	1.31	0.69	4.16	2.47	0.11	4.90	4.98	0.12	x	
2452	Long-chain fatty acid transport protein 4	Q6P1M0	14572.89	13260.28	10541.58	3319.23	2333.49	1927.75	2710.20	2166.76	1810.14	11846.01	10493.65	9330.09	11089.56	3528.67	2190.43	3327.59	1969.70	1873.18	5100.03	3283.65	3114.39	2.27	1.49	0.65	0.84	1.31	0.69	4.16	2.47	0.11	4.90	4.98	0.12	x	
2453	Long-chain-fatty-acid--CoA ligase 3	O95573	136557.42	131947.34	128070.06	105958.49	101843.22	101264.06	103764.79	102989.26	96026.21	138685.95	130456.83	122440.41	112418.64	109984.18	88673.91	136504.52	132128.65	129596.72	88772.60	87624.10	81293.45	81895.79	72701.84	63620.32	56473.52	53472.51	47742.53	65612.25	59638.87	56560.13	66396.18	62383.13	47231.53		
2454	Long-chain-fatty-acid--CoA ligase 4	O60488	62361.89	62049.88	59029.42	38317.68	38158.59	34915.42	52654.91	51375.16	53255.72	45726.48	43572.16	42490.54	48486.14	47519.11	40171.77	42202.87	40840.39	38608.91	49469.17	38062.97	39875.15	112486.99	105758.93	89874.33	71756.60	72193.27	59405.17	80722.38	76860.52	73109.25	92241.47	93253.98	54015.32		
2455	Low affinity immunoglobulin epsilon Fc receptor	P06734	15108.62	13342.28	10326.91	7781.06	7251.26	6000.16	11063.73	10145.49	9199.56	11021.39	9561.68	8098.00	9879.79	9520.24	6258.83	8645.55	8466.15	7367.12	9500.90	8992.85	8020.34	2.27	1.49	0.65	0.84	1.31	0.69	4.16	2.47	0.11	4.90	4.98	0.12	x	
2456	Low molecular weight phosphotyrosine protein phosphatase	P24666	35096.04	38281.28	29943.61	20353.55	20741.02	18539.43	26530.46	25435.64	23103.48	27687.00	25803.26	23988.67	25181.41	24198.36	20859.76	22599.34	21990.32	21907.98	19337.31	19135.16	18819.12	2.27	1.49	0.65	0.84	1.31	0.69	4.16	2.47	0.11	4.90	4.98	0.12	x	
2457	Low-density lipoprotein receptor-related protein 1B	Q9NZR2	0.14	2.53	0.81	1.07	2.77	0.68	0.96	0.36	0.63	0.35	0.75	0.38	2.13	0.97	0.60	4.64	1.07	0.47	2.22	0.19	0.06	5689.22	5760.31	4811.92	3625.53	3227.31	2775.63	3676.01	3735.89	3260.78	4068.74	3491.50	2168.79		x
2458	Low-density lipoprotein receptor-related protein 2	P98164	20995.53	19535.61	19490.14	19904.96	19616.22	19301.88	17766.48	17028.07	15694.26	28314.32	26449.24	24777.95	22203.53	18454.92	18024.41	22476.29	20634.23	20063.44	19105.91	17529.12	15988.05	2.27	1.49	0.65	0.84	1.31	0.69	4.16	2.47	0.11	4.90	4.98	0.12	x	
2459	Low-density lipoprotein receptor-related protein 4	O75096	1947.15	1676.17	1748.79	1342.47	1250.06	944.28	1651.48	1121.00	1504.69	1437.51	1778.62	1135.34	1707.64	859.62	806.01	1489.61	1318.05	1249.92	1333.51	1131.99	1196.13	2.27	1.49	0.65	0.84	1.31	0.69	4.16	2.47	0.11	4.90	4.98	0.12	x	
2460	LRP chaperone MESD	Q14696	42813.64	40300.50	38029.43	25163.34	23889.40	22035.51	27190.02	26360.75	26140.64	36265.47	33316.41	32259.74	34770.77	26241.53	19669.80	22707.85	23067.23	22396.99	26307.42	20486.13	20623.74	23107.84	22618.62	20570.97	14814.55	15128.68	14646.66	15058.54	15165.47	14687.65	16521.05	17267.30	22994.06		
2461	Luc7-like protein 3	O95232	101557.38	102676.55	93105.29	84779.58	83904.45	77872.81	102403.58	96053.27	90601.08	111592.68	106227.32	100752.20	94437.65	91425.44	75586.28	86894.60	80121.88	77684.87	81728.32	81611.25	78724.17	19884.32	18916.54	18437.17	17844.79	16867.54	15123.41	18561.07	18080.54	16187.32	18434.78	18523.44	13334.80		
2462	Lupus La protein	P05455	153487.62	142790.22	134953.12	107900.73	100578.97	100590.26	118213.19	114288.74	111557.91	129067.16	121410.25	114556.42	124495.84	118032.61	100445.59	101112.19	90611.26	94786.77	109560.13	103612.61	100493.33	182696.37	174824.39	160081.08	127312.95	125514.59	115068.22	145208.97	133751.11	127872.93	148474.85	140627.70	115766.36		
2463	L-xylulose reductase	Q7Z4W1	249456.15	240411.67	208567.36	181736.44	176880.74	160374.49	195569.17	187719.65	179745.44	211711.16	199880.27	176855.98	191027.08	172872.76	118789.75	203037.71	193746.24	192985.37	169379.34	157355.43	141366.02	7222.00	5717.25	7275.81	5359.83	5137.71	4278.65	6752.91	5820.00	6045.51	5883.30	5562.18	3584.53		
2464	Lymphocyte expansion molecule	Q3ZCV2	5215.91	4236.00	3679.33	2630.40	2568.72	2357.52	2764.07	2708.39	2573.63	3000.87	3043.91	2626.44	3800.74	3134.96	2072.49	2896.95	2993.94	2563.05	2482.81	2258.62	1952.94	2.27	1.49	0.65	0.84	1.31	0.69	4.16	2.47	0.11	4.90	4.98	0.12	x	
2465	Lymphocyte function-associated antigen 3	P19256	14913.23	15162.38	12935.67	10392.07	10145.38	9792.67	11021.68	10586.97	10009.66	12665.89	11278.24	10978.64	12051.40	12300.10	9680.14	9369.48	9050.48	8685.98	9971.88	8284.14	10206.52	2.27	1.49	0.65	0.84	1.31	0.69	4.16	2.47	0.11	4.90	4.98	0.12	x	
2466	Lymphoid-restricted membrane protein	Q12912	0.14	2.53	0.81	1.07	2.77	0.68	0.96	0.36	0.63	0.35	0.75	0.38	2.13	0.97	0.60	4.64	1.07	0.47	2.22	0.19	0.06	35049.60	35051.85	33355.26	17803.70	17217.56	14103.88	17780.32	16964.96	16472.40	16116.85	15381.50	9740.71		x
2467	Lymphokine-activated killer T-cell-originated protein kinase	Q96KB5	4425.62	4165.52	3950.32	2628.98	2703.61	2367.81	3280.67	2994.30	2944.87	2541.92	2626.63	2439.90	3440.71	3056.76	2268.02	1985.85	1891.62	2066.02	2637.98	2635.11	2858.57	5736.62	5030.20	4740.81	4245.07	4143.32	3682.57	5095.11	4714.89	4357.66	5046.46	4857.31	3584.55		
2468	Lys-63-specific deubiquitinase BRCC36	P46736	14994.58	12666.65	11231.61	8624.74	8469.48	7604.10	15100.12	15026.36	13162.70	11509.03	11049.28	10372.42	11293.20	10926.65	7347.63	9921.12	9643.35	8534.37	9356.35	8894.69	7719.23	2.27	1.49	0.65	0.84	1.31	0.69	4.16	2.47	0.11	4.90	4.98	0.12	x	
2469	Lysine-rich coiled-coil protein 1	Q9NPI7	2274.91	1764.19	1772.95	1534.72	1512.46	1520.76	2411.55	2132.38	1832.96	1837.21	1828.07	1660.80	1731.72	2020.93	1456.27	2119.41	1697.35	1469.15	1670.90	1500.06	1553.94	2.27	1.49	0.65	0.84	1.31	0.69	4.16	2.47	0.11	4.90	4.98	0.12	x	
2470	Lysine-specific demethylase 2B	Q8NHM5	503.36	690.01	606.77	415.24	369.06	367.92	370.08	380.19	338.13	493.90	370.88	383.94	728.38	520.01	377.24	433.57	266.87	430.90	474.58	308.68	296.33	12748.86	13571.58	9227.21	8092.92	7816.10	7132.19	10663.96	10434.45	9324.94	13340.08	10812.16	3890.68		
2471	Lysine-specific demethylase 3A	Q9Y4C1	0.14	2.53	0.81	1.07	2.77	0.68	0.96	0.36	0.63	0.35	0.75	0.38	2.13	0.97	0.60	4.64	1.07	0.47	2.22	0.19	0.06	2.27	1.49	0.65	37.05	24.77	28.91	613.46	651.32	592.62	194.57	173.26	136.31		x
2472	Lysine-specific demethylase 3B	Q7LBC6	288523.29	271710.08	220282.53	212251.14	212724.57	175144.44	239989.01	225217.51	204552.47	259042.46	252344.19	212607.69	237006.48	236750.07	126441.16	215582.60	203784.55	188624.91	224098.62	209810.74	175933.74	303459.92	267587.52	232645.48	190844.30	188265.93	136912.30	208072.55	191155.13	163997.87	252785.67	234371.41	151961.85		
2473	Lysine-specific demethylase 4C	Q9H3R0	2472.38	2241.03	2166.58	1702.06	1581.90	1379.12	2011.98	2367.55	2870.21	1927.45	1901.58	1813.35	2758.65	2001.68	1666.36	2717.79	2932.85	2761.56	3610.03	2222.83	2616.62	2.27	1.49	0.65	0.84	1.31	0.69	4.16	2.47	0.11	4.90	4.98	0.12	x	
2474	Lysine-specific demethylase 5D	Q9BY66	0.14	2.53	0.81	1.07	2.77	0.68	0.96	0.36	0.63	0.35	0.75	0.38	2.13	0.97	0.60	4.64	1.07	0.47	2.22	0.19	0.06	210094.09	187816.85	168051.74	119805.01	117109.63	98363.18	199857.37	204763.43	194557.69	149482.96	150558.87	100056.44		x
2475	Lysine-specific demethylase 7A	Q6ZMT4	0.14	2.53	0.81	1.07	2.77	0.68	0.96	0.36	0.63	0.35	0.75	0.38	2.13	0.97	0.60	4.64	1.07	0.47	2.22	0.19	0.06	30693.07	31354.31	24966.51	5708.45	5201.87	4987.83	5508.04	5363.14	5967.97	3910.70	4073.44	2420.31		x
2476	Lysine-specific demethylase 9	Q5VWQ0	4563.13	4375.58	4010.14	2866.64	2660.48	2567.84	2882.99	2555.25	2371.71	3209.00	3000.84	2915.88	3509.60	3314.37	2920.78	2413.47	2115.48	2161.47	2730.56	2834.35	2355.42	2.27	1.49	0.65	0.84	1.31	0.69	4.16	2.47	0.11	4.90	4.98	0.12	x	
2477	Lysine-specific demethylase hairless	O43593	40183.15	31834.49	24965.48	21567.78	19766.64	15010.62	21322.91	21191.37	17539.42	26028.70	23673.91	20058.04	26805.82	21822.68	8218.77	23006.52	20870.69	19535.23	20177.90	18422.48	14543.20	2.27	1.49	0.65	0.84	1.31	0.69	4.16	2.47	0.11	4.90	4.98	0.12	x	
2478	Lysine--tRNA ligase	Q15046	634303.00	617689.93	591640.33	440677.87	429445.34	400700.98	535383.94	510887.82	495213.49	523759.48	493421.27	468502.33	518146.34	497078.08	421692.30	437624.35	411289.94	407547.88	464672.65	425940.81	414266.36	362561.81	334675.27	323476.56	248391.72	234596.20	210987.70	300359.08	285114.15	278721.74	276658.10	267662.59	186421.71		
2479	LysM and putative peptidoglycan-binding domain-containing protein 2	Q8IV50	11313.00	9850.54	9456.69	6454.75	6472.81	5981.84	8437.28	8153.57	8146.29	9521.10	8838.43	8029.24	8553.52	8175.12	6766.18	8195.60	7456.65	7411.58	6500.67	6299.65	5941.14	2.27	1.49	0.65	0.84	1.31	0.69	4.16	2.47	0.11	4.90	4.98	0.12	x	
2480	Lysophosphatidylcholine acyltransferase 1	Q8NF37	0.14	2.53	0.81	1.07	2.77	0.68	0.96	0.36	0.63	0.35	0.75	0.38	2.13	0.97	0.60	4.64	1.07	0.47	2.22	0.19	0.06	937.06	851.81	740.42	385.71	390.87	61.04	439.97	377.41	245.12	587.95	576.37	200.80		x
2481	Lysophospholipase D GDPD3	Q7L5L3	29057.74	27368.20	25152.80	20152.99	18381.47	16809.31	19572.08	20662.72	19913.36	18406.32	17374.56	15948.62	20384.22	20225.88	17616.14	22168.53	21390.56	19380.76	26623.82	21623.53	20346.53	2.27	1.49	0.65	0.84	1.31	0.69	4.16	2.47	0.11	4.90	4.98	0.12	x	
2482	Lysophospholipase-like protein 1	Q5VWZ2	0.14	2.53	0.81	1.07	2.77	0.68	0.96	0.36	0.63	0.35	0.75	0.38	2.13	0.97	0.60	4.64	1.07	0.47	2.22	0.19	0.06	19032.48	18004.97	17601.49	11254.36	9953.94	8557.10	13747.38	12038.89	11226.66	11218.40	11537.35	8750.76		x
2483	Lysophospholipid acyltransferase 7	Q96N66	4936.37	4678.64	4902.20	3101.98	3159.09	2952.55	3533.33	3320.03	3150.34	5081.12	4973.59	5092.30	4478.22	3825.57	3468.48	3131.63	3037.44	3174.34	3715.35	3628.52	3586.13	7607.68	7350.68	6744.77	5186.33	4735.38	4488.57	6003.65	5796.63	5579.40	5880.86	6058.41	3869.63		
2484	Lysosomal acid phosphatase	P11117	2376.45	2352.48	1971.93	1683.19	1863.70	1524.51	1612.00	1588.84	1491.59	2427.05	2383.35	2150.58	2329.72	2003.86	1280.87	2327.45	1979.35	1888.39	1839.05	1656.11	1399.76	2.27	1.49	0.65	0.84	1.31	0.69	4.16	2.47	0.11	4.90	4.98	0.12	x	
2485	Lysosomal alpha-glucosidase	P10253	107573.00	98008.79	90369.76	73214.97	66714.41	65788.42	78820.98	77263.43	74258.52	93445.44	88196.80	84069.83	91266.65	86423.94	73313.33	76438.56	71092.97	69165.62	70203.56	66778.40	62886.79	2.27	1.49	0.65	0.84	1.31	0.69	4.16	2.47	0.11	4.90	4.98	0.12	x	
2486	Lysosomal protective protein	P10619	39502.78	35528.09	32138.68	24461.20	23048.50	20470.26	31561.34	31905.13	28648.90	34056.19	31765.57	29479.39	32926.18	31209.55	21759.69	30075.32	29024.03	26461.21	22952.04	21233.33	19523.05	58725.57	52888.27	48310.11	37213.00	36519.93	29765.75	46993.71	44177.87	41183.65	36754.05	38080.48	23607.80		
2487	Lysosome membrane protein 2	Q14108	95240.28	87782.63	75713.63	58291.01	56383.67	56977.99	72030.72	71448.91	66560.27	78419.27	74283.98	67105.12	69967.36	68070.45	55911.31	54516.12	49981.71	50553.83	55542.74	54726.10	49815.93	2.27	1.49	0.65	0.84	1.31	0.69	4.16	2.47	0.11	4.90	4.98	0.12	x	
2488	Lysosome-associated membrane glycoprotein 1	P11279	13899.55	10073.99	8520.68	5878.84	1044.08	754.80	1954.13	359.84	334.97	12681.59	9378.65	6625.77	11407.87	2254.94	582.03	2090.10	300.77	665.48	1256.11	6270.84	4207.81	22972.68	20171.50	22106.30	18555.34	19608.59	18833.78	22748.40	21798.89	21539.73	18869.06	20877.87	18513.02		
2489	Lysosome-associated membrane glycoprotein 2	P13473	8378.36	8793.30	7892.60	4702.29	4499.35	4642.81	8187.17	7806.72	6828.07	5964.26	5764.89	5422.70	5840.44	5547.44	4514.47	5546.23	5202.43	4978.70	4498.04	4750.47	4170.87	2.27	1.49	0.65	0.84	1.31	0.69	4.16	2.47	0.11	4.90	4.98	0.12	x	

Supplementary Table S1. Overview on the abundance of all relatively quantified 5180 proteins

			MCF-7																					MDA-MB-231													
	Protein name	UniProt	control 01	control 02	control 03	Dai IC20 01	Dai IC20 02	Dai IC20 03	Dai SC20 01	Dai SC20 02	Dai SC20 03	Gen IC20 01	Gen IC20 02	Gen IC20 03	Gen SC20 01	Gen SC20 02	Gen SC20 03	SSE IC20 01	SSE IC20 02	SSE IC20 03	SSE SC20 01	SSE SC20 02	SSE SC20 03	control 01	control 02	control 03	Dai IC20 01	Dai IC20 02	Dai IC20 03	Gen IC20 01	Gen IC20 02	Gen IC20 03	SSE IC20 01	SSE IC20 02	SSE IC20 03	unique to MCF-7	unique to MDA-MB-231
2490	Lysozyme-like protein 1	Q6UWQ	1838.48	1821.27	1636.80	1409.07	1587.64	1704.15	1493.24	1280.44	1405.38	2066.47	1894.18	1917.70	1480.58	1580.65	1354.38	1349.25	1282.74	1335.63	1546.98	1683.56	1535.36	2.27	1.49	0.65	0.84	1.31	0.69	4.16	2.47	0.11	4.90	4.98	0.12	x	
2491	Lysozyme-like protein 2	Q7Z4W2	3520.67	3393.75	3325.36	2111.95	1994.11	1763.27	2530.82	2434.95	2144.26	2743.66	2489.71	2320.57	2719.13	2326.75	1979.54	2021.53	1937.40	1900.24	2746.34	2503.28	2267.53	2.27	1.49	0.65	0.84	1.31	0.69	4.16	2.47	0.11	4.90	4.98	0.12	x	
2492	m7GpppN-mRNA hydrolase	Q8IU60	2059.88	1857.11	1798.91	1148.05	984.53	935.86	1192.98	1148.38	1181.60	1483.48	1479.93	1380.65	1522.28	1442.57	1048.82	1289.59	1211.25	1085.22	1170.84	991.85	914.76	2.27	1.49	0.65	0.84	1.31	0.69	4.16	2.47	0.11	4.90	4.98	0.12	x	
2493	m7GpppX diphosphatase	Q96C86	11431.25	16059.48	15909.06	9670.36	10028.95	7134.28	12430.54	10806.49	10357.29	13130.45	13080.47	12767.32	13055.33	9197.78	8404.84	9766.55	9167.22	8498.62	6704.72	11845.88	10156.54	2.27	1.49	0.65	0.84	1.31	0.69	4.16	2.47	0.11	4.90	4.98	0.12	x	
2494	m-AAA protease-interacting protein 1_ mitochondrial	Q8WWC	318.15	396.92	385.74	808.74	1127.60	828.15	1624.98	176.75	117.40	278.45	215.05	218.52	298.61	1509.15	519.57	487.88	419.56	938.19	375.09	419.20	209.95	2036.97	223.05	1356.52	808.01	883.45	554.38	1153.08	1015.95	591.21	908.89	668.16	400.67		
2495	Macrophage migration inhibitory factor	P14174	1567.73	1014.94	808.50	2480.17	2539.23	1698.16	2715.67	1229.63	388.09	949.94	780.54	718.85	1158.14	3542.88	793.77	2206.90	617.45	1814.43	5250.94	3315.77	1624.85	103927.04	106947.37	94935.11	19765.74	18892.63	18383.50	20694.83	20989.27	19914.76	14468.99	14938.62	10524.61		
2496	Macrophage-capping protein	P40121	137061.66	141615.79	128868.80	92660.94	83693.99	80450.10	107260.31	100758.95	100449.83	114704.51	110003.08	98345.64	136046.93	124149.71	107656.08	83860.32	75587.38	75854.48	95817.35	85568.78	80251.24	83600.72	82285.71	72666.02	62670.94	59930.37	53391.52	68428.88	63950.06	62419.30	65112.40	65337.43	45365.69		
2497	Maestro heat-like repeat-containing protein family member 1	Q8NDA8	633.51	586.24	608.06	75.31	139.56	88.68	109.13	138.57	110.23	482.55	384.41	384.83	258.91	13.30	8.42	202.64	406.23	403.62	240.23	239.44	197.82	2.27	1.49	0.65	0.84	1.31	0.69	4.16	2.47	0.11	4.90	4.98	0.12	x	
2498	Magnesium transporter protein 1	Q9H0U3	35051.16	35060.26	31878.69	23695.07	23678.68	23456.32	23914.03	26163.52	24164.81	33706.29	32121.47	30507.27	27815.52	25892.51	23464.00	21618.92	22290.00	22102.91	23597.89	21899.42	20564.42	25646.00	23204.63	21538.22	15663.63	15076.20	12649.26	19144.31	17669.89	16325.52	16739.31	15679.34	10557.59		
2499	MAGUK p55 subfamily member 3	Q13368	153.99	149.77	92.55	62.44	76.75	26.43	128.78	144.76	142.70	96.06	107.61	111.53	129.08	133.68	77.31	47.98	152.91	53.41	108.94	126.72	60.00	2.27	1.49	0.65	0.84	1.31	0.69	4.16	2.47	0.11	4.90	4.98	0.12	x	
2500	MAGUK p55 subfamily member 5	Q8N3R9	0.14	2.53	0.81	1.07	2.77	0.68	0.96	0.36	0.63	0.35	0.75	0.38	2.13	0.97	0.60	4.64	1.07	0.47	2.22	0.19	0.06	46787.62	47539.91	44085.14	39773.07	35152.70	36661.12	37027.55	35826.80	38389.65	44187.65	35783.54	24532.05		x
2501	MAGUK p55 subfamily member 7	Q5T2T1	38590.32	36130.59	37078.26	25707.65	24050.63	23384.85	29257.05	28511.92	27144.03	31662.44	30318.82	29061.73	30096.62	27240.92	22088.09	27930.70	25986.70	24626.68	26317.81	24031.59	22101.86	2.27	1.49	0.65	0.84	1.31	0.69	4.16	2.47	0.11	4.90	4.98	0.12	x	
2502	Major vault protein	Q14764	0.14	2.53	0.81	1.07	2.77	0.68	0.96	0.36	0.63	0.35	0.75	0.38	2.13	0.97	0.60	4.64	1.07	0.47	2.22	0.19	0.06	151318.01	143012.86	130335.20	91212.67	89710.41	78732.64	104712.55	99680.41	96419.52	106657.86	104552.73	74830.59		x
2503	Malate dehydrogenase_ cytoplasmic	P40925	362340.15	337828.01	316028.25	276272.60	274391.32	249899.91	352842.76	340747.62	332581.48	324943.50	311038.19	286894.31	309308.49	279418.34	222123.43	270391.03	253417.69	248753.45	262129.62	253154.12	232694.11	295565.97	272616.34	264342.93	193683.88	185729.63	154878.56	224463.19	210526.66	195923.76	226372.79	220731.47	155366.57		
2504	Malate dehydrogenase_ mitochondrial	P40926	786598.25	750172.19	723394.60	524763.81	517725.34	489226.05	803178.17	773596.67	747276.06	687964.95	647134.91	621673.52	663030.33	620498.75	506060.38	598894.47	567188.05	560899.10	554834.17	495180.88	440815.33	316160.69	296557.61	276302.85	162242.06	156200.23	140924.27	179506.58	168007.55	163452.42	156217.09	149870.65	105145.09		
2505	Malectin	Q14165	70896.26	65681.27	63336.47	49553.87	46693.41	42663.15	52202.23	50637.49	47703.43	65606.61	61902.53	56945.80	59023.61	52744.02	42379.63	49503.25	45665.10	44012.11	46862.31	44355.63	42135.44	5965.96	5760.02	5288.15	4798.05	4578.51	3662.01	5159.10	5315.92	5010.90	5642.91	5612.18	3468.23		
2506	Malignant T-cell-amplified sequence 1	Q9ULC4	96096.31	93690.91	95842.10	56660.04	56596.70	56207.01	73915.70	64775.53	65024.13	73666.76	72438.75	68842.99	73811.81	72802.78	58967.86	62141.15	54019.19	55184.08	56304.79	55574.87	51332.60	91914.43	75121.37	76368.65	60951.36	60017.26	50400.18	82198.10	80607.20	76531.64	68767.19	67129.59	48994.78		
2507	Malonyl-CoA decarboxylase_ mitochondrial	O95822	590.34	596.30	655.86	541.85	450.63	460.99	620.08	476.93	576.85	873.83	646.15	636.64	712.81	700.18	517.15	599.11	453.24	420.74	617.51	443.69	452.58	2.27	1.49	0.65	0.84	1.31	0.69	4.16	2.47	0.11	4.90	4.98	0.12	x	
2508	Maltase-glucoamylase_ intestinal	O43451	0.14	2.53	0.81	1.07	2.77	0.68	0.96	0.36	0.63	0.35	0.75	0.38	2.13	0.97	0.60	4.64	1.07	0.47	2.22	0.19	0.06	10671.74	10482.61	9262.10	6470.13	6148.08	3440.31	8811.12	8294.63	6956.05	9797.77	9432.23	9791.38		x
2509	Mannan-binding lectin serine protease 1	P48740	5409.37	4582.22	4112.57	2734.68	2784.87	2282.21	3739.66	3441.81	3278.95	4229.07	3931.89	3386.59	4090.66	3742.43	1859.25	3144.93	3325.83	3092.19	3598.71	3146.03	2696.74	2.27	1.49	0.65	0.84	1.31	0.69	4.16	2.47	0.11	4.90	4.98	0.12	x	
2510	Mannose-1-phosphate guanyltransferase beta	Q9Y5P6	22704.03	21346.62	21648.71	16256.70	15738.28	17274.39	18389.38	19021.85	17898.47	18805.96	18238.58	17788.74	17768.31	16172.79	15038.88	15738.15	14191.63	14418.22	15883.77	16094.30	15268.46	2.27	1.49	0.65	0.84	1.31	0.69	4.16	2.47	0.11	4.90	4.98	0.12	x	
2511	Mannose-6-phosphate isomerase	P34949	32176.72	32119.06	28176.13	22395.42	20542.08	17796.52	22448.60	21905.06	19874.55	24350.53	23803.12	20096.72	25944.99	24405.02	18555.96	25324.70	22537.72	22221.97	20877.10	18712.25	17483.15	2.27	1.49	0.65	0.84	1.31	0.69	4.16	2.47	0.11	4.90	4.98	0.12	x	
2512	Mannosyl-oligosaccharide 1_2-alpha-mannosidase IB	O60476	8476.09	8957.41	8072.97	8382.82	8269.88	7699.81	7574.97	6922.02	6584.70	9545.18	9059.30	8763.35	8628.92	7368.16	7409.48	8453.79	7485.73	6949.36	7212.66	7256.46	6430.35	2.27	1.49	0.65	0.84	1.31	0.69	4.16	2.47	0.11	4.90	4.98	0.12	x	
2513	Mannosyl-oligosaccharide glucosidase	Q13724	7652.47	8446.92	6117.12	6403.72	4811.72	5253.65	5588.39	3968.80	6074.11	8206.14	7651.85	6636.25	7914.59	5714.50	4374.48	5401.25	4347.33	4782.10	6185.90	4956.65	4385.48	2.27	1.49	0.65	0.84	1.31	0.69	4.16	2.47	0.11	4.90	4.98	0.12	x	
2514	MAP kinase-activated protein kinase 2	P49137	7874.93	7422.65	7392.46	5683.25	5247.96	5416.39	6266.74	5766.79	5924.57	7161.87	6726.39	6546.63	6562.77	6085.36	5188.65	5477.43	5063.68	5403.05	5151.64	4734.40	4799.22	2.27	1.49	0.65	0.84	1.31	0.69	4.16	2.47	0.11	4.90	4.98	0.12	x	
2515	MAP/microtubule affinity-regulating kinase 3	P27448	133988.77	140072.91	138613.91	112458.15	102351.22	101005.58	124733.62	116067.85	114029.74	120678.70	122768.12	119660.20	118997.62	122887.65	99714.31	106107.49	113324.17	100000.71	107037.55	101483.82	94474.96	2.27	1.49	0.65	0.84	1.31	0.69	4.16	2.47	0.11	4.90	4.98	0.12	x	
2516	MAP7 domain-containing protein 1	Q3KQU3	587.36	730.52	717.21	389.20	336.13	309.04	271.19	196.57	351.83	458.52	507.86	464.08	449.12	379.02	261.75	422.25	403.64	332.03	429.22	481.62	325.47	24487.17	24178.87	22530.99	20692.71	20776.74	19065.38	25016.41	25300.07	25620.93	24561.44	23678.98	16936.79		
2517	MARCKS-related protein	P49006	68796.09	68166.50	63589.68	47329.64	45271.09	42201.29	52067.86	47408.82	48009.88	59219.75	56156.68	54174.69	62396.17	55651.17	46711.71	39384.77	36442.67	35027.15	45553.00	45079.77	44449.13	41279.10	38034.40	36711.31	32801.48	32893.09	31610.34	35890.20	34632.26	33896.55	37499.41	35018.81	27619.96		
2518	Matrin-3	P43243	56845.18	54308.38	55134.72	36882.94	35764.79	34957.67	46694.44	46045.50	45790.60	45527.05	43524.74	40939.65	45551.44	40524.76	35775.02	38172.65	35212.09	36561.59	35878.19	33847.75	33961.86	12723.32	11960.46	12773.09	11876.57	12197.04	11260.40	10804.96	10708.84	11298.29	16573.64	17177.32	9034.56		
2519	Matrix metalloproteinase-15	P51511	9355.32	8211.62	8656.48	6060.69	6243.59	6276.62	8013.78	7868.41	8106.60	8459.83	8278.29	7734.41	9380.60	8051.72	6362.70	7991.20	5812.27	5834.91	6190.23	5773.24	5494.14	2.27	1.49	0.65	0.84	1.31	0.69	4.16	2.47	0.11	4.90	4.98	0.12	x	
2520	Matrix metalloproteinase-21	Q8N119	0.14	2.53	0.81	1.07	2.77	0.68	0.96	0.36	0.63	0.35	0.75	0.38	2.13	0.97	0.60	4.64	1.07	0.47	2.22	0.19	0.06	1966.67	2054.99	1613.35	903.39	805.59	587.19	1179.33	968.71	920.44	1028.51	1125.64	581.16		x
2521	Matrix-remodeling-associated protein 5	Q9NR99	11531.20	9915.83	10178.16	6883.40	6645.69	6746.35	10305.42	9582.04	9393.16	8356.44	7670.78	7186.38	9046.94	10015.70	7022.21	7890.80	8570.06	8227.95	8105.46	8046.72	7862.30	2.27	1.49	0.65	0.84	1.31	0.69	4.16	2.47	0.11	4.90	4.98	0.12	x	
2522	MBT domain-containing protein 1	Q05BQ5	1013.48	833.57	438.94	83.71	16.91	12.31	0.96	0.36	0.63	672.03	495.23	456.38	2.13	0.97	0.60	33.65	31.61	1.19	1826.14	34.57	32.65	2.27	1.49	0.65	0.84	1.31	0.69	4.16	2.47	0.11	4.90	4.98	0.12	x	
2523	Mdm2-binding protein	Q96DY7	17745.38	15661.55	12319.42	8070.14	7757.91	5951.96	9937.25	9283.10	7867.33	11123.48	9834.77	7401.48	9155.39	8738.17	4685.56	9161.73	7984.70	6468.49	7166.83	7100.83	5500.12	9413.42	8512.75	7939.60	5907.86	4607.88	3599.38	6733.16	6951.22	5660.89	6081.83	5642.68	3361.20		
2524	Mediator of DNA damage checkpoint protein 1	Q14676	10909.23	10244.89	10033.94	7184.79	6876.18	6594.62	7329.60	7083.05	7171.08	8353.48	8021.10	7963.38	8261.25	8091.30	5913.59	6914.07	6151.76	7353.16	8358.67	7377.11	7250.11	58987.37	55534.57	52149.12	44499.14	42540.75	34874.66	42996.54	42870.24	41005.71	50119.39	47839.83	32730.70		
2525	Mediator of RNA polymerase II transcription subunit 20	Q9H944	0.14	2.53	0.81	1.07	2.77	0.68	0.96	0.36	0.63	0.35	0.75	0.38	2.13	0.97	0.60	4.64	1.07	0.47	2.22	0.19	0.06	1927.07	1822.86	1887.54	1115.58	1178.80	1069.63	1193.44	1078.68	1176.97	1378.62	1319.37	800.25		x
2526	Mediator of RNA polymerase II transcription subunit 23	Q9ULK4	57037.80	55076.25	53863.66	20199.41	20066.66	19187.46	36008.06	35248.26	34577.16	34941.04	34458.77	32572.53	28327.26	28557.88	23443.74	35472.56	33155.87	34698.91	39980.37	39915.80	40465.34	2.27	1.49	0.65	0.84	1.31	0.69	4.16	2.47	0.11	4.90	4.98	0.12	x	
2527	Mediator of RNA polymerase II transcription subunit 24	O75448	114240.40	107677.94	99397.62	73170.31	70842.80	66809.43	80421.84	78666.66	78693.82	103384.76	97316.49	89424.03	93887.37	74764.83	59674.26	78669.08	77105.71	74196.25	78223.10	70731.31	67681.49	2.27	1.49	0.65	0.84	1.31	0.69	4.16	2.47	0.11	4.90	4.98	0.12	x	
2528	Medium-chain specific acyl-CoA dehydrogenase_ mitochondrial	P11310	0.14	2.53	0.81	1.07	2.77	0.68	0.96	0.36	0.63	0.35	0.75	0.38	2.13	0.97	0.60	4.64	1.07	0.47	2.22	0.19	0.06	3107.28	3211.05	3067.50	2181.20	2111.15	1752.12	2673.97	2592.46	2636.47	2969.32	3022.83	1759.35		x
2529	Megakaryocyte-associated tyrosine-protein kinase	P42679	0.14	2.53	0.81	1.07	2.77	0.68	0.96	0.36	0.63	0.35	0.75	0.38	2.13	0.97	0.60	4.64	1.07	0.47	2.22	0.19	0.06	2530.39	2408.01	2249.23	2011.27	1983.41	1849.53	1597.51	1788.11	1812.65	2487.63	2196.73	495.17		x
2530	Meiosis-specific coiled-coil domain-containing protein MEIOC	A2RUB1	236.24	145.36	98.52	3.28	39.45	77.47	76.47	66.88	32.17	455.88	528.96	525.92	308.61	55.21	39.80	41.36	180.78	203.88	148.66	206.82	166.91	2.27	1.49	0.65	0.84	1.31	0.69	4.16	2.47	0.11	4.90	4.98	0.12	x	
2531	Meiosis-specific nuclear structural protein 1	Q8NEH6	34288.62	31674.80	33217.09	26730.24	23686.15	25021.37	25552.27	26580.24	26149.51	35659.49	33348.00	33416.07	29963.16	28809.20	26258.59	26839.93	27491.33	27328.71	27310.58	24058.76	22827.78	1317.66	1254.76	1120.80	1354.52	1095.33	698.88	1417.03	1243.17	1272.21	1278.70	1339.36	819.83		
2532	Melanoma inhibitory activity protein 2	Q96PC5	7367.85	7139.19	5924.16	5262.61	5240.82	4300.17	5757.50	5548.98	5221.63	7166.95	7021.58	6491.69	6215.13	5645.78	3583.52	5271.31	4627.17	4672.05	5715.70	5424.40	4949.99	2.27	1.49	0.65	0.84	1.31	0.69	4.16	2.47	0.11	4.90	4.98	0.12	x	
2533	Melanoma-associated antigen B18	Q96M61	1145.10	961.59	959.60	1017.21	957.79	888.33	990.35	915.27	1075.29	1130.63	1260.26	1269.62	1359.08	1142.25	915.64	884.31	793.35	823.74	742.02	683.88	763.20	19191.22	17294.92	15121.35	11787.35	10998.58	9798.78	15908.75	14175.87	13287.16	15918.55	14242.50	9533.60		
2534	Melanoma-associated antigen D1	Q9Y5V3	9219.43	8669.59	9060.43	6763.93	6118.50	5929.74	7342.67	6979.36	6708.52	12817.44	12173.99	10891.12	8313.12	8486.28	7920.71	6422.85	6081.34	5740.89	6205.63	5717.79	5733.99	6623.98	6042.16	5773.53	4271.93	4218.00	3692.91	4309.80	4187.40	4167.79	4922.77	4941.51	3557.57		
2535	Melanoma-associated antigen D2	Q9UNF1	61690.58	60688.37	53595.25	41230.85	41153.89	36816.03	46360.46	44303.04	42793.43	58991.63	56779.52	52579.77	53683.78	49312.89	39137.16	42647.38	41787.23	38544.10	42458.84	40587.68	37910.88	27363.54	26065.01	23403.37	13902.58	12653.92	10132.40	17181.46	16421.32	15206.33	17725.87	16281.92	9739.11		
2536	Melanophilin	Q9BV36	21731.64	22089.77	24974.79	18692.06	17559.12	15560.80	17973.28	19071.58	19665.61	19736.17	18464.46	19099.97	22290.31	18716.61	17768.44	17130.41	16912.49	16186.81	17601.24	15183.20	16108.48	2.27	1.49	0.65	0.84	1.31	0.69	4.16	2.47	0.11	4.90	4.98	0.12	x	
2537	Melanoregulin	Q8N565	4100.17	4161.00	3718.39	2783.73	2547.76	1779.69	3063.12	2493.53	2569.45	3187.48	3008.34	2955.55	2791.04	3004.28	1977.16	2540.06	2487.69	2370.42	2192.53	1963.62	2227.70	2.27	1.49	0.65	0.84	1.31	0.69	4.16	2.47	0.11	4.90	4.98	0.12	x	
2538	Membrane magnesium transporter 1	Q8N4V1	23874.43	21731.19	21152.39	12269.75	10805.59	10232.00	14644.83	14723.85	13763.76	15123.04	16612.75	14144.97	14939.96	13769.70	11290.85	15429.29	13669.27	13417.08	13164.12	12155.22	11633.12	6789.79	6301.96	5422.33	3118.61	3162.35	2688.10	5638.11	5231.30	5156.41	4796.41	4729.90	3098.20		
2539	Membrane-associated progesterone receptor component 1	O00264	7100.48	7117.96	6888.64	7871.59	7288.73	7412.27	5414.57	5429.09	5024.97	10941.56	10959.90	10505.58	6762.60	7070.88	5759.71	6526.40	6232.23	5864.38	6939.52	6393.57	6321.99	2.27	1.49	0.65	0.84	1.31	0.69	4.16	2.47	0.11	4.90	4.98	0.12	x	
2540	Membrane-associated progesterone receptor component 2	O15173	132501.76	119898.21	113004.35	81415.44	77396.67	69074.58	92741.19	94293.47	90279.82	118132.19	111464.46	99653.52	114064.91	94788.65	78164.62	79703.93	78442.19	72468.56	76136.34	71081.39	66648.54	46403.72	29924.36	26363.25	31135.13	30683.42	23442.13	38037.67	34908.36	31423.90	51597.41	47118.37	28533.11		
2541	Merlin	P35240	1643.35	1225.92	1221.51	830.79	881.66	678.92	674.99	605.40	251.92	1263.51	1296.21	1260.33	1346.49	1046.14	249.74	421.43	574.63	622.98	1008.84	885.93	856.73	18199.33	16457.60	15886.30	17422.61	16433.38	14965.86	17879.76	17850.96	16584.21	20402.61	19872.16	11328.63		
2542	Mesencephalic astrocyte-derived neurotrophic factor	P55145	109882.84	106318.50	102744.69	75288.68	72312.24	69661.67	84729.45	83892.81	80700.25	92192.26	89006.82	84974.54	91871.53	89773.36	71597.83	73531.26	70151.42	65610.84	69339.37	69017.70	65516.28	32019.43	30183.73	27299.09	20111.14	20744.03	18018.55	26197.68	24771.06	23713.25	26971.24	25924.88	18390.24		
2543	Metabotropic glutamate receptor 1	Q13255	0.14	2.53	0.81	1.07	2.77	0.68	0.96	0.36	0.63	0.35	0.75	0.38	2.13	0.97	0.60	4.64	1.07	0.47	2.22	0.19	0.06	381.79	297.54	288.29	257.93	211.01	204.13	231.93	256.81	279.01	301.57	274.37	178.94		x
2544	Metallothionein-1E	P04732	0.14	2.53	0.81	1.07	2.77	0.68	0.96	0.36	0.63	0.35	0.75	0.38	2.13	0.97	0.60	4.64	1.07	0.47	2.22	0.19	0.06	20039.11	18387.25	17675.26	10029.80	10457.14	9803.19	12515.86	13542.01	12527.41	12540.99	13172.69	8075.14		x
2545	Metallothionein-1F	P04733	0.14	2.53	0.81	1.07	2.77	0.68	0.96	0.36	0.63	0.35	0.75	0.38	2.13	0.97	0.60	4.64	1.07	0.47	2.22	0.19	0.06	14856.08	12602.68	11131.52	9818.52	8427.06	8303.23	9455.52	8011.81	8838.29	9466.25	8128.51	2909.55		x
2546	Metallothionein-1M	Q8N339	51988.60	52651.62	49853.64	59549.40	54021.93	52048.46	46911.57	43038.73	42378.96	42979.75	46019.48	42536.23	62296.63	54473.48	276.00	62969.64	56901.74	56202.14	33244.13	33079.01	32411.10	2.27	1.49	0.65	0.84	1.31	0.69	4.16	2.47	0.11	4.90	4.98	0.12	x	
2547	Metallothionein-1X	P80297	0.14	2.53	0.81	1.07	2.77	0.68	0.96	0.36	0.63	0.35	0.75	0.38	2.13	0.97	0.60	4.64	1.07	0.47	2.22	0.19	0.06	301.31	343.52	286.63	60.17	83.82	103.49	147.40	227.81	134.68	92.84	114.96	52.95		x
2548	Metallothionein-2	P02795	0.14	2.53	0.81	1.07	2.77	0.68	0.96	0.36	0.63	0.35	0.75	0.38	2.13	0.97	0.60	4.64	1.07	0.47	2.22	0.19	0.06	87695.39	77604.36	80773.97	57460.89	57767.09	51404.97	52020.52	56080.83	57408.99	60155.28	61916.97	37178.74		x
2549	Metal-response element-binding transcription factor 2	Q9Y483	8584.53	7990.75	7347.75	5168.30	4900.25	4824.79	7024.58	6436.78	6584.86	7287.70	6826.37	6869.93	6835.83	6914.11	5223.18	5222.76	4971.29	5176.85	5402.87	5458.53	5265.88	5468.53	5450.77	5090.42	3621.37	3081.51	2608.13	3448.69	3312.50	3101.42	3395.15	3089.72	1775.26		
2550	Metastasis-associated protein MTA1	Q13330	228.49	260.55	156.50	176.59	172.31	59.61	63.43	76.35	23.23	120.96	167.95	78.67	137.04	85.54	25.27	73.51	51.64	62.35	177.35	145.32	64.70	507.46	155.82	146.05	312.24	252.26	91.16	676.87	537.11	405.38	594.30	571.26	215.73		
2551	Metastasis-associated protein MTA2	O94776	311894.97	307256.44	272579.33	211279.90	205213.89	191100.82	261304.00	242198.30	232445.16	297566.10	281621.52	274557.66	288143.47	267729.43	211185.86	203690.79	194932.66	190481.84	209432.49	208110.98	196271.50	21969.70	20475.22	19036.41	14808.83	14816.55	12724.04	17200.70	17117.35	16280.18	18499.96	18209.05	12926.46		
2552	Metastasis-associated protein MTA3	Q9BTC8	1998.76	1747.66	1783.36	860.92	934.24	713.57	751.24	762.43	775.26	1317.96	1377.13	1305.75	1812.39	1729.82	1703.73	598.74	625.31	469.57	767.46	760.36	739.21	2.27	1.49	0.65	0.84	1.31	0.69	4.16	2.47	0.11	4.90	4.98	0.12	x	
2553	Metaxin-1	Q13505	53831.53	55776.93	55208.03	41201.33	38513.26	37415.39	50684.11	49491.42	47788.28	51917.51	48797.61	46746.30	43210.89	46360.06	40804.55	39568.50	38205.04	37137.49	35811.53	35263.77	34008.41	2.27	1.49	0.65	0.84	1.31	0.69	4.16	2.47	0.11	4.90	4.98	0.12	x	
2554	Metaxin-2	O75431	0.14	2.53	0.81	1.07	2.77	0.68	0.96	0.36	0.63	0.35	0.75	0.38	2.13	0.97	0.60	4.64	1.07	0.47	2.22	0.19	0.06	2530.97	2744.90	3051.99	2287.95	2259.05	2191.95	2661.69	2182.38	2320.24	3013.44	2954.78	2160.87		x
2555	Methanethiol oxidase	Q13228	8089.97	7727.11	7582.97	5858.28	5791.01	5200.50	7540.31	7492.92	7151.13	7031.14	6752.07	6186.85	8195.60	7414.79	4720.37	6461.77	5962.93	5831.66	5477.96	5424.08	4562.61	2.27	1.49	0.65	0.84	1.31	0.69	4.16	2.47	0.11	4.90	4.98	0.12	x	
2556	Methionine adenosyltransferase 2 subunit beta	Q9NZL9	81185.16	81724.91	70288.29	59872.11	58210.59	54092.67	70024.08	68800.42	64138.41	72249.40	69348.39	65666.74	64274.13	61983.52	51405.45	59658.84	53357.20	53820.43	58972.18	53870.19	51734.17	37314.08	33661.63	30835.58	22764.63	21490.62	18655.74	30649.14	27623.04	23867.26	31401.95	29828.09	20486.03		
2557	Methionine aminopeptidase 1	P53582	2878.77	2882.00	2888.90	2155.09	2298.13	1860.70	2328.10	2020.35	2375.35	2213.55	2193.48	2345.39	2349.70	2282.53	1894.54	2040.92	1752.69	1694.91	1901.52	1847.64	1699.30	12777.56	11217.42	8598.66	8187.01	7647.45	6799.47	10292.85	10279.98	10354.28	8546.57	8741.99	4746.79		
2558	Methionine aminopeptidase 2	P50579	1457.21	1174.03	1194.59	1142.52	1189.55	1105.14	1187.22	1077.07	1040.99	1283.78	1535.15	1319.38	1538.66	1205.86	1228.90	1134.36	958.51	1061.91	1248.23	1176.49	1023.46	2.27	1.49	0.65	0.84	1.31	0.69	4.16	2.47	0.11	188.38	105.18	26.12		
2559	Methionine synthase reductase	Q9UBK8	15904.60	14130.07	13778.43	9607.96	9480.12	8250.64	10041.21	9319.93	10109.66	14317.62	13657.55	12924.80	12861.67	12962.60	11787.78	9204.89	9133.33	10766.27	11567.34	8964.49	9488.40	7169.43	8399.81	6420.69	6561.48	6278.27	5582.04	6323.14	6562.72	6612.12	9208.11	8295.91	3765.00		
2560	Methionine--tRNA ligase_ cytoplasmic	P56192	323825.91	327088.30	285824.65	258934.85	253464.72	233802.22	302970.56	279738.08	267334.59	326749.27	306790.29	286196.34	267222.82	256644.25	203266.74	264954.45	256642.59	252237.76	268159.86	269683.50	249821.71	138226.85	128655.16	116219.81	90846.68	85073.80	63191.75	101870.57	97166.55	86858.26	104029.74	98702.80	57686.91		
2561	Methionyl-tRNA formyltransferase_ mitochondrial	Q96DP5	0.14	2.53	0.81	1.07	2.77	0.68	0.96	0.36	0.63	0.35	0.75	0.38	2.13	0.97	0.60	4.64	1.07	0.47	2.22	0.19	0.06	19887.05	18737.67	16397.88	18714.85	19354.51	12042.01	18428.20	17777.15	15449.70	20577.35	18839.53	9726.43		x
2562	Methylated-DNA--protein-cysteine methyltransferase	P16455	31927.91	34681.02	33168.45	22561.15	21090.24	22461.36	24414.51	23954.70	24292.36	25907.22	23931.84	22138.12	26589.72	26556.21	20763.07	20227.56	19051.29	19904.38	20782.09	19798.15	19126.74	2.27	1.49	0.65	0.84	1.31	0.69	4.16	2.47	0.11	4.90	4.98	0.12	x	
2563	Methyl-CpG-binding domain protein 4	O95243	0.14	2.53	0.81	1.07	2.77	0.68	0.96	0.36	0.63	0.35	0.75	0.38	2.13	0.97	0.60	4.64	1.07	0.47	2.22	0.19	0.06	129.32	71.94	89.45	27.27	38.06	28.75	114.09	102.62	88.07	100.10	80.93	32.26		x
2564	Methyl-CpG-binding domain protein 5	Q9P267	6954.75	7090.82	6363.89	4902.47	4390.25	4297.08	5282.22	5115.83	4978.79	5070.69	5165.00	4700.89	5168.66	5353.64	4062.62	4755.48	4821.53	4181.16	5677.41	5065.74	4616.79	2.27	1.49	0.65	0.84	1.31	0.69	4.16	2.47	0.11	4.90	4.98	0.12	x	
2565	Methylcrotonoyl-CoA carboxylase beta chain_ mitochondrial	Q9HCC0	307413.00	299511.76	288135.96	193809.82	188128.62	174328.74	210567.33	199755.95	193643.50	268604.68	251328.85	243279.36	244585.60	228317.95	196130.11	193764.93	185023.45	175894.18	195123.71	186415.47	176460.69	55385.15	53220.04	50502.04	41429.70	40020.94	36873.42	51283.61	49000.09	46933.92	49468.60	48501.79	35004.08		
2566	Methylcrotonoyl-CoA carboxylase subunit alpha_ mitochondrial	Q96RQ3	50550.92	47198.51	44082.27	37804.94	36413.70	33809.41	39592.14	40231.90	37080.40	47730.28	42096.26	40703.88	42540.04	42205.16	33671.84	34192.64	32045.41	30103.73	32551.52	32886.92	30557.58	160835.28	146241.15	136975.39	128268.69	122181.14	106305.70	145130.84	131260.49	129319.41	141533.97	133839.03	97470.23		
2567	Methylcytosine dioxygenase TET2	Q6N021	36260.09	33542.41	32788.24	25791.92	24844.30	23906.11	25377.42	23742.36	21859.11	32403.86	30203.87	27591.16	24997.42	23378.58	20122.83	27688.17	24840.47	25303.93	23333.79	22304.64	21599.41	186.03	310.40	350.45	217.66	245.04	215.24	749.39	845.24	799.03	237.15	287.02	162.15		
2568	Methylmalonate-semialdehyde dehydrogenase [acylating]_ mitochondrial	Q02252	42880.80	40273.47	38075.13	26657.16	26404.10	25017.95	29736.12	29965.53	28750.53	33979.50	31904.31	30755.77	34912.87	32800.75	30414.15	27427.82	26066.71	24987.52	23424.34	22710.50	24555.48	2.27	1.49	0.65	0.84	1.31	0.69	4.16	2.47	0.11	4.90	4.98	0.12	x	
2569	Methylmalonyl-CoA mutase_ mitochondrial	P22033	36093.15	35110.82	30808.91	24301.27	22919.17	21257.76	28218.13	27577.25	27190.25	34439.92	33891.19	31637.24	32446.96	28922.33	24082.47	26721.45	23899.05	23688.34	25165.21	23513.18	23050.41	2.27	1.49	0.65	0.84	1.31	0.69	4.16	2.47	0.11	4.90	4.98	0.12	x	
2570	Methylosome protein 50	Q9BQA1	1529.07	1020.95	1025.47	1133.72	1064.07	660.16	1145.23	1252.18	1041.52	1309.13	1259.74	1168.30	1646.28	1273.36	328.94	956.09	697.88	587.51	980.41	766.92	635.31	19646.04	16804.65	14258.94	10589.84	10317.58	7793.03	14246.44	12077.98	13486.50	13599.95	13241.36	8558.52		
2571	Methylosome subunit pICln	P54105	2659.09	2981.58	2511.53	1676.21	1856.27	1656.41	2386.32	2202.06	2020.67	2231.15	2187.58	1886.12	2100.71	1860.64	1484.53	2017.47	1670.43	1659.45	1685.70	1684.66	1263.48	31682.48	31207.02	27721.68	22827.26	21985.04	20029.07	31389.05	30114.81	29929.46	28581.11	27672.16	20851.30		
2572	Methylsterol monooxygenase 1	Q15800	6954.24	6700.24	6522.77	7276.89	7187.94	6883.87	6589.10	6485.88	6176.81	8675.06	8077.19	7927.65	7300.48	6650.93	5578.18	6464.52	6477.89	6683.46	6491.28	6295.39	6084.12	2.27	1.49	0.65	0.84	1.31	0.69	4.16	2.47	0.11	4.90	4.98	0.12	x	
2573	Methylthioribose-1-phosphate isomerase	Q9BV20	271731.61	287296.42	234688.36	171146.49	160395.43	144850.64	214342.49	206939.09	190133.34	217229.42	206031.87	189629.63	199576.99	186284.93	152577.71	188851.73	174121.60	161352.46	174805.03	176990.69	153501.02	2.27	1.49	0.65	0.84	1.31	0.69	4.16	2.47	0.11	4.90	4.98	0.12	x	
2574	Methylthioribulose-1-phosphate dehydratase	Q96GX9	16270.51	15891.64	14406.34	12545.45	12565.01	10709.94	13646.32	14020.15	13826.58	15827.44	15059.01	13500.70	16589.72	16159.32	10456.44	11049.80	11013.26	10521.86	13684.22	13536.30	14362.92	19227.35	18679.81	18236.81	14974.80	14659.06	11394.61	17624.77	16252.06	15856.50	19375.44	18896.01	12055.13		
2575	Methyltransferase-like protein 2A	Q96IZ6	0.14	2.53	0.81	1.07	2.77	0.68	0.96	0.36	0.63	0.35	0.75	0.38	2.13	0.97	0.60	4.64	1.07	0.47	2.22	0.19	0.06	25971.32	24300.30	24377.60	21145.00	22382.51	20153.21	30144.74	27387.20	26859.49	9320.80	10132.18	7171.75		x
2576	Methyltransferase-like protein 2B	Q6P1Q9	5410.26	5439.91	4663.18	4533.48	3895.24	3459.21	4230.32	3886.61	3968.35	5347.31	4910.24	4697.20	4175.73	4006.22	3003.36	3547.17	3248.59	2906.39	3990.23	3890.67	3609.31	1142.96	1032.53	874.83	864.08	833.48	795.71	914.91	849.08	825.01	1032.98	945.82	650.74		
2577	MHC class I polypeptide-related sequence B	Q29980	0.14	2.53	0.81	1.07	2.77	0.68	0.96	0.36	0.63	0.35	0.75	0.38	2.13	0.97	0.60	4.64	1.07	0.47	2.22	0.19	0.06	18754.28	15756.27	15734.83	11958.50	12410.92	9877.73	14757.54	14780.59	12983.32	14271.11	15229.36	12995.50		x
2578	MICOS complex subunit MIC19	Q9NX63	77870.05	79188.24	71632.56	51939.13	51365.95	47610.06	59876.66	58774.40	57609.80	68602.29	64164.49	60523.89	57562.25	55544.23	47840.30	56242.99	52180.95	53304.37	54170.09	51508.20	51666.79	123930.15	117017.74	112842.99	85340.66	84633.39	77753.15	96596.62	93237.23	87060.50	97976.22	94311.71	70159.93		
2579	MICOS complex subunit MIC60	Q16891	297092.28	286449.54	281903.83	210800.14	203319.13	201890.15	248556.23	251148.82	241813.49	256053.98	244033.24	230502.71	227552.24	234953.87	209030.37	218379.75	210505.14	206949.10	229266.52	208562.56	203653.36	173978.89	158804.72	150130.19	126452.04	123076.91	113868.86	141944.74	138681.24	130822.49	144288.78	138970.44	91905.00		
2580	Microfibrillar-associated protein 1	P55081	1304.86	1281.11	1288.60	1818.77	1698.81	1665.63	1379.64	1563.50	1429.18	2785.66	2372.18	2412.35	1620.94	1474.97	1231.12	1801.45	1662.50	1480.69	1426.36	1400.63	1396.51	2.27	1.49	0.65	0.84	1.31	0.69	4.16	2.47	0.11	4.90	4.98	0.12	x	
2581	Microfibrillar-associated protein 3-like	O75121	18041.39	17850.02	17002.96	13345.96	13312.54	12131.18	12269.24	10658.07	11088.77	15059.84	13092.03	12517.25	18497.41	17081.45	14296.83	12944.05	11964.63	11316.77	12954.17	12281.35	11922.43	2.27	1.49	0.65	0.84	1.31	0.69	4.16	2.47	0.11	4.90	4.98	0.12	x	
2582	Microprocessor complex subunit DGCR8	Q8WYQ5	14037.30	12763.39	10925.97	10674.79	9684.55	8250.92	7309.99	7059.56	6585.75	11352.35	10947.45	9848.19	10452.48	9451.24	5890.83	8505.62	8298.42	7542.89	11098.53	9922.26	8641.58	2.27	1.49	0.65	0.84	1.31	0.69	4.16	2.47	0.11	4.90	4.98	0.12	x	
2583	Microsomal glutathione S-transferase 3	O14880	1602.88	1898.44	1519.91	747.57	711.37	691.74	1414.39	1071.63	1088.46	1598.63	1348.34	1347.08	706.64	652.65	526.65	1557.05	1482.40	1379.87	1046.79	1080.76	893.87	4960.50	4352.57	3933.09	3913.93	3652.56	2736.81	4300.07	4272.29	3971.46	4253.59	4152.04	2305.45		
2584	Microtubule cross-linking factor 1	Q9Y4B5	0.14	2.53	0.81	1.07	2.77	0.68	0.96	0.36	0.63	0.35	0.75	0.38	2.13	0.97	0.60	4.64	1.07	0.47	2.22	0.19	0.06	2755.39	2906.83	2195.81	2524.64	2250.84	2459.33	2461.75	1962.28	2091.58	3256.69	2581.49	1519.79		x
2585	Microtubule-actin cross-linking factor 1_ isoforms 1/2/3/5	Q9UPN3	892200.67	874997.48	834858.44	560165.89	559492.43	518021.65	589460.78	568374.36	537390.38	705034.59	678523.98	643642.01	643331.84	608733.08	529619.91	607928.63	578574.41	544422.76	591381.13	569181.99	527455.54	478734.23	441733.91	418710.56	308148.28	310206.41	268438.77	402980.65	393179.25	372090.59	415245.82	402269.08	279593.82		
2586	Microtubule-associated protein 1A	P78559	152.69	180.35	215.64	92.41	117.26	72.44	100.20	96.38	80.32	63.59	74.61	107.44	83.38	125.31	59.43	90.24	106.03	71.01	146.80	140.02	146.47	8237.16	7903.45	7831.25	5331.15	5232.41	4249.82	6168.36	5667.83	5295.28	6110.35	5928.50	2992.65		
2587	Microtubule-associated protein 1B	P46821	2051.35	1851.62	1502.39	612.14	672.29	680.26	845.20	825.95	664.11	1894.37	1699.85	1820.90	1592.42	1339.42	1133.60	633.49	542.62	635.39	901.54	839.31	771.91	256183.72	211857.13	200262.14	157584.23	154233.74	127537.72	188021.47	181875.19	169760.01	192281.82	184415.42	119845.91		
2588	Microtubule-associated protein 2	P11137	0.14	2.53	0.81	1.07	2.77	0.68	0.96	0.36	0.63	0.35	0.75	0.38	2.13	0.97	0.60	4.64	1.07	0.47	2.22	0.19	0.06	3564.50	4952.71	3338.73	3091.36	2632.95	2263.58	5618.08	5467.44	5255.15	3553.11	3510.17	1951.63		x
2589	Microtubule-associated protein 4	P27816	283472.62	264719.61	264818.40	183301.69	178862.95	161571.94	222259.92	205372.43	201578.24	252679.59	238366.97	224730.17	220747.77	219219.59	162220.57	178816.65	171206.69	164158.73	187215.68	187455.95	173124.04	571033.13	536905.22	504814.76	355478.33	346614.80	302396.38	419219.53	397298.83	377086.47	443531.55	429366.32	294305.84		
2590	Microtubule-associated protein 9	Q49MG5	0.14	2.53	0.81	1.07	2.77	0.68	0.96	0.36	0.63	0.35	0.75	0.38	2.13	0.97	0.60	4.64	1.07	0.47	2.22	0.19	0.06	19080.51	18255.09	20590.82	14589.62	14053.05	14113.62	14070.58	14137.04	15217.10	15597.44	13575.93	13578.15		x
2591	Microtubule-associated protein RP/EB family member 1	Q15691	286015.36	291869.44	276010.30	196883.15	190783.46	175083.36	221024.61	212240.46	202873.20	247353.67	234629.07	219217.09	232258.33	218996.38	189483.61	187358.95	183292.82	168811.86	198939.68	185780.45	179365.46	252445.45	240061.46	222973.86	156345.30	149744.76	132016.32	196351.41	191855.34	183318.63	196922.82	188974.92	133397.06		
2592	Microtubule-associated protein RP/EB family member 3	Q9UPY8	68279.02	62064.83	49660.35	58341.51	54853.29	46042.82	62439.46	61439.29	54245.44	66214.65	64790.15	55541.32	62384.16	53314.90	29770.83	53550.69	49556.55	46677.08	62646.17	56426.90	46331.15	2.27	1.49	0.65	0.84	1.31	0.69	4.16	2.47	0.11	4.90	4.98	0.12	x	
2593	Microtubule-associated protein tau	P10636	3230.44	3325.32	3006.84	2875.64	2789.10	2678.62	2577.60	2321.68	2457.18	4259.00	3818.03	3557.35	3330.74	3165.34	3448.19	2244.93	2202.19	2422.18	2308.65	2766.08	2664.15	141.20	111.04	105.28	100.39	119.80	88.73	786.23	832.19	771.61	329.88	338.14	164.90		
2594	Microtubule-associated proteins 1A/1B light chain 3A	Q9H492	6550.31	6257.46	5922.97	4760.03	5332.40	4846.10	5194.53	4868.74	5165.97	6849.71	6708.81	6547.42	6475.81	5572.14	5612.64	4528.79	3928.20	4040.80	4822.61	5105.79	5400.82	18651.40	18309.68	16370.81	13268.86	14035.65	13174.63	14665.65	14191.45	13186.40	14085.60	13762.99	11471.33		
2595	Microtubule-associated serine/threonine-protein kinase 4	O15021	161381.11	147901.15	134671.99	103978.94	99975.25	89050.20	112270.04	109063.58	100656.22	132742.97	127312.01	117180.32	129909.96	119486.33	86256.46	99017.42	94319.68	87411.06	102969.93	99943.85	92465.34	106541.70	100101.37	92872.16	76460.89	74569.93	66414.68	86505.11	82513.85	78578.52	98625.73	96121.14	63582.27		
2596	Microtubule-associated tumor suppressor candidate 2	Q5JR59	179716.48	173223.26	158878.55	127598.47	121468.81	111750.46	133049.16	127415.07	121445.30	158287.50	154547.70	139638.51	130808.45	132077.25	100987.06	126511.67	121907.04	123008.56	123768.93	117290.14	112329.17	13629.19	13276.43	12489.57	9714.00	9190.69	8755.15	10315.11	9888.43	9572.02	10781.35	10530.07	7686.77		
2597	Midkine	P21741	10279.92	9010.41	8136.54	7752.40	7639.55	7273.67	7036.60	7226.53	6900.18	11035.95	10697.13	10484.97	7750.61	7706.43	6211.11	7092.65	6341.37	6365.27	7466.16	7187.59	6581.97	2.27	1.49	0.65	0.84	1.31	0.69	4.16	2.47	0.11	4.90	4.98	0.12	x	
2598	MIF4G domain-containing protein	A9UHW6	7471.11	8096.66	6867.11	5432.68	5154.00	4569.69	6549.23	5580.27	5395.43	6817.82	6173.65	5120.25	6366.31	5829.65	4567.99	5557.11	5270.95	5284.33	5316.27	5014.90	4292.34	2.27	1.49	0.65	0.84	1.31	0.69	4.16	2.47	0.11	4.90	4.98	0.12	x	
2599	Mini-chromosome maintenance complex-binding protein	Q9BTE3	48052.22	47375.97	43917.26	38050.19	36068.98	33566.76	38402.12	34691.18	34321.39	43569.89	41502.45	39261.31	43917.58	37311.07	26566.76	36037.95	34180.97	33660.20	38194.02	36031.13	33892.87	30398.04	28164.05	26144.51	19938.78	19542.17	16698.26	26562.65	25394.32	25359.39	22995.36	22380.16	16274.18		
2600	Minor histocompatibility antigen H13	Q8TCT9	6123.47	6344.37	4898.32	7172.64	6772.61	5522.48	6628.66	5964.48	5675.08	10661.75	9315.80	8589.13	8132.66	7535.61	4423.64	7342.44	6845.91	6429.55	7246.31	6807.50	5304.85	72787.48	71462.49	71766.77	58649.93	59231.10	54563.97	62009.29	61372.03	59625.52	64748.59	68236.73	45795.91		
2601	Mirror-image polydactyly gene 1 protein	Q8TD10	11135.18	11098.08	9446.33	6970.01	6479.65	5675.50	8907.39	8285.87	7952.73	9006.55	9053.46	8045.11	8688.58	7748.62	4445.48	7805.83	7628.43	7104.78	7778.82	7678.37	6644.19	97909.16	96302.43	92067.69	60952.14	60692.21	57182.80	65009.74	65976.94	65492.83	66724.28	70719.71	45835.34		
2602	Mis18-binding protein 1	Q6P0N0	0.14	2.53	0.81	1.07	2.77	0.68	0.96	0.36	0.63	0.35	0.75	0.38	2.13	0.97	0.60	4.64	1.07	0.47	2.22	0.19	0.06	17242.28	16713.26	15463.53	16909.48	15401.99	14288.69	25316.19	25203.39	24757.32	17264.05	16456.14	10863.56		x
2603	Mismatch repair endonuclease PMS2	P54278	3040.00	3347.06	2963.90	2236.36	1951.65	2087.38	2329.16	2043.00	2084.89	3117.16	3178.81	3153.19	2576.37	2587.58	1926.25	2398.94	2198.12	2232.72	2202.99	2143.78	2251.43	2.27	1.49	0.65	0.84	1.31	0.69	4.16	2.47	0.11	4.90	4.98	0.12	x	
2604	Misshapen-like kinase 1	Q8N4C8	0.14	2.53	0.81	1.07	2.77	0.68	0.96	0.36	0.63	0.35	0.75	0.38	2.13	0.97	0.60	4.64	1.07	0.47	2.22	0.19	0.06	1438.74	1394.04	1424.88	564.43	521.96	524.52	972.57	826.93	826.58	990.88	914.51	485.09		x
2605	Mitochondrial 2-oxoglutarate/malate carrier protein	Q02978	205633.62	200853.75	189971.55	152187.86	146019.79	139348.16	145614.08	140396.87	136544.39	170378.51	168579.38	159040.90	174830.64	158212.91	143081.27	139090.16	132910.69	127970.05	160003.48	148862.86	143781.19	59410.32	61068.48	49935.53	37546.83	38667.75	34507.22	42400.46	41991.89	40586.01	46390.38	44614.47	31074.82		
2606	Mitochondrial antiviral-signaling protein	Q7Z434	6225.06	5739.29	5503.19	2071.72	2008.42	1945.53	3645.47	3281.94	3586.93	4395.19	3786.09	3996.44	3587.70	3754.15	3331.07	3507.05	3163.90	3066.21	3557.24	3264.85	3111.24	831.96	837.53	780.93	485.49	439.86	335.31	713.38	730.77	658.56	888.13	747.76	473.98		
2607	Mitochondrial cardiolipin hydrolase	Q8N2A8	6143.52	4956.65	4334.53	3177.79	3143.95	2485.00	5849.34	5725.56	5145.65	4391.35	4384.49	3651.80	4489.26	4148.82	2071.02	4083.42	4227.94	3662.38	3003.88	3013.34	2546.87	2.27	1.49	0.65	0.84	1.31	0.69	4.16	2.47	0.11	4.90	4.98	0.12	x	
2608	Mitochondrial carrier homolog 2	Q9Y6C9	187057.36	192112.54	177489.10	149853.90	144551.53	140182.73	153376.49	151836.10	150526.06	181176.71	174690.84	166596.90	162752.64	151266.95	133022.82	151777.77	149981.75	141747.09	156826.03	147101.84	138964.03	85448.79	81584.39	76744.01	61609.64	60303.73	56888.78	76321.74	74166.09	74062.75	73652.04	72469.09	55296.30		
2609	Mitochondrial chaperone BCS1	Q9Y276	0.14	2.53	0.81	1.07	2.77	0.68	0.96	0.36	0.63	0.35	0.75	0.38	2.13	0.97	0.60	4.64	1.07	0.47	2.22	0.19	0.06	18807.51	15886.35	15176.75	10643.93	10514.67	6779.73	15997.84	15257.99	12043.08	13122.77	13118.68	6896.56		x
2610	Mitochondrial dicarboxylate carrier	Q9UBX3	89318.95	89932.72	85663.78	65282.53	62504.09	59313.55	66170.31	66010.17	64795.66	76767.43	72183.16	69586.07	70883.13	70030.51	60491.32	61185.69	55889.63	55627.19	63189.34	60133.80	60069.71	4350.51	4031.35	3285.42	2713.04	2808.01	2635.37	3988.10	3618.23	3353.09	3380.68	3103.42	2325.98		
2611	Mitochondrial fission 1 protein	Q9Y3D6	57235.02	54860.18	51009.57	39768.31	41207.82	40061.90	41880.82	41162.42	38883.43	58925.41	57566.08	55031.23	50696.87	44892.89	42909.14	39670.84	37119.32	36773.67	40725.99	37776.95	36413.94	49452.91	45078.96	37715.37	29051.39	26893.70	23088.97	31882.17	29909.78	29027.47	32998.11	34652.90	22546.02		

Supplementary Table S1. Overview on the abundance of all relatively quantified 5180 proteins

			MCF-7																					MDA-MB-231													
	Protein name	UniProt	control 01	control 02	control 03	Dai IC20 01	Dai IC20 02	Dai IC20 03	Dai SC20 01	Dai SC20 02	Dai SC20 03	Gen IC20 01	Gen IC20 02	Gen IC20 03	Gen SC20 01	Gen SC20 02	Gen SC20 03	SSE IC20 01	SSE IC20 02	SSE IC20 03	SSE SC20 01	SSE SC20 02	SSE SC20 03	control 01	control 02	control 03	Dai IC20 01	Dai IC20 02	Dai IC20 03	Gen IC20 01	Gen IC20 02	Gen IC20 03	SSE IC20 01	SSE IC20 02	SSE IC20 03	unique to MCF-7	unique to MDA-MB-231
2612	Mitochondrial fission factor	Q9GZY8	26648.78	21389.42	19648.94	15148.05	14870.99	13597.57	17337.65	16560.58	15702.28	20803.28	21237.77	19257.96	16789.11	14778.32	9102.02	16725.69	14495.29	14559.29	15305.82	14758.76	13039.86	1942.55	1727.44	1105.63	858.55	1013.92	436.86	1668.25	1400.45	1242.47	2061.00	1943.52	800.88		
2613	Mitochondrial genome maintenance exonuclease 1	Q9BQP7	3402.22	3144.44	3151.99	2847.22	2666.74	2577.43	2634.19	2668.31	2599.07	2666.01	2751.64	2778.05	3069.71	3081.78	2418.28	2272.07	2000.93	2183.87	2729.80	2478.70	2448.19	3799.29	3487.21	3459.00	2383.29	2248.99	2026.69	2177.19	2069.90	2148.50	2564.53	2337.39	1856.11		
2614	Mitochondrial glutamate carrier 1	Q9H936	21044.34	21568.08	19579.96	13541.58	12119.18	13473.74	15198.95	14613.05	14691.45	15136.80	14300.37	13663.37	15883.91	15759.40	11520.34	13110.96	13654.88	14100.58	12149.38	11514.72	10929.73	2.27	1.49	0.65	0.84	1.31	0.69	4.16	2.47	0.11	4.90	4.98	0.12	x	
2615	Mitochondrial import inner membrane translocase subunit Tim10	P62072	31255.05	31209.33	32146.56	22802.82	20310.72	19584.02	24156.28	23765.75	21045.89	25937.16	24398.92	22960.06	25286.35	24352.01	18564.42	20612.47	19595.20	17942.38	24175.68	22724.75	20313.45	2.27	1.49	0.65	0.84	1.31	0.69	4.16	2.47	0.11	4.90	4.98	0.12	x	
2616	Mitochondrial import inner membrane translocase subunit Tim13	Q9Y5L4	32115.41	32751.54	29663.22	23093.60	21204.81	18854.65	31432.50	29519.76	27962.01	26625.36	26237.63	22413.97	25881.74	25384.87	20385.87	22510.91	22575.03	21622.48	20420.43	19715.61	18531.38	38909.60	39339.21	38951.56	19802.87	19281.13	16549.38	23993.09	21811.93	22626.09	18706.57	19479.79	13704.62		
2617	Mitochondrial import inner membrane translocase subunit TIM14	Q96DA6	4266.07	4251.08	4231.49	2606.56	2568.41	2340.39	3658.10	3557.84	3186.14	3434.33	3565.59	3316.74	3246.14	3110.24	2664.96	3321.46	3295.69	3346.99	2532.92	2395.42	2348.82	2.27	1.49	0.65	0.84	1.31	0.69	4.16	2.47	0.11	4.90	4.98	0.12	x	
2618	Mitochondrial import inner membrane translocase subunit Tim23	O14925	0.14	2.53	0.81	1.07	2.77	0.68	0.96	0.36	0.63	0.35	0.75	0.38	2.13	0.97	0.60	4.64	1.07	0.47	2.22	0.19	0.06	4778.25	4702.07	4241.17	3305.53	2807.41	1798.82	3920.13	3331.83	3029.19	3966.84	3572.16	1650.95		x
2619	Mitochondrial import inner membrane translocase subunit TIM44	O43615	100558.87	102389.44	94931.86	73976.78	73443.97	64694.16	84746.23	83968.14	81233.06	83382.25	79577.26	77074.30	83059.03	82396.32	79279.19	72029.83	72865.81	66388.93	72906.86	68409.49	64931.55	120832.05	113839.73	100350.11	81991.25	78307.53	67653.26	92438.37	88179.80	88411.64	86066.74	84509.37	60209.84		
2620	Mitochondrial import inner membrane translocase subunit TIM50	Q3ZCQ8	63617.95	60621.86	52388.73	44986.26	43599.96	39214.06	52201.69	49740.77	44334.11	52619.93	50232.89	46369.07	52185.22	47925.55	31933.17	48380.36	44077.49	42834.96	42557.41	41540.69	39591.63	33267.55	31957.16	28465.96	22336.58	21429.02	16292.57	25650.44	24079.46	21027.43	27380.59	25749.96	16284.17		
2621	Mitochondrial import inner membrane translocase subunit Tim8 A	O60220	27422.50	22393.48	23083.88	18541.52	17718.18	17283.58	20636.62	18412.00	18296.64	21294.45	20331.03	20032.16	18514.58	18245.53	14556.94	16124.56	15604.63	14760.58	17277.49	16908.15	14758.39	2.27	1.49	0.65	0.84	1.31	0.69	4.16	2.47	0.11	4.90	4.98	0.12	x	
2622	Mitochondrial import inner membrane translocase subunit Tim9	Q9Y5J7	71527.37	71285.03	66661.87	50900.19	51266.45	49302.60	57221.10	55446.72	52343.80	63768.42	59905.42	57391.45	57315.23	55598.58	50650.49	51036.25	48356.69	46909.95	51147.74	50686.04	49505.98	11709.91	9212.11	9659.60	8038.37	7931.94	7025.03	10315.25	10553.12	10371.53	10080.97	9693.11	7218.43		
2623	Mitochondrial import receptor subunit TOM22 homolog	Q9NS69	99003.17	97303.89	85180.06	62679.25	61627.91	56421.25	66733.79	64202.05	59526.83	80459.52	74415.79	68190.25	74670.95	62463.16	57325.09	61389.17	59038.94	55009.10	64781.22	60069.44	57252.33	43108.16	39734.74	37127.43	31085.61	30481.73	27647.33	36292.72	36267.64	32715.39	39047.81	39157.77	25114.09		
2624	Mitochondrial import receptor subunit TOM34	Q15785	67589.18	66297.64	61449.54	58221.59	56175.38	52088.34	55990.64	53751.53	54446.34	67623.22	62235.99	58505.19	62144.26	60962.03	48488.81	52570.30	49358.99	48751.40	52697.68	54479.96	52660.89	33861.91	31555.11	30955.12	22759.93	21335.32	18935.73	22989.66	20853.05	21353.06	22530.95	22170.76	17325.83		
2625	Mitochondrial import receptor subunit TOM40 homolog	O96008	30579.52	30825.29	28598.92	17545.86	16415.53	13931.75	25488.47	26015.42	24521.23	24132.03	22684.14	19914.68	19909.44	19278.99	14551.91	20120.79	20261.26	18716.41	21751.96	20480.63	19848.00	69615.18	68968.55	62346.89	49311.50	46470.76	40605.29	53073.74	52543.90	53173.29	56107.41	54644.72	38876.20		
2626	Mitochondrial import receptor subunit TOM7 homolog	Q9P0U1	7455.84	7281.85	7128.75	4332.62	4142.42	4353.21	5011.53	4553.17	4779.03	5472.90	5119.32	5107.92	4380.29	4219.97	3481.18	4554.83	4762.45	4475.23	4199.57	4703.84	4515.03	2.27	1.49	0.65	0.84	1.31	0.69	4.16	2.47	0.11	4.90	4.98	0.12	x	
2627	Mitochondrial import receptor subunit TOM70	O94826	285682.37	278319.48	253631.68	191379.30	188396.27	186359.09	232059.83	228453.23	207966.18	248231.53	233468.63	217673.83	239420.29	208437.15	163962.54	191690.42	188826.90	184050.40	195724.29	187002.06	180749.09	74242.07	70586.04	62002.25	46066.12	46171.53	40288.82	53928.99	52132.72	48036.96	55255.07	55620.97	27264.05		
2628	Mitochondrial inner membrane protein OXA1L	Q15070	41648.59	39050.03	37355.76	29486.06	28435.81	27673.47	33392.50	32327.82	32517.43	36673.18	35039.88	34081.81	35663.21	33993.36	27160.32	27150.49	24805.20	25755.70	27852.10	25858.69	27190.17	6543.83	6838.89	7084.00	4712.83	4675.78	4172.95	5598.67	4740.47	5358.13	5387.70	4755.37	3751.30		
2629	Mitochondrial ornithine transporter 1	Q9Y619	9270.11	7531.46	7325.96	6207.09	6121.19	5804.05	5373.15	6037.34	6114.12	8297.81	7747.09	7391.68	7577.26	7170.59	6467.64	5900.51	5530.19	5422.45	6260.36	5664.69	5790.00	2.27	1.49	0.65	0.84	1.31	0.69	4.16	2.47	0.11	4.90	4.98	0.12	x	
2630	Mitochondrial proton/calcium exchanger protein	O95202	92420.11	87802.74	78274.71	59101.06	58396.27	52523.72	77681.08	72718.05	69917.91	80928.67	78390.81	71913.61	79843.21	70833.56	53330.06	66779.98	62989.20	60143.40	64438.83	62551.16	59277.80	92053.74	83462.58	74525.82	56092.10	56068.80	44497.07	65799.34	61770.03	56187.34	70275.81	67748.60	45903.84		
2631	Mitochondrial Rho GTPase 2	Q8IXI1	61241.16	58419.69	52687.79	29745.67	31427.42	29229.21	36067.19	35591.49	34052.77	42930.15	40391.08	36968.17	42118.24	41257.10	39015.39	30666.19	32467.93	30662.06	31410.97	34176.68	29165.81	2.27	1.49	0.65	0.84	1.31	0.69	4.16	2.47	0.11	4.90	4.98	0.12	x	
2632	Mitochondrial ribonuclease P catalytic subunit	O15091	9792.52	7840.45	7959.00	6033.83	5886.32	5387.39	6946.90	6407.00	5675.28	7488.05	7282.84	6487.33	8208.79	7302.02	5106.29	5631.60	5527.49	5083.91	6566.15	6057.14	5169.05	294.55	250.43	229.86	184.59	203.66	67.42	157.44	123.89	116.58	321.65	262.85	106.64		
2633	Mitochondrial tRNA-specific 2-thiouridylase 1	O75648	414.04	389.02	367.40	211.93	203.25	297.50	348.73	328.59	341.37	336.77	283.75	340.32	346.69	273.09	277.38	259.22	258.41	244.77	255.40	306.44	250.07	9900.12	8645.45	7737.37	6052.54	6459.08	5847.87	8111.70	8009.07	7290.99	7609.78	7204.26	5615.65		
2634	Mitochondrial-processing peptidase subunit alpha	Q10713	190875.47	191293.71	183027.20	170080.48	165463.65	154968.22	182312.56	177337.54	175909.83	191343.23	177889.48	160613.60	159991.35	165256.90	145158.18	155569.49	147247.33	143998.24	138914.46	133521.22	135634.93	2.27	1.49	0.65	0.84	1.31	0.69	4.16	2.47	0.11	4.90	4.98	0.12	x	
2635	Mitochondrial-processing peptidase subunit beta	O75439	66516.34	64697.76	63799.85	43963.71	41510.01	40716.81	50314.14	48081.14	46348.83	56813.12	54637.46	50161.22	51451.67	52917.26	41892.21	44611.50	42281.46	40804.36	42669.36	41114.36	40159.88	2.27	1.49	0.65	0.84	1.31	0.69	4.16	2.47	0.11	4.90	4.98	0.12	x	
2636	Mitogen-activated protein kinase 1	P28482	11303.75	9585.43	8102.10	7280.07	7025.98	5566.40	9617.88	8558.09	6949.31	9382.59	8327.25	7425.13	8134.35	7525.58	3967.96	7478.37	7022.97	5971.09	6760.78	6672.54	5909.72	71761.41	66906.32	61141.72	43406.25	44347.69	40204.60	55127.98	52688.03	48877.23	52437.49	51171.36	37580.87		
2637	Mitogen-activated protein kinase 14	Q16539	23837.82	23894.59	22585.00	15454.45	16531.97	16109.43	17579.79	16455.21	16496.65	21751.06	19900.93	19457.08	21148.27	19564.43	17700.21	14975.63	13604.15	14632.37	15065.53	14584.02	13858.57	4334.01	4004.07	3675.37	2570.59	2545.89	1931.63	2693.69	2501.92	2532.58	3024.40	2822.11	1647.59		
2638	Mitogen-activated protein kinase 3	P27361	215496.16	212202.35	193566.07	122208.55	118401.08	111590.06	163083.81	157262.10	143012.22	150554.01	145227.63	134804.11	162063.14	155708.34	123311.54	134047.44	122789.93	115445.58	134477.25	127135.03	116083.96	5399.24	4596.69	4486.05	3055.54	2845.11	2648.47	3875.46	3630.97	3592.98	4279.02	3890.30	2577.61		
2639	Mitogen-activated protein kinase 9	P45984	1016.63	956.89	1068.89	618.17	580.26	693.74	751.76	669.14	626.20	764.90	597.40	752.99	694.88	626.06	748.49	619.31	564.40	495.30	660.91	561.60	617.17	2.27	1.49	0.65	0.84	1.31	0.69	4.16	2.47	0.11	4.90	4.98	0.12	x	
2640	Mitogen-activated protein kinase kinase kinase 1	Q13233	37277.83	31381.09	24032.21	20755.21	18986.46	16167.24	22416.27	21959.78	20600.77	34519.89	32478.17	28119.10	31900.27	24655.12	14229.08	18511.24	18041.45	15084.10	22407.31	19696.57	16092.10	2.27	1.49	0.65	0.84	1.31	0.69	4.16	2.47	0.11	4.90	4.98	0.12	x	
2641	Mitogen-activated protein kinase kinase kinase 12	Q12852	16194.23	14696.58	12633.86	10248.19	9417.94	8330.66	19002.77	18464.19	17627.60	13606.33	13205.38	12316.23	13858.05	12560.98	8834.29	10449.64	9190.79	9295.36	9816.13	9444.33	8774.55	46297.48	41836.41	38490.04	24828.84	22551.17	21950.83	29552.17	26447.33	26716.76	26482.54	25278.81	17976.11		
2642	Mitogen-activated protein kinase kinase kinase 13	O43283	3462.38	3353.09	2826.00	2924.52	2794.32	2092.19	6385.09	5987.57	4491.16	2698.88	2541.28	2261.97	1883.62	6617.70	1787.07	4206.95	4493.21	2714.60	5539.33	4220.19	4030.90	2.27	1.49	0.65	0.84	1.31	0.69	4.16	2.47	0.11	4.90	4.98	0.12	x	
2643	Mitogen-activated protein kinase kinase kinase 15	Q6ZN16	3285.53	2979.23	2962.39	2532.43	2377.00	2368.99	2799.28	2604.72	2638.76	2755.89	2672.33	2954.03	2914.91	2725.57	2008.41	2223.93	2307.98	2146.55	2165.12	1830.49	1614.98	2.27	1.49	0.65	0.84	1.31	0.69	4.16	2.47	0.11	4.90	4.98	0.12	x	
2644	Mitogen-activated protein kinase kinase kinase 2	Q9Y2U5	4725.76	4993.42	4586.07	3208.65	3313.91	3121.06	2703.78	2857.92	2581.62	4004.05	4071.03	3670.86	3536.22	3772.67	3215.83	2800.86	3065.81	2639.98	3667.96	3468.49	3336.19	2.27	1.49	0.65	0.84	1.31	0.69	4.16	2.47	0.11	4.90	4.98	0.12	x	
2645	Mitogen-activated protein kinase kinase kinase 20	Q9NYL2	0.14	2.53	0.81	1.07	2.77	0.68	0.96	0.36	0.63	0.35	0.75	0.38	2.13	0.97	0.60	4.64	1.07	0.47	2.22	0.19	0.06	4027.23	2681.65	414.16	2621.38	2590.35	1551.27	3836.29	3329.78	2948.48	3810.79	4224.22	2077.24		x
2646	Mitogen-activated protein kinase kinase kinase 3	Q99759	188734.47	166346.31	153512.47	102547.93	99068.24	90742.76	124881.84	122979.55	109321.18	141934.52	136015.78	117029.83	129648.79	115504.16	90253.54	118577.65	113117.92	103856.18	110316.61	96418.34	87070.16	65539.65	63368.26	58293.17	45786.32	46015.15	42476.21	57744.52	56324.09	53745.54	56912.29	53183.13	40889.62		
2647	Mitogen-activated protein kinase kinase kinase 4	Q9Y6R4	6047.58	5981.31	5357.34	4128.15	4024.57	3763.34	6008.12	5537.39	4766.00	4646.06	4638.74	4441.08	5395.26	4768.38	3555.71	4352.45	4321.54	3647.77	4690.06	4504.72	4163.92	29583.76	32069.82	26387.68	20272.72	20639.36	18658.67	23861.33	23534.98	22763.07	22348.01	23500.60	17532.23		
2648	Mitogen-activated protein kinase kinase kinase 7	O43318	57721.22	56042.31	50189.97	35389.72	34363.50	28691.87	44735.79	40563.44	36848.11	49350.71	49453.00	39022.32	48130.57	43223.12	28268.00	34605.21	32431.09	28905.70	38659.91	35152.71	31022.47	2.27	1.49	0.65	0.84	1.31	0.69	4.16	2.47	0.11	4.90	4.98	0.12	x	
2649	Mitotic checkpoint protein BUB3	O43684	316173.99	296709.83	291447.81	202065.87	197537.36	188802.44	242342.63	242100.41	231397.88	247247.45	233846.55	223161.50	246341.95	243846.25	208186.13	196887.57	187793.89	180242.07	206879.00	190691.12	185515.31	172210.16	166401.47	156002.07	110090.97	105720.30	95007.39	135056.01	132506.12	124290.24	127672.62	121805.52	93729.23		
2650	Mitotic spindle assembly checkpoint protein MAD1	Q9Y6D9	27313.88	27099.00	25288.69	19591.79	19099.08	18379.50	23105.52	23367.55	24460.91	25145.70	25369.31	23666.41	26641.87	20531.55	20076.70	19189.86	18198.93	18358.33	21166.16	20925.47	20216.74	27155.83	23038.73	20618.73	12485.87	12083.98	10291.17	19798.65	19721.29	18073.67	14396.96	13303.20	11924.80		
2651	Mitotic spindle assembly checkpoint protein MAD2A	Q13257	0.14	2.53	0.81	1.07	2.77	0.68	0.96	0.36	0.63	0.35	0.75	0.38	2.13	0.97	0.60	4.64	1.07	0.47	2.22	0.19	0.06	37105.83	34896.23	33811.84	24337.05	24145.22	21652.45	27371.62	26784.04	27130.68	28939.50	28758.79	20459.96		x
2652	Mixed lineage kinase domain-like protein	Q8NB16	0.14	2.53	0.81	1.07	2.77	0.68	0.96	0.36	0.63	0.35	0.75	0.38	2.13	0.97	0.60	4.64	1.07	0.47	2.22	0.19	0.06	4875.78	4748.45	4741.96	3446.81	3370.76	3156.39	3826.37	3640.29	3761.03	3896.18	3898.35	2697.59		x
2653	MMS19 nucleotide excision repair protein homolog	Q96T76	19355.80	20541.82	18234.77	13817.14	13953.91	12877.60	15208.50	14674.96	13792.27	16861.49	15166.40	15019.74	15301.52	15951.91	13392.61	12705.65	11778.18	12663.43	12459.32	12572.42	12530.49	2.27	1.49	0.65	0.84	1.31	0.69	4.16	2.47	0.11	4.90	4.98	0.12	x	
2654	MOB kinase activator 1B	Q7L9L4	18667.49	18049.09	17896.08	13398.09	13006.74	12884.23	13486.16	13723.21	13415.75	14260.59	14965.15	13915.22	15611.09	13854.84	13757.19	10894.87	10729.76	11019.28	12512.95	12317.88	11176.75	50841.00	46546.12	44727.16	30500.71	30161.23	24417.12	37214.47	37593.29	36017.06	37731.78	35455.19	25293.84		
2655	MOB-like protein phocein	Q9Y3A3	5016.85	4300.30	4824.34	3721.11	3570.25	4267.80	4373.63	4142.68	4348.80	3418.64	3516.59	3755.07	4158.75	3527.63	3056.98	3424.93	3265.89	3124.95	3236.20	3479.89	2831.30	9729.28	8680.32	7891.26	12620.71	11803.89	9747.86	15261.20	13853.92	13193.96	15450.54	14035.57	9764.09		
2656	Moesin	P26038	61239.56	59701.95	58535.34	44262.31	43345.41	40183.52	53854.12	53795.94	53598.53	55625.39	54414.08	52115.38	54086.71	49826.08	41779.70	47503.23	44169.82	43089.80	45983.02	41857.15	38456.89	689625.89	623318.40	603565.37	494212.35	479788.04	436390.11	535857.54	506140.90	483741.12	575959.59	569554.88	413900.69		
2657	Molybdopterin synthase catalytic subunit	O96007	35072.23	32598.02	26964.27	24242.65	21898.25	20469.91	27260.91	29197.11	24252.48	29574.91	27181.18	24161.78	28542.59	26103.75	18041.68	22644.88	21188.07	20814.30	25037.73	22529.05	20534.49	2.27	1.49	0.65	0.84	1.31	0.69	4.16	2.47	0.11	4.90	4.98	0.12	x	
2658	Monoacylglycerol lipase ABHD12	Q8N2K0	12881.81	13695.48	12766.05	9275.82	9344.57	9046.20	9773.80	8345.10	8481.16	10245.03	9520.53	9327.04	10494.95	10776.39	9153.94	8314.61	7839.13	8708.14	7897.23	8198.17	8133.82	2.27	1.49	0.65	0.84	1.31	0.69	4.16	2.47	0.11	4.90	4.98	0.12	x	
2659	Monocarboxylate transporter 1	P53985	0.14	2.53	0.81	1.07	2.77	0.68	0.96	0.36	0.63	0.35	0.75	0.38	2.13	0.97	0.60	4.64	1.07	0.47	2.22	0.19	0.06	6584.49	6628.89	5765.89	7344.89	6702.84	6215.14	7669.79	7584.21	7527.97	6820.27	6619.14	5439.76		x
2660	Monocarboxylate transporter 4	O15427	0.14	2.53	0.81	1.07	2.77	0.68	0.96	0.36	0.63	0.35	0.75	0.38	2.13	0.97	0.60	4.64	1.07	0.47	2.22	0.19	0.06	46710.13	43355.85	44982.41	33117.96	31246.27	29231.83	40975.37	38627.50	37088.29	42064.25	40023.96	28385.37		x
2661	Monofunctional C1-tetrahydrofolate synthase_ mitochondrial	Q6UB35	58650.65	54105.68	48390.17	41657.62	39459.39	37339.64	49855.10	46787.00	46580.51	51110.73	47454.68	44267.77	52324.19	44667.36	38045.45	41940.10	37419.66	37174.22	44841.76	42452.92	39307.03	49350.08	53256.78	43557.64	32822.13	32146.67	26832.12	43980.99	41226.33	43978.94	37821.57	36183.39	24205.61		
2662	MORC family CW-type zinc finger protein 4	Q8TE76	0.14	2.53	0.81	1.07	2.77	0.68	0.96	0.36	0.63	0.35	0.75	0.38	2.13	0.97	0.60	4.64	1.07	0.47	2.22	0.19	0.06	4739.24	4058.20	3538.49	2589.85	2375.90	1819.55	3341.11	2954.47	2727.71	3251.53	3236.31	1771.68		x
2663	MORN repeat-containing protein 2	Q502X0	6199.22	6563.18	5400.06	4748.46	4467.95	4298.58	5413.71	5514.89	5341.57	4661.91	4429.43	4442.79	6349.66	4958.66	4015.92	5055.31	4345.95	3983.66	5309.53	5469.41	4522.98	2.27	1.49	0.65	0.84	1.31	0.69	4.16	2.47	0.11	4.90	4.98	0.12	x	
2664	Mortality factor 4-like protein 2	Q15014	3505.90	3566.24	3535.57	2510.36	2620.50	2189.67	3916.83	3586.40	3088.95	2507.32	2573.00	2156.16	2879.35	2729.50	2387.64	2936.01	2472.83	2284.48	2343.74	2230.91	2130.52	2.27	1.49	0.65	0.84	1.31	0.69	4.16	2.47	0.11	4.90	4.98	0.12	x	
2665	Motilin receptor	O43193	0.14	2.53	0.81	68.51	74.61	56.52	45.92	82.62	93.82	52.46	30.39	60.92	107.57	114.11	61.51	51.36	20.98	38.03	30.17	58.02	56.62	2.27	1.49	0.65	0.84	1.31	0.69	4.16	2.47	0.11	4.90	4.98	0.12	x	
2666	M-phase inducer phosphatase 1	P30304	0.14	2.53	0.81	1.07	2.77	0.68	0.96	0.36	0.63	0.35	0.75	0.38	2.13	0.97	0.60	4.64	1.07	0.47	2.22	0.19	0.06	15571.10	14069.44	12384.94	19525.00	18591.49	17198.19	24941.06	24053.28	22906.83	17647.65	16489.28	13894.41		x
2667	M-phase inducer phosphatase 3	P30307	3727.40	3222.76	2822.16	1804.49	1373.37	1232.42	1540.21	1518.58	1540.47	3811.13	3342.48	2926.78	3633.30	1928.01	1455.58	1236.24	1361.67	1209.29	2080.54	1638.06	1403.27	2.27	1.49	0.65	0.84	1.31	0.69	4.16	2.47	0.11	4.90	4.98	0.12	x	
2668	M-phase phosphoprotein 9	Q99550	169575.53	164366.69	160165.63	108283.01	102121.93	99178.89	125291.62	127075.83	120549.82	100465.26	98973.83	90901.72	127390.33	113972.57	106641.92	98000.89	93807.31	90477.21	127003.04	118290.19	116205.73	24287.36	22246.46	22033.99	19197.31	19169.43	14352.33	23747.17	21449.44	19968.25	29448.76	28027.74	17056.38		
2669	MRG/MORF4L-binding protein	Q9NV56	5516.68	5055.91	4780.14	3210.78	3110.54	2695.01	3793.27	3811.33	3160.26	4364.03	4330.26	3763.80	4832.66	4671.17	3020.92	3240.58	2903.00	2839.13	3786.10	3652.29	3374.86	2.27	1.49	0.65	0.84	1.31	0.69	4.16	2.47	0.11	4.90	4.98	0.12	x	
2670	mRNA (2'-O-methyladenosine-N(6)-)-methyltransferase	Q9H4Z3	609.55	663.07	518.30	399.22	476.45	501.49	637.53	582.15	588.31	458.95	568.45	477.88	653.42	564.01	431.52	339.06	438.72	447.24	524.41	617.40	481.56	18445.18	16719.59	17063.35	13025.44	12774.68	12044.84	13802.61	13597.71	13378.34	14946.78	14161.03	12096.25		
2671	mRNA cap guanine-N7 methyltransferase	O43148	29218.29	26561.94	24154.76	18665.03	18613.62	17487.04	20029.14	21789.54	19974.01	24003.73	23289.99	22153.95	22598.15	22518.51	16595.48	17680.92	19859.05	17394.41	18084.90	18309.29	18090.39	35493.14	33205.89	31331.83	23942.17	22780.98	20506.51	23512.91	22539.98	22410.34	24101.35	22780.16	15679.61		
2672	mRNA export factor	P78406	38991.84	39103.37	34962.98	24813.32	24537.24	22790.98	28979.16	27724.93	27005.25	31096.69	29023.17	28354.27	28864.52	27037.24	24614.43	25429.95	23831.14	22230.23	24798.99	23171.26	22799.69	23875.50	24310.59	21062.93	12683.04	11921.00	9225.74	16079.58	14327.45	13793.32	16555.55	15952.16	9132.05		
2673	mRNA turnover protein 4 homolog	Q9UKD2	53684.27	51504.34	59926.90	37413.55	35556.14	35358.23	39012.44	38243.59	37324.45	53087.16	49566.30	48036.57	43204.67	45419.34	35348.46	34252.78	31252.71	31301.21	34958.50	33145.48	31725.55	109445.85	102750.18	98722.36	71669.09	72443.67	65209.39	87917.61	81919.83	79991.23	105263.34	98554.97	72948.31		
2674	mRNA-capping enzyme	O60942	6459.35	6254.22	5611.92	8498.33	7321.22	5930.62	13187.39	11272.89	11236.44	6203.29	5762.65	5164.25	8543.70	6125.88	8694.91	6456.44	7626.28	8066.77	5239.21	7345.64	7681.44	1020.69	960.68	818.60	683.52	601.89	317.80	812.87	602.15	519.85	893.90	889.28	340.65		
2675	Msx2-interacting protein	Q96T58	15177.90	14543.81	12654.82	8483.02	8451.91	6538.56	10753.34	10441.44	9323.34	11592.63	9826.61	8843.49	10319.84	10132.68	5549.63	9604.99	9250.00	8990.18	10052.66	9470.68	7797.81	2.27	1.49	0.65	0.84	1.31	0.69	4.16	2.47	0.11	4.90	4.98	0.12	x	
2676	Mth938 domain-containing protein	Q9H7C9	0.14	2.53	0.81	1.07	2.77	0.68	0.96	0.36	0.63	0.35	0.75	0.38	2.13	0.97	0.60	4.64	1.07	0.47	2.22	0.19	0.06	5377.24	4503.94	5401.76	3841.30	3831.70	3452.38	3924.45	3699.26	3657.00	3633.95	3564.48	2896.64		x
2677	Mucin-1	P15941	24820.68	24906.12	21032.98	17364.64	15770.00	14022.54	18986.48	18131.28	17539.57	16857.98	17342.95	15384.40	20208.12	18447.01	13207.47	16097.09	15373.85	13692.29	18718.53	18599.59	18052.06	2.27	1.49	0.65	0.84	1.31	0.69	4.16	2.47	0.11	4.90	4.98	0.12	x	
2678	Mucolipin-2	Q8IZK6	1326.74	1411.83	1602.19	1566.32	919.30	701.71	3608.46	3867.52	3708.71	1132.70	1648.93	1496.56	1028.89	942.12	1488.00	1630.51	1902.29	1573.28	1317.11	589.87	318.03	2.27	1.49	0.65	0.84	1.31	0.69	4.16	2.47	0.11	4.90	4.98	0.12	x	
2679	Multidrug resistance protein 1	P08183	14366.79	13644.47	12908.48	9954.07	9403.54	9029.96	10850.82	10023.17	10604.44	12949.14	12420.07	13196.15	11909.89	12157.33	9927.98	10116.54	9117.11	8962.54	9295.63	9068.56	8718.38	2.27	1.49	0.65	0.84	1.31	0.69	4.16	2.47	0.11	4.90	4.98	0.12	x	
2680	Multifunctional methyltransferase subunit TRM112-like protein	Q9UI30	39158.67	40850.28	38898.04	27463.69	25672.46	22678.30	32866.71	30148.53	27797.65	36426.17	33751.27	33000.29	31763.84	31785.30	21759.03	25391.26	25171.09	23378.63	27920.25	27370.39	24352.41	15257.43	14071.27	13460.45	10347.14	9748.91	7720.73	12580.55	12647.59	10929.18	11976.46	12495.35	7247.58		
2681	Multifunctional procollagen lysine hydroxylase and glycosyltransferase LH3	O60568	110149.98	107658.58	99762.43	73682.47	73486.82	66563.14	91159.72	87521.62	84607.82	98028.75	91355.65	88004.42	99000.64	92667.36	73819.75	77078.71	73624.37	70410.22	79933.81	75512.67	73075.62	54470.96	49010.40	46297.21	39603.82	35785.94	34635.89	43330.42	40985.27	39411.49	44684.01	43694.38	26797.03		
2682	Multifunctional protein ADE2	P22234	486955.12	468423.19	447554.18	342865.35	322287.42	298347.22	412571.89	398427.87	379467.08	431908.16	412988.61	388826.95	427687.14	374304.51	279294.95	313097.28	303042.69	292711.38	358795.53	327423.34	307137.84	373540.27	353237.22	326241.72	215484.18	210395.56	191706.82	261191.40	252658.42	246432.61	259805.01	254224.91	160963.60		
2683	Multivesicular body subunit 12A	Q96EY5	1215.93	1016.76	1021.09	345.60	288.43	268.97	596.44	634.12	455.15	820.69	840.29	642.35	1099.25	571.39	380.09	352.06	367.17	290.42	481.88	448.93	466.83	2.27	1.49	0.65	0.84	1.31	0.69	4.16	2.47	0.11	4.90	4.98	0.12	x	
2684	Muskelin	Q9UL63	17830.82	17231.89	17952.80	13258.06	13069.64	10391.78	14340.78	14189.87	12156.14	16388.16	18522.12	17937.03	17724.65	11958.13	5503.05	10101.58	9686.75	9423.50	12025.66	12653.82	10975.38	2.27	1.49	0.65	0.84	1.31	0.69	4.16	2.47	0.11	4.90	4.98	0.12	x	
2685	MutS protein homolog 4	O15457	0.14	2.53	0.81	1.07	2.77	0.68	0.96	0.36	0.63	0.35	0.75	0.38	2.13	0.97	0.60	4.64	1.07	0.47	2.22	0.19	0.06	14787.21	14245.86	13029.81	10065.17	10062.65	9309.96	8129.80	8801.42	8449.23	8270.35	7726.47	5663.36		x
2686	Myb/SANT-like DNA-binding domain-containing protein 4	Q8NCY6	40115.91	39775.84	35227.37	25060.43	23058.09	21443.33	29484.97	28742.49	26490.73	31675.51	30202.34	27370.53	32591.70	28934.54	22210.47	26049.36	24040.69	23448.42	25771.15	24833.09	21338.26	4539.57	4571.75	4108.07	2966.43	3036.01	2664.81	2873.02	2966.69	2776.79	3109.32	2915.52	2058.51		
2687	Myb-binding protein 1A	Q9BQG0	18201.70	16833.32	15313.85	15914.47	14922.19	14180.84	16834.16	16003.56	16890.01	21183.73	20525.45	20146.06	18439.81	15850.02	13799.34	12765.07	12777.87	12473.27	14148.97	13394.81	13287.24	27273.38	24469.83	22440.48	18333.92	17983.22	15394.92	20906.88	19572.90	17756.57	22769.85	20862.32	11770.80		
2688	Myb-related protein A	P10243	1515.21	1372.35	1463.13	1183.62	1088.37	1099.98	1031.62	866.13	891.65	1093.01	1222.16	1146.77	1439.32	1348.44	1107.46	1092.71	960.81	987.48	1006.32	821.39	1055.78	2.27	1.49	0.65	0.84	1.31	0.69	4.16	2.47	0.11	4.90	4.98	0.12	x	
2689	Myc box-dependent-interacting protein 1	O00499	0.14	2.53	0.81	1.07	2.77	0.68	0.96	0.36	0.63	0.35	0.75	0.38	2.13	0.97	0.60	4.64	1.07	0.47	2.22	0.19	0.06	14822.54	12626.26	12813.03	9505.46	9398.01	7779.58	8616.06	8636.85	8494.28	9437.15	9413.94	8067.94		x
2690	Mycophenolic acid acyl-glucuronide esterase_ mitochondrial	Q9NUJ1	9591.74	9461.62	8657.12	5350.69	4970.67	4473.90	6682.09	7400.82	6490.80	7047.39	7056.62	6136.87	6926.48	6400.08	4762.13	5510.29	4898.53	4823.34	6825.78	5473.29	5198.55	14486.17	10974.40	13355.54	8972.55	9110.68	7297.69	10989.05	10551.52	10620.13	11772.97	10736.54	8048.36		
2691	Myelin protein zero-like protein 1	O95297	0.14	2.53	0.81	1.07	2.77	0.68	0.96	0.36	0.63	0.35	0.75	0.38	2.13	0.97	0.60	4.64	1.07	0.47	2.22	0.19	0.06	8779.79	7786.24	7928.96	5429.56	5783.63	5169.08	6569.20	7115.57	6976.22	5466.36	5039.33	3861.01		x
2692	Myelin-oligodendrocyte glycoprotein	Q16653	4143.85	4135.63	3989.96	2760.68	2728.00	2644.23	3888.68	3737.44	3714.68	3427.55	3264.63	2987.14	3556.28	3183.96	2284.79	3047.39	2435.67	2523.13	2677.16	2247.20	2556.64	5908.09	6268.61	6190.72	3354.77	3357.16	3195.31	3200.92	3198.39	3111.68	2746.48	2323.05	1814.84		
2693	Myeloid leukemia factor 2	Q15773	26186.03	24763.76	28160.21	16594.21	16184.35	15681.96	18140.78	15479.63	14934.29	21404.73	20441.39	17912.41	17028.09	19064.08	16392.40	18491.91	15660.18	17035.44	13363.43	16766.69	13959.51	33512.14	33027.73	25754.66	19379.64	18984.89	12967.04	22620.88	22043.79	18719.03	26409.53	25265.36	14603.44		
2694	Myeloid-derived growth factor	Q969H8	123499.79	115622.71	109514.41	98358.00	89925.05	88274.24	103681.27	103091.59	97967.39	114076.11	113839.00	103694.47	102257.68	94261.81	86151.43	103296.12	95261.92	93666.97	88300.77	84138.76	80121.61	41148.41	38584.21	34837.24	27571.78	27144.67	23876.81	33931.14	32408.63	30876.38	34327.37	35394.27	23122.70		
2695	Myocardial zonula adherens protein	P0CAP1	0.14	2.53	0.81	1.07	2.77	0.68	0.96	0.36	0.63	0.35	0.75	0.38	2.13	0.97	0.60	4.64	1.07	0.47	2.22	0.19	0.06	826.91	861.10	954.68	595.27	726.38	616.18	1058.91	755.96	790.49	614.04	754.11	299.21		x
2696	Myocardin	Q8IZQ8	25950.64	19644.45	10597.66	9941.35	1141.59	607.11	1230.34	1448.78	305.52	10646.57	7476.07	3767.73	22309.97	5824.10	827.83	1412.45	1273.73	886.77	3792.27	3962.87	1833.28	2.27	1.49	0.65	0.84	1.31	0.69	4.16	2.47	0.11	4.90	4.98	0.12	x	
2697	Myocardin-related transcription factor B	Q9ULH7	27652.47	29832.62	28544.79	18199.00	16494.35	17044.50	19696.95	18919.42	19570.82	23088.91	20831.01	19629.85	23462.17	21051.44	17945.64	16677.66	16296.75	16555.85	18792.16	17561.13	16517.50	2.27	1.49	0.65	0.84	1.31	0.69	4.16	2.47	0.11	4.90	4.98	0.12	x	
2698	Myoferlin	Q9NZM1	187078.65	177135.13	157742.76	128533.75	118444.89	109303.33	139618.36	131890.93	127805.79	176953.22	167418.61	158042.57	153214.27	140992.78	110119.62	126617.61	120444.95	114872.44	121594.26	116307.34	107657.34	348698.18	321129.78	288294.95	226114.67	216547.79	187600.32	298376.61	282967.94	266793.36	277764.68	269619.86	185541.42		
2699	Myomegalin	Q5VU43	0.14	2.53	0.81	1.07	2.77	0.68	288.89	297.69	351.94	0.35	0.75	0.38	2.13	0.97	0.60	71.43	75.41	31.24	2.22	0.19	0.06	2.27	1.49	0.65	0.84	1.31	0.69	4.16	2.47	0.11	4.90	4.98	0.12	x	
2700	Myomesin-1	P52179	9291.96	7580.41	6670.17	7151.70	6869.75	6232.52	6896.34	5281.10	5789.10	7276.50	6921.89	6109.81	7101.24	6763.44	6079.15	5462.35	5148.87	5555.51	6860.40	5802.24	5876.49	42807.48	44568.87	39096.05	32282.27	32573.78	29644.70	34842.53	31738.02	32289.27	39624.13	39134.18	24617.53		
2701	Myosin light chain 1/3 skeletal muscle isoform	P05976	156240.58	146479.25	125604.07	95618.90	95135.18	74954.58	130887.41	127134.85	115731.69	125990.63	118583.71	104361.92	127937.62	116403.10	75186.41	102330.05	93017.08	84185.55	105813.04	100904.33	85571.64	2.27	1.49	0.65	0.84	1.31	0.69	4.16	2.47	0.11	4.90	4.98	0.12	x	
2702	Myosin light chain 3	P08590	37731.80	32515.08	26980.14	28044.13	25395.93	21580.28	27007.20	25472.55	23054.57	28736.14	28502.92	25259.29	27394.24	25079.85	12078.08	28513.40	26277.85	24940.97	26678.90	25243.68	21076.37	1296.94	1083.86	744.06	959.26	843.73	543.19	1046.52	976.32	1003.63	1281.96	1130.96	645.51		
2703	Myosin light chain 4	P12829	4062.72	3624.73	3565.64	2470.61	2410.62	2144.85	3767.47	3436.26	3416.08	3634.59	3981.30	3593.77	3538.06	2966.42	2412.49	2313.19	2311.78	2114.13	2973.46	2975.18	2841.29	2.27	1.49	0.65	0.84	1.31	0.69	4.16	2.47	0.11	4.90	4.98	0.12	x	
2704	Myosin light chain kinase_ smooth muscle	Q15746	21628.86	20343.57	19826.15	13338.49	12687.12	11543.39	15942.61	15864.53	15827.30	17898.70	17498.31	16789.31	17010.73	15011.39	10998.08	14887.39	13554.70	13101.53	14632.65	14434.11	12686.17	34632.41	32167.37	27933.75	24876.13	23591.19	19912.87	30236.72	29599.83	24798.45	38347.16	36163.55	23809.70		
2705	Myosin light polypeptide 6	P60660	365200.56	369105.86	352667.91	243557.97	233466.54	227668.14	260167.65	258143.17	245056.41	299873.71	285473.04	269720.29	270882.12	267432.05	234552.34	247100.07	220020.03	216618.10	236485.56	227393.50	216056.05	760261.95	718011.58	675764.23	473485.86	463356.79	419689.85	611626.88	571714.83	563209.72	510181.72	495080.35	355430.41		
2706	Myosin regulatory light chain 10	Q9BUA6	15086.32	14016.40	13307.15	10815.09	10534.37	9391.35	9924.66	9516.10	8939.94	14278.78	13353.23	12843.17	12814.16	10830.82	9697.88	11535.36	10871.69	9813.59	10192.11	9683.00	9471.79	2.27	1.49	0.65	0.84	1.31	0.69	4.16	2.47	0.11	4.90	4.98	0.12	x	
2707	Myosin regulatory light chain 12B	O14950	240726.72	224224.33	204253.05	139907.22	140286.40	125759.75	169061.50	157795.83	145722.44	186179.51	172106.62	160449.52	178215.12	165620.49	126079.39	145649.49	141656.10	133924.09	143468.61	134701.23	109520.23	289851.78	262428.74	238012.41	248811.67	238823.92	199055.77	342080.97	315114.74	296473.22	272256.64	256445.62	166903.78		
2708	Myosin regulatory light chain 2_ ventricular/cardiac muscle isoform	P10916	10598.69	9234.55	9050.98	5370.65	5264.07	5091.39	5898.69	7231.94	6299.91	8185.70	7094.73	6898.33	7644.03	7318.43	4908.01	5366.99	4904.08	5037.59	6444.02	6478.58	5910.57	2.27	1.49	0.65	0.84	1.31	0.69	4.16	2.47	0.11	4.90	4.98	0.12	x	
2709	Myosin regulatory light polypeptide 9	P24844	0.14	2.53	0.81	1.07	2.77	0.68	0.96	0.36	0.63	0.35	0.75	0.38	2.13	0.97	0.60	4.64	1.07	0.47	2.22	0.19	0.06	9027.01	8462.33	8423.33	6166.37	5851.77	4240.90	7602.09	7073.10	6467.98	6875.71	7162.26	4680.17		x
2710	Myosin-1	P12882	0.14	2.53	0.81	1.07	2.77	0.68	0.96	0.36	0.63	0.35	0.75	0.38	2.13	0.97	0.60	4.64	1.07	0.47	2.22	0.19	0.06	10144.86	9999.57	8881.22	8055.02	7680.24	7075.60	9297.55	8547.67	8433.98	8219.37	7606.30	4833.64		x
2711	Myosin-10	P35580	884072.93	876340.52	847417.99	654941.41	627843.81	609993.32	751998.94	727244.71	712628.18	782335.03	743949.99	711337.18	695685.23	723774.05	625194.50	631377.28	596303.64	588605.46	627505.44	614636.22	608484.44	235720.72	216767.35	190711.92	173175.99	166086.33	140053.83	204454.53	199153.42	179287.81	212795.37	203949.02	106759.67		
2712	Myosin-11	P35749	257854.99	244111.53	219086.84	172902.69	169188.23	152235.86	201895.47	188062.76	179435.00	233981.75	224661.19	217975.80	212085.21	197986.64	156189.89	176180.93	167078.78	160792.67	167491.25	164250.16	153967.21	151970.95	128772.60	115589.21	84139.91	81835.17	65464.19	108481.15	101584.88	89996.33	112004.60	107292.49	63654.54		
2713	Myosin-13	Q9UKX3	7385.74	7005.35	7256.50	4687.77	4770.51	4955.50	6092.60	6055.66	5786.09	6821.54	6556.21	6464.44	6223.30	6295.48	5208.61	4820.95	4782.40	4665.03	5412.64	5253.94	4862.48	46297.35	43450.54	39156.38	31384.88	31417.95	28344.93	45379.35	43461.15	40754.95	41729.80	39674.49	26271.94		
2714	Myosin-14	Q7Z406	260842.05	244984.03	215681.88	174423.30	171328.39	154233.40	215381.31	203299.31	194303.78	234473.94	228349.22	210533.44	217992.77	205859.79	147991.01	183046.77	172046.64	155877.60	187310.72	176211.83	164017.38	109009.30	103778.58	95008.56	78252.12	75400.44	69012.80	90101.54	88834.84	81662.34	94421.51	90055.32	67079.63		
2715	Myosin-15	Q9Y2K3	0.14	2.53	0.81	1.07	2.77	0.68	0.96	0.36	0.63	0.35	0.75	0.38	2.13	0.97	0.60	4.64	1.07	0.47	2.22	0.19	0.06	83193.21	77778.06	71204.40	54237.77	53149.27	47059.26	59685.86	57531.98	54566.50	66075.86	68316.26	46361.71		x
2716	Myosin-3	P11055	0.14	2.53	0.81	1.07	2.77	0.68	0.96	0.36	0.63	0.35	0.75	0.38	2.13	0.97	0.60	4.64	1.07	0.47	2.22	0.19	0.06	8853.82	7948.40	7310.99	4294.27	4033.41	3368.23	5852.01	5622.86	5122.85	4430.07	4435.07	2849.12		x
2717	Myosin-4	Q9Y623	0.14	2.53	0.81	1.07	2.77	0.68	0.96	0.36	0.63	0.35	0.75	0.38	2.13	0.97	0.60	4.64	1.07	0.47	2.22	0.19	0.06	1277.43	1091.95	1269.95	814.45	796.99	657.24	944.24	850.09	908.61	857.53	874.09	531.70		x
2718	Myosin-6	P13533	0.14	2.53	0.81	1.07	2.77	0.68	0.96	0.36	0.63	0.35	0.75	0.38	2.13	0.97	0.60	4.64	1.07	0.47	2.22	0.19	0.06	17831.70	16090.78	14454.72	11667.35	11486.49	8717.25	15495.64	14707.80	13379.01	18295.47	16823.99	11277.67		x
2719	Myosin-7B	A7E2Y1	125562.28	125281.05	120553.50	84361.40	82089.01	76014.95	105790.18	102328.07	99640.00	100259.13	97871.80	89553.94	103672.57	97258.31	82005.76	84817.03	82494.73	79575.30	84936.77	80584.63	74572.12	88689.51	79371.22	72156.79	71347.08	68673.42	62670.23	82470.02	76967.07	70647.35	101584.93	96967.92	67417.31		
2720	Myosin-8	P13535	0.14	2.53	0.81	1.07	2.77	0.68	0.96	0.36	0.63	0.35	0.75	0.38	2.13	0.97	0.60	4.64	1.07	0.47	2.22	0.19	0.06	20182.86	19184.57	19405.88	15511.60	15855.44	14954.51	17177.90	16328.88	16143.58	17249.20	16578.64	13973.22		x
2721	Myosin-9	P35579	1121907.08	1068005.42	1019198.22	745152.18	723574.34	689871.88	863999.46	841230.49	801487.95	950940.92	920406.73	877644.59	927662.00	858630.15	695754.87	740603.13	683295.75	665819.12	772049.97	741576.38	680962.22	2950422.35	2829516.56	2611457.50	2263125.48	2206356.83	1948315.60	2835614.36	2731772.29	2613046.26	2472699.13	2383871.49	1586408.82		
2722	Myotilin	Q9UBF9	865.86	912.54	867.72	553.36	508.70	454.77	637.12	512.40	489.34	481.78	526.22	498.62	758.53	577.00	562.82	466.64	490.34	471.48	464.77	424.84	404.15	6439.57	5709.65	5684.90	2243.34	2140.08	2150.36	1927.97	2025.71	1842.72	2150.02	2102.89	1707.88		
2723	Myotrophin	P58546	132621.05	125574.64	117833.66	96645.65	94782.28	87985.98	112373.04	106324.40	101901.20	126179.44	118626.34	113916.70	119252.51	107831.83	90529.67	95858.44	89312.37	84938.64	91257.04	87978.47	87619.77	55777.58	53732.61	48668.19	36452.82	34700.47	30407.54	43152.91	42079.21	40861.04	42294.69	39309.33	25406.72		
2724	Myotubularin-related protein 14	Q8NCE2	0.14	2.53	0.81	1.07	2.77	0.68	0.96	0.36	0.63	0.35	0.75	0.38	2.13	0.97	0.60	4.64	1.07	0.47	2.22	0.19	0.06	79128.41	72216.57	61421.32	47667.34	49668.99	29763.52	57777.85	50334.82	41835.36	71733.82	68187.97	39160.48		x

Supplementary Table S1. Overview on the abundance of all relatively quantified 5180 proteins

MCF-7

	Protein name	UniProt	control 01	control 02	control 03	Dai IC20 01	Dai IC20 02	Dai IC20 03	Dai SC20 01	Dai SC20 02	Dai SC20 03	Gen IC20 01	Gen IC20 02	Gen IC20 03
2725	Myotubularin-related protein 4	Q9NYA4	27427.51	28238.22	26161.01	18097.27	16905.07	16004.51	17827.10	16573.39	16396.78	21629.32	20993.57	20069.25
2726	Myotubularin-related protein 5	O95248	6533.13	6995.17	5841.98	4489.39	4547.47	3570.16	5769.46	5185.79	5112.84	5532.47	5413.30	4883.43
2727	Myotubularin-related protein 7	Q9Y216	12722.19	16753.06	13015.05	5751.91	4891.54	5429.11	9038.46	8847.20	9713.72	8160.31	7862.98	7036.86
2728	Myotubularin-related protein 8	Q96EF0	235.55	217.41	123.85	196.80	2659.44	2052.72	4350.93	2939.14	2534.64	157.29	194.56	32.88
2729	Myristoylated alanine-rich C-kinase substrate	P29966	0.14	2.53	0.81	1.07	2.77	0.68	0.96	0.36	0.63	0.35	0.75	0.38
2730	N(G)_N(G)-dimethylarginine dimethylaminohydrolase 1	O94760	0.14	2.53	0.81	1.07	2.77	0.68	0.96	0.36	0.63	0.35	0.75	0.38
2731	N(G)_N(G)-dimethylarginine dimethylaminohydrolase 2	O95865	16370.82	16465.64	15716.44	9098.33	9735.05	9049.09	11956.30	10864.65	10379.68	13649.80	12981.58	12504.28
2732	N6-adenosine-methyltransferase catalytic subunit	Q86U44	162279.61	134842.27	113110.33	100099.58	93810.88	81471.46	110162.33	103522.07	98285.20	137491.23	123676.60	112540.26
2733	N6-adenosine-methyltransferase non-catalytic subunit	Q9HCE5	0.14	2.53	0.81	361.36	507.37	299.71	485.34	647.21	508.12	0.35	0.75	0.38
2734	Na(+)/H(+) exchange regulatory cofactor NHE-RF1	O14745	636845.89	637495.61	604601.98	436745.50	422245.93	407811.03	532692.52	524545.25	506100.74	555279.91	528846.48	494384.04
2735	NAC-alpha domain-containing protein 1	O15069	16176.51	16218.62	13686.44	8085.51	7090.72	7715.73	9296.41	9952.58	9267.19	8034.81	7630.10	7661.46
2736	N-acetyl-D-glucosamine kinase	Q9UJ70	47746.29	40601.31	37492.55	30983.49	26937.49	23451.08	34906.89	32182.30	30699.34	35125.25	33973.39	30569.01
2737	N-acetylglucosamine-6-sulfatase	P15586	17664.47	15251.08	14375.99	12609.75	13369.69	11859.09	14452.08	13044.07	12446.78	17473.01	16651.56	15445.95
2738	NACHT domain- and WD repeat-containing protein 1	Q149M9	121106.68	109193.30	103443.90	72004.92	68486.69	63097.66	87692.82	82471.08	77969.60	96740.32	88487.76	83202.39
2739	NACHT_ LRR and PYD domains-containing protein 13	Q86W25	0.14	2.53	0.81	1.07	2.77	0.68	0.96	0.36	0.63	0.35	0.75	0.38
2740	NACHT_ LRR and PYD domains-containing protein 2	Q9NX02	50394.85	44592.61	45319.07	34663.15	34459.70	33618.26	37981.65	38137.98	37136.26	46741.03	45677.14	44248.54
2741	NACHT_ LRR and PYD domains-containing protein 3	Q96P20	0.14	2.53	0.81	1.07	2.77	0.68	0.96	0.36	0.63	0.35	0.75	0.38
2742	NACHT_ LRR and PYD domains-containing protein 5	P59047	51891.13	52265.77	43699.25	36501.43	35327.50	32189.46	38661.06	34195.03	33388.51	54387.59	48303.53	47303.71
2743	NACHT_ LRR and PYD domains-containing protein 7	Q8WX94	16944.32	15215.73	15079.93	15984.13	14165.03	14199.78	13355.63	13771.82	13414.34	18295.30	16965.48	16253.19
2744	NACHT_ LRR and PYD domains-containing protein 9	Q7RTR0	10570.47	10680.39	9993.33	7517.15	7041.48	6673.59	9226.26	9376.96	8835.64	11029.36	10041.98	9363.98
2745	N-acylneuraminate cytidylyltransferase	Q8NFW8	43971.86	41117.45	38621.78	31138.40	27867.43	28068.52	29137.44	30357.83	28870.60	41073.39	40436.26	37190.17
2746	N-acylneuraminate-9-phosphatase	Q8TBE9	7155.58	6962.80	6858.76	4751.59	4586.97	4585.07	5426.16	5013.68	5330.07	6346.19	6191.40	5814.69
2747	NAD kinase 2 mitochondrial	Q4G0N4	29273.99	28360.44	26884.81	20908.86	20909.73	19952.38	25627.65	23035.14	24638.12	27273.67	26560.28	25425.78
2748	NAD(P) transhydrogenase_ mitochondrial	Q13423	0.14	2.53	0.81	1.07	2.77	0.68	0.96	0.36	0.63	0.35	0.75	0.38
2749	NAD(P)H dehydrogenase [quinone] 1	P15559	344590.52	346579.18	316905.11	306750.23	310008.57	290656.17	277130.40	258965.80	249651.91	318121.90	293661.45	282927.62
2750	NAD(P)H-hydrate epimerase	Q8NCW5	14436.58	12518.78	12297.37	5906.16	7160.51	5761.68	9852.84	8401.28	8045.14	11631.27	10705.80	8891.00
2751	NAD-dependent malic enzyme_ mitochondrial	P23368	1083.44	1028.55	1099.45	658.06	729.07	682.35	907.70	912.30	908.79	1149.41	1038.36	1019.01
2752	NAD-dependent protein deacetylase sirtuin-2	Q8IXJ6	0.14	2.53	0.81	1.07	2.77	0.68	0.96	0.36	0.63	0.35	0.75	0.38
2753	NAD-dependent protein deacylase sirtuin-5_ mitochondrial	Q9NXA8	4404.34	4379.89	3629.99	3149.93	2829.07	2825.17	3218.79	3234.16	3073.46	4051.51	3784.66	3619.37
2754	NADH dehydrogenase [ubiquinone] 1 alpha subcomplex assembly factor 2	Q8N183	77722.12	75283.88	67465.03	53589.41	51749.50	48172.27	59563.40	55100.23	54427.05	67281.47	62415.78	60792.14
2755	NADH dehydrogenase [ubiquinone] 1 alpha subcomplex assembly factor 4	Q9P032	35369.12	31299.58	32171.05	25763.42	23913.06	24352.43	26261.72	27741.00	26368.40	29117.52	28734.98	26273.90
2756	NADH dehydrogenase [ubiquinone] 1 alpha subcomplex subunit 10_ mitochondrial	O95299	29647.58	28941.43	25980.12	19709.62	20047.28	18829.61	22536.75	21024.87	21496.12	25515.63	24400.35	23238.32
2757	NADH dehydrogenase [ubiquinone] 1 alpha subcomplex subunit 11	Q86Y39	26283.83	27014.84	26784.85	15861.79	15236.80	14016.86	19542.18	19523.72	19949.91	19156.64	17119.29	17588.06
2758	NADH dehydrogenase [ubiquinone] 1 alpha subcomplex subunit 12	Q9UI09	7320.78	7283.84	6997.34	5544.63	5639.68	5471.34	5646.21	5358.67	5096.46	7093.65	6338.99	6363.19
2759	NADH dehydrogenase [ubiquinone] 1 alpha subcomplex subunit 13	Q9P0J0	63158.74	65559.18	57875.85	43730.91	41646.03	36478.49	51000.76	49949.91	49021.03	55593.70	52990.50	49071.68
2760	NADH dehydrogenase [ubiquinone] 1 alpha subcomplex subunit 5	Q16718	64686.85	64472.09	60316.63	49071.93	48121.89	46035.15	73031.64	71479.48	69834.59	63910.99	61179.67	58794.21
2761	NADH dehydrogenase [ubiquinone] 1 alpha subcomplex subunit 6	P56556	6412.77	6221.05	5411.83	5179.36	4793.54	4263.59	5273.47	4932.18	4830.13	6285.17	5812.53	5649.23
2762	NADH dehydrogenase [ubiquinone] 1 alpha subcomplex subunit 7	O95182	146.37	244.54	375.95	167.63	164.34	204.24	148.73	178.42	305.62	181.51	289.67	153.59
2763	NADH dehydrogenase [ubiquinone] 1 alpha subcomplex subunit 8	P51970	18028.66	18825.14	16229.41	9298.18	8550.18	8391.69	14204.73	12970.66	12764.53	13352.43	12461.24	10865.61
2764	NADH dehydrogenase [ubiquinone] 1 alpha subcomplex subunit 9 mitochondrial	Q16795	54908.99	53368.24	51134.40	36988.09	34237.41	31728.99	41844.35	43975.50	39625.42	45970.10	46112.99	39137.36
2765	NADH dehydrogenase [ubiquinone] 1 beta subcomplex subunit 10	O96000	74624.41	71791.89	65054.34	49469.70	47333.76	44174.56	54119.10	53105.65	49215.12	62606.36	59317.80	55420.59
2766	NADH dehydrogenase [ubiquinone] 1 beta subcomplex subunit 11 mitochondrial	Q9NX14	2775.42	2999.28	2505.13	2599.25	2399.39	2260.78	2630.49	2501.13	2682.98	2894.95	2584.83	2816.24
2767	NADH dehydrogenase [ubiquinone] 1 beta subcomplex subunit 3	O43676	34107.80	27469.86	19508.50	15633.37	14396.72	9983.84	21693.95	19984.52	16687.76	27799.40	29299.49	23543.74
2768	NADH dehydrogenase [ubiquinone] 1 beta subcomplex subunit 4	O95168	49197.58	49632.26	46492.27	35781.46	34303.25	31339.22	38469.62	38422.13	36990.86	39744.35	38238.99	37622.28
2769	NADH dehydrogenase [ubiquinone] 1 beta subcomplex subunit 5_ mitochondrial	O43674	32290.95	26850.59	20417.29	16780.08	14782.53	11263.81	18854.29	17158.79	14490.16	23004.32	20740.07	16295.18
2770	NADH dehydrogenase [ubiquinone] 1 beta subcomplex subunit 6	O95139	41489.52	37439.65	34766.54	26649.22	24355.32	22025.97	29508.08	32923.35	25012.68	34708.59	34190.35	30254.30
2771	NADH dehydrogenase [ubiquinone] 1 beta subcomplex subunit 7	P17568	39027.69	40020.73	38247.92	29867.49	27226.52	27644.40	25777.44	26639.56	23495.80	38646.88	37795.64	42361.31
2772	NADH dehydrogenase [ubiquinone] 1 beta subcomplex subunit 8 mitochondrial	O95169	13107.48	10667.04	9315.58	7664.84	7333.48	5813.30	9251.71	8900.38	7787.31	10705.15	9820.86	7901.10
2773	NADH dehydrogenase [ubiquinone] 1 beta subcomplex subunit 9	Q9Y6M9	55135.50	51605.96	51164.65	35656.82	35914.00	32136.24	39208.92	37029.56	37048.76	48430.68	43777.21	42987.18
2774	NADH dehydrogenase [ubiquinone] 1 subunit C2	O95298	80571.88	72078.65	65810.14	35902.32	35927.44	34451.06	54232.48	54473.21	54511.83	53028.18	49424.07	51047.95
2775	NADH dehydrogenase [ubiquinone] flavoprotein 1_ mitochondrial	P49821	80962.41	77402.05	69779.47	52828.06	50352.16	47738.24	64136.04	57624.00	56397.67	64146.29	63150.72	56787.15
2776	NADH dehydrogenase [ubiquinone] flavoprotein 2_ mitochondrial	P19404	25598.84	23200.32	21188.68	17472.19	17955.99	14529.95	18438.90	16976.24	14530.17	20554.36	17864.09	17055.10
2777	NADH dehydrogenase [ubiquinone] iron-sulfur protein 2 mitochondrial	O75306	37817.05	35563.98	32911.77	24626.82	24936.33	22031.46	26976.72	26196.22	25054.31	32135.79	31364.76	29546.50
2778	NADH dehydrogenase [ubiquinone] iron-sulfur protein 3 mitochondrial	O75489	71234.74	64098.34	54199.14	29547.05	33186.73	26815.21	40124.36	37529.63	35263.70	51150.59	47519.48	39182.59
2779	NADH dehydrogenase [ubiquinone] iron-sulfur protein 4 mitochondrial	O43181	39865.69	38171.67	38057.14	24996.41	23874.25	23298.03	26161.67	25115.70	27635.15	30012.91	29313.23	28255.81
2780	NADH dehydrogenase [ubiquinone] iron-sulfur protein 5	O43920	17034.38	15724.56	14749.06	11561.85	10083.75	10069.04	13986.27	12688.63	12323.51	14426.75	13903.91	12597.75
2781	NADH dehydrogenase [ubiquinone] iron-sulfur protein 6_ mitochondrial	O75380	11816.55	10898.41	10727.03	9163.65	8403.19	8148.56	10430.86	10026.57	9491.60	10359.71	9847.03	9023.52
2782	NADH dehydrogenase [ubiquinone] iron-sulfur protein 8_ mitochondrial	O00217	89616.24	90002.79	90684.17	70800.88	65045.27	53946.27	56329.49	61270.06	53348.92	89037.91	90288.68	91764.33
2783	NADH-cytochrome b5 reductase 3	P00387	195128.83	182081.64	170409.80	127121.24	125222.88	113494.66	152415.16	151662.74	145709.90	156752.75	149405.26	140051.88
2784	NADH-ubiquinone oxidoreductase 75 kDa subunit mitochondrial	P28331	106693.65	100885.16	89294.65	76693.02	76376.25	69035.03	87923.05	81745.87	81916.77	88724.13	83919.84	79571.82
2785	NADH-ubiquinone oxidoreductase chain 4	P03905	26700.84	25218.27	25213.33	17922.23	17418.55	17937.48	18530.25	19192.12	19363.44	22715.75	21096.90	22947.76
2786	NADP-dependent malic enzyme	P48163	72717.53	66665.54	59333.18	52843.14	54984.22	51260.71	57062.77	54355.90	51505.52	66594.17	63974.72	57006.61
2787	NADP-dependent malic enzyme mitochondrial	Q16798	546.43	396.22	435.72	19.28	1154.01	578.32	1360.40	55.36	33.61	420.65	342.20	113.27
2788	NADPH:adrenodoxin oxidoreductase_ mitochondrial	P22570	65572.82	61247.92	57823.18	46298.82	43071.69	40916.31	53658.48	51160.98	47409.25	73155.80	72517.44	66187.53
2789	NADPH--cytochrome P450 reductase	P16435	128233.53	125118.42	119292.97	101809.37	99053.35	94106.97	120495.45	114732.46	109094.65	113495.38	110854.82	100226.59
2790	N-alpha-acetyltransferase 10	P41227	283144.73	271138.53	289261.95	208961.34	200516.80	187017.57	230049.02	224079.22	212319.54	284382.50	273070.77	253204.30
2791	N-alpha-acetyltransferase 11	Q9BSU3	7045.27	6326.82	5681.82	4212.06	4169.54	3935.74	4658.45	4556.21	4199.74	6335.03	6086.41	5938.26
2792	N-alpha-acetyltransferase 15_ NatA auxiliary subunit	Q9BXJ9	278619.59	271248.73	261240.44	194351.10	190499.49	180916.38	259559.22	244344.29	239650.42	233262.33	220526.39	211766.19
2793	N-alpha-acetyltransferase 16_ NatA auxiliary subunit	Q6N069	25804.61	22621.03	20936.95	18154.94	16946.25	15439.05	19971.05	16860.65	19057.13	22183.71	20884.46	21032.07
2794	N-alpha-acetyltransferase 20	P61599	1977.37	1680.08	1791.30	1160.73	1211.64	1038.85	1519.78	1504.73	1551.12	1591.39	1593.02	1601.88
2795	N-alpha-acetyltransferase 35_ NatC auxiliary subunit	Q5VZE5	10133.59	9664.88	9331.54	7218.47	6757.79	7012.54	6160.48	7037.94	6381.09	7998.67	7686.62	7079.65
2796	N-alpha-acetyltransferase 40	Q86UY6	436.31	575.31	545.02	936.82	662.14	1889.33	2586.18	2821.79	2187.13	547.76	882.96	373.41
2797	N-alpha-acetyltransferase 50	Q9GZZ1	114075.96	118712.84	108085.99	79398.92	75890.17	72414.23	93418.74	89504.89	86211.66	92630.35	91131.22	85185.77
2798	Nascent polypeptide-associated complex subunit alpha_ muscle-specific form	E9PAV3	294661.87	286976.88	274515.50	193706.78	186981.71	181633.65	225626.85	222463.87	214344.23	241507.42	235118.02	225713.00
2799	Natural cytotoxicity triggering receptor 2	O95944	12146.92	10969.80	11689.20	8814.73	8818.19	8620.57	9664.27	9420.47	9344.70	10409.13	9530.42	9650.11
2800	Nck-associated protein 1	Q9Y2A7	28499.32	26934.39	24372.45	26481.63	37412.68	28576.15	45905.18	39086.33	32904.15	23610.58	22691.71	20421.02
2801	Nck-associated protein 5-like	Q9HCH0	5643.36	5954.40	5380.48	4132.12	3829.49	3404.64	4091.83	4016.92	3695.41	3961.11	4089.23	3464.91
2802	Nebulette	O76041	41232.19	42619.80	39125.02	28677.94	27344.27	25849.47	32583.93	31304.17	35183.28	35561.98	31462.44	30614.18
2803	Nebulin	P20929	0.14	2.53	0.81	1.07	2.77	0.68	0.96	0.36	0.63	0.35	0.75	0.38
2804	Nebulin-related-anchoring protein	Q86VF7	118496.93	116577.71	105658.80	80834.69	80575.37	76789.95	93775.42	85892.24	88666.26	112448.05	107463.44	105717.10
2805	NEDD4-binding protein 1	O75113	0.14	2.53	0.81	1.07	2.77	0.68	0.96	0.36	0.63	0.35	0.75	0.38
2806	NEDD4-binding protein 2	Q86UW6	6546.67	6560.36	5419.23	4374.36	4049.49	3920.09	5901.08	4481.92	5594.01	6241.14	5757.96	5186.99
2807	NEDD8	Q15843	31813.57	31283.37	28246.34	23859.05	22793.25	20236.35	23322.31	22677.78	21456.24	29787.13	29573.46	27556.26
2808	NEDD8-activating enzyme E1 catalytic subunit	Q8TBC4	0.14	2.53	0.81	1.07	2.77	0.68	0.96	0.36	0.63	0.35	0.75	0.38
2809	NEDD8-activating enzyme E1 regulatory subunit	Q13564	298.90	367.37	308.38	332.65	6.82	13.17	36.58	1042.77	605.30	182.65	174.41	243.75
2810	NEDD8-conjugating enzyme Ubc12	P61081	46123.60	44061.40	42338.11	35307.43	35917.23	32590.01	39502.99	34748.72	34972.85	37452.75	35292.86	34015.95
2811	NEDD8-conjugating enzyme UBE2F	Q969M7	30216.91	30294.94	26876.00	30108.43	31332.21	28789.34	32019.62	30455.54	30919.80	38523.34	38693.28	34581.47
2812	Negative elongation factor A	Q9H3P2	38077.70	37753.52	34025.30	28985.39	28561.24	27068.78	28093.75	28050.22	27975.92	35588.76	33290.69	32103.63
2813	Negative elongation factor B	Q8WX92	16023.11	15271.87	13116.81	5426.75	7611.18	5074.62	9513.38	8663.77	9293.03	8773.72	8133.47	7724.47
2814	Negative elongation factor C/D	Q8IXH7	4899.49	4356.46	4457.66	3972.78	3102.13	2864.99	3395.99	3360.16	3323.84	3369.24	3613.05	3365.86
2815	Negative elongation factor E	P18615	34407.09	34457.59	35163.51	27405.19	25980.86	26574.37	28476.82	31559.76	29573.00	34870.72	33242.69	30837.03
2816	Nephrocystin-3	Q7Z494	6321.04	6724.51	6250.98	4120.68	4195.15	4315.98	4455.21	4393.30	4285.41	5573.96	5572.24	5600.92
2817	Nesprin-1	Q8NF91	362062.47	350770.92	326877.05	264881.44	254764.48	233830.51	287972.12	274714.72	267321.12	324436.58	311731.93	291650.76
2818	Nesprin-2	Q8WXH0	1014577.91	958527.02	927473.19	622008.42	592226.13	565827.99	749988.17	726069.87	702262.52	760014.17	724374.01	673303.93
2819	Nestin	P48681	240936.85	215623.93	191092.88	167580.52	151410.91	144666.38	178924.15	181374.98	166553.90	194772.32	183025.91	169968.23
2820	Netrin receptor UNC5D	Q6UXZ4	5779.75	5461.67	4634.17	3579.09	3614.14	3242.92	4055.74	4131.19	3912.75	4569.28	4340.25	3820.57
2821	Neudesin	Q9UMX5	0.14	2.53	0.81	1.07	2.77	0.68	0.96	0.36	0.63	0.35	0.75	0.38
2822	Neural retina-specific leucine zipper protein	P54845	103513.31	94412.83	83514.08	49949.81	51517.17	46325.50	72956.92	69975.53	65561.50	88520.14	84488.59	77297.80
2823	Neuralized-like protein 4	Q96JN8	4495.37	3933.60	4448.86	2854.92	2717.03	2886.78	3133.83	3023.10	3578.98	3815.55	3607.10	3869.36

MCF-7 (continued)

	Gen SC20 01	Gen SC20 02	Gen SC20 03	SSE IC20 01	SSE IC20 02	SSE IC20 03	SSE SC20 01	SSE SC20 02	SSE SC20 03
2725	22063.31	19671.09	15807.05	17160.06	15494.70	15389.81	20729.85	17263.37	16382.13
2726	5392.13	4876.83	3275.59	4645.44	4441.54	4288.38	5054.78	4253.07	4504.42
2727	8960.17	7859.62	6360.15	7781.52	7669.93	7214.33	6541.96	6627.52	5278.40
2728	182.97	432.25	76.52	2014.96	3556.58	3370.62	426.27	330.14	226.39
2729	2.13	0.97	0.60	4.64	1.07	0.47	2.22	0.19	0.06
2730	2.13	0.97	0.60	4.64	1.07	0.47	2.22	0.19	0.06
2731	14937.83	13977.66	12306.82	9473.45	8396.28	8161.15	9536.99	9100.02	9253.80
2732	116489.60	105411.03	67797.36	97395.30	94619.08	84880.57	103373.52	99560.70	86662.35
2733	2.13	0.97	0.60	187.73	420.04	70.94	105.04	0.37	82.01
2734	521243.95	506470.91	454077.01	432428.33	404329.08	392512.09	462130.34	438019.81	426871.77
2735	8998.87	9735.75	8612.31	8367.43	7705.44	7953.75	9564.23	9880.12	8224.26
2736	37476.56	34842.49	23965.46	35857.67	29079.28	28112.18	29526.34	26685.01	23602.36
2737	16456.79	15016.91	12015.48	13351.16	12629.03	11071.32	11970.11	10879.39	9493.15
2738	99209.79	86425.36	66037.98	76089.68	66357.44	66317.55	78529.02	75401.74	73286.32
2739	2.13	0.97	0.60	4.64	1.07	0.47	2.22	0.19	0.06
2740	44219.17	40531.36	35122.55	36992.91	34045.77	33445.74	35166.79	35044.33	32281.18
2741	2.13	0.97	0.60	4.64	1.07	0.47	2.22	0.19	0.06
2742	48660.17	40992.31	39666.39	47273.94	39626.51	40566.47	41574.36	42448.11	37667.69
2743	18084.30	15822.78	12951.61	14833.17	14457.95	14777.62	12477.04	11416.49	11609.92
2744	9129.59	8851.57	7231.40	7244.80	6704.63	6891.82	8009.31	7355.06	7388.01
2745	39242.69	34256.79	26572.86	28329.31	27920.16	27238.75	30707.89	28055.12	27472.44
2746	5937.23	5651.80	4553.31	5017.53	4544.04	4656.87	4117.83	4022.60	3806.49
2747	27868.14	25516.20	20083.39	19657.87	18930.90	19293.42	20753.51	20426.75	20209.71
2748	2.13	0.97	0.60	4.64	1.07	0.47	2.22	0.19	0.06
2749	232866.69	227831.86	195255.69	435089.92	402883.65	384284.69	226936.37	223014.74	209239.43
2750	12938.17	8309.02	4737.26	6390.27	8379.71	8463.67	6290.04	6761.09	5514.81
2751	959.93	898.87	833.22	836.44	744.27	781.66	721.05	821.94	686.42
2752	2.13	0.97	0.60	4.64	1.07	0.47	2.22	0.19	0.06
2753	3127.62	2863.74	2468.89	3823.35	2983.77	2919.13	3200.46	3225.79	3012.78
2754	64846.70	61271.86	50380.73	53188.03	44244.21	48522.31	52667.13	49980.22	47008.73
2755	28368.97	27913.17	24469.90	26315.84	25408.69	23788.53	24446.13	23895.20	22864.66
2756	26356.62	24223.26	20315.62	20530.70	23040.71	20200.14	19963.59	19711.84	17962.27
2757	18363.44	17295.54	14613.18	16828.33	16524.77	15672.92	16520.02	15373.34	13991.80
2758	6452.57	5436.76	5426.34	5373.95	5065.88	5186.71	5317.78	4973.42	4469.92
2759	50212.12	47796.75	40679.01	42863.25	41985.90	39444.89	42741.39	40674.95	39935.17
2760	53798.57	52329.22	43477.69	53171.50	51902.08	51656.97	43519.89	41499.99	41467.44
2761	4916.46	4699.91	4305.41	5173.86	4516.66	4316.25	4581.35	4578.45	4468.94
2762	285.92	226.76	155.11	221.80	236.24	209.74	131.68	160.13	145.44
2763	10340.04	9847.90	8130.24	13214.53	11843.08	11779.64	10779.55	10355.48	9569.30
2764	44459.53	40034.13	31002.69	38587.43	38241.14	34675.96	37014.47	33528.56	33735.70
2765	60006.86	56989.86	47017.69	49582.66	48586.12	44905.78	51771.96	46796.84	44575.23
2766	3008.89	2468.11	2183.25	2489.51	2443.63	2491.15	2353.71	1972.68	2066.55
2767	22839.85	17236.26	5629.01	17105.54	15426.72	12972.39	17994.91	14234.72	11808.93
2768	38087.73	37192.14	32231.29	35072.94	34091.17	31733.54	33649.52	31617.25	30194.84
2769	20593.67	16974.11	6259.79	16860.72	15657.38	13565.98	15333.70	13546.55	10078.69
2770	27223.21	29244.14	18118.87	29424.85	30102.62	26038.72	27895.02	26802.40	24460.25
2771	34883.11	58120.87	19026.49	20607.61	25343.99	26306.02	33472.84	29684.09	25629.37
2772	10027.72	9643.92	3965.85	7610.85	7392.85	6246.02	7764.84	7738.83	5898.16
2773	39127.42	37306.23	31007.30	34865.83	35654.70	33428.02	36470.60	34490.12	31890.06
2774	47743.91	46923.28	45040.31	68019.08	57808.83	62031.86	50032.88	46278.50	49901.15
2775	56835.22	53919.79	42326.77	53943.66	50154.19	46949.04	55485.00	51026.91	47538.88
2776	16138.62	19947.09	13019.59	19110.65	16295.56	16660.77	18643.68	17801.86	15954.37
2777	30475.92	28850.42	21819.56	24980.16	22927.24	22061.56	27769.14	26765.70	24899.38
2778	48503.15	35886.41	24716.11	37507.28	36772.71	36489.19	29554.22	27139.39	26862.87
2779	28048.79	28522.36	23039.21	25163.80	22854.98	23196.12	25306.89	26165.95	22649.97
2780	13270.38	11798.97	9999.60	11845.20	10881.68	10576.73	10959.46	10742.64	10374.97
2781	9460.10	8742.62	7897.04	7914.38	8078.97	8969.63	8295.24	7142.46	7081.62
2782	74759.71	78651.28	34463.46	48221.69	63907.03	70366.58	98243.60	87204.41	80166.16
2783	154593.68	144196.51	119571.19	138443.48	133447.73	128084.33	121710.20	115738.56	115614.67
2784	86548.26	80611.53	76118.06	75941.76	71793.23	68412.36	76587.93	72508.09	70323.32
2785	21819.56	17077.69	14733.06	14938.79	17345.82	16814.37	18494.35	18063.31	18344.93
2786	55879.51	46190.64	37980.92	61243.63	60786.63	58002.72	53948.71	47688.23	44290.26
2787	913.76	49.10	4.54	1342.35	742.14	569.79	4.44	6.69	4.47
2788	55003.62	50692.59	40802.70	51721.98	44973.71	43221.34	42481.93	42178.48	38940.48
2789	116037.81	99193.78	93107.98	106641.41	99159.67	98519.75	95361.49	96524.15	94106.65
2790	241753.34	238796.61	187048.67	199205.67	194073.48	188195.93	215759.65	206008.37	189474.79
2791	6514.35	5660.25	4823.28	3893.65	3512.27	3558.58	4019.82	4034.24	3723.56
2792	222037.71	217953.91	196935.85	186454.10	176449.01	171680.92	197504.09	191423.31	186324.03
2793	20008.24	20936.96	15392.30	16869.08	16505.63	14624.95	17672.36	17799.42	16056.90
2794	1581.20	1443.55	1211.93	1114.37	1032.56	969.11	1136.03	1119.12	1022.17
2795	8394.34	8940.92	6799.43	6562.74	6551.49	5890.48	6268.73	6142.85	6367.55
2796	711.67	1082.14	210.01	2016.94	852.41	2487.79	1306.67	518.79	399.65
2797	92530.36	83640.45	70491.87	71064.93	70628.47	65585.90	81535.11	80958.23	75304.80
2798	247811.47	226401.67	184606.48	198044.38	189382.65	181307.17	208323.26	196770.70	187479.98
2799	9512.91	10245.15	9285.62	8202.13	7766.51	8289.12	8508.19	8656.34	8695.27
2800	25034.35	19024.75	18702.24	37306.79	32446.22	27646.84	19300.77	18462.06	16283.16
2801	4206.44	3862.35	2323.58	3004.42	3397.82	3336.97	4018.31	4088.84	4011.05
2802	39374.16	34807.87	28990.91	33591.14	29167.80	31549.72	36911.54	28725.01	26414.74
2803	2.13	0.97	0.60	4.64	1.07	0.47	2.22	0.19	0.06
2804	117191.37	105828.15	82498.47	71280.48	70733.92	67860.71	77957.99	80060.14	73704.15
2805	2.13	0.97	0.60	4.64	1.07	0.47	2.22	0.19	0.06
2806	5664.93	4690.34	4077.16	4281.07	3896.30	4226.88	4399.54	4331.84	3668.09
2807	31102.94	26709.65	24709.11	21804.97	22375.71	18944.40	21615.82	22067.86	20878.71
2808	2.13	0.97	0.60	4.64	1.07	0.47	2.22	0.19	0.06
2809	2.13	0.97	0.60	66.47	65.80	21.20	2.22	0.19	0.06
2810	42808.20	37304.01	29533.01	29649.81	33644.40	32739.06	37063.11	31902.20	32057.49
2811	30736.47	25890.74	22682.72	30897.10	31685.70	29733.84	32515.69	30771.28	30002.89
2812	37046.66	33370.97	28682.36	22860.61	22370.63	22084.87	27256.87	26105.23	24823.76
2813	14054.61	13535.06	7296.05	8023.64	8143.42	7001.39	5629.94	5379.01	4818.14
2814	4076.09	4447.95	2903.29	2413.94	2448.49	2692.77	2983.73	2857.32	2616.87
2815	31163.68	30160.02	25779.67	26208.87	26637.95	24903.43	23899.48	26241.04	23909.17
2816	5096.70	5011.21	3872.39	4154.71	3658.30	3533.94	4075.18	3877.54	3830.17
2817	308281.33	279864.74	217594.61	255394.45	241659.60	235569.35	253705.24	242760.96	228911.13
2818	777425.64	746056.77	622327.63	627165.67	602424.48	586406.93	662506.44	628931.82	596351.30
2819	183218.70	176392.05	141506.61	164743.50	159350.95	149590.24	160012.15	152297.62	139619.33
2820	4234.05	3819.51	3435.88	3953.14	3803.79	3523.96	3486.32	3296.62	3013.09
2821	2.13	0.97	0.60	4.64	1.07	0.47	2.22	0.19	0.06
2822	89605.82	64378.74	57681.97	59463.13	58595.50	55007.87	63004.08	59296.59	55265.27
2823	4223.32	3562.14	2811.64	2830.33	2552.40	2942.09	2717.74	3180.21	2981.21

MDA-MB-231

	control 01	control 02	control 03	Dai IC20 01	Dai IC20 02	Dai IC20 03	Gen IC20 01	Gen IC20 02	Gen IC20 03	SSE IC20 01	SSE IC20 02	SSE IC20 03	unique to MCF-7	unique to MDA-MB-231
2725	29618.90	29847.52	26413.37	19928.69	20220.75	17247.17	22680.13	23015.90	24283.98	24133.68	24645.97	17008.74		
2726	34216.85	31164.90	27113.38	19548.33	18574.84	15605.56	25379.90	23864.42	22133.35	25602.54	24001.09	15204.92		
2727	2.27	1.49	0.65	0.84	1.31	0.69	4.16	2.47	0.11	4.90	4.98	0.12	x	
2728	2.27	1.49	0.65	0.84	1.31	0.69	4.16	2.47	0.11	4.90	4.98	0.12	x	
2729	9747.52	8936.48	8767.16	6803.55	6387.36	6358.00	6653.96	6430.48	6379.48	6463.92	7126.65	3968.53		x
2730	6712.42	5693.36	5319.80	3919.82	3407.14	3002.64	4109.83	4057.75	3633.73	6281.83	5951.88	1799.18		x
2731	15127.74	13999.34	13432.68	8476.36	8550.74	7528.61	9029.63	8982.74	8448.62	10414.68	9927.21	8579.77		
2732	12025.87	12013.03	10677.40	7203.58	7468.74	6652.83	9101.48	8747.74	8407.72	8953.43	8920.39	6476.76		
2733	2.27	1.49	0.65	0.84	1.31	0.69	4.16	2.47	0.11	4.90	4.98	0.12	x	
2734	39567.98	38172.73	34511.83	25961.52	23722.16	24437.49	29907.04	29020.43	29027.91	33092.74	31543.00	19962.46		
2735	2.27	1.49	0.65	0.84	1.31	0.69	4.16	2.47	0.11	4.90	4.98	0.12	x	
2736	23284.53	20964.12	16904.41	12612.10	11422.33	8474.81	14873.06	13799.36	12668.86	16338.87	15111.98	8586.70		
2737	22539.43	21179.63	17539.00	11334.53	11258.92	7816.18	14729.18	14124.03	12034.30	16289.83	15047.47	8150.64		
2738	28976.41	26931.74	25440.44	20168.59	19720.75	16927.33	23558.80	21899.88	19535.00	25019.64	24255.82	16400.26		
2739	1260.56	1072.07	1333.04	1195.89	1007.86	1165.53	1843.54	1740.68	1679.08	1758.02	1532.15	879.91		x
2740	2.27	1.49	0.65	0.84	1.31	0.69	4.16	2.47	0.11	4.90	4.98	0.12	x	
2741	6662.61	6165.04	5873.27	5143.70	4825.87	4115.72	5200.22	4752.77	5273.66	4540.80	4124.59	2499.72		x
2742	29595.28	27798.13	27739.30	18566.88	18565.20	16745.61	26718.92	26160.03	24954.58	27667.88	24473.82	18932.30		
2743	2.27	1.49	0.65	0.84	1.31	0.69	4.16	2.47	0.11	4.90	4.98	0.12	x	
2744	1741.66	1824.29	1735.68	1496.20	1517.84	1244.45	1608.96	1614.21	1246.00	1691.11	1732.21	1136.95		
2745	1120.33	6900.87	4637.85	479.41	391.86	198.44	618.95	530.79	392.06	878.50	679.26	229.36		
2746	2.27	1.49	0.65	0.84	1.31	0.69	4.16	2.47	0.11	4.90	4.98	0.12	x	
2747	2.27	1.49	0.65	0.84	1.31	0.69	4.16	2.47	0.11	4.90	4.98	0.12	x	
2748	51260.57	48744.74	44455.97	28102.19	26879.25	24559.57	34885.50	34245.91	31221.36	33147.98	31464.96	19080.31		x
2749	407368.59	378325.33	343287.43	276885.98	267644.92	242684.19	310489.20	298580.69	287887.38	329608.88	323341.49	242766.97		
2750	1115.29	460.55	582.90	394.85	422.21	112.10	1257.52	1189.31	795.80	844.22	800.42	267.35		
2751	74128.54	67400.87	63400.47	45538.66	45036.19	35871.12	57240.49	53385.81	49752.99	54736.08	51042.43	34060.55		
2752	274.50	1216.03	595.69	251.74	214.37	16.74	589.06	572.99	523.85	714.11	550.09	86.07		x
2753	2.27	1.49	0.65	0.84	1.31	0.69	4.16	2.47	0.11	4.90	4.98	0.12	x	
2754	38984.12	29070.95	24761.53	25227.89	27429.27	20531.66	32756.19	31225.75	28758.27	34835.64	34300.36	22114.74		
2755	3101.31	2964.18	2757.00	1955.36	1908.53	1534.00	2539.52	2351.40	2404.95	2026.08	1774.63	1176.43		
2756	2.27	1.49	0.65	0.84	1.31	0.69	4.16	2.47	0.11	4.90	4.98	0.12	x	
2757	2.27	1.49	0.65	0.84	1.31	0.69	4.16	2.47	0.11	4.90	4.98	0.12	x	
2758	2.27	1.49	0.65	0.84	1.31	0.69	4.16	2.47	0.11	4.90	4.98	0.12	x	
2759	2.27	1.49	0.65	0.84	1.31	0.69	4.16	2.47	0.11	4.90	4.98	0.12	x	
2760	10129.12	9403.47	8363.05	5439.33	5037.81	3801.02	4895.76	4388.69	3911.75	4366.84	4332.40	2250.24		
2761	2.27	1.49	0.65	0.84	1.31	0.69	4.16	2.47	0.11	4.90	4.98	0.12	x	
2762	2029.62	1876.81	1921.37	1466.94	1154.04	1339.25	1680.01	1612.59	1655.51	1953.34	1481.03	1314.18		
2763	183004.67	163856.23	163717.49	114080.59	110276.83	98225.68	120183.53	111564.40	107545.58	99879.74	89301.65	83822.32		
2764	11891.41	9791.35	10056.54	7299.22	6972.95	5188.46	8782.05	7686.17	6850.74	9420.94	8671.11	5429.89		
2765	20141.63	18816.97	17214.25	11303.08	10885.61	9352.43	12709.43	12691.17	11792.07	13852.57	12738.39	4680.75		
2766	2422.54	2594.96	2305.75	2641.29	2413.31	1722.21	2764.92	2900.89	2554.57	2822.79	2692.51	1719.98		
2767	2.27	1.49	0.65	0.84	1.31	0.69	4.16	2.47	0.11	4.90	4.98	0.12	x	
2768	13215.02	11072.36	9767.32	7183.02	6894.31	6431.26	8864.82	7856.64	7179.29	7722.75	7090.91	5104.30		
2769	6860.00	6096.52	4776.45	4335.17	4405.59	2616.15	6943.24	6166.69	4772.72	7998.73	7674.29	3616.44		
2770	2422.58	2291.25	2142.80	1569.59	1566.56	1051.39	2291.92	2203.73	1854.15	2360.52	2254.38	1145.20		
2771	2.27	1.49	0.65	0.84	1.31	0.69	4.16	2.47	0.11	4.90	4.98	0.12	x	
2772	2.27	1.49	0.65	0.84	1.31	0.69	4.16	2.47	0.11	4.90	4.98	0.12	x	
2773	3732.26	3078.04	2905.90	1829.79	1467.65	1285.12	2818.10	2646.98	2513.03	2906.36	2550.80	1679.72		
2774	2.27	1.49	0.65	0.84	1.31	0.69	4.16	2.47	0.11	4.90	4.98	0.12	x	
2775	15588.14	16613.62	14290.01	10877.91	9852.93	7618.64	13423.70	12559.88	12637.61	12609.11	11758.89	7458.19		
2776	6775.99	5727.79	4915.29	4194.42	4083.77	2917.82	5234.63	4776.71	4788.96	5435.68	5008.60	3009.56		
2777	13687.94	13033.90	12444.31	11491.89	11400.43	10770.30	14146.47	14007.17	13321.37	13095.98	13040.71	9424.92		
2778	315117.68	315304.44	288382.22	219697.93	209181.14	204461.58	288337.60	286036.33	260158.57	311371.50	300311.56	189342.57		
2779	2.27	1.49	0.65	0.84	1.31	0.69	4.16	2.47	0.11	4.90	4.98	0.12	x	
2780	6001.94	5168.98	5439.64	3980.99	3976.33	3375.23	5569.28	5157.54	5158.68	4859.21	4298.92	3136.45		
2781	2.27	1.49	0.65	0.84	1.31	0.69	4.16	2.47	0.11	4.90	4.98	0.12	x	
2782	721.13	795.10	745.10	664.01	732.18	728.24	689.02	749.78	749.51	828.21	901.85	739.06		
2783	30561.84	27936.46	24477.45	20046.24	18433.99	14573.75	26988.71	25493.86	22828.48	26785.46	24847.29	14629.00		
2784	81544.66	76393.50	68861.66	58636.12	57141.06	51163.50	64331.05	61557.79	58082.37	67155.33	65222.34	43103.54		
2785	2.27	1.49	0.65	0.84	1.31	0.69	4.16	2.47	0.11	4.90	4.98	0.12	x	
2786	27980.42	27528.15	24076.61	14562.95	15126.89	14249.20	18122.94	20390.19	19036.33	24950.86	23464.15	13297.77		
2787	2.27	1.49	0.65	0.84	1.31	0.69	4.16	2.47	0.11	4.90	4.98	0.12	x	
2788	40171.33	37269.97	34438.18	29457.95	28833.80	28255.65	36114.72	34896.23	32805.65	28607.25	23781.48	20839.63		
2789	26137.99	24357.38	22276.37	23751.50	22323.78	19650.16	22578.52	22547.39	21962.97	23455.66	23297.90	18028.13		
2790	115362.04	108793.48	98306.15	77087.10	73777.95	58308.81	92346.09	85061.72	80047.53	103210.96	98466.49	63330.67		
2791	2.27	1.49	0.65	0.84	1.31	0.69	4.16	2.47	0.11	4.90	4.98	0.12	x	
2792	402912.52	374399.24	359815.46	437354.15	437182.17	422761.36	605525.51	590599.05	580497.12	439841.15	435510.66	323078.21		
2793	2.27	1.49	0.65	0.84	1.31	0.69	4.16	2.47	0.11	4.90	4.98	0.12	x	
2794	2.27	1.49	0.65	0.84	1.31	0.69	4.16	2.47	0.11	4.90	4.98	0.12	x	
2795	2.27	1.49	0.65	0.84	1.31	0.69	4.16	2.47	0.11	4.90	4.98	0.12	x	
2796	2.27	1.49	0.65	0.84	1.31	0.69	4.16	2.47	0.11	4.90	4.98	0.12	x	
2797	24664.43	21811.64	21740.59	12351.23	12814.20	10874.53	18767.83	16921.47	15595.43	16493.66	16778.08	12043.57		
2798	188859.61	181902.42	163008.74	119703.09	118271.75	107473.47	164635.34	163040.72	155765.96	152139.42	148043.36	101927.84		
2799	2.27	1.49	0.65	0.84	1.31	0.69	4.16	2.47	0.11	4.90	4.98	0.12	x	
2800	71648.79	67953.90	61332.86	47595.04	45617.06	42134.00	56418.40	54454.68	51262.83	58280.84	55489.11	38215.52		
2801	2.27	1.49	0.65	0.84	1.31	0.69	4.16	2.47	0.11	4.90	4.98	0.12	x	
2802	2.27	1.49	0.65	0.84	1.31	0.69	4.16	2.47	0.11	4.90	4.98	0.12	x	
2803	110607.56	104409.24	97871.04	78107.68	76875.27	72073.26	82782.91	79761.37	77282.67	88578.68	79052.87	59231.41		x
2804	2.27	1.49	0.65	0.84	1.31	0.69	4.16	2.47	0.11	4.90	4.98	0.12	x	
2805	2.27	1.49	0.65	159.41	102.17	28.14	4.16	2.47	0.11	672.02	669.71	269.11		x
2806	2.27	1.49	0.65	0.84	1.31	0.69	4.16	2.47	0.11	4.90	4.98	0.12	x	
2807	32292.37	30111.87	26654.49	30356.33	29412.93	26246.01	35733.65	31541.21	30930.20	42222.98	40975.37	27448.89		
2808	8033.92	6932.70	5934.50	6598.25	6580.13	4866.43	8146.83	7538.16	6587.08	10724.00	10327.26	6088.77		x
2809	27535.48	25891.12	23366.27	20196.31	19855.05	17518.08	29396.60	27604.71	26055.48	24024.58	23706.83	15028.18		
2810	8113.78	7486.40	6319.80	5915.84	5895.64	5168.21	6012.69	5798.81	5270.69	5938.57	6263.51	5296.74		
2811	2.27	1.49	0.65	0.84	1.31	0.69	4.16	2.47	0.11	4.90	4.98	0.12	x	
2812	21581.77	19486.40	18897.53	17250.59	17081.21	14458.13	22183.01	21122.90	19772.87	25228.14	23137.33	14767.35		
2813	1145.11	67.17	19.70	457.70	539.23	346.30	612.18	512.14	303.58	512.83	532.03	229.91		
2814	923.57	1090.34	923.68	1056.83	953.56	1032.21	1143.83	1098.55	925.73	850.97	899.31	4.60		
2815	135668.04	121997.69	109735.30	115832.18	119387.09	75647.41	150692.95	139800.56	118118.15	98963.72	96712.97	47704.74		
2816	20335.05	21751.76	19006.44	12538.62	11932.63	14656.67	13136.89	10051.88	11186.68	22598.53	21340.26	4206.64		
2817	356536.09	318262.64	289791.08	225141.96	219407.58	185146.77	288729.76	266533.91	242440.53	291915.83	286517.27	194062.48		
2818	747079.23	691137.75	644368.81	524773.03	511445.92	460424.51	634923.40	589762.71	572085.83	619882.39	583691.88	423340.24		
2819	127674.26	119555.84	113345.30	87184.37	86426.08	73925.80	94603.89	89193.82	82235.88	106065.62	106392.55	77850.58		
2820	2.27	1.49	0.65	0.84	1.31	0.69	4.16	2.47	0.11	4.90	4.98	0.12	x	
2821	17350.83	16153.66	13940.56	12361.46	11679.05	9038.67	16813.86	14684.35	13781.44	15998.31	15450.77	8932.44		x
2822	2.27	1.49	0.65	0.84	1.31	0.69	4.16	2.47	0.11	4.90	4.98	0.12	x	
2823	2.27	1.49	0.65	0.84	1.31	0.69	4.16	2.47	0.11	4.90	4.98	0.12	x	

Supplementary Table S1. Overview on the abundance of all relatively quantified 5180 proteins

			MCF-7															
	Protein name	UniProt	control 01	control 02	control 03	Dai IC20 01	Dai IC20 02	Dai IC20 03	Dai SC20 01	Dai SC20 02	Dai SC20 03	Gen IC20 01	Gen IC20 02	Gen IC20 03	Gen SC20 01	Gen SC20 02	Gen SC20 03	SSE IC20 01
2824	Neuroblast differentiation-associated protein AHNAK	Q09666	1783851.69	1674987.94	1550419.96	1090831.45	1049154.10	962117.14	1341062.00	1293651.42	1227768.58	1438783.72	1379418.28	1269711.90	1511593.64	1407723.74	1115496.14	1060413.60
2825	Neuroblastoma breakpoint family member 12	Q5TAG4	13781.86	13997.15	13814.87	9400.42	8814.02	8286.14	11345.88	11336.20	10905.68	12146.36	11868.44	10599.59	12044.78	12008.25	9370.84	10222.47
2826	Neuroblastoma breakpoint family member 6	Q5VWK0	52749.05	50767.12	49006.05	42688.18	41047.87	40587.15	39944.02	39982.86	38192.89	50463.88	48458.55	45451.38	43090.63	44500.08	36904.03	40446.12
2827	Neuroblastoma-amplified sequence	A2RRP1	2986.43	2675.15	2507.37	2177.47	2078.10	2198.89	2115.71	2107.53	1813.16	2372.40	2075.84	2036.39	2208.38	2827.82	2004.41	2574.49
2828	Neurocalcin-delta	P61601	18755.50	19197.67	17218.90	10925.77	10439.62	10022.34	16962.38	16856.36	16204.24	13572.12	13476.91	11545.85	13388.87	13153.32	11912.56	12458.81
2829	Neuroendocrine convertase 1	P29120	28.88	9.69	1.62	207.70	996.98	374.38	1463.17	539.89	376.26	11.59	0.75	8.67	79.57	265.91	14.11	1036.76
2830	Neuroendocrine convertase 2	P16519	223145.27	224095.70	185049.08	109769.45	106052.42	90830.83	170192.19	157872.20	141400.64	157251.56	144147.97	129856.72	157134.80	146600.01	99946.53	142980.55
2831	Neurofascin	O94856	48996.93	48469.51	42845.81	39269.98	39280.82	33391.80	37868.88	36521.97	32144.36	46347.85	44313.47	40914.92	44656.54	44797.44	39726.49	34172.20
2832	Neurofibromin	P21359	81821.69	77857.78	72623.15	52607.58	47828.89	45920.69	50343.54	50683.77	46949.67	69921.78	65119.21	58219.34	59514.67	58015.85	51165.35	48319.91
2833	Neurofilament light polypeptide	P07196	0.14	2.53	0.81	1.07	2.77	0.68	0.96	0.36	0.63	0.35	0.75	0.38	2.13	0.97	0.60	4.64
2834	Neurofilament medium polypeptide	P07197	0.14	2.53	0.81	1.07	2.77	0.68	0.96	0.36	0.63	0.35	0.75	0.38	2.13	0.97	0.60	4.64
2835	Neurogenic differentiation factor 4	Q9HD90	0.14	2.53	0.81	1.07	2.77	0.68	0.96	0.36	0.63	0.35	0.75	0.38	2.13	0.97	0.60	4.64
2836	Neurogenic differentiation factor 6	Q96NK8	0.14	2.53	0.81	1.07	2.77	0.68	0.96	0.36	0.63	0.35	0.75	0.38	2.13	0.97	0.60	4.64
2837	Neurogenic locus notch homolog protein 1	P46531	0.14	2.53	0.81	1.07	2.77	0.68	0.96	0.36	0.63	0.35	0.75	0.38	2.13	0.97	0.60	4.64
2838	Neurogenic locus notch homolog protein 2	Q04721	19187.95	18864.92	18537.78	19685.37	20576.69	20614.64	18708.36	18350.74	17288.01	19892.72	18984.00	18960.54	18018.30	18042.06	16938.56	23658.55
2839	Neurolysin mitochondrial	Q9BYT8	102690.72	97576.24	90408.70	74981.46	67945.76	61949.65	86047.92	81134.33	75029.68	94659.27	89388.35	82521.91	85646.10	76120.39	59223.21	67274.18
2840	Neuronal acetylcholine receptor subunit alpha-7	P36544	1925.07	1797.13	1726.60	1124.80	1097.48	1085.39	1681.15	1715.25	1656.13	1510.63	1434.29	1423.19	1441.46	1494.18	1012.48	1490.36
2841	Neuronal calcium sensor 1	P62166	0.14	2.53	0.81	1.07	2.77	0.68	0.96	0.36	0.63	0.35	0.75	0.38	2.13	0.97	0.60	4.64
2842	Neuronal migration protein doublecortin	O43602	0.14	2.53	0.81	1.07	2.77	0.68	0.96	0.36	0.63	0.35	0.75	0.38	2.13	0.97	0.60	4.64
2843	Neuronal pentraxin-2	P47972	0.14	2.53	0.81	1.07	2.77	0.68	0.96	0.36	0.63	0.35	0.75	0.38	2.13	0.97	0.60	4.64
2844	Neuron-specific calcium-binding protein hippocalcin	P84074	12597.87	12806.99	10627.75	6697.00	5953.60	5817.61	10491.08	10661.07	10025.65	8781.54	8137.27	7893.59	8093.74	8006.42	6530.22	9686.92
2845	Neuropilin and tolloid-like protein 2	Q8NC67	0.14	2.53	0.81	1.07	2.77	0.68	0.96	0.36	0.63	0.35	0.75	0.38	2.13	0.97	0.60	4.64
2846	Neutral alpha-glucosidase AB	Q14697	1671268.45	1600680.42	1459596.83	1092284.59	1058825.50	972702.22	1299952.01	1251160.76	1169419.58	1290617.97	1235225.97	1150830.81	1274353.74	1189950.71	972206.19	1184750.74
2847	Neutral amino acid transporter A	P43007	1776.66	1710.79	1291.68	2005.93	3021.01	2469.26	2596.45	2350.18	2675.72	1620.81	1644.03	1503.74	1518.60	1759.29	1145.54	2571.08
2848	Neutral amino acid transporter B(0)	Q15758	245570.36	239657.06	226287.45	191185.27	188832.65	175935.46	219322.77	208481.06	199711.49	205524.35	198993.24	189926.71	208390.86	199203.44	176785.84	177282.54
2849	Neutral and basic amino acid transport protein rBAT	Q07837	0.14	2.53	0.81	1.07	2.77	0.68	0.96	0.36	0.63	0.35	0.75	0.38	2.13	0.97	0.60	4.64
2850	Neutral ceramidase	Q9NR71	0.14	2.53	0.81	1.07	2.77	0.68	0.96	0.36	0.63	0.35	0.75	0.38	2.13	0.97	0.60	4.64
2851	Neutral cholesterol ester hydrolase 1	Q6PIU2	0.14	2.53	0.81	1.07	2.77	0.68	0.96	0.36	0.63	0.35	0.75	0.38	2.13	0.97	0.60	4.64
2852	NFATC2-interacting protein	Q8NCF5	5938.40	5327.75	3361.97	2202.02	1127.34	1091.59	1411.11	528.31	965.72	3329.59	3037.28	1975.00	2392.37	935.16	317.01	1988.94
2853	NF-kappa-B essential modulator	Q9Y6K9	19366.64	18239.24	18771.56	7284.80	7218.53	6194.01	7771.76	7328.58	6662.97	14585.74	14072.88	12851.56	10386.76	9859.34	7612.52	12981.93
2854	NF-kappa-B inhibitor-like protein 1	Q9UBC1	0.14	2.53	0.81	1.07	2.77	0.68	0.96	0.36	0.63	0.35	0.75	0.38	2.13	0.97	0.60	4.64
2855	NF-kappa-B-activating protein	Q8N5F7	3093.18	2919.07	2225.77	1696.64	1647.44	1346.21	2423.63	2170.73	1776.99	2328.21	1845.22	1783.51	1592.73	1608.57	868.10	2586.22
2856	NF-X1-type zinc finger protein NFXL1	Q6ZNB6	26397.77	25642.82	22130.12	14439.10	15036.63	13013.00	19295.58	18683.13	16340.93	23923.99	21740.82	19551.95	20031.08	19380.83	13408.55	16727.57
2857	NFX1-type zinc finger-containing protein 1	Q9P2E3	8652.55	7547.39	8745.76	6954.18	6715.25	6551.22	7159.08	5943.34	6391.68	9724.65	8966.05	8402.36	9067.92	8390.07	8598.21	7581.46
2858	NGFI-A-binding protein 1	Q13506	18431.32	17849.43	16372.26	11755.93	11486.93	10566.56	13236.95	12934.25	12647.83	14330.34	13825.09	13662.67	12889.83	12198.12	10735.70	13401.71
2859	NGFI-A-binding protein 2	Q15742	88841.49	85117.46	82151.29	63152.82	61567.59	56767.05	67552.40	64104.85	61394.93	68907.71	67486.19	63842.83	74894.05	68767.92	61444.08	56960.30
2860	NHP2-like protein 1	P55769	4079.59	3942.60	3686.75	2581.89	2675.20	2637.80	2783.67	2446.11	2554.32	3095.23	2997.61	3413.63	2756.10	3299.07	3046.08	2659.54
2861	Niban-like protein 1	Q96TA1	180957.02	168256.12	143140.30	106573.90	103382.03	92336.48	121811.94	116185.11	102396.12	140920.54	138784.77	123060.52	137289.69	133344.48	92151.22	101823.07
2862	Nibrin	O60934	0.14	2.53	0.81	1.07	2.77	0.68	0.96	0.36	0.63	0.35	0.75	0.38	2.13	0.97	0.60	4.64
2863	Nicalin	Q969V3	143648.95	141053.00	134419.75	115727.22	111375.93	106363.09	124881.65	122711.20	117993.37	135834.03	133753.64	125696.54	125031.60	118065.02	89147.25	101048.26
2864	Nicolin-1	Q9BSH3	2325.17	1826.59	903.13	658.33	975.51	481.26	1090.63	2104.12	1473.56	1385.99	1283.44	767.12	1783.97	1015.38	86.45	940.73
2865	Nicotinamide N-methyltransferase	P40261	0.14	2.53	0.81	1.07	2.77	0.68	0.96	0.36	0.63	0.35	0.75	0.38	2.13	0.97	0.60	4.64
2866	Nicotinamide phosphoribosyltransferase	P43490	424064.34	405624.05	374410.95	277291.97	273837.43	246244.79	313829.49	316501.93	300023.70	350304.91	323042.15	318848.96	338808.08	306723.38	231916.32	282120.18
2867	Nicotinate phosphoribosyltransferase	Q6XQN6	0.14	2.53	0.81	1.07	2.77	0.68	0.96	0.36	0.63	0.35	0.75	0.38	2.13	0.97	0.60	4.64
2868	Nik-related protein kinase	Q7Z2Y5	19442.60	17921.99	17127.78	12917.55	13270.15	12441.34	14774.32	12847.74	13481.71	15542.04	14901.75	15048.67	15649.66	13796.09	10778.33	12477.63
2869	Ninein	Q8N4C6	268619.10	259699.44	252204.10	165777.34	164731.71	152140.37	206223.55	198405.84	187468.50	245383.03	234086.91	223081.63	209998.11	206353.80	167832.73	197403.34
2870	NIPA-like protein 2	Q9H841	3530.51	3416.25	3097.61	2218.51	1981.62	2013.93	2472.43	2459.59	2287.65	2403.46	2083.35	2063.95	2463.32	2396.88	1914.75	2109.23
2871	Nipped-B-like protein	Q6KC79	4691.89	5009.57	4515.11	2561.25	2765.46	2647.97	2704.97	2798.83	2583.72	3256.83	3201.06	3001.96	3911.98	3620.95	3352.92	2593.37
2872	Nitric oxide synthase-interacting protein	Q9Y314	14126.99	12481.65	11511.33	9191.03	5914.46	7490.85	9966.67	10242.74	9452.30	12611.19	12357.53	10879.34	10394.54	9105.65	7673.80	9347.69
2873	NLR family member X1	Q86UT6	3715.26	3355.51	2967.04	2878.03	2313.04	2035.23	2712.72	2528.88	2271.51	2791.14	2603.91	2597.64	2283.87	2125.96	1144.36	3165.17
2874	NmrA-like family domain-containing protein 1	Q9HBL8	13598.83	14077.02	13157.41	9796.92	10137.28	9532.15	11659.59	12002.30	11192.50	13879.20	13758.21	12659.23	11680.25	11179.06	9969.48	10082.86
2875	Nodal modulator 1	Q15155	166516.60	164806.99	150491.69	116353.69	109406.04	101160.79	123287.35	110454.22	106631.26	155093.55	147238.88	134408.97	138941.97	125512.43	91742.97	107013.59
2876	Nodal modulator 3	P69849	12559.76	11238.88	10284.37	8531.65	12659.34	10874.92	10029.56	8280.63	7747.64	11487.90	10632.35	10052.93	9966.97	10266.93	7406.26	13465.03
2877	Noelin-3	Q96PB7	0.14	2.53	0.81	1.07	2.77	0.68	0.96	0.36	0.63	0.35	0.75	0.38	2.13	0.97	0.60	4.64
2878	Non-histone chromosomal protein HMG-14	P05114	1046.18	1113.53	1126.01	568.19	799.68	395.16	136.46	271.13	1804.51	1199.96	1289.10	2318.11	996.10	6880.29	121.85	80.70
2879	Non-histone chromosomal protein HMG-17	P05204	0.14	2.53	0.81	1.07	2.77	0.68	0.96	0.36	0.63	0.35	0.75	0.38	2.13	0.97	0.60	4.64
2880	Non-POU domain-containing octamer-binding protein	Q15233	271387.27	268189.22	245770.22	211236.20	204802.91	198994.69	217205.31	204657.36	202458.22	252033.58	236035.09	229008.09	223637.50	214297.61	184594.09	198753.65
2881	Non-receptor tyrosine-protein kinase TYK2	P29597	2650.21	2101.49	1509.43	1654.62	1067.44	1026.33	1598.27	2330.01	1418.43	1990.91	1807.65	1840.61	2091.31	2513.71	625.69	1588.23
2882	Non-specific lipid-transfer protein	P22307	79258.10	76403.63	69039.72	47915.64	48399.26	44773.68	58901.44	61596.25	60391.72	65739.59	60991.71	55528.14	65540.89	59446.42	49392.29	47104.29
2883	Non-structural maintenance of chromosomes element 3 homolog	Q96MG7	19265.39	18767.23	18584.23	12213.45	11587.03	11315.76	18344.32	18591.11	17869.98	14180.48	14085.06	13520.59	14947.11	13178.20	11631.67	11665.34
2884	Nostrin	Q8IVI9	1837.17	1738.30	1700.65	1346.10	1194.25	1036.42	1529.41	1593.16	1468.91	1783.06	1960.72	1683.08	1676.68	1457.36	1195.96	1467.07
2885	Notch homolog 2 N-terminal-like protein C	P0DPK4	1399.46	1859.75	1948.33	1459.98	1227.02	1186.33	1681.03	1579.71	1677.83	1609.09	1241.72	1316.12	1860.90	1619.88	1222.98	1298.66
2886	Notchless protein homolog 1	Q9NVX2	0.14	2.53	0.81	1.07	2.77	0.68	0.96	0.36	0.63	0.35	0.75	0.38	2.13	0.97	0.60	4.64
2887	NPC intracellular cholesterol transporter 2	P61916	0.14	2.53	0.81	1.07	2.77	0.68	0.96	0.36	0.63	0.35	0.75	0.38	2.13	0.97	0.60	4.64
2888	NSFL1 cofactor p47	Q9UNZ2	48145.24	48766.71	47114.70	41991.99	40855.76	37932.11	42254.12	40400.31	39647.59	46987.96	44163.37	43312.31	42513.96	40889.66	34092.66	39654.12
2889	N-terminal kinase-like protein	Q96KG9	14789.44	14270.18	13224.52	8374.81	8173.23	7532.60	10796.93	10527.19	9481.40	11437.05	10587.06	10388.05	12072.04	11416.89	9637.34	8424.45
2890	N-terminal Xaa-Pro-Lys N-methyltransferase 1	Q9BV86	76430.32	77513.20	76370.30	54390.37	55472.41	52697.34	61211.71	57292.17	53510.35	73017.89	70256.34	64706.73	61850.61	61611.52	50009.66	45740.79
2891	Nuclear apoptosis-inducing factor 1	Q69YI7	508.53	380.17	179.52	1.07	2.77	0.68	0.96	0.36	0.63	120.98	102.00	44.43	2.13	0.97	0.60	4.64
2892	Nuclear autoantigen Sp-100	P23497	248644.93	248810.13	225520.63	167141.49	164469.61	160160.28	211388.37	203856.41	205370.76	182891.72	178007.64	172442.53	227523.89	190629.34	182846.07	194715.29
2893	Nuclear autoantigenic sperm protein	P49321	345650.38	335803.73	324075.10	261886.21	252654.35	239931.37	257218.96	245348.99	235553.49	286564.56	282203.06	260994.86	283360.53	278154.77	234626.67	231060.40
2894	Nuclear cap-binding protein subunit 1	Q09161	747686.89	717950.66	675726.78	499638.06	499004.30	452603.44	619041.62	602607.27	572389.43	612970.85	587897.91	533828.44	607205.81	577724.42	480125.94	523943.55
2895	Nuclear cap-binding protein subunit 2	P52298	1122.73	1179.93	950.17	772.20	758.16	606.69	457.33	505.94	424.43	883.56	772.66	923.89	1101.92	1152.31	8.23	405.36
2896	Nuclear distribution protein nudE homolog 1	Q9NXR1	0.14	2.53	0.81	1.07	2.77	0.68	0.96	0.36	0.63	0.35	0.75	0.38	2.13	0.97	0.60	4.64
2897	Nuclear distribution protein nudE-like 1	Q9GZM8	299.39	186.11	213.50	96.74	122.42	85.04	233.00	180.03	87.82	204.90	169.31	96.89	188.95	180.11	170.65	102.35
2898	Nuclear factor interleukin-3-regulated protein	Q16649	0.14	2.53	0.81	1.07	2.77	0.68	0.96	0.36	0.63	0.35	0.75	0.38	2.13	0.97	0.60	4.64
2899	Nuclear factor NF-kappa-B p100 subunit	Q00653	23410.25	21911.57	18568.14	13652.30	13176.50	12060.48	16257.77	15719.39	13921.32	18488.20	18156.83	15702.96	18282.41	15367.87	10818.65	14884.56
2900	Nuclear fragile X mental retardation-interacting protein 2	Q7Z417	31824.06	29771.42	26361.20	16883.23	16239.67	14319.44	19348.53	18194.05	16855.92	23390.71	22245.73	20127.01	21867.97	18621.41	14437.92	15114.06
2901	Nuclear GTPase SLIP-GC	Q68CJ6	0.14	2.53	0.81	1.07	2.77	0.68	0.96	0.36	0.63	0.35	0.75	0.38	2.13	0.97	0.60	4.64
2902	Nuclear migration protein nudC	Q9Y266	419958.29	407074.26	379786.25	290565.33	281558.66	264806.77	333651.97	325808.50	312280.11	357018.81	341985.58	324308.70	350086.37	332490.57	275552.71	278109.18
2903	Nuclear mitotic apparatus protein 1	Q14980	173081.16	161859.89	154984.85	128587.85	123579.66	113783.63	131318.12	129525.92	122107.21	148935.45	138090.79	130942.92	141836.19	141981.83	111092.59	118268.70
2904	Nuclear pore complex protein Nup107	P57740	20239.69	19071.13	17522.35	12726.56	12860.69	11511.30	14365.46	13959.79	13589.58	14270.48	13618.45	14062.96	13559.17	14608.20	12189.47	12856.22
2905	Nuclear pore complex protein Nup155	O75694	15632.26	14056.33	13002.11	7973.40	7165.17	6924.90	10555.94	10094.41	9921.44	12477.00	11203.14	10964.72	11020.09	10567.64	7470.94	11303.17
2906	Nuclear pore complex protein Nup50	Q9UKX7	6172.30	6103.99	5439.95	3581.20	3442.01	3031.40	3831.22	3474.37	3254.85	3248.68	3048.47	3175.09	3908.59	4313.31	3873.26	2932.21
2907	Nuclear pore complex protein Nup85	Q9BW27	0.14	2.53	0.81	1.07	2.77	0.68	0.96	0.36	0.63	0.35	0.75	0.38	2.13	0.97	0.60	4.64
2908	Nuclear pore complex protein Nup88	Q99567	0.14	2.53	0.81	1.07	2.77	0.68	0.96	0.36	0.63	0.35	0.75	0.38	2.13	0.97	0.60	4.64
2909	Nuclear pore complex protein Nup93	Q8N1F7	13.16	14.39	20.00	154.01	21.05	7.34	11.72	13.35	12.91	10.25	19.01	5.67	4.25	179.21	18.73	13.47
2910	Nuclear pore glycoprotein p62	P37198	0.14	2.53	0.81	1.07	2.77	0.68	0.96	0.36	0.63	0.35	0.75	0.38	2.13	0.97	0.60	4.64
2911	Nuclear pore membrane glycoprotein 210	Q8TEM1	9855.65	9249.49	7310.26	6614.08	6620.62	5523.12	6723.27	6477.45	6504.28	6694.26	6451.32	5912.06	8171.72	7805.34	4732.71	5322.12
2912	Nuclear protein localization protein 4 homolog	Q8TAT6	410264.04	406803.35	405596.57	311563.22	312665.59	306081.93	329700.16	318091.87	304491.20	370177.11	340829.72	322526.92	351356.73	348429.65	297297.44	296717.00
2913	Nuclear receptor 2C2-associated protein	Q86WQ0	15270.04	12832.33	8021.23	6207.62	5759.77	4129.93	6543.91	6005.15	4948.26	8765.82	7769.24	5962.45	9641.64	8539.47	3496.67	5440.85
2914	Nuclear receptor coactivator 2	Q15596	6464.76	6329.61	6704.02	5149.84	4901.65	4782.06	5498.21	5579.79	5478.83	6493.19	6327.12	5587.20	6676.38	6083.94	4610.96	5219.10
2915	Nuclear receptor coactivator 3	Q9Y6Q9	14243.84	13227.30	12003.79	10833.58	10790.94	10581.88	11015.73	11002.79	10216.37	11279.28	12009.37	12732.29	12704.76	12087.56	10717.03	10337.32
2916	Nuclear receptor coactivator 5	Q9HCD5	15340.85	14277.75	13907.69	10465.47	10707.43	9372.59	10091.26	9764.22	13009.90	14772.70	14864.91	14094.05	11696.15	12302.67	14316.97	7533.17
2917	Nuclear receptor coactivator 7	Q8NI08	0.14	2.53	0.81	1.07	2.77	0.68	0.96	0.36	0.63	0.35	0.75	0.38	2.13	0.97	0.60	4.64
2918	Nuclear receptor corepressor 2	Q9Y618	31676.53	30102.69	30544.64	24914.95	22904.20	22976.68	29962.64	29616.45	29099.62	29998.93	27807.76	27127.63	28580.62	26757.86	23099.02	25493.81
2919	Nuclear receptor subfamily 2 group F member 6	P10588	178.80	109.23	249.41	49.49	48.61	123.19	115.81	88.22	119.35	167.12	219.33	261.63	194.84	32.07	23.16	62.52
2920	Nuclear receptor subfamily 6 group A member 1	Q15406	25236.51	23024.52	23536.44	23243.28	21805.91	20944.07	19529.88	21796.00	20813.83	24291.04	23372.92	20062.51	17778.07	21989.23	20052.94	16935.85
2921	Nuclear receptor-binding protein	Q9UHY1	8113.49	7630.36	7666.45	6652.10	6009.57	5922.21	5830.84	5867.13	5672.20	8338.64	7874.29	7554.60	7386.13	7293.59	5985.72	5934.41
2922	Nuclear RNA export factor 2	Q9GZY0	10852.65	11192.90	11198.21	8899.25	7963.81	8069.53	9386.25	9407.37	8961.39	11281.22	10573.54	9810.16	10235.43	9770.04	8173.36	7789.20
2923	Nuclear RNA export factor 3	Q9H4D5	19688.24	19273.14	19696.16	13217.99	12540.43	12835.40	20779.16	17523.26	17025.63	15014.04	14741.19	13453.08	15652.03	14836.63	12377.49	14153.49
2924	Nuclear transport factor 2	P61970	180117.93	167560.90	150715.99	104333.26	101804.23	86928.20	138863.79	130696.70	122835.47	132729.28	126036.72	110787.16	131881.00	122395.13	89722.74	113521.79
2925	Nuclear ubiquitous casein and cyclin-dependent kinase substrate 1	Q9H1E3	124179.55	120323.98	112929.51	93192.37	86898.89	85361.49	90577.80	90390.65	84942.42	103399.94	97877.83	90398.99	107828.18	100418.96	61262.86	77718.08
2926	Nuclease-sensitive element-binding protein 1	P67809	328343.48	319626.46	297667.71	222520.56	212011.52	208251.72	241737.08	230392.76	221002.29	255136.42	243562.81	234385.36	242222.85	248509.01	215773.46	204675.46
2927	Nucleic acid dioxygenase ALKBH1	Q13686	7059.53	7040.54	7162.72	4589.41	4498.41	4354.81	5630.51	5477.41	5271.56	6937.73	6263.51	5980.78	6779.70	5180.32	4736.43	4589.29
2928	Nucleobindin-1	Q02818	8794.91	7590.69	7490.03	5332.28	5232.87	5444.27	6441.50	7392.86	5932.17	6619.57	7177.50	6903.71	6763.57	5452.59	3833.06	4845.34
2929	Nucleobindin-2	P80303	138766.04	127800.17	116678.28	86961.18	77019.61	64195.58	88826.11	85009.98	79226.05	119440.52	110177.38	101946.39	103135.39	96378.77	64484.75	77708.47
2930	Nucleolar GTP-binding protein 1	Q9BZE4	68572.89	64539.99	63365.42	52908.50	51310.05	50155.30	52462.17	53341.24	52060.84	60019.18	56055.27	52720.90	59554.44	59949.94	48366.68	52739.67
2931	Nucleolar MIF4G domain-containing protein 1	Q5C9Z4	20703.25	19613.37	17618.23	13072.20	13171.18	11357.10	16236.86	15486.51	13839.81	18545.75	16848.83	15988.17	16554.69	15115.52	10517.93	14441.43
2932	Nucleolar protein 16	Q9Y3C1	42738.43	40843.70	36525.84	29527.06	30293.02	27984.03	32343.96	30528.73	29841.69	38915.85	37058.24	36630.02	35333.60	31784.37	26605.15	29145.89
2933	Nucleolar protein 3	O60936	35710.62	35245.43	30570.25	23792.70	23013.15	20307.47	26938.04	25337.25	22676.22	32016.86	29519.87	26677.43	28171.00	25660.57	20444.70	25333.12
2934	Nucleolar protein 4	O94818	18435.56	16102.58	16967.85	12520.26	11583.13	11834.43	16961.89	15649.50	15131.69	15650.94	15157.85	14077.53	14711.23	14502.12	12293.80	13622.34
2935	Nucleolar protein 4-like	Q96MY1	5703.12	5401.15	6223.10	5304.41	4873.59	4694.88	4819.10	4062.69	3735.45	6713.02	5648.64	5304.89	5827.45	5322.89	5155.07	4139.28
2936	Nucleolar protein 58	Q9Y2X3	29908.49	28325.01	27104.69	17522.48	17758.69	17549.35	20950.60	19701.73	20234.83	23296.68	21149.78	21443.09	22070.53	21348.44	18222.34	17465.70
2937	Nucleolar protein 8	Q76FK4	34328.36	38429.08	33562.60	19221.64	18999.80	18675.77	20744.59	19282.71	19783.73	30303.52	29308.80	26172.10	24676.90	25897.11	21026.67	18531.99
2938	Nucleolar protein 9	Q86U38	0.14	2.53	0.81	1.07	2.77	0.68	0.96	0.36	0.63	0.35	0.75	0.38	2.13	0.97	0.60	4.64
2939	Nucleolar RNA helicase 2	Q9NR30	79684.12	79361.04	78185.98	49011.18	46095.71	48144.11	61350.62	58429.57	57576.42	58110.40	56027.86	52916.45	57992.37	56409.96	48019.92	49125.72
2940	Nucleolar transcription factor 1	P17480	93190.63	89073.01	87692.97	62174.68	60170.18	55824.18	77438.99	73485.33	70106.99	78076.43	76484.10	75380.55	76560.59	75404.05	63251.65	58192.98
2941	Nucleolin	P19338	2180418.52	2051338.74	1872251.36	1482780.67	1450192.72	1303847.06	1696273.08	1629063.14	1537407.21	1683445.36	1583570.17	1460344.35	1788423.06	1645519.83	1150355.55	1379557.45
2942	Nucleolysin TIA-1 isoform p40	P31483	37985.21	33880.22	34886.02	25821.44	24628.32	23838.26	28270.88	25733.16	25617.57	36852.85	34407.76	31924.82	31683.30	32339.57	21971.42	25838.48
2943	Nucleolysin TIAR	Q01085	520.20	601.39	406.50	66.84	577.96	322.57	543.55	330.76	164.60	296.69	235.54	274.84	299.03	121.33	4.96	443.03
2944	Nucleophosmin	P06748	1020244.23	931527.45	877419.90	636199.69	602590.43	551788.99	745686.66	726709.87	761202.40	774931.46	724985.47	673572.23	850568.69	763363.86	510706.28	706601.35
2945	Nucleoplasmin-3	O75607	3790.59	4499.70	3795.56	3598.39	3527.74	3102.63	3719.92	3549.26	3505.52	4135.73	3946.57	3570.04	4470.35	4025.74	3091.50	2720.67
2946	Nucleoporin NUP188 homolog	Q5SRE5	8208.81	7470.82	6469.94	5229.87	4910.14	3874.61	5996.78	5843.52	5147.27	6050.03	5724.87	5340.96	6321.49	5705.35	3543.97	5239.34
2947	Nucleoporin NUP35	Q8NFH5	0.14	2.53	0.81	1.07	2.77	0.68	0.96	0.36	0.63	0.35	0.75	0.38	2.13	0.97	0.60	4.64
2948	Nucleoporin Nup37	Q8NFH4	2772.36	3266.14	2666.36	2831.28	2402.47	2114.39	2245.63	2188.18	2299.54	2993.59	2827.14	2933.29	2527.02	3022.99	2174.83	2035.06
2949	Nucleoporin p54	Q7Z3B4	92787.00	90812.32	80391.36	88968.34	88932.54	77584.85	90793.77	86553.74	79281.28	87009.83	83474.93	74606.32	57858.83	53233.76	42518.57	135842.24
2950	Nucleoporin SEH1	Q96EE3	21233.40	19920.26	18428.29	17956.09	13421.88	11782.48	14366.28	17391.29	14619.46	16727.36	16056.19	15576.82	17726.12	29912.97	12867.56	13741.79
2951	Nucleoporin-like protein 2	O15504	2772.30	2904.57	2576.06	1759.14	1873.93	1847.78	1648.16	1316.70	804.72	2449.51	2686.96	2768.25	2168.45	1625.83	1023.58	1080.31
2952	Nucleoprotein TPR	P12270	251425.01	239388.27	225463.83	163079.21	159537.71	147612.08	176778.61	172238.16	171572.39	226645.36	215709.50	204855.21	200477.00	185511.67	160253.48	187510.99
2953	Nucleoredoxin	Q6DKJ4	0.14	2.53	0.81	1.07	2.77	0.68	0.96	0.36	0.63	0.35	0.75	0.38	2.13	0.97	0.60	4.64
2954	Nucleoredoxin-like protein 1	Q96CM4	7689.90	6520.52	4734.19	4474.24	28684.44	19380.78	37477.01	4357.15	4100.55	5682.15	4681.09	3865.71	5461.71	5879.29	2249.25	25890.24
2955	Nucleoredoxin-like protein 2	Q5VZ03	0.14	2.53	0.81	1.07	2.77	0.68	0.96	0.36	0.63	0.35	0.75	0.38	2.13	0.97	0.60	4.64
2956	Nucleoside diphosphate kinase 3	Q13232	1967.37	2218.69	2173.80	1753.51	2159.98	1953.13	2444.53	2352.67	1704.29	3231.79	3092.21	2628.46	1758.88	1988.84	1506.80	1849.46
2957	Nucleoside diphosphate kinase 7	Q9Y5B8	0.14	2.53	0.81	1.07	2.77	0.68	0.96	0.36	0.63	0.35	0.75	0.38	2.13	0.97	0.60	4.64
2958	Nucleoside diphosphate kinase A	P15531	431242.06	417146.51	365325.67	257202.67	244154.70	222701.01	336181.86	321046.91	307627.64	345871.98	336889.74	311035.16	317701.18	303134.53	269496.75	281596.20
2959	Nucleoside diphosphate kinase B	P22392	1947243.30	1891893.14	1762760.90	1293390.11	1234064.12	1185590.77	1620587.19	1631724.03	1518701.39	1635984.20	1605100.51	1532247.47	1447458.34	1445866.57	1185036.94	1326386.65
2960	Nucleoside diphosphate-linked moiety X motif 19	A8MXV4	9569.05	9754.69	7931.51	8773.83	7925.64	7168.49	8030.43	7322.25	6951.01	10154.38	9220.76	8667.99	8080.95	7203.93	5670.49	8989.67
2961	Nucleosome assembly protein 1-like 1	P55209	193824.10	178392.58	158508.71	142151.35	136473.69	123499.43	158289.76	152987.67	139375.06	170468.67	164392.87	155480.24	166081.55	157568.65	112276.79	138327.96

	MCF-7 (cont.)					MDA-MB-231													
	SSE IC20 02	SSE IC20 03	SSE SC20 01	SSE SC20 02	SSE SC20 03	control 01	control 02	control 03	Dai IC20 01	Dai IC20 02	Dai IC20 03	Gen IC20 01	Gen IC20 02	Gen IC20 03	SSE IC20 01	SSE IC20 02	SSE IC20 03	unique to MCF-7	unique to MDA-MB-231
2824	1018017.54	971212.01	1188977.07	1115448.23	1024976.66	4949613.62	4638296.97	4280682.65	3025689.56	2937570.61	2548596.63	3941676.97	3750367.32	3558957.57	3831047.15	3734277.12	2614411.09		
2825	9111.27	9567.64	10170.26	9341.54	8999.04	2.27	1.49	0.65	0.84	1.31	0.69	4.16	2.47	0.11	4.90	4.98	0.12	x	
2826	37109.46	36737.59	37347.38	36376.97	36446.43	2.27	1.49	0.65	0.84	1.31	0.69	4.16	2.47	0.11	4.90	4.98	0.12	x	
2827	2661.76	2390.23	4705.19	2076.03	4201.91	2.27	1.49	0.65	0.84	1.31	0.69	4.16	2.47	0.11	4.90	4.98	0.12	x	
2828	12402.68	11808.89	12279.19	11486.86	11136.24	1191.09	1258.44	1081.31	635.02	577.60	516.40	802.31	783.32	559.38	801.28	1003.55	353.49		
2829	640.02	390.73	31.08	5.75	11.62	2.27	1.49	0.65	0.84	1.31	0.69	4.16	2.47	0.11	4.90	4.98	0.12	x	
2830	137743.10	118723.78	141829.92	134413.72	121049.96	157453.24	147581.57	130198.10	85436.08	88200.53	67248.42	125482.52	110936.31	102079.10	112229.92	112014.25	69735.67		
2831	34314.57	31538.45	36963.53	34559.18	37102.82	2.27	1.49	0.65	0.84	1.31	0.69	4.16	2.47	0.11	4.90	4.98	0.12	x	
2832	47426.49	41845.95	66135.79	59777.25	59511.79	2.27	1.49	0.65	0.84	1.31	0.69	4.16	2.47	0.11	4.90	4.98	0.12	x	
2833	1.07	0.47	2.22	0.19	0.06	2195.72	1906.62	1806.57	1282.17	1129.27	859.40	1628.87	1610.55	1670.70	1392.89	1598.00	966.38		x
2834	1.07	0.47	2.22	0.19	0.06	855.24	904.05	682.64	600.35	682.85	478.66	733.00	767.16	700.03	1024.77	963.48	619.42		x
2835	1.07	0.47	2.22	0.19	0.06	688.56	418.66	173.75	509.36	326.53	64.82	628.11	492.73	198.60	1162.90	1048.73	179.05		x
2836	1.07	0.47	2.22	0.19	0.06	20268.33	19265.72	17887.33	13762.23	13344.01	9823.83	16490.68	15727.87	14351.89	14520.51	14077.90	8594.63		x
2837	1.07	0.47	2.22	0.19	0.06	8524.15	10235.35	7674.96	6802.70	6338.08	5913.80	7775.98	7952.77	7396.27	8213.44	7351.90	5268.96		x
2838	22166.02	22110.40	16914.19	17135.52	15646.53	2.27	1.49	0.65	0.84	1.31	0.69	4.16	2.47	0.11	4.90	4.98	0.12	x	
2839	66834.56	61373.40	87658.50	85145.88	74117.34	150662.20	140109.00	130134.98	86225.57	84237.10	72116.43	108607.43	101554.48	95731.97	103314.39	101155.80	67193.27		
2840	1384.82	1218.85	1261.32	1158.65	1068.12	2.27	1.49	0.65	0.84	1.31	0.69	4.16	2.47	0.11	4.90	4.98	0.12	x	
2841	1.07	0.47	2.22	0.19	0.06	8168.18	7707.88	7086.73	5425.35	4975.31	4516.84	6947.74	6642.10	5967.13	6369.82	5771.87	3297.80		x
2842	1.07	0.47	2.22	0.19	0.06	4783.10	4456.43	4187.87	2955.12	3086.84	2734.55	3791.42	3515.96	3242.18	3705.27	3543.11	2544.34		x
2843	1.07	0.47	2.22	0.19	0.06	611.77	691.31	701.84	319.02	383.45	301.32	460.06	422.30	409.69	368.77	370.34	174.76		x
2844	9164.52	9252.53	9025.64	7825.17	7710.88	2.27	1.49	0.65	0.84	1.31	0.69	4.16	2.47	0.11	4.90	4.98	0.12	x	
2845	1.07	0.47	2.22	0.19	0.06	1347.36	1120.59	893.60	744.08	739.58	667.95	1330.29	900.45	899.72	901.37	805.43	489.55		x
2846	1115613.88	1051722.33	1078258.26	1037693.74	953851.89	898135.74	864627.35	823076.62	566820.57	548369.07	501056.72	643470.90	612830.02	610482.72	613013.33	576253.29	448800.84		
2847	3099.42	2901.99	2209.26	2186.65	1814.61	2.27	1.49	0.65	0.84	1.31	0.69	4.16	2.47	0.11	4.90	4.98	0.12	x	
2848	165083.13	168081.02	196509.98	190166.06	177663.51	91340.38	84660.42	75030.61	67853.98	67682.62	64591.99	69142.04	65053.35	61881.29	76263.44	74485.51	38752.94		
2849	1.07	0.47	2.22	0.19	0.06	1812.51	1523.31	1478.40	1104.18	1014.64	844.92	1303.69	1402.55	1077.09	1209.70	1332.32	704.86		x
2850	1.07	0.47	2.22	0.19	0.06	6602.50	5584.50	4324.34	4891.66	5000.83	5076.33	5084.58	4843.76	4804.91	6779.59	7517.41	290.96		x
2851	1.07	0.47	2.22	0.19	0.06	62392.26	59887.76	54165.10	34241.82	35577.65	32935.84	50173.08	48057.13	42762.49	46811.39	43350.37	26787.95		x
2852	752.09	733.47	1260.62	1218.00	543.09	2.27	1.49	0.65	0.84	1.31	0.69	4.16	2.47	0.11	4.90	4.98	0.12	x	
2853	13136.74	12478.62	12681.01	10831.20	10882.45	2.27	1.49	0.65	0.84	1.31	0.69	4.16	2.47	0.11	4.90	4.98	0.12	x	
2854	1.07	0.47	2.22	0.19	0.06	15851.90	14284.88	15001.93	15602.97	15431.72	13455.25	18523.49	15577.56	16618.41	12922.76	15719.05	10203.60		x
2855	2018.57	2010.05	1785.21	2736.93	2329.56	2.27	1.49	0.65	0.84	1.31	0.69	4.16	2.47	0.11	4.90	4.98	0.12	x	
2856	15335.43	14297.30	16514.67	15661.61	14647.00	2.27	1.49	0.65	0.84	1.31	0.69	4.16	2.47	0.11	4.90	4.98	0.12	x	
2857	7205.03	6898.01	8477.42	7594.47	6418.52	16078.21	15982.73	13969.02	12822.62	13177.51	12029.74	10810.50	11536.17	11931.42	13783.12	13233.00	11305.38		
2858	13199.98	12508.62	12589.82	12160.05	11718.72	46232.97	43892.39	36547.79	12445.79	12233.82	7287.31	9817.18	8723.48	7175.14	11001.81	10374.47	5172.28		
2859	51796.37	51671.55	60509.25	59639.86	56973.39	2.27	1.49	0.65	0.84	1.31	0.69	4.16	2.47	0.11	4.90	4.98	0.12	x	
2860	1789.32	1908.46	2729.07	2560.54	2653.97	8789.71	8149.54	7714.77	5380.10	5146.76	4292.89	6786.80	6058.57	6501.90	7318.35	7358.72	4119.22		
2861	96329.15	92652.11	111938.23	109381.94	97722.90	129940.81	121173.71	104977.86	77678.02	76761.97	64276.85	94956.76	89862.55	80992.43	101701.78	93229.91	56682.29		
2862	1.07	0.47	2.22	0.19	0.06	5595.19	5470.17	5358.58	4461.14	3972.90	3892.31	4409.90	4103.21	4080.05	4851.60	4172.22	3262.66		x
2863	100666.94	101936.14	116890.80	105311.58	98432.54	10845.73	10381.53	9886.18	7343.53	7092.49	5854.02	9331.57	9033.11	8533.31	9555.44	9443.72	6326.77		
2864	978.52	568.00	454.58	494.90	141.36	2.27	1.49	0.65	0.84	1.31	0.69	4.16	2.47	0.11	4.90	4.98	0.12	x	
2865	1.07	0.47	2.22	0.19	0.06	128014.51	124022.84	113455.09	88482.69	85429.08	80851.32	100877.62	100904.03	96975.57	141820.41	135700.85	52406.49		x
2866	265625.85	259447.84	284456.18	262904.31	246090.73	23359.05	21517.27	20321.05	14512.61	12956.78	10258.69	20194.79	19146.43	16679.60	17048.99	16634.83	9930.65		
2867	1.07	0.47	2.22	0.19	0.06	9831.44	7439.34	8158.02	7697.63	6943.22	4907.09	8545.04	8168.34	5961.18	11117.11	9955.59	5607.96		x
2868	11519.92	10811.81	12518.26	11993.98	11596.24	2.27	1.49	0.65	0.84	1.31	0.69	4.16	2.47	0.11	4.90	4.98	0.12	x	
2869	186855.31	178961.49	182683.23	177800.55	164268.59	299164.35	285054.04	254493.90	290177.43	294552.25	289251.04	379549.52	382101.89	364986.64	283448.61	274588.14	207911.60		
2870	1973.37	1893.02	2234.60	2224.07	2046.41	2.27	1.49	0.65	0.84	1.31	0.69	4.16	2.47	0.11	4.90	4.98	0.12	x	
2871	2453.68	2434.91	2355.13	2537.66	2384.56	2.27	1.49	0.65	0.84	1.31	0.69	4.16	2.47	0.11	4.90	4.98	0.12	x	
2872	8261.94	8131.24	7789.45	7441.35	7225.82	3061.73	2696.88	2463.22	1962.26	1694.68	1709.18	2176.63	1984.21	1839.04	2498.68	2462.50	1456.50		
2873	3072.76	2628.00	2075.05	1993.90	1573.81	1144.55	1090.16	946.82	687.51	656.19	626.84	909.12	923.49	851.44	997.42	865.26	518.48		
2874	9999.17	9526.54	10613.79	9891.07	9825.24	2.27	1.49	0.65	0.84	1.31	0.69	4.16	2.47	0.11	4.90	4.98	0.12	x	
2875	99791.32	98801.32	112747.89	110783.59	98893.17	2.27	1.49	0.65	0.84	1.31	0.69	4.16	2.47	0.11	4.90	4.98	0.12	x	
2876	10936.64	11772.52	7532.64	7049.08	6562.52	2.27	1.49	0.65	0.84	1.31	0.69	4.16	2.47	0.11	4.90	4.98	0.12	x	
2877	1.07	0.47	2.22	0.19	0.06	3154.67	2951.89	2610.96	2264.14	1987.99	1228.01	2679.69	2422.79	2099.06	3992.38	3604.65	2054.96		x
2878	57.25	129.96	236.93	493.58	315.33	401.78	636.83	413.02	909.19	782.04	943.61	561.98	636.45	1073.27	1380.93	12115.19	253.97		
2879	1.07	0.47	2.22	0.19	0.06	5.32	151.12	42.26	1234.23	1171.08	1388.19	287.60	697.04	785.47	2326.68	2073.09	635.72		x
2880	183535.92	186179.96	192883.96	201445.65	181629.19	229745.15	215532.31	204396.66	174918.30	171842.09	162645.09	185619.77	175327.85	171283.53	203997.36	198362.01	141200.30		
2881	2038.08	950.53	1430.46	1391.77	1357.77	2.27	1.49	0.65	0.84	1.31	0.69	4.16	2.47	0.11	4.90	4.98	0.12	x	
2882	47062.10	45058.96	48536.10	45497.58	45126.95	23751.08	21435.53	20090.99	14808.23	14498.00	13028.82	17917.27	16580.92	15852.72	18349.40	17443.43	11756.95		
2883	11411.99	11225.65	12668.15	12192.06	12084.30	19059.10	18212.19	17117.09	10898.31	10281.56	8634.91	8477.04	8580.40	8103.38	6449.57	7138.63	4624.58		
2884	1275.01	1288.00	1745.02	1564.29	1367.24	2.27	1.49	0.65	0.84	1.31	0.69	4.16	2.47	0.11	4.90	4.98	0.12	x	
2885	1354.79	1285.95	1383.87	1215.34	1107.87	2.27	1.49	0.65	0.84	1.31	0.69	4.16	2.47	0.11	4.90	4.98	0.12	x	
2886	1.07	0.47	2.22	0.19	0.06	242.29	259.91	206.72	131.78	123.59	30.81	272.64	205.19	111.18	382.39	326.67	153.95		x
2887	1.07	0.47	2.22	0.19	0.06	4858.64	4600.78	4315.69	106.64	93.08	67.10	180.64	236.81	166.33	137.50	131.27	73.34		x
2888	39928.55	38298.48	38629.33	35736.86	35270.04	146019.08	146065.95	136887.51	106687.65	101783.78	92467.43	119471.45	114675.07	109744.42	126989.44	122222.77	78074.64		
2889	8362.58	8156.57	8695.62	8700.63	8219.90	1150.44	1205.11	850.11	1010.13	941.21	684.73	1075.55	972.78	990.98	1413.04	1469.24	835.21		
2890	55390.05	50357.97	65877.41	56011.91	57610.68	26453.93	22929.36	27776.96	17721.75	16255.03	15616.08	19965.21	20374.97	21908.36	20653.83	22359.21	14792.53		
2891	1.07	0.47	2.22	0.19	0.06	2.27	1.49	0.65	0.84	1.31	0.69	4.16	2.47	0.11	4.90	4.98	0.12	x	
2892	165683.89	168342.72	218530.60	204368.97	186547.80	28591.89	26823.43	23994.66	25619.09	23904.51	21924.21	28230.89	27691.82	27087.39	26587.10	25456.19	15008.16		
2893	217625.40	209929.79	264217.66	248278.63	232291.73	268008.59	244008.20	223626.88	173046.30	171983.91	150147.66	205929.91	197617.18	185305.20	210023.70	200428.00	140064.23		
2894	503161.30	479269.27	542974.83	513952.24	477893.76	47024.43	42729.61	38078.69	27577.34	25916.83	21266.02	34572.73	31160.68	29220.76	38961.53	35645.53	21696.60		
2895	564.70	393.22	1045.12	766.73	712.39	2.27	1.49	0.65	0.84	1.31	0.69	4.16	2.47	0.11	4.90	4.98	0.12	x	
2896	1.07	0.47	2.22	0.19	0.06	861.42	767.52	690.70	643.90	778.58	738.80	792.80	853.14	822.83	760.64	806.51	596.39		x
2897	104.47	27.10	86.36	100.76	97.37	6448.79	5976.85	5803.10	3814.79	3603.56	3035.15	5384.04	5083.25	5061.27	4822.91	4519.36	2631.23		
2898	1.07	0.47	2.22	0.19	0.06	10816.66	11127.08	10361.65	8076.09	8406.90	7666.47	8023.79	7508.16	7559.91	8345.57	8339.97	5031.25		x
2899	14503.66	13485.24	15294.43	13769.38	12578.66	6127.37	6036.58	5464.81	3647.90	3457.66	2979.85	4904.62	4895.06	4581.21	5182.13	4848.66	2827.73		
2900	14063.05	13087.96	20146.69	16758.18	16048.24	38390.55	36691.70	33011.01	27527.20	26961.34	25485.79	32205.81	30974.19	29646.55	31878.22	31623.37	24143.55		
2901	1.07	0.47	2.22	0.19	0.06	6331.49	5694.34	5511.54	3810.97	3637.07	3520.60	5002.71	4443.66	3805.61	3972.02	3681.39	3062.53		x
2902	264271.80	261375.35	309379.29	290352.09	273367.50	429532.94	406893.80	371684.30	296414.44	293387.75	270814.84	336051.85	325494.65	310732.14	331957.90	323225.48	242741.50		
2903	111046.34	108598.60	117701.27	113501.38	105082.57	43086.19	43417.47	43762.67	32545.09	32683.15	29491.76	40235.47	39234.51	36228.64	43427.34	38276.26	27959.77		
2904	10522.30	10269.99	13045.17	12273.10	12578.80	2.27	1.49	0.65	0.84	1.31	0.69	4.16	2.47	0.11	4.90	4.98	0.12	x	
2905	11275.38	11168.50	10403.38	9450.49	8940.83	54699.10	49568.02	46194.96	39422.82	38182.69	32700.91	57290.45	55420.62	50990.74	45441.09	44944.45	30475.84		
2906	2459.04	2361.95	3642.59	3995.29	4111.56	2562.06	2116.30	2130.55	2087.56	2036.19	1998.51	2327.23	2449.60	2219.10	2407.90	2497.52	1972.41		
2907	1.07	0.47	2.22	0.19	0.06	1907.74	1713.32	1682.38	1108.34	889.89	693.10	1450.98	1148.01	1163.82	1151.20	1213.74	646.43		x
2908	1.07	0.47	2.22	0.19	0.06	2238.72	2226.81	2038.79	1244.16	1185.97	971.93	1739.18	1941.73	1635.71	2179.11	2155.62	1331.58		x
2909	15.67	7.91	9.86	14.50	1.58	163302.82	155067.24	143154.98	107026.84	106816.74	94492.14	132067.79	129480.82	124655.80	136618.60	129758.23	88939.13		x
2910	1.07	0.47	2.22	0.19	0.06	1300.24	1010.14	766.46	461.52	441.93	167.09	764.15	680.12	394.15	1041.23	984.45	347.17		x
2911	4863.70	4595.39	6514.27	5700.36	5166.01	2.27	1.49	0.65	0.84	1.31	0.69	4.16	2.47	0.11	4.90	4.98	0.12	x	
2912	282652.33	283170.64	318457.25	295153.42	299718.22	44146.16	37997.76	34234.33	23663.16	23687.66	21100.66	32141.42	30802.75	30147.47	30695.89	29023.57	16676.65		
2913	4595.84	3918.58	6949.22	6369.43	5265.61	2.27	1.49	0.65	0.84	1.31	0.69	4.16	2.47	0.11	4.90	4.98	0.12	x	
2914	5045.87	5022.10	6042.24	5610.11	5151.14	2.27	1.49	0.65	0.84	1.31	0.69	4.16	2.47	0.11	4.90	4.98	0.12	x	
2915	10314.37	9367.14	10561.49	10373.02	8839.14	2.27	1.49	0.65	0.84	1.31	0.69	4.16	2.47	0.11	4.90	4.98	0.12	x	
2916	8059.58	8011.57	10934.07	10529.68	12404.56	2.27	1.49	0.65	0.84	1.31	0.69	4.16	2.47	0.11	4.90	4.98	0.12	x	
2917	1.07	0.47	2.22	0.19	0.06	11198.69	9849.65	8220.88	2969.46	3028.00	3022.80	5855.30	4589.87	3935.17	4658.08	4361.65	2615.23		x
2918	23931.83	27057.56	23527.87	21053.02	22772.52	2.27	1.49	0.65	0.84	1.31	0.69	4.16	2.47	0.11	4.90	4.98	0.12	x	
2919	116.45	95.34	41.63	172.50	178.80	2.27	1.49	0.65	0.84	1.31	0.69	4.16	2.47	0.11	4.90	4.98	0.12	x	
2920	17983.34	17743.10	21549.40	18807.80	20353.26	2.27	1.49	0.65	0.84	1.31	0.69	4.16	2.47	0.11	4.90	4.98	0.12	x	
2921	5546.69	5176.20	5841.46	5528.45	5525.82	2643.38	2482.13	3098.47	2598.42	2619.76	2499.67	2691.69	2363.23	2334.06	2917.38	2865.90	1942.52		
2922	7915.00	7453.12	8463.77	7875.97	7778.88	2.27	1.49	0.65	0.84	1.31	0.69	4.16	2.47	0.11	4.90	4.98	0.12	x	
2923	15911.90	13067.17	12933.09	12254.07	11803.33	2.27	1.49	0.65	0.84	1.31	0.69	4.16	2.47	0.11	4.90	4.98	0.12	x	
2924	103777.72	99155.31	106525.30	111164.45	99108.11	42874.95	38659.65	29607.40	22746.63	20790.50	14573.65	25710.38	22401.74	18658.55	28665.00	26549.98	14864.88		
2925	83887.98	79482.73	94494.74	95768.93	90351.78	20746.60	17997.12	17773.80	16238.81	16258.43	15221.14	17221.78	16617.66	16023.74	18011.16	15810.53	18214.49		
2926	193012.04	187991.24	226765.93	232394.79	223373.73	175572.07	166661.52	157067.92	149348.35	143730.45	129028.47	167328.39	158277.30	149753.06	197109.46	185460.54	132067.86		
2927	3794.43	3609.45	4522.86	4735.22	4542.23	2.27	1.49	0.65	0.84	1.31	0.69	4.16	2.47	0.11	4.90	4.98	0.12	x	
2928	4649.73	4499.31	5617.41	4691.70	4943.17	67375.02	60285.85	63656.64	40970.83	41029.02	40190.86	46650.73	44802.40	46037.40	50120.07	45274.68	32314.14		
2929	75259.06	77606.27	79937.11	78963.93	73912.04	3473.43	3505.28	2815.38	1905.59	1794.60	1565.13	2126.08	1843.78	1792.17	1826.83	1960.21	1284.13		
2930	47801.30	48306.38	45940.92	45991.84	44068.66	4156.57	2829.18	3241.91	2463.24	2235.80	2361.53	2385.32	2549.28	2448.85	2301.68	1808.20	1709.90		
2931	14008.33	13358.81	14150.21	13587.88	12269.46	2.27	1.49	0.65	0.84	1.31	0.69	4.16	2.47	0.11	4.90	4.98	0.12	x	
2932	28595.25	27374.11	31766.16	31023.94	28804.92	2.27	1.49	0.65	0.84	1.31	0.69	4.16	2.47	0.11	4.90	4.98	0.12	x	
2933	24354.89	22850.35	28033.03	26312.25	24760.83	2.27	1.49	0.65	0.84	1.31	0.69	4.16	2.47	0.11	4.90	4.98	0.12	x	
2934	12729.19	12662.33	14319.17	15190.11	13726.70	39936.02	37345.77	31756.81	29799.08	29951.91	26318.08	34104.67	31920.86	28985.79	32728.72	33237.22	25673.94		
2935	4590.52	4274.02	4662.92	4099.07	3948.90	36700.77	32666.43	29247.53	22660.67	21423.33	19827.18	26551.07	25100.46	21573.77	31821.07	32081.26	18456.91		
2936	18036.02	18059.27	19123.44	18280.90	17834.45	8014.80	6499.52	7047.19	5257.36	5278.97	4766.48	6811.28	6857.86	6740.29	6277.71	6267.74	4313.37		
2937	18306.40	19940.53	20884.37	21045.94	18803.82	24978.55	24608.39	24400.97	14087.56	15081.20	14098.78	21525.41	20737.39	19968.58	20788.71	20925.70	16280.40		
2938	1.07	0.47	2.22	0.19	0.06	9027.35	9483.24	8366.09	6389.74	6481.44	6263.95	5781.36	5450.79	5675.68	6031.99	5319.42	2599.65		x
2939	47663.96	43828.42	60577.14	53756.32	52575.50	47487.71	42626.27	38223.56	29106.19	28958.92	26507.39	37964.91	37669.68	36144.08	36669.59	35764.44	24671.88		
2940	53763.17	52917.98	62060.14	61212.97	55785.45	28978.51	27955.51	28109.69	18477.19	18464.08	16023.07	21482.35	20743.43	20314.71	19087.25	19568.35	14342.78		
2941	1366539.34	1273821.05	1734963.75	1630791.53	1486325.87	1188321.84	1136748.91	1020678.19	974810.73	941346.05	793907.40	964621.30	920893.21	844295.26	1162466.86	1109248.57	737068.77		
2942	22125.50	21537.36	27977.14	25635.12	23706.73	41508.17	36701.89	33391.63	24902.41	22593.99	19319.09	30434.79	28756.23	25835.31	28753.79	28218.40	18597.16		
2943	55.28	360.81	60.68	38.84	4.07	2.27	1.49	0.65	0.84	1.31	0.69	4.16	2.47	0.11	4.90	4.98	0.12	x	
2944	667662.02	645799.77	868898.39	724873.51	692255.72	400338.95	349845.26	293040.05	315080.63	315468.64	274472.25	348398.33	336540.79	309635.81	395990.42	377003.09	287490.40		
2945	2742.75	2924.82	3209.41	2527.63	2881.94	2.27	1.49	0.65	0.84	1.31	0.69	4.16	2.47	0.11	4.90	4.98	0.12	x	
2946	4419.40	3883.02	5248.64	5083.89	4479.77	2.27	1.49	0.65	0.84	1.31	0.69	4.16	2.47	0.11	4.90	4.98	0.12	x	
2947	1.07	0.47	2.22	0.19	0.06	47156.20	42487.77	39797.75	21058.45	20748.22	14620.84	20573.56	19123.94	16732.23	25131.52	22976.63	13438.65		x
2948	2092.81	1879.69	1995.83	1883.96	2253.49	2.27	1.49	0.65	0.84	1.31	0.69	4.16	2.47	0.11	4.90	4.98	0.12	x	
2949	128833.11	121012.77	82561.69	76056.58	70867.10	13228.18	15185.96	10183.65	9367.93	7954.29	8182.40	13135.53	9947.43	10380.36	9127.97	11403.17	1783.09		
2950	12210.55	11345.20	10220.72	13173.36	11640.33	20922.77	22799.74	19369.28	13517.60	12069.62	9165.88	21173.34	17869.45	14226.06	19123.85	17992.32	9119.47		
2951	1075.83	707.85	2096.72	1757.09	1642.71	2.27	1.49	0.65	0.84	1.31	0.69	4.16	2.47	0.11	4.90	4.98	0.12	x	
2952	178148.45	172728.13	176419.54	164449.85	163145.47	41248.54	42165.02	40587.46	28407.59	26315.67	24198.54	34374.21	31896.83	31187.40	33982.96	32172.51	23884.64		
2953	1.07	0.47	2.22	0.19	0.06	12680.60	11553.71	11743.08	10172.50	9159.36	8838.02	11490.23	10137.47	10386.49	11509.81	9863.25	7891.21		x
2954	24325.25	18948.59	4631.07	4250.46	3181.19	2.27	1.49	0.65	0.84	1.31	0.69	4.16	2.47	0.11	4.90	4.98	0.12	x	
2955	1.07	0.47	2.22	0.19	0.06	4652.94	4195.37	3925.05	2213.47	2541.26	1515.21	4404.46	3333.55	3030.53	3953.73	3961.08	1553.84		x
2956	1740.51	1974.05	1920.32	1814.71	1867.24	2.27	1.49	0.65	0.84	1.31	0.69	4.16	2.47	0.11	4.90	4.98	0.12	x	
2957	1.07	0.47	2.22	0.19	0.06	310.99	340.76	239.53	143.85	113.54	22.50	158.09	164.53	116.63	161.72	166.15	29.78		x
2958	278700.29	262702.03	294955.33	269244.09	262761.78	164382.37	158808.05	145881.05	122264.05	119902.04	108882.77	143233.24	138637.37	126831.50	129924.29	125988.75	91460.50		
2959	1274848.23	1230291.65	1363276.44	1316460.87	1245521.78	900485.95	863423.91	786760.87	469835.80	468387.14	437340.35	637695.29	612618.36	570140.73	565471.84	556994.99	419164.08		
2960	8001.68	7622.91	6972.14	6972.37	6203.12	3074.16	2617.47	2400.36	1434.13	1498.51	985.99	2235.56	1792.10	1757.62	2512.11	2383.06	1399.05		
2961	133400.77	129817.64	156433.04	151420.08	139893.13	79638.14	76056.77	69994.72	54171.97	55733.91	41515.50	71980.23	61895.54	60530.71	76759.42	73706.08	47983.87		

Supplementary Table S1. Overview on the abundance of all relatively quantified 5180 proteins

			MCF-7																					MDA-MB-231													
	Protein name	UniProt	control 01	control 02	control 03	Dai IC20 01	Dai IC20 02	Dai IC20 03	Dai SC20 01	Dai SC20 02	Dai SC20 03	Gen IC20 01	Gen IC20 02	Gen IC20 03	Gen SC20 01	Gen SC20 02	Gen SC20 03	SSE IC20 01	SSE IC20 02	SSE IC20 03	SSE SC20 01	SSE SC20 02	SSE SC20 03	control 01	control 02	control 03	Dai IC20 01	Dai IC20 02	Dai IC20 03	Gen IC20 01	Gen IC20 02	Gen IC20 03	SSE IC20 01	SSE IC20 02	SSE IC20 03	unique to MCF-7	unique to MDA-MB-231
2962	Nucleosome assembly protein 1-like 3	Q99457	0.14	2.53	0.81	1.07	2.77	0.68	0.96	0.36	0.63	0.35	0.75	0.38	2.13	0.97	0.60	4.64	1.07	0.47	2.22	0.19	0.06	75211.44	78308.41	67749.61	51931.15	51773.35	56570.99	42657.90	50256.24	49833.74	64863.04	58684.46	45381.28		x
2963	Nucleosome assembly protein 1-like 4	Q99733	184068.16	176276.29	163368.00	116993.23	107437.06	100679.24	134479.67	130188.80	124013.46	151013.34	144053.18	134105.00	150505.07	142825.00	99599.08	110149.44	108536.59	101522.50	122228.75	115683.09	106666.79	94745.16	82599.01	73534.58	56247.25	52835.54	48292.47	70283.51	66299.84	61792.25	69878.53	69388.59	46645.18		
2964	Nucleosome-remodeling factor subunit BPTF	Q12830	923.01	1038.09	866.05	789.95	722.05	679.70	611.67	668.73	654.31	1165.29	1026.30	974.84	705.47	540.07	607.22	615.78	692.75	714.18	926.37	864.41	917.98	2.27	1.49	0.65	0.84	1.31	0.69	4.16	2.47	0.11	4.90	4.98	0.12	x	
2965	Nucleus accumbens-associated protein 1	Q96RE7	81291.76	77805.94	72606.52	54475.65	49920.73	42656.98	67806.60	67589.05	60315.08	77138.53	73824.29	65192.33	70888.63	67671.99	43892.64	51222.53	51093.53	43723.35	58286.40	56234.84	47593.77	2.27	1.49	0.65	0.84	1.31	0.69	4.16	2.47	0.11	4.90	4.98	0.12	x	
2966	Nucleus accumbens-associated protein 2	Q96BF6	15473.96	17669.26	15355.79	13158.87	13091.22	11204.09	11464.81	10719.32	13952.17	14609.99	13053.83	12392.43	14465.72	12115.74	8882.85	12244.32	11718.84	11207.53	7884.30	12659.14	13061.29	2.27	1.49	0.65	0.84	1.31	0.69	4.16	2.47	0.11	4.90	4.98	0.12	x	
2967	NudC domain-containing protein 1	Q96RS6	55288.65	54681.47	51227.95	39225.47	38137.55	36412.36	42994.68	41568.58	38978.95	50110.19	49155.57	50680.59	44818.16	42853.67	33436.58	39473.03	39455.28	31681.08	34192.11	36436.61	34772.86	37121.47	35676.19	31346.57	21327.82	21113.23	18060.23	27012.27	26868.04	26142.94	27163.71	26434.84	18353.22		
2968	NudC domain-containing protein 2	Q8WVJ2	11545.67	10814.20	9309.43	8756.77	8603.77	7156.67	13366.27	12732.72	11717.58	12443.52	11807.65	9883.12	10643.98	9617.21	5961.81	10212.75	9069.44	8750.81	9793.41	9065.10	7551.37	10042.73	9909.31	9678.01	3818.62	3749.64	3457.65	3376.43	3031.20	2838.10	3959.24	3794.56	2122.03		
2969	NudC domain-containing protein 3	Q8IVD9	534.08	525.74	457.72	567.53	551.23	546.06	556.22	588.20	594.77	488.09	413.78	420.88	653.48	600.15	530.99	310.89	405.09	405.77	525.23	530.42	525.78	3530.92	3295.92	3186.00	2678.80	2648.42	2030.09	2968.21	2822.08	2551.07	3521.07	3252.01	2000.53		
2970	NXPE family member 1	Q8N323	1964.12	1999.33	1961.25	701.63	636.63	688.46	1628.95	1465.43	1563.68	880.10	980.03	815.75	1399.81	1269.57	1059.03	1065.69	1218.54	1088.48	1001.06	998.13	793.63	2.27	1.49	0.65	0.84	1.31	0.69	4.16	2.47	0.11	4.90	4.98	0.12	x	
2971	Obg-like ATPase 1	Q9NTK5	456789.95	449309.89	428680.55	337296.52	323973.56	310390.76	375499.88	352323.28	355050.76	415927.81	384416.22	369592.76	369396.08	364652.91	304565.00	329153.68	306146.12	303087.35	321440.66	308671.14	298726.60	246494.40	218877.16	212971.00	169081.12	160166.53	149703.98	191762.11	180800.73	171786.54	189887.47	182404.57	128301.66		
2972	Obscurin	Q5VST9	239948.76	231340.25	220058.88	173697.28	170965.49	164318.58	187976.98	195041.60	200268.19	203497.17	193077.10	185968.20	213628.03	188648.40	162265.27	206896.37	189671.67	186478.58	198640.92	182029.17	182601.93	221431.01	208433.56	175044.22	133917.44	132657.09	95569.35	160828.04	147841.24	128532.96	186763.74	179718.96	111058.66		
2973	Occludin	Q16625	83098.01	77713.13	70918.68	53468.50	51799.81	47727.74	57796.50	55273.39	51625.18	69084.44	63044.66	59975.11	66764.73	58787.73	51193.12	49638.38	47826.36	47841.01	55179.30	50884.26	48503.09	2.27	1.49	0.65	0.84	1.31	0.69	4.16	2.47	0.11	4.90	4.98	0.12	x	
2974	OCIA domain-containing protein 1	Q9NX40	41924.93	43076.59	36054.42	26324.81	26658.42	25478.62	28555.61	27417.79	26067.32	32332.63	30499.15	30142.82	31329.72	29662.43	25509.92	23903.49	21644.79	21595.52	23147.58	24742.54	23355.68	2.27	1.49	0.65	0.84	1.31	0.69	4.16	2.47	0.11	4.90	4.98	0.12	x	
2975	OCIA domain-containing protein 2	Q56VL3	4576.65	4691.69	3583.09	2798.24	2929.35	2663.97	2793.77	2862.68	3006.70	3812.09	3804.98	4120.59	3598.50	2955.60	2524.57	2553.72	2444.93	2794.79	3037.88	2792.65	2741.00	6113.95	6283.69	5783.80	3916.15	3853.12	3746.38	5230.54	5308.62	4924.59	5938.32	5357.55	2179.73		
2976	Oligoribonuclease mitochondrial	Q9Y3B8	0.14	2.53	0.81	1.07	2.77	0.68	0.96	0.36	0.63	0.35	0.75	0.38	2.13	0.97	0.60	4.64	1.07	0.47	2.22	0.19	0.06	14644.38	14187.02	13257.59	10288.31	10168.73	9203.19	11317.65	10747.27	10554.09	12967.19	12214.89	9051.34		x
2977	Oligosaccharyltransferase complex subunit OSTC	Q9NRP0	6133.79	6294.12	5573.15	3635.09	3742.27	3074.34	6965.66	6262.51	5986.42	5071.34	5044.18	4787.52	4227.69	4269.50	3051.19	5674.16	5498.60	5275.08	4156.37	4137.64	3470.25	3368.96	3361.83	3005.93	1522.15	1367.89	920.71	2444.20	2097.71	2116.76	1851.97	1649.30	903.68		
2978	Omega-amidase NIT2	Q9NQR4	45251.51	40644.90	29706.20	30264.22	19708.51	17794.84	20331.18	19321.31	18128.61	41878.76	38129.14	33181.69	31371.11	31226.78	16758.00	20511.34	18849.38	18656.51	19051.63	18283.73	17437.95	41601.75	33850.55	16830.35	29958.66	24960.54	15877.26	45852.85	39383.93	24898.35	47379.25	46738.37	22757.03		
2979	Oncomodulin-1	P0CE72	4176.98	2937.68	2286.95	2624.23	2331.74	1517.29	2372.29	2580.16	1831.25	3344.65	2824.64	1746.29	3532.50	2807.53	468.78	2364.87	2179.04	1832.53	2123.22	1856.85	1255.54	2.27	1.49	0.65	0.84	1.31	0.69	4.16	2.47	0.11	4.90	4.98	0.12	x	
2980	Opioid growth factor receptor	Q9NZT2	31117.09	29490.75	27414.99	19972.87	19999.39	19164.35	21838.09	20047.07	20647.58	30442.12	28799.74	28055.53	28359.79	25497.51	18300.78	18158.95	18095.66	17488.99	19857.29	20496.97	17464.23	23704.62	21896.94	20941.73	15954.07	16437.36	14541.90	19916.07	18385.87	17270.56	18469.21	18188.21	12732.29		
2981	Opioid-binding protein/cell adhesion molecule	Q14982	2946.47	1981.23	1208.11	616.56	681.15	423.23	1493.20	854.72	755.74	1419.92	1300.87	955.41	1516.06	807.12	117.54	875.35	955.11	529.23	2095.42	1895.14	1108.76	2.27	1.49	0.65	0.84	1.31	0.69	4.16	2.47	0.11	4.90	4.98	0.12	x	
2982	Oral-facial-digital syndrome 1 protein	O75665	0.14	2.53	0.81	1.07	2.77	0.68	0.96	0.36	0.63	0.35	0.75	0.38	2.13	0.97	0.60	4.64	1.07	0.47	2.22	0.19	0.06	7189.13	7660.91	6231.07	4604.35	4277.11	3485.98	6054.35	6047.14	5150.58	5682.99	5771.83	3287.42		x
2983	Ornithine aminotransferase_ mitochondrial	P04181	211674.24	200173.31	183003.83	135015.44	134988.39	123027.26	162316.30	153316.43	146554.55	170238.94	159571.97	149214.02	164214.46	150286.37	119801.92	149399.44	145514.99	143449.94	141575.35	134008.04	125480.67	83927.64	77058.73	68564.33	47062.48	44248.19	36303.14	57210.49	52508.59	48816.10	55656.68	54346.76	33875.88		
2984	Otoferlin	Q9HC10	25181.08	23872.91	21996.41	15893.62	16647.46	14752.51	19408.15	18762.83	18396.75	24762.36	24331.23	22257.52	20922.88	18710.33	13099.18	17753.64	17621.73	16244.18	16710.57	15845.88	15383.47	2.27	1.49	0.65	0.84	1.31	0.69	4.16	2.47	0.11	4.90	4.98	0.12	x	
2985	Otoraplin	Q9NRC9	4396.92	3699.89	3536.20	3259.67	2976.52	2537.21	3181.92	3196.30	2963.74	3413.32	3166.47	2764.46	3418.94	2886.08	2264.27	2866.65	2788.50	2712.89	3021.33	2705.52	2472.06	663.47	419.00	652.22	216.10	136.19	19.00	1307.18	1037.29	526.99	845.47	687.94	199.06		
2986	OTU domain-containing protein 7B	Q6GQQ9	50572.18	50235.92	46251.84	38318.32	36269.99	34546.84	44705.35	42706.56	43372.93	53251.73	50192.93	48360.59	47680.79	46276.10	35858.02	36634.95	35953.17	36255.24	39420.17	36691.51	37679.51	57735.75	52465.43	51510.69	35153.46	34971.39	33268.97	35381.86	34737.52	34235.50	35304.72	38380.35	33684.15		
2987	Outer dense fiber protein 2	Q5BJF6	74619.80	66927.63	63934.95	52714.32	51092.08	51864.28	59957.38	54943.86	56007.18	75044.00	73427.93	71184.77	66912.39	60036.30	45206.50	50586.97	47991.49	45797.33	50367.20	47906.14	46447.08	4424.03	5014.04	4199.24	4168.14	4005.25	3639.60	4560.51	4351.03	4270.92	4358.53	4242.77	3442.64		
2988	Overexpressed in colon carcinoma 1 protein	Q8TAD7	7399.70	6447.80	5027.68	3092.93	2634.32	2011.06	5799.47	998.87	4809.50	5128.88	5351.78	3781.52	4934.84	790.64	493.64	4137.03	1753.10	2984.08	3727.56	3202.33	2407.39	2.27	1.49	0.65	0.84	1.31	0.69	4.16	2.47	0.11	4.90	4.98	0.12	x	
2989	Oxidation resistance protein 1	Q8N573	2728.23	2589.87	2228.08	1476.32	1482.41	1499.35	1671.06	1497.50	1385.67	1692.65	1730.74	1619.81	2092.35	1496.91	1270.07	1168.77	997.03	1114.51	2083.50	1802.93	1602.58	2.27	1.49	0.65	0.84	1.31	0.69	4.16	2.47	0.11	4.90	4.98	0.12	x	
2990	Oxidative stress-induced growth inhibitor 1	Q9UJX0	11852.24	9629.45	6067.58	6128.27	5340.69	3953.26	5319.37	4221.30	3755.16	7724.80	7097.81	5455.50	6893.43	5624.21	892.13	6471.24	5883.61	4968.70	6530.54	5986.04	4363.68	2.27	1.49	0.65	0.84	1.31	0.69	4.16	2.47	0.11	4.90	4.98	0.12	x	
2991	Oxidoreductase HTATIP2	Q9BUP3	45731.55	44978.33	43159.91	36643.01	36036.85	34039.71	38958.62	39626.14	38733.20	48563.49	44836.63	43508.01	43440.51	41750.95	34377.46	32539.01	31092.01	32675.08	36403.81	33057.94	34394.60	2.27	1.49	0.65	0.84	1.31	0.69	4.16	2.47	0.11	4.90	4.98	0.12	x	
2992	Oxygen-dependent coproporphyrinogen-III oxidase_ mitochondrial	P36551	115965.24	100456.68	79243.40	25924.35	23383.22	19624.97	31270.72	30297.79	25762.64	80520.74	66284.15	55429.33	70736.16	33411.09	18539.20	26111.57	23202.28	21679.97	27343.36	25614.68	22171.46	83179.56	80289.66	72413.87	46185.64	46701.61	36227.66	49583.25	47397.16	44159.55	55139.22	52509.33	34411.88		
2993	Oxysterol-binding protein 1	P22059	78687.66	74026.94	66306.22	58189.60	56358.41	52568.40	62686.06	53607.14	59384.96	73495.24	73390.90	66700.66	71426.12	56338.44	41145.88	55036.91	50152.40	51780.07	61956.61	57002.77	52717.84	32403.01	33571.25	25772.68	20169.80	21783.22	16799.87	26186.33	22744.37	22547.10	26339.98	28097.66	19205.19		
2994	Oxysterol-binding protein-related protein 2	Q9H1P3	0.14	2.53	0.81	1.07	2.77	0.68	0.96	0.36	0.63	0.35	0.75	0.38	2.13	0.97	0.60	4.64	1.07	0.47	2.22	0.19	0.06	9597.68	8740.36	8289.19	7205.55	6985.14	6894.51	7996.54	7560.34	7471.62	8839.89	8389.79	6846.99		x
2995	Oxysterol-binding protein-related protein 3	Q9H4L5	45322.53	44220.40	42879.02	38010.83	37759.85	36607.69	39936.27	38606.83	37572.75	42738.70	40982.80	40716.58	38052.61	38812.41	31894.96	36399.30	35996.80	35550.02	33510.05	34682.90	31607.85	93791.81	86886.30	83749.80	69193.94	66431.25	57036.77	80299.13	75530.57	76053.78	82028.76	80035.21	55783.76		
2996	Oxysterol-binding protein-related protein 9	Q96SU4	0.14	2.53	0.81	1.07	2.77	0.68	0.96	0.36	0.63	0.35	0.75	0.38	2.13	0.97	0.60	4.64	1.07	0.47	2.22	0.19	0.06	18144.76	16163.10	15272.28	9153.80	9796.98	6810.05	14528.61	13460.47	11811.62	14602.08	16837.60	8905.74		x
2997	Oxytocin receptor	P30559	9748.14	8100.01	8302.21	4533.10	4648.61	3903.20	6984.96	6624.67	6257.09	7336.34	6661.43	6175.49	5750.36	6070.52	4197.47	5892.64	5413.15	5000.40	6845.54	6256.10	5740.23	2.27	1.49	0.65	0.84	1.31	0.69	4.16	2.47	0.11	4.90	4.98	0.12	x	
2998	Oxytocin-neurophysin 1	P01178	1853.48	2748.84	2224.36	1728.04	2508.21	2037.78	1980.32	1683.41	2412.78	3872.76	1988.68	2587.71	2116.77	2614.79	1982.64	1877.88	880.56	1530.67	1201.18	1526.42	2170.04	2.27	1.49	0.65	0.84	1.31	0.69	4.16	2.47	0.11	4.90	4.98	0.12	x	
2999	P antigen family member 2	Q7Z2X7	106917.95	108891.49	108145.93	87012.45	86041.30	82200.61	110482.81	104686.94	110224.80	101377.02	95345.32	88956.05	101126.33	94021.69	78585.05	79517.70	78283.49	82236.21	87225.42	79501.10	79013.84	2.27	1.49	0.65	0.84	1.31	0.69	4.16	2.47	0.11	4.90	4.98	0.12	x	
3000	P antigen family member 5	Q96GU1	0.14	2.53	0.81	116.86	86.64	54.42	45.24	22.47	41.21	176.86	243.35	184.11	93.16	83.76	15.78	88.06	88.44	97.20	63.03	63.76	49.63	2.27	1.49	0.65	0.84	1.31	0.69	4.16	2.47	0.11	4.90	4.98	1163.52	x	
3001	P2X purinoceptor 4	Q99571	12278.97	11461.82	10548.15	10157.93	9546.36	9358.02	11205.36	10786.62	9560.27	11168.41	12352.06	10791.75	11389.41	8006.54	9789.13	10890.06	9280.86	10041.36	11011.71	9529.65	9896.75	2.27	1.49	0.65	0.84	1.31	0.69	4.16	2.47	0.11	4.90	4.98	0.12	x	
3002	Pachytene checkpoint protein 2 homolog	Q15645	5514.22	6447.22	5977.32	3221.84	3889.37	2410.45	6179.74	5918.96	5341.76	5002.93	4817.59	3926.69	4381.50	5332.34	3214.61	4467.51	3966.51	2970.33	3832.65	2915.40	3785.42	83785.87	77614.58	74916.81	54501.32	52727.84	45933.90	75643.57	72028.68	67909.86	66259.25	63307.28	47274.68		
3003	PAK4-inhibitor INKA2	Q9NTI7	178888.88	148240.12	116822.58	85271.58	80568.86	62806.11	104879.55	106442.04	93018.19	103551.06	96017.12	78530.23	118327.34	111790.58	52077.05	90599.33	84228.87	73800.40	82593.49	75696.26	62450.65	2.27	1.49	0.65	0.84	1.31	0.69	4.16	2.47	0.11	4.90	4.98	0.12	x	
3004	Palmitoyl-protein thioesterase 1	P50897	27610.36	27971.20	24353.43	11936.10	13273.92	11167.75	16540.24	15192.93	14940.78	14968.08	13135.62	13403.25	18763.93	17611.13	12504.58	9805.05	9752.23	9894.75	13947.79	14000.39	13802.87	1268.05	1331.10	1003.92	776.13	783.43	364.60	745.95	659.21	624.59	856.16	973.63	487.22		
3005	Pancreatic alpha-amylase	P04746	14646.37	13652.76	13289.65	9218.65	9412.80	8191.98	11218.28	11439.78	9831.88	11290.39	10672.62	9734.39	11024.32	11243.39	7498.39	10453.88	9740.54	9067.46	10468.29	9706.03	8702.85	2.27	1.49	0.65	0.84	1.31	0.69	4.16	2.47	0.11	4.90	4.98	0.12	x	
3006	Pancreatic progenitor cell differentiation and proliferation factor	Q9H3Y8	1346.65	1288.50	1381.53	824.34	769.95	800.15	764.77	830.32	757.58	1448.05	1251.80	1262.30	2.13	0.97	0.60	250.73	520.25	520.47	643.19	599.67	509.36	2.27	1.49	0.65	0.84	1.31	0.69	4.16	2.47	0.11	4.90	4.98	0.12	x	
3007	Pancreatic prohormone	P01298	4498.06	3178.72	3519.45	3706.41	3625.82	3136.19	3800.25	3236.84	3765.76	4200.53	3738.37	3351.38	4306.73	3838.79	2319.22	3479.48	3004.54	3076.93	3531.06	3037.69	2874.23	2.27	1.49	0.65	0.84	1.31	0.69	4.16	2.47	0.11	4.90	4.98	0.12	x	
3008	Pantothenate kinase 2 mitochondrial	Q9BZ23	0.14	2.53	0.81	1.07	2.77	0.68	0.96	0.36	0.63	0.35	0.75	0.38	2.13	0.97	0.60	4.64	1.07	0.47	2.22	0.19	0.06	2893.82	2803.77	2296.93	1391.52	1304.23	1143.84	1563.25	1496.92	1474.29	1515.10	1262.20	964.72		x
3009	Pantothenate kinase 4	Q9NVE7	2497.96	2182.28	2142.19	2034.72	2232.85	2088.62	2032.16	1929.90	1982.70	2781.06	2551.83	2548.39	2501.93	2202.70	2158.67	1753.96	1761.99	1895.56	2102.20	1829.55	1785.89	2.27	1.49	0.65	0.84	1.31	0.69	4.16	2.47	0.11	4.90	4.98	0.12	x	
3010	Paraneoplastic antigen Ma1	Q8ND90	11257.56	10771.86	10131.51	7495.35	8129.49	7686.20	9358.99	9113.35	9008.34	12246.05	11574.76	11371.06	10025.84	9278.51	7315.48	7046.83	7369.15	7504.53	8622.09	7610.21	7464.66	2.27	1.49	0.65	0.84	1.31	0.69	4.16	2.47	0.11	4.90	4.98	0.12	x	
3011	Paraspeckle component 1	Q8WXF1	66174.97	60704.69	62570.93	40617.22	42542.95	38273.74	47721.35	43428.23	44647.06	59083.30	56881.66	51032.49	55656.45	49383.53	38749.06	43944.23	39282.46	38677.11	40395.39	40258.86	40272.71	52900.98	46310.54	40190.16	43633.06	40267.06	37211.18	50482.16	45930.14	44799.05	49832.23	47507.56	31524.79		
3012	Parathymosin	P20962	23129.01	21617.07	21268.48	16914.26	16474.23	13318.63	16464.75	14843.10	12568.07	17976.17	17665.69	16165.82	24630.49	18134.16	6132.18	12661.12	13646.45	13559.68	16959.05	16752.79	15614.64	220302.01	199015.39	195508.36	139151.18	137883.04	133755.33	141618.91	137538.35	133775.45	154446.80	156315.05	113956.80		
3013	Partitioning defective 3 homolog	Q8TEW0	36814.92	35499.38	35312.49	28312.06	27151.77	25820.44	27116.40	26575.02	23704.86	33025.67	31745.13	29853.51	32134.41	30159.00	25479.12	22779.31	22728.70	22480.90	23785.45	24080.30	24040.25	2.27	1.49	0.65	0.84	1.31	0.69	4.16	2.47	0.11	4.90	4.98	0.12	x	
3014	Partitioning defective 6 homolog beta	Q9BYG5	23953.62	22635.31	18828.91	15355.21	14462.80	12897.59	18378.83	16556.80	15214.54	19728.24	18250.72	16822.59	19802.87	17462.30	12990.67	15341.77	13502.03	13846.21	14953.41	14991.79	13675.63	2.27	1.49	0.65	0.84	1.31	0.69	4.16	2.47	0.11	4.90	4.98	0.12	x	
3015	Partner of Y14 and mago	Q9BRP8	137336.45	134254.51	129363.48	90040.33	85578.97	81158.77	92129.58	92367.77	87387.36	111066.77	106347.94	100591.79	111724.92	103251.99	86773.32	84760.79	80761.28	79696.53	87900.72	81621.39	79898.95	27398.28	24857.56	23797.73	17793.89	16599.55	15092.19	20375.38	19541.12	18761.48	23509.30	22512.19	14798.16		
3016	Paxillin	P49023	130116.88	119144.69	108426.88	89344.05	90212.31	82132.38	105737.61	96648.43	91482.56	112975.18	102737.88	95667.35	106895.36	96672.67	83436.78	94287.35	91971.09	85174.80	84482.04	76605.59	64143.65	54805.65	58122.94	46969.73	19193.03	20294.55	15653.87	25780.42	24359.52	22723.71	27715.36	25940.48	16279.23		
3017	PDZ and LIM domain protein 1	O00151	34720.89	32156.44	24588.58	19320.68	18956.87	15036.53	25192.50	23149.28	18857.20	29093.74	28285.24	25868.83	24147.70	24317.18	20510.60	19974.88	20386.43	19414.89	19925.26	19407.28	17965.35	103112.54	94802.41	89236.78	53024.90	53267.93	49253.42	76562.19	70954.92	69125.77	72493.26	68342.80	47455.77		
3018	PDZ and LIM domain protein 2	Q96JY6	0.14	2.53	0.81	1.07	2.77	0.68	0.96	0.36	0.63	0.35	0.75	0.38	2.13	0.97	0.60	4.64	1.07	0.47	2.22	0.19	0.06	46411.78	36719.60	39036.08	31368.52	31436.01	28223.45	34744.95	33651.14	33068.81	36525.15	38875.75	29153.17		x
3019	PDZ and LIM domain protein 5	Q96HC4	70579.10	68080.32	66951.39	49972.84	50829.90	45317.15	48225.46	49447.40	48690.01	63655.70	61757.86	61082.30	60743.18	52637.90	37875.89	47011.37	47312.06	45556.11	49167.70	46257.87	45398.72	28583.26	27031.98	24720.49	18015.90	17616.72	16358.42	22604.83	21387.69	21235.47	22720.61	21902.81	14231.74		
3020	PDZ and LIM domain protein 7	Q9NR12	1279.63	1252.60	999.19	1193.01	1176.40	960.78	3070.56	3152.98	4095.07	1378.16	1278.36	1074.09	991.58	828.13	442.38	1318.53	1252.04	1164.96	1591.73	1266.40	992.02	2715.23	2402.78	2113.68	2404.21	2119.11	1346.58	2486.59	2462.97	2090.86	2606.80	2595.02	1648.26		
3021	PDZ domain-containing protein 2	O15018	6476.21	6768.01	7126.25	4176.49	3891.57	3944.83	6253.69	6342.28	5849.61	5385.92	5661.86	4733.40	5283.07	4811.43	4218.81	4600.77	4702.39	4469.38	4316.91	3656.64	3529.70	2.27	1.49	0.65	0.84	1.31	0.69	4.16	2.47	0.11	4.90	4.98	0.12	x	
3022	PDZ domain-containing protein 4	Q76G19	12870.93	14995.59	12725.32	10251.04	10009.02	8985.72	11482.60	10410.56	9709.30	11624.22	10632.11	9703.11	11011.89	11193.63	8437.88	10117.34	9269.09	8482.85	9600.15	9142.78	8767.05	1340.53	1465.52	937.39	1128.80	919.53	463.30	1677.30	1429.39	969.80	1820.03	1690.10	774.75		
3023	PDZ domain-containing protein 8	Q8NEN9	10741.94	11613.97	11256.33	8707.33	8010.11	7956.66	9262.61	9039.13	8320.95	10374.47	9412.01	8659.99	8635.29	8739.42	8362.66	8249.34	7853.97	7856.28	8982.08	8605.04	8772.50	2.27	1.49	0.65	0.84	1.31	0.69	4.16	2.47	0.11	4.90	4.98	0.12	x	
3024	PDZ domain-containing protein GIPC1	O14908	84805.96	84583.36	80685.70	57503.49	53409.95	52624.75	64083.64	61698.65	58519.58	67018.28	66961.60	62402.15	63113.02	63769.40	53793.35	52125.03	47856.26	47274.05	55284.34	52407.64	50968.50	2.27	1.49	0.65	0.84	1.31	0.69	4.16	2.47	0.11	4.90	4.98	0.12	x	
3025	Pecanex-like protein 4	Q63HM2	50430.61	44332.19	34469.41	28581.57	28510.73	24803.46	29639.06	27482.03	27747.13	55343.68	54800.51	50744.52	37119.13	47554.22	20852.13	30575.53	29811.30	29518.42	28083.74	24005.69	22879.45	2.27	1.49	0.65	0.84	1.31	0.69	4.16	2.47	0.11	4.90	4.98	0.12	x	
3026	Peflin	Q9UBV8	3246.58	3762.92	4104.74	2261.11	3470.40	3777.49	3819.49	1201.24	1209.40	3451.06	4185.53	4109.35	4005.74	776.05	2155.37	732.08	4021.84	3024.90	3326.56	3055.73	2582.79	2.27	1.49	0.65	0.84	1.31	0.69	4.16	2.47	0.11	4.90	4.98	0.12	x	
3027	Pentatricopeptide repeat domain-containing protein 3 mitochondrial	Q96EY7	20776.28	19119.67	15508.02	12770.80	10581.99	7354.66	13221.74	13331.60	11536.46	19769.36	18503.33	16051.18	20058.77	17916.32	8315.23	10870.27	10688.34	9360.58	13772.75	11733.06	9328.03	20875.15	17076.93	14324.11	12502.95	11847.66	8066.70	14355.50	12872.00	10338.63	15507.54	13707.42	7158.43		
3028	Peptidase M20 domain-containing protein 2	Q8IYS1	0.14	2.53	0.81	1.07	2.77	0.68	0.96	0.36	0.63	0.35	0.75	0.38	2.13	0.97	0.60	4.64	1.07	0.47	2.22	0.19	0.06	12015.40	10450.30	8940.49	7180.18	7632.71	6311.66	9235.90	9207.40	9021.09	9227.31	8262.36	6293.09		x
3029	Peptide chain release factor 1 mitochondrial	O75570	0.14	2.53	0.81	1.07	2.77	0.68	0.96	0.36	0.63	0.35	0.75	0.38	2.13	0.97	0.60	4.64	1.07	0.47	2.22	0.19	0.06	187.24	492.47	199.57	336.74	298.98	364.67	42.90	99.46	113.97	266.82	53.11	297.20		x
3030	Peptide-N(4)-(N-acetyl-beta-glucosaminyl)asparagine amidase	Q96IV0	32329.56	29442.88	26774.68	21349.30	21554.89	18602.27	31989.04	30849.53	27599.40	26317.26	25544.45	24398.38	27660.74	26514.34	13126.57	20312.14	20855.47	18771.27	23165.13	24226.68	23226.87	2.27	1.49	0.65	0.84	1.31	0.69	4.16	2.47	0.11	4.90	4.98	0.12	x	
3031	Peptidyl-prolyl cis-trans isomerase A	P62937	1898511.12	1780030.40	1668712.74	1236079.04	1149202.04	1086090.65	1805146.74	1765920.47	1651028.87	1510296.97	1479031.87	1345204.51	1534229.20	1413741.56	1133493.70	1316366.25	1246124.01	1229530.14	1328637.28	1255214.06	1119351.64	2205524.44	2057515.97	1866606.54	1196117.55	1171084.63	1030228.24	1357681.49	1285117.78	1191210.05	1318013.36	1273966.81	912765.56		
3032	Peptidyl-prolyl cis-trans isomerase A-like 4A	Q9Y536	6243.04	5635.69	5606.99	3977.62	3460.25	3241.46	4543.97	4456.51	3666.99	5247.51	4852.78	4996.56	4819.50	4406.05	3523.96	4197.26	3834.82	3746.23	4869.25	4247.79	3692.88	3434.86	3135.01	2567.25	2546.85	2423.82	1351.07	3571.61	3121.48	2467.14	4284.85	4055.32	2039.54		
3033	Peptidyl-prolyl cis-trans isomerase A-like 4G	P0DN37	11427.36	10304.87	10333.13	7516.35	7481.99	7235.99	10898.46	10453.78	10331.46	10601.73	10335.53	9955.10	9855.34	9482.30	7665.83	7825.40	7872.62	7605.53	9239.44	8647.03	8027.14	4935.28	5643.59	4939.42	3351.70	3600.58	3724.09	3756.01	3732.11	3567.98	3352.46	3457.94	3401.27		
3034	Peptidyl-prolyl cis-trans isomerase A-like 4H	A0A075B	1955.69	1533.58	1548.02	491.73	497.33	453.31	875.06	680.82	707.31	661.05	625.18	474.92	996.96	482.12	554.17	679.76	635.49	610.47	601.84	605.31	461.37	2.27	1.49	0.65	0.84	1.31	0.69	4.16	2.47	0.11	4.90	4.98	0.12	x	
3035	Peptidyl-prolyl cis-trans isomerase B	P23284	978080.16	954704.02	911321.15	710659.03	694466.30	655144.36	910393.91	864414.55	823625.90	951676.50	897753.07	849341.36	775209.41	746097.24	629987.27	777528.08	742215.58	711532.37	688151.76	676594.80	640302.71	490585.02	469085.42	431693.99	282367.05	273953.52	237497.56	334155.42	318865.65	312524.06	317758.02	308743.77	218885.49		
3036	Peptidyl-prolyl cis-trans isomerase C	P45877	67349.92	60425.01	57983.62	35745.66	33520.13	32406.38	45441.66	44947.08	44817.63	49832.88	50032.26	44718.13	48517.73	44412.94	35117.19	39810.31	38238.57	39022.44	37291.04	35264.68	32704.12	2.27	1.49	0.65	0.84	1.31	0.69	4.16	2.47	0.11	4.90	4.98	0.12	x	
3037	Peptidyl-prolyl cis-trans isomerase D	Q08752	54512.44	50853.45	43659.97	31949.00	30748.18	25728.55	51931.51	48785.79	44463.67	42161.00	38900.74	33397.66	41399.03	38606.39	23888.94	35870.12	32786.00	31385.26	32772.02	31230.83	26621.68	105949.57	102346.98	91257.47	64020.85	63538.24	54372.19	82181.88	79832.47	74490.91	68120.73	71207.96	49154.94		
3038	Peptidyl-prolyl cis-trans isomerase F_ mitochondrial	P30405	73517.39	73516.48	70480.36	60304.23	59798.36	57524.72	55310.35	54755.56	54968.51	69543.20	65158.31	62692.43	63099.31	60546.91	46735.34	54856.28	54944.74	56232.41	55342.58	54407.83	54941.56	9919.23	9906.48	8452.90	5294.66	4915.81	4105.28	6144.54	5656.44	5058.13	6425.64	6245.55	3371.05		
3039	Peptidyl-prolyl cis-trans isomerase FKBP10	Q96AY3	116448.83	114552.77	104308.04	78660.65	75409.37	67077.27	95099.98	91997.97	89012.55	104476.39	97974.60	91082.82	101528.72	91046.53	66915.35	84916.66	77338.51	71354.39	84918.11	79130.41	70502.92	81092.77	78540.05	79419.36	57781.80	55140.22	51822.36	67942.20	60898.89	61804.78	60883.90	58042.32	46125.18		
3040	Peptidyl-prolyl cis-trans isomerase FKBP14	Q9NWM	917.74	824.22	758.26	707.54	611.26	642.30	722.19	555.04	709.52	1033.27	1270.43	1404.72	783.08	771.98	487.28	593.07	655.25	557.29	574.84	557.61	608.27	1686.28	1600.92	1336.79	940.80	1007.49	786.18	1253.01	1341.72	1073.13	1395.58	1322.03	772.87		
3041	Peptidyl-prolyl cis-trans isomerase FKBP1A	P62942	109329.97	108225.17	104685.49	79549.85	76191.03	71076.82	92956.98	91387.96	85410.39	104099.88	101574.49	96118.19	92465.54	92103.44	78281.46	80253.43	78003.26	74306.67	79308.09	76493.28	72639.80	202985.94	188736.70	156234.04	119364.76	115270.30	109253.45	136898.40	136185.00	129160.81	138222.95	134950.27	95125.02		
3042	Peptidyl-prolyl cis-trans isomerase FKBP2	P26885	44275.64	44996.49	39856.42	25167.89	24750.88	21735.44	36461.24	34187.75	33486.37	36318.34	34809.58	33460.68	34625.06	31843.85	25888.32	30834.88	28872.99	26370.62	24942.95	24035.63	21899.76	16421.45	15702.47	13955.32	8057.21	7629.41	6758.80	10191.93	10079.71	9860.48	8484.97	7890.81	5230.39		
3043	Peptidyl-prolyl cis-trans isomerase FKBP3	Q00688	361862.85	363313.45	346803.60	270962.82	260177.17	247826.06	310686.73	298856.90	296730.56	321752.29	294272.59	286487.68	313954.91	301272.50	254416.18	271652.54	259667.75	260176.36	272904.09	247164.14	238885.26	386956.77	368598.07	341104.79	238197.09	222982.04	191815.25	371974.07	350390.43	332894.40	313574.01	299157.56	209680.50		
3044	Peptidyl-prolyl cis-trans isomerase FKBP4	Q02790	1087440.90	1050555.53	998683.66	757225.98	740134.58	676980.11	924808.90	890434.28	860347.03	986374.59	940736.04	903035.94	939088.11	848470.02	704502.73	713621.40	686693.79	657831.13	839328.18	794184.38	749258.12	434288.74	395373.39	385337.24	273090.08	272141.94	242013.57	307560.88	294083.01	283051.35	319786.25	317652.57	224581.28		
3045	Peptidyl-prolyl cis-trans isomerase FKBP5	Q13451	99305.68	95070.34	89360.42	72729.66	69303.54	65619.52	81190.31	75491.43	71629.98	82759.55	81707.68	74709.11	82284.96	76966.75	68979.98	67781.80	65305.52	62530.52	65542.62	67096.43	59399.82	49310.75	51337.88	44650.56	42267.08	40005.17	37972.32	39788.93	38246.53	34370.82	47606.39	37666.94	13921.81		
3046	Peptidyl-prolyl cis-trans isomerase FKBP7	Q9Y680	1883.74	2191.57	2047.62	1570.92	1632.91	1101.61	1878.89	1830.99	1850.05	1729.51	1580.11	1568.65	1836.29	1629.26	1424.55	1533.03	2000.59	1291.28	1142.08	1126.34	1083.13	2.27	1.49	0.65	0.84	1.31	0.69	4.16	2.47	0.11	4.90	4.98	0.12	x	
3047	Peptidyl-prolyl cis-trans isomerase FKBP8	Q14318	118706.11	118057.19	110000.30	81380.33	72183.68	73062.12	91549.85	88083.81	90639.69	106918.02	101504.72	92939.42	104437.88	100319.37	81725.89	86937.29	81316.51	80929.37	90673.44	80327.54	78345.87	31111.10	29525.83	24615.97	16484.03	14574.97	11485.31	17953.66	16621.88	16294.97	18851.82	17503.78	12411.46		
3048	Peptidyl-prolyl cis-trans isomerase FKBP9	O95302	37712.42	35351.26	32611.78	19251.77	19734.65	17374.46	26310.85	25735.31	23959.46	26646.65	26197.33	24119.19	26169.38	23328.96	19328.31	22745.74	21039.61	19646.67	20615.28	19290.15	18217.76	2.27	1.49	0.65	0.84	1.31	0.69	4.16	2.47	0.11	4.90	4.98	0.12	x	
3049	Peptidyl-prolyl cis-trans isomerase H	O43447	0.14	2.53	0.81	1.07	2.77	0.68	0.96	0.36	0.63	0.35	0.75	0.38	2.13	0.97	0.60	4.64	1.07	0.47	2.22	0.19	0.06	16358.75	19162.98	17816.82	11515.09	11121.79	11249.70	13707.92	12226.92	11955.87	12915.20	12290.32	9494.40		x
3050	Peptidyl-prolyl cis-trans isomerase NIMA-interacting 1	Q13526	67772.41	66910.81	58838.54	43879.03	42120.71	38432.75	52682.89	47857.76	47419.25	60894.16	56534.06	54175.15	58776.24	52475.54	42129.17	47554.75	43470.81	40913.92	45392.59	43540.95	40915.52	5204.43	4839.62	3595.99	4201.73	3969.24	2886.40	5001.71	4653.64	4282.37	6237.06	6768.89	3703.88		
3051	Peptidyl-prolyl cis-trans isomerase NIMA-interacting 4	Q9Y237	90192.16	85940.53	78479.15	63934.27	61079.04	57025.35	68762.97	65122.40	61667.19	79883.59	77121.28	72842.93	79629.15	78783.46	73172.18	58337.00	56412.12	53120.59	63257.73	59314.93	59782.89	2.27	1.49	0.65	0.84	1.31	0.69	4.16	2.47	0.11	4.90	4.98	0.12	x	
3052	Peptidyl-prolyl cis-trans isomerase-like 1	Q9Y3C6	66531.10	69128.39	65664.58	47674.50	46953.52	45941.91	47757.46	46388.50	46186.75	57217.48	55489.13	51163.46	53202.44	53793.66	44829.28	44994.51	43956.42	43126.56	47571.83	46321.25	43280.43	19789.08	18811.40	17583.26	15620.75	14752.10	14368.68	16277.09	15742.89	14837.75	19269.59	17825.26	13601.16		
3053	Peptidylprolyl isomerase domain and WD repeat-containing protein 1	Q96BP3	5868.88	4757.75	4360.32	3460.15	3365.72	2763.17	4501.30	4551.24	4270.80	4495.08	4164.88	3657.06	4806.52	3774.10	2635.48	3492.21	3497.63	3113.95	3721.32	3434.14	2730.67	5278.72	4182.15	4607.04	3720.70	3319.36	2676.54	4109.01	3948.23	3641.05	4585.47	4358.25	2807.71		
3054	Peptidyl-tRNA hydrolase 2_ mitochondrial	Q9Y3E5	38202.55	34926.43	31155.49	18891.88	18270.90	16002.70	23424.35	24583.84	22021.72	27219.09	26147.77	23926.23	24302.89	22512.37	13950.25	21042.68	20706.52	19509.98	21674.75	20687.18	19526.90	2.27	1.49	0.65	0.84	1.31	0.69	4.16	2.47	0.11	4.90	4.98	0.12	x	
3055	Pericentrin	O95613	400608.58	375503.35	365523.66	270078.47	262984.74	251516.07	310773.39	299029.39	298557.56	355338.56	342209.01	336849.64	346613.54	311159.48	264582.91	286686.80	277288.68	277508.24	297196.66	284471.09	273847.24	94981.48	94320.47	86642.79	63804.02	65943.90	57349.18	71865.46	69149.68	68030.56	78658.48	76082.38	40651.65		
3056	Perilipin-3	O60664	193799.99	180444.52	169551.79	103248.84	102240.55	93619.32	117237.31	118329.65	113957.66	151525.72	139254.75	128889.51	155462.93	135390.03	115879.22	98888.61	96377.79	91337.70	107814.74	105866.49	100072.92	245987.57	225278.39	205012.22	141132.74	139990.60	122806.67	166557.36	167431.84	156070.26	186026.83	180283.52	129660.73		
3057	Periodic tryptophan protein 2 homolog	Q15269	0.14	2.53	0.81	42.44	20.39	45.54	0.96	0.36	0.63	3.45	13.65	6.40	8.57	1784.36	59.03	4.64	1.07	0.47	2.22	0.19	0.06	23763.07	22067.58	19868.85	15238.87	14531.72	10987.77	18758.24	17530.89	15931.44	18031.05	16674.11	0.12		
3058	Periostin	Q15063	0.14	2.53	0.81	1.07	2.77	0.68	0.96	0.36	0.63	0.35	0.75	0.38	2.13	0.97	0.60	4.64	1.07	0.47	2.22	0.19	0.06	41395.62	39525.52	32004.87	31077.05	30743.30	22537.63	33246.82	31188.46	28354.17	45622.82	44186.21	26993.69		x
3059	Peripheral plasma membrane protein CASK	O14936	0.14	2.53	0.81	1.07	2.77	0.68	0.96	0.36	0.63	0.35	0.75	0.38	2.13	0.97	0.60	4.64	1.07	0.47	2.22	0.19	0.06	758.82	354.35	426.43	442.63	336.70	257.30	1166.78	1054.80	317.46	959.23	917.23	351.55		x
3060	Peripherin	P41219	52963.65	50751.92	46518.24	40298.91	38495.44	36765.33	45186.12	42207.76	40955.54	49203.33	47044.58	43694.26	45987.52	46339.67	33864.76	37668.90	32835.16	32969.03	38616.89	35749.70	32974.37	58250.37	52046.08	51247.34	35904.95	38391.67	32882.20	34761.48	35816.23	34040.78	39223.23	36970.05	30990.63		
3061	Periplakin	O60437	184590.54	172225.44	163380.01	126760.45	128384.42	113810.06	142628.65	141628.43	137833.07	160938.76	149268.72	148167.73	161799.93	143682.89	116308.09	119395.37	117032.23	109977.17	126844.89	128303.05	123899.91	28852.87	27101.24	25353.42	17892.82	18512.91	17563.33	20686.26	19937.51	19215.57	21467.87	20135.07	17037.66		
3062	Peroxiredoxin-1	Q06830	1753463.55	1708869.90	1654503.78	1364364.03	1321495.52	1264342.08	1678404.12	1646582.42	1564680.14	1693574.71	1623742.04	1561151.16	1418102.92	1356873.11	1212091.78	1496791.26	1444936.90	1406243.58	1307441.47	1255500.69	1200212.44	1693652.67	1604002.36	1508651.73	1044604.73	1022887.03	934244.00	1159129.74	1119381.81	1081822.64	1139698.96	1113911.45	841166.81		
3063	Peroxiredoxin-2	P32119	710955.06	686345.46	659479.67	499090.29	469904.09	452810.68	603281.75	582433.49	579923.11	614731.22	589136.35	559450.98	611108.09	572959.98	500523.35	517352.62	476376.89	487519.70	504249.80	463541.77	450829.45	65103.10	61494.01	57200.84	45894.44	45158.14	42746.19	46313.63	44937.72	43622.63	46291.71	44775.37	29312.26		
3064	Peroxiredoxin-4	Q13162	229548.92	213702.75	211273.41	147538.73	139633.77	136666.99	185849.51	178506.89	167657.39	200539.38	190362.65	177579.52	175928.07	168317.65	136496.71	151606.14	140353.40	139270.93	141459.77	144179.47	119370.39	40447.01	42374.99	38758.58	24756.72	25178.11	21724.49	30418.24	32124.79	30598.01	28951.73	29494.28	20520.80		
3065	Peroxiredoxin-5 mitochondrial	P30044	363258.13	320414.55	287681.39	193440.78	174350.25	152881.93	242426.23	249303.35	220939.74	262552.15	249185.07	214681.98	258346.32	233554.91	152892.90	202347.90	188855.23	184292.89	192417.09	174146.65	153379.45	75502.86	70759.87	63261.46	36593.39	34381.42	27547.64	58896.13	53549.76	45254.79	57215.72	52044.01	31336.84		
3066	Peroxiredoxin-6	P30041	315849.23	299101.38	271677.58	254906.91	243407.10	223000.78	278079.28	294191.36	279986.46	322099.87	303150.27	277843.36	275218.50	261493.54	197072.27	258109.25	237102.44	226970.53	245682.95	228629.81	213702.93	324160.12	302431.31	276167.25	185856.16	184097.53	151167.80	224877.94	213988.08	197875.41	226850.96	216575.91	135784.75		
3067	Peroxiredoxin-like 2A	Q9BRX8	5676.48	4998.84	4566.88	2511.12	2848.96	2188.29	2981.92	3585.40	2782.81	3308.87	2840.73	2915.18	3487.58	4046.46	2435.14	3035.39	3100.71	2488.30	2362.39	2706.25	2248.93	2.27	1.49	0.65	0.84	1.31	0.69	4.16	2.47	0.11	4.90	4.98	0.12	x	
3068	Peroxisomal acyl-coenzyme A oxidase 1	Q15067	76655.92	63256.28	51534.50	28356.35	22225.36	21252.33	31261.40	33770.26	31969.43	56006.04	50575.28	45164.50	60261.42	31972.22	15954.52	27984.17	21923.26	22741.70	28299.65	26194.26	19043.71	2699.82	710.11	831.75	1838.37	1869.63	892.24	2543.16	1919.14	1520.76	2932.65	2977.53	1110.69		
3069	Peroxisomal biogenesis factor 19	P40855	36828.06	36544.74	35065.51	23056.64	22788.12	20694.49	30747.04	29371.80	27215.56	32586.84	30222.11	28651.92	29756.90	27425.17	22277.34	26311.18	23826.36	23453.28	25895.98	24042.50	22389.30	1968.30	1660.57	1892.52	1406.33	1653.12	1446.22	1786.46	1906.41	1983.30	1672.20	1614.49	1026.84		
3070	Peroxisomal membrane protein 11B	O96011	33949.68	33147.14	30191.37	25746.45	25628.58	24066.90	26470.66	25646.41	25569.39	34032.47	31543.60	30352.92	31483.09	30069.83	25608.59	24769.04	23738.34	23900.99	23947.13	23236.16	24349.63	2.27	1.49	0.65	0.84	1.31	0.69	4.16	2.47	0.11	4.90	4.98	0.12	x	
3071	Peroxisomal membrane protein PEX14	O75381	0.14	2.53	0.81	1.07	2.77	0.68	0.96	0.36	0.63	0.35	0.75	0.38	2.13	0.97	0.60	4.64	1.07	0.47	2.22	0.19	0.06	2973.97	2136.65	2899.07	2098.55	1963.06	1826.94	2731.86	2394.12	2366.92	2374.00	2384.77	1936.98		x
3072	Peroxisomal multifunctional enzyme type 2	P51659	373097.52	361314.73	334899.16	276003.40	257765.84	240646.87	294052.44	287333.25	271479.64	328443.38	310136.59	295449.12	313146.50	306030.17	250202.69	261398.11	253221.29	248482.29	261498.88	247563.90	243280.14	159322.74	150728.74	140681.53	106731.14	101161.75	96396.45	116728.57	111500.14	109827.99	119466.47	110895.70	77885.03		
3073	Peroxisomal targeting signal 1 receptor	P50542	9234.63	9312.29	8560.89	6355.99	6147.55	5699.86	7754.46	7240.93	6476.02	7440.34	7277.28	6786.09	7492.79	7198.53	5357.95	7134.77	6449.25	6093.68	6391.66	6343.30	5880.50	2.27	1.49	0.65	0.84	1.31	0.69	4.16	2.47	0.11	4.90	4.98	0.12	x	
3074	Peroxisome proliferator-activated receptor delta	Q03181	0.14	2.53	0.81	1.07	2.77	0.68	0.96	0.36	0.63	0.35	0.75	0.38	2.13	0.97	0.60	4.64	1.07	0.47	2.22	0.19	0.06	37008.38	34699.93	32589.97	15765.10	15908.41	15221.80	14264.08	14504.84	14184.80	11839.37	11523.51	7725.74		x
3075	Persulfide dioxygenase ETHE1_ mitochondrial	O95571	11968.25	10433.12	10879.10	9380.35	5380.79	5257.48	9800.92	9682.78	8743.97	10040.89	9323.35	8887.97	9557.46	8295.25	8002.59	7319.51	7395.98	6629.33	7106.81	6079.26	6316.17	23345.49	24141.14	19490.57	15845.69	13507.44	11386.73	14658.01	13763.63	14389.65	14302.48	14247.37	7946.05		
3076	Pescadillo homolog	O00541	3689.79	4305.72	4002.25	4425.71	4232.03	4352.94	3180.08	3654.34	3720.93	3245.31	3466.82	3344.74	4585.64	1361.21	1479.30	3661.82	2027.49	2346.86	1768.06	4065.70	3084.29	4404.08	3953.54	3686.18	3384.31	3362.50	3294.60	4138.80	3815.60	4160.12	4664.28	3051.62	2145.08		
3077	PEST proteolytic signal-containing nuclear protein	Q8WW1	44852.25	43230.74	41483.39	34321.02	32067.44	30555.06	35696.57	32526.11	32370.92	45506.95	42365.42	40028.74	39542.16	38599.55	34716.16	32365.06	28574.43	29802.23	32149.05	31442.06	31544.12	43651.71	42017.14	40825.99	34516.39	32184.02	29550.93	37500.88	35418.98	35467.43	40005.39	37219.93	18332.01		
3078	PH and SEC7 domain-containing protein 1	A5PKW4	35404.13	32377.02	31900.36	27249.43	25840.54	25531.12	33273.55	31538.75	30120.00	34004.13	32663.25	30551.50	29544.70	31300.64	25847.30	28927.34	27922.11	26505.65	28887.29	29009.46	27555.04	544.89	624.31	523.96	323.88	413.05	173.85	1392.72	971.46	553.53	884.04	668.45	365.06		
3079	PH and SEC7 domain-containing protein 4	Q8NDX1	149158.56	142835.89	140172.83	107199.33	102790.92	94585.80	89292.71	86026.02	85363.16	120170.19	117012.07	109563.52	118309.28	113181.17	90998.04	92648.74	90795.21	87973.59	97012.41	93551.12	89108.72	25274.70	24018.46	19708.76	15262.35	14268.03	10140.44	19490.85	17296.55	15728.61	20212.52	19875.97	12037.37		
3080	Phakinin	Q13515	0.14	2.53	0.81	1.07	2.77	0.68	0.96	0.36	0.63	0.35	0.75	0.38	2.13	0.97	0.60	4.64	1.07	0.47	2.22	0.19	0.06	423.46	348.61	280.72	310.97	294.67	187.90	300.38	275.72	246.04	212.22	190.75	105.95		x
3081	PHD finger protein 11	Q9UIL8	0.14	2.53	0.81	1.07	2.77	0.68	0.96	0.36	0.63	0.35	0.75	0.38	2.13	0.97	0.60	4.64	1.07	0.47	2.22	0.19	0.06	213.07	194.50	219.64	185.78	184.50	190.64	131.30	202.93	201.88	199.41	202.38	7.81		x
3082	PHD finger protein 6	Q8IWS0	35031.78	32469.09	31601.33	25612.71	25616.77	24336.01	22538.86	19794.04	19654.66	32790.34	31038.02	30702.14	28488.29	23885.34	17890.53	17691.33	21977.85	19929.48	25542.56	25527.40	25289.04	38439.82	34350.61	32034.14	24718.98	24718.13	20257.06	27300.11	26389.45	23791.64	31354.90	29651.98	20954.15		
3083	PHD finger-like domain-containing protein 5A	Q7RTV0	31730.11	32212.76	27939.40	17503.38	16678.10	15491.16	23670.04	23187.97	22058.98	20823.48	19982.68	19473.45	23512.68	21524.03	18269.52	20409.70	18495.33	17619.29	20464.73	18815.06	17866.79	13819.29	10716.16	11045.62	8426.26	7740.93	6910.66	10629.41	9351.28	9202.57	9589.94	8441.93	6405.38		
3084	Phenylalanine--tRNA ligase alpha subunit	Q9Y285	204170.95	189958.62	177881.34	130615.87	127263.98	120041.00	143537.59	140473.65	133875.31	176793.17	163888.04	156294.14	163764.24	152036.62	128883.77	125224.83	115208.04	112100.11	131435.54	126676.63	122532.18	51639.65	47282.04	46062.81	34770.64	32785.79	26576.92	43808.16	41915.59	37806.26	43428.80	40312.36	26806.57		
3085	Phenylalanine--tRNA ligase beta subunit	Q9NSD9	301179.80	292922.44	266781.87	218865.04	235286.10	203634.73	282366.90	273865.25	244565.32	255720.31	240913.93	219647.06	243251.24	230884.61	183812.94	231929.52	216636.77	204553.93	215724.04	197696.48	186718.35	41956.07	36118.34	35605.59	24260.88	22655.54	19272.01	30255.30	25249.51	25414.37	27978.99	27564.58	19148.81		
3086	PH-interacting protein	Q8WWQ	0.14	2.53	0.81	1.07	2.77	0.68	0.96	0.36	0.63	0.35	0.75	0.38	2.13	0.97	0.60	4.64	1.07	0.47	2.22	0.19	0.06	858.95	802.05	815.65	672.59	613.51	560.62	986.23	757.53	812.76	965.30	771.34	472.27		x
3087	Phosducin-like protein 2	Q8N4E4	0.14	2.53	0.81	1.07	2.77	0.68	0.96	0.36	0.63	0.35	0.75	0.38	2.13	0.97	0.60	4.64	1.07	0.47	2.22	0.19	0.06	3911.95	3460.36	3168.92	2285.01	2061.44	1572.52	2923.22	2663.21	2412.36	3059.70	2921.13	1649.99		x
3088	Phosducin-like protein	Q13371	10647.25	10138.80	10331.30	8173.13	7929.80	7622.90	9130.83	8756.60	7727.14	10578.34	10466.14	10383.18	10756.15	10472.65	9744.91	8246.04	7304.24	7691.82	9064.42	8284.41	8288.18	2.27	1.49	0.65	0.84	1.31	0.69	4.16	2.47	0.11	4.90	4.98	0.12	x	
3089	Phosphatase and actin regulator 4	Q8IZ21	62850.85	62606.21	59692.47	63076.53	60375.58	58312.76	62100.78	58786.37	56045.50	62612.61	60889.28	56939.64	66457.11	64612.47	61892.04	52376.32	51326.33	50318.73	58195.31	52882.43	53179.69	29051.42	27340.93	27271.55	22441.62	25460.40	23768.11	31927.48	31928.16	32339.73	28930.85	29030.82	23115.26		
3090	Phosphate carrier protein mitochondrial	Q00325	731052.94	727430.52	693269.54	490557.02	465985.68	432668.73	530571.93	524239.42	499195.71	617903.14	593250.88	588424.33	586304.43	570729.81	447276.72	466247.22	464002.27	444189.14	516726.27	487447.13	455721.03	188294.91	175797.65	161652.35	111994.12	109303.08	103969.86	142447.48	135999.75	129984.93	140104.89	136466.99	93780.71		
3091	Phosphate-regulating neutral endopeptidase PHEX	P78562	37037.54	34734.87	31682.11	20942.40	20480.93	17779.65	29402.98	27711.62	27633.58	26680.45	24893.98	22656.30	30770.40	28840.93	19813.68	24195.64	24492.55	22570.85	21034.19	19198.65	17199.17	25779.49	24990.19	22799.19	14052.47	14123.95	11609.22	16589.58	16992.09	15748.05	17456.84	16306.86	12045.74		
3092	Phosphatidylcholine translocator ABCB4	P21439	0.14	2.53	0.81	1.07	2.77	0.68	0.96	0.36	0.63	0.35	0.75	0.38	2.13	0.97	0.60	4.64	1.07	0.47	2.22	0.19	0.06	146.38	121.28	171.87	74.66	86.15	87.84	122.96	106.61	99.01	344.60	312.40	166.55		x
3093	Phosphatidylethanolamine-binding protein 1	P30086	554288.16	500554.91	494826.46	354029.11	328727.71	307523.62	421147.48	427506.79	397439.23	457400.86	436015.46	390326.17	423830.59	399452.18	316880.03	363320.01	341193.74	338051.77	360896.50	326997.97	319950.82	120138.17	111508.15	96029.08	72219.82	68065.48	51706.70	90833.69	84287.83	76619.66	96546.91	90941.71	55691.14		
3094	Phosphatidylinositide phosphatase SAC1	Q9NTJ5	5248.98	5354.14	4804.54	3850.26	4168.85	3616.38	4179.90	4171.44	4134.55	5269.91	4974.16	4890.75	4359.20	4311.97	3934.21	3624.43	3309.11	3257.81	3703.78	3609.43	3581.94	11718.86	11734.58	10817.19	7518.05	7136.52	6810.72	8749.02	8480.00	8441.58	9815.05	9479.07	6420.78		
3095	Phosphatidylinositol 3_4_5-trisphosphate-dependent Rac exchanger 1 protein	Q8TCU6	107222.54	102016.63	92060.24	75151.72	73557.38	65797.01	101995.57	98632.07	89967.33	106104.45	102083.96	96720.22	91303.33	83488.14	66814.45	78641.67	73930.16	70406.46	74985.69	72453.05	68103.87	2.27	1.49	0.65	0.84	1.31	0.69	4.16	2.47	0.11	4.90	4.98	0.12	x	
3096	Phosphatidylinositol 3_4_5-trisphosphate-dependent Rac exchanger 2 protein	Q70Z35	183314.46	172203.81	164963.99	114333.04	108184.43	100109.97	129000.68	123413.61	115280.41	139413.63	131685.81	125775.44	141703.37	137183.30	104209.53	112690.54	112371.93	104355.13	119470.30	114605.73	104009.39	37480.36	36846.61	31564.11	28550.31	28098.52	22479.83	30026.82	28155.62	24590.21	37785.29	39409.77	17919.50		

Supplementary Table S1. Overview on the abundance of all relatively quantified 5180 proteins

			MCF-7																					MDA-MB-231													
	Protein name	UniProt	control 01	control 02	control 03	Dai IC20 01	Dai IC20 02	Dai IC20 03	Dai SC20 01	Dai SC20 02	Dai SC20 03	Gen IC20 01	Gen IC20 02	Gen IC20 03	Gen SC20 01	Gen SC20 02	Gen SC20 03	SSE IC20 01	SSE IC20 02	SSE IC20 03	SSE SC20 01	SSE SC20 02	SSE SC20 03	control 01	control 02	control 03	Dai IC20 01	Dai IC20 02	Dai IC20 03	Gen IC20 01	Gen IC20 02	Gen IC20 03	SSE IC20 01	SSE IC20 02	SSE IC20 03	unique to MCF-7	unique to MDA-MB-231
3097	Phosphatidylinositol 3-kinase catalytic subunit type 3	Q8NEB9	382.43	304.32	430.10	236.66	280.27	170.55	359.85	338.64	296.08	257.50	217.66	182.28	437.86	156.93	174.67	204.33	140.95	183.09	215.47	158.91	226.16	2.27	1.49	0.65	0.84	1.31	0.69	4.16	2.47	0.11	4.90	4.98	0.12	x	
3098	Phosphatidylinositol 3-kinase regulatory subunit beta	O00459	1441.93	1028.81	862.30	669.20	637.29	588.93	1101.99	989.52	928.21	993.83	1094.60	1021.40	857.13	862.08	300.14	902.91	724.69	843.76	830.77	666.16	608.00	2.27	1.49	0.65	0.84	1.31	0.69	4.16	2.47	0.11	4.90	4.98	0.12	x	
3099	Phosphatidylinositol 4_5-bisphosphate 3-kinase catalytic subunit beta isoform	P42338	8242.50	8285.62	7347.15	5712.83	5666.14	5296.13	8334.11	7942.43	7840.19	7509.66	7628.72	7150.94	7532.38	6669.44	5920.68	6244.84	6033.24	5508.29	5333.29	5325.66	5238.14	2.27	1.49	0.65	0.84	1.31	0.69	4.16	2.47	0.11	4.90	4.98	0.12	x	
3100	Phosphatidylinositol 4-kinase alpha	P42356	139907.09	137485.91	128238.02	94283.66	89178.68	80374.88	112862.83	113002.98	107086.08	119319.36	122246.48	107718.65	114858.76	106974.55	84047.44	98179.56	94365.52	90626.85	108465.89	105084.54	93970.44	51326.15	52314.71	44189.36	33980.93	30950.77	25518.72	40644.14	36274.73	33952.73	39681.89	39044.36	29946.47		
3101	Phosphatidylinositol 4-kinase type 2-alpha	Q9BTU6	0.14	2.53	0.81	1.07	2.77	0.68	0.96	0.36	0.63	0.35	0.75	0.38	2.13	0.97	0.60	4.64	1.07	0.47	2.22	0.19	0.06	3627.12	2708.45	3101.50	1318.64	1352.90	1059.95	1566.82	1447.28	1231.43	1819.83	1559.89	956.77		x
3102	Phosphatidylinositol 4-phosphate 3-kinase C2 domain-containing subunit alpha	O00443	0.14	2.53	0.81	1.07	2.77	0.68	0.96	0.36	0.63	0.35	0.75	0.38	2.13	0.97	0.60	4.64	1.07	0.47	2.22	0.19	0.06	6511.53	7939.39	7706.74	6002.90	6029.39	5713.04	8444.83	8356.06	7827.14	6589.76	6404.36	4951.76		x
3103	Phosphatidylinositol 4-phosphate 5-kinase type-1 alpha	Q99755	0.14	2.53	0.81	1.07	2.77	0.68	0.96	0.36	0.63	0.35	0.75	0.38	2.13	0.97	0.60	4.64	1.07	0.47	2.22	0.19	0.06	11121.77	11560.43	9497.52	12419.71	12498.79	10199.99	15780.52	14804.68	13458.18	13418.25	12879.80	8885.71		x
3104	Phosphatidylinositol 5-phosphate 4-kinase type-2 beta	P78356	0.14	2.53	0.81	1.07	2.77	0.68	0.96	0.36	0.63	0.35	0.75	0.38	2.13	0.97	0.60	4.64	1.07	0.47	2.22	0.19	0.06	514.63	528.20	390.54	257.67	251.39	210.94	278.38	278.11	171.98	318.95	220.21	149.38		x
3105	Phosphatidylinositol 5-phosphate 4-kinase type-2 gamma	Q8TBX8	20763.70	19579.86	16637.38	17029.30	16162.31	14845.83	19052.85	16978.73	17352.48	22127.99	20705.52	17673.22	20094.42	17558.13	12712.48	16935.47	16411.59	15531.55	18057.73	16342.32	14814.64	2.27	1.49	0.65	0.84	1.31	0.69	4.16	2.47	0.11	4.90	4.98	0.12	x	
3106	Phosphatidylinositol transfer protein alpha isoform	Q00169	2079.47	1961.82	1729.47	1380.04	1231.69	1205.55	2349.12	2644.17	2284.49	1786.76	1846.19	1816.37	2256.73	2444.54	1716.26	1347.49	1251.02	1305.00	1486.72	1414.59	1363.35	107758.04	102873.36	93075.60	64998.61	63397.38	52114.60	86947.46	86009.13	80875.37	87668.33	85613.73	64460.52		
3107	Phosphatidylinositol transfer protein beta isoform	P48739	86276.57	80779.24	84177.18	58373.52	62701.43	52346.65	73792.06	62161.50	64229.23	77277.30	72012.62	70631.39	72509.73	68002.11	55028.37	64792.97	51527.76	56314.24	62662.41	60735.36	55703.27	89648.00	68082.96	74833.19	54457.76	53381.52	48982.03	68642.34	64891.88	62071.75	71349.81	67454.98	46829.90		
3108	Phosphatidylinositol-binding clathrin assembly protein	Q13492	19131.99	18405.87	17665.89	15686.01	16218.45	14000.71	16809.11	16686.58	15605.37	20425.78	19181.70	18948.09	18893.40	18918.12	16201.37	13158.37	14328.95	13580.39	16215.20	15750.90	16125.39	4754.86	4406.97	4003.48	3067.25	2963.93	2882.31	3533.34	3648.49	3363.86	3307.55	3103.59	2421.20		
3109	Phosphoacetylglucosamine mutase	O95394	78593.08	74648.43	72701.69	63792.07	60688.05	59082.08	59493.37	58526.21	57545.60	77875.23	74057.34	70185.19	67658.07	63205.27	52662.69	58235.00	57446.14	54933.29	60138.11	57082.00	54635.86	27365.46	26302.75	23005.87	23222.18	22073.47	19995.09	23809.69	25064.98	21916.70	31413.69	29508.56	18341.19		
3110	Phosphoenolpyruvate carboxykinase [GTP]_ mitochondrial	Q16822	555189.97	533246.91	481687.84	466014.39	447085.75	395502.07	483041.70	462787.03	427848.32	441025.85	413363.73	374852.61	427686.58	386103.45	289393.39	465989.05	440845.10	423112.56	407347.14	380394.18	351836.83	59357.75	54565.08	53180.08	45084.26	46022.82	42587.72	50358.79	49051.89	45772.54	58700.56	56282.10	39004.44		
3111	Phosphoenolpyruvate carboxykinase_ cytosolic [GTP]	P35558	45596.09	38005.26	31285.65	30777.10	30725.61	25174.19	33227.78	31486.28	28484.93	36910.59	35034.52	31089.18	34697.07	31911.26	20050.50	29618.69	29757.46	26986.39	31569.07	29546.49	25260.56	2.27	1.49	0.65	0.84	1.31	0.69	4.16	2.47	0.11	4.90	4.98	0.12	x	
3112	Phosphoglucomutase-1	P36871	1356.39	1270.17	1417.78	1417.33	1482.38	1020.37	2225.82	2346.12	1974.77	2014.03	1954.26	1767.56	1653.70	1519.29	1200.89	1579.83	1587.10	1501.07	1521.76	1404.52	1226.54	92591.12	85556.51	81500.07	57219.24	54869.75	47017.52	67676.22	64236.45	62667.82	64231.34	61838.76	39507.66		
3113	Phosphoglucomutase-2	Q96G03	85467.35	82343.41	80272.25	56089.53	52954.96	45364.53	63805.76	63687.34	61836.53	67459.94	64763.16	60211.18	69021.82	59656.52	50267.90	53951.81	52134.86	51197.46	56788.76	51386.03	48384.31	20666.51	20335.32	17665.15	16176.79	14633.62	12013.56	19062.02	18033.43	15697.69	19457.96	18302.84	12274.94		
3114	Phosphoglycerate kinase 1	P00558	1769861.87	1683194.05	1547088.84	1149649.41	1121696.41	1020484.02	1657140.63	1587996.76	1518323.44	1450184.19	1402066.33	1272346.52	1526998.14	1371906.77	981735.97	1179660.50	1129384.76	1086442.55	1258042.48	1183186.39	1088217.40	1713867.61	1616594.07	1491820.45	828162.20	815016.14	711444.15	947733.53	907815.79	850141.16	932427.38	897504.21	601759.72		
3115	Phosphoglycerate kinase 2	P07205	62054.56	59095.63	47096.65	40926.74	37669.73	31294.80	44540.26	41493.08	36320.02	50018.73	48183.57	41245.07	47143.63	38153.35	26285.73	52408.73	46084.62	40581.42	37493.45	33924.53	31486.07	10209.32	9423.78	7693.59	6145.05	5943.98	4884.93	7935.74	7667.77	6866.26	7963.21	7595.60	4357.90		
3116	Phosphoglycerate mutase 1	P18669	1725398.33	1649379.75	1473623.48	1053868.72	997701.49	902451.25	1335954.14	1276540.59	1169470.28	1338161.87	1274911.59	1142890.01	1390614.48	1263059.19	1055408.61	1065800.98	999435.94	938308.82	1128291.75	1054399.79	975760.02	1116265.88	1039301.16	937885.95	603924.22	583648.52	506105.38	740716.11	707110.68	659597.90	796765.49	775971.51	528214.66		
3117	Phosphoglycerate mutase 2	P15259	1352.96	1214.53	753.57	803.34	707.53	650.22	742.56	552.59	661.82	801.15	1054.70	696.14	910.35	558.81	98.19	875.45	567.76	680.10	600.52	799.87	564.70	232.10	203.12	205.82	80.96	91.36	79.72	118.26	119.37	119.92	176.22	191.04	125.63		
3118	Phosphoinositide 3-kinase adapter protein 1	Q6ZUJ8	0.14	2.53	0.81	1.07	2.77	0.68	0.96	0.36	0.63	0.35	0.75	0.38	2.13	0.97	0.60	4.64	1.07	0.47	2.22	0.19	0.06	2.27	1.49	0.65	1125.49	922.53	439.17	4.16	2.47	0.11	800.31	697.06	178.94		x
3119	Phosphoinositide 3-kinase regulatory subunit 6	Q5UE93	0.14	2.53	0.81	1.07	2.77	0.68	0.96	0.36	0.63	0.35	0.75	0.38	2.13	0.97	0.60	4.64	1.07	0.47	2.22	0.19	0.06	21501.06	19436.70	18698.23	16608.49	16534.67	16184.09	17337.00	16424.22	15610.07	17576.63	17142.89	13504.27		x
3120	Phospholipase A-2-activating protein	Q9Y263	73937.81	70395.17	67168.72	47788.04	43750.14	42385.11	55128.29	55004.03	52404.59	58028.33	55554.69	51726.40	59589.47	56004.20	47043.64	47770.00	44618.04	43554.08	53204.28	47766.09	43411.55	155098.75	147274.68	136692.93	101932.05	99573.30	95960.10	108558.40	104438.35	101891.82	112833.74	111878.09	83150.85		
3121	Phospholipase D1	Q13393	2482.33	2029.09	2036.65	1520.92	1194.93	1479.28	2359.99	2229.24	1791.60	2288.06	2264.30	1974.36	2009.41	1883.23	1424.41	1525.07	1436.16	1558.46	1755.37	1495.11	1441.52	5274.86	4924.34	4899.96	3653.06	3592.67	3160.73	4062.07	4354.11	3820.07	4705.22	4962.21	2485.02		
3122	Phospholipase D2	O14939	9428.08	7911.89	7515.41	5991.29	5119.70	5244.89	6259.32	6410.56	6486.29	7199.32	8035.99	7674.28	8696.33	8112.29	4906.42	6036.03	5467.27	5734.67	5980.05	5716.53	5187.08	2.27	1.49	0.65	0.84	1.31	0.69	4.16	2.47	0.11	4.90	4.98	0.12	x	
3123	Phospholipase DDHD2	O94830	1999.20	2238.33	1950.70	2606.67	2472.57	2550.94	1929.58	1717.26	1753.64	3337.47	3385.35	3036.76	2518.18	2308.49	1871.94	2374.64	2237.42	2320.07	1956.59	1849.74	1858.50	11059.39	10217.12	9492.23	7931.02	7295.50	7060.62	7273.51	7310.99	8141.36	8198.80	6952.97	3444.31		
3124	Phospholipid phosphatase-related protein type 4	Q7Z2D5	0.14	2.53	0.81	1.07	2.77	0.68	0.96	0.36	0.63	0.35	0.75	0.38	2.13	0.97	0.60	4.64	1.07	0.47	2.22	0.19	0.06	38063.88	28151.27	31616.25	30173.91	28876.64	25511.96	35923.49	35334.18	34812.56	41696.60	42404.52	32613.11		x
3125	Phospholipid-transporting ATPase IA	Q9Y2Q0	0.14	2.53	0.81	1.07	2.77	0.68	0.96	0.36	0.63	0.35	0.75	0.38	2.13	0.97	0.60	4.64	1.07	0.47	2.22	0.19	0.06	41659.63	11038.02	7882.99	24769.23	21565.03	12180.03	44504.54	36994.40	22139.78	50662.11	43255.45	19869.96		x
3126	Phosphomannomutase 1	Q92871	45561.89	41934.56	39512.54	28255.68	29372.23	27273.69	42398.78	39769.02	38196.45	39535.69	37906.59	35309.69	38195.69	35620.75	29378.59	29717.07	28640.37	28584.41	35807.11	30576.70	28250.02	1183.96	1151.75	1028.59	890.50	914.86	751.73	869.70	915.89	938.13	1025.07	870.91	669.27		
3127	Phosphomannomutase 2	O15305	30868.34	27868.51	26921.90	21396.87	20973.14	20130.56	23645.72	22967.43	21802.56	26872.69	26693.57	25187.58	24984.75	26543.44	22805.60	19916.09	18934.20	17909.48	22269.86	21217.19	19956.50	13166.24	12190.81	11230.35	9328.96	9876.08	7397.95	12522.91	11245.45	10815.54	12302.43	12300.90	8142.05		
3128	Phosphomevalonate kinase	Q15126	5151.38	4685.67	4014.87	2936.03	3070.34	2657.75	4321.45	3114.13	3543.67	3640.22	3534.64	3235.62	3928.01	3667.31	1820.87	3472.72	2523.57	2672.73	3662.34	3021.56	2745.66	13720.85	13132.22	12409.07	8671.22	8747.19	8230.21	12120.59	11302.50	10810.03	11216.75	10444.55	7559.16		
3129	Phosphoribosyl pyrophosphate synthase-associated protein 1	Q14558	55330.32	54018.23	67480.45	44521.96	42707.13	41729.93	45037.07	43445.35	40694.67	64717.47	62734.09	59794.85	46222.44	48298.92	38300.41	39484.84	38973.75	38468.88	39152.39	39447.13	37744.28	13044.34	12250.40	10601.26	8751.66	8554.78	6630.32	11335.86	11194.68	9612.52	11965.65	11735.80	15739.00		
3130	Phosphoribosyl pyrophosphate synthase-associated protein 2	O60256	27739.99	27953.73	26341.56	22051.25	21477.23	20542.20	23202.14	22105.70	20406.30	26136.96	23736.60	22663.11	25079.72	23536.80	21449.01	20677.40	17793.20	19093.54	20765.77	18961.85	18377.29	9294.09	9151.50	8018.00	4313.94	3891.17	2833.97	12991.51	12238.54	10699.39	7731.93	7336.60	6919.72		
3131	Phosphoribosylformylglycinamidine synthase	O15067	112303.04	107715.75	103552.99	82874.08	82084.49	78143.95	98777.14	95449.24	93461.30	100233.32	97038.13	91715.82	93689.34	89761.22	78567.58	81830.26	78172.49	74373.34	89237.21	77123.35	78026.54	256484.07	234177.49	218516.91	155709.66	152684.99	144811.70	191738.91	182572.20	177549.37	188084.93	178580.18	128240.71		
3132	Phosphoribosyltransferase domain-containing protein 1	Q9NRG1	0.14	2.53	0.81	1.07	2.77	0.68	0.96	0.36	0.63	0.35	0.75	0.38	2.13	0.97	0.60	4.64	1.07	0.47	2.22	0.19	0.06	463.93	336.87	296.39	55.28	92.20	8.90	226.58	98.63	39.93	250.67	204.66	11.89		x
3133	Phosphorylase b kinase gamma catalytic chain_ liver/testis isoform	P15735	14947.37	14998.35	13731.93	7738.49	6979.24	7040.45	10239.94	8184.83	9275.92	10503.53	10144.22	7835.17	11331.68	9173.01	8245.33	8964.88	7317.34	7602.06	8949.78	8034.12	6253.80	2.27	1.49	0.65	0.84	1.31	0.69	4.16	2.47	0.11	4.90	4.98	0.12	x	
3134	Phosphorylase b kinase gamma catalytic chain_ skeletal muscle/heart isoform	Q16816	0.14	2.53	0.81	1.07	2.77	0.68	0.96	0.36	0.63	0.35	0.75	0.38	2.13	0.97	0.60	4.64	1.07	0.47	2.22	0.19	0.06	2554.93	2604.82	2448.68	2829.81	2544.43	2074.58	8205.79	7871.07	7246.70	3250.69	2842.82	1902.04		x
3135	Phosphorylase b kinase regulatory subunit alpha_ liver isoform	P46019	31558.32	29604.31	26186.00	20572.34	20265.21	19085.67	24564.87	23469.99	22933.87	26918.63	25573.70	24203.13	25911.35	23561.63	18146.01	20454.82	20307.51	19054.75	22504.18	21764.66	20041.27	2.27	1.49	0.65	0.84	1.31	0.69	4.16	2.47	0.11	4.90	4.98	0.12	x	
3136	Phosphorylated adapter RNA export protein	Q9H814	40434.26	36766.11	35461.67	26578.77	26366.43	23707.30	27672.32	27340.23	28345.32	30096.92	29242.39	29453.04	29799.28	29023.05	22575.97	26175.01	26906.04	27061.51	28769.78	22876.15	21826.79	2.27	1.49	0.65	0.84	1.31	0.69	4.16	2.47	0.11	4.90	4.98	0.12	x	
3137	Phosphoserine aminotransferase	Q9Y617	131588.91	125049.88	112354.93	111895.17	110401.58	101007.86	120729.06	117275.47	111580.59	119506.47	111190.96	108829.17	104404.84	100591.84	85070.54	117096.04	111087.12	105948.15	95620.78	94273.92	91335.01	129523.95	120337.62	114908.78	92838.19	90452.79	81523.43	102189.45	102533.49	95625.27	100892.01	97522.13	70328.17		
3138	Phosphoserine phosphatase	P78330	19997.37	23001.02	20826.20	18782.91	20227.81	18363.12	20057.64	20424.66	14393.79	19244.11	20164.56	17636.63	18636.71	15750.46	34998.55	18381.36	12076.47	16760.96	12026.94	17852.78	16642.12	5032.06	4651.33	4485.24	2618.80	2665.23	2463.14	3589.58	3446.80	3709.69	3156.39	3258.78	2195.74		
3139	Phosphotriesterase-related protein	Q96BW5	0.14	2.53	0.81	1.07	2.77	0.68	0.96	0.36	0.63	0.35	0.75	0.38	2.13	0.97	0.60	4.64	1.07	0.47	2.22	0.19	0.06	1635.79	1738.35	1210.19	856.91	898.74	846.65	939.69	964.17	897.46	969.12	1189.27	1790.18		x
3140	Phylloquinone omega-hydroxylase CYP4F11	Q9HBI6	5910.82	4965.35	4496.78	3146.63	3229.03	3162.04	3533.91	3472.29	3535.72	4196.39	4336.67	4473.39	3483.33	3957.41	3628.25	3267.84	2909.81	3333.71	3232.65	3215.98	3087.05	2.27	1.49	0.65	0.84	1.31	0.69	4.16	2.47	0.11	4.90	4.98	0.12	x	
3141	Phytanoyl-CoA hydroxylase-interacting protein	Q92561	0.14	2.53	0.81	1.07	2.77	0.68	0.96	0.36	0.63	0.35	0.75	0.38	2.13	0.97	0.60	4.64	1.07	0.47	2.22	0.19	0.06	16981.65	16595.45	15403.95	21551.85	21618.70	21998.34	27796.13	29255.76	28377.17	17202.63	15461.85	5051.28		x
3142	Piezo-type mechanosensitive ion channel component 2	Q9H5I5	113830.72	108638.61	98815.62	81616.57	80563.89	74334.08	92565.26	86115.46	87482.81	98408.26	94800.90	88795.33	92350.83	86696.81	74861.56	81435.16	77263.94	75365.20	81315.99	81625.74	76977.00	2.27	1.49	0.65	0.84	1.31	0.69	4.16	2.47	0.11	4.90	4.98	0.12	x	
3143	PIH1 domain-containing protein 1	Q9NWS0	0.14	2.53	0.81	1.07	2.77	0.68	0.96	0.36	0.63	0.35	0.75	0.38	2.13	0.97	0.60	4.64	1.07	0.47	2.22	0.19	0.06	2131.13	2506.19	2390.60	771.97	780.43	636.43	611.65	671.15	729.02	553.46	592.35	404.34		x
3144	PIN2/TERF1-interacting telomerase inhibitor 1	Q96BK5	1524.66	1479.67	1423.93	1914.09	2012.39	1825.35	0.96	0.36	0.63	1754.47	1961.64	1693.84	1756.93	79.18	177.85	4.64	1.07	0.47	2.22	0.19	0.06	2.27	1.49	0.65	0.84	1.31	0.69	4.16	2.47	0.11	4.90	4.98	0.12	x	
3145	Pirin	O00625	47020.78	45399.11	45070.79	36593.42	35530.04	32624.01	35584.89	38086.01	34689.45	49264.04	48051.72	48116.56	45958.81	42691.22	26454.02	33462.14	39659.22	41734.37	42926.75	38783.39	35306.14	2.27	1.49	0.65	0.84	1.31	0.69	4.16	2.47	0.11	4.90	4.98	0.12	x	
3146	PITH domain-containing protein 1	Q9GZP4	23947.51	25810.03	24280.71	19526.80	19662.02	19521.33	21041.28	20728.80	20928.86	21745.58	20518.56	19512.62	22831.93	20535.46	18044.70	16732.83	19258.81	16803.03	20183.92	18947.04	19056.26	864.06	929.54	821.59	997.24	871.41	711.38	1190.88	1174.13	981.33	1114.49	1112.35	733.12		
3147	Pituitary homeobox 2	Q99697	1430.61	1236.67	1028.88	655.59	580.86	423.23	854.91	758.04	622.12	1019.67	959.84	843.24	888.63	827.21	327.38	923.28	811.54	923.91	702.16	682.51	639.97	2.27	1.49	0.65	0.84	1.31	0.69	4.16	2.47	0.11	4.90	4.98	0.12	x	
3148	Piwi-like protein 2	Q8TC59	11337.57	10587.05	10393.78	7777.52	7698.99	7696.87	9702.05	9401.46	8929.25	9024.41	6892.18	7790.20	8449.62	7938.40	6348.61	7595.50	6962.01	6889.70	7044.47	5713.31	6345.99	2.27	1.49	0.65	0.84	1.31	0.69	4.16	2.47	0.11	4.90	4.98	0.12	x	
3149	Piwi-like protein 4	Q7Z3Z4	0.14	2.53	0.81	1.07	2.77	0.68	0.96	0.36	0.63	0.35	0.75	0.38	2.13	0.97	0.60	4.64	1.07	0.47	2.22	0.19	0.06	33930.99	43184.88	40671.62	28658.39	28115.42	26930.81	34613.25	32745.66	33815.21	27552.96	26024.78	29044.80		x
3150	Plakophilin-3	Q9Y446	870.84	1025.31	826.05	751.43	507.57	557.38	873.50	1037.34	894.41	733.26	716.09	779.60	1056.59	833.16	758.28	612.72	656.84	636.79	1166.62	971.69	730.74	2.27	1.49	0.65	0.84	1.31	0.69	4.16	2.47	0.11	4.90	4.98	0.12	x	
3151	Plasma kallikrein	P03952	29741.90	27064.55	25234.85	39072.02	37215.82	37031.68	28880.14	28451.63	32223.64	52172.39	50131.23	49265.28	29106.61	27607.32	21543.62	41829.68	39362.83	39717.51	30971.15	29230.71	28936.02	2.27	1.49	0.65	0.84	1.31	0.69	4.16	2.47	0.11	4.90	4.98	0.12	x	
3152	Plasma membrane calcium-transporting ATPase 1	P20020	76070.68	72525.80	67943.14	44610.14	42525.44	39044.62	48862.56	48647.29	44857.64	69635.86	69221.56	54653.79	59943.39	51601.88	37412.96	46568.51	43095.56	42996.44	49810.54	45630.59	41901.20	2.27	1.49	0.65	0.84	1.31	0.69	4.16	2.47	0.11	4.90	4.98	0.12	x	
3153	Plasma membrane calcium-transporting ATPase 2	Q01814	37254.53	38111.66	32258.96	50847.81	50882.35	47978.33	58535.61	51436.38	49761.75	44824.18	41261.88	31017.44	50263.10	17423.80	7564.40	33982.13	37248.05	20053.28	15212.94	32144.37	52881.21	2.27	1.49	0.65	0.84	1.31	0.69	4.16	2.47	0.11	4.90	4.98	0.12	x	
3154	Plasma membrane calcium-transporting ATPase 3	Q16720	3681.97	3402.20	3129.06	2530.17	2713.43	2391.00	3452.69	3567.12	2862.82	3613.94	3071.74	2788.26	3065.21	2726.75	2072.49	2284.58	2037.47	1375.97	1553.06	2427.33	2216.86	2.27	1.49	0.65	0.84	1.31	0.69	4.16	2.47	0.11	4.90	4.98	0.12	x	
3155	Plasma membrane calcium-transporting ATPase 4	P23634	40593.90	37047.69	33851.46	21899.78	21889.06	20495.33	27335.51	27885.07	27033.45	31686.69	29318.70	25411.85	34379.66	25242.36	21413.51	24023.72	24267.36	21266.25	22370.14	21240.70	18840.74	223.49	189.94	126.02	65.30	128.53	33.27	87.05	94.33	36.71	230.79	258.41	18.77		
3156	Plasminogen activator inhibitor 1	P05121	0.14	2.53	0.81	1.07	2.77	0.68	0.96	0.36	0.63	0.35	0.75	0.38	2.13	0.97	0.60	4.64	1.07	0.47	2.22	0.19	0.06	11512.04	9795.93	8633.67	9059.97	8782.38	8383.82	9853.53	9480.63	9338.62	11026.85	10318.38	5998.81		x
3157	Plasminogen activator inhibitor 1 RNA-binding protein	Q8NC51	425513.80	419386.21	395995.82	320592.79	308588.85	297708.69	330231.16	326604.90	310775.09	360107.90	346989.80	350954.20	348511.13	318775.52	201437.58	296654.54	287283.18	280090.84	322543.98	320046.24	304279.11	265512.24	260540.37	228344.84	200662.70	196679.72	185479.25	212106.79	210792.74	203997.33	249672.60	233275.65	143170.81		
3158	Plasminogen	P00747	23276.71	22739.08	20712.33	15355.64	15337.25	14928.41	19543.99	19427.86	17207.29	18561.85	17910.15	18101.39	19197.20	17557.57	15985.66	12817.84	12696.65	12436.87	17028.77	16408.93	15594.39	2.27	1.49	0.65	0.84	1.31	0.69	4.16	2.47	0.11	4.90	4.98	0.12	x	
3159	Plastin-1	Q14651	1695.96	2280.44	1745.07	1860.93	1651.55	1811.77	1877.04	1280.10	1658.52	2358.57	2050.81	2150.10	1950.74	1803.77	1992.22	1698.65	1495.68	1562.67	1739.59	1581.22	1708.54	8874.27	7617.97	6771.53	4330.86	4810.72	3052.01	6378.97	5502.18	4806.39	6198.27	5973.59	2881.03		
3160	Plastin-2	P13796	45670.05	45723.07	43174.70	26092.12	24059.50	23898.86	33932.06	30037.75	29535.82	37577.42	35041.00	34789.09	35182.45	28792.53	25137.02	27668.46	25149.37	25550.19	24740.38	26925.46	23699.99	62089.00	55965.93	48266.88	37273.69	36347.33	27648.95	43290.13	37117.30	31957.04	55037.98	47751.04	28326.10		
3161	Plastin-3	P13797	167640.60	154321.38	144434.51	99761.66	92429.28	81031.10	112012.37	112280.29	103387.06	130428.43	127452.48	115350.81	124872.13	113241.47	89282.55	99939.12	98605.69	93090.05	98952.74	90885.09	86328.65	284385.57	259081.09	245041.89	174369.10	173182.04	155664.10	211033.80	203006.63	199155.51	217525.29	204935.30	154753.06		
3162	Platelet-activating factor acetylhydrolase IB subunit alpha	P43034	90164.17	89933.97	83090.40	64311.54	62596.05	62277.62	78623.81	74039.94	71397.22	79198.18	72582.50	69828.46	78226.04	72029.08	61852.08	64808.20	64667.43	58725.51	67542.77	62825.12	58649.37	207677.33	188870.13	186199.07	129877.86	125101.84	115272.07	146032.65	142757.53	135901.45	150485.58	143262.98	111082.32		
3163	Platelet-activating factor acetylhydrolase IB subunit gamma	Q15102	78138.24	82930.53	79774.19	61645.11	59785.91	53530.37	64664.58	61809.05	58233.25	65880.92	65600.22	60447.67	72174.56	70686.28	63954.20	52871.12	51842.87	48547.18	56398.50	55699.38	55412.39	36938.32	34478.99	30987.70	22244.94	21643.34	20377.68	27470.70	27228.31	25601.40	27108.02	27034.76	21049.21		
3164	Platelet-activating factor acetylhydrolase	Q13093	0.14	2.53	0.81	1.07	2.77	0.68	0.96	0.36	0.63	0.35	0.75	0.38	2.13	0.97	0.60	4.64	1.07	0.47	2.22	0.19	0.06	2.27	1.49	0.65	556.46	695.73	568.44	4.16	2.47	0.11	731.19	744.59	456.33		x
3165	Platelet-derived growth factor receptor alpha	P16234	3078.33	2540.91	2143.48	1636.30	1647.48	1394.90	2210.82	2256.77	1809.19	3652.96	3214.22	2525.21	2938.15	2574.48	1026.13	1621.02	1589.14	1378.57	2364.01	3171.21	4280.30	30519.43	27829.98	24738.16	20200.74	19588.63	16251.09	24545.07	22702.25	20061.34	24389.38	23754.59	13964.90		
3166	Pleckstrin homology domain-containing family A member 1	Q9HB21	26264.90	23430.43	18591.67	17855.91	16454.94	13958.15	18868.17	17985.88	18753.91	22054.93	21545.69	18994.85	20804.04	19151.97	11230.07	17273.67	16251.48	15489.78	19077.57	16532.15	14789.94	2.27	1.49	0.65	0.84	1.31	0.69	4.16	2.47	0.11	4.90	4.98	0.12	x	
3167	Pleckstrin homology domain-containing family B member 1	Q9UF11	355.61	381.66	424.95	259.08	243.23	255.37	195.19	168.91	145.58	400.86	452.74	498.58	317.22	261.34	316.32	128.20	166.71	186.94	149.05	132.52	179.92	2.27	1.49	0.65	0.84	1.31	0.69	4.16	2.47	0.11	4.90	4.98	0.12	x	
3168	Pleckstrin homology domain-containing family G member 3	A1L390	19407.45	20455.23	17205.80	15429.83	14850.09	14656.91	16425.38	16175.79	13953.58	18942.91	18275.71	17752.04	16523.28	16609.31	14849.34	14107.36	12600.74	13406.22	14726.56	14210.15	12894.98	2.27	1.49	0.65	0.84	1.31	0.69	4.16	2.47	0.11	4.90	4.98	0.12	x	
3169	Pleckstrin homology domain-containing family G member 4B	Q96PX9	0.14	2.53	0.81	1.07	2.77	0.68	0.96	0.36	0.63	0.35	0.75	0.38	2.13	0.97	0.60	4.64	1.07	0.47	2.22	0.19	0.06	30110.50	28804.29	26227.97	16506.43	16377.69	13638.37	21413.62	19858.12	17317.99	18530.15	17597.98	10197.26		x
3170	Pleckstrin homology domain-containing family H member 1	Q9ULM0	0.14	2.53	0.81	1.07	2.77	0.68	0.96	0.36	0.63	0.35	0.75	0.38	2.13	0.97	0.60	4.64	1.07	0.47	2.22	0.19	0.06	6161.03	6001.48	5381.42	4703.13	4626.26	4357.56	5589.74	5623.89	5338.92	5642.16	5240.49	3854.76		x
3171	Pleckstrin homology domain-containing family H member 2	Q8IVE3	4415.13	4559.94	3592.39	2413.82	2463.81	2321.99	3082.87	4464.55	4239.89	3583.81	3261.19	2875.88	3635.66	3185.11	2493.26	3198.41	2644.05	2505.47	3387.50	2537.21	2710.32	2.27	1.49	0.65	0.84	1.31	0.69	4.16	2.47	0.11	4.90	4.98	0.12	x	
3172	Pleckstrin homology domain-containing family H member 3	Q7Z736	28915.91	26885.24	26579.68	17905.66	18446.21	17902.80	18969.02	17805.55	17583.71	24699.74	22613.71	22455.74	26271.32	23334.56	22045.49	18149.10	15786.63	17158.98	19622.98	17768.94	16680.33	2.27	1.49	0.65	0.84	1.31	0.69	4.16	2.47	0.11	4.90	4.98	0.12	x	
3173	Pleckstrin homology domain-containing family M member 1	Q9Y4G2	2265.08	2150.01	2108.67	1506.07	1404.52	1542.35	1831.45	1809.42	1639.47	1951.75	1853.75	1619.37	2047.73	1887.63	1575.92	1627.84	1294.62	1362.53	1334.00	1496.73	1371.88	2.27	1.49	0.65	0.84	1.31	0.69	4.16	2.47	0.11	4.90	4.98	0.12	x	
3174	Pleckstrin homology-like domain family B member 2	Q86SQ0	37075.30	34967.53	29571.30	28857.34	27493.15	22461.56	32094.23	31096.71	26984.60	40316.44	38951.82	32535.17	37701.83	34299.36	17905.55	30247.00	27902.75	24746.82	30248.70	25999.86	21185.00	2.27	1.49	0.65	0.84	1.31	0.69	4.16	2.47	0.11	4.90	4.98	0.12	x	
3175	Pleckstrin-2	Q9NYT0	0.14	2.53	0.81	1.07	2.77	0.68	0.96	0.36	0.63	0.35	0.75	0.38	2.13	0.97	0.60	4.64	1.07	0.47	2.22	0.19	0.06	3634.35	3354.82	2616.02	1777.88	1699.59	1525.23	2302.98	2155.67	1877.48	2527.20	2423.55	1530.49		x
3176	Plectin	Q15149	1538262.17	1506695.94	1431180.36	1133237.22	1078926.88	1024363.51	1225519.77	1178098.85	1154399.37	1377555.48	1320783.61	1251432.72	1308435.95	1206861.13	1014492.66	1118091.33	1082871.08	1056810.09	1153866.43	1050980.13	1004804.65	1763189.55	1643623.47	1523470.66	1184358.78	1152566.50	1058796.21	1364602.77	1305767.65	1257719.89	1405544.57	1358006.99	955489.43		
3177	Plexin-A1	Q9UIW2	0.14	2.53	0.81	1.07	2.77	0.68	0.96	0.36	0.63	0.35	0.75	0.38	2.13	0.97	0.60	4.64	1.07	0.47	2.22	0.19	0.06	1315.20	1237.73	1265.24	976.19	942.91	729.03	1067.68	964.72	811.80	1277.96	1199.74	746.54		x
3178	Plexin-A3	P51805	0.14	2.53	0.81	1.07	2.77	0.68	0.96	0.36	0.63	0.35	0.75	0.38	2.13	0.97	0.60	4.64	1.07	0.47	2.22	0.19	0.06	4475.22	4486.59	3683.67	2774.76	2560.89	2538.25	2718.72	2850.90	2854.55	3707.16	3179.19	789.09		x
3179	PMS1 protein homolog 1	P54277	16840.55	16173.62	15745.71	11096.82	10213.76	10326.17	9709.70	9883.93	8507.76	11769.68	11798.99	11418.27	12454.11	12779.76	9745.98	9299.99	7603.97	8088.87	8251.07	9404.40	8308.45	1925.30	1990.57	1903.94	1350.83	1134.22	1184.92	1431.41	1385.42	1320.46	1204.31	1200.44	838.18		
3180	Poly [ADP-ribose] polymerase 1	P09874	417736.68	405243.99	387749.09	311531.47	299292.25	275680.57	337546.38	323586.84	308481.12	381038.85	363058.86	346221.97	343173.31	329884.60	260353.83	295451.11	288008.18	281987.50	318843.90	306148.23	283164.14	381149.24	366581.77	344543.74	264970.84	258946.94	225471.10	299297.33	283374.64	272480.51	308304.74	297553.73	219522.35		
3181	Poly [ADP-ribose] polymerase 2	Q9UGN5	25031.49	23007.86	20738.14	14622.91	14468.02	12695.88	19333.26	18145.81	16934.03	18692.73	17951.20	15427.32	18649.12	17057.49	13506.30	14669.33	14159.11	12717.08	14887.44	13958.56	11346.30	3038.62	3106.11	2473.99	2245.61	2141.46	2205.02	2974.08	2551.07	2626.03	2773.20	2662.29	2010.94		
3182	Poly(A) polymerase alpha	P51003	70521.32	63394.82	61893.91	47850.43	47262.09	44319.35	53831.74	51983.61	49166.31	60233.61	57178.57	53850.06	57928.43	53565.35	42907.14	46642.02	44049.80	45206.05	48261.13	43700.66	42475.50	1118.97	1036.73	942.59	423.11	415.60	298.58	592.92	515.69	508.76	414.30	456.62	240.16		
3183	Poly(A) polymerase beta	Q9NRJ5	4538.60	3982.06	3818.02	2654.36	2413.89	2330.21	3248.51	3542.92	2840.28	3121.62	2955.88	2595.40	3679.66	3636.90	1743.14	3026.35	2834.11	2642.70	2438.42	2107.92	2138.84	3489.58	3041.07	2573.64	1438.20	1548.08	781.04	2729.99	2459.03	1797.04	2718.60	2563.98	1163.32		
3184	Poly(A) RNA polymerase mitochondrial	Q9NVV4	7415.33	7042.99	6717.98	5886.52	6001.89	5845.10	5919.83	5578.26	5436.84	6787.53	6223.20	5926.42	6533.75	6199.74	5781.66	4776.84	4650.40	4356.37	5281.28	5045.51	4874.77	2.27	1.49	0.65	0.84	1.31	0.69	4.16	2.47	0.11	4.90	4.98	0.12	x	
3185	Poly(A)-specific ribonuclease PARN	O95453	0.14	2.53	0.81	1.07	2.77	0.68	0.96	0.36	0.63	0.35	0.75	0.38	2.13	0.97	0.60	4.64	1.07	0.47	2.22	0.19	0.06	1246.35	1324.76	1216.66	752.85	778.01	721.96	1202.77	1251.68	1170.95	1053.17	1092.14	792.75		
3186	Poly(rC)-binding protein 1	Q15365	893876.89	840051.88	781252.56	621081.97	599814.01	559385.03	695089.75	677672.66	641319.22	726257.46	699447.96	658897.08	692976.13	664826.83	571872.51	599203.63	566649.03	578833.97	608353.36	575435.61	545408.03	572666.71	543402.97	508709.23	364834.25	364077.00	342454.69	399785.83	382885.97	370984.44	419446.79	406480.58	322577.82		
3187	Poly(rC)-binding protein 2	Q15366	68983.75	66532.56	61922.87	33694.68	34993.06	31132.40	70782.55	66809.39	63213.99	48114.98	46263.19	42582.41	44683.42	44076.30	37048.39	43191.19	41979.24	38510.90	32767.10	32724.51	32460.58	154006.09	154415.46	143070.49	87908.09	84659.65	79035.52	115044.99	106001.18	102341.48	101527.17	94707.14	68689.64		
3188	Poly(rC)-binding protein 4	P57723	1944.96	1501.95	1369.41	1042.47	905.10	721.16	887.87	953.94	731.47	1388.62	1535.95	1387.45	1247.55	1297.25	658.59	956.09	1156.23	775.65	855.34	901.14	856.16	2.27	1.49	0.65	0.84	1.31	0.69	4.16	2.47	0.11	4.90	4.98	0.12	x	
3189	Poly(U)-binding-splicing factor PUF60	Q9UHX1	208788.19	193734.65	186708.29	119314.04	110157.59	104305.91	149418.96	142966.78	138639.59	149479.59	141328.29	131722.10	147399.19	131921.01	102206.30	113069.57	107923.31	103175.83	135833.50	125265.23	114984.23	78865.64	74333.60	69756.07	49235.38	46636.94	38837.14	56690.96	53813.57	48869.70	58533.14	55026.10	38753.77		
3190	Polyadenylate-binding protein 1	P11940	561566.60	564057.54	525061.62	367385.20	356085.27	334121.53	509606.46	492719.96	469818.01	433669.91	419333.40	391443.18	442345.47	418267.90	348171.33	364424.45	354393.51	330532.06	372013.04	357971.65	333936.45	380649.85	387542.26	345657.37	243960.34	234846.63	214007.28	292273.38	279035.12	273354.09	294610.79	278749.30	181673.83		
3191	Polyadenylate-binding protein 1-like 2	Q5JQF8	115470.75	109919.57	102809.90	86172.70	86726.75	84233.22	87025.46	87163.78	81527.49	115038.28	111392.89	105252.86	104147.90	102008.11	83501.64	75761.92	75448.45	72531.57	83771.02	78056.35	76107.86	41497.75	37038.78	36501.13	30023.48	29964.86	27938.03	36109.24	35093.47	31362.52	37290.67	33333.34	24723.69		
3192	Polyadenylate-binding protein 1-like	Q4VXU2	80942.59	88737.47	75436.67	54872.12	52667.85	49210.78	67055.72	62776.11	62412.02	71460.62	66907.97	65638.64	71045.56	63730.30	51801.01	58503.63	51777.68	54651.59	51308.73	53949.00	43715.26	30634.13	29323.63	28426.99	21743.01	22302.66	19601.07	26040.03	23756.37	22651.57	24749.04	23918.79	17945.76		
3193	Polyadenylate-binding protein 3	Q9H361	11265.37	9566.06	8589.58	5265.84	14003.72	11676.34	16470.31	5574.70	5360.38	8280.65	7612.54	6481.13	8015.32	6165.18	7647.73	15638.19	14919.32	14551.54	5773.76	5651.79	4527.37	29719.91	33181.83	28280.95	36006.08	36319.23	33459.21	33436.61	35955.93	38916.12	34358.29	38178.65	28715.27		
3194	Polyadenylate-binding protein 4	Q13310	165052.65	146961.41	125281.78	59879.44	55741.05	47811.18	69275.66	66226.52	60233.56	134901.18	123957.26	110331.54	125596.95	73145.17	49130.16	58189.10	58268.67	52695.33	61477.23	61289.69	55345.30	69531.25	59108.00	58617.52	49051.72	49034.12	41208.35	54385.50	52780.44	48829.07	59729.10	56333.50	39224.81		
3195	Polyadenylate-binding protein 4-like	P0CB38	18892.19	18049.29	17332.40	12649.24	12629.53	12202.59	18733.61	17463.72	17544.44	17700.87	16982.73	16667.52	17216.45	16559.41	14103.74	12973.56	13237.41	12232.89	11905.12	12109.37	11414.84	3064.14	2839.83	2699.86	1843.42	1745.95	1428.75	2317.19	2339.20	2034.57	2607.06	2390.30	1554.35		
3196	Polyadenylate-binding protein 5	Q96DU9	27439.05	27532.45	28834.14	20177.34	19263.57	17194.37	22156.26	21409.33	19589.50	25705.42	25465.05	22476.44	24790.28	23839.54	18174.73	18915.99	19732.10	17197.74	19291.08	19406.81	17927.10	210012.54	199286.87	179839.10	127725.28	124450.06	113027.30	133973.81	132015.20	129616.40	171053.88	165242.94	125788.90		
3197	Polyadenylate-binding protein-interacting protein 1	Q9H074	5757.66	5235.56	5378.39	4942.93	4511.14	4616.62	4694.94	4256.78	4490.42	5508.72	5720.98	4798.70	5491.79	4940.64	3796.97	4610.28	3547.75	4123.95	3513.28	3699.22	3937.64	23920.78	23888.33	20661.18	14626.30	13651.64	12658.58	21581.79	20344.72	20655.24	18275.16	16577.31	13731.49		
3198	Polyamine-modulated factor 1-binding protein 1	Q8TBY8	77355.59	74291.34	67274.46	56318.62	53911.23	52598.99	55619.67	58213.29	53878.88	65064.53	62855.16	59443.27	70406.74	72886.66	52069.07	46119.34	59453.01	57206.26	57552.53	58824.91	54425.15	16861.27	15028.97	14370.12	7096.54	7756.51	6851.52	9390.46	9738.77	8322.83	8498.69	9359.88	5486.98		
3199	Polycomb protein SCMH1	Q96GD3	0.14	2.53	0.81	1.07	2.77	0.68	0.96	0.36	0.63	0.35	0.75	0.38	2.13	0.97	0.60	4.64	1.07	0.47	2.22	0.19	0.06	3527.65	2971.25	2969.71	2978.38	2822.22	2336.78	4645.11	4412.18	4053.62	3501.13	3135.31	2481.60		x
3200	Polycystin-2	Q13563	59702.52	60012.18	60733.89	45635.11	44433.58	42267.19	53279.65	51314.59	50954.84	54815.33	51679.38	51893.75	53228.97	36716.15	30455.80	46345.50	43227.51	42078.57	43737.87	41328.11	39504.09	2.27	1.49	0.65	0.84	1.31	0.69	4.16	2.47	0.11	4.90	4.98	0.12	x	
3201	Polyglutamine-binding protein 1	O60828	0.14	2.53	0.81	1.07	2.77	0.68	0.96	0.36	0.63	0.35	0.75	0.38	2.13	0.97	0.60	4.64	1.07	0.47	2.22	0.19	0.06	1952.91	1949.91	1856.44	2458.28	2500.21	2320.88	2901.81	2886.18	2767.19	3685.23	3720.27	1793.32		x
3202	Polymerase delta-interacting protein 2	Q9Y2S7	23341.21	22812.60	20869.91	17787.47	16813.66	15401.80	17546.68	16480.00	15728.86	21587.77	20115.27	19401.32	20133.72	18489.30	16386.08	15530.90	14991.06	14846.75	16973.46	15008.75	14290.73	5257.90	4339.63	4145.85	3394.00	3381.03	2942.87	4641.37	4249.08	3970.36	4452.01	3761.68	2841.40		
3203	Polymeric immunoglobulin receptor	P01833	10241.68	9543.25	8830.78	7426.71	6875.56	7255.64	8221.72	7751.92	7203.08	9554.09	9184.79	8748.06	9118.16	8037.58	5529.06	7339.32	6930.30	6504.61	8037.20	7087.00	6546.42	4574.46	4860.42	4702.88	3074.18	2955.65	2605.35	4817.00	4716.95	4408.00	3992.95	3850.41	2755.48		
3204	Polypeptide N-acetylgalactosaminyltransferase 11	Q8NCW6	18127.91	17537.84	16821.20	12473.97	12679.21	11678.07	15886.79	14391.26	14333.68	15250.65	14569.20	14514.33	14028.08	14777.72	13280.35	11944.11	11525.99	11186.77	14468.75	14044.16	13599.71	2.27	1.49	0.65	0.84	1.31	0.69	4.16	2.47	0.11	4.90	4.98	0.12	x	

Supplementary Table S1. Overview on the abundance of all relatively quantified 5180 proteins

			MCF-7																					MDA-MB-231													
	Protein name	UniProt	control_01	control_02	control_03	Dai_IC20_01	Dai_IC20_02	Dai_IC20_03	Dai_SC20_01	Dai_SC20_02	Dai_SC20_03	Gen_IC20_01	Gen_IC20_02	Gen_IC20_03	Gen_SC20_01	Gen_SC20_02	Gen_SC20_03	SSE_IC20_01	SSE_IC20_02	SSE_IC20_03	SSE_SC20_01	SSE_SC20_02	SSE_SC20_03	control_01	control_02	control_03	Dai_IC20_01	Dai_IC20_02	Dai_IC20_03	Gen_IC20_01	Gen_IC20_02	Gen_IC20_03	SSE_IC20_01	SSE_IC20_02	SSE_IC20_03	unique to MCF-7	unique to MDA-MB-231
3205	Polypeptide N-acetylgalactosaminyltransferase 2	Q10471	48776.62	47082.10	46281.23	32100.62	31699.48	30454.69	39955.88	36939.81	37035.82	40354.98	37246.08	37254.30	39847.08	38052.71	32440.53	34314.89	31553.22	31079.08	33302.38	31094.02	29835.72	4100.26	3682.80	3798.64	3018.58	3017.69	2660.21	3884.95	3788.56	3451.49	3544.02	3146.58	2532.92		
3206	Polypeptide N-acetylgalactosaminyltransferase 3	Q14435	4724.72	4060.79	2662.91	727.12	1338.42	866.29	1697.97	620.47	1299.38	3221.44	3027.97	2182.59	2730.65	2630.36	405.45	898.37	1365.78	1476.78	2017.25	1115.32	687.95	2.27	1.49	0.65	0.84	1.31	0.69	4.16	2.47	0.11	4.90	4.98	0.12	x	
3207	Polypeptide N-acetylgalactosaminyltransferase 4	Q8N4A0	0.14	2.53	0.81	1.07	2.77	0.68	0.96	0.36	0.63	0.35	0.75	0.38	2.13	0.97	0.60	4.64	1.07	0.47	2.22	0.19	0.06	17446.49	18087.65	17672.40	15539.11	15000.21	12505.15	18567.07	17206.75	16837.56	22527.20	21520.51	14049.74		x
3208	Polypeptide N-acetylgalactosaminyltransferase 6	Q8NCL4	17974.38	15919.00	17045.16	10749.62	10924.38	10194.97	11650.69	12640.51	12489.68	14135.53	13694.91	12148.59	14850.78	14076.23	10683.28	12709.15	9654.91	10074.28	11379.94	10964.41	10010.48	2.27	1.49	0.65	0.84	1.31	0.69	4.16	2.47	0.11	4.90	4.98	0.12	x	
3209	Polypyrimidine tract-binding protein 1	P26599	104126.68	98680.53	89707.46	62928.12	58029.34	53470.25	72273.22	71912.37	66891.87	71201.86	69980.53	63747.32	80081.77	70161.46	46940.57	62158.95	61163.81	60307.87	63009.13	58977.43	54611.59	112422.30	100490.10	95647.31	75255.09	72375.19	65029.20	82042.82	81964.37	77383.89	92530.45	95551.51	53816.25		
3210	Polypyrimidine tract-binding protein 2	Q9UKA9	8753.32	8733.84	7930.68	5899.06	5646.72	5406.88	7022.65	6526.76	6290.91	8190.70	8250.76	7787.29	6508.15	5055.69	5004.68	7861.34	7692.66	6989.22	6961.27	6607.75	6252.48	15572.26	14715.03	13984.04	11941.70	11394.83	10306.61	12885.69	13059.72	12510.17	14570.82	14204.38	6388.78		
3211	Polypyrimidine tract-binding protein 3	O95758	92866.73	86289.07	76951.86	44137.21	42390.00	39368.48	61513.98	59579.68	59422.20	65551.11	63869.14	54337.52	60744.41	49032.00	43064.45	48324.61	45745.09	44032.27	46693.54	44232.77	41643.86	3147.82	3319.98	2874.43	2303.37	2158.00	1777.51	2716.69	2859.33	2756.04	3316.28	2890.38	1870.45		
3212	Polyribonucleotide nucleotidyltransferase 1_ mitochondrial	Q8TCS8	31579.85	29472.98	28375.56	27046.66	25460.73	24064.79	26363.46	26804.20	27275.22	31268.41	28476.60	28226.14	25699.44	25467.91	22064.48	25605.79	25312.09	24287.10	24338.95	23478.68	23169.11	24334.89	21824.60	21233.15	15188.29	13520.80	11603.34	18249.92	17727.77	16708.68	16807.28	15713.54	10088.62		
3213	Porphobilinogen deaminase	P08397	89669.48	85516.36	78495.83	70091.48	66544.14	65031.47	71970.44	70261.21	66685.58	90112.66	86617.53	82769.27	68846.76	67169.41	59302.25	64782.06	57181.93	59960.58	63451.09	64756.96	60934.22	2.27	1.49	0.65	0.84	1.31	0.69	4.16	2.47	0.11	4.90	4.98	0.12	x	
3214	Potassium voltage-gated channel subfamily A member 2	P16389	35653.90	28529.20	23323.13	22788.63	20612.81	16492.96	49039.76	45752.33	38958.63	29044.21	26151.96	22970.51	33802.15	27114.88	9267.02	20902.24	18561.56	16398.99	22903.75	21850.89	16594.98	2.27	1.49	0.65	0.84	1.31	0.69	4.16	2.47	0.11	4.90	4.98	0.12	x	
3215	Potassium voltage-gated channel subfamily A member 5	P22460	16002.35	16074.68	15793.46	8522.83	7902.08	7870.89	6064.16	5969.87	5787.01	10385.95	11723.50	9829.76	8672.38	8495.21	7309.52	9906.39	9194.72	9509.04	8127.83	8341.67	7819.14	2.27	1.49	0.65	0.84	1.31	0.69	4.16	2.47	0.11	4.90	4.98	0.12	x	
3216	Potassium voltage-gated channel subfamily G member 3	Q8TAE7	606.47	607.00	607.94	377.73	261.24	260.52	349.57	235.97	377.64	505.89	552.82	418.24	219.83	565.04	215.82	368.68	446.70	207.32	324.47	264.44	334.65	2.27	1.49	0.65	0.84	1.31	0.69	4.16	2.47	0.11	4.90	4.98	0.12	x	
3217	Potassium-transporting ATPase alpha chain 1	P20648	56090.75	56867.19	51197.75	37924.50	34873.77	34768.06	45029.09	44067.21	43015.36	42013.74	40633.85	40169.60	39029.70	39056.38	35149.31	39037.02	36269.30	35040.01	36241.80	36564.79	33522.86	7684.76	7470.07	7533.17	4436.07	4200.98	3622.40	6856.49	6152.23	5637.39	5716.09	5420.30	3737.29		
3218	Potassium-transporting ATPase alpha chain 2	P54707	66570.89	65029.55	60113.63	48937.96	50367.75	46071.86	57384.98	55040.03	50979.53	58445.97	57436.81	53499.18	57040.93	53938.32	53077.74	53403.54	45454.00	45300.14	45724.89	45944.00	39921.13	3738.71	3062.71	4361.65	16301.77	15805.24	12777.43	26888.11	25987.59	25243.22	26368.62	25328.55	16259.31		
3219	POTE ankyrin domain family member A	Q6S8J7	28655.37	28788.27	27031.86	25250.52	25080.43	23033.93	25446.26	23516.74	24190.93	25473.19	22275.93	21739.32	22478.77	21391.09	17466.16	27492.16	25063.66	27221.47	22166.26	21513.44	21565.22	45495.14	42022.31	38681.01	31850.35	29030.76	28485.98	34144.23	32549.70	32727.18	30619.06	29102.68	20581.78		
3220	POTE ankyrin domain family member E	Q6S8J3	2056.44	1729.21	1721.88	1423.96	1267.88	1337.59	1732.77	1430.80	1563.14	2394.37	2545.06	2285.89	1793.62	1641.49	1297.19	1091.78	1161.72	1000.76	1481.55	1181.08	1187.90	2.27	1.49	0.65	0.84	1.31	0.69	4.16	2.47	0.11	4.90	4.98	0.12	x	
3221	POTE ankyrin domain family member F	A5A3E0	248348.29	234255.06	214209.75	184147.34	174844.28	157279.23	199641.19	198904.30	185110.81	223223.83	219267.72	200754.99	214098.14	194435.67	144511.64	177482.33	171043.36	160544.54	181643.01	171124.64	159154.38	15382.70	12445.03	10724.21	10495.42	10082.66	8565.41	14557.25	13479.95	12259.66	12907.90	12218.03	7557.31		
3222	POTE ankyrin domain family member I	P0CG38	419059.70	410462.38	375765.25	306046.64	309237.39	287735.51	365070.28	353209.56	343839.22	380167.40	366657.82	351780.84	340617.49	321271.19	282333.99	328357.05	320717.82	291389.26	270268.19	267194.81	250106.29	622940.88	603003.07	570275.43	381624.54	376450.27	320060.33	465116.10	445636.82	435687.21	423422.62	416506.12	284699.44		
3223	POTE ankyrin domain family member J	P0CG39	0.14	2.53	0.81	1.07	2.77	0.68	0.96	0.36	0.63	0.35	0.75	0.38	2.13	0.97	0.60	4.64	1.07	0.47	2.22	0.19	0.06	7676.95	3567.88	2673.23	4275.33	3551.38	1635.35	8190.04	7227.60	3464.97	9023.35	8065.10	3089.91		x
3224	PR domain zinc finger protein 4	Q9UKN5	2539.78	2464.28	2280.84	1676.28	1612.85	1534.69	2000.75	1637.48	2104.44	1937.37	2112.56	1854.98	2587.54	2318.82	2029.17	2010.58	1853.22	1779.38	2259.31	1749.71	1593.30	2.27	1.49	0.65	0.84	1.31	0.69	4.16	2.47	0.11	4.90	4.98	0.12	x	
3225	PRA1 family protein 2	O60831	10083.71	7711.54	8860.55	5551.91	3809.38	4457.96	6081.08	6496.93	6385.44	9619.32	9292.70	7925.68	6099.79	4724.88	3999.79	6350.68	6027.57	5461.13	4082.98	4109.30	3924.30	2.27	1.49	0.65	0.84	1.31	0.69	4.16	2.47	0.11	4.90	4.98	0.12	x	
3226	PRA1 family protein 3	O75915	48148.55	44459.50	37518.19	22089.16	20824.50	17902.97	30034.69	27716.47	24209.73	41268.87	37929.82	34191.30	40215.19	26988.89	19102.79	22083.88	18272.57	18732.19	23760.23	22699.16	19701.91	29745.97	28885.63	25619.09	19866.55	18803.54	14408.99	22921.05	20580.74	19038.74	25820.74	22767.03	15240.55		
3227	PRAME family member 17	Q5VTA0	11308.54	9920.86	8620.43	6134.02	6003.27	4614.38	7383.35	6746.16	6041.07	8933.80	8054.26	7404.72	8535.46	7845.07	3476.37	6626.34	6346.26	5594.42	7592.47	6925.95	5637.75	638.15	656.56	542.44	379.41	355.69	260.46	497.46	475.74	428.95	373.76	359.30	213.58		
3228	PRAME family member 18	Q5VWM	11100.07	10772.26	11152.98	11593.58	11147.01	11211.50	12872.97	12011.99	12200.71	14150.07	13603.69	12392.23	12920.63	12067.91	9807.77	11510.70	11561.40	10989.09	11654.26	10564.81	10175.63	784.92	1883.22	862.67	1367.41	1215.56	1604.64	353.94	647.84	616.59	1491.48	1128.22	3.94		
3229	PRAME family member 19	Q5SWL8	0.14	2.53	0.81	1.07	2.77	0.68	0.96	0.36	0.63	0.35	0.75	0.38	2.13	0.97	0.60	4.64	1.07	0.47	2.22	0.19	0.06	1158.08	983.55	691.91	1089.26	877.42	522.80	1337.40	1169.64	1051.61	1178.68	1304.12	598.45		x
3230	PRAME family member 2	O60811	0.14	2.53	0.81	1.07	2.77	0.68	0.96	0.36	0.63	0.35	0.75	0.38	2.13	0.97	0.60	4.64	1.07	0.47	2.22	0.19	0.06	3112.32	3212.69	2740.54	373.02	352.47	298.11	163.34	254.98	309.45	234.32	202.27	135.29		x
3231	PRAME family member 33	A0A0G2J	40262.57	36254.61	33792.40	31980.47	47282.18	41390.93	53890.54	36127.64	34841.79	43855.17	41200.85	40749.23	36180.40	33658.13	28774.21	44490.41	40127.13	38699.51	29047.89	27573.03	25926.07	2.27	1.49	0.65	0.84	1.31	0.69	4.16	2.47	0.11	4.90	4.98	0.12	x	
3232	Pre-B-cell leukemia transcription factor 1	P40424	2376.11	2211.73	2229.77	1664.49	1485.56	1350.89	1790.76	2094.26	1815.89	1935.27	1921.67	1825.60	1983.31	1843.02	1566.57	1637.18	1538.35	1458.86	1496.91	1386.50	1342.09	2.27	1.49	0.65	0.84	1.31	0.69	4.16	2.47	0.11	4.90	4.98	0.12	x	
3233	Pre-B-cell leukemia transcription factor 4	Q9BYU1	0.14	2.53	0.81	1.07	2.77	0.68	0.96	0.36	0.63	0.35	0.75	0.38	2.13	0.97	0.60	4.64	1.07	0.47	2.22	0.19	0.06	1032.77	974.61	1042.62	1261.32	1257.21	1046.14	1106.77	1230.08	1150.10	2022.09	1924.93	1291.89		x
3234	Prefoldin subunit 1	O60925	7162.09	7238.37	6784.64	5138.36	4954.77	4790.76	5676.50	5417.38	5211.33	5870.91	5733.83	5547.73	5957.56	5841.03	4935.88	4876.30	4668.64	4644.53	4912.28	4823.05	4467.79	16029.90	18267.02	16692.56	13037.76	11728.05	12449.52	14146.47	13544.43	14270.26	13341.11	15038.98	11075.79		
3235	Prefoldin subunit 2	Q9UHV9	147201.62	130652.27	127509.04	96795.57	93619.94	92921.06	118232.16	112140.55	107418.28	108946.47	107518.27	109002.44	110649.13	101632.60	102324.59	94762.04	89254.17	87254.69	105498.10	98515.93	91940.43	98282.69	91170.30	87355.97	68926.72	63637.25	59239.03	78519.49	75975.32	74652.70	84439.99	82644.56	72638.88		
3236	Prefoldin subunit 3	P61758	41683.08	39753.59	36427.27	28721.97	27923.62	27353.12	32866.74	30243.64	28703.57	37428.02	35859.18	33504.12	34618.93	32702.48	28522.67	29063.72	26878.78	26374.05	28394.43	27094.86	26373.31	34938.25	34328.25	30667.58	20777.02	20623.96	18329.02	22039.04	21798.13	20866.66	21892.53	21327.87	15157.99		
3237	Prefoldin subunit 4	Q9NQP4	134066.22	131556.14	122135.32	103387.12	97430.05	87745.09	106650.58	100836.28	95566.05	115205.63	110496.00	105458.61	111103.47	97947.52	72516.03	89053.17	87480.02	83746.20	107255.61	98043.31	90804.35	64231.99	63371.51	58522.21	41990.49	39910.76	36695.09	43699.67	40821.34	40477.47	49855.32	46585.12	20428.77		
3238	Prefoldin subunit 5	Q99471	170521.93	163088.64	151617.33	127177.69	120933.40	117526.88	138771.07	136573.17	135626.56	145819.18	132872.85	130883.27	141565.13	140748.36	121611.18	125710.48	110942.48	110642.65	131814.29	120498.32	118518.80	57929.84	53288.99	50198.39	35631.84	33872.97	29511.68	44074.59	41130.42	39489.96	43124.03	40498.19	24109.38		
3239	Prefoldin subunit 6	O15212	70372.97	63529.63	61646.73	44656.49	45167.13	41217.61	50670.31	48669.39	47769.43	58384.43	55527.29	53513.19	56857.67	52147.19	43663.28	43456.97	42907.23	41277.41	48393.38	47117.39	42951.07	18794.39	18198.85	16158.25	14078.79	12613.40	10091.94	16395.73	16274.85	14790.22	17390.95	16775.66	9969.02		
3240	Pregnancy-specific beta-1-glycoprotein 5	Q15238	1500.70	1672.80	1375.93	783.92	976.73	855.84	1474.45	1433.51	1318.70	1152.34	982.99	928.35	801.05	858.61	666.24	1154.85	1085.13	1019.60	914.66	983.59	867.78	2.27	1.49	0.65	0.84	1.31	0.69	4.16	2.47	0.11	4.90	4.98	0.12	x	
3241	Pregnancy-specific beta-1-glycoprotein 9	Q00887	11525.57	10775.33	10807.13	8395.71	7959.86	8695.48	9998.17	10186.85	9211.34	10118.68	9831.53	9991.51	10659.59	9548.39	7559.34	7901.11	7684.58	8383.19	9381.30	9321.11	8705.94	9658.42	10249.75	9384.76	6956.17	6831.24	6148.34	6367.45	6824.39	6946.49	6102.30	6442.28	4975.72		
3242	Prelamin-A/C	P02545	903141.76	891188.68	875547.80	508714.73	490996.54	482211.35	642442.39	618492.85	618228.52	682164.26	649971.56	629733.67	664922.31	636200.33	563437.17	613994.05	577054.40	564369.63	590215.63	552902.26	529752.52	334961.57	312633.88	296114.28	280368.06	277601.93	254279.61	290640.02	284431.36	278678.41	437862.66	415010.23	318434.99		
3243	pre-mRNA 3' end processing protein WDR33	Q9C0J8	0.14	2.53	0.81	1.07	2.77	0.68	0.96	0.36	0.63	0.35	0.75	0.38	2.13	0.97	0.60	4.64	1.07	0.47	2.22	0.19	0.06	3398.18	4148.39	3295.36	3111.56	3202.88	3686.47	3263.49	2743.35	2688.46	3612.44	3327.02	2055.96		x
3244	Pre-mRNA-processing factor 17	O60508	0.14	2.53	0.81	1.07	2.77	0.68	0.96	0.36	0.63	0.35	0.75	0.38	2.13	0.97	0.60	4.64	1.07	0.47	2.22	0.19	0.06	987.20	883.41	753.85	1503.62	1426.79	1190.66	1757.58	1488.61	1424.72	1570.64	1517.07	5035.62		x
3245	Pre-mRNA-processing factor 19	Q9UMS4	188139.01	183938.61	172228.26	128283.25	129104.52	120352.23	146314.24	142064.22	131846.21	135636.75	131322.33	123069.04	146127.35	134478.58	125309.73	120915.62	116337.69	113737.27	126078.95	120591.09	112380.84	37219.29	36566.98	34592.24	26283.14	25305.79	21296.72	30567.69	28708.90	27961.47	31913.50	32107.31	21978.86		
3246	Pre-mRNA-processing factor 40 homolog A	O75400	24294.49	21171.08	18435.01	15154.69	15104.38	12853.55	18674.74	15135.92	15706.55	18525.50	17693.81	16089.00	19112.20	14886.80	11194.80	17630.35	13570.47	14967.53	17634.26	16629.39	14139.90	63919.51	57795.00	51583.25	40386.43	38388.88	32624.99	44719.07	40445.85	36809.29	46807.06	44206.19	28374.58		
3247	Pre-mRNA-processing factor 40 homolog B	Q6NWY9	58820.46	55717.44	53362.64	35642.77	34493.23	32506.08	38918.21	37851.83	36906.62	55912.19	53138.17	49786.32	40467.35	40343.72	32823.49	46127.02	47898.22	46372.92	42179.59	39552.09	39356.01	34323.41	32497.43	30725.14	33204.01	32524.35	29446.90	46579.51	45270.07	43931.37	29634.11	27178.06	20508.75		
3248	Pre-mRNA-processing factor 6	O94906	1503.80	1197.14	1184.08	629.03	637.56	684.24	687.88	791.25	733.18	557.51	624.46	661.43	915.98	880.39	744.98	760.59	637.57	596.83	1051.69	886.05	978.53	22435.89	21948.73	20272.75	15330.18	14885.20	13660.10	17507.52	16853.35	16695.77	17366.79	16850.63	11910.20		
3249	Pre-mRNA-processing-splicing factor 8	Q6P2Q9	0.14	2.53	0.81	1.07	2.77	0.68	0.96	0.36	0.63	0.35	0.75	0.38	2.13	0.97	0.60	4.64	1.07	0.47	2.22	0.19	0.06	123496.30	113244.61	99347.57	81147.39	79252.61	71028.16	112991.37	110013.23	96835.77	105639.92	100853.55	73563.91		x
3250	Pre-mRNA-splicing factor 38B	Q5VTL8	5471.58	5699.66	5375.84	4122.78	4139.99	3853.26	4118.11	3940.65	4042.80	4177.94	4017.57	3937.83	4649.26	2658.65	1252.06	3115.32	2695.76	2482.71	4458.13	4060.08	3868.73	547.48	541.70	513.48	304.66	339.35	326.48	403.56	407.43	382.65	424.96	394.94	249.10		
3251	Pre-mRNA-splicing factor ATP-dependent RNA helicase DHX15	O43143	31246.86	31876.90	30435.16	19096.05	19240.43	17201.53	25838.68	22833.21	23654.11	23352.22	23334.67	21872.77	25786.73	24611.31	18762.91	22640.60	19372.11	19544.17	20150.71	21089.52	18488.23	534734.47	492915.24	444925.74	424777.95	402547.39	326677.16	501396.34	479680.76	450802.01	531737.30	520975.76	337203.04		
3252	Pre-mRNA-splicing factor ATP-dependent RNA helicase PRP16	Q92620	0.14	2.53	0.81	1.07	2.77	0.68	0.96	0.36	0.63	0.35	0.75	0.38	2.13	0.97	0.60	4.64	1.07	0.47	2.22	0.19	0.06	15190.14	16354.78	41783.82	9727.61	8851.00	6348.77	12312.58	11950.83	9878.68	13355.62	12223.70	6609.72		x
3253	Pre-mRNA-splicing factor SLU7	O95391	0.14	2.53	0.81	1.07	2.77	0.68	0.96	0.36	0.63	0.35	0.75	0.38	2.13	0.97	0.60	4.64	1.07	0.47	2.22	0.19	0.06	24737.02	23535.04	22048.04	24752.06	25282.73	25475.61	31813.15	31210.04	31494.66	18901.71	18267.61	17574.86		x
3254	Pre-mRNA-splicing factor SPF27	O75934	76969.03	65087.43	68505.73	40740.28	39035.78	36528.68	52963.11	49127.35	46258.90	50098.22	46843.74	43791.17	48701.89	45204.47	37048.93	42362.26	41413.37	39134.70	42821.77	40445.52	37382.00	2.27	1.49	0.65	0.84	1.31	0.69	4.16	2.47	0.11	4.90	4.98	0.12	x	
3255	Prenylcysteine oxidase 1	Q9UHG3	50668.92	47267.25	54303.65	38148.05	38531.61	34280.58	39797.71	38529.95	36895.70	55175.70	55118.13	52872.03	42814.92	43555.76	33248.12	39453.28	38373.45	36571.85	36214.67	35485.64	32597.38	28718.21	25434.87	23058.33	26326.62	24592.94	20380.18	41240.13	40150.58	36073.56	39234.47	38164.49	23794.29		
3256	Pre-rRNA-processing protein TSR1 homolog	Q2NL82	35157.58	33191.40	33876.08	24434.68	23789.55	21783.80	26278.06	24302.66	23300.23	28336.57	27063.88	24651.35	27999.79	27433.42	22006.68	21199.29	19450.05	19856.38	24090.35	23638.83	22229.16	26951.81	25393.41	23786.46	18110.41	17684.29	15981.36	19507.76	18005.04	17204.17	21624.61	19934.50	15946.21		
3257	Pre-rRNA-processing protein TSR2 homolog	Q969E8	0.14	2.53	0.81	1.07	2.77	0.68	0.96	0.36	0.63	0.35	0.75	0.38	2.13	0.97	0.60	4.64	1.07	0.47	2.22	0.19	0.06	10449.19	8432.85	6979.63	4317.59	4696.61	2794.82	5417.51	4946.10	4131.16	7052.07	6603.93	3764.33		x
3258	Presequence protease_ mitochondrial	Q5JRX3	191314.51	179697.60	169054.85	126714.59	119580.07	113763.28	145401.06	140329.46	130682.39	157877.17	151096.53	143936.28	144552.91	134983.91	109696.80	134517.56	129090.92	121853.60	181470.06	171099.54	140799.21	25053.99	24453.33	20845.00	15881.84	15174.97	14126.69	18707.21	18388.32	17705.64	18759.69	17624.66	11339.56		
3259	Prion-like protein doppel	Q9UKY0	1493.76	1118.73	878.06	1138.80	1187.45	912.32	1392.04	1273.29	1118.24	2282.47	1928.58	1613.87	840.95	933.07	711.72	1969.53	1656.17	1469.43	1225.43	1065.01	982.65	2.27	1.49	0.65	0.84	1.31	0.69	4.16	2.47	0.11	4.90	4.98	0.12	x	
3260	PRKC apoptosis WT1 regulator protein	Q96IZ0	107159.76	102452.96	98394.58	78439.99	76749.52	69538.53	79317.07	79602.70	76787.95	106014.64	101412.04	100102.85	101420.24	91559.36	75087.49	72527.60	71598.10	69904.01	86611.96	77618.64	74829.75	15695.37	14281.84	13150.68	10122.71	9935.34	8887.33	13179.50	11770.57	11133.16	14254.75	13872.44	8102.61		
3261	PRKR-interacting protein 1	Q9H875	4820.26	4922.08	4624.62	2809.18	2862.63	2819.08	3237.49	2918.53	2877.61	3123.36	2756.04	2745.83	3621.22	3701.17	3680.41	2601.87	2875.43	2532.27	3107.54	2994.54	2616.94	2.27	1.49	0.65	0.84	1.31	0.69	4.16	2.47	0.11	4.90	4.98	0.12	x	
3262	Probable 28S rRNA (cytosine(4447)-C(5))-methyltransferase	P46087	0.14	2.53	0.81	1.07	2.77	0.68	0.96	0.36	0.63	0.35	0.75	0.38	2.13	0.97	0.60	4.64	1.07	0.47	2.22	0.19	0.06	85231.90	83385.46	74908.31	63405.34	62189.73	56290.70	59162.24	61952.57	62925.57	71632.81	69946.89	31497.82		x
3263	Probable 28S rRNA (cytosine-C(5))-methyltransferase	Q96P11	31913.48	27696.63	25702.25	18211.23	16989.76	15434.40	21115.45	20982.13	19611.15	22046.55	21672.80	18444.96	23697.25	21855.63	15452.00	17184.79	17116.78	15271.10	17573.72	16292.40	15422.50	2.27	1.49	0.65	0.84	1.31	0.69	4.16	2.47	0.11	4.90	4.98	0.12	x	
3264	Probable 2-oxoglutarate dehydrogenase E1 component DHKTD1_ mitochondrial	Q96HY7	149280.01	141645.52	135549.77	108828.35	100874.88	94808.50	118145.06	112184.93	115621.67	119685.55	116071.56	112748.13	124921.27	114737.14	98806.38	106717.35	105759.41	107260.94	110027.22	94434.62	90051.84	2.27	1.49	0.65	0.84	1.31	0.69	4.16	2.47	0.11	4.90	4.98	0.12	x	
3265	Probable aminopeptidase NPEPL1	Q8NDH3	55348.54	50258.22	45263.60	33043.68	33331.47	29526.18	36343.84	39032.64	33074.73	49002.44	45340.88	42219.89	46847.82	47020.85	34974.12	26446.29	28341.82	26569.64	39171.43	35418.18	33539.59	2.27	1.49	0.65	0.84	1.31	0.69	4.16	2.47	0.11	4.90	4.98	0.12	x	
3266	Probable ATP-dependent RNA helicase DDX17	Q92841	237133.92	226958.53	208748.05	153205.08	148566.42	138440.21	170685.01	160006.83	153073.01	179020.52	169918.01	160173.76	186747.35	174471.53	147696.35	150583.90	142685.22	137118.94	158082.05	150555.76	142403.40	159550.36	151376.11	136273.58	117146.87	111594.88	97759.39	136021.36	128511.79	120355.26	148466.58	142305.09	93394.61		
3267	Probable ATP-dependent RNA helicase DDX20	Q9UHI6	174828.86	175125.89	167842.45	124511.06	118629.47	111640.87	139424.04	136797.97	132104.47	159055.18	150518.73	146014.62	154709.86	143452.18	115757.58	117612.65	109827.24	110443.65	128819.08	116106.06	110864.85	7499.97	6817.24	7034.43	5315.59	5476.55	4523.28	6093.74	5619.15	5827.38	7077.82	7008.65	1659.48		
3268	Probable ATP-dependent RNA helicase DDX4	Q9NQI0	66468.32	62118.73	57536.58	39653.36	38363.33	33912.09	43989.78	44889.40	41030.71	52414.46	50315.72	45896.50	49405.62	41698.14	26525.91	39023.97	37185.48	35193.62	44546.24	40011.26	38196.82	19879.40	18765.78	17367.90	16165.67	15881.06	12562.86	18485.43	16347.86	15952.29	21938.85	21364.90	7601.96		
3269	Probable ATP-dependent RNA helicase DDX46	Q7L014	251767.33	247311.31	229915.28	160339.57	151536.34	138714.16	197746.91	181144.66	176237.36	197701.11	182255.12	177190.97	203136.21	194322.68	161095.16	156528.73	148685.99	142869.95	160670.06	158992.82	146849.72	380593.06	358811.04	340949.99	100653.22	103208.37	91184.47	128903.63	126413.27	125119.22	103722.76	100639.18	67342.93		
3270	Probable ATP-dependent RNA helicase DDX5	P17844	796715.39	775835.42	718575.75	517567.40	500459.61	471850.98	625294.03	601017.89	575898.53	604226.94	582071.60	552473.21	617799.94	586744.22	473513.57	530988.67	501292.81	484692.63	544337.77	519887.54	498083.82	387071.91	367030.74	340473.02	257366.94	250301.62	218602.70	279469.38	262514.07	253633.85	276390.62	273540.12	189800.67		
3271	Probable ATP-dependent RNA helicase DDX53	Q86TM3	0.14	2.53	0.81	1.07	2.77	0.68	0.96	0.36	0.63	0.35	0.75	0.38	2.13	0.97	0.60	4.64	1.07	0.47	2.22	0.19	0.06	46862.46	47059.77	42575.75	27759.93	27208.24	23983.41	41203.78	41315.92	40764.13	37555.95	36759.40	26596.35		x
3272	Probable ATP-dependent RNA helicase DDX6	P26196	212818.41	218123.27	196929.41	153810.35	147265.05	138530.83	175598.62	171206.84	160871.86	171048.60	163941.52	154006.81	185654.55	171711.83	153926.75	144237.54	139182.19	131752.32	147580.25	149583.47	139054.99	24254.82	24057.03	21253.02	13977.65	13462.76	12001.66	18568.87	18090.34	16779.69	16900.11	16432.45	11560.89		
3273	Probable bifunctional dTTP/UTP pyrophosphatase/methyltransferase protein	O95671	119977.95	115954.08	103597.07	94144.93	92535.51	89158.66	94402.26	101765.42	93059.71	109516.91	101650.83	95124.57	104712.34	94838.52	79913.70	74021.12	69794.47	68674.24	83506.41	78741.98	73893.72	24588.85	28415.36	26161.92	22886.14	21240.90	18672.09	38878.61	36219.97	36711.13	26678.83	25329.25	18135.62		
3274	Probable C-mannosyltransferase DPY19L4	Q7Z388	1562.24	1560.55	1344.64	1155.94	1073.35	1044.24	1772.39	1702.77	1538.80	1705.83	1511.84	1675.47	1291.08	1282.84	990.96	757.59	975.80	861.06	916.80	916.52	919.40	2.27	1.49	0.65	0.84	1.31	0.69	4.16	2.47	0.11	4.90	4.98	0.12	x	
3275	Probable cytosolic iron-sulfur protein assembly protein CIAO1	O76071	9526.59	8418.12	6398.49	4075.30	3859.48	2945.75	5985.95	4753.91	5107.75	5037.24	4656.39	3862.83	5425.56	4419.24	1892.23	4357.81	3814.48	3587.55	5083.59	4889.17	4167.05	2.27	1.49	0.65	0.84	1.31	0.69	4.16	2.47	0.11	4.90	4.98	0.12	x	
3276	Probable E3 SUMO-protein ligase RNF212	Q495C1	92.66	124.90	52.66	72.63	11.30	78.04	19.43	132.36	80.72	58.52	55.95	65.30	57.32	85.54	94.63	53.16	63.91	90.09	132.18	128.21	111.50	5005.72	4302.21	3879.34	3094.39	3108.64	1677.49	4340.71	3876.64	2882.43	5105.63	4920.09	2339.40		
3277	Probable E3 ubiquitin-protein ligase HECTD4	Q9Y4D8	200205.69	190562.54	171789.37	136649.62	134971.81	124404.71	157933.37	151270.17	144090.17	177150.96	166365.00	154153.80	162769.40	156872.53	123707.47	145436.27	136083.25	130019.14	132098.76	124752.26	116758.18	131005.44	121818.61	113268.98	95418.92	94393.37	81464.48	99255.77	94494.64	89631.13	93066.76	88944.92	65553.98		
3278	Probable E3 ubiquitin-protein ligase HERC3	Q15034	19719.62	19911.05	19182.29	15794.31	14731.26	15042.80	14980.05	14822.33	14555.71	19867.99	18081.18	18543.23	17113.07	17312.15	14955.81	14756.91	12772.31	12826.28	14953.11	13949.27	14147.34	2.27	1.49	0.65	0.84	1.31	0.69	4.16	2.47	0.11	4.90	4.98	0.12	x	
3279	Probable E3 ubiquitin-protein ligase HERC4	Q5GLZ8	19218.03	18230.35	18007.25	9532.33	8955.40	8923.44	12626.20	12193.68	11153.72	13674.20	13211.68	12439.54	14099.24	13328.40	11599.04	10944.30	9666.19	9710.19	12573.18	10908.70	9222.72	30429.24	24579.83	23981.14	18136.53	17051.38	14789.07	22090.81	21764.83	20113.44	21352.82	20458.67	14438.37		
3280	Probable E3 ubiquitin-protein ligase HERC6	Q8IVU3	0.14	2.53	0.81	1.07	2.77	0.68	0.96	0.36	0.63	0.35	0.75	0.38	2.13	0.97	0.60	4.64	1.07	0.47	2.22	0.19	0.06	24026.68	22916.62	22761.65	24838.75	24733.37	23110.37	32280.56	30107.77	30881.22	21096.88	20132.19	16624.74		x
3281	Probable E3 ubiquitin-protein ligase makorin-2	Q9H000	24202.95	22564.19	20754.63	17412.27	16838.87	14552.04	20604.61	19033.17	17624.69	26448.11	25432.14	23562.58	20880.66	19367.85	13823.23	18783.76	18245.15	16900.17	15281.04	15010.89	13209.73	4379.23	4338.36	4437.74	1219.05	1188.92	970.78	720.84	720.09	809.69	800.33	680.43	476.74		
3282	Probable E3 ubiquitin-protein ligase MARCH10	Q8NA82	7952.64	7498.98	7231.14	6308.90	6047.52	6690.10	8711.66	8601.42	7806.89	8693.45	7827.33	7957.18	7764.66	7816.02	7617.74	6021.72	5448.12	6062.00	6518.01	6285.34	6090.24	2.27	1.49	0.65	0.84	1.31	0.69	4.16	2.47	0.11	4.90	4.98	0.12	x	
3283	Probable ergosterol biosynthetic protein 28	Q9UKR5	23297.56	23885.40	23251.05	17784.67	17702.29	17492.44	20666.71	20622.37	19076.26	23408.44	22422.38	22120.84	18576.32	19372.48	15568.34	19640.06	18769.79	17774.01	16077.57	16983.67	15102.07	2.27	1.49	0.65	0.84	1.31	0.69	4.16	2.47	0.11	4.90	4.98	0.12	x	
3284	Probable global transcription activator SNF2L1	P28370	21993.32	20941.43	19671.47	13050.18	12470.59	12012.21	14891.94	14135.04	13436.31	17697.92	16063.10	15407.24	15171.00	16067.89	12786.18	12611.72	13176.43	13135.83	12803.92	12837.09	12661.11	13192.11	13333.96	12076.04	8637.68	9157.50	8577.81	11026.96	11604.99	11299.69	11413.27	10537.66	8288.93		
3285	Probable glutamate--tRNA ligase_ mitochondrial	Q5JPH6	104333.05	101412.39	90802.07	75000.28	71923.92	67829.88	92252.67	88799.08	84246.53	88267.47	88484.28	82975.32	99120.80	94335.33	68592.01	71928.28	71720.82	67665.62	83592.57	81979.98	73418.56	2.27	1.49	0.65	0.84	1.31	0.69	4.16	2.47	0.11	4.90	4.98	0.12	x	
3286	Probable G-protein coupled receptor 179	Q6PRD1	0.14	2.53	0.81	1.07	2.77	0.68	0.96	0.36	0.63	0.35	0.75	0.38	2.13	0.97	0.60	4.64	1.07	0.47	2.22	0.19	0.06	8479.20	9365.49	8593.51	5659.73	6051.21	5665.29	8091.86	7549.21	6765.54	6591.28	7119.03	5397.77		x
3287	Probable helicase senataxin	Q7Z333	157023.35	164510.38	164283.39	140430.29	137570.71	133555.35	147011.09	146540.04	144686.94	165186.74	145295.05	144500.83	141343.08	139724.67	120148.60	134737.07	125477.37	128332.27	124855.82	119357.02	118838.25	215630.06	203059.05	197974.43	146777.83	138891.49	129801.59	171544.34	153490.18	163521.03	161161.40	153402.46	104232.30		
3288	Probable histidine--tRNA ligase_ mitochondrial	P49590	0.14	2.53	0.81	1.07	2.77	0.68	0.96	0.36	0.63	0.35	0.75	0.38	2.13	0.97	0.60	4.64	1.07	0.47	2.22	0.19	0.06	242308.98	235399.14	219199.54	140471.75	130412.94	108010.09	147850.30	141865.75	138998.42	136349.65	130752.22	87076.92		x
3289	Probable JmjC domain-containing histone demethylation protein 2C	Q15652	258508.84	247591.90	226560.97	153760.04	152755.87	137856.79	194525.36	197067.75	188798.25	214706.53	207854.44	188131.21	198927.46	186791.05	146997.84	156146.15	153782.48	150684.15	178518.28	165922.72	158656.46	2942.50	2529.80	2332.44	2669.21	2236.63	2097.43	6418.74	5531.24	5992.45	3861.81	1472.57	109.12		
3290	Probable leucine--tRNA ligase_ mitochondrial	Q15031	0.14	2.53	0.81	1.07	2.77	0.68	0.96	0.36	0.63	0.35	0.75	0.38	2.13	0.97	0.60	4.64	1.07	0.47	2.22	0.19	0.06	15221.80	15207.93	13121.35	10478.54	10698.24	8548.49	12535.96	10874.22	10677.61	15995.10	15142.41	9345.52		x
3291	Probable maltase-glucoamylase 2	Q2M2H8	0.14	2.53	0.81	1.07	2.77	0.68	0.96	0.36	0.63	0.35	0.75	0.38	2.13	0.97	0.60	4.64	1.07	0.47	2.22	0.19	0.06	14515.13	13643.61	11964.17	15189.48	14667.60	11782.25	16038.17	15325.13	14176.52	17524.85	17225.97	10487.59		x
3292	Probable phosphoglycerate mutase 4	Q8N0Y7	12209.52	12911.44	9823.03	7429.05	8073.86	6701.16	10028.69	9336.45	9007.91	10556.34	10925.90	9452.49	10406.22	9924.08	7820.59	8467.66	8573.88	7229.87	7704.97	7792.22	7553.36	2.27	1.49	0.65	0.84	1.31	0.69	4.16	2.47	0.11	4.90	4.98	0.12	x	
3293	Probable ribonuclease ZC3H12C	Q9C0D7	0.14	2.53	0.81	1.07	2.77	0.68	0.96	0.36	0.63	0.35	0.75	0.38	2.13	0.97	0.60	4.64	1.07	0.47	2.22	0.19	0.06	5002.49	4871.55	4632.83	3636.33	3414.74	3155.90	5274.24	4730.81	4675.17	4480.65	4271.24	3244.97		x
3294	Probable ribonuclease ZC3H12D	A2A288	0.14	2.53	0.81	1.07	2.77	0.68	0.96	0.36	0.63	0.35	0.75	0.38	2.13	0.97	0.60	4.64	1.07	0.47	2.22	0.19	0.06	15400.52	14417.99	13116.02	9216.18	9268.88	7002.04	10645.40	10151.37	9254.48	11448.56	10933.98	7330.40		x
3295	Probable RNA-binding protein 46	Q8TBY0	12853.45	10554.34	10188.70	5435.53	5411.44	5265.14	8687.61	8356.14	7341.65	7068.92	7489.51	6831.13	9329.22	8185.14	5740.51	6751.98	6167.25	6340.06	6432.05	6281.29	5559.80	53999.42	48156.84	44192.77	30477.69	28785.29	24978.74	37539.99	35322.60	32441.67	34028.24	31966.08	18189.74		
3296	Probable RNA-binding protein EIF1AD	Q8N9N8	13689.40	14006.19	13116.96	9620.39	8948.90	9351.44	9961.84	9182.71	8267.46	11450.31	10270.52	10210.76	10684.58	9551.93	8692.13	8454.71	7268.29	8068.03	8702.80	7781.74	7639.10	3174.53	3469.64	2830.31	1972.63	2078.12	1595.64	2790.30	2733.08	2859.45	2288.39	2733.25	1767.42		
3297	Probable tRNA N6-adenosine threonylcarbamoyltransferase	Q9NPF4	16178.26	14362.05	12957.52	12224.47	12002.29	9852.93	13568.67	13172.38	11302.47	13211.27	12509.42	10948.58	13998.47	14418.46	6828.21	11205.29	12436.57	10252.88	5632.74	5615.85	4701.04	17196.73	9942.17	9116.80	12094.62	11752.96	7498.10	15328.98	13823.29	10065.79	16135.38	14464.44	7891.05		
3298	Probable ubiquitin carboxyl-terminal hydrolase FAF-X	Q93008	229020.35	221336.80	209949.04	161566.12	156293.79	140906.09	186555.70	184304.82	172607.28	206847.90	197014.50	182818.48	204844.99	188737.95	136941.50	160519.20	153779.07	146584.38	164955.59	154059.28	141176.68	250387.88	244733.20	217367.28	159267.12	158182.64	134208.54	171809.09	169112.20	156837.12	183873.20	173676.25	111210.13		
3299	Probable ubiquitin carboxyl-terminal hydrolase FAF-Y	O00507	0.14	2.53	0.81	1.07	2.77	0.68	0.96	0.36	0.63	0.35	0.75	0.38	2.13	0.97	0.60	4.64	1.07	0.47	2.22	0.19	0.06	2766.12	2514.18	2326.87	1478.52	1374.27	1124.74	1892.37	1846.58	1683.41	1796.50	1795.09	1097.74		x
3300	Pro-cathepsin H	P09668	1943.32	1583.24	1578.71	1093.50	1092.71	916.35	1336.29	1284.27	1394.91	962.06	985.63	1028.39	1201.38	1162.63	351.93	1144.05	932.04	933.11	1061.23	1240.10	1022.95	2.27	1.49	0.65	0.84	1.31	0.69	4.16	2.47	0.11	4.90	4.98	0.12	x	
3301	Procollagen galactosyltransferase 1	Q8NBJ5	0.14	2.53	0.81	1.07	2.77	0.68	0.96	0.36	0.63	0.35	0.75	0.38	2.13	0.97	0.60	4.64	1.07	0.47	2.22	0.19	0.06	235.50	262.06	125.06	311.79	259.42	234.59	329.16	337.41	345.78	213.24	222.15	115.93		x
3302	Procollagen-lysine_2-oxoglutarate 5-dioxygenase 1	Q02809	556907.68	540000.20	526239.60	414814.92	397274.19	385370.85	438216.58	441362.94	433121.18	485272.28	457502.61	447058.96	479991.80	461761.14	396110.73	379741.86	355857.84	363045.84	430766.27	410189.58	392132.46	12493.88	12012.16	11537.39	7548.45	7537.29	5596.46	11112.76	10186.47	9067.04	9573.39	9339.10	5887.06		
3303	Procollagen-lysine_2-oxoglutarate 5-dioxygenase 2	O00469	18934.79	17518.40	17253.04	14542.66	14033.40	13313.82	15487.32	15686.58	14166.03	20765.82	20718.99	19336.83	15909.47	15898.85	14247.49	15105.85	15228.98	14583.63	14078.40	12678.20	12259.67	3523.08	3254.39	2890.60	1729.98	1796.11	1710.48	2294.80	2533.05	2239.24	2219.16	2098.21	1477.99		
3304	Proenkephalin-A	P01210	3107.80	3054.72	2736.25	1540.57	1670.72	1736.85	2333.81	1959.51	1671.60	3198.99	2972.61	2928.95	2241.62	2094.58	1799.75	2346.61	1894.27	2291.49	1926.67	1630.82	1720.84	700.32	626.67	405.60	564.42	503.72	147.34	762.30	692.28	399.83	1258.61	1184.86	429.44		
3305	Proenkephalin-B	P01213	1853.47	1904.39	1775.12	1776.48	1668.52	1663.86	1797.06	1723.51	1915.17	3379.27	3194.33	2923.51	2252.53	1994.65	1189.65	2390.21	2252.97	2062.62	1807.95	1672.41	1590.55	2.27	1.49	0.65	0.84	1.31	0.69	4.16	2.47	0.11	4.90	4.98	0.12	x	
3306	Profilin-1	P07737	509678.06	475374.36	427030.97	295719.26	295234.89	257912.49	446001.84	436502.50	399016.08	380049.37	354876.21	322123.42	417185.72	380495.33	271073.19	340743.33	321704.75	300041.00	323781.99	311265.58	260711.71	1729732.72	1617791.52	1469255.81	872564.59	845795.24	692888.60	1351192.44	1241915.21	1155927.87	1188426.50	1145384.19	742589.83		
3307	Profilin-2	P35080	162725.13	151790.24	143610.56	128200.39	123400.36	117389.77	123057.71	120158.94	116982.45	156233.98	145469.86	146800.67	130048.51	132612.23	112654.92	127496.56	117755.93	111183.14	114789.86	111974.68	108653.80	48727.66	47201.47	43901.69	37117.71	35030.64	32891.20	40501.66	38500.29	36946.09	41868.28	41257.54	30571.97		
3308	Profilin-3	P60673	0.14	2.53	0.81	1.07	2.77	0.68	0.96	0.36	0.63	0.35	0.75	0.38	2.13	0.97	0.60	4.64	1.07	0.47	2.22	0.19	0.06	1643.32	2275.45	1624.05	1465.29	1606.21	1156.07	1806.90	2590.76	2172.71	2068.99	2092.12	1201.86		x
3309	Programmed cell death 6-interacting protein	Q8WUM	288536.25	287784.02	286457.61	205022.77	199670.92	189728.42	241593.57	228632.94	229923.39	249342.75	236392.58	223589.67	239124.74	225450.02	187863.32	214572.46	198185.72	197917.01	198485.89	190818.74	181267.82	321824.80	294614.71	279775.89	190353.02	183596.21	162818.31	227828.09	216092.20	210463.25	229476.48	219006.01	155079.98		
3310	Programmed cell death protein 10	Q9BUL8	0.14	2.53	0.81	1.07	2.77	0.68	0.96	0.36	0.63	0.35	0.75	0.38	2.13	0.97	0.60	4.64	1.07	0.47	2.22	0.19	0.06	3024.86	3728.33	2947.64	2593.04	2511.36	2139.95	2557.25	2731.59	3398.48	3166.90	2903.94	2507.48		x
3311	Programmed cell death protein 2-like	Q9BRP1	13013.33	11478.56	11082.19	9396.48	8515.07	8266.35	7902.91	7570.38	7558.79	10054.17	8831.23	10440.86	9100.21	8942.88	8768.94	8279.20	5459.30	6008.85	7721.24	7713.07	9066.11	1728.40	1881.24	1111.99	1060.40	1168.32	1126.76	1299.57	1338.75	1165.90	1308.99	1359.49	1181.67		
3312	Programmed cell death protein 4	Q53EL6	37760.92	29873.38	29821.60	17332.35	18225.86	14095.17	24304.32	23566.05	23241.86	22516.40	21138.61	20200.98	19361.00	16671.48	12951.72	25000.86	19379.87	19988.28	18413.62	17520.40	16105.92	1057.07	992.36	885.17	804.04	727.56	473.54	1001.24	928.87	876.64	1060.59	1027.73	500.32		
3313	Programmed cell death protein 5	O14737	125753.35	119603.26	119673.69	71576.38	67070.71	63516.95	85579.26	82417.26	81888.10	90982.54	88572.27	79975.99	85530.47	77841.10	62675.05	65595.72	66832.06	66744.42	81367.88	74851.57	71342.14	127106.29	120761.24	116125.36	73296.48	70908.23	62247.71	95368.18	93984.97	90728.06	96990.30	93905.70	57898.83		
3314	Programmed cell death protein 6	O75340	91704.11	90557.95	86451.84	56582.45	55436.18	50412.27	75663.63	72089.26	70877.29	74293.79	69420.40	65673.45	71257.90	70159.57	58116.24	57971.35	56005.68	53972.81	57710.49	53617.89	51176.51	42219.16	38039.33	34748.61	18408.90	17322.95	14922.60	25696.75	24350.71	22771.26	21261.35	20754.81	13089.68		
3315	Prohibitin	P35232	630786.60	611233.89	577741.83	428567.22	419360.26	393303.87	509953.19	487348.02	465026.42	510163.34	482027.43	456140.02	516208.48	477191.93	413591.48	421195.01	393938.93	388147.40	425241.78	408601.27	388221.61	389994.35	360425.62	327415.59	248278.44	234964.29	216782.26	287890.52	272611.12	264743.29	270026.91	257735.73	182012.13		
3316	Prohibitin-2	Q99623	651920.53	638809.21	602413.07	423816.11	405567.46	392615.13	525203.81	498465.73	490391.15	492333.88	463680.91	449637.38	500893.17	479494.54	406093.30	430968.57	421696.66	401019.57	439972.49	435367.50	419012.17	471784.49	454814.95	439983.86	327496.15	311554.97	275121.62	394536.13	366597.53	365909.21	370059.40	366058.02	213027.81		
3317	Prolactin regulatory element-binding protein	Q9HCU5	209.34	324.56	275.34	293.72	260.54	287.06	286.46	306.31	276.13	565.02	460.99	421.38	364.47	395.05	368.94	281.45	240.77	242.61	401.22	320.78	401.84	2.27	1.49	0.65	0.84	1.31	0.69	4.16	2.47	0.11	4.90	4.98	0.12	x	
3318	Proliferating cell nuclear antigen	P12004	287196.62	279381.37	262939.61	208177.01	204424.15	190914.87	257058.46	243621.98	235220.58	219098.81	210679.43	200200.19	248750.20	237966.65	208137.49	184736.39	177405.29	172610.44	191775.97	195820.12	186808.67	685981.82	652779.84	617767.08	422381.47	409932.32	375986.87	457870.57	432301.75	418159.37	451351.96	431035.14	300309.04		
3319	Proliferation-associated protein 2G4	Q9UQ80	1041124.88	1014470.14	958961.55	730267.91	707291.99	669364.28	823422.14	779650.46	768242.66	863968.76	820368.62	773228.66	848648.85	811616.95	697351.57	681049.55	652318.49	642793.94	772089.76	718071.44	697993.56	599444.06	573467.44	534310.34	418347.22	412130.53	384519.01	474052.42	463338.93	453563.03	500343.38	480949.26	346363.55		
3320	Proline-rich protein 18	Q8N4B5	1135.41	1147.47	1102.60	558.98	645.42	531.60	944.53	928.82	844.56	609.15	581.44	521.59	621.69	802.01	661.60	580.05	484.44	434.87	636.22	644.60	665.70	2.27	1.49	0.65	0.84	1.31	0.69	4.16	2.47	0.11	4.90	4.98	0.12	x	
3321	Proline-serine-threonine phosphatase-interacting protein 1	O43586	18768.35	17739.59	18449.97	13511.18	12607.50	12604.21	14685.14	14309.48	14204.33	16202.23	15134.75	14116.77	14858.36	15518.03	12796.72	13594.23	12954.18	12573.58	13511.84	12443.58	11127.32	2.27	1.49	0.65	0.84	1.31	0.69	4.16	2.47	0.11	4.90	4.98	0.12	x	
3322	Proline-serine-threonine phosphatase-interacting protein 2	Q9H939	0.14	2.53	0.81	1.07	2.77	0.68	0.96	0.36	0.63	0.35	0.75	0.38	2.13	0.97	0.60	4.64	1.07	0.47	2.22	0.19	0.06	3921.10	3015.97	3156.91	1283.01	1512.87	946.97	2000.88	1657.76	1550.96	3188.38	2048.82	912.28		x
3323	Prolyl 3-hydroxylase 1	Q32P28	0.14	2.53	0.81	1.07	2.77	0.68	0.96	0.36	0.63	0.35	0.75	0.38	2.13	0.97	0.60	4.64	1.07	0.47	2.22	0.19	0.06	1383.57	1362.49	1265.85	1048.73	1019.63	1000.45	1159.81	1124.08	1047.71	1177.34	1065.32	802.52		x
3324	Prolyl 3-hydroxylase 2	Q8IVL5	153.69	128.66	116.56	404.98	303.84	278.43	171.91	213.68	242.66	342.74	272.88	243.40	96.02	53.77	49.61	581.98	491.94	657.59	313.03	361.02	227.32	404.80	616.05	497.67	604.88	614.65	1095.73	162.84	324.29	280.90	1435.37	734.04	110.47		
3325	Prolyl 3-hydroxylase OGFOD1	Q8N543	0.14	2.53	0.81	1.07	2.77	0.68	0.96	0.36	0.63	0.35	0.75	0.38	2.13	0.97	0.60	4.64	1.07	0.47	2.22	0.19	0.06	28894.50	27238.34	26443.72	18410.36	17489.83	15260.55	22889.14	22581.24	19350.17	24347.07	22788.91	12839.01		x
3326	Prolyl 4-hydroxylase subunit alpha-1	P13674	105066.13	99612.40	100205.15	62448.60	60919.63	59288.44	72909.11	73068.39	66895.50	100422.60	97492.06	91328.67	98293.41	91670.71	80229.28	60722.85	60841.24	57308.95	66676.11	67077.02	61583.80	79055.60	72956.19	68418.69	46953.82	46442.13	41143.56	60219.35	57656.56	53468.19	51585.34	49134.12	36903.05		
3327	Prolyl 4-hydroxylase subunit alpha-2	O15460	0.14	2.53	0.81	1.07	2.77	0.68	0.96	0.36	0.63	0.35	0.75	0.38	2.13	0.97	0.60	4.64	1.07	0.47	2.22	0.19	0.06	53639.75	46722.60	40902.64	30618.77	28333.15	18580.01	51647.85	42813.59	33325.14	53871.01	48162.57	25489.84		x
3328	Prolyl endopeptidase FAP	Q12884	2885.43	2864.41	2703.31	2839.29	3102.07	2929.06	3000.54	3058.91	3418.96	2164.26	2066.08	2204.22	3031.69	2787.55	3898.72	3145.60	2544.01	2234.08	2535.24	2462.26	2285.12	43.34	41.13	497.65	44.08	9.78	1.38	85.61	48.59	11.82	99.20	94.71	13.84		
3329	Prolyl endopeptidase	P48147	45476.59	46324.90	40758.13	34065.78	31429.05	29674.87	36736.90	33206.85	36211.42	42046.90	38138.09	35747.03	39359.77	35662.09	30106.20	36529.87	32336.57	33083.37	36647.34	32392.50	31100.92	22647.91	22840.98	18921.81	15649.87	15501.15	14003.22	14525.81	14227.40	14423.62	19569.98	13542.90	11967.04		

Supplementary Table S1. Overview on the abundance of all relatively quantified 5180 proteins

			MCF-7																					MDA-MB-231																
	Protein name	UniProt	control 01	control 02	control 03	Dai IC20 01	Dai IC20 02	Dai IC20 03	Dai SC20 01	Dai SC20 02	Dai SC20 03	Gen IC20 01	Gen IC20 02	Gen IC20 03	Gen SC20 01	Gen SC20 02	Gen SC20 03	SSE IC20 01	SSE IC20 02	SSE IC20 03	SSE SC20 01	SSE SC20 02	SSE SC20 03	control 01	control 02	control 03	Dai IC20 01	Dai IC20 02	Dai IC20 03	Gen IC20 01	Gen IC20 02	Gen IC20 03	SSE IC20 01	SSE IC20 02	SSE IC20 03	unique to MCF-7	unique to MDA-MB-231
3330	Pro-opiomelanocortin	P01189	5245.32	5926.21	6700.82	5263.13	4876.70	4602.60	4530.30	3951.64	3266.97	5450.85	5206.81	4857.24	5728.22	3922.38	1911.57	3397.16	3838.29	3648.09	3778.80	4278.83	5199.75	2.27	1.49	0.65	0.84	1.31	0.69	4.16	2.47	0.11	4.90	4.98	0.12	x	
3331	Properdin	P27918	27202.10	30272.98	30730.52	19470.20	18288.75	17700.77	19069.24	20909.87	20350.66	24804.57	23538.07	20065.76	21377.55	23258.34	18193.54	19621.49	18771.84	19067.32	19591.98	16887.21	19269.23	2.27	1.49	0.65	0.84	1.31	0.69	4.16	2.47	0.11	4.90	4.98	0.12	x	
3332	Proprotein convertase subtilisin/kexin type 4	Q6UW60	723.10	686.91	727.40	693.79	605.86	531.63	572.16	470.69	549.63	663.48	638.12	630.87	565.86	640.29	567.30	570.98	504.81	449.49	460.32	481.36	461.69	8534.55	7657.49	6481.93	5276.44	4899.92	4246.66	6345.58	6664.11	5556.53	7330.00	7290.28	4241.23		
3333	Prospero homeobox protein 1	Q92786	50170.96	48389.74	44873.45	38093.33	37014.14	34398.66	39434.77	36205.73	33541.71	45483.41	44247.62	42005.68	42195.79	41221.85	36422.82	36783.23	33725.70	32978.31	39801.35	34922.44	34115.71	7726.90	7262.55	6602.28	3752.08	3860.79	3532.12	5513.60	5587.59	5341.22	5221.19	5024.89	3399.87		
3334	Prostaglandin E synthase 2	Q9H7Z7	18198.28	17493.37	15763.18	10490.97	9836.30	9518.28	13512.68	13131.52	12937.16	13571.39	12578.85	11756.16	13426.01	13047.84	9645.35	11483.61	10617.89	9794.62	10370.91	9926.17	8368.18	2.27	1.49	0.65	0.84	1.31	0.69	4.16	2.47	0.11	4.90	4.98	0.12	x	
3335	Prostaglandin E synthase 3	Q15185	372316.35	336717.82	291492.47	208619.93	204764.05	177593.91	297617.20	280224.89	250848.57	270491.90	249404.50	228320.49	259697.05	237971.78	175978.11	214633.10	209365.48	190764.47	241995.46	233077.60	205183.06	300761.39	287247.23	248767.33	166479.27	155170.30	137383.08	188825.65	176465.83	165071.65	205693.13	189556.81	122266.60		
3336	Prostaglandin F2 receptor negative regulator	Q9P2B2	105020.95	103047.85	101290.32	69856.93	68177.01	65633.49	82356.03	77685.46	75218.86	90298.26	83058.92	79174.57	86840.22	85967.35	72857.25	71942.77	67226.40	64796.80	68883.05	62539.26	64082.86	2.27	1.49	0.65	0.84	1.31	0.69	4.16	2.47	0.11	4.90	4.98	0.12	x	
3337	Prostaglandin reductase 1	Q14914	9257.43	9299.60	8260.17	8560.21	8254.29	7550.62	7508.75	7629.68	7335.25	10164.38	9974.72	10148.29	8585.43	7258.14	7126.75	7711.67	7266.38	7472.06	7403.62	7762.17	6753.63	94582.76	93825.85	85099.39	73778.08	69002.16	71874.68	81852.63	83250.64	81408.53	95621.53	87370.74	65388.18		
3338	Prostate and testis expressed protein 2	Q6UY27	1658.27	1859.22	1845.62	1636.09	1334.77	1469.23	1548.40	1388.04	1765.24	1733.01	1608.39	1108.17	1655.38	1590.43	1466.97	1443.83	1564.40	1878.41	2119.02	1226.95	1473.90	10742.32	7950.72	7582.71	7570.73	7649.01	5801.56	7678.10	7742.28	7313.74	11492.82	10993.42	7848.60		
3339	Prostate tumor-overexpressed gene 1 protein	Q86YD1	12164.16	8935.06	6692.52	7696.04	7641.79	6322.01	7033.96	3532.87	5628.78	9920.45	9300.02	7175.32	9543.49	4573.49	1778.22	6041.37	4094.75	6165.47	7338.29	7203.88	5009.06	2.27	1.49	0.65	0.84	1.31	0.69	4.16	2.47	0.11	4.90	4.98	0.12	x	
3340	Proteasomal ubiquitin receptor ADRM1	Q16186	40804.56	40280.92	38088.57	38348.47	37026.81	36034.35	38052.11	35213.63	33619.98	40253.10	38188.84	35408.38	40373.50	38200.14	29718.74	34761.65	33147.04	31734.97	35833.86	33117.56	32073.92	23527.23	25026.11	21334.19	17937.85	18286.62	18926.54	16101.97	17586.70	15343.15	23971.12	20527.47	16051.47		
3341	Proteasome activator complex subunit 1	Q06323	605189.75	584674.25	568443.76	363417.88	346399.66	340204.25	407551.36	398533.33	386869.58	445827.46	433858.17	402526.59	452938.64	427163.35	372043.77	365388.77	347432.87	340237.45	348925.94	331200.14	312764.74	134935.09	128144.16	116968.45	83490.20	81999.44	70429.28	102057.27	98220.52	93366.32	94623.41	93778.57	69342.66		
3342	Proteasome activator complex subunit 2	Q9UL46	520693.50	498012.70	467950.93	354776.95	359688.13	328929.54	405878.68	372538.62	353892.65	430540.42	414621.07	390500.37	430059.18	408153.67	359652.15	383623.76	342725.51	331640.95	359878.84	337799.30	314714.31	100659.29	86598.02	78442.24	60606.96	58952.28	51269.34	75544.97	68041.64	63636.09	72408.46	69625.52	50695.79		
3343	Proteasome activator complex subunit 3	P61289	15534.65	13738.05	12238.94	9515.00	9041.27	8491.53	10708.78	10031.79	9479.32	13621.02	13309.49	11836.79	11784.95	11514.71	8139.07	8987.96	7859.74	7818.93	10888.64	10100.47	9233.00	22522.87	20616.91	18134.90	15327.81	14891.99	11681.99	18714.46	16845.75	15169.21	20848.91	19725.37	11845.20		
3344	Proteasome activator complex subunit 4	Q14997	124790.87	116640.21	103281.34	79330.96	77564.55	68914.13	90731.09	88653.26	86172.86	102325.60	98288.22	88323.62	95538.03	87771.07	66460.35	82033.52	77361.01	74008.48	80769.10	75053.57	69658.53	2.27	1.49	0.65	0.84	1.31	0.69	4.16	2.47	0.11	4.90	4.98	0.12	x	
3345	Proteasome adapter and scaffold protein ECM29	Q5VYK3	127446.37	119456.70	114496.74	89650.61	86578.24	81443.82	104198.58	102176.47	95689.23	107544.91	102053.82	95506.80	104077.83	102417.32	84168.00	87546.27	81691.76	77856.66	91380.94	86178.67	80946.13	50882.71	43308.88	40161.11	32858.40	31763.79	28274.37	37552.01	34843.90	32422.58	39557.21	36915.29	20065.54		
3346	Proteasome assembly chaperone 1	O95456	84493.12	79424.46	74858.50	55872.55	55132.23	52252.28	71425.27	71072.92	68214.51	70700.87	68299.93	67895.42	68775.35	60271.19	54870.16	62179.06	58213.24	57635.57	62893.21	61833.76	58120.38	1562.79	1410.01	1395.91	903.99	860.03	816.09	1154.24	1007.61	1190.04	1762.21	1548.53	1031.87		
3347	Proteasome assembly chaperone 2	Q969U7	11008.76	8330.45	6603.49	6395.21	5984.63	4273.07	6375.85	5288.34	4858.31	9948.22	9140.27	7211.37	9597.65	7478.62	1564.08	6009.42	5641.19	4732.10	6805.19	6211.54	4870.63	196.08	149.95	200.32	108.43	80.81	85.77	389.28	370.86	348.47	148.18	154.96	107.01		
3348	Proteasome assembly chaperone 3	Q9BT73	2098.82	2149.91	1886.90	923.03	842.53	784.18	1461.97	1517.96	1403.08	1310.83	1163.77	1043.21	1508.83	1499.56	1267.14	1336.23	1148.89	1450.39	1001.71	1175.98	930.11	2.27	1.49	0.65	0.84	1.31	0.69	4.16	2.47	0.11	4.90	4.98	0.12	x	
3349	Proteasome assembly chaperone 4	Q5JS54	2288.35	2136.73	1948.53	1763.23	1967.89	1567.84	2235.38	2042.35	1820.78	2187.40	1899.64	1665.28	1928.85	2116.35	1202.99	1850.32	1742.12	1665.14	1864.31	1716.54	1644.16	1762.41	1136.55	62.86	362.39	333.89	64.29	850.24	522.62	361.37	658.52	683.25	143.27		
3350	Proteasome inhibitor PI31 subunit	Q92530	21635.76	16451.02	15813.96	8116.34	7480.36	6592.99	12308.45	11113.59	8906.40	11604.55	10833.33	9938.46	11294.67	10279.29	7167.04	8842.46	9038.80	8737.63	10271.37	9174.32	4059.83	257621.45	246101.30	222363.39	156030.16	150196.32	140815.39	206441.83	200445.80	190558.12	223965.19	216845.38	141044.02		
3351	Proteasome subunit alpha type-1	P25786	400947.58	400791.57	378804.52	261915.98	263538.50	241875.22	299414.40	283162.00	270094.17	327562.92	310282.14	298562.82	305881.71	299915.64	254729.76	266404.61	257091.13	247089.10	267715.46	262152.90	244615.90	225208.21	216363.57	197984.34	157784.32	151626.06	133959.40	189033.41	180584.39	174535.69	185346.87	180752.32	107607.27		
3352	Proteasome subunit alpha type-2	P25787	141678.97	123962.72	107317.67	85620.89	78928.36	64941.86	110337.77	103715.24	94614.95	104558.85	96829.64	82066.65	103636.02	99353.47	53766.25	90479.35	75445.80	71872.32	101228.46	88019.63	73310.04	63457.45	72724.43	63234.18	46745.65	46440.09	44108.79	53897.01	54240.05	51839.65	56109.90	53679.81	39306.57		
3353	Proteasome subunit alpha type-3	P25788	232626.67	261015.02	240727.18	187246.31	181719.35	180740.75	204426.40	196084.58	197730.55	216717.90	202995.76	199795.95	208410.33	178658.52	150153.86	177049.27	166973.20	167461.49	184122.07	169411.08	170120.40	110850.66	104965.27	99376.78	77140.04	73826.17	69059.74	83552.28	83244.74	79654.30	91632.59	86528.84	56363.20		
3354	Proteasome subunit alpha type-4	P25789	355506.12	345341.80	322682.66	237571.76	229892.40	221059.94	290697.07	274322.89	271601.18	292886.97	280874.22	260550.50	294246.81	276188.46	233024.59	232345.17	220813.00	218544.28	230943.64	224153.98	210702.03	168352.19	160651.75	144877.76	99447.45	98321.93	83915.34	110948.41	108199.02	101256.69	105129.63	101768.21	74020.19		
3355	Proteasome subunit alpha type-5	P28066	358361.66	354856.79	345102.90	247270.77	246681.20	232799.91	310861.90	284321.82	290025.27	307171.27	288270.17	281021.09	303019.49	286755.90	236606.83	248171.07	251983.36	241812.28	252918.42	246797.73	222889.33	252127.18	240991.45	232371.09	157466.11	150375.34	132394.73	209278.56	191632.21	195341.41	193292.25	186327.17	129272.16		
3356	Proteasome subunit alpha type-6	P60900	484367.11	470045.46	435862.44	307788.18	303486.94	286085.10	387272.24	375373.31	359177.74	378789.90	358202.78	340534.31	364466.11	357760.62	306596.18	313336.14	301006.34	290206.96	318215.80	306276.90	293130.91	164343.07	153672.62	140792.42	93221.31	87862.15	74677.61	113424.17	106756.67	104021.12	100446.69	95653.01	69306.02		
3357	Proteasome subunit alpha type-7	O14818	206480.21	199193.92	187757.61	141371.23	136044.73	124884.77	156966.65	157605.95	147383.91	163316.04	153775.42	142239.49	170464.26	160574.72	128478.82	137164.30	131863.19	129884.77	142601.09	135208.74	128880.37	126638.09	122659.75	114620.75	76575.19	77775.95	66739.40	105082.13	99724.93	93199.69	92055.47	90143.08	63716.31		
3358	Proteasome subunit alpha-type 8	Q8TAA3	0.14	2.53	0.81	1.07	2.77	0.68	0.96	0.36	0.63	0.35	0.75	0.38	2.13	0.97	0.60	4.64	1.07	0.47	2.22	0.19	0.06	315.18	255.84	245.09	205.69	129.58	17.42	274.31	187.99	133.68	316.15	274.23	61.41		x
3359	Proteasome subunit beta type-1	P20618	478530.68	463023.93	444514.65	332685.31	315754.90	301066.93	369985.99	357950.12	351911.16	394809.50	373304.21	350325.09	399593.81	371638.02	324867.39	326839.67	305380.59	304263.67	329816.26	307809.12	299097.33	152576.96	137433.92	135568.73	96392.21	94951.98	81901.45	126705.43	120938.77	116897.82	119096.78	119295.06	83780.56		
3360	Proteasome subunit beta type-2	P49721	143485.44	136980.19	140351.05	99557.75	94848.69	89820.58	112807.33	109002.54	105036.63	117210.57	105826.22	100845.68	110540.66	111109.54	88804.54	98809.81	94035.28	91870.54	95232.50	89947.05	88783.52	47757.63	43858.55	37723.57	27573.84	25465.88	21515.66	35526.53	30952.46	32643.06	29614.94	30655.18	19372.89		
3361	Proteasome subunit beta type-3	P49720	119413.85	119379.66	115574.05	72451.47	68531.28	64069.00	108490.65	106434.13	96370.93	88144.84	85407.78	79029.19	97304.50	96372.20	70908.09	75844.86	77539.03	72463.71	86900.56	78672.52	72093.34	41952.41	39054.71	34714.05	20626.50	20229.14	16617.18	25704.78	24017.23	22714.98	18238.50	18720.85	11815.40		
3362	Proteasome subunit beta type-4	P28070	227718.50	211421.80	182793.56	150999.86	148230.30	129349.51	168805.00	168407.53	167669.68	184527.60	174732.64	159134.57	192582.18	176678.64	136496.68	162197.41	151691.41	139698.56	164586.86	148433.47	145665.25	107889.72	97643.13	92994.49	71530.17	69278.29	61233.41	83541.62	76934.75	72203.23	84148.74	86935.09	59614.35		
3363	Proteasome subunit beta type-5	P28074	364436.23	365513.61	339248.61	246119.38	239402.76	223516.07	275202.35	266693.70	242104.84	298039.82	283005.80	271265.97	288568.26	292781.44	231764.40	233567.96	233017.45	229401.91	255360.18	245789.20	242785.80	279219.59	269607.39	206032.12	115411.94	112627.90	94815.19	130683.20	126690.73	116336.62	134467.72	125991.11	81582.18		
3364	Proteasome subunit beta type-6	P28072	179104.35	173143.44	162205.31	135518.30	124172.08	132923.26	155033.75	147560.23	148040.03	154782.02	147218.31	136147.56	162960.66	156544.20	151705.21	135229.61	127575.98	123796.57	149904.61	147682.54	141696.07	45154.38	44755.70	38079.02	31566.82	31928.17	29486.33	34317.72	32833.22	32347.38	38455.48	35492.37	34629.84		
3365	Proteasome subunit beta type-7	Q99436	130794.81	127101.43	118961.16	86924.96	87025.06	80202.02	109748.88	104370.74	104136.46	102259.44	96855.61	91559.64	109522.98	107542.05	81742.69	100175.24	91007.66	87207.36	98468.44	87558.75	78167.21	50714.75	45405.17	47379.86	26731.83	25832.82	24138.85	27530.10	26452.55	25001.29	28935.00	27496.60	25940.92		
3366	Proteasome subunit beta type-8	P28062	0.14	2.53	0.81	1.07	2.77	0.68	0.96	0.36	0.63	0.35	0.75	0.38	2.13	0.97	0.60	4.64	1.07	0.47	2.22	0.19	0.06	1806.96	1505.61	1483.14	1177.34	1135.94	1168.79	1323.78	1322.24	1340.62	1315.92	1257.04	967.92		x
3367	Protein AAR2 homolog	Q9Y312	0.14	2.53	0.81	1.07	2.77	0.68	0.96	0.36	0.63	0.35	0.75	0.38	2.13	0.97	0.60	4.64	1.07	0.47	2.22	0.19	0.06	7407.64	6728.15	6559.60	4354.62	4193.24	3617.21	5905.45	5546.23	4510.76	7073.17	6447.85	3695.49		x
3368	Protein AATF	Q9NY61	1375.89	897.02	818.02	648.42	620.08	667.30	591.04	672.51	685.89	664.78	791.09	870.45	1079.39	1002.28	780.15	517.28	463.96	395.45	782.38	676.40	831.89	2.27	1.49	0.65	0.84	1.31	0.69	4.16	2.47	0.11	4.90	4.98	0.12	x	
3369	Protein ABHD11	Q8NFV4	63688.66	57027.36	55123.87	33679.65	34056.24	32501.95	42704.32	39416.50	37903.65	41954.00	40981.90	39698.11	46470.62	40138.54	33706.19	38870.33	35730.61	37416.86	32485.36	30546.55	28229.70	2.27	1.49	0.65	0.84	1.31	0.69	4.16	2.47	0.11	4.90	4.98	0.12	x	
3370	Protein ABHD12B	Q7Z5M8	0.14	2.53	0.81	1.07	2.77	0.68	0.96	0.36	0.63	0.35	0.75	0.38	2.13	0.97	0.60	4.64	1.07	0.47	2.22	0.19	0.06	476.18	488.34	336.70	3579.00	3126.60	2603.59	8189.15	8977.70	8873.66	6838.17	6642.41	3836.96		x
3371	Protein ABHD14B	Q96IU4	67478.33	62037.37	62381.12	42429.08	39330.52	37806.58	49526.56	46650.77	44831.68	48189.19	47037.74	45868.97	47683.11	43965.68	38267.21	46710.25	43582.76	43100.01	38323.83	37460.49	34624.19	54511.18	52185.33	49409.81	40134.41	37234.21	33835.95	44704.96	42425.60	40933.54	43770.39	43393.78	35896.37		
3372	Protein AHNAK2	Q8IVF2	0.14	2.53	0.81	1.07	2.77	0.68	0.96	0.36	0.63	0.35	0.75	0.38	2.13	0.97	0.60	4.64	1.07	0.47	2.22	0.19	0.06	393003.96	365626.73	350016.64	235825.09	226049.99	198673.02	284775.67	277735.38	262543.09	267662.15	260297.51	189337.53		x
3373	Protein arginine N-methyltransferase 1	Q99873	524457.52	488002.71	468608.29	307138.67	298637.57	279802.07	444288.97	428350.39	407701.07	380623.71	362482.31	332017.02	386290.61	368935.05	302321.84	351685.66	331096.19	312292.70	330226.19	308470.12	275016.23	437064.82	420350.77	391060.36	275833.85	263694.39	229430.19	357739.52	344852.83	324387.46	330620.69	325073.83	228158.75		
3374	Protein arginine N-methyltransferase 5	O14744	68968.40	66921.72	64990.34	52947.56	52004.01	48875.77	59139.84	60401.40	55918.31	62502.01	63833.80	58496.38	61745.11	52818.11	39583.08	49557.66	46703.69	44898.01	53275.33	49422.08	52229.40	53762.79	50855.45	46814.51	38121.27	37583.80	35681.89	44202.11	42229.96	39381.24	41221.51	42501.54	31003.84		
3375	Protein arginine N-methyltransferase 6	Q96LA8	26110.15	22088.94	20341.48	15683.19	14921.38	13745.93	17765.09	15913.88	17174.93	19285.43	17597.18	16537.67	19698.67	16955.31	11940.28	14725.67	13762.42	12449.88	17291.59	15810.00	13991.97	2.27	1.49	0.65	0.84	1.31	0.69	4.16	2.47	0.11	4.90	4.98	0.12	x	
3376	Protein arginine N-methyltransferase 8	Q9NR22	45137.29	43828.79	40328.39	35914.12	37035.39	34890.10	39741.08	36577.97	35629.47	42100.34	37948.76	36724.96	38847.80	37192.33	29840.68	36869.26	36339.07	34528.36	35076.41	31946.01	30468.43	26834.71	25908.22	23675.20	20652.19	19388.04	16322.12	23289.56	22756.07	20853.25	26596.24	24296.74	16081.15		
3377	Protein argonaute-1	Q9UL18	28855.16	26171.74	25223.41	22198.67	21382.96	19394.08	22868.27	22625.24	20706.92	24762.70	23640.22	21954.20	23034.58	19596.33	15596.94	24630.03	22376.07	22313.81	20368.34	19614.83	18326.25	2.27	1.49	0.65	0.84	1.31	0.69	4.16	2.47	0.11	4.90	4.98	0.12	x	
3378	Protein argonaute-2	Q9UKV8	0.14	2.53	0.81	1.07	2.77	0.68	0.96	0.36	0.63	0.35	0.75	0.38	2.13	0.97	0.60	4.64	1.07	0.47	2.22	0.19	0.06	895.71	852.10	830.42	762.78	853.88	809.11	790.98	768.41	791.45	954.73	929.95	728.85		x
3379	Protein bicaudal D homolog 1	Q96G01	96912.51	94896.45	82392.47	60830.35	57598.64	56953.63	68190.47	64053.51	62924.89	80626.38	75009.27	71354.17	68890.02	65004.71	59965.29	63857.41	62970.07	58504.97	63197.11	61700.33	58757.02	24622.36	17398.27	24598.08	15740.15	15444.50	14027.31	17207.46	16541.33	16127.27	16379.98	13086.74	10388.45		
3380	Protein bicaudal D homolog 2	Q8TD16	45360.57	45409.49	40523.80	33198.52	31836.43	30226.34	34383.30	32093.20	30329.33	43039.33	40397.00	37258.04	35697.11	36932.43	33571.78	30203.00	28066.77	27311.58	30927.34	29812.69	29342.16	19772.24	16687.59	16363.59	12017.31	11653.16	8116.90	15332.17	14129.62	12815.05	14494.94	14620.23	8456.98		
3381	Protein BRICK1	Q8WUW	4266.23	4257.06	4782.06	2952.00	3078.06	2667.77	4057.80	4092.05	4065.99	3442.11	3414.02	3322.49	3909.27	3748.06	2849.37	3240.51	2906.00	2927.23	2777.89	2717.29	2714.74	2547.08	2637.77	2165.35	2281.67	2055.66	2258.24	2460.50	2319.51	2249.04	2855.98	3229.87	1805.86		
3382	Protein BUD31 homolog	P41223	61879.68	61704.37	58555.30	41218.04	39449.87	36321.61	44468.28	48906.90	47601.71	50423.61	49217.26	44727.38	50104.52	47136.52	35359.99	39824.79	40712.08	38342.71	39667.00	38896.38	35543.83	2.27	1.49	0.65	0.84	1.31	0.69	4.16	2.47	0.11	4.90	4.98	0.12	x	
3383	Protein C10	Q99622	25462.44	22983.88	20639.69	14808.38	14105.94	13376.38	17446.61	17084.03	16168.25	20532.72	20357.11	19941.53	20659.45	18869.29	14899.89	15268.51	14837.64	14037.17	16277.06	15566.41	15144.42	9378.87	9585.35	8520.08	6163.75	5765.80	4756.35	8604.18	7874.89	7623.10	7604.93	7578.25	4530.13		
3384	Protein C12orf4	Q9NQ89	0.14	2.53	0.81	1.07	2.77	0.68	0.96	0.36	0.63	0.35	0.75	0.38	2.13	0.97	0.60	4.64	1.07	0.47	2.22	0.19	0.06	2829.59	2712.59	2743.90	2147.19	2368.72	2337.81	2891.16	2874.13	2848.17	2737.57	2651.18	2063.69		x
3385	Protein canopy homolog 2	Q9Y2B0	170461.71	165655.27	150516.19	118649.07	116057.35	105802.57	135215.18	125288.21	118106.14	145324.82	136359.55	127109.44	132683.23	116548.36	98700.17	132067.73	122677.39	117568.38	125584.92	125212.22	113612.79	21946.17	20084.22	19074.57	11373.65	11318.53	10090.83	13893.32	13721.93	12460.47	12382.95	12184.44	8386.57		
3386	Protein CASP	Q13948	25317.81	26619.91	23387.42	17823.22	17587.87	17096.61	18739.94	16949.52	15760.45	23417.78	22378.35	21866.40	22160.96	19697.68	16092.36	15707.71	15848.55	14793.73	21116.14	18830.47	17676.46	2.27	1.49	0.65	0.84	1.31	0.69	4.16	2.47	0.11	4.90	4.98	0.12	x	
3387	Protein CBFA2T2	O43439	0.14	2.53	0.81	1.07	2.77	0.68	0.96	0.36	0.63	0.35	0.75	0.38	2.13	0.97	0.60	4.64	1.07	0.47	2.22	0.19	0.06	7548.16	7575.84	7182.72	6516.58	6419.10	5840.58	6774.82	6886.04	6657.55	7516.76	7037.96	4930.90		x
3388	Protein CDV3 homolog	Q9UKY7	93373.41	93821.06	83799.89	64592.41	61026.98	56588.74	90223.30	83650.74	80389.33	78172.62	73885.14	69097.47	74990.16	69195.63	57419.39	71919.79	66878.03	64248.23	73526.29	71244.16	67439.94	96808.20	88481.48	84314.33	58084.48	54696.33	50624.82	74293.27	69828.09	68315.33	67764.17	65605.35	46657.17		
3389	Protein C-ets-1	P14921	18162.49	16602.37	14526.31	12026.78	11898.13	11693.53	14122.19	13739.70	13554.43	17125.65	16295.92	15930.04	15486.15	14059.68	13698.76	12696.10	12044.62	11507.65	13691.82	12541.07	12689.03	2.27	1.49	0.65	0.84	1.31	0.69	4.16	2.47	0.11	4.90	4.98	0.12	x	
3390	Protein CIP2A	Q8TCG1	0.14	2.53	0.81	1.07	2.77	0.68	0.96	0.36	0.63	0.35	0.75	0.38	2.13	0.97	0.60	4.64	1.07	0.47	2.22	0.19	0.06	49501.19	45730.62	39199.73	31084.82	30012.00	22033.13	35452.32	31251.52	29670.65	38099.46	37203.77	23310.28		x
3391	Protein cordon-bleu	O75128	4055.20	4231.94	3771.02	3159.43	3029.13	2940.12	3387.34	3580.56	3282.33	4324.83	4650.55	4464.29	4321.90	3394.76	3030.22	3297.51	2823.49	3006.87	3124.51	3202.25	3103.07	3716.88	3485.10	3739.50	2427.54	2631.08	2476.75	2838.56	2820.19	2920.55	3247.32	3151.15	2508.04		
3392	Protein cornichon homolog 4	Q9P003	9471.20	9898.71	7902.29	4023.64	4433.16	3665.34	6990.77	6221.24	5865.72	6498.29	5543.88	5491.44	7021.43	6475.75	4389.42	6676.67	6034.43	5388.21	5065.71	4976.51	4547.83	7959.52	7588.65	6828.64	4964.99	4888.13	3965.01	6762.97	6629.96	5829.01	5611.08	5654.24	3637.95		
3393	Protein CUSTOS	Q96C57	0.14	2.53	0.81	1.07	2.77	0.68	0.96	0.36	0.63	0.35	0.75	0.38	2.13	0.97	0.60	4.64	1.07	0.47	2.22	0.19	0.06	145.03	161.36	133.69	133.03	154.04	129.40	119.54	101.52	89.42	125.58	84.79	142.08		x
3394	Protein CYR61	O00622	162.37	255.69	166.71	138.64	138.05	137.77	81.14	96.39	95.02	128.91	108.62	166.63	145.07	16.43	4.19	123.36	95.27	128.14	203.63	137.26	100.38	36079.40	33746.18	30081.08	27630.66	27504.27	23880.77	29649.88	30325.75	28311.90	38476.54	34695.45	21527.53		
3395	Protein Daple	Q9P219	0.14	2.53	0.81	1.07	2.77	0.68	0.96	0.36	0.63	0.35	0.75	0.38	2.13	0.97	0.60	4.64	1.07	0.47	2.22	0.19	0.06	22028.47	21507.42	20432.22	14073.16	13837.07	13208.74	16170.68	15508.51	15275.49	15907.14	15029.00	11334.89		x
3396	Protein DDI1 homolog 2	Q5TDH0	22091.64	19761.67	18196.00	11212.34	9846.61	10377.28	15245.43	14977.19	13326.14	13428.25	13846.02	12793.38	14581.30	13663.96	11467.54	12812.49	10463.10	11242.14	11294.99	11613.71	10852.90	2.27	1.49	0.65	0.84	1.31	0.69	4.16	2.47	0.11	4.90	4.98	0.12	x	
3397	Protein DEK	P35659	47399.79	45728.01	42957.32	29853.08	28117.95	26672.53	41119.42	41808.91	40833.97	36870.62	34696.65	35422.15	35842.68	33342.34	27232.23	33043.57	27193.51	27424.44	29167.81	28683.05	28193.37	37351.88	36492.79	35341.16	23191.57	21506.36	24390.04	25507.39	26160.73	24890.91	36586.36	25229.57	14683.66		
3398	Protein diaphanous homolog 1	O60610	367426.04	357562.70	335758.54	234488.10	229059.77	214014.70	283301.15	279238.76	264792.30	287072.50	270527.95	253534.76	296653.00	287294.47	233436.75	227142.90	217206.74	205807.53	253307.83	234181.45	221777.06	112690.50	97248.89	94536.57	76175.36	72885.55	63086.65	86711.53	83196.12	74260.12	88533.17	86406.16	67176.24		
3399	Protein disulfide-isomerase A3	P30101	1530069.57	1430536.53	1322712.98	1073448.24	1056319.49	988783.85	1227578.65	1170565.54	1121334.05	1360183.72	1295756.45	1207013.01	1224582.53	1159736.39	966720.32	1142282.58	1067029.03	1025237.54	1063191.33	1025288.11	955554.99	864600.84	798505.90	736389.65	518279.99	508152.01	448084.67	628084.97	588834.77	547759.19	632940.14	608788.00	422884.74		
3400	Protein disulfide-isomerase A4	P13667	1821593.60	1781717.24	1666958.71	1467320.85	1398786.93	1331557.42	1565194.64	1529407.70	1465434.98	1823110.09	1734503.35	1644726.79	1562767.70	1486839.56	1200005.22	1429939.91	1392822.11	1347868.87	1457600.23	1408159.70	1318832.55	637956.91	624575.26	587386.75	426484.16	413131.50	376891.27	466704.94	454918.79	428400.29	491070.26	463815.58	307136.88		
3401	Protein disulfide-isomerase A5	Q14554	500.55	451.12	625.79	161.69	134.22	116.88	119.08	164.85	86.89	380.01	357.66	507.20	343.27	285.68	20.31	93.76	215.41	220.00	374.33	275.42	137.80	2.27	1.49	0.65	0.84	1.31	0.69	4.16	2.47	0.11	4.90	4.98	0.12	x	
3402	Protein disulfide-isomerase A6	Q15084	739540.22	669888.09	633853.62	479602.25	460238.19	403607.95	591468.33	560877.17	513697.87	657745.72	613500.81	573643.80	600273.96	527300.80	408161.19	509632.22	468605.00	436764.42	506938.11	475780.77	408111.75	199587.54	188845.55	152483.17	121906.31	119434.15	105172.68	130188.58	131877.63	121442.85	156015.30	147422.30	95006.32		
3403	Protein disulfide-isomerase	P07237	2104582.71	2036420.12	1898589.02	1811682.05	1731950.69	1640193.62	1858250.03	1817965.34	1724282.81	2204824.11	2101335.75	2014787.42	1702107.24	1648941.26	1340274.86	1835004.48	1740109.46	1658951.75	1701778.08	1577070.17	1496549.62	781592.21	726974.81	693804.94	535537.47	516820.35	462888.32	598354.52	578538.28	539638.60	612084.72	589069.33	415380.38		
3404	Protein dopey-1	Q5JWR5	14374.35	16037.11	16599.03	13816.85	10772.21	11891.12	11593.93	14060.82	11898.89	12265.03	12032.64	12420.02	12788.94	11192.17	9052.71	12516.67	9960.78	10440.72	8219.34	8547.68	7024.95	12598.01	11950.50	10400.84	5411.08	5564.25	3192.75	9132.55	7845.08	6750.36	8624.80	8064.53	3500.86		
3405	Protein Dr1	Q01658	0.14	2.53	0.81	1.07	2.77	0.68	0.96	0.36	0.63	0.35	0.75	0.38	2.13	0.97	0.60	4.64	1.07	0.47	2.22	0.19	0.06	6092.68	6101.29	5600.86	4395.48	4262.13	3306.69	4171.89	4285.43	3907.94	4345.35	4156.05	2486.35		x
3406	Protein ECT2	Q9H8V3	16599.93	16478.82	13947.56	10503.67	9853.20	9783.26	13025.51	11583.59	11975.54	15053.68	13181.88	11887.50	13052.01	13350.10	10676.33	11595.36	11107.79	11411.07	12319.07	11633.97	9884.93	8246.03	10001.12	6765.54	5787.12	4884.86	3123.98	6794.96	6294.87	5881.06	6855.31	7054.74	3996.59		
3407	Protein EFR3 homolog B	Q9Y2G0	14224.72	11922.34	12189.55	7994.92	7740.03	7251.29	8671.76	8286.67	8145.80	13078.04	11358.05	10162.90	12693.30	11326.40	8601.31	8977.40	10029.24	8778.06	9409.26	9214.61	7944.52	2.27	1.49	0.65	0.84	1.31	0.69	4.16	2.47	0.11	4.90	4.98	0.12	x	
3408	Protein enabled homolog	Q8N8S7	67377.41	65823.58	64755.94	43391.31	43167.94	40765.51	46116.22	43188.04	41491.74	49112.88	46887.34	44143.34	52519.32	51772.89	43506.08	38740.87	39228.69	37504.01	48817.56	48497.67	42527.01	7407.06	6750.52	6125.80	5427.37	5910.51	5176.39	6253.85	5812.38	5396.05	9177.07	8504.24	5441.60		
3409	Protein ERGIC-53	P49257	173047.33	161551.51	156839.12	128138.27	122681.83	113915.04	139758.29	139455.86	129178.64	158590.84	153455.49	140602.82	147744.68	140147.93	109310.45	130722.88	119115.00	119884.17	127075.67	117118.97	113535.39	103893.55	96759.14	88091.40	72022.47	70933.05	64700.57	98524.76	88486.24	84259.38	100039.69	93464.78	64806.62		
3410	Protein eyes shut homolog	Q5T1H1	76886.71	78991.61	68006.07	53080.74	49846.63	49219.46	58728.11	55500.27	53697.32	62250.17	58719.94	57128.27	63033.99	61405.19	51032.71	50408.07	46886.02	45833.38	47598.93	45958.71	42396.52	2.27	1.49	0.65	0.84	1.31	0.69	4.16	2.47	0.11	4.90	4.98	0.12	x	
3411	Protein FAM102B	Q5T8I3	580.95	678.07	537.15	335.46	346.92	277.63	424.40	524.53	447.68	354.04	374.60	334.97	719.09	481.61	395.54	329.32	278.40	260.39	366.26	374.91	376.16	2.27	1.49	0.65	0.84	1.31	0.69	4.16	2.47	0.11	4.90	4.98	0.12	x	
3412	Protein FAM104B	Q5XKR9	796000.67	662147.64	476462.35	584389.43	576450.86	424936.81	615515.29	603663.00	508939.93	726683.62	687203.94	516808.47	642589.58	570672.99	177886.06	560317.56	552844.40	458671.51	525537.81	492071.31	372826.47	329294.50	277445.46	216023.91	250945.78	238609.12	144127.37	332183.23	302827.36	226211.11	374209.64	356975.66	185378.49		
3413	Protein FAM114A2	Q9NRY5	28983.16	27795.63	26948.63	20237.55	19678.43	19321.58	25774.61	24827.58	24185.93	26811.07	25266.97	24000.70	21473.76	20300.34	17573.29	20657.12	20274.70	19152.19	19073.56	18368.07	16743.42	2.27	1.49	0.65	0.84	1.31	0.69	4.16	2.47	0.11	4.90	4.98	0.12	x	
3414	Protein FAM122B	Q7Z309	21439.14	17678.27	13116.22	13831.48	13146.22	10132.65	15500.54	15203.71	12915.25	17419.20	15574.98	12703.33	17394.54	15424.87	7063.46	13361.86	12626.78	10720.61	13198.29	12977.07	10139.16	8838.61	8386.86	7889.00	6780.86	6596.90	5017.58	7275.64	6333.52	6191.61	9105.25	8764.59	5748.22		
3415	Protein FAM124B	Q9H5Z6	9708.89	9696.99	8507.43	7904.68	7789.40	7393.08	7960.56	7521.29	7385.24	11395.58	11371.75	10751.04	9401.83	7989.80	6173.56	7039.17	7978.05	7714.69	8909.13	8250.48	7833.19	2.27	1.49	0.65	0.84	1.31	0.69	4.16	2.47	0.11	4.90	4.98	0.12	x	
3416	Protein FAM135B	Q49AJ0	53698.96	55505.24	53938.77	31427.66	28377.30	24933.35	33190.17	45426.66	40411.41	46047.04	48288.91	42978.67	41314.59	31263.01	26610.81	42735.05	44164.67	41909.73	43121.53	39299.83	37998.73	2.27	1.49	0.65	0.84	1.31	0.69	4.16	2.47	0.11	4.90	4.98	0.12	x	
3417	Protein FAM136A	Q96C01	58309.71	54644.67	52376.52	38950.50	38173.03	35840.58	42719.16	42476.96	42137.16	48273.96	45814.44	45389.33	46814.55	45206.45	37372.91	39399.19	38249.85	35906.69	39205.22	35271.66	35843.81	75921.30	72237.19	64473.57	43512.81	41382.27	36029.44	54273.87	53418.83	47085.69	50581.94	50219.55	31601.82		
3418	Protein FAM160A1	Q05DH4	0.14	2.53	0.81	1.07	2.77	0.68	0.96	0.36	0.63	0.35	0.75	0.38	2.13	0.97	0.60	4.64	1.07	0.47	2.22	0.19	0.06	3912.17	3688.09	3707.45	3001.31	2988.65	2612.23	3758.08	3750.86	3466.70	3525.61	3596.49	2738.92		x
3419	Protein FAM162A	Q96A26	1383.23	1256.85	1296.91	797.11	832.84	641.49	1941.26	1874.53	1667.35	1090.29	812.46	808.87	1331.27	1140.49	559.98	999.49	1007.22	974.59	942.43	831.48	804.12	2.27	1.49	0.65	0.84	1.31	0.69	4.16	2.47	0.11	4.90	4.98	0.12	x	
3420	Protein FAM167B	Q9BTA0	0.14	2.53	0.81	1.07	2.77	0.68	0.96	0.36	0.63	0.35	0.75	0.38	2.13	0.97	0.60	4.64	1.07	0.47	2.22	0.19	0.06	13797.69	13472.09	11226.66	7658.68	8048.16	5456.25	8991.20	9054.64	7703.75	9015.69	8139.14	4966.36		x
3421	Protein FAM170A	A1A519	674.60	354.46	850.38	407.21	418.72	390.83	919.64	744.14	781.12	605.36	670.27	637.89	601.67	635.74	381.97	522.53	582.83	638.70	563.72	580.03	387.49	91.18	78.11	97.58	32.85	22.82	32.05	4.16	2.47	0.11	4.90	4.98	0.12		
3422	Protein FAM177A1	Q8N128	6841.45	6067.95	5513.35	4738.49	4633.46	4030.70	5805.30	5651.77	5609.11	5744.83	5668.61	5407.19	5113.32	5106.39	4710.07	4827.08	4635.74	4356.58	4891.92	4527.66	4321.57	2.27	1.49	0.65	0.84	1.31	0.69	4.16	2.47	0.11	4.90	4.98	0.12	x	
3423	Protein FAM183BP	Q6ZVS7	0.14	2.53	0.81	1.07	2.77	0.68	0.96	0.36	0.63	0.35	0.75	0.38	2.13	0.97	0.60	4.64	1.07	0.47	2.22	0.19	0.06	453.32	487.01	416.18	172.62	185.72	148.41	182.48	107.18	130.67	86.42	103.46	64.45		x
3424	Protein FAM186A	A6NE01	0.14	2.53	0.81	1.07	2.77	0.68	0.96	0.36	0.63	0.35	0.75	0.38	2.13	0.97	0.60	4.64	1.07	0.47	2.22	0.19	0.06	3151.67	2885.59	2862.82	2271.69	2319.43	1726.62	3121.04	2728.48	2508.90	3108.85	3183.87	1855.93		x
3425	Protein FAM193A	P78312	0.14	2.53	0.81	1.07	2.77	0.68	0.96	0.36	0.63	0.35	0.75	0.38	2.13	0.97	0.60	4.64	1.07	0.47	2.22	0.19	0.06	9082.87	8427.80	7557.42	6015.02	5736.68	4821.20	7726.82	6527.16	6236.32	7506.28	6579.81	2605.28		x
3426	Protein FAM204A	Q9H8W3	221.22	87.03	111.95	130.93	145.39	77.81	106.38	81.60	92.04	130.09	117.46	174.11	181.89	167.02	43.88	145.07	79.05	102.24	118.92	135.07	107.12	2.27	1.49	0.65	0.84	1.31	0.69	4.16	2.47	0.11	4.90	4.98	0.12	x	
3427	Protein FAM217A	Q8IXS0	28151.04	25952.31	22610.79	13473.42	13427.56	12689.85	17230.95	16750.72	15455.85	25060.58	24672.21	24305.78	18371.04	15518.91	11968.56	16588.27	15742.10	15297.09	12977.61	12591.79	11731.11	2.27	1.49	0.65	0.84	1.31	0.69	4.16	2.47	0.11	4.90	4.98	0.12	x	
3428	Protein FAM236D	A0A1B0C	190.33	126.93	99.82	187.61	227.92	128.88	2235.42	105.21	2040.25	241.25	341.21	267.88	2.13	0.97	0.60	455.55	318.95	358.87	263.61	191.37	116.85	2.27	1.49	0.65	0.84	1.31	0.69	4.16	2.47	0.11	4.90	4.98	0.12	x	
3429	Protein FAM3C	Q92520	1287.19	926.74	823.10	986.81	889.61	717.66	1061.38	1052.17	918.92	1363.16	1324.13	1013.20	1115.70	1003.12	355.09	931.63	1037.65	933.14	1121.52	1040.63	993.76	865.75	815.95	818.16	520.80	535.69	499.97	402.20	392.90	482.83	336.92	352.44	292.65		
3430	Protein FAM49A	Q9H0Q0	0.14	2.53	0.81	1.07	2.77	0.68	0.96	0.36	0.63	0.35	0.75	0.38	2.13	0.97	0.60	4.64	1.07	0.47	2.22	0.19	0.06	11708.53	12552.53	11859.35	8035.83	8320.32	7926.41	7871.67	7731.63	7919.87	8118.59	7639.24	5703.26		x
3431	Protein FAM49B	Q9NUQ9	131650.63	130748.75	122123.53	90095.78	88309.98	82474.72	98290.17	98273.44	91586.06	96127.40	91558.60	88439.14	101057.42	99076.38	84690.69	85748.72	83664.41	80094.77	81248.85	79231.58	72487.08	62867.33	59678.23	52497.98	41326.27	39285.09	39515.83	47583.74	43276.10	43707.95	52111.70	45300.96	28833.62		
3432	Protein FAM50A	Q14320	42616.43	38683.68	35078.16	28064.91	26112.75	23078.08	35167.60	33052.20	32568.55	36432.28	34273.59	31244.33	35305.78	33164.83	23245.59	30882.87	29105.69	27670.79	30167.40	29014.98	26243.47	156386.97	147937.22	145185.96	106108.76	106221.29	95846.19	121482.03	117586.16	114178.59	130925.25	128473.16	128586.35		
3433	Protein FAM50B	Q9Y247	143958.79	134772.22	131085.98	78905.83	78930.21	69067.42	90909.78	84122.69	78344.60	103586.76	99626.35	89588.57	100361.80	93341.19	78813.89	76667.71	75967.38	70443.72	93255.01	83780.75	83726.90	35193.61	33662.00	30465.16	25628.30	23938.59	21930.90	42486.52	40126.12	40063.63	38668.65	37464.62	21619.08		
3434	Protein FAM53B	Q14153	3337.67	2913.22	3138.53	2903.69	3052.72	2779.49	2929.37	2942.47	2943.54	3508.79	3766.09	2961.44	3580.87	2010.32	673.37	2178.63	2631.58	1954.14	3581.81	2946.85	3220.35	2.27	1.49	0.65	0.84	1.31	0.69	4.16	2.47	0.11	4.90	4.98	0.12	x	
3435	Protein FAM76B	Q5HYJ3	5823.90	4882.94	4897.61	3490.18	3490.33	3485.99	3953.52	4044.32	3765.97	4303.61	3795.23	3593.92	4158.53	3833.45	3247.67	3812.05	2912.93	3286.69	3235.04	3940.66	3259.54	7026.55	6744.30	6056.42	8818.08	8781.25	8537.05	10821.24	9810.30	9373.76	10880.95	10140.00	6293.02		
3436	Protein FAM83C	Q9BQN1	29.95	28.36	29.41	97.73	102.96	40.18	23.77	43.16	71.93	110.87	106.53	151.03	32.42	27.13	40.06	58.21	26.42	36.79	74.66	104.16	80.07	2.27	1.49	0.65	0.84	1.31	0.69	4.16	2.47	0.11	4.90	4.98	0.12	x	
3437	Protein FAM84B	Q96KN1	47555.14	46326.91	47688.32	35849.16	33672.69	34941.28	38418.39	39942.73	42682.85	40683.40	40982.36	41304.54	40459.54	47243.26	41750.64	34942.92	33841.48	38699.41	49622.77	32528.89	28733.03	2.27	1.49	0.65	0.84	1.31	0.69	4.16	2.47	0.11	4.90	4.98	0.12	x	
3438	Protein FAM86C1	Q9NVL1	69535.57	66237.17	63875.58	42914.65	42383.90	37202.81	54864.19	53139.25	47989.65	53456.33	49149.64	47424.42	54589.43	49921.65	44128.36	44448.04	40982.79	37892.73	48748.36	45434.07	42111.95	2.27	1.49	0.65	0.84	1.31	0.69	4.16	2.47	0.11	4.90	4.98	0.12	x	
3439	Protein FAM91A1	Q658Y4	13018.45	12897.55	11750.05	8684.80	8863.60	7972.28	10683.95	10595.76	9935.02	10838.61	9909.40	9680.12	10946.00	10569.03	8664.74	9340.29	9148.52	8378.67	7715.47	8199.97	7184.68	2.27	1.49	0.65	0.84	1.31	0.69	4.16	2.47	0.11	4.90	4.98	0.12	x	
3440	Protein FAM92B	Q6ZTR7	20885.36	14871.54	11794.87	609.48	430.05	212.15	880.05	1086.18	854.33	14171.10	13261.72	10516.61	18089.53	993.19	207.83	585.09	506.61	385.22	748.27	1109.60	360.11	2.27	1.49	0.65	0.84	1.31	0.69	4.16	2.47	0.11	4.90	4.98	775.31	x	
3441	Protein FAM98A	Q8NCA5	24349.91	23393.79	22275.33	19777.01	18058.51	17337.46	19316.60	17901.32	17578.19	20976.24	20092.55	19141.42	21529.47	22591.26	18222.42	14685.06	17312.27	15721.19	19251.88	17812.82	17469.91	2.27	1.49	0.65	0.84	1.31	0.69	4.16	2.47	0.11	4.90	4.98	0.12	x	
3442	Protein FAM98B	Q52LJ0	15272.93	11668.32	15035.85	9616.15	9134.89	8198.84	10880.28	10433.19	11108.09	12224.23	12251.79	10781.08	11936.18	10514.14	10469.04	8891.32	8318.82	8149.26	8306.33	7504.78	8349.18	40909.76	45130.59	40044.36	29777.57	29285.51	25858.94	32323.95	35852.58	33532.15	34773.85	33435.94	22940.79		
3443	Protein FAM9A	Q8IZU1	609.77	645.45	538.97	799.82	898.87	686.08	688.35	770.59	846.28	902.64	971.83	1050.42	779.58	782.86	579.86	861.46	907.49	946.90	769.06	717.00	709.02	6146.01	5062.68	4862.54	3796.16	4011.03	3322.42	4859.51	4851.87	5139.23	4679.43	4612.80	4266.84		
3444	Protein FAM9C	Q8IZT9	11377.14	11129.03	9530.38	8455.65	8088.38	7019.57	8454.32	7828.66	7519.08	8599.18	7624.75	7063.79	10768.79	10154.02	7973.76	10571.32	8756.27	8645.14	8679.65	8020.98	7325.94	2.27	1.49	0.65	330.01	308.27	188.40	205.52	235.59	272.13	4.90	4.98	0.12		
3445	Protein fantom	Q68CZ1	59120.22	53028.12	48346.62	41710.16	40340.32	38330.35	48341.33	45813.41	44293.99	58745.45	55444.02	53643.36	50421.46	46792.71	35732.71	42095.51	41045.65	39210.39	40525.24	38617.87	36565.15	25914.63	25167.37	20479.05	12111.98	11601.75	8968.38	12738.47	12321.48	11882.79	15688.74	15628.34	8452.32		
3446	Protein farnesyltransferase/geranylgeranyltransferase type-1 subunit alpha	P49354	15946.56	16366.70	14043.09	40400.35	37310.10	27960.66	50306.25	42365.34	31654.41	12847.81	12498.33	11731.01	13567.22	39583.52	15922.44	51981.26	38793.30	25445.30	21479.11	18330.92	14481.26	8902.41	8285.47	8992.89	6002.51	5785.08	4878.07	6624.49	6472.97	6375.62	7075.40	6428.55	4208.09		
3447	Protein fem-1 homolog B	Q9UK73	8100.24	7567.04	6738.71	4585.45	4590.85	4270.85	6914.55	6638.61	7754.04	6101.84	5886.21	4780.33	5804.89	5394.70	5538.65	4802.48	4617.98	4177.28	4584.54	4266.62	4096.54	2.27	1.49	0.65	0.84	1.31	0.69	4.16	2.47	0.11	4.90	4.98	0.12	x	
3448	Protein Flattop	Q5VTH2	2863.00	3544.33	3098.07	1768.65	1699.84	1347.42	2665.12	2482.25	2428.52	2331.58	2216.77	1965.99	2331.38	2309.44	1654.30	1828.71	2234.99	1722.74	1974.09	1872.54	1669.00	2.27	1.49	0.65	0.84	1.31	0.69	4.16	2.47	0.11	4.90	4.98	0.12	x	
3449	Protein flightless-1 homolog	Q13045	6902.04	7100.53	7447.65	4714.58	4887.40	4231.60	5489.13	5524.05	5176.79	6230.22	5758.34	5693.41	5979.23	5820.16	4776.65	4706.14	4653.48	4495.17	5246.50	4839.46	4657.63	91372.27	82282.30	78336.49	60282.77	58326.30	51396.78	74861.87	74535.21	73371.23	69359.62	68079.69	50105.38		
3450	Protein furry homolog	Q5TBA9	106883.44	99504.44	92172.57	87995.61	82076.18	77689.92	120921.32	125931.47	114961.47	94895.26	91595.13	85872.69	96718.32	90708.16	80197.23	88075.17	80813.87	78250.80	79621.95	77641.87	72216.54	2.27	1.49	0.65	0.84	1.31	0.69	4.16	2.47	0.11	4.90	4.98	0.12	x	
3451	Protein HEXIM1	O94992	9999.12	10245.00	9169.25	7166.66	5883.36	6214.67	8012.33	7563.23	7003.88	8057.87	7990.46	6763.42	8960.79	8375.80	6278.81	7576.40	6904.40	6791.49	6751.17	6135.35	5825.83	34117.49	27966.82	27634.28	16379.91	14668.21	12814.78	19207.17	18316.45	16840.97	18143.84	17388.89	11857.21		
3452	Protein HGH1 homolog	Q9BTY7	12700.87	12031.06	11607.93	9368.61	9245.11	8330.29	10403.22	8733.34	8265.35	10331.29	9807.31	9653.51	9764.96	9580.46	8257.73	9775.09	8278.71	8518.48	7833.93	7770.11	7531.14	2.27	1.49	0.65	0.84	1.31	0.69	4.16	2.47	0.11	4.90	4.98	0.12	x	
3453	Protein HID1	Q8IV36	59681.04	57386.60	54968.83	40347.49	39185.99	37554.74	43459.46	43452.67	41006.53	48553.23	47750.41	45391.77	50418.24	50228.99	49842.58	42818.39	40819.43	39197.19	37159.90	35804.53	33926.09	2.27	1.49	0.65	0.84	1.31	0.69	4.16	2.47	0.11	4.90	4.98	0.12	x	
3454	Protein hinderin	Q86T90	994.95	732.16	508.18	275.83	4109.44	3500.49	7295.42	953.14	652.86	733.91	634.52	499.59	784.47	802.66	2423.95	4420.31	4274.50	4316.56	81.89	99.73	87.57	2.27	1.49	0.65	0.84	1.31	0.69	4.16	2.47	0.11	4.90	4.98	0.12	x	
3455	Protein HIRA	P54198	56645.31	54572.73	46934.71	36149.94	35697.77	30614.64	37727.75	35132.25	31548.53	45875.53	42853.15	41027.90	45648.95	44727.57	25178.21	36558.01	36108.63	33858.77	44135.29	42595.52	40372.02	22250.94	20380.29	18371.44	13757.18	13049.91	10078.33	17185.20	15302.30	14538.32	17097.85	16150.63	9846.50		
3456	Protein Hook homolog 1	Q9UJC3	3525.95	3550.51	3414.33	2852.21	2377.62	2654.61	2976.27	2452.92	2475.30	3326.99	2509.93	2753.38	3046.34	2740.03	2632.70	3039.38	2609.09	2677.38	2887.08	2566.41	2555.80	4693.68	4171.68	3852.78	3765.36	3726.90	3326.39	4010.94	4114.44	4030.70	4247.54	4161.19	2829.52		
3457	Protein ITPRID1	Q6ZRS4	1192.35	1038.04	1001.08	858.29	938.74	736.29	884.69	722.78	663.20	1157.72	1104.61	1080.54	809.90	825.22	581.33	904.97	940.22	888.14	787.18	742.74	641.50	2.27	1.49	0.65	0.84	1.31	0.69	4.16	2.47	0.11	4.90	4.98	0.12	x	
3458	Protein IWS1 homolog	Q96ST2	4113.69	4568.46	3804.98	2899.57	2537.84	2644.96	3271.29	3009.28	2968.72	3058.66	2651.88	2595.15	3194.33	3434.57	2990.05	2633.07	2148.16	2203.10	3162.74	2910.41	2801.90	5991.36	5426.71	5490.81	4626.21	4710.17	4949.71	5370.05	4952.50	5069.38	5691.98	5715.93	4635.36		
3459	Protein jagunal homolog 1	Q8N5M9	40357.74	45519.97	42869.73	31910.50	29150.76	26919.16	35523.24	33012.66	31689.53	40433.74	39561.25	38019.12	38840.62	33623.48	18675.36	27705.01	26137.61	26946.17	32873.67	29630.36	28662.63	2.27	1.49	0.65	0.84	1.31	0.69	4.16	2.47	0.11	4.90	4.98	0.12	x	
3460	Protein Jumonji	Q92833	7637.55	7382.78	6800.75	4688.44	4490.28	4247.74	5451.35	5264.79	5245.57	6499.52	6729.73	6253.76	6174.76	5762.13	4569.98	4827.22	4389.59	4470.05	5130.28	4884.05	4506.43	1718.91	1676.14	1641.38	1822.25	1762.34	1487.16	1842.30	1770.30	1810.92	2419.39	2156.13	1521.65		
3461	Protein KIAA0100	Q14667	135311.12	135818.17	127551.62	75681.81	72321.65	70004.11	101584.11	96017.35	95933.11	105692.62	95772.30	96100.46	97006.53	92786.80	79943.55	108634.12	99623.78	101892.29	80337.03	82024.30	81182.44	2.27	1.49	0.65	0.84	1.31	0.69	4.16	2.47	0.11	4.90	4.98	0.12	x	
3462	Protein KIBRA	Q8IX03	3588.65	2713.91	1930.52	1901.12	1808.19	1207.32	2446.02	2765.12	2093.14	2555.57	2386.92	1779.98	2761.52	2519.38	527.62	1718.32	1811.91	1283.71	1816.16	1700.13	1196.50	2.27	1.49	0.65	0.84	1.31	0.69	4.16	2.47	0.11	4.90	4.98	0.12	x	
3463	Protein kinase C alpha type	P17252	0.14	2.53	0.81	1.07	2.77	0.68	0.96	0.36	0.63	0.35	0.75	0.38	2.13	0.97	0.60	4.64	1.07	0.47	2.22	0.19	0.06	23935.93	22724.38	21573.13	12749.00	12246.23	11803.72	19412.63	18912.02	18677.24	17168.43	16728.26	11360.46		x
3464	Protein kinase C and casein kinase substrate in neurons protein 1	Q9BY11	11699.74	12332.45	11389.04	11110.20	11384.26	10822.78	9322.24	8485.77	9287.76	11235.11	11112.84	9544.82	9837.90	11026.24	8385.45	9865.02	10071.89	11201.01	10025.78	8967.80	8950.56	2.27	1.49	0.65	0.84	1.31	0.69	4.16	2.47	0.11	4.90	4.98	0.12	x	
3465	Protein kinase C and casein kinase substrate in neurons protein 2	Q9UNF0	18372.30	17601.86	14609.54	10632.76	10090.30	7573.18	12554.89	11367.34	10299.18	13136.08	11576.55	10307.10	13555.26	11945.15	6589.24	10154.00	9864.02	8408.04	10780.57	9878.25	7987.13	2.27	1.49	0.65	0.84	1.31	0.69	4.16	2.47	0.11	4.90	4.98	0.12	x	
3466	Protein kinase C and casein kinase substrate in neurons protein 3	Q9UKS6	121085.46	113665.74	110947.60	87451.18	83792.44	83546.37	88304.39	87732.60	88320.11	100600.63	97240.64	101460.09	100801.26	96338.06	72266.87	79130.41	79787.43	78976.58	88737.47	85330.15	84548.40	21601.70	20148.53	17208.76	7646.55	6986.38	4895.85	9247.12	7736.70	6661.98	8056.27	9603.53	6001.66		
3467	Protein kinase C delta type	Q05655	5444.07	5292.40	4630.92	2222.70	2180.50	2211.32	2676.54	2512.92	2076.41	4346.35	4295.58	3392.71	4665.21	3660.97	3033.58	1965.58	1815.41	1924.80	2177.89	1955.83	2076.21	2.27	1.49	0.65	0.84	1.31	0.69	4.16	2.47	0.11	4.90	4.98	0.12	x	
3468	Protein kinase C gamma type	P05129	30455.82	26252.87	25826.17	17421.10	17071.50	16272.66	22678.51	19843.06	15983.50	25712.43	23717.47	22735.72	20302.67	22029.24	14407.09	17820.31	18526.24	16161.45	19047.90	18821.66	17279.93	4272.25	3837.51	3450.54	3592.68	3313.25	2882.89	4220.38	3857.94	3832.01	4225.21	4264.19	2564.74		
3469	Protein kinase C iota type	P41743	62900.06	57613.73	49848.11	46360.62	46179.61	37407.80	46905.19	42213.02	40719.60	59361.13	56868.50	48594.25	56403.86	46956.65	25885.55	42759.84	41611.27	39173.92	39196.15	42808.43	37638.19	1669.24	1766.43	1578.78	1159.82	1271.59	1169.54	1577.57	1413.80	1481.84	1761.84	1621.53	1310.82		
3470	Protein KRI1 homolog	Q8N9T8	9791.98	9673.29	10507.34	7744.19	7396.90	7217.97	8790.50	9113.11	9128.92	8716.26	8355.18	8178.49	8374.86	8308.19	6995.80	8080.29	8358.88	8121.38	7508.79	7186.91	7055.12	2.27	1.49	0.65	0.84	1.31	0.69	4.16	2.47	0.11	4.90	4.98	0.12	x	
3471	Protein lin-28 homolog B	Q6ZN17	0.14	2.53	0.81	1.07	2.77	0.68	0.96	0.36	0.63	0.35	0.75	0.38	2.13	0.97	0.60	4.64	1.07	0.47	2.22	0.19	0.06	10243.33	18844.00	10891.48	13403.40	13133.55	18829.62	7192.24	10073.23	9984.37	19513.41	14329.05	9509.47		x
3472	Protein lin-7 homolog A	O14910	24207.35	24479.34	21624.81	17207.51	17016.53	15451.71	18725.36	18203.74	17364.62	21382.13	20470.03	19106.08	18631.20	19845.98	18848.61	16450.68	15438.73	14886.59	16918.55	15428.89	14416.98	30507.10	26997.90	24949.70	20065.01	19671.05	17873.33	23082.40	21726.76	20751.71	24616.34	22395.28	17708.83		

Supplementary Table S1. Overview on the abundance of all relatively quantified 5180 proteins

			MCF-7																					MDA-MB-231													
	Protein name	UniProt	control 01	control 02	control 03	Dai IC20 01	Dai IC20 02	Dai IC20 03	Dai SC20 01	Dai SC20 02	Dai SC20 03	Gen IC20 01	Gen IC20 02	Gen IC20 03	Gen SC20 01	Gen SC20 02	Gen SC20 03	SSE IC20 01	SSE IC20 02	SSE IC20 03	SSE SC20 01	SSE SC20 02	SSE SC20 03	control 01	control 02	control 03	Dai IC20 01	Dai IC20 02	Dai IC20 03	Gen IC20 01	Gen IC20 02	Gen IC20 03	SSE IC20 01	SSE IC20 02	SSE IC20 03	unique to MCF-7	unique to MDA-MB-231
3473	Protein lin-9 homolog	Q5TKA1	117.21	96.46	118.56	105.43	106.99	149.65	125.37	98.93	107.60	172.44	235.95	179.24	98.39	94.83	71.08	128.10	150.73	122.16	80.32	71.98	141.64	2.27	1.49	0.65	0.84	1.31	0.69	4.16	2.47	0.11	4.90	4.98	0.12	x	
3474	Protein LLP homolog	Q9BRT6	33128.70	29638.88	26001.04	16912.85	15859.57	13867.01	22991.61	20402.21	19643.46	21951.72	20377.94	17654.32	22295.24	21069.70	15002.39	18492.26	16632.23	15557.10	19279.98	17925.15	15899.91	2.27	1.49	0.65	0.84	1.31	0.69	4.16	2.47	0.11	4.90	4.98	0.12	x	
3475	Protein LSM14 homolog A	Q8ND56	79587.35	77585.96	72967.21	49797.19	47052.42	44879.72	52076.15	50427.38	46396.38	70195.76	67272.97	63200.15	74439.88	50114.42	42176.72	45295.59	44254.62	43266.20	57757.35	52878.27	48375.22	191660.79	176134.73	160945.17	113644.88	116440.86	96218.80	125031.68	112093.13	121122.74	142576.47	135977.90	85133.05		
3476	Protein LTV1 homolog	Q96GA3	1541.38	1572.70	1531.32	1195.70	1229.33	1048.75	1157.50	1076.98	980.86	1344.62	1093.09	1159.49	1145.96	1011.74	1073.80	894.39	823.93	881.39	1037.97	923.15	968.50	1054.66	922.75	919.17	715.06	698.58	644.30	650.34	567.38	592.61	852.44	806.59	618.11		
3477	Protein LYRIC	Q86UE4	101705.89	94941.36	87607.56	72345.23	70727.13	66036.23	80820.24	78686.06	72686.82	97496.62	96693.56	92431.88	85570.47	79784.50	68159.68	73446.88	70165.81	67645.63	76067.54	74186.71	72067.58	13624.30	10502.37	14257.56	13886.18	16643.21	14199.57	17032.85	20094.03	18753.95	12907.85	12179.73	6758.70		
3478	Protein mago nashi homolog	P61326	37742.60	39118.20	33390.32	31340.81	29076.77	25921.20	34797.50	32900.21	29317.33	34374.15	32826.89	29626.43	36170.70	32693.88	22491.52	27873.30	26746.98	23435.79	34954.19	33378.11	30808.12	47798.54	43582.77	39273.07	35120.14	35110.50	23906.42	38789.01	34938.22	30125.20	48329.69	45503.73	24882.88		
3479	Protein MAK16 homolog	Q9BXY0	5573.19	5191.08	4847.40	3790.47	3637.36	3342.00	5568.71	4870.47	5064.79	4668.30	4563.12	4390.53	5274.09	4801.69	3779.96	4287.17	4105.80	3690.59	3872.32	3705.30	3493.78	14683.54	13521.05	12212.10	8552.91	8269.22	5136.61	10447.74	9588.53	7889.68	10221.30	10397.68	4537.41		
3480	Protein max	P61244	0.14	2.53	0.81	1.07	2.77	0.68	0.96	0.36	0.63	0.35	0.75	0.38	2.13	0.97	0.60	4.64	1.07	0.47	2.22	0.19	0.06	133.20	145.27	133.31	83.58	86.11	97.78	131.86	113.35	110.77	163.43	158.26	2.89		x
3481	Protein MB21D2	Q8IYB1	0.14	2.53	0.81	1.07	2.77	0.68	0.96	0.36	0.63	0.35	0.75	0.38	2.13	0.97	0.60	4.64	1.07	0.47	2.22	0.19	0.06	10980.76	10356.74	9671.84	5305.42	5571.39	4540.86	8074.94	7878.66	6762.51	7674.38	7821.95	5292.67		x
3482	Protein Mdm4	O15151	676.90	511.26	637.05	794.58	794.97	807.45	682.67	662.29	807.32	738.83	740.42	702.36	668.28	594.97	768.40	871.44	593.46	646.22	485.33	586.16	555.75	2360.90	3379.82	3401.20	1635.62	1706.62	1470.43	1531.29	1456.57	1445.15	2109.38	1389.36	1063.96		
3483	Protein MEMO1	Q9Y316	0.14	2.53	0.81	1.07	2.77	0.68	0.96	0.36	0.63	0.35	0.75	0.38	2.13	0.97	0.60	4.64	1.07	0.47	2.22	0.19	0.06	138.48	167.97	222.29	305.27	335.42	286.01	453.86	311.60	391.78	285.94	262.44	284.10		x
3484	Protein misato homolog 1	Q9BUK6	6333.80	4882.46	4626.83	2346.36	2316.57	2732.52	3478.47	3377.13	3018.39	4000.61	3453.61	2795.99	4001.56	3117.63	2178.39	2614.62	2194.48	2364.12	2373.53	2486.34	1835.99	2.27	1.49	0.65	0.84	1.31	0.69	4.16	2.47	0.11	4.90	4.98	0.12	x	
3485	Protein mono-ADP-ribosyltransferase TIPARP	Q7Z3E1	3705.65	6647.05	3788.34	5562.72	5238.27	5059.72	4224.73	5285.02	3752.89	3864.08	3433.03	5196.01	5516.27	4712.54	3377.32	3222.38	3990.20	2982.76	4917.87	3821.46	3424.83	2.27	1.49	0.65	0.84	1.31	0.69	4.16	2.47	0.11	4.90	4.98	0.12	x	
3486	Protein moonraker	Q2KHM9	22277.57	20660.17	20583.99	14155.05	14130.41	13962.74	16597.44	15711.86	15098.83	16916.83	16209.51	15279.74	16858.75	15989.46	14630.18	13866.39	13115.51	12569.97	17053.63	16472.48	16082.85	2.27	1.49	0.65	0.84	1.31	0.69	4.16	2.47	0.11	4.90	4.98	0.12	x	
3487	Protein NDRG1	Q92597	99465.35	97333.08	89876.81	60474.76	57983.61	54478.96	75369.60	71549.96	66335.37	74396.32	68880.63	67047.86	78365.57	72645.60	62542.59	60472.71	57452.71	55631.41	59477.94	60085.56	56470.83	4867.57	3914.45	5036.07	3113.99	3205.67	3061.92	3056.01	2905.79	2813.06	3532.33	3450.62	2550.26		
3488	Protein NDRG3	Q9UGV2	5448.99	3976.94	3223.95	318.42	275.95	251.70	875.93	1036.47	759.87	5653.72	4496.45	4114.20	4772.24	917.62	537.01	1115.44	938.99	961.55	2483.48	602.70	494.38	24375.56	25512.02	23788.12	13140.11	12773.48	11206.75	17828.76	16387.39	15518.77	16908.98	16198.87	0.23		
3489	Protein Niban	Q9BZQ8	0.14	2.53	0.81	1.07	2.77	0.68	0.96	0.36	0.63	0.35	0.75	0.38	2.13	0.97	0.60	4.64	1.07	0.47	2.22	0.19	0.06	1638.89	1756.12	1627.66	1583.05	1443.78	1210.20	1626.01	1623.96	1704.99	1955.49	1907.97	1128.18		x
3490	Protein NipSnap homolog 1	Q9BPW8	169603.17	163661.61	156561.36	114693.60	110719.45	106976.64	144990.11	140273.07	131663.83	144367.60	135377.91	129843.77	142904.22	134860.04	115423.01	116266.40	109647.32	104405.27	118156.77	115161.33	111688.61	38116.00	34471.20	32769.97	23217.35	21998.45	19958.26	26624.16	25251.81	23770.11	26078.48	24374.05	17568.38		
3491	Protein NipSnap homolog 2	O75323	0.14	2.53	0.81	1.07	2.77	0.68	0.96	0.36	0.63	0.35	0.75	0.38	2.13	0.97	0.60	4.64	1.07	0.47	2.22	0.19	0.06	18127.87	19717.14	16805.69	14225.12	14376.67	15035.38	12674.78	13723.62	12764.26	18884.24	16587.04	20993.36		x
3492	Protein NipSnap homolog 3A	Q9UFN0	19842.97	20457.63	16034.53	8032.82	9586.19	7706.42	14804.51	12753.86	10296.26	13434.75	12097.32	9989.11	11200.91	10638.76	7173.25	11469.61	12184.41	10954.15	12341.07	11169.44	10491.07	161.42	174.51	193.46	127.54	109.67	131.29	144.96	119.95	135.89	178.28	132.51	29.18		
3493	Protein NipSnap homolog 3B	Q9BS92	23515.48	23009.62	19465.41	13614.53	13510.04	12229.54	15435.38	14771.77	13448.74	16694.95	16432.97	15437.63	18206.94	16352.95	13884.04	14952.13	14137.19	12626.22	12351.81	11604.29	10865.48	2.27	1.49	0.65	0.84	1.31	0.69	4.16	2.47	0.11	4.90	4.98	0.12	x	
3494	Protein Njmu-R1	Q9HAS0	0.14	2.53	0.81	1.07	2.77	0.68	0.96	0.36	0.63	0.35	0.75	0.38	2.13	0.97	0.60	4.64	1.07	0.47	2.22	0.19	0.06	8031.52	7555.04	7647.93	5267.75	4658.30	4403.34	5742.63	5584.23	5750.54	7066.23	6227.94	3753.46		x
3495	Protein NOXP20	Q8IWE2	0.14	2.53	0.81	1.07	2.77	0.68	0.96	0.36	0.63	0.35	0.75	0.38	2.13	0.97	0.60	4.64	1.07	0.47	2.22	0.19	0.06	847.12	542.78	426.80	563.06	429.19	225.88	784.34	681.68	260.46	1669.65	1473.03	564.08		x
3496	Protein NRDE2 homolog	Q9H7Z3	113.53	205.91	153.01	203.31	235.19	146.20	240.94	227.98	177.15	568.08	441.33	577.56	212.63	149.61	142.32	79.39	140.03	98.92	104.28	89.59	126.42	2.27	1.49	0.65	0.84	1.31	0.69	4.16	2.47	0.11	4.90	4.98	0.12	x	
3497	Protein numb homolog	P49757	44193.25	43888.03	40116.80	31995.42	30268.03	29451.40	30116.59	30930.24	32099.20	40495.27	38379.47	37331.36	38546.71	36578.48	32897.25	30442.91	30761.09	30337.71	33397.30	33176.92	31496.12	12435.25	4212.21	4609.43	9542.11	8853.13	6916.59	9751.53	8703.06	7541.44	11575.19	11601.40	7270.64		
3498	Protein odr-4 homolog	Q5SWX8	796.28	789.74	954.73	935.34	765.79	669.53	877.78	824.28	631.42	1146.74	1152.42	927.13	1155.14	1070.56	874.69	752.39	829.19	785.50	646.54	598.38	642.08	2.27	1.49	0.65	0.84	1.31	0.69	4.16	2.47	0.11	4.90	4.98	0.12	x	
3499	Protein O-GlcNAcase	O60502	12.79	7.26	19.54	17.39	453.28	344.40	549.47	270.38	110.13	25.73	1.50	11.94	16.91	1189.41	167.45	419.85	5.90	361.03	624.94	80.02	243.26	2.27	1.49	0.65	0.84	1.31	0.69	4.16	2.47	0.11	4.90	4.98	0.12	x	
3500	Protein O-glucosyltransferase 2	Q6UW63	1397.71	737.56	922.55	998.27	927.21	792.58	1041.47	929.26	710.60	1123.40	1001.25	803.56	969.21	1023.82	594.82	826.15	870.45	550.63	1119.59	1045.57	890.91	2.27	1.49	0.65	0.84	1.31	0.69	4.16	2.47	0.11	4.90	4.98	0.12	x	
3501	Protein O-glucosyltransferase 3	Q7Z4H8	0.14	2.53	0.81	1.07	2.77	0.68	0.96	0.36	0.63	0.35	0.75	0.38	2.13	0.97	0.60	4.64	1.07	0.47	2.22	0.19	0.06	7215.23	4812.65	5164.34	991.43	971.12	872.25	628.60	776.06	718.53	984.36	823.96	411.68		x
3502	Protein O-mannosyl-transferase TMTC3	Q6ZXV5	0.14	2.53	0.81	1.07	2.77	0.68	0.96	0.36	0.63	0.35	0.75	0.38	2.13	0.97	0.60	4.64	1.07	0.47	2.22	0.19	0.06	14354.96	13578.19	12255.46	8843.54	8720.48	8352.82	9388.93	9310.54	8869.73	9395.11	8965.84	6729.80		x
3503	Protein OS-9	Q13438	68888.59	58165.00	50075.96	42141.08	38463.59	34236.53	60001.25	56959.12	55328.61	50751.83	48062.87	40107.70	49689.90	45752.42	30887.68	51601.25	48750.00	44206.78	39524.24	36909.46	34781.09	21458.49	20909.78	18717.97	16851.17	15652.14	12154.05	19172.75	18494.84	17189.29	21352.34	20157.57	12657.83		
3504	Protein PAT1 homolog 1	Q86TB9	2566.84	2799.64	2435.39	2139.85	2123.22	2018.87	1896.26	1938.47	2183.30	2327.59	2221.74	1961.15	2775.35	2232.38	2132.55	1952.14	1865.36	1852.59	1941.01	1951.50	2008.13	18635.38	16337.54	14532.02	10203.19	9957.29	6732.32	11656.40	10709.88	8763.70	14676.29	12952.77	5825.92		
3505	Protein PAXX	Q9BUH6	4731.99	4784.86	4823.79	3047.41	2833.17	2608.55	4102.99	4530.03	2646.75	3874.13	4031.98	3749.53	3902.65	3870.30	2553.80	3421.00	3205.74	3031.75	2692.86	2048.38	2631.91	2.27	1.49	0.65	0.84	1.31	0.69	4.16	2.47	0.11	4.90	4.98	0.12	x	
3506	Protein PBDC1	Q9BVG4	48975.08	48967.00	46587.59	36322.60	33373.19	32055.74	47338.79	45907.10	42425.86	42360.10	41785.38	38605.06	40756.66	43167.74	36481.43	37176.55	36213.13	32174.81	34560.11	32149.01	30495.16	110338.05	103756.48	97347.96	69675.76	67519.17	62836.52	83056.42	81274.53	79965.98	82774.13	78971.30	55365.36		
3507	Protein pelota homolog	Q9BRX2	1362.74	1240.17	1029.85	1116.84	1151.49	1049.18	1175.17	1155.60	1051.70	1412.20	1376.29	1363.84	1244.69	832.98	728.58	738.22	872.80	771.57	1170.52	1038.64	882.56	9179.75	6632.38	5473.67	6718.42	6346.64	4375.15	9293.47	7812.27	6516.90	10385.87	9214.10	4852.11		
3508	Protein phosphatase 1 regulatory subunit 12A	O14974	128570.37	120537.95	115434.20	86270.53	83698.29	80363.69	99061.67	95535.31	92838.78	110975.33	109189.92	104539.44	102528.96	102958.25	89535.79	82896.06	78486.92	74247.56	87092.32	80242.81	80293.41	16116.95	16058.62	14912.40	9985.61	10081.41	9617.90	13356.54	13537.82	12657.53	12880.98	12547.21	6678.45		
3509	Protein phosphatase 1 regulatory subunit 14B	Q96C90	76159.64	75489.50	66969.39	39925.54	40993.99	35854.16	48076.63	46151.00	41092.56	63648.11	59249.20	56431.00	60401.72	57146.46	48586.46	39324.38	37391.67	34401.22	46083.69	44378.22	39594.74	15962.89	14592.75	13220.85	8409.31	7614.53	6412.42	12248.30	11310.19	10801.43	12227.86	11319.94	7322.07		
3510	Protein phosphatase 1 regulatory subunit 26	Q5T8A7	5031.76	4814.43	4879.22	4605.42	4515.10	4254.86	4359.75	4110.42	3952.48	4648.75	4655.51	4260.32	4745.97	4476.44	3840.02	4007.83	3932.23	3668.53	3810.92	3505.29	3578.16	2.27	1.49	0.65	0.84	1.31	0.69	4.16	2.47	0.11	4.90	4.98	0.12	x	
3511	Protein phosphatase 1 regulatory subunit 3C	Q9UQK1	5947.33	5443.69	5411.89	3692.65	3494.52	3501.61	3740.28	3647.67	3775.16	5390.76	4799.11	4866.83	5824.19	5672.28	4666.77	3390.28	3475.48	3273.39	3290.41	3120.03	3144.43	2.27	1.49	0.65	0.84	1.31	0.69	4.16	2.47	0.11	4.90	4.98	0.12	x	
3512	Protein phosphatase 1 regulatory subunit 7	Q15435	94101.45	88245.63	79427.46	57069.67	54697.34	48900.49	72452.77	70131.26	64504.49	79732.69	75979.08	68676.15	74848.88	65850.00	50736.33	62429.01	59415.46	55438.01	57850.01	57351.68	46882.77	21095.02	20433.57	20859.03	13786.68	14227.30	12488.33	19436.77	17758.10	17214.33	18436.24	16542.63	11758.74		
3513	Protein phosphatase 1A	P35813	47885.46	48166.15	45806.93	32210.94	31443.96	30316.67	41499.32	40737.72	38302.66	44817.09	42846.90	39123.19	41423.88	39712.19	31629.39	32149.21	31682.90	31568.68	34458.91	31574.09	29527.85	2.27	1.49	0.65	0.84	1.31	0.69	4.16	2.47	0.11	4.90	4.98	0.12	x	
3514	Protein phosphatase 1B	O75688	22981.50	18804.15	18176.14	11918.17	12198.47	9810.20	18073.42	16971.34	13240.72	13319.61	12962.71	12977.21	17356.62	15668.86	14673.30	12738.36	11469.89	10205.02	15354.70	14229.74	13764.52	2.27	1.49	0.65	0.84	1.31	0.69	4.16	2.47	0.11	4.90	4.98	0.12	x	
3515	Protein phosphatase 1F	P49593	0.14	2.53	0.81	1.07	2.77	0.68	0.96	0.36	0.63	0.35	0.75	0.38	2.13	0.97	0.60	4.64	1.07	0.47	2.22	0.19	0.06	4163.17	3916.47	3835.60	3934.77	3796.24	4062.13	6128.34	5550.06	5649.73	4629.08	4527.89	3058.54		x
3516	Protein phosphatase 1G	O15355	290302.05	280758.07	251806.87	185378.12	181095.01	171923.25	214188.08	203669.40	190582.11	225186.04	210336.41	208604.01	237416.61	221870.34	182119.97	186959.52	171624.23	168679.66	204985.07	187869.67	180780.49	84356.19	87773.65	78524.58	65780.03	64997.70	61350.43	66950.49	67197.55	65648.24	78131.03	77423.91	45246.66		
3517	Protein phosphatase inhibitor 2 family member B	Q6NXS1	42975.55	32848.69	22791.39	10131.28	9890.87	8068.37	14838.26	6878.87	6385.67	38433.18	33746.33	25871.43	43267.15	12721.85	6136.13	10378.73	8810.29	8054.73	8030.27	6891.63	7095.80	2.27	1.49	0.65	0.84	1.31	0.69	4.16	2.47	0.11	4.90	4.98	0.12	x	
3518	Protein phosphatase inhibitor 2 family member C	O14990	4391.84	4593.13	3835.60	2554.40	2694.33	1693.61	3204.53	2767.08	2820.71	3378.33	3146.12	2969.80	4215.45	4098.05	2641.05	3379.36	2888.25	2530.66	3671.14	3812.29	3520.14	1394.74	1410.33	1082.61	941.21	892.98	981.32	938.17	914.82	928.65	4.90	4.98	0.12		
3519	Protein phosphatase inhibitor 2	P41236	8038.78	6595.93	5620.56	3912.42	3280.75	2621.51	5405.34	5163.05	4454.08	5870.23	5879.10	4168.39	4680.29	3956.48	1644.94	5326.37	5210.37	4448.42	4445.89	3942.73	3233.57	2883.69	3416.58	2533.28	2351.91	1620.09	1504.48	3026.91	2993.03	2642.13	2588.12	2555.25	1679.73		
3520	Protein phosphatase methylesterase 1	Q9Y570	77214.65	69723.29	61090.13	56617.24	39373.84	36269.77	46693.51	45883.14	45144.13	69461.51	64973.52	56090.80	71495.17	67932.66	43669.86	39328.44	36297.48	35734.24	41428.24	38043.73	39329.82	364268.23	336547.87	318482.69	214287.67	208455.58	182457.34	287822.42	273380.38	261887.21	285435.92	270136.81	196920.98		
3521	Protein piccolo	Q9Y6V0	115575.23	116547.65	111454.63	63360.48	61554.74	58567.71	68107.27	67477.61	62082.05	95843.90	91436.32	85375.19	78525.53	71492.80	70392.00	86203.50	83169.92	78148.68	87882.48	79073.66	76969.76	43151.10	39426.44	38360.14	18858.85	18492.16	15962.28	24719.52	23182.50	22316.28	22828.76	22385.89	15373.16		
3522	Protein PMS2CL	Q68D20	49217.90	46785.76	41491.92	33770.58	31546.38	30037.73	35053.85	32697.26	31303.87	34345.92	32481.84	27582.35	33721.81	32436.49	29289.46	30630.59	29416.32	29554.18	29458.28	27513.62	26181.16	2.27	1.49	0.65	0.84	1.31	0.69	4.16	2.47	0.11	4.90	4.98	0.12	x	
3523	Protein PPP4R3C	Q6ZMV5	17648.72	17741.49	14467.67	36209.93	42654.77	29623.71	44787.26	49957.55	45262.67	27455.79	23675.04	20198.75	28198.44	21247.41	11456.63	46739.18	47777.16	35877.24	11831.17	10068.26	8304.62	2.27	1.49	0.65	0.84	1.31	0.69	4.16	2.47	0.11	4.90	4.98	0.12	x	
3524	Protein PRRC2A	P48634	22466.38	16767.98	17701.64	14303.29	13909.37	13911.83	16970.41	14457.29	12159.00	19268.49	18928.12	16936.94	15423.41	18207.09	14906.99	12441.70	14260.93	12457.32	17812.25	17312.76	12767.13	2.27	1.49	0.65	0.84	1.31	0.69	4.16	2.47	0.11	4.90	4.98	0.12	x	
3525	Protein PRRC2B	Q5JSZ5	28691.74	25597.47	23000.67	16392.91	16079.57	12593.85	20262.38	18182.28	16809.45	23037.53	21916.64	17402.36	23496.46	19899.95	11568.20	17244.96	16517.76	15351.11	20482.68	18647.98	15831.55	2.27	1.49	0.65	0.84	1.31	0.69	4.16	2.47	0.11	4.90	4.98	0.12	x	
3526	Protein PRRC2C	Q9Y520	0.14	2.53	0.81	1.07	2.77	0.68	0.96	0.36	0.63	0.35	0.75	0.38	2.13	0.97	0.60	4.64	1.07	0.47	2.22	0.19	0.06	51110.46	43656.79	47739.22	28374.70	28503.49	26277.80	33804.49	31225.16	28206.13	41125.72	38772.52	24392.41		x
3527	Protein prune homolog 2	Q8WUY3	308.99	183.62	170.97	279.15	256.72	268.12	191.96	186.07	183.75	310.70	258.05	297.33	310.10	347.72	369.80	149.32	173.93	175.57	290.82	196.29	262.93	6854.50	5989.33	5645.39	3952.88	3764.44	2711.13	6280.40	5634.85	4464.94	5878.63	5589.74	2575.70		
3528	Protein quaking	Q96PU8	0.14	2.53	0.81	1.07	2.77	0.68	0.96	0.36	0.63	0.35	0.75	0.38	2.13	0.97	0.60	4.64	1.07	0.47	2.22	0.19	0.06	21350.76	20269.14	18816.38	13922.20	13379.29	13021.20	15788.60	16275.27	15918.94	12440.69	12012.39	8402.94		x
3529	Protein RCC2	Q9P258	152745.80	146242.96	139596.54	86338.57	81478.14	76316.00	144000.23	133564.97	126770.25	119761.95	112810.49	108987.98	117825.13	104939.06	86525.37	92847.57	84764.53	81323.30	99878.16	88491.58	82233.89	55062.36	51894.86	49478.14	24691.27	22571.10	19209.14	28762.79	26711.38	26864.39	25038.59	24425.72	16585.19		
3530	Protein Red	Q13123	0.14	2.53	0.81	1.07	2.77	0.68	0.96	0.36	0.63	0.35	0.75	0.38	2.13	0.97	0.60	4.64	1.07	0.47	2.22	0.19	0.06	15970.85	14895.68	14127.59	12980.21	12310.21	11947.66	13200.43	13335.53	13518.74	15613.40	13979.88	8093.35		x
3531	Protein regulator of cytokinesis 1	O43663	257.21	240.08	211.41	406.90	465.67	368.46	470.45	446.16	404.72	562.11	523.45	557.14	350.02	316.96	245.07	410.52	443.89	335.62	394.70	393.72	356.74	2.27	1.49	0.65	0.84	1.31	0.69	4.16	2.47	0.11	4.90	4.98	0.12	x	
3532	Protein RER1	O15258	23814.31	22536.45	21753.76	14448.03	14168.60	13508.50	20859.66	20439.89	19844.63	19264.89	18920.67	18043.78	18716.94	18086.48	15570.33	15923.91	15526.46	14841.59	16359.29	14800.85	14485.37	7731.75	7001.00	6585.25	6050.23	5593.91	5238.26	7284.66	7299.13	6647.46	7269.45	7024.72	3661.02		
3533	Protein RFT1 homolog	Q96AA3	0.14	2.53	0.81	1.07	2.77	0.68	0.96	0.36	0.63	0.35	0.75	0.38	2.13	0.97	0.60	4.64	1.07	0.47	2.22	0.19	0.06	367.91	302.97	319.64	236.56	236.89	88.74	257.72	306.84	268.71	415.61	364.40	109.61		x
3534	Protein RTF2 homolog	Q9BY42	161914.40	158513.85	150610.59	132443.19	134629.61	124068.13	135175.68	127363.14	125357.75	129248.43	126283.72	113869.35	149773.16	143452.70	137660.20	115138.47	105460.86	97964.60	119820.48	126775.09	118102.32	7130.08	6911.75	6482.43	5150.71	5368.29	5100.82	6324.31	5992.79	5974.91	6688.09	6561.06	4509.28		
3535	Protein RUFY3	Q7L099	3949.30	3125.20	2750.48	510.14	526.79	281.82	892.54	1011.23	954.05	2360.74	2141.06	1812.16	3116.32	1100.39	174.26	549.95	664.63	376.41	803.04	871.66	582.13	19056.30	18116.83	15288.96	14180.85	13629.76	9054.18	25166.45	21675.17	19334.41	19144.46	19396.03	171.28		
3536	Protein S100-A10	P60903	212183.47	206714.87	188327.14	161806.68	158517.54	143349.33	167653.58	164909.13	152727.00	231215.46	219072.09	201331.64	179349.10	163006.30	133645.09	176055.94	161942.66	159782.38	151788.99	144147.78	134113.05	610927.15	582682.73	526913.70	414410.22	399798.37	349996.02	428607.02	412896.85	389260.90	539250.29	508395.39	362290.03		
3537	Protein S100-A11	P31949	1546120.65	1507785.72	1451829.22	1285612.64	1227741.93	1175368.99	1368770.63	1338504.06	1302968.45	1411146.35	1382494.48	1292783.51	1479605.99	1401132.85	1240741.39	1167635.15	1099642.95	1083277.67	1286509.72	1207867.51	1183934.27	646804.61	603437.24	572254.37	435338.02	424312.42	409154.57	462604.06	456650.82	443952.22	485908.63	479628.24	354235.92		
3538	Protein S100-A13	Q99584	370242.83	359242.89	349678.52	223965.10	208775.76	205453.02	271647.24	265749.75	264568.67	270093.69	260480.61	248679.17	289738.80	280580.81	257658.71	228456.97	212583.76	216833.91	210818.34	204245.29	198893.92	63771.95	56031.12	52636.49	34214.15	31933.84	29028.96	45708.79	41373.28	38442.30	39725.63	38361.18	27610.28		
3539	Protein S100-A14	Q9HCY8	21432.29	21038.04	19249.90	20017.87	17741.74	18029.92	22157.14	20485.06	20214.15	22604.70	22823.59	20294.46	19608.52	15819.43	13929.48	25470.70	23590.81	23940.27	20226.80	19268.15	17300.55	2.27	1.49	0.65	0.84	1.31	0.69	4.16	2.47	0.11	4.90	4.98	0.12	x	
3540	Protein S100-A16	Q96FQ6	377348.67	366382.80	362016.41	234164.87	225145.86	222678.39	246923.44	240736.14	234078.37	305960.75	285254.62	270605.76	322318.52	309454.26	244627.00	226346.49	214686.86	213756.86	228308.35	213948.16	207075.08	43727.50	45334.33	38056.92	23237.42	24211.66	22381.39	27696.39	26936.74	25769.47	28905.17	29082.95	20494.13		
3541	Protein S100-A3	P33764	116.82	60.06	19.79	8.32	5.54	7.66	31.73	11.22	11.02	49.68	71.13	21.03	2.13	0.97	0.60	4.64	1.07	0.47	14.24	5.75	18.28	2.27	1.49	0.65	0.84	1.31	0.69	4.16	2.47	0.11	4.90	4.98	0.12	x	
3542	Protein S100-A6	P06703	169743.69	162574.92	159800.20	126423.46	123956.47	120776.03	160866.06	163556.60	181172.93	187335.10	183582.00	172481.31	184813.18	152878.20	150391.87	162039.57	142771.72	145396.69	151488.22	138639.13	144044.88	487916.06	457777.16	477494.96	284408.69	293325.80	286346.43	313291.28	328638.83	309005.79	309497.08	361593.42	234938.80		
3543	Protein S100-P	P25815	42750.87	41540.90	38611.96	28515.83	28394.28	28774.44	34883.01	33660.88	34231.51	37705.09	36268.92	35468.07	35749.14	30427.01	30545.02	34330.38	31425.84	32068.03	29498.17	28661.92	26190.09	2298.43	2067.48	1829.20	1546.32	1530.27	1485.65	1692.48	1533.39	1492.28	1861.84	1822.19	1464.19		
3544	Protein SAAL1	Q96ER3	5317.95	4767.00	4621.10	3673.51	3675.55	3127.10	4018.97	3862.58	3677.09	3741.82	3802.49	3515.96	4151.12	4416.51	2902.80	3544.33	3639.27	3443.68	4411.98	3387.71	3472.42	9746.07	8497.78	8782.60	7476.71	7738.24	6518.30	8519.47	8084.78	7975.73	10293.22	10034.87	3698.42		
3545	Protein salvador homolog 1	Q9H4B6	0.14	2.53	0.81	1.07	2.77	0.68	0.96	0.36	0.63	0.35	0.75	0.38	2.13	0.97	0.60	4.64	1.07	0.47	2.22	0.19	0.06	4959.59	4700.61	4935.67	2762.03	2605.38	2410.33	2889.67	2947.44	2861.75	3014.54	2695.75	2148.05		x
3546	Protein SCAI	Q8N9R8	17687.30	16210.14	15088.84	15203.83	14576.55	13191.84	14978.55	14237.38	13645.01	16204.49	14692.45	13866.00	15072.01	14017.44	10570.53	14658.58	13281.73	13281.83	12138.71	11472.99	10258.59	2.27	1.49	0.65	0.84	1.31	0.69	4.16	2.47	0.11	4.90	4.98	0.12	x	
3547	Protein SCO1 homolog mitochondrial	O75880	2269.76	2417.62	2762.29	1375.01	972.54	577.38	356.89	306.30	140.28	1494.86	2027.67	2985.83	2057.24	119.10	76.76	160.67	408.75	1229.19	3892.67	3494.93	3089.91	20182.26	17324.11	15385.95	9463.19	9120.24	7958.93	10959.46	11425.09	10669.44	11744.90	12089.57	7443.12		
3548	Protein scribble homolog	Q14160	35351.85	33279.45	28859.45	20653.99	20831.57	17297.38	26551.31	24483.93	23176.24	27487.28	26279.88	24031.55	25175.04	23882.07	20352.41	21469.85	20093.65	19652.95	22749.58	20750.85	19793.75	210.84	223.97	243.63	184.70	188.09	166.00	118.18	161.39	176.66	174.75	187.45	46.94		
3549	Protein SEC13 homolog	P55735	24986.66	23837.46	22574.74	18301.30	16989.97	15906.98	24108.59	22641.65	21171.23	23419.89	22185.95	20101.58	23799.84	21635.19	16006.27	18114.48	16643.65	16191.21	18395.28	17498.23	16250.74	5346.48	5053.61	4715.07	2774.49	2577.02	2230.91	2659.99	2656.79	2563.87	2885.50	2667.68	2312.64		
3550	Protein sel-1 homolog 1	Q9UBV2	67096.77	62490.76	58318.73	47372.15	45093.50	42092.80	44591.73	42424.28	45219.29	59646.43	57643.04	54508.17	60119.04	51007.24	39665.81	45659.12	43130.23	43114.75	61757.29	55914.00	50721.71	2.27	1.49	0.65	0.84	1.31	0.69	4.16	2.47	0.11	4.90	4.98	0.12	x	
3551	Protein SET	Q01105	661999.50	618730.89	575559.71	454947.12	446915.31	399037.94	563590.27	547914.52	508919.19	549189.53	513778.17	472453.01	576632.98	534927.73	412015.41	443479.96	419534.86	395880.28	498772.18	473522.42	429797.16	459183.25	428754.84	397195.99	283119.75	281357.04	246657.53	362646.80	340507.24	328818.90	360594.08	344996.82	238507.40		
3552	Protein SETSIP	P0DME0	17591.03	17084.23	14679.69	8650.43	9404.34	7919.08	12112.93	11718.67	10721.70	12413.32	12049.27	11177.61	12692.49	12110.58	8581.11	10209.57	9449.29	8450.82	10090.56	9744.65	8997.59	39.73	36.52	39.26	67.71	36.69	64.74	85.59	64.03	78.40	102.74	92.86	29.44		
3553	Protein SGT1 homolog	Q9Y2Z0	121733.70	119877.25	112938.05	86489.84	82906.82	81268.52	97950.98	96139.49	89029.82	108618.96	104335.77	100230.28	94231.15	96777.91	89379.98	94778.76	87826.99	84654.76	86681.97	83505.70	81133.92	79395.44	70802.72	68044.71	53998.95	52221.48	45867.61	67096.04	63242.55	59477.02	67515.60	63452.37	45777.69		
3554	Protein Smaug homolog 1	Q9UPU9	0.14	2.53	0.81	1.07	2.77	0.68	0.96	0.36	0.63	0.35	0.75	0.38	2.13	0.97	0.60	4.64	1.07	0.47	2.22	0.19	0.06	629.74	71.39	41.64	146.77	175.44	21.21	415.93	325.55	57.81	320.75	275.99	82.49		x
3555	Protein SMG8	Q8ND04	14802.04	13394.29	13535.18	10209.61	9110.71	8415.05	10751.03	10262.85	11376.32	11576.88	11096.37	10619.12	12052.99	10868.54	9070.24	11553.36	10322.63	10591.02	12750.16	9638.43	9717.87	2.27	1.49	0.65	0.84	1.31	0.69	4.16	2.47	0.11	4.90	4.98	0.12	x	
3556	Protein SOGA1	O94964	129271.50	119037.89	102015.46	67058.19	74794.42	67393.66	87834.62	82304.63	75689.12	96555.89	89703.29	81098.07	98007.98	77313.08	58891.65	83290.00	69637.52	66879.89	68574.81	64599.12	59686.89	390756.87	342684.70	305761.59	263262.88	267349.73	243264.58	351075.01	339428.55	292394.80	413141.89	395086.22	248508.19		
3557	Protein SOGA3	Q5TF21	0.14	2.53	0.81	1.07	2.77	0.68	0.96	0.36	0.63	0.35	0.75	0.38	2.13	0.97	0.60	4.64	1.07	0.47	2.22	0.19	0.06	15061.90	14647.27	12980.75	8791.54	8423.64	7697.71	10075.68	9524.61	9824.66	7186.49	6851.19	4778.76		x
3558	Protein SOX-15	O60248	107967.66	106990.54	109413.34	81399.58	75079.35	78349.67	80043.71	77944.77	76462.95	94463.04	98555.39	90406.05	98581.72	91771.57	80619.74	79362.13	69724.81	69424.95	79031.70	74391.00	70973.46	51623.47	44176.58	40283.01	34058.74	35226.42	31260.94	46793.17	43573.01	42757.81	44113.58	45207.69	33558.93		
3559	Protein spire homolog 2	Q8WWL2	5902.49	6279.90	5668.86	5311.16	4434.93	5267.63	5135.31	5250.36	5392.02	5536.68	5324.22	4938.06	5660.90	965.58	1346.50	5321.70	2556.93	4408.20	3931.25	5113.72	4873.24	2.27	1.49	0.65	0.84	1.31	0.69	4.16	2.47	0.11	4.90	4.98	0.12	x	
3560	Protein SPT2 homolog	Q68D10	0.14	2.53	0.81	1.07	2.77	0.68	0.96	0.36	0.63	0.35	0.75	0.38	2.13	0.97	0.60	4.64	1.07	0.47	2.22	0.19	0.06	8658.43	7553.79	6347.25	6048.22	6420.71	3242.79	8639.72	8238.43	6621.79	9759.16	9328.39	4584.02		x
3561	Protein SSX4	O60224	0.14	2.53	0.81	1.07	2.77	0.68	0.96	0.36	0.63	0.35	0.75	0.38	2.13	0.97	0.60	4.64	1.07	0.47	2.22	0.19	0.06	5077.55	4500.37	4624.41	3622.55	3610.12	3704.24	3864.08	3754.70	3494.28	4369.50	4074.87	3713.03		x
3562	Protein TALPID3	Q9BVV6	6239.13	6573.08	5223.44	3305.92	3602.19	3453.47	3364.71	3384.52	3188.51	4493.15	4338.16	4041.56	3667.97	4030.73	3596.15	3542.42	3060.54	3490.16	4020.69	3837.14	3493.22	6672.41	6028.30	5128.68	4254.81	3901.64	3814.64	5771.45	4583.02	5037.77	5161.39	4297.67	3280.36		
3563	Protein TBATA	Q96M53	0.14	2.53	0.81	1.07	2.77	0.68	0.96	0.36	0.63	0.35	0.75	0.38	2.13	0.97	0.60	4.64	1.07	0.47	2.22	0.19	0.06	23402.34	22950.29	19336.70	14316.22	14228.92	12324.36	13374.27	12226.20	12196.85	14459.07	13937.65	9222.27		x
3564	Protein TESPA1	A2RU30	0.14	2.53	0.81	1.07	2.77	0.68	0.96	0.36	0.63	0.35	0.75	0.38	2.13	0.97	0.60	4.64	1.07	0.47	2.22	0.19	0.06	560.92	545.84	465.34	431.34	419.68	269.87	532.76	435.11	423.75	713.91	596.99	246.76		x
3565	Protein TFG	Q92734	100078.96	97356.29	91934.59	75239.79	71481.51	68583.99	88329.55	89247.84	85638.07	95848.24	89889.74	84516.61	91247.14	86895.14	73209.37	79562.67	73181.56	73094.86	75761.77	70011.14	65268.02	40094.37	37820.00	35659.99	32108.07	31105.11	27309.47	33073.57	32025.22	29276.07	32738.94	33007.49	23571.82		
3566	Protein TMED8	Q6PL24	0.14	2.53	0.81	1.07	2.77	0.68	0.96	0.36	0.63	0.35	0.75	0.38	2.13	0.97	0.60	4.64	1.07	0.47	2.22	0.19	0.06	305.81	271.79	318.86	185.37	167.43	52.03	193.30	132.99	140.45	376.57	283.40	47.84		x
3567	Protein TOPAZ1	Q8N9V7	4992.28	5113.22	5268.67	3322.70	3126.50	2499.13	2531.54	3046.35	2419.97	5576.46	5899.79	6721.29	5482.60	3053.27	2615.48	2830.42	2840.56	3474.95	6659.52	4854.89	3780.37	2.27	1.49	0.65	0.84	1.31	0.69	4.16	2.47	0.11	4.90	4.98	0.12	x	
3568	Protein transport protein Sec16A	O15027	77910.59	71374.36	62679.05	42834.78	42646.05	37595.40	51943.58	50093.74	47811.94	55618.56	54138.19	49244.06	56238.23	52273.75	40624.23	47947.31	42602.09	40229.99	45964.11	43072.49	39413.24	5060.30	5142.38	4227.45	3520.99	3556.07	3665.26	2690.00	3056.13	2973.60	4049.17	3731.60	595.40		
3569	Protein transport protein Sec23A	Q15436	75613.88	70405.94	68112.89	49402.33	55067.22	52408.78	58477.38	41182.04	49578.74	65094.78	60254.46	56343.71	59639.41	47065.16	35627.52	59484.34	44602.20	53085.83	52501.73	46560.07	44555.83	82764.31	79272.75	73748.70	64591.83	62318.52	56230.50	69202.51	68534.57	65460.23	75965.19	72328.26	46415.64		
3570	Protein transport protein Sec23B	Q15437	113893.03	107693.71	97955.17	74084.28	71003.08	59641.27	86241.95	79889.74	75665.64	99960.10	97809.61	85538.78	92339.69	83642.15	60493.37	73873.23	72239.45	68228.37	79429.79	76107.10	67537.46	9560.49	8583.06	7483.94	4694.13	4507.96	3694.18	6119.18	5948.30	5506.33	6088.29	5624.85	3334.37		
3571	Protein transport protein Sec24B	O95487	4909.23	4758.61	4420.84	2785.18	2404.61	2459.02	3071.34	3345.12	3202.31	3287.27	3287.19	2753.22	3608.29	3130.09	2702.75	2451.67	2642.93	2312.64	2776.07	2224.15	2625.18	2.27	1.49	0.65	0.84	1.31	0.69	4.16	2.47	0.11	4.90	4.98	0.12	x	
3572	Protein transport protein Sec24C	P53992	182043.89	169958.93	161276.69	118735.93	115020.23	107396.73	136407.99	134807.60	130476.85	154007.23	149103.12	142900.10	147971.25	138576.22	119622.76	114038.17	110692.45	104178.85	115879.01	111791.11	106416.16	17652.28	15179.17	13482.12	9875.39	9968.89	9020.53	12015.26	11781.80	10455.54	12189.96	11507.87	7635.85		
3573	Protein transport protein Sec31A	O94979	66687.20	67566.92	62325.24	40071.41	39297.77	35529.96	46352.48	46930.71	44473.70	56441.81	52663.50	49532.15	54669.18	47966.58	39820.31	44729.02	40524.18	41415.52	36972.73	38770.74	34735.36	32643.85	31654.03	27992.09	21556.70	20309.11	17017.79	26458.21	23501.98	23597.26	25503.23	24194.31	16063.34		
3574	Protein transport protein Sec61 subunit alpha isoform 1	P61619	20743.64	20346.35	19090.02	15729.69	15306.25	15255.25	17529.38	16498.59	16667.97	20949.39	19146.58	18925.00	18755.06	19219.34	14756.91	15118.76	13957.67	14486.33	17514.81	15341.03	15333.20	25128.76	24111.72	21794.03	18511.53	17291.32	16854.37	20953.53	19675.24	19581.03	20373.19	19750.37	13640.41		
3575	Protein transport protein Sec61 subunit beta	P60468	95259.14	95007.18	90761.59	82901.30	77334.39	78618.97	96992.55	93767.82	90471.87	103628.65	94345.26	93598.30	86709.35	89808.10	80625.94	80186.93	73881.19	71900.83	80801.69	78931.15	78986.32	36892.32	32375.33	31677.20	29020.90	29513.71	27092.39	27205.64	25880.56	24495.34	25626.20	25146.65	10191.00		
3576	Protein tyrosine phosphatase domain-containing protein 1	A2A3K4	17528.68	17525.94	14893.71	12626.51	13235.23	11442.87	16609.75	17005.29	14068.52	17547.87	17498.86	14975.96	16182.26	15755.99	7580.32	13726.78	13959.13	12442.63	16645.92	14310.16	13233.24	5103.75	5168.42	4500.05	3126.41	2897.64	2876.36	3362.86	3297.13	2950.44	4157.49	4639.15	4566.22		
3577	Protein unc-119 homolog A	Q13432	56316.76	51980.14	46788.64	31435.99	32377.79	27875.40	39234.68	38199.71	32560.13	41622.57	38849.24	36186.79	37302.02	35683.37	29166.66	33925.10	34235.61	30198.08	30971.98	31563.01	29842.07	2.27	1.49	0.65	0.84	1.31	0.69	4.16	2.47	0.11	4.90	4.98	0.12	x	
3578	Protein unc-13 homolog A	Q9UPW8	956.97	764.19	617.81	497.78	526.79	378.38	702.27	562.13	509.83	1221.10	938.45	1009.97	997.70	679.39	456.94	504.90	427.92	335.62	550.02	487.01	453.38	2.27	1.49	0.65	0.84	1.31	0.69	4.16	2.47	0.11	4.90	4.98	0.12	x	
3579	Protein unc-13 homolog C	Q8NB66	61688.35	59401.45	63305.87	31781.98	30707.48	28861.44	39486.97	40538.46	37415.41	42937.94	41688.76	39431.35	41497.86	42210.29	33829.04	31996.70	31395.83	30161.61	35865.48	34556.19	32667.14	17195.70	15700.36	15618.26	10960.89	10933.60	10330.39	12992.74	12324.58	11610.55	18663.72	17982.71	11591.62		
3580	Protein unc-13 homolog D	Q70J99	1526.14	1334.23	1122.65	600.53	625.43	352.60	704.04	878.36	831.25	1034.76	1085.49	954.19	1340.50	1259.74	372.83	619.67	664.17	383.53	590.59	795.80	500.24	2.27	1.49	0.65	0.84	1.31	0.69	4.16	2.47	0.11	4.90	4.98	0.12	x	
3581	Protein unc-45 homolog A	Q9H3U1	124487.32	117498.13	109102.27	85665.04	82807.56	78779.59	98267.45	95889.79	91336.62	109174.61	104914.05	99768.69	107074.02	100345.46	80595.75	88034.99	82763.42	80343.58	87153.70	81468.41	77754.49	99856.24	86523.33	82198.86	59511.67	55327.23	53417.14	70899.39	66034.93	60535.76	71653.62	71245.84	41251.50		
3582	Protein unc-79 homolog	Q9P2D8	0.14	2.53	0.81	1.07	2.77	0.68	0.96	0.36	0.63	0.35	0.75	0.38	2.13	0.97	0.60	4.64	1.07	0.47	2.22	0.19	0.06	2538.23	1233.84	2502.37	2957.43	2700.55	2562.26	2349.75	2434.07	2447.04	3163.26	3872.15	2317.45		x
3583	Protein unc-80 homolog	Q8N2C7	822.36	739.84	837.04	615.30	624.17	534.44	765.95	680.60	788.50	931.37	866.10	797.20	1003.51	673.96	568.56	723.25	599.70	567.81	1037.00	884.54	775.34	2.27	1.49	0.65	21.85	45.82	41.00	4.16	2.47	0.11	81.43	51.45	46.01		
3584	Protein Wnt-11	O96014	6285.39	6552.29	5879.67	6226.86	6232.29	5753.55	5754.23	5891.59	5259.07	8854.91	7978.81	7765.05	7342.50	7279.57	6240.82	5682.49	5125.51	4769.91	6455.59	5970.97	6173.34	2.27	1.49	0.65	0.84	1.31	0.69	4.16	2.47	0.11	4.90	4.98	0.12	x	
3585	Protein Wnt-5b	Q9H1J7	0.14	2.53	0.81	1.07	2.77	0.68	0.96	0.36	0.63	0.35	0.75	0.38	2.13	0.97	0.60	4.64	1.07	0.47	2.22	0.19	0.06	1183.69	1113.59	1058.85	871.28	857.60	972.42	925.09	1019.02	1016.77	991.00	628.94	637.76		x
3586	Protein XRP2	O75695	0.14	2.53	0.81	1.07	2.77	0.68	0.96	0.36	0.63	0.35	0.75	0.38	2.13	0.97	0.60	4.64	1.07	0.47	2.22	0.19	0.06	2835.89	2317.40	1845.82	1532.41	1595.28	1273.81	2312.90	2034.41	1709.57	2055.59	2258.76	1578.31		x
3587	Protein yippee-like 5	P62699	0.14	2.53	0.81	1.07	2.77	0.68	0.96	0.36	0.63	0.35	0.75	0.38	2.13	0.97	0.60	4.64	1.07	0.47	2.22	0.19	0.06	1905.26	1900.48	1721.15	1438.42	1522.45	1292.09	1544.28	1490.90	1520.10	1804.18	1822.42	1145.83		x
3588	Protein zer-1 homolog	Q7Z7L7	0.14	2.53	0.81	1.07	2.77	0.68	0.96	0.36	0.63	0.35	0.75	0.38	2.13	0.97	0.60	4.64	1.07	0.47	2.22	0.19	0.06	6350.48	5330.21	2896.09	3292.36	3273.27	1625.04	3746.00	3569.72	2478.09	6390.96	5685.46	2570.75		x
3589	Protein/nucleic acid deglycase DJ-1	Q99497	392623.25	391962.38	363826.53	301855.93	284969.05	272253.82	381799.83	369352.25	362467.68	361414.73	344978.70	330562.66	335615.69	331850.95	275355.35	302521.83	283630.40	271574.58	301932.54	283630.44	270133.08	466611.52	430848.79	405331.10	244718.22	239879.07	210520.13	259861.65	247906.16	238703.33	275051.48	265394.73	186451.27		
3590	Protein-arginine deiminase type-2	Q9Y2J8	0.14	2.53	0.81	1.07	2.77	0.68	0.96	0.36	0.63	0.35	0.75	0.38	2.13	0.97	0.60	4.64	1.07	0.47	2.22	0.19	0.06	291495.40	267530.22	246089.29	122919.06	118495.72	104880.81	183074.84	173725.26	160017.25	168007.13	157587.12	108489.01		x
3591	Protein-arginine deiminase type-4	Q9UM07	2194.71	2231.51	2213.46	1006.07	972.77	1061.91	1490.77	1445.63	1485.59	1609.74	1357.99	1259.14	1457.73	1734.61	1065.90	1109.85	1060.56	1069.30	1071.76	1205.91	1141.05	2.27	1.49	0.65	0.84	1.31	0.69	4.16	2.47	0.11	4.90	4.98	0.12	x	
3592	Proteinase-activated receptor 1	P25116	0.14	2.53	0.81	1.07	2.77	0.68	0.96	0.36	0.63	0.35	0.75	0.38	2.13	0.97	0.60	4.64	1.07	0.47	2.22	0.19	0.06	905.40	712.31	593.13	736.28	681.36	700.43	1001.32	823.01	792.73	677.52	620.48	585.88		x
3593	Protein-glutamate O-methyltransferase	Q9H993	195034.28	181853.43	164848.06	74569.57	72074.82	62798.06	95741.50	95339.05	88044.70	109870.20	104957.95	92476.67	141288.13	129285.90	100958.69	72638.14	70669.67	67385.61	90489.86	83503.41	78610.47	2.27	1.49	0.65	0.84	1.31	0.69	4.16	2.47	0.11	4.90	4.98	0.12	x	
3594	Protein-glutamine gamma-glutamyltransferase 2	P21980	0.14	2.53	0.81	1.07	2.77	0.68	0.96	0.36	0.63	0.35	0.75	0.38	2.13	0.97	0.60	4.64	1.07	0.47	2.22	0.19	0.06	1964298.94	1824551.88	1693580.13	1171343.31	1143386.77	1048803.27	1451960.55	1379681.55	1324676.46	1405791.75	1363355.68	958068.34		x
3595	Protein-glutamine gamma-glutamyltransferase 4	P49221	0.14	2.53	0.81	1.07	2.77	0.68	0.96	0.36	0.63	0.35	0.75	0.38	2.13	0.97	0.60	4.64	1.07	0.47	2.22	0.19	0.06	233.25	165.86	176.23	354.06	252.51	80.86	416.97	366.75	277.56	525.06	472.15	139.62		x
3596	Protein-L-isoaspartate O-methyltransferase domain-containing protein 2	Q9NV79	605.31	523.84	501.31	388.33	302.67	226.48	551.69	456.67	419.92	573.14	580.39	522.84	482.95	620.02	120.59	459.18	514.71	305.07	389.54	276.74	314.05	2.27	1.49	0.65	0.84	1.31	0.69	4.16	2.47	0.11	4.90	4.98	0.12	x	
3597	Protein-L-isoaspartate(D-aspartate) O-methyltransferase	P22061	148482.38	142379.17	124128.04	78732.17	76870.37	67706.54	99712.35	95899.76	90780.12	102576.21	96153.73	86287.19	105688.74	100488.31	68649.48	84546.63	81401.58	79013.17	79217.76	76624.15	68508.65	6312.06	6528.20	6394.03	2675.41	2272.33	1600.41	4558.95	4832.98	3786.72	4627.30	4380.08	2068.28		
3598	Protein-tyrosine kinase 6	Q13882	129516.04	108543.77	95852.39	116330.82	110766.81	96883.61	102977.12	95348.58	84943.39	115422.10	112938.59	103138.14	117748.33	116797.28	66964.70	98369.93	97589.23	93475.76	114128.58	110670.30	91920.35	2.27	1.49	0.65	0.84	1.31	0.69	4.16	2.47	0.11	4.90	4.98	0.12	x	
3599	Proteolipid protein 2	Q04941	7030.69	6843.60	5812.85	4773.12	4269.66	3654.68	7285.75	6977.75	6122.31	6598.30	6448.84	5301.33	6741.72	6648.04	4193.64	5735.00	5363.70	4759.46	5986.27	5439.69	4588.34	118457.78	111148.58	113670.79	67993.52	67410.32	64669.92	57753.58	56008.79	54709.21	51089.26	49819.57	38675.74		
3600	Prothymosin alpha	P06454	308931.44	301405.25	295779.30	214263.29	205412.46	197700.50	241128.14	232657.65	232248.53	216566.40	211899.45	201292.69	330627.66	307474.43	295078.37	214602.10	198211.90	190935.15	255209.12	238258.53	241362.39	166050.18	147096.95	145924.88	121680.62	127498.78	116792.83	127964.35	123978.12	120659.85	146168.82	150332.16	108358.40		
3601	Protocadherin alpha-1	Q9Y5I3	0.14	2.53	0.81	1.07	2.77	0.68	0.96	0.36	0.63	0.35	0.75	0.38	2.13	0.97	0.60	4.64	1.07	0.47	2.22	0.19	0.06	7058.06	6305.02	6468.22	5179.66	4954.65	4342.99	6125.29	6073.88	5191.24	5454.18	4965.35	3981.81		x
3602	Protocadherin alpha-10	Q9Y5I2	0.14	2.53	0.81	1.07	2.77	0.68	0.96	0.36	0.63	0.35	0.75	0.38	2.13	0.97	0.60	4.64	1.07	0.47	2.22	0.19	0.06	2244.44	6.10	25.36	458.95	338.54	182.51	6850.33	4979.61	379.96	2929.19	2259.28	880.36		x
3603	Protocadherin alpha-9	Q9Y5H5	0.14	2.53	0.81	1.07	2.77	0.68	0.96	0.36	0.63	0.35	0.75	0.38	2.13	0.97	0.60	4.64	1.07	0.47	2.22	0.19	0.06	15987.41	15414.59	15789.62	9912.39	9913.83	9525.85	9364.78	9388.48	9412.28	7778.02	5420.49	3755.64		x
3604	Protocadherin beta-1	Q9Y5F3	0.14	2.53	0.81	1.07	2.77	0.68	0.96	0.36	0.63	0.35	0.75	0.38	2.13	0.97	0.60	4.64	1.07	0.47	2.22	0.19	0.06	749.55	576.14	488.70	520.05	527.28	248.39	2407.08	2184.12	1684.31	1515.78	1264.76	604.60		x
3605	Protocadherin beta-14	Q9Y5E9	33995.92	30982.81	25137.65	20128.74	19027.37	15520.64	24318.83	22549.30	21377.67	25745.12	24264.41	20387.60	25737.57	22227.96	11261.31	21207.81	19196.47	16864.42	20349.92	19024.07	16045.13	2.27	1.49	0.65	0.84	1.31	0.69	4.16	2.47	0.11	4.90	4.98	0.12	x	
3606	Protocadherin beta-6	Q9Y5E3	934.23	1191.14	797.01	487.06	511.61	652.89	754.02	606.46	501.73	730.79	891.00	1024.58	563.01	719.69	577.60	571.93	493.04	640.85	861.10	743.08	570.50	2.27	1.49	0.65	0.84	1.31	0.69	4.16	2.47	0.11	4.90	4.98	0.12	x	
3607	Protocadherin beta-8	Q9UN66	912.36	955.36	780.42	383.17	378.18	300.81	584.80	570.09	537.06	691.69	604.79	586.94	799.33	887.63	400.16	423.34	419.29	335.71	490.48	423.81	416.79	2.27	1.49	0.65	0.84	1.31	0.69	4.16	2.47	0.11	4.90	4.98	0.12	x	
3608	Protocadherin Fat 2	Q9NYQ8	8755.00	8097.27	7194.63	4572.73	4115.07	3841.00	6587.53	6330.76	6612.78	7305.95	6801.27	6502.97	6218.89	5659.22	4397.54	6578.67	6378.57	6007.27	5235.63	4992.26	5001.98	32307.22	33906.44	30996.15	25769.82	26454.08	22839.87	42442.90	38639.46	41652.67	29325.81	28374.98	19594.94		
3609	Protocadherin Fat 3	Q8TDW7	125861.03	114860.52	99262.48	71751.78	67442.80	61727.73	88008.04	87628.40	87101.32	94164.20	90525.13	80929.28	96022.54	84750.72	61591.87	78089.25	70499.22	69726.41	75679.05	71862.58	58967.56	6258.79	6259.64	5969.18	5004.63	4706.65	4256.58	5424.94	5455.43	4983.39	5572.27	5206.53	3723.41		
3610	Protocadherin gamma-C3	Q9UN70	0.14	2.53	0.81	1.07	2.77	0.68	0.96	0.36	0.63	0.35	0.75	0.38	2.13	0.97	0.60	4.64	1.07	0.47	2.22	0.19	0.06	3734.46	3409.30	2995.00	2211.67	2433.47	1896.90	3271.17	3129.84	2661.84	2673.89	2754.19	1643.08		x
3611	Proto-oncogene c-Rel	Q04864	1471.63	1582.85	1519.01	1087.42	983.98	1034.73	1098.30	976.57	912.07	1250.25	1060.05	1082.21	1293.21	76.66	54.98	1103.99	1005.52	895.38	969.37	982.54	840.93	3031.90	2944.87	3181.91	3469.56	3424.13	3061.73	3678.27	3418.84	3657.97	4155.96	3715.05	2909.79		
3612	Proto-oncogene DBL	P10911	215340.60	210220.36	185971.16	169939.51	170477.31	167357.92	188364.66	179251.23	176592.45	198943.52	190887.49	185381.69	176794.92	168720.20	157477.28	176145.13	177510.05	176089.64	161049.24	160627.22	132040.29	1674.41	1357.29	1562.08	1305.87	1049.82	896.38	931.67	1141.69	1759.86	1041.42	1128.57	294.99		

Supplementary Table S1. Overview on the abundance of all relatively quantified 5180 proteins

			MCF-7																					MDA-MB-231													
	Protein name	UniProt	control 01	control 02	control 03	Dai IC20 01	Dai IC20 02	Dai IC20 03	Dai SC20 01	Dai SC20 02	Dai SC20 03	Gen IC20 01	Gen IC20 02	Gen IC20 03	Gen SC20 01	Gen SC20 02	Gen SC20 03	SSE IC20 01	SSE IC20 02	SSE IC20 03	SSE SC20 01	SSE SC20 02	SSE SC20 03	control 01	control 02	control 03	Dai IC20 01	Dai IC20 02	Dai IC20 03	Gen IC20 01	Gen IC20 02	Gen IC20 03	SSE IC20 01	SSE IC20 02	SSE IC20 03	unique to MCF-7	unique to MDA-MB-231
3613	Proto-oncogene tyrosine-protein kinase receptor Ret	P07949	0.14	2.53	0.81	1.07	2.77	0.68	0.96	0.36	0.63	0.35	0.75	0.38	2.13	0.97	0.60	4.64	1.07	0.47	2.22	0.19	0.06	3901.13	3782.74	3470.81	2649.02	2573.86	2367.59	3329.42	3279.66	3287.54	3709.86	3757.49	2880.25		x
3614	Proto-oncogene tyrosine-protein kinase Src	P12931	3033.59	3077.79	2793.53	1461.24	1434.46	1407.14	1919.15	1893.08	1834.11	2851.34	2668.39	2592.60	2931.09	2359.05	2256.55	1310.37	1201.96	1265.14	1672.30	1645.07	1592.83	2.27	1.49	0.65	0.84	1.31	0.69	4.16	2.47	0.11	4.90	4.98	0.12	x	
3615	Pseudouridylate synthase 7 homolog	Q96PZ0	15842.57	15602.84	13548.24	10223.13	9458.27	9247.83	11935.58	11153.00	10586.67	12695.09	12620.39	11428.84	12054.47	12462.90	9246.91	9748.29	9374.18	8711.56	10019.52	10034.56	9022.75	40.33	14.29	49.32	76.53	45.14	54.43	61.18	64.69	78.27	99.29	45.31	89.94		
3616	Pterin-4-alpha-carbinolamine dehydratase 2	Q9H0N5	0.14	2.53	0.81	1.07	2.77	0.68	0.96	0.36	0.63	0.35	0.75	0.38	2.13	0.97	0.60	4.64	1.07	0.47	2.22	0.19	0.06	2.27	1.49	0.65	685.54	823.15	529.88	1070.14	1073.77	7.22	914.29	964.96	545.84		x
3617	Pterin-4-alpha-carbinolamine dehydratase	P61457	98219.81	93141.84	85737.22	70659.35	66863.45	65100.12	75247.71	73116.46	68993.13	90413.48	89861.91	84568.48	82178.67	72477.42	61762.60	74053.91	69751.72	68565.66	64197.76	62553.71	61234.74	7294.22	6784.45	5931.01	3093.27	3090.91	2989.39	5403.64	5369.75	4709.33	5228.10	5223.54	2877.26		
3618	Pulmonary surfactant-associated protein A2	Q8IWL1	0.14	2.53	0.81	1.07	2.77	0.68	0.96	0.36	0.63	0.35	0.75	0.38	2.13	0.97	0.60	4.64	1.07	0.47	2.22	0.19	0.06	9796.98	8315.55	8070.94	5317.82	5105.23	4287.18	5695.20	5526.91	5961.20	5423.41	5212.70	3932.57		x
3619	Pumilio homolog 1	Q14671	69238.93	65044.69	63597.13	51231.62	47489.65	46926.12	61370.52	60818.55	66496.54	62647.18	60654.21	57986.55	62996.06	56152.51	47572.92	55112.46	53148.63	52144.30	58169.88	50354.64	52104.84	10730.37	10234.98	8992.52	7378.24	6835.97	5833.71	8009.43	5839.72	6553.31	10512.26	10293.26	4989.86		
3620	Purine nucleoside phosphorylase	P00491	97564.27	91280.35	80634.96	63309.46	61484.17	55814.82	70672.12	73656.45	66495.38	76829.96	77024.62	71709.98	71400.46	61797.17	50074.20	61718.43	60053.99	58117.47	73353.72	66832.56	64099.48	58718.56	54539.42	48925.80	49209.06	46506.78	40903.63	53732.71	53673.12	49879.15	58322.80	55676.47	16986.73		
3621	Puromycin-sensitive aminopeptidase	P55786	371276.12	360065.25	339465.52	238436.99	230241.62	214510.24	287955.61	274500.21	256993.02	317788.05	305138.38	282803.69	302586.38	277597.86	237346.18	252460.33	230424.98	227049.26	237330.85	231174.81	216189.25	130608.84	124154.68	114062.67	80048.88	78719.87	72762.98	106683.52	102457.43	97737.23	101377.83	102239.23	73945.23		
3622	Puromycin-sensitive aminopeptidase-like protein	A6NEC2	22968.21	18862.07	21042.97	13020.61	12291.56	11039.99	19768.08	19238.53	17021.42	17103.85	16691.18	15706.62	17523.52	17037.21	14131.95	13370.52	13109.06	12654.29	14388.18	12201.93	13070.28	3252.89	362.69	195.10	1533.51	1307.84	619.63	3613.92	3028.97	267.35	3056.18	2885.55	1189.03		
3623	Putative 40S ribosomal protein S26-like 1	Q5JNZ5	16059.60	15146.18	14368.23	12336.73	11657.40	11364.90	12446.55	11767.53	10930.17	17292.90	17266.65	16538.49	14715.42	10636.59	5344.62	9391.93	11267.17	11063.82	14067.38	12274.68	12138.83	2.27	1.49	0.65	0.84	1.31	0.69	4.16	2.47	0.11	4.90	4.98	0.12	x	
3624	Putative 60S ribosomal protein L13a protein RPL13AP3	Q6NVV1	19969.60	19548.50	17157.16	11383.09	11193.58	10235.96	13659.33	12048.69	10910.20	16600.97	15300.18	13237.04	12226.89	8919.91	11177.90	10635.16	10760.11	10237.34	14119.57	12734.65	13134.35	2.27	1.49	0.65	0.84	1.31	0.69	4.16	2.47	0.11	4.90	4.98	0.12	x	
3625	Putative activator of 90 kDa heat shock protein ATPase homolog 2	Q719I0	0.14	2.53	0.81	1.07	2.77	0.68	0.96	0.36	0.63	0.35	0.75	0.38	2.13	0.97	0.60	4.64	1.07	0.47	2.22	0.19	0.06	1401.93	1331.91	1274.92	710.77	681.60	671.84	990.39	937.67	860.06	787.54	663.24	359.67		x
3626	Putative ankyrin repeat domain-containing protein 31	Q8N7Z5	186099.95	172248.72	163077.18	124664.11	113822.47	107308.16	141717.70	127203.78	120085.48	149438.04	141482.26	133774.26	142031.94	153139.29	114974.58	125552.19	117613.95	115736.53	127670.69	117392.60	107491.07	14424.94	14439.87	14401.46	12080.69	11704.18	11228.77	13537.52	12263.14	12314.11	13685.50	13841.15	12042.33		
3627	Putative ATP-dependent RNA helicase DDX12	Q92771	0.14	2.53	0.81	1.07	2.77	0.68	0.96	0.36	0.63	0.35	0.75	0.38	2.13	0.97	0.60	4.64	1.07	0.47	2.22	0.19	0.06	3344.92	3285.98	3093.11	2455.55	2489.80	2247.28	3719.98	3764.09	3824.43	2727.61	2456.60	1758.21		x
3628	Putative beta-actin-like protein 3	Q9BYX7	140790.92	137792.60	124529.22	75788.79	74622.74	62498.19	102954.51	97482.47	88807.48	100455.95	94736.57	82802.41	96029.61	86526.97	64706.33	102714.52	95208.01	87091.47	91235.64	87712.84	78393.25	71760.68	69040.00	66724.83	30611.10	29795.64	23664.71	30780.60	28793.11	28062.89	35472.41	34301.26	22844.86		
3629	Putative charged multivesicular body protein 4B-like protein CHMP4BP1	P59074	0.14	2.53	0.81	1.07	2.77	0.68	0.96	0.36	0.63	0.35	0.75	0.38	2.13	0.97	0.60	4.64	1.07	0.47	2.22	0.19	0.06	3062.78	2594.18	2516.62	2261.10	2416.87	2132.53	3188.27	3160.47	2968.66	3254.75	3004.35	2741.05		x
3630	Putative ciliary rootlet coiled-coil protein 2	H7BZ55	0.14	2.53	0.81	1.07	2.77	0.68	0.96	0.36	0.63	0.35	0.75	0.38	2.13	0.97	0.60	4.64	1.07	0.47	2.22	0.19	0.06	17049.29	16163.27	15570.93	12769.17	12714.02	10137.48	11804.34	11404.75	10199.93	15123.48	15152.44	10200.67		x
3631	Putative ciliary rootlet coiled-coil protein-like 2 protein	Q8IVE0	10757.65	10690.25	9390.55	8122.00	7569.66	7199.68	10090.65	9217.12	8694.36	9496.14	9677.82	8977.32	10044.30	8511.54	8260.42	9339.32	8540.22	7862.91	8566.79	8849.72	8011.03	5509.43	5383.43	4917.77	4752.89	4647.21	4291.03	4374.48	4762.75	4379.48	4670.76	5140.43	3710.23		
3632	Putative cleavage and polyadenylation specificity factor subunit 4-like protein	A6NMK7	0.14	2.53	0.81	1.07	2.77	0.68	0.96	0.36	0.63	0.35	0.75	0.38	2.13	0.97	0.60	4.64	1.07	0.47	2.22	0.19	0.06	8310.35	8090.26	7518.27	6545.27	6138.24	5608.55	5200.85	6115.39	6246.47	7236.88	7010.78	3929.46		x
3633	Putative CNGA1-overlapping antisense gene protein	Q8IZM0	11005.03	12285.48	12444.17	6376.09	6580.64	6410.21	8796.57	8022.99	8491.86	9908.23	8995.15	7809.26	8928.85	9350.64	6986.32	6808.84	7316.50	6942.13	7737.59	6969.42	7163.23	2.27	1.49	0.65	0.84	1.31	0.69	4.16	2.47	0.11	4.90	4.98	0.12	x	
3634	Putative coiled-coil domain-containing protein 144C	Q8IYA2	3301.61	3078.86	2472.95	2294.04	1070.80	1247.88	2544.48	231.70	1911.17	2709.26	2342.18	1826.32	2223.47	182.90	410.55	1900.30	677.56	1902.30	1043.38	2097.85	1059.71	6504.28	6145.39	6044.01	4921.41	5002.13	4201.55	5577.27	5161.58	4742.35	7037.69	6684.08	4526.25		
3635	Putative coiled-coil-helix-coiled-coil-helix domain-containing protein CHCHD2P9_ mitochondrial	Q5T1J5	0.14	2.53	0.81	1.07	2.77	0.68	0.96	0.36	0.63	0.35	0.75	0.38	2.13	0.97	0.60	4.64	1.07	0.47	2.22	0.19	0.06	2100.69	1981.85	1798.55	313.75	267.06	256.51	609.89	506.17	527.99	690.03	722.88	428.18		x
3636	Putative cTAGE family member 3	Q8IX95	1663.80	1300.60	1471.70	615.00	660.38	445.98	755.19	740.10	584.48	1208.29	954.75	862.47	1319.34	1231.59	757.56	800.15	832.60	530.63	702.66	587.33	526.10	2.27	1.49	0.65	0.84	1.31	0.69	4.16	2.47	0.11	4.90	4.98	0.12	x	
3637	Putative dehydrogenase/reductase SDR family member 4-like 1	P0CG22	2432.43	2951.20	3101.46	2995.38	3067.37	2930.08	2962.32	2680.99	2638.08	4344.31	4629.59	4132.72	2501.30	2684.75	2100.99	3877.04	3577.05	3511.50	3003.74	3007.63	2349.55	2.27	1.49	0.65	0.84	1.31	0.69	4.16	2.47	0.11	4.90	4.98	0.12	x	
3638	Putative deoxyribonuclease TATDN1	Q6P1N9	100096.97	95198.00	87794.20	81662.09	79247.79	75213.89	79019.71	75195.91	68437.38	99171.18	96184.19	90431.18	88739.70	85555.72	68287.91	71336.21	70344.82	67384.94	71136.11	73305.91	66982.94	46330.77	37309.62	32785.22	30184.22	29425.15	24404.80	36302.60	35332.05	29087.45	37724.84	36676.29	23798.72		
3639	Putative deoxyribonuclease TATDN2	Q93075	41286.10	37453.47	33666.41	28863.91	26363.02	24294.28	81521.85	76487.62	71993.99	38224.13	37638.57	31876.70	39408.05	36077.93	27323.02	34622.72	33645.55	29933.24	33219.59	30625.12	28843.60	3863.18	3199.04	3589.13	2667.81	2386.00	2111.45	2563.00	2311.35	2137.70	1841.69	1453.42	1420.64		
3640	Putative dimethylaniline monooxygenase [N-oxide-forming] 6	O60774	15661.66	12788.57	8556.50	1879.79	1733.24	968.88	2336.75	2875.78	1102.50	11444.09	10505.31	8719.04	11475.41	5580.56	720.30	1600.56	1498.74	1031.58	7539.41	1749.87	1339.66	2.27	1.49	0.65	0.84	1.31	0.69	4.16	2.47	0.11	4.90	4.98	5512.88	x	
3641	Putative E3 ubiquitin-protein ligase makorin-4	Q13434	3584.16	3016.47	2377.73	2386.31	2371.98	2070.94	2152.03	2042.74	2234.00	2502.24	2614.16	2376.19	2972.86	2971.98	2794.37	2606.93	2593.98	2229.77	2936.64	2751.45	2646.81	628.12	502.28	477.21	533.62	666.14	472.25	509.90	500.86	481.86	912.95	771.71	410.68		
3642	Putative E3 ubiquitin-protein ligase UBR7	Q8N806	6470.89	6272.17	5687.11	4707.02	3780.67	3962.05	5036.63	5338.22	4233.23	5037.40	5034.05	4449.59	5195.40	4095.03	3471.22	4448.72	3985.85	4301.81	4651.87	4622.52	4094.30	986.50	861.21	754.12	569.48	655.16	414.25	744.70	695.55	584.00	918.14	880.68	559.95		
3643	Putative elongation factor 1-alpha-like 3	Q5VTE0	3784314.98	3627712.47	3330817.05	2440403.36	2361681.09	2226360.83	2955558.73	2868103.12	2855673.54	2989273.42	2841975.18	2671898.19	2943870.87	2733866.50	2132153.45	2704066.19	2574182.86	2532437.63	2658010.53	2457100.84	2348114.51	330.67	327.96	131.01	15.12	35.42	7.09	102.32	75.55	35.60	84.44	67.02	5.26		
3644	Putative endoplasmin-like protein	Q58FF3	10392.70	9147.41	7045.81	7599.68	6727.81	5955.29	7306.23	7332.82	6048.64	10432.51	10050.27	9156.60	8675.27	10101.07	6519.39	7026.04	6671.45	5431.43	7010.66	7206.25	6928.80	21215.86	21242.56	19668.16	18816.20	18511.65	12668.81	22181.10	21185.74	17638.93	27673.52	26539.17	14635.48		
3645	Putative fatty acid-binding protein 5-like protein 3	A8MUU1	22045.24	21350.07	18254.38	12603.49	12341.58	10166.52	19334.01	17967.93	15932.62	17474.99	16885.80	15234.96	18169.13	18399.49	10259.04	13332.61	12388.98	11315.33	13620.35	12681.65	11083.68	16579.28	14623.04	12768.82	9376.77	9779.55	6957.35	12858.34	12067.80	10862.68	13357.97	13569.00	8331.42		
3646	Putative ferritin heavy polypeptide-like 19	P0C7X4	90071.21	81541.99	82801.55	60476.97	57628.73	56619.83	71764.62	67932.91	68358.10	70829.95	67656.30	64608.35	79026.30	72716.98	62743.40	59188.14	53660.52	53291.49	60696.89	55933.27	52522.10	2.27	1.49	0.65	0.84	1.31	0.69	4.16	2.47	0.11	4.90	4.98	0.12	x	
3647	Putative GED domain-containing protein DNM1P46	Q6ZS02	0.14	2.53	0.81	1.07	2.77	0.68	0.96	0.36	0.63	0.35	0.75	0.38	2.13	0.97	0.60	4.64	1.07	0.47	2.22	0.19	0.06	4790.61	4445.22	4186.77	855.42	935.51	533.03	2779.88	2672.61	2317.63	1631.84	1607.12	572.62		x
3648	Putative golgin subfamily A member 8I	A6NC78	0.14	2.53	0.81	1.07	2.77	0.68	0.96	0.36	0.63	0.35	0.75	0.38	2.13	0.97	0.60	4.64	1.07	0.47	2.22	0.19	0.06	20313.05	20045.95	18170.05	14497.97	14031.68	12599.64	13370.95	13288.36	12495.80	11901.16	11344.22	7855.59		x
3649	Putative heat shock 70 kDa protein 7	P48741	1414.53	1402.43	1186.37	355.98	350.02	354.91	793.71	613.71	642.76	551.78	471.40	580.40	896.29	748.68	528.33	407.56	369.79	382.83	553.76	581.22	494.18	2.27	1.49	0.65	0.84	1.31	0.69	4.16	2.47	0.11	4.90	4.98	0.12	x	
3650	Putative heat shock protein HSP 90-alpha A4	Q58FG1	87205.50	82769.40	80815.86	63620.05	61011.26	58649.43	74092.76	75636.59	68692.13	74266.53	74370.01	69282.16	73876.72	64706.82	51819.33	55931.57	58580.58	53784.35	67142.62	61901.63	59498.62	34466.92	31510.27	29839.47	17470.79	17136.79	14830.46	19901.33	21123.00	19748.70	19031.58	18429.77	12212.06		
3651	Putative heat shock protein HSP 90-alpha A5	Q58FG0	25824.66	22876.66	18956.85	14983.97	15455.79	15908.25	17830.81	18926.69	19626.77	18567.81	17994.61	17875.25	18630.68	15538.53	16539.21	17933.83	15839.07	14770.57	19035.68	17715.62	16115.22	793.44	957.17	554.16	278.51	281.68	226.26	536.37	584.82	459.70	448.39	589.11	305.15		
3652	Putative heat shock protein HSP 90-beta 2	Q58FF8	163259.32	153099.94	151335.17	125623.35	121518.56	117259.86	127389.00	123244.71	121425.97	145371.17	141782.15	136274.90	128871.76	131735.39	115901.18	120143.57	112350.18	109224.80	114197.34	116075.91	111950.07	70175.33	62426.87	62930.78	40542.97	40835.82	33315.10	52570.77	48592.58	50013.30	49024.28	45386.19	31519.28		
3653	Putative heat shock protein HSP 90-beta 4	Q58FF6	31868.96	29984.16	26617.05	18349.27	16723.52	16345.65	23129.76	22271.07	21820.06	25461.56	24932.29	22468.77	26038.24	22173.69	17459.49	20816.74	18965.23	19394.90	21821.45	18047.13	17144.66	17895.19	17931.10	16336.29	13090.53	13396.76	9794.01	19675.20	19898.02	16126.77	16951.49	18319.72	9813.68		
3654	Putative heat shock protein HSP 90-beta-3	Q58FF7	23487.39	24480.75	22358.95	18728.20	17916.30	17248.10	22293.38	24352.05	20389.85	23267.88	22869.83	21669.52	21023.68	19351.09	17213.62	19432.99	17196.00	17333.61	17289.06	16565.50	16988.47	9438.38	7914.14	7278.68	6558.87	6460.54	5509.95	7744.33	7247.84	6216.90	7109.23	7240.82	5134.43		
3655	Putative hexokinase HKDC1	Q2TB90	28228.24	27727.13	25488.19	21950.98	21861.32	20654.66	31143.11	30174.30	29356.16	28553.20	28276.57	25538.24	28054.77	24234.97	22797.31	23692.03	23214.44	20894.00	25973.20	24394.22	24865.96	2.27	1.49	0.65	0.84	1.31	0.69	4.16	2.47	0.11	4.90	4.98	0.12	x	
3656	Putative high mobility group protein B1-like 1	B2RPK0	67.38	130.88	20.61	142.23	115.03	143.99	48.34	49.93	58.96	146.62	154.00	166.65	100.61	88.11	139.33	143.43	74.03	51.48	31.22	94.03	49.74	4304.66	5933.52	4859.65	5554.45	5411.26	2845.44	6385.47	5113.42	5864.25	4064.87	5436.35	3048.24		
3657	Putative HLA class I histocompatibility antigen_ alpha chain H	P01893	21349.77	19836.56	21387.22	19766.12	19417.76	17559.28	20101.78	20175.77	19840.54	23379.95	21618.41	18268.52	20442.61	19905.17	17731.47	16532.18	14653.56	14666.50	15707.98	15398.04	16981.56	949.31	806.44	710.44	591.43	659.81	450.10	668.22	615.61	628.49	921.21	1035.46	462.05		
3658	Putative homeodomain transcription factor 1	Q9UMS5	15042.27	15617.78	14396.79	11177.19	10377.33	10433.95	13355.02	12600.41	12386.40	14508.56	13480.39	12100.88	12314.76	13470.18	11410.64	9334.52	9265.91	9222.85	11192.43	10809.57	9268.53	1225.09	1135.62	898.11	996.71	942.70	839.99	923.91	891.66	980.91	1225.10	1132.12	660.37		
3659	Putative hydrolase RBBP9	O75884	0.14	2.53	0.81	1.07	2.77	0.68	0.96	0.36	0.63	0.35	0.75	0.38	2.13	0.97	0.60	4.64	1.07	0.47	2.22	0.19	0.06	6957.65	7087.70	6567.96	4615.58	4066.67	3018.63	8452.37	8425.06	6979.67	6690.27	6546.16	3737.67		x
3660	Putative inactive cathepsin L-like protein CTSL3P	Q5NE16	115.30	203.70	141.93	106.56	83.08	83.27	138.85	130.63	124.75	156.74	137.47	103.07	79.00	91.75	19.41	173.24	162.84	139.08	113.45	145.25	53.38	2.27	1.49	0.65	0.84	1.31	0.69	4.16	2.47	0.11	4.90	4.98	0.12	x	
3661	Putative keratin-87 protein	A6NCN2	2650.99	2431.65	1915.18	1326.58	1108.61	876.36	2126.75	1783.75	1750.92	1776.30	1767.79	1480.14	1779.15	1430.79	866.52	1322.81	1363.71	1251.92	1250.38	1213.22	1078.08	2.27	1.49	0.65	0.84	1.31	0.69	4.16	2.47	0.11	4.90	4.98	0.12	x	
3662	Putative lipocalin 1-like protein 1	Q5VSP4	8725.34	8969.25	7944.96	21121.31	19125.89	21459.14	18839.22	21749.98	20727.53	8175.91	8850.23	8387.52	15095.29	16640.22	21829.37	19569.95	15126.21	15094.72	17674.06	18408.31	13430.08	2.27	1.49	0.65	0.84	1.31	0.69	4.16	2.47	0.11	4.90	4.98	0.12	x	
3663	Putative methyltransferase-like protein 15P1	P0C7V9	3092.94	2477.71	2060.49	2353.43	2614.42	2171.09	2063.58	1979.71	1957.36	5062.22	4790.76	5084.27	2857.35	1983.49	1690.62	2148.44	2153.48	1761.97	1468.71	1392.09	1270.24	2.27	1.49	0.65	0.84	1.31	0.69	4.16	2.47	0.11	4.90	4.98	0.12	x	
3664	Putative monooxygenase p33MONOX	Q96A73	1539.74	1608.93	1552.68	1058.65	970.39	976.89	1286.82	1075.56	1142.33	1040.65	1144.30	997.76	1318.65	1744.96	145.89	799.98	982.03	940.04	1351.14	1194.71	1154.06	2.27	1.49	0.65	0.84	1.31	0.69	4.16	2.47	0.11	4.90	4.98	0.12	x	
3665	Putative nascent polypeptide-associated complex subunit alpha-like protein	Q9BZK3	409.50	499.48	38.55	349.87	293.91	322.28	540.10	747.38	508.80	466.05	465.68	409.30	678.20	459.75	232.87	367.02	331.84	295.75	404.54	390.41	271.01	2.27	1.49	0.65	124.93	169.11	57.68	4.16	2.47	0.11	382.76	322.90	101.25		
3666	Putative oncomodulin-2	P0CE71	3177.68	2672.75	2029.73	1876.93	1677.42	1399.42	1920.22	1832.12	1652.90	3626.92	3698.24	3075.63	2691.43	2150.93	657.79	2100.06	1915.03	2028.40	2563.29	2263.50	1521.71	2.27	1.49	0.65	0.84	1.31	0.69	4.16	2.47	0.11	4.90	4.98	0.12	x	
3667	Putative peptidyl-tRNA hydrolase PTRHD1	Q6GMV3	16632.07	15361.45	14011.19	8508.04	9142.43	8260.89	9651.81	9452.17	8894.66	13907.99	13497.70	12055.78	13005.86	11028.00	9700.80	8202.21	8327.41	8473.06	9560.29	8111.05	8269.61	2.27	1.49	0.65	0.84	1.31	0.69	4.16	2.47	0.11	4.90	4.98	0.12	x	
3668	Putative PIP5K1A and PSMD4-like protein	A2A3N6	2409.12	2626.48	2171.63	1093.83	934.52	727.07	1508.98	1496.68	1365.99	1744.28	1633.25	1715.67	1806.92	1460.07	1203.54	1359.12	1265.81	1055.18	1626.21	1330.43	1274.73	33702.15	24779.12	21828.69	22000.25	19789.21	13157.61	29023.81	25539.93	18735.26	32070.60	31061.86	15975.14		
3669	Putative postmeiotic segregation increased 2-like protein 1	A4D2B8	387.84	391.44	392.16	330.14	286.83	243.91	158.64	219.83	14.58	274.15	314.13	268.42	337.38	13.69	10.18	125.90	205.38	273.58	343.39	332.08	327.60	12613.65	11556.38	11157.40	11344.59	12050.09	10988.19	12527.09	13203.87	13120.00	12426.59	11681.21	8659.50		
3670	Putative postmeiotic segregation increased 2-like protein 3	Q13401	557.04	519.70	501.26	257.49	284.89	319.38	179.80	128.81	173.35	651.49	637.37	556.16	487.53	465.05	296.79	250.62	243.72	262.82	106.70	141.37	188.31	2.27	1.49	0.65	668.71	425.17	182.93	4.16	2.47	0.11	771.02	678.99	317.98		
3671	Putative PRAME family member 13	Q5VWM	684.18	752.32	544.64	455.78	375.66	366.15	505.97	546.88	569.64	571.46	615.97	495.61	659.79	576.36	481.33	401.34	381.55	318.21	380.57	356.84	402.33	2.27	1.49	0.65	0.84	1.31	0.69	4.16	2.47	0.11	4.90	4.98	0.12	x	
3672	Putative protein FAM10A4	Q8IZP2	0.14	2.53	0.81	1.07	2.77	0.68	0.96	0.36	0.63	0.35	0.75	0.38	2.13	0.97	0.60	4.64	1.07	0.47	2.22	0.19	0.06	92536.03	81135.82	71694.74	48255.08	45539.40	38215.48	60471.01	56021.45	56751.16	52559.99	50642.05	33269.33		x
3673	Putative protein FAM10A5	Q8NFI4	42544.00	41707.23	40690.59	39080.91	37732.20	33231.22	30004.52	27363.69	28674.95	40100.07	39625.32	36368.42	39079.15	31999.25	17928.64	29940.88	30157.61	28270.57	29734.46	33171.25	34824.87	92991.50	83185.69	73635.94	63817.33	62645.57	54458.35	79739.80	76266.82	72526.71	76272.54	70370.52	52254.96		
3674	Putative protein FAM45B	Q6NSW5	4334.62	4920.11	4074.34	3144.23	3523.82	2871.09	3292.24	2958.87	3757.44	4455.03	3984.56	3997.09	3882.64	3679.65	2672.00	3570.27	3275.85	3186.48	3515.25	2825.65	3045.07	2.27	1.49	0.65	0.84	1.31	0.69	4.16	2.47	0.11	4.90	4.98	0.12	x	
3675	Putative protein PLEKHA9	O95397	0.14	2.53	0.81	1.07	2.77	0.68	0.96	0.36	0.63	0.35	0.75	0.38	2.13	0.97	0.60	4.64	1.07	0.47	2.22	0.19	0.06	4914.40	4204.98	3592.48	1673.40	1735.24	1113.83	2655.93	2545.28	1852.51	3311.11	2959.40	1462.39		x
3676	Putative protein SSX6	Q7RTT6	0.14	2.53	0.81	1.07	2.77	0.68	0.96	0.36	0.63	0.35	0.75	0.38	2.13	0.97	0.60	4.64	1.07	0.47	2.22	0.19	0.06	2165.46	2196.23	1854.51	1749.14	1840.24	1525.07	1975.29	2094.05	1889.06	1667.26	1505.96	932.74		x
3677	Putative protein SSX9	Q7RTT3	0.14	2.53	0.81	1.07	2.77	0.68	0.96	0.36	0.63	0.35	0.75	0.38	2.13	0.97	0.60	4.64	1.07	0.47	2.22	0.19	0.06	221.40	196.44	247.13	164.80	174.90	115.94	211.46	197.92	178.38	200.83	232.26	149.21		x
3678	Putative protein ZNF815	A8K554	0.14	2.53	0.81	1.07	2.77	0.68	0.96	0.36	0.63	0.35	0.75	0.38	2.13	0.97	0.60	4.64	1.07	0.47	2.22	0.19	0.06	892.09	799.33	667.48	422.65	394.95	252.14	552.90	534.96	475.31	673.65	647.59	319.86		x
3679	Putative pyridoxal-dependent decarboxylase domain-containing protein 2	Q6P474	55083.67	58228.81	53594.70	38682.37	37697.89	35832.71	52316.42	46938.69	46437.80	43402.35	41463.08	40125.14	40209.93	40746.95	34334.43	34212.69	36121.98	32282.77	37478.42	35531.47	32625.38	2.27	1.49	0.65	0.84	1.31	0.69	4.16	2.47	0.11	4.90	4.98	0.12	x	
3680	Putative Ras-related protein Rab-1C	Q92928	3454.97	3190.87	3683.81	3669.32	3667.03	3998.30	3925.69	3682.43	4127.05	3693.80	3357.95	3485.05	3555.08	3989.33	3391.12	3973.54	3452.62	3753.40	3217.88	3185.05	2798.47	2.27	1.49	0.65	0.84	1.31	0.69	4.16	2.47	0.11	4.90	4.98	0.12	x	
3681	Putative RNA polymerase II subunit B1 CTD phosphatase RPAP2	Q8IXW5	0.14	2.53	0.81	1.07	2.77	0.68	0.96	0.36	0.63	0.35	0.75	0.38	2.13	0.97	0.60	4.64	1.07	0.47	2.22	0.19	0.06	992.31	942.86	793.51	796.58	633.62	549.98	1272.15	1214.07	1142.01	1139.71	1006.71	501.84		x
3682	Putative RNA-binding protein Luc7-like 1	Q9NQ29	21196.06	21861.02	21509.70	17154.33	16697.95	15908.78	18637.25	19135.64	17269.68	21834.19	21703.06	19470.59	18289.62	17415.72	15267.64	17671.38	16842.41	16103.37	17153.87	15719.04	15085.92	2914.11	2832.79	2380.72	2095.60	1931.05	1907.07	2047.65	2027.63	2174.49	2515.49	2752.41	792.52		
3683	Putative RNA-binding protein Luc7-like 2	Q9Y383	245993.45	235962.29	223352.84	159285.06	149926.95	144905.55	184226.28	178194.76	176736.33	202715.94	191475.26	185707.12	200280.87	186947.01	167496.12	151923.55	144362.57	137812.33	162052.80	154831.35	147248.73	100782.04	89271.50	80777.21	55635.65	55546.03	43864.79	65139.58	59806.79	55662.16	75671.35	71546.70	49362.42		
3684	Putative small nuclear ribonucleoprotein G-like protein 15	A8MWD	6662.39	5229.34	3268.98	2428.98	1968.59	1347.87	3337.64	3120.80	2352.61	2820.49	2351.04	1782.59	3740.42	2650.51	559.42	2624.88	2274.93	1713.40	2179.17	1877.57	1115.70	2607.86	2101.22	1627.71	969.57	908.07	423.22	2333.78	1786.04	1340.75	1983.10	1737.54	703.13		
3685	Putative testis-expressed protein 13C	A0A0J9Y	2474.02	2358.40	2326.50	1606.91	1708.25	1489.76	1669.49	1546.64	1699.92	1636.72	1780.33	1757.35	1666.33	1874.78	1560.40	1553.98	1538.11	1409.67	1686.38	1542.00	1468.95	2.27	1.49	0.65	0.84	1.31	0.69	4.16	2.47	0.11	4.90	4.98	0.12	x	
3686	Putative trace amine-associated receptor 3	Q9P1P4	705.08	457.77	815.56	350.81	104.10	35.78	206.72	452.94	61.53	769.27	633.06	756.19	660.83	307.57	9.84	242.30	15.83	59.94	144.29	494.13	289.07	2.27	1.49	0.65	0.84	1.31	0.69	4.16	2.47	0.11	4.90	4.98	0.12	x	
3687	Putative tRNA pseudouridine synthase Pus10	Q3MIT2	9321.53	9702.54	8781.82	5961.99	6208.55	5716.06	9093.35	8753.68	8503.75	7447.71	7469.38	7341.05	7069.92	7345.97	6077.39	7226.77	6545.19	6631.92	5579.35	5180.46	4919.66	2092.30	1780.65	1353.47	1023.71	926.07	784.91	1307.18	1174.67	1216.09	1202.48	1183.57	830.06		
3688	Putative tubulin-like protein alpha-4B	Q9H853	4322.34	2910.71	2057.92	2599.35	2376.84	1643.21	2709.81	2440.33	2051.42	3059.13	2889.46	2325.39	3487.84	2847.88	790.36	1846.07	2018.98	1672.43	3027.36	2485.76	1786.58	2.27	1.49	0.65	0.84	1.31	0.69	4.16	2.47	0.11	4.90	4.98	0.12	x	
3689	Putative ubiquitin-conjugating enzyme E2 N-like	Q5JXB2	167040.77	152969.72	150012.82	124040.09	120322.01	114793.97	128627.07	127159.84	122395.04	146390.27	142938.13	134828.31	149645.63	140457.22	123009.95	113267.27	110097.56	114185.19	118481.69	112098.53	109924.74	52179.82	50095.81	47383.28	43186.11	41440.10	37523.06	46975.01	44847.50	43707.83	47656.49	48204.22	34181.94		
3690	Putative ubiquitin-conjugating enzyme E2Q2-like protein	H0YL09	9565.38	8978.35	8626.56	7677.37	8303.87	6971.75	8163.60	7406.05	6993.14	8759.43	8497.57	7563.42	8746.98	8656.66	5128.68	7236.46	6775.29	6685.63	6904.14	7548.04	6395.61	2.27	1.49	0.65	0.84	1.31	0.69	4.16	2.47	0.11	4.90	4.98	0.12	x	
3691	Putative uncharacterized protein C22orf34	Q6ZV56	0.14	2.53	0.81	1.07	2.77	0.68	0.96	0.36	0.63	0.35	0.75	0.38	2.13	0.97	0.60	4.64	1.07	0.47	2.22	0.19	0.06	1097.30	1260.19	1036.18	961.78	968.63	827.69	1219.15	1267.38	1202.50	1327.01	1289.24	915.62		x
3692	Putative uncharacterized protein C9orf129	Q5T035	2747.20	2909.09	2546.33	1793.88	1623.01	1514.61	1950.93	1786.27	1650.98	2069.34	2007.82	1962.52	2242.91	2314.17	1857.87	1642.98	1610.76	1643.00	1647.73	1526.90	1511.54	3300.68	3143.69	3056.93	2125.96	1973.58	1529.39	2794.80	2705.35	2347.13	2579.26	2594.80	1612.26		
3693	Putative uncharacterized protein encoded by LINC00114	Q6XXX2	24828.38	19694.04	13763.65	12857.18	11119.73	8037.57	13198.23	11418.33	10670.28	16824.39	15588.98	12061.05	18370.76	13103.20	3386.31	11276.61	10012.44	8242.19	13860.32	11940.50	8299.13	2.27	1.49	0.65	0.84	1.31	0.69	4.16	2.47	0.11	4.90	4.98	0.12	x	
3694	Putative uncharacterized protein encoded by LINC00471	Q8N535	56410.10	58819.15	46480.27	33932.07	31610.82	28064.36	41494.07	38758.50	36024.08	48788.33	44057.77	40167.66	41310.88	40214.90	28699.73	34246.89	33772.58	32140.36	41451.52	39142.66	35001.12	2.27	1.49	0.65	0.84	1.31	0.69	4.16	2.47	0.11	4.90	4.98	0.12	x	
3695	Putative uncharacterized protein encoded by MIR7-3HG	Q8N6C7	1042.68	627.95	293.52	1.07	2.77	0.68	0.96	0.36	0.63	1032.74	738.64	382.69	2.13	0.97	0.60	4.64	1.07	0.47	2.22	0.19	0.06	2.27	1.49	0.65	0.84	1.31	0.69	4.16	2.47	0.11	4.90	4.98	9627.69	x	
3696	Putative uncharacterized protein FLJ45721	Q6ZS92	365.69	383.05	297.29	310.65	329.59	310.07	409.99	321.25	295.27	331.86	393.38	244.55	230.17	177.29	281.67	237.08	220.65	275.76	326.46	260.88	290.36	2.27	1.49	0.65	0.84	1.31	0.69	4.16	2.47	0.11	4.90	4.98	0.12	x	
3697	Putative uncharacterized protein FLJ45831	Q6ZS49	0.14	2.53	0.81	1.07	2.77	0.68	0.96	0.36	0.63	0.35	0.75	0.38	2.13	0.97	0.60	4.64	1.07	0.47	2.22	0.19	0.06	997.96	963.29	840.75	832.33	767.36	386.47	1288.27	1005.65	884.93	1198.63	1371.97	401.88		x
3698	Putative uncharacterized protein KTN1-AS1	Q86SY8	0.14	2.53	0.81	1.07	2.77	0.68	0.96	0.36	0.63	0.35	0.75	0.38	2.13	0.97	0.60	4.64	1.07	0.47	2.22	0.19	0.06	1026.80	981.73	737.73	402.51	330.38	265.20	619.83	645.41	587.39	646.72	522.95	380.81		x
3699	Putative uncharacterized protein LOC100128429	Q6ZWC4	1379.91	1251.48	1335.76	959.58	1000.25	846.61	67.05	70.43	771.51	1091.20	981.24	947.65	1180.03	164.78	64.55	667.63	841.32	685.50	871.30	1064.57	89.24	2.27	1.49	0.65	0.84	1.31	0.69	4.16	2.47	0.11	4.90	4.98	0.12	x	
3700	Putative uncharacterized protein LOC100128554	Q0VFX4	0.14	2.53	0.81	1.07	2.77	0.68	0.96	0.36	0.63	0.35	0.75	0.38	2.13	0.97	0.60	4.64	1.07	0.47	2.22	0.19	0.06	62.35	53.81	68.14	87.05	61.92	59.67	26.33	38.02	41.53	105.49	104.29	33.30		x
3701	Putative uncharacterized protein MGC13053	Q9BRP9	0.14	2.53	0.81	1.07	2.77	0.68	0.96	0.36	0.63	10.70	24.79	54.79	2.13	0.97	0.60	4.64	1.07	0.47	31.00	33.90	30.53	2.27	1.49	0.65	0.84	1.31	0.69	4.16	2.47	0.11	4.90	4.98	0.12	x	
3702	Putative uncharacterized protein MRGPRG-AS1	Q2M3A8	2612.03	2364.24	2427.34	1812.04	1668.45	1402.70	2356.25	2641.69	2423.92	2680.59	2277.72	2286.67	2669.49	2488.50	1617.23	1962.97	1842.96	1256.77	1978.27	1915.59	1791.74	2.27	1.49	0.65	0.84	1.31	0.69	4.16	2.47	0.11	4.90	4.98	0.12	x	
3703	Putative uncharacterized protein MYH16	Q9H6N6	0.14	2.53	0.81	1.07	2.77	0.68	0.96	0.36	0.63	0.35	0.75	0.38	2.13	0.97	0.60	4.64	1.07	0.47	2.22	0.19	0.06	35270.82	35168.28	31611.17	21886.83	20564.07	14518.76	28635.85	25127.14	24468.52	33689.36	33126.75	19445.96		x
3704	Putative uncharacterized SMG1-like protein	Q6P435	2480.99	2275.57	2116.54	1594.02	1556.93	1275.41	2042.80	1550.00	1761.84	2265.43	2273.21	2143.36	1986.10	2089.09	1503.25	2218.51	2013.81	1880.97	1857.20	1865.46	1921.92	2.27	1.49	0.65	0.84	1.31	0.69	4.16	2.47	0.11	4.90	4.98	0.12	x	
3705	Putative UPF0607 protein ENSP00000383783	A8MUI8	2010.30	2309.84	1961.60	1085.53	1097.56	1079.95	1327.69	1205.91	1300.20	1275.84	1214.76	1228.26	1455.51	1354.26	1266.82	1133.88	1003.65	981.03	1447.35	1399.83	1090.76	11601.50	10508.60	10268.73	8313.99	7793.05	8215.22	7167.12	7347.99	6729.89	8645.75	8039.16	6549.31		
3706	Putative UPF0633 protein LOC554249	Q5XG85	353.25	307.67	336.48	403.86	314.87	289.93	255.97	285.31	295.52	488.32	433.25	411.04	406.52	251.37	325.68	350.37	253.62	313.46	290.68	288.99	280.98	2.27	1.49	0.65	0.84	1.31	0.69	4.16	2.47	0.11	4.90	4.98	0.12	x	
3707	Putative zinc finger and SCAN domain-containing protein 5C	A6NGD5	30651.14	29752.67	27985.58	17830.16	17287.34	15103.52	22547.43	22861.17	21437.27	22783.39	22414.17	19895.40	26362.84	23761.73	16771.58	18734.97	17535.25	17296.43	19029.94	17813.58	15923.68	2.27	1.49	0.65	0.84	1.31	0.69	4.16	2.47	0.11	4.90	4.98	0.12	x	
3708	PWWP domain-containing DNA repair factor 3B	Q5H9M0	65328.66	61577.32	56407.80	46440.47	50801.10	45377.93	61514.49	56455.18	50268.04	62328.31	57542.21	50888.28	61828.77	50599.10	39038.98	49670.71	46602.48	43525.67	37456.28	36898.51	35445.45	2.27	1.49	0.65	0.84	1.31	0.69	4.16	2.47	0.11	4.90	4.98	0.12	x	
3709	PX domain-containing protein kinase-like protein	Q7Z7A4	11945.98	11847.42	12525.65	9624.16	8073.87	8886.51	9044.79	10011.21	11544.52	12319.49	11667.51	9035.56	9702.12	11276.64	8649.75	8172.83	6886.56	7859.73	8555.47	8986.83	8326.15	4820.07	2872.84	5634.56	2505.26	4626.60	3311.09	3680.19	3892.13	3792.01	4043.21	3889.88	3193.73		
3710	Pygopus homolog 2	Q9BRQ0	1133.19	1139.39	1186.89	611.75	568.89	592.99	734.45	634.25	688.20	725.31	714.70	765.90	813.68	751.30	697.87	728.71	627.03	599.02	651.45	581.37	669.69	2.27	1.49	0.65	0.84	1.31	0.69	4.16	2.47	0.11	4.90	4.98	0.12	x	
3711	Pyridine nucleotide-disulfide oxidoreductase domain-containing protein 1	Q8WU10	0.14	2.53	0.81	1.07	2.77	0.68	0.96	0.36	0.63	0.35	0.75	0.38	2.13	0.97	0.60	4.64	1.07	0.47	2.22	0.19	0.06	491.58	341.95	475.33	396.27	373.77	365.30	507.51	448.83	390.26	323.66	337.27	92.62		x
3712	Pyridoxal kinase	O00764	65118.82	58070.65	56905.64	40698.31	38537.04	34612.35	44513.54	41913.73	41466.37	55263.67	52742.82	50462.64	51885.68	48130.34	37144.64	46885.15	40041.44	38880.17	41149.97	37926.87	35609.25	21092.34	19067.00	17265.19	10660.29	10589.42	8404.38	14322.95	15210.88	14299.69	16071.13	15656.47	9962.52		
3713	Pyridoxal phosphate homeostasis protein	O94903	488.56	555.49	504.52	348.71	350.56	348.17	327.60	265.74	318.39	451.03	557.68	455.07	415.38	477.43	428.76	354.24	388.06	400.46	398.59	386.59	299.68	2.27	1.49	0.65	0.84	1.31	0.69	4.16	2.47	0.11	4.90	4.98	0.12	x	
3714	Pyridoxal-dependent decarboxylase domain-containing protein 1	Q6P996	92399.52	85195.40	74078.07	58735.65	57385.07	51247.64	70260.81	67611.62	65221.27	80939.69	77832.05	71011.89	72077.19	67501.38	48189.83	60249.11	58093.77	55469.10	59503.45	56490.86	52236.85	2.27	1.49	0.65	0.84	1.31	0.69	4.16	2.47	0.11	4.90	4.98	0.12	x	
3715	Pyridoxine-5'-phosphate oxidase	Q9NVS9	52478.19	50901.53	45338.52	29108.93	29777.49	24454.75	38114.24	37401.98	35089.03	39184.86	37131.89	35034.40	38257.80	35503.09	26263.15	32847.66	31333.42	27887.44	31995.73	28899.92	25016.99	2.27	1.49	0.65	0.84	1.31	0.69	4.16	2.47	0.11	4.90	4.98	0.12	x	
3716	Pyrin domain-containing protein 5	W6CW81	11935.35	10843.30	9308.41	8229.43	7521.72	6476.62	9234.99	8914.87	8138.32	9125.49	8618.66	8510.87	9993.93	8898.02	6768.35	7432.74	7539.98	7253.55	8607.55	7780.30	7213.96	8178.14	7907.61	6806.26	3160.95	2695.78	2077.11	8477.42	7482.88	6244.61	6670.41	5932.40	3166.30		
3717	Pyroglutamyl-peptidase 1	Q9NXJ5	13423.15	12185.32	10806.95	7337.22	7139.23	5829.62	10347.31	8291.46	8238.05	12306.97	10099.62	8834.09	8683.85	7264.31	3504.27	8524.84	8323.09	7473.75	9022.10	8034.77	6715.31	2.27	1.49	0.65	0.84	1.31	0.69	4.16	2.47	0.11	4.90	4.98	0.12	x	
3718	Pyrroline-5-carboxylate reductase 1_ mitochondrial	P32322	287345.10	279346.53	265509.43	196778.54	195931.62	182336.03	223775.73	214397.97	198568.59	210677.83	203349.77	195816.53	205957.41	200131.10	183498.09	200379.17	192822.99	178319.22	182953.97	181103.98	173424.39	30258.05	29947.95	27360.37	22357.05	21895.40	19440.88	26140.53	24664.19	24115.60	27688.90	26849.25	19017.51		
3719	Pyrroline-5-carboxylate reductase 2	Q96C36	3337.63	4046.88	3330.13	2499.84	2724.14	2411.52	3664.42	3162.54	3260.39	3300.13	3458.90	3266.60	2865.23	2936.92	2202.19	2632.59	2973.34	2746.29	2754.47	2454.28	2516.86	751.07	678.87	578.97	839.59	793.54	696.74	838.23	720.27	843.73	898.22	857.79	564.01		
3720	Pyrroline-5-carboxylate reductase 3	Q53H96	107553.68	99075.32	93724.31	70425.18	69576.39	63694.62	76771.37	74274.29	69198.36	92097.04	88783.86	80646.93	80318.05	77287.97	58027.29	69348.41	66355.10	64531.31	68687.98	66306.45	61873.68	2.27	1.49	0.65	0.84	1.31	0.69	4.16	2.47	0.11	4.90	4.98	0.12	x	
3721	Pyruvate carboxylase_ mitochondrial	P11498	201107.15	187660.01	167831.09	129928.49	123145.50	116326.13	157473.30	149991.75	143063.59	159319.04	151266.94	140462.97	152831.20	140520.53	114824.81	141196.11	130628.88	124390.44	129166.27	127237.75	117767.81	2.27	1.49	0.65	0.84	1.31	0.69	4.16	2.47	0.11	4.90	4.98	0.12	x	
3722	Pyruvate dehydrogenase E1 component subunit alpha_ somatic form_ mitochondrial	P08559	129810.61	121568.09	113744.22	83154.05	78529.79	74048.84	97516.70	87806.56	89463.10	100715.46	97594.96	92337.04	99793.31	89258.16	69425.46	81541.38	78425.07	79089.69	83951.22	78263.98	72959.25	121984.86	118397.21	105715.40	79124.28	77159.10	70472.38	105137.23	98791.36	94511.93	99647.43	93168.42	64136.66		

Supplementary Table S1. Overview on the abundance of all relatively quantified 5180 proteins

			MCF-7																					MDA-MB-231													
	Protein name	UniProt	control 01	control 02	control 03	Dai IC20 01	Dai IC20 02	Dai IC20 03	Dai SC20 01	Dai SC20 02	Dai SC20 03	Gen IC20 01	Gen IC20 02	Gen IC20 03	Gen SC20 01	Gen SC20 02	Gen SC20 03	SSE IC20 01	SSE IC20 02	SSE IC20 03	SSE SC20 01	SSE SC20 02	SSE SC20 03	control 01	control 02	control 03	Dai IC20 01	Dai IC20 02	Dai IC20 03	Gen IC20 01	Gen IC20 02	Gen IC20 03	SSE IC20 01	SSE IC20 02	SSE IC20 03	unique to MCF-7	unique to MDA-MB-231
3723	Pyruvate dehydrogenase E1 component subunit alpha_ testis-specific form_ mitochondrial	P29803	0.14	2.53	0.81	1.07	2.77	0.68	0.96	0.36	0.63	0.35	0.75	0.38	2.13	0.97	0.60	4.64	1.07	0.47	2.22	0.19	0.06	19424.36	19581.20	17757.76	14451.90	14464.76	14078.74	13643.08	13795.52	14277.64	16387.30	15685.22	4222.86		x
3724	Pyruvate dehydrogenase E1 component subunit beta_ mitochondrial	P11177	149788.15	138546.48	124619.55	95635.20	94069.94	85003.83	113084.70	117290.41	105907.40	129002.57	122015.17	109958.23	119863.67	111756.35	77254.61	103386.50	99131.74	94264.07	101576.35	98089.92	84131.04	24878.31	22478.30	20083.77	14885.33	14194.11	9614.44	18743.39	16745.79	14881.33	19000.02	18392.88	9855.47		
3725	Pyruvate dehydrogenase phosphatase regulatory subunit_ mitochondrial	Q8NCN5	0.14	2.53	0.81	1.07	2.77	0.68	0.96	0.36	0.63	0.35	0.75	0.38	2.13	0.97	0.60	4.64	1.07	0.47	2.22	0.19	0.06	16778.64	13521.73	11977.18	9160.72	8599.15	7095.64	11601.00	12556.66	9605.44	12318.28	11194.79	7659.19		x
3726	Pyruvate dehydrogenase protein X component_ mitochondrial	O00330	0.14	2.53	0.81	1.07	2.77	0.68	0.96	0.36	0.63	0.35	0.75	0.38	2.13	0.97	0.60	4.64	1.07	0.47	2.22	0.19	0.06	6948.39	6576.20	5963.72	3512.35	3290.82	3028.08	4471.76	4522.76	4148.06	4017.12	3835.10	2506.50		x
3727	Pyruvate kinase PKLR	P30613	19929.46	18446.60	17557.35	9928.30	9176.53	8829.87	12496.01	12192.86	12283.51	14951.13	14845.16	13343.56	13497.58	12465.32	10341.75	10702.66	10472.03	10442.89	10800.61	10409.13	9909.30	14496.73	13625.12	12402.94	7032.41	7202.68	6121.33	9495.29	9308.55	8914.13	9295.43	8805.45	5348.36		
3728	Pyruvate kinase PKM	P14618	4423997.27	4126932.92	3753227.09	3010372.30	2909113.78	2618531.73	3729862.57	3566347.87	3324774.03	3934201.25	3685107.14	3366941.41	3847841.09	3627636.43	2590543.61	3031070.93	2847088.02	2750590.40	3114826.95	2920314.92	2650145.69	3479292.84	3173706.55	2921840.49	2093536.51	2049689.68	1709255.42	2683865.80	2521163.76	2301195.13	2599066.94	2532355.25	1713981.66		
3729	Queuine tRNA-ribosyltransferase accessory subunit 2	Q9H974	13769.03	12865.58	11265.17	11741.46	11581.54	10858.80	12407.44	11583.18	10966.70	14311.37	13804.65	12593.24	13086.10	11208.17	9392.52	11800.18	10440.58	10330.27	11735.38	11903.65	11031.77	2.27	1.49	0.65	0.84	1.31	0.69	4.16	2.47	0.11	4.90	4.98	0.12	x	
3730	Queuosine salvage protein	Q5T6V5	64868.17	51243.92	37034.98	25326.66	24251.33	18042.55	37893.46	39335.63	30698.28	48572.06	42851.29	32658.86	39515.52	34501.62	13113.61	49496.72	47613.41	38350.46	35191.85	31058.67	24127.47	2.27	1.49	0.65	0.84	1.31	0.69	4.16	2.47	0.11	4.90	4.98	0.12	x	
3731	Quinone oxidoreductase	Q08257	138627.37	128670.72	128914.11	74889.96	71236.67	70343.64	120792.86	112682.28	112302.42	113543.12	112357.62	104884.74	108174.20	95684.09	101404.95	122145.39	109119.61	111780.82	103908.23	108851.10	108443.73	24629.60	20287.23	21531.37	12371.34	12803.69	9882.75	13544.32	12385.83	12966.04	17532.48	16311.52	3799.66		
3732	Rab effector MyRIP	Q8NFW9	43460.01	47787.46	53144.88	27446.04	23737.93	20591.79	21138.71	23089.51	18724.42	43663.10	46794.18	57246.25	43956.47	40677.70	11234.41	16345.78	22480.45	26212.32	45269.05	40530.31	34002.85	2.27	1.49	0.65	0.84	1.31	0.69	4.16	2.47	0.11	4.90	4.98	0.12	x	
3733	Rab effector Noc2	Q9UNE2	0.14	2.53	0.81	1.07	2.77	0.68	0.96	0.36	0.63	0.35	0.75	0.38	2.13	0.97	0.60	4.64	1.07	0.47	2.22	0.19	0.06	2643.09	2434.37	2216.31	2250.83	2102.40	1871.73	3050.12	2779.80	2666.70	3247.63	3142.73	1899.90		x
3734	Rab GDP dissociation inhibitor alpha	P31150	237945.85	232192.67	206294.08	159269.71	157578.36	142617.54	204871.94	194060.22	181307.87	213513.66	198998.68	188632.17	198631.33	193343.69	154800.80	166247.18	157402.98	151504.03	166332.20	159729.44	151278.51	218778.62	205530.26	193696.07	126104.33	121073.01	102038.70	144563.76	136531.63	130958.09	138916.42	133805.28	92334.16		
3735	Rab GDP dissociation inhibitor beta	P50395	913823.98	865615.37	784358.46	561794.06	538604.47	500653.73	720327.73	682499.92	643748.90	723423.28	685388.12	629537.49	702919.05	660785.79	524586.22	592288.51	546762.39	536826.73	581163.27	551730.82	508542.02	290676.81	252163.27	245626.12	156307.00	150967.46	124661.30	184631.60	165775.23	154762.91	177608.35	169873.96	113960.43		
3736	Rab GTPase-activating protein 1	Q9Y3P9	169458.83	143719.59	120193.88	88605.79	85313.09	70942.66	121419.85	117487.37	100148.96	123891.43	113541.11	102901.03	121190.24	107647.04	62308.40	108077.32	98871.82	92167.64	97364.96	92523.02	78727.42	62822.18	57235.51	55154.38	38953.50	38058.39	31212.52	49292.19	46155.07	43645.12	40779.83	40956.53	29225.19		
3737	Rab GTPase-activating protein 1-like isoform 10	B7ZAP0	39830.78	39370.77	35919.34	25761.71	25282.70	22728.08	34867.84	35628.75	35205.57	38455.04	37363.43	36323.29	36281.73	33947.06	26953.84	26448.17	22540.05	23589.22	29612.21	25967.37	24734.41	2.27	1.49	0.65	0.84	1.31	0.69	4.16	2.47	0.11	4.90	4.98	0.12	x	
3738	Rab GTPase-binding effector protein 1	Q15276	495.99	444.27	561.73	487.99	483.69	447.01	683.15	681.54	653.64	638.22	640.67	621.06	472.65	463.22	421.49	300.24	383.97	367.28	346.31	311.90	334.85	2335.59	1896.69	1875.44	1546.22	1617.36	1448.65	2147.22	2044.03	1683.31	1914.75	2008.99	2693.77		
3739	Rab11 family-interacting protein 2	Q7L804	60462.86	59589.21	55869.21	47735.31	45003.15	44657.50	57631.48	53691.47	53259.35	57492.62	54073.45	50407.87	55537.30	48271.63	37124.21	47812.31	42800.05	44292.43	48237.22	45626.29	39383.97	8361.27	7937.09	7335.16	5507.77	5301.03	4369.40	6610.03	6650.86	5775.35	7307.61	6712.90	4240.41		
3740	Rab3 GTPase-activating protein catalytic subunit	Q15042	17080.77	16801.52	15104.23	13682.20	12914.00	12591.69	13997.29	13269.31	12572.25	17519.81	15855.34	16041.77	14583.94	14532.55	12386.83	13042.14	12315.16	11786.07	13356.03	12608.17	11924.47	34614.44	33570.36	31628.94	24292.17	24301.66	21356.93	29852.38	30439.61	27350.57	29513.51	28553.09	20553.90		
3741	Rab3 GTPase-activating protein non-catalytic subunit	Q9H2M9	13595.80	14444.91	13439.28	12978.60	12426.55	11098.53	13937.92	12998.63	12507.58	14026.26	13398.68	14093.22	11229.44	10097.09	5435.88	17209.63	14826.65	15346.69	12269.96	11026.73	7852.60	2.27	1.49	0.65	0.84	1.31	0.69	4.16	2.47	0.11	4.90	4.98	0.12	x	
3742	Rab9 effector protein with kelch motifs	Q7Z6M1	1387.54	1159.45	869.39	1161.28	973.23	1023.58	1312.09	1215.52	1266.40	1452.46	1192.32	1303.50	973.64	1052.08	829.79	1031.97	892.84	852.00	938.47	948.97	782.70	2.27	1.49	0.65	0.84	1.31	0.69	4.16	2.47	0.11	4.90	4.98	0.12	x	
3743	Rabankyrin-5	Q9P2R3	0.14	2.53	0.81	1.07	2.77	0.68	0.96	0.36	0.63	0.35	0.75	0.38	2.13	0.97	0.60	4.64	1.07	0.47	2.22	0.19	0.06	14207.79	13423.77	11927.05	9316.96	8758.22	7461.31	11483.10	11030.65	10260.22	12364.97	11383.95	6891.58		x
3744	Rab-interacting lysosomal protein	Q96NA2	1164.41	831.19	855.83	1020.55	908.25	874.83	810.46	786.00	754.24	1145.82	1106.66	1173.53	1122.68	877.30	937.72	821.27	751.77	771.14	878.65	791.26	896.53	2.27	1.49	0.65	0.84	1.31	0.69	4.16	2.47	0.11	4.90	4.98	0.12	x	
3745	Rab-like protein 3	Q5HYI8	32861.51	29565.28	30148.72	23164.78	22111.51	21420.06	23218.74	23652.34	23363.18	28759.06	28227.33	29453.37	28761.20	26726.54	20822.83	20099.47	19632.42	20246.09	26269.13	25368.13	24344.06	2.27	1.49	0.65	0.84	1.31	0.69	4.16	2.47	0.11	4.90	4.98	0.12	x	
3746	Rab-like protein 6	Q3YEC7	14544.54	14300.92	12422.49	9178.85	9258.61	8592.89	8799.05	8615.64	8225.93	12491.57	12232.21	11304.38	12286.13	10874.96	8896.90	8430.34	8074.51	7804.66	9554.62	8453.45	8064.14	2.27	1.49	0.65	0.84	1.31	0.69	4.16	2.47	0.11	4.90	4.98	0.12	x	
3747	RAC-alpha serine/threonine-protein kinase	P31749	106382.14	97385.02	86233.78	56922.18	54265.10	49816.27	57875.78	57112.07	55162.94	87963.32	83290.48	78424.92	92697.50	72757.89	59965.51	53141.86	52760.70	50554.34	56570.16	57973.44	53689.14	2.27	1.49	0.65	0.84	1.31	0.69	4.16	2.47	0.11	4.90	4.98	0.12	x	
3748	RAC-beta serine/threonine-protein kinase	P31751	107.47	76.11	158.28	162.63	129.04	169.17	115.98	151.96	69.45	161.07	186.51	96.27	31.65	64.80	114.82	168.97	216.61	216.06	140.97	128.81	160.38	5855.50	3003.75	2219.13	3749.95	3042.74	1408.90	5274.24	4548.26	2128.67	6442.51	5622.72	2569.64		
3749	RAC-gamma serine/threonine-protein kinase	Q9Y243	1365.80	1206.82	1008.47	772.55	497.38	407.16	661.36	618.40	561.16	800.48	850.71	706.20	553.45	745.12	428.62	493.32	577.69	455.04	668.83	708.32	614.59	18452.29	16782.66	14536.31	13108.31	11716.03	7557.81	14105.90	13268.44	11759.82	20097.29	20278.46	10137.47		
3750	RAD51-associated protein 2	Q09MP3	41103.90	39738.93	35434.42	29006.52	27954.05	26032.90	32564.17	30455.43	30135.46	31998.71	31104.34	28028.46	29743.92	29855.21	27507.37	26063.17	25581.94	26378.75	27318.53	25938.70	25088.28	2.27	1.49	0.65	0.84	1.31	0.69	4.16	2.47	0.11	4.90	4.98	0.12	x	
3751	Radial spoke head 1 homolog	Q8WYR4	15005.53	13947.36	14344.90	9727.21	9376.93	9004.75	12072.23	10290.64	11942.18	12699.84	12188.30	11635.75	11797.45	10932.83	8698.30	10432.47	9855.41	9672.82	9018.32	9280.78	8607.80	2.27	1.49	0.65	0.84	1.31	0.69	4.16	2.47	0.11	4.90	4.98	0.12	x	
3752	Radial spoke head protein 3 homolog	Q86UC2	4423.73	4319.31	3234.92	2813.09	3003.97	2548.96	3088.26	3052.85	2638.36	3766.71	3955.38	3081.45	3433.61	3398.49	2354.77	2748.80	2652.33	2604.02	2898.32	3090.76	2715.10	2.27	1.49	0.65	0.84	1.31	0.69	4.16	2.47	0.11	4.90	4.98	0.12	x	
3753	Radixin	P35241	647728.58	605002.92	556011.88	311344.04	303491.83	283965.72	380482.69	366813.33	350497.68	475722.44	452163.97	415447.47	474181.42	428673.18	366999.47	321324.62	302060.63	297123.89	361045.35	332204.38	311474.34	168867.14	155402.39	141834.43	117486.75	110567.55	94934.29	138870.04	128415.79	115040.56	136101.52	130665.97	95591.78		
3754	RAF proto-oncogene serine/threonine-protein kinase	P04049	20848.83	20409.24	20092.90	15882.49	15259.55	14194.50	14923.61	14527.65	14454.97	17402.37	16929.39	16292.56	18402.13	17490.21	16044.83	13287.80	13102.22	12015.26	13852.29	13479.81	13734.07	2.27	1.49	0.65	0.84	1.31	0.69	4.16	2.47	0.11	4.90	4.98	0.12	x	
3755	Raftlin	Q14699	0.14	2.53	0.81	1.07	2.77	0.68	0.96	0.36	0.63	0.35	0.75	0.38	2.13	0.97	0.60	4.64	1.07	0.47	2.22	0.19	0.06	2925.58	2743.90	3008.66	2106.36	2110.22	2001.69	3019.47	3077.59	2864.78	3242.51	3144.48	1887.03		x
3756	Ragulator complex protein LAMTOR1	Q6IAA8	14305.06	13019.32	11603.01	9517.94	9210.34	8271.04	10281.87	9738.41	9360.51	11648.10	11054.71	9956.89	12130.58	10182.98	7645.18	9569.12	8866.30	8550.60	10382.07	9350.28	8404.50	3684.43	3587.85	3345.92	2490.51	2435.94	1712.23	2691.16	2467.75	2393.09	3470.94	3209.66	1587.83		
3757	Ragulator complex protein LAMTOR5	O43504	26088.93	24716.14	22490.60	10958.54	11118.82	8795.57	14755.87	14572.51	12151.55	15026.26	13316.52	12807.57	13730.26	13530.36	9155.61	14965.91	14858.49	13078.68	12568.22	12005.45	10473.27	2.27	1.49	0.65	0.84	1.31	0.69	4.16	2.47	0.11	4.90	4.98	0.12	x	
3758	Ral GTPase-activating protein subunit alpha-2	Q2PPJ7	81124.98	73684.10	64673.57	45669.35	41731.51	34966.25	43600.07	46175.59	39347.09	65402.25	62281.93	57688.45	63818.30	56780.13	26949.97	42708.86	45162.78	41862.25	58907.99	51586.38	40965.89	116797.66	109149.10	102331.77	79468.25	78336.88	70885.41	89676.65	86089.91	80275.84	97580.71	93008.16	64650.19		
3759	Ran GTPase-activating protein 1	P46060	168818.71	162215.70	149647.55	108866.31	103890.20	96237.05	129753.48	125868.34	119659.18	136475.72	129362.63	119905.16	134360.40	126290.15	107181.62	109365.37	102200.56	99290.48	111267.04	108220.39	101335.90	9201.91	7778.01	6781.93	3246.11	3083.01	1841.13	4385.09	3920.53	3171.01	4381.79	3784.16	1870.76		
3760	Ran-binding protein 3	Q9H6Z4	398.53	421.98	318.12	262.01	315.53	290.55	281.31	289.34	298.60	340.50	300.04	301.71	321.62	343.21	234.74	230.09	204.85	213.34	252.08	225.69	261.47	1997.92	1985.31	2332.91	2220.21	2210.69	2084.68	2060.40	2053.74	2346.81	1939.98	1900.76	1734.88		
3761	Ran-specific GTPase-activating protein	P43487	354613.68	342197.48	316779.01	248384.25	238374.52	221016.41	284432.55	267036.90	273639.42	288644.49	274847.09	257366.42	318004.26	288062.63	240977.81	262758.39	244056.35	240785.21	279394.00	252766.15	239542.59	393520.40	365242.30	340195.96	247029.09	242339.70	214050.83	302425.89	289373.05	267758.36	295672.00	291587.37	203457.16		
3762	Rap guanine nucleotide exchange factor 2	Q9Y4G8	17724.68	15313.01	15162.10	10973.62	11125.65	10481.35	14234.21	13658.15	13804.37	17773.92	17162.95	16865.39	13984.92	13963.28	12314.06	13818.93	13134.94	12867.49	12940.29	11853.48	11128.80	2.27	1.49	0.65	0.84	1.31	0.69	4.16	2.47	0.11	4.90	4.98	0.12	x	
3763	Rap guanine nucleotide exchange factor 5	Q92565	48216.41	50228.08	46506.49	21949.07	22807.10	23773.13	34890.66	34136.63	32355.41	27564.24	25671.98	25765.21	33671.69	31285.85	26105.80	29236.97	24076.71	24555.69	23224.68	24859.46	22961.74	2.27	1.49	0.65	0.84	1.31	0.69	4.16	2.47	0.11	4.90	4.98	0.12	x	
3764	Rap guanine nucleotide exchange factor 6	Q8TEU7	0.14	2.53	0.81	1.07	2.77	0.68	0.96	0.36	0.63	0.35	0.75	0.38	2.13	0.97	0.60	4.64	1.07	0.47	2.22	0.19	0.06	5914.24	5757.77	5436.46	4186.43	4085.49	2916.92	4759.86	4745.06	4288.72	5797.45	5091.87	3423.99		x
3765	Rap1 GTPase-GDP dissociation stimulator 1	P52306	63990.81	58521.50	54922.72	42806.90	39994.72	35343.85	52713.11	47387.61	49179.29	56169.56	53399.48	47857.17	55813.73	45527.62	31410.24	42011.28	37052.32	38997.56	45720.25	40765.65	38058.76	4262.62	3855.59	3751.60	2829.28	2787.81	1962.17	3551.85	3368.66	2822.95	3486.45	3171.16	1723.87		
3766	Ras and EF-hand domain-containing protein	Q8IZ41	0.14	2.53	0.81	1.07	2.77	0.68	0.96	0.36	0.63	0.35	0.75	0.38	2.13	0.97	0.60	4.64	1.07	0.47	2.22	0.19	0.06	1630.61	1323.26	1337.51	786.05	769.53	577.42	1340.20	1260.66	1163.80	1263.39	1084.94	892.24		x
3767	Ras and Rab interactor-like protein	Q6ZS11	0.14	2.53	0.81	1.07	2.77	0.68	0.96	0.36	0.63	0.35	0.75	0.38	2.13	0.97	0.60	4.64	1.07	0.47	2.22	0.19	0.06	8757.27	7836.22	6723.51	5805.32	4782.92	3652.34	6934.60	6516.21	5040.90	7194.89	7165.50	3925.73		x
3768	Ras association domain-containing protein 2	P50749	4901.09	4860.45	3996.25	2505.85	2847.10	1965.17	3606.60	3461.16	2836.53	3531.58	3459.81	3411.15	3941.25	3897.48	3237.71	2758.81	2894.28	2899.93	2406.45	2439.12	2642.02	2.27	1.49	0.65	0.84	1.31	0.69	4.16	2.47	0.11	4.90	4.98	0.12	x	
3769	Ras association domain-containing protein 8	Q8NHQ8	37840.30	35693.87	32695.47	25063.90	24290.13	23086.07	28564.76	28198.73	30664.37	33686.77	32015.67	32249.02	34637.50	31937.08	28746.87	27199.72	26439.86	24898.98	39683.50	33856.00	35277.11	16651.86	16149.42	15377.05	17170.75	15939.93	15472.54	15735.93	14968.32	15850.90	16754.67	17549.67	2374.22		
3770	Ras GTPase-activating protein 1	P20936	0.14	2.53	0.81	1.07	2.77	0.68	0.96	0.36	0.63	0.35	0.75	0.38	2.13	0.97	0.60	4.64	1.07	0.47	2.22	0.19	0.06	2080.88	1861.80	1576.92	1113.45	1062.65	571.25	2017.41	1871.65	1702.19	1917.78	1790.47	669.37		x
3771	Ras GTPase-activating protein 3	Q14644	16660.03	15552.32	14186.63	11696.79	10031.07	8672.99	14055.45	13572.57	12496.96	13883.71	13988.94	13080.98	15293.15	16644.41	8879.60	10970.08	10371.53	9512.36	11622.01	11508.07	10338.18	29555.49	28273.62	24116.09	16158.11	15445.36	14450.19	22968.25	22245.97	22641.19	25173.84	25716.48	17394.28		
3772	Ras GTPase-activating protein-binding protein 1	Q13283	175964.74	174021.03	168023.20	117332.05	110576.47	105628.51	129484.56	127064.20	126288.56	146237.22	137418.23	127612.38	127696.60	122372.84	96865.57	120800.97	111595.47	112432.52	114725.32	113579.14	110074.85	153490.17	141059.73	136333.44	93935.37	86300.30	75573.27	123001.86	116792.32	108826.34	124646.72	116046.82	79026.23		
3773	Ras GTPase-activating protein-binding protein 2	Q9UN86	8505.02	7745.42	7065.68	4195.90	4602.68	4304.74	5348.94	4341.35	4095.07	7475.26	6727.60	6289.47	7054.75	5425.35	3642.36	4733.07	4348.67	4614.83	5066.63	4775.76	4239.18	53275.58	48536.97	47482.41	32304.49	30693.36	26031.79	36027.23	33269.34	32257.35	32347.88	29335.69	13534.37		
3774	Ras GTPase-activating-like protein IQGAP1	P46940	1137988.52	1072207.60	995264.49	718983.66	704757.35	650608.76	832095.63	786637.57	754856.55	906054.97	865951.94	799886.33	912892.10	844164.59	672743.48	710828.09	675611.00	672318.58	694672.83	670564.94	624913.33	1172671.32	1083508.04	1004387.31	710434.96	688639.58	580750.90	922199.83	877765.78	830713.39	891547.20	866858.90	573297.79		
3775	Ras GTPase-activating-like protein IQGAP2	Q13576	37803.78	34996.96	34098.67	21856.13	22356.05	21738.20	35500.42	33993.27	33361.25	30514.82	30286.24	29666.30	30434.35	26893.99	24711.96	26361.43	26279.48	25309.35	29674.86	27365.43	24621.42	152799.39	146233.21	130534.18	89941.52	91553.75	79449.01	108650.70	105422.72	97731.26	115119.35	110766.84	74266.47		
3776	Ras GTPase-activating-like protein IQGAP3	Q86VI3	0.14	2.53	0.81	1.07	2.77	0.68	0.96	0.36	0.63	0.35	0.75	0.38	2.13	0.97	0.60	4.64	1.07	0.47	2.22	0.19	0.06	15049.33	15586.44	13749.43	11199.70	11925.85	11084.06	14446.00	14079.60	13869.91	13208.41	12567.49	10421.56		x
3777	Ras suppressor protein 1	Q15404	0.14	2.53	0.81	1.07	2.77	0.68	0.96	0.36	0.63	0.35	0.75	0.38	2.13	0.97	0.60	4.64	1.07	0.47	2.22	0.19	0.06	24764.21	22970.92	18798.02	15223.86	14708.39	13621.65	18329.70	18116.58	17504.40	17591.92	17779.71	11673.98		x
3778	Ras/Rap GTPase-activating protein SynGAP	Q96PV0	15992.58	14626.81	13718.59	10680.93	9707.11	9000.87	13060.93	12922.42	12450.64	12825.94	12015.71	11030.63	14552.88	13132.26	9953.15	10062.49	9634.37	9717.74	11927.04	11533.65	10270.53	2.27	1.49	0.65	0.84	1.31	0.69	4.16	2.47	0.11	4.90	4.98	0.12	x	
3779	Ras-GEF domain-containing family member 1B	Q0VAM2	50410.92	47572.03	44487.15	42175.94	42590.51	39954.55	39965.41	37672.39	37675.25	51686.78	49559.52	47632.92	46759.09	40638.65	35442.24	41820.52	42465.16	40320.22	33891.67	33075.61	32478.31	2.27	1.49	0.65	0.84	1.31	0.69	4.16	2.47	0.11	4.90	4.98	0.12	x	
3780	Ras-GEF domain-containing family member 1C	Q8N431	2017.76	1769.51	2486.07	1552.63	1512.61	1406.19	2013.89	1855.31	1948.03	1664.27	1882.05	1711.59	1575.35	1698.54	1410.84	1538.11	1418.44	1385.12	1345.25	1292.08	1191.80	2.27	1.49	0.65	0.84	1.31	0.69	4.16	2.47	0.11	4.90	4.98	0.12	x	
3781	Ras-related C3 botulinum toxin substrate 1	P63000	178531.72	175825.75	172205.68	132124.08	129410.82	125860.95	156569.71	152368.28	149009.30	150073.24	146630.00	138566.97	140526.89	137241.02	122636.73	136643.89	131391.28	125092.59	125602.14	118926.07	113974.27	146832.21	137045.02	137709.21	84242.67	86241.56	74658.10	87306.44	85826.11	82783.64	85651.41	83560.98	60539.13		
3782	Ras-related GTP-binding protein B	Q5VZM2	0.14	2.53	0.81	1.07	2.77	0.68	0.96	0.36	0.63	0.35	0.75	0.38	2.13	0.97	0.60	4.64	1.07	0.47	2.22	0.19	0.06	19697.58	18541.58	15210.80	5525.12	6024.37	3226.17	15909.33	15955.18	13295.87	11251.59	10471.50	5250.43		x
3783	Ras-related protein Rab-10	P61026	64642.53	58164.64	55054.06	31738.23	30342.21	27905.07	40015.19	39500.89	37785.35	46751.75	43758.71	39579.37	48989.75	39303.99	30203.83	36568.26	34813.97	31501.36	33890.34	31570.12	29588.69	18724.07	17975.87	15013.33	12131.22	11694.49	10058.83	15759.66	14917.58	13732.98	14800.31	13640.42	8991.59		
3784	Ras-related protein Rab-11B	Q15907	402948.06	386846.74	363810.10	253434.05	248336.13	235978.10	291258.98	280036.66	278374.61	313643.53	301497.50	291550.86	313243.42	296795.98	265449.09	245714.75	232274.82	228314.27	243977.34	238889.50	224576.73	187184.05	179298.76	167198.72	123464.17	125245.55	116300.68	136735.35	138691.04	135333.19	149621.78	141501.10	108040.14		
3785	Ras-related protein Rab-12	Q6IQ22	127960.77	125144.85	118540.99	89774.42	85157.82	82913.31	104336.77	105689.76	96859.42	110902.73	107627.64	99278.00	105236.21	101124.97	82129.96	88458.19	85011.11	77959.72	81597.06	81111.21	77377.97	40995.31	40298.47	39819.55	27961.61	27581.94	24390.88	34664.61	33067.52	31792.35	27630.05	26983.43	20476.99		
3786	Ras-related protein Rab-13	P51153	59877.93	60157.15	53343.37	44213.43	45828.80	39415.85	67758.41	65698.57	57694.53	64096.48	62595.07	60431.88	53608.96	45797.59	42933.01	44233.00	41553.68	37782.78	40293.19	40739.39	37262.60	42477.26	42665.97	35886.07	28960.06	27863.03	20913.65	45923.95	43349.02	39785.05	40147.12	38056.26	24521.86		
3787	Ras-related protein Rab-14	P61106	309869.09	311695.71	282618.30	204458.94	196310.95	179287.83	233013.29	218612.02	201335.62	258095.03	239630.78	231148.31	248284.78	214415.51	164076.88	200381.37	202856.13	193290.96	214330.77	204714.32	168640.40	126530.62	115269.83	107127.08	82646.13	77469.85	69746.04	99191.59	94011.19	91971.61	99300.53	89101.46	53719.65		
3788	Ras-related protein Rab-15	P59190	10332.50	9385.24	7866.20	7203.71	9561.72	6977.41	12710.30	7295.17	6590.05	8745.08	8516.03	7614.69	8423.02	8448.93	3966.89	9948.28	8223.86	7940.23	6015.38	5603.44	5073.70	9046.01	8911.33	8448.98	6460.49	5972.32	5209.49	6564.84	5858.48	6128.35	5991.30	5397.40	3944.90		
3789	Ras-related protein Rab-17	Q9H0T7	2821.72	2300.71	2642.08	1649.84	1849.01	1528.99	2519.87	2406.60	2226.05	2455.41	2379.60	2162.80	2343.94	2140.78	1930.53	2079.91	1742.18	1657.59	1702.43	1331.85	1452.15	2.27	1.49	0.65	0.84	1.31	0.69	4.16	2.47	0.11	4.90	4.98	0.12	x	
3790	Ras-related protein Rab-18	Q9NP72	48144.90	47603.75	45574.67	33014.00	32359.15	28606.54	38602.74	36096.58	33688.59	41696.61	39518.59	37020.77	37204.24	34914.78	29071.00	33798.60	33537.80	32373.60	31221.40	31025.63	28827.67	35051.30	33566.42	31228.12	22014.27	20918.02	18280.85	27163.97	25955.49	24578.71	25919.30	24381.61	14769.14		
3791	Ras-related protein Rab-1A	P62820	1081820.04	1033749.83	982339.15	681412.68	683046.41	620555.48	832934.50	811964.99	743931.58	908577.26	873695.80	831563.02	875921.46	819098.97	667554.10	707912.18	695456.07	660674.32	688690.61	664339.69	621901.37	40245.23	43034.87	37101.80	33567.22	35374.43	31841.95	27331.44	31115.68	30307.90	38452.41	36851.63	28718.10		
3792	Ras-related protein Rab-1B	Q9H0U4	219243.87	199535.51	180771.14	139479.34	128460.84	116013.02	149004.34	142420.56	132623.07	178983.67	163806.68	152578.77	191763.95	176338.27	140176.40	130309.10	125580.63	128517.63	142022.16	128467.45	124774.93	534194.81	453687.30	434559.28	331099.95	322218.43	279123.59	388221.50	377629.64	364742.75	374742.81	367477.49	241148.07		
3793	Ras-related protein Rab-20	Q9NX57	3920.71	3846.66	3074.52	2894.84	2728.56	2560.72	2640.07	2587.57	2613.39	2787.83	2990.05	3186.67	3439.09	3389.08	2474.15	2822.74	2370.82	2139.32	3158.90	3201.79	2883.11	2.27	1.49	0.65	0.84	1.31	0.69	4.16	2.47	0.11	4.90	4.98	0.12	x	
3794	Ras-related protein Rab-21	Q9UL25	89427.64	82953.86	75851.25	62908.11	60348.47	56051.48	71296.76	68765.21	64684.71	76547.21	72219.06	69974.30	70948.17	67110.44	57055.67	61661.42	58096.54	54361.50	60748.62	58285.25	53938.63	43676.45	40123.01	35128.70	25112.44	25288.67	21973.08	31001.39	29430.22	27416.88	32938.32	31745.05	19692.26		
3795	Ras-related protein Rab-22A	Q9UL26	48266.40	46789.95	41204.33	31805.79	28892.41	24979.14	36338.77	34977.43	33857.54	48994.93	45066.16	39707.19	39176.29	36634.40	22862.16	35133.68	29902.85	30885.34	32595.93	32360.17	29429.52	10926.61	10480.60	13441.57	8595.16	7707.16	6103.10	7914.86	8888.63	8629.45	8430.69	8334.73	3324.21		
3796	Ras-related protein Rab-24	Q969Q5	4700.54	4702.85	5605.30	4140.16	4151.35	3642.72	5129.78	4095.93	4416.08	5104.11	4637.25	4542.12	4944.22	4623.69	3732.54	4220.29	3785.26	3756.12	3690.42	3378.69	3033.05	2.27	1.49	0.65	0.84	1.31	0.69	4.16	2.47	0.11	4.90	4.98	0.12	x	
3797	Ras-related protein Rab-25	P57735	29208.19	28066.37	26431.20	22903.87	22103.27	22150.91	21882.47	21810.42	21137.87	23757.07	22219.11	21811.77	23583.01	23061.24	31904.72	22143.48	19866.92	20046.36	21615.65	21303.62	21137.40	21468.18	18089.62	13230.85	13191.94	12520.63	12149.35	13820.44	14173.68	12069.46	12289.08	11506.51	8296.08		
3798	Ras-related protein Rab-2A	P61019	128545.97	120350.01	116690.02	80433.53	80479.44	73804.36	111393.09	104412.99	98638.40	111783.42	107075.41	99886.44	106252.76	96050.02	75404.79	89981.59	88403.28	84373.91	87104.60	91696.70	76390.74	48729.96	38710.85	38573.48	32462.19	30149.22	22541.81	38189.57	35776.11	33177.92	41200.73	38381.41	23949.25		
3799	Ras-related protein Rab-2B	Q8WUD1	9851.25	10754.05	9471.42	7546.48	6983.18	6444.21	7966.58	7937.75	7719.93	8897.18	8684.84	7270.79	8847.03	7697.44	4832.52	7440.90	6156.08	6087.68	7651.28	7152.23	6163.70	16978.34	16137.94	14083.45	11202.52	10485.02	6577.17	14687.45	13136.73	11174.46	15810.77	15068.63	7622.58		
3800	Ras-related protein Rab-30	Q15771	13697.46	13477.21	11734.44	10740.46	10681.55	8863.18	12608.77	13085.69	11923.60	12944.30	12859.28	12512.55	13442.17	12042.49	8808.08	11373.45	10066.43	9510.52	10610.38	9572.44	8199.71	10210.07	9200.07	6260.43	6314.13	5523.05	3588.41	8913.84	7013.19	6300.37	8689.46	8182.12	4304.49		
3801	Ras-related protein Rab-32	Q13637	11005.59	8222.71	11868.56	7984.06	5154.46	7419.80	8228.51	9479.89	8178.96	11144.28	10570.35	7565.64	8756.65	8282.98	6767.50	9849.46	7663.33	8013.52	6367.96	8148.88	7372.02	27066.99	25517.04	23202.68	17804.44	17576.58	14375.48	21582.46	20989.09	20078.53	22599.79	21041.07	14053.50		
3802	Ras-related protein Rab-33B	Q9H082	20351.56	18657.00	17649.71	10843.22	9938.12	9129.77	13793.07	12995.90	13081.37	14062.34	13310.93	12147.14	13974.39	12950.74	10341.97	11353.50	10695.45	10204.41	11714.44	11468.61	10146.46	8280.83	7258.95	6230.60	5902.31	5992.14	5486.05	10123.30	9795.62	8892.76	6485.97	6115.87	4260.04		
3803	Ras-related protein Rab-34	Q9BZG1	0.14	2.53	0.81	1.07	2.77	0.68	0.96	0.36	0.63	0.35	0.75	0.38	2.13	0.97	0.60	4.64	1.07	0.47	2.22	0.19	0.06	15637.84	15085.92	14556.45	11318.60	11206.99	9565.91	14016.58	13149.40	13445.56	13491.71	13549.60	8780.82		x
3804	Ras-related protein Rab-35	Q15286	22679.34	23308.05	21149.67	12415.43	12443.86	11384.41	17444.04	17463.36	16228.56	15138.07	14689.23	14095.37	17878.63	16753.10	14849.08	12652.85	12223.38	10353.89	15367.60	13202.77	13283.58	28455.64	25894.96	22746.34	15814.25	15566.70	13975.20	21137.73	19705.73	17598.86	20468.12	19314.80	13522.84		
3805	Ras-related protein Rab-37	Q96AX2	29124.35	28101.83	27055.91	24611.91	21080.03	21253.15	16620.47	15346.07	13280.60	25772.04	24876.63	25723.64	26242.90	26982.14	19233.49	13610.70	16253.54	17012.39	25780.32	21687.11	22378.65	11487.45	8703.76	8822.91	4879.84	4751.22	4380.15	6189.12	5256.94	4698.69	6131.54	5617.31	2466.52		
3806	Ras-related protein Rab-38	P57729	2162.88	2200.43	1999.96	1311.78	1222.76	1228.54	1635.48	1505.49	1675.53	2356.52	2067.32	2095.04	2062.44	1871.11	1371.73	1240.11	1254.42	1191.06	1399.06	1390.12	1512.42	2.27	1.49	0.65	0.84	1.31	0.69	4.16	2.47	0.11	4.90	4.98	0.12	x	
3807	Ras-related protein Rab-39A	Q14964	35895.57	33446.06	32614.73	24612.48	23736.64	20782.90	31018.01	29334.41	28062.72	32135.07	30607.14	27601.74	29882.63	26226.86	21586.84	28395.78	27044.37	25079.12	23796.67	22187.86	20616.57	12369.89	13029.53	11729.98	7796.21	7246.14	5127.80	10843.19	9147.74	9356.47	10023.44	10454.48	5791.79		
3808	Ras-related protein Rab-39B	Q96DA2	0.14	2.53	0.81	1.07	2.77	0.68	0.96	0.36	0.63	0.35	0.75	0.38	2.13	0.97	0.60	4.64	1.07	0.47	2.22	0.19	0.06	764.33	896.97	814.78	764.64	792.40	744.59	804.71	704.18	695.87	1205.53	1102.99	217.24		x
3809	Ras-related protein Rab-3A	P20336	9728.91	9363.40	8227.57	8130.04	7449.66	6408.41	7626.48	6980.52	6586.52	10019.16	9724.45	8592.11	8279.94	7903.39	5164.03	6820.55	6553.27	6316.58	7661.67	8121.96	7408.48	8575.80	7835.68	7685.59	8445.48	8078.95	5786.01	9666.85	9204.33	8521.28	10577.85	9345.20	5408.20		
3810	Ras-related protein Rab-3B	P20337	110104.89	103376.30	101931.30	74585.86	70735.68	66604.51	76888.76	76768.81	73911.15	89086.62	83383.34	78744.82	86871.55	84946.28	72291.03	68140.00	65744.88	63680.25	75147.37	71820.56	68795.47	17081.09	16166.18	13912.45	10541.40	9378.63	7296.07	13451.76	12402.26	11080.55	12540.40	11554.23	6777.62		
3811	Ras-related protein Rab-3C	Q96E17	10848.06	10203.20	7737.93	6801.49	5374.68	5314.69	7936.68	6168.41	7101.86	8582.57	8534.25	8125.19	9080.08	6516.33	4125.13	6568.49	4687.65	5316.91	7039.28	7048.69	5203.77	19765.56	16860.49	14314.89	14454.79	14571.63	11373.88	17585.78	16636.36	14713.57	19092.90	18705.49	11882.68		
3812	Ras-related protein Rab-3D	O95716	173522.06	161218.27	141295.00	108525.88	103718.65	94595.50	113357.62	113936.39	108855.90	133154.82	126729.47	119612.24	139748.45	131107.24	108924.66	100372.30	92094.30	89424.13	99570.59	98281.39	95007.35	28795.74	26521.77	21793.69	19525.53	19308.16	17103.75	25969.08	24079.27	22873.24	23486.16	22552.92	16268.64		
3813	Ras-related protein Rab-40C	Q96S21	12048.47	12015.12	12013.11	6446.72	5614.56	6036.34	6766.33	6793.70	6906.58	4913.08	5091.87	5228.16	6383.65	5928.49	5647.09	8241.41	8082.08	8094.55	5205.60	4618.82	4832.65	2.27	1.49	0.65	0.84	1.31	0.69	4.16	2.47	0.11	4.90	4.98	0.12	x	
3814	Ras-related protein Rab-41	Q5JT25	0.14	2.53	0.81	1.07	2.77	0.68	0.96	0.36	0.63	0.35	0.75	0.38	2.13	0.97	0.60	4.64	1.07	0.47	2.22	0.19	0.06	3169.07	3167.05	2420.64	2149.90	2104.93	1875.95	1995.45	1994.17	1911.78	2775.51	2438.95	63.88		x
3815	Ras-related protein Rab-43	Q86YS6	31110.83	29977.86	26367.58	21139.62	19811.23	18087.24	24614.75	21700.27	20961.33	25894.39	25355.19	22275.68	23724.67	20737.01	14238.05	22059.19	21127.98	21107.59	23822.53	21332.32	19420.89	9455.52	7982.81	7233.12	4475.39	4470.47	3772.35	5038.13	4164.22	4093.99	5786.82	5672.71	3247.03		
3816	Ras-related protein Rab-4A	P20338	16679.35	16140.50	13761.44	11445.40	11539.33	10060.19	14249.26	14224.49	13745.20	13442.23	12913.10	11244.09	13382.24	13024.94	9799.00	11175.44	10633.14	9968.44	11251.25	10595.15	9410.04	5679.90	4619.86	4131.38	3042.49	2930.90	2643.85	4137.45	3851.58	3698.18	3976.29	4125.74	2445.80		
3817	Ras-related protein Rab-4B	P61018	15806.01	16662.75	15435.72	11550.09	10541.14	10507.66	11940.37	11926.38	12373.06	13636.58	12305.42	11902.47	12909.02	13293.50	10511.77	11693.31	9683.55	10001.83	10537.06	9805.82	10348.45	203.42	207.67	254.47	162.28	151.58	125.86	174.12	169.62	213.99	235.67	183.86	97.40		
3818	Ras-related protein Rab-5A	P20339	26634.30	26108.20	25036.25	17489.19	17708.84	16761.68	19375.04	18479.49	18585.72	24907.57	24059.78	22261.98	23107.80	21692.15	18819.26	19613.93	17249.05	16719.64	16855.04	16298.51	14116.29	2541.54	3087.58	2105.05	2775.60	2783.65	2556.24	2101.06	2044.94	1761.18	4708.49	3879.83	942.57		
3819	Ras-related protein Rab-5B	P61020	9012.49	9644.03	9327.12	8927.69	8134.74	6939.24	6100.87	6557.39	5929.64	9600.99	8539.05	8227.57	8588.73	10373.62	5694.05	7310.20	6975.86	7273.17	6842.26	5559.72	5661.46	70794.69	67823.37	62076.07	48245.98	46095.52	40763.52	56842.70	52731.20	52132.90	52250.91	50838.94	41157.70		
3820	Ras-related protein Rab-5C	P51148	165597.18	160848.85	151331.47	100672.60	97794.96	91011.79	118457.40	114166.96	112552.35	125552.57	122885.04	113517.52	131949.82	120065.68	90084.80	99054.75	97459.42	93286.20	99649.42	95797.96	91308.93	56033.69	53407.48	48175.97	38250.37	37645.50	35844.98	41334.67	40166.95	39546.41	44718.74	42924.91	27884.26		
3821	Ras-related protein Rab-6A	P20340	71352.95	69189.45	67529.97	60742.99	62317.77	61109.22	61697.81	58018.38	61767.41	72544.68	74030.34	68262.98	66800.84	58559.99	53708.68	60292.98	54482.77	57964.90	54867.78	51042.56	54011.46	2.27	1.49	0.65	0.84	1.31	0.69	4.16	2.47	0.11	4.90	4.98	0.12	x	
3822	Ras-related protein Rab-6B	Q9NRW1	38301.48	36683.24	34052.45	26940.37	25854.68	24591.05	30280.85	28099.70	28177.23	31552.46	30465.77	30448.50	29851.22	29478.40	25678.17	24322.06	23375.47	21943.24	24976.56	24316.58	24471.98	59875.71	56880.08	53699.62	39002.95	37825.17	34368.33	46365.64	43354.38	41509.27	41590.66	40981.22	29398.28		
3823	Ras-related protein Rab-6D	Q53S08	11693.13	11112.64	9526.11	6874.31	6889.43	6072.24	7898.24	7445.91	6979.02	10527.04	10806.45	10595.27	10233.61	8905.04	6582.16	7330.61	6525.78	6484.48	6579.58	6446.12	5788.53	2587.33	3064.19	2595.56	1811.78	2010.75	1352.75	2749.01	2590.83	2537.50	3114.20	2593.38	1629.78		
3824	Ras-related protein Rab-7a	P51149	577621.70	550113.03	493984.65	347298.35	336433.97	303371.60	428603.28	404332.01	376667.12	421020.33	393582.63	366810.27	423102.50	398466.30	317541.72	365329.47	349458.49	329625.69	344298.18	340628.68	316593.74	155626.20	147053.98	129760.23	80177.87	79133.69	64789.67	103092.46	96484.56	94159.51	102620.79	98667.06	62731.71		
3825	Ras-related protein Rab-7L1	O14966	0.14	2.53	0.81	1.07	2.77	0.68	0.96	0.36	0.63	0.35	0.75	0.38	2.13	0.97	0.60	4.64	1.07	0.47	2.22	0.19	0.06	565.60	593.07	434.53	402.67	352.98	425.10	367.80	311.25	294.37	555.42	378.04	86.78		x
3826	Ras-related protein Rab-8A	P61006	79893.57	77987.46	73446.22	59081.36	57149.83	54102.36	58087.43	56700.10	52077.92	64273.19	64056.77	63076.92	64148.52	61157.80	56972.40	51349.25	48035.48	46491.94	58226.50	55130.19	50687.96	61487.19	64727.77	57856.01	48441.12	49237.37	46372.49	49710.09	48455.67	48593.00	57510.40	55924.94	39298.71		
3827	Ras-related protein Rab-8B	Q92930	56261.40	53126.90	48219.36	43754.08	41932.46	37808.37	43401.11	43198.69	39955.47	48490.00	47162.50	45315.81	45968.32	46837.03	37424.02	37650.38	35778.84	34175.57	41892.73	39260.23	37447.23	107363.78	104416.35	96960.83	70895.20	70393.21	62351.06	85197.54	80083.24	75954.11	83277.27	77987.04	55344.34		
3828	Ras-related protein Rab-9A	P51151	3914.25	2622.63	2221.53	9282.45	1981.02	1379.51	2954.92	8168.86	4596.89	2479.96	2088.32	1848.95	2607.15	14589.64	1614.17	2111.25	2549.96	1387.80	3027.86	3080.26	1921.64	2.27	1.49	0.65	0.84	1.31	0.69	4.16	2.47	0.11	4.90	4.98	0.12	x	
3829	Ras-related protein Ral-A	P11233	11402.33	10154.62	10947.75	7584.42	7871.94	6981.66	8511.40	8847.67	8677.85	11187.24	10590.80	10007.76	11291.82	9565.66	7616.58	9214.19	7833.16	8045.91	8638.58	7812.09	7259.00	43194.20	29912.85	27228.10	26847.23	25508.59	16732.85	34896.47	29324.80	22185.63	37844.23	33525.46	21699.21		
3830	Ras-related protein Ral-B	P11234	5119.71	4634.64	4960.59	3784.84	3541.94	3250.48	4556.80	4408.10	4499.98	4752.05	4472.89	4379.82	4433.63	4292.63	3965.24	3667.55	4016.43	3461.09	3455.07	3370.79	3357.46	58835.44	56359.44	53612.05	29596.68	28137.10	23744.00	34292.26	32942.11	31662.60	29786.03	28223.96	19325.27		
3831	Ras-related protein Rap-1A	P62834	0.14	2.53	0.81	1.07	2.77	0.68	0.96	0.36	0.63	0.35	0.75	0.38	2.13	0.97	0.60	4.64	1.07	0.47	2.22	0.19	0.06	6909.95	5059.61	4690.06	2526.52	2339.60	1562.95	3507.76	3127.53	2756.96	3376.81	3113.27	1523.07		x
3832	Ras-related protein Rap-1b	P61224	76737.10	74706.71	71490.41	45737.68	46126.25	43923.03	57746.56	59031.16	55745.52	63192.13	59703.84	58956.41	62218.08	59038.39	50943.45	46241.36	45167.05	44434.52	41316.41	45245.73	43529.10	52466.93	47294.85	44311.69	26953.86	25795.04	22301.54	32754.50	32194.79	28398.44	29694.13	28579.50	18752.81		
3833	Ras-related protein Rap-2b	P61225	160.67	133.34	130.32	166.95	188.44	139.06	1000.13	1005.37	919.92	370.71	360.61	246.69	265.61	156.87	171.98	354.23	351.48	237.09	226.01	206.49	136.61	1017.86	927.89	881.77	189.73	161.25	45.42	132.80	86.63	108.43	66.39	71.55	10.18		
3834	Ras-related protein R-Ras	P10301	373467.39	353316.45	340904.70	206186.73	200705.89	188021.79	160726.16	157370.74	149105.39	257183.15	248283.32	218513.53	229529.43	197737.33	169415.66	199184.51	189338.92	183711.21	195655.87	182272.01	159804.43	54451.10	52717.68	45832.15	69588.99	67141.31	67656.10	126564.37	128570.84	123920.68	99146.28	93455.85	55947.06		
3835	Ras-related protein R-Ras2	P62070	5470.80	5690.42	5004.01	4386.37	4208.49	3872.26	4765.10	4633.64	4605.79	5721.27	5448.08	5451.58	4889.62	5287.82	4923.66	4600.32	4151.10	4011.79	5251.33	4388.78	4186.72	28179.84	24961.14	28034.92	22156.48	22839.15	19718.63	23732.95	23493.90	22829.41	26428.88	28499.44	30232.18		
3836	Ras-specific guanine nucleotide-releasing factor RalGPS2	Q86X27	3900.40	3436.83	3098.12	4265.94	4156.28	4072.78	5729.29	5490.09	5632.68	5197.82	5102.55	4946.10	3790.47	3073.25	2567.64	4814.89	4326.67	4387.14	3690.31	3667.90	3665.98	2.27	1.49	0.65	0.84	1.31	0.69	4.16	2.47	0.11	4.90	4.98	0.12	x	
3837	RB1-inducible coiled-coil protein 1	Q8TDY2	0.14	2.53	0.81	1.07	2.77	0.68	0.96	0.36	0.63	0.35	0.75	0.38	2.13	0.97	0.60	4.64	1.07	0.47	2.22	0.19	0.06	5223.95	4400.02	4253.10	6207.85	6314.98	6057.36	6369.84	5953.49	5632.08	6707.03	6291.79	3953.86		x
3838	Reactive oxygen species modulator 1	P60602	12427.17	12532.06	11887.81	9015.35	9194.80	8172.59	11703.70	11232.27	10956.69	14130.47	12735.74	12761.98	9658.58	9556.80	7143.00	8719.50	8950.08	8642.18	7945.23	7570.60	7625.13	9146.62	8224.48	8762.72	4413.69	3925.38	3509.96	6173.99	6124.47	5788.16	4296.62	4157.67	2987.30		
3839	Receptor expression-enhancing protein 4	Q9H6H4	2799.17	2756.57	2338.87	3102.24	3039.85	2843.41	3386.16	3139.76	2723.67	3491.59	2756.32	3009.99	2731.94	2438.48	2712.47	1295.16	2205.93	1945.82	1574.39	2263.89	2077.36	2.27	1.49	0.65	0.84	1.31	0.69	4.16	2.47	0.11	4.90	4.98	0.12	x	
3840	Receptor expression-enhancing protein 5	Q00765	239727.08	239459.40	226923.12	169787.44	161794.09	160050.64	180785.30	184707.99	172247.53	189670.04	190461.14	176141.16	195762.90	190282.50	191157.02	172401.83	159567.94	156714.66	164995.03	162310.19	155113.96	124128.06	115720.56	108716.49	83917.63	80236.25	73930.92	98244.69	93875.35	90954.69	94772.47	96438.48	73636.13		
3841	Receptor of activated protein C kinase 1	P63244	990349.17	942976.22	908976.28	591038.12	565274.32	532628.85	788371.69	763289.73	727024.83	725660.33	700589.47	665236.76	732826.25	684324.04	597398.94	605000.15	578960.36	570578.66	629414.73	597774.30	557266.22	622133.88	577915.86	542283.30	358272.60	347527.11	313801.42	465915.85	448458.65	429577.85	435491.81	422000.73	351905.79		
3842	Receptor tyrosine-protein kinase erbB-2	P04626	16082.11	12226.73	9409.63	8108.73	7668.70	5736.14	10406.17	9703.02	8378.70	13125.07	11509.31	9017.10	9670.87	8903.01	3662.09	9664.49	8950.08	7934.77	9460.91	8019.96	6414.92	2.27	1.49	0.65	0.84	1.31	0.69	4.16	2.47	0.11	4.90	4.98	0.12	x	
3843	Receptor-interacting serine/threonine-protein kinase 1	Q13546	33637.07	32456.57	28488.63	20943.25	20263.87	17147.56	24135.78	24647.32	22520.41	27372.68	26906.09	23700.55	27682.21	23993.02	16297.70	22968.98	20745.55	20965.22	19437.80	20194.90	16801.25	6465.18	6643.34	8277.38	7151.64	6684.09	5311.70	6415.31	5222.53	5513.74	7754.92	7348.26	5058.00		
3844	Receptor-type tyrosine-protein kinase FLT3	P36888	0.14	2.53	0.81	1.07	2.77	0.68	0.96	0.36	0.63	0.35	0.75	0.38	2.13	0.97	0.60	4.64	1.07	0.47	2.22	0.19	0.06	38119.59	35610.79	31708.31	23411.40	22331.90	14213.83	25687.55	23234.18	19361.21	25680.69	24766.44	12503.61		x
3845	Receptor-type tyrosine-protein phosphatase alpha	P18433	2340.42	2452.63	2562.33	1738.10	1489.79	1497.88	1687.62	1594.29	1801.44	2312.26	2129.24	2264.68	2454.55	2422.05	1712.02	1649.60	1607.27	1665.02	1635.95	1681.33	1722.13	2.27	1.49	0.65	0.84	1.31	0.69	4.16	2.47	0.11	4.90	4.98	0.12	x	
3846	Receptor-type tyrosine-protein phosphatase F	P10586	0.14	2.53	0.81	1.07	2.77	0.68	0.96	0.36	0.63	0.35	0.75	0.38	2.13	0.97	0.60	4.64	1.07	0.47	2.22	0.19	0.06	71631.31	69946.60	66787.26	52241.17	50800.21	48001.05	65761.92	63444.72	59175.05	67945.16	62375.43	48738.01		x
3847	Receptor-type tyrosine-protein phosphatase kappa	Q15262	20827.26	20310.97	20984.25	14075.97	13197.30	11273.90	14741.71	14941.44	13886.28	20447.36	18619.65	17021.49	18405.18	18824.82	11097.42	13720.68	13097.94	13279.38	13530.80	13748.81	12453.14	2.27	1.49	0.65	0.84	1.31	0.69	4.16	2.47	0.11	4.90	4.98	0.12	x	
3848	Receptor-type tyrosine-protein phosphatase mu	P28827	0.14	2.53	0.81	1.07	2.77	0.68	0.96	0.36	0.63	0.35	0.75	0.38	2.13	0.97	0.60	4.64	1.07	0.47	2.22	0.19	0.06	270.15	44.50	146.73	49.63	48.68	25.32	160.16	130.46	7.37	105.20	79.17	37.08		x
3849	Receptor-type tyrosine-protein phosphatase N2	Q92932	23640.85	21605.64	22983.98	15750.52	15602.62	15303.66	20308.46	17849.82	17546.30	18143.62	18623.75	16952.77	20378.43	19405.31	14073.80	15248.57	12684.50	15030.44	18952.18	17297.87	14765.90	2.27	1.49	0.65	0.84	1.31	0.69	4.16	2.47	0.11	4.90	4.98	0.12	x	
3850	Receptor-type tyrosine-protein phosphatase S	Q13332	0.14	2.53	0.81	1.07	2.77	0.68	0.96	0.36	0.63	0.35	0.75	0.38	2.13	0.97	0.60	4.64	1.07	0.47	2.22	0.19	0.06	1303.69	1159.00	1123.23	737.61	734.75	601.37	753.22	781.28	795.46	831.63	801.36	528.91		x
3851	Regulating synaptic membrane exocytosis protein 1	Q86UR5	570.45	381.53	492.68	344.73	410.86	606.87	415.67	262.44	455.00	272.02	470.76	368.69	350.02	215.90	240.80	482.80	342.16	523.53	489.55	378.34	320.95	2.27	1.49	0.65	0.84	1.31	0.69	4.16	2.47	0.11	4.90	4.98	0.12	x	

Supplementary Table S1. Overview on the abundance of all relatively quantified 5180 proteins

			MCF-7																						MDA-MB-231													
	Protein name	UniProt	control_01	control_02	control_03	Dai_IC20_01	Dai_IC20_02	Dai_IC20_03	Dai_SC20_01	Dai_SC20_02	Dai_SC20_03	Gen_IC20_01	Gen_IC20_02	Gen_IC20_03	Gen_SC20_01	Gen_SC20_02	Gen_SC20_03	SSE_IC20_01	SSE_IC20_02	SSE_IC20_03	SSE_SC20_01	SSE_SC20_02	SSE_SC20_03	control_01	control_02	control_03	Dai_IC20_01	Dai_IC20_02	Dai_IC20_03	Gen_IC20_01	Gen_IC20_02	Gen_IC20_03	SSE_IC20_01	SSE_IC20_02	SSE_IC20_03	unique to MCF-7	unique to MDA-MB-231
3852	Regulation of nuclear pre-mRNA domain-containing protein 1A	Q96P16	7202.39	6779.87	7112.12	5900.41	5431.62	5272.52	6442.95	6029.47	6210.94	6761.00	7160.14	6170.76	7353.95	6374.21	4966.32	5563.38	4983.06	4712.89	5436.60	5085.24	4823.84	5002.69	4389.34	4527.85	3123.65	2859.81	2683.73	3672.65	3542.68	3716.11	4082.41	4078.92	147.54		
3853	Regulation of nuclear pre-mRNA domain-containing protein 1B	Q9NQG5	48438.69	44672.25	42157.13	28706.99	25493.78	25391.38	33212.01	31586.79	29938.33	37083.60	35136.89	34415.04	38052.63	32640.32	27314.81	29098.91	25916.29	26245.38	27061.10	29005.98	25940.82	19332.84	17403.06	15470.24	11197.50	10768.26	8186.86	15072.56	14408.37	12976.74	15451.82	14496.38	8174.27		
3854	Regulation of nuclear pre-mRNA domain-containing protein 2	Q5VT52	35069.42	34059.88	31768.29	26879.25	25903.61	24405.45	27725.97	27000.05	25602.52	32928.92	32598.20	30961.81	32378.63	29805.50	26866.61	26735.43	24874.68	23935.69	21590.62	20690.04	19905.30	2.27	1.49	0.65	0.84	1.31	0.69	4.16	2.47	0.11	4.90	4.98	0.12	x	
3855	Regulator of chromosome condensation	P18754	2225.31	2560.28	2463.12	1822.68	1885.42	1720.01	2211.56	2124.93	1991.23	2424.07	2519.69	2202.49	1793.65	1880.20	2109.29	2151.02	1913.76	1905.30	1778.17	1591.10	1541.46	43382.79	40722.66	43609.09	25977.96	24895.09	22699.72	27316.57	25968.39	25173.81	30495.62	28931.54	19496.86		
3856	Regulator of G-protein signaling 10	O43665	26503.44	22944.23	21098.93	14272.32	13355.15	11848.17	18936.06	16522.20	16039.23	19409.66	17907.38	16311.06	19099.52	17365.25	11149.18	16083.28	13927.92	14295.55	15388.67	13883.02	12944.02	27173.99	25888.49	24624.27	16838.18	16361.37	13709.53	21653.78	19621.99	17625.03	21373.43	19745.00	10099.56		
3857	Regulator of G-protein signaling 18	Q9NS28	0.14	2.53	0.81	1.07	2.77	0.68	0.96	0.36	0.63	0.35	0.75	0.38	2.13	0.97	0.60	4.64	1.07	0.47	2.22	0.19	0.06	10367.81	8187.71	7962.54	5921.41	5931.33	4028.18	7662.18	6829.61	6531.10	8116.75	8039.70	4162.11		x
3858	Regulator of G-protein signaling 19	P49795	9821.12	9600.90	9681.44	9687.02	9902.58	10523.71	8285.49	8583.17	8976.56	11591.15	11530.58	10738.27	10659.17	11848.45	12195.84	9950.80	9861.20	9498.42	10627.97	9819.91	10624.41	2.27	1.49	0.65	0.84	1.31	0.69	4.16	2.47	0.11	4.90	4.98	0.12	x	
3859	Regulator of G-protein signaling 2	P41220	38092.28	36665.91	32676.19	29305.30	27463.63	23731.02	30822.50	29450.91	26875.73	41294.40	38654.18	34838.45	33885.85	29846.04	18963.61	30849.05	29955.59	27580.85	27475.60	28636.45	21145.49	1124.59	1018.99	792.67	434.34	370.44	154.26	795.11	527.58	561.13	911.94	816.51	326.45		
3860	Regulator of G-protein signaling 21	Q2M5E4	5232.08	7155.45	6554.13	4994.67	6050.05	4838.37	7470.97	8653.68	7259.26	4629.04	4557.09	4654.02	3635.09	2608.72	3910.12	7820.10	8044.42	6042.93	2399.85	2314.32	1954.89	2.27	1.49	0.65	0.84	1.31	0.69	4.16	2.47	0.11	4.90	4.98	0.12	x	
3861	Regulator of G-protein signaling 4	P49798	0.14	2.53	0.81	1.07	2.77	0.68	0.96	0.36	0.63	0.35	0.75	0.38	2.13	0.97	0.60	4.64	1.07	0.47	2.22	0.19	0.06	116.54	77.91	121.90	72.89	92.95	74.47	139.32	111.33	112.49	100.12	94.48	52.34		x
3862	Regulator of G-protein signaling 7	P49802	42117.64	42803.62	38331.28	27867.83	26757.59	24131.98	34985.64	31375.99	33044.11	36974.72	34273.88	32290.89	34402.20	32138.43	24608.60	29415.08	29660.26	26665.12	27628.96	26713.96	25932.21	2.27	1.49	0.65	0.84	1.31	0.69	4.16	2.47	0.11	4.90	4.98	0.12	x	
3863	Regulator of microtubule dynamics protein 1	Q96DB5	59047.25	69681.45	43969.57	29278.55	29779.27	32178.30	41876.66	43036.91	32593.66	39630.04	36493.12	38583.45	40952.50	34878.54	26536.23	38795.46	29170.16	25793.71	26683.61	30636.28	25196.45	17618.87	17359.79	15093.86	11617.04	10782.04	8582.76	13290.19	13165.59	12200.47	12997.83	12486.11	7157.66		
3864	Regulator of nonsense transcripts 1	Q92900	212474.96	192295.80	179703.35	126398.10	119531.35	106972.08	143335.88	135609.39	131483.07	144299.29	138099.76	125380.57	159227.14	146231.14	109467.37	122844.45	112764.31	110577.81	132261.76	123830.39	112253.33	49827.74	48067.91	50634.77	37098.93	36643.95	34508.87	45343.99	42330.75	40568.07	39844.90	37228.19	26404.88		
3865	Regulator of nonsense transcripts 2	Q9HAU5	73257.55	67078.21	67848.90	49241.57	46292.94	45125.21	64196.23	62724.66	59577.66	63516.26	60474.80	55673.84	62120.46	56029.79	48221.72	49493.71	48648.32	47791.94	48622.37	43655.16	44560.28	2.27	1.49	0.65	0.84	1.31	0.69	4.16	2.47	0.11	4.90	4.98	0.12	x	
3866	RelA-associated inhibitor	Q8WUF5	35628.59	31979.82	29812.54	21238.42	20265.59	18296.82	22827.08	20673.56	20539.11	26760.80	24893.81	23920.32	29148.20	27862.19	25049.51	18806.13	18001.48	16658.18	20855.72	20474.87	20190.44	37161.34	35005.49	32453.68	17806.31	17096.57	15080.97	35172.67	31586.53	29347.34	31164.78	31890.45	13149.58		
3867	Remodeling and spacing factor 1	Q96T23	170826.65	157636.62	151653.45	130006.98	118227.68	119334.48	123731.07	126121.79	119537.58	159915.53	157699.90	143467.23	151456.97	145655.05	116850.60	124802.13	118590.06	118169.24	134008.58	117295.03	112486.16	295.94	361.07	315.33	301.39	234.94	271.21	454.77	427.19	383.18	395.94	419.37	305.19		
3868	Renin receptor	O75787	41243.20	41694.92	38893.62	26324.10	26252.34	23832.11	39336.09	38248.24	36606.66	29007.67	28885.64	28098.46	29333.60	28032.91	25325.94	24519.77	23714.24	23267.89	26501.85	25061.91	24550.40	2.27	1.49	0.65	0.84	1.31	0.69	4.16	2.47	0.11	4.90	4.98	0.12	x	
3869	Replication factor C subunit 2	P35250	101723.46	102561.80	92255.92	70985.47	68112.53	62434.52	80800.92	77721.47	72799.87	86237.12	81600.81	76316.50	87066.61	83396.57	66812.44	72246.92	67783.37	64003.06	73023.69	70697.04	68260.96	7966.16	7605.53	7299.88	5219.60	5442.94	5087.43	6584.84	6428.83	5737.66	6505.26	6469.21	4221.76		
3870	Replication factor C subunit 3	P40938	6664.69	5883.40	4986.06	3886.70	4279.11	3599.34	5358.16	4376.06	4380.32	4610.39	4273.30	3561.29	5050.89	4861.20	3944.60	3284.11	3539.09	3117.51	4479.87	4379.06	4453.25	52827.22	50717.72	46440.86	32867.44	30975.33	26168.86	38546.36	38657.88	36704.68	38513.35	38952.12	25328.53		
3871	Replication factor C subunit 4	P35249	19277.80	18407.92	13820.63	9847.06	10010.35	9650.67	14479.54	13428.44	13062.29	14502.89	13705.65	12228.22	14766.95	11977.05	9907.14	10448.68	10148.42	9880.61	10445.53	10075.29	9257.60	38631.54	36534.80	31964.58	30191.84	30012.25	28902.67	33348.91	31212.22	29659.17	32354.67	31280.06	23948.72		
3872	Replication factor C subunit 5	P40937	59009.15	52243.27	47622.83	40834.89	36846.94	34485.53	40903.05	39874.00	38976.37	45433.83	42476.32	38069.47	47748.08	39567.99	29908.84	32042.45	30089.98	30058.44	39675.15	37334.46	32051.53	2.27	1.49	0.65	0.84	1.31	0.69	4.16	2.47	0.11	4.90	4.98	0.12	x	
3873	Replication protein A 14 kDa subunit	P35244	0.14	2.53	0.81	1.07	2.77	0.68	0.96	0.36	0.63	0.35	0.75	0.38	2.13	0.97	0.60	4.64	1.07	0.47	2.22	0.19	0.06	9619.94	9629.36	7055.46	5096.93	4936.44	3424.30	8565.52	8108.74	5537.37	8486.22	8141.10	3779.66		x
3874	Replication protein A 30 kDa subunit	Q13156	0.14	2.53	0.81	1.07	2.77	0.68	0.96	0.36	0.63	0.35	0.75	0.38	2.13	0.97	0.60	4.64	1.07	0.47	2.22	0.19	0.06	2957.10	269.68	137.12	1684.78	1733.34	844.61	2948.24	2613.35	1782.10	3235.65	2924.69	1345.72		x
3875	Replication protein A 32 kDa subunit	P15927	371.58	181.15	101.50	308.92	290.22	191.00	185.71	259.97	181.35	286.87	158.08	277.41	278.44	266.09	162.95	343.26	229.14	210.51	375.69	340.54	260.81	45673.44	42129.50	38666.24	25076.82	25707.22	22839.79	30096.75	30413.88	29638.30	33646.28	30646.67	22271.18		
3876	Replication protein A 70 kDa DNA-binding subunit	P27694	62873.32	63096.96	58822.50	47982.72	45182.86	41643.58	49297.36	51397.40	48981.68	56847.52	51758.59	49083.95	54575.03	49611.27	37069.27	43073.15	44712.28	42082.90	46743.18	44684.88	45400.21	164273.77	151657.54	139908.96	102923.52	98101.31	88725.01	120423.65	111963.95	108011.10	117707.54	113114.21	67098.37		
3877	Replication stress response regulator SDE2	Q6IQ49	10538.00	9911.69	8682.75	7329.12	7517.99	7248.90	7669.75	7753.18	7174.96	11778.63	11437.46	11098.78	10388.54	9165.01	7487.45	6481.72	6792.88	6438.64	6676.60	7024.72	6666.49	2.27	1.49	0.65	0.84	1.31	0.69	4.16	2.47	0.11	4.90	4.98	0.12	x	
3878	Required for meiotic nuclear division protein 1 homolog	Q9NWS8	5145.71	5110.93	4534.95	2635.09	3666.44	2313.85	3214.14	2952.14	2799.86	4022.04	3851.56	3593.48	3802.81	3969.46	2613.14	2438.38	2828.70	2379.79	3312.43	3307.47	2577.49	2.27	1.49	0.65	0.84	1.31	0.69	4.16	2.47	0.11	4.90	4.98	0.12	x	
3879	Resistin	Q9HD89	636.96	670.77	890.04	715.79	650.78	773.91	497.31	493.81	478.08	875.20	920.14	936.66	686.18	681.85	804.71	572.23	722.20	581.35	549.75	629.06	507.90	2.27	1.49	0.65	0.84	1.31	0.69	4.16	2.47	0.11	4.90	4.98	0.12	x	
3880	REST corepressor 1	Q9UKL0	64170.88	56890.26	55826.31	40447.45	41889.78	40319.47	47338.28	47400.46	46134.34	55414.24	56150.64	53297.03	54462.49	48858.96	42370.61	41973.39	41047.41	41874.36	47434.10	40743.09	40264.04	2.27	1.49	0.65	0.84	1.31	0.69	4.16	2.47	0.11	4.90	4.98	0.12	x	
3881	Reticulocalbin-1	Q15293	304678.90	295413.40	273945.76	205752.23	202937.14	189838.97	225667.87	223748.68	210794.37	250834.16	234562.17	221523.36	245198.57	233076.14	194151.55	210299.60	200955.79	191922.32	216822.53	209765.76	197365.40	170385.71	157806.08	151067.32	118065.28	113078.55	101090.93	139290.60	131511.87	125809.50	145417.30	138188.39	99053.56		
3882	Reticulocalbin-2	Q14257	40391.52	39350.27	34564.91	30182.21	28549.31	24545.20	30461.26	30309.71	27409.63	35760.32	33676.11	28690.88	35390.66	30569.49	22447.45	33496.33	30229.06	28719.78	32201.28	28706.44	25844.26	20492.01	21812.12	16114.74	11114.80	11109.69	9281.43	14535.66	13984.93	13685.02	11688.11	12183.36	7892.25		
3883	Reticulocalbin-3	Q96D15	1531.27	1162.98	1455.07	1115.72	1029.45	1255.13	1106.72	966.01	1093.92	1313.45	1314.73	1350.47	1443.75	1329.02	989.19	963.85	994.45	888.99	1004.87	1069.70	970.85	2.27	1.49	0.65	0.84	1.31	0.69	4.16	2.47	0.11	4.90	4.98	0.12	x	
3884	Reticulon-1	Q16799	0.14	2.53	0.81	17.77	8.26	1.58	0.96	0.36	0.63	38.84	5.93	0.76	2.13	0.97	0.60	65.67	2.14	51.91	23.52	43.13	52.41	1680.85	955.31	300.06	2087.55	1922.66	1109.75	3742.99	3290.25	1852.28	3724.12	3354.42	1392.53		
3885	Reticulon-3	O95197	174291.47	166242.07	157266.07	107959.60	102341.48	97873.58	163396.06	153809.84	143618.13	139860.83	133392.12	128337.48	138885.29	126035.25	104834.11	130616.90	116346.36	118042.33	114469.38	106888.70	99083.50	30648.94	26913.53	27005.21	21880.31	21202.64	19474.90	26276.62	24855.79	24595.32	26084.95	25302.34	12439.47		
3886	Reticulon-4	Q9NQC3	101638.86	98463.69	94242.26	67775.34	64319.46	57569.56	70668.27	67140.51	62891.54	85318.93	82976.34	78393.67	92963.13	84656.55	68655.75	76332.33	69653.67	69569.17	63257.11	64533.89	57399.66	183573.90	170845.29	170662.46	131763.38	126521.34	113055.68	174024.40	159355.58	153660.57	169361.96	158983.81	111713.08		
3887	Reticulon-4 receptor-like 2	Q86UN3	58228.35	54523.62	51160.78	38221.39	37092.96	32211.81	77953.73	77894.04	73184.78	49968.83	49092.97	43410.30	53107.74	47513.11	40651.18	42282.34	39969.33	38163.86	38828.68	35362.49	32745.58	2.27	1.49	0.65	0.84	1.31	0.69	4.16	2.47	0.11	4.90	4.98	0.12	x	
3888	Retinal dehydrogenase 2	O94788	0.14	2.53	0.81	1.07	2.77	0.68	0.96	0.36	0.63	0.35	0.75	0.38	2.13	0.97	0.60	4.64	1.07	0.47	2.22	0.19	0.06	220.04	64.93	34.87	66.88	63.40	18.01	202.81	89.95	49.96	169.77	148.35	34.47		x
3889	Retinal rod rhodopsin-sensitive cGMP 3'_5'-cyclic phosphodiesterase subunit delta	O43924	0.14	2.53	0.81	1.07	2.77	0.68	0.96	0.36	0.63	0.35	0.75	0.38	2.13	0.97	0.60	4.64	1.07	0.47	2.22	0.19	0.06	1993.91	1962.05	1737.76	1843.85	1603.71	1050.13	1723.13	1579.02	1328.54	2740.81	2660.35	1591.76		x
3890	Retinaldehyde-binding protein 1	P12271	257.87	249.85	197.62	71.66	84.08	46.47	124.92	141.18	60.11	158.20	119.28	131.86	216.10	244.29	156.41	151.25	77.59	75.87	127.74	127.62	113.69	2.27	1.49	0.65	0.84	1.31	0.69	4.16	2.47	0.11	4.90	4.98	0.12	x	
3891	Retinoblastoma-associated protein	P06400	0.14	2.53	0.81	1.07	2.77	0.68	0.96	0.36	0.63	0.35	0.75	0.38	2.13	0.97	0.60	4.64	1.07	0.47	2.22	0.19	0.06	164233.93	162405.28	158171.77	118331.70	115633.26	109634.45	123628.75	116538.32	118694.30	131600.85	122238.67	97300.71		x
3892	Retinoic acid receptor alpha	P10276	7910.81	7657.27	7105.65	4999.47	4319.07	4475.59	5791.16	5001.94	5042.94	7771.75	7638.60	6845.03	6120.63	4851.67	4048.22	4872.49	4100.84	4564.87	5516.71	5003.73	4166.38	2.27	1.49	0.65	0.84	1.31	0.69	4.16	2.47	0.11	4.90	4.98	0.12	x	
3893	Retinoic acid receptor RXR-alpha	P19793	0.14	2.53	0.81	1.07	2.77	0.68	0.96	0.36	0.63	0.35	0.75	0.38	2.13	0.97	0.60	4.64	1.07	0.47	2.22	0.19	0.06	4931.59	4190.23	4226.59	3647.94	3628.80	3271.81	4422.14	4111.65	3849.68	4260.06	3906.54	3184.83		x
3894	Retinoic acid receptor RXR-gamma	P48443	0.14	2.53	0.81	1.07	2.77	0.68	0.96	0.36	0.63	0.35	0.75	0.38	2.13	0.97	0.60	4.64	1.07	0.47	2.22	0.19	0.06	4737.04	4618.49	4053.31	2493.30	2400.91	1630.43	4514.28	4122.08	4017.69	3712.63	3374.23	1855.35		x
3895	Retinoic acid-induced protein 3	Q8NFJ5	30204.41	31861.17	27006.22	13444.81	12565.74	11424.98	14789.88	13162.89	12651.61	18309.88	17178.15	16197.05	17675.29	16426.53	13450.85	17455.45	16326.09	15401.54	11599.70	11771.54	10950.95	9325.88	8732.08	7290.10	7084.66	6668.66	5246.70	7998.67	7473.27	7020.65	12025.59	12234.88	7472.30		
3896	Retinol dehydrogenase 11	Q8TC12	36808.67	36215.21	35508.68	26064.25	25217.64	23190.34	28677.95	26977.98	27384.42	32654.23	30014.00	31604.47	29736.83	28524.80	24538.55	27405.75	24861.98	24062.14	27237.12	24366.39	23435.55	7682.50	7403.82	5907.05	5221.29	5115.60	4276.52	6955.45	7125.08	6997.44	8991.64	8589.93	5443.55		
3897	Retinol-binding protein 1	P09455	0.14	2.53	0.81	1.07	2.77	0.68	0.96	0.36	0.63	0.35	0.75	0.38	2.13	0.97	0.60	4.64	1.07	0.47	2.22	0.19	0.06	3385.30	2966.66	2596.36	1827.70	1918.33	978.08	3237.30	2824.92	1977.86	3848.83	3457.20	1326.69		x
3898	Retinol-binding protein 2	P50120	0.14	2.53	0.81	1.07	2.77	0.68	0.96	0.36	0.63	0.35	0.75	0.38	2.13	0.97	0.60	4.64	1.07	0.47	2.22	0.19	0.06	6765.49	6037.68	5737.80	3666.17	3005.84	1485.87	5795.30	5121.35	4192.63	5407.20	5711.42	1803.79		x
3899	Retroelement silencing factor 1	Q9HCM1	13713.10	12857.42	12836.70	10766.38	12350.61	11113.89	11142.77	10856.90	11527.38	12533.81	12469.88	11072.19	12887.03	18519.07	21744.11	5946.10	8681.09	8188.09	12363.71	11183.01	10892.24	2.27	1.49	0.65	0.84	1.31	0.69	4.16	2.47	0.11	4.90	4.98	0.12	x	
3900	RGM domain family member B	Q6NW40	0.14	2.53	0.81	1.07	2.77	0.68	0.96	0.36	0.63	0.35	0.75	0.38	2.13	0.97	0.60	4.64	1.07	0.47	2.22	0.19	0.06	9768.41	9036.81	8176.00	4055.35	4016.33	2091.34	5831.57	5233.86	4160.61	7310.43	7263.39	2974.89		x
3901	Rho GDP-dissociation inhibitor 1	P52565	443842.91	425672.32	399152.56	278816.60	259222.53	244529.00	294149.20	278331.46	261692.77	322414.95	308473.48	295985.89	337585.18	326577.57	268011.59	262447.27	258405.13	246451.53	264290.44	259375.40	235852.74	303008.91	286438.60	253263.45	201694.95	200158.46	177947.18	240490.18	232798.80	224592.32	261126.62	254101.07	168492.79		
3902	Rho GTPase-activating protein 1	Q07960	265403.62	240219.63	217555.76	155139.94	149418.37	137411.71	168280.07	156784.65	151376.99	197528.16	185157.17	169531.21	190201.21	164422.27	137017.17	159239.55	153155.02	146153.09	158625.71	148176.77	141139.15	67149.69	57818.55	49977.17	39107.78	38455.31	34401.93	45212.50	45854.83	40876.59	45469.32	44034.37	31011.10		
3903	Rho GTPase-activating protein 15	Q53QZ3	0.14	2.53	0.81	1.07	2.77	0.68	0.96	0.36	0.63	0.35	0.75	0.38	2.13	0.97	0.60	4.64	1.07	0.47	2.22	0.19	0.06	50463.51	27348.75	27693.35	22595.43	20457.67	17408.12	27317.53	24435.54	22934.59	29074.18	27608.81	18750.89		x
3904	Rho GTPase-activating protein 17	Q68EM7	16000.67	15863.58	15184.38	9225.77	8711.28	7520.67	11841.70	10888.20	10225.97	12132.60	11385.38	10289.29	11966.72	10436.04	8138.17	10287.67	10005.46	9496.52	10071.33	9810.04	9044.16	2.27	1.49	0.65	0.84	1.31	0.69	4.16	2.47	0.11	4.90	4.98	0.12	x	
3905	Rho GTPase-activating protein 18	Q8N392	0.14	2.53	0.81	1.07	2.77	0.68	0.96	0.36	0.63	0.35	0.75	0.38	2.13	0.97	0.60	4.64	1.07	0.47	2.22	0.19	0.06	25031.30	20812.77	21514.31	11745.93	11337.02	10000.05	17062.67	16085.45	14483.53	15610.15	14753.53	9562.68		x
3906	Rho GTPase-activating protein 20	Q9P2F6	4167.73	3955.97	3343.35	4137.65	3584.43	3394.89	3593.90	4676.91	4212.38	5212.56	5283.59	5271.38	3924.51	2061.88	1479.80	3422.19	3368.33	2649.40	1787.55	3353.27	3541.41	1045.22	918.60	803.72	878.98	801.81	667.53	1211.92	1165.18	1169.44	927.00	842.23	606.17		
3907	Rho GTPase-activating protein 21	Q5T5U3	16167.73	15892.40	15674.08	12128.86	10983.32	10430.44	12753.86	13473.01	13080.40	13403.62	11868.27	11974.46	14191.89	12183.31	11358.40	11650.58	10865.04	10823.78	12022.28	11284.13	11608.80	2.27	1.49	0.65	0.84	1.31	0.69	4.16	2.47	0.11	4.90	4.98	0.12	x	
3908	Rho GTPase-activating protein 26	Q9UNA1	33078.33	29045.16	26189.53	23914.27	23932.58	20041.93	25172.70	25492.59	22203.96	29674.54	28067.92	24608.89	27341.40	25710.29	15754.71	23661.47	22611.66	21075.94	24221.77	23315.51	20886.65	2.27	1.49	0.65	0.84	1.31	0.69	4.16	2.47	0.11	4.90	4.98	0.12	x	
3909	Rho GTPase-activating protein 28	Q9P2N2	56648.65	45387.36	33925.54	3543.18	5490.71	3522.24	5644.18	4887.82	2643.71	49516.22	46335.07	35710.85	43334.08	6774.14	2095.94	4379.29	4584.96	3986.30	3297.56	4205.76	2107.21	2.27	1.49	0.65	0.84	1.31	0.69	4.16	2.47	0.11	4.90	4.98	2222.24	x	
3910	Rho GTPase-activating protein 29	Q52LW3	21734.27	20784.23	19334.53	15063.46	14567.97	13391.48	15554.41	15046.67	14585.78	17454.95	17473.17	16360.25	16904.60	14907.83	11938.78	13762.58	13720.06	12127.96	14393.54	14251.03	12816.33	105488.19	100780.98	91321.89	81709.57	79625.17	75228.20	90471.70	90339.17	88307.06	101574.62	94643.48	60705.41		
3911	Rho GTPase-activating protein 35	Q9NRY4	125896.63	119415.54	116090.29	93308.82	93436.88	85220.78	110219.42	105700.80	106180.04	110351.06	103181.62	96050.40	104420.86	93362.40	79556.83	101392.28	92442.29	95236.43	85689.99	83786.91	83668.93	166092.79	155595.82	155598.85	112199.12	114183.47	105667.22	114511.36	111568.79	109543.64	115161.30	112751.51	112526.77		
3912	Rho GTPase-activating protein 40	Q5TG30	9026.96	8567.32	7957.96	7052.91	7008.04	6650.49	7711.18	8046.51	7630.41	8542.25	7945.65	8004.02	8051.45	8394.21	6939.65	6347.62	5877.82	5711.91	7106.51	7021.23	7204.76	2.27	1.49	0.65	0.84	1.31	0.69	4.16	2.47	0.11	4.90	4.98	0.12	x	
3913	Rho GTPase-activating protein 44	Q17R89	395.76	306.11	395.94	188.35	181.96	151.14	194.27	154.61	149.81	242.48	255.60	183.92	234.17	223.38	179.14	216.63	169.89	128.45	178.47	127.71	160.16	2.27	1.49	0.65	0.84	1.31	0.69	4.16	2.47	0.11	4.90	4.98	0.12	x	
3914	Rho GTPase-activating protein 45	Q92619	48313.88	46828.38	46195.53	37188.14	36070.33	35766.45	37558.07	37203.11	38170.86	41258.47	40112.16	38125.74	40455.59	39614.55	32565.86	34604.20	34134.87	32283.57	30201.73	31161.36	29233.05	10023.81	9814.89	8911.89	7310.59	7211.27	6958.41	7499.69	7374.15	7416.38	7728.28	7656.22	8735.68		
3915	Rho GTPase-activating protein 5	Q13017	0.14	2.53	0.81	1.07	2.77	0.68	0.96	0.36	0.63	0.35	0.75	0.38	2.13	0.97	0.60	4.64	1.07	0.47	2.22	0.19	0.06	1975.01	1783.37	2278.13	2216.52	2513.41	2090.14	4160.53	4069.17	4005.98	2368.70	2500.32	1937.92		x
3916	Rho guanine nucleotide exchange factor 1	Q92888	86482.38	84168.35	75430.72	52501.54	51664.29	46411.43	70204.10	65826.05	62318.71	68177.23	65076.76	60045.28	68688.83	63508.60	49093.71	59966.55	55887.27	52572.62	54927.79	53190.31	48852.26	26313.48	24253.63	22077.86	15403.93	14859.93	13023.19	17907.05	18181.02	17642.87	18797.61	17748.15	12874.90		
3917	Rho guanine nucleotide exchange factor 11	O15085	37513.19	36012.68	28607.53	21599.29	19267.30	17522.18	28170.55	25551.96	23455.49	29891.47	27072.43	23894.14	28328.68	26781.51	17728.09	21513.64	21080.69	19083.43	21280.45	20401.68	18496.79	3610.56	3655.81	3744.60	2877.42	2621.56	2496.12	4013.47	3722.78	3451.71	4003.69	3730.07	2710.27		
3918	Rho guanine nucleotide exchange factor 16	Q5VV41	4875.44	3890.80	3552.22	3497.00	1491.02	2095.76	2179.36	2579.75	2658.49	4025.77	3892.74	3064.62	5531.49	2874.18	1545.76	2718.72	1409.52	1039.52	3074.03	2182.14	1133.62	14459.03	13753.63	12648.82	8375.64	8059.78	6963.11	9988.81	9299.51	8692.55	9514.40	9011.21	6975.03		
3919	Rho guanine nucleotide exchange factor 17	Q96PE2	14495.93	11652.18	10958.98	9689.97	9946.98	9007.63	9249.81	9810.08	7264.90	11788.64	12190.17	11896.77	11113.17	10753.10	9783.06	8480.19	7770.33	7013.67	10599.40	7458.90	8043.61	1143.91	794.48	659.86	2444.84	2104.37	1257.44	5218.57	4331.41	3200.66	3000.33	2970.55	1540.59		
3920	Rho guanine nucleotide exchange factor 18	Q6ZSZ5	104.43	103.62	48.42	12.24	29.98	20.06	56.03	37.96	32.24	109.77	36.37	56.54	69.97	96.21	45.14	13.39	20.29	49.53	2.22	0.19	0.06	2.27	1.49	0.65	0.84	1.31	0.69	4.16	2.47	0.11	4.90	4.98	0.12	x	
3921	Rho guanine nucleotide exchange factor 2	Q92974	0.14	2.53	0.81	1.07	2.77	0.68	0.96	0.36	0.63	0.35	0.75	0.38	2.13	0.97	0.60	4.64	1.07	0.47	2.22	0.19	0.06	13004.45	11761.89	10425.56	6019.73	6029.83	4873.70	6706.58	6712.92	6203.42	7975.05	7991.87	5098.50		x
3922	Rho guanine nucleotide exchange factor 28	Q8N1W1	62999.79	51574.12	44728.23	45515.86	43691.32	40023.84	48787.33	46928.71	44492.68	57161.47	53590.42	47257.12	50711.04	46271.37	30312.36	43800.16	43578.61	40055.37	43282.31	39492.21	35614.12	68622.46	65850.68	58474.96	53209.06	51672.70	49383.68	60216.31	57946.38	56051.38	60689.09	58893.85	37290.25		
3923	Rho guanine nucleotide exchange factor 4	Q9NR80	0.14	2.53	0.81	1.07	2.77	0.68	0.96	0.36	0.63	0.35	0.75	0.38	2.13	0.97	0.60	4.64	1.07	0.47	2.22	0.19	0.06	1238.13	1057.08	1184.66	1010.62	918.98	889.36	1224.34	1038.72	918.46	1107.59	937.89	743.64		x
3924	Rho guanine nucleotide exchange factor 7	Q14155	0.14	2.53	0.81	1.07	2.77	0.68	0.96	0.36	0.63	0.35	0.75	0.38	2.13	0.97	0.60	4.64	1.07	0.47	2.22	0.19	0.06	6781.72	7056.06	7015.21	5976.95	6237.18	5494.47	6201.01	5378.21	5937.73	6833.24	7032.40	7652.53		x
3925	Rho guanine nucleotide exchange factor 9	O43307	10097.90	9838.68	9257.47	7293.52	7554.39	6380.06	8601.78	8363.94	6901.26	9742.88	9522.08	8283.15	9511.28	7873.25	6771.22	8091.59	6863.46	6760.03	7442.54	6826.80	5997.11	2.27	1.49	0.65	0.84	1.31	0.69	4.16	2.47	0.11	4.90	4.98	0.12	x	
3926	Rho-associated protein kinase 1	Q13464	765.72	845.62	562.87	47.45	73.87	30.03	160.39	101.47	84.95	576.00	470.97	353.68	2.13	0.97	0.60	85.01	79.47	98.74	18.36	21.96	8.62	97826.38	97970.26	92428.02	63800.37	64334.82	60258.25	62101.63	61772.59	60912.10	76861.60	74820.97	0.12		
3927	Rho-associated protein kinase 2	O75116	2068.98	1930.99	1641.05	1559.02	1561.88	1247.21	1907.11	1778.36	1952.78	1909.13	1834.56	1708.67	2041.42	1673.79	1550.49	1042.38	1124.18	1083.66	1406.97	1444.10	1629.51	2.27	1.49	0.65	0.84	1.31	0.69	4.16	2.47	0.11	4.90	4.98	0.12	x	
3928	Rhophilin-2	Q8IUC4	9168.80	9536.42	7951.90	8894.81	7246.28	7237.91	8722.47	8604.43	7730.18	8814.56	7507.00	7790.65	9364.56	8376.96	5861.92	9026.90	8119.18	7488.72	9043.57	8234.18	6532.25	2.27	1.49	0.65	0.84	1.31	0.69	4.16	2.47	0.11	4.90	4.98	0.12	x	
3929	Rho-related GTP-binding protein RhoB	P62745	647.81	193.20	26.41	1.07	2.77	0.68	0.96	0.36	0.63	0.35	0.75	0.38	2.13	0.97	0.60	4.64	1.07	0.47	2.22	0.19	0.06	2.27	1.49	0.65	0.84	1.31	0.69	4.16	2.47	0.11	4.90	4.98	387.54	x	
3930	Rho-related GTP-binding protein RhoC	P08134	178193.37	164148.81	159006.23	107179.92	105960.07	97697.22	133767.79	126964.11	121334.78	134476.84	126306.15	120114.32	131807.88	122844.85	104749.03	113092.78	107471.44	100552.80	103678.61	97896.14	85971.06	52610.13	49356.51	41429.69	25809.01	24182.57	22336.65	32192.53	31611.46	29404.97	31831.59	31120.94	25634.37		
3931	Rho-related GTP-binding protein RhoG	P84095	54753.06	52022.70	49315.60	36927.21	35416.71	32845.79	42377.62	41294.91	39348.06	43326.99	43276.68	40306.92	41762.25	42313.54	35013.63	37452.90	37349.61	33556.35	35284.72	34529.43	32563.29	14604.24	14347.29	13104.20	9351.68	8665.94	6447.83	12666.15	11491.37	9527.45	16811.72	14655.39	8312.06		
3932	Rho-related GTP-binding protein RhoJ	Q9H4E5	0.14	2.53	0.81	1.07	2.77	0.68	0.96	0.36	0.63	0.35	0.75	0.38	2.13	0.97	0.60	4.64	1.07	0.47	2.22	0.19	0.06	1627.94	1490.19	958.67	969.88	785.10	646.80	1213.64	1130.25	1005.93	1175.55	1120.17	754.97		x
3933	Rhotekin	Q9BST9	16121.43	15231.57	14761.02	11140.23	11419.15	10604.53	12246.15	12099.69	11813.54	14119.68	13122.91	12708.15	14723.31	11640.94	5182.27	8756.03	10851.30	10902.74	13602.46	12475.40	11857.91	2.27	1.49	0.65	0.84	1.31	0.69	4.16	2.47	0.11	4.90	4.98	0.12	x	
3934	Ribonuclease inhibitor	P13489	101790.77	102914.41	95151.70	70142.23	67054.79	62368.63	92149.50	88440.68	86199.56	86796.53	83355.19	79726.71	85715.30	75157.07	62367.07	68386.88	66583.43	65407.20	68214.64	67329.41	63856.14	66727.84	62642.13	56414.53	36479.44	34441.95	31523.80	40475.07	38298.00	36699.65	41738.19	40353.13	26643.61		
3935	Ribonuclease P protein subunit p20	O75817	3026.57	3010.67	2317.05	2019.15	1793.87	1776.67	2234.54	2131.77	2046.55	2582.61	2454.96	2087.81	2269.05	2142.50	1521.28	2009.80	1921.24	1862.61	2055.04	2085.15	1749.74	2.27	1.49	0.65	0.84	1.31	0.69	4.16	2.47	0.11	4.90	4.98	0.12	x	
3936	Ribonuclease P protein subunit p40	O75818	153776.66	156602.07	140968.05	116397.40	118251.88	105128.22	148230.82	136332.05	124479.12	156582.19	147574.21	135708.27	130926.08	126771.83	100736.76	130952.08	127543.91	118853.90	122652.65	130462.72	143861.79	2.27	1.49	0.65	0.84	1.31	0.69	4.16	2.47	0.11	4.90	4.98	0.12	x	
3937	Ribonuclease T2	O00584	566.27	675.18	638.70	476.40	387.79	451.76	571.32	542.10	609.31	552.39	627.01	549.86	471.92	574.30	464.42	603.80	651.30	621.52	359.63	366.27	385.81	2.27	1.49	0.65	0.84	1.31	0.69	4.16	2.47	0.11	4.90	4.98	0.12	x	
3938	Ribonucleases P/MRP protein subunit POP1	Q99575	8626.89	8795.63	7093.74	5336.56	5621.74	5253.70	5797.49	5420.94	5450.24	7531.71	7190.28	6753.19	6979.14	6976.82	6077.25	4918.07	4497.30	4583.86	5994.43	6073.97	5389.16	2.27	1.49	0.65	0.84	1.31	0.69	4.16	2.47	0.11	4.90	4.98	0.12	x	
3939	Ribonucleoprotein PTB-binding 1	Q8IY67	11071.41	10898.19	10253.55	9982.71	8957.81	10233.25	10542.49	10855.78	9577.56	9796.98	9187.02	8871.39	10760.99	10622.09	9748.29	9863.26	8762.91	8519.70	10266.36	10427.01	9894.08	20813.55	19120.20	18219.02	15033.55	14680.49	14141.62	15501.55	15264.57	15405.71	16956.33	16664.52	5450.69		
3940	Ribonucleoside-diphosphate reductase large subunit	P23921	273607.12	262550.11	242473.19	205470.90	183801.19	177975.94	202986.55	194884.51	180985.10	215614.73	201249.27	192656.77	238972.64	241331.21	160456.53	171196.44	158970.12	154357.14	210133.49	208104.91	195696.40	40146.80	38247.25	34695.05	26566.74	25065.14	20851.71	32214.14	31511.08	28408.77	33788.97	32794.10	18563.05		
3941	Ribonucleoside-diphosphate reductase subunit M2 B	Q7LG56	8262.09	7901.95	7371.79	10457.77	6299.00	6243.03	6250.65	6842.34	6345.05	9294.68	8756.26	8479.25	7344.29	10442.93	6385.32	6248.59	6287.97	5972.53	6821.32	5752.45	5943.81	8485.39	7071.57	4810.92	4231.98	4111.00	2708.75	7692.34	6541.28	5688.89	6228.30	6155.05	3826.58		
3942	Ribonucleoside-diphosphate reductase subunit M2	P31350	200692.09	188167.06	178045.55	153191.95	146254.58	135643.81	177909.74	180224.19	171877.26	184100.79	172516.86	162656.75	167860.29	168950.81	134030.59	147626.91	138684.43	134855.47	135226.32	129097.50	126522.39	213612.18	201637.48	189849.35	131684.48	128545.29	114377.20	147356.23	143245.52	133528.09	133198.08	129345.03	93063.54		
3943	Ribose-5-phosphate isomerase	P49247	80392.82	69451.54	58304.29	1618.44	2745.32	1171.66	2426.10	23763.02	2253.41	62938.88	58541.02	50138.41	57132.83	13457.73	3653.69	1894.31	5862.53	2609.91	8407.40	2236.74	2592.56	2.27	1.49	0.65	0.84	1.31	0.69	4.16	2.47	0.11	4.90	4.98	0.12	x	
3944	Ribose-phosphate pyrophosphokinase 1	P60891	13483.53	14261.43	12437.65	9160.18	8705.39	8152.29	10021.56	10203.18	10069.22	12015.22	11782.06	11399.61	8630.24	8453.52	7103.28	10152.11	9120.08	8535.54	7857.19	7494.16	6586.22	23215.02	21997.79	21407.31	10644.77	10150.76	7996.06	13315.13	12916.05	12088.84	13539.08	13572.07	13462.79		
3945	Ribose-phosphate pyrophosphokinase 2	P11908	169390.66	160002.04	145728.25	101053.14	97167.65	90561.64	128058.07	123608.00	120006.57	127819.00	120330.38	117503.83	130939.67	122383.98	98342.93	104404.39	97152.01	91858.95	100254.14	100372.22	92283.43	679.31	706.72	499.96	1362.11	1314.63	495.96	4665.76	3615.72	2298.72	3886.17	3532.10	1224.02		
3946	Ribose-phosphate pyrophosphokinase 3	P21108	27434.51	28037.78	26642.91	14332.45	13889.44	12221.66	20864.24	20158.88	19919.47	18982.85	18013.50	16900.47	24062.41	22171.67	19374.96	15824.28	14732.66	13727.82	15522.59	15034.23	13877.89	106.00	88.39	98.01	52.57	18.20	0.69	362.69	298.23	54.90	204.44	122.38	13.76		
3947	Ribosomal biogenesis protein LAS1L	Q9Y4W2	17715.90	16345.18	14694.16	12469.84	11459.08	9922.54	15996.72	14627.58	13454.03	14738.92	14347.23	13007.54	14946.23	13027.16	8397.16	13743.70	11990.84	12001.31	13656.90	11849.57	10051.16	2.27	1.49	0.65	0.84	1.31	0.69	4.16	2.47	0.11	4.90	4.98	0.12	x	
3948	Ribosomal oxygenase 1	Q9H6W3	77717.53	71050.85	59861.47	23362.95	22752.42	20489.22	35675.33	35909.26	32631.77	57689.71	53067.75	46967.83	52933.08	39641.28	29637.61	48599.96	46954.35	44509.36	39009.82	33263.08	32842.93	2.27	1.49	0.65	0.84	1.31	0.69	4.16	2.47	0.11	4.90	4.98	0.12	x	
3949	Ribosomal protein S6 kinase alpha-1	Q15418	8051.42	6257.77	5338.23	3870.39	3586.19	2617.84	5847.28	7336.64	4830.28	5285.34	4931.72	4084.89	5116.93	11005.29	1807.65	4014.01	3902.26	4543.14	5164.36	4764.74	3438.16	27619.58	27225.45	24312.01	19197.57	18018.19	15002.42	22157.27	20849.15	20465.70	24988.94	24541.87	15587.50		
3950	Ribosomal protein S6 kinase alpha-2	Q15349	13056.39	12286.48	9888.93	9942.04	8437.49	6813.56	9180.40	9338.40	8317.26	10188.62	9494.06	8111.90	10748.40	10517.58	6147.90	9378.77	8153.67	7596.07	8641.60	7867.16	6646.73	18690.23	17901.51	16054.93	8875.73	8652.10	7321.70	11830.51	11230.37	10609.20	10231.63	10323.49	6954.35		
3951	Ribosomal protein S6 kinase alpha-3	P51812	335277.30	312053.44	290721.78	244063.01	225044.29	207919.70	518984.37	515312.19	497887.77	288289.46	280147.05	259963.44	279538.13	249095.80	200195.86	229416.87	220126.68	211353.63	249464.64	214536.29	207740.52	2.27	1.49	0.65	0.84	1.31	0.69	4.16	2.47	0.11	4.90	4.98	0.12	x	
3952	Ribosomal protein S6 kinase beta-1	P23443	197422.07	192818.91	171602.99	114554.46	115417.41	108049.77	160929.37	152724.16	142492.74	145787.04	138379.60	126850.02	138241.98	132305.02	117132.94	127211.38	124876.70	115508.68	123900.62	118747.74	113008.64	2.27	1.49	0.65	0.84	1.31	0.69	4.16	2.47	0.11	4.90	4.98	0.12	x	
3953	Ribosomal protein S6 kinase delta-1	Q96S38	114739.82	114358.46	106352.92	83861.06	79021.51	74688.97	96367.28	97192.79	87459.45	107764.65	103961.02	100733.73	88155.22	97894.41	76560.41	87429.28	84182.52	78469.31	86006.05	85110.80	82478.89	9124.63	8483.98	7700.72	5494.67	5101.28	3453.10	6764.52	6978.69	5113.90	7430.45	6599.09	3566.15		
3954	Ribosomal RNA processing protein 1 homolog A	P56182	615.70	394.89	335.08	199.25	178.43	185.47	486.78	143.87	323.52	527.23	491.28	329.84	544.56	83.24	9.95	323.40	279.63	369.62	431.82	284.25	202.07	307.93	357.42	323.44	144.72	169.08	114.96	465.33	406.82	289.28	229.10	242.63	138.22		
3955	Ribosomal RNA processing protein 36 homolog	Q96EU6	1452.67	1379.77	1196.49	847.49	790.02	814.01	1798.83	916.83	1239.62	1773.04	1619.11	1360.61	1621.76	902.81	862.63	924.64	891.78	725.44	1199.34	752.00	784.72	2.27	1.49	0.65	0.84	1.31	0.69	4.16	2.47	0.11	4.90	4.98	0.12	x	
3956	Ribosome biogenesis protein BMS1 homolog	Q14692	13360.97	12007.21	9437.26	8369.03	8400.58	7207.39	9164.30	8345.95	8133.25	11203.95	11093.18	9560.19	10271.23	8379.66	4570.23	9241.55	8260.96	8419.81	9358.50	9217.69	8015.61	82142.40	73376.22	65265.03	54331.79	52745.91	33965.87	63757.14	58876.71	50443.93	81441.19	76715.07	43698.82		
3957	Ribosome biogenesis protein BOP1	Q14137	50781.49	37690.25	25356.35	3124.56	8382.51	4495.28	10887.22	16514.65	15830.32	37147.48	27534.02	22283.08	38153.73	15153.86	7450.49	3268.06	11133.15	8295.53	2382.65	2608.48	1830.79	10119.26	2754.88	2658.04	6637.37	5984.44	2802.83	13238.56	11131.66	6689.49	13898.82	12531.73	25676.91		
3958	Ribosome biogenesis protein WDR12	Q9GZL7	77624.39	74138.23	69748.86	47971.42	44273.91	42135.19	59194.53	58005.68	57021.34	55196.64	52898.72	49820.14	59560.79	58506.42	48303.85	47042.41	43180.30	42688.10	46325.07	42449.64	42868.99	3775.21	3361.04	3100.82	1089.61	1117.08	653.82	1243.09	1056.78	735.13	1267.93	1285.47	556.23		
3959	Ribosome maturation protein SBDS	Q9Y3A5	0.14	2.53	0.81	1.07	2.77	0.68	0.96	0.36	0.63	0.35	0.75	0.38	2.13	0.97	0.60	4.64	1.07	0.47	2.22	0.19	0.06	10076.37	8961.64	9078.89	6501.37	6765.80	6294.62	7169.68	6961.21	6998.57	7339.02	6631.26	5439.48		x
3960	Ribosome-binding protein 1	Q9P2E9	340782.49	333480.37	306636.87	294958.43	281807.13	261831.60	293171.22	284532.72	271612.15	363530.69	353968.26	330704.81	304484.95	287353.06	227181.00	289439.78	279548.38	270615.41	273469.55	251204.19	239977.13	233014.11	222700.40	205675.58	166484.73	164072.90	148943.59	172483.45	165849.42	166839.45	197195.88	190797.60	116259.16		
3961	Ribosome-recycling factor_ mitochondrial	Q96E11	11483.65	10033.21	10270.07	9236.89	8322.81	9162.90	10968.98	11097.00	10612.30	9645.19	9504.74	8210.24	10673.62	13479.96	13087.51	10496.09	7825.35	9086.79	8131.62	9179.48	8802.64	3482.82	3338.43	2835.78	2004.43	2048.37	1338.27	2375.00	2392.45	2132.53	2557.77	2635.21	1149.97		
3962	Ribosyldihydronicotinamide dehydrogenase [quinone]	P16083	291.38	285.78	246.83	512.59	394.33	407.79	391.84	392.77	258.95	462.70	410.01	440.76	232.54	226.86	127.97	489.03	532.10	460.28	437.01	482.48	438.94	2.27	1.49	0.65	0.84	1.31	0.69	4.16	2.47	0.11	4.90	4.98	0.12	x	
3963	RILP-like protein 2	Q969X0	1855.11	2098.98	1983.30	1311.86	1290.95	1140.25	1381.73	1580.42	831.35	1506.01	1459.77	1280.22	1412.89	1494.62	1185.93	1007.91	1178.63	989.08	1418.80	1227.11	1250.54	2.27	1.49	0.65	0.84	1.31	0.69	4.16	2.47	0.11	4.90	4.98	0.12	x	
3964	RIMS-binding protein 3C	A6NJZ7	30314.28	21586.75	18221.24	11137.27	9977.34	9501.41	10124.23	6137.44	8893.28	19067.28	18411.96	13149.62	21582.33	8212.16	5060.83	12246.65	10408.88	10644.75	11787.09	11854.52	7866.66	2.27	1.49	0.65	0.84	1.31	0.69	4.16	2.47	0.11	4.90	4.98	0.12	x	
3965	RING finger and CHY zinc finger domain-containing protein 1	Q96PM5	11109.18	10422.88	8539.29	6714.86	6394.89	5640.00	8368.99	7879.83	6681.56	9657.58	9318.87	7678.47	8961.23	8468.04	5132.65	7003.29	6891.75	5970.60	6890.28	6914.33	5962.99	358.96	273.29	220.54	241.52	217.72	219.55	296.98	274.84	253.77	252.07	169.97	103.07		
3966	RING finger protein 122	Q9H9V4	1338.01	1162.14	1251.13	814.78	858.31	831.42	1029.38	1140.71	1037.16	1435.57	1491.04	1284.07	1220.81	1022.09	915.86	879.81	718.64	761.42	837.60	778.73	733.99	2.27	1.49	0.65	0.84	1.31	0.69	4.16	2.47	0.11	4.90	4.98	0.12	x	
3967	RING finger protein 212B	A8MTL3	56584.66	53156.70	52539.63	38734.78	35045.67	33309.85	41398.31	41313.03	38924.15	41708.54	41343.86	37377.94	46805.99	44616.19	37789.44	38179.42	36826.07	36177.11	40951.53	37443.36	34630.42	2.27	1.49	0.65	0.84	1.31	0.69	4.16	2.47	0.11	4.90	4.98	0.12	x	
3968	RING-box protein 2	Q9UBF6	3146.34	2461.62	2020.01	1484.90	1582.92	1308.99	1859.79	1747.52	1645.54	2908.01	2522.61	2493.92	1985.80	2168.42	1664.52	1521.50	1788.42	1794.41	1776.09	1737.81	1483.98	2.27	1.49	0.65	0.84	1.31	0.69	4.16	2.47	0.11	4.90	4.98	0.12	x	
3969	RNA binding motif protein_ X-linked-like-1	Q96E39	0.14	2.53	0.81	1.07	2.77	0.68	0.96	0.36	0.63	0.35	0.75	0.38	2.13	0.97	0.60	4.64	1.07	0.47	2.22	0.19	0.06	21616.84	20170.23	16970.49	12782.44	12014.98	8795.98	15497.02	12368.27	12082.18	15589.30	14965.00	10259.44		x
3970	RNA binding protein fox-1 homolog 1	Q9NWB1	2162.77	1626.76	1438.43	1285.32	1281.44	850.70	1040.76	1304.35	1178.13	2353.21	2155.57	1896.89	882.26	1251.59	497.60	1739.35	1579.60	1536.22	1588.45	1382.67	1292.30	2.27	1.49	0.65	0.84	1.31	0.69	4.16	2.47	0.11	4.90	4.98	0.12	x	
3971	RNA polymerase II subunit A C-terminal domain phosphatase SSU72	Q9NP77	0.14	2.53	0.81	1.07	2.77	0.68	0.96	0.36	0.63	0.35	0.75	0.38	2.13	0.97	0.60	4.64	1.07	0.47	2.22	0.19	0.06	4961.49	4111.66	4418.10	1901.34	1757.29	1666.13	1969.98	1785.09	1772.14	1960.37	1669.54	794.01		x
3972	RNA polymerase II-associated factor 1 homolog	Q8N7H5	1819.77	1822.98	1371.01	1982.04	1108.67	628.04	896.44	1487.52	1289.38	1465.39	1174.78	1183.89	1040.34	2752.85	1116.63	1104.65	1322.52	1173.04	1480.67	1035.19	986.34	2.27	1.49	0.65	0.84	1.31	0.69	4.16	2.47	0.11	4.90	4.98	0.12	x	
3973	RNA polymerase II-associated protein 3	Q9H6T3	85862.46	77045.31	64430.60	60870.92	60894.16	50794.35	69642.43	70338.80	63028.65	74011.12	70943.56	62323.24	73326.01	69751.61	39723.23	56470.00	54961.18	50042.43	67852.79	62017.32	54761.49	182159.80	171183.91	156753.93	124939.15	123598.25	112924.48	136082.49	129869.51	125310.09	140240.69	137158.21	89708.39		
3974	RNA polymerase-associated protein CTR9 homolog	Q6PD62	58150.74	57275.41	60732.67	48425.70	43033.76	33500.21	33045.92	35773.53	24929.98	57312.69	58362.06	58588.13	51342.41	51569.22	17068.14	22557.64	39444.93	46796.12	66756.67	59404.92	54364.09	100102.61	94847.47	86821.69	69719.69	67812.66	65860.83	70622.44	74379.80	72575.85	93801.82	90310.23	40184.98		
3975	RNA polymerase-associated protein RTF1 homolog	Q92541	11558.11	12187.99	9827.30	8823.71	8265.32	7583.09	10640.46	9434.47	9430.75	11172.50	10509.00	9871.71	10691.38	9909.52	7895.16	7537.42	8418.01	7153.68	8203.45	7792.42	7812.72	22424.72	22875.02	18443.48	13501.97	12781.08	7091.17	22973.32	20193.76	15484.36	23039.41	20526.27	9851.13		
3976	RNA transcription_ translation and transport factor protein	Q9Y224	63917.22	55661.28	49256.79	46909.70	46269.85	40663.41	51268.64	36450.99	43124.10	56818.50	54250.47	48925.91	46733.12	32253.87	30476.92	50756.36	43851.58	47228.46	42834.37	40518.78	37168.82	33546.45	31905.72	28276.80	21011.61	20449.75	16975.62	23723.36	22378.72	21538.37	22635.77	22468.87	14837.59		
3977	RNA-binding motif protein_ X chromosome	P38159	13315.25	13471.66	11489.94	8489.54	8594.35	7875.85	8998.01	7820.57	8076.56	12131.89	12083.36	10593.63	11023.02	10007.38	6597.66	8686.09	8047.55	7906.99	9457.70	8659.62	7607.45	2513.60	2305.04	2157.00	2056.86	2025.59	2027.03	2537.23	2382.60	2443.04	2707.08	2534.11	1717.19		
3978	RNA-binding motif protein_ X-linked-like-2	O75526	1454.19	1488.02	1246.18	737.10	788.79	703.95	1362.61	1267.09	1370.33	1026.19	1131.65	903.70	1432.35	1183.44	956.66	780.54	673.16	684.69	696.38	612.99	598.74	48248.32	39850.45	45602.18	34289.93	34443.27	32307.84	36825.76	34428.38	34527.97	41129.85	40401.77	17882.67		
3979	RNA-binding motif protein_ X-linked-like-3	Q8N7X1	0.14	2.53	0.81	1.07	2.77	0.68	0.96	0.36	0.63	0.35	0.75	0.38	2.13	0.97	0.60	4.64	1.07	0.47	2.22	0.19	0.06	5641.03	5384.84	4540.50	5667.38	5703.03	4902.04	7216.77	6990.11	6350.33	7800.46	7124.74	4634.09		x
3980	RNA-binding protein 12	Q9NTZ6	21386.11	22071.46	20120.65	13793.80	13393.98	12863.32	16096.90	15746.16	15091.04	17456.90	15947.64	15995.89	17400.75	17010.38	14684.87	12664.08	12247.69	12444.86	14495.45	13071.40	12004.61	26540.64	25886.67	22341.53	18172.59	17360.69	14859.09	20922.22	21180.57	21154.24	20156.76	19543.76	13468.36		
3981	RNA-binding protein 15	Q96T37	1469.73	1088.43	702.59	44071.66	1946.60	1255.58	1483.67	280.16	58.93	1082.36	777.21	330.83	949.12	32787.44	982.16	1922.13	189.21	1229.39	2726.28	2478.42	2555.97	238.78	287.82	215.19	322.01	365.70	270.58	205.47	163.26	196.00	380.14	361.97	81.60		
3982	RNA-binding protein 25	P49756	32143.76	29074.08	28691.37	18347.11	20807.31	18371.99	29185.95	27654.27	24511.09	24413.62	25595.86	20988.46	22728.30	22937.94	19081.84	22688.16	23085.05	20229.70	22397.13	20189.97	18627.02	14377.43	13496.59	11938.37	13229.67	12851.32	12010.02	16575.14	17762.84	16221.33	16474.70	15949.77	11884.65		
3983	RNA-binding protein 3	P98179	56690.50	47737.73	47841.32	28355.76	25434.42	25227.16	76780.77	74489.22	72950.86	37204.60	33523.06	31458.64	28804.46	32296.68	25924.49	28105.17	25986.25	24729.68	25220.70	25637.22	24536.87	108013.99	100418.33	90409.95	67800.33	65790.36	60461.95	76718.10	71286.88	68006.18	76862.54	78987.63	42026.95		

Supplementary Table S1. Overview on the abundance of all relatively quantified 5180 proteins

			MCF-7																					MDA-MB-231													
	Protein name	UniProt	control 01	control 02	control 03	Dai IC20 01	Dai IC20 02	Dai IC20 03	Dai SC20 01	Dai SC20 02	Dai SC20 03	Gen IC20 01	Gen IC20 02	Gen IC20 03	Gen SC20 01	Gen SC20 02	Gen SC20 03	SSE IC20 01	SSE IC20 02	SSE IC20 03	SSE SC20 01	SSE SC20 02	SSE SC20 03	control 01	control 02	control 03	Dai IC20 01	Dai IC20 02	Dai IC20 03	Gen IC20 01	Gen IC20 02	Gen IC20 03	SSE IC20 01	SSE IC20 02	SSE IC20 03	unique to MCF-7	unique to MDA-MB-231
3984	RNA-binding protein 39	Q14498	62828.38	59580.23	55116.63	47067.35	39233.74	33002.76	47442.86	45427.72	38101.01	49379.47	46571.10	43336.40	52613.41	45765.65	29070.44	41189.33	37835.13	35256.48	45116.93	41830.42	40354.40	33739.92	30991.53	28216.96	26626.10	26628.11	20547.81	34968.99	32959.60	28897.24	34214.87	30855.57	18481.71		
3985	RNA-binding protein 4	Q9BWF3	67810.97	68589.93	60174.11	36842.39	36260.63	32974.56	47059.68	43491.99	44681.77	44938.93	43461.78	39242.56	49713.24	47338.67	37977.26	37405.63	34695.98	34026.68	42961.88	40729.30	38431.07	32010.35	28434.25	28239.50	20058.22	19228.83	15254.69	24658.32	23266.05	21265.23	23360.37	23075.91	14981.58		
3986	RNA-binding protein 47	A0AV96	0.14	2.53	0.81	1.07	2.77	0.68	0.96	0.36	0.63	0.35	0.75	0.38	2.13	0.97	0.60	4.64	1.07	0.47	2.22	0.19	0.06	1826.64	1037.41	3082.06	457.58	320.55	161.21	1432.23	632.42	676.32	579.88	698.73	238.58		x
3987	RNA-binding protein 4B	Q9BQ04	2642.63	2410.16	2482.77	1460.71	1235.40	1144.76	2563.95	2093.60	2534.78	2097.03	2312.87	2102.87	2476.82	2253.29	1960.21	2093.59	1537.55	1603.26	1891.32	1944.77	1593.78	482.15	347.64	370.21	309.51	270.43	283.60	516.82	398.63	350.32	350.73	269.84	238.76		
3988	RNA-binding protein 8A	Q9Y5S9	86144.21	84733.02	86657.08	62354.37	59283.40	54596.10	56400.04	51073.24	53421.82	71274.10	70066.63	70864.88	73889.99	62605.49	38995.39	40111.17	45669.44	46191.28	62882.25	59107.40	56799.23	67176.27	65154.02	61689.00	48572.82	48669.31	44567.81	51499.46	52518.04	51470.57	57131.98	52476.29	27012.54		
3989	RNA-binding protein EWS	Q01844	299035.81	290586.60	270869.42	202205.52	187788.88	174641.17	237462.67	234711.32	226390.15	236209.20	228616.66	215603.19	246289.46	236770.27	202403.51	205496.46	198915.64	191610.53	206845.43	189946.75	180063.06	177607.42	168530.79	151723.01	107084.83	106733.61	95911.23	125311.21	122086.75	114720.46	129781.01	128764.18	86533.54		
3990	RNA-binding protein FUS	P35637	472622.95	443079.50	409478.79	290780.46	279110.98	261270.91	327707.03	323684.25	306407.02	347915.84	332046.44	306797.67	343302.99	324316.82	269852.95	308462.72	300482.20	295660.96	319936.92	306686.48	276653.03	209567.79	199835.75	181672.59	158760.58	154929.82	134812.93	183024.04	177074.82	166966.28	194736.29	188875.05	124441.54		
3991	RNA-binding protein NOB1	Q9ULX3	1001.24	893.23	889.20	647.10	626.52	659.86	800.89	834.79	924.46	957.41	985.51	1012.50	845.30	496.88	247.78	615.14	500.35	593.60	516.65	640.75	499.26	242.93	124.69	79.28	350.91	298.90	108.84	668.22	654.41	246.01	701.71	632.79	138.65		
3992	RNA-binding protein with multiple splicing	Q93062	5065.75	4948.26	4173.31	3559.38	3588.98	4100.24	4388.11	4256.20	4326.19	5470.69	5191.15	5047.42	4617.74	4039.91	3556.40	3574.12	3447.09	3418.44	3831.54	3799.54	3155.43	2.27	1.49	0.65	0.84	1.31	0.69	4.16	2.47	0.11	4.90	4.98	0.12	x	
3993	Rod cGMP-specific 3'_5'-cyclic phosphodiesterase subunit beta	P35913	0.14	2.53	0.81	1.07	2.77	0.68	0.96	0.36	0.63	0.35	0.75	0.38	2.13	0.97	0.60	4.64	1.07	0.47	2.22	0.19	0.06	4088.92	3184.07	3108.81	3173.59	2817.24	2646.95	2916.10	2822.90	2913.71	4136.40	3469.40	1081.26		x
3994	Rootletin	Q5TZA2	170656.24	164943.54	158890.16	114794.70	110660.93	105758.48	135255.47	131253.33	128465.84	155907.62	148877.38	147565.89	150008.97	123036.18	97987.90	122759.20	116447.47	113678.93	127512.61	121276.45	117628.91	202166.54	190318.31	171065.54	135036.90	131518.56	117613.05	149870.55	144461.19	134799.19	162582.51	153862.17	101047.90		
3995	Roquin-1	Q5TC82	313.47	264.04	309.94	115.80	60.50	63.27	100.61	163.46	154.45	221.75	225.11	271.19	197.73	97.84	111.24	156.89	156.99	152.78	180.68	80.31	190.38	2.27	1.49	0.65	0.84	1.31	0.69	4.16	2.47	0.11	4.90	4.98	0.12	x	
3996	rRNA 2'-O-methyltransferase fibrillarin	P22087	2641.22	2714.39	2312.73	1593.05	1551.77	1294.19	1563.34	1316.01	1259.11	1267.79	1188.32	1242.44	1309.84	1468.73	1069.63	977.07	1192.30	1011.15	1210.52	1317.12	1170.13	2.27	1.49	0.65	0.84	1.31	0.69	4.16	2.47	0.11	4.90	4.98	0.12	x	
3997	rRNA methyltransferase 1 mitochondrial	Q6IN84	1380.17	1327.62	1046.95	989.61	1106.00	1032.88	1026.29	1002.67	986.69	1496.46	1547.62	1564.59	1179.55	1197.76	978.40	1166.02	997.98	843.45	1124.04	1070.77	902.92	2.27	1.49	0.65	0.84	1.31	0.69	4.16	2.47	0.11	4.90	4.98	0.12	x	
3998	rRNA-processing protein UTP23 homolog	Q9BRU9	96983.46	93092.92	98706.24	91436.58	84065.16	82652.75	83733.30	80885.88	81720.24	95587.50	93216.28	87543.33	83983.39	90279.47	75751.35	89826.58	85751.70	84878.82	71806.11	68767.96	65723.71	2.27	1.49	0.65	0.84	1.31	0.69	4.16	2.47	0.11	4.90	4.98	0.12	x	
3999	RUN and FYVE domain-containing protein 1	Q96T51	206236.94	195357.68	177143.04	151118.30	148994.93	137197.23	160046.19	164183.71	150799.86	229411.48	231400.37	225092.07	184565.04	176198.82	150638.57	143510.76	142763.36	130822.84	154080.96	151554.37	147979.85	115993.86	106749.14	100550.39	70861.26	69281.98	61053.48	93615.66	91841.18	84875.04	90162.98	90235.64	60984.49		
4000	RUN and FYVE domain-containing protein 2	Q8WXA3	1704.18	1221.03	869.72	1475.45	1420.40	1610.64	921.20	998.26	840.59	1791.18	2613.03	2861.52	1536.62	1344.18	1509.39	1502.61	1145.79	1175.07	1352.20	1465.36	1494.24	13924.50	11698.83	13883.29	15193.71	15567.18	15030.27	14987.00	15836.36	14506.82	18692.03	18820.91	3851.02		
4001	Run domain Beclin-1-interacting and cysteine-rich domain-containing protein	Q92622	2503.82	2639.72	1901.99	2163.14	1634.27	1892.61	2014.01	1873.10	1846.05	2438.54	2639.12	2440.47	2382.63	1972.13	730.45	2305.63	2151.61	1921.97	1783.83	1833.31	1295.30	2.27	1.49	0.65	0.84	1.31	0.69	4.16	2.47	0.11	4.90	4.98	0.12	x	
4002	RUN domain-containing protein 3A	Q59EK9	37281.69	35236.52	34588.40	37231.90	35273.31	31492.45	31159.23	30403.35	30243.27	41958.21	39658.41	38284.15	32234.19	32151.87	27223.72	35045.18	32637.82	31716.09	28153.32	27749.88	26959.08	63173.92	58939.76	52655.39	50712.65	48930.46	36182.59	55475.43	53721.76	48436.59	59766.72	58343.01	35997.13		
4003	Runt-related transcription factor 2	Q13950	6017.65	5894.11	5696.37	2863.21	2760.76	2545.67	3485.30	3294.25	3331.89	4066.53	3815.20	3605.33	3919.69	3496.20	3055.06	3023.23	3012.06	2684.32	2926.77	2610.37	2532.21	2.27	1.49	0.65	0.84	1.31	0.69	4.16	2.47	0.11	4.90	4.98	0.12	x	
4004	RUS1 family protein C16orf58	Q96GQ5	10907.89	10412.03	8789.47	4494.52	4961.28	4070.23	6976.65	6151.16	5849.57	9533.31	8568.10	7854.51	7592.34	7012.23	4537.26	5694.72	5479.08	5946.44	6269.33	5324.04	5068.45	2.27	1.49	0.65	0.84	1.31	0.69	4.16	2.47	0.11	4.90	4.98	0.12	x	
4005	RuvB-like 1	Q9Y265	193630.50	183992.20	160065.81	135092.97	130323.55	115382.45	153674.25	147579.62	141894.69	163808.55	162791.32	148882.96	158955.68	145022.29	104661.93	130878.39	125380.34	122640.50	143338.17	131977.02	124450.85	247301.40	233298.56	215449.80	171632.96	167764.10	145358.45	188608.68	181859.87	173643.37	195849.48	190275.69	129823.14		
4006	RuvB-like 2	Q9Y230	668285.28	660632.06	626285.98	536007.93	519863.74	502662.68	578001.14	556943.75	530797.32	620790.89	620616.93	593138.64	564888.47	532659.49	480209.48	576537.12	533087.84	513757.37	467550.71	467981.51	437840.25	214160.26	204343.48	194915.40	148292.76	146498.30	137897.07	169861.96	165255.55	160426.32	168574.63	165199.67	120748.20		
4007	Ryanodine receptor 1	P21817	0.14	2.53	0.81	1.07	2.77	0.68	0.96	0.36	0.63	0.35	0.75	0.38	2.13	0.97	0.60	4.64	1.07	0.47	2.22	0.19	0.06	10086.46	8324.25	7753.48	5798.65	5482.77	4051.69	7609.37	7252.97	6847.66	7385.53	7046.34	4587.15		x
4008	Ryanodine receptor 2	Q92736	6282.05	6513.25	5996.93	3859.86	3556.34	3609.63	4320.23	4094.81	4290.97	5651.97	5443.08	4995.07	5602.25	5112.64	4162.55	3469.00	3748.45	3396.45	4030.01	3496.63	3384.06	15932.11	15774.36	13550.58	10313.15	10159.76	8558.71	13112.01	12590.85	11807.46	14260.45	13428.88	9200.22		
4009	Ryanodine receptor 3	Q15413	312610.66	285286.88	261793.57	242959.80	225703.41	200873.50	251659.14	244435.51	230367.58	290104.78	269040.07	246401.10	277853.23	245573.57	155338.47	213265.87	208227.03	190213.02	209747.02	197020.47	176649.42	202287.89	192825.14	167029.90	110888.59	106577.58	94318.41	129790.11	127686.86	120468.11	125838.53	123997.03	83544.94		
4010	Saccharopine dehydrogenase-like oxidoreductase	Q8NBX0	0.14	2.53	0.81	1.07	2.77	0.68	0.96	0.36	0.63	0.35	0.75	0.38	2.13	0.97	0.60	4.64	1.07	0.47	2.22	0.19	0.06	5240.94	5304.58	5282.87	4044.30	3920.75	3720.76	4645.59	4584.18	4208.81	4604.56	4641.43	4032.36		x
4011	Sacsin	Q9NZJ4	68248.04	63916.16	59513.02	46216.07	43377.56	39487.34	47636.06	43264.87	41570.34	65073.04	63389.03	59241.82	62389.71	40870.76	32248.71	45884.97	42811.18	41601.78	37054.05	40606.12	39455.73	8768.21	8823.10	8495.67	7267.36	6636.31	5119.41	8029.19	7444.69	6687.40	8212.50	7789.80	4014.94		
4012	S-adenosylhomocysteine hydrolase-like protein 1	O43865	119561.74	115022.34	110202.70	98112.21	92265.24	90148.44	106840.85	104848.13	104736.74	120945.76	112065.13	110699.05	102276.20	99468.31	87250.91	95921.46	90376.94	89176.53	81199.94	78971.09	78995.25	168044.39	159948.23	150998.26	99281.16	98482.16	89875.98	104950.76	100691.00	98326.54	88422.33	86251.07	65988.46		
4013	S-adenosyl-L-methionine-dependent tRNA 4-demethylwyosine synthase	Q9NV66	39078.63	40000.97	34019.91	26537.40	26570.25	25392.65	32378.80	32043.93	31139.95	31778.88	30891.12	28922.37	31927.84	29482.98	26029.19	26198.46	25824.64	24122.69	27943.30	26409.25	24389.39	2.27	1.49	0.65	0.84	1.31	0.69	4.16	2.47	0.11	4.90	4.98	0.12	x	
4014	S-adenosyl-L-methionine-dependent tRNA 4-demethylwyosine synthase	Q6NUM6	0.14	2.53	0.81	1.07	2.77	0.68	0.96	0.36	0.63	0.35	0.75	0.38	2.13	0.97	0.60	4.64	1.07	0.47	2.22	0.19	0.06	23432.30	24899.58	22398.80	4875.14	4540.08	3536.97	4957.10	5161.56	4403.37	3745.58	3132.98	2063.28		x
4015	S-adenosylmethionine synthase isoform type-1	Q00266	292.97	108.57	98.65	107.70	241.67	99.75	100.63	398.26	244.98	46.84	149.46	59.04	59.67	137.16	3.85	335.98	34.45	134.38	2.22	0.19	0.06	3379.62	2893.54	2612.16	1821.91	1833.36	1633.45	2975.67	2823.75	2452.02	2531.16	2294.48	1578.90		x
4016	S-adenosylmethionine synthase isoform type-2	P31153	171160.53	167472.22	147719.20	95047.26	93297.66	84688.14	117125.11	113037.75	106759.72	114395.78	106276.50	101674.64	118066.27	110928.31	96798.89	96563.34	90395.96	87490.64	99596.32	97762.69	90721.79	111502.69	96545.04	93925.71	57494.88	53531.47	49644.72	80018.66	71819.10	69841.39	75084.61	74150.10	51170.26		
4017	SAM domain-containing protein SAMSN-1	Q9NSI8	0.14	2.53	0.81	1.07	2.77	0.68	0.96	0.36	0.63	0.35	0.75	0.38	2.13	0.97	0.60	4.64	1.07	0.47	2.22	0.19	0.06	3813.52	3914.68	3613.85	4826.88	4262.90	4103.49	5415.50	5051.48	4679.23	5269.95	5125.18	4054.27		x
4018	SAM pointed domain-containing Ets transcription factor	O95238	1170.42	1048.05	889.87	657.37	751.79	709.75	773.31	756.00	850.29	1123.82	978.24	1100.04	909.75	906.84	645.31	805.47	776.90	701.14	839.68	774.44	612.38	2.27	1.49	0.65	0.84	1.31	0.69	4.16	2.47	0.11	4.90	4.98	0.12	x	
4019	SAP domain-containing ribonucleoprotein	P82979	0.14	2.53	0.81	1.07	2.77	0.68	0.96	0.36	0.63	0.35	0.75	0.38	2.13	0.97	0.60	4.64	1.07	0.47	2.22	0.19	0.06	25811.88	25010.10	24844.60	20985.23	21679.38	20240.00	23401.98	21337.20	21452.45	24670.67	25245.54	20639.28		x
4020	SAP30-binding protein	Q9UHR5	3972.17	3754.05	3017.04	1928.21	1972.29	1527.45	2423.15	1411.72	2000.27	2987.15	2519.75	2232.75	2491.89	1688.09	1025.85	1840.44	1666.07	1370.32	2075.32	2484.90	1848.35	4669.28	4146.98	3708.06	3268.98	3318.71	1873.90	4029.10	3529.53	2723.06	5100.56	4748.99	2443.00		
4021	Sarcolemmal membrane-associated protein	Q14BN4	4609.41	4303.05	4187.84	9123.89	3126.17	2900.69	4272.29	4269.10	3628.86	4982.48	4565.60	4054.02	4504.13	5699.42	4670.62	2704.39	2381.93	2811.40	3378.38	2993.51	3277.92	1516.18	1444.87	1502.38	1549.47	1408.70	1334.45	2004.79	1991.06	2014.06	2164.00	1871.04	1402.68		
4022	Sarcoplasmic/endoplasmic reticulum calcium ATPase 1	O14983	32010.75	32150.14	30693.94	19761.07	19711.45	17634.27	25408.45	24810.00	25618.78	25863.88	24252.19	22215.51	28446.24	29373.40	21448.24	20801.29	19650.41	19370.40	25045.21	20375.29	18113.61	19404.41	18985.52	16933.61	11259.72	10878.16	9598.53	12802.80	13011.29	11987.32	11027.74	10502.95	7663.46		
4023	Sarcoplasmic/endoplasmic reticulum calcium ATPase 2	P16615	669828.79	630835.92	582941.30	461821.50	448894.20	408405.20	523046.27	497466.21	469004.40	590235.00	556179.37	513829.60	563392.33	526527.18	417469.78	452847.38	425772.97	414753.91	477117.58	452289.26	413576.39	147057.48	138930.07	130139.31	82755.53	82772.38	74102.64	102391.02	99737.68	88823.89	111571.41	106088.48	70792.64		
4024	Sarcoplasmic/endoplasmic reticulum calcium ATPase 3	Q93084	23341.32	22233.43	19035.75	12140.75	11746.67	10898.23	13900.58	13175.68	12914.23	17432.54	18151.06	17616.60	19451.26	17235.39	11478.06	15194.28	12933.35	12486.51	11856.16	10474.54	9646.14	15228.55	14391.73	13143.49	9142.34	8871.72	7079.27	12707.85	11703.18	11615.13	10695.52	10042.06	6692.18		
4025	S-arrestin	P10523	0.14	2.53	0.81	1.07	2.77	0.68	0.96	0.36	0.63	0.35	0.75	0.38	2.13	0.97	0.60	4.64	1.07	0.47	2.22	0.19	0.06	1326.06	1010.16	952.17	776.75	806.36	394.84	959.85	949.27	816.74	1191.59	1233.05	528.61		x
4026	Scaffold attachment factor B1	Q15424	0.14	2.53	0.81	1.07	2.77	0.68	0.96	0.36	0.63	0.35	0.75	0.38	2.13	0.97	0.60	4.64	1.07	0.47	2.22	0.19	0.06	12850.29	12339.02	9172.31	5755.77	5321.60	4726.27	7390.66	7047.76	6428.58	7028.04	6375.00	4427.35		x
4027	Scaffold attachment factor B2	Q14151	744.98	559.95	449.40	598.56	560.44	490.31	456.76	485.99	579.86	581.41	575.03	629.12	697.39	115.83	18.02	251.31	636.79	346.19	659.40	510.66	420.66	2.27	1.49	0.65	0.84	1.31	0.69	4.16	2.47	0.11	4.90	4.98	0.12	x	
4028	Scavenger receptor class B member 1	Q8WTV0	31602.81	33204.30	32769.32	24511.49	23590.06	24203.56	23323.80	26083.56	23051.98	26204.09	26961.81	26321.50	22356.44	26934.68	23009.97	21390.16	22473.44	23289.46	26150.73	25000.46	25926.21	2.27	1.49	0.65	0.84	1.31	0.69	4.16	2.47	0.11	4.90	4.98	0.12	x	
4029	Scavenger receptor cysteine-rich type 1 protein M160	Q9NR16	1688.94	1653.68	1459.64	1204.05	988.69	1048.79	1565.50	1414.93	1392.51	1568.76	1464.87	1386.66	1757.07	1427.45	1459.86	1264.85	1283.67	1271.60	1468.90	1300.20	1323.63	2.27	1.49	0.65	0.84	1.31	0.69	4.16	2.47	0.11	4.90	4.98	0.12	x	
4030	Schlafen family member 11	Q7Z7L1	25119.56	23745.12	22793.24	17659.54	17614.44	17228.59	20598.02	21616.10	20010.01	22757.88	21452.95	21352.63	21844.78	22332.12	20337.17	16585.21	15836.38	16095.41	18460.66	16640.91	16753.55	7747.02	5988.75	6468.32	3871.91	3902.80	3330.71	4597.03	4344.22	3783.73	3933.75	3787.76	2966.17		
4031	Schwannomin-interacting protein 1	P0DPB3	0.14	2.53	0.81	1.07	2.77	0.68	0.96	0.36	0.63	0.35	0.75	0.38	2.13	0.97	0.60	4.64	1.07	0.47	2.22	0.19	0.06	10983.92	10788.76	10982.91	8229.18	7389.44	7445.48	9706.02	9444.49	9572.75	10954.43	10510.35	2243.09		x
4032	SCL-interrupting locus protein	Q15468	35876.58	35583.97	34779.78	27794.13	26099.36	24418.89	26903.97	26289.51	26158.53	28543.62	28620.14	27438.36	28480.75	29621.93	26419.24	27155.21	25642.65	23712.12	25835.73	24651.65	24228.74	2.27	1.49	0.65	0.84	1.31	0.69	4.16	2.47	0.11	4.90	4.98	0.12	x	
4033	SCY1-like protein 2	Q6P3W7	71931.30	61396.80	48776.97	38326.41	35845.07	30962.04	36227.73	39072.69	33050.26	64211.97	56978.01	48423.41	57042.94	44076.34	24758.21	31806.95	34961.06	33095.23	48375.78	43480.75	35919.21	2.27	1.49	0.65	0.84	1.31	0.69	4.16	2.47	0.11	4.90	4.98	0.12	x	
4034	Sec1 family domain-containing protein 1	Q8WVM8	762668.36	692375.65	670174.66	564405.70	546178.87	496012.34	646321.42	651718.45	574492.51	640734.05	644920.65	590373.03	606337.01	548100.51	438952.63	592982.76	591107.21	575537.59	567607.42	502807.09	486195.49	34917.23	25793.35	32354.97	22953.82	22450.66	20175.86	25669.89	24492.18	24096.28	28359.80	28179.43	32146.75		
4035	Sec1 family domain-containing protein 2	Q8WU76	0.14	2.53	0.81	1.07	2.77	0.68	0.96	0.36	0.63	0.35	0.75	0.38	2.13	0.97	0.60	4.64	1.07	0.47	2.22	0.19	0.06	201.03	192.42	152.71	167.34	192.62	98.42	164.50	202.39	146.52	216.24	260.67	133.04		x
4036	SEC23-interacting protein	Q9Y6Y8	53799.35	49234.93	44516.83	36210.93	35588.91	31172.46	44100.07	41050.23	37587.17	46328.18	43683.36	42124.29	41258.31	43251.48	30979.82	37495.10	34745.94	31941.03	33261.88	31797.04	32805.63	2.27	1.49	0.65	0.84	1.31	0.69	4.16	2.47	0.11	4.90	4.98	0.12	x	
4037	Secernin-1	Q12765	140816.23	129583.64	115791.31	90943.11	93712.40	79051.63	110683.89	103048.23	93456.32	122125.71	118269.20	105296.76	119826.47	116000.92	89291.06	89699.38	86516.13	84383.83	93387.14	95302.54	88949.10	80660.93	78101.18	69215.01	60573.24	58679.36	48354.54	66667.52	63427.62	57311.67	72175.23	69809.85	49565.29		
4038	Secernin-2	Q96FV2	983.94	1023.70	1149.09	640.13	621.46	731.76	435.35	520.27	383.97	1278.49	1153.02	1268.81	925.58	1074.60	378.82	322.38	605.00	831.09	1031.44	929.65	647.83	734.42	705.86	757.68	501.82	408.37	420.25	554.45	484.77	480.43	621.41	601.48	372.46		
4039	Secretogranin-1	P05060	161600.50	155646.05	167520.12	118364.25	114086.42	100507.91	93240.26	110389.98	87644.60	156784.61	162031.69	171377.93	158699.62	139213.37	38994.07	99976.18	141222.91	161192.71	166268.45	153636.88	128266.57	57304.43	50619.95	49065.04	35336.92	39890.82	33343.14	35661.02	37229.65	36932.31	50331.22	48048.42	14681.10		
4040	Secretogranin-2	P13521	0.14	2.53	0.81	1.07	2.77	0.68	0.96	0.36	0.63	0.35	0.75	0.38	2.13	0.97	0.60	4.64	1.07	0.47	2.22	0.19	0.06	8602.46	8288.67	7583.75	5277.25	5531.99	3655.66	6138.24	5425.74	5253.98	6725.60	6427.41	3625.45		x
4041	Secretory carrier-associated membrane protein 1	O15126	126142.19	125034.08	115788.16	85798.00	82894.10	77377.88	89790.15	86263.30	81248.45	107842.34	103340.31	100598.95	96401.35	94149.89	77257.45	81337.74	75150.68	72811.78	89387.76	83991.88	78640.21	22486.61	21729.97	20901.99	19548.00	19256.99	18430.36	22365.40	20402.85	19056.45	26033.91	24723.09	8799.92		
4042	Secretory carrier-associated membrane protein 2	O15127	549.31	520.86	521.79	241.42	159.33	159.07	314.21	295.87	239.86	421.24	437.91	275.86	379.45	468.08	69.79	375.83	271.70	308.53	325.57	431.24	352.45	2.27	1.49	0.65	0.84	1.31	0.69	4.16	2.47	0.11	4.90	4.98	0.12	x	
4043	Secretory carrier-associated membrane protein 3	O14828	111597.62	107357.48	94075.14	75182.54	73039.65	65719.15	86770.74	80857.92	73827.35	102132.06	92196.90	89100.26	86575.26	82540.22	63841.15	81894.37	77755.08	73821.51	77596.32	72926.12	70983.61	5040.68	4079.88	4337.37	3095.28	2685.19	2465.75	4636.62	4169.62	4154.45	3973.20	3684.05	2371.69		
4044	Secretory carrier-associated membrane protein 4	Q969E2	22825.47	21158.80	20820.91	17425.92	15982.25	15365.87	16317.10	16028.87	15095.99	21301.18	20050.23	18742.33	18936.38	19224.05	15588.77	16064.16	15377.38	14591.26	14921.02	15757.17	13631.23	21740.99	21678.12	19629.49	15491.15	15329.84	13049.47	18970.18	17061.19	17854.75	18311.14	17771.97	13264.73		
4045	Sedoheptulokinase	Q9UHJ6	0.14	2.53	0.81	1.07	2.77	0.68	0.96	0.36	0.63	0.35	0.75	0.38	2.13	0.97	0.60	4.64	1.07	0.47	2.22	0.19	0.06	37750.40	29180.92	29795.03	18881.18	19384.29	15875.84	23478.28	22152.36	21279.21	28084.45	25078.39	13845.14		x
4046	Segment polarity protein dishevelled homolog DVL-2	O14641	60292.99	57408.95	59331.39	56919.32	55056.49	50173.72	63399.56	59099.49	58032.47	65747.15	70074.11	70713.72	69438.99	60922.91	59030.18	62801.59	61076.96	53664.78	67377.10	61615.49	62464.19	110818.80	81066.88	72491.54	90649.40	88115.33	74105.08	100954.60	94290.52	87833.70	110642.05	105652.35	33361.99		
4047	Segment polarity protein dishevelled homolog DVL-3	Q92997	0.14	2.53	0.81	1.07	2.77	0.68	0.96	0.36	0.63	0.35	0.75	0.38	2.13	0.97	0.60	4.64	1.07	0.47	2.22	0.19	0.06	18852.53	16604.70	16135.65	14428.69	14536.92	10725.20	17547.95	16238.13	14261.48	18622.58	18770.07	11883.46		x
4048	Selenide_ water dikinase 1	P49903	26467.67	23787.20	20850.39	15072.26	14606.03	13603.35	17551.90	16873.36	16631.61	19273.03	18674.06	16286.89	19744.98	17452.14	14855.62	13831.65	13863.03	13576.24	16387.22	15897.87	15789.47	3176.97	3046.68	2653.13	1871.81	2002.78	1720.05	2771.79	2381.26	2369.51	2549.35	2456.16	2013.30		
4049	Separin	Q14674	929.03	1095.96	605.93	585.70	498.39	598.56	493.60	511.76	391.21	680.00	738.99	640.77	610.80	505.20	356.98	718.36	756.81	796.91	445.96	465.28	442.81	2705.09	2649.82	2382.75	1687.90	1731.20	1093.94	2543.26	2256.81	2015.61	2949.25	2814.12	1115.38		
4050	Sepiapterin reductase	P35270	42735.05	41214.61	36461.23	31275.27	30191.97	29244.70	32763.82	33073.85	30042.59	32447.23	32261.00	31601.47	36842.89	35863.25	32712.68	26905.75	27876.37	26901.25	33331.64	29427.91	29755.52	7067.19	6476.05	5911.59	3000.28	3058.26	2411.89	4589.54	4325.45	3738.86	5123.93	4737.17	3005.47		
4051	Septin-10	Q9P0V9	79806.87	75107.96	66326.45	14247.26	13548.12	10724.65	24169.29	23083.26	20891.04	71486.57	70975.14	55415.62	55970.98	21722.38	9538.26	26922.62	26633.06	22188.72	16664.96	18001.78	14078.37	53232.65	44911.56	44464.69	30792.57	29294.07	24680.41	37687.38	35584.28	35081.29	36605.21	36314.40	23866.86		
4052	Septin-11	Q9NVA2	63589.56	59385.71	53923.77	43456.03	39716.93	36069.55	46408.18	43689.07	41392.83	51174.22	51050.09	48911.32	56208.84	49232.27	43336.21	39932.58	40474.36	36639.50	41621.63	41511.67	37677.83	114619.34	114870.25	95347.10	71572.62	69913.68	66484.72	84172.57	82008.75	76537.22	93149.76	90411.27	55256.90		
4053	Septin-14	Q6ZU15	12683.57	10550.71	7377.44	6027.13	4993.66	3068.73	6489.29	5253.53	3850.04	8862.56	7298.22	4915.78	8366.15	5273.98	1081.57	6091.35	4998.98	3734.76	4969.43	4421.01	3016.30	3466.51	3388.05	2599.53	1795.11	1774.81	1476.94	2587.77	2372.95	2120.31	1907.66	2144.31	1432.39		
4054	Septin-2	Q15019	604487.67	593206.28	547576.58	386730.38	378589.00	336726.73	471949.34	446326.58	426120.87	514829.20	481644.18	451443.25	484038.23	446095.19	351959.39	413793.75	394604.11	363953.99	395512.27	382597.88	351948.13	277291.96	254181.47	243683.58	167295.53	157872.37	132274.94	218174.32	206993.72	194805.23	210911.10	196455.52	124293.27		
4055	Septin-4	O43236	29173.12	27811.08	28097.37	20917.61	20250.03	19039.16	20757.43	19538.79	19755.55	29321.00	27133.77	27382.45	25466.69	22969.28	20308.96	20434.04	20352.44	20067.33	19245.22	18371.47	18180.52	9903.97	9593.00	9012.94	5468.77	5215.38	4455.40	9268.06	10061.07	9595.25	7759.36	7419.87	4614.55		
4056	Septin-6	Q14141	33748.57	33446.26	32165.47	24590.38	23126.14	21615.32	26123.24	25056.20	25190.98	29049.16	28652.77	27597.23	29379.25	31198.59	26926.95	24387.98	24695.34	23954.40	23687.65	21209.59	21413.16	9662.20	9318.44	9121.72	7768.89	6938.13	6361.65	8778.22	8411.98	7859.24	8701.10	8618.77	5695.93		
4057	Septin-7	Q16181	247344.66	221146.80	203571.35	164448.53	152797.29	140477.30	183379.72	180610.49	166388.47	209903.01	198253.53	184493.33	205759.06	187526.72	120187.60	157103.68	146388.69	140981.24	163839.80	153485.43	140405.79	240562.81	230756.17	214033.38	158631.29	152945.18	138683.64	183317.81	174852.51	169870.94	180096.72	176220.13	98783.32		
4058	Septin-8	Q92599	80655.09	78176.85	68971.74	45155.10	45819.09	43076.97	51809.59	50143.38	55849.60	61253.89	54994.63	55461.18	62521.70	56357.81	49769.74	54269.59	48393.31	48497.05	49889.11	46495.86	44964.87	4012.83	3628.35	3431.26	2577.80	2344.22	1743.50	2971.43	2856.46	2600.71	3195.70	3138.85	1954.65		
4059	Septin-9	Q9UHD8	488453.16	476648.64	450696.41	331085.40	325992.17	310618.02	376648.67	365583.18	357830.97	440929.99	422253.86	401077.09	397830.07	377990.39	316228.29	340622.89	325869.25	323467.24	343478.34	322896.98	303883.80	344236.89	319300.98	284531.85	181450.09	180098.58	142620.88	226395.29	216670.20	195159.50	262303.06	245077.20	154689.40		
4060	Sequestosome-1	Q13501	44240.41	45053.88	41867.85	33006.00	30469.44	28700.42	30040.18	27260.38	28487.18	35178.57	32975.43	33092.91	28248.65	24153.54	21022.40	35552.13	31258.14	31949.76	28109.68	28043.85	26545.57	52028.44	47303.76	41836.93	44442.90	43805.80	37762.07	45849.51	44007.95	41194.46	57240.39	56345.94	48470.62		
4061	Serine dehydratase-like	Q96GA7	79528.11	76631.59	70271.53	59262.29	57309.36	54207.21	63418.91	60243.54	57305.15	78341.24	75123.18	71620.04	61955.98	58415.78	48149.20	62265.34	60260.46	58121.54	58958.64	58519.09	51027.03	2.27	1.49	0.65	0.84	1.31	0.69	4.16	2.47	0.11	4.90	4.98	0.12	x	
4062	Serine hydroxymethyltransferase cytosolic	P34896	40245.87	37909.34	33336.66	23130.15	22492.05	19125.58	28963.59	28593.58	26753.83	27015.80	25847.20	23016.09	27566.80	27322.42	18123.30	24670.82	23533.32	22232.53	25100.20	22980.54	21027.62	66656.88	67362.48	60145.47	42092.08	39494.16	33498.82	50732.48	47514.96	43710.51	51418.52	50912.68	30166.60		
4063	Serine hydroxymethyltransferase mitochondrial	P34897	528818.03	515817.19	479964.02	357467.39	350981.31	326083.32	450929.07	432107.74	413228.23	390811.63	373532.00	352434.35	418744.93	382749.93	324017.80	364093.15	348876.46	338552.38	356621.19	342760.28	323188.85	257459.23	234949.22	205692.24	137757.37	134244.32	91759.47	172441.18	155054.88	136851.14	193571.63	177683.79	107122.15		
4064	Serine palmitoyltransferase 1	O15269	40306.46	38357.82	37275.94	27351.61	26660.94	25935.35	27517.60	27211.84	26317.24	31745.93	29307.89	28210.89	30283.39	30098.29	26931.21	24434.79	23553.21	24172.12	26021.58	24509.78	23864.70	7547.66	6049.27	6784.30	5843.95	5992.58	5567.57	6062.78	5454.26	5562.42	6065.30	5721.54	2990.15		
4065	Serine palmitoyltransferase 2	O15270	112436.40	110247.10	97958.60	92999.37	88989.29	82062.02	91934.42	85164.24	81826.62	115865.25	109693.26	103564.12	105934.21	95118.08	75252.05	97382.39	90195.15	88722.95	78719.77	78275.08	73490.85	6939.13	6365.75	6024.74	3173.62	3148.24	2521.47	3461.93	3507.30	3344.64	3950.27	3487.34	2141.41		
4066	Serine protease 33	Q8NF86	0.14	2.53	0.81	1.07	2.77	0.68	0.96	0.36	0.63	0.35	0.75	0.38	2.13	0.97	0.60	4.64	1.07	0.47	2.22	0.19	0.06	19.73	53.58	35.83	52.07	57.02	65.58	64.76	72.47	110.06	45.80	64.52	26.90		x
4067	Serine protease 38	A1L453	1244.74	919.39	839.23	646.31	657.56	619.93	628.63	662.92	586.80	936.00	968.61	903.80	679.06	676.38	281.73	689.36	679.38	711.06	856.99	903.14	795.65	2.27	1.49	0.65	0.84	1.31	0.69	4.16	2.47	0.11	4.90	4.98	0.12	x	
4068	Serine protease HTRA1	Q92743	3273.30	4121.17	4151.52	2427.79	2396.97	2717.06	2758.08	2804.68	2459.99	3233.02	3148.83	2633.75	3562.95	2755.46	3266.60	2703.48	2263.65	2186.73	2521.10	2560.02	2473.26	1035.29	909.44	1115.00	795.41	903.62	802.95	835.56	815.74	795.72	984.13	904.04	774.93		
4069	Serine protease inhibitor Kazal-type 5	Q9NQ38	520.92	411.28	384.20	402.44	365.35	280.34	327.76	402.71	395.43	487.37	443.77	395.63	445.37	456.21	361.03	358.52	363.49	271.73	332.22	371.72	391.36	5574.87	5132.34	4603.27	2971.74	2916.19	2533.97	3082.25	2668.10	2730.63	3188.53	2955.97	1775.31		
4070	Serine protease inhibitor Kazal-type 8	P0C7L1	46.72	11.93	26.68	71.53	1905.79	956.04	637.22	329.77	256.37	5.05	27.26	23.06	2.13	0.97	0.60	3603.85	854.83	2030.50	2.22	0.19	0.06	2.27	1.49	0.65	0.84	1.31	0.69	4.16	2.47	0.11	4.90	4.98	0.12	x	
4071	Serine racemase	Q9GZT4	0.14	2.53	0.81	1.07	2.77	0.68	0.96	0.36	0.63	0.35	0.75	0.38	2.13	0.97	0.60	4.64	1.07	0.47	2.22	0.19	0.06	1012.81	1018.79	1087.55	1007.07	946.42	851.33	827.16	808.21	728.99	1028.91	1031.86	695.41		x
4072	Serine/arginine repetitive matrix protein 1	Q8IYB3	452.21	438.75	543.37	179.07	138.91	119.25	0.96	0.36	0.63	529.77	602.99	710.44	422.85	59.95	137.26	66.96	172.68	262.74	470.85	407.47	290.77	4703.97	5330.44	3218.34	2128.80	2791.02	2517.49	4975.32	4880.85	5837.89	4984.07	4968.45	2765.38		
4073	Serine/arginine repetitive matrix protein 2	Q9UQ35	172142.58	170655.44	158602.30	114199.67	112636.72	105697.95	130790.99	123869.65	120934.70	141341.97	135999.35	128438.89	139211.39	129139.19	115053.12	114963.08	105657.65	105252.77	132675.62	127160.64	122301.80	98570.00	89817.02	84707.37	71238.46	70760.78	66500.80	86409.48	80747.92	75785.27	85760.64	82955.95	51962.51		
4074	Serine/arginine-rich splicing factor 1	Q07955	192839.75	176697.86	176121.04	132944.69	120029.26	114283.37	141146.08	141097.11	129520.66	144871.19	140032.94	127846.35	146711.52	143189.76	108207.99	132084.75	122328.96	118961.82	154913.25	141307.97	133021.60	182761.27	172011.23	157763.19	128702.08	123438.30	106779.40	134644.05	125796.11	117836.34	140885.53	135003.95	93330.42		
4075	Serine/arginine-rich splicing factor 11	Q05519	0.14	2.53	0.81	1.07	2.77	0.68	0.96	0.36	0.63	0.35	0.75	0.38	2.13	0.97	0.60	4.64	1.07	0.47	2.22	0.19	0.06	1798.56	1742.96	1490.12	1044.82	1154.05	697.30	1544.52	1277.15	1228.10	1467.41	1566.96	706.94		x
4076	Serine/arginine-rich splicing factor 2	Q01130	133723.88	133367.25	125060.48	94151.18	89119.44	83153.04	111259.17	108301.39	103173.88	106560.08	107578.14	101079.11	114992.77	105641.86	80193.32	83179.46	80727.15	82847.25	108783.45	95473.42	86644.70	122304.32	114189.50	107095.26	89748.76	88614.42	80956.28	92875.30	93547.98	91093.56	99502.80	96233.34	73325.37		
4077	Serine/arginine-rich splicing factor 3	P84103	126299.20	121699.72	107964.64	79672.58	74670.58	67185.90	91027.68	86172.43	77739.46	93482.43	88976.85	82890.94	97136.91	93228.74	55905.24	82614.37	80723.75	75380.79	103384.89	101918.27	92251.50	76230.86	66098.89	64297.43	51870.98	50301.24	37111.41	51601.63	46540.71	42854.76	69775.20	68352.51	42869.87		
4078	Serine/arginine-rich splicing factor 4	Q08170	639.88	382.82	404.34	628.33	554.65	484.40	495.39	565.03	414.78	961.53	926.66	768.15	847.76	546.56	278.63	488.87	503.33	581.89	494.86	668.80	549.42	18698.02	17683.34	17311.70	9951.32	9687.47	7918.20	7662.93	7867.30	7694.80	7084.10	7140.41	4743.27		
4079	Serine/arginine-rich splicing factor 5	Q13243	12094.89	11064.53	9089.53	7864.85	8139.51	5640.69	8979.71	8457.18	7684.46	9338.25	9468.09	9287.08	10589.23	8632.01	6024.92	8112.41	6075.48	6638.03	9395.66	8164.11	8509.32	15719.87	14745.06	14497.19	9705.49	10684.82	8982.63	11975.62	11252.95	11206.59	13707.66	14465.49	9880.57		
4080	Serine/arginine-rich splicing factor 6	Q13247	277209.01	273388.10	258331.03	199481.15	196926.87	187324.37	226689.27	212238.09	203948.45	223339.53	207583.95	202180.95	227668.06	228336.87	186057.39	197491.59	187970.32	179519.55	199532.57	196342.35	191580.80	88304.47	83169.80	79088.10	60681.01	60334.95	57779.31	68253.51	64805.24	65633.40	68338.97	65890.42	52023.90		
4081	Serine/arginine-rich splicing factor 7	Q16629	107700.10	105396.02	91119.06	54663.82	53391.05	46677.52	64906.60	64494.26	59132.99	75444.69	75176.39	71476.86	74092.01	64530.87	46680.13	62224.79	55530.34	55106.86	81759.25	72027.70	68887.58	51174.09	48643.25	44646.80	45585.82	44732.24	37353.56	41900.32	42364.77	40627.05	58354.79	56835.50	33327.21		
4082	Serine/arginine-rich splicing factor 8	Q9BRL6	38515.48	42932.48	43366.71	38382.05	37736.85	35180.01	36514.31	39758.06	37235.49	40880.10	41789.98	39196.90	41425.76	66908.61	87106.25	31452.31	25586.35	28026.04	29333.83	34341.37	36556.38	1303.49	470.49	255.77	727.25	581.66	261.85	1211.31	901.31	203.47	1256.75	1160.31	504.74		
4083	Serine/arginine-rich splicing factor 9	Q13242	1405.94	1049.24	862.09	299.69	344.14	171.61	866.20	966.47	556.00	336.80	267.05	198.59	597.06	609.33	138.47	403.62	375.01	304.09	332.53	343.89	190.86	14087.56	12991.40	10349.32	8610.03	8124.99	7841.77	9053.33	8107.62	8134.47	10223.05	8064.00	4377.65		
4084	Serine/threonine-protein kinase 10	O94804	11119.63	11144.06	8438.29	5801.91	6037.62	5707.70	6301.83	6436.42	6283.80	7746.42	7898.51	7186.83	8081.63	7645.56	7278.70	6311.48	5938.30	5076.68	6253.21	6090.09	5637.70	2.27	1.49	0.65	0.84	1.31	0.69	4.16	2.47	0.11	4.90	4.98	0.12	x	
4085	Serine/threonine-protein kinase 17A	Q9UEE5	0.14	2.53	0.81	1.07	2.77	0.68	0.96	0.36	0.63	0.35	0.75	0.38	2.13	0.97	0.60	4.64	1.07	0.47	2.22	0.19	0.06	5510.82	5661.05	5155.36	3519.50	3345.97	3086.24	5909.08	5841.06	5144.95	4843.96	4515.60	3175.26		x
4086	Serine/threonine-protein kinase 19	P49842	1225.64	1072.39	859.27	663.35	765.43	765.91	804.51	772.71	933.55	1146.87	1133.36	1093.51	1281.77	1006.55	929.64	573.49	561.95	556.07	828.84	794.13	842.20	2.27	1.49	0.65	0.84	1.31	0.69	4.16	2.47	0.11	4.90	4.98	0.12	x	
4087	Serine/threonine-protein kinase 24	Q9Y6E0	24702.68	24836.78	20939.82	17600.89	16890.27	15001.79	23361.19	18286.84	17830.46	19236.42	18729.67	16765.75	20076.18	24977.15	16213.55	19445.46	17687.67	16741.98	15329.50	15833.09	14282.99	40493.99	36846.07	36611.71	30729.32	28598.12	25662.96	35357.09	34221.26	31969.75	32632.56	32607.45	22760.12		
4088	Serine/threonine-protein kinase 25	O00506	12124.73	9643.81	7037.38	5979.77	2008.51	1667.05	3343.54	2814.61	2832.54	8641.42	8053.97	5493.61	9349.50	9254.73	2218.24	2282.16	1998.88	1630.59	2573.21	1925.12	2004.58	7473.51	1213.26	1455.84	4458.55	3787.19	2176.11	8241.08	7498.72	1577.64	6985.58	6509.80	2854.47		
4089	Serine/threonine-protein kinase 26	Q9P289	9299.66	9965.74	9120.05	6403.91	6455.95	6417.06	7878.69	7946.62	7730.40	8019.79	7671.29	7915.83	7755.17	7053.19	6345.02	6657.28	6316.68	5996.16	7574.96	6897.85	6497.91	42850.90	38905.54	35237.67	23783.42	22249.76	20420.30	30756.46	29033.69	28228.39	30885.84	27862.29	12143.17		
4090	Serine/threonine-protein kinase 3	Q13188	0.14	2.53	0.81	1.07	2.77	0.68	0.96	0.36	0.63	0.35	0.75	0.38	2.13	0.97	0.60	4.64	1.07	0.47	2.22	0.19	0.06	278.37	2.98	5.70	116.84	115.26	49.25	162.80	114.92	0.22	144.42	134.69	54.43		x
4091	Serine/threonine-protein kinase 31	Q9BXU1	0.14	2.53	0.81	1.07	2.77	0.68	0.96	0.36	0.63	0.35	0.75	0.38	2.13	0.97	0.60	4.64	1.07	0.47	2.22	0.19	0.06	1831.68	2074.80	2115.36	8514.58	8376.58	7975.31	8801.00	8411.84	8154.05	6379.47	6168.90	4395.42		x
4092	Serine/threonine-protein kinase 32A	Q8WU08	0.14	2.53	0.81	1.07	2.77	0.68	0.96	0.36	0.63	0.35	0.75	0.38	2.13	0.97	0.60	4.64	1.07	0.47	2.22	0.19	0.06	833.60	1130.76	892.29	981.57	952.73	912.85	1435.37	1186.21	1177.97	1291.31	1105.91	1165.37		x
4093	Serine/threonine-protein kinase 38-like	Q9Y2H1	7603.17	6088.36	6469.35	4890.52	4675.62	4518.43	5054.70	5176.43	5374.32	6643.08	6653.46	6902.76	6367.62	5682.32	4816.96	5216.92	5623.44	5099.72	6201.77	4816.25	4586.21	2.27	1.49	0.65	0.84	1.31	0.69	4.16	2.47	0.11	4.90	4.98	0.12	x	
4094	Serine/threonine-protein kinase 4	Q13043	296.44	190.88	124.50	1.07	2.77	0.68	0.96	0.36	0.63	341.87	350.23	222.31	2.13	0.97	0.60	4.64	1.07	0.47	2.22	0.19	0.06	42226.08	38602.40	38361.81	27514.39	27190.89	25137.83	35625.82	33603.35	33358.67	37235.64	35012.79	0.12		
4095	Serine/threonine-protein kinase ATR	Q13535	312671.15	297575.04	249283.76	172171.22	169551.35	149338.41	214162.98	204228.54	188102.43	244950.74	231198.35	210331.20	223016.46	208857.16	144344.22	232167.55	219393.05	199409.03	203964.62	193681.45	173360.05	38744.40	37569.44	38555.71	30965.55	28259.01	26538.38	50571.81	47261.83	49331.03	43996.38	41921.66	29920.01		
4096	Serine/threonine-protein kinase Chk1	O14757	0.14	2.53	0.81	1.07	2.77	0.68	0.96	0.36	0.63	0.35	0.75	0.38	2.13	0.97	0.60	4.64	1.07	0.47	2.22	0.19	0.06	1188.95	1120.35	1231.76	875.16	800.60	594.31	1586.14	1284.84	1162.16	1008.33	929.31	415.95		x
4097	Serine/threonine-protein kinase greatwall	Q96GX5	2159.63	2218.85	2564.01	1567.91	1461.44	1361.69	2127.14	1877.84	1948.63	1443.18	1609.28	1438.89	1682.37	1596.37	1367.76	1553.22	1776.35	1519.57	1327.21	1416.38	1283.95	4842.61	4622.75	4795.50	3179.97	3185.91	2956.32	4391.01	4065.04	3994.95	3878.35	3567.35	2881.80		
4098	Serine/threonine-protein kinase ICK	Q9UPZ9	19921.56	20824.16	17073.86	13485.51	12341.34	11932.63	15630.93	14570.63	14067.71	21236.21	19849.68	18082.43	17795.02	16369.81	12352.15	12587.17	12065.28	12431.10	15346.24	15559.84	13862.24	2.27	1.49	0.65	0.84	1.31	0.69	4.16	2.47	0.11	4.90	4.98	0.12	x	
4099	Serine/threonine-protein kinase MAK	P20794	49278.43	36270.71	26369.84	22030.05	20855.42	16035.55	25881.80	27498.78	22213.23	29764.44	28446.37	20867.26	29542.81	25247.64	9129.42	22112.03	21485.55	18254.89	22473.46	20274.92	15646.80	17945.51	16615.66	15262.54	9574.80	9338.25	5835.00	14968.96	12973.49	11708.97	15185.15	14635.48	7481.75		
4100	Serine/threonine-protein kinase MARK1	Q9P0L2	111.86	46.10	53.26	1.07	2.77	0.68	0.96	0.36	0.63	105.15	72.62	52.05	2.13	0.97	0.60	4.64	1.07	0.47	2.22	0.19	0.06	2.27	1.49	0.65	0.84	1.31	0.69	4.16	2.47	0.11	4.90	4.98	0.12	x	
4101	Serine/threonine-protein kinase MARK2	Q7KZI7	78721.00	68940.49	59404.35	47695.84	44087.95	36067.00	46605.35	46144.98	40117.16	65062.28	61115.07	52546.15	68183.40	55882.93	31747.20	41217.54	40082.35	34713.26	46850.09	44087.56	36399.67	21502.90	19458.47	15589.58	13661.82	13524.80	9565.53	16162.67	15761.56	12841.47	19845.32	18345.35	9905.16		
4102	Serine/threonine-protein kinase MRCK beta	Q9Y5S2	6284.84	6339.15	5646.28	4923.49	4906.36	4356.43	4373.50	4134.02	3501.89	5949.65	5863.54	5086.71	5527.68	5054.97	4484.11	3788.90	3660.90	3950.29	4229.93	3684.49	3494.29	2.27	1.49	0.65	0.84	1.31	0.69	4.16	2.47	0.11	4.90	4.98	0.12	x	
4103	Serine/threonine-protein kinase N2	Q16513	0.14	2.53	0.81	1.07	2.77	0.68	0.96	0.36	0.63	0.35	0.75	0.38	2.13	0.97	0.60	4.64	1.07	0.47	2.22	0.19	0.06	33540.68	19213.42	16335.64	20000.55	17789.34	12158.48	33487.20	28896.70	21475.02	33664.18	30862.36	17437.64		x
4104	Serine/threonine-protein kinase Nek11	Q8NG66	7688.35	7792.36	7474.62	6915.68	6712.48	6337.74	7550.08	7175.51	6684.64	7602.71	7200.28	6744.07	6873.11	6717.70	6828.51	5355.57	5637.62	5177.71	6544.78	6103.47	6245.74	2.27	1.49	0.65	0.84	1.31	0.69	4.16	2.47	0.11	4.90	4.98	0.12	x	
4105	Serine/threonine-protein kinase Nek8	Q86SG6	1631.87	1602.32	1636.25	1158.73	1118.95	1304.46	1071.11	1136.47	1277.53	1739.93	1678.33	1651.72	1780.43	1586.98	1415.14	1014.46	1041.12	872.28	1144.28	931.03	965.63	2.27	1.49	0.65	0.84	1.31	0.69	4.16	2.47	0.11	4.90	4.98	0.12	x	
4106	Serine/threonine-protein kinase	O95747	45891.82	41444.39	37638.74	28324.31	26043.98	25707.18	34734.99	33130.70	30009.64	37315.56	37003.89	34501.36	36073.52	31186.76	25490.31	27564.45	25993.59	25884.94	27677.39	27910.50	23867.59	27021.29	23749.01	23050.61	17716.63	16905.93	15256.22	21455.74	19681.95	19242.68	20624.06	19014.92	12832.75		
4107	Serine/threonine-protein kinase PAK 1	Q13153	5727.60	5182.47	5059.46	3746.54	3974.26	3069.76	4315.64	4074.50	3823.89	4667.08	4067.41	3671.77	4478.93	4581.62	2989.45	4165.61	3769.78	3361.30	5372.04	4827.11	4287.02	2311.75	2193.54	2021.14	2251.12	1998.23	1745.94	1817.82	1943.90	1798.17	3212.50	2982.15	1857.02		
4108	Serine/threonine-protein kinase PAK 2	Q13177	245512.39	239687.50	222603.08	142092.23	141213.31	132647.59	184283.39	176079.16	168682.62	179470.45	171495.14	166213.73	192565.82	175537.82	155702.14	153102.97	141648.03	134545.43	147133.59	143047.46	131656.36	118936.56	107536.31	99973.18	66421.52	65712.53	56921.34	81961.90	79291.97	75931.60	78609.89	76239.90	51862.49		
4109	Serine/threonine-protein kinase PAK 3	O75914	12345.37	11868.59	11211.51	8863.11	8296.44	8591.86	10277.61	10238.01	9800.28	9685.98	9418.98	8922.62	10016.65	10192.51	8855.49	7212.75	7127.88	7364.13	7595.94	7411.57	6677.41	12949.98	11452.63	9430.56	4896.07	5084.24	4353.65	7551.64	7771.29	6980.13	6496.75	6241.51	4523.71		
4110	Serine/threonine-protein kinase PAK 4	O96013	7825.84	7663.05	7386.23	3959.49	3737.44	3523.68	5041.74	4784.29	4817.49	5606.23	5641.65	5518.15	5644.79	5265.60	4763.49	4448.83	4188.90	4010.85	5330.79	4427.84	4155.28	2.27	1.49	0.65	0.84	1.31	0.69	4.16	2.47	0.11	4.90	4.98	0.12	x	
4111	Serine/threonine-protein kinase PAK 6	Q9NQU5	9250.73	8643.98	7931.70	6588.77	7111.80	6446.34	8682.66	7985.00	7583.70	8593.01	8328.27	7878.84	8515.83	8066.01	6616.25	6625.91	6326.27	6078.01	6338.39	6965.20	6514.39	6159.76	6015.54	5641.13	4497.79	4317.92	3745.00	4064.90	3965.06	3974.83	4039.51	3705.66	2644.61		
4112	Serine/threonine-protein kinase PLK1	P53350	6041.92	5087.11	5200.74	4535.59	4322.26	3947.10	4915.78	4486.58	4474.62	6310.59	5759.12	5936.21	5581.37	4939.11	4370.03	4433.00	3905.30	3915.86	4289.08	4194.64	4174.34	2.27	1.49	0.65	0.84	1.31	0.69	4.16	2.47	0.11	4.90	4.98	0.12	x	
4113	Serine/threonine-protein kinase PLK2	Q9NYY3	4616.59	4805.86	4660.72	2864.27	2911.50	3005.36	3720.74	3825.65	4031.72	3680.55	3477.30	3554.02	3745.28	3320.01	2736.12	3028.40	3025.72	2843.13	3083.80	2830.58	2767.56	39537.40	37745.72	34924.74	22819.17	23174.51	19067.74	33160.84	30856.01	29511.85	30084.48	29108.36	19930.66		
4114	Serine/threonine-protein kinase PLK3	Q9H4B4	66254.69	69662.87	58512.15	42709.40	41972.98	35576.84	41929.58	40152.43	35493.21	59807.29	54766.23	48760.89	56681.96	51188.00	38313.31	42124.27	41426.50	38019.30	44628.90	44182.58	39385.97	1446.92	1585.68	1112.67	802.86	842.14	710.68	1162.97	1098.65	967.84	1136.84	1033.61	605.54		

Supplementary Table S1. Overview on the abundance of all relatively quantified 5180 proteins

			MCF-7																					MDA-MB-231													
	Protein name	UniProt	control 01	control 02	control 03	Dai IC20 01	Dai IC20 02	Dai IC20 03	Dai SC20 01	Dai SC20 02	Dai SC20 03	Gen IC20 01	Gen IC20 02	Gen IC20 03	Gen SC20 01	Gen SC20 02	Gen SC20 03	SSE IC20 01	SSE IC20 02	SSE IC20 03	SSE SC20 01	SSE SC20 02	SSE SC20 03	control 01	control 02	control 03	Dai IC20 01	Dai IC20 02	Dai IC20 03	Gen IC20 01	Gen IC20 02	Gen IC20 03	SSE IC20 01	SSE IC20 02	SSE IC20 03	unique to MCF-7	unique to MDA-MB-231
4115	Serine/threonine-protein kinase PRP4 homolog	Q13523	3309.60	2635.18	2742.33	1176.62	1474.43	1192.38	3146.11	2591.21	2326.13	2391.42	2246.52	2407.79	2061.20	1667.03	1227.82	1684.75	1239.10	1470.64	1476.47	1525.56	1412.71	78.81	44.85	38.15	212.72	155.28	71.17	159.35	181.13	136.25	317.87	278.15	95.52		
4116	Serine/threonine-protein kinase RIO1	Q9BRS2	3150.49	2365.70	1620.27	1690.35	1139.99	1070.41	1421.33	754.95	1116.17	2640.04	2508.91	1761.19	2073.70	585.80	358.68	2136.88	1361.83	1349.31	1814.46	1643.95	911.77	2.27	1.49	0.65	0.84	1.31	0.69	4.16	2.47	0.11	4.90	4.98	0.12	x	
4117	Serine/threonine-protein kinase RIO2	Q9BVS4	1589.20	1579.48	1843.75	1016.45	1008.75	914.95	1151.09	983.06	1163.40	1304.17	1303.32	1456.56	1429.85	1488.43	1010.99	1000.49	788.91	904.86	902.08	907.89	1047.90	2.27	1.49	0.65	0.84	1.31	0.69	4.16	2.47	0.11	4.90	4.98	0.12	x	
4118	Serine/threonine-protein kinase SIK3	Q9Y2K2	0.14	2.53	0.81	1.07	2.77	0.68	0.96	0.36	0.63	0.35	0.75	0.38	2.13	0.97	0.60	4.64	1.07	0.47	2.22	0.19	0.06	21670.38	31775.63	21788.39	28390.66	28443.08	26755.65	14633.59	19866.16	20178.29	33274.21	27942.15	4378.96		x
4119	Serine/threonine-protein kinase SMG1	Q96Q15	27598.18	25537.19	23710.78	20144.77	19670.66	18649.65	27634.70	28040.86	25009.59	24664.72	25734.93	23503.61	26054.92	23433.02	17573.43	20577.09	19981.98	19237.12	20809.86	20153.21	18312.40	10277.83	9558.42	8848.52	8105.39	8054.32	7073.79	8419.67	8480.23	8103.26	8081.96	7665.02	4916.09		
4120	Serine/threonine-protein kinase TAO1	Q7L7X3	3337.81	3338.03	3202.45	2917.94	2583.27	2472.02	2616.19	3423.42	2212.51	3342.70	2953.03	3374.76	3325.69	5705.19	3594.91	2198.69	1535.35	1599.10	1831.38	2486.38	2486.91	2.27	1.49	0.65	0.84	1.31	0.69	4.16	2.47	0.11	4.90	4.98	0.12	x	
4121	Serine/threonine-protein kinase TBK1	Q9UHD2	0.14	2.53	0.81	1.07	2.77	0.68	0.96	0.36	0.63	0.35	0.75	0.38	2.13	0.97	0.60	4.64	1.07	0.47	2.22	0.19	0.06	12627.63	11407.42	11056.27	9323.85	9122.96	8957.51	10549.86	10515.84	9865.30	10468.09	9965.93	7958.48		x
4122	Serine/threonine-protein kinase tousled-like 1	Q9UKI8	130.78	97.79	114.75	161.44	171.74	126.98	88.08	96.46	93.65	129.25	132.52	107.25	112.20	75.85	163.28	121.45	53.08	96.91	107.75	92.04	79.49	6485.02	5866.91	6259.41	4225.75	4289.10	3624.78	6934.49	6282.05	6511.52	6521.18	5890.21	4599.15		
4123	Serine/threonine-protein kinase tousled-like 2	Q86UE8	7016.27	6062.39	4727.16	3735.24	3367.19	2818.33	3860.93	3497.12	3177.46	4778.82	4473.80	3771.81	4614.64	4149.56	1782.90	4092.97	3966.83	3613.26	3869.90	3781.62	3077.42	850.40	789.13	767.84	338.23	287.60	262.98	451.83	441.70	375.72	313.59	309.48	153.76		
4124	Serine/threonine-protein kinase ULK3	Q6PHR2	3205.76	3417.09	4197.19	6680.66	8072.19	6478.67	6831.80	9084.99	8704.03	3165.33	3376.80	3776.19	2898.13	8498.54	3675.64	7992.73	9394.49	8211.49	12152.60	12385.67	10732.73	2.27	1.49	0.65	0.84	1.31	0.69	4.16	2.47	0.11	4.90	4.98	0.12	x	
4125	Serine/threonine-protein kinase WNK1	Q9H4A3	40911.70	42198.62	37954.35	27966.79	27293.06	26018.71	35192.24	34933.60	35098.38	35771.29	34348.55	32743.55	39416.72	35051.96	28204.44	32002.31	30385.57	30751.50	40778.31	33354.21	30329.87	121334.43	111581.02	100605.16	52355.33	49454.60	43207.23	65463.30	63156.78	61956.41	62904.66	60350.87	39000.49		
4126	Serine/threonine-protein kinase WNK2	Q9Y3S1	127802.18	103607.73	68623.51	87709.27	77272.45	58061.12	79755.34	73048.30	62407.07	120277.64	110686.71	79496.35	97327.95	77603.60	24638.50	79373.42	74314.16	62309.07	84409.71	72435.96	54481.99	3112.31	2998.79	2941.63	1919.09	1808.68	1986.45	2312.82	2238.11	2113.83	2868.27	2968.55	2564.34		
4127	Serine/threonine-protein kinase WNK3	Q9BYP7	25279.67	24347.84	24362.19	15239.65	14483.92	14194.30	20557.26	20055.86	19322.99	18892.83	17866.33	18027.69	17841.20	17323.07	14474.74	20696.93	18826.71	18515.93	16171.87	14984.30	14318.46	13293.32	13237.61	14224.24	9888.90	9138.10	9346.99	12194.97	12002.67	11533.07	11963.32	10706.46	7187.32		
4128	Serine/threonine-protein kinase/endoribonuclease IRE2	Q76MJ5	6736.81	6455.43	5671.44	3569.72	4074.48	3160.55	4790.13	4676.95	4275.66	4464.13	4480.59	3964.08	4925.41	4293.50	3095.18	3773.71	3975.37	3958.89	4336.25	4193.30	3417.80	2.27	1.49	0.65	0.84	1.31	0.69	4.16	2.47	0.11	4.90	4.98	0.12	x	
4129	Serine/threonine-protein phosphatase 1 regulatory subunit 10	Q96QC0	490277.03	481581.10	425120.79	252552.74	256684.20	239069.17	315479.30	308589.59	293175.30	339193.20	332089.20	313579.42	262115.59	262896.04	245387.48	332225.39	329789.67	308900.48	262406.54	265360.72	259180.81	235125.60	212278.78	194209.99	117780.09	114571.62	96594.23	186268.22	194564.56	189127.96	157061.74	161020.97	104625.25		
4130	Serine/threonine-protein phosphatase 2A 55 kDa regulatory subunit B alpha isoform	P63151	29837.27	28793.03	24299.91	20900.37	20513.29	18364.81	24702.00	23848.61	21272.24	29289.02	28793.18	27583.82	22602.32	23886.97	17019.95	22271.56	21119.33	19407.76	19763.93	20727.96	19261.30	32022.15	28164.47	29397.16	17407.60	17286.67	15176.46	22283.68	21493.89	20564.99	22744.93	21431.77	15984.63		
4131	Serine/threonine-protein phosphatase 2A 55 kDa regulatory subunit B beta isoform	Q00005	97707.76	103344.11	89577.47	74988.70	71701.92	68475.65	80586.49	71850.33	71511.13	91027.63	83642.87	77162.75	78321.67	83576.20	62152.63	69841.52	66295.45	65280.45	69621.02	67038.92	65210.21	2377.80	2320.40	1807.23	2101.42	1770.76	1778.17	2321.08	1988.70	2045.84	1820.68	1771.31	1043.79		
4132	Serine/threonine-protein phosphatase 2A 55 kDa regulatory subunit B delta isoform	Q66LE6	68.24	946.09	757.24	1.07	2.77	0.68	0.96	0.36	0.63	87.14	602.78	693.38	2.13	0.97	0.60	4.64	1.07	0.47	2.22	0.19	0.06	2433.24	2490.25	2443.74	1697.35	1591.44	1206.65	1858.00	1627.21	1462.32	2359.30	2223.21	0.12		
4133	Serine/threonine-protein phosphatase 2A 56 kDa regulatory subunit delta isoform	Q14738	49372.38	48207.91	47425.48	31548.21	31501.53	29707.14	35094.52	31711.23	33644.64	35838.99	34508.92	33353.14	37910.02	37863.70	28836.93	31897.49	28629.78	28025.47	30567.67	29540.92	28009.01	7885.95	7422.21	6845.63	3438.24	3503.83	2956.84	5313.70	5022.09	4772.87	4903.24	4817.30	2665.53		
4134	Serine/threonine-protein phosphatase 2A 56 kDa regulatory subunit epsilon isoform	Q16537	45347.14	38209.85	30934.94	28127.45	26274.27	19484.80	47267.95	37645.81	36854.05	36746.92	32910.54	28757.38	38326.56	29219.56	13854.36	26287.95	23990.83	22036.63	29411.09	27312.78	23164.37	62462.13	55859.17	45304.78	21177.10	20748.82	13545.80	23194.11	20652.82	16821.29	24441.66	23377.28	11877.61		
4135	Serine/threonine-protein phosphatase 2A 56 kDa regulatory subunit gamma isoform	Q13362	12101.12	12087.30	13429.85	6791.40	6389.79	6880.12	9577.41	10233.54	9710.46	9488.51	8734.83	7597.87	8557.27	9289.57	6707.69	8235.81	7682.53	7320.90	7001.16	6785.47	6218.63	2.27	1.49	0.65	0.84	1.31	0.69	4.16	2.47	0.11	4.90	4.98	0.12	x	
4136	Serine/threonine-protein phosphatase 2A 65 kDa regulatory subunit A alpha isoform	P30153	444712.07	424848.61	393362.14	279616.98	268282.11	249148.22	349668.79	318367.98	317795.20	342355.58	329568.95	307333.09	337730.90	311632.91	250865.86	298208.87	277046.26	274674.42	288863.38	269398.33	250380.81	349541.81	322232.09	302927.64	207011.20	203590.81	180064.91	259925.19	250404.49	238928.36	258342.48	256087.18	174333.91		
4137	Serine/threonine-protein phosphatase 2A 65 kDa regulatory subunit A beta isoform	P30154	28285.17	27359.40	25490.90	19155.18	18897.79	17170.35	21952.57	21076.53	21021.40	23121.68	23227.78	21587.82	23585.25	21100.74	18840.99	19755.59	18265.00	17599.30	18971.61	17831.40	15550.38	2034.00	1733.43	1340.32	1007.68	1067.11	779.90	1853.56	1671.27	1352.26	1930.73	1692.68	847.17		
4138	Serine/threonine-protein phosphatase 2A activator	Q15257	236544.43	223635.55	208496.39	145363.83	136055.12	126528.78	169625.29	166884.19	158675.70	179075.16	170328.77	155521.75	182574.59	168588.00	148595.67	157782.69	143492.27	144568.87	156995.39	136821.18	132187.90	70227.39	69337.95	61042.08	46411.30	45329.36	36339.72	58562.51	56312.82	52761.65	60123.95	59197.78	36196.61		
4139	Serine/threonine-protein phosphatase 2A catalytic subunit alpha isoform	P67775	217469.42	217157.35	194676.49	153766.56	143891.14	131258.29	182257.70	173639.69	158295.87	190440.22	184206.35	167849.81	185706.95	175046.68	127844.94	162226.81	151872.86	143119.90	147838.43	142856.98	127442.64	10853.33	10118.48	8945.93	6990.59	6618.83	4340.25	7374.70	6582.02	6076.93	9172.95	8599.50	4414.69		
4140	Serine/threonine-protein phosphatase 2A catalytic subunit beta isoform	P62714	2969.47	2830.64	2704.89	1812.42	1685.22	1668.02	2240.08	2304.89	2229.50	2097.42	1929.96	1891.04	2269.65	2148.34	1851.92	1753.98	1657.00	1646.05	2015.84	1707.55	1758.36	166042.20	154730.79	144315.92	118933.35	112128.77	94658.20	133792.04	125332.76	117888.20	158700.70	139635.71	91514.55		
4141	Serine/threonine-protein phosphatase 2A regulatory subunit B'' subunit gamma	Q969Q6	0.14	2.53	0.81	1.07	2.77	0.68	0.96	0.36	0.63	0.35	0.75	0.38	2.13	0.97	0.60	4.64	1.07	0.47	2.22	0.19	0.06	14030.50	11826.11	10563.94	9058.65	7929.82	6184.26	11595.72	10710.31	8937.78	11402.18	11497.01	6373.42		x
4142	Serine/threonine-protein phosphatase 2B catalytic subunit alpha isoform	Q08209	114600.39	106321.38	101197.01	81424.26	79573.97	77652.92	86897.79	84683.85	82525.00	97384.09	93064.31	89546.74	91471.73	92538.37	82103.51	79619.76	70128.73	73091.79	79594.78	72417.86	68834.51	2.27	1.49	0.65	0.84	1.31	0.69	4.16	2.47	0.11	4.90	4.98	0.12	x	
4143	Serine/threonine-protein phosphatase 2B catalytic subunit beta isoform	P16298	7112.42	6297.16	6223.43	6084.27	4931.76	5250.53	6288.05	6578.88	7085.99	10117.91	9335.08	9430.26	8668.84	7483.36	5970.00	5104.84	4431.86	5025.86	5416.05	4719.47	4815.47	14923.48	13024.72	13633.68	10978.63	10842.09	9689.39	12049.50	11118.19	10746.15	11004.20	10437.27	7649.77		
4144	Serine/threonine-protein phosphatase 2B catalytic subunit gamma isoform	P48454	561.64	597.24	700.02	643.44	811.02	593.09	704.28	2782.98	817.89	692.25	764.25	733.16	735.48	3268.11	2738.18	340.62	328.68	426.73	770.83	613.94	614.21	2.27	1.49	0.65	0.84	1.31	0.69	4.16	2.47	0.11	4.90	4.98	0.12	x	
4145	Serine/threonine-protein phosphatase 4 catalytic subunit	P60510	42695.29	42405.06	38961.96	32584.73	30446.97	28321.72	32320.68	30803.21	29968.69	42724.05	39494.88	35545.49	36832.89	37585.97	29609.72	29139.09	28500.06	27320.79	32211.72	29976.68	28161.33	9396.23	8636.35	7346.56	6095.91	5644.51	4282.48	7644.09	6863.48	5918.51	8244.32	7974.13	3913.33		
4146	Serine/threonine-protein phosphatase 4 regulatory subunit 1	Q8TF05	0.14	2.53	0.81	1.07	2.77	0.68	0.96	0.36	0.63	0.35	0.75	0.38	2.13	0.97	0.60	4.64	1.07	0.47	2.22	0.19	0.06	8297.98	6071.61	5697.77	4545.53	4259.24	3956.15	4375.35	3351.41	3873.14	4582.26	4571.10	1673.03		x
4147	Serine/threonine-protein phosphatase 4 regulatory subunit 2	Q9NY27	86633.25	83629.52	74195.23	55521.58	57123.95	46757.87	66067.28	63273.96	52438.42	73831.81	72004.52	61989.18	65142.31	61581.70	48298.63	56280.97	54086.96	48619.79	51168.05	51121.67	45151.84	5198.01	5462.23	3707.08	3633.11	3290.60	2621.05	3768.89	4385.10	4200.33	4507.06	5042.77	3294.16		
4148	Serine/threonine-protein phosphatase 4 regulatory subunit 3A	Q6IN85	34872.21	35715.71	32464.99	27862.87	27547.57	26261.35	33676.39	30803.70	29594.66	28674.93	28039.00	26172.54	30916.33	27346.73	22430.53	26575.12	25263.85	22354.84	27693.11	24485.44	26368.21	2.27	1.49	0.65	0.84	1.31	0.69	4.16	2.47	0.11	4.90	4.98	0.12	x	
4149	Serine/threonine-protein phosphatase 4 regulatory subunit 3B	Q5MIZ7	38204.81	35956.87	35590.58	26022.67	26015.20	23149.78	27633.42	25943.86	25885.55	32097.05	30293.02	29022.29	34602.82	28080.19	25347.44	23418.59	23346.86	22715.20	27919.40	25151.03	23495.34	2.27	1.49	0.65	0.84	1.31	0.69	4.16	2.47	0.11	4.90	4.98	0.12	x	
4150	Serine/threonine-protein phosphatase 5	P53041	38107.00	33689.38	30293.40	24080.07	22735.33	19099.23	23906.09	22169.82	20172.71	31399.50	29556.62	25467.87	28524.86	22532.77	14023.16	21378.76	20947.00	18993.92	25021.69	23656.55	20306.83	36608.60	32957.09	31037.60	23698.95	21994.91	18838.48	30946.98	27615.80	30806.60	27651.90	25963.61	16265.39		
4151	Serine/threonine-protein phosphatase 6 catalytic subunit	O00743	44350.90	45201.51	39513.93	31064.88	30190.81	28026.75	38474.43	36048.16	35485.93	40034.14	38671.11	36827.25	37875.79	35559.25	29822.58	31295.59	29805.87	28015.72	33024.22	29811.15	30084.09	17389.69	17782.75	16133.39	10237.92	10230.55	9050.87	12094.52	11470.18	11298.63	11429.44	10941.18	7478.84		
4152	Serine/threonine-protein phosphatase 6 regulatory subunit 2	O75170	2206.64	2271.85	2108.54	1530.60	1494.64	1411.73	1361.28	1613.74	1647.37	2219.24	1928.63	1859.19	2094.94	1897.66	1551.19	1524.29	1197.44	1331.91	1767.80	1697.40	1455.39	2.27	1.49	0.65	0.84	1.31	0.69	4.16	2.47	0.11	4.90	4.98	0.12	x	
4153	Serine/threonine-protein phosphatase 6 regulatory subunit 3	Q5H9R7	94225.15	86650.21	79638.92	58526.11	55684.86	50609.13	73138.49	70547.69	63647.11	70791.79	69405.98	61411.16	76650.71	70805.08	55250.28	59865.62	57271.60	53917.90	59488.30	55895.68	51959.48	7383.62	7096.90	6359.31	5626.47	5219.87	4779.24	6424.67	6298.78	5938.78	6795.74	6348.46	4239.82		
4154	Serine/threonine-protein phosphatase PGAM5_ mitochondrial	Q96HS1	64955.12	64126.72	59318.12	46328.12	44247.29	43856.00	55271.88	54050.15	51200.62	57603.45	53262.58	51606.52	51424.89	49493.96	41867.95	50796.78	45137.49	45639.35	49908.14	51321.84	49804.52	2.27	1.49	0.65	0.84	1.31	0.69	4.16	2.47	0.11	4.90	4.98	0.12	x	
4155	Serine/threonine-protein phosphatase PP1-alpha catalytic subunit	P62136	186439.96	169915.95	176848.00	117853.68	115934.93	107576.55	138374.38	137591.76	127831.55	138058.04	130573.39	124342.54	141125.01	135798.57	117744.02	117988.53	107734.78	106340.17	108328.78	114720.10	102769.79	15565.16	13306.50	13252.29	9379.94	9326.44	9017.64	11848.78	11196.76	11086.30	12900.23	12689.16	10578.70		
4156	Serine/threonine-protein phosphatase PP1-beta catalytic subunit	P62140	14617.77	15333.82	12840.41	6311.69	7476.71	6837.85	10427.11	9737.96	8947.10	9554.98	9698.10	7978.45	9764.45	10602.71	8340.00	8245.86	8732.17	7872.46	7777.54	7503.50	6875.57	60.07	70.44	65.66	63.45	68.46	70.63	69.46	67.30	44.38	102.71	99.05	45.86		
4157	Serine/threonine-protein phosphatase PP1-gamma catalytic subunit	P36873	31766.84	30184.17	30353.30	21446.55	21137.74	20569.59	25893.05	25036.26	24462.52	29868.68	28286.00	27306.26	26528.88	26727.57	22564.00	20886.11	19654.77	19251.02	22008.13	20528.31	20588.49	176543.08	158177.82	148848.71	98755.88	97359.19	84124.76	116968.01	113478.53	107408.75	114155.54	108140.98	75806.45		
4158	Serine-protein kinase ATM	Q13315	0.14	2.53	0.81	1.07	2.77	0.68	0.96	0.36	0.63	0.35	0.75	0.38	2.13	0.97	0.60	4.64	1.07	0.47	2.22	0.19	0.06	4127.91	3546.71	2909.07	2102.20	2218.76	1129.83	3181.91	3116.90	2216.41	3916.62	3758.25	1676.72		x
4159	Serine-threonine kinase receptor-associated protein	Q9Y3F4	243676.38	242455.94	224806.97	169135.42	162942.32	150471.62	230193.52	216089.60	213055.19	216826.69	203700.31	194179.96	195112.09	186834.94	148071.40	178971.06	170437.38	164013.27	183020.61	171120.33	163089.66	300044.49	282439.89	269703.88	206683.65	198650.90	169199.79	234480.47	224799.11	215255.65	228288.87	221562.80	155155.11		
4160	Serine--tRNA ligase cytoplasmic	P49591	289154.76	280968.97	264335.14	249522.06	239763.00	222840.64	280483.71	265586.76	257556.89	269216.22	249309.58	234852.41	261897.82	239401.73	191874.69	258426.18	241485.16	238401.31	239437.33	226731.64	210451.28	175039.44	162463.96	149121.31	122172.34	116056.65	98415.02	136519.22	127761.54	122145.16	146303.18	138683.16	95338.97		
4161	Serine--tRNA ligase mitochondrial	Q9NP81	42881.45	41922.24	41149.94	25895.91	25741.00	23646.78	29699.06	29984.61	28585.79	32504.45	30556.46	28789.29	31074.43	31471.51	26994.95	28797.57	26556.74	26097.30	26919.92	25672.13	23487.59	2406.73	2306.45	2511.88	2172.35	2066.02	2043.64	2493.82	2235.55	2322.78	2976.66	2407.17	1130.29		
4162	Serologically defined colon cancer antigen 8	Q86SQ7	3508.21	3367.62	3250.29	2136.58	2521.00	2542.13	4609.02	3844.03	3478.57	4203.01	4016.01	3930.48	3741.63	3411.87	2576.02	2903.00	2726.00	2665.18	2813.06	2449.16	2156.91	5764.59	5551.07	5080.45	4408.89	4503.40	4126.13	3890.60	3770.59	3588.60	4402.30	4405.28	2749.50		
4163	Serotransferrin	P02787	11128.75	10289.55	9969.17	6319.69	6237.44	6071.09	7138.66	6645.66	6903.90	7158.08	7191.76	6923.15	8402.84	7455.92	6643.83	6466.79	6078.73	5814.62	7141.13	7156.20	6227.34	2.27	1.49	0.65	0.84	1.31	0.69	4.16	2.47	0.11	4.90	4.98	0.12	x	
4164	Serpin B4	P48594	0.14	2.53	0.81	1.07	2.77	0.68	0.96	0.36	0.63	0.35	0.75	0.38	2.13	0.97	0.60	4.64	1.07	0.47	2.22	0.19	0.06	2053.63	1988.19	1773.33	1549.13	1604.63	1442.69	1801.46	1572.02	1763.46	1952.91	1599.47	1387.50		x
4165	Serpin B6	P35237	276076.41	260273.75	241764.59	165014.98	160604.24	143713.60	198774.32	183736.10	177443.87	217733.94	205818.54	189347.73	208586.35	186548.01	145102.52	172727.18	162511.40	153714.17	177037.28	167208.16	148251.63	92502.97	84890.53	78331.13	53235.37	49708.70	40585.93	58403.69	53401.11	49642.79	64876.69	61913.54	40882.83		
4166	Serpin B7	O75635	0.14	2.53	0.81	1.07	2.77	0.68	0.96	0.36	0.63	0.35	0.75	0.38	2.13	0.97	0.60	4.64	1.07	0.47	2.22	0.19	0.06	46109.68	44634.02	38851.47	36275.24	35146.43	35937.62	36933.68	36343.35	33721.05	42230.16	40422.00	28205.26		x
4167	Serpin B8	P50452	23935.11	21445.99	20041.08	14912.93	14838.90	13340.34	17279.67	17145.04	16479.38	19218.00	18391.25	17743.33	19099.07	18530.26	14169.66	14463.84	13228.41	13134.04	14752.68	14064.57	13727.09	196.91	230.79	147.36	72.09	88.99	35.05	45.80	70.71	61.52	56.84	140.23	4.41		
4168	Serpin H1	P50454	865077.39	814754.00	750641.74	541472.38	515804.45	481410.91	634306.17	603215.15	572854.54	644605.65	615365.40	573835.56	672788.49	637694.21	508165.59	525708.44	501387.15	483849.60	552822.65	521497.27	488628.44	97391.26	92055.66	84721.45	55440.17	53284.64	43310.45	77275.66	70833.05	66491.83	72602.51	66340.11	47369.57		
4169	Serrate RNA effector molecule homolog	Q9BXP5	229382.47	219119.00	203035.03	177457.02	165458.98	169638.46	182325.25	176421.87	175741.79	224417.51	210125.73	193690.59	194079.35	185315.72	156365.92	179705.24	173241.57	170421.11	177547.31	179752.13	175379.85	69591.10	72779.58	61995.03	55634.87	53639.40	55294.28	55250.35	54503.46	53509.31	70690.80	62841.21	36551.09		
4170	Serum albumin	P02768	1363083.87	1287654.37	1260831.72	817325.74	793317.16	760925.08	1015936.14	1001657.84	1002728.16	1097899.34	1042517.61	985908.58	963334.04	905942.73	816613.37	1101206.54	1032938.08	1012380.29	937748.04	892018.81	886512.72	738796.28	700948.20	675083.91	551930.89	543808.81	487720.35	803765.35	766415.55	730903.89	526180.83	517146.15	367323.26		
4171	S-formylglutathione hydrolase	P10768	104740.03	97659.14	92021.93	73603.78	71166.40	66646.29	85867.53	82544.19	74489.89	100026.15	94246.02	84664.27	90494.72	84410.19	66151.10	70858.10	64350.70	67160.64	74774.97	77895.25	72050.95	338821.84	333760.33	306628.20	257340.26	261338.10	251447.83	274617.94	275020.25	269178.19	289816.61	277370.26	139805.35		
4172	SH2 domain-containing protein 3C	Q8N5H7	0.14	2.53	0.81	1.07	2.77	0.68	0.96	0.36	0.63	0.35	0.75	0.38	2.13	0.97	0.60	4.64	1.07	0.47	2.22	0.19	0.06	53862.41	50668.70	49112.98	40277.86	39714.06	35950.28	47962.17	44936.35	44667.79	43417.73	40544.35	30385.74		x
4173	SH2 domain-containing protein 4A	Q9H788	2868.43	2748.09	2802.03	1411.82	1350.86	1271.25	1205.98	1248.52	1226.92	1318.41	1360.78	1388.83	2375.94	2321.98	1854.84	1171.27	980.77	1160.32	1380.71	1113.51	1107.65	2.27	1.49	0.65	0.84	1.31	0.69	4.16	2.47	0.11	4.90	4.98	0.12	x	
4174	SH2 domain-containing protein 4B	Q5SQS7	525.62	464.30	584.59	239.15	186.62	216.66	485.09	433.37	365.51	244.32	232.08	167.89	335.86	243.49	164.22	199.39	181.72	226.65	318.28	186.84	270.57	2.27	1.49	0.65	0.84	1.31	0.69	4.16	2.47	0.11	4.90	4.98	0.12	x	
4175	SH3 and cysteine-rich domain-containing protein	Q99469	5409.41	5041.76	5400.72	3887.34	3587.28	3999.00	4529.31	4003.91	4422.63	5245.64	4563.24	4717.39	4358.81	4191.12	3512.08	4121.77	3443.68	3811.78	4167.60	3670.49	3600.41	2.27	1.49	0.65	0.84	1.31	0.69	4.16	2.47	0.11	4.90	4.98	0.12	x	
4176	SH3 and PX domain-containing protein 2B	A1X283	0.14	2.53	0.81	1.07	2.77	0.68	0.96	0.36	0.63	0.35	0.75	0.38	2.13	0.97	0.60	4.64	1.07	0.47	2.22	0.19	0.06	20926.76	20661.78	18500.84	5602.66	5826.73	5798.04	6223.38	6412.41	6439.17	8350.59	7683.38	967.28		x
4177	SH3 domain-binding glutamic acid-rich-like protein 3	Q9H299	108578.42	100844.84	92064.61	78901.52	68732.57	70866.51	81520.33	78977.74	75108.43	104809.81	104898.09	93976.28	102210.92	93700.62	75273.36	78210.46	70901.88	71240.40	84554.39	80364.36	75846.97	111475.08	95211.74	91685.25	70273.02	66728.15	54151.15	80587.25	77544.52	73131.58	94962.25	95177.63	57279.06		
4178	SH3 domain-binding glutamic acid-rich-like protein	O75368	162152.66	156388.83	147263.71	97186.31	92102.48	87007.26	113771.04	108669.59	104051.84	122131.12	115849.45	102816.39	125445.08	112235.98	95615.93	106258.49	101157.55	95836.99	91428.90	85699.89	78943.51	33747.00	26496.07	22979.90	18396.02	17091.28	13019.00	24033.97	20494.71	18372.67	21970.43	20333.60	11915.36		
4179	SH3 domain-containing kinase-binding protein 1	Q96B97	0.14	2.53	0.81	1.07	2.77	0.68	0.96	0.36	0.63	0.35	0.75	0.38	2.13	0.97	0.60	4.64	1.07	0.47	2.22	0.19	0.06	216137.49	204360.70	188510.86	150716.09	152753.10	146296.19	167082.63	177154.09	170296.21	168392.84	169474.17	128916.73		x
4180	SHC SH2 domain-binding protein 1	Q8NEM2	44405.16	37953.49	38316.66	22263.76	22860.84	20595.57	34579.18	30657.11	28515.84	28592.43	26790.69	23920.15	26857.75	26792.97	21753.65	37850.94	35332.59	34368.90	27327.09	24838.64	22832.65	2.27	1.49	0.65	0.84	1.31	0.69	4.16	2.47	0.11	4.90	4.98	0.12	x	
4181	SHC-transforming protein 1	P29353	5062.93	4569.67	4367.61	2980.10	2797.67	2232.69	3881.81	3825.14	3251.90	3299.90	3247.39	2730.26	3613.25	3500.00	1926.33	3361.58	3116.09	2776.30	3527.10	3353.43	2925.50	8172.43	7500.42	7189.04	4978.52	4489.24	3462.16	6332.33	5409.33	4714.00	6613.26	7066.94	3807.34		
4182	Shootin-1	A0MZ66	136829.79	126800.22	121694.85	94901.63	92190.17	85949.72	99886.91	95572.36	93056.08	115388.17	110881.58	105675.46	116510.76	109482.90	93359.14	87670.49	85925.71	84959.28	96884.95	92174.35	84604.66	15705.64	12633.78	11773.80	10263.56	10466.26	8641.75	12006.87	12011.17	10877.98	12171.41	11404.58	8134.54		
4183	Short stature homeobox protein 2	O60902	0.14	2.53	0.81	1.07	2.77	0.68	0.96	0.36	0.63	0.35	0.75	0.38	2.13	0.97	0.60	4.64	1.07	0.47	2.22	0.19	0.06	350.97	333.97	303.83	396.43	407.84	253.92	382.73	370.47	334.58	603.92	575.82	345.84		x
4184	Short/branched chain specific acyl-CoA dehydrogenase mitochondrial	P45954	0.14	2.53	0.81	1.07	2.77	0.68	0.96	0.36	0.63	0.35	0.75	0.38	2.13	0.97	0.60	4.64	1.07	0.47	2.22	0.19	0.06	4389.66	4958.75	4605.63	4345.39	4546.52	4145.45	4138.61	4480.03	3979.66	4162.94	4360.89	3851.79		x
4185	Short-wave-sensitive opsin 1	P03999	0.14	2.53	0.81	1.07	2.77	0.68	0.96	0.36	0.63	0.35	0.75	0.38	2.13	0.97	0.60	4.64	1.07	0.47	2.22	0.19	0.06	147285.42	140621.01	132996.16	104774.74	101814.95	86549.73	119837.84	113412.98	113905.43	104660.27	101390.73	75134.21		x
4186	Shugoshin 2	Q562F6	0.14	2.53	0.81	1.07	2.77	0.68	0.96	0.36	0.63	0.35	0.75	0.38	2.13	0.97	0.60	4.64	1.07	0.47	2.22	0.19	0.06	52775.25	49855.16	44469.74	35650.75	32510.39	23626.98	41250.55	38523.15	33947.61	41143.04	39341.86	22918.50		x
4187	Sialic acid synthase	Q9NR45	572876.42	570249.61	526901.94	440211.89	435494.86	419224.77	491073.51	461199.64	437933.11	531658.57	516251.42	485478.19	499291.25	446061.81	395348.11	475300.40	465083.17	453243.43	464109.55	444312.69	416910.82	242376.80	212180.26	199722.94	168756.65	170094.18	153321.14	175189.87	171185.51	159487.16	228048.29	219913.03	102070.83		
4188	Sialidase-1	Q99519	4482.86	4260.66	3419.30	2724.86	2991.09	2341.39	4951.64	5536.01	5110.86	3706.83	4001.89	3633.54	3090.81	3317.00	2358.69	4087.59	4540.49	3502.36	2757.74	2376.32	2697.15	2.27	1.49	0.65	0.84	1.31	0.69	4.16	2.47	0.11	4.90	4.98	0.12	x	
4189	Sideroflexin-1	Q9H9B4	361240.23	343550.45	342912.32	244572.26	234478.28	221109.82	293413.02	286365.26	276034.26	282981.28	280259.13	257841.80	302237.74	284841.31	243295.83	240612.12	222248.36	219962.49	243537.03	230036.60	220221.21	51922.01	48497.09	39079.22	30760.42	28937.59	23824.44	34772.95	34430.90	32515.16	34398.29	34425.66	22114.89		
4190	Sideroflexin-2	Q96NB2	61658.73	55982.71	52941.66	30131.79	30194.34	26881.56	39012.29	37093.03	36347.13	49219.38	47527.92	45116.56	41131.91	39036.17	33194.88	50582.88	47925.96	48194.53	43630.85	34828.12	32969.93	20065.14	20123.99	16189.79	22990.26	21895.61	20306.26	37632.47	34406.73	31512.32	21684.96	21268.41	13235.68		
4191	Sideroflexin-3	Q9BWM	19886.57	17993.92	13798.31	7417.61	6374.68	4560.89	9339.00	9385.39	6843.22	14660.68	13691.02	10775.97	18351.70	11907.96	4580.75	7277.83	6104.19	5765.12	8293.83	8788.73	7169.57	5722.66	5517.91	5190.65	3359.25	3308.23	3060.45	4804.78	4095.23	3970.50	4135.25	3828.76	2867.01		
4192	Signal peptidase complex catalytic subunit SEC11A	P67812	82911.16	79977.82	73397.15	53479.70	51318.80	49235.11	58108.39	59501.48	57346.18	65501.55	62287.19	59375.31	57065.49	51848.14	43615.28	52322.60	54682.92	52910.03	53937.15	49664.84	46015.77	12107.41	11692.15	10202.97	7839.83	7450.35	6407.60	11387.38	10510.75	9921.46	8984.77	8983.84	6909.58		
4193	Signal peptidase complex subunit 1	Q9Y6A9	91326.27	90208.84	85874.32	68838.47	67776.44	64193.71	76653.43	71023.18	67031.38	88162.37	80481.67	77700.99	72424.26	73191.01	63003.99	73936.71	70323.61	66875.06	61890.30	62810.16	55936.10	2.27	1.49	0.65	0.84	1.31	0.69	4.16	2.47	0.11	4.90	4.98	0.12	x	
4194	Signal peptidase complex subunit 2	Q15005	109334.18	100921.85	92724.32	74461.99	70679.28	64995.88	93175.87	87412.60	81353.56	100531.50	97288.76	84945.77	86063.41	81718.08	56622.32	82373.07	77624.42	72088.07	74313.88	67303.29	58418.43	18052.90	17638.88	15309.80	11290.80	10460.12	8754.70	13853.61	12884.48	11706.01	13486.42	12872.21	7850.16		
4195	Signal peptidase complex subunit 3	P61009	75488.62	72712.71	65288.53	40131.28	39197.84	35033.48	53072.88	50906.28	49317.35	53818.10	50090.32	45263.50	52592.57	47443.39	39760.68	43335.11	41285.37	39794.50	40620.74	38993.12	36222.22	12714.90	12106.83	10993.16	7379.53	6928.15	5085.50	8412.55	7947.72	8430.67	8692.59	8424.66	5122.90		
4196	Signal peptide peptidase-like 2A	Q8TCT8	73.74	25.06	49.84	9.53	778.44	403.94	12.70	16.13	5.72	31.74	39.42	26.44	2.13	0.97	0.60	903.33	15.16	590.41	50.40	70.67	50.83	2.27	1.49	0.65	0.84	1.31	0.69	4.16	2.47	0.11	4.90	4.98	0.12	x	
4197	Signal recognition particle 14 kDa protein	P37108	77563.43	71398.29	65262.61	55193.75	52411.31	47443.02	65674.70	62297.18	58472.26	67531.08	64163.11	58866.37	68129.38	65699.79	43050.41	53098.07	51277.94	48300.52	53814.27	51686.57	47194.07	98748.36	90833.78	86907.07	59714.11	59943.46	55666.35	64697.31	58764.23	57509.97	64100.36	61593.93	46326.69		
4198	Signal recognition particle 19 kDa protein	P09132	25918.34	24151.35	24634.96	19327.72	17564.80	16221.30	18167.75	17959.99	18129.12	19589.74	18298.40	18342.24	22809.62	18855.66	16661.16	14060.92	14849.46	14923.19	20872.46	18830.10	18316.73	4664.08	4175.09	3971.40	3554.84	3399.93	3426.38	2102.14	2241.60	2402.33	3830.01	2319.17	1525.59		
4199	Signal recognition particle 54 kDa protein	P61011	101805.91	99787.48	93358.06	69769.58	70847.39	68189.53	76198.83	72066.36	75808.45	79864.46	75292.33	71005.02	88842.11	86238.39	76090.77	73734.08	63076.33	58641.78	70316.39	70284.25	70635.10	66055.03	63814.06	55889.75	37996.59	37093.36	29282.22	45026.47	43178.00	40180.01	49025.93	46047.56	29637.56		
4200	Signal recognition particle 9 kDa protein	P49458	275167.65	273904.61	246405.67	191696.43	183303.72	165870.77	237384.84	228403.08	212084.08	238031.39	220331.17	207202.55	242038.83	230335.40	173864.07	211693.14	199016.42	182368.87	208280.74	200953.76	185463.05	341542.70	317616.41	274563.44	233374.63	225667.09	174411.08	267012.93	252284.78	228764.52	269224.82	261972.17	171244.85		
4201	Signal recognition particle receptor subunit alpha	P08240	19979.88	18946.67	17421.14	12750.49	11256.68	10799.01	12045.81	11512.64	11368.96	17016.73	15921.18	14794.16	15174.42	13188.95	11839.68	11047.55	9932.71	11150.16	12250.28	11106.84	11088.78	23894.94	22782.36	21221.85	14723.08	14453.99	13832.74	17380.31	16421.53	16994.68	18513.80	17499.10	10376.14		
4202	Signal recognition particle receptor subunit beta	Q9Y5M8	239279.78	231637.07	227304.13	173140.18	163121.97	165557.51	200469.32	193168.58	189969.49	199655.39	190350.73	185092.12	197812.65	186035.51	164220.23	178940.43	167556.98	169324.50	169457.88	155836.72	148993.90	31732.85	31610.26	28297.39	23012.71	21660.65	19910.26	27454.14	24636.64	24042.88	26730.43	25906.57	14640.90		
4203	Signal recognition particle subunit SRP68	Q9UHB9	143244.13	139134.63	134065.18	102948.94	99259.71	95685.15	109944.43	101806.44	98881.52	121444.16	114207.49	107296.88	120961.88	114710.20	96223.05	91897.67	87741.95	87728.71	99143.61	93152.08	88213.61	69955.87	69735.69	62528.34	47132.78	45818.58	40653.68	51830.96	51681.23	47703.59	57082.93	55925.68	42358.08		
4204	Signal recognition particle subunit SRP72	O76094	191171.68	180703.30	176477.54	135810.33	135264.50	127716.11	148338.67	138957.17	134410.57	163767.24	156668.91	147161.77	156131.28	149404.72	127404.07	130966.22	127323.30	121765.12	132959.93	129151.53	123016.88	47070.01	43688.13	40527.49	34533.42	34244.96	34018.08	32141.64	31520.03	30393.68	37938.80	34856.18	28188.64		
4205	Signal transducer and activator of transcription 1-alpha/beta	P42224	393542.43	367364.22	332557.97	254560.69	246066.81	217013.82	297562.78	288284.24	267794.58	321171.80	304612.42	279171.11	314777.26	290905.84	218386.41	255712.93	242411.27	224543.02	255142.65	246774.46	218350.93	346752.52	320823.45	284894.90	180596.30	176475.76	140810.36	222120.90	203259.04	188444.75	176523.77	172531.71	107986.70		
4206	Signal transducer and activator of transcription 3	P40763	248797.62	223044.19	192173.68	179062.31	163413.33	149807.99	182568.45	168418.21	162273.20	245513.72	236989.83	212062.23	227747.08	210018.69	131793.28	165114.36	155234.23	154586.52	180960.08	171633.08	155460.82	50533.89	48843.05	39673.89	34062.58	32590.74	25677.90	42913.46	40007.13	35451.72	48743.43	44887.13	30586.05		
4207	Signal transducing adapter molecule 1	Q92783	32993.34	36061.91	23173.14	16080.81	15325.04	12984.88	18520.61	16558.37	12751.04	23951.48	20992.77	15398.54	28207.08	20424.35	13915.77	20703.52	17565.37	18036.10	15899.71	15815.92	14681.30	20286.31	18336.24	16954.16	12324.84	11644.78	8437.22	13685.03	13815.66	12012.67	14383.46	13701.43	7656.02		
4208	Signal transducing adapter molecule 2	O75886	2973.78	2751.37	2668.77	1651.07	1478.91	1374.65	2245.80	2135.40	1259.99	2070.57	1757.68	2250.04	1908.06	1934.74	1299.94	1703.98	1849.86	1597.22	1730.55	1740.46	1234.96	5965.39	4932.74	4931.10	3566.15	3161.95	2053.44	3471.06	2688.68	2952.52	4327.44	4100.93	2071.79		
4209	Signal-induced proliferation-associated 1-like protein 2	Q9P2F8	7706.48	7764.19	8132.48	6307.79	6367.28	6097.62	5905.45	5916.96	6036.36	7867.16	7264.11	6728.69	5744.69	6295.88	6088.67	5337.30	4950.23	5419.88	5924.29	5560.30	5829.79	2.27	1.49	0.65	0.84	1.31	0.69	4.16	2.47	0.11	4.90	4.98	0.12	x	
4210	Signal-induced proliferation-associated 1-like protein 3	O60292	7330.16	6429.93	5080.05	7247.11	4204.57	6041.83	4517.37	4742.82	4603.36	6100.88	6645.67	6520.41	5192.75	5969.95	5141.62	4712.37	4216.38	6178.70	3410.47	4671.60	4054.11	57396.12	67186.11	61431.91	40252.26	37391.63	34647.87	46521.11	43265.75	48000.97	55230.64	47566.71	34687.56		
4211	Signaling lymphocytic activation molecule	Q13291	0.14	2.53	0.81	1.07	2.77	0.68	0.96	0.36	0.63	0.35	0.75	0.38	2.13	0.97	0.60	4.64	1.07	0.47	2.22	0.19	0.06	696.02	654.89	691.93	610.03	662.82	567.43	744.07	772.12	836.36	797.34	755.87	465.59		x
4212	Single-stranded DNA-binding protein 2	P81877	553.56	576.47	391.03	340.03	247.49	229.18	199.43	177.41	128.01	293.61	106.16	280.89	388.30	478.91	345.35	77.98	214.29	149.94	272.29	349.43	300.69	2.27	1.49	0.65	0.84	1.31	0.69	4.16	2.47	0.11	4.90	4.98	0.12	x	
4213	Single-stranded DNA-binding protein_ mitochondrial	Q04837	115510.02	109083.00	104846.14	93802.18	91006.24	83536.06	101886.77	98390.39	92899.21	114819.97	109671.85	101185.80	98895.90	95300.38	81281.23	89155.79	82406.92	83883.25	87642.44	79681.65	79161.11	29149.04	26893.28	25889.17	22821.10	22639.01	19235.92	28727.49	24367.15	23865.80	28830.83	27122.97	19083.13		
4214	Sister chromatid cohesion protein PDS5 homolog A	Q29RF7	7462.09	7089.77	6795.54	5359.05	4985.69	4952.25	6163.85	5793.27	5636.67	7090.90	6028.48	6455.12	7048.86	6279.27	5908.47	4637.88	4379.73	4511.82	4997.63	4875.21	3414.09	101225.35	93784.05	86479.51	59743.94	59123.14	55927.13	72532.82	67307.34	64389.09	79742.02	81464.48	60267.68		
4215	Sjoegren syndrome/scleroderma autoantigen 1	O60232	9210.81	9581.96	9008.88	5323.99	5181.77	5026.75	9583.14	9057.77	8300.69	7360.33	6277.16	6408.88	8364.75	6377.22	4802.55	6551.30	5943.49	5663.12	6449.73	6269.58	6445.18	40869.81	39097.05	35470.73	28978.82	27799.85	26096.67	33240.30	30459.80	29407.00	29280.73	28904.24	21259.02		
4216	Ski-like protein	P12757	6278.99	5847.07	5674.03	4777.05	4589.50	4661.40	5254.90	4966.35	4895.76	5154.95	5055.07	4644.93	4535.30	5150.05	4198.35	4478.21	4407.18	4368.27	4582.28	4486.07	4357.09	2.27	1.49	0.65	0.84	1.31	0.69	4.16	2.47	0.11	4.90	4.98	0.12	x	
4217	SLAIN motif-containing protein 2	Q9P270	1125.27	1123.43	1208.21	842.24	876.36	791.36	988.31	831.98	822.73	1179.32	1242.73	1243.73	1047.88	1117.20	907.61	774.77	863.47	758.12	982.22	963.14	851.20	2.27	1.49	0.65	0.84	1.31	0.69	4.16	2.47	0.11	4.90	4.98	0.12	x	
4218	Slit homolog 2 protein	O94813	0.14	2.53	0.81	1.07	2.77	0.68	0.96	0.36	0.63	0.35	0.75	0.38	2.13	0.97	0.60	4.64	1.07	0.47	2.22	0.19	0.06	23857.49	22231.72	20899.16	14659.35	13587.93	11746.14	15458.36	14685.21	13368.72	15391.03	14433.71	11354.67		x

Supplementary Table S1. Overview on the abundance of all relatively quantified 5180 proteins

			MCF-7																					MDA-MB-231													
	Protein name	UniProt	control_01	control_02	control_03	Dai_IC20_01	Dai_IC20_02	Dai_IC20_03	Dai_SC20_01	Dai_SC20_02	Dai_SC20_03	Gen_IC20_01	Gen_IC20_02	Gen_IC20_03	Gen_SC20_01	Gen_SC20_02	Gen_SC20_03	SSE_IC20_01	SSE_IC20_02	SSE_IC20_03	SSE_SC20_01	SSE_SC20_02	SSE_SC20_03	control_01	control_02	control_03	Dai_IC20_01	Dai_IC20_02	Dai_IC20_03	Gen_IC20_01	Gen_IC20_02	Gen_IC20_03	SSE_IC20_01	SSE_IC20_02	SSE_IC20_03	unique to MCF-7	unique to MDA-MB-231
4219	SLIT-ROBO Rho GTPase-activating protein 1	Q7Z6B7	0.14	2.53	0.81	1.07	2.77	0.68	0.96	0.36	0.63	0.35	0.75	0.38	2.13	0.97	0.60	4.64	1.07	0.47	2.22	0.19	0.06	17090.88	16593.01	15192.40	12460.63	11928.64	9302.17	14570.77	13547.88	13049.07	15130.31	14778.17	9544.83		x
4220	SLIT-ROBO Rho GTPase-activating protein 2	O75044	0.14	2.53	0.81	1.07	2.77	0.68	0.96	0.36	0.63	0.35	0.75	0.38	2.13	0.97	0.60	4.64	1.07	0.47	2.22	0.19	0.06	12278.33	11105.95	8661.63	8304.28	8733.44	5854.96	9811.55	9059.08	7962.99	12294.92	11285.32	6699.45		x
4221	Small acidic protein	O00193	38993.24	36764.53	33645.00	23741.52	22368.40	19112.86	28071.73	26784.27	24549.49	30053.13	27960.08	26548.68	29196.84	26605.44	19091.72	26654.20	24387.64	23079.03	22282.33	20705.20	19468.90	426.09	381.01	351.25	681.97	636.26	517.30	617.38	721.97	701.96	616.96	561.27	446.77		
4222	Small EDRK-rich factor 2	P84101	9097.60	10538.67	8920.32	7955.46	6282.75	7965.17	5940.06	6720.36	6526.23	8098.69	8628.47	8997.99	7939.92	3352.84	3653.51	5112.48	5657.37	6285.42	8242.94	7379.22	6300.72	2.27	1.49	0.65	0.84	1.31	0.69	4.16	2.47	0.11	4.90	4.98	0.12	x	
4223	Small G protein signaling modulator 3	Q96HU1	58419.37	55581.63	45505.33	38718.29	35844.30	33227.35	39819.75	39427.88	34958.33	45685.03	43746.67	39924.27	42890.09	40583.12	33141.68	40960.32	39490.79	38638.91	39811.57	36838.79	34426.72	2.27	1.49	0.65	0.84	1.31	0.69	4.16	2.47	0.11	4.90	4.98	0.12	x	
4224	Small glutamine-rich tetratricopeptide repeat-containing protein alpha	O43765	0.14	2.53	0.81	1.07	2.77	0.68	0.96	0.36	0.63	0.35	0.75	0.38	2.13	0.97	0.60	4.64	1.07	0.47	2.22	0.19	0.06	53137.82	49384.86	40091.74	40436.96	39395.56	39545.92	41341.97	39954.78	38107.06	48794.76	52697.56	30452.46		x
4225	Small glutamine-rich tetratricopeptide repeat-containing protein beta	Q96EQ0	0.14	2.53	0.81	1.07	2.77	0.68	0.96	0.36	0.63	0.35	0.75	0.38	2.13	0.97	0.60	4.64	1.07	0.47	2.22	0.19	0.06	226.13	175.41	167.09	108.47	88.22	57.01	196.86	144.31	142.79	227.75	276.10	38.15		x
4226	Small nuclear ribonucleoprotein F	P62306	6443.13	5786.45	8909.05	4787.90	3334.02	3355.35	5498.92	5744.57	4781.07	4211.30	3088.05	4229.67	4301.03	4914.48	2911.43	5051.03	4534.03	4095.51	4043.66	4107.46	3234.48	2.27	1.49	0.65	0.84	1.31	0.69	4.16	2.47	0.11	4.90	4.98	0.12	x	
4227	Small nuclear ribonucleoprotein Sm D1	P62314	77320.11	70329.76	66978.74	46609.05	44763.50	40705.48	54902.02	54841.12	50915.40	60473.21	57648.16	50524.93	56155.38	46849.40	38041.02	49531.33	48296.25	42503.65	52673.73	49959.83	45158.57	26107.55	25135.43	22935.20	16786.98	16287.56	15054.11	28188.85	26672.87	24864.41	22531.75	22275.13	16672.11		
4228	Small nuclear ribonucleoprotein Sm D2	P62316	114812.83	111748.27	106560.72	79433.92	78665.94	75708.27	87968.57	83777.81	79800.55	87807.62	83404.38	80805.97	88942.61	88077.26	77694.58	76863.41	73941.22	70721.10	86719.59	82815.72	80265.56	33099.18	30551.54	28821.49	29149.67	30580.65	28332.44	30094.73	31859.61	31050.01	35014.71	34713.18	24479.87		
4229	Small nuclear ribonucleoprotein Sm D3	P62318	90607.86	88537.68	80352.06	60072.85	55077.50	54342.01	70994.02	67162.02	66521.82	69447.24	67181.90	60655.25	66141.03	62190.62	51613.11	61512.92	57758.17	56869.82	64157.84	60200.37	56772.94	2145.82	1879.26	1304.80	421.58	473.34	364.69	697.49	687.22	630.42	480.77	396.04	217.46		
4230	Small nuclear ribonucleoprotein-associated proteins B and B'	P14678	0.14	2.53	0.81	1.07	2.77	0.68	0.96	0.36	0.63	0.35	0.75	0.38	2.13	0.97	0.60	4.64	1.07	0.47	2.22	0.19	0.06	2847.83	2691.76	2283.58	359.43	361.66	302.84	465.85	430.40	438.74	440.89	389.99	258.54		x
4231	Small proline-rich protein 2A	P35326	0.14	2.53	0.81	1.07	2.77	0.68	0.96	0.36	0.63	0.35	0.75	0.38	2.13	0.97	0.60	4.64	1.07	0.47	2.22	0.19	0.06	17.37	3.04	8.89	12.67	10.13	5.72	16.38	4.94	0.85	9.80	13.42	748.95		x
4232	Small subunit processome component 20 homolog	O75691	11818.57	10506.63	9988.77	8638.02	8995.36	8328.13	9356.94	9095.04	9127.16	13753.85	13043.69	12626.90	11485.06	10290.21	7621.45	7948.17	7779.81	7633.17	8441.10	8301.36	7937.63	570.39	463.33	398.63	99.32	138.96	48.83	341.00	237.86	178.22	280.42	301.72	122.61		
4233	Small ubiquitin-related modifier 1	P63165	0.14	2.53	0.81	1.07	2.77	0.68	0.96	0.36	0.63	0.35	0.75	0.38	2.13	0.97	0.60	4.64	1.07	0.47	2.22	0.19	0.06	25846.63	23239.12	23922.16	15140.75	14868.15	13929.35	18257.80	17354.34	16745.31	19724.95	17124.21	12926.58		x
4234	Small ubiquitin-related modifier 2	P61956	233011.04	221388.31	217464.96	186000.52	176653.04	173953.30	199817.92	190572.83	191787.81	189903.04	178560.32	174456.71	201757.25	197311.60	184410.03	173220.37	162217.39	161972.57	182905.43	180404.59	178658.24	156803.73	150113.97	140983.81	107287.38	104352.85	99293.81	123398.42	117913.47	114803.72	111748.38	112019.60	66124.37		
4235	Small ubiquitin-related modifier 3	P55854	0.14	2.53	0.81	1.07	2.77	0.68	0.96	0.36	0.63	0.35	0.75	0.38	2.13	0.97	0.60	4.64	1.07	0.47	2.22	0.19	0.06	11947.27	12260.62	11619.79	10399.59	10590.27	9461.01	11389.49	11180.96	11080.14	10538.87	11407.29	7719.55		x
4236	S-methyl-5'-thioadenosine phosphorylase	Q13126	0.14	2.53	0.81	1.07	2.77	0.68	0.96	0.36	0.63	0.35	0.75	0.38	2.13	0.97	0.60	4.64	1.07	0.47	2.22	0.19	0.06	27369.18	26560.96	26188.23	16105.32	17741.26	15114.16	19665.31	19072.51	18111.63	19754.34	18940.02	13553.46		x
4237	snRNA-activating protein complex subunit 3	Q92966	0.14	2.53	0.81	1.07	2.77	0.68	0.96	0.36	0.63	0.35	0.75	0.38	2.13	0.97	0.60	4.64	1.07	0.47	2.22	0.19	0.06	12505.92	10952.01	9391.72	10475.12	10333.65	9209.49	18144.64	16953.43	15347.63	11289.00	11168.60	6341.56		x
4238	Snurportin-1	O95149	0.14	2.53	0.81	1.07	2.77	0.68	2422.87	2222.67	2204.32	0.35	0.75	0.38	2.13	0.97	0.60	4.64	1.07	0.47	2.22	0.19	0.06	2.27	1.49	0.65	0.84	1.31	0.69	4.16	2.47	0.11	4.90	4.98	0.12	x	
4239	SNW domain-containing protein 1	Q13573	22347.34	21300.56	20371.21	15083.62	14484.00	13364.94	16846.58	16248.89	14802.21	19970.52	18993.10	17752.59	19794.21	16990.36	13398.98	14121.37	12079.64	12102.42	14213.01	14312.14	13709.95	5860.27	5639.74	5560.83	4800.95	4462.58	4403.66	4914.70	4682.52	4672.32	5383.29	5266.60	3789.08		
4240	Sodium channel protein type 5 subunit alpha	Q14524	0.14	2.53	0.81	1.07	2.77	0.68	0.96	0.36	0.63	0.35	0.75	0.38	2.13	0.97	0.60	4.64	1.07	0.47	2.22	0.19	0.06	5585.22	5524.74	4918.46	3980.66	3533.21	2674.39	4496.55	4099.97	3792.09	4403.63	4390.21	2491.61		x
4241	Sodium/potassium-transporting ATPase subunit alpha-1	P05023	589393.45	580954.53	550780.16	427049.11	411347.60	387122.88	470093.65	456378.90	446501.04	529909.35	505398.94	483321.44	479226.13	437579.11	387166.76	454149.90	439854.92	426349.88	405750.19	392226.92	369578.72	268299.94	251957.53	238002.49	166917.01	164047.61	144945.39	205668.51	189884.26	184804.96	201982.08	190243.71	134364.86		
4242	Sodium/potassium-transporting ATPase subunit alpha-2	P50993	335320.04	321553.50	299895.75	233804.22	221429.12	210731.05	287397.56	280206.12	270173.30	288503.49	283832.42	267794.62	309453.83	277976.12	231188.70	225955.54	210360.83	211984.53	239977.91	224136.95	215890.92	151848.94	148022.86	134250.31	114634.17	111320.46	103380.15	114263.06	113549.93	112024.89	109860.09	105903.44	78623.91		
4243	Sodium/potassium-transporting ATPase subunit alpha-3	P13637	8382.49	8302.69	6956.45	5245.28	4583.23	4145.25	8030.35	8163.91	7032.57	7379.63	6449.34	6331.55	7049.52	6377.97	3577.81	6533.13	6348.83	5841.29	5755.05	5215.32	3950.45	37465.31	35419.89	33133.62	22362.39	22103.18	19564.24	27704.96	27396.20	25091.04	27510.24	24898.40	16665.21		
4244	Sodium/potassium-transporting ATPase subunit alpha-4	Q13733	57395.65	57262.11	55992.65	40613.78	40761.40	38312.48	48532.22	47485.22	45795.52	54311.19	53611.41	49660.20	49842.46	46128.73	35958.22	38955.24	37261.29	34726.58	37550.88	38440.40	36923.08	12337.08	11245.85	11153.96	6747.34	6355.47	5224.54	8465.21	7810.78	7920.44	7551.24	7184.79	4987.27		
4245	Sodium/potassium-transporting ATPase subunit beta-1	P05026	43829.46	42560.44	40649.23	30508.47	29046.02	27264.22	31059.16	30104.85	31314.34	38449.34	37310.26	36716.43	37542.77	35635.37	30555.13	29926.14	29595.96	28612.90	29207.46	28022.43	27943.12	12052.33	11222.48	10729.94	6328.60	6450.46	4717.52	9688.39	9457.63	7595.86	9848.46	9108.86	5147.83		
4246	Sodium/potassium-transporting ATPase subunit beta-3	P54709	131949.38	123251.63	118495.71	89861.46	84165.45	81154.09	99235.23	94969.13	88999.18	107943.92	105340.99	96877.70	94644.79	90045.93	79522.16	100939.56	94985.34	88875.07	88467.34	82777.70	65427.53	144759.63	132692.44	111547.54	58604.36	55251.71	43464.15	153971.60	137248.93	111071.36	105610.81	96698.77	58146.91		
4247	Sodium-coupled neutral amino acid transporter 2	Q96QD8	8230.43	7599.62	6311.14	4418.71	3973.21	3488.56	5937.81	5206.27	4821.39	7186.21	6484.25	5292.67	6423.81	5506.13	3501.05	4689.09	4450.17	4382.97	5882.77	5003.02	4668.73	2.27	1.49	0.65	0.84	1.31	0.69	4.16	2.47	0.11	4.90	4.98	0.12	x	
4248	Soluble calcium-activated nucleotidase 1	Q8WVQ1	5885.04	5810.73	5248.19	4775.13	4693.99	4203.67	5937.39	4933.69	4325.26	6552.82	6367.52	6175.91	5232.35	4922.14	4408.80	5079.18	5110.30	4515.90	5319.68	4475.45	4514.25	2.27	1.49	0.65	0.84	1.31	0.69	4.16	2.47	0.11	4.90	4.98	0.12	x	
4249	Solute carrier family 12 member 1	Q13621	0.14	2.53	0.81	1.07	2.77	0.68	0.96	0.36	0.63	0.35	0.75	0.38	2.13	0.97	0.60	4.64	1.07	0.47	2.22	0.19	0.06	28481.54	28330.59	25822.76	19175.33	19596.90	18057.65	21775.88	21808.26	21646.79	21441.87	19401.18	14367.26		x
4250	Solute carrier family 12 member 7	Q9Y666	0.14	2.53	0.81	1.07	2.77	0.68	0.96	0.36	0.63	0.35	0.75	0.38	2.13	0.97	0.60	4.64	1.07	0.47	2.22	0.19	0.06	63661.54	60869.23	56938.42	54698.06	53699.68	49805.44	73918.48	75243.59	73463.12	65398.45	63764.79	49427.97		x
4251	Solute carrier family 12 member 8	A0AV02	0.14	2.53	0.81	1.07	2.77	0.68	0.96	0.36	0.63	0.35	0.75	0.38	2.13	0.97	0.60	4.64	1.07	0.47	2.22	0.19	0.06	6829.30	6296.61	6969.67	4789.78	4351.40	4076.90	6251.75	6195.59	5744.32	5867.41	5651.57	4141.88		x
4252	Solute carrier family 2_ facilitated glucose transporter member 1	P11166	68413.34	67983.63	63805.73	56811.29	56828.20	48841.17	65651.19	62507.70	61876.83	89543.80	84359.51	80274.70	55712.97	52305.66	43021.42	51387.44	49001.39	45622.00	58166.71	56475.59	53401.57	105747.31	99661.26	87725.10	63473.78	57675.67	50916.32	72233.75	69447.07	65005.23	72710.33	70065.80	47986.18		
4253	Solute carrier family 2_ facilitated glucose transporter member 3	P11169	961.44	933.00	982.77	498.98	563.99	440.99	703.80	561.25	632.44	853.40	979.93	784.11	860.56	832.35	555.16	653.59	694.37	650.68	765.72	761.57	618.64	1310.26	1335.87	1028.28	1394.92	1194.32	964.89	2189.43	2007.52	1804.55	1829.83	1847.32	1029.66		
4254	Solute carrier family 27 member 3	Q5K4L6	13757.66	12409.68	12134.27	7970.35	7764.01	7025.78	8478.28	8094.97	7464.40	9604.48	9065.80	8529.69	9573.01	9977.65	6517.52	8506.17	7550.51	7212.87	7807.81	7553.03	6409.89	2.27	1.49	0.65	0.84	1.31	0.69	4.16	2.47	0.11	4.90	4.98	0.12	x	
4255	Sorbin and SH3 domain-containing protein 2	O94875	28020.52	29039.37	25112.24	19637.81	19605.36	18360.48	23425.50	22808.79	20728.34	27522.71	26498.42	25558.49	26225.74	23562.73	16572.51	19607.81	19448.93	16674.93	19834.42	18911.33	18720.67	2.27	1.49	0.65	0.84	1.31	0.69	4.16	2.47	0.11	4.90	4.98	0.12	x	
4256	Sorbitol dehydrogenase	Q00796	164464.44	153840.61	132316.16	100396.45	100155.25	91985.21	118986.37	117205.98	110153.11	130015.66	125249.55	114633.88	126749.62	120901.11	91631.11	96942.46	92795.52	90222.00	116683.84	106151.21	98233.45	3555.86	3502.02	3024.00	2828.35	2334.29	1985.38	3062.33	3021.70	2703.25	2962.72	2807.38	1788.00		
4257	Sorcin	P30626	30224.88	33257.58	30768.70	23801.38	21506.30	22414.65	29973.74	32607.14	30746.00	27051.14	28099.09	26910.45	26177.90	27148.69	19452.54	22510.72	27227.75	23951.66	22249.55	21154.18	20472.14	49002.55	46330.63	44015.34	32736.72	33206.44	30863.65	37876.97	36937.91	35930.25	35850.77	34727.50	25728.23		
4258	Sortilin	Q99523	20080.52	20647.29	17005.87	14126.91	15220.98	13695.46	15907.94	15546.26	14733.51	20277.35	20305.65	17830.90	18976.41	17770.30	13596.76	14228.90	13738.53	13006.70	14546.21	14184.83	14365.63	2.27	1.49	0.65	0.84	1.31	0.69	4.16	2.47	0.11	4.90	4.98	0.12	x	
4259	Sorting and assembly machinery component 50 homolog	Q9Y512	0.14	2.53	0.81	1.07	2.77	0.68	0.96	0.36	0.63	0.35	0.75	0.38	2.13	0.97	0.60	4.64	1.07	0.47	2.22	0.19	0.06	1001.15	32.95	7.72	622.24	299.82	111.64	4.16	2.47	0.11	669.34	848.58	263.23		x
4260	Sorting nexin-1	Q13596	143008.70	145017.45	136195.96	108949.38	103766.84	102165.80	110569.53	108555.27	102387.26	125514.06	121077.19	118178.49	123994.58	119091.64	101816.07	100717.70	99530.02	102554.15	112868.59	109428.55	100776.42	74477.76	67206.03	66792.87	50502.28	47831.88	43264.91	62500.60	59794.59	56058.98	60301.14	58211.98	43435.86		
4261	Sorting nexin-12	Q9UMY4	11864.92	11151.38	8991.09	6696.99	6889.30	5825.00	8242.20	7477.13	7416.90	10598.97	10636.74	8287.26	7923.04	7933.54	4606.27	7091.22	6420.87	6485.80	6945.70	6957.06	6057.88	19548.10	22441.42	18302.70	16472.95	15890.62	12799.40	14146.62	15209.55	13788.63	22477.70	21047.34	7640.68		
4262	Sorting nexin-13	Q9Y5W8	430160.42	444328.64	407575.31	363227.15	356908.71	323139.92	378488.62	350578.63	334731.76	425717.85	393394.59	389745.84	387716.40	355118.49	300971.66	385482.91	359010.20	346185.14	304611.81	306153.53	292695.33	2.27	1.49	0.65	0.84	1.31	0.69	4.16	2.47	0.11	4.90	4.98	0.12	x	
4263	Sorting nexin-2	O60749	284861.53	271605.04	255694.41	223053.42	211344.04	205894.72	223495.33	215230.18	210231.71	262240.09	249201.56	234042.34	248649.86	237386.16	215411.64	220082.61	208820.02	202361.47	224443.02	209010.81	214065.99	94835.92	88700.12	79657.97	63747.23	61672.07	51195.68	74459.97	74269.06	66898.37	79387.91	74288.25	49375.37		
4264	Sorting nexin-27	Q96L92	323049.43	315386.75	296415.57	224912.17	225856.63	208907.33	269991.23	252053.68	243709.75	287362.36	277949.01	257994.58	253616.17	242995.51	215080.18	239440.55	231812.86	221503.65	209545.23	213038.22	194176.72	2.27	1.49	0.65	0.84	1.31	0.69	4.16	2.47	0.11	4.90	4.98	0.12	x	
4265	Sorting nexin-3	O60493	55414.09	51263.37	47228.45	31899.25	31337.33	26943.05	39890.50	39087.69	36239.30	44498.49	41965.90	38640.90	39944.26	37751.16	27238.86	31087.44	31182.94	29172.37	34621.08	31430.64	28948.28	49021.78	44994.07	41071.58	27113.84	26687.85	20066.65	36900.27	34944.77	31505.64	36126.30	36040.91	21451.99		
4266	Sorting nexin-32	Q86XE0	0.14	2.53	0.81	1.07	2.77	0.68	0.96	0.36	0.63	0.35	0.75	0.38	2.13	0.97	0.60	4.64	1.07	0.47	2.22	0.19	0.06	706.21	646.81	591.97	684.57	652.82	497.75	844.39	809.68	747.60	1115.57	1011.16	671.87		x
4267	Sorting nexin-4	O95219	6082.17	6448.18	6292.43	4349.38	5095.61	4654.74	4825.29	4552.13	4680.41	5352.12	5140.73	5221.48	5461.06	5866.85	5066.02	4332.24	3886.06	4038.47	3976.72	3774.61	3343.27	2337.14	2268.87	2187.93	1933.09	1766.88	1584.07	2383.92	2278.15	2311.78	2607.13	2507.86	1642.09		
4268	Sorting nexin-5	Q9Y5X3	78543.80	75018.05	67902.44	48344.74	45855.46	41216.57	78680.64	75426.41	74504.25	58008.71	54594.69	51813.45	64126.79	55848.88	41885.65	54799.82	47255.11	48371.46	51469.15	49056.84	42952.42	29587.43	25798.13	22954.79	16680.70	16283.97	12431.64	18356.53	17155.48	15752.37	21010.10	19383.67	12454.06		
4269	Sorting nexin-6	Q9UNH7	139082.48	127630.13	119751.55	102443.97	99529.92	90761.58	100967.26	101503.46	96165.12	127979.99	124222.82	116247.10	104772.50	103092.13	86197.78	94180.20	92186.70	90780.72	89676.17	88027.33	83608.00	85644.61	78766.97	66621.34	45686.25	46380.45	37145.89	63048.55	57323.36	51928.69	65610.11	63056.97	32426.72		
4270	Sorting nexin-7	Q9UNH6	116218.90	110726.19	101331.18	81627.74	78275.25	73364.89	96854.58	95778.52	93278.59	97742.07	95000.07	89945.07	98240.95	94384.64	81995.43	81159.42	77422.58	71282.68	78480.86	76454.77	75508.70	2.27	1.49	0.65	0.84	1.31	0.69	4.16	2.47	0.11	4.90	4.98	0.12	x	
4271	Sorting nexin-8	Q9Y5X2	19697.56	21634.89	19284.41	18748.14	18126.46	15276.07	16314.12	14032.04	14765.61	22830.61	23312.00	21917.60	20135.87	17972.26	14266.24	13096.38	15305.58	14348.76	14656.41	15682.41	15559.93	9780.16	9508.04	8247.41	5207.62	5032.26	4210.26	6527.29	6401.37	6401.97	6772.08	6165.54	4054.00		
4272	Sorting nexin-9	Q9Y5X1	0.14	2.53	0.81	1.07	2.77	0.68	0.96	0.36	0.63	0.35	0.75	0.38	2.13	0.97	0.60	4.64	1.07	0.47	2.22	0.19	0.06	3206.73	2535.57	2576.88	1461.58	1541.45	1270.38	2590.17	2432.51	1882.83	2155.26	1767.91	1310.73		x
4273	SPATS2-like protein	Q9NUQ6	32705.84	32438.40	29146.89	23471.16	22469.18	20970.48	25162.80	24057.84	24153.61	35236.36	35527.65	32932.56	32668.58	30387.57	24817.84	21536.74	19969.17	19388.75	22716.54	21464.59	21131.71	50916.52	45128.16	41978.09	31553.23	30532.26	27920.98	38194.74	37200.68	33882.16	34910.01	34147.33	21486.83		
4274	Speckle-type P	Q6IQ16	251698.42	242772.17	236036.07	197827.77	204879.62	195928.70	264341.11	252369.88	250816.96	250344.87	245733.09	235392.80	214735.56	197114.44	180871.95	214392.94	207962.39	197095.86	190030.33	184516.05	171196.69	297556.68	286938.03	269968.47	170795.10	171321.58	160249.67	156046.47	160156.73	162431.04	143824.18	140285.39	111951.52		
4275	Spectrin alpha chain_ erythrocytic 1	P02549	99800.59	99370.73	89666.99	74846.17	67952.38	67225.65	73437.42	63953.01	63800.62	102910.57	97586.33	96487.94	91797.22	71737.96	53769.31	58728.98	58584.48	61452.22	79504.26	72886.80	61943.50	1710.37	1713.62	1861.45	1955.63	1776.87	1455.37	2311.97	2075.06	2360.83	1827.54	2297.74	1624.37		
4276	Spectrin alpha chain_ non-erythrocytic 1	Q13813	592387.05	573188.55	528339.77	440969.86	422596.62	386881.61	498718.24	479626.26	461986.57	524480.88	491864.53	467876.64	496047.51	470024.76	376076.49	416227.44	402742.21	400963.28	431887.83	404353.29	388723.83	571156.69	526227.12	466936.33	373802.41	361917.95	312659.29	473701.94	443116.71	410696.85	479888.16	461615.97	314255.51		
4277	Spectrin beta chain_ non-erythrocytic 1	Q01082	462837.56	461887.42	407541.04	299829.10	299700.42	260253.15	363895.48	336492.52	316748.38	393193.05	367027.70	343595.05	379518.86	351963.35	248505.69	323051.95	299125.50	287937.36	316232.89	306589.28	267005.85	615181.93	572556.93	523488.22	402581.21	394631.24	327745.45	501383.41	470018.83	444886.98	518497.51	498741.05	332230.67		
4278	Spectrin beta chain_ non-erythrocytic 2	O15020	975255.08	966007.98	909453.26	741042.40	731247.50	682949.88	898043.18	863256.00	839998.60	846766.09	831728.30	802896.34	759468.72	733932.44	642998.10	803424.90	781687.07	738721.32	644727.99	639714.70	587371.42	2.27	1.49	0.65	0.84	1.31	0.69	4.16	2.47	0.11	4.90	4.98	0.12	x	
4279	Spectrin beta chain_ non-erythrocytic 4	Q9H254	62406.56	56656.80	53686.70	34588.11	33508.87	31052.02	40623.06	39250.09	37451.98	54072.52	50478.17	47755.24	49279.11	37345.03	31083.40	36325.48	34497.28	33515.70	34597.86	34820.31	31002.17	187264.96	177209.32	161200.76	144177.25	134198.45	113292.29	184950.32	169186.38	172922.02	177369.30	177061.66	125936.71		
4280	Spectrin beta chain_ non-erythrocytic 5	Q9NRC6	133618.60	130975.06	123252.48	95737.43	92768.67	90333.91	107568.86	94827.28	92743.69	127675.84	123161.12	112684.46	128692.00	123914.39	104865.76	83811.83	78873.75	81675.40	94835.15	94572.98	87467.38	413.41	466.03	445.70	287.26	283.93	249.56	266.82	346.05	303.21	310.32	235.80	211.06		
4281	Speedy protein E1	Q8NFV5	87.08	71.95	58.27	19.19	44.89	43.15	6.14	46.71	10.97	123.87	78.82	35.30	59.73	85.91	58.11	68.04	18.63	53.40	160.07	99.89	57.07	2.27	1.49	0.65	0.84	1.31	0.69	4.16	2.47	0.11	4.90	4.98	0.12	x	
4282	Speedy protein E5	A6NIY4	947.11	536.22	655.62	104.06	157.65	60.12	289.28	149.42	94.90	521.42	420.83	237.89	914.62	200.01	84.08	299.99	319.44	219.98	146.96	149.52	131.90	2.27	1.49	0.65	0.84	1.31	0.69	4.16	2.47	0.11	4.90	4.98	0.12	x	
4283	Sperm flagellar protein 2	Q9C093	0.14	2.53	0.81	1.07	2.77	0.68	0.96	0.36	0.63	0.35	0.75	0.38	2.13	0.97	0.60	4.64	1.07	0.47	2.22	0.19	0.06	16795.00	14852.80	15479.99	11502.20	11281.82	9839.85	13253.82	12558.31	11980.53	14359.90	13559.40	9719.31		x
4284	Sperm-associated antigen 5	Q96R06	1219.87	1215.76	877.81	549.64	540.08	377.45	718.32	783.39	695.57	658.74	521.20	541.35	816.32	686.16	450.48	905.83	637.84	695.47	701.08	586.08	608.16	2.27	1.49	0.65	0.84	1.31	0.69	4.16	2.47	0.11	4.90	4.98	0.12	x	
4285	Spermatid perinuclear RNA-binding protein	Q96SI9	49697.26	50197.91	44021.33	29044.34	28735.93	24389.96	42506.22	40564.35	39322.78	41281.92	37474.88	35608.62	39665.92	36431.76	29283.14	32133.23	29773.47	27161.81	29783.16	27647.24	26888.72	58775.48	53268.47	51892.91	34112.51	33926.77	29389.42	37596.26	34335.71	30904.65	47299.66	44542.71	29679.43		
4286	Spermatid-specific manchette-related protein 1	Q8NCR6	0.14	2.53	0.81	1.07	2.77	0.68	0.96	0.36	0.63	0.35	0.75	0.38	2.13	0.97	0.60	4.64	1.07	0.47	2.22	0.19	0.06	12628.95	12501.21	10269.56	8724.20	8782.47	7432.22	9978.10	9289.17	8714.35	9418.85	9176.61	7467.37		x
4287	Spermatogenesis-associated protein 13	Q96N96	2309.51	1967.04	1737.62	2010.14	1462.84	1147.18	1411.26	1273.53	1340.37	2891.51	2805.54	1995.35	2333.13	1498.26	202.91	1215.60	1159.77	851.66	1296.27	971.37	649.49	2.27	1.49	0.65	0.84	1.31	0.69	4.16	2.47	0.11	4.90	4.98	0.12	x	
4288	Spermatogenesis-associated protein 17	Q96L03	26303.90	25380.82	23890.93	23107.50	22311.41	20911.61	23696.73	22947.63	21520.20	22763.10	21963.74	20343.53	24495.97	20573.35	6480.29	16170.54	21758.34	20228.05	25933.09	25142.78	23524.65	2328.88	2054.47	2379.82	1348.20	1272.77	1294.08	1630.60	1341.24	1388.57	1500.98	1250.74	1254.09		
4289	Spermatogenesis-associated protein 5-like protein 1	Q9BVQ7	56807.28	48489.97	45455.46	22969.09	21794.35	17857.44	23446.87	18598.03	20723.33	47325.65	43664.57	38296.57	43100.33	21885.80	17513.94	22168.72	23108.11	21465.01	26570.68	27365.82	21927.40	1391.94	1443.15	1434.84	1351.64	1288.52	1160.72	1700.72	1619.62	1709.28	1516.95	1485.64	396.33		
4290	Spermatogenesis-associated protein 8	Q6RVD6	0.14	2.53	0.81	1.07	2.77	0.68	0.96	0.36	0.63	0.35	0.75	0.38	2.13	0.97	0.60	4.64	1.07	0.47	2.22	0.19	0.06	1685.53	1702.40	1940.11	1925.43	1832.49	1743.69	1416.18	1733.59	1653.09	1059.94	2291.91	4220.57		x
4291	Spermatogenesis-associated serine-rich protein 2	Q86XZ4	54296.66	51634.65	51036.14	42433.72	36145.62	39489.34	40540.90	42780.47	38085.80	47228.48	45566.31	45846.95	48737.91	58685.93	47349.46	38874.43	35315.45	40238.34	56819.46	46617.25	41357.31	2983.04	2567.47	2232.28	2046.06	1993.42	1992.71	2141.48	1841.42	2006.93	2496.55	2544.08	1526.03		
4292	Spermatogenesis-defective protein 39 homolog	Q9H9C1	4253.79	4319.76	3393.69	2485.25	2251.00	2394.87	2819.82	2051.19	2361.24	2835.17	2485.08	2293.67	2555.60	2441.44	2181.66	2138.67	1792.14	2061.11	4019.17	2448.82	3071.46	2.27	1.49	0.65	0.84	1.31	0.69	4.16	2.47	0.11	4.90	4.98	0.12	x	
4293	Spermatogenic leucine zipper protein 1	Q9BXG8	0.14	2.53	0.81	1.07	2.77	0.68	0.96	0.36	0.63	0.35	0.75	0.38	2.13	0.97	0.60	4.64	1.07	0.47	2.22	0.19	0.06	5305.45	3290.70	4019.75	2856.88	2737.54	2351.37	3947.78	3638.47	3113.95	3404.71	3222.86	2423.11		x
4294	Spermidine synthase	P19623	65387.87	63797.24	62342.16	45819.21	42231.61	40865.97	49361.89	49458.98	48337.24	58877.83	59439.05	54826.18	51013.48	48086.56	39870.76	40858.18	40959.41	39910.14	48326.82	45138.65	45301.76	38738.95	36141.67	31611.95	25069.42	25615.42	22217.30	31222.05	29898.62	29185.29	28624.85	30114.75	19247.45		
4295	Spermine synthase	P52788	31620.38	38601.76	35875.57	28367.84	28019.75	23420.96	32025.77	35020.39	28182.42	35426.35	34037.56	34548.33	31733.67	18463.61	32557.23	24112.70	20892.30	22739.95	30628.16	25929.92	26030.61	50232.71	46265.09	41974.31	29283.23	27509.29	24487.76	33530.76	30692.20	29249.04	31505.61	32051.09	22487.31		
4296	S-phase kinase-associated protein 1	P63208	75682.70	69073.99	58463.05	46233.12	46238.79	39690.92	54987.83	55134.61	49642.59	68821.12	65040.68	56857.82	62946.15	59371.01	37383.67	48030.62	45764.99	43116.45	50379.57	47484.31	42615.12	38337.91	35593.57	32569.45	28510.21	28162.13	22157.40	38732.65	36479.89	31326.83	39062.08	37666.42	22484.88		
4297	Sphingomyelin phosphodiesterase 3	Q9NY59	3615.89	3345.14	2900.55	2784.16	2539.04	2391.79	3504.74	3254.60	3127.61	4907.10	4952.77	4077.05	3761.06	3612.21	2138.06	2912.92	2544.19	2445.13	2604.46	2461.91	2227.68	73.29	81.84	113.08	193.01	189.16	103.08	279.91	249.52	186.89	194.87	132.31	221.71		
4298	Sphingomyelin phosphodiesterase 4	Q9NXE4	0.14	2.53	0.81	1.07	2.77	0.68	0.96	0.36	0.63	0.35	0.75	0.38	2.13	0.97	0.60	4.64	1.07	0.47	2.22	0.19	0.06	1113.68	901.34	609.55	662.07	496.82	401.37	956.12	923.30	702.57	624.15	629.30	413.91		x
4299	Sphingomyelin phosphodiesterase	P17405	1884.29	1939.21	1659.28	1379.72	1286.16	1247.56	1582.78	1531.67	1680.03	1600.93	1586.52	1519.98	1555.34	1405.75	1181.69	1406.04	1194.14	1144.45	1432.02	1147.46	1206.79	2.27	1.49	0.65	0.84	1.31	0.69	4.16	2.47	0.11	4.90	4.98	0.12	x	
4300	Sphingosine-1-phosphate lyase 1	O95470	69221.40	69353.01	66264.65	50384.72	48677.61	46300.80	53725.01	51871.19	49364.19	60396.34	60002.99	57925.32	57049.16	58990.93	56120.62	45066.99	47449.95	45171.29	50044.96	48422.96	47660.14	2391.37	1973.52	2194.29	1744.80	1549.31	1322.85	2082.37	2114.07	1801.18	2112.73	1988.90	1361.00		
4301	Spindle assembly abnormal protein 6 homolog	Q6UVJ0	0.14	2.53	0.81	1.07	2.77	0.68	0.96	0.36	0.63	0.35	0.75	0.38	2.13	0.97	0.60	4.64	1.07	0.47	2.22	0.19	0.06	1538.92	1319.03	1207.10	1125.16	1448.60	1281.92	1230.51	1339.37	1491.01	1496.56	1027.91	1109.96		x
4302	Spindlin-2A	Q99865	2181.83	2433.49	2148.79	1340.19	1080.11	885.84	1059.80	1342.41	586.05	1839.69	1659.37	1527.59	2194.40	1783.02	507.31	1608.74	630.86	788.54	1154.68	1626.88	1389.59	2.27	1.49	0.65	0.84	1.31	0.69	4.16	2.47	0.11	4.90	4.98	0.12	x	
4303	Spliceosome RNA helicase DDX39B	Q13838	66923.30	65608.25	62045.58	56831.98	52962.75	50925.72	63535.13	61113.23	62689.56	67415.92	65059.77	60906.80	66774.60	58400.68	56370.63	62440.04	57651.07	58048.93	56877.24	57508.30	59527.80	89883.78	82718.94	79675.08	59930.44	57607.28	49585.48	70177.87	66875.38	62932.12	70829.60	69276.06	50789.88		
4304	Spliceosome-associated protein CWC27 homolog	Q6UX04	64200.32	58096.70	58626.51	31121.25	29953.41	31163.31	40535.35	40128.35	39366.15	50109.61	50367.92	45888.45	38937.39	33002.61	28016.47	43648.43	44553.47	47273.90	46419.56	43202.35	40236.46	34395.51	10971.81	8579.67	19119.22	17248.12	11398.05	23498.74	20345.35	12253.01	24553.81	21861.05	9450.47		
4305	Splicing factor 1	Q15637	71849.94	68960.57	66588.62	57813.55	52079.32	52422.87	62503.70	60218.90	58828.58	59638.87	56839.01	54275.81	61147.00	59639.69	53686.69	54014.34	48897.40	47752.84	57675.53	52811.02	54149.37	85093.43	81796.24	76927.67	63477.29	62388.55	59308.83	66714.07	63448.50	63345.69	70522.39	68213.52	46440.95		
4306	Splicing factor 3A subunit 1	Q15459	110928.47	106373.11	104179.03	79742.02	72211.12	77311.98	95162.21	91905.75	89804.85	92043.09	88288.84	84053.59	94220.34	87602.49	87430.57	86223.90	77867.69	78358.03	99508.60	92747.15	90665.01	64959.88	63084.96	59083.37	42101.68	40575.40	35936.91	54813.22	52914.29	49676.32	53657.53	52030.36	32005.33		
4307	Splicing factor 3A subunit 2	Q15428	0.14	2.53	0.81	1.07	2.77	0.68	0.96	0.36	0.63	0.35	0.75	0.38	2.13	0.97	0.60	4.64	1.07	0.47	2.22	0.19	0.06	7099.05	6370.80	5660.91	5112.46	4871.64	4652.84	5484.62	5366.55	5060.41	6916.96	6210.00	2203.03		x
4308	Splicing factor 3A subunit 3	Q12874	179469.71	169805.49	167860.48	131252.90	126319.51	120272.81	164175.68	156957.95	153658.91	156005.15	147943.35	141417.38	159836.54	152084.83	128538.20	123575.16	118476.20	126169.28	132242.43	119753.75	116261.50	157429.74	143582.20	134024.42	93557.90	90100.53	78934.56	103119.91	93292.85	95139.82	96394.10	93399.08	54549.51		
4309	Splicing factor 3B subunit 1	O75533	149890.75	135857.54	126106.26	90546.32	83462.35	77091.15	97845.03	94787.15	90004.18	99841.28	93425.12	88435.25	107988.40	105859.93	77595.50	85433.30	80618.60	77342.90	94307.57	91347.27	85392.42	75975.16	71625.36	71484.40	47942.55	46999.67	40154.54	54085.19	49188.37	46701.72	57799.74	55276.14	37128.74		
4310	Splicing factor 3B subunit 2	Q13435	179545.30	174746.00	161658.32	123777.87	123687.83	102757.07	139128.36	127832.48	127236.58	127066.95	121363.78	132285.95	143724.32	133605.97	122646.94	113984.62	108619.73	100044.02	120278.88	116248.93	111866.34	81240.74	76551.44	71446.14	56333.38	54718.88	46150.19	63862.75	61474.48	58890.43	76575.24	76521.28	50316.01		
4311	Splicing factor 3B subunit 3	Q15393	289488.81	277581.42	263384.59	195163.33	186495.06	176162.29	216898.10	205634.65	204504.40	227088.10	215592.99	202823.77	233286.99	217677.90	181048.72	181793.22	171560.73	165749.65	198838.68	186948.41	178983.44	208610.16	196700.12	179833.68	147444.84	140854.38	121579.94	176681.12	166281.49	155510.07	182045.10	179644.72	111264.75		
4312	Splicing factor 3B subunit 4	Q15427	15367.99	14103.12	13601.43	9445.65	9096.41	8520.90	10097.86	10571.48	9982.28	11638.85	11425.93	10447.86	10802.03	10377.34	9439.49	8473.41	8412.18	7927.17	8813.60	8366.40	8020.67	2.27	1.49	0.65	104.52	99.01	17.43	4.16	2.47	0.11	188.91	135.15	7.92		
4313	Splicing factor 3B subunit 5	Q9BWJ5	12015.55	12025.97	11747.84	9931.93	9453.89	9619.70	11349.20	11222.06	11328.74	11607.88	10535.63	10796.31	10495.65	10840.93	9419.44	8998.44	8123.35	8345.80	9468.65	9137.37	9285.49	8450.30	7750.94	7846.83	4606.15	4500.17	4125.96	5750.53	5206.88	5092.55	5267.03	5045.58	3759.75		
4314	Splicing factor 3B subunit 6	Q9Y3B4	18708.45	17719.83	15998.15	12433.56	12355.58	9516.35	16038.36	15350.07	13599.32	17847.08	17463.08	16576.47	15014.58	14022.51	9633.15	13480.64	11815.35	10413.39	12837.55	12619.48	10992.87	25359.52	23789.66	19507.22	14177.06	13007.72	9371.98	16181.70	15043.50	13837.07	17755.67	16481.61	8893.32		
4315	Splicing factor 45	Q96I25	9113.87	8743.89	7642.83	4780.18	4898.39	3997.99	5677.02	5461.80	4714.97	7442.60	6948.62	6147.09	7025.66	6363.36	6301.33	4199.37	5259.72	4093.89	4530.95	4160.30	4584.93	2.27	1.49	0.65	0.84	1.31	0.69	4.16	2.47	0.11	4.90	4.98	0.12	x	
4316	Splicing factor U2AF 26 kDa subunit	Q8WU68	69167.68	73303.42	63108.84	45916.80	43400.32	40700.52	55828.39	54649.05	56265.18	55148.67	57159.13	54970.36	53652.42	56832.89	40696.96	51205.51	41578.10	39734.95	44370.82	41647.46	36541.90	39442.77	34665.43	32879.32	30831.05	28522.40	24151.96	34556.18	31501.87	28207.93	36055.27	35712.93	24618.95		
4317	Splicing factor U2AF 35 kDa subunit-like protein	P0DN76	81967.15	80980.50	77635.46	54413.33	50876.02	50100.19	61257.40	59625.38	60167.32	65355.22	64303.78	62693.55	64295.39	63004.24	46340.01	52469.13	50554.60	49514.52	59744.68	57950.80	56322.72	45645.53	46245.53	41079.31	30596.40	30112.30	27627.10	33533.69	31085.86	30359.23	32362.85	31310.78	23884.04		
4318	Splicing factor U2AF 65 kDa subunit	P26368	32179.58	33997.19	27900.01	23942.04	20328.02	21261.48	30836.85	27812.04	26754.16	27032.96	25105.10	22273.61	26506.85	23034.12	16219.30	20519.26	20744.68	19841.97	21320.20	20997.50	18592.90	45203.46	41644.41	40267.98	16653.81	14458.34	13284.46	17849.37	17529.54	16491.55	13493.38	12233.87	7845.54		
4319	Splicing factor_ proline- and glutamine-rich	P23246	183991.11	177443.04	168176.39	125459.98	119538.36	112281.29	138655.73	130177.50	126550.64	139801.27	132724.95	129329.07	141536.78	139346.66	115953.69	121741.53	109143.98	109041.86	132568.13	128800.06	120688.61	85445.91	78482.62	80806.54	70415.32	69753.00	64311.60	76396.40	70724.68	67781.92	83724.62	83952.95	58283.32		
4320	SPOC domain-containing protein 1	Q6ZMY3	22224.71	23463.48	20731.71	15291.19	15041.18	14051.89	21108.26	20456.72	20141.44	18743.73	17800.27	17513.23	19022.96	17705.68	15254.13	14176.71	14121.42	13528.93	17050.83	15260.94	14552.96	2.27	1.49	0.65	0.84	1.31	0.69	4.16	2.47	0.11	4.90	4.98	0.12	x	
4321	Spondin-2	Q9BUD6	46933.01	44880.30	43751.67	35473.37	32705.97	32236.48	40974.85	40001.98	39044.30	41645.73	38028.21	38342.61	41799.27	40862.60	35260.40	31353.01	29219.02	29697.23	35917.71	34506.31	35761.08	2.27	1.49	0.65	0.84	1.31	0.69	4.16	2.47	0.11	4.90	4.98	0.12	x	
4322	Sprouty-related_ EVH1 domain-containing protein 1	Q7Z699	4949.27	4275.56	3981.96	2887.17	3051.68	2888.31	3238.75	2965.93	3080.19	4521.98	4455.89	4411.52	3611.26	3179.32	2738.81	2841.91	3082.63	2865.75	3102.12	2870.18	2651.74	2.27	1.49	0.65	0.84	1.31	0.69	4.16	2.47	0.11	4.90	4.98	0.12	x	
4323	Sprouty-related_ EVH1 domain-containing protein 2	Q7Z698	9506.06	8137.55	7356.96	6486.42	5916.98	5887.65	7442.95	7070.64	5656.36	8197.31	7810.06	7422.96	7473.20	7613.08	5959.03	5649.48	5037.93	5751.12	5095.59	6505.17	5576.28	2.27	1.49	0.65	0.84	1.31	0.69	4.16	2.47	0.11	4.90	4.98	0.12	x	
4324	SPRY domain-containing protein 3	Q8NCJ5	36121.34	35550.83	33438.51	24641.92	23107.51	22863.28	30437.95	27968.37	25989.00	27305.62	26933.78	25474.85	24685.39	28634.66	22910.20	25165.98	25250.54	23667.46	23562.65	26763.60	23566.96	2.27	1.49	0.65	0.84	1.31	0.69	4.16	2.47	0.11	4.90	4.98	0.12	x	
4325	SPRY domain-containing SOCS box protein 1	Q96BD6	11318.78	11150.00	9824.68	4606.66	4820.43	4280.67	7745.03	6994.99	6842.99	5747.55	5427.78	5284.49	7786.14	7571.72	5667.05	5796.41	5701.15	5781.75	5273.02	5150.49	4897.69	6364.46	5808.67	4538.06	3218.63	3078.87	2543.35	6078.38	5663.60	5741.95	4498.86	4125.33	2284.45		
4326	Squalene monooxygenase	Q14534	28404.61	25726.09	22807.00	16134.56	15700.96	15200.88	19653.78	19956.88	17326.56	19630.38	19126.71	16987.73	21771.25	20874.76	13036.07	20243.29	18321.38	16789.85	15032.34	14739.25	13499.97	2.27	1.49	0.65	0.84	1.31	0.69	4.16	2.47	0.11	4.90	4.98	0.12	x	
4327	Squalene synthase	P37268	13776.90	13121.85	14909.41	6568.78	5303.97	5209.47	9400.89	8428.82	8610.24	10345.93	10418.24	8507.76	8792.83	8122.35	6307.21	9842.81	10180.11	10466.41	10950.85	7830.19	7403.27	2.27	1.49	0.65	0.84	1.31	0.69	4.16	2.47	0.11	4.90	4.98	0.12	x	
4328	Squamous cell carcinoma antigen recognized by T-cells 3	Q15020	180398.27	175072.82	163930.61	137594.12	138880.24	119648.26	153892.15	135377.44	130389.41	148816.69	142986.55	136571.57	152874.25	155730.52	119045.95	136073.55	119953.12	124154.01	143697.15	136783.88	129156.18	37446.17	33292.33	31268.36	24968.62	23951.52	21868.26	29534.17	26929.16	25582.79	30945.95	30369.37	21755.34		
4329	SRA stem-loop-interacting RNA-binding protein_ mitochondrial	Q9GZT3	83640.72	76516.92	81462.67	61151.94	55518.87	56058.30	65402.41	66300.13	64494.04	72048.28	64488.28	61815.54	66202.84	65272.71	53154.94	56068.85	52156.15	54037.29	62020.90	57113.63	56173.86	43064.88	41052.34	39276.92	28732.41	25939.50	23287.08	31770.36	31570.61	30153.79	32911.67	29650.77	21591.05		
4330	Src substrate cortactin	Q14247	748629.66	763454.05	706263.17	548718.48	539892.45	523299.66	595303.13	579784.15	554746.87	698336.02	666175.96	620363.09	681680.11	617969.82	508801.60	502625.51	496148.96	482513.47	569739.53	533452.00	520124.58	240290.74	230160.98	212768.11	167647.95	165002.47	156648.86	178227.65	175764.76	170133.97	199171.79	197591.38	104907.72		
4331	SRSF protein kinase 1	Q96SB4	0.14	2.53	0.81	1.07	2.77	0.68	0.96	0.36	0.63	0.35	0.75	0.38	2.13	0.97	0.60	4.64	1.07	0.47	2.22	0.19	0.06	16323.99	15732.74	14567.69	12860.39	12267.92	11834.87	13812.73	13350.56	12889.71	14446.44	13458.75	11519.88		x
4332	SRSF protein kinase 2	P78362	20194.89	16757.24	13274.27	6130.05	5681.58	5643.00	7131.80	6370.79	6500.91	16149.09	14265.06	11828.88	13823.09	6403.94	3699.97	5817.96	6812.45	6901.77	8187.01	7129.10	5873.92	2.27	1.49	0.65	0.84	1.31	0.69	4.16	2.47	0.11	4.90	4.98	0.12	x	
4333	STAM-binding protein	O95630	3824.56	3482.28	3598.38	1316.81	1314.77	943.48	3391.21	3318.45	2133.73	1353.71	1065.03	1581.73	1901.82	1847.02	707.75	1909.96	1914.41	1391.06	1247.66	1414.16	1072.32	4532.85	4102.07	3290.71	2899.79	2957.62	2853.71	3464.55	3121.99	2998.62	3910.60	3812.40	2692.79		
4334	Staphylococcal nuclease domain-containing protein 1	Q7KZF4	649075.88	620078.03	587321.68	530084.90	504582.48	474681.55	566042.56	554380.47	537448.88	630312.41	598507.18	566159.18	554658.41	535664.30	426372.56	505203.31	472545.02	467171.94	496411.48	478796.12	465800.51	421133.19	397020.38	358676.35	282794.91	273027.23	238443.63	308182.11	291467.08	276665.87	319598.41	308312.47	217257.48		
4335	Starch-binding domain-containing protein 1	O95210	754.09	598.39	709.97	785.17	749.59	659.74	495.82	450.03	566.24	912.81	881.35	922.43	1247.87	967.64	1092.42	671.16	573.52	560.07	614.72	554.13	618.41	2.27	1.49	0.65	0.84	1.31	0.69	4.16	2.47	0.11	4.90	4.98	0.12	x	
4336	StAR-related lipid transfer protein 9	Q9P2P6	809757.99	762940.70	693315.81	395015.96	376249.79	335266.40	510716.63	497190.40	459325.07	592328.39	558495.46	516215.60	528665.24	479075.76	351077.66	586312.70	548493.17	519981.13	463834.81	434686.63	396886.46	332750.35	308363.08	281841.66	265489.89	263860.33	228805.92	409686.89	391887.03	368062.72	305342.17	300781.61	205403.47		
4337	START domain-containing protein 10	Q9Y365	193768.95	193821.13	180362.98	126681.15	117249.06	111096.56	150371.96	143427.63	141577.86	164639.31	151132.32	139500.37	156373.84	157262.53	128165.96	125937.72	118096.86	118261.84	143572.99	130368.36	128384.34	2.27	1.49	0.65	0.84	1.31	0.69	4.16	2.47	0.11	4.90	4.98	0.12	x	
4338	Stathmin domain-containing protein 1	H3BQB6	0.14	2.53	0.81	1.07	2.77	0.68	0.96	0.36	0.63	0.35	0.75	0.38	2.13	0.97	0.60	4.64	1.07	0.47	2.22	0.19	0.06	63703.76	59742.75	53204.01	30614.21	30813.88	27130.71	29433.57	28327.79	26971.82	46424.43	45948.41	34147.83		x
4339	Stathmin	P16949	247431.87	240230.88	235336.96	166233.72	157845.61	151366.25	212053.85	207532.13	206429.88	175336.27	174182.84	162701.14	219565.78	195689.76	179676.41	162829.46	151272.63	146535.04	187076.45	175443.70	176160.94	883651.19	805920.11	790057.56	528694.28	520085.55	461209.74	621484.09	604310.49	596942.13	649673.98	664677.12	447471.09		
4340	Stathmin-2	Q93045	7415.31	9335.79	9443.93	6416.94	5959.77	5399.69	7584.87	7044.77	7017.14	8334.78	8589.76	7418.39	6854.45	5466.42	6391.16	6594.08	6489.50	5929.20	7105.52	6568.79	6670.28	759.32	897.51	800.61	923.92	758.25	671.74	1085.53	990.19	840.24	942.63	899.34	628.82		
4341	Stathmin-3	Q9NZ72	0.14	2.53	0.81	1.07	2.77	0.68	0.96	0.36	0.63	0.35	0.75	0.38	2.13	0.97	0.60	4.64	1.07	0.47	2.22	0.19	0.06	7121.42	6156.58	6113.12	4145.84	4193.29	4470.22	5001.58	4572.42	4979.22	4676.89	4820.54	4389.87		x
4342	STE20-like serine/threonine-protein kinase	Q9H2G2	41380.20	35304.59	31255.81	20419.33	19675.77	18045.57	25627.14	23225.12	22326.28	29267.13	27660.50	25266.66	34033.11	25876.32	22100.80	22020.86	20931.83	20334.06	21268.79	20282.67	18800.60	56582.40	55576.29	50793.03	48271.34	46976.09	42255.69	69704.39	65486.14	65670.84	51360.42	49604.30	35908.02		

Supplementary Table S1. Overview on the abundance of all relatively quantified 5180 proteins

			MCF-7																					MDA-MB-231													
	Protein name	UniProt	control_01	control_02	control_03	Dai_IC20_01	Dai_IC20_02	Dai_IC20_03	Dai_SC20_01	Dai_SC20_02	Dai_SC20_03	Gen_IC20_01	Gen_IC20_02	Gen_IC20_03	Gen_SC20_01	Gen_SC20_02	Gen_SC20_03	SSE_IC20_01	SSE_IC20_02	SSE_IC20_03	SSE_SC20_01	SSE_SC20_02	SSE_SC20_03	control_01	control_02	control_03	Dai_IC20_01	Dai_IC20_02	Dai_IC20_03	Gen_IC20_01	Gen_IC20_02	Gen_IC20_03	SSE_IC20_01	SSE_IC20_02	SSE_IC20_03	unique to MCF-7	unique to MDA-MB-231
4343	Sterile alpha motif domain-containing protein 9	Q5K651	103428.76	93559.73	117976.85	66208.64	68605.97	52123.09	90552.99	100909.56	87381.05	95713.95	96959.32	83038.86	105527.19	90261.28	55037.13	77726.64	67118.43	76621.63	92134.92	75495.41	66663.51	98982.05	90038.67	86686.42	61348.75	61868.65	52481.64	75930.62	71423.42	66738.21	73939.80	73532.17	47047.20		
4344	Sterile alpha motif domain-containing protein 9-like	Q8IVG5	21941.00	25806.79	22778.91	18529.60	17887.43	16369.53	25285.98	22322.98	21321.51	24287.35	23145.53	21455.62	22253.98	18036.50	14465.90	20974.33	18835.78	17493.67	18071.37	16516.83	16335.54	112.67	171.49	200.22	363.42	345.68	286.62	357.06	404.97	352.68	425.49	314.48	305.82		
4345	Steroid receptor RNA activator 1	Q9HD15	148149.97	146632.34	141732.67	119437.90	114883.02	112411.67	128789.12	125078.30	122404.26	132107.33	121135.34	113608.90	116521.39	118675.33	107857.38	124072.49	119967.96	118731.25	121226.60	112731.21	109440.48	6547.22	5629.54	5290.59	4475.20	3959.50	3587.43	6454.76	5136.43	5033.67	4730.34	4375.26	2896.36		
4346	Sterol O-acyltransferase 1	P35610	0.14	2.53	0.81	1.07	2.77	0.68	0.96	0.36	0.63	0.35	0.75	0.38	2.13	0.97	0.60	4.64	1.07	0.47	2.22	0.19	0.06	9599.05	8935.54	8519.66	5505.84	5788.49	3845.71	8368.21	7726.91	7382.48	8401.32	7918.67	4363.46		x
4347	Sterol-4-alpha-carboxylate 3-dehydrogenase_ decarboxylating	Q15738	94608.09	92625.32	88157.17	60075.88	60318.20	52968.42	75403.38	71981.94	64469.15	79897.50	72971.93	68593.01	73648.54	70543.57	55434.03	63311.39	59205.80	55110.01	64481.37	62591.62	57457.86	94380.22	90122.51	83029.31	57818.34	57535.75	48562.11	70152.78	66826.47	64417.90	69919.78	68747.34	46926.13		
4348	Stomatin-like protein 2_ mitochondrial	Q9UJZ1	66597.68	60364.76	60130.72	42895.00	41175.08	37973.54	49971.21	45741.49	43356.22	56114.24	55592.01	50255.62	52578.53	48167.19	36908.78	44854.66	40879.29	40749.66	46849.21	43135.44	41504.42	58653.79	56311.54	48132.22	42386.64	42695.09	38182.37	52304.90	50609.73	47267.95	58425.80	54448.14	34035.31		
4349	Stress-70 protein_ mitochondrial	P38646	1999906.09	1954742.19	1839507.21	1467103.18	1420205.30	1332103.46	1673565.86	1598782.51	1536837.80	1657146.62	1603062.00	1492928.10	1638323.46	1521595.99	1327991.99	1437907.57	1363709.46	1343371.57	1470747.09	1399243.02	1323176.56	758815.01	726140.52	662323.30	531688.04	519548.19	481680.33	599509.06	563691.71	553057.66	633082.33	621864.02	511149.25		
4350	Stress-induced-phosphoprotein 1	P31948	1859649.60	1788593.20	1687626.98	1249527.32	1187542.73	1139981.75	1521452.59	1461534.14	1422288.65	1482014.36	1411243.49	1329322.99	1590674.83	1491985.79	1284121.66	1206787.49	1131103.67	1121551.80	1348377.91	1273940.20	1205795.24	1095160.55	1028006.87	955227.04	688040.81	672924.66	619050.37	782210.48	749246.13	730853.55	810619.98	791403.33	581829.36		
4351	Striated muscle preferentially expressed protein kinase	Q15772	101406.86	92819.30	93737.35	80326.86	73791.45	74008.50	94527.64	90778.51	89708.36	90334.69	85927.33	82171.36	87433.20	80851.68	68952.51	80586.25	75282.28	88349.53	75211.06	67963.70	72361.39	134357.02	124752.85	125642.89	98637.75	95516.50	91490.41	96805.17	88308.94	93549.91	90497.34	90280.47	71820.08		
4352	Striatin	O43815	3185.88	3145.35	2911.52	2301.30	1970.82	1681.98	2158.11	1950.33	1764.53	2869.17	2148.42	2133.86	3062.22	2272.79	1844.90	1827.55	1546.59	1674.88	1902.14	1611.43	1577.15	2.27	1.49	0.65	0.84	1.31	0.69	4.16	2.47	0.11	4.90	4.98	0.12	x	
4353	Striatin-3	Q13033	13630.77	13041.50	12736.38	10950.28	9808.13	9705.49	10184.45	9795.32	9528.93	13370.47	12977.26	12669.40	12844.61	12103.44	9490.03	9605.76	8599.16	9032.76	11729.13	10059.94	9758.89	53069.57	51461.03	47582.04	35576.65	36479.82	32909.71	41033.07	40714.85	38836.21	42882.88	40855.07	32603.53		
4354	Striatin-interacting protein 1	Q5VSL9	17314.17	16597.95	16837.50	11113.69	9756.91	9027.52	13781.52	12483.84	11940.36	13446.57	11928.04	11588.61	15437.35	15162.88	11217.17	9519.02	9308.05	8436.41	11861.67	11810.38	12433.24	2.27	1.49	0.65	0.84	1.31	0.69	4.16	2.47	0.11	4.90	4.98	0.12	x	
4355	Striatin-interacting protein 2	Q9ULQ0	19391.86	16424.44	13074.72	11262.45	11318.98	8799.06	14668.77	14466.79	13313.16	16500.14	15847.31	13615.04	16480.28	14824.18	6800.60	12102.03	10382.20	10283.14	12459.19	10671.88	9410.91	9304.13	10652.44	8201.33	5300.53	4920.85	3484.32	5464.99	5784.16	5292.99	6176.07	5570.15	3504.72		
4356	Stromal cell-derived factor 2	Q99470	57243.23	51534.66	46978.75	20132.82	19811.34	17537.83	22114.73	20392.80	19614.27	45351.22	41380.23	38029.68	24981.96	23325.71	18503.73	44166.38	42612.31	42087.13	28253.32	26302.92	23498.25	2.27	1.49	0.65	0.84	1.31	0.69	4.16	2.47	0.11	4.90	4.98	0.12	x	
4357	Stromal cell-derived factor 2-like protein 1	Q9HCN8	52467.16	50367.62	46338.63	34454.64	35195.85	30211.02	38380.40	36981.36	34746.08	42882.62	40789.65	36813.11	40752.13	38106.90	31996.65	34159.13	32873.74	30296.16	37489.36	35334.83	33636.68	26826.09	25769.85	23894.23	16515.58	15172.80	13219.14	19956.60	18545.91	17614.47	22183.19	20807.48	14497.59		
4358	Stromal interaction molecule 1	Q13586	9710.79	8853.66	8436.40	6287.86	5425.44	5357.36	8558.20	8355.14	8369.06	8134.77	8304.83	7503.68	8382.17	7815.96	6418.73	6130.05	5075.80	5114.88	6124.19	5933.65	6580.35	10165.45	13466.31	10713.39	7731.74	7805.17	7338.11	8933.28	8073.44	7960.55	8504.79	8217.88	5350.59		
4359	Stromelysin-2	P09238	12692.29	12045.93	11250.02	10109.03	9596.71	9155.66	14474.08	15042.85	14535.61	12249.25	11313.00	11735.77	11242.59	10662.37	9445.86	9941.06	9312.98	9441.70	9922.50	9849.48	10003.44	16303.92	16359.54	14515.86	9912.18	9568.44	8993.04	8079.41	8046.10	7254.47	6845.40	7026.36	5106.54		
4360	Structural maintenance of chromosomes protein 1A	Q14683	44896.32	41246.09	38363.71	26103.58	25499.61	22568.76	31152.54	30441.18	29216.61	34515.49	33478.79	30935.81	33708.90	32755.74	22497.28	27550.63	25448.18	25741.71	29439.09	26225.58	25001.25	49893.99	43439.39	36304.83	37301.77	34892.13	30814.66	45128.58	41615.29	37020.46	40532.06	37124.04	25374.68		
4361	Structural maintenance of chromosomes protein 1B	Q8NDV3	0.14	2.53	0.81	1.07	2.77	0.68	0.96	0.36	0.63	0.35	0.75	0.38	2.13	0.97	0.60	4.64	1.07	0.47	2.22	0.19	0.06	536.57	568.16	450.06	336.06	304.73	298.06	556.94	622.99	566.36	488.36	444.43	253.49		x
4362	Structural maintenance of chromosomes protein 2	O95347	148064.33	142793.94	139492.86	100952.56	97932.73	95153.23	111226.65	113048.76	110044.16	117443.12	110779.96	107207.53	125807.11	119545.99	102948.78	95663.85	90842.19	90705.93	100717.09	96060.33	93741.23	241578.67	212585.38	209110.94	164709.60	157427.39	143742.39	194858.88	181049.23	176494.93	210341.42	193524.95	136554.07		
4363	Structural maintenance of chromosomes protein 3	Q9UQE7	148610.23	144348.91	138645.31	108759.08	107135.21	104577.52	118326.26	109746.86	109655.82	124659.65	119014.58	115503.31	115662.05	116159.64	105503.24	101233.76	95837.23	95195.13	106177.81	103185.18	97934.42	217088.08	203617.75	186681.61	156230.70	148572.15	143722.24	166873.94	154870.31	151876.79	191657.83	181989.65	110810.91		
4364	Structural maintenance of chromosomes protein 4	Q9NTJ3	180679.31	178855.46	163439.20	101076.40	97043.11	90164.82	145980.87	140704.28	134542.10	128168.24	121808.86	116881.94	128644.05	125429.51	133743.75	135294.67	131533.11	126429.27	125280.69	124573.84	120100.44	320934.45	305160.80	291739.42	227628.29	224482.37	206185.55	270625.11	260435.75	249745.61	274990.58	274211.12	195965.82		
4365	Structural maintenance of chromosomes protein 5	Q8IY18	0.14	2.53	0.81	1.07	2.77	0.68	0.96	0.36	0.63	0.35	0.75	0.38	2.13	0.97	0.60	4.64	1.07	0.47	2.22	0.19	0.06	12539.72	11921.42	10804.88	8187.81	7204.65	6814.50	9056.24	8568.48	7953.13	8959.58	8984.56	6973.06		x
4366	Succinate dehydrogenase [ubiquinone] flavoprotein subunit_ mitochondrial	P31040	139097.14	126158.86	115978.11	90733.40	87608.56	84668.09	104834.80	96295.43	86742.03	107385.00	108264.22	105165.84	108622.06	101319.98	92296.17	77179.81	78821.10	80339.18	95006.36	88453.89	83130.61	176397.57	169090.36	146400.79	119542.77	115525.39	110153.62	132099.46	126726.99	119742.38	133042.33	130339.94	100980.79		
4367	Succinate dehydrogenase [ubiquinone] iron-sulfur subunit_ mitochondrial	P21912	8462.76	7882.30	7594.33	5651.16	5193.05	5265.61	6129.75	6043.19	6145.04	7768.02	8068.51	7151.54	8054.65	7067.28	5993.55	5175.44	4786.04	4792.49	5353.41	5241.93	5133.96	17174.21	17447.10	14995.23	15911.53	15031.15	12763.37	17234.80	15620.20	15116.27	18441.22	18189.45	10363.84		
4368	Succinate dehydrogenase assembly factor 1_ mitochondrial	A6NFY7	7463.91	7068.80	6288.42	5212.31	4494.74	5181.43	4619.17	4902.35	4924.13	6392.73	5875.68	5499.58	5256.12	5286.89	4653.58	4251.58	4016.95	4422.51	4776.65	4698.04	4337.34	2.27	1.49	0.65	0.84	1.31	0.69	4.16	2.47	0.11	4.90	4.98	0.12	x	
4369	Succinate--CoA ligase [ADP/GDP-forming] subunit alpha_ mitochondrial	P53597	41452.32	39744.25	36450.62	29190.35	25133.68	26113.14	29215.97	27286.57	27131.63	36098.76	34343.94	33045.98	36376.45	36257.14	33455.31	27434.15	26697.17	24787.26	27467.70	25700.05	26734.60	13483.01	12935.58	11513.55	4068.37	4080.82	3521.36	4287.12	4448.48	4472.10	4791.85	5216.50	3165.94		
4370	Succinate--CoA ligase [ADP-forming] subunit beta_ mitochondrial	Q9P2R7	9490.89	10627.51	8432.17	7621.19	7193.20	6802.76	7779.35	7452.07	7123.19	9268.08	8591.17	8060.70	8132.94	8442.38	7154.37	7126.83	6624.40	6542.57	7556.92	6504.82	6680.37	6883.64	6477.79	6704.05	4679.41	4761.27	4312.27	6058.24	5337.40	5380.15	6144.72	5479.41	5949.72		
4371	Succinate--CoA ligase [GDP-forming] subunit beta_ mitochondrial	Q96I99	139809.77	132582.21	118537.31	92503.80	91036.01	82924.62	106115.53	94836.94	90203.90	114902.97	107969.41	97815.11	111420.83	111689.38	92418.85	86605.59	86059.45	83485.64	92220.35	89983.49	84267.86	80006.92	73272.56	68296.23	49517.77	48639.42	43619.54	57081.82	54290.87	50051.63	56190.92	56052.49	38201.15		
4372	Succinyl-CoA:3-ketoacid coenzyme A transferase 1_ mitochondrial	P55809	0.14	2.53	0.81	1.07	2.77	0.68	0.96	0.36	0.63	0.35	0.75	0.38	2.13	0.97	0.60	4.64	1.07	0.47	2.22	0.19	0.06	54465.66	42529.67	37680.30	32970.65	32978.34	30237.41	33961.32	32435.79	30510.34	37197.40	36681.88	25551.07		x
4373	Sulfhydryl oxidase 2	Q6ZRP7	123.73	130.21	157.62	121.98	94.42	93.55	127.35	136.72	115.21	120.62	109.43	105.49	113.49	88.29	109.88	138.12	122.28	120.62	62.41	53.46	66.54	2.27	1.49	0.65	0.84	1.31	0.69	4.16	2.47	0.11	4.90	4.98	0.12	x	
4374	Sulfide:quinone oxidoreductase_ mitochondrial	Q9Y6N5	940.02	783.25	662.21	192.91	253.58	254.44	481.83	401.83	449.66	567.15	528.47	453.22	672.52	302.71	261.43	279.95	251.36	259.32	278.50	251.20	239.66	242413.85	234299.32	206171.07	203306.43	208052.63	189676.95	373679.60	382125.31	364678.46	232802.30	239416.60	180441.28		
4375	Sulfotransferase 1A1	P50225	121088.42	116856.34	108755.54	105130.61	106821.16	96571.86	118177.17	108263.17	104008.54	133508.92	123583.53	118709.19	102404.90	89974.68	73098.25	119348.89	112163.37	109140.94	93916.35	89790.11	82290.30	2.27	1.49	0.65	0.84	1.31	0.69	4.16	2.47	0.11	4.90	4.98	0.12	x	
4376	Sulfotransferase 1A2	P50226	24717.17	31674.90	16794.81	18501.55	21935.54	16809.03	20306.71	19220.91	15964.62	26358.54	21171.71	24457.30	27392.84	21866.11	22252.23	18787.36	19074.72	17163.37	23429.75	17029.55	18599.58	2.27	1.49	0.65	0.84	1.31	0.69	4.16	2.47	0.11	4.90	4.98	0.12	x	
4377	Sulfotransferase 1A3	P0DMM9	2461.67	2295.56	1963.25	1636.73	1518.05	1431.94	1851.58	2060.65	1480.37	2600.40	2493.61	2065.02	1946.23	2218.81	1348.06	1722.54	1605.38	1645.81	1994.26	1712.39	1740.24	2.27	1.49	0.65	0.84	1.31	0.69	4.16	2.47	0.11	4.90	4.98	0.12	x	
4378	Sulfotransferase 1C2	O00338	0.14	2.53	0.81	1.07	2.77	0.68	0.96	0.36	0.63	0.35	0.75	0.38	2.13	0.97	0.60	4.64	1.07	0.47	2.22	0.19	0.06	1665.20	1285.67	1267.50	900.58	761.16	634.20	1050.24	922.29	835.11	1113.59	1048.85	660.17		x
4379	Sulfotransferase family cytosolic 2B member 1	O00204	28.32	60.57	89.79	13.67	48.60	94.21	825.04	742.36	950.15	18.20	247.84	148.78	133.47	68.83	60.18	115.47	111.67	135.23	2.22	0.19	0.06	2.27	1.49	0.65	0.84	1.31	0.69	4.16	2.47	0.11	4.90	4.98	0.12	x	
4380	SUMO-activating enzyme subunit 1	Q9UBE0	178178.94	171089.09	142165.37	129529.13	121524.87	111815.24	162244.87	148374.30	140636.34	143297.18	138657.09	131141.90	156723.66	128518.14	71736.39	117393.00	110121.54	107306.17	117899.93	126532.94	115412.49	150476.93	119484.80	115712.15	82894.33	72872.13	64139.23	103739.96	86239.04	86504.35	96548.89	84794.46	55154.70		
4381	SUMO-activating enzyme subunit 2	Q9UBT2	68784.84	73793.06	60708.11	45348.75	45917.09	40161.89	53881.23	53777.85	50414.45	60048.39	56104.77	54929.88	56916.25	53606.68	43717.99	46383.15	43647.77	41748.03	42675.31	41618.76	40275.70	31676.91	31721.27	30041.50	19985.61	19067.47	14711.43	25832.80	25184.04	25056.34	21509.58	22151.18	13552.73		
4382	SUMO-conjugating enzyme UBC9	P63279	210497.02	198750.25	188692.84	127270.08	119887.67	114868.69	157280.45	153327.17	146967.85	154562.16	152738.39	140378.20	161603.67	156155.57	134801.35	127347.47	122281.30	115258.83	126025.34	122829.91	116298.22	66454.19	62939.62	55589.08	34722.59	34131.78	29711.97	44045.79	45864.81	43649.24	46106.04	45486.20	33743.54		
4383	SUMO-specific isopeptidase USPL1	Q5W0Q7	0.14	2.53	0.81	1.07	2.77	0.68	0.96	0.36	0.63	0.35	0.75	0.38	2.13	0.97	0.60	4.64	1.07	0.47	2.22	0.19	0.06	3399.97	3291.54	3082.77	2485.06	2655.06	2364.20	2909.22	2879.48	2842.04	2946.60	3316.22	1979.21		x
4384	Superoxide dismutase [Cu-Zn]	P00441	118354.68	102172.56	85966.04	81538.85	76891.32	67881.84	96151.40	91856.63	80845.43	104135.74	95637.91	82275.95	91432.75	82104.51	55632.29	69920.59	67719.39	62098.45	64918.69	61136.13	54068.34	31326.18	30208.53	23997.17	28119.25	26672.48	23810.66	30235.11	29143.63	26893.38	40409.47	36279.91	23496.80		
4385	Superoxide dismutase [Mn]_ mitochondrial	P04179	0.14	2.53	0.81	1.07	2.77	0.68	0.96	0.36	0.63	0.35	0.75	0.38	2.13	0.97	0.60	4.64	1.07	0.47	2.22	0.19	0.06	33411.28	31645.03	28228.84	22311.42	20722.63	19814.05	25257.69	23719.41	24047.34	22854.03	22478.59	14137.13		x
4386	Suppression of tumorigenicity 5 protein	P78524	42154.07	40859.64	38337.49	30125.77	29512.71	27306.88	33355.53	32350.48	29959.24	37417.63	36115.21	33336.51	34875.07	32943.87	27614.06	31810.43	29884.53	28862.06	31573.24	30320.55	28319.50	4378.08	4351.69	4186.12	2903.44	2999.60	2764.04	4432.39	4031.07	3874.16	3746.60	3441.59	2786.67		
4387	Suppressor of IKBKE 1	Q9BRV8	1484.28	1553.21	1255.18	1363.99	1361.36	1227.21	1069.44	1108.63	1018.92	1892.46	1470.20	1431.25	1278.82	1120.73	1121.29	1205.80	1060.28	892.29	1176.97	1173.02	1148.71	2.27	1.49	0.65	0.84	1.31	0.69	4.16	2.47	0.11	4.90	4.98	0.12	x	
4388	Surfeit locus protein 2	Q15527	30250.95	31622.61	28636.24	19055.28	18413.15	18442.56	23791.95	22743.64	22489.68	21987.95	21624.79	19639.38	24085.47	22732.94	17913.60	19893.83	20002.49	19142.25	20527.77	20125.69	18279.55	8097.71	7517.72	5376.05	4096.84	4503.10	2306.10	5439.61	4556.59	3285.27	6776.21	6822.46	3088.83		
4389	Surfeit locus protein 4	O15260	59598.87	49094.66	38306.82	32487.71	29563.22	22044.27	41438.82	39479.96	34055.42	43381.98	39080.57	29743.74	46475.98	39384.42	17039.45	32290.29	27865.67	23214.42	26309.61	24105.90	19868.63	39107.72	35501.33	31346.23	25707.07	21638.22	18048.21	33281.41	29849.03	28459.44	30216.64	29618.79	17511.00		
4390	Surfeit locus protein 6	O75683	813.09	1001.38	918.35	789.27	493.80	678.45	649.31	644.97	547.27	875.61	890.86	854.93	881.14	734.53	1079.51	428.56	516.12	586.76	673.73	703.63	768.07	2.27	1.49	0.65	0.84	1.31	0.69	4.16	2.47	0.11	4.90	4.98	0.12	x	
4391	SURP and G-patch domain-containing protein 1	Q8IWZ8	238685.11	239872.14	239868.02	180908.87	171283.16	169974.74	196167.18	201687.81	197532.75	205792.47	198733.75	192428.15	185609.13	181255.65	164414.96	197841.82	192292.00	187689.95	182968.67	172608.76	167218.45	290104.63	274772.31	261453.38	196223.89	189925.72	182950.40	244744.68	231412.85	231373.96	281617.78	270273.43	177691.53		
4392	Survival motor neuron protein	Q16637	343000.75	335873.34	339681.09	271025.51	259137.36	253164.76	274251.18	277398.46	266539.66	331788.93	294993.77	295426.01	296755.52	303854.23	272366.25	259772.60	234472.77	242523.79	254391.28	247537.02	247742.23	2.27	1.49	0.65	0.84	1.31	0.69	4.16	2.47	0.11	4.90	4.98	0.12	x	
4393	Survival of motor neuron-related-splicing factor 30	O75940	5136.96	5147.29	5094.78	2408.40	2444.90	2386.34	3743.16	3731.55	3563.28	3266.20	2676.15	2617.90	3066.97	3026.45	2568.70	2343.41	2129.51	2270.55	3495.92	2905.88	2935.94	6969.64	6952.31	6377.56	3856.61	3536.15	2936.46	6012.52	5648.18	5276.45	6913.56	6081.46	3884.38		
4394	Sushi repeat-containing protein SRPX2	O60687	4158.97	4119.59	3086.51	2514.48	2391.97	1762.57	3520.82	3475.38	3012.13	3425.93	3216.24	2891.13	3541.11	3389.93	1662.38	2449.10	2307.34	2167.05	2586.86	2385.69	2253.89	2.27	1.49	0.65	0.84	1.31	0.69	4.16	2.47	0.11	4.90	4.98	0.12	x	
4395	SUZ domain-containing protein 1	Q7Z422	2954.28	2427.15	2701.50	2335.50	2114.10	2129.77	2313.72	2360.58	2195.85	2460.51	2368.51	2296.88	2494.51	2554.74	2354.05	2073.82	1899.18	1980.07	2330.09	2192.79	1966.51	11808.56	9501.51	9545.42	6450.56	6190.59	5346.08	10285.86	8816.00	8450.25	9268.10	9624.84	6258.91		
4396	SWI/SNF complex subunit SMARCC1	Q92922	0.14	2.53	0.81	1.07	2.77	0.68	0.96	0.36	0.63	0.35	0.75	0.38	2.13	0.97	0.60	4.64	1.07	0.47	2.22	0.19	0.06	16566.24	16118.54	14614.27	7957.66	7747.21	6089.51	10583.54	10188.72	9704.03	10157.29	9671.74	6515.20		x
4397	SWI/SNF complex subunit SMARCC2	Q8TAQ2	0.14	2.53	0.81	1.07	2.77	0.68	0.96	0.36	0.63	0.35	0.75	0.38	2.13	0.97	0.60	4.64	1.07	0.47	2.22	0.19	0.06	7809.11	6991.08	7487.68	5257.41	5003.84	4318.54	6821.67	6641.31	5654.24	5930.02	6452.34	3477.12		x
4398	SWI/SNF-related matrix-associated actin-dependent regulator of chromatin subfamily A containing DEAD/H box 1	Q9H4L7	52326.69	47387.24	45056.28	31262.46	29956.49	27644.42	36551.93	35752.19	32654.24	43112.95	41020.37	38222.56	46561.49	38648.19	29873.77	30422.83	28360.68	28030.24	34669.87	29359.58	29949.93	81738.75	75451.02	69182.55	59889.46	59909.88	56256.40	65322.67	64160.11	63729.27	72290.69	72478.87	8854.63		
4399	SWI/SNF-related matrix-associated actin-dependent regulator of chromatin subfamily A member 5	O60264	57684.48	57766.74	52709.77	38565.70	38620.39	34651.97	45154.92	44419.76	42431.53	45531.91	44742.75	41786.48	45379.45	45305.50	37161.42	37709.04	36375.95	35763.64	44512.19	43308.95	39657.44	8659.64	17862.85	17771.91	6495.93	6239.72	5817.53	7268.87	6870.72	7123.10	7194.35	6909.22	5905.09		
4400	SWI/SNF-related matrix-associated actin-dependent regulator of chromatin subfamily A-like protein 1	Q9NZC9	29959.49	28156.82	26142.50	18728.72	19086.53	16964.92	25882.12	23418.54	21458.96	27175.75	24954.20	22749.58	22776.20	20206.34	18799.21	22326.71	20640.79	18261.87	21822.10	21516.96	13385.81	2.27	1.49	0.65	0.84	1.31	0.69	4.16	2.47	0.11	4.90	4.98	0.12	x	
4401	SWI/SNF-related matrix-associated actin-dependent regulator of chromatin subfamily D member 1	Q96GM5	11300.13	9984.44	10170.33	7879.87	7844.70	7345.32	8426.85	8574.46	8505.95	10110.12	9823.30	9916.09	9596.91	8940.50	7420.63	7810.38	7141.75	7136.86	7438.60	7378.47	7407.82	2.27	1.49	0.65	0.84	1.31	0.69	4.16	2.47	0.11	4.90	4.98	0.12	x	
4402	SWI/SNF-related matrix-associated actin-dependent regulator of chromatin subfamily D member 3	Q6STE5	394.17	267.01	262.66	183.04	234.55	218.86	281.30	170.79	222.94	198.28	218.60	210.71	189.05	266.03	183.44	255.15	325.23	286.15	225.88	148.71	206.57	44850.32	40001.63	37277.50	29863.66	27302.19	18629.87	35003.26	32068.86	27598.35	43668.81	40803.70	23905.53		
4403	SWI/SNF-related matrix-associated actin-dependent regulator of chromatin subfamily E member 1	Q969G3	0.14	2.53	0.81	1.07	2.77	0.68	0.96	0.36	0.63	0.35	0.75	0.38	2.13	0.97	0.60	4.64	1.07	0.47	2.22	0.19	0.06	13586.34	12987.86	12198.32	9811.60	9254.74	8663.81	12005.26	12017.90	10997.59	11049.77	11167.64	8073.16		x
4404	Synapse differentiation-inducing gene protein 1	Q9H7V2	6466.40	6272.42	5838.70	4826.53	4541.41	4554.07	4340.62	4185.13	4120.12	6232.83	5765.08	5715.89	5991.24	5432.81	4991.33	4425.85	3977.54	4627.53	4449.50	3936.23	3735.45	2.27	1.49	0.65	0.84	1.31	0.69	4.16	2.47	0.11	4.90	4.98	0.12	x	
4405	Synapse-associated protein 1	Q96A49	84985.28	84029.82	79873.21	72050.70	71001.30	65444.90	62067.76	58060.25	59648.46	87526.88	82108.26	76791.81	74951.02	75188.84	54888.28	62517.48	59076.92	59118.21	65169.25	61197.90	55452.74	4383.33	4470.85	3955.95	3280.43	3234.91	3231.24	4192.95	4163.30	4178.11	4334.93	4566.97	3444.20		
4406	Synapsin-3	O14994	2924.17	2114.63	1434.48	3113.33	3065.96	1768.74	2636.68	2330.35	1915.07	3886.31	3327.85	2122.02	2546.61	1887.95	388.43	2838.32	2715.33	2227.17	2638.96	2421.06	1424.46	433.94	802.71	267.13	173.97	126.02	46.36	497.69	392.97	279.78	651.31	609.08	169.37		
4407	Synaptic functional regulator FMR1	Q06787	247775.68	223865.29	196232.74	155895.40	149576.28	132001.41	195963.87	193968.95	174491.43	220008.93	209520.52	186448.83	222787.58	204299.51	117575.68	157610.47	153515.45	138214.62	154850.19	145482.13	131371.84	22078.38	16289.04	13772.67	12587.35	12227.41	8768.88	18273.51	16209.20	12508.86	19208.16	16154.87	10782.59		
4408	Synaptic vesicle 2-related protein	Q8N4V2	373.73	404.73	323.46	773.68	733.16	840.82	622.56	671.75	720.00	381.98	387.18	418.52	634.73	569.67	50.32	621.49	506.33	468.77	698.88	646.85	518.36	2.27	1.49	0.65	0.84	1.31	0.69	4.16	2.47	0.11	4.90	4.98	0.12	x	
4409	Synaptic vesicle membrane protein VAT-1 homolog	Q99536	4650.14	4632.29	4566.75	3652.01	3767.65	3394.37	4432.07	4391.01	4407.08	4650.26	4766.69	4543.14	3961.29	4517.44	3315.06	3945.44	3983.47	3609.80	3360.38	3634.57	3357.03	206531.51	196325.90	190304.87	144574.72	141792.00	124465.49	158411.35	148644.81	147824.25	172205.64	166625.81	119866.97		
4410	Synaptic vesicle membrane protein VAT-1 homolog-like	Q9HCJ6	0.14	2.53	0.81	1.07	2.77	0.68	0.96	0.36	0.63	0.35	0.75	0.38	2.13	0.97	0.60	4.64	1.07	0.47	2.22	0.19	0.06	1625.08	1639.75	1592.34	1056.29	1070.85	999.15	1433.05	1364.28	1302.29	1608.61	1586.28	1180.39		x
4411	Synaptobrevin homolog YKT6	O15498	36569.62	33689.35	33562.04	26182.99	25421.26	23835.61	33692.32	31258.59	31831.08	34094.26	33529.17	31855.77	35767.63	31623.18	29712.92	29507.75	26832.21	26863.93	32231.33	30997.97	28406.02	2.27	1.49	0.65	0.84	1.31	0.69	4.16	2.47	0.11	4.90	4.98	0.12	x	
4412	Synaptogyrin-1	O43759	0.14	2.53	0.81	1.07	2.77	0.68	0.96	0.36	0.63	0.35	0.75	0.38	2.13	0.97	0.60	4.64	1.07	0.47	2.22	0.19	0.06	29265.25	29669.56	25333.58	22056.11	21053.39	20208.44	23122.86	22184.54	22238.22	23696.00	21475.07	85.05		x
4413	Synaptogyrin-2	O43760	112070.25	102649.82	88346.98	68538.71	66752.62	56316.52	81010.47	77270.36	71023.44	85769.77	79256.99	71196.18	95813.83	86439.25	51777.72	62156.36	59874.90	53024.02	85926.99	70328.26	60564.94	9152.46	7341.11	7790.12	5510.72	5079.71	4864.57	6910.23	6546.04	6882.85	6046.38	6120.95	4952.02		
4414	Synaptojanin-2-binding protein	P57105	11502.86	10733.55	10163.52	8160.68	7701.11	7752.33	7585.79	7066.51	6575.84	10231.75	10204.42	8340.97	8425.34	8348.51	7435.19	7278.32	7107.59	7006.30	7175.42	7131.01	6273.06	3759.18	3518.23	3188.66	2284.90	2242.75	1761.59	3022.45	2807.14	2430.39	2740.93	2785.02	1570.55		
4415	Synaptonemal complex central element protein 1	Q8N0S2	11022.66	10781.66	9391.27	6811.70	6585.89	6441.65	7198.18	6915.11	7173.35	10187.73	9658.26	9705.16	9466.07	9130.56	6505.07	6372.99	6304.93	6141.28	7153.21	5524.96	6288.69	9665.55	14310.15	10464.10	9924.98	10471.11	10157.10	7898.14	9363.49	9429.91	13186.08	12319.04	6829.10		
4416	Synaptonemal complex central element protein 2	Q6PIF2	29855.31	29282.06	28789.39	20213.98	19056.13	19057.46	25876.58	24304.03	24413.39	23755.42	22938.78	21809.77	21667.08	22457.83	18748.46	19505.53	18633.92	18175.71	19480.17	18732.32	17927.14	2.27	1.49	0.65	0.84	1.31	0.69	4.16	2.47	0.11	4.90	4.98	0.12	x	
4417	Synaptonemal complex central element protein 3	A1L190	242544.50	217865.52	228937.04	168370.93	155429.91	152444.65	208010.60	185158.05	181829.85	207795.86	198998.98	188922.96	198688.47	186747.10	149289.39	175035.21	172001.31	161047.78	190195.90	182142.44	162745.98	2.27	1.49	0.65	0.84	1.31	0.69	4.16	2.47	0.11	4.90	4.98	0.12	x	
4418	Synaptonemal complex protein 2-like	Q5T4T6	0.14	2.53	0.81	1.07	2.77	0.68	0.96	0.36	0.63	0.35	0.75	0.38	2.13	0.97	0.60	4.64	1.07	0.47	2.22	0.19	0.06	26091.61	25657.59	20735.53	20395.68	19130.08	14419.37	22028.14	20794.37	18768.69	22390.94	21679.65	14214.71		x
4419	Synaptophysin-like protein 1	Q16563	127181.86	123247.74	120058.92	101753.93	95425.14	96666.82	94152.81	96541.80	90769.70	116854.38	109327.66	106019.11	102988.21	103751.44	91675.00	102300.48	93851.85	91916.53	82503.43	82449.66	79810.69	2.27	1.49	0.65	0.84	1.31	0.69	4.16	2.47	0.11	4.90	4.98	0.12	x	
4420	Synaptosomal-associated protein 23	O00161	11801.46	11298.21	10805.50	7511.35	8099.97	7550.84	8486.16	7879.15	7463.45	9376.17	8955.41	8586.28	8620.67	9171.54	6749.51	8695.07	7960.51	7978.11	7853.16	6900.14	5997.60	2293.60	2129.12	2099.41	1157.08	1304.78	972.04	1356.60	1367.42	1371.36	1930.08	1978.33	1279.07		
4421	Synaptosomal-associated protein 29	O95721	69809.90	66402.34	59052.07	51139.50	48412.77	43962.10	58002.02	54708.74	51722.42	61227.48	57858.28	54524.22	61836.28	57172.12	41350.01	50632.97	48549.20	48660.07	53480.89	52076.33	46687.50	11988.16	10924.48	10621.23	8032.21	8145.12	6586.60	9213.22	8542.85	8335.15	10875.98	10211.56	6788.38		
4422	Synaptotagmin-16	Q17RD7	375.30	618.04	442.67	358.66	282.40	262.78	578.88	433.76	291.70	345.94	268.93	303.53	207.22	283.09	183.85	416.39	437.15	560.74	528.39	443.04	406.92	2.27	1.49	0.65	0.84	1.31	0.69	4.16	2.47	0.11	4.90	4.98	0.12	x	
4423	Synaptotagmin-like protein 2	Q9HCH5	8666.13	8791.77	7833.52	1853.26	1925.76	1628.75	2086.14	2071.41	2157.82	3081.94	2940.09	2839.70	5848.25	4707.36	4105.76	1802.22	1835.18	1787.86	2278.23	2106.03	1888.04	2.27	1.49	0.65	0.84	1.31	0.69	4.16	2.47	0.11	4.90	4.98	0.12	x	
4424	Synaptotagmin-like protein 4	Q96C24	38423.71	38664.43	37994.77	25912.85	25937.06	23522.28	34497.35	32687.14	30696.61	30539.87	30246.83	28431.44	31048.24	30772.17	26997.46	24717.92	23619.30	24332.30	26505.23	25470.76	23368.14	2.27	1.49	0.65	0.84	1.31	0.69	4.16	2.47	0.11	4.90	4.98	0.12	x	
4425	Syndecan-1	P18827	29163.63	26947.67	26162.66	22791.24	20009.38	20121.07	18316.02	19921.74	18962.62	24524.84	22690.64	21063.61	23692.64	22992.01	20565.82	17072.68	14587.42	15961.54	19007.66	18195.00	17167.56	2.27	1.49	0.65	0.84	1.31	0.69	4.16	2.47	0.11	4.90	4.98	0.12	x	
4426	Syndetin	Q96JG6	0.14	2.53	0.81	1.07	2.77	0.68	0.96	0.36	0.63	0.35	0.75	0.38	2.13	0.97	0.60	4.64	1.07	0.47	2.22	0.19	0.06	10512.30	3717.72	1618.10	5500.72	4898.32	3499.58	5972.85	5592.78	3695.79	5458.91	4832.31	2886.20		x
4427	Synembryn-A	Q9NPQ8	145700.11	119475.31	92048.45	99483.68	99368.26	79310.25	111287.14	107792.13	94148.21	124552.68	119132.61	94701.51	130210.98	122463.87	60607.68	87108.73	86237.62	72907.47	110256.05	100910.71	86596.60	115830.38	108241.02	94586.68	70245.34	69679.50	47608.52	84695.98	76111.28	65889.69	91202.62	86803.76	51194.05		
4428	Synemin	O15061	35868.06	34026.16	31481.68	26125.51	26650.96	25260.78	31797.49	30142.22	29329.89	33283.07	31056.69	30199.19	31590.17	30382.86	26414.67	24838.32	24604.85	24238.51	25072.72	25283.28	25569.77	17196.56	16379.67	12084.83	10964.01	11083.85	10938.93	11711.81	10094.85	8958.23	20129.22	20125.95	13665.59		
4429	Syntabulin	Q9NX95	0.14	2.53	0.81	1.07	2.77	0.68	0.96	0.36	0.63	0.35	0.75	0.38	2.13	0.97	0.60	4.64	1.07	0.47	2.22	0.19	0.06	98002.59	92135.61	87429.57	127311.54	126873.01	121435.24	181480.91	173104.46	169597.27	102885.58	100609.23	80628.08		x
4430	Syntaxin-10	O60499	20425.86	18329.39	13534.78	1209.77	524.18	389.35	829.10	391.15	240.49	17870.04	15246.37	13908.42	16966.84	1446.21	140.85	705.32	303.93	410.41	1659.18	2038.56	1093.34	50105.99	16991.14	14917.58	35080.02	34391.99	18854.66	40409.01	37370.45	21989.98	52541.22	48939.70	0.12		
4431	Syntaxin-11	O75558	8321.33	8381.33	7544.39	8079.52	7682.18	7631.35	5897.33	6639.87	6127.97	9290.71	9738.43	9408.08	8133.82	6059.79	386.32	5370.28	8432.36	8713.55	8088.91	7194.32	6663.81	1097.57	962.40	1082.91	987.78	845.83	831.09	1068.98	963.19	981.21	969.67	1023.26	691.81		
4432	Syntaxin-12	Q86Y82	6183.39	6714.69	5628.40	4248.96	4119.81	3735.20	4590.42	4710.83	4630.19	5964.53	5833.83	5263.27	5202.87	4701.73	3983.19	4131.19	4549.09	4129.79	4223.66	3920.40	3473.52	2601.75	2326.87	1704.05	1387.91	1509.71	1194.07	1823.34	1963.10	1739.75	1951.04	1782.51	1099.64		
4433	Syntaxin-16	O14662	23604.44	21249.01	20212.49	14860.64	13919.44	12003.96	16984.26	16399.97	15549.70	19526.97	18400.08	16334.99	17602.69	15964.47	9465.28	16048.27	14798.96	14369.90	16787.94	15546.94	13435.31	2.27	1.49	0.65	0.84	1.31	0.69	4.16	2.47	0.11	4.90	4.98	0.12	x	
4434	Syntaxin-17	P56962	21291.30	21524.81	18757.16	17360.75	16423.13	15355.16	18664.72	17115.35	17528.12	20149.57	18134.68	18739.78	18504.16	18682.35	16912.13	16426.44	14644.38	15008.67	18775.83	17409.17	16791.65	2.27	1.49	0.65	0.84	1.31	0.69	4.16	2.47	0.11	4.90	4.98	0.12	x	
4435	Syntaxin-19	Q8N4C7	513.11	474.21	375.10	462.21	516.10	472.66	770.07	696.23	703.72	867.12	735.49	765.45	843.16	744.30	735.79	432.33	420.85	477.09	437.20	409.97	369.37	2.27	1.49	0.65	0.84	1.31	0.69	4.16	2.47	0.11	4.90	4.98	0.12	x	
4436	Syntaxin-1B	P61266	0.14	2.53	0.81	1.07	2.77	0.68	0.96	0.36	0.63	0.35	0.75	0.38	2.13	0.97	0.60	4.64	1.07	0.47	2.22	0.19	0.06	17439.61	18501.92	15712.44	14194.50	14113.79	13964.87	12025.56	12306.02	12040.57	12630.37	11931.46	3546.01		x
4437	Syntaxin-4	Q12846	91059.20	93019.95	86306.80	66822.24	62345.36	58801.74	64195.62	61334.46	62098.66	90668.24	85849.16	84483.02	82880.86	79821.06	68176.22	57232.66	58311.53	56484.13	58779.79	57942.67	55680.04	2.27	1.49	0.65	0.84	1.31	0.69	4.16	2.47	0.11	4.90	4.98	0.12	x	
4438	Syntaxin-5	Q13190	343965.52	318128.69	319496.33	350641.48	326686.82	314577.89	318494.30	298409.83	294092.42	333138.23	329668.07	301180.29	349391.12	322986.85	302163.19	327141.51	320076.27	309982.17	321911.60	321615.00	312421.30	2.27	1.49	0.65	0.84	1.31	0.69	4.16	2.47	0.11	4.90	4.98	0.12	x	
4439	Syntaxin-6	O43752	21417.06	22090.13	22098.25	17855.56	17378.27	17051.89	18698.23	17186.50	16324.66	24801.18	22854.04	20437.49	20567.53	19984.94	16684.20	17026.28	17705.23	17960.25	15385.18	18298.84	16166.07	2.27	1.49	0.65	0.84	1.31	0.69	4.16	2.47	0.11	4.90	4.98	0.12	x	
4440	Syntaxin-7	O15400	81892.31	78736.87	75882.89	58813.47	56430.83	53108.03	66152.05	64995.69	62813.01	74420.58	70803.42	66138.02	73465.83	68702.79	57218.79	58945.25	55962.87	53533.40	52730.65	49854.18	49254.92	35316.31	34504.29	32092.67	26081.46	25448.28	22786.22	28516.78	28569.47	26811.21	30932.36	29625.53	18465.19		
4441	Syntaxin-binding protein 1	P61764	444842.73	426813.21	374515.25	317898.78	347213.53	329709.98	351223.70	308807.66	300105.15	382121.75	377793.44	366208.76	339633.14	335227.84	362900.26	338378.69	329756.91	289454.02	361340.88	352011.48	337142.36	2.27	1.49	0.65	0.84	1.31	0.69	4.16	2.47	0.11	4.90	4.98	0.12	x	
4442	Syntaxin-binding protein 2	Q15833	43243.89	41206.58	34568.01	28920.11	28169.79	26168.04	38905.59	38402.61	38737.38	37414.68	35376.10	34090.02	34270.35	33119.80	26159.19	30739.35	30445.49	29231.87	27015.37	27127.93	26790.51	52451.24	49847.05	45871.24	35387.37	34630.21	29983.82	36481.75	35953.35	33954.61	32552.89	31870.26	21381.62		
4443	Syntaxin-binding protein 3	O00186	7209.43	6947.19	6303.35	4311.63	4262.37	4067.84	7018.42	7115.31	7337.78	5895.28	6026.53	4922.08	5480.01	5274.65	4514.02	4996.78	4902.59	4296.16	4490.02	4291.00	4084.06	2.27	1.49	0.65	0.84	1.31	0.69	4.16	2.47	0.11	4.90	4.98	0.12	x	
4444	Syntenin-1	O00560	15001.78	14084.99	13505.96	9741.96	9585.28	9518.62	13212.82	12631.12	12282.01	14618.32	14420.29	14212.15	12808.31	11445.25	8810.81	11049.93	10564.84	10433.13	12924.31	11918.08	11041.71	2.27	1.49	0.65	0.84	1.31	0.69	4.16	2.47	0.11	4.90	4.98	0.12	x	
4445	T cell receptor alpha constant	P01848	3476.03	3969.16	3475.64	2911.50	2810.92	2659.21	3276.57	3123.12	3241.58	3538.43	3556.80	3357.28	3279.07	2808.45	2634.49	2792.93	2609.93	2518.33	3004.90	2818.34	3060.69	2.27	1.49	0.65	0.84	1.31	0.69	4.16	2.47	0.11	4.90	4.98	0.12	x	
4446	T cell receptor alpha variable 12-2	A0A075B	0.14	2.53	0.81	1.07	2.77	0.68	0.96	0.36	0.63	0.35	0.75	0.38	2.13	0.97	0.60	4.64	1.07	0.47	2.22	0.19	0.06	263.63	267.85	260.01	43.29	31.09	12.82	38.46	58.96	37.98	37.08	17.49	9.85		x
4447	T cell receptor beta variable 12-4	A0A0B4J	0.14	2.53	0.81	1.07	2.77	0.68	0.96	0.36	0.63	0.35	0.75	0.38	2.13	0.97	0.60	29.73	18.44	2.60	2.22	0.19	0.06	2.27	1.49	0.65	0.84	1.31	0.69	4.16	2.47	0.11	4.90	4.98	0.12	x	
4448	T cell receptor beta variable 7-9	P04435	0.14	2.53	0.81	1.07	2.77	0.68	0.96	0.36	0.63	0.35	0.75	0.38	2.13	0.97	0.60	4.64	1.07	0.47	2.22	0.19	0.06	11624.49	3092.71	2094.94	6094.36	4641.75	2196.28	9569.42	7458.78	4472.51	10002.18	8706.96	4097.71		x
4449	T cell receptor gamma constant 2	P03986	0.14	2.53	0.81	1.07	2.77	0.68	0.96	0.36	0.63	0.35	0.75	0.38	2.13	0.97	0.60	4.64	1.07	0.47	2.22	0.19	0.06	49098.34	48080.85	45379.14	32709.01	33021.70	28948.61	41545.20	39424.49	37848.75	38728.97	35804.72	26939.02		x
4450	Tail-anchored protein insertion receptor WRB	O00258	0.14	2.53	0.81	1.07	2.77	0.68	0.96	0.36	0.63	0.35	0.75	0.38	2.13	0.97	0.60	4.64	1.07	0.47	2.22	0.19	0.06	123.91	133.98	111.86	0.84	1.31	0.69	90.25	37.06	12.84	4.90	4.98	0.12		x
4451	Talin-1	Q9Y490	301301.23	282355.36	257645.26	187085.27	184832.53	169117.50	219707.20	210186.05	204564.15	244852.88	236047.60	218759.14	233688.41	220101.36	176401.80	200332.96	193853.22	191236.57	197707.82	185903.72	174144.98	659845.26	612322.99	567763.92	422025.79	409357.96	373282.79	505255.61	484537.70	464593.63	489882.60	480742.86	307272.28		
4452	Talin-2	Q9Y4G6	0.14	2.53	0.81	1.07	2.77	0.68	0.96	0.36	0.63	0.35	0.75	0.38	2.13	0.97	0.60	4.64	1.07	0.47	2.22	0.19	0.06	43188.33	35560.64	31545.71	21479.05	20431.18	15797.70	43465.72	37902.53	24809.87	37182.54	32783.73	18751.70		x
4453	TAR DNA-binding protein 43	Q13148	68946.63	66549.67	65456.09	44078.48	40456.56	37006.17	48429.90	47205.84	43336.75	55302.81	54896.87	55385.92	58310.72	48281.43	39716.31	34277.95	33486.77	37751.44	50058.11	47370.53	44663.70	86252.38	84857.48	74635.45	66579.44	67591.57	65038.38	62879.50	65896.18	63728.94	73078.91	65214.48	60708.94		
4454	Target of EGR1 protein 1	Q96GM8	11220.10	10239.25	10982.18	9288.08	8874.59	8652.36	8142.88	8370.63	8280.65	11594.69	10656.10	10410.89	10130.37	8924.32	7022.06	7523.36	7144.64	7937.71	6467.16	6094.57	5688.02	2.27	1.49	0.65	0.84	1.31	0.69	4.16	2.47	0.11	4.90	4.98	0.12	x	

Supplementary Table S1. Overview on the abundance of all relatively quantified 5180 proteins

			MCF-7																					MDA-MB-231													
	Protein name	UniProt	control_01	control_02	control_03	Dai_IC20_01	Dai_IC20_02	Dai_IC20_03	Dai_SC20_01	Dai_SC20_02	Dai_SC20_03	Gen_IC20_01	Gen_IC20_02	Gen_IC20_03	Gen_SC20_01	Gen_SC20_02	Gen_SC20_03	SSE_IC20_01	SSE_IC20_02	SSE_IC20_03	SSE_SC20_01	SSE_SC20_02	SSE_SC20_03	control_01	control_02	control_03	Dai_IC20_01	Dai_IC20_02	Dai_IC20_03	Gen_IC20_01	Gen_IC20_02	Gen_IC20_03	SSE_IC20_01	SSE_IC20_02	SSE_IC20_03	unique to MCF-7	unique to MDA-MB-231
4455	Target of Myb protein 1	O60784	1700.78	1834.72	1515.17	1026.98	1129.15	891.15	1193.31	1185.90	1139.73	1632.17	1463.91	1647.54	1290.14	1224.34	1086.09	1538.26	1411.73	1242.22	1106.64	824.66	880.94	2.27	1.49	0.65	0.84	1.31	0.69	4.16	2.47	0.11	4.90	4.98	0.12	x	
4456	Target of rapamycin complex subunit LST8	Q9BVC4	13537.55	12495.59	12978.86	6808.50	6643.46	6876.18	8489.31	8234.10	7721.31	11078.18	9659.21	10296.74	9376.55	10212.52	8131.25	9813.85	9230.87	8996.95	9634.69	8178.62	8462.97	2.27	1.49	0.65	0.84	1.31	0.69	4.16	2.47	0.11	4.90	4.98	0.12	x	
4457	TATA element modulatory factor	P82094	258093.87	250698.89	238839.59	128641.98	125569.80	118460.48	223528.39	220503.27	214211.54	173567.70	179977.10	153856.90	180007.57	159126.50	147125.40	165169.10	161327.10	154298.46	156817.69	142621.75	130011.69	2.27	1.49	0.65	0.84	1.31	0.69	4.16	2.47	0.11	4.90	4.98	0.12	x	
4458	TATA-binding protein-associated factor 2N	Q92804	39144.82	38406.62	36446.89	24950.39	24266.36	22553.77	27526.58	25974.99	24707.68	37946.84	35614.32	32370.26	33268.62	30450.89	25953.27	30229.28	29929.13	26421.04	27421.80	27828.81	24514.88	15975.41	15706.99	14003.35	12573.12	12175.20	10799.13	17819.56	16893.72	15782.08	17905.61	15779.94	9678.49		
4459	Tau-tubulin kinase 1	Q5TCY1	0.14	2.53	0.81	1.07	2.77	0.68	0.96	0.36	0.63	0.35	0.75	0.38	2.13	0.97	0.60	4.64	1.07	0.47	2.22	0.19	0.06	5847.87	5692.95	5857.73	4797.08	4761.63	3998.56	5336.78	4675.72	4896.81	4233.51	5231.69	3076.19		x
4460	Tax1-binding protein 1	Q86VP1	8148.89	8102.03	8042.49	5111.44	5027.49	4730.07	5999.15	6660.29	6439.96	7255.95	6692.45	6286.45	6076.51	5674.91	4138.35	6363.51	6157.98	5588.70	6054.94	5728.85	5132.24	202.65	214.44	141.22	101.64	107.12	99.40	136.16	139.33	122.84	88.07	84.37	82.73		
4461	TBC domain-containing protein kinase-like protein	Q8TEA7	0.14	2.53	0.81	1.07	2.77	0.68	0.96	0.36	0.63	0.35	0.75	0.38	2.13	0.97	0.60	4.64	1.07	0.47	2.22	0.19	0.06	2359.84	1865.06	1874.49	1215.24	1175.73	742.71	2086.99	2012.96	1737.49	1897.88	1682.24	801.78		x
4462	TBC1 domain family member 1	Q86TI0	141640.27	138603.75	138990.59	103945.69	98854.53	96623.53	108439.92	107300.32	106869.12	126343.71	114757.83	111753.78	134544.03	131730.10	115722.49	94329.87	86666.45	85691.61	109343.33	102494.16	100328.41	1449.29	1225.98	1097.68	1004.82	1116.00	1017.47	1122.99	1078.18	1047.70	1467.08	1296.62	942.64		
4463	TBC1 domain family member 10A	Q9BXI6	5987.63	4824.92	5356.47	2646.93	2801.40	2339.60	3944.20	3744.19	3348.95	4468.56	4769.62	4344.63	3796.98	4308.68	3665.29	2679.68	3248.28	2819.38	3567.48	3451.80	3072.44	2.27	1.49	0.65	0.84	1.31	0.69	4.16	2.47	0.11	4.90	4.98	0.12	x	
4464	TBC1 domain family member 10B	Q4KMP7	36511.05	34641.21	32632.15	22891.27	21393.06	20903.57	25219.50	23899.23	23193.85	28651.74	28472.26	27409.79	29772.57	25176.16	22560.83	22609.33	20460.36	22776.20	26392.94	22623.63	20940.63	2.27	1.49	0.65	0.84	1.31	0.69	4.16	2.47	0.11	4.90	4.98	0.12	x	
4465	TBC1 domain family member 13	Q9NVG8	2733.42	2667.39	2753.33	2260.35	2693.52	2141.03	1813.01	2221.07	1775.53	2795.88	2633.41	2420.19	2406.65	2241.87	2705.95	1860.67	2184.12	1959.53	1829.67	1886.90	1765.15	3148.29	2390.63	2282.13	2406.77	2125.20	1196.65	4223.77	3275.07	2265.05	5480.03	5006.49	2088.14		
4466	TBC1 domain family member 15	Q8TC07	1961.62	1965.61	1919.33	1284.27	1189.17	1261.30	664.05	665.53	515.39	1620.19	1603.71	1512.90	1268.96	946.41	1035.85	913.18	917.49	1081.11	1271.48	1289.52	1204.74	3753.50	3609.65	3708.07	2195.54	2178.56	2076.37	2946.91	2569.15	2557.49	3337.61	2683.95	2031.86		
4467	TBC1 domain family member 16	Q8TBP0	110735.60	104994.87	93734.17	73224.41	70252.91	66175.89	75377.21	74971.54	69460.14	96313.92	90321.79	85152.44	84032.50	75851.83	59650.76	74007.17	65728.25	67812.81	72181.44	68949.26	64988.23	9699.52	8939.28	9670.53	7404.19	8462.17	8281.40	8489.12	9045.52	8284.05	10293.75	8574.42	3271.29		
4468	TBC1 domain family member 23	Q9NUY8	32617.19	26750.43	22433.51	11813.06	11687.64	10786.55	13260.47	13750.60	12077.11	21635.01	19374.90	16495.01	20671.30	13932.31	10422.21	11096.47	11227.83	10863.29	11774.66	10835.60	9819.00	23636.38	20426.99	16180.46	8206.66	7876.11	5285.27	10156.10	10174.97	8119.46	12225.65	11461.14	5635.28		
4469	TBC1 domain family member 2A	Q9BYX2	52491.62	49483.35	44981.15	30366.91	29284.96	27373.03	36085.90	36061.03	34600.13	37242.47	35773.96	33272.15	36342.79	33667.85	29580.09	33362.10	29875.19	29566.27	31893.69	29770.88	27627.53	4845.81	4703.39	4072.23	3364.78	3108.23	1913.65	4177.41	3496.83	2886.44	4372.57	4190.35	2102.72		
4470	TBC1 domain family member 30	Q9Y2I9	33663.98	33274.56	32145.84	23858.33	23323.03	20882.15	23968.74	22577.40	22296.80	29685.09	28800.56	26353.51	28240.78	29396.25	23455.37	21757.06	20997.99	19642.63	25208.92	24500.36	22371.12	2.27	1.49	0.65	0.84	1.31	0.69	4.16	2.47	0.11	4.90	4.98	0.12	x	
4471	TBC1 domain family member 3D	A0A087V	2525.97	2240.50	1891.16	1605.02	1311.59	1161.66	1733.31	1093.06	1356.11	1787.09	1564.00	1198.06	1880.28	1159.19	1037.62	1846.56	1751.48	1657.44	1419.61	1287.32	1063.35	2.27	1.49	0.65	0.84	1.31	0.69	4.16	2.47	0.11	4.90	4.98	0.12	x	
4472	TBC1 domain family member 4	O60343	71613.46	68865.96	66915.78	47267.52	43638.08	43146.12	58133.46	50615.18	45549.05	58626.25	55578.05	52488.62	57845.21	83458.44	57787.20	44124.91	55432.63	59432.01	55969.31	50268.37	43912.61	7799.39	7191.49	6220.82	4961.78	4468.44	4211.93	4868.86	4134.39	4229.57	4865.03	4434.83	4371.37		
4473	TBC1 domain family member 5	Q92609	470.58	419.74	226.38	382.76	296.13	378.45	198.70	203.85	194.56	324.45	345.51	310.29	2.13	0.97	0.60	385.47	283.29	265.85	343.52	357.03	329.26	2035.28	1771.14	1677.96	1048.90	1051.21	1010.02	1506.98	1433.66	1323.90	1639.27	1543.72	1013.12		
4474	TBC1 domain family member 8	O95759	60286.16	58115.53	55268.99	43487.36	40641.31	39960.40	42455.36	41696.57	42000.00	54798.38	52460.86	50337.35	41681.85	47603.85	40125.88	39323.08	36001.59	35510.73	42970.82	42858.77	41596.86	2.27	1.49	0.65	0.84	1.31	0.69	4.16	2.47	0.11	4.90	4.98	0.12	x	
4475	TBC1 domain family member 8B	Q0IIM8	0.14	2.53	0.81	1.07	2.77	0.68	0.96	0.36	0.63	0.35	0.75	0.38	2.13	0.97	0.60	4.64	1.07	0.47	2.22	0.19	0.06	3546.30	3613.93	3217.15	2626.17	2373.89	2266.48	2713.92	2695.20	2675.50	2547.92	2385.72	1469.65		x
4476	TBC1 domain family member 9B	Q66K14	0.14	2.53	0.81	1.07	2.77	0.68	0.96	0.36	0.63	0.35	0.75	0.38	2.13	0.97	0.60	4.64	1.07	0.47	2.22	0.19	0.06	798.11	841.97	798.21	551.21	604.37	533.30	643.55	566.10	537.97	531.24	496.53	316.35		x
4477	T-box transcription factor TBX15	Q96SF7	5094.38	4481.15	4481.71	3811.68	3863.10	3850.89	4271.04	3988.98	3904.56	6598.98	5713.61	5624.25	4854.84	4724.61	3307.71	3728.24	3260.09	3468.34	4000.95	3806.53	3546.86	2.27	1.49	0.65	0.84	1.31	0.69	4.16	2.47	0.11	4.90	4.98	0.12	x	
4478	T-cell activation Rho GTPase-activating protein	Q8N103	0.14	2.53	0.81	1.07	2.77	0.68	0.96	0.36	0.63	0.35	0.75	0.38	2.13	0.97	0.60	4.64	1.07	0.47	2.22	0.19	0.06	32988.04	32824.24	30137.77	25010.82	24525.34	24026.26	27232.95	27177.12	27024.56	28812.77	28384.88	27340.14		x
4479	T-complex protein 1 subunit alpha	P17987	387472.76	371323.26	351293.96	260135.67	247745.24	237910.65	310920.44	306769.97	297757.60	314140.18	297874.13	278035.52	333044.91	317291.42	256127.97	240890.43	240044.78	230814.20	262884.72	256254.54	237342.72	708556.25	667822.47	625070.48	448354.61	442381.38	394002.26	529780.01	495167.97	475258.03	506756.73	492522.07	362826.84		
4480	T-complex protein 1 subunit beta	P78371	1099863.71	1029441.09	971593.68	755084.99	731941.00	655930.02	874770.64	835892.72	803602.08	945820.12	904384.80	845347.36	943365.22	872739.51	647989.71	701745.84	702709.77	677774.97	781244.85	735522.18	668503.10	697985.70	659422.52	621054.43	445909.89	429084.21	376488.47	557355.92	528810.65	510148.68	559305.17	521500.10	367658.15		
4481	T-complex protein 1 subunit delta	P50991	692146.30	658471.98	617594.48	500413.17	478568.81	447360.60	584435.95	564404.50	536075.41	588045.85	561984.57	528863.83	603826.17	588736.52	437734.58	460782.40	454631.73	442946.41	522235.94	497678.58	462102.20	745497.08	713821.35	649148.60	433367.71	421405.37	382052.12	508749.47	488445.42	467718.83	519970.93	510884.71	376651.26		
4482	T-complex protein 1 subunit epsilon	P48643	779806.29	728433.97	683683.08	560343.88	531634.19	506126.03	644095.99	635665.79	610336.38	660817.55	630386.54	584248.58	638129.80	612175.17	494376.36	555390.80	523260.12	514249.53	569534.07	517203.04	489454.71	829797.22	787323.16	720668.45	502173.34	499623.80	451353.86	604324.88	568848.20	532366.02	595392.69	588735.27	449968.98		
4483	T-complex protein 1 subunit eta	Q99832	325181.35	313012.88	309237.58	246804.46	230553.70	221284.03	266467.29	264416.25	242595.49	296952.12	286669.73	274380.18	276399.18	248217.88	195468.66	230855.34	233744.39	219095.00	251868.68	238798.83	223084.28	469168.04	454901.90	416132.72	286803.84	275168.41	250264.32	352052.35	347225.56	323125.25	337866.21	318073.65	197337.96		
4484	T-complex protein 1 subunit gamma	P49368	678190.51	649033.04	609525.63	445290.04	436592.16	397465.58	551790.08	539315.34	503745.17	569765.96	549772.08	514748.39	549519.19	536149.81	430955.00	500537.24	481253.42	460938.29	505584.85	476806.49	447227.21	745393.44	705715.18	644947.76	417740.69	404421.87	345896.29	497319.58	479169.88	450334.85	492645.33	475696.13	319052.73		
4485	T-complex protein 1 subunit theta	P50990	899820.24	888719.66	849190.38	648973.36	614347.75	602525.80	821466.76	804134.81	772385.28	796410.00	766934.46	723887.37	775641.97	725355.33	636689.16	653607.88	617432.75	609001.44	682453.00	648492.18	621841.13	1094092.51	1020741.34	965399.40	682176.91	662483.92	585437.02	768828.28	739675.04	706630.83	711975.67	703112.92	469304.61		
4486	T-complex protein 1 subunit zeta	P40227	542082.17	518335.76	476506.17	353160.67	348228.61	315908.77	456969.67	445224.31	413277.14	415212.03	396483.58	358906.74	433430.25	417216.87	303488.64	348622.21	337511.45	318884.03	354771.95	339841.56	310854.76	875758.86	829136.58	764908.89	489883.44	474671.71	413681.37	586444.34	547769.72	528566.29	551199.60	538225.39	390788.93		
4487	T-complex protein 1 subunit zeta-2	Q92526	66409.64	58271.81	52166.89	32387.10	31779.98	23846.40	41583.86	35816.40	35471.63	48317.99	46122.64	41761.33	43647.01	33541.23	21147.23	33724.99	29782.14	35086.42	34883.19	34989.64	28032.79	17685.19	15909.43	15403.78	15746.95	15331.23	13703.43	21814.99	19751.66	17576.46	19071.54	19306.77	11313.88		
4488	T-complex protein 11-like protein 2	Q8N4U5	0.14	2.53	0.81	1.07	2.77	0.68	0.96	0.36	0.63	0.35	0.75	0.38	2.13	0.97	0.60	4.64	1.07	0.47	2.22	0.19	0.06	8299.69	8090.47	7080.64	6317.78	6116.81	5415.80	5300.52	5513.53	5041.98	4459.55	4348.56	3039.72		x
4489	Tctex1 domain-containing protein 1	Q8N7M0	0.14	2.53	0.81	1.07	2.77	0.68	0.96	0.36	0.63	0.35	0.75	0.38	2.13	0.97	0.60	4.64	1.07	0.47	2.22	0.19	0.06	250.62	249.12	228.56	160.23	102.28	35.12	193.05	122.34	42.07	156.41	179.04	6.65		x
4490	Teashirt homolog 3	Q63HK5	53891.24	53565.35	45852.73	38769.19	37436.75	34311.76	34003.30	32026.26	30566.01	41636.03	37872.96	36301.67	37911.72	35598.77	33399.42	36244.31	32619.73	29436.85	36315.17	33679.64	30469.01	3407.51	3448.40	3010.08	1899.69	1818.52	1578.02	1940.53	2103.85	1993.30	2201.02	2064.34	1447.70		
4491	Tectonin beta-propeller repeat-containing protein 1	Q7Z6L1	19246.96	18006.82	16125.82	11653.08	11687.95	11721.57	12384.64	12456.31	12040.08	16867.60	15076.02	13965.89	15899.35	14250.71	12277.11	11640.61	10760.55	10606.61	13244.63	12727.71	11011.05	2.27	1.49	0.65	0.84	1.31	0.69	4.16	2.47	0.11	4.90	4.98	0.12	x	
4492	Tektin-1	Q969V4	0.14	2.53	0.81	1.07	2.77	0.68	0.96	0.36	0.63	0.35	0.75	0.38	2.13	0.97	0.60	4.64	1.07	0.47	2.22	0.19	0.06	35739.95	31435.27	34044.87	20798.30	19466.01	18180.41	22270.02	21612.50	20417.35	27508.92	25850.39	20154.65		x
4493	Tektin-5	Q96M29	778.48	731.63	729.08	281.79	325.84	364.79	517.89	454.67	549.37	701.30	729.84	669.91	548.65	505.88	393.55	435.81	409.31	500.45	429.78	355.18	344.44	2.27	1.49	0.65	0.84	1.31	0.69	4.16	2.47	0.11	4.90	4.98	0.12	x	
4494	Telethonin	O15273	645.12	672.01	527.53	545.87	496.67	474.24	546.05	549.96	373.60	720.64	600.15	619.63	626.36	664.67	490.75	489.11	580.75	565.24	522.62	389.54	474.57	3122.60	2961.93	1902.29	1114.67	1278.18	646.88	941.15	959.04	674.32	1236.48	1013.68	405.89		
4495	Telomerase RNA component interacting RNase	Q9BQ61	4314.36	4088.12	3830.96	3756.49	3565.33	3422.21	3733.09	3916.72	3787.52	3902.33	3648.30	3794.43	4112.63	4445.73	3745.58	2906.19	2519.12	2437.38	3508.86	3371.60	3335.80	2.27	1.49	0.65	0.84	1.31	0.69	4.16	2.47	0.11	4.90	4.98	0.12	x	
4496	Telomere length and silencing protein 1 homolog	Q9NZ63	6149.00	5364.21	4209.80	4661.91	4587.36	3558.14	3854.61	3586.39	3568.74	5989.30	5295.34	4685.95	5568.09	5125.60	2199.59	4479.80	3254.38	3735.42	4592.90	4290.06	3695.36	298.90	195.12	51.17	161.12	129.28	51.94	230.88	43.58	72.95	169.70	265.82	63.50		
4497	Telomere-associated protein RIF1	Q5UIP0	9020.55	9052.39	8898.63	6667.45	6577.59	6479.37	6989.37	7208.86	6300.27	7270.64	7508.59	7529.10	7236.99	6199.10	6241.27	5267.80	5082.54	5112.62	7326.53	7273.19	5535.95	487.40	554.34	414.96	547.30	580.44	526.46	555.66	535.57	538.29	579.68	571.80	10.00		
4498	Telomeric repeat-binding factor 2-interacting protein 1	Q9NYB0	5886.44	5403.18	5463.63	3683.26	3678.84	2989.77	3902.50	3682.87	3625.17	4660.16	4144.09	4177.20	4747.09	4390.22	3582.86	3432.60	3302.78	3309.53	3303.45	3274.16	3236.68	944.45	739.47	717.18	917.58	666.78	545.85	844.76	713.40	794.92	1043.58	833.12	582.09		
4499	Tenascin-X	P22105	0.14	2.53	0.81	1.07	2.77	0.68	0.96	0.36	0.63	0.35	0.75	0.38	2.13	0.97	0.60	4.64	1.07	0.47	2.22	0.19	0.06	5801.08	5055.82	4659.21	3786.57	3577.41	3276.13	3579.98	3498.82	3508.70	5029.62	5272.76	3881.42		x
4500	Teneurin-3	Q9P273	4619.40	4336.61	4076.70	3029.71	2571.46	2610.97	3369.43	2728.91	2841.30	3620.88	3577.77	3245.42	3450.47	2956.39	2498.43	2496.68	2541.44	2232.23	3014.51	2683.03	2575.66	2.27	1.49	0.65	0.84	1.31	0.69	4.16	2.47	0.11	4.90	4.98	0.12	x	
4501	Tensin-3	Q68CZ2	13406.57	12654.00	11992.04	9774.42	10339.29	9830.77	11927.86	11320.46	11785.36	11849.92	10521.96	10244.02	12650.19	12724.45	10296.62	8177.32	8715.71	8236.06	10253.57	10352.51	10388.27	2.27	1.49	0.65	0.84	1.31	0.69	4.16	2.47	0.11	4.90	4.98	0.12	x	
4502	Terminal nucleotidyltransferase 5C	Q5VWP2	17328.00	16191.69	15447.68	13385.15	12714.62	12095.77	14830.24	13822.44	15201.41	16880.30	15537.35	15653.23	15649.86	13905.09	11742.84	12398.54	12025.32	11925.35	12310.85	11792.75	11658.37	2463.41	2800.91	2690.26	1124.96	1188.22	1133.56	1150.24	1380.24	1176.71	1049.29	964.71	675.23		
4503	Terminal uridylyltransferase 4	Q5TAX3	0.14	2.53	0.81	1.07	2.77	0.68	0.96	0.36	0.63	0.35	0.75	0.38	2.13	0.97	0.60	4.64	1.07	0.47	2.22	0.19	0.06	3478.47	4364.02	3279.00	3549.64	3675.98	3393.78	2759.09	3464.38	2762.66	4856.21	4907.63	5748.93		x
4504	Testican-2	Q92563	16201.10	14241.77	12120.49	8022.50	7593.64	5651.08	10486.49	8456.79	8637.18	12768.64	11613.57	10991.72	11081.66	8335.66	3664.14	7936.40	8515.12	8757.02	10051.25	9403.06	7349.13	63197.81	59351.42	50576.97	31180.77	30351.79	19393.05	49211.04	43396.48	39637.28	49726.55	49062.42	25687.85		
4505	Testican-3	Q9BQ16	13404.18	12679.30	12397.30	8024.51	8479.22	8113.61	9328.68	9056.79	8778.48	13507.84	13152.67	13065.46	10368.17	10718.04	9187.86	8666.44	7360.14	7328.64	9103.71	8557.92	8467.68	2.27	1.49	0.65	0.84	1.31	0.69	4.16	2.47	0.11	4.90	4.98	0.12	x	
4506	Testin	Q9UGI8	87527.97	84788.03	75197.79	56570.16	53531.54	47208.68	63332.19	60338.36	56762.87	70569.44	68391.17	61575.30	68081.47	63926.42	47013.60	53917.57	51418.37	49714.94	59666.10	56893.89	50837.77	6848.77	6058.87	5893.80	4233.21	4096.74	3771.61	4418.56	4178.15	3769.88	3657.54	3597.84	1989.46		
4507	Testis development-related protein	Q86YL5	9391.80	7047.31	8948.87	5638.51	4538.35	3659.29	7409.23	7176.01	5667.64	8309.11	7653.47	10282.08	7750.96	6720.36	4270.74	5665.90	5078.06	5807.76	6727.69	6804.42	4306.47	2.27	1.49	0.65	0.84	1.31	0.69	4.16	2.47	0.11	4.90	4.98	0.12	x	
4508	Testis-expressed protein 10	Q9NXF1	1560.79	1297.21	1221.71	673.14	699.01	639.60	712.82	733.07	695.41	1517.59	1065.05	1460.18	1357.28	1238.75	890.75	774.11	591.13	667.05	611.24	640.22	639.09	2.27	1.49	0.65	0.84	1.31	0.69	4.16	2.47	0.11	4.90	4.98	0.12	x	
4509	Testis-expressed protein 101	Q9BY14	408.59	304.88	145.79	217.08	573.03	311.34	553.38	418.47	349.45	110.81	200.83	134.37	392.36	1081.82	137.27	172.66	867.31	544.37	139.14	629.44	364.23	2.27	1.49	0.65	0.84	1.31	0.69	4.16	2.47	0.11	4.90	4.98	0.12	x	
4510	Testis-expressed protein 15	Q9BXT5	155353.12	135517.59	118697.98	92744.73	78949.09	78435.74	109529.98	102863.21	100863.59	113947.52	107983.09	100623.63	121613.35	115073.55	79393.07	86804.60	71701.68	76001.44	88600.47	86113.69	74460.74	77510.53	68761.80	63269.72	61798.52	59897.09	56102.57	82796.01	81841.06	79228.24	63440.38	63502.41	52212.07		
4511	Testis-expressed protein 264	Q9Y6I9	7044.42	6482.39	5979.03	5215.60	5199.60	5077.90	4652.36	4499.64	4528.47	6634.92	6889.75	6510.18	6002.78	5912.98	4410.01	4898.30	4593.62	5143.31	4926.01	4424.80	4267.29	2589.23	2407.64	2380.09	2407.65	2445.12	2303.24	2379.66	2352.60	2548.72	2654.99	2509.87	1860.57		
4512	Testis-expressed protein 48	A0A1B0G	0.14	2.53	0.81	1.07	2.77	0.68	0.96	0.36	0.63	0.35	0.75	0.38	2.13	0.97	0.60	4.64	1.07	0.47	2.22	0.19	0.06	30701.50	30110.32	28100.80	23697.21	23649.53	22276.90	24600.11	24273.58	23501.48	22267.55	21218.94	16028.73		x
4513	Testis-expressed protein 52	A6NCN8	1580.19	1339.79	1452.04	1216.71	1032.52	924.72	1303.20	1287.32	1159.40	1250.64	1163.85	1070.64	1815.01	1799.83	923.12	1035.69	1001.51	919.87	1631.70	1428.78	1110.32	2.27	1.49	0.65	0.84	1.31	0.69	4.16	2.47	0.11	4.90	4.98	0.12	x	
4514	Testis-expressed protein 54	A0A1B0G	0.14	2.53	0.81	1.07	2.77	0.68	0.96	0.36	0.63	0.35	0.75	0.38	2.13	0.97	0.60	4.64	1.07	0.47	2.22	0.19	0.06	7574.62	8072.11	7341.34	5672.44	5932.36	5732.84	6580.14	5894.45	6228.57	6031.31	6652.97	3279.29		x
4515	Testis-specific gene 10 protein	Q9BZW7	0.14	2.53	0.81	1.07	2.77	0.68	0.96	0.36	0.63	0.35	0.75	0.38	2.13	0.97	0.60	4.64	1.07	0.47	2.22	0.19	0.06	829.79	879.12	1130.05	821.06	832.61	796.14	804.73	854.41	882.16	889.70	967.06	766.86		x
4516	Testis-specific serine/threonine-protein kinase 3	Q96PN8	16771.51	16785.78	15743.21	10811.24	10927.54	10182.62	14307.93	15881.43	14571.41	13876.94	12679.78	12385.66	13999.04	14168.78	11676.69	10567.00	9318.93	9933.68	12015.31	10546.45	10890.60	2.27	1.49	0.65	0.84	1.31	0.69	4.16	2.47	0.11	4.90	4.98	0.12	x	
4517	Testis-specific serine/threonine-protein kinase 4	Q6SA08	29892.67	27214.80	24530.67	24609.50	22985.48	22928.59	23551.40	22334.84	23170.01	33295.97	32129.16	31789.94	26761.94	24584.08	21958.18	24961.71	23249.10	22068.57	24673.37	23839.08	23582.56	393.07	385.31	317.27	641.68	576.26	497.99	1182.05	1195.24	1335.16	479.58	266.84	171.76		
4518	Testis-specific Y-encoded-like protein 5	Q86VY4	16935.35	17249.90	15085.74	12133.48	12105.42	11218.04	12586.14	12214.81	11833.44	16972.18	15568.51	15283.38	16824.60	14123.22	11282.16	10113.92	10521.19	9817.72	13246.40	11909.73	11992.79	2.27	1.49	0.65	0.84	1.31	0.69	4.16	2.47	0.11	4.90	4.98	0.12	x	
4519	Tether containing UBX domain for GLUT4	Q9BZE9	30188.95	29552.83	29001.91	21099.84	20357.51	18855.55	23322.23	21788.49	21870.16	24960.18	23905.11	22885.45	25291.97	24424.98	21434.01	20575.28	20424.70	19196.01	20580.15	18814.52	18431.17	2.27	1.49	0.65	0.84	1.31	0.69	4.16	2.47	0.11	4.90	4.98	0.12	x	
4520	Tetraspanin-13	O95857	24686.89	23787.76	22618.93	19397.60	18099.03	17237.30	19387.66	19347.71	17592.03	18539.43	17073.25	16143.16	17220.83	16649.51	14493.75	17557.23	16559.95	16424.21	19218.41	18719.04	17405.23	2.27	1.49	0.65	0.84	1.31	0.69	4.16	2.47	0.11	4.90	4.98	0.12	x	
4521	Tetraspanin-15	O95858	2377.40	2351.95	2150.59	1234.87	1097.95	973.46	1648.47	1523.05	1491.54	1851.66	1694.67	1683.46	1732.66	1574.52	1135.51	1348.66	1325.57	1177.92	1687.25	1335.88	1403.87	2.27	1.49	0.65	0.84	1.31	0.69	4.16	2.47	0.11	4.90	4.98	0.12	x	
4522	Tetraspanin-31	Q12999	23211.27	21016.47	20859.72	15553.34	15093.80	14851.84	15428.74	15570.95	14974.81	19198.57	18840.88	18240.69	18547.22	18835.72	14776.93	13793.43	14567.70	12999.43	13587.16	14507.21	13112.14	337.22	374.99	354.58	318.22	336.55	289.27	257.47	208.38	259.36	309.97	206.41	55.38		
4523	Tetratricopeptide repeat protein 1	Q99614	46271.58	45130.72	44811.87	30227.48	29846.51	28025.42	36678.64	35195.11	34251.22	35900.44	34006.78	32135.02	36595.62	36289.93	28982.83	30828.42	28542.66	28440.15	29958.52	29385.90	27527.16	10353.90	9197.09	8689.52	4778.93	4289.51	3822.32	6569.12	5842.93	5918.12	6057.14	5469.29	3964.17		
4524	Tetratricopeptide repeat protein 13	Q8NBP0	110762.29	110142.32	107179.36	78730.22	73514.88	63288.41	77659.12	77908.65	67286.65	93083.22	91716.78	88769.69	92272.64	98478.13	59987.36	58336.90	71251.45	75001.30	100412.67	86037.14	81524.52	20442.07	20287.77	18495.62	15241.40	15856.75	12558.10	24150.88	22548.84	21057.55	18103.85	16949.62	9782.10		
4525	Tetratricopeptide repeat protein 14	Q96N46	1536.31	1509.98	1246.60	1365.47	1360.57	1399.63	1175.10	1161.19	1095.78	1348.60	1385.70	1480.36	1409.41	1201.26	1183.25	1338.36	1036.95	1278.54	1566.53	1754.28	1480.48	2.27	1.49	0.65	0.84	1.31	0.69	4.16	2.47	0.11	4.90	4.98	0.12	x	
4526	Tetratricopeptide repeat protein 21A	Q8NDW8	117637.18	111494.09	114795.17	98022.33	90441.27	94758.65	116708.51	89543.41	114981.67	122196.92	117254.82	110290.90	108382.91	93915.42	84838.80	95277.13	83966.62	89502.29	82134.49	79893.75	71223.36	56002.59	52039.29	46705.52	35546.19	34968.20	31327.40	43114.85	41172.26	38702.77	42187.29	42453.84	34693.31		
4527	Tetratricopeptide repeat protein 24	A2A3L6	11195.35	13057.89	13777.39	9888.68	9669.16	9470.00	11720.25	11057.10	11358.23	12978.47	12529.25	12280.79	10049.01	10320.74	9048.36	9468.31	9485.48	9427.13	8241.67	7545.66	7904.83	2.27	1.49	0.65	0.84	1.31	0.69	4.16	2.47	0.11	4.90	4.98	0.12	x	
4528	Tetratricopeptide repeat protein 25	Q96NG3	0.14	2.53	0.81	1.07	2.77	0.68	0.96	0.36	0.63	0.35	0.75	0.38	2.13	0.97	0.60	4.64	1.07	0.47	2.22	0.19	0.06	8192.10	8439.07	9274.33	5947.25	6442.08	5410.10	6294.73	6026.35	6313.10	5569.15	5534.07	3454.56		x
4529	Tetratricopeptide repeat protein 27	Q6P3X3	2627.94	2252.53	1681.37	1520.07	1425.10	949.91	1570.43	1353.12	1812.93	2063.74	2162.00	1610.80	2224.97	2014.88	842.75	1418.74	1365.49	1023.40	1961.57	1480.79	1298.42	2.27	1.49	0.65	0.84	1.31	0.69	4.16	2.47	0.11	4.90	4.98	0.12	x	
4530	Tetratricopeptide repeat protein 28	Q96AY4	0.14	2.53	0.81	1.07	2.77	0.68	0.96	0.36	0.63	0.35	0.75	0.38	2.13	0.97	0.60	4.64	1.07	0.47	2.22	0.19	0.06	3639.51	3646.52	3395.52	2815.83	2880.63	2278.53	3002.28	2855.56	2685.49	3913.63	3591.94	1845.55		x
4531	Tetratricopeptide repeat protein 30A	Q86WT1	7557.27	6135.02	4470.54	2520.42	1926.02	1541.95	2471.50	2330.34	2141.05	5021.76	4729.46	3807.65	4854.44	2680.58	846.17	2705.95	1899.19	1765.09	2980.66	2076.74	1342.29	2.27	1.49	0.65	0.84	1.31	0.69	4.16	2.47	0.11	4.90	4.98	0.12	x	
4532	Tetratricopeptide repeat protein 38	Q5R3I4	0.14	2.53	0.81	1.07	2.77	0.68	0.96	0.36	0.63	0.35	0.75	0.38	2.13	0.97	0.60	4.64	1.07	0.47	2.22	0.19	0.06	13870.73	11612.09	12430.66	14226.65	13422.75	11319.53	14970.04	15305.26	14815.18	14026.57	13535.87	6929.20		x
4533	Tetratricopeptide repeat protein 4	O95801	12810.33	9940.45	11317.98	9018.43	8186.35	7410.36	10469.44	11218.58	10525.29	11066.32	10463.34	9555.31	7542.42	9364.88	7167.88	8632.37	9209.17	6889.27	10504.66	8416.76	9240.11	22088.70	18375.85	17013.93	13472.95	12810.15	11495.82	17931.14	15797.82	12755.26	17441.81	17249.66	11521.88		
4534	Tetratricopeptide repeat protein 7A	Q9ULT0	2724.05	2490.65	2657.43	1615.75	1323.54	1176.00	2176.60	2401.88	2334.20	1922.15	1785.04	1704.71	1732.05	1842.71	1543.61	1835.16	1783.85	1575.32	1675.81	1485.70	1384.62	1327.18	1388.52	1385.09	1312.55	1321.44	1401.47	1589.90	1842.12	1481.34	1546.22	1125.69	1112.36		
4535	Tetratricopeptide repeat protein 7B	Q86TV6	70929.85	64225.64	60542.42	46913.36	47782.24	48362.28	55175.99	56133.87	53700.17	58943.55	56885.08	53984.26	55091.03	52705.53	49690.46	46488.81	44906.33	41970.11	50073.84	49526.91	47987.07	22446.52	21854.19	20174.86	14972.14	14682.68	14037.92	15703.66	15456.88	15280.23	15691.15	15151.07	9339.55		
4536	Tetratricopeptide repeat protein 8	Q8TAM2	24191.08	22069.28	21620.78	16545.17	15481.38	15756.43	18603.92	17497.16	17531.63	19006.15	17962.14	17513.37	19199.42	18122.40	15533.41	15484.26	14791.63	14496.22	17596.87	16952.29	16275.51	9510.23	9498.90	8731.57	5768.10	5939.12	5879.92	5787.72	5510.48	5478.64	6032.37	5591.21	3893.34		
4537	TGF-beta receptor type-1	P36897	0.14	2.53	0.81	1.07	2.77	0.68	0.96	0.36	0.63	0.35	0.75	0.38	2.13	0.97	0.60	4.64	1.07	0.47	2.22	0.19	0.06	10690.11	9695.41	9417.27	8948.29	9621.46	9005.89	8032.75	8736.82	8795.74	10274.20	9925.72	7703.18		x
4538	TGF-beta-activated kinase 1 and MAP3K7-binding protein 3	Q8N5C8	58689.00	57453.78	55364.05	47667.58	43207.51	41977.71	58106.79	56883.22	54341.92	59668.14	56574.95	53494.97	53480.25	53594.64	45484.39	48104.96	46442.05	43402.91	47276.18	43225.85	41984.64	2.27	1.49	0.65	0.84	1.31	0.69	4.16	2.47	0.11	4.90	4.98	0.12	x	
4539	Thimet oligopeptidase	P52888	45865.88	45605.58	41447.48	31637.32	29562.10	28591.22	39714.91	40157.32	36826.83	40099.84	39012.78	35874.63	40284.67	38434.72	31744.98	29771.27	28358.08	28039.45	31792.83	29288.12	27484.75	51215.82	49724.13	48111.96	39850.80	40434.22	37323.85	48802.48	45543.45	44480.03	46681.41	41793.04	32743.33		
4540	Thioredoxin domain-containing protein 12	O95881	24477.80	20215.11	16600.33	9896.65	8653.12	7957.24	9893.62	10384.13	9548.68	19668.36	16591.30	12824.10	18409.49	10524.43	7872.48	9831.05	9689.93	9638.46	9185.58	8394.25	7358.39	38969.51	36216.18	35403.16	25336.19	24690.80	21397.35	30282.18	28236.56	27464.96	30050.17	29031.69	21336.92		
4541	Thioredoxin domain-containing protein 17	Q9BRA2	52422.00	52553.76	48058.64	32391.00	31663.66	28566.04	44924.66	43632.63	41632.48	47425.74	45330.76	42866.52	41177.70	40282.11	30756.85	32234.59	32326.85	31108.37	33963.55	32206.08	30979.84	109169.00	100797.30	91049.41	61066.08	58044.88	46911.72	74325.64	69329.34	63328.91	74585.71	70250.50	44753.80		
4542	Thioredoxin domain-containing protein 5	Q8NBS9	421440.41	398644.81	378511.60	303130.02	286941.92	271413.17	339132.10	326150.63	320584.38	367625.60	352918.39	323577.60	329512.88	310811.36	266040.27	308011.14	289618.02	282561.16	295524.37	272913.83	260579.95	239428.00	219874.76	205980.92	112424.28	109281.94	99044.59	146942.32	139539.87	133104.40	143082.22	137892.39	89296.56		
4543	Thioredoxin domain-containing protein 6	Q86XW9	51.37	5.28	15.89	1.07	2.77	0.68	11.19	26.03	11.27	0.69	6.45	0.79	2.13	0.97	0.60	13.92	28.98	2.15	440.30	67.69	293.20	14080.78	13515.56	12929.86	10738.21	10980.61	10602.77	10584.91	9820.04	9607.21	12440.33	11464.94	0.12		
4544	Thioredoxin domain-containing protein 8	Q6A555	0.14	2.53	0.81	1.07	2.77	0.68	0.96	0.36	0.63	0.35	0.75	0.38	2.13	0.97	0.60	4.64	1.07	0.47	2.22	0.19	0.06	17345.97	24.54	5.21	6222.76	5211.17	2651.87	6982.20	5102.94	2516.69	5197.65	4321.53	1739.71		x
4545	Thioredoxin domain-containing protein 9	O14530	0.14	2.53	0.81	1.07	2.77	0.68	0.96	0.36	0.63	0.35	0.75	0.38	2.13	0.97	0.60	4.64	1.07	0.47	2.22	0.19	0.06	710.10	984.75	987.61	671.17	623.72	392.82	1698.78	1280.52	499.95	1518.79	1481.80	683.79		x
4546	Thioredoxin	P10599	320163.43	285124.76	264232.60	213662.22	212214.29	186901.13	250420.43	239807.76	224990.81	256710.10	237816.79	220749.63	246978.87	230353.03	165948.35	227740.36	216334.64	200147.95	201683.09	196398.52	174906.39	477018.32	456664.57	411023.63	294036.08	285821.46	256713.52	365486.48	338475.52	322509.51	351925.67	324557.43	160779.82		
4547	Thioredoxin reductase 1_ cytoplasmic	Q16881	56956.55	59629.05	47216.95	39948.99	39165.28	36577.84	43825.31	40438.45	42358.17	44413.02	41451.07	37561.71	41708.27	37578.70	27283.42	44938.16	41175.97	37367.70	38632.49	32785.64	31846.02	64369.86	62467.75	57597.80	43160.62	42099.06	41451.82	49304.92	47235.54	43724.31	53240.54	52614.97	34095.22		
4548	Thioredoxin reductase 2_ mitochondrial	Q9NNW7	0.14	2.53	0.81	1.07	2.77	0.68	0.96	0.36	0.63	0.35	0.75	0.38	2.13	0.97	0.60	4.64	1.07	0.47	2.22	0.19	0.06	11541.29	11025.75	10051.97	6222.07	6872.03	4688.31	7851.16	6541.78	6690.55	7664.98	7627.13	3984.82		x
4549	Thioredoxin-dependent peroxide reductase_ mitochondrial	P30048	163428.66	152169.63	142553.18	109047.79	106889.79	99926.06	141179.08	133468.55	129608.15	141180.07	132551.14	121657.67	132905.86	115658.46	104231.08	122237.55	117435.30	111533.41	119461.62	110218.46	106301.69	124225.04	112997.36	108496.99	70352.42	70533.18	58756.89	87004.65	80124.27	79193.86	89167.00	85224.01	50357.27		
4550	Thioredoxin-like protein 1	O43396	199799.99	194748.72	182617.46	145200.77	135611.09	127254.35	145445.02	144353.37	138307.18	192962.61	184040.31	172327.05	180272.05	157391.81	125351.02	141536.12	124416.89	123468.49	136813.40	134586.06	126842.83	104491.28	97054.76	85116.22	71755.59	69260.98	58861.44	92570.54	88363.57	79736.69	89673.59	86328.50	58323.73		
4551	Thioredoxin-related transmembrane protein 1	Q9H3N1	148213.56	149627.24	136518.90	109279.64	104815.32	101433.11	116508.13	112403.38	111627.78	130311.79	123283.27	108126.58	120724.74	120751.78	104095.21	104667.37	95367.77	96963.14	105527.17	99447.38	97812.03	84107.78	81725.81	79654.90	64041.26	61740.61	56762.12	70504.57	66128.80	65195.73	68977.06	66611.95	50690.76		
4552	Thioredoxin-related transmembrane protein 2	Q9Y320	540.44	524.67	463.72	108.21	124.32	105.94	282.68	335.17	228.91	205.62	197.76	185.70	358.52	331.12	257.61	271.37	247.60	213.10	154.16	144.79	146.22	2.27	1.49	0.65	0.84	1.31	0.69	4.16	2.47	0.11	4.90	4.98	0.12	x	
4553	Thiosulfate sulfurtransferase	Q16762	40789.05	36998.57	33136.10	27116.39	25525.36	23219.02	47727.94	43514.35	38712.24	35750.94	32360.19	31139.32	35236.89	30571.75	23633.04	33932.10	28552.64	25098.57	27824.59	26977.40	26528.92	2.27	1.49	0.65	0.84	1.31	0.69	4.16	2.47	0.11	4.90	4.98	0.12	x	
4554	Thiosulfate:glutathione sulfurtransferase	Q8NFU3	99805.31	95101.30	88567.12	47817.09	43510.09	40261.60	72155.30	64072.84	65685.02	65947.63	57306.81	57044.10	74142.87	68837.86	49110.29	74725.28	68259.93	66456.13	52217.56	52630.35	46573.35	2.27	1.49	0.65	0.84	1.31	0.69	4.16	2.47	0.11	4.90	4.98	0.12	x	
4555	THO complex subunit 4	Q86V81	11982.15	10752.03	9951.37	6201.02	5906.05	5637.11	7755.61	7222.57	6935.25	7347.29	6749.86	6340.68	8881.34	8154.65	4712.88	5771.56	5812.19	5645.82	7194.94	8109.73	7320.43	8559.89	8537.66	7674.27	6872.50	6196.18	4349.77	7139.23	6291.46	5694.73	10593.57	9867.03	6143.37		
4556	Threonine--tRNA ligase_ cytoplasmic	P26639	544338.98	511824.47	473114.72	308024.16	298547.46	265258.12	361260.65	353929.03	336539.52	392063.98	373624.98	340286.50	399242.12	378203.88	259294.44	351705.89	337050.27	315641.43	330360.86	309052.62	284165.90	543644.90	504713.13	472861.48	368655.26	356061.88	323001.41	399394.02	383480.15	366003.30	438676.28	423075.85	285292.42		
4557	Threonylcarbamoyladenosine tRNA methylthiotransferase	Q5VV42	0.14	2.53	0.81	1.07	2.77	0.68	0.96	0.36	0.63	0.35	0.75	0.38	2.13	0.97	0.60	4.64	1.07	0.47	2.22	0.19	0.06	25546.32	24117.75	24816.29	16507.78	17827.89	16112.32	18851.60	18358.42	17733.25	19010.16	18849.38	32578.26		x
4558	Thrombospondin type-1 domain-containing protein 4	Q6ZMP0	6272.98	6859.09	5994.81	4779.96	4755.18	4075.61	6446.64	5779.43	5391.17	6368.52	5526.37	5600.51	5803.85	5542.79	3989.97	5146.89	4566.36	4296.49	4339.17	4378.27	3681.64	2.27	1.49	0.65	0.84	1.31	0.69	4.16	2.47	0.11	4.90	4.98	0.12	x	
4559	Thrombospondin-1	P07996	158.61	145.08	119.81	98.37	90.32	130.85	149.27	105.46	75.74	149.12	189.14	122.51	2.13	0.97	0.60	147.81	109.24	103.08	105.94	135.79	117.19	14860.52	14813.11	13064.64	7427.67	7126.74	5888.53	12951.11	12574.44	11503.47	11005.43	10172.88	6415.18		
4560	Thrombospondin-4	P35443	16800.19	15187.84	12352.44	10370.23	11581.86	9486.01	13080.65	11972.68	10578.37	15985.05	14429.20	13044.73	15523.15	13186.92	7622.53	12225.71	11333.46	11598.84	10033.36	12173.30	11035.72	2.27	1.49	0.65	0.84	1.31	0.69	4.16	2.47	0.11	4.90	4.98	0.12	x	
4561	THUMP domain-containing protein 1	Q9NXG2	1079.98	1018.44	1286.29	386.26	418.18	444.83	920.42	994.82	801.40	573.42	704.23	644.01	1123.26	1095.35	819.40	817.02	643.38	731.90	1001.11	889.29	827.05	378.13	378.94	303.40	795.84	769.62	793.87	752.49	859.88	855.54	1256.53	1198.99	1.94		
4562	THUMP domain-containing protein 3	Q9BV44	27495.13	26628.28	23540.33	16857.29	16007.76	14581.80	22514.73	20305.77	20235.94	23560.31	22087.87	19081.52	21495.38	21539.24	14105.83	17608.31	16289.29	16230.18	18398.71	16526.81	15927.67	2907.86	2688.85	2618.37	2275.70	2355.99	2316.11	2710.37	2508.78	2527.50	2698.68	2591.28	2309.95		
4563	Thymidine kinase_ cytosolic	P04183	48040.08	49227.99	46613.05	42858.94	41745.27	37884.21	42753.34	41440.63	37751.47	41684.38	41383.86	37011.48	48270.77	41458.26	29475.00	34306.36	33936.90	32511.27	40219.56	39133.83	36459.22	25231.02	25423.46	23090.28	14464.02	13888.99	12799.54	19284.69	18020.63	18660.60	16708.86	16599.51	6590.09		
4564	Thymidylate kinase	P23919	145677.39	130022.96	132006.05	126528.52	124360.70	115552.73	143336.04	142711.75	139659.32	142428.52	143758.73	128266.43	145048.87	135386.09	104611.06	118713.87	114967.05	110805.28	125558.37	117831.90	109286.03	129650.35	120820.42	114452.40	82605.36	81071.50	73227.73	105034.82	100741.28	96940.37	98489.10	96363.33	69627.18		
4565	Thymidylate synthase	P04818	19874.01	18153.36	18502.40	12163.31	11296.89	11776.84	16138.19	15881.97	16887.26	12808.54	13190.38	13037.41	14466.21	13950.17	11320.05	15312.51	14437.42	14455.32	12155.44	12054.84	11308.08	51455.13	47360.16	40981.25	34193.27	34206.86	27933.09	50051.10	46724.93	41943.93	51920.12	50432.75	30035.72		
4566	Thymocyte nuclear protein 1	Q9P016	0.14	2.53	0.81	1.07	2.77	0.68	0.96	0.36	0.63	0.35	0.75	0.38	2.13	0.97	0.60	4.64	1.07	0.47	2.22	0.19	0.06	17234.04	15123.69	15083.29	12628.59	12756.46	12027.77	11873.42	12436.82	12273.40	14629.34	13379.70	12845.90		x
4567	Thymosin beta-10	P63313	22095.30	20722.29	17512.41	13998.03	13049.65	11978.28	16779.97	15963.66	15202.44	17502.09	16363.35	14993.00	16498.56	14676.54	11887.60	15058.55	13980.36	13730.66	14492.25	13980.27	13048.66	104698.62	94309.03	86463.97	63468.51	62074.21	50918.56	76735.81	72994.82	66022.73	79607.51	77418.89	47652.46		
4568	Thymosin beta-4	P62328	180409.30	177606.80	169435.21	205721.84	194025.48	188188.09	188947.51	182087.04	179405.91	226872.75	216527.05	200625.89	180873.19	170153.93	128593.41	187058.42	177760.52	175607.39	174558.24	164964.17	161135.39	132321.17	125639.92	118728.91	72362.17	70314.86	69664.85	84764.70	83821.30	82281.81	67536.67	64523.68	37506.55		
4569	Thyroid hormone receptor-associated protein 3	Q9Y2W1	37297.29	37758.62	34821.17	32529.87	31183.24	30320.29	29362.88	28995.75	30154.36	39999.47	39808.54	37274.29	37316.57	34628.24	29828.80	27757.87	28196.02	27281.29	35246.06	31957.97	30766.16	29368.58	29158.35	25026.92	29178.97	28089.50	28232.39	28926.15	28543.42	26534.13	40399.61	33156.08	29981.27		
4570	Thyroid peroxidase	P07202	30804.37	24885.75	23026.69	12502.78	12308.36	9262.87	17006.45	15349.94	11932.16	19071.94	14821.36	12942.40	22503.64	19545.63	8699.35	13455.35	13333.46	11771.71	11090.67	18664.15	14054.07	2.27	1.49	0.65	0.84	1.31	0.69	4.16	2.47	0.11	4.90	4.98	0.12	x	
4571	Thyroid receptor-interacting protein 11	Q15643	313009.64	311904.04	343804.61	185723.42	182072.42	170553.00	224944.67	210765.75	213801.74	255720.09	241739.68	235117.67	207546.12	201128.23	165715.57	213415.44	213362.70	203574.80	182050.06	184458.85	181777.24	2.27	1.49	0.65	0.84	1.31	0.69	4.16	2.47	0.11	4.90	4.98	0.12	x	
4572	Thyroid receptor-interacting protein 6	Q15654	111165.41	107349.49	99777.95	73561.10	69629.55	65464.25	79500.68	77369.32	74476.86	98579.34	96490.71	90871.67	95917.39	92868.40	80145.74	65476.64	59069.51	57494.88	71864.25	70222.00	66039.15	4726.24	4404.92	4213.76	2413.30	2287.54	2097.31	2864.41	2630.89	2749.80	2666.03	2500.60	1678.12		
4573	Thyrotropin-releasing hormone-degrading ectoenzyme	Q9UKU6	3249.55	3092.46	3108.53	2358.42	2231.89	2213.77	2214.98	2271.63	2259.09	2982.21	2727.77	2679.14	2848.03	2548.02	1939.41	2315.94	2004.80	2109.04	2345.96	2242.50	2283.29	76617.17	76535.90	72062.24	51016.84	53796.08	49475.07	65127.38	65798.24	63310.41	68444.83	62885.68	48687.83		
4574	Tigger transposable element-derived protein 5	Q53EQ6	23993.87	23429.61	21763.26	20594.36	19013.83	12930.75	19434.68	17811.28	16169.37	21202.11	19697.05	18280.32	19943.00	15224.32	12609.05	14783.94	18737.51	16987.59	19850.21	19214.46	16706.67	117.76	94.53	113.73	31.61	31.92	36.28	41.70	46.30	31.43	41.94	82.75	24.62		
4575	TIP41-like protein	O75663	456.68	749.08	291.78	586.97	926.43	854.00	2668.02	2314.02	2663.73	757.73	595.92	423.02	447.15	811.51	453.97	1467.95	2552.74	720.11	601.70	436.53	374.59	18157.18	15220.05	14116.39	13896.88	13159.57	12465.72	16214.53	14304.43	13477.64	16085.30	15626.15	8337.37		
4576	Titin	Q8WZ42	2712954.89	2598073.60	2424703.83	1808805.97	1756941.85	1645749.84	2253999.54	2156241.88	2074844.64	2338694.96	2234672.76	2132512.53	2216690.55	2073993.79	1700228.48	1827682.81	1758982.52	1695448.43	1877087.09	1797092.19	1700429.95	1281152.75	1210741.50	1093470.60	861005.01	836603.73	725367.25	999758.98	961787.38	906711.85	1013000.59	967964.84	652621.43		
4577	TNF receptor-associated factor 5	O00463	5072.68	4970.71	3986.73	3698.31	3705.25	3189.02	3656.70	3567.39	3267.77	3378.63	3269.54	3244.09	4036.60	3718.44	1800.36	2964.55	2838.51	3065.60	3847.23	3537.32	3121.18	48209.71	43991.75	35783.60	36135.71	35353.81	30330.80	39254.86	36855.58	32980.97	42935.58	41203.37	14861.28		
4578	TNF receptor-associated factor 6	Q9Y4K3	112.20	89.77	202.52	166.11	167.39	205.08	194.50	195.64	271.40	250.52	287.51	268.60	191.41	187.41	97.57	183.01	263.23	243.76	268.47	153.71	154.20	2.27	1.49	0.65	0.84	1.31	0.69	4.16	2.47	0.11	4.90	4.98	0.12	x	
4579	TNFAIP3-interacting protein 2	Q8NFZ5	3061.20	1959.24	1758.78	1364.01	927.73	1183.53	1498.67	1864.66	1524.59	1583.39	1089.27	1565.86	2096.15	1747.33	999.96	1639.63	1506.44	1606.73	1679.89	1513.73	1321.77	2059.56	1676.78	1859.92	1151.48	1182.83	1144.18	1542.32	1461.88	1403.19	1384.14	1567.50	784.10		
4580	TOG array regulator of axonemal microtubules protein 2	Q6ZUX3	0.14	2.53	0.81	1.07	2.77	0.68	0.96	0.36	0.63	0.35	0.75	0.38	2.13	0.97	0.60	4.64	1.07	0.47	2.22	0.19	0.06	71032.45	65527.12	62405.95	25913.05	26093.43	25497.72	27933.37	27098.35	26324.95	26796.23	25661.07	19986.78		x
4581	Toll-interacting protein	Q9H0E2	1039.33	798.14	774.53	544.45	705.85	572.29	989.87	2496.55	711.78	870.68	728.11	643.92	602.67	2490.16	1473.57	1422.51	1374.10	900.89	307.46	637.68	616.79	2.27	1.49	0.65	0.84	1.31	0.69	4.16	2.47	0.11	4.90	4.98	0.12	x	
4582	Toll-like receptor 5	O60602	0.14	2.53	0.81	1.07	2.77	0.68	0.96	0.36	0.63	0.35	0.75	0.38	2.13	0.97	0.60	4.64	1.07	0.47	2.22	0.19	0.06	10414.97	10147.31	9577.73	6651.13	6617.94	5636.84	7749.76	7271.27	6651.96	7860.36	7165.44	4819.90		x
4583	Tonsoku-like protein	Q96HA7	2173.41	1657.48	1638.55	217.68	96.29	72.28	205.08	249.42	220.16	1202.81	1162.49	1043.33	1619.95	234.22	66.23	173.84	92.73	150.62	197.12	252.89	163.89	2.27	1.49	0.65	0.84	1.31	0.69	4.16	2.47	0.11	4.90	4.98	10752.52	x	
4584	Torsin-1A	O14656	37273.36	35719.80	33377.05	25936.25	23495.99	23261.62	31317.96	29619.66	28837.95	31081.81	31083.78	27358.80	31763.17	30116.83	25868.75	26963.08	24036.09	23422.81	24348.66	22341.39	23266.76	2.27	1.49	0.65	0.84	1.31	0.69	4.16	2.47	0.11	4.90	4.98	0.12	x	
4585	Torsin-1A-interacting protein 1	Q5JTV8	92091.98	85064.19	83657.20	66034.72	59692.17	67206.00	69563.24	71966.74	70801.06	68864.69	66430.03	66255.76	79941.73	62775.23	55599.72	55330.22	64699.89	58694.28	83981.33	65051.88	61766.07	24967.81	23981.93	21881.79	16408.80	15808.12	15146.68	19857.48	19363.03	18709.89	19435.23	18669.71	10301.61		
4586	Torsin-1A-interacting protein 2	Q8NFQ8	64567.69	56142.20	54113.18	33867.52	31286.97	31470.52	36361.12	36340.04	35779.42	51495.56	46213.88	41415.69	42072.95	38594.78	31703.21	33888.13	29914.65	31354.49	34411.43	33609.69	31138.04	8636.24	7951.76	7631.93	5023.28	5113.64	4736.40	7422.05	7281.11	6819.99	7910.76	8042.40	3887.31		
4587	Torsin-3A	Q9H497	69244.23	58199.82	41006.75	36389.89	35247.03	25586.99	48785.70	41150.32	38524.93	45397.03	42487.31	33186.07	41958.41	36743.88	15311.91	46053.04	44619.16	40464.91	40135.69	33501.91	27905.46	2.27	1.49	0.65	0.84	1.31	0.69	4.16	2.47	0.11	4.90	4.98	0.12	x	
4588	TP53-regulated inhibitor of apoptosis 1	O43715	5418.26	4873.05	4284.17	2586.14	2526.67	1627.74	3163.77	2520.34	3005.64	4323.04	3771.54	2482.72	2560.30	3514.70	1993.61	1914.54	2711.85	1436.86	2688.08	2004.86	2710.29	308.85	271.38	215.33	231.25	271.75	175.25	275.06	306.86	263.43	309.84	403.72	123.90		
4589	TPR and ankyrin repeat-containing protein 1	O15050	10367.47	10034.36	8149.73	6255.22	5824.36	5188.89	6472.82	5957.19	5510.92	8115.94	7431.84	7248.73	7399.30	7125.04	4877.20	6916.39	6372.88	5766.35	6542.82	6223.50	5880.88	2.27	1.49	0.65	0.84	1.31	0.69	4.16	2.47	0.11	4.90	4.98	0.12	x	
4590	TPT1-like protein	Q56UQ5	126395.08	115813.92	100055.14	70277.93	68864.95	58843.97	94613.34	86912.77	81747.42	95319.17	89427.14	78860.90	91036.73	83279.58	58522.28	88161.49	82600.46	77951.99	78856.92	74054.94	65110.55	2.27	1.49	0.65	0.84	1.31	0.69	4.16	2.47	0.11	4.90	4.98	0.12	x	
4591	Trafficking protein particle complex subunit 13	A5PLN9	0.14	2.53	0.81	1.07	2.77	0.68	0.96	0.36	0.63	0.35	0.75	0.38	2.13	0.97	0.60	4.64	1.07	0.47	2.22	0.19	0.06	125.76	245.36	252.28	560.47	774.43	1910.31	144.50	322.84	265.32	1956.73	281.36	154.59		x

Supplementary Table S1. Overview on the abundance of all relatively quantified 5180 proteins

			MCF-7																					MDA-MB-231													
	Protein name	UniProt	control 01	control 02	control 03	Dai IC20 01	Dai IC20 02	Dai IC20 03	Dai SC20 01	Dai SC20 02	Dai SC20 03	Gen IC20 01	Gen IC20 02	Gen IC20 03	Gen SC20 01	Gen SC20 02	Gen SC20 03	SSE IC20 01	SSE IC20 02	SSE IC20 03	SSE SC20 01	SSE SC20 02	SSE SC20 03	control 01	control 02	control 03	Dai IC20 01	Dai IC20 02	Dai IC20 03	Gen IC20 01	Gen IC20 02	Gen IC20 03	SSE IC20 01	SSE IC20 02	SSE IC20 03	unique to MCF-7	unique to MDA-MB-231
4592	Trafficking protein particle complex subunit 2-like protein	Q9UL33	701.95	442.30	290.34	183.14	19.70	72.24	197.29	137.09	163.02	458.96	318.38	300.30	2.13	0.97	0.60	139.03	16.25	71.27	62.30	82.07	6.19	2.27	1.49	0.65	0.84	1.31	0.69	4.16	2.47	0.11	4.90	4.98	0.12	x	
4593	TRAF-type zinc finger domain-containing protein 1	O14545	2977.33	2523.03	3243.80	1853.88	1723.80	1105.01	1089.61	711.48	902.09	2702.88	2765.80	3367.20	2659.36	1621.63	967.97	1071.35	1251.46	1677.01	2504.57	2113.45	2052.10	2.27	1.49	0.65	0.84	1.31	0.69	4.16	2.47	0.11	4.90	4.98	0.12	x	
4594	Trans-acting T-cell-specific transcription factor GATA-3	P23771	12567.50	12510.89	11407.41	6732.68	6585.50	5756.93	9556.05	9139.61	8985.53	10658.62	10289.65	9550.87	9629.80	9677.83	8016.07	6528.76	6452.83	6295.67	5529.74	5469.90	5259.87	2.27	1.49	0.65	0.84	1.31	0.69	4.16	2.47	0.11	4.90	4.98	0.12	x	
4595	Transaldolase	P37837	900468.92	889113.55	855379.99	732470.08	694751.80	675614.09	748755.31	737959.39	714195.96	809709.42	777943.31	744302.29	777152.50	758334.11	680204.50	695360.07	656216.83	654809.47	655229.50	624241.46	609428.14	323935.29	323353.70	287451.44	230688.43	224765.03	225027.28	242838.90	238311.38	235002.27	264684.07	252211.25	185093.34		
4596	Transcobalamin-2	P20062	636.32	454.77	626.47	397.33	347.15	285.15	408.72	392.70	398.23	437.93	423.55	376.57	387.22	508.35	343.97	557.24	520.62	446.17	349.48	395.65	322.32	2.27	1.49	0.65	0.84	1.31	0.69	4.16	2.47	0.11	4.90	4.98	0.12	x	
4597	Transcription and mRNA export factor ENY2	Q9NPA8	54350.26	42020.05	33740.21	25442.90	23593.19	19597.88	28206.42	20011.87	23541.73	35036.69	32611.54	26947.53	36027.53	19868.61	10722.52	24702.26	20016.17	20150.86	29825.87	25110.53	19636.14	2.27	1.49	0.65	0.84	1.31	0.69	4.16	2.47	0.11	4.90	4.98	0.12	x	
4598	Transcription elongation factor A protein 1	P23193	185716.55	176579.70	166941.22	136663.00	129893.11	122409.39	145428.85	143064.13	140968.63	156879.69	151062.40	143659.87	148727.00	141291.91	120421.84	127896.72	121840.37	117966.21	130305.96	122605.07	120762.69	115853.83	112068.35	102384.90	85113.32	82196.76	73872.80	95662.02	90704.83	88052.25	99114.31	98091.56	68744.56		
4599	Transcription elongation factor A protein 2	Q15560	39314.28	29380.32	21650.92	10466.64	10581.49	8893.29	14083.79	30714.84	26619.15	28025.57	23437.29	18529.44	31409.78	12527.56	8493.39	10881.89	10876.96	9615.54	12059.07	11363.59	10032.37	9207.36	9464.34	8353.11	4778.89	4603.00	4462.32	3898.59	4538.27	4349.65	5006.42	4043.24	2512.71		
4600	Transcription elongation factor A protein 3	O75764	0.14	2.53	0.81	1.07	2.77	0.68	0.96	0.36	0.63	0.35	0.75	0.38	2.13	0.97	0.60	4.64	1.07	0.47	2.22	0.19	0.06	3248.06	2824.79	1764.24	2591.01	2268.90	1051.77	3225.20	2420.54	1825.61	4489.05	4292.84	1776.15		x
4601	Transcription elongation factor A protein-like 1	Q15170	3473.81	3481.09	2414.80	2836.46	2788.06	2350.29	3339.38	2988.15	3174.36	3684.12	3147.51	3072.63	3536.16	3233.69	2032.77	2750.97	2633.17	2328.72	3217.13	3051.22	2418.37	2.27	1.49	0.65	0.84	1.31	0.69	4.16	2.47	0.11	4.90	4.98	0.12	x	
4602	Transcription elongation factor A protein-like 2	Q9H3H9	5232.64	5464.92	5347.42	4085.04	4296.67	3670.38	5288.12	5146.77	4959.51	4865.64	4730.09	4570.70	4846.23	5310.99	3863.66	4280.08	4041.19	4089.21	3836.30	3583.18	3308.59	2.27	1.49	0.65	0.84	1.31	0.69	4.16	2.47	0.11	4.90	4.98	0.12	x	
4603	Transcription elongation factor A protein-like 4	Q96EI5	66735.15	61527.47	52659.70	38659.51	36024.28	32639.65	48069.36	43059.67	42388.11	49286.05	47179.07	43709.02	49377.23	45247.57	32441.61	36978.22	36008.08	35563.29	43790.26	40457.49	36417.17	9080.83	9815.19	9235.52	7306.37	7158.12	6789.58	8033.59	7930.62	7400.94	7843.80	7465.31	2784.69		
4604	Transcription elongation factor A protein-like 5	Q5H9L2	2677.79	2258.16	1961.57	3222.00	2923.05	2579.96	1657.67	1406.34	1284.66	2610.30	2495.57	2192.72	2039.72	1785.13	1340.27	2189.59	1874.12	1777.09	2600.56	2554.19	2269.86	2.27	1.49	0.65	0.84	1.31	0.69	4.16	2.47	0.11	4.90	4.98	0.12	x	
4605	Transcription elongation factor SPT5	O00267	11602.72	10947.17	10166.95	11020.70	10077.94	9555.50	9324.73	8872.67	7677.81	12503.07	12943.23	12113.72	10756.23	10919.94	5790.26	9650.88	10012.86	9930.66	9700.23	10485.84	8853.91	5750.56	5079.34	4759.66	2668.89	2296.31	1786.63	2602.79	2443.72	2144.27	2376.67	2160.30	1173.01		
4606	Transcription elongation regulator 1	O14776	87504.77	80211.64	78971.00	59573.88	56950.74	53073.19	71419.58	69991.12	65189.36	74196.38	69771.33	68448.72	68878.38	66891.23	53384.80	58991.78	57362.90	56459.32	58677.12	58477.60	54800.38	40605.32	37424.83	36239.64	31238.99	29071.05	26316.21	32908.73	33193.82	31678.19	36065.91	36088.75	25842.34		
4607	Transcription factor 15	Q12870	326.78	243.90	243.64	337.81	292.49	320.43	510.82	594.19	472.13	346.45	450.39	492.74	438.27	262.48	264.54	263.17	239.09	241.89	292.22	271.30	302.91	2.27	1.49	0.65	0.84	1.31	0.69	4.16	2.47	0.11	4.90	4.98	0.12	x	
4608	Transcription factor 20	Q9UGU0	0.14	2.53	0.81	1.07	2.77	0.68	0.96	0.36	0.63	0.35	0.75	0.38	2.13	0.97	0.60	4.64	1.07	0.47	2.22	0.19	0.06	4593.54	3869.82	3946.44	3299.56	3126.29	2953.56	3398.99	3468.84	3373.49	4380.62	3837.62	2544.74		x
4609	Transcription factor 25	Q9BQ70	33129.25	31931.35	31354.97	23523.09	21860.95	20892.53	28087.98	27432.84	27084.09	28597.30	27450.36	26222.93	30665.54	25845.60	25660.08	23944.93	22300.24	20811.56	25269.45	24358.27	23535.04	2.27	1.49	0.65	0.84	1.31	0.69	4.16	2.47	0.11	4.90	4.98	0.12	x	
4610	Transcription factor 7	P36402	0.14	2.53	0.81	1.07	2.77	0.68	0.96	0.36	0.63	0.35	0.75	0.38	2.13	0.97	0.60	4.64	1.07	0.47	2.22	0.19	0.06	3283.34	3000.06	3233.19	3224.07	2887.13	2284.57	7921.69	7310.67	7413.06	3214.73	3255.56	2218.29		x
4611	Transcription factor 7-like 2	Q9NQB0	5792.55	5697.28	5470.00	4711.07	4943.79	4780.71	4994.34	4890.09	5273.88	5515.27	5342.79	5229.66	5019.66	5104.52	4284.95	4470.41	4309.31	4242.47	4125.16	3801.71	3863.51	2.27	1.49	0.65	0.84	1.31	0.69	4.16	2.47	0.11	4.90	4.98	0.12	x	
4612	Transcription factor A_ mitochondrial	Q00059	57917.70	55134.40	56395.94	40740.18	39432.88	35163.79	44186.58	44949.42	40681.56	50948.83	48347.32	45130.52	48920.43	46736.29	41191.98	38997.01	39954.93	36639.14	36519.24	35259.37	33349.88	69819.75	61372.32	58050.63	49074.84	48283.57	44425.83	54853.57	52091.25	48885.36	58900.47	56306.53	41526.76		
4613	Transcription factor BTF3 homolog 4	Q96K17	0.14	2.53	0.81	1.07	2.77	0.68	0.96	0.36	0.63	0.35	0.75	0.38	2.13	0.97	0.60	4.64	1.07	0.47	2.22	0.19	0.06	2551.42	3734.74	2039.51	1168.72	1301.70	821.24	3290.19	1706.74	2047.24	2228.64	2193.54	935.68		x
4614	Transcription factor BTF3	P20290	12016.88	11291.10	8629.03	7391.66	7337.23	6885.36	7090.39	8093.25	7101.91	7219.06	7145.71	7009.45	7224.17	5957.93	7740.35	6942.85	6610.25	7263.07	7678.32	7413.18	6600.01	11784.99	8191.52	6629.53	5567.46	5721.48	6033.59	7614.65	7373.16	6730.07	8043.72	7873.56	1648.88		
4615	Transcription factor E2F8	A0AVK6	0.14	2.53	0.81	1.07	2.77	0.68	0.96	0.36	0.63	0.35	0.75	0.38	2.13	0.97	0.60	4.64	1.07	0.47	2.22	0.19	0.06	753.59	703.09	718.58	386.19	327.81	220.46	419.44	363.02	368.29	553.10	467.11	252.96		x
4616	Transcription factor ETV7	Q9Y603	11751.53	10247.29	9940.35	6404.48	6582.30	6320.26	9079.91	8323.35	8281.00	11384.13	11014.54	10355.51	8960.23	9225.64	7984.05	7640.80	7618.41	7475.43	9397.34	8275.04	7587.17	2.27	1.49	0.65	0.84	1.31	0.69	4.16	2.47	0.11	4.90	4.98	0.12	x	
4617	Transcription factor HIVEP2	P31629	2572.11	1918.31	1422.18	1245.42	1066.43	920.01	1563.70	1315.25	1188.51	1669.14	1608.97	1392.75	1608.86	1489.27	926.56	1414.58	1454.36	1458.59	1465.76	1557.35	1206.75	2.27	1.49	0.65	0.84	1.31	0.69	4.16	2.47	0.11	4.90	4.98	0.12	x	
4618	Transcription factor HIVEP3	Q5T1R4	29658.33	23828.78	18321.14	3257.90	3283.18	2697.72	3318.52	3895.95	3479.89	24027.45	21674.50	18370.50	23691.95	5114.89	2604.40	3062.80	3571.17	2885.69	3044.09	3110.78	2316.33	2.27	1.49	0.65	0.84	1.31	0.69	4.16	2.47	0.11	4.90	4.98	11990.45	x	
4619	Transcription factor jun-D	P17535	1077.25	910.75	841.72	584.01	540.61	419.08	610.63	406.37	485.17	973.58	762.61	657.04	705.36	517.42	182.83	615.51	557.77	621.00	633.46	547.84	364.63	2.27	1.49	0.65	0.84	1.31	0.69	4.16	2.47	0.11	4.90	4.98	0.12	x	
4620	Transcription factor p65	Q04206	716.91	788.79	531.02	464.56	635.00	466.89	608.34	550.64	630.29	883.34	798.38	827.54	714.90	607.87	316.25	605.46	574.73	469.81	680.20	588.24	655.31	747.38	837.72	720.22	1190.98	1206.95	1070.80	1003.68	932.48	956.29	1312.08	1221.05	785.12		
4621	Transcription factor RelB	Q01201	75266.89	70192.68	58260.01	59784.83	57775.68	54564.66	54786.60	49247.83	45833.62	84204.70	76421.22	67074.31	51279.75	47695.06	34546.28	72439.39	69720.63	67474.46	70932.55	72009.16	65666.81	2.27	1.49	0.65	0.84	1.31	0.69	4.16	2.47	0.11	4.90	4.98	0.12	x	
4622	Transcription factor Sp1	P08047	5243.73	6152.77	6097.95	3476.63	4274.68	3920.66	3445.07	3668.79	3956.82	4838.98	5051.37	5235.31	5608.00	4301.20	2101.73	2734.33	3294.85	2962.18	3915.63	4585.03	4413.72	2.27	1.49	0.65	0.84	1.31	0.69	4.16	2.47	0.11	4.90	4.98	0.12	x	
4623	Transcription factor TFIIIB component B'' homolog	A6H8Y1	7586.05	7138.45	6360.86	5981.99	5688.57	5503.32	10799.80	10881.66	10350.33	10318.35	9740.84	9813.10	7962.19	7548.75	5296.59	5461.71	5212.73	5016.90	6106.90	5549.37	5497.14	30985.33	28194.32	23932.56	19517.51	19102.18	12367.93	25537.27	22675.83	18716.35	30852.74	29054.42	15402.48		
4624	Transcription initiation factor IIB	Q00403	0.14	2.53	0.81	1.07	2.77	0.68	0.96	0.36	0.63	0.35	0.75	0.38	2.13	0.97	0.60	4.64	1.07	0.47	2.22	0.19	0.06	13298.78	12468.41	11869.37	8979.82	8463.79	8664.88	8120.78	6703.30	7096.89	9172.12	7905.97	4440.76		x
4625	Transcription initiation factor TFIID subunit 11	Q15544	0.14	2.53	0.81	1.07	2.77	0.68	0.96	0.36	0.63	0.35	0.75	0.38	2.13	0.97	0.60	4.64	1.07	0.47	2.22	0.19	0.06	2370.31	815.70	402.21	1856.30	1394.14	568.62	3402.47	2907.35	893.18	3094.95	2609.73	814.13		x
4626	Transcription intermediary factor 1-alpha	O15164	1441.47	1602.98	1456.48	1270.55	781.58	600.18	988.59	978.77	562.05	1688.83	1500.17	1513.62	1512.27	747.77	627.14	1252.91	679.01	857.63	1054.33	1310.50	1258.99	2.27	1.49	0.65	0.84	1.31	0.69	4.16	2.47	0.11	4.90	4.98	0.12	x	
4627	Transcription intermediary factor 1-beta	Q13263	622048.24	587833.43	546330.20	378198.29	360669.36	344231.88	490180.45	467145.48	453083.72	478231.31	451688.65	422083.10	453589.74	443653.47	354817.25	416704.57	389406.15	391152.49	443325.15	432500.41	398485.83	307121.14	286062.88	258346.47	191615.72	181072.18	157290.39	231801.91	211650.97	200951.63	236030.62	233068.30	149802.77		
4628	Transcription termination factor 1_ mitochondrial	Q99551	3780.64	3062.98	3230.00	1525.99	1474.23	1256.30	5352.27	1714.19	2193.63	2620.31	2321.85	2176.85	2182.07	2141.36	1597.51	2648.72	4653.29	2719.38	2131.38	1877.00	1890.58	2218.40	2095.08	1975.61	2575.97	2381.92	2218.66	4595.31	4097.86	4381.07	2456.92	2121.57	1951.49		
4629	Transcription termination factor 4_ mitochondrial	Q7Z6M4	1956.55	2228.29	1752.53	1416.49	1461.27	1091.93	2107.26	2243.44	1904.46	2058.68	2029.53	1661.34	2377.54	1918.95	1345.67	1536.80	1407.64	1322.61	1790.44	1678.28	1565.34	2.27	1.49	0.65	0.84	1.31	0.69	4.16	2.47	0.11	4.90	4.98	0.12	x	
4630	Transcriptional activator protein Pur-beta	Q96QR8	124551.34	118580.94	110052.03	68931.79	63660.76	63744.44	85056.33	81016.84	81011.55	88690.05	87046.66	80203.70	87178.07	78314.65	68938.27	78299.13	73603.59	70890.18	81253.63	73418.71	65203.26	7113.96	6891.77	6880.85	5432.89	5307.23	4605.77	4936.49	4811.01	4385.89	5142.58	4801.98	3641.80		
4631	Transcriptional adapter 2-alpha	O75478	0.14	2.53	0.81	1.07	2.77	0.68	0.96	0.36	0.63	0.35	0.75	0.38	2.13	0.97	0.60	4.64	1.07	0.47	2.22	0.19	0.06	249.41	253.76	229.39	201.14	275.84	145.47	156.91	166.28	116.29	233.29	191.31	59.64		x
4632	Transcriptional coactivator YAP1	P46937	81583.65	72002.27	54528.72	21722.93	19305.26	17813.08	19429.40	23288.92	20972.78	68546.85	61623.63	54286.99	70810.93	28976.44	19731.08	20672.62	17674.04	18227.85	51148.79	21464.66	18152.55	154903.72	140225.20	110030.82	70273.80	69529.75	48995.23	82069.43	73370.45	65383.27	79044.79	73308.78	45572.56		
4633	Transcriptional regulator ERG	P11308	12434.67	11564.58	11527.43	22714.64	21991.02	22646.06	18509.21	19740.40	19226.54	11533.77	11476.99	10870.41	17954.81	16965.38	21749.21	21832.82	19147.71	19357.37	19112.25	19433.24	17743.54	2.27	1.49	0.65	0.84	1.31	0.69	4.16	2.47	0.11	4.90	4.98	0.12	x	
4634	Transcriptional repressor CTCFL	Q8NI51	0.14	2.53	0.81	1.07	2.77	0.68	0.96	0.36	0.63	0.35	0.75	0.38	2.13	0.97	0.60	4.64	1.07	0.47	2.22	0.19	0.06	9778.71	8201.24	7213.15	4650.30	4923.56	3399.28	6480.64	5117.55	4613.02	6389.85	5916.56	3702.26		x
4635	Transcriptional repressor p66-alpha	Q86YP4	10481.79	9891.03	9521.87	6643.87	6381.61	6494.15	7768.11	7362.71	7188.09	7595.18	6973.80	6622.31	8228.66	7495.98	5824.82	6845.68	6010.28	5913.42	7504.43	7113.34	6581.52	1090.21	747.58	776.01	654.62	635.08	563.02	760.54	693.60	721.96	883.60	957.06	320.61		
4636	Transcriptional repressor p66-beta	Q8WXI9	135110.42	131399.92	125118.62	92811.95	86631.81	85668.53	103324.38	103119.07	101081.92	112757.01	101590.76	104247.83	95978.22	99234.85	86506.22	90623.84	83435.72	83765.94	81486.65	82997.07	81230.03	17692.11	16705.25	17027.48	14085.66	14698.18	13283.38	14706.08	13984.78	13582.84	16505.20	16384.10	10789.05		
4637	Transcriptional repressor protein YY1	P25490	15082.51	14421.74	16329.34	12862.74	11788.91	10676.37	13809.26	14628.12	14196.74	13145.99	14126.28	12378.77	16182.81	12149.41	11163.30	12659.72	10479.63	10895.84	13976.49	13239.34	10615.75	1950.31	1878.20	2240.81	1408.78	1382.67	1238.72	1883.87	1700.26	1720.60	1821.31	1651.41	1042.97		
4638	Transducin beta-like protein 2	Q9Y4P3	47953.60	46843.60	43330.01	31164.66	30038.70	29270.25	39907.55	37955.48	37480.76	41088.12	36718.53	36994.39	39811.02	35991.42	31860.70	34643.26	32464.70	31859.49	37977.56	37721.31	34493.33	1931.12	2037.56	2052.56	1217.21	1266.16	1151.45	2053.13	1997.90	2088.73	1775.19	1560.65	1087.32		
4639	Transferrin receptor protein 1	P02786	720324.61	691156.37	660840.81	575688.88	562779.33	524427.56	672990.81	641878.43	610769.23	587373.72	557436.31	527217.13	535263.01	498036.11	419688.13	550156.33	531957.74	512287.15	522497.27	491405.03	467237.50	100002.62	98610.72	91799.61	68643.88	64511.23	58239.24	83295.57	79970.08	76685.13	86226.46	77961.55	50170.56		
4640	Transferrin receptor protein 2	Q9UP52	1732.38	1629.46	1467.42	1231.10	1191.88	1155.12	1110.74	985.06	1002.21	1626.56	1790.62	1717.11	1363.85	1321.07	1218.06	1058.93	983.02	970.47	1193.71	1063.80	1062.30	2.27	1.49	0.65	0.84	1.31	0.69	4.16	2.47	0.11	4.90	4.98	0.12	x	
4641	Transformation/transcription domain-associated protein	Q9Y4A5	0.14	2.53	0.81	1.07	2.77	0.68	0.96	0.36	0.63	0.35	0.75	0.38	2.13	0.97	0.60	4.64	1.07	0.47	2.22	0.19	0.06	25519.81	25095.67	21195.49	14668.47	14329.99	11274.42	23549.25	20953.23	19019.66	23490.19	20736.11	12883.69		x
4642	Transformer-2 protein homolog alpha	Q13595	0.14	2.53	0.81	1.07	2.77	0.68	0.96	0.36	0.63	0.35	0.75	0.38	2.13	0.97	0.60	4.64	1.07	0.47	2.22	0.19	0.06	10384.29	10978.05	8963.54	7285.27	6909.14	5747.01	10227.60	10447.24	9086.59	9437.13	8795.41	6190.91		x
4643	Transformer-2 protein homolog beta	P62995	28033.03	26172.96	25185.11	15275.25	15003.55	13193.72	18147.83	17563.20	17250.45	19062.15	17782.88	16600.73	19591.26	18731.17	15308.18	15421.18	14221.45	14621.32	17423.99	17157.30	16491.54	25983.56	25085.83	22628.85	22845.40	21834.52	19658.49	31582.34	30065.52	28184.88	28737.46	28122.91	13543.50		
4644	Transforming acidic coiled-coil-containing protein 3	Q9Y6A5	1422.55	1727.48	1377.84	817.98	776.40	640.57	896.72	1015.44	987.77	874.56	835.41	640.67	846.45	823.87	540.07	568.70	554.42	543.92	1100.61	846.80	833.31	18375.31	21046.29	20284.50	16755.93	16079.23	13886.62	22921.25	18979.07	19505.30	20924.83	19696.97	9919.80		
4645	Transforming growth factor beta-1 proprotein	P01137	0.14	2.53	0.81	1.07	2.77	0.68	0.96	0.36	0.63	0.35	0.75	0.38	2.13	0.97	0.60	4.64	1.07	0.47	2.22	0.19	0.06	853097.50	823622.37	724585.83	642301.74	597051.72	495954.90	782245.84	755437.45	720632.47	1012764.08	973192.65	602550.86		x
4646	Transforming growth factor beta-3 proprotein	P10600	0.14	2.53	0.81	1.07	2.77	0.68	0.96	0.36	0.63	0.35	0.75	0.38	2.13	0.97	0.60	4.64	1.07	0.47	2.22	0.19	0.06	5781.86	5520.62	5578.03	4492.85	5127.20	4721.44	4912.76	4945.91	5128.78	5340.33	5297.09	5254.22		x
4647	Transforming protein RhoA	P61586	17428.18	16115.78	14074.49	8894.42	9521.94	8027.15	12229.79	11475.39	11330.04	11542.91	10277.45	13565.42	12265.05	11044.38	7411.41	10698.04	9308.54	9220.10	8745.50	8648.47	7967.66	202694.04	201895.89	189514.32	134143.85	134343.68	125534.87	140844.43	140809.03	135133.77	151283.40	144892.40	107725.48		
4648	Transgelin-2	P37802	1415292.86	1403165.83	1322223.93	829861.60	806815.06	755033.24	952785.37	915372.25	886016.36	1150758.05	1102669.94	1046655.37	1118435.93	1021070.32	857272.13	834041.49	793290.09	780151.00	911006.03	818059.16	784972.08	931009.38	895321.35	833946.26	552319.90	551371.08	495550.88	722957.06	697881.70	674544.41	703321.84	677576.32	499211.28		
4649	Transgelin-3	Q9UI15	0.14	2.53	0.81	1.07	2.77	0.68	0.96	0.36	0.63	0.35	0.75	0.38	2.13	0.97	0.60	4.64	1.07	0.47	2.22	0.19	0.06	731.50	193.29	154.44	690.70	694.80	278.98	173.89	439.15	768.57	1042.59	1255.10	581.05		x
4650	Trans-Golgi network integral membrane protein 2	O43493	17846.74	18286.53	18032.01	13496.53	13776.22	13165.46	13210.60	11693.37	12413.28	17897.22	16844.91	17338.79	16747.64	16042.12	15113.63	11979.95	11895.80	11988.95	14437.67	12777.21	11936.98	2354.83	2414.06	2249.64	1551.19	1703.48	1535.41	1860.24	1855.97	1876.90	1768.73	2140.82	528.28		
4651	Transient receptor potential cation channel subfamily A member 1	O75762	0.14	2.53	0.81	1.07	2.77	0.68	0.96	0.36	0.63	0.35	0.75	0.38	2.13	0.97	0.60	4.64	1.07	0.47	2.22	0.19	0.06	12974.82	11957.76	12058.70	4332.77	4187.53	3234.63	4421.53	4089.87	3529.13	4622.68	4245.67	2481.70		x
4652	Transient receptor potential cation channel subfamily M member 6	Q9BX84	15158.34	13764.00	12919.12	10988.96	11153.89	10212.96	12341.41	12367.39	12057.09	17337.82	16067.57	16525.06	12788.33	11086.49	9045.53	11430.49	10646.20	9816.01	8404.68	8307.45	7907.50	39092.09	38545.74	31927.10	30526.81	29685.53	22147.41	56502.74	51163.47	47418.76	37906.65	36624.20	22335.13		
4653	Transient receptor potential cation channel subfamily V member 2	Q9Y5S1	15317.58	13248.59	10946.54	6876.92	6599.50	5743.83	9383.43	8996.41	8454.18	8778.08	8365.98	7380.80	9803.43	8281.98	5571.19	7646.81	6464.69	7742.91	7633.68	6645.02	5902.31	2.27	1.49	0.65	0.84	1.31	0.69	4.16	2.47	0.11	4.90	4.98	0.12	x	
4654	Transient receptor potential cation channel subfamily V member 3	Q8NET8	0.14	2.53	0.81	1.07	2.77	0.68	0.96	0.36	0.63	0.35	0.75	0.38	2.13	0.97	0.60	4.64	1.07	0.47	2.22	0.19	0.06	1400.71	1050.73	96.69	325.47	311.53	92.01	696.29	422.26	289.93	493.73	423.43	99.36		x
4655	Transitional endoplasmic reticulum ATPase	P55072	816613.73	751610.18	713050.46	563842.09	533600.09	492127.18	591483.35	577949.20	530098.42	714414.78	691595.59	673331.80	703639.85	618318.19	386593.60	531744.83	530210.07	516860.87	593968.09	550478.09	498767.02	817236.01	755157.49	680652.78	526819.10	516930.21	423980.91	593722.44	553393.53	506133.14	654750.30	613521.33	403605.17		
4656	Transketolase	P29401	1638395.69	1579297.20	1497060.16	1248942.70	1202431.72	1148389.25	1447574.14	1424121.49	1357307.73	1446541.31	1408317.45	1309307.57	1370653.19	1260609.03	1066563.54	1346537.52	1281235.71	1251804.58	1224330.09	1153154.64	1091344.71	1593109.12	1491591.98	1379765.56	953656.84	966339.82	861427.81	1165136.88	1105405.73	1050085.85	1198011.54	1163766.12	856339.53		
4657	Translation initiation factor eIF-2B subunit alpha	Q14232	4093.30	3492.06	2768.36	2730.25	2425.84	1908.15	2848.83	3297.04	3259.28	3058.46	3084.32	2507.28	3384.06	3164.87	1587.64	2490.75	2550.95	2286.92	3055.75	2528.37	1907.92	5362.97	4970.43	3917.95	2530.90	2983.61	2782.34	3958.49	3683.75	2988.97	2917.85	2918.94	3194.04		
4658	Translation initiation factor eIF-2B subunit beta	P49770	28955.95	29557.99	27197.98	23415.81	22285.80	20234.20	22680.64	20798.55	21618.66	27405.36	26420.24	25245.64	25012.26	21335.28	17133.00	21206.96	20039.16	20543.55	21540.77	19870.67	18540.11	5308.51	5031.31	5252.47	4571.96	4219.54	4067.50	5379.42	5020.45	4656.79	5630.84	4606.23	2891.01		
4659	Translation initiation factor eIF-2B subunit delta	Q9UI10	11841.04	10206.26	9480.65	9428.77	9174.08	8095.68	10884.55	10161.35	9436.33	13307.20	12351.82	10888.07	10852.76	10629.57	6370.13	9531.52	8738.63	7819.51	9286.98	9563.44	9516.21	17171.63	16016.38	15001.65	10636.80	10534.28	10366.51	11704.86	11064.63	10774.15	11235.75	10407.72	8657.36		
4660	Translation initiation factor eIF-2B subunit gamma	Q9NR50	47487.46	46733.69	41979.89	30210.18	28689.78	27016.48	36141.53	32407.40	31452.37	36579.05	34088.83	31018.42	37710.31	33983.56	28578.70	30096.58	28256.47	28276.51	30485.27	28787.47	27473.36	2.27	1.49	0.65	0.84	1.31	0.69	4.16	2.47	0.11	4.90	4.98	0.12	x	
4661	Translation machinery-associated protein 16	Q96EY4	23109.98	22540.74	21544.35	16643.31	15022.23	14679.05	17420.82	16674.93	15834.61	22600.73	21436.76	20696.16	19798.68	19080.00	16546.24	16610.76	14735.80	14677.42	18414.25	16374.43	16386.91	2.27	1.49	0.65	0.84	1.31	0.69	4.16	2.47	0.11	4.90	4.98	0.12	x	
4662	Translation machinery-associated protein 7	Q9Y2S6	39143.26	37891.47	36111.76	31737.99	31743.43	29589.54	31277.49	29535.08	29292.85	38860.30	37795.53	37411.03	36862.23	34298.54	30481.01	29023.69	27066.84	26126.82	29013.96	28773.75	28510.87	5777.17	5380.99	4874.85	4255.18	4250.70	4588.50	5628.70	5134.52	4921.87	5559.82	5319.12	4169.79		
4663	Translational activator of cytochrome c oxidase 1	Q9BSH4	30689.87	28323.23	26467.10	18157.82	20304.50	18363.34	23380.97	20474.73	20379.77	25411.81	24066.05	22896.78	26583.79	25073.54	19555.55	17094.59	17392.78	17502.93	20369.53	19910.67	18319.98	2.27	1.49	0.65	0.84	1.31	0.69	4.16	2.47	0.11	4.90	4.98	0.12	x	
4664	Translationally-controlled tumor protein	P13693	356020.03	348487.45	316271.79	202170.23	199822.51	180195.76	258372.06	252968.05	236064.67	279041.35	267571.17	248391.99	252935.80	230378.76	203026.74	200729.68	188233.74	187773.87	235727.12	226812.55	205333.22	180162.14	173419.76	156260.09	103694.54	99698.61	87789.62	134734.78	128763.90	120553.59	143236.31	134669.30	94999.10		
4665	Translin	Q15631	102327.97	97413.52	91055.55	75618.02	74220.88	70895.97	78479.10	75079.40	71063.03	89086.77	85749.46	81276.56	88652.34	82980.49	72289.49	70622.84	68245.35	66615.07	75411.86	72250.95	65518.89	94519.55	86305.05	79698.49	59557.16	59849.84	55336.61	66328.77	67933.91	64195.58	70854.43	72316.35	53738.78		
4666	Translin-associated protein X	Q99598	28552.69	23180.15	18575.46	15552.93	13617.71	11115.11	16730.51	15587.76	13656.39	22722.17	21093.80	17244.69	21687.64	17878.26	7179.99	15931.34	14496.59	12569.13	17958.66	15779.18	12269.72	5260.69	4534.76	4391.86	3293.97	3611.08	3508.74	4704.95	4483.70	4389.56	4790.65	4821.42	3417.76		
4667	Translocation protein SEC62	Q99442	71773.12	71905.42	65920.25	69137.05	65718.63	61035.86	59102.17	62106.29	61249.23	72119.90	72115.42	68090.38	69836.31	54896.50	43632.22	62756.43	58484.09	54514.82	60154.97	57555.01	58107.89	7036.13	7170.98	5996.13	7230.69	6808.27	7671.19	6976.84	7217.66	7002.64	8799.38	8409.23	2450.73		
4668	Translocation protein SEC63 homolog	Q9UGP8	173845.42	178349.14	158126.52	135145.86	136258.40	126517.03	146632.16	142682.12	127314.19	168620.39	161099.29	154394.01	143385.04	143379.42	117778.28	122113.96	121956.03	117149.17	131110.16	127610.89	123415.74	11148.41	10532.15	9950.49	7198.46	7098.45	7175.99	8121.98	7484.41	7960.68	8583.97	8790.49	4106.82		
4669	Translocon-associated protein subunit alpha	P43307	60403.72	58315.76	55052.05	40893.41	38003.71	37258.22	43956.65	45225.17	43560.82	51700.40	49130.65	47068.37	48002.26	41409.91	37484.80	39074.63	35062.31	34264.96	46945.58	42308.19	36729.02	32654.32	26859.26	24687.37	23360.34	22206.64	18606.35	25971.14	23961.57	21821.04	27216.96	27218.79	17587.41		
4670	Translocon-associated protein subunit delta	P51571	130717.68	120936.78	114578.37	82781.78	81032.43	72126.69	108041.12	104213.84	97757.63	122415.54	115657.08	107397.63	99432.62	89479.12	59706.14	91034.49	86515.82	81630.03	78850.70	78824.60	71829.97	73918.50	65317.56	59454.82	46506.82	42958.50	33093.26	56351.06	52785.54	49475.58	49069.52	46184.48	30431.17		
4671	Translocon-associated protein subunit gamma	Q9UNL2	42861.66	40452.62	35148.02	36979.24	35039.90	29670.01	37546.11	35683.75	32815.57	44696.84	42497.15	38587.43	36921.02	34352.34	22760.44	36909.98	34374.81	31185.27	36029.06	35417.38	32970.43	2.27	1.49	0.65	0.84	1.31	0.69	4.16	2.47	0.11	4.90	4.98	0.12	x	
4672	Transmembrane 9 superfamily member 2	Q99805	70482.57	77182.64	62900.67	48595.47	55467.99	48031.88	61910.34	56913.44	57061.32	67275.04	64498.74	58608.18	57843.46	56600.26	49914.92	46454.51	50029.98	53321.44	48612.73	48069.86	52130.69	2.27	1.49	0.65	0.84	1.31	0.69	4.16	2.47	0.11	4.90	4.98	0.12	x	
4673	Transmembrane 9 superfamily member 3	Q9HD45	179060.13	165647.60	155852.47	115084.55	107673.69	102459.63	126139.12	123296.79	114368.54	141214.27	133514.64	118354.38	131327.49	128270.73	104940.50	110581.81	101812.03	97150.47	120801.84	109374.85	106136.49	2.27	1.49	0.65	0.84	1.31	0.69	4.16	2.47	0.11	4.90	4.98	0.12	x	
4674	Transmembrane 9 superfamily member 4	Q92544	97197.99	82136.76	71120.45	62129.61	57157.59	49887.08	62013.07	61308.14	54384.27	74952.12	66834.22	59962.12	70552.00	63207.82	40387.71	65061.12	59141.03	55081.10	68145.60	54951.28	51717.04	2654.66	2492.43	2419.12	1196.13	1126.37	867.69	2122.17	1765.44	1752.66	1657.08	1546.38	813.77		
4675	Transmembrane emp24 domain-containing protein 1	Q13445	16661.27	15757.39	14898.94	12516.26	12514.22	10292.01	12938.25	12842.04	11644.20	15019.96	14288.37	13448.66	13142.22	13070.22	9869.01	13020.44	12568.83	11867.37	10728.48	10587.93	9518.66	2.27	1.49	0.65	0.84	1.31	0.69	4.16	2.47	0.11	4.90	4.98	0.12	x	
4676	Transmembrane emp24 domain-containing protein 10	P49755	283263.82	260924.02	241475.53	173104.49	172369.86	154384.13	221917.17	199070.06	190780.47	242481.19	232597.54	207425.63	201747.10	193466.48	151100.55	206155.17	203516.27	192072.68	192270.97	181172.74	158507.60	34286.03	23770.71	21869.50	19930.43	20162.14	17561.57	29259.06	27514.52	25691.07	28104.29	25118.95	17558.26		
4677	Transmembrane emp24 domain-containing protein 2	Q15363	56.25	54.32	67.54	28.25	779.18	463.22	417.02	2497.46	1950.56	46.70	14.49	22.03	2.13	0.97	0.60	816.69	833.27	582.40	2.22	0.19	0.06	2.27	1.49	0.65	0.84	1.31	0.69	4.16	2.47	0.11	4.90	4.98	0.12	x	
4678	Transmembrane emp24 domain-containing protein 3	Q9Y3Q3	5522.27	4591.51	3952.02	2547.80	2017.64	1477.76	3456.49	3670.40	2687.14	3580.43	3249.00	2891.22	4073.04	3711.46	1201.46	2737.46	2716.70	2017.12	1993.91	1968.91	1591.84	2.27	1.49	0.65	0.84	1.31	0.69	4.16	2.47	0.11	4.90	4.98	0.12	x	
4679	Transmembrane emp24 domain-containing protein 4	Q7Z7H5	8432.70	6438.09	6857.32	6521.44	7091.35	7093.02	8832.43	8143.83	7555.18	7499.61	7596.44	7115.07	7389.89	7689.92	7681.56	7193.71	7611.77	6687.14	7305.81	5720.37	7049.17	2.27	1.49	0.65	0.84	1.31	0.69	4.16	2.47	0.11	4.90	4.98	0.12	x	
4680	Transmembrane emp24 domain-containing protein 5	Q9Y3A6	0.14	2.53	0.81	1.07	2.77	0.68	0.96	0.36	0.63	0.35	0.75	0.38	2.13	0.97	0.60	4.64	1.07	0.47	2.22	0.19	0.06	1225.27	976.82	1037.51	1021.35	998.62	739.22	1343.45	1194.77	1105.07	1189.52	913.95	579.06		x
4681	Transmembrane emp24 domain-containing protein 7	Q9Y3B3	138269.93	133352.90	124571.33	94231.36	93196.52	85787.42	112256.82	106454.59	102518.65	118497.80	114429.06	111365.38	112788.21	102891.58	90951.25	93552.42	89231.14	86820.90	94285.10	92362.31	86276.13	38805.38	33754.00	32626.53	38449.68	37836.68	32983.08	47634.32	50000.53	47512.89	51049.85	42860.48	32912.47		
4682	Transmembrane emp24 domain-containing protein 9	Q9BVK6	149021.63	141735.47	137963.10	118814.37	114557.93	112271.25	110474.16	114786.25	107997.40	148325.69	140090.73	127595.95	123765.48	120261.37	105822.10	113762.76	106844.55	104661.64	107218.55	110534.22	103554.09	129166.98	122851.87	106720.31	53446.32	52241.54	42257.45	68509.42	62164.67	57361.29	60523.63	60126.17	37719.90		
4683	Transmembrane prolyl 4-hydroxylase	Q9NXG6	1004.88	648.44	447.10	319.89	310.50	314.66	517.78	396.47	405.84	553.35	867.50	538.14	453.03	391.16	42.86	373.89	414.08	447.04	398.66	474.90	368.44	2.27	1.49	0.65	0.84	1.31	0.69	4.16	2.47	0.11	4.90	4.98	0.12	x	
4684	Transmembrane protease serine 11E	Q9UL52	34338.98	34041.18	30564.67	40350.99	38969.73	37129.04	33785.00	30672.69	30968.84	53648.54	49470.53	45624.65	34722.76	35828.07	30842.21	37984.62	30922.14	36957.12	35608.09	35854.53	34418.54	2.27	1.49	0.65	0.84	1.31	0.69	4.16	2.47	0.11	4.90	4.98	0.12	x	
4685	Transmembrane protease serine 12	Q86WS5	5215.91	4219.70	5173.41	3579.09	3333.48	3507.17	5007.22	4804.81	3557.96	4449.49	3619.37	4005.27	4732.29	4116.67	3019.78	4068.42	3066.02	3989.05	3114.98	3133.94	2610.88	2.27	1.49	0.65	0.84	1.31	0.69	4.16	2.47	0.11	4.90	4.98	0.12	x	
4686	Transmembrane protein 109	Q9BVC6	169217.69	172052.94	176818.79	98536.70	92058.73	88311.88	133179.69	121312.32	122627.93	120076.67	116206.63	108946.51	123887.76	118182.51	102621.43	98929.74	99852.38	95730.34	112856.46	98978.24	104512.79	66885.60	69346.05	60403.52	49130.31	45107.99	43180.49	50023.72	50068.95	49999.08	52826.63	50424.23	29963.17		
4687	Transmembrane protein 131-like	A2VDJ0	7818.22	8040.07	7699.46	7192.88	5356.00	5241.46	8234.26	7080.63	7262.15	8032.50	6943.08	6918.08	7530.87	7501.12	5788.91	5781.74	5792.46	5032.18	6209.42	6050.12	5827.33	9449.06	8742.98	8319.53	6576.65	6415.21	6204.64	5588.93	5511.72	5097.87	6146.51	5696.39	4214.96		
4688	Transmembrane protein 135	Q86UB9	10585.90	10369.30	9778.77	12246.93	13328.17	12764.37	9236.67	9249.11	9089.07	11097.20	10409.84	10442.86	8742.62	8116.79	7872.42	15792.32	15152.27	14493.06	8133.67	8140.23	7565.96	12918.52	12351.94	11250.75	10803.21	10902.54	10161.03	10117.12	10365.86	9963.80	12132.03	12020.55	8702.31		
4689	Transmembrane protein 151A	Q8N4L1	0.14	2.53	0.81	1.07	2.77	0.68	0.96	0.36	0.63	0.35	0.75	0.38	2.13	0.97	0.60	4.64	1.07	0.47	2.22	0.19	0.06	7576.64	7373.61	7016.66	4885.01	4738.29	4450.64	6155.60	5790.23	5695.96	5636.17	6060.74	4595.44		x
4690	Transmembrane protein 165	Q9HC07	19150.85	19049.58	18609.39	11948.38	11802.60	11944.62	12776.54	12069.67	12062.17	17461.57	16876.14	15720.94	14970.51	12971.98	11565.71	13256.45	11993.93	12177.29	10726.11	10479.75	9984.22	1273.60	1165.63	1087.55	679.18	597.78	490.14	1100.54	1034.44	967.91	1141.75	1078.89	640.63		
4691	Transmembrane protein 186	Q96B77	21714.23	20223.11	17937.49	13600.73	13183.09	12104.98	18252.55	17463.17	15888.46	17570.82	16714.40	14955.61	17809.42	17074.15	10757.65	15452.08	13980.58	13689.08	14838.35	14185.33	11697.40	2.27	1.49	0.65	0.84	1.31	0.69	4.16	2.47	0.11	4.90	4.98	0.12	x	
4692	Transmembrane protein 192	Q8IY95	2472.77	2700.89	2420.18	1508.00	1773.41	1666.00	2192.24	1697.06	1853.05	2580.89	2131.00	2371.41	2147.58	2182.15	1619.58	2033.09	1850.99	1879.46	1839.88	1788.63	1733.86	2.27	1.49	0.65	0.84	1.31	0.69	4.16	2.47	0.11	4.90	4.98	0.12	x	
4693	Transmembrane protein 205	Q6UW68	130888.75	132185.46	129498.36	95925.53	91940.99	87460.54	94202.64	87942.67	87206.54	122739.96	113932.62	102861.03	102774.14	106568.27	87391.25	89411.60	89520.41	90892.05	82796.97	79559.03	73049.28	2.27	1.49	0.65	0.84	1.31	0.69	4.16	2.47	0.11	4.90	4.98	0.12	x	
4694	Transmembrane protein 33	P57088	226934.77	206871.95	193249.01	142675.69	137050.73	129138.55	160916.62	157019.71	145411.60	169826.95	169227.45	149011.63	162515.24	148828.42	122390.89	146922.16	141256.79	136267.89	146700.76	134403.21	124573.55	771.27	860.13	908.99	163.97	150.07	137.79	1024.07	667.24	484.50	396.68	442.55	226.64		
4695	Transmembrane protein 40	Q8WWA	0.14	2.53	0.81	1.07	2.77	0.68	0.96	0.36	0.63	0.35	0.75	0.38	2.13	0.97	0.60	4.64	1.07	0.47	2.22	0.19	0.06	38778.24	39981.89	39855.80	29485.12	28846.32	25444.04	37393.27	34516.21	33916.36	36956.70	35729.95	23635.46		x
4696	Transmembrane protein 43	Q9BTV4	26606.65	27143.88	23377.50	19438.62	18594.14	17631.08	22100.14	23727.10	23015.27	23818.94	23392.78	21821.67	21611.18	21185.00	15718.82	19803.33	20745.74	18179.47	19145.21	18585.20	16421.96	8894.98	8017.87	9258.60	6674.71	5986.62	5853.06	7685.63	7020.72	6984.67	5878.51	5661.05	4178.14		
4697	Transmembrane protein 87A	Q8NBN3	4180.48	4759.94	4646.62	4404.91	4153.52	4405.71	3722.25	3576.90	2768.55	4507.67	4203.47	3842.91	4676.00	4461.98	4166.76	4806.96	3536.30	4179.27	4378.42	3619.28	3254.51	2.27	1.49	0.65	0.84	1.31	0.69	4.16	2.47	0.11	4.90	4.98	0.12	x	
4698	Transmembrane protein KIAA1109	Q2LD37	0.14	2.53	0.81	1.07	2.77	0.68	0.96	0.36	0.63	0.35	0.75	0.38	2.13	0.97	0.60	4.64	1.07	0.47	2.22	0.19	0.06	12647.68	11642.22	10684.88	11138.05	10033.04	8982.86	11210.58	10987.35	10376.88	9431.30	9991.56	8816.10		x
4699	Transport and Golgi organization protein 1 homolog	Q5JRA6	16924.23	16252.72	13950.65	10876.84	10597.82	8707.89	12611.81	12149.11	11499.10	15052.38	13970.53	13415.77	13186.18	12553.57	9420.16	12415.28	11711.41	11952.62	11915.99	11110.57	10396.53	2.27	1.49	0.65	0.84	1.31	0.69	4.16	2.47	0.11	4.90	4.98	0.12	x	
4700	Transportin-1	Q92973	99521.80	91720.74	81899.51	62902.34	60890.71	54768.65	73720.18	67807.84	65156.53	74982.32	70949.77	62179.20	81406.77	72147.16	53814.86	60418.27	54234.24	54894.79	69796.08	61435.38	58434.28	190062.41	162639.30	155845.33	115972.76	111251.28	96326.79	139882.19	128919.63	119533.85	142372.46	135710.82	83741.26		
4701	Transportin-2	O14787	9052.94	6852.65	4133.29	1180.12	1252.40	1153.19	1214.65	1890.52	1309.18	6934.57	6146.26	4097.49	4922.07	1384.15	1112.93	1381.34	1344.78	1203.35	750.05	1007.10	1014.67	5106.78	4945.24	4682.20	3585.70	3055.08	2853.05	3383.86	3271.25	3430.84	4266.09	4240.87	2381.28		
4702	Transportin-3	Q9Y5L0	0.14	2.53	0.81	1.07	2.77	0.68	0.96	0.36	0.63	0.35	0.75	0.38	2.13	0.97	0.60	4.64	1.07	0.47	2.22	0.19	0.06	511.52	382.79	387.42	314.11	254.52	291.44	403.56	335.42	341.47	488.03	382.57	95.63		x
4703	Treacle protein	Q13428	3819.33	4253.63	3513.41	2915.26	2952.74	2506.87	918.70	979.23	826.06	3610.12	3379.53	2798.68	3342.07	3032.97	1845.71	1456.28	1458.55	1260.68	3360.12	3494.55	2578.74	2.27	1.49	0.65	0.84	1.31	0.69	4.16	2.47	0.11	4.90	4.98	0.12	x	
4704	Trefoil factor 2	Q03403	3872.10	4106.80	3881.15	4183.63	3769.05	3608.86	4092.18	4084.83	4082.98	4660.74	4026.00	4046.28	4364.55	4334.24	3750.43	3661.71	3610.05	3612.56	3292.94	3429.32	3159.50	15808.61	15589.98	13923.71	14204.14	14365.46	13064.04	14979.72	14006.33	13481.33	12087.13	12266.36	10412.20		
4705	Treslin	Q7Z2Z1	26697.35	27309.21	23045.49	22305.53	23352.62	21159.68	31832.14	31130.85	27641.04	29090.33	28412.21	25158.67	25115.45	24773.46	23027.75	22336.52	22982.65	20819.83	23808.44	22876.34	21763.57	638.22	626.42	548.09	335.76	330.14	258.80	507.20	434.72	416.62	494.66	376.99	282.45		
4706	Tricarboxylate transport protein_ mitochondrial	P53007	57411.91	56077.76	53578.24	56332.39	52142.43	42659.66	56345.23	55284.07	50925.26	58582.72	59746.18	52428.52	49886.32	31647.85	27814.92	62679.24	60704.01	54924.38	47286.73	44357.66	44287.12	17675.81	17120.19	15642.76	11381.00	11240.05	9780.54	14158.37	13618.19	13153.51	19353.87	19262.81	9325.34		
4707	Trichohyalin	Q07283	1116.25	1115.24	940.84	296.64	349.07	314.54	484.45	443.14	471.41	719.66	549.14	667.23	429.41	430.17	537.65	1096.87	1111.09	980.74	618.23	524.50	475.31	83261.47	80600.85	69068.65	51120.31	47284.52	42821.11	58137.13	56494.19	52625.32	63082.67	62671.61	41056.24		
4708	Trifunctional enzyme subunit alpha_ mitochondrial	P40939	386636.23	352812.32	329780.72	257244.71	252336.56	239027.25	304330.52	282511.80	278874.91	345033.95	325374.30	300066.23	299419.69	265961.00	197244.16	280011.60	252269.96	253060.60	249055.66	238058.90	214263.44	90287.87	81501.65	71410.06	54284.78	52862.87	42457.95	70607.05	65227.46	58730.66	71029.64	66416.31	49074.17		
4709	Trifunctional enzyme subunit beta_ mitochondrial	P55084	229271.62	228093.87	211369.87	158722.69	152994.19	138910.05	176103.95	171898.63	165497.78	199869.06	190050.62	179625.84	185416.61	179499.60	143189.95	163835.46	153514.99	146140.86	158615.11	145457.21	132477.06	54118.14	53021.57	49048.33	40767.94	40311.54	35496.08	46897.85	45743.62	43045.70	45843.57	44842.81	33897.37		

Supplementary Table S1. Overview on the abundance of all relatively quantified 5180 proteins

			MCF-7																					MDA-MB-231													
	Protein name	UniProt	control_01	control_02	control_03	Dai_IC20_01	Dai_IC20_02	Dai_IC20_03	Dai_SC20_01	Dai_SC20_02	Dai_SC20_03	Gen_IC20_01	Gen_IC20_02	Gen_IC20_03	Gen_SC20_01	Gen_SC20_02	Gen_SC20_03	SSE_IC20_01	SSE_IC20_02	SSE_IC20_03	SSE_SC20_01	SSE_SC20_02	SSE_SC20_03	control_01	control_02	control_03	Dai_IC20_01	Dai_IC20_02	Dai_IC20_03	Gen_IC20_01	Gen_IC20_02	Gen_IC20_03	SSE_IC20_01	SSE_IC20_02	SSE_IC20_03	unique to MCF-7	unique to MDA-MB-231
4710	Trifunctional purine biosynthetic protein adenosine-3	P22102	332861.34	320680.76	306320.77	248335.77	239886.66	229064.95	283822.95	268580.52	256785.37	294372.55	278307.98	266141.37	272327.88	256354.92	221900.90	242244.42	230770.30	227894.95	236306.43	226867.70	214724.85	406573.14	382584.21	350724.32	245142.48	234273.66	216943.63	301929.02	284423.95	280432.63	308644.63	307950.15	205292.90		
4711	Trinucleotide repeat-containing gene 18 protein	O15417	252787.53	245916.98	232477.33	193440.21	189314.21	181873.27	208856.84	198739.27	195528.60	231621.40	218984.49	214922.71	214291.30	212264.49	186147.53	180646.68	172539.56	171625.20	180069.14	176833.29	172444.14	13335.64	11512.98	10794.04	9052.24	8519.92	8145.86	9674.35	9457.96	9265.92	10246.27	10077.42	6758.86		
4712	Trinucleotide repeat-containing gene 6A protein	Q8NDV7	0.14	2.53	0.81	1.07	2.77	0.68	0.96	0.36	0.63	0.35	0.75	0.38	2.13	0.97	0.60	4.64	1.07	0.47	2.22	0.19	0.06	9748.32	7151.80	9401.80	8073.34	7960.36	6808.23	9601.31	8748.21	9470.81	10804.09	9063.38	6649.67		x
4713	TRIO and F-actin-binding protein	Q9H2D6	0.14	2.53	0.81	1.07	2.77	0.68	0.96	0.36	0.63	0.35	0.75	0.38	2.13	0.97	0.60	4.64	1.07	0.47	2.22	0.19	0.06	11048.90	12420.18	10321.34	9294.46	9097.69	8821.40	8790.23	9559.74	9484.61	11470.76	10087.36	4804.81		x
4714	Triokinase/FMN cyclase	Q3LXA3	96657.42	89566.90	82713.59	53579.33	53959.97	49130.94	69584.94	66295.16	64929.98	74959.19	72611.33	65643.25	75183.33	70719.47	55365.44	54622.67	54318.44	52064.36	59923.72	55776.65	51470.33	6330.33	6113.20	5825.34	3890.28	3872.96	3241.53	4410.85	4405.36	3915.87	5032.85	4867.95	2804.46		
4715	Triosephosphate isomerase	P60174	1946306.61	1822256.08	1688975.17	1326484.28	1260918.50	1192890.49	1875844.90	1826150.68	1729510.64	1654056.49	1547873.24	1435322.08	1681175.19	1572798.56	1249874.25	1326853.11	1244882.70	1231513.41	1386590.15	1313486.00	1231288.45	2929581.51	2777485.90	2545980.25	1341371.94	1307597.18	1188827.74	1536753.95	1455144.17	1359730.95	1519023.52	1469287.17	924445.20		
4716	Tripartite motif-containing protein 10	Q9UDY6	1017.04	1088.64	1158.35	682.88	1244.60	1084.12	1004.61	1134.73	759.26	983.48	824.46	994.06	1071.41	751.86	729.15	1439.73	875.60	1494.17	692.11	633.24	577.04	2.27	1.49	0.65	0.84	1.31	0.69	4.16	2.47	0.11	4.90	4.98	0.12	x	
4717	Tripartite motif-containing protein 16-like protein	Q309B1	1101.54	989.31	603.80	577.65	527.95	338.17	552.64	523.90	398.34	777.35	697.49	572.73	687.07	649.00	253.81	502.44	664.21	490.56	667.34	587.38	555.91	2.27	1.49	0.65	0.84	1.31	0.69	4.16	2.47	0.11	4.90	4.98	0.12	x	
4718	Tripartite motif-containing protein 29	Q14134	0.14	2.53	0.81	1.07	2.77	0.68	0.96	0.36	0.63	0.35	0.75	0.38	2.13	0.97	0.60	4.64	1.07	0.47	2.22	0.19	0.06	33452.53	31721.40	31830.53	25098.97	24594.94	23301.81	21614.47	20655.64	21124.12	24442.33	23153.99	18446.91		x
4719	Tripartite motif-containing protein 34	Q9BYJ4	0.14	2.53	0.81	1.07	2.77	0.68	0.96	0.36	0.63	0.35	0.75	0.38	2.13	0.97	0.60	4.64	1.07	0.47	2.22	0.19	0.06	4961.74	5283.94	5085.23	3094.96	2670.33	2527.20	3895.65	3567.54	4070.12	3238.38	2998.46	1744.13		x
4720	Tripartite motif-containing protein 42	Q8IWZ5	3855.52	4019.69	3442.69	2753.84	2420.48	2623.13	3302.11	3107.58	3060.55	3106.82	3032.53	2817.21	3214.53	3022.30	1931.33	2876.70	2655.77	2903.30	3073.91	2993.94	2576.38	2.27	1.49	0.65	0.84	1.31	0.69	4.16	2.47	0.11	4.90	4.98	0.12	x	
4721	Tripartite motif-containing protein 45	Q9H8W5	0.14	2.53	0.81	1.07	2.77	0.68	0.96	0.36	0.63	0.35	0.75	0.38	2.13	0.97	0.60	4.64	1.07	0.47	2.22	0.19	0.06	9711.14	8569.25	8100.41	6988.30	6900.47	6679.75	9305.60	7890.88	7213.23	10172.53	9421.72	3282.96		x
4722	Tripartite motif-containing protein 51	Q9BSJ1	74550.33	70437.26	60203.50	50756.79	46000.21	41888.05	70394.71	51385.08	48646.90	55226.61	51093.23	45226.94	59833.71	67391.68	38979.37	54615.20	46418.86	47766.79	58948.18	55891.10	45087.03	73520.72	22337.02	2296.24	22749.64	21830.24	13344.38	74433.04	66507.10	47120.39	50430.40	48583.24	22893.97		
4723	Tripartite motif-containing protein 52	Q96A61	13207.43	14298.20	12194.71	9705.05	10045.22	9628.97	11794.15	10863.45	10805.34	12564.84	11964.70	11202.40	12203.56	11873.93	9191.80	8321.32	9015.56	8339.91	10258.28	9664.07	9909.59	2.27	1.49	0.65	0.84	1.31	0.69	4.16	2.47	0.11	4.90	4.98	0.12	x	
4724	Tripartite motif-containing protein 55	Q9BYV6	347455.74	331831.15	276334.82	213830.43	200456.85	167661.71	256366.40	246576.24	214705.06	292402.81	278936.32	229389.63	235676.54	207450.89	117596.25	266608.82	253302.91	227427.79	240032.78	226651.61	176910.41	2.27	1.49	0.65	0.84	1.31	0.69	4.16	2.47	0.11	4.90	4.98	0.12	x	
4725	Tripartite motif-containing protein 77	I1YAP6	0.14	2.53	0.81	1.07	2.77	0.68	0.96	0.36	0.63	0.35	0.75	0.38	2.13	0.97	0.60	4.64	1.07	0.47	2.22	0.19	0.06	1708.38	1524.07	1350.07	974.64	838.55	422.90	1563.26	1408.71	1045.33	1555.03	1404.23	505.65		x
4726	tRNA (cytosine(34)-C(5))-methyltransferase	Q08J23	60644.40	60785.66	52531.08	41428.43	39047.60	33787.99	54534.40	49332.56	48862.61	52301.14	50558.10	43809.45	50504.50	48295.11	34750.35	42789.92	39709.99	36383.42	47252.43	43793.19	42553.94	69801.76	63744.36	59932.90	41538.00	38829.06	33201.09	49783.54	47726.08	44661.09	48298.76	46082.94	28598.55		
4727	tRNA (guanine(10)-N2)-methyltransferase homolog	Q7Z4G4	603.94	667.95	493.08	227.30	300.56	286.76	787.75	1511.03	1286.89	434.66	348.46	290.18	547.82	1079.71	294.38	439.69	1435.58	273.53	812.21	304.88	340.60	2.27	1.49	0.65	0.84	1.31	0.69	4.16	2.47	0.11	4.90	4.98	0.12		
4728	tRNA methyltransferase 10 homolog C	Q7L0Y3	48583.80	44244.55	37698.16	29299.92	27965.76	25379.40	35532.76	35488.95	31380.87	43283.08	40270.43	38352.40	33733.14	32833.32	20412.15	31963.91	30599.02	29032.38	33035.09	30720.48	28336.10	9243.15	8758.13	8639.74	6031.28	5801.03	5422.29	7641.59	7753.40	7263.88	7667.01	7091.43	4931.20		
4729	tRNA pseudouridine synthase A	Q9Y606	38907.21	38804.71	35885.74	31156.11	31129.51	29375.67	35028.28	33434.82	32505.33	37182.87	36113.62	35085.29	32712.58	32100.96	26548.48	31065.83	29754.81	27919.99	28769.59	28840.56	27334.01	6868.99	6717.79	6072.02	2767.45	2557.88	2017.60	3486.53	3135.60	2858.39	2883.39	2710.76	1368.89		
4730	tRNA wybutosine-synthesizing protein 5	A2RUC4	0.14	2.53	0.81	1.07	2.77	0.68	0.96	0.36	0.63	0.35	0.75	0.38	2.13	0.97	0.60	4.64	1.07	0.47	2.22	0.19	0.06	348.50	622.98	631.82	609.14	649.37	561.12	529.87	660.47	702.55	466.74	431.51	467.00		x
4731	tRNA-dihydrouridine(20) synthase [NAD(P)+]-like	Q9NX74	4873.74	4723.62	4519.34	3137.70	2957.47	2715.85	3566.25	3402.12	2911.15	4102.31	3843.64	3601.09	4045.79	3692.22	3312.18	2763.28	2501.76	2548.45	3117.67	2992.00	2801.38	2.27	1.49	0.65	0.84	1.31	0.69	4.16	2.47	0.11	4.90	4.98	0.12	x	
4732	tRNA-dihydrouridine(20a/20b) synthase [NAD(P)+]-like	O95620	1579.04	940.45	404.19	949.45	959.95	429.22	970.79	1028.85	660.53	1573.16	1201.59	758.74	1498.52	1288.71	29.22	700.91	746.62	435.55	942.40	674.73	477.32	2.27	1.49	0.65	0.84	1.31	0.69	4.16	2.47	0.11	4.90	4.98	0.12	x	
4733	tRNA-specific adenosine deaminase 1	Q9BUB4	13547.92	12753.12	12832.65	8804.39	7893.73	8466.35	10393.90	9669.46	9313.05	11105.00	11007.91	10194.52	12331.39	10175.41	8974.62	7871.17	7379.97	7909.65	8750.54	9232.06	8051.92	2.27	1.49	0.65	0.84	1.31	0.69	4.16	2.47	0.11	4.90	4.98	0.12	x	
4734	tRNA-splicing endonuclease subunit Sen15	Q8WW0	3390.17	3162.84	3002.39	2550.75	2361.27	2273.71	2334.08	2366.97	2066.94	3006.08	3005.74	2828.43	3268.81	3142.89	2249.89	2003.73	2148.66	2194.22	2788.96	2683.33	2389.38	148.44	337.22	501.98	380.98	440.20	341.86	437.88	430.31	461.55	248.92	371.64	375.29		
4735	tRNA-splicing ligase RtcB homolog	Q9Y3I0	85912.69	76365.37	69093.37	45896.26	43565.05	37784.60	62881.69	85381.68	78042.28	64294.44	60252.63	53790.44	64288.64	55921.80	35099.62	47149.72	49939.02	44949.69	48901.54	44529.58	36407.10	55483.23	46917.92	40856.48	32175.83	29705.77	25026.71	40255.76	36695.50	32681.84	40443.52	35907.19	20426.03		
4736	Trophoblast glycoprotein	Q13641	42549.14	40714.77	39690.13	32998.54	32395.85	30943.77	35930.38	34767.68	34146.17	36723.50	35110.24	32989.33	29544.69	33053.22	29200.06	31411.07	29229.71	29313.59	27862.78	28031.26	27879.22	2.27	1.49	0.65	0.84	1.31	0.69	4.16	2.47	0.11	4.90	4.98	0.12	x	
4737	Tropomodulin-2	Q9NZR1	39070.64	32875.64	36563.24	30385.84	28830.40	26549.81	35912.61	34643.63	32038.12	33737.25	31476.08	30769.42	33466.35	35552.30	52304.99	33002.54	30379.30	27946.50	31730.52	30084.70	32682.75	57894.40	53817.73	52558.81	44094.58	43539.78	42424.83	55273.74	50882.83	51613.90	56108.33	53936.73	25722.61		
4738	Tropomodulin-3	Q9NYL9	150936.45	144200.11	132122.48	92216.72	89308.14	82622.93	117561.51	110494.31	105767.34	125717.26	119380.18	110085.94	120708.62	109850.97	83039.30	93388.92	89405.22	84946.10	92748.25	85500.35	79075.87	32246.47	29841.53	24560.88	20495.35	20004.41	16590.32	25115.67	24061.44	20754.51	28086.78	26026.92	16501.78		
4739	Tropomodulin-4	Q9NZQ9	9149.25	7257.86	5461.70	4150.74	3788.11	2886.17	5990.24	3210.81	5055.59	6306.21	6073.72	4737.49	6913.18	3920.43	1858.92	4323.00	3288.18	4086.41	4404.51	3976.36	3427.92	25777.90	23804.81	24438.20	17324.42	18469.03	17769.36	16278.33	16226.66	16088.00	20835.18	18711.45	17963.67		
4740	Tropomyosin alpha-1 chain	P09493	181.57	42.75	101.32	51.11	51.23	42.38	89.43	134.04	84.04	74.59	51.51	85.30	247.86	240.27	56.23	78.98	49.17	72.53	117.75	147.57	93.60	117959.74	116930.39	106232.29	82845.06	81488.34	76223.31	107440.10	103826.70	102820.32	106264.69	99709.67	73367.04		
4741	Tropomyosin alpha-3 chain	P06753	408322.28	389201.72	375745.15	305376.52	293716.88	278458.34	305960.02	295868.50	283251.78	335546.39	324913.07	307709.97	325663.92	309706.23	272370.41	292966.44	275460.53	271800.05	282438.78	269422.19	266000.47	84254.53	82900.31	76992.46	76553.27	78999.25	74792.14	86766.63	84376.37	83941.26	99767.73	99181.58	68803.04		
4742	Tropomyosin alpha-4 chain	P67936	341306.13	330360.26	304823.65	224082.24	217519.38	193833.57	245514.08	237849.33	228399.37	275744.04	262134.46	247134.07	266777.02	247268.03	208442.13	219299.50	210238.01	205486.27	217498.42	207353.15	194752.81	60047.33	57645.15	52759.72	39162.61	39002.37	34648.85	46987.08	45497.36	44298.17	52284.84	51745.09	35028.06		
4743	Tropomyosin beta chain	P07951	14430.51	13606.06	12996.93	9439.98	9208.30	8861.27	8727.34	9015.66	8741.36	12293.07	12162.93	11944.23	12110.58	11319.14	8989.38	9638.66	9229.44	9544.95	8890.02	8553.59	8695.52	543536.46	515384.85	473091.50	392249.93	382608.98	348541.72	501491.90	474872.02	440905.36	547973.81	509014.52	364599.38		
4744	Troponin C skeletal muscle	P02585	80524.31	79061.88	73955.09	61190.14	59104.16	52027.69	77957.67	70818.84	67730.42	69746.13	68744.71	55846.37	72571.04	67797.80	49853.61	62569.38	56853.61	52572.66	62911.50	57524.85	53470.95	17525.23	14944.50	13393.56	10315.13	9968.15	7745.49	11985.48	11615.10	10707.58	18581.25	15972.01	7217.36		
4745	TRPM8 channel-associated factor 1	Q9Y4C2	120490.98	114044.46	100268.11	65756.71	62374.40	55620.05	85962.56	83217.81	77628.62	86617.82	79558.27	74474.08	81974.90	73783.70	60544.01	70100.23	67421.89	64983.93	70483.64	67660.97	62500.85	2.27	1.49	0.65	0.84	1.31	0.69	4.16	2.47	0.11	4.90	4.98	0.12	x	
4746	Tryptophan 5-hydroxylase 1	P17752	9527.15	8830.95	8701.56	5338.54	5221.90	4762.37	6969.11	6875.44	6268.90	6838.36	6588.56	6239.25	7303.89	6615.33	4963.39	5883.48	5547.23	5216.46	6194.31	6072.34	5489.70	2.27	1.49	0.65	0.84	1.31	0.69	4.16	2.47	0.11	4.90	4.98	0.12	x	
4747	Tryptophan 5-hydroxylase 2	Q8IWU9	100315.77	93463.07	82199.29	56845.38	54043.23	49081.11	70738.96	67516.79	63484.19	72097.57	70948.61	65060.20	76996.08	70711.04	52792.62	63957.19	58268.79	55819.46	61013.00	60680.86	56256.12	2.27	1.49	0.65	0.84	1.31	0.69	4.16	2.47	0.11	4.90	4.98	0.12	x	
4748	Tryptophan--tRNA ligase_ cytoplasmic	P23381	315893.84	297765.25	273807.71	233911.31	227704.47	203991.94	283628.56	271637.16	255835.00	276257.76	263626.21	247239.92	262774.98	248082.30	201334.73	245373.40	234840.95	218347.20	228302.37	220222.33	202586.84	35799.54	33154.84	30606.29	20644.62	20571.97	18332.27	25837.38	23897.50	23087.72	23526.93	22396.87	12052.09		
4749	Tryptophan--tRNA ligase_ mitochondrial	Q9UGM	0.14	2.53	0.81	1.07	2.77	0.68	0.96	0.36	0.63	0.35	0.75	0.38	2.13	0.97	0.60	4.64	1.07	0.47	2.22	0.19	0.06	37398.29	35481.08	33337.53	25432.24	24174.31	22960.42	31495.33	30897.85	27656.86	28652.41	27723.64	19107.82		x
4750	Tubby-related protein 1	O00294	83051.80	77991.69	74632.60	75116.74	75428.42	71148.04	108982.72	103218.20	99620.51	97230.04	93366.51	87548.18	85427.02	77581.23	80220.11	97623.86	92184.67	83694.04	86213.73	81476.67	81221.27	168170.56	150585.47	146731.60	120748.19	120249.93	113459.12	108662.21	103729.50	101169.17	105303.17	100457.06	74676.13		
4751	Tuberin	P49815	60768.24	59915.04	53629.59	45914.75	46087.29	44399.80	53608.91	53494.76	56654.46	59108.50	57212.70	54609.23	54978.96	51692.99	46080.87	53575.17	49890.65	48144.13	58778.67	52308.51	53827.46	2.27	1.49	0.65	0.84	1.31	0.69	4.16	2.47	0.11	4.90	4.98	0.12	x	
4752	Tubulin alpha chain-like 3	A6NHL2	100909.20	95595.44	88061.86	35416.23	36567.43	34202.07	77360.17	81639.95	80793.35	73022.01	72662.76	65398.47	66416.24	57081.30	46753.25	81222.65	76763.78	80075.81	54301.22	50890.51	46727.92	92434.88	86561.21	78419.38	93497.59	91243.89	79228.56	133207.54	126607.57	116840.57	66722.80	65025.68	42300.41		
4753	Tubulin alpha-1A chain	Q71U36	0.14	2.53	0.81	1.07	2.77	0.68	0.96	0.36	0.63	0.35	0.75	0.38	2.13	0.97	0.60	4.64	1.07	0.47	2.22	0.19	0.06	4905131.72	4613792.03	4210092.03	2851432.14	2848130.09	2373046.09	3753392.08	3536755.79	3296210.07	3683622.73	3591777.08	2594335.72		x
4754	Tubulin alpha-1C chain	Q9BQE3	5255195.46	4986288.60	4527873.68	3353985.00	3238319.21	2955815.67	4331784.17	4269690.74	3984760.77	4069586.60	3877761.54	3547382.80	4222412.79	3912011.04	3023523.57	3521901.13	3345249.86	3130658.76	3747208.48	3565802.59	3279554.65	71745.47	65234.04	53448.67	36510.15	31585.24	18865.42	54000.42	48590.58	37273.01	56039.60	52320.74	26374.18		
4755	Tubulin alpha-3C chain	P0DPH7	371.83	473.55	307.95	380.34	277.44	284.48	126.11	48.96	6.16	820.90	824.87	806.48	863.27	471.16	51.57	209.42	173.20	122.02	648.50	558.69	382.16	2.27	1.49	0.65	0.84	1.31	0.69	4.16	2.47	0.11	4.90	4.98	0.12	x	
4756	Tubulin alpha-4A chain	P68366	400200.92	369246.83	283171.47	224628.20	209682.33	174325.62	350646.13	330268.20	293054.49	291796.29	269155.09	227884.61	300215.69	273278.86	142168.64	252529.12	244982.94	216434.17	280577.77	265052.30	225397.35	192775.38	177799.57	160963.25	89333.38	86150.50	64670.71	118339.46	103891.24	94554.23	94730.29	91946.91	57246.58		
4757	Tubulin alpha-8 chain	Q9NY65	31103.31	31181.54	31715.59	21743.80	20864.58	22531.83	28878.48	28130.10	27802.85	25541.64	24648.63	24271.11	27510.26	25734.94	22376.23	23468.38	22550.63	20875.80	20892.50	20534.78	19568.05	2.27	1.49	0.65	260.54	141.61	92.58	330.82	215.43	20.03	375.69	355.48	78.20		
4758	Tubulin beta chain	P07437	1244575.80	1097142.45	939662.78	754967.08	748288.17	635930.95	933971.02	911624.98	806784.21	930316.69	851303.36	725855.22	931305.76	875529.27	535126.32	693852.72	649544.33	592041.77	860136.60	800162.27	686120.64	4678039.72	4355567.98	4028591.05	2821762.28	2782506.63	2404440.26	3601673.83	3406403.96	3182265.53	3509365.98	3370486.46	2416968.08		
4759	Tubulin beta-1 chain	Q9H4B7	53798.33	50548.26	43154.20	33571.56	33737.83	30610.45	36523.42	34692.09	32090.01	39906.71	36661.46	34752.91	41647.57	35981.21	26715.85	38204.53	35117.77	34549.39	33407.31	33164.87	30447.44	65589.04	62102.95	61115.61	33087.80	32974.47	27784.27	38166.01	38264.92	35870.15	34847.11	32947.31	24142.80		
4760	Tubulin beta-2A chain	Q13885	126479.20	114796.71	96151.12	78592.95	76709.73	65875.62	79138.24	77079.46	71413.27	100193.14	97006.02	84326.36	103781.12	94264.10	53657.26	73403.17	67706.95	65689.00	92881.72	87995.17	77871.27	43727.10	41492.60	36249.88	27482.26	27068.20	21548.02	39628.11	36997.54	35781.49	37715.02	36354.04	22087.72		
4761	Tubulin beta-3 chain	Q13509	406463.19	391905.02	354038.85	307278.53	290661.14	267917.65	372810.91	355037.75	333830.91	385114.31	371824.83	339123.69	386866.68	367053.61	259639.19	290393.84	294111.15	274837.46	338719.39	334860.54	307542.42	386731.98	369575.92	344643.82	265641.89	262521.80	219248.92	307040.50	290209.02	271523.21	337842.13	320056.07	198499.19		
4762	Tubulin beta-4A chain	P04350	1836.80	1106.76	2234.12	862.28	953.63	576.79	919.33	1117.12	912.95	1242.99	1135.29	1112.72	884.90	1215.14	973.48	548.71	813.52	644.39	714.02	682.99	778.63	457826.53	414496.71	354682.26	389716.20	363242.20	267058.71	390504.09	342220.33	291090.91	499738.61	478768.54	282029.29		
4763	Tubulin beta-4B chain	P68371	4340055.70	4066281.47	3689368.24	2750170.75	2662968.51	2454704.25	3467581.21	3364434.70	3143940.77	3327212.91	3134787.88	2868952.66	3315694.47	3114746.06	2396666.68	2873950.69	2737749.14	2583118.14	2915773.77	2761067.15	2556399.33	2.27	1.49	0.65	0.84	1.31	0.69	4.16	2.47	0.11	4.90	4.98	0.12	x	
4764	Tubulin beta-6 chain	Q9BUF5	310944.87	293468.02	274011.52	215682.56	207966.10	193467.24	301967.91	257948.62	274645.86	310456.43	300805.90	275960.04	263164.34	240921.91	202390.56	219399.18	206175.28	196201.53	231022.54	219666.00	206034.78	404797.70	381011.17	341611.96	261050.80	250111.46	208883.95	347675.67	339455.82	308034.51	340261.86	330368.97	216297.23		
4765	Tubulin beta-8 chain	Q3ZCM7	67918.73	66829.18	59349.07	51209.48	48468.21	45694.22	55646.70	49801.75	46684.07	66263.31	64438.45	58818.04	57535.84	52722.05	46253.47	50832.72	48363.66	47918.37	49893.30	50806.58	44440.93	9689.38	7130.46	7122.12	5157.49	4898.84	3784.98	7147.99	6140.74	4858.56	6118.94	5748.73	3825.83		
4766	Tubulin beta-8 chain-like protein LOC260334	A6NNZ2	12334.88	11872.93	10971.94	7770.00	7053.23	5876.96	9344.19	8785.52	8463.20	9689.10	9694.39	8787.42	9676.26	8555.19	5826.65	8314.99	7790.80	7135.64	8790.32	8497.03	6901.39	10648.44	9712.26	8752.90	9499.60	9128.88	5080.83	9473.37	8580.30	7143.41	8980.68	8327.67	4119.78		
4767	Tubulin gamma-1 chain	P23258	1773.18	1385.00	632.56	1.07	2.77	0.68	0.96	0.36	0.63	264.89	210.56	43.84	2.13	0.97	0.60	126.37	18.59	7.64	2.22	0.19	0.06	1550.55	1520.78	1337.60	1264.49	1120.06	742.53	1367.53	1209.31	1149.80	1866.23	1589.12	0.12		
4768	Tubulin gamma-2 chain	Q9NRH3	27582.19	22735.47	15226.09	1397.06	806.24	386.43	1064.63	1141.57	573.31	23488.70	19297.78	15570.94	22505.49	2072.25	378.56	1518.82	723.71	623.75	15972.37	1174.43	997.54	37146.69	26407.41	21039.89	19373.61	18400.81	11263.11	22188.28	18738.65	15117.62	20028.85	18802.22	0.12		
4769	Tubulin-folding cofactor B	Q99426	40834.35	38458.25	36739.54	32965.37	31424.61	30694.46	33251.27	31877.60	29312.09	40640.42	39448.61	38197.89	31337.24	31064.43	29196.33	32153.63	30781.05	29109.55	32370.22	32031.05	30302.93	79854.15	73879.65	63971.82	57132.88	56245.13	53986.00	65980.45	63635.66	62092.55	67601.83	65583.35	45862.97		
4770	Tubulin-specific chaperone A	O75347	284198.68	276293.07	262110.70	218773.43	210242.90	199094.85	230419.08	217152.58	210764.21	260198.23	246166.42	229629.14	241105.68	236406.40	212880.28	208803.88	195771.59	193246.96	211388.65	199626.92	200900.57	111478.35	98830.07	95313.88	70649.11	71592.55	66028.00	74217.72	70791.82	72626.53	85072.91	79602.47	62866.98		
4771	Tubulin-specific chaperone C	Q15814	20022.60	16842.17	16743.74	13927.83	13139.44	12851.82	12489.35	12479.98	11909.73	19002.60	17417.15	16358.81	18046.03	15287.40	12509.85	13309.25	11678.79	12788.57	13016.30	10455.95	10971.82	2.27	1.49	0.65	0.84	1.31	0.69	4.16	2.47	0.11	4.90	4.98	0.12	x	
4772	Tubulin-specific chaperone D	Q9BTW9	256934.67	253991.88	237431.18	185763.52	184677.89	176739.66	200289.30	192433.74	193118.30	206719.14	201650.57	189790.34	224298.60	212753.19	192207.77	173838.11	165604.49	159933.21	205232.63	198037.59	192048.40	38021.55	36442.28	33847.11	19001.57	19116.45	17636.20	21365.47	20532.59	19416.80	21606.48	19777.84	14691.11		
4773	Tubulin-specific chaperone E	Q15813	0.14	2.53	0.81	1.07	2.77	0.68	0.96	0.36	0.63	0.35	0.75	0.38	2.13	0.97	0.60	4.64	1.07	0.47	2.22	0.19	0.06	71738.12	67256.98	64993.51	43137.01	42832.35	41006.13	46381.77	42644.58	43100.11	41536.68	42139.98	34192.48		x
4774	Tubulin--tyrosine ligase-like protein 12	Q14166	212549.68	201560.28	181604.94	141785.71	135045.44	126908.02	161965.26	158257.94	149605.43	182313.30	174422.72	159938.10	173310.22	162189.65	108170.26	142143.60	136582.19	129241.84	149945.76	141999.87	131189.71	5037.25	4993.47	4759.57	4471.81	4460.78	4174.42	5192.37	5201.58	5169.49	5559.31	5673.43	4236.55		
4775	Tubulinyl-Tyr carboxypeptidase 1	Q7L8A9	73741.37	75886.61	73004.08	66455.11	67971.85	64456.66	66381.24	71097.26	62411.75	78444.12	79557.55	76341.44	76669.24	33785.39	33487.09	47432.72	63857.13	64302.64	80711.58	70758.81	65348.87	58752.33	59281.85	52259.71	39637.17	38315.10	36311.74	40536.75	40546.60	40009.36	44060.35	42210.27	17195.22		
4776	Tudor and KH domain-containing protein	Q9Y2W6	3045.04	3177.18	3020.72	1222.45	1228.87	1058.18	2376.05	2181.66	1982.48	2233.72	1896.41	1915.83	1940.69	1652.55	1319.47	2103.68	1679.35	1796.99	1711.76	1592.86	1387.07	2.27	1.49	0.65	0.84	1.31	0.69	4.16	2.47	0.11	4.90	4.98	0.12	x	
4777	Tudor domain-containing protein 6	O60522	204929.57	203670.42	176352.31	142277.38	140886.59	121438.14	168781.90	158746.45	149735.07	177230.81	167914.94	154704.73	162555.42	153320.38	118556.80	145187.57	136682.91	131466.29	150924.78	143443.48	131045.44	71768.56	74328.64	60387.03	36982.68	34761.10	28262.46	37461.21	36812.28	35154.03	39871.37	37526.40	24595.08		
4778	Tuftelin-interacting protein 11	Q9UBB9	0.14	2.53	0.81	1.07	2.77	0.68	0.96	0.36	0.63	0.35	0.75	0.38	2.13	0.97	0.60	4.64	1.07	0.47	2.22	0.19	0.06	35422.00	33676.15	28982.43	19707.39	19574.40	15479.35	25570.19	26097.18	23991.66	20894.01	22275.10	13968.80		x
4779	Tumor necrosis factor alpha-induced protein 8	O95379	0.14	2.53	0.81	1.07	2.77	0.68	0.96	0.36	0.63	0.35	0.75	0.38	2.13	0.97	0.60	4.64	1.07	0.47	2.22	0.19	0.06	13077.96	12359.07	10290.70	8461.07	7954.46	6252.60	10210.17	10198.52	8364.68	10939.93	10630.85	6211.74		x
4780	Tumor necrosis factor receptor superfamily member EDAR	Q9UNE0	502.35	486.91	433.05	129.66	87.04	54.33	214.21	3372.20	2910.73	1030.61	842.17	557.76	449.74	144.36	19.00	41.52	482.35	80.44	1475.47	979.00	991.89	2.27	1.49	0.65	0.84	1.31	0.69	4.16	2.47	0.11	4.90	4.98	0.12	x	
4781	Tumor protein D52	P55327	165941.04	153855.35	149006.98	96234.20	94544.84	89964.20	113159.03	110992.49	101740.76	127564.39	125414.53	127138.30	123573.52	116537.59	94217.57	90238.98	88897.97	88241.52	104970.98	98634.44	92980.87	60218.57	57326.08	58671.75	38721.32	39543.40	34189.15	41787.30	43289.80	41097.00	49043.07	51730.53	26371.82		
4782	Tumor protein D53	Q16890	44403.03	44663.62	42852.99	26307.63	24733.97	24015.64	32286.34	31220.07	30375.62	32678.37	32448.59	30980.18	28614.88	29599.00	26037.52	30408.99	26033.38	26316.26	28949.37	29347.60	27486.77	2.27	1.49	0.65	0.84	1.31	0.69	4.16	2.47	0.11	4.90	4.98	0.12	x	
4783	Tumor protein D54	O43399	146282.85	141171.11	135650.69	106538.96	100846.98	95245.84	111951.63	114209.04	112074.16	120780.71	117208.54	115011.67	121257.83	111237.15	70942.12	92284.34	95529.20	91465.57	99643.41	102178.95	97756.42	175726.14	167573.39	158586.95	119947.11	116594.01	108956.27	130586.50	125838.55	120517.57	139759.80	138605.78	95432.38		
4784	Tumor protein p53-inducible protein 11	O14683	56914.44	56455.31	53068.93	45395.37	44882.41	43663.96	48242.47	47121.91	47192.61	57916.85	53512.64	52643.48	59149.64	57809.46	48562.23	41045.46	39377.73	39792.37	44562.37	41603.09	43159.85	2.27	1.49	0.65	0.84	1.31	0.69	4.16	2.47	0.11	4.90	4.98	0.12	x	
4785	Tumor suppressor candidate gene 1 protein	Q2TAM9	6250.69	6006.72	5665.09	4303.46	4196.64	4368.13	4472.64	4359.28	3758.83	5617.32	5434.50	5158.87	5461.76	4729.51	4548.03	4142.38	3677.10	3960.22	4250.06	4019.53	3798.48	2.27	1.49	0.65	0.84	1.31	0.69	4.16	2.47	0.11	4.90	4.98	0.12	x	
4786	Tumor susceptibility gene 101 protein	Q99816	2271.71	2183.91	2234.41	1611.35	1408.99	1446.91	2287.12	2331.52	2305.82	2458.60	2415.27	2432.42	2291.93	1826.12	1547.82	1491.37	1437.14	1444.91	1641.05	1548.27	1582.56	2.27	1.49	0.65	0.84	1.31	0.69	4.16	2.47	0.11	4.90	4.98	0.12	x	
4787	Tumor-associated calcium signal transducer 2	P09758	226670.35	218235.36	209327.86	183074.14	174309.50	172935.28	200428.57	197092.48	183168.60	199071.50	193876.70	182808.69	199416.43	181749.89	157498.31	174029.63	169144.77	158083.19	185184.20	175159.72	170568.74	2.27	1.49	0.65	0.84	1.31	0.69	4.16	2.47	0.11	4.90	4.98	0.12	x	
4788	Twinfilin-1	Q12792	92940.25	89635.16	86520.39	64517.31	62283.17	56990.51	74963.92	72996.27	70565.62	73124.93	71456.88	66311.30	75967.68	69837.32	57281.60	62937.27	63446.05	59213.61	59008.66	57939.09	57390.65	81082.85	77554.80	70943.38	55682.85	53441.53	46345.39	62566.33	61697.78	58317.16	64547.63	63184.19	46061.63		
4789	Twinfilin-2	Q6IBS0	32791.46	30911.68	28363.83	16478.45	15843.04	14254.75	61118.34	58626.57	51851.16	22504.53	21826.99	19950.76	26048.49	24027.51	17375.10	21277.55	19541.70	18821.25	18806.61	17163.75	15263.08	106330.12	96130.28	88394.91	34459.52	33762.76	27939.89	46733.20	42650.43	38448.08	44554.18	43990.94	27313.28		
4790	Twisted gastrulation protein homolog 1	Q9GZX9	0.14	2.53	0.81	1.07	2.77	0.68	0.96	0.36	0.63	0.35	0.75	0.38	2.13	0.97	0.60	4.64	1.07	0.47	2.22	0.19	0.06	4344.57	4305.16	3515.19	2442.11	1691.66	1349.94	3685.73	2911.56	2646.70	2953.34	3008.46	1704.70		x
4791	Twist-related protein 1	Q15672	6817.96	5733.73	6524.46	4641.91	4946.80	4090.96	11723.90	11083.20	10056.83	6289.86	6369.15	6607.85	6726.99	6126.18	5193.98	5718.20	5283.61	4891.65	4754.53	5048.04	4524.99	2.27	1.49	0.65	0.84	1.31	0.69	4.16	2.47	0.11	4.90	4.98	0.12	x	
4792	Type II inositol 1_4_5-trisphosphate 5-phosphatase	P32019	71903.27	65680.91	58359.32	41616.47	39411.03	35053.08	50506.12	46641.39	42847.66	50344.12	46106.71	42987.02	50371.70	44925.28	33488.04	45608.95	43361.28	41233.81	45055.94	39407.96	37106.05	627.95	470.91	489.29	468.55	416.43	389.79	636.91	632.29	562.31	645.41	545.84	440.21		
4793	Type II inositol 3_4-bisphosphate 4-phosphatase	O15327	9270.08	10902.20	9634.65	5344.56	4793.95	4759.38	5458.33	5542.79	5357.45	7646.31	7454.82	6945.88	8254.67	8190.86	6461.86	4926.51	4496.45	4384.04	5358.49	4939.21	4938.74	2.27	1.49	0.65	0.84	1.31	0.69	4.16	2.47	0.11	4.90	4.98	0.12	x	
4794	Type-1 angiotensin II receptor-associated protein	Q6RW13	0.14	2.53	0.81	1.07	2.77	0.68	0.96	0.36	0.63	0.35	0.75	0.38	2.13	0.97	0.60	4.64	1.07	0.47	2.22	0.19	0.06	987.79	770.50	662.84	901.64	903.73	599.72	871.90	902.21	732.45	1190.09	1145.18	498.11		x
4795	Tyrosine aminotransferase	P17735	0.14	2.53	0.81	1.07	2.77	0.68	0.96	0.36	0.63	0.35	0.75	0.38	2.13	0.97	0.60	4.64	1.07	0.47	2.22	0.19	0.06	1405.42	1271.29	1126.13	435.63	498.23	246.57	993.50	855.54	768.41	788.63	789.50	321.77		x
4796	Tyrosine-protein kinase BTK	Q06187	0.14	2.53	0.81	1.07	2.77	0.68	0.96	0.36	0.63	0.35	0.75	0.38	2.13	0.97	0.60	4.64	1.07	0.47	2.22	0.19	0.06	1304.72	1278.84	1252.33	994.17	1044.60	1022.75	1285.17	1316.74	1277.02	1699.59	1512.52	1222.41		x
4797	Tyrosine-protein kinase CSK	P41240	38279.75	35843.55	33504.49	24120.46	24060.27	20385.60	28391.08	27015.21	28093.62	29261.79	28904.82	26740.02	30373.99	27886.18	22470.52	25236.52	23328.02	22911.65	25653.44	24520.61	21574.41	2200.36	1823.84	1761.33	1222.20	1313.50	1364.84	1782.05	1554.58	1433.32	1883.74	1989.81	1057.71		
4798	Tyrosine-protein kinase Fer	P16591	17.72	13.68	10.31	296.86	343.40	396.24	14823.33	15192.23	14348.88	501.31	454.60	511.05	55.48	74.32	22.20	9.27	17.06	13.75	2.22	0.19	0.06	2.27	1.49	0.65	0.84	1.31	0.69	4.16	2.47	0.11	4.90	4.98	187.05	x	
4799	Tyrosine-protein kinase Fgr	P09769	1706.74	1740.38	1679.66	1336.69	1431.28	1229.02	1297.49	1258.82	1076.94	1482.98	1561.18	1497.89	1765.25	1476.85	1357.97	1225.85	1159.21	1092.74	1420.03	1247.65	1244.08	5581.87	5242.74	4797.01	2240.77	1932.26	1551.40	7453.74	7081.64	6557.39	4282.83	4151.31	2459.89		
4800	Tyrosine-protein kinase HCK	P08631	31360.89	31258.98	29315.70	22108.66	21310.19	20481.90	26436.78	24589.41	23071.10	27982.34	26166.30	25493.19	28680.77	25304.80	19844.81	19925.10	19806.84	18729.45	21761.28	19805.46	17276.93	10478.69	6255.78	4087.50	4885.65	5000.92	3077.18	7461.95	6952.78	4065.58	7480.13	7271.54	4319.28		
4801	Tyrosine-protein kinase JAK1	P23458	0.14	2.53	0.81	1.07	2.77	0.68	0.96	0.36	0.63	0.35	0.75	0.38	2.13	0.97	0.60	4.64	1.07	0.47	2.22	0.19	0.06	355.23	321.40	190.78	114.85	105.99	104.88	204.47	205.96	442.21	173.53	233.28	89.32		x
4802	Tyrosine-protein kinase Lyn	P07948	0.14	2.53	0.81	1.07	2.77	0.68	0.96	0.36	0.63	0.35	0.75	0.38	2.13	0.97	0.60	4.64	1.07	0.47	2.22	0.19	0.06	9932.37	9313.67	7997.37	5656.42	6157.27	5092.93	7304.06	7622.68	6974.07	7540.26	7526.99	5394.90		x
4803	Tyrosine-protein kinase receptor Tie-1	P35590	0.14	2.53	0.81	1.07	2.77	0.68	0.96	0.36	0.63	0.35	0.75	0.38	2.13	0.97	0.60	4.64	1.07	0.47	2.22	0.19	0.06	5165.55	4820.87	4066.44	3775.96	3579.59	3002.13	4731.29	3950.25	4495.42	4770.06	4092.99	2924.23		x
4804	Tyrosine-protein kinase receptor UFO	P30530	0.14	2.53	0.81	1.07	2.77	0.68	0.96	0.36	0.63	0.35	0.75	0.38	2.13	0.97	0.60	4.64	1.07	0.47	2.22	0.19	0.06	18814.31	17705.30	15893.02	12769.09	12526.26	10761.83	14266.80	12764.06	12414.96	15232.09	14761.63	10783.64		x
4805	Tyrosine-protein kinase transmembrane receptor ROR2	Q01974	0.14	2.53	0.81	1.07	2.77	0.68	0.96	0.36	0.63	0.35	0.75	0.38	2.13	0.97	0.60	4.64	1.07	0.47	2.22	0.19	0.06	10160.88	8046.86	5349.36	6505.32	5607.57	4387.46	8474.96	7648.83	7072.68	6092.68	7766.28	4639.80		x
4806	Tyrosine-protein kinase Yes	P07947	949.48	1282.60	1278.93	814.22	714.23	821.51	969.65	866.97	883.30	893.49	774.34	817.31	975.72	1147.51	1103.68	650.30	633.18	677.09	762.95	812.59	763.41	3511.87	3346.33	2812.77	1530.70	1597.93	1307.80	2637.34	2440.79	2370.76	2779.44	2620.48	1676.89		
4807	Tyrosine-protein kinase ZAP-70	P43403	459.44	855.34	373.23	495.77	454.49	305.12	675.11	602.80	639.28	687.90	575.52	615.51	481.33	458.39	364.19	357.65	445.86	365.85	515.14	554.37	535.35	9458.89	8823.19	7446.90	6451.24	6687.57	4690.89	7055.01	6774.75	5968.68	10339.50	9621.29	5288.74		
4808	Tyrosine-protein phosphatase non-receptor type 1	P18031	254989.52	254204.82	233945.98	194388.49	190955.97	186164.68	205552.18	211656.87	221577.23	213338.15	207084.55	197612.76	235888.62	201894.65	184913.56	216027.04	181085.81	192721.18	216469.87	204227.22	194514.95	62014.76	60511.37	54617.60	40100.41	38798.46	35622.05	48770.54	47615.20	46343.02	51136.95	48016.78	31716.36		
4809	Tyrosine-protein phosphatase non-receptor type 11	Q06124	65976.37	61391.61	52861.80	49893.75	48641.32	39740.34	52788.19	48194.13	43879.15	59932.12	56850.70	51589.92	56104.80	55793.89	34618.63	48436.95	44321.67	42313.84	46227.10	48832.20	41733.72	56926.77	51907.25	52934.96	36299.17	36705.57	32777.48	42384.71	39068.39	37715.57	44450.33	41466.78	26994.28		
4810	Tyrosine-protein phosphatase non-receptor type 12	Q05209	37.82	75.57	97.07	77.94	50.07	36.70	77.68	47.60	72.23	53.15	88.32	93.43	55.02	36.65	40.53	81.42	88.59	72.95	47.26	71.80	78.21	2999.89	3095.44	3133.65	2142.86	2194.78	1723.01	2950.25	2712.96	2377.29	2760.30	2293.15	1246.29		
4811	Tyrosine-protein phosphatase non-receptor type 13	Q12923	2180.01	2159.41	2144.80	1832.22	1705.71	1816.54	1866.55	1853.97	1930.44	1876.92	1747.50	1737.91	1952.35	1604.79	1584.98	2022.44	1655.41	1688.15	1383.50	1362.43	1399.56	2.27	1.49	0.65	0.84	1.31	0.69	4.16	2.47	0.11	4.90	4.98	0.12	x	
4812	Tyrosine-protein phosphatase non-receptor type 2	P17706	0.14	2.53	0.81	1.07	2.77	0.68	0.96	0.36	0.63	0.35	0.75	0.38	2.13	0.97	0.60	4.64	1.07	0.47	2.22	0.19	0.06	9172.16	9333.79	8464.97	3850.76	3673.51	2774.16	5789.59	5366.91	5397.17	4719.92	4793.46	2303.79		x
4813	Tyrosine-protein phosphatase non-receptor type 20	Q4JDL3	23205.98	22127.22	21556.42	14926.86	14710.08	14308.86	16675.24	16861.96	16507.31	16751.29	15942.96	15422.53	17281.22	16816.65	14506.01	14748.35	13491.77	13168.84	14066.11	13713.27	13053.92	2.27	1.49	0.65	0.84	1.31	0.69	4.16	2.47	0.11	4.90	4.98	0.12	x	
4814	Tyrosine-protein phosphatase non-receptor type 23	Q9H3S7	25540.48	26084.95	22924.53	15935.81	15669.46	13303.14	20474.34	20153.24	19184.40	20426.17	19341.23	18269.41	21295.54	19897.46	14153.62	15940.14	15864.76	15099.94	17854.77	16489.43	14972.71	3283.02	3677.04	3188.00	2976.67	2830.98	2245.95	3110.56	3513.67	3260.05	3525.33	3476.89	2263.69		
4815	Tyrosine-protein phosphatase non-receptor type 6	P29350	50584.05	47465.80	46087.53	34347.90	34036.02	31157.28	34498.95	33879.79	32979.22	47166.48	44893.39	42640.42	40847.97	41031.50	32629.65	34473.92	33662.86	31826.92	32530.46	31032.92	29652.15	2.27	1.49	0.65	0.84	1.31	0.69	4.16	2.47	0.11	4.90	4.98	0.12	x	
4816	Tyrosine--tRNA ligase_ cytoplasmic	P54577	330492.10	315961.80	299541.63	242610.22	232977.69	222231.84	267706.27	260256.99	245413.86	266533.12	252681.40	239411.21	265336.04	256379.18	218195.27	233552.61	220584.99	211690.39	227378.18	215990.20	205676.92	336307.80	326603.00	302909.95	245878.64	241360.95	214951.13	283230.91	274802.03	266255.13	283762.03	279435.87	191284.26		
4817	Tyrosine--tRNA ligase_ mitochondrial	Q9Y2Z4	22.21	26.94	16.94	48.68	354.48	256.30	136.48	83.34	91.37	0.35	0.75	0.38	6.06	1.93	24.34	330.58	93.06	353.42	2.22	0.19	0.06	41255.43	38793.72	35953.68	24159.74	23874.53	20068.31	26622.25	25654.35	22951.73	25877.12	24328.86	16039.15		
4818	Tyrosyl-DNA phosphodiesterase 1	Q9NUW	60.23	41.05	74.51	45.37	91.30	58.75	70.68	66.98	83.36	79.10	110.38	175.98	90.41	72.79	42.34	45.27	13.99	41.71	62.93	69.33	77.91	2.27	1.49	0.65	0.84	1.31	0.69	4.16	2.47	0.11	4.90	4.98	0.12	x	
4819	Tyrosyl-DNA phosphodiesterase 2	O95551	0.14	2.53	0.81	1.07	2.77	0.68	0.96	0.36	0.63	0.35	0.75	0.38	2.13	0.97	0.60	4.64	1.07	0.47	2.22	0.19	0.06	3700.78	4271.96	3486.13	2408.98	2632.03	2240.84	3443.64	3347.08	3124.91	2956.72	2615.46	1520.64		x
4820	U1 small nuclear ribonucleoprotein 70 kDa	P08621	132511.00	131307.19	122739.33	83622.62	81390.74	74985.31	109405.62	105735.10	100364.41	97185.90	94652.48	90180.79	104313.79	95601.57	86488.83	88762.78	81968.34	79970.86	105658.55	99388.52	92267.75	106182.46	99014.56	89242.81	89423.88	86734.50	78311.69	87795.37	85094.53	81253.56	91865.83	85029.30	54112.93		
4821	U1 small nuclear ribonucleoprotein A	P09012	90199.48	82484.09	78261.14	51823.41	48014.79	44196.75	58004.58	57761.93	56676.25	61693.02	59193.92	56232.30	63938.71	57644.48	51571.76	50659.42	46756.50	50938.99	61971.57	52719.47	48350.65	53485.55	50696.44	48280.24	45441.30	44149.93	41881.79	55115.97	52122.25	50016.14	55850.83	52662.91	40199.95		
4822	U1 small nuclear ribonucleoprotein C	P09234	24821.77	24554.57	21290.34	12051.97	11803.59	9523.03	18972.50	16267.20	14518.02	13621.87	12720.07	11710.44	14134.87	13291.99	10007.71	15241.49	14217.11	12616.69	14388.50	14678.97	12294.73	48291.02	43838.09	38635.08	32995.66	29194.26	25451.70	39724.86	36916.33	34891.39	39370.64	35516.42	21782.47		
4823	U11/U12 small nuclear ribonucleoprotein 35 kDa protein	Q16560	0.14	2.53	0.81	1.07	2.77	0.68	0.96	0.36	0.63	0.35	0.75	0.38	2.13	0.97	0.60	4.64	1.07	0.47	2.22	0.19	0.06	7205.84	6572.32	6425.10	4451.85	4212.23	3934.05	5311.89	4897.81	4888.74	4531.97	4113.26	3197.84		x
4824	U2 small nuclear ribonucleoprotein A'	P09661	40030.50	37710.21	35318.44	27359.77	30345.03	24889.93	35490.32	35403.88	35794.35	36894.08	34654.19	35842.14	35720.03	33694.80	26006.59	29148.76	27997.32	24654.62	28006.61	26923.28	25407.24	30795.56	32950.40	29078.10	25418.75	25419.46	25578.99	23454.34	25611.53	25002.44	30763.60	28704.38	16734.42		
4825	U2 small nuclear ribonucleoprotein B''	P08579	52493.94	48701.32	48750.06	37209.19	33563.33	38014.15	45632.91	44951.27	42963.33	42575.50	42908.34	40534.13	46152.45	47534.19	51038.02	34132.86	33247.43	34609.72	43351.52	40251.32	36705.34	1421.42	1199.38	1276.65	722.38	748.27	694.82	847.20	823.84	768.01	2255.67	920.49	2925.96		
4826	U3 small nucleolar ribonucleoprotein protein IMP3	Q9NV31	3264.08	3121.17	3240.46	2574.91	2468.47	2840.66	3478.35	2640.72	2799.61	3423.35	3665.69	3442.31	3251.86	3180.46	1647.62	1414.35	1881.65	1596.84	1971.69	1946.06	3001.16	2.27	1.49	0.65	0.84	1.31	0.69	4.16	2.47	0.11	4.90	4.98	0.12	x	
4827	U3 small nucleolar ribonucleoprotein protein IMP4	Q96G21	701.86	1419.32	879.28	653.05	751.56	477.91	641.22	514.44	503.50	1332.46	1034.24	850.62	1037.30	693.99	352.94	766.38	322.51	513.59	624.26	807.82	543.69	2.27	1.49	0.65	0.84	1.31	0.69	4.16	2.47	0.11	4.90	4.98	0.12	x	
4828	U3 small nucleolar RNA-associated protein 14 homolog A	Q9BVJ6	4452.86	5147.61	4691.62	3887.98	3833.42	3416.41	3373.39	3143.86	3126.19	5168.07	4641.75	4709.26	3937.43	1989.73	2119.51	3200.07	3493.69	3463.55	3966.98	3251.29	3331.93	11825.15	11255.18	10006.61	7763.84	6846.16	5919.34	10084.17	8531.77	9147.33	9532.08	8850.47	5566.89		
4829	U3 small nucleolar RNA-associated protein 18 homolog	Q9Y5J1	6540.98	5990.69	4932.01	3802.82	3741.82	2978.10	4754.73	4043.28	3855.70	5455.15	4695.50	4632.07	5123.61	4231.53	3420.44	4410.93	4228.23	3917.84	3486.48	3467.80	3288.93	4351.70	3492.20	3085.44	2825.37	2675.87	2397.71	3301.85	3178.50	3033.29	3585.70	3596.95	2152.86		
4830	U3 small nucleolar RNA-associated protein 6 homolog	Q9NYH9	0.14	2.53	0.81	1.07	2.77	0.68	0.96	0.36	0.63	0.35	0.75	0.38	2.13	0.97	0.60	4.64	1.07	0.47	2.22	0.19	0.06	4262.88	4138.73	3403.04	3141.08	3081.43	2823.19	2828.81	3108.32	2992.82	3557.03	3364.27	2325.53		x
4831	U4/U6 small nuclear ribonucleoprotein Prp3	O43395	9191.70	9656.50	8268.65	7230.73	6674.58	6800.40	8045.63	7036.10	6693.67	9218.11	9207.91	7965.41	7974.97	7682.40	6312.31	6829.76	6516.57	6712.86	6710.65	6566.56	6309.02	2.27	1.49	0.65	0.84	1.31	0.69	4.16	2.47	0.11	4.90	4.98	0.12	x	
4832	U4/U6 small nuclear ribonucleoprotein Prp31	Q8WWY	4984.81	4508.65	4762.46	3382.54	3270.66	3046.21	3730.14	3644.80	3862.25	3853.15	3728.79	3862.25	4097.68	3972.44	3351.36	3406.52	3087.24	3323.72	3632.13	3230.36	3575.72	3439.85	3458.59	3025.50	2272.66	2243.65	2017.83	3093.97	2972.22	2902.24	2880.26	2703.53	1877.33		

Supplementary Table S1. Overview on the abundance of all relatively quantified 5180 proteins

			MCF-7																					MDA-MB-231													
	Protein name	UniProt	control 01	control 02	control 03	Dai IC20 01	Dai IC20 02	Dai IC20 03	Dai SC20 01	Dai SC20 02	Dai SC20 03	Gen IC20 01	Gen IC20 02	Gen IC20 03	Gen SC20 01	Gen SC20 02	Gen SC20 03	SSE IC20 01	SSE IC20 02	SSE IC20 03	SSE SC20 01	SSE SC20 02	SSE SC20 03	control 01	control 02	control 03	Dai IC20 01	Dai IC20 02	Dai IC20 03	Gen IC20 01	Gen IC20 02	Gen IC20 03	SSE IC20 01	SSE IC20 02	SSE IC20 03	unique to MCF-7	unique to MDA-MB-231
4833	U4/U6 small nuclear ribonucleoprotein Prp4	O43172	37288.69	32181.83	28747.07	22562.38	22883.58	18687.57	29381.57	28802.52	25503.26	25480.89	24297.87	21707.11	25130.59	24595.26	16524.45	24123.94	23658.95	21209.40	27356.57	22899.08	21106.95	2.27	1.49	0.65	0.84	1.31	0.69	4.16	2.47	0.11	4.90	4.98	0.12	x	
4834	U4/U6.U5 tri-snRNP-associated protein 1	O43290	67872.74	61354.86	56417.43	38112.76	35827.90	31022.63	46902.28	45378.71	42020.29	52049.14	47731.99	45526.20	52911.35	46240.35	32520.02	37384.45	35751.64	34286.70	40238.81	38328.76	35456.16	29104.00	19748.85	23237.13	18021.42	17333.07	15356.80	20643.36	19146.40	16971.33	21802.90	22334.29	10207.46		
4835	U4/U6.U5 tri-snRNP-associated protein 2	Q53GS9	71253.11	69028.75	63272.86	47525.23	47694.21	41952.94	60123.24	53271.57	54929.78	62172.62	59844.78	57209.33	59200.83	57057.30	47005.08	48650.62	44326.46	45240.42	46588.01	45727.58	40930.55	24544.23	23863.08	19445.18	19235.08	17353.52	15468.41	23757.51	21576.71	20416.45	21732.41	21290.42	14282.37		
4836	U5 small nuclear ribonucleoprotein 200 kDa helicase	O75643	0.14	2.53	0.81	1.07	2.77	0.68	0.96	0.36	0.63	0.35	0.75	0.38	2.13	0.97	0.60	4.64	1.07	0.47	2.22	0.19	0.06	53606.92	57021.80	56571.78	56771.27	53781.89	42201.36	76152.05	69418.13	55679.07	91174.12	85303.22	54923.02		x
4837	U6 snRNA-associated Sm-like protein LSm2	Q9Y333	4728.54	3986.48	3828.16	2914.13	2767.55	2670.48	3014.69	2607.01	2203.96	3554.10	3919.98	3041.51	3743.19	3570.86	2023.90	2558.54	2776.28	2453.39	3628.56	3752.03	3225.16	10199.81	10471.51	8820.69	6760.03	6340.52	5015.65	8569.67	8293.14	7914.97	8634.56	8176.16	4965.35		
4838	U6 snRNA-associated Sm-like protein LSm4	Q9Y4Z0	60825.40	50526.77	45862.69	38211.24	37049.07	30683.53	42594.43	47093.08	39715.14	40919.59	39969.00	36538.30	47757.43	46414.53	25208.70	44232.86	47872.30	43293.17	38259.54	32626.86	29973.54	2.27	1.49	0.65	0.84	1.31	0.69	4.16	2.47	0.11	4.90	4.98	0.12	x	
4839	U6 snRNA-associated Sm-like protein LSm8	O95777	10759.20	8596.00	6279.58	5912.06	5343.42	3518.87	6927.77	6452.07	4558.13	7519.92	6479.59	4975.90	7755.63	6062.04	1952.42	5931.75	5063.94	3838.18	5620.34	5705.68	3893.57	2.27	1.49	0.65	0.84	1.31	0.69	4.16	2.47	0.11	4.90	4.98	0.12	x	
4840	Ubiquilin-1	Q9UMX0	11782.58	11096.46	10024.08	6578.33	6663.30	5633.49	9192.71	8726.36	8227.27	8825.67	8589.11	7552.72	9214.30	8420.09	5434.43	7490.01	7044.31	7012.27	7153.21	6791.47	6316.10	8605.71	6778.34	6027.39	4564.04	4728.37	3567.79	7128.09	6815.41	5776.95	6537.06	5898.73	3990.60		
4841	Ubiquilin-2	Q9UHD9	9979.96	9759.04	8689.44	7883.31	7837.59	7289.33	8618.25	8728.83	7604.47	9805.83	9463.95	8443.18	8434.05	8672.89	7241.08	8206.85	7713.46	7063.39	8302.74	8363.37	7840.88	37010.68	36652.04	28467.10	23900.45	24316.12	20808.72	29553.05	27464.39	25652.40	29371.37	30022.60	19707.88		
4842	Ubiquilin-4	Q9NRR5	92158.46	86121.76	79568.16	48197.60	46203.14	41259.87	63069.80	61395.38	60052.65	60097.90	57392.68	49947.43	62111.09	57073.42	44397.12	51993.35	50932.56	47495.88	52112.83	48768.27	45753.48	7391.26	8338.81	8393.91	6010.61	5802.21	5377.81	7669.60	8281.10	7336.35	6731.51	7298.00	4796.37		
4843	Ubiquinol-cytochrome-c reductase complex assembly factor 2	Q9BRT2	20142.99	17976.82	16783.92	14341.43	13142.33	12421.56	14075.61	14440.95	13505.47	21674.28	21437.16	20537.65	19793.14	16461.23	12246.80	13430.80	12067.16	12159.96	13094.71	12714.17	11738.97	16430.88	15689.17	14212.22	8693.85	8613.35	7631.95	12784.64	12709.70	11578.45	12790.76	12130.09	7571.10		
4844	Ubiquitin carboxyl-terminal hydrolase 10	Q14694	131937.83	124102.84	120475.82	94623.36	90872.01	80434.09	112019.48	111389.62	103498.24	108374.11	107306.86	101718.56	107653.35	98688.43	73526.86	68348.47	76011.31	70322.83	86941.84	88547.24	91071.03	40627.54	60272.67	35732.21	49891.89	52114.28	74108.65	35339.54	44668.99	38302.32	96737.01	70700.95	20196.80		
4845	Ubiquitin carboxyl-terminal hydrolase 11	P51784	0.14	2.53	0.81	1.07	2.77	0.68	0.96	0.36	0.63	0.35	0.75	0.38	2.13	0.97	0.60	4.64	1.07	0.47	2.22	0.19	0.06	5338.92	4805.36	4529.79	4632.65	4678.31	3268.47	5468.28	5191.99	4409.75	6466.14	5900.71	3848.57		x
4846	Ubiquitin carboxyl-terminal hydrolase 13	Q92995	23493.83	21914.84	18955.72	22203.52	21537.18	20552.23	23690.80	22383.72	22452.00	19934.04	20444.71	19605.18	21618.09	20176.12	17982.47	22074.85	20752.43	17119.21	16485.56	15849.29	13596.02	2.27	1.49	0.65	0.84	1.31	0.69	4.16	2.47	0.11	4.90	4.98	0.12	x	
4847	Ubiquitin carboxyl-terminal hydrolase 14	P54578	186019.99	182208.23	175083.66	132264.08	128482.56	119135.39	133457.96	131269.24	128584.46	158042.96	152657.69	146218.21	154641.22	149345.08	117238.99	132149.44	123306.76	121998.76	138420.12	128967.54	118725.96	118411.61	110625.49	100532.94	86981.97	84503.84	70166.36	103924.05	96885.94	91522.86	108818.53	106967.38	73814.48		
4848	Ubiquitin carboxyl-terminal hydrolase 15	Q9Y4E8	112286.07	109900.15	104556.95	83731.81	81856.76	73985.02	93324.81	91235.92	85839.40	103901.37	100152.76	95225.65	95145.50	92478.71	73421.86	83008.70	80868.65	77923.65	80044.52	74823.70	71211.17	11346.11	10985.26	8903.60	7889.87	7787.48	7431.10	10395.51	9597.32	10086.20	10295.66	9676.53	7476.02		
4849	Ubiquitin carboxyl-terminal hydrolase 16	Q9Y5T5	68912.76	67008.13	57576.24	49051.78	47790.17	44115.02	55187.36	57076.80	52404.00	68252.74	69140.47	63523.67	58025.94	55555.46	43451.34	51066.64	48174.14	45515.81	47844.06	45919.98	44179.67	2.27	1.49	0.65	0.84	1.31	0.69	4.16	2.47	0.11	4.90	4.98	0.12	x	
4850	Ubiquitin carboxyl-terminal hydrolase 19	O94966	0.14	2.53	0.81	1.07	2.77	0.68	0.96	0.36	0.63	0.35	0.75	0.38	2.13	0.97	0.60	4.64	1.07	0.47	2.22	0.19	0.06	4858.37	4291.65	3268.06	3675.74	3569.55	2288.47	4652.91	4218.99	3536.23	5815.50	5560.52	1893.69		x
4851	Ubiquitin carboxyl-terminal hydrolase 24	Q9UPU5	1216.32	1218.35	1156.78	1036.03	961.73	936.82	952.45	871.17	932.17	1037.35	1181.94	1123.53	1147.87	1026.88	1198.83	782.07	810.50	745.68	1215.77	1227.50	1189.70	9821.78	10292.93	10170.07	4089.53	3968.29	2900.17	4983.71	4138.54	4515.39	4342.26	5690.27	3080.99		
4852	Ubiquitin carboxyl-terminal hydrolase 29	Q9HBJ7	15300.93	14071.11	12910.16	9566.35	8698.86	7963.06	11462.31	10143.91	9268.09	12226.89	11129.32	9982.75	12279.37	11480.14	7741.89	9954.52	10080.83	8994.27	9531.39	8981.37	8027.45	16579.34	17465.81	14775.37	17931.71	16706.52	15518.53	27777.17	27036.74	25756.54	18105.34	17890.44	19148.57		
4853	Ubiquitin carboxyl-terminal hydrolase 30	Q70CQ3	0.14	2.53	0.81	1.07	2.77	0.68	0.96	0.36	0.63	0.35	0.75	0.38	2.13	0.97	0.60	4.64	1.07	0.47	2.22	0.19	0.06	18811.68	18520.82	17442.98	17477.73	17654.38	17586.42	14972.63	16064.26	15742.09	39646.73	38231.91	8656.58		x
4854	Ubiquitin carboxyl-terminal hydrolase 32	Q8NFA0	69792.74	65349.60	63157.24	46071.53	45395.80	46720.47	55564.76	54991.11	53807.73	60775.15	58366.87	56621.57	62801.97	55936.83	39513.49	47838.28	42789.07	44260.38	52407.04	45180.89	44505.30	2.27	1.49	0.65	0.84	1.31	0.69	4.16	2.47	0.11	4.90	4.98	0.12	x	
4855	Ubiquitin carboxyl-terminal hydrolase 34	Q70CQ2	444196.96	431971.67	374119.08	292980.04	284921.40	255458.13	346214.53	318807.38	299310.49	367376.63	341945.44	314346.10	360218.52	321626.37	279639.44	293519.92	263956.40	251428.48	323769.70	307634.32	282178.60	77427.45	73318.34	63141.02	46027.89	46821.49	36108.50	61356.60	58824.01	49658.12	65554.60	60727.42	44965.89		
4856	Ubiquitin carboxyl-terminal hydrolase 47	Q96K76	15179.98	13796.45	12749.68	10015.75	10437.16	9068.86	12993.60	11984.58	11654.40	14703.16	14235.75	12984.61	14235.81	14209.53	8486.09	11507.97	10445.40	9877.84	10873.30	10548.95	9127.59	13450.63	14209.44	13580.03	10321.83	10181.68	9988.71	14704.93	14176.51	13930.04	12089.37	13270.91	3551.40		
4857	Ubiquitin carboxyl-terminal hydrolase 48	Q86UV5	0.14	2.53	0.81	1.07	2.77	0.68	0.96	0.36	0.63	0.35	0.75	0.38	2.13	0.97	0.60	4.64	1.07	0.47	2.22	0.19	0.06	79430.97	76390.54	73298.53	56925.97	55880.58	52498.68	58222.60	55588.38	56071.11	55879.34	53872.12	38580.91		x
4858	Ubiquitin carboxyl-terminal hydrolase 49	Q70CQ1	22468.07	24639.05	20269.72	17049.06	17153.69	15837.28	18544.21	18346.64	16524.34	22115.08	20200.77	20017.03	18943.32	18801.42	15308.24	14932.18	15531.54	12859.05	13964.35	15240.73	14654.77	2.27	1.49	0.65	0.84	1.31	0.69	4.16	2.47	0.11	4.90	4.98	0.12	x	
4859	Ubiquitin carboxyl-terminal hydrolase 5	P45974	181046.66	172022.43	156998.35	127766.55	121478.92	111194.30	134607.07	128581.22	122467.61	159512.74	153941.84	143361.66	154059.40	143648.35	109339.38	125798.25	113923.63	109330.93	123583.62	117692.01	110112.88	149269.00	144136.23	135064.16	106390.51	104702.03	95104.88	127719.27	119551.63	114219.48	124481.31	121118.03	79923.87		
4860	Ubiquitin carboxyl-terminal hydrolase 7	Q93009	101931.67	91286.71	79644.83	65529.57	64821.93	55498.63	79190.52	78600.84	71451.31	74426.11	69620.05	63513.99	75668.70	74559.37	52867.82	65867.69	63427.38	59579.39	71050.42	65157.27	59927.58	6994.34	5124.81	5096.10	4215.59	4218.68	2824.02	6496.47	5798.93	4630.26	6357.36	6281.89	3352.69		
4861	Ubiquitin carboxyl-terminal hydrolase 8	P40818	68157.01	68860.30	59655.37	46073.50	45691.58	43218.51	46827.28	46121.33	44570.89	56880.16	55560.22	53101.75	52777.17	51880.25	46151.49	43380.74	41711.19	39199.73	45282.68	43258.19	42590.12	7767.99	7562.80	7048.04	5761.67	5961.78	5163.98	7129.53	6506.53	6507.31	7796.30	6985.55	5475.77		
4862	Ubiquitin carboxyl-terminal hydrolase isozyme L3	P15374	8687.14	8382.67	8048.77	8124.80	7274.19	6995.18	7844.08	7302.34	8678.82	9264.66	9732.90	8694.68	9779.71	8405.78	7465.02	9154.78	8655.31	7871.82	8103.13	7817.50	7692.08	17411.81	16845.86	14901.54	10323.84	9984.56	8578.04	14235.78	14280.09	12706.88	13798.05	13018.47	7135.48		
4863	Ubiquitin carboxyl-terminal hydrolase isozyme L5	Q9Y5K5	95142.12	96257.43	85208.77	61660.49	60845.70	56482.07	79205.31	73064.67	71555.39	74843.96	71567.11	68748.69	74447.18	68924.66	57337.20	61841.86	58987.87	57092.24	64820.69	63540.24	58552.83	24280.39	23392.10	20854.08	13318.18	12387.87	9321.38	18136.16	15887.58	14722.74	17381.45	16845.14	9720.80		
4864	Ubiquitin conjugation factor E4 A	Q14139	3587.56	3432.65	3099.73	3012.88	2693.34	2709.08	2720.54	2844.16	2546.78	3537.78	3481.84	3409.10	3588.42	3405.49	3123.45	2700.32	2512.03	2393.29	2889.33	2824.40	2710.90	2.27	1.49	0.65	0.84	1.31	0.69	4.16	2.47	0.11	4.90	4.98	0.12	x	
4865	Ubiquitin conjugation factor E4 B	O95155	0.14	2.53	0.81	1.07	2.77	0.68	0.96	0.36	0.63	0.35	0.75	0.38	2.13	0.97	0.60	4.64	1.07	0.47	2.22	0.19	0.06	12026.24	11051.26	10308.17	8972.34	8230.27	7991.53	9399.44	9050.03	8801.77	10546.03	9395.99	4437.03		x
4866	Ubiquitin domain-containing protein UBFD1	O14562	10127.12	10100.85	9487.89	7437.91	7457.77	7170.01	7786.03	7444.19	7371.79	8327.02	8212.43	8177.06	7557.11	7943.29	9214.08	6948.82	6706.18	6041.87	7506.60	7113.91	7358.27	2.27	1.49	0.65	0.84	1.31	0.69	4.16	2.47	0.11	4.90	4.98	0.12	x	
4867	Ubiquitin recognition factor in ER-associated degradation protein 1	Q92890	15595.55	16463.74	13175.98	10170.86	9836.47	8880.92	10067.39	7871.35	9471.76	12405.14	11030.38	10209.41	11790.80	11597.20	8563.41	8302.35	7838.77	7447.47	7077.75	8073.63	7969.66	528.52	437.37	442.80	198.16	232.46	84.92	330.88	269.25	210.35	102.39	120.91	18.13		
4868	Ubiquitin thioesterase	Q96FW1	190410.10	177001.75	172971.05	129669.68	128766.04	124223.77	143887.91	141363.22	133988.18	164297.28	160853.05	151228.81	155371.88	150559.49	137315.12	128401.22	126398.32	123126.50	124941.39	119915.24	115675.61	66852.19	62343.26	62114.95	39291.76	37743.28	34453.92	43554.08	42819.32	40383.82	45834.70	42040.44	30178.66		
4869	Ubiquitin thioesterase otulin	Q96BN8	49944.41	44662.03	41496.30	25285.05	24335.45	22188.15	31749.05	32335.52	28966.79	29815.54	28180.94	24994.94	32948.67	30037.85	22739.44	34492.89	30370.76	30634.21	23171.13	21327.52	18726.29	79962.76	78609.45	66117.67	49566.79	51085.03	42882.32	52176.47	51313.76	46962.29	61794.03	57648.80	39106.54		
4870	Ubiquitin-40S ribosomal protein S27a	P62979	1324981.13	1264297.18	1200273.01	1341474.39	1294167.40	1214715.28	1335471.40	1310963.23	1252977.11	1484489.71	1433147.79	1359357.92	1388160.73	1312566.92	1137209.72	1227174.26	1145522.54	1118144.22	1241308.72	1160112.44	1101470.37	612329.98	588672.26	527255.62	458721.45	445701.81	392449.51	456620.57	439096.34	426228.70	527842.63	520551.89	389781.28		
4871	Ubiquitin-associated and SH3 domain-containing protein B	Q8TF42	0.14	2.53	0.81	1.07	2.77	0.68	0.96	0.36	0.63	0.35	0.75	0.38	2.13	0.97	0.60	4.64	1.07	0.47	2.22	0.19	0.06	8538.30	8541.35	7263.26	6381.95	6087.48	5324.93	6445.53	6451.46	5964.14	7584.81	6761.79	2256.17		x
4872	Ubiquitin-associated domain-containing protein 2	Q8NBM4	1053.98	1047.57	894.96	812.24	836.67	875.63	724.56	832.73	708.56	1354.86	1413.72	1514.67	979.85	803.14	753.95	742.68	646.63	753.66	732.39	630.46	601.85	2.27	1.49	0.65	0.84	1.31	0.69	4.16	2.47	0.11	4.90	4.98	0.12	x	
4873	Ubiquitin-associated protein 2-like	Q14157	225904.10	220678.06	206165.57	168627.58	161507.25	153881.07	218548.53	211037.15	199042.19	201112.57	192068.67	180085.31	200982.76	188420.16	169652.22	162096.71	154829.04	149201.46	173685.78	166228.83	157761.17	31078.27	27891.37	28811.97	23276.86	22171.20	20760.02	24107.58	24696.32	22570.09	25609.39	24407.62	13849.25		
4874	Ubiquitin-conjugating enzyme E2 C	O00762	419.04	478.65	427.06	433.23	329.14	476.94	371.57	314.81	383.28	236.93	333.80	330.32	293.60	188.72	84.48	227.92	294.22	295.89	510.04	411.92	274.85	5530.10	4012.17	3215.31	2849.95	2692.88	1574.94	4295.59	3535.69	2149.00	4356.69	3821.35	1995.98		
4875	Ubiquitin-conjugating enzyme E2 D3	P61077	9851.78	9562.71	9298.19	4939.49	4486.18	4398.44	6523.75	6422.38	6049.84	6657.12	6779.44	6309.00	7962.84	7421.97	4489.50	4792.95	5713.68	6300.18	8189.62	6910.14	5945.65	1997.35	2112.06	1799.53	621.80	667.47	477.28	1507.47	1479.57	1261.42	842.02	863.02	321.72		
4876	Ubiquitin-conjugating enzyme E2 E1	P51965	73286.72	71049.11	65513.67	68868.33	65943.18	62530.26	74618.75	72026.53	71107.43	72295.54	71775.92	62572.37	77810.78	59063.33	56479.34	89155.43	80137.60	78691.36	69945.36	70143.61	63205.20	1024.27	877.15	808.58	304.47	243.48	288.02	403.92	359.96	387.18	411.94	468.06	187.17		
4877	Ubiquitin-conjugating enzyme E2 G2	P60604	2592.17	2200.28	1999.40	1752.10	1765.91	1509.11	1875.74	1552.94	1789.98	2876.25	2455.26	2162.57	2180.33	2032.20	1235.18	1814.60	1757.54	1852.29	1853.27	1978.74	1672.12	2.27	1.49	0.65	0.84	1.31	0.69	4.16	2.47	0.11	4.90	4.98	0.12	x	
4878	Ubiquitin-conjugating enzyme E2 J1	Q9Y385	185.77	124.35	62.63	124.63	150.29	149.23	110.85	221.89	228.38	112.20	191.07	154.78	79.46	145.55	53.39	108.53	122.43	142.93	141.56	187.66	111.57	2.27	1.49	0.65	0.84	1.31	0.69	4.16	2.47	0.11	4.90	4.98	0.12	x	
4879	Ubiquitin-conjugating enzyme E2 K	P61086	6233.27	6644.53	6293.69	6375.09	6314.34	6244.40	5824.55	6219.10	5586.14	6597.94	6487.71	6221.35	6650.16	6403.44	5169.34	5013.85	4223.48	4098.57	4907.51	4615.15	4703.35	11228.08	10629.70	9790.52	5769.98	5261.51	4927.75	7633.14	7458.64	6969.25	7719.97	7355.71	4537.64		
4880	Ubiquitin-conjugating enzyme E2 L3	P68036	219517.04	199057.32	193348.55	141608.69	137169.28	130694.00	173357.54	162514.70	158809.38	167670.24	155370.64	149253.28	175568.87	166110.39	138178.05	138379.29	124176.54	123615.12	134247.44	126635.94	124590.93	67846.97	65870.19	63362.88	51244.36	50836.56	51383.97	61669.76	59415.77	56386.20	64448.69	59578.85	47274.98		
4881	Ubiquitin-conjugating enzyme E2 N	P61088	34001.93	32879.69	30558.15	25522.79	24626.96	22819.55	27103.42	27364.10	25792.66	28628.70	27309.01	26155.05	27769.61	27695.35	21273.98	25854.39	24212.01	23002.55	24262.24	22220.66	22587.71	5179.56	4678.29	4790.73	2938.83	2997.85	2910.71	3532.52	3563.57	3587.82	4130.50	3875.61	2540.32		
4882	Ubiquitin-conjugating enzyme E2 S	Q16763	0.14	2.53	0.81	1.07	2.77	0.68	0.96	0.36	0.63	0.35	0.75	0.38	2.13	0.97	0.60	4.64	1.07	0.47	2.22	0.19	0.06	707.34	711.40	729.61	528.29	572.36	469.93	995.84	729.66	745.52	720.77	634.80	476.30		x
4883	Ubiquitin-conjugating enzyme E2 T	Q9NPD8	23148.63	19432.73	21422.95	14109.93	14060.17	14316.03	13068.94	12048.69	11554.11	15335.72	15279.42	15112.80	14917.52	13790.36	12340.17	10009.06	10351.86	9495.52	11988.78	12099.30	11729.38	5742.90	5138.47	5104.59	4255.21	4061.81	3462.33	4769.93	4517.91	4227.87	4926.83	4235.87	3454.26		
4884	Ubiquitin-conjugating enzyme E2 variant 1	Q13404	76737.86	74681.63	72339.97	55679.91	53686.38	50438.31	69481.94	65119.99	63126.47	69683.59	66898.41	65000.44	64935.43	61634.42	54029.78	56816.77	52767.46	50628.34	51236.32	50652.45	47515.13	198824.51	188876.30	175580.26	127495.42	122919.01	109507.23	173001.15	169182.00	163183.33	134513.41	131527.15	96466.89		
4885	Ubiquitin-conjugating enzyme E2 variant 2	Q15819	243416.93	240894.55	232315.61	172378.43	169494.14	163537.79	191744.49	178194.24	174128.03	207905.68	197341.09	181357.31	201692.86	189786.01	172400.85	163671.24	157363.36	155366.23	168026.04	161298.41	151976.74	3624.59	3594.84	3290.43	2065.12	2132.93	1821.87	2883.49	2960.94	2575.24	2737.26	2569.06	1848.23		
4886	Ubiquitin-conjugating enzyme E2 Z	Q9H832	141376.65	136572.43	124487.87	90107.81	86165.92	79350.02	116370.43	111021.00	104204.13	109178.17	101540.21	97126.87	116424.26	102399.89	92159.62	102536.70	95305.65	91365.19	104148.59	99128.37	94404.08	87135.23	82529.52	76398.08	58398.80	59114.21	53067.51	62978.20	59122.06	58996.23	67327.45	63219.03	57929.85		
4887	Ubiquitin-fold modifier 1	P61960	4157.83	4166.62	3835.94	2011.86	1996.17	1788.62	4046.90	3818.86	3513.02	2782.85	2589.90	2396.07	2276.61	2308.52	1995.51	3134.00	3164.77	3050.38	2341.89	2445.91	2340.25	3147.08	2821.93	2712.76	1335.11	1200.07	772.91	1840.85	1777.87	1599.81	1200.61	1153.44	565.60		
4888	Ubiquitin-fold modifier-conjugating enzyme 1	Q9Y3C8	21508.94	21244.13	21502.64	13797.99	14109.67	13132.92	15755.61	15757.78	15453.44	18965.55	17124.37	17720.25	18507.93	18462.23	16292.78	12590.66	12631.29	12943.21	13722.57	13278.87	13535.50	2.27	1.49	0.65	0.84	1.31	0.69	4.16	2.47	0.11	4.90	4.98	0.12	x	
4889	Ubiquitin-like domain-containing CTD phosphatase 1	Q8WVY7	15067.48	14576.35	12198.85	9525.05	9923.53	8202.88	11635.90	11164.49	10595.94	10390.82	10069.92	9070.13	10786.44	10495.96	8800.67	10241.53	9808.42	9750.21	8729.74	8705.48	5774.99	33795.33	30167.88	28602.68	23823.93	21616.82	20666.34	27827.05	25874.59	25571.64	23594.87	22240.86	15087.08		
4890	Ubiquitin-like modifier-activating enzyme 1	P22314	1167461.53	1133071.23	1094304.58	754180.35	738638.96	675009.73	893360.04	883920.15	843952.46	972422.66	940166.48	872079.49	975642.77	920301.81	737107.39	778899.82	753776.09	719448.33	765153.30	732637.44	683373.60	1044156.60	988323.69	901854.97	694812.72	675235.53	577216.97	827570.15	805152.32	753219.64	865699.91	827743.89	554651.72		
4891	Ubiquitin-like modifier-activating enzyme 5	Q9GZZ9	40983.13	37068.18	31292.69	22142.18	22587.64	19439.29	25142.80	21498.30	22763.47	35522.16	32594.71	30320.94	32956.44	23360.54	16488.39	23520.87	18993.93	21587.89	24377.03	19416.73	18217.72	4827.04	4542.59	4213.12	2581.89	2820.54	2230.54	2955.40	2863.05	2783.58	4407.29	3662.59	2372.19		
4892	Ubiquitin-like modifier-activating enzyme 6	A0AVT1	30907.45	30296.21	29771.27	21731.99	21032.00	20435.36	27362.66	25187.36	25673.60	27010.08	26507.33	24828.43	26999.30	27429.07	21369.84	24355.40	21489.84	20966.33	21812.18	21267.28	19966.23	34263.96	31484.40	35270.79	27485.09	26147.33	22337.40	35878.80	34355.20	28594.98	30428.39	29994.96	18172.16		
4893	Ubiquitin-like modifier-activating enzyme 7	P41226	99037.00	95267.92	87097.14	59482.18	57199.51	52695.72	71412.61	68545.38	66040.66	79150.07	76055.09	72861.68	77328.92	71161.14	60093.74	60438.43	57315.41	52862.89	64156.13	63080.70	58489.83	2.27	1.49	0.65	0.84	1.31	0.69	4.16	2.47	0.11	4.90	4.98	0.12	x	
4894	Ubiquitin-like modifier-activating enzyme ATG7	O95352	97141.16	88175.57	70710.46	61892.16	60354.52	49935.60	72746.68	70161.22	66582.19	79967.88	75790.89	66456.62	91735.06	85428.38	57087.69	83343.57	78374.33	72611.32	65554.59	59025.67	50750.60	6878.57	6804.00	5743.44	4085.43	3957.69	3398.41	4818.19	4782.37	4466.00	4629.27	4487.11	2859.16		
4895	Ubiquitin-like protein 5	Q9BZL1	11167.95	11159.35	9902.82	6947.65	6681.39	6097.12	7180.02	6852.16	6472.63	8647.02	8984.81	8252.14	8068.16	7518.99	6559.70	5624.03	5466.49	5127.58	6037.21	5919.92	5029.49	4156.11	3652.75	3484.40	1533.68	1501.76	1383.92	1605.18	1505.74	1444.70	1968.44	1875.14	1454.99		
4896	Ubiquitin-like protein ISG15	P05161	80847.57	84177.37	75990.36	53277.98	49980.90	47115.22	58748.65	63862.77	59924.05	68923.86	68642.04	65552.38	64480.81	65937.70	50061.17	58233.48	56203.71	53398.03	54651.69	53994.91	50865.96	2.27	1.49	0.65	0.84	1.31	0.69	4.16	2.47	0.11	4.90	4.98	0.12	x	
4897	Ubiquitin-like-conjugating enzyme ATG3	Q9NT62	18177.17	13582.47	10650.21	4655.00	9067.80	6012.30	11446.17	6811.91	5487.67	12432.07	10955.24	8949.84	12378.34	7603.48	2854.55	9518.24	9091.14	7291.53	5250.41	4676.59	4154.04	29437.36	29443.34	26295.67	20958.45	20362.41	19930.26	21368.15	21171.33	20984.93	25547.56	25095.63	20974.43		
4898	UBX domain-containing protein 1	Q04323	26127.95	30370.70	29377.98	23909.51	20857.54	18855.82	21195.57	21675.95	20154.74	28606.90	28189.43	27934.83	28378.41	25583.68	21429.55	20267.32	17876.67	18912.92	25641.03	23534.21	23361.98	26125.34	26244.85	23768.03	18791.30	18466.23	18154.90	19557.23	20553.33	20037.74	23503.75	21994.59	5310.92		
4899	UBX domain-containing protein 4	Q92575	462.38	477.22	480.43	263.12	261.39	256.98	412.39	384.02	422.83	474.32	379.05	411.69	643.77	584.93	593.60	342.07	321.33	302.71	252.92	207.62	235.09	2.27	1.49	0.65	0.84	1.31	0.69	4.16	2.47	0.11	4.90	4.98	0.12	x	
4900	UBX domain-containing protein 6	Q9BZV1	47069.24	43104.48	40944.21	37867.28	39261.42	34890.12	38338.41	34288.07	37418.30	42421.17	38926.55	38676.76	40194.06	42873.48	38117.92	36590.05	35598.34	32149.21	33135.87	32332.85	33770.28	17359.95	13830.99	16588.86	16025.35	14358.89	10123.76	14487.31	12959.17	13451.56	11525.22	13642.46	10148.41		
4901	UBX domain-containing protein 7	O94888	4014.32	4095.46	3314.66	1987.02	1845.60	1811.12	2567.46	2343.87	1983.16	3732.42	3892.64	3811.70	3507.32	2495.52	1596.38	2076.59	1774.83	1839.43	1867.76	1858.00	1600.46	2.27	1.49	0.65	0.84	1.31	0.69	4.16	2.47	0.11	4.90	4.98	0.12	x	
4902	UDP-glucose 4-epimerase	Q14376	22731.80	21378.53	17324.89	15253.02	16248.26	13024.26	17969.93	15858.25	14590.18	20802.76	19495.06	15223.23	19238.30	16011.57	10370.53	20500.20	16976.00	14258.17	13716.56	12209.21	11294.62	2.27	1.49	0.65	0.84	1.31	0.69	4.16	2.47	0.11	4.90	4.98	0.12	x	
4903	UDP-glucose 6-dehydrogenase	O60701	239135.82	227494.23	205588.18	184456.07	180826.52	164833.18	205356.79	197817.17	188676.23	228539.06	215031.27	203068.85	187683.92	178651.99	143876.77	181860.86	170942.39	161537.82	170725.13	163217.49	152134.53	30010.83	24076.60	25677.76	18794.35	18087.97	15733.22	23750.25	21167.72	18634.29	21592.09	19838.77	13367.47		
4904	UDP-glucose:glycoprotein glucosyltransferase 1	Q9NYU2	152040.80	139389.32	121638.35	99224.02	94801.47	82405.24	115157.49	107001.16	101074.97	128246.92	119448.37	108783.26	129393.92	116127.38	73924.99	99714.99	91755.14	87785.18	102322.89	98881.44	86446.99	121476.43	110366.64	92692.55	70161.73	68339.94	58160.71	84264.36	84818.43	73994.21	84637.94	81157.51	52977.24		
4905	UDP-glucuronosyltransferase 1-4	P22310	0.14	2.53	0.81	1.07	2.77	0.68	0.96	0.36	0.63	0.35	0.75	0.38	2.13	0.97	0.60	4.64	1.07	0.47	2.22	0.19	0.06	1510.66	1349.84	1268.89	1076.13	1081.18	988.96	993.68	965.62	942.55	1249.91	1270.75	762.73		x
4906	UDP-glucuronosyltransferase 3A2	Q3SY77	0.14	2.53	0.81	1.07	2.77	0.68	0.96	0.36	0.63	0.35	0.75	0.38	2.13	0.97	0.60	4.64	1.07	0.47	2.22	0.19	0.06	387.86	337.66	343.53	106.80	143.79	67.54	317.09	176.62	150.26	216.78	195.75	92.65		x
4907	UDP-N-acetylhexosamine pyrophosphorylase	Q16222	1568.97	1517.08	1578.05	1381.09	1386.11	1429.14	1307.39	1377.77	1344.72	1727.50	1709.27	1755.50	1603.64	1482.28	1121.15	1438.45	1012.97	1053.85	1408.30	1371.18	1402.91	209510.23	204278.20	182075.48	150511.41	148558.39	133682.76	179319.67	171959.37	160214.86	186351.18	180747.33	107972.45		
4908	UHRF1-binding protein 1	Q6BDS2	3848.04	3703.56	3696.80	4009.28	3756.11	3693.16	3568.27	3673.37	3651.51	5225.24	4678.27	4640.59	3648.60	3689.19	3096.79	3026.94	3099.66	3084.81	2724.45	2492.32	2622.08	7186.33	6577.05	7035.12	4610.39	4759.12	5065.21	4908.28	4689.50	4389.83	5685.00	5642.70	25660.42		
4909	UMP-CMP kinase	P30085	107023.56	106732.38	99122.28	68224.02	68907.02	63609.43	79842.59	75223.02	73803.97	87502.35	85229.79	82391.51	80724.53	81334.39	64795.52	69297.78	65662.42	65122.93	70183.36	67040.08	61945.54	71975.30	66614.02	63309.01	48547.87	49238.57	45351.06	56834.05	53901.84	51732.28	61270.27	58971.78	42482.28		
4910	Uncharacterized membrane protein C3orf80	F5H4A9	0.14	2.53	0.81	1.07	2.77	0.68	0.96	0.36	0.63	0.35	0.75	0.38	2.13	0.97	0.60	4.64	1.07	0.47	2.22	0.19	0.06	4089.84	3943.98	3663.33	1415.51	1300.52	1283.96	1565.45	1611.29	1919.97	2006.45	2007.03	1352.93		x
4911	Uncharacterized protein C10orf67_ mitochondrial	Q8IYJ2	36586.15	33862.65	27773.28	25509.15	25696.68	22204.41	28331.02	25540.61	24172.66	27204.48	24977.26	21218.74	27402.35	29859.53	19357.74	24146.88	21831.30	20476.61	22341.14	21680.05	18513.93	2.27	1.49	0.65	0.84	1.31	0.69	4.16	2.47	0.11	4.90	4.98	0.12	x	
4912	Uncharacterized protein C10orf82	Q8WW1	0.14	2.53	0.81	1.07	2.77	0.68	0.96	0.36	0.63	0.35	0.75	0.38	2.13	0.97	0.60	4.64	1.07	0.47	2.22	0.19	0.06	673.82	713.94	695.32	467.63	421.99	451.56	335.70	370.45	363.73	474.78	293.10	368.64		x
4913	Uncharacterized protein C11orf98	E9PRG8	6012.53	5820.82	5416.81	2320.84	2196.12	2206.31	4504.89	4543.07	3669.57	3193.85	2735.65	2676.84	3159.12	3338.20	2760.97	3242.20	3228.91	2781.79	2746.69	2267.70	2377.32	2.27	1.49	0.65	0.84	1.31	0.69	4.16	2.47	0.11	4.90	4.98	0.12	x	
4914	Uncharacterized protein C12orf71	A8MTZ7	0.14	2.53	0.81	1.07	2.77	0.68	0.96	0.36	0.63	0.35	0.75	0.38	2.13	0.97	0.60	4.64	1.07	0.47	2.22	0.19	0.06	3773.65	3406.95	3537.12	2451.39	2268.17	2562.35	3046.51	3050.82	2397.14	2253.31	2535.16	1858.34		x
4915	Uncharacterized protein C15orf62_ mitochondrial	A8K5M9	19440.16	19838.37	19384.47	14896.66	14029.67	13937.39	17144.52	15338.73	16135.25	19819.87	17320.48	17554.90	15909.68	17249.74	14304.79	14376.15	13289.12	12898.54	13695.40	12273.47	12504.90	2.27	1.49	0.65	0.84	1.31	0.69	4.16	2.47	0.11	4.90	4.98	0.12	x	
4916	Uncharacterized protein C16orf90	A8MZG2	62.94	112.60	67.54	25.35	31.15	18.31	85.42	12.28	63.18	15.76	82.98	45.20	28.42	33.13	32.68	21.33	58.07	25.00	38.16	33.76	10.02	2.27	1.49	0.65	0.84	1.31	0.69	4.16	2.47	0.11	4.90	4.98	0.12	x	
4917	Uncharacterized protein C17orf67	Q0P5P2	0.14	2.53	0.81	1.07	2.77	0.68	0.96	0.36	0.63	0.35	0.75	0.38	2.13	0.97	0.60	4.64	1.07	0.47	2.22	0.19	0.06	227.06	191.90	252.54	100.66	84.02	25.84	160.43	188.45	180.64	193.89	249.07	101.04		x
4918	Uncharacterized protein C19orf84	I3L1E1	0.14	2.53	0.81	1.07	2.77	0.68	0.96	0.36	0.63	0.35	0.75	0.38	2.13	0.97	0.60	4.64	1.07	0.47	2.22	0.19	0.06	203.40	104.68	90.73	282.69	251.05	100.17	305.96	172.12	61.22	594.93	493.58	131.15		x
4919	Uncharacterized protein C1orf21	Q9H246	38690.86	39749.19	34184.38	25039.42	26021.67	25743.69	29596.25	28321.89	26550.07	31493.58	27159.27	27095.94	28102.46	31060.14	25672.11	23751.16	22296.85	22013.09	26585.15	27061.28	26228.71	2.27	1.49	0.65	0.84	1.31	0.69	4.16	2.47	0.11	4.90	4.98	0.12	x	
4920	Uncharacterized protein C4orf36	Q96KX1	19278.79	16630.16	22316.11	8246.14	7856.65	6259.44	3481.79	2054.80	4402.39	21160.63	21099.62	36605.30	18407.36	9191.77	1983.43	2362.62	5200.12	5279.47	24223.20	8844.18	8576.50	2.27	1.49	0.65	0.84	1.31	0.69	4.16	2.47	0.11	4.90	4.98	0.12	x	
4921	Uncharacterized protein C4orf45	Q96LM5	0.14	2.53	0.81	1.07	2.77	0.68	0.96	0.36	0.63	0.35	0.75	0.38	2.13	0.97	0.60	4.64	1.07	0.47	2.22	0.19	0.06	1219.04	1100.03	989.83	817.75	795.52	682.64	1313.93	1247.11	1168.76	1133.02	1065.39	641.84		x
4922	Uncharacterized protein C5orf34	Q96MH7	14150.59	11703.71	9966.88	7616.74	7628.44	6336.13	9964.62	7403.98	8340.46	10834.51	9873.09	8780.43	9773.58	6982.90	3503.22	8188.70	7244.34	7385.74	8563.74	7820.26	6773.62	2.27	1.49	0.65	0.84	1.31	0.69	4.16	2.47	0.11	4.90	4.98	0.12	x	
4923	Uncharacterized protein C5orf67	F2Z3F1	10877.70	6958.83	3148.08	4715.76	4357.27	2334.56	5235.47	5003.70	3943.32	7679.52	6348.27	3016.41	6561.96	4652.55	269.98	5021.53	4779.14	2913.22	3650.06	2901.84	1646.34	2.27	1.49	0.65	0.84	1.31	0.69	4.16	2.47	0.11	4.90	4.98	0.12	x	
4924	Uncharacterized protein C6orf106	Q9H6K1	26384.54	25587.64	25067.90	16330.25	15963.08	14467.64	22844.54	23099.29	21073.03	22784.20	22111.32	20268.57	22034.62	19696.06	13371.63	21600.27	17599.87	17883.08	19867.21	19415.06	16458.35	2.27	1.49	0.65	0.84	1.31	0.69	4.16	2.47	0.11	4.90	4.98	0.12	x	
4925	Uncharacterized protein C7orf31	Q8N865	0.14	2.53	0.81	1.07	2.77	0.68	0.96	0.36	0.63	0.35	0.75	0.38	2.13	0.97	0.60	4.64	1.07	0.47	2.22	0.19	0.06	2695.70	2551.64	2499.24	1641.36	1650.08	1240.53	2337.81	2060.52	2281.70	2008.79	1981.72	1301.98		x
4926	Uncharacterized protein C7orf50	Q9BRJ6	14248.43	14309.59	12275.00	8384.71	8494.16	8219.87	10299.87	9143.06	9132.10	10334.88	9050.41	8191.64	13860.22	11763.84	10480.88	8639.32	7770.97	7747.74	8939.27	9415.32	8392.98	6662.67	5676.44	5752.40	4793.25	4305.67	3846.75	5635.99	5274.42	4872.27	5400.92	5276.16	3371.60		
4927	Uncharacterized protein C9orf153	Q5TBE3	1961.59	1518.50	954.36	809.00	574.89	393.30	979.57	298.22	833.04	1558.38	1483.74	997.66	1533.72	259.74	115.97	684.33	465.80	481.20	913.12	798.76	459.52	3131.05	1236.62	100.48	1538.77	1535.57	646.00	2852.82	2024.63	1516.21	3436.71	3353.29	1173.92		
4928	Uncharacterized protein encoded by LINC01600	Q96MT4	0.14	2.53	0.81	1.07	2.77	0.68	0.96	0.36	0.63	0.35	0.75	0.38	2.13	0.97	0.60	4.64	1.07	0.47	2.22	0.19	0.06	803.98	900.03	772.73	578.13	352.84	323.80	353.53	369.19	590.89	623.09	486.20	381.56		x
4929	Uncharacterized protein ENSP00000382033	A8MXK9	3841.78	2830.95	1841.25	1380.06	1170.26	618.54	1855.01	589.12	311.23	3156.69	2699.72	1879.92	3607.37	1559.06	67.02	1251.30	4331.46	854.46	2069.99	860.42	1739.17	2.27	1.49	0.65	0.84	1.31	0.69	4.16	2.47	0.11	4.90	4.98	0.12	x	
4930	Uncharacterized protein FLJ45252	Q6ZSR9	0.14	2.53	0.81	1.07	2.77	0.68	0.96	0.36	0.63	0.35	0.75	0.38	2.13	0.97	0.60	4.64	1.07	0.47	2.22	0.19	0.06	191.34	219.29	180.43	46.71	78.50	26.73	32.78	42.89	43.38	4.90	4.98	0.12		x
4931	Uncharacterized protein KIAA1143	Q96AT1	5844.44	5417.85	6106.89	2221.89	2433.89	2081.87	3609.25	3530.87	3198.17	3027.77	2934.96	2917.01	3222.85	3118.58	2399.85	2665.10	2000.16	2468.37	2857.39	2727.42	2364.53	2.27	1.49	0.65	0.84	1.31	0.69	4.16	2.47	0.11	4.90	4.98	0.12	x	
4932	Uncharacterized protein KIAA1257	Q9ULG3	18640.41	17963.33	16476.71	11740.63	11591.86	10264.65	17517.61	16275.89	16425.79	15626.93	14651.39	12890.25	14829.37	14028.40	10986.73	13895.34	12749.03	12724.26	12985.66	11673.63	10487.05	2.27	1.49	0.65	0.84	1.31	0.69	4.16	2.47	0.11	4.90	4.98	0.12	x	
4933	Uncharacterized protein KIAA1671	Q9BY89	14511.51	13478.24	13979.08	12382.03	11895.50	11611.87	6674.13	6589.05	6889.66	12825.41	12261.20	12056.26	12356.98	11071.76	9630.27	7814.12	6971.72	7415.13	11516.23	10996.94	10982.25	10991.89	10667.39	9685.43	6012.02	5727.53	4381.64	7101.72	6799.88	5437.09	6742.85	6104.09	3968.75		
4934	Uncharacterized protein KIAA1841	Q6NSI8	0.14	2.53	0.81	1.07	2.77	0.68	0.96	0.36	0.63	0.35	0.75	0.38	2.13	0.97	0.60	4.64	1.07	0.47	2.22	0.19	0.06	5331.83	5404.77	4774.69	2946.58	2962.68	2156.81	4703.73	4390.21	3900.35	4201.11	3809.51	2205.26		x
4935	Uncharacterized protein KIAA1958	Q8N8K9	0.14	2.53	0.81	1.07	2.77	0.68	0.96	0.36	0.63	0.35	0.75	0.38	2.13	0.97	0.60	4.64	1.07	0.47	2.22	0.19	0.06	481.53	499.53	373.95	355.34	297.68	318.70	571.96	491.03	423.82	587.00	472.86	265.25		x
4936	Uncharacterized protein KIAA2026	Q5HYC2	19426.00	19423.85	19604.53	16103.25	15344.67	14687.75	14526.59	15020.63	13295.51	17808.50	16537.52	14604.68	17076.73	16619.35	13766.68	12702.21	12457.42	12894.05	15204.93	13705.60	13254.22	2.27	1.49	0.65	0.84	1.31	0.69	4.16	2.47	0.11	4.90	4.98	0.12	x	
4937	Unconventional myosin-Ib	O43795	22358.41	23186.95	22742.51	16400.13	15165.84	15223.85	15971.84	15494.54	15471.18	15994.55	16014.80	15998.97	18401.88	16700.71	13770.60	16075.86	15634.93	15480.32	14965.43	14041.84	13285.61	2.27	1.49	0.65	0.84	1.31	0.69	4.16	2.47	0.11	4.90	4.98	0.12	x	
4938	Unconventional myosin-Ic	O00159	25727.41	23472.39	21085.26	16928.57	14012.73	12317.09	18112.86	16741.70	16313.04	20655.30	19116.92	18274.78	20355.24	17986.22	12096.62	16650.69	14180.60	13588.03	15123.42	15374.74	13301.68	426102.65	405144.13	363998.23	251338.06	250392.04	216253.26	305196.09	294946.93	280907.90	306833.23	299228.12	202456.65		
4939	Unconventional myosin-Id	O94832	19797.71	20191.02	19292.91	13838.61	12444.71	12388.37	16263.12	15450.84	14547.19	17046.81	16371.77	14291.10	16324.66	15475.90	12205.73	13786.56	13647.16	12961.98	15061.80	13909.30	12347.07	7938.72	7749.11	7874.26	5483.63	5350.38	4784.07	5500.30	5466.57	5335.87	5094.90	5180.27	3746.15		
4940	Unconventional myosin-Ie	Q12965	97.35	210.41	212.56	103.34	110.22	87.84	128.19	117.30	124.74	82.01	112.61	157.73	164.63	209.22	101.23	117.23	102.55	111.32	144.54	173.68	168.31	6172.57	5845.60	5585.13	4013.07	3646.62	2941.37	5987.48	5829.80	5632.63	4990.64	4708.47	2888.70		
4941	Unconventional myosin-Ih	Q8N1T3	0.14	2.53	0.81	1.07	2.77	0.68	0.96	0.36	0.63	0.35	0.75	0.38	2.13	0.97	0.60	4.64	1.07	0.47	2.22	0.19	0.06	74924.29	71328.70	61957.34	50108.58	47484.72	44288.26	49522.82	46410.59	48057.28	73402.51	62925.76	34381.03		x
4942	Unconventional myosin-IXa	B2RTY4	16381.36	17388.91	16161.87	12620.89	12735.54	12110.39	12883.47	12234.15	12787.59	14569.83	13033.61	12678.46	15821.71	15504.89	12834.48	11856.45	11004.43	11095.89	12065.14	10729.73	11359.46	2.27	1.49	0.65	0.84	1.31	0.69	4.16	2.47	0.11	4.90	4.98	0.12	x	
4943	Unconventional myosin-IXb	Q13459	0.14	2.53	0.81	1.07	2.77	0.68	0.96	0.36	0.63	0.35	0.75	0.38	2.13	0.97	0.60	4.64	1.07	0.47	2.22	0.19	0.06	27295.99	26616.41	24079.50	18698.43	18296.92	16383.43	24412.40	22910.69	21662.63	24650.36	23033.21	17692.19		x
4944	Unconventional myosin-Va	Q9Y4I1	1514.68	1576.48	1603.00	1353.17	1402.50	1367.71	1230.87	1224.70	1185.81	1408.12	1594.14	1428.10	1585.88	1387.68	1189.58	960.03	1033.51	1011.32	1147.42	1175.86	1146.56	2.27	1.49	0.65	0.84	1.31	0.69	4.16	2.47	0.11	4.90	4.98	0.12	x	
4945	Unconventional myosin-Vb	Q9ULV0	124258.97	124820.69	113965.48	93207.85	89023.57	80733.05	118833.58	111253.97	107473.18	119513.02	112285.45	100424.94	118878.54	112066.73	90341.98	93919.00	84244.76	82450.52	93855.25	95247.18	88549.88	27727.63	25878.51	22836.51	17414.61	15885.79	12597.25	19589.82	18845.86	17959.61	20631.09	20034.66	12505.82		
4946	Unconventional myosin-Vc	Q9NQX4	0.14	2.53	0.81	1.07	2.77	0.68	0.96	0.36	0.63	0.35	0.75	0.38	2.13	0.97	0.60	4.64	1.07	0.47	2.22	0.19	0.06	3097.97	3105.67	2998.34	2052.93	1904.09	1919.06	2336.26	2519.12	2477.69	2427.43	2392.67	1641.70		x
4947	Unconventional myosin-VI	Q9UM54	244013.46	235391.31	224041.50	152136.72	148105.25	139568.16	171039.72	171267.67	163555.11	189538.95	184749.93	177269.77	188782.54	178359.69	157141.00	164403.67	152823.12	148014.16	145120.76	137937.90	133111.90	25314.00	22758.72	20425.25	18183.12	17033.10	14981.93	22926.97	21043.68	19386.92	24336.67	22378.29	12150.82		
4948	Unconventional myosin-VIIb	Q6PIF6	0.14	2.53	0.81	1.07	2.77	0.68	0.96	0.36	0.63	0.35	0.75	0.38	2.13	0.97	0.60	4.64	1.07	0.47	2.22	0.19	0.06	61767.92	57136.46	48111.44	36697.38	36370.11	24799.42	52492.81	47740.20	39363.70	56695.44	53593.45	29488.61		x
4949	Unconventional myosin-XVIIIb	Q8IUG5	5847.75	4740.86	4113.43	2954.95	2967.19	2521.35	3875.34	3657.18	3255.05	4492.75	4390.28	3914.78	4757.11	4375.27	3000.26	2994.10	2831.33	2817.36	3322.24	2939.15	2746.22	2.27	1.49	0.65	0.84	1.31	0.69	4.16	2.47	0.11	4.90	4.98	0.12	x	
4950	UPF0160 protein MYG1_ mitochondrial	Q9HB07	60509.29	58898.46	57012.41	40692.93	44916.09	37328.92	50772.58	45562.86	41035.30	52168.23	45423.38	44131.11	50737.58	52441.64	43253.29	41278.31	39074.91	39705.24	30803.96	38703.03	35917.77	74635.73	71525.46	64318.22	41004.59	39877.65	36025.70	59720.69	59627.29	58769.24	53226.72	50008.80	35586.38		
4951	UPF0488 protein C8orf33	Q9H7E9	82184.75	80858.74	71942.70	66042.88	62741.67	53200.00	67567.70	59698.83	62635.28	65052.40	65687.61	55961.28	72972.76	59886.72	48414.35	53018.53	50815.54	54019.69	52619.29	49817.18	46967.36	1328.51	1352.89	1249.72	944.26	896.34	875.19	875.00	903.56	794.59	1217.22	1050.30	713.71		
4952	UPF0489 protein C5orf22	Q49AR2	925.45	970.67	736.11	793.43	664.44	678.35	674.94	626.30	655.26	1103.58	1019.68	899.68	699.70	749.02	655.00	601.58	572.31	446.15	742.47	682.32	727.32	2.27	1.49	0.65	0.84	1.31	0.69	4.16	2.47	0.11	4.90	4.98	0.12	x	
4953	UPF0600 protein C5orf51	A6NDU8	5446.52	4870.03	4309.96	3353.25	3333.93	2848.58	4556.50	3868.49	3712.78	5250.75	5003.00	4174.59	5188.62	5035.47	3738.35	3524.77	3421.81	3055.06	3381.91	3005.19	2564.33	4408.33	3606.18	3031.20	2197.30	2063.88	1426.44	2826.56	2553.05	1929.47	3057.91	3044.45	1783.35		
4954	UPF0687 protein C20orf27	Q9GZN8	19604.00	18133.17	18176.43	10535.45	9267.28	9081.47	13582.34	12942.40	12655.99	13552.50	13064.91	12205.63	13038.16	12932.44	9448.98	9951.53	8565.85	9280.74	10542.41	10331.84	9566.86	28964.85	26902.42	22706.95	18153.63	17249.20	14327.95	21678.35	20211.36	18655.26	21228.57	20348.84	14273.21		
4955	UPF0711 protein C18orf21	Q32NC0	0.14	2.53	0.81	1.07	2.77	0.68	0.96	0.36	0.63	0.35	0.75	0.38	2.13	0.97	0.60	4.64	1.07	0.47	2.22	0.19	0.06	4098.29	3805.04	3656.91	4922.61	5111.53	4352.38	9477.85	9041.04	8707.49	4308.19	4398.27	3216.95		x
4956	Up-regulated during skeletal muscle growth protein 5	Q96IX5	32866.75	29437.78	28327.18	21881.47	21515.11	19415.51	25966.89	26024.69	24682.55	29549.58	29695.10	28330.91	27029.17	24912.46	22497.33	25910.60	21355.46	20676.98	21662.59	21269.80	19360.94	33019.16	29179.99	29748.55	22163.24	21465.76	17788.01	26652.92	25743.61	26601.00	22138.42	21694.35	15317.62		
4957	Up-regulator of cell proliferation	Q8TCY9	44494.50	43711.55	39668.34	15281.86	15477.87	13086.42	23609.12	24311.58	22752.83	28543.02	29936.08	27947.89	23896.09	22010.25	20227.82	42247.13	39238.22	33860.46	24639.54	24210.39	24377.61	2.27	1.49	0.65	0.84	1.31	0.69	4.16	2.47	0.11	4.90	4.98	0.12	x	
4958	Upstream-binding factor 1-like protein 1	P0CB47	547.39	633.92	719.76	348.24	240.98	365.31	749.19	570.72	626.78	499.99	548.81	287.13	518.16	509.95	414.43	436.84	383.84	413.31	513.55	457.18	473.14	2.27	1.49	0.65	0.84	1.31	0.69	4.16	2.47	0.11	4.90	4.98	0.12	x	
4959	Uracil-DNA glycosylase	P13051	36774.19	36330.30	32919.33	18605.72	18574.40	15344.63	23734.21	23078.68	20673.91	24515.16	23444.40	21613.07	24824.59	24175.09	19809.28	18855.96	17682.02	15117.93	21388.15	20398.15	18392.19	2.27	1.49	0.65	0.84	1.31	0.69	4.16	2.47	0.11	4.90	4.98	0.12	x	
4960	Uridine 5'-monophosphate synthase	P11172	27012.76	25834.31	25927.25	13511.39	13356.54	13684.24	20622.56	19964.11	19276.75	15650.69	15422.05	15103.24	19311.18	17203.09	14708.35	15331.31	13673.15	13582.26	14838.72	13755.62	12610.68	4142.11	3432.22	4101.38	1870.08	2250.70	2087.57	3207.81	3116.90	3493.31	3040.42	2874.21	2230.33		
4961	Uridine phosphorylase 1	Q16831	0.14	2.53	0.81	1.07	2.77	0.68	0.96	0.36	0.63	0.35	0.75	0.38	2.13	0.97	0.60	4.64	1.07	0.47	2.22	0.19	0.06	11719.03	11869.63	10502.98	8550.47	8099.31	7461.71	9439.82	9776.35	9183.27	9169.86	8790.39	5935.20		x
4962	Uridine-cytidine kinase 2	Q9BZX2	3682.18	3751.58	3290.44	2078.24	2024.30	1551.26	2555.60	1886.06	2929.12	2834.25	2654.58	2643.44	2940.88	2705.51	2165.08	2351.88	2075.69	2080.09	2606.02	2313.35	1852.36	4609.49	4302.56	4130.36	2707.55	2599.37	2383.70	3290.10	3207.01	3048.91	3141.37	3200.59	2124.10		
4963	Uroporphyrinogen decarboxylase	P06132	42657.27	40904.19	33177.94	21844.27	21944.69	20950.08	25561.37	24229.67	23230.11	29848.44	28807.99	28259.71	26465.74	28191.36	26281.89	22125.94	22099.90	20629.74	24317.77	25529.39	22491.55	32179.18	32091.03	28817.08	18976.30	19626.48	16605.00	25581.47	24059.23	24688.04	22128.69	21165.13	15877.71		
4964	UTP--glucose-1-phosphate uridylyltransferase	Q16851	149899.18	131706.88	121688.87	85742.38	79748.57	70697.31	89865.15	84685.62	82198.90	117240.11	109122.51	103874.10	115222.75	104447.46	78758.60	84470.29	80347.88	77907.59	87005.60	82721.72	72694.49	44927.50	40223.84	36170.12	30549.07	29671.46	25065.26	34386.66	32280.01	30191.14	33934.56	34811.22	32474.89		
4965	Utrophin	P46939	0.14	2.53	0.81	1.07	2.77	0.68	0.96	0.36	0.63	0.35	0.75	0.38	2.13	0.97	0.60	4.64	1.07	0.47	2.22	0.19	0.06	240292.00	225632.40	214239.17	143114.94	139068.62	129305.22	160579.15	155786.70	150109.59	160822.13	153347.05	109218.01		x
4966	UV excision repair protein RAD23 homolog A	P54725	244.24	365.63	194.64	65.59	86.99	104.83	201.89	194.46	222.25	87.66	91.24	80.68	156.54	221.16	197.47	127.35	83.95	101.09	434.31	351.11	303.35	2.27	1.49	0.65	0.84	1.31	0.69	4.16	2.47	0.11	4.90	4.98	0.12	x	
4967	UV excision repair protein RAD23 homolog B	P54727	770178.94	756622.13	725030.51	502826.62	476076.66	456745.83	563502.20	545125.38	519456.63	614533.54	597601.17	572977.23	633992.34	610482.30	508858.42	490734.28	475654.13	457389.81	524770.07	497742.00	483496.84	196223.28	185784.82	173156.05	126511.50	128516.98	120992.42	142543.50	136836.86	128487.22	154528.88	150834.65	108668.61		

Supplementary Table S1. Overview on the abundance of all relatively quantified 5180 proteins

			MCF-7																					MDA-MB-231													
	Protein name	UniProt	control 01	control 02	control 03	Dai IC20 01	Dai IC20 02	Dai IC20 03	Dai SC20 01	Dai SC20 02	Dai SC20 03	Gen IC20 01	Gen IC20 02	Gen IC20 03	Gen SC20 01	Gen SC20 02	Gen SC20 03	SSE IC20 01	SSE IC20 02	SSE IC20 03	SSE SC20 01	SSE SC20 02	SSE SC20 03	control 01	control 02	control 03	Dai IC20 01	Dai IC20 02	Dai IC20 03	Gen IC20 01	Gen IC20 02	Gen IC20 03	SSE IC20 01	SSE IC20 02	SSE IC20 03	unique to MCF-7	unique to MDA-MB-231
4968	Uveal autoantigen with coiled-coil domains and ankyrin repeats	Q9BZF9	50471.79	48256.05	44268.39	34919.65	34543.66	32255.69	39701.61	38915.28	37174.47	47142.70	43998.54	42592.12	43043.09	39454.01	32017.93	33903.89	31223.25	29764.74	34091.98	32491.35	31964.83	2.27	1.49	0.65	0.84	1.31	0.69	4.16	2.47	0.11	4.90	4.98	0.12	x	
4969	Vacuolar fusion protein CCZ1 homolog B	P86790	3144.50	2942.77	2892.36	2070.45	2095.07	1797.04	2208.64	2206.83	2045.95	2714.18	2479.82	1928.65	2376.90	2715.29	1935.69	1791.93	1880.14	1800.43	1737.76	1956.77	1972.10	2.27	1.49	0.65	0.84	1.31	0.69	4.16	2.47	0.11	4.90	4.98	0.12	x	
4970	Vacuolar fusion protein MON1 homolog A	Q86VX9	0.14	2.53	0.81	1.07	2.77	0.68	0.96	0.36	0.63	0.35	0.75	0.38	2.13	0.97	0.60	4.64	1.07	0.47	2.22	0.19	0.06	3574.65	2902.61	3117.30	4297.52	3994.38	3234.72	4965.12	4306.05	4572.09	4302.52	4536.53	3333.47		x
4971	Vacuolar fusion protein MON1 homolog B	Q7L1V2	414.97	348.96	286.06	381.45	321.69	343.49	469.07	523.74	429.93	393.57	401.98	426.43	229.59	252.74	202.15	339.08	355.25	343.42	255.02	228.62	254.02	2.27	1.49	0.65	0.84	1.31	0.69	4.16	2.47	0.11	4.90	4.98	0.12	x	
4972	Vacuolar protein sorting-associated protein 11 homolog	Q9H270	14132.28	13603.54	12987.80	9781.89	9594.79	8599.89	12290.29	12657.63	11426.42	12449.65	12820.36	11442.36	12575.89	11723.59	8635.99	10420.47	9860.67	9334.11	10586.16	10056.79	8691.84	3667.48	3531.64	3451.27	2791.40	2774.30	2144.33	3071.95	2864.18	2734.25	3036.07	3054.77	2053.35		
4973	Vacuolar protein sorting-associated protein 13A	Q96RL7	46904.95	40976.51	37515.19	37103.09	34601.31	32701.71	31810.22	30243.27	27644.16	36056.20	35567.25	34500.39	40888.38	35985.70	21192.06	26925.87	26158.16	29101.08	42175.74	36543.72	30959.75	116494.52	110030.74	105891.02	32198.42	31083.64	27990.19	37472.07	35769.87	33431.21	31660.42	33821.16	21022.91		
4974	Vacuolar protein sorting-associated protein 13C	Q709C8	193656.72	180829.37	177189.64	129750.26	122552.15	116094.01	150347.07	145806.07	140312.75	152876.04	150894.05	138699.43	157300.40	149991.86	126658.90	122127.70	117145.95	112052.85	127553.36	120865.81	120038.46	143609.42	133926.96	132172.41	105166.86	102539.67	88612.73	125118.83	118357.94	113157.67	117345.68	113637.45	90887.81		
4975	Vacuolar protein sorting-associated protein 13D	Q5THJ4	100449.25	101220.58	94313.52	71637.74	70716.46	67308.72	77582.99	71005.86	72316.71	86309.66	82978.31	79295.59	92009.41	88576.36	74818.08	60317.51	60347.84	60006.73	71230.43	71152.48	68080.54	19580.85	17059.50	15248.28	12840.95	12794.29	10820.71	14470.67	14290.27	13417.14	15663.52	14875.88	7982.14		
4976	Vacuolar protein sorting-associated protein 18 homolog	Q9P253	14225.26	13115.97	12572.62	9627.52	9640.96	8973.08	10443.06	10146.41	9447.73	11157.66	11115.02	10368.05	11967.83	11689.48	7996.50	10095.27	9452.80	9553.12	9712.56	9428.91	8687.90	2.27	1.49	0.65	0.84	1.31	0.69	4.16	2.47	0.11	4.90	4.98	0.12	x	
4977	Vacuolar protein sorting-associated protein 26A	O75436	94621.74	91194.90	87701.58	63821.89	63012.03	57357.84	72402.12	69814.49	67250.38	71286.45	69054.96	63151.82	71709.69	63005.96	60502.75	66342.17	64492.64	60383.09	62940.96	58085.86	52095.64	83867.88	92796.11	76896.16	71643.63	70964.00	78894.10	79701.94	79868.02	75377.17	111281.03	93626.35	56430.15		
4978	Vacuolar protein sorting-associated protein 26B	Q4G0F5	18770.78	16997.06	17101.44	12700.76	11956.43	11180.13	13834.06	13512.29	13391.04	15116.24	14151.92	13849.00	13941.08	16464.04	11521.65	11764.91	11006.90	10783.59	12718.67	12247.71	11782.76	4153.67	3869.13	4088.32	3135.61	3245.87	3072.22	3847.89	3458.99	3457.18	3905.90	4134.21	3146.93		
4979	Vacuolar protein sorting-associated protein 29	Q9UBQ0	25703.62	26838.11	25415.38	16084.10	15837.05	16156.24	22307.67	18934.05	20498.71	21763.00	21181.75	22239.49	19957.02	21027.67	16808.79	17267.07	15579.82	16233.64	17120.51	16946.79	15331.89	45501.79	42653.93	41574.75	37774.92	34908.54	31302.57	40900.55	41288.54	36809.46	39024.75	37692.22	28502.28		
4980	Vacuolar protein sorting-associated protein 33B	Q9H267	13277.26	12800.96	12594.75	10124.10	8610.16	8505.88	10497.09	10039.50	9868.08	11462.59	10782.01	10133.29	11644.81	11524.85	8691.56	8868.99	8387.29	8341.17	9744.39	9569.01	9433.70	3469.69	3566.26	3304.00	2452.48	2229.29	1682.92	3094.24	2838.92	2997.16	2738.00	2475.41	1577.06		
4981	Vacuolar protein sorting-associated protein 35	Q96QK1	162180.99	153955.74	148042.56	122895.39	121909.99	108299.35	132842.03	124546.73	119480.13	142377.65	133199.66	127334.87	138920.02	136847.48	117132.13	119130.10	112883.50	111535.60	110909.37	110210.39	103327.67	222073.79	213539.16	198753.46	143211.67	143723.25	136856.20	165976.04	159359.97	153292.29	170262.76	159463.29	131799.79		
4982	Vacuolar protein sorting-associated protein 37A	Q8NEZ2	83651.23	77495.78	65405.25	42577.38	43070.75	38457.66	58207.70	61726.49	57583.11	64666.64	61561.04	54648.62	57182.95	53105.39	40414.36	45477.71	47644.94	45118.34	55317.18	52202.46	48484.99	3985.57	3590.54	3937.98	3042.92	2957.15	1564.77	5883.72	5160.00	4144.27	4004.85	3502.89	1720.79		
4983	Vacuolar protein sorting-associated protein 45	Q9NRW7	64025.94	60528.81	51959.74	44788.46	41397.42	40662.21	44182.18	40255.18	40226.66	56063.34	54699.16	50594.43	51924.59	52166.55	41525.73	41263.07	38796.03	41765.30	41367.12	40785.18	37576.65	2.27	1.49	0.65	0.84	1.31	0.69	4.16	2.47	0.11	4.90	4.98	0.12	x	
4984	Vacuolar protein sorting-associated protein 4A	Q9UN37	30148.56	30458.65	27401.27	15915.85	16530.88	14625.64	20662.92	19010.97	18507.70	21478.83	19729.78	19213.31	21164.05	20178.49	18080.12	17178.86	17270.26	16020.00	15857.18	15791.06	14380.21	18394.27	16474.99	17861.55	12186.89	11144.52	10617.78	16819.21	15722.49	16706.58	13916.57	13834.35	10070.13		
4985	Vacuolar protein sorting-associated protein 4B	O75351	26976.53	25979.83	23126.32	16896.02	13268.17	14794.96	19271.82	18241.16	20685.25	24496.62	22974.06	21412.63	20711.53	21988.26	16826.64	18469.75	15456.23	16588.60	16601.03	13555.62	14476.31	50822.20	50054.68	41734.57	34316.12	34466.38	31914.25	38390.11	37279.57	36023.68	43130.24	39785.27	45207.70		
4986	Vacuolar protein sorting-associated protein 8 homolog	Q8N3P4	560585.19	549346.72	467406.61	375504.32	353222.90	299297.79	450903.05	438668.40	384898.27	505311.26	492526.34	419200.02	419873.71	378465.05	211345.30	469681.35	446469.99	402389.41	416608.38	396995.29	316768.69	2.27	1.49	0.65	0.84	1.31	0.69	4.16	2.47	0.11	4.90	4.98	0.12	x	
4987	Vacuolar protein sorting-associated protein VTA1 homolog	Q9NP79	77937.70	75494.79	69379.63	55847.53	52975.07	49919.10	67169.36	65165.70	63097.52	62680.22	60585.16	56410.56	63991.96	59881.84	46152.52	56475.47	54739.24	51678.94	55874.25	52029.67	47776.42	9004.80	8337.03	7988.06	5454.30	4918.18	3699.62	6466.72	5768.84	5020.21	8205.62	7819.18	4487.53		
4988	Vacuolar protein-sorting-associated protein 25	Q9BRG1	1762.32	1578.43	1555.05	1518.13	1472.56	1469.19	1195.82	1562.03	1327.20	1835.30	1855.92	2005.83	1688.62	1524.98	1203.94	1245.88	1193.35	1401.16	1435.35	1532.24	1256.69	667.95	651.65	505.81	420.24	381.86	405.30	428.34	454.71	462.96	571.32	526.81	440.94		
4989	Vacuolar protein-sorting-associated protein 36	Q86VN1	3195.39	3063.86	2954.81	1938.79	1735.43	1497.54	2220.81	1993.40	2141.41	2904.25	2719.94	2735.48	2550.27	2361.81	1974.04	2512.65	2794.83	2335.38	2559.44	2379.14	2236.04	4703.99	4509.08	3745.72	2570.79	2320.56	2348.36	3539.04	3209.43	2809.76	3715.03	3981.69	2323.85		
4990	Vacuole membrane protein 1	Q96GC9	42368.70	42334.43	40591.85	27720.49	28428.66	25703.42	28586.30	29290.64	28354.59	33234.88	31928.48	31198.23	31901.21	31704.32	24156.45	26868.36	26847.79	28300.01	28466.15	25796.87	25075.66	2.27	1.49	0.65	0.84	1.31	0.69	4.16	2.47	0.11	4.90	4.98	0.12	x	
4991	Valine--tRNA ligase	P26640	344939.78	332013.95	310406.13	222887.19	215041.48	199294.54	248653.55	240766.22	233730.05	290338.23	275765.09	261393.84	265605.43	253806.50	220280.35	225144.61	216619.19	209534.68	230362.73	213190.42	201429.31	232842.27	218071.41	199796.63	144978.46	138067.17	120590.63	182222.80	174091.41	169488.79	161906.82	157031.00	103245.18		
4992	Vang-like protein 1	Q8TAA9	17706.99	18166.81	16924.04	11411.88	11042.38	10467.66	14141.30	13051.56	12961.17	15220.51	14569.69	13724.60	13054.07	12747.70	11248.09	10227.51	9728.92	8958.24	12519.80	11542.76	11839.28	2.27	1.49	0.65	0.84	1.31	0.69	4.16	2.47	0.11	4.90	4.98	0.12	x	
4993	Vang-like protein 2	Q9ULK5	2284.40	1803.05	1645.38	1941.25	1572.82	1279.87	683.89	641.26	638.27	1751.92	1876.85	1618.05	1479.08	1597.28	363.68	816.63	701.44	652.62	1359.42	1241.51	1076.12	2.27	1.49	0.65	0.84	1.31	0.69	4.16	2.47	0.11	4.90	4.98	0.12	x	
4994	Vascular endothelial growth factor C	P49767	0.14	2.53	0.81	1.07	2.77	0.68	0.96	0.36	0.63	0.35	0.75	0.38	2.13	0.97	0.60	4.64	1.07	0.47	2.22	0.19	0.06	559.84	523.54	443.10	925.91	993.12	855.33	1635.07	1603.14	1613.82	815.69	812.60	0.51		x
4995	Vascular endothelial growth factor receptor 3	P35916	0.14	2.53	0.81	1.07	2.77	0.68	0.96	0.36	0.63	0.35	0.75	0.38	2.13	0.97	0.60	4.64	1.07	0.47	2.22	0.19	0.06	8492.88	7719.80	6183.97	4729.98	4219.88	4215.76	7501.29	7042.09	6741.40	4973.44	3861.69	3582.00		x
4996	Vasculin-like protein 1	Q9HC44	0.14	2.53	0.81	1.07	2.77	0.68	0.96	0.36	0.63	0.35	0.75	0.38	2.13	0.97	0.60	4.64	1.07	0.47	2.22	0.19	0.06	3685.96	3511.51	3375.51	1927.84	1943.11	1623.57	1803.61	1690.94	1617.84	1577.14	1354.05	978.22		x
4997	Vasodilator-stimulated phosphoprotein	P50552	131653.70	131904.32	118285.57	84828.04	82823.60	75845.51	80927.64	79524.28	73706.27	111458.64	108059.14	103579.54	108285.74	92168.85	78286.85	77772.11	70988.68	69742.46	83756.79	80006.60	70639.01	101529.10	103150.39	90137.89	66673.30	65573.41	60487.69	77360.30	73372.43	71192.37	89157.70	82091.46	42486.37		
4998	Very large A-kinase anchor protein	Q68DQ2	336906.87	313853.83	309655.95	240594.47	231248.33	221036.59	285469.62	278021.96	278123.62	280791.60	266716.42	255980.46	300839.22	271427.38	229320.73	246400.52	232003.47	231884.97	267856.81	242312.50	255983.98	282204.93	277468.91	251385.26	207328.29	202191.69	203145.59	220237.64	214677.92	212145.53	239164.56	225442.52	183579.19		
4999	Very long-chain specific acyl-CoA dehydrogenase_ mitochondrial	P49748	154147.02	145799.40	124627.56	86503.38	84557.51	69336.38	94499.16	90789.51	86864.38	151558.20	140604.31	126503.39	137691.36	92395.38	84697.27	97810.42	94246.78	86991.62	90201.94	79247.19	70746.97	312708.26	289283.02	260843.57	210469.61	205275.33	186584.62	250759.64	243010.47	228946.26	254336.72	235730.20	155996.07		
5000	Very-long-chain (3R)-3-hydroxyacyl-CoA dehydratase 3	Q9P035	110018.54	105990.18	99176.18	71392.86	71332.64	65074.11	84943.71	79738.92	75636.20	88930.17	84205.37	77920.29	85883.42	87123.11	64681.22	70640.27	72675.97	66604.81	62772.62	62913.25	57954.92	21739.43	19349.60	17854.22	11904.54	12387.03	11055.71	16729.03	15761.37	13977.02	15791.77	14718.70	10090.01		
5001	Very-long-chain 3-oxoacyl-CoA reductase	Q53GQ0	25674.63	23136.27	21237.91	12829.50	11160.16	10922.85	15474.79	16470.64	14584.15	19414.43	18107.48	15294.97	19566.79	16161.74	9881.06	12072.75	11366.37	10589.00	15727.62	13150.91	10948.18	76093.79	66982.79	55450.08	44716.73	43346.82	34040.44	57280.46	53524.21	46505.77	57286.02	55019.75	35388.47		
5002	Very-long-chain enoyl-CoA reductase	Q9NZ01	86931.64	83913.79	80663.93	60306.04	56430.17	54024.09	65156.44	60837.28	56482.44	69507.26	63844.95	59577.57	67393.58	63739.24	55136.94	66202.78	62887.65	60599.66	58941.26	58747.18	50027.76	2.27	1.49	0.65	0.84	1.31	0.69	4.16	2.47	0.11	4.90	4.98	0.12	x	
5003	Vesicle transport protein SEC20	Q12981	16881.03	15987.15	15120.56	12481.26	13211.33	11827.05	17244.51	17520.10	16510.13	16094.19	15847.08	15094.30	14135.12	14104.82	12590.06	11566.31	10925.84	11507.43	12067.76	11138.38	11100.62	2.27	1.49	0.65	0.84	1.31	0.69	4.16	2.47	0.11	4.90	4.98	0.12	x	
5004	Vesicle transport through interaction with t-SNAREs homolog 1B	Q9UEU0	10563.14	9912.43	10670.60	6351.53	6778.34	5986.52	7202.17	7186.24	6593.67	8799.72	8094.87	7666.40	8166.72	8330.02	6905.01	5710.03	5867.00	5057.88	5960.57	5894.65	5757.37	2.27	1.49	0.65	0.84	1.31	0.69	4.16	2.47	0.11	4.90	4.98	0.12	x	
5005	Vesicle-associated membrane protein 2	P63027	0.14	2.53	0.81	1.07	2.77	0.68	0.96	0.36	0.63	0.35	0.75	0.38	2.13	0.97	0.60	4.64	1.07	0.47	2.22	0.19	0.06	28229.31	28361.00	25765.01	23115.97	22510.29	18981.47	24411.21	23269.16	21503.94	29029.19	29244.02	18747.86		x
5006	Vesicle-associated membrane protein 3	Q15836	55131.64	62215.62	58148.07	45540.22	42530.40	39332.48	41953.92	40449.51	35939.97	59238.21	58039.47	55484.24	52055.42	42661.32	35871.33	37543.32	39842.60	36109.16	46657.95	44078.95	44922.14	4986.05	4622.74	4190.27	4053.17	3288.95	2423.14	4035.90	3995.04	3262.66	5479.46	4830.37	3089.30		
5007	Vesicle-associated membrane protein 8	Q9BV40	21417.91	22136.71	18042.06	13318.97	13917.01	13600.75	11956.24	10113.89	11049.32	17276.29	17179.79	15533.68	16400.16	15964.03	15079.29	13149.87	12586.24	13478.42	14226.68	13033.11	12933.92	2.27	1.49	0.65	0.84	1.31	0.69	4.16	2.47	0.11	4.90	4.98	0.12	x	
5008	Vesicle-associated membrane protein-associated protein A	Q9P0L0	122685.42	117646.51	112164.21	85878.74	79662.58	74000.83	103548.89	99857.86	95029.73	94034.87	90145.77	86700.73	98024.03	96569.77	65996.57	81500.91	80576.42	77835.31	88636.74	84247.37	77036.92	87030.65	84215.06	70534.48	42036.71	41886.18	37546.86	46072.14	44548.46	42001.49	55898.95	50101.00	33734.66		
5009	Vesicle-associated membrane protein-associated protein B/C	O95292	416452.97	410668.23	409997.79	296349.24	293059.85	274595.31	318593.62	293211.55	293616.28	349829.19	336564.31	323543.70	342555.39	328463.44	284541.26	265792.05	261542.03	258583.90	277094.75	278478.79	269335.09	92242.63	87212.75	80683.20	65829.48	64675.93	60428.42	79409.15	77254.62	74093.93	73128.39	71320.68	51363.38		
5010	Vesicle-fusing ATPase	P46459	217280.95	208737.07	198143.55	125616.14	125201.40	122139.97	142586.51	142066.09	135065.19	172575.10	162444.71	150927.52	155493.57	152143.04	132133.95	135977.22	119009.60	119027.80	141557.03	132572.70	125214.36	123877.18	119065.03	109410.30	82410.75	79275.10	71556.34	91767.49	89139.15	87848.45	94843.92	89610.04	62127.19		
5011	Vesicle-trafficking protein SEC22a	Q96IW7	1116.25	1060.84	1115.04	773.11	789.03	695.37	822.26	810.74	676.17	1114.45	1005.34	892.70	896.77	876.94	589.80	981.94	994.57	875.58	1022.63	841.92	737.02	2.27	1.49	0.65	0.84	1.31	0.69	4.16	2.47	0.11	4.90	4.98	0.12	x	
5012	Vesicle-trafficking protein SEC22b	O75396	556221.15	531105.74	493613.42	377530.11	365627.43	339989.30	427968.50	407327.86	388158.32	473023.35	453531.28	418263.66	472239.41	406152.09	348578.62	392202.13	376223.11	365962.98	382608.55	362356.24	336371.80	31782.13	34147.00	29421.46	21337.44	20251.66	17048.27	26795.07	26137.46	22938.68	24922.41	24726.97	14868.84		
5013	Vesicular integral-membrane protein VIP36	Q12907	339279.96	331275.07	291546.25	243997.63	234707.31	207540.26	303324.41	284062.22	277258.83	303272.03	284656.48	260628.63	290179.75	266105.63	202269.68	243116.00	228777.64	212995.23	229571.98	213679.59	198799.58	54540.72	46038.85	39807.76	28838.72	27699.96	23120.04	37266.26	34767.69	28822.77	34670.89	34695.01	20331.94		
5014	Vigilin	Q00341	721030.56	691203.29	654587.00	487640.32	491884.71	449492.91	506963.16	480253.72	465705.34	624498.79	589878.07	554586.81	580753.50	542082.16	462735.05	464123.75	440060.03	426703.48	492709.88	472414.05	441108.52	113949.37	104663.85	99856.46	77889.07	76529.36	73517.15	84620.51	81536.60	76184.29	89675.12	84669.87	66549.77		
5015	Vimentin	P08670	4518.21	4596.30	5044.39	3939.80	4164.61	3672.39	3671.36	3197.41	3398.40	4115.83	3591.66	3458.18	3948.07	3151.70	3015.66	3718.08	3278.53	4189.94	3668.31	3030.32	2822.47	197010.65	181527.37	175107.48	182820.95	178930.52	161881.50	196129.01	190485.76	184993.08	579583.49	550124.75	408602.78		
5016	Vinculin	P18206	912014.21	868040.63	777163.92	629170.42	632824.73	569138.63	700755.68	669988.85	636437.08	788319.21	761178.70	686317.12	671504.97	678706.31	546800.84	651231.59	630155.45	619288.08	644494.79	604515.37	575544.75	704982.48	672741.13	618045.41	426656.35	418241.81	374610.36	520612.89	507387.64	484672.68	504409.15	486450.54	327487.07		
5017	Vitamin D-binding protein	P02774	2286.61	2295.99	2027.17	1728.84	1816.46	1593.52	1504.55	1411.17	1557.19	2701.47	2381.33	2458.73	2261.54	1937.71	1591.74	1436.91	1534.16	1460.23	1634.57	1720.91	1796.41	2.27	1.49	0.65	0.84	1.31	0.69	4.16	2.47	0.11	4.90	4.98	0.12	x	
5018	Vitamin K epoxide reductase complex subunit 1	Q9BQB6	52203.39	51281.62	49425.22	36314.06	35892.29	33777.45	39526.92	39739.95	38299.88	46054.89	45965.28	41539.35	44321.25	41139.33	36573.99	36547.69	32831.66	33716.81	30863.85	35062.41	33650.71	9110.57	9145.23	7770.83	5733.49	5103.17	4818.74	6234.58	6593.93	6456.11	6158.63	5959.20	4498.09		
5019	Vitronectin	P04004	0.14	2.53	0.81	1.07	2.77	0.68	0.96	0.36	0.63	0.35	0.75	0.38	2.13	0.97	0.60	4.64	1.07	0.47	2.22	0.19	0.06	3161.10	1203.59	1750.57	1163.42	1204.57	628.44	1915.24	1560.69	1110.76	1512.23	1355.97	494.71		x
5020	Voltage-dependent anion-selective channel protein 1	P21796	364578.64	342436.14	312863.35	238355.93	237416.73	208958.35	321866.21	321546.16	312741.02	299408.04	293296.53	285198.96	298453.67	269235.32	222566.17	291412.51	274535.51	259190.82	295980.89	289188.60	263381.12	281527.09	255845.50	226118.24	158851.36	152598.93	121326.50	224222.37	212207.24	194357.96	193166.18	181711.19	118797.06		
5021	Voltage-dependent anion-selective channel protein 2	P45880	236590.51	225729.36	202015.02	140659.36	138653.57	123713.43	190733.12	180381.08	174269.85	192066.29	178158.28	170609.26	171484.90	157000.71	121799.31	165297.35	155298.36	143753.61	159838.64	151873.72	141886.30	186605.15	175995.74	160442.92	107113.49	104201.99	92782.76	148109.31	135846.74	135965.92	122015.53	116361.72	76146.79		
5022	Voltage-dependent anion-selective channel protein 3	Q9Y277	60240.67	60452.04	55716.38	34683.64	34220.97	32200.29	43805.29	43673.97	42382.66	46631.95	43863.33	41911.42	42157.26	39723.13	31405.68	37928.03	38392.48	35763.97	39638.54	38155.74	34332.46	42892.05	42624.89	37485.73	26525.10	26611.83	22320.10	37475.53	36606.37	33558.30	33571.27	28843.87	23698.89		
5023	Voltage-dependent calcium channel subunit alpha-2/delta-3	Q8IZS8	155670.14	154514.42	135520.88	109228.70	108103.90	101154.48	129299.67	123910.54	119279.44	134598.19	130485.71	127415.99	123513.20	121614.59	104747.26	115499.62	105469.87	101718.80	113919.93	103028.47	99273.81	95139.83	98865.54	86116.87	51945.48	49448.41	46448.56	58261.76	54989.93	57979.69	59113.94	56447.00	38893.82		
5024	Voltage-dependent L-type calcium channel subunit alpha-1C	Q13936	77993.10	68478.15	68746.30	43903.31	43599.77	40118.37	52103.08	53287.00	48355.59	60912.72	63926.08	60835.16	58039.53	56852.41	44073.99	47006.39	44882.81	48408.99	44250.89	46735.54	43468.88	2.27	1.49	0.65	0.84	1.31	0.69	4.16	2.47	0.11	4.90	4.98	0.12	x	
5025	Voltage-dependent L-type calcium channel subunit beta-1	Q02641	0.14	2.53	0.81	1.07	2.77	0.68	0.96	0.36	0.63	0.35	0.75	0.38	2.13	0.97	0.60	4.64	1.07	0.47	2.22	0.19	0.06	7237.69	6782.43	5917.88	3691.00	3973.40	3623.91	5426.61	4742.07	4734.05	5560.02	5458.63	3975.24		x
5026	Voltage-dependent T-type calcium channel subunit alpha-1I	Q9P0X4	6747.00	6246.81	5720.15	6448.68	5880.46	6010.38	6709.91	6029.17	5654.88	6500.30	7055.24	6123.06	7116.39	5635.33	4296.24	6975.62	6562.38	6467.63	7582.49	5490.46	5175.45	2.27	1.49	0.65	0.84	1.31	0.69	4.16	2.47	0.11	4.90	4.98	0.12	x	
5027	von Willebrand factor A domain-containing protein 3B	Q502W6	4719.07	4597.97	3570.19	4358.23	4233.46	3724.50	4495.64	4717.86	4371.51	5557.87	5855.06	5058.55	4326.39	4226.71	2460.47	4454.21	4166.28	3857.90	4642.41	4412.87	3875.70	23697.23	22264.79	19429.08	17433.17	15740.18	12091.12	15422.26	16244.76	13953.55	18760.57	18238.88	18003.31		
5028	VPS9 domain-containing protein 1	Q9Y2B5	26891.07	31119.90	28885.20	24967.86	23917.49	22362.96	30501.30	28207.81	26097.47	33297.94	32688.19	32309.11	32535.37	34134.52	26717.43	22646.77	21985.31	21324.68	29575.06	26450.57	26459.25	29301.84	25934.66	24795.55	19930.55	19806.28	18405.43	17890.48	18270.15	17400.45	19343.94	18057.72	7139.47		
5029	V-set and transmembrane domain-containing protein 4	Q8IW00	15069.49	15023.79	16263.31	13146.83	12406.03	11713.05	10496.90	11638.56	10288.30	18315.19	18338.48	19582.14	14569.99	11598.88	5554.98	11225.73	11578.79	12764.04	15123.24	13001.16	11345.38	2.27	1.49	0.65	0.84	1.31	0.69	4.16	2.47	0.11	4.90	4.98	0.12	x	
5030	V-type proton ATPase 116 kDa subunit a isoform 1	Q93050	31689.40	30865.75	27833.59	29012.20	29267.43	24629.87	31388.92	66133.64	57748.90	26464.70	24818.66	22533.27	26133.75	31728.72	20830.14	34236.95	32541.72	28639.89	22959.46	20693.37	18943.57	2.27	1.49	0.65	0.84	1.31	0.69	4.16	2.47	0.11	4.90	4.98	0.12	x	
5031	V-type proton ATPase 116 kDa subunit a isoform 3	Q13488	2424.50	2263.15	1944.90	1680.10	1568.08	1366.19	1751.40	1606.35	1494.56	1907.35	1851.96	1868.86	1757.85	1717.68	1156.36	1585.46	1485.39	1392.95	1705.19	1626.93	1340.73	2.27	1.49	0.65	0.84	1.31	0.69	4.16	2.47	0.11	4.90	4.98	0.12	x	
5032	V-type proton ATPase catalytic subunit A	P38606	72674.18	73338.51	69627.31	53710.30	50001.66	46546.23	55610.73	52166.50	51160.47	63745.87	63563.26	60884.68	58223.42	59694.37	44287.16	56342.18	53458.49	50247.12	45531.05	48583.11	44668.04	63465.17	56856.20	52437.68	36287.36	34949.71	27285.50	52502.40	48461.33	42542.20	51780.93	49851.03	29042.44		
5033	V-type proton ATPase subunit B brain isoform	P21281	18912.03	18062.58	17914.30	14449.83	12902.27	12941.12	14799.42	14125.58	14167.36	16954.38	16218.68	15666.75	16709.78	15857.43	13348.79	13129.90	11559.19	11677.92	12968.11	11684.24	10515.51	23490.22	22742.59	19894.21	14750.63	14470.37	12020.26	17768.18	15241.69	15051.71	17942.80	17578.63	11432.57		
5034	V-type proton ATPase subunit E 1	P36543	16671.56	13850.11	11233.10	9841.94	10674.58	8721.07	10961.08	11394.53	10694.24	13172.27	12533.17	10216.48	13053.38	10385.16	4706.98	9410.13	8888.71	9926.82	10093.83	8771.65	7139.83	81820.88	73907.68	72193.05	41010.54	42865.76	37626.07	46776.93	45518.14	44467.18	55905.95	54425.40	43255.96		
5035	V-type proton ATPase subunit E 2	Q96A05	25126.54	27701.02	27875.13	18017.24	17641.59	16864.95	17154.18	17865.03	17159.18	26741.40	25441.22	24400.94	23847.67	21685.77	18883.10	19488.68	18424.83	19193.10	17026.02	16295.11	17551.61	2.27	1.49	0.65	0.84	1.31	0.69	4.16	2.47	0.11	4.90	4.98	0.12	x	
5036	V-type proton ATPase subunit G 1	O75348	5888.07	5502.84	5138.79	4253.67	4297.13	3821.04	4024.84	3798.91	3747.64	5464.99	5309.47	5087.75	4738.49	4686.30	3720.99	4691.43	4105.59	3923.55	4069.64	3776.33	3482.20	10078.05	9336.60	8544.05	6461.60	6646.95	6224.24	5392.59	5276.42	5360.67	9739.16	9791.85	6814.32		
5037	V-type proton ATPase subunit H	Q9UI12	27967.29	22254.21	21074.11	16359.37	13737.42	14276.54	16163.40	15688.33	16244.18	21964.45	20817.64	19240.57	21088.48	17471.67	12898.87	14663.38	12559.96	12901.39	15580.52	15994.03	15445.35	53988.86	48614.71	46083.20	41518.50	39393.70	36939.46	50078.80	44980.80	40370.05	48233.87	45522.89	34730.06		
5038	WAP four-disulfide core domain protein 8	Q8IUA0	0.14	2.53	0.81	1.07	2.77	0.68	0.96	0.36	0.63	0.35	0.75	0.38	2.13	0.97	0.60	4.64	1.07	0.47	2.22	0.19	0.06	1391.89	1362.90	1310.16	1790.52	1571.43	1193.15	4135.36	4277.47	3963.95	3510.10	3488.95	1619.73		x
5039	WAP_ Kazal_ immunoglobulin_ Kunitz and NTR domain-containing protein 2	Q8TEU8	37046.45	30991.10	22838.70	18043.65	16732.07	13578.28	25276.23	22204.88	19110.35	26466.79	23333.83	18591.76	23251.31	20942.16	7711.04	18972.98	18336.26	16213.39	23738.35	22973.63	17948.32	23212.62	20766.05	16984.49	12856.73	13021.68	7888.13	19354.70	17904.81	13705.07	22240.80	20549.19	11184.33		
5040	WASH complex subunit 1	A8K0Z3	0.14	2.53	0.81	1.07	2.77	0.68	0.96	0.36	0.63	0.35	0.75	0.38	2.13	0.97	0.60	4.64	1.07	0.47	2.22	0.19	0.06	1954.47	1864.25	1656.12	1483.58	1477.66	1433.02	2000.37	2297.85	2356.01	2171.77	2008.97	1394.04		x
5041	WASH complex subunit 2A	Q641Q2	20549.24	19658.43	17972.83	14018.91	11998.39	11733.14	14245.38	12987.32	12319.86	16780.56	16558.88	15337.91	16282.27	14287.24	10932.77	13378.44	12019.95	11482.46	15508.00	11496.42	11156.30	17649.75	14542.43	11151.68	11117.85	10897.75	8092.45	13866.26	13541.40	11324.17	15262.00	13925.47	8162.78		
5042	WASH complex subunit 2C	Q9Y4E1	3936.78	3119.90	2664.33	1840.89	1323.20	938.44	1709.85	1954.13	1643.20	2354.18	2141.46	1928.48	2826.48	2370.98	698.50	1569.04	1328.13	1031.99	2033.14	1904.37	1472.28	2.27	1.49	0.65	0.84	1.31	0.69	4.16	2.47	0.11	4.90	4.98	0.12	x	
5043	WASH complex subunit 2D	Q5SRD0	0.14	2.53	0.81	1.07	2.77	0.68	0.96	0.36	0.63	0.35	0.75	0.38	2.13	0.97	0.60	4.64	1.07	0.47	2.22	0.19	0.06	3034.95	2834.67	2444.87	1998.63	2006.17	1245.56	2693.49	2616.35	2197.79	3270.17	3012.04	1423.60		x
5044	WASH complex subunit 4	Q2M389	0.14	2.53	0.81	1.07	2.77	0.68	0.96	0.36	0.63	0.35	0.75	0.38	2.13	0.97	0.60	4.64	1.07	0.47	2.22	0.19	0.06	262.92	214.07	274.79	293.19	187.46	201.77	1253.63	1258.59	1307.61	396.50	346.89	161.14		x
5045	WASH complex subunit 5	Q12768	40211.89	43288.28	41390.97	34304.98	34205.19	32874.71	30987.95	32553.30	32191.88	39705.54	38053.52	37063.44	40437.18	37477.95	23473.21	26954.74	30526.16	33297.47	35838.60	34292.74	35310.92	2.27	1.49	0.65	0.84	1.31	0.69	4.16	2.47	0.11	4.90	4.98	0.12	x	
5046	WD repeat and FYVE domain-containing protein 2	Q96P53	4691.45	4278.13	3822.00	3797.83	3363.74	3320.20	3327.78	3200.85	2759.41	4414.47	4600.07	4689.51	4757.84	3545.14	2735.29	3569.26	3430.04	3509.76	3007.72	2497.79	2548.50	2.27	1.49	0.65	0.84	1.31	0.69	4.16	2.47	0.11	4.90	4.98	0.12	x	
5047	WD repeat and HMG-box DNA-binding protein 1	O75717	0.14	2.53	0.81	1.07	2.77	0.68	0.96	0.36	0.63	0.35	0.75	0.38	2.13	0.97	0.60	4.64	1.07	0.47	2.22	0.19	0.06	19455.00	17524.34	17252.93	12384.77	12110.66	10504.95	15489.79	14554.17	14693.55	15669.79	14502.82	11030.54		x
5048	WD repeat and SOCS box-containing protein 2	Q9NYS7	8688.86	7627.53	8059.33	6932.87	6424.47	5871.35	6860.97	6958.90	6885.99	7364.61	7010.34	6153.04	7661.80	7116.18	6515.44	4205.28	5364.89	5634.55	6318.65	6122.11	6932.14	2.27	1.49	0.65	0.84	1.31	0.69	4.16	2.47	0.11	4.90	4.98	0.12	x	
5049	WD repeat-containing protein 1	O75083	160927.44	140518.86	131015.03	94920.19	91239.81	84799.29	107437.34	109164.27	98840.52	132667.49	128213.65	116421.45	119736.24	113858.71	84766.05	97478.19	94905.98	89109.36	101068.74	94069.01	83664.35	252802.52	225559.09	208924.86	138597.42	130349.12	115464.72	182632.21	168536.18	160333.04	182887.82	175931.88	109276.61		
5050	WD repeat-containing protein 19	Q8NEZ3	0.14	2.53	0.81	1.07	2.77	0.68	0.96	0.36	0.63	0.35	0.75	0.38	2.13	0.97	0.60	4.64	1.07	0.47	2.22	0.19	0.06	32555.01	31803.12	27053.85	28965.39	27838.09	20373.06	31294.06	29493.32	25469.79	31713.04	31074.25	17666.37		x
5051	WD repeat-containing protein 44	Q5JSH3	18447.17	16030.95	16695.05	11406.53	11537.01	9437.57	15185.42	14259.94	14711.08	15332.34	14606.44	13220.93	14866.63	14125.11	11465.98	13003.90	11985.99	11271.04	11462.66	11078.36	10283.87	11810.30	9455.76	10799.42	7299.45	5709.70	5240.75	9792.35	7822.34	8461.57	10156.07	10211.62	6486.47		
5052	WD repeat-containing protein 46	O15213	0.14	2.53	0.81	1.07	2.77	0.68	0.96	0.36	0.63	0.35	0.75	0.38	2.13	0.97	0.60	4.64	1.07	0.47	2.22	0.19	0.06	264.08	97.53	43.61	0.84	1.31	0.69	143.78	28.81	3.90	389.01	199.71	7.96		x
5053	WD repeat-containing protein 5	P61964	34772.55	31614.05	33940.47	21565.56	24949.44	21815.50	20726.05	22391.33	25816.97	28521.64	28354.81	29763.40	25403.05	24763.37	20172.87	21189.03	24484.40	20606.84	21446.76	21570.20	21922.76	2.27	1.49	0.65	0.84	1.31	0.69	4.16	2.47	0.11	4.90	4.98	0.12	x	
5054	WD repeat-containing protein 55	Q9H6Y2	0.14	2.53	0.81	1.07	2.77	0.68	0.96	0.36	0.63	0.35	0.75	0.38	2.13	0.97	0.60	4.64	1.07	0.47	2.22	0.19	0.06	18331.41	15978.71	15942.46	11511.82	10373.01	9613.02	14564.38	13294.11	13100.50	16004.03	14260.17	10144.84		x
5055	WD repeat-containing protein 61	Q9GZS3	70046.66	57064.48	42288.04	32913.43	21738.92	16354.60	25660.74	23928.36	21972.66	50276.77	45259.49	32808.68	50763.59	45937.63	12423.80	20994.16	19889.64	16801.20	39029.00	32783.69	27116.30	6462.80	6469.78	6069.79	4292.33	4151.58	3505.80	5456.76	5113.59	4775.27	5946.05	5516.32	3636.64		
5056	WD repeat-containing protein 62	O43379	9207.67	8438.64	7871.40	4909.18	4857.88	4296.21	7198.14	6751.95	6283.30	6582.16	6187.05	5524.43	6145.21	5931.31	4671.39	6155.11	5700.99	5178.32	5405.78	5414.08	4992.97	2.27	1.49	0.65	0.84	1.31	0.69	4.16	2.47	0.11	4.90	4.98	0.12	x	
5057	WD repeat-containing protein 70	Q9NW82	52286.88	48492.81	47759.81	32221.38	30860.03	31334.14	43096.16	40917.09	39740.73	38761.14	39067.53	36280.23	48027.87	41676.17	45857.17	38875.54	33566.29	33142.29	40300.83	39196.26	36148.08	687.48	710.75	608.50	373.38	396.41	277.96	290.40	282.62	265.69	630.75	529.63	175.29		
5058	WD repeat-containing protein 81	Q562E7	34186.24	30157.15	28613.70	22819.76	21965.46	19993.74	22593.87	23331.28	21538.05	29106.27	28159.34	26191.71	28908.36	27293.47	19164.46	21074.39	20474.29	19548.65	22306.77	21267.13	19535.60	13734.28	12639.00	12232.15	9947.56	9996.20	8985.03	11419.25	11088.96	10967.66	11403.63	10702.12	12287.86		
5059	WD repeat-containing protein 87	Q6ZQQ6	163790.31	157621.77	151375.11	123082.59	111176.50	111774.86	133691.96	132878.40	128790.25	134064.96	126417.02	123383.80	137010.65	136842.67	112007.37	116540.04	107078.99	106993.71	122522.96	110335.33	107039.83	31971.88	27538.01	26070.73	18092.74	17101.37	14923.45	23706.46	20439.62	19037.98	21335.35	21858.70	16043.02		
5060	Wilms tumor protein 1-interacting protein	A6NIX2	0.14	2.53	0.81	1.07	2.77	0.68	0.96	0.36	0.63	0.35	0.75	0.38	2.13	0.97	0.60	4.64	1.07	0.47	2.22	0.19	0.06	25759.58	25818.83	22744.37	22425.71	21626.91	16082.27	25350.57	23469.02	21222.08	28299.15	30230.89	17865.56		x
5061	Wiskott-Aldrich syndrome protein family member 2	Q9Y6W5	4437.44	4225.20	4009.56	2319.76	2156.91	2137.18	2511.62	2572.98	2524.27	2393.15	2156.41	2146.58	2898.95	3023.38	2655.49	2234.16	1914.88	1916.56	2372.94	2354.69	2418.21	18935.87	18072.83	17744.22	11878.07	11521.74	10490.74	14422.80	11883.35	11834.94	15465.23	14964.11	9781.17		
5062	Wiskott-Aldrich syndrome protein	P42768	26585.09	24714.79	22864.59	18574.92	18869.43	17925.89	19187.93	17552.87	16762.68	24542.77	23753.39	23027.21	20950.60	21560.84	20112.70	18538.22	16116.47	15697.63	19360.18	18080.34	18022.62	2.27	1.49	0.65	0.84	1.31	0.69	4.16	2.47	0.11	4.90	4.98	0.12	x	
5063	WW domain-binding protein 11	Q9Y2W2	0.14	2.53	0.81	1.07	2.77	0.68	0.96	0.36	0.63	0.35	0.75	0.38	2.13	0.97	0.60	4.64	1.07	0.47	2.22	0.19	0.06	1581.70	1784.01	1444.81	1762.57	1549.98	1307.24	1740.91	1740.06	1524.01	2724.41	2500.90	1518.04		x
5064	WW domain-containing oxidoreductase	Q9NZC7	0.14	2.53	0.81	1.07	2.77	0.68	0.96	0.36	0.63	0.35	0.75	0.38	2.13	0.97	0.60	4.64	1.07	0.47	2.22	0.19	0.06	28885.36	26902.35	26493.42	18735.99	18479.10	17283.22	19463.81	18515.15	18202.34	19454.33	18204.12	14558.93		x
5065	X antigen family member 2	Q96GT9	11128.97	9352.12	8721.81	5800.17	5517.73	4809.39	7194.23	6525.12	6153.75	8023.10	7221.04	6544.30	7622.56	6656.52	5036.77	5311.56	5123.11	5221.81	6613.97	6020.86	5646.79	2.27	1.49	0.65	0.84	1.31	0.69	4.16	2.47	0.11	4.90	4.98	0.12	x	
5066	Xaa-Pro aminopeptidase 1	Q9NQW7	49059.47	46552.77	40479.09	34150.51	33730.84	31461.37	42245.12	41233.39	38763.32	41362.40	39381.98	37465.77	37359.72	38072.46	32984.75	36753.36	34686.79	33868.58	33248.92	33598.74	30693.02	64440.14	63145.97	59001.36	40306.42	38484.69	32735.80	48224.90	45968.98	45868.93	42342.34	43552.84	27154.74		
5067	Xaa-Pro aminopeptidase 3	Q9NQH7	73138.76	76502.46	72146.27	58661.01	54895.48	53977.54	58448.93	61494.90	55774.02	67680.85	67085.32	61188.43	64966.15	63177.50	52588.63	54470.33	49988.85	50678.45	54803.09	51132.48	49205.75	2.27	1.49	0.65	0.84	1.31	0.69	4.16	2.47	0.11	4.90	4.98	0.12		
5068	Xaa-Pro dipeptidase	P12955	0.14	2.53	0.81	1.07	2.77	0.68	0.96	0.36	0.63	0.35	0.75	0.38	2.13	0.97	0.60	4.64	1.07	0.47	2.22	0.19	0.06	69803.47	63713.67	55892.82	40546.46	38789.11	32212.37	70638.05	65024.51	60102.17	45175.16	46329.41	26294.34		x
5069	Xin actin-binding repeat-containing protein 2	A4UGR9	422399.12	408991.03	387440.99	302121.14	293386.28	271797.41	357865.10	334992.54	331619.07	378344.31	366796.42	345871.99	356921.50	323325.63	265660.65	314287.54	292581.68	285130.52	312069.50	300720.23	286683.91	240004.72	225043.89	217157.93	171835.29	160784.39	145824.94	204422.06	198760.16	191923.25	173089.52	164847.80	109767.70		
5070	X-linked interleukin-1 receptor accessory protein-like 2	Q9NP60	0.14	2.53	0.81	1.07	2.77	0.68	0.96	0.36	0.63	0.35	0.75	0.38	2.13	0.97	0.60	4.64	1.07	0.47	2.22	0.19	0.06	6752.29	6614.35	5966.63	5405.65	5307.65	3371.65	6768.00	5736.12	4393.17	8418.21	7310.37	4093.02		x
5071	X-ray repair cross-complementing protein 5	P13010	536061.60	524469.78	471929.23	390105.22	376288.07	348922.29	451618.85	435387.89	408394.64	433380.57	407082.50	381811.17	467825.75	445729.14	348024.20	378214.65	334851.00	337993.84	379500.78	354282.69	333447.39	393058.35	376790.37	337263.32	272771.33	259133.76	235590.33	289044.14	273897.30	258919.60	329841.88	314516.81	190919.46		
5072	X-ray repair cross-complementing protein 6	P12956	1066274.84	1021541.47	939447.89	691353.83	663695.95	614802.88	849293.96	799824.87	771310.38	827086.54	773945.98	730497.73	877440.30	828926.89	682175.34	638664.01	610813.68	576747.57	705789.68	685427.87	628596.05	522934.37	479617.03	460649.60	319814.79	325757.67	276586.53	418171.44	388916.29	365407.27	417745.47	404098.35	265430.27		
5073	Xyloside xylosyltransferase 1	Q8NBI6	1871.26	1767.42	1796.80	1379.61	1358.23	1336.26	1574.26	1820.24	1486.10	2225.06	2175.10	2473.73	1880.03	1624.55	1419.19	1470.94	1471.62	1453.29	1600.90	1503.33	1422.26	2.27	1.49	0.65	0.84	1.31	0.69	4.16	2.47	0.11	4.90	4.98	0.12	x	
5074	Xylosyltransferase 1	Q86Y38	1290.70	1272.07	1057.85	552.55	436.96	514.29	925.93	801.40	693.49	883.28	1000.39	888.35	994.86	849.97	771.45	762.17	792.67	669.05	467.69	540.41	497.49	2529.67	2391.54	2228.03	1882.70	1655.45	1377.19	2858.61	2824.38	2742.28	1832.17	1598.80	901.73		
5075	Y-box-binding protein 2	Q9Y2T7	18290.55	19186.41	17728.93	15144.86	14216.92	14109.63	14877.19	13890.44	13390.20	18351.02	17660.17	16418.27	15975.43	15480.52	10057.86	14097.19	13900.73	14023.42	13493.77	13758.11	11383.22	2.27	1.49	0.65	0.84	1.31	0.69	4.16	2.47	0.11	4.90	4.98	0.12	x	
5076	Y-box-binding protein 3	P16989	107071.82	102891.43	95027.85	70037.38	66123.36	61843.02	78148.45	77499.10	73887.12	83754.53	80625.03	78395.31	78706.12	77872.12	64429.70	64174.78	61323.30	61145.87	76038.01	72425.26	70500.44	11893.14	11090.95	9897.67	9998.82	10429.55	9027.37	10298.54	10722.80	9817.14	11442.45	11166.34	7690.73		
5077	YEATS domain-containing protein 2	Q9ULM3	0.14	2.53	0.81	1.07	2.77	0.68	0.96	0.36	0.63	0.35	0.75	0.38	2.13	0.97	0.60	4.64	1.07	0.47	2.22	0.19	0.06	662.87	758.59	679.53	252.23	221.37	196.37	281.68	238.71	238.62	459.32	401.02	295.92		x
5078	YjeF N-terminal domain-containing protein 3	A6XGL0	17224.90	16925.38	15684.36	13097.49	12900.45	11748.71	16386.34	16379.57	16134.08	14918.64	14457.51	13739.82	15727.51	15031.88	12939.77	12613.19	11857.04	11653.33	16306.38	14617.69	14524.81	23174.20	22278.57	20275.55	16517.11	16447.44	14731.24	16682.90	16828.87	16684.55	15978.14	15175.20	10889.22		
5079	YTH domain-containing family protein 1	Q9BYJ9	15054.46	15372.84	13134.65	11466.09	10119.16	9838.54	12669.09	11551.63	13123.85	13473.78	12796.25	12581.20	14204.87	12858.49	9698.72	11011.37	10277.09	9965.93	11073.44	11652.64	10334.68	8290.81	8137.59	7868.24	4051.42	3986.13	3213.38	4941.70	5038.38	4799.37	5099.96	4971.96	2830.12		
5080	YTH domain-containing family protein 2	Q9Y5A9	71536.06	65270.10	61593.49	43164.74	42031.10	37379.41	51238.23	48086.80	49813.16	56030.72	52691.62	50774.98	53693.69	50935.60	43535.74	41550.36	38861.17	36984.15	42149.69	41102.03	40915.73	66733.24	59225.35	64795.18	47510.73	49749.21	46571.70	52004.36	50798.03	48796.92	52441.22	54496.79	32667.33		
5081	ZBED6 C-terminal-like protein	Q96FA7	1074.44	1041.44	867.79	1558.71	1411.82	1341.74	1007.71	1041.50	909.70	1039.01	1154.80	1114.71	859.60	723.53	461.61	2123.59	2178.09	2298.59	1265.18	1159.99	919.45	2.27	1.49	0.65	0.84	1.31	0.69	4.16	2.47	0.11	4.90	4.98	0.12	x	

Supplementary Table S1. Overview on the abundance of all relatively quantified 5180 proteins

			MCF-7																					MDA-MB-231												
	Protein name	UniProt	control 01	control 02	control 03	Dai IC20 01	Dai IC20 02	Dai IC20 03	Dai SC20 01	Dai SC20 02	Dai SC20 03	Gen IC20 01	Gen IC20 02	Gen IC20 03	Gen SC20 01	Gen SC20 02	Gen SC20 03	SSE IC20 01	SSE IC20 02	SSE IC20 03	SSE SC20 01	SSE SC20 02	SSE SC20 03	control 01	control 02	control 03	Dai IC20 01	Dai IC20 02	Dai IC20 03	Gen IC20 01	Gen IC20 02	Gen IC20 03	SSE IC20 01	SSE IC20 02	SSE IC20 03	unique to MCF-7	unique to MDA-MB-231
5082	Zinc finger and BTB domain-containing protein 18	Q99592	204981.07	205707.76	155504.09	120159.40	112969.20	95180.81	131450.78	118773.86	105428.15	149456.58	138839.50	125472.83	147538.52	125489.00	108621.16	116526.52	103773.21	94886.66	143895.89	139056.66	124191.22	1239.98	1244.82	955.87	1780.81	1594.76	1338.42	1777.44	1586.71	1708.99	1569.40	1522.37	714.00		
5083	Zinc finger and BTB domain-containing protein 47	Q9UFB7	449.38	554.42	529.28	575.02	666.72	629.19	579.75	572.61	568.39	834.47	906.23	893.97	743.86	648.04	505.90	466.56	488.01	588.65	695.09	532.95	470.86	2.27	1.49	0.65	0.84	1.31	0.69	4.16	2.47	0.11	4.90	4.98	0.12	x	
5084	Zinc finger and SCAN domain-containing protein 23	Q3MJ62	9985.89	9438.76	9090.54	7670.89	7932.99	7413.14	9710.27	9326.55	9311.68	13683.00	12951.17	12919.30	10231.11	9706.00	8597.56	8665.95	8389.98	8292.54	8742.04	7704.98	7411.64	2.27	1.49	0.65	0.84	1.31	0.69	4.16	2.47	0.11	4.90	4.98	0.12	x	
5085	Zinc finger and SCAN domain-containing protein 30	Q86W11	34.88	73.44	48.37	1.07	2.77	0.68	0.96	0.36	0.63	148.83	94.89	57.96	2.13	0.97	0.60	4.64	1.07	0.47	2.22	0.19	0.06	2.27	1.49	0.65	0.84	1.31	0.69	4.16	2.47	0.11	4.90	4.98	0.12	x	
5086	Zinc finger C2HC domain-containing protein 1B	Q5TFG8	2519.52	2403.83	2057.31	1016.57	823.96	584.44	1071.04	1021.11	1033.48	1848.76	1675.73	1330.76	1585.26	1354.96	669.19	1152.34	1179.35	1035.50	1323.42	1234.54	1112.81	2.27	1.49	0.65	0.84	1.31	0.69	4.16	2.47	0.11	4.90	4.98	0.12	x	
5087	Zinc finger CCCH domain-containing protein 13	Q5T200	0.14	2.53	0.81	1.07	2.77	0.68	0.96	0.36	0.63	0.35	0.75	0.38	2.13	0.97	0.60	4.64	1.07	0.47	2.22	0.19	0.06	2179.71	2496.58	2036.57	2004.51	1834.44	2027.22	607.54	1038.11	1216.66	2595.61	2302.53	579.94		x
5088	Zinc finger CCCH domain-containing protein 15	Q8WU90	5922.29	5664.14	5189.10	4051.56	3890.59	3611.76	4117.50	4423.65	4231.31	4789.10	4225.32	4375.31	5257.67	4278.96	4162.36	3800.05	3206.89	3580.79	3507.66	3680.19	3232.95	45930.10	39893.50	32473.00	25979.51	24996.73	22437.26	34515.81	32224.20	28271.18	34998.57	33645.04	22592.35		
5089	Zinc finger CCCH domain-containing protein 18	Q86VM9	7380.02	7869.29	6524.44	4343.41	4207.74	4059.56	4842.63	5031.71	4417.09	5894.21	6120.46	5561.04	5637.60	6009.68	4272.09	4213.08	4034.87	4200.79	4667.36	4261.39	4254.95	10028.62	9845.41	8782.76	7507.81	7443.36	7470.98	7927.30	7667.30	7330.83	9092.31	7387.40	6183.36		
5090	Zinc finger CCCH domain-containing protein 4	Q9UPT8	8244.75	7938.97	8074.27	4594.36	4627.23	4392.02	5197.71	5233.46	5070.01	5307.51	5211.68	5071.77	6531.46	6012.92	5350.32	4722.12	4142.03	4016.33	5527.99	5391.95	4650.84	2.27	1.49	0.65	0.84	1.31	0.69	4.16	2.47	0.11	4.90	4.98	0.12	x	
5091	Zinc finger CCCH domain-containing protein 6	P61129	249.81	233.33	241.62	119.57	96.21	156.02	169.96	163.98	138.57	187.43	129.41	103.12	144.55	180.72	96.26	153.00	209.47	105.73	162.89	146.68	249.90	320.80	282.84	201.38	216.73	187.55	169.79	212.09	206.62	210.85	350.11	242.85	179.86		
5092	Zinc finger CCCH domain-containing protein 7A	Q8IWR0	3298.01	2938.82	1908.83	1618.18	1957.03	1202.56	2081.87	3137.01	2365.56	2290.62	2302.57	1540.10	3224.45	2567.05	1094.47	2572.87	1706.54	1392.85	17006.13	14222.60	8341.53	2.27	1.49	0.65	0.84	1.31	0.69	4.16	2.47	0.11	4.90	4.98	0.12	x	
5093	Zinc finger CCCH domain-containing protein 7B	Q9UGR2	105030.33	103128.94	91998.67	57335.38	57137.33	53176.09	72690.84	67832.36	66146.27	77391.53	73663.62	70493.99	75690.85	78519.00	64802.80	79919.83	76834.84	70393.00	63565.62	63521.31	61263.70	13122.85	12341.02	11153.18	9310.13	8687.34	6672.27	11621.18	10980.86	9961.24	12381.13	11595.79	6544.59		
5094	Zinc finger CCCH domain-containing protein 8	Q8N5P1	0.14	2.53	0.81	1.07	2.77	0.68	0.96	0.36	0.63	0.35	0.75	0.38	2.13	0.97	0.60	4.64	1.07	0.47	2.22	0.19	0.06	647.92	15.32	21.65	159.83	88.15	34.56	1897.41	1362.06	22.63	793.16	666.08	246.62		x
5095	Zinc finger CCCH-type antiviral protein 1	Q7Z2W4	118458.60	110974.61	97630.78	68007.57	67678.92	61042.55	77302.62	75299.14	68883.69	92462.56	88273.80	79656.83	86839.85	78354.74	60455.11	66600.74	64152.02	61690.34	70957.51	71147.15	63998.81	24422.45	21991.73	21208.50	16132.60	16107.05	13562.12	19234.27	18630.04	16483.58	22329.23	21177.00	14682.23		
5096	Zinc finger CCCH-type antiviral protein 1-like	Q96H79	0.14	2.53	0.81	1.07	2.77	0.68	0.96	0.36	0.63	0.35	0.75	0.38	2.13	0.97	0.60	4.64	1.07	0.47	2.22	0.19	0.06	2420.95	2410.36	2235.06	1603.32	1763.60	1678.86	2354.46	2254.72	2302.10	2479.44	2582.89	1880.42		x
5097	Zinc finger CCHC domain-containing protein 7	Q8N3Z6	0.14	2.53	0.81	1.07	2.77	0.68	0.96	0.36	0.63	0.35	0.75	0.38	2.13	0.97	0.60	4.64	1.07	0.47	2.22	0.19	0.06	3393.90	3009.84	2607.82	2704.24	2907.32	1596.61	3417.91	3013.93	2467.24	3619.36	3376.52	1541.66		x
5098	Zinc finger CCHC domain-containing protein 8	Q6NZY4	311907.86	292584.48	291306.50	153049.18	141789.02	134466.16	233516.36	235840.96	221981.67	195931.61	196082.81	180942.69	205053.59	186246.44	171646.77	178652.11	176897.98	169302.32	193526.51	175299.58	168293.32	2.27	1.49	0.65	0.84	1.31	0.69	4.16	2.47	0.11	4.90	4.98	0.12	x	
5099	Zinc finger FYVE domain-containing protein 16	Q7Z3T8	41631.47	39297.61	34406.09	24133.85	24404.11	22181.89	34273.05	32785.94	31528.34	36967.91	35122.56	34422.78	32967.28	29882.06	23682.80	27833.58	27702.45	24864.33	25446.45	24809.56	23265.73	2.27	1.49	0.65	0.84	1.31	0.69	4.16	2.47	0.11	4.90	4.98	0.12	x	
5100	Zinc finger FYVE domain-containing protein 21	Q9BQ24	32438.41	29040.32	28237.28	18874.23	19401.96	17067.28	24596.54	23376.05	23887.56	29626.17	28077.51	24909.29	25649.91	22643.73	16050.06	22549.19	22506.80	22519.52	22235.30	20479.34	19315.82	2.27	1.49	0.65	0.84	1.31	0.69	4.16	2.47	0.11	4.90	4.98	0.12	x	
5101	Zinc finger FYVE domain-containing protein 9	O95405	30451.48	27417.10	24385.06	17186.99	16770.58	15606.45	26898.74	26305.27	24943.01	29804.30	28709.43	27804.51	32057.39	26562.76	20327.64	17521.71	16329.57	15537.45	19027.89	17422.05	16551.85	2.27	1.49	0.65	0.84	1.31	0.69	4.16	2.47	0.11	4.90	4.98	0.12	x	
5102	Zinc finger HIT domain-containing protein 2	Q9UHR6	0.14	2.53	0.81	1.07	2.77	0.68	0.96	0.36	0.63	0.35	0.75	0.38	2.13	0.97	0.60	4.64	1.07	0.47	2.22	0.19	0.06	2.27	1.49	0.65	92.05	120.17	27.51	173.73	168.59	77.69	155.91	162.76	64.09		x
5103	Zinc finger homeobox protein 3	Q15911	0.14	2.53	0.81	1.07	2.77	0.68	0.96	0.36	0.63	0.35	0.75	0.38	2.13	0.97	0.60	4.64	1.07	0.47	2.22	0.19	0.06	74158.88	70287.67	62776.18	32521.87	29785.71	26130.60	39215.15	36514.36	37599.68	36681.87	34496.96	22774.33		x
5104	Zinc finger imprinted 3	Q96PE6	0.14	2.53	0.81	1.07	2.77	0.68	0.96	0.36	0.63	0.35	0.75	0.38	2.13	0.97	0.60	4.64	1.07	0.47	2.22	0.19	0.06	11262.04	7987.55	9310.97	7873.48	7248.75	6549.51	6767.37	6783.52	6963.45	7130.73	6519.89	1700.98		x
5105	Zinc finger matrin-type protein 2	Q96NC0	0.14	2.53	0.81	1.07	2.77	0.68	0.96	0.36	0.63	0.35	0.75	0.38	2.13	0.97	0.60	4.64	1.07	0.47	2.22	0.19	0.06	27808.36	21222.61	17457.97	16695.67	16408.06	13228.93	15328.38	12182.56	13429.70	17293.10	17144.00	12610.91		x
5106	Zinc finger MYM-type protein 2	Q9UBW7	48577.97	54524.70	54341.99	30187.79	28305.99	27094.93	31678.78	33746.03	30221.84	44742.20	46316.34	45592.71	40703.00	33584.86	22322.09	34328.33	35417.61	37779.61	40701.47	37035.48	34148.57	2.27	1.49	0.65	0.84	1.31	0.69	4.16	2.47	0.11	4.90	4.98	0.12	x	
5107	Zinc finger MYND domain-containing protein 15	Q9H091	493.45	563.79	352.08	149.04	143.33	153.90	241.82	157.51	136.36	368.82	453.36	314.46	314.00	234.73	180.16	203.64	263.29	172.36	155.42	187.26	167.33	2.27	1.49	0.65	0.84	1.31	0.69	4.16	2.47	0.11	4.90	4.98	0.12	x	
5108	Zinc finger protein 114	Q8NC26	853.44	754.05	806.02	521.53	555.50	492.98	560.12	506.59	524.56	557.46	625.57	522.89	594.76	697.88	440.61	421.44	408.28	393.86	557.68	512.86	532.28	2.27	1.49	0.65	0.84	1.31	0.69	4.16	2.47	0.11	4.90	4.98	0.12	x	
5109	Zinc finger protein 121	P58317	4715.11	4510.40	3644.07	3497.91	3754.86	3275.62	4302.17	3817.58	3505.48	4675.76	4784.49	4913.24	4655.91	3967.11	3717.31	2853.72	2570.81	2786.66	3550.88	3273.05	3123.20	2.27	1.49	0.65	0.84	1.31	0.69	4.16	2.47	0.11	4.90	4.98	0.12	x	
5110	Zinc finger protein 136	P52737	56265.70	55632.96	51884.61	49453.26	48541.11	46746.18	56549.09	55959.33	47626.77	58007.59	57116.21	55444.83	49236.68	50899.09	43859.72	50238.70	46916.66	46677.32	49271.76	45543.06	46266.01	2.27	1.49	0.65	0.84	1.31	0.69	4.16	2.47	0.11	4.90	4.98	0.12	x	
5111	Zinc finger protein 174	Q15697	0.14	2.53	0.81	1.07	2.77	0.68	0.96	0.36	0.63	0.35	0.75	0.38	2.13	0.97	0.60	4.64	1.07	0.47	2.22	0.19	0.06	43387.11	44612.73	39461.77	37792.79	35146.98	27440.75	41570.83	40006.46	36880.70	53114.13	50534.81	34066.15		x
5112	Zinc finger protein 185	O15231	9864.07	9444.79	8185.39	7261.23	6534.55	6477.40	7397.09	7394.85	7021.81	8864.93	8788.80	7855.87	8576.08	7863.74	6275.69	6905.39	6181.56	5754.68	7698.98	7539.17	6941.29	137404.85	130604.13	120588.55	104067.59	97188.12	80186.18	127486.66	121976.67	112316.56	111498.95	104227.16	68649.53		
5113	Zinc finger protein 2	Q9BSG1	0.14	2.53	0.81	1.07	2.77	0.68	0.96	0.36	0.63	0.35	0.75	0.38	2.13	0.97	0.60	4.64	1.07	0.47	2.22	0.19	0.06	49.97	79.48	75.85	187.56	185.77	142.27	83.44	116.82	82.47	345.79	305.74	155.53		x
5114	Zinc finger protein 248	Q8NDW4	0.14	2.53	0.81	1.07	2.77	0.68	0.96	0.36	0.63	0.35	0.75	0.38	2.13	0.97	0.60	4.64	1.07	0.47	2.22	0.19	0.06	3821.95	3471.17	3572.27	2247.83	2344.23	2046.94	2105.98	2089.48	2013.36	1766.20	1834.25	1532.43		x
5115	Zinc finger protein 263	O14978	0.14	2.53	0.81	1.07	2.77	0.68	0.96	0.36	0.63	0.35	0.75	0.38	2.13	0.97	0.60	4.64	1.07	0.47	2.22	0.19	0.06	16145.93	15503.90	14097.60	9038.98	8808.78	7029.41	12148.04	12122.32	11596.67	12500.07	11814.86	7901.48		x
5116	Zinc finger protein 276	Q8N554	310227.31	254608.62	196232.04	145830.65	143259.10	113998.92	185773.71	183214.18	161305.20	193040.04	180688.06	144553.13	172352.59	157038.20	76369.36	176519.22	166469.54	153703.95	160309.69	145862.01	116256.66	2.27	1.49	0.65	0.84	1.31	0.69	4.16	2.47	0.11	4.90	4.98	0.12	x	
5117	Zinc finger protein 318	Q5VUA4	8490.15	6829.92	9737.44	1248.21	1063.87	300.59	121.35	38.26	107.15	6608.80	6286.82	9742.08	5902.21	3007.71	113.54	380.15	514.61	1211.98	130.37	2851.25	1049.28	2.27	1.49	0.65	0.84	1.31	0.69	4.16	2.47	0.11	4.90	4.98	0.12	x	
5118	Zinc finger protein 324B	Q6AW86	0.14	2.53	0.81	1.07	2.77	0.68	0.96	0.36	0.63	0.35	0.75	0.38	2.13	0.97	0.60	4.64	1.07	0.47	2.22	0.19	0.06	439.12	491.50	470.71	336.62	410.62	228.01	592.99	670.31	416.79	682.98	555.66	267.88		x
5119	Zinc finger protein 330	Q9Y3S2	16107.82	15184.60	14945.23	11454.77	10980.71	10309.20	10048.24	10795.79	10016.68	15408.77	15320.66	16259.90	14883.65	11591.76	6421.97	8357.30	9145.93	9290.39	13055.45	11623.61	11553.02	2.27	1.49	0.65	0.84	1.31	0.69	4.16	2.47	0.11	4.90	4.98	0.12	x	
5120	Zinc finger protein 354C	Q86Y25	61624.09	59479.10	60819.61	40033.65	39572.92	33193.63	31946.87	31279.84	29546.47	44729.38	44352.96	43651.80	45088.75	41584.83	35505.16	32240.23	28246.10	27769.68	40837.45	40498.82	36066.56	2.27	1.49	0.65	0.84	1.31	0.69	4.16	2.47	0.11	4.90	4.98	0.12	x	
5121	Zinc finger protein 382	Q96SR6	11202.31	10610.05	9890.85	6262.39	7034.45	6304.25	10623.39	10163.43	9424.58	10848.71	10578.52	10552.51	9833.46	9634.85	6759.81	7585.79	7272.93	6635.86	6857.45	6803.37	6583.27	10901.62	10246.70	10301.30	6555.59	6427.96	5682.76	7958.58	7528.61	7467.41	8288.64	8671.61	2173.54		
5122	Zinc finger protein 383	Q8NA42	0.14	2.53	0.81	1.07	2.77	0.68	0.96	0.36	0.63	0.35	0.75	0.38	2.13	0.97	0.60	4.64	1.07	0.47	2.22	0.19	0.06	1722.23	1655.57	1268.59	1102.12	977.61	677.31	1451.34	1179.78	1148.15	1542.26	1428.81	762.34		x
5123	Zinc finger protein 394	Q53GI3	0.14	2.53	0.81	1.07	2.77	0.68	0.96	0.36	0.63	0.35	0.75	0.38	2.13	0.97	0.60	4.64	1.07	0.47	2.22	0.19	0.06	429.53	40.11	12.60	42.20	15.35	5.05	4.16	2.47	0.11	90.44	95.47	10.59		x
5124	Zinc finger protein 397	Q8NF99	13139.73	10458.37	6444.80	2893.79	1932.11	1512.11	2292.12	898.22	1208.88	8552.88	7487.17	5197.78	8682.51	3144.99	902.75	2864.48	1615.91	1891.86	5257.88	3658.46	2773.78	2.27	1.49	0.65	0.84	1.31	0.69	4.16	2.47	0.11	4.90	4.98	0.12	x	
5125	Zinc finger protein 40	P15822	15956.70	16121.36	15410.81	6948.41	6619.54	6045.71	11538.55	10338.62	10816.68	10107.54	9248.15	9498.96	18449.68	19576.51	15973.66	40021.18	38844.71	34966.97	7818.08	7618.32	7752.00	5177.92	7730.86	6535.31	2692.73	2416.23	2189.48	3492.41	3131.41	3165.60	3853.30	3681.37	2515.04		
5126	Zinc finger protein 42 homolog	Q96MM3	0.14	2.53	0.81	1.07	2.77	0.68	0.96	0.36	0.63	0.35	0.75	0.38	2.13	0.97	0.60	4.64	1.07	0.47	2.22	0.19	0.06	32798.44	31337.64	27517.17	17390.87	16894.85	13488.58	25254.45	23470.66	22610.96	24402.23	25118.22	15213.63		x
5127	Zinc finger protein 428	Q96B54	652.19	596.21	678.12	435.71	315.11	265.48	431.90	468.27	415.49	566.13	565.25	463.79	560.67	526.95	361.56	391.73	367.08	489.88	469.29	479.55	439.87	2134.30	1880.63	1985.25	1734.97	1848.93	1665.50	2024.21	1899.90	1913.05	2076.52	1709.80	754.70		
5128	Zinc finger protein 454	Q8N9F8	2817.71	2447.40	2869.09	2380.40	2150.41	2508.89	2180.47	1954.99	2536.29	2749.57	2479.53	3532.48	2417.33	1924.57	1721.39	1776.29	1684.46	1692.24	2024.75	2216.61	1782.52	2.27	1.49	0.65	0.84	1.31	0.69	4.16	2.47	0.11	4.90	4.98	0.12	x	
5129	Zinc finger protein 468	Q5VIY5	0.14	2.53	0.81	1.07	2.77	0.68	0.96	0.36	0.63	0.35	0.75	0.38	2.13	0.97	0.60	4.64	1.07	0.47	2.22	0.19	0.06	34862.22	33005.57	28390.27	24736.41	25442.63	19620.66	41690.11	39999.82	38260.11	35967.12	34336.58	20503.04		x
5130	Zinc finger protein 469	Q96JG9	262585.41	258093.04	241562.77	195764.72	201539.49	191906.26	213210.05	191253.40	177807.48	223744.89	212333.66	201363.69	215902.25	229153.89	180969.79	192143.56	172678.81	170118.09	182641.76	186051.43	178525.36	50353.24	46725.01	42198.44	37331.78	36242.32	32588.67	47843.90	45119.84	42700.49	49506.78	49211.76	30619.37		
5131	Zinc finger protein 484	Q5JVG2	656.87	635.63	532.43	367.71	390.30	211.89	334.85	436.07	328.23	506.20	275.23	327.47	362.72	492.31	174.16	327.79	258.63	226.44	472.66	406.90	294.05	2.27	1.49	0.65	0.84	1.31	0.69	4.16	2.47	0.11	4.90	4.98	0.12	x	
5132	Zinc finger protein 496	Q96IT1	21470.25	22252.25	21811.36	14920.21	14236.92	14266.31	14568.97	14055.52	13791.48	19280.13	17945.96	16683.61	16697.90	18517.66	15015.75	14161.15	13321.89	12767.16	15462.45	14701.13	13971.03	2.27	1.49	0.65	0.84	1.31	0.69	4.16	2.47	0.11	4.90	4.98	0.12	x	
5133	Zinc finger protein 511	Q8NB15	0.14	2.53	0.81	1.07	2.77	0.68	0.96	0.36	0.63	0.35	0.75	0.38	2.13	0.97	0.60	4.64	1.07	0.47	2.22	0.19	0.06	2759.42	2595.30	2260.63	2249.41	2025.64	1952.58	2494.13	2429.37	2383.98	2580.93	2643.21	2827.73		x
5134	Zinc finger protein 518A	Q6AHZ1	17921.07	17400.06	13761.79	9556.98	9453.07	8201.36	11303.37	10179.45	8397.14	12330.68	12000.35	10622.92	11607.58	10689.12	6683.09	10338.30	9556.83	8673.76	10325.59	9874.61	9140.28	2.27	1.49	0.65	0.84	1.31	0.69	4.16	2.47	0.11	4.90	4.98	0.12	x	
5135	Zinc finger protein 541	Q9H0D2	10547.02	9417.02	7374.64	6472.29	6062.42	5611.57	8418.04	7839.35	6361.48	7348.70	6973.89	6110.94	7215.75	5810.18	4585.67	7086.89	6475.35	6047.02	5759.40	6402.49	6608.40	2.27	1.49	0.65	0.84	1.31	0.69	4.16	2.47	0.11	4.90	4.98	0.12	x	
5136	Zinc finger protein 552	Q9H707	0.14	2.53	0.81	1.07	2.77	0.68	0.96	0.36	0.63	0.35	0.75	0.38	2.13	0.97	0.60	4.64	1.07	0.47	2.22	0.19	0.06	41522.52	36766.70	35019.45	22029.82	24077.21	25629.80	23638.22	25505.86	23097.91	22347.14	21059.64	15050.38		x
5137	Zinc finger protein 57 homolog	Q9NU63	15651.52	16523.61	14455.25	9455.29	9264.04	8553.77	12777.07	12369.37	12658.93	11951.02	10694.17	10483.96	13002.81	11898.84	9887.67	10907.20	10379.10	9352.51	10276.42	9722.57	8991.04	2.27	1.49	0.65	0.84	1.31	0.69	4.16	2.47	0.11	4.90	4.98	0.12	x	
5138	Zinc finger protein 57	Q68EA5	32178.62	32656.92	29876.48	21033.65	19871.26	18096.61	28004.21	27615.35	27626.14	24636.41	24791.31	22021.47	23500.32	22804.81	19370.84	20889.71	21138.34	20022.50	23589.61	22506.41	21194.31	2.27	1.49	0.65	0.84	1.31	0.69	4.16	2.47	0.11	4.90	4.98	0.12	x	
5139	Zinc finger protein 578	Q96N58	0.14	2.53	0.81	1.07	2.77	0.68	0.96	0.36	0.63	0.35	0.75	0.38	2.13	0.97	0.60	4.64	1.07	0.47	2.22	0.19	0.06	19701.00	17357.26	15047.57	11673.01	11071.00	10398.42	16338.62	15022.86	15192.65	16414.76	14837.23	9429.79		x
5140	Zinc finger protein 616	Q08AN1	31361.11	30299.65	27831.49	20982.96	20278.91	18557.93	24682.02	25388.01	22550.01	29046.79	27426.81	27036.56	26268.55	23117.85	22161.90	20026.09	19998.08	19727.09	24522.32	23306.62	24666.58	2.27	1.49	0.65	0.84	1.31	0.69	4.16	2.47	0.11	4.90	4.98	0.12	x	
5141	Zinc finger protein 620	Q6ZNG0	10936.74	9723.19	9121.39	9277.77	8369.38	8679.66	9252.51	9204.21	8265.00	10952.40	10510.34	10131.78	11049.02	10875.63	8510.27	7821.12	6599.65	6776.11	8337.72	8895.54	8945.75	2.27	1.49	0.65	0.84	1.31	0.69	4.16	2.47	0.11	4.90	4.98	0.12	x	
5142	Zinc finger protein 648	Q5T619	4971.99	4390.63	5986.99	2009.66	1913.78	1848.16	2588.90	2464.06	2679.72	4432.27	4241.35	4104.98	3923.97	3999.43	2980.26	1835.28	1788.74	1786.91	2475.59	2524.13	2185.63	2.27	1.49	0.65	0.84	1.31	0.69	4.16	2.47	0.11	4.90	4.98	0.12	x	
5143	Zinc finger protein 674	Q2M3X9	20246.91	20972.91	19082.25	15723.61	15483.53	14209.29	15994.17	15646.92	15667.89	18959.59	17969.56	16152.43	17286.68	17186.60	15026.23	13802.57	13985.04	13581.10	14894.98	12766.18	14140.70	2.27	1.49	0.65	0.84	1.31	0.69	4.16	2.47	0.11	4.90	4.98	0.12	x	
5144	Zinc finger protein 678	Q5SXM1	630.73	456.66	184.14	220.14	11144.81	6684.71	13444.69	221.24	331.23	602.76	552.58	250.61	511.41	448.59	1021.22	5975.92	5655.98	7273.34	315.63	46.06	325.72	2.27	1.49	0.65	0.84	1.31	0.69	4.16	2.47	0.11	4.90	4.98	0.12	x	
5145	Zinc finger protein 705A	Q6ZN79	0.14	2.53	0.81	1.07	2.77	0.68	0.96	0.36	0.63	0.35	0.75	0.38	2.13	0.97	0.60	4.64	1.07	0.47	2.22	0.19	0.06	2853.93	3255.20	2749.21	3612.68	3714.86	3700.92	5060.70	5212.85	4944.45	3804.42	3977.41	712.93		x
5146	Zinc finger protein 763	Q0D2J5	3264.40	3043.57	2953.37	2508.04	2334.61	2411.00	2798.71	2651.63	2833.84	2671.19	2256.97	2239.82	2650.75	2457.77	2104.76	2612.22	2559.30	2506.61	2462.48	2295.40	2363.00	2.27	1.49	0.65	0.84	1.31	0.69	4.16	2.47	0.11	4.90	4.98	0.12	x	
5147	Zinc finger protein 781	Q8N8C0	1823.03	1037.79	1354.67	1028.89	923.24	1164.63	1081.73	999.36	1095.19	1794.58	1851.81	1657.28	1814.49	1619.95	1346.05	1003.80	1243.31	1048.18	1395.55	1143.32	1062.35	2.27	1.49	0.65	0.84	1.31	0.69	4.16	2.47	0.11	4.90	4.98	0.12	x	
5148	Zinc finger protein 789	Q5FWF6	0.14	2.53	0.81	1.07	2.77	0.68	0.96	0.36	0.63	0.35	0.75	0.38	2.13	0.97	0.60	4.64	1.07	0.47	2.22	0.19	0.06	1460.86	2441.12	1607.65	3474.90	3509.04	1535.82	8565.54	6385.94	3839.83	11039.07	9008.46	4266.63		x
5149	Zinc finger protein 791	Q3KP31	278.07	344.73	342.94	282.99	238.34	270.19	260.57	267.14	248.63	330.91	292.55	328.22	325.82	328.42	283.13	294.16	183.68	179.94	216.51	307.70	226.60	2.27	1.49	0.65	0.84	1.31	0.69	4.16	2.47	0.11	4.90	4.98	0.12	x	
5150	Zinc finger protein 8	P17098	0.14	2.53	0.81	1.07	2.77	0.68	0.96	0.36	0.63	0.35	0.75	0.38	2.13	0.97	0.60	4.64	1.07	0.47	2.22	0.19	0.06	36675.85	37548.36	34458.72	25837.58	25536.79	22357.06	29894.05	28301.79	27755.57	28444.24	26614.32	18918.32		x
5151	Zinc finger protein 816	Q0VGE8	1024.91	1054.76	827.74	494.51	516.56	405.11	559.94	533.82	385.40	1186.21	1114.61	823.25	469.43	203.09	486.28	518.16	494.11	352.44	796.84	713.93	722.94	2.27	1.49	0.65	0.84	1.31	0.69	4.16	2.47	0.11	4.90	4.98	0.12	x	
5152	Zinc finger protein 830	Q96NB3	368.07	334.47	341.44	218.04	221.68	242.04	377.22	344.94	376.39	279.65	334.09	268.71	341.44	318.82	260.70	288.89	362.13	320.43	318.95	326.28	286.58	2.27	1.49	0.65	0.84	1.31	0.69	4.16	2.47	0.11	4.90	4.98	0.12	x	
5153	Zinc finger protein 837	Q96EG3	1477.00	1432.04	1296.60	922.80	870.03	866.45	832.48	761.59	1046.24	1318.44	1141.62	1110.82	1211.45	964.02	1008.79	1316.95	1105.54	1061.66	651.81	822.38	645.33	2.27	1.49	0.65	0.84	1.31	0.69	4.16	2.47	0.11	4.90	4.98	0.12	x	
5154	Zinc finger protein 891	A8MT65	16.89	5.05	18.64	223.51	27.96	10.17	11.02	5.40	3.69	23.47	3.25	8.81	14.36	709.44	281.21	480.66	6.81	12.00	53.49	45.41	37.01	2.27	1.49	0.65	0.84	1.31	0.69	4.16	2.47	0.11	4.90	4.98	0.12	x	
5155	Zinc finger protein Aiolos	Q9UKT9	1250.81	1311.27	1070.77	591.85	695.63	533.84	1167.03	1182.73	937.51	672.85	821.62	799.23	1008.59	772.93	385.39	1060.79	851.08	842.32	840.48	703.88	686.00	2.27	1.49	0.65	0.84	1.31	0.69	4.16	2.47	0.11	4.90	4.98	0.12	x	
5156	Zinc finger protein basonuclin-2	Q6ZN30	0.14	2.53	0.81	1.07	2.77	0.68	0.96	0.36	0.63	0.35	0.75	0.38	2.13	0.97	0.60	4.64	1.07	0.47	2.22	0.19	0.06	9151.21	6882.48	7558.50	10309.32	9126.51	7626.82	11776.63	10370.55	10170.05	10156.90	8477.20	6582.88		x
5157	Zinc finger protein castor homolog 1	Q86V15	28532.52	26527.60	24916.26	22131.03	20947.54	20062.89	23576.94	22032.91	22494.87	24877.24	24366.84	24204.36	25030.49	23250.68	18874.26	19174.87	19180.45	18360.97	19934.22	17637.86	16960.35	2.27	1.49	0.65	0.84	1.31	0.69	4.16	2.47	0.11	4.90	4.98	0.12	x	
5158	Zinc finger protein DZIP1	Q86YF9	0.14	2.53	0.81	1.07	2.77	0.68	0.96	0.36	0.63	0.35	0.75	0.38	2.13	0.97	0.60	4.64	1.07	0.47	2.22	0.19	0.06	57193.65	47670.38	52937.84	52560.05	53334.04	50814.79	65919.85	67197.35	66214.93	64786.77	63967.83	46826.16		x
5159	Zinc finger protein Eos	Q9H2S9	830.13	744.51	614.12	524.32	576.10	482.69	613.14	574.03	860.68	896.67	670.10	601.56	620.79	551.54	846.59	507.57	384.64	453.24	684.31	741.05	845.10	2.27	1.49	0.65	0.84	1.31	0.69	4.16	2.47	0.11	4.90	4.98	0.12	x	
5160	Zinc finger protein GLI1	P08151	11621.11	11298.71	10095.16	7821.71	7828.22	7108.38	8315.39	8034.77	8425.66	10276.89	10178.43	10453.23	10912.96	10109.33	8241.51	6775.85	6853.35	6112.55	7557.78	7374.48	6844.03	2.27	1.49	0.65	0.84	1.31	0.69	4.16	2.47	0.11	4.90	4.98	0.12	x	
5161	Zinc finger protein Helios	Q9UKS7	85278.92	65924.32	40455.75	1975.36	3275.95	1067.66	2067.73	3196.04	2372.52	40542.70	36083.15	25910.58	50984.62	7069.69	1838.37	1407.49	3584.06	2459.43	4666.86	4033.98	1771.65	14577.43	14264.18	12886.47	9788.23	9336.43	8585.56	13264.99	12322.32	11958.84	12682.79	12676.71	0.12		
5162	Zinc finger protein neuro-d4	Q92782	10784.69	11001.69	9137.06	7072.91	6872.32	5736.61	8956.39	8336.43	7453.44	9278.91	8779.84	7583.75	8801.37	8472.90	5423.61	7178.00	6833.82	6494.11	7618.97	7300.80	6431.77	2.27	1.49	0.65	0.84	1.31	0.69	4.16	2.47	0.11	4.90	4.98	0.12	x	
5163	Zinc finger protein Rlf	Q13129	64072.75	65676.29	56034.70	56430.47	56124.30	52166.93	60052.84	59754.00	53172.73	74784.36	69134.91	65806.75	60302.42	50003.80	45537.71	53275.13	53055.35	49047.06	56354.97	51218.46	51470.52	28039.06	25082.74	24704.59	19442.10	18689.47	18550.85	25019.89	22910.23	23104.90	23211.56	21630.41	17548.87		
5164	Zinc finger protein ubi-d4	Q92785	0.14	2.53	0.81	1.07	2.77	0.68	0.96	0.36	0.63	0.35	0.75	0.38	2.13	0.97	0.60	4.64	1.07	0.47	2.22	0.19	0.06	25586.09	24083.89	22565.79	15703.65	13140.77	13573.61	20473.15	10449.19	18033.27	16439.44	18765.89	12058.36		x
5165	Zinc finger protein with KRAB and SCAN domains 1	P17029	778.89	775.45	633.85	231.55	75.38	62.30	233.59	455.07	523.13	676.52	546.80	314.04	698.84	225.50	15.97	137.19	148.46	368.97	28.55	51.30	74.41	2.27	1.49	0.65	0.84	1.31	0.69	4.16	2.47	0.11	4.90	4.98	0.12	x	
5166	Zinc finger protein with KRAB and SCAN domains 3	Q9BRR0	830.61	869.18	879.11	572.20	641.51	781.20	821.13	696.53	729.05	505.79	662.86	525.29	729.69	833.31	644.03	800.88	449.49	567.50	585.26	641.97	547.12	3925.64	3523.12	3096.53	2067.22	2121.65	1823.08	3032.21	3137.47	2844.40	3353.32	3242.67	1206.85		
5167	Zinc finger protein with KRAB and SCAN domains 4	Q969J2	0.14	2.53	0.81	1.07	2.77	0.68	0.96	0.36	0.63	0.35	0.75	0.38	2.13	0.97	0.60	4.64	1.07	0.47	2.22	0.19	0.06	7424.58	6947.69	6450.49	5547.14	5401.51	4795.44	5297.10	5014.71	4922.39	5577.45	5302.88	2676.20		x
5168	Zinc finger protein ZPR1	O75312	6906.14	7230.54	5795.64	5219.88	4977.46	4593.31	5017.87	4924.30	4805.12	7130.78	6451.61	6388.55	5076.13	5120.69	5515.48	4783.24	4251.08	4218.18	5023.50	4667.52	4182.50	10712.61	11220.71	10645.32	6971.33	6381.79	4895.76	8593.10	8010.58	7437.14	8199.49	7172.39	4585.23		
5169	Zinc finger protein-like 1	O95159	79987.91	79198.67	71640.06	56232.23	52223.00	48328.14	59953.71	57929.80	54983.45	71610.30	69132.40	61413.21	66852.03	63376.23	43400.36	54930.63	51693.38	46600.17	56942.25	53640.86	48971.20	6194.28	5304.35	4871.52	4468.12	4054.05	3571.62	5000.61	4750.97	4349.68	5549.20	5043.74	3284.66		
5170	Zinc finger Ran-binding domain-containing protein 2	O95218	15079.53	14791.19	14720.18	9356.58	9789.32	9252.72	10655.75	11047.40	10447.25	13677.93	12700.21	11523.71	13094.57	11067.54	9467.10	8920.40	7881.93	10054.49	10400.96	9232.43	8807.23	57476.00	51232.18	50898.48	34648.61	34321.40	30222.22	41336.43	39343.43	38816.10	42518.09	40591.01	34867.79		
5171	Zinc finger SWIM domain-containing protein 1	Q9BR11	25598.85	24650.85	23053.37	18390.88	17738.74	15598.47	18303.45	18673.38	17769.95	21784.04	20697.30	19874.64	21518.78	20627.73	6229.89	13649.36	16527.45	16969.33	18230.09	17591.81	16989.69	1450.48	1312.26	1179.65	932.40	759.44	463.38	1225.74	1127.03	890.03	1479.35	1440.28	780.21		
5172	Zinc finger SWIM domain-containing protein 4	Q9H7M6	3945.53	3425.45	2930.69	2155.04	2158.55	1584.84	6566.42	5877.15	6169.80	2785.90	2848.92	2451.44	2988.72	2431.48	1410.11	2598.73	10930.80	1519.78	2357.94	2247.86	2000.96	2.27	1.49	0.65	0.84	1.31	0.69	4.16	2.47	0.11	4.90	4.98	0.12	x	
5173	Zinc finger SWIM domain-containing protein 5	Q9P217	8179.48	7747.30	7036.91	5022.21	4645.87	4059.65	6470.74	5938.30	5687.59	6679.13	6264.20	5655.18	6622.58	5967.15	4359.00	5832.11	5273.62	4645.26	4881.09	4661.92	4250.98	8330.08	8470.69	7919.24	6810.17	6563.33	5599.97	7257.20	7499.99	6990.67	7675.59	5414.09	4071.52		
5174	Zinc finger SWIM domain-containing protein 6	Q9HCJ5	2313.07	2111.61	1731.29	1518.84	1451.68	1387.00	2139.98	2449.43	2092.31	2483.16	2390.44	2314.96	2293.51	2165.16	1767.33	1497.55	1456.92	1479.78	1316.36	1211.76	1198.25	2.27	1.49	0.65	0.84	1.31	0.69	4.16	2.47	0.11	4.90	4.98	0.12	x	
5175	Zinc finger transcription factor Trps1	Q9UHF7	0.14	2.53	0.81	1.07	2.77	0.68	0.96	0.36	0.63	0.35	0.75	0.38	2.13	0.97	0.60	4.64	1.07	0.47	2.22	0.19	0.06	127.16	136.59	121.93	96.33	100.98	77.20	119.98	146.86	128.36	141.84	85.82	54.46		x
5176	Zinc finger-containing ubiquitin peptidase 1	Q96AP4	73719.88	67850.62	65589.27	52254.08	50000.26	47296.06	59185.74	58626.05	56295.06	65775.24	59774.25	56124.64	55442.93	54954.12	45436.04	53039.37	49435.41	48070.86	47968.47	43290.44	44133.66	461.64	156.80	38.05	669.08	561.66	272.12	921.19	718.68	85.41	887.96	814.04	402.54		
5177	Zinc phosphodiesterase ELAC protein 2	Q9BQ52	4667.60	3805.81	3887.43	2858.90	2461.83	2669.34	4099.22	3929.17	3716.11	4105.24	4100.91	3473.73	4165.51	3515.71	2718.94	2794.05	2660.91	2511.99	3076.77	2839.89	2870.89	32087.43	30698.34	26777.87	21144.02	20528.72	17808.43	24687.75	22997.68	22082.16	24134.14	23343.64	17229.24		
5178	Zona pellucida sperm-binding protein 1	P60852	0.14	2.53	0.81	1.07	2.77	0.68	0.96	0.36	0.63	0.35	0.75	0.38	2.13	0.97	0.60	4.64	1.07	0.47	2.22	0.19	0.06	15055.02	13837.74	14569.99	17575.06	16690.20	15464.13	13269.73	13766.61	13338.77	18723.07	18911.06	2916.89		x
5179	Zygote arrest protein 1	Q86SH2	4159.54	4652.75	4472.85	3483.42	3094.52	3186.38	4927.46	4665.02	4623.52	3814.07	3410.81	3819.35	4166.12	4076.61	3093.59	3727.93	3264.09	3537.06	3437.52	3687.30	3313.38	2.27	1.49	0.65	0.84	1.31	0.69	4.16	2.47	0.11	4.90	4.98	0.12	x	
5180	Zyxin	Q15942	88507.92	85038.20	79790.01	56038.21	57096.21	54918.52	61232.07	59801.65	58056.09	82749.42	76635.62	72584.78	68089.00	59891.00	55113.76	60595.02	55063.90	53194.05	54222.29	51100.83	49746.45	186006.34	172306.35	167466.34	97263.75	96065.75	86751.01	131736.48	124313.61	121017.31	136562.09	129362.33	81413.53		
