# Supplementary material for: From Proteomics to Personalized Medicine: The Importance of Isoflavone Dose and Estrogen Receptor Status in Breast Cancer Cells
Source: J Pers Med. 2020 Dec 19;10(4):292. doi: 10.3390/jpm10040292 (PMC7766658; doi:10.3390/jpm10040292)
Supplement: Supplementary file 1 [file jpm-10-00292-s001.zip › Supplementary Table S2. Overview on all relatively quantified 5180 proteins statistical analysis.pdf]

Supplemetary Table S2. Overview on all relatively quantified 5180 proteins statistical analysis

			MCF-7																		MDA-MB-231								
			Dai SC20 vs control			Gen SC20 vs control			SSE SC20 vs control			Dai IC20 vs control			Gen IC20 vs control			SSE IC20 vs control			Dai IC20 vs control			Gen IC20 vs control			SSE IC20 vs control		
	**Protein name**	**UniProt**	**p value**	**BH q value**	**log2FC**	**p value**	**BH q value**	**log2FC**	**p value**	**BH q value**	**log2FC**	**p value**	**BH q value**	**log2FC**	**p value**	**BH q value**	**log2FC**	**p value**	**BH q value**	**log2FC**	**p value**	**BH q value**	**log2FC**	**p value**	**BH q value**	**log2FC**	**p value**	**BH q value**	**log2FC**
1	(E2-independent) E3 ubiquitin-conjugating enzyme FATS	Q96M02	0.0773	0.1902	0.41	0.1128	0.6351	0.48	0.0737	0.1625	0.55	0.0689	0.1462	0.51	0.0049	0.0523	1.20	0.0356	0.1219	0.56	0.3736	0.5739	-2.55	0.8708	1.1668	-0.33	0.0651	0.1780	2.06
2	(E3-independent) E2 ubiquitin-conjugating enzyme	Q9C0C9	0.8335	0.8671	0.01	0.6141	0.7147	0.06	0.6940	0.7418	0.03	0.1917	0.3213	0.10	0.5710	0.6512	-0.04	0.3245	0.5269	-0.07	0.5684	0.6184	1.14	0.3048	0.6503	2.22	0.3995	0.4712	1.73
3	[F-actin]-monooxygenase MICAL1	Q8TDZ2	0.4108	0.6320	-3.31	0.5752	0.8844	-1.75	0.5661	0.7889	-1.19	0.5388	0.7455	0.89	0.2938	0.5239	-1.88	0.9510	1.1856	0.09	0.5023	0.5560	-0.05	0.0308	0.1589	0.18	0.2837	0.3557	0.05
4	[F-actin]-monooxygenase MICAL3	Q7RTP6	0.0040	0.0402	0.21	0.0104	0.4452	0.18	0.0006	0.0211	0.34	0.0006	0.0137	0.35	0.0080	0.0650	0.17	0.0015	0.0518	0.38	0.0231	0.1366	0.34	0.2311	0.5446	0.07	0.3660	0.4375	0.05
5	[Protein ADP-ribosylarginine] hydrolase-like protein 1	Q8NDY3	0.0628	0.1658	0.13	0.3789	0.7259	0.09	0.0762	0.1663	0.11	0.0090	0.0381	0.26	0.9313	0.9480	0.00	0.0138	0.0779	0.23	0.1930	0.4007	0.27	0.7380	1.1810	0.04	0.2351	0.3050	0.11
6	[Pyruvate dehydrogenase [acetyl-transferring]]-phosphatase 1_ mitochondrial	Q9P0J1	0.0119	0.0631	9.34	0.5752	0.8854	-1.75	0.5661	0.7896	-1.19	0.1000	0.1942	8.82	0.2938	0.5251	-1.88	0.0293	0.1097	10.39	0.0233	0.1365	0.27	0.1931	0.4872	0.12	0.8947	0.9165	0.01
7	[Pyruvate dehydrogenase [acetyl-transferring]]-phosphatase 2_ mitochondrial	Q9P2J9	0.4108	0.6318	-3.31	0.5752	0.8841	-1.75	0.5661	0.7887	-1.19	0.5388	0.7453	0.89	0.2938	0.5237	-1.88	0.9510	1.1853	0.09	0.0618	0.1959	-0.27	0.9248	0.9447	-0.01	0.1310	0.1911	-0.20
8	1_25-dihydroxyvitamin D(3) 24-hydroxylase_ mitochondrial	Q07973	0.2555	0.4432	-0.05	0.6260	0.7230	0.06	0.0237	0.0754	0.14	0.0980	0.1911	0.08	0.0351	0.1358	-0.11	0.6833	0.9225	-0.02	0.0275	0.1436	0.32	0.5850	1.0172	0.07	0.1309	0.1911	-0.17
9	1 2-dihydroxy-3-keto-5-methylthiopentene dioxygenase	Q9BV57	0.0140	0.0695	0.19	0.1457	0.6551	0.24	0.0050	0.0345	0.31	0.0038	0.0251	0.20	0.4451	0.5389	0.02	0.0064	0.0620	0.21	0.1108	0.2737	0.12	0.1590	0.4312	-0.10	0.2799	0.3517	-0.07
10	1 4-alpha-glucan-branching enzyme	Q04446	0.3606	0.5732	-0.04	0.5462	0.8613	-0.12	0.0310	0.0899	0.15	0.9625	0.9675	0.00	0.0873	0.2313	-0.09	0.0681	0.1829	-0.14	0.0457	0.1714	0.31	0.5706	0.9996	0.02	0.0628	0.2240	0.08
11	10 kDa heat shock protein mitochondrial	P61604	0.0035	0.0385	0.26	0.3477	0.7035	0.15	0.0027	0.0285	0.28	0.0074	0.0351	0.21	0.6200	0.6931	-0.02	0.0109	0.0715	0.24	0.1061	0.2664	0.27	0.2874	0.6266	-0.12	0.2413	0.3115	-0.12
12	116 kDa U5 small nuclear ribonucleoprotein component	Q15029	0.8671	0.8949	0.00	0.5976	0.7011	0.09	0.0291	0.0866	0.13	0.0324	0.0848	0.11	0.0655	0.1949	-0.08	0.7465	0.9849	0.01	0.0776	0.2216	0.16	0.2017	0.5006	0.08	0.0222	0.1266	-0.08
13	11-cis retinol dehydrogenase	Q92781	0.8178	0.8532	0.02	0.2966	0.6715	0.12	0.5202	0.7413	-0.06	0.7472	0.7821	0.03	0.5667	0.6480	-0.04	0.6189	0.8549	0.04	0.3736	0.4506	-2.55	0.8708	0.9419	-0.33	0.0651	0.1197	2.06
14	14 kDa phosphohistidine phosphatase	Q9NRX4	0.0514	0.1469	0.15	0.0995	0.6347	0.22	0.0059	0.0373	0.26	0.0011	0.0157	0.35	0.0001	0.0110	0.27	0.1299	0.2807	0.47	0.0007	0.0422	-0.53	0.0003	0.0216	-0.48	0.0030	0.0555	-0.61
15	14-3-3 protein beta/alpha	P31946	0.0170	0.0762	0.28	0.1689	0.6608	0.36	0.0043	0.0325	0.41	0.0120	0.0440	0.37	0.2299	0.4398	0.09	0.0090	0.0691	0.40	0.0268	0.1421	0.66	0.1623	0.4383	0.18	0.1947	0.2626	0.11
16	14-3-3 protein epsilon	P62258	0.0307	0.1082	0.17	0.2332	0.6623	0.30	0.0044	0.0327	0.38	0.0014	0.0171	0.43	0.1801	0.3720	0.08	0.0118	0.0737	0.33	0.0437	0.1688	0.54	0.1718	0.4538	0.18	0.5038	0.5705	0.06
17	14-3-3 protein eta	Q04917	0.0119	0.0630	0.20	0.1863	0.6669	0.30	0.0019	0.0244	0.38	0.0024	0.0211	0.32	0.2006	0.4012	0.06	0.0152	0.0820	0.24	0.0509	0.1792	0.34	0.9856	0.9900	0.00	0.8010	0.8377	0.01
18	14-3-3 protein gamma	P61981	0.1033	0.2299	0.14	0.1619	0.6619	0.27	0.0080	0.0421	0.35	0.0045	0.0272	0.41	0.0580	0.1808	0.18	0.0336	0.1182	0.24	0.0440	0.1686	0.50	0.7784	1.2222	0.03	0.5821	0.6443	0.05
19	14-3-3 protein sigma	P31947	0.0193	0.0817	0.10	0.1047	0.6328	0.25	0.0037	0.0320	0.20	0.0063	0.0319	0.22	0.0183	0.0964	0.10	0.0250	0.1006	0.13	0.0325	0.1510	0.39	0.7030	1.1448	-0.03	0.3593	0.4306	0.06
20	14-3-3 protein theta	P27348	0.0039	0.0399	0.30	0.2056	0.6590	0.32	0.0030	0.0299	0.40	0.0057	0.0304	0.30	0.8031	0.8464	0.01	0.0051	0.0610	0.40	0.1012	0.2575	0.30	0.6266	1.0625	0.04	0.7123	0.7627	-0.03
21	14-3-3 protein zeta/delta	P63104	0.0052	0.0443	0.31	0.1491	0.6601	0.36	0.0026	0.0280	0.39	0.0004	0.0121	0.45	0.0123	0.0795	0.20	0.0010	0.0463	0.45	0.4700	0.5249	0.13	0.0185	0.1160	-0.35	0.0368	0.1646	-0.30
22	182 kDa tankyrase-1-binding protein	Q9C0C2	0.0064	0.0482	0.14	0.1016	0.6318	0.21	0.0010	0.0219	0.29	0.0005	0.0130	0.28	0.0048	0.0522	0.13	0.0163	0.0848	0.22	0.0243	0.1379	-0.32	0.0451	0.1970	0.26	0.0089	0.0843	-0.37
23	1-aminocyclopropane-1-carboxylate synthase-like protein 1	Q96QU6	0.6043	0.6694	0.31	0.2718	0.6651	0.83	0.8027	0.8393	0.14	0.7175	0.7556	0.24	0.1012	0.2550	1.41	0.6047	0.8418	0.33	0.3736	0.4522	-2.55	0.8708	0.9449	-0.33	0.0651	0.1203	2.06
24	1-phosphatidylinositol 3-phosphate 5-kinase	Q9Y2I7	0.4108	0.6316	-3.31	0.5752	0.8839	-1.75	0.5661	0.7885	-1.19	0.5388	0.7451	0.89	0.2938	0.5235	-1.88	0.9510	1.1850	0.09	0.0560	0.1875	-8.02	0.4407	0.8421	0.83	0.2622	0.3343	1.26
25	1-phosphatidylinositol 4_5-bisphosphate phosphodiesterase beta-2	Q00722	0.1320	0.2741	-0.19	0.0108	0.4337	0.57	0.2209	0.3779	0.18	0.0096	0.0393	0.41	0.0017	0.0323	1.04	0.0733	0.1921	0.23	0.0317	0.1502	0.35	0.0806	0.2798	0.25	0.1440	0.2057	0.49
26	1-phosphatidylinositol 4_5-bisphosphate phosphodiesterase beta-3	Q01970	0.0000	0.0000	-0.65	0.0260	0.4951	-0.38	0.0168	0.0615	-0.13	0.0001	0.0075	-0.39	0.0008	0.0232	-0.32	0.0022	0.0548	-0.27	0.0606	0.1947	-0.22	0.4231	0.8169	-0.04	0.9547	0.9646	0.00
27	1-phosphatidylinositol 4_5-bisphosphate phosphodiesterase beta-4	Q15147	0.0394	0.1250	0.24	0.4673	0.7978	0.07	0.0726	0.1611	0.09	0.2491	0.3983	0.05	0.9765	0.9820	0.00	0.0220	0.0951	0.17	0.0046	0.0726	-3.06	0.0000	0.0000	-2.33	0.0252	0.1347	-3.70
28	1-phosphatidylinositol 4_5-bisphosphate phosphodiesterase gamma-1	P19174	0.0067	0.0494	0.16	0.2411	0.6608	0.16	0.0029	0.0295	0.19	0.0051	0.0289	0.16	0.4542	0.5468	0.02	0.0093	0.0688	0.15	0.0451	0.1703	-0.14	0.0183	0.1155	-0.11	0.0097	0.0874	-0.15
29	2' 3'-cyclic-nucleotide 3'-phosphodiesterase	P09543	0.3877	0.6045	0.04	0.0290	0.5092	0.29	0.0170	0.0618	0.31	0.0032	0.0231	0.30	0.0021	0.0358	0.63	0.0667	0.1812	0.11	0.0411	0.1666	0.38	0.1559	0.4268	0.14	0.2777	0.3496	0.03
30	2 4-dienoyl-CoA reductase mitochondrial	Q16698	0.0186	0.0803	-0.16	0.0334	0.5259	-0.31	0.8003	0.8370	-0.01	0.0227	0.0660	-0.15	0.0752	0.2108	-0.10	0.1883	0.3611	-0.06	0.5252	0.5781	-0.03	0.2760	0.6118	-0.07	0.5259	0.5923	0.04
31	26S proteasome non-ATPase regulatory subunit 1	Q99460	0.0158	0.0731	0.21	0.1722	0.6617	0.23	0.0016	0.0235	0.41	0.0058	0.0307	0.29	0.3375	0.4346	0.04	0.0034	0.0585	0.35	0.0421	0.1678	0.39	0.3299	0.6860	0.06	0.0904	0.1438	0.10
32	26S proteasome non-ATPase regulatory subunit 10	O75832	0.2881	0.4847	0.02	0.8589	0.8981	-0.01	0.0485	0.1225	0.05	0.0032	0.0229	0.10	0.0051	0.0536	0.08	0.1693	0.3342	0.10	0.0037	0.0663	-2.83	0.0001	0.0130	-2.86	0.0314	0.1507	-3.63
33	26S proteasome non-ATPase regulatory subunit 11	O00231	0.0091	0.0569	0.09	0.2848	0.6645	0.18	0.0406	0.1076	0.16	0.0290	0.0785	0.22	0.0492	0.1633	-0.05	0.0817	0.2057	0.21	0.6797	0.7230	0.04	0.4572	0.8625	0.07	0.2925	0.3641	-0.09
34	26S proteasome non-ATPase regulatory subunit 12	O00232	0.0046	0.0432	0.18	0.2364	0.6637	0.22	0.0315	0.0906	0.19	0.0013	0.0164	0.20	0.1745	0.3636	-0.04	0.0125	0.0756	0.21	0.0138	0.1112	0.32	0.1343	0.3863	0.10	0.0685	0.1159	0.12
35	26S proteasome non-ATPase regulatory subunit 13	Q9UNM6	0.0049	0.0436	0.11	0.3639	0.7129	0.09	0.0157	0.0591	0.10	0.0096	0.0394	0.08	0.0077	0.0636	-0.11	0.0968	0.2295	0.13	0.1256	0.2983	0.21	0.5335	0.9582	-0.05	0.1014	0.1572	-0.12
36	26S proteasome non-ATPase regulatory subunit 14	O00487	0.0028	0.0359	0.27	0.6460	0.7385	-0.04	0.5577	0.7816	0.02	0.6335	0.6793	0.03	0.0077	0.0632	-0.21	0.1382	0.2914	0.31	0.0752	0.2182	0.10	0.4012	0.7860	0.09	0.9992	0.9994	0.00
37	26S proteasome non-ATPase regulatory subunit 2	Q13200	0.0107	0.0599	0.09	0.4625	0.7946	0.10	0.0100	0.0465	0.11	0.0045	0.0272	0.14	0.0074	0.0628	-0.12	0.1456	0.3011	0.21	0.0726	0.2133	0.35	0.1257	0.3697	0.15	0.4254	0.4966	0.05
38	26S proteasome non-ATPase regulatory subunit 3	O43242	0.0326	0.1126	0.19	0.2352	0.6621	0.26	0.0058	0.0369	0.32	0.0063	0.0318	0.30	0.7111	0.7717	0.02	0.0220	0.0952	0.28	0.0189	0.1276	0.33	0.0684	0.2524	0.18	0.2311	0.3013	0.08
39	26S proteasome non-ATPase regulatory subunit 4	P55036	0.0340	0.1153	0.12	0.5219	0.8409	0.10	0.0311	0.0901	0.14	0.0070	0.0338	0.17	0.0380	0.1424	-0.10	0.0121	0.0745	0.18	0.0272	0.1430	0.25	0.6175	1.0515	-0.04	0.6425	0.6992	0.02
40	26S proteasome non-ATPase regulatory subunit 5	Q16401	0.0284	0.1025	-0.11	0.0488	0.5745	-0.17	0.0022	0.0268	-0.23	0.0089	0.0383	-0.22	0.0120	0.0786	-0.14	0.0118	0.0742	-0.16	0.0062	0.0841	-0.26	0.1766	0.4615	-0.11	0.0442	0.1838	-0.17
41	26S proteasome non-ATPase regulatory subunit 6	Q15008	0.3327	0.5399	0.07	0.5741	0.8856	0.09	0.2306	0.3900	0.10	0.0511	0.1166	0.20	0.7364	0.7937	0.02	0.0689	0.1844	0.19	0.1755	0.3746	0.24	0.4707	0.8815	0.06	0.8677	0.8927	-0.01
42	26S proteasome non-ATPase regulatory subunit 7	P51665	0.7601	0.8052	0.03	0.8560	0.8965	-0.05	0.3567	0.5529	0.13	0.2783	0.4363	0.16	0.9518	0.9626	0.01	0.5365	0.7683	0.10	0.7351	0.7741	0.02	0.1880	0.4781	-0.07	0.0738	0.1232	0.28
43	26S proteasome non-ATPase regulatory subunit 8	P48556	0.0198	0.0826	-0.20	0.5036	0.8247	0.13	0.0454	0.1172	0.17	0.0420	0.1020	0.16	0.0789	0.2165	0.12	0.2636	0.4561	0.10	0.1398	0.3203	0.27	0.9676	0.9772	0.00	0.1609	0.2251	-0.09
44	26S proteasome non-ATPase regulatory subunit 9	O00233	0.1265	0.2653	0.07	0.5198	0.8404	0.09	0.0210	0.0702	0.19	0.0076	0.0352	0.23	0.5376	0.6222	-0.02	0.0129	0.0763	0.22	0.0786	0.2227	0.32	0.1765	0.4615	0.12	0.5734	0.6363	0.03
45	26S proteasome regulatory subunit 10B	P62333	0.0058	0.0462	0.19	0.1534	0.6589	0.17	0.0019	0.0244	0.25	0.0061	0.0315	0.25	0.5632	0.6456	0.02	0.0031	0.0558	0.27	0.0338	0.1532	0.29	0.3400	0.6978	0.06	0.0987	0.1541	0.13
46	26S proteasome regulatory subunit 4	P62191	0.0351	0.1171	0.12	0.3427	0.7000	0.11	0.0016	0.0230	0.25	0.0013	0.0168	0.25	0.8947	0.9194	0.00	0.0042	0.0598	0.25	0.0037	0.0665	0.33	0.0110	0.0859	0.28	0.0205	0.1230	0.22
47	26S proteasome regulatory subunit 6A	P17980	0.0350	0.1170	0.15	0.2371	0.6628	0.21	0.0234	0.0753	0.21	0.0155	0.0515	0.23	0.7734	0.8228	-0.01	0.0215	0.0945	0.25	0.0615	0.1954	0.23	0.0199	0.1221	0.25	0.0113	0.0928	0.13
48	26S proteasome regulatory subunit 6B	P43686	0.0082	0.0538	0.16	0.0989	0.6364	0.15	0.0008	0.0217	0.33	0.0005	0.0132	0.31	0.2461	0.4619	0.04	0.0435	0.1372	0.30	0.0236	0.1370	0.26	0.1644	0.4412	0.10	0.0687	0.1161	0.16
49	26S proteasome regulatory subunit 7	P35998	0.1403	0.2858	0.07	0.2039	0.6597	0.19	0.0014	0.0226	0.32	0.0039	0.0254	0.30	0.3302	0.4275	0.04	0.0086	0.0677	0.23	0.0306	0.1479	0.30	0.7276	1.1734	0.03	0.4103	0.4820	-0.05
50	26S proteasome regulatory subunit 8	P62195	0.0070	0.0500	0.14	0.1383	0.6519	0.10	0.0012	0.0228	0.19	0.0039	0.0255	0.19	0.0231	0.1104	0.02	0.1090	0.2483	0.23	0.1789	0.3784	0.12	0.2984	0.6419	-0.09	0.0504	0.1964	-0.21
51	28 kDa heat- and acid-stable phosphoprotein	Q13442	0.0190	0.0813	0.08	0.1396	0.6538	0.19	0.0002	0.0188	0.22	0.0018	0.0190	0.13	0.0081	0.0653	-0.09	0.0896	0.2183	0.06	0.3706	0.6527	0.07	0.3324	0.6890	0.06	0.0624	0.2232	0.16
52	28S ribosomal protein S14 mitochondrial	O60783	0.0476	0.1402	0.20	0.0071	0.4086	0.34	0.1925	0.3399	0.14	0.0277	0.0758	0.25	0.0855	0.2279	0.17	0.6106	0.8480	-0.05	0.3768	0.4326	-1.28	0.1296	0.3767	-0.96	0.1000	0.1556	0.28
53	28S ribosomal protein S16 mitochondrial	Q9Y3D3	0.7024	0.7555	0.01	0.8477	0.8905	-0.02	0.0038	0.0313	-0.07	0.1083	0.2060	-0.04	0.0478	0.1606	-0.14	0.3769	0.5856	-0.09	0.5806	0.6294	0.06	0.9843	0.9893	0.00	0.7679	0.8091	-0.03
54	28S ribosomal protein S17 mitochondrial	Q9Y2R5	0.2232	0.4010	-0.23	0.2605	0.6641	-0.52	0.1837	0.3285	-0.29	0.0419	0.1018	-0.53	0.6034	0.6787	-0.09	0.0365	0.1234	-0.63	0.0049	0.0736	-1.47	0.0000	0.0000	-2.59	0.0000	0.0000	-2.49
55	28S ribosomal protein S18b mitochondrial	Q9Y676	0.0783	0.1919	-0.10	0.2167	0.6603	0.13	0.0108	0.0484	0.14	0.1349	0.2434	0.04	0.3639	0.4612	0.02	0.9913	0.9928	0.00	0.0472	0.1736	0.34	0.9457	0.9605	0.01	0.0264	0.1384	0.43
56	28S ribosomal protein S2 mitochondrial	Q9Y399	0.5937	0.6597	-0.04	0.2389	0.6607	0.19	0.3363	0.5287	0.10	0.0962	0.1883	0.15	0.0450	0.1561	0.23	0.7766	1.0141	-0.02	0.1374	0.3170	0.20	0.2152	0.5209	-0.11	0.0865	0.1390	0.11
57	28S ribosomal protein S21 mitochondrial	P82921	0.4108	0.6314	-3.31	0.5752	0.8836	-1.75	0.5661	0.7883	-1.19	0.5388	0.7449	0.89	0.2938	0.5233	-1.88	0.9510	1.1847	0.09	0.0235	0.1366	-0.71	0.0033	0.0472	-0.70	0.0195	0.1211	-0.95
58	28S ribosomal protein S22 mitochondrial	P82650	0.1434	0.2903	-0.10	0.9211	0.9457	0.01	0.0066	0.0391	0.25	0.0436	0.1045	0.12	0.6309	0.7012	0.02	0.1305	0.2812	0.08	0.1164	0.2837	0.11	0.0490	0.2075	-0.15	0.5599	0.6241	0.03
59	28S ribosomal protein S23 mitochondrial	Q9Y3D9	0.4722	0.5481	0.04	0.8615	0.8999	0.01	0.1067	0.2149	0.07	0.7013	0.7405	0.01	0.2784	0.5037	-0.04	0.2743	0.4686	0.07	0.3607	0.6392	0.34	0.3174	0.6670	0.38	0.7600	0.8020	0.10
60	28S ribosomal protein S25 mitochondrial	P82663	0.4235	0.5010	-0.24	0.1980	0.6682	0.40	0.3625	0.5602	0.23	0.6495	0.6935	-0.15	0.3336	0.4303	0.24	0.3725	0.5805	-0.43	0.3736	0.4714	-2.55	0.8708	0.9808	-0.33	0.0651	0.1284	2.06
61	28S ribosomal protein S26 mitochondrial	Q9BYN8	0.1070	0.2357	0.17	0.1533	0.6590	0.13	0.6322	0.6855	0.04	0.0809	0.1657	0.18	0.3366	0.4335	0.08	0.8605	1.0963	0.01	0.3736	0.4355	-2.55	0.8708	0.9135	-0.33	0.0651	0.1137	2.06
62	28S ribosomal protein S27 mitochondrial	Q92552	0.0068	0.0495	0.23	0.0570	0.5835	0.13	0.4865	0.7063	0.03	0.0128	0.0458	0.21	0.2349	0.4467	-0.06	0.1008	0.2348	0.24	0.0514	0.1797	0.32	0.9755	0.9825	0.00	0.4484	0.5177	0.07
63	28S ribosomal protein S29 mitochondrial	P51398	0.1767	0.3391	-0.05	0.0118	0.4274	-0.18	0.0897	0.1878	-0.06	0.0185	0.0575	-0.13	0.0441	0.1554	-0.14	0.0826	0.2073	0.05	0.0339	0.1534	-0.20	0.0722	0.2608	-0.13	0.1746	0.2405	-0.09
64	28S ribosomal protein S31 mitochondrial	Q92665	0.3336	0.5404	0.18	0.2963	0.6717	-0.21	0.1032	0.2096	-0.37	0.0735	0.1534	-0.43	0.0580	0.1807	-0.51	0.3133	0.5142	-0.19	0.3736	0.4795	-2.55	0.8708	0.9957	-0.33	0.0651	0.1318	2.06
65	28S ribosomal protein S33 mitochondrial	Q9Y291	0.8077	0.8451	0.01	0.1004	0.6342	0.33	0.0293	0.0871	0.22	0.0009	0.0148	0.40	0.1606	0.3438	0.12	0.6765	0.9152	0.03	0.3736	0.4462	-2.55	0.8708	0.9337	-0.33	0.0651	0.1179	2.06
66	28S ribosomal protein S34 mitochondrial	P82930	0.3368	0.5442	-0.04	0.3577	0.7080	0.15	0.0381	0.1029	0.08	0.5567	0.6076	0.01	0.1567	0.3379	0.04	0.4566	0.6793	0.02	0.0071	0.0865	-0.35	0.0522	0.2168	-0.18	0.0592	0.2176	-0.27
67	28S ribosomal protein S35 mitochondrial	P82673	0.0445	0.1343	0.16	0.0840	0.6216	0.28	0.2014	0.3532	0.11	0.0109	0.0418	0.25	0.8588	0.8917	0.01	0.0919	0.2217	0.12	0.1278	0.3010	0.22	0.9184	0.9406	-0.01	0.0664	0.1130	-0.24
68	28S ribosomal protein S36 mitochondrial	P82909	0.2961	0.4945	0.25	0.5866	0.6922	-0.12	0.4278	0.6375	-0.03	0.0209	0.0622	-0.29	0.7241	0.7837	-0.03	0.0194	0.0896	0.35	0.3736	0.5944	-2.55	0.8708	1.2029	-0.33	0.0651	0.1896	2.06
69	28S ribosomal protein S5 mitochondrial	P82675	0.4108	0.6313	-3.31	0.5752	0.8833	-1.75	0.5661	0.7881	-1.19	0.5388	0.7447	0.89	0.2938	0.5232	-1.88	0.9510	1.1845	0.09	0.3812	0.4371	0.08	0.5896	1.0225	0.07	0.7750	0.8150	0.04

Supplemetary Table S2. Overview on all relatively quantified 5180 proteins statistical analysis

			MCF-7																		MDA-MB-231								
			Dai SC20 vs control			Gen SC20 vs control			SSE SC20 vs control			Dai IC20 vs control			Gen IC20 vs control			SSE IC20 vs control			Dai IC20 vs control			Gen IC20 vs control			SSE IC20 vs control		
	**Protein name**	**UniProt**	**p value**	**BH q value**	**log2FC**	**p value**	**BH q value**	**log2FC**	**p value**	**BH q value**	**log2FC**	**p value**	**BH q value**	**log2FC**	**p value**	**BH q value**	**log2FC**	**p value**	**BH q value**	**log2FC**	**p value**	**BH q value**	**log2FC**	**p value**	**BH q value**	**log2FC**	**p value**	**BH q value**	**log2FC**
70	28S ribosomal protein S6 mitochondrial	P82932	0.0208	0.0852	0.18	0.0704	0.5998	0.12	0.7982	0.8351	-0.01	0.4985	0.7021	0.03	0.0342	0.1353	0.17	0.2946	0.4916	-0.06	0.1125	0.2764	0.33	0.6379	1.0756	0.07	0.1197	0.1780	0.30
71	28S ribosomal protein S7 mitochondrial	Q9Y2R9	0.4895	0.5636	-0.03	0.4505	0.7847	-0.08	0.9313	0.9465	0.00	0.1622	0.2816	-0.10	0.0427	0.1525	-0.15	0.0470	0.1441	0.17	0.7386	0.7765	0.03	0.0032	0.0464	0.35	0.0322	0.1530	-0.19
72	28S ribosomal protein S9 mitochondrial	P82933	0.0104	0.0593	0.11	0.4618	0.7942	0.07	0.0040	0.0320	0.28	0.0009	0.0152	0.22	0.0628	0.1900	0.08	0.0791	0.2015	0.27	0.0001	0.0273	0.33	0.0322	0.1623	0.07	0.0004	0.0266	0.28
73	2'-deoxynucleoside 5'-phosphate N-hydrolase 1	O43598	0.7746	0.8165	0.01	0.1256	0.6429	-0.13	0.7740	0.8141	-0.01	0.9530	0.9593	0.00	0.1776	0.3680	-0.06	0.5792	0.8135	0.03	0.3736	0.4536	-2.55	0.8708	0.9476	-0.33	0.0651	0.1210	2.06
74	2-iminobutanoate/2-iminopropanoate deaminase	P52758	0.4108	0.6311	-3.31	0.5752	0.8831	-1.75	0.5661	0.7879	-1.19	0.5388	0.7445	0.89	0.2938	0.5230	-1.88	0.9510	1.1842	0.09	0.2153	0.4350	-0.28	0.3652	0.7347	0.08	0.1089	0.1658	0.12
75	2-methoxy-6-polyprenyl-1_4-benzoquinol methylase_ mitochondrial	Q5HYK3	0.3708	0.5852	1.31	0.5752	0.8847	-1.75	0.5661	0.7891	-1.19	0.0920	0.1820	5.89	0.2938	0.5253	-1.88	0.3432	0.5470	5.15	0.3736	0.4748	-2.55	0.8708	0.9870	-0.33	0.0651	0.1298	2.06
76	2-oxoglutarate dehydrogenase mitochondrial	Q02218	0.0165	0.0752	0.14	0.3140	0.6806	0.23	0.0013	0.0222	0.30	0.0047	0.0279	0.21	0.2684	0.4904	0.04	0.0061	0.0617	0.28	0.0097	0.0985	0.22	0.8198	1.2642	-0.01	0.4087	0.4804	0.03
77	2-oxoisovalerate dehydrogenase subunit alpha_ mitochondrial	P12694	0.0266	0.0988	-0.27	0.0831	0.6185	-0.27	0.0135	0.0545	-0.33	0.0208	0.0621	-0.78	0.7537	0.8073	0.05	0.1161	0.2594	-0.36	0.3736	0.5288	-2.55	0.8708	1.0859	-0.33	0.0651	0.1545	2.06
78	3'-5' RNA helicase YTHDC2	Q9H6S0	0.1023	0.2287	0.37	0.1495	0.6602	0.30	0.0583	0.1387	0.48	0.0891	0.1779	0.42	0.2965	0.3946	0.19	0.3145	0.5154	0.19	0.1331	0.3101	-0.29	0.4071	0.7931	-0.07	0.6232	0.6813	0.02
79	39S ribosomal protein L1 mitochondrial	Q9BYD6	0.4501	0.5277	-0.05	0.1861	0.6671	-0.13	0.4961	0.7180	-0.05	0.0469	0.1101	-0.23	0.0557	0.1763	-0.23	0.6002	0.8367	-0.04	0.3736	0.4525	-2.55	0.8708	0.9455	-0.33	0.0651	0.1205	2.06
80	39S ribosomal protein L10 mitochondrial	Q7Z7H8	0.9827	0.9878	-0.01	0.1000	0.6340	0.67	0.1143	0.2271	0.62	0.0409	0.1002	1.01	0.0752	0.2107	0.81	0.1683	0.3331	0.53	0.3736	0.5099	-2.55	0.8708	1.0517	-0.33	0.0651	0.1455	2.06
81	39S ribosomal protein L11 mitochondrial	Q9Y3B7	0.0647	0.1684	0.13	0.4357	0.7719	0.10	0.6243	0.6777	0.02	0.7643	0.7968	-0.02	0.5718	0.6518	-0.03	0.0439	0.1380	0.17	0.6280	0.6741	-0.08	0.3965	0.7806	-0.15	0.8920	0.9146	-0.02
82	39S ribosomal protein L12 mitochondrial	P52815	0.1277	0.2676	0.05	0.1171	0.6319	-0.12	0.9301	0.9458	0.00	0.5097	0.7145	0.02	0.0003	0.0139	-0.22	0.3383	0.5425	-0.03	0.0377	0.1611	-0.15	0.0511	0.2133	-0.13	0.0287	0.1448	-0.18
83	39S ribosomal protein L13 mitochondrial	Q9BYD1	0.2354	0.4186	-0.05	0.3860	0.7300	-0.04	0.4704	0.6881	0.03	0.9732	0.9772	0.00	0.0532	0.1712	-0.13	0.5051	0.7306	-0.03	0.3736	0.4595	-2.55	0.8708	0.9585	-0.33	0.0651	0.1233	2.06
84	39S ribosomal protein L14 mitochondrial	Q6P1L8	0.9724	0.9805	-0.01	0.7241	0.7986	0.10	0.0125	0.0523	0.85	0.8088	0.8361	-0.05	0.0895	0.2351	0.45	0.3680	0.5750	0.18	0.8083	0.8381	0.03	0.0171	0.1106	-0.26	0.0800	0.1310	-0.17
85	39S ribosomal protein L15 mitochondrial	Q9P015	0.9799	0.9864	0.00	0.6080	0.7101	-0.05	0.9375	0.9518	0.00	0.1630	0.2825	-0.06	0.2605	0.4818	0.06	0.6546	0.8916	-0.04	0.6134	0.6607	-0.05	0.5601	0.9872	0.05	0.7405	0.7860	-0.03
86	39S ribosomal protein L16 mitochondrial	Q9NX20	0.0459	0.1370	-0.14	0.0008	0.2302	-0.43	0.1103	0.2210	-0.10	0.0665	0.1424	-0.30	0.0042	0.0476	-0.41	0.1324	0.2834	0.10	0.3736	0.5232	-2.55	0.8708	1.0758	-0.33	0.0651	0.1518	2.06
87	39S ribosomal protein L17 mitochondrial	Q9NRX2	0.1427	0.2892	0.14	0.1975	0.6704	0.17	0.0067	0.0395	0.28	0.0560	0.1253	0.34	0.1880	0.3834	0.08	0.0042	0.0599	0.46	0.0292	0.1467	0.36	0.4467	0.8479	0.08	0.1648	0.2299	0.16
88	39S ribosomal protein L19 mitochondrial	P49406	0.0601	0.1622	-0.21	0.7042	0.7824	0.05	0.7566	0.7979	-0.02	0.0394	0.0974	0.26	0.9377	0.9520	-0.01	0.2550	0.4450	0.10	0.3736	0.4889	-2.55	0.8708	1.0132	-0.33	0.0651	0.1359	2.06
89	39S ribosomal protein L2 mitochondrial	Q5T653	0.0683	0.1739	-0.18	0.5026	0.8241	0.05	0.1450	0.2716	0.12	0.0746	0.1553	0.16	0.4124	0.5073	0.06	0.6697	0.9081	0.05	0.9116	0.9277	-0.01	0.1806	0.4680	-0.08	0.0055	0.0712	0.49
90	39S ribosomal protein L21 mitochondrial	Q7Z2W9	0.0288	0.1035	-0.18	0.3838	0.7306	0.10	0.1823	0.3268	-0.09	0.6470	0.6912	0.03	0.9333	0.9491	0.01	0.1514	0.3090	-0.13	0.0229	0.1365	-0.25	0.7391	1.1813	-0.02	0.0476	0.1910	-0.19
91	39S ribosomal protein L22 mitochondrial	Q9NWU5	0.0906	0.2107	-0.19	0.3863	0.7300	-0.10	0.1724	0.3135	-0.14	0.6347	0.6801	-0.04	0.2565	0.4764	0.11	0.5399	0.7717	0.06	0.2192	0.4406	0.21	0.5203	0.9424	0.09	0.9735	0.9786	0.00
92	39S ribosomal protein L24 mitochondrial	Q96A35	0.1518	0.3027	-0.20	0.4498	0.7842	-0.12	0.6052	0.6610	0.06	0.6576	0.7008	0.05	0.4722	0.5628	-0.08	0.7203	0.9602	0.04	0.1631	0.3569	0.20	0.0661	0.2478	0.15	0.0078	0.0794	0.23
93	39S ribosomal protein L28 mitochondrial	Q13084	0.1342	0.2776	0.07	0.7626	0.8283	0.02	0.0860	0.1827	0.11	0.2524	0.4020	0.05	0.0280	0.1215	-0.14	0.5942	0.8312	-0.02	0.9238	0.9366	0.01	0.2121	0.5153	-0.14	0.0700	0.1178	-0.13
94	39S ribosomal protein L3 mitochondrial	P09001	0.5538	0.6232	-0.09	0.4103	0.7510	-0.33	0.3703	0.5682	-0.11	0.1754	0.2992	-0.34	0.3666	0.4635	-0.10	0.6828	0.9220	-0.10	0.3736	0.4458	-2.55	0.8708	0.9329	-0.33	0.0651	0.1178	2.06
95	39S ribosomal protein L30 mitochondrial	Q8TCC3	0.8473	0.8785	-0.02	0.7922	0.8505	-0.04	0.9877	0.9896	0.00	0.8098	0.8363	0.04	0.0673	0.1973	0.21	0.4988	0.7240	0.09	0.3736	0.4601	-2.55	0.8708	0.9597	-0.33	0.0651	0.1236	2.06
96	39S ribosomal protein L32 mitochondrial	Q9BYC8	0.0064	0.0481	-0.72	0.6925	0.7748	0.07	0.2852	0.4627	-0.18	0.5433	0.5944	0.08	0.1902	0.3865	-0.22	0.0527	0.1551	-0.37	0.3736	0.5590	-2.55	0.8708	1.1402	-0.33	0.0651	0.1699	2.06
97	39S ribosomal protein L37 mitochondrial	Q9BZE1	0.0437	0.1326	0.16	0.2206	0.6594	0.26	0.0116	0.0504	0.27	0.0070	0.0338	0.34	0.0681	0.1990	0.14	0.0159	0.0838	0.26	0.0395	0.1640	0.26	0.1868	0.4771	0.15	0.0939	0.1482	0.21
98	39S ribosomal protein L38 mitochondrial	Q96DV4	0.4108	0.6309	-3.31	0.5752	0.8828	-1.75	0.5661	0.7876	-1.19	0.5388	0.7443	0.89	0.2938	0.5228	-1.88	0.9510	1.1839	0.09	0.1027	0.2603	-5.95	0.1528	0.4228	-3.74	0.2601	0.3322	-1.35
99	39S ribosomal protein L39 mitochondrial	Q9NYK5	0.0555	0.1542	0.13	0.0703	0.5999	0.12	0.0165	0.0610	0.20	0.0031	0.0228	0.33	0.3338	0.4304	0.05	0.0266	0.1043	0.19	0.3736	0.5843	-2.55	0.8708	1.1852	-0.33	0.0651	0.1838	2.06
100	39S ribosomal protein L4 mitochondrial	Q9BYD3	0.9931	0.9944	0.00	0.2602	0.6643	-0.11	0.5301	0.7515	-0.05	0.0223	0.0652	-0.30	0.0243	0.1125	-0.29	0.0370	0.1246	-0.37	0.9060	0.9237	-0.02	0.0039	0.0501	-0.30	0.7273	0.7752	0.05
101	39S ribosomal protein L40 mitochondrial	Q9NQ50	0.1676	0.3260	0.09	0.1720	0.6624	0.17	0.3502	0.5443	-0.05	0.9130	0.9244	0.01	0.3530	0.4506	-0.06	0.4321	0.6533	0.10	0.0005	0.0381	8.37	0.0201	0.1228	7.27	0.0028	0.0539	8.29
102	39S ribosomal protein L41 mitochondrial	Q8IXM3	0.0554	0.1542	-12.68	0.0453	0.5641	-11.12	0.0160	0.0598	-10.56	0.0007	0.0135	-8.48	0.0534	0.1715	-1.24	0.0087	0.0682	-9.28	0.3736	0.6165	-2.55	0.8708	1.2416	-0.33	0.0651	0.2029	2.06
103	39S ribosomal protein L43 mitochondrial	Q8N983	0.0034	0.0385	-0.42	0.2791	0.6647	-0.15	0.4677	0.6850	-0.08	0.1024	0.1976	-0.11	0.0163	0.0911	-0.20	0.1351	0.2872	-0.18	0.4483	0.5036	0.15	0.7536	1.1960	-0.06	0.5258	0.5924	-0.13
104	39S ribosomal protein L44 mitochondrial	Q9H9J2	0.0822	0.1976	0.15	0.4241	0.7609	-0.07	0.0266	0.0817	-0.10	0.5929	0.6410	0.04	0.0001	0.0077	0.28	0.0207	0.0933	0.18	0.4603	0.5150	-0.10	0.4652	0.8731	-0.08	0.5310	0.5970	-0.07
105	39S ribosomal protein L45 mitochondrial	Q9BRJ2	0.0010	0.0232	-1.03	0.1795	0.6646	-0.55	0.0045	0.0331	-1.13	0.0025	0.0213	-1.08	0.4288	0.5229	-0.15	0.0044	0.0600	-1.09	0.3736	0.6322	-2.55	0.8708	1.2688	-0.33	0.0651	0.2129	2.06
106	39S ribosomal protein L46 mitochondrial	Q9H2W6	0.4108	0.6307	-3.31	0.5752	0.8826	-1.75	0.5661	0.7874	-1.19	0.5388	0.7441	0.89	0.2938	0.5226	-1.88	0.9510	1.1836	0.09	0.0219	0.1349	0.21	0.7589	1.2011	0.02	0.7700	0.8109	0.01
107	39S ribosomal protein L47 mitochondrial	Q9HD33	0.9909	0.9926	0.00	0.2776	0.6651	0.16	0.1213	0.2389	-0.12	0.1681	0.2891	-0.25	0.2498	0.4668	-0.17	0.7090	0.9505	-0.25	0.2197	0.4411	-0.16	0.0269	0.1479	-0.54	0.0670	0.1138	-0.55
108	39S ribosomal protein L48 mitochondrial	Q96GC5	0.1449	0.2923	0.11	0.2815	0.6646	0.11	0.0933	0.1935	0.13	0.1687	0.2900	0.12	0.4583	0.5506	0.04	0.1521	0.3096	0.10	0.0448	0.1701	0.33	0.9924	0.9951	0.00	0.9136	0.9307	0.01
109	39S ribosomal protein L49 mitochondrial	Q13405	0.0687	0.1743	0.11	0.3406	0.6993	0.06	0.0086	0.0437	0.22	0.0366	0.0921	0.11	0.3546	0.4521	-0.03	0.0056	0.0613	0.25	0.0658	0.2017	0.31	0.9295	0.9487	-0.01	0.0392	0.1708	0.26
110	39S ribosomal protein L50 mitochondrial	Q8N5N7	0.0879	0.2063	-0.03	0.6352	0.7315	0.03	0.9580	0.9662	0.00	0.4263	0.6180	-0.02	0.1766	0.3671	-0.03	0.3326	0.5356	0.01	0.0422	0.1675	0.30	0.1536	0.4228	-0.12	0.1302	0.1901	0.14
111	39S ribosomal protein L52 mitochondrial	Q86TS9	0.1899	0.3561	-0.18	0.0298	0.5061	0.10	0.6515	0.7037	-0.03	0.5110	0.7156	0.03	0.0284	0.1224	0.09	0.3568	0.5630	0.03	0.3736	0.4736	-2.55	0.8708	0.9849	-0.33	0.0651	0.1293	2.06
112	39S ribosomal protein L53 mitochondrial	Q96EL3	0.0065	0.0484	0.30	0.3493	0.7049	0.13	0.0574	0.1371	0.15	0.0061	0.0313	0.36	0.0341	0.1351	0.19	0.0455	0.1412	0.32	0.0213	0.1339	2.84	0.1131	0.3438	1.66	0.0261	0.1382	2.72
113	39S ribosomal protein L54 mitochondrial	Q6P161	0.0018	0.0293	0.23	0.2114	0.6621	0.14	0.1603	0.2951	0.13	0.1493	0.2642	0.09	0.0775	0.2140	0.07	0.6207	0.8567	0.03	0.3736	0.4504	-2.55	0.8708	0.9415	-0.33	0.0651	0.1196	2.06
114	39S ribosomal protein L9 mitochondrial	Q9BYD2	0.0527	0.1490	-0.22	0.3004	0.6733	-0.13	0.0789	0.1706	-0.24	0.4168	0.6089	-0.06	0.9429	0.9558	-0.01	0.1053	0.2419	-0.37	0.2550	0.4931	-0.54	0.0129	0.0946	0.55	0.0014	0.0456	0.60
115	39S ribosomal protein S30 mitochondrial	Q9NP92	0.0087	0.0552	-0.93	0.3194	0.6825	-0.71	0.0101	0.0468	-0.96	0.0025	0.0211	-1.26	0.0595	0.1839	-0.48	0.0066	0.0628	-1.15	0.1205	0.2906	-0.18	0.0202	0.1230	-0.49	0.1004	0.1561	-0.20
116	3-hydroxy-3-methylglutaryl-coenzyme A reductase	P04035	0.2391	0.4237	-0.11	0.1293	0.6428	-0.15	0.3973	0.6002	-0.07	0.0804	0.1649	-0.26	0.0372	0.1400	-0.29	0.3873	0.5985	-0.07	0.3736	0.4704	-2.55	0.8708	0.9789	-0.33	0.0651	0.1279	2.06
117	3-hydroxyacyl-CoA dehydrogenase type-2	Q99714	0.1953	0.3638	-0.05	0.9331	0.9549	0.01	0.9679	0.9732	0.00	0.0700	0.1479	-0.08	0.0249	0.1138	-0.15	0.0289	0.1091	0.13	0.8181	0.8459	-0.03	0.3396	0.6972	0.10	0.2609	0.3329	-0.11
118	3-hydroxybutyrate dehydrogenase type 2	Q9BUT1	0.2135	0.3876	-0.06	0.3510	0.7042	0.13	0.5849	0.6412	0.04	0.0663	0.1421	0.11	0.3644	0.4614	-0.04	0.1814	0.3511	-0.08	0.0003	0.0389	0.91	0.0002	0.0203	1.45	0.0106	0.0909	0.53
119	3-hydroxyisobutyrate dehydrogenase mitochondrial	P31937	0.7929	0.8321	0.01	0.7542	0.8218	0.02	0.1086	0.2180	0.12	0.2198	0.3586	0.08	0.0758	0.2119	0.16	0.1000	0.2338	0.13	0.4237	0.4794	0.14	0.5197	0.9423	-0.09	0.1704	0.2361	-0.23
120	3-hydroxyisobutyryl-CoA hydrolase mitochondrial	Q6NVY1	0.4108	0.6305	-3.31	0.5752	0.8823	-1.75	0.5661	0.7872	-1.19	0.5388	0.7439	0.89	0.2938	0.5224	-1.88	0.9510	1.1833	0.09	0.6937	0.7350	0.08	0.7543	1.1967	-0.05	0.7234	0.7717	-0.09
121	3-ketoacyl-CoA thiolase mitochondrial	P42765	0.8746	0.9012	-0.01	0.0855	0.6212	0.06	0.0009	0.0216	0.23	0.0007	0.0141	0.25	0.0521	0.1693	0.07	0.0160	0.0839	0.12	0.3646	0.6450	0.09	0.9723	0.9801	0.00	0.8981	0.9190	-0.01
122	3-ketoacyl-CoA thiolase peroxisomal	P09110	0.0356	0.1180	-0.13	0.9744	0.9827	0.00	0.3860	0.5870	-0.04	0.0850	0.1719	0.11	0.3644	0.4613	-0.04	0.0849	0.2118	0.17	0.3908	0.4466	-0.08	0.5971	1.0306	-0.05	0.1091	0.1659	0.20
123	3-mercaptopyruvate sulfurtransferase	P25325	0.1916	0.3582	-0.19	0.2673	0.6631	-0.19	0.0477	0.1210	-0.35	0.0790	0.1625	-0.29	0.1118	0.2725	0.22	0.0526	0.1551	-0.55	0.1650	0.3594	1.54	0.2507	0.5739	1.29	0.0922	0.1460	2.25
124	3-phosphoinositide-dependent protein kinase 1	O15530	0.3328	0.5397	0.09	0.5418	0.8572	0.06	0.4216	0.6299	0.07	0.3952	0.5836	-0.04	0.0029	0.0406	0.17	0.4147	0.6322	-0.12	0.3736	0.4677	-2.55	0.8708	0.9738	-0.33	0.0651	0.1268	2.06
125	40S ribosomal protein S10	P46783	0.3314	0.5381	-0.10	0.6271	0.7240	-0.07	0.0944	0.1953	0.22	0.2510	0.4003	0.12	0.7328	0.7908	-0.03	0.3970	0.6104	0.08	0.0967	0.2498	0.33	0.5079	0.9264	0.07	0.5609	0.6250	-0.04
126	40S ribosomal protein S11	P62280	0.0818	0.1972	0.14	0.3113	0.6792	0.24	0.0038	0.0316	0.38	0.0115	0.0431	0.31	0.8330	0.8706	0.01	0.0105	0.0703	0.32	0.0739	0.2158	0.55	0.9892	0.9926	0.00	0.0932	0.1472	0.20
127	40S ribosomal protein S12	P25398	0.0010	0.0231	0.27	0.7175	0.7930	0.05	0.0220	0.0723	0.13	0.0888	0.1775	0.07	0.0149	0.0871	-0.16	0.0160	0.0840	0.16	0.7788	0.8132	-0.02	0.0538	0.2210	-0.15	0.0085	0.0825	-0.27
128	40S ribosomal protein S13	P62277	0.1598	0.3149	0.10	0.4411	0.7769	0.13	0.0070	0.0396	0.29	0.0108	0.0417	0.25	0.6378	0.7075	-0.02	0.0447	0.1397	0.17	0.1267	0.3000	0.25	0.3057	0.6517	-0.11	0.1853	0.2528	-0.12
129	40S ribosomal protein S14	P62263	0.0216	0.0876	0.24	0.2336	0.6616	0.31	0.0018	0.0239	0.50	0.0045	0.0274	0.39	0.6326	0.7024	0.03	0.0099	0.0702	0.39	0.0262	0.1411	0.51	0.5430	0.9686	0.07	0.0974	0.1526	0.19
130	40S ribosomal protein S15	P62841	0.1115	0.2426	0.08	0.3104	0.6796	0.22	0.0083	0.0428	0.32	0.0011	0.0156	0.34	0.7686	0.8199	0.01	0.0165	0.0850	0.18	0.0557	0.1874	0.52	0.4112	0.7987	-0.07	0.0748	0.1246	0.14
131	40S ribosomal protein S15a	P62244	0.0080	0.0530	0.13	0.9545	0.9699	0.01	0.0207	0.0694	0.26	0.0142	0.0484	0.17	0.1308	0.2987	-0.09	0.0055	0.0617	0.23	0.3017	0.5597	0.18	0.0945	0.3065	-0.16	0.2393	0.3096	-0.09
132	40S ribosomal protein S16	P62249	0.3808	0.5965	0.06	0.7934	0.8505	0.05	0.0296	0.0873	0.22	0.1001	0.1943	0.13	0.0506	0.1661	-0.19	0.0424	0.1359	0.22	0.3897	0.4457	0.13	0.5551	0.9810	0.03	0.1453	0.2073	-0.10
133	40S ribosomal protein S17	P08708	0.0853	0.2020	0.14	0.2510	0.6610	0.14	0.6921	0.7401	0.03	0.2395	0.3856	0.08	0.1023	0.2564	-0.12	0.1349	0.2871	0.12	0.0059	0.0815	-0.50	0.0012	0.0293	-0.91	0.0028	0.0554	-0.78
134	40S ribosomal protein S18	P62269	0.0400	0.1260	0.21	0.4424	0.7779	0.17	0.0086	0.0436	0.32	0.0083	0.0369	0.33	0.3757	0.4725	-0.06	0.0302	0.1116	0.28	0.1111	0.2743	0.34	0.6012	1.0350	-0.05	0.2692	0.3409	-0.09
135	40S ribosomal protein S19	P39019	0.0209	0.0855	0.27	0.7915	0.8501	0.04	0.0728	0.1614	0.14	0.4932	0.6961	0.04	0.0670	0.1971	-0.15	0.0415	0.1341	0.20	0.1382	0.3182	0.18	0.1311	0.3796	-0.17	0.3667	0.4383	-0.09
136	40S ribosomal protein S2	P15880	0.3155	0.5180	0.06	0.6098	0.7118	0.09	0.0114	0.0496	0.25	0.0338	0.0873	0.17	0.2267	0.4361	-0.07	0.0702	0.1862	0.15	0.0913	0.2429	0.35	0.8964	0.9224	-0.01	0.6602	0.7150	-0.03
137	40S ribosomal protein S20	P60866	0.0042	0.0409	0.41	0.4041	0.7476	0.20	0.0052	0.0351	0.38	0.0090	0.0383	0.33	0.8868	0.9134	-0.01	0.0195	0.0899	0.35	0.7730	0.8073	0.04	0.0269	0.1476	-0.33	0.0035	0.0583	-0.50
138	40S ribosomal protein S21	P63220	0.1004	0.2255	0.14	0.8297	0.8776	0.04	0.1767	0.3193	0.11	0.0493	0.1140	0.17	0.1378	0.3087	-0.11	0.0593	0.1682	0.18	0.1233	0.2957	0.32	0.1503	0.4184	0.15	0.9845	0.9881	0.00
139	40S ribosomal protein S23	P62266	0.1404	0.2859	0.21	0.5183	0.8398	-0.11	0.0049	0.0341	-0.72	0.0070	0.0339	-0.72	0.0144	0.0855	-0.83	0.4301	0.6512	0.09	0.0063	0.0833	-1.30	0.0088	0.0778	0.54	0.1607	0.2250	-0.16
140	40S ribosomal protein S24	P62847	0.0001	0.0173	0.23	0.3776	0.7250	-0.06	0.0009	0.0206	0.19	0.7766	0.8081	-0.01	0.0011	0.0256	-0.12	0.0010	0.0471	0.22	0.1078	0.2682	-0.27	0.0012	0.0284	-0.49	0.0045	0.0666	-0.46
141	40S ribosomal protein S25	P62851	0.0554	0.1541	0.20	0.4322	0.7680	0.16	0.0093	0.0449	0.36	0.0246	0.0699	0.26	0.2601	0.4812	-0.09	0.0397	0.1302	0.27	0.1808	0.3815	0.27	0.6399	1.0779	-0.05	0.2089	0.2776	-0.12
142	40S ribosomal protein S26	P62854	0.0030	0.0372	0.30	0.1133	0.6345	0.14	0.0001	0.0173	0.28	0.0025	0.0215	0.24	0.6908	0.7537	-0.01	0.3299	0.5329	0.12	0.1155	0.2822	0.20	0.0008	0.0245	-0.40	0.0358	0.1620	-0.30
143	40S ribosomal protein S27	P42677	0.4885	0.5626	0.03	0.6738	0.7611	-0.08	0.1803	0.3240	0.10	0.2639	0.4180	0.07	0.0066	0.0589	-0.33	0.0303	0.1119	-0.16	0.3343	0.6038	0.17	0.3969	0.7811	-0.10	0.0342	0.1580	-0.26
144	40S ribosomal protein S27-like	Q71UM5	0.0044	0.0421	0.24	0.5698	0.8832	0.08	0.0604	0.1422	0.13	0.5596	0.6100	0.01	0.2145	0.4191	-0.03	0.0588	0.1672	0.10	0.3736	0.5559	-2.55	0.8708	1.1348	-0.33	0.0651	0.1683	2.06
145	40S ribosomal protein S28	P62857	0.0057	0.0457	0.27	0.3595	0.7083	0.20	0.0052	0.0352	0.40	0.0018	0.0187	0.38	0.2482	0.4650	0.06	0.0045	0.0596	0.35	0.0496	0.1778	0.34	0.2276	0.5401	-0.12	0.3327	0.4038	-0.07
146	40S ribosomal protein S29	P62273	0.2670	0.4587	0.10	0.6989	0.7782	-0.03	0.0689	0.1553	0.19	0.0753	0.1564	0.18	0.5173	0.6054	-0.05	0.4014	0.6163	0.06	0.4828	0.5374	-0.08	0.0080	0.0733	-0.41	0.0305	0.1477	-0.34
147	40S ribosomal protein S3	P23396	0.0229	0.0906	0.20	0.3533	0.7050	0.18	0.0055	0.0358	0.33	0.0056	0.0300	0.30	0.3423	0.4395	-0.05	0.0135	0.0772	0.28	0.0915	0.2432	0.29	0.7924	1.2356	-0.03	0.1075	0.1639	-0.17
148	40S ribosomal protein S30	P62861	0.3752	0.5906	-0.06	0.2323	0.6626	-0.14	0.0457	0.1177	-0.11	0.0042	0.0263	-0.41	0.0205	0.1029	-0.38	0.3042	0.5023	-0.04	0.0003	0.0379	-0.81	0.0014	0.0307	-0.54	0.0074	0.0787	-0.51
149	40S ribosomal protein S3a	P61247	0.0352	0.1172	0.21	0.3069	0.6779	0.14	0.0050	0.0344	0.37	0.0065	0.0324	0.32	0.5946	0.6710	0.03	0.0239	0.0988	0.30	0.1278	0.3013	0.30	0.0959	0.3091	-0.23	0.1099	0.1669	-0.20
150	40S ribosomal protein S4 X isoform	P62701	0.0137	0.0686	0.17	0.3422	0.7001	0.18	0.0179	0.0638	0.26	0.0027	0.0217	0.26	0.3233	0.4205	-0.04	0.0171	0.0852	0.19	0.0531	0.1824	0.36	0.1557	0.4267	-0.11	0.0594	0.2179	-0.16
151	40S ribosomal protein S5	P46782	0.0002	0.0173	0.26	0.1861	0.6676	0.17	0.0003	0.0202	0.45	0.0005	0.0136	0.29	0.2769	0.5019	-0.06	0.0235	0.0980	0.34	0.1261	0.2989	0.31	0.2997	0.6431	-0.10	0.6725	0.7259	-0.03

Supplemetary Table S2. Overview on all relatively quantified 5180 proteins statistical analysis

			MCF-7																		MDA-MB-231								
			Dai SC20 vs control			Gen SC20 vs control			SSE SC20 vs control			Dai IC20 vs control			Gen IC20 vs control			SSE IC20 vs control			Dai IC20 vs control			Gen IC20 vs control			SSE IC20 vs control		
	**Protein name**	**UniProt**	**p value**	**BH q value**	**log2FC**	**p value**	**BH q value**	**log2FC**	**p value**	**BH q value**	**log2FC**	**p value**	**BH q value**	**log2FC**	**p value**	**BH q value**	**log2FC**	**p value**	**BH q value**	**log2FC**	**p value**	**BH q value**	**log2FC**	**p value**	**BH q value**	**log2FC**	**p value**	**BH q value**	**log2FC**
152	40S ribosomal protein S6	P62753	0.0521	0.1479	0.11	0.3417	0.6999	0.14	0.0025	0.0273	0.29	0.0055	0.0299	0.26	0.1695	0.3569	-0.07	0.0177	0.0870	0.24	0.1074	0.2676	0.18	0.0808	0.2792	-0.20	0.9819	0.9857	0.00
153	40S ribosomal protein S7	P62081	0.0047	0.0436	0.26	0.2285	0.6598	0.25	0.0023	0.0266	0.38	0.0042	0.0265	0.26	0.5390	0.6235	-0.02	0.0034	0.0583	0.45	0.0261	0.1408	0.77	0.0033	0.0471	0.51	0.1067	0.1630	0.14
154	40S ribosomal protein S8	P62241	0.0109	0.0602	0.23	0.2954	0.6714	0.20	0.0055	0.0359	0.36	0.0067	0.0330	0.28	0.6230	0.6949	-0.02	0.0158	0.0835	0.27	0.0715	0.2116	0.34	0.3985	0.7837	-0.09	0.3868	0.4579	-0.08
155	40S ribosomal protein S9	P46781	0.0307	0.1081	0.25	0.4552	0.7878	0.15	0.0163	0.0604	0.30	0.0156	0.0516	0.29	0.3335	0.4305	-0.07	0.0317	0.1146	0.28	0.0804	0.2256	0.38	0.4371	0.8380	-0.08	0.1586	0.2224	-0.14
156	40S ribosomal protein SA	P08865	0.0041	0.0408	0.19	0.5178	0.8395	0.09	0.0105	0.0477	0.26	0.0155	0.0515	0.19	0.0424	0.1520	-0.09	0.0083	0.0669	0.22	0.2293	0.4563	0.15	0.1810	0.4681	0.14	0.8034	0.8392	-0.02
157	45 kDa calcium-binding protein	Q9BRK5	0.0008	0.0218	-0.28	0.5098	0.8320	0.11	0.3587	0.5555	-0.05	0.0749	0.1557	-0.10	0.4959	0.5851	0.04	0.0357	0.1219	-0.11	0.0790	0.2234	-0.15	0.6622	1.1015	-0.04	0.0443	0.1837	-0.23
158	4-aminobutyrate aminotransferase mitochondrial	P80404	0.0070	0.0499	0.17	0.1079	0.6308	0.16	0.0055	0.0361	0.25	0.0028	0.0219	0.20	0.0040	0.0470	0.26	0.0196	0.0903	0.20	0.6210	0.6675	0.08	0.3899	0.7718	0.14	0.2019	0.2701	-0.23
159	4F2 cell-surface antigen heavy chain	P08195	0.0003	0.0173	0.69	0.1600	0.6641	0.35	0.0002	0.0182	0.90	0.0001	0.0133	0.89	0.0020	0.0360	0.53	0.0010	0.0467	1.06	0.0061	0.0834	0.62	0.0334	0.1651	0.28	0.0071	0.0776	0.30
160	4-trimethylaminobutyraldehyde dehydrogenase	P49189	0.0000	0.0000	0.28	0.0145	0.4498	0.15	0.0107	0.0483	0.35	0.0079	0.0360	0.24	0.0754	0.2111	0.08	0.1150	0.2579	0.20	0.0593	0.1933	0.13	0.7174	1.1624	0.02	0.9089	0.9275	0.00
161	5'-3' exoribonuclease 1	Q8IZH2	0.2544	0.4421	-0.06	0.7656	0.8304	0.05	0.3238	0.5115	0.05	0.8441	0.8665	-0.01	0.0587	0.1820	-0.14	0.4373	0.6591	-0.08	0.3736	0.4657	-2.55	0.8708	0.9701	-0.33	0.0651	0.1259	2.06
162	5'-3' exoribonuclease 2	Q9H0D6	0.0946	0.2168	-0.10	0.6066	0.7088	0.09	0.0019	0.0240	0.39	0.3635	0.5436	0.04	0.6220	0.6942	-0.02	0.0476	0.1451	0.22	0.9237	0.9369	-0.01	0.1431	0.4037	-0.12	0.0509	0.1981	-0.14
163	5'-AMP-activated protein kinase catalytic subunit alpha-1	Q13131	0.4108	0.6303	-3.31	0.5752	0.8820	-1.75	0.5661	0.7870	-1.19	0.5388	0.7437	0.89	0.2938	0.5223	-1.88	0.9510	1.1830	0.09	0.2525	0.4891	0.32	0.1674	0.4467	0.42	0.2150	0.2838	0.36
164	5'-AMP-activated protein kinase subunit beta-1	Q9Y478	0.0637	0.1672	-0.13	0.3574	0.7088	0.13	0.0064	0.0385	0.30	0.1132	0.2135	0.15	0.9283	0.9462	0.00	0.0158	0.0834	-0.24	0.3736	0.6010	-2.55	0.8708	1.2145	-0.33	0.0651	0.1935	2.06
165	5'-AMP-activated protein kinase subunit beta-2	O43741	0.4108	0.6301	-3.31	0.5752	0.8818	-1.75	0.5661	0.7868	-1.19	0.5388	0.7435	0.89	0.2938	0.5221	-1.88	0.9510	1.1828	0.09	0.0043	0.0703	0.44	0.0184	0.1160	0.25	0.0037	0.0593	0.48
166	5'-AMP-activated protein kinase subunit gamma-1	P54619	0.4108	0.6299	-3.31	0.5752	0.8815	-1.75	0.5661	0.7866	-1.19	0.5388	0.7433	0.89	0.2938	0.5219	-1.88	0.9510	1.1825	0.09	0.3776	0.4333	0.18	0.4259	0.8207	-0.16	0.0832	0.1348	-0.47
167	5-methylcytosine rRNA methyltransferase NSUN4	Q96CB9	0.1760	0.3382	0.07	0.5401	0.8556	0.07	0.8106	0.8466	0.02	0.1533	0.2698	-0.07	0.0593	0.1835	-0.15	0.4626	0.6852	0.04	0.3736	0.4628	-2.55	0.8708	0.9647	-0.33	0.0651	0.1247	2.06
168	5'-nucleotidase domain-containing protein 1	Q5TFE4	0.1086	0.2381	-0.15	0.5854	0.6915	-0.05	0.0157	0.0590	0.18	0.4737	0.6736	0.03	0.1499	0.3272	0.07	0.0280	0.1070	0.26	0.0314	0.1496	0.25	0.1939	0.4888	0.11	0.0298	0.1472	0.31
169	5'-nucleotidase domain-containing protein 3	Q86UY8	0.1578	0.3120	-0.25	0.7666	0.8308	-0.05	0.1233	0.2416	-0.27	0.0323	0.0846	-0.47	0.2127	0.4164	0.20	0.0964	0.2291	-0.41	0.3736	0.5356	-2.55	0.8708	1.0983	-0.33	0.0651	0.1579	2.06
170	60 kDa heat shock protein mitochondrial	P10809	0.0001	0.0167	0.38	0.1033	0.6340	0.33	0.0001	0.0179	0.57	0.0001	0.0148	0.47	0.0154	0.0884	0.13	0.0002	0.0334	0.51	0.0102	0.1018	0.49	0.2800	0.6175	0.10	0.1414	0.2028	0.13
171	60 kDa SS-A/Ro ribonucleoprotein	P10155	0.0398	0.1259	0.26	0.2729	0.6652	0.09	0.8840	0.9078	0.01	0.4077	0.5989	0.07	0.5669	0.6480	-0.04	0.1170	0.2607	0.17	0.0239	0.1369	0.28	0.0787	0.2753	0.16	0.2818	0.3535	0.06
172	60S acidic ribosomal protein P0	P05388	0.0003	0.0171	0.39	0.2470	0.6588	0.21	0.0034	0.0310	0.33	0.0144	0.0489	0.27	0.0709	0.2030	-0.07	0.0018	0.0524	0.28	0.1289	0.3030	-0.22	0.0011	0.0275	-0.61	0.0005	0.0301	-0.73
173	60S acidic ribosomal protein P0-like	Q8NHW5	0.1295	0.2706	-0.26	0.3547	0.7064	-0.21	0.3085	0.4926	-0.14	0.4748	0.6749	0.09	0.8465	0.8819	0.02	0.7016	0.9435	0.05	0.3736	0.4455	-2.55	0.8708	0.9324	-0.33	0.0651	0.1177	2.06
174	60S acidic ribosomal protein P1	P05386	0.3330	0.5399	-0.31	0.3843	0.7303	0.06	0.0074	0.0406	-0.19	0.0052	0.0291	-0.21	0.0007	0.0208	-0.32	0.7040	0.9462	-0.19	0.0254	0.1397	-1.31	0.7948	1.2375	0.08	0.1310	0.1911	-0.68
175	60S acidic ribosomal protein P2	P05387	0.0153	0.0717	0.28	0.2581	0.6628	0.20	0.0037	0.0316	0.39	0.0188	0.0580	0.27	0.9843	0.9883	0.00	0.0144	0.0798	0.33	0.0207	0.1332	0.20	0.2467	0.5682	-0.10	0.2544	0.3259	-0.05
176	60S ribosomal export protein NMD3	Q96D46	0.0671	0.1718	-0.07	0.5574	0.8713	-0.09	0.0005	0.0184	-0.22	0.2577	0.4092	-0.07	0.0043	0.0485	-0.12	0.4071	0.6235	-0.11	0.0182	0.1252	-0.34	0.0372	0.1760	-0.21	0.0047	0.0669	-0.49
177	60S ribosomal protein L10	P27635	0.0328	0.1130	0.12	0.0225	0.4757	0.15	0.0009	0.0221	0.34	0.0042	0.0266	0.27	0.2779	0.5032	0.07	0.0074	0.0648	0.23	0.0043	0.0707	0.50	0.2566	0.5825	-0.06	0.0139	0.1020	0.26
178	60S ribosomal protein L10a	P62906	0.0460	0.1371	0.19	0.6471	0.7390	0.09	0.0371	0.1010	0.20	0.1036	0.1991	0.14	0.0820	0.2211	-0.16	0.0915	0.2211	0.16	0.1746	0.3734	0.20	0.1991	0.4963	-0.14	0.0724	0.1213	-0.20
179	60S ribosomal protein L10-like	Q96L21	0.1820	0.3457	-0.57	0.1404	0.6528	-0.52	0.1255	0.2449	-0.18	0.1192	0.2220	-0.48	0.0040	0.0468	-0.46	0.7484	0.9864	0.03	0.3933	0.4491	0.15	0.5862	1.0186	0.09	0.8580	0.8848	0.03
180	60S ribosomal protein L11	P62913	0.0920	0.2130	0.19	0.2570	0.6640	0.19	0.0047	0.0334	0.39	0.0318	0.0837	0.21	0.3236	0.4207	-0.07	0.0158	0.0837	0.33	0.0792	0.2231	0.35	0.7912	1.2345	-0.03	0.4372	0.5078	-0.10
181	60S ribosomal protein L12	P30050	0.0011	0.0234	0.36	0.0194	0.4652	-0.15	0.8532	0.8829	0.01	0.0181	0.0567	-0.13	0.0073	0.0623	-0.22	0.7255	0.9658	0.01	0.0217	0.1346	-0.37	0.0012	0.0288	-0.57	0.0036	0.0596	-0.79
182	60S ribosomal protein L13	P26373	0.2089	0.3817	0.10	0.3808	0.7273	0.17	0.0208	0.0697	0.29	0.0629	0.1367	0.19	0.4511	0.5443	-0.06	0.0980	0.2307	0.16	0.0512	0.1793	0.48	0.7631	1.2051	-0.04	0.6564	0.7118	-0.04
183	60S ribosomal protein L13a	P40429	0.0652	0.1690	0.13	0.3986	0.7427	0.19	0.0196	0.0674	0.31	0.0112	0.0426	0.28	0.6873	0.7503	-0.02	0.0357	0.1218	0.15	0.0505	0.1788	0.39	0.8771	0.9069	0.01	0.5829	0.6449	0.04
184	60S ribosomal protein L14	P50914	0.0086	0.0551	0.25	0.4098	0.7506	0.15	0.0078	0.0415	0.25	0.0083	0.0370	0.25	0.6237	0.6954	-0.02	0.0291	0.1092	0.22	0.1660	0.3604	0.26	0.2101	0.5126	-0.14	0.0585	0.2160	-0.25
185	60S ribosomal protein L15	P61313	0.0787	0.1922	0.14	0.3164	0.6803	0.21	0.0096	0.0456	0.29	0.0186	0.0577	0.23	0.9903	0.9932	0.00	0.0585	0.1668	0.16	0.1193	0.2886	0.32	0.1530	0.4229	-0.15	0.0977	0.1529	-0.13
186	60S ribosomal protein L17	P18621	0.0350	0.1170	0.14	0.3626	0.7107	0.18	0.0062	0.0381	0.30	0.0040	0.0258	0.27	0.1111	0.2717	-0.08	0.0391	0.1288	0.16	0.0394	0.1639	0.45	0.6376	1.0755	-0.04	0.4686	0.5370	-0.04
187	60S ribosomal protein L18	Q07020	0.0371	0.1212	0.21	0.5461	0.8614	0.11	0.0202	0.0683	0.26	0.0948	0.1863	0.14	0.1765	0.3670	-0.11	0.0980	0.2309	0.15	0.3569	0.6340	0.12	0.0092	0.0784	-0.43	0.0217	0.1257	-0.34
188	60S ribosomal protein L18a	Q02543	0.0148	0.0708	0.33	0.1890	0.6696	0.26	0.0122	0.0518	0.30	0.0447	0.1061	0.19	0.9006	0.9241	-0.01	0.0165	0.0849	0.36	0.2471	0.4825	0.19	0.1349	0.3874	-0.23	0.0737	0.1231	-0.29
189	60S ribosomal protein L19	P84098	0.0546	0.1529	0.15	0.2164	0.6602	0.26	0.0063	0.0384	0.30	0.0136	0.0472	0.24	0.3172	0.4145	-0.06	0.0540	0.1575	0.19	0.0547	0.1858	0.46	0.3385	0.6966	-0.09	0.7313	0.7785	-0.02
190	60S ribosomal protein L21	P46778	0.5610	0.6298	0.03	0.4056	0.7485	0.13	0.0398	0.1062	0.16	0.1584	0.2765	0.06	0.0613	0.1868	-0.09	0.0606	0.1707	0.09	0.9881	0.9910	0.00	0.5575	0.9833	-0.04	0.0669	0.1137	-0.07
191	60S ribosomal protein L22	P35268	0.0267	0.0990	0.18	0.2687	0.6634	0.20	0.0005	0.0188	0.30	0.0053	0.0295	0.23	0.1830	0.3765	-0.04	0.0061	0.0618	0.23	0.5407	0.5923	0.06	0.0009	0.0255	-0.18	0.2817	0.3535	-0.13
192	60S ribosomal protein L22-like 1	Q6P5R6	0.0071	0.0498	0.22	0.3304	0.6893	0.14	0.0109	0.0483	0.40	0.0009	0.0149	0.46	0.0054	0.0547	-0.26	0.1689	0.3339	-0.24	0.3736	0.5097	-2.55	0.8708	1.0512	-0.33	0.0651	0.1454	2.06
193	60S ribosomal protein L23	P62829	0.0325	0.1123	0.22	0.2645	0.6641	0.19	0.0107	0.0482	0.30	0.0363	0.0915	0.19	0.2205	0.4267	-0.08	0.0850	0.2119	0.15	0.0927	0.2445	0.21	0.5674	0.9953	-0.05	0.0400	0.1730	-0.23
194	60S ribosomal protein L23a	P62750	0.0163	0.0745	0.31	0.2469	0.6599	0.24	0.0054	0.0358	0.39	0.0127	0.0457	0.30	0.8556	0.8891	0.01	0.0182	0.0871	0.30	0.1237	0.2961	0.27	0.0415	0.1874	-0.28	0.0504	0.1966	-0.27
195	60S ribosomal protein L24	P83731	0.0130	0.0667	0.16	0.6864	0.7708	-0.07	0.4464	0.6595	-0.02	0.0039	0.0252	-0.16	0.0013	0.0277	-0.34	0.1867	0.3589	-0.06	0.3812	0.4372	-0.09	0.0131	0.0954	-0.27	0.0048	0.0670	-0.47
196	60S ribosomal protein L26	P61254	0.0217	0.0878	0.22	0.3217	0.6838	0.22	0.0075	0.0410	0.31	0.0033	0.0234	0.39	0.5792	0.6578	0.03	0.0269	0.1049	0.27	0.0911	0.2429	0.70	0.0756	0.2679	0.21	0.2294	0.2998	0.26
197	60S ribosomal protein L27	P61353	0.0226	0.0896	0.15	0.4695	0.7992	0.12	0.0144	0.0564	0.28	0.0391	0.0970	0.13	0.0806	0.2188	-0.10	0.0183	0.0874	0.19	0.0242	0.1376	0.34	0.4785	0.8900	0.04	0.2913	0.3632	0.07
198	60S ribosomal protein L27a	P46776	0.0353	0.1174	0.21	0.4714	0.8006	0.12	0.0273	0.0829	0.24	0.0396	0.0978	0.20	0.2017	0.4023	-0.09	0.1072	0.2452	0.14	0.0729	0.2136	0.28	0.2785	0.6160	-0.13	0.0941	0.1484	-0.22
199	60S ribosomal protein L28	P46779	0.0256	0.0969	0.26	0.2107	0.6623	0.31	0.0045	0.0331	0.43	0.0063	0.0318	0.35	0.9529	0.9633	0.00	0.0166	0.0846	0.30	0.0570	0.1898	0.44	0.3953	0.7795	-0.11	0.3260	0.3974	-0.11
200	60S ribosomal protein L29	P47914	0.5218	0.5935	-0.03	0.6177	0.7171	0.09	0.0120	0.0512	0.32	0.3208	0.4919	0.07	0.1159	0.2787	-0.11	0.3429	0.5472	0.05	0.1147	0.2807	0.36	0.1118	0.3415	-0.22	0.8504	0.8791	0.01
201	60S ribosomal protein L3	P39023	0.0071	0.0498	0.21	0.3257	0.6855	0.22	0.0006	0.0190	0.43	0.0025	0.0215	0.32	0.3427	0.4397	-0.04	0.0129	0.0764	0.23	0.0627	0.1972	0.32	0.1110	0.3400	-0.19	0.2223	0.2922	-0.11
202	60S ribosomal protein L30	P62888	0.0047	0.0435	0.42	0.1467	0.6562	0.25	0.0097	0.0460	0.31	0.0445	0.1058	0.19	0.1686	0.3552	-0.11	0.0634	0.1756	0.18	0.0143	0.1117	-0.41	0.0010	0.0271	-0.53	0.0161	0.1090	-0.59
203	60S ribosomal protein L31	P62899	0.0119	0.0630	0.19	0.2428	0.6595	0.26	0.0107	0.0483	0.26	0.0025	0.0211	0.30	0.7750	0.8238	0.01	0.0278	0.1067	0.18	0.0691	0.2071	0.44	0.1202	0.3589	-0.17	0.3091	0.3802	-0.07
204	60S ribosomal protein L32	P62910	0.9371	0.9524	0.00	0.8314	0.8785	0.02	0.0005	0.0182	0.17	0.1016	0.1964	0.06	0.0015	0.0302	-0.18	0.2064	0.3853	0.04	0.8769	0.8977	0.02	0.0524	0.2170	-0.28	0.2938	0.3653	-0.11
205	60S ribosomal protein L34	P49207	0.0089	0.0562	-1.20	0.2124	0.6628	-0.63	0.3404	0.5329	0.27	0.0541	0.1218	-0.62	0.5764	0.6553	0.12	0.3806	0.5897	-0.30	0.0381	0.1618	0.42	0.5275	0.9507	-0.09	0.2979	0.3693	-0.10
206	60S ribosomal protein L35	P42766	0.3806	0.5965	-0.05	0.8110	0.8642	0.02	0.0219	0.0721	0.17	0.0065	0.0323	0.19	0.0119	0.0785	-0.17	0.1106	0.2509	0.10	0.1387	0.3189	0.24	0.6518	1.0916	-0.05	0.0802	0.1310	0.26
207	60S ribosomal protein L35a	P18077	0.0435	0.1324	0.23	0.2613	0.6645	0.22	0.0132	0.0537	0.32	0.0284	0.0772	0.26	0.3180	0.4150	-0.07	0.0692	0.1849	0.20	0.0635	0.1986	0.40	0.4448	0.8465	-0.07	0.7704	0.8109	0.03
208	60S ribosomal protein L36	Q9Y3U8	0.0004	0.0180	0.46	0.6559	0.7464	0.07	0.0820	0.1760	0.22	0.0442	0.1054	0.11	0.0088	0.0669	-0.16	0.0675	0.1822	0.13	0.0868	0.2361	0.16	0.6132	1.0466	0.02	0.0964	0.1514	0.36
209	60S ribosomal protein L36a-like	Q969Q0	0.0103	0.0592	0.21	0.2782	0.6650	0.28	0.0020	0.0249	0.42	0.0024	0.0211	0.32	0.5886	0.6659	0.02	0.0081	0.0664	0.30	0.2494	0.4853	0.20	0.0043	0.0523	-0.38	0.0103	0.0898	-0.32
210	60S ribosomal protein L37	P61927	0.4288	0.5063	0.08	0.2502	0.6602	0.16	0.0017	0.0234	0.39	0.3654	0.5463	0.06	0.3251	0.4224	-0.05	0.1906	0.3642	0.12	0.4291	0.4851	0.15	0.0429	0.1911	-0.27	0.9610	0.9690	0.01
211	60S ribosomal protein L37a	P61513	0.0454	0.1361	0.20	0.2911	0.6693	0.12	0.0651	0.1490	0.21	0.9131	0.9243	0.00	0.0232	0.1103	-0.14	0.0448	0.1399	0.09	0.0392	0.1642	-0.51	0.0003	0.0177	-1.04	0.0015	0.0480	-1.00
212	60S ribosomal protein L38	P63173	0.0206	0.0848	0.15	0.1269	0.6426	-0.18	0.3057	0.4889	0.04	0.0064	0.0319	-0.31	0.0017	0.0325	-0.46	0.0045	0.0605	-0.26	0.0458	0.1717	-0.26	0.0964	0.3098	-0.23	0.0439	0.1834	-0.25
213	60S ribosomal protein L39	P62891	0.0030	0.0371	-0.17	0.0423	0.5677	-0.40	0.4255	0.6352	-0.03	0.0047	0.0275	-0.43	0.0235	0.1109	-0.51	0.4061	0.6226	-0.07	0.3736	0.4689	-2.55	0.8708	0.9761	-0.33	0.0651	0.1273	2.06
214	60S ribosomal protein L3-like	Q92901	0.0041	0.0407	-0.24	0.1272	0.6428	0.33	0.0070	0.0401	0.19	0.0644	0.1392	0.24	0.0091	0.0674	-0.26	0.0171	0.0857	-0.19	0.3736	0.5986	-2.55	0.8708	1.2103	-0.33	0.0651	0.1920	2.06
215	60S ribosomal protein L4	P36578	0.1110	0.2421	0.12	0.5645	0.8781	0.09	0.0188	0.0655	0.26	0.1145	0.2152	0.12	0.0686	0.1992	-0.17	0.1006	0.2348	0.14	0.0791	0.2232	0.33	0.3998	0.7848	-0.08	0.3674	0.4387	-0.06
216	60S ribosomal protein L5	P46777	0.0601	0.1621	0.18	0.2204	0.6595	0.21	0.0056	0.0363	0.39	0.0097	0.0396	0.33	0.4465	0.5401	0.05	0.0344	0.1195	0.25	0.0862	0.2349	0.34	0.9555	0.9682	0.01	0.8157	0.8509	-0.02
217	60S ribosomal protein L6	Q02878	0.0315	0.1100	0.18	0.4753	0.8025	0.18	0.0039	0.0315	0.34	0.0118	0.0436	0.25	0.1807	0.3729	-0.08	0.0370	0.1245	0.21	0.0888	0.2398	0.41	0.1215	0.3611	-0.20	0.0682	0.1156	-0.20
218	60S ribosomal protein L7	P18124	0.0463	0.1376	0.21	0.2919	0.6693	0.25	0.0072	0.0401	0.36	0.0109	0.0420	0.31	0.6672	0.7318	-0.03	0.0297	0.1105	0.28	0.0611	0.1950	0.43	0.4725	0.8842	0.07	0.4755	0.5443	0.05
219	60S ribosomal protein L7a	P62424	0.0299	0.1061	0.17	0.3696	0.7179	0.21	0.0055	0.0358	0.34	0.0098	0.0398	0.25	0.1224	0.2882	-0.10	0.0376	0.1255	0.18	0.0727	0.2135	0.33	0.2880	0.6271	-0.10	0.1476	0.2099	-0.10
220	60S ribosomal protein L8	P62917	0.2593	0.4482	0.09	0.2957	0.6715	0.25	0.0354	0.0981	0.29	0.0112	0.0426	0.30	0.3759	0.4725	-0.06	0.0949	0.2272	0.16	0.0437	0.1684	0.51	0.4744	0.8859	0.09	0.4287	0.5000	0.08
221	60S ribosomal protein L9	P32969	0.0009	0.0225	0.20	0.4963	0.8187	0.05	0.5494	0.7723	0.02	0.1414	0.2531	-0.03	0.0093	0.0686	-0.22	0.3499	0.5551	0.07	0.1564	0.3476	-0.15	0.0042	0.0518	-0.45	0.0044	0.0653	-0.56
222	6-phosphofructo-2-kinase/fructose-2_6-bisphosphatase 1	P16118	0.4108	0.6298	-3.31	0.5752	0.8813	-1.75	0.5661	0.7864	-1.19	0.5388	0.7431	0.89	0.2938	0.5217	-1.88	0.9510	1.1822	0.09	0.6198	0.6668	-0.05	0.0343	0.1678	-0.30	0.6533	0.7093	-0.04
223	6-phosphofructo-2-kinase/fructose-2_6-bisphosphatase 2	O60825	0.0166	0.0754	0.69	0.0604	0.5926	0.38	0.0649	0.1488	0.37	0.2419	0.3887	0.28	0.0430	0.1530	0.47	0.1567	0.3161	0.36	0.3677	0.6492	-0.10	0.2233	0.5325	-0.06	0.0017	0.0484	-0.35
224	6-phosphofructo-2-kinase/fructose-2_6-bisphosphatase 3	Q16875	0.3850	0.6018	0.04	0.7571	0.8239	0.04	0.5805	0.6377	0.02	0.0836	0.1695	0.15	0.0497	0.1642	-0.08	0.0617	0.1728	-0.08	0.8769	0.8979	-0.10	0.8958	0.9220	0.11	0.1864	0.2538	0.98
225	6-phosphofructo-2-kinase/fructose-2_6-bisphosphatase 4	Q16877	0.1038	0.2305	0.45	0.8339	0.8805	0.11	0.0524	0.1294	0.30	0.0100	0.0402	0.69	0.0004	0.0164	0.92	0.2063	0.3852	0.75	0.1085	0.2694	0.21	0.1296	0.3769	0.12	0.3998	0.4714	0.06
226	6-phosphogluconate dehydrogenase decarboxylating	P52209	0.0056	0.0455	0.55	0.2096	0.6616	0.28	0.0059	0.0373	0.35	0.0009	0.0151	0.63	0.0453	0.1569	0.22	0.0065	0.0626	0.60	0.3798	0.4356	0.11	0.2363	0.5521	-0.11	0.0876	0.1404	-0.18
227	6-phosphogluconolactonase	O95336	0.0051	0.0440	0.05	0.6929	0.7740	0.02	0.0896	0.1877	-0.12	0.1947	0.3249	0.08	0.0345	0.1353	-0.04	0.0097	0.0703	0.13	0.1449	0.3282	-0.35	0.2598	0.5866	-0.25	0.4460	0.5158	-0.14
228	7 8-dihydro-8-oxoguanine triphosphatase	P36639	0.8853	0.9099	0.00	0.4553	0.7877	0.10	0.0167	0.0612	0.08	0.0196	0.0597	0.16	0.0272	0.1194	-0.07	0.1661	0.3295	0.13	0.2613	0.5015	0.06	0.0068	0.0671	0.21	0.7888	0.8273	0.01
229	7-dehydrocholesterol reductase	Q9UBM7	0.0283	0.1023	0.16	0.5689	0.8828	0.09	0.0624	0.1453	0.15	0.0143	0.0487	0.19	0.8423	0.8788	-0.01	0.0207	0.0929	0.24	0.2691	0.5117	0.10	0.0223	0.1301	-0.31	0.0621	0.2228	-0.29
230	7SK snRNA methylphosphate capping enzyme	Q7L2J0	0.1009	0.2265	0.09	0.4399	0.7759	0.07	0.0100	0.0466	0.23	0.1132	0.2135	0.08	0.8338	0.8711	0.01	0.0305	0.1118	0.18	0.3736	0.5810	-2.55	0.8708	1.1793	-0.33	0.0651	0.1819	2.06

Supplemetary Table S2. Overview on all relatively quantified 5180 proteins statistical analysis

			MCF-7																		MDA-MB-231								
			Dai SC20 vs control			Gen SC20 vs control			SSE SC20 vs control			Dai IC20 vs control			Gen IC20 vs control			SSE IC20 vs control			Dai IC20 vs control			Gen IC20 vs control			SSE IC20 vs control		
	**Protein name**	**UniProt**	**p value**	**BH q value**	**log2FC**	**p value**	**BH q value**	**log2FC**	**p value**	**BH q value**	**log2FC**	**p value**	**BH q value**	**log2FC**	**p value**	**BH q value**	**log2FC**	**p value**	**BH q value**	**log2FC**	**p value**	**BH q value**	**log2FC**	**p value**	**BH q value**	**log2FC**	**p value**	**BH q value**	**log2FC**
231	A disintegrin and metalloproteinase with thrombospondin motifs 12	P58397	0.4108	0.6296	-3.31	0.5752	0.8810	-1.75	0.5661	0.7862	-1.19	0.5388	0.7429	0.89	0.2938	0.5216	-1.88	0.9510	1.1819	0.09	0.3121	0.5729	-0.42	0.9218	0.9427	0.04	0.5918	0.6529	-0.20
232	A disintegrin and metalloproteinase with thrombospondin motifs 17	Q8TE56	0.0260	0.0976	-0.11	0.5746	0.8861	-0.06	0.0431	0.1128	-0.12	0.1009	0.1953	-0.08	0.1731	0.3618	-0.10	0.0041	0.0598	-0.24	0.3736	0.6341	-2.55	0.8708	1.2721	-0.33	0.0651	0.2141	2.06
233	A disintegrin and metalloproteinase with thrombospondin motifs 19	Q8TE59	0.0039	0.0398	0.41	0.1872	0.6665	0.26	0.0031	0.0302	0.31	0.0085	0.0374	0.24	0.3192	0.4163	0.05	0.0130	0.0766	0.30	0.0020	0.0540	-0.60	0.1388	0.3953	0.14	0.2324	0.3025	-0.23
234	ABI gene family member 3	Q9P2A4	0.6937	0.7483	-0.07	0.3086	0.6782	-0.36	0.9219	0.9388	0.02	0.1863	0.3137	-0.27	0.9097	0.9320	-0.02	0.6177	0.8537	-0.11	0.3736	0.4507	-2.55	0.8708	0.9421	-0.33	0.0651	0.1198	2.06
235	Abl interactor 1	Q8IZP0	0.7642	0.8080	0.03	0.7133	0.7900	0.06	0.5727	0.6304	0.09	0.9511	0.9578	0.01	0.4525	0.5457	-0.08	0.3201	0.5227	-0.12	0.1645	0.3586	0.14	0.1052	0.3287	0.14	0.3012	0.3725	-0.04
236	Abl interactor 2	Q9NYB9	0.4108	0.6294	-3.31	0.5752	0.8807	-1.75	0.5661	0.7860	-1.19	0.5388	0.7427	0.89	0.2938	0.5214	-1.88	0.9510	1.1816	0.09	0.0111	0.1030	-0.50	0.9619	0.9728	0.01	0.3736	0.4455	0.12
237	Abnormal spindle-like microcephaly-associated protein	Q8IZT6	0.0310	0.1088	0.17	0.0490	0.5743	0.35	0.0055	0.0362	0.35	0.0030	0.0225	0.36	0.0498	0.1644	0.14	0.0213	0.0943	0.31	0.4031	0.4590	0.06	0.0226	0.1312	-0.16	0.1427	0.2041	-0.09
238	Acetoacetyl-CoA synthetase	Q86V21	0.4011	0.6209	-0.04	0.4489	0.7837	0.14	0.1321	0.2540	0.12	0.0084	0.0370	0.23	0.0610	0.1862	0.10	0.0540	0.1576	0.14	0.3736	0.5584	-2.55	0.8708	1.1391	-0.33	0.0651	0.1695	2.06
239	Acetolactate synthase-like protein	A1L0T0	0.1073	0.2359	-0.22	0.9365	0.9570	0.01	0.0273	0.0828	-0.36	0.0026	0.0214	-0.43	0.0485	0.1619	-0.35	0.1884	0.3612	-0.37	0.3736	0.5050	-2.55	0.8708	1.0427	-0.33	0.0651	0.1432	2.06
240	Acetyl-CoA acetyltransferase cytosolic	Q9BWD1	0.3851	0.6016	-0.17	0.4866	0.8100	-0.26	0.0265	0.0815	-0.55	0.0053	0.0292	-0.72	0.0976	0.2490	-0.25	0.1655	0.3291	-0.50	0.1172	0.2850	-0.20	0.0496	0.2096	0.30	0.3361	0.4070	0.10
241	Acetyl-CoA acetyltransferase mitochondrial	P24752	0.4465	0.5239	0.02	0.9156	0.9416	-0.01	0.0003	0.0192	0.27	0.0138	0.0478	0.27	0.0137	0.0839	0.12	0.0003	0.0370	0.32	0.0912	0.2430	0.29	0.6099	1.0420	0.06	0.1024	0.1583	-0.23
242	Acetyl-CoA carboxylase 1	Q13085	0.0253	0.0962	0.07	0.0856	0.6210	0.21	0.0059	0.0374	0.36	0.0005	0.0125	0.38	0.0446	0.1560	0.18	0.0207	0.0932	0.32	0.0228	0.1367	0.13	0.1741	0.4578	-0.04	0.0046	0.0664	0.22
243	Acetyl-CoA carboxylase 2	O00763	0.2165	0.3916	-0.19	0.2014	0.6603	-0.27	0.9372	0.9517	-0.01	0.1653	0.2854	-0.07	0.2397	0.4530	-0.08	0.2388	0.4246	-0.19	0.3736	0.4928	-2.55	0.8708	1.0203	-0.33	0.0651	0.1376	2.06
244	Acidic fibroblast growth factor intracellular-binding protein	O43427	0.7436	0.7917	-0.01	0.8279	0.8761	0.02	0.8382	0.8694	0.01	0.0348	0.0890	0.20	0.0319	0.1305	0.13	0.0158	0.0838	0.25	0.8067	0.8367	0.03	0.0161	0.1075	-0.49	0.7582	0.8005	-0.04
245	Acidic leucine-rich nuclear phosphoprotein 32 family member A	P39687	0.0038	0.0396	0.33	0.1105	0.6332	0.30	0.0038	0.0319	0.34	0.0105	0.0412	0.29	0.5640	0.6459	-0.03	0.0203	0.0920	0.25	0.8473	0.8720	-0.03	0.0288	0.1527	-0.34	0.0636	0.2261	-0.27
246	Acidic leucine-rich nuclear phosphoprotein 32 family member B	Q92688	0.0990	0.2237	0.24	0.6598	0.7490	-0.15	0.0042	0.0320	0.62	0.0053	0.0293	0.48	0.0532	0.1713	-0.23	0.0425	0.1361	0.29	0.0245	0.1384	-0.35	0.0010	0.0268	-0.48	0.0050	0.0687	-0.55
247	Acidic leucine-rich nuclear phosphoprotein 32 family member C	O43423	0.1633	0.3201	0.50	0.1048	0.6320	0.64	0.1370	0.2606	0.55	0.0773	0.1597	0.72	0.0300	0.1261	1.27	0.2840	0.4803	0.37	0.0508	0.1790	-1.71	0.1302	0.3776	-0.55	0.1033	0.1594	0.63
248	Acidic leucine-rich nuclear phosphoprotein 32 family member D	O95626	0.5163	0.5887	-0.17	0.6526	0.7436	-0.19	0.2709	0.4441	0.33	0.2295	0.3727	0.37	0.8815	0.9091	-0.04	0.2613	0.4531	0.34	0.7385	0.7766	-0.11	0.7864	1.2307	-0.09	0.9972	0.9978	0.00
249	Acidic leucine-rich nuclear phosphoprotein 32 family member E	Q9BTT0	0.0061	0.0471	-0.22	0.0142	0.4540	-0.34	0.0063	0.0387	-0.42	0.0001	0.0082	-0.78	0.0001	0.0130	-0.78	0.0009	0.0481	-0.64	0.0602	0.1942	-0.18	0.0333	0.1649	-0.25	0.0057	0.0717	-0.49
250	Acid-sensing ion channel 1	P78348	0.9800	0.9861	0.00	0.4641	0.7958	-0.43	0.6335	0.6867	-0.11	0.7764	0.8081	0.06	0.4045	0.5008	0.18	0.6598	0.8973	-0.08	0.3736	0.4471	-2.55	0.8708	0.9355	-0.33	0.0651	0.1183	2.06
251	Aconitate hydratase mitochondrial	Q99798	0.0756	0.1874	0.12	0.2950	0.6717	0.20	0.0160	0.0598	0.22	0.0063	0.0317	0.27	0.2664	0.4881	0.06	0.0144	0.0799	0.31	0.1436	0.3261	0.16	0.0513	0.2136	0.13	0.6264	0.6843	0.01
252	Acrosin-binding protein	Q8NEB7	0.5829	0.6503	-0.03	0.2151	0.6613	-0.09	0.3776	0.5768	-0.06	0.1134	0.2137	-0.14	0.4015	0.4978	-0.07	0.7413	0.9806	-0.03	0.3736	0.4422	-2.55	0.8708	0.9262	-0.33	0.0651	0.1164	2.06
253	Actin alpha skeletal muscle	P68133	0.0063	0.0480	0.15	0.6255	0.7229	-0.04	0.0004	0.0180	0.38	0.0062	0.0316	0.16	0.9749	0.9806	0.00	0.0278	0.1066	0.46	0.0302	0.1476	0.47	0.0042	0.0523	0.27	0.0146	0.1045	0.28
254	Actin aortic smooth muscle	P62736	0.0721	0.1806	-0.59	0.8488	0.8913	0.03	0.0059	0.0370	-1.20	0.1075	0.2052	-0.43	0.7283	0.7874	-0.06	0.7654	1.0035	-0.11	0.3736	0.4409	-2.55	0.8708	0.9238	-0.33	0.0651	0.1158	2.06
255	Actin cytoplasmic 1	P60709	0.0095	0.0576	0.34	0.2653	0.6633	0.24	0.0013	0.0224	0.56	0.0022	0.0205	0.50	0.2648	0.4862	0.08	0.0045	0.0595	0.68	0.0399	0.1643	0.43	0.1827	0.4715	0.13	0.0456	0.1867	0.26
256	Actin cytoplasmic 2	P63261	0.0050	0.0438	0.25	0.3748	0.7223	-0.18	0.4417	0.6535	0.04	0.8453	0.8676	0.01	0.1782	0.3689	0.08	0.0104	0.0700	0.19	0.0944	0.2471	-0.30	0.0635	0.2422	-0.14	0.0617	0.2218	-0.11
257	Actin gamma-enteric smooth muscle	P63267	0.1594	0.3143	-0.32	0.4824	0.8071	0.13	0.0325	0.0927	-0.80	0.0121	0.0441	-1.01	0.0479	0.1607	-0.64	0.1555	0.3144	0.74	0.3736	0.5140	-2.55	0.8708	1.0591	-0.33	0.0651	0.1474	2.06
258	Actin-binding LIM protein 3	O94929	0.4108	0.6292	-3.31	0.5752	0.8805	-1.75	0.5661	0.7857	-1.19	0.5388	0.7425	0.89	0.2938	0.5212	-1.88	0.9510	1.1813	0.09	0.0299	0.1469	-0.69	0.0013	0.0293	-1.77	0.0235	0.1303	-0.84
259	Actin-histidine N-methyltransferase	Q86TU7	0.0740	0.1845	0.15	0.0578	0.5871	0.27	0.0089	0.0442	0.28	0.0289	0.0783	0.20	0.0520	0.1691	0.17	0.1266	0.2754	0.11	0.3736	0.5255	-2.55	0.8708	1.0799	-0.33	0.0651	0.1529	2.06
260	Actin-like protein 6A	O96019	0.9839	0.9885	0.00	0.0105	0.4422	-0.31	0.0315	0.0908	-0.16	0.0411	0.1004	-0.17	0.0084	0.0659	-0.27	0.5008	0.7260	-0.04	0.0419	0.1671	-0.18	0.0168	0.1099	0.32	0.1037	0.1597	0.13
261	Actin-like protein 7A	Q9Y615	0.7483	0.7950	0.10	0.5931	0.6976	0.14	0.6145	0.6690	0.13	0.6800	0.7200	0.10	0.7593	0.8120	0.08	0.8415	1.0766	-0.05	0.3736	0.4360	-2.55	0.8708	0.9144	-0.33	0.0651	0.1139	2.06
262	Actin-like protein 7B	Q9Y614	0.0096	0.0577	10.36	0.0395	0.5637	4.42	0.5661	0.7906	-1.19	0.0240	0.0686	5.40	0.2938	0.5257	-1.88	0.2586	0.4495	7.13	0.5978	0.6466	0.11	0.1631	0.4393	0.26	0.0045	0.0657	0.61
263	Actin-related protein 2	P61160	0.0001	0.0162	0.18	0.6929	0.7745	0.03	0.4831	0.7029	0.03	0.2344	0.3787	0.02	0.9283	0.9460	0.00	0.3322	0.5351	0.09	0.9614	0.9681	-0.01	0.0101	0.0827	-0.32	0.0240	0.1321	-0.28
264	Actin-related protein 2/3 complex subunit 1A	Q92747	0.4108	0.6290	-3.31	0.5752	0.8802	-1.75	0.5661	0.7855	-1.19	0.5388	0.7423	0.89	0.2938	0.5210	-1.88	0.9510	1.1811	0.09	0.0555	0.1869	0.33	0.1945	0.4898	0.15	0.7369	0.7832	0.03
265	Actin-related protein 2/3 complex subunit 1B	O15143	0.1941	0.3618	-0.07	0.7620	0.8278	0.05	0.2329	0.3934	0.07	0.7320	0.7688	-0.02	0.1261	0.2930	-0.09	0.4871	0.7124	0.04	0.0846	0.2324	0.42	0.9030	0.9277	-0.01	0.0212	0.1244	0.20
266	Actin-related protein 2/3 complex subunit 2	O15144	0.2025	0.3737	0.05	0.2047	0.6590	0.28	0.0063	0.0385	0.22	0.0027	0.0219	0.34	0.0088	0.0674	0.18	0.0121	0.0747	0.25	0.0144	0.1118	0.43	0.2396	0.5566	0.12	0.1123	0.1696	0.18
267	Actin-related protein 2/3 complex subunit 3	O15145	0.1016	0.2276	-0.11	0.9508	0.9672	0.00	0.0235	0.0753	0.07	0.0217	0.0639	0.14	0.7767	0.8246	-0.01	0.8057	1.0434	0.02	0.1844	0.3855	0.15	0.8930	0.9200	-0.01	0.1988	0.2673	0.13
268	Actin-related protein 2/3 complex subunit 4	P59998	0.2703	0.4627	0.08	0.4888	0.8121	-0.10	0.0340	0.0956	-0.24	0.0257	0.0720	-0.23	0.0232	0.1104	-0.31	0.2683	0.4614	-0.08	0.0676	0.2042	-0.21	0.0651	0.2454	-0.20	0.0078	0.0795	-0.47
269	Actin-related protein 2/3 complex subunit 5	O15511	0.0166	0.0754	0.27	0.8265	0.8752	-0.01	0.0300	0.0879	0.19	0.7709	0.8030	0.02	0.0796	0.2177	-0.11	0.0053	0.0613	0.50	0.1372	0.3167	-0.12	0.1521	0.4213	-0.08	0.0900	0.1433	0.11
270	Actin-related protein 2/3 complex subunit 5-like protein	Q9BPX5	0.0021	0.0320	-0.16	0.7927	0.8508	0.01	0.1838	0.3285	0.03	0.0170	0.0544	-0.16	0.3069	0.4042	-0.04	0.0428	0.1364	-0.05	0.3736	0.5679	-2.55	0.8708	1.1560	-0.33	0.0651	0.1746	2.06
271	Actin-related protein 3	P61158	0.2427	0.4281	0.04	0.0082	0.4084	0.15	0.0023	0.0271	0.24	0.0155	0.0516	0.18	0.1946	0.3927	0.07	0.0391	0.1287	0.14	0.0470	0.1740	0.19	0.0779	0.2738	-0.14	0.3204	0.3919	0.06
272	Actin-related protein 3B	Q9P1U1	0.0984	0.2227	-0.12	0.0861	0.6186	-0.33	0.0013	0.0234	-0.44	0.0314	0.0829	-0.71	0.0010	0.0244	-0.55	0.1493	0.3063	-0.56	0.8602	0.8839	0.01	0.0580	0.2299	-0.15	0.1168	0.1747	-0.11
273	Actin-related protein 8	Q9H981	0.4108	0.6288	-3.31	0.5752	0.8800	-1.75	0.5661	0.7853	-1.19	0.5388	0.7421	0.89	0.2938	0.5208	-1.88	0.9510	1.1808	0.09	0.0668	0.2031	0.37	0.3981	0.7832	0.08	0.1378	0.1988	0.15
274	Activated RNA polymerase II transcriptional coactivator p15	P53999	0.3764	0.5917	0.03	0.5975	0.7012	0.08	0.0294	0.0870	0.12	0.0566	0.1260	0.09	0.7615	0.8136	0.01	0.0696	0.1854	0.10	0.0297	0.1469	0.46	0.0337	0.1661	0.25	0.1741	0.2400	0.10
275	Activating signal cointegrator 1	Q15650	0.4108	0.6286	-3.31	0.5752	0.8797	-1.75	0.5661	0.7851	-1.19	0.5388	0.7419	0.89	0.2938	0.5207	-1.88	0.9510	1.1805	0.09	0.0190	0.1277	0.89	0.0716	0.2595	0.48	0.0033	0.0574	1.63
276	Activating signal cointegrator 1 complex subunit 3	Q8N3C0	0.4108	0.6285	-3.31	0.5752	0.8794	-1.75	0.5661	0.7849	-1.19	0.5388	0.7417	0.89	0.2938	0.5205	-1.88	0.9510	1.1802	0.09	0.0197	0.1298	0.24	0.1661	0.4447	-0.10	0.7047	0.7553	-0.02
277	Activator of 90 kDa heat shock protein ATPase homolog 1	O95433	0.0086	0.0551	0.18	0.1454	0.6566	0.26	0.0030	0.0301	0.32	0.0027	0.0220	0.24	0.1732	0.3619	0.05	0.0084	0.0671	0.20	0.1536	0.3425	0.14	0.5134	0.9328	0.05	0.3945	0.4659	-0.05
278	Active breakpoint cluster region-related protein	Q12979	0.4108	0.6283	-3.31	0.5752	0.8792	-1.75	0.5661	0.7847	-1.19	0.5388	0.7415	0.89	0.2938	0.5203	-1.88	0.9510	1.1799	0.09	0.1410	0.3218	-1.50	0.0126	0.0934	-1.02	0.1395	0.2008	-0.43
279	Activin receptor type-2B	Q13705	0.4108	0.6281	-3.31	0.5752	0.8789	-1.75	0.5661	0.7845	-1.19	0.5388	0.7413	0.89	0.2938	0.5201	-1.88	0.9510	1.1796	0.09	0.5426	0.5938	0.06	0.5987	1.0317	-0.07	0.3266	0.3979	-0.18
280	Activity-dependent neuroprotector homeobox protein 2	Q6IQ32	0.5770	0.6446	0.10	0.0949	0.6310	-0.38	0.5097	0.7312	-0.13	0.8092	0.8363	-0.04	0.0918	0.2390	-0.36	0.7113	0.9521	0.06	0.3736	0.4448	-2.55	0.8708	0.9310	-0.33	0.0651	0.1174	2.06
281	Acyl carrier protein mitochondrial	O14561	0.2328	0.4147	-0.09	0.4074	0.7502	-0.06	0.0471	0.1203	0.10	0.3172	0.4873	-0.02	0.0011	0.0258	-0.17	0.4430	0.6649	-0.05	0.3736	0.4649	-2.55	0.8708	0.9686	-0.33	0.0651	0.1256	2.06
282	Acylamino-acid-releasing enzyme	P13798	0.0081	0.0534	0.23	0.0517	0.5759	0.21	0.0033	0.0310	0.30	0.0238	0.0683	0.19	0.8649	0.8964	0.01	0.0432	0.1369	0.15	0.0252	0.1396	0.23	0.2156	0.5211	-0.07	0.1741	0.2400	0.04
283	Acyl-CoA dehydrogenase family member 9_ mitochondrial	Q9H845	0.1852	0.3500	0.09	0.4037	0.7476	-0.15	0.5140	0.7347	-0.04	0.0035	0.0237	-0.37	0.0801	0.2181	-0.13	0.0195	0.0900	0.31	0.0930	0.2447	-0.09	0.1869	0.4767	-0.08	0.0169	0.1112	0.20
284	Acyl-CoA desaturase	O00767	0.0002	0.0170	0.30	0.2170	0.6597	0.08	0.0035	0.0313	0.15	0.0001	0.0086	0.64	0.0065	0.0591	-0.12	0.0000	0.0000	1.01	0.0112	0.1036	0.48	0.0468	0.2020	0.25	0.0210	0.1239	0.34
285	Acyl-CoA synthetase family member 2 mitochondrial	Q96CM8	0.4882	0.5625	0.02	0.3377	0.6966	0.14	0.0019	0.0241	0.17	0.0018	0.0188	0.19	0.0609	0.1860	0.05	0.0053	0.0614	0.22	0.0971	0.2504	0.38	0.2282	0.5410	-0.10	0.1174	0.1754	0.15
286	Acyl-CoA-binding protein	P07108	0.0640	0.1676	0.17	0.4666	0.7979	0.11	0.0428	0.1123	0.18	0.0246	0.0699	0.25	0.9099	0.9320	-0.01	0.0058	0.0616	0.40	0.2207	0.4428	0.14	0.8433	1.2871	-0.02	0.2890	0.3610	0.06
287	Acyl-coenzyme A synthetase ACSM5 mitochondrial	Q6NUN0	0.0726	0.1816	0.14	0.0943	0.6303	0.17	0.0174	0.0630	0.26	0.1147	0.2155	0.10	0.0154	0.0881	0.21	0.3022	0.5001	0.08	0.0080	0.0911	1.48	0.0160	0.1075	1.58	0.0182	0.1157	1.25
288	Acyl-coenzyme A thioesterase 2 mitochondrial	P49753	0.4108	0.6279	-3.31	0.5752	0.8787	-1.75	0.5661	0.7843	-1.19	0.5388	0.7411	0.89	0.2938	0.5199	-1.88	0.9510	1.1794	0.09	0.0898	0.2406	0.38	0.1533	0.4231	0.28	0.1942	0.2622	0.24
289	Acyl-coenzyme A thioesterase 8	O14734	0.0720	0.1806	-0.13	0.2469	0.6596	0.13	0.0473	0.1205	0.11	0.0136	0.0472	0.18	0.2948	0.3928	0.03	0.2355	0.4207	0.04	0.4429	0.4981	0.08	0.1831	0.4721	-0.15	0.2376	0.3076	0.13
290	Acyl-coenzyme A thioesterase 9 mitochondrial	Q9Y305	0.4108	0.6277	-3.31	0.5752	0.8784	-1.75	0.5661	0.7841	-1.19	0.5388	0.7409	0.89	0.2938	0.5198	-1.88	0.9510	1.1791	0.09	0.1202	0.2901	0.27	0.9229	0.9433	-0.01	0.4935	0.5611	-0.09
291	Acylglycerol kinase mitochondrial	Q53H12	0.8562	0.8860	0.01	0.0877	0.6215	0.20	0.1780	0.3212	0.10	0.0852	0.1722	0.15	0.0234	0.1106	0.12	0.0391	0.1288	0.19	0.1063	0.2667	-0.18	0.0010	0.0266	0.69	0.9159	0.9315	0.01
292	Acylphosphatase-1	P07311	0.4108	0.6275	-3.31	0.5752	0.8781	-1.75	0.5661	0.7839	-1.19	0.5388	0.7407	0.89	0.2938	0.5196	-1.88	0.9510	1.1788	0.09	0.1981	0.4083	-0.53	0.7871	1.2307	-0.05	0.3059	0.3770	0.20
293	Acyl-protein thioesterase 1	O75608	0.3786	0.5945	0.03	0.5534	0.8689	0.08	0.0283	0.0851	0.10	0.1039	0.1995	0.06	0.0269	0.1192	0.10	0.8475	1.0826	0.01	0.0003	0.0353	-0.56	0.0019	0.0362	-0.47	0.0032	0.0570	-0.56
294	Acyl-protein thioesterase 2	O95372	0.0598	0.1619	-0.30	0.7309	0.8040	-0.04	0.0206	0.0692	-0.45	0.0409	0.1002	-0.30	0.4892	0.5787	-0.08	0.0786	0.2006	-0.36	0.0242	0.1375	-0.35	0.9879	0.9915	0.00	0.9925	0.9940	0.00

Supplemetary Table S2. Overview on all relatively quantified 5180 proteins statistical analysis

			MCF-7																		MDA-MB-231								
			Dai SC20 vs control			Gen SC20 vs control			SSE SC20 vs control			Dai IC20 vs control			Gen IC20 vs control			SSE IC20 vs control			Dai IC20 vs control			Gen IC20 vs control			SSE IC20 vs control		
	Protein name	UniProt	p value	BH q value	log2FC	p value	BH q value	log2FC	p value	BH q value	log2FC	p value	BH q value	log2FC	p value	BH q value	log2FC	p value	BH q value	log2FC	p value	BH q value	log2FC	p value	BH q value	log2FC	p value	BH q value	log2FC
295	Acylpyruvase FAHD1 mitochondrial	Q6P587	0.1441	0.2911	0.13	0.1506	0.6589	-0.21	0.0400	0.1065	-0.31	0.0026	0.0214	-0.24	0.0033	0.0432	-0.27	0.0057	0.0620	-0.25	0.0222	0.1356	-0.54	0.0088	0.0775	-0.34	0.0069	0.0769	-0.40
296	Adapter molecule crk	P46108	0.1030	0.2297	0.08	0.3745	0.7220	0.12	0.0211	0.0703	0.15	0.0184	0.0574	0.16	0.8147	0.8557	-0.01	0.1075	0.2455	0.09	0.0330	0.1521	0.41	0.2698	0.6016	0.09	0.5706	0.6343	-0.03
297	Adaptin ear-binding coat-associated protein 2	Q9NVZ3	0.3167	0.5195	-0.14	0.2236	0.6577	-0.60	0.1304	0.2515	-0.29	0.0353	0.0899	-0.53	0.0280	0.1216	-0.43	0.0275	0.1066	-0.50	0.0243	0.1377	0.38	0.2305	0.5440	0.18	0.6157	0.6747	-0.02
298	Adenine phosphoribosyltransferase	P07741	0.0108	0.0601	0.31	0.2964	0.6716	0.22	0.0132	0.0536	0.21	0.0163	0.0531	0.25	0.1077	0.2658	0.11	0.0130	0.0767	0.28	0.2150	0.4345	0.17	0.2797	0.6171	-0.11	0.0339	0.1573	-0.20
299	Adenomatous polyposis coli protein	P25054	0.0130	0.0666	0.36	0.6739	0.7610	0.05	0.1427	0.2688	0.10	0.0105	0.0410	0.25	0.0272	0.1196	0.19	0.0164	0.0850	0.42	0.1709	0.3678	0.23	0.0499	0.2098	-0.20	0.0407	0.1751	-0.26
300	Adenomatous polyposis coli protein 2	O95996	0.4108	0.6273	-3.31	0.5752	0.8779	-1.75	0.5661	0.7836	-1.19	0.5388	0.7405	0.89	0.2938	0.5194	-1.88	0.9510	1.1785	0.09	0.0227	0.1364	-0.49	0.0886	0.2955	-0.23	0.7125	0.7627	-0.04
301	Adenosine 3'-phospho 5'-phosphosulfate transporter 1	Q8TB61	0.0399	0.1260	-0.17	0.3590	0.7084	-0.17	0.9476	0.9591	-0.01	0.7265	0.7643	0.02	0.2283	0.4378	0.07	0.0206	0.0930	-0.26	0.3736	0.5935	-2.55	0.8708	1.2013	-0.33	0.0651	0.1890	2.06
302	Adenosine deaminase domain-containing protein 2	Q8NCV1	0.4108	0.6272	-3.31	0.5752	0.8776	-1.75	0.5661	0.7834	-1.19	0.5388	0.7403	0.89	0.2938	0.5192	-1.88	0.9510	1.1782	0.09	0.0034	0.0640	-0.49	0.0005	0.0231	-1.00	0.0005	0.0320	-1.27
303	Adenosine kinase	P55263	0.0436	0.1325	-0.10	0.4561	0.7878	0.13	0.7406	0.7832	0.02	0.0360	0.0911	0.10	0.5518	0.6350	0.02	0.8168	1.0533	0.01	0.0657	0.2015	0.27	0.1802	0.4674	0.17	0.4126	0.4841	0.09
304	Adenosylhomocysteinase	P23526	0.0013	0.0255	0.25	0.4372	0.7732	0.12	0.0213	0.0705	0.21	0.0067	0.0330	0.30	0.0126	0.0801	0.13	0.0007	0.0427	0.38	0.0570	0.1900	0.42	0.1088	0.3359	0.17	0.5556	0.6205	0.04
305	Adenosylhomocysteinase 3	Q96HN2	0.0947	0.2169	-0.30	0.1521	0.6610	-0.72	0.0593	0.1408	-0.37	0.0451	0.1069	-0.55	0.6157	0.6894	0.08	0.1032	0.2382	-0.28	0.3736	0.5327	-2.55	0.8708	1.0930	-0.33	0.0651	0.1564	2.06
306	Adenylate cyclase type 6	O43306	0.1647	0.3219	-0.16	0.1574	0.6618	-0.09	0.0201	0.0684	0.22	0.1625	0.2820	-0.11	0.2495	0.4667	0.07	0.4458	0.6674	-0.09	0.3736	0.4644	-2.55	0.8708	0.9678	-0.33	0.0651	0.1254	2.06
307	Adenylate cyclase type 8	P40145	0.4108	0.6270	-3.31	0.5752	0.8774	-1.75	0.5661	0.7832	-1.19	0.5388	0.7401	0.89	0.2938	0.5191	-1.88	0.9510	1.1779	0.09	0.5841	0.6330	0.30	0.2281	0.5410	0.69	0.1168	0.1746	1.09
308	Adenylate kinase 2 mitochondrial	P54819	0.0003	0.0169	0.52	0.1025	0.6328	0.39	0.0009	0.0215	0.56	0.0001	0.0098	0.67	0.0038	0.0462	0.48	0.0016	0.0518	0.75	0.7378	0.7762	0.03	0.3788	0.7553	-0.06	0.0040	0.0617	-0.15
309	Adenylate kinase 4 mitochondrial	P27144	0.0140	0.0695	-0.20	0.1540	0.6604	-0.29	0.3204	0.5075	-0.04	0.0005	0.0117	-0.58	0.0008	0.0229	-0.41	0.0281	0.1072	-0.13	0.9184	0.9334	-0.01	0.3278	0.6828	0.09	0.1414	0.2028	0.12
310	Adenylate kinase isoenzyme 1	P00568	0.1655	0.3230	-0.10	0.3175	0.6813	-0.13	0.4820	0.7019	0.04	0.3815	0.5666	0.05	0.0412	0.1497	0.19	0.1248	0.2725	0.18	0.5213	0.5745	-0.09	0.1809	0.4681	0.08	0.5942	0.6550	-0.16
311	Adenylosuccinate lyase	P30566	0.2896	0.4864	0.04	0.0112	0.4297	0.12	0.0896	0.1879	0.20	0.0014	0.0168	0.18	0.0021	0.0359	0.21	0.0767	0.1976	0.14	0.0118	0.1041	0.31	0.0203	0.1233	0.29	0.0300	0.1477	0.28
312	Adenylosuccinate synthetase isozyme 2	P30520	0.0098	0.0583	0.18	0.0156	0.4591	0.20	0.0039	0.0321	0.39	0.0102	0.0406	0.28	0.0246	0.1135	0.13	0.0046	0.0599	0.30	0.0313	0.1497	-0.16	0.0023	0.0404	-0.32	0.0177	0.1142	-0.21
313	Adenylyl cyclase-associated protein 1	Q01518	0.1869	0.3526	0.07	0.2594	0.6636	0.18	0.0576	0.1375	0.13	0.0389	0.0966	0.16	0.4616	0.5534	-0.04	0.0411	0.1334	0.19	0.1775	0.3768	0.23	0.1270	0.3715	-0.20	0.0926	0.1465	-0.20
314	Adenylyl cyclase-associated protein 2	P40123	0.2154	0.3901	-2.68	0.3368	0.6953	-6.34	0.0151	0.0577	-0.94	0.0022	0.0201	-1.48	0.5450	0.6296	0.10	0.0076	0.0653	-1.27	0.8605	0.8839	0.09	0.5445	0.9699	0.31	0.1929	0.2609	0.81
315	Adhesion G protein-coupled receptor B3	O60242	0.0666	0.1710	0.15	0.0339	0.5289	0.23	0.0038	0.0322	0.39	0.0478	0.1115	0.16	0.0091	0.0675	0.32	0.0438	0.1379	0.19	0.3736	0.5659	-2.55	0.8708	1.1525	-0.33	0.0651	0.1736	2.06
316	Adhesion G-protein coupled receptor G6	Q86SQ4	0.0064	0.0480	-0.39	0.9843	0.9887	0.00	0.0005	0.0180	-0.51	0.1085	0.2062	-0.09	0.0641	0.1920	-0.12	0.2481	0.4365	-0.14	0.3736	0.4906	-2.55	0.8708	1.0162	-0.33	0.0651	0.1366	2.06
317	Adhesion G-protein coupled receptor G7	Q96K78	0.0166	0.0753	0.12	0.3577	0.7083	0.12	0.0178	0.0636	0.13	0.0328	0.0856	0.17	0.0234	0.1107	0.11	0.2447	0.4322	0.18	0.3736	0.4913	-2.55	0.8708	1.0175	-0.33	0.0651	0.1370	2.06
318	Adipocyte plasma membrane-associated protein	Q9HDC9	0.3817	0.5975	0.04	0.2441	0.6572	0.14	0.5554	0.7799	0.03	0.0147	0.0496	0.18	0.4129	0.5076	0.03	0.0241	0.0992	0.17	0.4776	0.5322	-0.09	0.6003	1.0341	-0.06	0.0224	0.1264	-0.51
319	ADP/ATP translocase 1	P12235	0.2047	0.3768	0.17	0.0437	0.5688	-0.27	0.1120	0.2236	-0.18	0.1945	0.3248	-0.17	0.3577	0.4549	-0.09	0.9775	0.9821	0.00	0.3736	0.4302	-2.55	0.8708	0.9034	-0.33	0.0651	0.1117	2.06
320	ADP/ATP translocase 2	P05141	0.0314	0.1098	0.22	0.5339	0.8504	0.12	0.0131	0.0535	0.30	0.0386	0.0960	0.20	0.2341	0.4453	-0.09	0.0179	0.0869	0.37	0.2302	0.4576	0.23	0.2740	0.6092	0.11	0.6114	0.6714	-0.04
321	ADP/ATP translocase 3	P12236	0.1261	0.2651	0.08	0.4704	0.7997	0.06	0.0112	0.0490	0.20	0.3234	0.4947	-0.04	0.0862	0.2292	-0.10	0.1083	0.2469	0.12	0.0078	0.0906	-0.71	0.0134	0.0967	0.15	0.0820	0.1333	-0.28
322	ADP/ATP translocase 4	Q9H0C2	0.2249	0.4037	0.04	0.0782	0.6110	0.19	0.0758	0.1657	0.08	0.0208	0.0621	0.15	0.0392	0.1446	0.09	0.0216	0.0948	0.12	0.1660	0.3605	0.27	0.8837	0.9120	0.02	0.8782	0.9015	-0.02
323	ADP-ribose glycohydrolase ARH3	Q9NX46	0.0349	0.1169	0.24	0.2611	0.6649	0.12	0.0276	0.0836	0.27	0.6378	0.6826	0.03	0.3697	0.4667	-0.07	0.0311	0.1132	0.28	0.7546	0.7906	0.04	0.0097	0.0812	-0.26	0.0476	0.1913	-0.16
324	ADP-ribose glycohydrolase MACROD1	Q9BQ69	0.0009	0.0224	-0.39	0.0016	0.2125	-0.27	0.0004	0.0247	-0.45	0.0020	0.0199	-0.66	0.0000	0.0000	-0.91	0.0248	0.1002	-0.45	0.3736	0.5866	-2.55	0.8708	1.1892	-0.33	0.0651	0.1851	2.06
325	ADP-ribose glycohydrolase OARD1	Q9Y530	0.1876	0.3534	-0.35	0.9203	0.9453	0.03	0.8795	0.9046	0.03	0.5956	0.6438	-0.12	0.4687	0.5599	-0.16	0.2212	0.4032	-0.31	0.1181	0.2864	0.38	0.2484	0.5706	0.25	0.2505	0.3214	0.24
326	ADP-ribose pyrophosphatase mitochondrial	Q9BW91	0.1015	0.2275	0.27	0.1194	0.6357	0.25	0.5258	0.7468	0.08	0.0993	0.1932	0.28	0.1392	0.3108	0.24	0.1212	0.2664	0.31	0.3736	0.5266	-2.55	0.8708	1.0820	-0.33	0.0651	0.1534	2.06
327	ADP-ribosyl cyclase/cyclic ADP-ribose hydrolase 2	Q10588	0.1873	0.3531	0.13	0.0789	0.6137	0.22	0.0514	0.1273	0.24	0.0979	0.1910	0.18	0.6137	0.6878	0.04	0.3516	0.5570	0.08	0.8129	0.8418	-0.01	0.0003	0.0169	0.55	0.0910	0.1446	0.15
328	ADP-ribosylation factor 1	P84077	0.0413	0.1287	0.12	0.0967	0.6349	0.19	0.0031	0.0303	0.17	0.0011	0.0159	0.24	0.0342	0.1354	0.06	0.0080	0.0669	0.21	0.3736	0.6191	-2.55	0.8708	1.2461	-0.33	0.0651	0.2045	2.06
329	ADP-ribosylation factor 3	P61204	0.4108	0.6268	-3.31	0.5752	0.8771	-1.75	0.5661	0.7830	-1.19	0.5388	0.7399	0.89	0.2938	0.5189	-1.88	0.9510	1.1777	0.09	0.0103	0.1014	0.41	0.0173	0.1113	0.27	0.0734	0.1226	0.18
330	ADP-ribosylation factor 4	P18085	0.3121	0.5132	0.06	0.7972	0.8530	0.03	0.9678	0.9732	0.00	0.0815	0.1662	0.13	0.4924	0.5821	0.04	0.0498	0.1496	0.19	0.0229	0.1367	0.37	0.1742	0.4578	0.14	0.4220	0.4933	0.06
331	ADP-ribosylation factor 5	P84085	0.0010	0.0230	0.24	0.1173	0.6323	0.37	0.0005	0.0204	0.34	0.0331	0.0861	0.23	0.7769	0.8247	0.01	0.0030	0.0549	0.22	0.1171	0.2849	0.26	0.3335	0.6910	0.09	0.5609	0.6251	0.04
332	ADP-ribosylation factor 6	P62330	0.1375	0.2822	-0.12	0.5288	0.8473	0.11	0.6520	0.7039	-0.03	0.3983	0.5873	0.06	0.0135	0.0831	-0.29	0.0968	0.2294	-0.14	0.0894	0.2404	0.23	0.1610	0.4353	0.13	0.2525	0.3238	0.05
333	ADP-ribosylation factor GTPase-activating protein 1	Q8N6T3	0.0041	0.0406	0.38	0.2771	0.6645	0.08	0.0070	0.0397	0.32	0.0129	0.0461	0.30	0.0500	0.1649	0.19	0.0201	0.0921	0.33	0.0039	0.0676	0.37	0.0133	0.0962	0.27	0.0538	0.2042	0.19
334	ADP-ribosylation factor GTPase-activating protein 2	Q8N6H7	0.0668	0.1713	-0.03	0.0834	0.6198	-0.09	0.1355	0.2585	-0.10	0.0623	0.1358	-0.13	0.0223	0.1075	-0.12	0.0156	0.0833	-0.16	0.3377	0.6080	0.06	0.1813	0.4686	-0.05	0.0517	0.1993	0.21
335	ADP-ribosylation factor GTPase-activating protein 3	Q9NP61	0.2459	0.4316	0.08	0.5524	0.8682	0.04	0.0658	0.1502	0.18	0.6301	0.6762	0.03	0.8256	0.8641	0.02	0.1788	0.3482	0.16	0.3736	0.5081	-2.55	0.8708	1.0483	-0.33	0.0651	0.1446	2.06
336	ADP-ribosylation factor-like protein 1	P40616	0.2419	0.4274	0.07	0.0281	0.5054	0.22	0.0023	0.0271	0.43	0.0043	0.0267	0.41	0.0203	0.1023	0.22	0.0041	0.0603	0.44	0.9607	0.9676	0.02	0.4949	0.9081	0.28	0.0975	0.1527	0.88
337	ADP-ribosylation factor-like protein 2	P36404	0.4108	0.6266	-3.31	0.5752	0.8768	-1.75	0.5661	0.7828	-1.19	0.5388	0.7397	0.89	0.2938	0.5187	-1.88	0.9510	1.1774	0.09	0.3136	0.5746	-0.20	0.0009	0.0256	-0.89	0.2812	0.3531	-0.78
338	ADP-ribosylation factor-like protein 3	P36405	0.3313	0.5381	0.06	0.4519	0.7853	0.14	0.0149	0.0574	0.27	0.1801	0.3050	0.08	0.4425	0.5368	-0.04	0.0331	0.1171	0.17	0.1908	0.3969	-0.12	0.2082	0.5099	-0.08	0.3930	0.4645	0.05
339	ADP-ribosylation factor-like protein 6-interacting protein 1	Q15041	0.0399	0.1259	-0.19	0.2977	0.6722	-0.83	0.6672	0.7182	-0.02	0.1003	0.1945	0.11	0.7739	0.8232	-0.01	0.2985	0.4954	0.12	0.1483	0.3333	0.33	0.1575	0.4292	-0.18	0.1265	0.1862	-0.13
340	ADP-ribosylation factor-like protein 6-interacting protein 4	Q66PJ3	0.2530	0.4404	-0.12	0.5551	0.8698	-0.04	0.0382	0.1031	-0.18	0.1672	0.2878	-0.08	0.0023	0.0372	-0.35	0.0239	0.0990	-0.22	0.3736	0.5884	-2.55	0.8708	1.1924	-0.33	0.0651	0.1861	2.06
341	ADP-ribosylation factor-like protein 8A	Q96BM9	0.4108	0.6264	-3.31	0.5752	0.8766	-1.75	0.5661	0.7826	-1.19	0.5388	0.7395	0.89	0.2938	0.5185	-1.88	0.9510	1.1771	0.09	0.0940	0.2464	0.14	0.3045	0.6504	0.07	0.3269	0.3980	0.15
342	ADP-ribosylation factor-like protein 8B	Q9NVJ2	0.0350	0.1169	0.17	0.3160	0.6806	0.15	0.1833	0.3280	0.10	0.0193	0.0590	0.21	0.5597	0.6424	0.03	0.0434	0.1371	0.19	0.0004	0.0414	-0.79	0.0065	0.0660	-0.34	0.0035	0.0587	-0.47
343	ADP-sugar pyrophosphatase	Q9UKK9	0.0140	0.0694	0.09	0.0048	0.3552	0.14	0.0014	0.0235	0.18	0.0257	0.0720	0.15	0.2477	0.4642	0.05	0.1442	0.2996	0.12	0.0011	0.0438	0.60	0.0001	0.0140	0.97	0.0091	0.0852	0.58
344	Adrenocortical dysplasia protein homolog	Q96AP0	0.0026	0.0344	3.65	0.4125	0.7524	-1.40	0.2216	0.3787	0.95	0.5340	0.7412	0.91	0.0749	0.2102	-1.35	0.0877	0.2156	3.63	0.3736	0.5394	-2.55	0.8708	1.1050	-0.33	0.0651	0.1597	2.06
345	Advillin	O75366	0.7544	0.8003	-0.03	0.6091	0.7111	0.06	0.1805	0.3242	0.03	0.4553	0.6522	0.02	0.1554	0.3360	0.06	0.0766	0.1975	0.06	0.3736	0.5459	-2.55	0.8708	1.1168	-0.33	0.0651	0.1631	2.06
346	AF4/FMR2 family member 3	P51826	0.0295	0.1053	-0.15	0.2893	0.6693	0.20	0.5989	0.6548	-0.05	0.8941	0.9097	0.01	0.0966	0.2470	-0.08	0.8859	1.1231	0.03	0.3736	0.4346	-2.55	0.8708	0.9118	-0.33	0.0651	0.1134	2.06
347	Afadin	P55196	0.3732	0.5879	-0.07	0.0007	0.2133	0.36	0.3155	0.5024	-0.12	0.0320	0.0840	0.16	0.0002	0.0130	0.63	0.3578	0.5637	-0.08	0.4472	0.5026	-0.34	0.3000	0.6435	0.32	0.3417	0.4125	0.24
348	Afamin	P43652	0.3658	0.5791	0.04	0.0064	0.4144	0.10	0.6083	0.6639	0.01	0.1875	0.3151	-0.06	0.0452	0.1566	-0.09	0.1452	0.3006	0.10	0.0044	0.0712	0.74	0.0050	0.0570	0.73	0.0076	0.0792	0.83
349	AFG3-like protein 2	Q9Y4W6	0.3259	0.5317	0.03	0.3050	0.6769	0.07	0.0033	0.0306	0.12	0.0421	0.1022	0.06	0.0123	0.0793	-0.09	0.0160	0.0841	0.12	0.0305	0.1477	0.53	0.0661	0.2479	0.15	0.0424	0.1791	0.12
350	Aflatoxin B1 aldehyde reductase member 2	O43488	0.0063	0.0479	-0.19	0.6931	0.7741	-0.03	0.0140	0.0554	-0.35	0.0006	0.0125	-0.28	0.9401	0.9541	0.00	0.6207	0.8565	-0.03	0.1418	0.3234	-0.12	0.4686	0.8785	0.06	0.0027	0.0544	-0.27
351	Aflatoxin B1 aldehyde reductase member 4	Q8NHP1	0.1153	0.2488	-0.21	0.0666	0.5948	-0.31	0.0693	0.1560	-0.25	0.0584	0.1289	-0.44	0.0252	0.1140	-0.42	0.1384	0.2917	-0.19	0.0854	0.2336	-0.35	0.1507	0.4186	0.09	0.1861	0.2536	0.05
352	Agrin	O00468	0.0639	0.1675	0.29	0.6471	0.7388	0.10	0.2547	0.4226	0.16	0.1327	0.2402	0.22	0.0264	0.1177	0.51	0.0082	0.0669	0.81	0.3736	0.6185	-2.55	0.8708	1.2450	-0.33	0.0651	0.2041	2.06
353	AH receptor-interacting protein	O00170	0.0683	0.1739	0.14	0.0049	0.3575	0.14	0.0045	0.0333	0.12	0.0052	0.0292	0.23	0.0090	0.0678	0.09	0.0385	0.1277	0.12	0.1556	0.3461	-0.18	0.0680	0.2512	-0.29	0.2420	0.3124	-0.14
354	A-kinase anchor protein 12	Q02952	0.6696	0.7282	-0.06	0.9246	0.9480	-0.01	0.2558	0.4240	-0.09	0.5698	0.6199	-0.04	0.6566	0.7227	0.04	0.2171	0.3984	-0.10	0.0251	0.1394	0.45	0.0280	0.1517	0.25	0.0265	0.1388	0.25
355	A-kinase anchor protein 3	O75969	0.0004	0.0179	-0.68	0.4165	0.7552	-1.05	0.4196	0.6276	0.11	0.0193	0.0589	-0.23	0.2432	0.4571	0.18	0.1265	0.2754	-0.60	0.3736	0.5256	-2.55	0.8708	1.0802	-0.33	0.0651	0.1529	2.06
356	A-kinase anchor protein 8-like	Q9ULX6	0.0186	0.0802	0.11	0.1502	0.6610	0.13	0.0238	0.0756	0.18	0.0130	0.0462	0.11	0.9049	0.9282	0.00	0.3496	0.5548	0.04	0.3736	0.4743	-2.55	0.8708	0.9862	-0.33	0.0651	0.1296	2.06
357	A-kinase anchor protein 9	Q99996	0.0108	0.0600	0.16	0.1358	0.6513	0.08	0.0036	0.0317	0.23	0.0070	0.0339	0.17	0.5813	0.6593	0.02	0.0158	0.0836	0.17	0.0082	0.0917	0.79	0.3838	0.7626	0.20	0.2653	0.3370	0.34
358	A-kinase anchor protein SPHKAP	Q2M3C7	0.0184	0.0798	-0.40	0.4542	0.7871	-0.11	0.1952	0.3440	0.39	0.0292	0.0788	-0.38	0.3935	0.4902	0.12	0.4955	0.7206	-0.08	0.3736	0.4604	-2.55	0.8708	0.9603	-0.33	0.0651	0.1237	2.06
359	Alanine aminotransferase 2	Q8TD30	0.0379	0.1224	-0.11	0.4228	0.7597	-0.03	0.0234	0.0752	0.13	0.2433	0.3903	-0.07	0.0335	0.1336	-0.13	0.0893	0.2180	-0.15	0.3736	0.5383	-2.55	0.8708	1.1031	-0.33	0.0651	0.1592	2.06
360	Alanine--tRNA ligase cytoplasmic	P49588	0.0038	0.0395	0.25	0.2649	0.6635	0.19	0.0030	0.0298	0.30	0.0011	0.0160	0.34	0.0730	0.2066	-0.10	0.0027	0.0553	0.38	0.0198	0.1303	0.47	0.0557	0.2251	0.12	0.0434	0.1819	0.11
361	Alanine--tRNA ligase mitochondrial	Q5JTZ9	0.5113	0.5841	0.10	0.4391	0.7755	0.12	0.4328	0.6431	-0.11	0.9959	0.9961	0.00	0.2050	0.4065	-0.21	0.2260	0.4089	-0.20	0.3736	0.4961	-2.55	0.8708	1.0263	-0.33	0.0651	0.1391	2.06
362	Alanyl-tRNA editing protein Aarsd1	Q9BTE6	0.4637	0.5404	-0.05	0.3820	0.7294	0.14	0.0046	0.0332	0.29	0.0147	0.0497	0.24	0.0158	0.0895	0.21	0.0123	0.0748	0.14	0.0042	0.0704	-1.30	0.0008	0.0254	-0.88	0.0025	0.0549	-0.77
363	Alcohol dehydrogenase class-3	P11766	0.0349	0.1169	0.08	0.2712	0.6645	0.17	0.0022	0.0263	0.21	0.0088	0.0381	0.13	0.1725	0.3613	0.04	0.0040	0.0594	0.21	0.0287	0.1458	0.33	0.0636	0.2422	0.20	0.0131	0.0989	0.18
364	Aldehyde dehydrogenase family 1 member A3	P47895	0.4108	0.6262	-3.31	0.5752	0.8763	-1.75	0.5661	0.7824	-1.19	0.5388	0.7393	0.89	0.2938	0.5184	-1.88	0.9510	1.1768	0.09	0.6653	0.7093	-0.16	0.6021	1.0355	-0.19	0.6905	0.7430	-0.14
365	Aldehyde dehydrogenase family 16 member A1	Q8IZ83	0.0076	0.0515	0.78	0.0673	0.5899	0.46	0.0732	0.1618	0.39	0.0250	0.0706	0.55	0.2112	0.4149	0.22	0.3194	0.5219	0.17	0.1498	0.3358	0.20	0.2903	0.6297	-0.11	0.1617	0.2261	-0.16
366	Aldehyde dehydrogenase X mitochondrial	P30837	0.2900	0.4868	-0.08	0.2840	0.6642	0.14	0.9839	0.9871	0.00	0.2873	0.4484	-0.08	0.6765	0.7402	0.03	0.1411	0.2948	-0.14	0.0647	0.2003	-0.09	0.0643	0.2442	0.08	0.0020	0.0498	0.20
367	Aldo-keto reductase family 1 member A1	P14550	0.0011	0.0234	-2.55	0.6358	0.7320	-0.43	0.5066	0.7283	0.13	0.2125	0.3490	-1.79	0.0447	0.1557	0.46	0.0512	0.1527	-2.43	0.1201	0.2900	0.22	0.0357	0.1722	0.34	0.0368	0.1645	0.43
368	Aldose reductase	P15121	0.4108	0.6261	-3.31	0.5752	0.8761	-1.75	0.5661	0.7822	-1.19	0.5388	0.7391	0.89	0.2938	0.5182	-1.88	0.9510	1.1765	0.09	0.5066	0.5600	-0.12	0.0074	0.0707	-0.38	0.0097	0.0877	-0.47
369	Alkyldihydroxyacetonephosphate synthase_ peroxisomal	O00116	0.4108	0.6259	-3.31	0.5752	0.8758	-1.75	0.5661	0.7820	-1.19	0.5388	0.7389	0.89	0.2938	0.5180	-1.88	0.9510	1.1763	0.09	0.2011	0.4135	0.08	0.3105	0.6586	0.06	0.1272	0.1868	0.10
370	Allograft inflammatory factor 1-like	Q9BQI0	0.0947	0.2168	0.21	0.1224	0.6391	0.21	0.3053	0.4886	-0.34	0.5747	0.6238	-0.05	0.8488	0.8834	-0.02	0.8118	1.0484	-0.02	0.3736	0.4378	-2.55	0.8708	0.9179	-0.33	0.0651	0.1146	2.06
371	Alpha N-terminal protein methyltransferase 1B	Q5VVY1	0.0136	0.0683	-0.32	0.0555	0.5843	-0.49	0.0003	0.0222	-0.77	0.0001	0.0123	-0.67	0.0000	0.0000	-0.84	0.0026	0.0573	-0.45	0.3736	0.6417	-2.55	0.8708	1.2851	-0.33	0.0651	0.2191	2.06
372	Alpha/beta hydrolase domain-containing protein 17A	Q96GS6	0.2296	0.4105	-0.23	0.5720	0.8847	0.11	0.3986	0.6018	0.15	0.6713	0.7130	0.07	0.9263	0.9447	0.01	0.5898	0.8266	0.09	0.3736	0.4533	-2.55	0.8708	0.9470	-0.33	0.0651	0.1208	2.06
373	Alpha-1 3/1 6-mannosyltransferase ALG2	Q9H553	0.0777	0.1908	0.06	0.1371	0.6515	0.08	0.0033	0.0309	0.31	0.0868	0.1745	0.09	0.0263	0.1173	0.13	0.0276	0.1065	0.11	0.5899	0.6387	0.07	0.3221	0.6741	-0.07	0.0836	0.1352	-0.23
374	Alpha-2-HS-glycoprotein	P02765	0.0048	0.0437	0.24	0.4346	0.7710	0.13	0.0353	0.0978	0.23	0.1854	0.3126	0.07	0.1280	0.2955	0.08	0.0065	0.0624	0.39	0.0213	0.1342	0.63	0.0266	0.1471	0.30	0.0242	0.1324	-0.40
375	Alpha-2-macroglobulin	P01023	0.1840	0.3489	-0.06	0.1009	0.6335	0.14	0.3003	0.4825	-0.08	0.7556	0.7893	-0.01	0.0062	0.0571	0.22	0.6195	0.8555	0.04	0.0011	0.0428	0.88	0.0033	0.0477	0.91	0.0111	0.0924	0.63

Supplemetary Table S2. Overview on all relatively quantified 5180 proteins statistical analysis

			MCF-7																		MDA-MB-231								
			Dai SC20 vs control			Gen SC20 vs control			SSE SC20 vs control			Dai IC20 vs control			Gen IC20 vs control			SSE IC20 vs control			Dai IC20 vs control			Gen IC20 vs control			SSE IC20 vs control		
	**Protein name**	**UniProt**	**p value**	**BH q value**	**log2FC**	**p value**	**BH q value**	**log2FC**	**p value**	**BH q value**	**log2FC**	**p value**	**BH q value**	**log2FC**	**p value**	**BH q value**	**log2FC**	**p value**	**BH q value**	**log2FC**	**p value**	**BH q value**	**log2FC**	**p value**	**BH q value**	**log2FC**	**p value**	**BH q value**	**log2FC**
376	Alpha-2-macroglobulin receptor-associated protein	P30533	0.0315	0.1099	-0.15	0.1312	0.6454	0.19	0.4095	0.6156	0.03	0.1539	0.2704	0.06	0.0012	0.0269	0.31	0.0904	0.2191	0.08	0.3736	0.5374	-2.55	0.8708	1.1015	-0.33	0.0651	0.1588	2.06
377	Alpha-actinin-1	P12814	0.0060	0.0471	0.19	0.2000	0.6637	0.27	0.0004	0.0192	0.44	0.0023	0.0208	0.26	0.1774	0.3682	0.06	0.0153	0.0824	0.22	0.0282	0.1448	0.47	0.0521	0.2168	0.23	0.0466	0.1890	0.25
378	Alpha-actinin-2	P35609	0.1308	0.2727	0.08	0.2743	0.6646	0.19	0.0105	0.0478	0.12	0.0018	0.0186	0.22	0.3900	0.4871	0.02	0.0413	0.1337	0.10	0.0011	0.0442	0.43	0.0191	0.1186	0.28	0.0139	0.1021	0.24
379	Alpha-actinin-3	Q08043	0.0872	0.2053	0.18	0.1361	0.6510	0.28	0.0188	0.0657	0.34	0.0004	0.0114	0.51	0.0001	0.0118	0.15	0.0100	0.0709	0.54	0.7868	0.8200	0.02	0.6039	1.0375	0.04	0.0100	0.0878	0.43
380	Alpha-actinin-4	O43707	0.0680	0.1733	0.11	0.5314	0.8506	0.10	0.0036	0.0313	0.29	0.0523	0.1188	0.13	0.0906	0.2373	-0.10	0.0113	0.0730	0.25	0.0337	0.1529	0.49	0.2023	0.5009	0.14	0.1680	0.2332	0.12
381	Alpha-aminoadipic semialdehyde dehydrogenase	P49419	0.1728	0.3341	0.05	0.3477	0.7030	0.14	0.0185	0.0653	0.15	0.0031	0.0227	0.21	0.0632	0.1904	0.08	0.0045	0.0599	0.23	0.1029	0.2606	0.23	0.0442	0.1950	0.19	0.0919	0.1457	-0.09
382	Alpha-centractin	P61163	0.1488	0.2977	0.09	0.1085	0.6315	0.23	0.0108	0.0484	0.27	0.0109	0.0419	0.33	0.0231	0.1103	0.24	0.0188	0.0888	0.30	0.0387	0.1631	0.25	0.0244	0.1389	0.12	0.0413	0.1762	0.08
383	Alpha-endosulfine	O43768	0.1863	0.3516	0.09	0.6325	0.7289	0.09	0.4507	0.6644	-0.06	0.8568	0.8766	-0.01	0.3142	0.4113	-0.07	0.6958	0.9367	0.04	0.1066	0.2669	0.24	0.7328	1.1763	-0.03	0.8358	0.8669	0.01
384	Alpha-enolase	P06733	0.0002	0.0167	0.43	0.0820	0.6174	0.44	0.0004	0.0220	0.51	0.0001	0.0113	0.54	0.0198	0.1008	0.15	0.0005	0.0381	0.58	0.1390	0.3193	0.23	0.8025	1.2461	0.02	0.2890	0.3611	-0.09
385	Alpha-galactosidase A	P06280	0.9401	0.9548	0.00	0.2801	0.6646	-0.33	0.0212	0.0705	-0.26	0.0442	0.1054	-0.31	0.4484	0.5419	-0.05	0.8959	1.1327	-0.02	0.3736	0.4340	-2.55	0.8708	0.9107	-0.33	0.0651	0.1131	2.06
386	Alpha-internexin	Q16352	0.4108	0.6257	-3.31	0.5752	0.8756	-1.75	0.5661	0.7818	-1.19	0.5388	0.7387	0.89	0.2938	0.5178	-1.88	0.9510	1.1760	0.09	0.1424	0.3239	0.22	0.0365	0.1739	0.42	0.3401	0.4111	0.22
387	Alpha-ketoglutarate-dependent dioxygenase alkB homolog 3	Q96Q83	0.1047	0.2318	0.14	0.9575	0.9714	0.00	0.5810	0.6380	-0.04	0.1857	0.3128	-0.10	0.0209	0.1038	-0.26	0.0873	0.2155	-0.17	0.3736	0.5401	-2.55	0.8708	1.1064	-0.33	0.0651	0.1601	2.06
388	Alpha-ketoglutarate-dependent dioxygenase alkB homolog 4	Q9NXW9	0.0032	0.0372	-2.64	0.3076	0.6777	-2.15	0.0171	0.0621	-1.39	0.0059	0.0309	-3.24	0.2309	0.4410	-0.19	0.0064	0.0622	-3.33	0.3736	0.6235	-2.55	0.8708	1.2537	-0.33	0.0651	0.2073	2.06
389	Alpha-ketoglutarate-dependent dioxygenase FTO	Q9C0B1	0.4108	0.6255	-3.31	0.5752	0.8753	-1.75	0.5661	0.7816	-1.19	0.5388	0.7386	0.89	0.2938	0.5176	-1.88	0.9510	1.1757	0.09	0.3256	0.5922	0.12	0.0205	0.1235	0.22	0.8002	0.8370	-0.02
390	Alpha-N-acetylgalactosaminidase	P17050	0.0067	0.0493	0.27	0.0072	0.4098	0.27	0.0043	0.0327	-0.28	0.0015	0.0175	-0.39	0.2476	0.4642	-0.06	0.0090	0.0690	-0.28	0.0642	0.1995	-0.16	0.0220	0.1288	-0.36	0.0478	0.1912	-0.16
391	Alpha-N-acetylglucosaminidase	P54802	0.0283	0.1022	0.29	0.5913	0.6960	-0.05	0.7949	0.8323	-0.02	0.4692	0.6684	-0.05	0.5670	0.6479	-0.05	0.5657	0.8004	0.04	0.3736	0.4551	-2.55	0.8708	0.9504	-0.33	0.0651	0.1216	2.06
392	Alpha-parvin	Q9NVD7	0.9431	0.9573	0.00	0.1103	0.6327	0.13	0.0178	0.0637	-0.24	0.1147	0.2154	-0.12	0.9132	0.9347	0.01	0.1398	0.2934	-0.11	0.0912	0.2428	-0.15	0.4823	0.8929	-0.05	0.0616	0.2217	-0.19
393	Alpha-protein kinase 2	Q86TB3	0.4108	0.6253	-3.31	0.5752	0.8750	-1.75	0.5661	0.7813	-1.19	0.5388	0.7384	0.89	0.2938	0.5175	-1.88	0.9510	1.1754	0.09	0.0118	0.1034	1.05	0.0000	0.0000	1.10	0.0002	0.0334	0.72
394	Alpha-S1-casein	P47710	0.5135	0.5861	0.04	0.4777	0.8037	-0.51	0.0011	0.0215	0.37	0.5141	0.7186	-0.04	0.5750	0.6548	0.04	0.4841	0.7090	-0.04	0.3736	0.4617	-2.55	0.8708	0.9626	-0.33	0.0651	0.1243	2.06
395	Alpha-soluble NSF attachment protein	P54920	0.0892	0.2085	-0.12	0.8068	0.8608	0.01	0.1113	0.2226	-0.10	0.2664	0.4214	-0.07	0.5873	0.6647	-0.03	0.0518	0.1537	-0.17	0.0559	0.1875	-0.07	0.0897	0.2977	-0.09	0.0381	0.1684	-0.10
396	Alpha-taxilin	P40222	0.5840	0.6511	0.04	0.0109	0.4343	0.32	0.0108	0.0486	0.34	0.0147	0.0497	0.32	0.0031	0.0415	0.46	0.7639	1.0023	-0.02	0.0577	0.1912	0.43	0.6767	1.1160	0.04	0.1220	0.1809	0.10
397	Amidophosphoribosyltransferase	Q06203	0.1231	0.2605	0.06	0.8978	0.9275	0.01	0.0369	0.1007	0.11	0.3279	0.4997	-0.05	0.1881	0.3833	-0.05	0.0274	0.1064	-0.13	0.0133	0.1094	-0.28	0.0097	0.0816	-0.21	0.0628	0.2242	-0.32
398	Amine oxidase [flavin-containing] A	P21397	0.9862	0.9896	0.00	0.9790	0.9857	0.00	0.1783	0.3213	0.12	0.7358	0.7723	0.03	0.1722	0.3613	-0.12	0.8208	1.0571	-0.02	0.3736	0.4371	-2.55	0.8708	0.9166	-0.33	0.0651	0.1143	2.06
399	Aminoacyl tRNA synthase complex-interacting multifunctional protein 1	Q12904	0.0091	0.0568	0.16	0.2996	0.6736	0.14	0.0049	0.0342	0.29	0.0044	0.0269	0.25	0.0232	0.1106	0.12	0.0061	0.0624	0.24	0.1443	0.3273	0.10	0.0678	0.2509	-0.12	0.1881	0.2556	-0.05
400	Aminoacyl tRNA synthase complex-interacting multifunctional protein 2	Q13155	0.0010	0.0229	0.28	0.1253	0.6420	0.14	0.0009	0.0213	0.24	0.0034	0.0238	0.17	0.3388	0.4359	0.03	0.0180	0.0868	0.18	0.0002	0.0324	-0.74	0.0007	0.0263	-0.86	0.0005	0.0312	-0.74
401	Aminopeptidase B	Q9H4A4	0.3504	0.5600	-0.04	0.3152	0.6809	0.12	0.0070	0.0396	0.25	0.0038	0.0250	0.21	0.0348	0.1356	0.10	0.0101	0.0706	0.17	0.0068	0.0855	-0.69	0.0003	0.0185	-1.03	0.0019	0.0510	-1.14
402	Amphiregulin	P15514	0.0010	0.0228	0.85	0.3790	0.7258	-0.18	0.0073	0.0404	0.69	0.0114	0.0429	0.40	0.0061	0.0570	0.72	0.2397	0.4257	0.11	0.3736	0.4923	-2.55	0.8708	1.0194	-0.33	0.0651	0.1374	2.06
403	Amyloid-beta A4 precursor protein-binding family B member 2	Q92870	0.4108	0.6251	-3.31	0.5752	0.8748	-1.75	0.5661	0.7811	-1.19	0.5388	0.7382	0.89	0.2938	0.5173	-1.88	0.9510	1.1751	0.09	0.2107	0.4280	-0.34	0.0083	0.0748	0.48	0.0184	0.1165	0.61
404	Amyloid-beta A4 precursor protein-binding family B member 3	O95704	0.4108	0.6249	-3.31	0.5752	0.8745	-1.75	0.5661	0.7809	-1.19	0.5388	0.7380	0.89	0.2938	0.5171	-1.88	0.9510	1.1749	0.09	0.0089	0.0939	0.35	0.0707	0.2574	0.15	0.0252	0.1346	0.28
405	Amyloid-beta precursor protein	P05067	0.6059	0.6709	-0.03	0.0528	0.5819	-0.38	0.0487	0.1228	-0.50	0.6031	0.6504	0.06	0.0316	0.1299	0.42	0.0245	0.1000	0.17	0.3736	0.5875	-2.55	0.8708	1.1908	-0.33	0.0651	0.1856	2.06
406	Amyotrophic lateral sclerosis 2 chromosomal region candidate gene 12 protein	Q96Q35	0.4108	0.6248	-3.31	0.5752	0.8743	-1.75	0.5661	0.7807	-1.19	0.5388	0.7378	0.89	0.2938	0.5169	-1.88	0.9510	1.1746	0.09	0.0335	0.1524	0.30	0.2456	0.5664	-0.14	0.7657	0.8073	-0.03
407	AN1-type zinc finger protein 1	Q8TCF1	0.0163	0.0744	-0.37	0.6954	0.7755	-0.07	0.7406	0.7831	0.03	0.2784	0.4363	-0.18	0.0314	0.1297	-0.29	0.3766	0.5855	-0.25	0.3736	0.4711	-2.55	0.8708	0.9802	-0.33	0.0651	0.1282	2.06
408	AN1-type zinc finger protein 6	Q6FIF0	0.2190	0.3950	-0.09	0.8433	0.8875	0.06	0.5810	0.6382	0.07	0.0931	0.1838	-0.09	0.6936	0.7562	-0.03	0.0276	0.1064	0.15	0.3736	0.5829	-2.55	0.8708	1.1827	-0.33	0.0651	0.1830	2.06
409	Anamorsin	Q6FI81	0.1206	0.2563	0.06	0.5352	0.8512	0.09	0.0018	0.0235	0.17	0.0034	0.0236	0.16	0.0199	0.1011	0.06	0.1545	0.3130	0.20	0.0249	0.1391	0.44	0.0551	0.2237	0.18	0.1090	0.1659	0.13
410	Androglobin	Q8N7X0	0.4108	0.6246	-3.31	0.5752	0.8740	-1.75	0.5661	0.7805	-1.19	0.5388	0.7376	0.89	0.2938	0.5168	-1.88	0.9510	1.1743	0.09	0.1026	0.2601	0.24	0.6676	1.1063	0.05	0.8965	0.9178	0.02
411	Angio-associated migratory cell protein	Q13685	0.1370	0.2818	-0.25	0.1083	0.6318	-0.70	0.2582	0.4268	-0.17	0.0018	0.0185	-1.06	0.1381	0.3093	-0.24	0.1411	0.2950	0.31	0.3736	0.5194	-2.55	0.8708	1.0689	-0.33	0.0651	0.1499	2.06
412	Angiopoietin-1	Q15389	0.0255	0.0966	-0.29	0.7795	0.8412	0.04	0.5885	0.6449	-0.04	0.4204	0.6122	-0.08	0.8748	0.9047	-0.02	0.5949	0.8313	0.06	0.3736	0.4530	-2.55	0.8708	0.9464	-0.33	0.0651	0.1207	2.06
413	Angiopoietin-2	O15123	0.4108	0.6244	-3.31	0.5752	0.8738	-1.75	0.5661	0.7803	-1.19	0.5388	0.7374	0.89	0.2938	0.5166	-1.88	0.9510	1.1740	0.09	0.2418	0.4741	0.15	0.2274	0.5401	-0.11	0.4494	0.5185	-0.06
414	Anillin	Q9NQW6	0.0019	0.0305	-0.86	0.1015	0.6319	-0.61	0.0064	0.0389	-0.79	0.0474	0.1108	-0.40	0.0318	0.1305	-0.91	0.0007	0.0407	-1.49	0.0306	0.1480	0.48	0.2428	0.5615	0.16	0.0422	0.1786	0.31
415	Anion exchange protein 3	P48751	0.1886	0.3544	-0.25	0.1980	0.6690	-0.26	0.3884	0.5898	0.14	0.7868	0.8168	-0.05	0.4481	0.5417	0.12	0.7533	0.9909	0.04	0.6820	0.7251	-0.14	0.9246	0.9448	-0.03	0.6683	0.7224	-0.15
416	Ankycorbin	Q9P0K7	0.4108	0.6242	-3.31	0.5752	0.8735	-1.75	0.5661	0.7801	-1.19	0.5388	0.7372	0.89	0.2938	0.5164	-1.88	0.9510	1.1737	0.09	0.0007	0.0407	-0.38	0.0168	0.1102	-0.16	0.0013	0.0443	-0.37
417	Ankyrin and armadillo repeat-containing protein	Q7Z5J8	0.8031	0.8411	0.06	0.6419	0.7360	-0.18	0.2781	0.4524	0.28	0.7496	0.7841	0.08	0.2112	0.4147	0.35	0.9881	0.9910	0.00	0.3736	0.4298	-2.55	0.8708	0.9027	-0.33	0.0651	0.1115	2.06
418	Ankyrin repeat and KH domain-containing protein 1	Q8IWZ3	0.6383	0.7002	0.02	0.3806	0.7275	0.07	0.2999	0.4820	0.07	0.2325	0.3765	0.07	0.0695	0.2007	0.12	0.1003	0.2342	0.11	0.0367	0.1591	0.44	0.0310	0.1593	0.18	0.2766	0.3484	0.06
419	Ankyrin repeat and LEM domain-containing protein 1	Q8NAG6	0.0101	0.0587	-1.28	0.2795	0.6654	-1.23	0.2335	0.3940	-0.51	0.0286	0.0776	-0.82	0.3051	0.4029	0.52	0.0418	0.1347	-1.36	0.3736	0.5687	-2.55	0.8708	1.1575	-0.33	0.0651	0.1751	2.06
420	Ankyrin repeat and MYND domain-containing protein 2	Q8IV38	0.4108	0.6240	-3.31	0.5752	0.8733	-1.75	0.5661	0.7799	-1.19	0.5388	0.7370	0.89	0.2938	0.5162	-1.88	0.9510	1.1735	0.09	0.1067	0.2670	-0.12	0.0316	0.1602	-0.25	0.0289	0.1452	-0.23
421	Ankyrin repeat domain-containing protein 11	Q6UB99	0.4108	0.6238	-3.31	0.5752	0.8730	-1.75	0.5661	0.7797	-1.19	0.5388	0.7368	0.89	0.2938	0.5161	-1.88	0.9510	1.1732	0.09	0.0384	0.1621	0.26	0.7514	1.1936	-0.03	0.3770	0.4483	-0.13
422	Ankyrin repeat domain-containing protein 12	Q6UB98	0.4108	0.6237	-3.31	0.5752	0.8727	-1.75	0.5661	0.7795	-1.19	0.5388	0.7366	0.89	0.2938	0.5159	-1.88	0.9510	1.1729	0.09	0.5574	0.6077	0.04	0.0469	0.2018	0.15	0.6544	0.7101	0.04
423	Ankyrin repeat domain-containing protein 16	Q6P6B7	0.0480	0.1410	-0.50	0.1743	0.6610	-0.20	0.0790	0.1708	-0.54	0.3349	0.5087	-0.10	0.1292	0.2971	-0.12	0.0329	0.1166	-0.22	0.3736	0.5775	-2.55	0.8708	1.1731	-0.33	0.0651	0.1799	2.06
424	Ankyrin repeat domain-containing protein 17	O75179	0.4108	0.6235	-3.31	0.5752	0.8725	-1.75	0.5661	0.7793	-1.19	0.5388	0.7364	0.89	0.2938	0.5157	-1.88	0.9510	1.1726	0.09	0.1185	0.2871	0.15	0.4531	0.8572	0.06	0.0212	0.1245	-0.28
425	Ankyrin repeat domain-containing protein 18A	Q8IVF6	0.1538	0.3052	-0.30	0.3237	0.6855	0.19	0.1202	0.2369	-0.37	0.4059	0.5970	-0.15	0.8043	0.8473	0.04	0.8799	1.1166	-0.02	0.4150	0.4708	0.16	0.4011	0.7861	0.16	0.9012	0.9211	-0.03
426	Ankyrin repeat domain-containing protein 20A3	Q5VUR7	0.1483	0.2969	0.03	0.6454	0.7382	0.03	0.0112	0.0490	0.09	0.3198	0.4908	0.05	0.0056	0.0553	0.07	0.0221	0.0954	0.16	0.3736	0.5906	-2.55	0.8708	1.1962	-0.33	0.0651	0.1873	2.06
427	Ankyrin repeat domain-containing protein 20A4	Q4UJ75	0.4108	0.6233	-3.31	0.5752	0.8722	-1.75	0.5661	0.7791	-1.19	0.5388	0.7362	0.89	0.2938	0.5155	-1.88	0.9510	1.1723	0.09	0.0302	0.1473	0.23	0.5054	0.9231	-0.04	0.6681	0.7225	-0.02
428	Ankyrin repeat domain-containing protein 20B	Q5CZ79	0.0187	0.0804	0.17	0.0657	0.5950	0.30	0.0006	0.0206	0.39	0.0005	0.0133	0.38	0.0217	0.1059	0.13	0.0088	0.0684	0.20	0.0020	0.0542	0.87	0.0007	0.0250	0.81	0.8644	0.8900	-0.01
429	Ankyrin repeat domain-containing protein 26	Q9UPS8	0.3879	0.6047	-0.05	0.7666	0.8306	0.04	0.9010	0.9216	0.01	0.1198	0.2227	0.12	0.8306	0.8683	0.01	0.8877	1.1248	0.01	0.9857	0.9889	0.00	0.5516	0.9775	0.03	0.4022	0.4738	-0.06
430	Ankyrin repeat domain-containing protein 35	Q8N283	0.8871	0.9108	0.01	0.3357	0.6950	0.05	0.5101	0.7313	-0.02	0.1389	0.2495	-0.05	0.0061	0.0567	-0.16	0.5180	0.7458	-0.03	0.2103	0.4275	0.22	0.4120	0.7999	0.04	0.0759	0.1258	-0.20
431	Ankyrin repeat domain-containing protein 36C	Q5JPF3	0.1764	0.3387	0.25	0.3024	0.6755	-0.74	0.1210	0.2384	-0.70	0.0787	0.1621	-0.89	0.1970	0.3966	-0.87	0.0100	0.0705	0.88	0.3736	0.6132	-2.55	0.8708	1.2358	-0.33	0.0651	0.2008	2.06
432	Ankyrin repeat domain-containing protein 50	Q9ULJ7	0.6440	0.7057	0.02	0.1676	0.6607	0.45	0.0445	0.1154	0.38	0.0112	0.0425	0.28	0.1421	0.3151	0.09	0.2367	0.4225	0.22	0.3736	0.4938	-2.55	0.8708	1.0221	-0.33	0.0651	0.1381	2.06
433	Ankyrin repeat domain-containing protein 54	Q6NXT1	0.0003	0.0167	0.42	0.0019	0.2289	0.36	0.4274	0.6375	0.08	0.0019	0.0190	0.18	0.0027	0.0398	0.53	0.0386	0.1278	0.27	0.3049	0.5641	0.22	0.6368	1.0752	-0.08	0.4711	0.5397	-0.12
434	Ankyrin repeat domain-containing protein 62	A6NC57	0.5168	0.5891	0.03	0.0027	0.2498	0.45	0.4283	0.6377	0.04	0.0116	0.0430	0.20	0.0151	0.0876	0.27	0.0038	0.0600	0.47	0.0467	0.1732	0.34	0.9596	0.9714	0.00	0.8208	0.8553	-0.02
435	Ankyrin-2	Q01484	0.0672	0.1718	0.12	0.0998	0.6351	0.26	0.0087	0.0440	0.26	0.0048	0.0279	0.22	0.0074	0.0627	0.19	0.0212	0.0942	0.21	0.0058	0.0812	-0.26	0.0764	0.2698	0.26	0.1354	0.1962	-0.06
436	Annexin A1	P04083	0.4108	0.6231	-3.31	0.5752	0.8720	-1.75	0.5661	0.7789	-1.19	0.5388	0.7360	0.89	0.2938	0.5154	-1.88	0.9510	1.1721	0.09	0.2659	0.5079	0.13	0.3712	0.7435	-0.07	0.2434	0.3136	-0.09
437	Annexin A11	P50995	0.8816	0.9068	0.01	0.8178	0.8700	0.03	0.0589	0.1400	-0.10	0.2305	0.3737	0.05	0.1375	0.3082	-0.07	0.0203	0.0922	0.16	0.1238	0.2959	0.23	0.4216	0.8146	0.09	0.2469	0.3175	-0.09
438	Annexin A13	P27216	0.1106	0.2414	-0.16	0.6107	0.7123	-0.06	0.3046	0.4877	-0.09	0.1629	0.2824	-0.13	0.0419	0.1514	-0.25	0.2792	0.4751	-0.11	0.3736	0.4856	-2.55	0.8708	1.0071	-0.33	0.0651	0.1345	2.06
439	Annexin A2	P07355	0.0020	0.0311	0.22	0.1905	0.6690	0.26	0.0022	0.0265	0.33	0.0001	0.0091	0.42	0.0013	0.0289	0.33	0.0004	0.0406	0.46	0.0230	0.1368	0.55	0.5006	0.9169	0.07	0.6035	0.6637	0.05
440	Annexin A3	P12429	0.4108	0.6229	-3.31	0.5752	0.8717	-1.75	0.5661	0.7787	-1.19	0.5388	0.7358	0.89	0.2938	0.5152	-1.88	0.9510	1.1718	0.09	0.3131	0.5739	-0.10	0.0356	0.1719	-0.22	0.0071	0.0774	-0.39
441	Annexin A4	P09525	0.0842	0.2005	-0.06	0.1503	0.6598	0.17	0.0182	0.0647	0.11	0.0228	0.0663	0.14	0.0116	0.0772	0.14	0.0106	0.0706	0.14	0.2404	0.4722	0.14	0.1638	0.4408	-0.11	0.0155	0.1071	-0.14
442	Annexin A5	P08758	0.0042	0.0408	0.14	0.2020	0.6606	0.18	0.0016	0.0234	0.17	0.0009	0.0154	0.20	0.0323	0.1313	-0.07	0.0026	0.0557	0.28	0.0921	0.2438	0.32	0.2204	0.5276	-0.11	0.1574	0.2212	-0.12
443	Annexin A6	P08133	0.1108	0.2418	0.07	0.1568	0.6620	0.25	0.0029	0.0295	0.22	0.0057	0.0304	0.19	0.1273	0.2948	0.06	0.0106	0.0704	0.20	0.0706	0.2097	0.26	0.4766	0.8884	0.07	0.5836	0.6454	0.04
444	Annexin A7	P20073	0.0276	0.1007	0.25	0.2102	0.6623	0.22	0.0255	0.0794	0.24	0.0077	0.0354	0.35	0.2200	0.4262	0.09	0.0253	0.1009	0.33	0.0862	0.2350	0.32	0.9800	0.9859	0.00	0.8189	0.8537	-0.02
445	Annexin A8	P13928	0.0000	0.0000	5.34	0.1346	0.6492	2.90	0.0010	0.0218	4.19	0.1751	0.2988	3.06	0.0211	0.1045	-1.95	0.3788	0.5875	3.35	0.8374	0.8631	-0.02	0.9006	0.9258	-0.04	0.4596	0.5280	0.15
446	Annexin A9	O76027	0.0084	0.0545	-0.52	0.0260	0.4933	-0.41	0.0012	0.0231	-0.89	0.0054	0.0296	-0.78	0.0090	0.0677	-0.54	0.0052	0.0619	-0.79	0.3736	0.6293	-2.55	0.8708	1.2639	-0.33	0.0651	0.2110	2.06
447	Anosmin-1	P23352	0.4108	0.6228	-3.31	0.5752	0.8715	-1.75	0.5661	0.7784	-1.19	0.5388	0.7356	0.89	0.2938	0.5150	-1.88	0.9510	1.1715	0.09	0.8053	0.8360	0.05	0.5782	1.0084	0.13	0.9158	0.9316	-0.02
448	Anterior gradient protein 2 homolog	O95994	0.0723	0.1810	0.10	0.1503	0.6604	0.27	0.0078	0.0417	0.37	0.0008	0.0145	0.42	0.0139	0.0843	0.24	0.0008	0.0446	0.53	0.3736	0.6505	-2.55	0.8708	1.3003	-0.33	0.0651	0.2251	2.06
449	AP-1 complex subunit beta-1	Q10567	0.5998	0.6659	0.05	0.1925	0.6679	0.17	0.4836	0.7035	0.07	0.6425	0.6869	0.04	0.1139	0.2757	0.21	0.4829	0.7080	0.07	0.0889	0.2397	-0.06	0.0035	0.0483	-0.25	0.0056	0.0722	-0.21

Supplemetary Table S2. Overview on all relatively quantified 5180 proteins statistical analysis

			MCF-7																		MDA-MB-231								
			Dai SC20 vs control			Gen SC20 vs control			SSE SC20 vs control			Dai IC20 vs control			Gen IC20 vs control			SSE IC20 vs control			Dai IC20 vs control			Gen IC20 vs control			SSE IC20 vs control		
	Protein name	UniProt	p value	BH q value	log2FC	p value	BH q value	log2FC	p value	BH q value	log2FC	p value	BH q value	log2FC	p value	BH q value	log2FC	p value	BH q value	log2FC	p value	BH q value	log2FC	p value	BH q value	log2FC	p value	BH q value	log2FC
450	AP-1 complex subunit gamma-1	O43747	0.0458	0.1370	0.14	0.2271	0.6598	0.15	0.0650	0.1489	0.16	0.0406	0.0996	0.17	0.8137	0.8551	-0.01	0.0280	0.1069	0.19	0.1207	0.2905	-0.14	0.2673	0.5973	-0.10	0.0262	0.1383	-0.29
451	AP-1 complex subunit gamma-like 2	O75843	0.0421	0.1302	-0.18	0.3212	0.6836	-0.62	0.1232	0.2415	0.25	0.0199	0.0601	-0.24	0.0924	0.2397	-0.21	0.6132	0.8502	-0.05	0.3736	0.4514	-2.55	0.8708	0.9435	-0.33	0.0651	0.1200	2.06
452	AP-1 complex subunit mu-1	Q9BXS5	0.9452	0.9587	0.00	0.3207	0.6842	0.08	0.4391	0.6508	0.05	0.0072	0.0343	0.04	0.0656	0.1951	0.04	0.0975	0.2303	0.07	0.0350	0.1548	0.31	0.1203	0.3590	0.17	0.7347	0.7811	0.02
453	AP-1 complex subunit mu-2	Q9Y6Q5	0.0048	0.0436	0.22	0.1664	0.6595	0.35	0.0346	0.0968	0.34	0.0005	0.0120	0.51	0.0014	0.0291	0.31	0.0014	0.0526	0.39	0.3736	0.6470	-2.55	0.8708	1.2943	-0.33	0.0651	0.2227	2.06
454	AP-1 complex subunit sigma-1A	P61966	0.1426	0.2893	0.12	0.5798	0.6876	-0.06	0.2956	0.4761	-0.07	0.7979	0.8261	-0.02	0.0697	0.2010	-0.15	0.4096	0.6268	0.06	0.0999	0.2555	-0.23	0.0083	0.0749	-0.45	0.0528	0.2023	-0.40
455	AP-2 complex subunit alpha-1	O95782	0.3327	0.5397	-0.04	0.8210	0.8724	0.01	0.0993	0.2029	0.07	0.4413	0.6353	-0.03	0.6523	0.7194	0.02	0.7134	0.9541	0.01	0.6313	0.6770	-0.05	0.0296	0.1553	-0.27	0.0490	0.1938	-0.24
456	AP-2 complex subunit alpha-2	O94973	0.4108	0.6226	-3.31	0.5752	0.8712	-1.75	0.5661	0.7782	-1.19	0.5388	0.7354	0.89	0.2938	0.5148	-1.88	0.9510	1.1712	0.09	0.0016	0.0518	0.47	0.0138	0.0981	0.35	0.0302	0.1476	0.18
457	AP-2 complex subunit beta	P63010	0.0822	0.1975	0.08	0.0516	0.5773	0.15	0.0015	0.0233	0.30	0.0063	0.0319	0.18	0.0101	0.0726	0.19	0.0086	0.0680	0.19	0.2436	0.4767	0.13	0.1680	0.4474	0.11	0.1151	0.1727	-0.05
458	AP-2 complex subunit mu	Q96CW1	0.8956	0.9183	0.01	0.2750	0.6641	0.17	0.9390	0.9528	0.01	0.0857	0.1729	0.19	0.5172	0.6056	0.05	0.2846	0.4810	0.10	0.0504	0.1787	-0.30	0.0031	0.0457	-0.75	0.0046	0.0667	-1.00
459	AP2-associated protein kinase 1	Q2M2I8	0.0328	0.1130	-0.51	0.7715	0.8340	-0.05	0.0983	0.2014	-0.36	0.6419	0.6866	-0.08	0.2268	0.4361	-0.21	0.4804	0.7047	-0.12	0.0384	0.1622	-0.55	0.0194	0.1198	-0.60	0.0097	0.0872	-1.16
460	AP-3 complex subunit beta-1	O00203	0.2423	0.4278	0.06	0.2877	0.6686	0.24	0.3624	0.5604	0.10	0.0354	0.0902	0.22	0.2611	0.4822	-0.05	0.0597	0.1688	0.12	0.5649	0.6150	0.05	0.1838	0.4727	0.11	0.5031	0.5701	-0.04
461	AP-3 complex subunit delta-1	O14617	0.4455	0.5230	0.05	0.0596	0.5926	0.20	0.0140	0.0556	0.19	0.0091	0.0383	0.28	0.5639	0.6460	0.02	0.3019	0.4998	0.14	0.1168	0.2844	0.17	0.2803	0.6176	-0.10	0.1756	0.2415	0.13
462	AP-3 complex subunit mu-1	Q9Y2T2	0.0458	0.1369	0.16	0.5504	0.8658	-0.03	0.1263	0.2459	0.11	0.1535	0.2700	-0.09	0.7782	0.8259	-0.02	0.0378	0.1259	0.21	0.0078	0.0908	-0.50	0.0007	0.0247	-0.80	0.0446	0.1844	-0.25
463	AP-4 complex subunit beta-1	Q9Y6B7	0.0010	0.0227	0.17	0.6929	0.7744	0.06	0.0274	0.0831	-0.06	0.1125	0.2127	0.05	0.3849	0.4825	-0.03	0.0122	0.0744	0.09	0.3736	0.6080	-2.55	0.8708	1.2267	-0.33	0.0651	0.1977	2.06
464	Apolipoprotein A-I	P02647	0.6907	0.7462	0.02	0.3829	0.7303	-0.14	0.0108	0.0482	-0.44	0.0004	0.0115	-0.53	0.1290	0.2967	0.10	0.0204	0.0924	-0.16	0.3736	0.5936	-2.55	0.8708	1.2016	-0.33	0.0651	0.1891	2.06
465	Apolipoprotein A-V	Q6Q788	0.0810	0.1961	0.14	0.3002	0.6735	0.21	0.1817	0.3258	0.10	0.0260	0.0725	0.23	0.2219	0.4289	-0.09	0.3614	0.5675	0.06	0.3736	0.4727	-2.55	0.8708	0.9832	-0.33	0.0651	0.1289	2.06
466	Apolipoprotein B receptor	Q0VD83	0.0313	0.1096	-0.14	0.4422	0.7778	0.07	0.0077	0.0415	0.31	0.0001	0.0078	0.68	0.0000	0.0000	0.86	0.0050	0.0621	0.27	0.3736	0.6304	-2.55	0.8708	1.2656	-0.33	0.0651	0.2117	2.06
467	Apolipoprotein B-100	P04114	0.0515	0.1471	0.08	0.2541	0.6628	0.14	0.0042	0.0322	0.27	0.0002	0.0102	0.21	0.0026	0.0397	0.08	0.0357	0.1221	0.20	0.0023	0.0559	-0.43	0.0171	0.1109	-0.23	0.0037	0.0595	-0.41
468	Apoptogenic protein 1 mitochondrial	Q96IL0	0.0021	0.0319	0.22	0.0295	0.5094	0.25	0.0080	0.0422	0.10	0.1656	0.2858	0.04	0.0001	0.0080	0.46	0.7376	0.9772	0.03	0.3736	0.4428	-2.55	0.8708	0.9274	-0.33	0.0651	0.1166	2.06
469	Apoptosis inhibitor 5	Q9BZZ5	0.0215	0.0874	0.14	0.3864	0.7297	0.17	0.0287	0.0858	0.24	0.0028	0.0216	0.38	0.4411	0.5356	-0.03	0.0032	0.0566	0.32	0.0303	0.1475	0.31	0.0160	0.1074	0.18	0.0679	0.1152	0.11
470	Apoptosis regulator BAX	Q07812	0.0028	0.0358	0.21	0.1392	0.6525	0.24	0.0186	0.0655	0.15	0.0159	0.0522	0.24	0.0002	0.0131	0.40	0.0903	0.2191	0.19	0.0153	0.1147	0.29	0.5295	0.9534	0.04	0.4181	0.4895	0.05
471	Apoptosis-associated speck-like protein containing a CARD	Q9ULZ3	0.0818	0.1971	0.37	0.4179	0.7564	0.10	0.2357	0.3969	0.13	0.0030	0.0222	0.29	0.0169	0.0932	0.24	0.1196	0.2642	0.29	0.3736	0.5272	-2.55	0.8708	1.0830	-0.33	0.0651	0.1537	2.06
472	Apoptosis-inducing factor 1 mitochondrial	O95831	0.6884	0.7441	0.01	0.5430	0.8573	0.07	0.0161	0.0600	0.12	0.0158	0.0520	0.08	0.2567	0.4766	0.02	0.0086	0.0679	0.15	0.0382	0.1621	0.38	0.4413	0.8426	0.07	0.1483	0.2109	0.13
473	Arachidonate 15-lipoxygenase	P16050	0.6789	0.7360	0.06	0.6513	0.7429	0.07	0.5090	0.7306	-0.08	0.3167	0.4868	-0.12	0.9880	0.9914	0.00	0.5730	0.8074	-0.07	0.3736	0.4544	-2.55	0.8708	0.9490	-0.33	0.0651	0.1213	2.06
474	Arfaptin-1	P53367	0.6218	0.6852	-0.02	0.4071	0.7499	0.11	0.1492	0.2782	0.07	0.0498	0.1148	0.15	0.4531	0.5458	-0.03	0.1500	0.3076	0.08	0.0104	0.1020	-0.48	0.7842	1.2284	-0.04	0.3866	0.4577	0.13
475	Arfaptin-2	P53365	0.1254	0.2638	-0.11	0.9096	0.9373	0.01	0.4330	0.6432	0.05	0.9224	0.9319	0.01	0.0371	0.1399	-0.22	0.0740	0.1936	-0.15	0.3736	0.5479	-2.55	0.8708	1.1204	-0.33	0.0651	0.1641	2.06
476	Arf-GAP domain and FG repeat-containing protein 1	P52594	0.4400	0.5174	0.05	0.6149	0.7153	0.05	0.6023	0.6582	0.04	0.1577	0.2754	0.10	0.1285	0.2958	-0.11	0.6559	0.8929	-0.04	0.1829	0.3842	-0.10	0.0342	0.1676	0.18	0.0102	0.0891	0.33
477	Arf-GAP with coiled-coil_ ANK repeat and PH domain-containing protein 1	Q15027	0.4108	0.6224	-3.31	0.5752	0.8710	-1.75	0.5661	0.7780	-1.19	0.5388	0.7352	0.89	0.2938	0.5147	-1.88	0.9510	1.1709	0.09	0.0184	0.1257	-11.26	0.2978	0.6411	-2.13	0.3765	0.4480	-1.68
478	Arf-GAP with coiled-coil_ ANK repeat and PH domain-containing protein 2	Q15057	0.0128	0.0664	-0.10	0.0752	0.6154	0.24	0.0169	0.0616	0.20	0.4803	0.6807	0.03	0.0311	0.1292	0.10	0.1194	0.2639	-0.12	0.3736	0.5273	-2.55	0.8708	1.0833	-0.33	0.0651	0.1538	2.06
479	Arf-GAP with dual PH domain-containing protein 1	O75689	0.6916	0.7470	-0.04	0.6936	0.7743	0.05	0.9703	0.9746	0.00	0.8560	0.8760	0.02	0.4700	0.5608	0.08	0.4240	0.6437	-0.33	0.0018	0.0518	-0.65	0.0025	0.0418	-0.35	0.0015	0.0477	-0.36
480	Arf-GAP with GTPase_ ANK repeat and PH domain-containing protein 11	Q8TF27	0.0542	0.1523	-0.34	0.1983	0.6666	-0.41	0.0569	0.1364	-0.37	0.0060	0.0310	-0.90	0.2173	0.4228	-0.48	0.0052	0.0612	-0.60	0.3736	0.6289	-2.55	0.8708	1.2632	-0.33	0.0651	0.2108	2.06
481	Arf-GAP with GTPase_ ANK repeat and PH domain-containing protein 5	A6NIR3	0.1401	0.2855	0.19	0.7665	0.8308	-0.16	0.3070	0.4907	-0.11	0.2652	0.4197	-0.23	0.3465	0.4437	-0.07	0.1381	0.2913	-0.33	0.3736	0.5209	-2.55	0.8708	1.0717	-0.33	0.0651	0.1507	2.06
482	Arf-GAP with GTPase_ ANK repeat and PH domain-containing protein 9	Q5VTM2	0.4108	0.6222	-3.31	0.5752	0.8707	-1.75	0.5661	0.7778	-1.19	0.5388	0.7350	0.89	0.2938	0.5145	-1.88	0.9510	1.1707	0.09	0.1056	0.2657	-0.15	0.1854	0.4750	0.15	0.1406	0.2020	-0.13
483	Arginase-1	P05089	0.0553	0.1540	-0.94	0.2440	0.6579	-1.82	0.1285	0.2492	-0.88	0.0482	0.1121	-0.93	0.4662	0.5575	-0.52	0.0340	0.1188	-1.19	0.3736	0.5758	-2.55	0.8708	1.1701	-0.33	0.0651	0.1790	2.06
484	Arginine and glutamate-rich protein 1	Q9NWB6	0.0178	0.0783	1.40	0.2037	0.6599	0.41	0.3199	0.5072	-0.25	0.3573	0.5355	0.24	0.5769	0.6558	0.13	0.3755	0.5841	-0.22	0.8723	0.8944	0.01	0.0828	0.2831	-0.23	0.1487	0.2113	-0.13
485	Arginine--tRNA ligase cytoplasmic	P54136	0.2347	0.4176	0.09	0.3935	0.7372	0.15	0.0877	0.1854	0.16	0.0628	0.1365	0.19	0.9378	0.9520	0.01	0.0468	0.1437	0.23	0.0437	0.1686	0.36	0.3338	0.6914	0.10	0.7411	0.7865	0.02
486	Argininosuccinate lyase	P04424	0.0109	0.0601	-0.18	0.0211	0.4752	-0.32	0.0009	0.0222	-0.40	0.0007	0.0141	-0.53	0.0025	0.0390	-0.39	0.4072	0.6235	-0.03	0.0024	0.0563	0.50	0.0289	0.1531	0.25	0.0377	0.1673	0.47
487	Argininosuccinate synthase	P00966	0.0001	0.0157	0.65	0.0481	0.5728	0.46	0.0001	0.0235	0.62	0.0000	0.0000	0.68	0.0068	0.0601	0.23	0.0004	0.0384	0.60	0.3736	0.6531	-2.55	0.8708	1.3048	-0.33	0.0651	0.2269	2.06
488	Arginyl-tRNA--protein transferase 1	O95260	0.0252	0.0959	-0.50	0.6043	0.7072	-0.15	0.3885	0.5898	0.07	0.4507	0.6465	0.16	0.1235	0.2897	-0.13	0.5393	0.7713	-0.04	0.3736	0.4577	-2.55	0.8708	0.9553	-0.33	0.0651	0.1226	2.06
489	ARL14 effector protein	Q8N8R7	0.0073	0.0505	-1.21	0.2073	0.6588	-1.09	0.1092	0.2191	-1.26	0.0062	0.0314	-1.71	0.7431	0.7989	0.07	0.0316	0.1143	-1.58	0.3736	0.5794	-2.55	0.8708	1.1765	-0.33	0.0651	0.1810	2.06
490	Armadillo repeat protein deleted in velo-cardio-facial syndrome	O00192	0.4108	0.6220	-3.31	0.5752	0.8704	-1.75	0.5661	0.7776	-1.19	0.5388	0.7349	0.89	0.2938	0.5143	-1.88	0.9510	1.1704	0.09	0.0067	0.0844	1.18	0.0005	0.0227	1.07	0.0281	0.1434	0.33
491	Armadillo repeat-containing protein 10	Q8N2F6	0.0518	0.1473	0.66	0.3914	0.7357	0.04	0.3697	0.5676	0.05	0.0337	0.0871	0.26	0.4556	0.5482	-0.04	0.2602	0.4515	0.10	0.1671	0.3617	-2.09	0.1873	0.4770	-1.02	0.3859	0.4572	0.43
492	Armadillo repeat-containing protein 2	Q8NEN0	0.0017	0.0284	0.41	0.1002	0.6337	0.34	0.0017	0.0239	0.41	0.0016	0.0179	0.40	0.0006	0.0201	0.37	0.0022	0.0551	0.39	0.3736	0.6436	-2.55	0.8708	1.2884	-0.33	0.0651	0.2204	2.06
493	Armadillo repeat-containing protein 6	Q6NXE6	0.8157	0.8517	-0.07	0.5541	0.8690	-0.27	0.4448	0.6576	0.24	0.3389	0.5130	-0.32	0.4612	0.5531	0.23	0.8700	1.1059	-0.05	0.3736	0.4352	-2.55	0.8708	0.9129	-0.33	0.0651	0.1136	2.06
494	Armadillo repeat-containing X-linked protein 3	Q9UH62	0.0120	0.0633	0.41	0.0626	0.5917	0.23	0.0048	0.0338	0.53	0.0075	0.0352	0.47	0.0078	0.0636	0.54	0.0224	0.0961	0.37	0.2093	0.4258	0.17	0.9259	0.9452	0.01	0.0832	0.1348	-0.30
495	Arrestin domain-containing protein 5	A6NEK1	0.4108	0.6218	-3.31	0.5752	0.8702	-1.75	0.5661	0.7774	-1.19	0.5388	0.7347	0.89	0.2938	0.5142	-1.88	0.9510	1.1701	0.09	0.6704	0.7140	-0.23	0.0201	0.1226	2.46	0.0148	0.1050	3.77
496	Asialoglycoprotein receptor 1	P07306	0.4108	0.6217	-3.31	0.5752	0.8699	-1.75	0.5661	0.7772	-1.19	0.5388	0.7345	0.89	0.2938	0.5140	-1.88	0.9510	1.1698	0.09	0.0047	0.0725	-0.59	0.0330	0.1640	-0.43	0.0303	0.1477	-0.41
497	ASNSD1 upstream open reading frame protein	L0R819	0.6110	0.6754	-0.02	0.2383	0.6622	0.20	0.6302	0.6835	0.03	0.0305	0.0811	0.11	0.0202	0.1021	-0.11	0.6052	0.8423	0.05	0.1370	0.3164	2.00	0.1176	0.3533	2.20	0.0885	0.1414	2.62
498	Asparagine synthetase [glutamine-hydrolyzing]	P08243	0.0007	0.0216	0.44	0.5027	0.8240	0.11	0.0005	0.0185	0.50	0.0001	0.0108	0.82	0.0143	0.0856	0.23	0.0007	0.0422	1.12	0.0016	0.0531	0.28	0.9698	0.9785	0.00	0.2015	0.2696	0.04
499	Asparagine--tRNA ligase cytoplasmic	O43776	0.0108	0.0600	0.14	0.5241	0.8431	0.08	0.0056	0.0360	0.22	0.0135	0.0471	0.13	0.0676	0.1981	-0.07	0.0023	0.0552	0.26	0.0611	0.1951	0.16	0.1570	0.4289	-0.10	0.6287	0.6863	0.03
500	Aspartate aminotransferase cytoplasmic	P17174	0.0042	0.0407	0.22	0.3434	0.7009	0.13	0.0365	0.1002	0.14	0.0080	0.0361	0.21	0.0914	0.2389	-0.07	0.1794	0.3487	0.23	0.7367	0.7753	0.04	0.0169	0.1100	-0.25	0.0380	0.1682	-0.20
501	Aspartate aminotransferase mitochondrial	P00505	0.0096	0.0576	0.21	0.1927	0.6681	0.20	0.0111	0.0489	0.17	0.0022	0.0202	0.25	0.1735	0.3621	0.05	0.0098	0.0705	0.27	0.9235	0.9371	0.01	0.0132	0.0956	-0.41	0.0050	0.0682	-0.50
502	Aspartate-rich protein 1	Q6PGQ1	0.4108	0.6215	-3.31	0.5752	0.8697	-1.75	0.5661	0.7770	-1.19	0.5388	0.7343	0.89	0.2938	0.5138	-1.88	0.9510	1.1696	0.09	0.0233	0.1364	0.52	0.0793	0.2764	0.33	0.1054	0.1618	0.29
503	Aspartate--tRNA ligase cytoplasmic	P14868	0.0372	0.1213	0.19	0.2245	0.6596	0.26	0.0016	0.0233	0.33	0.0054	0.0296	0.27	0.6028	0.6785	0.02	0.0048	0.0601	0.32	0.0641	0.1994	0.38	0.5489	0.9744	0.07	0.8531	0.8808	-0.02
504	Aspartate--tRNA ligase mitochondrial	Q6PI48	0.0637	0.1672	-0.21	0.2820	0.6640	-0.23	0.3712	0.5692	0.07	0.3741	0.5575	-0.10	0.3432	0.4402	-0.08	0.2723	0.4663	-0.25	0.1000	0.2556	-0.17	0.0682	0.2518	-0.34	0.0164	0.1096	-0.35
505	Aspartyl aminopeptidase	Q9ULA0	0.2397	0.4243	0.06	0.2410	0.6609	0.18	0.5091	0.7305	0.03	0.4669	0.6661	0.04	0.2186	0.4243	-0.07	0.7565	0.9941	-0.01	0.0003	0.0361	-0.53	0.7491	1.1903	0.01	0.0616	0.2216	-0.18
506	Aspartyl/asparaginyl beta-hydroxylase	Q12797	0.5983	0.6644	0.03	0.2007	0.6622	0.10	0.3245	0.5123	0.08	0.0114	0.0428	0.18	0.1918	0.3879	0.04	0.4517	0.6739	0.02	0.3736	0.4639	-2.55	0.8708	0.9667	-0.33	0.0651	0.1252	2.06
507	Astrocytic phosphoprotein PEA-15	Q15121	0.9051	0.9262	-0.01	0.1472	0.6573	0.23	0.0560	0.1352	0.14	0.0048	0.0278	0.32	0.1436	0.3177	0.09	0.0281	0.1070	0.20	0.0577	0.1916	0.44	0.3120	0.6610	0.08	0.1752	0.2410	0.07
508	Astrotactin-1	O14525	0.0756	0.1873	-0.05	0.2648	0.6636	0.27	0.0399	0.1064	0.18	0.1591	0.2774	0.11	0.3499	0.4475	-0.04	0.6524	0.8893	-0.05	0.4524	0.5073	0.48	0.1835	0.4727	0.96	0.0198	0.1215	1.56
509	Ataxin-10	Q9UBB4	0.0896	0.2093	-0.06	0.4144	0.7532	0.08	0.0873	0.1848	0.06	0.1784	0.3031	0.04	0.9510	0.9621	0.00	0.1299	0.2804	0.04	0.8299	0.8567	0.00	0.7037	1.1452	0.02	0.0151	0.1054	0.10
510	Ataxin-2-like protein	Q8WWM7	0.1158	0.2495	-0.05	0.9773	0.9853	0.00	0.0293	0.0870	0.12	0.8069	0.8343	0.01	0.0422	0.1517	-0.08	0.0623	0.1737	-0.07	0.0508	0.1791	0.37	0.9340	0.9520	-0.01	0.2607	0.3327	0.13
511	Ataxin-3	P54252	0.4914	0.5655	-0.03	0.0379	0.5530	0.10	0.0037	0.0321	0.29	0.8180	0.8429	-0.01	0.5481	0.6320	0.02	0.0131	0.0766	-0.11	0.3736	0.6069	-2.55	0.8708	1.2247	-0.33	0.0651	0.1970	2.06
512	Atlastin-2	Q8NHH9	0.6562	0.7170	0.07	0.2334	0.6617	0.21	0.4666	0.6839	-0.11	0.6972	0.7367	-0.06	0.3862	0.4833	-0.14	0.2396	0.4256	-0.21	0.3736	0.4924	-2.55	0.8708	1.0196	-0.33	0.0651	0.1375	2.06
513	Atlastin-3	Q6DD88	0.0959	0.2186	0.13	0.1562	0.6621	0.24	0.0512	0.1272	0.16	0.2285	0.3713	0.08	0.6949	0.7573	-0.02	0.8208	1.0569	0.01	0.3035	0.5619	0.13	0.1900	0.4822	0.18	0.2284	0.2988	0.16
514	ATP synthase F(0) complex subunit B1 mitochondrial	P24539	0.0078	0.0525	0.12	0.2872	0.6677	0.23	0.0023	0.0265	0.26	0.0026	0.0215	0.30	0.0235	0.1110	0.10	0.0054	0.0619	0.24	0.0279	0.1445	0.26	0.5540	0.9798	0.05	0.1337	0.1939	0.09
515	ATP synthase mitochondrial F1 complex assembly factor 2	Q8N5M1	0.1537	0.3052	-0.20	0.1507	0.6588	-0.57	0.1034	0.2097	-0.25	0.0695	0.1471	-0.44	0.4806	0.5706	-0.10	0.0365	0.1237	-0.33	0.3736	0.5724	-2.55	0.8708	1.1641	-0.33	0.0651	0.1771	2.06
516	ATP synthase subunit a	P00846	0.1338	0.2770	-0.03	0.2740	0.6648	0.19	0.0368	0.1006	0.13	0.0043	0.0265	0.14	0.0843	0.2256	0.04	0.2665	0.4592	0.11	0.0208	0.1324	0.45	0.0037	0.0495	0.21	0.0096	0.0874	0.18
517	ATP synthase subunit alpha mitochondrial	P25705	0.0065	0.0484	0.27	0.1873	0.6664	0.32	0.0023	0.0268	0.35	0.0009	0.0153	0.43	0.0288	0.1234	0.18	0.0028	0.0549	0.47	0.1464	0.3306	0.24	0.6902	1.1321	-0.04	0.1100	0.1669	-0.15
518	ATP synthase subunit beta mitochondrial	P06576	0.0002	0.0164	0.19	0.3856	0.7311	0.11	0.0313	0.0904	0.16	0.0087	0.0377	0.11	0.2556	0.4754	-0.02	0.0047	0.0597	0.31	0.2315	0.4589	0.13	0.2546	0.5792	0.07	0.4148	0.4861	-0.04
519	ATP synthase subunit d mitochondrial	O75947	0.0954	0.2179	0.08	0.1533	0.6595	0.07	0.0095	0.0455	0.19	0.0987	0.1921	0.14	0.8728	0.9031	-0.01	0.0147	0.0805	0.15	0.0067	0.0846	0.43	0.0017	0.0340	0.16	0.0287	0.1450	0.29
520	ATP synthase subunit delta mitochondrial	P30049	0.2016	0.3722	0.13	0.5270	0.8457	0.12	0.2006	0.3521	0.13	0.2489	0.3981	0.12	0.4116	0.5067	0.07	0.2381	0.4240	0.12	0.3736	0.4932	-2.55	0.8708	1.0210	-0.33	0.0651	0.1378	2.06
521	ATP synthase subunit e mitochondrial	P56385	0.3262	0.5320	0.25	0.2724	0.6656	0.35	0.2949	0.4754	0.27	0.1224	0.2260	0.46	0.5367	0.6218	0.15	0.1233	0.2701	0.68	0.3736	0.5263	-2.55	0.8708	1.0815	-0.33	0.0651	0.1533	2.06
522	ATP synthase subunit epsilon mitochondrial	P56381	0.4204	0.4979	-0.16	0.5352	0.8509	0.12	0.0987	0.2019	-0.52	0.8295	0.8525	0.04	0.3410	0.4382	0.20	0.6119	0.8489	-0.10	0.0140	0.1109	-0.31	0.1096	0.3375	-0.23	0.0513	0.1988	-0.19

Supplemetary Table S2. Overview on all relatively quantified 5180 proteins statistical analysis

			MCF-7																		MDA-MB-231								
			Dai SC20 vs control			Gen SC20 vs control			SSE SC20 vs control			Dai IC20 vs control			Gen IC20 vs control			SSE IC20 vs control			Dai IC20 vs control			Gen IC20 vs control			SSE IC20 vs control		
	**Protein name**	**UniProt**	**p value**	**BH q value**	**log2FC**	**p value**	**BH q value**	**log2FC**	**p value**	**BH q value**	**log2FC**	**p value**	**BH q value**	**log2FC**	**p value**	**BH q value**	**log2FC**	**p value**	**BH q value**	**log2FC**	**p value**	**BH q value**	**log2FC**	**p value**	**BH q value**	**log2FC**	**p value**	**BH q value**	**log2FC**
523	ATP synthase subunit f mitochondrial	P56134	0.0082	0.0538	-2.71	0.2217	0.6589	-1.90	0.1366	0.2602	-2.15	0.0261	0.0727	-2.85	0.0634	0.1906	-0.33	0.0438	0.1378	-2.55	0.5196	0.5728	-0.28	0.2902	0.6298	0.50	0.6297	0.6871	0.20
524	ATP synthase subunit g mitochondrial	O75964	0.0285	0.1027	-0.27	0.0768	0.6139	-0.27	0.3641	0.5617	-0.08	0.0142	0.0484	-0.33	0.5868	0.6644	-0.05	0.0529	0.1553	-0.32	0.3270	0.5943	-0.17	0.0149	0.1030	-0.46	0.0061	0.0740	-0.64
525	ATP synthase subunit gamma mitochondrial	P36542	0.0417	0.1295	-0.11	0.2270	0.6602	-0.14	0.6242	0.6777	0.03	0.0945	0.1859	-0.16	0.7439	0.7995	-0.02	0.3909	0.6028	-0.03	0.0813	0.2273	0.29	0.4426	0.8441	-0.10	0.1020	0.1579	-0.14
526	ATP synthase subunit O mitochondrial	P48047	0.9233	0.9413	0.01	0.5401	0.8553	0.12	0.1908	0.3378	0.12	0.1157	0.2170	0.16	0.5778	0.6566	0.04	0.0827	0.2075	0.21	0.0724	0.2131	0.26	0.2957	0.6374	0.07	0.8764	0.8999	-0.01
527	ATP synthase-coupling factor 6 mitochondrial	P18859	0.5461	0.6158	-0.02	0.5208	0.8412	-0.09	0.5816	0.6384	0.02	0.0844	0.1709	0.08	0.4172	0.5120	-0.03	0.0969	0.2293	0.20	0.0378	0.1612	0.25	0.6615	1.1007	0.03	0.2114	0.2801	0.08
528	ATPase ASNA1	O43681	0.1072	0.2359	-0.12	0.3493	0.7046	-0.19	0.0731	0.1616	-0.16	0.0506	0.1159	-0.21	0.0648	0.1937	-0.18	0.3252	0.5276	-0.10	0.8733	0.8949	0.08	0.5261	0.9502	0.33	0.2340	0.3040	0.69
529	ATPase family AAA domain-containing protein 1	Q8NBU5	0.1383	0.2830	0.08	0.9498	0.9666	0.00	0.0175	0.0630	0.28	0.0039	0.0253	0.27	0.1591	0.3417	0.11	0.0014	0.0514	0.44	0.0397	0.1643	0.61	0.0042	0.0522	0.74	0.0458	0.1872	1.02
530	ATPase family AAA domain-containing protein 2	Q6PL18	0.0040	0.0402	-3.20	0.0286	0.5039	-14.11	0.0010	0.0222	-6.51	0.0051	0.0290	-5.05	0.2142	0.4190	-0.78	0.0421	0.1353	-3.82	0.8149	0.8436	0.14	0.5179	0.9393	0.32	0.9123	0.9295	0.19
531	ATPase family AAA domain-containing protein 2B	Q9ULI0	0.0515	0.1471	-0.29	0.4625	0.7943	0.09	0.0502	0.1255	-0.13	0.0085	0.0372	-0.08	0.5655	0.6472	-0.04	0.9220	1.1592	0.00	0.3736	0.4329	-2.55	0.8708	0.9087	-0.33	0.0651	0.1127	2.06
532	ATPase family AAA domain-containing protein 3A	Q9NVI7	0.2275	0.4073	0.06	0.4062	0.7488	0.14	0.0355	0.0983	0.18	0.0063	0.0317	0.28	0.0056	0.0557	0.36	0.0391	0.1289	0.15	0.0259	0.1400	0.68	0.1182	0.3547	0.18	0.0277	0.1426	0.31
533	ATPase family AAA domain-containing protein 3B	Q5T9A4	0.1413	0.2871	-0.12	0.7173	0.7929	0.03	0.2379	0.4000	-0.09	0.4538	0.6504	0.06	0.9848	0.9886	0.00	0.2499	0.4393	-0.11	0.1127	0.2765	0.16	0.0106	0.0842	0.34	0.0045	0.0660	0.85
534	ATPase family protein 2 homolog	Q8NB90	0.1034	0.2300	0.06	0.1918	0.6677	0.13	0.0629	0.1459	0.12	0.0011	0.0153	0.26	0.0418	0.1513	0.09	0.0042	0.0601	0.31	0.3028	0.5614	-0.19	0.2266	0.5387	-0.18	0.0922	0.1460	0.17
535	ATPase inhibitor mitochondrial	Q9UII2	0.4108	0.6213	-3.31	0.5752	0.8694	-1.75	0.5661	0.7768	-1.19	0.5388	0.7341	0.89	0.2938	0.5136	-1.88	0.9510	1.1693	0.09	0.2655	0.5075	-0.14	0.1242	0.3668	-0.23	0.0759	0.1259	-0.48
536	ATPase MORC2	Q9Y6X9	0.2804	0.4739	0.18	0.8253	0.8746	-0.08	0.4392	0.6508	0.14	0.9287	0.9372	0.04	0.7562	0.8090	-0.07	0.5579	0.7933	-0.10	0.3736	0.4563	-2.55	0.8708	0.9526	-0.33	0.0651	0.1220	2.06
537	ATP-binding cassette sub-family A member 12	Q86UK0	0.0544	0.1527	0.22	0.2073	0.6584	0.20	0.0084	0.0431	0.26	0.0393	0.0973	0.14	0.9406	0.9544	0.00	0.0122	0.0743	0.28	0.0285	0.1454	0.53	0.1555	0.4266	0.18	0.0550	0.2070	0.29
538	ATP-binding cassette sub-family B member 6_ mitochondrial	Q9NP58	0.1722	0.3332	0.32	0.4644	0.7960	-0.64	0.0759	0.1658	0.48	0.7267	0.7643	0.08	0.0859	0.2289	0.46	0.0717	0.1889	0.51	0.3736	0.5487	-2.55	0.8708	1.1218	-0.33	0.0651	0.1645	2.06
539	ATP-binding cassette sub-family C member 11	Q96J66	0.3798	0.5956	-0.08	0.6641	0.7522	0.04	0.4542	0.6690	-0.06	0.0621	0.1354	-0.23	0.5604	0.6428	-0.06	0.1479	0.3041	-0.15	0.3736	0.5170	-2.55	0.8708	1.0646	-0.33	0.0651	0.1488	2.06
540	ATP-binding cassette sub-family D member 3	P28288	0.3111	0.5122	-0.06	0.0697	0.6017	-0.22	0.1039	0.2102	-0.12	0.1633	0.2828	-0.13	0.3055	0.4032	-0.07	0.7434	0.9824	-0.05	0.0144	0.1115	0.28	0.1334	0.3850	0.10	0.3468	0.4181	0.03
541	ATP-binding cassette sub-family E member 1	P61221	0.0760	0.1881	0.20	0.2343	0.6610	0.13	0.0124	0.0523	0.28	0.0165	0.0536	0.25	0.2569	0.4766	0.07	0.0333	0.1176	0.24	0.0247	0.1385	0.30	0.0964	0.3100	0.19	0.2945	0.3660	0.11
542	ATP-binding cassette sub-family F member 1	Q8NE71	0.0026	0.0344	0.28	0.2556	0.6627	0.18	0.0036	0.0315	0.41	0.0022	0.0204	0.30	0.0316	0.1302	0.13	0.0057	0.0616	0.39	0.0055	0.0791	0.34	0.0073	0.0707	0.28	0.0150	0.1056	0.23
543	ATP-binding cassette sub-family F member 2	Q9UG63	0.2415	0.4270	-0.04	0.6763	0.7629	0.06	0.0648	0.1486	0.08	0.1170	0.2185	0.06	0.2729	0.4967	-0.04	0.3258	0.5282	-0.04	0.0772	0.2213	0.29	0.7252	1.1703	-0.03	0.2728	0.3447	0.14
544	ATP-binding cassette sub-family F member 3	Q9NUQ8	0.4149	0.4928	0.03	0.2769	0.6647	0.17	0.3667	0.5640	0.04	0.6791	0.7197	0.03	0.0153	0.0881	-0.18	0.9090	1.1462	0.01	0.3736	0.4336	-2.55	0.8708	0.9100	-0.33	0.0651	0.1130	2.06
545	ATP-binding cassette sub-family G member 1	P45844	0.0001	0.0152	0.77	0.0717	0.6049	0.81	0.0029	0.0296	0.88	0.0079	0.0361	0.76	0.0086	0.0664	0.31	0.0041	0.0605	0.90	0.3736	0.6343	-2.55	0.8708	1.2724	-0.33	0.0651	0.2142	2.06
546	ATP-citrate synthase	P53396	0.8985	0.9204	-0.01	0.9977	0.9983	0.00	0.1482	0.2766	0.10	0.6414	0.6862	0.03	0.8640	0.8956	0.01	0.0227	0.0964	0.31	0.0890	0.2397	0.21	0.0672	0.2497	0.20	0.6674	0.7219	-0.04
547	ATP-dependent 6-phosphofructokinase liver type	P17858	0.0484	0.1415	0.09	0.6331	0.7294	0.06	0.0076	0.0411	0.11	0.1344	0.2426	0.06	0.0129	0.0808	-0.13	0.0057	0.0618	0.19	0.9330	0.9445	0.01	0.0007	0.0235	-0.16	0.0020	0.0508	-0.22
548	ATP-dependent 6-phosphofructokinase muscle type	P08237	0.0424	0.1304	-0.06	0.0009	0.2454	-0.19	0.6710	0.7213	-0.01	0.0360	0.0911	-0.08	0.0460	0.1579	-0.13	0.3554	0.5609	0.03	0.0381	0.1619	0.16	0.7808	1.2249	-0.01	0.2484	0.3193	0.11
549	ATP-dependent 6-phosphofructokinase platelet type	Q01813	0.0104	0.0592	0.29	0.2196	0.6606	0.26	0.0066	0.0393	0.31	0.0161	0.0527	0.24	0.9490	0.9609	0.00	0.0395	0.1297	0.22	0.0877	0.2380	0.28	0.3897	0.7717	0.10	0.4186	0.4900	-0.06
550	ATP-dependent Clp protease ATP-binding subunit clpX-like_ mitochondrial	O76031	0.0458	0.1368	-0.19	0.1241	0.6416	-0.18	0.1275	0.2475	-0.24	0.0108	0.0416	-0.31	0.0030	0.0410	-0.43	0.0304	0.1119	-0.24	0.0210	0.1333	0.32	0.0045	0.0540	0.49	0.0080	0.0802	0.63
551	ATP-dependent Clp protease proteolytic subunit_ mitochondrial	Q16740	0.0039	0.0398	0.15	0.1504	0.6597	0.22	0.0013	0.0231	0.18	0.0617	0.1346	0.08	0.1993	0.3997	-0.03	0.6133	0.8499	0.03	0.1676	0.3623	-0.32	0.0023	0.0403	-0.39	0.1811	0.2480	-0.18
552	ATP-dependent DNA helicase DDX11	Q96FC9	0.0602	0.1622	0.19	0.3927	0.7362	0.15	0.0229	0.0744	0.30	0.1632	0.2827	0.12	0.3174	0.4146	-0.08	0.0880	0.2158	0.17	0.3736	0.5389	-2.55	0.8708	1.1042	-0.33	0.0651	0.1595	2.06
553	ATP-dependent DNA helicase Q1	P46063	0.0105	0.0593	0.10	0.3261	0.6858	0.21	0.0023	0.0264	0.25	0.1129	0.2131	0.38	0.1313	0.2992	-0.06	0.2368	0.4225	0.22	0.0766	0.2203	0.28	0.7379	1.1812	-0.04	0.3189	0.3902	-0.08
554	ATP-dependent RNA helicase A	Q08211	0.0173	0.0771	0.14	0.1473	0.6572	0.16	0.0005	0.0196	0.37	0.0082	0.0367	0.17	0.0817	0.2209	-0.08	0.0174	0.0863	0.19	0.0244	0.1380	0.53	0.2506	0.5739	0.09	0.0041	0.0623	0.27
555	ATP-dependent RNA helicase DDX1	Q92499	0.5054	0.5784	0.05	0.9571	0.9714	0.01	0.2705	0.4436	0.09	0.4941	0.6972	0.05	0.2815	0.5084	-0.08	0.5564	0.7916	0.05	0.0687	0.2067	0.20	0.1758	0.4609	0.17	0.1112	0.1685	-0.06
556	ATP-dependent RNA helicase DDX18	Q9NVP1	0.4108	0.6211	-3.31	0.5752	0.8692	-1.75	0.5661	0.7766	-1.19	0.5388	0.7339	0.89	0.2938	0.5135	-1.88	0.9510	1.1690	0.09	0.3345	0.6039	0.09	0.0269	0.1478	-0.27	0.0287	0.1449	-0.29
557	ATP-dependent RNA helicase DDX19A	Q9NUU7	0.6982	0.7524	-0.02	0.6041	0.7072	0.02	0.3374	0.5298	-0.03	0.1035	0.1990	0.04	0.4693	0.5604	-0.03	0.0241	0.0991	-0.07	0.2708	0.5140	0.10	0.7162	1.1623	-0.02	0.9527	0.9631	0.00
558	ATP-dependent RNA helicase DDX19B	Q9UMR2	0.0511	0.1466	-13.44	0.0419	0.5712	-11.88	0.2063	0.3596	-1.76	0.1324	0.2399	-1.86	0.0611	0.1864	-0.40	0.2907	0.4876	-3.48	0.6252	0.6713	0.69	0.5342	0.9585	0.89	0.4117	0.4835	1.23
559	ATP-dependent RNA helicase DDX39A	O00148	0.2073	0.3801	0.07	0.6383	0.7335	0.06	0.1931	0.3408	0.08	0.1599	0.2785	0.08	0.1172	0.2804	-0.10	0.2096	0.3905	0.09	0.0168	0.1205	0.23	0.9542	0.9673	0.00	0.0178	0.1141	0.24
560	ATP-dependent RNA helicase DDX3X	O00571	0.4757	0.5513	-0.04	0.1057	0.6301	-0.18	0.0712	0.1592	0.14	0.1801	0.3049	0.08	0.1412	0.3139	-0.10	0.4647	0.6870	-0.04	0.0493	0.1771	0.33	0.6415	1.0796	0.04	0.6124	0.6724	-0.03
561	ATP-dependent RNA helicase DDX3Y	O15523	0.0054	0.0448	0.17	0.6176	0.7171	0.06	0.0160	0.0597	0.12	0.1031	0.1984	0.10	0.0161	0.0907	0.12	0.2575	0.4482	0.16	0.0216	0.1343	0.32	0.1606	0.4344	0.11	0.6190	0.6776	0.03
562	ATP-dependent RNA helicase DDX42	Q86XP3	0.0085	0.0548	-0.17	0.0084	0.4144	-0.20	0.0041	0.0324	-0.18	0.0274	0.0752	-0.25	0.0027	0.0395	-0.23	0.0908	0.2200	-0.27	0.1770	0.3764	-0.27	0.2616	0.5894	0.27	0.1521	0.2153	0.31
563	ATP-dependent RNA helicase DDX50	Q9BQ39	0.3174	0.5201	0.14	0.1026	0.6319	0.24	0.6497	0.7019	0.06	0.2730	0.4301	0.14	0.2510	0.4689	0.15	0.2206	0.4024	-0.16	0.0267	0.1420	0.26	0.0774	0.2724	0.19	0.1135	0.1710	0.31
564	ATP-dependent RNA helicase DDX54	Q8TDD1	0.4108	0.6209	-3.31	0.5752	0.8689	-1.75	0.5661	0.7764	-1.19	0.5388	0.7337	0.89	0.2938	0.5133	-1.88	0.9510	1.1687	0.09	0.3736	0.4309	-2.55	0.8708	0.9049	-0.33	0.0034	0.0577	8.68
565	ATP-dependent zinc metalloprotease YME1L1	Q96TA2	0.1495	0.2987	0.20	0.2040	0.6592	0.28	0.0617	0.1442	0.48	0.0574	0.1273	0.31	0.0433	0.1538	0.34	0.7545	0.9922	0.06	0.3736	0.4413	-2.55	0.8708	0.9245	-0.33	0.0651	0.1160	2.06
566	Atrial natriuretic peptide-converting enzyme	Q9Y5Q5	0.0011	0.0233	1.85	0.0250	0.4832	1.56	0.0001	0.0259	2.12	0.0007	0.0143	2.45	0.0237	0.1112	0.27	0.0028	0.0551	2.22	0.3736	0.6400	-2.55	0.8708	1.2822	-0.33	0.0651	0.2180	2.06
567	AT-rich interactive domain-containing protein 3B	Q8IVW6	0.2300	0.4111	-0.24	0.2617	0.6632	-0.22	0.1597	0.2942	-0.31	0.1641	0.2838	-0.30	0.1073	0.2649	-0.37	0.0794	0.2018	-0.44	0.3736	0.5439	-2.55	0.8708	1.1132	-0.33	0.0651	0.1620	2.06
568	AT-rich interactive domain-containing protein 4A	P29374	0.0276	0.1006	0.46	0.3047	0.6768	0.11	0.5385	0.7605	0.06	0.7790	0.8100	-0.03	0.4491	0.5425	0.08	0.5778	0.8124	0.05	0.3736	0.4540	-2.55	0.8708	0.9482	-0.33	0.0651	0.1211	2.06
569	AT-rich interactive domain-containing protein 4B	Q4LE39	0.0005	0.0192	0.56	0.3346	0.6936	0.18	0.2927	0.4731	0.05	0.0348	0.0891	0.15	0.5653	0.6471	0.02	0.0848	0.2117	0.10	0.0265	0.1422	0.35	0.0013	0.0294	0.29	0.0348	0.1600	0.19
570	Aurora kinase B	Q96GD4	0.4108	0.6208	-3.31	0.5752	0.8687	-1.75	0.5661	0.7762	-1.19	0.5388	0.7335	0.89	0.2938	0.5131	-1.88	0.9510	1.1684	0.09	0.0024	0.0560	-0.25	0.8394	1.2838	0.03	0.5035	0.5705	-0.09
571	Autophagy protein 5	Q9H1Y0	0.4108	0.6206	-3.31	0.5752	0.8684	-1.75	0.5661	0.7760	-1.19	0.5388	0.7333	0.89	0.2938	0.5129	-1.88	0.9510	1.1682	0.09	0.3597	0.6379	0.28	0.2133	0.5178	0.41	0.2134	0.2822	0.41
572	Autophagy-related protein 101	Q9BSB4	0.2531	0.4404	0.15	0.0356	0.5392	0.36	0.2507	0.4177	0.21	0.0272	0.0748	0.50	0.0076	0.0635	0.70	0.0797	0.2025	0.26	0.3736	0.5438	-2.55	0.8708	1.1129	-0.33	0.0651	0.1620	2.06
573	Axin interactor dorsalization-associated protein	Q96BJ3	0.4108	0.6204	-3.31	0.5752	0.8682	-1.75	0.5661	0.7758	-1.19	0.5388	0.7331	0.89	0.2938	0.5128	-1.88	0.9510	1.1679	0.09	0.0137	0.1107	-1.02	0.0041	0.0517	-0.67	0.0003	0.0263	-0.94
574	B box and SPRY domain-containing protein	Q5W0U4	0.4113	0.4893	0.05	0.1464	0.6554	-0.77	0.4382	0.6496	0.08	0.5717	0.6214	0.03	0.0788	0.2164	0.15	0.0638	0.1763	-0.17	0.3736	0.5532	-2.55	0.8708	1.1299	-0.33	0.0651	0.1669	2.06
575	Baculoviral IAP repeat-containing protein 6	Q9NR09	0.5868	0.6537	0.04	0.7972	0.8528	0.04	0.0173	0.0627	0.28	0.0705	0.1488	0.15	0.8172	0.8574	-0.01	0.0043	0.0600	0.46	0.4672	0.5222	0.09	0.3602	0.7283	0.08	0.1699	0.2354	0.10
576	BAG family molecular chaperone regulator 1	Q99933	0.0112	0.0607	0.22	0.3377	0.6969	-0.82	0.0295	0.0871	0.47	0.0007	0.0133	0.42	0.0008	0.0226	0.39	0.0341	0.1190	0.31	0.3736	0.5756	-2.55	0.8708	1.1698	-0.33	0.0651	0.1789	2.06
577	BAG family molecular chaperone regulator 2	O95816	0.4108	0.6202	-3.31	0.5752	0.8679	-1.75	0.5661	0.7756	-1.19	0.5388	0.7329	0.89	0.2938	0.5126	-1.88	0.9510	1.1676	0.09	0.1405	0.3213	0.13	0.0662	0.2478	-0.16	0.8301	0.8624	0.01
578	BAG family molecular chaperone regulator 3	O95817	0.0030	0.0370	-0.18	0.5362	0.8520	0.09	0.6468	0.6996	-0.02	0.0094	0.0389	-0.12	0.0171	0.0938	-0.15	0.0124	0.0750	-0.18	0.3736	0.6076	-2.55	0.8708	1.2261	-0.33	0.0651	0.1974	2.06
579	BAI1-associated protein 3	O94812	0.4108	0.6200	-3.31	0.5752	0.8677	-1.75	0.5661	0.7754	-1.19	0.5388	0.7327	0.89	0.2938	0.5124	-1.88	0.9510	1.1673	0.09	0.1298	0.3042	-0.86	0.4839	0.8946	0.32	0.2319	0.3020	0.56
580	Band 3 anion transport protein	P02730	0.0592	0.1611	-4.96	0.1933	0.6680	-2.33	0.0700	0.1573	-2.67	0.0576	0.1275	-3.45	0.0110	0.0749	-1.75	0.0787	0.2007	-3.38	0.0721	0.2128	-7.70	0.0832	0.2839	-5.48	0.0108	0.0905	-3.09
581	Band 4.1-like protein 2	O43491	0.6096	0.6740	-0.02	0.0306	0.5097	0.15	0.2880	0.4671	-0.05	0.0076	0.0351	0.15	0.0049	0.0527	0.32	0.4206	0.6399	0.08	0.2806	0.5293	0.10	0.8957	0.9220	0.01	0.5562	0.6205	-0.02
582	Band 4.1-like protein 4A	Q9HCS5	0.4108	0.6198	-3.31	0.5752	0.8674	-1.75	0.5661	0.7752	-1.19	0.5388	0.7325	0.89	0.2938	0.5122	-1.88	0.9510	1.1671	0.09	0.5994	0.6478	0.04	0.1737	0.4572	-0.32	0.0032	0.0564	0.34
583	Band 4.1-like protein 5	Q9HCM4	0.0024	0.0339	-0.24	0.6927	0.7747	0.04	0.0009	0.0203	-0.21	0.0091	0.0384	-0.11	0.0025	0.0388	-0.19	0.0926	0.2232	-0.14	0.0009	0.0416	-0.55	0.0003	0.0210	-0.68	0.0006	0.0331	-0.93
584	Bardet-Biedl syndrome 10 protein	Q8TAM1	0.0056	0.0454	-1.43	0.0219	0.4807	-0.95	0.0201	0.0684	-0.71	0.0095	0.0391	-1.15	0.0052	0.0540	-1.20	0.0337	0.1183	-0.87	0.3736	0.5767	-2.55	0.8708	1.1716	-0.33	0.0651	0.1795	2.06
585	Bargin	Q6ZT62	0.7215	0.7723	0.07	0.7821	0.8430	-0.05	0.0873	0.1847	-0.46	0.0342	0.0880	-0.76	0.0011	0.0259	-1.77	0.0324	0.1161	-0.69	0.3736	0.5789	-2.55	0.8708	1.1756	-0.33	0.0651	0.1807	2.06
586	Barrier-to-autointegration factor	O75531	0.0511	0.1466	0.14	0.0449	0.5673	0.14	0.2546	0.4226	0.06	0.0455	0.1077	0.17	0.1869	0.3822	-0.08	0.0228	0.0966	0.31	0.1332	0.3102	0.20	0.1451	0.4069	-0.12	0.2059	0.2745	-0.09
587	Basal body-orientation factor 1	Q8ND07	0.4108	0.6197	-3.31	0.5752	0.8672	-1.75	0.5661	0.7749	-1.19	0.5388	0.7323	0.89	0.2938	0.5121	-1.88	0.9510	1.1668	0.09	0.1031	0.2608	0.26	0.1430	0.4037	-0.18	0.2787	0.3505	-0.12
588	Basal cell adhesion molecule	P50895	0.4108	0.6195	-3.31	0.5752	0.8669	-1.75	0.5661	0.7747	-1.19	0.5388	0.7322	0.89	0.2938	0.5119	-1.88	0.9510	1.1665	0.09	0.0427	0.1674	-0.34	0.1407	0.3989	-0.26	0.0325	0.1539	-0.30
589	Basic leucine zipper and W2 domain-containing protein 1	Q7L1Q6	0.0760	0.1880	-0.15	0.8808	0.9143	0.01	0.0776	0.1685	0.15	0.2323	0.3763	0.08	0.2251	0.4338	-0.09	0.2571	0.4480	0.08	0.9528	0.9613	0.00	0.0012	0.0281	-0.16	0.0025	0.0546	-0.20
590	Basic leucine zipper and W2 domain-containing protein 2	Q9Y6E2	0.2660	0.4578	0.09	0.0620	0.5893	0.35	0.0234	0.0754	0.28	0.0223	0.0652	0.32	0.5407	0.6250	0.06	0.5689	0.8036	0.06	0.4177	0.4735	-0.44	0.6421	1.0799	-0.08	0.0109	0.0911	0.52
591	Basic leucine zipper transcriptional factor ATF-like	Q16520	0.0014	0.0260	2.79	0.4776	0.8040	0.77	0.0004	0.0182	2.97	0.3967	0.5854	0.17	0.4945	0.5836	0.27	0.0030	0.0551	2.62	0.3736	0.6383	-2.55	0.8708	1.2793	-0.33	0.0651	0.2169	2.06
592	Basigin	P35613	0.0078	0.0525	0.23	0.5054	0.8272	0.06	0.0006	0.0188	0.25	0.0000	0.0000	0.35	0.0024	0.0387	0.21	0.0002	0.0398	0.46	0.0148	0.1126	0.33	0.5667	0.9947	0.04	0.1331	0.1933	0.07
593	B-cell lymphoma/leukemia 10	O95999	0.4108	0.6193	-3.31	0.5752	0.8666	-1.75	0.5661	0.7745	-1.19	0.5388	0.7320	0.89	0.2938	0.5117	-1.88	0.9510	1.1662	0.09	0.4572	0.5120	-0.14	0.0390	0.1794	-0.43	0.0326	0.1542	-0.67
594	B-cell receptor-associated protein 31	P51572	0.1412	0.2872	0.10	0.3643	0.7129	0.16	0.0185	0.0652	0.25	0.0051	0.0287	0.39	0.0947	0.2434	0.11	0.0119	0.0740	0.38	0.0795	0.2237	0.30	0.5636	0.9910	0.06	0.2201	0.2896	0.11
595	Bcl-2 homologous antagonist/killer	Q16611	0.0004	0.0177	0.62	0.1035	0.6345	0.31	0.0113	0.0494	0.47	0.0207	0.0619	0.62	0.0523	0.1695	0.40	0.0819	0.2061	0.77	0.3736	0.5430	-2.55	0.8708	1.1116	-0.33	0.0651	0.1616	2.06
596	BCL2/adenovirus E1B 19 kDa protein-interacting protein 3	Q12983	0.4108	0.6191	-3.31	0.5752	0.8664	-1.75	0.5661	0.7743	-1.19	0.5388	0.7318	0.89	0.2938	0.5116	-1.88	0.9510	1.1660	0.09	0.4201	0.4759	0.14	0.0359	0.1722	0.35	0.5366	0.6024	-0.74
597	Bcl2-associated agonist of cell death	Q92934	0.4108	0.6189	-3.31	0.5752	0.8661	-1.75	0.5661	0.7741	-1.19	0.5388	0.7316	0.89	0.2938	0.5114	-1.88	0.9510	1.1657	0.09	0.0004	0.0370	0.79	0.0027	0.0434	0.55	0.0030	0.0553	0.57
598	Bcl-2-associated transcription factor 1	Q9NYF8	0.0805	0.1952	-0.13	0.5867	0.6921	-0.07	0.0841	0.1794	0.11	0.1358	0.2448	-0.19	0.0029	0.0407	-0.25	0.1772	0.3465	-0.20	0.0166	0.1199	0.62	0.0550	0.2243	0.25	0.0569	0.2124	0.23
599	Bcl-2-like protein 15	Q5TBC7	0.0036	0.0388	0.90	0.1201	0.6368	0.17	0.0138	0.0552	0.37	0.1761	0.2999	0.13	0.3058	0.4034	-0.09	0.1705	0.3359	0.50	0.3736	0.5095	-2.55	0.8708	1.0510	-0.33	0.0651	0.1453	2.06
600	BCL-6 corepressor	Q6W2J9	0.0090	0.0564	0.20	0.1267	0.6428	0.30	0.0118	0.0509	0.37	0.0002	0.0092	0.45	0.0075	0.0630	0.20	0.0314	0.1140	0.30	0.0186	0.1266	0.47	0.4406	0.8422	0.09	0.2054	0.2741	0.14
601	BEN domain-containing protein 5	Q7L4P6	0.4108	0.6188	-3.31	0.5752	0.8659	-1.75	0.5661	0.7739	-1.19	0.5388	0.7314	0.89	0.2938	0.5112	-1.88	0.9510	1.1654	0.09	0.4421	0.4977	-0.06	0.0121	0.0919	-0.25	0.0000	0.0000	-8.94

Supplemetary Table S2. Overview on all relatively quantified 5180 proteins statistical analysis

			MCF-7																		MDA-MB-231								
			Dai_SC20 vs control			Gen_SC20 vs control			SSE_SC20 vs control			Dai_IC20 vs control			Gen_IC20 vs control			SSE_IC20 vs control			Dai_IC20 vs control			Gen_IC20 vs control			SSE_IC20 vs control		
	**Protein name**	**UniProt**	**p value**	**BH q value**	**log2FC**	**p value**	**BH q value**	**log2FC**	**p value**	**BH q value**	**log2FC**	**p value**	**BH q value**	**log2FC**	**p value**	**BH q value**	**log2FC**	**p value**	**BH q value**	**log2FC**	**p value**	**BH q value**	**log2FC**	**p value**	**BH q value**	**log2FC**	**p value**	**BH q value**	**log2FC**
602	Beta-1_3-galactosyl-O-glycosyl-glycoprotein beta-1_6-N-acetylglucosaminyltransferase	Q02742	0.0709	0.1785	-0.19	0.8414	0.8862	0.02	0.0210	0.0701	-0.31	0.0265	0.0734	-0.28	0.5498	0.6333	-0.06	0.0210	0.0937	-0.38	0.3736	0.5925	-2.55	0.8708	1.1997	-0.33	0.0651	0.1885	2.06
603	Beta-1_3-galactosyl-O-glycosyl-glycoprotein beta-1_6-N-acetylglucosaminyltransferase 4	Q9P109	0.0004	0.0176	0.30	0.2797	0.6652	0.21	0.0022	0.0263	0.41	0.0003	0.0111	0.47	0.0003	0.0173	0.46	0.0007	0.0448	0.52	0.0860	0.2347	0.24	0.0013	0.0290	-0.31	0.1166	0.1745	-0.39
604	Beta-1_3-galactosyltransferase 2	O43825	0.0639	0.1674	-0.12	0.5767	0.6858	-0.08	0.3231	0.5107	0.08	0.2497	0.3990	-0.05	0.5283	0.6147	-0.02	0.0048	0.0602	0.30	0.4566	0.5114	-0.08	0.5386	0.9637	-0.06	0.1251	0.1847	0.07
605	Beta-actin-like protein 2	Q562R1	0.0069	0.0498	0.14	0.1089	0.6303	-0.15	0.0006	0.0202	0.31	0.0142	0.0486	-0.11	0.0046	0.0505	0.27	0.0002	0.0370	0.74	0.0376	0.1608	0.41	0.7766	1.2198	-0.02	0.5115	0.5780	0.05
606	Beta-adrenergic receptor kinase 2	P35626	0.4108	0.6186	-3.31	0.5752	0.8656	-1.75	0.5661	0.7737	-1.19	0.5388	0.7312	0.89	0.2938	0.5110	-1.88	0.9510	1.1651	0.09	0.3866	0.4426	0.44	0.3374	0.6949	0.47	0.0939	0.1482	1.13
607	Beta-arrestin-1	P49407	0.0046	0.0431	-0.63	0.6575	0.7476	-0.07	0.0064	0.0385	-0.51	0.0062	0.0316	-0.55	0.0204	0.1025	-0.45	0.0161	0.0842	-0.57	0.3736	0.6008	-2.55	0.8708	1.2142	-0.33	0.0651	0.1934	2.06
608	Beta-catenin-like protein 1	Q8WYA6	0.4108	0.6184	-3.31	0.5752	0.8654	-1.75	0.5661	0.7735	-1.19	0.5388	0.7310	0.89	0.2938	0.5109	-1.88	0.9510	1.1649	0.09	0.1313	0.3071	0.29	0.1063	0.3305	0.13	0.0613	0.2216	0.27
609	Beta-centractin	P42025	0.0314	0.1098	0.11	0.1060	0.6311	0.19	0.0183	0.0648	0.31	0.0146	0.0494	0.55	0.0041	0.0471	0.43	0.0001	0.0288	0.52	0.0003	0.0409	0.67	0.7109	1.1555	-0.02	0.0009	0.0361	0.82
610	Beta-crystallin A3	P05813	0.1871	0.3528	-1.48	0.1683	0.6605	-2.26	0.1399	0.2647	-1.02	0.0264	0.0733	-1.05	0.2204	0.4266	-0.37	0.0188	0.0884	-1.26	0.3736	0.5953	-2.55	0.8708	1.2045	-0.33	0.0651	0.1901	2.06
611	Beta-defensin 116	Q30KQ4	0.1474	0.2957	0.30	0.1163	0.6361	0.33	0.0559	0.1351	0.50	0.3320	0.5052	0.17	0.5319	0.6174	0.10	0.4344	0.6555	0.13	0.3736	0.4660	-2.55	0.8708	0.9707	-0.33	0.0651	0.1261	2.06
612	Beta-defensin 119	Q8N690	0.5438	0.6141	-0.04	0.5558	0.8701	-0.08	0.2663	0.4374	0.07	0.0050	0.0284	-0.25	0.0041	0.0474	-0.42	0.0247	0.1002	-0.14	0.3736	0.5872	-2.55	0.8708	1.1902	-0.33	0.0651	0.1854	2.06
613	Beta-defensin 132	Q7Z7B7	0.4108	0.6182	-3.31	0.5752	0.8651	-1.75	0.5661	0.7733	-1.19	0.5388	0.7308	0.89	0.2938	0.5107	-1.88	0.9510	1.1646	0.09	0.2516	0.4876	0.21	0.1575	0.4289	0.30	0.3438	0.4148	0.16
614	Beta-enolase	P13929	0.0114	0.0613	0.25	0.0445	0.5678	0.26	0.0199	0.0680	0.29	0.0012	0.0158	0.46	0.0091	0.0677	0.21	0.0103	0.0703	0.44	0.8088	0.8384	-0.03	0.0555	0.2248	-0.26	0.2915	0.3633	0.10
615	Beta-hexosaminidase subunit alpha	P06865	0.6609	0.7209	0.01	0.3327	0.6916	-0.03	0.1327	0.2548	-0.04	0.6658	0.7083	-0.02	0.2833	0.5111	0.05	0.2944	0.4918	0.11	0.3254	0.5921	0.14	0.0169	0.1101	0.13	0.0032	0.0566	0.30
616	Beta-hexosaminidase subunit beta	P07686	0.0291	0.1042	-0.17	0.6037	0.7070	-0.02	0.2277	0.3867	-0.06	0.2501	0.3994	-0.09	0.1888	0.3844	0.07	0.0530	0.1555	0.14	0.0000	0.0000	0.67	0.0000	0.0000	1.17	0.0004	0.0300	0.48
617	Beta-klotho	Q86Z14	0.1684	0.3271	-0.10	0.0580	0.5879	-0.31	0.2108	0.3652	0.09	0.1749	0.2985	-0.10	0.1157	0.2786	-0.12	0.2015	0.3789	0.09	0.2410	0.4732	-0.16	0.1774	0.4627	0.07	0.0491	0.1936	0.14
618	Beta-parvin	Q9HBI1	0.0056	0.0453	-0.28	0.1164	0.6360	-0.19	0.0299	0.0878	-0.22	0.0085	0.0373	-0.49	0.0163	0.0912	-0.33	0.0792	0.2017	-0.27	0.3736	0.5444	-2.55	0.8708	1.1140	-0.33	0.0651	0.1623	2.06
619	Beta-soluble NSF attachment protein	Q9H115	0.2100	0.3826	-0.15	0.0337	0.5290	0.33	0.0196	0.0675	0.22	0.0326	0.0852	0.30	0.0216	0.1060	0.28	0.3593	0.5654	0.16	0.0598	0.1937	0.24	0.9942	0.9959	0.00	0.0366	0.1641	-0.29
620	Beta-ureidopropionase	Q9UBR1	0.5731	0.6405	1.27	0.1244	0.6412	1.62	0.6062	0.6618	0.46	0.0009	0.0144	3.58	0.2612	0.4822	-0.76	0.0014	0.0507	3.95	0.3736	0.6466	-2.55	0.8708	1.2936	-0.33	0.0651	0.2224	2.06
621	BH3-interacting domain death agonist	P55957	0.4456	0.5230	-0.08	0.3158	0.6805	0.11	0.7279	0.7717	0.04	0.5098	0.7143	0.09	0.7732	0.8228	0.03	0.9588	0.9661	-0.01	0.0496	0.1777	0.30	0.1985	0.4953	0.10	0.1293	0.1889	0.17
622	Bifunctional 3'-phosphoadenosine 5'-phosphosulfate synthase 1	O43252	0.0024	0.0338	-0.20	0.9971	0.9981	0.00	0.0530	0.1303	-0.05	0.1206	0.2238	-0.07	0.0090	0.0673	0.24	0.2250	0.4081	-0.03	0.0580	0.1916	0.28	0.6674	1.1063	0.02	0.0011	0.0407	0.25
623	Bifunctional 3'-phosphoadenosine 5'-phosphosulfate synthase 2	O95340	0.0150	0.0711	-0.29	0.9879	0.9913	0.00	0.0042	0.0320	-0.32	0.0095	0.0392	-0.29	0.0425	0.1522	-0.17	0.0087	0.0683	-0.30	0.0325	0.1514	0.36	0.0624	0.2391	0.30	0.3125	0.3839	0.11
624	Bifunctional arginine demethylase and lysyl-hydroxylase JMJD6	Q6NYC1	0.2378	0.4221	0.17	0.0122	0.4358	0.37	0.0133	0.0539	0.50	0.0037	0.0246	0.21	0.0030	0.0411	0.26	0.0508	0.1518	0.43	0.3736	0.5601	-2.55	0.8708	1.1422	-0.33	0.0651	0.1705	2.06
625	Bifunctional coenzyme A synthase	Q13057	0.7365	0.7858	-0.02	0.1750	0.6617	0.12	0.5850	0.6412	0.09	0.5125	0.7167	0.07	0.6373	0.7070	-0.03	0.7487	0.9866	-0.04	0.3736	0.4417	-2.55	0.8708	0.9253	-0.33	0.0651	0.1162	2.06
626	Bifunctional glutamate/proline--tRNA ligase	P07814	0.0261	0.0979	0.12	0.4683	0.7985	0.10	0.0038	0.0314	0.25	0.0045	0.0273	0.19	0.2121	0.4159	0.04	0.0038	0.0593	0.31	0.0279	0.1447	0.57	0.0605	0.2349	0.23	0.0311	0.1497	0.27
627	Bifunctional methylenetetrahydrofolate dehydrogenase/cyclohydrolase_ mitochondrial	P13995	0.0085	0.0547	0.40	0.3327	0.6921	0.19	0.0369	0.1008	0.45	0.0003	0.0104	0.67	0.0013	0.0287	0.19	0.0089	0.0690	0.74	0.0054	0.0786	0.54	0.0026	0.0425	0.29	0.0016	0.0490	0.50
628	Bifunctional purine biosynthesis protein PURH	P31939	0.0653	0.1691	0.08	0.1364	0.6512	0.21	0.0051	0.0350	0.20	0.0020	0.0196	0.23	0.0861	0.2292	0.07	0.0123	0.0747	0.20	0.0832	0.2305	0.10	0.9348	0.9525	0.00	0.2778	0.3496	0.05
629	Biliverdin reductase A	P53004	0.0424	0.1303	0.21	0.2008	0.6621	0.23	0.0139	0.0553	0.25	0.0090	0.0382	0.39	0.0270	0.1192	0.23	0.0070	0.0646	0.35	0.0516	0.1800	0.22	0.8180	1.2630	0.02	0.8703	0.8947	-0.01
630	Biogenesis of lysosome-related organelles complex 1 subunit 1	P78537	0.0148	0.0707	-4.36	0.0495	0.5749	-9.86	0.0125	0.0526	-9.31	0.0021	0.0200	-7.22	0.7609	0.8132	0.30	0.0036	0.0585	-8.03	0.3736	0.6360	-2.55	0.8708	1.2753	-0.33	0.0651	0.2153	2.06
631	Biogenesis of lysosome-related organelles complex 1 subunit 2	Q6QNY1	0.8111	0.8479	0.05	0.6385	0.7335	-0.08	0.1693	0.3089	0.27	0.4537	0.6505	-0.15	0.2102	0.4135	0.24	0.1784	0.3478	0.29	0.3736	0.5083	-2.55	0.8708	1.0488	-0.33	0.0651	0.1447	2.06
632	Biorientation of chromosomes in cell division protein 1-like 1	Q8NFC6	0.0001	0.0148	-0.21	0.9107	0.9379	0.02	0.3991	0.6022	0.25	0.1867	0.3141	0.16	0.6496	0.7173	-0.02	0.4213	0.6405	-0.05	0.2014	0.4140	0.15	0.5502	0.9764	0.06	0.1007	0.1564	0.23
633	Bleomycin hydrolase	Q13867	0.0667	0.1711	-2.56	0.0669	0.5934	-9.23	0.0992	0.2028	-1.18	0.0133	0.0468	-2.36	0.0239	0.1118	-2.13	0.1951	0.3701	-3.26	0.2230	0.4465	2.66	0.6056	1.0374	1.22	0.1770	0.2431	3.16
634	Blood vessel epicardial substance	Q8NE79	0.0601	0.1621	-0.11	0.5007	0.8231	-0.27	0.0008	0.0202	0.48	0.0004	0.0126	0.49	0.0003	0.0165	0.57	0.0422	0.1355	0.32	0.3736	0.5685	-2.55	0.8708	1.1572	-0.33	0.0651	0.1750	2.06
635	Bloom syndrome protein	P54132	0.1634	0.3201	-0.14	0.3762	0.7242	0.08	0.1743	0.3161	0.25	0.1183	0.2206	0.18	0.0380	0.1421	-0.21	0.9722	0.9775	0.01	0.0189	0.1273	-0.17	0.0071	0.0691	-0.36	0.0006	0.0308	-0.25
636	BMP-binding endothelial regulator protein	Q8N8U9	0.0280	0.1017	0.16	0.4160	0.7548	0.11	0.3642	0.5615	0.04	0.2650	0.4195	0.06	0.1283	0.2955	-0.07	0.0614	0.1722	0.12	0.3736	0.5555	-2.55	0.8708	1.1339	-0.33	0.0651	0.1680	2.06
637	BolA-like protein 1	Q9Y3E2	0.0423	0.1303	-0.40	0.1424	0.6545	-0.33	0.6689	0.7193	-0.06	0.1319	0.2391	-0.24	0.0447	0.1558	-0.39	0.1582	0.3180	-0.59	0.3736	0.5128	-2.55	0.8708	1.0569	-0.33	0.0651	0.1468	2.06
638	BolA-like protein 2	Q9H3K6	0.0446	0.1345	0.12	0.0578	0.5882	0.18	0.0013	0.0235	0.21	0.0424	0.1025	0.13	0.5686	0.6490	0.01	0.0137	0.0777	0.16	0.0018	0.0539	0.40	0.0000	0.0000	0.38	0.0182	0.1161	0.35
639	Bone marrow proteoglycan	P13727	0.8767	0.9028	0.15	0.4547	0.7875	0.50	0.0015	0.0225	2.66	0.1788	0.3036	0.68	0.5585	0.6413	0.04	0.3597	0.5655	0.22	0.3736	0.4732	-2.55	0.8708	0.9840	-0.33	0.0651	0.1291	2.06
640	Bone morphogenetic protein 5	P22003	0.4108	0.6180	-3.31	0.5752	0.8649	-1.75	0.5661	0.7731	-1.19	0.5388	0.7306	0.89	0.2938	0.5105	-1.88	0.9510	1.1643	0.09	0.0006	0.0393	-0.33	0.3361	0.6936	0.03	0.0237	0.1310	-0.12
641	Bone morphogenetic protein receptor type-1A	P36894	0.0049	0.0435	1.22	0.2169	0.6601	0.41	0.0137	0.0549	1.29	0.0040	0.0256	1.41	0.2284	0.4379	0.45	0.0116	0.0734	1.07	0.3736	0.6095	-2.55	0.8708	1.2294	-0.33	0.0651	0.1986	2.06
642	Box C/D snoRNA protein 1	Q9NWK9	0.4108	0.6179	-3.31	0.5752	0.8646	-1.75	0.5661	0.7729	-1.19	0.5388	0.7304	0.89	0.2938	0.5104	-1.88	0.9510	1.1640	0.09	0.1923	0.3996	0.20	0.0720	0.2604	-0.14	0.1323	0.1926	-0.09
643	Brain acid soluble protein 1	P80723	0.0068	0.0494	-0.49	0.3130	0.6807	0.25	0.0664	0.1512	-0.21	0.1291	0.2351	0.12	0.0421	0.1518	0.21	0.2226	0.4054	-0.08	0.0090	0.0942	1.07	0.0100	0.0824	0.47	0.0037	0.0597	0.72
644	Brain-specific angiogenesis inhibitor 1-associated protein 2	Q9UQB8	0.0486	0.1418	0.12	0.4508	0.7849	0.04	0.0041	0.0320	0.26	0.0075	0.0352	0.22	0.0624	0.1892	0.12	0.0215	0.0946	0.25	0.0291	0.1465	-0.32	0.0482	0.2052	0.24	0.6984	0.7504	-0.05
645	Brain-specific angiogenesis inhibitor 1-associated protein 2-like protein 1	Q9UHR4	0.0263	0.0983	0.15	0.1315	0.6457	0.12	0.0033	0.0310	0.35	0.0026	0.0215	0.32	0.8456	0.8812	0.01	0.0129	0.0762	0.28	0.4749	0.5296	0.16	0.1446	0.4058	-0.35	0.9249	0.9392	-0.02
646	Branched-chain-amino-acid aminotransferase_ mitochondrial	O15382	0.0843	0.2005	0.19	0.4093	0.7508	-0.17	0.6132	0.6680	-0.05	0.1627	0.2822	-0.13	0.0252	0.1139	-0.27	0.1242	0.2716	-0.20	0.3736	0.5262	-2.55	0.8708	1.0812	-0.33	0.0651	0.1532	2.06
647	BRCA1-A complex subunit RAP80	Q96RL1	0.8381	0.8707	-0.02	0.3247	0.6857	0.15	0.3889	0.5902	-0.11	0.0497	0.1146	-0.28	0.0861	0.2291	-0.32	0.3049	0.5030	-0.45	0.3736	0.4809	-2.55	0.8708	0.9984	-0.33	0.0651	0.1324	2.06
648	BRCA1-associated ATM activator 1	Q6PJG6	0.2202	0.3963	-0.19	0.0203	0.4715	0.35	0.4504	0.6641	-0.11	0.5576	0.6085	0.07	0.1034	0.2584	0.22	0.0447	0.1398	-0.18	0.3736	0.5655	-2.55	0.8708	1.1519	-0.33	0.0651	0.1734	2.06
649	BRCA1-associated protein	Q7Z569	0.4108	0.6177	-3.31	0.5752	0.8644	-1.75	0.5661	0.7727	-1.19	0.5388	0.7302	0.89	0.2938	0.5102	-1.88	0.9510	1.1638	0.09	0.8104	0.8396	0.06	0.3600	0.7284	0.09	0.0017	0.0481	0.56
650	BRCA2 and CDKN1A-interacting protein	Q9P287	0.7473	0.7944	0.05	0.6060	0.7086	-0.20	0.6218	0.6760	-0.10	0.1144	0.2151	-0.37	0.5359	0.6216	-0.11	0.2773	0.4727	-0.22	0.0588	0.1924	-0.58	0.9935	0.9960	0.00	0.8886	0.9113	0.01
651	Breast cancer anti-estrogen resistance protein 1	P56945	0.6678	0.7272	0.02	0.5932	0.6976	0.04	0.0232	0.0750	-0.20	0.3351	0.5089	-0.05	0.4184	0.5130	-0.04	0.6903	0.9305	-0.02	0.1194	0.2887	0.17	0.0459	0.1991	0.17	0.0707	0.1188	-0.13
652	Breast cancer anti-estrogen resistance protein 3	O75815	0.5469	0.6161	-0.02	0.7382	0.8096	0.05	0.0118	0.0508	0.28	0.0263	0.0731	0.18	0.4771	0.5674	-0.04	0.0177	0.0869	0.20	0.1480	0.3329	-0.08	0.5529	0.9795	0.04	0.1231	0.1822	0.10
653	Breast carcinoma-amplified sequence 1	O75363	0.0379	0.1223	-0.14	0.1176	0.6319	0.31	0.8132	0.8486	-0.02	0.0885	0.1771	0.11	0.0089	0.0674	0.22	0.5478	0.7813	-0.04	0.3736	0.4571	-2.55	0.8708	0.9540	-0.33	0.0651	0.1224	2.06
654	Brefeldin A-inhibited guanine nucleotide-exchange protein 1	Q9Y6D6	0.0000	0.0000	-0.97	0.0671	0.5911	-0.21	0.9691	0.9740	0.00	0.0021	0.0196	-0.29	0.0545	0.1739	-0.09	0.4669	0.6892	0.04	0.3736	0.4625	-2.55	0.8708	0.9642	-0.33	0.0651	0.1246	2.06
655	Brefeldin A-inhibited guanine nucleotide-exchange protein 2	Q9Y6D5	0.0023	0.0331	-1.29	0.4994	0.8220	-0.91	0.0438	0.1140	0.48	0.0320	0.0841	-0.54	0.1127	0.2739	0.44	0.2135	0.3948	-0.44	0.4344	0.4901	-0.47	0.9310	0.9497	0.01	0.9445	0.9558	0.02
656	BRI3-binding protein	Q8WY22	0.0186	0.0802	-0.11	0.0860	0.6196	-0.11	0.1760	0.3184	0.04	0.1707	0.2926	-0.09	0.1837	0.3773	-0.11	0.2651	0.4577	0.13	0.3736	0.4877	-2.55	0.8708	1.0109	-0.33	0.0651	0.1354	2.06
657	BRISC and BRCA1-A complex member 2	Q9NXR7	0.0135	0.0680	0.70	0.1035	0.6337	0.29	0.5102	0.7313	-0.09	0.0133	0.0467	-0.59	0.2153	0.4202	0.18	0.1344	0.2865	-0.60	0.0029	0.0594	-2.07	0.0024	0.0410	-2.72	0.0461	0.1873	-2.77
658	BRISC complex subunit Abraxas 2	Q15018	0.5694	0.6373	-0.01	0.0597	0.5924	0.18	0.0023	0.0269	0.19	0.2125	0.3491	0.11	0.0022	0.0363	0.24	0.1404	0.2941	0.19	0.6585	0.7030	0.05	0.1929	0.4870	-0.16	0.3295	0.4007	-0.27
659	BRO1 domain-containing protein BROX	Q5VW32	0.1374	0.2822	0.16	0.1524	0.6595	0.19	0.0175	0.0630	0.28	0.5375	0.7452	0.04	0.7990	0.8435	-0.01	0.0067	0.0631	0.39	0.2515	0.4879	-0.09	0.0488	0.2069	-0.37	0.0069	0.0765	-0.49
660	Bromodomain adjacent to zinc finger domain protein 2B	Q9UIF8	0.4108	0.6175	-3.31	0.5752	0.8641	-1.75	0.5661	0.7725	-1.19	0.5388	0.7301	0.89	0.2938	0.5100	-1.88	0.9510	1.1635	0.09	0.0023	0.0549	0.37	0.0247	0.1403	0.22	0.1332	0.1934	0.12
661	Bromodomain-containing protein 2	P25440	0.4108	0.6173	-3.31	0.5752	0.8639	-1.75	0.5661	0.7723	-1.19	0.5388	0.7299	0.89	0.2938	0.5098	-1.88	0.9510	1.1632	0.09	0.2938	0.5482	-0.57	0.0947	0.3070	-1.10	0.0114	0.0928	-3.75
662	Bromodomain-containing protein 3	Q15059	0.4108	0.6172	-3.31	0.5752	0.8636	-1.75	0.5661	0.7721	-1.19	0.5388	0.7297	0.89	0.2938	0.5097	-1.88	0.9510	1.1629	0.09	0.3334	0.6028	0.13	0.3917	0.7741	-0.06	0.6858	0.7389	-0.02
663	Bromodomain-containing protein 4	O60885	0.4108	0.6170	-3.31	0.5752	0.8634	-1.75	0.5661	0.7719	-1.19	0.5388	0.7295	0.89	0.2938	0.5095	-1.88	0.9510	1.1627	0.09	0.0812	0.2272	-0.48	0.2190	0.5262	-0.18	0.1516	0.2147	-0.41
664	Bromodomain-containing protein 7	Q9NPI1	0.4108	0.6168	-3.31	0.5752	0.8631	-1.75	0.5661	0.7717	-1.19	0.5388	0.7293	0.89	0.2938	0.5093	-1.88	0.9510	1.1624	0.09	0.8933	0.9120	0.03	0.0091	0.0780	-0.35	0.1121	0.1694	-0.32
665	BTB/POZ domain-containing adapter for CUL3-mediated RhoA degradation protein 1	Q8WZ19	0.4108	0.6166	-3.31	0.5752	0.8629	-1.75	0.5661	0.7715	-1.19	0.5388	0.7291	0.89	0.2938	0.5092	-1.88	0.9510	1.1621	0.09	0.7527	0.7891	-0.04	0.0042	0.0516	0.31	0.0018	0.0493	-0.30
666	BTB/POZ domain-containing protein 9	Q96Q07	0.4108	0.6164	-3.31	0.5752	0.8626	-1.75	0.5661	0.7713	-1.19	0.5388	0.7289	0.89	0.2938	0.5090	-1.88	0.9510	1.1618	0.09	0.0327	0.1514	-0.48	0.0038	0.0501	-0.37	0.1136	0.1711	-0.30

Supplemetary Table S2. Overview on all relatively quantified 5180 proteins statistical analysis

			MCF-7															MDA-MB-231		
			Dai SC20 vs control			Gen SC20 vs control			SSE SC20 vs control			Dai IC20 vs control			Gen IC20 vs control			SSE IC20 vs control		
																		Dai IC20 vs control		
	**Protein name**	**UniProt**	**p value**	**BH q value**	**log2FC**	**p value**	**BH q value**	**log2FC**	**p value**	**BH q value**	**log2FC**	**p value**	**BH q value**	**log2FC**	**p value**	**BH q value**	**log2FC**	**p value**	**BH q value**	**log2FC**
667	BTB/POZ domain-containing protein KCTD15	Q96SI1	0.2131	0.3873	0.22	0.4182	0.7566	0.13	0.2469	0.4127	0.20	0.8696	0.8881	0.03	0.9911	0.9936	0.00	0.5145	0.7415	-0.11
668	BTB/POZ domain-containing protein KCTD2	Q14681	0.6931	0.7478	0.07	0.5493	0.8649	-0.22	0.9392	0.9528	-0.01	0.8386	0.8612	0.03	0.5853	0.6630	0.09	0.7677	1.0057	0.07

Supplemetary Table S2. Overview on all relatively quantified 5180 proteins statistical analysis

			MCF-7															MDA-MB-231		
			Dai SC20 vs control			Gen SC20 vs control			SSE SC20 vs control			Dai IC20 vs control			Gen IC20 vs control			SSE IC20 vs control		
	**Protein name**	**UniProt**	**p value**	**BH q value**	**log2FC**	**p value**	**BH q value**	**log2FC**	**p value**	**BH q value**	**log2FC**	**p value**	**BH q value**	**log2FC**	**p value**	**BH q value**	**log2FC**	**p value**	**BH q value**	**log2FC**
739	cAMP-dependent protein kinase type II-beta regulatory subunit	P31323	0.0009	0.0223	0.67	0.0096	0.4521	0.78	0.0032	0.0306	0.75	0.0002	0.0106	0.97	0.0000	0.0000	0.98	0.0335	0.1180	0.69
740	cAMP-regulated phosphoprotein 19	P56211	0.2787	0.4724	-0.07	0.3466	0.7027	-0.12	0.7244	0.7685	-0.02	0.1276	0.2331	-0.12	0.0292	0.1244	-0.20	0.7200	0.9600	-0.02
741	cAMP-specific 3' 5'-cyclic phosphodiesterase 4A	P27815	0.0782	0.1917	-2.82	0.7843	0.8448	0.44	0.5999	0.6557	-0.20	0.7578	0.7911	0.25	0.0716	0.2039	-0.62	0.5072	0.7335	0.16
742	Cannabinoid receptor 2	P34972	0.4108	0.6131	-3.31	0.5752	0.8579	-1.75	0.5661	0.7674	-1.19	0.5388	0.7253	0.89	0.2938	0.5058	-1.88	0.9510	1.1569	0.09
743	CAP-Gly domain-containing linker protein 1	P30622	0.0172	0.0768	0.18	0.1106	0.6317	0.26	0.0038	0.0319	0.27	0.0006	0.0128	0.45	0.0214	0.1056	0.21	0.0052	0.0616	0.41
744	CAP-Gly domain-containing linker protein 2	Q9UDT6	0.8888	0.9122	0.01	0.5494	0.8647	0.10	0.2454	0.4111	0.10	0.4114	0.6032	0.04	0.0974	0.2487	-0.10	0.3650	0.5717	0.06
745	Caprin-1	Q14444	0.1222	0.2591	0.08	0.4260	0.7617	0.14	0.0054	0.0356	0.24	0.0100	0.0403	0.22	0.2179	0.4235	-0.06	0.1755	0.3434	0.07
746	Caprin-2	Q6IMN6	0.0026	0.0343	0.11	0.0093	0.4461	0.35	0.0013	0.0239	0.20	0.0310	0.0821	0.22	0.0005	0.0176	0.23	0.1234	0.2702	0.07
747	Carbamoyl-phosphate synthase [ammonia]_ mitochondrial	P31327	0.0043	0.0414	-0.75	0.6533	0.7442	-0.05	0.0599	0.1416	-0.37	0.0062	0.0315	-0.49	0.1013	0.2551	-0.19	0.2255	0.4084	-0.39
748	Carbohydrate sulfotransferase 1	O43916	0.7571	0.8023	-0.13	0.4622	0.7946	-0.38	0.2919	0.4721	0.51	0.4587	0.6560	0.33	0.2560	0.4760	0.56	0.8345	1.0689	0.09
749	Carbohydrate sulfotransferase 12	Q9NRB3	0.0920	0.2129	-0.12	0.0213	0.4756	-0.22	0.0978	0.2007	-0.11	0.0076	0.0352	-0.28	0.0020	0.0351	-0.41	0.0811	0.2049	-0.13
750	Carbonic anhydrase 12	O43570	0.0099	0.0581	0.23	0.3861	0.7299	0.17	0.0028	0.0290	0.56	0.1951	0.3255	0.10	0.0718	0.2042	0.22	0.1412	0.2949	-0.13
751	Carbonyl reductase [NADPH] 1	P16152	0.0181	0.0791	0.20	0.1888	0.6694	0.21	0.0017	0.0235	0.36	0.0038	0.0252	0.36	0.0192	0.0993	0.16	0.0064	0.0626	0.33
752	Carbonyl reductase [NADPH] 3	O75828	0.0010	0.0226	0.36	0.0058	0.3852	0.29	0.0001	0.0345	0.44	0.0005	0.0138	0.36	0.0009	0.0233	0.21	0.0004	0.0423	0.39
753	Carbonyl reductase family member 4	Q8N4T8	0.4108	0.6129	-3.31	0.5752	0.8577	-1.75	0.5661	0.7672	-1.19	0.5388	0.7251	0.89	0.2938	0.5056	-1.88	0.9510	1.1567	0.09
754	Carboxylesterase 3	Q6UWW8	0.1305	0.2722	-0.45	0.7541	0.8218	0.06	0.4633	0.6800	0.14	0.6199	0.6668	0.09	0.0476	0.1603	0.56	0.3614	0.5676	-0.23
755	Carboxymethylenebutenolidase homolog	Q96DG6	0.4108	0.6127	-3.31	0.5752	0.8574	-1.75	0.5661	0.7670	-1.19	0.5388	0.7249	0.89	0.2938	0.5054	-1.88	0.9510	1.1564	0.09
756	Carboxy-terminal domain RNA polymerase II polypeptide A small phosphatase 1	Q9GZU7	0.6484	0.7096	0.02	0.1369	0.6518	0.21	0.0017	0.0237	0.30	0.0042	0.0265	0.21	0.0256	0.1154	0.09	0.0166	0.0851	0.33
757	Carnitine O-palmitoyltransferase 1 liver isoform	P50416	0.3404	0.5485	-0.06	0.5213	0.8407	0.11	0.5123	0.7333	0.05	0.1329	0.2404	0.11	0.0762	0.2126	-0.16	0.6133	0.8501	0.03
758	Carnitine O-palmitoyltransferase 2 mitochondrial	P23786	0.0932	0.2149	0.15	0.8666	0.9032	0.02	0.0625	0.1452	0.20	0.5912	0.6395	0.04	0.1024	0.2565	-0.14	0.1735	0.3408	0.11
759	Cartilage oligomeric matrix protein	P49747	0.0014	0.0259	0.22	0.3709	0.7185	0.08	0.1735	0.3151	0.04	0.0729	0.1525	0.12	0.0016	0.0314	0.23	0.0052	0.0621	0.25
760	Cartilage-associated protein	O75718	0.0007	0.0215	0.25	0.0596	0.5937	0.25	0.0127	0.0528	0.20	0.0401	0.0987	0.17	0.0085	0.0660	0.24	0.2037	0.3816	0.20
761	Casein kinase I isoform alpha	P48729	0.0868	0.2049	-0.16	0.0190	0.4621	-0.38	0.0058	0.0370	-0.35	0.0057	0.0303	-0.60	0.0061	0.0572	-0.55	0.0074	0.0654	-0.38
762	Casein kinase II subunit alpha	P68400	0.4108	0.6125	-3.31	0.5752	0.8572	-1.75	0.5661	0.7668	-1.19	0.5388	0.7247	0.89	0.2938	0.5053	-1.88	0.9510	1.1561	0.09
763	Casein kinase II subunit alpha'	P19784	0.6061	0.6710	0.04	0.9663	0.9767	0.00	0.3633	0.5606	-0.09	0.0905	0.1798	-0.14	0.3051	0.4028	-0.10	0.1092	0.2486	-0.13
764	Casein kinase II subunit alpha 3	Q8NEV1	0.8476	0.8786	0.02	0.2315	0.6618	0.19	0.0641	0.1476	0.35	0.1016	0.1963	0.31	0.2168	0.4222	0.20	0.2781	0.4739	0.16
765	Casein kinase II subunit beta	P67870	0.4108	0.6124	-3.31	0.5752	0.8569	-1.75	0.5661	0.7666	-1.19	0.5388	0.7246	0.89	0.2938	0.5051	-1.88	0.9510	1.1558	0.09
766	Caseinolytic peptidase B protein homolog	Q9H078	0.0517	0.1473	0.14	0.2512	0.6609	0.17	0.0108	0.0483	0.22	0.0087	0.0378	0.24	0.1162	0.2791	0.10	0.0239	0.0989	0.21
767	CASP8 and FADD-like apoptosis regulator	O15519	0.4108	0.6122	-3.31	0.5752	0.8567	-1.75	0.5661	0.7664	-1.19	0.5388	0.7244	0.89	0.2938	0.5049	-1.88	0.9510	1.1556	0.09
768	CASP8-associated protein 2	Q9UKL3	0.0007	0.0213	0.23	0.1048	0.6327	0.27	0.0004	0.0209	0.30	0.0005	0.0135	0.34	0.0094	0.0693	0.12	0.0006	0.0404	0.37
769	Caspase recruitment domain-containing protein 11	Q9BXL7	0.0051	0.0439	-0.89	0.1237	0.6414	-0.56	0.0136	0.0547	-0.77	0.0076	0.0353	-0.74	0.3197	0.4165	-0.15	0.0062	0.0622	-0.93
770	Caspase-3	P42574	0.4108	0.6120	-3.31	0.5752	0.8564	-1.75	0.5661	0.7662	-1.19	0.5388	0.7242	0.89	0.2938	0.5048	-1.88	0.9510	1.1553	0.09
771	Caspase-6	P55212	0.0001	0.0140	-2.40	0.0679	0.5911	-1.85	0.0008	0.0220	-2.56	0.0004	0.0127	-2.70	0.6485	0.7164	-0.09	0.0004	0.0340	-2.54
772	Caspase-7	P55210	0.0080	0.0529	-0.37	0.2027	0.6604	-0.11	0.0877	0.1855	-0.15	0.0188	0.0579	-0.19	0.0275	0.1200	-0.22	0.0874	0.2154	-0.09
773	Caspase-8	Q14790	0.7639	0.8079	0.10	0.3518	0.7039	0.39	0.4795	0.6987	0.26	0.3776	0.5616	0.33	0.3250	0.4224	0.37	0.5617	0.7963	0.20
774	Catalase	P04040	0.0001	0.0136	0.20	0.2525	0.6619	0.15	0.1185	0.2342	0.16	0.0001	0.0066	0.31	0.0120	0.0788	0.12	0.0493	0.1491	0.45
775	Catechol O-methyltransferase	P21964	0.0000	0.0000	-0.36	0.4004	0.7437	-0.04	0.0002	0.0162	-0.37	0.0038	0.0252	-0.31	0.0120	0.0787	-0.25	0.0151	0.0818	-0.19
776	Catenin alpha-1	P35221	0.1774	0.3397	0.04	0.3582	0.7082	0.11	0.0374	0.1016	0.13	0.0146	0.0494	0.12	0.3572	0.4551	-0.02	0.0159	0.0837	0.16
777	Catenin alpha-2	P26232	0.0626	0.1658	0.08	0.3710	0.7184	0.14	0.0161	0.0600	0.24	0.0165	0.0535	0.19	0.2388	0.4519	-0.02	0.0016	0.0502	0.15
778	Catenin beta-1	P35222	0.2034	0.3750	0.08	0.7246	0.7988	0.04	0.0040	0.0317	0.23	0.0528	0.1198	0.19	0.0526	0.1700	0.07	0.0104	0.0701	0.25
779	Catenin delta-1	O60716	0.3625	0.5753	0.04	0.3182	0.6805	0.18	0.0270	0.0823	0.18	0.0055	0.0297	0.31	0.2539	0.4731	0.06	0.0140	0.0785	0.29
780	Catenin delta-2	Q9UQB3	0.4048	0.6252	-0.04	0.0637	0.5913	0.13	0.0081	0.0424	0.29	0.0311	0.0822	0.17	0.2252	0.4338	-0.07	0.0641	0.1764	0.15
781	Cathepsin B	P07858	0.4108	0.6118	-3.31	0.5752	0.8562	-1.75	0.5661	0.7660	-1.19	0.5388	0.7240	0.89	0.2938	0.5046	-1.88	0.9510	1.1550	0.09
782	Cathepsin D	P07339	0.0136	0.0682	0.32	0.3395	0.6990	0.19	0.0287	0.0859	0.24	0.8221	0.8459	-0.01	0.0289	0.1235	0.25	0.4542	0.6769	-0.04
783	Cathepsin L1	P07711	0.4108	0.6117	-3.31	0.5752	0.8559	-1.75	0.5661	0.7658	-1.19	0.5388	0.7238	0.89	0.2938	0.5044	-1.88	0.9510	1.1548	0.09
784	Cathepsin L2	O60911	0.2153	0.3901	0.30	0.9783	0.9855	-0.01	0.1438	0.2702	-0.47	0.0574	0.1273	-0.58	0.1059	0.2622	-0.42	0.7519	0.9898	-0.18
785	Cathepsin Z	Q9UBR2	0.4108	0.6115	-3.31	0.5752	0.8557	-1.75	0.5661	0.7656	-1.19	0.5388	0.7236	0.89	0.2938	0.5043	-1.88	0.9510	1.1545	0.09
786	Cation-dependent mannose-6-phosphate receptor	P20645	0.4641	0.5406	-0.12	0.5727	0.8848	0.09	0.3994	0.6025	-0.15	0.5659	0.6161	-0.09	0.9222	0.9418	-0.02	0.3260	0.5280	-0.17
787	Cationic amino acid transporter 2	P52569	0.0053	0.0446	0.46	0.0074	0.3993	0.35	0.0006	0.0213	0.69	0.0003	0.0117	0.81	0.0003	0.0175	0.67	0.0539	0.1575	0.58
788	Cation-independent mannose-6-phosphate receptor	P11717	0.4108	0.6113	-3.31	0.5752	0.8555	-1.75	0.5661	0.7654	-1.19	0.5388	0.7234	0.89	0.2938	0.5041	-1.88	0.9510	1.1542	0.09
789	Caveolae-associated protein 1	Q6NZI2	0.4108	0.6111	-3.31	0.5752	0.8552	-1.75	0.5661	0.7652	-1.19	0.5388	0.7232	0.89	0.2938	0.5039	-1.88	0.9510	1.1539	0.09
790	Caveolin-1	Q03135	0.4108	0.6110	-3.31	0.5752	0.8550	-1.75	0.5661	0.7650	-1.19	0.5388	0.7231	0.89	0.2938	0.5038	-1.88	0.9510	1.1537	0.09
791	Caveolin-2	P51636	0.4108	0.6108	-3.31	0.5752	0.8547	-1.75	0.5661	0.7648	-1.19	0.5388	0.7229	0.89	0.2938	0.5036	-1.88	0.9510	1.1534	0.09
792	Caveolin-3	P56539	0.4108	0.6106	-3.31	0.5752	0.8545	-1.75	0.5661	0.7646	-1.19	0.5388	0.7227	0.89	0.2938	0.5034	-1.88	0.9510	1.1531	0.09
793	CCA tRNA nucleotidyltransferase 1 mitochondrial	Q96Q11	0.0147	0.0705	0.17	0.2389	0.6611	0.29	0.0384	0.1034	0.22	0.0017	0.0181	0.34	0.0115	0.0770	-0.15	0.1016	0.2362	0.30
794	CCR4-NOT transcription complex subunit 1	A5YKK6	0.0190	0.0812	-1.01	0.1454	0.6561	-0.70	0.0074	0.0407	-1.14	0.0183	0.0572	-1.18	0.1192	0.2834	-0.43	0.0190	0.0888	-1.21
795	CCR4-NOT transcription complex subunit 10	Q9H9A5	0.4108	0.6104	-3.31	0.5752	0.8542	-1.75	0.5661	0.7644	-1.19	0.5388	0.7225	0.89	0.2938	0.5033	-1.88	0.9510	1.1529	0.09
796	CCR4-NOT transcription complex subunit 2	Q9NZN8	0.0065	0.0483	-0.17	0.6926	0.7747	0.04	0.2699	0.4427	-0.06	0.0473	0.1106	-0.12	0.0080	0.0646	-0.27	0.0278	0.1067	-0.25
797	CCR4-NOT transcription complex subunit 3	O75175	0.0060	0.0470	-0.15	0.1113	0.6315	-0.14	0.6973	0.7451	-0.01	0.0354	0.0901	-0.08	0.2659	0.4874	0.03	0.0100	0.0704	-0.15
798	CD151 antigen	P48509	0.4108	0.6103	-3.31	0.5752	0.8540	-1.75	0.5661	0.7642	-1.19	0.5388	0.7223	0.89	0.2938	0.5031	-1.88	0.9510	1.1526	0.09
799	CD164 sialomucin-like 2 protein	Q6UWJ8	0.2719	0.4647	0.05	0.1460	0.6554	0.30	0.0145	0.0567	0.28	0.0002	0.0089	0.27	0.4261	0.5206	-0.02	0.0648	0.1775	0.16
800	CD166 antigen	Q13740	0.4108	0.6101	-3.31	0.5752	0.8537	-1.75	0.5661	0.7640	-1.19	0.5388	0.7221	0.89	0.2938	0.5029	-1.88	0.9510	1.1523	0.09
801	CD2 antigen cytoplasmic tail-binding protein 2	O95400	0.0305	0.1077	0.06	0.3246	0.6860	0.19	0.0068	0.0396	0.32	0.0061	0.0314	0.25	0.0060	0.0567	0.08	0.0390	0.1287	0.12
802	CD2-associated protein	Q9Y5K6	0.0617	0.1647	-0.15	0.7546	0.8219	-0.03	0.3791	0.5784	-0.05	0.7329	0.7696	-0.02	0.0514	0.1678	-0.12	0.8976	1.1346	0.00
803	CD44 antigen	P16070	0.4108	0.6099	-3.31	0.5752	0.8535	-1.75	0.5661	0.7638	-1.19	0.5388	0.7219	0.89	0.2938	0.5028	-1.88	0.9510	1.1521	0.09
804	CD5 antigen-like	O43866	0.4108	0.6097	-3.31	0.5752	0.8532	-1.75	0.5661	0.7636	-1.19	0.5388	0.7217	0.89	0.2938	0.5026	-1.88	0.9510	1.1518	0.09
805	CD59 glycoprotein	P13987	0.4108	0.6096	-3.31	0.5752	0.8530	-1.75	0.5661	0.7634	-1.19	0.5388	0.7216	0.89	0.2938	0.5024	-1.88	0.9510	1.1515	0.09
806	CD63 antigen	P08962	0.0813	0.1965	0.09	0.2829	0.6643	0.16	0.5129	0.7337	0.03	0.0077	0.0354	0.20	0.0118	0.0782	0.20	0.2280	0.4107	0.07
807	CD9 antigen	P21926	0.0149	0.0710	-0.10	0.3708	0.7186	0.19	0.3717	0.5698	0.06	0.0663	0.1422	0.14	0.0268	0.1191	-0.08	0.0498	0.1497	0.08
808	CD97 antigen	P48960	0.0934	0.2150	-0.07	0.1014	0.6328	0.30	0.0921	0.1914	0.07	0.0021	0.0197	0.43	0.0011	0.0264	0.38	0.0035	0.0598	0.26
809	CD99 antigen	P14209	0.8758	0.9023	0.01	0.0642	0.5917	0.25	0.2185	0.3750	0.11	0.0042	0.0262	0.31	0.0310	0.1290	0.26	0.9753	0.9802	-0.01
810	CD99 antigen-like protein 2	Q8TCZ2	0.0486	0.1418	0.38	0.1142	0.6334	0.25	0.5480	0.7712	0.16	0.5911	0.6396	-0.07	0.5257	0.6124	0.08	0.3985	0.6122	0.11
811	Cdc42 effector protein 4	Q9H3Q1	0.0000	0.0000	-0.42	0.0143	0.4544	-0.35	0.0015	0.0234	-0.16	0.0002	0.0110	-0.51	0.0000	0.0000	-0.63	0.0001	0.0518	-0.50
812	Cdc42-interacting protein 4	Q15642	0.4108	0.6094	-3.31	0.5752	0.8528	-1.75	0.5661	0.7632	-1.19	0.5388	0.7214	0.89	0.2938	0.5023	-1.88	0.9510	1.1512	0.09
813	CDGSH iron-sulfur domain-containing protein 1	Q9NZ45	0.4269	0.5043	-0.05	0.4671	0.7978	-0.08	0.0758	0.1657	-0.17	0.0263	0.0731	-0.30	0.0144	0.0853	-0.29	0.3672	0.5741	-0.11
814	CDGSH iron-sulfur domain-containing protein 2	Q8N5K1	0.4770	0.5524	0.05	0.5518	0.8675	-0.05	0.7973	0.8343	-0.02	0.4446	0.6388	0.05	0.1867	0.3821	-0.10	0.2738	0.4681	0.08
815	CDK5 and ABL1 enzyme substrate 1	Q8TDN4	0.2698	0.4623	-1.32	0.2050	0.6596	-2.03	0.0266	0.0818	-1.55	0.1234	0.2271	-0.87	0.6201	0.6930	-0.22	0.2097	0.3905	-0.64
816	CDK5 regulatory subunit-associated protein 3	Q96JB5	0.7556	0.8012	0.01	0.2055	0.6591	0.18	0.8627	0.8907	0.01	0.0034	0.0234	0.25	0.0345	0.1356	0.13	0.0299	0.1109	0.16
817	CDK-activating kinase assembly factor MAT1	P51948	0.0025	0.0341	0.57	0.9692	0.9792	0.01	0.0015	0.0223	0.32	0.5986	0.6467	0.03	0.3962	0.4929	0.16	0.4250	0.6448	0.11
818	CDKN2A-interacting protein	Q9NXV6	0.7644	0.8081	-0.02	0.1322	0.6479	0.14	0.0109	0.0485	0.51	0.0132	0.0466	0.38	0.0002	0.0133	0.66	0.0005	0.0411	0.44

			MDA-MB-231								
			Dai IC20 vs control			Gen IC20 vs control			SSE IC20 vs control		
	**Protein name**	**UniProt**	**p value**	**BH q value**	**log2FC**	**p value**	**BH q value**	**log2FC**	**p value**	**BH q value**	**log2FC**
739	cAMP-dependent protein kinase type II-beta regulatory subunit	P31323	0.0104	0.1022	0.74	0.1480	0.4135	0.18	0.0137	0.1015	0.30
740	cAMP-regulated phosphoprotein 19	P56211	0.3736	0.4443	-2.55	0.8708	0.9301	-0.33	0.0651	0.1172	2.06
741	cAMP-specific 3' 5'-cyclic phosphodiesterase 4A	P27815	0.2584	0.4974	0.27	0.5534	0.9794	-0.12	0.5476	0.6123	-0.12
742	Cannabinoid receptor 2	P34972	0.0031	0.0611	0.41	0.0006	0.0237	0.36	0.0025	0.0551	0.29
743	CAP-Gly domain-containing linker protein 1	P30622	0.0911	0.2431	0.20	0.4833	0.8941	0.04	0.0392	0.1709	0.12
744	CAP-Gly domain-containing linker protein 2	Q9UDT6	0.0031	0.0613	-0.32	0.0005	0.0233	-0.47	0.0274	0.1418	-0.40
745	Caprin-1	Q14444	0.0586	0.1924	0.34	0.4937	0.9065	0.06	0.1596	0.2236	-0.11
746	Caprin-2	Q6IMN6	0.0282	0.1446	0.44	0.0146	0.1014	0.59	0.0226	0.1272	0.26
747	Carbamoyl-phosphate synthase [ammonia]_ mitochondrial	P31327	0.3736	0.4965	-2.55	0.8708	1.0270	-0.33	0.0651	0.1393	2.06
748	Carbohydrate sulfotransferase 1	O43916	0.6851	0.7268	0.05	0.0963	0.3098	-0.25	0.6126	0.6723	-0.05
749	Carbohydrate sulfotransferase 12	Q9NRB3	0.4376	0.4933	-0.08	0.5869	1.0192	-0.02	0.0149	0.1050	0.22
750	Carbonic anhydrase 12	O43570	0.3736	0.5193	-2.55	0.8708	1.0686	-0.33	0.0651	0.1499	2.06
751	Carbonyl reductase [NADPH] 1	P16152	0.1078	0.2681	0.10	0.2878	0.6272	-0.06	0.5844	0.6460	-0.03
752	Carbonyl reductase [NADPH] 3	O75828	0.2398	0.4714	0.14	0.6087	1.0406	-0.04	0.6906	0.7430	-0.02
753	Carbonyl reductase family member 4	Q8N4T8	0.0221	0.1352	-0.20	0.0426	0.1906	-0.29	0.0097	0.0871	-0.64
754	Carboxylesterase 3	Q6UWW8	0.3736	0.4728	-2.55	0.8708	0.9834	-0.33	0.0651	0.1290	2.06
755	Carboxymethylenebutenolidase homolog	Q96DG6	0.5477	0.5988	0.08	0.1014	0.3205	-0.27	0.0914	0.1451	-0.41
756	Carboxy-terminal domain RNA polymerase II polypeptide A small phosphatase 1	Q9GZU7	0.3736	0.5997	-2.55	0.8708	1.2122	-0.33	0.0651	0.1927	2.06
757	Carnitine O-palmitoyltransferase 1 liver isoform	P50416	0.0310	0.1487	0.46	0.7567	1.1994	0.03	0.8466	0.8760	0.01
758	Carnitine O-palmitoyltransferase 2 mitochondrial	P23786	0.3736	0.5089	-2.55	0.8708	1.0497	-0.33	0.0651	0.1450	2.06
759	Cartilage oligomeric matrix protein	P49747	0.1119	0.2755	0.20	0.0286	0.1527	-0.16	0.2972	0.3686	-0.05
760	Cartilage-associated protein	O75718	0.0387	0.1630	-0.22	0.9904	0.9933	0.00	0.0765	0.1266	-0.13
761	Casein kinase I isoform alpha	P48729	0.2076	0.4230	-0.38	0.1054	0.3289	-0.20	0.0224	0.1267	-0.81
762	Casein kinase II subunit alpha	P68400	0.9102	0.9267	0.00	0.0127	0.0936	0.26	0.1051	0.1614	0.09
763	Casein kinase II subunit alpha'	P19784	0.7926	0.8251	0.02	0.1977	0.4935	-0.10	0.0259	0.1375	-0.18
764	Casein kinase II subunit alpha 3	Q8NEV1	0.3736	0.4859	-2.55	0.8708	1.0075	-0.33	0.0651	0.1346	2.06
765	Casein kinase II subunit beta	P67870	0.0138	0.1110	-0.50	0.0005	0.0218	-1.00	0.0004	0.0284	-1.06
766	Caseinolytic peptidase B protein homolog	Q9H078	0.3736	0.5882	-2.55	0.8708	1.1921	-0.33	0.0651	0.1860	2.06
767	CASP8 and FADD-like apoptosis regulator	O15519	0.3331	0.6027	-0.14	0.4390	0.8401	-0.10	0.1424	0.2038	-0.24
768	CASP8-associated protein 2	Q9UKL3	0.0726	0.2134	0.10	0.0422	0.1896	-0.12	0.0171	0.1117	-0.18
769	Caspase recruitment domain-containing protein 11	Q9BXL7	0.3736	0.6249	-2.55	0.8708	1.2561	-0.33	0.0651	0.2082	2.06
770	Caspase-3	P42574	0.0035	0.0645	-2.43	0.0003	0.0192	-2.95	0.0141	0.1029	-3.28
771	Caspase-6	P55212	0.3736	0.6525	-2.55	0.8708	1.3037	-0.33	0.0651	0.2265	2.06
772	Caspase-7	P55210	0.3736	0.5398	-2.55	0.8708	1.1058	-0.33	0.0651	0.1600	2.06
773	Caspase-8	Q14790	0.3736	0.4556	-2.55	0.8708	0.9512	-0.33	0.0651	0.1217	2.06
774	Catalase	P04040	0.1340	0.3117	0.13	0.0073	0.0704	-0.19	0.5650	0.6289	0.02
775	Catechol O-methyltransferase	P21964	0.0014	0.0493	-0.84	0.0283	0.1527	-0.79	0.2625	0.3345	-0.43
776	Catenin alpha-1	P35221	0.0108	0.1025	0.35	0.0582	0.2300	0.22	0.0341	0.1579	0.15
777	Catenin alpha-2	P26232	0.8751	0.8964	0.03	0.0605	0.2347	-0.33	0.0335	0.1568	-0.45
778	Catenin beta-1	P35222	0.6822	0.7250	-0.05	0.0764	0.2696	-0.17	0.9325	0.9460	0.01
779	Catenin delta-1	O60716	0.0108	0.1026	0.45	0.8671	1.3095	0.02	0.6167	0.6757	0.04
780	Catenin delta-2	Q9UQB3	0.3736	0.5521	-2.55	0.8708	1.1280	-0.33	0.0651	0.1663	2.06
781	Cathepsin B	P07858	0.0071	0.0863	-1.19	0.0002	0.0170	-2.71	0.0049	0.0680	-2.53
782	Cathepsin D	P07339	0.0011	0.0432	-0.50	0.0041	0.0519	-0.54	0.0020	0.0503	-0.46
783	Cathepsin L1	P07711	0.0123	0.1046	-1.66	0.0008	0.0250	-1.53	0.0003	0.0283	-1.05
784	Cathepsin L2	O60911	0.0039	0.0669	1.31	0.0061	0.0629	1.15	0.0071	0.0779	1.75
785	Cathepsin Z	Q9UBR2	0.0433	0.1680	0.31	0.2989	0.6419	0.10	0.9145	0.9312	0.01
786	Cation-dependent mannose-6-phosphate receptor	P20645	0.0924	0.2440	-0.16	0.0279	0.1516	-0.29	0.0104	0.0898	-0.44
787	Cationic amino acid transporter 2	P52569	0.3736	0.5585	-2.55	0.8708	1.1394	-0.33	0.0651	0.1696	2.06
788	Cation-independent mannose-6-phosphate receptor	P11717	0.0010	0.0418	0.89	0.0011	0.0282	1.16	0.0219	0.1263	0.42
789	Caveolae-associated protein 1	Q6NZI2	0.2022	0.4153	0.12	0.5347	0.9591	-0.06	0.3782	0.4494	0.07
790	Caveolin-1	Q03135	0.0296	0.1470	0.28	0.0981	0.3135	0.07	0.2590	0.3309	0.17
791	Caveolin-2	P51636	0.0006	0.0389	1.88	0.0007	0.0257	1.57	0.1792	0.2457	1.54
792	Caveolin-3	P56539	0.0144	0.1112	-0.34	0.0486	0.2062	-0.13	0.0279	0.1432	-0.19
793	CCA tRNA nucleotidyltransferase 1 mitochondrial	Q96Q11	0.3736	0.5337	-2.55	0.8708	1.0948	-0.33	0.0651	0.1569	2.06
794	CCR4-NOT transcription complex subunit 1	A5YKK6	0.1421	0.3236	0.14	0.5714	1.0000	0.06	0.3084	0.3795	-0.07
795	CCR4-NOT transcription complex subunit 10	Q9H9A5	0.0395	0.1637	-0.59	0.0057	0.0608	-0.39	0.1397	0.2011	-0.13
796	CCR4-NOT transcription complex subunit 2	Q9NZN8	0.3736	0.5826	-2.55	0.8708	1.1821	-0.33	0.0651	0.1828	2.06
797	CCR4-NOT transcription complex subunit 3	O75175	0.6364	0.6821	0.20	0.3209	0.6719	0.42	0.8843	0.9072	0.05
798	CD151 antigen	P48509	0.2748	0.5197	-0.13	0.3446	0.7047	-0.13	0.0378	0.1675	-0.42
799	CD164 sialomucin-like 2 protein	Q6UWJ8	0.3736	0.5517	-2.55	0.8708	1.1271	-0.33	0.0651	0.1660	2.06
800	CD166 antigen	Q13740	0.4425	0.4979	0.14	0.0402	0.1833	-0.26	0.0002	0.0220	1.25
801	CD2 antigen cytoplasmic tail-binding protein 2	O95400	0.0004	0.0423	-0.62	0.0305	0.1578	-0.48	0.1854	0.2529	-0.39
802	CD2-associated protein	Q9Y5K6	0.4423	0.4979	-0.08	0.9009	0.9259	-0.01	0.3945	0.4658	0.06
803	CD44 antigen	P16070	0.0082	0.0912	0.57	0.1992	0.4963	0.13	0.0517	0.1991	0.23
804	CD5 antigen-like	O43866	0.0021	0.0541	-0.85	0.0006	0.0241	-1.06	0.0026	0.0552	-1.23
805	CD59 glycoprotein	P13987	0.0017	0.0521	0.96	0.0007	0.0254	0.56	0.0027	0.0557	0.90
806	CD63 antigen	P08962	0.3736	0.4955	-2.55	0.8708	1.0252	-0.33	0.0651	0.1388	2.06
807	CD9 antigen	P21926	0.0186	0.1264	0.32	0.1520	0.4213	0.15	0.0326	0.1541	0.16
808	CD97 antigen	P48960	0.0596	0.1939	-0.22	0.1484	0.4142	-0.13	0.2236	0.2935	0.11
809	CD99 antigen	P14209	0.1125	0.2763	0.88	0.2985	0.6419	-0.50	0.4496	0.5186	0.33
810	CD99 antigen-like protein 2	Q8TCZ2	0.3736	0.4692	-2.55	0.8708	0.9766	-0.33	0.0651	0.1274	2.06
811	Cdc42 effector protein 4	Q9H3Q1	0.1954	0.4041	-1.16	0.8248	1.2704	0.20	0.9559	0.9656	-0.04
812	Cdc42-interacting protein 4	Q15642	0.9283	0.9408	0.00	0.2893	0.6289	-0.06	0.0284	0.1444	0.17
813	CDGSH iron-sulfur domain-containing protein 1	Q9NZ45	0.3736	0.4721	-2.55	0.8708	0.9821	-0.33	0.0651	0.1287	2.06
814	CDGSH iron-sulfur domain-containing protein 2	Q8N5K1	0.3198	0.5837	-0.09	0.5638	0.9910	-0.07	0.6575	0.7127	-0.04
815	CDK5 and ABL1 enzyme substrate 1	Q8TDN4	0.1242	0.2966	-1.80	0.2418	0.5599	-0.69	0.1412	0.2026	-0.60
816	CDK5 regulatory subunit-associated protein 3	Q96JB5	0.7149	0.7556	0.23	0.7849	1.2291	0.17	0.8179	0.8530	0.14
817	CDK-activating kinase assembly factor MAT1	P51948	0.3736	0.4668	-2.55	0.8708	0.9721	-0.33	0.0651	0.1264	2.06
818	CDKN2A-interacting protein	Q9NXV6	0.3450	0.6177	0.10	0.0928	0.3031	0.19	0.0153	0.1061	0.27

Supplemetary Table S2. Overview on all relatively quantified 5180 proteins statistical analysis

			MCF-7															MDA-MB-231														
			Dai SC20 vs control			Gen SC20 vs control			SSE SC20 vs control			Dai IC20 vs control			Gen IC20 vs control			SSE IC20 vs control			Dai IC20 vs control			Gen IC20 vs control			SSE IC20 vs control		
	**Protein name**	**UniProt**	**p value**	**BH q value**	**log2FC**	**p value**	**BH q value**	**log2FC**	**p value**	**BH q value**	**log2FC**	**p value**	**BH q value**	**log2FC**	**p value**	**BH q value**	**log2FC**	**p value**	**BH q value**	**log2FC**	**p value**	**BH q value**	**log2FC**	**p value**	**BH q value**	**log2FC**	**p value**	**BH q value**	**log2FC**
819	Cell cycle and apoptosis regulator protein 2	Q8N163	0.2064	0.3790	-0.05	0.7343	0.8065	-0.04	0.6977	0.7453	-0.03	0.5556	0.6067	0.05	0.0059	0.0561	-0.20	0.4802	0.7049	-0.02	0.0909	0.2427	0.25	0.3919	0.7742	-0.11	0.9297	0.9434	-0.01
820	Cell cycle checkpoint control protein RAD9A	Q99638	0.1710	0.3313	-0.13	0.8709	0.9066	0.01	0.1416	0.2674	-0.13	0.8710	0.8894	-0.02	0.8781	0.9068	0.01	0.0223	0.0957	-0.26	0.3736	0.5902	-2.55	0.8708	1.1955	-0.33	0.0651	0.1871	2.06
821	Cell cycle progression protein 1	Q9ULG6	0.8607	0.8897	-0.02	0.4117	0.7520	0.12	0.7579	0.7991	0.05	0.3241	0.4955	-0.06	0.0604	0.1850	0.30	0.4259	0.6456	-0.12	0.0081	0.0908	-0.21	0.0883	0.2951	-0.13	0.0060	0.0731	-0.54
822	Cell death activator CIDE-B	Q9UHD4	0.0063	0.0479	0.50	0.0014	0.2198	0.75	0.0017	0.0241	0.73	0.0010	0.0156	0.78	0.0006	0.0203	0.90	0.0029	0.0558	0.56	0.3736	0.6393	-2.55	0.8708	1.2811	-0.33	0.0651	0.2176	2.06
823	Cell division control protein 42 homolog	P60953	0.0002	0.0162	0.62	0.2386	0.6624	0.13	0.5334	0.7541	-0.04	0.1231	0.2266	0.35	0.9767	0.9820	0.00	0.0061	0.0616	0.33	0.6074	0.6551	0.10	0.0018	0.0344	-0.64	0.0475	0.1910	-0.28
824	Cell division cycle 5-like protein	Q99459	0.8376	0.8705	-0.01	0.7508	0.8193	0.09	0.4788	0.6981	0.05	0.9284	0.9371	0.00	0.6863	0.7494	0.01	0.0814	0.2052	0.34	0.0080	0.0915	0.33	0.1269	0.3716	0.12	0.0514	0.1987	0.29
825	Cell division cycle and apoptosis regulator protein 1	Q8IX12	0.0665	0.1710	0.21	0.3477	0.7033	-0.48	0.0069	0.0397	0.46	0.0148	0.0499	0.39	0.4459	0.5395	0.07	0.1617	0.3232	0.19	0.3736	0.5116	-2.55	0.8708	1.0547	-0.33	0.0651	0.1462	2.06
826	Cell division cycle protein 123 homolog	O75794	0.1176	0.2523	0.03	0.3402	0.6990	0.12	0.0159	0.0595	0.39	0.0061	0.0313	0.29	0.0050	0.0531	0.34	0.0594	0.1684	0.41	0.0280	0.1443	0.28	0.0267	0.1474	0.27	0.0765	0.1266	0.19
827	Cell division cycle protein 20 homolog B	Q86Y33	0.2946	0.4926	-0.06	0.5575	0.8711	0.10	0.7051	0.7518	0.02	0.4890	0.6913	0.05	0.3874	0.4846	-0.05	0.3062	0.5047	-0.06	0.3736	0.4806	-2.55	0.8708	0.9977	-0.33	0.0651	0.1322	2.06
828	Cell division cycle protein 23 homolog	Q9UJX2	0.4108	0.6092	-3.31	0.5752	0.8525	-1.75	0.5661	0.7630	-1.19	0.5388	0.7212	0.89	0.2938	0.5021	-1.88	0.9510	1.1510	0.09	0.0288	0.1458	0.34	0.0796	0.2769	0.18	0.6318	0.6884	0.08
829	Cell division cycle protein 27 homolog	P30260	0.4108	0.6090	-3.31	0.5752	0.8523	-1.75	0.5661	0.7629	-1.19	0.5388	0.7210	0.89	0.2938	0.5019	-1.88	0.9510	1.1507	0.09	0.0064	0.0839	0.36	0.2404	0.5577	0.09	0.1259	0.1854	0.28
830	Cell division cycle-associated 7-like protein	Q96GN5	0.4108	0.6089	-3.31	0.5752	0.8520	-1.75	0.5661	0.7627	-1.19	0.5388	0.7208	0.89	0.2938	0.5018	-1.88	0.9510	1.1504	0.09	0.1792	0.3787	-0.28	0.0009	0.0258	-0.60	0.0019	0.0502	-0.69
831	Cell division cycle-associated protein 2	Q69YH5	0.2590	0.4480	-0.04	0.3145	0.6799	0.06	0.0635	0.1468	0.16	0.0320	0.0841	0.11	0.4635	0.5551	0.03	0.1921	0.3661	0.15	0.0626	0.1970	0.19	0.2176	0.5238	0.09	0.0476	0.1911	0.21
832	Cell division cycle-associated protein 7	Q9BWT1	0.0118	0.0628	0.44	0.2012	0.6605	0.21	0.0207	0.0695	0.28	0.0608	0.1331	0.29	0.0282	0.1217	0.28	0.2877	0.4840	0.08	0.3736	0.4838	-2.55	0.8708	1.0037	-0.33	0.0651	0.1337	2.06
833	Cell growth regulator with EF hand domain protein 1	Q99674	0.0302	0.1069	-0.58	0.2234	0.6579	-0.31	0.0303	0.0885	-0.57	0.0184	0.0574	-0.81	0.1142	0.2762	-0.36	0.1025	0.2375	-1.40	0.3736	0.5330	-2.55	0.8708	1.0935	-0.33	0.0651	0.1566	2.06
834	Cell growth-regulating nucleolar protein	Q9NX58	0.4108	0.6087	-3.31	0.5752	0.8518	-1.75	0.5661	0.7625	-1.19	0.5388	0.7206	0.89	0.2938	0.5016	-1.88	0.9510	1.1502	0.09	0.0003	0.0345	-0.64	0.3169	0.6673	-0.28	0.0056	0.0711	0.40
835	Cell surface glycoprotein MUC18	P43121	0.4108	0.6085	-3.31	0.5752	0.8515	-1.75	0.5661	0.7623	-1.19	0.5388	0.7204	0.89	0.2938	0.5014	-1.88	0.9510	1.1499	0.09	0.0466	0.1730	0.37	0.3444	0.7046	0.14	0.5801	0.6428	0.05
836	Cellular nucleic acid-binding protein	P62633	0.3976	0.6163	-0.04	0.5911	0.6959	0.03	0.0167	0.0611	0.20	0.0345	0.0886	0.16	0.0339	0.1347	-0.18	0.9234	1.1604	0.00	0.3166	0.5791	0.16	0.0984	0.3141	-0.22	0.1335	0.1938	-0.16
837	Cellular retinoic acid-binding protein 1	P29762	0.3121	0.5131	-0.08	0.9965	0.9977	0.00	0.7439	0.7864	-0.02	0.0942	0.1855	-0.15	0.0825	0.2222	-0.18	0.2836	0.4799	0.08	0.3736	0.4845	-2.55	0.8708	1.0051	-0.33	0.0651	0.1340	2.06
838	Cellular retinoic acid-binding protein 2	P29373	0.7733	0.8157	0.02	0.4676	0.7976	0.15	0.7185	0.7636	-0.02	0.3217	0.4926	0.07	0.0797	0.2179	-0.15	0.4016	0.6164	0.05	0.1656	0.3603	0.26	0.1423	0.4021	-0.10	0.0872	0.1400	-0.14
839	Centrin-1	Q12798	0.0324	0.1121	-2.45	0.3866	0.7293	-2.24	0.2682	0.4402	-4.48	0.0598	0.1315	-4.40	0.9633	0.9716	0.01	0.1668	0.3307	-5.64	0.0000	0.0000	-0.92	0.0000	0.0000	-1.40	0.0001	0.0398	-1.37
840	Centrin-2	P41208	0.4108	0.6083	-3.31	0.5752	0.8513	-1.75	0.5661	0.7621	-1.19	0.5388	0.7203	0.89	0.2938	0.5013	-1.88	0.9510	1.1496	0.09	0.0355	0.1558	0.30	0.2739	0.6092	-0.10	0.1679	0.2331	0.12
841	Centriolin	Q7Z7A1	0.0053	0.0446	0.21	0.0942	0.6304	0.28	0.0018	0.0242	0.30	0.0005	0.0127	0.38	0.0007	0.0216	0.51	0.0295	0.1102	0.29	0.0062	0.0836	0.70	0.0033	0.0475	1.18	0.0504	0.1970	0.35
842	Centromere protein F	P49454	0.4108	0.6082	-3.31	0.5752	0.8511	-1.75	0.5661	0.7619	-1.19	0.5388	0.7201	0.89	0.2938	0.5011	-1.88	0.9510	1.1494	0.09	0.0284	0.1454	0.53	0.5688	0.9974	0.06	0.2142	0.2831	0.15
843	Centromere protein J	Q9HC77	0.4108	0.6080	-3.31	0.5752	0.8508	-1.75	0.5661	0.7617	-1.19	0.5388	0.7199	0.89	0.2938	0.5009	-1.88	0.9510	1.1491	0.09	0.0393	0.1640	-0.48	0.0604	0.2352	-0.22	0.0492	0.1938	-0.35
844	Centromere protein R	Q13352	0.0025	0.0340	0.35	0.0457	0.5623	0.26	0.0005	0.0214	0.36	0.0027	0.0221	0.25	0.9997	1.0001	0.00	0.0311	0.1131	0.43	0.3736	0.5799	-2.55	0.8708	1.1774	-0.33	0.0651	0.1813	2.06
845	Centromere protein V	Q7Z7K6	0.4108	0.6078	-3.31	0.5752	0.8506	-1.75	0.5661	0.7615	-1.19	0.5388	0.7197	0.89	0.2938	0.5008	-1.88	0.9510	1.1488	0.09	0.0138	0.1108	0.27	0.1929	0.4872	-0.07	0.0492	0.1937	0.16
846	Centromere protein X	A8MT69	0.0994	0.2241	0.15	0.0113	0.4304	0.36	0.0258	0.0800	0.25	0.0423	0.1024	0.21	0.0105	0.0732	0.49	0.2172	0.3984	0.14	0.3736	0.4986	-2.55	0.8708	1.0310	-0.33	0.0651	0.1403	2.06
847	Centromere/kinetochore protein zw10 homolog	O43264	0.0263	0.0982	-0.21	0.2305	0.6615	-0.28	0.0295	0.0872	-0.25	0.0934	0.1841	-0.13	0.4936	0.5828	0.04	0.1862	0.3582	-0.06	0.8258	0.8530	-0.06	0.4858	0.8965	0.20	0.2679	0.3395	0.32
848	Centromere-associated protein E	Q02224	0.0031	0.0370	0.26	0.2104	0.6621	0.17	0.0031	0.0301	0.25	0.0015	0.0177	0.26	0.1353	0.3051	0.05	0.0024	0.0550	0.32	0.1393	0.3197	0.24	0.1535	0.4227	0.13	0.1381	0.1992	0.13
849	Centrosomal protein of 128 kDa	Q6ZU80	0.4108	0.6076	-3.31	0.5752	0.8503	-1.75	0.5661	0.7613	-1.19	0.5388	0.7195	0.89	0.2938	0.5006	-1.88	0.9510	1.1486	0.09	0.4522	0.5073	0.09	0.0918	0.3012	-0.16	0.9158	0.9320	-0.01
850	Centrosomal protein of 135 kDa	Q66GS9	0.0648	0.1684	0.10	0.0845	0.6200	0.21	0.1832	0.3280	0.11	0.0035	0.0237	0.32	0.0095	0.0696	0.14	0.0755	0.1951	0.12	0.1047	0.2638	-0.28	0.2732	0.6079	-0.16	0.0459	0.1874	-0.29
851	Centrosomal protein of 152 kDa	O94986	0.4108	0.6075	-3.31	0.5752	0.8501	-1.75	0.5661	0.7611	-1.19	0.5388	0.7193	0.89	0.2938	0.5005	-1.88	0.9510	1.1483	0.09	0.5350	0.5868	-0.30	0.7693	1.2120	0.13	0.7141	0.7643	0.18
852	Centrosomal protein of 162 kDa	Q5TB80	0.1544	0.3062	-0.13	0.3459	0.7027	-0.12	0.3986	0.6020	0.05	0.0080	0.0360	-0.10	0.0407	0.1486	0.19	0.9710	0.9767	0.00	0.3736	0.4303	-2.55	0.8708	0.9038	-0.33	0.0651	0.1117	2.06
853	Centrosomal protein of 170 kDa	Q5SW79	0.0002	0.0159	0.25	0.0663	0.5942	0.31	0.1197	0.2361	0.11	0.3170	0.4871	0.05	0.1868	0.3822	0.03	0.4343	0.6557	0.04	0.0015	0.0508	0.31	0.0337	0.1663	0.14	0.0026	0.0550	0.35
854	Centrosomal protein of 192 kDa	Q8TEP8	0.2676	0.4593	0.12	0.4652	0.7963	0.10	0.0896	0.1878	0.22	0.4215	0.6131	0.08	0.9652	0.9731	0.00	0.0459	0.1419	0.30	0.3736	0.5644	-2.55	0.8708	1.1498	-0.33	0.0651	0.1728	2.06
855	Centrosomal protein of 290 kDa	O15078	0.0129	0.0665	0.31	0.2009	0.6620	0.20	0.0036	0.0316	0.38	0.0067	0.0330	0.32	0.0646	0.1932	0.17	0.0136	0.0776	0.38	0.0019	0.0538	0.42	0.0208	0.1248	0.24	0.2469	0.3176	0.07
856	Centrosomal protein of 295 kDa	Q9C0D2	0.8792	0.9052	0.02	0.6695	0.7569	0.07	0.3489	0.5431	0.15	0.7089	0.7476	0.06	0.5463	0.6303	0.10	0.8746	1.1109	0.03	0.3736	0.4350	-2.55	0.8708	0.9126	-0.33	0.0651	0.1135	2.06
857	Centrosomal protein of 41 kDa	Q9BYV8	0.1807	0.3445	0.08	0.3902	0.7342	0.15	0.0915	0.1904	0.12	0.0811	0.1659	0.13	0.4322	0.5263	0.04	0.0481	0.1462	0.16	0.0987	0.2535	0.11	0.6253	1.0620	-0.04	0.2098	0.2784	-0.08
858	Centrosomal protein of 83 kDa	Q9Y592	0.1249	0.2631	0.08	0.0834	0.6189	0.22	0.0018	0.0242	0.28	0.0026	0.0216	0.21	0.0657	0.1951	0.08	0.2398	0.4255	0.12	0.0384	0.1624	0.38	0.2356	0.5515	0.12	0.4043	0.4760	0.10
859	Centrosomal protein of 85 kDa-like	Q5SZL2	0.4108	0.6073	-3.31	0.5752	0.8498	-1.75	0.5661	0.7609	-1.19	0.5388	0.7191	0.89	0.2938	0.5003	-1.88	0.9510	1.1480	0.09	0.4876	0.5415	-0.20	0.0503	0.2108	0.30	0.0026	0.0547	0.86
860	Centrosomal protein of 95 kDa	Q96GE4	0.5623	0.6305	-0.25	0.7671	0.8309	-0.14	0.6882	0.7372	-0.17	0.5795	0.6285	-0.24	0.1430	0.3167	0.87	0.6824	0.9222	-0.18	0.3736	0.4459	-2.55	0.8708	0.9331	-0.33	0.0651	0.1178	2.06
861	Centrosome and spindle pole-associated protein 1	Q1MSJ5	0.0610	0.1636	-0.83	0.3712	0.7183	-0.71	0.0365	0.1001	-1.00	0.0266	0.0736	-1.05	0.8197	0.8597	0.07	0.0213	0.0940	-1.47	0.3736	0.5924	-2.55	0.8708	1.1993	-0.33	0.0651	0.1884	2.06
862	Centrosome-associated protein 350	Q5VT06	0.2554	0.4432	0.09	0.8555	0.8963	-0.03	0.0060	0.0375	0.45	0.4324	0.6253	0.09	0.3180	0.4149	-0.10	0.9915	0.9926	0.00	0.0593	0.1932	0.46	0.8956	0.9223	0.01	0.0424	0.1789	0.29
863	Centrosome-associated protein CEP250	Q9BV73	0.4108	0.6071	-3.31	0.5752	0.8496	-1.75	0.5661	0.7607	-1.19	0.5388	0.7190	0.89	0.2938	0.5001	-1.88	0.9510	1.1478	0.09	0.6768	0.7203	0.15	0.3071	0.6536	0.52	0.0027	0.0546	-2.96
864	Ceramide synthase 2	Q96G23	0.1938	0.3615	-0.04	0.4660	0.7974	-0.08	0.0031	0.0298	-0.21	0.0094	0.0390	-0.19	0.0058	0.0561	-0.22	0.2358	0.4210	0.04	0.0004	0.0398	1.01	0.0009	0.0273	1.18	0.4457	0.5156	-0.06
865	cGMP-gated cation channel alpha-1	P29973	0.4108	0.6069	-3.31	0.5752	0.8494	-1.75	0.5661	0.7605	-1.19	0.5388	0.7188	0.89	0.2938	0.5000	-1.88	0.9510	1.1475	0.09	0.0128	0.1075	-0.44	0.0010	0.0273	-0.62	0.0021	0.0516	-0.60
866	Charged multivesicular body protein 1a	Q9HD42	0.2064	0.3789	-0.04	0.2386	0.6620	0.12	0.0019	0.0241	-0.16	0.0893	0.1781	-0.06	0.0347	0.1355	-0.08	0.0035	0.0592	-0.18	0.5743	0.6241	-0.06	0.5111	0.9303	-0.07	0.5677	0.6313	-0.11
867	Charged multivesicular body protein 2b	Q9UQN3	0.2605	0.4498	-0.10	0.1224	0.6385	0.20	0.4306	0.6408	0.03	0.0002	0.0084	0.32	0.0083	0.0658	0.15	0.3238	0.5261	0.15	0.3736	0.4775	-2.55	0.8708	0.9920	-0.33	0.0651	0.1309	2.06
868	Charged multivesicular body protein 3	Q9Y3E7	0.0571	0.1568	-0.36	0.0849	0.6203	-0.20	0.0002	0.0203	-0.30	0.0466	0.1097	-0.21	0.0000	0.0000	-0.22	0.3141	0.5149	-0.19	0.3736	0.4793	-2.55	0.8708	0.9953	-0.33	0.0651	0.1317	2.06
869	Charged multivesicular body protein 4a	Q9BY43	0.0058	0.0461	0.13	0.1738	0.6610	0.11	0.0016	0.0235	0.33	0.0079	0.0361	0.35	0.0110	0.0750	0.12	0.0013	0.0506	0.29	0.0254	0.1404	0.27	0.1229	0.3640	0.08	0.0452	0.1861	0.20
870	Charged multivesicular body protein 4b	Q9H444	0.0086	0.0550	0.17	0.1982	0.6671	0.26	0.0101	0.0468	0.22	0.0046	0.0275	0.18	0.0468	0.1593	0.09	0.0234	0.0981	0.14	0.1070	0.2671	0.19	0.3187	0.6686	-0.11	0.0774	0.1278	-0.21
871	Charged multivesicular body protein 4c	Q96CF2	0.0041	0.0405	0.61	0.0086	0.4203	0.46	0.0046	0.0337	0.47	0.0017	0.0183	0.95	0.0003	0.0154	1.13	0.0021	0.0541	1.01	0.3736	0.6440	-2.55	0.8708	1.2892	-0.33	0.0651	0.2207	2.06
872	Charged multivesicular body protein 5	Q9NZZ3	0.3507	0.5602	-0.04	0.1772	0.6632	0.21	0.0403	0.1072	0.14	0.0705	0.1487	0.11	0.3639	0.4613	-0.04	0.1266	0.2753	0.09	0.0207	0.1327	-0.60	0.0026	0.0422	-0.86	0.0196	0.1213	-0.80
873	Charged multivesicular body protein 6	Q96FZ7	0.3397	0.5475	0.16	0.1420	0.6544	0.27	0.9496	0.9604	0.01	0.4438	0.6379	0.12	0.5370	0.6219	0.10	0.3910	0.6028	-0.13	0.3736	0.4699	-2.55	0.8708	0.9780	-0.33	0.0651	0.1277	2.06
874	Chitinase domain-containing protein 1	Q9BWS9	0.3503	0.5602	0.14	0.0194	0.4631	0.56	0.2057	0.3589	0.28	0.0374	0.0937	0.48	0.0830	0.2230	0.34	0.0216	0.0947	0.63	0.3736	0.5920	-2.55	0.8708	1.1987	-0.33	0.0651	0.1882	2.06
875	Chloride channel CLIC-like protein 1	Q96S66	0.0511	0.1465	0.22	0.0507	0.5734	0.18	0.0514	0.1274	0.18	0.0042	0.0263	0.43	0.1209	0.2860	0.16	0.0232	0.0975	0.29	0.0355	0.1560	0.27	0.7293	1.1747	0.03	0.0370	0.1651	0.27
876	Chloride intracellular channel protein 1	O00299	0.2522	0.4391	0.05	0.2604	0.6645	0.26	0.0176	0.0632	0.16	0.0139	0.0479	0.17	0.2085	0.4114	-0.05	0.1659	0.3294	0.09	0.1623	0.3559	0.25	0.3408	0.6989	-0.10	0.2290	0.2994	-0.11
877	Chloride intracellular channel protein 2	O15247	0.4108	0.6068	-3.31	0.5752	0.8491	-1.75	0.5661	0.7603	-1.19	0.5388	0.7186	0.89	0.2938	0.4998	-1.88	0.9510	1.1472	0.09	0.3100	0.5704	0.30	0.1540	0.4236	0.44	0.1184	0.1765	1.18
878	Chloride intracellular channel protein 3	O95833	0.0000	0.0000	-0.46	0.0046	0.3556	0.25	0.0004	0.0244	-0.60	0.0001	0.0118	-0.39	0.0198	0.1009	0.11	0.0403	0.1318	-0.51	0.3736	0.5700	-2.55	0.8708	1.1599	-0.33	0.0651	0.1758	2.06
879	Chloride intracellular channel protein 4	Q9Y696	0.1163	0.2503	0.10	0.1216	0.6388	0.38	0.0047	0.0335	0.31	0.0020	0.0197	0.40	0.8498	0.8843	-0.01	0.0239	0.0986	0.26	0.0519	0.1803	0.25	0.5017	0.9183	0.05	0.5310	0.5969	0.02
880	Chloride intracellular channel protein 5	Q9NZA1	0.6836	0.7402	-0.44	0.2573	0.6628	-1.55	0.3174	0.5043	-0.49	0.4734	0.6733	-0.31	0.8339	0.8711	-0.08	0.2523	0.4426	-0.86	0.1069	0.2670	-1.51	0.3551	0.7208	-0.46	0.8635	0.8893	-0.04
881	Choline O-acetyltransferase	P28329	0.4108	0.6066	-3.31	0.5752	0.8489	-1.75	0.5661	0.7601	-1.19	0.5388	0.7184	0.89	0.2938	0.4996	-1.88	0.9510	1.1470	0.09	0.0406	0.1655	0.46	0.1376	0.3927	-0.29	0.4259	0.4971	0.25
882	Choline transporter-like protein 1	Q8WWI5	0.4108	0.6064	-3.31	0.5752	0.8486	-1.75	0.5661	0.7599	-1.19	0.5388	0.7182	0.89	0.2938	0.4995	-1.88	0.9510	1.1467	0.09	0.0002	0.0305	0.81	0.0099	0.0818	0.58	0.2002	0.2686	0.46
883	Choline-phosphate cytidylyltransferase A	P49585	0.0021	0.0318	-0.09	0.9118	0.9384	-0.01	0.0070	0.0395	0.22	0.7872	0.8168	-0.02	0.1354	0.3052	-0.06	0.3947	0.6078	0.05	0.0219	0.1351	0.19	0.8754	0.9056	-0.01	0.0241	0.1320	0.16
884	Choline-phosphate cytidylyltransferase B	Q9Y5K3	0.1261	0.2650	-0.08	0.5319	0.8501	0.10	0.0068	0.0394	0.23	0.0599	0.1316	0.12	0.5061	0.5949	-0.03	0.2295	0.4126	0.07	0.3736	0.4951	-2.55	0.8708	1.0245	-0.33	0.0651	0.1387	2.06
885	Chondrolectin	Q9H9P2	0.9225	0.9407	-0.01	0.0860	0.6187	0.16	0.0063	0.0386	0.32	0.0323	0.0847	0.23	0.3049	0.4027	0.06	0.0764	0.1972	0.15	0.3736	0.5461	-2.55	0.8708	1.1171	-0.33	0.0651	0.1631	2.06
886	Chromatin accessibility complex protein 1	Q9NRG0	0.4108	0.6063	-3.31	0.5752	0.8484	-1.75	0.5661	0.7597	-1.19	0.5388	0.7180	0.89	0.2938	0.4993	-1.88	0.9510	1.1464	0.09	0.0647	0.2002	-0.40	0.0366	0.1741	0.26	0.0912	0.1449	-0.08
887	Chromatin complexes subunit BAP18	Q8IXM2	0.4108	0.6061	-3.31	0.5752	0.8481	-1.75	0.5661	0.7595	-1.19	0.5388	0.7178	0.89	0.2938	0.4991	-1.88	0.9510	1.1462	0.09	0.1228	0.2950	-0.20	0.0582	0.2296	-0.27	0.0411	0.1759	-0.38
888	Chromatin target of PRMT1 protein	Q9Y3Y2	0.4108	0.6059	-3.31	0.5752	0.8479	-1.75	0.5661	0.7593	-1.19	0.5388	0.7177	0.89	0.2938	0.4990	-1.88	0.9510	1.1459	0.09	0.2828	0.5321	0.33	0.4914	0.9030	0.19	0.0294	0.1460	1.29
889	Chromobox protein homolog 1	P83916	0.0624	0.1657	-0.13	0.0264	0.4955	-0.33	0.9992	0.9994	0.00	0.1137	0.2141	-0.10	0.0105	0.0731	0.26	0.0185	0.0881	0.22	0.1643	0.3585	0.15	0.7652	1.2074	-0.03	0.0090	0.0845	0.42
890	Chromobox protein homolog 3	Q13185	0.0409	0.1279	0.10	0.3400	0.6989	0.17	0.0030	0.0298	0.28	0.0022	0.0204	0.27	0.1004	0.2537	-0.08	0.0261	0.1033	0.14	0.0476	0.1747	0.22	0.2059	0.5062	0.11	0.2397	0.3099	-0.08
891	Chromobox protein homolog 5	P45973	0.1307	0.2726	0.04	0.3787	0.7260	-0.05	0.2065	0.3596	0.03	0.2040	0.3376	0.05	0.0049	0.0522	-0.23	0.1604	0.3214	0.09	0.2433	0.4763	-0.15	0.4061	0.7917	-0.04	0.3574	0.4286	0.06
892	Chromodomain-helicase-DNA-binding protein 1	O14646	0.0113	0.0612	-0.21	0.0858	0.6199	-0.25	0.0716	0.1596	0.30	0.7922	0.8210	0.01	0.1051	0.2607	0.09	0.0744	0.1940	0.11	0.3736	0.5476	-2.55	0.8708	1.1198	-0.33	0.0651	0.1639	2.06
893	Chromodomain-helicase-DNA-binding protein 1-like	Q86WJ1	0.3763	0.5918	-0.02	0.3510	0.7039	-0.10	0.0973	0.1999	0.09	0.0833	0.1691	0.10	0.4874	0.5775	0.02	0.0080	0.0665	0.17	0.0333	0.1525	0.76	0.7301	1.1749	-0.07	0.1008	0.1564	0.39
894	Chromodomain-helicase-DNA-binding protein 3	Q12873	0.4108	0.6057	-3.31	0.5752	0.8477	-1.75	0.5661	0.7591	-1.19	0.5388	0.7175	0.89	0.2938	0.4988	-1.88	0.9510	1.1456	0.09	0.3071	0.5667	0.18	0.7382	1.1809	-0.02	0.7901	0.8283	0.02
895	Chromodomain-helicase-DNA-binding protein 4	Q14839	0.1426	0.2892	0.09	0.3639	0.7127	0.12	0.0105	0.0477	0.20	0.2972	0.4611	0.06	0.0657	0.1949	-0.11	0.1877	0.3604	0.07	0.0106	0.1024	0.39	0.3641	0.7336	-0.07	0.3251	0.3965	0.03
896	Chromosome-associated kinesin KIF4A	O95239	0.4108	0.6056	-3.31	0.5752	0.8474	-1.75	0.5661	0.7589	-1.19	0.5388	0.7173	0.89	0.2938	0.4987	-1.88	0.9510	1.1454	0.09	0.3816	0.4373	-0.04	0.0057	0.0606	0.47	0.0143	0.1032	0.22
897	Chromosome-associated kinesin KIF4B	Q2VIQ3	0.0008	0.0217	0.18	0.0939	0.6309	0.24	0.0466	0.1194	0.09	0.0005	0.0120	0.30	0.0944	0.2432	0.05	0.2240	0.4070	0.11	0.3736	0.4971	-2.55	0.8708	1.0282	-0.33	0.0651	0.1396	2.06
898	Chymotrypsin-C	Q99895	0.4108	0.6054	-3.31	0.5752	0.8472	-1.75	0.5661	0.7587	-1.19	0.5388	0.7171	0.89	0.2938	0.4985	-1.88	0.9510	1.1451	0.09	0.2355	0.4649	-0.17	0.3040	0.6499	0.14	0.4315	0.5029	-0.12
899	Chymotrypsin-like elastase family member 2A	P08217	0.2172	0.3924	-0.06	0.0116	0.4323	-0.29	0.0097	0.0460	-0.48	0.0061	0.0314	-0.69	0.0008	0.0217	-0.42	0.0783	0.2002	-0.21	0.3736	0.5447	-2.55	0.8708	1.1146	-0.33	0.0651	0.1624	2.06
900	Cilia- and flagella-associated protein 161	Q6P656	0.4108	0.6052	-3.31	0.5752	0.8469	-1.75	0.5661	0.7585	-1.19	0.5388	0.7169	0.89	0.2938	0.4983	-1.88	0.9510	1.1448	0.09	0.0004	0.0364	-0.75	0.0091	0.0784	-0.42	0.0151	0.1056	-0.34
901	Cilia- and flagella-associated protein 20	Q9Y6A4	0.4108	0.6050	-3.31	0.5752	0.8467	-1.75	0.5661	0.7583	-1.19	0.5388	0.7167	0.89	0.2938	0.4982	-1.88	0.9510	1.1446	0.09	0.2396	0.4712	0.18	0.7610	1.2033	0.03	0.1735	0.2394	-0.14
902	Cilia- and flagella-associated protein 298	P57076	0.3525	0.5625	-0.15	0.3726	0.7204	-0.18	0.6487	0.7009	-0.08	0.2736	0.4308	-0.21	0.7821	0.8290	0.05	0.3317	0.5349	-0.27	0.0146	0.1119	0.47	0.0238	0.1365	0.40	0.0024	0.0534	0.76

Supplemetary Table S2. Overview on all relatively quantified 5180 proteins statistical analysis

			MCF-7																		MDA-MB-231								
			Dai SC20 vs control			Gen SC20 vs control			SSE SC20 vs control			Dai IC20 vs control			Gen IC20 vs control			SSE IC20 vs control			Dai IC20 vs control			Gen IC20 vs control			SSE IC20 vs control		
	**Protein name**	**UniProt**	**p value**	**BH q value**	**log2FC**	**p value**	**BH q value**	**log2FC**	**p value**	**BH q value**	**log2FC**	**p value**	**BH q value**	**log2FC**	**p value**	**BH q value**	**log2FC**	**p value**	**BH q value**	**log2FC**	**p value**	**BH q value**	**log2FC**	**p value**	**BH q value**	**log2FC**	**p value**	**BH q value**	**log2FC**
903	Cilia- and flagella-associated protein 43	Q8NDM7	0.4108	0.6049	-3.31	0.5752	0.8465	-1.75	0.5661	0.7581	-1.19	0.5388	0.7166	0.89	0.2938	0.4980	-1.88	0.9510	1.1443	0.09	0.5145	0.5679	0.11	0.1576	0.4290	0.30	0.7396	0.7856	0.05
904	Cilia- and flagella-associated protein 52	Q8N1V2	0.3446	0.5530	0.11	0.3611	0.7096	-0.12	0.1246	0.2434	-0.22	0.1018	0.1966	-0.28	0.2095	0.4126	-0.17	0.8994	1.1366	-0.01	0.0455	0.1712	-0.34	0.0374	0.1763	-0.14	0.0066	0.0758	-0.28
905	Cilia- and flagella-associated protein 54	Q96N23	0.4108	0.6047	-3.31	0.5752	0.8462	-1.75	0.5661	0.7579	-1.19	0.5388	0.7164	0.89	0.2938	0.4978	-1.88	0.9510	1.1440	0.09	0.2951	0.5505	0.06	0.2600	0.5868	-0.08	0.1373	0.1983	-0.10
906	Cilia- and flagella-associated protein 58	Q5T655	0.0324	0.1120	-0.47	0.2986	0.6728	-0.07	0.2485	0.4147	-0.08	0.1651	0.2852	-0.14	0.0335	0.1337	-0.24	0.0171	0.0855	-0.28	0.0915	0.2431	0.34	0.0387	0.1795	-0.22	0.0088	0.0839	-0.23
907	Cilia- and flagella-associated protein 69	A5D8W1	0.0165	0.0751	0.16	0.1381	0.6527	0.25	0.0190	0.0661	0.25	0.5422	0.5937	0.02	0.0163	0.0910	0.12	0.0063	0.0628	0.20	0.3736	0.6243	-2.55	0.8708	1.2551	-0.33	0.0651	0.2078	2.06
908	Cilia- and flagella-associated protein 74	Q9C0B2	0.0030	0.0369	0.11	0.6425	0.7365	0.04	0.0007	0.0198	0.28	0.0207	0.0620	-0.07	0.6467	0.7147	0.01	0.0618	0.1727	0.43	0.0008	0.0423	0.59	0.0001	0.0157	0.81	0.1840	0.2514	0.15
909	Cilia- and flagella-associated protein 91	Q7Z4T9	0.0238	0.0927	0.20	0.1423	0.6552	0.18	0.2458	0.4115	0.08	0.9105	0.9223	-0.01	0.8348	0.8717	-0.01	0.0816	0.2056	0.14	0.6117	0.6592	0.09	0.4376	0.8386	0.14	0.6367	0.6932	0.08
910	Cilia- and flagella-associated protein 97	Q9P2B7	0.8595	0.8888	-0.20	0.2210	0.6591	1.04	0.0103	0.0472	1.81	0.0040	0.0257	2.18	0.7293	0.7882	0.11	0.2328	0.4168	1.11	0.0841	0.2318	-0.29	0.0611	0.2365	-0.10	0.1471	0.2095	-0.09
911	Cingulin	Q9P2M7	0.0510	0.1464	0.10	0.2471	0.6588	0.16	0.0260	0.0804	0.18	0.0052	0.0291	0.22	0.1270	0.2945	0.07	0.0126	0.0755	0.21	0.3723	0.6544	0.09	0.1631	0.4396	-0.20	0.1023	0.1582	-0.23
912	Citrate synthase mitochondrial	O75390	0.0237	0.0925	0.20	0.1043	0.6341	0.21	0.0011	0.0219	0.35	0.0012	0.0164	0.27	0.0452	0.1567	0.08	0.0953	0.2277	0.24	0.1673	0.3620	0.15	0.4729	0.8843	-0.04	0.2998	0.3711	-0.07
913	C-Jun-amino-terminal kinase-interacting protein 4	O60271	0.0178	0.0782	0.21	0.3140	0.6803	0.16	0.0149	0.0576	0.21	0.0124	0.0451	0.25	0.3419	0.4391	0.05	0.0163	0.0847	0.26	0.0607	0.1945	0.39	0.2658	0.5953	0.09	0.1616	0.2260	0.13
914	Clathrin heavy chain 1	Q00610	0.0799	0.1940	0.13	0.2905	0.6688	0.23	0.0112	0.0491	0.25	0.0185	0.0576	0.22	0.5206	0.6083	0.03	0.0169	0.0854	0.30	0.0908	0.2426	0.33	0.3697	0.7414	0.10	0.7926	0.8306	0.02
915	Clathrin heavy chain 2	P53675	0.0468	0.1385	0.12	0.3959	0.7395	0.15	0.0040	0.0320	0.28	0.0018	0.0188	0.31	0.3304	0.4277	0.04	0.0181	0.0871	0.19	0.0751	0.2181	0.33	0.1802	0.4672	0.11	0.1416	0.2030	0.12
916	Clathrin interactor 1	Q14677	0.9010	0.9224	-0.01	0.5192	0.8399	0.06	0.0181	0.0644	0.16	0.0867	0.1745	0.09	0.1200	0.2845	-0.07	0.1892	0.3626	0.07	0.2306	0.4578	0.09	0.4201	0.8123	-0.04	0.1672	0.2324	-0.08
917	Clathrin light chain A	P09496	0.6285	0.6911	0.09	0.2141	0.6621	0.25	0.2064	0.3596	0.26	0.5439	0.5949	0.10	0.2116	0.4152	0.25	0.6172	0.8532	0.08	0.0842	0.2318	0.42	0.6937	1.1361	-0.04	0.7005	0.7522	-0.03
918	Clathrin light chain B	P09497	0.3318	0.5386	0.05	0.2127	0.6625	0.33	0.0059	0.0372	0.30	0.0011	0.0157	0.38	0.1514	0.3292	0.07	0.0060	0.0619	0.27	0.5006	0.5544	0.15	0.1650	0.4422	-0.16	0.4336	0.5046	-0.16
919	Claudin-1	O95832	0.4108	0.6045	-3.31	0.5752	0.8460	-1.75	0.5661	0.7577	-1.19	0.5388	0.7162	0.89	0.2938	0.4977	-1.88	0.9510	1.1438	0.09	0.1237	0.2960	-0.29	0.4193	0.8120	-0.03	0.2915	0.3632	0.13
920	Claudin-3	O15551	0.0068	0.0493	0.15	0.3112	0.6793	0.19	0.0004	0.0188	0.22	0.0034	0.0238	0.33	0.0021	0.0357	0.25	0.0047	0.0604	0.13	0.3736	0.6310	-2.55	0.8708	1.2667	-0.33	0.0651	0.2121	2.06
921	Cleavage and polyadenylation specificity factor subunit 5	O43809	0.0060	0.0469	-0.15	0.2777	0.6650	0.09	0.5408	0.7629	-0.02	0.0831	0.1688	-0.08	0.0037	0.0452	-0.18	0.0754	0.1951	-0.17	0.2161	0.4362	0.15	0.1346	0.3869	-0.16	0.1372	0.1982	-0.12
922	Cleavage and polyadenylation specificity factor subunit 6	Q16630	0.6039	0.6691	-0.02	0.1828	0.6650	-0.05	0.1873	0.3329	0.07	0.0997	0.1938	-0.08	0.0477	0.1604	-0.13	0.8219	1.0570	-0.01	0.2120	0.4300	-0.06	0.9491	0.9632	0.00	0.2849	0.3566	-0.03
923	Cleavage and polyadenylation specificity factor subunit 7	Q8N684	0.3928	0.6103	-0.08	0.8969	0.9271	0.01	0.0945	0.1954	0.20	0.0799	0.1640	0.21	0.6232	0.6950	0.04	0.8432	1.0785	0.02	0.0116	0.1041	0.41	0.0860	0.2908	0.18	0.1862	0.2537	-0.10
924	Cleavage stimulation factor subunit 1	Q05048	0.0050	0.0437	0.14	0.2735	0.6651	0.21	0.0359	0.0992	0.29	0.0105	0.0411	0.11	0.0195	0.0998	-0.10	0.0484	0.1470	0.07	0.3736	0.5621	-2.55	0.8708	1.1457	-0.33	0.0651	0.1715	2.06
925	Cleavage stimulation factor subunit 2	P33240	0.3423	0.5500	0.10	0.6462	0.7384	0.06	0.2456	0.4113	0.13	0.3419	0.5165	0.10	0.6033	0.6788	-0.05	0.2102	0.3908	0.15	0.0037	0.0661	0.43	0.2174	0.5235	0.09	0.0210	0.1236	0.39
926	Cleft lip and palate transmembrane protein 1	O96005	0.0022	0.0325	-0.19	0.2886	0.6683	0.15	0.0018	0.0237	0.18	0.0712	0.1496	0.07	0.0143	0.0852	0.11	0.2134	0.3948	0.03	0.0024	0.0565	-0.48	0.0156	0.1056	-0.37	0.1186	0.1767	-0.39
927	Cleft lip and palate transmembrane protein 1-like protein	Q96KA5	0.4108	0.6044	-3.31	0.5752	0.8457	-1.75	0.5661	0.7575	-1.19	0.5388	0.7160	0.89	0.2938	0.4975	-1.88	0.9510	1.1435	0.09	0.3067	0.5666	-0.06	0.0569	0.2283	0.11	0.5469	0.6116	-0.02
928	CLIP-associating protein 1	Q7Z460	0.0769	0.1894	0.10	0.5787	0.6874	0.07	0.8855	0.9092	-0.01	0.0134	0.0469	0.23	0.1774	0.3680	-0.07	0.0264	0.1041	0.17	0.0095	0.0978	0.27	0.0104	0.0840	0.22	0.0130	0.0992	0.16
929	CLIP-associating protein 2	O75122	0.4108	0.6042	-3.31	0.5752	0.8455	-1.75	0.5661	0.7573	-1.19	0.5388	0.7158	0.89	0.2938	0.4973	-1.88	0.9510	1.1432	0.09	0.1742	0.3727	0.16	0.2597	0.5869	0.12	0.9496	0.9604	0.01
930	Clustered mitochondria protein homolog	O75153	0.8856	0.9098	0.01	0.4967	0.8186	0.09	0.7100	0.7561	-0.02	0.8844	0.9009	0.01	0.0965	0.2468	-0.15	0.1791	0.3485	-0.11	0.0509	0.1790	0.31	0.4788	0.8899	0.09	0.9416	0.9532	0.01
931	Clusterin	P10909	0.0101	0.0587	-0.14	0.0293	0.5110	0.29	0.0093	0.0452	-0.12	0.0926	0.1829	0.15	0.5129	0.6020	0.03	0.2269	0.4098	0.21	0.3736	0.4958	-2.55	0.8708	1.0259	-0.33	0.0651	0.1390	2.06
932	Clusterin-associated protein 1	Q96AJ1	0.1169	0.2512	0.15	0.0107	0.4399	-0.38	0.0237	0.0757	-0.35	0.1414	0.2532	-0.17	0.0099	0.0716	-0.37	0.2271	0.4100	-0.11	0.0475	0.1746	-0.07	0.1228	0.3643	0.20	0.5590	0.6233	-0.07
933	CMP-sialic acid transporter	P78382	0.0024	0.0337	-0.35	0.1334	0.6494	0.26	0.1860	0.3312	-0.13	0.0869	0.1746	-0.10	0.2040	0.4055	0.06	0.0025	0.0558	-0.45	0.3736	0.6419	-2.55	0.8708	1.2855	-0.33	0.0651	0.2193	2.06
934	C-myc promoter-binding protein	Q7Z401	0.0079	0.0527	-1.72	0.1860	0.6677	-1.25	0.1681	0.3070	-0.56	0.0061	0.0312	-1.38	0.8726	0.9031	0.04	0.0044	0.0597	-1.65	0.0101	0.1010	0.99	0.0006	0.0230	0.86	0.0009	0.0376	0.74
935	c-Myc-binding protein	Q99417	0.0426	0.1308	-0.22	0.1644	0.6622	0.06	0.0485	0.1226	0.13	0.0578	0.1278	0.09	0.0604	0.1851	0.09	0.0456	0.1414	-0.12	0.1515	0.3383	0.18	0.4103	0.7972	0.06	0.3018	0.3731	-0.06
936	CNK3/IPCEF1 fusion protein	G9CGD6	0.4108	0.6040	-3.31	0.0909	0.6278	3.23	0.5661	0.7902	-1.19	0.5388	0.7463	0.89	0.2938	0.5246	-1.88	0.9510	1.1865	0.09	0.3736	0.4312	-2.55	0.8708	0.9054	-0.33	0.0651	0.1120	2.06
937	Coactosin-like protein	Q14019	0.0414	0.1289	0.27	0.3860	0.7303	0.12	0.0734	0.1619	0.23	0.0510	0.1164	0.24	0.5238	0.6108	0.05	0.0345	0.1197	0.34	0.0525	0.1813	0.46	0.1388	0.3950	0.16	0.4074	0.4790	0.06
938	Coagulation factor X	P00742	0.0217	0.0877	0.23	0.0168	0.4629	0.25	0.0030	0.0302	0.62	0.0006	0.0130	0.64	0.0158	0.0897	0.26	0.0081	0.0670	0.52	0.3736	0.6187	-2.55	0.8708	1.2454	-0.33	0.0651	0.2043	2.06
939	Coatomer subunit alpha	P53621	0.0808	0.1959	0.14	0.3609	0.7100	0.17	0.0373	0.1014	0.18	0.0196	0.0596	0.23	0.3869	0.4841	0.05	0.0304	0.1118	0.26	0.0439	0.1683	0.35	0.7853	1.2293	0.02	0.6135	0.6729	0.03
940	Coatomer subunit beta	P53618	0.1921	0.3587	0.09	0.4402	0.7761	0.11	0.0669	0.1521	0.15	0.0425	0.1024	0.19	0.7714	0.8217	-0.02	0.0396	0.1299	0.20	0.1784	0.3780	0.15	0.0055	0.0600	0.32	0.1331	0.1934	0.08
941	Coatomer subunit beta'	P35606	0.0269	0.0995	0.14	0.2450	0.6582	0.19	0.0093	0.0450	0.19	0.0036	0.0241	0.26	0.0964	0.2468	0.09	0.0074	0.0649	0.26	0.0322	0.1514	0.29	0.4301	0.8264	0.06	0.8291	0.8615	0.01
942	Coatomer subunit delta	P48444	0.0613	0.1638	0.21	0.1865	0.6667	0.21	0.0082	0.0426	0.39	0.0091	0.0384	0.42	0.0814	0.2204	0.19	0.0179	0.0870	0.41	0.0246	0.1385	0.55	0.2950	0.6362	0.10	0.1518	0.2150	0.11
943	Coatomer subunit epsilon	O14579	0.4639	0.5405	-0.01	0.3213	0.6835	0.07	0.0017	0.0233	0.17	0.0009	0.0153	0.16	0.0118	0.0784	0.09	0.0007	0.0437	0.23	0.0214	0.1340	0.16	0.0952	0.3078	-0.09	0.0111	0.0921	0.19
944	Coatomer subunit gamma-1	Q9Y678	0.0605	0.1628	0.14	0.3327	0.6918	0.17	0.0102	0.0470	0.23	0.0292	0.0789	0.19	0.4576	0.5502	0.03	0.0181	0.0870	0.23	0.0323	0.1514	0.36	0.0949	0.3072	0.16	0.0899	0.1432	0.13
945	Coatomer subunit gamma-2	Q9UBF2	0.0098	0.0583	0.21	0.1856	0.6667	0.21	0.0024	0.0270	0.27	0.0011	0.0161	0.33	0.0744	0.2095	0.09	0.0046	0.0596	0.35	0.0975	0.2509	0.25	0.3608	0.7286	0.11	0.2423	0.3127	0.12
946	Coatomer subunit zeta-1	P61923	0.5932	0.6594	0.02	0.8921	0.9235	0.01	0.1713	0.3119	0.07	0.0779	0.1607	0.09	0.9314	0.9479	0.00	0.0460	0.1421	0.13	0.0051	0.0757	0.49	0.0036	0.0492	0.37	0.0142	0.1033	0.30
947	Cofilin-1	P23528	0.0006	0.0203	0.34	0.1074	0.6308	0.30	0.0046	0.0336	0.29	0.0004	0.0118	0.34	0.0362	0.1384	0.11	0.0014	0.0522	0.30	0.2327	0.4604	0.17	0.1175	0.3533	-0.17	0.0179	0.1146	-0.34
948	Cofilin-2	Q9Y281	0.1883	0.3544	0.14	0.2068	0.6592	0.13	0.0546	0.1330	0.28	0.0300	0.0804	0.31	0.9952	0.9969	0.00	0.0540	0.1574	0.30	0.0127	0.1071	-0.35	0.0051	0.0572	-0.47	0.0065	0.0755	-0.56
949	Cohesin subunit SA-2	Q8N3U4	0.1195	0.2551	0.32	0.8008	0.8562	0.13	0.5974	0.6535	0.08	0.0273	0.0750	0.60	0.2673	0.4891	-0.25	0.1483	0.3047	0.34	0.3736	0.5169	-2.55	0.8708	1.0644	-0.33	0.0651	0.1488	2.06
950	Coiled-coil alpha-helical rod protein 1	Q8TD31	0.4108	0.6038	-3.31	0.5752	0.8453	-1.75	0.5661	0.7571	-1.19	0.5388	0.7156	0.89	0.2938	0.4972	-1.88	0.9510	1.1430	0.09	0.0610	0.1950	0.87	0.5780	1.0084	-0.27	0.1547	0.2180	0.57
951	Coiled-coil and C2 domain-containing protein 1A	Q6P1N0	0.1153	0.2486	0.13	0.2818	0.6641	0.19	0.0171	0.0622	0.26	0.0262	0.0729	0.25	0.8434	0.8792	-0.01	0.1809	0.3503	0.11	0.3736	0.5067	-2.55	0.8708	1.0458	-0.33	0.0651	0.1440	2.06
952	Coiled-coil domain-containing glutamate-rich protein 2	I3L3R5	0.4108	0.6037	-3.31	0.5752	0.8450	-1.75	0.5661	0.7569	-1.19	0.5388	0.7155	0.89	0.2938	0.4970	-1.88	0.9510	1.1427	0.09	0.1366	0.3156	1.07	0.6145	1.0481	0.43	0.0117	0.0931	2.38
953	Coiled-coil domain-containing protein 102A	Q96A19	0.1226	0.2597	-0.81	0.3542	0.7057	-0.64	0.0083	0.0428	0.69	0.0665	0.1425	0.34	0.0009	0.0226	1.18	0.5494	0.7831	0.13	0.3736	0.4570	-2.55	0.8708	0.9538	-0.33	0.0651	0.1223	2.06
954	Coiled-coil domain-containing protein 121	Q6ZUS5	0.0013	0.0254	-0.98	0.3859	0.7309	-1.34	0.0678	0.1534	0.31	0.0662	0.1423	-0.27	0.3643	0.4614	0.12	0.1358	0.2879	-0.68	0.0369	0.1596	0.50	0.2083	0.5099	-0.22	0.0071	0.0771	0.27
955	Coiled-coil domain-containing protein 122	Q5T0U0	0.4108	0.6035	-3.31	0.5752	0.8448	-1.75	0.5661	0.7567	-1.19	0.5388	0.7153	0.89	0.2938	0.4969	-1.88	0.9510	1.1424	0.09	0.7328	0.7718	-0.05	0.0046	0.0543	-0.16	0.9026	0.9224	-0.01
956	Coiled-coil domain-containing protein 124	Q96CT7	0.0232	0.0912	0.09	0.1678	0.6605	0.11	0.0167	0.0613	0.11	0.0033	0.0233	0.14	0.0036	0.0447	0.15	0.0175	0.0866	0.11	0.1396	0.3201	0.95	0.7206	1.1657	-0.17	0.3018	0.3730	0.57
957	Coiled-coil domain-containing protein 136	Q96JN2	0.0483	0.1413	0.10	0.2368	0.6630	0.14	0.0098	0.0460	0.21	0.0347	0.0890	0.14	0.1151	0.2778	-0.07	0.9859	0.9893	0.00	0.0007	0.0390	-0.77	0.0004	0.0223	-0.97	0.0019	0.0507	-1.10
958	Coiled-coil domain-containing protein 14	Q49A88	0.0602	0.1622	-12.02	0.0494	0.5750	-10.47	0.0163	0.0604	-9.91	0.0006	0.0125	-7.83	0.1719	0.3608	-1.96	0.0054	0.0615	-8.63	0.3736	0.6281	-2.55	0.8708	1.2617	-0.33	0.0651	0.2102	2.06
959	Coiled-coil domain-containing protein 141	Q6ZP82	0.2205	0.3967	-0.07	0.0757	0.6166	0.09	0.4199	0.6277	0.04	0.0505	0.1157	0.09	0.0055	0.0552	0.16	0.9688	0.9748	0.00	0.3736	0.4304	-2.55	0.8708	0.9040	-0.33	0.0651	0.1118	2.06
960	Coiled-coil domain-containing protein 144A	A2RUR9	0.5558	0.6249	0.06	0.4227	0.7597	0.14	0.1062	0.2141	0.19	0.0283	0.0771	0.29	0.2901	0.5209	-0.10	0.0290	0.1090	0.36	0.0418	0.1677	0.40	0.1907	0.4828	0.18	0.2343	0.3043	0.14
961	Coiled-coil domain-containing protein 144B	Q3MJ40	0.0423	0.1303	-0.17	0.1296	0.6437	-0.13	0.0084	0.0431	-0.27	0.0043	0.0266	-0.33	0.0102	0.0726	-0.34	0.0428	0.1366	-0.18	0.0051	0.0759	0.82	0.0003	0.0187	0.67	0.0002	0.0296	1.03
962	Coiled-coil domain-containing protein 146	Q8IYE0	0.1012	0.2269	-0.25	0.0026	0.2494	-0.84	0.8977	0.9192	-0.01	0.0103	0.0407	-0.69	0.1913	0.3872	0.17	0.0952	0.2276	0.24	0.3736	0.5361	-2.55	0.8708	1.0991	-0.33	0.0651	0.1581	2.06
963	Coiled-coil domain-containing protein 150	Q8NCX0	0.0054	0.0448	0.46	0.0543	0.5872	0.56	0.0011	0.0226	0.61	0.0007	0.0139	0.68	0.0609	0.1861	0.19	0.0056	0.0619	0.53	0.1723	0.3699	0.15	0.0111	0.0865	-0.27	0.0183	0.1160	-0.22
964	Coiled-coil domain-containing protein 153	Q494R4	0.0008	0.0216	0.68	0.1056	0.6316	0.59	0.0047	0.0336	0.53	0.0002	0.0100	1.30	0.0013	0.0288	0.60	0.0304	0.1120	1.12	0.2167	0.4368	0.53	0.2025	0.5012	0.50	0.0374	0.1663	1.35
965	Coiled-coil domain-containing protein 168	Q8NDH2	0.4108	0.6033	-3.31	0.5752	0.8445	-1.75	0.5661	0.7566	-1.19	0.5388	0.7151	0.89	0.2938	0.4967	-1.88	0.9510	1.1422	0.09	0.3169	0.5794	0.83	0.2760	0.6120	0.92	0.1166	0.1745	1.66
966	Coiled-coil domain-containing protein 170	Q8IYT3	0.0796	0.1936	-0.15	0.4671	0.7980	0.06	0.0181	0.0644	0.27	0.1890	0.3172	0.14	0.1450	0.3195	0.11	0.4087	0.6256	-0.06	0.3093	0.5696	0.18	0.1276	0.3728	-0.13	0.1767	0.2428	-0.16
967	Coiled-coil domain-containing protein 171	Q6TFL3	0.1322	0.2742	0.10	0.0933	0.6293	0.28	0.0466	0.1194	0.22	0.0125	0.0452	0.14	0.0530	0.1707	0.09	0.0526	0.1552	0.14	0.1390	0.3192	0.37	0.2815	0.6194	-0.15	0.6312	0.6883	-0.06
968	Coiled-coil domain-containing protein 175	P0C221	0.3381	0.5459	0.18	0.3425	0.7001	0.15	0.2233	0.3806	0.20	0.0901	0.1794	-0.33	0.0815	0.2205	-0.32	0.5357	0.7676	0.13	0.3736	0.4582	-2.55	0.8708	0.9561	-0.33	0.0651	0.1228	2.06
969	Coiled-coil domain-containing protein 180	Q9P1Z9	0.4108	0.6032	-3.31	0.5752	0.8443	-1.75	0.5661	0.7564	-1.19	0.5388	0.7149	0.89	0.2938	0.4965	-1.88	0.9510	1.1419	0.09	0.1012	0.2573	0.28	0.3955	0.7796	0.15	0.0957	0.1504	0.26
970	Coiled-coil domain-containing protein 181	Q5TID7	0.0515	0.1470	-0.16	0.7900	0.8488	0.05	0.0532	0.1306	-0.20	0.3751	0.5585	0.05	0.3479	0.4452	-0.09	0.0221	0.0952	-0.29	0.3736	0.5904	-2.55	0.8708	1.1958	-0.33	0.0651	0.1872	2.06
971	Coiled-coil domain-containing protein 186	Q7Z3E2	0.4108	0.6030	-3.31	0.5752	0.8441	-1.75	0.5661	0.7562	-1.19	0.5388	0.7147	0.89	0.2938	0.4964	-1.88	0.9510	1.1416	0.09	0.0537	0.1839	0.23	0.0913	0.3005	-0.05	0.6257	0.6836	-0.02
972	Coiled-coil domain-containing protein 22	O60826	0.0386	0.1235	-0.22	0.2233	0.6580	0.10	0.5084	0.7299	0.05	0.7075	0.7464	-0.03	0.1799	0.3717	0.11	0.0993	0.2329	-0.16	0.3109	0.5715	-0.05	0.0119	0.0908	-0.17	0.0451	0.1859	-0.25
973	Coiled-coil domain-containing protein 25	Q86WR0	0.0008	0.0215	0.56	0.0631	0.5889	0.28	0.0103	0.0473	0.21	0.0088	0.0380	0.25	0.0001	0.0098	0.33	0.0433	0.1372	0.29	0.3736	0.5669	-2.55	0.8708	1.1542	-0.33	0.0651	0.1741	2.06
974	Coiled-coil domain-containing protein 30	Q5VVM6	0.4108	0.6028	-3.31	0.5752	0.8438	-1.75	0.5661	0.7560	-1.19	0.5388	0.7145	0.89	0.2938	0.4962	-1.88	0.9510	1.1414	0.09	0.0279	0.1441	0.45	0.1189	0.3562	0.13	0.0233	0.1295	0.23
975	Coiled-coil domain-containing protein 39	Q9UFE4	0.3886	0.6052	-4.80	0.1083	0.6310	-6.67	0.0384	0.1035	-6.12	0.6286	0.6747	1.01	0.8817	0.9091	-0.17	0.7077	0.9495	-0.55	0.3736	0.4451	-2.55	0.8708	0.9316	-0.33	0.0651	0.1175	2.06
976	Coiled-coil domain-containing protein 43	Q96MW1	0.7837	0.8246	-0.02	0.1778	0.6631	0.17	0.4755	0.6934	0.04	0.1621	0.2815	0.09	0.6901	0.7530	-0.02	0.2764	0.4716	0.07	0.3736	0.4861	-2.55	0.8708	1.0080	-0.33	0.0651	0.1347	2.06
977	Coiled-coil domain-containing protein 47	Q96A33	0.1665	0.3245	0.02	0.8830	0.9161	0.02	0.0878	0.1854	0.07	0.0059	0.0308	0.17	0.0027	0.0397	-0.11	0.0247	0.1003	0.18	0.5986	0.6473	0.03	0.0034	0.0475	-0.11	0.1357	0.1965	-0.21
978	Coiled-coil domain-containing protein 50	Q8IVM0	0.4108	0.6026	-3.31	0.5752	0.8436	-1.75	0.5661	0.7558	-1.19	0.5388	0.7144	0.89	0.2938	0.4961	-1.88	0.9510	1.1411	0.09	0.2567	0.4956	0.55	0.6395	1.0780	0.19	0.2866	0.3584	0.51
979	Coiled-coil domain-containing protein 51	Q96ER9	0.1997	0.3702	-0.26	0.7332	0.8057	0.06	0.8595	0.8880	-0.03	0.9029	0.9165	0.02	0.6459	0.7140	0.09	0.1971	0.3723	-0.27	0.3736	0.5029	-2.55	0.8708	1.0389	-0.33	0.0651	0.1422	2.06
980	Coiled-coil domain-containing protein 58	Q4VC31	0.3705	0.5849	-0.07	0.1147	0.6334	-0.20	0.0140	0.0556	-0.39	0.0216	0.0638	-0.38	0.0345	0.1355	-0.26	0.0519	0.1539	-0.26	0.3736	0.5598	-2.55	0.8708	1.1417	-0.33	0.0651	0.1703	2.06
981	Coiled-coil domain-containing protein 6	Q16204	0.2675	0.4593	0.12	0.2220	0.6579	0.19	0.0057	0.0366	0.29	0.0361	0.0913	0.15	0.1612	0.3442	-0.07	0.0070	0.0642	0.27	0.0658	0.2016	0.21	0.0554	0.2245	0.10	0.0878	0.1405	0.11
982	Coiled-coil domain-containing protein 60	Q8IWA6	0.2093	0.3819	5.32	0.0251	0.4815	6.07	0.5661	0.7908	-1.19	0.5388	0.7465	0.89	0.2938	0.5248	-1.88	0.9510	1.1867	0.09	0.3736	0.4313	-2.55	0.8708	0.9056	-0.33	0.0651	0.1121	2.06
983	Coiled-coil domain-containing protein 62	Q6P9F0	0.1040	0.2306	-0.07	0.1935	0.6678	0.22	0.0133	0.0539	0.22	0.0075	0.0351	0.20	0.0465	0.1586	0.11	0.0912	0.2204	0.07	0.3736	0.5368	-2.55	0.8708	1.1004	-0.33	0.0651	0.1585	2.06

Supplemetary Table S2. Overview on all relatively quantified 5180 proteins statistical analysis

			MCF-7																		MDA-MB-231								
			Dai SC20 vs control			Gen SC20 vs control			SSE SC20 vs control			Dai IC20 vs control			Gen IC20 vs control			SSE IC20 vs control			Dai IC20 vs control			Gen IC20 vs control			SSE IC20 vs control		
	**Protein name**	**UniProt**	**p value**	**BH q value**	**log2FC**	**p value**	**BH q value**	**log2FC**	**p value**	**BH q value**	**log2FC**	**p value**	**BH q value**	**log2FC**	**p value**	**BH q value**	**log2FC**	**p value**	**BH q value**	**log2FC**	**p value**	**BH q value**	**log2FC**	**p value**	**BH q value**	**log2FC**	**p value**	**BH q value**	**log2FC**
984	Coiled-coil domain-containing protein 66	A2RUB6	0.0126	0.0655	-0.10	0.0143	0.4517	-0.30	0.0781	0.1693	-0.10	0.0139	0.0479	-0.45	0.0195	0.0997	-0.29	0.0471	0.1443	-0.31	0.3736	0.5631	-2.55	0.8708	1.1475	-0.33	0.0651	0.1721	2.06
985	Coiled-coil domain-containing protein 68	Q9H2F9	0.4108	0.6025	-3.31	0.5752	0.8433	-1.75	0.5661	0.7556	-1.19	0.5388	0.7142	0.89	0.2938	0.4959	-1.88	0.9510	1.1408	0.09	0.0213	0.1336	0.65	0.0213	0.1267	0.41	0.0380	0.1681	0.23
986	Coiled-coil domain-containing protein 7	Q96M83	0.0425	0.1306	-0.19	0.5509	0.8663	-0.08	0.3498	0.5440	0.07	0.0095	0.0390	-0.28	0.0380	0.1422	-0.16	0.0058	0.0619	0.38	0.0111	0.1036	0.52	0.0086	0.0764	0.44	0.0079	0.0796	0.56
987	Coiled-coil domain-containing protein 83	Q8IWF9	0.0375	0.1217	0.21	0.2934	0.6698	-0.27	0.0229	0.0745	0.29	0.1126	0.2128	0.16	0.4627	0.5544	-0.05	0.0464	0.1430	0.21	0.1183	0.2868	-0.53	0.9456	0.9606	-0.01	0.2643	0.3363	0.35
988	Coiled-coil domain-containing protein 85B	Q15834	0.2391	0.4236	0.13	0.1159	0.6367	0.21	0.3174	0.5045	0.10	0.2230	0.3631	0.16	0.1454	0.3200	0.18	0.3484	0.5536	0.11	0.2393	0.4708	0.21	0.0392	0.1800	0.34	0.0302	0.1474	0.41
989	Coiled-coil domain-containing protein 91	Q7Z6B0	0.4108	0.6023	-3.31	0.5752	0.8431	-1.75	0.5661	0.7554	-1.19	0.5388	0.7140	0.89	0.2938	0.4957	-1.88	0.9510	1.1406	0.09	0.0431	0.1677	0.42	0.7292	1.1749	-0.04	0.0015	0.0463	0.49
990	Coiled-coil domain-containing protein 93	Q567U6	0.4108	0.6021	-3.31	0.5752	0.8429	-1.75	0.5661	0.7552	-1.19	0.5388	0.7138	0.89	0.2938	0.4956	-1.88	0.9510	1.1403	0.09	0.0089	0.0937	0.47	0.7181	1.1628	0.04	0.9167	0.9320	0.01
991	Coiled-coil domain-containing protein 97	Q96F63	0.3220	0.5260	0.13	0.7050	0.7828	0.07	0.2012	0.3529	0.20	0.1263	0.2313	0.25	0.1858	0.3809	0.20	0.1165	0.2599	0.27	0.0165	0.1199	1.13	0.0566	0.2275	0.70	0.0157	0.1073	1.36
992	Coiled-coil-helix-coiled-coil-helix domain-containing protein 5	Q9BSY4	0.0217	0.0877	0.38	0.0134	0.4421	0.62	0.0028	0.0294	0.79	0.0076	0.0354	0.51	0.0003	0.0144	1.23	0.1258	0.2743	0.19	0.3736	0.5259	-2.55	0.8708	1.0807	-0.33	0.0651	0.1531	2.06
993	Cold shock domain-containing protein E1	O75534	0.0131	0.0669	0.35	0.8931	0.9241	0.01	0.0093	0.0448	0.29	0.0028	0.0218	0.44	0.5152	0.6039	0.04	0.0102	0.0700	0.36	0.3040	0.5626	0.15	0.9450	0.9604	0.01	0.1688	0.2340	-0.13
994	Cold-inducible RNA-binding protein	Q14011	0.0141	0.0695	-0.27	0.2771	0.6642	-0.12	0.0602	0.1419	-0.11	0.8104	0.8366	-0.01	0.0043	0.0483	-0.28	0.0964	0.2292	-0.10	0.0156	0.1164	0.45	0.1545	0.4246	0.16	0.0638	0.2262	0.27
995	Collagen alpha-1(V) chain	P20908	0.0450	0.1353	0.21	0.1457	0.6546	0.20	0.0011	0.0217	0.46	0.0047	0.0279	0.34	0.0000	0.0000	0.27	0.0033	0.0581	0.59	0.3736	0.6372	-2.55	0.8708	1.2775	-0.33	0.0651	0.2162	2.06
996	Collagen alpha-1(XVIII) chain	P39060	0.0268	0.0992	-0.18	0.9442	0.9632	0.00	0.3454	0.5394	0.03	0.3817	0.5665	0.06	0.3795	0.4766	-0.03	0.1447	0.2998	-0.07	0.0042	0.0697	-1.18	0.0018	0.0352	-0.90	0.0062	0.0749	-1.12
997	Collagen alpha-1(XXIII) chain	Q86Y22	0.4328	0.5101	-0.04	0.2829	0.6640	0.17	0.0849	0.1808	0.13	0.1239	0.2276	0.09	0.9428	0.9559	0.00	0.8798	1.1167	-0.01	0.3736	0.4348	-2.55	0.8708	0.9122	-0.33	0.0651	0.1134	2.06
998	Collagen alpha-1(XXVIII) chain	Q2UY09	0.1980	0.3675	0.16	0.0184	0.4627	0.33	0.1244	0.2432	0.27	0.0108	0.0417	0.38	0.0034	0.0433	0.56	0.0464	0.1432	0.29	0.3736	0.5640	-2.55	0.8708	1.1492	-0.33	0.0651	0.1726	2.06
999	Collagen alpha-2(XI) chain	P13942	0.0319	0.1110	5.48	0.0278	0.5053	5.44	0.0237	0.0756	5.83	0.0220	0.0646	6.40	0.0210	0.1042	7.06	0.0250	0.1005	6.89	0.3736	0.5861	-2.55	0.8708	1.1883	-0.33	0.0651	0.1848	2.06
1000	Collagen alpha-3(VI) chain	P12111	0.6725	0.7306	-0.15	0.7550	0.8221	0.07	0.6277	0.6809	-0.22	0.1485	0.2630	0.19	0.4286	0.5228	0.06	0.5685	0.8037	0.25	0.3736	0.4549	-2.55	0.8708	0.9500	-0.33	0.0651	0.1215	2.06
1001	Collagen alpha-5(VI) chain	A8TX70	0.4108	0.6020	-3.31	0.5752	0.8426	-1.75	0.5661	0.7550	-1.19	0.5388	0.7136	0.89	0.2938	0.4954	-1.88	0.9510	1.1401	0.09	0.0163	0.1193	-0.31	0.2533	0.5775	-0.07	0.0016	0.0474	-0.70
1002	Collagen alpha-6(VI) chain	A6NMZ7	0.4108	0.6018	-3.31	0.5752	0.8424	-1.75	0.5661	0.7548	-1.19	0.5388	0.7134	0.89	0.2938	0.4952	-1.88	0.9510	1.1398	0.09	0.1944	0.4028	0.61	0.1943	0.4895	0.62	0.0544	0.2057	1.26
1003	Collagen triple helix repeat-containing protein 1	Q96CG8	0.0031	0.0369	0.60	0.0100	0.4466	0.38	0.5044	0.7264	0.05	0.2300	0.3731	0.11	0.0296	0.1253	0.25	0.8659	1.1021	0.03	0.3736	0.4353	-2.55	0.8708	0.9131	-0.33	0.0651	0.1136	2.06
1004	Collagen type IV alpha-3-binding protein	Q9Y5P4	0.0635	0.1669	-11.56	0.0521	0.5791	-10.00	0.0158	0.0594	-9.44	0.0008	0.0143	-7.36	0.4747	0.5653	-0.69	0.0038	0.0591	-8.17	0.3736	0.6353	-2.55	0.8708	1.2742	-0.33	0.0651	0.2149	2.06
1005	Collectrin	Q9HBJ8	0.0330	0.1135	0.07	0.3340	0.6932	0.15	0.0187	0.0654	0.47	0.0019	0.0192	0.36	0.0078	0.0637	0.34	0.0552	0.1601	0.49	0.3736	0.5575	-2.55	0.8708	1.1376	-0.33	0.0651	0.1691	2.06
1006	COMM domain-containing protein 1	Q8N668	0.4108	0.6016	-3.31	0.5752	0.8422	-1.75	0.5661	0.7546	-1.19	0.5388	0.7133	0.89	0.2938	0.4951	-1.88	0.9510	1.1395	0.09	0.0298	0.1472	0.23	0.1586	0.4306	0.08	0.0387	0.1700	0.15
1007	COMM domain-containing protein 3	Q9UBI1	0.9583	0.9690	0.01	0.5317	0.8501	-0.17	0.0612	0.1436	0.18	0.0601	0.1320	0.20	0.1053	0.2610	0.15	0.2154	0.3964	0.09	0.3736	0.4990	-2.55	0.8708	1.0317	-0.33	0.0651	0.1404	2.06
1008	Complement C1q tumor necrosis factor-related protein 6	Q9BXI9	0.0000	0.0000	3.75	0.0046	0.3504	1.71	0.0343	0.0963	1.32	0.0152	0.0508	0.84	0.0175	0.0949	0.90	0.2264	0.4095	0.86	0.0752	0.2181	0.23	0.7568	1.1992	-0.05	0.0580	0.2144	0.29
1009	Complement C2	P06681	0.2451	0.4307	-0.02	0.6029	0.7064	0.09	0.0263	0.0809	0.11	0.0039	0.0253	0.36	0.0011	0.0263	0.16	0.0063	0.0630	0.21	0.3736	0.6245	-2.55	0.8708	1.2554	-0.33	0.0651	0.2079	2.06
1010	Complement C3	P01024	0.4108	0.6015	-3.31	0.5752	0.8419	-1.75	0.5661	0.7544	-1.19	0.5388	0.7131	0.89	0.2938	0.4949	-1.88	0.9510	1.1393	0.09	0.0500	0.1783	0.38	0.0188	0.1173	0.55	0.0387	0.1702	0.45
1011	Complement C5	P01031	0.4108	0.6013	-3.31	0.5752	0.8417	-1.75	0.5661	0.7542	-1.19	0.5388	0.7129	0.89	0.2938	0.4948	-1.88	0.9510	1.1390	0.09	0.0227	0.1363	-2.01	0.0988	0.3149	-0.64	0.0887	0.1416	-0.81
1012	Complement component 1 Q subcomponent-binding protein_ mitochondrial	Q07021	0.0305	0.1076	0.10	0.0079	0.4052	0.20	0.0002	0.0247	0.38	0.0013	0.0169	0.29	0.0162	0.0910	0.12	0.0152	0.0822	0.27	0.0010	0.0435	0.73	0.0060	0.0623	0.52	0.0000	0.0000	0.64
1013	Complement component C7	P10643	0.4108	0.6011	-3.31	0.5752	0.8414	-1.75	0.5661	0.7540	-1.19	0.5388	0.7127	0.89	0.2938	0.4946	-1.88	0.9510	1.1387	0.09	0.6846	0.7265	-0.05	0.5419	0.9676	-0.07	0.0599	0.2185	0.36
1014	Complement component receptor 1-like protein	Q2VPA4	0.3252	0.5307	0.05	0.6198	0.7178	0.08	0.0061	0.0377	0.45	0.0187	0.0578	0.22	0.0144	0.0854	0.49	0.0473	0.1446	0.14	0.3736	0.5626	-2.55	0.8708	1.1466	-0.33	0.0651	0.1718	2.06
1015	Complement factor H	P08603	0.0924	0.2137	0.28	0.5427	0.8576	-0.08	0.5450	0.7678	0.09	0.4980	0.7016	-0.08	0.8616	0.8940	-0.04	0.9145	1.1515	0.03	0.0025	0.0566	0.35	0.0339	0.1668	0.17	0.0206	0.1228	-0.19
1016	Complex I assembly factor TIMMDC1 mitochondrial	Q9NPL8	0.0047	0.0434	-0.17	0.3947	0.7384	-0.08	0.3613	0.5590	0.04	0.0133	0.0467	0.12	0.7291	0.7881	-0.01	0.3014	0.4993	0.03	0.3736	0.4816	-2.55	0.8708	0.9997	-0.33	0.0651	0.1327	2.06
1017	Complex I intermediate-associated protein 30_ mitochondrial	Q9Y375	0.0644	0.1681	0.17	0.8613	0.9000	-0.01	0.7827	0.8217	0.02	0.8955	0.9106	-0.01	0.1453	0.3199	0.11	0.3099	0.5098	-0.07	0.3736	0.4802	-2.55	0.8708	0.9971	-0.33	0.0651	0.1321	2.06
1018	Complex III assembly factor LYRM7	Q5U5X0	0.9608	0.9711	0.00	0.6367	0.7326	0.05	0.1351	0.2580	0.34	0.0306	0.0813	0.15	0.0039	0.0462	-0.23	0.4599	0.6824	0.38	0.3736	0.4629	-2.55	0.8708	0.9649	-0.33	0.0651	0.1248	2.06
1019	Complexin-1	O14810	0.4108	0.6009	-3.31	0.5752	0.8412	-1.75	0.5661	0.7538	-1.19	0.5388	0.7125	0.89	0.2938	0.4944	-1.88	0.9510	1.1385	0.09	0.0028	0.0592	-1.00	0.0132	0.0960	-0.95	0.0055	0.0718	1.33
1020	Complexin-4	Q7Z7G2	0.0003	0.0164	0.32	0.3753	0.7230	0.11	0.0004	0.0183	0.30	0.0111	0.0424	0.12	0.0446	0.1559	0.17	0.1634	0.3259	0.23	0.3736	0.5112	-2.55	0.8708	1.0539	-0.33	0.0651	0.1460	2.06
1021	Condensin complex subunit 1	Q15021	0.1888	0.3546	0.06	0.5741	0.8859	-0.03	0.4851	0.7053	0.02	0.9392	0.9469	0.00	0.0063	0.0577	-0.16	0.0026	0.0561	0.25	0.0149	0.1125	0.32	0.0221	0.1292	0.29	0.0351	0.1608	0.30
1022	Condensin complex subunit 2	Q15003	0.4108	0.6008	-3.31	0.5752	0.8410	-1.75	0.5661	0.7536	-1.19	0.5388	0.7123	0.89	0.2938	0.4943	-1.88	0.9510	1.1382	0.09	0.0246	0.1382	0.43	0.0111	0.0866	0.39	0.0006	0.0299	0.30
1023	Condensin complex subunit 3	Q9BPX3	0.0165	0.0750	-0.15	0.2038	0.6598	-0.17	0.8788	0.9041	-0.01	0.0107	0.0413	-0.21	0.0181	0.0960	-0.18	0.0800	0.2030	-0.07	0.0599	0.1938	-0.19	0.2925	0.6329	-0.05	0.0615	0.2215	-0.10
1024	Condensin-2 complex subunit H2	Q6IBW4	0.4108	0.6006	-3.31	0.5752	0.8407	-1.75	0.5661	0.7534	-1.19	0.5388	0.7122	0.89	0.2938	0.4941	-1.88	0.9510	1.1379	0.09	0.0727	0.2134	1.16	0.5118	0.9312	-0.33	0.0022	0.0516	-7.85
1025	Connector enhancer of kinase suppressor of ras 1	Q969H4	0.1426	0.2891	0.06	0.1289	0.6433	0.33	0.2116	0.3663	0.10	0.0701	0.1481	0.25	0.0319	0.1304	0.11	0.0708	0.1872	0.07	0.1252	0.2978	-0.20	0.0042	0.0519	0.69	0.0592	0.2178	0.32
1026	Connector enhancer of kinase suppressor of ras 2	Q8WXI2	0.0246	0.0947	0.44	0.0107	0.4470	0.63	0.3364	0.5285	-0.13	0.0185	0.0575	0.59	0.9318	0.9481	0.01	0.3222	0.5253	0.28	0.3736	0.4781	-2.55	0.8708	0.9931	-0.33	0.0651	0.1312	2.06
1027	Conserved oligomeric Golgi complex subunit 1	Q8WTW3	0.2460	0.4315	-0.12	0.2524	0.6620	-0.15	0.0827	0.1771	-0.24	0.0320	0.0841	-0.33	0.0447	0.1559	-0.29	0.4781	0.7026	-0.12	0.3736	0.4621	-2.55	0.8708	0.9634	-0.33	0.0651	0.1244	2.06
1028	Conserved oligomeric Golgi complex subunit 2	Q14746	0.3907	0.6076	-0.05	0.0277	0.5088	0.33	0.0157	0.0591	0.27	0.3386	0.5127	0.06	0.6548	0.7212	0.03	0.0151	0.0817	-0.34	0.3736	0.6025	-2.55	0.8708	1.2171	-0.33	0.0651	0.1944	2.06
1029	Conserved oligomeric Golgi complex subunit 4	Q9H9E3	0.9927	0.9942	0.00	0.6613	0.7504	0.05	0.5277	0.7487	0.05	0.4716	0.6709	0.06	0.8533	0.8869	0.01	0.1822	0.3522	0.13	0.3736	0.5063	-2.55	0.8708	1.0451	-0.33	0.0651	0.1438	2.06
1030	Conserved oligomeric Golgi complex subunit 5	Q9UP83	0.0142	0.0697	-0.25	0.0129	0.4425	-0.24	0.0477	0.1212	-0.23	0.0040	0.0255	-0.35	0.0164	0.0912	-0.28	0.4567	0.6792	-0.09	0.3736	0.4634	-2.55	0.8708	0.9659	-0.33	0.0651	0.1250	2.06
1031	Constitutive coactivator of peroxisome proliferator-activated receptor gamma	Q96EK7	0.1536	0.3052	0.08	0.1384	0.6511	0.21	0.0233	0.0753	0.17	0.0069	0.0336	0.15	0.0009	0.0231	0.23	0.0028	0.0564	0.18	0.3736	0.6406	-2.55	0.8708	1.2833	-0.33	0.0651	0.2184	2.06
1032	Constitutive coactivator of PPAR-gamma-like protein 1	Q9NZB2	0.1200	0.2556	0.12	0.1839	0.6634	0.27	0.0090	0.0443	0.31	0.0115	0.0431	0.29	0.2776	0.5028	0.07	0.0441	0.1384	0.21	0.0537	0.1840	0.25	0.0815	0.2807	0.11	0.3754	0.4470	0.04
1033	Contactin-associated protein-like 2	Q9UHC6	0.4108	0.6004	-3.31	0.5752	0.8405	-1.75	0.5661	0.7532	-1.19	0.5388	0.7120	0.89	0.2938	0.4940	-1.88	0.9510	1.1377	0.09	0.8260	0.8530	0.04	0.6749	1.1137	-0.04	0.0697	0.1174	0.24
1034	Contactin-associated protein-like 3	Q9BZ76	0.0007	0.0212	-0.79	0.8733	0.9084	0.03	0.0035	0.0310	0.60	0.0080	0.0363	0.40	0.1282	0.2954	0.15	0.3443	0.5483	0.08	0.3736	0.4747	-2.55	0.8708	0.9868	-0.33	0.0651	0.1297	2.06
1035	Contactin-associated protein-like 4	Q9C0A0	0.0120	0.0632	0.31	0.1660	0.6599	0.27	0.0642	0.1478	0.18	0.0047	0.0276	0.42	0.0327	0.1321	0.24	0.0226	0.0964	0.33	0.0438	0.1684	-0.26	0.4340	0.8333	-0.06	0.8283	0.8610	0.02
1036	COP9 signalosome complex subunit 1	Q13098	0.0374	0.1216	0.15	0.2162	0.6607	0.14	0.0695	0.1564	0.12	0.0383	0.0954	0.18	0.3911	0.4878	0.04	0.2972	0.4942	0.05	0.0250	0.1392	0.49	0.1245	0.3675	0.15	0.0526	0.2020	0.20
1037	COP9 signalosome complex subunit 2	P61201	0.1133	0.2454	0.09	0.4045	0.7475	0.09	0.0739	0.1627	0.12	0.4290	0.6213	0.04	0.1390	0.3105	-0.08	0.1539	0.3121	0.08	0.6851	0.7269	-0.05	0.6065	1.0382	-0.06	0.2392	0.3095	-0.17
1038	COP9 signalosome complex subunit 3	Q9UNS2	0.1914	0.3579	0.13	0.4122	0.7524	0.09	0.1341	0.2565	0.16	0.6695	0.7115	-0.04	0.7528	0.8068	0.02	0.1836	0.3542	0.13	0.0209	0.1328	0.38	0.0264	0.1463	0.39	0.1119	0.1692	0.19
1039	COP9 signalosome complex subunit 4	Q9BT78	0.8667	0.8947	0.01	0.9857	0.9897	0.00	0.1650	0.3019	0.10	0.3559	0.5339	0.05	0.4659	0.5574	-0.04	0.4955	0.7204	0.03	0.3649	0.6453	0.07	0.0886	0.2951	0.14	0.3758	0.4474	-0.05
1040	COP9 signalosome complex subunit 5	Q92905	0.1785	0.3414	-0.06	0.0385	0.5571	0.12	0.8121	0.8478	-0.01	0.9500	0.9568	0.00	0.7489	0.8038	0.02	0.9866	0.9897	0.00	0.3736	0.4299	-2.55	0.8708	0.9029	-0.33	0.0651	0.1116	2.06
1041	COP9 signalosome complex subunit 6	Q7L5N1	0.0295	0.1052	-0.15	0.0108	0.4371	-0.25	0.0032	0.0305	-0.29	0.0030	0.0226	-0.32	0.0874	0.2315	-0.18	0.1936	0.3677	-0.21	0.4603	0.5149	-0.10	0.2374	0.5532	-0.05	0.0652	0.1114	-0.23
1042	COP9 signalosome complex subunit 7a	Q9UBW8	0.6692	0.7281	-0.03	0.7457	0.8158	0.03	0.4090	0.6150	-0.06	0.6508	0.6947	-0.03	0.9234	0.9427	-0.01	0.4749	0.6989	0.05	0.0057	0.0805	-0.55	0.1601	0.4335	-0.07	0.0066	0.0753	-0.31
1043	COP9 signalosome complex subunit 8	Q99627	0.7604	0.8053	0.00	0.6781	0.7641	-0.04	0.6677	0.7183	0.02	0.0250	0.0705	-0.19	0.8970	0.9214	-0.01	0.5173	0.7452	-0.05	0.0065	0.0842	0.42	0.0101	0.0829	0.38	0.0113	0.0929	0.45
1044	Copine-1	Q99829	0.4108	0.6003	-3.31	0.5752	0.8403	-1.75	0.5661	0.7531	-1.19	0.5388	0.7118	0.89	0.2938	0.4938	-1.88	0.9510	1.1374	0.09	0.1065	0.2669	0.14	0.0498	0.2097	0.09	0.0160	0.1085	-0.20
1045	Copine-2	Q96FN4	0.4108	0.6001	-3.31	0.5752	0.8400	-1.75	0.5661	0.7529	-1.19	0.5388	0.7116	0.89	0.2938	0.4936	-1.88	0.9510	1.1372	0.09	0.0636	0.1986	0.23	0.9059	0.9300	0.01	0.7042	0.7551	0.02
1046	Copine-3	O75131	0.0069	0.0497	0.25	0.4288	0.7641	0.10	0.4000	0.6030	0.05	0.0113	0.0426	0.25	0.2638	0.4849	0.05	0.0067	0.0632	0.31	0.0522	0.1810	0.33	0.5536	0.9794	0.06	0.2959	0.3676	-0.09
1047	Copine-5	Q9HCH3	0.7031	0.7559	0.12	0.1937	0.6680	-0.80	0.0027	0.0285	-1.35	0.0709	0.1492	-0.63	0.1341	0.3036	-0.43	0.0147	0.0807	-0.66	0.6922	0.7337	-0.03	0.1098	0.3375	-0.13	0.0901	0.1434	-0.14
1048	Copine-8	Q86YQ8	0.4108	0.5999	-3.31	0.5752	0.8398	-1.75	0.5661	0.7527	-1.19	0.5388	0.7114	0.89	0.2938	0.4935	-1.88	0.9510	1.1369	0.09	0.0005	0.0370	-0.31	0.0269	0.1481	-0.38	0.0016	0.0488	-0.38
1049	Copper transport protein ATOX1	O00244	0.0079	0.0527	0.08	0.6455	0.7381	0.03	0.0400	0.1065	0.08	0.1870	0.3145	0.07	0.0289	0.1236	0.09	0.3715	0.5796	0.08	0.5626	0.6128	-0.04	0.0037	0.0494	-0.30	0.0657	0.1121	-0.21
1050	Cordon-bleu protein-like 1	Q53SF7	0.9990	0.9992	0.00	0.9646	0.9757	-0.01	0.1256	0.2450	0.41	0.1717	0.2940	0.33	0.1456	0.3203	0.37	0.2377	0.4236	0.27	0.3736	0.4934	-2.55	0.8708	1.0215	-0.33	0.0651	0.1379	2.06
1051	Coronin-1A	P31146	0.0275	0.1005	0.25	0.7211	0.7959	-0.05	0.0305	0.0888	0.23	0.8266	0.8502	0.03	0.0218	0.1061	-0.22	0.8522	1.0873	0.01	0.1549	0.3448	-0.58	0.0615	0.2369	-0.22	0.0414	0.1765	-0.19
1052	Coronin-1B	Q9BR76	0.0045	0.0425	-0.26	0.4745	0.8019	-0.06	0.2525	0.4199	-0.05	0.4341	0.6274	-0.04	0.0064	0.0584	-0.25	0.6975	0.9385	0.01	0.0250	0.1391	0.34	0.1511	0.4192	0.15	0.8350	0.8664	0.02
1053	Coronin-1C	Q9ULV4	0.4108	0.5998	-3.31	0.5752	0.8395	-1.75	0.5661	0.7525	-1.19	0.5388	0.7113	0.89	0.2938	0.4933	-1.88	0.9510	1.1366	0.09	0.0178	0.1236	0.57	0.0856	0.2896	0.14	0.0000	0.0000	0.89
1054	Coronin-2A	Q92828	0.4108	0.5996	-3.31	0.5752	0.8393	-1.75	0.5661	0.7523	-1.19	0.5388	0.7111	0.89	0.2938	0.4932	-1.88	0.9510	1.1364	0.09	0.3982	0.4543	-0.12	0.2122	0.5153	-0.12	0.0493	0.1939	0.39
1055	Coronin-7	P57737	0.0436	0.1325	0.08	0.0818	0.6168	0.14	0.1183	0.2340	0.14	0.0365	0.0919	0.10	0.0142	0.0852	0.13	0.1744	0.3422	0.13	0.0364	0.1584	-0.30	0.4379	0.8389	-0.07	0.0205	0.1226	-0.34
1056	Corrinoid adenosyltransferase	Q96EY8	0.6646	0.7242	-0.08	0.5687	0.8831	-0.08	0.3958	0.5984	-0.13	0.3843	0.5691	-0.13	0.2893	0.5196	-0.17	0.2936	0.4908	-0.17	0.3736	0.4828	-2.55	0.8708	1.0019	-0.33	0.0651	0.1332	2.06
1057	Costars family protein ABRACL	Q9P1F3	0.3005	0.4997	0.10	0.9562	0.9710	-0.01	0.9064	0.9262	0.01	0.9699	0.9740	0.00	0.4228	0.5173	0.08	0.1006	0.2347	0.20	0.3736	0.5339	-2.55	0.8708	1.0951	-0.33	0.0651	0.1570	2.06
1058	COX assembly mitochondrial protein 2 homolog	Q9NRP2	0.0454	0.1360	-2.32	0.2425	0.6601	-0.57	0.5167	0.7369	0.18	0.0177	0.0561	-0.42	0.9358	0.9512	0.02	0.0631	0.1751	-0.76	0.3736	0.5539	-2.55	0.8708	1.1311	-0.33	0.0651	0.1672	2.06
1059	Coxsackievirus and adenovirus receptor	P78310	0.4108	0.5994	-3.31	0.5752	0.8391	-1.75	0.5661	0.7521	-1.19	0.5388	0.7109	0.89	0.2938	0.4930	-1.88	0.9510	1.1361	0.09	0.0633	0.1982	-0.31	0.3341	0.6911	0.13	0.8402	0.8711	-0.02
1060	Craniofacial development protein 1	Q9UEE9	0.0070	0.0499	-0.21	0.0354	0.5409	-0.32	0.0195	0.0672	-0.27	0.0000	0.0000	-0.48	0.0018	0.0337	-0.50	0.0136	0.0777	-0.36	0.1640	0.3581	0.12	0.9895	0.9926	0.00	0.0313	0.1504	-0.20
1061	Creatine kinase B-type	P12277	0.4108	0.5993	-3.31	0.5752	0.8388	-1.75	0.5661	0.7519	-1.19	0.5388	0.7107	0.89	0.2938	0.4928	-1.88	0.9510	1.1358	0.09	0.4380	0.4937	0.14	0.3796	0.7560	0.16	0.1607	0.2249	0.30

Supplemetary Table S2. Overview on all relatively quantified 5180 proteins statistical analysis

			MCF-7																		MDA-MB-231								
			Dai SC20 vs control			Gen SC20 vs control			SSE SC20 vs control			Dai IC20 vs control			Gen IC20 vs control			SSE IC20 vs control			Dai IC20 vs control			Gen IC20 vs control			SSE IC20 vs control		
	**Protein name**	**UniProt**	**p value**	**BH q value**	**log2FC**	**p value**	**BH q value**	**log2FC**	**p value**	**BH q value**	**log2FC**	**p value**	**BH q value**	**log2FC**	**p value**	**BH q value**	**log2FC**	**p value**	**BH q value**	**log2FC**	**p value**	**BH q value**	**log2FC**	**p value**	**BH q value**	**log2FC**	**p value**	**BH q value**	**log2FC**
1062	Creatine kinase U-type mitochondrial	P12532	0.5304	0.6016	0.04	0.9321	0.9544	0.01	0.0321	0.0918	0.20	0.0385	0.0958	0.20	0.4111	0.5063	0.05	0.0360	0.1225	0.22	0.3736	0.5732	-2.55	0.8708	1.1656	-0.33	0.0651	0.1776	2.06
1063	Crk-like protein	P46109	0.1374	0.2821	-0.10	0.3288	0.6882	-0.05	0.0895	0.1878	-0.08	0.0202	0.0609	-0.13	0.1186	0.2825	-0.05	0.1408	0.2946	-0.18	0.0004	0.0406	0.16	0.3630	0.7319	-0.05	0.2548	0.3262	0.02
1064	Crooked neck-like protein 1	Q9BZJ0	0.4108	0.5991	-3.31	0.5752	0.8386	-1.75	0.5661	0.7517	-1.19	0.5388	0.7105	0.89	0.2938	0.4927	-1.88	0.9510	1.1356	0.09	0.0012	0.0450	0.30	0.0044	0.0533	0.18	0.1046	0.1607	0.07
1065	Cryptochrome-2	Q49AN0	0.4108	0.5989	-3.31	0.5752	0.8384	-1.75	0.5661	0.7515	-1.19	0.5388	0.7104	0.89	0.2938	0.4925	-1.88	0.9510	1.1353	0.09	0.1932	0.4008	0.21	0.0303	0.1573	-0.30	0.0203	0.1224	-0.41
1066	CST complex subunit STN1	Q9H668	0.3965	0.6151	-0.06	0.2452	0.6581	-0.08	0.3054	0.4886	-0.03	0.1230	0.2266	-0.13	0.9493	0.9610	0.00	0.3308	0.5341	0.06	0.3736	0.4765	-2.55	0.8708	0.9903	-0.33	0.0651	0.1305	2.06
1067	cTAGE family member 2	Q96RT6	0.2325	0.4143	-1.00	0.3713	0.7182	-0.92	0.8406	0.8716	0.15	0.5118	0.7165	-0.56	0.6037	0.6789	0.37	0.6307	0.8670	0.34	0.3736	0.4493	-2.55	0.8708	0.9395	-0.33	0.0651	0.1192	2.06
1068	C-terminal-binding protein 1	Q13363	0.1900	0.3562	-0.36	0.3793	0.7258	-0.37	0.0598	0.1415	-0.48	0.4245	0.6168	-0.17	0.1311	0.2990	-0.29	0.3431	0.5470	-0.28	0.3736	0.4749	-2.55	0.8708	0.9872	-0.33	0.0651	0.1298	2.06
1069	C-terminal-binding protein 2	P56545	0.7069	0.7591	-0.02	0.8334	0.8801	0.02	0.1304	0.2514	0.11	0.7432	0.7788	-0.01	0.0689	0.1999	-0.08	0.3214	0.5245	0.04	0.3736	0.4784	-2.55	0.8708	0.9938	-0.33	0.0651	0.1313	2.06
1070	CTP synthase 1	P17812	0.0895	0.2091	0.19	0.3229	0.6852	0.21	0.1106	0.2215	0.16	0.2628	0.4164	0.10	0.8518	0.8860	-0.01	0.2719	0.4659	0.10	0.0956	0.2491	0.29	0.0738	0.2631	0.17	0.8331	0.8652	0.01
1071	CTP synthase 2	Q9NRF8	0.2046	0.3768	-0.07	0.2815	0.6643	-0.08	0.0344	0.0965	-0.14	0.0422	0.1023	-0.23	0.0511	0.1674	-0.21	0.0132	0.0766	-0.19	0.3736	0.6067	-2.55	0.8708	1.2244	-0.33	0.0651	0.1969	2.06
1072	CTTNBP2 N-terminal-like protein	Q9P2B4	0.4108	0.5987	-3.31	0.5752	0.8381	-1.75	0.5661	0.7513	-1.19	0.5388	0.7102	0.89	0.2938	0.4924	-1.88	0.9510	1.1351	0.09	0.5288	0.5812	-0.05	0.0442	0.1949	-0.22	0.4441	0.5143	-0.12
1073	C-type lectin domain family 12 member A	Q5QGZ9	0.4108	0.5986	-3.31	0.5752	0.8379	-1.75	0.5661	0.7511	-1.19	0.5388	0.7100	0.89	0.2938	0.4922	-1.88	0.9510	1.1348	0.09	0.0345	0.1543	-0.80	0.1410	0.3996	0.25	0.1025	0.1584	0.16
1074	C-type lectin domain family 2 member B	Q92478	0.4108	0.5984	-3.31	0.5752	0.8377	-1.75	0.5661	0.7509	-1.19	0.5388	0.7098	0.89	0.2938	0.4920	-1.88	0.9510	1.1345	0.09	0.9525	0.9612	0.03	0.0184	0.1155	-0.53	0.0360	0.1623	1.01
1075	C-type lectin domain family 2 member D	Q9UHP7	0.0317	0.1105	-0.29	0.1506	0.6594	-0.98	0.0165	0.0609	-0.78	0.0247	0.0700	-0.64	0.0064	0.0585	-0.59	0.4389	0.6607	-0.84	0.3736	0.4654	-2.55	0.8708	0.9696	-0.33	0.0651	0.1258	2.06
1076	C-type lectin domain family 4 member A	Q9UMR7	0.0945	0.2167	0.11	0.4078	0.7496	0.07	0.0164	0.0606	0.28	0.0033	0.0233	0.40	0.0223	0.1078	0.20	0.0021	0.0533	0.66	0.3736	0.6438	-2.55	0.8708	1.2888	-0.33	0.0651	0.2205	2.06
1077	C-type lectin domain family 4 member D	Q8WXI8	0.6220	0.6852	0.02	0.0857	0.6209	-0.16	0.4675	0.6849	-0.09	0.6375	0.6824	-0.02	0.1035	0.2585	-0.13	0.1321	0.2830	0.09	0.3736	0.5233	-2.55	0.8708	1.0760	-0.33	0.0651	0.1518	2.06
1078	C-type lectin domain family 4 member G	Q6UXB4	0.2871	0.4835	-0.21	0.4300	0.7660	-0.50	0.1615	0.2966	-0.31	0.1197	0.2226	-0.46	0.1102	0.2702	-0.39	0.3886	0.6000	-0.26	0.3736	0.4703	-2.55	0.8708	0.9787	-0.33	0.0651	0.1279	2.06
1079	C-type mannose receptor 2	Q9UBG0	0.4108	0.5982	-3.31	0.5752	0.8374	-1.75	0.5661	0.7507	-1.19	0.5388	0.7096	0.89	0.2938	0.4919	-1.88	0.9510	1.1343	0.09	0.0000	0.0000	-1.04	0.0024	0.0419	-1.61	0.0002	0.0259	-1.66
1080	CUB domain-containing protein 1	Q9H5V8	0.4108	0.5981	-3.31	0.5752	0.8372	-1.75	0.5661	0.7505	-1.19	0.5388	0.7095	0.89	0.2938	0.4917	-1.88	0.9510	1.1340	0.09	0.9918	0.9935	0.00	0.2018	0.5004	-0.14	0.0990	0.1544	-0.20
1081	CUE domain-containing protein 1	Q9NWM3	0.2881	0.4845	0.15	0.1211	0.6388	-0.45	0.0712	0.1591	0.19	0.0010	0.0150	-1.08	0.0127	0.0805	0.46	0.0108	0.0712	0.93	0.3736	0.6107	-2.55	0.8708	1.2314	-0.33	0.0651	0.1993	2.06
1082	CUGBP Elav-like family member 1	Q92879	0.0021	0.0317	1.15	0.0708	0.6002	0.23	0.3729	0.5711	0.15	0.2169	0.3548	0.12	0.1100	0.2700	-0.18	0.9281	1.1655	0.01	0.0123	0.1048	-0.54	0.0010	0.0274	-1.33	0.0031	0.0561	-1.48
1083	Cullin-1	Q13616	0.0766	0.1889	-0.10	0.1148	0.6333	0.25	0.0013	0.0233	0.38	0.3038	0.4695	0.20	0.3857	0.4831	0.04	0.0201	0.0917	-0.17	0.0301	0.1476	0.24	0.5710	0.9996	0.04	0.3902	0.4615	0.06
1084	Cullin-2	Q13617	0.0153	0.0717	0.15	0.7703	0.8334	-0.03	0.3726	0.5709	0.04	0.2039	0.3376	0.04	0.0153	0.0882	-0.12	0.0399	0.1306	0.17	0.2512	0.4875	0.06	0.7881	1.2311	-0.01	0.0428	0.1801	-0.15
1085	Cullin-3	Q13618	0.0345	0.1166	0.14	0.3341	0.6931	0.08	0.0201	0.0682	0.16	0.3917	0.5789	-0.02	0.1202	0.2847	-0.03	0.1164	0.2599	0.28	0.0046	0.0722	0.74	0.0004	0.0207	0.86	0.7893	0.8276	-0.02
1086	Cullin-4A	Q13619	0.0036	0.0387	1.36	0.3562	0.7078	0.13	0.9770	0.9808	0.00	0.1875	0.3150	0.84	0.5393	0.6236	0.02	0.0166	0.0848	1.22	0.5288	0.5813	0.10	0.7327	1.1765	-0.05	0.5743	0.6372	0.09
1087	Cullin-4B	Q13620	0.7008	0.7547	0.03	0.0781	0.6120	0.19	0.0513	0.1273	0.22	0.0669	0.1429	0.22	0.1110	0.2716	0.16	0.1142	0.2569	0.16	0.2738	0.5186	0.13	0.6632	1.1018	-0.03	0.7988	0.8363	-0.02
1088	Cullin-5	Q93034	0.4108	0.5979	-3.31	0.5752	0.8369	-1.75	0.5661	0.7504	-1.19	0.5388	0.7093	0.89	0.2938	0.4916	-1.88	0.9510	1.1338	0.09	0.0137	0.1105	-0.37	0.1947	0.4893	-0.16	0.0346	0.1596	-0.31
1089	Cullin-associated NEDD8-dissociated protein 1	Q86VP6	0.0196	0.0823	0.20	0.2930	0.6698	0.20	0.0092	0.0448	0.24	0.0164	0.0533	0.20	0.4268	0.5211	-0.04	0.0114	0.0729	0.27	0.0419	0.1676	0.33	0.1493	0.4165	0.11	0.0535	0.2038	0.20
1090	Cullin-associated NEDD8-dissociated protein 2	O75155	0.5203	0.5921	0.07	0.0376	0.5549	-0.46	0.0477	0.1211	-0.20	0.0003	0.0103	-0.81	0.0241	0.1125	0.21	0.0004	0.0370	-1.04	0.3736	0.6529	-2.55	0.8708	1.3044	-0.33	0.0651	0.2268	2.06
1091	CWF19-like protein 2	Q2TBE0	0.0320	0.1111	0.36	0.0736	0.6139	-0.36	0.9986	0.9990	0.00	0.0219	0.0643	-0.55	0.0152	0.0879	-0.68	0.0203	0.0923	-0.29	0.3736	0.5938	-2.55	0.8708	1.2019	-0.33	0.0651	0.1892	2.06
1092	CXXC motif containing zinc binding protein	Q9NWV4	0.2249	0.4035	-0.05	0.1240	0.6417	-0.08	0.9504	0.9608	0.00	0.0445	0.1057	0.08	0.1116	0.2723	-0.09	0.0235	0.0982	0.17	0.0456	0.1714	0.46	0.2497	0.5726	0.17	0.0359	0.1620	0.54
1093	CXXC-type zinc finger protein 1	Q9P0U4	0.8934	0.9166	-0.01	0.3671	0.7157	0.15	0.6370	0.6899	0.01	0.5614	0.6116	-0.02	0.2046	0.4064	-0.07	0.1299	0.2805	0.04	0.3736	0.5242	-2.55	0.8708	1.0776	-0.33	0.0651	0.1522	2.06
1094	Cyclic AMP-responsive element-binding protein 3-like protein 4	Q8TEY5	0.4108	0.5977	-3.31	0.5752	0.8367	-1.75	0.5661	0.7502	-1.19	0.5388	0.7091	0.89	0.2938	0.4914	-1.88	0.9510	1.1335	0.09	0.0929	0.2446	0.38	0.1235	0.3654	0.28	0.1128	0.1701	0.31
1095	Cyclic nucleotide-gated cation channel alpha-3	Q16281	0.0351	0.1170	0.24	0.0551	0.5861	0.18	0.6919	0.7400	0.07	0.0269	0.0742	0.22	0.0422	0.1519	0.21	0.2856	0.4822	0.18	0.3311	0.5997	-0.12	0.1955	0.4906	-0.12	0.0666	0.1132	-0.13
1096	Cyclic nucleotide-gated cation channel alpha-4	Q8IV77	0.0206	0.0848	0.73	0.3295	0.6888	-0.38	0.3348	0.5268	-0.29	0.0567	0.1261	-0.51	0.0898	0.2358	-0.43	0.7474	0.9856	0.06	0.3736	0.4418	-2.55	0.8708	0.9255	-0.33	0.0651	0.1162	2.06
1097	Cyclin-dependent kinase 1	P06493	0.1070	0.2356	0.10	0.4823	0.8072	-0.08	0.0281	0.0847	0.16	0.2139	0.3509	0.07	0.0060	0.0564	-0.29	0.9610	0.9679	0.00	0.4142	0.4701	0.10	0.5703	0.9994	-0.05	0.0697	0.1175	-0.19
1098	Cyclin-dependent kinase 12	Q9NYV4	0.4108	0.5976	-3.31	0.5752	0.8365	-1.75	0.5661	0.7500	-1.19	0.5388	0.7089	0.89	0.2938	0.4912	-1.88	0.9510	1.1332	0.09	0.1230	0.2952	0.34	0.1632	0.4394	-0.14	0.0515	0.1988	0.04
1099	Cyclin-dependent kinase 13	Q14004	0.0000	0.0000	0.42	0.1683	0.6610	0.24	0.0025	0.0276	0.69	0.0001	0.0089	0.64	0.0006	0.0207	0.64	0.0137	0.0781	0.69	0.0009	0.0428	-0.56	0.0031	0.0463	0.61	0.2746	0.3465	0.06
1100	Cyclin-dependent kinase 15	Q96Q40	0.0054	0.0447	0.28	0.6567	0.7470	0.02	0.6038	0.6596	0.02	0.0762	0.1579	-0.15	0.6297	0.7004	-0.04	0.2171	0.3985	-0.06	0.3736	0.4988	-2.55	0.8708	1.0313	-0.33	0.0651	0.1403	2.06
1101	Cyclin-dependent kinase 16	Q00536	0.4194	0.4970	0.09	0.2189	0.6600	-0.20	0.6991	0.7464	0.06	0.3030	0.4684	-0.23	0.3916	0.4881	0.09	0.2672	0.4601	-0.11	0.3736	0.4872	-2.55	0.8708	1.0100	-0.33	0.0651	0.1352	2.06
1102	Cyclin-dependent kinase 17	Q00537	0.4108	0.5974	-3.31	0.5752	0.8362	-1.75	0.5661	0.7498	-1.19	0.5388	0.7087	0.89	0.2938	0.4911	-1.88	0.9510	1.1330	0.09	0.0178	0.1234	0.38	0.0092	0.0786	0.45	0.0802	0.1312	0.48
1103	Cyclin-dependent kinase 18	Q07002	0.1597	0.3148	-0.94	0.3458	0.7030	0.49	0.2047	0.3576	0.66	0.6332	0.6791	0.23	0.5450	0.6295	-0.65	0.9205	1.1576	0.04	0.3736	0.4330	-2.55	0.8708	0.9089	-0.33	0.0651	0.1127	2.06
1104	Cyclin-dependent kinase 2	P24941	0.8417	0.8736	-0.02	0.1443	0.6540	0.21	0.4727	0.6901	-0.09	0.1785	0.3032	-0.17	0.8510	0.8854	0.02	0.2550	0.4452	-0.22	0.0079	0.0911	-0.33	0.3931	0.7757	-0.11	0.0169	0.1111	-0.24
1105	Cyclin-dependent kinase 3	Q00526	0.3788	0.5946	0.03	0.6444	0.7375	0.04	0.2943	0.4748	-0.04	0.0857	0.1730	0.08	0.0296	0.1254	-0.14	0.0116	0.0735	0.25	0.4068	0.4627	-0.08	0.0098	0.0812	0.34	0.6044	0.6644	-0.04
1106	Cyclin-dependent kinase 4	P11802	0.0104	0.0591	-0.77	0.0241	0.4876	-0.69	0.0061	0.0380	-0.86	0.0064	0.0321	-0.88	0.6297	0.7006	0.07	0.0452	0.1405	-0.87	0.1261	0.2988	-0.91	0.9630	0.9735	-0.02	0.4932	0.5610	0.13
1107	Cyclin-dependent kinase 6	Q00534	0.0186	0.0801	-1.41	0.7114	0.7884	0.11	0.0363	0.0998	-1.01	0.0128	0.0459	-1.27	0.0411	0.1497	-1.81	0.7126	0.9533	-0.16	0.3249	0.5913	0.37	0.0511	0.2131	1.23	0.6026	0.6632	0.18
1108	Cyclin-dependent kinase 9	P50750	0.0475	0.1400	-0.20	0.0628	0.5893	-0.20	0.8823	0.9072	0.01	0.0156	0.0515	-0.34	0.0663	0.1959	-0.20	0.0343	0.1194	-0.28	0.1095	0.2713	0.57	0.4457	0.8475	0.20	0.1078	0.1643	0.57
1109	Cyclin-dependent kinase inhibitor 1	P38936	0.0008	0.0214	-1.21	0.1171	0.6332	-0.38	0.4863	0.7064	0.08	0.2160	0.3539	0.18	0.2280	0.4376	-0.16	0.1290	0.2794	-0.26	0.0001	0.0259	-2.08	0.0045	0.0543	-2.64	0.0079	0.0798	-2.49
1110	Cyclin-dependent kinase inhibitor 3	Q16667	0.8249	0.8598	-0.01	0.0702	0.6011	0.35	0.0967	0.1992	0.15	0.0089	0.0381	0.28	0.0001	0.0096	0.20	0.3265	0.5287	0.07	0.0227	0.1367	0.54	0.0244	0.1390	0.32	0.0122	0.0952	0.37
1111	Cyclin-dependent kinase-like 5	O76039	0.0173	0.0770	-0.25	0.2701	0.6637	0.07	0.4170	0.6243	-0.05	0.3723	0.5554	-0.06	0.2014	0.4020	0.18	0.0216	0.0947	-0.29	0.0878	0.2381	0.31	0.6268	1.0624	0.04	0.8288	0.8614	0.01
1112	Cyclin-dependent kinases regulatory subunit 1	P61024	0.1536	0.3051	0.23	0.1611	0.6649	0.36	0.0341	0.0958	0.43	0.0193	0.0591	0.54	0.2739	0.4982	0.16	0.2298	0.4127	0.18	0.3736	0.4948	-2.55	0.8708	1.0240	-0.33	0.0651	0.1385	2.06
1113	Cyclin-dependent-like kinase 5	Q00535	0.2664	0.4582	0.07	0.2811	0.6655	-0.12	0.2760	0.4503	-0.07	0.0068	0.0333	-0.30	0.0059	0.0564	-0.32	0.0901	0.2190	-0.27	0.0017	0.0530	-1.16	0.0168	0.1100	-0.37	0.1183	0.1764	-0.86
1114	Cyclin-K	O75909	0.0008	0.0213	-0.36	0.0202	0.4735	-0.34	0.0646	0.1483	-0.26	0.0004	0.0112	-0.61	0.0010	0.0247	-0.36	0.0002	0.0432	-0.70	0.3736	0.6542	-2.55	0.8708	1.3067	-0.33	0.0651	0.2277	2.06
1115	Cystathionine beta-synthase	P35520	0.4108	0.5972	-3.31	0.5752	0.8360	-1.75	0.5661	0.7496	-1.19	0.5388	0.7086	0.89	0.2938	0.4909	-1.88	0.9510	1.1327	0.09	0.0333	0.1522	0.45	0.7130	1.1581	0.04	0.9092	0.9275	-0.01
1116	Cystathionine beta-synthase-like protein	P0DN79	0.0149	0.0709	0.35	0.0881	0.6201	0.23	0.0594	0.1410	0.19	0.0099	0.0399	0.27	0.3096	0.4075	0.06	0.0072	0.0645	0.48	0.3736	0.6215	-2.55	0.8708	1.2502	-0.33	0.0651	0.2060	2.06
1117	Cystatin-B	P04080	0.4050	0.6253	0.02	0.4766	0.8034	-0.10	0.8208	0.8550	-0.01	0.5298	0.7369	-0.02	0.0086	0.0662	-0.06	0.0000	0.0000	0.27	0.3266	0.5938	-0.11	0.1381	0.3937	0.12	0.2300	0.3003	-0.21
1118	Cystatin-C	P01034	0.0129	0.0664	-0.40	0.0162	0.4586	0.32	0.0127	0.0529	-0.29	0.4836	0.6846	0.07	0.2567	0.4764	-0.08	0.8088	1.0458	-0.03	0.3736	0.4381	-2.55	0.8708	0.9185	-0.33	0.0651	0.1147	2.06
1119	Cysteine and glycine-rich protein 1	P21291	0.5006	0.5742	-0.04	0.4189	0.7576	0.08	0.7902	0.8279	0.02	0.0973	0.1901	0.15	0.3468	0.4439	0.06	0.0476	0.1453	0.20	0.6047	0.6524	0.05	0.3291	0.6846	-0.10	0.0780	0.1285	-0.20
1120	Cysteine and glycine-rich protein 2	Q16527	0.3552	0.5658	0.06	0.4114	0.7517	0.10	0.4320	0.6425	-0.07	0.4631	0.6610	-0.06	0.4845	0.5746	-0.05	0.7167	0.9568	-0.02	0.0017	0.0524	0.46	0.1196	0.3577	0.19	0.0811	0.1321	0.12
1121	Cysteine and histidine-rich domain-containing protein 1	Q9UHD1	0.1561	0.3091	0.06	0.2735	0.6648	-0.04	0.1920	0.3393	0.05	0.9898	0.9913	0.00	0.2174	0.4229	-0.06	0.1914	0.3653	0.08	0.8728	0.8947	0.01	0.7732	1.2166	-0.02	0.7001	0.7519	0.02
1122	Cysteine and histidine-rich protein 1	Q6ZMK1	0.9976	0.9980	0.00	0.1571	0.6616	-0.37	0.2634	0.4336	-0.26	0.1293	0.2354	-0.47	0.1228	0.2886	-0.42	0.3774	0.5860	-0.21	0.3736	0.4710	-2.55	0.8708	0.9800	-0.33	0.0651	0.1282	2.06
1123	Cysteine dioxygenase type 1	Q16878	0.4108	0.5971	-3.31	0.5752	0.8358	-1.75	0.5661	0.7494	-1.19	0.5388	0.7084	0.89	0.2938	0.4908	-1.88	0.9510	1.1325	0.09	0.2400	0.4716	-0.15	0.0372	0.1758	0.18	0.3456	0.4168	-0.05
1124	Cysteine protease ATG4B	Q9Y4P1	0.0345	0.1165	0.07	0.0222	0.4812	0.19	0.3712	0.5694	0.03	0.5177	0.7228	-0.05	0.1213	0.2866	-0.06	0.0303	0.1118	-0.08	0.2043	0.4176	-0.55	0.7616	1.2039	-0.08	0.0460	0.1870	0.42
1125	Cysteine protease ATG4C	Q96DT6	0.4108	0.5969	-3.31	0.5752	0.8355	-1.75	0.5661	0.7492	-1.19	0.5388	0.7082	0.89	0.2938	0.4906	-1.88	0.9510	1.1322	0.09	0.0438	0.1681	0.67	0.0010	0.0267	0.40	0.0201	0.1218	0.34
1126	Cysteine protease ATG4D	Q86TL0	0.9785	0.9857	0.00	0.9490	0.9660	0.02	0.5464	0.7693	-0.06	0.6734	0.7145	0.06	0.5968	0.6731	-0.06	0.3805	0.5898	-0.16	0.0015	0.0511	0.61	0.0053	0.0585	0.55	0.0028	0.0549	0.69
1127	Cysteine/serine-rich nuclear protein 3	Q8WYN3	0.4108	0.5967	-3.31	0.5752	0.8353	-1.75	0.5661	0.7490	-1.19	0.5388	0.7080	0.89	0.2938	0.4905	-1.88	0.9510	1.1319	0.09	0.6817	0.7249	0.05	0.0074	0.0706	0.43	0.6168	0.6756	-0.04
1128	Cysteine-rich protein 1	P50238	0.4108	0.5966	-3.31	0.5752	0.8351	-1.75	0.5661	0.7488	-1.19	0.5388	0.7078	0.89	0.2938	0.4903	-1.88	0.9510	1.1317	0.09	0.1327	0.3095	0.24	0.0208	0.1247	-0.27	0.0539	0.2044	-0.18
1129	Cysteine-rich protein 2	P52943	0.6844	0.7404	-0.03	0.2396	0.6612	0.27	0.1302	0.2512	0.12	0.0244	0.0695	0.16	0.0948	0.2435	0.09	0.1819	0.3518	0.07	0.0131	0.1084	-0.19	0.0089	0.0777	-0.27	0.0111	0.0923	-0.25
1130	Cysteine-rich with EGF-like domain protein 2	Q6UXH1	0.0330	0.1134	0.15	0.1302	0.6448	0.16	0.0071	0.0400	0.20	0.0435	0.1043	0.12	0.0143	0.0853	0.17	0.0442	0.1386	0.12	0.3736	0.5657	-2.55	0.8708	1.1522	-0.33	0.0651	0.1735	2.06
1131	Cysteine--tRNA ligase cytoplasmic	P49589	0.0828	0.1981	0.25	0.1353	0.6501	0.22	0.0004	0.0199	0.34	0.0001	0.0110	0.40	0.0161	0.0909	0.15	0.0408	0.1329	0.52	0.0126	0.1068	0.32	0.0352	0.1706	0.20	0.0988	0.1542	0.14
1132	Cytochrome b-245 light chain	P13498	0.1529	0.3040	0.09	0.0976	0.6335	-0.19	0.0038	0.0320	0.12	0.0001	0.0085	0.59	0.0000	0.0000	0.45	0.0001	0.0576	1.21	0.0009	0.0440	0.83	0.0009	0.0269	0.39	0.0002	0.0280	0.90
1133	Cytochrome b5	P00167	0.2813	0.4746	0.11	0.1170	0.6333	0.30	0.3417	0.5347	0.09	0.0561	0.1254	0.29	0.0691	0.2003	0.27	0.1031	0.2383	0.20	0.3570	0.6340	-0.07	0.0234	0.1347	0.28	0.1277	0.1873	0.15
1134	Cytochrome b5 type B	O43169	0.2907	0.4878	-0.05	0.6542	0.7448	0.05	0.2726	0.4463	0.05	0.9091	0.9214	0.01	0.0180	0.0956	-0.17	0.0983	0.2312	0.09	0.0933	0.2451	0.19	0.0475	0.2032	0.27	0.0299	0.1475	0.33
1135	Cytochrome b561	P49447	0.0011	0.0232	-0.46	0.9505	0.9671	0.00	0.0047	0.0333	-0.33	0.0186	0.0577	-0.26	0.0143	0.0854	-0.22	0.0625	0.1739	-0.49	0.3736	0.5544	-2.55	0.8708	1.1319	-0.33	0.0651	0.1674	2.06
1136	Cytochrome b-c1 complex subunit 1 mitochondrial	P31930	0.0402	0.1264	0.14	0.3109	0.6792	0.15	0.0050	0.0344	0.25	0.0209	0.0623	0.16	0.8863	0.9131	0.01	0.0201	0.0916	0.22	0.0507	0.1791	0.33	0.2515	0.5744	0.11	0.0638	0.2260	0.18
1137	Cytochrome b-c1 complex subunit 2 mitochondrial	P22695	0.0350	0.1168	0.13	0.4314	0.7677	0.12	0.0201	0.0681	0.16	0.0414	0.1009	0.12	0.5455	0.6296	0.02	0.0365	0.1235	0.16	0.0869	0.2363	0.22	0.3043	0.6503	0.08	0.1846	0.2520	0.06
1138	Cytochrome b-c1 complex subunit 6 mitochondrial	P07919	0.0008	0.0211	-0.26	0.3456	0.7029	-0.39	0.0001	0.0144	0.38	0.0960	0.1882	0.14	0.0010	0.0243	0.28	0.2278	0.4107	0.11	0.2190	0.4406	0.09	0.0181	0.1148	-0.26	0.1707	0.2364	-0.27
1139	Cytochrome b-c1 complex subunit 6-like mitochondrial	A0A096LP55	0.1143	0.2472	0.09	0.3046	0.6775	0.17	0.0043	0.0323	0.29	0.0119	0.0438	0.22	0.0455	0.1572	0.14	0.0531	0.1557	0.15	0.3736	0.5588	-2.55	0.8708	1.1399	-0.33	0.0651	0.1698	2.06
1140	Cytochrome b-c1 complex subunit 7	P14927	0.0643	0.1680	0.07	0.4053	0.7485	0.10	0.0029	0.0293	0.25	0.0494	0.1141	0.13	0.5928	0.6693	0.02	0.0121	0.0742	0.15	0.0192	0.1285	0.54	0.4642	0.8725	0.06	0.0784	0.1290	0.21
1141	Cytochrome b-c1 complex subunit 9	Q9UDW1	0.4580	0.5349	0.02	0.6298	0.7266	0.06	0.0065	0.0388	0.25	0.0052	0.0292	0.20	0.1910	0.3872	0.05	0.2308	0.4141	0.15	0.0139	0.1113	0.41	0.0284	0.1526	0.23	0.0075	0.0785	0.29
1142	Cytochrome b-c1 complex subunit Rieske mitochondrial	P47985	0.8435	0.8751	0.01	0.8022	0.8571	-0.02	0.0839	0.1791	0.12	0.4438	0.6380	0.04	0.2326	0.4433	0.07	0.1164	0.2598	0.10	0.0641	0.1993	0.25	0.0136	0.0974	0.35	0.1523	0.2154	0.12
1143	Cytochrome c	P99999	0.0075	0.0511	0.26	0.3221	0.6841	0.04	0.0016	0.0231	0.29	0.0008	0.0148	0.37	0.0036	0.0449	0.24	0.0015	0.0525	0.54	0.0116	0.1045	0.38	0.0118	0.0902	0.34	0.1904	0.2583	0.11

Supplemetary Table S2. Overview on all relatively quantified 5180 proteins statistical analysis

			MCF-7																		MDA-MB-231								
			Dai SC20 vs control			Gen SC20 vs control			SSE SC20 vs control			Dai IC20 vs control			Gen IC20 vs control			SSE IC20 vs control			Dai IC20 vs control			Gen IC20 vs control			SSE IC20 vs control		
	**Protein name**	**UniProt**	**p value**	**BH q value**	**log2FC**	**p value**	**BH q value**	**log2FC**	**p value**	**BH q value**	**log2FC**	**p value**	**BH q value**	**log2FC**	**p value**	**BH q value**	**log2FC**	**p value**	**BH q value**	**log2FC**	**p value**	**BH q value**	**log2FC**	**p value**	**BH q value**	**log2FC**	**p value**	**BH q value**	**log2FC**
1144	Cytochrome c oxidase assembly factor 6 homolog	Q5JTJ3	0.2786	0.4724	-0.18	0.3020	0.6749	0.10	0.5717	0.6294	-0.07	0.2508	0.4001	0.09	0.0703	0.2019	-0.18	0.8879	1.1248	0.01	0.4049	0.4607	0.12	0.1149	0.3475	0.13	0.1154	0.1730	-0.12
1145	Cytochrome c oxidase assembly factor 7	Q96BR5	0.1314	0.2734	-0.25	0.0702	0.6020	-0.33	0.0559	0.1352	-0.40	0.0565	0.1260	-0.40	0.0554	0.1756	-0.36	0.0750	0.1949	-0.33	0.5008	0.5545	0.09	0.0018	0.0345	-0.42	0.0019	0.0500	-0.54
1146	Cytochrome c oxidase assembly protein COX16 homolog_ mitochondrial	Q9P0S2	0.0327	0.1128	0.20	0.7142	0.7905	0.04	0.2133	0.3682	0.08	0.0612	0.1338	0.16	0.5601	0.6426	0.03	0.2257	0.4086	0.20	0.3736	0.4963	-2.55	0.8708	1.0268	-0.33	0.0651	0.1392	2.06
1147	Cytochrome c oxidase assembly protein COX20_ mitochondrial	Q5RI15	0.0669	0.1715	-0.22	0.2696	0.6628	0.10	0.1038	0.2101	0.17	0.7758	0.8076	-0.02	0.1192	0.2831	0.18	0.2384	0.4242	-0.13	0.1755	0.3744	0.09	0.0806	0.2796	0.15	0.1474	0.2098	-0.12
1148	Cytochrome c oxidase copper chaperone	Q14061	0.7049	0.7574	0.04	0.9513	0.9676	-0.01	0.1729	0.3143	0.20	0.3082	0.4753	0.14	0.9544	0.9645	0.01	0.1268	0.2755	0.25	0.0948	0.2476	0.35	0.4698	0.8805	0.08	0.2907	0.3627	0.10
1149	Cytochrome c oxidase subunit 2	P00403	0.8710	0.8980	0.02	0.7713	0.8339	-0.04	0.2134	0.3681	-0.17	0.0364	0.0917	-0.39	0.1729	0.3619	-0.19	0.0656	0.1789	-0.34	0.0006	0.0420	-0.30	0.0002	0.0192	-0.71	0.0001	0.0259	-0.89
1150	Cytochrome c oxidase subunit 4 isoform 1_ mitochondrial	P13073	0.4176	0.4952	0.03	0.8041	0.8586	0.05	0.0057	0.0365	0.23	0.0078	0.0358	0.20	0.1215	0.2869	-0.08	0.0089	0.0688	0.23	0.2809	0.5297	0.12	0.4084	0.7950	-0.07	0.0851	0.1372	-0.11
1151	Cytochrome c oxidase subunit 4 isoform 2_ mitochondrial	Q96KJ9	0.4108	0.5964	-3.31	0.5752	0.8348	-1.75	0.5661	0.7486	-1.19	0.5388	0.7077	0.89	0.2938	0.4901	-1.88	0.9510	1.1314	0.09	0.0260	0.1404	-0.99	0.0059	0.0617	-1.57	0.0059	0.0729	-0.55
1152	Cytochrome c oxidase subunit 5A mitochondrial	P20674	0.0523	0.1480	-0.12	0.0006	0.2072	-0.32	0.0014	0.0236	-0.25	0.0010	0.0156	-0.32	0.0017	0.0327	-0.35	0.1958	0.3710	0.04	0.1363	0.3153	-0.14	0.6622	1.1012	-0.04	0.0655	0.1118	-0.23
1153	Cytochrome c oxidase subunit 5B mitochondrial	P10606	0.0977	0.2214	-0.15	0.7198	0.7947	-0.06	0.5232	0.7441	0.05	0.2482	0.3971	0.09	0.0754	0.2109	-0.19	0.6866	0.9264	0.03	0.0117	0.1038	0.57	0.0084	0.0753	0.39	0.0143	0.1030	0.35
1154	Cytochrome c oxidase subunit 6B1	P14854	0.0109	0.0601	-0.39	0.2258	0.6601	-0.28	0.5768	0.6344	0.09	0.0133	0.0467	-0.35	0.6038	0.6789	0.08	0.0242	0.0993	-0.38	0.0698	0.2082	0.47	0.3517	0.7156	-0.19	0.3320	0.4032	0.15
1155	Cytochrome c oxidase subunit 6C	P09669	0.0757	0.1874	-0.03	0.0706	0.6005	-0.11	0.1714	0.3120	0.03	0.8525	0.8727	0.00	0.0178	0.0953	-0.13	0.9016	1.1385	-0.01	0.0958	0.2489	0.25	0.5264	0.9501	0.06	0.7053	0.7558	-0.04
1156	Cytochrome c oxidase subunit 7A2 mitochondrial	P14406	0.0160	0.0738	0.12	0.0156	0.4565	0.13	0.0007	0.0212	0.40	0.0512	0.1167	0.12	0.4235	0.5179	-0.02	0.0147	0.0806	0.16	0.3736	0.6033	-2.55	0.8708	1.2185	-0.33	0.0651	0.1948	2.06
1157	Cytochrome c oxidase subunit NDUFA4	O00483	0.0037	0.0390	-0.11	0.0021	0.2314	-0.23	0.0007	0.0215	-0.22	0.0067	0.0331	-0.37	0.0001	0.0102	-0.33	0.0019	0.0544	-0.17	0.1605	0.3535	0.17	0.1020	0.3214	-0.12	0.0304	0.1476	-0.18
1158	Cytochrome c1 heme protein mitochondrial	P08574	0.0117	0.0625	-0.19	0.0047	0.3528	-0.33	0.1160	0.2299	0.09	0.0330	0.0859	-0.14	0.0524	0.1696	-0.13	0.2886	0.4852	0.05	0.0600	0.1938	0.08	0.0108	0.0852	0.10	0.2725	0.3444	0.04
1159	Cytochrome c-type heme lyase	P53701	0.0715	0.1795	0.07	0.5840	0.6913	0.05	0.2915	0.4717	0.04	0.4977	0.7013	-0.02	0.0064	0.0583	-0.11	0.4396	0.6612	-0.02	0.0705	0.2096	0.50	0.0438	0.1934	0.15	0.0235	0.1302	0.31
1160	Cytochrome P450 1A1	P04798	0.4108	0.5962	-3.31	0.5752	0.8346	-1.75	0.5661	0.7484	-1.19	0.5388	0.7075	0.89	0.2938	0.4900	-1.88	0.9510	1.1312	0.09	0.0001	0.0207	-0.94	0.4510	0.8545	0.05	0.0169	0.1110	-0.48
1161	Cytochrome P450 20A1	Q6UW02	0.0416	0.1293	0.20	0.3597	0.7082	-0.06	0.4120	0.6181	0.05	0.2960	0.4593	0.07	0.1524	0.3309	0.10	0.1347	0.2870	0.11	0.0304	0.1477	0.57	0.0792	0.2763	0.17	0.0741	0.1237	0.34
1162	Cytochrome P450 26C1	Q6V0L0	0.1294	0.2706	-0.26	0.3695	0.7179	-0.13	0.1745	0.3164	-0.21	0.5122	0.7167	-0.08	0.1504	0.3276	-0.25	0.0859	0.2134	-0.29	0.3736	0.5415	-2.55	0.8708	1.1088	-0.33	0.0651	0.1608	2.06
1163	Cytochrome P450 2C9	P11712	0.1275	0.2673	-0.18	0.9162	0.9420	0.01	0.0480	0.1215	-0.35	0.0210	0.0624	0.22	0.0177	0.0952	0.26	0.1516	0.3092	0.07	0.3736	0.5157	-2.55	0.8708	1.0621	-0.33	0.0651	0.1482	2.06
1164	Cytochrome P450 4X1	Q8N118	0.0223	0.0891	0.38	0.3283	0.6879	0.16	0.3686	0.5661	0.08	0.1576	0.2755	0.14	0.6253	0.6961	0.04	0.1553	0.3142	0.15	0.3736	0.5143	-2.55	0.8708	1.0596	-0.33	0.0651	0.1475	2.06
1165	Cytochrome P450 4Z1	Q86W10	0.0121	0.0637	0.34	0.0693	0.6003	0.28	0.0547	0.1331	0.20	0.0043	0.0265	0.45	0.0198	0.1007	0.35	0.0118	0.0741	0.46	0.3736	0.6090	-2.55	0.8708	1.2284	-0.33	0.0651	0.1982	2.06
1166	Cytohesin-2	Q99418	0.0912	0.2116	0.55	0.0485	0.5736	0.13	0.0025	0.0280	0.20	0.0139	0.0480	0.15	0.0196	0.1001	0.32	0.2298	0.4130	0.31	0.3736	0.4949	-2.55	0.8708	1.0242	-0.33	0.0651	0.1386	2.06
1167	Cytohesin-3	O43739	0.6679	0.7271	-0.02	0.1088	0.6304	-0.11	0.1330	0.2553	-0.10	0.0632	0.1371	-0.14	0.0123	0.0792	-0.32	0.5109	0.7378	-0.04	0.3736	0.4594	-2.55	0.8708	0.9583	-0.33	0.0651	0.1233	2.06
1168	Cytohesin-4	Q9UIA0	0.0002	0.0157	3.66	0.9360	0.9567	0.18	0.0051	0.0348	2.30	0.3559	0.5341	1.88	0.1046	0.2601	-0.62	0.0172	0.0854	3.52	0.3736	0.5979	-2.55	0.8708	1.2090	-0.33	0.0651	0.1916	2.06
1169	Cytokine receptor-like factor 3	Q8IUI8	0.4108	0.5961	-3.31	0.5752	0.8344	-1.75	0.5661	0.7483	-1.19	0.5388	0.7073	0.89	0.2938	0.4898	-1.88	0.9510	1.1309	0.09	0.0344	0.1543	0.22	0.7584	1.2010	0.02	0.1360	0.1967	0.11
1170	Cytoplasmic aconitate hydratase	P21399	0.1729	0.3342	-0.35	0.2077	0.6584	-0.85	0.0910	0.1900	-0.63	0.2373	0.3827	-0.29	0.8088	0.8514	0.05	0.4433	0.6652	-0.17	0.0512	0.1792	0.55	0.0295	0.1551	0.35	0.0307	0.1483	0.24
1171	Cytoplasmic dynein 1 heavy chain 1	Q14204	0.0093	0.0573	0.18	0.2104	0.6617	0.21	0.0058	0.0370	0.31	0.0015	0.0175	0.30	0.0505	0.1661	0.12	0.0037	0.0593	0.46	0.0187	0.1266	0.50	0.0592	0.2323	0.25	0.1724	0.2383	0.14
1172	Cytoplasmic dynein 1 intermediate chain 2	Q13409	0.2165	0.3914	0.10	0.1113	0.6301	0.17	0.0121	0.0515	0.30	0.0100	0.0402	0.29	0.0018	0.0333	0.37	0.0009	0.0466	0.46	0.0050	0.0746	0.67	0.0128	0.0942	0.34	0.0159	0.1081	0.41
1173	Cytoplasmic dynein 1 light intermediate chain 1	Q9Y6G9	0.9544	0.9660	0.00	0.6587	0.7479	-0.04	0.9911	0.9921	0.00	0.0438	0.1047	-0.14	0.1575	0.3392	-0.08	0.4370	0.6588	-0.04	0.0441	0.1687	0.20	0.5804	1.0119	0.04	0.1993	0.2678	0.06
1174	Cytoplasmic dynein 1 light intermediate chain 2	O43237	0.6840	0.7403	0.02	0.6564	0.7468	0.04	0.0094	0.0452	-0.08	0.7807	0.8114	0.02	0.0677	0.1981	-0.04	0.6024	0.8393	-0.04	0.0880	0.2383	0.30	0.3447	0.7046	0.13	0.4892	0.5575	0.09
1175	Cytoplasmic dynein 2 heavy chain 1	Q8NCM8	0.0421	0.1301	0.19	0.2499	0.6601	0.22	0.0193	0.0668	0.28	0.0162	0.0528	0.28	0.3025	0.4004	0.07	0.0181	0.0871	0.28	0.5539	0.6051	0.23	0.6232	1.0598	0.20	0.6095	0.6695	0.20
1176	Cytoplasmic FMR1-interacting protein 1	Q7L576	0.3777	0.5934	-0.07	0.4394	0.7758	0.05	0.0389	0.1044	-0.13	0.1619	0.2812	-0.08	0.9464	0.9584	0.00	0.2676	0.4607	-0.13	0.1317	0.3077	0.14	0.4379	0.8386	0.04	0.0206	0.1227	-0.20
1177	Cytoplasmic protein NCK1	P16333	0.0550	0.1535	-0.51	0.1831	0.6647	-0.82	0.0141	0.0557	-0.76	0.0255	0.0717	-1.26	0.0351	0.1359	-0.76	0.0471	0.1442	-0.96	0.3736	0.5629	-2.55	0.8708	1.1472	-0.33	0.0651	0.1720	2.06
1178	Cytoplasmic tRNA 2-thiolation protein 2	Q2VPK5	0.7610	0.8055	0.04	0.9929	0.9954	0.00	0.5663	0.6243	-0.05	0.1159	0.2171	-0.22	0.2309	0.4409	-0.18	0.0287	0.1085	-0.40	0.3736	0.5817	-2.55	0.8708	1.1805	-0.33	0.0651	0.1823	2.06
1179	Cytoskeleton-associated protein 4	Q07065	0.2416	0.4270	0.05	0.2401	0.6605	0.17	0.0511	0.1273	0.15	0.0144	0.0488	0.17	0.0426	0.1525	0.12	0.0320	0.1154	0.16	0.3288	0.5970	0.05	0.0922	0.3017	-0.16	0.0832	0.1349	-0.11
1180	Cytoskeleton-associated protein 5	Q14008	0.3850	0.6016	0.03	0.7822	0.8429	0.05	0.0108	0.0482	0.14	0.2393	0.3854	0.04	0.1524	0.3310	-0.05	0.0285	0.1080	0.11	0.1509	0.3372	0.18	0.6848	1.1265	-0.04	0.7784	0.8175	-0.02
1181	Cytosol aminopeptidase	P28838	0.1111	0.2422	0.09	0.2487	0.6586	0.18	0.0573	0.1370	0.15	0.0166	0.0537	0.20	0.1388	0.3103	0.08	0.0742	0.1936	0.12	0.0888	0.2397	0.26	0.0450	0.1969	0.24	0.3791	0.4503	0.05
1182	Cytosolic 5'-nucleotidase 1A	Q9BXI3	0.4070	0.6276	0.06	0.4537	0.7868	0.12	0.1589	0.2928	0.11	0.0487	0.1131	0.16	0.2094	0.4126	-0.08	0.5946	0.8313	0.03	0.3736	0.4531	-2.55	0.8708	0.9466	-0.33	0.0651	0.1207	2.06
1183	Cytosolic acyl coenzyme A thioester hydrolase	O00154	0.3781	0.5939	-0.08	0.2313	0.6631	-0.08	0.2432	0.4078	-0.06	0.0234	0.0676	-0.18	0.0400	0.1468	-0.10	0.0374	0.1251	-0.10	0.7899	0.8228	-0.03	0.0497	0.2096	0.27	0.8702	0.8947	-0.01
1184	Cytosolic beta-glucosidase	Q9H227	0.2745	0.4677	-0.49	0.4155	0.7544	0.21	0.5019	0.7244	0.16	0.7776	0.8088	-0.06	0.0277	0.1207	0.82	0.4978	0.7227	-0.31	0.3736	0.4602	-2.55	0.8708	0.9599	-0.33	0.0651	0.1237	2.06
1185	Cytosolic carboxypeptidase 2	Q5U5Z8	0.0002	0.0155	0.35	0.1601	0.6640	0.12	0.0003	0.0204	0.26	0.0003	0.0119	0.27	0.0012	0.0276	0.31	0.0004	0.0414	0.40	0.4143	0.4701	0.05	0.1928	0.4872	-0.10	0.0124	0.0962	-0.42
1186	Cytosolic carboxypeptidase 3	Q8NEM8	0.4108	0.5959	-3.31	0.5752	0.8341	-1.75	0.5661	0.7481	-1.19	0.5388	0.7071	0.89	0.2938	0.4897	-1.88	0.9510	1.1306	0.09	0.0018	0.0515	0.33	0.0218	0.1280	0.15	0.0053	0.0700	0.47
1187	Cytosolic Fe-S cluster assembly factor NUBP1	P53384	0.3807	0.5965	-0.08	0.1616	0.6623	-0.30	0.1882	0.3342	0.12	0.4586	0.6560	-0.06	0.8898	0.9154	-0.01	0.0458	0.1418	0.24	0.3736	0.5647	-2.55	0.8708	1.1504	-0.33	0.0651	0.1729	2.06
1188	Cytosolic Fe-S cluster assembly factor NUBP2	Q9Y5Y2	0.0286	0.1030	0.08	0.6032	0.7066	-0.05	0.2022	0.3540	0.05	0.0817	0.1666	-0.09	0.0094	0.0691	-0.17	0.5655	0.8006	0.02	0.3736	0.4552	-2.55	0.8708	0.9506	-0.33	0.0651	0.1216	2.06
1189	Cytosolic iron-sulfur assembly component 2A	Q9H5X1	0.0109	0.0600	0.32	0.1362	0.6508	0.22	0.0019	0.0246	0.20	0.0028	0.0222	0.27	0.0121	0.0789	0.21	0.1313	0.2823	0.34	0.3736	0.5239	-2.55	0.8708	1.0771	-0.33	0.0651	0.1521	2.06
1190	Cytosolic iron-sulfur assembly component 2B	Q9Y3D0	0.4528	0.5303	0.14	0.7933	0.8508	-0.09	0.5153	0.7357	0.13	0.9009	0.9150	0.02	0.4454	0.5392	0.15	0.4343	0.6555	0.14	0.0023	0.0554	0.39	0.0049	0.0563	0.48	0.0005	0.0288	0.79
1191	Cytosolic non-specific dipeptidase	Q96KP4	0.0538	0.1514	-0.07	0.3177	0.6812	-0.08	0.1503	0.2799	0.08	0.0778	0.1606	0.13	0.7416	0.7977	-0.01	0.0268	0.1048	0.07	0.0323	0.1513	0.34	0.0409	0.1858	0.14	0.0304	0.1477	0.12
1192	Cytosolic phospholipase A2	P47712	0.0057	0.0456	-1.66	0.3426	0.7001	-1.11	0.0080	0.0419	-2.16	0.0164	0.0533	-1.08	0.8166	0.8571	0.06	0.0314	0.1138	-1.61	0.3736	0.5796	-2.55	0.8708	1.1768	-0.33	0.0651	0.1811	2.06
1193	Cytosolic purine 5'-nucleotidase	P49902	0.0904	0.2107	0.10	0.2199	0.6600	0.22	0.0112	0.0491	0.21	0.0168	0.0541	0.19	0.2416	0.4554	-0.06	0.0302	0.1117	0.18	0.5349	0.5868	0.10	0.4976	0.9121	0.07	0.6218	0.6800	-0.08
1194	Cytospin-B	Q5M775	0.4108	0.5957	-3.31	0.5752	0.8339	-1.75	0.5661	0.7479	-1.19	0.5388	0.7069	0.89	0.2938	0.4895	-1.88	0.9510	1.1304	0.09	0.0045	0.0719	0.38	0.0522	0.2170	0.15	0.0115	0.0934	0.39
1195	D-3-phosphoglycerate dehydrogenase	O43175	0.0023	0.0330	0.47	0.2134	0.6631	0.30	0.0042	0.0323	0.40	0.0010	0.0154	0.56	0.8488	0.8836	-0.01	0.0039	0.0591	0.59	0.0419	0.1677	0.48	0.6523	1.0921	-0.04	0.5655	0.6293	-0.05
1196	D-aminoacyl-tRNA deacylase 1	Q8TEA8	0.4978	0.5716	-0.03	0.3826	0.7300	0.12	0.0079	0.0417	0.15	0.1364	0.2458	0.09	0.4065	0.5025	-0.02	0.0010	0.0443	0.10	0.4195	0.4754	0.02	0.0152	0.1039	0.08	0.2649	0.3366	0.10
1197	DAZ-associated protein 1	Q96EP5	0.0132	0.0670	-0.23	0.0562	0.5846	-0.19	0.2137	0.3684	0.68	0.4252	0.6171	-0.11	0.1003	0.2536	-0.11	0.0211	0.0940	-0.24	0.5419	0.5933	0.06	0.0975	0.3123	-0.09	0.0615	0.2217	0.11
1198	D-beta-hydroxybutyrate dehydrogenase mitochondrial	Q02338	0.0000	0.0000	0.47	0.0711	0.6018	0.31	0.0041	0.0324	0.53	0.0006	0.0127	0.24	0.0053	0.0546	0.17	0.1178	0.2618	0.28	0.1002	0.2558	0.19	0.5883	1.0206	-0.09	0.0648	0.2285	0.11
1199	DCC-interacting protein 13-alpha	Q9UKG1	0.0206	0.0847	-0.64	0.5112	0.8332	-0.16	0.3921	0.5937	-0.10	0.6569	0.7002	0.06	0.4178	0.5124	-0.07	0.3744	0.5828	-0.19	0.3568	0.6340	0.11	0.0330	0.1639	0.17	0.1070	0.1633	0.08
1200	DCC-interacting protein 13-beta	Q8NEU8	0.0113	0.0611	6.95	0.5752	0.8857	-1.75	0.0151	0.0576	6.62	0.0094	0.0390	7.17	0.1964	0.3957	2.13	0.0156	0.0832	7.15	0.3736	0.6018	-2.55	0.8708	1.2158	-0.33	0.0651	0.1939	2.06
1201	DCN1-like protein 1	Q96GG9	0.1217	0.2584	0.15	0.0898	0.6227	0.18	0.0732	0.1617	0.16	0.0113	0.0427	0.20	0.2027	0.4038	0.06	0.0063	0.0623	0.33	0.3736	0.6241	-2.55	0.8708	1.2547	-0.33	0.0651	0.2076	2.06
1202	DCN1-like protein 2	Q6PH85	0.0516	0.1472	0.59	0.0457	0.5610	0.66	0.0491	0.1235	0.59	0.0355	0.0903	0.65	0.0826	0.2223	0.50	0.0811	0.2048	0.53	0.3736	0.5432	-2.55	0.8708	1.1118	-0.33	0.0651	0.1617	2.06
1203	DCN1-like protein 5	Q9BTE7	0.4328	0.5100	-0.04	0.2192	0.6598	0.21	0.0950	0.1961	0.10	0.0206	0.0617	0.17	0.0327	0.1320	0.15	0.2195	0.4008	0.07	0.1545	0.3444	-0.09	0.1691	0.4490	-0.09	0.9633	0.9708	0.00
1204	dCTP pyrophosphatase 1	Q9H773	0.3409	0.5489	-0.03	0.3617	0.7102	-0.08	0.0153	0.0581	0.14	0.1704	0.2922	-0.05	0.0037	0.0451	-0.25	0.1798	0.3491	-0.05	0.8764	0.8975	-0.01	0.3389	0.6966	-0.04	0.0398	0.1725	0.19
1205	DDB1- and CUL4-associated factor 11	Q8TEB1	0.1031	0.2297	-0.09	0.2493	0.6595	0.10	0.0432	0.1130	-0.12	0.0262	0.0729	-0.19	0.0791	0.2169	0.07	0.1466	0.3027	-0.14	0.0983	0.2526	-0.28	0.0314	0.1595	0.34	0.7864	0.8249	0.09
1206	DDB1- and CUL4-associated factor 12	Q5T6F0	0.6585	0.7190	0.03	0.0669	0.5914	0.26	0.1993	0.3502	0.11	0.0266	0.0735	0.26	0.1232	0.2892	0.13	0.7466	0.9848	-0.02	0.3736	0.4419	-2.55	0.8708	0.9257	-0.33	0.0651	0.1162	2.06
1207	DDB1- and CUL4-associated factor 17	Q5H9S7	0.4108	0.5956	-3.31	0.5752	0.8337	-1.75	0.5661	0.7477	-1.19	0.5388	0.7068	0.89	0.2938	0.4894	-1.88	0.9510	1.1301	0.09	0.2031	0.4158	-0.59	0.0096	0.0809	1.16	0.0362	0.1628	0.85
1208	DDB1- and CUL4-associated factor 5	Q96JK2	0.0699	0.1765	-0.17	0.1404	0.6540	-0.22	0.0954	0.1968	-0.17	0.0204	0.0613	-0.26	0.0350	0.1359	-0.21	0.1297	0.2805	-0.19	0.3736	0.5246	-2.55	0.8708	1.0784	-0.33	0.0651	0.1524	2.06
1209	DDB1- and CUL4-associated factor 7	P61962	0.8465	0.8779	0.01	0.7526	0.8206	0.02	0.0823	0.1765	0.21	0.0054	0.0294	-0.15	0.8995	0.9234	-0.01	0.9072	1.1448	0.01	0.3736	0.4337	-2.55	0.8708	0.9102	-0.33	0.0651	0.1130	2.06
1210	DDB1- and CUL4-associated factor 8	Q5TAQ9	0.6197	0.6833	0.04	0.3692	0.7176	-0.22	0.1827	0.3274	0.09	0.2020	0.3347	0.08	0.8740	0.9042	0.01	0.1405	0.2941	-0.18	0.2030	0.4158	-0.12	0.0431	0.1918	-0.27	0.3845	0.4557	-0.29
1211	D-dopachrome decarboxylase	P30046	0.0868	0.2048	-0.13	0.1678	0.6600	-0.37	0.0150	0.0576	-0.29	0.0099	0.0400	-0.58	0.0281	0.1217	-0.27	0.0618	0.1729	-0.16	0.3736	0.5551	-2.55	0.8708	1.1334	-0.33	0.0651	0.1679	2.06
1212	D-dopachrome decarboxylase-like protein	A6NHG4	0.4108	0.5954	-3.31	0.5752	0.8334	-1.75	0.5661	0.7475	-1.19	0.5388	0.7066	0.89	0.2938	0.4892	-1.88	0.9510	1.1299	0.09	0.0415	0.1673	-10.61	0.0402	0.1836	-8.39	0.0002	0.0225	-6.00
1213	DDRGK domain-containing protein 1	Q96HY6	0.0087	0.0552	-0.12	0.6204	0.7180	0.08	0.3769	0.5761	0.04	0.0067	0.0329	0.19	0.0448	0.1560	0.13	0.0359	0.1223	0.10	0.0502	0.1785	0.34	0.5328	0.9573	-0.05	0.1278	0.1874	0.14
1214	Death domain-containing protein 1	Q6ZMT9	0.4108	0.5952	-3.31	0.5752	0.8332	-1.75	0.5661	0.7473	-1.19	0.5388	0.7064	0.89	0.2938	0.4890	-1.88	0.9510	1.1296	0.09	0.3360	0.6064	0.17	0.1051	0.3286	0.34	0.0991	0.1544	0.38
1215	Death-associated protein kinase 1	P53355	0.0123	0.0644	0.25	0.2006	0.6627	0.21	0.0329	0.0935	0.12	0.0049	0.0281	0.40	0.0081	0.0651	0.30	0.0015	0.0515	0.40	0.0700	0.2085	-0.53	0.0409	0.1855	-0.31	0.0246	0.1330	-0.51
1216	Death-inducer obliterator 1	Q9BTC0	0.0113	0.0610	-0.18	0.2579	0.6627	0.18	0.3664	0.5637	0.07	0.0407	0.0998	-0.05	0.1444	0.3188	0.07	0.0376	0.1254	-0.12	0.1728	0.3703	-0.07	0.0679	0.2511	0.12	0.0194	0.1209	0.55
1217	Dedicator of cytokinesis protein 11	Q5JSL3	0.2778	0.4720	0.03	0.4734	0.8014	0.10	0.0237	0.0755	0.24	0.0001	0.0072	0.44	0.0124	0.0796	0.14	0.0005	0.0387	0.45	0.3736	0.6523	-2.55	0.8708	1.3033	-0.33	0.0651	0.2263	2.06
1218	Dedicator of cytokinesis protein 6	Q96HP0	0.4108	0.5951	-3.31	0.5752	0.8330	-1.75	0.5661	0.7471	-1.19	0.5388	0.7062	0.89	0.2938	0.4889	-1.88	0.9510	1.1293	0.09	0.9384	0.9492	-0.01	0.0005	0.0207	-0.62	0.0020	0.0510	-0.80
1219	Dedicator of cytokinesis protein 7	Q96N67	0.4108	0.5949	-3.31	0.5752	0.8327	-1.75	0.5661	0.7469	-1.19	0.5388	0.7060	0.89	0.0342	0.1352	5.06	0.0965	0.2292	3.15	0.1565	0.3476	0.15	0.0124	0.0928	0.35	0.1418	0.2032	0.39
1220	Dedicator of cytokinesis protein 8	Q8NF50	0.4108	0.5947	-3.31	0.5752	0.8325	-1.75	0.5661	0.7467	-1.19	0.5388	0.7059	0.89	0.2938	0.4887	-1.88	0.9510	1.1291	0.09	0.2636	0.5052	-0.13	0.0055	0.0599	-0.24	0.0030	0.0547	-0.51
1221	Dehydrogenase/reductase SDR family member 1	Q96LJ7	0.0095	0.0576	0.33	0.9010	0.9301	-0.03	0.0017	0.0232	0.53	0.0008	0.0147	0.56	0.0071	0.0614	0.38	0.1318	0.2829	0.47	0.0129	0.1076	0.42	0.0122	0.0924	0.38	0.0280	0.1435	0.23
1222	Dehydrogenase/reductase SDR family member 7	Q9Y394	0.0046	0.0430	0.33	0.1977	0.6698	0.34	0.0034	0.0313	0.37	0.0110	0.0422	0.28	0.5214	0.6090	0.03	0.0192	0.0890	0.29	0.0628	0.1973	0.22	0.3304	0.6865	0.02	0.2629	0.3349	0.08

Supplemetary Table S2. Overview on all relatively quantified 5180 proteins statistical analysis

			MCF-7																		MDA-MB-231								
			Dai_SC20 vs control			Gen_SC20 vs control			SSE_SC20 vs control			Dai_IC20 vs control			Gen_IC20 vs control			SSE_IC20 vs control			Dai_IC20 vs control			Gen_IC20 vs control			SSE_IC20 vs control		
	Protein name	UniProt	p value	BH q value	log2FC	p value	BH q value	log2FC	p value	BH q value	log2FC	p value	BH q value	log2FC	p value	BH q value	log2FC	p value	BH q value	log2FC	p value	BH q value	log2FC	p value	BH q value	log2FC	p value	BH q value	log2FC
1223	Dehydrogenase/reductase SDR family member 7B	Q6IAN0	0.6020	0.6677	0.02	0.1948	0.6691	0.19	0.0332	0.0942	0.12	0.0038	0.0249	0.24	0.3160	0.4135	0.04	0.1772	0.3464	0.09	0.3736	0.5085	-2.55	0.8708	1.0490	-0.33	0.0651	0.1448	2.06
1224	Delphilin	A4D2P6	0.4375	0.5152	-0.07	0.2471	0.6581	-0.52	0.0018	0.0238	0.72	0.0170	0.0545	0.36	0.0249	0.1139	0.32	0.0200	0.0920	0.41	0.1601	0.3531	-0.13	0.0020	0.0366	-0.37	0.0160	0.1086	-0.22
1225	Delta(14)-sterol reductase	Q14739	0.4108	0.5946	-3.31	0.5752	0.8323	-1.75	0.5661	0.7465	-1.19	0.5388	0.7057	0.89	0.2938	0.4886	-1.88	0.9510	1.1288	0.09	0.5064	0.5599	0.06	0.0693	0.2542	-0.13	0.0012	0.0429	-0.37
1226	Delta(24)-sterol reductase	Q15392	0.7753	0.8169	0.27	0.0970	0.6336	-1.41	0.0026	0.0282	-1.89	0.7434	0.7789	0.31	0.0130	0.0810	-0.94	0.0092	0.0691	1.37	0.3736	0.6159	-2.55	0.8708	1.2406	-0.33	0.0651	0.2025	2.06
1227	Delta(3_5)-Delta(2_4)-dienoyl-CoA isomerase_ mitochondrial	Q13011	0.1139	0.2465	0.04	0.3993	0.7430	0.09	0.1424	0.2684	-0.05	0.0415	0.1010	0.06	0.5408	0.6250	-0.02	0.2129	0.3941	0.19	0.9940	0.9953	0.00	0.3395	0.6973	-0.07	0.0093	0.0860	-0.24
1228	Delta-1-pyrroline-5-carboxylate dehydrogenase_ mitochondrial	P30038	0.0010	0.0225	-0.47	0.0077	0.4029	-0.26	0.0025	0.0280	-0.60	0.0006	0.0130	-0.52	0.0024	0.0386	-0.47	0.0099	0.0708	-0.30	0.1091	0.2705	0.37	0.7238	1.1691	0.02	0.4465	0.5163	0.04
1229	Delta-1-pyrroline-5-carboxylate synthase	P54886	0.1143	0.2471	-0.14	0.4756	0.8025	0.05	0.0336	0.0949	0.22	0.7301	0.7671	0.02	0.7443	0.7997	-0.03	0.5935	0.8307	-0.04	0.2415	0.4739	0.15	0.7481	1.1894	0.04	0.0326	0.1538	0.47
1230	Delta-aminolevulinic acid dehydratase	P13716	0.1217	0.2583	-0.11	0.3318	0.6911	0.09	0.1845	0.3293	-0.09	0.5627	0.6127	-0.03	0.0813	0.2203	-0.13	0.7595	0.9973	-0.02	0.2738	0.5184	0.14	0.5906	1.0235	0.06	0.1044	0.1606	0.23
1231	DENN domain-containing protein 2A	Q9ULE3	0.0796	0.1935	0.68	0.2066	0.6598	0.14	0.0085	0.0436	0.19	0.0000	0.0000	0.40	0.0527	0.1702	0.08	0.0157	0.0835	0.51	0.0379	0.1613	0.47	0.3288	0.6843	-0.10	0.0991	0.1543	-0.16
1232	DENN domain-containing protein 2C	Q68D51	0.1413	0.2870	-0.30	0.2994	0.6737	0.17	0.0654	0.1495	0.40	0.6079	0.6552	0.08	0.0999	0.2529	0.33	0.7787	1.0160	0.07	0.3736	0.4400	-2.55	0.8708	0.9221	-0.33	0.0651	0.1155	2.06
1233	DENN domain-containing protein 2D	Q9H6A0	0.0014	0.0258	-0.41	0.0434	0.5720	0.28	0.1458	0.2730	-0.21	0.0109	0.0419	-0.22	0.2005	0.4013	0.07	0.1540	0.3121	-0.32	0.3736	0.5146	-2.55	0.8708	1.0601	-0.33	0.0651	0.1476	2.06
1234	DENN domain-containing protein 4C	Q5VZ89	0.1483	0.2968	0.10	0.2181	0.6607	0.08	0.4573	0.6724	0.07	0.0181	0.0567	-0.23	0.0154	0.0882	-0.24	0.5596	0.7953	-0.15	0.3736	0.4562	-2.55	0.8708	0.9524	-0.33	0.0651	0.1220	2.06
1235	Density-regulated protein	O43583	0.2545	0.4421	0.20	0.5169	0.8386	-0.32	0.0646	0.1483	-0.74	0.1850	0.3124	-0.66	0.3893	0.4865	-0.13	0.8104	1.0471	-0.03	0.0026	0.0568	-5.14	0.0051	0.0573	-1.93	0.0117	0.0938	-2.87
1236	Deoxycytidine kinase	P27707	0.0103	0.0591	-0.56	0.0154	0.4585	-0.23	0.0211	0.0704	-0.19	0.0030	0.0224	-0.58	0.0003	0.0148	-0.60	0.0061	0.0627	-0.43	0.0328	0.1516	-0.95	0.0028	0.0441	-0.87	0.0048	0.0679	-0.69
1237	Deoxycytidylate deaminase	P32321	0.0035	0.0384	-0.46	0.0179	0.4707	-0.40	0.0069	0.0399	-0.52	0.0138	0.0477	-0.54	0.0012	0.0274	-0.62	0.0093	0.0693	-0.47	0.1443	0.3271	-0.12	0.0279	0.1515	-0.35	0.0415	0.1765	-0.20
1238	Deoxyhypusine synthase	P49366	0.0068	0.0493	-0.35	0.5428	0.8575	-0.07	0.0040	0.0318	-0.45	0.0476	0.1112	-0.17	0.0156	0.0889	-0.30	0.7438	0.9826	-0.02	0.3736	0.4421	-2.55	0.8708	0.9260	-0.33	0.0651	0.1163	2.06
1239	Deoxynucleoside triphosphate triphosphohydrolase SAMHD1	Q9Y3Z3	0.0436	0.1324	-0.05	0.1919	0.6671	-0.06	0.1859	0.3311	0.02	0.0040	0.0255	-0.12	0.1026	0.2569	-0.06	0.2338	0.4179	-0.05	0.1207	0.2904	0.14	0.3965	0.7809	0.03	0.1496	0.2123	-0.04
1240	Deoxyribonuclease-2-alpha	O00115	0.9952	0.9958	0.00	0.0003	0.1943	0.24	0.0148	0.0573	0.30	0.0046	0.0275	0.20	0.0013	0.0276	0.27	0.0574	0.1648	0.22	0.3736	0.5569	-2.55	0.8708	1.1365	-0.33	0.0651	0.1688	2.06
1241	Deoxyribose-phosphate aldolase	Q9Y315	0.4108	0.5944	-3.31	0.5752	0.8320	-1.75	0.5661	0.7463	-1.19	0.5388	0.7055	0.89	0.2938	0.4884	-1.88	0.9510	1.1286	0.09	0.9563	0.9643	-0.02	0.4532	0.8568	0.19	0.0165	0.1097	0.43
1242	Deoxyuridine 5'-triphosphate nucleotidohydrolase_ mitochondrial	P33316	0.1150	0.2484	0.10	0.4737	0.8014	0.07	0.0171	0.0621	0.21	0.0574	0.1274	0.14	0.0653	0.1945	-0.13	0.8510	1.0860	-0.01	0.1726	0.3702	0.22	0.2113	0.5143	-0.19	0.5396	0.6051	-0.07
1243	Derlin-1	Q9BUN8	0.3008	0.4999	-0.02	0.6381	0.7335	0.04	0.0896	0.1878	-0.08	0.7673	0.7997	-0.01	0.0043	0.0484	-0.13	0.0624	0.1739	0.10	0.3736	0.5547	-2.55	0.8708	1.1325	-0.33	0.0651	0.1676	2.06
1244	Desmin	P17661	0.5019	0.5754	-0.03	0.2438	0.6584	0.14	0.5812	0.6381	0.05	0.8731	0.8910	-0.01	0.7336	0.7910	0.02	0.4299	0.6511	-0.04	0.2787	0.5263	-1.22	0.4571	0.8626	-0.76	0.7411	0.7863	-0.32
1245	Desmoglein-1	Q02413	0.0750	0.1862	0.14	0.4575	0.7897	-0.03	0.0476	0.1210	0.18	0.0028	0.0216	0.22	0.0600	0.1847	0.05	0.0346	0.1200	0.12	0.0010	0.0432	-1.91	0.0001	0.0144	-2.59	0.0168	0.1111	-2.37
1246	Desmoglein-2	Q14126	0.1801	0.3436	-1.02	0.3131	0.6806	-0.35	0.2348	0.3957	0.24	0.0616	0.1344	-0.92	0.2184	0.4242	0.25	0.4763	0.7003	0.12	0.1759	0.3748	-0.43	0.2057	0.5064	-0.24	0.0354	0.1611	0.62
1247	Desmoplakin	P15924	0.0029	0.0364	0.20	0.0299	0.5062	0.13	0.0189	0.0658	0.34	0.0006	0.0124	0.30	0.0145	0.0857	0.12	0.0022	0.0538	0.26	0.3736	0.6432	-2.55	0.8708	1.2877	-0.33	0.0651	0.2201	2.06
1248	Destrin	P60981	0.0337	0.1147	0.21	0.4472	0.7823	0.12	0.2584	0.4270	0.10	0.0530	0.1201	0.15	0.4820	0.5719	0.04	0.0872	0.2154	0.13	0.2592	0.4980	0.16	0.1335	0.3848	-0.14	0.0053	0.0699	-0.34
1249	Deuterosome assembly protein 1	Q05D60	0.1682	0.3269	-0.05	0.0620	0.5904	0.33	0.0724	0.1608	0.04	0.6762	0.7169	0.01	0.0474	0.1601	0.09	0.1021	0.2368	-0.03	0.0281	0.1445	0.36	0.1142	0.3459	0.11	0.0016	0.0476	0.34
1250	Developmentally-regulated GTP-binding protein 1	Q9Y295	0.6253	0.6887	-0.06	0.2650	0.6628	-0.19	0.0539	0.1318	-0.38	0.0456	0.1078	-0.44	0.3364	0.4334	-0.15	0.0854	0.2127	-0.33	0.6734	0.7170	0.03	0.0021	0.0376	-0.19	0.0587	0.2166	-0.18
1251	Developmentally-regulated GTP-binding protein 2	P55039	0.4108	0.5942	-3.31	0.5752	0.8318	-1.75	0.5661	0.7462	-1.19	0.5388	0.7053	0.89	0.2938	0.4883	-1.88	0.9510	1.1283	0.09	0.0560	0.1874	-0.52	0.4749	0.8865	-0.08	0.7504	0.7941	0.03
1252	Diablo homolog_ mitochondrial	Q9NR28	0.2759	0.4693	0.05	0.4196	0.7579	0.09	0.0490	0.1233	0.12	0.0216	0.0638	0.07	0.0024	0.0384	0.12	0.0143	0.0799	-0.08	0.1756	0.3745	0.24	0.0317	0.1605	-0.30	0.0100	0.0881	-0.43
1253	Diacylglycerol kinase theta	P52824	0.4108	0.5941	-3.31	0.5752	0.8316	-1.75	0.5661	0.7460	-1.19	0.5388	0.7052	0.89	0.2938	0.4881	-1.88	0.9510	1.1280	0.09	0.5314	0.5838	0.06	0.0543	0.2224	-0.24	0.1070	0.1633	-0.16
1254	Diacylglycerol O-acyltransferase 2	Q96PD7	0.0081	0.0533	1.46	0.9951	0.9970	0.01	0.0236	0.0754	0.88	0.0053	0.0292	1.45	0.8604	0.8932	-0.06	0.0991	0.2326	1.61	0.3736	0.5345	-2.55	0.8708	1.0962	-0.33	0.0651	0.1573	2.06
1255	Diamine acetyltransferase 1	P21673	0.1638	0.3205	-0.65	0.3558	0.7078	-1.26	0.0797	0.1721	-1.01	0.0421	0.1021	-1.18	0.6302	0.7005	-0.19	0.0697	0.1854	-1.11	0.3736	0.5495	-2.55	0.8708	1.1232	-0.33	0.0651	0.1649	2.06
1256	Differentially expressed in FDCP 6 homolog	Q9H4E7	0.1378	0.2826	0.08	0.2329	0.6629	0.22	0.0071	0.0399	0.32	0.0035	0.0238	0.33	0.0430	0.1531	0.11	0.1415	0.2954	0.08	0.0017	0.0527	0.59	0.0113	0.0876	0.34	0.0296	0.1467	0.28
1257	Digestive organ expansion factor homolog	Q68CQ4	0.4108	0.5939	-3.31	0.5752	0.8313	-1.75	0.5661	0.7458	-1.19	0.5388	0.7050	0.89	0.2938	0.4879	-1.88	0.9510	1.1278	0.09	0.1961	0.4053	-0.64	0.3010	0.6448	-0.25	0.1269	0.1865	0.26
1258	Dihydrofolate reductase	P00374	0.4758	0.5513	-0.20	0.7072	0.7848	-0.12	0.2458	0.4114	0.13	0.8836	0.9005	0.01	0.4933	0.5827	-0.10	0.0241	0.0990	-0.47	0.4534	0.5082	0.09	0.2896	0.6292	0.11	0.6606	0.7153	0.04
1259	Dihydrolipoyl dehydrogenase_ mitochondrial	P09622	0.0864	0.2044	0.19	0.1027	0.6311	0.32	0.0290	0.0865	0.29	0.0179	0.0564	0.37	0.0562	0.1771	0.26	0.0309	0.1128	0.36	0.3301	0.5983	0.12	0.0056	0.0602	-0.17	0.2710	0.3430	-0.16
1260	Dihydrolipoyllysine-residue acetyltransferase component of pyruvate dehydrogenase complex_ mitochondrial	P10515	0.4108	0.5937	-3.31	0.5752	0.8311	-1.75	0.5661	0.7456	-1.19	0.5388	0.7048	0.89	0.2938	0.4878	-1.88	0.9510	1.1275	0.09	0.1845	0.3854	-0.16	0.9466	0.9613	0.00	0.0637	0.2260	-0.26
1261	Dihydrolipoyllysine-residue succinyltransferase component of 2-oxoglutarate dehydrogenase complex_ mitochondrial	P36957	0.1993	0.3696	0.06	0.3248	0.6856	0.08	0.0155	0.0586	0.17	0.0656	0.1412	0.11	0.6646	0.7295	-0.02	0.0252	0.1008	0.15	0.9075	0.9241	0.01	0.6818	1.1219	-0.04	0.0194	0.1206	-0.33
1262	Dihydroorotate dehydrogenase (quinone)_ mitochondrial	Q02127	0.3997	0.6192	-0.06	0.2287	0.6600	-0.10	0.0087	0.0438	-0.33	0.6615	0.7042	0.03	0.0596	0.1839	-0.16	0.3015	0.4993	-0.06	0.0645	0.2001	-0.16	0.6360	1.0742	0.04	0.0747	0.1245	-0.16
1263	Dihydropteridine reductase	P09417	0.4108	0.5936	-3.31	0.5752	0.8309	-1.75	0.5661	0.7454	-1.19	0.5388	0.7046	0.89	0.2938	0.4876	-1.88	0.9510	1.1273	0.09	0.0200	0.1308	0.33	0.5263	0.9502	-0.05	0.0171	0.1116	0.39
1264	Dihydropyrimidinase-related protein 1	Q14194	0.0507	0.1457	-0.73	0.4133	0.7522	-1.57	0.0289	0.0863	-0.43	0.0499	0.1149	-1.11	0.9343	0.9499	-0.01	0.2419	0.4281	-0.97	0.1833	0.3847	5.54	0.2197	0.5269	4.84	0.1400	0.2014	6.61
1265	Dihydropyrimidinase-related protein 2	Q16555	0.1889	0.3547	0.14	0.9442	0.9630	-0.01	0.0568	0.1362	0.25	0.1200	0.2230	0.18	0.2967	0.3948	0.11	0.0982	0.2311	0.20	0.7540	0.7902	0.02	0.0175	0.1119	-0.15	0.0742	0.1238	-0.10
1266	Dihydropyrimidinase-related protein 3	Q14195	0.4108	0.5934	-3.31	0.5752	0.8306	-1.75	0.5661	0.7452	-1.19	0.5388	0.7044	0.89	0.2938	0.4875	-1.88	0.9510	1.1270	0.09	0.0025	0.0563	-0.52	0.0021	0.0382	-0.46	0.0058	0.0726	-0.39
1267	Dihydropyrimidinase-related protein 4	O14531	0.9360	0.9516	-0.01	0.0763	0.6156	0.43	0.1987	0.3495	0.18	0.0537	0.1213	0.32	0.0034	0.0432	0.75	0.1648	0.3282	0.27	0.3736	0.5108	-2.55	0.8708	1.0532	-0.33	0.0651	0.1459	2.06
1268	Dihydropyrimidinase-related protein 5	Q9BPU6	0.1651	0.3224	-0.07	0.6441	0.7375	-0.07	0.1287	0.2494	-0.10	0.0307	0.0815	-0.17	0.0041	0.0470	-0.33	0.0418	0.1347	-0.13	0.3736	0.5689	-2.55	0.8708	1.1578	-0.33	0.0651	0.1752	2.06
1269	Dipeptidase 2	Q9H4A9	0.4860	0.5604	-0.17	0.5084	0.8302	-0.40	0.4196	0.6275	0.23	0.6752	0.7161	-0.11	0.4644	0.5558	-0.19	0.4570	0.6791	-0.23	0.3736	0.4631	-2.55	0.8708	0.9653	-0.33	0.0651	0.1248	2.06
1270	Dipeptidyl peptidase 1	P53634	0.6346	0.6964	-0.04	0.4277	0.7629	0.08	0.3363	0.5285	0.09	0.3399	0.5139	0.07	0.3434	0.4402	0.07	0.1153	0.2581	0.14	0.3736	0.5293	-2.55	0.8708	1.0869	-0.33	0.0651	0.1548	2.06
1271	Dipeptidyl peptidase 2	Q9UHL4	0.0347	0.1166	0.20	0.0043	0.3480	0.23	0.2546	0.4227	0.10	0.1027	0.1978	0.12	0.0681	0.1988	0.14	0.0820	0.2063	0.08	0.3736	0.5428	-2.55	0.8708	1.1113	-0.33	0.0651	0.1615	2.06
1272	Dipeptidyl peptidase 3	Q9NY33	0.0196	0.0822	0.14	0.1948	0.6696	0.22	0.0048	0.0337	0.24	0.0013	0.0167	0.31	0.0344	0.1357	0.15	0.0047	0.0603	0.29	0.1229	0.2951	0.16	0.5067	0.9245	-0.05	0.3878	0.4589	-0.06
1273	Diphosphomevalonate decarboxylase	P53602	0.4319	0.5094	0.06	0.8288	0.8769	-0.03	0.5629	0.7872	0.03	0.3077	0.4747	-0.06	0.3228	0.4200	0.07	0.4692	0.6918	0.06	0.1882	0.3920	0.08	0.2609	0.5886	0.05	0.1885	0.2561	0.05
1274	DIS3-like exonuclease 1	Q8TF46	0.0194	0.0818	0.20	0.5014	0.8230	-0.05	0.1791	0.3225	0.12	0.0626	0.1363	-0.14	0.4008	0.4972	0.04	0.2129	0.3943	-0.07	0.0037	0.0659	0.74	0.0098	0.0816	0.66	0.0166	0.1102	0.53
1275	DIS3-like exonuclease 2	Q8IYB7	0.4124	0.4904	-0.09	0.1693	0.6619	0.11	0.2082	0.3618	0.27	0.1012	0.1957	0.13	0.8299	0.8681	-0.02	0.3544	0.5599	-0.06	0.8065	0.8369	-0.03	0.1269	0.3714	0.28	0.1207	0.1793	0.31
1276	Discoidin_ CUB and LCCL domain-containing protein 2	Q96PD2	0.4108	0.5932	-3.31	0.5752	0.8304	-1.75	0.5661	0.7450	-1.19	0.5388	0.7043	0.89	0.2938	0.4873	-1.88	0.9510	1.1268	0.09	0.0850	0.2330	-0.14	0.0048	0.0555	-0.29	0.0168	0.1110	-0.28
1277	Disco-interacting protein 2 homolog B	Q9P265	0.2712	0.4639	-0.04	0.4992	0.8220	-0.07	0.9747	0.9789	0.00	0.0143	0.0486	-0.19	0.1446	0.3191	-0.19	0.8229	1.0575	-0.01	0.3736	0.4367	-2.55	0.8708	0.9157	-0.33	0.0651	0.1142	2.06
1278	Disheveled-associated activator of morphogenesis 1	Q9Y4D1	0.0820	0.1973	0.12	0.1293	0.6434	-0.51	0.0004	0.0201	-0.71	0.0014	0.0174	-0.78	0.0006	0.0202	-0.56	0.2268	0.4098	-0.37	0.3736	0.4960	-2.55	0.8708	1.0261	-0.33	0.0651	0.1391	2.06
1279	Disheveled-associated activator of morphogenesis 2	Q86T65	0.0022	0.0324	0.36	0.0422	0.5722	0.52	0.0014	0.0231	0.55	0.0319	0.0839	0.39	0.0328	0.1322	0.42	0.2035	0.3815	0.33	0.3736	0.5019	-2.55	0.8708	1.0370	-0.33	0.0651	0.1417	2.06
1280	Disintegrin and metalloproteinase domain-containing protein 10	O14672	0.4108	0.5931	-3.31	0.5752	0.8302	-1.75	0.5661	0.7448	-1.19	0.5388	0.7041	0.89	0.2938	0.4872	-1.88	0.9510	1.1265	0.09	0.2261	0.4512	-0.15	0.0011	0.0274	-0.40	0.0330	0.1553	-0.13
1281	Disintegrin and metalloproteinase domain-containing protein 17	P78536	0.0959	0.2185	-9.28	0.9593	0.9724	0.08	0.0318	0.0913	-7.16	0.0861	0.1736	1.74	0.5999	0.6755	-0.46	0.6169	0.8531	1.11	0.3736	0.4508	-2.55	0.8708	0.9423	-0.33	0.0651	0.1198	2.06
1282	Disintegrin and metalloproteinase domain-containing protein 22	Q9P0K1	0.5040	0.5771	0.05	0.2877	0.6683	-0.45	0.0110	0.0486	0.32	0.0018	0.0186	0.46	0.0003	0.0151	-0.76	0.1301	0.2807	0.11	0.0081	0.0910	0.55	0.0106	0.0847	0.61	0.0378	0.1676	0.55
1283	Disintegrin and metalloproteinase domain-containing protein 7	Q9H2U9	0.0334	0.1143	-1.19	0.7651	0.8300	0.17	0.0299	0.0877	-0.85	0.3019	0.4671	-0.24	0.0835	0.2239	0.47	0.0832	0.2086	-1.03	0.3736	0.5422	-2.55	0.8708	1.1102	-0.33	0.0651	0.1612	2.06
1284	Disintegrin and metalloproteinase domain-containing protein 9	Q13443	0.4108	0.5929	-3.31	0.5752	0.8300	-1.75	0.5661	0.7446	-1.19	0.5388	0.7039	0.89	0.2938	0.4870	-1.88	0.9510	1.1262	0.09	0.0008	0.0402	-0.65	0.0001	0.0152	-1.05	0.0004	0.0296	-1.00
1285	Disks large homolog 1	Q12959	0.1527	0.3038	0.12	0.1899	0.6696	0.19	0.8363	0.8681	0.02	0.3546	0.5323	0.06	0.5196	0.6077	0.04	0.5158	0.7432	-0.04	0.0186	0.1261	0.39	0.0407	0.1851	0.33	0.0268	0.1394	0.45
1286	Disks large homolog 2	Q15700	0.3103	0.5114	0.30	0.4835	0.8084	0.29	0.3939	0.5961	-0.23	0.2786	0.4365	0.31	0.1976	0.3973	0.39	0.8784	1.1155	0.04	0.3736	0.4349	-2.55	0.8708	0.9124	-0.33	0.0651	0.1135	2.06
1287	Disks large homolog 5	Q8TDM6	0.0149	0.0709	0.25	0.0072	0.4054	0.19	0.0007	0.0213	0.32	0.8751	0.8929	0.00	0.1312	0.2991	0.06	0.0180	0.0870	0.16	0.3736	0.5962	-2.55	0.8708	1.2061	-0.33	0.0651	0.1906	2.06

Supplemetary Table S2. Overview on all relatively quantified 5180 proteins statistical analysis

			MCF-7																		MDA-MB-231								
			Dai SC20 vs control			Gen SC20 vs control			SSE SC20 vs control			Dai IC20 vs control			Gen IC20 vs control			SSE IC20 vs control			Dai IC20 vs control			Gen IC20 vs control			SSE IC20 vs control		
	**Protein name**	**UniProt**	**p value**	**BH q value**	**log2FC**	**p value**	**BH q value**	**log2FC**	**p value**	**BH q value**	**log2FC**	**p value**	**BH q value**	**log2FC**	**p value**	**BH q value**	**log2FC**	**p value**	**BH q value**	**log2FC**	**p value**	**BH q value**	**log2FC**	**p value**	**BH q value**	**log2FC**	**p value**	**BH q value**	**log2FC**
1288	Disks large-associated protein 3	O95886	0.0009	0.0222	-0.46	0.0430	0.5697	-0.50	0.0570	0.1366	-0.19	0.0001	0.0070	-0.73	0.0027	0.0402	-0.29	0.0024	0.0563	0.38	0.3736	0.6425	-2.55	0.8708	1.2866	-0.33	0.0651	0.2197	2.06
1289	Disks large-associated protein 5	Q15398	0.4108	0.5927	-3.31	0.5752	0.8297	-1.75	0.5661	0.7445	-1.19	0.5388	0.7037	0.89	0.2938	0.4868	-1.88	0.9510	1.1260	0.09	0.0433	0.1679	-0.17	0.0078	0.0722	0.28	0.6015	0.6624	0.03
1290	Dixin	Q155Q3	0.0158	0.0731	0.23	0.4242	0.7609	0.04	0.1296	0.2503	-0.08	0.2384	0.3842	-0.08	0.0178	0.0954	-0.11	0.4099	0.6267	0.06	0.3736	0.4686	-2.55	0.8708	0.9755	-0.33	0.0651	0.1272	2.06
1291	DNA (cytosine-5)-methyltransferase 1	P26358	0.0005	0.0190	0.19	0.1257	0.6428	0.15	0.0005	0.0198	0.16	0.0008	0.0150	0.22	0.2168	0.4223	-0.02	0.0016	0.0505	0.33	0.0117	0.1045	0.56	0.0027	0.0433	0.58	0.0415	0.1766	0.30
1292	DNA (cytosine-5)-methyltransferase 3-like	Q9UJW3	0.4108	0.5926	-3.31	0.5752	0.8295	-1.75	0.5661	0.7443	-1.19	0.5388	0.7036	0.89	0.2938	0.4867	-1.88	0.9510	1.1257	0.09	0.5266	0.5794	0.78	0.6116	1.0442	0.80	0.1017	0.1576	2.17
1293	DNA damage-binding protein 1	Q16531	0.1104	0.2413	0.07	0.1705	0.6606	0.21	0.0211	0.0703	0.15	0.0034	0.0234	0.26	0.9987	0.9995	0.00	0.0514	0.1528	0.19	0.0141	0.1112	0.21	0.0035	0.0485	0.22	0.0495	0.1943	0.10
1294	DNA damage-induced apoptosis suppressor protein	Q8IXT1	0.0010	0.0224	-0.62	0.0506	0.5735	-0.20	0.0583	0.1388	-0.14	0.0079	0.0359	-0.30	0.0002	0.0138	-0.32	0.0018	0.0539	-0.61	0.3736	0.6457	-2.55	0.8708	1.2921	-0.33	0.0651	0.2219	2.06
1295	DNA dC->dU-editing enzyme APOBEC-3B	Q9UH17	0.4108	0.5924	-3.31	0.5752	0.8293	-1.75	0.5661	0.7441	-1.19	0.5388	0.7034	0.89	0.2938	0.4865	-1.88	0.9510	1.1255	0.09	0.1788	0.3783	0.24	0.1987	0.4956	0.22	0.2656	0.3373	0.18
1296	DNA dC->dU-editing enzyme APOBEC-3C	Q9NRW3	0.0005	0.0189	2.29	0.3418	0.6998	0.89	0.1472	0.2752	1.33	0.0047	0.0276	1.84	0.6212	0.6936	0.08	0.0876	0.2156	1.69	0.3299	0.5981	0.22	0.5479	0.9733	0.13	0.0636	0.2260	0.55
1297	DNA dC->dU-editing enzyme APOBEC-3G	Q9HC16	0.4108	0.5922	-3.31	0.5752	0.8290	-1.75	0.5661	0.7439	-1.19	0.5388	0.7032	0.89	0.2938	0.4864	-1.88	0.9510	1.1252	0.09	0.0051	0.0753	-0.48	0.0188	0.1175	0.26	0.0970	0.1521	-0.38
1298	DNA endonuclease RBBP8	Q99708	0.1049	0.2321	0.97	0.0316	0.5099	1.11	0.0091	0.0446	1.23	0.0332	0.0863	0.81	0.0205	0.1026	0.95	0.7894	1.0266	0.13	0.3736	0.4392	-2.55	0.8708	0.9206	-0.33	0.0651	0.1152	2.06
1299	DNA excision repair protein ERCC-6	Q03468	0.0528	0.1491	0.16	0.2901	0.6685	0.21	0.0282	0.0849	0.21	0.0117	0.0433	0.32	0.6206	0.6933	0.03	0.0222	0.0955	0.27	0.0147	0.1123	0.47	0.7643	1.2063	0.03	0.1608	0.2250	0.13
1300	DNA excision repair protein ERCC-6-like	Q2NKX8	0.4108	0.5921	-3.31	0.5752	0.8288	-1.75	0.5661	0.7437	-1.19	0.5388	0.7030	0.89	0.2938	0.4862	-1.88	0.9510	1.1250	0.09	0.2482	0.4842	0.21	0.0887	0.2953	-0.35	0.3821	0.4535	-0.13
1301	DNA fragmentation factor subunit alpha	O00273	0.0753	0.1867	0.09	0.6027	0.7063	0.07	0.2573	0.4257	0.09	0.3242	0.4955	0.04	0.0736	0.2075	-0.09	0.2178	0.3994	0.05	0.0657	0.2016	0.24	0.0388	0.1795	0.17	0.2227	0.2926	0.07
1302	DNA helicase MCM8	Q9UJA3	0.1236	0.2611	0.10	0.2346	0.6612	-0.10	0.7740	0.8139	-0.03	0.8816	0.8990	0.01	0.0242	0.1124	-0.19	0.2125	0.3938	0.07	0.0056	0.0797	0.64	0.0063	0.0644	0.50	0.0030	0.0545	-0.50
1303	DNA ligase 1	P18858	0.4108	0.5919	-3.31	0.5752	0.8286	-1.75	0.5661	0.7435	-1.19	0.5388	0.7028	0.89	0.2938	0.4861	-1.88	0.9510	1.1247	0.09	0.4307	0.4868	-0.11	0.1748	0.4587	-0.21	0.0702	0.1181	-0.35
1304	DNA methyltransferase 1-associated protein 1	Q9NPF5	0.3612	0.5739	0.08	0.1016	0.6310	0.29	0.0011	0.0220	0.53	0.0141	0.0484	0.14	0.0232	0.1105	0.12	0.0140	0.0787	0.17	0.0422	0.1676	0.40	0.4045	0.7904	-0.07	0.0614	0.2216	0.21
1305	DNA mismatch repair protein Msh2	P43246	0.0422	0.1303	0.21	0.0464	0.5629	0.24	0.0184	0.0651	0.34	0.0112	0.0427	0.24	0.0286	0.1229	0.18	0.0144	0.0800	0.33	0.1843	0.3854	0.20	0.4784	0.8901	0.06	0.7328	0.7798	0.03
1306	DNA mismatch repair protein Msh3	P20585	0.4108	0.5918	-3.31	0.5752	0.8283	-1.75	0.5661	0.7433	-1.19	0.5388	0.7027	0.89	0.2938	0.4859	-1.88	0.9510	1.1244	0.09	0.1872	0.3901	0.40	0.1784	0.4641	0.41	0.0776	0.1281	0.68
1307	DNA mismatch repair protein Msh6	P52701	0.0566	0.1558	0.08	0.2812	0.6648	0.18	0.0755	0.1652	0.15	0.0136	0.0472	0.14	0.1660	0.3520	0.05	0.0464	0.1431	0.10	0.1198	0.2896	0.20	0.5025	0.9188	0.06	0.5479	0.6125	-0.05
1308	DNA polymerase alpha catalytic subunit	P09884	0.9325	0.9486	-0.01	0.8427	0.8872	0.03	0.5333	0.7544	0.09	0.2988	0.4629	0.15	0.3850	0.4825	-0.12	0.7511	0.9892	0.04	0.3736	0.4416	-2.55	0.8708	0.9251	-0.33	0.0651	0.1161	2.06
1309	DNA polymerase beta	P06746	0.4108	0.5916	-3.31	0.5752	0.8281	-1.75	0.5661	0.7431	-1.19	0.5388	0.7025	0.89	0.2938	0.4858	-1.88	0.9510	1.1242	0.09	0.7859	0.8194	-0.02	0.0296	0.1552	-0.37	0.0077	0.0790	-0.48
1310	DNA polymerase delta catalytic subunit	P28340	0.4108	0.5914	-3.31	0.5752	0.8279	-1.75	0.5661	0.7429	-1.19	0.5388	0.7023	0.89	0.2938	0.4856	-1.88	0.9510	1.1239	0.09	0.0286	0.1454	-0.38	0.0181	0.1149	-0.36	0.0096	0.0869	-0.33
1311	DNA polymerase delta subunit 2	P49005	0.0825	0.1979	-0.10	0.8498	0.8922	0.02	0.2017	0.3535	0.09	0.2764	0.4343	-0.08	0.0630	0.1901	-0.17	0.0089	0.0689	-0.28	0.9308	0.9432	-0.01	0.0551	0.2235	0.42	0.3932	0.4646	0.16
1312	DNA polymerase delta subunit 3	Q15054	0.4108	0.5913	-3.31	0.5752	0.8276	-1.75	0.5661	0.7428	-1.19	0.5388	0.7021	0.89	0.2938	0.4854	-1.88	0.9510	1.1237	0.09	0.0889	0.2396	0.13	0.9855	0.9901	0.00	0.2830	0.3549	-0.16
1313	DNA polymerase epsilon catalytic subunit A	Q07864	0.0150	0.0710	0.15	0.0355	0.5393	0.17	0.0000	0.0000	0.39	0.0073	0.0348	0.33	0.0102	0.0725	0.21	0.0568	0.1635	0.35	0.3736	0.5574	-2.55	0.8708	1.1374	-0.33	0.0651	0.1690	2.06
1314	DNA polymerase epsilon subunit 3	Q9NRF9	0.0006	0.0202	0.56	0.0914	0.6288	0.32	0.1036	0.2100	0.40	0.0028	0.0216	0.42	0.0168	0.0928	0.22	0.0648	0.1776	0.81	0.7851	0.8188	0.05	0.9570	0.9695	-0.01	0.9718	0.9773	-0.01
1315	DNA polymerase epsilon subunit 4	Q9NR33	0.0249	0.0953	-0.57	0.2076	0.6585	-0.60	0.2264	0.3853	0.22	0.1701	0.2918	-0.26	0.7765	0.8246	-0.05	0.0638	0.1761	-0.44	0.3736	0.5529	-2.55	0.8708	1.1294	-0.33	0.0651	0.1667	2.06
1316	DNA polymerase kappa	Q9UBT6	0.2107	0.3836	-0.04	0.0010	0.2252	0.38	0.0743	0.1632	0.10	0.3821	0.5668	0.03	0.0006	0.0191	0.29	0.3791	0.5878	-0.05	0.0578	0.1913	0.44	0.5932	1.0256	0.08	0.1510	0.2141	0.21
1317	DNA polymerase zeta catalytic subunit	O60673	0.4108	0.5911	-3.31	0.5752	0.8274	-1.75	0.5661	0.7426	-1.19	0.5388	0.7020	0.89	0.2938	0.4853	-1.88	0.9510	1.1234	0.09	0.0355	0.1557	0.32	0.0434	0.1925	0.20	0.1367	0.1976	0.12
1318	DNA primase small subunit	P49642	0.1557	0.3084	-0.14	0.6181	0.7172	0.06	0.0474	0.1207	-0.23	0.9982	0.9982	0.00	0.0652	0.1944	-0.22	0.0353	0.1212	-0.28	0.9207	0.9350	-0.03	0.3883	0.7692	0.28	0.8743	0.8984	0.09
1319	DNA repair and recombination protein RAD54-like	Q92698	0.2621	0.4521	-0.12	0.3584	0.7083	-0.13	0.4885	0.7084	-0.09	0.0059	0.0307	-0.60	0.2111	0.4148	0.14	0.0166	0.0850	0.48	0.0063	0.0843	0.46	0.0102	0.0833	0.47	0.2013	0.2696	0.12
1320	DNA repair protein complementing XP-G cells	P28715	0.0608	0.1634	-0.18	0.0059	0.3869	-0.24	0.0002	0.0253	-0.36	0.0004	0.0131	-0.57	0.0003	0.0167	-0.41	0.0640	0.1765	-0.24	0.3736	0.5528	-2.55	0.8708	1.1291	-0.33	0.0651	0.1666	2.06
1321	DNA repair protein RAD50	Q92878	0.9632	0.9730	-0.01	0.5183	0.8393	-0.25	0.2058	0.3589	-0.21	0.2094	0.3451	-0.23	0.9842	0.9884	0.00	0.5088	0.7354	-0.09	0.0179	0.1236	0.55	0.0920	0.3014	0.23	0.1532	0.2162	0.18
1322	DNA repair protein RAD51 homolog 1	Q06609	0.0590	0.1607	0.60	0.4366	0.7727	-0.16	0.1786	0.3217	-0.21	0.4173	0.6094	0.31	0.0179	0.0955	-0.54	0.1067	0.2447	0.70	0.3736	0.5317	-2.55	0.8708	1.0911	-0.33	0.0651	0.1559	2.06
1323	DNA repair protein REV1	Q9UBZ9	0.0028	0.0357	0.24	0.0707	0.6004	0.31	0.0135	0.0545	0.33	0.0002	0.0090	0.63	0.0001	0.0123	0.54	0.0003	0.0444	0.62	0.0683	0.2058	0.26	0.0151	0.1037	0.29	0.0535	0.2041	-0.47
1324	DNA repair protein XRCC1	P18887	0.0004	0.0173	-0.28	0.5570	0.8714	-0.02	0.3860	0.5872	0.01	0.3586	0.5369	0.05	0.1100	0.2699	0.06	0.0052	0.0615	-0.11	0.3736	0.6291	-2.55	0.8708	1.2635	-0.33	0.0651	0.2109	2.06
1325	DNA repair protein XRCC2	O43543	0.4108	0.5909	-3.31	0.5752	0.8272	-1.75	0.5661	0.7424	-1.19	0.5388	0.7018	0.89	0.2938	0.4851	-1.88	0.9510	1.1232	0.09	0.0095	0.0974	0.48	0.0013	0.0298	0.53	0.0474	0.1908	0.15
1326	DNA repair protein XRCC4	Q13426	0.4108	0.5908	-3.31	0.5752	0.8270	-1.75	0.5661	0.7422	-1.19	0.5388	0.7016	0.89	0.2938	0.4850	-1.88	0.9510	1.1229	0.09	0.3874	0.4432	-0.07	0.0010	0.0262	-0.55	0.0002	0.0273	-0.79
1327	DNA replication ATP-dependent helicase/nuclease DNA2	P51530	0.0063	0.0478	0.15	0.0642	0.5928	0.56	0.7687	0.8095	0.01	0.0007	0.0132	0.44	0.0000	0.0000	0.61	0.0024	0.0568	0.24	0.0394	0.1641	-0.42	0.0000	0.0000	-0.78	0.0647	0.2283	-0.78
1328	DNA replication complex GINS protein PSF1	Q14691	0.0259	0.0974	0.37	0.0271	0.5031	0.36	0.0254	0.0793	0.41	0.0250	0.0706	0.39	0.0229	0.1096	0.41	0.0230	0.0972	0.35	0.0000	0.0000	0.83	0.0008	0.0267	0.29	0.0005	0.0308	0.68
1329	DNA replication complex GINS protein PSF2	Q9Y248	0.4217	0.4991	-0.09	0.0623	0.5900	-0.28	0.2819	0.4578	-0.13	0.0409	0.1001	-0.32	0.0121	0.0786	-0.53	0.4510	0.6734	-0.08	0.8953	0.9138	-0.01	0.1084	0.3352	-0.16	0.0546	0.2061	0.24
1330	DNA replication complex GINS protein PSF3	Q9BRX5	0.0684	0.1738	-0.25	0.2541	0.6624	-2.98	0.4526	0.6670	0.06	0.0646	0.1395	-0.13	0.0003	0.0141	-0.70	0.0045	0.0609	-0.50	0.0014	0.0500	0.45	0.0035	0.0486	0.33	0.0000	0.0000	-7.50
1331	DNA replication complex GINS protein SLD5	Q9BRT9	0.4108	0.5906	-3.31	0.5752	0.8267	-1.75	0.5661	0.7420	-1.19	0.5388	0.7014	0.89	0.2938	0.4848	-1.88	0.9510	1.1226	0.09	0.7485	0.7855	-0.03	0.4861	0.8964	-0.06	0.8464	0.8762	-0.02
1332	DNA replication licensing factor MCM2	P49736	0.0742	0.1848	0.07	0.2141	0.6625	0.12	0.0034	0.0312	0.19	0.0627	0.1364	0.07	0.0138	0.0841	-0.20	0.0375	0.1254	-0.10	0.0043	0.0705	0.30	0.0260	0.1447	0.26	0.0000	0.0000	0.27
1333	DNA replication licensing factor MCM3	P25205	0.1173	0.2519	-0.09	0.9574	0.9715	0.00	0.3455	0.5394	0.04	0.1340	0.2421	-0.09	0.0043	0.0486	-0.30	0.0731	0.1917	-0.15	0.0591	0.1928	0.16	0.9966	0.9974	0.00	0.1221	0.1810	-0.11
1334	DNA replication licensing factor MCM4	P33991	0.8318	0.8656	0.01	0.2551	0.6620	0.15	0.0005	0.0189	0.27	0.0005	0.0134	0.26	0.1717	0.3605	0.04	0.5745	0.8089	-0.01	0.0481	0.1756	0.20	0.0567	0.2277	0.14	0.4047	0.4762	-0.04
1335	DNA replication licensing factor MCM5	P33992	0.4254	0.5029	0.01	0.2151	0.6616	0.15	0.0053	0.0356	0.22	0.0060	0.0311	0.15	0.0000	0.0000	-0.17	0.0003	0.0457	0.12	0.0238	0.1374	0.13	0.0513	0.2138	0.10	0.1876	0.2551	0.05
1336	DNA replication licensing factor MCM6	Q14566	0.1117	0.2428	0.08	0.1179	0.6309	0.29	0.0024	0.0272	0.44	0.0006	0.0131	0.46	0.0102	0.0728	0.18	0.0027	0.0555	0.43	0.0318	0.1506	0.43	0.1026	0.3231	0.19	0.1404	0.2018	0.15
1337	DNA replication licensing factor MCM7	P33993	0.1308	0.2725	0.09	0.3858	0.7310	0.12	0.0054	0.0356	0.32	0.2850	0.4452	0.06	0.7890	0.8343	0.01	0.0073	0.0646	0.43	0.0497	0.1779	0.37	0.0740	0.2635	0.17	0.1985	0.2670	0.07
1338	DNA topoisomerase 1	P11387	0.4803	0.5550	0.03	0.4778	0.8033	0.11	0.0041	0.0319	0.23	0.0837	0.1696	0.07	0.0228	0.1093	-0.16	0.0251	0.1008	0.12	0.0216	0.1345	0.30	0.8540	1.2965	-0.01	0.4906	0.5585	-0.05
1339	DNA topoisomerase 2-alpha	P11388	0.4108	0.5904	-3.31	0.5752	0.8265	-1.75	0.5661	0.7418	-1.19	0.5388	0.7013	0.89	0.2938	0.4847	-1.88	0.9510	1.1224	0.09	0.0652	0.2010	0.13	0.0781	0.2741	0.11	0.0751	0.1250	-0.20
1340	DNA topoisomerase 2-beta	Q02880	0.4108	0.5903	-3.31	0.5752	0.8263	-1.75	0.5661	0.7416	-1.19	0.5388	0.7011	0.89	0.2938	0.4845	-1.88	0.9510	1.1221	0.09	0.6394	0.6847	0.08	0.0040	0.0508	-0.32	0.0139	0.1023	-0.33
1341	DNA topoisomerase 3-alpha	Q13472	0.3646	0.5777	0.12	0.8716	0.9072	0.02	0.1716	0.3122	-0.13	0.7293	0.7666	0.02	0.3758	0.4725	-0.07	0.4107	0.6277	0.07	0.3736	0.4685	-2.55	0.8708	0.9753	-0.33	0.0651	0.1271	2.06
1342	DNA topoisomerase I mitochondrial	Q969P6	0.1455	0.2932	0.08	0.5578	0.8711	0.09	0.0214	0.0707	0.18	0.0169	0.0543	0.19	0.2232	0.4312	-0.06	0.0180	0.0869	0.21	0.0382	0.1619	-1.02	0.3690	0.7406	0.22	0.1280	0.1875	0.37
1343	DNA-(apurinic or apyrimidinic site) lyase	P27695	0.0009	0.0221	0.28	0.1866	0.6666	0.32	0.0014	0.0227	0.41	0.0006	0.0134	0.27	0.0305	0.1274	0.06	0.0132	0.0768	0.32	0.0107	0.1025	0.72	0.0248	0.1406	0.33	0.0353	0.1612	0.35
1344	DNA-3-methyladenine glycosylase	P29372	0.0236	0.0924	-0.30	0.0024	0.2438	0.43	0.8175	0.8520	-0.01	0.0020	0.0194	0.24	0.0001	0.0106	0.52	0.4780	0.7026	-0.08	0.3736	0.4622	-2.55	0.8708	0.9636	-0.33	0.0651	0.1245	2.06
1345	DNA-binding protein SATB2	Q9UPW6	0.0041	0.0405	0.97	0.8986	0.9282	0.03	0.8496	0.8798	0.03	0.0012	0.0157	1.19	0.0003	0.0155	1.43	0.0006	0.0426	1.55	0.0112	0.1034	0.49	0.0358	0.1723	0.26	0.5817	0.6441	-0.06
1346	DNA-dependent protein kinase catalytic subunit	P78527	0.0030	0.0368	0.16	0.1623	0.6615	0.20	0.0014	0.0229	0.26	0.0004	0.0123	0.28	0.3178	0.4150	0.02	0.0026	0.0552	0.25	0.0172	0.1219	0.40	0.0322	0.1621	0.15	0.0336	0.1568	0.19
1347	DNA-directed RNA polymerase II subunit RPB2	P30876	0.0040	0.0401	0.59	0.2730	0.6652	0.11	0.0384	0.1034	0.36	0.0401	0.0987	0.38	0.0274	0.1198	0.23	0.1959	0.3710	0.48	0.3398	0.6103	0.08	0.8695	1.3116	-0.01	0.3109	0.3823	-0.08
1348	DNA-directed RNA polymerase III subunit RPC5	Q9NVU0	0.4108	0.5901	-3.31	0.5752	0.8260	-1.75	0.5661	0.7414	-1.19	0.5388	0.7009	0.89	0.2938	0.4844	-1.88	0.9510	1.1219	0.09	0.0484	0.1761	-0.20	0.0409	0.1857	-0.26	0.1511	0.2141	-0.15
1349	DNA-directed RNA polymerase III subunit RPC9	O75575	0.0007	0.0211	-0.40	0.3575	0.7084	-0.16	0.0508	0.1268	-0.16	0.0203	0.0611	-0.45	0.0069	0.0603	-0.34	0.0029	0.0554	-0.58	0.3736	0.6389	-2.55	0.8708	1.2804	-0.33	0.0651	0.2173	2.06
1350	DNA-directed RNA polymerases I and III subunit RPAC1	O15160	0.3511	0.5606	-0.21	0.6778	0.7641	-0.08	0.8096	0.8459	0.05	0.4988	0.7021	-0.14	0.3188	0.4159	0.23	0.4401	0.6617	-0.17	0.1250	0.2976	0.22	0.8327	1.2758	-0.02	0.1906	0.2583	0.06
1351	DNA-directed RNA polymerases I_ II_ and III subunit RPABC1	P19388	0.0568	0.1563	-12.68	0.0473	0.5685	-11.12	0.0192	0.0666	-10.57	0.0032	0.0230	-8.48	0.9432	0.9557	0.02	0.0300	0.1112	-9.29	0.0046	0.0724	7.48	0.0010	0.0277	8.55	0.0029	0.0552	9.31
1352	DNA-directed RNA polymerases I_ II_ and III subunit RPABC3	P52434	0.0039	0.0397	0.15	0.2929	0.6704	0.25	0.0012	0.0225	0.27	0.0029	0.0223	0.34	0.5286	0.6149	0.01	0.9914	0.9927	0.00	0.0202	0.1318	0.33	0.1799	0.4673	0.16	0.1216	0.1804	0.17
1353	DnaJ homolog subfamily A member 1	P31689	0.1343	0.2777	0.09	0.5484	0.8642	0.06	0.0818	0.1757	0.13	0.1562	0.2738	0.08	0.2337	0.4447	0.07	0.9114	1.1487	0.01	0.0392	0.1640	-0.13	0.0997	0.3164	-0.12	0.0060	0.0730	-0.20
1354	DnaJ homolog subfamily A member 2	O60884	0.9300	0.9472	0.00	0.6998	0.7787	0.02	0.0544	0.1327	-0.07	0.3756	0.5588	-0.03	0.0135	0.0832	-0.13	0.6702	0.9086	-0.02	0.2117	0.4297	0.19	0.2882	0.6273	-0.06	0.4900	0.5582	-0.04
1355	DnaJ homolog subfamily A member 3 mitochondrial	Q96EY1	0.4108	0.5899	-3.31	0.5752	0.8258	-1.75	0.5661	0.7413	-1.19	0.5388	0.7007	0.89	0.2938	0.4842	-1.88	0.9510	1.1216	0.09	0.0023	0.0547	-0.66	0.0106	0.0849	-0.49	0.0015	0.0474	-0.67
1356	DnaJ homolog subfamily A member 4	Q8WW22	0.2244	0.4030	-0.24	0.2187	0.6606	-2.34	0.8775	0.9029	-0.03	0.1795	0.3045	-0.29	0.8001	0.8439	0.05	0.1465	0.3026	-0.32	0.3736	0.5177	-2.55	0.8708	1.0659	-0.33	0.0651	0.1491	2.06
1357	DnaJ homolog subfamily B member 1	P25685	0.5034	0.5768	0.01	0.6936	0.7745	0.02	0.3505	0.5446	0.06	0.1210	0.2243	0.04	0.0463	0.1584	-0.15	0.1511	0.3089	0.07	0.8569	0.8811	0.32	0.5671	0.9951	1.04	0.3565	0.4283	1.81
1358	DnaJ homolog subfamily B member 11	Q9UBS4	0.0232	0.0911	0.20	0.0790	0.6126	0.28	0.0052	0.0353	0.36	0.0009	0.0152	0.45	0.0124	0.0795	0.27	0.0044	0.0606	0.45	0.0009	0.0436	0.32	0.1266	0.3716	0.15	0.0011	0.0410	0.19
1359	DnaJ homolog subfamily B member 12	Q9NXW2	0.2553	0.4432	-0.09	0.3518	0.7041	-0.11	0.8592	0.8878	-0.02	0.9455	0.9525	0.00	0.0101	0.0723	-0.20	0.7882	1.0256	0.01	0.3736	0.4394	-2.55	0.8708	0.9209	-0.33	0.0651	0.1152	2.06
1360	DnaJ homolog subfamily B member 3	Q8WWF6	0.4108	0.5898	-3.31	0.5752	0.8256	-1.75	0.5661	0.7411	-1.19	0.5388	0.7005	0.89	0.2938	0.4841	-1.88	0.9510	1.1214	0.09	0.0007	0.0403	-0.74	0.0374	0.1764	-1.96	0.0002	0.0230	-1.48
1361	DnaJ homolog subfamily B member 4	Q9UDY4	0.3552	0.5656	0.11	0.6516	0.7431	-0.22	0.2199	0.3767	0.19	0.6869	0.7269	0.04	0.3105	0.4079	0.10	0.4201	0.6393	-0.08	0.0184	0.1259	-0.84	0.0922	0.3019	-0.18	0.2970	0.3684	-0.63
1362	DnaJ homolog subfamily B member 6	O75190	0.4108	0.5896	-3.31	0.5752	0.8254	-1.75	0.5661	0.7409	-1.19	0.5388	0.7004	0.89	0.2938	0.4839	-1.88	0.9510	1.1211	0.09	0.0025	0.0561	0.55	0.0032	0.0470	0.39	0.0873	0.1401	0.23
1363	DnaJ homolog subfamily B member 9	Q9UBS3	0.4108	0.5895	-3.31	0.5752	0.8251	-1.75	0.5661	0.7407	-1.19	0.5388	0.7002	0.89	0.2938	0.4838	-1.88	0.9510	1.1209	0.09	0.1568	0.3480	0.20	0.4964	0.9105	0.08	0.3152	0.3865	0.23
1364	DnaJ homolog subfamily C member 10	Q8IXB1	0.2223	0.3998	-0.09	0.4282	0.7633	-0.06	0.1589	0.2929	0.09	0.3371	0.5112	0.07	0.3572	0.4548	0.05	0.2688	0.4618	-0.16	0.0221	0.1353	0.23	0.0692	0.2544	0.11	0.3670	0.4383	-0.04
1365	DnaJ homolog subfamily C member 13	O75165	0.1809	0.3446	0.12	0.4478	0.7826	0.11	0.1392	0.2637	0.15	0.3115	0.4799	0.08	0.6417	0.7107	-0.03	0.2538	0.4442	0.10	0.3736	0.4897	-2.55	0.8708	1.0146	-0.33	0.0651	0.1362	2.06
1366	DnaJ homolog subfamily C member 2	Q99543	0.0033	0.0379	0.26	0.1070	0.6306	0.24	0.0120	0.0513	0.35	0.0019	0.0192	0.34	0.0031	0.0417	0.29	0.0012	0.0489	0.47	0.0409	0.1663	0.27	0.1057	0.3292	0.19	0.9070	0.9261	0.01
1367	DnaJ homolog subfamily C member 21	Q5F1R6	0.9519	0.9646	0.00	0.3196	0.6824	0.17	0.2022	0.3541	0.12	0.0328	0.0856	0.17	0.5228	0.6098	0.03	0.5474	0.7809	0.04	0.0120	0.1038	0.46	0.4428	0.8442	-0.06	0.0085	0.0821	0.48

Supplemetary Table S2. Overview on all relatively quantified 5180 proteins statistical analysis

			MCF-7																		MDA-MB-231								
			Dai SC20 vs control			Gen SC20 vs control			SSE SC20 vs control			Dai IC20 vs control			Gen IC20 vs control			SSE IC20 vs control			Dai IC20 vs control			Gen IC20 vs control			SSE IC20 vs control		
	**Protein name**	**UniProt**	**p value**	**BH q value**	**log2FC**	**p value**	**BH q value**	**log2FC**	**p value**	**BH q value**	**log2FC**	**p value**	**BH q value**	**log2FC**	**p value**	**BH q value**	**log2FC**	**p value**	**BH q value**	**log2FC**	**p value**	**BH q value**	**log2FC**	**p value**	**BH q value**	**log2FC**	**p value**	**BH q value**	**log2FC**
1368	DnaJ homolog subfamily C member 3	Q13217	0.4108	0.5893	-3.31	0.5752	0.8249	-1.75	0.5661	0.7405	-1.19	0.5388	0.7000	0.89	0.2938	0.4836	-1.88	0.9510	1.1206	0.09	0.0964	0.2496	-0.53	0.7008	1.1434	-0.16	0.5091	0.5755	-0.33
1369	DnaJ homolog subfamily C member 7	Q99615	0.0389	0.1240	0.07	0.1046	0.6330	0.25	0.0015	0.0231	0.27	0.0002	0.0109	0.26	0.0107	0.0742	0.10	0.0129	0.0765	0.12	0.0399	0.1642	0.28	0.2911	0.6301	0.10	0.6600	0.7149	-0.04
1370	DnaJ homolog subfamily C member 8	O75937	0.0720	0.1805	0.15	0.1944	0.6687	0.18	0.1529	0.2839	0.10	0.2717	0.4283	0.07	0.3802	0.4773	0.05	0.5959	0.8322	-0.05	0.2342	0.4629	0.18	0.7734	1.2166	0.03	0.2068	0.2755	0.09
1371	DnaJ homolog subfamily C member 9	Q8WXX5	0.0005	0.0188	0.43	0.2432	0.6603	0.28	0.0070	0.0398	0.43	0.0001	0.0080	0.67	0.0023	0.0377	0.29	0.0004	0.0398	0.65	0.0121	0.1043	0.26	0.4464	0.8476	0.04	0.4908	0.5586	0.03
1372	DNL-type zinc finger protein	Q5SXM8	0.0850	0.2015	0.05	0.2960	0.6719	0.11	0.0721	0.1603	0.09	0.0010	0.0149	0.28	0.0001	0.0113	0.38	0.1567	0.3162	0.03	0.3736	0.5133	-2.55	0.8708	1.0579	-0.33	0.0651	0.1471	2.06
1373	Docking protein 5	Q9P104	0.4108	0.5891	-3.31	0.5752	0.8247	-1.75	0.5661	0.7403	-1.19	0.5388	0.6998	0.89	0.2938	0.4834	-1.88	0.9510	1.1204	0.09	0.0066	0.0838	0.28	0.0961	0.3094	0.10	0.0098	0.0875	0.27
1374	Dolichol-phosphate mannosyltransferase subunit 1	O60762	0.0023	0.0329	0.21	0.3978	0.7420	0.15	0.3657	0.5629	0.08	0.0029	0.0220	0.25	0.6090	0.6831	-0.01	0.0090	0.0684	0.34	0.0322	0.1512	-0.78	0.0051	0.0571	-0.59	0.0361	0.1625	-0.61
1375	Dolichol-phosphate mannosyltransferase subunit 3	Q9P2X0	0.6144	0.6789	0.05	0.1641	0.6636	0.23	0.0380	0.1027	0.30	0.0378	0.0943	0.29	0.9989	0.9995	0.00	0.0671	0.1818	0.28	0.0640	0.1994	0.34	0.3373	0.6953	0.13	0.1913	0.2591	0.20
1376	Dolichyl-diphosphooligosaccharide--protein glycosyltransferase 48 kDa subunit	P39656	0.0149	0.0708	0.20	0.3702	0.7185	-0.07	0.0262	0.0808	-0.17	0.1963	0.3270	-0.07	0.0765	0.2126	-0.11	0.1109	0.2513	0.13	0.0344	0.1544	-0.43	0.0867	0.2918	0.14	0.1049	0.1611	-0.16
1377	Dolichyl-diphosphooligosaccharide--protein glycosyltransferase subunit 1	P04843	0.0047	0.0433	0.22	0.2295	0.6612	0.26	0.0013	0.0227	0.34	0.0003	0.0113	0.42	0.0355	0.1365	0.12	0.0034	0.0587	0.40	0.2001	0.4120	0.19	0.1293	0.3769	-0.12	0.0424	0.1790	-0.21
1378	Dolichyl-diphosphooligosaccharide--protein glycosyltransferase subunit 2	P04844	0.0016	0.0274	0.14	0.0975	0.6337	0.11	0.0001	0.0192	0.27	0.0036	0.0243	0.25	0.0086	0.0665	0.11	0.0179	0.0872	0.34	0.0107	0.1023	0.30	0.0067	0.0670	0.27	0.0162	0.1093	0.20
1379	Dolichyl-diphosphooligosaccharide--protein glycosyltransferase subunit DAD1	P61803	0.7990	0.8371	0.02	0.0447	0.5675	-0.23	0.3867	0.5879	-0.07	0.0104	0.0408	-0.37	0.3968	0.4929	-0.08	0.3502	0.5551	0.08	0.0414	0.1673	-0.15	0.9720	0.9799	0.00	0.0213	0.1244	-0.19
1380	Dolichyl-diphosphooligosaccharide--protein glycosyltransferase subunit STT3A	P46977	0.0093	0.0572	0.21	0.2163	0.6602	0.21	0.0034	0.0311	0.28	0.0089	0.0382	0.31	0.2611	0.4823	0.05	0.0646	0.1772	0.32	0.1362	0.3152	0.22	0.3052	0.6509	0.07	0.3828	0.4539	-0.03
1381	Dolichyl-diphosphooligosaccharide--protein glycosyltransferase subunit STT3B	Q8TCJ2	0.4108	0.5890	-3.31	0.5752	0.8244	-1.75	0.5661	0.7401	-1.19	0.5388	0.6997	0.89	0.2938	0.4833	-1.88	0.9510	1.1201	0.09	0.0359	0.1567	0.25	0.5382	0.9640	0.04	0.2853	0.3571	-0.06
1382	Dolichyl-phosphate beta-glucosyltransferase	Q9Y673	0.1657	0.3232	-0.15	0.6466	0.7386	0.07	0.0455	0.1174	0.24	0.1315	0.2385	0.16	0.2430	0.4569	0.11	0.0660	0.1798	0.21	0.3736	0.5512	-2.55	0.8708	1.1263	-0.33	0.0651	0.1658	2.06
1383	Dol-P-Man:Man(5)GlcNAc(2)-PP-Dol alpha-1_3-mannosyltransferase	Q92685	0.4236	0.5010	0.11	0.2689	0.6630	0.13	0.0201	0.0682	0.94	0.1253	0.2298	0.41	0.0139	0.0839	-0.32	0.0013	0.0503	0.81	0.3736	0.6475	-2.55	0.8708	1.2951	-0.33	0.0651	0.2230	2.06
1384	Doublecortin domain-containing protein 1	M0R2J8	0.4108	0.5888	-3.31	0.5752	0.8242	-1.75	0.5661	0.7399	-1.19	0.5388	0.6995	0.89	0.2938	0.4831	-1.88	0.9510	1.1198	0.09	0.0239	0.1368	0.18	0.8879	0.9155	0.01	0.1148	0.1724	0.11
1385	Doublecortin domain-containing protein 2B	A2VCK2	0.4108	0.5886	-3.31	0.5752	0.8240	-1.75	0.5661	0.7398	-1.19	0.5388	0.6993	0.89	0.2938	0.4830	-1.88	0.9510	1.1196	0.09	0.1773	0.3766	3.02	0.2064	0.5072	2.69	0.1423	0.2037	3.51
1386	Doublecortin domain-containing protein 2C	A8MYV0	0.4108	0.5885	-3.31	0.5752	0.8238	-1.75	0.5661	0.7396	-1.19	0.5388	0.6991	0.89	0.2938	0.4828	-1.88	0.9510	1.1193	0.09	0.0028	0.0590	8.53	0.0028	0.0442	8.73	0.0037	0.0601	8.54
1387	Double-strand break repair protein MRE11	P49959	0.4108	0.5883	-3.31	0.5752	0.8235	-1.75	0.5661	0.7394	-1.19	0.5388	0.6990	0.89	0.2938	0.4827	-1.88	0.9510	1.1191	0.09	0.1656	0.3600	0.23	0.2305	0.5437	0.19	0.0751	0.1249	0.37
1388	Double-stranded RNA-binding protein Staufen homolog 1	O95793	0.2389	0.4237	-0.04	0.2574	0.6624	0.04	0.1236	0.2419	0.07	0.2354	0.3801	0.05	0.0679	0.1986	0.08	0.1882	0.3612	-0.05	0.2506	0.4867	0.10	0.4702	0.8809	-0.04	0.0216	0.1256	-0.13
1389	Double-stranded RNA-binding protein Staufen homolog 2	Q9NUL3	0.8040	0.8417	0.01	0.9324	0.9543	0.00	0.8896	0.9120	0.01	0.5046	0.7085	0.04	0.1667	0.3527	-0.09	0.3879	0.5991	-0.05	0.3523	0.6284	0.30	0.2654	0.5949	-0.36	0.8988	0.9194	0.07
1390	Double-stranded RNA-specific adenosine deaminase	P55265	0.9201	0.9391	-0.01	0.1460	0.6548	0.14	0.9677	0.9735	0.00	0.9734	0.9772	0.00	0.1777	0.3680	-0.13	0.1507	0.3083	-0.47	0.3736	0.5163	-2.55	0.8708	1.0634	-0.33	0.0651	0.1485	2.06
1391	Drebrin	Q16643	0.5328	0.6035	0.04	0.4582	0.7901	0.12	0.0990	0.2025	0.14	0.0155	0.0514	0.25	0.0444	0.1556	0.16	0.2097	0.3906	0.16	0.0399	0.1638	0.43	0.0426	0.1902	0.23	0.0006	0.0296	0.52
1392	Drebrin-like protein	Q9UJU6	0.0157	0.0729	0.17	0.1116	0.6304	0.21	0.0247	0.0780	0.18	0.0039	0.0253	0.27	0.0900	0.2362	0.10	0.0188	0.0885	0.23	0.0060	0.0824	0.53	0.0314	0.1601	0.23	0.0541	0.2050	0.19
1393	DTW domain-containing protein 2	Q8NBA8	0.0210	0.0858	-0.19	0.3520	0.7040	-0.15	0.3083	0.4924	-0.05	0.0009	0.0145	-0.44	0.0156	0.0891	-0.25	0.0408	0.1330	0.19	0.3736	0.5695	-2.55	0.8708	1.1590	-0.33	0.0651	0.1755	2.06
1394	Dual oxidase 2	Q9NRD8	0.4108	0.5882	-3.31	0.5752	0.8233	-1.75	0.5661	0.7392	-1.19	0.5388	0.6988	0.89	0.2938	0.4825	-1.88	0.9510	1.1188	0.09	0.3393	0.6098	-0.85	0.0468	0.2017	-0.42	0.2345	0.3044	-0.26
1395	Dual serine/threonine and tyrosine protein kinase	Q6XUX3	0.5273	0.5987	-0.06	0.2731	0.6651	0.16	0.0352	0.0977	0.20	0.0088	0.0379	0.27	0.2676	0.4893	0.08	0.2819	0.4783	0.20	0.3736	0.4849	-2.55	0.8708	1.0057	-0.33	0.0651	0.1341	2.06
1396	Dual specificity mitogen-activated protein kinase kinase 1	Q02750	0.1893	0.3553	-0.09	0.9087	0.9365	0.01	0.5735	0.6311	0.04	0.9857	0.9878	0.00	0.6187	0.6920	-0.03	0.9326	1.1686	0.00	0.3736	0.4323	-2.55	0.8708	0.9074	-0.33	0.0651	0.1124	2.06
1397	Dual specificity mitogen-activated protein kinase kinase 2	P36507	0.0036	0.0386	-0.38	0.6685	0.7561	-0.03	0.2428	0.4074	-0.10	0.0361	0.0913	-0.18	0.6439	0.7125	-0.03	0.0239	0.0987	-0.25	0.0033	0.0633	0.41	0.0004	0.0211	0.42	0.0025	0.0553	0.52
1398	Dual specificity mitogen-activated protein kinase kinase 3	P46734	0.0132	0.0670	-0.12	0.0412	0.5676	0.29	0.0739	0.1628	0.06	0.0370	0.0929	0.08	0.0179	0.0956	0.16	0.5974	0.8339	0.03	0.0069	0.0861	0.52	0.0105	0.0843	0.45	0.0280	0.1436	0.42
1399	Dual specificity mitogen-activated protein kinase kinase 4	P45985	0.1113	0.2424	0.05	0.9525	0.9686	0.01	0.4753	0.6933	0.02	0.7176	0.7555	0.01	0.0177	0.0949	-0.11	0.4172	0.6356	0.02	0.3736	0.4676	-2.55	0.8708	0.9736	-0.33	0.0651	0.1267	2.06
1400	Dual specificity protein kinase TTK	P33981	0.4108	0.5880	-3.31	0.5752	0.8231	-1.75	0.5661	0.7390	-1.19	0.5388	0.6986	0.89	0.2938	0.4824	-1.88	0.9510	1.1186	0.09	0.0354	0.1557	0.31	0.0704	0.2570	0.22	0.0634	0.2257	0.27
1401	Dual specificity protein phosphatase 3	P51452	0.0132	0.0669	3.16	0.0799	0.6123	-8.09	0.0078	0.0418	3.25	0.3266	0.4985	1.24	0.0072	0.0615	-8.22	0.0448	0.1400	2.16	0.0811	0.2271	0.19	0.0395	0.1808	0.12	0.0802	0.1311	-0.03
1402	Dynactin subunit 1	Q14203	0.0070	0.0498	0.21	0.1258	0.6426	0.33	0.0068	0.0398	0.30	0.0007	0.0136	0.39	0.0007	0.0215	0.24	0.0012	0.0493	0.28	0.0174	0.1225	0.39	0.1783	0.4641	0.13	0.0966	0.1516	0.16
1403	Dynactin subunit 2	Q13561	0.0181	0.0790	0.19	0.0118	0.4305	0.19	0.0179	0.0639	0.17	0.0029	0.0221	0.27	0.0246	0.1136	0.15	0.0121	0.0746	0.26	0.0437	0.1687	-0.40	0.0039	0.0504	-0.58	0.0102	0.0893	-0.45
1404	Dynactin subunit 4	Q9UJW0	0.0349	0.1168	-0.13	0.4495	0.7840	-0.08	0.0751	0.1646	-0.10	0.1514	0.2672	-0.07	0.0186	0.0973	-0.19	0.0733	0.1920	-0.10	0.0649	0.2006	0.33	0.2634	0.5922	0.10	0.8333	0.8652	-0.02
1405	Dynactin subunit 5	Q9BTE1	0.1198	0.2556	0.21	0.5321	0.8499	0.05	0.0141	0.0556	-0.89	0.0038	0.0250	-0.67	0.0162	0.0909	-0.27	0.0068	0.0638	-0.81	0.1440	0.3267	0.54	0.3141	0.6627	0.33	0.5509	0.6155	0.17
1406	Dynamin-1	Q05193	0.0098	0.0582	0.32	0.6089	0.7110	-0.08	0.1684	0.3075	-0.09	0.1958	0.3264	-0.07	0.0560	0.1769	-0.15	0.5003	0.7259	0.06	0.2351	0.4643	-1.53	0.3596	0.7285	-1.09	0.5557	0.6205	-0.65
1407	Dynamin-1-like protein	O00429	0.0053	0.0445	0.29	0.4076	0.7500	0.13	0.0075	0.0409	0.32	0.0047	0.0278	0.31	0.2090	0.4123	0.07	0.0051	0.0613	0.43	0.1624	0.3560	0.17	0.0904	0.2992	-0.10	0.3482	0.4195	0.04
1408	Dynamin-2	P50570	0.3096	0.5106	0.04	0.1838	0.6644	0.21	0.0213	0.0707	0.15	0.0423	0.1024	0.11	0.4197	0.5142	-0.03	0.0555	0.1606	0.12	0.0611	0.1949	0.31	0.8249	1.2702	0.01	0.0117	0.0930	0.14
1409	Dynamin-3	Q9UQ16	0.0071	0.0497	9.51	0.5752	0.8862	-1.75	0.5661	0.7900	-1.19	0.5388	0.7461	0.89	0.2938	0.5244	-1.88	0.9510	1.1862	0.09	0.3736	0.4311	-2.55	0.8708	0.9052	-0.33	0.0651	0.1120	2.06
1410	Dynamin-like 120 kDa protein mitochondrial	O60313	0.0086	0.0549	0.16	0.1899	0.6705	0.24	0.0015	0.0227	0.29	0.0009	0.0154	0.31	0.0893	0.2348	0.07	0.0019	0.0515	0.32	0.0544	0.1854	0.41	0.1685	0.4481	0.10	0.2699	0.3417	0.14
1411	Dynein assembly factor 1 axonemal	Q8NEP3	0.1349	0.2785	-0.27	0.2258	0.6590	-0.77	0.4357	0.6465	-0.14	0.0534	0.1208	-0.28	0.0374	0.1407	-0.36	0.3411	0.5453	0.09	0.7806	0.8144	0.10	0.0947	0.3068	-0.54	0.9199	0.9345	-0.01
1412	Dynein assembly factor 5 axonemal	Q86Y56	0.0030	0.0367	0.44	0.1786	0.6646	0.36	0.0055	0.0360	0.39	0.0153	0.0511	0.31	0.1490	0.3259	-0.11	0.3009	0.4988	0.22	0.0035	0.0652	-0.50	0.0394	0.1808	-0.27	0.0106	0.0906	-0.47
1413	Dynein heavy chain 1 axonemal	Q9P2D7	0.4108	0.5878	-3.31	0.5752	0.8228	-1.75	0.5661	0.7388	-1.19	0.5388	0.6984	0.89	0.2938	0.4822	-1.88	0.9510	1.1183	0.09	0.3579	0.6353	-0.10	0.5425	0.9680	-0.06	0.1662	0.2314	-0.22
1414	Dynein heavy chain 10 axonemal	Q8IVF4	0.1101	0.2408	0.12	0.2787	0.6647	0.13	0.0061	0.0378	0.28	0.1081	0.2058	0.22	0.3329	0.4299	0.05	0.8864	1.1235	-0.01	0.0823	0.2293	0.36	0.4774	0.8889	-0.05	0.6136	0.6728	-0.03
1415	Dynein heavy chain 12 axonemal	Q6ZR08	0.0824	0.1978	0.08	0.1417	0.6536	-0.09	0.0119	0.0511	-0.12	0.1409	0.2525	-0.10	0.0025	0.0384	-0.25	0.2753	0.4702	-0.09	0.3736	0.4864	-2.55	0.8708	1.0084	-0.33	0.0651	0.1348	2.06
1416	Dynein heavy chain 14 axonemal	Q0VDD8	0.0433	0.1321	0.19	0.1472	0.6579	0.10	0.0025	0.0277	0.39	0.0190	0.0584	0.23	0.0347	0.1358	0.16	0.3237	0.5261	0.10	0.0119	0.1036	0.33	0.0056	0.0606	0.28	0.0151	0.1057	0.30
1417	Dynein heavy chain 17 axonemal	Q9UFH2	0.0180	0.0788	-0.50	0.2573	0.6631	-0.35	0.0186	0.0654	-0.50	0.0183	0.0572	-0.49	0.5873	0.6648	-0.07	0.0293	0.1097	-0.46	0.7115	0.7523	0.02	0.3065	0.6528	0.11	0.1562	0.2197	0.08
1418	Dynein heavy chain 2 axonemal	Q9P225	0.0089	0.0561	0.21	0.2745	0.6644	0.13	0.0339	0.0954	0.15	0.0055	0.0297	0.25	0.0948	0.2436	0.09	0.0057	0.0615	0.30	0.3736	0.6269	-2.55	0.8708	1.2596	-0.33	0.0651	0.2095	2.06
1419	Dynein heavy chain 3 axonemal	Q8TD57	0.4108	0.5877	-3.31	0.5752	0.8226	-1.75	0.5661	0.7386	-1.19	0.5388	0.6983	0.89	0.2938	0.4821	-1.88	0.9510	1.1181	0.09	0.0001	0.0199	0.56	0.0012	0.0297	0.28	0.0018	0.0496	0.32
1420	Dynein heavy chain 5 axonemal	Q8TE73	0.1883	0.3543	0.12	0.0637	0.5924	0.12	0.0762	0.1664	-0.18	0.1122	0.2122	-0.15	0.0184	0.0967	0.26	0.0322	0.1158	-0.19	0.8073	0.8372	0.03	0.0187	0.1170	-0.38	0.0120	0.0943	-0.56
1421	Dynein heavy chain 6 axonemal	Q9C0G6	0.5448	0.6144	-0.03	0.6063	0.7086	0.05	0.0713	0.1592	0.10	0.0064	0.0320	0.19	0.0111	0.0754	0.18	0.0035	0.0594	0.33	0.3736	0.6366	-2.55	0.8708	1.2764	-0.33	0.0651	0.2158	2.06
1422	Dynein heavy chain 7 axonemal	Q8WXX0	0.1814	0.3450	-1.46	0.2304	0.6616	-0.71	0.0183	0.0648	-1.00	0.0142	0.0485	-1.29	0.0313	0.1296	-0.75	0.3163	0.5180	-2.10	0.3736	0.4791	-2.55	0.8708	0.9951	-0.33	0.0651	0.1316	2.06
1423	Dynein heavy chain 8 axonemal	Q96JB1	0.0004	0.0171	-0.33	0.0001	0.2590	-0.42	0.3730	0.5711	-0.03	0.0000	0.0000	-0.87	0.2556	0.4756	-0.05	0.0014	0.0511	0.45	0.0531	0.1825	0.26	0.0109	0.0855	0.31	0.2534	0.3247	0.09
1424	Dynein heavy chain 9 axonemal	Q9NYC9	0.4108	0.5875	-3.31	0.5752	0.8224	-1.75	0.5661	0.7385	-1.19	0.5388	0.6981	0.89	0.2938	0.4819	-1.88	0.9510	1.1178	0.09	0.0645	0.1999	0.60	0.9304	0.9495	-0.01	0.0423	0.1789	0.33
1425	Dynein heavy chain domain-containing protein 1	Q96M86	0.3639	0.5768	0.10	0.1985	0.6647	0.17	0.5560	0.7799	-0.06	0.7297	0.7669	0.04	0.5224	0.6095	-0.06	0.6305	0.8670	-0.05	0.3736	0.4494	-2.55	0.8708	0.9397	-0.33	0.0651	0.1192	2.06
1426	Dynein light chain 1 cytoplasmic	P63167	0.0052	0.0442	0.18	0.0408	0.5681	0.40	0.0013	0.0236	0.22	0.0012	0.0164	0.35	0.0128	0.0808	0.14	0.0028	0.0556	0.26	0.0005	0.0405	0.63	0.0060	0.0624	0.23	0.0084	0.0824	0.63
1427	Dynein light chain 2 cytoplasmic	Q96FJ2	0.0555	0.1541	0.07	0.0038	0.3281	-0.33	0.0005	0.0221	-0.28	0.0004	0.0130	-0.34	0.0564	0.1774	-0.25	0.0033	0.0574	0.14	0.3736	0.6368	-2.55	0.8708	1.2767	-0.33	0.0651	0.2159	2.06
1428	Dynein light chain roadblock-type 1	Q9NP97	0.4108	0.5873	-3.31	0.5752	0.8222	-1.75	0.5661	0.7383	-1.19	0.5388	0.6979	0.89	0.2938	0.4818	-1.88	0.9510	1.1176	0.09	0.0116	0.1040	-0.44	0.0062	0.0635	-0.31	0.0023	0.0534	-0.63
1429	Dynein light chain roadblock-type 2	Q8TF09	0.0057	0.0456	0.37	0.0368	0.5509	0.13	0.0320	0.0917	0.14	0.0362	0.0915	0.15	0.0121	0.0787	0.19	0.0080	0.0671	0.29	0.3736	0.6193	-2.55	0.8708	1.2464	-0.33	0.0651	0.2046	2.06
1430	Dynein light chain Tctex-type 1	P63172	0.0610	0.1635	-0.56	0.7611	0.8272	0.05	0.1910	0.3380	-0.16	0.0360	0.0912	0.41	0.0213	0.1052	-0.31	0.7994	1.0365	0.04	0.5700	0.6199	-0.13	0.2929	0.6332	0.28	0.8027	0.8388	0.06
1431	Dynein light chain Tctex-type 3	P51808	0.4108	0.5872	-3.31	0.5752	0.8219	-1.75	0.5661	0.7381	-1.19	0.5388	0.6977	0.89	0.2938	0.4816	-1.88	0.9510	1.1173	0.09	0.0220	0.1349	-0.24	0.0735	0.2633	-0.15	0.4784	0.5467	-0.13
1432	Dynein regulatory complex protein 8	Q5VUJ9	0.3025	0.5021	0.06	0.7577	0.8244	-0.02	0.0016	0.0228	-0.41	0.0006	0.0132	-0.55	0.0149	0.0872	-0.46	0.2823	0.4785	-0.06	0.3736	0.4848	-2.55	0.8708	1.0055	-0.33	0.0651	0.1341	2.06
1433	Dynein regulatory complex protein 9	Q9H095	0.0185	0.0801	-0.25	0.5780	0.6869	0.09	0.2215	0.3787	-0.24	0.0382	0.0952	-0.17	0.2171	0.4226	-0.08	0.0098	0.0704	-0.40	0.3736	0.6146	-2.55	0.8708	1.2382	-0.33	0.0651	0.2017	2.06
1434	Dystonin	Q03001	0.4108	0.5870	-3.31	0.5752	0.8217	-1.75	0.5661	0.7379	-1.19	0.5388	0.6976	0.89	0.2938	0.4815	-1.88	0.9510	1.1170	0.09	0.0217	0.1345	0.52	0.0564	0.2270	0.39	0.0302	0.1473	0.54
1435	Dystrobrevin alpha	Q9Y4J8	0.0613	0.1638	-1.52	0.1879	0.6676	-1.32	0.1971	0.3469	-0.54	0.1132	0.2134	-1.49	0.0949	0.2435	-0.81	0.0143	0.0797	-1.77	0.3736	0.6042	-2.55	0.8708	1.2201	-0.33	0.0651	0.1954	2.06
1436	Dystrobrevin beta	O60941	0.4108	0.5869	-3.31	0.5752	0.8215	-1.75	0.5661	0.7377	-1.19	0.5388	0.6974	0.89	0.2938	0.4813	-1.88	0.9510	1.1168	0.09	0.8354	0.8613	0.02	0.0256	0.1435	-0.10	0.0143	0.1029	0.44
1437	Dystroglycan	Q14118	0.5868	0.6535	-0.04	0.2420	0.6598	-0.22	0.0145	0.0566	0.47	0.0676	0.1440	-0.21	0.0936	0.2418	-0.19	0.4616	0.6841	0.06	0.0013	0.0471	0.38	0.0024	0.0413	0.41	0.0206	0.1229	0.30
1438	Dystrophin	P11532	0.9445	0.9582	0.00	0.3865	0.7294	0.11	0.0081	0.0423	0.17	0.0046	0.0276	0.22	0.8303	0.8684	0.01	0.0037	0.0588	0.22	0.0327	0.1516	-0.29	0.1872	0.4770	0.13	0.1757	0.2416	-0.15

Supplemetary Table S2. Overview on all relatively quantified 5180 proteins statistical analysis

			MCF-7																		MDA-MB-231								
			Dai SC20 vs control			Gen SC20 vs control			SSE SC20 vs control			Dai IC20 vs control			Gen IC20 vs control			SSE IC20 vs control			Dai IC20 vs control			Gen IC20 vs control			SSE IC20 vs control		
	**Protein name**	**UniProt**	**p value**	**BH q value**	**log2FC**	**p value**	**BH q value**	**log2FC**	**p value**	**BH q value**	**log2FC**	**p value**	**BH q value**	**log2FC**	**p value**	**BH q value**	**log2FC**	**p value**	**BH q value**	**log2FC**	**p value**	**BH q value**	**log2FC**	**p value**	**BH q value**	**log2FC**	**p value**	**BH q value**	**log2FC**
1439	E1A-binding protein p400	Q96L91	0.3134	0.5149	-0.11	0.2900	0.6685	0.17	0.0329	0.0935	0.28	0.2982	0.4622	0.13	0.1019	0.2559	0.19	0.5410	0.7729	0.09	0.3736	0.4575	-2.55	0.8708	0.9549	-0.33	0.0651	0.1225	2.06
1440	E2F-associated phosphoprotein	Q56P03	0.0164	0.0748	0.34	0.1312	0.6460	0.34	0.0267	0.0818	0.30	0.0161	0.0527	0.31	0.7182	0.7786	0.02	0.0463	0.1429	0.23	0.3736	0.5642	-2.55	0.8708	1.1495	-0.33	0.0651	0.1727	2.06
1441	E3 SUMO-protein ligase PIAS3	Q9Y6X2	0.0045	0.0425	0.39	0.0217	0.4783	0.34	0.8821	0.9071	-0.01	0.0045	0.0271	0.45	0.0261	0.1168	0.24	0.0251	0.1007	0.28	0.3736	0.5859	-2.55	0.8708	1.1880	-0.33	0.0651	0.1847	2.06
1442	E3 SUMO-protein ligase RanBP2	P49792	0.2505	0.4368	0.03	0.3178	0.6808	0.13	0.0046	0.0333	0.27	0.0084	0.0371	0.17	0.1356	0.3054	0.05	0.0056	0.0616	0.23	0.0508	0.1793	0.37	0.9861	0.9899	0.00	0.2923	0.3641	0.04
1443	E3 ubiquitin/ISG15 ligase TRIM25	Q14258	0.0189	0.0810	0.20	0.3924	0.7362	0.12	0.0393	0.1053	0.14	0.0103	0.0408	0.24	0.5753	0.6548	-0.02	0.0130	0.0764	0.23	0.0992	0.2544	0.24	0.2217	0.5292	-0.13	0.6698	0.7234	-0.04
1444	E3 ubiquitin-protein ligase ARIH1	Q9Y4X5	0.5926	0.6592	-0.07	0.0296	0.5077	-0.37	0.0742	0.1631	-0.28	0.5726	0.6222	-0.06	0.2799	0.5061	-0.13	0.3217	0.5249	0.21	0.3736	0.4783	-2.55	0.8708	0.9936	-0.33	0.0651	0.1313	2.06
1445	E3 ubiquitin-protein ligase ARIH2	O95376	0.0242	0.0935	-0.21	0.1279	0.6414	-0.26	0.0013	0.0232	-0.50	0.1003	0.1946	-0.24	0.5564	0.6398	-0.13	0.1126	0.2539	-0.37	0.3736	0.5305	-2.55	0.8708	1.0890	-0.33	0.0651	0.1553	2.06
1446	E3 ubiquitin-protein ligase BRE1A	Q5VTR2	0.0025	0.0339	0.21	0.2161	0.6616	0.19	0.0002	0.0170	0.51	0.0020	0.0200	0.29	0.0008	0.0220	0.28	0.0009	0.0476	0.59	0.1610	0.3543	0.22	0.2288	0.5419	0.12	0.3348	0.4059	-0.08
1447	E3 ubiquitin-protein ligase BRE1B	O75150	0.0304	0.1075	0.16	0.0561	0.5847	0.20	0.0108	0.0486	0.28	0.0051	0.0288	0.36	0.0004	0.0170	0.59	0.0533	0.1562	0.14	0.1011	0.2573	0.35	0.4792	0.8900	-0.08	0.5360	0.6019	-0.09
1448	E3 ubiquitin-protein ligase CBL	P22681	0.4108	0.5867	-3.31	0.5752	0.8213	-1.75	0.5661	0.7375	-1.19	0.5388	0.6972	0.89	0.2938	0.4812	-1.88	0.9510	1.1165	0.09	0.0446	0.1697	0.31	0.0032	0.0467	0.82	0.9119	0.9295	-0.01
1449	E3 ubiquitin-protein ligase CHIP	Q9UNE7	0.1042	0.2310	0.06	0.4397	0.7758	-0.03	0.0038	0.0314	0.18	0.0521	0.1185	0.15	0.0174	0.0948	0.12	0.0373	0.1249	0.24	0.0586	0.1925	3.13	0.3116	0.6604	1.85	0.0412	0.1759	3.99
1450	E3 ubiquitin-protein ligase DCST1	Q5T197	0.9551	0.9665	0.00	0.4714	0.8003	0.13	0.0918	0.1910	0.11	0.0945	0.1860	0.11	0.3313	0.4285	-0.05	0.9921	0.9931	0.00	0.3736	0.4295	-2.55	0.8708	0.9021	-0.33	0.0651	0.1114	2.06
1451	E3 ubiquitin-protein ligase HECTD1	Q9ULT8	0.3624	0.5753	-0.05	0.1991	0.6645	-0.21	0.0039	0.0318	-0.32	0.0076	0.0353	-0.26	0.0097	0.0707	-0.28	0.0276	0.1066	-0.23	0.3736	0.5831	-2.55	0.8708	1.1830	-0.33	0.0651	0.1831	2.06
1452	E3 ubiquitin-protein ligase HECTD3	Q5T447	0.2494	0.4359	0.40	0.5570	0.8712	0.85	0.0053	0.0355	1.88	0.8282	0.8515	0.29	0.1263	0.2932	-0.56	0.5596	0.7950	-0.43	0.3736	0.4561	-2.55	0.8708	0.9522	-0.33	0.0651	0.1220	2.06
1453	E3 ubiquitin-protein ligase HERC2	O95714	0.0001	0.0133	0.49	0.6372	0.7328	-0.06	0.0065	0.0388	0.22	0.0001	0.0081	0.62	0.0001	0.0126	0.53	0.0396	0.1300	0.43	0.0166	0.1201	0.27	0.0725	0.2610	0.13	0.0144	0.1032	0.25
1454	E3 ubiquitin-protein ligase HUWE1	Q7Z6Z7	0.0554	0.1540	0.14	0.1543	0.6606	0.25	0.0082	0.0427	0.27	0.0051	0.0288	0.29	0.0435	0.1540	0.16	0.0159	0.0839	0.26	0.0332	0.1523	0.32	0.0633	0.2416	0.20	0.0103	0.0895	0.45
1455	E3 ubiquitin-protein ligase Itchy homolog	Q96J02	0.4108	0.5865	-3.31	0.5752	0.8210	-1.75	0.5661	0.7373	-1.19	0.5388	0.6970	0.89	0.2938	0.4810	-1.88	0.9510	1.1163	0.09	0.0231	0.1361	-0.25	0.0141	0.0992	-0.30	0.0467	0.1891	-0.23
1456	E3 ubiquitin-protein ligase LRSAM1	Q6UWE0	0.0784	0.1920	0.18	0.3093	0.6783	0.08	0.0427	0.1121	0.22	0.0115	0.0430	0.35	0.0706	0.2025	0.19	0.0750	0.1948	0.19	0.0075	0.0891	0.48	0.2631	0.5918	0.11	0.0849	0.1370	0.22
1457	E3 ubiquitin-protein ligase MARCH11	A6NNE9	0.0033	0.0378	0.19	0.1327	0.6479	0.19	0.1299	0.2507	0.14	0.0074	0.0349	0.18	0.0004	0.0166	0.29	0.0626	0.1740	0.17	0.3736	0.5542	-2.55	0.8708	1.1316	-0.33	0.0651	0.1674	2.06
1458	E3 ubiquitin-protein ligase MARCH5	Q9NX47	0.6315	0.6938	0.02	0.1307	0.6448	0.19	0.4281	0.6376	0.04	0.0428	0.1030	0.10	0.0371	0.1401	0.12	0.0767	0.1975	0.08	0.3736	0.5456	-2.55	0.8708	1.1162	-0.33	0.0651	0.1629	2.06
1459	E3 ubiquitin-protein ligase MARCH7	Q9H992	0.0905	0.2108	0.14	0.0941	0.6306	0.30	0.0019	0.0247	0.33	0.0021	0.0202	0.29	0.0041	0.0476	0.33	0.0699	0.1859	0.11	0.3736	0.5493	-2.55	0.8708	1.1229	-0.33	0.0651	0.1648	2.06
1460	E3 ubiquitin-protein ligase Mdm2	Q00987	0.2227	0.4003	0.07	0.5491	0.8648	0.08	0.1058	0.2133	0.25	0.9577	0.9635	0.00	0.0991	0.2516	-0.12	0.3553	0.5609	0.06	0.3736	0.4737	-2.55	0.8708	0.9851	-0.33	0.0651	0.1294	2.06
1461	E3 ubiquitin-protein ligase MGRN1	O60291	0.4108	0.5864	-3.31	0.5752	0.8208	-1.75	0.5661	0.7372	-1.19	0.5388	0.6969	0.89	0.2938	0.4808	-1.88	0.9510	1.1160	0.09	0.0025	0.0558	-1.61	0.0012	0.0296	0.95	0.0758	0.1259	-0.39
1462	E3 ubiquitin-protein ligase MIB1	Q86YT6	0.1772	0.3395	0.19	0.8240	0.8738	0.03	0.8192	0.8536	0.02	0.8191	0.8439	-0.03	0.7817	0.8289	-0.03	0.6447	0.8814	-0.05	0.3736	0.4479	-2.55	0.8708	0.9368	-0.33	0.0651	0.1186	2.06
1463	E3 ubiquitin-protein ligase NEDD4	P46934	0.0152	0.0717	-0.51	0.0010	0.2355	-0.27	0.0031	0.0307	-0.31	0.0170	0.0545	-0.64	0.0240	0.1121	-0.53	0.0722	0.1898	-0.13	0.3736	0.5482	-2.55	0.8708	1.1210	-0.33	0.0651	0.1643	2.06
1464	E3 ubiquitin-protein ligase NEDD4-like	Q96PU5	0.4108	0.5862	-3.31	0.5752	0.8206	-1.75	0.5661	0.7370	-1.19	0.5388	0.6967	0.89	0.2938	0.4807	-1.88	0.9510	1.1158	0.09	0.0358	0.1564	0.28	0.1701	0.4512	0.12	0.6201	0.6782	0.02
1465	E3 ubiquitin-protein ligase PDZRN3	Q9UPQ7	0.4108	0.5860	-3.31	0.5752	0.8204	-1.75	0.5661	0.7368	-1.19	0.5388	0.6965	0.89	0.2938	0.4805	-1.88	0.9510	1.1155	0.09	0.7466	0.7841	0.06	0.7399	1.1822	-0.06	0.0138	0.1017	0.73
1466	E3 ubiquitin-protein ligase pellino homolog 3	Q8N2H9	0.4108	0.5859	-3.31	0.5752	0.8201	-1.75	0.5661	0.7366	-1.19	0.5388	0.6964	0.89	0.2938	0.4804	-1.88	0.9510	1.1153	0.09	0.4372	0.4930	0.11	0.0748	0.2656	0.26	0.7030	0.7544	0.05
1467	E3 ubiquitin-protein ligase PPP1R11	O60927	0.0072	0.0502	0.49	0.4342	0.7708	-0.42	0.0086	0.0437	0.47	0.1169	0.2184	0.44	0.2189	0.4245	0.12	0.3279	0.5305	0.38	0.3736	0.4769	-2.55	0.8708	0.9909	-0.33	0.0651	0.1307	2.06
1468	E3 ubiquitin-protein ligase RAD18	Q9NS91	0.4108	0.5857	-3.31	0.5752	0.8199	-1.75	0.5661	0.7364	-1.19	0.5388	0.6962	0.89	0.2938	0.4802	-1.88	0.9510	1.1150	0.09	0.0128	0.1073	0.38	0.0057	0.0609	0.22	0.0013	0.0449	0.44
1469	E3 ubiquitin-protein ligase RBBP6	Q7Z6E9	0.0175	0.0775	0.12	0.4546	0.7876	0.18	0.0191	0.0663	0.35	0.0021	0.0199	0.34	0.8443	0.8800	0.01	0.0030	0.0547	0.27	0.3736	0.6381	-2.55	0.8708	1.2789	-0.33	0.0651	0.2167	2.06
1470	E3 ubiquitin-protein ligase RBX1	P62877	0.0144	0.0700	-0.97	0.6375	0.7330	-0.06	0.1173	0.2323	-0.25	0.5405	0.5920	-0.09	0.2040	0.4053	-0.17	0.0347	0.1200	-0.45	0.3736	0.5749	-2.55	0.8708	1.1686	-0.33	0.0651	0.1785	2.06
1471	E3 ubiquitin-protein ligase RNF113A	O15541	0.3975	0.6163	-0.08	0.4895	0.8124	0.07	0.4470	0.6602	-0.12	0.4949	0.6981	0.06	0.1125	0.2736	-0.18	0.3423	0.5466	-0.09	0.0073	0.0875	-0.47	0.0137	0.0975	-0.75	0.1730	0.2390	-1.29
1472	E3 ubiquitin-protein ligase RNF114	Q9Y508	0.0389	0.1239	0.16	0.2767	0.6651	0.19	0.0026	0.0278	0.36	0.0038	0.0251	0.32	0.4719	0.5627	0.04	0.0242	0.0993	0.24	0.0117	0.1041	0.40	0.1469	0.4109	0.11	0.1474	0.2098	0.10
1473	E3 ubiquitin-protein ligase RNF123	Q5XPI4	0.1234	0.2609	1.80	0.5248	0.8440	-0.88	0.4852	0.7052	-0.60	0.2144	0.3515	2.22	0.2863	0.5160	-0.93	0.2148	0.3962	2.68	0.3736	0.4995	-2.55	0.8708	1.0327	-0.33	0.0651	0.1407	2.06
1474	E3 ubiquitin-protein ligase RNF126	Q9BV68	0.5717	0.6393	-0.06	0.6939	0.7743	0.07	0.0553	0.1342	0.34	0.3568	0.5351	0.11	0.2574	0.4774	0.15	0.8856	1.1233	0.01	0.0607	0.1943	0.23	0.1604	0.4341	0.15	0.0026	0.0545	0.81
1475	E3 ubiquitin-protein ligase RNF14	Q9UBS8	0.4108	0.5856	-3.31	0.5752	0.8197	-1.75	0.5661	0.7362	-1.19	0.5388	0.6960	0.89	0.2938	0.4801	-1.88	0.9510	1.1148	0.09	0.0012	0.0447	1.05	0.0000	0.0000	0.76	0.0000	0.0000	1.26
1476	E3 ubiquitin-protein ligase RNF187	Q5TA31	0.2314	0.4130	0.12	0.4059	0.7488	0.15	0.0894	0.1878	0.20	0.0897	0.1787	0.20	0.7119	0.7724	-0.03	0.8256	1.0596	-0.02	0.3736	0.4365	-2.55	0.8708	0.9153	-0.33	0.0651	0.1141	2.06
1477	E3 ubiquitin-protein ligase RNF213	Q63HN8	0.4108	0.5854	-3.31	0.5752	0.8195	-1.75	0.5661	0.7360	-1.19	0.5388	0.6958	0.89	0.2938	0.4799	-1.88	0.9510	1.1145	0.09	0.9197	0.9341	0.05	0.6325	1.0693	-0.24	0.6548	0.7103	-0.27
1478	E3 ubiquitin-protein ligase RNF216	Q9NWF9	0.7702	0.8134	-0.27	0.7785	0.8403	0.25	0.7291	0.7725	0.30	0.7107	0.7493	0.33	0.3965	0.4929	0.82	0.9140	1.1511	0.11	0.3736	0.4334	-2.55	0.8708	0.9096	-0.33	0.0651	0.1129	2.06
1479	E3 ubiquitin-protein ligase RNF220	Q5VTB9	0.0625	0.1657	-2.28	0.2187	0.6609	-3.46	0.0172	0.0623	-11.24	0.2426	0.3894	-2.68	0.0344	0.1354	-0.92	0.0020	0.0523	-1.80	0.0425	0.1674	0.28	0.6506	1.0899	-0.06	0.3824	0.4536	-0.06
1480	E3 ubiquitin-protein ligase RNF34	Q969K3	0.2109	0.3839	-0.14	0.0202	0.4713	-0.31	0.0644	0.1481	-0.21	0.0141	0.0484	-0.35	0.0447	0.1560	-0.28	0.0862	0.2140	-0.20	0.3736	0.5412	-2.55	0.8708	1.1083	-0.33	0.0651	0.1607	2.06
1481	E3 ubiquitin-protein ligase RNF6	Q9Y252	0.0226	0.0896	0.23	0.4794	0.8047	0.09	0.1294	0.2503	0.11	0.0305	0.0812	0.20	0.9155	0.9368	0.01	0.0348	0.1201	0.25	0.3736	0.5748	-2.55	0.8708	1.1683	-0.33	0.0651	0.1784	2.06
1482	E3 ubiquitin-protein ligase TRIM13	O60858	0.0110	0.0603	0.28	0.0898	0.6235	0.27	0.0005	0.0211	0.60	0.0017	0.0185	0.37	0.0247	0.1138	0.14	0.0102	0.0704	0.22	0.3736	0.6126	-2.55	0.8708	1.2348	-0.33	0.0651	0.2005	2.06
1483	E3 ubiquitin-protein ligase TRIM17	Q9Y577	0.4108	0.5852	-3.31	0.5752	0.8192	-1.75	0.5661	0.7359	-1.19	0.5388	0.6957	0.89	0.2938	0.4798	-1.88	0.9510	1.1143	0.09	0.1611	0.3542	-1.69	0.1178	0.3537	-0.55	0.8528	0.8807	0.04
1484	E3 ubiquitin-protein ligase TRIM33	Q9UPN9	0.0116	0.0621	0.08	0.3335	0.6924	0.08	0.0010	0.0209	0.22	0.0054	0.0297	0.10	0.6405	0.7098	0.01	0.0038	0.0589	0.15	0.3736	0.6351	-2.55	0.8708	1.2739	-0.33	0.0651	0.2148	2.06
1485	E3 ubiquitin-protein ligase TRIM41	Q8WV44	0.4108	0.5851	-3.31	0.5752	0.8190	-1.75	0.5661	0.7357	-1.19	0.5388	0.6955	0.89	0.2938	0.4796	-1.88	0.9510	1.1140	0.09	0.3374	0.6081	-0.13	0.2043	0.5042	-0.19	0.7154	0.7653	0.04
1486	E3 ubiquitin-protein ligase TRIM56	Q9BRZ2	0.4108	0.5849	-3.31	0.5752	0.8188	-1.75	0.5661	0.7355	-1.19	0.5388	0.6953	0.89	0.2938	0.4795	-1.88	0.9510	1.1138	0.09	0.0782	0.2221	0.39	0.3432	0.7027	0.09	0.0152	0.1058	0.47
1487	E3 ubiquitin-protein ligase TRIM58	Q8NG06	0.0965	0.2193	0.43	0.3268	0.6859	-0.95	0.0005	0.0186	0.53	0.0235	0.0678	0.57	0.0036	0.0446	0.28	0.0587	0.1672	0.39	0.1500	0.3361	-0.06	0.0075	0.0712	-0.09	0.0755	0.1255	-0.05
1488	E3 ubiquitin-protein ligase TRIM63	Q969Q1	0.0474	0.1399	0.19	0.5332	0.8498	0.07	0.0076	0.0412	0.35	0.0105	0.0411	0.36	0.0035	0.0440	0.47	0.0361	0.1228	0.25	0.3736	0.5731	-2.55	0.8708	1.1653	-0.33	0.0651	0.1775	2.06
1489	E3 ubiquitin-protein ligase TRIM69	Q86WT6	0.1500	0.2994	-0.28	0.4367	0.7726	-0.43	0.1261	0.2456	-0.30	0.0186	0.0576	-0.65	0.0497	0.1643	-0.48	0.1199	0.2646	-0.33	0.1389	0.3192	0.56	0.5243	0.9479	0.33	0.0245	0.1328	1.24
1490	E3 ubiquitin-protein ligase TRIM7	Q9C029	0.0599	0.1619	-0.07	0.6133	0.7146	0.06	0.1439	0.2703	-0.07	0.0054	0.0294	-0.14	0.9936	0.9955	0.00	0.1978	0.3731	-0.18	0.0478	0.1749	0.42	0.1120	0.3417	0.27	0.6039	0.6640	0.09
1491	E3 ubiquitin-protein ligase TRIP12	Q14669	0.0843	0.2004	0.13	0.6636	0.7523	0.04	0.2769	0.4509	-0.07	0.0349	0.0891	0.19	0.0774	0.2142	0.13	0.3772	0.5859	0.24	0.0288	0.1460	0.37	0.3831	0.7615	0.11	0.1136	0.1710	0.42
1492	E3 ubiquitin-protein ligase UBR4	Q5T4S7	0.6035	0.6688	0.02	0.0926	0.6287	0.35	0.0047	0.0337	0.29	0.0059	0.0309	0.20	0.1723	0.3613	0.05	0.0013	0.0499	0.34	0.0472	0.1741	0.34	0.0574	0.2291	0.22	0.2357	0.3056	0.10
1493	E3 ubiquitin-protein ligase UHRF1	Q96T88	0.6725	0.7305	-0.14	0.2533	0.6623	-0.28	0.1864	0.3316	0.36	0.4954	0.6987	0.07	0.4635	0.5550	-0.06	0.0539	0.1574	-0.20	0.0133	0.1092	-0.42	0.2068	0.5079	-0.10	0.9348	0.9478	-0.02
1494	E3 ubiquitin-protein ligase ZFP91	Q96JP5	0.4108	0.5848	-3.31	0.5752	0.8186	-1.75	0.5661	0.7353	-1.19	0.5388	0.6951	0.89	0.2938	0.4793	-1.88	0.9510	1.1135	0.09	0.0393	0.1639	0.53	0.2352	0.5510	0.16	0.1001	0.1557	0.19
1495	E3 ubiquitin-protein ligase ZNF598	Q86UK7	0.0004	0.0170	1.27	0.3347	0.6932	0.06	0.0319	0.0915	-0.19	0.0013	0.0163	0.71	0.0074	0.0629	0.50	0.2412	0.4272	-0.07	0.0201	0.1313	0.28	0.0540	0.2215	-0.12	0.1423	0.2037	-0.16
1496	E3 ubiquitin-protein transferase MAEA	Q7L5Y9	0.0489	0.1418	0.14	0.2650	0.6631	0.25	0.0038	0.0317	0.42	0.0025	0.0214	0.33	0.0045	0.0501	0.34	0.0051	0.0622	0.31	0.3736	0.6300	-2.55	0.8708	1.2649	-0.33	0.0651	0.2114	2.06
1497	E3 UFM1-protein ligase 1	O94874	0.7717	0.8148	0.02	0.1348	0.6495	0.18	0.0103	0.0473	0.37	0.0711	0.1494	0.20	0.1940	0.3919	0.11	0.4359	0.6575	0.06	0.3736	0.4659	-2.55	0.8708	0.9705	-0.33	0.0651	0.1260	2.06
1498	Early endosome antigen 1	Q15075	0.2346	0.4176	-0.06	0.6158	0.7160	0.09	0.0500	0.1251	0.18	0.0041	0.0260	0.28	0.8715	0.9025	-0.01	0.0211	0.0939	0.23	0.8412	0.8663	0.11	0.7320	1.1757	0.19	0.3165	0.3879	0.60
1499	EARP and GARP complex-interacting protein 1	Q53HC9	0.7512	0.7975	0.02	0.0964	0.6337	0.30	0.1739	0.3156	0.17	0.0099	0.0399	0.19	0.0059	0.0563	0.21	0.8100	1.0469	0.02	0.3736	0.4380	-2.55	0.8708	0.9183	-0.33	0.0651	0.1147	2.06
1500	Echinoderm microtubule-associated protein-like 2	O95834	0.3415	0.5494	0.06	0.2571	0.6632	0.26	0.8375	0.8690	-0.04	0.0330	0.0859	0.21	0.8206	0.8601	0.01	0.0469	0.1438	0.17	0.3736	0.5632	-2.55	0.8708	1.1478	-0.33	0.0651	0.1721	2.06
1501	Echinoderm microtubule-associated protein-like 3	Q32P44	0.5185	0.5907	-0.04	0.0607	0.5921	-0.16	0.0267	0.0818	-0.22	0.0267	0.0737	-0.21	0.0399	0.1466	-0.27	0.6708	0.9089	0.07	0.3736	0.4465	-2.55	0.8708	0.9343	-0.33	0.0651	0.1181	2.06
1502	Echinoderm microtubule-associated protein-like 4	Q9HC35	0.1482	0.2969	0.08	0.4267	0.7624	-0.05	0.5493	0.7724	-0.03	0.9060	0.9186	0.00	0.8953	0.9198	0.01	0.0411	0.1333	0.14	0.2259	0.4511	0.07	0.0031	0.0456	0.34	0.0460	0.1875	0.16
1503	Echinoderm microtubule-associated protein-like 6	Q6ZMW3	0.0290	0.1041	0.13	0.3162	0.6805	0.11	0.1699	0.3095	0.15	0.0161	0.0526	0.15	0.7295	0.7882	-0.01	0.1038	0.2394	0.16	0.1692	0.3649	-0.35	0.1566	0.4285	0.29	0.1143	0.1719	1.08
1504	Ecto-NOX disulfide-thiol exchanger 1	Q8TC92	0.1181	0.2530	-0.10	0.2954	0.6711	0.09	0.0762	0.1663	0.13	0.0079	0.0359	0.27	0.0395	0.1455	0.17	0.0133	0.0766	0.31	0.3736	0.6061	-2.55	0.8708	1.2234	-0.33	0.0651	0.1965	2.06
1505	eEF1A lysine and N-terminal methyltransferase	Q8N6R0	0.2487	0.4354	-0.74	0.1827	0.6655	-1.67	0.3271	0.5159	-0.72	0.0491	0.1138	-1.25	0.9463	0.9585	0.01	0.0608	0.1711	-1.15	0.3736	0.5556	-2.55	0.8708	1.1342	-0.33	0.0651	0.1681	2.06
1506	EEF1A lysine methyltransferase 1	Q8WVE0	0.0038	0.0394	-0.36	0.2668	0.6632	-0.69	0.0929	0.1930	-0.37	0.1225	0.2261	-0.49	0.3331	0.4301	-0.21	0.0004	0.0351	-0.74	0.0087	0.0939	0.88	0.0007	0.0243	1.53	0.0157	0.1079	0.71
1507	EEF1A lysine methyltransferase 2	Q5JPI9	0.4108	0.5846	-3.31	0.5752	0.8183	-1.75	0.5661	0.7351	-1.19	0.5388	0.6950	0.89	0.2938	0.4792	-1.88	0.9510	1.1133	0.09	0.0328	0.1514	-12.01	0.0299	0.1564	-9.80	0.0001	0.0207	-7.41
1508	EEF1AKMT4-ECE2 readthrough transcript protein	P0DPD8	0.7023	0.7555	-0.03	0.2065	0.6603	0.11	0.2772	0.4513	-0.14	0.9848	0.9871	0.00	0.4676	0.5589	-0.05	0.0543	0.1581	-0.20	0.3736	0.5582	-2.55	0.8708	1.1388	-0.33	0.0651	0.1695	2.06
1509	EF-hand domain-containing protein 1	Q5JVL4	0.0145	0.0701	0.27	0.8269	0.8754	0.06	0.0144	0.0564	0.38	0.0092	0.0385	0.31	0.1884	0.3838	0.12	0.2436	0.4305	0.34	0.3736	0.4914	-2.55	0.8708	1.0178	-0.33	0.0651	0.1370	2.06
1510	EF-hand domain-containing protein D1	Q9BUP0	0.4108	0.5844	-3.31	0.5752	0.8181	-1.75	0.5661	0.7349	-1.19	0.5388	0.6948	0.89	0.2938	0.4790	-1.88	0.9510	1.1130	0.09	0.0431	0.1676	-0.14	0.2908	0.6303	0.06	0.0836	0.1353	-0.17
1511	EF-hand domain-containing protein D2	Q96C19	0.0019	0.0304	0.50	0.0499	0.5757	-0.14	0.0587	0.1395	-0.47	0.0369	0.0927	-0.58	0.0502	0.1652	-0.33	0.0084	0.0673	-0.45	0.2695	0.5121	0.14	0.0650	0.2454	-0.24	0.1458	0.2079	-0.17
1512	EH domain-binding protein 1-like protein 1	Q8N3D4	0.4108	0.5843	-3.31	0.5752	0.8179	-1.75	0.5661	0.7348	-1.19	0.5388	0.6946	0.89	0.2938	0.4789	-1.88	0.9510	1.1128	0.09	0.0172	0.1215	0.37	0.0182	0.1153	0.43	0.0409	0.1758	0.54
1513	EH domain-containing protein 1	Q9H4M9	0.0712	0.1790	0.06	0.4255	0.7616	0.06	0.0055	0.0357	0.13	0.0043	0.0267	0.15	0.0116	0.0773	0.15	0.0052	0.0618	0.24	0.0088	0.0936	0.28	0.0510	0.2132	0.15	0.0875	0.1403	0.13
1514	EH domain-containing protein 2	Q9NZN4	0.1244	0.2623	-0.09	0.2732	0.6650	0.11	0.0165	0.0609	0.20	0.3239	0.4954	0.06	0.1431	0.3168	-0.11	0.6706	0.9089	0.02	0.0236	0.1367	0.21	0.3100	0.6578	0.04	0.0025	0.0540	0.15
1515	EH domain-containing protein 3	Q9NZN3	0.4108	0.5841	-3.31	0.5752	0.8177	-1.75	0.5661	0.7346	-1.19	0.5388	0.6944	0.89	0.2938	0.4787	-1.88	0.9510	1.1125	0.09	0.6202	0.6671	-0.06	0.4612	0.8678	-0.13	0.9632	0.9709	0.00
1516	EH domain-containing protein 4	Q9H223	0.2166	0.3915	-0.04	0.0674	0.5898	0.15	0.0901	0.1884	-0.06	0.8981	0.9125	0.01	0.0282	0.1216	0.08	0.0273	0.1061	-0.11	0.8915	0.9105	0.01	0.5877	1.0199	0.03	0.0103	0.0892	0.28
1517	eIF-2-alpha kinase activator GCN1	Q92616	0.0131	0.0669	0.12	0.2321	0.6624	0.20	0.0206	0.0693	0.19	0.0024	0.0208	0.22	0.3613	0.4584	0.02	0.0075	0.0651	0.17	0.0451	0.1704	0.27	0.0015	0.0312	0.41	0.4479	0.5175	0.04
1518	eIF-2-alpha kinase GCN2	Q9P2K8	0.0345	0.1164	0.21	0.0444	0.5693	0.19	0.1358	0.2589	0.15	0.0834	0.1692	0.26	0.0516	0.1683	0.17	0.0339	0.1188	0.25	0.3736	0.5763	-2.55	0.8708	1.1710	-0.33	0.0651	0.1793	2.06
1519	EKC/KEOPS complex subunit GON7	Q9BXV9	0.6765	0.7339	-0.23	0.2595	0.6632	-1.29	0.0055	0.0360	-1.17	0.0668	0.1429	-0.86	0.5140	0.6029	0.11	0.7554	0.9929	-0.13	0.3736	0.4411	-2.55	0.8708	0.9241	-0.33	0.0651	0.1159	2.06
1520	EKC/KEOPS complex subunit LAGE3	Q14657	0.9522	0.9647	0.01	0.2313	0.6623	0.20	0.5099	0.7313	0.09	0.0936	0.1844	0.28	0.5977	0.6736	-0.07	0.2463	0.4344	0.16	0.6093	0.6567	0.14	0.9858	0.9900	0.00	0.8760	0.8996	-0.05
1521	EKC/KEOPS complex subunit TP53RK	Q96S44	0.4108	0.5840	-3.31	0.5752	0.8174	-1.75	0.5661	0.7344	-1.19	0.5388	0.6943	0.89	0.2938	0.4786	-1.88	0.9510	1.1123	0.09	0.0143	0.1112	0.39	0.7019	1.1441	0.08	0.0152	0.1057	0.36
1522	EKC/KEOPS complex subunit TPRKB	Q9Y3C4	0.1481	0.2969	-0.11	0.5011	0.8227	-0.15	0.1765	0.3190	0.11	0.0291	0.0787	-0.14	0.0033	0.0427	0.17	0.0061	0.0626	0.10	0.3736	0.6253	-2.55	0.8708	1.2568	-0.33	0.0651	0.2084	2.06

Supplemetary Table S2. Overview on all relatively quantified 5180 proteins statistical analysis

			MCF-7																											
			Dai SC20 vs control			Gen SC20 vs control			SSE SC20 vs control			Dai IC20 vs control			Gen IC20 vs control			SSE IC20 vs control			MDA-MB-231<br>Dai IC20 vs control			Gen IC20 vs control			SSE IC20 vs control		
	**Protein name**	**UniProt**	**p value**	**BH q value**	**log2FC**	**p value**	**BH q value**	**log2FC**	**p value**	**BH q value**	**log2FC**	**p value**	**BH q value**	**log2FC**	**p value**	**BH q value**	**log2FC**	**p value**	**BH q value**	**log2FC**	**p value**	**BH q value**	**log2FC**	**p value**	**BH q value**	**log2FC**	**p value**	**BH q value**	**log2FC**
1523	ELAV-like protein 1	Q15717	0.0484	0.1414	0.10	0.6132	0.7146	0.01	0.0311	0.0901	0.17	0.8455	0.8676	0.00	0.0597	0.1841	-0.14	0.4903	0.7158	0.05	0.0196	0.1295	0.21	0.0591	0.2323	-0.20	0.0293	0.1461	0.21
1524	Electrogenic sodium bicarbonate cotransporter 1	Q9Y6R1	0.4108	0.5838	-3.31	0.5752	0.8172	-1.75	0.5661	0.7342	-1.19	0.5388	0.6941	0.89	0.2938	0.4784	-1.88	0.9510	1.1120	0.09	0.9071	0.9240	-0.03	0.7627	1.2049	-0.07	0.6146	0.6738	0.12
1525	Electron transfer flavoprotein subunit alpha_ mitochondrial	P13804	0.5648	0.6327	0.03	0.1672	0.6606	0.15	0.1659	0.3033	0.09	0.0859	0.1733	0.13	0.5252	0.6119	0.03	0.0485	0.1471	0.17	0.2121	0.4300	0.18	0.3018	0.6460	-0.12	0.0684	0.1158	-0.26
1526	Electron transfer flavoprotein subunit beta	P38117	0.5580	0.6267	-0.04	0.6657	0.7537	0.03	0.8724	0.8991	-0.01	0.1781	0.3027	0.11	0.6708	0.7346	0.03	0.2121	0.3934	0.10	0.0451	0.1700	0.18	0.1852	0.4747	0.06	0.2407	0.3110	0.15
1527	ELKS/Rab6-interacting/CAST family member 1	Q8IUD2	0.4108	0.5836	-3.31	0.5752	0.8170	-1.75	0.5661	0.7340	-1.19	0.5388	0.6939	0.89	0.2938	0.4783	-1.88	0.9510	1.1118	0.09	0.0666	0.2031	0.18	0.1267	0.3712	-0.22	0.0039	0.0607	-0.37
1528	Ellis-van Creveld syndrome protein	P57679	0.0131	0.0668	-0.19	0.5562	0.8704	-0.15	0.0015	0.0224	-0.47	0.0086	0.0376	-0.37	0.0072	0.0620	-0.38	0.3054	0.5037	0.10	0.3736	0.4808	-2.55	0.8708	0.9982	-0.33	0.0651	0.1323	2.06
1529	Elongation factor 1-alpha 1	P68104	0.4108	0.5835	-3.31	0.5752	0.8168	-1.75	0.5661	0.7338	-1.19	0.5388	0.6938	0.89	0.2938	0.4781	-1.88	0.9510	1.1115	0.09	0.1119	0.2752	0.34	0.0325	0.1625	0.34	0.5348	0.6007	0.06
1530	Elongation factor 1-alpha 2	Q05639	0.1523	0.3033	0.08	0.1958	0.6690	0.15	0.0357	0.0987	-0.13	0.4964	0.6999	-0.03	0.0233	0.1104	-0.12	0.1266	0.2755	0.06	0.3658	0.6463	-0.14	0.1356	0.3889	-0.13	0.0061	0.0738	-0.47
1531	Elongation factor 1-beta	P24534	0.0014	0.0257	0.11	0.5464	0.8613	0.05	0.0022	0.0261	0.15	0.0171	0.0546	0.11	0.3912	0.4877	-0.02	0.0005	0.0370	0.19	0.0230	0.1366	0.37	0.0134	0.0965	0.21	0.0294	0.1464	0.19
1532	Elongation factor 1-delta	P29692	0.0422	0.1302	0.20	0.3866	0.7290	0.17	0.0097	0.0458	0.33	0.0080	0.0361	0.35	0.1241	0.2905	0.13	0.0129	0.0765	0.44	0.0655	0.2014	0.36	0.1112	0.3402	0.19	0.3504	0.4219	0.08
1533	Elongation factor 1-gamma	P26641	0.0095	0.0575	0.24	0.2500	0.6600	0.25	0.0013	0.0225	0.44	0.0032	0.0232	0.34	0.0569	0.1783	0.16	0.0061	0.0621	0.42	0.0517	0.1802	0.44	0.0434	0.1923	0.28	0.4609	0.5293	0.06
1534	Elongation factor 2	P13639	0.0086	0.0549	0.31	0.2781	0.6654	0.24	0.0014	0.0223	0.46	0.0027	0.0221	0.38	0.1605	0.3437	0.09	0.0117	0.0738	0.38	0.0911	0.2430	0.37	0.3602	0.7286	0.11	0.7739	0.8140	-0.02
1535	Elongation factor G mitochondrial	Q96RP9	0.4032	0.6231	-0.08	0.7882	0.8476	0.03	0.8761	0.9019	0.01	0.5992	0.6470	0.04	0.4301	0.5243	-0.07	0.6824	0.9220	-0.04	0.5275	0.5801	-0.06	0.6290	1.0648	-0.04	0.6029	0.6633	-0.05
1536	Elongation factor Ts mitochondrial	P43897	0.2980	0.4968	0.04	0.0489	0.5744	0.19	0.0290	0.0865	0.16	0.0597	0.1314	0.14	0.9247	0.9436	-0.01	0.1573	0.3167	0.12	0.8873	0.9067	-0.02	0.1579	0.4296	-0.18	0.0916	0.1453	-0.17
1537	Elongation factor Tu mitochondrial	P49411	0.0086	0.0548	0.26	0.2035	0.6609	0.26	0.0080	0.0421	0.42	0.0023	0.0205	0.40	0.0826	0.2224	0.13	0.0058	0.0617	0.44	0.0903	0.2417	0.32	0.5623	0.9890	0.06	0.7518	0.7949	-0.02
1538	Elongation of very long chain fatty acids protein 1	Q9BW60	0.4108	0.5833	-3.31	0.5752	0.8165	-1.75	0.5661	0.7336	-1.19	0.5388	0.6936	0.89	0.2938	0.4780	-1.88	0.9510	1.1113	0.09	0.5117	0.5650	0.07	0.4647	0.8728	0.07	0.6742	0.7274	0.04
1539	Elongator complex protein 1	O95163	0.6923	0.7474	-0.13	0.8344	0.8803	-0.11	0.2098	0.3638	-0.42	0.4165	0.6088	-0.30	0.5793	0.6578	0.16	0.6224	0.8577	-0.18	0.9065	0.9238	0.02	0.0056	0.0601	-0.71	0.0169	0.1115	-0.56
1540	Elongator complex protein 5	Q8TE02	0.4108	0.5832	-3.31	0.5752	0.8163	-1.75	0.5661	0.7335	-1.19	0.5388	0.6934	0.89	0.2938	0.4778	-1.88	0.9510	1.1110	0.09	0.0171	0.1215	-0.69	0.1396	0.3967	-0.30	0.0846	0.1366	-0.42
1541	Elongin-B	Q15370	0.8818	0.9067	-0.01	0.4466	0.7821	0.15	0.5711	0.6289	0.05	0.1027	0.1977	0.20	0.5057	0.5945	0.06	0.1639	0.3268	0.15	0.8338	0.8600	0.03	0.2101	0.5124	-0.17	0.0879	0.1406	-0.25
1542	Elongin-C	Q15369	0.0121	0.0636	0.21	0.0450	0.5672	0.23	0.0002	0.0230	0.59	0.0023	0.0209	0.50	0.4279	0.5221	0.02	0.0675	0.1819	0.45	0.1212	0.2915	0.18	0.2218	0.5292	-0.10	0.1146	0.1722	-0.16
1543	Embryonic stem cell-specific 5-hydroxymethylcytosine-binding protein	Q96FZ2	0.3391	0.5472	0.08	0.6522	0.7435	0.03	0.6807	0.7303	0.03	0.3842	0.5691	0.06	0.7195	0.7797	-0.02	0.1928	0.3668	0.10	0.3736	0.5040	-2.55	0.8708	1.0408	-0.33	0.0651	0.1427	2.06
1544	Embryonic testis differentiation protein homolog B	P0DPP9	0.4108	0.5830	-3.31	0.5752	0.8161	-1.75	0.5661	0.7333	-1.19	0.5388	0.6932	0.89	0.2938	0.4777	-1.88	0.9510	1.1108	0.09	0.0120	0.1036	0.47	0.0005	0.0219	0.98	0.0135	0.1011	0.43
1545	Emerin	P50402	0.0491	0.1422	-0.46	0.0438	0.5686	-11.60	0.3836	0.5844	-0.14	0.1226	0.2262	-0.33	0.5317	0.6173	-0.10	0.0783	0.2001	-0.43	0.1761	0.3751	0.15	0.1249	0.3678	-0.16	0.7373	0.7834	-0.02
1546	Ena/VASP-like protein	Q9UI08	0.0257	0.0970	-0.44	0.3242	0.6855	-0.16	0.2139	0.3685	-0.13	0.0145	0.0492	-0.39	0.1441	0.3186	-0.18	0.2290	0.4123	-0.12	0.3736	0.4953	-2.55	0.8708	1.0249	-0.33	0.0651	0.1388	2.06
1547	Endogenous Bornavirus-like nucleoprotein 2	Q6P2I7	0.4108	0.5828	-3.31	0.5752	0.8159	-1.75	0.5661	0.7331	-1.19	0.5388	0.6931	0.89	0.2938	0.4775	-1.88	0.9510	1.1105	0.09	0.8615	0.8847	-0.02	0.0027	0.0429	-0.62	0.0107	0.0912	-0.73
1548	Endogenous retrovirus group 3 member 1 Env polyprotein	Q14264	0.4108	0.5827	-3.31	0.5752	0.8156	-1.75	0.5661	0.7329	-1.19	0.5388	0.6929	0.89	0.2938	0.4774	-1.88	0.9510	1.1103	0.09	0.0010	0.0414	0.59	0.0006	0.0245	0.51	0.0012	0.0438	0.58
1549	Endogenous retrovirus group K member 104 Rec protein	P61576	0.0545	0.1528	-0.53	0.2624	0.6634	-0.43	0.0125	0.0524	-0.81	0.1288	0.2348	-0.33	0.2318	0.4421	-0.24	0.4096	0.6266	-0.15	0.3736	0.4687	-2.55	0.8708	0.9757	-0.33	0.0651	0.1272	2.06
1550	Endonuclease 8-like 2	Q969S2	0.5232	0.5947	0.08	0.7003	0.7789	-0.12	0.9840	0.9870	-0.01	0.5127	0.7168	0.08	0.1702	0.3578	0.20	0.6370	0.8741	-0.17	0.3736	0.4487	-2.55	0.8708	0.9384	-0.33	0.0651	0.1189	2.06
1551	Endonuclease 8-like 3	Q8TAT5	0.2850	0.4806	-0.24	0.1864	0.6668	-0.89	0.0476	0.1211	-0.50	0.0148	0.0499	-0.72	0.0629	0.1899	-0.43	0.2394	0.4254	-0.27	0.3736	0.4926	-2.55	0.8708	1.0198	-0.33	0.0651	0.1375	2.06
1552	Endonuclease/exonuclease/phosphatase family domain-containing protein 1	Q7L9B9	0.4108	0.5825	-3.31	0.5752	0.8154	-1.75	0.5661	0.7327	-1.19	0.5388	0.6927	0.89	0.2938	0.4772	-1.88	0.9510	1.1100	0.09	0.3769	0.4326	0.95	0.2293	0.5424	1.44	0.2499	0.3209	1.35
1553	Endophilin-A1	Q99962	0.4108	0.5824	-3.31	0.5752	0.8152	-1.75	0.5661	0.7326	-1.19	0.5388	0.6926	0.89	0.2938	0.4771	-1.88	0.9510	1.1097	0.09	0.1113	0.2745	0.56	0.1004	0.3181	0.57	0.1456	0.2077	0.48
1554	Endophilin-A2	Q99961	0.0582	0.1590	-0.25	0.1759	0.6627	-0.08	0.0253	0.0790	-0.14	0.0304	0.0810	-0.14	0.0082	0.0653	-0.25	0.0167	0.0850	-0.29	0.2910	0.5436	0.07	0.1646	0.4415	0.11	0.3207	0.3920	0.06
1555	Endophilin-B2	Q9NR46	0.1469	0.2952	0.12	0.1992	0.6644	0.28	0.0064	0.0388	0.28	0.0159	0.0523	0.23	0.3382	0.4352	-0.05	0.0145	0.0802	0.25	0.2229	0.4465	0.19	0.2323	0.5470	-0.19	0.1145	0.1721	-0.29
1556	Endoplasmic reticulum aminopeptidase 1	Q9NZ08	0.4108	0.5822	-3.31	0.5752	0.8150	-1.75	0.5661	0.7324	-1.19	0.5388	0.6924	0.89	0.2938	0.4769	-1.88	0.9510	1.1095	0.09	0.7705	0.8048	0.03	0.2183	0.5247	0.18	0.2635	0.3355	0.11
1557	Endoplasmic reticulum chaperone BiP	P11021	0.0032	0.0372	0.42	0.2011	0.6610	0.29	0.0009	0.0210	0.55	0.0010	0.0158	0.61	0.0129	0.0810	0.32	0.0030	0.0555	0.77	0.0114	0.1041	0.83	0.0835	0.2846	0.24	0.0365	0.1640	0.32
1558	Endoplasmic reticulum junction formation protein lunapark	Q9C0E8	0.0991	0.2239	-0.17	0.6429	0.7368	-0.08	0.1606	0.2954	-0.15	0.1302	0.2367	0.14	0.1043	0.2600	0.15	0.0584	0.1667	0.21	0.8543	0.8786	0.01	0.9614	0.9725	0.00	0.2992	0.3706	0.06
1559	Endoplasmic reticulum metallopeptidase 1	Q7Z2K6	0.0312	0.1093	-0.22	0.7397	0.8108	0.05	0.0930	0.1931	-0.19	0.0349	0.0892	-0.17	0.0090	0.0674	-0.27	0.0651	0.1780	-0.20	0.3736	0.5515	-2.55	0.8708	1.1268	-0.33	0.0651	0.1660	2.06
1560	Endoplasmic reticulum protein SC65	Q92791	0.0745	0.1850	-2.58	0.3092	0.6787	-2.13	0.0333	0.0943	-2.25	0.0291	0.0787	-3.55	0.8184	0.8585	-0.06	0.0141	0.0789	-3.90	0.3736	0.6046	-2.55	0.8708	1.2208	-0.33	0.0651	0.1956	2.06
1561	Endoplasmic reticulum resident protein 29	P30040	0.0106	0.0596	0.23	0.3092	0.6790	0.13	0.0052	0.0352	0.29	0.0014	0.0170	0.39	0.0441	0.1556	0.16	0.0019	0.0518	0.47	0.1902	0.3960	0.16	0.4730	0.8842	-0.05	0.0703	0.1183	-0.14
1562	Endoplasmic reticulum resident protein 44	Q9BS26	0.0028	0.0356	0.34	0.1662	0.6602	0.38	0.0105	0.0479	0.22	0.0051	0.0288	0.34	0.0946	0.2433	0.11	0.0060	0.0620	0.36	0.1290	0.3030	0.25	0.2363	0.5526	0.09	0.5840	0.6457	0.03
1563	Endoplasmic reticulum-Golgi intermediate compartment protein 1	Q969X5	0.0632	0.1665	-0.10	0.2002	0.6635	0.08	0.0566	0.1361	-0.09	0.5270	0.7334	-0.03	0.0868	0.2306	-0.07	0.9097	1.1468	0.00	0.3736	0.4335	-2.55	0.8708	0.9098	-0.33	0.0651	0.1129	2.06
1564	Endoplasmic reticulum-Golgi intermediate compartment protein 2	Q96RQ1	0.4108	0.5820	-3.31	0.5752	0.8147	-1.75	0.5661	0.7322	-1.19	0.5388	0.6922	0.89	0.2938	0.4768	-1.88	0.9510	1.1093	0.09	0.0025	0.0556	-1.53	0.0034	0.0480	-1.50	0.0005	0.0291	-1.75
1565	Endoplasmic reticulum-Golgi intermediate compartment protein 3	Q9Y282	0.0216	0.0875	-0.16	0.2839	0.6642	0.14	0.0039	0.0317	-0.07	0.3422	0.5168	0.03	0.0018	0.0331	0.09	0.7876	1.0258	0.01	0.3736	0.4396	-2.55	0.8708	0.9213	-0.33	0.0651	0.1153	2.06
1566	Endoplasmin	P14625	0.0049	0.0434	0.38	0.1982	0.6675	0.31	0.0014	0.0227	0.52	0.0021	0.0203	0.49	0.0411	0.1498	0.22	0.0076	0.0654	0.55	0.0466	0.1733	0.46	0.3859	0.7656	0.10	0.8429	0.8731	0.02
1567	Endoribonuclease LACTB2	Q53H82	0.1194	0.2549	-0.33	0.7552	0.8222	0.05	0.6104	0.6655	-0.02	0.3116	0.4800	-0.07	0.7333	0.7909	-0.04	0.0434	0.1370	0.16	0.3736	0.5662	-2.55	0.8708	1.1531	-0.33	0.0651	0.1737	2.06
1568	Endothelial differentiation-related factor 1	O60869	0.2489	0.4354	0.07	0.3303	0.6893	0.17	0.0359	0.0991	0.16	0.0456	0.1079	0.15	0.2137	0.4182	-0.07	0.4374	0.6590	0.04	0.0891	0.2399	0.36	0.1777	0.4630	0.17	0.0773	0.1277	-0.26
1569	Endothelin-converting enzyme 1	P42892	0.4108	0.5819	-3.31	0.5752	0.8145	-1.75	0.5661	0.7320	-1.19	0.5388	0.6920	0.89	0.2938	0.4766	-1.88	0.9510	1.1090	0.09	0.1107	0.2736	0.25	0.0309	0.1593	0.41	0.5009	0.5681	0.10
1570	Enhancer of filamentation 1	Q14511	0.4108	0.5817	-3.31	0.5752	0.8143	-1.75	0.5661	0.7318	-1.19	0.5388	0.6919	0.89	0.2938	0.4765	-1.88	0.9510	1.1088	0.09	0.2288	0.4555	0.19	0.1528	0.4226	-0.17	0.0498	0.1948	-0.25
1571	Enhancer of mRNA-decapping protein 4	Q6P2E9	0.3183	0.5211	0.02	0.3699	0.7182	-0.07	0.0088	0.0439	0.05	0.2430	0.3899	-0.08	0.0248	0.1137	-0.11	0.2782	0.4739	0.11	0.0636	0.1987	-0.14	0.6016	1.0353	0.04	0.8050	0.8407	0.02
1572	Enhancer of rudimentary homolog	P84090	0.0184	0.0798	-0.38	0.9895	0.9924	0.00	0.8454	0.8757	0.02	0.9203	0.9300	0.01	0.4421	0.5366	-0.07	0.4562	0.6789	-0.08	0.0019	0.0529	0.64	0.0195	0.1203	0.25	0.0119	0.0941	0.36
1573	Enolase-phosphatase E1	Q9UHY7	0.1466	0.2948	-0.26	0.0871	0.6189	-0.37	0.0628	0.1457	-0.39	0.0196	0.0596	-0.56	0.3697	0.4669	-0.14	0.0496	0.1493	-0.48	0.3736	0.5611	-2.55	0.8708	1.1440	-0.33	0.0651	0.1710	2.06
1574	Enoyl-CoA delta isomerase 1 mitochondrial	P42126	0.0309	0.1086	0.28	0.6255	0.7228	0.05	0.2185	0.3749	0.10	0.1121	0.2121	0.14	0.2618	0.4830	-0.08	0.0995	0.2331	0.16	0.1256	0.2982	0.20	0.2292	0.5424	-0.12	0.0261	0.1381	0.35
1575	Enoyl-CoA delta isomerase 2 mitochondrial	O75521	0.1037	0.2303	0.12	0.3867	0.7289	0.06	0.1249	0.2439	0.15	0.1305	0.2372	0.11	0.7984	0.8430	0.01	0.8347	1.0686	0.01	0.0433	0.1681	0.25	0.9531	0.9665	0.00	0.7465	0.7906	-0.04
1576	Enoyl-CoA hydratase domain-containing protein 2_ mitochondrial	Q86YB7	0.0613	0.1637	0.14	0.3119	0.6800	0.22	0.0122	0.0518	0.26	0.0095	0.0392	0.27	0.4562	0.5488	-0.04	0.1272	0.2762	0.09	0.3736	0.5253	-2.55	0.8708	1.0796	-0.33	0.0651	0.1528	2.06
1577	Enoyl-CoA hydratase mitochondrial	P30084	0.0035	0.0383	0.16	0.0500	0.5743	0.24	0.8569	0.8862	-0.01	0.5342	0.7413	0.02	0.0074	0.0623	-0.12	0.0409	0.1330	0.07	0.0289	0.1462	-0.17	0.0036	0.0487	-0.16	0.0022	0.0523	-0.34
1578	Ensconsin	Q14244	0.0144	0.0700	0.31	0.1918	0.6686	0.31	0.0010	0.0212	0.60	0.0049	0.0282	0.39	0.3288	0.4259	0.07	0.0138	0.0780	0.42	0.3736	0.6055	-2.55	0.8708	1.2224	-0.33	0.0651	0.1962	2.06
1579	Envoplakin	Q92817	0.6023	0.6679	-0.02	0.2491	0.6593	0.17	0.3359	0.5284	-0.04	0.9942	0.9946	0.00	0.2685	0.4904	0.05	0.1148	0.2577	-0.13	0.3947	0.4505	0.08	0.1582	0.4299	0.11	0.7442	0.7885	0.02
1580	Envoplakin-like protein	A8MZ36	0.4108	0.5816	-3.31	0.5752	0.8141	-1.75	0.5661	0.7316	-1.19	0.5388	0.6917	0.89	0.2938	0.4763	-1.88	0.9510	1.1085	0.09	0.7273	0.7665	0.04	0.8180	1.2626	0.03	0.7309	0.7782	0.04
1581	Ephrin type-A receptor 2	P29317	0.4108	0.5814	-3.31	0.5752	0.8139	-1.75	0.5661	0.7315	-1.19	0.5388	0.6915	0.89	0.2938	0.4762	-1.88	0.9510	1.1083	0.09	0.0131	0.1082	0.30	0.1098	0.3379	0.16	0.7221	0.7706	-0.02
1582	Ephrin type-A receptor 4	P54764	0.0135	0.0679	1.41	0.0135	0.4426	1.24	0.0118	0.0509	1.54	0.0302	0.0808	0.95	0.0138	0.0842	1.52	0.0171	0.0857	1.61	0.3736	0.5984	-2.55	0.8708	1.2100	-0.33	0.0651	0.1919	2.06
1583	Ephrin type-A receptor 6	Q9UF33	0.4108	0.5812	-3.31	0.5752	0.8136	-1.75	0.5661	0.7313	-1.19	0.5388	0.6914	0.89	0.2938	0.4760	-1.88	0.9510	1.1080	0.09	0.0848	0.2328	0.47	0.0025	0.0410	0.53	0.0002	0.0247	0.80
1584	Ephrin type-A receptor 7	Q15375	0.4108	0.5811	-3.31	0.5752	0.8134	-1.75	0.5661	0.7311	-1.19	0.5388	0.6912	0.89	0.2938	0.4759	-1.88	0.9510	1.1078	0.09	0.1242	0.2965	1.25	0.0061	0.0628	3.81	0.0090	0.0851	3.91
1585	Ephrin type-B receptor 3	P54753	0.2978	0.4967	0.07	0.2476	0.6581	0.20	0.0291	0.0867	0.26	0.0185	0.0576	0.26	0.9363	0.9514	0.00	0.0752	0.1950	0.17	0.3736	0.5465	-2.55	0.8708	1.1179	-0.33	0.0651	0.1634	2.06
1586	Ephrin type-B receptor 4	P54760	0.0930	0.2147	0.35	0.1066	0.6304	0.31	0.6183	0.6726	0.08	0.6620	0.7044	-0.06	0.2585	0.4789	0.18	0.4544	0.6770	0.12	0.3736	0.4636	-2.55	0.8708	0.9663	-0.33	0.0651	0.1251	2.06
1587	Epidermal growth factor receptor	P00533	0.4108	0.5809	-3.31	0.5752	0.8132	-1.75	0.5661	0.7309	-1.19	0.5388	0.6910	0.89	0.2938	0.4757	-1.88	0.9510	1.1075	0.09	0.0833	0.2305	0.34	0.2390	0.5559	0.13	0.8352	0.8665	-0.02
1588	Epidermal growth factor receptor kinase substrate 8	Q12929	0.4108	0.5808	-3.31	0.5752	0.8130	-1.75	0.5661	0.7307	-1.19	0.5388	0.6908	0.89	0.2938	0.4756	-1.88	0.9510	1.1073	0.09	0.8689	0.8918	0.05	0.2856	0.6237	-0.39	0.8507	0.8790	0.06
1589	Epidermal growth factor receptor kinase substrate 8-like protein 1	Q8TE68	0.1490	0.2980	-7.40	0.1842	0.6635	-5.85	0.1746	0.3163	-5.29	0.3472	0.5228	-3.21	0.1388	0.3102	-5.98	0.7513	0.9893	-0.96	0.3736	0.4415	-2.55	0.8708	0.9249	-0.33	0.0651	0.1161	2.06
1590	Epidermal growth factor receptor kinase substrate 8-like protein 2	Q9H6S3	0.1205	0.2562	0.07	0.0661	0.5934	-0.08	0.0088	0.0440	-0.35	0.0000	0.0000	-0.24	0.5759	0.6552	-0.05	0.0420	0.1350	-0.17	0.0030	0.0609	-0.66	0.0161	0.1077	-0.47	0.7701	0.8108	-0.03
1591	Epidermal growth factor receptor substrate 15	P42566	0.5998	0.6657	0.08	0.0325	0.5180	0.37	0.4325	0.6429	0.17	0.0748	0.1557	0.25	0.0292	0.1245	0.35	0.0523	0.1545	0.32	0.3736	0.5592	-2.55	0.8708	1.1405	-0.33	0.0651	0.1700	2.06
1592	Epidermal growth factor receptor substrate 15-like 1	Q9UBC2	0.0119	0.0629	-0.42	0.1724	0.6610	-0.22	0.3516	0.5458	-0.08	0.0156	0.0516	-0.40	0.0476	0.1604	-0.23	0.0061	0.0622	-0.66	0.3736	0.6251	-2.55	0.8708	1.2565	-0.33	0.0651	0.2083	2.06
1593	Epididymal sperm-binding protein 1	Q96BH3	0.0250	0.0955	-3.91	0.2742	0.6646	-3.81	0.2302	0.3896	-2.78	0.0500	0.1149	-3.14	0.2516	0.4697	0.53	0.0967	0.2294	-5.44	0.4311	0.4872	1.59	0.4050	0.7905	1.70	0.2332	0.3031	2.74
1594	Epiplakin	P58107	0.4445	0.5221	0.04	0.1731	0.6598	0.21	0.0185	0.0653	0.20	0.0064	0.0320	0.26	0.4688	0.5599	-0.03	0.0241	0.0992	0.21	0.0208	0.1325	0.44	0.0531	0.2186	0.34	0.0067	0.0759	0.47

Supplemetary Table S2. Overview on all relatively quantified 5180 proteins statistical analysis

			MCF-7																		MDA-MB-231								
			Dai SC20 vs control			Gen SC20 vs control			SSE SC20 vs control			Dai IC20 vs control			Gen IC20 vs control			SSE IC20 vs control			Dai IC20 vs control			Gen IC20 vs control			SSE IC20 vs control		
	**Protein name**	**UniProt**	**p value**	**BH q value**	**log2FC**	**p value**	**BH q value**	**log2FC**	**p value**	**BH q value**	**log2FC**	**p value**	**BH q value**	**log2FC**	**p value**	**BH q value**	**log2FC**	**p value**	**BH q value**	**log2FC**	**p value**	**BH q value**	**log2FC**	**p value**	**BH q value**	**log2FC**	**p value**	**BH q value**	**log2FC**
1595	Epithelial cell adhesion molecule	P16422	0.0151	0.0713	0.18	0.4832	0.8082	0.09	0.0010	0.0207	0.37	0.0014	0.0172	0.31	0.0118	0.0783	0.27	0.0058	0.0622	0.38	0.3736	0.6267	-2.55	0.8708	1.2593	-0.33	0.0651	0.2093	2.06
1596	Epithelial splicing regulatory protein 1	Q6NXG1	0.4108	0.5806	-3.31	0.5752	0.8127	-1.75	0.5661	0.7305	-1.19	0.5388	0.6907	0.89	0.2938	0.4754	-1.88	0.9510	1.1070	0.09	0.0128	0.1071	-0.51	0.0040	0.0512	0.68	0.9517	0.9623	0.01
1597	Epithelial splicing regulatory protein 2	Q9H6T0	0.0003	0.0162	-1.55	0.0001	0.5180	-1.59	0.0065	0.0391	-1.30	0.0003	0.0109	-3.74	0.0006	0.0206	-0.66	0.1815	0.3512	0.18	0.3736	0.5066	-2.55	0.8708	1.0456	-0.33	0.0651	0.1439	2.06
1598	Epithelial-stromal interaction protein 1	Q96J88	0.7097	0.7614	-0.03	0.6862	0.7709	0.04	0.0247	0.0779	-0.29	0.0137	0.0475	-0.36	0.0209	0.1039	-0.37	0.5263	0.7558	0.04	0.3736	0.4585	-2.55	0.8708	0.9567	-0.33	0.0651	0.1229	2.06
1599	Epoxide hydrolase 1	P07099	0.0674	0.1721	0.22	0.4434	0.7794	-0.07	0.1358	0.2588	0.16	0.2902	0.4522	0.19	0.0119	0.0782	0.43	0.0070	0.0648	0.61	0.0261	0.1407	0.30	0.0314	0.1598	0.24	0.1904	0.2582	0.11
1600	Eppin	O95925	0.0532	0.1499	0.36	0.0222	0.4792	0.24	0.0178	0.0638	0.43	0.0028	0.0218	0.24	0.0100	0.0720	0.18	0.0040	0.0602	0.51	0.3736	0.6347	-2.55	0.8708	1.2731	-0.33	0.0651	0.2145	2.06
1601	Epsilon-sarcoglycan	O43556	0.0624	0.1656	4.69	0.5772	0.6862	0.94	0.0741	0.1629	1.61	0.4183	0.6102	1.02	0.5373	0.6219	-0.60	0.1599	0.3207	1.04	0.3736	0.5122	-2.55	0.8708	1.0559	-0.33	0.0651	0.1466	2.06
1602	Epsin-1	Q9Y6I3	0.4108	0.5805	-3.31	0.5752	0.8125	-1.75	0.5661	0.7304	-1.19	0.5388	0.6905	0.89	0.2938	0.4753	-1.88	0.9510	1.1068	0.09	0.2261	0.4510	-0.19	0.3235	0.6762	-0.12	0.0716	0.1200	-0.24
1603	Epsin-2	O95208	0.4108	0.5803	-3.31	0.5752	0.8123	-1.75	0.5661	0.7302	-1.19	0.5388	0.6903	0.89	0.2938	0.4751	-1.88	0.9510	1.1065	0.09	0.0140	0.1107	-0.52	0.0191	0.1185	-0.55	0.0912	0.1449	-0.58
1604	Equilibrative nucleoside transporter 1	Q99808	0.0864	0.2043	0.10	0.3293	0.6886	0.19	0.0310	0.0900	0.21	0.0030	0.0223	0.28	0.1387	0.3102	0.07	0.0497	0.1495	0.13	0.0789	0.2232	0.47	0.0258	0.1439	0.13	0.0305	0.1475	0.22
1605	ER lumen protein-retaining receptor 1	P24390	0.0039	0.0396	-0.42	0.1024	0.6337	-0.25	0.0071	0.0400	-0.35	0.0063	0.0318	-0.33	0.0126	0.0802	-0.40	0.0902	0.2191	-0.32	0.3736	0.5377	-2.55	0.8708	1.1021	-0.33	0.0651	0.1589	2.06
1606	ER membrane protein complex subunit 1	Q8N766	0.8315	0.8654	0.01	0.5947	0.6990	-0.04	0.5058	0.7278	-0.04	0.4206	0.6122	0.08	0.5205	0.6083	-0.05	0.8468	1.0820	-0.01	0.0099	0.0996	-0.16	0.0143	0.1001	-0.13	0.3568	0.4284	-0.03
1607	ER membrane protein complex subunit 2	Q15006	0.5296	0.6009	0.05	0.8330	0.8799	-0.03	0.0234	0.0751	0.29	0.2548	0.4051	0.14	0.2290	0.4387	0.12	0.6376	0.8747	0.06	0.9073	0.9241	0.02	0.9059	0.9298	-0.02	0.1929	0.2608	0.23
1608	ER membrane protein complex subunit 3	Q9P0I2	0.0197	0.0824	0.27	0.0105	0.4458	-0.25	0.5179	0.7384	-0.03	0.2193	0.3579	-0.18	0.0598	0.1843	-0.17	0.2148	0.3964	0.14	0.0577	0.1915	-0.54	0.0598	0.2338	0.14	0.6280	0.6857	0.04
1609	ER membrane protein complex subunit 4	Q5J8M3	0.0484	0.1413	-0.08	0.1462	0.6551	0.13	0.1462	0.2737	0.05	0.1940	0.3241	0.05	0.0345	0.1354	-0.11	0.0850	0.2120	0.06	0.0087	0.0933	-0.58	0.0002	0.0164	-0.72	0.0005	0.0328	-0.99
1610	ER membrane protein complex subunit 7	Q9NPA0	0.0089	0.0560	-0.70	0.0607	0.5933	-0.27	0.0101	0.0469	-0.23	0.0131	0.0464	-0.21	0.0033	0.0429	-0.31	0.0091	0.0690	-0.33	0.3736	0.6163	-2.55	0.8708	1.2413	-0.33	0.0651	0.2028	2.06
1611	ER membrane protein complex subunit 8	O43402	0.2858	0.4814	0.05	0.9111	0.9381	0.01	0.6149	0.6692	-0.02	0.2116	0.3479	0.06	0.0253	0.1143	-0.15	0.7361	0.9759	0.03	0.3736	0.4429	-2.55	0.8708	0.9276	-0.33	0.0651	0.1166	2.06
1612	Erbin	Q96RT1	0.4108	0.5801	-3.31	0.5752	0.8121	-1.75	0.5661	0.7300	-1.19	0.5388	0.6902	0.89	0.2938	0.4750	-1.88	0.9510	1.1063	0.09	0.0039	0.0667	0.71	0.0005	0.0229	0.67	0.0026	0.0554	0.52
1613	ERC protein 2	O15083	0.4108	0.5800	-3.31	0.5752	0.8119	-1.75	0.5661	0.7298	-1.19	0.5388	0.6900	0.89	0.2938	0.4748	-1.88	0.9510	1.1060	0.09	0.5450	0.5960	-0.02	0.2106	0.5129	-0.12	0.1007	0.1564	0.07
1614	Erlin-1	O75477	0.0295	0.1052	0.17	0.2816	0.6642	0.15	0.0102	0.0471	0.26	0.0224	0.0654	0.22	0.0442	0.1552	0.15	0.0211	0.0941	0.21	0.0424	0.1675	-1.56	0.0008	0.0247	-2.08	0.1037	0.1597	-0.58
1615	Erlin-2	O94905	0.0270	0.0996	0.17	0.2627	0.6635	0.15	0.0177	0.0635	0.15	0.8457	0.8676	0.01	0.1666	0.3527	0.06	0.2028	0.3806	0.06	0.0323	0.1511	0.17	0.4039	0.7898	0.04	0.7557	0.7987	0.02
1616	ERO1-like protein alpha	Q96HE7	0.1025	0.2290	0.12	0.1616	0.6628	0.15	0.0166	0.0610	0.24	0.0358	0.0908	0.23	0.1294	0.2972	0.11	0.0467	0.1436	0.20	0.2293	0.4561	0.08	0.3317	0.6884	-0.04	0.5169	0.5832	-0.03
1617	ERO1-like protein beta	Q86YB8	0.0580	0.1586	0.15	0.2109	0.6625	0.15	0.0082	0.0426	0.19	0.0539	0.1215	0.15	0.7031	0.7642	-0.01	0.3865	0.5975	0.07	0.0205	0.1324	0.40	0.0759	0.2687	0.15	0.0351	0.1606	0.19
1618	Erythrocyte band 7 integral membrane protein	P27105	0.0000	0.0000	3.07	0.9453	0.9637	-0.01	0.6535	0.7049	-0.02	0.0001	0.0064	1.05	0.0049	0.0529	0.47	0.0042	0.0606	0.25	0.0728	0.2135	0.31	0.0043	0.0524	0.19	0.0007	0.0333	0.62
1619	Erythroid differentiation-related factor 1	Q3B7T1	0.4108	0.5798	-3.31	0.5752	0.8116	-1.75	0.5661	0.7296	-1.19	0.5388	0.6898	0.89	0.2938	0.4747	-1.88	0.9510	1.1058	0.09	0.0867	0.2360	0.36	0.0097	0.0813	0.38	0.2230	0.2929	-0.03
1620	ESF1 homolog	Q9H501	0.0332	0.1137	0.42	0.0800	0.6112	0.32	0.0004	0.0223	0.51	0.0189	0.0582	0.21	0.0769	0.2131	0.10	0.0032	0.0572	0.34	0.3736	0.6374	-2.55	0.8708	1.2778	-0.33	0.0651	0.2163	2.06
1621	Ester hydrolase C11orf54	Q9H0W9	0.4108	0.5797	-3.31	0.5752	0.8114	-1.75	0.5661	0.7295	-1.19	0.5388	0.6896	0.89	0.2938	0.4746	-1.88	0.9510	1.1055	0.09	0.2994	0.5563	-0.18	0.0026	0.0420	-0.69	0.0288	0.1450	-0.71
1622	Esterase OVCA2	Q8WZ82	0.0935	0.2151	0.38	0.0180	0.4685	0.68	0.4932	0.7142	0.13	0.5871	0.6361	0.12	0.1475	0.3233	0.33	0.3782	0.5867	-0.17	0.3736	0.4707	-2.55	0.8708	0.9795	-0.33	0.0651	0.1281	2.06
1623	Estrogen receptor beta	Q92731	0.3415	0.5492	0.09	0.1646	0.6604	0.28	0.0083	0.0430	0.46	0.0005	0.0124	0.47	0.0054	0.0548	0.20	0.0937	0.2250	0.37	0.3736	0.5364	-2.55	0.8708	1.0996	-0.33	0.0651	0.1582	2.06
1624	Ethanolamine-phosphate cytidylyltransferase	Q99447	0.1350	0.2786	-0.13	0.2028	0.6599	-0.13	0.1047	0.2116	0.10	0.4686	0.6680	-0.09	0.0223	0.1074	-0.19	0.2193	0.4007	-0.07	0.9562	0.9644	-0.03	0.8497	1.2911	0.17	0.3206	0.3920	0.56
1625	Ethylmalonyl-CoA decarboxylase	Q9NTX5	0.4108	0.5795	-3.31	0.5752	0.8112	-1.75	0.5661	0.7293	-1.19	0.5388	0.6895	0.89	0.2938	0.4744	-1.88	0.9510	1.1053	0.09	0.0015	0.0505	7.24	0.0016	0.0329	7.97	0.0017	0.0495	8.70
1626	ETS domain-containing protein Elk-4	P28324	0.1770	0.3393	-0.26	0.4439	0.7795	-0.13	0.2767	0.4510	0.32	0.6797	0.7200	-0.07	0.2804	0.5068	0.19	0.8616	1.0974	-0.03	0.3736	0.4354	-2.55	0.8708	0.9133	-0.33	0.0651	0.1137	2.06
1627	ETS translocation variant 3	P41162	0.2015	0.3722	-0.26	0.0052	0.3741	-0.24	0.3391	0.5316	-0.11	0.0005	0.0117	-0.44	0.0050	0.0532	-0.35	0.0663	0.1805	-0.19	0.3736	0.5510	-2.55	0.8708	1.1260	-0.33	0.0651	0.1657	2.06
1628	ETS translocation variant 5	P41161	0.2674	0.4593	0.06	0.3515	0.7046	0.10	0.0788	0.1705	0.12	0.5184	0.7234	0.04	0.2048	0.4065	0.07	0.8277	1.0615	-0.01	0.7805	0.8145	0.04	0.6070	1.0387	0.09	0.9142	0.9311	0.01
1629	ETS-related transcription factor Elf-4	Q99607	0.0269	0.0994	-0.25	0.1006	0.6332	0.38	0.7255	0.7695	0.03	0.1159	0.2170	0.27	0.2178	0.4235	0.16	0.1323	0.2833	-0.13	0.1352	0.3133	-0.81	0.5123	0.9315	-0.15	0.7621	0.8038	-0.03
1630	Eukaryotic initiation factor 4A-I	P60842	0.0197	0.0823	0.21	0.2934	0.6701	0.22	0.0042	0.0321	0.33	0.0097	0.0397	0.27	0.5493	0.6331	-0.03	0.0161	0.0842	0.27	0.0558	0.1873	0.35	0.0657	0.2472	0.25	0.4785	0.5467	0.06
1631	Eukaryotic initiation factor 4A-II	Q14240	0.7008	0.7546	-0.04	0.1813	0.6646	-0.19	0.0937	0.1941	-0.26	0.1301	0.2366	-0.23	0.2523	0.4706	-0.15	0.6393	0.8765	-0.05	0.8739	0.8953	0.01	0.0533	0.2191	0.15	0.2301	0.3003	0.06
1632	Eukaryotic initiation factor 4A-III	P38919	0.0698	0.1764	0.12	0.3450	0.7028	0.16	0.0245	0.0774	0.22	0.0074	0.0349	0.24	0.2143	0.4191	-0.06	0.0563	0.1622	0.13	0.0008	0.0427	0.35	0.0000	0.0000	0.49	0.0009	0.0382	0.49
1633	Eukaryotic peptide chain release factor GTP-binding subunit ERF3A	P15170	0.4719	0.5480	0.04	0.4847	0.8091	0.12	0.0691	0.1557	0.15	0.0928	0.1833	0.12	0.0901	0.2362	-0.13	0.1606	0.3216	0.10	0.1486	0.3337	0.38	0.6090	1.0408	-0.07	0.7425	0.7872	0.08
1634	Eukaryotic peptide chain release factor GTP-binding subunit ERF3B	Q8IYD1	0.4566	0.5338	0.26	0.9925	0.9952	0.01	0.0638	0.1471	-0.96	0.1960	0.3267	-0.54	0.4729	0.5635	-0.26	0.4663	0.6887	0.26	0.2856	0.5364	0.08	0.1364	0.3904	-0.12	0.7486	0.7923	-0.02
1635	Eukaryotic peptide chain release factor subunit 1	P62495	0.0078	0.0523	0.17	0.2371	0.6632	0.19	0.0129	0.0532	0.25	0.0091	0.0384	0.17	0.0017	0.0324	-0.09	0.0042	0.0608	0.16	0.3075	0.5673	0.13	0.9023	0.9272	0.02	0.2082	0.2771	-0.16
1636	Eukaryotic translation elongation factor 1 epsilon-1	O43324	0.5200	0.5919	0.04	0.2162	0.6603	0.15	0.0190	0.0661	0.23	0.0122	0.0445	0.29	0.3534	0.4509	0.05	0.2531	0.4432	0.06	0.0596	0.1938	0.39	0.0997	0.3163	0.13	0.0310	0.1494	0.18
1637	Eukaryotic translation initiation factor 1A_ X-chromosomal	P47813	0.0193	0.0817	0.19	0.1867	0.6665	0.23	0.0072	0.0402	0.38	0.0015	0.0174	0.41	0.1873	0.3829	0.07	0.0201	0.0918	0.25	0.0266	0.1420	0.32	0.1336	0.3849	0.14	0.5073	0.5739	0.09
1638	Eukaryotic translation initiation factor 1b	O60739	0.0011	0.0231	0.30	0.9601	0.9725	0.00	0.0421	0.1111	0.10	0.0867	0.1745	0.07	0.1259	0.2930	0.07	0.1892	0.3624	0.24	0.0850	0.2331	0.15	0.2833	0.6213	0.18	0.1000	0.1556	0.14
1639	Eukaryotic translation initiation factor 2 subunit 1	P05198	0.0022	0.0323	0.22	0.4483	0.7832	0.13	0.0368	0.1006	0.24	0.0085	0.0373	0.23	0.1200	0.2846	0.05	0.0071	0.0644	0.24	0.5228	0.5758	0.06	0.0124	0.0926	-0.24	0.0086	0.0828	-0.29
1640	Eukaryotic translation initiation factor 2 subunit 2	P20042	0.0469	0.1387	0.16	0.2616	0.6633	0.14	0.0051	0.0349	0.32	0.0344	0.0885	0.20	0.4093	0.5047	0.04	0.0372	0.1246	0.21	0.2383	0.4694	0.13	0.1807	0.4680	-0.18	0.7762	0.8161	0.03
1641	Eukaryotic translation initiation factor 2 subunit 3	P41091	0.0033	0.0377	0.29	0.2072	0.6597	0.27	0.0098	0.0461	0.30	0.0064	0.0321	0.31	0.2193	0.4251	0.06	0.0055	0.0615	0.30	0.0982	0.2524	-0.26	0.0041	0.0515	-0.58	0.0041	0.0625	-0.72
1642	Eukaryotic translation initiation factor 2 subunit 3B	Q2VIR3	0.4108	0.5793	-3.31	0.5752	0.8110	-1.75	0.5661	0.7291	-1.19	0.5388	0.6893	0.89	0.2938	0.4743	-1.88	0.9510	1.1050	0.09	0.0016	0.0515	0.74	0.0425	0.1904	0.24	0.2104	0.2790	0.44
1643	Eukaryotic translation initiation factor 2A	Q9BY44	0.0083	0.0541	0.18	0.2953	0.6715	0.14	0.0035	0.0312	0.27	0.0034	0.0235	0.22	0.0547	0.1745	0.09	0.0081	0.0668	0.24	0.1037	0.2616	0.30	0.6173	1.0515	-0.03	0.2143	0.2832	0.10
1644	Eukaryotic translation initiation factor 2-alpha kinase 3	Q9NZJ5	0.0254	0.0963	-1.98	0.1553	0.6626	-1.71	0.1619	0.2971	-1.40	0.0049	0.0280	-2.04	0.3132	0.4107	-0.32	0.0302	0.1117	-1.26	0.3736	0.5812	-2.55	0.8708	1.1796	-0.33	0.0651	0.1820	2.06
1645	Eukaryotic translation initiation factor 2D	P41214	0.8512	0.8815	0.02	0.9096	0.9371	0.02	0.1419	0.2678	0.14	0.2403	0.3866	0.10	0.8216	0.8608	0.02	0.3540	0.5596	0.08	0.3736	0.4740	-2.55	0.8708	0.9855	-0.33	0.0651	0.1295	2.06
1646	Eukaryotic translation initiation factor 3 subunit A	Q14152	0.0905	0.2107	0.11	0.2811	0.6658	0.24	0.0083	0.0429	0.29	0.0101	0.0404	0.27	0.5831	0.6609	0.03	0.0281	0.1069	0.20	0.0958	0.2491	0.26	0.4542	0.8584	0.09	0.5915	0.6527	-0.05
1647	Eukaryotic translation initiation factor 3 subunit B	P55884	0.0580	0.1585	0.16	0.2682	0.6631	0.15	0.0089	0.0442	0.26	0.0134	0.0470	0.24	0.7833	0.8298	0.01	0.1660	0.3295	0.09	0.6404	0.6854	0.05	0.1869	0.4764	-0.11	0.0533	0.2036	-0.19
1648	Eukaryotic translation initiation factor 3 subunit C	Q99613	0.4108	0.5792	-3.31	0.5752	0.8108	-1.75	0.5661	0.7289	-1.19	0.5388	0.6891	0.89	0.2938	0.4741	-1.88	0.9510	1.1048	0.09	0.0534	0.1831	0.35	0.1365	0.3904	0.15	0.5878	0.6495	0.03
1649	Eukaryotic translation initiation factor 3 subunit C-like protein	B5ME19	0.0448	0.1348	0.12	0.4734	0.8016	0.12	0.0252	0.0788	0.17	0.0071	0.0342	0.25	0.2638	0.4851	-0.05	0.0637	0.1761	0.11	0.3736	0.5534	-2.55	0.8708	1.1302	-0.33	0.0651	0.1669	2.06
1650	Eukaryotic translation initiation factor 3 subunit D	O15371	0.0671	0.1717	0.08	0.3838	0.7304	0.09	0.0083	0.0427	0.16	0.0399	0.0983	0.10	0.3724	0.4694	-0.03	0.1061	0.2434	0.07	0.4125	0.4686	0.08	0.0898	0.2976	-0.07	0.0514	0.1988	-0.10
1651	Eukaryotic translation initiation factor 3 subunit E	P60228	0.5823	0.6498	0.03	0.6575	0.7474	-0.06	0.7650	0.8058	-0.02	0.2853	0.4455	-0.07	0.0667	0.1965	-0.18	0.4641	0.6867	-0.04	0.2040	0.4173	0.10	0.0300	0.1562	0.17	0.4447	0.5146	0.04
1652	Eukaryotic translation initiation factor 3 subunit F	O00303	0.0521	0.1478	-0.12	0.0480	0.5729	-0.12	0.1019	0.2072	0.14	0.0444	0.1056	0.16	0.3817	0.4790	-0.04	0.6003	0.8366	-0.05	0.1044	0.2632	0.24	0.0705	0.2572	0.21	0.1537	0.2169	0.09
1653	Eukaryotic translation initiation factor 3 subunit G	O75821	0.0190	0.0811	0.18	0.2531	0.6628	0.18	0.0025	0.0274	0.38	0.0032	0.0232	0.31	0.1841	0.3778	0.07	0.0134	0.0770	0.23	0.0500	0.1784	0.47	0.0649	0.2452	0.23	0.0918	0.1456	0.19
1654	Eukaryotic translation initiation factor 3 subunit H	O15372	0.0578	0.1582	-0.07	0.2914	0.6694	0.11	0.3046	0.4879	0.08	0.1338	0.2418	0.05	0.5288	0.6149	0.02	0.4644	0.6867	0.02	0.3109	0.5713	0.09	0.2879	0.6271	0.09	0.9027	0.9223	0.01
1655	Eukaryotic translation initiation factor 3 subunit I	Q13347	0.0962	0.2189	0.15	0.5217	0.8411	0.09	0.0895	0.1878	0.15	0.1283	0.2341	0.12	0.8065	0.8493	-0.01	0.1480	0.3042	0.12	0.1317	0.3079	0.22	0.6691	1.1073	0.03	0.1458	0.2078	-0.10
1656	Eukaryotic translation initiation factor 3 subunit J	O75822	0.0549	0.1534	0.16	0.0871	0.6198	0.31	0.1623	0.2977	0.19	0.0204	0.0613	0.22	0.3106	0.4079	-0.06	0.0328	0.1163	0.19	0.0867	0.2361	0.28	0.0267	0.1473	0.32	0.4845	0.5527	0.05
1657	Eukaryotic translation initiation factor 3 subunit K	Q9UBQ5	0.0110	0.0602	0.22	0.0228	0.4762	0.13	0.0795	0.1718	0.03	0.0814	0.1662	0.11	0.0479	0.1606	-0.05	0.1804	0.3497	0.06	0.2551	0.4931	0.12	0.8768	0.9069	0.01	0.1872	0.2546	-0.13
1658	Eukaryotic translation initiation factor 3 subunit L	Q9Y262	0.1075	0.2361	0.08	0.2314	0.6622	0.13	0.0061	0.0378	0.37	0.0222	0.0650	0.17	0.2417	0.4554	0.05	0.0337	0.1182	0.14	0.1837	0.3846	0.14	0.8444	1.2880	-0.01	0.7328	0.7796	0.02
1659	Eukaryotic translation initiation factor 3 subunit M	Q7L2H7	0.0054	0.0446	0.25	0.2199	0.6603	0.16	0.1111	0.2223	0.12	0.0025	0.0210	0.20	0.8631	0.8952	0.01	0.0388	0.1283	0.23	0.0249	0.1393	0.46	0.0803	0.2790	0.17	0.0349	0.1603	0.19
1660	Eukaryotic translation initiation factor 4 gamma 1	Q04637	0.0794	0.1934	0.10	0.3557	0.7078	0.17	0.0068	0.0395	0.32	0.0100	0.0402	0.24	0.3003	0.3983	0.05	0.0135	0.0773	0.24	0.0400	0.1639	0.31	0.3789	0.7552	0.08	0.1750	0.2410	0.11
1661	Eukaryotic translation initiation factor 4 gamma 2	P78344	0.1379	0.2827	0.11	0.3823	0.7297	0.17	0.0068	0.0395	0.35	0.0025	0.0213	0.21	0.3908	0.4876	0.03	0.0027	0.0546	0.39	0.2360	0.4654	0.17	0.2363	0.5524	0.12	0.1323	0.1926	0.12
1662	Eukaryotic translation initiation factor 4 gamma 3	O43432	0.0739	0.1844	0.13	0.3031	0.6762	0.19	0.0020	0.0248	0.39	0.0029	0.0223	0.31	0.6527	0.7195	0.02	0.0097	0.0702	0.29	0.5329	0.5850	0.03	0.5367	0.9623	0.04	0.5621	0.6260	0.03
1663	Eukaryotic translation initiation factor 4B	P23588	0.3297	0.5360	0.06	0.2548	0.6626	0.14	0.0064	0.0387	0.34	0.0110	0.0421	0.25	0.4211	0.5157	0.04	0.1990	0.3750	0.08	0.1322	0.3086	0.27	0.3027	0.6477	0.11	0.7173	0.7669	-0.03
1664	Eukaryotic translation initiation factor 4E	P06730	0.1950	0.3633	-0.12	0.0165	0.4571	-0.26	0.0013	0.0238	-0.23	0.0574	0.1274	-0.26	0.0025	0.0385	-0.24	0.1863	0.3582	0.04	0.0041	0.0692	-0.61	0.4050	0.7908	-0.11	0.0267	0.1390	-0.33
1665	Eukaryotic translation initiation factor 4E type 2	O60573	0.4108	0.5790	-3.31	0.5752	0.8105	-1.75	0.5661	0.7287	-1.19	0.5388	0.6890	0.89	0.2938	0.4740	-1.88	0.9510	1.1045	0.09	0.7877	0.8208	0.19	0.6073	1.0386	0.38	0.2751	0.3468	0.92
1666	Eukaryotic translation initiation factor 4E-binding protein 1	Q13541	0.1908	0.3571	-0.36	0.4491	0.7838	0.19	0.6367	0.6898	-0.11	0.7396	0.7754	0.07	0.9567	0.9662	0.01	0.6073	0.8447	-0.12	0.3736	0.4521	-2.55	0.8708	0.9447	-0.33	0.0651	0.1203	2.06
1667	Eukaryotic translation initiation factor 4H	Q15056	0.0519	0.1476	0.15	0.2401	0.6608	0.25	0.0213	0.0706	0.25	0.0157	0.0518	0.21	0.2416	0.4556	0.06	0.0371	0.1245	0.18	0.0142	0.1118	0.56	0.0250	0.1411	0.27	0.0089	0.0844	0.40
1668	Eukaryotic translation initiation factor 5	P55010	0.0620	0.1650	0.16	0.9291	0.9523	0.01	0.0149	0.0573	0.26	0.0864	0.1741	0.14	0.0077	0.0633	-0.45	0.6487	0.8857	-0.03	0.6748	0.7183	0.05	0.0237	0.1361	-0.14	0.1531	0.2163	-0.06
1669	Eukaryotic translation initiation factor 5A-1	P63241	0.0182	0.0792	0.22	0.2358	0.6631	0.24	0.0080	0.0420	0.20	0.0301	0.0806	0.14	0.1090	0.2681	-0.08	0.0380	0.1265	0.13	0.0615	0.1953	0.27	0.1353	0.3883	0.16	0.1962	0.2643	-0.09
1670	Eukaryotic translation initiation factor 5A-2	Q9GZV4	0.0592	0.1611	-0.30	0.5634	0.8769	-0.22	0.0739	0.1626	0.39	0.5385	0.7464	0.07	0.9178	0.9385	0.01	0.4681	0.6904	0.08	0.3425	0.6143	1.09	0.2552	0.5795	1.38	0.1320	0.1923	2.16
1671	Eukaryotic translation initiation factor 5B	O60841	0.4835	0.5581	0.03	0.1517	0.6598	0.19	0.0037	0.0318	0.33	0.0080	0.0362	0.23	0.2623	0.4835	0.05	0.0113	0.0728	0.24	0.0111	0.1034	0.50	0.0095	0.0803	0.36	0.0797	0.1306	0.15

Supplemetary Table S2. Overview on all relatively quantified 5180 proteins statistical analysis

			MCF-7																		MDA-MB-231								
			Dai SC20 vs control			Gen SC20 vs control			SSE SC20 vs control			Dai IC20 vs control			Gen IC20 vs control			SSE IC20 vs control			Dai IC20 vs control			Gen IC20 vs control			SSE IC20 vs control		
	**Protein name**	**UniProt**	**p value**	**BH q value**	**log2FC**	**p value**	**BH q value**	**log2FC**	**p value**	**BH q value**	**log2FC**	**p value**	**BH q value**	**log2FC**	**p value**	**BH q value**	**log2FC**	**p value**	**BH q value**	**log2FC**	**p value**	**BH q value**	**log2FC**	**p value**	**BH q value**	**log2FC**	**p value**	**BH q value**	**log2FC**
1672	Eukaryotic translation initiation factor 6	P56537	0.0027	0.0351	0.22	0.1880	0.6675	0.25	0.0039	0.0318	0.20	0.0015	0.0176	0.29	0.0457	0.1575	0.09	0.0081	0.0669	0.19	0.4160	0.4718	-0.09	0.0163	0.1082	-0.26	0.0140	0.1024	-0.29
1673	EVI5-like protein	Q96CN4	0.0198	0.0826	0.28	0.0534	0.5848	0.37	0.0025	0.0279	0.44	0.0034	0.0236	0.41	0.0058	0.0563	0.30	0.0326	0.1163	0.22	0.3736	0.5782	-2.55	0.8708	1.1744	-0.33	0.0651	0.1803	2.06
1674	Exocyst complex component 1	Q9NV70	0.2793	0.4726	-0.07	0.3135	0.6803	-0.17	0.9395	0.9529	-0.01	0.4314	0.6240	-0.02	0.8533	0.8870	0.01	0.1280	0.2774	0.02	0.3736	0.5250	-2.55	0.8708	1.0791	-0.33	0.0651	0.1527	2.06
1675	Exocyst complex component 4	Q96A65	0.0031	0.0368	0.15	0.2666	0.6633	0.22	0.0004	0.0190	0.36	0.0062	0.0316	0.18	0.5789	0.6576	0.01	0.0249	0.1005	0.28	0.0336	0.1527	0.34	0.9969	0.9975	0.00	0.5366	0.6023	0.05
1676	Exocyst complex component 5	O00471	0.1470	0.2951	0.06	0.4865	0.8106	0.04	0.1466	0.2741	-0.16	0.4409	0.6351	0.03	0.2143	0.4189	-0.06	0.1506	0.3082	-0.06	0.3736	0.5165	-2.55	0.8708	1.0636	-0.33	0.0651	0.1486	2.06
1677	Exocyst complex component 6B	Q9Y2D4	0.4108	0.5789	-3.31	0.5752	0.8103	-1.75	0.5661	0.7285	-1.19	0.5388	0.6888	0.89	0.2938	0.4738	-1.88	0.9510	1.1043	0.09	0.0304	0.1476	0.49	0.0067	0.0667	0.91	0.0096	0.0872	0.35
1678	Exocyst complex component 7	Q9UPT5	0.9528	0.9647	0.00	0.8342	0.8804	-0.02	0.4990	0.7212	-0.06	0.1502	0.2655	0.12	0.0130	0.0812	-0.22	0.0265	0.1044	0.18	0.3736	0.5847	-2.55	0.8708	1.1858	-0.33	0.0651	0.1840	2.06
1679	Exocyst complex component 8	Q8IYI6	0.0108	0.0599	-0.19	0.3500	0.7049	-0.19	0.0038	0.0315	0.22	0.0051	0.0289	-0.29	0.0113	0.0761	-0.14	0.0479	0.1459	0.43	0.0241	0.1373	0.56	0.2239	0.5335	0.16	0.0107	0.0907	0.27
1680	Exonuclease 3'-5' domain-containing protein 2	Q9NVH0	0.0355	0.1177	-0.26	0.0372	0.5506	-0.27	0.1466	0.2742	-0.15	0.5272	0.7335	-0.05	0.0748	0.2101	-0.19	0.5441	0.7766	-0.05	0.3736	0.4572	-2.55	0.8708	0.9543	-0.33	0.0651	0.1224	2.06
1681	Exosome complex component CSL4	Q9Y3B2	0.0236	0.0923	0.43	0.6138	0.7145	0.06	0.0040	0.0317	0.23	0.3366	0.5107	0.03	0.0026	0.0392	-0.38	0.2074	0.3870	-0.16	0.1769	0.3763	-0.17	0.6772	1.1165	-0.05	0.0579	0.2142	-0.34
1682	Exosome complex component MTR3	Q5RKV6	0.4108	0.5787	-3.31	0.5752	0.8101	-1.75	0.5661	0.7284	-1.19	0.5388	0.6886	0.89	0.2938	0.4737	-1.88	0.9510	1.1040	0.09	0.0417	0.1674	0.25	0.1573	0.4293	-0.23	0.4895	0.5578	0.09
1683	Exosome complex component RRP42	Q15024	0.0527	0.1489	0.14	0.6638	0.7524	-0.06	0.0177	0.0635	0.21	0.4485	0.6437	0.05	0.9812	0.9860	0.00	0.4380	0.6597	0.09	0.5748	0.6243	0.23	0.2764	0.6121	0.49	0.1835	0.2509	0.66
1684	Exosome complex component RRP45	Q06265	0.4108	0.5786	-3.31	0.5752	0.8099	-1.75	0.5661	0.7282	-1.19	0.5388	0.6885	0.89	0.2938	0.4735	-1.88	0.9510	1.1038	0.09	0.9586	0.9659	0.00	0.4180	0.8100	0.12	0.9422	0.9536	-0.01
1685	Exosome complex exonuclease RRP44	Q9Y2L1	0.0777	0.1908	-0.08	0.0016	0.2072	0.21	0.0013	0.0240	0.32	0.0076	0.0354	0.15	0.0109	0.0746	0.28	0.0104	0.0702	0.14	0.2224	0.4457	0.04	0.1494	0.4165	0.09	0.0223	0.1268	0.02
1686	Exosome component 10	Q01780	0.0050	0.0436	0.24	0.5607	0.8748	-0.07	0.0276	0.0836	0.15	0.2326	0.3765	0.11	0.0602	0.1846	0.12	0.0226	0.0963	0.24	0.4092	0.4649	0.03	0.0711	0.2581	-0.08	0.0441	0.1838	-0.36
1687	Exosome RNA helicase MTR4	P42285	0.1760	0.3380	-0.06	0.7642	0.8295	-0.03	0.2301	0.3895	0.07	0.2940	0.4572	0.04	0.0279	0.1213	-0.10	0.7854	1.0237	-0.02	0.0316	0.1503	0.58	0.2032	0.5024	0.15	0.0095	0.0869	0.39
1688	Exostosin-like 3	O43909	0.0053	0.0444	-0.15	0.1110	0.6325	0.28	0.1440	0.2704	0.19	0.5026	0.7063	-0.02	0.8347	0.8717	-0.01	0.0026	0.0545	-0.23	0.3736	0.6412	-2.55	0.8708	1.2844	-0.33	0.0651	0.2188	2.06
1689	Exportin-1	O14980	0.0593	0.1610	0.06	0.1834	0.6643	0.18	0.0086	0.0438	0.13	0.0072	0.0344	0.13	0.0380	0.1423	-0.07	0.1677	0.3322	0.04	0.0256	0.1397	0.25	0.0523	0.2169	0.17	0.5414	0.6070	0.03
1690	Exportin-2	P55060	0.0098	0.0581	0.14	0.2475	0.6585	0.21	0.0086	0.0438	0.22	0.0031	0.0228	0.31	0.2216	0.4285	0.04	0.0714	0.1885	0.20	0.0640	0.1992	0.39	0.4581	0.8632	0.09	0.6877	0.7408	-0.03
1691	Exportin-5	Q9HAV4	0.0068	0.0492	0.22	0.1990	0.6650	0.27	0.0006	0.0193	0.38	0.0023	0.0208	0.27	0.6539	0.7207	0.02	0.0235	0.0979	0.15	0.9062	0.9237	-0.01	0.6986	1.1419	0.02	0.0038	0.0600	0.48
1692	Exportin-7	Q9UIA9	0.1130	0.2448	0.12	0.0920	0.6304	0.15	0.2191	0.3757	0.06	0.0446	0.1059	0.09	0.6507	0.7184	0.02	0.7136	0.9539	-0.01	0.3736	0.4446	-2.55	0.8708	0.9306	-0.33	0.0651	0.1173	2.06
1693	Exportin-T	O43592	0.0111	0.0606	0.14	0.1807	0.6638	0.13	0.0153	0.0582	0.12	0.0124	0.0450	0.15	0.0391	0.1446	-0.09	0.3540	0.5597	0.03	0.0008	0.0410	-1.73	0.0038	0.0503	-0.58	0.0749	0.1247	-1.21
1694	Extended synaptotagmin-1	Q9BSJ8	0.0098	0.0581	0.16	0.2119	0.6632	0.19	0.0074	0.0407	0.19	0.0397	0.0980	0.12	0.1088	0.2679	0.07	0.0803	0.2037	0.21	0.1290	0.3029	0.12	0.4187	0.8111	0.05	0.3252	0.3965	0.06
1695	Extended synaptotagmin-2	A0FGR8	0.1458	0.2936	-0.08	0.5538	0.8690	-0.03	0.2721	0.4456	-0.06	0.5314	0.7390	-0.03	0.2687	0.4906	-0.06	0.9906	0.9925	0.00	0.1012	0.2572	-0.18	0.0862	0.2909	0.15	0.0819	0.1332	0.18
1696	Ezrin	P15311	0.2490	0.4355	-0.07	0.2910	0.6694	0.16	0.0878	0.1855	0.13	0.1535	0.2701	0.09	0.1277	0.2953	-0.11	0.1921	0.3662	0.09	0.0398	0.1641	0.53	0.1869	0.4769	0.17	0.6200	0.6783	0.04
1697	FACT complex subunit SPT16	Q9Y5B9	0.0010	0.0223	0.31	0.1438	0.6540	0.27	0.0062	0.0382	0.30	0.0011	0.0157	0.30	0.0430	0.1532	0.10	0.0040	0.0597	0.25	0.0246	0.1388	0.40	0.0623	0.2389	0.20	0.8426	0.8729	0.01
1698	FACT complex subunit SSRP1	Q08945	0.0277	0.1008	0.07	0.1658	0.6606	0.16	0.0010	0.0213	0.18	0.0084	0.0372	0.10	0.0450	0.1562	-0.05	0.0352	0.1210	0.07	0.1258	0.2985	0.19	0.9220	0.9427	0.01	0.0389	0.1702	-0.26
1699	F-actin-capping protein subunit alpha-1	P52907	0.8353	0.8687	0.01	0.5636	0.8770	0.05	0.3627	0.5602	0.05	0.3899	0.5766	-0.04	0.1397	0.3114	-0.08	0.1616	0.3231	0.08	0.0638	0.1988	0.20	0.2369	0.5530	-0.08	0.8465	0.8761	-0.01
1700	F-actin-capping protein subunit alpha-2	P47755	0.8277	0.8620	0.22	0.9554	0.9704	-0.06	0.5547	0.7791	-0.62	0.7855	0.8156	-0.27	0.1729	0.3617	1.82	0.7483	0.9866	-0.32	0.0142	0.1116	-0.32	0.0076	0.0720	-0.42	0.0220	0.1265	-0.39
1701	F-actin-capping protein subunit beta	P47756	0.0997	0.2244	-0.05	0.2120	0.6623	0.20	0.0076	0.0414	0.14	0.0031	0.0228	0.17	0.1091	0.2682	0.05	0.0283	0.1076	0.13	0.0784	0.2224	0.31	0.0836	0.2847	0.17	0.0277	0.1428	0.24
1702	FAD synthase	Q8NFF5	0.0162	0.0743	0.21	0.2656	0.6627	0.18	0.0293	0.0870	0.14	0.0008	0.0142	0.43	0.0083	0.0657	0.22	0.0011	0.0471	0.49	0.3736	0.6485	-2.55	0.8708	1.2969	-0.33	0.0651	0.2238	2.06
1703	FAD-linked sulfhydryl oxidase ALR	P55789	0.0098	0.0580	-0.24	0.1555	0.6619	0.31	0.0315	0.0907	0.32	0.0052	0.0290	0.43	0.0997	0.2528	0.12	0.9300	1.1673	0.01	0.3736	0.4327	-2.55	0.8708	0.9081	-0.33	0.0651	0.1126	2.06
1704	Fanconi anemia group A protein	O15360	0.4108	0.5784	-3.31	0.5752	0.8097	-1.75	0.5661	0.7280	-1.19	0.5388	0.6883	0.89	0.2938	0.4734	-1.88	0.9510	1.1035	0.09	0.1697	0.3658	-0.27	0.1863	0.4766	0.36	0.0157	0.1071	0.50
1705	Fanconi anemia group C protein	Q00597	0.8870	0.9109	0.00	0.0668	0.5935	-0.07	0.4830	0.7032	-0.02	0.2328	0.3767	0.04	0.3572	0.4550	-0.05	0.0167	0.0848	0.28	0.0148	0.1121	0.48	0.2533	0.5778	0.23	0.1713	0.2371	0.82
1706	Fanconi anemia group I protein	Q9NVI1	0.0391	0.1244	0.07	0.1307	0.6460	0.35	0.0102	0.0471	0.34	0.0021	0.0200	0.46	0.0004	0.0176	0.28	0.1094	0.2489	0.34	0.0664	0.2027	0.40	0.2986	0.6418	-0.17	0.2270	0.2973	-0.21
1707	Fanconi anemia group M protein	Q8IYD8	0.3826	0.5988	0.02	0.5849	0.6914	0.07	0.0013	0.0220	0.20	0.0010	0.0156	0.15	0.3575	0.4550	0.01	0.0020	0.0518	0.14	0.0023	0.0576	-0.36	0.8650	1.3075	-0.03	0.4511	0.5197	-0.03
1708	Far upstream element-binding protein 1	Q96AE4	0.0646	0.1682	0.11	0.3731	0.7209	0.17	0.0089	0.0441	0.28	0.0111	0.0424	0.20	0.6249	0.6961	-0.02	0.0413	0.1336	0.15	0.0953	0.2487	0.36	0.8313	1.2740	-0.02	0.4634	0.5318	-0.06
1709	Far upstream element-binding protein 2	Q92945	0.3246	0.5301	0.05	0.3319	0.6910	0.19	0.0141	0.0556	0.21	0.0140	0.0482	0.21	0.6359	0.7058	-0.02	0.0664	0.1806	0.14	0.1587	0.3510	0.21	0.0257	0.1438	0.22	0.1190	0.1771	0.14
1710	Far upstream element-binding protein 3	Q96I24	0.1208	0.2567	0.06	0.1846	0.6645	0.23	0.0079	0.0419	0.19	0.0025	0.0212	0.22	0.8885	0.9146	0.01	0.0238	0.0987	0.14	0.0547	0.1859	0.40	0.4431	0.8445	0.06	0.2712	0.3431	0.04
1711	Farnesyl pyrophosphate synthase	P14324	0.0107	0.0599	0.34	0.2186	0.6610	0.32	0.0025	0.0275	0.42	0.0025	0.0214	0.47	0.1307	0.2988	0.11	0.0059	0.0619	0.39	0.0485	0.1762	0.36	0.6916	1.1341	0.04	0.4975	0.5646	-0.06
1712	FAS-associated death domain protein	Q13158	0.0001	0.0130	-5.18	0.0272	0.5032	-14.78	0.0014	0.0232	-3.87	0.0328	0.0857	-5.73	0.0722	0.2052	-0.66	0.0288	0.1088	-5.77	0.6007	0.6485	-0.17	0.1823	0.4707	0.54	0.3033	0.3745	0.38
1713	FAS-associated factor 1	Q9UNN5	0.4108	0.5782	-3.31	0.5752	0.8094	-1.75	0.5661	0.7278	-1.19	0.5388	0.6881	0.89	0.2938	0.4732	-1.88	0.9510	1.1033	0.09	0.3998	0.4557	0.07	0.5912	1.0239	-0.04	0.1649	0.2300	0.13
1714	FAS-associated factor 2	Q96CS3	0.0794	0.1933	-0.08	0.0869	0.6209	-0.08	0.4614	0.6778	0.04	0.2383	0.3842	-0.05	0.0600	0.1846	-0.09	0.4097	0.6266	0.03	0.3491	0.6240	0.10	0.0420	0.1889	0.12	0.1706	0.2363	0.06
1715	Fascin	Q16658	0.0230	0.0906	0.10	0.7783	0.8403	0.07	0.0995	0.2032	0.26	0.1737	0.2967	0.04	0.4217	0.5163	0.05	0.4920	0.7173	0.04	0.2669	0.5092	-0.17	0.0041	0.0514	-0.52	0.0034	0.0583	-0.45
1716	Fat storage-inducing transmembrane protein 2	Q8N6M3	0.0491	0.1422	-0.15	0.6181	0.7174	-0.04	0.1231	0.2414	-0.10	0.2806	0.4387	-0.08	0.2669	0.4889	-0.06	0.6098	0.8473	0.03	0.3736	0.4518	-2.55	0.8708	0.9443	-0.33	0.0651	0.1202	2.06
1717	Fatty acid synthase	P49327	0.0353	0.1173	0.17	0.3462	0.7027	0.18	0.0134	0.0541	0.25	0.0023	0.0204	0.40	0.1023	0.2565	-0.12	0.0054	0.0617	0.57	0.0346	0.1544	0.40	0.4829	0.8937	0.07	0.1952	0.2632	0.14
1718	Fatty acid-binding protein 5	Q01469	0.0024	0.0336	0.60	0.1281	0.6417	0.43	0.0022	0.0266	0.57	0.0021	0.0202	0.48	0.0081	0.0652	0.26	0.0056	0.0620	0.46	0.1158	0.2828	-0.26	0.0021	0.0378	-0.78	0.0036	0.0594	-0.89
1719	Fatty acyl-CoA reductase 1	Q8WVX9	0.4336	0.5108	-0.04	0.7155	0.7916	-0.03	0.4061	0.6113	-0.05	0.1405	0.2519	0.08	0.0612	0.1866	0.10	0.0080	0.0667	0.25	0.0009	0.0432	1.18	0.0218	0.1282	0.56	0.0116	0.0937	0.65
1720	Fatty aldehyde dehydrogenase	P51648	0.0488	0.1416	-0.09	0.2229	0.6583	0.17	0.0108	0.0484	0.14	0.0011	0.0154	0.30	0.0493	0.1635	0.09	0.0308	0.1125	0.23	0.9946	0.9958	0.00	0.0177	0.1128	-0.30	0.0150	0.1054	-0.31
1721	Fatty-acid amide hydrolase 1	O00519	0.1598	0.3147	0.36	0.6228	0.7201	-0.11	0.2081	0.3617	0.34	0.1268	0.2320	0.32	0.6975	0.7590	-0.06	0.0868	0.2149	0.50	0.3736	0.5406	-2.55	0.8708	1.1072	-0.33	0.0651	0.1604	2.06
1722	F-box only protein 15	Q8NCQ5	0.4108	0.5781	-3.31	0.5752	0.8092	-1.75	0.5661	0.7276	-1.19	0.5388	0.6879	0.89	0.2938	0.4731	-1.88	0.9510	1.1030	0.09	0.9896	0.9921	0.01	0.2475	0.5690	-0.64	0.4010	0.4727	-0.48
1723	F-box only protein 22	Q8NEZ5	0.2497	0.4359	0.05	0.2871	0.6678	-0.08	0.0975	0.2003	0.18	0.3513	0.5281	-0.04	0.0216	0.1058	0.14	0.0228	0.0966	0.21	0.3736	0.5893	-2.55	0.8708	1.1940	-0.33	0.0651	0.1866	2.06
1724	F-box only protein 43	Q4G163	0.0626	0.1657	-0.17	0.7844	0.8447	-0.03	0.1922	0.3394	-0.15	0.8819	0.8991	-0.01	0.3269	0.4240	0.07	0.7915	1.0289	-0.02	0.0073	0.0873	0.41	0.7034	1.1451	0.02	0.0603	0.2193	0.70
1725	F-box only protein 48	Q5FWF7	0.0409	0.1278	0.10	0.0013	0.2245	0.18	0.1437	0.2701	0.05	0.2141	0.3511	0.02	0.0260	0.1165	0.10	0.6138	0.8501	-0.04	0.3736	0.4513	-2.55	0.8708	0.9433	-0.33	0.0651	0.1200	2.06
1726	F-box/WD repeat-containing protein 7	Q969H0	0.5621	0.6304	-0.04	0.7478	0.8170	0.05	0.9957	0.9965	0.00	0.7380	0.7740	0.03	0.0619	0.1883	-0.17	0.9883	0.9910	0.00	0.3736	0.4297	-2.55	0.8708	0.9025	-0.33	0.0651	0.1115	2.06
1727	F-box-like/WD repeat-containing protein TBL1XR1	Q9BZK7	0.4108	0.5779	-3.31	0.5752	0.8090	-1.75	0.5661	0.7275	-1.19	0.5388	0.6878	0.89	0.2938	0.4729	-1.88	0.9510	1.1028	0.09	0.0456	0.1713	2.25	0.0038	0.0498	6.29	0.0059	0.0731	5.47
1728	Fer-1-like protein 5	A0AVI2	0.4108	0.5778	-3.31	0.5752	0.8088	-1.75	0.5661	0.7273	-1.19	0.5388	0.6876	0.89	0.2938	0.4728	-1.88	0.9510	1.1025	0.09	0.1342	0.3120	0.31	0.6253	1.0616	0.04	0.4121	0.4838	-0.06
1729	Fer-1-like protein 6	Q2WGJ9	0.0013	0.0253	0.30	0.4076	0.7503	-0.17	0.3647	0.5619	0.16	0.9165	0.9274	0.01	0.9632	0.9716	0.00	0.4927	0.7175	-0.28	0.2582	0.4972	3.01	0.2785	0.6157	2.83	0.2087	0.2775	3.53
1730	FERM and PDZ domain-containing protein 2	Q68DX3	0.0846	0.2008	-0.11	0.8308	0.8785	0.03	0.3500	0.5441	-0.06	0.3523	0.5294	-0.04	0.0262	0.1170	-0.17	0.9304	1.1672	0.00	0.0228	0.1364	0.34	0.9381	0.9547	-0.01	0.0866	0.1391	0.21
1731	FERM domain-containing protein 4B	Q9Y2L6	0.4108	0.5776	-3.31	0.5752	0.8086	-1.75	0.5661	0.7271	-1.19	0.5388	0.6874	0.89	0.2938	0.4726	-1.88	0.9510	1.1023	0.09	0.2507	0.4867	0.11	0.0622	0.2387	0.20	0.0285	0.1446	0.33
1732	FERM domain-containing protein 8	Q9BZ67	0.2010	0.3719	-0.14	0.3575	0.7082	0.15	0.6061	0.6618	-0.05	0.0719	0.1510	0.23	0.0413	0.1498	0.31	0.0987	0.2318	0.20	0.3736	0.5346	-2.55	0.8708	1.0964	-0.33	0.0651	0.1574	2.06
1733	FERM_ ARHGEF and pleckstrin domain-containing protein 1	Q9Y4F1	0.2745	0.4676	-0.07	0.3676	0.7161	-1.06	0.5459	0.7688	-0.82	0.1067	0.2037	0.31	0.0000	0.0000	1.10	0.3650	0.5721	0.17	0.7407	0.7784	-0.03	0.3172	0.6668	-0.09	0.3705	0.4420	-0.12
1734	FERM_ ARHGEF and pleckstrin domain-containing protein 2	O94887	0.0459	0.1369	-0.20	0.5549	0.8700	-0.05	0.2833	0.4599	0.07	0.2249	0.3660	-0.08	0.4754	0.5658	0.04	0.2186	0.4000	0.08	0.9421	0.9520	0.01	0.0119	0.0907	-0.17	0.1582	0.2221	-0.26
1735	Fermitin family homolog 2	Q96AC1	0.3663	0.5795	-0.09	0.1751	0.6611	-0.26	0.9802	0.9838	0.00	0.6718	0.7132	-0.05	0.0469	0.1590	-0.22	0.3296	0.5327	-0.10	0.4549	0.5098	-0.19	0.2665	0.5961	-0.29	0.0660	0.1125	0.30
1736	Fermitin family homolog 3	Q86UX7	0.0826	0.1980	0.61	0.5628	0.8765	-0.32	0.0129	0.0532	0.92	0.0450	0.1067	0.81	0.6982	0.7596	0.02	0.0625	0.1738	0.82	0.5547	0.6057	-0.06	0.0124	0.0924	-0.40	0.2996	0.3709	-0.28
1737	Ferritin heavy chain	P02794	0.7727	0.8154	-0.02	0.6175	0.7172	0.03	0.0355	0.0982	0.24	0.0021	0.0199	0.42	0.2393	0.4524	0.07	0.0255	0.1013	0.23	0.0011	0.0445	0.84	0.0083	0.0753	0.46	0.0114	0.0931	0.50
1738	Ferrochelatase mitochondrial	P22830	0.4108	0.5775	-3.31	0.5752	0.8083	-1.75	0.5661	0.7269	-1.19	0.5388	0.6873	0.89	0.2938	0.4725	-1.88	0.9510	1.1021	0.09	0.0116	0.1038	0.38	0.0174	0.1115	0.46	0.0024	0.0538	0.78
1739	FGFR1 oncogene partner	O95684	0.4108	0.5773	-3.31	0.5752	0.8081	-1.75	0.5661	0.7267	-1.19	0.5388	0.6871	0.89	0.2938	0.4723	-1.88	0.9510	1.1018	0.09	0.9189	0.9337	0.02	0.8447	1.2881	0.03	0.0263	0.1384	0.71
1740	Fibrinogen beta chain	P02675	0.2772	0.4711	-0.03	0.2330	0.6628	-0.18	0.1374	0.2611	0.11	0.1440	0.2564	0.12	0.0683	0.1988	0.15	0.0892	0.2178	0.26	0.0668	0.2032	0.24	0.5729	1.0022	-0.05	0.1738	0.2398	-0.10
1741	Fibrinogen C domain-containing protein 1	Q8N539	0.0025	0.0338	0.39	0.2054	0.6592	0.28	0.0361	0.0994	0.22	0.0015	0.0172	0.48	0.0127	0.0803	0.25	0.0055	0.0622	0.41	0.4851	0.5397	0.09	0.0258	0.1437	-0.32	0.0085	0.0826	-0.76
1742	Fibrinogen gamma chain	P02679	0.4108	0.5771	-3.31	0.5752	0.8079	-1.75	0.5661	0.7266	-1.19	0.5388	0.6869	0.89	0.2938	0.4722	-1.88	0.9510	1.1016	0.09	0.2274	0.4531	-0.48	0.3764	0.7516	0.10	0.2007	0.2691	0.20
1743	Fibroblast growth factor receptor 2	P21802	0.4108	0.5770	-3.31	0.5752	0.8077	-1.75	0.5661	0.7264	-1.19	0.5388	0.6868	0.89	0.2938	0.4720	-1.88	0.9510	1.1013	0.09	0.0304	0.1474	0.21	0.8354	1.2792	0.01	0.0139	0.1018	0.15
1744	Fibroblast growth factor receptor 4	P22455	0.4108	0.5768	-3.31	0.5752	0.8075	-1.75	0.5661	0.7262	-1.19	0.5388	0.6866	0.89	0.2938	0.4719	-1.88	0.9510	1.1011	0.09	0.3124	0.5728	-0.18	0.4636	0.8717	0.05	0.0479	0.1909	0.30
1745	Fibrocystin	P08F94	0.4108	0.5767	-3.31	0.5752	0.8072	-1.75	0.5661	0.7260	-1.19	0.5388	0.6864	0.89	0.2938	0.4718	-1.88	0.9510	1.1008	0.09	0.1801	0.3803	-0.32	0.3611	0.7287	0.14	0.1876	0.2550	0.16
1746	Fibrocystin-L	Q86WI1	0.1243	0.2622	0.06	0.0150	0.4571	0.12	0.0436	0.1136	0.24	0.0071	0.0341	0.15	0.0048	0.0521	0.16	0.0064	0.0629	0.26	0.3736	0.6239	-2.55	0.8708	1.2544	-0.33	0.0651	0.2075	2.06
1747	Fibronectin type III and SPRY domain-containing protein 1	Q9BTV5	0.0069	0.0496	0.31	0.0013	0.2405	0.49	0.6165	0.6708	0.03	0.0347	0.0889	0.22	0.0012	0.0273	0.50	0.2000	0.3766	-0.08	0.3736	0.5025	-2.55	0.8708	1.0381	-0.33	0.0651	0.1420	2.06
1748	Fibronectin type III and SPRY domain-containing protein 2	A1L4K1	0.0035	0.0382	-0.21	0.2984	0.6729	-0.06	0.5071	0.7284	0.05	0.0468	0.1100	-0.18	0.0346	0.1356	-0.10	0.1310	0.2819	-0.21	0.0509	0.1791	0.93	0.0660	0.2477	0.83	0.0321	0.1527	1.13
1749	Fibronectin type III domain-containing protein 5	Q8NAU1	0.9270	0.9443	-0.02	0.0003	0.2220	0.90	0.0013	0.0241	0.66	0.0005	0.0129	0.82	0.0001	0.0115	1.17	0.2987	0.4956	0.31	0.3736	0.4818	-2.55	0.8708	0.9999	-0.33	0.0651	0.1328	2.06
1750	Fibrosin-1-like protein	Q9HCM7	0.7433	0.7919	-0.02	0.2145	0.6614	0.16	0.2515	0.4185	0.13	0.0553	0.1241	0.18	0.7786	0.8260	0.02	0.6514	0.8882	0.03	0.0200	0.1310	-0.30	0.0428	0.1910	-0.14	0.0468	0.1892	-0.12
1751	Filaggrin	P20930	0.4108	0.5765	-3.31	0.5752	0.8070	-1.75	0.5661	0.7258	-1.19	0.5388	0.6863	0.89	0.2938	0.4716	-1.88	0.9510	1.1006	0.09	0.0349	0.1545	0.75	0.9362	0.9537	0.02	0.0435	0.1819	-0.65

Supplemetary Table S2. Overview on all relatively quantified 5180 proteins statistical analysis

			MCF-7																		MDA-MB-231								
			Dai SC20 vs control			Gen SC20 vs control			SSE SC20 vs control			Dai IC20 vs control			Gen IC20 vs control			SSE IC20 vs control			Dai IC20 vs control			Gen IC20 vs control			SSE IC20 vs control		
	**Protein name**	**UniProt**	**p value**	**BH q value**	**log2FC**	**p value**	**BH q value**	**log2FC**	**p value**	**BH q value**	**log2FC**	**p value**	**BH q value**	**log2FC**	**p value**	**BH q value**	**log2FC**	**p value**	**BH q value**	**log2FC**	**p value**	**BH q value**	**log2FC**	**p value**	**BH q value**	**log2FC**	**p value**	**BH q value**	**log2FC**
1752	Filamin A-interacting protein 1-like	Q4L180	0.4108	0.5764	-3.31	0.5752	0.8068	-1.75	0.5661	0.7257	-1.19	0.5388	0.6861	0.89	0.2938	0.4715	-1.88	0.9510	1.1003	0.09	0.3230	0.5885	-0.09	0.0014	0.0297	-0.49	0.0047	0.0676	-0.62
1753	Filamin-A	P21333	0.0014	0.0256	0.41	0.1575	0.6617	0.36	0.0008	0.0216	0.52	0.0002	0.0115	0.62	0.0030	0.0418	0.39	0.0037	0.0595	0.59	0.0804	0.2257	0.38	0.5964	1.0298	0.06	0.9860	0.9891	0.00
1754	Filamin-A-interacting protein 1	Q7Z7B0	0.2593	0.4480	-0.19	0.4044	0.7476	-0.44	0.0298	0.0876	-0.53	0.0585	0.1291	-0.41	0.8304	0.8683	0.03	0.6481	0.8851	0.09	0.0229	0.1363	0.30	0.0106	0.0845	0.24	0.3262	0.3976	0.05
1755	Filamin-B	O75369	0.0144	0.0699	0.26	0.1790	0.6647	0.27	0.0036	0.0317	0.36	0.0034	0.0235	0.36	0.0225	0.1081	0.24	0.0114	0.0733	0.37	0.0431	0.1682	0.47	0.2809	0.6184	0.13	0.6135	0.6730	0.04
1756	Filamin-binding LIM protein 1	Q8WUP2	0.4108	0.5762	-3.31	0.5752	0.8066	-1.75	0.5661	0.7255	-1.19	0.5388	0.6859	0.89	0.2938	0.4713	-1.88	0.9510	1.1001	0.09	0.0493	0.1770	0.42	0.2854	0.6238	0.12	0.2738	0.3458	0.10
1757	Filamin-C	Q14315	0.4108	0.5761	-3.31	0.5752	0.8064	-1.75	0.5661	0.7253	-1.19	0.5388	0.6857	0.89	0.2938	0.4712	-1.88	0.9510	1.0998	0.09	0.0010	0.0411	0.40	0.0046	0.0550	0.31	0.0053	0.0704	0.24
1758	FK506-binding protein 15	Q5T1M5	0.2533	0.4406	0.12	0.0747	0.6123	0.24	0.0526	0.1297	0.24	0.1469	0.2608	0.15	0.5568	0.6398	0.05	0.4061	0.6224	0.07	0.3736	0.4688	-2.55	0.8708	0.9759	-0.33	0.0651	0.1273	2.06
1759	Flap endonuclease 1	P39748	0.4423	0.5199	0.03	0.2279	0.6602	0.10	0.0057	0.0365	0.22	0.0303	0.0809	0.15	0.0123	0.0794	-0.18	0.5121	0.7389	0.02	0.0396	0.1640	0.18	0.8775	0.9071	-0.01	0.0661	0.1126	0.14
1760	Flavin reductase (NADPH)	P30043	0.0001	0.0126	0.39	0.1168	0.6355	0.29	0.1898	0.3364	0.10	0.0152	0.0508	0.27	0.8487	0.8837	0.00	0.0334	0.1177	0.33	0.1400	0.3206	-0.18	0.0058	0.0612	-0.46	0.0048	0.0676	-0.65
1761	Flotillin-1	O75955	0.4108	0.5759	-3.31	0.5752	0.8062	-1.75	0.5661	0.7251	-1.19	0.5388	0.6856	0.89	0.2938	0.4710	-1.88	0.9510	1.0996	0.09	0.0004	0.0357	-1.12	0.0011	0.0285	-1.42	0.0010	0.0395	-1.51
1762	FLYWCH family member 2	Q96CP2	0.0240	0.0931	-0.16	0.5193	0.8398	0.11	0.3420	0.5351	0.05	0.6759	0.7167	-0.02	0.0041	0.0469	-0.21	0.0298	0.1108	-0.12	0.0777	0.2218	0.23	0.0688	0.2533	0.12	0.1488	0.2113	0.09
1763	FLYWCH-type zinc finger-containing protein 1	Q4VC44	0.0029	0.0363	-0.55	0.0111	0.4291	0.28	0.0047	0.0338	0.32	0.0101	0.0404	0.40	0.2496	0.4668	0.07	0.1314	0.2823	-0.18	0.3736	0.5237	-2.55	0.8708	1.0768	-0.33	0.0651	0.1520	2.06
1764	Folate receptor beta	P14207	0.0950	0.2172	0.25	0.0279	0.5036	0.51	0.0360	0.0994	0.39	0.8506	0.8711	0.03	0.2036	0.4049	0.37	0.1511	0.3086	-0.19	0.3736	0.5159	-2.55	0.8708	1.0626	-0.33	0.0651	0.1483	2.06
1765	Folliculin-interacting protein 1	Q8TF40	0.0592	0.1610	0.15	0.0658	0.5938	0.23	0.0251	0.0787	0.18	0.1037	0.1992	0.12	0.0860	0.2290	0.12	0.4733	0.6969	0.04	0.3736	0.4624	-2.55	0.8708	0.9640	-0.33	0.0651	0.1246	2.06
1766	Folliculin-interacting protein 2	Q9P278	0.3672	0.5808	0.28	0.2042	0.6594	0.45	0.1029	0.2090	0.65	0.5055	0.7092	0.23	0.0496	0.1642	0.87	0.6359	0.8733	0.14	0.3736	0.4490	-2.55	0.8708	0.9390	-0.33	0.0651	0.1191	2.06
1767	Follistatin	P19883	0.0031	0.0367	-0.17	0.4227	0.7600	-0.14	0.0700	0.1572	-0.16	0.3256	0.4975	0.06	0.0008	0.0211	-0.14	0.3823	0.5920	-0.07	0.1113	0.2747	-0.19	0.0384	0.1786	-0.30	0.0001	0.0199	-6.56
1768	Follistatin-related protein 1	Q12841	0.4108	0.5757	-3.31	0.5752	0.8059	-1.75	0.5661	0.7249	-1.19	0.5388	0.6854	0.89	0.2938	0.4709	-1.88	0.9510	1.0993	0.09	0.0104	0.1018	-0.51	0.0165	0.1092	-0.56	0.0003	0.0239	-0.78
1769	Follitropin subunit beta	P01225	0.3832	0.5995	0.07	0.2659	0.6625	0.20	0.9089	0.9281	-0.01	0.1216	0.2252	0.12	0.3140	0.4113	-0.09	0.5609	0.7958	0.03	0.0200	0.1311	-7.28	0.0908	0.3002	-4.10	0.1002	0.1558	-4.68
1770	Folylpolyglutamate synthase mitochondrial	Q05932	0.4108	0.5756	-3.31	0.5752	0.8057	-1.75	0.5661	0.7248	-1.19	0.5388	0.6852	0.89	0.2938	0.4707	-1.88	0.9510	1.0991	0.09	0.3873	0.4433	0.11	0.0601	0.2344	0.15	0.5310	0.5972	-0.03
1771	Forkhead box protein D4-like 1	Q9NU39	0.0297	0.1057	0.24	0.8444	0.8883	0.02	0.0008	0.0199	0.46	0.4247	0.6169	0.13	0.0794	0.2174	-0.20	0.3967	0.6101	0.09	0.3736	0.4695	-2.55	0.8708	0.9772	-0.33	0.0651	0.1275	2.06
1772	Forkhead box protein M1	Q08050	0.4108	0.5754	-3.31	0.5752	0.8055	-1.75	0.5661	0.7246	-1.19	0.5388	0.6851	0.89	0.2938	0.4706	-1.88	0.9510	1.0989	0.09	0.2074	0.4230	-0.19	0.0046	0.0544	-0.62	0.0065	0.0757	-0.92
1773	Formin-1	Q68DA7	0.0345	0.1163	0.16	0.8584	0.8977	-0.01	0.0023	0.0263	0.58	0.0002	0.0108	0.38	0.0033	0.0435	0.36	0.0213	0.0944	0.23	0.0526	0.1815	-0.24	0.0012	0.0285	-0.21	0.0081	0.0805	0.13
1774	Formin-2	Q9NZ56	0.0767	0.1891	-10.53	0.0661	0.5944	-8.98	0.0230	0.0746	-8.42	0.0014	0.0169	-6.34	0.0058	0.0564	-9.11	0.0075	0.0653	-7.14	0.0030	0.0612	8.72	0.0029	0.0454	9.17	0.0030	0.0551	9.67
1775	Four and a half LIM domains protein 1	Q13642	0.4108	0.5753	-3.31	0.5752	0.8053	-1.75	0.5661	0.7244	-1.19	0.5388	0.6849	0.89	0.2938	0.4704	-1.88	0.9510	1.0986	0.09	0.0781	0.2222	0.20	0.8157	1.2605	-0.01	0.1230	0.1821	-0.08
1776	Four and a half LIM domains protein 2	Q14192	0.4108	0.5751	-3.31	0.5752	0.8051	-1.75	0.5661	0.7242	-1.19	0.5388	0.6847	0.89	0.2938	0.4703	-1.88	0.9510	1.0984	0.09	0.0128	0.1069	0.50	0.0807	0.2794	0.21	0.0051	0.0686	0.69
1777	Four and a half LIM domains protein 5	Q5TD97	0.0671	0.1716	0.12	0.0875	0.6209	0.36	0.0103	0.0473	0.44	0.0005	0.0123	0.44	0.0027	0.0401	0.40	0.0069	0.0645	0.26	0.3736	0.6225	-2.55	0.8708	1.2519	-0.33	0.0651	0.2066	2.06
1778	Fragile X mental retardation syndrome-related protein 1	P51114	0.0058	0.0460	0.17	0.1622	0.6616	0.20	0.0008	0.0215	0.34	0.0047	0.0279	0.17	0.3677	0.4647	0.02	0.0186	0.0881	0.25	0.0239	0.1373	0.34	0.6312	1.0678	0.03	0.5380	0.6037	0.03
1779	Fragile X mental retardation syndrome-related protein 2	P51116	0.2745	0.4674	-0.07	0.3996	0.7432	0.15	0.0158	0.0592	0.18	0.8493	0.8700	-0.01	0.0022	0.0362	0.26	0.0509	0.1519	-0.05	0.1853	0.3866	0.17	0.0737	0.2635	0.25	0.0557	0.2091	0.33
1780	Free fatty acid receptor 2	O15552	0.2850	0.4804	-0.06	0.7380	0.8096	-0.05	0.0752	0.1647	0.17	0.2098	0.3456	0.08	0.0660	0.1955	-0.14	0.1506	0.3083	0.09	0.3736	0.5166	-2.55	0.8708	1.0639	-0.33	0.0651	0.1486	2.06
1781	Fructose-1 6-bisphosphatase 1	P09467	0.7514	0.7976	-0.01	0.2433	0.6588	0.25	0.4875	0.7076	-0.04	0.2689	0.4247	-0.06	0.0330	0.1327	-0.19	0.2117	0.3932	-0.07	0.3736	0.5003	-2.55	0.8708	1.0341	-0.33	0.0651	0.1410	2.06
1782	Fructose-1 6-bisphosphatase isozyme 2	O00757	0.3094	0.5104	-0.04	0.2510	0.6607	0.22	0.5057	0.7281	0.05	0.4435	0.6380	0.03	0.0235	0.1107	-0.17	0.2254	0.4085	-0.10	0.3736	0.4966	-2.55	0.8708	1.0273	-0.33	0.0651	0.1393	2.06
1783	Fructose-2 6-bisphosphatase TIGAR	Q9NQ88	0.0025	0.0337	0.33	0.1265	0.6431	0.45	0.0011	0.0217	0.67	0.0002	0.0113	0.54	0.0568	0.1784	0.11	0.0043	0.0604	0.47	0.0657	0.2017	0.40	0.8026	1.2459	0.02	0.1862	0.2536	0.10
1784	Fructose-bisphosphate aldolase A	P04075	0.0098	0.0579	0.36	0.1565	0.6623	0.28	0.0039	0.0319	0.43	0.0016	0.0177	0.56	0.0404	0.1479	0.21	0.0044	0.0605	0.72	0.0541	0.1847	0.47	0.9423	0.9584	0.01	0.9163	0.9318	-0.01
1785	Fructose-bisphosphate aldolase B	P05062	0.4605	0.5374	-0.02	0.9350	0.9562	-0.01	0.0199	0.0679	0.25	0.1794	0.3044	0.13	0.1912	0.3872	0.09	0.2837	0.4799	0.10	0.3909	0.4466	0.11	0.1580	0.4296	-0.12	0.0955	0.1502	-0.38
1786	Fructose-bisphosphate aldolase C	P09972	0.0087	0.0551	0.23	0.1956	0.6688	0.30	0.0013	0.0230	0.39	0.0002	0.0111	0.62	0.0126	0.0803	0.21	0.0007	0.0432	0.67	0.0049	0.0740	0.36	0.2215	0.5292	0.11	0.0227	0.1274	0.29
1787	Fumarate hydratase mitochondrial	P07954	0.0044	0.0420	-0.38	0.2395	0.6617	-0.19	0.0046	0.0334	-0.45	0.0084	0.0372	-0.39	0.0984	0.2505	-0.16	0.0061	0.0620	-0.42	0.0343	0.1542	0.27	0.2834	0.6210	0.10	0.2235	0.2935	0.12
1788	Fumarylacetoacetate hydrolase domain-containing protein 2A	Q96GK7	0.3406	0.5486	0.18	0.1976	0.6699	-0.32	0.0731	0.1618	0.36	0.2035	0.3371	0.30	0.0419	0.1512	0.48	0.1883	0.3613	0.67	0.3736	0.5052	-2.55	0.8708	1.0429	-0.33	0.0651	0.1433	2.06
1789	Fumarylacetoacetate hydrolase domain-containing protein 2B	Q6P2I3	0.4108	0.5750	-3.31	0.5752	0.8048	-1.75	0.5661	0.7240	-1.19	0.5388	0.6846	0.89	0.2938	0.4702	-1.88	0.9510	1.0981	0.09	0.3457	0.6188	0.24	0.8607	1.3025	0.04	0.2113	0.2800	0.35
1790	FXYD domain-containing ion transport regulator 3	Q14802	0.0238	0.0926	-0.17	0.1767	0.6623	0.19	0.0141	0.0558	-0.27	0.1010	0.1954	0.10	0.0083	0.0655	0.36	0.0900	0.2190	0.12	0.3736	0.5379	-2.55	0.8708	1.1023	-0.33	0.0651	0.1590	2.06
1791	FYVE and coiled-coil domain-containing protein 1	Q9BQS8	0.0196	0.0821	-0.26	0.4954	0.8175	0.10	0.0031	0.0297	0.42	0.4502	0.6460	0.05	0.0138	0.0838	0.43	0.7261	0.9661	0.03	0.0139	0.1111	0.48	0.0377	0.1769	0.35	0.0574	0.2134	0.32
1792	FYVE RhoGEF and PH domain-containing protein 5	Q6ZNL6	0.1118	0.2429	0.07	0.2784	0.6652	0.14	0.2359	0.3971	0.06	0.5621	0.6122	0.02	0.0008	0.0210	0.47	0.0748	0.1946	-0.10	0.3736	0.5470	-2.55	0.8708	1.1187	-0.33	0.0651	0.1636	2.06
1793	G protein-activated inward rectifier potassium channel 3	Q92806	0.4108	0.5748	-3.31	0.5752	0.8046	-1.75	0.5661	0.7239	-1.19	0.5388	0.6844	0.89	0.2938	0.4700	-1.88	0.9510	1.0979	0.09	0.0065	0.0840	-0.58	0.0151	0.1040	-0.61	0.0004	0.0273	-0.81
1794	G protein-coupled receptor kinase 4	P32298	0.2782	0.4723	-0.16	0.5132	0.8357	-0.30	0.3032	0.4861	-0.21	0.1386	0.2491	-0.37	0.6440	0.7125	0.06	0.2954	0.4925	-0.14	0.3736	0.4825	-2.55	0.8708	1.0013	-0.33	0.0651	0.1331	2.06
1795	G2/M phase-specific E3 ubiquitin-protein ligase	Q7L622	0.0084	0.0544	-0.69	0.4101	0.7509	-0.40	0.0089	0.0440	-0.79	0.0035	0.0238	-0.86	0.5759	0.6551	0.06	0.0162	0.0843	-0.59	0.3736	0.6004	-2.55	0.8708	1.2135	-0.33	0.0651	0.1931	2.06
1796	G2/mitotic-specific cyclin-B1	P14635	0.0061	0.0470	0.32	0.3888	0.7321	0.10	0.0100	0.0466	0.32	0.0008	0.0144	0.30	0.6419	0.7108	-0.02	0.0050	0.0617	0.29	0.7555	0.7912	-0.04	0.9801	0.9858	0.00	0.4215	0.4929	0.13
1797	Galactokinase	P51570	0.8376	0.8704	-0.01	0.4817	0.8065	0.10	0.1434	0.2696	0.08	0.0285	0.0774	0.11	0.2990	0.3970	0.03	0.0754	0.1950	0.09	0.9325	0.9442	-0.01	0.2173	0.5235	-0.24	0.5020	0.5691	-0.11
1798	Galactosylgalactosylxylosylprotein 3-beta-glucuronosyltransferase 3	O94766	0.0011	0.0230	0.68	0.0026	0.2541	-0.34	0.1369	0.2605	-0.20	0.0492	0.1139	-0.32	0.3060	0.4035	-0.12	0.1569	0.3164	-0.19	0.3736	0.5132	-2.55	0.8708	1.0576	-0.33	0.0651	0.1470	2.06
1799	Galectin-1	P09382	0.0007	0.0210	0.40	0.3018	0.6753	0.17	0.0055	0.0359	0.25	0.0002	0.0098	0.50	0.0052	0.0542	0.37	0.0016	0.0515	0.63	0.8525	0.8769	0.03	0.0336	0.1659	-0.34	0.0155	0.1072	-0.55
1800	Galectin-3	P17931	0.7283	0.7788	-0.02	0.4321	0.7681	-0.14	0.2092	0.3629	-0.09	0.3988	0.5879	0.06	0.9603	0.9693	0.00	0.0154	0.0825	0.33	0.3736	0.6019	-2.55	0.8708	1.2162	-0.33	0.0651	0.1940	2.06
1801	Galectin-3-binding protein	Q08380	0.1044	0.2313	-0.15	0.1916	0.6688	0.11	0.0550	0.1336	-0.36	0.2756	0.4331	0.09	0.1943	0.3922	-0.10	0.2080	0.3878	-0.10	0.3736	0.5008	-2.55	0.8708	1.0350	-0.33	0.0651	0.1413	2.06
1802	Gametocyte-specific factor 1	Q8WW33	0.6628	0.7228	0.04	0.2826	0.6642	0.16	0.2619	0.4315	0.10	0.1388	0.2494	0.15	0.1879	0.3833	-0.12	0.3113	0.5118	0.09	0.3736	0.4800	-2.55	0.8708	0.9966	-0.33	0.0651	0.1320	2.06
1803	Gamma-aminobutyric acid receptor-associated protein-like 2	P60520	0.2945	0.4926	0.05	0.7918	0.8502	-0.03	0.1036	0.2099	0.11	0.0567	0.1262	-0.06	0.0002	0.0125	-0.32	0.2519	0.4420	-0.08	0.2082	0.4238	0.18	0.0724	0.2610	-0.24	0.0347	0.1598	-0.25
1804	Gamma-crystallin A	P11844	0.1900	0.3561	0.10	0.0558	0.5839	0.37	0.0035	0.0315	0.54	0.0015	0.0177	0.56	0.0717	0.2041	0.18	0.0340	0.1189	0.26	0.3736	0.5760	-2.55	0.8708	1.1704	-0.33	0.0651	0.1791	2.06
1805	Gamma-crystallin S	P22914	0.0057	0.0455	-0.27	0.2138	0.6624	-0.16	0.0061	0.0378	-0.30	0.0116	0.0432	-0.24	0.3313	0.4286	-0.05	0.2039	0.3819	-0.17	0.3736	0.5016	-2.55	0.8708	1.0365	-0.33	0.0651	0.1416	2.06
1806	Gamma-enolase	P09104	0.2339	0.4165	0.17	0.1085	0.6308	0.49	0.0069	0.0399	0.28	0.0024	0.0210	0.61	0.0020	0.0354	0.24	0.0019	0.0532	0.38	0.0554	0.1874	-0.21	0.7336	1.1765	-0.03	0.2105	0.2791	-0.11
1807	Gamma-glutamyl hydrolase	Q92820	0.0691	0.1749	-0.26	0.2496	0.6597	-0.32	0.5315	0.7528	-0.04	0.6791	0.7195	-0.04	0.0746	0.2099	-0.12	0.2895	0.4861	0.04	0.3736	0.4836	-2.55	0.8708	1.0033	-0.33	0.0651	0.1336	2.06
1808	Gamma-glutamylcyclotransferase	O75223	0.0070	0.0497	0.23	0.1070	0.6298	0.27	0.0021	0.0259	0.29	0.0011	0.0161	0.34	0.0634	0.1908	0.10	0.0186	0.0882	0.21	0.9308	0.9430	0.01	0.9628	0.9735	0.00	0.1260	0.1855	-0.16
1809	Gamma-soluble NSF attachment protein	Q99747	0.0044	0.0419	0.31	0.6929	0.7742	-0.03	0.0053	0.0354	0.28	0.0016	0.0177	0.45	0.1573	0.3391	0.09	0.0032	0.0570	0.42	0.1199	0.2897	0.16	0.1556	0.4267	0.13	0.7510	0.7944	0.03
1810	Gamma-synuclein	O76070	0.4646	0.5411	0.31	0.3832	0.7306	-0.38	0.7871	0.8255	-0.11	0.3283	0.5002	-0.44	0.2065	0.4087	-0.60	0.9181	1.1554	-0.04	0.3736	0.4333	-2.55	0.8708	0.9094	-0.33	0.0651	0.1129	2.06
1811	Gamma-taxilin	Q9NUQ3	0.1736	0.3350	-0.04	0.7938	0.8506	-0.01	0.0611	0.1435	0.22	0.0529	0.1200	0.09	0.0063	0.0579	0.19	0.4891	0.7145	-0.03	0.3736	0.4611	-2.55	0.8708	0.9616	-0.33	0.0651	0.1240	2.06
1812	Gamma-tubulin complex component 5	Q96RT8	0.4108	0.5747	-3.31	0.5752	0.8044	-1.75	0.5661	0.7237	-1.19	0.5388	0.6842	0.89	0.2938	0.4699	-1.88	0.9510	1.0976	0.09	0.1275	0.3008	-0.44	0.2757	0.6116	-0.26	0.0435	0.1820	-0.77
1813	GAS2-like protein 2	Q8NHY3	0.0060	0.0469	0.60	0.2372	0.6617	-0.14	0.0915	0.1905	-0.28	0.5460	0.5971	-0.06	0.9506	0.9619	0.01	0.7300	0.9698	-0.04	0.3736	0.4436	-2.55	0.8708	0.9287	-0.33	0.0651	0.1169	2.06
1814	Gastrin/cholecystokinin type B receptor	P32239	0.9315	0.9480	0.01	0.2386	0.6613	0.22	0.9462	0.9578	0.01	0.0724	0.1517	0.15	0.0147	0.0864	0.28	0.0877	0.2157	0.15	0.3736	0.5395	-2.55	0.8708	1.1053	-0.33	0.0651	0.1598	2.06
1815	GATA zinc finger domain-containing protein 1	Q8WUU5	0.4108	0.5745	-3.31	0.5752	0.8042	-1.75	0.5661	0.7235	-1.19	0.5388	0.6841	0.89	0.2938	0.4697	-1.88	0.9510	1.0974	0.09	0.0375	0.1607	0.64	0.0025	0.0411	0.30	0.0002	0.0253	0.38
1816	GATOR complex protein DEPDC5	O75140	0.6857	0.7415	0.01	0.3062	0.6784	0.10	0.9702	0.9747	0.00	0.9182	0.9284	0.00	0.0056	0.0551	-0.17	0.0124	0.0751	-0.15	0.3736	0.6078	-2.55	0.8708	1.2264	-0.33	0.0651	0.1976	2.06
1817	GATOR complex protein WDR24	Q96S15	0.4108	0.5743	-3.31	0.5752	0.8040	-1.75	0.5661	0.7233	-1.19	0.5388	0.6839	0.89	0.2938	0.4696	-1.88	0.9510	1.0971	0.09	0.0145	0.1116	-0.49	0.5021	0.9184	-0.06	0.2660	0.3376	-0.20
1818	GC-rich sequence DNA-binding factor 2	P16383	0.7488	0.7953	-0.02	0.0123	0.4364	0.25	0.0072	0.0404	0.24	0.0566	0.1261	0.21	0.0030	0.0408	0.47	0.5189	0.7466	0.01	0.2902	0.5425	0.29	0.2609	0.5884	0.29	0.0709	0.1190	0.62
1819	GDNF family receptor alpha-1	P56159	0.2473	0.4332	0.12	0.4898	0.8127	0.14	0.0567	0.1362	0.25	0.0305	0.0812	0.31	0.7493	0.8041	0.03	0.0240	0.0989	0.43	0.3736	0.5880	-2.55	0.8708	1.1917	-0.33	0.0651	0.1859	2.06
1820	GDNF family receptor alpha-2	O00451	0.4108	0.5742	-3.31	0.5752	0.8038	-1.75	0.5661	0.7232	-1.19	0.5388	0.6837	0.89	0.2938	0.4694	-1.88	0.9510	1.0969	0.09	0.1212	0.2913	0.23	0.5602	0.9867	0.05	0.8079	0.8436	0.02
1821	GDNF-inducible zinc finger protein 1	Q9H116	0.0034	0.0384	0.46	0.0766	0.6142	0.36	0.0303	0.0885	0.54	0.0066	0.0327	0.63	0.3382	0.4354	0.08	0.0450	0.1403	0.73	0.3736	0.5654	-2.55	0.8708	1.1516	-0.33	0.0651	0.1733	2.06
1822	GDP-fucose protein O-fucosyltransferase 1	Q9H488	0.2091	0.3818	0.10	0.3110	0.6792	-0.10	0.2973	0.4786	0.09	0.6228	0.6693	0.04	0.3026	0.4004	-0.08	0.3812	0.5905	0.07	0.0005	0.0387	-0.37	0.0002	0.0195	-0.47	0.0013	0.0461	-0.71
1823	GDP-fucose protein O-fucosyltransferase 2	Q9Y2G5	0.0169	0.0761	-0.13	0.3916	0.7355	0.07	0.2938	0.4744	-0.06	0.8094	0.8364	0.01	0.0667	0.1964	0.04	0.1114	0.2522	-0.14	0.3736	0.5308	-2.55	0.8708	1.0896	-0.33	0.0651	0.1555	2.06
1824	GDP-L-fucose synthase	Q13630	0.3588	0.5705	0.07	0.3923	0.7363	0.07	0.6527	0.7045	-0.03	0.0815	0.1663	-0.18	0.9973	0.9985	0.00	0.2157	0.3966	-0.13	0.0313	0.1494	0.12	0.0214	0.1267	-0.10	0.3768	0.4483	0.02
1825	GDP-mannose 4 6 dehydratase	O60547	0.4108	0.5740	-3.31	0.5752	0.8035	-1.75	0.5661	0.7230	-1.19	0.5388	0.6836	0.89	0.2938	0.4693	-1.88	0.9510	1.0967	0.09	0.4874	0.5416	0.12	0.1054	0.3287	0.33	0.2296	0.3000	0.21
1826	Gelsolin	P06396	0.0684	0.1737	-0.04	0.2210	0.6594	0.11	0.7043	0.7511	0.01	0.1917	0.3212	0.04	0.0000	0.0000	0.60	0.0001	0.0471	0.36	0.1505	0.3366	0.23	0.1758	0.4606	-0.12	0.0620	0.2227	0.10
1827	Gem-associated protein 4	P57678	0.0739	0.1843	0.08	0.1041	0.6337	0.25	0.1344	0.2570	0.09	0.1080	0.2057	0.07	0.3750	0.4723	0.03	0.6722	0.9103	-0.01	0.0423	0.1673	-0.18	0.3719	0.7444	-0.17	0.8080	0.8435	0.03
1828	Gem-associated protein 5	Q8TEQ6	0.0413	0.1286	0.15	0.1801	0.6649	0.20	0.0121	0.0515	0.25	0.0071	0.0342	0.23	0.6055	0.6805	0.02	0.0223	0.0959	0.18	0.3229	0.5885	0.09	0.0563	0.2268	0.24	0.2006	0.2690	-0.14
1829	Gem-associated protein 6	Q8WXD5	0.3065	0.5076	-0.18	0.2310	0.6626	0.25	0.2485	0.4148	-0.21	0.4375	0.6309	0.13	0.7691	0.8202	0.04	0.2518	0.4420	-0.29	0.3736	0.4903	-2.55	0.8708	1.0157	-0.33	0.0651	0.1365	2.06
1830	General transcription factor 3C polypeptide 4	Q9UKN8	0.0006	0.0201	0.32	0.1002	0.6345	0.24	0.0011	0.0221	0.32	0.0007	0.0139	0.30	0.0003	0.0160	0.40	0.0060	0.0627	0.30	0.3736	0.6257	-2.55	0.8708	1.2575	-0.33	0.0651	0.2087	2.06
1831	General transcription factor IIF subunit 1	P35269	0.4601	0.5370	0.04	0.0223	0.4773	0.24	0.0030	0.0301	0.32	0.2910	0.4532	0.02	0.0621	0.1884	0.09	0.2958	0.4928	-0.09	0.0267	0.1417	0.31	0.0079	0.0728	0.46	0.0115	0.0935	0.64

Supplemetary Table S2. Overview on all relatively quantified 5180 proteins statistical analysis

			MCF-7																		MDA-MB-231								
			Dai SC20 vs control			Gen SC20 vs control			SSE SC20 vs control			Dai IC20 vs control			Gen IC20 vs control			SSE IC20 vs control			Dai IC20 vs control			Gen IC20 vs control			SSE IC20 vs control		
	**Protein name**	**UniProt**	**p value**	**BH q value**	**log2FC**	**p value**	**BH q value**	**log2FC**	**p value**	**BH q value**	**log2FC**	**p value**	**BH q value**	**log2FC**	**p value**	**BH q value**	**log2FC**	**p value**	**BH q value**	**log2FC**	**p value**	**BH q value**	**log2FC**	**p value**	**BH q value**	**log2FC**	**p value**	**BH q value**	**log2FC**
1832	General transcription factor IIF subunit 2	P13984	0.4998	0.5735	-0.04	0.4936	0.8166	0.07	0.0776	0.1685	-0.14	0.3029	0.4684	-0.06	0.1650	0.3504	-0.11	0.9074	1.1447	0.01	0.0837	0.2312	0.50	0.7444	1.1872	-0.09	0.0925	0.1464	0.49
1833	General transcription factor II-I	P78347	0.9213	0.9400	0.01	0.9531	0.9688	-0.01	0.1288	0.2494	-0.12	0.4781	0.6785	0.04	0.0864	0.2296	-0.15	0.4234	0.6430	0.05	0.9568	0.9646	-0.01	0.7150	1.1607	-0.02	0.3728	0.4446	0.11
1834	General transcription factor II-I repeat domain-containing protein 2B	Q6EKJ0	0.0038	0.0394	0.29	0.1499	0.6603	0.13	0.0361	0.0995	0.16	0.0129	0.0460	0.25	0.0350	0.1360	0.13	0.1458	0.3014	0.18	0.3736	0.5179	-2.55	0.8708	1.0661	-0.33	0.0651	0.1492	2.06
1835	General vesicular transport factor p115	O60763	0.0050	0.0435	0.27	0.1562	0.6627	0.24	0.0009	0.0211	0.40	0.0009	0.0155	0.40	0.0059	0.0565	0.39	0.0036	0.0596	0.45	0.0462	0.1727	0.33	0.6313	1.0676	-0.05	0.8742	0.8985	0.01
1836	Geranylgeranyl transferase type-2 subunit alpha	Q92696	0.1102	0.2410	0.09	0.6183	0.7172	0.08	0.0446	0.1155	0.18	0.0015	0.0172	0.23	0.7994	0.8435	-0.01	0.1135	0.2556	0.11	0.3736	0.5302	-2.55	0.8708	1.0885	-0.33	0.0651	0.1552	2.06
1837	Geranylgeranyl transferase type-2 subunit beta	P53611	0.1331	0.2760	-0.11	0.4703	0.7998	0.10	0.0238	0.0756	0.16	0.0118	0.0436	0.19	0.0256	0.1152	0.23	0.5523	0.7862	0.06	0.3736	0.4566	-2.55	0.8708	0.9532	-0.33	0.0651	0.1222	2.06
1838	Germ cell-less protein-like 2	Q8NEA9	0.4108	0.5739	-3.31	0.5752	0.8033	-1.75	0.5661	0.7228	-1.19	0.5388	0.6834	0.89	0.2938	0.4691	-1.88	0.9510	1.0964	0.09	0.0106	0.1021	0.89	0.2045	0.5044	0.17	0.1747	0.2406	0.31
1839	Germinal-center associated nuclear protein	O60318	0.0763	0.1883	-1.32	0.4881	0.8117	-0.43	0.0814	0.1751	-1.23	0.0811	0.1660	-1.18	0.4084	0.5041	-0.45	0.0430	0.1366	-1.55	0.0252	0.1398	-0.35	0.1110	0.3402	-0.18	0.0386	0.1699	-0.33
1840	Gigaxonin	Q9H2C0	0.0001	0.0123	-5.53	0.2189	0.6604	-4.12	0.0087	0.0439	-4.18	0.0024	0.0210	-5.38	0.9930	0.9953	0.00	0.0016	0.0496	-6.08	0.3736	0.6459	-2.55	0.8708	1.2925	-0.33	0.0651	0.2220	2.06
1841	Girdin	Q3V6T2	0.0417	0.1294	0.14	0.1055	0.6318	0.18	0.0073	0.0405	0.25	0.0048	0.0279	0.28	0.0922	0.2394	0.11	0.0255	0.1014	0.19	0.0579	0.1915	0.34	0.4260	0.8206	0.10	0.9182	0.9333	0.01
1842	Glia maturation factor beta	P60983	0.6390	0.7008	0.02	0.1938	0.6679	0.08	0.0046	0.0334	0.25	0.0224	0.0654	0.18	0.0426	0.1524	0.14	0.0897	0.2185	0.22	0.0108	0.1019	0.47	0.0043	0.0527	0.17	0.0003	0.0259	0.36
1843	Glial fibrillary acidic protein	P14136	0.0223	0.0891	0.38	0.9428	0.9621	-0.01	0.1289	0.2494	0.17	0.0148	0.0498	0.42	0.5247	0.6116	0.07	0.0556	0.1608	0.53	0.0934	0.2452	0.38	0.0310	0.1591	0.43	0.0100	0.0882	0.71
1844	GLIPR1-like protein 2	Q4G1C9	0.1737	0.3351	0.99	0.3164	0.6801	0.68	0.1721	0.3130	1.01	0.2536	0.4036	0.79	0.2749	0.4989	0.73	0.1684	0.3331	1.02	0.3736	0.5098	-2.55	0.8708	1.0515	-0.33	0.0651	0.1454	2.06
1845	Glucocorticoid modulatory element-binding protein 2	Q9UKD1	0.7433	0.7918	-0.13	0.9866	0.9904	0.01	0.5115	0.7325	0.29	0.8960	0.9108	-0.06	0.2080	0.4106	0.60	0.4132	0.6308	0.40	0.3736	0.4681	-2.55	0.8708	0.9747	-0.33	0.0651	0.1270	2.06
1846	Glucosamine 6-phosphate N-acetyltransferase	Q96EK6	0.0267	0.0989	0.14	0.1511	0.6594	0.08	0.6389	0.6918	0.02	0.2102	0.3459	0.06	0.2238	0.4322	0.05	0.0087	0.0680	0.24	0.0296	0.1474	-0.86	0.0598	0.2340	-0.19	0.0039	0.0608	-0.28
1847	Glucosamine-6-phosphate isomerase 1	P46926	0.0142	0.0697	0.25	0.0073	0.4023	-0.25	0.0088	0.0440	-0.30	0.0027	0.0218	-0.52	0.0016	0.0320	-0.40	0.4491	0.6718	-0.04	0.0410	0.1664	0.33	0.9247	0.9448	-0.01	0.0016	0.0471	-0.27
1848	Glucose-6-phosphate 1-dehydrogenase	P11413	0.0032	0.0371	0.39	0.2549	0.6625	0.24	0.0017	0.0234	0.47	0.0005	0.0128	0.66	0.0121	0.0791	0.33	0.0011	0.0467	0.84	0.0147	0.1125	0.54	0.0762	0.2694	0.22	0.0987	0.1542	0.16
1849	Glucose-6-phosphate isomerase	P06744	0.0428	0.1312	0.18	0.1735	0.6608	0.29	0.0105	0.0478	0.29	0.0101	0.0404	0.28	0.4530	0.5458	0.04	0.0252	0.1009	0.28	0.2047	0.4183	0.26	0.3636	0.7329	0.09	0.1937	0.2617	-0.11
1850	Glucose-fructose oxidoreductase domain-containing protein 2	Q3B7J2	0.4108	0.5737	-3.31	0.5752	0.8031	-1.75	0.5661	0.7226	-1.19	0.5388	0.6832	0.89	0.2938	0.4690	-1.88	0.9510	1.0962	0.09	0.1920	0.3991	-0.68	0.8272	1.2707	-0.08	0.5718	0.6348	0.22
1851	Glucose-induced degradation protein 8 homolog	Q9NWU2	0.1588	0.3135	-0.03	0.9370	0.9573	0.02	0.4747	0.6927	0.08	0.7880	0.8175	0.03	0.7993	0.8436	-0.02	0.5892	0.8262	-0.12	0.0527	0.1815	-1.38	0.1368	0.3909	0.27	0.7192	0.7680	0.05
1852	Glucosidase 2 subunit beta	P14314	0.0144	0.0698	0.26	0.2812	0.6651	0.24	0.0286	0.0857	0.21	0.0074	0.0349	0.32	0.7722	0.8222	0.02	0.0131	0.0764	0.34	0.2120	0.4301	0.16	0.6478	1.0870	-0.04	0.0792	0.1300	-0.20
1853	Glutamate dehydrogenase 1 mitochondrial	P00367	0.2304	0.4115	0.06	0.2944	0.6715	0.17	0.0444	0.1152	0.14	0.0410	0.1003	0.14	0.3542	0.4518	-0.04	0.0682	0.1830	0.13	0.1068	0.2670	0.22	0.6693	1.1073	-0.04	0.0484	0.1917	-0.14
1854	Glutamate dehydrogenase 2 mitochondrial	P49448	0.4829	0.5575	0.03	0.3522	0.7041	0.10	0.0066	0.0392	0.19	0.1064	0.2034	0.06	0.4441	0.5382	-0.02	0.0084	0.0669	0.17	0.0222	0.1354	-0.16	0.0009	0.0260	-0.77	0.0049	0.0679	-0.98
1855	Glutamate receptor ionotropic NMDA 2A	Q12879	0.3084	0.5092	0.08	0.3271	0.6860	-0.08	0.0347	0.0969	-0.25	0.0423	0.1023	-0.25	0.0307	0.1280	-0.34	0.4038	0.6192	-0.08	0.0419	0.1672	-0.29	0.0285	0.1525	-0.80	0.0195	0.1210	-1.05
1856	Glutamate--cysteine ligase regulatory subunit	P48507	0.4108	0.5736	-3.31	0.5752	0.8029	-1.75	0.5661	0.7224	-1.19	0.5388	0.6831	0.89	0.2938	0.4688	-1.88	0.9510	1.0959	0.09	0.0138	0.1107	0.51	0.9777	0.9842	0.00	0.0220	0.1263	0.08
1857	Glutamate-rich protein 4	A6NGS2	0.4261	0.5036	-0.02	0.0992	0.6360	-0.23	0.0078	0.0417	0.16	0.3402	0.5142	-0.06	0.1280	0.2953	-0.05	0.0101	0.0702	0.16	0.5409	0.5924	-0.06	0.0956	0.3083	0.13	0.0516	0.1990	0.17
1858	Glutamate-rich protein 6B	Q5W0A0	0.4843	0.5588	-0.24	0.4854	0.8095	-0.52	0.3680	0.5655	-0.31	0.2983	0.4622	-0.61	0.4036	0.4999	0.27	0.1342	0.2864	-0.65	0.3736	0.5225	-2.55	0.8708	1.0745	-0.33	0.0651	0.1514	2.06
1859	Glutamate-rich WD repeat-containing protein 1	Q9BQ67	0.1702	0.3298	0.13	0.1068	0.6308	-0.23	0.0258	0.0800	0.27	0.0885	0.1771	-0.18	0.0347	0.1356	0.25	0.0071	0.0645	0.63	0.0813	0.2274	0.18	0.8478	1.2890	-0.02	0.8608	0.8870	0.01
1860	Glutaminase kidney isoform mitochondrial	O94925	0.5286	0.5999	0.04	0.7233	0.7978	-0.03	0.9606	0.9681	0.00	0.2455	0.3935	-0.08	0.0830	0.2229	-0.15	0.2821	0.4785	0.07	0.0765	0.2202	0.19	0.0369	0.1752	0.20	0.1684	0.2336	0.16
1861	Glutamine amidotransferase-like class 1 domain-containing protein 3B_ mitochondrial	A0A0B4J2D5	0.0836	0.1994	-0.16	0.0169	0.4583	-0.27	0.3258	0.5142	-0.07	0.1650	0.2851	-0.11	0.0139	0.0837	-0.28	0.2943	0.4918	0.08	0.3736	0.4827	-2.55	0.8708	1.0017	-0.33	0.0651	0.1332	2.06
1862	Glutamine--fructose-6-phosphate aminotransferase [isomerizing] 1	Q06210	0.0217	0.0876	0.16	0.2066	0.6602	0.21	0.0064	0.0387	0.23	0.0083	0.0370	0.23	0.2711	0.4941	0.05	0.0190	0.0887	0.24	0.4514	0.5066	0.08	0.1689	0.4487	0.16	0.3401	0.4110	-0.09
1863	Glutamine--fructose-6-phosphate aminotransferase [isomerizing] 2	O94808	0.3676	0.5811	0.03	0.2749	0.6651	0.19	0.0644	0.1481	0.21	0.0020	0.0194	0.25	0.6397	0.7093	0.02	0.2139	0.3953	0.05	0.1151	0.2815	-0.17	0.0026	0.0421	-0.26	0.6455	0.7019	0.02
1864	Glutamine-rich protein 1	Q2TAL8	0.4108	0.5734	-3.31	0.5752	0.8027	-1.75	0.5661	0.7223	-1.19	0.5388	0.6829	0.89	0.2938	0.4687	-1.88	0.9510	1.0957	0.09	0.0530	0.1823	0.46	0.1434	0.4041	0.18	0.6884	0.7412	-0.55
1865	Glutamine--tRNA ligase	P47897	0.2538	0.4413	0.05	0.4652	0.7966	0.10	0.1403	0.2653	0.09	0.0220	0.0645	0.15	0.4930	0.5825	0.03	0.0398	0.1304	0.15	0.0200	0.1313	0.56	0.1903	0.4825	0.19	0.0370	0.1652	0.28
1866	Glutamyl-tRNA(Gln) amidotransferase subunit A_ mitochondrial	Q9H0R6	0.4108	0.5733	-3.31	0.5752	0.8025	-1.75	0.5661	0.7221	-1.19	0.5388	0.6827	0.89	0.2938	0.4686	-1.88	0.9510	1.0954	0.09	0.0472	0.1738	-0.42	0.5300	0.9536	-0.15	0.9295	0.9433	-0.03
1867	Glutamyl-tRNA(Gln) amidotransferase subunit C_ mitochondrial	O43716	0.4108	0.5731	-3.31	0.5752	0.8022	-1.75	0.5661	0.7219	-1.19	0.5388	0.6826	0.89	0.2938	0.4684	-1.88	0.9510	1.0952	0.09	0.0944	0.2470	-0.29	0.8071	1.2506	-0.03	0.9922	0.9939	0.00
1868	Glutaredoxin-3	O76003	0.0001	0.0120	0.17	0.3413	0.6999	0.09	0.0074	0.0406	0.16	0.0002	0.0094	0.11	0.1424	0.3156	-0.06	0.1308	0.2817	0.14	0.0019	0.0535	0.75	0.0007	0.0252	0.90	0.0064	0.0757	0.65
1869	Glutaredoxin-related protein 5 mitochondrial	Q86SX6	0.9794	0.9863	0.00	0.6676	0.7556	-0.07	0.0877	0.1853	-0.27	0.5121	0.7167	-0.08	0.1733	0.3618	-0.19	0.4413	0.6634	0.13	0.1068	0.2669	0.22	0.0589	0.2317	-0.17	0.1257	0.1852	0.04
1870	Glutathione peroxidase 2	P18283	0.2837	0.4785	0.12	0.2878	0.6679	0.13	0.9148	0.9321	-0.01	0.9279	0.9368	-0.01	0.6769	0.7405	0.04	0.2830	0.4792	0.14	0.3736	0.4847	-2.55	0.8708	1.0053	-0.33	0.0651	0.1340	2.06
1871	Glutathione peroxidase 6	P59796	0.0007	0.0208	6.70	0.8641	0.9017	-0.12	0.0770	0.1676	-0.84	0.0025	0.0210	3.07	0.0029	0.0414	2.66	0.3104	0.5104	-0.89	0.3736	0.4801	-2.55	0.8708	0.9968	-0.33	0.0651	0.1320	2.06
1872	Glutathione reductase mitochondrial	P00390	0.0199	0.0829	0.20	0.2432	0.6596	0.20	0.0249	0.0783	0.20	0.0208	0.0621	0.20	0.3194	0.4162	0.05	0.0494	0.1489	0.17	0.9688	0.9744	0.00	0.8430	1.2870	0.02	0.7192	0.7678	0.05
1873	Glutathione S-transferase kappa 1	Q9Y2Q3	0.2742	0.4675	-0.06	0.9380	0.9580	0.01	0.5631	0.7873	-0.04	0.5875	0.6363	-0.03	0.2212	0.4279	-0.07	0.2254	0.4084	0.07	0.1486	0.3335	0.09	0.7522	1.1941	0.02	0.1724	0.2382	0.06
1874	Glutathione S-transferase LANCL1	O43813	0.0971	0.2205	0.14	0.0964	0.6345	0.20	0.3643	0.5615	0.04	0.0166	0.0536	0.16	0.1316	0.2998	0.06	0.3429	0.5470	0.02	0.1870	0.3898	-0.51	0.2085	0.5099	-0.47	0.1137	0.1711	-0.67
1875	Glutathione S-transferase Mu 1	P09488	0.0345	0.1163	0.44	0.2528	0.6624	-0.16	0.2886	0.4675	-0.15	0.0424	0.1024	-0.40	0.8146	0.8557	0.04	0.0219	0.0949	-0.57	0.3736	0.5907	-2.55	0.8708	1.1965	-0.33	0.0651	0.1874	2.06
1876	Glutathione S-transferase Mu 2	P28161	0.0466	0.1383	-0.18	0.1342	0.6497	0.23	0.9179	0.9350	-0.01	0.9263	0.9357	0.00	0.1392	0.3107	0.06	0.0383	0.1273	-0.26	0.3736	0.5712	-2.55	0.8708	1.1620	-0.33	0.0651	0.1765	2.06
1877	Glutathione S-transferase Mu 3	P21266	0.0001	0.0118	0.60	0.2470	0.6592	0.24	0.1631	0.2991	0.30	0.0027	0.0216	0.40	0.1518	0.3300	-0.06	0.0234	0.0981	0.46	0.0793	0.2232	0.22	0.0287	0.1529	-0.08	0.9189	0.9337	0.01
1878	Glutathione S-transferase Mu 5	P46439	0.0322	0.1116	0.36	0.4647	0.7963	0.13	0.3876	0.5890	-0.08	0.3719	0.5550	0.09	0.1195	0.2836	-0.19	0.1391	0.2927	0.18	0.3736	0.5205	-2.55	0.8708	1.0709	-0.33	0.0651	0.1505	2.06
1879	Glutathione S-transferase omega-1	P78417	0.0207	0.0850	0.21	0.1788	0.6644	0.25	0.0045	0.0332	0.27	0.0093	0.0387	0.25	0.3262	0.4236	0.05	0.0218	0.0950	0.20	0.2320	0.4596	0.17	0.2150	0.5209	-0.11	0.0257	0.1365	-0.34
1880	Glutathione S-transferase P	P09211	0.4108	0.5730	-3.31	0.5752	0.8020	-1.75	0.5661	0.7217	-1.19	0.5388	0.6824	0.89	0.2938	0.4683	-1.88	0.9510	1.0949	0.09	0.0157	0.1170	0.15	0.0016	0.0325	0.31	0.0143	0.1036	0.24
1881	Glutathione S-transferase theta-4	A0A1W2PR19	0.2728	0.4658	-0.32	0.3620	0.7098	-0.26	0.0850	0.1810	-0.32	0.0494	0.1140	-0.32	0.0754	0.2110	-0.35	0.2648	0.4575	-0.27	0.3736	0.4878	-2.55	0.8708	1.0112	-0.33	0.0651	0.1354	2.06
1882	Glutathione synthetase	P48637	0.0775	0.1905	0.16	0.4259	0.7618	0.14	0.4671	0.6845	0.05	0.0260	0.0724	0.22	0.5675	0.6482	-0.03	0.1904	0.3639	0.09	0.0510	0.1790	0.29	0.8642	1.3070	0.01	0.6233	0.6813	-0.04
1883	Glyceraldehyde-3-phosphate dehydrogenase	P04406	0.0000	0.0000	0.53	0.1523	0.6607	0.16	0.0010	0.0215	0.34	0.0000	0.0000	0.45	0.0122	0.0790	0.16	0.0184	0.0878	0.66	0.1689	0.3645	-0.18	0.0300	0.1563	-0.28	0.0100	0.0884	-0.56
1884	Glyceraldehyde-3-phosphate dehydrogenase_ testis-specific	O14556	0.1003	0.2255	-8.96	0.0912	0.6290	-7.40	0.0313	0.0905	-6.84	0.0054	0.0294	-4.76	0.0068	0.0599	-7.53	0.0092	0.0692	-5.56	0.0057	0.0809	-0.68	0.0026	0.0424	-0.91	0.0473	0.1907	-0.86
1885	Glycerol-3-phosphate dehydrogenase [NAD(+)]_ cytoplasmic	P21695	0.4108	0.5728	-3.31	0.5752	0.8018	-1.75	0.5661	0.7216	-1.19	0.5388	0.6822	0.89	0.2938	0.4681	-1.88	0.9510	1.0947	0.09	0.0760	0.2193	0.14	0.2892	0.6289	-0.13	0.0478	0.1911	-0.21
1886	Glycerol-3-phosphate dehydrogenase mitochondrial	P43304	0.4990	0.5727	-0.04	0.7213	0.7960	-0.05	0.6905	0.7389	-0.02	0.0259	0.0722	-0.19	0.0182	0.0962	-0.27	0.5220	0.7503	0.03	0.0105	0.1020	-0.24	0.0304	0.1576	0.22	0.0595	0.2180	-0.13
1887	Glycerol-3-phosphate phosphatase	A6NDG6	0.0461	0.1372	0.09	0.7258	0.7999	0.06	0.0152	0.0579	0.32	0.0022	0.0201	0.24	0.0692	0.2003	0.10	0.0117	0.0739	0.20	0.3736	0.6093	-2.55	0.8708	1.2291	-0.33	0.0651	0.1985	2.06
1888	Glycine cleavage system H protein mitochondrial	P23434	0.0382	0.1228	0.13	0.9450	0.9636	-0.02	0.0244	0.0772	0.50	0.0010	0.0151	0.58	0.0042	0.0479	0.41	0.0022	0.0540	0.51	0.0081	0.0916	-0.91	0.0154	0.1050	-1.14	0.0241	0.1325	-1.57
1889	Glycine receptor subunit alpha-2	P23416	0.4108	0.5726	-3.31	0.5752	0.8016	-1.75	0.5661	0.7214	-1.19	0.5388	0.6821	0.89	0.2938	0.4680	-1.88	0.9510	1.0945	0.09	0.2251	0.4504	0.35	0.0341	0.1673	-0.89	0.4933	0.5610	-0.23
1890	Glycine receptor subunit alpha-3	O75311	0.0063	0.0477	0.12	0.9118	0.9382	0.01	0.0004	0.0179	-0.11	0.0036	0.0242	-0.12	0.0280	0.1217	-0.12	0.7780	1.0154	0.01	0.3736	0.4401	-2.55	0.8708	0.9223	-0.33	0.0651	0.1155	2.06
1891	Glycine--tRNA ligase	P41250	0.0061	0.0470	0.27	0.2282	0.6600	0.17	0.0014	0.0225	0.41	0.0016	0.0180	0.45	0.1299	0.2979	0.09	0.0066	0.0626	0.40	0.0193	0.1290	0.58	0.0065	0.0659	0.21	0.0006	0.0311	0.28
1892	Glycogen [starch] synthase muscle	P13807	0.1018	0.2278	0.09	0.1916	0.6692	0.24	0.0186	0.0655	0.19	0.0424	0.1024	0.14	0.1371	0.3076	-0.08	0.7528	0.9905	-0.01	0.3736	0.4414	-2.55	0.8708	0.9247	-0.33	0.0651	0.1160	2.06
1893	Glycogen debranching enzyme	P35573	0.4108	0.5725	-3.31	0.5752	0.8014	-1.75	0.5661	0.7212	-1.19	0.5388	0.6819	0.89	0.2938	0.4678	-1.88	0.9510	1.0942	0.09	0.1402	0.3208	0.39	0.0833	0.2841	0.48	0.1667	0.2319	0.35
1894	Glycogen phosphorylase brain form	P11216	0.0229	0.0905	0.20	0.2605	0.6644	0.17	0.0426	0.1121	0.16	0.0156	0.0516	0.23	0.1072	0.2648	0.11	0.0182	0.0872	0.30	0.0307	0.1482	0.34	0.7602	1.2024	-0.02	0.2609	0.3328	-0.09
1895	Glycogen phosphorylase liver form	P06737	0.0284	0.1024	0.22	0.2800	0.6650	0.22	0.0109	0.0484	0.29	0.0104	0.0410	0.33	0.2277	0.4372	0.08	0.0190	0.0887	0.31	0.0318	0.1504	0.39	0.3139	0.6626	0.11	0.4506	0.5193	-0.06
1896	Glycogen phosphorylase muscle form	P11217	0.8089	0.8460	0.02	0.9837	0.9885	0.00	0.1149	0.2280	0.14	0.2956	0.4589	0.08	0.0804	0.2186	-0.18	0.0666	0.1810	0.19	0.0415	0.1676	-0.12	0.4764	0.8883	0.02	0.0391	0.1706	0.25
1897	Glycogen synthase kinase-3 alpha	P49840	0.0002	0.0152	0.60	0.2584	0.6630	0.21	0.0002	0.0167	0.60	0.0003	0.0120	0.61	0.0009	0.0235	0.34	0.0047	0.0606	0.71	0.7694	0.8040	-0.02	0.2963	0.6385	0.07	0.0471	0.1900	-0.20
1898	Glycogen synthase kinase-3 beta	P49841	0.0119	0.0628	0.14	0.7948	0.8512	0.03	0.4615	0.6778	0.05	0.0094	0.0389	0.15	0.2956	0.3936	-0.03	0.0266	0.1044	0.12	0.3384	0.6089	-0.12	0.3660	0.7357	-0.10	0.0196	0.1212	-0.31
1899	Glycogenin-1	P46976	0.0004	0.0168	-0.68	0.0242	0.4859	-0.20	0.0005	0.0216	-0.38	0.0009	0.0157	-0.67	0.0096	0.0701	-0.45	0.0005	0.0392	-0.58	0.6225	0.6687	-0.09	0.8066	1.2506	0.05	0.3371	0.4079	0.20
1900	Glycophorin-A	P02724	0.4108	0.5723	-3.31	0.5752	0.8012	-1.75	0.5661	0.7210	-1.19	0.5388	0.6817	0.89	0.2938	0.4677	-1.88	0.9510	1.0940	0.09	0.0031	0.0608	-0.96	0.0034	0.0479	-0.84	0.0243	0.1324	-1.33
1901	Glycosaminoglycan xylosylkinase	O75063	0.7433	0.7916	0.04	0.2948	0.6718	0.16	0.7719	0.8125	-0.04	0.8955	0.9108	0.02	0.8136	0.8552	0.03	0.8126	1.0492	0.03	0.3736	0.4377	-2.55	0.8708	0.9178	-0.33	0.0651	0.1146	2.06
1902	Glycylpeptide N-tetradecanoyltransferase 1	P30419	0.5174	0.5897	-0.09	0.1853	0.6666	0.30	0.1232	0.2416	0.21	0.6816	0.7214	0.07	0.9778	0.9829	0.00	0.4791	0.7036	-0.18	0.0008	0.0406	0.52	0.0856	0.2898	0.20	0.1113	0.1686	0.27
1903	Glycylpeptide N-tetradecanoyltransferase 2	O60551	0.1851	0.3499	0.11	0.0466	0.5640	0.22	0.8738	0.9002	-0.02	0.0634	0.1374	0.20	0.0872	0.2313	0.17	0.1151	0.2580	0.14	0.0691	0.2070	-0.16	0.0082	0.0748	-0.33	0.0173	0.1122	-0.38
1904	Glyoxalase domain-containing protein 4	Q9HC38	0.0080	0.0529	0.21	0.3739	0.7216	0.08	0.0561	0.1353	0.08	0.0482	0.1121	0.09	0.0850	0.2271	-0.09	0.0677	0.1824	0.12	0.0680	0.2053	0.11	0.1012	0.3202	-0.09	0.1276	0.1872	0.08
1905	Glyoxylate reductase/hydroxypyruvate reductase	Q9UBQ7	0.4108	0.5722	-3.31	0.5752	0.8010	-1.75	0.5661	0.7208	-1.19	0.5388	0.6816	0.89	0.2938	0.4676	-1.88	0.9510	1.0937	0.09	0.0781	0.2220	0.15	0.1458	0.4082	0.20	0.5381	0.6037	0.13

Supplemetary Table S2. Overview on all relatively quantified 5180 proteins statistical analysis

			MCF-7																		MDA-MB-231								
			Dai SC20 vs control			Gen SC20 vs control			SSE SC20 vs control			Dai IC20 vs control			Gen IC20 vs control			SSE IC20 vs control			Dai IC20 vs control			Gen IC20 vs control			SSE IC20 vs control		
	**Protein name**	**UniProt**	**p value**	**BH q value**	**log2FC**	**p value**	**BH q value**	**log2FC**	**p value**	**BH q value**	**log2FC**	**p value**	**BH q value**	**log2FC**	**p value**	**BH q value**	**log2FC**	**p value**	**BH q value**	**log2FC**	**p value**	**BH q value**	**log2FC**	**p value**	**BH q value**	**log2FC**	**p value**	**BH q value**	**log2FC**
1906	GMP reductase 2	Q9P2T1	0.4108	0.5720	-3.31	0.5752	0.8007	-1.75	0.5661	0.7207	-1.19	0.5388	0.6814	0.89	0.2938	0.4674	-1.88	0.9510	1.0935	0.09	0.0006	0.0384	-0.68	0.0050	0.0572	-0.58	0.0302	0.1477	-0.60
1907	GMP synthase [glutamine-hydrolyzing]	P49915	0.0373	0.1214	0.12	0.1215	0.6396	0.22	0.0007	0.0207	0.32	0.0043	0.0268	0.20	0.2274	0.4368	0.05	0.0214	0.0943	0.15	0.1641	0.3582	0.16	0.7172	1.1624	-0.03	0.2175	0.2865	0.14
1908	Golgi apparatus protein 1	Q92896	0.0661	0.1704	0.14	0.2284	0.6599	0.44	0.0136	0.0546	0.32	0.0000	0.0000	0.37	0.0005	0.0199	0.13	0.0000	0.0000	0.29	0.0010	0.0439	-0.71	0.1295	0.3773	-0.19	0.0043	0.0640	-0.51
1909	Golgi membrane protein 1	Q8NBJ4	0.0261	0.0978	-1.48	0.2641	0.6638	-2.25	0.0014	0.0224	-5.05	0.0266	0.0736	-1.56	0.7481	0.8031	-0.16	0.0200	0.0918	-1.76	0.3736	0.5942	-2.55	0.8708	1.2025	-0.33	0.0651	0.1894	2.06
1910	Golgi phosphoprotein 3	Q9H4A6	0.3849	0.6018	-0.54	0.8574	0.8971	-0.11	0.5033	0.7254	-0.39	0.4372	0.6308	-0.47	0.1461	0.3208	1.12	0.3720	0.5799	-0.56	0.0021	0.0547	0.40	0.8947	0.9216	-0.01	0.0783	0.1288	0.20
1911	Golgi reassembly-stacking protein 2	Q9H8Y8	0.7636	0.8077	0.02	0.7314	0.8042	0.05	0.5042	0.7263	-0.04	0.6675	0.7097	0.02	0.0317	0.1302	-0.22	0.3345	0.5376	0.06	0.2787	0.5265	-0.11	0.0124	0.0927	0.36	0.1326	0.1929	0.25
1912	Golgi resident protein GCP60	Q9H3P7	0.0382	0.1227	0.17	0.1443	0.6545	0.23	0.0034	0.0313	0.23	0.0033	0.0235	0.26	0.0261	0.1169	0.12	0.0055	0.0621	0.35	0.2689	0.5117	0.15	0.2862	0.6247	-0.11	0.4482	0.5175	0.05
1913	Golgi SNAP receptor complex member 2	O14653	0.2589	0.4479	0.71	0.9832	0.9882	0.03	0.2133	0.3681	0.87	0.0890	0.1779	1.29	0.1460	0.3207	1.03	0.2762	0.4714	0.68	0.3736	0.4862	-2.55	0.8708	1.0082	-0.33	0.0651	0.1347	2.06
1914	Golgi to ER traffic protein 4 homolog	Q7L5D6	0.1388	0.2834	0.06	0.2416	0.6594	-0.09	0.0012	0.0226	-0.30	0.0045	0.0275	-0.34	0.0012	0.0275	-0.30	0.1176	0.2614	-0.19	0.0912	0.2429	4.66	0.8060	1.2500	0.54	0.0927	0.1465	4.66
1915	Golgin subfamily A member 1	Q92805	0.7216	0.7723	-0.02	0.5251	0.8439	0.11	0.1213	0.2388	0.08	0.0790	0.1625	0.11	0.2487	0.4658	-0.06	0.8224	1.0573	-0.01	0.3736	0.4369	-2.55	0.8708	0.9161	-0.33	0.0651	0.1142	2.06
1916	Golgin subfamily A member 2	Q08379	0.0019	0.0303	0.23	0.2058	0.6593	0.15	0.0153	0.0581	0.22	0.0008	0.0144	0.27	0.0435	0.1541	0.08	0.0028	0.0545	0.24	0.4313	0.4873	-0.15	0.0383	0.1784	-0.58	0.0003	0.0232	-7.49
1917	Golgin subfamily A member 3	Q08378	0.0486	0.1417	0.08	0.3619	0.7104	-0.06	0.0093	0.0449	0.18	0.1718	0.2941	0.05	0.0192	0.0991	0.11	0.0868	0.2150	0.22	0.0555	0.1873	0.20	0.0027	0.0430	0.29	0.0007	0.0336	0.58
1918	Golgin subfamily A member 4	Q13439	0.0207	0.0850	0.05	0.2281	0.6601	0.16	0.0445	0.1154	0.14	0.0007	0.0133	0.16	0.0114	0.0767	0.07	0.0581	0.1662	0.04	0.9664	0.9722	0.00	0.1717	0.4538	-0.15	0.6442	0.7009	-0.04
1919	Golgin subfamily A member 5	Q8TBA6	0.2011	0.3718	0.04	0.1089	0.6296	0.22	0.0125	0.0525	0.16	0.1011	0.1956	0.13	0.0583	0.1812	0.07	0.0618	0.1728	0.07	0.3736	0.5550	-2.55	0.8708	1.1331	-0.33	0.0651	0.1678	2.06
1920	Golgin subfamily A member 8C	A6NN73	0.4108	0.5719	-3.31	0.5752	0.8005	-1.75	0.5661	0.7205	-1.19	0.5388	0.6812	0.89	0.2938	0.4673	-1.88	0.9510	1.0932	0.09	0.0509	0.1788	1.12	0.0783	0.2744	0.92	0.0733	0.1225	1.01
1921	Golgin subfamily A member 8R	I6L899	0.4108	0.5717	-3.31	0.5752	0.8003	-1.75	0.5661	0.7203	-1.19	0.5388	0.6811	0.89	0.2938	0.4671	-1.88	0.9510	1.0930	0.09	0.1205	0.2903	-0.62	0.0702	0.2564	-0.33	0.2101	0.2787	0.14
1922	Golgin subfamily B member 1	Q14789	0.0013	0.0252	0.22	0.0801	0.6111	0.25	0.0001	0.0216	0.38	0.0001	0.0152	0.35	0.0025	0.0394	0.17	0.0008	0.0455	0.33	0.0584	0.1927	0.18	0.1977	0.4938	0.11	0.4661	0.5345	0.05
1923	Golgi-specific brefeldin A-resistance guanine nucleotide exchange factor 1	Q92538	0.0106	0.0596	-0.13	0.3389	0.6986	-0.07	0.7456	0.7880	-0.01	0.0046	0.0274	-0.17	0.2096	0.4127	-0.06	0.6959	0.9365	0.04	0.2573	0.4962	0.17	0.4270	0.8223	-0.08	0.3955	0.4669	-0.09
1924	GON-4-like protein	Q3T8J9	0.0647	0.1683	0.23	0.3788	0.7259	0.08	0.1549	0.2868	0.16	0.8099	0.8362	-0.02	0.0774	0.2141	-0.22	0.4570	0.6795	0.09	0.3736	0.4633	-2.55	0.8708	0.9657	-0.33	0.0651	0.1249	2.06
1925	G-patch domain and KOW motifs-containing protein	Q92917	0.0041	0.0404	-0.39	0.0837	0.6203	-0.43	0.0623	0.1451	-0.30	0.0047	0.0276	-0.38	0.0165	0.0916	-0.24	0.0028	0.0554	-0.48	0.3217	0.5868	0.08	0.0650	0.2452	0.15	0.3451	0.4163	0.15
1926	GPI transamidase component PIG-T	Q969N2	0.2914	0.4888	-0.13	0.2645	0.6638	0.14	0.3834	0.5843	-0.12	0.0772	0.1596	0.28	0.3825	0.4797	0.10	0.9064	1.1440	0.01	0.2797	0.5278	-0.13	0.0053	0.0583	-0.24	0.0009	0.0370	-0.57
1927	GPN-loop GTPase 1	Q9HCN4	0.2964	0.4948	-2.20	0.2754	0.6638	-1.99	0.3883	0.5899	0.08	0.2281	0.3707	-0.13	0.7835	0.8296	0.03	0.0079	0.0664	-0.61	0.5820	0.6308	0.09	0.2241	0.5337	-0.12	0.4982	0.5653	-2.06
1928	GPN-loop GTPase 3	Q9UHW5	0.4948	0.5692	-0.44	0.2539	0.6629	-1.48	0.1793	0.3226	0.32	0.3269	0.4986	0.22	0.2100	0.4133	0.28	0.1008	0.2349	-1.00	0.0029	0.0596	-0.68	0.0006	0.0239	-0.79	0.0002	0.0288	-1.29
1929	G-protein-signaling modulator 1	Q86YR5	0.4108	0.5716	-3.31	0.5752	0.8001	-1.75	0.5661	0.7201	-1.19	0.5388	0.6809	0.89	0.2938	0.4670	-1.88	0.9510	1.0928	0.09	0.0228	0.1362	-0.35	0.1137	0.3452	-0.18	0.0008	0.0342	-0.85
1930	G-protein-signaling modulator 2	P81274	0.0629	0.1660	-0.39	0.2649	0.6632	-0.62	0.0224	0.0733	0.51	0.0224	0.0653	-0.50	0.3481	0.4453	-0.13	0.1524	0.3098	0.24	0.3736	0.5152	-2.55	0.8708	1.0614	-0.33	0.0651	0.1480	2.06
1931	Grancalcin	P28676	0.0060	0.0468	-0.25	0.7683	0.8319	-0.04	0.0044	0.0326	-0.25	0.0086	0.0375	-0.23	0.7702	0.8211	0.01	0.0395	0.1298	-0.16	0.3736	0.5704	-2.55	0.8708	1.1605	-0.33	0.0651	0.1760	2.06
1932	GRB10-interacting GYF protein 2	Q6Y7W6	0.6269	0.6899	0.03	0.3984	0.7426	0.11	0.8691	0.8966	-0.01	0.0904	0.1798	0.14	0.6210	0.6936	0.03	0.4948	0.7198	0.04	0.3736	0.4606	-2.55	0.8708	0.9606	-0.33	0.0651	0.1238	2.06
1933	GRB2-related adapter protein	Q13588	0.0067	0.0492	-3.31	0.1965	0.6692	-2.24	0.0456	0.1176	-1.42	0.0229	0.0665	-2.27	0.5638	0.6460	-0.09	0.0004	0.0345	-2.77	0.3736	0.6527	-2.55	0.8708	1.3041	-0.33	0.0651	0.2266	2.06
1934	G-rich sequence factor 1	Q12849	0.0000	0.0000	1.00	0.0294	0.5093	0.20	0.0052	0.0354	0.13	0.0176	0.0559	0.13	0.1137	0.2755	0.07	0.1318	0.2828	0.06	0.3736	0.5236	-2.55	0.8708	1.0765	-0.33	0.0651	0.1520	2.06
1935	GRIP1-associated protein 1	Q4V328	0.2078	0.3804	-0.06	0.7477	0.8171	0.02	0.3025	0.4854	-0.05	0.1023	0.1974	0.09	0.3068	0.4042	0.05	0.0347	0.1199	0.17	0.0250	0.1395	0.53	0.0004	0.0203	0.79	0.0137	0.1018	0.34
1936	Growth arrest and DNA damage-inducible proteins-interacting protein 1	Q8TAE8	0.3021	0.5016	0.06	0.9571	0.9715	0.00	0.0597	0.1413	0.15	0.1194	0.2221	0.11	0.1475	0.3235	0.09	0.2397	0.4255	0.07	0.1132	0.2775	0.10	0.1148	0.3474	0.07	0.1575	0.2213	0.06
1937	Growth factor receptor-bound protein 2	P62993	0.0558	0.1545	0.14	0.1909	0.6691	0.20	0.0043	0.0325	0.26	0.0028	0.0219	0.29	0.0715	0.2038	0.11	0.0141	0.0790	0.26	0.0061	0.0832	0.58	0.0012	0.0292	0.76	0.0224	0.1269	0.18
1938	Growth hormone-inducible transmembrane protein	Q9H3K2	0.4108	0.5714	-3.31	0.5752	0.7999	-1.75	0.5661	0.7200	-1.19	0.5388	0.6807	0.89	0.2938	0.4668	-1.88	0.9510	1.0925	0.09	0.2603	0.4999	0.14	0.2629	0.5916	-0.07	0.0199	0.1218	-0.11
1939	Growth/differentiation factor 5	P43026	0.4279	0.5054	-0.08	0.4859	0.8101	-0.10	0.2616	0.4311	0.13	0.0460	0.1086	-0.30	0.4900	0.5795	-0.13	0.3976	0.6110	-0.11	0.3736	0.4693	-2.55	0.8708	0.9768	-0.33	0.0651	0.1274	2.06
1940	GrpE protein homolog 1 mitochondrial	Q9HAV7	0.8384	0.8708	0.01	0.5525	0.8680	-0.07	0.0047	0.0334	0.10	0.6009	0.6483	0.03	0.0254	0.1146	-0.15	0.9348	1.1710	-0.01	0.0269	0.1418	0.28	0.1723	0.4542	0.08	0.0301	0.1474	0.18
1941	GTP:AMP phosphotransferase AK3 mitochondrial	Q9UIJ7	0.1907	0.3570	-0.07	0.8020	0.8571	0.06	0.6229	0.6767	0.02	0.4296	0.6219	-0.06	0.4276	0.5219	-0.04	0.1578	0.3173	-0.08	0.3736	0.5129	-2.55	0.8708	1.0571	-0.33	0.0651	0.1469	2.06
1942	GTPase HRas	P01112	0.0490	0.1420	0.17	0.4683	0.7982	0.05	0.3491	0.5432	0.06	0.1075	0.2050	0.13	0.8128	0.8545	-0.02	0.1116	0.2526	0.25	0.0030	0.0607	-0.48	0.0004	0.0214	-0.74	0.0008	0.0360	-1.24
1943	GTPase IMAP family member 4	Q9NUV9	0.2096	0.3823	-0.39	0.6739	0.7609	-0.09	0.0347	0.0968	-0.25	0.4602	0.6574	-0.08	0.3137	0.4112	0.13	0.0217	0.0949	-0.96	0.3736	0.5918	-2.55	0.8708	1.1984	-0.33	0.0651	0.1881	2.06
1944	GTPase IMAP family member 5	Q96F15	0.4108	0.5713	-3.31	0.5752	0.7997	-1.75	0.5661	0.7198	-1.19	0.5388	0.6806	0.89	0.2938	0.4667	-1.88	0.9510	1.0923	0.09	0.0902	0.2416	-0.82	0.0002	0.0152	-0.89	0.0774	0.1278	-0.12
1945	GTPase KRas	P01116	0.6811	0.7379	0.03	0.5388	0.8546	-0.09	0.2550	0.4230	0.08	0.1758	0.2996	-0.11	0.0605	0.1851	-0.18	0.4754	0.6992	0.06	0.3736	0.4623	-2.55	0.8708	0.9638	-0.33	0.0651	0.1245	2.06
1946	GTPase NRas	P01111	0.0409	0.1277	0.12	0.7380	0.8098	0.04	0.3200	0.5071	-0.04	0.0112	0.0425	-0.18	0.0258	0.1158	-0.15	0.9939	0.9945	0.00	0.4077	0.4636	-0.40	0.0598	0.2336	-0.27	0.5692	0.6329	0.07
1947	GTPase-activating protein and VPS9 domain-containing protein 1	Q14C86	0.0113	0.0610	0.20	0.2200	0.6599	0.26	0.0009	0.0209	0.45	0.0021	0.0203	0.36	0.2373	0.4499	0.06	0.0176	0.0867	0.33	0.3736	0.5971	-2.55	0.8708	1.2077	-0.33	0.0651	0.1912	2.06
1948	GTP-binding nuclear protein Ran	P62826	0.0083	0.0540	0.34	0.2744	0.6645	0.28	0.0096	0.0457	0.31	0.0239	0.0686	0.24	0.2425	0.4563	-0.08	0.0314	0.1139	0.25	0.3994	0.4553	0.13	0.2713	0.6044	-0.12	0.0217	0.1253	-0.36
1949	GTP-binding protein Rheb	Q15382	0.0339	0.1151	0.17	0.3238	0.6854	0.09	0.0162	0.0602	0.17	0.0246	0.0699	0.16	0.9288	0.9463	0.00	0.0412	0.1336	0.16	0.0126	0.1066	0.19	0.2476	0.5690	0.07	0.5748	0.6376	0.02
1950	GTP-binding protein Rhes	Q96D21	0.4108	0.5711	-3.31	0.5752	0.7994	-1.75	0.5661	0.7196	-1.19	0.5388	0.6804	0.89	0.2938	0.4665	-1.88	0.9510	1.0920	0.09	0.0130	0.1079	0.47	0.8078	1.2513	-0.03	0.1054	0.1617	0.22
1951	GTP-binding protein Rit1	Q92963	0.4108	0.5710	-3.31	0.5752	0.7992	-1.75	0.5661	0.7194	-1.19	0.5388	0.6802	0.89	0.2938	0.4664	-1.88	0.9510	1.0918	0.09	0.0012	0.0444	7.48	0.0784	0.2746	4.42	0.0031	0.0560	8.97
1952	GTP-binding protein SAR1a	Q9NR31	0.2805	0.4739	-0.04	0.3658	0.7140	-0.13	0.2746	0.4484	0.08	0.0032	0.0229	-0.26	0.0659	0.1954	-0.15	0.9836	0.9874	0.00	0.6697	0.7134	-0.08	0.7481	1.1891	-0.06	0.5869	0.6486	-0.10
1953	GTP-binding protein SAR1b	Q9Y6B6	0.0547	0.1531	0.07	0.0169	0.4607	0.12	0.0212	0.0706	0.08	0.0106	0.0413	0.09	0.0024	0.0385	0.12	0.0063	0.0629	0.14	0.2592	0.4982	-0.04	0.0455	0.1982	-0.11	0.0264	0.1388	-0.13
1954	Guanidinoacetate N-methyltransferase	Q14353	0.4108	0.5708	-3.31	0.5752	0.7990	-1.75	0.5661	0.7193	-1.19	0.5388	0.6801	0.89	0.2938	0.4663	-1.88	0.9510	1.0916	0.09	0.5107	0.5643	0.18	0.7793	1.2229	0.03	0.0033	0.0572	0.73
1955	Guanine nucleotide exchange factor DBS	O15068	0.1685	0.3271	-0.08	0.8854	0.9184	0.01	0.0705	0.1580	-0.12	0.0773	0.1598	-0.12	0.0457	0.1574	-0.15	0.3529	0.5583	-0.06	0.4524	0.5075	-0.27	0.2637	0.5924	-0.27	0.0439	0.1831	0.42
1956	Guanine nucleotide exchange factor VAV2	P52735	0.1412	0.2871	0.23	0.0378	0.5547	0.42	0.0001	0.0247	0.60	0.0003	0.0122	0.52	0.0090	0.0680	0.17	0.0963	0.2290	0.38	0.6826	0.7253	0.06	0.0388	0.1793	0.17	0.0092	0.0856	-0.20
1957	Guanine nucleotide-binding protein G(i) subunit alpha-1	P63096	0.0159	0.0734	-0.60	0.1112	0.6323	-0.56	0.0106	0.0480	-0.65	0.0058	0.0306	-0.88	0.2315	0.4417	-0.18	0.0209	0.0934	-0.57	0.0294	0.1467	0.52	0.6054	1.0377	0.08	0.3523	0.4237	0.15
1958	Guanine nucleotide-binding protein G(i) subunit alpha-2	P04899	0.0711	0.1789	0.16	0.1579	0.6617	0.12	0.0101	0.0468	0.38	0.0294	0.0792	0.21	0.0401	0.1471	0.20	0.2917	0.4890	0.13	0.0431	0.1679	0.38	0.0351	0.1704	0.13	0.0036	0.0590	0.29
1959	Guanine nucleotide-binding protein G(I)/G(S)/G(O) subunit gamma-12	Q9UBI6	0.4108	0.5706	-3.31	0.5752	0.7988	-1.75	0.5661	0.7191	-1.19	0.5388	0.6799	0.89	0.2938	0.4661	-1.88	0.9510	1.0913	0.09	0.0415	0.1672	0.42	0.4813	0.8917	-0.07	0.1250	0.1846	0.21
1960	Guanine nucleotide-binding protein G(I)/G(S)/G(T) subunit beta-1	P62873	0.0359	0.1184	0.10	0.2915	0.6693	0.18	0.1649	0.3018	0.08	0.0560	0.1252	0.08	0.4577	0.5502	-0.03	0.3505	0.5554	0.03	0.7357	0.7746	-0.04	0.0074	0.0705	-0.30	0.0237	0.1312	-0.26
1961	Guanine nucleotide-binding protein G(I)/G(S)/G(T) subunit beta-2	P62879	0.0127	0.0659	0.15	0.3439	0.7011	0.17	0.1518	0.2822	0.11	0.0755	0.1567	0.08	0.0376	0.1412	-0.11	0.2844	0.4808	0.05	0.0308	0.1484	-0.32	0.0000	0.0000	-0.64	0.0663	0.1129	-0.65
1962	Guanine nucleotide-binding protein G(I)/G(S)/G(T) subunit beta-3	P16520	0.6980	0.7523	-0.05	0.0859	0.6197	0.30	0.3031	0.4861	0.17	0.2100	0.3457	0.18	0.2973	0.3952	0.14	0.4470	0.6690	-0.09	0.0633	0.1984	0.47	0.6501	1.0895	0.03	0.2982	0.3695	0.17
1963	Guanine nucleotide-binding protein G(k) subunit alpha	P08754	0.0809	0.1960	-0.22	0.0107	0.4434	-0.14	0.2313	0.3909	0.05	0.0473	0.1107	-0.22	0.0300	0.1262	-0.24	0.7877	1.0255	0.04	0.0025	0.0568	0.53	0.0118	0.0903	0.46	0.0067	0.0761	0.62
1964	Guanine nucleotide-binding protein G(o) subunit alpha	P09471	0.0376	0.1219	0.19	0.8615	0.9001	0.02	0.0408	0.1080	0.18	0.7500	0.7844	0.03	0.8572	0.8902	0.01	0.1689	0.3338	0.12	0.3612	0.6397	-0.26	0.3002	0.6436	-0.28	0.4900	0.5581	0.15
1965	Guanine nucleotide-binding protein G(olf) subunit alpha	P38405	0.0157	0.0729	0.24	0.0116	0.4354	0.18	0.0647	0.1484	0.08	0.0209	0.0623	0.11	0.0008	0.0216	0.28	0.8183	1.0544	0.01	0.6205	0.6673	0.06	0.0109	0.0853	-0.27	0.1734	0.2393	-0.22
1966	Guanine nucleotide-binding protein G(s) subunit alpha isoforms XLas	Q5JWF2	0.1757	0.3378	0.06	0.3256	0.6856	0.21	0.0220	0.0724	0.15	0.0630	0.1368	0.11	0.5056	0.5946	0.02	0.0298	0.1107	0.15	0.0643	0.1996	0.26	0.0964	0.3096	0.14	0.0598	0.2188	0.26
1967	Guanine nucleotide-binding protein G(t) subunit alpha-1	P11488	0.1629	0.3196	-0.03	0.1822	0.6661	0.20	0.0406	0.1077	0.18	0.0105	0.0410	0.10	0.0051	0.0535	0.29	0.0000	0.0000	0.07	0.3736	0.6558	-2.55	0.8708	1.3094	-0.33	0.0651	0.2288	2.06
1968	Guanine nucleotide-binding protein G(t) subunit alpha-2	P19087	0.4381	0.5158	-0.18	0.4108	0.7514	-0.47	0.3302	0.5200	-0.25	0.2677	0.4233	-0.29	0.9649	0.9730	0.01	0.7088	0.9504	-0.08	0.0014	0.0483	-0.21	0.0039	0.0508	-0.40	0.1068	0.1631	-0.34
1969	Guanine nucleotide-binding protein G(t) subunit alpha-3	A8MTJ3	0.0003	0.0160	0.65	0.0225	0.4777	0.47	0.2154	0.3703	0.16	0.0311	0.0822	0.35	0.0006	0.0192	0.70	0.0014	0.0529	0.42	0.9761	0.9805	0.00	0.2160	0.5216	-0.06	0.8649	0.8903	0.01
1970	Guanine nucleotide-binding protein subunit alpha-11	P29992	0.0659	0.1702	0.33	0.0189	0.4662	0.59	0.0131	0.0536	0.59	0.0905	0.1798	0.29	0.1002	0.2534	0.30	0.0582	0.1664	0.39	0.0072	0.0867	1.18	0.0004	0.0205	1.04	0.0117	0.0943	0.40
1971	Guanine nucleotide-binding protein subunit alpha-12	Q03113	0.4108	0.5705	-3.31	0.5752	0.7986	-1.75	0.5661	0.7189	-1.19	0.5388	0.6797	0.89	0.2938	0.4660	-1.88	0.9510	1.0911	0.09	0.0207	0.1322	-0.22	0.1295	0.3771	0.22	0.1942	0.2622	-0.09
1972	Guanine nucleotide-binding protein subunit alpha-13	Q14344	0.0476	0.1401	0.14	0.3045	0.6775	0.15	0.0046	0.0333	0.31	0.0174	0.0554	0.20	0.2749	0.4995	-0.06	0.0292	0.1094	0.19	0.1143	0.2799	0.11	0.0483	0.2054	0.13	0.4340	0.5050	-0.05
1973	Guanine nucleotide-binding protein subunit alpha-14	O95837	0.4108	0.5703	-3.31	0.5752	0.7984	-1.75	0.5661	0.7187	-1.19	0.5388	0.6796	0.89	0.2938	0.4658	-1.88	0.9510	1.0908	0.09	0.0088	0.0934	-0.18	0.1432	0.4038	0.07	0.0908	0.1444	-0.25
1974	Guanine nucleotide-binding protein subunit beta-4	Q9HAV0	0.1045	0.2314	-0.24	0.7938	0.8508	-0.04	0.1006	0.2049	0.26	0.6592	0.7022	0.05	0.2643	0.4855	0.14	0.6110	0.8483	0.06	0.0000	0.0000	-0.48	0.0038	0.0506	-0.15	0.0347	0.1599	-0.31
1975	Guanine nucleotide-binding protein subunit beta-5	O14775	0.1586	0.3132	-0.09	0.1232	0.6401	0.13	0.5698	0.6276	0.09	0.5395	0.5913	-0.02	0.4111	0.5062	-0.01	0.0402	0.1315	-0.15	0.3736	0.5702	-2.55	0.8708	1.1602	-0.33	0.0651	0.1759	2.06

Supplemetary Table S2. Overview on all relatively quantified 5180 proteins statistical analysis

			MCF-7																			MDA-MB-231								
			Dai SC20 vs control			Gen SC20 vs control			SSE SC20 vs control			Dai IC20 vs control			Gen IC20 vs control			SSE IC20 vs control			Dai IC20 vs control			Gen IC20 vs control			SSE IC20 vs control		
	**Protein name**	**UniProt**	**p value**	**BH q value**	**log2FC**	**p value**	**BH q value**	**log2FC**	**p value**	**BH q value**	**log2FC**	**p value**	**BH q value**	**log2FC**	**p value**	**BH q value**	**log2FC**	**p value**	**BH q value**	**log2FC**	**p value**	**BH q value**	**log2FC**	**p value**	**BH q value**	**log2FC**	**p value**	**BH q value**	**log2FC**
1976	Guanine nucleotide-binding protein-like 1	P36915	0.0079	0.0526	0.23	0.1870	0.6671	0.28	0.0008	0.0210	0.48	0.0059	0.0310	0.26	0.0491	0.1632	0.13	0.0069	0.0642	0.31	0.7887	0.8217	0.03	0.8683	1.3109	0.02	0.2624	0.3345	0.19
1977	Guanylate kinase	Q16774	0.0642	0.1679	0.16	0.7042	0.7823	0.06	0.0233	0.0751	0.21	0.0345	0.0886	0.19	0.1281	0.2954	0.12	0.0459	0.1420	0.20	0.3736	0.5645	-2.55	0.8708	1.1501	-0.33	0.0651	0.1728	2.06
1978	Guanylate-binding protein 2	P32456	0.0336	0.1145	0.26	0.9807	0.9868	-0.01	0.0140	0.0554	0.56	0.4764	0.6765	0.08	0.9307	0.9475	-0.01	0.1030	0.2382	0.18	0.1175	0.2852	3.58	0.1071	0.3324	3.91	0.1254	0.1850	3.58
1979	Guanylate-binding protein 7	Q8N8V2	0.2090	0.3817	-0.18	0.4149	0.7536	-0.10	0.0266	0.0817	-0.46	0.0089	0.0382	-0.56	0.0456	0.1574	-0.37	0.6507	0.8875	-0.11	0.3736	0.4475	-2.55	0.8708	0.9360	-0.33	0.0651	0.1184	2.06
1980	Guanylyl cyclase-activating protein 2	Q9UMX6	0.5257	0.5972	-0.12	0.3132	0.6800	-0.15	0.0213	0.0705	-0.26	0.0337	0.0872	-0.37	0.0245	0.1133	-0.27	0.1761	0.3445	-0.18	0.6176	0.6646	-0.10	0.5271	0.9507	0.14	0.0947	0.1492	0.40
1981	Haloacid dehalogenase-like hydrolase domain-containing 5	Q9BXW7	0.0026	0.0342	0.33	0.2601	0.6644	0.14	0.0023	0.0265	0.37	0.0465	0.1095	0.34	0.5488	0.6327	0.02	0.2637	0.4561	0.28	0.7700	0.8045	-0.02	0.6043	1.0372	0.06	0.1026	0.1585	-0.12
1982	Hamartin	Q92574	0.9682	0.9765	0.01	0.9763	0.9845	0.01	0.5504	0.7735	0.17	0.9039	0.9174	-0.06	0.6072	0.6820	0.16	0.4417	0.6636	0.18	0.3736	0.4651	-2.55	0.8708	0.9690	-0.33	0.0651	0.1257	2.06
1983	HAUS augmin-like complex subunit 5	O94927	0.5284	0.5998	-0.10	0.5316	0.8507	-0.17	0.0086	0.0436	-0.82	0.0253	0.0712	-0.75	0.0156	0.0890	-0.85	0.6665	0.9047	-0.06	0.9399	0.9504	0.01	0.0010	0.0260	1.45	0.0204	0.1227	0.50
1984	HBS1-like protein	Q9Y450	0.1027	0.2293	0.13	0.4915	0.8144	0.08	0.5018	0.7244	0.05	0.5210	0.7259	0.04	0.3041	0.4019	0.07	0.7114	0.9520	-0.03	0.1265	0.2996	0.16	0.1228	0.3641	-0.11	0.0481	0.1912	-0.12
1985	HCLS1-associated protein X-1	O00165	0.6582	0.7188	0.03	0.1043	0.6334	0.23	0.0937	0.1942	0.11	0.0076	0.0352	0.33	0.3600	0.4572	0.05	0.1515	0.3091	0.13	0.3736	0.5158	-2.55	0.8708	1.0624	-0.33	0.0651	0.1482	2.06
1986	HD domain-containing protein 2	Q7Z4H3	0.2698	0.4622	0.07	0.2722	0.6654	0.17	0.1151	0.2283	0.11	0.1527	0.2690	0.09	0.6299	0.7005	0.03	0.3234	0.5260	0.07	0.6839	0.7262	-0.12	0.1402	0.3977	-0.54	0.5296	0.5960	-0.19
1987	HEAT repeat-containing protein 3	Q7Z4Q2	0.4108	0.5702	-3.31	0.5752	0.7982	-1.75	0.5661	0.7185	-1.19	0.5388	0.6794	0.89	0.2938	0.4657	-1.88	0.9510	1.0906	0.09	0.0036	0.0650	0.28	0.2583	0.5853	0.05	0.0714	0.1197	0.11
1988	HEAT repeat-containing protein 6	Q6AI08	0.1745	0.3363	-0.03	0.1553	0.6621	0.11	0.0012	0.0227	0.22	0.0005	0.0130	0.23	0.0005	0.0192	0.15	0.0025	0.0566	0.17	0.3736	0.6421	-2.55	0.8708	1.2858	-0.33	0.0651	0.2194	2.06
1989	Heat shock 70 kDa protein 14	Q0VDF9	0.0879	0.2062	-0.16	0.0342	0.5304	-0.24	0.0327	0.0930	-0.24	0.0975	0.1904	-0.17	0.0285	0.1227	-0.26	0.9860	0.9892	0.00	0.3736	0.4300	-2.55	0.8708	0.9031	-0.33	0.0651	0.1116	2.06
1990	Heat shock 70 kDa protein 1A	P0DMV8	0.0111	0.0605	0.37	0.1709	0.6606	0.34	0.0069	0.0397	0.45	0.0114	0.0429	0.36	0.8101	0.8522	-0.02	0.0112	0.0726	0.40	0.0143	0.1122	0.58	0.5283	0.9519	0.07	0.7590	0.8011	0.03
1991	Heat shock 70 kDa protein 1-like	P34931	0.0025	0.0336	0.77	0.1274	0.6413	0.88	0.0006	0.0199	0.82	0.0077	0.0355	1.16	0.5155	0.6041	0.01	0.0304	0.1118	0.93	0.0279	0.1444	0.58	0.5385	0.9639	0.06	0.1267	0.1863	0.38
1992	Heat shock 70 kDa protein 4	P34932	0.0031	0.0367	0.28	0.1099	0.6325	0.36	0.0002	0.0192	0.49	0.0005	0.0137	0.43	0.0018	0.0335	0.26	0.0019	0.0535	0.39	0.0163	0.1196	0.47	0.3224	0.6745	0.08	0.0177	0.1139	0.27
1993	Heat shock 70 kDa protein 4L	O95757	0.0228	0.0902	0.17	0.1613	0.6636	0.25	0.0081	0.0424	0.27	0.0027	0.0219	0.35	0.0404	0.1478	0.15	0.0120	0.0744	0.28	0.0325	0.1513	0.48	0.0170	0.1104	0.20	0.0095	0.0871	0.19
1994	Heat shock 70 kDa protein 6	P17066	0.5613	0.6297	-0.04	0.0096	0.4480	-0.33	0.1241	0.2428	-0.12	0.1452	0.2582	-0.12	0.1417	0.3148	-0.12	0.1364	0.2886	0.12	0.9365	0.9478	0.01	0.2728	0.6073	-0.05	0.1101	0.1671	-0.27
1995	Heat shock cognate 71 kDa protein	P11142	0.0142	0.0696	0.32	0.3066	0.6784	0.24	0.0038	0.0317	0.45	0.0054	0.0295	0.40	0.2262	0.4354	0.09	0.0135	0.0774	0.45	0.0414	0.1674	0.50	0.2348	0.5506	0.16	0.7175	0.7668	0.03
1996	Heat shock protein 105 kDa	Q92598	0.0242	0.0935	0.22	0.2519	0.6624	0.21	0.0029	0.0294	0.39	0.0066	0.0327	0.31	0.4142	0.5090	0.05	0.0243	0.0993	0.28	0.0571	0.1897	0.26	0.9764	0.9832	0.00	0.3267	0.3979	-0.08
1997	Heat shock protein 75 kDa mitochondrial	Q12931	0.0067	0.0492	0.20	0.3032	0.6761	0.16	0.0014	0.0222	0.29	0.0024	0.0211	0.25	0.3912	0.4878	-0.03	0.0102	0.0701	0.25	0.0519	0.1806	0.17	0.0249	0.1407	0.11	0.0985	0.1539	0.09
1998	Heat shock protein beta-1	P04792	0.0269	0.0993	0.17	0.0923	0.6308	0.28	0.0031	0.0304	0.33	0.0435	0.1044	0.14	0.7839	0.8297	0.01	0.0238	0.0988	0.23	0.0193	0.1288	-0.34	0.0151	0.1039	-0.48	0.0083	0.0820	-0.46
1999	Heat shock protein HSP 90-alpha	P07900	0.0106	0.0595	0.34	0.2111	0.6623	0.29	0.0026	0.0279	0.45	0.0037	0.0247	0.41	0.1294	0.2974	0.12	0.0099	0.0704	0.49	0.0317	0.1505	0.65	0.2388	0.5557	0.15	0.4642	0.5325	0.06
2000	Heat shock protein HSP 90-alpha A2	Q14568	0.6032	0.6686	0.04	0.4838	0.8084	0.06	0.1431	0.2694	-0.16	0.6093	0.6566	-0.04	0.3624	0.4597	-0.07	0.2730	0.4672	-0.09	0.0096	0.0979	-0.57	0.0084	0.0754	-0.18	0.0989	0.1543	-0.34
2001	Heat shock protein HSP 90-beta	P08238	0.0070	0.0497	0.34	0.1933	0.6684	0.36	0.0023	0.0267	0.51	0.0021	0.0201	0.45	0.1772	0.3679	0.09	0.0123	0.0746	0.42	0.0367	0.1592	0.61	0.1765	0.4618	0.20	0.3152	0.3866	0.11
2002	Heat shock-related 70 kDa protein 2	P54652	0.9884	0.9915	0.00	0.2538	0.6630	0.11	0.0672	0.1525	0.21	0.0443	0.1055	0.25	0.0672	0.1973	0.21	0.0679	0.1828	0.21	0.0119	0.1039	0.78	0.0036	0.0491	0.55	0.0001	0.0216	0.75
2003	Helicase MOV-10	Q9HCE1	0.5325	0.6034	-0.03	0.1794	0.6647	0.19	0.3189	0.5064	0.04	0.4673	0.6665	0.10	0.5172	0.6054	-0.02	0.1747	0.3425	0.05	0.0691	0.2069	0.40	0.0868	0.2920	0.37	0.0320	0.1524	0.60
2004	Helicase SKI2W	Q15477	0.1768	0.3392	-0.09	0.2235	0.6578	-0.10	0.5555	0.7796	-0.03	0.4349	0.6282	-0.06	0.0927	0.2401	0.14	0.2948	0.4916	0.12	0.0076	0.0895	-0.56	0.0192	0.1188	-0.47	0.0332	0.1558	-0.42
2005	Helicase SRCAP	Q6ZRS2	0.4108	0.5700	-3.31	0.5752	0.7979	-1.75	0.5661	0.7184	-1.19	0.5388	0.6792	0.89	0.2938	0.4656	-1.88	0.9510	1.0903	0.09	0.0040	0.0679	0.55	0.0344	0.1681	0.30	0.1126	0.1699	0.17
2006	Helicase with zinc finger domain 2	Q9BYK8	0.0401	0.1262	0.32	0.0811	0.6142	0.28	0.0064	0.0389	0.55	0.0081	0.0364	0.60	0.1502	0.3275	0.17	0.0235	0.0981	0.38	0.0297	0.1474	0.35	0.8495	1.2912	0.02	0.7444	0.7885	0.03
2007	Helicase-like transcription factor	Q14527	0.5494	0.6185	0.05	0.0386	0.5570	-0.25	0.1522	0.2829	-0.20	0.1242	0.2279	-0.37	0.0221	0.1074	-0.29	0.0977	0.2305	-0.12	0.3736	0.5349	-2.55	0.8708	1.0970	-0.33	0.0651	0.1575	2.06
2008	Helix-loop-helix protein 2	Q02577	0.4108	0.5699	-3.31	0.5752	0.7977	-1.75	0.5661	0.7182	-1.19	0.5388	0.6791	0.89	0.2938	0.4654	-1.88	0.9510	1.0901	0.09	0.0323	0.1510	-0.32	0.0000	0.0000	-0.86	0.0018	0.0501	-1.00
2009	Hematopoietically-expressed homeobox protein HHEX	Q03014	0.0000	0.0000	0.64	0.1477	0.6573	0.30	0.0144	0.0565	0.43	0.0080	0.0361	0.20	0.0041	0.0472	0.28	0.0000	0.0000	0.35	0.9416	0.9517	-0.01	0.0201	0.1225	-0.54	0.0654	0.1117	-0.58
2010	Heme oxygenase 1	P09601	0.4108	0.5697	-3.31	0.5752	0.7975	-1.75	0.5661	0.7180	-1.19	0.5388	0.6789	0.89	0.2938	0.4653	-1.88	0.9510	1.0899	0.09	0.0829	0.2301	-0.64	0.1252	0.3685	-0.36	0.3618	0.4332	0.10
2011	Heme oxygenase 2	P30519	0.0506	0.1455	0.15	0.2180	0.6611	0.30	0.0223	0.0731	0.19	0.0190	0.0583	0.20	0.2637	0.4851	-0.06	0.1100	0.2500	0.11	0.4322	0.4880	0.09	0.8463	1.2886	0.01	0.9642	0.9713	0.00
2012	Heme-binding protein 1	Q9NRV9	0.0516	0.1471	-0.26	0.1991	0.6650	-0.33	0.0615	0.1441	-0.25	0.0337	0.0872	-0.30	0.0358	0.1373	-0.29	0.4970	0.7217	-0.06	0.0295	0.1471	0.55	0.2801	0.6174	0.10	0.0876	0.1403	0.14
2013	Heme-binding protein 2	Q9Y5Z4	0.0556	0.1540	0.13	0.0073	0.3980	0.26	0.0725	0.1609	-0.12	0.0553	0.1242	-0.13	0.0683	0.1989	-0.13	0.1117	0.2527	0.12	0.0021	0.0552	0.42	0.0089	0.0779	0.40	0.0038	0.0604	0.41
2014	Hemicentin-2	Q8NDA2	0.0402	0.1264	0.13	0.4243	0.7608	0.05	0.0194	0.0670	0.48	0.0179	0.0564	0.24	0.0084	0.0660	0.28	0.0150	0.0814	0.34	0.3736	0.6027	-2.55	0.8708	1.2175	-0.33	0.0651	0.1945	2.06
2015	Hemogen	Q9BXL5	0.8150	0.8513	0.02	0.8141	0.8670	0.02	0.2560	0.4241	-0.08	0.5463	0.5973	-0.04	0.2538	0.4731	-0.07	0.2696	0.4627	-0.09	0.0269	0.1422	-0.32	0.0460	0.1992	-0.25	0.0325	0.1542	-0.34
2016	Hemoglobin subunit alpha	P69905	0.0070	0.0496	0.56	0.0830	0.6204	0.62	0.1835	0.3282	0.38	0.0819	0.1669	0.25	0.2259	0.4350	0.15	0.0127	0.0756	0.57	0.0220	0.1352	0.62	0.0083	0.0752	0.44	0.2330	0.3030	0.12
2017	Hemoglobin subunit delta	P02042	0.1601	0.3150	1.17	0.3609	0.7097	0.65	0.9297	0.9456	0.06	0.5992	0.6469	-0.39	0.5462	0.6303	0.40	0.4395	0.6612	0.55	0.3736	0.4652	-2.55	0.8708	0.9692	-0.33	0.0651	0.1257	2.06
2018	Heparan-sulfate 6-O-sulfotransferase 1	O60243	0.4108	0.5696	-3.31	0.5752	0.7973	-1.75	0.5661	0.7178	-1.19	0.5388	0.6787	0.89	0.2938	0.4651	-1.88	0.9510	1.0896	0.09	0.6773	0.7207	0.06	0.0167	0.1096	-0.28	0.0056	0.0714	-0.50
2019	Heparan-sulfate 6-O-sulfotransferase 2	Q96MM7	0.1629	0.3195	0.14	0.3082	0.6782	0.11	0.7525	0.7944	-0.04	0.0645	0.1393	0.21	0.4163	0.5110	0.07	0.0773	0.1983	0.23	0.3736	0.5453	-2.55	0.8708	1.1157	-0.33	0.0651	0.1628	2.06
2020	Hepatocyte growth factor-regulated tyrosine kinase substrate	O14964	0.0002	0.0150	0.84	0.0097	0.4486	0.27	0.0004	0.0238	0.74	0.0127	0.0457	0.25	0.0243	0.1126	0.23	0.0230	0.0971	0.28	0.0423	0.1675	0.37	0.6740	1.1129	0.02	0.1719	0.2376	-0.04
2021	Hepatocyte nuclear factor 4-gamma	Q14541	0.4108	0.5694	-3.31	0.5752	0.7971	-1.75	0.5661	0.7177	-1.19	0.5388	0.6786	0.89	0.2938	0.4650	-1.88	0.9510	1.0894	0.09	0.2101	0.4273	0.19	0.2283	0.5410	-0.09	0.4250	0.4963	0.05
2022	Hepatoma-derived growth factor	P51858	0.3074	0.5079	0.05	0.2836	0.6644	0.16	0.0260	0.0804	0.18	0.3128	0.4817	0.06	0.1021	0.2562	-0.11	0.2571	0.4481	0.06	0.3339	0.6033	0.11	0.3466	0.7082	-0.05	0.2974	0.3687	-0.06
2023	Hepatoma-derived growth factor-like protein 1	Q5TGJ6	0.9394	0.9543	0.00	0.1113	0.6294	-0.26	0.6117	0.6665	0.04	0.7125	0.7509	0.02	0.5910	0.6678	-0.02	0.0117	0.0737	-0.34	0.1375	0.3171	0.16	0.0075	0.0713	-0.31	0.0087	0.0836	-0.29
2024	Hepatoma-derived growth factor-related protein 2	Q7Z4V5	0.4053	0.6256	-0.31	0.9810	0.9869	0.01	0.9091	0.9281	0.04	0.7526	0.7866	-0.11	0.1812	0.3735	0.60	0.4661	0.6886	-0.27	0.0373	0.1605	0.49	0.0387	0.1796	0.34	0.3131	0.3844	0.08
2025	Hermansky-Pudlak syndrome 3 protein	Q969F9	0.2447	0.4303	-0.22	0.1709	0.6602	-0.12	0.0715	0.1595	-0.19	0.6800	0.7202	-0.03	0.0528	0.1703	-0.21	0.0771	0.1983	-0.22	0.3736	0.5454	-2.55	0.8708	1.1160	-0.33	0.0651	0.1628	2.06
2026	Heterogeneous nuclear ribonucleoprotein A/B	Q99729	0.1036	0.2302	-0.17	0.2217	0.6585	-0.12	0.6859	0.7350	0.03	0.1218	0.2254	-0.17	0.0051	0.0534	-0.51	0.0102	0.0706	-0.50	0.0011	0.0449	0.58	0.0284	0.1528	0.18	0.0013	0.0440	0.59
2027	Heterogeneous nuclear ribonucleoprotein A0	Q13151	0.0555	0.1540	-0.13	0.6477	0.7393	0.07	0.1010	0.2056	0.12	0.6720	0.7133	-0.02	0.0189	0.0980	-0.21	0.0693	0.1849	-0.13	0.0167	0.1203	0.48	0.0033	0.0474	0.54	0.0039	0.0614	0.30
2028	Heterogeneous nuclear ribonucleoprotein A1	P09651	0.0190	0.0811	0.09	0.3195	0.6825	0.10	0.0010	0.0211	0.46	0.0014	0.0172	0.21	0.0023	0.0376	-0.16	0.0294	0.1100	0.27	0.0009	0.0424	0.99	0.0014	0.0306	0.56	0.0037	0.0605	0.84
2029	Heterogeneous nuclear ribonucleoprotein A1-like 2	Q32P51	0.6274	0.6902	0.09	0.2614	0.6638	0.23	0.1367	0.2603	0.36	0.1575	0.2754	0.32	0.2525	0.4708	0.24	0.2231	0.4058	0.27	0.7945	0.8267	0.04	0.3597	0.7284	-0.15	0.6125	0.6723	-0.08
2030	Heterogeneous nuclear ribonucleoprotein A3	P51991	0.3215	0.5257	-0.05	0.6587	0.7481	0.05	0.0522	0.1289	0.14	0.7905	0.8198	0.01	0.0300	0.1260	-0.21	0.2899	0.4866	0.06	0.0008	0.0414	0.80	0.0098	0.0819	0.42	0.0028	0.0547	0.71
2031	Heterogeneous nuclear ribonucleoprotein C-like 2	B2RXH8	0.0046	0.0429	-0.35	0.0898	0.6244	-0.21	0.4112	0.6172	-0.08	0.0154	0.0513	-0.43	0.0197	0.1005	-0.62	0.0444	0.1391	-0.19	0.0029	0.0598	-0.65	0.0003	0.0197	-0.97	0.0057	0.0720	-0.90
2032	Heterogeneous nuclear ribonucleoprotein C-like 3	B7ZW38	0.4108	0.5693	-3.31	0.5752	0.7969	-1.75	0.5661	0.7175	-1.19	0.5388	0.6784	0.89	0.2938	0.4648	-1.88	0.9510	1.0891	0.09	0.2009	0.4133	0.38	0.4524	0.8568	0.19	0.0596	0.2182	0.69
2033	Heterogeneous nuclear ribonucleoprotein D0	Q14103	0.2382	0.4226	-0.05	0.7391	0.8104	0.04	0.0435	0.1134	0.15	0.9130	0.9246	-0.01	0.0115	0.0768	-0.17	0.0941	0.2258	-0.08	0.1036	0.2615	0.24	0.1050	0.3286	-0.17	0.3279	0.3991	-0.07
2034	Heterogeneous nuclear ribonucleoprotein D-like	O14979	0.6406	0.7024	0.04	0.5332	0.8496	0.07	0.1854	0.3306	0.06	0.0574	0.1272	-0.21	0.0596	0.1840	-0.09	0.7791	1.0163	0.03	0.0094	0.0970	0.59	0.0189	0.1178	0.31	0.0105	0.0905	0.33
2035	Heterogeneous nuclear ribonucleoprotein F	P52597	0.0176	0.0776	-0.15	0.4732	0.8018	-0.06	0.0291	0.0866	-0.18	0.0120	0.0440	-0.25	0.0039	0.0464	-0.50	0.0952	0.2278	-0.28	0.0624	0.1966	0.21	0.2598	0.5869	-0.10	0.2084	0.2772	-0.11
2036	Heterogeneous nuclear ribonucleoprotein H	P31943	0.0346	0.1164	0.09	0.7804	0.8417	0.03	0.0033	0.0306	0.31	0.0203	0.0611	0.14	0.2842	0.5126	-0.03	0.0086	0.0678	0.21	0.0217	0.1348	0.45	0.1103	0.3387	0.14	0.0288	0.1448	0.17
2037	Heterogeneous nuclear ribonucleoprotein H2	P55795	0.7693	0.8128	0.01	0.1061	0.6310	-0.09	0.3151	0.5021	0.04	0.1503	0.2656	-0.10	0.7324	0.7905	-0.02	0.2275	0.4103	0.14	0.0605	0.1947	-0.27	0.0353	0.1707	-0.24	0.4879	0.5564	-0.05
2038	Heterogeneous nuclear ribonucleoprotein H3	P31942	0.0009	0.0220	-0.16	0.1437	0.6547	-0.16	0.0042	0.0323	-0.18	0.0041	0.0261	-0.13	0.0277	0.1208	-0.16	0.0299	0.1109	-0.06	0.0373	0.1606	0.35	0.0299	0.1563	0.20	0.0782	0.1288	0.14
2039	Heterogeneous nuclear ribonucleoprotein K	P61978	0.0141	0.0694	0.14	0.3740	0.7215	0.15	0.0075	0.0409	0.24	0.0035	0.0238	0.22	0.0194	0.0998	-0.12	0.0267	0.1045	0.13	0.0390	0.1637	0.44	0.3126	0.6617	0.11	0.6993	0.7512	0.03
2040	Heterogeneous nuclear ribonucleoprotein L	P14866	0.7549	0.8007	0.02	0.9942	0.9963	0.00	0.0135	0.0543	0.19	0.3865	0.5719	0.04	0.0818	0.2207	-0.11	0.1241	0.2715	0.08	0.0042	0.0700	0.86	0.0169	0.1103	0.35	0.0010	0.0392	0.89
2041	Heterogeneous nuclear ribonucleoprotein L-like	Q8WVV9	0.3103	0.5112	-0.08	0.8310	0.8783	0.02	0.1597	0.2941	-0.08	0.0179	0.0564	-0.21	0.0175	0.0948	-0.21	0.4923	0.7173	-0.09	0.0167	0.1200	-0.47	0.0005	0.0216	-0.18	0.1860	0.2535	-0.20
2042	Heterogeneous nuclear ribonucleoprotein M	P52272	0.0007	0.0207	0.25	0.1825	0.6657	0.25	0.0128	0.0532	0.19	0.0049	0.0282	0.15	0.1545	0.3342	-0.04	0.0115	0.0733	0.16	0.0586	0.1927	0.30	0.6055	1.0375	-0.04	0.4740	0.5429	0.05
2043	Heterogeneous nuclear ribonucleoprotein Q	O60506	0.0220	0.0883	0.04	0.3765	0.7242	0.05	0.0000	0.0000	0.30	0.0002	0.0125	0.17	0.0030	0.0419	-0.10	0.0003	0.0389	0.22	0.0113	0.1040	0.54	0.0210	0.1253	0.31	0.0615	0.2218	0.21
2044	Heterogeneous nuclear ribonucleoprotein R	O43390	0.0596	0.1616	0.15	0.2407	0.6607	0.25	0.0129	0.0532	0.30	0.0049	0.0281	0.27	0.0421	0.1519	0.14	0.0508	0.1518	0.17	0.0086	0.0936	0.40	0.1236	0.3654	0.12	0.0043	0.0642	0.45
2045	Heterogeneous nuclear ribonucleoprotein U	Q00839	0.0205	0.0847	-0.09	0.1137	0.6340	-0.08	0.0063	0.0386	0.17	0.0022	0.0202	-0.28	0.0014	0.0290	-0.32	0.0370	0.1247	-0.10	0.0123	0.1051	0.65	0.0025	0.0412	0.42	0.0008	0.0354	0.54
2046	Heterogeneous nuclear ribonucleoprotein U-like protein 1	Q9BUJ2	0.2323	0.4141	0.07	0.2439	0.6580	0.17	0.0015	0.0226	0.41	0.0015	0.0178	0.33	0.0127	0.0804	0.18	0.0165	0.0851	0.30	0.0348	0.1547	0.43	0.1113	0.3403	0.13	0.0654	0.1117	0.18
2047	Heterogeneous nuclear ribonucleoprotein U-like protein 2	Q1KMD3	0.6693	0.7280	-0.08	0.0185	0.4629	-0.32	0.5203	0.7412	-0.11	0.0170	0.0543	-0.31	0.0067	0.0595	-0.53	0.4304	0.6513	-0.07	0.3736	0.4663	-2.55	0.8708	0.9713	-0.33	0.0651	0.1262	2.06
2048	Heterogeneous nuclear ribonucleoproteins A2/B1	P22626	0.4679	0.5442	-0.04	0.9352	0.9562	-0.01	0.0038	0.0313	0.32	0.6199	0.6669	-0.02	0.0045	0.0498	-0.43	0.5684	0.8038	-0.03	0.0028	0.0594	1.02	0.0192	0.1190	0.36	0.0052	0.0696	0.79
2049	Heterogeneous nuclear ribonucleoproteins C1/C2	P07910	0.6157	0.6800	-0.02	0.5907	0.6957	-0.04	0.0829	0.1773	-0.04	0.0120	0.0439	-0.09	0.0029	0.0409	-0.24	0.1003	0.2344	-0.05	0.1382	0.3180	0.17	0.2045	0.5042	0.10	0.0137	0.1014	0.31
2050	Hexokinase-1	P19367	0.0007	0.0206	0.36	0.3398	0.6990	0.13	0.0119	0.0510	0.16	0.0473	0.1108	0.10	0.4254	0.5198	0.03	0.0085	0.0674	0.23	0.8132	0.8420	-0.02	0.1568	0.4286	-0.12	0.2046	0.2734	-0.10
2051	Hexokinase-2	P52789	0.3973	0.6162	0.12	0.7078	0.7853	-0.12	0.2801	0.4553	0.16	0.1044	0.2001	0.29	0.3319	0.4292	0.16	0.2201	0.4016	0.21	0.0561	0.1876	-0.13	0.0046	0.0545	-0.28	0.0309	0.1490	-0.14
2052	Hexokinase-3	P52790	0.0079	0.0525	-0.31	0.0022	0.2279	-0.61	0.0053	0.0357	-0.62	0.0002	0.0099	-0.83	0.0020	0.0357	-0.56	0.2244	0.4073	0.12	0.3736	0.4970	-2.55	0.8708	1.0280	-0.33	0.0651	0.1395	2.06
2053	HHIP-like protein 1	Q96JK4	0.0003	0.0159	-2.00	0.5556	0.8703	-0.36	0.1631	0.2990	-0.27	0.4102	0.6018	-0.13	0.0699	0.2014	0.34	0.0185	0.0880	-0.61	0.4390	0.4946	-0.05	0.0189	0.1175	-0.21	0.1284	0.1879	-0.03
2054	High affinity cAMP-specific 3'_5'-cyclic phosphodiesterase 7A	Q13946	0.0247	0.0949	-0.34	0.0451	0.5643	0.10	0.1540	0.2855	-0.32	0.3347	0.5087	-0.06	0.0308	0.1283	-0.17	0.1060	0.2433	0.16	0.3736	0.5318	-2.55	0.8708	1.0914	-0.33	0.0651	0.1560	2.06

Supplemetary Table S2. Overview on all relatively quantified 5180 proteins statistical analysis

			MCF-7																		MDA-MB-231								
			Dai_SC20 vs control			Gen_SC20 vs control			SSE_SC20 vs control			Dai_IC20 vs control			Gen_IC20 vs control			SSE_IC20 vs control			Dai_IC20 vs control			Gen_IC20 vs control			SSE_IC20 vs control		
	**Protein name**	**UniProt**	**p value**	**BH q value**	**log2FC**	**p value**	**BH q value**	**log2FC**	**p value**	**BH q value**	**log2FC**	**p value**	**BH q value**	**log2FC**	**p value**	**BH q value**	**log2FC**	**p value**	**BH q value**	**log2FC**	**p value**	**BH q value**	**log2FC**	**p value**	**BH q value**	**log2FC**	**p value**	**BH q value**	**log2FC**
2055	High affinity cAMP-specific and IBMX-insensitive 3'_5'-cyclic phosphodiesterase 8A	O60658	0.1064	0.2347	-0.57	0.8436	0.8876	-0.04	0.3468	0.5409	-0.27	0.5210	0.7261	-0.14	0.7905	0.8357	0.05	0.7329	0.9719	-0.10	0.3736	0.4431	-2.55	0.8708	0.9278	-0.33	0.0651	0.1167	2.06
2056	High mobility group nucleosome-binding domain-containing protein 5	P82970	0.0013	0.0251	0.46	0.0071	0.4132	0.85	0.0001	0.0324	0.96	0.0003	0.0123	0.67	0.0007	0.0218	1.19	0.0034	0.0589	0.50	0.0109	0.1025	0.40	0.0944	0.3068	0.17	0.2067	0.2755	-0.12
2057	High mobility group protein B1	P09429	0.8800	0.9055	0.00	0.6305	0.7269	0.11	0.1064	0.2144	0.15	0.8883	0.9047	0.00	0.0003	0.0138	-0.38	0.0675	0.1823	-0.05	0.1969	0.4064	0.22	0.6861	1.1279	0.04	0.4615	0.5297	-0.05
2058	High mobility group protein B2	P26583	0.0253	0.0961	0.16	0.2496	0.6600	0.28	0.0116	0.0504	0.31	0.0034	0.0235	0.29	0.0703	0.2021	-0.11	0.0221	0.0953	0.18	0.0544	0.1853	0.47	0.0470	0.2019	0.19	0.1284	0.1879	0.11
2059	High mobility group protein B3	O15347	0.0633	0.1666	0.15	0.2859	0.6665	0.27	0.0117	0.0506	0.28	0.0537	0.1213	0.18	0.0587	0.1821	-0.17	0.1478	0.3041	0.10	0.3984	0.4545	0.11	0.4650	0.8730	-0.08	0.1335	0.1938	-0.12
2060	High mobility group protein HMG-I/HMG-Y	P17096	0.4108	0.5691	-3.31	0.5752	0.7967	-1.75	0.5661	0.7173	-1.19	0.5388	0.6782	0.89	0.2938	0.4647	-1.88	0.9510	1.0889	0.09	0.1134	0.2779	0.39	0.5104	0.9293	-0.08	0.3210	0.3922	-0.07
2061	Hippocalcin-like protein 1	P37235	0.0049	0.0433	0.40	0.2372	0.6620	0.05	0.0138	0.0551	0.08	0.0092	0.0386	0.12	0.8780	0.9069	-0.01	0.4010	0.6158	0.40	0.3173	0.5798	-0.37	0.1297	0.3766	0.44	0.0116	0.0936	0.51
2062	Hippocalcin-like protein 4	Q9UM19	0.5777	0.6451	-0.33	0.8855	0.9183	-0.11	0.0091	0.0446	-3.16	0.7900	0.8194	0.29	0.3325	0.4296	0.47	0.6632	0.9014	-0.20	0.3736	0.4470	-2.55	0.8708	0.9353	-0.33	0.0651	0.1183	2.06
2063	Histidine triad nucleotide-binding protein 1	P49773	0.0092	0.0571	0.11	0.3367	0.6954	0.21	0.0158	0.0593	0.26	0.0025	0.0211	0.30	0.4334	0.5275	0.02	0.0069	0.0641	0.22	0.0551	0.1865	0.37	0.0339	0.1664	0.31	0.1146	0.1722	0.13
2064	Histidine triad nucleotide-binding protein 2_ mitochondrial	Q9BX68	0.0442	0.1337	-0.09	0.1037	0.6335	-0.18	0.4638	0.6806	-0.04	0.0299	0.0802	-0.12	0.5810	0.6593	-0.02	0.7042	0.9462	0.01	0.2626	0.5034	0.11	0.0142	0.0995	0.31	0.4610	0.5293	-0.11
2065	Histidine--tRNA ligase_ cytoplasmic	P12081	0.0559	0.1546	0.14	0.2257	0.6609	0.16	0.0162	0.0602	0.24	0.0276	0.0756	0.22	0.3219	0.4190	0.06	0.0758	0.1957	0.13	0.0948	0.2478	0.24	0.7470	1.1880	-0.02	0.0576	0.2137	-0.15
2066	Histone acetyltransferase KAT8	Q9H7Z6	0.3703	0.5848	-0.05	0.3175	0.6810	-0.08	0.0031	0.0300	0.32	0.5174	0.7226	-0.04	0.0155	0.0886	-0.21	0.3571	0.5633	0.07	0.0586	0.1922	0.23	0.2053	0.5057	-0.09	0.1912	0.2590	-0.07
2067	Histone acetyltransferase p300	Q09472	0.4108	0.5690	-3.31	0.5752	0.7965	-1.75	0.5661	0.7171	-1.19	0.5388	0.6781	0.89	0.2938	0.4646	-1.88	0.9510	1.0887	0.09	0.0932	0.2451	0.33	0.6897	1.1320	-0.03	0.0410	0.1758	-0.16
2068	Histone acetyltransferase type B catalytic subunit	O14929	0.0248	0.0951	0.59	0.8466	0.8895	-0.09	0.2069	0.3600	-0.25	0.0565	0.1259	0.86	0.4875	0.5775	-0.13	0.0131	0.0767	0.82	0.1406	0.3214	0.08	0.0611	0.2364	-0.10	0.7225	0.7709	-0.02
2069	Histone chaperone ASF1A	Q9Y294	0.9426	0.9570	-0.01	0.1983	0.6661	-0.57	0.1839	0.3286	-0.22	0.0410	0.1002	-0.47	0.1188	0.2828	-0.23	0.7408	0.9802	-0.03	0.3736	0.4423	-2.55	0.8708	0.9264	-0.33	0.0651	0.1164	2.06
2070	Histone chaperone ASF1B	Q9NVP2	0.0245	0.0946	-0.59	0.0526	0.5822	-0.56	0.0000	0.0000	-0.51	0.0152	0.0509	-0.71	0.0017	0.0320	-1.11	0.0426	0.1363	-1.24	0.3736	0.5682	-2.55	0.8708	1.1566	-0.33	0.0651	0.1748	2.06
2071	Histone deacetylase 1	Q13547	0.0008	0.0210	-0.15	0.8397	0.8850	-0.02	0.4207	0.6287	-0.02	0.0412	0.1005	-0.09	0.0008	0.0214	-0.19	0.3065	0.5050	0.03	0.0119	0.1038	0.68	0.0039	0.0505	0.42	0.0047	0.0673	0.39
2072	Histone deacetylase 11	Q96DB2	0.0152	0.0716	-0.09	0.4594	0.7914	-0.07	0.5004	0.7230	0.05	0.5473	0.5981	-0.02	0.1478	0.3239	0.09	0.4301	0.6511	-0.12	0.3736	0.4664	-2.55	0.8708	0.9715	-0.33	0.0651	0.1263	2.06
2073	Histone deacetylase 2	Q92769	0.1571	0.3108	-0.09	0.0401	0.5645	-0.17	0.8872	0.9106	-0.01	0.0118	0.0435	-0.26	0.0031	0.0416	-0.35	0.1072	0.2453	-0.16	0.2586	0.4974	0.12	0.1532	0.4230	0.09	0.9750	0.9797	0.00
2074	Histone deacetylase 7	Q8WUI4	0.0374	0.1215	-0.56	0.0024	0.2391	-0.74	0.0083	0.0431	-0.65	0.0040	0.0257	-2.05	0.0913	0.2387	-0.30	0.0775	0.1986	0.40	0.3736	0.5450	-2.55	0.8708	1.1151	-0.33	0.0651	0.1626	2.06
2075	Histone H1.0	P07305	0.3013	0.5004	0.21	0.3092	0.6784	-0.39	0.0085	0.0435	0.48	0.1006	0.1950	0.19	0.2314	0.4417	-0.15	0.9540	0.9620	0.01	0.0013	0.0468	1.08	0.5176	0.9391	0.14	0.4755	0.5442	0.41
2076	Histone H1.2	P16403	0.0233	0.0914	-0.46	0.0980	0.6346	-0.22	0.0193	0.0668	-0.39	0.0055	0.0297	-0.66	0.0032	0.0425	-0.84	0.0674	0.1821	-0.91	0.0278	0.1444	0.60	0.9203	0.9416	-0.01	0.0173	0.1120	0.37
2077	Histone H1.4	P10412	0.3216	0.5257	-0.07	0.7413	0.8122	0.07	0.0511	0.1270	0.19	0.7371	0.7732	0.02	0.0030	0.0405	-0.58	0.2867	0.4830	-0.07	0.3736	0.4841	-2.55	0.8708	1.0042	-0.33	0.0651	0.1338	2.06
2078	Histone H1.5	P16401	0.1052	0.2327	0.15	0.4963	0.8185	0.14	0.0212	0.0704	0.31	0.0270	0.0744	0.25	0.0348	0.1354	-0.25	0.1743	0.3421	0.11	0.0514	0.1795	0.51	0.8367	1.2804	0.02	0.0025	0.0535	0.49
2079	Histone H1t	P22492	0.3812	0.5969	-0.07	0.8188	0.8704	0.03	0.7219	0.7668	0.03	0.8114	0.8374	0.02	0.1509	0.3286	-0.12	0.9185	1.1557	-0.01	0.3736	0.4332	-2.55	0.8708	0.9092	-0.33	0.0651	0.1128	2.06
2080	Histone H1x	Q92522	0.0060	0.0467	-0.14	0.0934	0.6291	-0.17	0.0348	0.0970	-0.11	0.0003	0.0105	-0.29	0.0000	0.0000	-0.67	0.0695	0.1853	-0.27	0.1599	0.3529	0.11	0.1798	0.4673	0.07	0.1994	0.2679	0.06
2081	Histone H2A type 1	P0C0S8	0.6261	0.6895	-0.03	0.3850	0.7302	-0.14	0.6485	0.7009	-0.03	0.0416	0.1012	0.17	0.0035	0.0438	-0.56	0.0632	0.1753	0.16	0.0048	0.0736	0.66	0.0130	0.0951	0.49	0.0002	0.0235	1.89
2082	Histone H2A.Z	P0C0S5	0.4108	0.5688	-3.31	0.5752	0.7962	-1.75	0.5661	0.7170	-1.19	0.5388	0.6779	0.89	0.2938	0.4644	-1.88	0.9510	1.0884	0.09	0.2770	0.5237	-0.14	0.2374	0.5534	-0.14	0.3778	0.4491	0.06
2083	Histone H2B type 1-K	O60814	0.3657	0.5791	-0.14	0.2091	0.6613	-0.51	0.1196	0.2360	0.27	0.1159	0.2172	0.29	0.3857	0.4832	-0.12	0.2574	0.4482	0.17	0.3736	0.4887	-2.55	0.8708	1.0127	-0.33	0.0651	0.1358	2.06
2084	Histone H2B type 1-L	Q99880	0.4108	0.5687	-3.31	0.5752	0.7960	-1.75	0.5661	0.7168	-1.19	0.5388	0.6778	0.89	0.2938	0.4643	-1.88	0.9510	1.0882	0.09	0.0217	0.1343	0.56	0.2534	0.5775	0.15	0.0005	0.0282	1.48
2085	Histone H3.1	P68431	0.1751	0.3369	0.08	0.4473	0.7822	0.12	0.9397	0.9529	-0.01	0.0273	0.0749	0.20	0.0357	0.1370	-0.19	0.6652	0.9037	-0.02	0.0207	0.1324	0.53	0.8993	0.9246	0.02	0.0040	0.0615	1.28
2086	Histone H4	P62805	0.9822	0.9877	0.00	0.6348	0.7312	0.09	0.5982	0.6543	-0.03	0.0015	0.0170	0.37	0.0005	0.0190	-0.83	0.6378	0.8747	-0.02	0.0206	0.1329	0.67	0.0138	0.0978	0.36	0.0003	0.0243	1.85
2087	Histone PARylation factor 1	Q9NWY4	0.2784	0.4724	-0.06	0.8264	0.8752	-0.02	0.1808	0.3245	-0.08	0.1850	0.3123	-0.25	0.0301	0.1264	-0.24	0.2539	0.4442	-0.15	0.3736	0.4896	-2.55	0.8708	1.0143	-0.33	0.0651	0.1362	2.06
2088	Histone-arginine methyltransferase CARM1	Q86X55	0.0908	0.2109	0.15	0.2885	0.6686	0.17	0.7121	0.7577	0.03	0.8836	0.9003	0.01	0.3900	0.4869	-0.06	0.9776	0.9820	0.00	0.3736	0.4301	-2.55	0.8708	0.9032	-0.33	0.0651	0.1116	2.06
2089	Histone-binding protein RBBP4	Q09028	0.0013	0.0250	0.22	0.0019	0.2237	0.16	0.0007	0.0216	0.39	0.0036	0.0242	0.25	0.0181	0.0961	0.26	0.0010	0.0454	0.41	0.4507	0.5059	0.18	0.7898	1.2326	-0.09	0.0659	0.1124	0.61
2090	Histone-binding protein RBBP7	Q16576	0.0036	0.0385	0.18	0.5167	0.8393	0.07	0.0048	0.0336	0.27	0.0004	0.0117	0.19	0.0552	0.1755	-0.09	0.0343	0.1195	0.27	0.9449	0.9543	0.01	0.9964	0.9974	0.00	0.0505	0.1967	-0.18
2091	Histone-lysine N-methyltransferase 2D	O14686	0.4108	0.5685	-3.31	0.5752	0.7958	-1.75	0.5661	0.7166	-1.19	0.5388	0.6776	0.89	0.2938	0.4641	-1.88	0.9510	1.0879	0.09	0.3475	0.6216	0.14	0.5975	1.0310	0.14	0.2098	0.2785	0.30
2092	Histone-lysine N-methyltransferase ASH1L	Q9NR48	0.1154	0.2488	0.12	0.1142	0.6327	0.14	0.0027	0.0287	0.29	0.0028	0.0221	0.33	0.0000	0.0000	0.18	0.0006	0.0432	0.29	0.3736	0.6520	-2.55	0.8708	1.3029	-0.33	0.0651	0.2262	2.06
2093	Histone-lysine N-methyltransferase EZH2	Q15910	0.9082	0.9285	-0.02	0.9223	0.9462	-0.06	0.3630	0.5603	0.42	0.0538	0.1214	-0.56	0.3794	0.4765	-0.42	0.5145	0.7418	0.07	0.3736	0.4592	-2.55	0.8708	0.9581	-0.33	0.0651	0.1233	2.06
2094	Histone-lysine N-methyltransferase KMT5B	Q4FZB7	0.0152	0.0716	0.17	0.2997	0.6735	-0.22	0.9864	0.9887	0.00	0.0017	0.0180	-0.36	0.4865	0.5765	0.03	0.0627	0.1741	-0.15	0.3736	0.5540	-2.55	0.8708	1.1314	-0.33	0.0651	0.1673	2.06
2095	Histone-lysine N-methyltransferase SETD7	Q8WTS6	0.3560	0.5665	-0.03	0.1640	0.6637	-0.04	0.1879	0.3339	0.11	0.0228	0.0662	0.08	0.4341	0.5282	0.07	0.2874	0.4837	0.05	0.3736	0.4839	-2.55	0.8708	1.0039	-0.33	0.0651	0.1337	2.06
2096	Histone-lysine N-methyltransferase SUV39H1	O43463	0.0579	0.1584	0.36	0.0556	0.5842	0.43	0.0280	0.0845	0.41	0.0172	0.0548	0.50	0.0497	0.1644	0.37	0.0572	0.1644	0.37	0.0010	0.0428	0.57	0.0004	0.0218	0.40	0.0149	0.1052	0.67
2097	HIV Tat-specific factor 1	O43719	0.0031	0.0366	0.24	0.0571	0.5834	0.28	0.0018	0.0243	0.32	0.0006	0.0131	0.33	0.2066	0.4088	0.04	0.0218	0.0951	0.28	0.2125	0.4307	0.26	0.1266	0.3711	0.33	0.0524	0.2015	0.59
2098	HLA class I histocompatibility antigen_ A-1 alpha chain	P30443	0.4108	0.5684	-3.31	0.5752	0.7956	-1.75	0.5661	0.7164	-1.19	0.5388	0.6774	0.89	0.2938	0.4640	-1.88	0.9510	1.0877	0.09	0.0048	0.0727	0.30	0.2087	0.5102	0.06	0.0769	0.1271	0.07
2099	HLA class I histocompatibility antigen_ A-2 alpha chain	P01892	0.0471	0.1393	-0.59	0.1237	0.6408	-0.80	0.4277	0.6375	-0.10	0.0613	0.1339	-0.51	0.6151	0.6889	-0.14	0.0072	0.0644	-0.33	0.3736	0.6213	-2.55	0.8708	1.2499	-0.33	0.0651	0.2059	2.06
2100	HLA class I histocompatibility antigen_ A-23 alpha chain	P30447	0.8280	0.8621	0.03	0.4511	0.7852	0.12	0.1894	0.3359	0.31	0.7769	0.8083	0.05	0.8892	0.9150	0.01	0.7759	1.0137	0.02	0.3736	0.4404	-2.55	0.8708	0.9228	-0.33	0.0651	0.1156	2.06
2101	HLA class I histocompatibility antigen_ A-24 alpha chain	P05534	0.0476	0.1400	-0.16	0.8224	0.8731	-0.03	0.3164	0.5034	0.13	0.7845	0.8149	-0.01	0.0077	0.0631	-0.25	0.1540	0.3122	-0.23	0.3736	0.5147	-2.55	0.8708	1.0604	-0.33	0.0651	0.1477	2.06
2102	HLA class I histocompatibility antigen_ A-3 alpha chain	P04439	0.7976	0.8360	0.05	0.5234	0.8423	0.24	0.0347	0.0968	0.61	0.0091	0.0383	1.06	0.0158	0.0896	0.81	0.0171	0.0856	1.02	0.4386	0.4942	-0.06	0.0083	0.0746	-0.17	0.2799	0.3518	-0.07
2103	HLA class I histocompatibility antigen_ A-30 alpha chain	P16188	0.4108	0.5682	-3.31	0.5752	0.7954	-1.75	0.5661	0.7163	-1.19	0.5388	0.6773	0.89	0.2938	0.4638	-1.88	0.9510	1.0875	0.09	0.0603	0.1944	0.37	0.8864	0.9141	0.01	0.5558	0.6205	0.04
2104	HLA class I histocompatibility antigen_ A-32 alpha chain	P10314	0.4108	0.5681	-3.31	0.5752	0.7952	-1.75	0.5661	0.7161	-1.19	0.5388	0.6771	0.89	0.2938	0.4637	-1.88	0.9510	1.0872	0.09	0.0554	0.1871	-0.85	0.0573	0.2290	-0.91	0.0389	0.1703	-1.02
2105	HLA class I histocompatibility antigen_ A-68 alpha chain	P01891	0.4108	0.5679	-3.31	0.5752	0.7950	-1.75	0.5661	0.7159	-1.19	0.5388	0.6769	0.89	0.2938	0.4636	-1.88	0.9510	1.0870	0.09	0.0021	0.0539	0.21	0.3470	0.7088	0.05	0.4943	0.5618	-0.08
2106	HLA class I histocompatibility antigen_ alpha chain E	P13747	0.0022	0.0322	-1.28	0.2030	0.6601	-1.22	0.0007	0.0204	-1.74	0.0015	0.0179	-1.25	0.2971	0.3951	0.17	0.0100	0.0703	-1.27	0.3426	0.6143	0.12	0.1302	0.3774	0.15	0.0156	0.1075	0.30
2107	HLA class I histocompatibility antigen_ alpha chain F	P30511	0.4108	0.5678	-3.31	0.5752	0.7948	-1.75	0.5661	0.7157	-1.19	0.5388	0.6768	0.89	0.2938	0.4634	-1.88	0.9510	1.0867	0.09	0.0096	0.0977	3.91	0.0077	0.0725	5.16	0.0051	0.0690	5.66
2108	HLA class I histocompatibility antigen_ alpha chain G	P17693	0.0444	0.1341	-0.12	0.2522	0.6625	0.17	0.0298	0.0876	0.26	0.0046	0.0275	0.29	0.0099	0.0718	0.18	0.6136	0.8501	0.06	0.3460	0.6191	0.13	0.0288	0.1532	-0.40	0.3611	0.4326	-0.10
2109	HLA class I histocompatibility antigen_ B-13 alpha chain	P30461	0.0031	0.0365	-0.43	0.7188	0.7939	-0.21	0.0104	0.0475	0.31	0.3017	0.4669	0.11	0.3089	0.4066	0.13	0.1191	0.2635	-0.25	0.2890	0.5408	0.06	0.0376	0.1766	0.08	0.0747	0.1245	0.06
2110	HLA class I histocompatibility antigen_ B-27 alpha chain	P03989	0.4108	0.5676	-3.31	0.5752	0.7945	-1.75	0.5661	0.7156	-1.19	0.5388	0.6766	0.89	0.2938	0.4633	-1.88	0.9510	1.0865	0.09	0.1270	0.3004	-0.28	0.0310	0.1590	0.12	0.1844	0.2518	0.19
2111	HLA class I histocompatibility antigen_ B-45 alpha chain	P30483	0.4108	0.5675	-3.31	0.5752	0.7943	-1.75	0.5661	0.7154	-1.19	0.5388	0.6764	0.89	0.2938	0.4631	-1.88	0.9510	1.0863	0.09	0.1777	0.3771	0.21	0.2296	0.5426	0.14	0.5205	0.5869	0.07
2112	HLA class I histocompatibility antigen_ B-49 alpha chain	P30487	0.4320	0.5094	-0.04	0.2211	0.6590	0.26	0.7745	0.8143	-0.01	0.1576	0.2753	0.08	0.0025	0.0383	0.20	0.7050	0.9471	-0.02	0.3736	0.4454	-2.55	0.8708	0.9322	-0.33	0.0651	0.1176	2.06
2113	HLA class I histocompatibility antigen_ B-53 alpha chain	P30491	0.4108	0.5673	-3.31	0.5752	0.7941	-1.75	0.5661	0.7152	-1.19	0.5388	0.6763	0.89	0.2938	0.4630	-1.88	0.9510	1.0860	0.09	0.0424	0.1673	0.23	0.4790	0.8900	0.05	0.0205	0.1225	0.35
2114	HLA class I histocompatibility antigen_ B-7 alpha chain	P01889	0.0845	0.2007	0.12	0.7146	0.7908	0.04	0.2638	0.4341	-0.11	0.5740	0.6232	-0.02	0.0949	0.2434	-0.10	0.3905	0.6024	-0.04	0.3736	0.4701	-2.55	0.8708	0.9783	-0.33	0.0651	0.1278	2.06
2115	HLA class I histocompatibility antigen_ B-8 alpha chain	P30460	0.3916	0.6088	-0.10	0.2292	0.6607	0.19	0.7583	0.7993	0.03	0.1730	0.2957	0.18	0.8679	0.8991	0.02	0.8297	1.0638	-0.03	0.3736	0.4363	-2.55	0.8708	0.9150	-0.33	0.0651	0.1140	2.06
2116	HLA class I histocompatibility antigen_ Cw-12 alpha chain	P30508	0.4108	0.5671	-3.31	0.5752	0.7939	-1.75	0.5661	0.7150	-1.19	0.5388	0.6761	0.89	0.2938	0.4629	-1.88	0.9510	1.0858	0.09	0.5635	0.6136	0.04	0.0604	0.2347	0.09	0.4249	0.4963	0.09
2117	HLA class I histocompatibility antigen_ Cw-16 alpha chain	Q29960	0.4108	0.5670	-3.31	0.5752	0.7937	-1.75	0.5661	0.7149	-1.19	0.5388	0.6759	0.89	0.2938	0.4627	-1.88	0.9510	1.0855	0.09	0.4011	0.4569	0.10	0.4195	0.8114	0.10	0.2881	0.3600	0.20
2118	HLA class I histocompatibility antigen_ Cw-17 alpha chain	Q95604	0.4108	0.5668	-3.31	0.5752	0.7935	-1.75	0.5661	0.7147	-1.19	0.5388	0.6758	0.89	0.2938	0.4626	-1.88	0.9510	1.0853	0.09	0.0157	0.1168	-0.52	0.0885	0.2954	-0.36	0.0013	0.0437	-0.94
2119	HLA class I histocompatibility antigen_ Cw-2 alpha chain	P30501	0.0794	0.1932	-0.16	0.4629	0.7948	-0.10	0.7494	0.7916	0.02	0.2797	0.4376	-0.10	0.0239	0.1117	-0.24	0.3545	0.5599	-0.06	0.3736	0.4739	-2.55	0.8708	0.9853	-0.33	0.0651	0.1294	2.06
2120	HLA class I histocompatibility antigen_ Cw-4 alpha chain	P30504	0.4108	0.5667	-3.31	0.5752	0.7933	-1.75	0.5661	0.7145	-1.19	0.5388	0.6756	0.89	0.2938	0.4624	-1.88	0.9510	1.0851	0.09	0.2884	0.5403	0.10	0.8746	0.9050	-0.01	0.0794	0.1302	0.20
2121	HLA class I histocompatibility antigen_ Cw-7 alpha chain	P10321	0.9796	0.9863	0.00	0.9526	0.9685	-0.01	0.0948	0.1958	0.20	0.0662	0.1422	0.25	0.1342	0.3037	0.17	0.3031	0.5013	0.10	0.3736	0.4815	-2.55	0.8708	0.9995	-0.33	0.0651	0.1327	2.06

Supplemetary Table S2. Overview on all relatively quantified 5180 proteins statistical analysis

			MCF-7																		MDA-MB-231								
			Dai SC20 vs control			Gen SC20 vs control			SSE SC20 vs control			Dai IC20 vs control			Gen IC20 vs control			SSE IC20 vs control			Dai IC20 vs control			Gen IC20 vs control			SSE IC20 vs control		
	**Protein name**	**UniProt**	**p value**	**BH q value**	**log2FC**	**p value**	**BH q value**	**log2FC**	**p value**	**BH q value**	**log2FC**	**p value**	**BH q value**	**log2FC**	**p value**	**BH q value**	**log2FC**	**p value**	**BH q value**	**log2FC**	**p value**	**BH q value**	**log2FC**	**p value**	**BH q value**	**log2FC**	**p value**	**BH q value**	**log2FC**
2122	HLA class II histocompatibility antigen_ DRB1-10 beta chain	Q30167	0.4108	0.5665	-3.31	0.5752	0.7931	-1.75	0.5661	0.7143	-1.19	0.5388	0.6755	0.89	0.2938	0.4623	-1.88	0.9510	1.0848	0.09	0.0038	0.0665	-0.46	0.0057	0.0610	-0.54	0.0578	0.2143	-0.48
2123	Holliday junction recognition protein	Q8NCD3	0.4108	0.5664	-3.31	0.5752	0.7929	-1.75	0.5661	0.7142	-1.19	0.5388	0.6753	0.89	0.2938	0.4622	-1.88	0.9510	1.0846	0.09	0.1666	0.3612	-0.17	0.1549	0.4252	-0.11	0.8264	0.8596	0.01
2124	Homeobox protein aristaless-like 4	Q9H161	0.0500	0.1442	0.15	0.4192	0.7574	-0.34	0.0008	0.0203	0.49	0.0039	0.0255	0.44	0.0265	0.1180	0.18	0.0126	0.0756	0.25	0.3736	0.6070	-2.55	0.8708	1.2251	-0.33	0.0651	0.1971	2.06
2125	Homeobox protein cut-like 1	P39880	0.6927	0.7475	-0.02	0.7772	0.8393	0.03	0.0304	0.0887	0.17	0.0991	0.1928	-0.11	0.0272	0.1192	-0.18	0.2895	0.4863	-0.06	0.3736	0.4837	-2.55	0.8708	1.0035	-0.33	0.0651	0.1336	2.06
2126	Homeobox protein cut-like 2	O14529	0.1264	0.2653	0.21	0.1431	0.6542	0.18	0.2012	0.3531	0.29	0.0214	0.0633	0.33	0.0665	0.1963	0.21	0.1703	0.3357	0.43	0.0100	0.1002	0.61	0.0206	0.1239	0.32	0.0803	0.1311	0.15
2127	Homeobox protein engrailed-2	P19622	0.0626	0.1656	0.12	0.0762	0.6177	0.21	0.6358	0.6890	0.01	0.0001	0.0065	0.42	0.5297	0.6156	0.02	0.0011	0.0460	0.28	0.3736	0.6481	-2.55	0.8708	1.2962	-0.33	0.0651	0.2235	2.06
2128	Homeobox protein Hox-A13	P31271	0.0384	0.1231	-1.40	0.3611	0.7099	-1.91	0.1763	0.3188	-3.10	0.1159	0.2171	-1.63	0.3713	0.4684	-0.63	0.1621	0.3237	-1.00	0.3736	0.5114	-2.55	0.8708	1.0544	-0.33	0.0651	0.1462	2.06
2129	Homeobox protein Hox-B5	P09067	0.2585	0.4474	0.10	0.4597	0.7911	-0.06	0.7102	0.7559	-0.03	0.8475	0.8686	0.02	0.4848	0.5748	-0.06	0.0751	0.1948	0.20	0.3736	0.5467	-2.55	0.8708	1.1182	-0.33	0.0651	0.1635	2.06
2130	Homeobox protein Hox-C11	O43248	0.7623	0.8067	-0.07	0.7815	0.8427	0.07	0.9668	0.9728	-0.01	0.7929	0.8216	0.06	0.6293	0.7003	-0.16	0.3828	0.5923	-0.19	0.3736	0.4706	-2.55	0.8708	0.9793	-0.33	0.0651	0.1280	2.06
2131	Homeobox protein Hox-C5	Q00444	0.0033	0.0377	-0.22	0.4737	0.8016	0.10	0.5907	0.6469	-0.03	0.0027	0.0215	-0.23	0.2390	0.4522	-0.08	0.0084	0.0670	-0.30	0.3736	0.6171	-2.55	0.8708	1.2426	-0.33	0.0651	0.2033	2.06
2132	Homeobox protein Hox-C6	P09630	0.0115	0.0617	-0.34	0.7584	0.8250	0.06	0.0078	0.0415	-0.38	0.0189	0.0582	-0.23	0.0659	0.1953	-0.15	0.0985	0.2315	-0.33	0.7997	0.8310	-0.03	0.2949	0.6362	0.11	0.0282	0.1435	0.39
2133	Homeobox protein Nkx-2.8	O15522	0.9188	0.9380	0.00	0.0187	0.4657	0.17	0.8284	0.8615	0.03	0.0487	0.1130	0.13	0.0329	0.1325	0.19	0.8225	1.0572	-0.03	0.3736	0.4368	-2.55	0.8708	0.9159	-0.33	0.0651	0.1142	2.06
2134	Homeobox protein Nkx-6.1	P78426	0.2664	0.4580	0.51	0.1657	0.6618	0.42	0.0826	0.1770	0.59	0.1184	0.2207	-0.51	0.5288	0.6150	0.14	0.5004	0.7257	0.36	0.3736	0.4599	-2.55	0.8708	0.9593	-0.33	0.0651	0.1235	2.06
2135	Homeobox protein SIX1	Q15475	0.0182	0.0791	1.37	0.1775	0.6629	0.69	0.0557	0.1348	0.93	0.0651	0.1404	0.87	0.3142	0.4114	0.36	0.0355	0.1217	1.25	0.3736	0.5743	-2.55	0.8708	1.1674	-0.33	0.0651	0.1781	2.06
2136	Host cell factor 1	P51610	0.4108	0.5662	-3.31	0.5752	0.7926	-1.75	0.5661	0.7140	-1.19	0.5388	0.6751	0.89	0.2938	0.4620	-1.88	0.9510	1.0843	0.09	0.6399	0.6850	-0.02	0.0291	0.1538	0.20	0.0718	0.1203	0.14
2137	Hsc70-interacting protein	P50502	0.0061	0.0469	0.19	0.1413	0.6529	0.24	0.0010	0.0216	0.27	0.0010	0.0157	0.28	0.0150	0.0874	0.13	0.0647	0.1774	0.21	0.3736	0.5518	-2.55	0.8708	1.1274	-0.33	0.0651	0.1661	2.06
2138	Hsp70-binding protein 1	Q9NZL4	0.0102	0.0590	0.50	0.6712	0.7586	0.07	0.2267	0.3857	-0.13	0.2396	0.3857	-0.12	0.6252	0.6963	-0.04	0.2984	0.4954	0.72	0.0672	0.2034	-0.27	0.2204	0.5281	-0.14	0.1870	0.2544	-0.16
2139	Hsp90 co-chaperone Cdc37	Q16543	0.5372	0.6078	0.02	0.0436	0.5703	0.12	0.0029	0.0297	0.17	0.6537	0.6972	0.02	0.2268	0.4359	0.03	0.7603	0.9981	-0.01	0.1249	0.2975	0.23	0.6440	1.0824	-0.04	0.3865	0.4577	0.04
2140	Huntingtin-associated protein 1	P54257	0.4108	0.5661	-3.31	0.5752	0.7924	-1.75	0.5661	0.7138	-1.19	0.5388	0.6750	0.89	0.2938	0.4619	-1.88	0.9510	1.0841	0.09	0.0466	0.1729	0.27	0.1390	0.3954	-0.08	0.4148	0.4862	-0.09
2141	Huntingtin-interacting protein 1-related protein	O75146	0.3876	0.6046	0.03	0.5177	0.8396	-0.04	0.0097	0.0457	0.29	0.0198	0.0600	0.18	0.5452	0.6295	0.03	0.1007	0.2348	0.38	0.6949	0.7361	0.07	0.3380	0.6959	0.13	0.0146	0.1042	0.65
2142	Hydroxyacyl-coenzyme A dehydrogenase_ mitochondrial	Q16836	0.1775	0.3398	-0.08	0.5556	0.8700	-0.05	0.9024	0.9227	0.01	0.9447	0.9519	0.00	0.0472	0.1596	-0.13	0.3393	0.5436	0.06	0.3736	0.4754	-2.55	0.8708	0.9881	-0.33	0.0651	0.1300	2.06
2143	Hydroxyacylglutathione hydrolase mitochondrial	Q16775	0.7839	0.8247	0.05	0.2615	0.6637	0.22	0.1331	0.2554	0.26	0.1804	0.3052	0.22	0.1903	0.3866	0.21	0.2648	0.4574	0.17	0.2219	0.4448	0.25	0.6881	1.1297	0.06	0.7691	0.8101	0.08
2144	Hydroxymethylglutaryl-CoA lyase mitochondrial	P35914	0.3179	0.5206	-0.18	0.8072	0.8611	0.04	0.5150	0.7355	0.08	0.4368	0.6304	-0.11	0.1254	0.2925	-0.23	0.3260	0.5282	0.14	0.3899	0.4456	0.08	0.1721	0.4541	0.11	0.2668	0.3382	-0.08
2145	Hydroxymethylglutaryl-CoA synthase cytoplasmic	Q01581	0.0258	0.0972	0.08	0.2574	0.6627	0.18	0.0072	0.0401	0.22	0.0671	0.1433	0.13	0.0462	0.1584	-0.05	0.0009	0.0444	0.12	0.1637	0.3576	0.16	0.0345	0.1684	0.24	0.1040	0.1601	0.13
2146	Hydroxyproline dehydrogenase	Q9UF12	0.8490	0.8796	0.02	0.9775	0.9853	0.00	0.0046	0.0331	0.41	0.2558	0.4066	0.13	0.0130	0.0811	0.24	0.3392	0.5436	0.09	0.0119	0.1034	-0.21	0.0068	0.0673	-0.29	0.0003	0.0255	-0.67
2147	Hydroxysteroid dehydrogenase-like protein 2	Q6YN16	0.0495	0.1431	0.08	0.3667	0.7152	0.07	0.0535	0.1310	-0.12	0.0025	0.0210	0.26	0.2608	0.4821	0.06	0.0492	0.1489	0.10	0.0130	0.1083	-0.33	0.5844	1.0165	-0.08	0.4974	0.5647	-0.07
2148	Hypoxanthine-guanine phosphoribosyltransferase	P00492	0.0000	0.0000	0.46	0.1025	0.6336	0.20	0.0001	0.0185	0.28	0.0039	0.0255	0.25	0.5260	0.6126	0.02	0.1970	0.3724	0.19	0.5800	0.6289	0.04	0.0015	0.0310	-0.40	0.0077	0.0791	-0.38
2149	Hypoxia up-regulated protein 1	Q9Y4L1	0.0006	0.0199	0.33	0.1911	0.6684	0.26	0.0002	0.0179	0.48	0.0001	0.0130	0.51	0.0098	0.0713	0.21	0.0004	0.0377	0.52	0.0371	0.1603	0.44	0.5619	0.9887	0.05	0.0796	0.1305	0.12
2150	Hypoxia-inducible factor 1-alpha inhibitor	Q9NWT6	0.0292	0.1045	1.24	0.0396	0.5635	1.28	0.0533	0.1307	1.06	0.0415	0.1011	1.27	0.0391	0.1445	1.34	0.0472	0.1444	1.24	0.3736	0.5627	-2.55	0.8708	1.1469	-0.33	0.0651	0.1719	2.06
2151	Iduronate 2-sulfatase	P22304	0.3858	0.6023	0.03	0.2830	0.6639	0.10	0.0066	0.0393	0.25	0.0013	0.0166	0.23	0.0147	0.0865	0.15	0.0143	0.0798	0.28	0.3736	0.6044	-2.55	0.8708	1.2204	-0.33	0.0651	0.1955	2.06
2152	Immunity-related GTPase family Q protein	Q8WZA9	0.4108	0.5659	-3.31	0.5752	0.7922	-1.75	0.5661	0.7137	-1.19	0.5388	0.6748	0.89	0.2938	0.4617	-1.88	0.9510	1.0839	0.09	0.5501	0.6013	-0.15	0.6603	1.1005	-0.08	0.3070	0.3780	0.22
2153	Immunoglobulin heavy constant alpha 1	P01876	0.3191	0.5221	0.34	0.8213	0.8725	-0.07	0.2246	0.3825	-0.37	0.4182	0.6102	-0.22	0.1790	0.3701	0.42	0.1803	0.3497	-0.42	0.3736	0.5071	-2.55	0.8708	1.0466	-0.33	0.0651	0.1442	2.06
2154	Immunoglobulin heavy variable 1-69D	A0A0B4J2H0	0.0680	0.1733	2.26	0.0549	0.5851	2.18	0.1387	0.2629	0.77	0.0060	0.0310	2.82	0.5052	0.5944	-0.29	0.1564	0.3157	2.78	0.3736	0.5135	-2.55	0.8708	1.0581	-0.33	0.0651	0.1471	2.06
2155	Immunoglobulin heavy variable 3-43	A0A0B4J1X8	0.1166	0.2507	-2.70	0.0476	0.5694	-10.93	0.1216	0.2390	-2.75	0.0535	0.1210	-1.43	0.5111	0.6003	0.29	0.7681	1.0058	-0.24	0.3736	0.4407	-2.55	0.8708	0.9234	-0.33	0.0651	0.1158	2.06
2156	Immunoglobulin superfamily member 3	O75054	0.0041	0.0403	-0.24	0.0207	0.4745	0.20	0.0659	0.1504	-0.19	0.6918	0.7315	-0.02	0.3351	0.4319	-0.03	0.0451	0.1404	-0.10	0.3736	0.5650	-2.55	0.8708	1.1510	-0.33	0.0651	0.1731	2.06
2157	Importin subunit alpha-1	P52292	0.0250	0.0954	-0.15	0.0865	0.6206	-0.22	0.0605	0.1424	-0.12	0.1906	0.3197	-0.06	0.0362	0.1381	-0.16	0.0131	0.0768	-0.22	0.0556	0.1871	0.31	0.0184	0.1158	0.40	0.1152	0.1728	0.20
2158	Importin subunit alpha-3	O00629	0.1032	0.2298	-0.17	0.2485	0.6584	-1.09	0.1255	0.2449	0.22	0.6708	0.7126	0.04	0.0133	0.0823	0.44	0.2932	0.4909	-0.09	0.0070	0.0861	0.62	0.0727	0.2615	0.52	0.0126	0.0974	0.68
2159	Importin subunit alpha-5	P52294	0.6265	0.6896	0.04	0.5123	0.8348	0.06	0.1228	0.2410	0.17	0.7996	0.8276	0.02	0.1180	0.2819	0.17	0.0895	0.2183	0.20	0.0240	0.1369	0.41	0.1747	0.4587	0.14	0.0385	0.1697	0.20
2160	Importin subunit alpha-6	O15131	0.0847	0.2010	-0.10	0.2124	0.6624	0.10	0.0567	0.1363	-0.14	0.0008	0.0141	-0.43	0.0206	0.1027	-0.18	0.0246	0.1002	-0.34	0.3736	0.5873	-2.55	0.8708	1.1905	-0.33	0.0651	0.1855	2.06
2161	Importin subunit alpha-7	O60684	0.9061	0.9269	0.01	0.1484	0.6587	0.21	0.0176	0.0633	0.25	0.0221	0.0647	0.31	0.0185	0.0970	0.31	0.0266	0.1045	0.26	0.7671	0.8021	-0.02	0.4634	0.8716	0.03	0.0095	0.0868	-0.16
2162	Importin subunit beta-1	Q14974	0.0587	0.1600	0.17	0.1828	0.6654	0.27	0.0068	0.0397	0.35	0.0116	0.0432	0.30	0.2953	0.3933	-0.07	0.0459	0.1419	0.21	0.0449	0.1703	0.42	0.0574	0.2292	0.25	0.0856	0.1379	0.19
2163	Importin-4	Q8TEX9	0.0002	0.0148	0.38	0.5942	0.6986	0.06	0.3774	0.5767	0.04	0.1844	0.3115	-0.04	0.0683	0.1986	-0.07	0.0972	0.2298	-0.06	0.6555	0.7000	0.05	0.8130	1.2579	0.02	0.0336	0.1565	-0.21
2164	Importin-5	O00410	0.2810	0.4744	-0.05	0.3728	0.7206	0.04	0.0740	0.1628	-0.11	0.1982	0.3295	-0.07	0.4172	0.5117	-0.05	0.5516	0.7858	-0.04	0.4976	0.5514	0.05	0.3582	0.7262	0.07	0.2739	0.3458	-0.07
2165	Importin-7	O95373	0.3559	0.5666	0.04	0.0249	0.4831	0.16	0.0082	0.0428	0.21	0.0334	0.0867	0.15	0.6659	0.7306	0.02	0.0670	0.1818	0.11	0.0191	0.1280	0.36	0.0162	0.1080	0.30	0.3752	0.4469	0.07
2166	Importin-9	Q96P70	0.0146	0.0701	-0.14	0.4802	0.8055	-0.03	0.0009	0.0204	-0.29	0.0012	0.0164	-0.25	0.1221	0.2879	-0.08	0.0047	0.0598	-0.24	0.0223	0.1357	0.17	0.0211	0.1256	0.20	0.0119	0.0940	0.24
2167	Inactive C-alpha-formylglycine-generating enzyme 2	Q8NBJ7	0.0030	0.0367	0.44	0.1167	0.6363	0.27	0.0154	0.0584	0.31	0.0040	0.0256	0.43	0.1147	0.2770	0.13	0.0072	0.0646	0.43	0.1816	0.3824	0.14	0.2098	0.5121	0.10	0.3428	0.4137	0.03
2168	Inactive peptidyl-prolyl cis-trans isomerase FKBP6	O75344	0.5233	0.5947	-0.07	0.8565	0.8968	0.05	0.1245	0.2433	0.21	0.5202	0.7251	0.12	0.0710	0.2029	0.27	0.6423	0.8786	0.10	0.3736	0.4481	-2.55	0.8708	0.9372	-0.33	0.0651	0.1187	2.06
2169	Inactive polypeptide N-acetylgalactosaminyltransferase-like protein 5	Q7Z4T8	0.2934	0.4914	-0.46	0.1815	0.6644	-0.79	0.1141	0.2268	-0.79	0.0986	0.1921	-1.05	0.8022	0.8456	0.09	0.3232	0.5261	-0.59	0.4487	0.5039	-0.38	0.9479	0.9624	0.02	0.1181	0.1762	0.56
2170	Inactive rhomboid protein 2	Q6PJF5	0.4108	0.5658	-3.31	0.5752	0.7920	-1.75	0.5661	0.7135	-1.19	0.5388	0.6746	0.89	0.2938	0.4616	-1.88	0.9510	1.0836	0.09	0.1647	0.3589	0.23	0.5532	0.9793	0.08	0.3798	0.4510	0.12
2171	Inactive tyrosine-protein kinase 7	Q13308	0.4108	0.5656	-3.31	0.5752	0.7918	-1.75	0.5661	0.7133	-1.19	0.5388	0.6745	0.89	0.2938	0.4615	-1.88	0.9510	1.0834	0.09	0.0024	0.0557	-0.39	0.0019	0.0359	-0.26	0.2295	0.2999	-0.21
2172	Inactive tyrosine-protein kinase PEAK1	Q9H792	0.4108	0.5655	-3.31	0.5752	0.7916	-1.75	0.5661	0.7131	-1.19	0.5388	0.6743	0.89	0.2938	0.4613	-1.88	0.9510	1.0832	0.09	0.3111	0.5715	0.17	0.3852	0.7648	0.13	0.5761	0.6386	0.11
2173	Inactive ubiquitin thioesterase OTULINL	Q9NUU6	0.0016	0.0274	0.38	0.0017	0.2148	0.38	0.0031	0.0306	0.48	0.0001	0.0088	0.74	0.0003	0.0177	0.93	0.0014	0.0533	0.71	0.3736	0.6472	-2.55	0.8708	1.2947	-0.33	0.0651	0.2229	2.06
2174	Influenza virus NS1A-binding protein	Q9Y6Y0	0.0026	0.0341	0.37	0.0298	0.5078	0.34	0.0227	0.0741	-0.15	0.0077	0.0355	-0.27	0.0250	0.1140	-0.14	0.0700	0.1859	0.28	0.9535	0.9619	-0.01	0.3153	0.6642	-0.20	0.2619	0.3340	-0.23
2175	Inhibitor of Bruton tyrosine kinase	Q9P2D0	0.1076	0.2361	-0.13	0.4445	0.7802	-0.05	0.6635	0.7148	-0.02	0.2684	0.4240	-0.07	0.1990	0.3992	0.11	0.8212	1.0568	0.01	0.0040	0.0682	0.30	0.2058	0.5064	0.09	0.0711	0.1193	0.26
2176	Inhibitor of nuclear factor kappa-B kinase subunit beta	O14920	0.8067	0.8442	0.01	0.3657	0.7140	0.77	0.0073	0.0404	-0.28	0.7209	0.7588	0.36	0.0002	0.0122	-0.48	0.7182	0.9581	-0.03	0.9180	0.9331	-0.01	0.8636	1.3065	0.02	0.8926	0.9150	0.01
2177	Inhibitor of nuclear factor kappa-B kinase subunit epsilon	Q14164	0.7843	0.8247	0.01	0.8099	0.8634	0.02	0.7073	0.7536	0.02	0.9934	0.9942	0.00	0.7458	0.8010	-0.02	0.2604	0.4517	-0.08	0.3736	0.4883	-2.55	0.8708	1.0121	-0.33	0.0651	0.1356	2.06
2178	Inhibitor of nuclear factor kappa-B kinase-interacting protein	Q70UQ0	0.4108	0.5653	-3.31	0.5752	0.7914	-1.75	0.5661	0.7130	-1.19	0.5388	0.6742	0.89	0.2938	0.4612	-1.88	0.9510	1.0829	0.09	0.0377	0.1610	0.59	0.5093	0.9283	0.22	0.3905	0.4617	0.15
2179	Inner nuclear membrane protein Man1	Q9Y2U8	0.4385	0.5159	0.06	0.5808	0.6886	0.05	0.0162	0.0601	0.32	0.1659	0.2862	0.13	0.0121	0.0784	-0.14	0.5893	0.8261	0.07	0.3736	0.4534	-2.55	0.8708	0.9472	-0.33	0.0651	0.1209	2.06
2180	Inorganic pyrophosphatase	Q15181	0.0010	0.0222	0.21	0.1307	0.6466	0.19	0.0898	0.1879	0.13	0.0031	0.0226	0.17	0.0165	0.0917	0.07	0.0191	0.0888	0.23	0.1269	0.3003	0.14	0.1674	0.4470	0.11	0.3226	0.3937	0.05
2181	Inorganic pyrophosphatase 2 mitochondrial	Q9H2U2	0.2477	0.4338	0.04	0.6441	0.7373	0.07	0.0019	0.0239	0.22	0.0377	0.0942	0.12	0.0666	0.1965	-0.08	0.0346	0.1199	0.11	0.1523	0.3399	0.18	0.0078	0.0720	0.22	0.0189	0.1188	0.24
2182	Inosine triphosphate pyrophosphatase	Q9BY32	0.4108	0.5652	-3.31	0.5752	0.7912	-1.75	0.5661	0.7128	-1.19	0.5388	0.6740	0.89	0.2938	0.4610	-1.88	0.9510	1.0827	0.09	0.0661	0.2020	-2.93	0.0071	0.0690	-2.88	0.1567	0.2203	-2.06
2183	Inosine-5'-monophosphate dehydrogenase 1	P20839	0.0815	0.1967	-0.91	0.4942	0.8171	-0.25	0.1851	0.3302	-0.23	0.1235	0.2270	-0.36	0.0054	0.0544	0.98	0.9022	1.1390	-0.05	0.3641	0.6444	0.09	0.7255	1.1704	0.05	0.0665	0.1131	-0.24
2184	Inosine-5'-monophosphate dehydrogenase 2	P12268	0.0169	0.0761	0.15	0.3841	0.7301	0.17	0.0009	0.0205	0.31	0.0017	0.0185	0.27	0.0730	0.2065	0.08	0.0059	0.0620	0.27	0.0554	0.1873	0.41	0.0388	0.1796	0.12	0.1743	0.2402	0.06
2185	Inositol 1 4 5-trisphosphate receptor type 1	Q14643	0.6686	0.7276	0.10	0.1291	0.6430	-0.48	0.8424	0.8733	0.04	0.0456	0.1078	0.53	0.1029	0.2573	0.39	0.0292	0.1095	0.68	0.0416	0.1674	-0.88	0.3181	0.6676	-0.32	0.5974	0.6583	-0.17
2186	Inositol 1 4 5-trisphosphate receptor type 2	Q14571	0.4108	0.5650	-3.31	0.5752	0.7910	-1.75	0.5661	0.7126	-1.19	0.5388	0.6738	0.89	0.2938	0.4609	-1.88	0.9510	1.0824	0.09	0.2490	0.4851	-0.12	0.0251	0.1413	-0.28	0.4038	0.4755	-0.07
2187	Inositol monophosphatase 1	P29218	0.4108	0.5649	-3.31	0.5752	0.7907	-1.75	0.5661	0.7124	-1.19	0.5388	0.6737	0.89	0.2938	0.4608	-1.88	0.9510	1.0822	0.09	0.2127	0.4309	0.14	0.0737	0.2633	0.12	0.0894	0.1425	-0.14
2188	Inositol monophosphatase 3	Q9NX62	0.7410	0.7901	-0.04	0.2181	0.6603	-0.12	0.0806	0.1737	-0.20	0.5525	0.6035	-0.04	0.0269	0.1190	-0.21	0.1825	0.3525	-0.15	0.3736	0.5062	-2.55	0.8708	1.0449	-0.33	0.0651	0.1437	2.06
2189	Inositol polyphosphate 1-phosphatase	P49441	0.3107	0.5117	-0.08	0.7005	0.7788	-0.08	0.0068	0.0394	0.37	0.0191	0.0585	0.15	0.0001	0.0086	0.28	0.0264	0.1043	0.07	0.3736	0.5848	-2.55	0.8708	1.1861	-0.33	0.0651	0.1841	2.06
2190	Inositol polyphosphate 5-phosphatase OCRL-1	Q01968	0.8392	0.8713	0.01	0.5617	0.8756	0.07	0.2272	0.3862	0.07	0.1865	0.3140	-0.05	0.6784	0.7418	-0.01	0.0224	0.0957	0.17	0.0013	0.0474	0.18	0.0024	0.0414	0.30	0.0019	0.0497	0.22
2191	Inositol-3-phosphate synthase 1	Q9NPH2	0.6136	0.6781	-0.03	0.9208	0.9456	-0.01	0.0871	0.1845	-0.12	0.7600	0.7928	-0.02	0.6939	0.7564	-0.02	0.3454	0.5498	0.05	0.1233	0.2956	0.30	0.5981	1.0317	-0.04	0.0779	0.1284	-0.11
2192	Inositol-tetrakisphosphate 1-kinase	Q13572	0.0477	0.1402	0.53	0.7870	0.8467	-0.11	0.1949	0.3436	0.28	0.7986	0.8267	-0.05	0.1897	0.3860	0.28	0.4272	0.6472	-0.27	0.3736	0.4665	-2.55	0.8708	0.9717	-0.33	0.0651	0.1263	2.06
2193	Insulin	P01308	0.4108	0.5647	-3.31	0.5752	0.7905	-1.75	0.5661	0.7123	-1.19	0.5388	0.6735	0.89	0.2938	0.4606	-1.88	0.9510	1.0820	0.09	0.7973	0.8290	0.06	0.1185	0.3554	-0.40	0.0383	0.1690	0.34
2194	Insulin receptor substrate 1	P35568	0.0145	0.0700	0.44	0.1930	0.6683	0.18	0.6776	0.7276	0.03	0.7087	0.7475	0.03	0.1437	0.3178	-0.16	0.1079	0.2463	0.20	0.3736	0.5312	-2.55	0.8708	1.0903	-0.33	0.0651	0.1557	2.06
2195	Insulin-degrading enzyme	P14735	0.2196	0.3958	0.07	0.5252	0.8438	0.08	0.0486	0.1226	0.14	0.1421	0.2537	0.09	0.5307	0.6165	-0.03	0.0881	0.2160	0.12	0.1349	0.3131	0.23	0.1900	0.4825	0.31	0.0335	0.1565	0.51
2196	Insulin-like growth factor 1 receptor	P08069	0.0015	0.0266	-0.31	0.1488	0.6599	-0.29	0.8336	0.8659	0.01	0.0007	0.0132	-0.40	0.0279	0.1214	-0.13	0.0020	0.0529	0.43	0.3736	0.6444	-2.55	0.8708	1.2899	-0.33	0.0651	0.2210	2.06
2197	Insulin-like growth factor 2 mRNA-binding protein 1	Q9NZI8	0.4108	0.5646	-3.31	0.5752	0.7903	-1.75	0.5661	0.7121	-1.19	0.5388	0.6733	0.89	0.2938	0.4605	-1.88	0.9510	1.0817	0.09	0.2002	0.4120	0.15	0.6484	1.0873	0.04	0.0801	0.1311	0.26
2198	Insulin-like growth factor 2 mRNA-binding protein 2	Q9Y6M1	0.4108	0.5644	-3.31	0.5752	0.7901	-1.75	0.5661	0.7119	-1.19	0.5388	0.6732	0.89	0.2938	0.4603	-1.88	0.9510	1.0815	0.09	0.0426	0.1673	0.38	0.6300	1.0661	-0.04	0.6723	0.7258	-0.02
2199	Insulin-like growth factor 2 mRNA-binding protein 3	O00425	0.1900	0.3559	0.19	0.1009	0.6328	0.08	0.1866	0.3318	0.32	0.6356	0.6808	0.15	0.0685	0.1990	-0.10	0.0923	0.2226	0.32	0.1700	0.3663	0.17	0.0826	0.2828	-0.25	0.0548	0.2064	-0.14

Supplemetary Table S2. Overview on all relatively quantified 5180 proteins statistical analysis

			MCF-7																		MDA-MB-231								
			Dai_SC20 vs control			Gen_SC20 vs control			SSE_SC20 vs control			Dai_IC20 vs control			Gen_IC20 vs control			SSE_IC20 vs control			Dai_IC20 vs control			Gen_IC20 vs control			SSE_IC20 vs control		
	**Protein name**	**UniProt**	**p value**	**BH q value**	**log2FC**	**p value**	**BH q value**	**log2FC**	**p value**	**BH q value**	**log2FC**	**p value**	**BH q value**	**log2FC**	**p value**	**BH q value**	**log2FC**	**p value**	**BH q value**	**log2FC**	**p value**	**BH q value**	**log2FC**	**p value**	**BH q value**	**log2FC**	**p value**	**BH q value**	**log2FC**
2200	Insulin-like growth factor-binding protein 7	Q16270	0.4108	0.5643	-3.31	0.5752	0.7899	-1.75	0.5661	0.7117	-1.19	0.5388	0.6730	0.89	0.2938	0.4602	-1.88	0.9510	1.0813	0.09	0.0046	0.0718	-0.38	0.0078	0.0724	-0.33	0.0062	0.0745	-0.39
2201	Integrator complex subunit 1	Q8N201	0.0014	0.0255	0.19	0.0553	0.5858	0.47	0.0029	0.0296	0.31	0.0002	0.0107	0.32	0.0065	0.0590	0.18	0.1045	0.2406	0.27	0.2191	0.4406	0.26	0.2495	0.5724	0.17	0.2663	0.3378	0.13
2202	Integrator complex subunit 13	Q9NVM9	0.1335	0.2767	-0.10	0.2443	0.6567	0.11	0.0487	0.1228	0.16	0.5427	0.5940	0.03	0.0162	0.0908	-0.24	0.5753	0.8096	-0.04	0.3736	0.4541	-2.55	0.8708	0.9484	-0.33	0.0651	0.1211	2.06
2203	Integrator complex subunit 3	Q68E01	0.8839	0.9086	0.01	0.5718	0.8849	0.03	0.0011	0.0214	0.51	0.0160	0.0525	0.31	0.9370	0.9517	0.00	0.0076	0.0652	0.51	0.3736	0.6199	-2.55	0.8708	1.2474	-0.33	0.0651	0.2050	2.06
2204	Integrator complex subunit 5	Q6P9B9	0.4108	0.5641	-3.31	0.5752	0.7897	-1.75	0.5661	0.7116	-1.19	0.5388	0.6729	0.89	0.2938	0.4601	-1.88	0.9510	1.0810	0.09	0.1701	0.3664	1.08	0.1679	0.4474	1.08	0.1057	0.1621	1.44
2205	Integrin alpha-2	P17301	0.0070	0.0495	0.33	0.1005	0.6341	0.22	0.0004	0.0214	0.28	0.0091	0.0385	0.34	0.0061	0.0571	0.23	0.0059	0.0625	0.26	0.3736	0.6263	-2.55	0.8708	1.2586	-0.33	0.0651	0.2091	2.06
2206	Integrin alpha-3	P26006	0.7083	0.7603	-0.02	0.2998	0.6732	0.10	0.0270	0.0823	0.09	0.0013	0.0165	0.40	0.0008	0.0224	0.48	0.0011	0.0475	0.51	0.0102	0.1016	0.60	0.0348	0.1694	0.22	0.0068	0.0764	0.29
2207	Integrin alpha-E	P38570	0.0094	0.0574	-0.66	0.2432	0.6599	-0.44	0.0039	0.0317	-1.20	0.0004	0.0119	-1.62	0.1171	0.2803	-0.23	0.1173	0.2610	-0.50	0.3035	0.5621	0.15	0.0065	0.0655	0.39	0.5037	0.5706	0.09
2208	Integrin alpha-V	P06756	0.4108	0.5640	-3.31	0.5752	0.7895	-1.75	0.5661	0.7114	-1.19	0.5388	0.6727	0.89	0.2938	0.4599	-1.88	0.9510	1.0808	0.09	0.0087	0.0931	-0.35	0.0052	0.0578	-0.54	0.0200	0.1217	-0.97
2209	Integrin alpha-X	P20702	0.4108	0.5638	-3.31	0.5752	0.7893	-1.75	0.5661	0.7112	-1.19	0.5388	0.6725	0.89	0.2938	0.4598	-1.88	0.9510	1.0805	0.09	0.1565	0.3475	-5.02	0.4255	0.8206	-2.35	0.4322	0.5034	-2.31
2210	Integrin beta-1	P05556	0.5920	0.6586	-0.03	0.5065	0.8282	0.17	0.0616	0.1442	0.15	0.1086	0.2063	0.12	0.1624	0.3460	0.10	0.0189	0.0885	0.29	0.0231	0.1363	0.44	0.0200	0.1226	0.26	0.3822	0.4535	0.05
2211	Integrin beta-5	P18084	0.9351	0.9509	0.00	0.2884	0.6687	-0.07	0.2143	0.3689	-0.09	0.3816	0.5665	-0.06	0.0256	0.1151	0.24	0.0753	0.1951	-0.16	0.0464	0.1729	0.64	0.5450	0.9698	0.06	0.3339	0.4050	0.08
2212	Integrin beta-like protein 1	O95965	0.1588	0.3134	0.04	0.3569	0.7083	0.18	0.0221	0.0725	0.29	0.0226	0.0658	0.41	0.0016	0.0316	0.11	0.0834	0.2090	0.19	0.3736	0.5421	-2.55	0.8708	1.1099	-0.33	0.0651	0.1611	2.06
2213	Integrin-linked kinase-associated serine/threonine phosphatase 2C	Q9H0C8	0.4108	0.5637	-3.31	0.5752	0.7891	-1.75	0.5661	0.7111	-1.19	0.5388	0.6724	0.89	0.2938	0.4596	-1.88	0.9510	1.0803	0.09	0.9173	0.9330	-0.03	0.2510	0.5740	-0.36	0.2552	0.3265	-0.36
2214	Integrin-linked protein kinase	Q13418	0.0020	0.0310	0.48	0.0647	0.5890	0.77	0.0004	0.0225	0.82	0.0130	0.0463	0.88	0.0017	0.0321	1.16	0.0180	0.0873	0.55	0.3709	0.6526	0.05	0.8197	1.2645	-0.01	0.1384	0.1995	0.05
2215	Interferon alpha-5	P01569	0.4108	0.5635	-3.31	0.5752	0.7889	-1.75	0.5661	0.7109	-1.19	0.5388	0.6722	0.89	0.2938	0.4595	-1.88	0.9510	1.0801	0.09	0.0490	0.1764	0.31	0.0389	0.1794	0.30	0.0875	0.1404	0.26
2216	Interferon regulatory factor 2-binding protein 2	Q7Z5L9	0.2895	0.4864	0.09	0.2184	0.6608	0.16	0.0742	0.1630	0.22	0.8148	0.8399	0.02	0.0539	0.1722	0.27	0.0621	0.1734	-0.24	0.3736	0.5548	-2.55	0.8708	1.1328	-0.33	0.0651	0.1677	2.06
2217	Interferon regulatory factor 2-binding protein-like	Q9H1B7	0.3174	0.5200	-0.21	0.8510	0.8932	0.04	0.7964	0.8336	0.05	0.9365	0.9445	-0.01	0.2397	0.4528	0.26	0.4925	0.7174	-0.13	0.0669	0.2033	-0.25	0.0074	0.0709	-0.32	0.0662	0.1127	-0.16
2218	Interferon regulatory factor 4	Q15306	0.0768	0.1893	0.28	0.9594	0.9724	-0.01	0.4913	0.7119	0.07	0.0748	0.1556	-0.25	0.1894	0.3855	-0.19	0.2971	0.4942	-0.15	0.3736	0.4821	-2.55	0.8708	1.0006	-0.33	0.0651	0.1329	2.06
2219	Interferon-induced helicase C domain-containing protein 1	Q9BYX4	0.0517	0.1472	0.16	0.1662	0.6597	0.21	0.0201	0.0683	0.22	0.0203	0.0611	0.35	0.5888	0.6658	0.02	0.0120	0.0742	0.25	0.0638	0.1990	-0.14	0.0022	0.0390	-0.49	0.0038	0.0606	-0.50
2220	Interferon-induced protein with tetratricopeptide repeats 3	O14879	0.6264	0.6896	0.05	0.6625	0.7514	0.07	0.1195	0.2359	0.20	0.1798	0.3047	0.16	0.4605	0.5524	-0.07	0.2546	0.4449	0.13	0.3736	0.4893	-2.55	0.8708	1.0139	-0.33	0.0651	0.1361	2.06
2221	Interferon-induced protein with tetratricopeptide repeats 5	Q13325	0.4108	0.5634	-3.31	0.5752	0.7887	-1.75	0.5661	0.7107	-1.19	0.5388	0.6720	0.89	0.2938	0.4594	-1.88	0.9510	1.0798	0.09	0.0127	0.1070	-1.34	0.0790	0.2759	-0.58	0.2804	0.3523	-0.23
2222	Interferon-induced transmembrane protein 3	Q01628	0.5905	0.6571	-0.30	0.5320	0.8500	-0.38	0.4418	0.6535	-0.44	0.4398	0.6337	-0.44	0.1630	0.3470	1.00	0.5199	0.7477	-0.36	0.3736	0.4588	-2.55	0.8708	0.9573	-0.33	0.0651	0.1231	2.06
2223	Interferon-induced_ double-stranded RNA-activated protein kinase	P19525	0.0024	0.0335	0.09	0.0990	0.6363	-0.06	0.0473	0.1206	-0.11	0.0374	0.0938	-0.17	0.0012	0.0271	-0.10	0.0042	0.0609	-0.10	0.0378	0.1613	-0.15	0.1160	0.3498	-0.09	0.1844	0.2518	-0.10
2224	Interferon-inducible double-stranded RNA-dependent protein kinase activator A	O75569	0.0003	0.0157	0.85	0.0102	0.4440	0.30	0.5231	0.7442	0.04	0.0733	0.1530	0.15	0.0293	0.1247	0.34	0.0493	0.1490	0.17	0.3736	0.5614	-2.55	0.8708	1.1446	-0.33	0.0651	0.1712	2.06
2225	Interferon-stimulated gene 20 kDa protein	Q96AZ6	0.0106	0.0594	0.50	0.8448	0.8882	-0.02	0.3456	0.5394	-0.09	0.0480	0.1118	0.26	0.0366	0.1391	0.33	0.0416	0.1343	0.34	0.3736	0.5690	-2.55	0.8708	1.1581	-0.33	0.0651	0.1753	2.06
2226	Interleukin enhancer-binding factor 2	Q12905	0.0011	0.0229	0.27	0.0809	0.6154	0.21	0.0007	0.0211	0.40	0.0004	0.0128	0.35	0.1776	0.3681	0.04	0.0010	0.0450	0.36	0.0674	0.2039	0.16	0.2303	0.5437	-0.10	0.0057	0.0715	0.14
2227	Interleukin enhancer-binding factor 3	Q12906	0.1663	0.3242	0.08	0.3771	0.7245	0.16	0.0262	0.0807	0.17	0.1376	0.2476	0.09	0.0438	0.1548	-0.15	0.7640	1.0022	0.01	0.1186	0.2871	0.22	0.0918	0.3013	-0.17	0.4966	0.5639	-0.05
2228	Interleukin-1 receptor accessory protein-like 1	Q9NZN1	0.5632	0.6313	-0.28	0.3962	0.7398	-2.24	0.5067	0.7283	-0.36	0.2880	0.4493	-0.64	0.4144	0.5090	-0.48	0.0970	0.2294	-0.92	0.3736	0.5352	-2.55	0.8708	1.0975	-0.33	0.0651	0.1577	2.06
2229	Interleukin-1 receptor-associated kinase 3	Q9Y616	0.2505	0.4366	-0.03	0.2980	0.6723	0.17	0.0489	0.1231	0.18	0.0356	0.0904	0.21	0.0023	0.0373	0.15	0.2011	0.3782	0.10	0.3736	0.5021	-2.55	0.8708	1.0374	-0.33	0.0651	0.1419	2.06
2230	Interleukin-1 receptor-like 2	Q9HB29	0.4108	0.5632	-3.31	0.5752	0.7884	-1.75	0.5661	0.7105	-1.19	0.5388	0.6719	0.89	0.2938	0.4592	-1.88	0.9510	1.0796	0.09	0.4695	0.5245	0.05	0.3585	0.7265	-0.08	0.0019	0.0487	0.46
2231	Interleukin-12 receptor subunit beta-1	P42701	0.4108	0.5631	-3.31	0.5752	0.7882	-1.75	0.0975	0.2002	3.41	0.0391	0.0969	8.88	0.2938	0.5255	-1.88	0.9510	1.1870	0.09	0.3736	0.4314	-2.55	0.8708	0.9058	-0.33	0.0651	0.1121	2.06
2232	Interleukin-13 receptor subunit alpha-1	P78552	0.4108	0.5629	-3.31	0.5752	0.7880	-1.75	0.5661	0.7104	-1.19	0.5388	0.6717	0.89	0.2938	0.4591	-1.88	0.9510	1.0794	0.09	0.0257	0.1401	0.42	0.0014	0.0300	1.51	0.0771	0.1274	0.34
2233	Interleukin-17 receptor B	Q9NRM6	0.4268	0.5043	-0.12	0.0882	0.6191	0.27	0.8993	0.9201	0.01	0.0676	0.1439	0.42	0.1231	0.2891	0.22	0.6199	0.8558	-0.11	0.3736	0.4505	-2.55	0.8708	0.9417	-0.33	0.0651	0.1197	2.06
2234	Interleukin-17B	Q9UHF5	0.4108	0.5628	-3.31	0.5752	0.7878	-1.75	0.5661	0.7102	-1.19	0.5388	0.6716	0.89	0.2938	0.4590	-1.88	0.9510	1.0791	0.09	0.0040	0.0677	-0.49	0.3729	0.7461	-0.08	0.2128	0.2815	-0.12
2235	Interleukin-6 receptor subunit beta	P40189	0.4108	0.5627	-3.31	0.5752	0.7876	-1.75	0.5661	0.7100	-1.19	0.5388	0.6714	0.89	0.2938	0.4588	-1.88	0.9510	1.0789	0.09	0.0779	0.2217	0.20	0.9970	0.9972	0.00	0.0079	0.0793	0.25
2236	Intermediate filament family orphan 1	Q0D2I5	0.4108	0.5625	-3.31	0.5752	0.7874	-1.75	0.5661	0.7099	-1.19	0.5388	0.6712	0.89	0.2938	0.4587	-1.88	0.9510	1.0786	0.09	0.0290	0.1463	-0.66	0.1148	0.3476	-0.30	0.3169	0.3883	-0.17
2237	Intersectin-1	Q15811	0.0819	0.1971	0.11	0.1731	0.6603	0.22	0.0465	0.1193	0.15	0.0033	0.0232	0.30	0.0912	0.2386	0.10	0.0100	0.0706	0.33	0.4935	0.5474	-0.06	0.0687	0.2531	-0.24	0.5426	0.6081	0.06
2238	Intracellular hyaluronan-binding protein 4	Q5JVS0	0.2714	0.4641	0.38	0.4784	0.8041	0.25	0.0785	0.1699	0.76	0.3015	0.4668	0.41	0.1527	0.3314	0.55	0.0740	0.1935	0.80	0.3736	0.5478	-2.55	0.8708	1.1201	-0.33	0.0651	0.1640	2.06
2239	Intraflagellar transport protein 122 homolog	Q9HBG6	0.0005	0.0186	0.15	0.4465	0.7822	0.04	0.4320	0.6423	0.01	0.3785	0.5628	-0.03	0.0320	0.1305	-0.05	0.0040	0.0599	0.16	0.0830	0.2302	-0.64	0.8353	1.2794	-0.04	0.7604	0.8022	0.05
2240	Intraflagellar transport protein 172 homolog	Q9UG01	0.0140	0.0693	-2.82	0.4086	0.7498	-1.02	0.0149	0.0575	-2.67	0.1634	0.2829	-2.43	0.6736	0.7374	-0.20	0.0106	0.0702	-3.55	0.3736	0.6111	-2.55	0.8708	1.2321	-0.33	0.0651	0.1995	2.06
2241	Intraflagellar transport protein 74 homolog	Q96LB3	0.1164	0.2504	-0.08	0.2728	0.6653	-0.14	0.0031	0.0300	-0.42	0.0053	0.0294	-0.26	0.0004	0.0168	-0.39	0.0782	0.2001	0.24	0.0203	0.1319	0.56	0.0132	0.0959	0.79	0.3137	0.3851	0.38
2242	Inverted formin-2	Q27J81	0.0026	0.0340	-0.15	0.1414	0.6528	0.27	0.0032	0.0305	0.12	0.0091	0.0384	-0.17	0.8062	0.8491	-0.01	0.0432	0.1370	-0.27	0.3736	0.5670	-2.55	0.8708	1.1545	-0.33	0.0651	0.1742	2.06
2243	Iporin	Q8N2Y8	0.7655	0.8091	0.02	0.6506	0.7423	0.02	0.0139	0.0553	0.23	0.0273	0.0751	0.17	0.0661	0.1955	0.15	0.0101	0.0704	0.39	0.8023	0.8332	-0.04	0.0377	0.1767	0.50	0.1319	0.1922	0.31
2244	IQ domain-containing protein E	Q6IPM2	0.4108	0.5624	-3.31	0.5752	0.7872	-1.75	0.5661	0.7097	-1.19	0.5388	0.6711	0.89	0.2938	0.4585	-1.88	0.9510	1.0784	0.09	0.0107	0.1019	0.78	0.0667	0.2489	0.24	0.0002	0.0216	1.36
2245	IQ motif and SEC7 domain-containing protein 1	Q6DN90	0.3583	0.5698	0.06	0.0158	0.4547	0.29	0.8991	0.9201	-0.01	0.0058	0.0305	0.45	0.1280	0.2956	0.12	0.0319	0.1152	0.23	0.5559	0.6066	-0.03	0.0579	0.2300	-0.13	0.4169	0.4885	-0.03
2246	Iron-sulfur cluster assembly enzyme ISCU_ mitochondrial	Q9H1K1	0.9901	0.9922	0.00	0.5610	0.8748	-0.25	0.4102	0.6164	0.33	0.9321	0.9405	-0.04	0.3254	0.4227	0.41	0.5387	0.7706	-0.35	0.3736	0.4578	-2.55	0.8708	0.9555	-0.33	0.0651	0.1227	2.06
2247	Islet cell autoantigen 1	Q05084	0.0185	0.0800	-0.17	0.1134	0.6344	-0.16	0.1034	0.2098	-0.09	0.0112	0.0425	-0.29	0.0126	0.0800	-0.21	0.0224	0.0960	-0.18	0.3736	0.5900	-2.55	0.8708	1.1952	-0.33	0.0651	0.1870	2.06
2248	Isobutyryl-CoA dehydrogenase_ mitochondrial	Q9UKU7	0.4108	0.5622	-3.31	0.5752	0.7870	-1.75	0.5661	0.7095	-1.19	0.5388	0.6709	0.89	0.2938	0.4584	-1.88	0.9510	1.0782	0.09	0.0345	0.1542	0.58	0.0470	0.2020	0.31	0.0980	0.1532	0.48
2249	Isochorismatase domain-containing protein 1	Q96CN7	0.0637	0.1671	-0.22	0.2020	0.6597	0.12	0.1288	0.2495	-0.16	0.4154	0.6075	-0.04	0.0921	0.2393	-0.15	0.1793	0.3486	-0.17	0.3736	0.5078	-2.55	0.8708	1.0478	-0.33	0.0651	0.1445	2.06
2250	Isochorismatase domain-containing protein 2	Q96AB3	0.1920	0.3587	-0.06	0.1538	0.6601	-0.09	0.0027	0.0286	-0.27	0.0055	0.0299	-0.26	0.0029	0.0410	-0.29	0.0366	0.1237	-0.15	0.3736	0.5722	-2.55	0.8708	1.1638	-0.33	0.0651	0.1770	2.06
2251	Isocitrate dehydrogenase [NAD] subunit alpha_ mitochondrial	P50213	0.0705	0.1778	0.10	0.2765	0.6649	0.14	0.0285	0.0856	0.19	0.0013	0.0164	0.30	0.0422	0.1520	0.07	0.0454	0.1410	0.16	0.0065	0.0848	0.34	0.8508	1.2924	0.01	0.0971	0.1522	-0.14
2252	Isocitrate dehydrogenase [NAD] subunit beta_ mitochondrial	O43837	0.0268	0.0992	0.09	0.4944	0.8169	0.08	0.8357	0.8677	0.01	0.1079	0.2056	0.06	0.9116	0.9334	0.00	0.1800	0.3493	0.09	0.3736	0.5074	-2.55	0.8708	1.0471	-0.33	0.0651	0.1443	2.06
2253	Isocitrate dehydrogenase [NADP] cytoplasmic	O75874	0.0162	0.0743	0.24	0.6855	0.7708	0.02	0.0027	0.0284	0.31	0.0010	0.0155	0.51	0.0298	0.1258	0.16	0.0020	0.0526	0.71	0.0087	0.0937	0.51	0.3606	0.7285	0.09	0.1956	0.2635	0.08
2254	Isocitrate dehydrogenase [NADP]_ mitochondrial	P48735	0.0055	0.0449	-0.19	0.9644	0.9759	0.00	0.0194	0.0670	-0.13	0.0706	0.1488	-0.08	0.0044	0.0492	-0.26	0.5855	0.8213	-0.03	0.0269	0.1419	0.55	0.1708	0.4523	0.18	0.0512	0.1987	0.25
2255	Isoleucine--tRNA ligase_ cytoplasmic	P41252	0.0096	0.0576	0.15	0.1983	0.6670	0.18	0.0004	0.0194	0.28	0.0012	0.0165	0.29	0.2294	0.4391	0.03	0.0010	0.0447	0.31	0.0371	0.1601	0.12	0.8059	1.2502	0.01	0.1123	0.1695	0.06
2256	Isoleucine--tRNA ligase_ mitochondrial	Q9NSE4	0.6080	0.6725	0.05	0.2951	0.6713	0.13	0.3099	0.4944	0.12	0.2943	0.4574	0.12	0.0787	0.2163	0.29	0.5040	0.7297	0.07	0.0746	0.2171	-0.26	0.1722	0.4542	0.19	0.0684	0.1158	-0.29
2257	Isopentenyl-diphosphate Delta-isomerase 1	Q13907	0.0265	0.0987	0.31	0.0182	0.4644	0.13	0.0087	0.0440	0.47	0.0036	0.0241	0.40	0.0055	0.0553	0.17	0.0038	0.0602	0.62	0.9519	0.9608	0.01	0.9653	0.9753	0.00	0.0819	0.1332	-0.23
2258	Isopentenyl-diphosphate delta-isomerase 2	Q9BXS1	0.0694	0.1755	0.11	0.6541	0.7448	0.04	0.2974	0.4786	0.19	0.1420	0.2536	0.10	0.4172	0.5119	-0.04	0.1930	0.3670	0.07	0.3736	0.5038	-2.55	0.8708	1.0405	-0.33	0.0651	0.1426	2.06
2259	IST1 homolog	P53990	0.5618	0.6302	0.04	0.6258	0.7229	0.05	0.0165	0.0608	0.17	0.0102	0.0405	0.27	0.8527	0.8868	0.01	0.0806	0.2041	0.10	0.0843	0.2318	0.35	0.9489	0.9632	0.01	0.5561	0.6206	-0.05
2260	Izumo sperm-egg fusion protein 2	Q6UXV1	0.1114	0.2425	-0.10	0.7437	0.8146	0.03	0.8029	0.8392	-0.02	0.0198	0.0599	0.18	0.1071	0.2647	0.09	0.1070	0.2449	0.09	0.3736	0.5314	-2.55	0.8708	1.0906	-0.33	0.0651	0.1558	2.06
2261	Izumo sperm-egg fusion protein 3	Q5VZ72	0.4108	0.5621	-3.31	0.5752	0.7868	-1.75	0.5661	0.7093	-1.19	0.5388	0.6707	0.89	0.2938	0.4583	-1.88	0.9510	1.0779	0.09	0.5930	0.6418	-0.12	0.6572	1.0982	-0.11	0.8379	0.8689	-0.04
2262	Jerky protein homolog	O75564	0.2778	0.4718	0.10	0.6523	0.7434	0.04	0.9067	0.9264	-0.01	0.9182	0.9286	-0.01	0.3715	0.4686	-0.08	0.7197	0.9598	-0.03	0.3736	0.4444	-2.55	0.8708	0.9302	-0.33	0.0651	0.1172	2.06
2263	Junction plakoglobin	P14923	0.0791	0.1929	-0.08	0.8735	0.9084	0.02	0.2799	0.4551	-0.10	0.0141	0.0483	-0.15	0.0033	0.0428	-0.29	0.9567	0.9643	0.00	0.1948	0.4031	-0.19	0.6018	1.0353	-0.06	0.1098	0.1668	0.25
2264	Junctional protein associated with coronary artery disease	Q9P266	0.0870	0.2050	0.06	0.0002	0.2590	0.31	0.0090	0.0444	0.76	0.0905	0.1799	0.10	0.1583	0.3404	-0.20	0.0157	0.0833	0.47	0.3736	0.6012	-2.55	0.8708	1.2149	-0.33	0.0651	0.1936	2.06
2265	Junctophilin-3	Q8WXH2	0.0037	0.0389	3.45	0.1688	0.6609	1.89	0.0607	0.1428	0.82	0.0947	0.1862	2.34	0.0462	0.1583	0.89	0.0026	0.0554	3.41	0.0185	0.1261	0.36	0.0282	0.1525	0.41	0.0120	0.0942	0.46
2266	Jupiter microtubule associated homolog 1	Q9UK76	0.3349	0.5414	0.01	0.2636	0.6632	0.16	0.0009	0.0207	0.15	0.0102	0.0407	0.18	0.0022	0.0365	0.11	0.0947	0.2270	0.18	0.6478	0.6924	-0.06	0.0455	0.1981	-0.27	0.0070	0.0771	-0.37
2267	Jupiter microtubule associated homolog 2	Q9H910	0.0771	0.1898	-0.12	0.4803	0.8054	0.12	0.5357	0.7569	0.03	0.4249	0.6169	0.04	0.0500	0.1648	-0.15	0.7976	1.0352	0.01	0.1726	0.3704	0.12	0.1573	0.4291	0.13	0.1767	0.2427	0.10
2268	Kalirin	O60229	0.4108	0.5619	-3.31	0.5752	0.7866	-1.75	0.5661	0.7092	-1.19	0.5388	0.6706	0.89	0.2938	0.4581	-1.88	0.9510	1.0777	0.09	0.4233	0.4791	0.13	0.5505	0.9759	0.08	0.4144	0.4859	0.12
2269	Kallikrein-14	Q9P0G3	0.0000	0.0000	2.37	0.0040	0.3397	0.48	0.3480	0.5418	0.16	0.0215	0.0636	-0.22	0.2413	0.4552	0.08	0.0064	0.0621	0.32	0.3736	0.6233	-2.55	0.8708	1.2533	-0.33	0.0651	0.2071	2.06
2270	Kanadaptin	Q9BWU0	0.0133	0.0673	-0.14	0.9299	0.9527	-0.01	0.0321	0.0919	0.16	0.4039	0.5945	0.03	0.7097	0.7704	-0.01	0.0695	0.1852	-0.08	0.0114	0.1038	-0.30	0.5200	0.9421	0.04	0.1854	0.2528	0.16
2271	Katanin p60 ATPase-containing subunit A1	O75449	0.4108	0.5618	-3.31	0.5752	0.7864	-1.75	0.5661	0.7090	-1.19	0.5388	0.6704	0.89	0.2938	0.4580	-1.88	0.9510	1.0775	0.09	0.4005	0.4564	-0.25	0.0200	0.1225	-0.36	0.0936	0.1478	0.52

Supplemetary Table S2. Overview on all relatively quantified 5180 proteins statistical analysis

			MCF-7																			MDA-MB-231								
			Dai SC20 vs control			Gen SC20 vs control			SSE SC20 vs control			Dai IC20 vs control			Gen IC20 vs control			SSE IC20 vs control			Dai IC20 vs control			Gen IC20 vs control			SSE IC20 vs control		
	Protein name	UniProt	p value	BH q value	log2FC	p value	BH q value	log2FC	p value	BH q value	log2FC	p value	BH q value	log2FC	p value	BH q value	log2FC	p value	BH q value	log2FC	p value	BH q value	log2FC	p value	BH q value	log2FC	p value	BH q value	log2FC
2272	Katanin p60 ATPase-containing subunit A-like 1	Q9BW62	0.4108	0.5616	-3.31	0.5752	0.7862	-1.75	0.5661	0.7088	-1.19	0.5388	0.6703	0.89	0.2938	0.4578	-1.88	0.9510	1.0772	0.09	0.9882	0.9909	0.00	0.5646	0.9921	0.20	0.4763	0.5449	0.24
2273	Katanin p60 ATPase-containing subunit A-like 2	Q8IYT4	0.0031	0.0364	-0.41	0.2382	0.6627	-0.15	0.0764	0.1666	-0.10	0.0057	0.0302	0.19	0.0005	0.0181	-0.17	0.0079	0.0666	0.12	0.3736	0.6195	-2.55	0.8708	1.2468	-0.33	0.0651	0.2047	2.06
2274	Kazrin	Q674X7	0.4108	0.5615	-3.31	0.5752	0.7859	-1.75	0.5661	0.7087	-1.19	0.5388	0.6701	0.89	0.2938	0.4577	-1.88	0.9510	1.0770	0.09	0.0922	0.2435	-0.16	0.0047	0.0547	-0.46	0.0273	0.1414	-0.31
2275	Kelch domain-containing protein 4	Q8TBB5	0.0727	0.1818	-0.47	0.5422	0.8573	-0.11	0.3641	0.5615	-0.21	0.6723	0.7135	-0.07	0.4737	0.5643	-0.13	0.1441	0.2997	-0.32	0.3736	0.5187	-2.55	0.8708	1.0676	-0.33	0.0651	0.1496	2.06
2276	Kelch domain-containing protein 8A	Q8IYD2	0.4108	0.5613	-3.31	0.5752	0.7857	-1.75	0.5661	0.7085	-1.19	0.5388	0.6699	0.89	0.2938	0.4576	-1.88	0.9510	1.0768	0.09	0.2880	0.5397	0.13	0.0711	0.2583	0.21	0.2847	0.3566	0.10
2277	Kelch domain-containing protein 9	Q8NEP7	0.0265	0.0986	-0.44	0.0639	0.5921	-0.30	0.0323	0.0923	-0.43	0.0309	0.0819	-0.44	0.0101	0.0725	-0.50	0.0184	0.0878	-0.56	0.3736	0.5960	-2.55	0.8708	1.2058	-0.33	0.0651	0.1905	2.06
2278	Kelch repeat and BTB domain-containing protein 11	O94819	0.4108	0.5612	-3.31	0.5752	0.7855	-1.75	0.5661	0.7083	-1.19	0.5388	0.6698	0.89	0.2938	0.4574	-1.88	0.9510	1.0765	0.09	0.2145	0.4337	0.11	0.1324	0.3829	-0.10	0.4432	0.5135	-0.12
2279	Kelch-like protein 13	Q9P2N7	0.8421	0.8738	-0.08	0.3412	0.7000	-1.50	0.8692	0.8965	-0.06	0.6936	0.7332	0.07	0.1252	0.2921	0.40	0.0679	0.1827	0.50	0.3736	0.5501	-2.55	0.8708	1.1243	-0.33	0.0651	0.1652	2.06
2280	Kelch-like protein 15	Q96M94	0.4107	0.6320	-0.53	0.2655	0.6628	1.01	0.5008	0.7232	0.39	0.3577	0.5358	1.35	0.2250	0.4338	0.77	0.4388	0.6608	0.43	0.3736	0.4655	-2.55	0.8708	0.9698	-0.33	0.0651	0.1259	2.06
2281	Kelch-like protein 20	Q9Y2M5	0.0001	0.0115	0.48	0.2930	0.6704	0.08	0.0028	0.0291	0.16	0.0140	0.0482	0.10	0.7785	0.8260	-0.01	0.0066	0.0625	0.16	0.2589	0.4978	0.13	0.6586	1.0994	0.04	0.1812	0.2481	0.17
2282	Kelch-like protein 24	Q6TFL4	0.1056	0.2333	-0.19	0.2571	0.6636	-0.27	0.0971	0.1997	-0.19	0.0088	0.0379	-0.50	0.3671	0.4640	-0.08	0.2959	0.4927	-0.10	0.3736	0.4822	-2.55	0.8708	1.0008	-0.33	0.0651	0.1330	2.06
2283	Kelch-like protein 25	Q9H0H3	0.1286	0.2692	0.19	0.1676	0.6612	0.22	0.0887	0.1869	0.25	0.0745	0.1552	0.24	0.4800	0.5700	0.07	0.3415	0.5456	0.11	0.3736	0.4750	-2.55	0.8708	0.9875	-0.33	0.0651	0.1299	2.06
2284	Kelch-like protein 26	Q53HC5	0.4108	0.5610	-3.31	0.5752	0.7853	-1.75	0.5661	0.7081	-1.19	0.5388	0.6696	0.89	0.2938	0.4573	-1.88	0.9510	1.0763	0.09	0.1655	0.3604	0.15	0.2583	0.5850	-0.04	0.1895	0.2572	-0.13
2285	Kelch-like protein 31	Q9H511	0.4108	0.5609	-3.31	0.5752	0.7851	-1.75	0.5661	0.7080	-1.19	0.5388	0.6695	0.89	0.2938	0.4572	-1.88	0.9510	1.0761	0.09	0.0279	0.1439	-1.61	0.9249	0.9446	-0.01	0.0278	0.1429	-0.34
2286	Kelch-like protein 5	Q96PQ7	0.2441	0.4296	-0.08	0.0951	0.6299	0.16	0.2806	0.4559	-0.11	0.0891	0.1779	-0.12	0.0353	0.1358	0.26	0.1188	0.2634	-0.11	0.3736	0.5279	-2.55	0.8708	1.0843	-0.33	0.0651	0.1541	2.06
2287	Keratin type I cuticular Ha1	Q15323	0.0022	0.0321	-0.83	0.0614	0.5934	-1.85	0.0013	0.0235	-1.09	0.0284	0.0772	-2.97	0.0226	0.1085	-0.43	0.0243	0.0996	1.17	0.0163	0.1194	0.51	0.0880	0.2947	0.28	0.3410	0.4118	0.24
2288	Keratin type I cuticular Ha2	Q14532	0.1235	0.2610	0.11	0.0795	0.6146	0.21	0.2961	0.4768	0.06	0.0339	0.0874	0.20	0.1101	0.2700	0.12	0.0587	0.1671	0.18	0.3736	0.5561	-2.55	0.8708	1.1351	-0.33	0.0651	0.1684	2.06
2289	Keratin type I cuticular Ha3-II	Q14525	0.0466	0.1382	0.24	0.2686	0.6635	0.10	0.3373	0.5298	-0.15	0.3375	0.5113	0.10	0.0793	0.2172	0.20	0.3245	0.5271	0.09	0.2796	0.5278	0.20	0.2078	0.5097	-0.15	0.0269	0.1398	0.35
2290	Keratin type I cuticular Ha5	Q92764	0.7285	0.7787	0.02	0.4730	0.8017	0.15	0.1053	0.2125	0.12	0.0214	0.0634	0.19	0.9742	0.9801	0.00	0.0940	0.2256	0.11	0.9423	0.9520	-0.01	0.0103	0.0835	-0.28	0.3018	0.3732	-0.19
2291	Keratin type I cuticular Ha6	O76013	0.0022	0.0320	0.40	0.0147	0.4506	0.25	0.2206	0.3775	0.05	0.0654	0.1409	0.11	0.0052	0.0537	0.26	0.9650	0.9714	0.00	0.0000	0.0000	-2.44	0.0000	0.0000	-3.20	0.0001	0.0370	-3.53
2292	Keratin type I cuticular Ha7	O76014	0.0006	0.0198	-0.27	0.1834	0.6648	-0.40	0.2892	0.4683	0.44	0.0340	0.0876	-0.42	0.2154	0.4203	-0.05	0.7817	1.0192	-0.11	0.3736	0.4399	-2.55	0.8708	0.9219	-0.33	0.0651	0.1154	2.06
2293	Keratin type I cuticular Ha8	O76015	0.0047	0.0432	-0.52	0.2996	0.6739	-0.40	0.1551	0.2870	-0.15	0.3324	0.5057	-0.13	0.2385	0.4517	-0.13	0.1270	0.2758	-0.18	0.1737	0.3720	-0.20	0.0047	0.0546	-0.29	0.0056	0.0718	-0.30
2294	Keratin type I cytoskeletal 12	Q99456	0.0008	0.0209	-0.36	0.9637	0.9754	0.01	0.0796	0.1719	0.15	0.3752	0.5585	0.03	0.0666	0.1964	-0.08	0.0243	0.0994	-0.17	0.0001	0.0288	2.21	0.0001	0.0185	2.73	0.0015	0.0483	2.50
2295	Keratin type I cytoskeletal 13	P13646	0.6887	0.7443	0.03	0.5140	0.8365	0.09	0.7492	0.7915	-0.03	0.8896	0.9059	-0.01	0.3721	0.4691	-0.07	0.1032	0.2383	0.18	0.0194	0.1290	0.28	0.2372	0.5532	0.08	0.0630	0.2244	0.18
2296	Keratin type I cytoskeletal 14	P02533	0.0250	0.0954	0.20	0.2695	0.6629	0.13	0.0013	0.0224	0.50	0.1561	0.2737	0.11	0.1016	0.2556	0.13	0.0155	0.0829	0.30	0.2687	0.5117	0.15	0.0247	0.1401	0.32	0.8538	0.8814	-0.01
2297	Keratin type I cytoskeletal 15	P19012	0.0361	0.1190	0.15	0.1634	0.6633	0.15	0.0032	0.0304	0.28	0.0104	0.0410	0.27	0.0281	0.1216	0.18	0.0078	0.0663	0.33	0.7326	0.7718	0.04	0.5336	0.9581	0.05	0.1843	0.2518	0.09
2298	Keratin type I cytoskeletal 16	P08779	0.0796	0.1934	0.14	0.2136	0.6621	0.25	0.0302	0.0883	0.28	0.0098	0.0397	0.25	0.0727	0.2060	0.15	0.0493	0.1488	0.16	0.0022	0.0559	0.81	0.0005	0.0238	1.25	0.0246	0.1332	0.34
2299	Keratin type I cytoskeletal 17	Q04695	0.0000	0.0000	1.03	0.0108	0.4405	0.33	0.0079	0.0420	0.20	0.0059	0.0309	0.33	0.0020	0.0352	0.29	0.0351	0.1209	0.17	0.6222	0.6685	0.06	0.3178	0.6673	-0.13	0.0802	0.1311	-0.30
2300	Keratin type I cytoskeletal 18	P05783	0.0039	0.0395	0.31	0.1190	0.6348	0.30	0.0084	0.0432	0.27	0.0000	0.0000	0.64	0.0302	0.1267	0.12	0.0023	0.0557	0.48	0.1480	0.3330	0.22	0.0890	0.2959	-0.19	0.9997	0.9997	0.00
2301	Keratin type I cytoskeletal 19	P08727	0.0065	0.0482	0.38	0.1960	0.6693	0.31	0.0545	0.1328	0.18	0.0014	0.0168	0.53	0.0589	0.1825	0.18	0.0108	0.0711	0.43	0.0171	0.1218	-0.37	0.0012	0.0289	-0.57	0.0029	0.0550	-0.66
2302	Keratin type I cytoskeletal 20	P35900	0.5547	0.6240	-0.08	0.1906	0.6685	0.14	0.5738	0.6313	-0.07	0.1616	0.2809	0.07	0.0084	0.0658	0.19	0.1277	0.2770	0.11	0.3736	0.5252	-2.55	0.8708	1.0794	-0.33	0.0651	0.1527	2.06
2303	Keratin type I cytoskeletal 24	Q2M2I5	0.3633	0.5760	0.03	0.3328	0.6915	0.13	0.0907	0.1896	0.12	0.0068	0.0333	0.13	0.1610	0.3441	-0.05	0.4669	0.6890	-0.03	0.2622	0.5028	-0.08	0.5384	0.9640	-0.04	0.8018	0.8380	0.02
2304	Keratin type I cytoskeletal 25	Q7Z3Z0	0.0284	0.1024	-0.66	0.2621	0.6636	-0.92	0.0525	0.1295	-0.91	0.0167	0.0538	-1.15	0.1201	0.2846	-0.40	0.0212	0.0941	-0.85	0.2640	0.5056	-0.60	0.0831	0.2838	-0.40	0.4111	0.4829	0.14
2305	Keratin type I cytoskeletal 26	Q7Z3Y9	0.7728	0.8153	0.02	0.1931	0.6682	0.17	0.6906	0.7388	0.04	0.1227	0.2263	0.13	0.5496	0.6332	0.04	0.4434	0.6650	0.05	0.1452	0.3286	0.80	0.1257	0.3695	0.78	0.4362	0.5071	0.40
2306	Keratin type I cytoskeletal 28	Q7Z3Y7	0.0673	0.1719	0.11	0.3782	0.7256	0.05	0.0920	0.1913	0.07	0.0015	0.0171	0.15	0.1677	0.3544	-0.06	0.0952	0.2277	0.12	0.0000	0.0000	-1.78	0.0000	0.0000	-2.28	0.0040	0.0620	-1.53
2307	Keratin type I cytoskeletal 39	Q6A163	0.4108	0.5607	-3.31	0.5752	0.7849	-1.75	0.5661	0.7078	-1.19	0.5388	0.6693	0.89	0.2938	0.4570	-1.88	0.9510	1.0758	0.09	0.2062	0.4208	-0.12	0.0019	0.0355	-0.39	0.0013	0.0452	-0.58
2308	Keratin type II cuticular Hb2	Q9NSB4	0.0003	0.0155	0.22	0.1730	0.6604	0.26	0.0001	0.0162	0.33	0.0000	0.0000	0.45	0.0003	0.0179	0.23	0.0084	0.0675	0.43	0.3736	0.6175	-2.55	0.8708	1.2433	-0.33	0.0651	0.2035	2.06
2309	Keratin type II cuticular Hb4	Q9NSB2	0.0433	0.1320	0.15	0.2614	0.6641	0.20	0.0138	0.0551	0.22	0.0157	0.0518	0.26	0.1556	0.3361	0.09	0.0203	0.0921	0.26	0.0519	0.1804	0.23	0.0872	0.2929	0.10	0.0260	0.1379	0.13
2310	Keratin type II cuticular Hb5	P78386	0.5970	0.6630	-0.03	0.2421	0.6597	0.10	0.0649	0.1487	-0.07	0.0169	0.0542	-0.11	0.0117	0.0778	-0.10	0.0080	0.0672	-0.14	0.2050	0.4187	0.80	0.2610	0.5883	0.68	0.1121	0.1694	1.18
2311	Keratin type II cuticular Hb6	O43790	0.3392	0.5472	-0.07	0.3391	0.6987	0.21	0.8399	0.8710	0.02	0.0186	0.0576	0.26	0.3140	0.4114	-0.05	0.0051	0.0612	0.37	0.0212	0.1342	-0.21	0.4168	0.8080	-0.07	0.0075	0.0783	0.31
2312	Keratin type II cytoskeletal 3	P12035	0.0818	0.1970	0.23	0.6889	0.7721	0.05	0.0481	0.1217	0.08	0.1419	0.2536	0.05	0.1541	0.3336	-0.05	0.0420	0.1351	0.10	0.3183	0.5812	0.18	0.7182	1.1622	0.06	0.9275	0.9417	0.01
2313	Keratin type II cytoskeletal 4	P19013	0.0473	0.1397	0.10	0.7369	0.8089	-0.01	0.0349	0.0970	0.10	0.0353	0.0900	0.12	0.1272	0.2947	-0.08	0.1428	0.2975	0.08	0.9434	0.9530	0.01	0.6026	1.0360	0.06	0.2345	0.3043	-0.15
2314	Keratin type II cytoskeletal 5	P13647	0.0004	0.0167	1.48	0.1639	0.6638	0.18	0.3821	0.5828	0.02	0.0000	0.0000	0.39	0.0150	0.0876	0.40	0.7261	0.9659	0.02	0.0001	0.0216	0.49	0.0006	0.0247	0.34	0.0006	0.0327	0.61
2315	Keratin type II cytoskeletal 6A	P02538	0.0215	0.0873	0.21	0.1842	0.6640	0.25	0.0001	0.0157	0.27	0.0119	0.0438	0.36	0.0009	0.0229	0.20	0.0773	0.1986	0.33	0.0423	0.1674	-0.18	0.0894	0.2969	0.12	0.3568	0.4283	-0.06
2316	Keratin type II cytoskeletal 6B	P04259	0.2389	0.4235	0.05	0.9377	0.9579	-0.01	0.0129	0.0531	0.27	0.0274	0.0751	0.12	0.2923	0.5239	-0.04	0.0333	0.1175	0.12	0.1814	0.3823	0.22	0.0525	0.2172	0.23	0.6930	0.7452	0.02
2317	Keratin type II cytoskeletal 7	P08729	0.4399	0.5174	-0.02	0.5886	0.6942	0.05	0.0204	0.0687	0.08	0.0154	0.0513	0.09	0.6842	0.7472	-0.01	0.1341	0.2863	0.04	0.3759	0.4317	0.12	0.1019	0.3213	-0.18	0.0915	0.1452	-0.15
2318	Keratin type II cytoskeletal 71	Q3SY84	0.0271	0.0999	0.28	0.2847	0.6649	0.23	0.0128	0.0530	0.36	0.0277	0.0758	0.27	0.7400	0.7966	0.02	0.0277	0.1065	0.36	0.1802	0.3804	0.29	0.7461	1.1884	-0.05	0.4398	0.5103	-0.18
2319	Keratin type II cytoskeletal 72	Q14CN4	0.0272	0.1000	0.15	0.3687	0.7175	0.05	0.0056	0.0362	0.25	0.0136	0.0472	0.19	0.0888	0.2339	0.10	0.0106	0.0705	0.27	0.0234	0.1365	-0.17	0.0234	0.1350	-0.07	0.9797	0.9839	0.00
2320	Keratin type II cytoskeletal 73	Q86Y46	0.0026	0.0339	0.26	0.1839	0.6643	0.23	0.0046	0.0335	0.20	0.0014	0.0171	0.25	0.9667	0.9740	0.00	0.0064	0.0619	0.20	0.0929	0.2448	-0.49	0.2513	0.5742	-0.08	0.0083	0.0819	0.43
2321	Keratin type II cytoskeletal 74	Q7RTS7	0.0007	0.0205	0.45	0.0317	0.5100	0.28	0.0061	0.0379	0.59	0.0009	0.0150	0.23	0.0000	0.0000	0.14	0.0212	0.0943	0.47	0.1048	0.2639	1.95	0.9163	0.9388	0.09	0.1012	0.1570	2.04
2322	Keratin type II cytoskeletal 75	O95678	0.0095	0.0574	0.34	0.1839	0.6638	0.22	0.3920	0.5937	0.06	0.0027	0.0216	0.46	0.6931	0.7558	0.02	0.0148	0.0810	0.36	0.0427	0.1676	-0.13	0.0531	0.2185	-0.17	0.0547	0.2062	-0.13
2323	Keratin type II cytoskeletal 78	Q8N1N4	0.7564	0.8017	0.01	0.1397	0.6531	0.17	0.0669	0.1521	0.21	0.9868	0.9887	0.00	0.4507	0.5439	-0.03	0.0693	0.1848	-0.11	0.3057	0.5653	0.07	0.1119	0.3416	0.14	0.0013	0.0432	0.95
2324	Keratin type II cytoskeletal 79	Q5XKE5	0.0056	0.0453	0.06	0.0437	0.5702	0.20	0.0031	0.0305	0.10	0.0004	0.0120	0.16	0.0005	0.0193	0.16	0.1962	0.3713	0.05	0.0896	0.2406	-0.11	0.2366	0.5526	0.06	0.1591	0.2230	-0.08
2325	Keratin type II cytoskeletal 8	P05787	0.0058	0.0459	0.47	0.1548	0.6616	0.39	0.0054	0.0360	0.43	0.0008	0.0146	0.74	0.1054	0.2611	0.15	0.0070	0.0643	0.65	0.0800	0.2249	0.41	0.3924	0.7749	-0.10	0.3669	0.4383	0.08
2326	Keratin type II cytoskeletal 80	Q6KB66	0.0050	0.0435	0.13	0.2482	0.6583	0.17	0.1266	0.2463	0.12	0.0032	0.0229	0.20	0.0980	0.2498	0.05	0.0122	0.0746	0.14	0.3736	0.6082	-2.55	0.8708	1.2271	-0.33	0.0651	0.1978	2.06
2327	Keratin-like protein KRT222	Q8N1A0	0.0398	0.1258	0.18	0.5100	0.8321	0.07	0.0498	0.1247	0.41	0.0563	0.1258	0.15	0.0766	0.2126	0.08	0.4140	0.6313	0.13	0.3736	0.4678	-2.55	0.8708	0.9740	-0.33	0.0651	0.1268	2.06
2328	Keratinocyte-associated protein 2	Q8N6L1	0.1646	0.3219	-0.11	0.0428	0.5685	-0.47	0.0091	0.0446	-0.37	0.0085	0.0373	-0.38	0.0703	0.2020	-0.20	0.1847	0.3559	0.10	0.3736	0.5057	-2.55	0.8708	1.0439	-0.33	0.0651	0.1435	2.06
2329	KH domain-containing_ RNA-binding_ signal transduction-associated protein 1	Q07666	0.5845	0.6515	-0.05	0.2005	0.6628	-0.23	0.0013	0.0228	-0.22	0.5912	0.6396	0.04	0.0132	0.0819	-0.21	0.9888	0.9911	0.00	0.0265	0.1420	0.53	0.0602	0.2346	0.16	0.0495	0.1941	0.23
2330	KH domain-containing_ RNA-binding_ signal transduction-associated protein 2	Q5VWX1	0.4108	0.5606	-3.31	0.5752	0.7847	-1.75	0.5661	0.7076	-1.19	0.5388	0.6691	0.89	0.2938	0.4569	-1.88	0.9510	1.0756	0.09	0.7470	0.7844	0.06	0.5502	0.9757	0.09	0.8511	0.8791	-0.03
2331	KICSTOR complex protein SZT2	Q5T011	0.4108	0.5604	-3.31	0.5752	0.7845	-1.75	0.5661	0.7075	-1.19	0.5388	0.6690	0.89	0.2938	0.4567	-1.88	0.9510	1.0754	0.09	0.1714	0.3686	-0.10	0.2571	0.5833	0.08	0.5665	0.6303	-0.04
2332	KIF1-binding protein	Q96EK5	0.1685	0.3270	0.13	0.1898	0.6706	0.27	0.0104	0.0476	0.41	0.0097	0.0395	0.43	0.1401	0.3120	0.15	0.0425	0.1361	0.30	0.0297	0.1471	0.29	0.2755	0.6114	0.09	0.1421	0.2035	-0.12
2333	Killer cell immunoglobulin-like receptor 2DL3	P43628	0.0530	0.1495	-0.43	0.0130	0.4430	-0.46	0.0601	0.1418	-0.23	0.0009	0.0146	-0.72	0.0905	0.2371	-0.18	0.0099	0.0705	0.42	0.3736	0.6140	-2.55	0.8708	1.2372	-0.33	0.0651	0.2013	2.06
2334	Killer cell lectin-like receptor subfamily B member 1	Q12918	0.2433	0.4287	0.51	0.4699	0.7996	0.44	0.0404	0.1073	0.32	0.0225	0.0655	1.04	0.0441	0.1553	-0.34	0.0118	0.0740	1.07	0.3736	0.6088	-2.55	0.8708	1.2281	-0.33	0.0651	0.1981	2.06
2335	Kinase D-interacting substrate of 220 kDa	Q9ULH0	0.2196	0.3957	-0.20	0.6195	0.7176	0.09	0.8897	0.9119	-0.03	0.5967	0.6449	-0.08	0.3280	0.4250	-0.17	0.1805	0.3498	-0.23	0.3736	0.5070	-2.55	0.8708	1.0463	-0.33	0.0651	0.1441	2.06
2336	Kinectin	Q86UP2	0.0218	0.0878	0.10	0.2770	0.6646	0.14	0.0120	0.0512	0.22	0.0023	0.0204	0.21	0.3964	0.4929	0.02	0.0094	0.0690	0.19	0.0368	0.1594	0.38	0.0875	0.2934	0.16	0.1059	0.1623	0.14
2337	Kinesin heavy chain isoform 5A	Q12840	0.3526	0.5625	0.05	0.1909	0.6686	0.11	0.0597	0.1414	0.15	0.0338	0.0873	0.06	0.0634	0.1904	0.04	0.2049	0.3832	0.09	0.0725	0.2133	0.09	0.1947	0.4889	-0.08	0.0091	0.0849	0.24
2338	Kinesin heavy chain isoform 5C	O60282	0.0001	0.0113	0.34	0.0968	0.6347	0.39	0.0005	0.0209	0.37	0.0099	0.0401	0.48	0.0800	0.2180	0.06	0.0773	0.1984	0.32	0.0098	0.0990	0.58	0.0328	0.1635	0.30	0.0357	0.1618	0.27
2339	Kinesin light chain 1	Q07866	0.0393	0.1248	-0.04	0.3366	0.6955	0.19	0.0201	0.0681	0.24	0.0004	0.0113	0.25	0.0056	0.0559	0.12	0.0004	0.0391	0.22	0.0237	0.1370	0.33	0.0023	0.0405	0.38	0.0526	0.2021	0.14
2340	Kinesin light chain 2	Q9H0B6	0.0204	0.0845	-0.17	0.9584	0.9717	-0.01	0.3840	0.5845	0.12	0.9796	0.9823	0.00	0.0482	0.1612	-0.08	0.4423	0.6643	-0.09	0.0102	0.1014	0.82	0.0771	0.2715	0.15	0.0001	0.0192	0.74
2341	Kinesin light chain 4	Q9NSK0	0.1602	0.3150	0.07	0.0865	0.6197	0.20	0.0039	0.0320	0.26	0.0021	0.0201	0.34	0.0030	0.0414	0.28	0.0015	0.0529	0.44	0.2028	0.4155	-0.41	0.0806	0.2795	-0.51	0.2968	0.3683	-0.24
2342	Kinesin-1 heavy chain	P33176	0.2581	0.4468	0.09	0.2005	0.6632	0.20	0.0652	0.1492	0.21	0.1075	0.2051	0.16	0.1611	0.3441	0.13	0.1124	0.2536	0.15	0.0292	0.1461	0.44	0.0690	0.2538	0.16	0.0442	0.1839	0.17
2343	Kinesin-like protein KIF11	P52732	0.4108	0.5603	-3.31	0.5752	0.7843	-1.75	0.5661	0.7073	-1.19	0.5388	0.6688	0.89	0.2938	0.4566	-1.88	0.9510	1.0751	0.09	0.9377	0.9489	-0.01	0.8568	1.2985	-0.02	0.8242	0.8580	-0.03
2344	Kinesin-like protein KIF13B	Q9NQT8	0.4108	0.5601	-3.31	0.5752	0.7841	-1.75	0.5661	0.7071	-1.19	0.5388	0.6687	0.89	0.2938	0.4565	-1.88	0.9510	1.0749	0.09	0.0570	0.1896	0.46	0.2590	0.5859	0.09	0.6801	0.7333	0.02
2345	Kinesin-like protein KIF15	Q9NS87	0.4108	0.5600	-3.31	0.5752	0.7839	-1.75	0.5661	0.7069	-1.19	0.5388	0.6685	0.89	0.2938	0.4563	-1.88	0.9510	1.0746	0.09	0.2571	0.4960	0.13	0.4759	0.8877	-0.04	0.2157	0.2845	0.06
2346	Kinesin-like protein KIF16B	Q96L93	0.9138	0.9336	0.01	0.3909	0.7350	0.16	0.1000	0.2040	0.20	0.3456	0.5212	0.10	0.4089	0.5044	-0.04	0.0998	0.2335	0.11	0.2984	0.5556	0.23	0.3130	0.6620	0.20	0.9618	0.9697	-0.01
2347	Kinesin-like protein KIF18A	Q8NI77	0.4139	0.4919	-0.06	0.4348	0.7708	-0.07	0.3830	0.5839	0.07	0.0782	0.1612	-0.22	0.0464	0.1584	-0.28	0.1583	0.3181	-0.14	0.3736	0.5126	-2.55	0.8708	1.0566	-0.33	0.0651	0.1467	2.06
2348	Kinesin-like protein KIF1A	Q12756	0.4108	0.5598	-3.31	0.5752	0.7837	-1.75	0.5661	0.7068	-1.19	0.5388	0.6683	0.89	0.2938	0.4562	-1.88	0.9510	1.0744	0.09	0.6935	0.7349	-0.06	0.0550	0.2235	0.23	0.0066	0.0756	0.42
2349	Kinesin-like protein KIF1B	O60333	0.2563	0.4442	0.08	0.1648	0.6607	0.17	0.1483	0.2767	0.21	0.0028	0.0216	0.24	0.0442	0.1554	0.07	0.1218	0.2675	0.35	0.1620	0.3554	0.09	0.4407	0.8418	-0.03	0.0111	0.0920	-0.23
2350	Kinesin-like protein KIF27	Q86VH2	0.0005	0.0185	0.34	0.0628	0.5915	0.26	0.0054	0.0360	0.42	0.0012	0.0161	0.47	0.0003	0.0157	0.44	0.0036	0.0600	0.43	0.0076	0.0899	0.70	0.0577	0.2294	0.18	0.0131	0.0991	0.35
2351	Kinesin-like protein KIF28P	B7ZC32	0.0871	0.2052	0.07	0.1020	0.6320	0.16	0.0004	0.0211	0.17	0.0125	0.0453	0.13	0.0049	0.0526	0.11	0.2462	0.4344	0.13	0.0758	0.2191	0.36	0.0708	0.2574	-0.25	0.0646	0.2281	-0.22

Supplemetary Table S2. Overview on all relatively quantified 5180 proteins statistical analysis

			MCF-7																		MDA-MB-231								
			Dai SC20 vs control			Gen SC20 vs control			SSE SC20 vs control			Dai IC20 vs control			Gen IC20 vs control			SSE IC20 vs control			Dai IC20 vs control			Gen IC20 vs control			SSE IC20 vs control		
	**Protein name**	**UniProt**	**p value**	**BH q value**	**log2FC**	**p value**	**BH q value**	**log2FC**	**p value**	**BH q value**	**log2FC**	**p value**	**BH q value**	**log2FC**	**p value**	**BH q value**	**log2FC**	**p value**	**BH q value**	**log2FC**	**p value**	**BH q value**	**log2FC**	**p value**	**BH q value**	**log2FC**	**p value**	**BH q value**	**log2FC**
2352	Kinesin-like protein KIF2A	O00139	0.0889	0.2079	0.13	0.3041	0.6769	0.10	0.0089	0.0441	0.29	0.0863	0.1739	0.14	0.7714	0.8215	-0.01	0.3656	0.5723	0.04	0.0309	0.1488	0.45	0.3178	0.6676	0.12	0.2893	0.3613	-0.11
2353	Kinesin-like protein KIF2C	Q99661	0.1260	0.2650	0.11	0.9819	0.9874	0.00	0.0012	0.0223	0.55	0.0015	0.0179	0.34	0.4665	0.5577	-0.03	0.0005	0.0365	0.58	0.1421	0.3238	0.15	0.8927	0.9199	-0.01	0.6831	0.7363	-0.02
2354	Kinesin-like protein KIF3B	O15066	0.4108	0.5597	-3.31	0.5752	0.7835	-1.75	0.5661	0.7066	-1.19	0.5388	0.6682	0.89	0.2938	0.4561	-1.88	0.9510	1.0742	0.09	0.0449	0.1701	0.42	0.6269	1.0623	0.05	0.1251	0.1846	0.16
2355	Kinesin-like protein KIF3C	O14782	0.0012	0.0241	1.10	0.1774	0.6630	0.66	0.0037	0.0318	0.96	0.0002	0.0104	1.32	0.3021	0.4002	0.12	0.0029	0.0544	1.10	0.2451	0.4791	0.21	0.0364	0.1738	-0.35	0.0170	0.1115	-0.66
2356	Kinesin-like protein KIF7	Q2M1P5	0.4108	0.5595	-3.31	0.5752	0.7833	-1.75	0.5661	0.7064	-1.19	0.5388	0.6680	0.89	0.2938	0.4559	-1.88	0.9510	1.0739	0.09	0.2642	0.5057	-0.14	0.0323	0.1620	-0.45	0.0186	0.1175	-0.40
2357	Kinesin-like protein KIF9	Q9HAQ2	0.0487	0.1416	0.16	0.3518	0.7044	0.06	0.0056	0.0362	0.33	0.0092	0.0386	0.28	0.0018	0.0334	0.30	0.0083	0.0671	0.25	0.3736	0.6179	-2.55	0.8708	1.2440	-0.33	0.0651	0.2038	2.06
2358	Kinesin-like protein KIFC1	Q9BW19	0.0290	0.1040	0.19	0.1050	0.6317	0.22	0.0009	0.0214	0.51	0.0887	0.1774	0.14	0.5260	0.6124	-0.03	0.0167	0.0847	0.32	0.6907	0.7323	0.03	0.0121	0.0918	-0.20	0.2428	0.3132	-0.05
2359	Kinetochore protein NDC80 homolog	O14777	0.4108	0.5594	-3.31	0.5752	0.7831	-1.75	0.5661	0.7063	-1.19	0.5388	0.6679	0.89	0.2938	0.4558	-1.88	0.9510	1.0737	0.09	0.0364	0.1583	0.13	0.0005	0.0212	-0.26	0.0456	0.1869	0.10
2360	Kinetochore protein Spc24	Q8NBT2	0.0007	0.0204	1.85	0.0419	0.5727	0.38	0.2122	0.3669	0.10	0.0168	0.0541	0.30	0.0148	0.0869	0.28	0.0019	0.0521	0.73	0.3736	0.6449	-2.55	0.8708	1.2906	-0.33	0.0651	0.2213	2.06
2361	Kinetochore protein Spc25	Q9HBM1	0.8692	0.8964	0.05	0.6982	0.7778	-0.41	0.4358	0.6465	-0.31	0.4749	0.6749	-0.30	0.7376	0.7945	-0.11	0.2735	0.4677	-0.38	0.3736	0.4865	-2.55	0.8708	1.0087	-0.33	0.0651	0.1348	2.06
2362	Klotho	Q9UEF7	0.0820	0.1972	0.50	0.0195	0.4633	0.85	0.0195	0.0673	0.87	0.0386	0.0960	0.81	0.0101	0.0724	1.25	0.0700	0.1860	0.59	0.3736	0.5492	-2.55	0.8708	1.1226	-0.33	0.0651	0.1647	2.06
2363	Krueppel-like factor 11	O14901	0.0191	0.0812	0.18	0.1871	0.6670	0.22	0.0235	0.0754	0.25	0.0237	0.0681	0.17	0.4187	0.5131	0.03	0.0431	0.1368	0.13	0.3736	0.5672	-2.55	0.8708	1.1548	-0.33	0.0651	0.1743	2.06
2364	Kunitz-type protease inhibitor 1	O43278	0.3196	0.5227	0.07	0.2153	0.6603	0.17	0.2429	0.4075	0.09	0.0326	0.0852	0.22	0.6695	0.7338	-0.03	0.0643	0.1769	0.19	0.3736	0.5520	-2.55	0.8708	1.1277	-0.33	0.0651	0.1662	2.06
2365	Kunitz-type protease inhibitor 2	O43291	0.0916	0.2122	0.10	0.0569	0.5860	-0.13	0.0038	0.0321	-0.30	0.0684	0.1454	-0.12	0.2029	0.4041	-0.06	0.7999	1.0366	-0.05	0.3736	0.4385	-2.55	0.8708	0.9192	-0.33	0.0651	0.1149	2.06
2366	Kunitz-type protease inhibitor 4	Q6UDR6	0.4108	0.5592	-3.31	0.5752	0.7829	-1.75	0.5661	0.7061	-1.19	0.5388	0.6677	0.89	0.2938	0.4557	-1.88	0.9510	1.0735	0.09	0.3375	0.6079	0.07	0.2542	0.5785	0.07	0.0119	0.0938	-0.28
2367	Kv channel-interacting protein 1	Q9NZI2	0.2290	0.4097	0.05	0.1793	0.6648	0.08	0.0004	0.0195	0.36	0.0062	0.0317	0.24	0.5708	0.6511	0.02	0.0137	0.0776	0.26	0.3736	0.6057	-2.55	0.8708	1.2228	-0.33	0.0651	0.1963	2.06
2368	Kv channel-interacting protein 2	Q9NS61	0.6902	0.7458	-0.03	0.3025	0.6754	-0.12	0.0532	0.1307	-0.23	0.0133	0.0468	-0.42	0.0778	0.2144	-0.22	0.1119	0.2528	-0.28	0.0186	0.1263	0.34	0.1956	0.4904	0.11	0.2253	0.2955	-0.09
2369	Kynureninase	Q16719	0.0042	0.0406	-0.53	0.0548	0.5877	-0.24	0.0000	0.0000	-0.51	0.0025	0.0215	-0.61	0.0161	0.0908	-0.58	0.1299	0.2806	-0.56	0.3736	0.5243	-2.55	0.8708	1.0778	-0.33	0.0651	0.1523	2.06
2370	L-2-hydroxyglutarate dehydrogenase mitochondrial	Q9H9P8	0.0458	0.1367	-0.15	0.4777	0.8039	0.04	0.4716	0.6889	0.04	0.0189	0.0581	-0.23	0.0638	0.1913	-0.16	0.2661	0.4590	-0.10	0.3736	0.4876	-2.55	0.8708	1.0107	-0.33	0.0651	0.1353	2.06
2371	Laccase domain-containing protein 1	Q8IV20	0.3067	0.5077	-0.36	0.8787	0.9127	-0.05	0.6840	0.7333	0.13	0.6110	0.6581	-0.16	0.9965	0.9980	0.00	0.8307	1.0648	-0.07	0.3736	0.4362	-2.55	0.8708	0.9148	-0.33	0.0651	0.1140	2.06
2372	Lactotransferrin	P02788	0.1713	0.3317	0.31	0.5027	0.8235	-0.19	0.2602	0.4294	0.16	0.7359	0.7723	-0.04	0.5755	0.6549	-0.04	0.0792	0.2016	0.15	0.0000	0.0000	0.55	0.0000	0.0000	1.09	0.0011	0.0419	0.71
2373	Lactoylglutathione lyase	Q04760	0.0415	0.1290	0.21	0.6760	0.7627	0.05	0.2214	0.3786	0.08	0.6888	0.7286	0.03	0.1342	0.3036	-0.11	0.0691	0.1848	0.16	0.8873	0.9069	-0.02	0.1089	0.3360	-0.21	0.0210	0.1238	-0.43
2374	Ladinin-1	O00515	0.0003	0.0154	-0.62	0.0155	0.4588	-0.26	0.0068	0.0399	-0.18	0.0069	0.0336	-0.19	0.0002	0.0140	-0.38	0.1456	0.3013	-0.19	0.3736	0.5180	-2.55	0.8708	1.0664	-0.33	0.0651	0.1493	2.06
2375	Lambda-crystallin homolog	Q9Y2S2	0.0007	0.0203	-1.17	0.0933	0.6309	-0.27	0.1048	0.2117	-0.25	0.0404	0.0992	-0.35	0.0553	0.1755	-0.36	0.0172	0.0855	-0.54	0.3736	0.5980	-2.55	0.8708	1.2093	-0.33	0.0651	0.1917	2.06
2376	Lamina-associated polypeptide 2 isoform alpha	P42166	0.0389	0.1238	0.09	0.3127	0.6806	0.16	0.0193	0.0667	0.15	0.0356	0.0905	0.09	0.0020	0.0349	-0.29	0.4135	0.6311	0.03	0.5223	0.5754	0.04	0.0822	0.2818	0.18	0.2488	0.3197	0.15
2377	Lamina-associated polypeptide 2 isoforms beta/gamma	P42167	0.0959	0.2185	-0.16	0.0297	0.5077	-0.28	0.0480	0.1216	-0.22	0.0140	0.0481	-0.39	0.0248	0.1139	-0.35	0.0209	0.0935	-0.38	0.1207	0.2907	0.18	0.9330	0.9514	0.01	0.2805	0.3523	0.09
2378	Lamin-B1	P20700	0.2073	0.3800	0.09	0.3291	0.6885	0.16	0.0140	0.0555	0.26	0.0283	0.0772	0.21	0.6816	0.7450	0.02	0.0588	0.1671	0.18	0.0121	0.1039	0.37	0.4870	0.8977	0.06	0.0148	0.1046	0.47
2379	Lamin-B2	Q03252	0.4108	0.5591	-3.31	0.5752	0.7826	-1.75	0.5661	0.7059	-1.19	0.5388	0.6675	0.89	0.2938	0.4555	-1.88	0.9510	1.0732	0.09	0.0139	0.1109	0.73	0.1086	0.3354	0.32	0.1453	0.2074	0.29
2380	Laminin subunit alpha-5	O15230	0.2112	0.3841	0.05	0.1994	0.6638	0.15	0.0079	0.0419	0.19	0.0025	0.0212	0.24	0.4928	0.5824	0.02	0.0104	0.0699	0.17	0.0014	0.0497	-1.67	0.0093	0.0791	-1.16	0.0552	0.2077	-1.09
2381	Laminin subunit beta-1	P07942	0.0028	0.0355	0.16	0.0090	0.4357	0.26	0.2990	0.4809	0.05	0.8157	0.8407	-0.01	0.0002	0.0120	0.27	0.2319	0.4158	-0.16	0.1773	0.3769	0.59	0.0065	0.0656	0.83	0.0135	0.1009	0.94
2382	L-aminoadipate-semialdehyde dehydrogenase-phosphopantetheinyl transferase	Q9NRN7	0.0015	0.0265	-0.45	0.1516	0.6605	-0.10	0.0723	0.1606	0.13	0.9276	0.9366	-0.01	0.4694	0.5603	0.04	0.1894	0.3627	-0.08	0.0578	0.1914	0.52	0.0171	0.1107	0.32	0.1097	0.1668	0.16
2383	LanC-like protein 2	Q9NS86	0.4108	0.5590	-3.31	0.5752	0.7824	-1.75	0.5661	0.7058	-1.19	0.5388	0.6674	0.89	0.2938	0.4554	-1.88	0.9510	1.0730	0.09	0.4081	0.4638	-0.05	0.7175	1.1622	-0.02	0.7398	0.7856	0.02
2384	Lanosterol 14-alpha demethylase	Q16850	0.0073	0.0504	-0.12	0.2257	0.6613	-0.12	0.0076	0.0413	0.17	0.5169	0.7221	-0.03	0.0081	0.0649	0.12	0.3914	0.6032	0.02	0.0086	0.0934	0.32	0.0246	0.1399	0.23	0.0062	0.0743	0.35
2385	Lanosterol synthase	P48449	0.4108	0.5588	-3.31	0.5752	0.7822	-1.75	0.5661	0.7056	-1.19	0.5388	0.6672	0.89	0.2938	0.4552	-1.88	0.9510	1.0728	0.09	0.0273	0.1431	0.39	0.0632	0.2414	0.26	0.0964	0.1514	0.10
2386	La-related protein 1	Q6PKG0	0.0063	0.0476	0.13	0.0496	0.5735	0.20	0.0023	0.0270	0.31	0.0205	0.0615	0.20	0.0262	0.1171	0.08	0.0040	0.0601	0.21	0.2303	0.4576	0.12	0.4086	0.7951	0.06	0.1156	0.1732	-0.16
2387	La-related protein 1B	Q659C4	0.0375	0.1216	0.26	0.0450	0.5658	0.36	0.0028	0.0293	0.58	0.0035	0.0239	0.52	0.0733	0.2069	0.19	0.0074	0.0650	0.54	0.3736	0.6205	-2.55	0.8708	1.2485	-0.33	0.0651	0.2054	2.06
2388	La-related protein 4	Q71RC2	0.2400	0.4246	0.11	0.7177	0.7930	0.04	0.3399	0.5324	0.08	0.1065	0.2035	0.18	0.5566	0.6397	-0.05	0.3823	0.5917	0.08	0.3096	0.5699	0.79	0.5766	1.0070	0.49	0.4022	0.4737	0.62
2389	La-related protein 4B	Q92615	0.4381	0.5156	-0.13	0.2377	0.6616	0.48	0.2509	0.4178	0.22	0.4226	0.6146	0.29	0.1441	0.3184	-0.31	0.2602	0.4517	-0.23	0.1075	0.2677	0.22	0.0562	0.2265	0.30	0.1425	0.2039	0.20
2390	La-related protein 7	Q4G0J3	0.0593	0.1609	-1.50	0.1787	0.6645	-1.62	0.0599	0.1417	0.22	0.0171	0.0545	0.54	0.0157	0.0893	-0.67	0.1782	0.3475	-0.25	0.7089	0.7499	-0.02	0.0006	0.0227	-0.58	0.0003	0.0288	-0.78
2391	Large neutral amino acids transporter small subunit 1	Q01650	0.0013	0.0249	0.70	0.0676	0.5905	0.50	0.0000	0.0000	1.00	0.0000	0.0000	1.02	0.0006	0.0210	0.75	0.0001	0.0398	1.07	0.0070	0.0870	0.81	0.0088	0.0777	0.47	0.1890	0.2567	0.52
2392	Large proline-rich protein BAG6	P46379	0.0092	0.0571	0.12	0.1791	0.6646	0.27	0.2075	0.3609	0.09	0.0080	0.0360	0.13	0.0584	0.1814	0.06	0.1444	0.2998	0.16	0.6043	0.6521	-0.10	0.9251	0.9446	0.02	0.3365	0.4074	0.22
2393	Large subunit GTPase 1 homolog	Q9H089	0.3063	0.5074	0.12	0.0643	0.5906	-0.20	0.1441	0.2704	0.07	0.0044	0.0269	-0.32	0.0529	0.1705	-0.17	0.0020	0.0521	-0.16	0.3736	0.6442	-2.55	0.8708	1.2895	-0.33	0.0651	0.2208	2.06
2394	Lariat debranching enzyme	Q9UK59	0.5023	0.5756	0.10	0.9663	0.9769	-0.01	0.1370	0.2605	0.28	0.2738	0.4310	0.18	0.6309	0.7010	0.07	0.7661	1.0039	0.04	0.0779	0.2218	0.93	0.9315	0.9500	-0.07	0.2305	0.3008	0.86
2395	Lateral signaling target protein 2 homolog	Q9HCC9	0.5185	0.5906	0.05	0.0174	0.4670	0.35	0.4427	0.6546	-0.08	0.1242	0.2278	0.16	0.0133	0.0824	0.52	0.9452	1.1815	0.01	0.0109	0.1021	-1.07	0.0894	0.2970	-0.46	0.8424	0.8729	-0.04
2396	Latexin	Q9BS40	0.0011	0.0228	-0.70	0.1599	0.6642	-0.26	0.0002	0.0185	-1.09	0.0006	0.0139	-1.11	0.0009	0.0232	-0.81	0.0007	0.0442	-0.82	0.3736	0.6509	-2.55	0.8708	1.3011	-0.33	0.0651	0.2254	2.06
2397	Legumain	Q99538	0.3331	0.5399	0.02	0.5270	0.8454	0.02	0.0509	0.1269	0.08	0.0279	0.0761	0.08	0.1028	0.2571	-0.05	0.1055	0.2422	0.12	0.3736	0.5320	-2.55	0.8708	1.0917	-0.33	0.0651	0.1560	2.06
2398	Leiomodin-2	Q6P5Q4	0.4108	0.5587	-3.31	0.5752	0.7820	-1.75	0.5661	0.7054	-1.19	0.5388	0.6671	0.89	0.2938	0.4551	-1.88	0.9510	1.0725	0.09	0.0904	0.2418	-0.15	0.2492	0.5719	-0.14	0.0553	0.2079	-0.27
2399	Leptin receptor	P48357	0.0180	0.0788	-1.97	0.3166	0.6802	-1.18	0.0118	0.0509	-1.45	0.0440	0.1051	-1.18	0.3643	0.4615	-0.36	0.0165	0.0850	-1.47	0.3736	0.5999	-2.55	0.8708	1.2126	-0.33	0.0651	0.1928	2.06
2400	Lethal(2) giant larvae protein homolog 1	Q15334	0.4108	0.5585	-3.31	0.5752	0.7818	-1.75	0.5661	0.7052	-1.19	0.5388	0.6669	0.89	0.2938	0.4550	-1.88	0.9510	1.0723	0.09	0.1031	0.2606	0.08	0.0737	0.2637	-0.09	0.0732	0.1224	-0.09
2401	Lethal(3)malignant brain tumor-like protein 4	Q8NA19	0.4108	0.5584	-3.31	0.5752	0.7816	-1.75	0.5661	0.7051	-1.19	0.5388	0.6667	0.89	0.2938	0.4548	-1.88	0.9510	1.0721	0.09	0.9104	0.9267	0.13	0.2653	0.5949	1.47	0.3068	0.3779	1.31
2402	Leucine rich adaptor protein 1	Q96LR2	0.4108	0.5582	-3.31	0.5752	0.7814	-1.75	0.5661	0.7049	-1.19	0.5388	0.6666	0.89	0.2938	0.4547	-1.88	0.9510	1.0718	0.09	0.5574	0.6076	-0.04	0.1030	0.3234	-0.17	0.3821	0.4534	-0.06
2403	Leucine zipper protein 1	Q86V48	0.0034	0.0383	-0.14	0.3795	0.7257	0.12	0.7284	0.7721	-0.01	0.8941	0.9096	0.00	0.7728	0.8225	-0.01	0.1488	0.3055	-0.16	0.0123	0.1050	0.58	0.9100	0.9332	0.01	0.0157	0.1070	0.33
2404	Leucine zipper protein 4	Q9P127	0.0865	0.2044	-0.29	0.8259	0.8749	0.03	0.2300	0.3895	-0.20	0.2273	0.3696	-0.13	0.1910	0.3871	0.20	0.1093	0.2488	-0.35	0.3736	0.5311	-2.55	0.8708	1.0901	-0.33	0.0651	0.1556	2.06
2405	Leucine zipper putative tumor suppressor 1	Q9Y250	0.4108	0.5581	-3.31	0.5752	0.7812	-1.75	0.5661	0.7047	-1.19	0.5388	0.6664	0.89	0.2938	0.4546	-1.88	0.9510	1.0716	0.09	0.0612	0.1951	0.36	0.0991	0.3151	0.20	0.0266	0.1388	0.36
2406	Leucine zipper putative tumor suppressor 2	Q9BRK4	0.4108	0.5579	-3.31	0.5752	0.7810	-1.75	0.5661	0.7046	-1.19	0.5388	0.6663	0.89	0.2938	0.4544	-1.88	0.9510	1.0714	0.09	0.0105	0.1019	0.83	0.0059	0.0619	0.85	0.0013	0.0446	0.35
2407	Leucine zipper transcription factor-like protein 1	Q9NQ48	0.4108	0.5578	-3.31	0.5752	0.7808	-1.75	0.5661	0.7044	-1.19	0.5388	0.6661	0.89	0.2938	0.4543	-1.88	0.9510	1.0711	0.09	0.0699	0.2083	0.46	0.5019	0.9183	0.13	0.4585	0.5268	0.13
2408	Leucine-rich PPR motif-containing protein_ mitochondrial	P42704	0.0039	0.0395	0.12	0.2247	0.6598	0.17	0.0025	0.0274	0.18	0.0103	0.0408	0.15	0.6245	0.6960	-0.01	0.0024	0.0548	0.14	0.0619	0.1960	0.41	0.0580	0.2302	0.20	0.2152	0.2840	0.09
2409	Leucine-rich repeat flightless-interacting protein 1	Q32MZ4	0.0131	0.0667	0.15	0.1455	0.6560	0.32	0.0019	0.0245	0.35	0.0012	0.0163	0.24	0.0456	0.1575	0.07	0.0170	0.0857	0.25	0.0266	0.1423	0.41	0.2191	0.5262	0.10	0.2044	0.2732	0.08
2410	Leucine-rich repeat flightless-interacting protein 2	Q9Y608	0.6724	0.7307	-0.07	0.2894	0.6686	-0.61	0.4850	0.7053	-0.14	0.2002	0.3326	-0.27	0.2884	0.5187	-0.21	0.3574	0.5632	-0.18	0.0079	0.0907	-0.36	0.1751	0.4592	-0.16	0.4545	0.5228	-0.05
2411	Leucine-rich repeat serine/threonine-protein kinase 1	Q38SD2	0.0645	0.1682	0.30	0.7647	0.8299	0.07	0.0631	0.1462	-0.32	0.8805	0.8980	-0.02	0.5574	0.6403	-0.07	0.1907	0.3642	-0.21	0.0293	0.1465	0.35	0.8262	1.2711	-0.02	0.1661	0.2313	0.11
2412	Leucine-rich repeat transmembrane protein FLRT1	Q9NZU1	0.4108	0.5576	-3.31	0.5752	0.7806	-1.75	0.5661	0.7042	-1.19	0.5388	0.6659	0.89	0.2938	0.4542	-1.88	0.9510	1.0709	0.09	0.8159	0.8444	0.03	0.0448	0.1965	0.36	0.0176	0.1137	0.48
2413	Leucine-rich repeat-containing G-protein coupled receptor 5	O75473	0.4108	0.5575	-3.31	0.5752	0.7804	-1.75	0.5661	0.7041	-1.19	0.5388	0.6658	0.89	0.2938	0.4540	-1.88	0.9510	1.0707	0.09	0.0176	0.1229	0.32	0.5575	0.9836	0.05	0.1258	0.1853	-0.15
2414	Leucine-rich repeat-containing protein 1	Q9BTT6	0.0692	0.1751	-0.29	0.5858	0.6917	-0.08	0.0622	0.1449	0.29	0.7353	0.7720	0.03	0.7858	0.8316	0.03	0.2631	0.4555	-0.27	0.3539	0.6304	0.33	0.3474	0.7093	0.33	0.0947	0.1492	0.82
2415	Leucine-rich repeat-containing protein 14	Q15048	0.0188	0.0807	-0.20	0.5211	0.8409	0.08	0.3663	0.5637	-0.04	0.0090	0.0381	0.15	0.0332	0.1330	0.10	0.0051	0.0617	0.25	0.3736	0.6298	-2.55	0.8708	1.2646	-0.33	0.0651	0.2113	2.06
2416	Leucine-rich repeat-containing protein 23	Q53EV4	0.0623	0.1656	0.17	0.0779	0.6123	0.25	0.2404	0.4039	0.09	0.0629	0.1366	0.21	0.4963	0.5855	-0.04	0.2945	0.4916	0.08	0.3736	0.4826	-2.55	0.8708	1.0015	-0.33	0.0651	0.1331	2.06
2417	Leucine-rich repeat-containing protein 40	Q9H9A6	0.4108	0.5573	-3.31	0.5752	0.7802	-1.75	0.5661	0.7039	-1.19	0.5388	0.6656	0.89	0.2938	0.4539	-1.88	0.9510	1.0704	0.09	0.0613	0.1950	-7.47	0.0994	0.3159	-5.26	0.3349	0.4059	2.18
2418	Leucine-rich repeat-containing protein 42	Q9Y546	0.4108	0.5572	-3.31	0.5752	0.7800	-1.75	0.5661	0.7037	-1.19	0.5388	0.6655	0.89	0.2938	0.4538	-1.88	0.9510	1.0702	0.09	0.0766	0.2201	0.12	0.0001	0.0110	1.31	0.0030	0.0559	0.50
2419	Leucine-rich repeat-containing protein 47	Q8N1G4	0.3619	0.5747	0.05	0.0325	0.5196	0.12	0.9565	0.9649	0.00	0.2218	0.3614	-0.06	0.8841	0.9110	0.01	0.9602	0.9673	0.00	0.0852	0.2333	0.08	0.0113	0.0874	-0.11	0.6527	0.7088	-0.05
2420	Leucine-rich repeat-containing protein 59	Q96AG4	0.0006	0.0197	0.23	0.1145	0.6330	0.19	0.0004	0.0207	0.26	0.0036	0.0242	0.26	0.1458	0.3206	0.09	0.0143	0.0796	0.28	0.0042	0.0702	-0.66	0.0006	0.0235	-0.72	0.0019	0.0505	-0.94
2421	Leucine--tRNA ligase cytoplasmic	Q9P2J5	0.1875	0.3533	0.07	0.3523	0.7041	0.10	0.0231	0.0748	0.20	0.0357	0.0906	0.18	0.3229	0.4200	0.05	0.0413	0.1338	0.17	0.0292	0.1466	0.33	0.0378	0.1770	0.21	0.0405	0.1745	0.12
2422	Leucine-zipper-like transcriptional regulator 1	Q8N653	0.0486	0.1416	0.51	0.1706	0.6605	0.39	0.0784	0.1699	0.46	0.2358	0.3806	0.23	0.3163	0.4137	0.18	0.3031	0.5011	0.19	0.3736	0.4814	-2.55	0.8708	0.9993	-0.33	0.0651	0.1326	2.06
2423	Leucyl-cystinyl aminopeptidase	Q9UIQ6	0.8593	0.8888	-0.02	0.9953	0.9970	0.00	0.7832	0.8219	0.03	0.8248	0.8486	0.02	0.5294	0.6154	-0.07	0.7520	0.9897	0.04	0.1084	0.2693	0.19	0.0049	0.0562	0.59	0.0200	0.1219	0.60
2424	Leukocyte elastase inhibitor	P30740	0.1813	0.3449	-0.07	0.6887	0.7722	0.03	0.0358	0.0990	-0.17	0.8458	0.8676	-0.01	0.0428	0.1528	-0.13	0.1646	0.3279	0.08	0.3736	0.5109	-2.55	0.8708	1.0534	-0.33	0.0651	0.1459	2.06
2425	Leukocyte immunoglobulin-like receptor subfamily B member 1	Q8NHL6	0.0161	0.0741	-0.18	0.1340	0.6493	0.15	0.9996	0.9996	0.00	0.2265	0.3685	-0.08	0.4357	0.5297	0.02	0.2156	0.3966	-0.18	0.0749	0.2177	0.53	0.3169	0.6670	0.23	0.2717	0.3437	0.26
2426	Leukotriene A-4 hydrolase	P09960	0.0002	0.0146	0.18	0.2537	0.6631	0.13	0.0089	0.0442	0.29	0.0011	0.0155	0.25	0.0372	0.1401	0.06	0.0955	0.2279	0.34	0.0080	0.0913	0.55	0.0197	0.1213	0.33	0.0028	0.0541	0.48
2427	LIM and cysteine-rich domains protein 1	Q9NZU5	0.4108	0.5571	-3.31	0.5752	0.7798	-1.75	0.5661	0.7036	-1.19	0.5388	0.6653	0.89	0.2938	0.4536	-1.88	0.9510	1.0700	0.09	0.0964	0.2494	-0.20	0.0028	0.0440	-0.47	0.0062	0.0747	-0.55
2428	LIM and senescent cell antigen-like-containing domain protein 1	P48059	0.8794	0.9053	-0.01	0.3104	0.6793	0.14	0.1033	0.2097	0.09	0.0512	0.1167	0.06	0.3170	0.4143	-0.04	0.4509	0.6735	0.05	0.5285	0.5811	-0.04	0.0379	0.1772	-0.20	0.0187	0.1178	-0.15

Supplemetary Table S2. Overview on all relatively quantified 5180 proteins statistical analysis

			MCF-7																		MDA-MB-231								
			Dai SC20 vs control			Gen SC20 vs control			SSE SC20 vs control			Dai IC20 vs control			Gen IC20 vs control			SSE IC20 vs control			Dai IC20 vs control			Gen IC20 vs control			SSE IC20 vs control		
	**Protein name**	**UniProt**	**p value**	**BH q value**	**log2FC**	**p value**	**BH q value**	**log2FC**	**p value**	**BH q value**	**log2FC**	**p value**	**BH q value**	**log2FC**	**p value**	**BH q value**	**log2FC**	**p value**	**BH q value**	**log2FC**	**p value**	**BH q value**	**log2FC**	**p value**	**BH q value**	**log2FC**	**p value**	**BH q value**	**log2FC**
2429	LIM and senescent cell antigen-like-containing domain protein 2	Q7Z4I7	0.4108	0.5569	-3.31	0.5752	0.7796	-1.75	0.5661	0.7034	-1.19	0.5388	0.6652	0.89	0.2938	0.4535	-1.88	0.9510	1.0697	0.09	0.7976	0.8291	0.07	0.4461	0.8474	0.20	0.3468	0.4180	0.30
2430	LIM and SH3 domain protein 1	Q14847	0.0061	0.0468	0.36	0.0778	0.6125	0.31	0.0011	0.0223	0.57	0.0013	0.0168	0.51	0.0146	0.0861	0.31	0.0045	0.0604	0.64	0.0652	0.2013	0.35	0.8244	1.2702	0.02	0.4553	0.5235	0.05
2431	LIM domain and actin-binding protein 1	Q9UHB6	0.0005	0.0184	-0.29	0.2199	0.6607	-0.16	0.0034	0.0311	-0.30	0.7213	0.7591	0.02	0.6914	0.7541	0.01	0.0097	0.0701	0.17	0.3736	0.6150	-2.55	0.8708	1.2389	-0.33	0.0651	0.2019	2.06
2432	LIM domain kinase 1	P53667	0.4108	0.5568	-3.31	0.5752	0.7794	-1.75	0.5661	0.7032	-1.19	0.5388	0.6650	0.89	0.2938	0.4533	-1.88	0.9510	1.0695	0.09	0.0226	0.1363	-0.20	0.2620	0.5898	0.06	0.6031	0.6634	0.02
2433	LIM domain kinase 2	P53671	0.0128	0.0663	-0.33	0.5164	0.8391	-0.14	0.1345	0.2571	-0.15	0.5414	0.5929	-0.03	0.0552	0.1754	-0.09	0.2484	0.4369	-0.25	0.3736	0.4904	-2.55	0.8708	1.0159	-0.33	0.0651	0.1366	2.06
2434	LIM domain only protein 7	Q8WWI1	0.4315	0.5090	-0.01	0.2440	0.6576	0.13	0.0001	0.0152	0.16	0.0044	0.0270	0.15	0.0455	0.1573	-0.12	0.0610	0.1714	0.23	0.5247	0.5777	-0.09	0.0057	0.0605	-0.28	0.1151	0.1728	-0.06
2435	LIM/homeobox protein Lhx4	Q969G2	0.0486	0.1415	-0.24	0.3485	0.7041	-0.06	0.0236	0.0755	0.27	0.7713	0.8032	-0.02	0.4119	0.5069	-0.05	0.2079	0.3878	-0.09	0.3736	0.5010	-2.55	0.8708	1.0353	-0.33	0.0651	0.1413	2.06
2436	Limbin	Q86UK5	0.4108	0.5566	-3.31	0.5752	0.7792	-1.75	0.5661	0.7030	-1.19	0.5388	0.6648	0.89	0.2938	0.4532	-1.88	0.9510	1.0693	0.09	0.0004	0.0351	0.84	0.0007	0.0261	0.87	0.0060	0.0738	0.97
2437	LINE-1 retrotransposable element ORF1 protein	Q9UN81	0.7812	0.8221	-0.01	0.3531	0.7051	0.17	0.0220	0.0723	0.19	0.1041	0.1996	0.10	0.5392	0.6236	-0.02	0.8708	1.1064	-0.01	0.6526	0.6971	-0.06	0.8468	1.2890	0.02	0.9584	0.9666	-0.01
2438	LINE-1 type transposase domain-containing protein 1	Q5T7N2	0.0417	0.1293	0.13	0.3101	0.6795	0.08	0.0079	0.0418	0.30	0.0125	0.0452	0.21	0.0389	0.1442	0.15	0.0064	0.0624	0.27	0.4079	0.4638	0.09	0.0871	0.2928	-0.14	0.4342	0.5051	-0.05
2439	Lipid droplet-associated hydrolase	Q9H6V9	0.7306	0.7808	0.01	0.3574	0.7085	0.17	0.1082	0.2173	0.18	0.5907	0.6393	0.07	0.1406	0.3130	0.09	0.0468	0.1438	-0.13	0.3736	0.5634	-2.55	0.8708	1.1481	-0.33	0.0651	0.1722	2.06
2440	Lipocalin-15	Q6UWW0	0.4108	0.5565	-3.31	0.5752	0.7790	-1.75	0.5661	0.7029	-1.19	0.5388	0.6647	0.89	0.2938	0.4531	-1.88	0.9510	1.0690	0.09	0.3585	0.6362	-0.26	0.3315	0.6882	-0.10	0.0841	0.1359	0.22
2441	Lipolysis-stimulated lipoprotein receptor	Q86X29	0.1685	0.3269	0.12	0.4770	0.8035	0.11	0.0249	0.0783	0.26	0.0069	0.0335	0.39	0.6128	0.6869	0.03	0.0279	0.1068	0.33	0.3151	0.5770	0.08	0.1155	0.3489	-0.06	0.5175	0.5838	-0.02
2442	Lipoma-preferred partner	Q93052	0.0010	0.0221	-0.31	0.6863	0.7708	0.06	0.0471	0.1202	-0.08	0.0003	0.0104	0.10	0.0005	0.0195	0.13	0.1070	0.2451	0.06	0.0118	0.1038	-0.18	0.0503	0.2111	-0.14	0.0488	0.1931	-0.10
2443	Lipopolysaccharide-responsive and beige-like anchor protein	P50851	0.0648	0.1683	0.10	0.1113	0.6308	0.23	0.0272	0.0827	0.17	0.0023	0.0204	0.30	0.0070	0.0608	0.27	0.0103	0.0704	0.21	0.3736	0.6120	-2.55	0.8708	1.2338	-0.33	0.0651	0.2001	2.06
2444	Liprin-alpha-1	Q13136	0.0049	0.0432	0.22	0.0774	0.6121	0.23	0.0583	0.1388	0.22	0.0001	0.0069	0.28	0.0112	0.0757	0.13	0.0003	0.0379	0.24	0.0050	0.0749	0.71	0.0142	0.0997	0.58	0.0161	0.1087	0.85
2445	Liprin-alpha-3	O75145	0.0784	0.1919	0.12	0.2276	0.6605	0.23	0.0043	0.0323	0.33	0.0050	0.0285	0.35	0.0926	0.2401	0.13	0.0081	0.0666	0.40	0.0086	0.0938	0.35	0.0025	0.0414	0.23	0.0281	0.1433	0.35
2446	Liprin-beta-1	Q86W92	0.0000	0.0000	-0.33	0.1953	0.6682	-0.21	0.0054	0.0359	-0.12	0.0054	0.0295	-0.27	0.6066	0.6815	-0.02	0.0009	0.0436	-0.20	0.0454	0.1710	-0.22	0.0070	0.0687	-0.39	0.2481	0.3190	-0.18
2447	L-lactate dehydrogenase A chain	P00338	0.0035	0.0382	0.46	0.1143	0.6326	0.49	0.0020	0.0251	0.56	0.0016	0.0179	0.58	0.0418	0.1512	0.23	0.0127	0.0759	0.48	0.0670	0.2034	0.39	0.1945	0.4893	0.17	0.9881	0.9906	0.00
2448	L-lactate dehydrogenase B chain	P07195	0.0068	0.0491	-0.22	0.1780	0.6633	-0.18	0.0639	0.1473	-0.26	0.0266	0.0735	-0.16	0.0000	0.0000	-0.35	0.0020	0.0537	-0.15	0.0857	0.2341	0.41	0.5562	0.9820	0.07	0.2489	0.3198	-0.12
2449	L-lactate dehydrogenase C chain	P07864	0.1453	0.2930	-0.22	0.0820	0.6156	-0.27	0.1410	0.2665	-0.21	0.1499	0.2651	-0.21	0.5123	0.6015	-0.08	0.6601	0.8975	-0.08	0.3406	0.6111	-0.11	0.0666	0.2489	0.16	0.7549	0.7980	0.03
2450	LON peptidase N-terminal domain and RING finger protein 2	Q1L5Z9	0.2574	0.4458	0.04	0.2063	0.6601	-0.06	0.0213	0.0706	0.14	0.1656	0.2857	-0.07	0.0332	0.1329	-0.12	0.0188	0.0887	0.18	0.3736	0.5955	-2.55	0.8708	1.2048	-0.33	0.0651	0.1902	2.06
2451	Lon protease homolog mitochondrial	P36776	0.0052	0.0442	0.29	0.1411	0.6532	0.38	0.0025	0.0277	0.46	0.0061	0.0314	0.40	0.1812	0.3737	0.08	0.0065	0.0622	0.36	0.3736	0.6231	-2.55	0.8708	1.2530	-0.33	0.0651	0.2070	2.06
2452	Long-chain fatty acid transport protein 4	Q6P1M0	0.0006	0.0195	-2.09	0.1829	0.6649	-1.08	0.0130	0.0534	-1.15	0.0060	0.0311	-1.74	0.6523	0.7195	-0.05	0.0006	0.0393	-2.07	0.3736	0.6514	-2.55	0.8708	1.3018	-0.33	0.0651	0.2257	2.06
2453	Long-chain-fatty-acid--CoA ligase 3	O95573	0.3292	0.5359	0.05	0.2522	0.6621	0.16	0.1184	0.2341	0.10	0.0038	0.0249	0.36	0.0251	0.1143	0.24	0.0029	0.0548	0.74	0.0305	0.1478	0.38	0.0426	0.1904	0.12	0.0123	0.0957	0.12
2454	Long-chain-fatty-acid--CoA ligase 4	O60488	0.0454	0.1359	0.19	0.6641	0.7524	0.06	0.2297	0.3892	0.14	0.4176	0.6097	-0.04	0.0221	0.1073	-0.21	0.1369	0.2896	0.09	0.0207	0.1325	0.26	0.5768	1.0070	-0.03	0.0988	0.1542	0.16
2455	Low affinity immunoglobulin epsilon Fc receptor	P06734	0.8672	0.8948	0.02	0.2086	0.6601	-0.18	0.6657	0.7169	0.05	0.0884	0.1770	-0.28	0.2122	0.4159	-0.20	0.5961	0.8323	-0.08	0.3736	0.4528	-2.55	0.8708	0.9460	-0.33	0.0651	0.1206	2.06
2456	Low molecular weight phosphotyrosine protein phosphatase	P24666	0.4490	0.5267	-0.06	0.5313	0.8507	-0.08	0.0755	0.1652	-0.19	0.1540	0.2705	-0.14	0.1154	0.2782	-0.16	0.6419	0.8785	0.03	0.3736	0.4483	-2.55	0.8708	0.9376	-0.33	0.0651	0.1188	2.06
2457	Low-density lipoprotein receptor-related protein 1B	Q9NZR2	0.4108	0.5563	-3.31	0.5752	0.7788	-1.75	0.5661	0.7027	-1.19	0.5388	0.6645	0.89	0.2938	0.4529	-1.88	0.9510	1.0688	0.09	0.0824	0.2294	-0.15	0.0082	0.0747	-0.34	0.1149	0.1725	-0.32
2458	Low-density lipoprotein receptor-related protein 2	P98164	0.0709	0.1785	0.12	0.0738	0.6126	0.39	0.0027	0.0288	0.41	0.0004	0.0120	0.57	0.0010	0.0248	0.60	0.0028	0.0562	0.65	0.3736	0.6404	-2.55	0.8708	1.2829	-0.33	0.0651	0.2183	2.06
2459	Low-density lipoprotein receptor-related protein 4	O75096	0.7518	0.7979	-0.06	0.2395	0.6613	-0.41	0.3198	0.5072	-0.09	0.3369	0.5110	-0.15	0.4776	0.5677	-0.14	0.8997	1.1367	0.01	0.3736	0.4339	-2.55	0.8708	0.9105	-0.33	0.0651	0.1131	2.06
2460	LRP chaperone MESD	Q14696	0.0051	0.0438	-0.19	0.1658	0.6617	-0.15	0.1296	0.2504	-0.19	0.0097	0.0395	-0.11	0.8791	0.9073	0.00	0.0100	0.0701	-0.14	0.2990	0.5563	0.17	0.0425	0.1903	-0.25	0.2063	0.2750	-0.14
2461	Luc7-like protein 3	O95232	0.0042	0.0405	0.38	0.0925	0.6296	0.31	0.0050	0.0346	0.41	0.0011	0.0158	0.43	0.0069	0.0605	0.35	0.0046	0.0602	0.41	0.0023	0.0573	0.56	0.0429	0.1912	0.22	0.0468	0.1891	0.21
2462	Lupus La protein	P05455	0.0207	0.0849	0.12	0.2468	0.6600	0.19	0.0058	0.0369	0.27	0.0156	0.0517	0.25	0.2637	0.4852	0.03	0.1779	0.3472	0.14	0.0433	0.1683	0.42	0.4799	0.8904	0.05	0.4384	0.5089	0.05
2463	L-xylulose reductase	Q7Z4W1	0.0015	0.0264	0.15	0.7699	0.8331	-0.01	0.0008	0.0200	0.18	0.0000	0.0000	0.33	0.3265	0.4239	0.04	0.0025	0.0556	0.53	0.2898	0.5419	0.20	0.3170	0.6667	0.19	0.9582	0.9666	-0.01
2464	Lymphocyte expansion molecule	Q3ZCV2	0.0617	0.1646	-0.36	0.2473	0.6583	-0.20	0.0251	0.0787	-0.44	0.1085	0.2062	-0.26	0.0416	0.1508	-0.37	0.5251	0.7543	-0.10	0.3736	0.4586	-2.55	0.8708	0.9569	-0.33	0.0651	0.1230	2.06
2465	Lymphocyte function-associated antigen 3	P19256	0.1127	0.2444	-0.08	0.3849	0.7303	0.08	0.9691	0.9738	-0.01	0.0720	0.1510	0.09	0.1630	0.3472	-0.07	0.1324	0.2833	-0.07	0.3736	0.5230	-2.55	0.8708	1.0755	-0.33	0.0651	0.1517	2.06
2466	Lymphoid-restricted membrane protein	Q12912	0.4108	0.5562	-3.31	0.5752	0.7786	-1.75	0.5661	0.7025	-1.19	0.5388	0.6644	0.89	0.2938	0.4528	-1.88	0.9510	1.0686	0.09	0.0342	0.1539	-0.36	0.0024	0.0409	-0.72	0.0060	0.0736	-0.92
2467	Lymphokine-activated killer T-cell-originated protein kinase	Q96KB5	0.0198	0.0825	-0.12	0.0243	0.4823	-0.16	0.3330	0.5241	-0.11	0.0074	0.0348	-0.19	0.0006	0.0195	-0.50	0.0825	0.2071	-0.53	0.0135	0.1100	0.28	0.0030	0.0454	0.16	0.0071	0.0781	0.17
2468	Lys-63-specific deubiquitinase BRCC36	P46736	0.0073	0.0504	0.50	0.9074	0.9354	0.01	0.9145	0.9321	0.01	0.4562	0.6532	-0.06	0.8904	0.9159	-0.01	0.3667	0.5737	0.11	0.3736	0.4722	-2.55	0.8708	0.9823	-0.33	0.0651	0.1287	2.06
2469	Lysine-rich coiled-coil protein 1	Q9NPI7	0.0275	0.1004	0.40	0.2560	0.6624	0.17	0.1738	0.3156	0.17	0.2787	0.4365	0.13	0.5916	0.6684	0.06	0.2206	0.4022	0.20	0.3736	0.4979	-2.55	0.8708	1.0296	-0.33	0.0651	0.1399	2.06
2470	Lysine-specific demethylase 2B	Q8NHM5	0.0762	0.1882	-0.46	0.6943	0.7746	0.09	0.2017	0.3536	-0.36	0.2329	0.3768	-0.23	0.1159	0.2788	-0.36	0.4511	0.6734	-0.39	0.6380	0.6835	0.08	0.4938	0.9064	0.11	0.2269	0.2973	0.29
2471	Lysine-specific demethylase 3A	Q9Y4C1	0.4108	0.5560	-3.31	0.5752	0.7784	-1.75	0.5661	0.7024	-1.19	0.5388	0.6642	0.89	0.2938	0.4527	-1.88	0.9510	1.0684	0.09	0.0073	0.0871	4.99	0.0032	0.0468	9.52	0.0047	0.0674	7.60
2472	Lysine-specific demethylase 3B	Q7LBC6	0.0186	0.0800	0.25	0.3479	0.7031	0.12	0.0022	0.0262	0.41	0.0026	0.0215	0.39	0.0471	0.1595	0.18	0.0061	0.0623	0.40	0.0008	0.0432	0.28	0.0024	0.0416	-0.11	0.0262	0.1385	0.19
2473	Lysine-specific demethylase 4C	Q9H3R0	0.1841	0.3488	0.34	0.1577	0.6620	0.21	0.0528	0.1301	0.73	0.2604	0.4130	-0.08	0.0525	0.1699	-0.10	0.0011	0.0463	0.80	0.3736	0.6483	-2.55	0.8708	1.2966	-0.33	0.0651	0.2236	2.06
2474	Lysine-specific demethylase 5D	Q9BY66	0.4108	0.5559	-3.31	0.5752	0.7781	-1.75	0.5661	0.7022	-1.19	0.5388	0.6640	0.89	0.2938	0.4525	-1.88	0.9510	1.0681	0.09	0.1436	0.3260	0.13	0.0239	0.1368	0.52	0.6937	0.7457	-0.02
2475	Lysine-specific demethylase 7A	Q6ZMT4	0.4108	0.5557	-3.31	0.5752	0.7779	-1.75	0.5661	0.7020	-1.19	0.5388	0.6639	0.89	0.2938	0.4524	-1.88	0.9510	1.0679	0.09	0.0000	0.0000	-1.87	0.0003	0.0222	-2.16	0.0001	0.0235	-2.76
2476	Lysine-specific demethylase 9	Q5VWQ0	0.0142	0.0695	-0.40	0.6573	0.7475	-0.04	0.0563	0.1356	-0.19	0.0247	0.0699	-0.16	0.0007	0.0205	-0.29	0.0310	0.1131	-0.46	0.3736	0.5803	-2.55	0.8708	1.1780	-0.33	0.0651	0.1815	2.06
2477	Lysine-specific demethylase hairless	O43593	0.1656	0.3231	-0.27	0.3249	0.6855	-0.44	0.2595	0.4285	-0.21	0.4911	0.6935	-0.13	0.2582	0.4785	-0.20	0.8880	1.1247	0.02	0.3736	0.4345	-2.55	0.8708	0.9116	-0.33	0.0651	0.1133	2.06
2478	Lysine--tRNA ligase	Q15046	0.0215	0.0873	0.24	0.2626	0.6635	0.23	0.0067	0.0394	0.32	0.0133	0.0469	0.28	0.9584	0.9676	0.00	0.0208	0.0933	0.27	0.0314	0.1498	0.40	0.1125	0.3426	0.19	0.9696	0.9760	0.00
2479	LysM and putative peptidoglycan-binding domain-containing protein 2	Q8IV50	0.4038	0.6238	0.04	0.7302	0.8036	0.03	0.0620	0.1447	-0.13	0.0869	0.1747	-0.12	0.9907	0.9934	0.00	0.0584	0.1666	0.14	0.3736	0.5564	-2.55	0.8708	1.1356	-0.33	0.0651	0.1685	2.06
2480	Lysophosphatidylcholine acyltransferase 1	Q8NF37	0.4108	0.5556	-3.31	0.5752	0.7777	-1.75	0.5661	0.7019	-1.19	0.5388	0.6637	0.89	0.2938	0.4523	-1.88	0.9510	1.0677	0.09	0.2071	0.4225	-1.68	0.0325	0.1623	-1.14	0.0130	0.0989	-0.38
2481	Lysophospholipase D GDPD3	Q7L5L3	0.4116	0.4896	-0.05	0.8277	0.8761	-0.03	0.0302	0.0883	0.37	0.2076	0.3424	0.07	0.0015	0.0301	-0.40	0.1361	0.2885	0.24	0.3736	0.5218	-2.55	0.8708	1.0732	-0.33	0.0651	0.1511	2.06
2482	Lysophospholipase-like protein 1	Q5VWZ2	0.4108	0.5555	-3.31	0.5752	0.7775	-1.75	0.5661	0.7017	-1.19	0.5388	0.6636	0.89	0.2938	0.4521	-1.88	0.9510	1.0674	0.09	0.0481	0.1755	-0.20	0.0224	0.1302	-0.26	0.0104	0.0899	-0.46
2483	Lysophospholipid acyltransferase 7	Q96N66	0.0511	0.1464	-0.21	0.4810	0.8063	0.07	0.1661	0.3036	0.11	0.1315	0.2384	-0.13	0.0251	0.1141	0.25	0.2967	0.4937	-0.09	0.5166	0.5701	0.05	0.5900	1.0228	-0.03	0.4446	0.5146	-0.06
2484	Lysosomal acid phosphatase	P11117	0.0341	0.1154	-0.21	0.7465	0.8165	0.05	0.8243	0.8581	0.02	0.3664	0.5473	0.07	0.0159	0.0899	0.23	0.0107	0.0706	0.29	0.3736	0.6109	-2.55	0.8708	1.2318	-0.33	0.0651	0.1994	2.06
2485	Lysosomal alpha-glucosidase	P10253	0.0512	0.1464	0.07	0.1368	0.6519	0.27	0.0074	0.0408	0.14	0.0302	0.0808	0.18	0.0176	0.0953	0.11	0.0042	0.0604	0.25	0.3736	0.6337	-2.55	0.8708	1.2713	-0.33	0.0651	0.2138	2.06
2486	Lysosomal protective protein	P10619	0.0092	0.0570	0.17	0.0285	0.5039	0.13	0.0579	0.1381	-0.10	0.8666	0.8854	-0.01	0.1045	0.2600	0.07	0.0478	0.1456	0.31	0.0152	0.1143	0.17	0.0587	0.2311	0.09	0.1380	0.1991	-0.25
2487	Lysosome membrane protein 2	Q14108	0.0273	0.1002	0.13	0.4750	0.8022	0.09	0.6959	0.7437	0.02	0.1307	0.2373	0.11	0.4469	0.5404	0.03	0.4320	0.6534	-0.03	0.3736	0.4662	-2.55	0.8708	0.9711	-0.33	0.0651	0.1262	2.06
2488	Lysosome-associated membrane glycoprotein 1	P11279	0.0392	0.1247	-3.61	0.2595	0.6635	-1.66	0.2528	0.4203	-1.12	0.1397	0.2508	-2.06	0.9583	0.9676	0.02	0.0764	0.1971	-3.89	0.0278	0.1443	0.58	0.0316	0.1603	0.37	0.4500	0.5188	0.13
2489	Lysosome-associated membrane glycoprotein 2	P13473	0.0241	0.0933	0.19	0.0128	0.4480	-0.25	0.0036	0.0319	-0.34	0.0089	0.0382	-0.29	0.0092	0.0681	-0.32	0.0548	0.1590	-0.13	0.3736	0.5577	-2.55	0.8708	1.1379	-0.33	0.0651	0.1692	2.06
2490	Lysozyme-like protein 1	Q6UWQ5	0.4381	0.5155	-0.06	0.5260	0.8443	0.07	0.0232	0.0751	0.30	0.1202	0.2232	0.28	0.0013	0.0273	0.31	0.3852	0.5956	0.05	0.3736	0.4705	-2.55	0.8708	0.9791	-0.33	0.0651	0.1280	2.06
2491	Lysozyme-like protein 2	Q7Z4W2	0.0158	0.0730	-0.21	0.0162	0.4561	-0.19	0.2807	0.4560	0.05	0.0064	0.0319	-0.30	0.0098	0.0712	-0.24	0.0069	0.0643	-0.30	0.3736	0.6221	-2.55	0.8708	1.2512	-0.33	0.0651	0.2064	2.06
2492	m7GpppN-mRNA hydrolase	Q8IU60	0.0007	0.0201	-0.40	0.0216	0.4802	-0.18	0.0125	0.0526	-0.42	0.0126	0.0454	-0.42	0.0074	0.0626	-0.21	0.1349	0.2872	-0.24	0.3736	0.5222	-2.55	0.8708	1.0740	-0.33	0.0651	0.1513	2.06
2493	m7GpppX diphosphatase	Q96C86	0.9893	0.9920	0.00	0.7301	0.8036	-0.08	0.9427	0.9554	-0.03	0.6788	0.7195	-0.10	0.7207	0.7807	0.08	0.7376	0.9769	-0.07	0.3736	0.4427	-2.55	0.8708	0.9272	-0.33	0.0651	0.1166	2.06
2494	m-AAA protease-interacting protein 1 mitochondrial	Q8WWC4	0.9760	0.9834	0.04	0.2786	0.6650	0.99	0.6102	0.6654	0.17	0.0010	0.0149	1.65	0.0387	0.1436	-0.47	0.2893	0.4861	1.14	0.7956	0.8277	0.30	0.8004	1.2443	0.30	0.9683	0.9749	0.05
2495	Macrophage migration inhibitory factor	P14174	0.7737	0.8159	0.25	0.3326	0.6922	0.73	0.0312	0.0903	1.88	0.0095	0.0391	1.42	0.3959	0.4926	-0.25	0.6906	0.9306	0.41	0.0003	0.0370	-1.75	0.0000	0.0000	-2.06	0.0001	0.0345	-2.66
2496	Macrophage-capping protein	P40121	0.4440	0.5216	0.04	0.0766	0.6133	0.36	0.1639	0.3002	0.09	0.3393	0.5133	0.06	0.2986	0.3966	-0.06	0.2180	0.3994	-0.07	0.0136	0.1104	0.41	0.3009	0.6449	0.09	0.9856	0.9888	0.00
2497	Maestro heat-like repeat-containing protein family member 1	Q8NDA8	0.0011	0.0227	-2.04	0.1127	0.6352	-3.92	0.0005	0.0206	-1.02	0.0120	0.0440	-2.20	0.0312	0.1295	-0.39	0.0452	0.1406	-0.17	0.3736	0.5649	-2.55	0.8708	1.1507	-0.33	0.0651	0.1730	2.06
2498	Magnesium transporter protein 1	Q9H0U3	0.3895	0.6063	-0.06	0.6496	0.7413	0.06	0.5146	0.7351	0.02	0.0918	0.1818	0.12	0.0173	0.0946	0.15	0.0854	0.2128	0.06	0.4047	0.4605	0.03	0.0129	0.0945	-0.08	0.0074	0.0785	-0.30
2499	MAGUK p55 subfamily member 3	Q13368	0.2916	0.4890	0.28	0.9672	0.9774	-0.01	0.6986	0.7460	-0.15	0.1308	0.2374	-1.04	0.4856	0.5756	-0.17	0.8398	1.0749	-0.19	0.3736	0.4361	-2.55	0.8708	0.9146	-0.33	0.0651	0.1139	2.06
2500	MAGUK p55 subfamily member 5	Q8N3R9	0.4108	0.5553	-3.31	0.5752	0.7773	-1.75	0.5661	0.7015	-1.19	0.5388	0.6634	0.89	0.2938	0.4520	-1.88	0.9510	1.0672	0.09	0.0445	0.1697	0.51	0.7683	1.2108	0.04	0.7436	0.7880	0.06
2501	MAGUK p55 subfamily member 7	Q5T2T1	0.9994	0.9994	0.00	0.6911	0.7737	-0.03	0.7309	0.7742	0.02	0.5821	0.6309	0.04	0.4457	0.5394	-0.05	0.1376	0.2905	0.12	0.3736	0.5212	-2.55	0.8708	1.0722	-0.33	0.0651	0.1508	2.06
2502	Major vault protein	Q14764	0.4108	0.5552	-3.31	0.5752	0.7771	-1.75	0.5661	0.7014	-1.19	0.5388	0.6633	0.89	0.2938	0.4519	-1.88	0.9510	1.0670	0.09	0.1740	0.3724	0.16	0.1963	0.4912	-0.11	0.0785	0.1290	-0.13
2503	Malate dehydrogenase cytoplasmic	P40925	0.0002	0.0144	0.48	0.1175	0.6320	0.23	0.0005	0.0202	0.34	0.0003	0.0118	0.43	0.0151	0.0877	0.15	0.0018	0.0533	0.39	0.0285	0.1456	0.28	0.8771	0.9070	0.01	0.9713	0.9771	0.00

Supplemetary Table S2. Overview on all relatively quantified 5180 proteins statistical analysis

			MCF-7																		MDA-MB-231								
			Dai SC20 vs control			Gen SC20 vs control			SSE SC20 vs control			Dai IC20 vs control			Gen IC20 vs control			SSE IC20 vs control			Dai IC20 vs control			Gen IC20 vs control			SSE IC20 vs control		
	**Protein name**	**UniProt**	**p value**	**BH q value**	**log2FC**	**p value**	**BH q value**	**log2FC**	**p value**	**BH q value**	**log2FC**	**p value**	**BH q value**	**log2FC**	**p value**	**BH q value**	**log2FC**	**p value**	**BH q value**	**log2FC**	**p value**	**BH q value**	**log2FC**	**p value**	**BH q value**	**log2FC**	**p value**	**BH q value**	**log2FC**
2504	Malate dehydrogenase mitochondrial	P40926	0.0015	0.0263	0.53	0.1724	0.6615	0.25	0.0223	0.0731	0.23	0.0100	0.0402	0.27	0.1429	0.3166	0.10	0.0063	0.0624	0.45	0.6903	0.7320	-0.05	0.0068	0.0670	-0.41	0.0060	0.0733	-0.68
2505	Malectin	Q14165	0.7477	0.7946	0.01	0.2140	0.6626	0.11	0.0470	0.1202	0.10	0.0119	0.0437	0.16	0.0203	0.1022	0.13	0.0235	0.0983	0.15	0.0033	0.0635	0.25	0.0875	0.2936	0.16	0.0098	0.0877	0.22
2506	Malignant T-cell-amplified sequence 1	Q9ULC4	0.5097	0.5825	-0.06	0.8166	0.8689	0.03	0.2739	0.4476	-0.10	0.6226	0.6692	-0.04	0.1652	0.3507	-0.14	0.4907	0.7162	-0.06	0.0103	0.1016	0.34	0.0090	0.0781	0.38	0.5904	0.6518	0.03
2507	Malonyl-CoA decarboxylase mitochondrial	O95822	0.5309	0.6018	0.09	0.0369	0.5493	0.33	0.5642	0.7884	0.09	0.6703	0.7122	0.05	0.0882	0.2326	0.34	0.4843	0.7091	-0.07	0.3736	0.4615	-2.55	0.8708	0.9624	-0.33	0.0651	0.1242	2.06
2508	Maltase-glucoamylase intestinal	O43451	0.4108	0.5550	-3.31	0.5752	0.7769	-1.75	0.5661	0.7012	-1.19	0.5388	0.6631	0.89	0.2938	0.4517	-1.88	0.9510	1.0667	0.09	0.2389	0.4702	-0.34	0.4839	0.8943	-0.04	0.0457	0.1870	0.17
2509	Mannan-binding lectin serine protease 1	P48740	0.2605	0.4496	-0.10	0.3836	0.7305	-0.23	0.5925	0.6487	-0.06	0.0207	0.0619	-0.32	0.3963	0.4929	-0.08	0.9443	1.1807	0.01	0.3736	0.4317	-2.55	0.8708	0.9063	-0.33	0.0651	0.1122	2.06
2510	Mannose-1-phosphate guanyltransferase beta	Q9Y5P6	0.0988	0.2234	0.12	0.8766	0.9113	0.02	0.0863	0.1831	0.14	0.1223	0.2260	0.20	0.6175	0.6910	-0.03	0.5708	0.8057	0.03	0.3736	0.4546	-2.55	0.8708	0.9494	-0.33	0.0651	0.1213	2.06
2511	Mannose-6-phosphate isomerase	P34949	0.0055	0.0449	-0.13	0.6040	0.7072	0.02	0.1259	0.2454	-0.06	0.6522	0.6960	0.03	0.0511	0.1671	-0.19	0.0039	0.0598	0.21	0.3736	0.6349	-2.55	0.8708	1.2735	-0.33	0.0651	0.2147	2.06
2512	Mannosyl-oligosaccharide 1 2-alpha-mannosidase IB	O60476	0.2568	0.4449	0.06	0.1529	0.6584	0.27	0.0078	0.0417	0.27	0.0056	0.0300	0.48	0.0144	0.0856	0.30	0.0116	0.0735	0.34	0.3736	0.6097	-2.55	0.8708	1.2298	-0.33	0.0651	0.1987	2.06
2513	Mannosyl-oligosaccharide glucosidase	Q13724	0.4795	0.5543	-0.17	0.6876	0.7716	0.06	0.8267	0.8602	0.03	0.4653	0.6640	0.12	0.1270	0.2943	0.22	0.4527	0.6748	-0.11	0.3736	0.4638	-2.55	0.8708	0.9665	-0.33	0.0651	0.1251	2.06
2514	MAP kinase-activated protein kinase 2	P49137	0.9898	0.9921	0.00	0.5587	0.8720	0.04	0.2107	0.3651	-0.07	0.2517	0.4010	0.08	0.2420	0.4558	0.05	0.5734	0.8078	0.05	0.3736	0.4543	-2.55	0.8708	0.9488	-0.33	0.0651	0.1212	2.06
2515	MAP/microtubule affinity-regulating kinase 3	P27448	0.0965	0.2192	0.22	0.1985	0.6651	0.24	0.0621	0.1448	0.28	0.0296	0.0796	0.34	0.3945	0.4912	0.09	0.0375	0.1252	0.39	0.3736	0.5715	-2.55	0.8708	1.1626	-0.33	0.0651	0.1766	2.06
2516	MAP7 domain-containing protein 1	Q3KQU3	0.0326	0.1125	-1.06	0.0216	0.4782	-0.62	0.1561	0.2885	-0.32	0.0242	0.0691	-0.56	0.0943	0.2430	-0.34	0.0983	0.2311	-0.45	0.0113	0.1034	0.54	0.0199	0.1223	0.45	0.0351	0.1609	0.29
2517	MARCKS-related protein	P49006	0.6069	0.6716	-0.02	0.1242	0.6408	0.20	0.1518	0.2823	0.12	0.0434	0.1042	0.11	0.6634	0.7287	0.01	0.0160	0.0838	-0.18	0.0317	0.1504	0.56	0.0510	0.2130	0.21	0.0389	0.1705	0.18
2518	Matrin-3	P43243	0.1011	0.2268	0.14	0.7910	0.8497	0.03	0.9389	0.9529	-0.01	0.5124	0.7168	0.05	0.2110	0.4148	-0.10	0.3332	0.5364	0.08	0.0076	0.0893	0.65	0.3902	0.7721	0.13	0.0071	0.0769	0.71
2519	Matrix metalloproteinase-15	P51511	0.0440	0.1333	0.21	0.0346	0.5318	0.24	0.7003	0.7475	-0.02	0.4456	0.6401	0.06	0.1908	0.3870	0.11	0.9430	1.1793	0.00	0.3736	0.4318	-2.55	0.8708	0.9065	-0.33	0.0651	0.1123	2.06
2520	Matrix metalloproteinase-21	Q8N119	0.4108	0.5549	-3.31	0.5752	0.7767	-1.75	0.5661	0.7010	-1.19	0.5388	0.6629	0.89	0.2938	0.4516	-1.88	0.9510	1.0665	0.09	0.0060	0.0822	-0.84	0.0031	0.0460	-0.67	0.0222	0.1268	-0.64
2521	Matrix-remodeling-associated protein 5	Q9NR99	0.0351	0.1169	0.23	0.2985	0.6729	0.12	0.0571	0.1367	0.18	0.3851	0.5701	-0.06	0.0302	0.1265	-0.22	0.0263	0.1040	0.27	0.3736	0.5850	-2.55	0.8708	1.1864	-0.33	0.0651	0.1842	2.06
2522	MBT domain-containing protein 1	Q05BQ5	0.0551	0.1536	-12.77	0.0452	0.5642	-11.21	0.3827	0.5836	-2.06	0.0240	0.0687	-4.29	0.4984	0.5877	-0.26	0.2188	0.4002	-6.33	0.3736	0.4983	-2.55	0.8708	1.0303	-0.33	0.0651	0.1401	2.06
2523	Mdm2-binding protein	Q96DY7	0.0359	0.1184	-0.37	0.0340	0.5289	-0.62	0.0082	0.0427	-0.60	0.0246	0.0700	-0.46	0.0578	0.1804	-0.45	0.1213	0.2665	-0.44	0.1690	0.3646	-0.27	0.1309	0.3792	-0.13	0.0439	0.1832	-0.35
2524	Mediator of DNA damage checkpoint protein 1	Q14676	0.0122	0.0640	-0.17	0.1098	0.6327	-0.09	0.0433	0.1131	0.13	0.5328	0.7403	-0.02	0.0182	0.0960	-0.14	0.8798	1.1170	-0.03	0.0047	0.0727	0.36	0.6810	1.1209	-0.03	0.1551	0.2184	0.10
2525	Mediator of RNA polymerase II transcription subunit 20	Q9H944	0.4108	0.5547	-3.31	0.5752	0.7765	-1.75	0.5661	0.7009	-1.19	0.5388	0.6628	0.89	0.2938	0.4515	-1.88	0.9510	1.0663	0.09	0.0741	0.2159	-0.22	0.0076	0.0718	-0.49	0.0339	0.1576	-0.31
2526	Mediator of RNA polymerase II transcription subunit 23	Q9ULK4	0.0128	0.0662	-0.23	0.0205	0.4720	-0.54	0.0731	0.1619	0.21	0.0003	0.0102	-0.79	0.0051	0.0538	-0.43	0.9688	0.9750	0.00	0.3736	0.4305	-2.55	0.8708	0.9041	-0.33	0.0651	0.1118	2.06
2527	Mediator of RNA polymerase II transcription subunit 24	O75448	0.9054	0.9263	0.01	0.9787	0.9855	0.00	0.0128	0.0529	0.14	0.0129	0.0461	0.11	0.0058	0.0559	0.12	0.0006	0.0409	0.25	0.3736	0.6518	-2.55	0.8708	1.3026	-0.33	0.0651	0.2260	2.06
2528	Medium-chain specific acyl-CoA dehydrogenase_ mitochondrial	P11310	0.4108	0.5546	-3.31	0.5752	0.7763	-1.75	0.5661	0.7007	-1.19	0.5388	0.6626	0.89	0.2938	0.4513	-1.88	0.9510	1.0660	0.09	0.4342	0.4900	-0.07	0.8547	1.2964	0.02	0.1664	0.2316	0.15
2529	Megakaryocyte-associated tyrosine-protein kinase	P42679	0.4108	0.5544	-3.31	0.5752	0.7761	-1.75	0.5661	0.7005	-1.19	0.5388	0.6625	0.89	0.2938	0.4512	-1.88	0.9510	1.0658	0.09	0.0321	0.1510	0.29	0.1907	0.4830	-0.23	0.2329	0.3030	0.17
2530	Meiosis-specific coiled-coil domain-containing protein MEIOC	A2RUB1	0.0647	0.1682	-1.22	0.5846	0.6915	-0.54	0.2496	0.4163	0.50	0.2136	0.3506	-2.43	0.0238	0.1116	1.77	0.1605	0.3215	0.67	0.3736	0.5121	-2.55	0.8708	1.0556	-0.33	0.0651	0.1465	2.06
2531	Meiosis-specific nuclear structural protein 1	Q8NEH6	0.5567	0.6255	0.05	0.2045	0.6596	0.24	0.0499	0.1249	0.22	0.0500	0.1150	0.24	0.0324	0.1313	0.27	0.0190	0.0889	0.40	0.3680	0.6495	0.25	0.0086	0.0763	0.35	0.0953	0.1500	0.29
2532	Melanoma inhibitory activity protein 2	Q96PC5	0.5563	0.6252	0.03	0.6888	0.7721	-0.04	0.0427	0.1120	0.20	0.3286	0.5005	0.08	0.0323	0.1312	0.22	0.6139	0.8500	0.03	0.3736	0.4512	-2.55	0.8708	0.9431	-0.33	0.0651	0.1200	2.06
2533	Melanoma-associated antigen B18	Q96M61	0.1104	0.2412	0.21	0.0045	0.3532	0.44	0.5986	0.6546	-0.05	0.0151	0.0506	0.33	0.0151	0.0875	0.40	0.1904	0.3641	0.11	0.4362	0.4919	0.05	0.0609	0.2361	0.08	0.4515	0.5201	0.06
2534	Melanoma-associated antigen D1	Q9Y5V3	0.8173	0.8529	-0.01	0.1761	0.6615	0.27	0.5157	0.7361	-0.05	0.5716	0.6214	0.04	0.0019	0.0343	0.59	0.8404	1.0754	-0.01	0.4944	0.5482	0.03	0.0260	0.1445	-0.27	0.1043	0.1605	-0.10
2535	Melanoma-associated antigen D2	Q9UNF1	0.3727	0.5875	0.02	0.1063	0.6300	0.17	0.0036	0.0318	0.14	0.0045	0.0274	0.12	0.0004	0.0171	0.18	0.1314	0.2824	0.16	0.0009	0.0413	-0.38	0.0011	0.0283	-0.34	0.0102	0.0894	-0.36
2536	Melanophilin	Q9BV36	0.5194	0.5913	0.10	0.2555	0.6627	0.21	0.4041	0.6087	0.13	0.2094	0.3450	0.20	0.8358	0.8725	-0.03	0.2572	0.4480	0.18	0.3736	0.4888	-2.55	0.8708	1.0130	-0.33	0.0651	0.1359	2.06
2537	Melanoregulin	Q8N565	0.0649	0.1685	-0.24	0.0667	0.5937	-0.27	0.0349	0.0971	-0.39	0.2401	0.3864	-0.26	0.0032	0.0423	-0.19	0.0167	0.0851	-0.17	0.3736	0.5991	-2.55	0.8708	1.2113	-0.33	0.0651	0.1924	2.06
2538	Membrane magnesium transporter 1	Q8N4V1	0.0034	0.0382	-0.24	0.0181	0.4688	-0.29	0.0042	0.0326	-0.23	0.0057	0.0303	-0.38	0.0195	0.0999	-0.29	0.1470	0.3031	-0.06	0.0005	0.0392	-0.45	0.1358	0.3893	0.09	0.0226	0.1271	-0.16
2539	Membrane-associated progesterone receptor component 1	O00264	0.1602	0.3149	-0.08	0.0738	0.6117	0.27	0.0009	0.0217	0.43	0.0004	0.0125	0.63	0.0003	0.0164	0.78	0.0053	0.0621	0.35	0.3736	0.6287	-2.55	0.8708	1.2628	-0.33	0.0651	0.2106	2.06
2540	Membrane-associated progesterone receptor component 2	O15173	0.3470	0.5560	0.05	0.1452	0.6569	0.16	0.1807	0.3244	-0.05	0.2492	0.3983	0.04	0.0327	0.1319	0.12	0.2822	0.4785	0.07	0.0775	0.2214	0.57	0.1234	0.3653	0.43	0.0301	0.1475	0.88
2541	Merlin	P35240	0.0907	0.2108	-1.21	0.4319	0.7680	-0.61	0.4045	0.6091	-0.11	0.0823	0.1675	-0.32	0.5351	0.6208	0.08	0.0121	0.0743	-0.69	0.0029	0.0601	0.72	0.0031	0.0461	0.40	0.0014	0.0459	0.53
2542	Mesencephalic astrocyte-derived neurotrophic factor	P55145	0.1732	0.3345	0.08	0.2085	0.6606	0.17	0.2943	0.4749	0.07	0.0338	0.0872	0.16	0.8709	0.9021	0.01	0.1318	0.2827	0.10	0.1603	0.3533	0.15	0.1506	0.4187	0.09	0.0758	0.1258	0.09
2543	Metabotropic glutamate receptor 1	Q13255	0.4108	0.5543	-3.31	0.5752	0.7759	-1.75	0.5661	0.7004	-1.19	0.5388	0.6623	0.89	0.2938	0.4511	-1.88	0.9510	1.0656	0.09	0.3962	0.4522	-0.11	0.4093	0.7962	-0.14	0.9361	0.9486	-0.01
2544	Metallothionein-1E	P04732	0.4108	0.5542	-3.31	0.5752	0.7757	-1.75	0.5661	0.7002	-1.19	0.5388	0.6622	0.89	0.2938	0.4509	-1.88	0.9510	1.0654	0.09	0.2023	0.4153	-0.19	0.0913	0.3003	-0.23	0.0826	0.1341	-0.31
2545	Metallothionein-1F	P04733	0.4108	0.5540	-3.31	0.5752	0.7755	-1.75	0.5661	0.7000	-1.19	0.5388	0.6620	0.89	0.2938	0.4508	-1.88	0.9510	1.0651	0.09	0.2061	0.4208	0.16	0.0853	0.2892	-0.25	0.1216	0.1804	-0.32
2546	Metallothionein-1M	Q8N339	0.0337	0.1146	0.18	0.5038	0.8248	-1.91	0.7232	0.7675	0.03	0.0002	0.0084	0.75	0.7820	0.8291	0.02	0.0019	0.0513	0.82	0.3736	0.6447	-2.55	0.8708	1.2903	-0.33	0.0651	0.2211	2.06
2547	Metallothionein-1X	P80297	0.4108	0.5539	-3.31	0.5752	0.7753	-1.75	0.5661	0.6999	-1.19	0.5388	0.6618	0.89	0.2938	0.4507	-1.88	0.9510	1.0649	0.09	0.0243	0.1376	-1.68	0.0738	0.2633	-0.76	0.0358	0.1618	-1.53
2548	Metallothionein-2	P02795	0.4108	0.5537	-3.31	0.5752	0.7751	-1.75	0.5661	0.6997	-1.19	0.5388	0.6617	0.89	0.2938	0.4505	-1.88	0.9510	1.0647	0.09	0.0759	0.2193	0.28	0.2489	0.5715	-0.21	0.2042	0.2730	-0.15
2549	Metal-response element-binding transcription factor 2	Q9Y483	0.0943	0.2164	0.08	0.3766	0.7239	0.06	0.8368	0.8685	-0.01	0.0258	0.0720	-0.12	0.4579	0.5503	0.02	0.3423	0.5464	-0.07	0.0784	0.2223	-0.19	0.0068	0.0672	-0.45	0.0059	0.0728	-0.54
2550	Metastasis-associated protein MTA1	Q13330	0.0528	0.1490	-1.85	0.1516	0.6599	-1.39	0.3615	0.5591	-0.49	0.4601	0.6575	-0.45	0.1295	0.2973	-0.69	0.0073	0.0648	-1.51	0.8326	0.8590	0.18	0.1053	0.3288	1.50	0.1045	0.1606	1.60
2551	Metastasis-associated protein MTA2	O94776	0.0013	0.0248	0.19	0.0673	0.5909	0.33	0.0231	0.0749	0.24	0.0009	0.0148	0.22	0.0008	0.0225	0.22	0.0073	0.0652	0.19	0.0414	0.1675	0.19	0.4525	0.8564	0.05	0.0569	0.2122	0.10
2552	Metastasis-associated protein MTA3	Q9BTC8	0.0001	0.0110	-0.95	0.1411	0.6538	0.26	0.0004	0.0197	-0.80	0.0060	0.0312	-0.67	0.0090	0.0676	-0.28	0.0841	0.2101	-1.25	0.3736	0.5419	-2.55	0.8708	1.1097	-0.33	0.0651	0.1610	2.06
2553	Metaxin-1	Q13505	0.0612	0.1637	0.25	0.4755	0.8026	0.15	0.7727	0.8130	0.03	0.1078	0.2054	0.18	0.3322	0.4294	0.09	0.1446	0.3000	0.16	0.3736	0.5183	-2.55	0.8708	1.0669	-0.33	0.0651	0.1494	2.06
2554	Metaxin-2	O75431	0.4108	0.5536	-3.31	0.5752	0.7749	-1.75	0.5661	0.6995	-1.19	0.5388	0.6615	0.89	0.2938	0.4504	-1.88	0.9510	1.0644	0.09	0.1833	0.3846	0.30	0.8108	1.2548	0.04	0.1661	0.2314	0.32
2555	Methanethiol oxidase	Q13228	0.0044	0.0418	0.25	0.2017	0.6600	0.15	0.3961	0.5987	-0.05	0.0973	0.1902	0.08	0.5835	0.6611	-0.02	0.0249	0.1004	0.17	0.3736	0.5863	-2.55	0.8708	1.1886	-0.33	0.0651	0.1849	2.06
2556	Methionine adenosyltransferase 2 subunit beta	Q9NZL9	0.0036	0.0384	0.22	0.4098	0.7509	0.11	0.0043	0.0322	0.19	0.0020	0.0198	0.26	0.0563	0.1773	0.09	0.0132	0.0765	0.19	0.2868	0.5381	0.06	0.4011	0.7864	0.03	0.0023	0.0525	0.12
2557	Methionine aminopeptidase 1	P53582	0.5462	0.6157	-0.06	0.4435	0.7793	-0.05	0.0600	0.1417	-0.17	0.6883	0.7282	0.03	0.0836	0.2240	-0.16	0.0326	0.1162	-0.22	0.2078	0.4233	0.18	0.1016	0.3207	0.27	0.5058	0.5727	-0.08
2558	Methionine aminopeptidase 2	P50579	0.4975	0.5715	0.06	0.0495	0.5736	0.36	0.0369	0.1008	0.29	0.0482	0.1122	0.29	0.0711	0.2030	0.27	0.3405	0.5445	0.13	0.3736	0.4751	-2.55	0.8708	0.9877	-0.33	0.0021	0.0504	7.19
2559	Methionine synthase reductase	Q9UBK8	0.0480	0.1409	-0.20	0.2524	0.6623	0.21	0.6893	0.7380	0.04	0.1101	0.2088	-0.09	0.0625	0.1893	0.12	0.7055	0.9475	0.07	0.0509	0.1789	0.43	0.4043	0.7903	0.14	0.0331	0.1556	0.51
2560	Methionine--tRNA ligase cytoplasmic	P56192	0.0016	0.0273	0.33	0.2120	0.6627	0.19	0.0016	0.0233	0.52	0.0004	0.0123	0.45	0.0070	0.0609	0.26	0.0015	0.0521	0.52	0.0164	0.1198	0.17	0.3927	0.7752	-0.04	0.3504	0.4218	-0.04
2561	Methionyl-tRNA formyltransferase mitochondrial	Q96DP5	0.4108	0.5534	-3.31	0.5752	0.7747	-1.75	0.5661	0.6994	-1.19	0.5388	0.6614	0.89	0.2938	0.4503	-1.88	0.9510	1.0642	0.09	0.0327	0.1512	0.60	0.0014	0.0298	0.25	0.0182	0.1160	0.37
2562	Methylated-DNA--protein-cysteine methyltransferase	P16455	0.5088	0.5818	-0.06	0.8362	0.8818	0.02	0.3223	0.5098	-0.09	0.6423	0.6869	0.05	0.0798	0.2180	-0.22	0.3529	0.5582	-0.09	0.3736	0.4741	-2.55	0.8708	0.9857	-0.33	0.0651	0.1295	2.06
2563	Methyl-CpG-binding domain protein 4	O95243	0.4108	0.5533	-3.31	0.5752	0.7745	-1.75	0.5661	0.6992	-1.19	0.5388	0.6612	0.89	0.2938	0.4501	-1.88	0.9510	1.0640	0.09	0.0105	0.1017	-1.40	0.3654	0.7348	0.27	0.8560	0.8831	0.05
2564	Methyl-CpG-binding domain protein 5	Q9P267	0.0665	0.1709	-0.07	0.2371	0.6624	-0.10	0.0488	0.1229	0.13	0.4430	0.6374	-0.04	0.0020	0.0348	-0.23	0.8237	1.0582	-0.03	0.3736	0.4366	-2.55	0.8708	0.9155	-0.33	0.0651	0.1141	2.06
2565	Methylcrotonoyl-CoA carboxylase beta chain_ mitochondrial	Q9HCC0	0.1732	0.3344	-0.09	0.4138	0.7526	0.14	0.1617	0.2968	0.10	0.1698	0.2914	0.09	0.3126	0.4101	0.06	0.1701	0.3354	0.09	0.0279	0.1442	0.39	0.0257	0.1439	0.26	0.0891	0.1421	0.17
2566	Methylcrotonoyl-CoA carboxylase subunit alpha_ mitochondrial	Q96RQ3	0.0168	0.0759	0.12	0.1397	0.6537	0.21	0.0948	0.1959	0.10	0.0001	0.0066	0.27	0.0539	0.1723	0.12	0.1391	0.2928	0.09	0.0048	0.0731	0.59	0.0166	0.1094	0.28	0.0263	0.1387	0.21
2567	Methylcytosine dioxygenase TET2	Q6N021	0.0265	0.0985	-0.13	0.3317	0.6912	-0.12	0.4398	0.6513	0.04	0.0102	0.0405	0.18	0.2633	0.4849	0.05	0.0153	0.0823	0.23	0.7199	0.7596	0.14	0.0257	0.1436	1.85	0.8490	0.8783	0.08
2568	Methylmalonate-semialdehyde dehydrogenase [acylating]_ mitochondrial	Q02252	0.2055	0.3780	-0.05	0.3411	0.7000	0.16	0.3021	0.4849	-0.12	0.4607	0.6580	0.02	0.0314	0.1296	-0.08	0.3337	0.5368	0.03	0.3736	0.4761	-2.55	0.8708	0.9894	-0.33	0.0651	0.1303	2.06
2569	Methylmalonyl-CoA mutase mitochondrial	P22033	0.0466	0.1381	0.10	0.0916	0.6293	0.20	0.0333	0.0944	0.14	0.0485	0.1127	0.07	0.0003	0.0143	0.21	0.0019	0.0538	0.16	0.3736	0.6453	-2.55	0.8708	1.2914	-0.33	0.0651	0.2216	2.06
2570	Methylosome protein 50	Q9BQA1	0.2800	0.4735	0.23	0.8806	0.9143	-0.10	0.5326	0.7536	-0.14	0.7265	0.7644	0.10	0.2561	0.4758	0.24	0.1250	0.2727	-0.40	0.1146	0.2805	-0.13	0.8648	1.3075	-0.02	0.2633	0.3354	-0.08
2571	Methylosome subunit pICln	P54105	0.9569	0.9677	0.00	0.0501	0.5742	-0.23	0.0557	0.1349	-0.33	0.1040	0.1996	-0.16	0.0799	0.2178	-0.18	0.0839	0.2099	-0.19	0.0399	0.1639	0.29	0.0108	0.0853	0.38	0.0636	0.2263	0.17
2572	Methylsterol monooxygenase 1	Q15800	0.0054	0.0445	0.25	0.0093	0.4420	0.33	0.0003	0.0232	0.44	0.0001	0.0126	0.61	0.0004	0.0180	0.47	0.0063	0.0631	0.53	0.3736	0.6247	-2.55	0.8708	1.2558	-0.33	0.0651	0.2080	2.06
2573	Methylthioribose-1-phosphate isomerase	Q9BV20	0.1647	0.3218	0.09	0.9720	0.9811	0.00	0.0985	0.2018	0.12	0.5192	0.7241	0.04	0.2246	0.4335	-0.08	0.0745	0.1941	0.15	0.3736	0.5475	-2.55	0.8708	1.1196	-0.33	0.0651	0.1639	2.06
2574	Methylthioribulose-1-phosphate dehydratase	Q96GX9	0.0426	0.1307	0.19	0.0339	0.5305	0.28	0.0325	0.0928	0.43	0.0177	0.0561	0.21	0.0351	0.1360	0.15	0.0810	0.2048	0.10	0.0476	0.1745	0.27	0.1203	0.3588	0.16	0.0414	0.1764	0.29
2575	Methyltransferase-like protein 2A	Q96IZ6	0.4108	0.5531	-3.31	0.5752	0.7743	-1.75	0.5661	0.6990	-1.19	0.5388	0.6611	0.89	0.2938	0.4500	-1.88	0.9510	1.0637	0.09	0.0157	0.1167	0.55	0.0036	0.0488	0.54	0.0096	0.0875	-1.17
2576	Methyltransferase-like protein 2B	Q6P1Q9	0.4567	0.5338	-0.03	0.0546	0.5868	-0.10	0.0669	0.1522	0.10	0.2192	0.3578	0.14	0.0206	0.1026	0.14	0.1732	0.3404	-0.19	0.0421	0.1675	0.24	0.9894	0.9927	0.00	0.1374	0.1983	0.15
2577	MHC class I polypeptide-related sequence B	Q29980	0.4108	0.5530	-3.31	0.5752	0.7741	-1.75	0.5661	0.6989	-1.19	0.5388	0.6609	0.89	0.2938	0.4499	-1.88	0.9510	1.0635	0.09	0.0418	0.1675	0.16	0.1676	0.4470	0.09	0.5439	0.6090	0.07
2578	MICOS complex subunit MIC19	Q9NX63	0.3102	0.5114	0.05	0.9659	0.9766	-0.01	0.0702	0.1575	0.15	0.0777	0.1604	0.09	0.6435	0.7123	0.02	0.0290	0.1089	0.20	0.0554	0.1872	0.36	0.6996	1.1421	0.03	0.9720	0.9773	0.00
2579	MICOS complex subunit MIC60	Q16891	0.0221	0.0886	0.24	0.4040	0.7479	0.19	0.0078	0.0416	0.34	0.0156	0.0517	0.28	0.4783	0.5684	0.04	0.0201	0.0917	0.34	0.0311	0.1489	0.50	0.0755	0.2677	0.18	0.0965	0.1515	0.12

Supplemetary Table S2. Overview on all relatively quantified 5180 proteins statistical analysis

			MCF-7												MDA-MB-231								
			Dai SC20 vs control			Gen SC20 vs control			SSE SC20 vs control			Dai IC20 vs control			Gen IC20 vs control			SSE IC20 vs control			Dai IC20 vs control			Gen IC20 vs control			SSE IC20 vs control		
	**Protein name**	**UniProt**	**p value**	**BH q value**	**log2FC**	**p value**	**BH q value**	**log2FC**	**p value**	**BH q value**	**log2FC**	**p value**	**BH q value**	**log2FC**	**p value**	**BH q value**	**log2FC**	**p value**	**BH q value**	**log2FC**	**p value**	**BH q value**	**log2FC**	**p value**	**BH q value**	**log2FC**	**p value**	**BH q value**	**log2FC**
2580	Microfibrillar-associated protein 1	P55081	0.0082	0.0537	0.43	0.0014	0.2133	0.45	0.0004	0.0250	0.56	0.0001	0.0120	0.84	0.0001	0.0133	1.07	0.0245	0.1001	0.73	0.3736	0.5877	-2.55	0.8708	1.1911	-0.33	0.0651	0.1857	2.06
2581	Microfibrillar-associated protein 3-like	O75121	0.0129	0.0664	-0.26	0.0255	0.4874	0.33	0.0775	0.1684	0.09	0.0344	0.0884	0.15	0.0620	0.1884	-0.15	0.4424	0.6642	0.03	0.3736	0.4650	-2.55	0.8708	0.9688	-0.33	0.0651	0.1256	2.06
2582	Microprocessor complex subunit DGCR8	Q8WYQ5	0.0084	0.0543	-0.47	0.2086	0.6605	-0.16	0.0262	0.0808	0.23	0.0981	0.1913	0.18	0.9189	0.9394	-0.01	0.5630	0.7975	-0.05	0.3736	0.4554	-2.55	0.8708	0.9508	-0.33	0.0651	0.1217	2.06
2583	Microsomal glutathione S-transferase 3	O14880	0.1963	0.3651	-0.21	0.0056	0.3817	-1.05	0.0612	0.1436	-0.27	0.0092	0.0385	-0.74	0.6514	0.7188	-0.05	0.0736	0.1926	0.26	0.0446	0.1699	0.25	0.0072	0.0698	0.22	0.0219	0.1262	0.15
2584	Microtubule cross-linking factor 1	Q9Y4B5	0.4108	0.5529	-3.31	0.5752	0.7739	-1.75	0.5661	0.6987	-1.19	0.5388	0.6607	0.89	0.2938	0.4497	-1.88	0.9510	1.0633	0.09	0.0375	0.1605	0.50	0.9520	0.9656	-0.01	0.1893	0.2570	0.38
2585	Microtubule-actin cross-linking factor 1_ isoforms 1/2/3/5	Q9UPN3	0.1721	0.3331	-0.10	0.7443	0.8146	0.06	0.0270	0.0822	0.22	0.0509	0.1163	0.17	0.4916	0.5813	-0.04	0.0225	0.0961	0.25	0.0398	0.1643	0.38	0.0497	0.2098	0.25	0.0584	0.2158	0.21
2586	Microtubule-associated protein 1A	P78559	0.0273	0.1002	-0.75	0.0620	0.5915	-0.83	0.9748	0.9788	-0.01	0.0583	0.1287	-0.64	0.0273	0.1195	-1.01	0.1780	0.3473	-0.71	0.4138	0.4699	-0.06	0.0666	0.2487	-0.20	0.0777	0.1282	-0.20
2587	Microtubule-associated protein 1B	P46821	0.0016	0.0272	-0.89	0.4692	0.7990	-0.08	0.0123	0.0519	-0.62	0.0010	0.0153	-0.96	0.1512	0.3289	0.18	0.0191	0.0887	-1.10	0.0143	0.1119	0.31	0.1484	0.4144	0.11	0.1034	0.1595	0.08
2588	Microtubule-associated protein 2	P11137	0.4108	0.5527	-3.31	0.5752	0.7737	-1.75	0.5661	0.6985	-1.19	0.5388	0.6606	0.89	0.2938	0.4496	-1.88	0.9510	1.0631	0.09	0.8593	0.8832	0.04	0.0462	0.1999	0.81	0.7476	0.7916	0.07
2589	Microtubule-associated protein 4	P27816	0.1854	0.3500	0.10	0.3537	0.7055	0.11	0.0396	0.1058	0.20	0.1171	0.2186	0.13	0.1862	0.3814	0.10	0.1435	0.2988	0.12	0.0667	0.2031	0.29	0.9956	0.9969	0.00	0.6687	0.7227	0.03
2590	Microtubule-associated protein 9	Q49MG5	0.4108	0.5526	-3.31	0.5752	0.7735	-1.75	0.5661	0.6984	-1.19	0.5388	0.6604	0.89	0.2938	0.4495	-1.88	0.9510	1.0628	0.09	0.1596	0.3527	0.30	0.6032	1.0367	-0.10	0.3342	0.4052	-0.17
2591	Microtubule-associated protein RP/EB family member 1	Q15691	0.4790	0.5538	0.05	0.4215	0.7592	0.14	0.0627	0.1456	0.17	0.0814	0.1663	0.17	0.9266	0.9448	0.01	0.1750	0.3429	0.12	0.1203	0.2902	0.20	0.3617	0.7296	0.09	0.9350	0.9478	0.01
2592	Microtubule-associated protein RP/EB family member 3	Q9UPY8	0.0181	0.0789	0.39	0.4537	0.7871	0.12	0.0039	0.0315	0.55	0.0051	0.0289	0.50	0.0266	0.1184	0.30	0.0180	0.0872	0.40	0.3736	0.5964	-2.55	0.8708	1.2064	-0.33	0.0651	0.1907	2.06
2593	Microtubule-associated protein tau	P10636	0.2355	0.4186	-0.07	0.1374	0.6512	0.41	0.2368	0.3983	0.19	0.0015	0.0170	0.30	0.0022	0.0370	0.45	0.5923	0.8294	0.06	0.3495	0.6243	0.15	0.0001	0.0108	3.11	0.0012	0.0441	1.72
2594	Microtubule-associated proteins 1A/1B light chain 3A	Q9H492	0.5658	0.6337	0.03	0.1351	0.6504	0.30	0.1235	0.2418	0.25	0.0560	0.1253	0.21	0.0001	0.0082	0.29	0.2242	0.4072	-0.08	0.0869	0.2362	0.34	0.6925	1.1348	-0.02	0.1032	0.1593	-0.11
2595	Microtubule-associated serine/threonine-protein kinase 4	O15021	0.5341	0.6046	-0.01	0.0793	0.6140	0.11	0.0102	0.0472	0.15	0.0028	0.0218	0.13	0.0799	0.2181	0.04	0.2741	0.4684	0.07	0.0222	0.1353	0.40	0.1597	0.4329	0.11	0.0169	0.1114	0.27
2596	Microtubule-associated tumor suppressor candidate 2	Q5JR59	0.1963	0.3650	0.03	0.7442	0.8148	0.04	0.0098	0.0460	0.21	0.0005	0.0123	0.24	0.0143	0.0855	0.10	0.0103	0.0702	0.30	0.1340	0.3118	0.19	0.2004	0.4986	-0.10	0.2932	0.3648	-0.07
2597	Midkine	P21741	0.7082	0.7603	-0.02	0.4595	0.7910	0.07	0.0491	0.1234	0.20	0.0162	0.0528	0.29	0.0068	0.0598	0.43	0.2526	0.4429	0.07	0.3736	0.4902	-2.55	0.8708	1.0155	-0.33	0.0651	0.1365	2.06
2598	MIF4G domain-containing protein	A9UHW6	0.7852	0.8255	-0.02	0.5749	0.8860	-0.03	0.3420	0.5349	-0.07	0.8121	0.8380	-0.01	0.3578	0.4549	-0.10	0.2373	0.4231	0.08	0.3736	0.4936	-2.55	0.8708	1.0217	-0.33	0.0651	0.1380	2.06
2599	Mini-chromosome maintenance complex-binding protein	Q9BTE3	0.4582	0.5350	0.03	0.0796	0.6118	0.08	0.0008	0.0219	0.29	0.0008	0.0149	0.29	0.0681	0.1991	0.08	0.0066	0.0627	0.24	0.0471	0.1740	0.16	0.0573	0.2292	0.22	0.1357	0.1965	-0.06
2600	Minor histocompatibility antigen H13	Q8TCT9	0.0166	0.0752	0.39	0.0243	0.4841	0.54	0.0039	0.0320	0.67	0.0023	0.0207	0.69	0.0007	0.0212	0.88	0.0036	0.0598	0.75	0.0220	0.1353	0.53	0.3109	0.6592	0.14	0.2432	0.3134	0.17
2601	Mirror-image polydactyly gene 1 protein	Q8TD10	0.6078	0.6724	0.02	0.2169	0.6597	-0.24	0.2445	0.4097	0.05	0.0602	0.1320	-0.15	0.2189	0.4247	-0.06	0.2041	0.3820	0.08	0.3150	0.5770	0.16	0.2158	0.5214	-0.18	0.1810	0.2480	-0.20
2602	Mis18-binding protein 1	Q6P0N0	0.4108	0.5524	-3.31	0.5752	0.7733	-1.75	0.5661	0.6982	-1.19	0.5388	0.6603	0.89	0.2938	0.4493	-1.88	0.9510	1.0626	0.09	0.0026	0.0566	0.67	0.0008	0.0256	0.99	0.0180	0.1151	0.29
2603	Mismatch repair endonuclease PMS2	P54278	0.0300	0.1064	-0.22	0.4760	0.8026	-0.05	0.9688	0.9739	0.00	0.4010	0.5908	-0.08	0.0423	0.1517	0.19	0.6313	0.8676	0.03	0.3736	0.4492	-2.55	0.8708	0.9393	-0.33	0.0651	0.1192	2.06
2604	Misshapen-like kinase 1	Q8N4C8	0.4108	0.5523	-3.31	0.5752	0.7731	-1.75	0.5661	0.6980	-1.19	0.5388	0.6601	0.89	0.2938	0.4492	-1.88	0.9510	1.0624	0.09	0.0006	0.0379	-0.97	0.0038	0.0505	-0.49	0.0108	0.0910	-0.42
2605	Mitochondrial 2-oxoglutarate/malate carrier protein	Q02978	0.4540	0.5315	-0.04	0.2662	0.6626	0.21	0.0020	0.0250	0.35	0.0033	0.0235	0.30	0.6174	0.6910	0.02	0.0311	0.1131	0.18	0.1837	0.3848	0.19	0.4022	0.7877	-0.09	0.5755	0.6382	-0.04
2606	Mitochondrial antiviral-signaling protein	Q7Z434	0.0118	0.0628	-0.39	0.1044	0.6332	-0.31	0.0005	0.0207	-0.27	0.0000	0.0000	-0.98	0.0154	0.0885	-0.30	0.0009	0.0448	-0.33	0.0185	0.1263	-0.54	0.9844	0.9892	0.00	0.3404	0.4113	0.17
2607	Mitochondrial cardiolipin hydrolase	Q8N2A8	0.0372	0.1213	0.43	0.4256	0.7615	-0.21	0.0679	0.1536	-0.30	0.0932	0.1839	-0.27	0.4318	0.5259	-0.10	0.2678	0.4609	0.18	0.3736	0.4871	-2.55	0.8708	1.0098	-0.33	0.0651	0.1351	2.06
2608	Mitochondrial carrier homolog 2	Q9Y6C9	0.0545	0.1527	0.16	0.2404	0.6606	0.22	0.0041	0.0321	0.41	0.0033	0.0234	0.38	0.0519	0.1690	0.18	0.0049	0.0612	0.43	0.0490	0.1772	0.41	0.0524	0.2171	0.27	0.1151	0.1727	0.14
2609	Mitochondrial chaperone BCS1	Q9Y276	0.4108	0.5521	-3.31	0.5752	0.7729	-1.75	0.5661	0.6979	-1.19	0.5388	0.6600	0.89	0.2938	0.4491	-1.88	0.9510	1.0621	0.09	0.2606	0.5003	-0.18	0.1997	0.4973	0.12	0.1065	0.1628	-0.10
2610	Mitochondrial dicarboxylate carrier	Q9UBX3	0.9674	0.9761	0.00	0.5008	0.8228	0.11	0.0629	0.1458	0.17	0.0317	0.0835	0.20	0.7949	0.8398	-0.01	0.2375	0.4233	0.07	0.4081	0.4639	0.10	0.0706	0.2572	0.20	0.5450	0.6101	-0.05
2611	Mitochondrial fission 1 protein	Q9Y3D6	0.7371	0.7863	-0.01	0.2189	0.6596	0.24	0.0077	0.0416	0.17	0.0298	0.0801	0.24	0.0001	0.0085	0.31	0.0037	0.0599	0.15	0.5509	0.6020	0.03	0.0286	0.1526	-0.19	0.3215	0.3927	-0.11
2612	Mitochondrial fission factor	Q9GZY8	0.5393	0.6097	-0.06	0.0615	0.5921	-0.31	0.8070	0.8433	-0.02	0.9663	0.9710	0.00	0.3394	0.4366	0.10	0.7657	1.0036	0.03	0.1936	0.4015	-0.62	0.5834	1.0151	0.13	0.0963	0.1513	0.59
2613	Mitochondrial genome maintenance exonuclease 1	Q9BQP7	0.8185	0.8538	0.01	0.0439	0.5685	0.17	0.0202	0.0684	0.15	0.0049	0.0281	0.23	0.4051	0.5013	-0.05	0.3776	0.5861	-0.10	0.0896	0.2405	-0.11	0.0048	0.0554	-0.51	0.0213	0.1248	-0.36
2614	Mitochondrial glutamate carrier 1	Q9H936	0.0652	0.1690	-0.10	0.1580	0.6616	-0.09	0.0070	0.0399	-0.23	0.5835	0.6323	-0.05	0.0074	0.0622	-0.28	0.2935	0.4909	0.07	0.3736	0.4830	-2.55	0.8708	1.0022	-0.33	0.0651	0.1333	2.06
2615	Mitochondrial import inner membrane translocase subunit Tim10	P62072	0.4524	0.5299	-0.07	0.8096	0.8633	-0.02	0.1776	0.3205	0.14	0.6358	0.6809	0.04	0.2151	0.4200	-0.12	0.4270	0.6471	-0.08	0.3736	0.4667	-2.55	0.8708	0.9719	-0.33	0.0651	0.1263	2.06
2616	Mitochondrial import inner membrane translocase subunit Tim13	Q9Y5L4	0.0054	0.0445	0.29	0.5627	0.8766	0.06	0.2574	0.4257	-0.05	0.3601	0.5390	0.05	0.1831	0.3765	-0.09	0.0187	0.0884	0.16	0.0490	0.1770	-0.35	0.0207	0.1244	-0.46	0.0056	0.0713	-0.82
2617	Mitochondrial import inner membrane translocase subunit TIM14	Q96DA6	0.7442	0.7919	0.02	0.1713	0.6612	-0.13	0.0100	0.0467	-0.29	0.0241	0.0689	-0.24	0.1169	0.2801	-0.11	0.0364	0.1236	0.18	0.3736	0.5727	-2.55	0.8708	1.1647	-0.33	0.0651	0.1773	2.06
2618	Mitochondrial import inner membrane translocase subunit Tim23	O14925	0.4108	0.5520	-3.31	0.5752	0.7727	-1.75	0.5661	0.6977	-1.19	0.5388	0.6598	0.89	0.2938	0.4489	-1.88	0.9510	1.0619	0.09	0.2993	0.5567	-0.26	0.0881	0.2946	-0.16	0.3886	0.4598	-0.07
2619	Mitochondrial import inner membrane translocase subunit TIM44	O43615	0.0281	0.1019	0.17	0.3424	0.7002	0.23	0.0288	0.0861	0.18	0.0277	0.0758	0.21	0.4052	0.5012	-0.04	0.0554	0.1606	0.23	0.0204	0.1324	0.31	0.4294	0.8256	0.08	0.0779	0.1285	-0.10
2620	Mitochondrial import inner membrane translocase subunit TIM50	Q3ZCQ8	0.0153	0.0716	0.14	0.3016	0.6751	0.04	0.0198	0.0678	0.17	0.0019	0.0191	0.21	0.5682	0.6487	0.01	0.0037	0.0590	0.27	0.0757	0.2192	0.10	0.1597	0.4327	-0.07	0.4944	0.5617	0.03
2621	Mitochondrial import inner membrane translocase subunit Tim8 A	O60220	0.6462	0.7078	0.04	0.5984	0.7019	-0.06	0.5235	0.7444	0.05	0.0948	0.1862	0.18	0.9841	0.9885	0.00	0.8162	1.0528	-0.02	0.3736	0.4374	-2.55	0.8708	0.9172	-0.33	0.0651	0.1145	2.06
2622	Mitochondrial import inner membrane translocase subunit Tim9	Q9Y5J7	0.1470	0.2950	0.07	0.4418	0.7776	0.14	0.0297	0.0875	0.22	0.0104	0.0409	0.22	0.2923	0.5241	0.05	0.0328	0.1164	0.17	0.0322	0.1511	0.29	0.0234	0.1348	0.37	0.0932	0.1473	0.21
2623	Mitochondrial import receptor subunit TOM22 homolog	Q9NS69	0.0051	0.0437	-0.12	0.8447	0.8884	-0.03	0.0440	0.1144	0.08	0.0187	0.0577	0.07	0.0996	0.2527	-0.07	0.5730	0.8077	0.03	0.0334	0.1523	0.37	0.0306	0.1580	0.17	0.0221	0.1266	0.25
2624	Mitochondrial import receptor subunit TOM34	Q15785	0.0248	0.0950	0.16	0.0745	0.6116	0.29	0.0172	0.0624	0.39	0.0004	0.0114	0.44	0.0030	0.0417	0.19	0.0026	0.0571	0.31	0.1304	0.3054	0.15	0.0453	0.1975	-0.24	0.0263	0.1386	-0.28
2625	Mitochondrial import receptor subunit TOM40 homolog	O96008	0.0266	0.0988	0.14	0.0082	0.4124	-0.29	0.0676	0.1532	0.10	0.0185	0.0576	-0.27	0.0266	0.1183	-0.19	0.4660	0.6887	0.04	0.0320	0.1508	0.26	0.7924	1.2360	0.03	0.9401	0.9519	0.00
2626	Mitochondrial import receptor subunit TOM7 homolog	Q9P0U1	0.0074	0.0507	-0.26	0.0032	0.2858	-0.45	0.2046	0.3576	-0.15	0.0368	0.0925	-0.21	0.0054	0.0545	-0.26	0.1795	0.3488	-0.08	0.3736	0.5077	-2.55	0.8708	1.0475	-0.33	0.0651	0.1444	2.06
2627	Mitochondrial import receptor subunit TOM70	O94826	0.0038	0.0393	0.18	0.2100	0.6621	0.12	0.0153	0.0581	0.23	0.0239	0.0685	0.24	0.0838	0.2244	0.06	0.0063	0.0625	0.26	0.1235	0.2958	0.18	0.2822	0.6207	-0.06	0.5628	0.6265	-0.03
2628	Mitochondrial inner membrane protein OXA1L	Q15070	0.0358	0.1184	0.13	0.1219	0.6385	0.17	0.2229	0.3803	0.11	0.0078	0.0357	0.19	0.0449	0.1561	0.08	0.4436	0.6649	0.05	0.7440	0.7816	0.04	0.5295	0.9530	-0.10	0.1924	0.2604	-0.22
2629	Mitochondrial ornithine transporter 1	Q9Y619	0.3746	0.5898	-0.11	0.1455	0.6554	0.22	0.2300	0.3896	0.12	0.1530	0.2694	0.15	0.1257	0.2928	0.17	0.6423	0.8783	0.04	0.3736	0.4480	-2.55	0.8708	0.9370	-0.33	0.0651	0.1187	2.06
2630	Mitochondrial proton/calcium exchanger protein	O95202	0.0013	0.0248	0.19	0.0460	0.5607	0.14	0.0169	0.0616	0.23	0.0003	0.0107	0.10	0.0072	0.0621	0.10	0.0301	0.1114	0.25	0.0218	0.1348	0.15	0.0314	0.1599	-0.07	0.3205	0.3919	0.02
2631	Mitochondrial Rho GTPase 2	Q8IXI1	0.0041	0.0402	-0.28	0.9959	0.9974	0.00	0.0864	0.1832	-0.18	0.0232	0.0671	-0.25	0.0070	0.0607	-0.25	0.1043	0.2403	-0.15	0.3736	0.5324	-2.55	0.8708	1.0925	-0.33	0.0651	0.1563	2.06
2632	Mitochondrial ribonuclease P catalytic subunit	O15091	0.4101	0.6317	-0.08	0.4716	0.8004	0.07	0.7091	0.7553	0.03	0.9901	0.9914	0.00	0.5704	0.6508	-0.05	0.3677	0.5747	-0.09	0.3707	0.6525	-0.57	0.0055	0.0597	-0.84	0.2049	0.2736	0.34
2633	Mitochondrial tRNA-specific 2-thiouridylase 1	O75648	0.4975	0.5714	0.03	0.2979	0.6724	-0.12	0.2126	0.3672	-0.16	0.2090	0.3446	-0.36	0.2390	0.4520	-0.14	0.0323	0.1159	-0.24	0.2255	0.4507	0.16	0.0174	0.1114	0.15	0.7940	0.8319	-0.01
2634	Mitochondrial-processing peptidase subunit alpha	Q10713	0.0052	0.0441	0.37	0.2480	0.6585	0.27	0.0299	0.0878	0.28	0.0036	0.0241	0.53	0.0496	0.1644	0.18	0.0140	0.0787	0.41	0.3736	0.6050	-2.55	0.8708	1.2214	-0.33	0.0651	0.1958	2.06
2635	Mitochondrial-processing peptidase subunit beta	O75439	0.8213	0.8563	-0.01	0.5784	0.6872	0.07	0.6406	0.6935	0.03	0.3919	0.5790	0.06	0.7832	0.8298	-0.01	0.2624	0.4547	0.07	0.3736	0.4882	-2.55	0.8708	1.0118	-0.33	0.0651	0.1356	2.06
2636	Mitogen-activated protein kinase 1	P28482	0.3478	0.5567	0.13	0.3516	0.7043	-0.21	0.9554	0.9643	-0.01	0.8359	0.8589	0.02	0.9005	0.9242	0.01	0.8345	1.0687	0.03	0.2164	0.4367	0.18	0.7214	1.1663	0.02	0.0926	0.1464	-0.09
2637	Mitogen-activated protein kinase 14	Q16539	0.0898	0.2096	-0.09	0.2437	0.6592	0.17	0.1698	0.3095	-0.07	0.3546	0.5326	0.07	0.5090	0.5981	0.03	0.4331	0.6541	-0.08	0.0481	0.1757	-0.20	0.0128	0.0940	-0.38	0.0599	0.2188	-0.26
2638	Mitogen-activated protein kinase 3	P27361	0.2647	0.4563	0.04	0.6978	0.7775	0.04	0.2570	0.4253	0.04	0.1326	0.2402	-0.06	0.0078	0.0635	-0.23	0.9382	1.1745	0.00	0.0748	0.2176	-0.16	0.0988	0.3148	-0.11	0.6811	0.7343	-0.03
2639	Mitogen-activated protein kinase 9	P45984	0.0337	0.1145	-0.30	0.3071	0.6775	-0.24	0.0426	0.1120	-0.29	0.1210	0.2243	-0.25	0.0682	0.1989	-0.35	0.0339	0.1187	-0.48	0.3736	0.5761	-2.55	0.8708	1.1707	-0.33	0.0651	0.1792	2.06
2640	Mitogen-activated protein kinase kinase kinase 1	Q13233	0.4968	0.5710	-0.10	0.9473	0.9652	0.01	0.8829	0.9072	-0.02	0.6139	0.6610	-0.07	0.1227	0.2886	0.28	0.2959	0.4928	-0.21	0.3736	0.4824	-2.55	0.8708	1.0011	-0.33	0.0651	0.1330	2.06
2641	Mitogen-activated protein kinase kinase kinase 12	Q12852	0.0035	0.0381	0.68	0.1441	0.6548	0.10	0.5242	0.7452	-0.03	0.5607	0.6109	-0.04	0.2519	0.4700	0.07	0.7101	0.9515	-0.02	0.3898	0.4456	-0.11	0.0294	0.1548	-0.28	0.0004	0.0269	-0.46
2642	Mitogen-activated protein kinase kinase kinase 13	O43283	0.0067	0.0491	1.06	0.7960	0.8519	0.17	0.0043	0.0322	0.99	0.2411	0.3876	0.18	0.0528	0.1704	-0.16	0.3117	0.5122	0.64	0.3736	0.4799	-2.55	0.8708	0.9964	-0.33	0.0651	0.1319	2.06
2643	Mitogen-activated protein kinase kinase kinase 15	Q6ZN16	0.0612	0.1637	0.10	0.1590	0.6637	0.07	0.0842	0.1796	-0.23	0.0235	0.0677	0.15	0.5660	0.6475	0.05	0.3777	0.5861	0.05	0.3736	0.4709	-2.55	0.8708	0.9797	-0.33	0.0651	0.1281	2.06
2644	Mitogen-activated protein kinase kinase kinase 2	Q9Y2U5	0.0009	0.0219	-0.47	0.6161	0.7162	-0.06	0.2055	0.3587	0.07	0.4101	0.6018	-0.04	0.1638	0.3485	-0.08	0.2546	0.4451	-0.19	0.3736	0.4894	-2.55	0.8708	1.0141	-0.33	0.0651	0.1361	2.06
2645	Mitogen-activated protein kinase kinase kinase 20	Q9NYL2	0.4108	0.5519	-3.31	0.5752	0.7725	-1.75	0.5661	0.6975	-1.19	0.5388	0.6597	0.89	0.2938	0.4488	-1.88	0.9510	1.0617	0.09	0.4046	0.4605	1.05	0.3047	0.6503	1.36	0.2553	0.3265	1.57

Supplemetary Table S2. Overview on all relatively quantified 5180 proteins statistical analysis

			MCF-7																		MDA-MB-231									
			Dai SC20 vs control			Gen SC20 vs control			SSE SC20 vs control			Dai IC20 vs control			Gen IC20 vs control			SSE IC20 vs control			Dai IC20 vs control			Gen IC20 vs control			SSE IC20 vs control		
	**Protein name**	**UniProt**	**p value**	**BH q value**	**log2FC**	**p value**	**BH q value**	**log2FC**	**p value**	**BH q value**	**log2FC**	**p value**	**BH q value**	**log2FC**	**p value**	**BH q value**	**log2FC**	**p value**	**BH q value**	**log2FC**	**p value**	**BH q value**	**log2FC**	**p value**	**BH q value**	**log2FC**	**p value**	**BH q value**	**log2FC**
2646	Mitogen-activated protein kinase kinase kinase 3	Q99759	0.2360	0.4192	-0.05	0.3707	0.7189	-0.07	0.3474	0.5412	-0.05	0.2224	0.3623	-0.05	0.1867	0.3823	-0.08	0.1189	0.2633	0.14	0.0605	0.1948	0.35	0.0423	0.1899	0.22	0.1732	0.2391	0.10
2647	Mitogen-activated protein kinase kinase kinase 4	Q9Y6R4	0.0625	0.1656	0.22	0.4244	0.7607	0.02	0.0052	0.0351	0.15	0.5975	0.6456	-0.01	0.0026	0.0390	-0.13	0.8935	1.1302	0.02	0.1767	0.3761	0.20	0.8820	0.9110	0.02	0.4248	0.4963	-0.10
2648	Mitogen-activated protein kinase kinase kinase 7	O43318	0.7344	0.7840	-0.01	0.9482	0.9653	0.00	0.4631	0.6799	0.03	0.2827	0.4417	-0.06	0.9703	0.9771	0.00	0.3080	0.5070	-0.12	0.3736	0.4803	-2.55	0.8708	0.9973	-0.33	0.0651	0.1321	2.06
2649	Mitotic checkpoint protein BUB3	O43684	0.0742	0.1847	0.13	0.3414	0.6998	0.18	0.0675	0.1530	0.14	0.0499	0.1149	0.16	0.2780	0.5032	-0.06	0.1352	0.2871	0.10	0.1274	0.3008	0.21	0.5032	0.9198	0.06	0.2392	0.3096	-0.11
2650	Mitotic spindle assembly checkpoint protein MAD1	Q9Y6D9	0.0655	0.1694	0.21	0.2433	0.6591	0.19	0.0077	0.0416	0.27	0.0127	0.0456	0.15	0.0177	0.0954	0.13	0.0375	0.1253	0.12	0.0020	0.0537	-0.32	0.4293	0.8258	0.04	0.0020	0.0496	-0.54
2651	Mitotic spindle assembly checkpoint protein MAD2A	Q13257	0.4108	0.5517	-3.31	0.5752	0.7723	-1.75	0.5661	0.6974	-1.19	0.5388	0.6595	0.89	0.2938	0.4487	-1.88	0.9510	1.0614	0.09	0.1383	0.3181	0.18	0.7219	1.1668	-0.04	0.6594	0.7144	-0.03
2652	Mixed lineage kinase domain-like protein	Q8NB16	0.4108	0.5516	-3.31	0.5752	0.7721	-1.75	0.5661	0.6972	-1.19	0.5388	0.6593	0.89	0.2938	0.4485	-1.88	0.9510	1.0612	0.09	0.3784	0.4341	0.09	0.4160	0.8068	-0.08	0.2895	0.3614	-0.09
2653	MMS19 nucleotide excision repair protein homolog	Q96T76	0.4533	0.5308	-0.04	0.6684	0.7561	0.06	0.8248	0.8584	-0.02	0.1438	0.2562	0.09	0.2242	0.4329	-0.07	0.7931	1.0307	-0.02	0.3736	0.4390	-2.55	0.8708	0.9202	-0.33	0.0651	0.1151	2.06
2654	MOB kinase activator 1B	Q7L9L4	0.3688	0.5828	-0.05	0.5541	0.8692	0.10	0.9303	0.9458	0.00	0.0730	0.1525	0.13	0.1170	0.2802	-0.11	0.1399	0.2934	-0.11	0.5320	0.5843	0.03	0.9772	0.9838	0.00	0.1059	0.1622	-0.11
2655	MOB-like protein phocein	Q9Y3A3	0.1320	0.2739	0.18	0.7857	0.8456	-0.03	0.6673	0.7180	-0.05	0.1851	0.3122	0.23	0.1307	0.2986	-0.20	0.9120	1.1489	-0.01	0.0000	0.0000	1.13	0.0001	0.0192	1.05	0.0201	0.1219	1.04
2656	Moesin	P26038	0.0140	0.0693	0.25	0.1705	0.6611	0.18	0.0514	0.1273	0.17	0.0464	0.1094	0.18	0.1333	0.3022	0.10	0.0289	0.1090	0.25	0.0224	0.1360	0.57	0.2418	0.5602	0.12	0.0788	0.1294	0.19
2657	Molybdopterin synthase catalytic subunit	O96007	0.0971	0.2204	0.16	0.3486	0.7037	0.06	0.0576	0.1374	0.17	0.0978	0.1909	0.14	0.7998	0.8438	0.02	0.2015	0.3787	0.09	0.3736	0.5020	-2.55	0.8708	1.0372	-0.33	0.0651	0.1418	2.06
2658	Monoacylglycerol lipase ABHD12	Q8N2K0	0.0457	0.1368	-0.20	0.6992	0.7784	0.05	0.2584	0.4268	-0.11	0.2769	0.4348	0.08	0.0465	0.1584	-0.21	0.6801	0.9193	-0.05	0.3736	0.4460	-2.55	0.8708	0.9333	-0.33	0.0651	0.1179	2.06
2659	Monocarboxylate transporter 1	P53985	0.4108	0.5514	-3.31	0.5752	0.7719	-1.75	0.5661	0.6970	-1.19	0.5388	0.6592	0.89	0.2938	0.4484	-1.88	0.9510	1.0610	0.09	0.0007	0.0398	0.80	0.0024	0.0417	0.59	0.0178	0.1144	0.33
2660	Monocarboxylate transporter 4	O15427	0.4108	0.5513	-3.31	0.5752	0.7717	-1.75	0.5661	0.6969	-1.19	0.5388	0.6590	0.89	0.2938	0.4483	-1.88	0.9510	1.0608	0.09	0.0928	0.2446	0.27	0.2038	0.5032	0.15	0.2263	0.2967	0.14
2661	Monofunctional C1-tetrahydrofolate synthase_ mitochondrial	Q6UB35	0.0018	0.0292	0.24	0.0972	0.6341	0.22	0.0004	0.0216	0.32	0.0012	0.0166	0.23	0.0384	0.1429	0.08	0.0033	0.0576	0.18	0.3058	0.5653	0.12	0.2046	0.5042	0.19	0.2646	0.3364	-0.13
2662	MORC family CW-type zinc finger protein 4	Q8TE76	0.4108	0.5511	-3.31	0.5752	0.7715	-1.75	0.5661	0.6967	-1.19	0.5388	0.6589	0.89	0.2938	0.4481	-1.88	0.9510	1.0605	0.09	0.0275	0.1435	-0.30	0.0527	0.2177	-0.18	0.1205	0.1790	-0.13
2663	MORN repeat-containing protein 2	Q502X0	0.0487	0.1415	0.17	0.2032	0.6603	0.12	0.0128	0.0531	0.29	0.1229	0.2266	0.11	0.0343	0.1354	-0.21	0.7105	0.9513	0.03	0.3736	0.4449	-2.55	0.8708	0.9312	-0.33	0.0651	0.1174	2.06
2664	Mortality factor 4-like protein 2	Q15014	0.0264	0.0985	0.29	0.6050	0.7076	-0.04	0.0774	0.1682	-0.15	0.6263	0.6727	-0.03	0.0089	0.0673	-0.35	0.5613	0.7961	-0.04	0.3736	0.4558	-2.55	0.8708	0.9516	-0.33	0.0651	0.1218	2.06
2665	Motilin receptor	O43193	0.0229	0.0904	6.49	0.0237	0.4852	6.86	0.0273	0.0829	5.99	0.0288	0.0781	6.49	0.0298	0.1257	5.83	0.0323	0.1160	5.30	0.3736	0.5791	-2.55	0.8708	1.1759	-0.33	0.0651	0.1808	2.06
2666	M-phase inducer phosphatase 1	P30304	0.4108	0.5510	-3.31	0.5752	0.7713	-1.75	0.5661	0.6965	-1.19	0.5388	0.6587	0.89	0.2938	0.4480	-1.88	0.9510	1.0603	0.09	0.0028	0.0587	1.20	0.0003	0.0199	1.17	0.0013	0.0458	0.56
2667	M-phase inducer phosphatase 3	P30307	0.0027	0.0350	-0.74	0.5015	0.8229	-0.21	0.0480	0.1216	-0.43	0.0249	0.0704	-0.64	0.0919	0.2390	0.22	0.0077	0.0659	-0.79	0.3736	0.6197	-2.55	0.8708	1.2471	-0.33	0.0651	0.2049	2.06
2668	M-phase phosphoprotein 9	Q99550	0.4608	0.5376	0.05	0.8663	0.9031	0.03	0.0094	0.0451	0.29	0.3070	0.4739	0.07	0.0058	0.0558	-0.47	0.3288	0.5316	-0.06	0.0065	0.0844	0.36	0.0224	0.1304	0.27	0.0058	0.0724	0.62
2669	MRG/MORF4L-binding protein	Q9NV56	0.0740	0.1844	-0.19	0.5299	0.8488	0.05	0.3624	0.5602	0.02	0.0126	0.0454	-0.24	0.1168	0.2801	-0.10	0.0043	0.0602	-0.27	0.3736	0.6331	-2.55	0.8708	1.2703	-0.33	0.0651	0.2134	2.06
2670	mRNA (2'-O-methyladenosine-N(6)-)-methyltransferase	Q9H4Z3	0.0610	0.1634	0.25	0.1974	0.6705	0.15	0.1050	0.2120	0.25	0.9097	0.9216	0.02	0.4719	0.5626	-0.10	0.9236	1.1604	-0.01	0.0818	0.2282	0.26	0.6917	1.1339	-0.04	0.8269	0.8599	-0.01
2671	mRNA cap guanine-N7 methyltransferase	O43148	0.7949	0.8339	0.02	0.3154	0.6810	0.07	0.2979	0.4792	0.08	0.0422	0.1022	0.09	0.2546	0.4742	0.04	0.3025	0.5005	0.14	0.0778	0.2218	0.19	0.0508	0.2127	-0.22	0.0148	0.1049	-0.26
2672	mRNA export factor	P78406	0.2459	0.4315	-0.03	0.8988	0.9280	-0.02	0.5935	0.6497	-0.02	0.8635	0.8826	0.00	0.0156	0.0888	-0.10	0.5899	0.8265	-0.03	0.0115	0.1038	-0.35	0.0130	0.0950	-0.34	0.0420	0.1780	-0.27
2673	mRNA turnover protein 4 homolog	Q9UKD2	0.4506	0.5282	-0.11	0.6903	0.7733	0.07	0.7207	0.7656	-0.05	0.6308	0.6768	0.07	0.4225	0.5171	0.12	0.4703	0.6929	-0.10	0.0824	0.2295	0.29	0.4310	0.8278	0.07	0.0337	0.1568	0.28
2674	mRNA-capping enzyme	O60942	0.0010	0.0220	1.24	0.1135	0.6335	0.71	0.0937	0.1941	0.65	0.0193	0.0590	0.76	0.1350	0.3047	0.10	0.0311	0.1133	0.91	0.2866	0.5379	-0.42	0.1414	0.4005	-0.35	0.1079	0.1643	0.12
2675	Msx2-interacting protein	Q96T58	0.0379	0.1222	-0.11	0.1352	0.6503	-0.32	0.5104	0.7314	-0.05	0.0728	0.1523	-0.26	0.0673	0.1974	-0.26	0.7879	1.0255	-0.01	0.3736	0.4395	-2.55	0.8708	0.9211	-0.33	0.0651	0.1153	2.06
2676	Mth938 domain-containing protein	Q9H7C9	0.4108	0.5509	-3.31	0.5752	0.7711	-1.75	0.5661	0.6964	-1.19	0.5388	0.6586	0.89	0.2938	0.4479	-1.88	0.9510	1.0601	0.09	0.2708	0.5138	0.18	0.2948	0.6363	-0.16	0.1223	0.1813	-0.30
2677	Mucin-1	P15941	0.7495	0.7959	0.01	0.6157	0.7161	-0.02	0.0095	0.0454	0.27	0.5736	0.6230	0.03	0.0036	0.0444	-0.26	0.6399	0.8769	-0.04	0.3736	0.4485	-2.55	0.8708	0.9380	-0.33	0.0651	0.1189	2.06
2678	Mucolipin-2	Q8IZK6	0.0018	0.0291	1.58	0.9567	0.9713	-0.02	0.3050	0.4882	-0.74	0.8368	0.8595	-0.07	0.5751	0.6547	0.13	0.0325	0.1160	0.72	0.3736	0.5784	-2.55	0.8708	1.1747	-0.33	0.0651	0.1804	2.06
2679	Multidrug resistance protein 1	P08183	0.7357	0.7851	-0.02	0.2299	0.6609	0.15	0.8769	0.9025	-0.01	0.0919	0.1820	0.06	0.1088	0.2677	0.13	0.2168	0.3982	0.02	0.3736	0.4989	-2.55	0.8708	1.0315	-0.33	0.0651	0.1404	2.06
2680	Multifunctional methyltransferase subunit TRM112-like protein	Q9UI30	0.9168	0.9365	0.01	0.7192	0.7942	-0.03	0.3155	0.5022	0.07	0.9939	0.9945	0.00	0.5643	0.6461	0.04	0.7697	1.0073	-0.02	0.1718	0.3691	0.06	0.1715	0.4537	0.08	0.7993	0.8363	0.02
2681	Multifunctional procollagen lysine hydroxylase and glycosyltransferase LH3	O60568	0.0073	0.0503	0.16	0.0852	0.6207	0.24	0.0060	0.0376	0.24	0.0213	0.0631	0.14	0.0805	0.2188	0.07	0.0065	0.0625	0.19	0.0586	0.1929	0.37	0.1808	0.4680	0.09	0.0464	0.1884	0.10
2682	Multifunctional protein ADE2	P22234	0.0145	0.0699	0.25	0.0616	0.5920	0.18	0.0068	0.0398	0.30	0.0102	0.0405	0.26	0.1052	0.2609	0.11	0.0384	0.1274	0.19	0.2668	0.5092	0.17	0.6158	1.0500	-0.05	0.1236	0.1829	-0.14
2683	Multivesicular body subunit 12A	Q96EY5	0.0177	0.0780	-0.67	0.2787	0.6650	-0.48	0.0008	0.0206	-0.77	0.0004	0.0126	-1.39	0.0593	0.1836	-0.33	0.0521	0.1541	-1.25	0.3736	0.5593	-2.55	0.8708	1.1408	-0.33	0.0651	0.1701	2.06
2684	Muskelin	Q9UL63	0.8384	0.8707	-0.02	0.4328	0.7688	-0.31	0.7164	0.7617	0.03	0.5255	0.7315	0.06	0.1069	0.2643	0.21	0.0568	0.1635	-0.25	0.3736	0.5572	-2.55	0.8708	1.1371	-0.33	0.0651	0.1689	2.06
2685	MutS protein homolog 4	O15457	0.4108	0.5507	-3.31	0.5752	0.7709	-1.75	0.5661	0.6962	-1.19	0.5388	0.6584	0.89	0.2938	0.4477	-1.88	0.9510	1.0598	0.09	0.1345	0.3126	0.19	0.0287	0.1528	-0.44	0.0007	0.0324	-0.61
2686	Myb/SANT-like DNA-binding domain-containing protein 4	Q8NCY6	0.0827	0.1981	-0.04	0.9152	0.9414	0.00	0.4223	0.6308	-0.03	0.1206	0.2237	-0.07	0.0173	0.0945	-0.12	0.7454	0.9840	-0.01	0.9468	0.9560	0.00	0.0063	0.0641	-0.36	0.0069	0.0770	-0.36
2687	Myb-binding protein 1A	Q9BQG0	0.0123	0.0643	0.34	0.0323	0.5180	0.35	0.0060	0.0376	0.30	0.0002	0.0097	0.43	0.0000	0.0000	0.50	0.0051	0.0623	0.22	0.0331	0.1521	0.23	0.1193	0.3570	-0.02	0.2645	0.3365	0.08
2688	Myb-related protein A	P10243	0.0111	0.0605	-0.36	0.0868	0.6210	0.15	0.3759	0.5751	-0.15	0.2798	0.4376	0.08	0.1236	0.2898	-0.15	0.2010	0.3782	-0.10	0.3736	0.5023	-2.55	0.8708	1.0377	-0.33	0.0651	0.1419	2.06
2689	Myc box-dependent-interacting protein 1	O00499	0.4108	0.5506	-3.31	0.5752	0.7707	-1.75	0.5661	0.6960	-1.19	0.5388	0.6583	0.89	0.2938	0.4476	-1.88	0.9510	1.0596	0.09	0.1484	0.3334	0.10	0.0155	0.1051	-0.35	0.0131	0.0992	-0.28
2690	Mycophenolic acid acyl-glucuronide esterase_ mitochondrial	Q9NUJ1	0.2709	0.4636	-0.08	0.0001	0.1727	-0.22	0.2996	0.4817	-0.11	0.0083	0.0369	-0.34	0.0192	0.0992	-0.23	0.0008	0.0441	-0.33	0.5975	0.6464	0.08	0.7306	1.1753	0.05	0.7568	0.7997	0.05
2691	Myelin protein zero-like protein 1	O95297	0.4108	0.5504	-3.31	0.5752	0.7705	-1.75	0.5661	0.6959	-1.19	0.5388	0.6581	0.89	0.2938	0.4475	-1.88	0.9510	1.0594	0.09	0.4822	0.5368	0.08	0.6438	1.0824	0.06	0.0053	0.0695	-0.44
2692	Myelin-oligodendrocyte glycoprotein	Q16653	0.0096	0.0575	0.19	0.1544	0.6604	-0.09	0.1269	0.2468	-0.20	0.1081	0.2057	-0.08	0.0233	0.1106	-0.14	0.0528	0.1552	-0.18	0.0949	0.2478	-0.29	0.0082	0.0745	-0.71	0.0072	0.0774	-1.13
2693	Myeloid leukemia factor 2	Q15773	0.0683	0.1738	-0.31	0.5188	0.8395	-0.13	0.2323	0.3925	-0.21	0.5321	0.7397	-0.08	0.2372	0.4499	-0.16	0.8313	1.0653	-0.03	0.2859	0.5368	-0.13	0.0743	0.2642	-0.21	0.8182	0.8531	0.02
2694	Myeloid-derived growth factor	Q969H8	0.0018	0.0290	0.24	0.2680	0.6639	0.21	0.0028	0.0291	0.25	0.0028	0.0221	0.38	0.0034	0.0435	0.19	0.0016	0.0521	0.45	0.0518	0.1803	0.24	0.0826	0.2830	0.12	0.1910	0.2588	0.14
2695	Myocardial zonula adherens protein	P0CAP1	0.4108	0.5503	-3.31	0.5752	0.7703	-1.75	0.5661	0.6957	-1.19	0.5388	0.6579	0.89	0.2938	0.4473	-1.88	0.9510	1.0592	0.09	0.8163	0.8445	0.04	0.3258	0.6800	0.20	0.4287	0.5002	-0.21
2696	Myocardin	Q8IZQ8	0.0152	0.0715	-3.90	0.3538	0.7054	-1.43	0.0122	0.0517	-1.89	0.1539	0.2705	-2.55	0.0918	0.2391	-1.11	0.0036	0.0594	-3.34	0.3736	0.6362	-2.55	0.8708	1.2757	-0.33	0.0651	0.2155	2.06
2697	Myocardin-related transcription factor B	Q9ULH7	0.1184	0.2532	-0.17	0.9215	0.9456	-0.01	0.4318	0.6424	-0.07	0.3265	0.4985	-0.10	0.0921	0.2394	-0.19	0.1825	0.3523	-0.14	0.3736	0.5061	-2.55	0.8708	1.0446	-0.33	0.0651	0.1437	2.06
2698	Myoferlin	Q9NZM1	0.0426	0.1307	0.07	0.1671	0.6607	0.16	0.0150	0.0576	0.15	0.0206	0.0618	0.19	0.0000	0.0000	0.22	0.0428	0.1367	0.22	0.0327	0.1515	0.34	0.0180	0.1145	0.26	0.0698	0.1175	0.10
2699	Myomegalin	Q5VU43	0.0167	0.0756	8.54	0.5752	0.8849	-1.75	0.5661	0.7893	-1.19	0.5388	0.7457	0.89	0.2938	0.5241	-1.88	0.0207	0.0930	6.07	0.3736	0.5929	-2.55	0.8708	1.2003	-0.33	0.0651	0.1887	2.06
2700	Myomesin-1	P52179	0.7418	0.7905	-0.04	0.2583	0.6630	0.17	0.1128	0.2248	0.21	0.0517	0.1177	0.34	0.9618	0.9704	0.00	0.7283	0.9681	0.04	0.0352	0.1550	0.38	0.9678	0.9772	0.00	0.1224	0.1814	0.18
2701	Myosin light chain 1/3 skeletal muscle isoform	P05976	0.0025	0.0335	0.25	0.1277	0.6410	0.07	0.0070	0.0400	0.18	0.5816	0.6305	0.04	0.6974	0.7591	-0.02	0.3319	0.5351	0.08	0.3736	0.4763	-2.55	0.8708	0.9898	-0.33	0.0651	0.1304	2.06
2702	Myosin light chain 3	P08590	0.6789	0.7359	0.04	0.4127	0.7522	-0.20	0.0720	0.1603	0.23	0.0552	0.1241	0.27	0.8652	0.8965	0.02	0.0377	0.1257	0.34	0.7519	0.7886	0.09	0.3358	0.6933	0.24	0.1293	0.1889	0.45
2703	Myosin light chain 4	P12829	0.0069	0.0496	0.22	0.7605	0.8269	0.02	0.0252	0.0788	0.15	0.0169	0.0543	-0.17	0.0469	0.1594	0.18	0.0780	0.1997	-0.22	0.3736	0.5448	-2.55	0.8708	1.1149	-0.33	0.0651	0.1625	2.06
2704	Myosin light chain kinase smooth muscle	Q15746	0.9933	0.9945	0.00	0.0551	0.5849	-0.10	0.2560	0.4242	0.05	0.0444	0.1056	-0.10	0.7151	0.7756	-0.01	0.6220	0.8578	0.02	0.0021	0.0544	0.30	0.0104	0.0842	0.19	0.0003	0.0251	0.53
2705	Myosin light polypeptide 6	P60660	0.6982	0.7522	-0.03	0.6878	0.7717	0.08	0.1776	0.3207	0.11	0.0813	0.1661	0.16	0.4790	0.5691	-0.05	0.2183	0.3999	0.10	0.0842	0.2319	0.34	0.2082	0.5102	0.15	0.1062	0.1625	-0.19
2706	Myosin regulatory light chain 10	Q9BUA6	0.0024	0.0334	-0.21	0.4513	0.7853	0.07	0.1574	0.2906	0.06	0.0078	0.0357	0.12	0.0027	0.0396	0.15	0.1938	0.3679	0.17	0.3736	0.5033	-2.55	0.8708	1.0396	-0.33	0.0651	0.1424	2.06
2707	Myosin regulatory light chain 12B	O14950	0.1061	0.2342	-0.04	0.7260	0.8000	0.03	0.5785	0.6360	-0.03	0.0330	0.0859	0.04	0.0222	0.1076	-0.08	0.0841	0.2100	0.10	0.0018	0.0530	0.76	0.0003	0.0204	0.73	0.0036	0.0598	0.34
2708	Myosin regulatory light chain 2_ ventricular/cardiac muscle isoform	P10916	0.1388	0.2833	-0.22	0.0812	0.6140	-0.15	0.3778	0.5769	-0.05	0.0066	0.0326	-0.30	0.0571	0.1788	-0.16	0.0067	0.0633	-0.35	0.3736	0.6229	-2.55	0.8708	1.2526	-0.33	0.0651	0.2069	2.06
2709	Myosin regulatory light polypeptide 9	P24844	0.4108	0.5501	-3.31	0.5752	0.7701	-1.75	0.5661	0.6955	-1.19	0.5388	0.6578	0.89	0.2938	0.4472	-1.88	0.9510	1.0589	0.09	0.6537	0.6982	-0.04	0.9719	0.9800	0.00	0.4122	0.4838	-0.08
2710	Myosin-1	P12882	0.4108	0.5500	-3.31	0.5752	0.7699	-1.75	0.5661	0.6954	-1.19	0.5388	0.6576	0.89	0.2938	0.4471	-1.88	0.9510	1.0587	0.09	0.0157	0.1165	0.35	0.0359	0.1725	0.17	0.2964	0.3680	-0.06
2711	Myosin-10	P35580	0.0290	0.1040	0.26	0.3164	0.6806	0.25	0.0188	0.0656	0.34	0.0084	0.0370	0.37	0.2547	0.4742	0.09	0.0265	0.1043	0.33	0.0066	0.0844	0.51	0.0066	0.0663	0.28	0.0051	0.0692	0.29
2712	Myosin-11	P35749	0.0032	0.0370	0.12	0.1644	0.6627	0.19	0.0315	0.0907	0.19	0.0000	0.0000	0.22	0.0062	0.0575	0.19	0.0064	0.0628	0.25	0.0671	0.2036	0.08	0.6710	1.1091	-0.01	0.1499	0.2126	0.04
2713	Myosin-13	Q9UKX3	0.3542	0.5644	0.06	0.3503	0.7041	0.10	0.3165	0.5034	0.07	0.6748	0.7158	-0.04	0.2695	0.4914	0.08	0.5710	0.8057	-0.03	0.0634	0.1984	0.30	0.0017	0.0336	0.39	0.0136	0.1012	0.20
2714	Myosin-14	Q7Z406	0.0001	0.0108	0.23	0.0653	0.5934	0.20	0.0011	0.0225	0.31	0.0002	0.0114	0.23	0.0002	0.0150	0.18	0.1230	0.2696	0.25	0.0330	0.1519	0.40	0.0840	0.2859	0.15	0.0856	0.1379	0.14
2715	Myosin-15	Q9Y2K3	0.4108	0.5499	-3.31	0.5752	0.7697	-1.75	0.5661	0.6952	-1.19	0.5388	0.6575	0.89	0.2938	0.4470	-1.88	0.9510	1.0585	0.09	0.0664	0.2026	0.25	0.3276	0.6829	-0.07	0.3621	0.4335	0.09
2716	Myosin-3	P11055	0.4108	0.5497	-3.31	0.5752	0.7695	-1.75	0.5661	0.6950	-1.19	0.5388	0.6573	0.89	0.2938	0.4468	-1.88	0.9510	1.0583	0.09	0.0005	0.0365	-0.44	0.0007	0.0259	-0.26	0.0229	0.1282	-0.67
2717	Myosin-4	Q9Y623	0.4108	0.5496	-3.31	0.5752	0.7693	-1.75	0.5661	0.6949	-1.19	0.5388	0.6572	0.89	0.2938	0.4467	-1.88	0.9510	1.0580	0.09	0.1817	0.3824	-0.19	0.1583	0.4300	-0.21	0.0652	0.1115	-0.32
2718	Myosin-6	P13533	0.4108	0.5494	-3.31	0.5752	0.7691	-1.75	0.5661	0.6947	-1.19	0.5388	0.6570	0.89	0.2938	0.4466	-1.88	0.9510	1.0578	0.09	0.0652	0.2009	0.10	0.0004	0.0201	0.18	0.0455	0.1868	0.39
2719	Myosin-7B	A7E2Y1	0.0564	0.1555	0.16	0.3840	0.7305	0.12	0.1856	0.3308	0.09	0.1489	0.2636	0.11	0.1838	0.3774	-0.09	0.0879	0.2158	0.15	0.0190	0.1278	0.63	0.0009	0.0262	0.33	0.0003	0.0278	0.63
2720	Myosin-8	P13535	0.4108	0.5493	-3.31	0.5752	0.7689	-1.75	0.5661	0.6946	-1.19	0.5388	0.6569	0.89	0.2938	0.4464	-1.88	0.9510	1.0576	0.09	0.0422	0.1674	0.41	0.3902	0.7718	0.09	0.6607	0.7152	0.04
2721	Myosin-9	P35579	0.0385	0.1233	0.16	0.1940	0.6682	0.23	0.0054	0.0359	0.30	0.0069	0.0337	0.28	0.1331	0.3020	0.09	0.0296	0.1103	0.23	0.0105	0.1022	0.75	0.0138	0.0979	0.48	0.1523	0.2154	0.18
2722	Myotilin	Q9UBF9	0.0279	0.1014	-0.43	0.1607	0.6638	-0.18	0.0006	0.0197	-0.60	0.0060	0.0312	-0.38	0.0006	0.0197	-0.62	0.0037	0.0597	-0.43	0.0055	0.0794	-0.90	0.0001	0.0123	-1.42	0.0004	0.0288	-1.34

Supplemetary Table S2. Overview on all relatively quantified 5180 proteins statistical analysis

			MCF-7																		MDA-MB-231									
			Dai_SC20 vs control			Gen_SC20 vs control			SSE_SC20 vs control			Dai_IC20 vs control			Gen_IC20 vs control			SSE_IC20 vs control			Dai_IC20 vs control			Gen_IC20 vs control			SSE_IC20 vs control		
	**Protein name**	**UniProt**	**p value**	**BH q value**	**log2FC**	**p value**	**BH q value**	**log2FC**	**p value**	**BH q value**	**log2FC**	**p value**	**BH q value**	**log2FC**	**p value**	**BH q value**	**log2FC**	**p value**	**BH q value**	**log2FC**	**p value**	**BH q value**	**log2FC**	**p value**	**BH q value**	**log2FC**	**p value**	**BH q value**	**log2FC**
2723	Myotrophin	P58546	0.0037	0.0388	0.21	0.1056	0.6309	0.27	0.0370	0.1009	0.23	0.0006	0.0123	0.29	0.0053	0.0545	0.20	0.0040	0.0604	0.23	0.0969	0.2502	0.16	0.6635	1.1019	0.04	0.1283	0.1878	-0.10
2724	Myotubularin-related protein 14	Q8NCE2	0.4108	0.5491	-3.31	0.5752	0.7687	-1.75	0.5661	0.6944	-1.19	0.5388	0.6567	0.89	0.2938	0.4463	-1.88	0.9510	1.0573	0.09	0.8114	0.8404	0.03	0.1028	0.3233	-0.15	0.0066	0.0755	0.28
2725	Myotubularin-related protein 4	Q9NYA4	0.0070	0.0495	-0.29	0.3361	0.6950	-0.06	0.6368	0.6898	0.03	0.3343	0.5083	-0.05	0.0635	0.1906	-0.14	0.0485	0.1472	-0.15	0.0955	0.2491	0.17	0.7296	1.1744	0.05	0.7174	0.7668	0.03
2726	Myotubularin-related protein 5	O95248	0.3341	0.5407	0.06	0.1384	0.6506	-0.15	0.5225	0.7438	0.05	0.4096	0.6012	-0.08	0.2124	0.4161	-0.08	0.8829	1.1201	0.01	0.2659	0.5081	-0.04	0.3199	0.6706	-0.03	0.1470	0.2094	-0.05
2727	Myotubularin-related protein 7	Q9Y216	0.1811	0.3449	-0.24	0.0643	0.5916	-0.44	0.0235	0.0754	-0.60	0.0088	0.0380	-0.79	0.0310	0.1289	-0.62	0.1338	0.2858	-0.29	0.3736	0.5226	-2.55	0.8708	1.0748	-0.33	0.0651	0.1515	2.06
2728	Myotubularin-related protein 8	Q96EF0	0.0003	0.0152	4.14	0.7974	0.8529	0.19	0.0326	0.0928	1.08	0.1416	0.2534	2.74	0.4422	0.5366	-0.73	0.0028	0.0543	4.48	0.3736	0.6398	-2.55	0.8708	1.2818	-0.33	0.0651	0.2178	2.06
2729	Myristoylated alanine-rich C-kinase substrate	P29966	0.4108	0.5490	-3.31	0.5752	0.7685	-1.75	0.5661	0.6942	-1.19	0.5388	0.6565	0.89	0.2938	0.4462	-1.88	0.9510	1.0571	0.09	0.1944	0.4026	0.19	0.0389	0.1793	-0.21	0.2546	0.3260	-0.21
2730	N(G)_N(G)-dimethylarginine dimethylaminohydrolase 1	O94760	0.4108	0.5489	-3.31	0.5752	0.7683	-1.75	0.5661	0.6941	-1.19	0.5388	0.6564	0.89	0.2938	0.4460	-1.88	0.9510	1.0569	0.09	0.0546	0.1857	-0.17	0.0019	0.0358	-0.32	0.0050	0.0685	0.29
2731	N(G)_N(G)-dimethylarginine dimethylaminohydrolase 2	O95865	0.0196	0.0821	-0.18	0.1697	0.6624	0.19	0.0336	0.0950	-0.20	0.0218	0.0641	-0.20	0.1475	0.3236	-0.08	0.0101	0.0701	-0.33	0.1854	0.3866	-0.11	0.0024	0.0405	-0.39	0.0134	0.1007	-0.25
2732	N6-adenosine-methyltransferase catalytic subunit	Q86U44	0.5072	0.5802	0.06	0.9780	0.9856	0.00	0.0914	0.1904	0.24	0.1638	0.2835	0.16	0.2019	0.4024	0.14	0.1840	0.3548	0.17	0.7521	0.7886	-0.03	0.1713	0.4534	-0.10	0.0729	0.1220	-0.15
2733	N6-adenosine-methyltransferase non-catalytic subunit	Q9HCE5	0.0138	0.0689	9.31	0.5752	0.8852	-1.75	0.2310	0.3905	4.37	0.0138	0.0477	8.96	0.2938	0.5259	-1.88	0.0280	0.1070	7.86	0.3736	0.5820	-2.55	0.8708	1.1811	-0.33	0.0651	0.1825	2.06
2734	Na(+)/H(+) exchange regulatory cofactor NHE-RF1	O14745	0.0274	0.1002	0.23	0.2977	0.6725	0.25	0.0109	0.0483	0.32	0.0204	0.0613	0.25	0.4019	0.4982	0.05	0.0560	0.1616	0.21	0.3004	0.5575	0.18	0.9733	0.9807	0.00	0.2909	0.3628	0.06
2735	NAC-alpha domain-containing protein 1	O15069	0.0064	0.0480	-0.31	0.1548	0.6611	-0.31	0.0701	0.1574	-0.14	0.0174	0.0553	-0.40	0.0001	0.0089	-0.72	0.0126	0.0759	-0.34	0.3736	0.6072	-2.55	0.8708	1.2254	-0.33	0.0651	0.1972	2.06
2736	N-acetyl-D-glucosamine kinase	Q9UJ70	0.4848	0.5593	0.04	0.3019	0.6749	0.07	0.9651	0.9717	0.00	0.7814	0.8120	0.03	0.2350	0.4467	-0.08	0.0948	0.2270	0.14	0.0682	0.2056	-0.22	0.0545	0.2230	-0.24	0.1776	0.2438	-0.12
2737	N-acetylglucosamine-6-sulfatase	P15586	0.0877	0.2063	0.12	0.0076	0.4017	0.29	0.4594	0.6753	0.05	0.0083	0.0368	0.27	0.0140	0.0842	0.28	0.1708	0.3364	0.21	0.0250	0.1394	-0.34	0.0089	0.0776	-0.27	0.0817	0.1330	-0.13
2738	NACHT domain- and WD repeat-containing protein 1	Q149M9	0.6836	0.7400	0.01	0.1326	0.6480	0.09	0.0339	0.0955	0.16	0.8489	0.8699	0.01	0.2673	0.4889	-0.04	0.7447	0.9833	0.01	0.0165	0.1197	0.24	0.7948	1.2378	0.01	0.0612	0.2214	0.13
2739	NACHT_ LRR and PYD domains-containing protein 13	Q86W25	0.4108	0.5487	-3.31	0.5752	0.7681	-1.75	0.5661	0.6939	-1.19	0.5388	0.6562	0.89	0.2938	0.4459	-1.88	0.9510	1.0567	0.09	0.0876	0.2378	0.43	0.0214	0.1265	0.81	0.0243	0.1321	0.65
2740	NACHT_ LRR and PYD domains-containing protein 2	Q9NX02	0.1785	0.3413	0.10	0.1167	0.6357	0.24	0.0272	0.0827	0.21	0.0255	0.0716	0.21	0.0368	0.1395	0.20	0.0404	0.1320	0.22	0.3736	0.5699	-2.55	0.8708	1.1596	-0.33	0.0651	0.1757	2.06
2741	NACHT_ LRR and PYD domains-containing protein 3	Q96P20	0.4108	0.5486	-3.31	0.5752	0.7679	-1.75	0.5661	0.6937	-1.19	0.5388	0.6561	0.89	0.2938	0.4458	-1.88	0.9510	1.0564	0.09	0.0016	0.0512	0.24	0.9377	0.9547	-0.01	0.0496	0.1943	-0.33
2742	NACHT_ LRR and PYD domains-containing protein 5	P59047	0.2225	0.4001	-0.06	0.1624	0.6613	0.28	0.0010	0.0214	0.38	0.0267	0.0737	0.16	0.0037	0.0453	0.26	0.0057	0.0619	0.40	0.4441	0.4992	0.09	0.0561	0.2265	0.22	0.2270	0.2974	0.14
2743	NACHT_ LRR and PYD domains-containing protein 7	Q8WX94	0.0564	0.1554	0.14	0.0027	0.2543	0.40	0.0294	0.0872	0.18	0.0017	0.0182	0.50	0.0041	0.0477	0.33	0.0019	0.0529	0.51	0.3736	0.6451	-2.55	0.8708	1.2910	-0.33	0.0651	0.2214	2.06
2744	NACHT_ LRR and PYD domains-containing protein 9	Q7RTR0	0.0143	0.0696	0.16	0.2682	0.6635	0.09	0.0704	0.1578	0.11	0.7396	0.7755	0.01	0.0191	0.0988	0.17	0.7648	1.0030	-0.01	0.0680	0.2051	0.24	0.9153	0.9381	-0.01	0.1672	0.2324	0.15
2745	N-acylneuraminate cytidylyltransferase	Q8NFW8	0.1758	0.3379	-0.08	0.0262	0.4935	0.15	0.0267	0.0819	0.13	0.0797	0.1637	0.15	0.0009	0.0225	0.18	0.0177	0.0872	0.11	0.0615	0.1956	-3.00	0.0932	0.3040	-2.62	0.1452	0.2073	-2.02
2746	N-acylneuraminate-9-phosphatase	Q8TBE9	0.2555	0.4431	-0.08	0.8871	0.9196	0.01	0.0069	0.0396	-0.26	0.4128	0.6051	-0.04	0.7728	0.8227	0.01	0.5687	0.8036	-0.03	0.3736	0.4548	-2.55	0.8708	0.9498	-0.33	0.0651	0.1214	2.06
2747	NAD kinase 2_ mitochondrial	Q4G0N4	0.0565	0.1556	0.17	0.0181	0.4665	0.23	0.0543	0.1325	0.17	0.0064	0.0320	0.18	0.0163	0.0913	0.14	0.0935	0.2247	0.10	0.3736	0.5365	-2.55	0.8708	1.0999	-0.33	0.0651	0.1583	2.06
2748	NAD(P) transhydrogenase_ mitochondrial	Q13423	0.4108	0.5484	-3.31	0.5752	0.7677	-1.75	0.5661	0.6936	-1.19	0.5388	0.6559	0.89	0.2938	0.4456	-1.88	0.9510	1.0562	0.09	0.3701	0.6525	-0.09	0.0238	0.1364	-0.18	0.0105	0.0903	-0.32
2749	NAD(P)H dehydrogenase [quinone] 1	P15559	0.0705	0.1777	0.12	0.7640	0.8295	-0.05	0.0331	0.0940	0.17	0.0002	0.0087	0.62	0.0705	0.2023	0.12	0.0004	0.0364	1.03	0.0387	0.1632	0.45	0.2899	0.6294	0.10	0.0805	0.1314	0.13
2750	NAD(P)H-hydrate epimerase	Q8NCW5	0.0399	0.1259	-0.21	0.3766	0.7241	-0.28	0.0034	0.0308	-0.48	0.0166	0.0537	-0.47	0.2922	0.5241	-0.11	0.7418	0.9807	-0.02	0.2650	0.5071	-0.96	0.1011	0.3201	0.94	0.3005	0.3718	0.49
2751	NAD-dependent malic enzyme_ mitochondrial	P23368	0.6871	0.7429	0.03	0.5725	0.8847	0.05	0.3431	0.5364	-0.10	0.0575	0.1273	-0.20	0.0962	0.2464	0.15	0.6546	0.8919	-0.04	0.0526	0.1814	0.11	0.7462	1.1879	0.01	0.0857	0.1380	-0.09
2752	NAD-dependent protein deacetylase sirtuin-2	Q8IXJ6	0.4108	0.5483	-3.31	0.5752	0.7675	-1.75	0.5661	0.6934	-1.19	0.5388	0.6558	0.89	0.2938	0.4455	-1.88	0.9510	1.0560	0.09	0.2137	0.4327	-2.37	0.8365	1.2805	0.16	0.7303	0.7777	0.27
2753	NAD-dependent protein deacylase sirtuin-5_ mitochondrial	Q9NXA8	0.3071	0.5077	-0.06	0.0553	0.5834	-0.18	0.0891	0.1875	0.12	0.7279	0.7653	0.02	0.2108	0.4146	0.08	0.3220	0.5252	0.06	0.3736	0.4782	-2.55	0.8708	0.9933	-0.33	0.0651	0.1312	2.06
2754	NADH dehydrogenase [ubiquinone] 1 alpha subcomplex assembly factor 2	Q8N183	0.1852	0.3499	0.04	0.2014	0.6607	0.17	0.0119	0.0510	0.13	0.0014	0.0170	0.17	0.0569	0.1784	0.05	0.5832	0.8182	0.06	0.0333	0.1524	0.46	0.0639	0.2430	0.39	0.0450	0.1856	0.47
2755	NADH dehydrogenase [ubiquinone] 1 alpha subcomplex assembly factor 4	Q9P032	0.2454	0.4311	0.09	0.2935	0.6697	0.17	0.0565	0.1359	0.17	0.0310	0.0820	0.22	0.8760	0.9054	0.01	0.0245	0.0999	0.25	0.0081	0.0912	-0.15	0.8577	1.2991	-0.01	0.0868	0.1394	-0.46
2756	NADH dehydrogenase [ubiquinone] 1 alpha subcomplex subunit 10_ mitochondrial	O95299	0.7913	0.8308	0.01	0.1215	0.6390	0.19	0.0332	0.0942	0.08	0.0375	0.0938	0.10	0.0571	0.1787	0.03	0.1719	0.3382	0.28	0.3736	0.5093	-2.55	0.8708	1.0505	-0.33	0.0651	0.1452	2.06
2757	NADH dehydrogenase [ubiquinone] 1 alpha subcomplex subunit 11	Q86Y39	0.5341	0.6045	-0.05	0.1036	0.6336	-0.21	0.1047	0.2117	-0.17	0.0828	0.1683	-0.20	0.0188	0.0978	-0.32	0.3951	0.6080	-0.07	0.3736	0.4696	-2.55	0.8708	0.9774	-0.33	0.0651	0.1276	2.06
2758	NADH dehydrogenase [ubiquinone] 1 alpha subcomplex subunit 12	Q9UI09	0.0942	0.2163	-0.09	0.5964	0.7005	0.07	0.9291	0.9452	0.00	0.0180	0.0565	0.17	0.1597	0.3427	0.07	0.1522	0.3097	0.08	0.3736	0.5154	-2.55	0.8708	1.0616	-0.33	0.0651	0.1480	2.06
2759	NADH dehydrogenase [ubiquinone] 1 alpha subcomplex subunit 13	Q9P0J0	0.0884	0.2072	0.10	0.6267	0.7237	0.06	0.1741	0.3159	0.09	0.2298	0.3730	0.06	0.7541	0.8074	0.01	0.1018	0.2363	0.10	0.3736	0.5333	-2.55	0.8708	1.0940	-0.33	0.0651	0.1567	2.06
2760	NADH dehydrogenase [ubiquinone] 1 alpha subcomplex subunit 5	Q16718	0.0002	0.0142	0.57	0.2932	0.6699	0.14	0.1685	0.3076	0.10	0.0031	0.0226	0.27	0.0194	0.0999	0.20	0.0023	0.0559	0.42	0.0225	0.1363	-0.35	0.0003	0.0181	-0.83	0.0005	0.0324	-0.92
2761	NADH dehydrogenase [ubiquinone] 1 alpha subcomplex subunit 6	P56556	0.0952	0.2175	0.07	0.8770	0.9113	0.02	0.0547	0.1330	0.13	0.0297	0.0798	0.19	0.0060	0.0565	0.17	0.0308	0.1127	0.12	0.3736	0.5806	-2.55	0.8708	1.1787	-0.33	0.0651	0.1817	2.06
2762	NADH dehydrogenase [ubiquinone] 1 alpha subcomplex subunit 7	O95182	0.9461	0.9594	-0.04	0.8429	0.8873	0.09	0.4555	0.6703	-0.37	0.8600	0.8795	-0.08	0.8199	0.8596	-0.12	0.5947	0.8312	0.25	0.9157	0.9315	-0.02	0.8555	1.2973	0.01	0.9917	0.9936	0.00
2763	NADH dehydrogenase [ubiquinone] 1 alpha subcomplex subunit 8	P51970	0.3990	0.6183	-0.04	0.0069	0.4156	-0.46	0.0300	0.0879	-0.18	0.0010	0.0151	-0.40	0.0110	0.0752	-0.29	0.2516	0.4418	0.05	0.1063	0.2665	0.23	0.0643	0.2440	-0.21	0.0058	0.0722	-0.58
2764	NADH dehydrogenase [ubiquinone] 1 alpha subcomplex subunit 9_ mitochondrial	Q16795	0.2756	0.4691	0.06	0.9231	0.9469	0.01	0.3280	0.5171	0.06	0.4140	0.6065	0.04	0.6085	0.6828	-0.03	0.1427	0.2975	0.16	0.5038	0.5571	-0.05	0.0880	0.2945	-0.15	0.8788	0.9020	0.01
2765	NADH dehydrogenase [ubiquinone] 1 beta subcomplex subunit 10	O96000	0.3739	0.5889	-0.01	0.3123	0.6806	0.12	0.0214	0.0707	0.12	0.0107	0.0415	0.10	0.6215	0.6938	0.01	0.1777	0.3471	0.13	0.0341	0.1539	-0.14	0.0097	0.0814	-0.29	0.0315	0.1508	-0.26
2766	NADH dehydrogenase [ubiquinone] 1 beta subcomplex subunit 11_ mitochondrial	Q9NX14	0.0387	0.1237	0.21	0.0378	0.5563	0.22	0.2135	0.3682	0.11	0.0107	0.0414	0.30	0.0629	0.1900	0.18	0.0094	0.0692	0.35	0.0254	0.1403	0.47	0.0082	0.0749	0.45	0.0267	0.1391	0.39
2767	NADH dehydrogenase [ubiquinone] 1 beta subcomplex subunit 3	O43676	0.7683	0.8119	-0.06	0.3315	0.6910	-0.55	0.3244	0.5123	-0.23	0.1727	0.2953	-0.37	0.2672	0.4891	0.25	0.3254	0.5277	-0.23	0.3736	0.4772	-2.55	0.8708	0.9916	-0.33	0.0651	0.1308	2.06
2768	NADH dehydrogenase [ubiquinone] 1 beta subcomplex subunit 4	O95168	0.2988	0.4977	0.05	0.7467	0.8165	0.04	0.2474	0.4134	0.06	0.0453	0.1073	0.14	0.1607	0.3438	-0.08	0.0767	0.1974	0.14	0.5798	0.6288	-0.05	0.0304	0.1575	-0.21	0.0080	0.0797	-0.40
2769	NADH dehydrogenase [ubiquinone] 1 beta subcomplex subunit 5_ mitochondrial	O43674	0.1807	0.3444	-0.24	0.2591	0.6641	-0.54	0.0881	0.1858	-0.39	0.2214	0.3609	-0.25	0.4075	0.5033	-0.15	0.3336	0.5368	-0.17	0.8174	0.8453	-0.05	0.0797	0.2771	0.32	0.0162	0.1091	0.68
2770	NADH dehydrogenase [ubiquinone] 1 beta subcomplex subunit 6	O95139	0.8968	0.9188	0.01	0.1796	0.6636	-0.16	0.0210	0.0702	0.13	0.8017	0.8296	0.01	0.2653	0.4870	0.05	0.1978	0.3734	0.25	0.2327	0.4602	-0.20	0.0611	0.2362	0.15	0.0016	0.0482	0.22
2771	NADH dehydrogenase [ubiquinone] 1 beta subcomplex subunit 7	P17568	0.0253	0.0960	-0.22	0.5384	0.8544	0.25	0.0260	0.0803	0.24	0.0575	0.1274	0.18	0.0730	0.2064	0.25	0.2803	0.4764	0.09	0.3736	0.4854	-2.55	0.8708	1.0066	-0.33	0.0651	0.1343	2.06
2772	NADH dehydrogenase [ubiquinone] 1 beta subcomplex subunit 8_ mitochondrial	O95169	0.9213	0.9398	0.01	0.5974	0.7012	-0.17	0.7013	0.7484	-0.05	0.4988	0.7023	-0.09	1.0000	1.0000	0.00	0.6152	0.8514	-0.08	0.3736	0.4510	-2.55	0.8708	0.9427	-0.33	0.0651	0.1199	2.06
2773	NADH dehydrogenase [ubiquinone] 1 beta subcomplex subunit 9	Q9Y6M9	0.2423	0.4276	-0.07	0.5160	0.8389	-0.07	0.3189	0.5061	0.05	0.2471	0.3957	0.06	0.6075	0.6820	0.03	0.1471	0.3031	0.10	0.0128	0.1076	-0.56	0.8312	1.2746	-0.01	0.6884	0.7410	-0.04
2774	NADH dehydrogenase [ubiquinone] 1 subunit C2	O95298	0.8991	0.9208	0.01	0.5182	0.8399	-0.13	0.2710	0.4441	0.12	0.0011	0.0152	-0.34	0.0081	0.0654	-0.23	0.0640	0.1764	0.44	0.3736	0.5526	-2.55	0.8708	1.1288	-0.33	0.0651	0.1665	2.06
2775	NADH dehydrogenase [ubiquinone] flavoprotein 1_ mitochondrial	P49821	0.1261	0.2649	0.07	0.3704	0.7186	-0.08	0.0075	0.0410	0.13	0.0391	0.0970	0.10	0.1458	0.3204	-0.05	0.2217	0.4039	0.08	0.7153	0.7559	-0.04	0.5871	1.0192	0.06	0.2761	0.3479	-0.11
2776	NADH dehydrogenase [ubiquinone] flavoprotein 2_ mitochondrial	P19404	0.2085	0.3812	-0.11	0.6163	0.7161	-0.08	0.0073	0.0403	0.20	0.1062	0.2031	0.13	0.1531	0.3321	-0.10	0.0518	0.1538	0.15	0.8311	0.8576	-0.02	0.4358	0.8364	0.07	0.3499	0.4214	0.08
2777	NADH dehydrogenase [ubiquinone] iron-sulfur protein 2_ mitochondrial	O75306	0.0319	0.1109	-0.04	0.2621	0.6633	0.06	0.0008	0.0207	0.22	0.0106	0.0414	0.08	0.0131	0.0816	0.05	0.2171	0.3986	0.03	0.0184	0.1261	0.51	0.0031	0.0459	0.42	0.0157	0.1077	0.25
2778	NADH dehydrogenase [ubiquinone] iron-sulfur protein 3_ mitochondrial	O75489	0.0175	0.0774	-0.31	0.0529	0.5806	-0.34	0.0018	0.0244	-0.48	0.0072	0.0345	-0.39	0.0976	0.2489	-0.19	0.3379	0.5422	-0.06	0.0782	0.2222	0.41	0.0433	0.1924	0.30	0.0491	0.1939	0.33

Supplemetary Table S2. Overview on all relatively quantified 5180 proteins statistical analysis

			MCF-7																		MDA-MB-231								
			Dai SC20 vs control			Gen SC20 vs control			SSE SC20 vs control			Dai IC20 vs control			Gen IC20 vs control			SSE IC20 vs control			Dai IC20 vs control			Gen IC20 vs control			SSE IC20 vs control		
	**Protein name**	**UniProt**	**p value**	**BH q value**	**log2FC**	**p value**	**BH q value**	**log2FC**	**p value**	**BH q value**	**log2FC**	**p value**	**BH q value**	**log2FC**	**p value**	**BH q value**	**log2FC**	**p value**	**BH q value**	**log2FC**	**p value**	**BH q value**	**log2FC**	**p value**	**BH q value**	**log2FC**	**p value**	**BH q value**	**log2FC**
2779	NADH dehydrogenase [ubiquinone] iron-sulfur protein 4_ mitochondrial	O43181	0.1568	0.3104	-0.15	0.5353	0.8508	-0.07	0.9534	0.9633	0.00	0.5774	0.6265	-0.03	0.0641	0.1919	-0.15	0.3138	0.5146	-0.06	0.3736	0.4794	-2.55	0.8708	0.9955	-0.33	0.0651	0.1317	2.06
2780	NADH dehydrogenase [ubiquinone] iron-sulfur protein 5	O43920	0.0635	0.1668	0.08	0.8572	0.8970	-0.01	0.4263	0.6362	0.03	0.8195	0.8441	0.01	0.7538	0.8073	0.01	0.0387	0.1280	0.07	0.3293	0.5975	0.07	0.0347	0.1691	0.24	0.5464	0.6112	-0.06
2781	NADH dehydrogenase [ubiquinone] iron-sulfur protein 6_ mitochondrial	O75380	0.0224	0.0891	0.19	0.6162	0.7162	0.06	0.9529	0.9631	0.00	0.0113	0.0425	0.19	0.6103	0.6843	0.02	0.2244	0.4071	0.21	0.3736	0.4969	-2.55	0.8708	1.0277	-0.33	0.0651	0.1395	2.06
2782	NADH dehydrogenase [ubiquinone] iron-sulfur protein 8_ mitochondrial	O00217	0.0618	0.1647	-0.22	0.6430	0.7367	-0.11	0.0055	0.0357	0.66	0.1235	0.2272	0.18	0.0439	0.1550	0.26	0.0943	0.2261	0.31	0.0548	0.1860	0.43	0.2173	0.5238	0.18	0.0591	0.2176	0.39
2783	NADH-cytochrome b5 reductase 3	P00387	0.0110	0.0602	0.17	0.3066	0.6787	0.14	0.1465	0.2742	0.12	0.0101	0.0403	0.16	0.5810	0.6594	-0.01	0.0011	0.0456	0.30	0.0504	0.1789	0.09	0.0017	0.0337	0.21	0.0393	0.1711	0.17
2784	NADH-ubiquinone oxidoreductase 75 kDa subunit_ mitochondrial	P28331	0.0141	0.0694	0.19	0.2866	0.6672	0.22	0.0125	0.0523	0.27	0.0000	0.0000	0.29	0.0319	0.1307	0.03	0.0178	0.0871	0.25	0.0266	0.1422	0.41	0.2338	0.5492	0.07	0.0704	0.1184	0.10
2785	NADH-ubiquinone oxidoreductase chain 4	P03905	0.5248	0.5963	-0.05	0.3548	0.7063	-0.09	0.1338	0.2561	0.14	0.2457	0.3937	0.09	0.7301	0.7886	0.03	0.2383	0.4242	0.07	0.3736	0.4931	-2.55	0.8708	1.0208	-0.33	0.0651	0.1378	2.06
2786	NADP-dependent malic enzyme	P48163	0.0214	0.0871	0.13	0.7612	0.8271	-0.02	0.0028	0.0288	0.24	0.0020	0.0198	0.36	0.0082	0.0655	0.17	0.0002	0.0345	0.56	0.3702	0.6523	-0.13	0.3259	0.6799	-0.14	0.0759	0.1259	0.13
2787	NADP-dependent malic enzyme mitochondrial	Q16798	0.4557	0.5329	-1.46	0.3311	0.6905	-2.64	0.0001	0.0148	-5.97	0.7839	0.8144	-0.56	0.3405	0.4377	-0.67	0.0586	0.1670	0.89	0.3736	0.5563	-2.55	0.8708	1.1353	-0.33	0.0651	0.1684	2.06
2788	NADPH:adrenodoxin oxidoreductase mitochondrial	P22570	0.0099	0.0581	0.13	0.1637	0.6640	0.15	0.0468	0.1198	0.10	0.0073	0.0347	0.17	0.0001	0.0100	0.43	0.0019	0.0541	0.23	0.0651	0.2011	0.43	0.0090	0.0780	0.26	0.2343	0.3043	-0.27
2789	NADPH--cytochrome P450 reductase	P16435	0.0105	0.0592	0.32	0.2151	0.6620	0.24	0.0156	0.0589	0.34	0.0017	0.0182	0.38	0.1899	0.3862	0.07	0.0036	0.0588	0.42	0.0022	0.0561	0.64	0.0625	0.2393	0.23	0.0156	0.1076	0.21
2790	N-alpha-acetyltransferase 10	P41227	0.2723	0.4651	0.13	0.2135	0.6626	0.21	0.0652	0.1491	0.30	0.0825	0.1678	0.27	0.1223	0.2882	0.22	0.0986	0.2316	0.25	0.0178	0.1238	0.22	0.2788	0.6161	0.06	0.0291	0.1456	0.21
2791	N-alpha-acetyltransferase 11	Q9BSU3	0.0377	0.1218	-0.16	0.0245	0.4825	0.21	0.0429	0.1125	-0.14	0.1615	0.2808	-0.08	0.0550	0.1751	0.15	0.0100	0.0708	-0.27	0.3736	0.6136	-2.55	0.8708	1.2365	-0.33	0.0651	0.2011	2.06
2792	N-alpha-acetyltransferase 15 NatA auxiliary subunit	Q9BXJ9	0.0070	0.0494	0.33	0.3501	0.7043	0.20	0.0149	0.0576	0.27	0.0133	0.0469	0.25	0.9331	0.9492	0.00	0.0592	0.1680	0.17	0.0082	0.0915	1.23	0.0007	0.0245	1.14	0.0059	0.0733	0.56
2793	N-alpha-acetyltransferase 16 NatA auxiliary subunit	Q6N069	0.4780	0.5533	0.07	0.1918	0.6672	0.14	0.0157	0.0591	0.19	0.0265	0.0735	0.16	0.0617	0.1878	0.12	0.4362	0.6578	0.08	0.3736	0.4658	-2.55	0.8708	0.9703	-0.33	0.0651	0.1260	2.06
2794	N-alpha-acetyltransferase 20	P61599	0.6697	0.7282	0.03	0.6547	0.7452	-0.03	0.0439	0.1142	-0.26	0.0532	0.1205	-0.21	0.9029	0.9263	-0.01	0.0145	0.0799	-0.36	0.3736	0.6036	-2.55	0.8708	1.2191	-0.33	0.0651	0.1950	2.06
2795	N-alpha-acetyltransferase 35 NatC auxiliary subunit	Q5VZE5	0.0752	0.1866	-0.22	0.2442	0.6568	0.13	0.4189	0.6270	-0.06	0.1889	0.3172	0.09	0.0094	0.0690	-0.14	0.5811	0.8155	-0.05	0.3736	0.4535	-2.55	0.8708	0.9474	-0.33	0.0651	0.1209	2.06
2796	N-alpha-acetyltransferase 40	Q86UY6	0.0004	0.0166	2.43	0.6362	0.7322	0.33	0.2759	0.4503	0.70	0.0864	0.1740	1.39	0.5216	0.6088	0.26	0.2400	0.4258	1.87	0.3736	0.4922	-2.55	0.8708	1.0191	-0.33	0.0651	0.1374	2.06
2797	N-alpha-acetyltransferase 50	Q9GZZ1	0.1584	0.3129	0.09	0.6966	0.7765	0.04	0.0277	0.0837	0.20	0.0746	0.1554	0.13	0.2630	0.4845	-0.07	0.7943	1.0317	0.02	0.0454	0.1709	-0.22	0.1650	0.4424	-0.09	0.0491	0.1937	-0.21
2798	Nascent polypeptide-associated complex subunit alpha_ muscle-specific form	E9PAV3	0.1242	0.2621	0.10	0.2269	0.6603	0.17	0.0117	0.0507	0.24	0.0396	0.0978	0.17	0.9066	0.9292	0.01	0.0370	0.1244	0.19	0.1041	0.2625	0.27	0.0391	0.1797	0.28	0.3322	0.4034	0.06
2799	Natural cytotoxicity triggering receptor 2	O95944	0.4311	0.5087	0.06	0.4170	0.7550	0.15	0.1614	0.2966	0.15	0.0874	0.1755	0.17	0.8230	0.8618	-0.02	0.4514	0.6737	0.07	0.3736	0.4640	-2.55	0.8708	0.9669	-0.33	0.0651	0.1252	2.06
2800	Nck-associated protein 1	Q9Y2A7	0.0101	0.0586	0.90	0.5081	0.8300	0.10	0.0393	0.1052	0.07	0.0302	0.0807	0.81	0.5025	0.5918	-0.02	0.0741	0.1936	0.82	0.0906	0.2422	0.26	0.3389	0.6969	0.06	0.3169	0.3882	0.05
2801	Nck-associated protein 5-like	Q9HCH0	0.0238	0.0926	-0.19	0.0810	0.6152	-0.36	0.4723	0.6897	0.05	0.4592	0.6565	-0.05	0.0061	0.0566	-0.34	0.0384	0.1276	-0.18	0.3736	0.5710	-2.55	0.8708	1.1617	-0.33	0.0651	0.1764	2.06
2802	Nebulette	O76041	0.4215	0.4989	0.09	0.0741	0.6112	0.20	0.1182	0.2339	0.22	0.2211	0.3605	0.07	0.1763	0.3669	-0.08	0.1183	0.2627	0.25	0.3736	0.5283	-2.55	0.8708	1.0851	-0.33	0.0651	0.1543	2.06
2803	Nebulin	P20929	0.4108	0.5482	-3.31	0.5752	0.7673	-1.75	0.5661	0.6932	-1.19	0.5388	0.6556	0.89	0.2938	0.4454	-1.88	0.9510	1.0558	0.09	0.0515	0.1798	0.41	0.9794	0.9857	0.00	0.7176	0.7667	-0.03
2804	Nebulin-related-anchoring protein	Q86VF7	0.1282	0.2684	0.09	0.0236	0.4851	0.34	0.0571	0.1367	0.16	0.0077	0.0354	0.20	0.0020	0.0350	0.20	0.1478	0.3042	0.04	0.3736	0.5172	-2.55	0.8708	1.0649	-0.33	0.0651	0.1489	2.06
2805	NEDD4-binding protein 1	O75113	0.4108	0.5480	-3.31	0.5752	0.7671	-1.75	0.5661	0.6931	-1.19	0.5388	0.6555	0.89	0.2938	0.4453	-1.88	0.9510	1.0555	0.09	0.0034	0.0638	6.49	0.8708	1.3105	-0.33	0.0033	0.0570	9.58
2806	NEDD4-binding protein 2	Q86UW6	0.4682	0.5441	0.11	0.7693	0.8326	0.02	0.5471	0.7701	-0.04	0.4753	0.6753	-0.04	0.2029	0.4039	0.09	0.7324	0.9715	-0.03	0.3736	0.4432	-2.55	0.8708	0.9279	-0.33	0.0651	0.1167	2.06
2807	NEDD8	Q15843	0.0405	0.1269	-0.04	0.1054	0.6326	0.30	0.0866	0.1835	0.14	0.0079	0.0359	0.18	0.0009	0.0230	0.16	0.4909	0.7163	0.10	0.0051	0.0755	0.76	0.0018	0.0351	0.51	0.0001	0.0225	0.79
2808	NEDD8-activating enzyme E1 catalytic subunit	Q8TBC4	0.4108	0.5479	-3.31	0.5752	0.7669	-1.75	0.5661	0.6929	-1.19	0.5388	0.6553	0.89	0.2938	0.4451	-1.88	0.9510	1.0553	0.09	0.0030	0.0598	0.46	0.0071	0.0693	0.41	0.0037	0.0599	0.88
2809	NEDD8-activating enzyme E1 regulatory subunit	Q13564	0.9813	0.9872	0.04	0.0564	0.5831	-10.13	0.0238	0.0757	-9.58	0.2201	0.3590	-2.97	0.0561	0.1769	-0.55	0.1793	0.3488	-2.69	0.0195	0.1292	0.35	0.0003	0.0183	0.48	0.0153	0.1062	0.17
2810	NEDD8-conjugating enzyme Ubc12	P61081	0.0653	0.1690	0.12	0.0284	0.5055	0.18	0.0126	0.0526	0.26	0.0027	0.0218	0.30	0.1541	0.3337	-0.06	0.0093	0.0689	0.28	0.0083	0.0919	0.31	0.2372	0.5535	-0.07	0.7296	0.7772	-0.03
2811	NEDD8-conjugating enzyme UBE2F	Q969M7	0.0009	0.0218	0.47	0.0559	0.5838	0.30	0.0002	0.0225	0.72	0.0001	0.0140	0.67	0.0001	0.0136	0.57	0.0036	0.0602	0.73	0.3736	0.6364	-2.55	0.8708	1.2760	-0.33	0.0651	0.2156	2.06
2812	Negative elongation factor A	Q9H3P2	0.7749	0.8167	0.01	0.0670	0.5912	0.31	0.0068	0.0398	0.16	0.0005	0.0125	0.27	0.0051	0.0537	0.12	0.1343	0.2865	-0.04	0.0012	0.0457	0.46	0.0007	0.0255	0.43	0.0028	0.0558	0.56
2813	Negative elongation factor B	Q8WX92	0.0096	0.0574	-0.31	0.8955	0.9263	0.02	0.0001	0.0133	-0.87	0.0430	0.1034	-0.70	0.0002	0.0128	-0.59	0.1456	0.3012	-0.34	0.2885	0.5403	2.52	0.3199	0.6703	2.31	0.2969	0.3684	2.47
2814	Negative elongation factor C/D	Q8IXH7	0.0875	0.2059	-0.12	0.4077	0.7500	0.09	0.0513	0.1272	-0.17	0.7503	0.7845	0.04	0.0287	0.1231	-0.20	0.1201	0.2650	-0.28	0.0176	0.1230	0.61	0.0600	0.2342	0.36	0.9147	0.9312	0.01
2815	Negative elongation factor E	P18615	0.1404	0.2858	0.17	0.1910	0.6685	0.20	0.1899	0.3364	0.15	0.0393	0.0973	0.26	0.1351	0.3048	0.16	0.0528	0.1551	0.24	0.0165	0.1200	0.63	0.0001	0.0113	0.57	0.3398	0.4109	-0.03
2816	Nephrocystin-3	Q7Z494	0.0330	0.1134	-0.22	0.2557	0.6623	-0.08	0.0512	0.1271	-0.17	0.4209	0.6124	-0.07	0.9998	1.0000	0.00	0.0249	0.1003	-0.26	0.7662	0.8015	0.08	0.0165	0.1089	-0.54	0.0388	0.1703	0.38
2817	Nesprin-1	Q8NF91	0.0172	0.0767	0.15	0.1355	0.6505	0.18	0.0037	0.0319	0.26	0.0030	0.0226	0.32	0.0479	0.1608	0.13	0.0105	0.0705	0.28	0.0302	0.1477	0.33	0.0088	0.0774	0.15	0.0192	0.1201	0.18
2818	Nesprin-2	Q8WXH0	0.1345	0.2780	0.10	0.3652	0.7133	0.17	0.0186	0.0654	0.23	0.0654	0.1409	0.14	0.1493	0.3265	-0.10	0.0518	0.1539	0.19	0.0252	0.1395	0.54	0.0448	0.1967	0.25	0.1271	0.1867	0.14
2819	Nestin	P48681	0.0116	0.0620	0.16	0.2393	0.6615	0.17	0.0042	0.0322	0.24	0.0053	0.0294	0.27	0.2292	0.4389	0.05	0.0052	0.0611	0.31	0.0215	0.1342	0.33	0.3992	0.7848	-0.06	0.1544	0.2176	0.12
2820	Netrin receptor UNC5D	Q6UXZ4	0.3032	0.5029	-0.06	0.4040	0.7477	-0.08	0.0559	0.1351	-0.16	0.2424	0.3892	-0.07	0.1416	0.3147	-0.11	0.6470	0.8841	0.03	0.3736	0.4477	-2.55	0.8708	0.9364	-0.33	0.0651	0.1185	2.06
2821	Neudesin	Q9UMX5	0.4108	0.5477	-3.31	0.5752	0.7667	-1.75	0.5661	0.6927	-1.19	0.5388	0.6552	0.89	0.2938	0.4450	-1.88	0.9510	1.0551	0.09	0.0082	0.0910	0.19	0.0025	0.0415	0.27	0.0089	0.0847	0.25
2822	Neural retina-specific leucine zipper protein	P54845	0.8935	0.9165	0.00	0.5395	0.8549	0.08	0.1745	0.3163	0.05	0.0024	0.0207	-0.21	0.0331	0.1328	0.10	0.5790	0.8137	0.02	0.3736	0.4539	-2.55	0.8708	0.9480	-0.33	0.0651	0.1210	2.06
2823	Neuralized-like protein 4	Q96JN8	0.5385	0.6090	-0.09	0.4745	0.8017	0.07	0.8880	0.9112	-0.02	0.4350	0.6282	-0.09	0.9504	0.9619	0.01	0.5415	0.7734	-0.10	0.3736	0.4574	-2.55	0.8708	0.9547	-0.33	0.0651	0.1225	2.06
2824	Neuroblast differentiation-associated protein AHNAK	Q09666	0.0098	0.0579	0.16	0.1266	0.6429	0.31	0.0012	0.0229	0.29	0.0058	0.0307	0.19	0.2819	0.5090	0.04	0.0114	0.0730	0.18	0.0422	0.1678	0.44	0.1093	0.3370	0.22	0.3034	0.3746	0.10
2825	Neuroblastoma breakpoint family member 12	Q5TAG4	0.4567	0.5337	0.05	0.2923	0.6694	0.10	0.5157	0.7359	0.04	0.3667	0.5476	-0.06	0.5096	0.5987	-0.04	0.5613	0.7959	0.05	0.3736	0.4557	-2.55	0.8708	0.9514	-0.33	0.0651	0.1218	2.06
2826	Neuroblastoma breakpoint family member 6	Q5VWK0	0.3940	0.6120	0.04	0.2681	0.6638	0.18	0.0464	0.1193	0.20	0.0018	0.0185	0.37	0.0362	0.1383	0.16	0.0135	0.0774	0.24	0.3736	0.6059	-2.55	0.8708	1.2231	-0.33	0.0651	0.1964	2.06
2827	Neuroblastoma-amplified sequence	A2RRP1	0.0803	0.1949	-0.13	0.4624	0.7947	0.12	0.1537	0.2851	0.82	0.0902	0.1794	0.15	0.0627	0.1898	-0.14	0.0546	0.1587	0.40	0.3736	0.5580	-2.55	0.8708	1.1385	-0.33	0.0651	0.1694	2.06
2828	Neurocalcin-delta	P61601	0.0034	0.0381	0.22	0.5866	0.6923	-0.08	0.2132	0.3681	-0.05	0.0047	0.0275	-0.20	0.0094	0.0692	-0.28	0.3823	0.5918	0.03	0.0030	0.0602	-0.57	0.0388	0.1798	-0.54	0.4410	0.5114	-0.23
2829	Neuroendocrine convertase 1	P29120	0.0169	0.0760	6.28	0.1262	0.6434	3.09	0.5618	0.7863	0.85	0.0184	0.0573	5.81	0.5207	0.6083	-1.84	0.0255	0.1012	6.05	0.3736	0.5854	-2.55	0.8708	1.1870	-0.33	0.0651	0.1844	2.06
2830	Neuroendocrine convertase 2	P16519	0.4675	0.5438	0.03	0.0453	0.5614	-0.12	0.0970	0.1996	0.09	0.0027	0.0217	-0.28	0.0049	0.0528	-0.25	0.5270	0.7566	0.08	0.9572	0.9648	0.00	0.4794	0.8897	0.03	0.2245	0.2946	-0.07
2831	Neurofascin	O94856	0.8414	0.8734	0.01	0.1264	0.6432	0.35	0.0572	0.1368	0.29	0.0020	0.0195	0.33	0.0013	0.0279	0.15	0.1185	0.2630	0.19	0.3736	0.5282	-2.55	0.8708	1.0848	-0.33	0.0651	0.1542	2.06
2832	Neurofibromin	P21359	0.0014	0.0254	-0.22	0.7869	0.8467	0.04	0.0049	0.0340	0.36	0.5233	0.7289	0.03	0.8790	0.9074	-0.01	0.5313	0.7619	-0.06	0.3736	0.4583	-2.55	0.8708	0.9563	-0.33	0.0651	0.1228	2.06
2833	Neurofilament light polypeptide	P07196	0.4108	0.5476	-3.31	0.5752	0.7665	-1.75	0.5661	0.6926	-1.19	0.5388	0.6550	0.89	0.2938	0.4449	-1.88	0.9510	1.0549	0.09	0.0590	0.1928	-0.35	0.8822	0.9110	-0.01	0.3288	0.4000	-0.22
2834	Neurofilament medium polypeptide	P07197	0.4108	0.5475	-3.31	0.5752	0.7663	-1.75	0.5661	0.6924	-1.19	0.5388	0.6549	0.89	0.2938	0.4447	-1.88	0.9510	1.0546	0.09	0.9622	0.9686	0.01	0.4805	0.8908	0.09	0.0290	0.1453	0.49
2835	Neurogenic differentiation factor 4	Q9HD90	0.4108	0.5473	-3.31	0.5752	0.7661	-1.75	0.5661	0.6923	-1.19	0.5388	0.6547	0.89	0.2938	0.4446	-1.88	0.9510	1.0544	0.09	0.7640	0.7993	-0.35	0.6948	1.1371	0.31	0.0785	0.1290	1.84
2836	Neurogenic differentiation factor 6	Q96NK8	0.4108	0.5472	-3.31	0.5752	0.7659	-1.75	0.5661	0.6921	-1.19	0.5388	0.6545	0.89	0.2938	0.4445	-1.88	0.9510	1.0542	0.09	0.3235	0.5892	0.06	0.5908	1.0235	0.02	0.0316	0.1509	-0.18
2837	Neurogenic locus notch homolog protein 1	P46531	0.4108	0.5470	-3.31	0.5752	0.7658	-1.75	0.5661	0.6919	-1.19	0.5388	0.6544	0.89	0.2938	0.4443	-1.88	0.9510	1.0540	0.09	0.2387	0.4700	0.21	0.4422	0.8437	0.12	0.7036	0.7547	0.06
2838	Neurogenic locus notch homolog protein 2	Q04721	0.0154	0.0719	0.30	0.1436	0.6548	0.34	0.0019	0.0245	0.41	0.0014	0.0171	0.70	0.0130	0.0813	0.25	0.0010	0.0458	0.85	0.3736	0.6492	-2.55	0.8708	1.2981	-0.33	0.0651	0.2242	2.06
2839	Neurolysin mitochondrial	Q9BYT8	0.0011	0.0226	0.16	0.1781	0.6632	0.10	0.0000	0.0000	0.46	0.0236	0.0679	0.20	0.0024	0.0383	0.13	0.1536	0.3117	0.15	0.4580	0.5126	0.06	0.2596	0.5870	-0.07	0.0433	0.1818	-0.17
2840	Neuronal acetylcholine receptor subunit alpha-7	P36544	0.0075	0.0510	0.17	0.1596	0.6640	-0.15	0.0093	0.0451	-0.18	0.0015	0.0174	-0.25	0.0027	0.0400	-0.14	0.9623	0.9690	0.00	0.3736	0.4306	-2.55	0.8708	0.9043	-0.33	0.0651	0.1118	2.06
2841	Neuronal calcium sensor 1	P62166	0.4108	0.5469	-3.31	0.5752	0.7656	-1.75	0.5661	0.6918	-1.19	0.5388	0.6542	0.89	0.2938	0.4442	-1.88	0.9510	1.0537	0.09	0.6837	0.7262	0.02	0.0696	0.2546	0.07	0.2122	0.2809	-0.12
2842	Neuronal migration protein doublecortin	O43602	0.4108	0.5467	-3.31	0.5752	0.7654	-1.75	0.5661	0.6916	-1.19	0.5388	0.6541	0.89	0.2938	0.4441	-1.88	0.9510	1.0535	0.09	0.9237	0.9367	-0.01	0.0309	0.1591	-0.08	0.0171	0.1118	-0.10
2843	Neuronal pentraxin-2	P47972	0.4108	0.5466	-3.31	0.5752	0.7652	-1.75	0.5661	0.6914	-1.19	0.5388	0.6539	0.89	0.2938	0.4440	-1.88	0.9510	1.0533	0.09	0.0206	0.1327	-0.59	0.0621	0.2385	-0.45	0.0233	0.1296	-0.73
2844	Neuron-specific calcium-binding protein hippocalcin	P84074	0.0419	0.1299	0.14	0.0483	0.5738	-0.24	0.6250	0.6783	0.03	0.0033	0.0231	-0.37	0.0077	0.0637	-0.30	0.0154	0.0828	0.23	0.3736	0.6021	-2.55	0.8708	1.2165	-0.33	0.0651	0.1941	2.06
2845	Neuropilin and tolloid-like protein 2	Q8NC67	0.4108	0.5465	-3.31	0.5752	0.7650	-1.75	0.5661	0.6913	-1.19	0.5388	0.6538	0.89	0.2938	0.4438	-1.88	0.9510	1.0531	0.09	0.3838	0.4396	-0.13	0.5447	0.9696	0.13	0.2175	0.2866	-0.21
2846	Neutral alpha-glucosidase AB	Q14697	0.0073	0.0502	0.19	0.3505	0.7043	0.17	0.0038	0.0315	0.25	0.0012	0.0162	0.28	0.3450	0.4420	-0.03	0.0014	0.0504	0.38	0.0918	0.2432	0.34	0.8695	1.3120	-0.02	0.1329	0.1932	-0.21
2847	Neutral amino acid transporter A	P43007	0.0021	0.0316	0.93	0.2204	0.6599	0.20	0.0048	0.0337	0.82	0.0111	0.0424	1.08	0.2118	0.4154	0.17	0.0023	0.0549	1.38	0.3736	0.6427	-2.55	0.8708	1.2869	-0.33	0.0651	0.2198	2.06
2848	Neutral amino acid transporter B(0)	Q15758	0.0099	0.0580	0.27	0.2224	0.6579	0.26	0.0009	0.0208	0.42	0.0017	0.0185	0.40	0.5368	0.6218	0.03	0.0102	0.0699	0.29	0.0451	0.1702	0.54	0.6777	1.1169	0.02	0.0137	0.1012	0.15
2849	Neutral and basic amino acid transport protein rBAT	Q07837	0.4108	0.5463	-3.31	0.5752	0.7648	-1.75	0.5661	0.6911	-1.19	0.5388	0.6536	0.89	0.2938	0.4437	-1.88	0.9510	1.0528	0.09	0.0268	0.1419	-0.19	0.3132	0.6622	-0.12	0.3246	0.3961	-0.16
2850	Neutral ceramidase	Q9NR71	0.4108	0.5462	-3.31	0.5752	0.7646	-1.75	0.5661	0.6910	-1.19	0.5388	0.6535	0.89	0.2938	0.4436	-1.88	0.9510	1.0526	0.09	0.0334	0.1522	0.55	0.3072	0.6535	0.15	0.0289	0.1451	0.65
2851	Neutral cholesterol ester hydrolase 1	Q6PIU2	0.4108	0.5460	-3.31	0.5752	0.7644	-1.75	0.5661	0.6908	-1.19	0.5388	0.6533	0.89	0.2938	0.4434	-1.88	0.9510	1.0524	0.09	0.9040	0.9218	0.02	0.4501	0.8531	0.04	0.1008	0.1564	-0.11
2852	NFATC2-interacting protein	Q8NCF5	0.0205	0.0847	-2.00	0.0964	0.6353	-1.98	0.0203	0.0686	-1.77	0.0357	0.0907	-1.21	0.1006	0.2538	-0.58	0.0082	0.0666	-2.08	0.3736	0.6183	-2.55	0.8708	1.2447	-0.33	0.0651	0.2040	2.06
2853	NF-kappa-B essential modulator	Q9Y6K9	0.0004	0.0164	-0.97	0.0016	0.2181	-0.57	0.2050	0.3580	-0.11	0.0005	0.0118	-0.82	0.0455	0.1574	-0.20	0.2479	0.4363	0.08	0.3736	0.4907	-2.55	0.8708	1.0164	-0.33	0.0651	0.1367	2.06

Supplemetary Table S2. Overview on all relatively quantified 5180 proteins statistical analysis

			MCF-7																		MDA-MB-231								
			Dai SC20 vs control			Gen SC20 vs control			SSE SC20 vs control			Dai IC20 vs control			Gen IC20 vs control			SSE IC20 vs control			Dai IC20 vs control			Gen IC20 vs control			SSE IC20 vs control		
	**Protein name**	**UniProt**	**p value**	**BH q value**	**log2FC**	**p value**	**BH q value**	**log2FC**	**p value**	**BH q value**	**log2FC**	**p value**	**BH q value**	**log2FC**	**p value**	**BH q value**	**log2FC**	**p value**	**BH q value**	**log2FC**	**p value**	**BH q value**	**log2FC**	**p value**	**BH q value**	**log2FC**	**p value**	**BH q value**	**log2FC**
2854	NF-kappa-B inhibitor-like protein 1	Q9UBC1	0.4108	0.5459	-3.31	0.5752	0.7642	-1.75	0.5661	0.6906	-1.19	0.5388	0.6532	0.89	0.2938	0.4433	-1.88	0.9510	1.0522	0.09	0.0015	0.0501	0.74	0.0078	0.0725	0.51	0.5024	0.5695	0.17
2855	NF-kappa-B-activating protein	Q8N5F7	0.6476	0.7091	-0.07	0.0343	0.5288	-0.69	0.4130	0.6192	0.21	0.0625	0.1361	-0.31	0.1129	0.2740	-0.27	0.4888	0.7142	0.08	0.3736	0.4612	-2.55	0.8708	0.9618	-0.33	0.0651	0.1241	2.06
2856	NF-X1-type zinc finger protein NFXL1	Q6ZNB6	0.2007	0.3716	-0.06	0.2364	0.6634	-0.06	0.2333	0.3938	-0.03	0.0065	0.0323	-0.17	0.4129	0.5077	0.05	0.2146	0.3962	-0.08	0.3736	0.4997	-2.55	0.8708	1.0329	-0.33	0.0651	0.1407	2.06
2857	NFX1-type zinc finger-containing protein 1	Q9P2E3	0.8936	0.9164	-0.02	0.0759	0.6172	0.46	0.0294	0.0872	0.39	0.1027	0.1979	0.25	0.0668	0.1966	0.31	0.0601	0.1697	0.34	0.0207	0.1329	0.46	0.4487	0.8514	-0.11	0.2863	0.3582	0.06
2858	NGFI-A-binding protein 1	Q13506	0.0606	0.1630	-0.07	0.3395	0.6987	-0.12	0.1042	0.2108	0.07	0.0161	0.0526	-0.03	0.0205	0.1028	-0.10	0.0645	0.1772	0.18	0.0093	0.0963	-1.38	0.0001	0.0120	-2.09	0.0001	0.0273	-1.81
2859	NGFI-A-binding protein 2	Q15742	0.6286	0.6910	0.02	0.2798	0.6648	0.18	0.0349	0.0970	0.17	0.0220	0.0645	0.20	0.1145	0.2766	-0.09	0.7314	0.9712	0.01	0.3736	0.4435	-2.55	0.8708	0.9285	-0.33	0.0651	0.1168	2.06
2860	NHP2-like protein 1	P55769	0.0238	0.0925	-0.27	0.9736	0.9823	0.01	0.4251	0.6348	-0.05	0.3260	0.4980	-0.05	0.3178	0.4149	-0.10	0.0662	0.1803	-0.53	0.0419	0.1675	-0.10	0.5425	0.9683	-0.06	0.2214	0.2911	0.07
2861	Niban-like protein 1	Q96TA1	0.1354	0.2792	-0.07	0.2469	0.6592	0.08	0.0296	0.0873	0.12	0.2660	0.4209	0.04	0.8764	0.9056	-0.01	0.7433	0.9825	-0.01	0.0888	0.2396	0.15	0.3278	0.6830	-0.04	0.7805	0.8194	0.01
2862	Nibrin	O60934	0.4108	0.5458	-3.31	0.5752	0.7640	-1.75	0.5661	0.6905	-1.19	0.5388	0.6530	0.89	0.2938	0.4432	-1.88	0.9510	1.0519	0.09	0.0916	0.2430	0.22	0.2105	0.5131	-0.11	0.5715	0.6346	-0.07
2863	Nicalin	Q969V3	0.0103	0.0590	0.24	0.0958	0.6322	0.17	0.0050	0.0346	0.34	0.0019	0.0194	0.39	0.0306	0.1277	0.18	0.0078	0.0662	0.30	0.3763	0.4321	0.05	0.1379	0.3933	0.10	0.1059	0.1623	0.11
2864	Nicolin-1	Q9BSH3	0.6558	0.7167	0.21	0.4253	0.7618	-1.20	0.0580	0.1382	-1.81	0.1691	0.2905	-0.74	0.5034	0.5926	-0.31	0.3519	0.5573	-0.58	0.3736	0.4742	-2.55	0.8708	0.9860	-0.33	0.0651	0.1295	2.06
2865	Nicotinamide N-methyltransferase	P40261	0.4108	0.5456	-3.31	0.5752	0.7638	-1.75	0.5661	0.6903	-1.19	0.5388	0.6529	0.89	0.2938	0.4431	-1.88	0.9510	1.0517	0.09	0.0696	0.2077	0.36	0.3324	0.6893	0.10	0.0113	0.0926	0.51
2866	Nicotinamide phosphoribosyltransferase	P43490	0.0531	0.1497	0.11	0.2689	0.6633	0.10	0.0031	0.0301	0.19	0.0090	0.0383	0.20	0.5434	0.6279	0.02	0.0090	0.0687	0.22	0.1749	0.3736	-0.09	0.0375	0.1763	0.11	0.0372	0.1657	-0.12
2867	Nicotinate phosphoribosyltransferase	Q6XQN6	0.4108	0.5455	-3.31	0.5752	0.7636	-1.75	0.5661	0.6901	-1.19	0.5388	0.6527	0.89	0.2938	0.4429	-1.88	0.9510	1.0515	0.09	0.1049	0.2640	0.28	0.4038	0.7899	0.13	0.0120	0.0940	0.58
2868	Nik-related protein kinase	Q7Z2Y5	0.5438	0.6140	-0.04	0.6408	0.7352	-0.01	0.6905	0.7390	0.01	0.0298	0.0800	0.11	0.4317	0.5259	-0.03	0.2326	0.4166	-0.06	0.3736	0.4944	-2.55	0.8708	1.0233	-0.33	0.0651	0.1384	2.06
2869	Ninein	Q8N4C6	0.2682	0.4600	0.07	0.4024	0.7460	0.13	0.0305	0.0889	0.19	0.2352	0.3799	0.07	0.0989	0.2513	0.13	0.0180	0.0871	0.29	0.0175	0.1228	1.06	0.0023	0.0407	0.90	0.0134	0.1009	0.33
2870	NIPA-like protein 2	Q9H841	0.0007	0.0200	-0.16	0.0189	0.4684	-0.21	0.0047	0.0338	-0.12	0.0503	0.1154	-0.19	0.0102	0.0721	-0.41	0.0149	0.0812	-0.26	0.3736	0.6029	-2.55	0.8708	1.2178	-0.33	0.0651	0.1946	2.06
2871	Nipped-B-like protein	Q6KC79	0.0014	0.0254	-0.48	0.9481	0.9656	-0.01	0.0047	0.0333	-0.43	0.0097	0.0397	-0.30	0.0095	0.0695	-0.37	0.0093	0.0691	-0.40	0.3736	0.6155	-2.55	0.8708	1.2399	-0.33	0.0651	0.2023	2.06
2872	Nitric oxide synthase-interacting protein	Q9Y314	0.9892	0.9921	0.00	0.3224	0.6844	-0.07	0.0214	0.0707	-0.17	0.4191	0.6108	-0.18	0.0600	0.1845	0.13	0.7318	0.9712	-0.01	0.6519	0.6965	-0.05	0.0035	0.0482	-0.20	0.1105	0.1675	0.06
2873	NLR family member X1	Q86UT6	0.1576	0.3117	-0.11	0.0869	0.6200	-0.54	0.0346	0.0968	-0.33	0.8941	0.9099	0.02	0.0870	0.2310	-0.13	0.1624	0.3242	0.29	0.0374	0.1605	-0.20	0.7944	1.2376	-0.01	0.9090	0.9274	-0.01
2874	NmrA-like family domain-containing protein 1	Q9HBL8	0.0684	0.1736	0.13	0.4129	0.7523	0.11	0.0408	0.1080	0.16	0.0857	0.1731	0.12	0.0293	0.1246	0.19	0.0624	0.1738	0.14	0.3736	0.5545	-2.55	0.8708	1.1322	-0.33	0.0651	0.1675	2.06
2875	Nodal modulator 1	Q15155	0.2487	0.4352	-0.05	0.1570	0.6623	0.08	0.0092	0.0450	0.16	0.0063	0.0317	0.18	0.0185	0.0971	0.13	0.0665	0.1808	0.08	0.3736	0.5509	-2.55	0.8708	1.1257	-0.33	0.0651	0.1656	2.06
2876	Nodal modulator 3	P69849	0.6815	0.7382	-0.04	0.2004	0.6633	0.10	0.0731	0.1617	-0.11	0.1144	0.2152	0.47	0.0344	0.1355	0.13	0.0337	0.1183	0.60	0.3736	0.5765	-2.55	0.8708	1.1713	-0.33	0.0651	0.1794	2.06
2877	Noelin-3	Q96PB7	0.4108	0.5453	-3.31	0.5752	0.7634	-1.75	0.5661	0.6900	-1.19	0.5388	0.6526	0.89	0.2938	0.4428	-1.88	0.9510	1.0513	0.09	0.5141	0.5676	-0.15	0.7591	1.2010	-0.02	0.0289	0.1449	0.62
2878	Non-histone chromosomal protein HMG-14	P05114	0.4153	0.4931	-1.12	0.9711	0.9804	0.06	0.0505	0.1261	-1.26	0.1848	0.3121	-0.51	0.1826	0.3758	0.62	0.1135	0.2557	-3.15	0.0095	0.0976	1.47	0.1198	0.3579	0.88	0.2833	0.3552	3.47
2879	Non-histone chromosomal protein HMG-17	P05204	0.4108	0.5452	-3.31	0.5752	0.7632	-1.75	0.5661	0.6898	-1.19	0.5388	0.6524	0.89	0.2938	0.4427	-1.88	0.9510	1.0510	0.09	0.0528	0.1817	6.07	0.0815	0.2809	4.45	0.0473	0.1905	6.54
2880	Non-POU domain-containing octamer-binding protein	Q15233	0.0245	0.0945	0.14	0.2616	0.6636	0.21	0.0123	0.0520	0.32	0.0012	0.0160	0.41	0.0195	0.1000	0.15	0.0076	0.0656	0.30	0.0287	0.1456	0.59	0.2211	0.5285	0.13	0.0588	0.2168	0.21
2881	Non-receptor tyrosine-protein kinase TYK2	P29597	0.8264	0.8608	0.06	0.8402	0.8851	-0.12	0.7312	0.7744	-0.07	0.3832	0.5681	-0.26	0.7679	0.8195	0.06	0.9400	1.1761	-0.05	0.3736	0.4320	-2.55	0.8708	0.9069	-0.33	0.0651	0.1123	2.06
2882	Non-specific lipid-transfer protein	P22307	0.1780	0.3406	0.11	0.2674	0.6631	0.13	0.9144	0.9322	0.00	0.3260	0.4978	0.02	0.2727	0.4965	-0.04	0.2596	0.4509	0.03	0.2449	0.4789	0.11	0.2459	0.5669	-0.05	0.2072	0.2760	-0.03
2883	Non-structural maintenance of chromosomes element 3 homolog	Q96MG7	0.0053	0.0444	0.31	0.4616	0.7941	-0.07	0.9129	0.9314	-0.01	0.1977	0.3289	-0.08	0.0285	0.1226	-0.20	0.1460	0.3017	-0.10	0.0321	0.1512	-0.18	0.0003	0.0179	-0.84	0.0293	0.1465	-1.24
2884	Nostrin	Q8IVI9	0.0905	0.2106	0.08	0.3270	0.6861	0.03	0.0290	0.0864	0.28	0.3055	0.4718	-0.10	0.0537	0.1718	0.20	0.3719	0.5799	0.03	0.3736	0.4716	-2.55	0.8708	0.9810	-0.33	0.0651	0.1284	2.06
2885	Notch homolog 2 N-terminal-like protein C	P0DPK4	0.3531	0.5631	0.21	0.4340	0.7707	0.17	0.9143	0.9323	-0.02	0.8474	0.8687	0.04	0.5362	0.6216	-0.14	0.6272	0.8631	0.10	0.3736	0.4496	-2.55	0.8708	0.9401	-0.33	0.0651	0.1193	2.06
2886	Notchless protein homolog 1	Q9NVX2	0.4108	0.5451	-3.31	0.5752	0.7630	-1.75	0.5661	0.6897	-1.19	0.5388	0.6523	0.89	0.2938	0.4425	-1.88	0.9510	1.0508	0.09	0.2027	0.4155	-1.30	0.6582	1.0991	-0.19	0.0221	0.1264	0.77
2887	NPC intracellular cholesterol transporter 2	P61916	0.4108	0.5449	-3.31	0.5752	0.7628	-1.75	0.5661	0.6895	-1.19	0.5388	0.6521	0.89	0.2938	0.4424	-1.88	0.9510	1.0506	0.09	0.0006	0.0374	-5.61	0.0009	0.0271	-4.59	0.0000	0.0000	-5.18
2888	NSFL1 cofactor p47	Q9UNZ2	0.0660	0.1703	0.16	0.1995	0.6637	0.18	0.0150	0.0576	0.27	0.0121	0.0442	0.40	0.0924	0.2398	0.14	0.0114	0.0731	0.40	0.0371	0.1600	0.38	0.4850	0.8957	0.08	0.3246	0.3960	0.10
2889	N-terminal kinase-like protein	Q96KG9	0.0376	0.1218	-0.09	0.4458	0.7817	0.07	0.0456	0.1175	-0.13	0.0000	0.0000	-0.22	0.0039	0.0468	-0.15	0.0009	0.0453	-0.15	0.2143	0.4336	0.24	0.2826	0.6208	0.18	0.0224	0.1265	0.66
2890	N-terminal Xaa-Pro-Lys N-methyltransferase 1	Q9BV86	0.9897	0.9922	0.00	0.5279	0.8463	0.08	0.0168	0.0613	0.33	0.0697	0.1475	0.19	0.2183	0.4242	0.11	0.1022	0.2370	0.19	0.5570	0.6076	0.11	0.8794	0.9089	0.03	0.9926	0.9939	0.00
2891	Nuclear apoptosis-inducing factor 1	Q69YI7	0.0640	0.1675	-11.70	0.0535	0.5847	-10.14	0.0189	0.0658	-9.58	0.0009	0.0149	-7.50	0.0353	0.1361	-1.77	0.0084	0.0674	-8.31	0.3736	0.6173	-2.55	0.8708	1.2430	-0.33	0.0651	0.2034	2.06
2892	Nuclear autoantigen Sp-100	P23497	0.0098	0.0578	0.24	0.2112	0.6618	0.27	0.0018	0.0240	0.51	0.0166	0.0537	0.21	0.0207	0.1030	-0.14	0.0109	0.0716	0.27	0.0081	0.0914	0.64	0.0136	0.0977	0.43	0.0074	0.0784	0.25
2893	Nuclear autoantigenic sperm protein	P49321	0.5578	0.6266	0.03	0.2560	0.6620	0.22	0.0070	0.0398	0.35	0.0048	0.0280	0.36	0.7544	0.8076	0.02	0.0599	0.1693	0.17	0.0446	0.1700	0.36	0.1947	0.4891	0.10	0.2000	0.2685	0.06
2894	Nuclear cap-binding protein subunit 1	Q09161	0.0075	0.0509	0.24	0.2373	0.6616	0.23	0.0019	0.0242	0.35	0.0078	0.0358	0.27	0.8528	0.8867	0.01	0.0056	0.0611	0.33	0.6680	0.7120	-0.01	0.0418	0.1884	-0.08	0.2369	0.3068	0.08
2895	Nuclear cap-binding protein subunit 2	P52298	0.0004	0.0163	-0.93	0.4669	0.7982	-1.98	0.7521	0.7943	0.05	0.1595	0.2779	-0.17	0.2019	0.4026	-0.16	0.1919	0.3660	-0.73	0.3736	0.5045	-2.55	0.8708	1.0417	-0.33	0.0651	0.1429	2.06
2896	Nuclear distribution protein nudE homolog 1	Q9NXR1	0.4108	0.5448	-3.31	0.5752	0.7626	-1.75	0.5661	0.6893	-1.19	0.5388	0.6519	0.89	0.2938	0.4423	-1.88	0.9510	1.0504	0.09	0.0977	0.2513	0.42	0.0270	0.1480	0.34	0.1390	0.2002	0.20
2897	Nuclear distribution protein nudE-like 1	Q9GZM8	0.4781	0.5533	-0.35	0.6432	0.7368	-0.10	0.0203	0.0685	-0.91	0.0225	0.0656	-0.83	0.2627	0.4841	-0.46	0.3137	0.5146	-1.72	0.0170	0.1213	-0.20	0.3520	0.7159	0.06	0.0242	0.1320	-0.17
2898	Nuclear factor interleukin-3-regulated protein	Q16649	0.4108	0.5446	-3.31	0.5752	0.7624	-1.75	0.5661	0.6892	-1.19	0.5388	0.6518	0.89	0.2938	0.4422	-1.88	0.9510	1.0501	0.09	0.0764	0.2200	0.27	0.0996	0.3163	-0.19	0.1671	0.2324	-0.14
2899	Nuclear factor NF-kappa-B p100 subunit	Q00653	0.1310	0.2729	-0.09	0.1664	0.6590	-0.10	0.9825	0.9859	0.00	0.1265	0.2315	-0.09	0.4147	0.5093	-0.05	0.4589	0.6815	0.05	0.0173	0.1219	-0.20	0.8957	0.9222	-0.01	0.8146	0.8499	-0.01
2900	Nuclear fragile X mental retardation-interacting protein 2	Q7Z417	0.0003	0.0151	-0.29	0.0022	0.2326	-0.23	0.2099	0.3639	-0.10	0.0022	0.0200	-0.25	0.0069	0.0604	-0.17	0.0206	0.0932	-0.43	0.0606	0.1946	0.36	0.0819	0.2811	0.14	0.1329	0.1933	0.09
2901	Nuclear GTPase SLIP-GC	Q68CJ6	0.4108	0.5445	-3.31	0.5752	0.7622	-1.75	0.5661	0.6890	-1.19	0.5388	0.6516	0.89	0.2938	0.4420	-1.88	0.9510	1.0499	0.09	0.5795	0.6287	-0.05	0.1204	0.3586	-0.13	0.0098	0.0874	-0.42
2902	Nuclear migration protein nudC	Q9Y266	0.0142	0.0695	0.17	0.2035	0.6605	0.23	0.0015	0.0227	0.32	0.0031	0.0229	0.26	0.1911	0.3871	0.06	0.0120	0.0743	0.22	0.0435	0.1684	0.49	0.2339	0.5492	0.12	0.6585	0.7136	0.03
2903	Nuclear mitotic apparatus protein 1	Q14980	0.0619	0.1649	0.09	0.1751	0.6616	0.21	0.0072	0.0403	0.20	0.0032	0.0231	0.31	0.2452	0.4605	0.05	0.0194	0.0897	0.20	0.0667	0.2032	0.34	0.1945	0.4896	0.19	0.2211	0.2908	0.19
2904	Nuclear pore complex protein Nup107	P57740	0.0397	0.1256	-0.06	0.7140	0.7905	-0.05	0.6474	0.7000	0.03	0.6718	0.7134	-0.01	0.0274	0.1197	-0.20	0.0002	0.0324	-0.22	0.3736	0.6536	-2.55	0.8708	1.3056	-0.33	0.0651	0.2272	2.06
2905	Nuclear pore complex protein Nup155	O75694	0.0331	0.1136	-0.11	0.0221	0.4810	-0.14	0.5063	0.7281	0.02	0.0029	0.0220	-0.35	0.1322	0.3007	-0.07	0.0062	0.0625	0.28	0.0089	0.0941	0.36	0.0006	0.0232	0.51	0.0403	0.1740	0.13
2906	Nuclear pore complex protein Nup50	Q9UKX7	0.0034	0.0380	-0.40	0.3860	0.7308	-0.15	0.6458	0.6987	-0.05	0.0075	0.0350	-0.28	0.0007	0.0207	-0.67	0.0004	0.0441	-0.71	0.0179	0.1240	0.45	0.0104	0.0839	0.32	0.0275	0.1419	0.33
2907	Nuclear pore complex protein Nup85	Q9BW27	0.4108	0.5444	-3.31	0.5752	0.7620	-1.75	0.5661	0.6888	-1.19	0.5388	0.6515	0.89	0.2938	0.4419	-1.88	0.9510	1.0497	0.09	0.0579	0.1914	-0.51	0.0685	0.2525	-0.27	0.0660	0.1125	-0.42
2908	Nuclear pore complex protein Nup88	Q99567	0.4108	0.5442	-3.31	0.5752	0.7618	-1.75	0.5661	0.6887	-1.19	0.5388	0.6513	0.89	0.2938	0.4418	-1.88	0.9510	1.0495	0.09	0.0011	0.0425	-0.43	0.6334	1.0705	-0.04	0.0200	0.1215	0.20
2909	Nuclear pore complex protein Nup93	Q8N1F7	0.4161	0.4939	-0.19	0.6737	0.7611	0.74	0.3471	0.5412	-1.17	0.4995	0.7029	1.02	0.4051	0.5012	-0.51	0.6402	0.8771	-0.30	0.0577	0.1913	0.31	0.1640	0.4406	0.15	0.1930	0.2609	0.10
2910	Nuclear pore glycoprotein p62	P37198	0.4108	0.5441	-3.31	0.5752	0.7616	-1.75	0.5661	0.6885	-1.19	0.5388	0.6512	0.89	0.2938	0.4416	-1.88	0.9510	1.0492	0.09	0.0963	0.2495	-1.25	0.1549	0.4254	-0.58	0.3548	0.4266	0.20
2911	Nuclear pore membrane glycoprotein 210	Q8TEM1	0.4736	0.5493	-0.07	0.8674	0.9037	0.02	0.7016	0.7486	-0.04	0.4567	0.6535	0.07	0.0749	0.2103	-0.24	0.0466	0.1434	-0.29	0.3736	0.5637	-2.55	0.8708	1.1486	-0.33	0.0651	0.1724	2.06
2912	Nuclear protein localization protein 4 homolog	Q8TAT6	0.2080	0.3806	0.12	0.2152	0.6608	0.27	0.0150	0.0575	0.37	0.0111	0.0423	0.40	0.5199	0.6079	0.05	0.0365	0.1236	0.31	0.9708	0.9763	0.00	0.5929	1.0254	0.04	0.0129	0.0986	-0.11
2913	Nuclear receptor 2C2-associated protein	Q86WQ0	0.0809	0.1959	-0.63	0.3464	0.7026	-0.39	0.2507	0.4176	-0.33	0.1051	0.2012	-0.55	0.1820	0.3747	-0.41	0.0433	0.1369	-0.83	0.3736	0.5664	-2.55	0.8708	1.1533	-0.33	0.0651	0.1738	2.06
2914	Nuclear receptor coactivator 2	Q15596	0.3118	0.5129	0.09	0.0976	0.6328	0.20	0.0370	0.1009	0.32	0.1566	0.2743	0.14	0.2380	0.4509	0.11	0.0978	0.2306	0.20	0.3736	0.5347	-2.55	0.8708	1.0967	-0.33	0.0651	0.1574	2.06
2915	Nuclear receptor coactivator 3	Q9Y6Q9	0.0419	0.1298	0.07	0.1112	0.6316	0.26	0.0160	0.0598	0.17	0.0047	0.0277	0.29	0.4041	0.5004	0.09	0.1466	0.3025	0.19	0.3736	0.5176	-2.55	0.8708	1.0656	-0.33	0.0651	0.1491	2.06
2916	Nuclear receptor coactivator 5	Q9HCD5	0.7768	0.8182	-0.05	0.4518	0.7853	0.24	0.1988	0.3496	0.22	0.0749	0.1558	0.08	0.0030	0.0407	0.22	0.0069	0.0644	-0.23	0.3736	0.6223	-2.55	0.8708	1.2516	-0.33	0.0651	0.2065	2.06
2917	Nuclear receptor coactivator 7	Q8NI08	0.4108	0.5440	-3.31	0.5752	0.7614	-1.75	0.5661	0.6884	-1.19	0.5388	0.6510	0.89	0.2938	0.4415	-1.88	0.9510	1.0490	0.09	0.0035	0.0643	-1.12	0.0093	0.0790	-0.78	0.0012	0.0432	-0.93
2918	Nuclear receptor corepressor 2	Q9Y618	0.0096	0.0574	0.32	0.1382	0.6526	0.22	0.1130	0.2250	0.18	0.0192	0.0588	0.25	0.1418	0.3147	0.11	0.1138	0.2562	0.39	0.3736	0.5301	-2.55	0.8708	1.0882	-0.33	0.0651	0.1551	2.06
2919	Nuclear receptor subfamily 2 group F member 6	P10588	0.3158	0.5183	-0.46	0.2333	0.6622	-1.43	0.7144	0.7597	-0.31	0.1574	0.2754	-1.01	0.3588	0.4558	0.42	0.4248	0.6447	-0.35	0.3736	0.4669	-2.55	0.8708	0.9724	-0.33	0.0651	0.1264	2.06
2920	Nuclear receptor subfamily 6 group A member 1	Q15406	0.1359	0.2800	0.17	0.5008	0.8230	0.18	0.0147	0.0571	0.37	0.0024	0.0208	0.49	0.1009	0.2543	0.14	0.0372	0.1247	0.23	0.3736	0.5717	-2.55	0.8708	1.1629	-0.33	0.0651	0.1767	2.06
2921	Nuclear receptor-binding protein	Q9UHY1	0.1237	0.2612	-0.09	0.0721	0.6073	0.21	0.1782	0.3213	0.08	0.0158	0.0520	0.21	0.0219	0.1065	0.22	0.5100	0.7369	0.04	0.0555	0.1872	0.53	0.5401	0.9654	0.12	0.1963	0.2642	0.30
2922	Nuclear RNA export factor 2	Q9GZY0	0.2959	0.4943	0.09	0.1495	0.6596	0.17	0.2150	0.3700	0.11	0.1231	0.2267	0.16	0.1500	0.3272	0.14	0.3714	0.5796	0.07	0.3736	0.4717	-2.55	0.8708	0.9812	-0.33	0.0651	0.1285	2.06
2923	Nuclear RNA export factor 3	Q9H4D5	0.0274	0.1002	0.27	0.8640	0.9018	-0.02	0.4560	0.6709	-0.05	0.9353	0.9435	0.01	0.0619	0.1882	-0.20	0.3296	0.5325	0.20	0.3736	0.4767	-2.55	0.8708	0.9905	-0.33	0.0651	0.1306	2.06
2924	Nuclear transport factor 2	P61970	0.0008	0.0208	0.11	0.8056	0.8599	-0.01	0.1911	0.3381	0.09	0.4824	0.6831	-0.02	0.0403	0.1476	-0.15	0.0700	0.1859	0.07	0.1549	0.3450	-0.21	0.0187	0.1171	-0.41	0.1950	0.2630	-0.15
2925	Nuclear ubiquitous casein and cyclin-dependent kinase substrate 1	Q9H1E3	0.6939	0.7484	0.01	0.3507	0.7044	0.07	0.0068	0.0396	0.37	0.0043	0.0266	0.29	0.5130	0.6019	-0.02	0.0086	0.0676	0.21	0.0232	0.1363	0.52	0.0582	0.2298	0.16	0.3749	0.4467	0.10
2926	Nuclease-sensitive element-binding protein 1	P67809	0.4547	0.5320	0.03	0.4912	0.8142	0.14	0.0281	0.0847	0.31	0.0137	0.0474	0.22	0.1360	0.3058	-0.07	0.0902	0.2189	0.09	0.0055	0.0789	0.69	0.0113	0.0875	0.35	0.0107	0.0909	0.53
2927	Nucleic acid dioxygenase ALKBH1	Q13686	0.5383	0.6090	-0.04	0.7888	0.8481	0.03	0.3911	0.5931	-0.07	0.1730	0.2958	-0.11	0.4746	0.5653	0.05	0.0145	0.0801	-0.36	0.3736	0.6040	-2.55	0.8708	1.2198	-0.33	0.0651	0.1953	2.06
2928	Nucleobindin-1	Q02818	0.5395	0.6098	0.06	0.1151	0.6336	-0.20	0.3097	0.4942	-0.09	0.8200	0.8443	-0.02	0.9359	0.9511	0.01	0.0344	0.1194	-0.21	0.3547	0.6310	0.17	0.3716	0.7441	-0.12	0.2083	0.2772	-0.15
2929	Nucleobindin-2	P80303	0.0060	0.0467	-0.15	0.2685	0.6636	-0.04	0.7848	0.8233	0.01	0.7455	0.7806	-0.03	0.0798	0.2179	0.06	0.6413	0.8779	0.02	0.0264	0.1419	-0.34	0.0042	0.0524	-0.53	0.0181	0.1156	-0.61
2930	Nucleolar GTP-binding protein 1	Q9BZE4	0.1383	0.2829	0.10	0.1241	0.6409	0.25	0.0636	0.1469	0.15	0.0027	0.0217	0.33	0.4430	0.5373	0.04	0.0090	0.0688	0.27	0.6782	0.7215	0.08	0.2825	0.6209	-0.20	0.1035	0.1595	-0.55
2931	Nucleolar MIF4G domain-containing protein 1	Q5C9Z4	0.4860	0.5603	0.02	0.2629	0.6637	-0.04	0.0049	0.0343	0.08	0.6558	0.6991	-0.01	0.1701	0.3579	0.05	0.0385	0.1276	0.14	0.3736	0.5707	-2.55	0.8708	1.1611	-0.33	0.0651	0.1762	2.06
2932	Nucleolar protein 16	Q9Y3C1	0.1911	0.3575	0.03	0.2800	0.6647	0.11	0.0045	0.0330	0.26	0.0095	0.0392	0.20	0.0104	0.0730	0.15	0.0368	0.1243	0.17	0.3736	0.5721	-2.55	0.8708	1.1635	-0.33	0.0651	0.1769	2.06
2933	Nucleolar protein 3	O60936	0.3082	0.5091	-0.04	0.9176	0.9431	0.01	0.0003	0.0190	0.28	0.1757	0.2995	0.05	0.4153	0.5099	0.04	0.0595	0.1686	0.16	0.3736	0.5558	-2.55	0.8708	1.1345	-0.33	0.0651	0.1682	2.06
2934	Nucleolar protein 4	O94818	0.0403	0.1264	0.25	0.3496	0.7046	0.12	0.0109	0.0483	0.34	0.3218	0.4926	0.08	0.6834	0.7465	0.03	0.0722	0.1897	0.20	0.0143	0.1116	0.46	0.0222	0.1296	0.16	0.1279	0.1875	0.14
2935	Nucleolar protein 4-like	Q96MY1	0.3439	0.5522	-0.13	0.1263	0.6433	0.29	0.4831	0.7031	0.08	0.0761	0.1578	0.31	0.1566	0.3379	0.21	0.2058	0.3846	0.18	0.2112	0.4289	0.14	0.0311	0.1592	-0.09	0.0920	0.1457	0.24

Supplemetary Table S2. Overview on all relatively quantified 5180 proteins statistical analysis

			MCF-7																		MDA-MB-231								
			Dai SC20 vs control			Gen SC20 vs control			SSE SC20 vs control			Dai IC20 vs control			Gen IC20 vs control			SSE IC20 vs control			Dai IC20 vs control			Gen IC20 vs control			SSE IC20 vs control		
	**Protein name**	**UniProt**	**p value**	**BH q value**	**log2FC**	**p value**	**BH q value**	**log2FC**	**p value**	**BH q value**	**log2FC**	**p value**	**BH q value**	**log2FC**	**p value**	**BH q value**	**log2FC**	**p value**	**BH q value**	**log2FC**	**p value**	**BH q value**	**log2FC**	**p value**	**BH q value**	**log2FC**	**p value**	**BH q value**	**log2FC**
2936	Nucleolar protein 58	Q9Y2X3	0.0938	0.2156	-0.10	0.9063	0.9348	-0.01	0.8576	0.8865	0.01	0.3483	0.5242	-0.06	0.0251	0.1139	-0.13	0.7456	0.9840	0.01	0.1656	0.3601	0.16	0.0765	0.2698	0.23	0.7047	0.7555	0.03
2937	Nucleolar protein 8	Q76FK4	0.0104	0.0591	-0.42	0.4840	0.8085	-0.10	0.1225	0.2405	-0.16	0.0413	0.1007	-0.25	0.4345	0.5285	-0.06	0.1552	0.3142	-0.21	0.7546	0.7905	-0.05	0.4082	0.7949	0.09	0.9077	0.9267	0.01
2938	Nucleolar protein 9	Q86U38	0.4108	0.5438	-3.31	0.5752	0.7613	-1.75	0.5661	0.6882	-1.19	0.5388	0.6509	0.89	0.2938	0.4414	-1.88	0.9510	1.0488	0.09	0.2255	0.4505	0.19	0.0154	0.1048	-0.39	0.0243	0.1322	-0.46
2939	Nucleolar RNA helicase 2	Q9NR30	0.9362	0.9516	0.01	0.7502	0.8188	-0.04	0.0567	0.1360	0.18	0.7278	0.7653	-0.03	0.0499	0.1646	-0.23	0.4610	0.6835	-0.06	0.1713	0.3685	0.19	0.1120	0.3415	0.17	0.2015	0.2698	0.03
2940	Nucleolar transcription factor 1	P17480	0.0830	0.1984	0.14	0.2754	0.6641	0.17	0.1070	0.2154	0.11	0.1309	0.2375	0.10	0.5656	0.6472	0.03	0.7754	1.0133	-0.01	0.5853	0.6341	0.06	0.3093	0.6572	-0.11	0.0431	0.1811	-0.32
2941	Nucleolin	P19338	0.0018	0.0290	0.21	0.0820	0.6165	0.21	0.0001	0.0207	0.56	0.0006	0.0139	0.36	0.3009	0.3989	-0.03	0.0098	0.0703	0.31	0.0026	0.0576	0.77	0.0933	0.3041	0.18	0.0219	0.1260	0.40
2942	Nucleolysin TIA-1 isoform p40	P31483	0.6720	0.7304	-0.03	0.1527	0.6586	0.13	0.0641	0.1477	0.18	0.1193	0.2220	0.12	0.0533	0.1713	0.18	0.6909	0.9308	-0.02	0.6685	0.7122	0.02	0.0318	0.1609	-0.05	0.0831	0.1348	-0.12
2943	Nucleolysin TIAR	Q01085	0.4326	0.5100	-0.45	0.2343	0.6607	-2.82	0.0706	0.1581	-4.10	0.5184	0.7236	-0.71	0.0132	0.0820	-0.73	0.4840	0.7092	-1.40	0.3736	0.4618	-2.55	0.8708	0.9628	-0.33	0.0651	0.1243	2.06
2944	Nucleophosmin	P06748	0.0993	0.2240	0.17	0.0132	0.4411	0.16	0.0014	0.0232	0.53	0.0077	0.0355	0.19	0.1684	0.3552	-0.06	0.0054	0.0616	0.37	0.0105	0.1026	0.80	0.0052	0.0577	0.39	0.0028	0.0551	0.51
2945	Nucleoplasmin-3	O75607	0.2091	0.3817	0.15	0.0698	0.6016	0.29	0.8616	0.8898	0.02	0.0672	0.1433	0.27	0.2301	0.4400	0.14	0.7545	0.9920	0.03	0.3736	0.4412	-2.55	0.8708	0.9243	-0.33	0.0651	0.1160	2.06
2946	Nucleoporin NUP188 homolog	Q5SRE5	0.5093	0.5821	-0.04	0.2814	0.6650	-0.15	0.6473	0.7000	-0.03	0.3460	0.5215	-0.11	0.0620	0.1883	-0.15	0.1379	0.2910	-0.24	0.3736	0.5211	-2.55	0.8708	1.0719	-0.33	0.0651	0.1507	2.06
2947	Nucleoporin NUP35	Q8NFH5	0.4108	0.5437	-3.31	0.5752	0.7611	-1.75	0.5661	0.6880	-1.19	0.5388	0.6507	0.89	0.2938	0.4413	-1.88	0.9510	1.0486	0.09	0.0078	0.0904	-0.49	0.0000	0.0000	-0.91	0.0093	0.0863	-0.61
2948	Nucleoporin Nup37	Q8NFH4	0.5395	0.6096	-0.06	0.2784	0.6649	0.17	0.9489	0.9598	-0.01	0.1159	0.2169	0.24	0.1223	0.2881	0.19	0.8149	1.0516	-0.03	0.3736	0.4375	-2.55	0.8708	0.9174	-0.33	0.0651	0.1145	2.06
2949	Nucleoporin p54	Q7Z3B4	0.0000	0.0000	0.37	0.0509	0.5719	-0.27	0.0000	0.0000	0.49	0.0000	0.0000	0.64	0.0137	0.0841	0.15	0.0046	0.0600	1.22	0.5747	0.6244	0.11	0.5243	0.9476	0.12	0.7663	0.8076	-0.08
2950	Nucleoporin SEH1	Q96EE3	0.9313	0.9480	0.01	0.3139	0.6806	0.36	0.3681	0.5655	-0.16	0.4794	0.6800	0.12	0.0057	0.0555	-0.07	0.1971	0.3725	-0.11	0.1811	0.3818	-0.18	0.6206	1.0557	0.07	0.4791	0.5472	0.07
2951	Nucleoporin-like protein 2	O15504	0.0825	0.1978	-0.86	0.1103	0.6334	-0.48	0.2540	0.4220	-0.10	0.2008	0.3334	-0.10	0.2723	0.4960	0.12	0.1554	0.3143	-1.12	0.3736	0.5141	-2.55	0.8708	1.0594	-0.33	0.0651	0.1474	2.06
2952	Nucleoprotein TPR	P12270	0.8674	0.8949	0.01	0.3475	0.7037	0.15	0.0151	0.0577	0.25	0.0142	0.0485	0.16	0.0340	0.1350	0.13	0.0053	0.0617	0.35	0.3524	0.6284	0.12	0.9939	0.9958	0.00	0.5707	0.6340	-0.06
2953	Nucleoredoxin	Q6DKJ4	0.4108	0.5435	-3.31	0.5752	0.7609	-1.75	0.5661	0.6879	-1.19	0.5388	0.6506	0.89	0.2938	0.4411	-1.88	0.9510	1.0483	0.09	0.0291	0.1463	0.36	0.1295	0.3769	0.15	0.5879	0.6493	0.07
2954	Nucleoredoxin-like protein 1	Q96CM4	0.5036	0.5768	0.79	0.6209	0.7184	-0.18	0.6222	0.6762	-0.10	0.1790	0.3038	1.64	0.3756	0.4725	-0.19	0.0040	0.0592	2.32	0.3736	0.6345	-2.55	0.8708	1.2728	-0.33	0.0651	0.2144	2.06
2955	Nucleoredoxin-like protein 2	Q5VZ03	0.4108	0.5434	-3.31	0.5752	0.7607	-1.75	0.5661	0.6877	-1.19	0.5388	0.6504	0.89	0.2938	0.4410	-1.88	0.9510	1.0481	0.09	0.0848	0.2327	-0.50	0.9162	0.9389	0.01	0.1364	0.1972	0.11
2956	Nucleoside diphosphate kinase 3	Q13232	0.1459	0.2936	0.29	0.6586	0.7481	0.06	0.0555	0.1346	0.29	0.0535	0.1210	0.35	0.0048	0.0520	0.63	0.1330	0.2842	0.30	0.3736	0.5228	-2.55	0.8708	1.0750	-0.33	0.0651	0.1516	2.06
2957	Nucleoside diphosphate kinase 7	Q9Y5B8	0.4108	0.5433	-3.31	0.5752	0.7605	-1.75	0.5661	0.6875	-1.19	0.5388	0.6503	0.89	0.2938	0.4409	-1.88	0.9510	1.0479	0.09	0.1727	0.3703	-1.80	0.0090	0.0778	-0.89	0.0233	0.1298	-0.74
2958	Nucleoside diphosphate kinase A	P15531	0.0024	0.0333	0.15	0.5027	0.8238	0.13	0.0190	0.0660	0.24	0.0589	0.1298	0.05	0.3663	0.4632	0.01	0.0323	0.1159	0.26	0.0220	0.1350	0.49	0.0473	0.2027	0.21	0.8585	0.8852	0.01
2959	Nucleoside diphosphate kinase B	P22392	0.0046	0.0429	0.30	0.3934	0.7373	0.18	0.0040	0.0321	0.37	0.0059	0.0310	0.29	0.1128	0.2739	0.10	0.0118	0.0738	0.35	0.5443	0.5955	0.11	0.6752	1.1139	-0.04	0.0395	0.1717	-0.27
2960	Nucleoside diphosphate-linked moiety X motif 19	A8MXV4	0.3714	0.5858	0.06	0.7496	0.8187	0.02	0.0947	0.1957	0.13	0.0055	0.0296	0.37	0.0169	0.0931	0.24	0.0082	0.0670	0.37	0.0345	0.1546	-0.54	0.0640	0.2432	-0.24	0.2229	0.2928	0.07
2961	Nucleosome assembly protein 1-like 1	P55209	0.0006	0.0194	0.21	0.0315	0.5115	0.23	0.0002	0.0235	0.50	0.0001	0.0144	0.34	0.0027	0.0404	0.16	0.0017	0.0524	0.35	0.0154	0.1153	0.24	0.0646	0.2448	0.16	0.0223	0.1271	0.30
2962	Nucleosome assembly protein 1-like 3	Q99457	0.4108	0.5431	-3.31	0.5752	0.7603	-1.75	0.5661	0.6874	-1.19	0.5388	0.6501	0.89	0.2938	0.4407	-1.88	0.9510	1.0477	0.09	0.1787	0.3783	0.38	0.2106	0.5131	-0.28	0.7801	0.8192	0.03
2963	Nucleosome assembly protein 1-like 4	Q99733	0.3532	0.5631	0.03	0.1141	0.6342	0.10	0.0062	0.0382	0.14	0.2014	0.3341	0.06	0.7756	0.8243	-0.01	0.3004	0.4983	0.05	0.1810	0.3818	0.17	0.3070	0.6536	0.04	0.4941	0.5616	0.03
2964	Nucleosome-remodeling factor subunit BPTF	Q12830	0.0297	0.1056	-0.27	0.1370	0.6517	-0.30	0.0113	0.0493	0.36	0.4850	0.6864	0.06	0.0182	0.0961	0.31	0.7317	0.9713	0.03	0.3736	0.4434	-2.55	0.8708	0.9283	-0.33	0.0651	0.1168	2.06
2965	Nucleus accumbens-associated protein 1	Q96RE7	0.0038	0.0392	0.17	0.0678	0.5913	0.12	0.0108	0.0485	0.17	0.6113	0.6583	0.03	0.0200	0.1013	0.15	0.8503	1.0854	0.02	0.3736	0.4359	-2.55	0.8708	0.9142	-0.33	0.0651	0.1138	2.06
2966	Nucleus accumbens-associated protein 2	Q96BF6	0.7203	0.7715	-0.06	0.6109	0.7124	-0.04	0.9051	0.9251	0.04	0.0705	0.1486	0.22	0.5748	0.6547	-0.05	0.1937	0.3678	0.13	0.3736	0.5034	-2.55	0.8708	1.0398	-0.33	0.0651	0.1425	2.06
2967	NudC domain-containing protein 1	Q96RS6	0.4663	0.5426	0.03	0.4560	0.7879	0.06	0.4927	0.7137	0.06	0.0117	0.0433	0.17	0.0702	0.2019	0.14	0.5981	0.8346	0.10	0.6144	0.6615	-0.03	0.6785	1.1175	-0.03	0.0644	0.2277	-0.12
2968	NudC domain-containing protein 2	Q8WVJ2	0.0008	0.0207	0.59	0.4002	0.7441	0.10	0.0149	0.0576	0.30	0.0348	0.0891	0.20	0.0222	0.1075	0.31	0.0059	0.0622	0.36	0.0008	0.0436	-0.84	0.0008	0.0261	-1.48	0.0028	0.0556	-1.19
2969	NudC domain-containing protein 3	Q8IVD9	0.0043	0.0413	0.42	0.0005	0.2590	0.49	0.0010	0.0223	0.44	0.0006	0.0135	0.51	0.4876	0.5775	-0.05	0.1146	0.2573	0.09	0.0522	0.1809	0.14	0.9550	0.9679	0.00	0.0285	0.1445	0.24
2970	NXPE family member 1	Q8N323	0.3756	0.5908	-0.06	0.0068	0.4193	-0.33	0.0045	0.0333	-0.61	0.0002	0.0101	-1.06	0.0007	0.0220	-0.93	0.0910	0.2203	-0.28	0.3736	0.5371	-2.55	0.8708	1.1010	-0.33	0.0651	0.1586	2.06
2971	Obg-like ATPase 1	Q9NTK5	0.0382	0.1226	0.18	0.2828	0.6644	0.21	0.0135	0.0544	0.28	0.0055	0.0298	0.34	0.1418	0.3148	0.11	0.0186	0.0882	0.29	0.0442	0.1690	0.43	0.2420	0.5601	0.10	0.5878	0.6494	0.03
2972	Obscurin	Q5VST9	0.0812	0.1965	0.21	0.1526	0.6593	0.24	0.0024	0.0271	0.46	0.0029	0.0222	0.32	0.3928	0.4895	0.04	0.0037	0.0592	0.50	0.0339	0.1535	0.14	0.1777	0.4633	-0.07	0.0287	0.1446	0.18
2973	Occludin	Q16625	0.0079	0.0525	-0.06	0.3946	0.7384	0.10	0.0212	0.0704	0.11	0.0016	0.0176	0.10	0.7368	0.7940	-0.01	0.2055	0.3842	0.04	0.3736	0.5012	-2.55	0.8708	1.0358	-0.33	0.0651	0.1415	2.06
2974	OCIA domain-containing protein 1	Q9NX40	0.0446	0.1344	-0.15	0.9299	0.9525	-0.01	0.2624	0.4321	-0.10	0.5031	0.7068	0.03	0.0475	0.1602	-0.12	0.0166	0.0851	-0.20	0.3736	0.5995	-2.55	0.8708	1.2119	-0.33	0.0651	0.1926	2.06
2975	OCIA domain-containing protein 2	Q56VL3	0.0778	0.1908	-0.23	0.2370	0.6632	-0.13	0.5401	0.7621	-0.06	0.3743	0.5576	-0.09	0.5214	0.6088	0.07	0.3543	0.5599	-0.16	0.7864	0.8198	-0.03	0.5008	0.9170	0.06	0.2327	0.3028	0.12
2976	Oligoribonuclease mitochondrial	Q9Y3B8	0.4108	0.5430	-3.31	0.5752	0.7601	-1.75	0.5661	0.6872	-1.19	0.5388	0.6500	0.89	0.2938	0.4406	-1.88	0.9510	1.0475	0.09	0.0788	0.2231	0.20	0.4259	0.8211	-0.06	0.1740	0.2400	0.09
2977	Oligosaccharyltransferase complex subunit OSTC	Q9NRP0	0.0008	0.0206	0.41	0.0114	0.4310	-0.26	0.2035	0.3559	-0.08	0.0235	0.0676	-0.24	0.1007	0.2540	-0.07	0.0009	0.0440	0.40	0.0214	0.1339	-0.88	0.0091	0.0783	-0.30	0.0177	0.1140	-0.73
2978	Omega-amidase NIT2	Q9NQR4	0.0381	0.1227	-0.56	0.5629	0.8764	-0.11	0.0681	0.1540	-0.39	0.5991	0.6471	-0.13	0.1819	0.3746	0.22	0.1018	0.2364	-0.33	0.3124	0.5730	0.45	0.1626	0.4385	0.69	0.0878	0.1405	1.03
2979	Oncomodulin-1	P0CE72	0.6002	0.6659	-0.13	0.5808	0.6885	-0.51	0.2795	0.4546	-0.33	0.9058	0.9186	-0.04	0.8429	0.8789	-0.07	0.7407	0.9803	-0.08	0.3736	0.4424	-2.55	0.8708	0.9266	-0.33	0.0651	0.1164	2.06
2980	Opioid growth factor receptor	Q9NZT2	0.0936	0.2152	-0.10	0.0007	0.2266	0.14	0.5345	0.7554	0.03	0.1340	0.2420	0.06	0.0001	0.0081	0.22	0.0340	0.1190	-0.05	0.0750	0.2179	0.24	0.1655	0.4433	0.07	0.5824	0.6445	-0.02
2981	Opioid-binding protein/cell adhesion molecule	Q14982	0.1900	0.3558	-0.64	0.2570	0.6636	-1.51	0.5776	0.6351	0.24	0.0335	0.0868	-1.27	0.2689	0.4906	-0.47	0.2059	0.3846	-0.92	0.3736	0.5011	-2.55	0.8708	1.0355	-0.33	0.0651	0.1414	2.06
2982	Oral-facial-digital syndrome 1 protein	O75665	0.4108	0.5428	-3.31	0.5752	0.7599	-1.75	0.5661	0.6871	-1.19	0.5388	0.6498	0.89	0.2938	0.4405	-1.88	0.9510	1.0472	0.09	0.1702	0.3664	-0.15	0.9687	0.9778	0.00	0.4501	0.5188	-0.07
2983	Ornithine aminotransferase mitochondrial	P04181	0.0033	0.0376	0.09	0.4358	0.7718	0.08	0.0049	0.0341	0.18	0.0008	0.0146	0.15	0.1359	0.3057	-0.03	0.0002	0.0314	0.33	0.2144	0.4337	-0.04	0.0034	0.0476	-0.17	0.0039	0.0612	-0.20
2984	Otoferlin	Q9HC10	0.0972	0.2205	0.05	0.7994	0.8548	-0.01	0.1698	0.3094	0.06	0.2650	0.4197	0.04	0.0016	0.0313	0.23	0.1534	0.3114	0.17	0.3736	0.5148	-2.55	0.8708	1.0606	-0.33	0.0651	0.1478	2.06
2985	Otoraplin	Q9NRC9	0.9848	0.9888	0.00	0.2822	0.6642	-0.08	0.9148	0.9323	0.01	0.3215	0.4926	0.10	0.2113	0.4148	-0.12	0.4956	0.7203	0.05	0.1459	0.3297	-2.57	0.1135	0.3448	0.92	0.1035	0.1596	0.62
2986	OTU domain-containing protein 7B	Q6GQQ9	0.0078	0.0523	0.23	0.0435	0.5705	0.28	0.0198	0.0679	0.29	0.0020	0.0195	0.23	0.0036	0.0448	0.28	0.0056	0.0621	0.26	0.3031	0.5617	0.16	0.0320	0.1617	-0.29	0.1485	0.2111	-0.29
2987	Outer dense fiber protein 2	Q5BJF6	0.0259	0.0974	0.15	0.0076	0.4059	0.22	0.0147	0.0573	0.18	0.0155	0.0515	0.28	0.0009	0.0227	0.34	0.0126	0.0758	0.17	0.0255	0.1399	0.44	0.1056	0.3291	0.25	0.2724	0.3444	0.14
2988	Overexpressed in colon carcinoma 1 protein	Q8TAD7	0.4745	0.5501	-0.68	0.1614	0.6635	-1.88	0.0537	0.1314	-0.47	0.0170	0.0544	-0.73	0.3127	0.4102	-0.19	0.2530	0.4432	-0.86	0.3736	0.4899	-2.55	0.8708	1.0150	-0.33	0.0651	0.1364	2.06
2989	Oxidation resistance protein 1	Q8N573	0.0029	0.0362	-0.42	0.0850	0.6201	-0.30	0.7934	0.8309	0.02	0.0096	0.0393	-0.26	0.0034	0.0434	-0.38	0.0433	0.1371	-0.73	0.3736	0.5667	-2.55	0.8708	1.1539	-0.33	0.0651	0.1740	2.06
2990	Oxidative stress-induced growth inhibitor 1	Q9UJX0	0.0792	0.1930	-0.64	0.3177	0.6809	-1.02	0.6932	0.7411	-0.11	0.4200	0.6118	-0.24	0.5122	0.6015	-0.18	0.6655	0.9036	-0.12	0.3736	0.4468	-2.55	0.8708	0.9349	-0.33	0.0651	0.1182	2.06
2991	Oxidoreductase HTATIP2	Q9BUP3	0.0179	0.0786	0.21	0.0645	0.5903	0.30	0.0175	0.0631	0.29	0.0040	0.0255	0.33	0.0090	0.0679	0.27	0.0824	0.2070	0.21	0.3736	0.5424	-2.55	0.8708	1.1105	-0.33	0.0651	0.1613	2.06
2992	Oxygen-dependent coproporphyrinogen-III oxidase_ mitochondrial	P36551	0.0010	0.0219	-1.26	0.1207	0.6380	-0.93	0.0026	0.0281	-1.23	0.0003	0.0110	-1.35	0.1304	0.2986	-0.27	0.0018	0.0521	-1.35	0.2677	0.5104	-0.07	0.0030	0.0450	-0.39	0.0250	0.1343	-0.28
2993	Oxysterol-binding protein 1	P22059	0.2730	0.4659	0.10	0.1989	0.6651	0.09	0.0000	0.0000	0.33	0.0003	0.0124	0.30	0.0035	0.0442	0.22	0.1106	0.2511	0.21	0.3174	0.5797	0.11	0.8815	0.9109	-0.01	0.4319	0.5032	0.11
2994	Oxysterol-binding protein-related protein 2	Q9H1P3	0.4108	0.5427	-3.31	0.5752	0.7597	-1.75	0.5661	0.6869	-1.19	0.5388	0.6497	0.89	0.2938	0.4404	-1.88	0.9510	1.0470	0.09	0.0722	0.2126	0.35	0.1517	0.4207	0.10	0.0029	0.0544	0.20
2995	Oxysterol-binding protein-related protein 3	Q9H4L5	0.0181	0.0789	0.21	0.2319	0.6626	0.18	0.0126	0.0526	0.25	0.0012	0.0159	0.42	0.0363	0.1384	0.15	0.0078	0.0661	0.38	0.0070	0.0867	0.40	0.0934	0.3043	0.20	0.0696	0.1174	0.17
2996	Oxysterol-binding protein-related protein 9	Q96SU4	0.4108	0.5426	-3.31	0.5752	0.7595	-1.75	0.5661	0.6867	-1.19	0.5388	0.6495	0.89	0.2938	0.4402	-1.88	0.9510	1.0468	0.09	0.0517	0.1801	-0.28	0.9681	0.9773	0.00	0.4137	0.4852	0.18
2997	Oxytocin receptor	P30559	0.5004	0.5741	-0.05	0.0232	0.4807	-0.31	0.2767	0.4512	0.09	0.0060	0.0310	-0.43	0.1051	0.2609	-0.15	0.1363	0.2886	-0.15	0.3736	0.5216	-2.55	0.8708	1.0730	-0.33	0.0651	0.1510	2.06
2998	Oxytocin-neurophysin 1	P01178	0.6342	0.6962	0.12	0.2141	0.6617	0.32	0.9477	0.9590	-0.02	0.2052	0.3389	0.35	0.2401	0.4531	0.43	0.4562	0.6791	-0.44	0.3736	0.4635	-2.55	0.8708	0.9661	-0.33	0.0651	0.1250	2.06
2999	P antigen family member 2	Q7Z2X7	0.0088	0.0557	0.43	0.1091	0.6300	0.26	0.0248	0.0782	0.31	0.0198	0.0599	0.36	0.3506	0.4482	0.08	0.0344	0.1196	0.32	0.3736	0.5753	-2.55	0.8708	1.1692	-0.33	0.0651	0.1787	2.06
3000	P antigen family member 5	Q96GU1	0.0334	0.1142	5.50	0.0162	0.4536	6.00	0.0304	0.0887	6.32	0.0215	0.0636	6.79	0.0193	0.0995	7.85	0.0253	0.1011	6.98	0.3736	0.5857	-2.55	0.8708	1.1877	-0.33	0.0651	0.1846	2.06
3001	P2X purinoceptor 4	Q99571	0.0114	0.0613	0.22	0.4836	0.8083	0.17	0.0131	0.0535	0.40	0.0027	0.0218	0.33	0.0579	0.1806	0.21	0.1143	0.2568	0.36	0.3736	0.5296	-2.55	0.8708	1.0875	-0.33	0.0651	0.1549	2.06
3002	Pachytene checkpoint protein 2 homolog	Q15645	0.0785	0.1920	0.28	0.5169	0.8388	-0.11	0.2435	0.4082	-0.23	0.1239	0.2275	-0.39	0.1998	0.4005	-0.18	0.4258	0.6457	-0.23	0.0711	0.2108	0.20	0.0205	0.1236	0.25	0.9909	0.9930	0.00
3003	PAK4-inhibitor INKA2	Q9NTI7	0.5553	0.6245	-0.07	0.4165	0.7549	-0.19	0.1147	0.2278	-0.25	0.1895	0.3180	-0.21	0.0489	0.1628	-0.37	0.4434	0.6652	-0.11	0.3736	0.4648	-2.55	0.8708	0.9684	-0.33	0.0651	0.1255	2.06
3004	Palmitoyl-protein thioesterase 1	P50897	0.0003	0.0149	-0.38	0.0010	0.2467	-0.27	0.0170	0.0619	-0.30	0.0034	0.0234	-0.50	0.0004	0.0173	-0.67	0.0005	0.0405	-0.76	0.2415	0.4740	-0.52	0.0133	0.0961	-0.63	0.2747	0.3465	-0.22
3005	Pancreatic alpha-amylase	P04746	0.9757	0.9833	0.00	0.2971	0.6715	-0.08	0.1476	0.2757	0.06	0.2592	0.4112	-0.04	0.0105	0.0729	-0.16	0.3249	0.5273	0.06	0.3736	0.4774	-2.55	0.8708	0.9918	-0.33	0.0651	0.1309	2.06
3006	Pancreatic progenitor cell differentiation and proliferation factor	Q9H3Y8	0.0039	0.0394	-0.48	0.0410	0.5694	-12.05	0.0009	0.0220	-0.74	0.0193	0.0591	-0.30	0.1481	0.3242	0.14	0.0038	0.0595	-0.88	0.3736	0.6355	-2.55	0.8708	1.2746	-0.33	0.0651	0.2151	2.06
3007	Pancreatic prohormone	P01298	0.1600	0.3150	0.26	0.2631	0.6638	0.22	0.1483	0.2766	0.27	0.0667	0.1428	0.41	0.2356	0.4474	0.20	0.1730	0.3402	0.25	0.3736	0.5091	-2.55	0.8708	1.0502	-0.33	0.0651	0.1451	2.06
3008	Pantothenate kinase 2 mitochondrial	Q9BZ23	0.4108	0.5424	-3.31	0.5752	0.7593	-1.75	0.5661	0.6866	-1.19	0.5388	0.6494	0.89	0.2938	0.4401	-1.88	0.9510	1.0466	0.09	0.0057	0.0802	-0.53	0.0017	0.0341	-0.59	0.0528	0.2024	-0.79
3009	Pantothenate kinase 4	Q9NVE7	0.1391	0.2837	0.09	0.0556	0.5830	0.34	0.0179	0.0639	0.22	0.0047	0.0277	0.37	0.0032	0.0426	0.37	0.1442	0.2997	0.19	0.3736	0.5186	-2.55	0.8708	1.0674	-0.33	0.0651	0.1495	2.06
3010	Paraneoplastic antigen Ma1	Q8ND90	0.0154	0.0719	0.12	0.0405	0.5685	0.13	0.0473	0.1206	0.13	0.1618	0.2812	0.11	0.0000	0.0000	0.33	0.1790	0.3484	0.07	0.3736	0.5079	-2.55	0.8708	1.0480	-0.33	0.0651	0.1445	2.06
3011	Paraspeckle component 1	Q8WXF1	0.4103	0.6316	-0.06	0.3381	0.6972	0.08	0.6818	0.7312	0.04	0.5559	0.6069	0.04	0.3360	0.4330	0.07	0.7367	0.9765	0.02	0.0106	0.1022	0.64	0.0029	0.0452	0.41	0.0041	0.0626	0.37
3012	Parathymosin	P20962	0.0812	0.1964	-0.21	0.6678	0.7556	-0.21	0.0096	0.0456	0.19	0.2150	0.3523	0.11	0.0517	0.1684	-0.11	0.3006	0.4984	-0.04	0.1288	0.3029	0.33	0.1203	0.3585	-0.18	0.2849	0.3568	-0.10
3013	Partitioning defective 3 homolog	Q8TEW0	0.2132	0.3872	-0.08	0.1977	0.6693	0.16	0.4499	0.6636	0.07	0.0210	0.0624	0.24	0.3510	0.4486	0.05	0.7905	1.0278	0.01	0.3736	0.4391	-2.55	0.8708	0.9204	-0.33	0.0651	0.1151	2.06
3014	Partitioning defective 6 homolog beta	Q9BYG5	0.9614	0.9715	0.00	0.3793	0.7255	0.05	0.4912	0.7119	0.04	0.9138	0.9249	0.01	0.7511	0.8055	-0.02	0.7708	1.0085	-0.02	0.3736	0.4406	-2.55	0.8708	0.9232	-0.33	0.0651	0.1157	2.06
3015	Partner of Y14 and mago	Q9BRP8	0.0995	0.2242	-0.11	0.4138	0.7529	0.11	0.4708	0.6879	0.04	0.1720	0.2942	0.08	0.2970	0.3951	-0.06	0.5609	0.7960	0.03	0.1846	0.3854	0.12	0.4822	0.8930	-0.04	0.0556	0.2089	0.12
3016	Paxillin	P49023	0.0035	0.0380	0.15	0.2097	0.6615	0.20	0.4117	0.6178	0.05	0.0002	0.0085	0.27	0.0965	0.2470	0.07	0.0416	0.1342	0.33	0.0023	0.0557	-0.83	0.0052	0.0579	-0.82	0.0078	0.0797	-0.76

Supplemetary Table S2. Overview on all relatively quantified 5180 proteins statistical analysis

			MCF-7																		MDA-MB-231								
			Dai SC20 vs control			Gen SC20 vs control			SSE SC20 vs control			Dai IC20 vs control			Gen IC20 vs control			SSE IC20 vs control			Dai IC20 vs control			Gen IC20 vs control			SSE IC20 vs control		
	**Protein name**	**UniProt**	**p value**	**BH q value**	**log2FC**	**p value**	**BH q value**	**log2FC**	**p value**	**BH q value**	**log2FC**	**p value**	**BH q value**	**log2FC**	**p value**	**BH q value**	**log2FC**	**p value**	**BH q value**	**log2FC**	**p value**	**BH q value**	**log2FC**	**p value**	**BH q value**	**log2FC**	**p value**	**BH q value**	**log2FC**
3017	PDZ and LIM domain protein 1	O00151	0.7021	0.7555	-0.05	0.6631	0.7519	0.06	0.8276	0.8608	-0.02	0.2974	0.4612	-0.13	0.3172	0.4144	0.11	0.5120	0.7390	0.07	0.6417	0.6865	-0.06	0.6641	1.1019	-0.03	0.0452	0.1857	-0.16
3018	PDZ and LIM domain protein 2	Q96JY6	0.4108	0.5423	-3.31	0.5752	0.7591	-1.75	0.5661	0.6864	-1.19	0.5388	0.6492	0.89	0.2938	0.4400	-1.88	0.9510	1.0463	0.09	0.0273	0.1430	0.38	0.3781	0.7547	0.09	0.2311	0.3012	0.17
3019	PDZ and LIM domain protein 5	Q96HC4	0.3476	0.5566	-0.07	0.6189	0.7175	0.03	0.0706	0.1580	0.14	0.0401	0.0986	0.19	0.1039	0.2591	0.11	0.0611	0.1716	0.16	0.2958	0.5516	0.12	0.5763	1.0068	0.04	0.8492	0.8784	-0.01
3020	PDZ and LIM domain protein 7	Q9NR12	0.0030	0.0366	1.74	0.2210	0.6598	-0.39	0.0539	0.1318	0.53	0.0229	0.0664	0.35	0.0877	0.2318	0.23	0.0096	0.0700	0.50	0.3031	0.5615	0.24	0.0122	0.0921	0.24	0.0064	0.0752	0.33
3021	PDZ domain-containing protein 2	O15018	0.1620	0.3181	0.18	0.3450	0.7025	-0.12	0.0626	0.1454	-0.28	0.1169	0.2184	-0.21	0.2050	0.4067	-0.16	0.9522	0.9605	0.01	0.3736	0.4308	-2.55	0.8708	0.9047	-0.33	0.0651	0.1119	2.06
3022	PDZ domain-containing protein 4	Q76G19	0.9842	0.9886	0.00	0.9120	0.9383	0.01	0.7382	0.7812	0.03	0.2699	0.4257	0.11	0.2594	0.4801	-0.12	0.9115	1.1485	0.01	0.7178	0.7582	-0.15	0.2246	0.5344	0.37	0.0427	0.1798	0.74
3023	PDZ domain-containing protein 8	Q8NEN9	0.8625	0.8912	0.01	0.8520	0.8936	0.03	0.0668	0.1520	0.22	0.2042	0.3378	0.13	0.7597	0.8122	-0.03	0.3206	0.5234	0.09	0.3736	0.4785	-2.55	0.8708	0.9940	-0.33	0.0651	0.1314	2.06
3024	PDZ domain-containing protein GIPC1	O14908	0.7875	0.8276	-0.01	0.8181	0.8702	0.03	0.4704	0.6879	0.04	0.2012	0.3338	0.09	0.1785	0.3694	-0.08	0.2197	0.4010	-0.07	0.3736	0.4980	-2.55	0.8708	1.0299	-0.33	0.0651	0.1400	2.06
3025	Pecanex-like protein 4	Q63HM2	0.1850	0.3499	-0.18	0.6538	0.7447	0.11	0.3596	0.5565	-0.11	0.8822	0.8992	0.02	0.0249	0.1136	0.55	0.2096	0.3907	0.17	0.3736	0.5007	-2.55	0.8708	1.0348	-0.33	0.0651	0.1412	2.06
3026	Peflin	Q9UBV8	0.2891	0.4859	-0.72	0.4733	0.8017	-0.58	0.2947	0.4753	0.19	0.4458	0.6402	0.25	0.2154	0.4201	0.26	0.1957	0.3709	0.44	0.3736	0.5032	-2.55	0.8708	1.0393	-0.33	0.0651	0.1423	2.06
3027	Pentatricopeptide repeat domain-containing protein 3_ mitochondrial	Q96EY7	0.1189	0.2541	-0.16	0.7512	0.8195	0.08	0.6113	0.6664	-0.07	0.2910	0.4531	-0.27	0.0765	0.2125	0.20	0.1372	0.2901	-0.23	0.9720	0.9773	0.00	0.2037	0.5032	-0.16	0.9640	0.9713	0.00
3028	Peptidase M20 domain-containing protein 2	Q8IYS1	0.4108	0.5422	-3.31	0.5752	0.7589	-1.75	0.5661	0.6863	-1.19	0.5388	0.6491	0.89	0.2938	0.4399	-1.88	0.9510	1.0461	0.09	0.1986	0.4090	0.11	0.1742	0.4576	0.13	0.8016	0.8380	-0.02
3029	Peptide chain release factor 1 mitochondrial	O75570	0.4108	0.5420	-3.31	0.5752	0.7587	-1.75	0.5661	0.6861	-1.19	0.5388	0.6489	0.89	0.2938	0.4397	-1.88	0.9510	1.0459	0.09	0.2271	0.4526	0.81	0.0737	0.2631	-1.67	0.5344	0.6003	-1.08
3030	Peptide-N(4)-(N-acetyl-beta-glucosaminyl)asparagine amidase	Q96IV0	0.0002	0.0140	0.40	0.9463	0.9645	-0.01	0.0268	0.0820	0.31	0.0249	0.0704	0.11	0.3520	0.4498	0.03	0.3393	0.5435	0.09	0.3736	0.4753	-2.55	0.8708	0.9879	-0.33	0.0651	0.1299	2.06
3031	Peptidyl-prolyl cis-trans isomerase A	P62937	0.0005	0.0182	0.49	0.1980	0.6695	0.24	0.0036	0.0316	0.35	0.0045	0.0274	0.26	0.4589	0.5510	0.03	0.0029	0.0542	0.40	0.1909	0.3970	0.22	0.0575	0.2293	-0.21	0.0264	0.1387	-0.31
3032	Peptidyl-prolyl cis-trans isomerase A-like 4A	Q9Y536	0.1580	0.3123	-0.14	0.1291	0.6436	-0.08	0.3582	0.5550	0.08	0.0765	0.1584	-0.18	0.8480	0.8833	-0.01	0.2278	0.4104	-0.06	0.9955	0.9963	0.00	0.0955	0.3084	0.28	0.0055	0.0714	0.71
3033	Peptidyl-prolyl cis-trans isomerase A-like 4G	P0DN37	0.0046	0.0428	0.32	0.0924	0.6298	0.15	0.0163	0.0604	0.26	0.3656	0.5464	0.05	0.0448	0.1559	0.16	0.0572	0.1645	0.13	0.6649	0.7090	0.09	0.1570	0.4287	-0.22	0.0435	0.1822	-0.41
3034	Peptidyl-prolyl cis-trans isomerase A-like 4H	A0A075B767	0.0025	0.0335	-0.84	0.0494	0.5776	-1.01	0.0009	0.0219	-1.11	0.0032	0.0233	-1.31	0.0015	0.0305	-1.28	0.0060	0.0623	-0.92	0.3736	0.6255	-2.55	0.8708	1.2572	-0.33	0.0651	0.2085	2.06
3035	Peptidyl-prolyl cis-trans isomerase B	P23284	0.0107	0.0598	0.37	0.3180	0.6807	0.20	0.0079	0.0418	0.34	0.0047	0.0278	0.38	0.0383	0.1426	0.23	0.0074	0.0651	0.50	0.2516	0.4878	0.14	0.3490	0.7112	-0.10	0.0478	0.1909	-0.25
3036	Peptidyl-prolyl cis-trans isomerase C	P45877	0.4686	0.5445	-0.04	0.4721	0.8010	-0.05	0.0321	0.0919	-0.13	0.0101	0.0403	-0.18	0.0568	0.1783	-0.10	0.4498	0.6726	0.04	0.3736	0.4643	-2.55	0.8708	0.9676	-0.33	0.0651	0.1254	2.06
3037	Peptidyl-prolyl cis-trans isomerase D	Q08752	0.0009	0.0217	0.36	0.3834	0.7307	-0.07	0.3220	0.5095	-0.05	0.2166	0.3545	-0.08	0.1443	0.3188	-0.12	0.1117	0.2526	0.08	0.2106	0.4280	0.12	0.5470	0.9724	0.04	0.1043	0.1605	-0.24
3038	Peptidyl-prolyl cis-trans isomerase F mitochondrial	P30405	0.7159	0.7675	0.02	0.1917	0.6687	0.13	0.0254	0.0792	0.29	0.0028	0.0217	0.39	0.1183	0.2821	0.11	0.0090	0.0693	0.34	0.0075	0.0893	-0.36	0.0039	0.0506	-0.48	0.0143	0.1033	-0.36
3039	Peptidyl-prolyl cis-trans isomerase FKBP10	Q96AY3	0.0049	0.0432	0.15	0.0293	0.5128	0.12	0.0034	0.0315	0.20	0.0132	0.0467	0.11	0.0441	0.1555	0.07	0.0995	0.2332	0.16	0.0916	0.2431	0.30	0.7027	1.1446	0.05	0.2435	0.3136	-0.15
3040	Peptidyl-prolyl cis-trans isomerase FKBP14	Q9NWM8	0.5870	0.6536	-0.08	0.8626	0.9009	-0.02	0.2952	0.4758	-0.10	0.4191	0.6107	0.07	0.0367	0.1394	0.68	0.8671	1.1033	-0.02	0.0228	0.1365	-0.25	0.3249	0.6789	-0.10	0.8797	0.9027	0.01
3041	Peptidyl-prolyl cis-trans isomerase FKBP1A	P62942	0.0560	0.1548	0.17	0.2387	0.6609	0.21	0.0233	0.0752	0.22	0.0366	0.0920	0.20	0.0631	0.1903	0.17	0.0230	0.0969	0.25	0.1842	0.3854	0.23	0.6290	1.0651	-0.04	0.0687	0.1161	-0.11
3042	Peptidyl-prolyl cis-trans isomerase FKBP2	P26885	0.0452	0.1356	0.09	0.8523	0.8935	-0.01	0.0093	0.0448	-0.20	0.0046	0.0275	-0.19	0.1594	0.3422	-0.05	0.4667	0.6891	0.05	0.0030	0.0600	-0.38	0.0199	0.1224	-0.31	0.0008	0.0345	-0.71
3043	Peptidyl-prolyl cis-trans isomerase FKBP3	Q00688	0.0273	0.1001	0.23	0.1918	0.6681	0.26	0.0151	0.0578	0.28	0.0117	0.0434	0.32	0.4888	0.5785	0.04	0.0122	0.0743	0.36	0.1079	0.2682	0.19	0.0088	0.0771	0.39	0.3636	0.4351	0.07
3044	Peptidyl-prolyl cis-trans isomerase FKBP4	Q02790	0.0092	0.0569	0.28	0.1603	0.6643	0.27	0.0015	0.0229	0.45	0.0104	0.0410	0.32	0.0492	0.1634	0.17	0.0230	0.0970	0.26	0.0668	0.2033	0.34	0.7295	1.1746	-0.03	0.7766	0.8161	-0.02
3045	Peptidyl-prolyl cis-trans isomerase FKBP5	Q13451	0.0211	0.0861	0.11	0.2817	0.6642	0.19	0.0345	0.0966	0.14	0.0012	0.0158	0.25	0.5332	0.6187	0.02	0.0074	0.0646	0.18	0.0135	0.1101	0.56	0.8396	1.2837	-0.02	0.6778	0.7312	0.08
3046	Peptidyl-prolyl cis-trans isomerase FKBP7	Q9Y680	0.2193	0.3954	0.15	0.9186	0.9439	0.01	0.0376	0.1020	-0.39	0.7571	0.7907	-0.06	0.2247	0.4333	-0.14	0.7061	0.9478	0.15	0.3736	0.4453	-2.55	0.8708	0.9320	-0.33	0.0651	0.1176	2.06
3047	Peptidyl-prolyl cis-trans isomerase FKBP8	Q14318	0.2414	0.4269	0.08	0.1383	0.6513	0.23	0.0070	0.0400	0.24	0.1866	0.3140	0.10	0.1850	0.3794	0.06	0.0101	0.0700	0.24	0.0105	0.1024	-0.30	0.0017	0.0339	-0.43	0.0033	0.0578	-0.41
3048	Peptidyl-prolyl cis-trans isomerase FKBP9	O95302	0.0030	0.0365	-0.08	0.1215	0.6403	-0.15	0.0004	0.0203	-0.21	0.0008	0.0150	-0.25	0.0004	0.0177	-0.20	0.1683	0.3333	-0.11	0.3736	0.5101	-2.55	0.8708	1.0519	-0.33	0.0651	0.1455	2.06
3049	Peptidyl-prolyl cis-trans isomerase H	O43447	0.4108	0.5419	-3.31	0.5752	0.7585	-1.75	0.5661	0.6859	-1.19	0.5388	0.6488	0.89	0.2938	0.4396	-1.88	0.9510	1.0457	0.09	0.7632	0.7987	0.06	0.3142	0.6624	-0.18	0.1940	0.2620	-0.27
3050	Peptidyl-prolyl cis-trans isomerase NIMA-interacting 1	Q13526	0.3503	0.5600	0.03	0.1301	0.6449	0.14	0.0166	0.0611	0.11	0.0633	0.1373	0.05	0.0118	0.0781	0.08	0.1009	0.2349	0.11	0.0689	0.2068	0.33	0.0812	0.2800	0.35	0.0117	0.0934	0.79
3051	Peptidyl-prolyl cis-trans isomerase NIMA-interacting 4	Q9Y237	0.0114	0.0612	0.05	0.1604	0.6642	0.36	0.0449	0.1161	0.22	0.0015	0.0172	0.21	0.0008	0.0223	0.11	0.1298	0.2806	0.10	0.3736	0.5245	-2.55	0.8708	1.0781	-0.33	0.0651	0.1524	2.06
3052	Peptidyl-prolyl cis-trans isomerase-like 1	Q9Y3C6	0.2163	0.3915	-0.10	0.5705	0.8837	0.08	0.1272	0.2471	0.13	0.0761	0.1579	0.17	0.5598	0.6424	-0.04	0.2266	0.4096	0.09	0.0397	0.1640	0.42	0.2651	0.5950	0.07	0.0142	0.1032	0.25
3053	Peptidylprolyl isomerase domain and WD repeat-containing protein 1	Q96BP3	0.1822	0.3460	0.16	0.6182	0.7172	-0.07	0.5612	0.7857	-0.07	0.3354	0.5092	-0.11	0.4814	0.5713	-0.08	0.7722	1.0096	-0.03	0.4882	0.5421	0.07	0.8456	1.2883	0.02	0.1865	0.2539	0.16
3054	Peptidyl-tRNA hydrolase 2 mitochondrial	Q9Y3E5	0.0213	0.0869	-0.17	0.0315	0.5099	-0.34	0.0333	0.0944	-0.10	0.0009	0.0147	-0.32	0.0056	0.0558	-0.18	0.0784	0.2003	-0.11	0.3736	0.5445	-2.55	0.8708	1.1143	-0.33	0.0651	0.1624	2.06
3055	Pericentrin	O95613	0.0521	0.1477	0.15	0.1612	0.6643	0.25	0.0033	0.0308	0.37	0.0102	0.0406	0.25	0.0464	0.1585	0.15	0.0053	0.0616	0.37	0.0660	0.2019	0.29	0.8023	1.2465	-0.03	0.6172	0.6759	0.04
3056	Perilipin-3	O60664	0.0195	0.0821	-0.17	0.3856	0.7308	0.11	0.3998	0.6029	-0.04	0.0193	0.0589	-0.11	0.0419	0.1515	-0.09	0.0154	0.0827	-0.16	0.2021	0.4153	0.16	0.4856	0.8964	-0.06	0.7316	0.7786	0.01
3057	Periodic tryptophan protein 2 homolog	Q15269	0.4108	0.5417	-3.31	0.0645	0.5893	6.96	0.5661	0.7904	-1.19	0.0300	0.0804	5.55	0.0901	0.2363	3.19	0.9510	1.1873	0.09	0.5986	0.6472	0.02	0.9222	0.9428	0.00	0.1492	0.2119	-0.09
3058	Periostin	Q15063	0.4108	0.5416	-3.31	0.5752	0.7583	-1.75	0.5661	0.6858	-1.19	0.5388	0.6486	0.89	0.2938	0.4395	-1.88	0.9510	1.0455	0.09	0.0057	0.0800	0.36	0.2728	0.6075	0.07	0.0066	0.0751	0.56
3059	Peripheral plasma membrane protein CASK	O14936	0.4108	0.5415	-3.31	0.5752	0.7582	-1.75	0.5661	0.6856	-1.19	0.5388	0.6485	0.89	0.2938	0.4393	-1.88	0.9510	1.0452	0.09	0.8161	0.8445	-0.09	0.2979	0.6411	0.84	0.0605	0.2196	1.17
3060	Peripherin	P41219	0.0010	0.0219	0.18	0.0796	0.6145	0.21	0.0001	0.0225	0.18	0.0026	0.0217	0.29	0.0003	0.0149	0.14	0.0836	0.2094	0.10	0.1278	0.3012	0.21	0.0414	0.1873	-0.28	0.0280	0.1433	-0.24
3061	Periplakin	O60437	0.0240	0.0931	0.15	0.1012	0.6323	0.22	0.0269	0.0822	0.29	0.0045	0.0271	0.24	0.0477	0.1605	0.09	0.0253	0.1010	0.17	0.2994	0.5565	0.16	0.1342	0.3862	-0.11	0.0477	0.1909	-0.13
3062	Peroxiredoxin-1	Q06830	0.0061	0.0467	0.46	0.2831	0.6639	0.27	0.0047	0.0334	0.43	0.0030	0.0226	0.50	0.0357	0.1371	0.25	0.0072	0.0649	0.66	0.0808	0.2265	0.39	0.6902	1.1325	-0.05	0.1685	0.2337	-0.16
3063	Peroxiredoxin-2	P32119	0.0103	0.0590	0.28	0.1878	0.6677	0.30	0.0092	0.0449	0.29	0.0084	0.0371	0.29	0.1972	0.3969	0.09	0.0092	0.0689	0.36	0.0637	0.1988	0.37	0.3046	0.6504	-0.09	0.0603	0.2195	-0.16
3064	Peroxiredoxin-4	Q13162	0.0672	0.1717	0.16	0.4799	0.8053	0.09	0.3497	0.5440	0.06	0.0874	0.1754	0.13	0.2202	0.4264	0.08	0.0644	0.1771	0.15	0.9981	0.9983	0.00	0.7752	1.2187	-0.04	0.1127	0.1700	-0.22
3065	Peroxiredoxin-5 mitochondrial	P30044	0.4542	0.5316	0.04	0.2161	0.6612	-0.05	0.0404	0.1073	-0.11	0.1274	0.2329	-0.11	0.0829	0.2228	-0.12	0.3741	0.5826	0.03	0.0030	0.0605	-0.32	0.2966	0.6388	-0.05	0.2431	0.3133	-0.08
3066	Peroxiredoxin-6	P30041	0.0152	0.0714	0.41	0.0754	0.6151	0.27	0.0003	0.0219	0.41	0.0000	0.0000	0.47	0.0001	0.0148	0.30	0.0004	0.0471	0.46	0.1632	0.3570	0.12	0.1956	0.4902	-0.09	0.0969	0.1520	-0.13
3067	Peroxiredoxin-like 2A	Q9BRX8	0.0464	0.1377	-0.37	0.1493	0.6604	-0.25	0.0072	0.0403	-0.52	0.0125	0.0452	-0.48	0.0013	0.0275	-0.52	0.2583	0.4491	-0.31	0.3736	0.4885	-2.55	0.8708	1.0123	-0.33	0.0651	0.1357	2.06
3068	Peroxisomal acyl-coenzyme A oxidase 1	Q15067	0.0155	0.0721	-0.52	0.2919	0.6699	-0.51	0.0107	0.0482	-0.68	0.0069	0.0336	-0.71	0.6060	0.6809	-0.06	0.0082	0.0665	-0.76	0.2585	0.4974	0.91	0.2079	0.5097	1.04	0.1076	0.1640	1.61
3069	Peroxisomal biogenesis factor 19	P40855	0.1645	0.3218	0.08	0.8971	0.9270	0.01	0.2416	0.4057	0.06	0.2747	0.4321	-0.06	0.9363	0.9512	0.00	0.2162	0.3973	0.07	0.1290	0.3028	0.27	0.0909	0.3001	0.31	0.7710	0.8114	0.03
3070	Peroxisomal membrane protein 11B	O96011	0.1263	0.2652	0.07	0.0930	0.6297	0.29	0.1073	0.2159	0.20	0.0005	0.0119	0.27	0.0004	0.0179	0.21	0.0219	0.0952	0.23	0.3736	0.5915	-2.55	0.8708	1.1978	-0.33	0.0651	0.1879	2.06
3071	Peroxisomal membrane protein PEX14	O75381	0.4108	0.5413	-3.31	0.5752	0.7580	-1.75	0.5661	0.6855	-1.19	0.5388	0.6483	0.89	0.2938	0.4392	-1.88	0.9510	1.0450	0.09	0.4657	0.5207	0.15	0.3805	0.7569	0.19	0.7989	0.8362	0.05
3072	Peroxisomal multifunctional enzyme type 2	P51659	0.0140	0.0692	0.15	0.1760	0.6621	0.26	0.0130	0.0535	0.28	0.0010	0.0152	0.32	0.0629	0.1901	0.10	0.0055	0.0619	0.32	0.0897	0.2406	0.32	0.8067	1.2504	-0.02	0.2255	0.2957	-0.09
3073	Peroxisomal targeting signal 1 receptor	P50542	0.9305	0.9475	0.00	0.2681	0.6635	-0.04	0.5128	0.7338	0.02	0.6616	0.7041	-0.01	0.0199	0.1009	-0.12	0.1923	0.3664	0.06	0.3736	0.5044	-2.55	0.8708	1.0415	-0.33	0.0651	0.1429	2.06
3074	Peroxisome proliferator-activated receptor delta	Q03181	0.4108	0.5412	-3.31	0.5752	0.7578	-1.75	0.5661	0.6853	-1.19	0.5388	0.6482	0.89	0.2938	0.4391	-1.88	0.9510	1.0448	0.09	0.0470	0.1738	-0.44	0.0012	0.0286	-1.00	0.0005	0.0298	-1.39
3075	Persulfide dioxygenase ETHE1 mitochondrial	O95571	0.1632	0.3200	0.11	0.7105	0.7878	0.05	0.0634	0.1467	-0.19	0.4892	0.6914	-0.21	0.7653	0.8174	-0.02	0.5579	0.7931	-0.06	0.9741	0.9790	0.00	0.0329	0.1637	-0.33	0.0165	0.1099	-0.41
3076	Pescadillo homolog	O00541	0.4008	0.6207	0.12	0.3586	0.7084	-0.55	0.9856	0.9881	0.01	0.0080	0.0359	0.62	0.6019	0.6776	-0.06	0.1445	0.2999	-0.33	0.0606	0.1944	0.37	0.0696	0.2548	0.30	0.7415	0.7866	0.13
3077	PEST proteolytic signal-containing nuclear protein	Q8WW12	0.4101	0.6315	0.04	0.1773	0.6631	0.27	0.0424	0.1117	0.21	0.0040	0.0255	0.24	0.0082	0.0654	0.22	0.1587	0.3188	0.12	0.0164	0.1195	0.41	0.2114	0.5141	0.14	0.1460	0.2081	0.15
3078	PH and SEC7 domain-containing protein 1	A5PKW4	0.0093	0.0571	0.31	0.1701	0.6615	0.25	0.0017	0.0236	0.41	0.0034	0.0237	0.30	0.0242	0.1126	0.20	0.0024	0.0553	0.38	0.2278	0.4537	-0.58	0.1098	0.3377	0.96	0.1382	0.1992	0.65
3079	PH and SEC7 domain-containing protein 4	Q8NDX1	0.0104	0.0590	-0.27	0.4282	0.7635	0.10	0.1386	0.2628	0.10	0.0294	0.0792	0.22	0.4586	0.5508	-0.04	0.2102	0.3910	0.08	0.3301	0.5981	-0.10	0.2947	0.6363	-0.06	0.2930	0.3647	0.06
3080	Phakinin	Q13515	0.4108	0.5410	-3.31	0.5752	0.7576	-1.75	0.5661	0.6851	-1.19	0.5388	0.6480	0.89	0.2938	0.4390	-1.88	0.9510	1.0446	0.09	0.9916	0.9937	0.00	0.3404	0.6983	-0.16	0.0209	0.1236	-0.67
3081	PHD finger protein 11	Q9UIL8	0.4108	0.5409	-3.31	0.5752	0.7574	-1.75	0.5661	0.6850	-1.19	0.5388	0.6479	0.89	0.2938	0.4388	-1.88	0.9510	1.0443	0.09	0.1020	0.2587	0.25	0.7872	1.2304	-0.08	0.4169	0.4884	0.08
3082	PHD finger protein 6	Q8IWS0	0.0047	0.0432	-0.28	0.2169	0.6605	-0.06	0.0254	0.0791	0.26	0.0028	0.0217	0.25	0.0119	0.0786	0.17	0.8085	1.0460	0.02	0.0204	0.1321	0.18	0.0258	0.1440	-0.10	0.0295	0.1464	0.07
3083	PHD finger-like domain-containing protein 5A	Q7RTV0	0.4727	0.5485	-0.02	0.4236	0.7606	-0.08	0.2084	0.3619	-0.04	0.0021	0.0197	-0.24	0.0013	0.0278	-0.35	0.0652	0.1782	-0.09	0.4925	0.5464	0.06	0.7140	1.1594	0.03	0.2038	0.2725	-0.16
3084	Phenylalanine--tRNA ligase alpha subunit	Q9Y285	0.7948	0.8339	0.01	0.2750	0.6644	0.17	0.0426	0.1120	0.16	0.0069	0.0335	0.15	0.0402	0.1474	0.08	0.1570	0.3164	0.04	0.0671	0.2033	0.16	0.0953	0.3080	0.13	0.3050	0.3763	0.07
3085	Phenylalanine--tRNA ligase beta subunit	Q9NSD9	0.0002	0.0138	0.36	0.2506	0.6606	0.16	0.0011	0.0216	0.25	0.0021	0.0203	0.38	0.4005	0.4969	0.02	0.0025	0.0553	0.36	0.6413	0.6862	-0.03	0.0977	0.3124	-0.15	0.0221	0.1265	-0.19
3086	PH-interacting protein	Q8WWQ0	0.4108	0.5408	-3.31	0.5752	0.7572	-1.75	0.5661	0.6848	-1.19	0.5388	0.6477	0.89	0.2938	0.4387	-1.88	0.9510	1.0441	0.09	0.3092	0.5696	0.06	0.0886	0.2953	0.28	0.3359	0.4068	0.25
3087	Phosducin-like protein	Q13371	0.3506	0.5602	0.06	0.0737	0.6138	0.39	0.0096	0.0457	0.29	0.0479	0.1116	0.18	0.0236	0.1109	0.22	0.1742	0.3421	0.13	0.3736	0.5087	-2.55	0.8708	1.0495	-0.33	0.0651	0.1449	2.06
3088	Phosducin-like protein 2	Q8N4E4	0.4108	0.5406	-3.31	0.5752	0.7570	-1.75	0.5661	0.6847	-1.19	0.5388	0.6476	0.89	0.2938	0.4386	-1.88	0.9510	1.0439	0.09	0.0597	0.1936	-0.28	0.0115	0.0884	-0.14	0.3509	0.4223	-0.02
3089	Phosphatase and actin regulator 4	Q8IZ21	0.0118	0.0627	0.34	0.0814	0.6147	0.54	0.0011	0.0223	0.50	0.0003	0.0114	0.64	0.0313	0.1298	0.21	0.0082	0.0668	0.43	0.0490	0.1771	0.57	0.0083	0.0750	0.58	0.0227	0.1275	0.33
3090	Phosphate carrier protein mitochondrial	Q00325	0.5670	0.6348	0.04	0.3040	0.6770	0.16	0.0199	0.0680	0.27	0.0493	0.1141	0.20	0.4446	0.5386	0.05	0.0513	0.1527	0.21	0.2428	0.4757	0.20	0.6180	1.0520	0.04	0.4546	0.5228	-0.04
3091	Phosphate-regulating neutral endopeptidase PHEX	P78562	0.0339	0.1150	0.11	0.1273	0.6421	0.06	0.0093	0.0452	-0.20	0.0199	0.0601	-0.16	0.0112	0.0756	-0.23	0.1969	0.3724	0.13	0.0296	0.1471	-0.18	0.0243	0.1386	-0.26	0.0089	0.0846	-0.30
3092	Phosphatidylcholine translocator ABCB4	P21439	0.4108	0.5405	-3.31	0.5752	0.7568	-1.75	0.5661	0.6845	-1.19	0.5388	0.6474	0.89	0.2938	0.4385	-1.88	0.9510	1.0437	0.09	0.0916	0.2428	-0.50	0.2665	0.5963	-0.27	0.0103	0.0894	1.38
3093	Phosphatidylethanolamine-binding protein 1	P30086	0.0376	0.1217	0.18	0.3452	0.7023	0.13	0.0341	0.0958	0.19	0.0465	0.1095	0.16	0.5745	0.6545	0.03	0.0246	0.1000	0.25	0.1162	0.2837	0.06	0.8859	0.9138	0.00	0.0704	0.1183	0.08
3094	Phosphatidylinositide phosphatase SAC1	Q9NTJ5	0.6045	0.6695	0.02	0.4810	0.8061	0.09	0.5312	0.7526	0.03	0.0641	0.1388	0.12	0.0065	0.0588	0.17	0.0562	0.1620	-0.09	0.9932	0.9947	0.00	0.2199	0.5271	-0.12	0.8654	0.8907	-0.01

Supplemetary Table S2. Overview on all relatively quantified 5180 proteins statistical analysis

			MCF-7																		MDA-MB-231								
			Dai SC20 vs control			Gen SC20 vs control			SSE SC20 vs control			Dai IC20 vs control			Gen IC20 vs control			SSE IC20 vs control			Dai IC20 vs control			Gen IC20 vs control			SSE IC20 vs control		
	**Protein name**	**UniProt**	**p value**	**BH q value**	**log2FC**	**p value**	**BH q value**	**log2FC**	**p value**	**BH q value**	**log2FC**	**p value**	**BH q value**	**log2FC**	**p value**	**BH q value**	**log2FC**	**p value**	**BH q value**	**log2FC**	**p value**	**BH q value**	**log2FC**	**p value**	**BH q value**	**log2FC**	**p value**	**BH q value**	**log2FC**
3095	Phosphatidylinositol 3_4_5-trisphosphate-dependent Rac exchanger 1 protein	Q8TCU6	0.0004	0.0162	0.37	0.1310	0.6457	0.18	0.0128	0.0532	0.23	0.0001	0.0076	0.22	0.0000	0.0000	0.27	0.0359	0.1224	0.27	0.3736	0.5736	-2.55	0.8708	1.1662	-0.33	0.0651	0.1778	2.06
3096	Phosphatidylinositol 3_4_5-trisphosphate-dependent Rac exchanger 2 protein	Q70Z35	0.2792	0.4728	-0.04	0.4113	0.7518	0.08	0.0431	0.1129	0.12	0.2174	0.3554	0.05	0.0682	0.1988	-0.11	0.1344	0.2866	0.10	0.0070	0.0865	0.36	0.8035	1.2469	-0.01	0.0366	0.1643	0.42
3097	Phosphatidylinositol 3-kinase catalytic subunit type 3	Q8NEB9	0.7401	0.7893	0.06	0.4103	0.7507	-0.41	0.0863	0.1831	-0.52	0.2293	0.3725	-0.34	0.0422	0.1518	-0.60	0.0717	0.1890	-0.80	0.3736	0.5489	-2.55	0.8708	1.1221	-0.33	0.0651	0.1646	2.06
3098	Phosphatidylinositol 3-kinase regulatory subunit beta	O00459	0.4799	0.5546	0.15	0.3102	0.6794	-0.52	0.3650	0.5622	-0.20	0.1774	0.3017	-0.34	0.6480	0.7160	0.09	0.9358	1.1717	-0.02	0.3736	0.4322	-2.55	0.8708	0.9072	-0.33	0.0651	0.1124	2.06
3099	Phosphatidylinositol 4_5-bisphosphate 3-kinase catalytic subunit beta isoform	P42338	0.0002	0.0136	0.34	0.1664	0.6600	0.15	0.5974	0.6537	-0.03	0.1576	0.2754	0.04	0.0183	0.0963	0.11	0.2331	0.4171	0.12	0.3736	0.4942	-2.55	0.8708	1.0228	-0.33	0.0651	0.1383	2.06
3100	Phosphatidylinositol 4-kinase alpha	P42356	0.0171	0.0764	0.16	0.2919	0.6696	0.11	0.0032	0.0302	0.32	0.0498	0.1148	0.11	0.2746	0.4991	0.05	0.0115	0.0732	0.22	0.3726	0.6545	0.07	0.4066	0.7924	-0.06	0.4779	0.5464	-0.05
3101	Phosphatidylinositol 4-kinase type 2-alpha	Q9BTU6	0.4108	0.5404	-3.31	0.5752	0.7566	-1.75	0.5661	0.6843	-1.19	0.5388	0.6473	0.89	0.2938	0.4383	-1.88	0.9510	1.0435	0.09	0.0089	0.0935	-0.83	0.0042	0.0526	-0.94	0.0163	0.1095	-0.73
3102	Phosphatidylinositol 4-phosphate 3-kinase C2 domain-containing subunit alpha	O00443	0.4108	0.5402	-3.31	0.5752	0.7564	-1.75	0.5661	0.6842	-1.19	0.5388	0.6471	0.89	0.2938	0.4382	-1.88	0.9510	1.0432	0.09	0.1478	0.3329	0.36	0.0925	0.3023	0.48	0.8138	0.8492	0.04
3103	Phosphatidylinositol 4-phosphate 5-kinase type-1 alpha	Q99755	0.4108	0.5401	-3.31	0.5752	0.7562	-1.75	0.5661	0.6840	-1.19	0.5388	0.6470	0.89	0.2938	0.4381	-1.88	0.9510	1.0430	0.09	0.0019	0.0526	0.87	0.0060	0.0622	0.81	0.0129	0.0990	0.56
3104	Phosphatidylinositol 5-phosphate 4-kinase type-2 beta	P78356	0.4108	0.5400	-3.31	0.5752	0.7560	-1.75	0.5661	0.6839	-1.19	0.5388	0.6468	0.89	0.2938	0.4380	-1.88	0.9510	1.0428	0.09	0.0258	0.1401	-0.59	0.0363	0.1738	-0.85	0.1867	0.2540	-0.73
3105	Phosphatidylinositol 5-phosphate 4-kinase type-2 gamma	Q8TBX8	0.0058	0.0459	0.27	0.0110	0.4350	0.23	0.0014	0.0233	0.39	0.0030	0.0227	0.36	0.0159	0.0900	0.30	0.0029	0.0550	0.39	0.3736	0.6387	-2.55	0.8708	1.2800	-0.33	0.0651	0.2171	2.06
3106	Phosphatidylinositol transfer protein alpha isoform	Q00169	0.0031	0.0363	0.60	0.0099	0.4459	0.46	0.4005	0.6036	0.03	0.0874	0.1753	-0.12	0.1217	0.2872	0.09	0.2030	0.3809	-0.09	0.2190	0.4407	0.08	0.1506	0.4185	0.13	0.2431	0.3134	0.07
3107	Phosphatidylinositol transfer protein beta isoform	P48739	0.3297	0.5359	0.09	0.2795	0.6650	0.13	0.0746	0.1637	0.21	0.1151	0.2160	0.16	0.4048	0.5010	0.07	0.4328	0.6538	0.10	0.1035	0.2614	0.28	0.2678	0.5979	0.14	0.2645	0.3364	0.14
3108	Phosphatidylinositol-binding clathrin assembly protein	Q13492	0.0059	0.0464	0.19	0.0618	0.5917	0.39	0.0112	0.0492	0.40	0.0007	0.0136	0.33	0.0011	0.0265	0.30	0.0081	0.0672	0.23	0.6064	0.6541	0.05	0.4607	0.8672	-0.04	0.0355	0.1612	-0.26
3109	Phosphoacetylglucosamine mutase	O95394	0.2792	0.4726	0.06	0.1701	0.6610	0.19	0.0035	0.0314	0.29	0.0013	0.0167	0.39	0.0239	0.1119	0.22	0.0072	0.0650	0.30	0.0065	0.0850	0.55	0.0352	0.1707	0.24	0.0009	0.0367	0.54
3110	Phosphoenolpyruvate carboxykinase [GTP]_ mitochondrial	Q16822	0.0019	0.0302	0.29	0.4580	0.7903	0.06	0.0002	0.0159	0.34	0.0000	0.0000	0.54	0.1304	0.2987	-0.05	0.0001	0.0247	0.57	0.0286	0.1458	0.51	0.0669	0.2490	0.17	0.0214	0.1248	0.34
3111	Phosphoenolpyruvate carboxykinase cytosolic [GTP]	P35558	0.3613	0.5739	0.11	0.7442	0.8147	0.04	0.0996	0.2033	0.25	0.0914	0.1812	0.25	0.4020	0.4982	0.10	0.0909	0.2201	0.26	0.3736	0.5373	-2.55	0.8708	1.1013	-0.33	0.0651	0.1587	2.06
3112	Phosphoglucomutase-1	P36871	0.0009	0.0216	0.93	0.0312	0.5098	0.41	0.0095	0.0456	0.47	0.0894	0.1782	0.37	0.0069	0.0602	0.64	0.0071	0.0649	0.65	0.1601	0.3532	0.12	0.5851	1.0171	-0.04	0.0563	0.2107	-0.18
3113	Phosphoglucomutase-2	Q96G03	0.5119	0.5846	0.04	0.8116	0.8647	0.02	0.5138	0.7346	0.03	0.8688	0.8875	0.01	0.1261	0.2929	-0.10	0.3014	0.4994	0.06	0.0071	0.0867	0.28	0.0333	0.1651	0.18	0.0241	0.1324	0.21
3114	Phosphoglycerate kinase 1	P00558	0.0002	0.0135	0.45	0.0517	0.5772	0.24	0.0005	0.0212	0.38	0.0022	0.0205	0.28	0.1741	0.3631	0.05	0.0045	0.0598	0.34	0.9739	0.9790	0.00	0.0136	0.0973	-0.39	0.0165	0.1101	-0.48
3115	Phosphoglycerate kinase 2	P07205	0.5998	0.6656	-0.04	0.1693	0.6614	-0.13	0.6982	0.7457	-0.03	0.5163	0.7215	0.06	0.8876	0.9139	-0.01	0.0607	0.1709	0.34	0.6442	0.6889	-0.03	0.6738	1.1130	0.03	0.8602	0.8867	0.01
3116	Phosphoglycerate mutase 1	P18669	0.0051	0.0437	0.18	0.2280	0.6602	0.24	0.0017	0.0235	0.26	0.0019	0.0195	0.17	0.1908	0.3871	-0.03	0.0250	0.1005	0.19	0.2701	0.5131	0.14	0.2431	0.5614	-0.10	0.4813	0.5496	-0.04
3117	Phosphoglycerate mutase 2	P15259	0.1501	0.2995	-0.45	0.2863	0.6671	-1.21	0.3189	0.5063	-0.29	0.5589	0.6098	-0.15	0.5189	0.6070	-0.18	0.2770	0.4723	-0.33	0.0007	0.0417	-1.05	0.0003	0.0213	-0.71	0.4476	0.5173	-0.09
3118	Phosphoinositide 3-kinase adapter protein 1	Q6ZUJ8	0.4108	0.5398	-3.31	0.5752	0.7558	-1.75	0.5661	0.6837	-1.19	0.5388	0.6467	0.89	0.2938	0.4378	-1.88	0.9510	1.0426	0.09	0.0003	0.0338	10.14	0.8708	1.3120	-0.33	0.0027	0.0542	9.75
3119	Phosphoinositide 3-kinase regulatory subunit 6	Q5UE93	0.4108	0.5397	-3.31	0.5752	0.7557	-1.75	0.5661	0.6835	-1.19	0.5388	0.6465	0.89	0.2938	0.4377	-1.88	0.9510	1.0424	0.09	0.0475	0.1745	0.49	0.2548	0.5794	0.06	0.1849	0.2523	0.06
3120	Phospholipase A-2-activating protein	Q9Y263	0.3116	0.5127	0.04	0.3707	0.7186	0.11	0.0199	0.0680	0.13	0.5367	0.7443	0.03	0.0651	0.1944	-0.09	0.2192	0.4007	0.05	0.1119	0.2754	0.33	0.3340	0.6912	-0.09	0.2428	0.3131	-0.09
3121	Phospholipase D1	Q13393	0.0996	0.2243	0.24	0.6805	0.7665	0.03	0.9619	0.9690	0.00	0.3203	0.4913	-0.16	0.1079	0.2662	0.17	0.7150	0.9551	-0.04	0.2358	0.4653	0.08	0.7467	1.1879	-0.03	0.1834	0.2509	0.16
3122	Phospholipase D2	O14939	0.7329	0.7828	-0.03	0.2437	0.6585	0.16	0.9428	0.9553	0.00	0.6429	0.6872	-0.04	0.3432	0.4400	0.09	0.7405	0.9803	0.03	0.3736	0.4425	-2.55	0.8708	0.9268	-0.33	0.0651	0.1165	2.06
3123	Phospholipase DDHD2	O94830	0.2688	0.4607	0.09	0.0121	0.4353	0.43	0.0123	0.0521	0.34	0.0006	0.0126	0.76	0.0013	0.0284	0.80	0.0027	0.0562	0.63	0.1115	0.2749	0.22	0.3828	0.7612	-0.14	0.2600	0.3321	-0.22
3124	Phospholipid phosphatase-related protein type 4	Q7Z2D5	0.4108	0.5395	-3.31	0.5752	0.7555	-1.75	0.5661	0.6834	-1.19	0.5388	0.6464	0.89	0.2938	0.4376	-1.88	0.9510	1.0421	0.09	0.0118	0.1033	0.60	0.0189	0.1177	0.50	0.0121	0.0947	0.68
3125	Phospholipid-transporting ATPase IA	Q9Y2Q0	0.4108	0.5394	-3.31	0.5752	0.7553	-1.75	0.5661	0.6832	-1.19	0.5388	0.6462	0.89	0.2938	0.4374	-1.88	0.9510	1.0419	0.09	0.2501	0.4861	1.08	0.1424	0.4022	1.53	0.0990	0.1544	1.97
3126	Phosphomannomutase 1	Q92871	0.0005	0.0181	0.31	0.1618	0.6625	0.17	0.0250	0.0785	0.22	0.1054	0.2017	0.08	0.0395	0.1454	0.07	0.0126	0.0755	0.13	0.0281	0.1447	0.12	0.4841	0.8943	-0.08	0.6063	0.6662	-0.07
3127	Phosphomannomutase 2	O15305	0.1448	0.2922	0.06	0.2119	0.6628	0.25	0.0072	0.0402	0.20	0.0082	0.0367	0.18	0.0347	0.1359	0.11	0.4188	0.6375	0.03	0.0255	0.1398	0.23	0.0038	0.0502	0.24	0.0275	0.1420	0.27
3128	Phosphomevalonate kinase	Q15126	0.8796	0.9053	-0.02	0.3509	0.7042	-0.25	0.7227	0.7673	-0.04	0.0867	0.1744	-0.15	0.0469	0.1593	-0.20	0.0440	0.1382	-0.27	0.5074	0.5608	0.08	0.0806	0.2793	0.12	0.4904	0.5584	-0.04
3129	Phosphoribosyl pyrophosphate synthase-associated protein 1	Q14558	0.8523	0.8825	-0.03	0.7106	0.7877	0.08	0.6608	0.7122	0.08	0.2790	0.4368	0.22	0.1663	0.3525	0.33	0.5474	0.7811	0.11	0.0499	0.1784	0.09	0.0130	0.0948	0.16	0.0045	0.0658	0.24
3130	Phosphoribosyl pyrophosphate synthase-associated protein 2	O60256	0.2137	0.3879	0.06	0.1980	0.6686	0.22	0.0436	0.1135	0.13	0.0055	0.0296	0.27	0.2755	0.4999	0.05	0.3645	0.5716	0.09	0.0092	0.0959	-0.69	0.0005	0.0221	0.79	0.9581	0.9667	0.00
3131	Phosphoribosylformylglycinamidine synthase	O15067	0.0048	0.0435	0.26	0.2533	0.6630	0.21	0.0090	0.0442	0.30	0.0028	0.0219	0.30	0.0916	0.2393	0.10	0.0090	0.0695	0.25	0.1551	0.3451	0.27	0.5446	0.9698	0.05	0.2863	0.3581	-0.06
3132	Phosphoribosyltransferase domain-containing protein 1	Q9NRG1	0.4108	0.5393	-3.31	0.5752	0.7551	-1.75	0.5661	0.6831	-1.19	0.5388	0.6461	0.89	0.2938	0.4373	-1.88	0.9510	1.0417	0.09	0.0957	0.2490	-3.21	0.1261	0.3705	-1.83	0.0802	0.1311	-0.55
3133	Phosphorylase b kinase gamma catalytic chain_ liver/testis isoform	P15735	0.0688	0.1744	-0.29	0.1272	0.6422	-0.18	0.0730	0.1617	-0.33	0.0043	0.0265	-0.40	0.0517	0.1685	-0.39	0.0359	0.1223	-0.34	0.3736	0.5734	-2.55	0.8708	1.1659	-0.33	0.0651	0.1777	2.06
3134	Phosphorylase b kinase gamma catalytic chain_ skeletal muscle/heart isoform	Q16816	0.4108	0.5391	-3.31	0.5752	0.7549	-1.75	0.5661	0.6829	-1.19	0.5388	0.6459	0.89	0.2938	0.4372	-1.88	0.9510	1.0415	0.09	0.0017	0.0518	0.57	0.0003	0.0207	2.00	0.0356	0.1615	0.49
3135	Phosphorylase b kinase regulatory subunit alpha_ liver isoform	P46019	0.0193	0.0816	0.09	0.1169	0.6347	0.08	0.0015	0.0230	0.19	0.0161	0.0528	0.09	0.0875	0.2315	0.05	0.1151	0.2579	0.10	0.3736	0.5295	-2.55	0.8708	1.0872	-0.33	0.0651	0.1548	2.06
3136	Phosphorylated adapter RNA export protein	Q9H814	0.6065	0.6713	-0.03	0.9338	0.9552	-0.01	0.7707	0.8114	0.03	0.0662	0.1421	0.09	0.0875	0.2314	-0.09	0.0085	0.0676	0.20	0.3736	0.6169	-2.55	0.8708	1.2423	-0.33	0.0651	0.2031	2.06
3137	Phosphoserine aminotransferase	Q9Y617	0.0013	0.0247	0.35	0.2886	0.6686	0.17	0.0213	0.0705	0.33	0.0002	0.0088	0.52	0.0102	0.0729	0.15	0.0160	0.0841	0.57	0.0251	0.1395	0.42	0.2392	0.5559	0.11	0.9942	0.9954	0.00
3138	Phosphoserine phosphatase	P78330	0.4200	0.4975	0.14	0.4528	0.7860	0.47	0.5897	0.6461	0.14	0.0166	0.0536	0.45	0.5171	0.6056	0.07	0.8333	1.0676	0.07	0.0447	0.1699	-0.28	0.2840	0.6215	-0.13	0.0391	0.1708	-0.37
3139	Phosphotriesterase-related protein	Q96BW5	0.4108	0.5390	-3.31	0.5752	0.7547	-1.75	0.5661	0.6827	-1.19	0.5388	0.6458	0.89	0.2938	0.4371	-1.88	0.9510	1.0413	0.09	0.1345	0.3124	-0.30	0.0539	0.2212	-0.48	0.2735	0.3455	-0.33
3140	Phylloquinone omega-hydroxylase CYP4F11	Q9HBI6	0.0720	0.1804	-0.20	0.6811	0.7668	-0.08	0.1425	0.2685	-0.15	0.1429	0.2550	-0.15	0.7345	0.7918	-0.03	0.3693	0.5767	-0.15	0.3736	0.4719	-2.55	0.8708	0.9817	-0.33	0.0651	0.1286	2.06
3141	Phytanoyl-CoA hydroxylase-interacting protein	Q92561	0.4108	0.5389	-3.31	0.5752	0.7545	-1.75	0.5661	0.6826	-1.19	0.5388	0.6456	0.89	0.2938	0.4369	-1.88	0.9510	1.0410	0.09	0.0086	0.0932	1.22	0.0014	0.0301	1.20	0.0477	0.1912	0.26
3142	Piezo-type mechanosensitive ion channel component 2	Q9H5I5	0.0344	0.1163	0.16	0.2820	0.6643	0.17	0.0198	0.0678	0.29	0.0001	0.0073	0.27	0.0035	0.0443	0.08	0.0009	0.0457	0.26	0.3736	0.6496	-2.55	0.8708	1.2988	-0.33	0.0651	0.2245	2.06
3143	PIH1 domain-containing protein 1	Q9NWS0	0.4108	0.5387	-3.31	0.5752	0.7543	-1.75	0.5661	0.6824	-1.19	0.5388	0.6455	0.89	0.2938	0.4368	-1.88	0.9510	1.0408	0.09	0.0092	0.0957	-1.24	0.0009	0.0263	-1.65	0.0012	0.0435	-1.95
3144	PIN2/TERF1-interacting telomerase inhibitor 1	Q96BK5	0.0492	0.1424	-13.74	0.2520	0.6623	-1.96	0.0166	0.0609	-11.63	0.0000	0.0000	0.81	0.0137	0.0840	0.43	0.0357	0.1220	-10.35	0.3736	0.5737	-2.55	0.8708	1.1665	-0.33	0.0651	0.1779	2.06
3145	Pirin	O00625	0.4127	0.4905	0.06	0.0906	0.6274	0.17	0.0047	0.0337	0.42	0.0259	0.0723	0.26	0.0067	0.0594	0.31	0.0106	0.0708	0.51	0.3736	0.6115	-2.55	0.8708	1.2328	-0.33	0.0651	0.1998	2.06
3146	PITH domain-containing protein 1	Q9GZP4	0.1492	0.2983	0.14	0.1950	0.6685	0.17	0.0281	0.0848	0.27	0.0247	0.0700	0.29	0.7194	0.7798	-0.03	0.1867	0.3587	0.20	0.0070	0.0863	0.50	0.0022	0.0393	0.61	0.0086	0.0830	0.56
3147	Pituitary homeobox 2	Q99697	0.0336	0.1144	-0.44	0.1944	0.6691	-0.64	0.0459	0.1181	-0.41	0.0255	0.0716	-0.71	0.1396	0.3113	-0.20	0.8270	1.0609	-0.03	0.3736	0.4364	-2.55	0.8708	0.9151	-0.33	0.0651	0.1140	2.06
3148	Piwi-like protein 2	Q8TC59	0.0357	0.1182	0.14	0.1168	0.6349	-0.10	0.1431	0.2695	-0.18	0.1327	0.2403	0.09	0.1309	0.2987	-0.23	0.3471	0.5522	-0.03	0.3736	0.4746	-2.55	0.8708	0.9866	-0.33	0.0651	0.1297	2.06
3149	Piwi-like protein 4	Q7Z3Z4	0.4108	0.5386	-3.31	0.5752	0.7541	-1.75	0.5661	0.6823	-1.19	0.5388	0.6453	0.89	0.2938	0.4367	-1.88	0.9510	1.0406	0.09	0.2551	0.4929	0.30	0.5407	0.9658	0.14	0.2549	0.3263	-0.30
3150	Plakophilin-3	Q9Y446	0.0603	0.1623	0.29	0.0768	0.6130	0.25	0.0723	0.1607	0.47	0.3981	0.5873	-0.17	0.2926	0.5243	-0.12	0.6639	0.9022	-0.04	0.3736	0.4469	-2.55	0.8708	0.9351	-0.33	0.0651	0.1182	2.06
3151	Plasma kallikrein	P03952	0.0282	0.1021	0.49	0.0131	0.4406	0.37	0.0005	0.0219	0.74	0.0002	0.0119	1.08	0.0000	0.0000	1.07	0.0002	0.0471	1.18	0.3736	0.6547	-2.55	0.8708	1.3075	-0.33	0.0651	0.2280	2.06
3152	Plasma membrane calcium-transporting ATPase 1	P20020	0.0022	0.0319	-0.18	0.0529	0.5818	-0.06	0.2303	0.3896	0.03	0.0240	0.0687	-0.09	0.3729	0.4699	0.09	0.5003	0.7257	-0.02	0.3736	0.4600	-2.55	0.8708	0.9595	-0.33	0.0651	0.1236	2.06
3153	Plasma membrane calcium-transporting ATPase 2	Q01814	0.0000	0.0000	0.93	0.5232	0.8422	-0.46	0.5827	0.6392	0.37	0.0000	0.0000	1.10	0.0945	0.2432	0.33	0.6212	0.8569	0.29	0.3736	0.4503	-2.55	0.8708	0.9413	-0.33	0.0651	0.1196	2.06
3154	Plasma membrane calcium-transporting ATPase 3	Q16720	0.0624	0.1655	0.25	0.3918	0.7356	-0.03	0.4064	0.6116	-0.23	0.1126	0.2127	0.08	0.4468	0.5404	0.07	0.3187	0.5214	-0.48	0.3736	0.4789	-2.55	0.8708	0.9947	-0.33	0.0651	0.1315	2.06
3155	Plasma membrane calcium-transporting ATPase 4	P23634	0.4410	0.5185	-0.04	0.8755	0.9103	-0.01	0.0022	0.0260	-0.18	0.0057	0.0304	-0.14	0.1365	0.3064	-0.12	0.8169	1.0531	-0.02	0.1736	0.3719	-1.16	0.0791	0.2761	-1.30	0.0785	0.1291	0.65
3156	Plasminogen	P00747	0.0304	0.1074	0.12	0.4313	0.7677	0.10	0.0077	0.0415	0.17	0.1523	0.2685	0.07	0.2005	0.4012	-0.05	0.0029	0.0546	-0.17	0.3736	0.6385	-2.55	0.8708	1.2796	-0.33	0.0651	0.2170	2.06
3157	Plasminogen activator inhibitor 1	P05121	0.4108	0.5384	-3.31	0.5752	0.7539	-1.75	0.5661	0.6821	-1.19	0.5388	0.6452	0.89	0.2938	0.4366	-1.88	0.9510	1.0404	0.09	0.0139	0.1108	0.53	0.0170	0.1105	0.27	0.0065	0.0753	0.36
3158	Plasminogen activator inhibitor 1 RNA-binding protein	Q8NC51	0.0774	0.1904	0.12	0.6843	0.7697	0.02	0.0041	0.0319	0.40	0.0028	0.0220	0.37	0.3031	0.4007	0.07	0.0202	0.0919	0.29	0.0301	0.1475	0.58	0.1965	0.4915	0.16	0.0347	0.1596	0.27
3159	Plastin-1	Q14651	0.8878	0.9114	0.03	0.1706	0.6600	0.35	0.1417	0.2675	0.29	0.0852	0.1723	0.37	0.0916	0.2390	0.36	0.2955	0.4925	0.18	0.0971	0.2502	-0.33	0.0556	0.2248	-0.20	0.0826	0.1341	-0.12
3160	Plastin-2	P13796	0.0700	0.1766	-0.12	0.2420	0.6601	-0.12	0.0804	0.1734	-0.17	0.0198	0.0599	-0.20	0.1664	0.3525	-0.07	0.0673	0.1819	-0.13	0.0487	0.1767	0.07	0.0366	0.1743	-0.22	0.2363	0.3062	0.18
3161	Plastin-3	P13797	0.1176	0.2522	-0.05	0.8766	0.9111	0.01	0.6096	0.6649	-0.02	0.4690	0.6683	-0.03	0.1730	0.3618	-0.04	0.1043	0.2402	0.08	0.1007	0.2567	0.28	0.5745	1.0047	0.05	0.9747	0.9796	0.00
3162	Platelet-activating factor acetylhydrolase	Q13093	0.4108	0.5383	-3.31	0.5752	0.7537	-1.75	0.5661	0.6820	-1.19	0.5388	0.6450	0.89	0.2938	0.4364	-1.88	0.9510	1.0402	0.09	0.0023	0.0544	9.76	0.8708	1.3109	-0.33	0.0031	0.0554	9.73

Supplemetary Table S2. Overview on all relatively quantified 5180 proteins statistical analysis

			MCF-7																		MDA-MB-231								
			Dai SC20 vs control			Gen SC20 vs control			SSE SC20 vs control			Dai IC20 vs control			Gen IC20 vs control			SSE IC20 vs control			Dai IC20 vs control			Gen IC20 vs control			SSE IC20 vs control		
	**Protein name**	**UniProt**	**p value**	**BH q value**	**log2FC**	**p value**	**BH q value**	**log2FC**	**p value**	**BH q value**	**log2FC**	**p value**	**BH q value**	**log2FC**	**p value**	**BH q value**	**log2FC**	**p value**	**BH q value**	**log2FC**	**p value**	**BH q value**	**log2FC**	**p value**	**BH q value**	**log2FC**	**p value**	**BH q value**	**log2FC**
3163	Platelet-activating factor acetylhydrolase IB subunit alpha	P43034	0.0142	0.0694	0.19	0.2136	0.6625	0.19	0.0088	0.0439	0.22	0.0171	0.0546	0.22	0.6974	0.7593	0.01	0.0811	0.2047	0.23	0.1280	0.3014	0.25	0.5469	0.9725	-0.06	0.2396	0.3099	-0.10
3164	Platelet-activating factor acetylhydrolase IB subunit gamma	Q15102	0.6184	0.6820	0.04	0.1963	0.6690	0.28	0.1332	0.2554	0.17	0.0770	0.1593	0.23	0.4614	0.5533	-0.06	0.5382	0.7703	0.05	0.4337	0.4896	0.09	0.9439	0.9596	0.00	0.1751	0.2410	-0.07
3165	Platelet-derived growth factor receptor alpha	P16234	0.9943	0.9951	0.00	0.9433	0.9624	-0.03	0.1013	0.2061	0.78	0.1365	0.2458	-0.23	0.0513	0.1677	0.43	0.1376	0.2906	-0.27	0.0083	0.0917	0.18	0.0817	0.2808	0.04	0.0341	0.1577	0.06
3166	Pleckstrin homology domain-containing family A member 1	Q9HB21	0.3885	0.6052	0.09	0.9644	0.9757	-0.01	0.1420	0.2679	0.19	0.2952	0.4585	0.12	0.3007	0.3988	0.11	0.2233	0.4059	0.14	0.3736	0.4972	-2.55	0.8708	1.0284	-0.33	0.0651	0.1396	2.06
3167	Pleckstrin homology domain-containing family B member 1	Q9UF11	0.0028	0.0355	-0.94	0.5963	0.7006	-0.11	0.0087	0.0437	-0.95	0.1307	0.2374	-0.24	0.0872	0.2312	0.34	0.0140	0.0786	-0.73	0.3736	0.6048	-2.55	0.8708	1.2211	-0.33	0.0651	0.1957	2.06
3168	Pleckstrin homology domain-containing family G member 3	A1L390	0.2469	0.4327	0.07	0.2720	0.6652	0.19	0.0528	0.1300	0.16	0.0075	0.0350	0.26	0.0423	0.1520	0.17	0.3283	0.5309	0.09	0.3736	0.4768	-2.55	0.8708	0.9907	-0.33	0.0651	0.1306	2.06
3169	Pleckstrin homology domain-containing family G member 4B	Q96PX9	0.4108	0.5382	-3.31	0.5752	0.7535	-1.75	0.5661	0.6818	-1.19	0.5388	0.6449	0.89	0.2938	0.4363	-1.88	0.9510	1.0399	0.09	0.0372	0.1603	-0.15	0.0125	0.0929	-0.22	0.0048	0.0672	-0.42
3170	Pleckstrin homology domain-containing family H member 1	Q9ULM0	0.4108	0.5380	-3.31	0.5752	0.7534	-1.75	0.5661	0.6816	-1.19	0.5388	0.6447	0.89	0.2938	0.4362	-1.88	0.9510	1.0397	0.09	0.0471	0.1739	0.30	0.0262	0.1455	0.22	0.0750	0.1248	0.12
3171	Pleckstrin homology domain-containing family H member 2	Q8IVE3	0.3869	0.6037	0.20	0.3948	0.7383	-0.07	0.8241	0.8581	-0.03	0.0397	0.0979	-0.27	0.1239	0.2903	-0.17	0.1262	0.2749	-0.15	0.3736	0.5257	-2.55	0.8708	1.0804	-0.33	0.0651	0.1530	2.06
3172	Pleckstrin homology domain-containing family H member 3	Q7Z736	0.0128	0.0662	-0.21	0.1737	0.6611	0.25	0.6114	0.6663	0.02	0.6153	0.6624	0.03	0.9521	0.9627	0.00	0.4939	0.7191	-0.07	0.3736	0.4608	-2.55	0.8708	0.9610	-0.33	0.0651	0.1239	2.06
3173	Pleckstrin homology domain-containing family M member 1	Q9Y4G2	0.6430	0.7048	-0.02	0.0656	0.5951	0.09	0.1376	0.2614	-0.16	0.3836	0.5685	-0.07	0.2166	0.4221	-0.09	0.0972	0.2297	-0.21	0.3736	0.5350	-2.55	0.8708	1.0972	-0.33	0.0651	0.1576	2.06
3174	Pleckstrin homology-like domain family B member 2	Q86SQ0	0.0128	0.0661	0.22	0.2898	0.6687	0.21	0.0931	0.1932	0.24	0.0283	0.0771	0.27	0.0090	0.0675	0.36	0.0880	0.2159	0.31	0.3736	0.5391	-2.55	0.8708	1.1045	-0.33	0.0651	0.1596	2.06
3175	Pleckstrin-2	Q9NYT0	0.4108	0.5379	-3.31	0.5752	0.7532	-1.75	0.5661	0.6815	-1.19	0.5388	0.6446	0.89	0.2938	0.4361	-1.88	0.9510	1.0395	0.09	0.0340	0.1535	-0.38	0.0414	0.1875	-0.35	0.1914	0.2591	-0.16
3176	Plectin	Q15149	0.0358	0.1183	0.20	0.1912	0.6683	0.28	0.0043	0.0324	0.39	0.0041	0.0260	0.40	0.0942	0.2430	0.14	0.0122	0.0747	0.43	0.0390	0.1640	0.54	0.2006	0.4986	0.15	0.2075	0.2763	0.12
3177	Plexin-A1	Q9UIW2	0.4108	0.5378	-3.31	0.5752	0.7530	-1.75	0.5661	0.6813	-1.19	0.5388	0.6444	0.89	0.2938	0.4359	-1.88	0.9510	1.0393	0.09	0.7293	0.7685	-0.03	0.0722	0.2604	-0.21	0.0850	0.1372	0.15
3178	Plexin-A3	P51805	0.4108	0.5376	-3.31	0.5752	0.7528	-1.75	0.5661	0.6812	-1.19	0.5388	0.6443	0.89	0.2938	0.4358	-1.88	0.9510	1.0391	0.09	0.4564	0.5113	-0.08	0.0404	0.1841	-0.33	0.5176	0.5838	-0.09
3179	PMS1 protein homolog 1	P54277	0.0015	0.0263	-0.42	0.5211	0.8412	-0.05	0.0432	0.1129	-0.30	0.6577	0.7007	-0.02	0.0038	0.0457	-0.24	0.0427	0.1365	-0.41	0.3281	0.5961	-0.13	0.0479	0.2044	-0.26	0.0106	0.0905	-0.53
3180	Poly [ADP-ribose] polymerase 1	P09874	0.0517	0.1471	0.16	0.2209	0.6599	0.19	0.0034	0.0310	0.37	0.0087	0.0378	0.34	0.0731	0.2066	0.14	0.0152	0.0821	0.33	0.0231	0.1364	0.41	0.4777	0.8891	0.07	0.4962	0.5635	0.06
3181	Poly [ADP-ribose] polymerase 2	Q9UGN5	0.1885	0.3544	0.04	0.4205	0.7590	-0.04	0.1120	0.2235	-0.16	0.0294	0.0792	-0.09	0.0581	0.1808	-0.16	0.2889	0.4856	-0.11	0.1778	0.3772	0.22	0.0904	0.2990	0.20	0.1904	0.2581	0.13
3182	Poly(A) polymerase alpha	P51003	0.1335	0.2766	0.08	0.1702	0.6609	0.14	0.0325	0.0927	0.14	0.0138	0.0477	0.19	0.1979	0.3978	0.06	0.0494	0.1489	0.17	0.0080	0.0917	-1.06	0.0012	0.0290	-0.76	0.0468	0.1894	-1.14
3183	Poly(A) polymerase beta	Q9NRJ5	0.6273	0.6902	-0.04	0.5537	0.8691	-0.15	0.0039	0.0314	-0.36	0.0135	0.0472	-0.22	0.0085	0.0661	-0.30	0.4804	0.7049	-0.04	0.0757	0.2189	-0.82	0.4195	0.8117	-0.14	0.8699	0.8946	0.01
3184	Poly(A) RNA polymerase mitochondrial	Q9NVV4	0.5611	0.6297	0.01	0.2110	0.6624	0.20	0.0557	0.1348	0.07	0.0068	0.0333	0.29	0.2380	0.4511	0.04	0.2379	0.4238	-0.07	0.3736	0.4933	-2.55	0.8708	1.0212	-0.33	0.0651	0.1379	2.06
3185	Poly(A)-specific ribonuclease PARN	O95453	0.4108	0.5375	-3.31	0.5752	0.7526	-1.75	0.5661	0.6810	-1.19	0.5388	0.6441	0.89	0.2938	0.4357	-1.88	0.9510	1.0388	0.09	0.0488	0.1768	-0.26	0.0950	0.3074	0.18	0.5207	0.5870	-0.06
3186	Poly(rC)-binding protein 1	Q15365	0.0048	0.0435	0.19	0.3007	0.6737	0.22	0.0035	0.0311	0.30	0.0006	0.0129	0.34	0.2080	0.4108	0.04	0.0171	0.0853	0.33	0.0921	0.2437	0.39	0.5557	0.9814	-0.06	0.4416	0.5119	-0.06
3187	Poly(rC)-binding protein 2	Q15366	0.0008	0.0205	0.43	0.2999	0.6731	-0.15	0.0234	0.0753	-0.32	0.0004	0.0113	-0.29	0.0007	0.0217	-0.26	0.8643	1.1006	0.01	0.8991	0.9173	0.02	0.3605	0.7286	-0.10	0.0433	0.1816	-0.34
3188	Poly(rC)-binding protein 4	P57723	0.0142	0.0693	-0.59	0.2659	0.6628	-0.30	0.0492	0.1236	-0.40	0.0762	0.1579	-0.39	0.8423	0.8786	0.03	0.5039	0.7297	-0.26	0.3736	0.4598	-2.55	0.8708	0.9591	-0.33	0.0651	0.1235	2.06
3189	Poly(U)-binding-splicing factor PUF60	Q9UHX1	0.8118	0.8485	0.01	0.2575	0.6623	-0.09	0.0755	0.1653	0.10	0.2018	0.3345	-0.07	0.0230	0.1100	-0.19	0.0704	0.1866	-0.10	0.2252	0.4504	0.08	0.1016	0.3205	-0.12	0.1276	0.1872	-0.11
3190	Polyadenylate-binding protein 1	P11940	0.0102	0.0589	0.32	0.4251	0.7617	0.13	0.0360	0.0992	0.18	0.0431	0.1035	0.17	0.1616	0.3445	-0.10	0.0514	0.1528	0.17	0.1260	0.2988	0.26	0.8251	1.2701	0.03	0.5706	0.6340	-0.06
3191	Polyadenylate-binding protein 1-like	Q4VXU2	0.3026	0.5021	0.08	0.3287	0.6882	0.10	0.7953	0.8326	-0.02	0.4270	0.6189	0.05	0.9986	0.9996	0.00	0.2455	0.4334	0.12	0.0431	0.1680	0.29	0.4786	0.8899	0.05	0.7710	0.8112	-0.02
3192	Polyadenylate-binding protein 1-like 2	Q5JQF8	0.0584	0.1593	0.08	0.0949	0.6319	0.32	0.0065	0.0390	0.25	0.0052	0.0292	0.36	0.0022	0.0368	0.27	0.0044	0.0611	0.19	0.0402	0.1646	0.41	0.0279	0.1518	0.19	0.1412	0.2027	0.15
3193	Polyadenylate-binding protein 3	Q9H361	0.9421	0.9567	0.04	0.9472	0.9653	-0.02	0.0249	0.0782	-0.31	0.3586	0.5370	0.52	0.1318	0.3000	-0.17	0.0027	0.0551	1.17	0.0018	0.0542	1.06	0.0323	0.1623	0.63	0.0510	0.1983	0.55
3194	Polyadenylate-binding protein 4	Q13310	0.0019	0.0301	-0.68	0.1974	0.6710	-0.38	0.0013	0.0229	-0.54	0.0005	0.0128	-0.67	0.5814	0.6593	0.03	0.0090	0.0686	-0.60	0.0052	0.0765	0.41	0.1247	0.3676	0.11	0.0397	0.1722	0.19
3195	Polyadenylate-binding protein 4-like	P0CB38	0.0005	0.0180	0.34	0.1017	0.6309	0.25	0.8705	0.8975	-0.01	0.1223	0.2259	0.07	0.0107	0.0739	0.14	0.1417	0.2957	0.13	0.0025	0.0570	-0.23	0.0549	0.2243	-0.11	0.9896	0.9919	0.00
3196	Polyadenylate-binding protein 5	Q96DU9	0.7969	0.8356	-0.02	0.2960	0.6716	0.11	0.4980	0.7202	0.07	0.5101	0.7145	0.06	0.6229	0.6949	0.05	0.6279	0.8637	0.06	0.1584	0.3505	0.21	0.1369	0.3909	-0.18	0.1818	0.2488	0.09
3197	Polyadenylate-binding protein-interacting protein 1	Q9H074	0.5491	0.6183	0.04	0.0630	0.5901	0.16	0.8537	0.8832	-0.02	0.0075	0.0350	0.31	0.0818	0.2209	0.16	0.7693	1.0071	0.05	0.8384	0.8639	-0.02	0.0618	0.2377	0.22	0.1195	0.1778	-0.15
3198	Polyamine-modulated factor 1-binding protein 1	Q8TBY8	0.4172	0.4949	0.04	0.0548	0.5853	0.31	0.0159	0.0596	0.33	0.0124	0.0450	0.26	0.0096	0.0700	0.04	0.0025	0.0563	0.40	0.0395	0.1641	-0.43	0.0033	0.0470	-0.46	0.0993	0.1546	-0.58
3199	Polycomb protein SCMH1	Q96GD3	0.4108	0.5374	-3.31	0.5752	0.7524	-1.75	0.5661	0.6808	-1.19	0.5388	0.6440	0.89	0.2938	0.4356	-1.88	0.9510	1.0386	0.09	0.0053	0.0773	0.39	0.0020	0.0370	0.79	0.0601	0.2189	0.29
3200	Polycystin-2	Q13563	0.0887	0.2077	0.19	0.3541	0.7057	-0.13	0.1610	0.2959	0.14	0.0673	0.1435	0.23	0.4533	0.5459	0.06	0.0873	0.2153	0.21	0.3736	0.5400	-2.55	0.8708	1.1061	-0.33	0.0651	0.1600	2.06
3201	Polyglutamine-binding protein 1	O60828	0.4108	0.5372	-3.31	0.5752	0.7522	-1.75	0.5661	0.6807	-1.19	0.5388	0.6438	0.89	0.2938	0.4354	-1.88	0.9510	1.0384	0.09	0.0008	0.0398	0.97	0.0002	0.0188	0.88	0.0002	0.0314	1.21
3202	Polymerase delta-interacting protein 2	Q9Y2S7	0.0266	0.0987	-0.05	0.2442	0.6571	0.16	0.0929	0.1930	0.08	0.0039	0.0252	0.19	0.0046	0.0507	0.09	0.0150	0.0814	0.07	0.0937	0.2458	0.12	0.0346	0.1688	0.20	0.6696	0.7235	0.06
3203	Polymeric immunoglobulin receptor	P01833	0.1493	0.2984	0.04	0.4983	0.8207	0.04	0.0367	0.1005	0.16	0.1009	0.1954	0.16	0.0061	0.0568	0.15	0.1778	0.3472	0.09	0.3334	0.6030	-0.12	0.0841	0.2860	0.27	0.5926	0.6537	-0.06
3204	Polypeptide N-acetylgalactosaminyltransferase 11	Q8NCW6	0.0246	0.0947	0.13	0.5060	0.8279	0.12	0.0028	0.0289	0.28	0.0492	0.1140	0.09	0.6548	0.7214	-0.02	0.7252	0.9657	0.01	0.3736	0.4440	-2.55	0.8708	0.9295	-0.33	0.0651	0.1170	2.06
3205	Polypeptide N-acetylgalactosaminyltransferase 2	Q10471	0.1749	0.3367	0.08	0.4068	0.7496	0.11	0.2277	0.3868	0.07	0.2310	0.3743	0.07	0.3193	0.4163	-0.06	0.1354	0.2874	0.10	0.0417	0.1676	0.20	0.0370	0.1755	0.23	0.9847	0.9881	0.00
3206	Polypeptide N-acetylgalactosaminyltransferase 3	Q14435	0.0622	0.1654	-1.37	0.3209	0.6841	-0.99	0.0841	0.1795	-1.15	0.0125	0.0452	-1.43	0.4067	0.5027	-0.22	0.0361	0.1227	-0.83	0.3736	0.5729	-2.55	0.8708	1.1650	-0.33	0.0651	0.1774	2.06
3207	Polypeptide N-acetylgalactosaminyltransferase 4	Q8N4A0	0.4108	0.5371	-3.31	0.5752	0.7520	-1.75	0.5661	0.6805	-1.19	0.5388	0.6437	0.89	0.2938	0.4353	-1.88	0.9510	1.0382	0.09	0.0387	0.1628	0.43	0.0581	0.2299	0.32	0.0245	0.1330	0.59
3208	Polypeptide N-acetylgalactosaminyltransferase 6	Q8NCL4	0.3486	0.5578	-0.10	0.4789	0.8046	0.06	0.4983	0.7204	-0.05	0.3725	0.5556	-0.07	0.1916	0.3877	-0.12	0.1511	0.3088	-0.15	0.3736	0.5161	-2.55	0.8708	1.0629	-0.33	0.0651	0.1484	2.06
3209	Polypyrimidine tract-binding protein 1	P26599	0.1666	0.3246	-0.03	0.0771	0.6116	-0.09	0.2113	0.3659	-0.02	0.3422	0.5166	-0.04	0.0016	0.0312	-0.23	0.0149	0.0812	0.06	0.0436	0.1685	0.33	0.7004	1.1431	0.03	0.1538	0.2170	0.17
3210	Polypyrimidine tract-binding protein 2	Q9UKA9	0.5089	0.5818	-0.02	0.1795	0.6637	-0.21	0.0007	0.0205	0.20	0.3436	0.5185	-0.02	0.0046	0.0503	0.14	0.0743	0.1938	0.37	0.0147	0.1121	0.32	0.1861	0.4763	0.12	0.0248	0.1337	0.22
3211	Polypyrimidine tract-binding protein 3	O95758	0.1443	0.2913	-0.07	0.1063	0.6307	-0.24	0.0007	0.0208	-0.24	0.0001	0.0102	-0.31	0.0356	0.1368	-0.21	0.0017	0.0515	-0.18	0.8881	0.9074	-0.01	0.3125	0.6618	0.11	0.1692	0.2345	0.21
3212	Polyribonucleotide nucleotidyltransferase 1_ mitochondrial	Q8TCS8	0.0347	0.1166	0.23	0.2826	0.6645	0.17	0.0070	0.0397	0.30	0.0002	0.0095	0.40	0.0033	0.0434	0.21	0.0008	0.0450	0.40	0.6209	0.6676	-0.03	0.6483	1.0875	-0.03	0.0113	0.0925	-0.23
3213	Porphobilinogen deaminase	P08397	0.0012	0.0240	0.14	0.4475	0.7823	0.13	0.0267	0.0817	0.27	0.0048	0.0278	0.36	0.0000	0.0000	0.28	0.1426	0.2975	0.20	0.3736	0.5191	-2.55	0.8708	1.0684	-0.33	0.0651	0.1498	2.06
3214	Potassium voltage-gated channel subfamily A member 2	P16389	0.0050	0.0434	0.98	0.9215	0.9458	-0.04	0.4191	0.6271	0.13	0.5468	0.5977	0.09	0.5370	0.6217	0.09	0.7225	0.9626	-0.05	0.3736	0.4441	-2.55	0.8708	0.9297	-0.33	0.0651	0.1171	2.06
3215	Potassium voltage-gated channel subfamily A member 5	P22460	0.0009	0.0215	-1.02	0.0129	0.4455	-0.52	0.0042	0.0326	-0.37	0.0042	0.0264	-0.37	0.0166	0.0919	-0.35	0.1046	0.2407	-0.14	0.3736	0.5322	-2.55	0.8708	1.0922	-0.33	0.0651	0.1562	2.06
3216	Potassium voltage-gated channel subfamily G member 3	Q8TAE7	0.0791	0.1928	-0.68	0.2339	0.6614	-0.71	0.0305	0.0889	-0.58	0.0522	0.1186	-0.63	0.2495	0.4669	-0.16	0.4786	0.7031	-0.57	0.3736	0.4620	-2.55	0.8708	0.9632	-0.33	0.0651	0.1244	2.06
3217	Potassium-transporting ATPase alpha chain 1	P20648	0.0555	0.1539	0.10	0.7870	0.8465	-0.04	0.3218	0.5093	0.05	0.2703	0.4262	0.06	0.0119	0.0783	-0.16	0.0697	0.1855	0.09	0.0514	0.1798	-0.27	0.9434	0.9593	0.01	0.0768	0.1270	-0.23
3218	Potassium-transporting ATPase alpha chain 2	P54707	0.0073	0.0502	0.18	0.2633	0.6637	0.26	0.0162	0.0602	0.14	0.0008	0.0143	0.28	0.0430	0.1533	0.07	0.0028	0.0547	0.22	0.0046	0.0731	2.86	0.0020	0.0371	3.30	0.0037	0.0603	3.20
3219	POTE ankyrin domain family member A	Q6S8J7	0.0225	0.0894	0.17	0.8987	0.9281	-0.01	0.0179	0.0638	0.26	0.0048	0.0279	0.42	0.4246	0.5191	-0.04	0.0495	0.1491	0.55	0.1002	0.2559	0.30	0.8985	0.9242	0.01	0.0071	0.0766	-0.24
3220	POTE ankyrin domain family member E	Q6S8J3	0.5263	0.5977	0.06	0.1972	0.6707	0.10	0.6036	0.6595	-0.05	0.8364	0.8593	0.02	0.0020	0.0346	0.54	0.1550	0.3139	-0.27	0.3736	0.5144	-2.55	0.8708	1.0599	-0.33	0.0651	0.1476	2.06
3221	POTE ankyrin domain family member F	A5A3E0	0.0024	0.0332	0.20	0.0581	0.5878	0.20	0.0004	0.0230	0.31	0.0002	0.0122	0.32	0.0008	0.0230	0.16	0.0254	0.1012	0.31	0.0374	0.1608	0.32	0.0296	0.1555	0.41	0.0820	0.1332	0.23
3222	POTE ankyrin domain family member I	P0CG38	0.0015	0.0262	0.29	0.2852	0.6652	0.22	0.0285	0.0855	0.18	0.0007	0.0134	0.37	0.0121	0.0790	0.16	0.0272	0.1058	0.44	0.1068	0.2671	0.24	0.8406	1.2841	0.02	0.1336	0.1939	-0.19
3223	POTE ankyrin domain family member J	P0CG39	0.4108	0.5370	-3.31	0.5752	0.7518	-1.75	0.5661	0.6804	-1.19	0.5388	0.6435	0.89	0.2938	0.4352	-1.88	0.9510	1.0380	0.09	0.8789	0.8994	0.09	0.1970	0.4925	0.82	0.0782	0.1287	1.32
3224	PR domain zinc finger protein 4	Q9UKN5	0.6974	0.7518	-0.05	0.0312	0.5082	0.26	0.5070	0.7285	0.09	0.0120	0.0439	-0.11	0.1385	0.3099	-0.12	0.1231	0.2697	0.09	0.3736	0.5265	-2.55	0.8708	1.0817	-0.33	0.0651	0.1534	2.06
3225	PRA1 family protein 2	O60831	0.3293	0.5359	-0.13	0.0295	0.5077	-0.44	0.0236	0.0755	-0.55	0.1115	0.2112	-0.39	0.1608	0.3438	0.21	0.7997	1.0366	-0.03	0.3736	0.4386	-2.55	0.8708	0.9194	-0.33	0.0651	0.1149	2.06
3226	PRA1 family protein 3	O75915	0.0166	0.0752	-0.25	0.3346	0.6933	-0.17	0.0064	0.0386	-0.31	0.0021	0.0200	-0.43	0.4088	0.5044	0.05	0.0023	0.0547	-0.52	0.2312	0.4587	0.07	0.1003	0.3180	-0.10	0.5883	0.6496	0.05
3227	PRAME family member 17	Q5VTA0	0.0511	0.1463	-0.21	0.3927	0.7365	-0.27	0.9967	0.9973	0.00	0.0796	0.1636	-0.26	0.3901	0.4868	-0.07	0.2946	0.4915	-0.13	0.0273	0.1433	-0.48	0.0754	0.2675	-0.19	0.0076	0.0786	-0.61
3228	PRAME family member 18	Q5VWM3	0.0052	0.0440	0.50	0.0041	0.3371	0.47	0.0059	0.0374	0.53	0.0018	0.0187	0.60	0.0085	0.0664	0.47	0.0044	0.0609	0.63	0.1459	0.3299	0.95	0.1857	0.4755	-0.88	0.4103	0.4822	0.46
3229	PRAME family member 19	Q5SWL8	0.4108	0.5368	-3.31	0.5752	0.7516	-1.75	0.5661	0.6802	-1.19	0.5388	0.6434	0.89	0.2938	0.4351	-1.88	0.9510	1.0377	0.09	0.3988	0.4547	0.30	0.0581	0.2297	0.62	0.0512	0.1988	0.63
3230	PRAME family member 2	O60811	0.4108	0.5367	-3.31	0.5752	0.7515	-1.75	0.5661	0.6801	-1.19	0.5388	0.6432	0.89	0.2938	0.4349	-1.88	0.9510	1.0375	0.09	0.0000	0.0000	-2.83	0.0070	0.0688	-3.65	0.0024	0.0541	-3.81
3231	PRAME family member 33	A0A0G2JMD5	0.0776	0.1907	0.53	0.0776	0.6118	0.30	0.0022	0.0267	0.23	0.0520	0.1183	0.75	0.0002	0.0126	0.42	0.0002	0.0450	0.77	0.3736	0.6545	-2.55	0.8708	1.3071	-0.33	0.0651	0.2279	2.06
3232	Pre-B-cell leukemia transcription factor 1	P40424	0.7417	0.7905	0.03	0.9854	0.9896	0.00	0.0102	0.0470	-0.21	0.1510	0.2667	-0.12	0.1229	0.2887	-0.08	0.1017	0.2362	-0.10	0.3736	0.5334	-2.55	0.8708	1.0943	-0.33	0.0651	0.1568	2.06
3233	Pre-B-cell leukemia transcription factor 4	Q9BYU1	0.4108	0.5365	-3.31	0.5752	0.7513	-1.75	0.5661	0.6799	-1.19	0.5388	0.6431	0.89	0.2938	0.4348	-1.88	0.9510	1.0373	0.09	0.0071	0.0861	0.79	0.0153	0.1044	0.45	0.0027	0.0551	1.20
3234	Prefoldin subunit 1	O60925	0.2616	0.4514	-0.04	0.5739	0.8858	0.05	0.4159	0.6228	-0.03	0.3266	0.4983	0.04	0.0633	0.1906	-0.09	0.4644	0.6869	-0.02	0.2032	0.4159	0.27	0.7837	1.2279	0.04	0.9184	0.9334	-0.02
3235	Prefoldin subunit 2	Q9UHV9	0.0317	0.1104	0.18	0.4778	0.8036	0.16	0.0088	0.0438	0.27	0.0207	0.0619	0.21	0.5455	0.6298	-0.04	0.0522	0.1542	0.15	0.0501	0.1784	0.32	0.2637	0.5926	0.11	0.0826	0.1340	0.15

Supplemetary Table S2. Overview on all relatively quantified 5180 proteins statistical analysis

			MCF-7																		MDA-MB-231								
			Dai SC20 vs control			Gen SC20 vs control			SSE SC20 vs control			Dai IC20 vs control			Gen IC20 vs control			SSE IC20 vs control			Dai IC20 vs control			Gen IC20 vs control			SSE IC20 vs control		
	**Protein name**	**UniProt**	**p value**	**BH q value**	**log2FC**	**p value**	**BH q value**	**log2FC**	**p value**	**BH q value**	**log2FC**	**p value**	**BH q value**	**log2FC**	**p value**	**BH q value**	**log2FC**	**p value**	**BH q value**	**log2FC**	**p value**	**BH q value**	**log2FC**	**p value**	**BH q value**	**log2FC**	**p value**	**BH q value**	**log2FC**
3236	Prefoldin subunit 3	P61758	0.1671	0.3252	0.04	0.2549	0.6618	0.17	0.0567	0.1362	0.13	0.0336	0.0870	0.16	0.0004	0.0161	0.10	0.0006	0.0420	0.12	0.9134	0.9294	0.01	0.0146	0.1012	-0.30	0.0108	0.0908	-0.38
3237	Prefoldin subunit 4	Q9NQP4	0.0826	0.1979	0.08	0.3594	0.7087	0.04	0.0020	0.0248	0.33	0.0013	0.0168	0.29	0.2421	0.4559	0.04	0.0144	0.0799	0.17	0.2079	0.4233	0.16	0.0548	0.2240	-0.23	0.2662	0.3377	-0.09
3238	Prefoldin subunit 5	Q99471	0.0157	0.0728	0.21	0.2103	0.6622	0.26	0.0035	0.0313	0.35	0.0024	0.0210	0.33	0.3277	0.4249	0.03	0.0032	0.0568	0.23	0.2568	0.4956	0.09	0.8080	1.2513	-0.01	0.0976	0.1528	-0.10
3239	Prefoldin subunit 6	O15212	0.8135	0.8501	0.01	0.2735	0.6642	0.13	0.0126	0.0525	0.18	0.0557	0.1248	0.11	0.3894	0.4865	0.03	0.0891	0.2177	0.09	0.0276	0.1438	0.18	0.0233	0.1346	0.17	0.0250	0.1342	0.21
3240	Pregnancy-specific beta-1-glycoprotein 5	Q15238	0.0974	0.2208	0.17	0.0018	0.2220	-0.62	0.0301	0.0881	-0.26	0.0485	0.1127	-0.34	0.0128	0.0807	-0.38	0.5425	0.7746	-0.05	0.3736	0.4573	-2.55	0.8708	0.9545	-0.33	0.0651	0.1224	2.06
3241	Pregnancy-specific beta-1-glycoprotein 9	Q00887	0.0229	0.0903	0.17	0.0389	0.5582	0.14	0.0042	0.0325	0.30	0.1333	0.2410	0.17	0.2303	0.4402	0.07	0.2727	0.4668	0.14	0.3102	0.5706	0.12	0.1398	0.3970	-0.25	0.0278	0.1430	-0.43
3242	Prelamin-A/C	P02545	0.9039	0.9252	0.01	0.6599	0.7490	0.09	0.1007	0.2050	0.17	0.9741	0.9777	0.00	0.1903	0.3864	-0.12	0.0615	0.1724	0.24	0.0114	0.1040	0.77	0.0501	0.2105	0.29	0.0054	0.0706	0.80
3243	pre-mRNA 3' end processing protein WDR33	Q9C0J8	0.4108	0.5364	-3.31	0.5752	0.7511	-1.75	0.5661	0.6797	-1.19	0.5388	0.6429	0.89	0.2938	0.4347	-1.88	0.9510	1.0371	0.09	0.0956	0.2490	0.53	0.7367	1.1804	-0.05	0.3292	0.4004	0.16
3244	Pre-mRNA-processing factor 17	O60508	0.4108	0.5363	-3.31	0.5752	0.7509	-1.75	0.5661	0.6796	-1.19	0.5388	0.6428	0.89	0.2938	0.4346	-1.88	0.9510	1.0369	0.09	0.0011	0.0422	1.25	0.0002	0.0185	1.13	0.0030	0.0557	1.06
3245	Pre-mRNA-processing factor 19	Q9UMS4	0.1166	0.2506	0.08	0.5391	0.8548	0.11	0.0228	0.0743	0.15	0.0062	0.0315	0.22	0.0223	0.1077	-0.19	0.0547	0.1589	0.13	0.0695	0.2075	0.20	0.6880	1.1299	0.03	0.2922	0.3640	0.09
3246	Pre-mRNA-processing factor 40 homolog A	O75400	0.9070	0.9276	0.01	0.3968	0.7407	-0.08	0.0393	0.1052	0.21	0.4714	0.6710	0.05	0.4884	0.5781	-0.05	0.5398	0.7718	0.07	0.0502	0.1784	0.17	0.0005	0.0211	-0.15	0.0271	0.1407	-0.07
3247	Pre-mRNA-processing factor 40 homolog B	Q6NWY9	0.0137	0.0685	-0.14	0.5203	0.8409	-0.08	0.0206	0.0692	0.20	0.3456	0.5213	-0.03	0.0187	0.0975	0.17	0.0021	0.0536	0.45	0.0031	0.0615	0.78	0.0005	0.0235	0.88	0.3123	0.3838	0.07
3248	Pre-mRNA-processing factor 6	O94906	0.0084	0.0543	-0.51	0.0553	0.5846	-0.28	0.7613	0.8022	0.03	0.0074	0.0348	-0.53	0.0022	0.0366	-0.86	0.0093	0.0692	-0.58	0.1334	0.3106	0.18	0.8838	0.9120	-0.01	0.2094	0.2782	-0.09
3249	Pre-mRNA-processing-splicing factor 8	Q6P2Q9	0.4108	0.5361	-3.31	0.5752	0.7507	-1.75	0.5661	0.6794	-1.19	0.5388	0.6426	0.89	0.2938	0.4345	-1.88	0.9510	1.0367	0.09	0.0561	0.1875	0.33	0.0011	0.0278	0.33	0.0095	0.0874	0.19
3250	Pre-mRNA-splicing factor 38B	Q5VTL8	0.0844	0.2005	-0.12	0.1984	0.6656	-0.75	0.0982	0.2013	0.11	0.1857	0.3129	0.08	0.0245	0.1132	-0.24	0.0071	0.0646	-0.52	0.0935	0.2454	-0.30	0.0051	0.0569	-0.23	0.0293	0.1464	-0.24
3251	Pre-mRNA-splicing factor ATP-dependent RNA helicase DHX15	O43143	0.7996	0.8376	0.02	0.8575	0.8970	0.01	0.8886	0.9115	-0.01	0.1275	0.2330	-0.11	0.0370	0.1399	-0.20	0.8687	1.1048	-0.01	0.0006	0.0398	0.65	0.0056	0.0607	0.42	0.0022	0.0520	0.46
3252	Pre-mRNA-splicing factor ATP-dependent RNA helicase PRP16	Q92620	0.4108	0.5360	-3.31	0.5752	0.7505	-1.75	0.5661	0.6793	-1.19	0.5388	0.6425	0.89	0.2938	0.4343	-1.88	0.9510	1.0364	0.09	0.3091	0.5696	-0.77	0.3650	0.7345	-0.66	0.4307	0.5021	-0.56
3253	Pre-mRNA-splicing factor SLU7	O95391	0.4108	0.5359	-3.31	0.5752	0.7503	-1.75	0.5661	0.6791	-1.19	0.5388	0.6423	0.89	0.2938	0.4342	-1.88	0.9510	1.0362	0.09	0.0195	0.1290	0.91	0.0030	0.0453	0.81	0.1620	0.2264	-0.09
3254	Pre-mRNA-splicing factor SPF27	O75934	0.3393	0.5472	-0.08	0.1423	0.6546	-0.19	0.2287	0.3882	-0.11	0.1222	0.2261	-0.16	0.0359	0.1374	-0.31	0.3764	0.5853	-0.07	0.3736	0.4712	-2.55	0.8708	0.9804	-0.33	0.0651	0.1283	2.06
3255	Prenylcysteine oxidase 1	Q9UHG3	0.9483	0.9613	0.01	0.4081	0.7494	0.12	0.3627	0.5603	0.12	0.1833	0.3099	0.21	0.0780	0.2146	0.34	0.1259	0.2744	0.26	0.0006	0.0414	0.68	0.0005	0.0242	1.01	0.0017	0.0498	0.90
3256	Pre-rRNA-processing protein TSR1 homolog	Q2NL82	0.2876	0.4840	-0.07	0.5846	0.6914	0.06	0.1963	0.3457	0.10	0.2035	0.3370	0.10	0.1615	0.3444	-0.11	0.1523	0.3097	-0.11	0.0958	0.2490	0.19	0.0326	0.1627	-0.15	0.5061	0.5728	-0.04
3257	Pre-rRNA-processing protein TSR2 homolog	Q969E8	0.4108	0.5357	-3.31	0.5752	0.7501	-1.75	0.5661	0.6790	-1.19	0.5388	0.6422	0.89	0.2938	0.4341	-1.88	0.9510	1.0360	0.09	0.0489	0.1770	-0.54	0.0121	0.0920	-0.56	0.4209	0.4923	-0.10
3258	Presequence protease mitochondrial	Q5JRX3	0.0663	0.1705	0.08	0.6008	0.7044	0.06	0.0007	0.0199	0.60	0.0094	0.0390	0.16	0.3559	0.4536	0.03	0.0060	0.0618	0.26	0.3448	0.6176	0.10	0.7171	1.1626	-0.03	0.0835	0.1352	-0.12
3259	Prion-like protein doppel	Q9UKY0	0.1346	0.2781	0.40	0.5004	0.8229	-0.14	0.1578	0.2912	0.36	0.1699	0.2915	0.34	0.0173	0.0944	0.88	0.0227	0.0965	0.90	0.3736	0.5895	-2.55	0.8708	1.1943	-0.33	0.0651	0.1867	2.06
3260	PRKC apoptosis WT1 regulator protein	Q96IZ0	0.3419	0.5495	0.05	0.0548	0.5865	0.30	0.0015	0.0232	0.34	0.0150	0.0503	0.25	0.0046	0.0504	0.25	0.0234	0.0982	0.21	0.1610	0.3541	0.13	0.1026	0.3229	0.06	0.0007	0.0318	0.22
3261	PRKR-interacting protein 1	Q9H875	0.0013	0.0246	-0.34	0.9874	0.9910	0.00	0.0095	0.0453	-0.20	0.0090	0.0383	-0.23	0.0005	0.0180	-0.52	0.1392	0.2928	-0.27	0.3736	0.5204	-2.55	0.8708	1.0707	-0.33	0.0651	0.1504	2.06
3262	Probable 28S rRNA (cytosine(4447)-C(5))-methyltransferase	P46087	0.4108	0.5356	-3.31	0.5752	0.7499	-1.75	0.5661	0.6788	-1.19	0.5388	0.6420	0.89	0.2938	0.4340	-1.88	0.9510	1.0358	0.09	0.0225	0.1362	0.43	0.8200	1.2642	-0.03	0.2100	0.2786	0.09
3263	Probable 28S rRNA (cytosine-C(5))-methyltransferase	Q96P11	0.2052	0.3776	-0.07	0.3972	0.7412	-0.04	0.0516	0.1276	-0.15	0.0884	0.1771	-0.11	0.0286	0.1227	-0.21	0.2260	0.4090	-0.14	0.3736	0.4962	-2.55	0.8708	1.0266	-0.33	0.0651	0.1392	2.06
3264	Probable 2-oxoglutarate dehydrogenase E1 component DHKTD1 mitochondrial	Q96HY7	0.0644	0.1681	0.14	0.2412	0.6607	0.19	0.0178	0.0636	0.19	0.0043	0.0266	0.24	0.7174	0.7779	-0.01	0.0045	0.0592	0.35	0.3736	0.6316	-2.55	0.8708	1.2678	-0.33	0.0651	0.2125	2.06
3265	Probable aminopeptidase NPEPL1	Q8NDH3	0.3414	0.5494	-0.06	0.0610	0.5939	0.24	0.0038	0.0321	0.19	0.5339	0.7412	0.02	0.0280	0.1214	0.10	0.0430	0.1367	-0.16	0.3736	0.5675	-2.55	0.8708	1.1554	-0.33	0.0651	0.1745	2.06
3266	Probable ATP-dependent RNA helicase DDX17	Q92841	0.6526	0.7135	-0.01	0.3614	0.7099	0.14	0.0097	0.0458	0.18	0.0037	0.0246	0.15	0.0234	0.1108	-0.11	0.0110	0.0719	0.12	0.0112	0.1032	0.44	0.0310	0.1595	0.19	0.0205	0.1229	0.28
3267	Probable ATP-dependent RNA helicase DDX20	Q9UHI6	0.1461	0.2939	0.11	0.1474	0.6565	0.20	0.0381	0.1030	0.20	0.0428	0.1030	0.19	0.2030	0.4040	0.09	0.1031	0.2382	0.13	0.0796	0.2238	0.17	0.9193	0.9409	0.01	0.0527	0.2022	0.22
3268	Probable ATP-dependent RNA helicase DDX4	Q9NQI0	0.0431	0.1319	-0.10	0.0950	0.6309	-0.21	0.0565	0.1360	0.08	0.0956	0.1876	-0.05	0.0361	0.1381	-0.06	0.1856	0.3573	-0.06	0.0032	0.0621	0.41	0.0156	0.1055	0.19	0.0018	0.0491	0.49
3269	Probable ATP-dependent RNA helicase DDX46	Q7L014	0.1386	0.2832	0.07	0.3100	0.6796	0.16	0.0602	0.1419	0.12	0.1451	0.2581	0.07	0.0742	0.2090	-0.10	0.1600	0.3207	0.07	0.0018	0.0527	-1.04	0.0005	0.0240	-1.16	0.0013	0.0455	-1.59
3270	Probable ATP-dependent RNA helicase DDX5	P17844	0.0217	0.0875	0.16	0.3652	0.7136	0.14	0.0079	0.0418	0.28	0.0084	0.0371	0.21	0.1259	0.2931	-0.08	0.0169	0.0855	0.24	0.0325	0.1511	0.36	0.6546	1.0945	-0.04	0.2690	0.3408	-0.09
3271	Probable ATP-dependent RNA helicase DDX53	Q86TM3	0.4108	0.5355	-3.31	0.5752	0.7498	-1.75	0.5661	0.6786	-1.19	0.5388	0.6419	0.89	0.2938	0.4338	-1.88	0.9510	1.0356	0.09	0.8393	0.8647	-0.02	0.0975	0.3120	0.22	0.7354	0.7817	-0.02
3272	Probable ATP-dependent RNA helicase DDX6	P26196	0.0482	0.1412	0.15	0.2270	0.6599	0.24	0.0207	0.0694	0.23	0.0112	0.0426	0.24	0.1980	0.3978	-0.07	0.0352	0.1209	0.16	0.2325	0.4602	-0.10	0.4193	0.8116	-0.05	0.0326	0.1539	-0.24
3273	Probable bifunctional dTTP/UTP pyrophosphatase/methyltransferase protein	O95671	0.0562	0.1552	0.20	0.1437	0.6541	0.23	0.0046	0.0335	0.19	0.0045	0.0273	0.41	0.0075	0.0629	0.12	0.0137	0.0780	0.05	0.0671	0.2035	0.44	0.0096	0.0810	0.89	0.2001	0.2686	0.25
3274	Probable C-mannosyltransferase DPY19L4	Q7Z388	0.0006	0.0193	0.43	0.8883	0.9201	-0.01	0.0125	0.0523	-0.24	0.9055	0.9186	0.01	0.0194	0.0996	0.29	0.1807	0.3500	-0.22	0.3736	0.5069	-2.55	0.8708	1.0461	-0.33	0.0651	0.1440	2.06
3275	Probable cytosolic iron-sulfur protein assembly protein CIAO1	O76071	0.1383	0.2828	-0.26	0.1236	0.6415	-0.75	0.1900	0.3365	-0.21	0.0178	0.0562	-0.59	0.0145	0.0856	-0.59	0.0344	0.1197	-0.52	0.3736	0.5755	-2.55	0.8708	1.1695	-0.33	0.0651	0.1788	2.06
3276	Probable E3 SUMO-protein ligase RNF212	Q495C1	0.7312	0.7813	-0.33	0.8732	0.9085	0.07	0.1289	0.2495	0.80	0.4816	0.6824	-0.79	0.3631	0.4604	-0.38	0.7754	1.0135	0.13	0.4497	0.5049	-0.21	0.9187	0.9407	0.01	0.0071	0.0765	0.43
3277	Probable E3 ubiquitin-protein ligase HECTD4	Q9Y4D8	0.0009	0.0214	0.14	0.1904	0.6696	0.19	0.0109	0.0484	0.16	0.0009	0.0150	0.24	0.0046	0.0508	0.10	0.0126	0.0757	0.29	0.0113	0.1036	0.45	0.7818	1.2257	0.02	0.0809	0.1320	-0.14
3278	Probable E3 ubiquitin-protein ligase HERC3	Q15034	0.5546	0.6240	-0.03	0.2475	0.6581	0.18	0.0613	0.1437	0.16	0.0190	0.0583	0.24	0.0413	0.1499	0.16	0.6234	0.8586	0.03	0.3736	0.4498	-2.55	0.8708	0.9405	-0.33	0.0651	0.1194	2.06
3279	Probable E3 ubiquitin-protein ligase HERC4	Q5GLZ8	0.0092	0.0569	-0.25	0.5326	0.8497	-0.07	0.1384	0.2625	-0.16	0.0012	0.0157	-0.40	0.0164	0.0913	-0.26	0.0064	0.0627	-0.29	0.2314	0.4589	0.08	0.5246	0.9478	0.04	0.2364	0.3063	-0.07
3280	Probable E3 ubiquitin-protein ligase HERC6	Q8IVU3	0.4108	0.5353	-3.31	0.5752	0.7496	-1.75	0.5661	0.6785	-1.19	0.5388	0.6418	0.89	0.2938	0.4337	-1.88	0.9510	1.0353	0.09	0.0033	0.0631	0.86	0.0012	0.0295	0.80	0.3370	0.4079	0.08
3281	Probable E3 ubiquitin-protein ligase makorin-2	Q9H000	0.0193	0.0815	0.14	0.0069	0.4205	0.10	0.5441	0.7667	-0.01	0.0364	0.0917	0.15	0.0000	0.0000	0.38	0.0681	0.1830	0.29	0.0018	0.0533	-1.50	0.0001	0.0136	-2.43	0.0019	0.0513	-2.51
3282	Probable E3 ubiquitin-protein ligase MARCH10	Q8NA82	0.0001	0.0106	0.47	0.1072	0.6303	0.42	0.0012	0.0229	0.28	0.0595	0.1310	0.29	0.0022	0.0364	0.30	0.2794	0.4753	0.18	0.3736	0.4855	-2.55	0.8708	1.0069	-0.33	0.0651	0.1344	2.06
3283	Probable ergosterol biosynthetic protein 28	Q9UKR5	0.0973	0.2207	0.15	0.7054	0.7831	0.05	0.3673	0.5647	0.07	0.0398	0.0981	0.21	0.0723	0.2053	0.17	0.0218	0.0952	0.28	0.3736	0.5916	-2.55	0.8708	1.1981	-0.33	0.0651	0.1880	2.06
3284	Probable global transcription activator SNF2L1	P28370	0.0017	0.0283	-0.18	0.5773	0.6862	-0.07	0.2170	0.3728	-0.09	0.0134	0.0469	-0.12	0.0272	0.1195	-0.11	0.3874	0.5985	-0.02	0.3218	0.5867	0.15	0.2668	0.5965	0.14	0.9158	0.9318	0.01
3285	Probable glutamate--tRNA ligase mitochondrial	Q5JPH6	0.0000	0.0000	0.26	0.0208	0.4746	0.31	0.0003	0.0225	0.39	0.0018	0.0190	0.24	0.0522	0.1693	0.07	0.0382	0.1271	0.24	0.3736	0.5714	-2.55	0.8708	1.1623	-0.33	0.0651	0.1766	2.06
3286	Probable G-protein coupled receptor 179	Q6PRD1	0.4108	0.5352	-3.31	0.5752	0.7494	-1.75	0.5661	0.6783	-1.19	0.5388	0.6416	0.89	0.2938	0.4336	-1.88	0.9510	1.0351	0.09	0.7258	0.7652	0.06	0.6062	1.0381	0.06	0.3579	0.4290	-0.14
3287	Probable helicase senataxin	Q7Z333	0.0520	0.1477	0.29	0.2176	0.6603	0.25	0.0406	0.1077	0.31	0.0149	0.0501	0.49	0.1601	0.3431	0.17	0.0252	0.1010	0.42	0.0671	0.2034	0.34	0.5824	1.0144	0.07	0.3571	0.4285	-0.09
3288	Probable histidine--tRNA ligase mitochondrial	P49590	0.4108	0.5351	-3.31	0.5752	0.7492	-1.75	0.5661	0.6782	-1.19	0.5388	0.6415	0.89	0.2938	0.4335	-1.88	0.9510	1.0349	0.09	0.9584	0.9659	0.00	0.0320	0.1616	-0.31	0.0165	0.1100	-0.52
3289	Probable JmjC domain-containing histone demethylation protein 2C	Q15652	0.0531	0.1497	0.13	0.4492	0.7837	0.08	0.0056	0.0361	0.22	0.0906	0.1799	0.04	0.3268	0.4239	0.02	0.0125	0.0755	0.12	0.0117	0.1043	0.45	0.0006	0.0229	1.56	0.9277	0.9417	0.08
3290	Probable leucine--tRNA ligase mitochondrial	Q15031	0.4108	0.5349	-3.31	0.5752	0.7490	-1.75	0.5661	0.6780	-1.19	0.5388	0.6413	0.89	0.2938	0.4333	-1.88	0.9510	1.0347	0.09	0.0621	0.1963	0.15	0.5381	0.9645	-0.04	0.0115	0.0932	0.36
3291	Probable maltase-glucoamylase 2	Q2M2H8	0.4108	0.5348	-3.31	0.5752	0.7488	-1.75	0.5661	0.6779	-1.19	0.5388	0.6412	0.89	0.2938	0.4332	-1.88	0.9510	1.0345	0.09	0.0006	0.0370	0.81	0.0001	0.0162	0.53	0.0002	0.0324	0.66
3292	Probable phosphoglycerate mutase 4	Q8N0Y7	0.5007	0.5742	0.06	0.3046	0.6772	0.10	0.9072	0.9265	-0.01	0.4768	0.6768	-0.07	0.6094	0.6834	0.05	0.7318	0.9710	0.05	0.3736	0.4433	-2.55	0.8708	0.9281	-0.33	0.0651	0.1168	2.06
3293	Probable ribonuclease ZC3H12C	Q9C0D7	0.4108	0.5347	-3.31	0.5752	0.7486	-1.75	0.5661	0.6777	-1.19	0.5388	0.6410	0.89	0.2938	0.4331	-1.88	0.9510	1.0343	0.09	0.1747	0.3733	0.11	0.0055	0.0601	0.31	0.2332	0.3032	0.07
3294	Probable ribonuclease ZC3H12D	A2A288	0.4108	0.5345	-3.31	0.5752	0.7484	-1.75	0.5661	0.6775	-1.19	0.5388	0.6409	0.89	0.2938	0.4330	-1.88	0.9510	1.0340	0.09	0.1172	0.2849	-0.08	0.0017	0.0335	-0.21	0.0172	0.1119	-0.12
3295	Probable RNA-binding protein 46	Q8TBY0	0.2449	0.4305	-0.10	0.1619	0.6614	-0.13	0.0345	0.0967	-0.29	0.0087	0.0377	-0.47	0.0089	0.0676	-0.41	0.0541	0.1576	-0.22	0.7180	0.7581	-0.02	0.0098	0.0811	-0.13	0.0066	0.0763	-0.31
3296	Probable RNA-binding protein EIF1AD	Q8N9N8	0.0168	0.0759	-0.21	0.5156	0.8388	-0.07	0.0233	0.0752	-0.17	0.5703	0.6204	0.04	0.0508	0.1667	-0.13	0.2194	0.4007	-0.21	0.1288	0.3030	-0.19	0.4209	0.8135	0.10	0.5426	0.6080	-0.14
3297	Probable tRNA N6-adenosine threonylcarbamoyltransferase	Q9NPF4	0.0500	0.1441	0.16	0.8343	0.8804	0.05	0.0001	0.0136	-0.84	0.0284	0.0773	0.23	0.7333	0.7910	-0.02	0.2559	0.4462	0.26	0.1186	0.2872	0.54	0.1437	0.4048	0.49	0.0859	0.1381	0.67
3298	Probable ubiquitin carboxyl-terminal hydrolase FAF-X	Q93008	0.0192	0.0815	0.18	0.0262	0.4953	0.21	0.0157	0.0592	0.23	0.0169	0.0543	0.23	0.0815	0.2206	0.11	0.0103	0.0698	0.24	0.0538	0.1841	0.26	0.2178	0.5240	-0.12	0.2055	0.2742	-0.10
3299	Probable ubiquitin carboxyl-terminal hydrolase FAF-Y	O00507	0.4108	0.5344	-3.31	0.5752	0.7483	-1.75	0.5661	0.6774	-1.19	0.5388	0.6407	0.89	0.2938	0.4328	-1.88	0.9510	1.0338	0.09	0.0065	0.0838	-0.41	0.0008	0.0251	-0.24	0.0351	0.1610	-0.32
3300	Pro-cathepsin H	P09668	0.5525	0.6219	-0.06	0.2364	0.6630	-0.76	0.2198	0.3766	-0.15	0.0454	0.1075	-0.25	0.0033	0.0426	-0.57	0.0290	0.1091	-0.37	0.3736	0.5815	-2.55	0.8708	1.1802	-0.33	0.0651	0.1822	2.06
3301	Procollagen galactosyltransferase 1	Q8NBJ5	0.4108	0.5343	-3.31	0.5752	0.7481	-1.75	0.5661	0.6772	-1.19	0.5388	0.6406	0.89	0.2938	0.4327	-1.88	0.9510	1.0336	0.09	0.0969	0.2501	0.90	0.0785	0.2748	1.01	0.4540	0.5226	0.29
3302	Procollagen-lysine 2-oxoglutarate 5-dioxygenase 1	Q02809	0.0563	0.1553	0.19	0.1803	0.6647	0.30	0.0041	0.0322	0.41	0.0059	0.0310	0.37	0.2671	0.4891	0.08	0.0246	0.1001	0.26	0.0818	0.2283	-0.15	0.3424	0.7013	0.06	0.1360	0.1968	-0.12
3303	Procollagen-lysine 2-oxoglutarate 5-dioxygenase 2	O00469	0.0381	0.1226	0.12	0.2291	0.6608	0.21	0.0250	0.0784	0.14	0.0061	0.0313	0.24	0.0017	0.0326	0.39	0.0024	0.0565	0.36	0.0619	0.1961	-0.33	0.1014	0.3207	-0.20	0.0013	0.0434	-0.39
3304	Proenkephalin-A	P01210	0.1160	0.2498	-0.28	0.0536	0.5845	-0.18	0.0426	0.1121	-0.25	0.0476	0.1111	-0.34	0.0008	0.0213	0.21	0.9514	0.9599	0.01	0.7067	0.7480	-0.27	0.4046	0.7903	0.31	0.0199	0.1217	1.36
3305	Proenkephalin-B	P01213	0.0492	0.1423	0.25	0.2218	0.6580	0.25	0.0013	0.0228	0.33	0.0010	0.0157	0.34	0.0000	0.0000	0.91	0.0149	0.0813	0.70	0.3736	0.6031	-2.55	0.8708	1.2181	-0.33	0.0651	0.1947	2.06
3306	Profilin-1	P07737	0.0001	0.0104	0.34	0.0763	0.6166	0.16	0.0242	0.0767	0.15	0.0021	0.0199	0.07	0.0211	0.1046	-0.11	0.0635	0.1758	0.25	0.7495	0.7862	0.03	0.1927	0.4872	0.12	0.2013	0.2696	-0.11
3307	Profilin-2	P35080	0.0431	0.1318	0.10	0.2425	0.6604	0.24	0.0130	0.0534	0.28	0.0003	0.0108	0.42	0.0035	0.0441	0.24	0.0026	0.0566	0.35	0.0299	0.1472	0.40	0.2659	0.5952	0.09	0.1602	0.2244	0.11
3308	Profilin-3	P60673	0.4108	0.5341	-3.31	0.5752	0.7479	-1.75	0.5661	0.6771	-1.19	0.5388	0.6404	0.89	0.2938	0.4326	-1.88	0.9510	1.0334	0.09	0.4573	0.5120	0.17	0.1156	0.3488	0.53	0.1526	0.2157	0.40
3309	Programmed cell death 6-interacting protein	Q8WUM4	0.1136	0.2459	0.17	0.3569	0.7086	0.15	0.1001	0.2041	0.18	0.0565	0.1260	0.23	0.9227	0.9422	0.01	0.0474	0.1448	0.27	0.1629	0.3569	0.19	0.6605	1.1005	-0.04	0.1838	0.2512	-0.10
3310	Programmed cell death protein 10	Q9BUL8	0.4108	0.5340	-3.31	0.5752	0.7477	-1.75	0.5661	0.6769	-1.19	0.5388	0.6403	0.89	0.2938	0.4325	-1.88	0.9510	1.0332	0.09	0.2993	0.5565	0.18	0.6613	1.1008	0.11	0.4445	0.5146	0.12
3311	Programmed cell death protein 2-like	Q9BRP1	0.0049	0.0431	-0.26	0.9214	0.9459	0.02	0.7782	0.8178	0.04	0.0522	0.1186	0.14	0.5818	0.6596	-0.06	0.0808	0.2044	-0.43	0.7484	0.7856	0.08	0.8714	0.9020	-0.04	0.9369	0.9490	-0.02

Supplemetary Table S2. Overview on all relatively quantified 5180 proteins statistical analysis

			MCF-7																		MDA-MB-231								
			Dai SC20 vs control			Gen SC20 vs control			SSE SC20 vs control			Dai IC20 vs control			Gen IC20 vs control			SSE IC20 vs control			Dai IC20 vs control			Gen IC20 vs control			SSE IC20 vs control		
	**Protein name**	**UniProt**	**p value**	**BH q value**	**log2FC**	**p value**	**BH q value**	**log2FC**	**p value**	**BH q value**	**log2FC**	**p value**	**BH q value**	**log2FC**	**p value**	**BH q value**	**log2FC**	**p value**	**BH q value**	**log2FC**	**p value**	**BH q value**	**log2FC**	**p value**	**BH q value**	**log2FC**	**p value**	**BH q value**	**log2FC**
3312	Programmed cell death protein 4	Q53EL6	0.5641	0.6322	-0.05	0.0136	0.4431	-0.52	0.0769	0.1675	-0.25	0.0328	0.0856	-0.32	0.0412	0.1498	-0.34	0.6694	0.9082	-0.04	0.6314	0.6770	-0.10	0.0065	0.0654	0.18	0.0056	0.0716	0.29
3313	Programmed cell death protein 5	O14737	0.1770	0.3392	-0.10	0.1320	0.6475	-0.17	0.5267	0.7477	0.04	0.1118	0.2116	-0.13	0.0410	0.1496	-0.21	0.1694	0.3343	-0.10	0.8013	0.8325	0.03	0.9332	0.9514	0.01	0.4776	0.5461	-0.06
3314	Programmed cell death protein 6	O75340	0.0610	0.1633	0.13	0.5841	0.6913	0.07	0.6668	0.7179	-0.02	0.6228	0.6692	-0.02	0.1336	0.3027	-0.09	0.4640	0.6867	0.04	0.0041	0.0690	-0.46	0.0033	0.0476	-0.33	0.0004	0.0276	-0.64
3315	Prohibitin	P35232	0.0308	0.1083	0.18	0.2760	0.6650	0.21	0.0117	0.0506	0.24	0.0072	0.0344	0.27	0.6626	0.7279	-0.02	0.0231	0.0972	0.23	0.0791	0.2233	0.33	0.6200	1.0551	0.04	0.0792	0.1300	-0.13
3316	Prohibitin-2	Q99623	0.0314	0.1097	0.17	0.4410	0.7770	0.14	0.0209	0.0699	0.27	0.0275	0.0754	0.19	0.0985	0.2506	-0.11	0.0189	0.0886	0.24	0.0349	0.1546	0.39	0.2332	0.5483	0.16	0.8497	0.8787	0.02
3317	Prolactin regulatory element-binding protein	Q9HCU5	0.2255	0.4043	0.32	0.0398	0.5633	0.73	0.0286	0.0858	0.82	0.1484	0.2629	0.41	0.0215	0.1057	0.93	0.3588	0.5649	0.22	0.3736	0.4734	-2.55	0.8708	0.9844	-0.33	0.0651	0.1292	2.06
3318	Proliferating cell nuclear antigen	P12004	0.0055	0.0448	0.29	0.1795	0.6642	0.29	0.0384	0.1035	0.24	0.0031	0.0227	0.31	0.0883	0.2328	-0.10	0.0469	0.1439	0.15	0.1165	0.2839	0.30	0.2069	0.5079	-0.15	0.0810	0.1321	-0.24
3319	Proliferation-associated protein 2G4	Q9UQ80	0.0387	0.1236	0.17	0.2541	0.6631	0.25	0.0034	0.0309	0.38	0.0048	0.0281	0.33	0.6323	0.7024	0.02	0.0224	0.0958	0.26	0.0423	0.1676	0.51	0.2414	0.5595	0.15	0.2087	0.2776	0.13
3320	Proline-rich protein 18	Q8N4B5	0.2253	0.4041	-0.05	0.1307	0.6454	-0.38	0.0132	0.0538	-0.36	0.0086	0.0375	-0.52	0.0002	0.0136	-0.78	0.0276	0.1067	-0.83	0.3736	0.5833	-2.55	0.8708	1.1833	-0.33	0.0651	0.1832	2.06
3321	Proline-serine-threonine phosphatase-interacting protein 1	O43586	0.7458	0.7931	0.02	0.5317	0.8503	0.08	0.6223	0.6762	0.03	0.2298	0.3729	0.10	0.5129	0.6019	-0.05	0.1842	0.3551	0.11	0.3736	0.5058	-2.55	0.8708	1.0442	-0.33	0.0651	0.1436	2.06
3322	Proline-serine-threonine phosphatase-interacting protein 2	Q9H939	0.4108	0.5339	-3.31	0.5752	0.7475	-1.75	0.5661	0.6768	-1.19	0.5388	0.6401	0.89	0.2938	0.4324	-1.88	0.9510	1.0330	0.09	0.0088	0.0932	-0.95	0.0021	0.0380	-0.73	0.6449	0.7014	-0.20
3323	Prolyl 3-hydroxylase 1	Q32P28	0.4108	0.5337	-3.31	0.5752	0.7473	-1.75	0.5661	0.6766	-1.19	0.5388	0.6400	0.89	0.2938	0.4322	-1.88	0.9510	1.0327	0.09	0.1815	0.3823	0.15	0.3945	0.7782	-0.03	0.3551	0.4268	-0.08
3324	Prolyl 3-hydroxylase 2	Q8IVL5	0.0205	0.0846	0.80	0.0603	0.5938	-0.81	0.0051	0.0350	1.43	0.0017	0.0183	1.57	0.0020	0.0355	1.16	0.0321	0.1157	2.38	0.0885	0.2395	1.12	0.1170	0.3522	-0.87	0.1945	0.2625	1.27
3325	Prolyl 3-hydroxylase OGFOD1	Q8N543	0.4108	0.5336	-3.31	0.5752	0.7471	-1.75	0.5661	0.6764	-1.19	0.5388	0.6398	0.89	0.2938	0.4321	-1.88	0.9510	1.0325	0.09	0.5347	0.5867	0.05	0.7867	1.2304	-0.02	0.6020	0.6628	0.03
3326	Prolyl 4-hydroxylase subunit alpha-1	P13674	0.2517	0.4384	-0.08	0.1010	0.6326	0.33	0.3379	0.5304	0.07	0.6983	0.7376	-0.03	0.0693	0.2003	0.18	0.5909	0.8277	-0.03	0.3114	0.5718	0.11	0.9034	0.9278	0.01	0.0162	0.1090	-0.28
3327	Prolyl 4-hydroxylase subunit alpha-2	O15460	0.4108	0.5335	-3.31	0.5752	0.7469	-1.75	0.5661	0.6763	-1.19	0.5388	0.6397	0.89	0.2938	0.4320	-1.88	0.9510	1.0323	0.09	0.3685	0.6501	-0.13	0.1973	0.4930	0.21	0.0458	0.1871	0.42
3328	Prolyl endopeptidase	P48147	0.3058	0.5067	0.08	0.2340	0.6609	0.13	0.0030	0.0299	0.25	0.0093	0.0388	0.18	0.2436	0.4577	0.05	0.0196	0.0902	0.26	0.0704	0.2095	0.23	0.0549	0.2241	-0.26	0.6501	0.7061	-0.15
3329	Prolyl endopeptidase FAP	Q12884	0.0321	0.1114	0.45	0.1981	0.6681	0.53	0.0021	0.0258	0.27	0.0043	0.0268	0.55	0.0335	0.1339	-0.20	0.1700	0.3353	0.27	0.1584	0.3506	-3.54	0.4404	0.8421	-1.35	0.9327	0.9460	0.12
3330	Pro-opiomelanocortin	P01189	0.1992	0.3696	-0.27	0.3592	0.7086	-0.37	0.6898	0.7384	0.10	0.2098	0.3454	0.26	0.9898	0.9931	0.00	0.5603	0.7956	-0.10	0.3736	0.4560	-2.55	0.8708	0.9520	-0.33	0.0651	0.1219	2.06
3331	Properdin	P27918	0.2941	0.4922	-0.15	0.8368	0.8823	-0.03	0.8583	0.8871	-0.03	0.7868	0.8166	-0.03	0.3572	0.4547	-0.13	0.7892	1.0266	0.03	0.3736	0.4393	-2.55	0.8708	0.9207	-0.33	0.0651	0.1152	2.06
3332	Proprotein convertase subtilisin/kexin type 4	Q6UW60	0.1649	0.3221	-0.17	0.8656	0.9029	0.02	0.0396	0.1058	-0.20	0.1663	0.2865	0.17	0.9057	0.9288	0.01	0.2305	0.4137	-0.15	0.9176	0.9329	-0.01	0.8744	0.9050	0.01	0.0331	0.1554	0.20
3333	Prospero homeobox protein 1	Q92786	0.7316	0.7815	0.01	0.2313	0.6627	0.22	0.0080	0.0420	0.26	0.0002	0.0093	0.27	0.0054	0.0550	0.12	0.0070	0.0649	0.18	0.0407	0.1657	-0.34	0.1906	0.4830	-0.10	0.0026	0.0543	-0.28
3334	Prostaglandin E synthase 2	Q9H7Z7	0.7523	0.7982	-0.01	0.1284	0.6426	-0.09	0.0260	0.0805	-0.24	0.0170	0.0545	-0.18	0.0102	0.0723	-0.20	0.2274	0.4104	-0.11	0.3736	0.4957	-2.55	0.8708	1.0256	-0.33	0.0651	0.1389	2.06
3335	Prostaglandin E synthase 3	Q15185	0.0052	0.0439	0.20	0.8399	0.8850	-0.01	0.0066	0.0392	0.23	0.4148	0.6070	0.03	0.0344	0.1353	-0.11	0.2556	0.4458	0.09	0.6640	0.7082	0.04	0.0104	0.0837	-0.26	0.0304	0.1479	-0.19
3336	Prostaglandin F2 receptor negative regulator	Q9P2B2	0.5305	0.6016	0.04	0.3011	0.6743	0.17	0.5280	0.7489	0.05	0.1535	0.2699	0.11	0.6664	0.7310	-0.03	0.1731	0.3403	0.10	0.3736	0.5090	-2.55	0.8708	1.0500	-0.33	0.0651	0.1450	2.06
3337	Prostaglandin reductase 1	Q14914	0.0623	0.1655	0.09	0.2546	0.6627	0.18	0.0106	0.0479	0.27	0.0001	0.0077	0.42	0.0025	0.0392	0.37	0.0290	0.1092	0.31	0.0618	0.1960	0.52	0.0935	0.3042	0.24	0.0245	0.1329	0.31
3338	Prostate and testis expressed protein 2	Q6UY27	0.5928	0.6592	0.08	0.2895	0.6686	0.14	0.3273	0.5161	0.27	0.2016	0.3343	0.18	0.5778	0.6565	-0.12	0.1446	0.2997	0.42	0.0435	0.1683	0.37	0.3481	0.7105	0.12	0.0195	0.1208	0.64
3339	Prostate tumor-overexpressed gene 1 protein	Q86YD1	0.2737	0.4668	-0.43	0.3437	0.7009	-0.66	0.7537	0.7953	0.08	0.3288	0.5006	0.24	0.5052	0.5942	0.16	0.5727	0.8079	-0.24	0.3736	0.4545	-2.55	0.8708	0.9492	-0.33	0.0651	0.1213	2.06
3340	Proteasomal ubiquitin receptor ADRM1	Q16186	0.0071	0.0496	0.23	0.0171	0.4613	0.31	0.0006	0.0210	0.41	0.0002	0.0116	0.55	0.0180	0.0959	0.17	0.0021	0.0539	0.39	0.0965	0.2496	0.43	0.1444	0.4061	-0.19	0.2154	0.2841	0.19
3341	Proteasome activator complex subunit 1	Q06323	0.4014	0.6212	-0.06	0.5909	0.6958	0.10	0.9533	0.9633	0.00	0.3154	0.4852	0.08	0.1015	0.2555	-0.14	0.2122	0.3934	0.09	0.1070	0.2670	0.17	0.8147	1.2594	0.02	0.0799	0.1309	-0.14
3342	Proteasome activator complex subunit 2	Q9UL46	0.0713	0.1792	0.10	0.2324	0.6622	0.27	0.0040	0.0321	0.25	0.0022	0.0203	0.29	0.3654	0.4622	0.04	0.0105	0.0704	0.29	0.0845	0.2322	0.21	0.4587	0.8637	0.02	0.1014	0.1572	-0.03
3343	Proteasome activator complex subunit 3	P61289	0.1685	0.3268	-0.09	0.8520	0.8938	0.01	0.0821	0.1761	0.13	0.6663	0.7087	-0.02	0.0917	0.2389	0.12	0.0428	0.1365	-0.19	0.0016	0.0528	0.18	0.0743	0.2643	0.06	0.0010	0.0384	0.26
3344	Proteasome activator complex subunit 4	Q14997	0.0947	0.2167	0.07	0.4912	0.8145	0.04	0.0023	0.0264	0.11	0.0020	0.0198	0.11	0.5252	0.6120	0.02	0.0092	0.0688	0.15	0.3736	0.6157	-2.55	0.8708	1.2402	-0.33	0.0651	0.2024	2.06
3345	Proteasome adapter and scaffold protein ECM29	Q5VYK3	0.0141	0.0693	0.18	0.2192	0.6601	0.20	0.0037	0.0317	0.23	0.0042	0.0265	0.23	0.4604	0.5524	0.03	0.0171	0.0851	0.16	0.0505	0.1789	0.27	0.9731	0.9807	0.00	0.1913	0.2590	0.05
3346	Proteasome assembly chaperone 1	O95456	0.0022	0.0318	0.24	0.4131	0.7524	0.12	0.0029	0.0293	0.31	0.0083	0.0370	0.15	0.1764	0.3670	0.05	0.0016	0.0508	0.27	0.0212	0.1344	-0.26	0.2940	0.6353	-0.15	0.0952	0.1498	0.39
3347	Proteasome assembly chaperone 2	Q969U7	0.2065	0.3789	-0.28	0.5968	0.7008	-0.41	0.8335	0.8659	0.04	0.7540	0.7878	-0.07	0.2730	0.4967	0.24	0.5655	0.8004	-0.12	0.0269	0.1420	-0.68	0.0132	0.0958	1.28	0.4783	0.5467	-0.13
3348	Proteasome assembly chaperone 3	Q9BT73	0.0071	0.0496	-0.19	0.0671	0.5901	-0.18	0.0218	0.0718	-0.50	0.0003	0.0106	-0.78	0.0056	0.0560	-0.60	0.5212	0.7493	-0.16	0.3736	0.4587	-2.55	0.8708	0.9571	-0.33	0.0651	0.1230	2.06
3349	Proteasome assembly chaperone 4	Q5JS54	0.0400	0.1260	0.21	0.8971	0.9272	0.02	0.0032	0.0301	0.18	0.0840	0.1702	0.20	0.8079	0.8506	0.02	0.0119	0.0739	0.18	0.6133	0.6608	-0.98	0.8396	1.2833	0.36	0.7216	0.7702	0.63
3350	Proteasome inhibitor PI31 subunit	Q92530	0.0596	0.1615	-0.35	0.0283	0.5072	-0.47	0.1989	0.3496	-0.68	0.0091	0.0382	-0.65	0.0296	0.1256	-0.47	0.0614	0.1723	-0.36	0.1947	0.4031	0.22	0.1623	0.4381	0.14	0.0567	0.2118	0.19
3351	Proteasome subunit alpha type-1	P25786	0.7794	0.8206	0.02	0.5256	0.8440	0.11	0.0398	0.1062	0.18	0.0518	0.1179	0.17	0.5505	0.6338	-0.03	0.0526	0.1550	0.19	0.0230	0.1365	0.39	0.0945	0.3067	0.18	0.2310	0.3013	0.09
3352	Proteasome subunit alpha type-2	P25787	0.0547	0.1530	0.17	0.6137	0.7147	-0.07	0.0633	0.1466	0.21	0.7853	0.8155	0.02	0.1962	0.3955	-0.12	0.7945	1.0317	0.01	0.1587	0.3509	0.29	0.7276	1.1730	0.05	0.9794	0.9838	0.00
3353	Proteasome subunit alpha type-3	P25788	0.1788	0.3415	0.17	0.5183	0.8395	0.09	0.0636	0.1467	0.27	0.0363	0.0915	0.34	0.6795	0.7429	0.04	0.0911	0.2203	0.25	0.0582	0.1921	0.35	0.7631	1.2048	0.03	0.4309	0.5023	0.05
3354	Proteasome subunit alpha type-4	P25789	0.0121	0.0636	0.18	0.2387	0.6612	0.21	0.0256	0.0795	0.16	0.0095	0.0391	0.21	0.9899	0.9930	0.00	0.0178	0.0868	0.19	0.2193	0.4406	0.12	0.0595	0.2330	-0.18	0.0150	0.1053	-0.33
3355	Proteasome subunit alpha type-5	P28066	0.0387	0.1235	0.21	0.2331	0.6627	0.20	0.0373	0.1014	0.23	0.0293	0.0790	0.24	0.7278	0.7871	0.02	0.0277	0.1066	0.31	0.1631	0.3571	0.19	0.2835	0.6209	0.13	0.6327	0.6893	-0.04
3356	Proteasome subunit alpha type-6	P60900	0.0107	0.0597	0.18	0.4514	0.7849	0.14	0.0202	0.0683	0.20	0.0134	0.0470	0.17	0.1678	0.3545	-0.06	0.0185	0.0879	0.19	0.8195	0.8471	0.02	0.1894	0.4812	-0.11	0.0121	0.0945	-0.36
3357	Proteasome subunit alpha type-7	O14818	0.0707	0.1781	0.10	0.2224	0.6576	0.16	0.0087	0.0438	0.20	0.0146	0.0495	0.19	0.0942	0.2429	-0.08	0.0188	0.0885	0.19	0.2497	0.4855	0.14	0.2474	0.5691	0.10	0.1714	0.2370	-0.13
3358	Proteasome subunit alpha-type 8	Q8TAA3	0.4108	0.5333	-3.31	0.5752	0.7468	-1.75	0.5661	0.6761	-1.19	0.5388	0.6395	0.89	0.2938	0.4319	-1.88	0.9510	1.0321	0.09	0.3087	0.5691	-1.56	0.3392	0.6970	-0.35	0.1267	0.1864	0.28
3359	Proteasome subunit beta type-1	P20618	0.0832	0.1988	0.13	0.2471	0.6584	0.23	0.0166	0.0610	0.24	0.0118	0.0436	0.25	0.8814	0.9091	-0.01	0.0212	0.0940	0.24	0.0655	0.2012	0.24	0.0941	0.3060	0.18	0.5168	0.5832	0.05
3360	Proteasome subunit beta type-2	P49721	0.3358	0.5427	0.08	0.5661	0.8798	0.08	0.2287	0.3880	0.11	0.1116	0.2113	0.16	0.2473	0.4638	-0.10	0.1123	0.2536	0.17	0.5023	0.5561	-0.03	0.7343	1.1772	-0.03	0.1049	0.1611	-0.26
3361	Proteasome subunit beta type-3	P49720	0.0333	0.1140	0.24	0.3988	0.7426	0.08	0.1001	0.2041	0.14	0.3128	0.4815	-0.07	0.0511	0.1672	-0.21	0.1928	0.3669	0.10	0.0011	0.0435	-0.28	0.0009	0.0268	-0.35	0.0183	0.1162	-0.84
3362	Proteasome subunit beta type-4	P28070	0.0504	0.1451	0.16	0.0733	0.6124	0.23	0.0042	0.0324	0.32	0.0015	0.0176	0.22	0.4072	0.5031	0.02	0.0448	0.1397	0.28	0.0411	0.1667	0.29	0.6934	1.1359	0.02	0.4043	0.4759	0.08
3363	Proteasome subunit beta type-5	P28074	0.5175	0.5897	0.03	0.3255	0.6857	0.17	0.0217	0.0716	0.26	0.0257	0.0720	0.19	0.5555	0.6389	-0.03	0.0347	0.1198	0.20	0.0179	0.1238	-0.35	0.0029	0.0450	-0.63	0.0084	0.0826	-0.67
3364	Proteasome subunit beta type-6	P28072	0.0044	0.0417	0.25	0.1643	0.6628	0.40	0.0020	0.0251	0.51	0.0296	0.0797	0.35	0.2330	0.4439	0.05	0.0062	0.0624	0.33	0.0604	0.1946	0.34	0.9141	0.9371	-0.01	0.3180	0.3893	0.07
3365	Proteasome subunit beta type-7	Q99436	0.0082	0.0536	0.19	0.1559	0.6630	0.17	0.0069	0.0398	0.20	0.0130	0.0462	0.15	0.0656	0.1950	-0.10	0.0044	0.0603	0.26	0.3085	0.5689	-0.14	0.0077	0.0720	-0.54	0.0191	0.1198	-0.52
3366	Proteasome subunit beta type-8	P28062	0.4108	0.5332	-3.31	0.5752	0.7466	-1.75	0.5661	0.6760	-1.19	0.5388	0.6394	0.89	0.2938	0.4317	-1.88	0.9510	1.0319	0.09	0.5005	0.5544	0.08	0.8262	1.2707	-0.02	0.0850	0.1371	-0.13
3367	Protein AAR2 homolog	Q9Y312	0.4108	0.5331	-3.31	0.5752	0.7464	-1.75	0.5661	0.6758	-1.19	0.5388	0.6393	0.89	0.2938	0.4316	-1.88	0.9510	1.0317	0.09	0.0199	0.1308	-0.14	0.2392	0.5561	-0.10	0.0650	0.2290	0.20
3368	Protein AATF	Q9NY61	0.1880	0.3540	-0.35	0.3647	0.7132	0.22	0.8984	0.9197	0.03	0.3875	0.5732	-0.20	0.4185	0.5130	-0.21	0.0393	0.1293	-0.76	0.3736	0.5705	-2.55	0.8708	1.1608	-0.33	0.0651	0.1761	2.06
3369	Protein ABHD11	Q8NFV4	0.0319	0.1108	-0.13	0.4671	0.7983	-0.06	0.0110	0.0485	-0.26	0.0425	0.1025	-0.13	0.0049	0.0524	-0.25	0.6257	0.8613	0.03	0.3736	0.4497	-2.55	0.8708	0.9403	-0.33	0.0651	0.1194	2.06
3370	Protein ABHD12B	Q7Z5M8	0.4108	0.5329	-3.31	0.5752	0.7462	-1.75	0.5661	0.6757	-1.19	0.5388	0.6391	0.89	0.2938	0.4315	-1.88	0.9510	1.0314	0.09	0.0004	0.0345	3.61	0.0000	0.0000	4.85	0.0010	0.0398	4.39
3371	Protein ABHD14B	Q96IU4	0.6170	0.6810	-0.03	0.5850	0.6912	-0.07	0.1128	0.2247	-0.11	0.9884	0.9901	0.00	0.0405	0.1481	-0.18	0.0684	0.1833	0.15	0.0271	0.1427	0.32	0.3501	0.7131	0.08	0.7922	0.8304	0.02
3372	Protein AHNAK2	Q8IVF2	0.4108	0.5328	-3.31	0.5752	0.7460	-1.75	0.5661	0.6755	-1.19	0.5388	0.6390	0.89	0.2938	0.4314	-1.88	0.9510	1.0312	0.09	0.1505	0.3365	0.19	0.9690	0.9779	0.00	0.1044	0.1605	-0.17
3373	Protein arginine N-methyltransferase 1	Q99873	0.0070	0.0493	0.27	0.5428	0.8572	0.09	0.0892	0.1874	0.11	0.1919	0.3213	0.07	0.0416	0.1507	-0.15	0.0119	0.0742	0.23	0.0770	0.2210	0.25	0.1625	0.4384	0.15	0.7973	0.8350	-0.02
3374	Protein arginine N-methyltransferase 5	O14744	0.0138	0.0688	0.22	0.1429	0.6551	0.09	0.0277	0.0838	0.31	0.0081	0.0363	0.30	0.0577	0.1803	0.13	0.0408	0.1328	0.17	0.0648	0.2004	0.37	0.1330	0.3840	0.10	0.9697	0.9759	0.00
3375	Protein arginine N-methyltransferase 6	Q96LA8	0.5954	0.6614	-0.04	0.3430	0.7003	-0.07	0.2764	0.4508	0.08	0.9776	0.9808	0.00	0.1494	0.3265	-0.12	0.1797	0.3490	-0.14	0.3736	0.5075	-2.55	0.8708	1.0473	-0.33	0.0651	0.1444	2.06
3376	Protein arginine N-methyltransferase 8	Q9NR22	0.0015	0.0261	0.19	0.1405	0.6527	0.18	0.0010	0.0217	0.25	0.0028	0.0222	0.39	0.0412	0.1500	0.10	0.0043	0.0605	0.40	0.0046	0.0729	0.32	0.0437	0.1933	0.15	0.0202	0.1221	0.27
3377	Protein argonaute-1	Q9UL18	0.0368	0.1208	0.10	0.6192	0.7174	-0.02	0.0093	0.0449	0.17	0.0024	0.0209	0.27	0.2150	0.4199	0.05	0.0012	0.0478	0.40	0.3736	0.6479	-2.55	0.8708	1.2958	-0.33	0.0651	0.2233	2.06
3378	Protein argonaute-2	Q9UKV8	0.4108	0.5327	-3.31	0.5752	0.7458	-1.75	0.5661	0.6754	-1.19	0.5388	0.6388	0.89	0.2938	0.4313	-1.88	0.9510	1.0310	0.09	0.0510	0.1789	0.44	0.2325	0.5472	0.10	0.0072	0.0772	0.32
3379	Protein bicaudal D homolog 1	Q96G01	0.0840	0.2001	-0.05	0.9332	0.9548	0.01	0.0526	0.1296	0.13	0.2267	0.3687	0.06	0.7920	0.8371	-0.01	0.1079	0.2462	0.15	0.4217	0.4774	0.20	0.7356	1.1790	-0.08	0.2354	0.3053	-0.35
3380	Protein bicaudal D homolog 2	Q8TD16	0.2073	0.3798	-0.03	0.3958	0.7396	0.17	0.0891	0.1875	0.12	0.0019	0.0190	0.19	0.0107	0.0741	0.12	0.2104	0.3911	0.03	0.5918	0.6407	-0.05	0.9603	0.9719	0.00	0.6394	0.6960	-0.03
3381	Protein BRICK1	Q8WUW1	0.1846	0.3495	0.19	0.8522	0.8936	0.02	0.2122	0.3666	-0.17	0.4411	0.6352	-0.09	0.2012	0.4019	-0.18	0.6163	0.8527	-0.06	0.0555	0.1870	0.45	0.0604	0.2351	0.21	0.0836	0.1353	0.54
3382	Protein BUD31 homolog	P41223	0.6300	0.6923	0.04	0.7766	0.8388	0.02	0.9069	0.9264	0.01	0.4084	0.5996	0.04	0.1666	0.3528	-0.08	0.1357	0.2880	0.09	0.3736	0.5219	-2.55	0.8708	1.0735	-0.33	0.0651	0.1512	2.06
3383	Protein C10	Q99622	0.1746	0.3363	-0.06	0.1378	0.6525	0.09	0.1797	0.3231	0.07	0.0946	0.1860	-0.08	0.2457	0.4613	0.05	0.6909	0.9305	-0.02	0.2997	0.5566	-0.08	0.1544	0.4245	0.12	0.5261	0.5924	-0.04
3384	Protein C12orf4	Q9NQ89	0.4108	0.5325	-3.31	0.5752	0.7456	-1.75	0.5661	0.6752	-1.19	0.5388	0.6387	0.89	0.2938	0.4311	-1.88	0.9510	1.0308	0.09	0.1237	0.2958	0.33	0.0143	0.1000	0.34	0.0979	0.1532	0.17
3385	Protein canopy homolog 2	Q9Y2B0	0.0144	0.0698	0.09	0.6965	0.7766	0.04	0.0026	0.0278	0.32	0.0017	0.0184	0.22	0.2587	0.4791	0.03	0.0005	0.0375	0.34	0.0722	0.2129	-0.20	0.0019	0.0357	-0.30	0.0006	0.0320	-0.51
3386	Protein CASP	Q13948	0.0307	0.1080	-0.16	0.3776	0.7247	0.06	0.0090	0.0442	0.23	0.0921	0.1822	0.11	0.1584	0.3405	0.08	0.3686	0.5758	-0.05	0.3736	0.4720	-2.55	0.8708	0.9819	-0.33	0.0651	0.1286	2.06
3387	Protein CBFA2T2	O43439	0.4108	0.5324	-3.31	0.5752	0.7454	-1.75	0.5661	0.6750	-1.19	0.5388	0.6385	0.89	0.2938	0.4310	-1.88	0.9510	1.0306	0.09	0.0080	0.0909	0.43	0.1229	0.3642	0.17	0.0657	0.1121	0.20
3388	Protein CDV3 homolog	Q9UKY7	0.0009	0.0213	0.33	0.4944	0.8172	0.07	0.0012	0.0224	0.35	0.0131	0.0464	0.13	0.3701	0.4671	-0.03	0.0026	0.0550	0.27	0.3497	0.6244	0.11	0.6396	1.0778	0.04	0.0620	0.2226	-0.15
3389	Protein C-ets-1	P14921	0.0582	0.1589	0.12	0.2027	0.6600	0.24	0.0086	0.0438	0.26	0.0494	0.1141	0.13	0.0170	0.0935	0.22	0.0347	0.1202	0.15	0.3736	0.5751	-2.55	0.8708	1.1689	-0.33	0.0651	0.1786	2.06
3390	Protein CIP2A	Q8TCG1	0.4108	0.5323	-3.31	0.5752	0.7453	-1.75	0.5661	0.6749	-1.19	0.5388	0.6384	0.89	0.2938	0.4309	-1.88	0.9510	1.0304	0.09	0.1752	0.3741	0.07	0.0248	0.1404	-0.13	0.4474	0.5172	0.02
3391	Protein cordon-bleu	O75128	0.1753	0.3372	0.08	0.0973	0.6332	0.18	0.0518	0.1281	0.16	0.0469	0.1101	0.11	0.0076	0.0634	0.33	0.3930	0.6053	0.07	0.6845	0.7266	0.06	0.4905	0.9023	-0.09	0.8555	0.8829	0.02
3392	Protein cornichon homolog 4	Q9P003	0.0803	0.1948	-0.16	0.0434	0.5706	-0.22	0.0229	0.0746	-0.33	0.0014	0.0169	-0.59	0.0057	0.0559	-0.40	0.4744	0.6983	-0.07	0.0941	0.2466	-0.06	0.0604	0.2349	0.08	0.0422	0.1787	-0.19
3393	Protein CUSTOS	Q96C57	0.4108	0.5321	-3.31	0.5752	0.7451	-1.75	0.5661	0.6747	-1.19	0.5388	0.6382	0.89	0.2938	0.4308	-1.88	0.9510	1.0302	0.09	0.0465	0.1732	0.31	0.0394	0.1805	-0.37	0.3825	0.4536	-0.40

Supplemetary Table S2. Overview on all relatively quantified 5180 proteins statistical analysis

			MCF-7 Dai SC20 vs control			Gen SC20 vs control			SSE SC20 vs control			Dai IC20 vs control			Gen IC20 vs control			SSE IC20 vs control			MDA-MB-231 Dai IC20 vs control			Gen IC20 vs control			SSE IC20 vs control		
	**Protein name**	**UniProt**	**p value**	**BH q value**	**log2FC**	**p value**	**BH q value**	**log2FC**	**p value**	**BH q value**	**log2FC**	**p value**	**BH q value**	**log2FC**	**p value**	**BH q value**	**log2FC**	**p value**	**BH q value**	**log2FC**	**p value**	**BH q value**	**log2FC**	**p value**	**BH q value**	**log2FC**	**p value**	**BH q value**	**log2FC**
3394	Protein CYR61	O00622	0.0335	0.1144	-0.84	0.1747	0.6610	-2.85	0.7556	0.7970	-0.11	0.5589	0.6096	-0.14	0.2289	0.4387	-0.38	0.2501	0.4395	-0.44	0.0114	0.1036	0.45	0.0910	0.3002	0.18	0.0352	0.1609	0.42
3395	Protein Daple	Q9P219	0.4108	0.5320	-3.31	0.5752	0.7449	-1.75	0.5661	0.6746	-1.19	0.5388	0.6381	0.89	0.2938	0.4306	-1.88	0.9510	1.0299	0.09	0.4850	0.5397	0.09	0.1849	0.4741	-0.13	0.0510	0.1982	-0.23
3396	Protein DDI1 homolog 2	Q5TDH0	0.0940	0.2159	-0.08	0.1529	0.6589	-0.15	0.0177	0.0635	-0.21	0.0218	0.0642	-0.31	0.0011	0.0260	-0.33	0.1295	0.2803	-0.23	0.3736	0.5247	-2.55	0.8708	1.0786	-0.33	0.0651	0.1525	2.06
3397	Protein DEK	P35659	0.0094	0.0573	0.26	0.7650	0.8301	-0.02	0.9538	0.9635	0.00	0.4999	0.7033	-0.02	0.0763	0.2125	-0.09	0.3414	0.5457	-0.03	0.6286	0.6746	0.11	0.1476	0.4126	-0.18	0.9964	0.9974	0.00
3398	Protein diaphanous homolog 1	O60610	0.0524	0.1482	0.12	0.2671	0.6630	0.18	0.0108	0.0483	0.20	0.0345	0.0886	0.14	0.1248	0.2917	-0.09	0.1203	0.2649	0.09	0.0147	0.1120	0.34	0.2084	0.5099	0.07	0.1401	0.2014	0.08
3399	Protein disulfide-isomerase	P07237	0.0024	0.0332	0.37	0.2144	0.6615	0.27	0.0005	0.0192	0.55	0.0002	0.0118	0.66	0.0038	0.0461	0.38	0.0017	0.0521	0.68	0.0278	0.1446	0.48	0.3737	0.7468	0.09	0.5286	0.5950	0.05
3400	Protein disulfide-isomerase A3	P30101	0.0017	0.0282	0.24	0.2152	0.6612	0.27	0.0022	0.0264	0.38	0.0005	0.0126	0.41	0.0171	0.0939	0.17	0.0015	0.0511	0.46	0.0817	0.2281	0.31	0.9446	0.9602	0.01	0.6846	0.7377	-0.02
3401	Protein disulfide-isomerase A4	P13667	0.0054	0.0444	0.32	0.1474	0.6571	0.31	0.0009	0.0212	0.54	0.0007	0.0140	0.55	0.0152	0.0880	0.30	0.0058	0.0621	0.56	0.0587	0.1923	0.39	0.8816	0.9108	-0.02	0.6931	0.7452	-0.04
3402	Protein disulfide-isomerase A5	Q14554	0.0057	0.0454	-1.82	0.2838	0.6643	-1.74	0.2004	0.3520	-0.71	0.0023	0.0203	-1.52	0.4565	0.5490	-0.19	0.0325	0.1161	-0.84	0.3736	0.5785	-2.55	0.8708	1.1750	-0.33	0.0651	0.1805	2.06
3403	Protein disulfide-isomerase A6	Q15084	0.0157	0.0727	0.20	0.1573	0.6619	0.17	0.0033	0.0309	0.27	0.0060	0.0312	0.22	0.0252	0.1141	0.16	0.0103	0.0700	0.28	0.0704	0.2096	0.26	0.3029	0.6478	-0.09	0.2873	0.3592	0.07
3404	Protein dopey-1	Q5JWR5	0.8135	0.8499	0.04	0.5102	0.8319	-0.09	0.0546	0.1329	-0.38	0.2122	0.3487	0.22	0.3752	0.4724	-0.12	0.9476	1.1839	0.01	0.0507	0.1790	-0.74	0.0352	0.1704	-0.27	0.0064	0.0748	-0.26
3405	Protein Dr1	Q01658	0.4108	0.5319	-3.31	0.5752	0.7447	-1.75	0.5661	0.6744	-1.19	0.5388	0.6379	0.89	0.2938	0.4305	-1.88	0.9510	1.0297	0.09	0.4705	0.5253	0.05	0.0173	0.1110	-0.26	0.0255	0.1359	-0.28
3406	Protein ECT2	Q9H8V3	0.9206	0.9395	0.01	0.4189	0.7574	0.08	0.0772	0.1679	0.12	0.4205	0.6122	-0.04	0.9281	0.9462	-0.01	0.0590	0.1676	0.15	0.3294	0.5974	-0.26	0.6040	1.0374	-0.09	0.9147	0.9311	-0.02
3407	Protein EFR3 homolog B	Q9Y2G0	0.0440	0.1332	-0.24	0.0944	0.6301	0.17	0.4482	0.6616	0.05	0.1177	0.2195	-0.15	0.5005	0.5900	0.06	0.2278	0.4106	0.17	0.3736	0.4956	-2.55	0.8708	1.0254	-0.33	0.0651	0.1389	2.06
3408	Protein enabled homolog	Q8N8S7	0.0592	0.1609	-0.17	0.6167	0.7164	0.07	0.0448	0.1159	0.18	0.4257	0.6177	0.05	0.0381	0.1422	-0.23	0.3177	0.5200	-0.06	0.0483	0.1758	0.38	0.0018	0.0348	0.08	0.0233	0.1299	0.65
3409	Protein ERGIC-53	P49257	0.0193	0.0815	0.18	0.1102	0.6336	0.21	0.0043	0.0327	0.28	0.0055	0.0298	0.30	0.0399	0.1467	0.15	0.0047	0.0601	0.32	0.0462	0.1725	0.39	0.0027	0.0432	0.31	0.0041	0.0628	0.31
3410	Protein eyes shut homolog	Q5T1H1	0.7694	0.8127	0.01	0.3073	0.6774	0.14	0.5286	0.7496	-0.02	0.0463	0.1092	0.14	0.1481	0.3241	-0.06	0.2791	0.4753	0.04	0.3736	0.4858	-2.55	0.8708	1.0073	-0.33	0.0651	0.1345	2.06
3411	Protein FAM102B	Q5T8I3	0.4027	0.6227	-0.12	0.7085	0.7859	0.07	0.0532	0.1307	-0.28	0.0100	0.0401	-0.50	0.0088	0.0671	-0.57	0.0054	0.0622	-0.72	0.3736	0.6285	-2.55	0.8708	1.2625	-0.33	0.0651	0.2105	2.06
3412	Protein FAM104B	Q5XKR9	0.1017	0.2277	0.35	0.8872	0.9195	-0.06	0.0910	0.1899	0.36	0.0314	0.0828	0.54	0.1372	0.3077	0.31	0.0559	0.1615	0.53	0.0226	0.1364	0.55	0.0103	0.0838	0.51	0.0090	0.0849	0.80
3413	Protein FAM114A2	Q9NRY5	0.0084	0.0542	0.22	0.7139	0.7905	-0.04	0.9136	0.9320	0.00	0.0409	0.1001	0.13	0.0818	0.2208	0.09	0.0284	0.1078	0.16	0.3736	0.5819	-2.55	0.8708	1.1808	-0.33	0.0651	0.1824	2.06
3414	Protein FAM122B	Q7Z309	0.4630	0.5399	0.13	0.9101	0.9374	-0.03	0.5848	0.6412	0.10	0.5098	0.7145	0.12	0.7885	0.8339	0.05	0.6788	0.9178	0.08	0.0126	0.1065	0.21	0.3565	0.7231	-0.05	0.0084	0.0818	0.34
3415	Protein FAM124B	Q9H5Z6	0.0737	0.1840	0.06	0.0125	0.4405	0.14	0.0001	0.0273	0.40	0.0004	0.0134	0.29	0.0001	0.0120	0.46	0.0007	0.0453	0.34	0.3736	0.6512	-2.55	0.8708	1.3014	-0.33	0.0651	0.2256	2.06
3416	Protein FAM135B	Q49AJ0	0.7724	0.8152	-0.05	0.0666	0.5938	-0.25	0.0470	0.1203	0.23	0.0281	0.0766	-0.27	0.9713	0.9775	0.00	0.0188	0.0882	0.36	0.3736	0.5951	-2.55	0.8708	1.2041	-0.33	0.0651	0.1900	2.06
3417	Protein FAM136A	Q96C01	0.4691	0.5450	0.04	0.3157	0.6811	0.13	0.1783	0.3214	0.09	0.0194	0.0591	0.12	0.7940	0.8390	0.01	0.0538	0.1573	0.13	0.8791	0.8994	-0.01	0.1064	0.3306	-0.10	0.0163	0.1092	-0.22
3418	Protein FAM160A1	Q05DH4	0.4108	0.5317	-3.31	0.5752	0.7445	-1.75	0.5661	0.6743	-1.19	0.5388	0.6378	0.89	0.2938	0.4304	-1.88	0.9510	1.0295	0.09	0.0316	0.1502	0.22	0.0230	0.1333	0.24	0.1392	0.2005	0.13
3419	Protein FAM162A	Q96A26	0.0002	0.0133	0.72	0.6137	0.7149	-0.15	0.0378	0.1023	-0.17	0.0362	0.0914	-0.35	0.0740	0.2086	-0.37	0.2661	0.4589	0.06	0.3736	0.4875	-2.55	0.8708	1.0105	-0.33	0.0651	0.1353	2.06
3420	Protein FAM167B	Q9BTA0	0.4108	0.5316	-3.31	0.5752	0.7443	-1.75	0.5661	0.6741	-1.19	0.5388	0.6376	0.89	0.2938	0.4303	-1.88	0.9510	1.0293	0.09	0.1059	0.2660	-0.22	0.0084	0.0752	-0.28	0.0212	0.1246	-0.36
3421	Protein FAM170A	A1A519	0.2068	0.3793	0.68	0.7893	0.8484	0.12	0.7189	0.7639	0.16	0.7415	0.7772	-0.14	0.5716	0.6517	0.25	0.3442	0.5483	0.46	0.0079	0.0909	-1.42	0.0610	0.2363	-6.69	0.0006	0.0293	-4.30
3422	Protein FAM177A1	Q8N128	0.0174	0.0772	0.22	0.5276	0.8461	0.09	0.0867	0.1837	0.12	0.1981	0.3294	0.08	0.2075	0.4101	0.07	0.1023	0.2371	0.12	0.3736	0.5331	-2.55	0.8708	1.0938	-0.33	0.0651	0.1566	2.06
3423	Protein FAM183BP	Q6ZVS7	0.4108	0.5315	-3.31	0.5752	0.7441	-1.75	0.5661	0.6740	-1.19	0.5388	0.6375	0.89	0.2938	0.4302	-1.88	0.9510	1.0291	0.09	0.0002	0.0296	-1.08	0.0127	0.0940	-1.63	0.0209	0.1239	-2.23
3424	Protein FAM186A	A6NE01	0.4108	0.5313	-3.31	0.5752	0.7440	-1.75	0.5661	0.6738	-1.19	0.5388	0.6374	0.89	0.2938	0.4300	-1.88	0.9510	1.0289	0.09	0.3070	0.5667	0.08	0.0271	0.1484	0.18	0.0251	0.1346	0.30
3425	Protein FAM193A	P78312	0.4108	0.5312	-3.31	0.5752	0.7438	-1.75	0.5661	0.6736	-1.19	0.5388	0.6372	0.89	0.2938	0.4299	-1.88	0.9510	1.0286	0.09	0.0333	0.1521	0.06	0.9375	0.9546	0.00	0.8222	0.8566	-0.02
3426	Protein FAM204A	Q9H8W3	0.5223	0.5940	-0.28	0.9708	0.9803	-0.03	0.6557	0.7070	0.19	0.8046	0.8322	0.12	0.6447	0.7131	0.21	0.6566	0.8936	-0.20	0.3736	0.4474	-2.55	0.8708	0.9358	-0.33	0.0651	0.1184	2.06
3427	Protein FAM217A	Q8IXS0	0.0108	0.0598	-0.24	0.0021	0.2266	-0.30	0.0018	0.0245	-0.40	0.0026	0.0217	-0.31	0.0114	0.0766	0.18	0.2143	0.3959	-0.06	0.3736	0.4998	-2.55	0.8708	1.0332	-0.33	0.0651	0.1408	2.06
3428	Protein FAM236D	A0A1B0GTK5	0.1975	0.3667	2.63	0.0699	0.6015	-8.94	0.1383	0.2624	0.72	0.0949	0.1863	0.69	0.0202	0.1020	1.12	0.0114	0.0734	1.62	0.3736	0.6101	-2.55	0.8708	1.2304	-0.33	0.0651	0.1989	2.06
3429	Protein FAM3C	Q92520	0.2013	0.3720	0.27	0.7800	0.8416	-0.13	0.0766	0.1670	0.50	0.3164	0.4866	0.21	0.0803	0.2185	0.44	0.0886	0.2170	0.43	0.0461	0.1725	-0.22	0.0251	0.1415	-0.80	0.0029	0.0548	-1.17
3430	Protein FAM49A	Q9H0Q0	0.4108	0.5311	-3.31	0.5752	0.7436	-1.75	0.5661	0.6735	-1.19	0.5388	0.6371	0.89	0.2938	0.4298	-1.88	0.9510	1.0284	0.09	0.4700	0.5248	0.12	0.0575	0.2291	-0.33	0.0480	0.1911	-0.40
3431	Protein FAM49B	Q9NUQ9	0.5445	0.6144	0.03	0.5713	0.8847	0.09	0.9314	0.9464	0.00	0.0244	0.0694	0.16	0.0214	0.1054	-0.19	0.0646	0.1774	0.11	0.1645	0.3588	0.28	0.8263	1.2705	-0.02	0.8756	0.8994	0.02
3432	Protein FAM50A	Q14320	0.0034	0.0380	0.19	0.0284	0.5073	0.11	0.0078	0.0419	0.21	0.2515	0.4010	0.06	0.1767	0.3672	0.05	0.0052	0.0614	0.23	0.0584	0.1926	0.35	0.6563	1.0970	0.05	0.3263	0.3976	0.10
3433	Protein FAM50B	Q9Y247	0.0101	0.0585	-0.24	0.6186	0.7173	-0.06	0.2340	0.3947	0.08	0.0637	0.1380	-0.12	0.0137	0.0837	-0.20	0.0932	0.2242	-0.13	0.0342	0.1542	0.30	0.0020	0.0367	0.69	0.0035	0.0591	0.49
3434	Protein FAM53B	Q14153	0.0547	0.1529	0.21	0.4461	0.7817	-0.52	0.0054	0.0355	0.54	0.0135	0.0471	0.39	0.0462	0.1585	0.29	0.8211	1.0570	0.06	0.3736	0.4370	-2.55	0.8708	0.9164	-0.33	0.0651	0.1143	2.06
3435	Protein FAM76B	Q5HYJ3	0.2936	0.4915	-0.08	0.2804	0.6651	-0.09	0.6799	0.7299	-0.05	0.5738	0.6231	-0.04	0.0457	0.1573	-0.21	0.2655	0.4581	-0.19	0.0064	0.0841	1.14	0.0000	0.0000	0.95	0.0003	0.0317	0.95
3436	Protein FAM83C	Q9BQN1	0.3051	0.5059	0.63	0.2777	0.6647	0.31	0.0058	0.0368	1.72	0.0665	0.1425	1.51	0.0057	0.0556	2.03	0.4207	0.6398	0.31	0.3736	0.4673	-2.55	0.8708	0.9732	-0.33	0.0651	0.1266	2.06
3437	Protein FAM84B	Q96KN1	0.1840	0.3487	0.17	0.2212	0.6585	0.34	0.2739	0.4477	0.27	0.0610	0.1334	0.22	0.6033	0.6789	0.04	0.1607	0.3216	0.31	0.3736	0.5120	-2.55	0.8708	1.0554	-0.33	0.0651	0.1464	2.06
3438	Protein FAM86C1	Q9NVL1	0.1897	0.3559	0.06	0.6203	0.7180	0.07	0.0433	0.1131	0.13	0.6034	0.6506	-0.02	0.0242	0.1125	-0.15	0.5579	0.7935	-0.03	0.3736	0.4564	-2.55	0.8708	0.9528	-0.33	0.0651	0.1221	2.06
3439	Protein FAM91A1	Q658Y4	0.0095	0.0574	0.08	0.2866	0.6675	0.09	0.1049	0.2118	-0.12	0.3073	0.4742	0.02	0.0350	0.1358	-0.08	0.2913	0.4885	0.09	0.3736	0.4834	-2.55	0.8708	1.0031	-0.33	0.0651	0.1335	2.06
3440	Protein FAM92B	Q6ZTR7	0.0003	0.0148	-3.54	0.2385	0.6624	-2.83	0.0063	0.0384	-3.85	0.0030	0.0225	-4.64	0.7517	0.8060	-0.07	0.0010	0.0435	-4.41	0.3736	0.6488	-2.55	0.8708	1.2973	-0.33	0.0651	0.2239	2.06
3441	Protein FAM98A	Q8NCA5	0.4291	0.5065	0.02	0.1196	0.6361	0.27	0.0026	0.0281	0.26	0.0040	0.0258	0.27	0.5609	0.6432	0.02	0.1787	0.3481	0.15	0.3736	0.5082	-2.55	0.8708	1.0485	-0.33	0.0651	0.1447	2.06
3442	Protein FAM98B	Q52LJ0	0.9873	0.9906	0.00	0.6864	0.7706	0.08	0.3032	0.4859	-0.19	0.7906	0.8196	-0.04	0.8914	0.9165	-0.02	0.4324	0.6534	-0.13	0.1677	0.3624	0.22	0.7808	1.2245	0.04	0.7587	0.8009	-0.04
3443	Protein FAM9A	Q8IZU1	0.0200	0.0831	0.58	0.0014	0.2072	0.51	0.0017	0.0241	0.68	0.0034	0.0237	0.78	0.0030	0.0412	0.81	0.0006	0.0414	1.03	0.1827	0.3839	0.10	0.1766	0.4613	0.19	0.7180	0.7670	0.02
3444	Protein FAM9C	Q8IZT9	0.0984	0.2226	-0.07	0.0072	0.4010	0.25	0.0126	0.0526	0.16	0.0592	0.1304	0.13	0.0205	0.1025	-0.23	0.0065	0.0627	0.30	0.0017	0.0540	8.47	0.0025	0.0419	8.03	0.0651	0.2292	2.06
3445	Protein fantom	Q68CZ1	0.0083	0.0539	0.19	0.0309	0.5081	0.20	0.0044	0.0328	0.20	0.0018	0.0188	0.26	0.0033	0.0433	0.31	0.0026	0.0568	0.29	0.0049	0.0738	-0.45	0.0007	0.0248	-0.66	0.0091	0.0851	-0.37
3446	Protein farnesyltransferase/geranylgeranyltransferase type-1 subunit alpha	P49354	0.0058	0.0458	1.70	0.2260	0.6592	0.81	0.0120	0.0512	0.79	0.0024	0.0209	1.73	0.0822	0.2215	-0.09	0.1068	0.2448	1.62	0.9756	0.9801	0.00	0.2853	0.6238	-0.14	0.2398	0.3100	-0.15
3447	Protein fem-1 homolog B	Q9UK73	0.1057	0.2334	0.26	0.9271	0.9504	-0.02	0.0025	0.0272	-0.23	0.0123	0.0447	-0.18	0.1022	0.2564	-0.20	0.1589	0.3190	-0.18	0.3736	0.5125	-2.55	0.8708	1.0564	-0.33	0.0651	0.1467	2.06
3448	Protein Flattop	Q5VTH2	0.8728	0.8997	-0.02	0.1269	0.6419	-0.24	0.1014	0.2062	-0.28	0.0235	0.0677	-0.48	0.0616	0.1876	-0.34	0.5186	0.7464	-0.16	0.3736	0.4589	-2.55	0.8708	0.9575	-0.33	0.0651	0.1231	2.06
3449	Protein flightless-1 homolog	Q13045	0.4783	0.5534	-0.07	0.8668	0.9032	0.02	0.9438	0.9560	0.01	0.3981	0.5872	-0.09	0.4710	0.5619	-0.07	0.4837	0.7090	-0.07	0.0424	0.1674	0.27	0.1033	0.3239	0.21	0.9356	0.9482	0.00
3450	Protein furry homolog	Q5TBA9	0.0010	0.0218	0.69	0.1142	0.6340	0.35	0.0017	0.0239	0.33	0.0007	0.0137	0.43	0.0062	0.0574	0.13	0.0002	0.0357	0.42	0.3736	0.6538	-2.55	0.8708	1.3059	-0.33	0.0651	0.2274	2.06
3451	Protein HEXIM1	O94992	0.3461	0.5550	-0.03	0.0739	0.6115	0.08	0.0621	0.1448	-0.08	0.6347	0.6803	-0.04	0.0791	0.2168	-0.15	0.0873	0.2154	0.08	0.0063	0.0841	-0.32	0.0040	0.0513	-0.40	0.0065	0.0758	-0.52
3452	Protein HGH1 homolog	Q9BTY7	0.4806	0.5552	-0.06	0.8421	0.8868	0.02	0.2050	0.3579	-0.07	0.0111	0.0423	0.15	0.0775	0.2141	-0.06	0.1965	0.3718	0.08	0.3736	0.5031	-2.55	0.8708	1.0391	-0.33	0.0651	0.1423	2.06
3453	Protein HID1	Q8IV36	0.7338	0.7836	-0.01	0.2647	0.6640	0.29	0.8338	0.8659	-0.01	0.0362	0.0913	0.12	0.5162	0.6047	-0.02	0.0145	0.0800	0.19	0.3736	0.6038	-2.55	0.8708	1.2194	-0.33	0.0651	0.1951	2.06
3454	Protein hinderin	Q86T90	0.3007	0.4999	1.40	0.2750	0.6647	0.96	0.0034	0.0308	-2.54	0.3479	0.5237	1.52	0.8165	0.8572	-0.07	0.0056	0.0612	2.95	0.3736	0.6271	-2.55	0.8708	1.2600	-0.33	0.0651	0.2096	2.06
3455	Protein HIRA	P54198	0.0125	0.0651	-0.18	0.8901	0.9216	-0.02	0.0007	0.0197	0.35	0.2478	0.3965	0.05	0.2939	0.3918	-0.03	0.0875	0.2154	0.12	0.5033	0.5568	-0.02	0.1704	0.4515	-0.05	0.1324	0.1926	-0.04
3456	Protein Hook homolog 1	Q9UJC3	0.2600	0.4491	-0.10	0.6944	0.7745	0.04	0.0737	0.1624	0.11	0.4163	0.6086	0.09	0.4077	0.5034	-0.10	0.0751	0.1949	0.12	0.0096	0.0983	0.41	0.0737	0.2638	0.23	0.0010	0.0387	0.21
3457	Protein ITPRID1	Q6ZRS4	0.1072	0.2358	-0.24	0.0346	0.5303	-0.24	0.0961	0.1980	-0.14	0.4375	0.6311	0.08	0.0390	0.1443	0.20	0.0266	0.1045	0.22	0.3736	0.5845	-2.55	0.8708	1.1855	-0.33	0.0651	0.1839	2.06
3458	Protein IWS1 homolog	Q96ST2	0.1790	0.3418	-0.11	0.9972	0.9980	0.00	0.7135	0.7589	0.02	0.2477	0.3965	-0.11	0.0076	0.0632	-0.38	0.0110	0.0719	-0.38	0.0927	0.2446	0.42	0.0918	0.3015	0.16	0.0203	0.1223	0.25
3459	Protein jagunal homolog 1	Q8N5M9	0.6920	0.7473	0.04	0.6047	0.7074	-0.09	0.2005	0.3521	0.16	0.3456	0.5210	0.11	0.3029	0.4006	0.12	0.9981	0.9981	0.00	0.3736	0.4293	-2.55	0.8708	0.9018	-0.33	0.0651	0.1113	2.06
3460	Protein Jumonji	Q92833	0.0331	0.1135	-0.11	0.6406	0.7351	-0.01	0.0008	0.0201	-0.04	0.0075	0.0352	-0.15	0.2679	0.4897	0.04	0.1180	0.2621	-0.13	0.0059	0.0817	0.59	0.0077	0.0727	0.38	0.0133	0.1003	0.67
3461	Protein KIAA0100	Q14667	0.9478	0.9610	0.00	0.7943	0.8508	-0.04	0.8273	0.8607	0.02	0.0555	0.1244	-0.14	0.0511	0.1673	-0.14	0.0070	0.0644	0.36	0.3736	0.6219	-2.55	0.8708	1.2509	-0.33	0.0651	0.2062	2.06
3462	Protein KIBRA	Q8IX03	0.5326	0.6034	0.16	0.5688	0.8829	-0.43	0.3114	0.4965	-0.29	0.4240	0.6163	-0.23	0.7668	0.8186	-0.08	0.4391	0.6608	-0.28	0.3736	0.4653	-2.55	0.8708	0.9694	-0.33	0.0651	0.1258	2.06
3463	Protein kinase C alpha type	P17252	0.4108	0.5309	-3.31	0.5752	0.7434	-1.75	0.5661	0.6733	-1.19	0.5388	0.6369	0.89	0.2938	0.4297	-1.88	0.9510	1.0282	0.09	0.1903	0.3960	-0.18	0.3787	0.7554	0.08	0.0479	0.1910	-0.18
3464	Protein kinase C and casein kinase substrate in neurons protein 1	Q9BY11	0.6840	0.7402	-0.03	0.3398	0.6988	0.13	0.0160	0.0597	0.23	0.0015	0.0173	0.48	0.3860	0.4833	0.06	0.0766	0.1974	0.45	0.3736	0.5458	-2.55	0.8708	1.1165	-0.33	0.0651	0.1630	2.06
3465	Protein kinase C and casein kinase substrate in neurons protein 2	Q9UNF0	0.0373	0.1214	-0.19	0.1981	0.6676	-0.28	0.0736	0.1623	-0.22	0.1400	0.2511	-0.24	0.0233	0.1105	-0.29	0.2040	0.3819	-0.25	0.3736	0.5015	-2.55	0.8708	1.0362	-0.33	0.0651	0.1416	2.06
3466	Protein kinase C and casein kinase substrate in neurons protein 3	Q9UKS6	0.4233	0.5008	0.05	0.1388	0.6513	0.14	0.0109	0.0485	0.30	0.0090	0.0382	0.28	0.3852	0.4827	0.06	0.0215	0.0945	0.21	0.0062	0.0839	-0.99	0.0020	0.0369	-1.07	0.0980	0.1533	-0.96
3467	Protein kinase C delta type	Q05655	0.0069	0.0495	-0.73	0.3516	0.7046	-0.07	0.0016	0.0230	-0.76	0.0004	0.0122	-0.67	0.2071	0.4096	-0.15	0.0175	0.0864	-0.88	0.3736	0.5973	-2.55	0.8708	1.2080	-0.33	0.0651	0.1913	2.06
3468	Protein kinase C gamma type	P05129	0.3790	0.5947	-0.12	0.3342	0.6930	-0.10	0.3523	0.5467	0.05	0.2698	0.4257	-0.06	0.4206	0.5152	0.04	0.9853	0.9889	0.00	0.0004	0.0329	0.39	0.0037	0.0499	0.34	0.0161	0.1089	0.37
3469	Protein kinase C iota type	P41743	0.5833	0.6506	0.02	0.9111	0.9379	0.02	0.0941	0.1948	0.17	0.0142	0.0484	0.28	0.0296	0.1255	0.20	0.0137	0.0778	0.22	0.5999	0.6479	0.06	0.3665	0.7361	0.08	0.0621	0.2226	0.22
3470	Protein KRI1 homolog	Q8N9T8	0.1373	0.2821	0.19	0.6061	0.7086	0.07	0.3381	0.5304	0.10	0.2242	0.3650	0.14	0.7410	0.7972	-0.03	0.0638	0.1762	0.31	0.3736	0.5531	-2.55	0.8708	1.1297	-0.33	0.0651	0.1668	2.06
3471	Protein lin-28 homolog B	Q6ZN17	0.4108	0.5308	-3.31	0.5752	0.7432	-1.75	0.5661	0.6732	-1.19	0.5388	0.6368	0.89	0.2938	0.4295	-1.88	0.9510	1.0280	0.09	0.0895	0.2405	0.99	0.6139	1.0474	-0.21	0.1788	0.2452	0.66
3472	Protein lin-7 homolog A	O14910	0.7464	0.7936	0.01	0.4740	0.8013	0.15	0.2931	0.4736	0.03	0.0283	0.0770	0.12	0.3298	0.4271	0.03	0.3597	0.5656	0.03	0.0841	0.2317	0.25	0.7995	1.2437	0.01	0.5480	0.6124	0.04

Supplemetary Table S2. Overview on all relatively quantified 5180 proteins statistical analysis

			MCF-7																		MDA-MB-231								
			Dai SC20 vs control			Gen SC20 vs control			SSE SC20 vs control			Dai IC20 vs control			Gen IC20 vs control			SSE IC20 vs control			Dai IC20 vs control			Gen IC20 vs control			SSE IC20 vs control		
	**Protein name**	**UniProt**	**p value**	**BH q value**	**log2FC**	**p value**	**BH q value**	**log2FC**	**p value**	**BH q value**	**log2FC**	**p value**	**BH q value**	**log2FC**	**p value**	**BH q value**	**log2FC**	**p value**	**BH q value**	**log2FC**	**p value**	**BH q value**	**log2FC**	**p value**	**BH q value**	**log2FC**	**p value**	**BH q value**	**log2FC**
3473	Protein lin-9 homolog	Q5TKA1	0.2763	0.4699	0.17	0.3619	0.7098	-0.13	0.8833	0.9073	0.05	0.1547	0.2716	0.39	0.0085	0.0659	0.88	0.0751	0.1950	0.59	0.3736	0.5468	-2.55	0.8708	1.1185	-0.33	0.0651	0.1635	2.06
3474	Protein LLP homolog	Q9BRT6	0.1366	0.2812	-0.10	0.0405	0.5670	-0.15	0.1133	0.2255	-0.10	0.0073	0.0346	-0.28	0.0109	0.0747	-0.31	0.0308	0.1126	-0.20	0.3736	0.5805	-2.55	0.8708	1.1784	-0.33	0.0651	0.1816	2.06
3475	Protein LSM14 homolog A	Q8ND56	0.0124	0.0648	-0.20	0.9692	0.9790	0.00	0.0163	0.0603	0.16	0.9094	0.9215	0.00	0.1565	0.3378	0.06	0.1028	0.2380	-0.08	0.0903	0.2416	0.19	0.2165	0.5226	-0.17	0.3742	0.4460	-0.04
3476	Protein LTV1 homolog	Q96GA3	0.0102	0.0588	-0.25	0.2592	0.6637	-0.19	0.0269	0.0821	-0.21	0.5993	0.6469	0.03	0.0927	0.2400	-0.19	0.0386	0.1278	-0.38	0.9262	0.9389	0.00	0.0035	0.0480	-0.48	0.2845	0.3565	-0.05
3477	Protein LYRIC	Q86UE4	0.0011	0.0225	0.14	0.1805	0.6650	0.22	0.0120	0.0513	0.35	0.0002	0.0091	0.26	0.0014	0.0292	0.27	0.0003	0.0420	0.28	0.0120	0.1041	1.00	0.0155	0.1054	0.92	0.3121	0.3836	0.23
3478	Protein mago nashi homolog	P61326	0.0100	0.0583	0.20	0.0179	0.4683	0.17	0.0024	0.0274	0.49	0.0039	0.0254	0.29	0.2514	0.4694	0.05	0.3190	0.5218	0.13	0.0425	0.1670	0.31	0.6161	1.0501	0.02	0.0005	0.0278	0.40
3479	Protein MAK16 homolog	Q9BXY0	0.0142	0.0693	0.30	0.0005	0.2355	0.19	0.1118	0.2233	0.03	0.7943	0.8227	-0.01	0.8092	0.8516	0.00	0.2632	0.4555	0.14	0.2198	0.4411	-0.25	0.0458	0.1992	-0.24	0.1520	0.2152	-0.15
3480	Protein max	P61244	0.4108	0.5307	-3.31	0.5752	0.7430	-1.75	0.5661	0.6730	-1.19	0.5388	0.6366	0.89	0.2938	0.4294	-1.88	0.9510	1.0278	0.09	0.1533	0.3420	-0.29	0.5405	0.9658	-0.06	0.0254	0.1355	0.37
3481	Protein MB21D2	Q8IYB1	0.4108	0.5305	-3.31	0.5752	0.7428	-1.75	0.5661	0.6729	-1.19	0.5388	0.6365	0.89	0.2938	0.4293	-1.88	0.9510	1.0276	0.09	0.0028	0.0585	-0.38	0.0148	0.1025	-0.16	0.0677	0.1149	-0.19
3482	Protein Mdm4	O15151	0.0460	0.1370	0.47	0.1638	0.6639	0.43	0.2230	0.3804	0.23	0.0158	0.0520	0.78	0.0806	0.2189	0.40	0.0527	0.1552	0.44	0.2747	0.5197	-0.37	0.0776	0.2729	-0.81	0.2160	0.2848	-0.65
3483	Protein MEMO1	Q9Y316	0.4108	0.5304	-3.31	0.5752	0.7427	-1.75	0.5661	0.6727	-1.19	0.5388	0.6363	0.89	0.2938	0.4292	-1.88	0.9510	1.0274	0.09	0.0298	0.1470	1.32	0.0141	0.0991	1.40	0.0752	0.1250	0.84
3484	Protein misato homolog 1	Q9BUK6	0.0608	0.1634	-0.33	0.0484	0.5737	-0.41	0.0068	0.0399	-0.70	0.0212	0.0630	-0.55	0.0519	0.1689	-0.41	0.0119	0.0741	-0.63	0.3736	0.6086	-2.55	0.8708	1.2277	-0.33	0.0651	0.1980	2.06
3485	Protein mono-ADP-ribosyltransferase TIPARP	Q7Z3E1	0.4350	0.5123	0.26	0.3181	0.6806	0.33	0.3200	0.5072	0.33	0.1025	0.1977	0.72	0.8869	0.9133	0.05	0.6735	0.9114	0.15	0.3736	0.4463	-2.55	0.8708	0.9339	-0.33	0.0651	0.1180	2.06
3486	Protein moonraker	Q2KHM9	0.3411	0.5491	-0.04	0.8566	0.8968	0.02	0.0072	0.0400	0.25	0.6277	0.6739	0.03	0.0484	0.1616	-0.15	0.1717	0.3379	-0.07	0.3736	0.5094	-2.55	0.8708	1.0507	-0.33	0.0651	0.1452	2.06
3487	Protein NDRG1	Q92597	0.8457	0.8772	0.01	0.5347	0.8507	0.08	0.8990	0.9201	0.01	0.5199	0.7249	-0.02	0.0048	0.0519	-0.17	0.6493	0.8860	-0.01	0.7358	0.7745	0.06	0.1083	0.3351	-0.40	0.3185	0.3898	-0.19
3488	Protein NDRG3	Q9UGV2	0.0013	0.0245	-1.83	0.2632	0.6638	-1.23	0.1312	0.2526	-1.64	0.0005	0.0119	-3.29	0.1407	0.3131	0.37	0.0095	0.0694	-1.54	0.0571	0.1898	-0.29	0.0901	0.2984	-0.25	0.0593	0.2177	-0.34
3489	Protein Niban	Q9BZQ8	0.4108	0.5303	-3.31	0.5752	0.7425	-1.75	0.5661	0.6726	-1.19	0.5388	0.6362	0.89	0.2938	0.4291	-1.88	0.9510	1.0271	0.09	0.0356	0.1559	0.31	0.0969	0.3108	0.24	0.0299	0.1474	0.42
3490	Protein NipSnap homolog 1	Q9BPW8	0.0230	0.0905	0.21	0.2258	0.6597	0.21	0.0187	0.0655	0.23	0.0200	0.0604	0.18	0.6494	0.7172	0.02	0.0295	0.1100	0.17	0.4317	0.4876	0.07	0.0293	0.1544	-0.14	0.0076	0.0791	-0.23
3491	Protein NipSnap homolog 2	O75323	0.4108	0.5301	-3.31	0.5752	0.7423	-1.75	0.5661	0.6724	-1.19	0.5388	0.6360	0.89	0.2938	0.4289	-1.88	0.9510	1.0269	0.09	0.0955	0.2490	0.42	0.2362	0.5526	-0.17	0.1412	0.2027	0.22
3492	Protein NipSnap homolog 3A	Q9UFN0	0.2005	0.3713	-0.20	0.0028	0.2545	-0.52	0.1883	0.3343	-0.11	0.0069	0.0335	-0.53	0.0199	0.1010	-0.42	0.5802	0.8147	-0.05	0.4207	0.4764	-0.16	0.1849	0.4744	-0.25	0.8015	0.8381	-0.07
3493	Protein NipSnap homolog 3B	Q9BS92	0.0061	0.0467	-0.21	0.9787	0.9857	0.00	0.0066	0.0391	-0.29	0.0497	0.1147	-0.12	0.0148	0.0867	-0.20	0.4947	0.7200	-0.06	0.3736	0.4607	-2.55	0.8708	0.9608	-0.33	0.0651	0.1238	2.06
3494	Protein Njmu-R1	Q9HAS0	0.4108	0.5300	-3.31	0.5752	0.7421	-1.75	0.5661	0.6723	-1.19	0.5388	0.6359	0.89	0.2938	0.4288	-1.88	0.9510	1.0267	0.09	0.5345	0.5866	-0.06	0.1715	0.4535	-0.16	0.9967	0.9975	0.00
3495	Protein NOXP20	Q8IWE2	0.4108	0.5299	-3.31	0.5752	0.7419	-1.75	0.5661	0.6721	-1.19	0.5388	0.6358	0.89	0.2938	0.4287	-1.88	0.9510	1.0265	0.09	0.7073	0.7485	-0.17	0.9387	0.9549	0.05	0.0147	0.1045	1.69
3496	Protein NRDE2 homolog	Q9H7Z3	0.0993	0.2239	0.64	0.3161	0.6805	0.32	0.5022	0.7246	-0.22	0.1139	0.2144	0.62	0.0070	0.0606	1.81	0.8899	1.1265	-0.05	0.3736	0.4343	-2.55	0.8708	0.9113	-0.33	0.0651	0.1132	2.06
3497	Protein numb homolog	P49757	0.4987	0.5725	-0.05	0.2126	0.6626	0.22	0.0072	0.0401	0.27	0.0103	0.0408	0.17	0.0217	0.1058	0.10	0.0063	0.0626	0.20	0.1241	0.2965	1.16	0.2153	0.5209	0.81	0.1199	0.1782	1.19
3498	Protein odr-4 homolog	Q5SWX8	0.5211	0.5929	0.12	0.0417	0.5714	0.56	0.9558	0.9646	-0.01	0.1405	0.2518	0.30	0.0368	0.1394	0.47	0.0861	0.2138	0.37	0.3736	0.5413	-2.55	0.8708	1.1086	-0.33	0.0651	0.1607	2.06
3499	Protein O-GlcNAcase	O60502	0.0072	0.0501	4.30	0.1646	0.6615	3.62	0.0201	0.0683	4.30	0.1281	0.2338	3.60	0.6783	0.7419	-0.57	0.6103	0.8478	2.04	0.3736	0.4517	-2.55	0.8708	0.9441	-0.33	0.0651	0.1202	2.06
3500	Protein O-glucosyltransferase 2	Q6UW63	0.7352	0.7847	0.10	0.8054	0.8598	0.07	0.1863	0.3316	0.46	0.3391	0.5132	0.30	0.6569	0.7229	0.13	0.8958	1.1329	-0.06	0.3736	0.4341	-2.55	0.8708	0.9109	-0.33	0.0651	0.1132	2.06
3501	Protein O-glucosyltransferase 3	Q7Z4H8	0.4108	0.5297	-3.31	0.5752	0.7417	-1.75	0.5661	0.6720	-1.19	0.5388	0.6356	0.89	0.2938	0.4286	-1.88	0.9510	1.0263	0.09	0.0027	0.0580	-2.15	0.0001	0.0133	-2.90	0.0009	0.0379	-2.58
3502	Protein O-mannosyl-transferase TMTC3	Q6ZXV5	0.4108	0.5296	-3.31	0.5752	0.7415	-1.75	0.5661	0.6718	-1.19	0.5388	0.6355	0.89	0.2938	0.4285	-1.88	0.9510	1.0261	0.09	0.5897	0.6386	0.06	0.0209	0.1249	-0.24	0.0024	0.0536	-0.32
3503	Protein OS-9	Q13438	0.0273	0.1000	0.37	0.8519	0.8938	-0.01	0.8144	0.8495	0.02	0.5033	0.7069	0.06	0.3988	0.4952	-0.09	0.0231	0.0973	0.37	0.0058	0.0810	0.28	0.0218	0.1279	0.18	0.0106	0.0908	0.29
3504	Protein PAT1 homolog 1	Q86TB9	0.5142	0.5867	-0.07	0.1078	0.6310	0.21	0.3322	0.5230	0.08	0.0611	0.1336	0.17	0.2986	0.3967	-0.08	0.6182	0.8542	0.03	0.1364	0.3153	-0.22	0.0139	0.0982	-0.38	0.9120	0.9294	-0.01
3505	Protein PAXX	Q9BUH6	0.7916	0.8309	-0.06	0.2296	0.6611	-0.12	0.0684	0.1546	-0.44	0.0302	0.0806	-0.23	0.2055	0.4071	-0.10	0.3590	0.5651	-0.07	0.3736	0.4733	-2.55	0.8708	0.9842	-0.33	0.0651	0.1292	2.06
3506	Protein PBDC1	Q9BVG4	0.0136	0.0681	0.30	0.2655	0.6634	0.21	0.1274	0.2474	0.09	0.0375	0.0937	0.16	0.7701	0.8211	0.01	0.1492	0.3062	0.20	0.1604	0.3534	0.21	0.7245	1.1699	0.03	0.2647	0.3365	-0.07
3507	Protein pelota homolog	Q9BRX2	0.1379	0.2826	0.17	0.6215	0.7189	-0.08	0.1241	0.2427	0.20	0.0447	0.1061	0.31	0.0362	0.1382	0.35	0.4694	0.6919	-0.09	0.1015	0.2579	0.39	0.0593	0.2325	0.49	0.0241	0.1322	0.76
3508	Protein phosphatase 1 regulatory subunit 12A	O14974	0.0552	0.1538	0.10	0.2831	0.6636	0.21	0.0451	0.1165	0.16	0.0106	0.0412	0.18	0.0480	0.1608	0.10	0.0901	0.2189	0.09	0.7807	0.8143	0.04	0.4102	0.7973	0.07	0.3639	0.4353	-0.07
3509	Protein phosphatase 1 regulatory subunit 14B	Q96C90	0.0020	0.0309	-0.26	0.4467	0.7820	0.10	0.0470	0.1202	-0.06	0.0009	0.0147	-0.21	0.2864	0.5160	-0.02	0.0652	0.1781	-0.29	0.0065	0.0846	-0.31	0.3136	0.6622	-0.03	0.2409	0.3111	-0.07
3510	Protein phosphatase 1 regulatory subunit 26	Q5T8A7	0.2184	0.3943	0.07	0.0681	0.5919	0.20	0.1922	0.3396	0.09	0.0095	0.0390	0.38	0.2051	0.4066	0.07	0.0512	0.1526	0.18	0.3736	0.5600	-2.55	0.8708	1.1420	-0.33	0.0651	0.1704	2.06
3511	Protein phosphatase 1 regulatory subunit 3C	Q9UQK1	0.0085	0.0546	-0.25	0.0182	0.4667	0.31	0.0048	0.0339	-0.27	0.0598	0.1315	-0.12	0.4315	0.5258	0.04	0.0355	0.1216	-0.17	0.3736	0.5741	-2.55	0.8708	1.1671	-0.33	0.0651	0.1780	2.06
3512	Protein phosphatase 1 regulatory subunit 7	Q15435	0.0011	0.0224	0.09	0.4240	0.7610	0.04	0.8572	0.8863	0.01	0.9783	0.9813	0.00	0.1807	0.3728	0.04	0.1374	0.2904	0.13	0.4195	0.4753	0.10	0.2429	0.5612	0.14	0.9682	0.9750	0.00
3513	Protein phosphatase 1A	P35813	0.0482	0.1411	0.16	0.2003	0.6634	0.14	0.1432	0.2694	0.09	0.2342	0.3785	0.07	0.1712	0.3596	0.08	0.0932	0.2241	0.12	0.3736	0.5367	-2.55	0.8708	1.1002	-0.33	0.0651	0.1584	2.06
3514	Protein phosphatase 1B	O75688	0.6639	0.7237	0.05	0.4406	0.7766	0.11	0.1286	0.2493	0.15	0.0710	0.1493	-0.20	0.0177	0.0950	-0.35	0.1051	0.2415	-0.23	0.3736	0.5321	-2.55	0.8708	1.0919	-0.33	0.0651	0.1561	2.06
3515	Protein phosphatase 1F	P49593	0.4108	0.5295	-3.31	0.5752	0.7414	-1.75	0.5661	0.6716	-1.19	0.5388	0.6353	0.89	0.2938	0.4283	-1.88	0.9510	1.0259	0.09	0.0351	0.1550	0.64	0.0002	0.0157	0.87	0.0057	0.0722	0.44
3516	Protein phosphatase 1G	O15355	0.0906	0.2106	0.04	0.2251	0.6599	0.19	0.0070	0.0398	0.25	0.0149	0.0501	0.16	0.1039	0.2592	-0.06	0.0035	0.0591	0.12	0.0329	0.1518	0.48	0.6807	1.1208	0.05	0.1329	0.1932	0.19
3517	Protein phosphatase inhibitor 2	P41236	0.4851	0.5595	-0.08	0.1505	0.6596	-0.69	0.1197	0.2360	-0.25	0.0411	0.1004	-0.49	0.4419	0.5365	-0.14	0.5334	0.7645	0.10	0.5110	0.5644	-0.15	0.1642	0.4409	0.26	0.9084	0.9272	0.02
3518	Protein phosphatase inhibitor 2 family member B	Q6NXS1	0.0335	0.1143	-1.43	0.4419	0.7775	-0.61	0.0128	0.0529	-1.44	0.0212	0.0629	-1.11	0.3452	0.4422	0.25	0.0190	0.0886	-1.22	0.3736	0.5949	-2.55	0.8708	1.2038	-0.33	0.0651	0.1899	2.06
3519	Protein phosphatase inhibitor 2 family member C	O14990	0.0249	0.0953	-0.22	0.2753	0.6642	0.11	0.0064	0.0386	0.29	0.1479	0.2622	-0.39	0.0165	0.0915	-0.23	0.3752	0.5838	-0.11	0.6844	0.7266	0.07	0.0808	0.2790	-0.25	0.0001	0.0185	-8.33
3520	Protein phosphatase methylesterase 1	Q9Y570	0.0205	0.0845	-0.16	0.0067	0.4181	0.28	0.1781	0.3212	-0.11	0.9413	0.9488	0.01	0.1005	0.2537	0.13	0.0201	0.0920	-0.21	0.1634	0.3571	0.18	0.2143	0.5195	0.12	0.6313	0.6883	0.03
3521	Protein piccolo	Q9Y6V0	0.0138	0.0687	-0.35	0.5690	0.8822	-0.11	0.0255	0.0793	0.22	0.0469	0.1102	-0.18	0.3328	0.4299	-0.06	0.0238	0.0989	0.24	0.0023	0.0565	-0.46	0.0015	0.0316	-0.46	0.0027	0.0555	-0.60
3522	Protein PMS2CL	Q68D20	0.0139	0.0692	-0.06	0.7733	0.8357	-0.04	0.0624	0.1451	-0.06	0.0216	0.0637	0.13	0.0320	0.1306	-0.28	0.1393	0.2927	0.06	0.3736	0.5202	-2.55	0.8708	1.0704	-0.33	0.0651	0.1503	2.06
3523	Protein PPP4R3C	Q6ZMV5	0.0001	0.0102	1.79	0.0845	0.6217	0.61	0.2937	0.4744	-0.13	0.0015	0.0170	1.68	0.0053	0.0544	0.70	0.0501	0.1503	1.92	0.3736	0.5608	-2.55	0.8708	1.1434	-0.33	0.0651	0.1708	2.06
3524	Protein PRRC2A	P48634	0.9183	0.9377	-0.02	0.3301	0.6892	0.21	0.0658	0.1503	0.35	0.1955	0.3260	0.18	0.1975	0.3973	0.18	0.3585	0.5646	0.14	0.3736	0.4735	-2.55	0.8708	0.9847	-0.33	0.0651	0.1293	2.06
3525	Protein PRRC2B	Q5JSZ5	0.1380	0.2827	-0.10	0.5329	0.8499	-0.09	0.0980	0.2010	0.13	0.1698	0.2915	-0.15	0.4813	0.5713	-0.07	0.6143	0.8504	-0.03	0.3736	0.4511	-2.55	0.8708	0.9429	-0.33	0.0651	0.1199	2.06
3526	Protein PRRC2C	Q9Y520	0.4108	0.5293	-3.31	0.5752	0.7412	-1.75	0.5661	0.6715	-1.19	0.5388	0.6352	0.89	0.2938	0.4282	-1.88	0.9510	1.0256	0.09	0.9879	0.9910	0.00	0.0875	0.2932	-0.28	0.8228	0.8570	0.02
3527	Protein prune homolog 2	Q8WUY3	0.9522	0.9645	0.02	0.0391	0.5595	0.89	0.1394	0.2638	0.53	0.1005	0.1948	0.65	0.1343	0.3037	0.53	0.8152	1.0517	0.06	0.0914	0.2430	-0.23	0.3362	0.6936	0.11	0.0084	0.0819	0.13
3528	Protein quaking	Q96PU8	0.4108	0.5292	-3.31	0.5752	0.7410	-1.75	0.5661	0.6713	-1.19	0.5388	0.6350	0.89	0.2938	0.4281	-1.88	0.9510	1.0254	0.09	0.2888	0.5407	0.15	0.9606	0.9719	0.00	0.0047	0.0667	-0.50
3529	Protein RCC2	Q9P258	0.0051	0.0436	0.32	0.8631	0.9010	0.02	0.4415	0.6534	0.03	0.0551	0.1239	-0.11	0.1166	0.2798	-0.08	0.3097	0.5096	-0.04	0.0020	0.0534	-0.50	0.0016	0.0328	-0.61	0.0023	0.0537	-0.85
3530	Protein Red	Q13123	0.4108	0.5291	-3.31	0.5752	0.7408	-1.75	0.5661	0.6712	-1.19	0.5388	0.6349	0.89	0.2938	0.4280	-1.88	0.9510	1.0252	0.09	0.0309	0.1486	0.46	0.1963	0.4915	0.16	0.0847	0.1368	0.23
3531	Protein regulator of cytokinesis 1	O43663	0.0001	0.0100	1.07	0.0010	0.2590	0.58	0.0007	0.0217	1.01	0.0006	0.0136	1.12	0.0000	0.0000	1.28	0.0966	0.2293	1.05	0.3736	0.5355	-2.55	0.8708	1.0980	-0.33	0.0651	0.1578	2.06
3532	Protein RER1	O15258	0.0030	0.0364	0.22	0.5764	0.6857	0.06	0.3292	0.5188	0.04	0.0879	0.1761	-0.07	0.2366	0.4489	-0.04	0.1042	0.2402	0.07	0.0315	0.1500	0.33	0.0073	0.0705	0.31	0.0017	0.0487	0.25
3533	Protein RFT1 homolog	Q96AA3	0.4108	0.5289	-3.31	0.5752	0.7406	-1.75	0.5661	0.6710	-1.19	0.5388	0.6347	0.89	0.2938	0.4279	-1.88	0.9510	1.0250	0.09	0.3235	0.5890	-0.59	0.6264	1.0625	-0.06	0.0536	0.2037	0.41
3534	Protein RTF2 homolog	Q9BY42	0.0272	0.0999	0.16	0.1567	0.6626	0.40	0.0153	0.0582	0.36	0.0010	0.0152	0.46	0.1859	0.3809	-0.07	0.0683	0.1832	0.15	0.1102	0.2726	0.27	0.1030	0.3236	0.13	0.0325	0.1540	0.19
3535	Protein RUFY3	Q7L099	0.0008	0.0204	-1.41	0.2806	0.6652	-1.56	0.0013	0.0223	-1.59	0.0056	0.0301	-2.39	0.0348	0.1355	-0.42	0.0980	0.2310	-2.13	0.1983	0.4086	0.19	0.0003	0.0173	0.70	0.0093	0.0862	0.41
3536	Protein S100-A10	P60903	0.0067	0.0490	0.13	0.1924	0.6684	0.18	0.0008	0.0209	0.25	0.0018	0.0190	0.36	0.0003	0.0152	0.37	0.0004	0.0450	0.45	0.0240	0.1372	0.43	0.5918	1.0242	-0.05	0.0627	0.2240	0.21
3537	Protein S100-A11	P31949	0.0081	0.0532	0.35	0.1046	0.6337	0.49	0.0016	0.0237	0.57	0.0016	0.0180	0.58	0.0673	0.1975	0.18	0.0114	0.0732	0.45	0.0653	0.2010	0.48	0.8448	1.2878	0.02	0.9705	0.9765	0.00
3538	Protein S100-A13	Q99584	0.4512	0.5288	0.05	0.4077	0.7497	0.18	0.7722	0.8127	-0.02	0.6469	0.6912	0.03	0.0709	0.2028	-0.16	0.2526	0.4428	0.08	0.4242	0.4799	-0.07	0.0163	0.1081	-0.10	0.0020	0.0500	-0.30
3539	Protein S100-A14	Q9HCY8	0.0002	0.0131	0.39	0.2846	0.6650	0.10	0.0000	0.0000	0.48	0.0093	0.0388	0.46	0.0028	0.0405	0.31	0.0055	0.0623	0.84	0.3736	0.6277	-2.55	0.8708	1.2610	-0.33	0.0651	0.2100	2.06
3540	Protein S100-A16	Q96FQ6	0.1387	0.2833	-0.13	0.1658	0.6601	0.22	0.7482	0.7906	0.02	0.2681	0.4237	0.09	0.3938	0.4905	-0.06	0.4917	0.7171	0.05	0.4874	0.5417	-0.10	0.0200	0.1223	-0.33	0.0335	0.1569	-0.29
3541	Protein S100-A3	P33764	0.1524	0.3034	-1.50	0.0846	0.6198	-7.65	0.0951	0.1963	-1.89	0.0554	0.1242	-2.57	0.8281	0.8666	-0.20	0.0082	0.0664	-5.82	0.3736	0.6181	-2.55	0.8708	1.2443	-0.33	0.0651	0.2039	2.06
3542	Protein S100-A6	P06703	0.0247	0.0948	0.47	0.0771	0.6125	0.50	0.0031	0.0304	0.55	0.0058	0.0307	0.33	0.0088	0.0672	0.40	0.0019	0.0524	0.58	0.2972	0.5538	0.25	0.3286	0.6841	-0.16	0.4330	0.5043	-0.19
3543	Protein S100-P	P25815	0.0460	0.1369	0.14	0.4867	0.8099	0.12	0.0088	0.0438	0.11	0.1163	0.2175	0.13	0.0314	0.1298	0.08	0.0200	0.0919	0.32	0.2493	0.4853	0.13	0.0255	0.1431	-0.15	0.3976	0.4691	0.03
3544	Protein SAAL1	Q96ER3	0.4957	0.5700	-0.02	0.9890	0.9921	0.00	0.2775	0.4516	0.13	0.5251	0.7312	0.03	0.0066	0.0588	-0.20	0.1320	0.2830	0.08	0.0066	0.0840	0.37	0.0676	0.2503	0.18	0.0130	0.0987	0.43
3545	Protein salvador homolog 1	Q9H4B6	0.4108	0.5288	-3.31	0.5752	0.7404	-1.75	0.5661	0.6709	-1.19	0.5388	0.6346	0.89	0.2938	0.4277	-1.88	0.9510	1.0248	0.09	0.0302	0.1472	-0.33	0.0081	0.0741	-0.50	0.0094	0.0866	-0.59
3546	Protein SCAI	Q8N9R8	0.0064	0.0479	0.17	0.0165	0.4595	0.11	0.0486	0.1227	0.07	0.0001	0.0071	0.40	0.0702	0.2020	0.09	0.0008	0.0432	0.33	0.3736	0.6503	-2.55	0.8708	1.2999	-0.33	0.0651	0.2250	2.06
3547	Protein SCO1 homolog mitochondrial	O75880	0.0109	0.0599	-2.91	0.1728	0.6606	-2.80	0.0087	0.0439	0.95	0.0900	0.1792	-0.93	0.8618	0.8941	-0.06	0.3486	0.5536	-1.27	0.0042	0.0693	-0.31	0.0159	0.1070	-0.37	0.0412	0.1762	-0.33
3548	Protein scribble homolog	Q14160	0.9642	0.9738	0.00	0.8182	0.8701	-0.02	0.5185	0.7391	0.02	0.1570	0.2748	-0.09	0.0681	0.1987	-0.08	0.3430	0.5470	-0.03	0.6287	0.6745	0.06	0.1801	0.4674	-0.44	0.2337	0.3037	-0.20
3549	Protein SEC13 homolog	P55735	0.0003	0.0147	0.30	0.0014	0.2266	0.21	0.0052	0.0354	0.17	0.0153	0.0511	0.14	0.0316	0.1301	0.11	0.0180	0.0871	0.12	0.0002	0.0334	-0.42	0.0010	0.0282	-0.70	0.0057	0.0718	-0.69
3550	Protein sel-1 homolog 1	Q9UBV2	0.2699	0.4622	-0.08	0.0384	0.5572	0.15	0.0000	0.0000	0.51	0.0009	0.0158	0.20	0.0084	0.0661	0.12	0.0081	0.0671	0.18	0.3736	0.6189	-2.55	0.8708	1.2457	-0.33	0.0651	0.2044	2.06
3551	Protein SET	Q01105	0.0014	0.0253	0.30	0.0831	0.6194	0.29	0.0002	0.0207	0.41	0.0047	0.0280	0.30	0.2770	0.5019	0.03	0.0047	0.0595	0.26	0.0737	0.2154	0.30	0.2152	0.5211	0.12	0.4542	0.5226	0.05
3552	Protein SETSIP	P0DME0	0.0130	0.0665	-0.14	0.0157	0.4569	-0.14	0.0142	0.0559	-0.17	0.0061	0.0313	-0.32	0.0046	0.0506	-0.23	0.1317	0.2828	-0.24	0.1252	0.2976	0.84	0.0067	0.0666	1.17	0.0022	0.0518	1.52
3553	Protein SGT1 homolog	Q9Y2Z0	0.0699	0.1765	0.11	0.4221	0.7595	0.18	0.0155	0.0586	0.22	0.0116	0.0431	0.22	0.1111	0.2716	0.09	0.0094	0.0691	0.30	0.0258	0.1405	0.31	0.0233	0.1347	0.18	0.0477	0.1911	0.14
3554	Protein Smaug homolog 1	Q9UPU9	0.4108	0.5287	-3.31	0.5752	0.7403	-1.75	0.5661	0.6707	-1.19	0.5388	0.6345	0.89	0.2938	0.4276	-1.88	0.9510	1.0246	0.09	0.8715	0.8939	-0.27	0.5832	1.0151	0.91	0.3409	0.4118	1.49
3555	Protein SMG8	Q8ND04	0.9557	0.9669	0.00	0.6401	0.7349	0.03	0.1604	0.2952	0.20	0.9639	0.9688	0.00	0.1304	0.2985	-0.10	0.0294	0.1099	0.20	0.3736	0.5813	-2.55	0.8708	1.1799	-0.33	0.0651	0.1821	2.06
3556	Protein SOGA1	O94964	0.1538	0.3051	-0.06	0.2188	0.6605	-0.08	0.0370	0.1008	-0.13	0.8142	0.8398	-0.02	0.0552	0.1753	-0.11	0.7583	0.9962	-0.01	0.0472	0.1739	0.55	0.0033	0.0468	0.37	0.0000	0.0000	0.58

Supplemetary Table S2. Overview on all relatively quantified 5180 proteins statistical analysis

			MCF-7																		MDA-MB-231								
			Dai SC20 vs control			Gen SC20 vs control			SSE SC20 vs control			Dai IC20 vs control			Gen IC20 vs control			SSE IC20 vs control			Dai IC20 vs control			Gen IC20 vs control			SSE IC20 vs control		
	**Protein name**	**UniProt**	**p value**	**BH q value**	**log2FC**	**p value**	**BH q value**	**log2FC**	**p value**	**BH q value**	**log2FC**	**p value**	**BH q value**	**log2FC**	**p value**	**BH q value**	**log2FC**	**p value**	**BH q value**	**log2FC**	**p value**	**BH q value**	**log2FC**	**p value**	**BH q value**	**log2FC**	**p value**	**BH q value**	**log2FC**
3557	Protein SOGA3	Q5TF21	0.4108	0.5286	-3.31	0.5752	0.7401	-1.75	0.5661	0.6706	-1.19	0.5388	0.6343	0.89	0.2938	0.4275	-1.88	0.9510	1.0244	0.09	0.2714	0.5146	-0.09	0.0585	0.2304	-0.23	0.0009	0.0364	-0.83
3558	Protein SOX-15	O60248	0.7209	0.7719	-0.03	0.1769	0.6626	0.25	0.1135	0.2258	0.18	0.0602	0.1321	0.24	0.4438	0.5381	0.07	0.2487	0.4373	0.11	0.0504	0.1788	0.38	0.0136	0.0976	0.35	0.0956	0.1503	0.27
3559	Protein spire homolog 2	Q8WWL2	0.1001	0.2251	0.15	0.2218	0.6577	-1.19	0.3848	0.5856	0.17	0.1033	0.1987	0.28	0.6267	0.6975	0.02	0.6365	0.8739	-0.26	0.3736	0.4489	-2.55	0.8708	0.9388	-0.33	0.0651	0.1190	2.06
3560	Protein SPT2 homolog	Q68D10	0.4108	0.5284	-3.31	0.5752	0.7399	-1.75	0.5661	0.6704	-1.19	0.5388	0.6342	0.89	0.2938	0.4274	-1.88	0.9510	1.0242	0.09	0.7907	0.8234	0.07	0.0102	0.0831	0.38	0.0080	0.0798	0.61
3561	Protein SSX4	O60224	0.4108	0.5283	-3.31	0.5752	0.7397	-1.75	0.5661	0.6703	-1.19	0.5388	0.6340	0.89	0.2938	0.4273	-1.88	0.9510	1.0239	0.09	0.1597	0.3526	0.26	0.2355	0.5515	-0.08	0.4363	0.5071	0.05
3562	Protein TALPID3	Q9BVV6	0.0092	0.0568	-0.50	0.1727	0.6612	-0.27	0.1653	0.3023	-0.12	0.0349	0.0892	-0.25	0.0389	0.1441	-0.27	0.1572	0.3166	-0.30	0.5218	0.5750	0.07	0.4810	0.8914	0.08	0.4843	0.5526	-0.12
3563	Protein TBATA	Q96M53	0.4108	0.5282	-3.31	0.5752	0.7395	-1.75	0.5661	0.6701	-1.19	0.5388	0.6339	0.89	0.2938	0.4271	-1.88	0.9510	1.0237	0.09	0.5680	0.6181	0.04	0.0015	0.0311	-0.50	0.0117	0.0941	-0.39
3564	Protein TESPA1	A2RU30	0.4108	0.5280	-3.31	0.5752	0.7393	-1.75	0.5661	0.6700	-1.19	0.5388	0.6337	0.89	0.2938	0.4270	-1.88	0.9510	1.0235	0.09	0.7060	0.7476	-0.07	0.7560	1.1990	0.03	0.1090	0.1658	0.51
3565	Protein TFG	Q92734	0.0030	0.0363	0.29	0.1075	0.6306	0.30	0.0065	0.0389	0.25	0.0023	0.0206	0.28	0.0243	0.1128	0.16	0.0025	0.0561	0.34	0.0045	0.0724	0.47	0.1877	0.4775	0.08	0.3456	0.4167	0.07
3566	Protein TMED8	Q6PL24	0.4108	0.5279	-3.31	0.5752	0.7392	-1.75	0.5661	0.6698	-1.19	0.5388	0.6336	0.89	0.2938	0.4269	-1.88	0.9510	1.0233	0.09	0.2167	0.4366	-1.04	0.0151	0.1036	-0.82	0.3578	0.4290	0.29
3567	Protein TOPAZ1	Q8N9V7	0.0082	0.0536	-0.60	0.5341	0.8500	-0.16	0.1095	0.2196	0.47	0.0803	0.1647	-0.26	0.0391	0.1444	0.42	0.5044	0.7298	-0.15	0.3736	0.4596	-2.55	0.8708	0.9587	-0.33	0.0651	0.1234	2.06
3568	Protein transport protein Sec16A	O15027	0.0924	0.2136	-0.07	0.8227	0.8733	-0.02	0.3768	0.5761	-0.03	0.0538	0.1214	-0.09	0.0128	0.0806	-0.14	0.3373	0.5414	-0.04	0.2416	0.4739	0.21	0.0338	0.1664	-0.47	0.2567	0.3282	-0.10
3569	Protein transport protein Sec23A	Q15436	0.4967	0.5710	-0.11	0.1085	0.6301	-0.12	0.0509	0.1269	0.11	0.0657	0.1414	0.24	0.5924	0.6690	0.02	0.4319	0.6534	0.17	0.0121	0.1038	0.49	0.1125	0.3428	0.17	0.0546	0.2060	0.21
3570	Protein transport protein Sec23B	Q15437	0.1030	0.2296	0.03	0.1205	0.6376	0.06	0.0001	0.0167	0.19	0.2458	0.3937	0.07	0.0929	0.2403	0.09	0.0812	0.2049	0.15	0.0044	0.0714	-0.37	0.0025	0.0416	-0.26	0.0112	0.0927	-0.33
3571	Protein transport protein Sec24B	O95487	0.0549	0.1533	-0.22	0.0420	0.5710	-0.20	0.0700	0.1574	-0.36	0.0251	0.0707	-0.35	0.0205	0.1027	-0.38	0.1390	0.2927	-0.37	0.3736	0.5206	-2.55	0.8708	1.0712	-0.33	0.0651	0.1505	2.06
3572	Protein transport protein Sec24C	P53992	0.0592	0.1608	0.10	0.2611	0.6646	0.19	0.0479	0.1214	0.12	0.0104	0.0409	0.15	0.0775	0.2142	0.07	0.0629	0.1746	0.11	0.7920	0.8246	0.02	0.0419	0.1886	-0.12	0.0327	0.1541	-0.14
3573	Protein transport protein Sec31A	O94979	0.1112	0.2423	-0.09	0.8189	0.8703	0.02	0.0720	0.1603	-0.14	0.1201	0.2231	-0.09	0.2749	0.4991	-0.05	0.4417	0.6638	0.04	0.0959	0.2490	0.10	0.8264	1.2703	0.01	0.3107	0.3821	-0.05
3574	Protein transport protein Sec61 subunit alpha isoform 1	P61619	0.0259	0.0973	0.12	0.0782	0.6119	0.24	0.0075	0.0411	0.28	0.0117	0.0434	0.23	0.0030	0.0409	0.19	0.1228	0.2694	0.14	0.0712	0.2110	0.33	0.2020	0.5004	0.10	0.6879	0.7408	0.02
3575	Protein transport protein Sec61 subunit beta	P60468	0.0048	0.0434	0.42	0.1513	0.6597	0.37	0.0043	0.0326	0.46	0.0023	0.0207	0.46	0.0080	0.0648	0.30	0.0128	0.0761	0.37	0.0203	0.1321	0.57	0.4973	0.9119	-0.04	0.0353	0.1611	-0.15
3576	Protein tyrosine phosphatase domain-containing protein 1	A2A3K4	0.0179	0.0785	0.29	0.9482	0.9655	0.01	0.0029	0.0292	0.41	0.0101	0.0405	0.18	0.0180	0.0957	0.22	0.1030	0.2383	0.29	0.2834	0.5329	-0.13	0.0091	0.0782	-0.36	0.7147	0.7647	0.05
3577	Protein unc-119 homolog A	Q13432	0.1836	0.3482	-0.08	0.2919	0.6690	-0.12	0.2653	0.4361	-0.07	0.0437	0.1046	-0.08	0.0032	0.0424	-0.15	0.8088	1.0461	0.02	0.3736	0.4382	-2.55	0.8708	0.9187	-0.33	0.0651	0.1148	2.06
3578	Protein unc-13 homolog A	Q9UPW8	0.4897	0.5637	-0.13	0.6879	0.7716	0.11	0.2484	0.4148	-0.22	0.1587	0.2769	-0.31	0.0298	0.1256	0.58	0.0969	0.2294	-0.57	0.3736	0.5353	-2.55	0.8708	1.0978	-0.33	0.0651	0.1577	2.06
3579	Protein unc-13 homolog C	Q8NB66	0.0885	0.2073	-0.23	0.2878	0.6682	-0.16	0.1803	0.3241	-0.15	0.0492	0.1139	-0.33	0.0608	0.1859	-0.30	0.0680	0.1829	-0.28	0.3392	0.6099	0.12	0.2349	0.5506	-0.08	0.0110	0.0918	0.45
3580	Protein unc-13 homolog D	Q70J99	0.0399	0.1258	-0.43	0.5894	0.6948	-0.30	0.0515	0.1275	-0.64	0.0475	0.1110	-0.91	0.1332	0.3021	-0.19	0.2415	0.4275	-0.90	0.3736	0.4917	-2.55	0.8708	1.0182	-0.33	0.0651	0.1371	2.06
3581	Protein unc-45 homolog A	Q9H3U1	0.0030	0.0362	0.14	0.1198	0.6358	0.22	0.0040	0.0322	0.21	0.0028	0.0220	0.21	0.0111	0.0755	0.11	0.0029	0.0552	0.23	0.2653	0.5075	0.17	0.0898	0.2978	-0.07	0.4263	0.4975	-0.04
3582	Protein unc-79 homolog	Q9P2D8	0.4108	0.5278	-3.31	0.5752	0.7390	-1.75	0.5661	0.6697	-1.19	0.5388	0.6335	0.89	0.2938	0.4268	-1.88	0.9510	1.0231	0.09	0.0878	0.2380	1.11	0.2552	0.5798	0.56	0.0835	0.1353	1.07
3583	Protein unc-80 homolog	Q8N2C7	0.2423	0.4275	0.14	0.4022	0.7459	0.15	0.0067	0.0394	0.56	0.7934	0.8220	-0.02	0.0550	0.1750	0.25	0.8267	1.0608	-0.02	0.0023	0.0552	5.23	0.8708	1.3113	-0.33	0.0029	0.0542	6.00
3584	Protein Wnt-11	O96014	0.0144	0.0697	0.18	0.0176	0.4699	0.53	0.0015	0.0233	0.52	0.0033	0.0235	0.49	0.0002	0.0142	0.57	0.0327	0.1164	0.23	0.3736	0.5780	-2.55	0.8708	1.1741	-0.33	0.0651	0.1802	2.06
3585	Protein Wnt-5b	Q9H1J7	0.4108	0.5276	-3.31	0.5752	0.7388	-1.75	0.5661	0.6695	-1.19	0.5388	0.6333	0.89	0.2938	0.4267	-1.88	0.9510	1.0229	0.09	0.3003	0.5575	0.21	0.6369	1.0750	0.05	0.4818	0.5501	-0.35
3586	Protein XRP2	O75695	0.4108	0.5275	-3.31	0.5752	0.7386	-1.75	0.5661	0.6693	-1.19	0.5388	0.6332	0.89	0.2938	0.4265	-1.88	0.9510	1.0227	0.09	0.4751	0.5297	-0.10	0.6851	1.1266	0.06	0.5270	0.5933	0.11
3587	Protein yippee-like 5	P62699	0.4108	0.5274	-3.31	0.5752	0.7384	-1.75	0.5661	0.6692	-1.19	0.5388	0.6330	0.89	0.2938	0.4264	-1.88	0.9510	1.0225	0.09	0.0404	0.1652	0.18	0.6593	1.0999	-0.03	0.0354	0.1610	0.18
3588	Protein zer-1 homolog	Q7Z7L7	0.4108	0.5272	-3.31	0.5752	0.7382	-1.75	0.5661	0.6690	-1.19	0.5388	0.6329	0.89	0.2938	0.4263	-1.88	0.9510	1.0222	0.09	0.5725	0.6222	-0.24	0.4835	0.8942	-0.26	0.1542	0.2175	0.64
3589	Protein/nucleic acid deglycase DJ-1	Q99497	0.0014	0.0252	0.42	0.1806	0.6649	0.28	0.0024	0.0271	0.36	0.0023	0.0208	0.37	0.0576	0.1801	0.14	0.0078	0.0660	0.36	0.7795	0.8136	0.03	0.0090	0.0777	-0.40	0.0167	0.1106	-0.38
3590	Protein-arginine deiminase type-2	Q9Y2J8	0.4108	0.5271	-3.31	0.5752	0.7381	-1.75	0.5661	0.6689	-1.19	0.5388	0.6327	0.89	0.2938	0.4262	-1.88	0.9510	1.0220	0.09	0.0351	0.1549	-0.35	0.0100	0.0822	-0.24	0.0045	0.0662	-0.43
3591	Protein-arginine deiminase type-4	Q9UM07	0.0088	0.0556	-0.28	0.1039	0.6339	-0.32	0.0100	0.0468	-0.47	0.0023	0.0205	-0.64	0.0108	0.0744	-0.46	0.0033	0.0578	-0.55	0.3736	0.6370	-2.55	0.8708	1.2771	-0.33	0.0651	0.2160	2.06
3592	Proteinase-activated receptor 1	P25116	0.4108	0.5270	-3.31	0.5752	0.7379	-1.75	0.5661	0.6687	-1.19	0.5388	0.6326	0.89	0.2938	0.4261	-1.88	0.9510	1.0218	0.09	0.0408	0.1660	0.48	0.0363	0.1736	0.51	0.9819	0.9859	0.00
3593	Protein-glutamate O-methyltransferase	Q9H993	0.0002	0.0130	-0.48	0.8693	0.9051	-0.01	0.0005	0.0181	-0.34	0.0005	0.0134	-0.61	0.0022	0.0371	-0.51	0.0044	0.0613	-0.59	0.3736	0.6328	-2.55	0.8708	1.2699	-0.33	0.0651	0.2133	2.06
3594	Protein-glutamine gamma-glutamyltransferase 2	P21980	0.4108	0.5268	-3.31	0.5752	0.7377	-1.75	0.5661	0.6686	-1.19	0.5388	0.6324	0.89	0.2938	0.4259	-1.88	0.9510	1.0216	0.09	0.0946	0.2474	0.37	0.4492	0.8520	0.08	0.6022	0.6629	-0.04
3595	Protein-glutamine gamma-glutamyltransferase 4	P49221	0.4108	0.5267	-3.31	0.5752	0.7375	-1.75	0.5661	0.6684	-1.19	0.5388	0.6323	0.89	0.2938	0.4258	-1.88	0.9510	1.0214	0.09	0.5689	0.6188	0.44	0.0039	0.0503	1.12	0.0019	0.0495	1.62
3596	Protein-L-isoaspartate O-methyltransferase domain-containing protein 2	Q9NV79	0.7203	0.7714	0.04	0.5610	0.8750	-0.43	0.0968	0.1993	-0.35	0.1354	0.2441	-0.46	0.0469	0.1592	0.18	0.9264	1.1636	-0.04	0.3736	0.4328	-2.55	0.8708	0.9085	-0.33	0.0651	0.1127	2.06
3597	Protein-L-isoaspartate(D-aspartate) O-methyltransferase	P22061	0.0035	0.0379	-0.08	0.1093	0.6305	-0.08	0.0006	0.0201	-0.15	0.0012	0.0165	-0.16	0.0067	0.0596	-0.25	0.2848	0.4812	-0.01	0.0058	0.0808	-1.05	0.0657	0.2473	-0.28	0.0637	0.2262	-0.31
3598	Protein-tyrosine kinase 6	Q13882	0.0600	0.1620	0.20	0.0354	0.5393	0.32	0.0023	0.0270	0.63	0.0032	0.0232	0.67	0.0460	0.1580	0.26	0.0101	0.0705	0.53	0.3736	0.6128	-2.55	0.8708	1.2351	-0.33	0.0651	0.2006	2.06
3599	Proteolipid protein 2	Q04941	0.0022	0.0317	0.37	0.1131	0.6347	0.19	0.0364	0.1000	0.24	0.3467	0.5222	-0.09	0.2912	0.5227	0.10	0.1774	0.3466	0.19	0.6821	0.7251	0.07	0.0042	0.0517	-0.69	0.0073	0.0781	-0.95
3600	Prothymosin alpha	P06454	0.1676	0.3259	0.11	0.0780	0.6122	0.59	0.0042	0.0324	0.47	0.0335	0.0869	0.22	0.0445	0.1558	-0.22	0.0753	0.1950	0.18	0.0307	0.1481	0.59	0.3194	0.6698	0.10	0.0545	0.2059	0.27
3601	Protocadherin alpha-1	Q9Y5I3	0.4108	0.5266	-3.31	0.5752	0.7373	-1.75	0.5661	0.6683	-1.19	0.5388	0.6322	0.89	0.2938	0.4257	-1.88	0.9510	1.0212	0.09	0.0405	0.1654	0.20	0.1721	0.4544	0.10	0.1551	0.2185	-0.13
3602	Protocadherin alpha-10	Q9Y5I2	0.4108	0.5265	-3.31	0.5752	0.7371	-1.75	0.5661	0.6681	-1.19	0.5388	0.6320	0.89	0.2938	0.4256	-1.88	0.9510	1.0210	0.09	0.4165	0.4723	2.68	0.1573	0.4288	5.56	0.1646	0.2298	5.65
3603	Protocadherin alpha-9	Q9Y5H5	0.4108	0.5263	-3.31	0.5752	0.7370	-1.75	0.5661	0.6680	-1.19	0.5388	0.6319	0.89	0.2938	0.4255	-1.88	0.9510	1.0208	0.09	0.9236	0.9370	0.01	0.0151	0.1035	-0.46	0.1144	0.1720	-1.10
3604	Protocadherin beta-1	Q9Y5F3	0.4108	0.5262	-3.31	0.5752	0.7368	-1.75	0.5661	0.6678	-1.19	0.5388	0.6317	0.89	0.2938	0.4253	-1.88	0.9510	1.0205	0.09	0.8015	0.8325	-0.10	0.0003	0.0171	2.14	0.0043	0.0651	1.46
3605	Protocadherin beta-14	Q9Y5E9	0.9756	0.9834	0.00	0.3596	0.7083	-0.22	0.4964	0.7183	-0.06	0.4367	0.6305	-0.08	0.2938	0.5250	-0.11	0.6117	0.8488	-0.06	0.3736	0.4516	-2.55	0.8708	0.9439	-0.33	0.0651	0.1201	2.06
3606	Protocadherin beta-6	Q9Y5E3	0.1281	0.2683	-0.38	0.1836	0.6646	-0.32	0.9899	0.9910	0.00	0.1458	0.2592	-0.38	0.9190	0.9393	0.02	0.3120	0.5126	-0.32	0.3736	0.4797	-2.55	0.8708	0.9962	-0.33	0.0651	0.1319	2.06
3607	Protocadherin beta-8	Q9UN66	0.0173	0.0769	-0.37	0.6812	0.7668	-0.12	0.0016	0.0228	-0.56	0.0018	0.0189	-0.88	0.0125	0.0798	-0.32	0.0887	0.2171	-0.77	0.3736	0.5386	-2.55	0.8708	1.1037	-0.33	0.0651	0.1594	2.06
3608	Protocadherin Fat 2	Q9NYQ8	0.4598	0.5368	0.04	0.0193	0.4672	-0.17	0.1126	0.2245	-0.10	0.0033	0.0232	-0.38	0.7800	0.8273	-0.01	0.0190	0.0885	0.21	0.0212	0.1339	0.41	0.0046	0.0547	0.72	0.3631	0.4346	0.10
3609	Protocadherin Fat 3	Q8TDW7	0.2321	0.4139	0.07	0.7313	0.8043	0.01	0.9883	0.9900	0.00	0.4051	0.5961	-0.03	0.1152	0.2779	-0.08	0.1450	0.3003	0.07	0.0340	0.1537	0.24	0.3797	0.7559	0.07	0.7246	0.7726	0.03
3610	Protocadherin gamma-C3	Q9UN70	0.4108	0.5261	-3.31	0.5752	0.7366	-1.75	0.5661	0.6677	-1.19	0.5388	0.6316	0.89	0.2938	0.4252	-1.88	0.9510	1.0203	0.09	0.4681	0.5231	-0.05	0.0730	0.2619	0.11	0.1946	0.2626	-0.11
3611	Proto-oncogene c-Rel	Q04864	0.0113	0.0609	-0.33	0.1747	0.6615	-2.72	0.0224	0.0733	-0.26	0.2335	0.3776	-0.10	0.0308	0.1284	-0.25	0.1426	0.2974	-0.20	0.0062	0.0832	0.77	0.0137	0.0977	0.53	0.0134	0.1006	0.60
3612	Proto-oncogene DBL	P10911	0.0025	0.0334	0.28	0.2667	0.6632	0.26	0.0087	0.0438	0.32	0.0076	0.0353	0.48	0.0015	0.0304	0.19	0.0003	0.0398	0.58	0.8966	0.9150	0.02	0.8656	1.3080	-0.07	0.0819	0.1332	-0.33
3613	Proto-oncogene tyrosine-protein kinase receptor Ret	P07949	0.4108	0.5259	-3.31	0.5752	0.7364	-1.75	0.5661	0.6675	-1.19	0.5388	0.6314	0.89	0.2938	0.4251	-1.88	0.9510	1.0201	0.09	0.5386	0.5902	0.04	0.2113	0.5141	0.11	0.0294	0.1463	0.23
3614	Proto-oncogene tyrosine-protein kinase Src	P12931	0.0004	0.0161	-0.34	0.3250	0.6855	0.11	0.0004	0.0185	-0.36	0.0000	0.0000	-0.54	0.1123	0.2732	0.05	0.0201	0.0919	-0.73	0.3736	0.5940	-2.55	0.8708	1.2022	-0.33	0.0651	0.1893	2.06
3615	Pseudouridylate synthase 7 homolog	Q96PZ0	0.1066	0.2351	-0.05	0.9292	0.9522	0.01	0.3192	0.5064	-0.03	0.3496	0.5257	-0.04	0.0871	0.2311	-0.06	0.1750	0.3430	-0.10	0.1500	0.3359	1.27	0.1399	0.3971	1.35	0.2274	0.2977	1.29
3616	Pterin-4-alpha-carbinolamine dehydratase	P61457	0.0054	0.0443	0.08	0.2549	0.6622	0.14	0.0820	0.1760	0.15	0.0094	0.0389	0.25	0.0016	0.0319	0.19	0.0002	0.0414	0.32	0.0226	0.1366	-0.53	0.0722	0.2610	-0.08	0.0431	0.1812	-0.13
3617	Pterin-4-alpha-carbinolamine dehydratase 2	Q9H0N5	0.4108	0.5258	-3.31	0.5752	0.7362	-1.75	0.5661	0.6674	-1.19	0.5388	0.6313	0.89	0.2938	0.4250	-1.88	0.9510	1.0199	0.09	0.0019	0.0524	9.92	0.0861	0.2907	7.77	0.0027	0.0548	10.10
3618	Pulmonary surfactant-associated protein A2	Q8IWL1	0.4108	0.5257	-3.31	0.5752	0.7361	-1.75	0.5661	0.6672	-1.19	0.5388	0.6312	0.89	0.2938	0.4249	-1.88	0.9510	1.0197	0.09	0.0135	0.1098	-0.20	0.0669	0.2491	-0.32	0.0029	0.0546	-0.51
3619	Pumilio homolog 1	Q14671	0.0363	0.1195	0.34	0.0984	0.6348	0.23	0.0073	0.0405	0.38	0.0097	0.0396	0.24	0.0602	0.1847	0.13	0.0075	0.0652	0.39	0.0613	0.1953	0.09	0.1415	0.4005	-0.28	0.0023	0.0530	0.31
3620	Purine nucleoside phosphorylase	P00491	0.1420	0.2883	0.08	0.4590	0.7910	-0.05	0.0014	0.0221	0.30	0.0030	0.0227	0.13	0.6452	0.7135	0.01	0.0036	0.0586	0.14	0.0055	0.0796	0.59	0.0140	0.0987	0.34	0.0034	0.0581	0.37
3621	Puromycin-sensitive aminopeptidase	P55786	0.1176	0.2521	0.09	0.3067	0.6784	0.17	0.0440	0.1144	0.14	0.0389	0.0966	0.14	0.2742	0.4985	0.05	0.0285	0.1079	0.18	0.2025	0.4156	0.19	0.1444	0.4059	0.13	0.7284	0.7760	0.02
3622	Puromycin-sensitive aminopeptidase-like protein	A6NEC2	0.1237	0.2611	0.20	0.5680	0.8822	0.07	0.6809	0.7304	-0.05	0.1670	0.2876	-0.17	0.3336	0.4304	-0.11	0.5984	0.8346	-0.05	0.3706	0.6525	1.41	0.4297	0.8259	1.53	0.1676	0.2328	2.60
3623	Putative 40S ribosomal protein S26-like 1	Q5JNZ5	0.6212	0.6848	-0.01	0.4213	0.7593	-0.26	0.0056	0.0361	0.34	0.0042	0.0264	0.22	0.0001	0.0104	0.37	0.0068	0.0639	0.18	0.3736	0.6227	-2.55	0.8708	1.2523	-0.33	0.0651	0.2068	2.06
3624	Putative 60S ribosomal protein L13a protein RPL13AP3	Q6NVV1	0.0363	0.1194	-0.25	0.2283	0.6599	-0.36	0.1478	0.2760	0.11	0.0045	0.0271	-0.17	0.2197	0.4258	-0.10	0.0164	0.0848	-0.20	0.3736	0.6001	-2.55	0.8708	1.2129	-0.33	0.0651	0.1929	2.06
3625	Putative activator of 90 kDa heat shock protein ATPase homolog 2	Q719I0	0.4108	0.5255	-3.31	0.5752	0.7359	-1.75	0.5661	0.6671	-1.19	0.5388	0.6310	0.89	0.2938	0.4248	-1.88	0.9510	1.0195	0.09	0.0134	0.1095	-0.48	0.0022	0.0392	-0.31	0.0829	0.1345	-0.75
3626	Putative ankyrin repeat domain-containing protein 31	Q8N7Z5	0.4435	0.5212	0.03	0.2761	0.6646	0.18	0.0097	0.0459	0.18	0.0235	0.0677	0.15	0.6052	0.6803	-0.01	0.0090	0.0685	0.21	0.0302	0.1479	0.42	0.2440	0.5632	0.14	0.1562	0.2198	0.18
3627	Putative ATP-dependent RNA helicase DDX12	Q92771	0.4108	0.5254	-3.31	0.5752	0.7357	-1.75	0.5661	0.6669	-1.19	0.5388	0.6309	0.89	0.2938	0.4246	-1.88	0.9510	1.0193	0.09	0.0894	0.2403	0.16	0.0099	0.0817	0.52	0.1989	0.2673	-0.13
3628	Putative beta-actin-like protein 3	Q9BYX7	0.2152	0.3900	-0.03	0.0239	0.4874	-0.19	0.0204	0.0688	0.08	0.0187	0.0578	-0.19	0.0121	0.0788	-0.25	0.1089	0.2482	0.20	0.0130	0.1081	-0.56	0.0007	0.0242	-0.94	0.0075	0.0788	-0.75
3629	Putative charged multivesicular body protein 4B-like protein CHMP4BP1	P59074	0.4108	0.5253	-3.31	0.5752	0.7355	-1.75	0.5661	0.6668	-1.19	0.5388	0.6307	0.89	0.2938	0.4245	-1.88	0.9510	1.0191	0.09	0.0238	0.1371	0.34	0.0005	0.0214	0.49	0.0063	0.0752	0.43
3630	Putative ciliary rootlet coiled-coil protein 2	H7BZ55	0.7631	0.8074	0.03	0.4881	0.8114	-0.30	0.3362	0.5287	0.11	0.8293	0.8525	0.02	0.1495	0.3266	-0.20	0.5198	0.7477	0.07	0.2430	0.4759	0.10	0.0724	0.2608	-0.13	0.1988	0.2673	0.11
3631	Putative ciliary rootlet coiled-coil protein-like 2 protein	Q8IVE0	0.0052	0.0439	0.20	0.2012	0.6609	0.20	0.0054	0.0358	0.29	0.0129	0.0461	0.14	0.0465	0.1585	0.08	0.0710	0.1876	0.27	0.0085	0.0933	0.45	0.5231	0.9464	0.06	0.4349	0.5058	0.12
3632	Putative cleavage and polyadenylation specificity factor subunit 4-like protein	A6NMK7	0.4108	0.5252	-3.31	0.5752	0.7353	-1.75	0.5661	0.6666	-1.19	0.5388	0.6306	0.89	0.2938	0.4244	-1.88	0.9510	1.0189	0.09	0.0157	0.1163	0.28	0.4002	0.7852	-0.16	0.2325	0.3026	0.07
3633	Putative CNGA1-overlapping antisense gene protein	Q8IZM0	0.3012	0.5004	-0.14	0.5091	0.8311	-0.09	0.3392	0.5315	-0.12	0.0807	0.1654	-0.30	0.1795	0.3710	-0.20	0.3236	0.5261	-0.13	0.3736	0.4776	-2.55	0.8708	0.9922	-0.33	0.0651	0.1310	2.06

Supplemetary Table S2. Overview on all relatively quantified 5180 proteins statistical analysis

			MCF-7																		MDA-MB-231								
			Dai SC20 vs control			Gen SC20 vs control			SSE SC20 vs control			Dai IC20 vs control			Gen IC20 vs control			SSE IC20 vs control			Dai IC20 vs control			Gen IC20 vs control			SSE IC20 vs control		
	**Protein name**	**UniProt**	**p value**	**BH q value**	**log2FC**	**p value**	**BH q value**	**log2FC**	**p value**	**BH q value**	**log2FC**	**p value**	**BH q value**	**log2FC**	**p value**	**BH q value**	**log2FC**	**p value**	**BH q value**	**log2FC**	**p value**	**BH q value**	**log2FC**	**p value**	**BH q value**	**log2FC**	**p value**	**BH q value**	**log2FC**
3634	Putative coiled-coil domain-containing protein 144C	Q8IYA2	0.3884	0.6053	-1.15	0.1751	0.6606	-2.00	0.1812	0.3251	-0.64	0.2452	0.3931	-0.51	0.3166	0.4140	-0.18	0.4621	0.6847	-0.83	0.0159	0.1175	0.24	0.8000	1.2441	0.01	0.0108	0.0904	0.38
3635	Putative coiled-coil-helix-coiled-coil-helix domain-containing protein CHCHD2P9_ mitochondrial	Q5T1J5	0.4108	0.5250	-3.31	0.5752	0.7351	-1.75	0.5661	0.6665	-1.19	0.5388	0.6304	0.89	0.2938	0.4243	-1.88	0.9510	1.0186	0.09	0.0004	0.0340	-2.49	0.0010	0.0280	-1.70	0.0193	0.1205	-1.36
3636	Putative cTAGE family member 3	Q8IX95	0.0037	0.0387	-0.79	0.4468	0.7819	-0.13	0.0028	0.0290	-0.82	0.0090	0.0384	-0.91	0.0661	0.1957	-0.37	0.2576	0.4481	-0.66	0.3736	0.4886	-2.55	0.8708	1.0125	-0.33	0.0651	0.1358	2.06
3637	Putative dehydrogenase/reductase SDR family member 4-like 1	P0CG22	0.2004	0.3713	0.26	0.4674	0.7975	0.13	0.0615	0.1441	0.46	0.0510	0.1164	0.57	0.0236	0.1108	0.78	0.0270	0.1052	0.83	0.3736	0.5836	-2.55	0.8708	1.1839	-0.33	0.0651	0.1834	2.06
3638	Putative deoxyribonuclease TATDN1	Q6P1N9	0.0150	0.0710	0.08	0.0913	0.6289	0.27	0.0186	0.0655	0.28	0.0003	0.0106	0.44	0.0003	0.0171	0.27	0.0013	0.0510	0.28	0.0148	0.1124	0.33	0.0988	0.3151	0.16	0.0541	0.2049	0.23
3639	Putative deoxyribonuclease TATDN2	Q93075	0.0001	0.0098	1.38	0.0020	0.2302	0.32	0.0008	0.0224	0.37	0.0287	0.0779	0.15	0.0441	0.1552	0.17	0.0649	0.1777	0.44	0.8729	0.8947	0.02	0.0424	0.1902	-0.36	0.0536	0.2040	-0.97
3640	Putative dimethylaniline monooxygenase [N-oxide-forming] 6	O60774	0.0154	0.0718	-2.18	0.3297	0.6889	-1.30	0.1496	0.2788	-1.59	0.0025	0.0209	-2.40	0.9426	0.9559	-0.02	0.0125	0.0754	-2.61	0.3736	0.6074	-2.55	0.8708	1.2257	-0.33	0.0651	0.1973	2.06
3641	Putative E3 ubiquitin-protein ligase makorin-4	Q13434	0.3270	0.5330	-0.15	0.1165	0.6359	0.33	0.0751	0.1646	0.40	0.4338	0.6272	0.11	0.7027	0.7639	-0.05	0.2434	0.4303	0.22	0.0230	0.1363	0.55	0.2511	0.5740	0.12	0.0233	0.1294	0.87
3642	Putative E3 ubiquitin-protein ligase UBR7	Q8N806	0.8996	0.9211	-0.01	0.1176	0.6306	-0.15	0.0561	0.1354	0.07	0.7579	0.7910	-0.03	0.0474	0.1600	-0.13	0.9906	0.9927	0.00	0.2665	0.5088	-0.22	0.1496	0.4169	-0.15	0.0452	0.1860	0.24
3643	Putative elongation factor 1-alpha-like 3	Q5VTE0	0.0134	0.0675	0.25	0.2621	0.6639	0.20	0.0020	0.0250	0.40	0.0026	0.0216	0.32	0.7309	0.7893	0.01	0.0021	0.0531	0.48	0.0148	0.1122	-3.92	0.0419	0.1887	-1.83	0.0481	0.1914	-1.64
3644	Putative endoplasmin-like protein	Q58FF3	0.9656	0.9746	-0.01	0.0951	0.6291	0.32	0.1295	0.2504	0.25	0.2336	0.3777	0.18	0.0677	0.1982	0.37	0.7959	1.0333	0.05	0.0144	0.1113	0.42	0.0177	0.1129	0.31	0.0079	0.0795	0.68
3645	Putative fatty acid-binding protein 5-like protein 3	A8MUU1	0.0382	0.1225	0.16	0.9932	0.9955	0.00	0.0563	0.1356	-0.11	0.0466	0.1096	-0.20	0.0944	0.2430	-0.08	0.1576	0.3170	-0.14	0.3404	0.6112	-0.08	0.4770	0.8888	0.03	0.1189	0.1770	0.13
3646	Putative ferritin heavy polypeptide-like 19	P0C7X4	0.0921	0.2131	0.13	0.1334	0.6482	0.25	0.1433	0.2695	0.11	0.0645	0.1394	0.16	0.3525	0.4503	-0.06	0.2805	0.4764	0.07	0.3736	0.4851	-2.55	0.8708	1.0062	-0.33	0.0651	0.1342	2.06
3647	Putative GED domain-containing protein DNM1P46	Q6ZS02	0.4108	0.5249	-3.31	0.5752	0.7350	-1.75	0.5661	0.6663	-1.19	0.5388	0.6303	0.89	0.2938	0.4242	-1.88	0.9510	1.0184	0.09	0.0076	0.0891	-2.16	0.0002	0.0167	-0.55	0.0000	0.0000	-1.33
3648	Putative golgin subfamily A member 8I	A6NC78	0.4108	0.5248	-3.31	0.5752	0.7348	-1.75	0.5661	0.6662	-1.19	0.5388	0.6302	0.89	0.2938	0.4240	-1.88	0.9510	1.0182	0.09	0.0499	0.1783	0.22	0.0144	0.1005	-0.27	0.0066	0.0761	-0.53
3649	Putative heat shock 70 kDa protein 7	P48741	0.0095	0.0573	-0.67	0.0183	0.4647	-0.57	0.0002	0.0241	-0.83	0.0000	0.0000	-1.44	0.0028	0.0409	-1.10	0.0002	0.0384	-1.33	0.3736	0.6540	-2.55	0.8708	1.3063	-0.33	0.0651	0.2275	2.06
3650	Putative heat shock protein HSP 90-alpha A4	Q58FG1	0.0111	0.0604	0.22	0.2643	0.6640	0.09	0.0050	0.0345	0.28	0.0079	0.0360	0.24	0.2613	0.4822	0.06	0.0917	0.2213	0.16	0.0239	0.1371	-0.23	0.0380	0.1775	-0.33	0.0023	0.0532	-0.54
3651	Putative heat shock protein HSP 90-alpha A5	Q58FG0	0.2988	0.4975	0.12	0.8302	0.8780	0.04	0.0542	0.1323	0.27	0.4302	0.6225	0.08	0.4353	0.5293	-0.07	0.2830	0.4794	0.10	0.0286	0.1457	-1.15	0.2712	0.6045	-0.32	0.3039	0.3751	-0.40
3652	Putative heat shock protein HSP 90-beta 2	Q58FF8	0.0978	0.2215	0.12	0.3141	0.6799	0.22	0.0225	0.0735	0.28	0.0032	0.0230	0.37	0.0832	0.2233	0.13	0.0235	0.0982	0.28	0.7180	0.7583	0.03	0.9588	0.9710	-0.01	0.0606	0.2198	-0.20
3653	Putative heat shock protein HSP 90-beta 4	Q58FF6	0.8760	0.9023	0.00	0.7155	0.7914	0.01	0.9842	0.9871	0.00	0.0430	0.1033	-0.14	0.2306	0.4406	-0.04	0.3234	0.5261	0.05	0.0628	0.1972	0.18	0.0042	0.0520	0.44	0.1320	0.1923	0.28
3654	Putative heat shock protein HSP 90-beta-3	Q58FF7	0.0170	0.0762	0.30	0.2558	0.6622	0.15	0.0972	0.1998	0.15	0.0122	0.0444	0.23	0.0406	0.1483	0.17	0.0206	0.0931	0.21	0.0059	0.0819	0.27	0.1445	0.4057	0.09	0.5148	0.5814	0.04
3655	Putative hexokinase HKDC1	Q2TB90	0.0000	0.0000	0.52	0.1026	0.6327	0.33	0.0060	0.0375	0.51	0.0004	0.0116	0.29	0.0009	0.0234	0.24	0.0878	0.2158	0.35	0.3736	0.5392	-2.55	0.8708	1.1048	-0.33	0.0651	0.1597	2.06
3656	Putative high mobility group protein B1-like 1	B2RPK0	0.9622	0.9721	0.04	0.2690	0.6629	1.08	0.8722	0.8991	0.15	0.1763	0.3001	1.46	0.1733	0.3620	1.48	0.6557	0.8929	0.38	0.1951	0.4036	0.47	0.0873	0.2931	0.52	0.7056	0.7559	0.13
3657	Putative HLA class I histocompatibility antigen_ alpha chain H	P01893	0.0328	0.1129	0.31	0.0896	0.6238	0.33	0.1319	0.2538	0.23	0.0220	0.0645	0.46	0.0765	0.2128	0.23	0.1945	0.3691	0.13	0.6327	0.6783	-0.07	0.1503	0.4181	-0.15	0.0883	0.1411	0.45
3658	Putative homeodomain transcription factor 1	Q9UMS5	0.0650	0.1687	0.13	0.3835	0.7306	0.15	0.2711	0.4441	0.06	0.1434	0.2556	0.10	0.4128	0.5077	0.05	0.1823	0.3522	-0.07	0.0599	0.1937	0.31	0.8600	1.3018	0.02	0.0482	0.1915	0.32
3659	Putative hydrolase RBBP9	O75884	0.4108	0.5246	-3.31	0.5752	0.7346	-1.75	0.5661	0.6660	-1.19	0.5388	0.6300	0.89	0.2938	0.4239	-1.88	0.9510	1.0180	0.09	0.1233	0.2954	-0.22	0.0030	0.0452	0.53	0.0942	0.1485	0.18
3660	Putative inactive cathepsin L-like protein CTSL3P	Q5NE16	0.9940	0.9950	0.00	0.1725	0.6609	-1.27	0.5305	0.7518	-0.33	0.2109	0.3469	-0.41	0.7973	0.8420	-0.08	0.2933	0.4909	0.33	0.3736	0.4831	-2.55	0.8708	1.0024	-0.33	0.0651	0.1333	2.06
3661	Putative keratin-87 protein	A6NCN2	0.9661	0.9749	0.00	0.0916	0.6285	-0.47	0.0259	0.0802	-0.48	0.0300	0.0804	-0.60	0.0708	0.2028	-0.28	0.0574	0.1647	-0.31	0.3736	0.5567	-2.55	0.8708	1.1362	-0.33	0.0651	0.1687	2.06
3662	Putative lipocalin 1-like protein 1	Q5VSP4	0.0016	0.0271	1.54	0.0503	0.5739	1.41	0.0024	0.0273	1.47	0.0011	0.0161	1.79	0.0465	0.1587	0.19	0.0000	0.0000	1.38	0.3736	0.6556	-2.55	0.8708	1.3090	-0.33	0.0651	0.2286	2.06
3663	Putative methyltransferase-like protein 15P1	P0C7V9	0.8260	0.8606	-0.03	0.5575	0.8709	0.10	0.0831	0.1777	-0.37	0.0604	0.1323	0.40	0.0056	0.0554	1.12	0.4923	0.7175	0.14	0.3736	0.4609	-2.55	0.8708	0.9612	-0.33	0.0651	0.1239	2.06
3664	Putative monooxygenase p33MONOX	Q96A73	0.1363	0.2807	-0.14	0.4888	0.8123	-0.84	0.1407	0.2660	0.10	0.0312	0.0824	-0.19	0.0057	0.0557	-0.38	0.0236	0.0983	-0.22	0.3736	0.5888	-2.55	0.8708	1.1930	-0.33	0.0651	0.1863	2.06
3665	Putative nascent polypeptide-associated complex subunit alpha-like protein	Q9BZK3	0.2559	0.4436	1.71	0.3681	0.7168	1.26	0.4109	0.6171	1.12	0.4539	0.6504	1.00	0.3748	0.4721	1.23	0.4607	0.6832	0.99	0.0007	0.0412	7.01	0.8708	1.3116	-0.33	0.0031	0.0556	8.59
3666	Putative oncomodulin-2	P0CE71	0.2430	0.4284	-0.22	0.5250	0.8440	-0.37	0.5135	0.7344	0.16	0.3664	0.5474	-0.17	0.0347	0.1354	0.57	0.4800	0.7048	0.12	0.3736	0.4619	-2.55	0.8708	0.9630	-0.33	0.0651	0.1243	2.06
3667	Putative peptidyl-tRNA hydrolase PTRHD1	Q6GMV3	0.0010	0.0217	-0.34	0.7766	0.8390	-0.02	0.0367	0.1005	-0.23	0.0113	0.0427	-0.23	0.9255	0.9441	0.00	0.0206	0.0930	-0.24	0.3736	0.5933	-2.55	0.8708	1.2009	-0.33	0.0651	0.1889	2.06
3668	Putative PIP5K1A and PSMD4-like protein	A2A3N6	0.0092	0.0567	-0.41	0.0101	0.4472	-0.35	0.0363	0.0999	-0.29	0.0135	0.0470	-0.90	0.0129	0.0809	-0.30	0.0930	0.2240	-0.54	0.2892	0.5410	0.19	0.2473	0.5691	0.22	0.0345	0.1593	0.55
3669	Putative postmeiotic segregation increased 2-like protein 1	A4D2B8	0.2320	0.4138	-1.99	0.1759	0.6622	-3.09	0.0430	0.1127	0.14	0.4895	0.6917	-0.09	0.0269	0.1191	-0.30	0.3596	0.5657	-0.33	0.0143	0.1114	0.70	0.0284	0.1524	0.48	0.0048	0.0668	0.29
3670	Putative postmeiotic segregation increased 2-like protein 3	Q13401	0.0098	0.0578	-1.43	0.5253	0.8437	-0.09	0.0297	0.0874	-1.47	0.0488	0.1132	-0.48	0.0102	0.0722	0.35	0.0474	0.1449	-0.64	0.0003	0.0398	8.99	0.8708	1.3124	-0.33	0.0028	0.0535	9.70
3671	Putative PRAME family member 13	Q5VWM6	0.8243	0.8593	-0.03	0.5126	0.8350	0.08	0.0464	0.1192	-0.38	0.0787	0.1622	-0.31	0.5951	0.6714	-0.07	0.0753	0.1949	-0.48	0.3736	0.5464	-2.55	0.8708	1.1176	-0.33	0.0651	0.1633	2.06
3672	Putative protein FAM10A4	Q8IZP2	0.4108	0.5245	-3.31	0.5752	0.7344	-1.75	0.5661	0.6658	-1.19	0.5388	0.6299	0.89	0.2938	0.4238	-1.88	0.9510	1.0178	0.09	0.1308	0.3060	-0.09	0.1916	0.4849	-0.13	0.0001	0.0173	-0.40
3673	Putative protein FAM10A5	Q8NFI4	0.0901	0.2101	-0.13	0.6047	0.7076	-0.09	0.1324	0.2545	0.30	0.0023	0.0203	0.46	0.0725	0.2057	0.13	0.0423	0.1357	0.17	0.0289	0.1461	0.39	0.0283	0.1525	0.27	0.1291	0.1888	0.11
3674	Putative protein FAM45B	Q6NSW5	0.5338	0.6044	-0.09	0.7849	0.8451	-0.02	0.9126	0.9313	0.01	0.7108	0.7493	0.03	0.2881	0.5184	0.09	0.3056	0.5038	0.09	0.3736	0.4807	-2.55	0.8708	0.9980	-0.33	0.0651	0.1323	2.06
3675	Putative protein PLEKHA9	O95397	0.4108	0.5244	-3.31	0.5752	0.7342	-1.75	0.5661	0.6657	-1.19	0.5388	0.6297	0.89	0.2938	0.4237	-1.88	0.9510	1.0176	0.09	0.0075	0.0889	-1.00	0.0183	0.1157	-0.62	0.0878	0.1406	-0.23
3676	Putative protein SSX6	Q7RTT6	0.4108	0.5243	-3.31	0.5752	0.7341	-1.75	0.5661	0.6655	-1.19	0.5388	0.6296	0.89	0.2938	0.4236	-1.88	0.9510	1.0174	0.09	0.0106	0.1019	0.30	0.0469	0.2019	0.21	0.0883	0.1411	-0.20
3677	Putative protein SSX9	Q7RTT3	0.4108	0.5241	-3.31	0.5752	0.7339	-1.75	0.5661	0.6654	-1.19	0.5388	0.6295	0.89	0.2938	0.4235	-1.88	0.9510	1.0172	0.09	0.3693	0.6513	-0.19	0.9938	0.9959	0.00	0.5938	0.6547	0.11
3678	Putative protein ZNF815	A8K554	0.4108	0.5240	-3.31	0.5752	0.7337	-1.75	0.5661	0.6652	-1.19	0.5388	0.6293	0.89	0.2938	0.4233	-1.88	0.9510	1.0170	0.09	0.0382	0.1617	-0.76	0.0213	0.1265	-0.39	0.3789	0.4502	-0.08
3679	Putative pyridoxal-dependent decarboxylase domain-containing protein 2	Q6P474	0.0368	0.1207	0.21	0.7500	0.8188	-0.05	0.8109	0.8467	0.01	0.1798	0.3046	0.10	0.0892	0.2347	-0.16	0.9561	0.9639	0.01	0.3736	0.4307	-2.55	0.8708	0.9045	-0.33	0.0651	0.1119	2.06
3680	Putative Ras-related protein Rab-1C	Q92928	0.0169	0.0759	0.47	0.0545	0.5869	0.43	0.0617	0.1444	0.33	0.0071	0.0341	0.63	0.1394	0.3110	0.21	0.0157	0.0834	0.58	0.3736	0.6014	-2.55	0.8708	1.2152	-0.33	0.0651	0.1937	2.06
3681	Putative RNA polymerase II subunit B1 CTD phosphatase RPAP2	Q8IXW5	0.4108	0.5239	-3.31	0.5752	0.7335	-1.75	0.5661	0.6651	-1.19	0.5388	0.6292	0.89	0.2938	0.4232	-1.88	0.9510	1.0168	0.09	0.8812	0.9014	0.02	0.0043	0.0522	0.68	0.0416	0.1768	0.44
3682	Putative RNA-binding protein Luc7-like 1	Q9NQ29	0.1295	0.2705	0.14	0.4541	0.7872	0.10	0.0801	0.1728	0.18	0.0488	0.1132	0.24	0.0748	0.2102	0.18	0.0420	0.1352	0.26	0.2269	0.4524	0.13	0.3256	0.6798	-0.12	0.2814	0.3533	0.17
3683	Putative RNA-binding protein Luc7-like 2	Q9Y383	0.1609	0.3162	0.08	0.3063	0.6783	0.20	0.0248	0.0781	0.16	0.0471	0.1105	0.13	0.8981	0.9223	0.00	0.1657	0.3291	0.06	0.6002	0.6481	0.02	0.0073	0.0703	-0.22	0.7280	0.7758	0.00
3684	Putative small nuclear ribonucleoprotein G-like protein 15	A8MWD9	0.2281	0.4083	-0.40	0.2623	0.6634	-1.05	0.0423	0.1115	-1.00	0.0578	0.1279	-0.84	0.0482	0.1614	-0.85	0.0962	0.2290	-0.73	0.0692	0.2071	-1.08	0.9100	0.9331	0.03	0.8416	0.8722	0.03
3685	Putative testis-expressed protein 13C	A0A0J9YWL9	0.0345	0.1162	-0.24	0.3237	0.6858	-0.14	0.0182	0.0647	-0.13	0.0850	0.1720	-0.09	0.0288	0.1233	-0.27	0.1157	0.2587	-0.19	0.3736	0.5289	-2.55	0.8708	1.0861	-0.33	0.0651	0.1545	2.06
3686	Putative trace amine-associated receptor 3	Q9P1P4	0.1848	0.3497	-1.54	0.3619	0.7101	-2.04	0.2580	0.4266	-0.81	0.1384	0.2488	-2.11	0.3648	0.4616	0.30	0.1326	0.2835	-3.88	0.3736	0.5229	-2.55	0.8708	1.0753	-0.33	0.0651	0.1516	2.06
3687	Putative tRNA pseudouridine synthase Pus10	Q3MIT2	0.0105	0.0592	0.26	0.7598	0.8263	-0.03	0.0089	0.0439	-0.26	0.1660	0.2862	-0.07	0.0759	0.2119	-0.10	0.0900	0.2189	0.10	0.0694	0.2073	-0.42	0.1832	0.4721	-0.25	0.1066	0.1629	-0.36
3688	Putative tubulin-like protein alpha-4B	Q9H853	0.9799	0.9862	-0.01	0.7274	0.8012	-0.22	0.5937	0.6498	0.18	0.8905	0.9066	0.04	0.8031	0.8462	0.07	0.5983	0.8347	-0.16	0.3736	0.4527	-2.55	0.8708	0.9458	-0.33	0.0651	0.1206	2.06
3689	Putative ubiquitin-conjugating enzyme E2 N-like	Q5JXB2	0.0481	0.1411	0.13	0.1178	0.6310	0.34	0.0090	0.0443	0.27	0.0025	0.0212	0.34	0.0684	0.1988	0.12	0.0264	0.1042	0.29	0.0071	0.0869	0.53	0.0433	0.1925	0.23	0.0470	0.1898	0.23
3690	Putative ubiquitin-conjugating enzyme E2Q2-like protein	H0YL09	0.1290	0.2699	0.07	0.4641	0.7960	0.09	0.0771	0.1677	0.18	0.0089	0.0381	0.31	0.1190	0.2830	0.08	0.0098	0.0706	0.16	0.3736	0.6148	-2.55	0.8708	1.2385	-0.33	0.0651	0.2018	2.06
3691	Putative uncharacterized protein C22orf34	Q6ZV56	0.4108	0.5237	-3.31	0.5752	0.7333	-1.75	0.5661	0.6649	-1.19	0.5388	0.6290	0.89	0.2938	0.4231	-1.88	0.9510	1.0165	0.09	0.1317	0.3076	0.23	0.0363	0.1735	0.38	0.0484	0.1920	0.41
3692	Putative uncharacterized protein C9orf129	Q5T035	0.0048	0.0433	-0.30	0.9476	0.9653	0.00	0.0034	0.0307	-0.31	0.0136	0.0473	-0.24	0.0175	0.0950	-0.24	0.0137	0.0779	-0.23	0.0491	0.1765	-0.21	0.7574	1.1998	-0.02	0.1419	0.2033	-0.10
3693	Putative uncharacterized protein encoded by LINC00114	Q6XXX2	0.2257	0.4044	-0.30	0.4221	0.7592	-0.57	0.5621	0.7865	-0.15	0.3752	0.5583	-0.24	0.5802	0.6587	-0.13	0.1571	0.3165	-0.41	0.3736	0.5131	-2.55	0.8708	1.0574	-0.33	0.0651	0.1469	2.06
3694	Putative uncharacterized protein encoded by LINC00471	Q8N535	0.3917	0.6088	-0.06	0.3238	0.6852	-0.08	0.0659	0.1503	0.19	0.1662	0.2864	-0.11	0.7272	0.7866	-0.02	0.9507	1.1875	0.00	0.3736	0.4316	-2.55	0.8708	0.9061	-0.33	0.0651	0.1122	2.06
3695	Putative uncharacterized protein encoded by MIR7-3HG	Q8N6C7	0.0560	0.1547	-12.47	0.0453	0.5627	-10.91	0.0144	0.0566	-10.36	0.0005	0.0121	-8.27	0.6075	0.6822	0.33	0.0044	0.0598	-9.08	0.3736	0.6320	-2.55	0.8708	1.2685	-0.33	0.0651	0.2128	2.06
3696	Putative uncharacterized protein FLJ45721	Q6ZS92	0.2742	0.4674	0.18	0.3261	0.6856	-0.34	0.3951	0.5976	0.11	0.0877	0.1759	0.23	0.9711	0.9777	0.01	0.6333	0.8699	-0.11	0.3736	0.4491	-2.55	0.8708	0.9392	-0.33	0.0651	0.1191	2.06
3697	Putative uncharacterized protein FLJ45831	Q6ZS49	0.4108	0.5236	-3.31	0.5752	0.7332	-1.75	0.5661	0.6648	-1.19	0.5388	0.6289	0.89	0.2938	0.4230	-1.88	0.9510	1.0163	0.09	0.7939	0.8263	-0.09	0.0617	0.2374	0.42	0.1007	0.1565	0.67
3698	Putative uncharacterized protein KTN1-AS1	Q86SY8	0.4108	0.5235	-3.31	0.5752	0.7330	-1.75	0.5661	0.6646	-1.19	0.5388	0.6287	0.89	0.2938	0.4229	-1.88	0.9510	1.0161	0.09	0.0028	0.0582	-1.07	0.0648	0.2450	-0.35	0.1032	0.1593	-0.50
3699	Putative uncharacterized protein LOC100128429	Q6ZWC4	0.1397	0.2848	-2.72	0.1924	0.6680	-2.14	0.4073	0.6126	-1.14	0.4471	0.6419	-0.06	0.0321	0.1307	-0.22	0.2151	0.3962	-0.33	0.3736	0.4993	-2.55	0.8708	1.0322	-0.33	0.0651	0.1406	2.06
3700	Putative uncharacterized protein LOC100128554	Q0VFX4	0.4108	0.5234	-3.31	0.5752	0.7328	-1.75	0.5661	0.6645	-1.19	0.5388	0.6286	0.89	0.2938	0.4227	-1.88	0.9510	1.0159	0.09	0.0674	0.2037	0.50	0.0744	0.2643	-0.73	0.0163	0.1094	0.94
3701	Putative uncharacterized protein MGC13053	Q9BRP9	0.4108	0.5232	-3.31	0.5752	0.7326	-1.75	0.0409	0.1082	5.46	0.5388	0.7467	0.89	0.0293	0.1245	4.97	0.9510	1.1876	0.09	0.3736	0.4315	-2.55	0.8708	0.9060	-0.33	0.0651	0.1121	2.06
3702	Putative uncharacterized protein MRGPRG-AS1	Q2M3A8	0.0213	0.0868	0.29	0.1571	0.6621	0.18	0.1277	0.2477	0.10	0.2368	0.3820	-0.12	0.1081	0.2665	0.14	0.6029	0.8397	-0.19	0.3736	0.4524	-2.55	0.8708	0.9453	-0.33	0.0651	0.1204	2.06
3703	Putative uncharacterized protein MYH16	Q9H6N6	0.4108	0.5231	-3.31	0.5752	0.7324	-1.75	0.5661	0.6643	-1.19	0.5388	0.6285	0.89	0.2938	0.4226	-1.88	0.9510	1.0157	0.09	0.2474	0.4829	-0.12	0.5400	0.9656	-0.04	0.0336	0.1567	0.25
3704	Putative uncharacterized SMG1-like protein	Q6P435	0.5562	0.6252	-0.07	0.6418	0.7360	0.03	0.0677	0.1533	0.19	0.1273	0.2328	-0.16	0.0139	0.0838	0.14	0.0311	0.1134	0.26	0.3736	0.5801	-2.55	0.8708	1.1777	-0.33	0.0651	0.1814	2.06

Supplemetary Table S2. Overview on all relatively quantified 5180 proteins statistical analysis

			MCF-7																		MDA-MB-231								
			Dai SC20 vs control			Gen SC20 vs control			SSE SC20 vs control			Dai IC20 vs control			Gen IC20 vs control			SSE IC20 vs control			Dai IC20 vs control			Gen IC20 vs control			SSE IC20 vs control		
	**Protein name**	**UniProt**	**p value**	**BH q value**	**log2FC**	**p value**	**BH q value**	**log2FC**	**p value**	**BH q value**	**log2FC**	**p value**	**BH q value**	**log2FC**	**p value**	**BH q value**	**log2FC**	**p value**	**BH q value**	**log2FC**	**p value**	**BH q value**	**log2FC**	**p value**	**BH q value**	**log2FC**	**p value**	**BH q value**	**log2FC**
3705	Putative UPF0607 protein ENSP00000383783	A8MUI8	0.0101	0.0585	-0.41	0.0592	0.5909	-0.27	0.1382	0.2623	-0.20	0.0107	0.0414	-0.46	0.0100	0.0719	-0.55	0.0131	0.0769	-0.57	0.1579	0.3498	0.29	0.0070	0.0685	-0.32	0.0447	0.1846	-0.15
3706	Putative UPF0633 protein LOC554249	Q5XG85	0.7605	0.8051	-0.03	0.4084	0.7496	0.21	0.1234	0.2417	0.15	0.0811	0.1658	0.35	0.0028	0.0402	0.52	0.5373	0.7693	0.14	0.3736	0.4580	-2.55	0.8708	0.9559	-0.33	0.0651	0.1228	2.06
3707	Putative zinc finger and SCAN domain-containing protein 5C	A6NGD5	0.6845	0.7404	-0.01	0.2970	0.6718	0.03	0.0158	0.0593	-0.11	0.0074	0.0350	-0.18	0.0060	0.0566	-0.19	0.0427	0.1365	-0.09	0.3736	0.5680	-2.55	0.8708	1.1563	-0.33	0.0651	0.1747	2.06
3708	PWWP domain-containing DNA repair factor 3B	Q5H9M0	0.0162	0.0742	0.28	0.0332	0.5275	0.19	0.3034	0.4861	-0.05	0.0182	0.0569	0.31	0.0700	0.2016	0.14	0.0632	0.1752	0.27	0.3736	0.5537	-2.55	0.8708	1.1308	-0.33	0.0651	0.1671	2.06
3709	PX domain-containing protein kinase-like protein	Q7Z7A4	0.5694	0.6372	0.10	0.4773	0.8038	0.12	0.3737	0.5720	0.09	0.3212	0.4924	0.12	0.6323	0.7023	0.06	0.5009	0.7260	-0.11	0.5573	0.6078	0.29	0.7788	1.2225	0.11	0.7774	0.8167	0.11
3710	Pygopus homolog 2	Q9BRQ0	0.0044	0.0417	-0.47	0.0278	0.5071	-0.29	0.0175	0.0631	-0.42	0.0029	0.0221	-0.52	0.0042	0.0477	-0.46	0.0099	0.0707	-0.45	0.3736	0.6144	-2.55	0.8708	1.2379	-0.33	0.0651	0.2016	2.06
3711	Pyridine nucleotide-disulfide oxidoreductase domain-containing protein 1	Q8WU10	0.4108	0.5230	-3.31	0.5752	0.7323	-1.75	0.5661	0.6642	-1.19	0.5388	0.6283	0.89	0.2938	0.4225	-1.88	0.9510	1.0155	0.09	0.2546	0.4925	0.27	0.2655	0.5948	0.27	0.2966	0.3682	-0.25
3712	Pyridoxal kinase	O00764	0.1593	0.3142	-0.07	0.2548	0.6629	0.08	0.5035	0.7255	0.03	0.7022	0.7413	0.02	0.1836	0.3773	0.07	0.0872	0.2156	0.11	0.0010	0.0425	-0.27	0.4998	0.9158	-0.06	0.2448	0.3151	-0.02
3713	Pyridoxal phosphate homeostasis protein	O94903	0.0140	0.0691	-0.51	0.7374	0.8093	0.05	0.3127	0.4984	-0.13	0.0963	0.1885	-0.17	0.6572	0.7231	0.06	0.8180	1.0543	0.02	0.3736	0.4373	-2.55	0.8708	0.9170	-0.33	0.0651	0.1144	2.06
3714	Pyridoxal-dependent decarboxylase domain-containing protein 1	Q6P996	0.0236	0.0923	0.12	0.1722	0.6612	0.07	0.0254	0.0792	0.12	0.0347	0.0889	0.11	0.0187	0.0977	0.13	0.0103	0.0701	0.17	0.3736	0.6118	-2.55	0.8708	1.2335	-0.33	0.0651	0.2000	2.06
3715	Pyridoxine-5'-phosphate oxidase	Q9NVS9	0.2444	0.4300	-0.02	0.0622	0.5901	-0.10	0.0858	0.1825	-0.12	0.0552	0.1240	-0.17	0.0001	0.0084	-0.16	0.6478	0.8849	-0.04	0.3736	0.4476	-2.55	0.8708	0.9362	-0.33	0.0651	0.1185	2.06
3716	Pyrin domain-containing protein 5	W6CW81	0.2981	0.4968	0.07	0.2415	0.6598	0.08	0.0898	0.1879	0.13	0.6019	0.6493	0.04	0.3101	0.4078	-0.06	0.2471	0.4357	0.08	0.0048	0.0729	-0.99	0.0394	0.1806	0.26	0.5461	0.6110	-0.05
3717	Pyroglutamyl-peptidase 1	Q9NXJ5	0.4061	0.6264	-0.08	0.1343	0.6489	-0.57	0.6536	0.7049	-0.04	0.0324	0.0848	-0.26	0.9301	0.9471	-0.01	0.9400	1.1759	-0.01	0.3736	0.4319	-2.55	0.8708	0.9067	-0.33	0.0651	0.1123	2.06
3718	Pyrroline-5-carboxylate reductase 1 mitochondrial	P32322	0.1612	0.3167	0.08	0.7521	0.8204	0.06	0.0889	0.1872	0.14	0.0104	0.0408	0.24	0.0433	0.1539	-0.15	0.0275	0.1065	0.23	0.0234	0.1362	0.31	0.1681	0.4475	0.12	0.0839	0.1356	0.17
3719	Pyrroline-5-carboxylate reductase 2	Q96C36	0.1186	0.2535	0.22	0.5924	0.6969	-0.06	0.7274	0.7713	0.04	0.8474	0.8689	0.02	0.4003	0.4968	0.10	0.1303	0.2810	0.21	0.0038	0.0667	0.75	0.0205	0.1241	0.51	0.0068	0.0767	0.60
3720	Pyrroline-5-carboxylate reductase 3	Q53H96	0.6445	0.7061	-0.01	0.7486	0.8177	0.02	0.0308	0.0896	0.10	0.0122	0.0444	0.15	0.0764	0.2127	0.06	0.0233	0.0979	0.13	0.3736	0.5891	-2.55	0.8708	1.1936	-0.33	0.0651	0.1865	2.06
3721	Pyruvate carboxylase mitochondrial	P11498	0.0003	0.0145	0.15	0.4536	0.7872	0.09	0.0258	0.0799	0.18	0.0135	0.0470	0.16	0.1400	0.3119	-0.02	0.0191	0.0887	0.24	0.3736	0.5945	-2.55	0.8708	1.2032	-0.33	0.0651	0.1897	2.06
3722	Pyruvate dehydrogenase E1 component subunit alpha_ testis-specific form_ mitochondrial	P29803	0.4108	0.5228	-3.31	0.5752	0.7321	-1.75	0.5661	0.6640	-1.19	0.5388	0.6282	0.89	0.2938	0.4224	-1.88	0.9510	1.0153	0.09	0.0791	0.2230	0.34	0.3386	0.6966	-0.13	0.8933	0.9154	0.01
3723	Pyruvate dehydrogenase E1 component subunit alpha_ somatic form mitochondrial	P08559	0.4823	0.5569	0.03	0.8689	0.9049	0.01	0.0197	0.0676	0.09	0.0329	0.0857	0.09	0.0799	0.2179	-0.05	0.0414	0.1339	0.13	0.1006	0.2567	0.25	0.0541	0.2217	0.18	0.5452	0.6102	0.04
3724	Pyruvate dehydrogenase E1 component subunit beta_ mitochondrial	P11177	0.0358	0.1183	0.15	0.0692	0.6004	0.08	0.0038	0.0320	0.19	0.0045	0.0273	0.14	0.0549	0.1749	0.08	0.0098	0.0707	0.26	0.3540	0.6301	-0.11	0.0530	0.2186	-0.10	0.3705	0.4421	-0.01
3725	Pyruvate dehydrogenase phosphatase regulatory subunit_ mitochondrial	Q8NCN5	0.4108	0.5227	-3.31	0.5752	0.7319	-1.75	0.5661	0.6639	-1.19	0.5388	0.6280	0.89	0.2938	0.4223	-1.88	0.9510	1.0151	0.09	0.3445	0.6173	-0.08	0.9654	0.9752	0.00	0.9052	0.9247	-0.01
3726	Pyruvate dehydrogenase protein X component_ mitochondrial	O00330	0.4108	0.5226	-3.31	0.5752	0.7317	-1.75	0.5661	0.6637	-1.19	0.5388	0.6279	0.89	0.2938	0.4222	-1.88	0.9510	1.0149	0.09	0.0089	0.0933	-0.39	0.0062	0.0636	-0.30	0.0002	0.0241	-0.54
3727	Pyruvate kinase PKLR	P30613	0.0083	0.0539	-0.22	0.0770	0.6127	-0.18	0.0011	0.0224	-0.23	0.0008	0.0149	-0.39	0.0100	0.0722	-0.14	0.0033	0.0579	-0.19	0.0088	0.0938	-0.34	0.0173	0.1115	-0.25	0.0007	0.0330	-0.36
3728	Pyruvate kinase PKM	P14618	0.0006	0.0192	0.35	0.0631	0.5900	0.36	0.0002	0.0216	0.43	0.0003	0.0121	0.41	0.0084	0.0662	0.18	0.0024	0.0555	0.43	0.0294	0.1469	0.40	0.1168	0.3520	0.15	0.3773	0.4486	0.07
3729	Queuine tRNA-ribosyltransferase accessory subunit 2	Q9H974	0.0048	0.0432	0.23	0.0205	0.4741	0.24	0.0005	0.0218	0.45	0.0017	0.0186	0.43	0.0013	0.0281	0.31	0.0050	0.0620	0.33	0.3736	0.6302	-2.55	0.8708	1.2653	-0.33	0.0651	0.2116	2.06
3730	Queuosine salvage protein	Q5T6V5	0.7438	0.7916	-0.06	0.2986	0.6725	-0.44	0.7325	0.7756	-0.07	0.0776	0.1603	-0.48	0.8829	0.9100	-0.03	0.1132	0.2552	0.47	0.3736	0.5304	-2.55	0.8708	1.0888	-0.33	0.0651	0.1553	2.06
3731	Quinone oxidoreductase	Q08257	0.0196	0.0820	0.24	0.5394	0.8550	0.15	0.0217	0.0715	0.42	0.0847	0.1715	-0.14	0.8165	0.8574	0.01	0.0035	0.0587	0.50	0.0585	0.1926	-0.23	0.0087	0.0770	-0.47	0.1570	0.2207	-0.14
3732	Rab effector MyRIP	Q8NFW9	0.0172	0.0767	-0.76	0.5317	0.8506	-0.34	0.0935	0.1939	0.38	0.1150	0.2159	-0.34	0.2642	0.4855	0.26	0.2348	0.4195	-0.28	0.3736	0.4939	-2.55	0.8708	1.0224	-0.33	0.0651	0.1381	2.06
3733	Rab effector Noc2	Q9UNE2	0.4108	0.5225	-3.31	0.5752	0.7315	-1.75	0.5661	0.6636	-1.19	0.5388	0.6278	0.89	0.2938	0.4220	-1.88	0.9510	1.0147	0.09	0.0031	0.0606	0.37	0.0003	0.0194	0.51	0.0002	0.0305	0.63
3734	Rab GDP dissociation inhibitor alpha	P31150	0.0020	0.0308	0.24	0.1678	0.6610	0.23	0.0086	0.0439	0.26	0.0008	0.0145	0.20	0.0028	0.0406	0.11	0.0005	0.0398	0.25	0.4875	0.5416	0.06	0.0654	0.2464	-0.19	0.0301	0.1478	-0.31
3735	Rab GDP dissociation inhibitor beta	P50395	0.0015	0.0260	0.18	0.3069	0.6782	0.16	0.0038	0.0316	0.20	0.0042	0.0264	0.16	0.5161	0.6047	-0.01	0.0014	0.0500	0.23	0.6589	0.7032	0.03	0.0145	0.1011	-0.25	0.0213	0.1247	-0.30
3736	Rab GTPase-activating protein 1	Q9Y3P9	0.3053	0.5061	0.10	0.4850	0.8094	-0.09	0.5490	0.7721	0.05	0.4019	0.5918	-0.08	0.5049	0.5943	-0.06	0.1102	0.2503	0.18	0.1330	0.3101	0.10	0.6053	1.0379	0.03	0.0213	0.1245	-0.25
3737	Rab GTPase-activating protein 1-like isoform 10	B7ZAP0	0.0168	0.0758	0.27	0.0644	0.5904	0.21	0.0343	0.0962	0.13	0.6535	0.6971	0.01	0.0011	0.0257	0.20	0.4915	0.7170	-0.05	0.3736	0.4610	-2.55	0.8708	0.9614	-0.33	0.0651	0.1240	2.06
3738	Rab GTPase-binding effector protein 1	Q15276	0.0318	0.1107	0.64	0.3954	0.7391	0.13	0.2221	0.3793	-0.20	0.1260	0.2310	0.30	0.0572	0.1789	0.47	0.9644	0.9710	-0.01	0.1130	0.2771	0.17	0.0571	0.2288	0.21	0.1731	0.2390	0.15
3739	Rab11 family-interacting protein 2	Q7L804	0.0013	0.0244	0.31	0.0278	0.5088	0.15	0.0021	0.0260	0.27	0.0034	0.0236	0.32	0.0299	0.1259	0.13	0.0316	0.1145	0.28	0.9229	0.9366	0.00	0.6612	1.1009	-0.02	0.3221	0.3932	0.06
3740	Rab3 GTPase-activating protein catalytic subunit	Q15042	0.0069	0.0494	0.07	0.1994	0.6642	0.19	0.0002	0.0176	0.23	0.0049	0.0283	0.27	0.0088	0.0673	0.23	0.0166	0.0853	0.19	0.0444	0.1696	0.26	0.0883	0.2949	0.16	0.3389	0.4099	0.07
3741	Rab3 GTPase-activating protein non-catalytic subunit	Q9H2M9	0.0257	0.0970	0.28	0.2251	0.6603	-0.27	0.3971	0.6001	0.15	0.0068	0.0332	0.39	0.0343	0.1355	0.21	0.0015	0.0501	0.73	0.3736	0.6462	-2.55	0.8708	1.2928	-0.33	0.0651	0.2221	2.06
3742	Rab9 effector protein with kelch motifs	Q7Z6M1	0.0932	0.2149	0.43	0.6810	0.7669	0.07	0.5802	0.6376	0.10	0.1327	0.2401	0.34	0.1067	0.2639	0.38	0.5791	0.8136	0.10	0.3736	0.4538	-2.55	0.8708	0.9478	-0.33	0.0651	0.1210	2.06
3743	Rabankyrin-5	Q9P2R3	0.4108	0.5223	-3.31	0.5752	0.7314	-1.75	0.5661	0.6634	-1.19	0.5388	0.6276	0.89	0.2938	0.4219	-1.88	0.9510	1.0145	0.09	0.1578	0.3498	0.06	0.2250	0.5351	0.05	0.1605	0.2248	0.09
3744	Rab-interacting lysosomal protein	Q96NA2	0.9836	0.9884	0.00	0.1080	0.6307	0.35	0.1368	0.2604	0.28	0.0663	0.1423	0.40	0.0628	0.1899	0.42	0.3744	0.5829	0.14	0.3736	0.4713	-2.55	0.8708	0.9806	-0.33	0.0651	0.1283	2.06
3745	Rab-like protein 3	Q5HYI8	0.9222	0.9405	-0.01	0.0631	0.5879	0.17	0.0054	0.0360	0.35	0.0508	0.1162	0.16	0.1159	0.2790	0.14	0.5400	0.7716	0.04	0.3736	0.4576	-2.55	0.8708	0.9551	-0.33	0.0651	0.1226	2.06
3746	Rab-like protein 6	Q3YEC7	0.0012	0.0239	-0.31	0.3361	0.6953	0.05	0.1264	0.2460	-0.07	0.4857	0.6870	-0.02	0.3267	0.4239	0.03	0.0059	0.0617	-0.17	0.3736	0.6259	-2.55	0.8708	1.2579	-0.33	0.0651	0.2088	2.06
3747	RAC-alpha serine/threonine-protein kinase	P31749	0.0006	0.0191	-0.32	0.1892	0.6699	0.13	0.2660	0.4371	-0.07	0.0089	0.0381	-0.13	0.1261	0.2933	0.05	0.0093	0.0690	-0.15	0.3736	0.6153	-2.55	0.8708	1.2396	-0.33	0.0651	0.2022	2.06
3748	RAC-beta serine/threonine-protein kinase	P31751	0.7604	0.8052	0.14	0.4499	0.7841	-0.58	0.1504	0.2799	0.66	0.1162	0.2174	0.76	0.3102	0.4077	0.47	0.0535	0.1567	1.27	0.7537	0.7900	0.17	0.4576	0.8629	0.42	0.0864	0.1389	1.10
3749	RAC-gamma serine/threonine-protein kinase	Q9Y243	0.0055	0.0447	-0.66	0.0308	0.5081	-0.74	0.0230	0.0746	-0.39	0.0951	0.1867	-0.68	0.0176	0.0952	-0.41	0.0840	0.2100	-0.73	0.7574	0.7931	0.05	0.3997	0.7849	-0.03	0.0083	0.0817	0.57
3750	RAD51-associated protein 2	Q09MP3	0.0481	0.1410	0.08	0.7355	0.8077	0.06	0.0909	0.1899	0.08	0.0013	0.0162	0.16	0.0337	0.1341	-0.10	0.2036	0.3816	0.11	0.3736	0.5017	-2.55	0.8708	1.0367	-0.33	0.0651	0.1417	2.06
3751	Radial spoke head 1 homolog	Q8WYR4	0.8335	0.8670	0.02	0.5136	0.8361	-0.04	0.2138	0.3684	-0.09	0.6002	0.6477	-0.03	0.6894	0.7524	-0.02	0.3612	0.5675	0.06	0.3736	0.4729	-2.55	0.8708	0.9836	-0.33	0.0651	0.1290	2.06
3752	Radial spoke head protein 3 homolog	Q86UC2	0.3301	0.5364	-0.12	0.8610	0.8999	-0.02	0.5818	0.6385	0.06	0.9594	0.9648	0.01	0.7221	0.7820	0.05	0.6421	0.8785	-0.05	0.3736	0.4482	-2.55	0.8708	0.9374	-0.33	0.0651	0.1187	2.06
3753	Radixin	P35241	0.0010	0.0216	-0.21	0.6106	0.7124	0.08	0.4278	0.6373	-0.02	0.0037	0.0246	-0.18	0.0108	0.0742	-0.11	0.0071	0.0648	-0.14	0.0116	0.1043	0.36	0.0571	0.2289	0.11	0.1060	0.1623	0.09
3754	RAF proto-oncogene serine/threonine-protein kinase	P04049	0.1126	0.2442	-0.10	0.2398	0.6614	0.20	0.6479	0.7004	0.03	0.0437	0.1046	0.17	0.3900	0.4870	-0.04	0.4219	0.6411	-0.06	0.3736	0.4671	-2.55	0.8708	0.9728	-0.33	0.0651	0.1265	2.06
3755	Raftlin	Q14699	0.4108	0.5222	-3.31	0.5752	0.7312	-1.75	0.5661	0.6633	-1.19	0.5388	0.6275	0.89	0.2938	0.4218	-1.88	0.9510	1.0142	0.09	0.4319	0.4877	0.10	0.0498	0.2096	0.33	0.0571	0.2125	0.36
3756	Ragulator complex protein LAMTOR1	Q6IAA8	0.3086	0.5094	-0.04	0.6370	0.7328	0.02	0.0660	0.1505	0.11	0.1970	0.3280	0.06	0.5364	0.6217	-0.03	0.3031	0.5010	0.04	0.3154	0.5773	-0.12	0.0063	0.0642	-0.24	0.0904	0.1439	0.13
3757	Ragulator complex protein LAMTOR5	O43504	0.0152	0.0714	-0.43	0.0055	0.3850	-0.56	0.0022	0.0268	-0.43	0.0106	0.0413	-0.62	0.0035	0.0439	-0.57	0.2984	0.4956	-0.15	0.3736	0.4819	-2.55	0.8708	1.0002	-0.33	0.0651	0.1328	2.06
3758	Ral GTPase-activating protein subunit alpha-2	Q2PPJ7	0.0065	0.0482	-0.33	0.4866	0.8102	-0.16	0.2293	0.3887	0.14	0.1280	0.2337	-0.15	0.5673	0.6481	0.02	0.7417	0.9809	-0.02	0.0487	0.1765	0.35	0.7010	1.1433	0.03	0.1678	0.2330	0.10
3759	Ran GTPase-activating protein 1	P46060	0.0175	0.0773	0.09	0.3473	0.7038	0.14	0.0174	0.0629	0.16	0.0140	0.0482	0.10	0.1640	0.3487	-0.04	0.0254	0.1011	0.11	0.0234	0.1363	-1.02	0.0021	0.0379	-0.81	0.0240	0.1323	-0.77
3760	Ran-binding protein 3	Q9H6Z4	0.2367	0.4203	-0.15	0.5374	0.8531	-0.08	0.1161	0.2300	-0.24	0.8959	0.9108	-0.02	0.3139	0.4113	-0.12	0.0251	0.1006	-0.46	0.0259	0.1402	0.66	0.1841	0.4730	0.30	0.6907	0.7429	0.07
3761	Ran-specific GTPase-activating protein	P43487	0.0309	0.1085	0.18	0.1163	0.6368	0.29	0.0004	0.0205	0.38	0.0017	0.0186	0.26	0.7761	0.8245	-0.01	0.0024	0.0545	0.34	0.0647	0.2004	0.31	0.3512	0.7148	0.07	0.9546	0.9647	0.00
3762	Rap guanine nucleotide exchange factor 2	Q9Y4G8	0.0486	0.1414	0.15	0.2725	0.6655	0.17	0.0446	0.1156	0.17	0.5053	0.7091	0.04	0.0104	0.0728	0.32	0.0175	0.0865	0.32	0.3736	0.5975	-2.55	0.8708	1.2083	-0.33	0.0651	0.1914	2.06
3763	Rap guanine nucleotide exchange factor 5	Q92565	0.1442	0.2912	-0.10	0.1268	0.6427	-0.19	0.0112	0.0492	-0.35	0.0178	0.0563	-0.41	0.0016	0.0318	-0.60	0.0140	0.0788	-0.29	0.3736	0.6051	-2.55	0.8708	1.2218	-0.33	0.0651	0.1959	2.06
3764	Rap guanine nucleotide exchange factor 6	Q8TEU7	0.4108	0.5221	-3.31	0.5752	0.7310	-1.75	0.5661	0.6631	-1.19	0.5388	0.6273	0.89	0.2938	0.4217	-1.88	0.9510	1.0140	0.09	0.9193	0.9339	-0.01	0.5986	1.0319	-0.03	0.2284	0.2988	0.16
3765	Rap1 GTPase-GDP dissociation stimulator 1	P52306	0.0402	0.1263	0.16	0.5862	0.6920	0.03	0.0061	0.0377	0.16	0.1020	0.1969	0.09	0.0801	0.2180	0.08	0.3650	0.5719	0.08	0.4744	0.5292	-0.08	0.7407	1.1831	-0.02	0.5153	0.5818	-0.04
3766	Ras and EF-hand domain-containing protein	Q8IZ41	0.4108	0.5219	-3.31	0.5752	0.7308	-1.75	0.5661	0.6630	-1.19	0.5388	0.6272	0.89	0.2938	0.4216	-1.88	0.9510	1.0138	0.09	0.0045	0.0717	-0.54	0.4051	0.7904	0.06	0.4491	0.5182	-0.11
3767	Ras and Rab interactor-like protein	Q6ZS11	0.4108	0.5218	-3.31	0.5752	0.7306	-1.75	0.5661	0.6628	-1.19	0.5388	0.6270	0.89	0.2938	0.4215	-1.88	0.9510	1.0136	0.09	0.4946	0.5483	-0.09	0.6413	1.0796	-0.05	0.0697	0.1175	0.13
3768	Ras association domain-containing protein 2	P50749	0.1682	0.3268	-0.15	0.4687	0.7986	0.06	0.0390	0.1046	-0.34	0.0694	0.1470	-0.39	0.0442	0.1550	-0.19	0.1438	0.2992	-0.11	0.3736	0.5188	-2.55	0.8708	1.0679	-0.33	0.0651	0.1497	2.06
3769	Ras association domain-containing protein 8	Q8NHQ8	0.2667	0.4584	0.11	0.1169	0.6334	0.30	0.0069	0.0398	0.67	0.0247	0.0701	0.10	0.0368	0.1397	0.12	0.0685	0.1835	0.21	0.0052	0.0763	0.78	0.0612	0.2364	0.29	0.0419	0.1778	0.36
3770	Ras GTPase-activating protein 1	P20936	0.4108	0.5217	-3.31	0.5752	0.7305	-1.75	0.5661	0.6627	-1.19	0.5388	0.6269	0.89	0.2938	0.4213	-1.88	0.9510	1.0134	0.09	0.1457	0.3296	-0.57	0.0285	0.1524	0.29	0.0445	0.1844	0.22
3771	Ras GTPase-activating protein 3	Q14644	0.0010	0.0215	0.15	0.2971	0.6717	0.19	0.0049	0.0342	0.12	0.8270	0.8505	-0.02	0.1224	0.2881	0.04	0.8694	1.1054	-0.01	0.3566	0.6339	-0.10	0.4440	0.8459	0.07	0.1022	0.1581	0.17
3772	Ras GTPase-activating protein-binding protein 1	Q13283	0.7765	0.8180	0.02	0.6769	0.7632	-0.05	0.1412	0.2667	0.13	0.1418	0.2536	0.11	0.3879	0.4850	-0.05	0.0685	0.1834	0.15	0.2564	0.4952	0.11	0.2549	0.5794	0.09	0.5072	0.5739	0.04
3773	Ras GTPase-activating protein-binding protein 2	Q9UN86	0.0297	0.1055	-0.41	0.2733	0.6650	-0.18	0.0093	0.0450	-0.17	0.0181	0.0568	-0.27	0.6726	0.7364	0.02	0.1165	0.2598	-0.21	0.6228	0.6689	0.03	0.0288	0.1529	-0.21	0.0073	0.0780	-0.45
3774	Ras GTPase-activating-like protein IQGAP1	P46940	0.0558	0.1544	0.09	0.2158	0.6611	0.21	0.0224	0.0733	0.17	0.0029	0.0220	0.23	0.8721	0.9030	0.00	0.0050	0.0612	0.23	0.0425	0.1673	0.31	0.1349	0.3876	0.16	0.5058	0.5726	0.05
3775	Ras GTPase-activating-like protein IQGAP2	Q13576	0.0014	0.0251	0.33	0.5404	0.8555	0.08	0.0040	0.0319	0.25	0.4143	0.6066	-0.05	0.9252	0.9440	0.00	0.0112	0.0725	0.21	0.2181	0.4393	0.15	0.2991	0.6421	-0.07	0.4056	0.4771	-0.05
3776	Ras GTPase-activating-like protein IQGAP3	Q86VI3	0.4108	0.5216	-3.31	0.5752	0.7303	-1.75	0.5661	0.6625	-1.19	0.5388	0.6268	0.89	0.2938	0.4212	-1.88	0.9510	1.0132	0.09	0.0692	0.2070	0.35	0.0370	0.1754	0.27	0.5576	0.6220	0.04
3777	Ras suppressor protein 1	Q15404	0.4108	0.5214	-3.31	0.5752	0.7301	-1.75	0.5661	0.6624	-1.19	0.5388	0.6266	0.89	0.2938	0.4211	-1.88	0.9510	1.0130	0.09	0.2426	0.4755	0.13	0.6263	1.0626	0.04	0.3337	0.4048	-0.07
3778	Ras/Rap GTPase-activating protein SynGAP	Q96PV0	0.0041	0.0401	0.15	0.0036	0.3161	0.17	0.0017	0.0240	0.20	0.9910	0.9921	0.00	0.0762	0.2124	-0.08	0.6468	0.8840	0.01	0.3736	0.4478	-2.55	0.8708	0.9366	-0.33	0.0651	0.1186	2.06

Supplemetary Table S2. Overview on all relatively quantified 5180 proteins statistical analysis

			MCF-7																		MDA-MB-231								
			Dai SC20 vs control			Gen SC20 vs control			SSE SC20 vs control			Dai IC20 vs control			Gen IC20 vs control			SSE IC20 vs control			Dai IC20 vs control			Gen IC20 vs control			SSE IC20 vs control		
	**Protein name**	**UniProt**	**p value**	**BH q value**	**log2FC**	**p value**	**BH q value**	**log2FC**	**p value**	**BH q value**	**log2FC**	**p value**	**BH q value**	**log2FC**	**p value**	**BH q value**	**log2FC**	**p value**	**BH q value**	**log2FC**	**p value**	**BH q value**	**log2FC**	**p value**	**BH q value**	**log2FC**	**p value**	**BH q value**	**log2FC**
3779	Ras-GEF domain-containing family member 1B	Q0VAM2	0.0386	0.1234	0.10	0.1011	0.6325	0.25	0.0721	0.1604	0.15	0.0003	0.0102	0.47	0.0003	0.0169	0.30	0.0027	0.0564	0.49	0.3736	0.6410	-2.55	0.8708	1.2840	-0.33	0.0651	0.2187	2.06
3780	Ras-GEF domain-containing family member 1C	Q8N431	0.3891	0.6058	0.18	0.7448	0.8150	-0.07	0.3161	0.5030	-0.22	0.9811	0.9836	0.00	0.7534	0.8072	-0.06	0.7286	0.9682	-0.07	0.3736	0.4437	-2.55	0.8708	0.9289	-0.33	0.0651	0.1169	2.06
3781	Ras-related C3 botulinum toxin substrate 1	P63000	0.0258	0.0971	0.24	0.4661	0.7974	0.14	0.0493	0.1237	0.19	0.0128	0.0459	0.30	0.9374	0.9519	0.00	0.0185	0.0878	0.32	0.5449	0.5960	0.08	0.0284	0.1523	-0.35	0.0242	0.1322	-0.46
3782	Ras-related GTP-binding protein B	Q5VZM2	0.4108	0.5213	-3.31	0.5752	0.7299	-1.75	0.5661	0.6622	-1.19	0.5388	0.6265	0.89	0.2938	0.4210	-1.88	0.9510	1.0128	0.09	0.0189	0.1275	-1.31	0.2234	0.5325	0.09	0.0032	0.0562	-0.49
3783	Ras-related protein Rab-10	P61026	0.0058	0.0457	-0.18	0.0858	0.6207	-0.12	0.0048	0.0338	-0.22	0.0015	0.0175	-0.30	0.0077	0.0639	-0.19	0.2291	0.4123	-0.12	0.1247	0.2973	0.10	0.0944	0.3066	0.11	0.6063	0.6664	-0.03
3784	Ras-related protein Rab-11B	Q15907	0.3947	0.6129	0.04	0.3937	0.7373	0.17	0.1535	0.2849	0.09	0.0264	0.0732	0.15	0.2867	0.5164	-0.05	0.0891	0.2178	0.09	0.0851	0.2331	0.36	0.8397	1.2831	0.02	0.8344	0.8660	0.01
3785	Ras-related protein Rab-12	Q6IQ22	0.0224	0.0890	0.16	0.2753	0.6645	0.15	0.1759	0.3184	0.09	0.0148	0.0498	0.19	0.3278	0.4249	0.04	0.1010	0.2350	0.15	0.1720	0.3694	0.18	0.5129	0.9322	0.07	0.0569	0.2120	-0.32
3786	Ras-related protein Rab-13	P51153	0.0002	0.0128	0.53	0.2465	0.6595	0.20	0.0434	0.1133	0.13	0.0013	0.0162	0.25	0.0002	0.0148	0.35	0.1514	0.3091	0.17	0.1271	0.3004	0.13	0.0061	0.0627	0.47	0.0442	0.1840	0.24
3787	Ras-related protein Rab-14	P61106	0.8956	0.9181	0.01	0.8250	0.8745	0.02	0.0383	0.1033	0.15	0.0415	0.1011	0.14	0.6644	0.7295	-0.02	0.0137	0.0776	0.21	0.0636	0.1988	0.26	0.2315	0.5453	0.10	0.8509	0.8791	-0.01
3788	Ras-related protein Rab-15	P59190	0.4635	0.5403	0.23	0.7605	0.8267	-0.07	0.1097	0.2199	-0.15	0.0998	0.1939	0.33	0.3806	0.4777	0.07	0.0170	0.0854	0.41	0.3059	0.5653	0.07	0.0525	0.2170	-0.23	0.0148	0.1047	-0.43
3789	Ras-related protein Rab-17	Q9H0T7	0.1816	0.3451	0.17	0.5362	0.8517	0.07	0.0714	0.1594	-0.30	0.2695	0.4254	-0.14	0.7448	0.8001	0.03	0.4136	0.6311	-0.09	0.3736	0.4680	-2.55	0.8708	0.9745	-0.33	0.0651	0.1269	2.06
3790	Ras-related protein Rab-18	Q9NP72	0.6465	0.7080	0.02	0.9413	0.9610	-0.01	0.5514	0.7747	0.03	0.1797	0.3046	0.07	0.8428	0.8789	-0.01	0.0342	0.1193	0.18	0.5173	0.5707	0.05	0.7461	1.1881	-0.02	0.0628	0.2239	-0.15
3791	Ras-related protein Rab-1A	P62820	0.0617	0.1645	0.14	0.2362	0.6635	0.21	0.0209	0.0700	0.20	0.0197	0.0598	0.21	0.2013	0.4020	0.07	0.0113	0.0729	0.29	0.0161	0.1185	0.56	0.5757	1.0061	-0.10	0.1543	0.2175	0.19
3792	Ras-related protein Rab-1B	Q9H0U4	0.1685	0.3267	-0.03	0.0715	0.6042	0.29	0.0228	0.0744	0.15	0.0509	0.1164	0.11	0.7497	0.8044	0.01	0.1049	0.2413	0.14	0.0364	0.1586	0.37	0.2764	0.6124	0.11	0.6442	0.7007	-0.02
3793	Ras-related protein Rab-20	Q9NX57	0.1358	0.2799	-0.14	0.1580	0.6611	0.13	0.0350	0.0972	0.28	0.2301	0.3732	0.11	0.5215	0.6088	-0.07	0.2178	0.3992	-0.14	0.3736	0.4985	-2.55	0.8708	1.0308	-0.33	0.0651	0.1402	2.06
3794	Ras-related protein Rab-21	Q9UL25	0.0019	0.0300	0.15	0.2930	0.6701	0.15	0.0014	0.0222	0.18	0.0003	0.0112	0.23	0.0060	0.0569	0.08	0.0786	0.2007	0.18	0.4941	0.5479	0.06	0.0612	0.2362	-0.09	0.2787	0.3506	-0.02
3795	Ras-related protein Rab-22A	Q9UL26	0.1294	0.2705	0.03	0.7318	0.8045	-0.02	0.0155	0.0585	0.13	0.8715	0.8895	-0.01	0.0381	0.1421	0.21	0.1861	0.3581	0.12	0.8823	0.9022	0.03	0.5390	0.9641	-0.15	0.3219	0.3931	-0.24
3796	Ras-related protein Rab-24	Q969Q5	0.3139	0.5155	0.17	0.2591	0.6638	0.19	0.7614	0.8021	-0.04	0.2613	0.4142	0.19	0.4323	0.5263	0.12	0.3645	0.5715	0.15	0.3736	0.4725	-2.55	0.8708	0.9827	-0.33	0.0651	0.1288	2.06
3797	Ras-related protein Rab-25	P57735	0.5896	0.6562	0.02	0.4012	0.7449	0.35	0.0295	0.0871	0.24	0.0082	0.0366	0.31	0.0526	0.1701	-0.06	0.0170	0.0857	0.18	0.1670	0.3616	0.28	0.7248	1.1700	-0.05	0.1370	0.1980	-0.31
3798	Ras-related protein Rab-2A	P61019	0.0190	0.0810	0.22	0.2212	0.6589	0.11	0.0312	0.0903	0.20	0.1177	0.2196	0.08	0.1684	0.3550	0.07	0.0083	0.0668	0.26	0.0358	0.1565	0.21	0.1141	0.3460	0.13	0.0445	0.1843	0.21
3799	Ras-related protein Rab-2B	Q8WUD1	0.9842	0.9884	0.00	0.3445	0.7020	-0.12	0.5259	0.7468	0.04	0.4798	0.6802	0.05	0.4642	0.5557	-0.06	0.1598	0.3206	-0.11	0.6089	0.6564	-0.09	0.5046	0.9220	0.04	0.0090	0.0846	0.23
3800	Ras-related protein Rab-30	Q15771	0.0021	0.0315	0.30	0.0020	0.2252	0.21	0.1272	0.2472	0.12	0.0290	0.0785	0.22	0.0025	0.0387	0.19	0.0217	0.0950	0.21	0.6627	0.7071	-0.10	0.5699	0.9990	0.11	0.2399	0.3101	0.25
3801	Ras-related protein Rab-32	Q13637	0.6633	0.7232	0.09	0.8680	0.9041	0.03	0.7601	0.8011	0.07	0.9082	0.9206	-0.03	0.6299	0.7003	0.12	0.3641	0.5714	0.21	0.0213	0.1337	0.13	0.3099	0.6582	0.07	0.2899	0.3618	0.05
3802	Ras-related protein Rab-33B	Q9H082	0.0170	0.0761	-0.13	0.0425	0.5689	-0.17	0.0038	0.0322	-0.15	0.0046	0.0276	-0.31	0.0011	0.0261	-0.28	0.0066	0.0631	-0.21	0.0239	0.1374	0.36	0.0013	0.0295	0.75	0.5935	0.6545	0.03
3803	Ras-related protein Rab-34	Q9BZG1	0.4108	0.5212	-3.31	0.5752	0.7297	-1.75	0.5661	0.6621	-1.19	0.5388	0.6263	0.89	0.2938	0.4209	-1.88	0.9510	1.0126	0.09	0.0393	0.1638	0.21	0.1173	0.3529	0.17	0.2665	0.3379	0.09
3804	Ras-related protein Rab-35	Q15286	0.7176	0.7691	-0.01	0.9620	0.9742	0.01	0.3215	0.5090	-0.06	0.0115	0.0429	-0.26	0.0056	0.0555	-0.36	0.1656	0.3290	-0.34	0.6951	0.7362	-0.03	0.0406	0.1848	-0.07	0.0128	0.0985	-0.12
3805	Ras-related protein Rab-37	Q96AX2	0.0036	0.0384	-0.50	0.0527	0.5821	0.22	0.0119	0.0511	0.35	0.0243	0.0693	0.29	0.1467	0.3220	0.09	0.2628	0.4553	-0.09	0.0148	0.1119	-0.41	0.0047	0.0548	-0.58	0.0113	0.0931	-0.51
3806	Ras-related protein Rab-38	P57729	0.2102	0.3829	-0.11	0.2870	0.6679	0.06	0.3837	0.5844	-0.09	0.0035	0.0237	-0.28	0.0212	0.1049	0.20	0.0099	0.0706	-0.29	0.3736	0.6142	-2.55	0.8708	1.2375	-0.33	0.0651	0.2014	2.06
3807	Ras-related protein Rab-39A	Q14964	0.0208	0.0852	0.18	0.4054	0.7484	0.06	0.4286	0.6380	0.03	0.1140	0.2145	0.08	0.1804	0.3724	0.06	0.0213	0.0941	0.29	0.0993	0.2545	-0.25	0.7330	1.1759	-0.03	0.7082	0.7584	-0.04
3808	Ras-related protein Rab-39B	Q96DA2	0.4108	0.5210	-3.31	0.5752	0.7296	-1.75	0.5661	0.6619	-1.19	0.5388	0.6262	0.89	0.2938	0.4208	-1.88	0.9510	1.0124	0.09	0.0409	0.1662	0.42	0.6696	1.1074	0.06	0.0147	0.1046	0.69
3809	Ras-related protein Rab-3A	P20336	0.5707	0.6385	-0.02	0.7633	0.8289	0.02	0.0118	0.0507	0.32	0.0442	0.1055	0.24	0.0063	0.0580	0.25	0.0278	0.1068	0.09	0.0057	0.0807	0.57	0.0013	0.0302	0.51	0.0317	0.1512	0.57
3810	Ras-related protein Rab-3B	P20337	0.5488	0.6181	-0.03	0.3772	0.7245	0.14	0.0362	0.0996	0.16	0.0583	0.1288	0.14	0.2877	0.5178	-0.06	0.3482	0.5534	0.05	0.1564	0.3477	-0.12	0.3343	0.6913	-0.04	0.0644	0.2279	-0.15
3811	Ras-related protein Rab-3C	Q96E17	0.5596	0.6284	-0.08	0.3863	0.7298	-0.19	0.9455	0.9573	-0.01	0.3347	0.5086	-0.14	0.7265	0.7860	0.04	0.1170	0.2606	-0.33	0.0096	0.0981	0.41	0.0300	0.1567	0.29	0.0108	0.0907	0.44
3812	Ras-related protein Rab-3D	O95716	0.2889	0.4857	-0.04	0.1977	0.6689	0.20	0.4640	0.6807	0.04	0.0071	0.0341	0.11	0.1137	0.2756	-0.04	0.2706	0.4640	-0.02	0.0236	0.1369	0.30	0.0104	0.0838	0.28	0.1351	0.1959	0.11
3813	Ras-related protein Rab-40C	Q96S21	0.0029	0.0361	-0.45	0.0226	0.4759	-0.57	0.0006	0.0209	-0.70	0.0101	0.0405	-0.41	0.0002	0.0135	-0.98	0.5563	0.7917	0.04	0.3736	0.4565	-2.55	0.8708	0.9530	-0.33	0.0651	0.1221	2.06
3814	Ras-related protein Rab-41	Q5JT25	0.4108	0.5209	-3.31	0.5752	0.7294	-1.75	0.5661	0.6618	-1.19	0.5388	0.6261	0.89	0.2938	0.4206	-1.88	0.9510	1.0122	0.09	0.4717	0.5265	0.08	0.0470	0.2017	-0.32	0.7021	0.7538	0.05
3815	Ras-related protein Rab-43	Q86YS6	0.8255	0.8602	0.01	0.0508	0.5721	-0.14	0.0096	0.0457	0.19	0.0960	0.1881	0.07	0.7835	0.8298	-0.01	0.0050	0.0614	0.20	0.0034	0.0648	-0.33	0.0015	0.0315	-0.63	0.0108	0.0914	-0.30
3816	Ras-related protein Rab-4A	P20338	0.0048	0.0432	0.22	0.3151	0.6809	0.06	0.5678	0.6257	0.02	0.0523	0.1187	0.10	0.1999	0.4006	-0.08	0.4551	0.6778	0.04	0.1747	0.3735	-0.13	0.8525	1.2946	-0.01	0.7958	0.8336	-0.02
3817	Ras-related protein Rab-4B	P61018	0.6406	0.7023	-0.04	0.6895	0.7726	0.04	0.6084	0.6639	-0.04	0.5325	0.7401	0.04	0.1219	0.2875	-0.11	0.3321	0.5352	-0.07	0.2622	0.5030	-0.23	0.7181	1.1624	-0.08	0.8404	0.8712	0.05
3818	Ras-related protein Rab-5A	P20339	0.1428	0.2893	-0.08	0.2367	0.6631	0.16	0.0848	0.1807	-0.09	0.3218	0.4924	0.05	0.0691	0.2002	0.10	0.2618	0.4539	0.05	0.0267	0.1419	0.72	0.4912	0.9029	-0.13	0.0147	0.1047	1.00
3819	Ras-related protein Rab-5B	P61020	0.0437	0.1325	-0.24	0.3486	0.7040	0.17	0.4811	0.7008	-0.07	0.0223	0.0652	0.33	0.2010	0.4017	0.12	0.0804	0.2039	0.20	0.0419	0.1673	0.25	0.4547	0.8587	0.06	0.1416	0.2030	-0.10
3820	Ras-related protein Rab-5C	P51148	0.7193	0.7708	-0.02	0.5826	0.6900	0.03	0.9221	0.9388	0.00	0.6500	0.6939	0.02	0.0483	0.1614	-0.12	0.3521	0.5572	0.04	0.0921	0.2435	0.32	0.7929	1.2356	-0.02	0.7516	0.7949	0.01
3821	Ras-related protein Rab-6A	P20340	0.0387	0.1234	0.21	0.1456	0.6553	0.27	0.0295	0.0873	0.30	0.0018	0.0186	0.50	0.0068	0.0600	0.29	0.0328	0.1165	0.41	0.3736	0.5777	-2.55	0.8708	1.1735	-0.33	0.0651	0.1800	2.06
3822	Ras-related protein Rab-6B	Q9NRW1	0.1148	0.2481	0.06	0.4632	0.7950	0.11	0.2482	0.4146	0.09	0.0043	0.0265	0.15	0.8112	0.8530	0.01	0.9924	0.9932	0.00	0.1352	0.3132	0.19	0.7892	1.2321	-0.02	0.0459	0.1872	-0.20
3823	Ras-related protein Rab-6D	Q53S08	0.0195	0.0820	-0.17	0.1950	0.6689	0.06	0.0154	0.0583	-0.20	0.0409	0.1003	-0.13	0.0177	0.0951	0.19	0.0597	0.1689	-0.12	0.4420	0.4977	-0.13	0.1959	0.4907	0.21	0.2124	0.2811	0.26
3824	Ras-related protein Rab-7a	P51149	0.0135	0.0678	0.07	0.5794	0.6874	0.07	0.0891	0.1874	0.12	0.0021	0.0198	0.10	0.0005	0.0188	-0.14	0.0372	0.1248	0.18	0.1378	0.3175	-0.10	0.0670	0.2491	-0.17	0.0200	0.1216	-0.23
3825	Ras-related protein Rab-7L1	O14966	0.4108	0.5208	-3.31	0.5752	0.7292	-1.75	0.5661	0.6616	-1.19	0.5388	0.6259	0.89	0.2938	0.4205	-1.88	0.9510	1.0120	0.09	0.8662	0.8892	0.03	0.0223	0.1299	-0.53	0.8981	0.9192	-0.05
3826	Ras-related protein Rab-8A	P61006	0.2434	0.4287	-0.05	0.4121	0.7524	0.16	0.0149	0.0575	0.19	0.0030	0.0224	0.25	0.8215	0.8609	-0.01	0.4894	0.7147	0.02	0.0348	0.1545	0.49	0.7460	1.1886	0.04	0.1757	0.2415	0.17
3827	Ras-related protein Rab-8B	Q92930	0.0191	0.0812	0.09	0.1871	0.6666	0.20	0.0051	0.0349	0.26	0.0013	0.0166	0.31	0.0108	0.0745	0.09	0.0706	0.1870	0.12	0.0779	0.2220	0.25	0.7517	1.1937	0.03	0.3841	0.4553	-0.07
3828	Ras-related protein Rab-9A	P51151	0.1199	0.2555	1.02	0.4594	0.7911	0.80	0.2280	0.3871	0.37	0.5793	0.6284	0.53	0.3716	0.4686	-0.22	0.8919	1.1285	-0.07	0.3736	0.4342	-2.55	0.8708	0.9111	-0.33	0.0651	0.1132	2.06
3829	Ras-related protein Ral-A	P11233	0.7149	0.7665	0.03	0.0723	0.6060	0.20	0.2085	0.3619	0.11	0.6176	0.6646	0.04	0.1085	0.2673	0.17	0.1310	0.2818	0.15	0.2305	0.4578	0.23	0.4645	0.8727	0.14	0.0683	0.1157	0.41
3830	Ras-related protein Ral-B	P11234	0.0656	0.1695	0.19	0.2637	0.6631	0.16	0.9561	0.9647	0.00	0.5334	0.7408	0.05	0.3019	0.4002	0.08	0.3079	0.5070	0.15	0.0183	0.1257	-0.29	0.0046	0.0548	-0.44	0.0052	0.0698	-0.72
3831	Ras-related protein Rap-1A	P62834	0.4108	0.5207	-3.31	0.5752	0.7290	-1.75	0.5661	0.6615	-1.19	0.5388	0.6258	0.89	0.2938	0.4204	-1.88	0.9510	1.0117	0.09	0.0092	0.0955	-0.84	0.0216	0.1274	-0.56	0.0238	0.1314	-0.57
3832	Ras-related protein Rap-1b	P61224	0.3285	0.5351	0.05	0.3980	0.7421	0.12	0.4551	0.6701	-0.08	0.6669	0.7092	-0.02	0.4693	0.5603	-0.03	0.9571	0.9645	0.00	0.0429	0.1676	-0.18	0.0013	0.0292	-0.29	0.0021	0.0513	-0.48
3833	Ras-related protein Rap-2b	P61225	0.0002	0.0126	2.86	0.0588	0.5914	0.65	0.0545	0.1329	0.69	0.0202	0.0609	0.51	0.0081	0.0650	1.24	0.1094	0.2488	1.33	0.0495	0.1776	-2.85	0.0033	0.0467	-3.10	0.0108	0.0911	-3.82
3834	Ras-related protein R-Ras	P10301	0.0009	0.0212	-0.68	0.1135	0.6342	-0.27	0.0269	0.0822	-0.20	0.3149	0.4846	-0.05	0.0079	0.0642	-0.25	0.1210	0.2660	-0.10	0.0082	0.0913	1.32	0.0005	0.0223	1.78	0.0005	0.0316	1.26
3835	Ras-related protein R-Ras2	P62070	0.0402	0.1262	0.11	0.2002	0.6639	0.28	0.0195	0.0672	0.29	0.0189	0.0581	0.15	0.0052	0.0539	0.23	0.0247	0.1001	0.16	0.0296	0.1473	0.45	0.3484	0.7108	0.13	0.1252	0.1847	0.29
3836	Ras-specific guanine nucleotide-releasing factor RalGPS2	Q86X27	0.0002	0.0125	0.97	0.0428	0.5699	0.20	0.0010	0.0221	0.58	0.0009	0.0155	0.75	0.0032	0.0427	0.71	0.0009	0.0462	0.84	0.3736	0.6498	-2.55	0.8708	1.2992	-0.33	0.0651	0.2247	2.06
3837	RB1-inducible coiled-coil protein 1	Q8TDY2	0.4108	0.5205	-3.31	0.5752	0.7288	-1.75	0.5661	0.6613	-1.19	0.5388	0.6256	0.89	0.2938	0.4203	-1.88	0.9510	1.0115	0.09	0.0044	0.0710	1.14	0.0003	0.0190	0.70	0.0003	0.0299	0.75
3838	Reactive oxygen species modulator 1	P60602	0.0128	0.0660	0.23	0.2401	0.6612	-0.07	0.1832	0.3279	-0.09	0.0854	0.1725	0.10	0.0024	0.0380	0.31	0.0528	0.1553	0.13	0.0062	0.0834	-0.54	0.0572	0.2290	-0.25	0.0071	0.0773	-0.87
3839	Receptor expression-enhancing protein 4	Q9H6H4	0.0032	0.0369	0.50	0.1612	0.6648	0.34	0.7832	0.8221	0.06	0.0027	0.0215	0.66	0.0260	0.1166	0.39	0.2323	0.4162	0.17	0.3736	0.4946	-2.55	0.8708	1.0235	-0.33	0.0651	0.1384	2.06
3840	Receptor expression-enhancing protein 5	Q00765	0.2768	0.4706	0.07	0.3493	0.7043	0.26	0.0301	0.0881	0.21	0.0187	0.0578	0.24	0.3587	0.4557	-0.05	0.0365	0.1237	0.22	0.0606	0.1945	0.32	0.2941	0.6353	0.09	0.8623	0.8882	0.01
3841	Receptor of activated protein C kinase 1	P63244	0.0340	0.1152	0.19	0.5550	0.8699	0.11	0.0386	0.1038	0.18	0.1525	0.2688	0.10	0.1102	0.2700	-0.12	0.0579	0.1657	0.17	0.1979	0.4083	0.20	0.4918	0.9034	0.07	0.1935	0.2615	-0.11
3842	Receptor tyrosine-protein kinase erbB-2	P04626	0.9181	0.9376	-0.02	0.2807	0.6652	-0.42	0.7790	0.8183	-0.06	0.3572	0.5355	-0.20	0.7471	0.8022	0.07	0.7258	0.9660	0.07	0.3736	0.4439	-2.55	0.8708	0.9293	-0.33	0.0651	0.1170	2.06
3843	Receptor-interacting serine/threonine-protein kinase 1	Q13546	0.6606	0.7209	-0.01	0.3989	0.7425	-0.05	0.1352	0.2581	-0.10	0.3000	0.4646	-0.06	0.3705	0.4675	-0.04	0.1207	0.2655	0.08	0.1102	0.2725	0.52	0.9066	0.9303	-0.03	0.2267	0.2971	0.33
3844	Receptor-type tyrosine-protein kinase FLT3	P36888	0.4108	0.5204	-3.31	0.5752	0.7287	-1.75	0.5661	0.6612	-1.19	0.5388	0.6255	0.89	0.2938	0.4202	-1.88	0.9510	1.0113	0.09	0.4901	0.5441	-0.10	0.0122	0.0920	-0.31	0.0075	0.0786	-0.23
3845	Receptor-type tyrosine-protein phosphatase alpha	P18433	0.0947	0.2166	-0.23	0.1595	0.6642	0.16	0.5822	0.6388	-0.06	0.1919	0.3212	-0.16	0.6405	0.7097	0.05	0.4503	0.6730	-0.08	0.3736	0.4642	-2.55	0.8708	0.9673	-0.33	0.0651	0.1253	2.06
3846	Receptor-type tyrosine-protein phosphatase F	P10586	0.4108	0.5203	-3.31	0.5752	0.7285	-1.75	0.5661	0.6610	-1.19	0.5388	0.6254	0.89	0.2938	0.4201	-1.88	0.9510	1.0111	0.09	0.0532	0.1825	0.37	0.0562	0.2267	0.24	0.0919	0.1456	0.20
3847	Receptor-type tyrosine-protein phosphatase kappa	Q15262	0.1555	0.3081	-0.13	0.7540	0.8219	0.04	0.6845	0.7336	-0.03	0.3790	0.5633	-0.08	0.3677	0.4646	0.08	0.9311	1.1675	-0.01	0.3736	0.4325	-2.55	0.8708	0.9078	-0.33	0.0651	0.1125	2.06
3848	Receptor-type tyrosine-protein phosphatase mu	P28827	0.4108	0.5202	-3.31	0.5752	0.7283	-1.75	0.5661	0.6609	-1.19	0.5388	0.6252	0.89	0.2938	0.4199	-1.88	0.9510	1.0109	0.09	0.2074	0.4228	-1.40	0.5648	0.9921	-1.10	0.7419	0.7869	-0.30
3849	Receptor-type tyrosine-protein phosphatase N2	Q92932	0.3464	0.5554	0.08	0.3106	0.6794	0.09	0.0778	0.1688	0.19	0.3933	0.5809	0.07	0.2271	0.4363	-0.11	0.7102	0.9513	-0.07	0.3736	0.4450	-2.55	0.8708	0.9314	-0.33	0.0651	0.1175	2.06
3850	Receptor-type tyrosine-protein phosphatase S	Q13332	0.4108	0.5200	-3.31	0.5752	0.7281	-1.75	0.5661	0.6607	-1.19	0.5388	0.6251	0.89	0.2938	0.4198	-1.88	0.9510	1.0107	0.09	0.0005	0.0360	-0.31	0.0280	0.1519	-0.41	0.0007	0.0327	-0.39
3851	Regulating synaptic membrane exocytosis protein 1	Q86UR5	0.6761	0.7336	-0.13	0.0741	0.6102	-0.56	0.6766	0.7267	0.09	0.4394	0.6333	0.27	0.4439	0.5381	-0.24	0.5954	0.8318	0.23	0.3736	0.4529	-2.55	0.8708	0.9462	-0.33	0.0651	0.1207	2.06
3852	Regulation of nuclear pre-mRNA domain-containing protein 1A	Q96P16	0.0911	0.2114	0.15	0.0604	0.5914	0.20	0.2599	0.4290	0.09	0.0503	0.1154	0.20	0.1536	0.3330	0.13	0.5925	0.8295	0.04	0.3321	0.6011	-0.08	0.4733	0.8844	-0.07	0.6299	0.6872	0.03
3853	Regulation of nuclear pre-mRNA domain-containing protein 1B	Q9NQG5	0.0251	0.0957	-0.10	0.9634	0.9755	0.00	0.4106	0.6169	-0.06	0.1560	0.2736	-0.10	0.0205	0.1024	-0.09	0.0936	0.2249	-0.09	0.0685	0.2062	-0.11	0.1739	0.4575	0.03	0.2780	0.3498	0.03
3854	Regulation of nuclear pre-mRNA domain-containing protein 2	Q5VT52	0.0550	0.1534	0.06	0.1313	0.6453	0.27	0.1964	0.3458	-0.05	0.0005	0.0118	0.25	0.0021	0.0361	0.17	0.0022	0.0545	0.21	0.3736	0.6434	-2.55	0.8708	1.2880	-0.33	0.0651	0.2203	2.06
3855	Regulator of chromosome condensation	P18754	0.3902	0.6070	0.09	0.9200	0.9452	0.02	0.4650	0.6820	-0.08	0.5423	0.5936	0.06	0.2158	0.4207	0.15	0.1985	0.3742	0.16	0.8166	0.8446	-0.03	0.0477	0.2037	-0.38	0.1170	0.1749	-0.27
3856	Regulator of G-protein signaling 10	O43665	0.3093	0.5104	-0.07	0.1359	0.6512	-0.15	0.1025	0.2083	-0.11	0.0216	0.0637	-0.21	0.0536	0.1718	-0.15	0.2576	0.4482	-0.08	0.9992	0.9992	0.00	0.2579	0.5849	-0.07	0.2344	0.3043	-0.08
3857	Regulator of G-protein signaling 18	Q9NS28	0.4108	0.5199	-3.31	0.5752	0.7280	-1.75	0.5661	0.6606	-1.19	0.5388	0.6249	0.89	0.2938	0.4197	-1.88	0.9510	1.0105	0.09	0.3865	0.4425	-0.11	0.6683	1.1071	-0.03	0.1815	0.2485	0.11
3858	Regulator of G-protein signaling 19	P49795	0.1198	0.2555	0.17	0.0923	0.6299	0.64	0.0027	0.0287	0.65	0.0053	0.0294	0.60	0.0091	0.0676	0.41	0.0044	0.0608	0.58	0.3736	0.6326	-2.55	0.8708	1.2696	-0.33	0.0651	0.2132	2.06

Supplemetary Table S2. Overview on all relatively quantified 5180 proteins statistical analysis

			MCF-7																		MDA-MB-231								
			Dai SC20 vs control			Gen SC20 vs control			SSE SC20 vs control			Dai IC20 vs control			Gen IC20 vs control			SSE IC20 vs control			Dai IC20 vs control			Gen IC20 vs control			SSE IC20 vs control		
	**Protein name**	**UniProt**	**p value**	**BH q value**	**log2FC**	**p value**	**BH q value**	**log2FC**	**p value**	**BH q value**	**log2FC**	**p value**	**BH q value**	**log2FC**	**p value**	**BH q value**	**log2FC**	**p value**	**BH q value**	**log2FC**	**p value**	**BH q value**	**log2FC**	**p value**	**BH q value**	**log2FC**	**p value**	**BH q value**	**log2FC**
3859	Regulator of G-protein signaling 2	P41220	0.0203	0.0842	0.09	0.5444	0.8590	0.05	0.2069	0.3601	0.16	0.0321	0.0842	0.22	0.0066	0.0591	0.32	0.0701	0.1860	0.36	0.0825	0.2294	-1.36	0.0911	0.3004	-0.45	0.9580	0.9668	0.01
3860	Regulator of G-protein signaling 21	Q2M5E4	0.0407	0.1273	0.62	0.2063	0.6605	-0.50	0.0198	0.0679	-0.94	0.2056	0.3395	0.28	0.2821	0.5092	-0.23	0.0866	0.2147	0.71	0.3736	0.5409	-2.55	0.8708	1.1077	-0.33	0.0651	0.1605	2.06
3861	Regulator of G-protein signaling 4	P49798	0.4108	0.5198	-3.31	0.5752	0.7278	-1.75	0.5661	0.6605	-1.19	0.5388	0.6248	0.89	0.2938	0.4196	-1.88	0.9510	1.0103	0.09	0.8603	0.8838	-0.05	0.2073	0.5087	0.40	0.9103	0.9282	0.03
3862	Regulator of G-protein signaling 7	P49802	0.1852	0.3497	0.09	0.6449	0.7379	0.03	0.4265	0.6363	0.04	0.7777	0.8088	0.01	0.8802	0.9083	-0.01	0.2150	0.3962	0.14	0.3736	0.4994	-2.55	0.8708	1.0324	-0.33	0.0651	0.1406	2.06
3863	Regulator of microtubule dynamics protein 1	Q96DB5	0.5084	0.5815	-0.12	0.2092	0.6612	-0.25	0.1075	0.2163	-0.36	0.2795	0.4374	-0.21	0.1607	0.3437	-0.30	0.1358	0.2881	-0.33	0.9911	0.9934	0.00	0.3852	0.7645	-0.05	0.0639	0.2262	-0.15
3864	Regulator of nonsense transcripts 1	Q92900	0.1668	0.3247	-0.05	0.6019	0.7056	0.03	0.0553	0.1343	0.09	0.4598	0.6572	0.02	0.0016	0.0310	-0.22	0.7091	0.9504	-0.01	0.0766	0.2202	0.35	0.2830	0.6212	0.15	0.4481	0.5175	-0.10
3865	Regulator of nonsense transcripts 2	Q9HAU5	0.0256	0.0969	0.25	0.2277	0.6600	0.16	0.2884	0.4674	0.08	0.1078	0.2055	0.12	0.4580	0.5503	0.04	0.0519	0.1538	0.19	0.3736	0.5596	-2.55	0.8708	1.1414	-0.33	0.0651	0.1702	2.06
3866	RelA-associated inhibitor	Q8WUF5	0.0048	0.0431	-0.20	0.2181	0.6611	0.21	0.7805	0.8197	-0.02	0.1364	0.2457	-0.06	0.0251	0.1140	-0.12	0.0503	0.1507	-0.23	0.0085	0.0935	-0.34	0.0098	0.0815	0.23	0.1751	0.2410	0.13
3867	Remodeling and spacing factor 1	Q96T23	0.1829	0.3472	0.07	0.0844	0.6219	0.30	0.0016	0.0237	0.33	0.0072	0.0345	0.35	0.0083	0.0656	0.21	0.0027	0.0557	0.34	0.4176	0.4734	0.16	0.0288	0.1530	0.61	0.0340	0.1575	0.51
3868	Renin receptor	O75787	0.0077	0.0519	0.30	0.5793	0.6876	-0.08	0.6745	0.7247	-0.02	0.6604	0.7033	-0.02	0.0062	0.0572	-0.24	0.1030	0.2384	-0.10	0.3736	0.5328	-2.55	0.8708	1.0932	-0.33	0.0651	0.1565	2.06
3869	Replication factor C subunit 2	P35250	0.1093	0.2393	0.07	0.1741	0.6607	0.18	0.0125	0.0525	0.22	0.0172	0.0548	0.15	0.6824	0.7457	-0.01	0.0229	0.0968	0.16	0.3502	0.6251	0.12	0.9030	0.9275	0.01	0.8577	0.8847	-0.01
3870	Replication factor C subunit 3	P40938	0.8663	0.8944	0.02	0.5844	0.6915	0.05	0.1587	0.2927	0.15	0.7159	0.7540	-0.03	0.0523	0.1694	-0.28	0.1245	0.2721	-0.24	0.4862	0.5406	0.04	0.6161	1.0498	-0.04	0.1962	0.2642	-0.10
3871	Replication factor C subunit 4	P35249	0.6317	0.6939	0.05	0.6079	0.7102	-0.06	0.1949	0.3437	-0.17	0.1678	0.2888	-0.18	0.3611	0.4582	-0.11	0.2870	0.4833	-0.13	0.0382	0.1618	0.54	0.0164	0.1086	0.17	0.0514	0.1990	0.11
3872	Replication factor C subunit 5	P40937	0.8476	0.8785	0.01	0.5903	0.6956	0.02	0.0411	0.1087	0.13	0.0278	0.0760	0.17	0.1671	0.3533	-0.08	0.0680	0.1828	-0.10	0.3736	0.5499	-2.55	0.8708	1.1240	-0.33	0.0651	0.1651	2.06
3873	Replication protein A 14 kDa subunit	P35244	0.4108	0.5196	-3.31	0.5752	0.7276	-1.75	0.5661	0.6603	-1.19	0.5388	0.6247	0.89	0.2938	0.4195	-1.88	0.9510	1.0101	0.09	0.0586	0.1921	-0.35	0.8094	1.2530	0.05	0.2184	0.2876	0.18
3874	Replication protein A 30 kDa subunit	Q13156	0.4108	0.5195	-3.31	0.5752	0.7274	-1.75	0.5661	0.6602	-1.19	0.5388	0.6245	0.89	0.2938	0.4194	-1.88	0.9510	1.0099	0.09	0.2489	0.4851	2.14	0.1793	0.4663	2.71	0.1527	0.2158	3.03
3875	Replication protein A 32 kDa subunit	P15927	0.5932	0.6593	0.31	0.4168	0.7549	0.49	0.1493	0.2783	1.07	0.2535	0.4035	0.75	0.5173	0.6053	0.40	0.3740	0.5827	0.54	0.9069	0.9240	-0.01	0.1900	0.4820	-0.14	0.1239	0.1832	-0.13
3876	Replication protein A 70 kDa DNA-binding subunit	P27694	0.1200	0.2555	0.11	0.1382	0.6520	0.08	0.0294	0.0871	0.24	0.0088	0.0380	0.22	0.5875	0.6648	0.02	0.0173	0.0858	0.21	0.1073	0.2675	0.23	0.6639	1.1019	-0.03	0.1068	0.1630	-0.10
3877	Replication stress response regulator SDE2	Q6IQ49	0.6669	0.7264	-0.02	0.0040	0.3342	0.28	0.4274	0.6373	0.06	0.0185	0.0575	0.17	0.0013	0.0274	0.43	0.3048	0.5030	0.05	0.3736	0.4810	-2.55	0.8708	0.9986	-0.33	0.0651	0.1325	2.06
3878	Required for meiotic nuclear division protein 1 homolog	Q9NWS8	0.0007	0.0199	-0.39	0.1618	0.6620	-0.15	0.1585	0.2924	-0.16	0.2505	0.3998	-0.27	0.0019	0.0344	-0.16	0.1802	0.3496	-0.37	0.3736	0.5073	-2.55	0.8708	1.0468	-0.33	0.0651	0.1442	2.06
3879	Resistin	Q9HD89	0.2258	0.4044	-0.31	0.3421	0.7001	0.28	0.8732	0.8998	0.03	0.1590	0.2773	0.37	0.1127	0.2738	0.46	0.3387	0.5430	0.26	0.3736	0.4755	-2.55	0.8708	0.9883	-0.33	0.0651	0.1300	2.06
3880	REST corepressor 1	Q9UKL0	0.1502	0.2996	0.09	0.1620	0.6613	0.20	0.0197	0.0677	0.21	0.0604	0.1324	0.15	0.0337	0.1340	0.15	0.0247	0.1000	0.20	0.3736	0.5870	-2.55	0.8708	1.1899	-0.33	0.0651	0.1853	2.06
3881	Reticulocalbin-1	Q15293	0.1204	0.2561	0.07	0.2896	0.6685	0.17	0.0032	0.0303	0.29	0.0032	0.0232	0.23	0.6637	0.7288	-0.01	0.0050	0.0615	0.24	0.0290	0.1464	0.37	0.1559	0.4271	0.13	0.1290	0.1887	0.14
3882	Reticulocalbin-2	Q14257	0.3271	0.5330	0.02	0.0110	0.4284	0.08	0.0088	0.0440	0.25	0.0355	0.0903	0.19	0.7382	0.7950	0.02	0.0108	0.0710	0.31	0.1852	0.3865	-0.19	0.2908	0.6300	-0.14	0.0265	0.1385	-0.47
3883	Reticulocalbin-3	Q96D15	0.5021	0.5755	-0.11	0.3078	0.6779	0.17	0.9481	0.9592	0.01	0.3851	0.5699	0.16	0.4633	0.5550	0.11	0.6232	0.8586	-0.08	0.3736	0.4500	-2.55	0.8708	0.9407	-0.33	0.0651	0.1195	2.06
3884	Reticulon-1	Q16799	0.4108	0.5194	-3.31	0.5752	0.7272	-1.75	0.0288	0.0860	5.70	0.1094	0.2077	3.13	0.2093	0.4125	2.93	0.2983	0.4956	3.91	0.1275	0.3009	1.71	0.0734	0.2631	2.22	0.0712	0.1195	2.49
3885	Reticulon-3	O95197	0.0051	0.0435	0.33	0.4211	0.7595	0.10	0.0459	0.1181	0.10	0.2576	0.4092	0.05	0.4105	0.5057	-0.03	0.0094	0.0689	0.27	0.0324	0.1513	0.34	0.0583	0.2298	0.19	0.1006	0.1563	0.13
3886	Reticulon-4	Q9NQC3	0.0762	0.1881	-0.12	0.0762	0.6167	0.24	0.4476	0.6609	0.04	0.1614	0.2807	0.07	0.7748	0.8240	0.01	0.0098	0.0702	0.25	0.0258	0.1404	0.41	0.0365	0.1741	0.31	0.0991	0.1545	0.22
3887	Reticulon-4 receptor-like 2	Q86UN3	0.0000	0.0000	0.86	0.0958	0.6330	0.26	0.0617	0.1443	0.06	0.1209	0.2242	0.06	0.2185	0.4242	0.05	0.0041	0.0597	0.22	0.3736	0.6339	-2.55	0.8708	1.2717	-0.33	0.0651	0.2140	2.06
3888	Retinal dehydrogenase 2	O94788	0.4108	0.5193	-3.31	0.5752	0.7271	-1.75	0.5661	0.6600	-1.19	0.5388	0.6244	0.89	0.2938	0.4193	-1.88	0.9510	1.0097	0.09	0.5551	0.6060	-0.65	0.6580	1.0991	0.47	0.2636	0.3355	1.19
3889	Retinal rod rhodopsin-sensitive cGMP 3'_5'-cyclic phosphodiesterase subunit delta	O43924	0.4108	0.5191	-3.31	0.5752	0.7269	-1.75	0.5661	0.6599	-1.19	0.5388	0.6242	0.89	0.2938	0.4191	-1.88	0.9510	1.0095	0.09	0.4045	0.4605	0.18	0.4379	0.8383	-0.06	0.0017	0.0479	0.74
3890	Retinaldehyde-binding protein 1	P12271	0.1086	0.2380	-0.95	0.8308	0.8783	0.03	0.0130	0.0533	-0.58	0.0154	0.0513	-1.47	0.0105	0.0733	-0.63	0.0043	0.0607	-1.23	0.3736	0.6333	-2.55	0.8708	1.2706	-0.33	0.0651	0.2136	2.06
3891	Retinoblastoma-associated protein	P06400	0.4108	0.5190	-3.31	0.5752	0.7267	-1.75	0.5661	0.6597	-1.19	0.5388	0.6241	0.89	0.2938	0.4190	-1.88	0.9510	1.0093	0.09	0.0619	0.1959	0.40	0.7456	1.1887	-0.04	0.6193	0.6778	-0.05
3892	Retinoic acid receptor alpha	P10276	0.0573	0.1572	-0.18	0.0612	0.5937	-0.21	0.3442	0.5380	-0.08	0.1112	0.2107	-0.16	0.0181	0.0959	0.17	0.2431	0.4299	-0.22	0.3736	0.4916	-2.55	0.8708	1.0180	-0.33	0.0651	0.1371	2.06
3893	Retinoic acid receptor RXR-alpha	P19793	0.4108	0.5189	-3.31	0.5752	0.7265	-1.75	0.5661	0.6596	-1.19	0.5388	0.6240	0.89	0.2938	0.4189	-1.88	0.9510	1.0090	0.09	0.0145	0.1114	0.30	0.0383	0.1786	0.18	0.2095	0.2783	0.09
3894	Retinoic acid receptor RXR-gamma	P48443	0.4108	0.5188	-3.31	0.5752	0.7264	-1.75	0.5661	0.6594	-1.19	0.5388	0.6238	0.89	0.2938	0.4188	-1.88	0.9510	1.0088	0.09	0.0392	0.1639	-0.50	0.0079	0.0727	0.21	0.1594	0.2234	-0.13
3895	Retinoic acid-induced protein 3	Q8NFJ5	0.0002	0.0123	-0.72	0.0057	0.3835	-0.44	0.0002	0.0259	-0.72	0.0004	0.0132	-0.60	0.0025	0.0391	-0.52	0.0164	0.0851	-0.23	0.0108	0.1023	0.25	0.0667	0.2487	0.14	0.0016	0.0479	0.80
3896	Retinol dehydrogenase 11	Q8TC12	0.8741	0.9009	0.01	0.5651	0.8788	0.07	0.1108	0.2218	0.11	0.1431	0.2553	0.10	0.5668	0.6480	0.04	0.1193	0.2638	0.11	0.1662	0.3607	0.13	0.0282	0.1523	0.33	0.0103	0.0897	0.59
3897	Retinol-binding protein 1	P09455	0.4108	0.5186	-3.31	0.5752	0.7262	-1.75	0.5661	0.6593	-1.19	0.5388	0.6237	0.89	0.2938	0.4187	-1.88	0.9510	1.0086	0.09	0.2164	0.4365	-0.44	0.6373	1.0753	0.09	0.0141	0.1027	0.53
3898	Retinol-binding protein 2	P50120	0.4108	0.5185	-3.31	0.5752	0.7260	-1.75	0.5661	0.6591	-1.19	0.5388	0.6235	0.89	0.2938	0.4186	-1.88	0.9510	1.0084	0.09	0.1617	0.3549	-0.72	0.8283	1.2720	-0.02	0.4768	0.5453	0.07
3899	Retroelement silencing factor 1	Q9HCM1	0.1535	0.3051	0.12	0.1679	0.6599	0.80	0.0015	0.0228	0.38	0.0142	0.0486	0.37	0.1341	0.3037	0.09	0.6370	0.8743	-0.02	0.3736	0.4488	-2.55	0.8708	0.9386	-0.33	0.0651	0.1190	2.06
3900	RGM domain family member B	Q6NW40	0.4108	0.5184	-3.31	0.5752	0.7258	-1.75	0.5661	0.6590	-1.19	0.5388	0.6234	0.89	0.2938	0.4184	-1.88	0.9510	1.0082	0.09	0.0654	0.2012	-0.90	0.0205	0.1238	-0.57	0.1346	0.1952	-0.08
3901	Rho GDP-dissociation inhibitor 1	P52565	0.0629	0.1659	-0.11	0.4304	0.7664	0.13	0.2189	0.3755	0.06	0.0698	0.1476	0.10	0.0273	0.1194	-0.14	0.0732	0.1919	0.09	0.0421	0.1676	0.40	0.1417	0.4007	0.15	0.0249	0.1339	0.20
3902	Rho GTPase-activating protein 1	Q07960	0.0066	0.0488	-0.13	0.9239	0.9475	-0.01	0.0851	0.1811	0.08	0.0685	0.1455	0.06	0.0194	0.0997	-0.09	0.0157	0.0836	0.12	0.1481	0.3330	0.17	0.5271	0.9510	-0.04	0.0715	0.1199	-0.10
3903	Rho GTPase-activating protein 15	Q53QZ3	0.4108	0.5183	-3.31	0.5752	0.7257	-1.75	0.5661	0.6588	-1.19	0.5388	0.6233	0.89	0.2938	0.4183	-1.88	0.9510	1.0080	0.09	0.9846	0.9880	0.00	0.6977	1.1408	-0.10	0.9563	0.9658	0.01
3904	Rho GTPase-activating protein 17	Q68EM7	0.0245	0.0944	-0.15	0.0101	0.4434	-0.20	0.0698	0.1570	-0.10	0.0085	0.0372	-0.29	0.0058	0.0562	-0.24	0.2336	0.4178	-0.06	0.3736	0.4941	-2.55	0.8708	1.0226	-0.33	0.0651	0.1382	2.06
3905	Rho GTPase-activating protein 18	Q8N392	0.4108	0.5181	-3.31	0.5752	0.7255	-1.75	0.5661	0.6587	-1.19	0.5388	0.6231	0.89	0.2938	0.4182	-1.88	0.9510	1.0078	0.09	0.0114	0.1034	-0.33	0.0818	0.2810	-0.18	0.0223	0.1269	-0.33
3906	Rho GTPase-activating protein 20	Q9P2F6	0.0669	0.1714	0.42	0.3262	0.6855	-0.36	0.8936	0.9153	0.05	0.0043	0.0264	0.46	0.0005	0.0185	0.63	0.4448	0.6663	0.18	0.0172	0.1217	0.28	0.0010	0.0270	0.63	0.2056	0.2743	0.12
3907	Rho GTPase-activating protein 21	Q5T5U3	0.2783	0.4723	0.08	0.4889	0.8120	0.09	0.0955	0.1969	0.14	0.2050	0.3388	0.08	0.0776	0.2142	-0.13	0.2190	0.4004	0.07	0.3736	0.4981	-2.55	0.8708	1.0301	-0.33	0.0651	0.1400	2.06
3908	Rho GTPase-activating protein 26	Q9UNA1	0.1016	0.2275	0.11	0.5187	0.8396	0.06	0.0182	0.0646	0.27	0.0099	0.0400	0.25	0.0774	0.2143	0.13	0.0219	0.0952	0.24	0.3736	0.5913	-2.55	0.8708	1.1974	-0.33	0.0651	0.1878	2.06
3909	Rho GTPase-activating protein 28	Q9P2N2	0.0034	0.0379	-2.89	0.2251	0.6606	-1.87	0.0014	0.0225	-3.12	0.0006	0.0133	-2.72	0.3042	0.4020	0.21	0.0006	0.0398	-2.63	0.3736	0.6516	-2.55	0.8708	1.3022	-0.33	0.0651	0.2259	2.06
3910	Rho GTPase-activating protein 29	Q52LW3	0.0109	0.0599	-0.07	0.1838	0.6649	-0.07	0.1525	0.2833	0.04	0.0030	0.0222	0.09	0.1361	0.3057	-0.04	0.7504	0.9886	-0.03	0.0286	0.1455	0.55	0.0675	0.2505	0.25	0.0129	0.0987	0.29
3911	Rho GTPase-activating protein 35	Q9NRY4	0.0062	0.0474	0.27	0.3200	0.6830	0.13	0.0511	0.1272	0.21	0.0090	0.0382	0.31	0.3528	0.4505	0.04	0.0087	0.0679	0.39	0.0729	0.2137	0.37	0.3265	0.6809	-0.12	0.1280	0.1876	-0.19
3912	Rho GTPase-activating protein 40	Q5TG30	0.0256	0.0968	0.21	0.1077	0.6311	0.27	0.0380	0.1028	0.30	0.0013	0.0163	0.25	0.0170	0.0936	0.14	0.0537	0.1572	0.03	0.3736	0.5587	-2.55	0.8708	1.1397	-0.33	0.0651	0.1697	2.06
3913	Rho GTPase-activating protein 44	Q17R89	0.0064	0.0478	-0.88	0.0538	0.5855	-0.51	0.0103	0.0474	-0.86	0.0157	0.0518	-0.69	0.0412	0.1499	-0.53	0.0640	0.1763	-0.90	0.3736	0.5525	-2.55	0.8708	1.1285	-0.33	0.0651	0.1664	2.06
3914	Rho GTPase-activating protein 45	Q92619	0.2943	0.4924	0.08	0.2712	0.6642	0.14	0.7400	0.7828	0.02	0.0086	0.0374	0.28	0.8922	0.9170	0.01	0.0350	0.1206	0.19	0.0962	0.2494	0.27	0.4448	0.8461	-0.07	0.1292	0.1888	-0.09
3915	Rho GTPase-activating protein 5	Q13017	0.4108	0.5180	-3.31	0.5752	0.7253	-1.75	0.5661	0.6585	-1.19	0.5388	0.6230	0.89	0.2938	0.4181	-1.88	0.9510	1.0076	0.09	0.0196	0.1293	0.80	0.0077	0.0724	1.36	0.0704	0.1184	0.50
3916	Rho guanine nucleotide exchange factor 1	Q92888	0.0008	0.0203	0.11	0.4557	0.7879	0.05	0.0687	0.1551	0.05	0.4537	0.6507	-0.01	0.0059	0.0562	-0.08	0.0930	0.2241	0.14	0.7223	0.7620	-0.02	0.2754	0.6115	-0.11	0.0053	0.0697	-0.16
3917	Rho guanine nucleotide exchange factor 11	O15085	0.9558	0.9668	0.00	0.5915	0.6960	-0.04	0.2064	0.3595	-0.11	0.1168	0.2183	-0.15	0.3336	0.4305	-0.09	0.4253	0.6451	-0.07	0.2739	0.5184	0.14	0.0727	0.2612	0.31	0.0689	0.1164	0.30
3918	Rho guanine nucleotide exchange factor 16	Q5VV41	0.0555	0.1538	-0.39	0.7857	0.8458	-0.12	0.2880	0.4669	-0.53	0.4133	0.6056	-0.35	0.8298	0.8682	0.03	0.0692	0.1848	-1.19	0.0399	0.1640	-0.13	0.0053	0.0584	-0.25	0.0030	0.0549	-0.34
3919	Rho guanine nucleotide exchange factor 17	Q96PE2	0.3444	0.5528	-0.14	0.2334	0.6621	0.20	0.6684	0.7189	0.07	0.0975	0.1904	0.21	0.1317	0.2999	0.17	0.2945	0.4918	-0.12	0.0037	0.0656	1.79	0.0004	0.0209	2.70	0.0070	0.0773	2.12
3920	Rho guanine nucleotide exchange factor 18	Q6ZSZ5	0.1248	0.2630	-0.78	0.8810	0.9144	-0.07	0.0320	0.0917	-7.70	0.0243	0.0692	-1.74	0.6248	0.6962	-0.29	0.3174	0.5196	-1.04	0.3736	0.4790	-2.55	0.8708	0.9949	-0.33	0.0651	0.1316	2.06
3921	Rho guanine nucleotide exchange factor 2	Q92974	0.4108	0.5179	-3.31	0.5752	0.7251	-1.75	0.5661	0.6584	-1.19	0.5388	0.6228	0.89	0.2938	0.4180	-1.88	0.9510	1.0074	0.09	0.0000	0.0000	-0.42	0.0014	0.0310	-0.56	0.0197	0.1216	-0.33
3922	Rho guanine nucleotide exchange factor 28	Q8N1W1	0.1000	0.2250	0.23	0.2232	0.6584	0.14	0.0814	0.1752	0.25	0.0394	0.0975	0.38	0.0763	0.2126	0.24	0.0295	0.1101	0.37	0.0335	0.1525	0.53	0.0324	0.1623	0.23	0.0315	0.1507	0.19
3923	Rho guanine nucleotide exchange factor 4	Q9NR80	0.4108	0.5177	-3.31	0.5752	0.7249	-1.75	0.5661	0.6582	-1.19	0.5388	0.6227	0.89	0.2938	0.4179	-1.88	0.9510	1.0072	0.09	0.0878	0.2379	0.23	0.4025	0.7880	0.10	0.9566	0.9659	-0.01
3924	Rho guanine nucleotide exchange factor 7	Q14155	0.4108	0.5176	-3.31	0.5752	0.7248	-1.75	0.5661	0.6581	-1.19	0.5388	0.6226	0.89	0.2938	0.4178	-1.88	0.9510	1.0070	0.09	0.0233	0.1362	0.44	0.7732	1.2170	0.04	0.1256	0.1851	0.23
3925	Rho guanine nucleotide exchange factor 9	O43307	0.5336	0.6043	0.05	0.1508	0.6586	0.12	0.4404	0.6520	0.04	0.0823	0.1674	0.10	0.0780	0.2148	0.12	0.0330	0.1168	0.08	0.3736	0.5773	-2.55	0.8708	1.1728	-0.33	0.0651	0.1798	2.06
3926	Rho-associated protein kinase 1	Q13464	0.0031	0.0362	-2.34	0.0467	0.5639	-11.20	0.0029	0.0297	-5.05	0.0050	0.0285	-3.44	0.1051	0.2610	-0.47	0.0036	0.0592	-2.54	0.1836	0.3847	0.24	0.0673	0.2499	-0.28	0.5453	0.6102	-0.06
3927	Rho-associated protein kinase 2	O75116	0.0221	0.0885	0.28	0.0571	0.5822	0.23	0.3380	0.5304	0.14	0.3546	0.5324	0.09	0.1239	0.2901	0.13	0.0277	0.1067	-0.27	0.3736	0.5827	-2.55	0.8708	1.1824	-0.33	0.0651	0.1829	2.06
3928	Rhophilin-2	Q8IUC4	0.0108	0.0598	0.25	0.0224	0.4775	0.20	0.0287	0.0859	0.38	0.0278	0.0760	0.36	0.3753	0.4724	0.06	0.0115	0.0731	0.40	0.3736	0.6099	-2.55	0.8708	1.2301	-0.33	0.0651	0.1988	2.06
3929	Rho-related GTP-binding protein RhoB	P62745	0.0633	0.1665	-10.63	0.0502	0.5740	-9.08	0.0132	0.0538	-8.52	0.0201	0.0606	-6.44	0.0052	0.0538	-9.20	0.0168	0.0852	-7.24	0.3736	0.5990	-2.55	0.8708	1.2109	-0.33	0.0651	0.1923	2.06
3930	Rho-related GTP-binding protein RhoC	P08134	0.2015	0.3721	0.06	0.7013	0.7794	0.05	0.2019	0.3537	-0.06	0.2411	0.3875	0.05	0.0713	0.2034	-0.11	0.1241	0.2716	0.09	0.0829	0.2303	-0.23	0.0077	0.0721	-0.28	0.0043	0.0646	-0.35
3931	Rho-related GTP-binding protein RhoG	P84095	0.0761	0.1882	0.06	0.5102	0.8321	0.09	0.1609	0.2959	0.06	0.0246	0.0698	0.10	0.1881	0.3835	-0.04	0.2144	0.3959	0.15	0.1825	0.3838	-0.12	0.8295	1.2731	-0.02	0.0537	0.2039	0.43
3932	Rho-related GTP-binding protein RhoJ	Q9H4E5	0.4108	0.5175	-3.31	0.5752	0.7246	-1.75	0.5661	0.6579	-1.19	0.5388	0.6224	0.89	0.2938	0.4176	-1.88	0.9510	1.0068	0.09	0.3335	0.6028	-0.25	0.9495	0.9633	-0.01	0.8995	0.9197	-0.03
3933	Rhotekin	Q9BST9	0.7068	0.7591	0.01	0.4737	0.8011	-0.25	0.0009	0.0204	0.31	0.0219	0.0644	0.12	0.6158	0.6894	0.02	0.0258	0.1022	0.12	0.3736	0.5852	-2.55	0.8708	1.1867	-0.33	0.0651	0.1843	2.06
3934	Ribonuclease inhibitor	P13489	0.0078	0.0522	0.26	0.4460	0.7818	0.08	0.0829	0.1774	0.12	0.0628	0.1366	0.12	0.9210	0.9410	0.00	0.0544	0.1582	0.15	0.4869	0.5412	-0.07	0.0040	0.0509	-0.34	0.0056	0.0720	-0.33
3935	Ribonuclease P protein subunit p20	O75817	0.4728	0.5485	-0.07	0.1985	0.6642	-0.15	0.9426	0.9555	-0.01	0.4436	0.6379	-0.08	0.7226	0.7824	-0.04	0.7272	0.9669	-0.03	0.3736	0.4438	-2.55	0.8708	0.9291	-0.33	0.0651	0.1170	2.06
3936	Ribonuclease P protein subunit p40	O75818	0.0028	0.0354	0.29	0.1855	0.6668	0.19	0.0508	0.1268	0.54	0.0055	0.0296	0.32	0.0110	0.0751	0.23	0.0028	0.0558	0.48	0.3736	0.6402	-2.55	0.8708	1.2826	-0.33	0.0651	0.2181	2.06
3937	Ribonuclease T2	O00584	0.3770	0.5925	0.11	0.8568	0.8968	-0.03	0.0426	0.1119	-0.35	0.4342	0.6274	-0.12	0.8139	0.8552	0.03	0.0364	0.1235	0.43	0.3736	0.5726	-2.55	0.8708	1.1644	-0.33	0.0651	0.1772	2.06
3938	Ribonucleases P/MRP protein subunit POP1	Q99575	0.0412	0.1285	-0.20	0.3791	0.7257	0.11	0.3026	0.4854	0.07	0.6200	0.6667	-0.03	0.7032	0.7641	0.02	0.0247	0.0999	-0.25	0.3736	0.5868	-2.55	0.8708	1.1895	-0.33	0.0651	0.1852	2.06
3939	Ribonucleoprotein PTB-binding 1	Q8IY67	0.0034	0.0378	0.28	0.0995	0.6355	0.35	0.0018	0.0241	0.49	0.0373	0.0936	0.42	0.8485	0.8836	0.00	0.0029	0.0540	0.28	0.0714	0.2115	0.34	0.9495	0.9634	-0.01	0.2816	0.3534	0.05
3940	Ribonucleoside-diphosphate reductase large subunit	P23921	0.1841	0.3487	0.04	0.0507	0.5722	0.24	0.0035	0.0316	0.42	0.0084	0.0372	0.31	0.0848	0.2267	-0.06	0.0596	0.1687	0.08	0.0490	0.1768	0.12	0.2906	0.6301	0.06	0.1102	0.1672	0.09
3941	Ribonucleoside-diphosphate reductase subunit M2	P31350	0.0017	0.0281	0.35	0.1479	0.6576	0.26	0.0244	0.0773	0.21	0.0006	0.0124	0.36	0.0206	0.1028	0.15	0.0060	0.0622	0.32	0.1364	0.3154	0.21	0.2010	0.4994	-0.12	0.0239	0.1318	-0.33

Supplemetary Table S2. Overview on all relatively quantified 5180 proteins statistical analysis

			MCF-7																		MDA-MB-231								
			Dai SC20 vs control			Gen SC20 vs control			SSE SC20 vs control			Dai IC20 vs control			Gen IC20 vs control			SSE IC20 vs control			Dai IC20 vs control			Gen IC20 vs control			SSE IC20 vs control		
	**Protein name**	**UniProt**	**p value**	**BH q value**	**log2FC**	**p value**	**BH q value**	**log2FC**	**p value**	**BH q value**	**log2FC**	**p value**	**BH q value**	**log2FC**	**p value**	**BH q value**	**log2FC**	**p value**	**BH q value**	**log2FC**	**p value**	**BH q value**	**log2FC**	**p value**	**BH q value**	**log2FC**	**p value**	**BH q value**	**log2FC**
3942	Ribonucleoside-diphosphate reductase subunit M2 B	Q7LG56	0.3693	0.5834	0.06	0.1594	0.6643	0.40	0.0658	0.1504	0.20	0.1649	0.2850	0.47	0.0000	0.0000	0.36	0.0501	0.1504	0.22	0.2989	0.5563	-0.26	0.2205	0.5276	0.31	0.5250	0.5916	0.14
3943	Ribose-5-phosphate isomerase	P49247	0.0944	0.2166	-3.22	0.2051	0.6595	-1.75	0.0233	0.0752	-3.47	0.0039	0.0253	-4.53	0.8887	0.9147	-0.01	0.1016	0.2361	-3.35	0.3736	0.5336	-2.55	0.8708	1.0946	-0.33	0.0651	0.1568	2.06
3944	Ribose-phosphate pyrophosphokinase 1	P60891	0.4491	0.5267	-0.04	0.0422	0.5678	-0.30	0.0057	0.0366	-0.28	0.4260	0.6178	-0.04	0.5145	0.6032	0.03	0.7710	1.0085	0.02	0.0055	0.0787	-0.55	0.0030	0.0456	-0.50	0.0048	0.0677	-0.49
3945	Ribose-phosphate pyrophosphokinase 2	P11908	0.0293	0.1047	0.10	0.4133	0.7525	0.09	0.3391	0.5315	0.04	0.2885	0.4499	0.02	0.0102	0.0719	-0.09	0.3502	0.5553	0.03	0.1019	0.2587	1.19	0.0024	0.0406	2.84	0.0006	0.0317	2.91
3946	Ribose-phosphate pyrophosphokinase 3	P21108	0.4462	0.5236	-0.04	0.3169	0.6806	0.12	0.0235	0.0753	-0.25	0.0038	0.0250	-0.39	0.0114	0.0765	-0.35	0.0167	0.0849	-0.28	0.2962	0.5521	-5.13	0.3373	0.6950	1.11	0.2523	0.3236	0.87
3947	Ribosomal biogenesis protein LAS1L	Q9Y4W2	0.0082	0.0535	0.21	0.7474	0.8170	-0.03	0.1883	0.3342	0.13	0.3277	0.4996	0.07	0.6429	0.7117	0.01	0.0054	0.0613	0.19	0.3736	0.6279	-2.55	0.8708	1.2614	-0.33	0.0651	0.2101	2.06
3948	Ribosomal oxygenase 1	Q9H6W3	0.0017	0.0280	-0.55	0.0310	0.5082	-0.30	0.0092	0.0450	-0.29	0.0012	0.0160	-0.93	0.0890	0.2343	-0.13	0.0856	0.2129	0.12	0.3736	0.5418	-2.55	0.8708	1.1094	-0.33	0.0651	0.1610	2.06
3949	Ribosomal protein S6 kinase alpha-1	Q15418	0.3629	0.5756	0.20	0.9071	0.9355	-0.08	0.9425	0.9556	-0.01	0.0808	0.1656	-0.41	0.1873	0.3827	-0.23	0.8071	1.0444	-0.05	0.0734	0.2148	0.14	0.7865	1.2305	0.02	0.0490	0.1936	0.18
3950	Ribosomal protein S6 kinase alpha-2	Q15349	0.7010	0.7546	-0.03	0.8899	0.9216	0.02	0.7901	0.8280	-0.02	0.5595	0.6100	0.09	0.2578	0.4780	-0.11	0.6241	0.8593	0.04	0.0005	0.0375	-0.42	0.0010	0.0278	-0.34	0.0038	0.0608	-0.56
3951	Ribosomal protein S6 kinase alpha-3	P51812	0.0001	0.0096	1.14	0.1633	0.6634	0.18	0.0067	0.0395	0.29	0.0013	0.0165	0.30	0.0180	0.0958	0.11	0.0015	0.0508	0.28	0.3736	0.6464	-2.55	0.8708	1.2932	-0.33	0.0651	0.2223	2.06
3952	Ribosomal protein S6 kinase beta-1	P23443	0.0050	0.0433	0.15	0.9581	0.9716	0.01	0.0911	0.1899	0.09	0.5596	0.6099	0.02	0.0009	0.0224	-0.16	0.1289	0.2793	0.15	0.3736	0.5249	-2.55	0.8708	1.0789	-0.33	0.0651	0.1526	2.06
3953	Ribosomal protein S6 kinase delta-1	Q96S38	0.0154	0.0717	0.18	0.3860	0.7305	0.16	0.0097	0.0458	0.31	0.0080	0.0362	0.22	0.0243	0.1127	0.16	0.0111	0.0723	0.29	0.1656	0.3604	-0.25	0.2692	0.6005	-0.15	0.6288	0.6863	-0.04
3954	Ribosomal RNA processing protein 1 homolog A	P56182	0.5441	0.6140	-0.37	0.2907	0.6690	-2.19	0.6140	0.6687	-0.19	0.0581	0.1284	-0.81	0.6078	0.6822	0.16	0.9150	1.1518	-0.03	0.0061	0.0829	-0.88	0.1169	0.3521	0.42	0.0536	0.2039	-0.36
3955	Ribosomal RNA processing protein 36 homolog	Q96EU6	0.5446	0.6143	0.19	0.9648	0.9757	0.01	0.5276	0.7488	-0.14	0.0089	0.0380	-0.26	0.0251	0.1142	0.38	0.2858	0.4822	-0.26	0.3736	0.4844	-2.55	0.8708	1.0048	-0.33	0.0651	0.1339	2.06
3956	Ribosome biogenesis protein BMS1 homolog	Q14692	0.5056	0.5785	-0.07	0.3451	0.7024	-0.23	0.1449	0.2716	0.20	0.6276	0.6739	0.05	0.3967	0.4929	0.10	0.2405	0.4263	0.14	0.2705	0.5136	0.15	0.4553	0.8595	0.02	0.0025	0.0537	0.41
3957	Ribosome biogenesis protein BOP1	Q14137	0.0374	0.1215	-0.93	0.3060	0.6783	-0.68	0.0008	0.0204	-3.30	0.0168	0.0541	-2.20	0.7013	0.7627	-0.11	0.0275	0.1064	-1.22	0.2820	0.5314	0.88	0.0956	0.3085	1.63	0.0780	0.1286	1.98
3958	Ribosome biogenesis protein WDR12	Q9GZL7	0.1054	0.2329	0.08	0.5211	0.8406	0.08	0.3476	0.5414	-0.06	0.4781	0.6783	-0.03	0.0090	0.0671	-0.22	0.1173	0.2611	-0.05	0.0167	0.1201	-1.42	0.0124	0.0931	-1.62	0.0050	0.0683	-1.28
3959	Ribosome maturation protein SBDS	Q9Y3A5	0.4108	0.5174	-3.31	0.5752	0.7244	-1.75	0.5661	0.6578	-1.19	0.5388	0.6223	0.89	0.2938	0.4175	-1.88	0.9510	1.0066	0.09	0.2536	0.4909	0.15	0.2136	0.5180	-0.12	0.0705	0.1185	-0.21
3960	Ribosome-binding protein 1	Q9P2E9	0.0023	0.0328	0.26	0.0933	0.6301	0.29	0.0004	0.0218	0.42	0.0002	0.0120	0.55	0.0020	0.0358	0.37	0.0020	0.0531	0.56	0.0299	0.1474	0.47	0.8592	1.3010	0.02	0.1409	0.2023	0.13
3961	Ribosome-recycling factor mitochondrial	Q96E11	0.0056	0.0452	0.38	0.1225	0.6384	0.62	0.0686	0.1550	0.28	0.0298	0.0800	0.32	0.9718	0.9778	0.00	0.2336	0.4177	0.27	0.1174	0.2851	-0.31	0.0105	0.0842	-0.23	0.2003	0.2687	-0.11
3962	Ribosyldihydronicotinamide dehydrogenase [quinone]	P16083	0.0904	0.2106	0.51	0.2658	0.6629	-0.28	0.0002	0.0164	1.05	0.0053	0.0295	0.99	0.0003	0.0147	0.77	0.0227	0.0965	1.19	0.3736	0.5897	-2.55	0.8708	1.1946	-0.33	0.0651	0.1868	2.06
3963	RILP-like protein 2	Q969X0	0.2504	0.4367	-0.40	0.1149	0.6332	-0.20	0.2037	0.3561	-0.14	0.1050	0.2011	-0.19	0.0365	0.1388	-0.29	0.1188	0.2633	-0.37	0.3736	0.5277	-2.55	0.8708	1.0841	-0.33	0.0651	0.1540	2.06
3964	RIMS-binding protein 3C	A6NJZ7	0.0173	0.0769	-1.06	0.2202	0.6601	-0.78	0.0703	0.1576	-0.51	0.0647	0.1396	-0.53	0.3527	0.4504	-0.21	0.0910	0.2202	-0.46	0.3736	0.5370	-2.55	0.8708	1.1007	-0.33	0.0651	0.1585	2.06
3965	RING finger and CHY zinc finger domain-containing protein 1	Q96PM5	0.6567	0.7174	-0.04	0.7524	0.8205	-0.04	0.6800	0.7299	-0.03	0.2045	0.3382	-0.10	0.6748	0.7385	0.04	0.7617	0.9996	-0.04	0.5561	0.6067	0.12	0.3879	0.7687	0.18	0.4680	0.5365	-0.30
3966	RING finger protein 122	Q9H9V4	0.6180	0.6818	0.04	0.4724	0.8013	0.06	0.0494	0.1239	-0.23	0.1306	0.2373	-0.14	0.0144	0.0852	0.32	0.0369	0.1245	-0.29	0.3736	0.5719	-2.55	0.8708	1.1632	-0.33	0.0651	0.1768	2.06
3967	RING finger protein 212B	A8MTL3	0.9265	0.9440	0.00	0.2761	0.6643	0.15	0.0504	0.1259	0.15	0.2540	0.4040	0.07	0.0304	0.1271	-0.17	0.0692	0.1850	0.14	0.3736	0.5496	-2.55	0.8708	1.1235	-0.33	0.0651	0.1650	2.06
3968	RING-box protein 2	Q9UBF6	0.2494	0.4357	-0.21	0.8864	0.9190	-0.02	0.5209	0.7419	-0.10	0.1471	0.2610	-0.29	0.1968	0.3964	0.25	0.8591	1.0947	0.03	0.3736	0.4356	-2.55	0.8708	0.9137	-0.33	0.0651	0.1137	2.06
3969	RNA binding motif protein X-linked-like-1	Q96E39	0.4108	0.5172	-3.31	0.5752	0.7242	-1.75	0.5661	0.6576	-1.19	0.5388	0.6222	0.89	0.2938	0.4174	-1.88	0.9510	1.0064	0.09	0.1778	0.3770	-0.12	0.0503	0.2110	-0.25	0.0878	0.1405	-0.11
3970	RNA binding protein fox-1 homolog 1	Q9NWB1	0.1918	0.3584	-0.26	0.1176	0.6313	-0.72	0.2922	0.4724	0.18	0.4974	0.7011	-0.15	0.0509	0.1669	0.46	0.1169	0.2606	0.34	0.3736	0.5286	-2.55	0.8708	1.0856	-0.33	0.0651	0.1544	2.06
3971	RNA polymerase II subunit A C-terminal domain phosphatase SSU72	Q9NP77	0.4108	0.5171	-3.31	0.5752	0.7241	-1.75	0.5661	0.6575	-1.19	0.5388	0.6220	0.89	0.2938	0.4173	-1.88	0.9510	1.0062	0.09	0.0009	0.0405	-0.80	0.0008	0.0259	-1.08	0.0124	0.0963	-1.17
3972	RNA polymerase II-associated factor 1 homolog	Q8N7H5	0.5228	0.5944	-0.18	0.7497	0.8186	0.16	0.7101	0.7559	-0.07	0.8204	0.8445	-0.11	0.1684	0.3549	-0.20	0.5992	0.8355	0.07	0.3736	0.4526	-2.55	0.8708	0.9456	-0.33	0.0651	0.1205	2.06
3973	RNA polymerase II-associated protein 3	Q9H6T3	0.0233	0.0914	0.26	0.2704	0.6638	0.14	0.0083	0.0430	0.39	0.0126	0.0455	0.30	0.1181	0.2820	0.13	0.0727	0.1910	0.20	0.0470	0.1739	0.41	0.8297	1.2731	0.02	0.8452	0.8751	0.01
3974	RNA polymerase-associated protein CTR9 homolog	Q6PD62	0.0348	0.1167	-0.49	0.5689	0.8826	-0.23	0.0089	0.0439	0.69	0.2947	0.4579	0.16	0.0893	0.2347	0.23	0.2687	0.4618	0.26	0.0777	0.2216	0.39	0.9536	0.9669	0.01	0.0209	0.1234	0.28
3975	RNA polymerase-associated protein RTF1 homolog	Q92541	0.0661	0.1703	0.16	0.0617	0.5919	0.17	0.2615	0.4311	0.08	0.1059	0.2026	0.13	0.1249	0.2918	0.13	0.5693	0.8040	0.08	0.2820	0.5312	-0.30	0.1696	0.4501	0.21	0.0412	0.1761	0.31
3976	RNA transcription_ translation and transport factor protein	Q9Y224	0.7975	0.8361	0.04	0.3476	0.7036	-0.15	0.0511	0.1271	0.19	0.0120	0.0439	0.34	0.0447	0.1561	0.17	0.0443	0.1388	0.40	0.1580	0.3499	0.07	0.0550	0.2240	-0.14	0.0117	0.0937	-0.22
3977	RNA-binding motif protein X chromosome	P38159	0.0129	0.0663	-0.25	0.3268	0.6862	-0.08	0.8515	0.8813	0.01	0.4150	0.6071	-0.03	0.1446	0.3190	0.08	0.1690	0.3339	-0.06	0.0539	0.1843	0.41	0.0219	0.1283	0.36	0.0403	0.1738	0.39
3978	RNA-binding motif protein X-linked-like-2	O75526	0.0356	0.1179	0.20	0.3126	0.6807	0.07	0.0003	0.0194	-0.67	0.0015	0.0179	-0.45	0.0502	0.1653	-0.27	0.0018	0.0527	-0.56	0.0543	0.1852	0.41	0.8834	0.9119	0.02	0.2843	0.3563	0.15
3979	RNA-binding motif protein X-linked-like-3	Q8N7X1	0.4108	0.5170	-3.31	0.5752	0.7239	-1.75	0.5661	0.6573	-1.19	0.5388	0.6219	0.89	0.2938	0.4172	-1.88	0.9510	1.0060	0.09	0.0002	0.0288	0.75	0.0009	0.0274	0.73	0.0018	0.0499	0.79
3980	RNA-binding protein 12	Q9NTZ6	0.2160	0.3911	-0.06	0.5765	0.6857	0.07	0.1760	0.3183	-0.07	0.2914	0.4534	-0.05	0.0434	0.1540	-0.13	0.0557	0.1610	-0.13	0.0292	0.1463	0.18	0.3520	0.7156	0.10	0.1836	0.2510	-0.08
3981	RNA-binding protein 15	Q96T37	0.3533	0.5631	-1.52	0.3711	0.7183	1.83	0.0188	0.0656	1.71	0.2387	0.3846	2.56	0.4226	0.5171	-0.49	0.7147	0.9551	-0.64	0.0069	0.0859	0.84	0.2524	0.5760	-0.22	0.0217	0.1255	0.78
3982	RNA-binding protein 25	P49756	0.0076	0.0514	0.23	0.9042	0.9330	-0.01	0.1326	0.2548	0.08	0.9179	0.9285	-0.01	0.2319	0.4421	-0.10	0.2026	0.3804	0.19	0.0111	0.1032	0.69	0.0078	0.0723	0.71	0.0006	0.0305	0.56
3983	RNA-binding protein 3	P98179	0.0007	0.0198	0.93	0.1400	0.6539	-0.31	0.0099	0.0464	-0.33	0.0182	0.0570	-0.27	0.0124	0.0794	-0.31	0.0220	0.0953	-0.29	0.1293	0.3032	0.23	0.1335	0.3850	-0.09	0.4247	0.4963	-0.07
3984	RNA-binding protein 39	Q14498	0.6606	0.7207	-0.03	0.6676	0.7557	-0.03	0.0077	0.0414	0.20	0.4704	0.6698	0.09	0.0063	0.0578	-0.09	0.6848	0.9242	0.02	0.0001	0.0235	0.45	0.0001	0.0179	0.43	0.0601	0.2191	0.35
3985	RNA-binding protein 4	Q9BWF3	0.0643	0.1680	-0.11	0.5847	0.6913	-0.05	0.9891	0.9904	0.00	0.0125	0.0451	-0.20	0.0004	0.0163	-0.35	0.0115	0.0735	-0.21	0.5026	0.5562	0.04	0.6864	1.1280	-0.02	0.1585	0.2223	-0.09
3986	RNA-binding protein 47	A0AV96	0.4108	0.5169	-3.31	0.5752	0.7237	-1.75	0.5661	0.6572	-1.19	0.5388	0.6217	0.89	0.2938	0.4171	-1.88	0.9510	1.0058	0.09	0.0255	0.1395	-2.33	0.2303	0.5440	-0.90	0.1008	0.1565	-1.40
3987	RNA-binding protein 4B	Q9BQ04	0.1469	0.2951	0.21	0.0563	0.5844	0.17	0.9279	0.9443	0.01	0.0112	0.0424	-0.48	0.6955	0.7578	-0.03	0.0740	0.1934	-0.17	0.8309	0.8576	-0.03	0.1725	0.4545	0.29	0.4135	0.4850	-0.23
3988	RNA-binding protein 8A	Q9Y5S9	0.0482	0.1411	-0.24	0.3865	0.7296	-0.10	0.1117	0.2231	0.17	0.1512	0.2669	0.15	0.8609	0.8935	-0.01	0.1267	0.2754	-0.16	0.0415	0.1674	0.38	0.6690	1.1075	0.05	0.6154	0.6745	0.04
3989	RNA-binding protein EWS	Q01844	0.0153	0.0715	0.17	0.2439	0.6584	0.22	0.0076	0.0413	0.20	0.0142	0.0485	0.16	0.2688	0.4906	-0.04	0.0096	0.0699	0.26	0.1676	0.3625	0.20	0.3961	0.7804	-0.07	0.3311	0.4022	-0.06
3990	RNA-binding protein FUS	P35637	0.4740	0.5497	0.02	0.6100	0.7118	0.08	0.0006	0.0187	0.25	0.0104	0.0411	0.13	0.0174	0.0949	-0.12	0.0017	0.0518	0.27	0.0085	0.0937	0.53	0.0275	0.1504	0.25	0.0224	0.1268	0.28
3991	RNA-binding protein NOB1	Q9ULX3	0.2592	0.4481	0.13	0.2315	0.6622	-0.66	0.0805	0.1735	-0.33	0.1767	0.3007	-0.10	0.0187	0.0976	0.24	0.2322	0.4162	-0.32	0.1510	0.3373	1.21	0.0300	0.1565	2.13	0.0265	0.1387	2.59
3992	RNA-binding protein with multiple splicing	Q93062	0.0199	0.0828	0.19	0.0946	0.6307	0.15	0.1104	0.2211	0.12	0.2051	0.3389	0.19	0.0105	0.0730	0.33	0.1376	0.2907	0.09	0.3736	0.5213	-2.55	0.8708	1.0725	-0.33	0.0651	0.1509	2.06
3993	Rod cGMP-specific 3'_5'-cyclic phosphodiesterase subunit beta	P35913	0.4108	0.5167	-3.31	0.5752	0.7235	-1.75	0.5661	0.6570	-1.19	0.5388	0.6216	0.89	0.2938	0.4170	-1.88	0.9510	1.0055	0.09	0.0176	0.1227	0.36	0.8566	1.2986	0.02	0.1159	0.1736	0.37
3994	Rootletin	Q5TZA2	0.0662	0.1704	0.12	0.2425	0.6597	0.10	0.0053	0.0355	0.30	0.0335	0.0869	0.16	0.0458	0.1575	0.14	0.0226	0.0964	0.26	0.0445	0.1699	0.36	0.8881	0.9155	0.01	0.2528	0.3241	0.06
3995	Roquin-1	Q5TC82	0.0486	0.1414	-0.86	0.0602	0.5951	-0.91	0.2180	0.3743	-0.70	0.0237	0.0681	-1.57	0.2961	0.3942	-0.17	0.0219	0.0950	-0.55	0.3736	0.5909	-2.55	0.8708	1.1968	-0.33	0.0651	0.1876	2.06
3996	rRNA 2'-O-methyltransferase fibrillarin	P22087	0.0048	0.0430	-0.57	0.0067	0.4232	-0.63	0.0014	0.0234	-0.55	0.0180	0.0566	-0.30	0.0001	0.0088	-0.83	0.0719	0.1893	-0.69	0.3736	0.5485	-2.55	0.8708	1.1215	-0.33	0.0651	0.1644	2.06
3997	rRNA methyltransferase 1 mitochondrial	Q6IN84	0.7025	0.7554	-0.03	0.1806	0.6640	0.16	0.1696	0.3092	0.16	0.1377	0.2477	0.18	0.0138	0.0840	0.45	0.8704	1.1062	0.02	0.3736	0.4351	-2.55	0.8708	0.9127	-0.33	0.0651	0.1135	2.06
3998	rRNA-processing protein UTP23 homolog	Q9BRU9	0.1113	0.2423	0.20	0.1894	0.6697	0.29	0.0911	0.1901	0.22	0.0092	0.0384	0.53	0.1300	0.2980	0.19	0.0174	0.0862	0.56	0.3736	0.5977	-2.55	0.8708	1.2087	-0.33	0.0651	0.1915	2.06
3999	RUN and FYVE domain-containing protein 1	Q96T51	0.0252	0.0958	0.17	0.1138	0.6339	0.35	0.0168	0.0614	0.40	0.0001	0.0074	0.34	0.0041	0.0478	0.51	0.0870	0.2152	0.29	0.1503	0.3365	0.17	0.1029	0.3234	0.13	0.5431	0.6084	0.04
4000	RUN and FYVE domain-containing protein 2	Q8WXA3	0.6159	0.6801	-0.13	0.1154	0.6346	0.56	0.0835	0.1784	0.66	0.0691	0.1465	0.73	0.0272	0.1193	1.08	0.2274	0.4103	0.38	0.0042	0.0695	0.99	0.0242	0.1384	0.55	0.0152	0.1060	0.80
4001	Run domain Beclin-1-interacting and cysteine-rich domain-containing protein	Q92622	0.9381	0.9532	0.01	0.5499	0.8653	-0.28	0.7994	0.8362	-0.04	0.3272	0.4989	0.17	0.0971	0.2481	0.27	0.0884	0.2166	0.30	0.3736	0.5388	-2.55	0.8708	1.1040	-0.33	0.0651	0.1594	2.06
4002	RUN domain-containing protein 3A	Q59EK9	0.0218	0.0877	0.17	0.1626	0.6616	0.23	0.0086	0.0439	0.27	0.0003	0.0109	0.59	0.0057	0.0560	0.39	0.0039	0.0601	0.52	0.0002	0.0357	0.46	0.0035	0.0487	0.23	0.0017	0.0492	0.32
4003	Runt-related transcription factor 2	Q13950	0.0002	0.0122	-0.45	0.0098	0.4492	-0.36	0.0001	0.0288	-0.58	0.0002	0.0123	-0.56	0.0005	0.0196	-0.40	0.0616	0.1726	-0.47	0.3736	0.5553	-2.55	0.8708	1.1336	-0.33	0.0651	0.1679	2.06
4004	RUS1 family protein C16orf58	Q96GQ5	0.0062	0.0473	-0.30	0.0454	0.5613	-0.26	0.0131	0.0536	-0.28	0.0015	0.0173	-0.57	0.9809	0.9858	0.00	0.1558	0.3148	-0.21	0.3736	0.5137	-2.55	0.8708	1.0586	-0.33	0.0651	0.1473	2.06
4005	RuvB-like 1	Q9Y265	0.0012	0.0238	0.17	0.0641	0.5929	0.12	0.0006	0.0207	0.32	0.0003	0.0116	0.25	0.0104	0.0732	0.10	0.0012	0.0482	0.25	0.0181	0.1248	0.40	0.5154	0.9358	0.06	0.4488	0.5180	0.05
4006	RuvB-like 2	Q9Y230	0.0203	0.0841	0.26	0.2406	0.6608	0.28	0.0106	0.0480	0.31	0.0015	0.0173	0.49	0.0269	0.1194	0.21	0.0068	0.0637	0.54	0.0591	0.1929	0.42	0.3598	0.7283	0.10	0.8603	0.8867	0.01
4007	Ryanodine receptor 1	P21817	0.4108	0.5166	-3.31	0.5752	0.7234	-1.75	0.5661	0.6569	-1.19	0.5388	0.6215	0.89	0.2938	0.4168	-1.88	0.9510	1.0053	0.09	0.1494	0.3352	-0.14	0.4681	0.8779	0.04	0.4479	0.5174	-0.04
4008	Ryanodine receptor 2	Q92736	0.0133	0.0672	-0.23	0.4060	0.7487	0.04	0.0103	0.0472	-0.24	0.0130	0.0462	-0.22	0.6205	0.6933	-0.02	0.0873	0.2152	-0.24	0.3394	0.6098	0.06	0.3785	0.7553	0.05	0.0837	0.1354	0.13
4009	Ryanodine receptor 3	Q15413	0.0005	0.0179	0.23	0.0141	0.4537	0.17	0.0053	0.0357	0.21	0.0009	0.0151	0.41	0.0020	0.0356	0.19	0.0705	0.1868	0.29	0.7606	0.7961	0.03	0.0763	0.2696	-0.18	0.0078	0.0792	-0.29
4010	Saccharopine dehydrogenase-like oxidoreductase	Q8NBX0	0.4108	0.5165	-3.31	0.5752	0.7232	-1.75	0.5661	0.6568	-1.19	0.5388	0.6213	0.89	0.2938	0.4167	-1.88	0.9510	1.0051	0.09	0.1470	0.3315	0.20	0.6238	1.0605	0.05	0.7986	0.8362	0.02
4011	Sacsin	Q9NZJ4	0.0181	0.0788	-0.11	0.6779	0.7640	-0.06	0.8166	0.8514	-0.02	0.0224	0.0653	0.11	0.0006	0.0193	0.22	0.0082	0.0671	0.12	0.0859	0.2346	0.20	0.4584	0.8635	0.06	0.2408	0.3111	0.11
4012	S-adenosylhomocysteine hydrolase-like protein 1	O43865	0.0041	0.0401	0.30	0.1996	0.6636	0.26	0.0599	0.1416	0.19	0.0010	0.0150	0.41	0.0060	0.0568	0.25	0.0051	0.0620	0.39	0.3215	0.5866	0.14	0.0349	0.1697	-0.28	0.0069	0.0767	-0.61
4013	S-adenosyl-L-methionine-dependent tRNA 4-demethylwyosine synthase	Q9NV66	0.0175	0.0773	0.15	0.4525	0.7858	0.10	0.0447	0.1157	0.13	0.0351	0.0896	0.13	0.2031	0.4040	-0.05	0.1508	0.3084	0.09	0.3736	0.5162	-2.55	0.8708	1.0631	-0.33	0.0651	0.1484	2.06
4014	S-adenosyl-L-methionine-dependent tRNA 4-demethylwyosine synthase	Q6NUM6	0.4108	0.5164	-3.31	0.5752	0.7230	-1.75	0.5661	0.6566	-1.19	0.5388	0.6212	0.89	0.2938	0.4166	-1.88	0.9510	1.0049	0.09	0.0003	0.0331	-1.91	0.0002	0.0199	-2.09	0.0021	0.0518	-2.69

Supplemetary Table S2. Overview on all relatively quantified 5180 proteins statistical analysis

			MCF-7																		MDA-MB-231								
			Dai SC20 vs control			Gen SC20 vs control			SSE SC20 vs control			Dai IC20 vs control			Gen IC20 vs control			SSE IC20 vs control			Dai IC20 vs control			Gen IC20 vs control			SSE IC20 vs control		
	**Protein name**	**UniProt**	**p value**	**BH q value**	**log2FC**	**p value**	**BH q value**	**log2FC**	**p value**	**BH q value**	**log2FC**	**p value**	**BH q value**	**log2FC**	**p value**	**BH q value**	**log2FC**	**p value**	**BH q value**	**log2FC**	**p value**	**BH q value**	**log2FC**	**p value**	**BH q value**	**log2FC**	**p value**	**BH q value**	**log2FC**
4015	S-adenosylmethionine synthase isoform type-1	Q00266	0.3832	0.5993	0.71	0.3140	0.6800	-1.95	0.0229	0.0744	-8.50	0.7364	0.7726	0.21	0.2867	0.5162	-0.81	0.5728	0.8076	-0.76	0.0399	0.1636	-0.18	0.0339	0.1666	0.17	0.2742	0.3461	-0.09
4016	S-adenosylmethionine synthase isoform type-2	P31153	0.0093	0.0571	-0.07	0.7802	0.8416	-0.04	0.7527	0.7944	-0.01	0.0097	0.0394	-0.09	0.0000	0.0000	-0.30	0.0097	0.0706	-0.08	0.4473	0.5026	-0.08	0.2840	0.6213	-0.07	0.0452	0.1858	-0.15
4017	SAM domain-containing protein SAMSN-1	Q9NSI8	0.4108	0.5162	-3.31	0.5752	0.7228	-1.75	0.5661	0.6565	-1.19	0.5388	0.6210	0.89	0.2938	0.4165	-1.88	0.9510	1.0047	0.09	0.0008	0.0395	0.89	0.0058	0.0614	0.73	0.0064	0.0755	0.71
4018	SAM pointed domain-containing Ets transcription factor	O95238	0.3342	0.5406	-0.11	0.7096	0.7869	-0.04	0.6692	0.7195	-0.05	0.3063	0.4729	-0.11	0.0997	0.2527	0.20	0.7765	1.0142	-0.03	0.3736	0.4403	-2.55	0.8708	0.9226	-0.33	0.0651	0.1156	2.06
4019	SAP domain-containing ribonucleoprotein	P82979	0.4108	0.5161	-3.31	0.5752	0.7227	-1.75	0.5661	0.6563	-1.19	0.5388	0.6209	0.89	0.2938	0.4164	-1.88	0.9510	1.0045	0.09	0.0238	0.1370	0.51	0.1848	0.4744	0.15	0.0651	0.2291	0.25
4020	SAP30-binding protein	Q9UHR5	0.1039	0.2306	-0.57	0.0740	0.6114	-0.74	0.1475	0.2756	-0.23	0.0124	0.0450	-0.47	0.0769	0.2132	-0.27	0.0617	0.1727	-0.69	0.9656	0.9718	-0.01	0.8473	1.2890	-0.02	0.0026	0.0541	0.48
4021	Sarcolemmal membrane-associated protein	Q14BN4	0.0368	0.1206	0.20	0.0870	0.6199	0.54	0.3782	0.5774	0.08	0.4186	0.6103	0.50	0.0319	0.1303	0.23	0.3521	0.5574	-0.21	0.0039	0.0671	0.51	0.0014	0.0303	0.72	0.0248	0.1338	0.66
4022	Sarcoplasmic/endoplasmic reticulum calcium ATPase 1	O14983	0.3394	0.5472	0.07	0.0994	0.6357	0.18	0.6240	0.6776	0.05	0.1410	0.2526	-0.09	0.0444	0.1555	-0.15	0.6071	0.8447	-0.02	0.1779	0.3771	-0.10	0.0202	0.1228	-0.24	0.0035	0.0585	-0.56
4023	Sarcoplasmic/endoplasmic reticulum calcium ATPase 2	P16615	0.0094	0.0572	0.16	0.1367	0.6520	0.26	0.0003	0.0213	0.33	0.0010	0.0160	0.31	0.0198	0.1008	0.12	0.0053	0.0618	0.28	0.5967	0.6457	0.06	0.1057	0.3290	-0.14	0.3978	0.4693	-0.06
4024	Sarcoplasmic/endoplasmic reticulum calcium ATPase 3	Q93084	0.0030	0.0361	-0.30	0.8811	0.9143	-0.01	0.0011	0.0213	-0.39	0.0069	0.0337	-0.27	0.4714	0.5623	-0.04	0.0543	0.1580	-0.11	0.0521	0.1809	-0.09	0.2405	0.5576	0.07	0.0055	0.0710	-0.23
4025	S-arrestin	P10523	0.4108	0.5160	-3.31	0.5752	0.7225	-1.75	0.5661	0.6562	-1.19	0.5388	0.6208	0.89	0.2938	0.4163	-1.88	0.9510	1.0043	0.09	0.3852	0.4412	-0.32	0.7888	1.2318	-0.03	0.0493	0.1938	0.36
4026	Scaffold attachment factor B1	Q15424	0.4108	0.5159	-3.31	0.5752	0.7223	-1.75	0.5661	0.6560	-1.19	0.5388	0.6206	0.89	0.2938	0.4162	-1.88	0.9510	1.0041	0.09	0.0241	0.1372	-0.48	0.0443	0.1950	-0.43	0.0157	0.1076	-0.56
4027	Scaffold attachment factor B2	Q14151	0.7908	0.8307	0.06	0.2843	0.6646	-2.02	0.3176	0.5045	0.25	0.1797	0.3047	0.32	0.3607	0.4579	0.19	0.8200	1.0564	0.12	0.3736	0.4372	-2.55	0.8708	0.9168	-0.33	0.0651	0.1144	2.06
4028	Scavenger receptor class B member 1	Q8WTV0	0.7039	0.7566	-0.04	0.8956	0.9262	0.03	0.0352	0.0976	0.30	0.0833	0.1691	0.21	0.5634	0.6457	-0.05	0.1405	0.2942	0.16	0.3736	0.5197	-2.55	0.8708	1.0694	-0.33	0.0651	0.1501	2.06
4029	Scavenger receptor cysteine-rich type 1 protein M160	Q9NR16	0.0223	0.0890	0.14	0.1140	0.6343	0.27	0.0136	0.0547	0.22	0.2916	0.4536	-0.11	0.1938	0.3917	0.05	0.0184	0.0877	0.15	0.3736	0.5958	-2.55	0.8708	1.2054	-0.33	0.0651	0.1904	2.06
4030	Schlafen family member 11	Q7Z7L1	0.0237	0.0924	0.17	0.1806	0.6644	0.29	0.0376	0.1020	0.15	0.0197	0.0598	0.17	0.0269	0.1193	0.10	0.1529	0.3106	0.07	0.0757	0.2191	-0.24	0.0293	0.1546	-0.40	0.0166	0.1101	-0.61
4031	Schwannomin-interacting protein 1	P0DPB3	0.4108	0.5157	-3.31	0.5752	0.7221	-1.75	0.5661	0.6559	-1.19	0.5388	0.6205	0.89	0.2938	0.4160	-1.88	0.9510	1.0039	0.09	0.2459	0.4805	0.18	0.3170	0.6670	0.12	0.1179	0.1759	0.22
4032	SCL-interrupting locus protein	Q15468	0.6747	0.7324	-0.02	0.4634	0.7951	0.13	0.0816	0.1754	0.14	0.0307	0.0815	0.20	0.2078	0.4105	-0.08	0.0872	0.2155	0.15	0.3736	0.5403	-2.55	0.8708	1.1067	-0.33	0.0651	0.1602	2.06
4033	SCY1-like protein 2	Q6P3W7	0.0598	0.1618	-0.31	0.5974	0.7014	-0.11	0.2027	0.3547	0.17	0.4142	0.6066	-0.10	0.2636	0.4852	0.15	0.3902	0.6021	-0.10	0.3736	0.4702	-2.55	0.8708	0.9785	-0.33	0.0651	0.1278	2.06
4034	Sec1 family domain-containing protein 1	Q8WVM8	0.0036	0.0383	0.31	0.2362	0.6639	0.17	0.0019	0.0242	0.38	0.0047	0.0278	0.42	0.0556	0.1760	0.13	0.0041	0.0602	0.58	0.1834	0.3845	0.27	0.8705	1.3123	0.03	0.4171	0.4885	0.14
4035	Sec1 family domain-containing protein 2	Q8WU76	0.4108	0.5156	-3.31	0.5752	0.7220	-1.75	0.5661	0.6557	-1.19	0.5388	0.6204	0.89	0.2938	0.4159	-1.88	0.9510	1.0037	0.09	0.7963	0.8281	0.08	0.6254	1.0615	0.08	0.1188	0.1770	0.56
4036	SEC23-interacting protein	Q9Y6Y8	0.0103	0.0589	0.14	0.2691	0.6628	0.11	0.3295	0.5191	0.08	0.0048	0.0277	0.15	0.0258	0.1159	0.09	0.1487	0.3054	0.14	0.3736	0.5168	-2.55	0.8708	1.0641	-0.33	0.0651	0.1487	2.06
4037	Secernin-1	Q12765	0.0213	0.0867	0.11	0.0806	0.6140	0.26	0.0271	0.0825	0.25	0.0091	0.0383	0.17	0.0223	0.1076	0.11	0.0074	0.0653	0.17	0.0023	0.0567	0.40	0.1501	0.4180	0.09	0.0399	0.1728	0.20
4038	Secernin-2	Q96FV2	0.0035	0.0378	-0.95	0.5858	0.6918	-0.23	0.5481	0.7711	0.13	0.1851	0.3123	-0.23	0.0469	0.1591	0.37	0.7171	0.9569	-0.11	0.0839	0.2315	-0.28	0.0289	0.1529	-0.34	0.3138	0.3851	-0.10
4039	Secretogranin-1	P05060	0.0915	0.2121	-0.28	0.6454	0.7383	-0.23	0.0105	0.0476	0.61	0.1570	0.2748	0.19	0.0780	0.2147	0.28	0.0356	0.1218	0.67	0.1115	0.2748	0.28	0.2337	0.5493	-0.16	0.0294	0.1462	0.20
4040	Secretogranin-2	P13521	0.4108	0.5155	-3.31	0.5752	0.7218	-1.75	0.5661	0.6556	-1.19	0.5388	0.6202	0.89	0.2938	0.4158	-1.88	0.9510	1.0035	0.09	0.2833	0.5329	-0.15	0.0038	0.0497	-0.26	0.0916	0.1453	-0.09
4041	Secretory carrier-associated membrane protein 1	O15126	0.1475	0.2958	-0.07	0.6293	0.7262	0.06	0.0118	0.0508	0.18	0.0229	0.0665	0.14	0.3643	0.4616	0.04	0.3644	0.5717	0.03	0.0154	0.1151	0.59	0.0343	0.1679	0.27	0.0114	0.0930	0.50
4042	Secretory carrier-associated membrane protein 2	O15127	0.0147	0.0704	-0.65	0.3560	0.7079	-0.90	0.3872	0.5885	-0.14	0.0237	0.0681	-1.12	0.1941	0.3920	-0.35	0.1187	0.2633	-0.46	0.3736	0.5280	-2.55	0.8708	1.0846	-0.33	0.0651	0.1541	2.06
4043	Secretory carrier-associated membrane protein 3	O14828	0.0869	0.2050	0.06	0.3846	0.7303	0.08	0.0200	0.0682	0.21	0.0002	0.0089	0.16	0.0217	0.1061	0.12	0.0182	0.0873	0.29	0.2676	0.5104	-0.11	0.0449	0.1968	0.24	0.8558	0.8831	-0.01
4044	Secretory carrier-associated membrane protein 4	Q969E2	0.1438	0.2907	-0.07	0.2016	0.6601	0.17	0.2886	0.4676	0.07	0.0103	0.0407	0.20	0.0459	0.1578	0.12	0.0537	0.1571	0.12	0.0375	0.1608	0.21	0.2891	0.6290	0.11	0.6091	0.6692	0.03
4045	Sedoheptulokinase	Q9UHJ6	0.4108	0.5154	-3.31	0.5752	0.7216	-1.75	0.5661	0.6554	-1.19	0.5388	0.6201	0.89	0.2938	0.4157	-1.88	0.9510	1.0033	0.09	0.3163	0.5787	-0.09	0.0795	0.2768	-0.19	0.8995	0.9199	-0.01
4046	Segment polarity protein dishevelled homolog DVL-2	O14641	0.0145	0.0699	0.43	0.0458	0.5595	0.57	0.0008	0.0222	0.78	0.0116	0.0433	0.54	0.0085	0.0663	0.45	0.0145	0.0802	0.66	0.0035	0.0655	0.85	0.0216	0.1276	0.53	0.0209	0.1237	0.64
4047	Segment polarity protein dishevelled homolog DVL-3	Q92997	0.4108	0.5152	-3.31	0.5752	0.7214	-1.75	0.5661	0.6553	-1.19	0.5388	0.6199	0.89	0.2938	0.4156	-1.88	0.9510	1.0031	0.09	0.0026	0.0564	0.34	0.0046	0.0549	0.23	0.0107	0.0910	0.39
4048	Selenide water dikinase 1	P49903	0.1435	0.2904	-0.09	0.9814	0.9871	0.00	0.3390	0.5316	0.06	0.1549	0.2719	-0.09	0.0559	0.1767	-0.15	0.0855	0.2128	-0.13	0.2359	0.4653	-0.10	0.6545	1.0947	0.03	0.2994	0.3708	-0.04
4049	Separin	Q14674	0.0851	0.2017	-0.60	0.1185	0.6328	-0.51	0.1421	0.2680	-0.49	0.4888	0.6912	-0.18	0.5586	0.6413	-0.15	0.2857	0.4822	0.30	0.1979	0.4081	-0.27	0.2274	0.5403	0.08	0.0025	0.0542	0.38
4050	Sepiapterin reductase	P35270	0.0608	0.1633	0.07	0.1830	0.6648	0.28	0.0212	0.0705	0.27	0.0072	0.0344	0.24	0.1153	0.2781	-0.07	0.0038	0.0596	0.14	0.0013	0.0478	-0.63	0.0054	0.0593	-0.36	0.1098	0.1668	-0.18
4051	Septin-10	Q9P0V9	0.0000	0.0000	-1.23	0.1642	0.6635	-1.17	0.0017	0.0237	-1.46	0.0016	0.0179	-1.80	0.3824	0.4797	0.09	0.0813	0.2050	-0.85	0.6297	0.6755	0.03	0.5952	1.0281	-0.04	0.0942	0.1485	-0.12
4052	Septin-11	Q9NVA2	0.3754	0.5907	-0.01	0.1427	0.6547	0.23	0.0270	0.0824	0.13	0.0913	0.1811	0.11	0.3111	0.4084	0.03	0.3123	0.5129	0.10	0.1965	0.4058	0.22	0.6475	1.0869	-0.04	0.4061	0.4775	0.06
4053	Septin-14	Q6ZU15	0.0690	0.1748	-0.60	0.2899	0.6686	-1.03	0.0375	0.1018	-0.71	0.1426	0.2545	-0.56	0.3025	0.4003	-0.31	0.1248	0.2724	-0.59	0.0522	0.1807	-0.35	0.2011	0.4994	-0.15	0.0800	0.1309	-0.45
4054	Septin-2	Q15019	0.0538	0.1513	0.12	0.3108	0.6796	0.13	0.0124	0.0523	0.19	0.0425	0.1025	0.15	0.3861	0.4833	0.04	0.0275	0.1065	0.24	0.1251	0.2977	0.15	0.2674	0.5973	0.09	0.6195	0.6779	-0.03
4055	Septin-4	O43236	0.1311	0.2729	-0.11	0.2726	0.6654	0.14	0.7059	0.7524	0.02	0.1026	0.1978	0.13	0.0316	0.1300	0.21	0.0671	0.1820	0.17	0.0107	0.1021	-0.28	0.0463	0.2002	0.34	0.1360	0.1968	-0.10
4056	Septin-6	Q14141	0.8037	0.8416	0.01	0.1905	0.6686	0.27	0.3585	0.5553	0.06	0.0662	0.1422	0.12	0.6564	0.7227	0.02	0.0246	0.0999	0.23	0.0297	0.1472	0.26	0.1128	0.3431	0.14	0.1531	0.2162	0.12
4057	Septin-7	Q16181	0.0174	0.0772	0.12	0.0271	0.5050	0.12	0.0067	0.0396	0.21	0.0038	0.0251	0.20	0.0382	0.1424	0.10	0.0132	0.0767	0.16	0.0664	0.2028	0.31	0.7277	1.1728	0.03	0.4880	0.5564	-0.05
4058	Septin-8	Q92599	0.3467	0.5557	-0.10	0.6116	0.7129	0.06	0.7888	0.8270	0.01	0.1222	0.2259	-0.07	0.0352	0.1358	-0.14	0.0956	0.2278	0.08	0.1764	0.3756	-0.17	0.0005	0.0209	-0.13	0.4500	0.5189	-0.01
4059	Septin-9	Q9UHD8	0.0717	0.1799	0.12	0.2717	0.6651	0.20	0.0085	0.0435	0.26	0.0088	0.0381	0.25	0.0719	0.2044	0.13	0.0104	0.0701	0.30	0.9622	0.9687	0.00	0.0248	0.1402	-0.16	0.9363	0.9486	0.00
4060	Sequestosome-1	Q13501	0.0225	0.0893	-0.20	0.0266	0.4974	-0.36	0.9433	0.9557	0.00	0.0372	0.0934	0.15	0.0625	0.1894	-0.12	0.0213	0.0942	0.22	0.0066	0.0846	0.67	0.0127	0.0938	0.27	0.0049	0.0677	0.58
4061	Serine dehydratase-like	Q96GA7	0.0074	0.0506	0.10	0.5286	0.8472	0.07	0.0015	0.0225	0.26	0.0006	0.0133	0.28	0.0006	0.0204	0.24	0.0001	0.0370	0.37	0.3736	0.6551	-2.55	0.8708	1.3082	-0.33	0.0651	0.2283	2.06
4062	Serine hydroxymethyltransferase cytosolic	P34896	0.9235	0.9413	0.00	0.0611	0.5938	-0.16	0.1816	0.3257	-0.04	0.0532	0.1205	-0.13	0.0028	0.0403	-0.29	0.7103	0.9512	-0.01	0.6039	0.6518	0.04	0.2788	0.6159	-0.09	0.4335	0.5046	-0.06
4063	Serine hydroxymethyltransferase mitochondrial	P34897	0.0072	0.0501	0.25	0.3872	0.7293	0.14	0.0089	0.0440	0.23	0.0057	0.0303	0.25	0.0506	0.1662	-0.14	0.0133	0.0765	0.28	0.2913	0.5438	-0.08	0.0038	0.0496	-0.19	0.7401	0.7858	-0.01
4064	Serine palmitoyltransferase 1	O15269	0.0487	0.1414	-0.11	0.6927	0.7745	0.06	0.7788	0.8183	0.01	0.0564	0.1259	0.11	0.0385	0.1431	-0.13	0.8491	1.0844	-0.01	0.0212	0.1338	0.46	0.6994	1.1421	0.04	0.8275	0.8604	0.02
4065	Serine palmitoyltransferase 2	O15270	0.0024	0.0331	0.12	0.0448	0.5674	0.28	0.0178	0.0637	0.24	0.0000	0.0000	0.43	0.0008	0.0233	0.29	0.0001	0.0345	0.49	0.0000	0.0000	-0.55	0.0028	0.0444	-0.66	0.0595	0.2181	-0.61
4066	Serine protease 33	Q8NF86	0.4108	0.5151	-3.31	0.5752	0.7213	-1.75	0.5661	0.6551	-1.19	0.5388	0.6198	0.89	0.2938	0.4155	-1.88	0.9510	1.0029	0.09	0.1163	0.2838	1.14	0.0635	0.2420	1.46	0.2187	0.2879	0.83
4067	Serine protease 38	A1L453	0.0877	0.2062	-0.38	0.1593	0.6644	-0.64	0.2421	0.4064	0.21	0.2771	0.4350	-0.19	0.5976	0.6737	0.08	0.7135	0.9540	-0.06	0.3736	0.4447	-2.55	0.8708	0.9308	-0.33	0.0651	0.1173	2.06
4068	Serine protease HTRA1	Q92743	0.2928	0.4907	-0.20	0.6839	0.7695	0.10	0.5905	0.6468	-0.09	0.6061	0.6534	-0.10	0.3966	0.4929	-0.16	0.2299	0.4128	-0.24	0.2142	0.4336	0.23	0.5230	0.9466	-0.10	0.6469	0.7032	0.07
4069	Serine protease inhibitor Kazal-type 5	Q9NQ38	0.9436	0.9577	0.01	0.1523	0.6596	0.21	0.4460	0.6591	0.12	0.8059	0.8334	0.04	0.2656	0.4872	0.15	0.7800	1.0172	-0.07	0.0037	0.0654	-0.27	0.0048	0.0556	-0.61	0.0197	0.1215	-0.55
4070	Serine protease inhibitor Kazal-type 8	P0C7L1	0.0056	0.0451	3.91	0.1129	0.6350	-6.65	0.0443	0.1150	-6.09	0.0733	0.1531	4.47	0.5300	0.6158	-0.63	0.0121	0.0744	5.84	0.3736	0.6084	-2.55	0.8708	1.2274	-0.33	0.0651	0.1979	2.06
4071	Serine racemase	Q9GZT4	0.4108	0.5150	-3.31	0.5752	0.7211	-1.75	0.5661	0.6550	-1.19	0.5388	0.6197	0.89	0.2938	0.4154	-1.88	0.9510	1.0027	0.09	0.0481	0.1753	0.38	0.1839	0.4728	-0.19	0.2057	0.2743	0.17
4072	Serine/arginine repetitive matrix protein 1	Q8IYB3	0.0609	0.1635	-12.21	0.2164	0.6598	-1.34	0.7843	0.8229	0.06	0.0027	0.0215	-1.32	0.0634	0.1907	0.48	0.2118	0.3932	-0.75	0.4394	0.4949	-0.22	0.0738	0.2629	0.59	0.1401	0.2015	0.44
4073	Serine/arginine repetitive matrix protein 2	Q9UQ35	0.4074	0.6281	0.04	0.4003	0.7437	0.14	0.0024	0.0270	0.35	0.0254	0.0714	0.15	0.5281	0.6146	-0.02	0.0582	0.1663	0.11	0.0438	0.1683	0.48	0.0109	0.0857	0.22	0.0281	0.1431	0.19
4074	Serine/arginine-rich splicing factor 1	Q07955	0.3950	0.6132	0.05	0.4893	0.8124	0.07	0.0057	0.0365	0.39	0.0435	0.1043	0.17	0.1130	0.2740	-0.12	0.0508	0.1517	0.19	0.0132	0.1089	0.39	0.4882	0.8993	-0.04	0.9107	0.9284	-0.01
4075	Serine/arginine-rich splicing factor 11	Q05519	0.4108	0.5149	-3.31	0.5752	0.7209	-1.75	0.5661	0.6548	-1.19	0.5388	0.6195	0.89	0.2938	0.4152	-1.88	0.9510	1.0025	0.09	0.1784	0.3778	-0.32	0.3706	0.7429	-0.08	0.6316	0.6883	0.05
4076	Serine/arginine-rich splicing factor 2	Q01130	0.0376	0.1217	0.16	0.1435	0.6549	0.12	0.0046	0.0336	0.29	0.0292	0.0789	0.17	0.4362	0.5302	-0.04	0.2019	0.3793	0.09	0.0275	0.1437	0.48	0.4421	0.8438	0.08	0.2659	0.3376	0.07
4077	Serine/arginine-rich splicing factor 3	P84103	0.1885	0.3543	-0.04	0.5168	0.8389	-0.05	0.0018	0.0236	0.46	0.2749	0.4322	0.04	0.0001	0.0078	-0.14	0.1197	0.2643	0.15	0.0063	0.0837	0.24	0.0173	0.1116	-0.20	0.0055	0.0716	0.30
4078	Serine/arginine-rich splicing factor 4	Q08170	0.2784	0.4722	0.30	0.4048	0.7478	0.38	0.0594	0.1409	0.66	0.0725	0.1518	0.62	0.0248	0.1138	1.02	0.0773	0.1985	0.61	0.0302	0.1474	-0.29	0.0007	0.0240	-0.95	0.0004	0.0280	-1.15
4079	Serine/arginine-rich splicing factor 5	Q13243	0.9735	0.9815	0.00	0.7689	0.8324	0.03	0.0319	0.0915	0.27	0.9444	0.9517	-0.01	0.7369	0.7939	0.03	0.2670	0.4599	-0.14	0.4424	0.4979	0.08	0.3691	0.7405	-0.07	0.2048	0.2736	0.16
4080	Serine/arginine-rich splicing factor 6	Q13247	0.0679	0.1732	0.13	0.2400	0.6613	0.21	0.0139	0.0553	0.31	0.0052	0.0291	0.30	0.2659	0.4876	-0.06	0.0166	0.0849	0.25	0.0825	0.2295	0.37	0.6740	1.1126	0.04	0.5956	0.6564	-0.03
4081	Serine/arginine-rich splicing factor 7	Q16629	0.0003	0.0144	-0.24	0.0006	0.2391	-0.22	0.0044	0.0329	0.26	0.0006	0.0127	-0.26	0.0034	0.0436	-0.17	0.0078	0.0666	-0.12	0.0004	0.0432	0.65	0.1445	0.4059	0.16	0.0023	0.0527	0.56
4082	Serine/arginine-rich splicing factor 8	Q9BRL6	0.1176	0.2520	0.26	0.1652	0.6608	1.03	0.1554	0.2873	0.33	0.0418	0.1016	0.48	0.1834	0.3770	0.20	0.7060	0.9479	0.05	0.7092	0.7500	0.32	0.7209	1.1659	0.40	0.1650	0.2301	1.41
4083	Serine/arginine-rich splicing factor 9	Q13242	0.4747	0.5502	-0.21	0.1697	0.6619	-1.21	0.0098	0.0461	-1.49	0.0135	0.0471	-1.59	0.0021	0.0355	-1.80	0.0120	0.0744	-1.20	0.4426	0.4979	0.09	0.0358	0.1722	-0.26	0.3603	0.4317	-0.22
4084	Serine/threonine-protein kinase	O95747	0.1962	0.3652	0.05	0.5986	0.7020	0.03	0.8922	0.9141	0.01	0.6986	0.7378	0.02	0.1724	0.3614	0.05	0.7149	0.9552	0.01	0.0875	0.2377	0.18	0.3917	0.7744	0.05	0.2701	0.3419	-0.06
4085	Serine/threonine-protein kinase 10	O94804	0.0402	0.1261	-0.32	0.9537	0.9692	0.01	0.1392	0.2636	-0.19	0.0924	0.1827	-0.22	0.1243	0.2908	-0.19	0.1394	0.2928	-0.28	0.3736	0.5201	-2.55	0.8708	1.0702	-0.33	0.0651	0.1503	2.06
4086	Serine/threonine-protein kinase 17A	Q9UEE5	0.4108	0.5147	-3.31	0.5752	0.7207	-1.75	0.5661	0.6547	-1.19	0.5388	0.6194	0.89	0.2938	0.4151	-1.88	0.9510	1.0023	0.09	0.2581	0.4972	-0.10	0.0160	0.1072	0.35	0.9644	0.9713	0.00
4087	Serine/threonine-protein kinase 19	P49842	0.7265	0.7771	-0.05	0.0645	0.5882	0.33	0.5058	0.7280	0.09	0.5871	0.6360	-0.08	0.1250	0.2918	0.26	0.0503	0.1505	-0.44	0.3736	0.5603	-2.55	0.8708	1.1425	-0.33	0.0651	0.1706	2.06
4088	Serine/threonine-protein kinase 24	Q9Y6E0	0.2967	0.4951	0.12	0.2331	0.6623	0.22	0.8906	0.9126	-0.01	0.0461	0.1088	0.11	0.0359	0.1375	-0.12	0.0132	0.0767	0.20	0.0118	0.1043	0.37	0.0473	0.2028	0.19	0.4949	0.5622	0.05
4089	Serine/threonine-protein kinase 25	O00506	0.0100	0.0583	-1.25	0.6386	0.7335	-0.29	0.0028	0.0288	-1.52	0.1466	0.2604	-1.20	0.5565	0.6397	-0.15	0.0039	0.0592	-1.75	0.2852	0.5358	1.16	0.3080	0.6547	1.30	0.1528	0.2159	1.82
4090	Serine/threonine-protein kinase 26	Q9P289	0.2683	0.4600	0.07	0.8663	0.9033	-0.02	0.1088	0.2184	0.12	0.8988	0.9131	0.01	0.4494	0.5428	-0.05	0.6712	0.9092	-0.03	0.5184	0.5717	-0.06	0.3129	0.6621	-0.06	0.1788	0.2453	-0.15
4091	Serine/threonine-protein kinase 3	Q13188	0.4108	0.5146	-3.31	0.5752	0.7206	-1.75	0.5661	0.6546	-1.19	0.5388	0.6193	0.89	0.2938	0.4150	-1.88	0.9510	1.0021	0.09	0.3067	0.5664	2.85	0.9951	0.9966	0.03	0.2528	0.3240	3.34
4092	Serine/threonine-protein kinase 31	Q9BXU1	0.4108	0.5145	-3.31	0.5752	0.7204	-1.75	0.5661	0.6544	-1.19	0.5388	0.6191	0.89	0.2938	0.4149	-1.88	0.9510	1.0019	0.09	0.0001	0.0192	2.85	0.0014	0.0309	2.48	0.0046	0.0669	1.95
4093	Serine/threonine-protein kinase 32A	Q8WU08	0.4108	0.5144	-3.31	0.5752	0.7202	-1.75	0.5661	0.6543	-1.19	0.5388	0.6190	0.89	0.2938	0.4148	-1.88	0.9510	1.0017	0.09	0.0652	0.2008	0.55	0.0402	0.1835	0.69	0.0682	0.1156	0.54
4094	Serine/threonine-protein kinase 38-like	Q9Y2H1	0.7739	0.8160	-0.03	0.2335	0.6617	0.13	0.2384	0.4007	0.16	0.7154	0.7537	0.03	0.0973	0.2485	0.20	0.0797	0.2024	0.25	0.3736	0.5436	-2.55	0.8708	1.1127	-0.33	0.0651	0.1619	2.06
4095	Serine/threonine-protein kinase 4	Q13043	0.0727	0.1817	-10.98	0.0626	0.5907	-9.42	0.0241	0.0764	-8.87	0.0019	0.0191	-6.79	0.1307	0.2989	0.73	0.0162	0.0844	-7.59	0.1470	0.3316	0.20	0.1527	0.4228	0.14	0.1255	0.1851	0.14
4096	Serine/threonine-protein kinase ATR	Q13535	0.4578	0.5348	-0.02	0.5002	0.8231	-0.03	0.0136	0.0545	0.19	0.4056	0.5967	-0.03	0.4283	0.5225	-0.03	0.0407	0.1328	0.36	0.0418	0.1676	0.37	0.0040	0.0510	0.75	0.0386	0.1700	0.46
4097	Serine/threonine-protein kinase Chk1	O14757	0.4108	0.5142	-3.31	0.5752	0.7200	-1.75	0.5661	0.6541	-1.19	0.5388	0.6188	0.89	0.2938	0.4147	-1.88	0.9510	1.0015	0.09	0.2713	0.5146	-0.17	0.0242	0.1382	0.44	0.3209	0.3921	-0.12
4098	Serine/threonine-protein kinase greatwall	Q96GX5	0.6168	0.6810	0.07	0.1760	0.6616	-0.23	0.1082	0.2174	-0.30	0.2583	0.4099	-0.18	0.0471	0.1594	-0.43	0.9464	1.1827	0.01	0.9745	0.9792	0.00	0.3646	0.7340	0.09	0.1708	0.2364	-0.15

Supplemetary Table S2. Overview on all relatively quantified 5180 proteins statistical analysis

			MCF-7																		MDA-MB-231								
			Dai SC20 vs control			Gen SC20 vs control			SSE SC20 vs control			Dai IC20 vs control			Gen IC20 vs control			SSE IC20 vs control			Dai IC20 vs control			Gen IC20 vs control			SSE IC20 vs control		
	**Protein name**	**UniProt**	**p value**	**BH q value**	**log2FC**	**p value**	**BH q value**	**log2FC**	**p value**	**BH q value**	**log2FC**	**p value**	**BH q value**	**log2FC**	**p value**	**BH q value**	**log2FC**	**p value**	**BH q value**	**log2FC**	**p value**	**BH q value**	**log2FC**	**p value**	**BH q value**	**log2FC**	**p value**	**BH q value**	**log2FC**
4099	Serine/threonine-protein kinase ICK	Q9UPZ9	0.8646	0.8929	-0.01	0.1109	0.6327	0.11	0.0147	0.0572	0.24	0.9796	0.9824	0.00	0.0123	0.0790	0.25	0.8859	1.1234	-0.01	0.3736	0.4347	-2.55	0.8708	0.9120	-0.33	0.0651	0.1134	2.06
4100	Serine/threonine-protein kinase MAK	P20794	0.5883	0.6549	-0.12	0.3280	0.6876	-0.45	0.3169	0.5038	-0.25	0.3412	0.5156	-0.24	0.3957	0.4925	-0.22	0.4602	0.6827	-0.18	0.1072	0.2674	-0.38	0.7435	1.1861	-0.02	0.0304	0.1473	0.10
4101	Serine/threonine-protein kinase MARK1	Q9P0L2	0.0933	0.2150	-9.52	0.0844	0.6228	-7.96	0.0337	0.0951	-7.41	0.0033	0.0233	-5.32	0.5886	0.6657	0.26	0.0178	0.0871	-6.13	0.3736	0.5966	-2.55	0.8708	1.2067	-0.33	0.0651	0.1908	2.06
4102	Serine/threonine-protein kinase MARK2	Q7KZI7	0.0352	0.1171	-0.21	0.8875	0.9196	0.02	0.8823	0.9070	-0.01	0.9367	0.9445	-0.01	0.5310	0.6167	0.05	0.2834	0.4797	-0.15	0.3723	0.6542	0.09	0.9205	0.9416	-0.01	0.0297	0.1468	0.29
4103	Serine/threonine-protein kinase MRCK beta	Q9Y5S2	0.0381	0.1225	-0.27	0.2768	0.6650	0.11	0.0540	0.1319	-0.14	0.0026	0.0213	0.17	0.1660	0.3521	0.08	0.3322	0.5352	-0.11	0.3736	0.4762	-2.55	0.8708	0.9896	-0.33	0.0651	0.1304	2.06
4104	Serine/threonine-protein kinase N2	Q16513	0.4108	0.5141	-3.31	0.5752	0.7199	-1.75	0.5661	0.6540	-1.19	0.5388	0.6187	0.89	0.2938	0.4146	-1.88	0.9510	1.0013	0.09	0.3169	0.5792	0.32	0.0822	0.2820	0.70	0.0681	0.1155	0.86
4105	Serine/threonine-protein kinase Nek11	Q8NG66	0.0160	0.0737	0.23	0.2902	0.6684	0.23	0.0104	0.0475	0.27	0.0075	0.0351	0.34	0.0829	0.2230	0.11	0.3191	0.5218	0.08	0.3736	0.4788	-2.55	0.8708	0.9944	-0.33	0.0651	0.1315	2.06
4106	Serine/threonine-protein kinase Nek8	Q86SG6	0.1722	0.3331	-0.20	0.0128	0.4450	0.29	0.0625	0.1453	-0.22	0.9268	0.9360	0.01	0.0348	0.1353	0.22	0.2101	0.3909	-0.28	0.3736	0.5005	-2.55	0.8708	1.0343	-0.33	0.0651	0.1411	2.06
4107	Serine/threonine-protein kinase PAK 1	Q13153	0.1467	0.2949	-0.06	0.5737	0.8860	-0.05	0.0031	0.0297	0.38	0.6471	0.6911	-0.04	0.1182	0.2820	-0.16	0.8091	1.0460	-0.02	0.0036	0.0652	0.47	0.7317	1.1756	0.03	0.0093	0.0859	0.75
4108	Serine/threonine-protein kinase PAK 2	Q13177	0.2492	0.4357	0.05	0.4916	0.8141	0.11	0.6577	0.7090	0.02	0.9694	0.9737	0.00	0.0165	0.0914	-0.16	0.4435	0.6649	0.03	0.5378	0.5895	0.05	0.2897	0.6292	-0.08	0.0098	0.0872	-0.21
4109	Serine/threonine-protein kinase PAK 3	O75914	0.0060	0.0466	0.13	0.3590	0.7087	0.13	0.0031	0.0299	-0.13	0.1285	0.2344	0.12	0.0124	0.0793	-0.11	0.2025	0.3803	-0.09	0.0016	0.0525	-0.61	0.0177	0.1131	-0.30	0.0090	0.0848	-0.61
4110	Serine/threonine-protein kinase PAK 4	O96013	0.0012	0.0237	-0.29	0.2366	0.6632	-0.14	0.0996	0.2034	-0.17	0.0002	0.0086	-0.47	0.0034	0.0437	-0.23	0.0024	0.0560	-0.31	0.3736	0.6423	-2.55	0.8708	1.2862	-0.33	0.0651	0.2195	2.06
4111	Serine/threonine-protein kinase PAK 6	Q9NQU5	0.0019	0.0299	0.24	0.0412	0.5691	0.24	0.1217	0.2392	0.18	0.0233	0.0674	0.20	0.0050	0.0530	0.15	0.0178	0.0872	0.12	0.0674	0.2038	0.13	0.0165	0.1090	-0.30	0.0074	0.0781	-0.43
4112	Serine/threonine-protein kinase PLK1	P53350	0.2431	0.4285	0.09	0.0467	0.5626	0.24	0.0778	0.1688	0.17	0.0678	0.1443	0.18	0.0123	0.0791	0.33	0.2665	0.4594	0.08	0.3736	0.4873	-2.55	0.8708	1.0102	-0.33	0.0651	0.1352	2.06
4113	Serine/threonine-protein kinase PLK2	Q9NYY3	0.6947	0.7491	0.03	0.0722	0.6062	-0.15	0.0560	0.1352	-0.17	0.1103	0.2091	-0.16	0.0427	0.1526	-0.19	0.1188	0.2632	-0.13	0.5997	0.6479	-0.03	0.1759	0.4606	0.09	0.2015	0.2697	-0.08
4114	Serine/threonine-protein kinase PLK3	Q9H4B4	0.0047	0.0431	-0.30	0.2557	0.6626	0.07	0.1507	0.2804	0.08	0.8650	0.8839	-0.01	0.9422	0.9557	0.00	0.8250	1.0591	0.01	0.1246	0.2972	-0.31	0.4494	0.8521	-0.12	0.3302	0.4013	-0.16
4115	Serine/threonine-protein kinase PRP4 homolog	Q13523	0.2080	0.3805	0.18	0.0144	0.4493	-0.47	0.0147	0.0573	-0.47	0.0062	0.0315	-0.67	0.3137	0.4113	-0.10	0.0855	0.2127	-0.57	0.0357	0.1562	1.82	0.0155	0.1052	1.87	0.0074	0.0782	2.80
4116	Serine/threonine-protein kinase RIO1	Q9BRS2	0.0777	0.1907	-0.79	0.1812	0.6647	-1.22	0.5334	0.7543	-0.23	0.2723	0.4291	-0.36	0.5898	0.6668	0.16	0.3703	0.5781	-0.25	0.3736	0.4718	-2.55	0.8708	0.9814	-0.33	0.0651	0.1285	2.06
4117	Serine/threonine-protein kinase RIO2	Q9BVS4	0.0887	0.2076	-0.31	0.7831	0.8437	-0.04	0.0883	0.1862	-0.34	0.1041	0.1996	-0.30	0.4101	0.5055	-0.12	0.0572	0.1643	-0.49	0.3736	0.5571	-2.55	0.8708	1.1368	-0.33	0.0651	0.1689	2.06
4118	Serine/threonine-protein kinase SIK3	Q9Y2K2	0.4108	0.5140	-3.31	0.5752	0.7197	-1.75	0.5661	0.6538	-1.19	0.5388	0.6186	0.89	0.2938	0.4145	-1.88	0.9510	1.0011	0.09	0.0268	0.1418	0.99	0.7102	1.1547	-0.13	0.0972	0.1523	0.59
4119	Serine/threonine-protein kinase SMG1	Q96Q15	0.0006	0.0190	0.44	0.0003	0.2590	0.23	0.0003	0.0235	0.25	0.0005	0.0136	0.23	0.0217	0.1060	0.17	0.0008	0.0436	0.27	0.0103	0.1022	0.39	0.1668	0.4456	0.11	0.0953	0.1499	-0.05
4120	Serine/threonine-protein kinase TAO1	Q7L7X3	0.9081	0.9285	0.02	0.1252	0.6421	0.65	0.8154	0.8504	-0.05	0.0403	0.0990	0.18	0.1563	0.3375	0.15	0.0091	0.0687	-0.54	0.3736	0.6161	-2.55	0.8708	1.2409	-0.33	0.0651	0.2027	2.06
4121	Serine/threonine-protein kinase TBK1	Q9UHD2	0.4108	0.5139	-3.31	0.5752	0.7195	-1.75	0.5661	0.6537	-1.19	0.5388	0.6184	0.89	0.2938	0.4143	-1.88	0.9510	1.0008	0.09	0.0721	0.2127	0.35	0.0707	0.2572	0.14	0.2728	0.3447	0.04
4122	Serine/threonine-protein kinase tousled-like 1	Q9UKI8	0.4294	0.5068	-0.11	0.6756	0.7626	0.19	0.9661	0.9725	-0.01	0.0106	0.0411	0.70	0.2059	0.4077	0.21	0.5642	0.7989	-0.35	0.9002	0.9183	0.01	0.0149	0.1029	0.40	0.0810	0.1321	0.23
4123	Serine/threonine-protein kinase tousled-like 2	Q86UE8	0.0500	0.1440	-0.40	0.2413	0.6603	-0.46	0.2412	0.4051	-0.18	0.1027	0.1978	-0.29	0.1654	0.3510	-0.23	0.6313	0.8674	-0.06	0.0022	0.0556	-1.06	0.0013	0.0303	-0.76	0.0000	0.0000	-1.27
4124	Serine/threonine-protein kinase ULK3	Q6PHR2	0.0027	0.0349	1.44	0.2848	0.6648	0.67	0.0039	0.0316	2.17	0.0035	0.0239	1.45	0.5549	0.6383	0.12	0.0026	0.0547	1.79	0.3736	0.6414	-2.55	0.8708	1.2847	-0.33	0.0651	0.2190	2.06
4125	Serine/threonine-protein kinase WNK1	Q9H4A3	0.0226	0.0895	0.19	0.0332	0.5259	0.22	0.0123	0.0521	0.42	0.1235	0.2271	0.08	0.7681	0.8195	0.01	0.0063	0.0622	0.28	0.0113	0.1038	-0.40	0.0123	0.0925	-0.45	0.0015	0.0471	-0.59
4126	Serine/threonine-protein kinase WNK2	Q9Y3S1	0.9793	0.9863	-0.01	0.6137	0.7145	-0.25	0.3717	0.5696	0.22	0.2536	0.4035	0.30	0.2333	0.4441	0.32	0.3573	0.5632	0.23	0.5716	0.6215	-0.09	0.0383	0.1783	-0.19	0.1189	0.1771	0.16
4127	Serine/threonine-protein kinase WNK3	Q9BYP7	0.2356	0.4187	0.07	0.2825	0.6646	-0.12	0.2248	0.3827	-0.08	0.0909	0.1803	-0.13	0.0329	0.1324	-0.19	0.0305	0.1120	0.25	0.3320	0.6011	0.18	0.3792	0.7555	0.13	0.8448	0.8749	-0.03
4128	Serine/threonine-protein kinase/endoribonuclease IRE2	Q76MJ5	0.0149	0.0707	-0.12	0.0176	0.4675	-0.24	0.1694	0.3090	-0.12	0.0657	0.1413	-0.26	0.0017	0.0319	-0.33	0.0405	0.1323	-0.09	0.3736	0.5697	-2.55	0.8708	1.1593	-0.33	0.0651	0.1756	2.06
4129	Serine/threonine-protein phosphatase 1 regulatory subunit 10	Q96QC0	0.0150	0.0709	-0.11	0.3117	0.6798	-0.26	0.8306	0.8636	-0.02	0.0819	0.1668	-0.09	0.0012	0.0270	-0.19	0.0150	0.0816	0.30	0.2716	0.5148	-0.09	0.1265	0.3715	0.25	0.2299	0.3003	-0.12
4130	Serine/threonine-protein phosphatase 2A 55 kDa regulatory subunit B alpha isoform	P63151	0.0297	0.1054	0.14	0.5023	0.8239	0.06	0.0677	0.1533	0.16	0.0257	0.0719	0.16	0.0053	0.0541	0.28	0.0588	0.1673	0.22	0.2823	0.5316	-0.11	0.1533	0.4228	-0.15	0.1018	0.1577	-0.19
4131	Serine/threonine-protein phosphatase 2A 55 kDa regulatory subunit B beta isoform	Q00005	0.3494	0.5590	0.06	0.3569	0.7081	0.13	0.0348	0.0969	0.18	0.0093	0.0387	0.27	0.3244	0.4217	0.06	0.0445	0.1393	0.19	0.0292	0.1464	0.39	0.0729	0.2617	0.24	0.4542	0.5227	-0.07
4132	Serine/threonine-protein phosphatase 2A 55 kDa regulatory subunit B delta isoform	Q66LE6	0.0509	0.1462	-11.86	0.0379	0.5546	-10.31	0.0083	0.0430	-9.75	0.0099	0.0401	-7.67	0.9967	0.9980	-0.01	0.0091	0.0686	-8.47	0.1402	0.3209	-0.18	0.0213	0.1264	-0.35	0.3025	0.3736	0.10
4133	Serine/threonine-protein phosphatase 2A 56 kDa regulatory subunit delta isoform	Q14738	0.1440	0.2910	-0.12	0.9980	0.9984	0.00	0.3913	0.5932	-0.05	0.6978	0.7372	0.02	0.0330	0.1326	-0.22	0.2588	0.4497	-0.07	0.0004	0.0391	-0.57	0.0008	0.0264	-0.28	0.0005	0.0305	-0.40
4134	Serine/threonine-protein phosphatase 2A 56 kDa regulatory subunit epsilon isoform	Q16537	0.0180	0.0787	0.48	0.6653	0.7534	-0.13	0.2665	0.4375	0.14	0.9025	0.9163	0.02	0.7656	0.8175	0.04	0.8004	1.0370	-0.03	0.0047	0.0729	-0.87	0.0002	0.0176	-1.15	0.0028	0.0560	-0.96
4135	Serine/threonine-protein phosphatase 2A 56 kDa regulatory subunit gamma isoform	Q13362	0.9537	0.9654	0.01	0.1892	0.6694	-0.20	0.0816	0.1755	-0.32	0.0640	0.1386	-0.32	0.0652	0.1945	-0.31	0.3274	0.5300	-0.13	0.3736	0.4770	-2.55	0.8708	0.9912	-0.33	0.0651	0.1307	2.06
4136	Serine/threonine-protein phosphatase 2A 65 kDa regulatory subunit A alpha isoform	P30153	0.0286	0.1029	0.13	0.5218	0.8410	0.08	0.0076	0.0412	0.15	0.0121	0.0442	0.13	0.1094	0.2687	-0.06	0.0039	0.0594	0.23	0.1424	0.3241	0.21	0.6244	1.0608	0.04	0.7763	0.8160	-0.02
4137	Serine/threonine-protein phosphatase 2A 65 kDa regulatory subunit A beta isoform	P30154	0.2247	0.4034	0.04	0.4166	0.7548	0.10	0.8891	0.9116	0.00	0.0341	0.0878	0.07	0.4889	0.5785	-0.02	0.0340	0.1188	0.07	0.0933	0.2452	-0.32	0.2497	0.5723	0.20	0.1153	0.1729	0.31
4138	Serine/threonine-protein phosphatase 2A activator	Q15257	0.3331	0.5397	0.03	0.4505	0.7849	0.12	0.0688	0.1552	0.11	0.1976	0.3289	0.05	0.0135	0.0833	-0.11	0.0222	0.0956	0.17	0.0689	0.2069	0.16	0.1534	0.4229	0.12	0.1243	0.1837	0.12
4139	Serine/threonine-protein phosphatase 2A catalytic subunit alpha isoform	P67775	0.0113	0.0608	0.16	0.0888	0.6224	0.16	0.0099	0.0464	0.16	0.0040	0.0257	0.20	0.0952	0.2440	0.07	0.0051	0.0614	0.28	0.4602	0.5150	-0.11	0.0007	0.0239	-0.29	0.1064	0.1627	0.07
4140	Serine/threonine-protein phosphatase 2A catalytic subunit beta isoform	P62714	0.4880	0.5624	-0.03	0.2409	0.6609	-0.09	0.1117	0.2232	-0.14	0.0088	0.0379	-0.22	0.0007	0.0206	-0.32	0.0004	0.0432	-0.26	0.0053	0.0776	0.38	0.1836	0.4727	0.10	0.0732	0.1224	0.26
4141	Serine/threonine-protein phosphatase 2A regulatory subunit B" subunit gamma	Q969Q6	0.4108	0.5137	-3.31	0.5752	0.7193	-1.75	0.5661	0.6535	-1.19	0.5388	0.6183	0.89	0.2938	0.4142	-1.88	0.9510	1.0006	0.09	0.8219	0.8493	0.02	0.1955	0.4904	0.10	0.0531	0.2031	0.17
4142	Serine/threonine-protein phosphatase 2B catalytic subunit alpha isoform	Q08209	0.0511	0.1462	0.09	0.2668	0.6628	0.24	0.0065	0.0389	0.17	0.0048	0.0280	0.28	0.0987	0.2510	0.06	0.1205	0.2652	0.17	0.3736	0.5267	-2.55	0.8708	1.0822	-0.33	0.0651	0.1535	2.06
4143	Serine/threonine-protein phosphatase 2B catalytic subunit beta isoform	P16298	0.0380	0.1224	0.34	0.0005	0.2158	0.53	0.0634	0.1467	0.15	0.0720	0.1511	0.26	0.0002	0.0123	0.73	0.5016	0.7268	0.10	0.0227	0.1366	0.32	0.7756	1.2186	0.02	0.1499	0.2126	-0.14
4144	Serine/threonine-protein phosphatase 2B catalytic subunit gamma isoform	P48454	0.2007	0.3714	1.12	0.1276	0.6411	1.84	0.0378	0.1024	0.49	0.0383	0.0954	0.52	0.0789	0.2166	0.37	0.3343	0.5376	-0.30	0.3736	0.4760	-2.55	0.8708	0.9892	-0.33	0.0651	0.1303	2.06
4145	Serine/threonine-protein phosphatase 4 catalytic subunit	P60510	0.6682	0.7273	-0.01	0.1488	0.6594	0.21	0.0038	0.0318	0.19	0.0028	0.0220	0.21	0.0241	0.1124	0.16	0.0194	0.0898	0.12	0.7474	0.7847	-0.02	0.7739	1.2170	-0.02	0.0353	0.1610	0.18
4146	Serine/threonine-protein phosphatase 4 regulatory subunit 1	Q8TF05	0.4108	0.5136	-3.31	0.5752	0.7192	-1.75	0.5661	0.6534	-1.19	0.5388	0.6182	0.89	0.2938	0.4141	-1.88	0.9510	1.0004	0.09	0.9968	0.9974	0.00	0.0275	0.1503	-0.52	0.0800	0.1310	-0.33
4147	Serine/threonine-protein phosphatase 4 regulatory subunit 2	Q9NY27	0.9439	0.9578	0.00	0.8526	0.8937	0.01	0.3465	0.5406	-0.03	0.1918	0.3212	0.08	0.5721	0.6520	0.03	0.4558	0.6787	0.07	0.8504	0.8749	0.03	0.6624	1.1012	0.09	0.2661	0.3377	0.24
4148	Serine/threonine-protein phosphatase 4 regulatory subunit 3A	Q6IN85	0.0048	0.0429	0.25	0.2172	0.6599	0.10	0.0496	0.1243	0.25	0.0022	0.0200	0.30	0.1581	0.3401	-0.07	0.2548	0.4451	0.14	0.3736	0.4892	-2.55	0.8708	1.0137	-0.33	0.0651	0.1360	2.06
4149	Serine/threonine-protein phosphatase 4 regulatory subunit 3B	Q5MIZ7	0.2177	0.3932	-0.07	0.2708	0.6642	0.14	0.0595	0.1411	0.12	0.1096	0.2080	0.10	0.6588	0.7244	-0.02	0.7446	0.9834	0.01	0.3736	0.4420	-2.55	0.8708	0.9259	-0.33	0.0651	0.1163	2.06

Supplemetary Table S2. Overview on all relatively quantified 5180 proteins statistical analysis

			MCF-7 Dai SC20 vs control			Gen SC20 vs control			SSE SC20 vs control			Dai IC20 vs control			Gen IC20 vs control			SSE IC20 vs control			MDA-MB-231 Dai IC20 vs control			Gen IC20 vs control			SSE IC20 vs control		
	**Protein name**	**UniProt**	**p value**	**BH q value**	**log2FC**	**p value**	**BH q value**	**log2FC**	**p value**	**BH q value**	**log2FC**	**p value**	**BH q value**	**log2FC**	**p value**	**BH q value**	**log2FC**	**p value**	**BH q value**	**log2FC**	**p value**	**BH q value**	**log2FC**	**p value**	**BH q value**	**log2FC**	**p value**	**BH q value**	**log2FC**
4150	Serine/threonine-protein phosphatase 5	P53041	0.0111	0.0603	-0.22	0.1728	0.6601	-0.23	0.1752	0.3172	0.08	0.8523	0.8727	0.01	0.9656	0.9733	0.00	0.3233	0.5261	-0.09	0.0597	0.1938	0.12	0.2135	0.5180	0.19	0.1062	0.1625	-0.06
4151	Serine/threonine-protein phosphatase 6 catalytic subunit	O00743	0.0074	0.0506	0.17	0.2324	0.6625	0.15	0.0285	0.0857	0.18	0.0256	0.0718	0.13	0.0709	0.2029	0.09	0.0672	0.1819	0.12	0.2982	0.5554	-0.10	0.0381	0.1778	-0.25	0.0251	0.1345	-0.39
4152	Serine/threonine-protein phosphatase 6 regulatory subunit 2	O75170	0.1812	0.3450	-0.22	0.0789	0.6146	0.08	0.3839	0.5845	0.05	0.0708	0.1491	-0.09	0.5314	0.6170	0.04	0.1559	0.3148	-0.29	0.3736	0.5136	-2.55	0.8708	1.0584	-0.33	0.0651	0.1472	2.06
4153	Serine/threonine-protein phosphatase 6 regulatory subunit 3	Q5H9R7	0.0117	0.0624	0.10	0.1605	0.6640	0.13	0.0244	0.0772	0.06	0.1230	0.2267	0.04	0.0423	0.1518	-0.10	0.1108	0.2512	0.10	0.0256	0.1399	0.24	0.0329	0.1639	0.14	0.0293	0.1462	0.15
4154	Serine/threonine-protein phosphatase PGAM5_ mitochondrial	Q96HS1	0.0074	0.0505	0.18	0.4967	0.8189	0.09	0.0190	0.0660	0.36	0.0179	0.0565	0.19	0.2358	0.4476	0.04	0.0085	0.0675	0.24	0.3736	0.6167	-2.55	0.8708	1.2419	-0.33	0.0651	0.2030	2.06
4155	Serine/threonine-protein phosphatase PP1-alpha catalytic subunit	P62136	0.4923	0.5664	0.05	0.5484	0.8640	0.10	0.6889	0.7378	0.04	0.2447	0.3924	0.10	0.1355	0.3053	-0.15	0.4948	0.7200	0.05	0.4485	0.5037	0.10	0.8146	1.2600	0.02	0.0764	0.1265	0.12
4156	Serine/threonine-protein phosphatase PP1-beta catalytic subunit	P62140	0.0223	0.0889	-0.19	0.2790	0.6648	-0.15	0.0116	0.0503	-0.35	0.0178	0.0563	-0.45	0.0071	0.0613	-0.41	0.1684	0.3332	-0.16	0.0523	0.1810	0.38	0.9800	0.9861	-0.01	0.0135	0.1006	0.78
4157	Serine/threonine-protein phosphatase PP1-gamma catalytic subunit	P36873	0.1634	0.3200	0.09	0.2611	0.6643	0.17	0.2193	0.3759	0.09	0.1874	0.3151	0.09	0.1119	0.2726	0.12	0.8648	1.1009	0.01	0.3722	0.6544	0.09	0.1319	0.3817	-0.13	0.0156	0.1073	-0.25
4158	Serine-protein kinase ATM	Q13315	0.4108	0.5135	-3.31	0.5752	0.7190	-1.75	0.5661	0.6532	-1.19	0.5388	0.6180	0.89	0.2938	0.4140	-1.88	0.9510	1.0002	0.09	0.1966	0.4059	-0.46	0.7411	1.1834	-0.05	0.0460	0.1872	0.36
4159	Serine-threonine kinase receptor-associated protein	Q9Y3F4	0.0021	0.0314	0.34	0.3595	0.7086	0.12	0.0031	0.0298	0.30	0.0184	0.0575	0.20	0.1681	0.3550	0.07	0.0106	0.0703	0.29	0.0178	0.1239	0.37	0.4138	0.8028	0.08	0.7578	0.8003	-0.02
4160	Serine--tRNA ligase cytoplasmic	P49591	0.0016	0.0270	0.40	0.0849	0.6212	0.27	0.0008	0.0218	0.46	0.0007	0.0142	0.53	0.0434	0.1541	0.14	0.0017	0.0512	0.59	0.0078	0.0910	0.36	0.3254	0.6797	0.07	0.0825	0.1340	0.12
4161	Serine--tRNA ligase mitochondrial	Q9NP81	0.1174	0.2520	-0.11	0.9028	0.9318	-0.02	0.2498	0.4165	-0.07	0.1912	0.3205	-0.08	0.0371	0.1400	-0.20	0.7368	0.9764	0.02	0.0347	0.1547	0.40	0.1102	0.3386	0.24	0.1635	0.2283	0.37
4162	Serologically defined colon cancer antigen 8	Q86SQ7	0.0216	0.0875	0.51	0.0004	0.2302	0.28	0.5584	0.7824	0.04	0.9558	0.9617	0.01	0.0003	0.0136	0.43	0.0060	0.0625	0.20	0.0391	0.1640	0.32	0.0024	0.0408	-0.28	0.0858	0.1381	-0.10
4163	Serotransferrin	P02787	0.0078	0.0521	-0.24	0.4568	0.7887	-0.07	0.3019	0.4848	-0.04	0.0067	0.0329	-0.18	0.0004	0.0167	-0.33	0.0056	0.0622	-0.21	0.3736	0.6273	-2.55	0.8708	1.2603	-0.33	0.0651	0.2097	2.06
4164	Serpin B4	P48594	0.4108	0.5134	-3.31	0.5752	0.7188	-1.75	0.5661	0.6531	-1.19	0.5388	0.6179	0.89	0.2938	0.4139	-1.88	0.9510	1.0000	0.09	0.0446	0.1696	0.23	0.4800	0.8902	0.08	0.7154	0.7652	0.06
4165	Serpin B6	P35237	0.8918	0.9151	0.00	0.8353	0.8811	0.02	0.0242	0.0767	0.11	0.1627	0.2822	0.04	0.0927	0.2402	-0.05	0.0583	0.1665	0.09	0.5665	0.6166	-0.02	0.0016	0.0324	-0.31	0.0354	0.1613	-0.15
4166	Serpin B7	O75635	0.4108	0.5133	-3.31	0.5752	0.7187	-1.75	0.5661	0.6529	-1.19	0.5388	0.6177	0.89	0.2938	0.4138	-1.88	0.9510	0.9998	0.09	0.0541	0.1846	0.55	0.2215	0.5290	0.08	0.0280	0.1432	0.22
4167	Serpin B8	P50452	0.6086	0.6730	0.02	0.1740	0.6613	0.10	0.9865	0.9886	0.00	0.5914	0.6396	0.02	0.8565	0.8898	-0.01	0.0968	0.2293	-0.07	0.0568	0.1895	-1.38	0.0050	0.0566	-1.62	0.3801	0.4513	-1.00
4168	Serpin H1	P50454	0.0343	0.1161	0.09	0.2750	0.6650	0.18	0.0063	0.0383	0.20	0.0044	0.0269	0.18	0.0535	0.1717	-0.09	0.0127	0.0758	0.16	0.5109	0.5644	-0.04	0.6719	1.1102	0.02	0.1267	0.1863	-0.12
4169	Serrate RNA effector molecule homolog	Q9BXP5	0.0194	0.0818	0.17	0.1762	0.6614	0.26	0.0138	0.0552	0.46	0.0116	0.0431	0.41	0.0014	0.0288	0.22	0.0008	0.0460	0.45	0.0524	0.1811	0.54	0.6646	1.1023	0.05	0.0654	0.1117	0.26
4170	Serum albumin	P02768	0.0992	0.2239	0.15	0.6912	0.7736	0.08	0.0147	0.0570	0.34	0.0915	0.1813	0.15	0.9607	0.9695	0.00	0.0078	0.0659	0.55	0.0164	0.1197	0.61	0.0050	0.0568	0.60	0.3301	0.4013	-0.10
4171	S-formylglutathione hydrolase	P10768	0.0047	0.0430	0.15	0.0880	0.6210	0.21	0.0184	0.0650	0.32	0.0010	0.0151	0.23	0.0066	0.0594	0.18	0.1362	0.2886	0.17	0.0374	0.1606	0.63	0.2173	0.5240	0.17	0.2366	0.3065	0.12
4172	SH2 domain-containing protein 3C	Q8N5H7	0.4108	0.5131	-3.31	0.5752	0.7185	-1.75	0.5661	0.6528	-1.19	0.5388	0.6176	0.89	0.2938	0.4137	-1.88	0.9510	0.9996	0.09	0.0214	0.1337	0.41	0.0619	0.2379	0.21	0.7993	0.8364	-0.02
4173	SH2 domain-containing protein 4A	Q9H788	0.0001	0.0094	-0.86	0.7929	0.8509	-0.02	0.0027	0.0284	-0.73	0.0022	0.0204	-0.56	0.0002	0.0144	-0.82	0.0716	0.1888	-0.86	0.3736	0.5490	-2.55	0.8708	1.1224	-0.33	0.0651	0.1647	2.06
4174	SH2 domain-containing protein 4B	Q5SQS7	0.7040	0.7566	-0.06	0.0301	0.5046	-0.83	0.0784	0.1699	-0.65	0.0048	0.0278	-0.89	0.0045	0.0500	-1.10	0.0433	0.1370	-0.94	0.3736	0.5665	-2.55	0.8708	1.1536	-0.33	0.0651	0.1739	2.06
4175	SH3 and cysteine-rich domain-containing protein	Q99469	0.7456	0.7931	0.03	0.8592	0.8982	-0.02	0.4862	0.7065	0.06	0.5669	0.6171	0.06	0.4263	0.5207	0.07	0.9196	1.1568	0.01	0.3736	0.4331	-2.55	0.8708	0.9091	-0.33	0.0651	0.1128	2.06
4176	SH3 and PX domain-containing protein 2B	A1X283	0.4108	0.5130	-3.31	0.5752	0.7183	-1.75	0.5661	0.6527	-1.19	0.5388	0.6175	0.89	0.2938	0.4136	-1.88	0.9510	0.9994	0.09	0.0063	0.0830	-1.19	0.0006	0.0234	-1.42	0.0003	0.0293	-1.14
4177	SH3 domain-binding glutamic acid-rich-like protein	O75368	0.1600	0.3149	-0.06	0.7178	0.7930	0.04	0.0389	0.1044	-0.12	0.7028	0.7417	-0.01	0.0142	0.0851	-0.17	0.0438	0.1378	0.12	0.6587	0.7031	-0.04	0.5443	0.9699	-0.06	0.2500	0.3209	-0.12
4178	SH3 domain-binding glutamic acid-rich-like protein 3	Q9H299	0.0063	0.0476	0.08	0.0458	0.5609	0.34	0.0011	0.0227	0.38	0.0551	0.1240	0.24	0.0024	0.0381	0.27	0.0221	0.0955	0.25	0.0076	0.0897	0.20	0.7833	1.2277	0.02	0.0280	0.1430	0.24
4179	SH3 domain-containing kinase-binding protein 1	Q96B97	0.4108	0.5129	-3.31	0.5752	0.7181	-1.75	0.5661	0.6525	-1.19	0.5388	0.6173	0.89	0.2938	0.4134	-1.88	0.9510	0.9992	0.09	0.0624	0.1965	0.49	0.2403	0.5577	0.17	0.5435	0.6087	0.05
4180	SHC SH2 domain-binding protein 1	Q8NEM2	0.6290	0.6913	0.03	0.1441	0.6542	-0.20	0.6185	0.6726	-0.03	0.0402	0.0988	-0.21	0.0068	0.0597	-0.34	0.0106	0.0707	0.48	0.3736	0.6113	-2.55	0.8708	1.2324	-0.33	0.0651	0.1997	2.06
4181	SHC-transforming protein 1	P29353	0.5771	0.6445	-0.04	0.1817	0.6647	-0.30	0.8746	0.9009	0.01	0.0720	0.1511	-0.29	0.0112	0.0755	-0.38	0.3275	0.5300	-0.11	0.0613	0.1952	-0.21	0.0722	0.2606	-0.20	0.5144	0.5810	0.07
4182	Shootin-1	A0MZ66	0.4785	0.5534	0.03	0.1644	0.6617	0.24	0.0070	0.0399	0.23	0.0053	0.0293	0.23	0.1771	0.3678	0.06	0.0176	0.0868	0.17	0.0173	0.1221	0.27	0.1156	0.3490	0.13	0.3090	0.3802	0.07
4183	Short stature homeobox protein 2	O60902	0.4108	0.5128	-3.31	0.5752	0.7180	-1.75	0.5661	0.6524	-1.19	0.5388	0.6172	0.89	0.2938	0.4133	-1.88	0.9510	0.9990	0.09	0.0854	0.2334	0.53	0.0002	0.0155	0.35	0.0001	0.0247	1.05
4184	Short/branched chain specific acyl-CoA dehydrogenase_ mitochondrial	P45954	0.4108	0.5126	-3.31	0.5752	0.7178	-1.75	0.5661	0.6522	-1.19	0.5388	0.6171	0.89	0.2938	0.4132	-1.88	0.9510	0.9988	0.09	0.0171	0.1213	0.56	0.3489	0.7113	0.14	0.5224	0.5888	0.09
4185	Short-wave-sensitive opsin 1	P03999	0.4108	0.5125	-3.31	0.5752	0.7176	-1.75	0.5661	0.6521	-1.19	0.5388	0.6169	0.89	0.2938	0.4131	-1.88	0.9510	0.9986	0.09	0.0140	0.1105	0.36	0.2838	0.6213	0.12	0.1203	0.1788	-0.16
4186	Shugoshin 2	Q562F6	0.4108	0.5124	-3.31	0.5752	0.7174	-1.75	0.5661	0.6519	-1.19	0.5388	0.6168	0.89	0.2938	0.4130	-1.88	0.9510	0.9984	0.09	0.2911	0.5436	0.09	0.5948	1.0280	-0.02	0.7059	0.7561	-0.01
4187	Sialic acid synthase	Q9NR45	0.0229	0.0903	0.23	0.2068	0.6596	0.26	0.0008	0.0208	0.47	0.0014	0.0173	0.44	0.0353	0.1360	0.18	0.0045	0.0601	0.57	0.0348	0.1549	0.53	0.5602	0.9870	0.04	0.0028	0.0537	0.37
4188	Sialidase-1	Q99519	0.0021	0.0313	0.66	0.3298	0.6889	-0.10	0.3511	0.5452	-0.11	0.4758	0.6758	-0.08	0.3060	0.4034	0.10	0.1668	0.3308	0.51	0.3736	0.5102	-2.55	0.8708	1.0522	-0.33	0.0651	0.1456	2.06
4189	Sideroflexin-1	Q9H9B4	0.0627	0.1658	0.18	0.2340	0.6613	0.22	0.0533	0.1307	0.19	0.0505	0.1157	0.20	0.4369	0.5308	-0.05	0.0895	0.2182	0.17	0.5193	0.5726	0.04	0.2540	0.5786	-0.10	0.0727	0.1217	-0.16
4190	Sideroflexin-2	Q96NB2	0.0038	0.0391	-0.18	0.4266	0.7625	-0.10	0.5388	0.7607	0.05	0.0026	0.0213	-0.28	0.7227	0.7824	-0.01	0.0009	0.0432	0.46	0.0008	0.0441	1.02	0.0017	0.0343	1.29	0.0117	0.0940	0.47
4191	Sideroflexin-3	Q9BWM7	0.0148	0.0707	-0.62	0.5070	0.8287	-0.33	0.0204	0.0687	-0.47	0.0160	0.0525	-0.88	0.2931	0.5250	-0.16	0.0077	0.0658	-0.87	0.1306	0.3057	-0.15	0.2428	0.5612	-0.08	0.0158	0.1075	-0.26
4192	Signal peptidase complex catalytic subunit SEC11A	P67812	0.8961	0.9183	-0.01	0.2710	0.6644	-0.13	0.1036	0.2100	0.04	0.0643	0.1391	0.08	0.0270	0.1191	-0.07	0.0018	0.0530	0.18	0.5588	0.6089	0.03	0.0060	0.0620	0.23	0.0936	0.1478	-0.10
4193	Signal peptidase complex subunit 1	Q9Y6A9	0.0908	0.2108	0.11	0.4036	0.7477	0.15	0.0536	0.1312	0.13	0.0056	0.0299	0.29	0.0487	0.1622	0.14	0.0048	0.0605	0.35	0.3736	0.6306	-2.55	0.8708	1.2660	-0.33	0.0651	0.2118	2.06
4194	Signal peptidase complex subunit 2	Q15005	0.0002	0.0120	0.22	0.1998	0.6639	0.06	0.0672	0.1525	0.11	0.0012	0.0157	0.18	0.0271	0.1194	0.16	0.0458	0.1419	0.31	0.3383	0.6089	-0.05	0.1292	0.3770	-0.09	0.0713	0.1196	-0.13
4195	Signal peptidase complex subunit 3	P61009	0.0593	0.1608	-0.05	0.2950	0.6720	-0.11	0.0042	0.0321	-0.19	0.0007	0.0137	-0.21	0.0129	0.0811	-0.25	0.0389	0.1285	-0.08	0.0476	0.1746	-0.25	0.0935	0.3044	-0.23	0.0087	0.0833	-0.26
4196	Signal peptide peptidase-like 2A	Q8TCT8	0.0353	0.1172	-1.88	0.0935	0.6290	-7.47	0.3237	0.5115	0.55	0.4375	0.6307	1.88	0.4660	0.5573	-0.38	0.7061	0.9476	1.29	0.3736	0.4452	-2.55	0.8708	0.9318	-0.33	0.0651	0.1175	2.06
4197	Signal recognition particle 14 kDa protein	P37108	0.0020	0.0307	0.22	0.0279	0.5053	0.19	0.0004	0.0233	0.20	0.0009	0.0158	0.22	0.0132	0.0821	0.09	0.0352	0.1211	0.20	0.2311	0.4587	0.19	0.0184	0.1157	-0.25	0.0257	0.1367	-0.28
4198	Signal recognition particle 19 kDa protein	P09132	0.2856	0.4813	-0.08	0.4275	0.7628	0.08	0.0168	0.0614	0.25	0.1066	0.2036	0.13	0.0554	0.1755	-0.17	0.2161	0.3972	-0.08	0.0802	0.2253	0.33	0.0283	0.1524	-0.69	0.5309	0.5972	-0.33
4199	Signal recognition particle 54 kDa protein	P61011	0.5724	0.6400	0.04	0.1824	0.6658	0.27	0.0645	0.1482	0.23	0.0155	0.0514	0.21	0.0521	0.1692	-0.11	0.3474	0.5525	0.05	0.3539	0.6302	-0.05	0.0267	0.1471	-0.18	0.0952	0.1499	-0.11
4200	Signal recognition particle 9 kDa protein	P49458	0.0102	0.0588	0.23	0.0830	0.6195	0.24	0.0006	0.0203	0.35	0.0053	0.0295	0.21	0.3577	0.4550	0.03	0.0326	0.1161	0.33	0.0009	0.0420	0.38	0.0662	0.2480	0.11	0.0665	0.1131	0.10
4201	Signal recognition particle receptor subunit alpha	P08240	0.0019	0.0298	-0.30	0.6113	0.7127	-0.05	0.0986	0.2018	-0.10	0.2056	0.3394	-0.09	0.6672	0.7316	-0.01	0.2959	0.4925	-0.19	0.5546	0.6057	0.07	0.3521	0.7155	-0.09	0.1584	0.2222	-0.08
4202	Signal recognition particle receptor subunit beta	Q9Y5M8	0.0369	0.1209	0.20	0.3031	0.6765	0.19	0.0371	0.1010	0.20	0.0180	0.0566	0.28	0.9128	0.9344	0.01	0.0118	0.0736	0.33	0.0497	0.1778	0.26	0.3062	0.6525	0.08	0.4603	0.5287	0.05
4203	Signal recognition particle subunit SRP68	Q9UHB9	0.6478	0.7091	0.02	0.2257	0.6602	0.19	0.0377	0.1021	0.16	0.0099	0.0400	0.24	0.8907	0.9160	-0.01	0.1073	0.2452	0.10	0.0790	0.2233	0.22	0.5101	0.9291	-0.06	0.7041	0.7551	0.03
4204	Signal recognition particle subunit SRP72	O76094	0.1972	0.3664	0.07	0.2691	0.6625	0.19	0.0134	0.0542	0.23	0.0064	0.0321	0.28	0.3531	0.4506	0.05	0.0218	0.0949	0.23	0.0694	0.2074	0.46	0.1288	0.3761	-0.14	0.9568	0.9659	0.00
4205	Signal transducer and activator of transcription 1-alpha/beta	P42224	0.0005	0.0177	0.12	0.1701	0.6620	0.15	0.0008	0.0214	0.19	0.0005	0.0131	0.18	0.1598	0.3428	0.02	0.1014	0.2359	0.19	0.5791	0.6283	-0.03	0.0058	0.0611	-0.23	0.0006	0.0314	-0.58
4206	Signal transducer and activator of transcription 3	P40763	0.0958	0.2185	0.10	0.0044	0.3506	0.29	0.0061	0.0381	0.37	0.0025	0.0213	0.33	0.0030	0.0413	0.34	0.0075	0.0654	0.29	0.0568	0.1896	0.20	0.1141	0.3458	0.13	0.0137	0.1017	0.31
4207	Signal transducing adapter molecule 1	Q92783	0.0430	0.1317	-0.53	0.5064	0.8283	-0.13	0.1380	0.2620	-0.32	0.0907	0.1800	-0.39	0.1502	0.3273	-0.35	0.5752	0.8097	-0.09	0.2682	0.5110	-0.09	0.0278	0.1514	-0.18	0.0011	0.0413	-0.16
4208	Signal transducing adapter molecule 2	O75886	0.3123	0.5132	-0.31	0.0243	0.4804	-0.36	0.0971	0.1998	-0.35	0.0073	0.0347	-0.40	0.1345	0.3040	-0.27	0.2863	0.4826	-0.18	0.2025	0.4154	-0.29	0.0166	0.1093	-0.55	0.1176	0.1757	-0.11
4209	Signal-induced proliferation-associated 1-like protein 2	Q9P2F8	0.5149	0.5874	-0.06	0.9162	0.9418	0.02	0.3153	0.5022	0.11	0.0810	0.1658	0.22	0.3415	0.4387	0.09	0.8588	1.0946	-0.02	0.3736	0.4357	-2.55	0.8708	0.9138	-0.33	0.0651	0.1138	2.06
4210	Signal-induced proliferation-associated 1-like protein 3	O60292	0.4551	0.5323	-0.09	0.3461	0.7028	0.19	0.6672	0.7181	-0.09	0.2166	0.3544	0.41	0.1184	0.2822	0.24	0.4967	0.7217	0.28	0.6836	0.7262	0.07	0.7112	1.1556	-0.07	0.9868	0.9895	0.00
4211	Signaling lymphocytic activation molecule	Q13291	0.4108	0.5123	-3.31	0.5752	0.7173	-1.75	0.5661	0.6518	-1.19	0.5388	0.6167	0.89	0.2938	0.4129	-1.88	0.9510	0.9982	0.09	0.0139	0.1106	0.35	0.0325	0.1627	0.45	0.0186	0.1174	0.37
4212	Single-stranded DNA-binding protein 2	P81877	0.0040	0.0400	-1.31	0.8342	0.8806	-0.04	0.1540	0.2853	-0.32	0.0707	0.1489	-0.50	0.1330	0.3019	-1.08	0.0862	0.2138	-1.05	0.3736	0.5410	-2.55	0.8708	1.1080	-0.33	0.0651	0.1606	2.06
4213	Single-stranded DNA-binding protein mitochondrial	Q04837	0.0099	0.0579	0.26	0.1704	0.6612	0.25	0.0051	0.0349	0.29	0.0030	0.0225	0.41	0.0102	0.0727	0.24	0.0059	0.0624	0.35	0.0025	0.0576	0.43	0.0137	0.0979	0.26	0.0096	0.0871	0.31
4214	Sister chromatid cohesion protein PDS5 homolog A	Q29RF7	0.0660	0.1703	0.05	0.1328	0.6471	0.24	0.3549	0.5504	-0.15	0.1660	0.2861	0.07	0.3067	0.4041	0.08	0.1574	0.3168	-0.10	0.3108	0.5715	0.16	0.1612	0.4356	-0.09	0.3571	0.4286	0.07
4215	Sjoegren syndrome/scleroderma autoantigen 1	O60232	0.0089	0.0559	0.29	0.2375	0.6614	-0.13	0.7062	0.7525	0.03	0.0115	0.0429	-0.27	0.0188	0.0980	-0.25	0.1896	0.3629	-0.08	0.0623	0.1965	0.31	0.4772	0.8889	0.04	0.0443	0.1839	-0.15
4216	Ski-like protein	P12757	0.0295	0.1051	0.09	0.8459	0.8892	0.03	0.0275	0.0834	0.13	0.0230	0.0666	0.19	0.1045	0.2602	-0.06	0.0187	0.0885	0.13	0.3736	0.5956	-2.55	0.8708	1.2051	-0.33	0.0651	0.1903	2.06
4217	SLAIN motif-containing protein 2	Q9P270	0.2655	0.4572	-0.12	0.2386	0.6616	0.14	0.2116	0.3662	0.12	0.7487	0.7835	-0.03	0.0577	0.1802	0.24	0.6725	0.9103	-0.05	0.3736	0.4464	-2.55	0.8708	0.9341	-0.33	0.0651	0.1180	2.06
4218	Slit homolog 2 protein	O94813	0.4108	0.5121	-3.31	0.5752	0.7171	-1.75	0.5661	0.6516	-1.19	0.5388	0.6165	0.89	0.2938	0.4128	-1.88	0.9510	0.9980	0.09	0.5762	0.6256	-0.03	0.0050	0.0563	-0.31	0.0027	0.0553	-0.35
4219	SLIT-ROBO Rho GTPase-activating protein 1	Q7Z6B7	0.4108	0.5120	-3.31	0.5752	0.7169	-1.75	0.5661	0.6515	-1.19	0.5388	0.6164	0.89	0.2938	0.4127	-1.88	0.9510	0.9978	0.09	0.0351	0.1547	0.17	0.2120	0.5153	0.08	0.0758	0.1258	0.13
4220	SLIT-ROBO Rho GTPase-activating protein 2	O75044	0.4108	0.5119	-3.31	0.5752	0.7168	-1.75	0.5661	0.6514	-1.19	0.5388	0.6162	0.89	0.2938	0.4125	-1.88	0.9510	0.9976	0.09	0.1833	0.3844	0.19	0.5198	0.9421	0.06	0.0241	0.1321	0.41
4221	Small acidic protein	O00193	0.0392	0.1246	-0.06	0.0014	0.2014	-0.10	0.0015	0.0235	-0.16	0.1645	0.2844	-0.10	0.0065	0.0587	-0.12	0.2121	0.3935	0.06	0.0001	0.0247	1.19	0.0078	0.0727	1.08	0.0100	0.0887	0.81
4222	Small EDRK-rich factor 2	P84101	0.1121	0.2435	-0.22	0.1743	0.6605	-0.62	0.1337	0.2560	0.18	0.2903	0.4523	0.19	0.5834	0.6611	0.06	0.5698	0.8045	-0.08	0.3736	0.4547	-2.55	0.8708	0.9496	-0.33	0.0651	0.1214	2.06

Supplemetary Table S2. Overview on all relatively quantified 5180 proteins statistical analysis

			MCF-7																		MDA-MB-231								
			Dai SC20 vs control			Gen SC20 vs control			SSE SC20 vs control			Dai IC20 vs control			Gen IC20 vs control			SSE IC20 vs control			Dai IC20 vs control			Gen IC20 vs control			SSE IC20 vs control		
	**Protein name**	**UniProt**	**p value**	**BH q value**	**log2FC**	**p value**	**BH q value**	**log2FC**	**p value**	**BH q value**	**log2FC**	**p value**	**BH q value**	**log2FC**	**p value**	**BH q value**	**log2FC**	**p value**	**BH q value**	**log2FC**	**p value**	**BH q value**	**log2FC**	**p value**	**BH q value**	**log2FC**	**p value**	**BH q value**	**log2FC**
4223	Small G protein signaling modulator 3	Q96HU1	0.2999	0.4989	-0.06	0.7359	0.8080	0.03	0.0663	0.1510	0.15	0.1159	0.2173	0.11	0.4603	0.5524	-0.04	0.0271	0.1055	0.26	0.3736	0.5834	-2.55	0.8708	1.1836	-0.33	0.0651	0.1833	2.06
4224	Small glutamine-rich tetratricopeptide repeat-containing protein alpha	O43765	0.4108	0.5118	-3.31	0.5752	0.7166	-1.75	0.5661	0.6512	-1.19	0.5388	0.6161	0.89	0.2938	0.4124	-1.88	0.9510	0.9974	0.09	0.0398	0.1640	0.58	0.1639	0.4406	0.12	0.0923	0.1461	0.40
4225	Small glutamine-rich tetratricopeptide repeat-containing protein beta	Q96EQ0	0.4108	0.5116	-3.31	0.5752	0.7164	-1.75	0.5661	0.6511	-1.19	0.5388	0.6160	0.89	0.2938	0.4123	-1.88	0.9510	0.9972	0.09	0.0501	0.1782	-0.91	0.6992	1.1422	-0.07	0.1114	0.1687	0.58
4226	Small nuclear ribonucleoprotein F	P62306	0.8581	0.8877	-0.05	0.1992	0.6640	-0.41	0.2745	0.4484	-0.32	0.2953	0.4585	-0.33	0.0853	0.2276	-0.63	0.6697	0.9084	-0.11	0.3736	0.4466	-2.55	0.8708	0.9345	-0.33	0.0651	0.1181	2.06
4227	Small nuclear ribonucleoprotein Sm D1	P62314	0.8596	0.8888	0.01	0.1430	0.6544	-0.12	0.0237	0.0756	0.15	0.7589	0.7918	-0.01	0.1277	0.2952	-0.09	0.5242	0.7532	0.07	0.2836	0.5330	0.12	0.0010	0.0263	0.47	0.0756	0.1256	0.12
4228	Small nuclear ribonucleoprotein Sm D2	P62316	0.4616	0.5384	0.03	0.4727	0.8015	0.13	0.0044	0.0327	0.30	0.0129	0.0461	0.20	0.0563	0.1772	-0.13	0.0485	0.1473	0.13	0.0182	0.1254	0.74	0.0493	0.2085	0.37	0.0016	0.0485	0.47
4229	Small nuclear ribonucleoprotein Sm D3	P62318	0.0449	0.1351	0.08	0.8214	0.8724	-0.02	0.0018	0.0235	0.18	0.1439	0.2563	0.09	0.0072	0.0616	-0.13	0.0051	0.0619	0.14	0.0039	0.0678	-1.67	0.0172	0.1111	-1.20	0.0028	0.0543	-1.92
4230	Small nuclear ribonucleoprotein-associated proteins B and B'	P14678	0.4108	0.5115	-3.31	0.5752	0.7162	-1.75	0.5661	0.6509	-1.19	0.5388	0.6158	0.89	0.2938	0.4122	-1.88	0.9510	0.9970	0.09	0.0000	0.0000	-2.60	0.0000	0.0000	-2.44	0.0034	0.0585	-2.60
4231	Small proline-rich protein 2A	P35326	0.4108	0.5114	-3.31	0.5752	0.7161	-1.75	0.5661	0.6508	-1.19	0.5388	0.6157	0.89	0.2938	0.4121	-1.88	0.9510	0.9968	0.09	0.6682	0.7121	0.40	0.5822	1.0144	-0.93	0.4833	0.5516	0.65
4232	Small subunit processome component 20 homolog	O75691	0.0430	0.1316	0.12	0.0110	0.4317	0.25	0.0111	0.0489	0.19	0.0033	0.0234	0.26	0.0026	0.0394	0.48	0.0579	0.1658	0.12	0.0324	0.1512	-2.19	0.0659	0.2477	-0.80	0.0192	0.1200	-0.58
4233	Small ubiquitin-related modifier 1	P63165	0.4108	0.5113	-3.31	0.5752	0.7159	-1.75	0.5661	0.6506	-1.19	0.5388	0.6156	0.89	0.2938	0.4120	-1.88	0.9510	0.9966	0.09	0.9768	0.9808	0.00	0.1197	0.3578	-0.16	0.2360	0.3059	-0.16
4234	Small ubiquitin-related modifier 2	P61956	0.0140	0.0691	0.25	0.1985	0.6655	0.34	0.0061	0.0379	0.44	0.0022	0.0202	0.43	0.7011	0.7626	-0.02	0.0112	0.0727	0.33	0.0756	0.2190	0.37	0.4907	0.9023	0.07	0.1805	0.2474	-0.12
4235	Small ubiquitin-related modifier 3	P55854	0.4108	0.5112	-3.31	0.5752	0.7157	-1.75	0.5661	0.6505	-1.19	0.5388	0.6154	0.89	0.2938	0.4119	-1.88	0.9510	0.9964	0.09	0.0099	0.0994	0.48	0.0781	0.2743	0.23	0.4048	0.4762	0.12
4236	S-methyl-5'-thioadenosine phosphorylase	Q13126	0.4108	0.5110	-3.31	0.5752	0.7155	-1.75	0.5661	0.6503	-1.19	0.5388	0.6153	0.89	0.2938	0.4118	-1.88	0.9510	0.9962	0.09	0.8485	0.8731	0.02	0.1245	0.3673	-0.17	0.0887	0.1415	-0.22
4237	snRNA-activating protein complex subunit 3	Q92966	0.4108	0.5109	-3.31	0.5752	0.7154	-1.75	0.5661	0.6502	-1.19	0.5388	0.6152	0.89	0.2938	0.4117	-1.88	0.9510	0.9960	0.09	0.0018	0.0512	0.60	0.0018	0.0353	0.99	0.0108	0.0913	0.30
4238	Snurportin-1	O95149	0.0100	0.0582	11.31	0.5752	0.8860	-1.75	0.5661	0.7898	-1.19	0.5388	0.7459	0.89	0.2938	0.5242	-1.88	0.9510	1.1859	0.09	0.3736	0.4310	-2.55	0.8708	0.9050	-0.33	0.0651	0.1120	2.06
4239	SNW domain-containing protein 1	Q13573	0.2058	0.3784	-0.04	0.1105	0.6325	0.07	0.7367	0.7798	0.02	0.2187	0.3573	0.04	0.1154	0.2780	0.05	0.0081	0.0665	-0.17	0.0429	0.1677	0.33	0.7169	1.1627	0.03	0.1374	0.1984	0.13
4240	Sodium channel protein type 5 subunit alpha	Q14524	0.4108	0.5108	-3.31	0.5752	0.7152	-1.75	0.5661	0.6501	-1.19	0.5388	0.6150	0.89	0.2938	0.4115	-1.88	0.9510	0.9958	0.09	0.5606	0.6107	-0.06	0.1050	0.3284	-0.10	0.2173	0.2864	-0.07
4241	Sodium/potassium-transporting ATPase subunit alpha-1	P05023	0.0447	0.1346	0.17	0.3459	0.7029	0.18	0.0136	0.0546	0.25	0.0076	0.0352	0.32	0.1091	0.2681	0.12	0.0101	0.0703	0.45	0.0973	0.2506	0.25	0.7681	1.2112	0.03	0.4300	0.5014	-0.06
4242	Sodium/potassium-transporting ATPase subunit alpha-2	P50993	0.0015	0.0259	0.28	0.0881	0.6209	0.32	0.0035	0.0315	0.29	0.0025	0.0214	0.26	0.0379	0.1423	0.10	0.0102	0.0704	0.22	0.0259	0.1403	0.50	0.6987	1.1417	0.04	0.1429	0.2043	-0.13
4243	Sodium/potassium-transporting ATPase subunit alpha-3	P13637	0.0049	0.0430	0.30	0.4709	0.8003	-0.13	0.3915	0.5933	-0.12	0.0692	0.1466	-0.20	0.7531	0.8070	-0.02	0.0575	0.1649	0.21	0.7253	0.7649	0.03	0.3313	0.6881	-0.06	0.0748	0.1246	-0.18
4244	Sodium/potassium-transporting ATPase subunit alpha-4	Q13733	0.0934	0.2149	0.14	0.2434	0.6587	0.10	0.3453	0.5396	0.08	0.0672	0.1434	0.16	0.1120	0.2728	0.13	0.4851	0.7098	0.05	0.0168	0.1204	-0.28	0.0313	0.1597	-0.23	0.0068	0.0766	-0.44
4245	Sodium/potassium-transporting ATPase subunit beta-1	P05026	0.4090	0.6302	-0.05	0.2226	0.6578	0.17	0.2230	0.3802	0.08	0.0477	0.1113	0.11	0.1360	0.3056	0.07	0.0305	0.1119	0.15	0.0087	0.0935	-0.33	0.4904	0.9024	-0.05	0.6133	0.6729	-0.03
4246	Sodium/potassium-transporting ATPase subunit beta-3	P54709	0.2976	0.4965	0.04	0.8798	0.9137	0.02	0.5006	0.7231	0.05	0.0149	0.0500	0.17	0.9221	0.9419	0.00	0.0070	0.0641	0.32	0.0017	0.0537	-0.51	0.0045	0.0542	0.46	0.2723	0.3444	-0.06
4247	Sodium-coupled neutral amino acid transporter 2	Q96QD8	0.1787	0.3416	-0.13	0.2703	0.6639	-0.16	0.6148	0.6692	0.04	0.0163	0.0530	-0.34	0.8751	0.9048	-0.02	0.1190	0.2634	-0.15	0.3736	0.5276	-2.55	0.8708	1.0838	-0.33	0.0651	0.1539	2.06
4248	Soluble calcium-activated nucleotidase 1	Q8WVQ1	0.2499	0.4360	0.15	0.1904	0.6691	0.16	0.0295	0.0872	0.28	0.0011	0.0154	0.22	0.0000	0.0000	0.36	0.1389	0.2926	0.32	0.3736	0.5208	-2.55	0.8708	1.0714	-0.33	0.0651	0.1506	2.06
4249	Solute carrier family 12 member 1	Q13621	0.4108	0.5107	-3.31	0.5752	0.7150	-1.75	0.5661	0.6499	-1.19	0.5388	0.6149	0.89	0.2938	0.4114	-1.88	0.9510	0.9956	0.09	0.1447	0.3279	0.22	0.9747	0.9819	0.00	0.0812	0.1322	-0.19
4250	Solute carrier family 12 member 7	Q9Y666	0.4108	0.5105	-3.31	0.5752	0.7149	-1.75	0.5661	0.6498	-1.19	0.5388	0.6148	0.89	0.2938	0.4113	-1.88	0.9510	0.9954	0.09	0.0110	0.1029	0.66	0.0047	0.0550	0.70	0.0113	0.0932	0.39
4251	Solute carrier family 12 member 8	A0AV02	0.4108	0.5104	-3.31	0.5752	0.7147	-1.75	0.5661	0.6496	-1.19	0.5388	0.6146	0.89	0.2938	0.4112	-1.88	0.9510	0.9952	0.09	0.7985	0.8299	0.03	0.2452	0.5658	0.16	0.9794	0.9840	0.00
4252	Solute carrier family 2_ facilitated glucose transporter member 1	P11166	0.0017	0.0280	0.33	0.4560	0.7881	0.08	0.0007	0.0201	0.43	0.0013	0.0169	0.37	0.0025	0.0389	0.57	0.0254	0.1011	0.22	0.4138	0.4700	0.06	0.0705	0.2570	-0.13	0.0266	0.1389	-0.17
4253	Solute carrier family 2_ facilitated glucose transporter member 3	P11169	0.0370	0.1210	-0.33	0.4939	0.8169	-0.08	0.9496	0.9602	-0.01	0.0083	0.0368	-0.51	0.8786	0.9071	0.02	0.3766	0.5853	-0.07	0.0136	0.1102	0.50	0.0041	0.0518	1.01	0.0060	0.0735	0.82
4254	Solute carrier family 27 member 3	Q5K4L6	0.0023	0.0327	-0.30	0.1335	0.6481	-0.15	0.0093	0.0451	-0.23	0.0178	0.0564	-0.16	0.0047	0.0513	-0.26	0.0162	0.0846	-0.17	0.3736	0.6006	-2.55	0.8708	1.2139	-0.33	0.0651	0.1932	2.06
4255	Sorbin and SH3 domain-containing protein 2	O94875	0.0878	0.2063	0.09	0.0425	0.5674	0.12	0.0818	0.1757	0.12	0.0436	0.1044	0.12	0.0216	0.1059	0.18	0.6405	0.8773	0.06	0.3736	0.4484	-2.55	0.8708	0.9378	-0.33	0.0651	0.1188	2.06
4256	Sorbitol dehydrogenase	Q00796	0.0570	0.1566	0.07	0.2483	0.6582	0.11	0.0013	0.0226	0.25	0.0150	0.0503	0.12	0.8920	0.9170	0.00	0.0976	0.2304	0.06	0.4140	0.4700	0.08	0.1667	0.4456	0.08	0.7425	0.7873	-0.01
4257	Sorcin	P30626	0.0161	0.0740	0.36	0.4865	0.8108	0.07	0.3757	0.5749	0.08	0.1471	0.2610	0.16	0.6453	0.7135	0.04	0.0493	0.1487	0.37	0.1077	0.2681	0.28	0.7663	1.2087	0.02	0.1078	0.1642	-0.13
4258	Sortilin	Q99523	0.3170	0.5198	0.05	0.0133	0.4416	0.23	0.0525	0.1296	0.19	0.0272	0.0749	0.19	0.0111	0.0752	0.24	0.0894	0.2181	0.12	0.3736	0.5382	-2.55	0.8708	1.1029	-0.33	0.0651	0.1591	2.06
4259	Sorting and assembly machinery component 50 homolog	Q9Y512	0.4108	0.5103	-3.31	0.5752	0.7145	-1.75	0.5661	0.6495	-1.19	0.5388	0.6145	0.89	0.2938	0.4111	-1.88	0.9510	0.9950	0.09	0.3373	0.6081	2.67	0.0911	0.3000	-6.17	0.2053	0.2741	3.93
4260	Sorting nexin-1	Q13596	0.4214	0.4989	0.05	0.2143	0.6615	0.22	0.0076	0.0414	0.33	0.0078	0.0357	0.30	0.3505	0.4482	0.05	0.0200	0.0918	0.28	0.0527	0.1816	0.27	0.0973	0.3119	0.15	0.4399	0.5103	0.05
4261	Sorting nexin-12	Q9UMY4	0.2319	0.4138	-0.11	0.1273	0.6415	-0.26	0.2570	0.4255	-0.10	0.1464	0.2602	-0.14	0.4264	0.5207	0.09	0.2148	0.3961	-0.11	0.0957	0.2491	0.32	0.2870	0.6260	-0.16	0.0645	0.2279	0.39
4262	Sorting nexin-13	Q9Y5W8	0.0450	0.1352	0.21	0.1567	0.6621	0.27	0.0196	0.0674	0.29	0.0086	0.0375	0.49	0.0375	0.1410	0.21	0.0074	0.0652	0.55	0.3736	0.6207	-2.55	0.8708	1.2488	-0.33	0.0651	0.2055	2.06
4263	Sorting nexin-2	O60749	0.0187	0.0803	0.14	0.1721	0.6618	0.34	0.0098	0.0461	0.44	0.0010	0.0153	0.42	0.0294	0.1248	0.16	0.0042	0.0603	0.41	0.0073	0.0877	0.27	0.1590	0.4314	0.10	0.0605	0.2198	0.10
4264	Sorting nexin-27	Q96L92	0.0219	0.0880	0.18	0.3938	0.7372	0.17	0.0352	0.0977	0.18	0.0048	0.0278	0.27	0.0917	0.2392	0.10	0.0051	0.0616	0.35	0.3736	0.6296	-2.55	0.8708	1.2642	-0.33	0.0651	0.2112	2.06
4265	Sorting nexin-3	O60493	0.7096	0.7615	-0.01	0.0895	0.6248	-0.08	0.3015	0.4843	-0.03	0.0502	0.1153	-0.10	0.1192	0.2832	-0.04	0.2819	0.4785	-0.06	0.0171	0.1217	-0.12	0.2171	0.5238	-0.04	0.3066	0.3778	-0.05
4266	Sorting nexin-32	Q86XE0	0.4108	0.5102	-3.31	0.5752	0.7143	-1.75	0.5661	0.6493	-1.19	0.5388	0.6143	0.89	0.2938	0.4110	-1.88	0.9510	0.9948	0.09	0.0231	0.1360	0.41	0.0008	0.0248	0.55	0.0200	0.1220	0.93
4267	Sorting nexin-4	O95219	0.2897	0.4864	-0.09	0.2613	0.6641	0.18	0.0529	0.1302	-0.22	0.2943	0.4575	0.12	0.4761	0.5666	-0.05	0.2865	0.4828	-0.09	0.0175	0.1227	0.21	0.0112	0.0871	0.31	0.0095	0.0873	0.39
4268	Sorting nexin-5	Q9Y5X3	0.0025	0.0333	0.45	0.3578	0.7079	0.03	0.0446	0.1155	0.06	0.5051	0.7091	-0.02	0.0003	0.0140	-0.16	0.1983	0.3739	0.10	0.0920	0.2436	-0.06	0.0009	0.0259	-0.29	0.0985	0.1540	-0.12
4269	Sorting nexin-6	Q9UNH7	0.1020	0.2281	0.07	0.4080	0.7494	0.13	0.0333	0.0943	0.16	0.0013	0.0163	0.32	0.0052	0.0541	0.20	0.0026	0.0564	0.27	0.5790	0.6284	-0.03	0.1892	0.4809	-0.05	0.3769	0.4483	0.03
4270	Sorting nexin-7	Q9UNH6	0.0096	0.0573	0.23	0.1886	0.6691	0.25	0.0540	0.1319	0.21	0.0011	0.0153	0.22	0.0179	0.0957	0.05	0.1175	0.2613	0.19	0.3736	0.5285	-2.55	0.8708	1.0854	-0.33	0.0651	0.1543	2.06
4271	Sorting nexin-8	Q9Y5X2	0.5391	0.6096	-0.05	0.0441	0.5683	0.21	0.1116	0.2231	0.21	0.0072	0.0343	0.38	0.0111	0.0753	0.38	0.0622	0.1735	0.19	0.0079	0.0913	-0.30	0.0523	0.2171	-0.22	0.0314	0.1506	-0.29
4272	Sorting nexin-9	Q9Y5X1	0.4108	0.5101	-3.31	0.5752	0.7142	-1.75	0.5661	0.6492	-1.19	0.5388	0.6142	0.89	0.2938	0.4109	-1.88	0.9510	0.9946	0.09	0.0086	0.0930	-0.42	0.9057	0.9299	-0.01	0.1962	0.2641	-0.32
4273	SPATS2-like protein	Q9NUQ6	0.4126	0.4905	0.03	0.0342	0.5288	0.34	0.0693	0.1561	0.11	0.0021	0.0198	0.14	0.0002	0.0146	0.36	0.9090	1.1465	0.00	0.1617	0.3551	0.18	0.5914	1.0239	0.02	0.0100	0.0879	-0.15
4274	Speckle-type P	Q6IQ16	0.0012	0.0236	0.52	0.2504	0.6604	0.24	0.0054	0.0357	0.34	0.0021	0.0201	0.47	0.0135	0.0833	0.28	0.0030	0.0553	0.53	0.3674	0.6489	0.15	0.0370	0.1752	-0.45	0.0066	0.0760	-0.73
4275	Spectrin alpha chain erythrocytic 1	P02549	0.1388	0.2832	-0.09	0.3036	0.6764	0.07	0.0068	0.0397	0.27	0.0142	0.0486	0.24	0.0006	0.0194	0.29	0.4171	0.6357	0.06	0.0218	0.1346	0.55	0.0115	0.0885	0.64	0.2290	0.2995	0.43
4276	Spectrin alpha chain non-erythrocytic 1	Q13813	0.0021	0.0313	0.26	0.1675	0.6613	0.24	0.0025	0.0276	0.34	0.0011	0.0160	0.37	0.0464	0.1586	0.11	0.0015	0.0498	0.36	0.0282	0.1449	0.41	0.0173	0.1112	0.21	0.0199	0.1220	0.20
4277	Spectrin beta chain non-erythrocytic 1	Q01082	0.0366	0.1203	0.10	0.1301	0.6455	0.11	0.0028	0.0292	0.22	0.0106	0.0413	0.17	0.3395	0.4366	0.03	0.0056	0.0615	0.24	0.0140	0.1112	0.38	0.0714	0.2590	0.18	0.0614	0.2218	0.18
4278	Spectrin beta chain non-erythrocytic 2	O15020	0.0050	0.0432	0.37	0.3807	0.7274	0.19	0.0193	0.0667	0.24	0.0031	0.0227	0.44	0.1215	0.2870	0.11	0.0031	0.0560	0.56	0.3736	0.6379	-2.55	0.8708	1.2786	-0.33	0.0651	0.2166	2.06
4279	Spectrin beta chain non-erythrocytic 4	Q9H254	0.0162	0.0741	-0.14	0.3560	0.7076	-0.08	0.0425	0.1119	-0.10	0.0206	0.0618	-0.12	0.0696	0.2009	0.07	0.1785	0.3479	-0.05	0.0021	0.0549	0.48	0.0146	0.1017	0.43	0.0100	0.0885	0.34
4280	Spectrin beta chain non-erythrocytic 5	Q9NRC6	0.3713	0.5858	0.05	0.0771	0.6107	0.39	0.0078	0.0418	0.24	0.0065	0.0323	0.25	0.0236	0.1110	0.17	0.2852	0.4817	0.08	0.1020	0.2589	-0.28	0.1085	0.3353	-0.36	0.1497	0.2124	-0.59
4281	Speedy protein E1	Q8NFV5	0.1317	0.2735	-2.04	0.6906	0.7735	0.09	0.2170	0.3727	0.69	0.1693	0.2908	-0.81	0.9060	0.9290	0.07	0.4439	0.6651	-0.88	0.3736	0.4646	-2.55	0.8708	0.9682	-0.33	0.0651	0.1255	2.06
4282	Speedy protein E5	A6NIY4	0.0347	0.1165	-1.80	0.3158	0.6808	-1.17	0.0113	0.0492	-1.84	0.0109	0.0419	-2.35	0.1358	0.3057	-0.70	0.0891	0.2179	-0.94	0.3736	0.5385	-2.55	0.8708	1.1034	-0.33	0.0651	0.1593	2.06
4283	Sperm flagellar protein 2	Q9C093	0.4108	0.5099	-3.31	0.5752	0.7140	-1.75	0.5661	0.6490	-1.19	0.5388	0.6141	0.89	0.2938	0.4108	-1.88	0.9510	0.9944	0.09	0.0887	0.2397	0.18	1.0000	1.0000	0.00	0.3477	0.4190	0.08
4284	Sperm-associated antigen 5	Q96R06	0.0991	0.2238	-0.30	0.0568	0.5861	-0.46	0.0674	0.1529	-0.35	0.0181	0.0568	-0.73	0.0076	0.0636	-0.74	0.1525	0.3099	-0.25	0.3736	0.5151	-2.55	0.8708	1.0611	-0.33	0.0651	0.1479	2.06
4285	Spermatid perinuclear RNA-binding protein	Q96SI9	0.0054	0.0443	0.17	0.7556	0.8224	0.02	0.0464	0.1192	-0.10	0.0273	0.0750	-0.14	0.0771	0.2136	-0.08	0.4713	0.6942	-0.05	0.6517	0.6965	0.04	0.0156	0.1052	-0.34	0.6496	0.7057	0.02
4286	Spermatid-specific manchette-related protein 1	Q8NCR6	0.4108	0.5098	-3.31	0.5752	0.7138	-1.75	0.5661	0.6489	-1.19	0.5388	0.6139	0.89	0.2938	0.4107	-1.88	0.9510	0.9942	0.09	0.0463	0.1727	0.19	0.6465	1.0859	-0.03	0.1651	0.2301	-0.11
4287	Spermatogenesis-associated protein 13	Q96N96	0.0432	0.1319	-0.27	0.4759	0.8027	-0.82	0.1193	0.2356	-0.61	0.8028	0.8305	0.06	0.0490	0.1630	0.50	0.2227	0.4053	-0.50	0.3736	0.4976	-2.55	0.8708	1.0291	-0.33	0.0651	0.1398	2.06
4288	Spermatogenesis-associated protein 17	Q96L03	0.0053	0.0443	0.22	0.5340	0.8501	-0.31	0.0000	0.0000	0.60	0.0001	0.0162	0.43	0.4502	0.5436	0.02	0.0245	0.0999	0.39	0.1408	0.3216	-0.25	0.0359	0.1723	-0.41	0.0534	0.2038	-0.56
4289	Spermatogenesis-associated protein 5-like protein 1	Q9BVQ7	0.0030	0.0361	-0.82	0.1803	0.6652	-0.48	0.0117	0.0507	-0.31	0.0023	0.0205	-0.59	0.6704	0.7343	0.03	0.0046	0.0594	-0.46	0.0275	0.1439	0.39	0.0089	0.0775	0.51	0.0730	0.1221	0.27
4290	Spermatogenesis-associated protein 8	Q6RVD6	0.4108	0.5097	-3.31	0.5752	0.7137	-1.75	0.5661	0.6488	-1.19	0.5388	0.6138	0.89	0.2938	0.4105	-1.88	0.9510	0.9940	0.09	0.0233	0.1361	0.64	0.6273	1.0626	0.10	0.9983	0.9987	0.00
4291	Spermatogenesis-associated serine-rich protein 2	Q86XZ4	0.5463	0.6157	0.04	0.1250	0.6424	0.45	0.0063	0.0385	0.53	0.0692	0.1467	0.25	0.2428	0.4567	0.07	0.2540	0.4442	0.23	0.1205	0.2905	0.23	0.2796	0.6171	-0.12	0.0816	0.1328	0.18
4292	Spermatogenesis-defective protein 39 homolog	Q9H9C1	0.0542	0.1522	-0.40	0.0250	0.4814	-0.35	0.4864	0.7064	0.16	0.0626	0.1362	-0.22	0.0077	0.0634	-0.43	0.0774	0.1985	-0.50	0.3736	0.5451	-2.55	0.8708	1.1154	-0.33	0.0651	0.1627	2.06
4293	Spermatogenic leucine zipper protein 1	Q9BXG8	0.4108	0.5096	-3.31	0.5752	0.7135	-1.75	0.5661	0.6486	-1.19	0.5388	0.6137	0.89	0.2938	0.4104	-1.88	0.9510	0.9938	0.09	0.8096	0.8389	-0.05	0.7584	1.2006	0.06	0.5759	0.6385	-0.11
4294	Spermidine synthase	P19623	0.5466	0.6159	0.04	0.8236	0.8737	0.02	0.0266	0.0816	0.21	0.1188	0.2214	0.11	0.1189	0.2829	0.11	0.3137	0.5147	0.06	0.0717	0.2121	0.23	0.1456	0.4079	0.12	0.9374	0.9493	-0.01
4295	Spermine synthase	P52788	0.1812	0.3448	0.23	0.8662	0.9033	0.07	0.1230	0.2413	0.28	0.1774	0.3018	0.23	0.2092	0.4125	0.21	0.9231	1.1603	-0.01	0.9225	0.9366	0.01	0.0048	0.0553	-0.23	0.0624	0.2234	-0.28
4296	S-phase kinase-associated protein 1	P63208	0.1972	0.3663	0.08	0.1496	0.6595	0.11	0.0601	0.1418	0.16	0.2309	0.3742	0.07	0.0456	0.1573	0.16	0.0931	0.2241	0.12	0.0008	0.0419	0.35	0.0004	0.0220	0.37	0.0027	0.0559	0.40
4297	Sphingomyelin phosphodiesterase	P17405	0.2924	0.4902	0.09	0.1615	0.6629	-0.07	0.4552	0.6701	-0.07	0.6694	0.7116	-0.02	0.3580	0.4551	-0.04	0.0327	0.1163	-0.15	0.3736	0.5779	-2.55	0.8708	1.1738	-0.33	0.0651	0.1801	2.06
4298	Sphingomyelin phosphodiesterase 3	Q9NY59	0.0103	0.0588	0.30	0.1481	0.6579	0.26	0.2533	0.4209	0.07	0.0614	0.1340	0.15	0.0016	0.0315	0.65	0.0805	0.2040	0.13	0.0238	0.1373	1.25	0.0067	0.0669	1.67	0.0886	0.1415	1.04
4299	Sphingomyelin phosphodiesterase 4	Q9NXE4	0.4108	0.5094	-3.31	0.5752	0.7133	-1.75	0.5661	0.6485	-1.19	0.5388	0.6135	0.89	0.2938	0.4103	-1.88	0.9510	0.9936	0.09	0.3562	0.6334	-0.28	0.3687	0.7403	0.25	0.3057	0.3769	-0.28

Supplemetary Table S2. Overview on all relatively quantified 5180 proteins statistical analysis

			MCF-7																		MDA-MB-231								
			Dai SC20 vs control			Gen SC20 vs control			SSE SC20 vs control			Dai IC20 vs control			Gen IC20 vs control			SSE IC20 vs control			Dai IC20 vs control			Gen IC20 vs control			SSE IC20 vs control		
	**Protein name**	**UniProt**	**p value**	**BH q value**	**log2FC**	**p value**	**BH q value**	**log2FC**	**p value**	**BH q value**	**log2FC**	**p value**	**BH q value**	**log2FC**	**p value**	**BH q value**	**log2FC**	**p value**	**BH q value**	**log2FC**	**p value**	**BH q value**	**log2FC**	**p value**	**BH q value**	**log2FC**	**p value**	**BH q value**	**log2FC**
4300	Sphingosine-1-phosphate lyase 1	O95470	0.7446	0.7922	0.02	0.3234	0.6854	0.24	0.0347	0.0970	0.20	0.0309	0.0819	0.19	0.3347	0.4315	0.06	0.0522	0.1543	0.16	0.6397	0.6849	0.05	0.2274	0.5398	0.13	0.2937	0.3653	0.11
4301	Spindle assembly abnormal protein 6 homolog	Q6UVJ0	0.4108	0.5093	-3.31	0.5752	0.7131	-1.75	0.5661	0.6483	-1.19	0.5388	0.6134	0.89	0.2938	0.4102	-1.88	0.9510	0.9934	0.09	0.0842	0.2316	0.49	0.2092	0.5112	0.26	0.8506	0.8791	0.06
4302	Spindlin-2A	Q99865	0.0980	0.2219	-0.93	0.4415	0.7774	-0.49	0.2955	0.4761	-0.23	0.0422	0.1023	-0.56	0.0322	0.1310	-0.24	0.0672	0.1820	-1.15	0.3736	0.5506	-2.55	0.8708	1.1252	-0.33	0.0651	0.1655	2.06
4303	Spliceosome RNA helicase DDX39B	Q13838	0.0036	0.0382	0.35	0.1130	0.6349	0.38	0.0169	0.0616	0.51	0.0007	0.0135	0.40	0.0150	0.0875	0.24	0.0007	0.0417	0.54	0.0326	0.1513	0.24	0.5553	0.9811	0.04	0.6977	0.7498	0.02
4304	Spliceosome-associated protein CWC27 homolog	Q6UX04	0.0419	0.1297	-0.17	0.0161	0.4582	-0.36	0.0415	0.1096	0.20	0.0212	0.0629	-0.28	0.4068	0.5027	-0.05	0.0448	0.1398	0.32	0.2689	0.5115	0.84	0.3734	0.7468	0.65	0.2318	0.3020	0.95
4305	Splicing factor 1	Q15637	0.0089	0.0559	0.22	0.2276	0.6609	0.24	0.0081	0.0423	0.35	0.0068	0.0334	0.33	0.5968	0.6732	-0.02	0.0265	0.1042	0.20	0.0406	0.1657	0.46	0.6636	1.1017	0.04	0.4200	0.4914	0.06
4306	Splicing factor 3A subunit 1	Q15459	0.0202	0.0838	0.21	0.2946	0.6717	0.26	0.0008	0.0205	0.52	0.0674	0.1436	0.22	0.7876	0.8331	-0.01	0.0106	0.0701	0.29	0.1716	0.3688	0.15	0.1768	0.4616	0.12	0.5708	0.6340	0.04
4307	Splicing factor 3A subunit 2	Q15428	0.4108	0.5092	-3.31	0.5752	0.7130	-1.75	0.5661	0.6482	-1.19	0.5388	0.6133	0.89	0.2938	0.4101	-1.88	0.9510	0.9932	0.09	0.0610	0.1949	0.28	0.4870	0.8974	0.03	0.0889	0.1418	0.28
4308	Splicing factor 3A subunit 3	Q12874	0.0090	0.0563	0.32	0.1249	0.6425	0.29	0.0145	0.0567	0.25	0.0110	0.0421	0.29	0.3150	0.4123	0.06	0.0653	0.1782	0.28	0.1822	0.3833	0.14	0.0863	0.2910	-0.20	0.0074	0.0779	-0.33
4309	Splicing factor 3B subunit 1	O75533	0.0256	0.0967	-0.09	0.8241	0.8737	0.02	0.0210	0.0701	0.13	0.6414	0.6863	0.02	0.0018	0.0328	-0.26	0.3345	0.5378	-0.03	0.2815	0.5306	0.12	0.1061	0.3301	-0.19	0.3268	0.3979	-0.10
4310	Splicing factor 3B subunit 2	Q13435	0.1406	0.2861	0.06	0.3845	0.7304	0.17	0.0279	0.0843	0.18	0.0153	0.0510	0.18	0.1452	0.3198	-0.15	0.3477	0.5528	0.06	0.0118	0.1036	0.29	0.4365	0.8371	0.06	0.0128	0.0984	0.30
4311	Splicing factor 3B subunit 3	Q15393	0.2111	0.3841	0.07	0.3069	0.6776	0.16	0.0097	0.0459	0.21	0.0109	0.0419	0.20	0.1702	0.3580	-0.07	0.0954	0.2277	0.10	0.0118	0.1040	0.40	0.0412	0.1867	0.18	0.0303	0.1475	0.21
4312	Splicing factor 3B subunit 4	Q15427	0.0587	0.1600	-0.12	0.6382	0.7335	-0.06	0.0044	0.0327	-0.18	0.0589	0.1299	-0.08	0.0115	0.0769	-0.13	0.0270	0.1052	-0.19	0.0077	0.0900	5.99	0.8708	1.3101	-0.33	0.0021	0.0506	7.39
4313	Splicing factor 3B subunit 5	Q9BWJ5	0.0094	0.0572	0.27	0.2326	0.6624	0.19	0.0310	0.0900	0.22	0.0107	0.0414	0.27	0.1577	0.3395	0.09	0.2700	0.4633	0.07	0.0437	0.1683	-0.23	0.0122	0.0923	-0.31	0.0130	0.0990	-0.44
4314	Splicing factor 3B subunit 6	Q9Y3B4	0.0396	0.1255	0.14	0.4951	0.8173	-0.03	0.0567	0.1361	0.08	0.8626	0.8818	-0.02	0.0001	0.0076	0.20	0.8439	1.0791	-0.02	0.0790	0.2231	-0.22	0.0209	0.1250	-0.29	0.0515	0.1989	-0.17
4315	Splicing factor 45	Q96I25	0.0085	0.0546	-0.33	0.8102	0.8635	0.03	0.0291	0.0865	-0.37	0.0131	0.0464	-0.33	0.1500	0.3273	-0.10	0.3366	0.5405	-0.28	0.3736	0.4756	-2.55	0.8708	0.9885	-0.33	0.0651	0.1301	2.06
4316	Splicing factor U2AF 26 kDa subunit	Q8WU68	0.1431	0.2898	0.12	0.6939	0.7745	0.04	0.2938	0.4743	-0.06	0.5888	0.6375	0.03	0.5856	0.6632	-0.03	0.5770	0.8115	-0.03	0.0010	0.0421	0.43	0.0058	0.0613	0.17	0.0080	0.0800	0.29
4317	Splicing factor U2AF 35 kDa subunit-like protein	P0DN76	0.7896	0.8296	0.02	0.8126	0.8656	0.02	0.0174	0.0628	0.23	0.2912	0.4533	0.07	0.2997	0.3979	-0.06	0.3317	0.5351	0.05	0.1464	0.3304	0.20	0.1442	0.4057	-0.14	0.0686	0.1160	-0.22
4318	Splicing factor U2AF 65 kDa subunit	P26368	0.0155	0.0721	0.24	0.2233	0.6583	-0.08	0.7731	0.8133	0.02	0.2360	0.3808	0.12	0.2008	0.4014	-0.09	0.4062	0.6223	0.05	0.0009	0.0409	-0.83	0.0001	0.0148	-1.01	0.0004	0.0292	-1.54
4319	Splicing factor proline- and glutamine-rich	P23246	0.4239	0.5012	0.03	0.4521	0.7853	0.12	0.0036	0.0313	0.27	0.0144	0.0489	0.17	0.0535	0.1716	-0.11	0.0962	0.2289	0.09	0.0115	0.1040	0.61	0.1187	0.3558	0.20	0.0367	0.1643	0.34
4320	SPOC domain-containing protein 1	Q6ZMY3	0.0086	0.0547	0.26	0.4231	0.7599	0.09	0.0826	0.1770	0.11	0.4425	0.6369	0.04	0.2550	0.4746	-0.06	0.5521	0.7861	-0.03	0.3736	0.4567	-2.55	0.8708	0.9534	-0.33	0.0651	0.1222	2.06
4321	Spondin-2	Q9BUD6	0.0093	0.0570	0.22	0.1402	0.6543	0.27	0.0256	0.0795	0.30	0.0113	0.0428	0.22	0.3546	0.4522	0.05	0.1820	0.3519	0.08	0.3736	0.5065	-2.55	0.8708	1.0454	-0.33	0.0651	0.1439	2.06
4322	Sprouty-related EVH1 domain-containing protein 1	Q7Z699	0.0434	0.1322	-0.18	0.2131	0.6626	-0.10	0.1890	0.3353	-0.09	0.4081	0.5994	-0.06	0.0325	0.1314	0.21	0.8114	1.0481	-0.02	0.3736	0.4379	-2.55	0.8708	0.9181	-0.33	0.0651	0.1147	2.06
4323	Sprouty-related EVH1 domain-containing protein 2	Q7Z698	0.7988	0.8371	0.03	0.1554	0.6620	0.15	0.9023	0.9228	0.02	0.1840	0.3110	0.11	0.1700	0.3578	0.12	0.8007	1.0372	-0.04	0.3736	0.4384	-2.55	0.8708	0.9191	-0.33	0.0651	0.1149	2.06
4324	SPRY domain-containing protein 3	Q8NCJ5	0.1124	0.2440	0.07	0.9964	0.9977	0.00	0.2218	0.3789	0.13	0.1803	0.3051	0.07	0.0174	0.0947	-0.15	0.0437	0.1378	0.16	0.3736	0.5660	-2.55	0.8708	1.1528	-0.33	0.0651	0.1736	2.06
4325	SPRY domain-containing SOCS box protein 1	Q96BD6	0.0046	0.0427	-0.22	0.0098	0.4453	-0.21	0.0001	0.0305	-0.49	0.0001	0.0157	-0.65	0.0000	0.0000	-0.72	0.0112	0.0728	-0.30	0.0599	0.1939	-0.32	0.0229	0.1328	0.39	0.2127	0.2814	-0.15
4326	Squalene monooxygenase	Q14534	0.3805	0.5965	-0.05	0.5889	0.6944	-0.04	0.0138	0.0550	-0.19	0.1398	0.2509	-0.08	0.0076	0.0633	-0.22	0.1852	0.3566	0.11	0.3736	0.5054	-2.55	0.8708	1.0434	-0.33	0.0651	0.1434	2.06
4327	Squalene synthase	P37268	0.0832	0.1987	-0.29	0.0441	0.5697	-0.42	0.5555	0.7798	-0.10	0.0061	0.0312	-0.69	0.0811	0.2198	-0.28	0.1925	0.3665	0.18	0.3736	0.5041	-2.55	0.8708	1.0410	-0.33	0.0651	0.1428	2.06
4328	Squamous cell carcinoma antigen recognized by T-cells 3	Q15020	0.0334	0.1141	0.14	0.1386	0.6509	0.24	0.0006	0.0198	0.40	0.0014	0.0173	0.34	0.9431	0.9558	0.00	0.0370	0.1243	0.27	0.0744	0.2166	0.25	0.3642	0.7335	0.03	0.0207	0.1231	0.12
4329	SRA stem-loop-interacting RNA-binding protein_ mitochondrial	Q9GZT3	0.2189	0.3950	0.12	0.4391	0.7758	0.11	0.0609	0.1431	0.23	0.0766	0.1585	0.21	0.7763	0.8245	-0.02	0.2106	0.3913	0.13	0.2486	0.4847	0.11	0.5441	0.9699	-0.05	0.1566	0.2203	-0.14
4330	Src substrate cortactin	Q14247	0.0838	0.1998	0.15	0.1217	0.6381	0.29	0.0053	0.0356	0.38	0.0055	0.0298	0.37	0.1017	0.2557	0.15	0.0316	0.1144	0.28	0.0567	0.1894	0.45	0.7928	1.2358	0.03	0.1805	0.2474	0.12
4331	SRSF protein kinase 1	Q96SB4	0.4108	0.5091	-3.31	0.5752	0.7128	-1.75	0.5661	0.6480	-1.19	0.5388	0.6131	0.89	0.2938	0.4100	-1.88	0.9510	0.9930	0.09	0.0378	0.1610	0.40	0.1532	0.4232	0.11	0.1436	0.2052	0.09
4332	SRSF protein kinase 2	P78362	0.0097	0.0578	-0.91	0.1659	0.6600	-0.81	0.0132	0.0537	-0.62	0.0105	0.0411	-0.88	0.9295	0.9467	-0.01	0.0266	0.1041	-0.62	0.3736	0.5840	-2.55	0.8708	1.1845	-0.33	0.0651	0.1836	2.06
4333	STAM-binding protein	O95630	0.9117	0.9318	-0.02	0.0881	0.6192	-1.03	0.0025	0.0278	-1.03	0.0071	0.0342	-1.10	0.0150	0.0873	-1.21	0.2034	0.3815	-0.61	0.2295	0.4564	0.17	0.6972	1.1404	-0.03	0.1239	0.1832	0.19
4334	Staphylococcal nuclease domain-containing protein 1	Q7KZF4	0.0024	0.0330	0.33	0.1343	0.6496	0.29	0.0034	0.0314	0.45	0.0005	0.0126	0.51	0.0175	0.0951	0.25	0.0031	0.0563	0.46	0.0259	0.1405	0.40	0.9964	0.9976	0.00	0.9486	0.9595	0.00
4335	Starch-binding domain-containing protein 1	O95210	0.2632	0.4539	-0.19	0.0070	0.4120	0.94	0.1841	0.3288	0.20	0.0249	0.0704	0.49	0.0231	0.1102	0.53	0.2719	0.4661	0.15	0.3736	0.4867	-2.55	0.8708	1.0091	-0.33	0.0651	0.1349	2.06
4336	StAR-related lipid transfer protein 9	Q9P2P6	0.0048	0.0429	-0.11	0.1326	0.6486	-0.15	0.2076	0.3610	0.03	0.0008	0.0141	-0.19	0.0059	0.0566	-0.12	0.0019	0.0526	0.37	0.0081	0.0918	0.69	0.0005	0.0225	0.81	0.0079	0.0799	0.32
4337	START domain-containing protein 10	Q9Y365	0.1939	0.3616	0.07	0.3059	0.6786	0.17	0.0095	0.0454	0.25	0.2137	0.3506	0.07	0.4489	0.5424	-0.04	0.0903	0.2190	0.11	0.3736	0.5376	-2.55	0.8708	1.1018	-0.33	0.0651	0.1588	2.06
4338	Stathmin	P16949	0.0188	0.0806	0.25	0.2095	0.6617	0.26	0.0105	0.0478	0.33	0.0679	0.1444	0.16	0.0449	0.1560	-0.20	0.1654	0.3290	0.11	0.0999	0.2557	0.29	0.9581	0.9705	0.01	0.9092	0.9273	0.01
4339	Stathmin domain-containing protein 1	H3BQB6	0.4108	0.5090	-3.31	0.5752	0.7126	-1.75	0.5661	0.6479	-1.19	0.5388	0.6130	0.89	0.2938	0.4099	-1.88	0.9510	0.9928	0.09	0.0853	0.2334	-0.22	0.0004	0.0199	-0.74	0.1363	0.1971	-0.08
4340	Stathmin-2	Q93045	0.6794	0.7363	0.07	0.7704	0.8333	-0.07	0.3093	0.4937	0.20	0.9759	0.9793	0.01	0.5584	0.6414	0.10	0.5753	0.8094	0.10	0.0342	0.1540	0.45	0.0276	0.1507	0.49	0.0797	0.1306	0.36
4341	Stathmin-3	Q9NZ72	0.4108	0.5088	-3.31	0.5752	0.7125	-1.75	0.5661	0.6478	-1.19	0.5388	0.6129	0.89	0.2938	0.4098	-1.88	0.9510	0.9926	0.09	0.8042	0.8350	0.04	0.2584	0.5850	-0.13	0.0626	0.2238	-0.24
4342	STE20-like serine/threonine-protein kinase	Q9H2G2	0.0627	0.1657	-0.19	0.5827	0.6899	0.06	0.0859	0.1827	-0.18	0.0507	0.1161	-0.23	0.1273	0.2946	-0.14	0.1916	0.3656	-0.11	0.0056	0.0799	0.59	0.0015	0.0313	0.71	0.0812	0.1323	0.18
4343	Sterile alpha motif domain-containing protein 9	Q5K651	0.2148	0.3895	0.25	0.4710	0.8002	0.13	0.1897	0.3363	0.27	0.8095	0.8361	-0.04	0.6614	0.7268	0.07	0.3499	0.5550	0.20	0.0861	0.2349	0.19	0.8390	1.2835	0.01	0.5995	0.6603	-0.03
4344	Sterile alpha motif domain-containing protein 9-like	Q8IVG5	0.0451	0.1354	0.33	0.5653	0.8788	0.06	0.2152	0.3701	0.15	0.1434	0.2557	0.20	0.1540	0.3336	0.19	0.0706	0.1869	0.28	0.0332	0.1522	1.57	0.0321	0.1619	1.50	0.0308	0.1487	1.43
4345	Steroid receptor RNA activator 1	Q9HD15	0.0237	0.0924	0.23	0.4113	0.7520	0.18	0.0041	0.0320	0.38	0.0035	0.0239	0.39	0.7078	0.7686	0.02	0.0075	0.0649	0.48	0.1831	0.3845	0.09	0.0816	0.2807	0.22	0.0761	0.1261	-0.14
4346	Sterol O-acyltransferase 1	P35610	0.4108	0.5087	-3.31	0.5752	0.7123	-1.75	0.5661	0.6476	-1.19	0.5388	0.6127	0.89	0.2938	0.4097	-1.88	0.9510	0.9924	0.09	0.1397	0.3202	-0.22	0.0564	0.2268	0.10	0.0808	0.1319	0.09
4347	Sterol-4-alpha-carboxylate 3-dehydrogenase_ decarboxylating	Q15738	0.3160	0.5185	0.05	0.7068	0.7845	0.04	0.0488	0.1229	0.13	0.4147	0.6070	0.04	0.3106	0.4078	-0.05	0.3382	0.5425	0.06	0.1758	0.3748	0.12	0.6419	1.0799	-0.04	0.2299	0.3002	-0.08
4348	Stomatin-like protein 2 mitochondrial	Q9UJZ1	0.8066	0.8442	-0.01	0.5849	0.6913	0.03	0.0297	0.0874	0.17	0.2405	0.3868	0.06	0.3754	0.4723	0.05	0.1081	0.2466	0.10	0.0272	0.1429	0.42	0.0092	0.0785	0.26	0.0039	0.0610	0.35
4349	Stress-70 protein mitochondrial	P38646	0.0149	0.0707	0.27	0.2542	0.6624	0.27	0.0026	0.0279	0.42	0.0028	0.0221	0.43	0.4124	0.5074	0.05	0.0090	0.0689	0.42	0.0369	0.1597	0.54	0.2521	0.5755	0.13	0.1182	0.1763	0.16
4350	Stress-induced-phosphoprotein 1	P31948	0.0095	0.0572	0.26	0.1721	0.6623	0.34	0.0022	0.0266	0.40	0.0056	0.0301	0.31	0.9415	0.9551	0.00	0.0148	0.0809	0.27	0.0735	0.2150	0.40	0.8990	0.9245	0.01	0.8934	0.9153	-0.01
4351	Striated muscle preferentially expressed protein kinase	Q15772	0.0066	0.0488	0.35	0.1597	0.6639	0.22	0.0334	0.0945	0.29	0.0051	0.0287	0.37	0.1555	0.3360	0.11	0.1295	0.2802	0.50	0.0428	0.1675	0.46	0.5210	0.9433	-0.08	0.0880	0.1407	-0.22
4352	Striatin	O43815	0.0191	0.0811	-0.34	0.7219	0.7965	-0.04	0.0174	0.0629	-0.36	0.2691	0.4249	-0.15	0.2332	0.4441	-0.19	0.0998	0.2336	-0.41	0.3736	0.5342	-2.55	0.8708	1.0956	-0.33	0.0651	0.1571	2.06
4353	Striatin-3	Q13033	0.1806	0.3444	-0.06	0.0151	0.4548	0.21	0.0101	0.0470	0.26	0.0197	0.0599	0.21	0.0107	0.0740	0.20	0.5728	0.8078	0.04	0.0891	0.2398	0.27	0.7754	1.2186	0.02	0.9327	0.9458	0.00
4354	Striatin-interacting protein 1	Q5VSL9	0.4877	0.5621	-0.04	0.1006	0.6324	0.14	0.3103	0.4949	0.11	0.0890	0.1778	-0.16	0.0235	0.1108	-0.22	0.0416	0.1343	-0.30	0.3736	0.5692	-2.55	0.8708	1.1584	-0.33	0.0651	0.1754	2.06
4355	Striatin-interacting protein 2	Q9ULQ0	0.2301	0.4111	0.17	0.9575	0.9712	-0.01	0.8703	0.8975	0.02	0.8197	0.8441	-0.03	0.2971	0.3950	0.14	0.9350	1.1710	-0.01	0.0379	0.1612	-0.44	0.0253	0.1423	-0.49	0.0339	0.1575	-0.47
4356	Stromal cell-derived factor 2	Q99470	0.0000	0.0000	-0.88	0.0016	0.2240	-0.72	0.0009	0.0223	-0.32	0.0001	0.0100	-0.75	0.1901	0.3865	-0.06	0.0018	0.0518	0.41	0.3736	0.6455	-2.55	0.8708	1.2917	-0.33	0.0651	0.2217	2.06
4357	Stromal cell-derived factor 2-like protein 1	Q9HCN8	0.1068	0.2353	-0.03	0.6358	0.7319	0.05	0.0049	0.0340	0.18	0.0454	0.1075	0.09	0.1349	0.3046	-0.06	0.4594	0.6821	0.05	0.4787	0.5333	-0.05	0.0841	0.2859	-0.12	0.8668	0.8919	0.01
4358	Stromal interaction molecule 1	Q13586	0.0030	0.0360	0.24	0.0845	0.6209	0.14	0.7754	0.8151	0.03	0.2163	0.3542	-0.10	0.3265	0.4238	0.04	0.0050	0.0618	-0.22	0.6144	0.6617	0.11	0.4580	0.8633	-0.15	0.3164	0.3878	-0.22
4359	Stromelysin-2	P09238	0.0032	0.0368	0.62	0.1522	0.6609	0.21	0.0337	0.0950	0.30	0.0018	0.0185	0.26	0.0270	0.1193	0.18	0.0389	0.1284	0.25	0.7487	0.7856	-0.03	0.0007	0.0237	-0.74	0.0011	0.0416	-1.00
4360	Structural maintenance of chromosomes protein 1A	Q14683	0.0829	0.1983	-0.05	0.4948	0.8170	-0.03	0.3762	0.5754	0.03	0.0186	0.0576	-0.09	0.0190	0.0984	-0.08	0.8903	1.1267	0.00	0.0032	0.0619	0.49	0.0205	0.1242	0.31	0.1071	0.1634	0.13
4361	Structural maintenance of chromosomes protein 1B	Q8NDV3	0.4108	0.5086	-3.31	0.5752	0.7121	-1.75	0.5661	0.6475	-1.19	0.5388	0.6126	0.89	0.2938	0.4095	-1.88	0.9510	0.9922	0.09	0.0416	0.1673	-0.30	0.0204	0.1237	0.40	0.9215	0.9360	0.01
4362	Structural maintenance of chromosomes protein 2	O95347	0.2507	0.4368	0.08	0.2229	0.6579	0.22	0.0534	0.1308	0.16	0.0345	0.0885	0.18	0.2052	0.4066	-0.08	0.1228	0.2693	0.11	0.0345	0.1545	0.42	0.1214	0.3610	0.15	0.0530	0.2029	0.19
4363	Structural maintenance of chromosomes protein 3	Q9UQE7	0.1351	0.2787	0.09	0.4159	0.7549	0.17	0.0104	0.0475	0.24	0.0056	0.0300	0.30	0.8233	0.8619	0.01	0.0349	0.1204	0.18	0.0506	0.1789	0.49	0.5548	0.9808	0.05	0.0445	0.1841	0.20
4364	Structural maintenance of chromosomes protein 4	Q9NTJ3	0.0177	0.0779	0.14	0.6857	0.7707	0.11	0.0255	0.0793	0.24	0.0310	0.0821	-0.11	0.0089	0.0675	-0.22	0.0026	0.0559	0.34	0.0353	0.1554	0.47	0.1125	0.3424	0.20	0.1291	0.1887	0.17
4365	Structural maintenance of chromosomes protein 5	Q8IY18	0.4108	0.5085	-3.31	0.5752	0.7120	-1.75	0.5661	0.6473	-1.19	0.5388	0.6125	0.89	0.2938	0.4094	-1.88	0.9510	0.9920	0.09	0.9225	0.9364	0.01	0.0084	0.0750	-0.16	0.0425	0.1791	-0.16
4366	Succinate dehydrogenase [ubiquinone] flavoprotein subunit_ mitochondrial	P31040	0.3854	0.6019	0.04	0.3209	0.6838	0.19	0.0012	0.0224	0.21	0.0098	0.0398	0.19	0.5360	0.6216	0.03	0.1504	0.3081	0.09	0.0719	0.2125	0.40	0.7464	1.1878	0.02	0.8029	0.8389	-0.01
4367	Succinate dehydrogenase [ubiquinone] iron-sulfur subunit_ mitochondrial	P21912	0.3674	0.5809	-0.04	0.0370	0.5492	0.20	0.3078	0.4918	-0.05	0.7601	0.7927	-0.02	0.0323	0.1311	0.14	0.0082	0.0667	-0.15	0.0025	0.0573	0.56	0.0157	0.1058	0.29	0.0097	0.0875	0.42
4368	Succinate dehydrogenase assembly factor 1_ mitochondrial	A6NFY7	0.0787	0.1921	-0.18	0.6414	0.7357	-0.05	0.1641	0.3005	-0.04	0.5680	0.6181	0.06	0.5810	0.6591	-0.02	0.3231	0.5261	-0.14	0.3736	0.4777	-2.55	0.8708	0.9925	-0.33	0.0651	0.1310	2.06
4369	Succinate--CoA ligase [ADP/GDP-forming] subunit alpha_ mitochondrial	P53597	0.0423	0.1302	-0.09	0.1716	0.6614	0.32	0.2469	0.4128	0.10	0.2500	0.3993	0.10	0.0036	0.0445	0.06	0.2705	0.4640	0.08	0.0000	0.0000	-1.12	0.0021	0.0383	-1.29	0.0340	0.1577	-1.17
4370	Succinate--CoA ligase [ADP-forming] subunit beta_ mitochondrial	Q9P2R7	0.9833	0.9883	0.00	0.3229	0.6849	0.14	0.2728	0.4465	0.11	0.1211	0.2244	0.17	0.3879	0.4851	0.07	0.4181	0.6366	0.07	0.3740	0.4297	0.10	0.7314	1.1755	0.03	0.8754	0.8994	0.02
4371	Succinate--CoA ligase [GDP-forming] subunit beta_ mitochondrial	Q96I99	0.5734	0.6407	0.02	0.2356	0.6629	0.21	0.0285	0.0856	0.17	0.0007	0.0134	0.17	0.6659	0.7308	-0.01	0.0093	0.0686	0.14	0.1473	0.3320	0.18	0.0917	0.3014	-0.09	0.0856	0.1378	-0.12

Supplemetary Table S2. Overview on all relatively quantified 5180 proteins statistical analysis

			MCF-7																		MDA-MB-231								
			Dai SC20 vs control			Gen SC20 vs control			SSE SC20 vs control			Dai IC20 vs control			Gen IC20 vs control			SSE IC20 vs control			Dai IC20 vs control			Gen IC20 vs control			SSE IC20 vs control		
	**Protein name**	**UniProt**	**p value**	**BH q value**	**log2FC**	**p value**	**BH q value**	**log2FC**	**p value**	**BH q value**	**log2FC**	**p value**	**BH q value**	**log2FC**	**p value**	**BH q value**	**log2FC**	**p value**	**BH q value**	**log2FC**	**p value**	**BH q value**	**log2FC**	**p value**	**BH q value**	**log2FC**	**p value**	**BH q value**	**log2FC**
4372	Succinyl-CoA:3-ketoacid coenzyme A transferase 1_ mitochondrial	P55809	0.4108	0.5083	-3.31	0.5752	0.7118	-1.75	0.5661	0.6472	-1.19	0.5388	0.6123	0.89	0.2938	0.4093	-1.88	0.9510	0.9918	0.09	0.0523	0.1809	0.33	0.2464	0.5678	-0.11	0.9537	0.9639	0.00
4373	Sulfhydryl oxidase 2	Q6ZRP7	0.6603	0.7207	0.07	0.4217	0.7593	-0.18	0.0073	0.0403	-0.85	0.5590	0.6096	-0.11	0.3168	0.4142	-0.17	0.3657	0.5723	0.15	0.3736	0.4724	-2.55	0.8708	0.9825	-0.33	0.0651	0.1288	2.06
4374	Sulfide:quinone oxidoreductase mitochondrial	Q9Y6N5	0.0142	0.0692	-0.55	0.1407	0.6531	-0.75	0.0039	0.0319	-1.18	0.0027	0.0219	-1.32	0.0290	0.1238	-0.44	0.0045	0.0602	-1.17	0.0151	0.1139	0.78	0.0013	0.0297	1.20	0.0285	0.1447	0.39
4375	Sulfotransferase 1A1	P50225	0.0003	0.0143	0.36	0.2277	0.6604	0.12	0.0006	0.0191	0.33	0.0001	0.0106	0.54	0.0005	0.0198	0.37	0.0010	0.0475	0.68	0.3736	0.6494	-2.55	0.8708	1.2984	-0.33	0.0651	0.2244	2.06
4376	Sulfotransferase 1A2	P50226	0.9315	0.9478	0.02	0.1645	0.6616	0.44	0.2463	0.4120	0.34	0.2883	0.4497	0.30	0.3769	0.4735	0.24	0.3352	0.5384	0.27	0.3736	0.4757	-2.55	0.8708	0.9888	-0.33	0.0651	0.1301	2.06
4377	Sulfotransferase 1A3	P0DMM9	0.8152	0.8514	-0.03	0.8147	0.8673	0.03	0.0619	0.1446	0.17	0.3043	0.4701	-0.07	0.0470	0.1592	0.26	0.4969	0.7218	0.04	0.3736	0.4603	-2.55	0.8708	0.9601	-0.33	0.0651	0.1237	2.06
4378	Sulfotransferase 1C2	O00338	0.4108	0.5082	-3.31	0.5752	0.7116	-1.75	0.5661	0.6470	-1.19	0.5388	0.6122	0.89	0.2938	0.4092	-1.88	0.9510	0.9916	0.09	0.0266	0.1419	-0.39	0.0236	0.1357	-0.37	0.1196	0.1779	-0.20
4379	Sulfotransferase family cytosolic 2B member 1	O00204	0.0107	0.0597	3.95	0.2593	0.6636	0.76	0.0326	0.0928	-7.15	0.8585	0.8782	-0.18	0.5760	0.6550	0.76	0.0869	0.2151	1.42	0.3736	0.5404	-2.55	0.8708	1.1069	-0.33	0.0651	0.1603	2.06
4380	SUMO-activating enzyme subunit 1	Q9UBE0	0.0021	0.0312	0.32	0.9782	0.9856	0.00	0.0314	0.0905	0.29	0.0019	0.0191	0.30	0.3881	0.4851	0.03	0.0240	0.0990	0.19	0.5995	0.6478	0.04	0.2803	0.6173	-0.09	0.1120	0.1693	-0.21
4381	SUMO-activating enzyme subunit 2	Q9UBT2	0.3473	0.5563	0.06	0.4041	0.7473	0.09	0.8380	0.8694	-0.01	0.3425	0.5169	0.06	0.8199	0.8597	0.01	0.3487	0.5536	0.06	0.4267	0.4825	-0.07	0.6590	1.0997	0.05	0.0565	0.2113	-0.27
4382	SUMO-conjugating enzyme UBC9	P63279	0.1073	0.2358	0.07	0.4213	0.7596	0.14	0.4006	0.6036	0.04	0.4907	0.6932	0.03	0.0270	0.1190	-0.13	0.3146	0.5154	0.04	0.1773	0.3767	-0.12	0.3135	0.6623	-0.11	0.0251	0.1343	-0.16
4383	SUMO-specific isopeptidase USPL1	Q5W0Q7	0.4108	0.5081	-3.31	0.5752	0.7114	-1.75	0.5661	0.6469	-1.19	0.5388	0.6121	0.89	0.2938	0.4091	-1.88	0.9510	0.9914	0.09	0.0813	0.2271	0.22	0.2204	0.5278	0.09	0.4013	0.4730	0.15
4384	Superoxide dismutase [Cu-Zn]	P00441	0.0431	0.1317	0.25	0.3676	0.7164	0.07	0.5586	0.7825	-0.04	0.0328	0.0855	0.28	0.1538	0.3333	0.15	0.2753	0.4700	0.10	0.0014	0.0490	0.69	0.0107	0.0849	0.38	0.0028	0.0545	0.73
4385	Superoxide dismutase [Mn] mitochondrial	P04179	0.4108	0.5080	-3.31	0.5752	0.7113	-1.75	0.5661	0.6468	-1.19	0.5388	0.6119	0.89	0.2938	0.4090	-1.88	0.9510	0.9912	0.09	0.1574	0.3492	0.20	0.8973	0.9231	-0.01	0.0081	0.0804	-0.21
4386	Suppression of tumorigenicity 5 protein	P78524	0.1067	0.2352	0.05	0.3091	0.6790	0.12	0.0013	0.0222	0.22	0.0080	0.0363	0.17	0.0840	0.2249	0.06	0.0039	0.0596	0.22	0.7677	0.8026	0.03	0.0442	0.1947	0.22	0.4749	0.5438	-0.06
4387	Suppressor of IKBKE 1	Q9BRV8	0.1152	0.2486	-0.14	0.7275	0.8011	0.04	0.0891	0.1873	0.16	0.0217	0.0639	0.33	0.0707	0.2027	0.31	0.6120	0.8488	-0.08	0.3736	0.4515	-2.55	0.8708	0.9437	-0.33	0.0651	0.1201	2.06
4388	Surfeit locus protein 2	Q15527	0.9854	0.9890	0.00	0.6581	0.7477	-0.03	0.7024	0.7493	0.02	0.3822	0.5668	-0.06	0.0108	0.0741	-0.26	0.3832	0.5927	0.04	0.2375	0.4680	-0.38	0.1212	0.3606	-0.40	0.2147	0.2836	0.22
4389	Surfeit locus protein 4	O15260	0.6181	0.6818	0.07	0.6616	0.7506	-0.14	0.0624	0.1452	-0.38	0.4680	0.6673	-0.13	0.4829	0.5728	-0.13	0.2791	0.4751	-0.22	0.4728	0.5276	0.05	0.0213	0.1262	0.15	0.2749	0.3466	0.03
4390	Surfeit locus protein 6	O75683	0.0790	0.1928	-0.30	0.4197	0.7578	0.27	0.6216	0.6759	0.07	0.7536	0.7875	-0.08	0.4397	0.5340	0.10	0.1609	0.3219	-0.28	0.3736	0.5118	-2.55	0.8708	1.0551	-0.33	0.0651	0.1464	2.06
4391	SURP and G-patch domain-containing protein 1	Q8IWZ8	0.1008	0.2263	0.19	0.5739	0.8861	0.11	0.0312	0.0902	0.30	0.0303	0.0808	0.30	0.8172	0.8576	0.02	0.0223	0.0958	0.46	0.0775	0.2216	0.40	0.1216	0.3612	0.20	0.0357	0.1616	0.34
4392	Survival motor neuron protein	Q16637	0.1337	0.2769	0.16	0.2045	0.6600	0.34	0.0232	0.0751	0.34	0.0131	0.0464	0.40	0.1614	0.3445	0.14	0.0321	0.1156	0.31	0.3736	0.5792	-2.55	0.8708	1.1762	-0.33	0.0651	0.1809	2.06
4393	Survival of motor neuron-related-splicing factor 30	O75940	0.0627	0.1656	-0.15	0.0081	0.4114	-0.44	0.0603	0.1420	-0.19	0.0009	0.0146	-0.55	0.0038	0.0459	-0.62	0.0097	0.0705	-0.65	0.0045	0.0722	-0.38	0.5950	1.0281	0.03	0.1927	0.2607	0.17
4394	Sushi repeat-containing protein SRPX2	O60687	0.2789	0.4726	0.13	0.6846	0.7699	-0.10	0.2851	0.4627	-0.13	0.1678	0.2887	-0.26	0.6520	0.7194	-0.05	0.1363	0.2885	-0.21	0.3736	0.5215	-2.55	0.8708	1.0727	-0.33	0.0651	0.1509	2.06
4395	SUZ domain-containing protein 1	Q7Z422	0.4971	0.5712	0.06	0.1861	0.6667	0.22	0.1540	0.2854	0.17	0.1192	0.2219	0.19	0.9518	0.9628	0.01	0.6722	0.9101	0.04	0.1348	0.3130	-0.12	0.0855	0.2897	0.15	0.2282	0.2987	0.12
4396	SWI/SNF complex subunit SMARCC1	Q92922	0.4108	0.5079	-3.31	0.5752	0.7111	-1.75	0.5661	0.6466	-1.19	0.5388	0.6118	0.89	0.2938	0.4089	-1.88	0.9510	0.9910	0.09	0.0024	0.0555	-0.47	0.0030	0.0457	-0.34	0.0051	0.0693	-0.45
4397	SWI/SNF complex subunit SMARCC2	Q8TAQ2	0.4108	0.5077	-3.31	0.5752	0.7109	-1.75	0.5661	0.6465	-1.19	0.5388	0.6117	0.89	0.2938	0.4088	-1.88	0.9510	0.9908	0.09	0.7384	0.7766	0.03	0.4392	0.8401	0.07	0.7476	0.7915	-0.04
4398	SWI/SNF-related matrix-associated actin-dependent regulator of chromatin subfamily A containing DEAD/H box 1	Q9H4L7	0.1375	0.2821	-0.05	0.0422	0.5693	0.12	0.5254	0.7467	0.04	0.2739	0.4310	-0.04	0.7638	0.8159	0.01	0.0837	0.2096	-0.07	0.0438	0.1682	0.50	0.1778	0.4630	0.15	0.0300	0.1474	0.24
4399	SWI/SNF-related matrix-associated actin-dependent regulator of chromatin subfamily A member 5	O60264	0.1182	0.2531	0.06	0.4947	0.8171	0.09	0.0016	0.0229	0.27	0.0699	0.1478	0.09	0.0536	0.1716	-0.09	0.0955	0.2278	0.07	0.3385	0.6088	-0.54	0.2402	0.5577	-0.72	0.2078	0.2766	-0.80
4400	SWI/SNF-related matrix-associated actin-dependent regulator of chromatin subfamily A-like protein 1	Q9NZC9	0.0654	0.1692	0.13	0.9423	0.9618	0.01	0.8867	0.9102	0.03	0.5021	0.7060	0.01	0.1982	0.3981	0.06	0.3127	0.5134	0.13	0.3736	0.4796	-2.55	0.8708	0.9960	-0.33	0.0651	0.1318	2.06
4401	SWI/SNF-related matrix-associated actin-dependent regulator of chromatin subfamily D member 1	Q96GM5	0.4646	0.5409	0.05	0.1523	0.6602	0.13	0.3472	0.5412	0.07	0.0994	0.1933	0.12	0.0713	0.2035	0.13	0.3975	0.6110	0.05	0.3736	0.4694	-2.55	0.8708	0.9770	-0.33	0.0651	0.1275	2.06
4402	SWI/SNF-related matrix-associated actin-dependent regulator of chromatin subfamily D member 3	Q6STE5	0.4074	0.6279	-0.23	0.3059	0.6783	-0.26	0.2729	0.4465	-0.30	0.4567	0.6537	-0.16	0.1243	0.2907	-0.38	0.1277	0.2769	0.38	0.6163	0.6633	0.06	0.4859	0.8964	-0.02	0.0031	0.0558	0.34
4403	SWI/SNF-related matrix-associated actin-dependent regulator of chromatin subfamily E member 1	Q969G3	0.4108	0.5076	-3.31	0.5752	0.7108	-1.75	0.5661	0.6463	-1.19	0.5388	0.6115	0.89	0.2938	0.4087	-1.88	0.9510	0.9906	0.09	0.0760	0.2192	0.22	0.0314	0.1596	0.18	0.7255	0.7734	0.02
4404	Synapse differentiation-inducing gene protein 1	Q9H7V2	0.0009	0.0211	-0.22	0.1453	0.6568	0.20	0.1353	0.2582	-0.08	0.0633	0.1373	0.12	0.0114	0.0764	0.13	0.8068	1.0445	0.04	0.3736	0.4383	-2.55	0.8708	0.9189	-0.33	0.0651	0.1148	2.06
4405	Synapse-associated protein 1	Q96A49	0.4884	0.5626	-0.04	0.0789	0.6127	0.20	0.0229	0.0746	0.24	0.0045	0.0272	0.43	0.0172	0.0942	0.24	0.0128	0.0760	0.24	0.1433	0.3257	0.23	0.0366	0.1739	0.26	0.0681	0.1155	0.29
4406	Synapsin-3	O14994	0.2201	0.3963	0.41	0.6227	0.7202	-0.41	0.2238	0.3813	0.47	0.0801	0.1644	0.77	0.1019	0.2560	0.69	0.0771	0.1982	0.73	0.0632	0.1982	-1.91	0.9165	0.9388	-0.06	0.2849	0.3567	0.67
4407	Synaptic functional regulator FMR1	Q06787	0.0037	0.0386	0.22	0.0970	0.6352	0.20	0.0348	0.0970	0.13	0.0119	0.0437	0.15	0.0102	0.0724	0.16	0.1350	0.2872	0.19	0.5750	0.6244	0.09	0.2825	0.6211	0.19	0.1487	0.2113	0.31
4408	Synaptic vesicle 2-related protein	Q8N4V2	0.0007	0.0197	1.06	0.8488	0.8915	-0.23	0.0009	0.0202	1.09	0.0003	0.0115	1.43	0.0566	0.1779	0.24	0.0023	0.0554	0.78	0.3736	0.6429	-2.55	0.8708	1.2873	-0.33	0.0651	0.2200	2.06
4409	Synaptic vesicle membrane protein VAT-1 homolog	Q99536	0.0094	0.0571	0.24	0.3033	0.6760	0.13	0.2290	0.3884	0.10	0.0395	0.0976	0.16	0.0184	0.0968	0.20	0.0673	0.1820	0.26	0.0283	0.1450	0.38	0.8544	1.2964	0.02	0.3550	0.4268	0.09
4410	Synaptic vesicle membrane protein VAT-1 homolog-like	Q9HCJ6	0.4108	0.5075	-3.31	0.5752	0.7106	-1.75	0.5661	0.6462	-1.19	0.5388	0.6114	0.89	0.2938	0.4086	-1.88	0.9510	0.9904	0.09	0.2576	0.4964	-0.11	0.9642	0.9745	0.00	0.0897	0.1429	0.18
4411	Synaptobrevin homolog YKT6	O15498	0.0050	0.0432	0.28	0.0909	0.6287	0.36	0.0046	0.0337	0.46	0.0305	0.0813	0.18	0.0347	0.1357	0.17	0.0076	0.0655	0.31	0.3736	0.6201	-2.55	0.8708	1.2478	-0.33	0.0651	0.2051	2.06
4412	Synaptogyrin-1	O43759	0.4108	0.5074	-3.31	0.5752	0.7104	-1.75	0.5661	0.6460	-1.19	0.5388	0.6113	0.89	0.2938	0.4084	-1.88	0.9510	0.9902	0.09	0.0476	0.1744	0.36	0.8017	1.2460	0.02	0.4578	0.5263	-0.06
4413	Synaptogyrin-2	O43760	0.3651	0.5784	0.04	0.3131	0.6803	0.09	0.0986	0.2019	0.22	0.3374	0.5115	0.05	0.1358	0.3056	-0.08	0.4114	0.6284	-0.09	0.9838	0.9874	0.00	0.6498	1.0893	0.05	0.0907	0.1443	-0.19
4414	Synaptojanin-2-binding protein	P57105	0.0017	0.0279	-0.25	0.9740	0.9825	0.00	0.0442	0.1148	-0.08	0.0726	0.1519	0.12	0.6193	0.6926	0.04	0.5808	0.8153	-0.01	0.0215	0.1340	-0.17	0.1416	0.4006	-0.08	0.1389	0.2001	-0.14
4415	Synaptonemal complex central element protein 1	Q8N0S2	0.0236	0.0922	-0.19	0.0896	0.6247	0.08	0.2732	0.4469	-0.15	0.0779	0.1606	-0.08	0.0102	0.0720	0.13	0.0166	0.0852	-0.13	0.0989	0.2539	0.57	0.8691	1.3118	-0.05	0.1826	0.2498	0.43
4416	Synaptonemal complex central element protein 2	Q6PIF2	0.0575	0.1576	0.14	0.8464	0.8895	-0.03	0.8328	0.8654	-0.01	0.5020	0.7060	0.04	0.1141	0.2761	-0.12	0.9318	1.1678	0.00	0.3736	0.4324	-2.55	0.8708	0.9076	-0.33	0.0651	0.1125	2.06
4417	Synaptonemal complex central element protein 3	A1L190	0.0832	0.1986	0.20	0.2257	0.6605	0.17	0.0263	0.0810	0.39	0.0569	0.1264	0.22	0.3901	0.4869	0.07	0.0266	0.1042	0.33	0.3736	0.5841	-2.55	0.8708	1.1849	-0.33	0.0651	0.1837	2.06
4418	Synaptonemal complex protein 2-like	Q5T4T6	0.4108	0.5073	-3.31	0.5752	0.7103	-1.75	0.5661	0.6459	-1.19	0.5388	0.6111	0.89	0.2938	0.4083	-1.88	0.9510	0.9900	0.09	0.0114	0.1032	0.32	0.1838	0.4730	0.11	0.1315	0.1917	0.13
4419	Synaptophysin-like protein 1	Q16563	0.4679	0.5440	0.04	0.3139	0.6809	0.21	0.1449	0.2717	0.13	0.0046	0.0274	0.38	0.1209	0.2858	0.11	0.0153	0.0825	0.34	0.3736	0.6023	-2.55	0.8708	1.2168	-0.33	0.0651	0.1943	2.06
4420	Synaptosomal-associated protein 23	O00161	0.0047	0.0429	-0.15	0.4982	0.8208	-0.06	0.1331	0.2553	-0.14	0.6163	0.6633	0.03	0.0251	0.1138	-0.11	0.0276	0.1065	0.10	0.0169	0.1207	-0.42	0.0080	0.0735	-0.45	0.5113	0.5779	0.04
4421	Synaptosomal-associated protein 29	O95721	0.0000	0.0000	0.17	0.0151	0.4574	0.19	0.0003	0.0229	0.32	0.0036	0.0243	0.24	0.0007	0.0211	0.09	0.0237	0.0986	0.30	0.0481	0.1758	0.12	0.2910	0.6302	-0.06	0.0264	0.1386	0.16
4422	Synaptotagmin-16	Q17RD7	0.8510	0.8815	0.07	0.0413	0.5675	-0.78	0.2362	0.3975	0.34	0.3374	0.5113	-0.26	0.1419	0.3148	-0.46	0.1931	0.3671	0.47	0.3736	0.5037	-2.55	0.8708	1.0403	-0.33	0.0651	0.1426	2.06
4423	Synaptotagmin-like protein 2	Q9HCH5	0.0001	0.0093	-1.60	0.0144	0.4521	-0.38	0.0000	0.0000	-1.42	0.0000	0.0000	-1.63	0.0001	0.0152	-1.24	0.0000	0.0000	-1.60	0.3736	0.6560	-2.55	0.8708	1.3097	-0.33	0.0651	0.2289	2.06
4424	Synaptotagmin-like protein 4	Q96C24	0.0955	0.2180	0.16	0.5419	0.8571	0.09	0.5411	0.7631	0.04	0.5358	0.7433	0.04	0.1511	0.3289	-0.12	0.9307	1.1673	0.01	0.3736	0.4326	-2.55	0.8708	0.9080	-0.33	0.0651	0.1126	2.06
4425	Syndecan-1	P18827	0.1380	0.2825	-0.13	0.3108	0.6793	0.16	0.3474	0.5414	0.03	0.0268	0.0739	0.24	0.4310	0.5253	-0.03	0.2387	0.4246	-0.18	0.3736	0.4929	-2.55	0.8708	1.0205	-0.33	0.0651	0.1377	2.06
4426	Syndetin	Q96JG6	0.4108	0.5071	-3.31	0.5752	0.7101	-1.75	0.5661	0.6458	-1.19	0.5388	0.6110	0.89	0.2938	0.4082	-1.88	0.9510	0.9898	0.09	0.3587	0.6363	0.87	0.4794	0.8901	0.64	0.4920	0.5599	0.62
4427	Synembryn-A	Q9NPQ8	0.1458	0.2935	0.26	0.2337	0.6615	0.27	0.0485	0.1226	0.47	0.0684	0.1453	0.37	0.2386	0.4517	0.20	0.3040	0.5021	0.19	0.4562	0.5112	0.06	0.0265	0.1467	-0.12	0.2606	0.3327	0.04
4428	Synemin	O15061	0.0003	0.0141	0.23	0.1404	0.6546	0.26	0.0785	0.1700	0.23	0.0085	0.0372	0.26	0.0014	0.0289	0.14	0.0003	0.0409	0.21	0.1659	0.3605	0.27	0.0966	0.3100	-0.26	0.0127	0.0980	0.70
4429	Syntabulin	Q9NX95	0.4108	0.5070	-3.31	0.5752	0.7099	-1.75	0.5661	0.6456	-1.19	0.5388	0.6109	0.89	0.2938	0.4081	-1.88	0.9510	0.9896	0.09	0.0043	0.0700	1.39	0.0001	0.0126	1.38	0.0078	0.0798	0.45
4430	Syntaxin-10	O60499	0.0072	0.0500	-4.77	0.2375	0.6618	-3.01	0.0018	0.0238	-2.81	0.0090	0.0384	-4.09	0.5679	0.6485	0.08	0.0079	0.0665	-4.88	0.1421	0.3237	1.11	0.2209	0.5283	0.85	0.0972	0.1523	1.46
4431	Syntaxin-11	O75558	0.6372	0.6992	-0.04	0.4365	0.7728	-1.19	0.0013	0.0221	0.40	0.0007	0.0138	0.50	0.0039	0.0467	0.42	0.0127	0.0760	0.66	0.0590	0.1929	0.29	0.1465	0.4100	0.18	0.3667	0.4382	0.11
4432	Syntaxin-12	Q86Y82	0.2931	0.4910	-0.07	0.6829	0.7685	-0.03	0.0981	0.2012	-0.13	0.2790	0.4369	-0.07	0.2355	0.4473	0.08	0.5028	0.7283	0.06	0.3987	0.4547	-0.14	0.9342	0.9520	0.01	0.8342	0.8660	-0.03
4433	Syntaxin-16	O14662	0.3438	0.5522	-0.03	0.1929	0.6684	-0.19	0.0799	0.1725	0.10	0.3487	0.5245	-0.06	0.5502	0.6336	-0.03	0.0857	0.2130	0.08	0.3736	0.5416	-2.55	0.8708	1.1091	-0.33	0.0651	0.1609	2.06
4434	Syntaxin-17	P56962	0.0184	0.0797	0.16	0.2009	0.6616	0.25	0.0002	0.0173	0.39	0.0007	0.0142	0.28	0.0594	0.1837	0.12	0.0493	0.1489	0.17	0.3736	0.5613	-2.55	0.8708	1.1443	-0.33	0.0651	0.1711	2.06
4435	Syntaxin-19	Q8N4C7	0.0067	0.0490	0.88	0.0013	0.2494	1.02	0.1327	0.2549	0.22	0.0209	0.0622	0.47	0.0028	0.0404	0.90	0.0794	0.2019	0.38	0.3736	0.5441	-2.55	0.8708	1.1135	-0.33	0.0651	0.1621	2.06
4436	Syntaxin-1B	P61266	0.4108	0.5069	-3.31	0.5752	0.7098	-1.75	0.5661	0.6455	-1.19	0.5388	0.6107	0.89	0.2938	0.4080	-1.88	0.9510	0.9894	0.09	0.0427	0.1673	0.46	0.1382	0.3938	-0.19	0.0671	0.1140	-0.26
4437	Syntaxin-4	Q12846	0.1636	0.3203	-0.09	0.1404	0.6534	0.27	0.3989	0.6021	0.06	0.0307	0.0814	0.18	0.0247	0.1135	0.20	0.1651	0.3286	0.09	0.3736	0.5105	-2.55	0.8708	1.0527	-0.33	0.0651	0.1457	2.06
4438	Syntaxin-5	Q13190	0.0099	0.0579	0.36	0.0796	0.6136	0.54	0.0010	0.0220	0.73	0.0005	0.0131	0.78	0.0302	0.1266	0.26	0.0046	0.0597	0.75	0.3736	0.6312	-2.55	0.8708	1.2671	-0.33	0.0651	0.2122	2.06
4439	Syntaxin-6	O43752	0.5865	0.6535	0.04	0.1014	0.6321	0.24	0.1562	0.2886	0.21	0.0334	0.0867	0.28	0.0352	0.1359	0.27	0.0243	0.0995	0.35	0.3736	0.5879	-2.55	0.8708	1.1914	-0.33	0.0651	0.1858	2.06
4440	Syntaxin-7	O15400	0.0369	0.1208	0.13	0.1123	0.6337	0.24	0.3454	0.5396	0.06	0.0134	0.0469	0.20	0.1049	0.2607	0.09	0.0170	0.0855	0.20	0.0246	0.1387	0.32	0.4276	0.8231	0.07	0.2012	0.2696	0.10
4441	Syntaxin-binding protein 1	P61764	0.0714	0.1794	0.11	0.3567	0.7085	0.31	0.0069	0.0396	0.55	0.0209	0.0623	0.47	0.0331	0.1327	0.15	0.1107	0.2511	0.41	0.3736	0.5309	-2.55	0.8708	1.0898	-0.33	0.0651	0.1555	2.06
4442	Syntaxin-binding protein 2	Q15833	0.0028	0.0353	0.36	0.2269	0.6607	0.12	0.2091	0.3629	0.10	0.0471	0.1104	0.14	0.1117	0.2724	0.09	0.0090	0.0694	0.28	0.0364	0.1587	0.23	0.1237	0.3655	-0.13	0.0062	0.0742	-0.37

Supplemetary Table S2. Overview on all relatively quantified 5180 proteins statistical analysis

			MCF-7																		MDA-MB-231								
			Dai SC20 vs control			Gen SC20 vs control			SSE SC20 vs control			Dai IC20 vs control			Gen IC20 vs control			SSE IC20 vs control			Dai IC20 vs control			Gen IC20 vs control			SSE IC20 vs control		
	**Protein name**	**UniProt**	**p value**	**BH q value**	**log2FC**	**p value**	**BH q value**	**log2FC**	**p value**	**BH q value**	**log2FC**	**p value**	**BH q value**	**log2FC**	**p value**	**BH q value**	**log2FC**	**p value**	**BH q value**	**log2FC**	**p value**	**BH q value**	**log2FC**	**p value**	**BH q value**	**log2FC**	**p value**	**BH q value**	**log2FC**
4443	Syntaxin-binding protein 3	O00186	0.0170	0.0760	0.39	0.7263	0.8001	-0.03	0.0036	0.0312	-0.12	0.0043	0.0267	-0.14	0.3583	0.4553	-0.08	0.9737	0.9788	0.00	0.3736	0.4302	-2.55	0.8708	0.9036	-0.33	0.0651	0.1117	2.06
4444	Syntenin-1	O00560	0.0025	0.0332	0.19	0.1435	0.6555	0.04	0.0003	0.0210	0.34	0.5187	0.7236	0.03	0.0018	0.0329	0.23	0.0073	0.0651	0.18	0.3736	0.6211	-2.55	0.8708	1.2495	-0.33	0.0651	0.2057	2.06
4445	T cell receptor alpha constant	P01848	0.1884	0.3544	0.13	0.7292	0.8028	0.04	0.0960	0.1979	0.21	0.2003	0.3327	0.12	0.2002	0.4010	0.12	0.7161	0.9563	0.03	0.3736	0.4445	-2.55	0.8708	0.9304	-0.33	0.0651	0.1173	2.06
4446	T cell receptor alpha variable 12-2	A0A075B6T6	0.4108	0.5068	-3.31	0.5752	0.7096	-1.75	0.5661	0.6453	-1.19	0.5388	0.6106	0.89	0.2938	0.4079	-1.88	0.9510	0.9892	0.09	0.0254	0.1395	-3.25	0.0056	0.0603	-2.56	0.1020	0.1579	-3.43
4447	T cell receptor beta variable 12-4	A0A0B4J2E0	0.4108	0.5067	-3.31	0.5752	0.7094	-1.75	0.5661	0.6452	-1.19	0.5388	0.6105	0.89	0.2938	0.4078	-1.88	0.1897	0.3630	3.31	0.3736	0.5049	-2.55	0.8708	1.0425	-0.33	0.0651	0.1431	2.06
4448	T cell receptor beta variable 7-9	P04435	0.4108	0.5065	-3.31	0.5752	0.7092	-1.75	0.5661	0.6451	-1.19	0.5388	0.6103	0.89	0.2938	0.4077	-1.88	0.9510	0.9890	0.09	0.5327	0.5849	0.56	0.2765	0.6121	1.04	0.1761	0.2420	1.44
4449	T cell receptor gamma constant 2	P03986	0.4108	0.5064	-3.31	0.5752	0.7091	-1.75	0.5661	0.6449	-1.19	0.5388	0.6102	0.89	0.2938	0.4076	-1.88	0.9510	0.9888	0.09	0.1096	0.2714	0.20	0.2833	0.6210	0.10	0.3050	0.3762	-0.09
4450	Tail-anchored protein insertion receptor WRB	O00258	0.4108	0.5063	-3.31	0.5752	0.7089	-1.75	0.5661	0.6448	-1.19	0.5388	0.6101	0.89	0.2938	0.4075	-1.88	0.9510	0.9886	0.09	0.0521	0.1808	-9.40	0.1686	0.4481	-1.77	0.0001	0.0179	-4.80
4451	Talin-1	Q9Y490	0.0687	0.1742	0.07	0.3173	0.6811	0.13	0.0054	0.0357	0.18	0.0011	0.0158	0.14	0.1282	0.2955	0.03	0.0004	0.0357	0.27	0.0622	0.1965	0.39	0.2829	0.6212	0.11	0.9714	0.9771	0.00
4452	Talin-2	Q9Y4G6	0.4108	0.5062	-3.31	0.5752	0.7087	-1.75	0.5661	0.6446	-1.19	0.5388	0.6099	0.89	0.2938	0.4074	-1.88	0.9510	0.9884	0.09	0.0348	0.1543	-0.19	0.2541	0.5786	0.28	0.1136	0.1710	0.21
4453	TAR DNA-binding protein 43	Q13148	0.1201	0.2556	-0.11	0.7952	0.8514	0.02	0.0363	0.0998	0.18	0.4922	0.6949	-0.04	0.7560	0.8089	-0.02	0.2802	0.4763	-0.19	0.0394	0.1638	0.57	0.8312	1.2742	0.02	0.6184	0.6771	0.04
4454	Target of EGR1 protein 1	Q96GM8	0.6725	0.7303	-0.03	0.3286	0.6883	0.08	0.0571	0.1366	-0.25	0.0306	0.0813	0.29	0.0672	0.1974	0.21	0.5383	0.7703	0.08	0.3736	0.4579	-2.55	0.8708	0.9557	-0.33	0.0651	0.1227	2.06
4455	Target of Myb protein 1	O60784	0.0433	0.1319	-0.23	0.1223	0.6399	-0.15	0.0475	0.1209	-0.38	0.0427	0.1028	-0.27	0.3753	0.4723	0.08	0.2931	0.4909	0.14	0.3736	0.4832	-2.55	0.8708	1.0026	-0.33	0.0651	0.1334	2.06
4456	Target of rapamycin complex subunit LST8	Q9BVC4	0.0262	0.0980	-0.31	0.5813	0.6889	-0.07	0.8748	0.9009	0.01	0.0091	0.0382	-0.35	0.2075	0.4102	-0.11	0.1898	0.3631	0.10	0.3736	0.5048	-2.55	0.8708	1.0422	-0.33	0.0651	0.1431	2.06
4457	TATA element modulatory factor	P82094	0.0056	0.0450	0.27	0.6994	0.7784	-0.07	0.4707	0.6880	-0.03	0.0113	0.0426	-0.23	0.0099	0.0717	-0.26	0.0520	0.1540	0.14	0.3736	0.5595	-2.55	0.8708	1.1411	-0.33	0.0651	0.1701	2.06
4458	TATA-binding protein-associated factor 2N	Q92804	0.0345	0.1161	-0.14	0.3070	0.6776	0.12	0.0313	0.0904	0.13	0.6456	0.6900	-0.02	0.0296	0.1252	0.13	0.1434	0.2987	0.25	0.0069	0.0863	0.36	0.0013	0.0299	0.49	0.0455	0.1866	0.41
4459	Tau-tubulin kinase 1	Q5TCY1	0.4108	0.5061	-3.31	0.5752	0.7086	-1.75	0.5661	0.6445	-1.19	0.5388	0.6098	0.89	0.2938	0.4072	-1.88	0.9510	0.9882	0.09	0.0597	0.1935	0.28	0.5576	0.9831	0.06	0.7185	0.7674	-0.09
4460	Tax1-binding protein 1	Q86VP1	0.9247	0.9423	-0.01	0.0241	0.4858	-0.22	0.5801	0.6376	0.03	0.0744	0.1551	-0.15	0.3610	0.4582	-0.05	0.2295	0.4128	0.12	0.0832	0.2303	-0.50	0.1945	0.4891	-0.31	0.0242	0.1321	-1.01
4461	TBC domain-containing protein kinase-like protein	Q8TEA7	0.4108	0.5059	-3.31	0.5752	0.7084	-1.75	0.5661	0.6443	-1.19	0.5388	0.6097	0.89	0.2938	0.4071	-1.88	0.9510	0.9880	0.09	0.0791	0.2229	-0.48	0.0693	0.2544	0.20	0.8929	0.9152	0.01
4462	TBC1 domain family member 1	Q86TI0	0.4139	0.4917	0.07	0.1062	0.6309	0.38	0.0187	0.0655	0.30	0.0359	0.0910	0.24	0.7437	0.7994	0.02	0.3651	0.5717	0.07	0.0739	0.2157	0.28	0.6658	1.1040	0.03	0.0558	0.2093	0.34
4463	TBC1 domain family member 10A	Q9BXI6	0.1039	0.2305	-0.22	0.6885	0.7721	-0.07	0.2203	0.3771	-0.14	0.0115	0.0430	-0.51	0.6560	0.7224	-0.05	0.1308	0.2816	-0.27	0.3736	0.5240	-2.55	0.8708	1.0773	-0.33	0.0651	0.1522	2.06
4464	TBC1 domain family member 10B	Q4KMP7	0.0054	0.0442	-0.12	0.7170	0.7928	0.04	0.2460	0.4116	0.07	0.5397	0.5914	-0.02	0.1324	0.3009	-0.05	0.9941	0.9945	0.00	0.3736	0.4294	-2.55	0.8708	0.9020	-0.33	0.0651	0.1114	2.06
4465	TBC1 domain family member 13	Q9NVG8	0.1815	0.3450	-0.19	0.4454	0.7813	0.20	0.2564	0.4246	-0.08	0.0553	0.1241	0.27	0.1266	0.2937	0.12	0.2827	0.4790	0.12	0.7961	0.8281	0.07	0.0975	0.3121	0.58	0.0014	0.0453	1.29
4466	TBC1 domain family member 15	Q8TC07	0.0024	0.0329	-1.34	0.0524	0.5812	-0.50	0.0183	0.0648	-0.16	0.0551	0.1239	-0.18	0.0547	0.1744	-0.12	0.1501	0.3077	-0.47	0.1174	0.2852	-0.21	0.0807	0.2792	-0.20	0.5992	0.6601	-0.10
4467	TBC1 domain family member 16	Q8TBP0	0.0744	0.1850	-0.05	0.8882	0.9202	0.01	0.0309	0.0898	0.13	0.0130	0.0461	0.15	0.0066	0.0593	0.08	0.1961	0.3713	0.12	0.0826	0.2295	0.47	0.1874	0.4770	0.18	0.2272	0.2975	0.24
4468	TBC1 domain family member 23	Q9NUY8	0.0122	0.0639	-0.64	0.0586	0.5906	-0.43	0.0150	0.0577	-0.68	0.0234	0.0676	-0.59	0.1045	0.2601	-0.25	0.0213	0.0941	-0.61	0.0034	0.0645	-0.87	0.0020	0.0373	-0.80	0.0182	0.1158	-0.53
4469	TBC1 domain family member 2A	Q9BYX2	0.1787	0.3414	-0.05	0.5386	0.8545	-0.08	0.0086	0.0435	-0.05	0.0127	0.0456	-0.08	0.0001	0.0093	-0.20	0.2475	0.4362	-0.02	0.4906	0.5445	-0.15	0.3862	0.7659	-0.11	0.0466	0.1889	0.14
4470	TBC1 domain family member 30	Q9Y2I9	0.0619	0.1648	-0.13	0.2432	0.6592	0.16	0.0340	0.0957	0.18	0.1193	0.2221	0.10	0.7410	0.7973	0.02	0.6395	0.8766	-0.03	0.3736	0.4486	-2.55	0.8708	0.9382	-0.33	0.0651	0.1189	2.06
4471	TBC1 domain family member 3D	A0A087WVF3	0.1481	0.2968	-0.38	0.1259	0.6425	-0.39	0.0336	0.0950	-0.34	0.1585	0.2766	-0.23	0.0980	0.2497	-0.36	0.2152	0.3961	0.13	0.3736	0.4992	-2.55	0.8708	1.0320	-0.33	0.0651	0.1405	2.06
4472	TBC1 domain family member 4	O60343	0.8619	0.8908	-0.01	0.2072	0.6589	0.41	0.0145	0.0566	0.21	0.3399	0.5141	0.06	0.2767	0.5017	-0.05	0.0429	0.1366	0.45	0.9325	0.9443	0.01	0.0073	0.0702	-0.41	0.0226	0.1274	-0.40
4473	TBC1 domain family member 5	Q92609	0.1667	0.3246	-0.59	0.0538	0.5842	-10.26	0.3784	0.5775	0.31	0.3527	0.5299	0.34	0.9563	0.9660	0.02	0.9698	0.9756	0.01	0.0450	0.1701	-0.30	0.0389	0.1796	-0.12	0.8236	0.8575	-0.01
4474	TBC1 domain family member 8	O95759	0.4031	0.6231	-0.04	0.7469	0.8166	0.06	0.0257	0.0798	0.23	0.0141	0.0484	0.19	0.0491	0.1631	0.11	0.6047	0.8420	0.02	0.3736	0.4523	-2.55	0.8708	0.9451	-0.33	0.0651	0.1204	2.06
4475	TBC1 domain family member 8B	Q0IIM8	0.4108	0.5058	-3.31	0.5752	0.7082	-1.75	0.5661	0.6442	-1.19	0.5388	0.6095	0.89	0.2938	0.4070	-1.88	0.9510	0.9878	0.09	0.3701	0.6523	0.08	0.2690	0.6004	-0.10	0.0134	0.1005	-0.30
4476	TBC1 domain family member 9B	Q66K14	0.4108	0.5057	-3.31	0.5752	0.7081	-1.75	0.5661	0.6441	-1.19	0.5388	0.6094	0.89	0.2938	0.4069	-1.88	0.9510	0.9876	0.09	0.6077	0.6553	-0.05	0.0311	0.1590	-0.28	0.0076	0.0789	-0.53
4477	T-box transcription factor TBX15	Q96SF7	0.0962	0.2188	0.11	0.0152	0.4551	0.22	0.0323	0.0923	0.21	0.0148	0.0499	0.23	0.0010	0.0245	0.52	0.4117	0.6287	0.07	0.3736	0.4682	-2.55	0.8708	0.9749	-0.33	0.0651	0.1270	2.06
4478	T-cell activation Rho GTPase-activating protein	Q8N103	0.4108	0.5056	-3.31	0.5752	0.7079	-1.75	0.5661	0.6439	-1.19	0.5388	0.6093	0.89	0.2938	0.4068	-1.88	0.9510	0.9874	0.09	0.0596	0.1937	0.40	0.3152	0.6643	0.11	0.2088	0.2775	0.10
4479	T-complex protein 1 subunit alpha	P17987	0.0124	0.0647	0.20	0.1381	0.6533	0.26	0.0069	0.0398	0.23	0.0093	0.0387	0.21	0.5983	0.6740	-0.02	0.0274	0.1063	0.18	0.0616	0.1955	0.36	0.7701	1.2129	0.03	0.3203	0.3919	-0.09
4480	T-complex protein 1 subunit beta	P78371	0.0080	0.0528	0.21	0.0764	0.6155	0.26	0.0025	0.0278	0.34	0.0073	0.0347	0.32	0.0664	0.1961	0.11	0.0092	0.0690	0.31	0.0511	0.1791	0.33	0.2179	0.5240	0.14	0.5390	0.6046	0.05
4481	T-complex protein 1 subunit delta	P50991	0.0038	0.0391	0.27	0.0797	0.6116	0.30	0.0006	0.0204	0.41	0.0010	0.0159	0.36	0.1193	0.2832	0.08	0.0090	0.0692	0.32	0.1845	0.3855	0.22	0.4035	0.7896	-0.09	0.2007	0.2690	-0.12
4482	T-complex protein 1 subunit epsilon	P48643	0.0023	0.0326	0.29	0.1765	0.6620	0.26	0.0008	0.0213	0.35	0.0009	0.0156	0.37	0.0885	0.2332	0.08	0.0031	0.0561	0.38	0.1010	0.2573	0.32	0.9191	0.9409	-0.01	0.4634	0.5317	-0.06
4483	T-complex protein 1 subunit eta	Q99832	0.0675	0.1722	0.18	0.2010	0.6611	0.15	0.0099	0.0463	0.37	0.0098	0.0398	0.33	0.1050	0.2609	0.14	0.0134	0.0770	0.33	0.1463	0.3305	0.24	0.6781	1.1172	0.04	0.1954	0.2633	-0.13
4484	T-complex protein 1 subunit gamma	P49368	0.0105	0.0591	0.22	0.2128	0.6624	0.23	0.0010	0.0211	0.38	0.0125	0.0453	0.22	0.1878	0.3834	0.06	0.0036	0.0590	0.41	0.2506	0.4869	0.14	0.2328	0.5476	-0.12	0.0682	0.1155	-0.20
4485	T-complex protein 1 subunit theta	P50990	0.0079	0.0524	0.36	0.2020	0.6602	0.30	0.0043	0.0324	0.40	0.0080	0.0362	0.34	0.1725	0.3615	0.11	0.0168	0.0851	0.36	0.0586	0.1926	0.35	0.8472	1.2892	-0.02	0.0858	0.1380	-0.20
4486	T-complex protein 1 subunit zeta	P40227	0.0015	0.0258	0.26	0.1823	0.6659	0.15	0.0028	0.0292	0.20	0.0035	0.0239	0.21	0.0385	0.1432	-0.08	0.0080	0.0668	0.21	0.2576	0.4966	0.15	0.2192	0.5259	-0.13	0.0405	0.1744	-0.27
4487	T-complex protein 1 subunit zeta-2	Q92526	0.0194	0.0817	-0.22	0.0591	0.5921	-0.40	0.0645	0.1482	-0.17	0.0541	0.1218	-0.33	0.0854	0.2278	-0.11	0.4635	0.6862	-0.14	0.0034	0.0643	0.63	0.0002	0.0182	0.63	0.0079	0.0801	0.51
4488	T-complex protein 11-like protein 2	Q8N4U5	0.4108	0.5054	-3.31	0.5752	0.7077	-1.75	0.5661	0.6438	-1.19	0.5388	0.6091	0.89	0.2938	0.4067	-1.88	0.9510	0.9872	0.09	0.0085	0.0931	0.28	0.0146	0.1015	-0.29	0.0019	0.0492	-0.64
4489	Tctex1 domain-containing protein 1	Q8N7M0	0.4108	0.5053	-3.31	0.5752	0.7076	-1.75	0.5661	0.6436	-1.19	0.5388	0.6090	0.89	0.2938	0.4066	-1.88	0.9510	0.9870	0.09	0.1931	0.4007	-1.27	0.2154	0.5209	-1.18	0.1631	0.2278	-0.42
4490	Teashirt homolog 3	Q63HK5	0.0036	0.0381	-0.24	0.8514	0.8935	-0.03	0.0894	0.1877	0.06	0.0049	0.0280	0.20	0.0093	0.0687	-0.14	0.8456	1.0807	-0.01	0.0099	0.0998	-0.35	0.0114	0.0880	-0.47	0.0036	0.0592	-0.45
4491	Tectonin beta-propeller repeat-containing protein 1	Q7Z6L1	0.0111	0.0603	-0.15	0.2963	0.6720	0.10	0.0879	0.1855	0.07	0.9591	0.9647	0.00	0.8565	0.8896	0.01	0.0234	0.0980	-0.10	0.3736	0.5889	-2.55	0.8708	1.1933	-0.33	0.0651	0.1864	2.06
4492	Tektin-1	Q969V4	0.4108	0.5052	-3.31	0.5752	0.7074	-1.75	0.5661	0.6435	-1.19	0.5388	0.6089	0.89	0.2938	0.4065	-1.88	0.9510	0.9868	0.09	0.7252	0.7649	-0.04	0.0437	0.1931	-0.33	0.4434	0.5136	-0.08
4493	Tektin-5	Q96M29	0.0723	0.1809	-0.30	0.0011	0.2374	-0.34	0.0088	0.0441	-0.57	0.0235	0.0678	-0.78	0.1816	0.3742	0.06	0.3046	0.5028	-0.29	0.3736	0.4812	-2.55	0.8708	0.9988	-0.33	0.0651	0.1325	2.06
4494	Telethonin	O15273	0.6151	0.6795	-0.10	0.0721	0.6063	0.22	0.9137	0.9319	-0.02	0.2422	0.3890	0.12	0.0878	0.2319	0.21	0.0455	0.1411	0.32	0.0413	0.1673	-0.94	0.0038	0.0500	-1.45	0.0164	0.1098	-1.08
4495	Telomerase RNA component interacting RNase	Q9BQ61	0.0322	0.1116	0.21	0.0752	0.6144	0.37	0.0110	0.0486	0.25	0.0011	0.0154	0.32	0.1228	0.2887	0.08	0.0073	0.0649	-0.17	0.3736	0.6209	-2.55	0.8708	1.2492	-0.33	0.0651	0.2056	2.06
4496	Telomere length and silencing protein 1 homolog	Q9NZ63	0.2434	0.4286	-0.17	0.9842	0.9888	-0.01	0.1614	0.2965	0.21	0.1558	0.2734	0.23	0.1578	0.3396	0.22	0.9302	1.1673	-0.01	0.8840	0.9037	-0.14	0.6359	1.0744	-0.54	0.4519	0.5204	0.74
4497	Telomere-associated protein RIF1	Q5UIP0	0.4056	0.6259	-0.05	0.7391	0.8103	-0.05	0.2737	0.4475	0.13	0.1217	0.2253	0.11	0.4053	0.5012	-0.06	0.0258	0.1023	-0.22	0.0098	0.0991	0.70	0.0531	0.2188	0.40	0.0563	0.2109	0.44
4498	Telomeric repeat-binding factor 2-interacting protein 1	Q9NYB0	0.0133	0.0671	-0.24	0.7007	0.7789	-0.02	0.0130	0.0533	-0.23	0.0944	0.1859	-0.16	0.0318	0.1304	-0.16	0.0239	0.0990	-0.19	0.1964	0.4058	0.31	0.1327	0.3836	0.21	0.1660	0.2313	0.42
4499	Tenascin-X	P22105	0.4108	0.5051	-3.31	0.5752	0.7072	-1.75	0.5661	0.6434	-1.19	0.5388	0.6087	0.89	0.2938	0.4064	-1.88	0.9510	0.9866	0.09	0.2260	0.4511	0.08	0.0281	0.1521	-0.28	0.1405	0.2019	0.23
4500	Teneurin-3	Q9P273	0.0862	0.2040	-0.22	0.0277	0.5106	-0.18	0.0337	0.0951	-0.14	0.1419	0.2537	-0.15	0.0189	0.0981	-0.12	0.1520	0.3095	-0.32	0.3736	0.5155	-2.55	0.8708	1.0619	-0.33	0.0651	0.1481	2.06
4501	Tensin-3	Q68CZ2	0.0205	0.0845	0.23	0.0542	0.5874	0.31	0.0360	0.0993	0.28	0.0219	0.0643	0.23	0.9156	0.9368	0.00	0.4218	0.6411	0.03	0.3736	0.4672	-2.55	0.8708	0.9730	-0.33	0.0651	0.1266	2.06
4502	Terminal nucleotidyltransferase 5C	Q5VWP2	0.0784	0.1918	0.20	0.0893	0.6251	0.17	0.0396	0.1059	0.15	0.0008	0.0142	0.24	0.0040	0.0471	0.19	0.0039	0.0603	0.18	0.0107	0.1026	-0.70	0.0047	0.0551	-0.90	0.0043	0.0648	-1.27
4503	Terminal uridylyltransferase 4	Q5TAX3	0.4108	0.5050	-3.31	0.5752	0.7071	-1.75	0.5661	0.6432	-1.19	0.5388	0.6086	0.89	0.2938	0.4063	-1.88	0.9510	0.9864	0.09	0.0319	0.1505	0.59	0.8542	1.2964	-0.04	0.0372	0.1655	0.65
4504	Testican-2	Q92563	0.0582	0.1588	-0.25	0.1976	0.6703	-0.58	0.4980	0.7204	-0.08	0.0478	0.1114	-0.41	0.6414	0.7105	-0.04	0.3223	0.5253	-0.08	0.0689	0.2067	-0.37	0.4712	0.8821	-0.03	0.2107	0.2793	0.06
4505	Testican-3	Q9BQ16	0.0241	0.0932	-0.14	0.5793	0.6875	0.07	0.5622	0.7864	0.03	0.3332	0.5067	-0.06	0.0024	0.0379	0.25	0.0133	0.0768	-0.19	0.3736	0.6065	-2.55	0.8708	1.2241	-0.33	0.0651	0.1967	2.06
4506	Testin	Q9UGI8	0.0504	0.1450	-0.03	0.6395	0.7343	0.02	0.0001	0.0140	0.13	0.2528	0.4026	0.04	0.1841	0.3777	-0.04	0.0656	0.1788	0.03	0.9593	0.9664	0.00	0.0029	0.0444	-0.34	0.0003	0.0268	-0.60
4507	Testis development-related protein	Q86YL5	0.9311	0.9479	0.01	0.6984	0.7778	-0.07	0.9032	0.9233	0.03	0.1718	0.2940	-0.32	0.2844	0.5128	0.25	0.8214	1.0568	-0.04	0.3736	0.4369	-2.55	0.8708	0.9163	-0.33	0.0651	0.1143	2.06
4508	Testis-expressed protein 10	Q9NXF1	0.0067	0.0489	-0.63	0.4272	0.7628	0.08	0.0021	0.0257	-0.64	0.0102	0.0406	-0.55	0.4791	0.5691	0.14	0.0332	0.1173	-0.63	0.3736	0.5770	-2.55	0.8708	1.1722	-0.33	0.0651	0.1797	2.06
4509	Testis-expressed protein 101	Q9BY14	0.1316	0.2736	0.90	0.4342	0.7705	0.77	0.4693	0.6869	0.61	0.2768	0.4348	0.70	0.2125	0.4162	-0.70	0.0430	0.1367	1.72	0.3736	0.5674	-2.55	0.8708	1.1551	-0.33	0.0651	0.1744	2.06
4510	Testis-expressed protein 15	Q9BXT5	0.2757	0.4692	0.07	0.0774	0.6112	0.13	0.7232	0.7677	0.02	0.7738	0.8057	0.02	0.3062	0.4036	-0.06	0.2683	0.4613	-0.11	0.0254	0.1398	0.63	0.0106	0.0843	0.63	0.1115	0.1688	0.15
4511	Testis-expressed protein 264	Q9Y6I9	0.0072	0.0499	-0.17	0.0494	0.5763	0.12	0.4879	0.7079	0.02	0.0053	0.0291	0.21	0.0053	0.0543	0.23	0.3440	0.5481	0.15	0.0093	0.0962	0.57	0.1080	0.3348	0.25	0.0069	0.0764	0.28
4512	Testis-expressed protein 48	A0A1B0GUV7	0.4108	0.5049	-3.31	0.5752	0.7069	-1.75	0.5661	0.6431	-1.19	0.5388	0.6085	0.89	0.2938	0.4062	-1.88	0.9510	0.9862	0.09	0.0347	0.1546	0.43	0.6071	1.0386	0.05	0.0674	0.1144	-0.20
4513	Testis-expressed protein 52	A6NCN8	0.5113	0.5840	0.05	0.2533	0.6627	0.30	0.0715	0.1594	0.36	0.8762	0.8938	-0.02	0.1296	0.2973	-0.15	0.2227	0.4052	-0.12	0.3736	0.4975	-2.55	0.8708	1.0289	-0.33	0.0651	0.1398	2.06
4514	Testis-expressed protein 54	A0A1B0GVG6	0.4108	0.5047	-3.31	0.5752	0.7067	-1.75	0.5661	0.6429	-1.19	0.5388	0.6083	0.89	0.2938	0.4061	-1.88	0.9510	0.9860	0.09	0.1428	0.3247	0.26	0.9711	0.9794	0.00	0.7165	0.7662	-0.05
4515	Testis-specific gene 10 protein	Q9BZW7	0.4108	0.5046	-3.31	0.5752	0.7066	-1.75	0.5661	0.6428	-1.19	0.5388	0.6082	0.89	0.2938	0.4059	-1.88	0.9510	0.9858	0.09	0.2460	0.4805	0.32	0.7385	1.1811	0.08	0.5159	0.5823	0.16
4516	Testis-specific serine/threonine-protein kinase 3	Q96PN8	0.0499	0.1440	0.22	0.3210	0.6837	0.12	0.5362	0.7574	0.04	0.4609	0.6581	-0.03	0.0369	0.1396	-0.11	0.2137	0.3951	-0.14	0.3736	0.4999	-2.55	0.8708	1.0334	-0.33	0.0651	0.1409	2.06
4517	Testis-specific serine/threonine-protein kinase 4	Q6SA08	0.0522	0.1479	0.14	0.0999	0.6349	0.29	0.0024	0.0273	0.45	0.0016	0.0178	0.42	0.0001	0.0108	0.47	0.0015	0.0532	0.39	0.0002	0.0370	1.18	0.0005	0.0244	2.09	0.8117	0.8472	0.13
4518	Testis-specific Y-encoded-like protein 5	Q86VY4	0.1253	0.2638	-0.06	0.0130	0.4401	0.19	0.0188	0.0657	0.19	0.0253	0.0712	0.12	0.0054	0.0546	0.17	0.2981	0.4954	-0.06	0.3736	0.4820	-2.55	0.8708	1.0004	-0.33	0.0651	0.1329	2.06
4519	Tether containing UBX domain for GLUT4	Q9BZE9	0.7436	0.7916	-0.02	0.3507	0.7041	0.13	0.7544	0.7959	0.02	0.2060	0.3399	0.08	0.2592	0.4799	-0.07	0.1848	0.3560	0.09	0.3736	0.5056	-2.55	0.8708	1.0437	-0.33	0.0651	0.1434	2.06
4520	Tetraspanin-13	O95857	0.2123	0.3860	0.04	0.4124	0.7525	-0.10	0.0010	0.0208	0.26	0.0020	0.0199	0.24	0.0022	0.0369	-0.21	0.0178	0.0873	0.13	0.3736	0.5967	-2.55	0.8708	1.2070	-0.33	0.0651	0.1910	2.06
4521	Tetraspanin-15	O95858	0.0019	0.0297	-0.26	0.0232	0.4826	-0.30	0.1797	0.3232	-0.16	0.0129	0.0459	-0.57	0.0065	0.0589	-0.21	0.1202	0.2650	-0.37	0.3736	0.5269	-2.55	0.8708	1.0825	-0.33	0.0651	0.1536	2.06
4522	Tetraspanin-31	Q12999	0.0573	0.1571	-0.12	0.2728	0.6656	0.12	0.5575	0.7816	-0.04	0.0998	0.1938	0.10	0.5978	0.6736	0.02	0.9316	1.1679	-0.01	0.0705	0.2095	0.27	0.0675	0.2503	-0.39	0.4208	0.4923	-0.37
4523	Tetratricopeptide repeat protein 1	Q99614	0.4784	0.5534	0.04	0.6404	0.7350	0.05	0.8233	0.8574	0.01	0.5751	0.6241	0.03	0.0768	0.2131	-0.16	0.6898	0.9300	0.02	0.0035	0.0648	-0.52	0.0139	0.0984	-0.34	0.0519	0.1997	-0.51
4524	Tetratricopeptide repeat protein 13	Q8NBP0	0.1313	0.2733	-0.12	0.3890	0.7322	0.09	0.0037	0.0316	0.41	0.1939	0.3240	0.10	0.9108	0.9328	0.01	0.1218	0.2673	0.17	0.0122	0.1045	0.29	0.0036	0.0489	0.55	0.2551	0.3264	0.08
4525	Tetratricopeptide repeat protein 14	Q96N46	0.5185	0.5904	-0.04	0.2402	0.6604	0.14	0.0021	0.0258	0.60	0.0054	0.0296	0.39	0.1671	0.3534	0.14	0.4874	0.7126	0.16	0.3736	0.4613	-2.55	0.8708	0.9620	-0.33	0.0651	0.1241	2.06
4526	Tetratricopeptide repeat protein 21A	Q8NDW8	0.1182	0.2530	0.32	0.1642	0.6629	0.25	0.1261	0.2457	0.15	0.0084	0.0370	0.43	0.0463	0.1585	0.28	0.0370	0.1245	0.35	0.1164	0.2839	0.20	0.5274	0.9509	0.03	0.8303	0.8624	-0.01

Supplemetary Table S2. Overview on all relatively quantified 5180 proteins statistical analysis

			MCF-7																		MDA-MB-231								
			Dai SC20 vs control			Gen SC20 vs control			SSE SC20 vs control			Dai IC20 vs control			Gen IC20 vs control			SSE IC20 vs control			Dai IC20 vs control			Gen IC20 vs control			SSE IC20 vs control		
	**Protein name**	**UniProt**	**p value**	**BH q value**	**log2FC**	**p value**	**BH q value**	**log2FC**	**p value**	**BH q value**	**log2FC**	**p value**	**BH q value**	**log2FC**	**p value**	**BH q value**	**log2FC**	**p value**	**BH q value**	**log2FC**	**p value**	**BH q value**	**log2FC**	**p value**	**BH q value**	**log2FC**	**p value**	**BH q value**	**log2FC**
4527	Tetratricopeptide repeat protein 24	A2A3L6	0.2720	0.4647	0.20	0.7756	0.8380	0.05	0.5685	0.6263	-0.09	0.2774	0.4352	0.19	0.2620	0.4831	0.20	0.2860	0.4824	0.19	0.3736	0.4843	-2.55	0.8708	1.0046	-0.33	0.0651	0.1339	2.06
4528	Tetratricopeptide repeat protein 25	Q96NG3	0.4108	0.5045	-3.31	0.5752	0.7064	-1.75	0.5661	0.6426	-1.19	0.5388	0.6081	0.89	0.2938	0.4058	-1.88	0.9510	0.9856	0.09	0.4856	0.5401	0.12	0.3099	0.6579	-0.19	0.0810	0.1320	-0.44
4529	Tetratricopeptide repeat protein 27	Q6P3X3	0.4547	0.5319	-0.16	0.7328	0.8054	-0.13	0.9678	0.9734	0.01	0.2814	0.4398	-0.28	0.9063	0.9291	0.02	0.2693	0.4624	-0.36	0.3736	0.4869	-2.55	0.8708	1.0093	-0.33	0.0651	0.1350	2.06
4530	Tetratricopeptide repeat protein 28	Q96AY4	0.4108	0.5044	-3.31	0.5752	0.7062	-1.75	0.5661	0.6425	-1.19	0.5388	0.6079	0.89	0.2938	0.4057	-1.88	0.9510	0.9854	0.09	0.0456	0.1712	0.18	0.3510	0.7147	-0.06	0.0190	0.1193	0.30
4531	Tetratricopeptide repeat protein 30A	Q86WT1	0.0222	0.0889	-0.99	0.2221	0.6574	-1.00	0.0512	0.1271	-0.99	0.0113	0.0427	-1.04	0.3762	0.4728	-0.19	0.0156	0.0831	-1.11	0.3736	0.6016	-2.55	0.8708	1.2155	-0.33	0.0651	0.1938	2.06
4532	Tetratricopeptide repeat protein 38	Q5R3I4	0.4108	0.5043	-3.31	0.5752	0.7061	-1.75	0.5661	0.6424	-1.19	0.5388	0.6078	0.89	0.2938	0.4056	-1.88	0.9510	0.9852	0.09	0.0038	0.0663	0.79	0.0037	0.0498	0.60	0.0297	0.1469	0.39
4533	Tetratricopeptide repeat protein 4	O95801	0.0928	0.2143	0.27	0.6767	0.7632	-0.08	0.0811	0.1747	0.30	0.3906	0.5774	0.11	0.4769	0.5672	0.09	0.7014	0.9435	0.10	0.1503	0.3363	0.12	0.8842	0.9122	0.01	0.0598	0.2186	0.12
4534	Tetratricopeptide repeat protein 7A	Q9ULT0	0.2800	0.4734	0.11	0.0694	0.6002	-0.26	0.0139	0.0554	-0.30	0.0306	0.0814	-0.45	0.0148	0.0868	-0.34	0.2929	0.4907	-0.13	0.0318	0.1503	0.55	0.0134	0.0964	0.53	0.6480	0.7041	0.14
4535	Tetratricopeptide repeat protein 7B	Q86TV6	0.0174	0.0771	0.17	0.3643	0.7132	0.18	0.0142	0.0559	0.28	0.0510	0.1165	0.23	0.1361	0.3056	0.05	0.0749	0.1948	0.13	0.2156	0.4354	0.18	0.1361	0.3899	-0.15	0.0335	0.1566	-0.24
4536	Tetratricopeptide repeat protein 8	Q8TAM2	0.3730	0.5878	0.04	0.4548	0.7874	0.08	0.0077	0.0415	0.20	0.1128	0.2130	0.11	0.1037	0.2589	-0.08	0.5075	0.7337	0.02	0.9801	0.9839	0.00	0.0029	0.0443	-0.45	0.0045	0.0664	-0.47
4537	TGF-beta receptor type-1	P36897	0.4108	0.5041	-3.31	0.5752	0.7059	-1.75	0.5661	0.6422	-1.19	0.5388	0.6077	0.89	0.2938	0.4055	-1.88	0.9510	0.9850	0.09	0.0290	0.1461	0.61	0.5168	0.9380	0.09	0.0117	0.0932	0.27
4538	TGF-beta-activated kinase 1 and MAP3K7-binding protein 3	Q8N5C8	0.0040	0.0399	0.38	0.1191	0.6347	0.31	0.0041	0.0323	0.30	0.0048	0.0280	0.30	0.0249	0.1140	0.23	0.0059	0.0621	0.36	0.3736	0.6261	-2.55	0.8708	1.2582	-0.33	0.0651	0.2089	2.06
4539	Thimet oligopeptidase	P52888	0.0020	0.0307	0.21	0.1444	0.6538	0.20	0.0423	0.1116	0.07	0.0599	0.1316	0.09	0.1904	0.3865	0.04	0.1503	0.3080	0.04	0.0254	0.1401	0.48	0.0390	0.1796	0.27	0.3055	0.3767	0.11
4540	Thioredoxin	P10599	0.0139	0.0691	0.19	0.1613	0.6642	0.11	0.0064	0.0388	0.17	0.0066	0.0327	0.27	0.7130	0.7735	0.01	0.0178	0.0870	0.34	0.0973	0.2505	0.28	0.6006	1.0343	0.04	0.2652	0.3369	-0.08
4541	Thioredoxin domain-containing protein 12	O95881	0.0160	0.0737	-0.62	0.2216	0.6593	-0.36	0.0131	0.0536	-0.66	0.0129	0.0460	-0.57	0.6253	0.6963	-0.09	0.0520	0.1539	-0.41	0.1393	0.3196	0.14	0.8023	1.2462	-0.02	0.3760	0.4475	-0.06
4542	Thioredoxin domain-containing protein 17	Q9BRA2	0.0118	0.0626	0.17	0.7441	0.8149	0.03	0.7051	0.7517	0.02	0.1605	0.2795	-0.05	0.1017	0.2556	0.07	0.5687	0.8038	0.02	0.3291	0.5973	-0.04	0.0077	0.0719	-0.17	0.0098	0.0878	-0.19
4543	Thioredoxin domain-containing protein 5	Q8NBS9	0.0102	0.0587	0.20	0.3530	0.7052	0.17	0.0039	0.0316	0.26	0.0019	0.0194	0.31	0.0881	0.2325	0.10	0.0081	0.0667	0.35	0.1943	0.4028	-0.18	0.0162	0.1079	-0.28	0.0118	0.0935	-0.37
4544	Thioredoxin domain-containing protein 6	Q86XW9	0.9825	0.9878	-0.02	0.1251	0.6422	-6.09	0.0347	0.0970	3.76	0.0378	0.0943	-3.45	0.0748	0.2103	-3.19	0.7302	0.9699	-0.85	0.0587	0.1922	0.40	0.1050	0.3288	-0.13	0.3523	0.4238	0.06
4545	Thioredoxin domain-containing protein 8	Q6A555	0.4108	0.5040	-3.31	0.5752	0.7057	-1.75	0.5661	0.6421	-1.19	0.5388	0.6075	0.89	0.2938	0.4054	-1.88	0.9510	0.9848	0.09	0.2444	0.4781	6.00	0.2639	0.5925	5.65	0.2635	0.3354	5.67
4546	Thioredoxin domain-containing protein 9	O14530	0.4108	0.5039	-3.31	0.5752	0.7055	-1.75	0.5661	0.6419	-1.19	0.5388	0.6074	0.89	0.2938	0.4053	-1.88	0.9510	0.9846	0.09	0.4737	0.5285	-0.23	0.4755	0.8873	0.46	0.0460	0.1873	0.99
4547	Thioredoxin reductase 1 cytoplasmic	Q16881	0.5439	0.6139	0.05	0.1054	0.6319	-0.14	0.8825	0.9070	0.01	0.0708	0.1490	0.18	0.0917	0.2391	-0.14	0.0601	0.1697	0.23	0.1479	0.3330	0.28	0.6110	1.0435	-0.03	0.3559	0.4276	0.06
4548	Thioredoxin reductase 2 mitochondrial	Q9NNW7	0.4108	0.5038	-3.31	0.5752	0.7054	-1.75	0.5661	0.6418	-1.19	0.5388	0.6073	0.89	0.2938	0.4052	-1.88	0.9510	0.9844	0.09	0.0921	0.2434	-0.24	0.0161	0.1076	-0.35	0.0047	0.0671	-0.29
4549	Thioredoxin-dependent peroxide reductase_ mitochondrial	P30048	0.0005	0.0176	0.26	0.3501	0.7046	0.15	0.0022	0.0261	0.29	0.0016	0.0178	0.20	0.0727	0.2061	0.06	0.0015	0.0492	0.35	0.5311	0.5836	0.05	0.2017	0.5004	-0.11	0.1268	0.1864	-0.12
4550	Thioredoxin-like protein 1	O43396	0.5308	0.6018	0.03	0.0824	0.6168	0.20	0.0138	0.0552	0.21	0.0040	0.0256	0.25	0.0228	0.1094	0.20	0.0269	0.1051	0.15	0.0131	0.1086	0.34	0.0016	0.0326	0.26	0.0120	0.0945	0.18
4551	Thioredoxin-related transmembrane protein 1	Q9H3N1	0.1027	0.2292	0.09	0.3072	0.6774	0.19	0.0187	0.0655	0.21	0.0041	0.0260	0.27	0.8723	0.9030	0.01	0.0304	0.1117	0.18	0.0263	0.1415	0.43	0.3852	0.7651	0.10	0.8950	0.9166	0.01
4552	Thioredoxin-related transmembrane protein 2	Q9Y320	0.0442	0.1336	-0.61	0.0012	0.2486	-0.41	0.0000	0.0000	-1.36	0.0002	0.0126	-1.75	0.0000	0.0000	-1.17	0.0641	0.1765	-0.73	0.3736	0.5523	-2.55	0.8708	1.1283	-0.33	0.0651	0.1664	2.06
4553	Thiosulfate sulfurtransferase	Q16762	0.0007	0.0196	0.60	0.0211	0.4773	0.14	0.0215	0.0709	0.20	0.0443	0.1055	0.10	0.0809	0.2195	0.08	0.1826	0.3524	0.22	0.3736	0.5059	-2.55	0.8708	1.0444	-0.33	0.0651	0.1436	2.06
4554	Thiosulfate:glutathione sulfurtransferase	Q8NFU3	0.2874	0.4838	-0.06	0.1057	0.6308	-0.07	0.0067	0.0396	-0.19	0.0023	0.0206	-0.39	0.0041	0.0475	-0.37	0.0043	0.0610	0.25	0.3736	0.6335	-2.55	0.8708	1.2710	-0.33	0.0651	0.2137	2.06
4555	THO complex subunit 4	Q86V81	0.0069	0.0494	-0.21	0.2162	0.6611	-0.22	0.6088	0.6642	0.05	0.0021	0.0197	-0.30	0.0004	0.0174	-0.44	0.0058	0.0623	-0.31	0.3541	0.6301	0.13	0.2002	0.4983	-0.09	0.0018	0.0488	0.57
4556	Threonine--tRNA ligase cytoplasmic	P26639	0.2348	0.4177	-0.04	0.9597	0.9725	0.00	0.0315	0.0905	0.08	0.9683	0.9728	0.00	0.0051	0.0533	-0.15	0.0228	0.0967	0.21	0.0366	0.1591	0.45	0.7375	1.1813	0.03	0.2114	0.2800	0.10
4557	Threonylcarbamoyladenosine tRNA methylthiotransferase	Q5VV42	0.4108	0.5037	-3.31	0.5752	0.7052	-1.75	0.5661	0.6417	-1.19	0.5388	0.6071	0.89	0.2938	0.4051	-1.88	0.9510	0.9843	0.09	0.2545	0.4925	0.18	0.3076	0.6541	-0.11	0.2001	0.2685	-0.15
4558	Thrombospondin type-1 domain-containing protein 4	Q6ZMP0	0.0358	0.1182	0.20	0.4160	0.7545	0.05	0.2605	0.4297	-0.08	0.4795	0.6799	0.05	0.3423	0.4393	0.07	0.5040	0.7295	0.04	0.3736	0.4597	-2.55	0.8708	0.9589	-0.33	0.0651	0.1234	2.06
4559	Thrombospondin-1	P07996	0.5034	0.5767	-0.21	0.0700	0.6013	-9.00	0.6516	0.7036	0.07	0.6341	0.6798	-0.11	0.3323	0.4295	0.21	0.4598	0.6825	-0.08	0.0103	0.1018	-0.42	0.1082	0.3352	0.12	0.0367	0.1645	-0.20
4560	Thrombospondin-4	P35443	0.5836	0.6508	0.05	0.6189	0.7174	0.08	0.2491	0.4156	0.18	0.3176	0.4877	0.10	0.0939	0.2425	0.19	0.0492	0.1490	0.26	0.3736	0.5616	-2.55	0.8708	1.1449	-0.33	0.0651	0.1713	2.06
4561	THUMP domain-containing protein 1	Q9NXG2	0.7583	0.8034	-0.05	0.3840	0.7302	0.14	0.4687	0.6862	0.12	0.0053	0.0292	-0.98	0.0214	0.1055	-0.62	0.2264	0.4093	-0.25	0.0002	0.0314	1.74	0.0008	0.0266	1.51	0.0009	0.0373	2.07
4562	THUMP domain-containing protein 3	Q9BV44	0.0834	0.1990	0.08	0.8223	0.8732	-0.01	0.5597	0.7838	0.02	0.0374	0.0936	-0.08	0.6383	0.7079	-0.03	0.6489	0.8857	-0.01	0.0707	0.2099	0.36	0.0277	0.1510	0.19	0.0221	0.1268	0.16
4563	Thymidine kinase cytosolic	P04183	0.0655	0.1693	0.16	0.0513	0.5752	0.17	0.0078	0.0419	0.35	0.0107	0.0415	0.42	0.8331	0.8706	-0.01	0.0603	0.1700	0.17	0.2653	0.5073	-0.12	0.5082	0.9266	-0.07	0.0208	0.1234	-0.33
4564	Thymidylate kinase	P23919	0.0020	0.0306	0.49	0.0118	0.4335	0.42	0.0061	0.0380	0.51	0.0042	0.0263	0.57	0.0153	0.0883	0.28	0.0088	0.0685	0.49	0.1029	0.2605	0.24	0.1773	0.4627	0.12	0.6473	0.7035	-0.03
4565	Thymidylate synthase	P04818	0.1092	0.2392	0.16	0.4145	0.7531	-0.07	0.3230	0.5107	-0.06	0.3611	0.5403	-0.07	0.0090	0.0672	-0.29	0.0209	0.0936	0.25	0.0072	0.0869	0.26	0.0014	0.0302	0.37	0.0005	0.0294	0.44
4566	Thymocyte nuclear protein 1	Q9P016	0.4108	0.5035	-3.31	0.5752	0.7050	-1.75	0.5661	0.6415	-1.19	0.5388	0.6070	0.89	0.2938	0.4050	-1.88	0.9510	0.9841	0.09	0.0448	0.1700	0.39	0.6045	1.0372	-0.06	0.2962	0.3679	0.07
4567	Thymosin beta-10	P63313	0.3578	0.5692	0.05	0.4438	0.7795	-0.05	0.1774	0.3204	0.08	0.8209	0.8449	-0.01	0.2423	0.4561	-0.07	0.1073	0.2453	0.11	0.0428	0.1676	0.14	0.4128	0.8012	-0.03	0.5811	0.6437	0.01
4568	Thymosin beta-4	P62328	0.0010	0.0214	0.50	0.0159	0.4550	0.38	0.0004	0.0235	0.66	0.0001	0.0115	0.88	0.0051	0.0539	0.54	0.0018	0.0536	0.78	0.8821	0.9021	0.02	0.0989	0.3147	-0.21	0.0073	0.0778	-0.67
4569	Thyroid hormone receptor-associated protein 3	Q9Y2W1	0.2680	0.4598	0.08	0.0588	0.5903	0.34	0.0004	0.0228	0.47	0.0006	0.0138	0.42	0.0030	0.0416	0.32	0.0092	0.0693	0.28	0.0145	0.1118	0.85	0.0043	0.0525	0.37	0.0741	0.1237	0.69
4570	Thyroid peroxidase	P07202	0.0367	0.1205	-0.43	0.3760	0.7240	-0.28	0.4337	0.6441	-0.23	0.0102	0.0405	-0.57	0.0351	0.1361	-0.50	0.0410	0.1332	-0.39	0.3736	0.5694	-2.55	0.8708	1.1587	-0.33	0.0651	0.1755	2.06
4571	Thyroid receptor-interacting protein 11	Q15643	0.4949	0.5692	-0.09	0.3246	0.6857	-0.19	0.8097	0.8458	-0.03	0.6362	0.6812	-0.06	0.4533	0.5458	-0.10	0.2330	0.4170	0.19	0.3736	0.4943	-2.55	0.8708	1.0231	-0.33	0.0651	0.1383	2.06
4572	Thyroid receptor-interacting protein 6	Q15654	0.4719	0.5478	-0.02	0.1727	0.6607	0.26	0.0616	0.1443	0.10	0.0230	0.0666	0.10	0.0182	0.0963	0.11	0.0324	0.1161	-0.11	0.0036	0.0657	-0.40	0.0094	0.0797	-0.45	0.0008	0.0348	-0.61
4573	Thyrotropin-releasing hormone-degrading ectoenzyme	Q9UKU6	0.0271	0.0998	-0.18	0.5820	0.6894	-0.02	0.5144	0.7351	0.04	0.6238	0.6701	0.02	0.7672	0.8189	0.01	0.2598	0.4511	-0.09	0.1291	0.3029	0.29	0.1938	0.4887	0.17	0.3567	0.4284	0.09
4574	Tigger transposable element-derived protein 5	Q53EQ6	0.9486	0.9614	0.00	0.2427	0.6596	-0.11	0.0005	0.0190	0.30	0.3486	0.5245	0.19	0.8390	0.8755	0.01	0.1006	0.2346	0.28	0.0007	0.0427	-1.50	0.0031	0.0464	-1.40	0.3650	0.4366	-0.81
4575	TIP41-like protein	O75663	0.0144	0.0696	2.59	0.3211	0.6836	0.50	0.4151	0.6218	0.37	0.0667	0.1427	1.10	0.3576	0.4550	0.43	0.2440	0.4311	1.91	0.0194	0.1288	0.48	0.0056	0.0604	0.23	0.0100	0.0889	0.27
4576	Titin	Q8WZ42	0.0047	0.0429	0.29	0.2007	0.6626	0.28	0.0033	0.0307	0.38	0.0026	0.0214	0.34	0.0558	0.1765	0.13	0.0094	0.0693	0.37	0.0212	0.1341	0.49	0.1663	0.4450	0.15	0.2764	0.3482	0.09
4577	TNF receptor-associated factor 5	O00463	0.2714	0.4640	-0.09	0.3732	0.7208	-0.25	0.2224	0.3796	0.11	0.1777	0.3021	0.12	0.0351	0.1357	-0.28	0.3395	0.5435	-0.11	0.0026	0.0571	0.49	0.1041	0.3262	0.14	0.0301	0.1476	0.27
4578	TNF receptor-associated factor 6	Q9Y4K3	0.1023	0.2286	0.92	0.3503	0.7044	0.45	0.1340	0.2564	0.83	0.1481	0.2625	0.78	0.0795	0.2175	1.14	0.0653	0.1783	1.28	0.3736	0.5514	-2.55	0.8708	1.1266	-0.33	0.0651	0.1659	2.06
4579	TNFAIP3-interacting protein 2	Q8NFZ5	0.5709	0.6386	-0.14	0.5713	0.8844	-0.16	0.7522	0.7942	-0.07	0.1519	0.2679	-0.45	0.1573	0.3389	-0.45	0.9823	0.9863	0.00	0.2694	0.5121	-0.15	0.3303	0.6866	-0.10	0.3892	0.4604	-0.16
4580	TOG array regulator of axonemal microtubules protein 2	Q6ZUX3	0.4108	0.5034	-3.31	0.5752	0.7049	-1.75	0.5661	0.6414	-1.19	0.5388	0.6069	0.89	0.2938	0.4049	-1.88	0.9510	0.9839	0.09	0.0309	0.1485	-0.61	0.0002	0.0159	-0.99	0.0014	0.0462	-1.12
4581	Toll-interacting protein	Q9H0E2	0.2983	0.4970	0.71	0.2991	0.6733	0.88	0.4139	0.6202	-0.37	0.5533	0.6043	-0.09	0.6963	0.7584	-0.06	0.1932	0.3671	0.79	0.3736	0.5036	-2.55	0.8708	1.0401	-0.33	0.0651	0.1425	2.06
4582	Toll-like receptor 5	O60602	0.4108	0.5033	-3.31	0.5752	0.7047	-1.75	0.5661	0.6412	-1.19	0.5388	0.6067	0.89	0.2938	0.4048	-1.88	0.9510	0.9837	0.09	0.8347	0.8608	-0.01	0.0576	0.2292	-0.19	0.0562	0.2106	-0.20
4583	Tonsoku-like protein	Q96HA7	0.0000	0.0000	-2.62	0.2027	0.6608	-2.24	0.0005	0.0193	-2.64	0.0135	0.0472	-3.44	0.0282	0.1218	-0.47	0.0501	0.1502	-3.38	0.3736	0.5606	-2.55	0.8708	1.1431	-0.33	0.0651	0.1707	2.06
4584	Torsin-1A	O14656	0.0019	0.0296	0.15	0.2052	0.6594	0.18	0.5227	0.7438	0.04	0.1288	0.2347	0.10	0.8637	0.8955	-0.01	0.0190	0.0884	0.10	0.3736	0.5947	-2.55	0.8708	1.2035	-0.33	0.0651	0.1898	2.06
4585	Torsin-1A-interacting protein 1	Q5JTV8	0.1161	0.2500	0.13	0.4214	0.7593	0.09	0.0297	0.0875	0.38	0.0879	0.1762	0.26	0.0949	0.2436	-0.10	0.0649	0.1776	0.24	0.2318	0.4593	0.16	0.5147	0.9348	0.05	0.2454	0.3157	-0.06
4586	Torsin-1A-interacting protein 2	Q8NFQ8	0.0070	0.0493	-0.26	0.1684	0.6603	-0.15	0.0498	0.1247	-0.13	0.0412	0.1006	-0.17	0.2998	0.3979	-0.07	0.0715	0.1887	-0.21	0.5585	0.6087	-0.06	0.0362	0.1735	0.14	0.0484	0.1921	0.22
4587	Torsin-3A	Q9H497	0.7949	0.8337	0.05	0.2382	0.6623	-0.44	0.8452	0.8756	-0.04	0.6201	0.6667	-0.10	0.3466	0.4437	-0.19	0.1443	0.2997	0.33	0.3736	0.5184	-2.55	0.8708	1.0671	-0.33	0.0651	0.1495	2.06
4588	TP53-regulated inhibitor of apoptosis 1	O43715	0.0347	0.1164	-0.41	0.0940	0.6307	-0.49	0.0910	0.1901	-0.45	0.0689	0.1462	-0.60	0.2916	0.5232	-0.28	0.3420	0.5463	-0.73	0.3830	0.4388	0.18	0.0931	0.3039	0.30	0.1714	0.2371	0.61
4589	TPR and ankyrin repeat-containing protein 1	O15050	0.0131	0.0667	-0.31	0.0811	0.6151	-0.16	0.5320	0.7529	-0.04	0.0896	0.1786	-0.15	0.1923	0.3888	-0.10	0.5941	0.8313	-0.05	0.3736	0.4532	-2.55	0.8708	0.9468	-0.33	0.0651	0.1208	2.06
4590	TPT1-like protein	Q56UQ5	0.1499	0.2993	0.06	0.2053	0.6593	-0.05	0.1174	0.2324	0.07	0.1690	0.2905	-0.07	0.1004	0.2536	-0.10	0.0072	0.0648	0.25	0.3736	0.6217	-2.55	0.8708	1.2506	-0.33	0.0651	0.2061	2.06
4591	Trafficking protein particle complex subunit 13	A5PLN9	0.4108	0.5032	-3.31	0.5752	0.7045	-1.75	0.5661	0.6411	-1.19	0.5388	0.6066	0.89	0.2938	0.4046	-1.88	0.9510	0.9835	0.09	0.0310	0.1485	2.89	0.4885	0.8995	0.42	0.3684	0.4398	2.16
4592	Trafficking protein particle complex subunit 2-like protein	Q9UL33	0.0472	0.1395	-1.17	0.0504	0.5725	-10.57	0.0889	0.1873	-3.38	0.1083	0.2059	-2.38	0.6221	0.6942	-0.19	0.1753	0.3432	-3.26	0.3736	0.5086	-2.55	0.8708	1.0493	-0.33	0.0651	0.1449	2.06
4593	TRAF-type zinc finger domain-containing protein 1	O14545	0.0035	0.0378	-1.35	0.2221	0.6578	-0.48	0.5254	0.7465	0.10	0.1509	0.2666	-0.43	0.3448	0.4419	0.19	0.2226	0.4053	-0.48	0.3736	0.4977	-2.55	0.8708	1.0294	-0.33	0.0651	0.1399	2.06
4594	Trans-acting T-cell-specific transcription factor GATA-3	P23771	0.1295	0.2704	-0.04	0.9986	0.9986	0.00	0.0005	0.0179	-0.57	0.0014	0.0173	-0.35	0.1264	0.2933	-0.04	0.0014	0.0518	-0.31	0.3736	0.6468	-2.55	0.8708	1.2940	-0.33	0.0651	0.2226	2.06
4595	Transaldolase	P37837	0.0273	0.0999	0.24	0.1984	0.6660	0.35	0.0095	0.0455	0.35	0.0024	0.0209	0.50	0.1330	0.3022	0.13	0.0087	0.0681	0.45	0.0709	0.2103	0.50	0.7561	1.1988	0.04	0.5416	0.6071	0.05
4596	Transcobalamin-2	P20062	0.2498	0.4360	-0.25	0.4269	0.7625	-0.18	0.2151	0.3700	-0.28	0.1638	0.2834	-0.34	0.1947	0.3927	-0.30	0.4175	0.6359	0.18	0.3736	0.4675	-2.55	0.8708	0.9734	-0.33	0.0651	0.1267	2.06
4597	Transcription and mRNA export factor ENY2	Q9NPA8	0.0786	0.1921	-0.43	0.1833	0.6649	-0.62	0.4793	0.6986	-0.13	0.2011	0.3338	-0.24	0.3021	0.4001	-0.18	0.0956	0.2277	-0.38	0.3736	0.5358	-2.55	0.8708	1.0986	-0.33	0.0651	0.1579	2.06
4598	Transcription elongation factor A protein 1	P23193	0.0282	0.1020	0.15	0.3132	0.6802	0.16	0.0141	0.0557	0.24	0.0012	0.0159	0.30	0.1983	0.3981	0.05	0.0122	0.0745	0.22	0.0203	0.1323	0.42	0.1869	0.4772	0.12	0.1059	0.1622	0.14
4599	Transcription elongation factor A protein 2	Q15560	0.9678	0.9763	0.02	0.3393	0.6988	-0.50	0.0511	0.1271	-0.74	0.0324	0.0848	-0.90	0.6699	0.7341	-0.10	0.0307	0.1124	-0.83	0.0395	0.1638	-0.34	0.0101	0.0828	-0.83	0.0690	0.1165	-0.82
4600	Transcription elongation factor A protein 3	O75764	0.4108	0.5031	-3.31	0.5752	0.7044	-1.75	0.5661	0.6410	-1.19	0.5388	0.6065	0.89	0.2938	0.4045	-1.88	0.9510	0.9833	0.09	0.8058	0.8363	0.11	0.5055	0.9227	0.21	0.0388	0.1702	1.05
4601	Transcription elongation factor A protein-like 1	Q15170	0.1028	0.2293	0.33	0.2111	0.6619	0.24	0.0670	0.1522	0.39	0.1447	0.2575	0.27	0.1480	0.3242	0.27	0.2550	0.4453	0.20	0.3736	0.4891	-2.55	0.8708	1.0134	-0.33	0.0651	0.1360	2.06
4602	Transcription elongation factor A protein-like 2	Q9H3H9	0.0414	0.1288	0.25	0.1526	0.6598	0.17	0.4495	0.6632	-0.05	0.1711	0.2931	0.11	0.7706	0.8213	0.02	0.0897	0.2183	0.16	0.3736	0.5380	-2.55	0.8708	1.1026	-0.33	0.0651	0.1591	2.06
4603	Transcription elongation factor A protein-like 4	Q96EI5	0.7101	0.7617	-0.02	0.4382	0.7744	-0.03	0.0861	0.1829	0.10	0.1961	0.3267	-0.07	0.0832	0.2232	-0.10	0.4500	0.6727	-0.03	0.0966	0.2497	0.28	0.7620	1.2041	0.03	0.5513	0.6159	-0.07
4604	Transcription elongation factor A protein-like 5	Q5H9L2	0.0335	0.1142	-0.36	0.4454	0.7816	-0.08	0.0150	0.0575	0.58	0.0020	0.0196	0.81	0.0672	0.1976	0.26	0.1529	0.3105	0.18	0.3736	0.5150	-2.55	0.8708	1.0609	-0.33	0.0651	0.1478	2.06
4605	Transcription elongation factor SPT5	O00267	0.8629	0.8915	0.01	0.5621	0.8760	0.10	0.0161	0.0599	0.39	0.0032	0.0231	0.47	0.0037	0.0455	0.40	0.0001	0.0305	0.47	0.0159	0.1177	-0.67	0.0001	0.0115	-0.89	0.0217	0.1260	-1.04
4606	Transcription elongation regulator 1	O14776	0.0372	0.1212	0.17	0.3360	0.6954	0.11	0.0268	0.0820	0.18	0.0350	0.0894	0.16	0.3396	0.4366	0.05	0.0286	0.1082	0.20	0.0117	0.1040	0.39	0.1823	0.4710	0.13	0.0382	0.1687	0.20
4607	Transcription factor 15	Q12870	0.0017	0.0278	1.13	0.1132	0.6346	0.45	0.0368	0.1007	0.44	0.0180	0.0566	0.57	0.0247	0.1136	0.76	0.2119	0.3933	0.20	0.3736	0.5002	-2.55	0.8708	1.0339	-0.33	0.0651	0.1410	2.06

Supplemetary Table S2. Overview on all relatively quantified 5180 proteins statistical analysis

			MCF-7																		MDA-MB-231								
			Dai SC20 vs control			Gen SC20 vs control			SSE SC20 vs control			Dai IC20 vs control			Gen IC20 vs control			SSE IC20 vs control			Dai IC20 vs control			Gen IC20 vs control			SSE IC20 vs control		
	**Protein name**	**UniProt**	**p value**	**BH q value**	**log2FC**	**p value**	**BH q value**	**log2FC**	**p value**	**BH q value**	**log2FC**	**p value**	**BH q value**	**log2FC**	**p value**	**BH q value**	**log2FC**	**p value**	**BH q value**	**log2FC**	**p value**	**BH q value**	**log2FC**	**p value**	**BH q value**	**log2FC**	**p value**	**BH q value**	**log2FC**
4608	Transcription factor 20	Q9UGU0	0.4108	0.5029	-3.31	0.5752	0.7042	-1.75	0.5661	0.6408	-1.19	0.5388	0.6063	0.89	0.2938	0.4044	-1.88	0.9510	0.9831	0.09	0.0559	0.1874	0.22	0.9822	0.9873	0.00	0.1576	0.2214	0.21
4609	Transcription factor 25	Q9BQ70	0.0302	0.1069	0.16	0.2413	0.6606	0.22	0.0094	0.0452	0.24	0.1135	0.2138	0.10	0.8013	0.8450	0.01	0.1642	0.3273	0.09	0.3736	0.5110	-2.55	0.8708	1.0537	-0.33	0.0651	0.1460	2.06
4610	Transcription factor 7	P36402	0.4108	0.5028	-3.31	0.5752	0.7040	-1.75	0.5661	0.6407	-1.19	0.5388	0.6062	0.89	0.2938	0.4043	-1.88	0.9510	0.9829	0.09	0.0132	0.1087	0.42	0.0001	0.0118	1.62	0.0691	0.1166	0.24
4611	Transcription factor 7-like 2	Q9NQB0	0.1075	0.2360	0.16	0.2204	0.6592	0.14	0.8451	0.8759	0.01	0.0074	0.0348	0.30	0.0256	0.1153	0.12	0.0238	0.0989	0.15	0.3736	0.5886	-2.55	0.8708	1.1927	-0.33	0.0651	0.1862	2.06
4612	Transcription factor A mitochondrial	Q00059	0.7019	0.7554	0.03	0.2912	0.6692	0.17	0.8558	0.8852	-0.01	0.1710	0.2930	0.12	0.7504	0.8049	0.02	0.1403	0.2940	0.14	0.0425	0.1672	0.42	0.1078	0.3344	0.09	0.0198	0.1217	0.16
4613	Transcription factor BTF3	P20290	0.2467	0.4326	-0.15	0.5366	0.8521	-0.18	0.7914	0.8290	0.03	0.8140	0.8398	0.02	0.0560	0.1768	-0.33	0.9797	0.9839	0.00	0.7267	0.7660	0.09	0.7989	1.2431	0.05	0.5559	0.6205	0.12
4614	Transcription factor BTF3 homolog 4	Q96K17	0.4108	0.5027	-3.31	0.5752	0.7039	-1.75	0.5661	0.6405	-1.19	0.5388	0.6061	0.89	0.2938	0.4042	-1.88	0.9510	0.9827	0.09	0.0619	0.1958	-0.83	0.9859	0.9899	0.01	0.7571	0.7999	-0.09
4615	Transcription factor E2F8	A0AVK6	0.4108	0.5026	-3.31	0.5752	0.7037	-1.75	0.5661	0.6404	-1.19	0.5388	0.6059	0.89	0.2938	0.4041	-1.88	0.9510	0.9825	0.09	0.0392	0.1638	-0.86	0.0003	0.0175	-0.75	0.1475	0.2098	-0.37
4616	Transcription factor ETV7	Q9Y603	0.4633	0.5402	0.04	0.3762	0.7239	0.13	0.0133	0.0538	0.23	0.0443	0.1056	-0.15	0.0218	0.1062	0.24	0.1070	0.2450	0.11	0.3736	0.5315	-2.55	0.8708	1.0909	-0.33	0.0651	0.1558	2.06
4617	Transcription factor HIVEP2	P31629	0.3897	0.6064	-0.21	0.4094	0.7504	-0.21	0.9342	0.9490	0.02	0.1879	0.3156	-0.36	0.5996	0.6753	-0.12	0.6667	0.9048	0.10	0.3736	0.4467	-2.55	0.8708	0.9347	-0.33	0.0651	0.1182	2.06
4618	Transcription factor HIVEP3	Q5T1R4	0.0005	0.0175	-2.25	0.2298	0.6613	-1.31	0.0003	0.0199	-2.39	0.0011	0.0163	-2.26	0.6144	0.6883	0.08	0.0028	0.0541	-2.17	0.3736	0.6395	-2.55	0.8708	1.2815	-0.33	0.0651	0.2177	2.06
4619	Transcription factor jun-D	P17535	0.0370	0.1209	-0.64	0.1962	0.6691	-0.89	0.1295	0.2503	-0.47	0.0374	0.0937	-0.45	0.5904	0.6673	-0.09	0.1655	0.3290	-0.22	0.3736	0.5104	-2.55	0.8708	1.0524	-0.33	0.0651	0.1457	2.06
4620	Transcription factor p65	Q04206	0.6518	0.7128	0.07	0.7674	0.8311	-0.09	0.1124	0.2242	0.33	0.8918	0.9077	0.03	0.0690	0.2001	0.45	0.7876	1.0256	0.06	0.0012	0.0454	1.17	0.0071	0.0694	0.59	0.0033	0.0576	0.94
4621	Transcription factor RelB	Q01201	0.7559	0.8014	-0.02	0.0852	0.6199	-0.12	0.0002	0.0199	0.71	0.0035	0.0240	0.44	0.0039	0.0465	0.40	0.0029	0.0556	0.73	0.3736	0.6391	-2.55	0.8708	1.2807	-0.33	0.0651	0.2174	2.06
4622	Transcription factor Sp1	P08047	0.0877	0.2061	-0.32	0.4314	0.7674	-0.26	0.5571	0.7812	0.10	0.7583	0.7913	-0.05	0.9674	0.9744	-0.01	0.0821	0.2063	-0.33	0.3736	0.5425	-2.55	0.8708	1.1107	-0.33	0.0651	0.1613	2.06
4623	Transcription factor TFIIIB component B" homolog	A6H8Y1	0.0000	0.0000	0.91	0.0014	0.2339	0.34	0.0031	0.0306	0.25	0.0011	0.0159	0.25	0.0000	0.0000	0.68	0.0192	0.0890	0.12	0.9378	0.9488	0.01	0.7682	1.2110	0.02	0.0021	0.0508	0.40
4624	Transcription initiation factor IIB	Q00403	0.4108	0.5025	-3.31	0.5752	0.7036	-1.75	0.5661	0.6403	-1.19	0.5388	0.6058	0.89	0.2938	0.4040	-1.88	0.9510	0.9823	0.09	0.2869	0.5381	0.17	0.0094	0.0796	-0.50	0.1178	0.1759	-0.34
4625	Transcription initiation factor TFIID subunit 11	Q15544	0.4108	0.5023	-3.31	0.5752	0.7034	-1.75	0.5661	0.6401	-1.19	0.5388	0.6057	0.89	0.2938	0.4039	-1.88	0.9510	0.9821	0.09	0.3718	0.6540	0.88	0.1864	0.4766	1.49	0.1122	0.1695	1.92
4626	Transcription intermediary factor 1-alpha	O15164	0.1264	0.2652	-0.58	0.2761	0.6649	-0.41	0.4125	0.6186	0.13	0.3182	0.4885	-0.37	0.0386	0.1433	0.22	0.1925	0.3666	-0.49	0.3736	0.5042	-2.55	0.8708	1.0413	-0.33	0.0651	0.1428	2.06
4627	Transcription intermediary factor 1-beta	Q13263	0.0053	0.0442	0.18	0.5229	0.8420	0.10	0.0014	0.0220	0.35	0.0359	0.0910	0.12	0.1109	0.2715	-0.07	0.0053	0.0611	0.27	0.0591	0.1930	0.24	0.8272	1.2711	0.01	0.4220	0.4932	0.04
4628	Transcription termination factor 1 mitochondrial	Q99551	0.9901	0.9920	0.01	0.0189	0.4640	-0.40	0.0465	0.1194	-0.26	0.0017	0.0180	-0.72	0.0319	0.1306	-0.30	0.3405	0.5447	0.60	0.0016	0.0521	0.81	0.0015	0.0317	1.40	0.1522	0.2153	0.34
4629	Transcription termination factor 4 mitochondrial	Q7Z6M4	0.0222	0.0888	0.35	0.1541	0.6602	0.22	0.0862	0.1830	0.23	0.4072	0.5984	-0.11	0.2945	0.3925	0.12	0.6871	0.9269	-0.04	0.3736	0.4457	-2.55	0.8708	0.9327	-0.33	0.0651	0.1177	2.06
4630	Transcriptional activator protein Pur-beta	Q96QR8	0.1229	0.2603	-0.07	0.5958	0.7001	-0.07	0.3270	0.5159	0.03	0.0932	0.1838	-0.12	0.0014	0.0287	-0.18	0.0708	0.1873	0.05	0.0642	0.1994	0.21	0.0374	0.1761	-0.29	0.0359	0.1621	-0.28
4631	Transcriptional adapter 2-alpha	O75478	0.4108	0.5022	-3.31	0.5752	0.7032	-1.75	0.5661	0.6400	-1.19	0.5388	0.6056	0.89	0.2938	0.4038	-1.88	0.9510	0.9819	0.09	0.6629	0.7071	0.12	0.0414	0.1871	-0.61	0.6785	0.7318	-0.07
4632	Transcriptional coactivator YAP1	P46937	0.0009	0.0210	-1.22	0.3180	0.6810	-0.49	0.2505	0.4175	-0.63	0.0027	0.0216	-1.09	0.4659	0.5575	0.09	0.0035	0.0589	-1.18	0.0232	0.1364	-0.28	0.0102	0.0832	-0.51	0.0064	0.0753	-0.55
4633	Transcriptional regulator ERG	P11308	0.0003	0.0140	1.01	0.0603	0.5950	1.06	0.0000	0.0000	1.22	0.0001	0.0167	1.47	0.0381	0.1424	0.14	0.0001	0.0259	1.29	0.3736	0.6549	-2.55	0.8708	1.3078	-0.33	0.0651	0.2282	2.06
4634	Transcriptional repressor CTCFL	Q8NI51	0.4108	0.5021	-3.31	0.5752	0.7031	-1.75	0.5661	0.6398	-1.19	0.5388	0.6054	0.89	0.2938	0.4037	-1.88	0.9510	0.9817	0.09	0.0297	0.1468	-0.34	0.0379	0.1773	-0.37	0.0360	0.1622	-0.23
4635	Transcriptional repressor p66-alpha	Q86YP4	0.0475	0.1400	-0.07	0.0450	0.5644	-0.07	0.0662	0.1509	0.07	0.4167	0.6089	-0.04	0.0012	0.0268	-0.27	0.0182	0.0874	-0.14	0.9406	0.9509	0.01	0.8904	0.9177	-0.02	0.1656	0.2308	0.29
4636	Transcriptional repressor p66-beta	Q8WXI9	0.1371	0.2819	0.09	0.7818	0.8428	0.05	0.5025	0.7249	0.06	0.0374	0.0936	0.16	0.6542	0.7209	-0.02	0.0611	0.1715	0.12	0.0204	0.1323	0.45	0.6628	1.1015	0.04	0.1023	0.1582	0.20
4637	Transcriptional repressor protein YY1	P25490	0.1052	0.2326	0.25	0.2258	0.6593	0.19	0.0686	0.1549	0.30	0.1475	0.2616	0.21	0.9094	0.9319	0.01	0.3895	0.6012	0.11	0.7788	0.8130	-0.04	0.7047	1.1465	0.06	0.8499	0.8787	-0.03
4638	Transducin beta-like protein 2	Q9Y4P3	0.0072	0.0499	0.14	0.3847	0.7302	0.11	0.0007	0.0203	0.33	0.2115	0.3478	0.05	0.6589	0.7243	-0.02	0.0092	0.0687	0.18	0.1683	0.3634	-0.20	0.0727	0.2613	0.30	0.5339	0.5999	-0.09
4639	Transferrin receptor protein 1	P02786	0.0066	0.0487	0.39	0.6146	0.7151	0.08	0.0037	0.0315	0.34	0.0015	0.0177	0.50	0.9668	0.9739	0.00	0.0071	0.0643	0.46	0.0774	0.2216	0.25	0.2781	0.6154	0.11	0.5706	0.6341	0.05
4640	Transferrin receptor protein 2	Q9UP52	0.0026	0.0338	-0.35	0.7931	0.8507	0.03	0.1298	0.2506	-0.08	0.4275	0.6194	0.03	0.0320	0.1304	0.25	0.0111	0.0722	-0.23	0.3736	0.6105	-2.55	0.8708	1.2311	-0.33	0.0651	0.1992	2.06
4641	Transformation/transcription domain-associated protein	Q9Y4A5	0.4108	0.5020	-3.31	0.5752	0.7029	-1.75	0.5661	0.6397	-1.19	0.5388	0.6053	0.89	0.2938	0.4036	-1.88	0.9510	0.9815	0.09	0.0998	0.2555	-0.12	0.0453	0.1977	0.16	0.2027	0.2711	0.15
4642	Transformer-2 protein homolog alpha	Q13595	0.4108	0.5019	-3.31	0.5752	0.7027	-1.75	0.5661	0.6396	-1.19	0.5388	0.6052	0.89	0.2938	0.4035	-1.88	0.9510	0.9813	0.09	0.4692	0.5243	0.06	0.0333	0.1648	0.30	0.3324	0.4035	0.09
4643	Transformer-2 protein homolog beta	P62995	0.0034	0.0377	-0.19	0.2457	0.6584	-0.11	0.9104	0.9292	-0.01	0.0016	0.0176	-0.24	0.0005	0.0189	-0.32	0.0409	0.1331	-0.21	0.0039	0.0673	0.59	0.0002	0.0179	0.66	0.0022	0.0525	0.49
4644	Transforming acidic coiled-coil-containing protein 3	Q9Y6A5	0.0421	0.1300	-0.35	0.0053	0.3761	-0.71	0.1309	0.2521	-0.25	0.0097	0.0395	-0.56	0.0072	0.0619	-0.74	0.0086	0.0681	-0.96	0.0962	0.2495	0.39	0.0911	0.3002	0.39	0.1669	0.2322	0.29
4645	Transforming growth factor beta-1 proprotein	P01137	0.4108	0.5018	-3.31	0.5752	0.7026	-1.75	0.5661	0.6394	-1.19	0.5388	0.6050	0.89	0.2938	0.4034	-1.88	0.9510	0.9811	0.09	0.0027	0.0578	0.56	0.0152	0.1040	0.39	0.0053	0.0702	0.69
4646	Transforming growth factor beta-3 proprotein	P10600	0.4108	0.5016	-3.31	0.5752	0.7024	-1.75	0.5661	0.6393	-1.19	0.5388	0.6049	0.89	0.2938	0.4033	-1.88	0.9510	0.9809	0.09	0.0622	0.1963	0.42	0.3339	0.6913	0.12	0.1399	0.2013	0.14
4647	Transforming protein RhoA	P61586	0.1435	0.2902	-0.07	0.0293	0.5093	-0.22	0.0031	0.0305	-0.30	0.0090	0.0384	-0.24	0.2806	0.5070	-0.20	0.0406	0.1325	-0.14	0.1121	0.2756	0.32	0.3533	0.7174	-0.12	0.2746	0.3464	-0.12
4648	Transgelin-2	P37802	0.4166	0.4943	-0.05	0.3905	0.7345	0.15	0.0768	0.1674	0.15	0.2505	0.3999	0.08	0.9935	0.9956	0.00	0.1676	0.3321	0.10	0.1380	0.3178	0.28	0.3485	0.7107	0.11	0.8254	0.8587	-0.02
4649	Transgelin-3	Q9UI15	0.4108	0.5015	-3.31	0.5752	0.7022	-1.75	0.5661	0.6391	-1.19	0.5388	0.6048	0.89	0.2938	0.4031	-1.88	0.9510	0.9807	0.09	0.1665	0.3612	1.40	0.5055	0.9230	0.72	0.0696	0.1174	2.32
4650	Trans-Golgi network integral membrane protein 2	O43493	0.0946	0.2167	-0.16	0.1900	0.6695	0.25	0.1349	0.2578	0.13	0.0668	0.1428	0.18	0.0846	0.2262	0.16	0.5783	0.8129	0.04	0.9392	0.9498	0.01	0.4246	0.8195	-0.08	0.7342	0.7809	-0.08
4651	Transient receptor potential cation channel subfamily A member 1	O75762	0.4108	0.5014	-3.31	0.5752	0.7021	-1.75	0.5661	0.6390	-1.19	0.5388	0.6046	0.89	0.2938	0.4030	-1.88	0.9510	0.9805	0.09	0.0002	0.0280	-1.08	0.0001	0.0167	-1.40	0.0003	0.0311	-1.32
4652	Transient receptor potential cation channel subfamily M member 6	Q9BX84	0.0093	0.0569	0.17	0.2072	0.6593	0.07	0.0106	0.0480	-0.17	0.0029	0.0221	0.22	0.0005	0.0186	0.46	0.1051	0.2416	0.17	0.0063	0.0839	0.37	0.0011	0.0281	0.91	0.0217	0.1259	0.32
4653	Transient receptor potential cation channel subfamily V member 2	Q9Y5S1	0.1524	0.3033	-0.18	0.0388	0.5583	-0.33	0.0239	0.0759	-0.37	0.0188	0.0580	-0.43	0.0177	0.0953	-0.44	0.2721	0.4661	-0.26	0.3736	0.4866	-2.55	0.8708	1.0089	-0.33	0.0651	0.1349	2.06
4654	Transient receptor potential cation channel subfamily V member 3	Q8NET8	0.4108	0.5013	-3.31	0.5752	0.7019	-1.75	0.5661	0.6389	-1.19	0.5388	0.6045	0.89	0.2938	0.4029	-1.88	0.9510	0.9803	0.09	0.5423	0.5936	-0.94	0.9810	0.9863	-0.03	0.9860	0.9889	-0.02
4655	Transitional endoplasmic reticulum ATPase	P55072	0.0956	0.2182	0.08	0.0247	0.4846	0.14	0.0044	0.0328	0.36	0.0020	0.0197	0.31	0.0127	0.0802	0.17	0.0078	0.0665	0.34	0.0121	0.1041	0.39	0.9938	0.9961	0.00	0.1321	0.1924	0.10
4656	Transketolase	P29401	0.0036	0.0381	0.37	0.2036	0.6604	0.27	0.0019	0.0243	0.43	0.0012	0.0162	0.48	0.0693	0.2004	0.14	0.0047	0.0600	0.60	0.0830	0.2300	0.38	0.6530	1.0929	0.04	0.7427	0.7872	0.03
4657	Translation initiation factor eIF-2B subunit alpha	Q14232	0.2811	0.4745	0.18	0.8540	0.8949	-0.05	0.8887	0.9114	0.03	0.7905	0.8196	-0.04	0.6958	0.7580	-0.06	0.8216	1.0568	0.03	0.3605	0.6391	-0.17	0.2094	0.5114	-0.15	0.0150	0.1051	-0.51
4658	Translation initiation factor eIF-2B subunit beta	P49770	0.8638	0.8922	-0.01	0.8783	0.9125	0.01	0.0596	0.1413	0.11	0.0058	0.0306	0.25	0.0683	0.1990	0.12	0.0434	0.1372	0.17	0.0247	0.1383	0.37	0.0810	0.2795	0.24	0.3357	0.4067	0.20
4659	Translation initiation factor eIF-2B subunit delta	Q9UI10	0.0121	0.0635	0.29	0.1650	0.6610	0.19	0.0078	0.0416	0.42	0.0079	0.0360	0.33	0.0033	0.0431	0.41	0.0838	0.2097	0.26	0.4799	0.5344	0.10	0.0156	0.1054	-0.22	0.0064	0.0750	-0.35
4660	Translation initiation factor eIF-2B subunit gamma	Q9NR50	0.2799	0.4735	-0.04	0.7342	0.8066	0.03	0.6261	0.6793	0.01	0.9605	0.9657	0.00	0.0150	0.0872	-0.17	0.5659	0.8005	0.01	0.3736	0.4550	-2.55	0.8708	0.9502	-0.33	0.0651	0.1215	2.06
4661	Translation machinery-associated protein 16	Q96EY4	0.2163	0.3913	-0.05	0.2437	0.6589	0.16	0.0111	0.0488	0.22	0.1755	0.2992	0.08	0.0202	0.1019	0.17	0.2638	0.4561	0.04	0.3736	0.4881	-2.55	0.8708	1.0116	-0.33	0.0651	0.1355	2.06
4662	Translation machinery-associated protein 7	Q9Y2S6	0.1253	0.2637	0.07	0.1049	0.6318	0.30	0.0249	0.0784	0.26	0.0009	0.0148	0.36	0.0026	0.0395	0.24	0.0150	0.0815	0.18	0.1409	0.3217	0.36	0.0018	0.0347	0.27	0.0003	0.0273	0.26
4663	Translational activator of cytochrome c oxidase 1	Q9BSH4	0.5929	0.6592	-0.02	0.0603	0.5927	0.18	0.0153	0.0583	0.09	0.5073	0.7116	0.05	0.9832	0.9878	0.00	0.2478	0.4366	-0.04	0.3736	0.4911	-2.55	0.8708	1.0171	-0.33	0.0651	0.1369	2.06
4664	Translationally-controlled tumor protein	P13693	0.3184	0.5211	0.03	0.9731	0.9820	-0.01	0.0046	0.0332	0.17	0.4258	0.6177	-0.02	0.1063	0.2631	-0.06	0.4036	0.6191	-0.03	0.5181	0.5715	0.07	0.8659	1.3081	-0.01	0.8233	0.8574	0.01
4665	Translin	Q15631	0.0815	0.1966	0.06	0.1699	0.6617	0.25	0.0014	0.0228	0.26	0.0012	0.0163	0.31	0.0437	0.1545	0.08	0.0073	0.0650	0.22	0.1232	0.2956	0.27	0.8573	1.2989	-0.02	0.8857	0.9085	0.01
4666	Translin-associated protein X	Q99598	0.2026	0.3737	-0.21	0.4911	0.8146	-0.26	0.9312	0.9465	0.01	0.3213	0.4924	-0.17	0.7832	0.8300	0.04	0.4670	0.6890	-0.12	0.2909	0.5436	0.19	0.0108	0.0850	0.23	0.0172	0.1116	0.25
4667	Translocation protein SEC62	Q99442	0.0479	0.1407	0.21	0.0878	0.6213	0.15	0.0065	0.0390	0.43	0.0002	0.0096	0.58	0.0021	0.0363	0.27	0.0064	0.0630	0.41	0.0299	0.1471	0.82	0.0150	0.1035	0.39	0.0071	0.0778	0.61
4668	Translocation protein SEC63 homolog	Q9UGP8	0.0249	0.0952	0.15	0.2586	0.6631	0.19	0.0059	0.0372	0.32	0.0012	0.0161	0.38	0.0215	0.1058	0.19	0.0101	0.0707	0.27	0.4210	0.4767	0.12	0.2381	0.5546	-0.13	0.4761	0.5448	-0.05
4669	Translocon-associated protein subunit alpha	P43307	0.6179	0.6819	0.03	0.8031	0.8577	0.03	0.0149	0.0574	0.20	0.0925	0.1828	0.09	0.4769	0.5674	0.02	0.3668	0.5737	-0.03	0.0018	0.0521	0.38	0.0827	0.2829	0.12	0.0184	0.1164	0.24
4670	Translocon-associated protein subunit delta	P51571	0.0102	0.0586	0.19	0.0930	0.6305	-0.07	0.2621	0.4317	0.05	0.0473	0.1107	0.09	0.0072	0.0617	0.18	0.0107	0.0707	0.22	0.0483	0.1759	0.10	0.2585	0.5850	0.05	0.0223	0.1267	-0.20
4671	Translocon-associated protein subunit gamma	Q9UNL2	0.0016	0.0269	0.23	0.0703	0.6009	0.11	0.0002	0.0211	0.47	0.0044	0.0270	0.42	0.0005	0.0184	0.32	0.0490	0.1485	0.41	0.3736	0.5619	-2.55	0.8708	1.1454	-0.33	0.0651	0.1714	2.06
4672	Transmembrane 9 superfamily member 2	Q99805	0.0847	0.2009	0.16	0.4080	0.7497	0.14	0.1830	0.3278	0.19	0.0567	0.1262	0.22	0.1754	0.3653	0.11	0.0578	0.1656	0.28	0.3736	0.5566	-2.55	0.8708	1.1359	-0.33	0.0651	0.1686	2.06
4673	Transmembrane 9 superfamily member 3	Q9HD45	0.8358	0.8688	-0.01	0.5817	0.6892	0.07	0.0143	0.0562	0.17	0.0266	0.0736	0.12	0.1449	0.3195	-0.07	0.2692	0.4624	0.04	0.3736	0.4870	-2.55	0.8708	1.0096	-0.33	0.0651	0.1350	2.06
4674	Transmembrane 9 superfamily member 4	Q92544	0.4822	0.5569	-0.06	0.5894	0.6947	-0.05	0.1219	0.2395	0.18	0.1595	0.2780	0.14	0.6397	0.7091	-0.04	0.0787	0.2006	0.19	0.0021	0.0555	-0.76	0.0669	0.2493	-0.18	0.0025	0.0544	-0.49
4675	Transmembrane emp24 domain-containing protein 1	Q13445	0.3339	0.5407	0.03	0.6302	0.7267	0.03	0.3899	0.5914	-0.02	0.0653	0.1408	0.17	0.0161	0.0907	0.07	0.0249	0.1005	0.25	0.3736	0.5864	-2.55	0.8708	1.1889	-0.33	0.0651	0.1850	2.06
4676	Transmembrane emp24 domain-containing protein 10	P49755	0.0276	0.1005	0.11	0.8144	0.8671	0.03	0.0011	0.0213	0.20	0.0136	0.0473	0.12	0.0436	0.1543	0.08	0.0039	0.0599	0.40	0.1057	0.2658	0.32	0.0665	0.2487	0.44	0.1262	0.1858	0.30
4677	Transmembrane emp24 domain-containing protein 2	Q15363	0.0291	0.1042	4.40	0.0894	0.6250	-7.84	0.0400	0.1066	-7.28	0.2895	0.4513	2.09	0.1305	0.2986	-1.12	0.0164	0.0849	3.75	0.3736	0.6003	-2.55	0.8708	1.2132	-0.33	0.0651	0.1930	2.06
4678	Transmembrane emp24 domain-containing protein 3	Q9Y3Q3	0.2422	0.4278	-0.19	0.4013	0.7448	-0.44	0.0025	0.0273	-0.79	0.0481	0.1120	-0.70	0.0478	0.1607	-0.31	0.2370	0.4227	-0.43	0.3736	0.4937	-2.55	0.8708	1.0219	-0.33	0.0651	0.1380	2.06
4679	Transmembrane emp24 domain-containing protein 4	Q7Z7H5	0.0240	0.0930	0.50	0.1000	0.6348	0.46	0.0511	0.1273	0.43	0.0164	0.0533	0.48	0.1173	0.2805	0.24	0.0207	0.0931	0.55	0.3736	0.5931	-2.55	0.8708	1.2006	-0.33	0.0651	0.1888	2.06
4680	Transmembrane emp24 domain-containing protein 5	Q9Y3A6	0.4108	0.5012	-3.31	0.5752	0.7017	-1.75	0.5661	0.6387	-1.19	0.5388	0.6044	0.89	0.2938	0.4028	-1.88	0.9510	0.9801	0.09	0.0477	0.1746	0.29	0.0124	0.0930	0.43	0.5796	0.6423	0.14
4681	Transmembrane emp24 domain-containing protein 7	Q9Y3B3	0.0145	0.0698	0.14	0.3474	0.7038	0.15	0.0100	0.0467	0.19	0.0062	0.0315	0.19	0.0875	0.2316	0.07	0.0170	0.0856	0.18	0.0017	0.0534	0.89	0.0055	0.0602	0.87	0.0697	0.1175	0.72
4682	Transmembrane emp24 domain-containing protein 9	Q9BVK6	0.2187	0.3947	0.08	0.2464	0.6596	0.23	0.0128	0.0530	0.31	0.0016	0.0176	0.41	0.0172	0.0943	0.22	0.0132	0.0769	0.33	0.0005	0.0398	-0.48	0.0008	0.0262	-0.58	0.0006	0.0324	-0.73
4683	Transmembrane prolyl 4-hydroxylase	Q9NXG6	0.3517	0.5614	-0.34	0.2787	0.6644	-1.44	0.4340	0.6442	-0.28	0.1445	0.2572	-0.66	0.8100	0.8523	0.09	0.5620	0.7965	-0.20	0.3736	0.4555	-2.55	0.8708	0.9510	-0.33	0.0651	0.1217	2.06
4684	Transmembrane protease serine 11E	Q9UL52	0.0024	0.0328	0.33	0.0660	0.5946	0.48	0.0023	0.0268	0.73	0.0000	0.0000	0.86	0.0001	0.0144	0.79	0.1248	0.2727	0.70	0.3736	0.5260	-2.55	0.8708	1.0809	-0.33	0.0651	0.1531	2.06
4685	Transmembrane protease serine 12	Q86WS5	0.3448	0.5531	0.18	0.6689	0.7564	0.06	0.2243	0.3821	-0.19	0.7572	0.7906	0.04	0.6199	0.6931	-0.07	0.7713	1.0087	0.08	0.3736	0.4405	-2.55	0.8708	0.9230	-0.33	0.0651	0.1157	2.06
4686	Transmembrane protein 109	Q9BVC6	0.9649	0.9741	0.00	0.7656	0.8302	-0.05	0.7815	0.8206	0.03	0.2164	0.3543	-0.15	0.0728	0.2062	-0.29	0.6952	0.9361	-0.04	0.0719	0.2123	0.31	0.8460	1.2885	-0.02	0.4394	0.5100	-0.07
4687	Transmembrane protein 131-like	A2VDJ0	0.0145	0.0697	0.26	0.0359	0.5390	0.21	0.0367	0.1006	0.17	0.4015	0.5913	0.13	0.1987	0.3988	0.09	0.7269	0.9667	0.04	0.1450	0.3283	0.21	0.0004	0.0197	-0.44	0.0077	0.0793	-0.37

Supplemetary Table S2. Overview on all relatively quantified 5180 proteins statistical analysis

			MCF-7																		MDA-MB-231								
			Dai SC20 vs control			Gen SC20 vs control			SSE SC20 vs control			Dai IC20 vs control			Gen IC20 vs control			SSE IC20 vs control			Dai IC20 vs control			Gen IC20 vs control			SSE IC20 vs control		
	**Protein name**	**UniProt**	**p value**	**BH q value**	**log2FC**	**p value**	**BH q value**	**log2FC**	**p value**	**BH q value**	**log2FC**	**p value**	**BH q value**	**log2FC**	**p value**	**BH q value**	**log2FC**	**p value**	**BH q value**	**log2FC**	**p value**	**BH q value**	**log2FC**	**p value**	**BH q value**	**log2FC**	**p value**	**BH q value**	**log2FC**
4688	Transmembrane protein 135	Q86UB9	0.0089	0.0558	0.19	0.5223	0.8413	0.10	0.0040	0.0319	0.20	0.0022	0.0203	0.87	0.0008	0.0219	0.26	0.0000	0.0000	1.11	0.0208	0.1327	0.53	0.5243	0.9483	0.05	0.0071	0.0768	0.24
4689	Transmembrane protein 151A	Q8N4L1	0.4108	0.5010	-3.31	0.5752	0.7016	-1.75	0.5661	0.6386	-1.19	0.5388	0.6042	0.89	0.2938	0.4027	-1.88	0.9510	0.9799	0.09	0.9953	0.9963	0.00	0.7025	1.1447	-0.03	0.3905	0.4616	-0.11
4690	Transmembrane protein 165	Q9HC07	0.0152	0.0713	-0.24	0.3987	0.7426	-0.09	0.0121	0.0514	-0.25	0.3958	0.5843	-0.06	0.4229	0.5173	0.04	0.8676	1.1037	-0.01	0.0161	0.1183	-0.55	0.0029	0.0451	0.05	0.1129	0.1702	0.10
4691	Transmembrane protein 186	Q96B77	0.0059	0.0464	0.16	0.8240	0.8739	0.02	0.4110	0.6171	0.05	0.7683	0.8004	-0.01	0.2765	0.5015	-0.05	0.0324	0.1159	0.12	0.3736	0.5787	-2.55	0.8708	1.1753	-0.33	0.0651	0.1806	2.06
4692	Transmembrane protein 192	Q8IY95	0.3626	0.5753	-0.11	0.8392	0.8846	-0.01	0.7890	0.8270	-0.02	0.2515	0.4009	-0.13	0.4368	0.5308	0.07	0.3034	0.5013	0.07	0.3736	0.4813	-2.55	0.8708	0.9991	-0.33	0.0651	0.1326	2.06
4693	Transmembrane protein 205	Q6UW68	0.2444	0.4299	-0.10	0.4925	0.8151	0.12	0.8750	0.9009	-0.01	0.0568	0.1263	0.21	0.4529	0.5458	0.06	0.0500	0.1501	0.22	0.3736	0.5609	-2.55	0.8708	1.1437	-0.33	0.0651	0.1709	2.06
4694	Transmembrane protein 33	P57088	0.3216	0.5255	0.03	0.9304	0.9528	0.01	0.0095	0.0454	0.13	0.0088	0.0381	0.14	0.0995	0.2525	-0.07	0.0055	0.0618	0.20	0.0023	0.0570	-2.20	0.8254	1.2702	-0.08	0.0130	0.0986	-0.90
4695	Transmembrane protein 40	Q8WWA1	0.4108	0.5009	-3.31	0.5752	0.7014	-1.75	0.5661	0.6384	-1.19	0.5388	0.6041	0.89	0.2938	0.4026	-1.88	0.9510	0.9797	0.09	0.0999	0.2554	0.28	0.2030	0.5022	0.19	0.2934	0.3650	0.15
4696	Transmembrane protein 43	Q9BTV4	0.0498	0.1438	0.21	0.4791	0.8047	0.04	0.0294	0.0871	0.12	0.0115	0.0430	0.16	0.0935	0.2417	0.07	0.1620	0.3236	0.25	0.3022	0.5605	0.17	0.8234	1.2690	0.03	0.0762	0.1262	-0.39
4697	Transmembrane protein 87A	Q8NBN3	0.4176	0.4951	-0.12	0.0737	0.6128	0.34	0.1214	0.2389	0.23	0.0197	0.0597	0.44	0.4945	0.5838	0.08	0.1978	0.3733	0.31	0.3736	0.5027	-2.55	0.8708	1.0384	-0.33	0.0651	0.1421	2.06
4698	Transmembrane protein KIAA1109	Q2LD37	0.4108	0.5008	-3.31	0.5752	0.7012	-1.75	0.5661	0.6383	-1.19	0.5388	0.6040	0.89	0.2938	0.4025	-1.88	0.9510	0.9796	0.09	0.0068	0.0853	0.50	0.0233	0.1349	0.22	0.7644	0.8061	-0.03
4699	Transport and Golgi organization protein 1 homolog	Q5JRA6	0.7720	0.8150	-0.01	0.9701	0.9797	0.00	0.0532	0.1305	0.10	0.5093	0.7142	-0.05	0.0981	0.2498	0.07	0.0177	0.0871	0.22	0.3736	0.5969	-2.55	0.8708	1.2074	-0.33	0.0651	0.1911	2.06
4700	Transportin-1	Q92973	0.2494	0.4356	0.03	0.0615	0.5932	0.09	0.0110	0.0487	0.18	0.0119	0.0437	0.09	0.0654	0.1947	-0.12	0.8493	1.0844	0.01	0.0548	0.1859	0.24	0.8159	1.2605	0.01	0.5669	0.6306	0.02
4701	Transportin-2	O14787	0.0076	0.0513	-1.74	0.1287	0.6429	-1.27	0.0037	0.0319	-2.20	0.0190	0.0583	-1.83	0.9559	0.9658	0.02	0.0178	0.0869	-1.72	0.7194	0.7594	-0.03	0.0459	0.1990	-0.28	0.9010	0.9211	0.01
4702	Transportin-3	Q9Y5L0	0.4108	0.5007	-3.31	0.5752	0.7011	-1.75	0.5661	0.6382	-1.19	0.5388	0.6038	0.89	0.2938	0.4024	-1.88	0.9510	0.9794	0.09	0.4262	0.4820	-0.14	0.7457	1.1885	-0.04	0.4414	0.5117	0.19
4703	Treacle protein	Q13428	0.0000	0.0000	-1.71	0.3019	0.6752	-0.17	0.1632	0.2990	0.20	0.5894	0.6381	0.04	0.6137	0.6876	-0.05	0.0169	0.0853	-0.95	0.3736	0.5988	-2.55	0.8708	1.2106	-0.33	0.0651	0.1921	2.06
4704	Trefoil factor 2	Q03403	0.0060	0.0465	0.35	0.0190	0.4642	0.42	0.0183	0.0649	0.24	0.0020	0.0195	0.46	0.0136	0.0836	0.28	0.0102	0.0705	0.40	0.0071	0.0872	0.63	0.0126	0.0935	0.24	0.3083	0.3795	-0.07
4705	Treslin	Q7Z2Z1	0.0002	0.0119	0.59	0.1202	0.6366	0.37	0.0005	0.0201	0.45	0.0006	0.0138	0.42	0.0017	0.0329	0.32	0.0327	0.1163	0.42	0.0026	0.0573	-0.57	0.0292	0.1542	-0.22	0.3154	0.3867	-0.34
4706	Tricarboxylate transport protein mitochondrial	P53007	0.0048	0.0428	0.36	0.3434	0.7006	-0.17	0.0032	0.0302	0.37	0.0074	0.0350	0.51	0.0041	0.0473	0.27	0.0110	0.0720	0.75	0.3373	0.6084	0.07	0.7313	1.1757	0.02	0.0021	0.0511	0.47
4707	Trichohyalin	Q07283	0.0001	0.0091	-0.88	0.0545	0.5881	-0.84	0.0066	0.0393	-0.53	0.0004	0.0116	-1.26	0.0272	0.1197	-0.53	0.0603	0.1701	0.43	0.3122	0.5729	0.10	0.1327	0.3834	-0.11	0.7241	0.7723	-0.02
4708	Trifunctional enzyme subunit alpha mitochondrial	P40939	0.0060	0.0465	0.17	0.3364	0.6954	0.06	0.0048	0.0336	0.18	0.0020	0.0197	0.27	0.0100	0.0721	0.15	0.0028	0.0560	0.33	0.0160	0.1181	0.12	0.0285	0.1527	0.06	0.1238	0.1831	0.05
4709	Trifunctional enzyme subunit beta mitochondrial	P55084	0.1316	0.2734	0.08	0.3068	0.6783	0.14	0.0476	0.1210	0.14	0.0255	0.0716	0.19	0.2749	0.4993	0.05	0.0132	0.0764	0.22	0.0144	0.1117	0.40	0.0920	0.3016	0.17	0.3069	0.3780	0.08
4710	Trifunctional purine biosynthetic protein adenosine-3	P22102	0.0209	0.0854	0.22	0.2797	0.6649	0.20	0.0059	0.0371	0.28	0.0023	0.0206	0.36	0.1255	0.2926	0.09	0.0091	0.0688	0.34	0.1576	0.3495	0.24	0.7376	1.1811	0.03	0.7374	0.7834	0.02
4711	Trinucleotide repeat-containing gene 18 protein	O15417	0.0162	0.0741	0.18	0.2040	0.6596	0.29	0.0145	0.0566	0.30	0.0013	0.0165	0.39	0.0341	0.1353	0.15	0.0070	0.0645	0.30	0.1007	0.2568	0.23	0.8289	1.2726	-0.01	0.5588	0.6232	0.02
4712	Trinucleotide repeat-containing gene 6A protein	Q8NDV7	0.4108	0.5006	-3.31	0.5752	0.7009	-1.75	0.5661	0.6380	-1.19	0.5388	0.6037	0.89	0.2938	0.4023	-1.88	0.9510	0.9792	0.09	0.0786	0.2226	0.50	0.0988	0.3146	0.42	0.1034	0.1595	0.44
4713	TRIO and F-actin-binding protein	Q9H2D6	0.4108	0.5005	-3.31	0.5752	0.7007	-1.75	0.5661	0.6379	-1.19	0.5388	0.6036	0.89	0.2938	0.4022	-1.88	0.9510	0.9790	0.09	0.0449	0.1700	0.40	0.8301	1.2733	0.03	0.2242	0.2942	0.18
4714	Triokinase/FMN cyclase	Q3LXA3	0.6590	0.7194	0.01	0.3687	0.7169	0.08	0.2768	0.4509	0.02	0.0141	0.0483	-0.07	0.0442	0.1553	-0.07	0.6094	0.8470	-0.01	0.0898	0.2408	-0.12	0.0112	0.0870	-0.25	0.1786	0.2451	-0.09
4715	Triosephosphate isomerase	P60174	0.0001	0.0089	0.52	0.1106	0.6323	0.35	0.0011	0.0218	0.41	0.0011	0.0162	0.35	0.0663	0.1960	0.10	0.0015	0.0495	0.37	0.8201	0.8476	-0.04	0.0085	0.0757	-0.46	0.0135	0.1008	-0.54
4716	Tripartite motif-containing protein 10	Q9UDY6	0.7049	0.7572	0.07	0.6855	0.7706	-0.06	0.0458	0.1179	-0.34	0.4301	0.6225	0.27	0.6676	0.7319	-0.06	0.4025	0.6176	0.52	0.3736	0.4690	-2.55	0.8708	0.9764	-0.33	0.0651	0.1273	2.06
4717	Tripartite motif-containing protein 16-like protein	Q309B1	0.1032	0.2297	-0.56	0.2741	0.6647	-0.53	0.6644	0.7157	-0.11	0.1945	0.3247	-0.46	0.4772	0.5673	-0.19	0.6221	0.8577	-0.16	0.3736	0.4502	-2.55	0.8708	0.9411	-0.33	0.0651	0.1195	2.06
4718	Tripartite motif-containing protein 29	Q14134	0.4108	0.5003	-3.31	0.5752	0.7006	-1.75	0.5661	0.6378	-1.19	0.5388	0.6035	0.89	0.2938	0.4021	-1.88	0.9510	0.9788	0.09	0.0465	0.1730	0.37	0.0481	0.2051	-0.29	0.1100	0.1670	-0.19
4719	Tripartite motif-containing protein 34	Q9BYJ4	0.4108	0.5002	-3.31	0.5752	0.7004	-1.75	0.5661	0.6376	-1.19	0.5388	0.6033	0.89	0.2938	0.4020	-1.88	0.9510	0.9786	0.09	0.0599	0.1936	-0.30	0.3764	0.7519	-0.14	0.0191	0.1196	-0.53
4720	Tripartite motif-containing protein 42	Q8IWZ5	0.2552	0.4432	0.05	0.2677	0.6635	-0.13	0.0733	0.1618	0.12	0.7453	0.7806	-0.03	0.0434	0.1539	-0.14	0.4137	0.6310	0.09	0.3736	0.4679	-2.55	0.8708	0.9742	-0.33	0.0651	0.1269	2.06
4721	Tripartite motif-containing protein 45	Q9H8W5	0.4108	0.5001	-3.31	0.5752	0.7002	-1.75	0.5661	0.6375	-1.19	0.5388	0.6032	0.89	0.2938	0.4019	-1.88	0.9510	0.9784	0.09	0.0761	0.2192	0.33	0.0509	0.2130	0.19	0.0215	0.1253	0.41
4722	Tripartite motif-containing protein 51	Q9BSJ1	0.3952	0.6133	0.13	0.2735	0.6645	0.15	0.0147	0.0572	0.32	0.0702	0.1482	0.12	0.0325	0.1315	-0.17	0.0555	0.1607	0.19	0.5356	0.5873	1.08	0.2295	0.5426	2.47	0.2938	0.3652	2.05
4723	Tripartite motif-containing protein 52	Q96A61	0.1302	0.2717	0.11	0.0796	0.6127	0.16	0.0620	0.1447	0.17	0.0657	0.1413	0.15	0.2885	0.5187	0.07	0.9011	1.1382	0.01	0.3736	0.4338	-2.55	0.8708	0.9103	-0.33	0.0651	0.1130	2.06
4724	Tripartite motif-containing protein 55	Q9BYV6	0.1740	0.3354	0.07	0.0879	0.6212	-0.25	0.0670	0.1522	0.20	0.3111	0.4795	0.07	0.6182	0.6916	0.04	0.0476	0.1452	0.41	0.3736	0.5624	-2.55	0.8708	1.1463	-0.33	0.0651	0.1717	2.06
4725	Tripartite motif-containing protein 77	I1YAP6	0.4108	0.5000	-3.31	0.5752	0.7001	-1.75	0.5661	0.6373	-1.19	0.5388	0.6031	0.89	0.2938	0.4018	-1.88	0.9510	0.9782	0.09	0.0224	0.1359	0.26	0.0263	0.1459	-0.24	0.0781	0.1286	0.15
4726	tRNA (cytosine(34)-C(5))-methyltransferase	Q08J23	0.0030	0.0359	0.22	0.1224	0.6398	0.09	0.0017	0.0238	0.29	0.2129	0.3495	0.07	0.8774	0.9064	0.01	0.2243	0.4072	0.10	0.6155	0.6626	0.03	0.1676	0.4468	-0.08	0.0296	0.1466	-0.18
4727	tRNA (guanine(10)-N2)-methyltransferase homolog	Q7Z4G4	0.0444	0.1340	1.18	0.6970	0.7768	0.21	0.9662	0.9724	-0.02	0.0165	0.0535	-0.70	0.0371	0.1402	-0.55	0.7393	0.9789	0.51	0.3736	0.4426	-2.55	0.8708	0.9270	-0.33	0.0651	0.1165	2.06
4728	tRNA methyltransferase 10 homolog C	Q7L0Y3	0.3251	0.5307	0.06	0.1578	0.6619	-0.13	0.0585	0.1391	0.16	0.9016	0.9156	0.01	0.0604	0.1849	0.15	0.0525	0.1550	0.15	0.7393	0.7771	0.03	0.3862	0.7656	0.07	0.5311	0.5969	-0.04
4729	tRNA pseudouridine synthase A	Q9Y606	0.0045	0.0424	0.22	0.2563	0.6625	0.15	0.0123	0.0520	0.23	0.0006	0.0126	0.34	0.0085	0.0665	0.17	0.0104	0.0703	0.29	0.0002	0.0345	-0.88	0.0001	0.0173	-0.82	0.0001	0.0288	-1.07
4730	tRNA wybutosine-synthesizing protein 5	A2RUC4	0.4108	0.4999	-3.31	0.5752	0.6999	-1.75	0.5661	0.6372	-1.19	0.5388	0.6029	0.89	0.2938	0.4017	-1.88	0.9510	0.9780	0.09	0.1606	0.3536	0.75	0.2749	0.6106	0.52	0.8964	0.9178	-0.05
4731	tRNA-dihydrouridine(20) synthase [NAD(P)+]-like	Q9NX74	0.0599	0.1619	-0.19	0.8026	0.8574	0.02	0.0131	0.0534	-0.14	0.0115	0.0431	-0.16	0.0344	0.1356	-0.09	0.0083	0.0670	-0.34	0.3736	0.6177	-2.55	0.8708	1.2437	-0.33	0.0651	0.2036	2.06
4732	tRNA-dihydrouridine(20a/20b) synthase [NAD(P)+]-like	O95620	0.6275	0.6901	0.29	0.6759	0.7628	-0.85	0.8830	0.9072	0.09	0.7519	0.7860	0.21	0.4024	0.4985	0.55	0.8578	1.0936	-0.12	0.3736	0.4358	-2.55	0.8708	0.9140	-0.33	0.0651	0.1138	2.06
4733	tRNA-specific adenosine deaminase 1	Q9BUB4	0.2997	0.4987	-0.06	0.3085	0.6783	0.09	0.9546	0.9641	0.00	0.5428	0.5939	-0.05	0.3043	0.4020	-0.05	0.1876	0.3603	-0.15	0.3736	0.5053	-2.55	0.8708	1.0432	-0.33	0.0651	0.1433	2.06
4734	tRNA-splicing endonuclease subunit Sen15	Q8WW01	0.0247	0.0948	-0.19	0.0184	0.4649	0.19	0.0018	0.0245	0.21	0.0725	0.1519	0.09	0.0185	0.0969	0.07	0.4844	0.7090	-0.03	0.2732	0.5176	0.88	0.2927	0.6331	0.83	0.7839	0.8228	0.20
4735	tRNA-splicing ligase RtcB homolog	Q9Y3I0	0.1348	0.2784	0.37	0.2143	0.6619	-0.11	0.1305	0.2515	-0.14	0.0234	0.0676	-0.16	0.0716	0.2040	-0.11	0.6828	0.9223	0.03	0.2529	0.4897	0.07	0.5502	0.9760	-0.03	0.5722	0.6351	-0.05
4736	Trophoblast glycoprotein	Q13641	0.0217	0.0875	0.16	0.7930	0.8508	0.05	0.2350	0.3959	0.10	0.0033	0.0232	0.30	0.7091	0.7699	0.01	0.0166	0.0847	0.20	0.3736	0.5993	-2.55	0.8708	1.2116	-0.33	0.0651	0.1925	2.06
4737	Tropomodulin-2	Q9NZR1	0.0632	0.1664	0.30	0.2634	0.6633	0.59	0.0192	0.0665	0.44	0.0592	0.1304	0.30	0.5050	0.5943	0.07	0.0318	0.1149	0.37	0.0431	0.1681	0.50	0.0216	0.1273	0.32	0.0329	0.1549	0.30
4738	Tropomodulin-3	Q9NYL9	0.0063	0.0475	0.09	0.3158	0.6810	0.07	0.9253	0.9418	0.00	0.0870	0.1747	0.04	0.6326	0.7023	0.01	0.1142	0.2568	0.06	0.0490	0.1766	0.16	0.5122	0.9316	0.04	0.0393	0.1709	0.18
4739	Tropomodulin-4	Q9NZQ9	0.4026	0.6227	-0.29	0.2964	0.6713	-0.54	0.1734	0.3151	-0.31	0.0892	0.1780	-0.44	0.5494	0.6331	-0.12	0.2129	0.3940	-0.38	0.1548	0.3449	0.29	0.0447	0.1964	-0.29	0.4673	0.5358	-0.07
4740	Tropomyosin alpha-1 chain	P09493	0.6771	0.7342	0.28	0.3589	0.7088	0.85	0.3892	0.5905	0.62	0.3679	0.5492	-0.65	0.6576	0.7234	-0.30	0.6409	0.8776	-0.33	0.0605	0.1945	0.37	0.0459	0.1995	0.28	0.1272	0.1868	0.16
4741	Tropomyosin alpha-3 chain	P06753	0.1962	0.3651	0.08	0.3241	0.6855	0.20	0.0174	0.0629	0.27	0.0030	0.0224	0.37	0.6700	0.7341	0.02	0.0131	0.0765	0.31	0.0140	0.1111	0.81	0.0106	0.0846	0.47	0.0048	0.0674	0.61
4742	Tropomyosin alpha-4 chain	P67936	0.5053	0.5784	0.02	0.4140	0.7527	0.12	0.0277	0.0838	0.13	0.0157	0.0517	0.16	0.5218	0.6089	-0.02	0.0182	0.0871	0.17	0.0964	0.2497	0.21	0.5617	0.9887	0.05	0.0570	0.2123	0.16
4743	Tropomyosin beta chain	P07951	0.0095	0.0572	-0.26	0.2076	0.6589	0.07	0.3564	0.5526	-0.06	0.6611	0.7039	0.01	0.1787	0.3697	0.05	0.2631	0.4557	0.07	0.0254	0.1400	0.55	0.0114	0.0879	0.34	0.0154	0.1066	0.40
4744	Troponin C skeletal muscle	P02585	0.0013	0.0243	0.30	0.0234	0.4829	0.18	0.0038	0.0323	0.26	0.0027	0.0220	0.25	0.8988	0.9229	-0.01	0.0437	0.1377	0.22	0.6218	0.6682	-0.02	0.0719	0.2603	-0.10	0.0875	0.1403	0.45
4745	TRPM8 channel-associated factor 1	Q9Y4C2	0.8816	0.9066	0.00	0.2836	0.6641	-0.11	0.4395	0.6510	-0.02	0.0127	0.0456	-0.14	0.0039	0.0463	-0.20	0.7589	0.9967	0.00	0.3736	0.4410	-2.55	0.8708	0.9240	-0.33	0.0651	0.1159	2.06
4746	Tryptophan 5-hydroxylase 1	P17752	0.0815	0.1965	-0.08	0.0411	0.5692	-0.13	0.2517	0.4187	-0.04	0.0066	0.0326	-0.25	0.0119	0.0784	-0.24	0.0466	0.1435	-0.15	0.3736	0.5639	-2.55	0.8708	1.1489	-0.33	0.0651	0.1725	2.06
4747	Tryptophan 5-hydroxylase 2	Q8IWU9	0.4198	0.4974	-0.02	0.4817	0.8067	0.04	0.1258	0.2453	0.08	0.0316	0.0833	-0.08	0.0038	0.0458	-0.13	0.0807	0.2042	0.06	0.3736	0.5433	-2.55	0.8708	1.1121	-0.33	0.0651	0.1617	2.06
4748	Tryptophan--tRNA ligase cytoplasmic	P23381	0.0004	0.0159	0.33	0.1656	0.6619	0.23	0.0007	0.0206	0.33	0.0006	0.0136	0.36	0.0164	0.0914	0.11	0.0203	0.0922	0.43	0.8916	0.9104	0.01	0.0693	0.2541	-0.11	0.0051	0.0688	-0.29
4749	Tryptophan--tRNA ligase mitochondrial	Q9UGM6	0.4108	0.4998	-3.31	0.5752	0.6998	-1.75	0.5661	0.6371	-1.19	0.5388	0.6028	0.89	0.2938	0.4016	-1.88	0.9510	0.9778	0.09	0.1243	0.2966	0.23	0.1082	0.3350	0.11	0.2500	0.3209	-0.07
4750	Tubby-related protein 1	O00294	0.0001	0.0088	0.80	0.1051	0.6316	0.53	0.0007	0.0210	0.76	0.0001	0.0104	0.60	0.0027	0.0403	0.48	0.0115	0.0734	0.88	0.0404	0.1653	0.52	0.0831	0.2839	-0.19	0.0249	0.1341	-0.31
4751	Tuberin	P49815	0.0384	0.1230	0.31	0.1412	0.6531	0.29	0.0052	0.0353	0.59	0.0053	0.0293	0.32	0.0005	0.0182	0.22	0.0001	0.0432	0.46	0.3736	0.6553	-2.55	0.8708	1.3086	-0.33	0.0651	0.2285	2.06
4752	Tubulin alpha chain-like 3	A6NHL2	0.0961	0.2188	0.18	0.0613	0.5935	-0.23	0.0009	0.0218	-0.19	0.0006	0.0135	-0.70	0.0059	0.0567	-0.16	0.0531	0.1558	0.46	0.0018	0.0536	0.94	0.0000	0.0000	0.99	0.0768	0.1271	-0.10
4753	Tubulin alpha-1A chain	Q71U36	0.4108	0.4996	-3.31	0.5752	0.6996	-1.75	0.5661	0.6369	-1.19	0.5388	0.6027	0.89	0.2938	0.4014	-1.88	0.9510	0.9776	0.09	0.0500	0.1781	0.37	0.2192	0.5262	0.14	0.5140	0.5807	0.06
4754	Tubulin alpha-1C chain	Q9BQE3	0.0008	0.0202	0.34	0.1754	0.6613	0.26	0.0006	0.0194	0.46	0.0009	0.0156	0.32	0.9168	0.9376	0.00	0.0021	0.0528	0.39	0.1420	0.3238	-0.42	0.3996	0.7850	-0.09	0.2925	0.3642	0.06
4755	Tubulin alpha-3C chain	P0DPH7	0.1217	0.2582	-3.15	0.8655	0.9030	-0.21	0.0307	0.0893	0.80	0.7049	0.7438	0.08	0.0146	0.0860	1.19	0.0867	0.2149	-0.98	0.3736	0.5407	-2.55	0.8708	1.1075	-0.33	0.0651	0.1604	2.06
4756	Tubulin alpha-4A chain	P68366	0.0263	0.0982	0.37	0.7546	0.8220	-0.05	0.0325	0.0926	0.34	0.9512	0.9577	0.01	0.3101	0.4077	-0.11	0.1102	0.2504	0.23	0.0048	0.0733	-0.31	0.0010	0.0276	-0.38	0.0007	0.0339	-0.66
4757	Tubulin alpha-8 chain	Q9NY65	0.0554	0.1540	0.23	0.3084	0.6783	0.14	0.8451	0.8757	0.02	0.3377	0.5115	0.11	0.2936	0.5257	-0.09	0.1691	0.3339	0.14	0.0006	0.0404	7.55	0.0217	0.1277	6.88	0.0040	0.0619	8.65
4758	Tubulin beta chain	P07437	0.0137	0.0684	0.22	0.1345	0.6493	0.10	0.0041	0.0323	0.37	0.0113	0.0428	0.24	0.3954	0.4922	-0.06	0.2425	0.4290	0.09	0.0461	0.1724	0.44	0.1847	0.4743	0.16	0.5834	0.6453	0.05
4759	Tubulin beta-1 chain	Q9H4B7	0.0645	0.1681	-0.10	0.3741	0.7215	-0.03	0.1567	0.2894	0.07	0.1048	0.2008	0.08	0.0201	0.1017	-0.14	0.0179	0.0871	0.21	0.0531	0.1823	-0.24	0.0103	0.0836	-0.41	0.0092	0.0857	-0.65
4760	Tubulin beta-2A chain	Q13885	0.1182	0.2529	-0.12	0.6920	0.7744	0.04	0.0150	0.0574	0.33	0.1131	0.2134	0.11	0.8222	0.8611	0.01	0.8529	1.0879	0.01	0.0606	0.1943	0.09	0.0107	0.0846	0.26	0.0322	0.1529	0.15
4761	Tubulin beta-3 chain	Q13509	0.0005	0.0174	0.35	0.0209	0.4748	0.36	0.0013	0.0237	0.55	0.0001	0.0094	0.37	0.0006	0.0209	0.22	0.0147	0.0808	0.39	0.0143	0.1121	0.40	0.3526	0.7163	0.09	0.1166	0.1744	0.17
4762	Tubulin beta-4A chain	P04350	0.2655	0.4571	-0.46	0.3784	0.7257	-0.36	0.1332	0.2553	-0.72	0.1740	0.2971	-0.62	0.3995	0.4959	-0.32	0.1395	0.2929	-0.70	0.0000	0.0000	0.72	0.0117	0.0898	0.17	0.0003	0.0247	0.63
4763	Tubulin beta-4B chain	P68371	0.0006	0.0188	0.29	0.2451	0.6582	0.21	0.0016	0.0232	0.38	0.0007	0.0138	0.31	0.4591	0.5511	-0.02	0.0010	0.0439	0.38	0.3736	0.6490	-2.55	0.8708	1.2977	-0.33	0.0651	0.2241	2.06
4764	Tubulin beta-6 chain	Q9BUF5	0.0125	0.0651	0.38	0.1741	0.6612	0.24	0.0006	0.0195	0.35	0.0010	0.0158	0.26	0.0021	0.0360	0.29	0.0022	0.0543	0.27	0.0174	0.1223	0.30	0.0163	0.1084	0.26	0.0494	0.1940	0.17
4765	Tubulin beta-8 chain	Q3ZCM7	0.1797	0.3430	0.06	0.2441	0.6575	0.18	0.0071	0.0398	0.26	0.0010	0.0154	0.26	0.0003	0.0159	0.21	0.0004	0.0460	0.29	0.2214	0.4440	-0.15	0.4524	0.8565	-0.11	0.1513	0.2144	-0.20
4766	Tubulin beta-8 chain-like protein LOC260334	A6NNZ2	0.0271	0.0998	-0.05	0.0980	0.6338	-0.16	0.6332	0.6865	0.03	0.1299	0.2364	-0.19	0.0369	0.1397	-0.10	0.5362	0.7681	-0.04	0.2322	0.4598	0.34	0.2309	0.5444	0.09	0.2314	0.3015	0.07
4767	Tubulin gamma-1 chain	P23258	0.0496	0.1433	-13.41	0.0397	0.5634	-11.85	0.0133	0.0539	-11.30	0.0005	0.0122	-9.22	0.0474	0.1602	-2.80	0.0207	0.0932	-6.03	0.9761	0.9803	-0.01	0.9363	0.9536	0.00	0.1178	0.1759	0.44
4768	Tubulin gamma-2 chain	Q9NRH3	0.0006	0.0187	-4.05	0.2372	0.6624	-2.53	0.1947	0.3435	-2.34	0.0080	0.0360	-4.10	0.7300	0.7886	0.08	0.0004	0.0334	-4.25	0.8067	0.8369	-0.06	0.2580	0.5849	-0.25	0.2436	0.3137	-0.26
4769	Tubulin-folding cofactor B	Q99426	0.0300	0.1063	0.10	0.4916	0.8144	0.13	0.0018	0.0237	0.35	0.0010	0.0155	0.36	0.0013	0.0283	0.26	0.0045	0.0607	0.31	0.0550	0.1863	0.48	0.0693	0.2546	0.20	0.0076	0.0787	0.17
4770	Tubulin-specific chaperone A	O75347	0.0420	0.1300	0.15	0.2153	0.6607	0.30	0.0111	0.0489	0.34	0.0019	0.0193	0.38	0.0778	0.2145	0.12	0.0102	0.0703	0.31	0.0998	0.2557	0.31	0.3134	0.6623	-0.11	0.7030	0.7543	-0.02
4771	Tubulin-specific chaperone C	Q15814	0.0785	0.1919	-0.16	0.0463	0.5630	0.19	0.7289	0.7724	-0.03	0.0535	0.1209	0.18	0.0442	0.1551	0.20	0.4450	0.6664	0.09	0.3736	0.4645	-2.55	0.8708	0.9680	-0.33	0.0651	0.1255	2.06

Supplemetary Table S2. Overview on all relatively quantified 5180 proteins statistical analysis

			MCF-7																		MDA-MB-231								
			Dai SC20 vs control			Gen SC20 vs control			SSE SC20 vs control			Dai IC20 vs control			Gen IC20 vs control			SSE IC20 vs control			Dai IC20 vs control			Gen IC20 vs control			SSE IC20 vs control		
	**Protein name**	**UniProt**	**p value**	**BH q value**	**log2FC**	**p value**	**BH q value**	**log2FC**	**p value**	**BH q value**	**log2FC**	**p value**	**BH q value**	**log2FC**	**p value**	**BH q value**	**log2FC**	**p value**	**BH q value**	**log2FC**	**p value**	**BH q value**	**log2FC**	**p value**	**BH q value**	**log2FC**	**p value**	**BH q value**	**log2FC**
4772	Tubulin-specific chaperone D	Q9BTW9	0.0933	0.2149	0.11	0.1970	0.6705	0.30	0.0023	0.0267	0.43	0.0035	0.0240	0.31	0.4624	0.5542	-0.03	0.0324	0.1158	0.19	0.1273	0.3007	-0.22	0.0011	0.0277	-0.51	0.0024	0.0545	-0.57
4773	Tubulin-specific chaperone E	Q15813	0.4108	0.4995	-3.31	0.5752	0.6994	-1.75	0.5661	0.6368	-1.19	0.5388	0.6025	0.89	0.2938	0.4013	-1.88	0.9510	0.9774	0.09	0.3933	0.4490	0.13	0.0305	0.1577	-0.28	0.0084	0.0821	-0.45
4774	Tubulin--tyrosine ligase-like protein 12	Q14166	0.0098	0.0577	0.12	0.0769	0.6128	0.09	0.0018	0.0243	0.26	0.0083	0.0371	0.19	0.0086	0.0663	0.08	0.0504	0.1507	0.21	0.0097	0.0983	0.48	0.0125	0.0930	0.38	0.0084	0.0823	0.43
4775	Tubulinyl-Tyr carboxypeptidase 1	Q7L8A9	0.0261	0.0978	0.26	0.4813	0.8063	-0.24	0.0021	0.0257	0.64	0.0029	0.0222	0.52	0.0144	0.0857	0.32	0.0066	0.0630	0.51	0.1378	0.3177	0.24	0.2422	0.5603	-0.14	0.1674	0.2326	-0.13
4776	Tudor and KH domain-containing protein	Q9Y2W6	0.0322	0.1115	-0.19	0.0006	0.2220	-0.55	0.0014	0.0235	-0.48	0.0001	0.0093	-0.88	0.0045	0.0502	-0.41	0.0548	0.1591	-0.30	0.3736	0.5579	-2.55	0.8708	1.1382	-0.33	0.0651	0.1693	2.06
4777	Tudor domain-containing protein 6	O60522	0.0074	0.0504	0.16	0.3216	0.6839	0.10	0.0004	0.0187	0.29	0.0010	0.0159	0.22	0.0732	0.2067	0.05	0.0016	0.0512	0.25	0.0540	0.1845	-0.27	0.0034	0.0477	-0.58	0.0118	0.0936	-0.58
4778	Tuftelin-interacting protein 11	Q9UBB9	0.4108	0.4994	-3.31	0.5752	0.6993	-1.75	0.5661	0.6366	-1.19	0.5388	0.6024	0.89	0.2938	0.4012	-1.88	0.9510	0.9772	0.09	0.0548	0.1858	-0.10	0.6687	1.1074	-0.03	0.1059	0.1622	-0.36
4779	Tumor necrosis factor alpha-induced protein 8	O95379	0.4108	0.4993	-3.31	0.5752	0.6991	-1.75	0.5661	0.6365	-1.19	0.5388	0.6023	0.89	0.2938	0.4011	-1.88	0.9510	0.9770	0.09	0.7585	0.7941	0.02	0.9703	0.9788	0.00	0.1245	0.1839	0.11
4780	Tumor necrosis factor receptor superfamily member EDAR	Q9UNE0	0.3295	0.5361	1.61	0.2704	0.6635	-1.84	0.0083	0.0429	1.60	0.0251	0.0708	-2.05	0.0637	0.1911	0.83	0.6222	0.8576	-0.85	0.3736	0.4501	-2.55	0.8708	0.9409	-0.33	0.0651	0.1195	2.06
4781	Tumor protein D52	P55327	0.1548	0.3069	-0.07	0.7304	0.8036	0.04	0.1305	0.2514	0.08	0.9913	0.9923	0.00	0.7044	0.7653	-0.02	0.3394	0.5435	-0.04	0.2515	0.4877	0.16	0.3350	0.6922	-0.13	0.6620	0.7165	0.06
4782	Tumor protein D53	Q16890	0.1848	0.3496	-0.08	0.3646	0.7132	-0.16	0.5440	0.7668	0.04	0.0538	0.1213	-0.15	0.0315	0.1299	-0.20	0.3606	0.5667	-0.05	0.3736	0.4731	-2.55	0.8708	0.9838	-0.33	0.0651	0.1291	2.06
4783	Tumor protein D54	O43399	0.1129	0.2447	0.12	0.8447	0.8883	0.01	0.0403	0.1071	0.23	0.0085	0.0373	0.24	0.7709	0.8215	0.01	0.0321	0.1155	0.17	0.0652	0.2012	0.36	0.8456	1.2887	-0.02	0.6240	0.6819	0.04
4784	Tumor protein p53-inducible protein 11	O14683	0.0179	0.0784	0.18	0.0443	0.5694	0.46	0.0204	0.0688	0.31	0.0017	0.0182	0.35	0.0077	0.0638	0.22	0.0118	0.0743	0.22	0.3736	0.6091	-2.55	0.8708	1.2288	-0.33	0.0651	0.1984	2.06
4785	Tumor suppressor candidate gene 1 protein	Q2TAM9	0.0522	0.1478	-0.18	0.4127	0.7525	0.10	0.0710	0.1588	-0.03	0.3747	0.5581	0.06	0.0250	0.1139	0.05	0.4324	0.6536	-0.08	0.3736	0.4661	-2.55	0.8708	0.9709	-0.33	0.0651	0.1261	2.06
4786	Tumor susceptibility gene 101 protein	Q99816	0.0043	0.0412	0.33	0.3145	0.6802	0.08	0.8630	0.8907	-0.01	0.1976	0.3288	-0.11	0.0074	0.0624	0.29	0.0821	0.2064	-0.13	0.3736	0.5427	-2.55	0.8708	1.1110	-0.33	0.0651	0.1614	2.06
4787	Tumor-associated calcium signal transducer 2	P09758	0.0104	0.0589	0.28	0.1658	0.6612	0.26	0.0014	0.0229	0.45	0.0016	0.0181	0.45	0.1160	0.2788	0.10	0.0044	0.0601	0.38	0.3736	0.6324	-2.55	0.8708	1.2692	-0.33	0.0651	0.2130	2.06
4788	Twinfilin-1	Q12792	0.0539	0.1515	0.13	0.3914	0.7354	0.09	0.3058	0.4889	0.08	0.0457	0.1079	0.15	0.1412	0.3140	-0.08	0.0328	0.1165	0.19	0.0247	0.1386	0.27	0.5753	1.0058	0.04	0.6661	0.7208	0.02
4789	Twinfilin-2	Q6IBS0	0.0003	0.0139	1.24	0.6865	0.7705	-0.01	0.0147	0.0570	-0.20	0.0012	0.0159	-0.34	0.0013	0.0282	-0.26	0.7230	0.9630	-0.01	0.0001	0.0225	-0.85	0.0002	0.0207	-0.86	0.0003	0.0305	-0.89
4790	Twisted gastrulation protein homolog 1	Q9GZX9	0.4108	0.4992	-3.31	0.5752	0.6989	-1.75	0.5661	0.6364	-1.19	0.5388	0.6022	0.89	0.2938	0.4010	-1.88	0.9510	0.9768	0.09	0.0687	0.2065	-0.65	0.2831	0.6211	-0.14	0.0371	0.1654	-0.24
4791	Twist-related protein 1	Q15672	0.0034	0.0376	1.07	0.0504	0.5738	0.30	0.2692	0.4417	0.13	0.5800	0.6289	0.06	0.1139	0.2758	0.21	0.0874	0.2153	0.24	0.3736	0.5397	-2.55	0.8708	1.1056	-0.33	0.0651	0.1599	2.06
4792	Type II inositol 1 4 5-trisphosphate 5-phosphatase	P32019	0.1366	0.2811	-0.06	0.0176	0.4651	-0.12	0.9554	0.9645	0.00	0.1236	0.2271	-0.07	0.0029	0.0408	-0.22	0.0513	0.1528	0.10	0.2342	0.4630	0.16	0.0350	0.1701	0.45	0.1117	0.1690	0.35
4793	Type II inositol 3 4-bisphosphate 4-phosphatase	O15327	0.0192	0.0814	-0.50	0.8187	0.8705	0.02	0.0309	0.0898	-0.39	0.0212	0.0629	-0.42	0.1276	0.2952	-0.21	0.0219	0.0951	-0.55	0.3736	0.5911	-2.55	0.8708	1.1971	-0.33	0.0651	0.1877	2.06
4794	Type-1 angiotensin II receptor-associated protein	Q6RW13	0.4108	0.4990	-3.31	0.5752	0.6988	-1.75	0.5661	0.6362	-1.19	0.5388	0.6020	0.89	0.2938	0.4009	-1.88	0.9510	0.9766	0.09	0.0424	0.1671	0.51	0.1015	0.3206	0.30	0.0217	0.1256	0.77
4795	Tyrosine aminotransferase	P17735	0.4108	0.4989	-3.31	0.5752	0.6986	-1.75	0.5661	0.6361	-1.19	0.5388	0.6019	0.89	0.2938	0.4008	-1.88	0.9510	0.9764	0.09	0.0355	0.1556	-1.35	0.0183	0.1156	-0.33	0.0010	0.0389	-0.53
4796	Tyrosine-protein kinase BTK	Q06187	0.4108	0.4988	-3.31	0.5752	0.6984	-1.75	0.5661	0.6360	-1.19	0.5388	0.6018	0.89	0.2938	0.4007	-1.88	0.9510	0.9763	0.09	0.1616	0.3550	0.21	0.0208	0.1246	0.27	0.0232	0.1295	0.54
4797	Tyrosine-protein kinase CSK	P41240	0.5610	0.6297	0.03	0.5331	0.8499	0.04	0.0431	0.1128	0.06	0.9051	0.9184	0.00	0.0065	0.0586	-0.09	0.0596	0.1688	0.05	0.9176	0.9331	-0.02	0.6536	1.0936	-0.03	0.1160	0.1737	0.21
4798	Tyrosine-protein kinase Fer	P16591	0.0003	0.0138	9.80	0.0431	0.5695	1.78	0.0708	0.1584	-5.29	0.0001	0.0067	4.72	0.0007	0.0221	4.98	0.2644	0.4570	0.34	0.3736	0.4880	-2.55	0.8708	1.0114	-0.33	0.0651	0.1355	2.06
4799	Tyrosine-protein kinase Fgr	P09769	0.0376	0.1216	-0.21	0.0973	0.6340	0.16	0.2639	0.4341	0.07	0.1341	0.2421	0.10	0.9712	0.9776	0.00	0.0888	0.2173	-0.12	0.0054	0.0784	-0.93	0.0000	0.0000	0.76	0.0224	0.1271	-0.09
4800	Tyrosine-protein kinase HCK	P08631	0.1105	0.2413	0.07	0.0487	0.5746	0.12	0.8310	0.8637	-0.01	0.0423	0.1023	0.11	0.4105	0.5058	0.03	0.9887	0.9912	0.00	0.9496	0.9587	0.02	0.6698	1.1074	0.19	0.3128	0.3841	0.44
4801	Tyrosine-protein kinase JAK1	P23458	0.4108	0.4987	-3.31	0.5752	0.6983	-1.75	0.5661	0.6358	-1.19	0.5388	0.6016	0.89	0.2938	0.4006	-1.88	0.9510	0.9761	0.09	0.0468	0.1734	-1.05	0.8197	1.2648	0.12	0.3944	0.4659	-0.35
4802	Tyrosine-protein kinase Lyn	P07948	0.4108	0.4986	-3.31	0.5752	0.6981	-1.75	0.5661	0.6357	-1.19	0.5388	0.6015	0.89	0.2938	0.4005	-1.88	0.9510	0.9759	0.09	0.6877	0.7294	-0.03	0.8831	0.9118	-0.01	0.4124	0.4840	-0.04
4803	Tyrosine-protein kinase receptor Tie-1	P35590	0.4108	0.4985	-3.31	0.5752	0.6979	-1.75	0.5661	0.6355	-1.19	0.5388	0.6014	0.89	0.2938	0.4004	-1.88	0.9510	0.9757	0.09	0.0391	0.1639	0.19	0.1639	0.4408	0.20	0.3349	0.4058	0.14
4804	Tyrosine-protein kinase receptor UFO	P30530	0.4108	0.4983	-3.31	0.5752	0.6978	-1.75	0.5661	0.6354	-1.19	0.5388	0.6012	0.89	0.2938	0.4003	-1.88	0.9510	0.9755	0.09	0.0299	0.1468	0.18	0.0865	0.2915	-0.09	0.3041	0.3752	0.03
4805	Tyrosine-protein kinase transmembrane receptor ROR2	Q01974	0.4108	0.4982	-3.31	0.5752	0.6976	-1.75	0.5661	0.6353	-1.19	0.5388	0.6011	0.89	0.2938	0.4002	-1.88	0.9510	0.9753	0.09	0.4859	0.5404	0.18	0.2387	0.5557	0.34	0.7855	0.8243	0.09
4806	Tyrosine-protein kinase Yes	P07947	0.6517	0.7128	-0.09	0.4483	0.7829	0.20	0.5060	0.7279	-0.13	0.5334	0.7410	-0.13	0.1957	0.3946	-0.31	0.1519	0.3094	-0.36	0.0006	0.0409	-0.59	0.1032	0.3238	-0.11	0.3740	0.4459	-0.05
4807	Tyrosine-protein kinase ZAP-70	P43403	0.2648	0.4563	0.49	0.9804	0.9867	-0.01	0.3402	0.5327	0.40	0.9555	0.9616	0.02	0.3684	0.4653	0.37	0.9539	0.9621	0.02	0.2166	0.4367	0.12	0.1509	0.4189	-0.08	0.0019	0.0490	0.48
4808	Tyrosine-protein phosphatase non-receptor type 1	P18031	0.0742	0.1846	0.24	0.1651	0.6609	0.29	0.0005	0.0195	0.49	0.0022	0.0205	0.38	0.5923	0.6690	0.02	0.0513	0.1526	0.39	0.1669	0.3616	0.18	0.5302	0.9536	0.06	0.6669	0.7215	0.03
4809	Tyrosine-protein phosphatase non-receptor type 11	Q06124	0.0842	0.2004	0.10	0.1027	0.6318	0.16	0.0108	0.0485	0.28	0.0149	0.0501	0.29	0.0137	0.0838	0.15	0.0053	0.0620	0.24	0.1667	0.3613	0.19	0.3433	0.7026	-0.09	0.4891	0.5576	-0.06
4810	Tyrosine-protein phosphatase non-receptor type 12	Q05209	0.7910	0.8308	0.13	0.4379	0.7742	-0.39	0.6527	0.7044	0.23	0.9057	0.9186	-0.06	0.5690	0.6494	0.30	0.2976	0.4947	0.56	0.7065	0.7479	-0.04	0.5706	0.9992	0.06	0.5815	0.6440	-0.10
4811	Tyrosine-protein phosphatase non-receptor type 13	Q12923	0.2074	0.3798	0.09	0.9820	0.9873	0.00	0.0599	0.1415	-0.17	0.0493	0.1140	0.19	0.1103	0.2701	-0.09	0.0669	0.1816	0.12	0.3736	0.5507	-2.55	0.8708	1.1254	-0.33	0.0651	0.1655	2.06
4812	Tyrosine-protein phosphatase non-receptor type 2	P17706	0.4108	0.4981	-3.31	0.5752	0.6975	-1.75	0.5661	0.6351	-1.19	0.5388	0.6010	0.89	0.2938	0.4001	-1.88	0.9510	0.9751	0.09	0.0005	0.0355	-0.82	0.0047	0.0552	-0.43	0.0017	0.0489	-0.73
4813	Tyrosine-protein phosphatase non-receptor type 20	Q4JDL3	0.4451	0.5227	-0.04	0.8963	0.9267	-0.01	0.0859	0.1826	-0.09	0.7681	0.8004	0.01	0.0134	0.0828	-0.23	0.0948	0.2271	-0.09	0.3736	0.5362	-2.55	0.8708	1.0994	-0.33	0.0651	0.1582	2.06
4814	Tyrosine-protein phosphatase non-receptor type 23	Q9H3S7	0.1093	0.2392	0.06	0.9635	0.9754	0.00	0.4072	0.6126	0.03	0.0826	0.1679	-0.11	0.0303	0.1268	-0.12	0.5108	0.7379	-0.02	0.0836	0.2311	0.27	0.1337	0.3850	0.25	0.0912	0.1448	0.27
4815	Tyrosine-protein phosphatase non-receptor type 6	P29350	0.0662	0.1704	-0.10	0.2655	0.6631	0.14	0.3917	0.5935	0.04	0.0462	0.1090	0.13	0.0389	0.1440	0.15	0.0332	0.1174	0.14	0.3736	0.5772	-2.55	0.8708	1.1725	-0.33	0.0651	0.1798	2.06
4816	Tyrosine--tRNA ligase cytoplasmic	P54577	0.0190	0.0809	0.18	0.2815	0.6649	0.20	0.0073	0.0404	0.23	0.0016	0.0177	0.34	0.5473	0.6313	-0.02	0.0093	0.0687	0.27	0.0191	0.1282	0.50	0.1151	0.3479	0.20	0.1901	0.2579	0.13
4817	Tyrosine--tRNA ligase mitochondrial	Q9Y2Z4	0.0011	0.0223	2.25	0.2894	0.6692	-1.51	0.0580	0.1383	-5.92	0.0687	0.1458	3.05	0.0222	0.1074	-6.61	0.1744	0.3421	3.21	0.8569	0.8809	-0.01	0.0052	0.0576	-0.30	0.0035	0.0589	-0.38
4818	Tyrosyl-DNA phosphodiesterase 1	Q9NUW8	0.1730	0.3343	0.50	0.3599	0.7083	0.36	0.1536	0.2850	0.53	0.3812	0.5663	0.37	0.0694	0.2005	1.06	0.4229	0.6424	-0.96	0.3736	0.4670	-2.55	0.8708	0.9726	-0.33	0.0651	0.1265	2.06
4819	Tyrosyl-DNA phosphodiesterase 2	O95551	0.4108	0.4980	-3.31	0.5752	0.6973	-1.75	0.5661	0.6350	-1.19	0.5388	0.6009	0.89	0.2938	0.4000	-1.88	0.9510	0.9749	0.09	0.6392	0.6847	-0.06	0.5610	0.9878	0.07	0.1236	0.1829	-0.26
4820	U1 small nuclear ribonucleoprotein 70 kDa	P08621	0.0393	0.1247	0.15	0.5795	0.6874	0.09	0.0040	0.0318	0.34	0.3739	0.5574	0.04	0.0249	0.1137	-0.17	0.1110	0.2514	0.09	0.0108	0.1017	0.68	0.0647	0.2450	0.18	0.0317	0.1511	0.15
4821	U1 small nuclear ribonucleoprotein A	P09012	0.0651	0.1689	-0.11	0.8254	0.8745	-0.03	0.2551	0.4230	0.07	0.0502	0.1153	-0.10	0.0087	0.0668	-0.23	0.7039	0.9463	-0.04	0.0152	0.1141	0.62	0.0037	0.0497	0.43	0.0143	0.1035	0.39
4822	U1 small nuclear ribonucleoprotein C	P09234	0.2099	0.3826	-0.13	0.0002	0.2072	-0.46	0.0455	0.1174	-0.15	0.0129	0.0460	-0.45	0.0001	0.0091	-0.63	0.2516	0.4419	-0.16	0.0450	0.1703	0.21	0.0303	0.1574	0.13	0.4366	0.5072	0.06
4823	U11/U12 small nuclear ribonucleoprotein 35 kDa protein	Q16560	0.4108	0.4979	-3.31	0.5752	0.6971	-1.75	0.5661	0.6349	-1.19	0.5388	0.6007	0.89	0.2938	0.3999	-1.88	0.9510	0.9747	0.09	0.5267	0.5794	-0.05	0.0628	0.2401	-0.14	0.0198	0.1218	-0.45
4824	U2 small nuclear ribonucleoprotein A'	P09661	0.0147	0.0704	0.30	0.0475	0.5696	0.21	0.0052	0.0353	0.16	0.0565	0.1261	0.19	0.0334	0.1336	0.16	0.2654	0.4581	0.16	0.0463	0.1728	0.51	0.9095	0.9329	0.02	0.1214	0.1802	0.21
4825	U2 small nuclear ribonucleoprotein B''	P08579	0.0190	0.0809	0.23	0.2134	0.6627	0.42	0.0128	0.0531	0.34	0.1467	0.2605	0.20	0.9863	0.9899	0.00	0.1204	0.2651	0.14	0.0146	0.1120	-0.36	0.0127	0.0937	-0.47	0.6901	0.7427	0.35
4826	U3 small nucleolar ribonucleoprotein protein IMP3	Q9NV31	0.2059	0.3785	0.18	0.8628	0.9009	0.04	0.9851	0.9878	-0.01	0.1493	0.2641	0.20	0.0104	0.0729	0.30	0.1321	0.2831	-0.36	0.3736	0.5235	-2.55	0.8708	1.0763	-0.33	0.0651	0.1519	2.06
4827	U3 small nucleolar ribonucleoprotein protein IMP4	Q96G21	0.2037	0.3754	-0.52	0.5435	0.8578	-0.29	0.7100	0.7560	-0.13	0.5726	0.6221	-0.21	0.4446	0.5385	0.28	0.2046	0.3827	-0.77	0.3736	0.5014	-2.55	0.8708	1.0360	-0.33	0.0651	0.1415	2.06
4828	U3 small nucleolar RNA-associated protein 14 homolog A	Q9BVJ6	0.0878	0.2062	-0.24	0.1561	0.6628	-0.51	0.4730	0.6904	0.08	0.1734	0.2962	0.16	0.1026	0.2567	0.21	0.3471	0.5524	0.10	0.6369	0.6825	-0.03	0.5321	0.9564	0.06	0.5010	0.5681	-0.03
4829	U3 small nucleolar RNA-associated protein 18 homolog	Q9Y5J1	0.2200	0.3962	-0.13	0.4093	0.7505	-0.07	0.0642	0.1477	-0.22	0.1263	0.2313	-0.19	0.7199	0.7800	-0.03	0.5275	0.7571	0.06	0.1928	0.4004	0.15	0.3736	0.7469	0.09	0.1036	0.1596	0.21
4830	U3 small nucleolar RNA-associated protein 6 homolog	Q9NYH9	0.4108	0.4978	-3.31	0.5752	0.6970	-1.75	0.5661	0.6347	-1.19	0.5388	0.6006	0.89	0.2938	0.3998	-1.88	0.9510	0.9745	0.09	0.0350	0.1547	0.24	0.2988	0.6420	-0.13	0.6278	0.6856	0.03
4831	U4/U6 small nuclear ribonucleoprotein Prp3	O43395	0.6923	0.7473	0.02	0.2543	0.6623	0.10	0.1078	0.2168	0.09	0.0375	0.0938	0.17	0.0381	0.1423	0.16	0.0747	0.1944	0.14	0.3736	0.5471	-2.55	0.8708	1.1190	-0.33	0.0651	0.1637	2.06
4832	U4/U6 small nuclear ribonucleoprotein Prp31	Q8WWY3	0.7798	0.8208	-0.02	0.5528	0.8683	0.05	0.4693	0.6867	0.07	0.6101	0.6573	-0.03	0.1600	0.3430	-0.11	0.8440	1.0790	-0.02	0.7667	0.8018	-0.02	0.0794	0.2766	0.13	0.4428	0.5131	-0.04
4833	U4/U6 small nuclear ribonucleoprotein Prp4	O43172	0.0640	0.1674	0.16	0.1429	0.6545	-0.12	0.0769	0.1674	0.18	0.6913	0.7311	0.03	0.0351	0.1356	-0.20	0.2232	0.4058	0.13	0.3736	0.4974	-2.55	0.8708	1.0287	-0.33	0.0651	0.1397	2.06
4834	U4/U6.U5 tri-snRNP-associated protein 1	O43290	0.1518	0.3025	-0.05	0.3257	0.6853	-0.03	0.5026	0.7248	-0.02	0.0514	0.1171	-0.14	0.0325	0.1316	-0.09	0.0285	0.1081	-0.10	0.1839	0.3849	0.26	0.9969	0.9973	0.00	0.3659	0.4375	0.16
4835	U4/U6.U5 tri-snRNP-associated protein 2	Q53GS9	0.0609	0.1634	0.14	0.2216	0.6589	0.17	0.0248	0.0781	0.07	0.0032	0.0230	0.11	0.0177	0.0955	0.07	0.1153	0.2582	0.12	0.0066	0.0842	0.38	0.0137	0.0976	0.31	0.0504	0.1967	0.20
4836	U5 small nuclear ribonucleoprotein 200 kDa helicase	O75643	0.4108	0.4976	-3.31	0.5752	0.6968	-1.75	0.5661	0.6346	-1.19	0.5388	0.6005	0.89	0.2938	0.3997	-1.88	0.9510	0.9743	0.09	0.0284	0.1452	0.71	0.0194	0.1199	0.66	0.0138	0.1018	0.99
4837	U6 snRNA-associated Sm-like protein LSm2	Q9Y333	0.0431	0.1316	-0.36	0.5587	0.8722	-0.10	0.0186	0.0653	0.27	0.3575	0.5357	-0.06	0.5928	0.6691	-0.06	0.2934	0.4909	-0.13	0.4639	0.5188	-0.05	0.4444	0.8463	0.06	0.9067	0.9260	0.01
4838	U6 snRNA-associated Sm-like protein LSm4	Q9Y4Z0	0.2001	0.3708	0.13	0.8017	0.8570	0.04	0.6863	0.7353	0.03	0.2582	0.4099	0.10	0.1238	0.2902	-0.15	0.0102	0.0702	0.50	0.3736	0.6124	-2.55	0.8708	1.2345	-0.33	0.0651	0.2004	2.06
4839	U6 snRNA-associated Sm-like protein LSm8	O95777	0.5156	0.5880	-0.16	0.4017	0.7453	-0.48	0.4485	0.6619	-0.18	0.4006	0.5904	-0.23	0.3986	0.4950	-0.20	0.2924	0.4900	-0.33	0.3736	0.4833	-2.55	0.8708	1.0028	-0.33	0.0651	0.1334	2.06
4840	Ubiquilin-1	Q9UMX0	0.2809	0.4744	0.02	0.2120	0.6619	-0.13	0.0093	0.0451	-0.12	0.0167	0.0539	-0.22	0.0286	0.1228	-0.17	0.1326	0.2836	-0.03	0.3485	0.6231	-0.10	0.1190	0.3563	0.20	0.7023	0.7538	0.04
4841	Ubiquilin-2	Q9UHD9	0.0379	0.1222	0.15	0.1960	0.6688	0.18	0.0037	0.0317	0.35	0.0002	0.0103	0.26	0.0213	0.1053	0.17	0.1162	0.2596	0.23	0.0839	0.2317	0.21	0.5531	0.9795	0.05	0.4195	0.4910	0.08
4842	Ubiquilin-4	Q9NRR5	0.1628	0.3196	-0.05	0.0992	0.6367	-0.15	0.0047	0.0336	-0.11	0.0012	0.0161	-0.22	0.0080	0.0649	-0.34	0.2735	0.4679	-0.06	0.3404	0.6110	0.18	0.1924	0.4866	0.26	0.8694	0.8943	0.03
4843	Ubiquinol-cytochrome-c reductase complex assembly factor 2	Q9BRT2	0.7911	0.8307	-0.01	0.0066	0.4221	0.23	0.1554	0.2874	0.06	0.0271	0.0746	0.14	0.0008	0.0215	0.42	0.2886	0.4854	0.04	0.0406	0.1656	-0.22	0.9456	0.9608	0.00	0.1212	0.1799	-0.07
4844	Ubiquitin carboxyl-terminal hydrolase 10	Q14694	0.0104	0.0589	0.23	0.2509	0.6611	0.08	0.1081	0.2173	0.22	0.0118	0.0435	0.22	0.5623	0.6447	0.03	0.7678	1.0056	-0.02	0.0466	0.1732	1.27	0.5486	0.9742	0.19	0.0314	0.1505	1.24
4845	Ubiquitin carboxyl-terminal hydrolase 11	P51784	0.4108	0.4975	-3.31	0.5752	0.6966	-1.75	0.5661	0.6344	-1.19	0.5388	0.6003	0.89	0.2938	0.3995	-1.88	0.9510	0.9741	0.09	0.0336	0.1525	0.41	0.0077	0.0723	0.34	0.0390	0.1705	0.59
4846	Ubiquitin carboxyl-terminal hydrolase 13	Q92995	0.0004	0.0158	0.45	0.0760	0.6171	0.33	0.0386	0.1039	0.13	0.0002	0.0086	0.61	0.0430	0.1534	0.13	0.1467	0.3026	0.46	0.3736	0.5174	-2.55	0.8708	1.0654	-0.33	0.0651	0.1490	2.06
4847	Ubiquitin carboxyl-terminal hydrolase 14	P54578	0.8801	0.9054	-0.01	0.2262	0.6590	0.16	0.0237	0.0755	0.25	0.0247	0.0700	0.23	0.5666	0.6480	0.05	0.0303	0.1117	0.22	0.0023	0.0562	0.42	0.0118	0.0904	0.22	0.0063	0.0750	0.28
4848	Ubiquitin carboxyl-terminal hydrolase 15	Q9Y4E8	0.0362	0.1192	0.16	0.1808	0.6637	0.18	0.0176	0.0633	0.18	0.0167	0.0539	0.26	0.0568	0.1782	0.14	0.0110	0.0718	0.29	0.0840	0.2317	0.26	0.0433	0.1922	0.28	0.0611	0.2213	0.19
4849	Ubiquitin carboxyl-terminal hydrolase 16	Q9Y5T5	0.0132	0.0668	0.18	0.1182	0.6319	0.18	0.0113	0.0494	0.20	0.0019	0.0193	0.23	0.0006	0.0199	0.30	0.0103	0.0705	0.26	0.3736	0.6122	-2.55	0.8708	1.2341	-0.33	0.0651	0.2002	2.06
4850	Ubiquitin carboxyl-terminal hydrolase 19	O94966	0.4108	0.4974	-3.31	0.5752	0.6965	-1.75	0.5661	0.6343	-1.19	0.5388	0.6002	0.89	0.2938	0.3994	-1.88	0.9510	0.9739	0.09	0.2687	0.5115	0.22	0.0866	0.2917	0.30	0.0204	0.1226	0.73
4851	Ubiquitin carboxyl-terminal hydrolase 24	Q9UPU5	0.1081	0.2371	-0.11	0.3254	0.6857	0.22	0.0010	0.0210	0.45	0.0162	0.0529	0.14	0.5019	0.5913	0.06	0.1517	0.3093	-0.16	0.0028	0.0597	-0.90	0.0024	0.0412	-0.91	0.1303	0.1902	-0.84
4852	Ubiquitin carboxyl-terminal hydrolase 29	Q9HBJ7	0.2037	0.3752	-0.09	0.6464	0.7385	-0.02	0.0348	0.0969	-0.08	0.1262	0.2312	-0.10	0.1161	0.2789	-0.12	0.5490	0.7828	0.06	0.0014	0.0487	0.81	0.0004	0.0216	1.12	0.0200	0.1222	0.42

Supplemetary Table S2. Overview on all relatively quantified 5180 proteins statistical analysis

			MCF-7																		MDA-MB-231								
			Dai_SC20 vs control			Gen_SC20 vs control			SSE_SC20 vs control			Dai_IC20 vs control			Gen_IC20 vs control			SSE_IC20 vs control			Dai_IC20 vs control			Gen_IC20 vs control			SSE_IC20 vs control		
	**Protein name**	**UniProt**	**p value**	**BH q value**	**log2FC**	**p value**	**BH q value**	**log2FC**	**p value**	**BH q value**	**log2FC**	**p value**	**BH q value**	**log2FC**	**p value**	**BH q value**	**log2FC**	**p value**	**BH q value**	**log2FC**	**p value**	**BH q value**	**log2FC**	**p value**	**BH q value**	**log2FC**	**p value**	**BH q value**	**log2FC**
4853	Ubiquitin carboxyl-terminal hydrolase 30	Q70CQ3	0.4108	0.4973	-3.31	0.5752	0.6963	-1.75	0.5661	0.6342	-1.19	0.5388	0.6001	0.89	0.2938	0.3993	-1.88	0.9510	0.9737	0.09	0.0204	0.1319	0.72	0.4360	0.8365	0.10	0.0015	0.0468	1.42
4854	Ubiquitin carboxyl-terminal hydrolase 32	Q8NFA0	0.0257	0.0969	0.14	0.0399	0.5632	0.14	0.0199	0.0681	0.20	0.0968	0.1894	0.16	0.0929	0.2404	0.08	0.1437	0.2991	0.12	0.3736	0.5190	-2.55	0.8708	1.0681	-0.33	0.0651	0.1497	2.06
4855	Ubiquitin carboxyl-terminal hydrolase 34	Q70CQ2	0.0034	0.0376	0.11	0.2782	0.6653	0.19	0.0003	0.0197	0.34	0.0004	0.0130	0.21	0.5513	0.6346	0.01	0.0146	0.0804	0.15	0.1350	0.3130	0.08	0.4052	0.7903	0.04	0.0623	0.2232	0.12
4856	Ubiquitin carboxyl-terminal hydrolase 47	Q96K76	0.0049	0.0429	0.17	0.1427	0.6553	0.20	0.0166	0.0611	0.14	0.0557	0.1247	0.09	0.0019	0.0345	0.22	0.0258	0.1023	0.17	0.1303	0.3053	0.28	0.0323	0.1621	0.39	0.4334	0.5046	0.13
4857	Ubiquitin carboxyl-terminal hydrolase 48	Q86UV5	0.4108	0.4972	-3.31	0.5752	0.6962	-1.75	0.5661	0.6340	-1.19	0.5388	0.6000	0.89	0.2938	0.3992	-1.88	0.9510	0.9736	0.09	0.0544	0.1851	0.37	0.5478	0.9734	-0.07	0.0852	0.1374	-0.21
4858	Ubiquitin carboxyl-terminal hydrolase 49	Q70CQ1	0.5123	0.5849	0.04	0.3871	0.7294	0.10	0.9645	0.9714	0.00	0.0679	0.1444	0.19	0.1518	0.3298	0.12	0.9290	1.1663	-0.01	0.3736	0.4327	-2.55	0.8708	0.9083	-0.33	0.0651	0.1126	2.06
4859	Ubiquitin carboxyl-terminal hydrolase 5	P45974	0.0223	0.0889	0.05	0.0970	0.6344	0.19	0.0045	0.0332	0.20	0.0004	0.0118	0.24	0.0026	0.0396	0.12	0.0030	0.0557	0.17	0.0346	0.1545	0.41	0.1266	0.3713	0.15	0.3139	0.3851	0.08
4860	Ubiquitin carboxyl-terminal hydrolase 7	Q93009	0.0235	0.0921	0.18	0.3404	0.6992	0.07	0.0167	0.0612	0.23	0.0302	0.0807	0.15	0.0583	0.1813	-0.12	0.0284	0.1079	0.18	0.9918	0.9937	0.00	0.0923	0.3018	0.28	0.0479	0.1912	0.40
4861	Ubiquitin carboxyl-terminal hydrolase 8	P40818	0.0451	0.1354	-0.09	0.5011	0.8230	0.11	0.1157	0.2294	0.10	0.0110	0.0421	0.14	0.6970	0.7590	0.01	0.5516	0.7856	0.02	0.0255	0.1396	0.26	0.0626	0.2395	0.15	0.0860	0.1383	0.22
4862	Ubiquitin carboxyl-terminal hydrolase isozyme L3	P15374	0.1150	0.2483	0.25	0.0201	0.4733	0.41	0.0008	0.0223	0.46	0.0037	0.0247	0.38	0.0029	0.0412	0.33	0.0343	0.1196	0.56	0.1826	0.3839	-0.08	0.2037	0.5034	0.07	0.2914	0.3633	-0.05
4863	Ubiquitin carboxyl-terminal hydrolase isozyme L5	Q9Y5K5	0.0195	0.0819	0.12	0.6999	0.7787	0.04	0.0154	0.0583	0.14	0.0671	0.1432	0.08	0.0553	0.1754	-0.09	0.0879	0.2159	0.07	0.0108	0.1021	-0.28	0.0167	0.1098	-0.17	0.0316	0.1510	-0.17
4864	Ubiquitin conjugation factor E4 A	Q14139	0.7841	0.8247	-0.01	0.0569	0.5848	0.35	0.0014	0.0230	0.24	0.0258	0.0722	0.23	0.0004	0.0162	0.22	0.1352	0.2873	0.07	0.3736	0.5221	-2.55	0.8708	1.0737	-0.33	0.0651	0.1512	2.06
4865	Ubiquitin conjugation factor E4 B	O95155	0.4108	0.4971	-3.31	0.5752	0.6960	-1.75	0.5661	0.6339	-1.19	0.5388	0.5998	0.89	0.2938	0.3991	-1.88	0.9510	0.9734	0.09	0.0717	0.2120	0.29	0.7417	1.1836	0.02	0.4018	0.4735	0.08
4866	Ubiquitin domain-containing protein UBFD1	O14562	0.2088	0.3816	-0.05	0.6250	0.7225	0.14	0.1131	0.2252	0.13	0.0162	0.0528	0.14	0.2401	0.4533	-0.05	0.6274	0.8632	-0.04	0.3736	0.4495	-2.55	0.8708	0.9399	-0.33	0.0651	0.1193	2.06
4867	Ubiquitin recognition factor in ER-associated degradation protein 1	Q92890	0.0598	0.1618	-0.35	0.3050	0.6772	-0.08	0.0547	0.1330	-0.36	0.4716	0.6711	-0.05	0.0521	0.1691	-0.19	0.0138	0.0780	-0.34	0.1010	0.2572	-1.24	0.0416	0.1877	-0.66	0.0246	0.1329	-2.04
4868	Ubiquitin thioesterase	Q96FW1	0.1315	0.2735	0.09	0.2633	0.6634	0.25	0.0407	0.1078	0.16	0.0095	0.0391	0.25	0.1178	0.2816	0.09	0.0218	0.0951	0.25	0.8637	0.8868	0.02	0.0485	0.2059	-0.25	0.0397	0.1724	-0.28
4869	Ubiquitin thioesterase otulin	Q96BN8	0.0201	0.0835	-0.14	0.0070	0.4168	-0.19	0.0004	0.0241	-0.43	0.0036	0.0242	-0.25	0.0008	0.0218	-0.44	0.0370	0.1248	0.12	0.1030	0.2606	0.18	0.0253	0.1421	-0.22	0.4584	0.5268	-0.04
4870	Ubiquitin-40S ribosomal protein S27a	P62979	0.0006	0.0186	0.55	0.0601	0.5953	0.62	0.0002	0.0220	0.74	0.0001	0.0136	0.87	0.0028	0.0407	0.48	0.0027	0.0559	0.74	0.0118	0.1045	0.60	0.5866	1.0190	0.06	0.0532	0.2034	0.21
4871	Ubiquitin-associated and SH3 domain-containing protein B	Q8TF42	0.4108	0.4970	-3.31	0.5752	0.6958	-1.75	0.5661	0.6338	-1.19	0.5388	0.5997	0.89	0.2938	0.3990	-1.88	0.9510	0.9732	0.09	0.0223	0.1356	0.22	0.2856	0.6240	-0.07	0.5618	0.6258	0.05
4872	Ubiquitin-associated domain-containing protein 2	Q8NBM4	0.1768	0.3391	-0.14	0.4649	0.7964	0.06	0.0530	0.1304	-0.18	0.0813	0.1662	0.18	0.0039	0.0460	0.65	0.6884	0.9284	-0.06	0.3736	0.4456	-2.55	0.8708	0.9325	-0.33	0.0651	0.1177	2.06
4873	Ubiquitin-associated protein 2-like	Q14157	0.0031	0.0362	0.39	0.1667	0.6597	0.31	0.0013	0.0231	0.36	0.0015	0.0178	0.32	0.0941	0.2429	0.09	0.0091	0.0689	0.28	0.0310	0.1488	0.37	0.6441	1.0822	0.04	0.6683	0.7226	0.03
4874	Ubiquitin-conjugating enzyme E2 C	O00762	0.5296	0.6008	-0.08	0.1174	0.6322	-1.10	0.5033	0.7256	0.18	0.2747	0.4320	0.26	0.1279	0.2955	-0.41	0.0967	0.2295	-0.18	0.3375	0.6081	-0.28	0.7387	1.1810	-0.11	0.3572	0.4285	0.20
4875	Ubiquitin-conjugating enzyme E2 D3	P61077	0.0104	0.0588	-0.24	0.2426	0.6597	-0.16	0.3214	0.5090	0.10	0.0006	0.0123	-0.48	0.0015	0.0306	-0.31	0.5141	0.7414	-0.08	0.0004	0.0377	-1.33	0.0374	0.1766	-0.24	0.0007	0.0321	-1.08
4876	Ubiquitin-conjugating enzyme E2 E1	P51965	0.0004	0.0157	0.46	0.0998	0.6359	0.36	0.0002	0.0195	0.63	0.0000	0.0000	0.59	0.0083	0.0659	0.22	0.0001	0.0273	0.89	0.0066	0.0848	-1.32	0.0002	0.0173	-1.07	0.0513	0.1989	-0.92
4877	Ubiquitin-conjugating enzyme E2 G2	P60604	0.4769	0.5524	-0.08	0.9403	0.9601	-0.01	0.1002	0.2042	0.18	0.5715	0.6214	0.05	0.0505	0.1660	0.30	0.1143	0.2569	0.18	0.3736	0.5298	-2.55	0.8708	1.0877	-0.33	0.0651	0.1550	2.06
4878	Ubiquitin-conjugating enzyme E2 J1	Q9Y385	0.2059	0.3783	0.80	0.7402	0.8111	-0.19	0.2605	0.4296	0.63	0.2731	0.4301	0.60	0.3644	0.4612	0.49	0.3224	0.5252	0.53	0.3736	0.4778	-2.55	0.8708	0.9927	-0.33	0.0651	0.1311	2.06
4879	Ubiquitin-conjugating enzyme E2 K	P61086	0.0435	0.1323	0.20	0.0145	0.4471	0.30	0.1857	0.3309	0.11	0.0025	0.0209	0.51	0.0586	0.1819	0.20	0.4518	0.6739	-0.05	0.0183	0.1256	-0.35	0.0050	0.0567	-0.23	0.0046	0.0666	-0.27
4880	Ubiquitin-conjugating enzyme E2 L3	P68036	0.0292	0.1044	0.15	0.2414	0.6602	0.19	0.1864	0.3317	0.09	0.0177	0.0561	0.17	0.1185	0.2824	-0.08	0.1519	0.3095	0.07	0.0661	0.2021	0.48	0.0614	0.2368	0.23	0.0855	0.1378	0.20
4881	Ubiquitin-conjugating enzyme E2 N	P61088	0.0219	0.0879	0.11	0.2577	0.6625	0.11	0.0728	0.1614	0.14	0.0004	0.0111	0.22	0.7598	0.8122	-0.01	0.0143	0.0796	0.21	0.3494	0.6243	-0.12	0.1110	0.3398	-0.19	0.2607	0.3328	-0.08
4882	Ubiquitin-conjugating enzyme E2 S	Q16763	0.4108	0.4968	-3.31	0.5752	0.6957	-1.75	0.5661	0.6336	-1.19	0.5388	0.5996	0.89	0.2938	0.3989	-1.88	0.9510	0.9730	0.09	0.8425	0.8675	0.02	0.0464	0.2005	0.43	0.4827	0.5510	0.09
4883	Ubiquitin-conjugating enzyme E2 T	Q9NPD8	0.0281	0.1018	-0.41	0.2046	0.6595	-0.19	0.0911	0.1900	-0.21	0.7801	0.8109	0.03	0.0759	0.2118	-0.24	0.0164	0.0850	-0.45	0.0169	0.1209	0.19	0.3323	0.6894	0.04	0.9576	0.9666	-0.01
4884	Ubiquitin-conjugating enzyme E2 variant 1	Q13404	0.0185	0.0799	0.23	0.2662	0.6629	0.18	0.1037	0.2100	0.11	0.0229	0.0664	0.20	0.1045	0.2604	0.10	0.0298	0.1107	0.20	0.0809	0.2266	0.25	0.0459	0.1993	0.26	0.0542	0.2051	-0.20
4885	Ubiquitin-conjugating enzyme E2 variant 2	Q15819	0.3113	0.5124	0.07	0.3267	0.6863	0.20	0.0417	0.1101	0.19	0.0165	0.0535	0.26	0.9538	0.9640	0.00	0.0576	0.1651	0.19	0.0120	0.1039	-0.24	0.3553	0.7209	-0.06	0.0201	0.1216	-0.21
4886	Ubiquitin-conjugating enzyme E2 Z	Q9H832	0.0070	0.0492	0.16	0.3498	0.7048	0.15	0.0036	0.0314	0.29	0.0195	0.0594	0.07	0.0045	0.0499	-0.11	0.0012	0.0486	0.24	0.0628	0.1974	0.32	0.4098	0.7968	-0.07	0.3966	0.4681	-0.05
4887	Ubiquitin-fold modifier 1	P61960	0.0038	0.0390	0.21	0.0212	0.4754	-0.50	0.0245	0.0774	-0.25	0.0000	0.0000	-0.54	0.0011	0.0266	-0.43	0.0060	0.0624	0.15	0.0313	0.1496	-0.94	0.0000	0.0000	-0.50	0.0000	0.0000	-1.16
4888	Ubiquitin-fold modifier-conjugating enzyme 1	Q9Y3C8	0.3339	0.5405	-0.07	0.2968	0.6717	0.17	0.5836	0.6401	-0.05	0.6526	0.6963	-0.03	0.7330	0.7909	-0.02	0.2389	0.4247	-0.10	0.3736	0.4927	-2.55	0.8708	1.0201	-0.33	0.0651	0.1376	2.06
4889	Ubiquitin-like domain-containing CTD phosphatase 1	Q8WVY7	0.4152	0.4931	0.04	0.6299	0.7265	-0.05	0.1959	0.3452	-0.27	0.9175	0.9283	-0.01	0.0067	0.0593	-0.26	0.0917	0.2214	0.11	0.0760	0.2194	0.29	0.1108	0.3400	0.13	0.0068	0.0762	-0.18
4890	Ubiquitin-like modifier-activating enzyme 1	P22314	0.0869	0.2049	0.15	0.1903	0.6697	0.24	0.0317	0.0911	0.22	0.0471	0.1104	0.21	0.5727	0.6526	0.04	0.0282	0.1073	0.28	0.0195	0.1293	0.44	0.1366	0.3905	0.17	0.1351	0.1958	0.15
4891	Ubiquitin-like modifier-activating enzyme 5	Q9GZZ9	0.0288	0.1035	-0.24	0.2894	0.6689	-0.16	0.1498	0.2790	-0.17	0.1365	0.2457	-0.11	0.1878	0.3833	0.09	0.3571	0.5631	-0.15	0.0330	0.1518	-0.25	0.0029	0.0447	-0.41	0.7768	0.8162	0.04
4892	Ubiquitin-like modifier-activating enzyme 6	A0AVT1	0.0411	0.1283	0.16	0.1407	0.6525	0.18	0.1156	0.2293	0.11	0.1094	0.2077	0.11	0.6613	0.7268	0.02	0.0713	0.1883	0.15	0.0722	0.2127	0.37	0.0960	0.3093	0.33	0.4364	0.5071	0.11
4893	Ubiquitin-like modifier-activating enzyme 7	P41226	0.4189	0.4965	-0.02	0.5339	0.8502	0.07	0.0435	0.1135	0.11	0.2181	0.3564	-0.02	0.0817	0.2208	-0.03	0.6589	0.8965	-0.02	0.3736	0.4472	-2.55	0.8708	0.9356	-0.33	0.0651	0.1184	2.06
4894	Ubiquitin-like modifier-activating enzyme ATG7	O95352	0.1519	0.3026	0.15	0.0219	0.4787	0.35	0.1216	0.2392	0.16	0.1759	0.2996	0.13	0.4444	0.5385	0.06	0.0055	0.0614	0.56	0.0629	0.1973	-0.14	0.0322	0.1619	-0.18	0.0205	0.1228	-0.30
4895	Ubiquitin-like protein 5	Q9BZL1	0.0007	0.0195	-0.29	0.2399	0.6614	-0.12	0.0041	0.0321	-0.35	0.0046	0.0276	-0.13	0.0649	0.1939	-0.09	0.0188	0.0886	-0.41	0.0015	0.0515	-0.83	0.0000	0.0000	-1.11	0.0001	0.0324	-0.81
4896	Ubiquitin-like protein ISG15	P05161	0.7477	0.7945	0.02	0.5002	0.8228	0.08	0.1332	0.2552	0.10	0.7460	0.7810	0.02	0.7570	0.8097	0.02	0.0351	0.1208	0.18	0.3736	0.5746	-2.55	0.8708	1.1680	-0.33	0.0651	0.1783	2.06
4897	Ubiquitin-like-conjugating enzyme ATG3	Q9NT62	0.2061	0.3786	-0.49	0.2768	0.6647	-0.66	0.0200	0.0682	-0.95	0.1927	0.3224	-0.52	0.5017	0.5912	-0.14	0.5626	0.7971	-0.14	0.1086	0.2694	0.29	0.3805	0.7572	-0.09	0.1998	0.2683	0.10
4898	UBX domain-containing protein 1	Q04323	0.6557	0.7167	-0.06	0.1231	0.6402	0.26	0.0555	0.1347	0.38	0.2048	0.3386	0.19	0.1757	0.3658	0.21	0.8247	1.0590	0.03	0.1092	0.2706	0.30	0.9647	0.9749	-0.01	0.2141	0.2831	0.10
4899	UBX domain-containing protein 4	Q92575	0.8853	0.9097	0.01	0.0116	0.4292	0.61	0.0048	0.0339	-0.64	0.0057	0.0303	-0.47	0.7759	0.8244	-0.03	0.0428	0.1363	-0.20	0.3736	0.5677	-2.55	0.8708	1.1557	-0.33	0.0651	0.1745	2.06
4900	UBX domain-containing protein 6	Q9BZV1	0.1212	0.2574	0.15	0.1582	0.6614	0.36	0.0635	0.1468	0.26	0.0001	0.0068	0.42	0.0177	0.0956	0.12	0.0779	0.1996	0.32	0.0366	0.1589	0.48	0.4900	0.9020	0.11	0.6538	0.7097	-0.10
4901	UBX domain-containing protein 7	O94888	0.0148	0.0706	-0.40	0.2610	0.6650	-0.28	0.0016	0.0232	-0.58	0.0034	0.0237	-0.49	0.0485	0.1618	0.19	0.0038	0.0598	-0.52	0.3736	0.6358	-2.55	0.8708	1.2749	-0.33	0.0651	0.2152	2.06
4902	UDP-glucose 4-epimerase	Q14376	0.6714	0.7299	0.03	0.8232	0.8736	-0.02	0.2120	0.3667	-0.10	0.1300	0.2365	0.15	0.5365	0.6217	0.08	0.2098	0.3905	0.25	0.3736	0.5006	-2.55	0.8708	1.0346	-0.33	0.0651	0.1412	2.06
4903	UDP-glucose 6-dehydrogenase	O60701	0.0003	0.0136	0.27	0.2998	0.6734	0.14	0.0035	0.0312	0.29	0.0000	0.0000	0.41	0.0001	0.0140	0.22	0.0201	0.0921	0.36	0.1632	0.3568	0.15	0.9377	0.9545	0.01	0.2755	0.3472	-0.11
4904	UDP-glucose:glycoprotein glucosyltransferase 1	Q9NYU2	0.0406	0.1271	0.09	0.0663	0.5932	0.12	0.0036	0.0318	0.21	0.0144	0.0489	0.15	0.1330	0.3020	0.06	0.0121	0.0744	0.15	0.1361	0.3152	0.13	0.5771	1.0072	-0.03	0.0875	0.1402	-0.09
4905	UDP-glucuronosyltransferase 1-4	P22310	0.4108	0.4967	-3.31	0.5752	0.6955	-1.75	0.5661	0.6335	-1.19	0.5388	0.5994	0.89	0.2938	0.3988	-1.88	0.9510	0.9728	0.09	0.0886	0.2395	0.15	0.0050	0.0564	-0.29	0.2989	0.3703	0.06
4906	UDP-glucuronosyltransferase 3A2	Q3SY77	0.4108	0.4966	-3.31	0.5752	0.6953	-1.75	0.5661	0.6333	-1.19	0.5388	0.5993	0.89	0.2938	0.3987	-1.88	0.9510	0.9726	0.09	0.0343	0.1541	-1.53	0.1667	0.4458	-0.66	0.0175	0.1132	-0.68
4907	UDP-N-acetylhexosamine pyrophosphorylase	Q16222	0.3792	0.5949	0.06	0.0669	0.5924	0.16	0.0120	0.0514	0.29	0.0132	0.0466	0.30	0.0088	0.0670	0.31	0.1753	0.3433	-0.11	0.0265	0.1421	0.46	0.0550	0.2242	0.19	0.0576	0.2139	0.21
4908	UHRF1-binding protein 1	Q6BDS2	0.0098	0.0576	0.25	0.0628	0.5904	0.25	0.8514	0.8813	-0.01	0.0004	0.0111	0.52	0.0003	0.0162	0.53	0.0133	0.0769	0.25	0.5366	0.5883	0.12	0.0550	0.2236	-0.30	0.4449	0.5148	-0.07
4909	UMP-CMP kinase	P30085	0.7246	0.7752	-0.01	0.7156	0.7914	0.05	0.1914	0.3384	0.06	0.1419	0.2536	0.07	0.5539	0.6373	-0.02	0.1350	0.2871	0.08	0.0729	0.2135	0.34	0.4221	0.8152	0.06	0.1018	0.1576	0.12
4910	Uncharacterized membrane protein C3orf80	F5H4A9	0.4108	0.4965	-3.31	0.5752	0.6952	-1.75	0.5661	0.6332	-1.19	0.5388	0.5992	0.89	0.2938	0.3986	-1.88	0.9510	0.9724	0.09	0.0017	0.0515	-1.04	0.0211	0.1258	-0.99	0.0003	0.0235	-0.80
4911	Uncharacterized protein C10orf67 mitochondrial	Q8IYJ2	0.3662	0.5796	0.06	0.4207	0.7590	0.09	0.9584	0.9664	0.00	0.0392	0.0971	0.22	0.1165	0.2796	-0.17	0.4629	0.6855	0.05	0.3736	0.4626	-2.55	0.8708	0.9645	-0.33	0.0651	0.1247	2.06
4912	Uncharacterized protein C10orf82	Q8WW14	0.4108	0.4964	-3.31	0.5752	0.6950	-1.75	0.5661	0.6331	-1.19	0.5388	0.5991	0.89	0.2938	0.3985	-1.88	0.9510	0.9722	0.09	0.2233	0.4469	-0.18	0.0024	0.0404	-0.80	0.2578	0.3295	-0.78
4913	Uncharacterized protein C11orf98	E9PRG8	0.2399	0.4246	-0.11	0.0284	0.5038	-0.50	0.0084	0.0432	-0.68	0.0011	0.0156	-0.81	0.0042	0.0478	-0.77	0.1556	0.3145	-0.37	0.3736	0.5139	-2.55	0.8708	1.0589	-0.33	0.0651	0.1473	2.06
4914	Uncharacterized protein C12orf71	A8MTZ7	0.4108	0.4963	-3.31	0.5752	0.6949	-1.75	0.5661	0.6329	-1.19	0.5388	0.5989	0.89	0.2938	0.3984	-1.88	0.9510	0.9720	0.09	0.8363	0.8621	0.04	0.4251	0.8201	-0.08	0.1283	0.1879	-0.40
4915	Uncharacterized protein C15orf62 mitochondrial	A8K5M9	0.2305	0.4116	0.10	0.4190	0.7573	0.13	0.9891	0.9906	0.00	0.0787	0.1620	0.15	0.1450	0.3196	0.12	0.3637	0.5709	0.06	0.3736	0.4726	-2.55	0.8708	0.9829	-0.33	0.0651	0.1289	2.06
4916	Uncharacterized protein C16orf90	A8MZG2	0.4647	0.5409	-0.76	0.0307	0.5081	-1.09	0.1189	0.2349	-1.44	0.0133	0.0468	-1.38	0.3306	0.4278	-0.86	0.4111	0.6282	-0.74	0.3736	0.4684	-2.55	0.8708	0.9751	-0.33	0.0651	0.1271	2.06
4917	Uncharacterized protein C17orf67	Q0P5P2	0.4108	0.4961	-3.31	0.5752	0.6947	-1.75	0.5661	0.6328	-1.19	0.5388	0.5988	0.89	0.2938	0.3983	-1.88	0.9510	0.9718	0.09	0.1115	0.2746	-1.65	0.4006	0.7857	-0.17	0.6730	0.7263	0.12
4918	Uncharacterized protein C19orf84	I3L1E1	0.4108	0.4960	-3.31	0.5752	0.6945	-1.75	0.5661	0.6327	-1.19	0.5388	0.5987	0.89	0.2938	0.3982	-1.88	0.9510	0.9716	0.09	0.1790	0.3785	-0.65	0.7950	1.2374	0.04	0.1583	0.2222	0.15
4919	Uncharacterized protein C1orf21	Q9H246	0.6507	0.7119	-0.02	0.7122	0.7891	0.06	0.0843	0.1797	0.16	0.2071	0.3416	0.10	0.0580	0.1806	-0.14	0.1201	0.2648	-0.07	0.3736	0.5270	-2.55	0.8708	1.0828	-0.33	0.0651	0.1536	2.06
4920	Uncharacterized protein C4orf36	Q96KX1	0.0108	0.0597	-2.15	0.3144	0.6803	-1.02	0.9281	0.9443	-0.05	0.0241	0.0689	-0.76	0.1614	0.3443	0.60	0.0147	0.0807	-1.22	0.3736	0.6034	-2.55	0.8708	1.2188	-0.33	0.0651	0.1949	2.06
4921	Uncharacterized protein C4orf45	Q96LM5	0.4108	0.4959	-3.31	0.5752	0.6944	-1.75	0.5661	0.6325	-1.19	0.5388	0.5985	0.89	0.2938	0.3981	-1.88	0.9510	0.9714	0.09	0.4959	0.5496	-0.02	0.0032	0.0466	0.43	0.0189	0.1190	0.19
4922	Uncharacterized protein C5orf34	Q96MH7	0.4513	0.5288	-0.12	0.1952	0.6683	-0.50	0.7134	0.7590	-0.04	0.2574	0.4090	-0.14	0.6524	0.7193	-0.05	0.4576	0.6798	-0.08	0.3736	0.4630	-2.55	0.8708	0.9651	-0.33	0.0651	0.1248	2.06
4923	Uncharacterized protein C5orf67	F2Z3F1	0.8958	0.9181	-0.07	0.4513	0.7850	-1.22	0.2662	0.4373	-0.70	0.6937	0.7332	-0.22	0.9660	0.9735	-0.03	0.7932	1.0305	-0.16	0.3736	0.4389	-2.55	0.8708	0.9200	-0.33	0.0651	0.1151	2.06
4924	Uncharacterized protein C6orf106	Q9H6K1	0.0251	0.0956	0.17	0.3124	0.6805	-0.06	0.0372	0.1012	0.15	0.1269	0.2321	-0.09	0.8753	0.9048	-0.01	0.0754	0.1949	0.12	0.3736	0.5462	-2.55	0.8708	1.1174	-0.33	0.0651	0.1632	2.06
4925	Uncharacterized protein C7orf31	Q8N865	0.4108	0.4958	-3.31	0.5752	0.6942	-1.75	0.5661	0.6324	-1.19	0.5388	0.5984	0.89	0.2938	0.3980	-1.88	0.9510	0.9713	0.09	0.0266	0.1418	-0.24	0.6467	1.0859	0.05	0.0286	0.1447	-0.19
4926	Uncharacterized protein C7orf50	Q9BRJ6	0.0271	0.0997	-0.15	0.0765	0.6153	0.23	0.7243	0.7685	-0.02	0.0580	0.1282	-0.11	0.0189	0.0982	-0.33	0.0095	0.0695	-0.19	0.0570	0.1899	0.16	0.1453	0.4073	0.10	0.3688	0.4402	0.05
4927	Uncharacterized protein C9orf153	Q5TBE3	0.2293	0.4101	-0.87	0.2320	0.6625	-1.62	0.1881	0.3341	-0.57	0.0793	0.1630	-0.85	0.8369	0.8735	0.07	0.0453	0.1408	-1.09	0.4925	0.5465	1.25	0.3420	0.7008	1.85	0.2306	0.3008	2.55
4928	Uncharacterized protein encoded by LINC01600	Q96MT4	0.4108	0.4957	-3.31	0.5752	0.6940	-1.75	0.5661	0.6323	-1.19	0.5388	0.5983	0.89	0.2938	0.3979	-1.88	0.9510	0.9711	0.09	0.1126	0.2764	-0.60	0.1138	0.3453	-0.78	0.1891	0.2568	-0.44
4929	Uncharacterized protein ENSP00000382033	A8MXK9	0.1383	0.2827	-1.60	0.4347	0.7709	-1.54	0.4362	0.6469	-0.40	0.0833	0.1690	-0.93	0.8221	0.8612	0.07	0.9910	0.9927	0.02	0.3736	0.4296	-2.55	0.8708	0.9023	-0.33	0.0651	0.1114	2.06
4930	Uncharacterized protein FLJ45252	Q6ZSR9	0.4108	0.4956	-3.31	0.5752	0.6939	-1.75	0.5661	0.6321	-1.19	0.5388	0.5982	0.89	0.2938	0.3978	-1.88	0.9510	0.9709	0.09	0.0490	0.1763	-1.88	0.0011	0.0279	-2.29	0.0002	0.0266	-5.50
4931	Uncharacterized protein KIAA1143	Q96AT1	0.0331	0.1135	-0.41	0.0139	0.4500	-0.59	0.0109	0.0486	-0.58	0.0033	0.0234	-0.81	0.0108	0.0743	-0.73	0.0728	0.1911	-0.80	0.3736	0.5481	-2.55	0.8708	1.1207	-0.33	0.0651	0.1642	2.06
4932	Uncharacterized protein KIAA1257	Q9ULG3	0.0073	0.0501	0.28	0.7934	0.8507	0.01	0.8665	0.8941	0.01	0.0740	0.1544	-0.05	0.2464	0.4623	-0.07	0.0336	0.1181	0.16	0.3736	0.5768	-2.55	0.8708	1.1719	-0.33	0.0651	0.1796	2.06
4933	Uncharacterized protein KIAA1671	Q9BY89	0.0007	0.0194	-0.67	0.4555	0.7878	0.07	0.0186	0.0653	0.26	0.0090	0.0382	0.36	0.4936	0.5830	0.04	0.0334	0.1178	-0.33	0.0036	0.0654	-0.33	0.0085	0.0758	-0.43	0.0146	0.1043	-0.50

Supplemetary Table S2. Overview on all relatively quantified 5180 proteins statistical analysis

			MCF-7																		MDA-MB-231								
			Dai SC20 vs control			Gen SC20 vs control			SSE SC20 vs control			Dai IC20 vs control			Gen IC20 vs control			SSE IC20 vs control			Dai IC20 vs control			Gen IC20 vs control			SSE IC20 vs control		
	**Protein name**	**UniProt**	**p value**	**BH q value**	**log2FC**	**p value**	**BH q value**	**log2FC**	**p value**	**BH q value**	**log2FC**	**p value**	**BH q value**	**log2FC**	**p value**	**BH q value**	**log2FC**	**p value**	**BH q value**	**log2FC**	**p value**	**BH q value**	**log2FC**	**p value**	**BH q value**	**log2FC**	**p value**	**BH q value**	**log2FC**
4934	Uncharacterized protein KIAA1841	Q6NSI8	0.4108	0.4954	-3.31	0.5752	0.6937	-1.75	0.5661	0.6320	-1.19	0.5388	0.5980	0.89	0.2938	0.3977	-1.88	0.9510	0.9707	0.09	0.0111	0.1029	-0.37	0.6602	1.1007	0.03	0.1097	0.1667	-0.16
4935	Uncharacterized protein KIAA1958	Q8N8K9	0.4108	0.4953	-3.31	0.5752	0.6936	-1.75	0.5661	0.6318	-1.19	0.5388	0.5979	0.89	0.2938	0.3976	-1.88	0.9510	0.9705	0.09	0.8244	0.8517	-0.03	0.0555	0.2246	0.36	0.1495	0.2122	0.41
4936	Uncharacterized protein KIAA2026	Q5HYC2	0.3415	0.5490	-0.07	0.2669	0.6628	0.13	0.1355	0.2584	0.13	0.0351	0.0895	0.26	0.7021	0.7634	-0.03	0.7992	1.0365	0.02	0.3736	0.4387	-2.55	0.8708	0.9196	-0.33	0.0651	0.1150	2.06
4937	Unconventional myosin-Ib	O43795	0.1074	0.2359	-0.16	0.6306	0.7269	-0.04	0.3630	0.5605	-0.07	0.3836	0.5684	0.07	0.0297	0.1255	-0.26	0.2410	0.4269	0.10	0.3736	0.4918	-2.55	0.8708	1.0185	-0.33	0.0651	0.1372	2.06
4938	Unconventional myosin-Ic	O00159	0.0842	0.2003	-0.07	0.3463	0.7026	-0.06	0.2223	0.3795	-0.06	0.4695	0.6687	-0.08	0.2887	0.5189	-0.04	0.0480	0.1460	-0.10	0.1248	0.2974	0.21	0.8712	0.9020	-0.01	0.2792	0.3510	-0.07
4939	Unconventional myosin-Id	O94832	0.7857	0.8259	0.01	0.9880	0.9913	0.00	0.1842	0.3289	0.08	0.9198	0.9297	-0.01	0.2294	0.4390	-0.08	0.2553	0.4454	0.07	0.5777	0.6271	0.06	0.0614	0.2366	-0.25	0.0202	0.1220	-0.41
4940	Unconventional myosin-Ie	Q12965	0.6330	0.6950	-0.21	0.8157	0.8682	0.11	0.5160	0.7361	0.29	0.4064	0.5975	-0.39	0.4502	0.5435	-0.39	0.5321	0.7629	-0.28	0.0774	0.2215	-0.13	0.0065	0.0658	0.29	0.2144	0.2832	-0.06
4941	Unconventional myosin-Ih	Q8N1T3	0.4108	0.4952	-3.31	0.5752	0.6934	-1.75	0.5661	0.6317	-1.19	0.5388	0.5978	0.89	0.2938	0.3975	-1.88	0.9510	0.9703	0.09	0.0834	0.2307	0.28	0.1567	0.4286	-0.17	0.1555	0.2189	0.27
4942	Unconventional myosin-IXa	B2RTY4	0.6817	0.7383	-0.03	0.0765	0.6144	0.24	0.5397	0.7618	0.05	0.0499	0.1148	0.18	0.2488	0.4658	-0.08	0.5299	0.7604	0.04	0.3736	0.4584	-2.55	0.8708	0.9565	-0.33	0.0651	0.1229	2.06
4943	Unconventional myosin-IXb	Q13459	0.4108	0.4951	-3.31	0.5752	0.6932	-1.75	0.5661	0.6316	-1.19	0.5388	0.5976	0.89	0.2938	0.3974	-1.88	0.9510	0.9701	0.09	0.0737	0.2153	0.20	0.0374	0.1760	0.17	0.0690	0.1165	0.14
4944	Unconventional myosin-Va	Q9Y4I1	0.2993	0.4982	-0.09	0.1372	0.6508	0.14	0.8144	0.8497	0.02	0.0316	0.0833	0.26	0.4013	0.4977	0.08	0.1613	0.3226	-0.13	0.3736	0.5117	-2.55	0.8708	1.0549	-0.33	0.0651	0.1463	2.06
4945	Unconventional myosin-Vb	Q9ULV0	0.0033	0.0375	0.32	0.0581	0.5867	0.33	0.0055	0.0361	0.33	0.0058	0.0306	0.25	0.0314	0.1299	0.13	0.0181	0.0872	0.22	0.7089	0.7500	-0.01	0.0983	0.3139	-0.11	0.0535	0.2039	-0.07
4946	Unconventional myosin-Vc	Q9NQX4	0.4108	0.4950	-3.31	0.5752	0.6931	-1.75	0.5661	0.6314	-1.19	0.5388	0.5975	0.89	0.2938	0.3973	-1.88	0.9510	0.9699	0.09	0.5252	0.5780	-0.07	0.5657	0.9933	-0.07	0.1102	0.1671	-0.16
4947	Unconventional myosin-VI	Q9UM54	0.8687	0.8960	-0.01	0.4791	0.8045	0.12	0.8886	0.9117	0.01	0.1239	0.2274	0.08	0.2247	0.4335	-0.06	0.0440	0.1381	0.16	0.0213	0.1341	0.30	0.0002	0.0162	0.23	0.0604	0.2196	0.31
4948	Unconventional myosin-VIIb	Q6PIF6	0.4108	0.4949	-3.31	0.5752	0.6929	-1.75	0.5661	0.6313	-1.19	0.5388	0.5974	0.89	0.2938	0.3972	-1.88	0.9510	0.9697	0.09	0.9971	0.9975	0.00	0.1392	0.3957	0.10	0.0076	0.0784	0.28
4949	Unconventional myosin-XVIIIb	Q8IUG5	0.3719	0.5864	-0.11	0.4875	0.8109	0.09	0.2139	0.3684	-0.17	0.0973	0.1903	-0.26	0.9289	0.9463	0.01	0.1409	0.2947	-0.22	0.3736	0.5195	-2.55	0.8708	1.0692	-0.33	0.0651	0.1500	2.06
4950	UPF0160 protein MYG1 mitochondrial	Q9HB07	0.4703	0.5462	0.05	0.2196	0.6602	0.21	0.6806	0.7304	-0.07	0.0701	0.1480	0.15	0.3208	0.4178	-0.06	0.0671	0.1819	0.13	0.6421	0.6868	-0.05	0.2243	0.5339	0.14	0.0400	0.1731	-0.17
4951	UPF0488 protein C8orf33	Q9H7E9	0.1231	0.2604	0.11	0.1814	0.6645	0.11	0.1393	0.2637	0.04	0.0113	0.0426	0.32	0.2351	0.4467	-0.07	0.2298	0.4129	0.15	0.8451	0.8700	-0.02	0.0029	0.0446	-0.40	0.7734	0.8136	-0.04
4952	UPF0489 protein C5orf22	Q49AR2	0.1967	0.3656	-0.16	0.9358	0.9567	-0.01	0.2542	0.4222	0.14	0.3317	0.5049	0.12	0.0335	0.1335	0.35	0.2479	0.4365	-0.34	0.3736	0.4908	-2.55	0.8708	1.0166	-0.33	0.0651	0.1367	2.06
4953	UPF0600 protein C5orf51	A6NDU8	0.5407	0.6109	0.05	0.0103	0.4446	0.29	0.0944	0.1954	-0.18	0.2188	0.3573	-0.09	0.1144	0.2765	0.17	0.7169	0.9569	-0.03	0.0490	0.1765	-0.41	0.0787	0.2751	-0.35	0.6314	0.6883	-0.05
4954	UPF0687 protein C20orf27	Q9GZN8	0.0568	0.1562	-0.14	0.0158	0.4572	-0.23	0.0109	0.0485	-0.26	0.0060	0.0311	-0.34	0.0139	0.0842	-0.28	0.0555	0.1608	-0.42	0.1155	0.2823	0.08	0.3609	0.7286	-0.04	0.1444	0.2062	-0.08
4955	UPF0711 protein C18orf21	Q32NC0	0.4108	0.4948	-3.31	0.5752	0.6928	-1.75	0.5661	0.6312	-1.19	0.5388	0.5973	0.89	0.2938	0.3970	-1.88	0.9510	0.9695	0.09	0.0004	0.0334	1.00	0.0000	0.0000	1.61	0.0216	0.1257	0.41
4956	Up-regulated during skeletal muscle growth protein 5	Q96IX5	0.0175	0.0772	0.14	0.2572	0.6632	0.17	0.0563	0.1357	0.10	0.0573	0.1273	0.11	0.0082	0.0652	0.18	0.0297	0.1106	0.15	0.0741	0.2161	0.17	0.2746	0.6102	0.13	0.0443	0.1840	-0.24
4957	Up-regulator of cell proliferation	Q8TCY9	0.0014	0.0250	-0.43	0.0570	0.5847	-0.46	0.1273	0.2472	-0.14	0.0003	0.0101	-0.87	0.0086	0.0666	-0.30	0.1140	0.2565	0.46	0.3736	0.5299	-2.55	0.8708	1.0880	-0.33	0.0651	0.1550	2.06
4958	Upstream-binding factor 1-like protein 1	P0CB47	0.2073	0.3797	0.27	0.5324	0.8499	-0.11	0.9613	0.9686	0.01	0.0789	0.1624	-0.60	0.2796	0.5057	-0.39	0.2313	0.4149	-0.24	0.3736	0.4947	-2.55	0.8708	1.0238	-0.33	0.0651	0.1385	2.06
4959	Uracil-DNA glycosylase	P13051	0.0014	0.0249	-0.25	0.1998	0.6634	-0.15	0.0034	0.0312	-0.17	0.0093	0.0388	-0.36	0.0001	0.0094	-0.35	0.1348	0.2871	-0.42	0.3736	0.5223	-2.55	0.8708	1.0742	-0.33	0.0651	0.1514	2.06
4960	Uridine 5'-monophosphate synthase	P11172	0.8196	0.8547	-0.01	0.1230	0.6403	-0.17	0.0166	0.0612	-0.30	0.0106	0.0413	-0.32	0.0026	0.0393	-0.51	0.0209	0.0937	-0.28	0.1435	0.3260	-0.34	0.8701	1.3121	0.03	0.2205	0.2900	-0.20
4961	Uridine phosphorylase 1	Q16831	0.4108	0.4946	-3.31	0.5752	0.6926	-1.75	0.5661	0.6310	-1.19	0.5388	0.5971	0.89	0.2938	0.3969	-1.88	0.9510	0.9693	0.09	0.0829	0.2300	0.19	0.5826	1.0144	0.05	0.1646	0.2297	-0.11
4962	Uridine-cytidine kinase 2	Q9BZX2	0.3387	0.5467	-0.24	0.0356	0.5376	-0.10	0.2203	0.3772	-0.17	0.0426	0.1026	-0.42	0.0039	0.0461	-0.20	0.0035	0.0596	-0.24	0.0500	0.1785	-0.17	0.0107	0.0847	-0.19	0.0334	0.1566	-0.26
4963	Uroporphyrinogen decarboxylase	P06132	0.0310	0.1087	-0.26	0.8315	0.8785	-0.04	0.6760	0.7262	-0.03	0.0424	0.1023	-0.18	0.0653	0.1946	-0.16	0.0646	0.1773	-0.17	0.9037	0.9217	-0.01	0.8475	1.2889	0.02	0.0354	0.1609	-0.28
4964	UTP--glucose-1-phosphate uridylyltransferase	Q16851	0.0126	0.0655	-0.19	0.2480	0.6581	0.08	0.9451	0.9571	0.00	0.4179	0.6100	-0.04	0.7872	0.8329	-0.01	0.8545	1.0897	0.01	0.0193	0.1287	0.28	0.3707	0.7428	0.03	0.5913	0.6527	0.04
4965	Utrophin	P46939	0.4108	0.4945	-3.31	0.5752	0.6924	-1.75	0.5661	0.6309	-1.19	0.5388	0.5970	0.89	0.2938	0.3968	-1.88	0.9510	0.9691	0.09	0.2494	0.4851	0.18	0.1761	0.4609	-0.15	0.0599	0.2187	-0.23
4966	UV excision repair protein RAD23 homolog A	P54725	0.6765	0.7337	-0.12	0.5725	0.8850	-0.19	0.0575	0.1373	0.80	0.0170	0.0544	-1.25	0.0247	0.1137	-1.38	0.0279	0.1069	-1.11	0.3736	0.5824	-2.55	0.8708	1.1818	-0.33	0.0651	0.1827	2.06
4967	UV excision repair protein RAD23 homolog B	P54727	0.5138	0.5864	0.04	0.2455	0.6582	0.23	0.0221	0.0725	0.25	0.0472	0.1106	0.18	0.7398	0.7965	-0.02	0.0621	0.1733	0.19	0.1062	0.2665	0.35	0.5439	0.9698	-0.05	0.6566	0.7118	0.03
4968	Uveal autoantigen with coiled-coil domains and ankyrin repeats	Q9BZF9	0.0093	0.0569	0.10	0.1561	0.6633	0.15	0.0746	0.1637	0.13	0.0015	0.0171	0.17	0.0008	0.0222	0.15	0.1591	0.3193	0.05	0.3736	0.5124	-2.55	0.8708	1.0561	-0.33	0.0651	0.1466	2.06
4969	Vacuolar fusion protein CCZ1 homolog B	P86790	0.0064	0.0478	-0.17	0.9323	0.9544	-0.01	0.2276	0.3868	-0.17	0.0932	0.1838	-0.10	0.3029	0.4007	-0.15	0.0100	0.0702	-0.18	0.3736	0.6130	-2.55	0.8708	1.2355	-0.33	0.0651	0.2007	2.06
4970	Vacuolar fusion protein MON1 homolog A	Q86VX9	0.4108	0.4944	-3.31	0.5752	0.6923	-1.75	0.5661	0.6308	-1.19	0.5388	0.5969	0.89	0.2938	0.3967	-1.88	0.9510	0.9690	0.09	0.0019	0.0521	0.92	0.0009	0.0266	0.85	0.0069	0.0772	0.71
4971	Vacuolar fusion protein MON1 homolog B	Q7L1V2	0.0133	0.0671	0.65	0.0800	0.6121	-0.33	0.3927	0.5944	-0.13	0.0650	0.1402	0.36	0.0710	0.2030	0.35	0.0746	0.1943	0.39	0.3736	0.5473	-2.55	0.8708	1.1193	-0.33	0.0651	0.1638	2.06
4972	Vacuolar protein sorting-associated protein 11 homolog	Q9H270	0.0067	0.0488	0.19	0.0223	0.4793	0.10	0.0481	0.1217	0.11	0.1609	0.2801	0.04	0.1496	0.3267	0.07	0.0560	0.1617	0.12	0.1066	0.2670	0.12	0.5811	1.0128	-0.03	0.7681	0.8092	-0.02
4973	Vacuolar protein sorting-associated protein 13A	Q96RL7	0.1747	0.3364	-0.07	0.5791	0.6875	0.07	0.0074	0.0406	0.45	0.0019	0.0193	0.39	0.7958	0.8406	0.01	0.4750	0.6988	0.10	0.0004	0.0384	-1.13	0.0003	0.0219	-1.34	0.0035	0.0592	-1.56
4974	Vacuolar protein sorting-associated protein 13C	Q709C8	0.0827	0.1980	0.12	0.2635	0.6632	0.19	0.0619	0.1446	0.16	0.0339	0.0874	0.16	0.4749	0.5654	-0.04	0.1123	0.2535	0.10	0.0134	0.1097	0.42	0.0860	0.2906	0.20	0.4992	0.5663	0.06
4975	Vacuolar protein sorting-associated protein 13D	Q5THJ4	0.8354	0.8686	0.01	0.1196	0.6354	0.29	0.0253	0.0790	0.22	0.0133	0.0469	0.21	0.7403	0.7967	0.01	0.4840	0.7090	0.03	0.0151	0.1137	0.22	0.5564	0.9820	0.03	0.0592	0.2175	0.08
4976	Vacuolar protein sorting-associated protein 18 homolog	Q9P253	0.1224	0.2594	-0.05	0.2416	0.6597	0.06	0.0636	0.1468	0.07	0.0313	0.0826	0.09	0.0634	0.1905	-0.07	0.0307	0.1125	0.13	0.3736	0.5808	-2.55	0.8708	1.1790	-0.33	0.0651	0.1818	2.06
4977	Vacuolar protein sorting-associated protein 26A	O75436	0.3295	0.5359	0.04	0.8940	0.9249	0.02	0.3505	0.5444	0.04	0.0601	0.1319	0.13	0.0335	0.1338	-0.15	0.0264	0.1042	0.19	0.0594	0.1934	0.69	0.0852	0.2890	0.29	0.0286	0.1448	0.59
4978	Vacuolar protein sorting-associated protein 26B	Q4G0F5	0.9131	0.9331	-0.01	0.5404	0.8553	0.09	0.1748	0.3166	0.08	0.4104	0.6019	0.04	0.2399	0.4530	-0.06	0.2796	0.4755	-0.06	0.0915	0.2429	0.27	0.2794	0.6169	0.11	0.1314	0.1916	0.21
4979	Vacuolar protein sorting-associated protein 29	Q9UBQ0	0.6214	0.6849	0.04	0.8925	0.9237	0.02	0.6232	0.6769	-0.03	0.4066	0.5977	-0.07	0.7836	0.8296	-0.02	0.5311	0.7619	-0.05	0.0047	0.0731	0.49	0.0301	0.1565	0.24	0.2150	0.2839	0.10
4980	Vacuolar protein sorting-associated protein 33B	Q9H267	0.8160	0.8519	0.01	0.0980	0.6330	0.13	0.0477	0.1211	0.15	0.3726	0.5556	0.07	0.4101	0.5054	-0.04	0.8068	1.0443	-0.01	0.2189	0.4407	-0.14	0.5337	0.9579	0.06	0.0789	0.1295	-0.21
4981	Vacuolar protein sorting-associated protein 35	Q96QK1	0.0395	0.1252	0.14	0.1699	0.6622	0.28	0.0142	0.0559	0.22	0.0092	0.0385	0.34	0.1682	0.3550	0.07	0.0080	0.0666	0.31	0.1179	0.2861	0.33	0.9807	0.9862	0.00	0.4692	0.5376	-0.06
4982	Vacuolar protein sorting-associated protein 37A	Q8NEZ2	0.2653	0.4570	0.08	0.2066	0.6594	-0.09	0.0391	0.1048	0.16	0.0388	0.0964	-0.16	0.2975	0.3953	-0.06	0.5114	0.7383	0.03	0.7685	0.8032	-0.08	0.0046	0.0542	0.70	0.2118	0.2805	0.18
4983	Vacuolar protein sorting-associated protein 45	Q9NRW7	0.0888	0.2078	-0.08	0.1606	0.6639	0.21	0.0237	0.0755	0.12	0.0126	0.0454	0.20	0.0241	0.1123	0.12	0.2186	0.4003	0.17	0.3736	0.4984	-2.55	0.8708	1.0306	-0.33	0.0651	0.1402	2.06
4984	Vacuolar protein sorting-associated protein 4A	Q9UN37	0.0027	0.0348	-0.20	0.4590	0.7912	-0.10	0.0007	0.0200	-0.29	0.0011	0.0162	-0.26	0.0007	0.0213	-0.29	0.0977	0.2306	-0.15	0.5554	0.6062	0.08	0.1205	0.3587	0.24	0.3618	0.4333	-0.10
4985	Vacuolar protein sorting-associated protein 4B	O75351	0.9820	0.9877	0.00	0.4254	0.7617	0.09	0.1850	0.3301	-0.14	0.3217	0.4927	-0.13	0.0203	0.1021	0.09	0.9767	0.9814	0.00	0.0769	0.2208	0.31	0.9195	0.9409	0.01	0.2448	0.3150	0.08
4986	Vacuolar protein sorting-associated protein 8 homolog	Q8N3P4	0.0039	0.0393	0.18	0.2923	0.6697	-0.11	0.0140	0.0555	0.32	0.0243	0.0693	0.19	0.0486	0.1620	0.14	0.0451	0.1405	0.54	0.3736	0.5652	-2.55	0.8708	1.1513	-0.33	0.0651	0.1732	2.06
4987	Vacuolar protein sorting-associated protein VTA1 homolog	Q9NP79	0.0008	0.0201	0.23	0.2343	0.6614	0.10	0.0032	0.0304	0.17	0.0017	0.0183	0.20	0.0725	0.2058	-0.05	0.0179	0.0871	0.24	0.0688	0.2067	-0.24	0.0088	0.0773	-0.28	0.0228	0.1278	0.16
4988	Vacuolar protein-sorting-associated protein 25	Q9BRG1	0.9333	0.9492	0.01	0.0248	0.4829	0.16	0.0339	0.0955	0.24	0.0017	0.0181	0.32	0.0154	0.0883	0.37	0.4475	0.6696	0.14	0.3525	0.6283	-0.13	0.1292	0.3768	-0.23	0.8248	0.8584	0.02
4989	Vacuolar protein-sorting-associated protein 36	Q86VN1	0.0305	0.1075	-0.22	0.1714	0.6611	-0.07	0.0041	0.0322	0.13	0.0391	0.0970	-0.34	0.1586	0.3407	0.04	0.2680	0.4611	0.25	0.0886	0.2397	-0.25	0.0475	0.2033	-0.17	0.6339	0.6904	0.05
4990	Vacuole membrane protein 1	Q96GC9	0.0761	0.1881	-0.13	0.5789	0.6875	-0.05	0.9552	0.9645	0.00	0.4229	0.6148	0.04	0.0777	0.2143	-0.13	0.3066	0.5050	0.09	0.3736	0.4804	-2.55	0.8708	0.9975	-0.33	0.0651	0.1322	2.06
4991	Valine--tRNA ligase	P26640	0.4680	0.5440	0.03	0.4220	0.7595	0.15	0.0115	0.0500	0.17	0.0175	0.0556	0.15	0.3097	0.4074	0.04	0.0188	0.0883	0.19	0.0805	0.2258	0.22	0.2618	0.5896	0.11	0.0742	0.1237	-0.14
4992	Vang-like protein 1	Q8TAA9	0.5822	0.6498	-0.03	0.5704	0.8838	-0.07	0.4705	0.6879	0.05	0.1611	0.2803	-0.08	0.2999	0.3979	-0.05	0.0312	0.1133	-0.27	0.3736	0.5798	-2.55	0.8708	1.1771	-0.33	0.0651	0.1812	2.06
4993	Vang-like protein 2	Q9ULK5	0.0053	0.0441	-1.20	0.3685	0.7173	-0.65	0.2341	0.3947	-0.16	0.3166	0.4868	0.20	0.6359	0.7056	0.06	0.0045	0.0593	-0.97	0.3736	0.6318	-2.55	0.8708	1.2681	-0.33	0.0651	0.2126	2.06
4994	Vascular endothelial growth factor C	P49767	0.4108	0.4943	-3.31	0.5752	0.6921	-1.75	0.5661	0.6306	-1.19	0.5388	0.5967	0.89	0.2938	0.3966	-1.88	0.9510	0.9688	0.09	0.0000	0.0000	1.45	0.0000	0.0000	2.01	0.0018	0.0504	0.89
4995	Vascular endothelial growth factor receptor 3	P35916	0.4108	0.4942	-3.31	0.5752	0.6919	-1.75	0.5661	0.6305	-1.19	0.5388	0.5966	0.89	0.2938	0.3965	-1.88	0.9510	0.9686	0.09	0.3428	0.6144	-0.12	0.0550	0.2238	0.25	0.1436	0.2053	-0.57
4996	Vasculin-like protein 1	Q9HC44	0.4108	0.4941	-3.31	0.5752	0.6918	-1.75	0.5661	0.6304	-1.19	0.5388	0.5965	0.89	0.2938	0.3964	-1.88	0.9510	0.9684	0.09	0.0020	0.0531	-0.39	0.0003	0.0202	-0.83	0.0313	0.1505	-1.13
4997	Vasodilator-stimulated phosphoprotein	P50552	0.0033	0.0374	-0.25	0.5690	0.8825	0.06	0.4893	0.7094	0.02	0.0763	0.1580	0.08	0.3279	0.4249	0.03	0.0609	0.1713	-0.09	0.1326	0.3094	0.24	0.7312	1.1759	-0.03	0.2895	0.3614	0.10
4998	Very large A-kinase anchor protein	Q68DQ2	0.0103	0.0588	0.28	0.1163	0.6375	0.29	0.0101	0.0469	0.45	0.0074	0.0350	0.31	0.5201	0.6080	0.04	0.0088	0.0683	0.35	0.0597	0.1939	0.55	0.4288	0.8251	0.09	0.2679	0.3395	0.10
4999	Very long-chain specific acyl-CoA dehydrogenase_ mitochondrial	P49748	0.0045	0.0423	-0.18	0.6460	0.7384	0.07	0.1149	0.2281	-0.09	0.1935	0.3235	-0.09	0.0033	0.0425	0.24	0.1304	0.2811	0.13	0.0459	0.1719	0.43	0.0783	0.2746	0.17	0.1126	0.1699	0.09
5000	Very-long-chain (3R)-3-hydroxyacyl-CoA dehydratase 3	Q9P035	0.1929	0.3601	0.04	0.3687	0.7172	0.10	0.2116	0.3661	-0.06	0.0258	0.0721	0.11	0.1115	0.2723	-0.06	0.1251	0.2728	0.15	0.8096	0.8391	-0.02	0.1296	0.3765	-0.02	0.1128	0.1701	-0.12
5001	Very-long-chain 3-oxoacyl-CoA reductase	Q53GQ0	0.0204	0.0844	-0.20	0.1899	0.6701	-0.22	0.1545	0.2861	-0.20	0.0086	0.0374	-0.37	0.1001	0.2533	-0.17	0.0103	0.0697	-0.42	0.1253	0.2977	0.11	0.4726	0.8841	0.04	0.3310	0.4022	0.06
5002	Very-long-chain enoyl-CoA reductase	Q9NZ01	0.4163	0.4940	-0.04	0.6190	0.7173	0.07	0.0689	0.1554	0.11	0.0362	0.0914	0.14	0.0561	0.1770	-0.12	0.0100	0.0707	0.29	0.3736	0.6134	-2.55	0.8708	1.2362	-0.33	0.0651	0.2010	2.06
5003	Vesicle transport protein SEC20	Q12981	0.0004	0.0156	0.44	0.2455	0.6586	0.19	0.0609	0.1431	0.11	0.0047	0.0277	0.24	0.0007	0.0210	0.19	0.2479	0.4366	0.11	0.3736	0.4909	-2.55	0.8708	1.0168	-0.33	0.0651	0.1368	2.06
5004	Vesicle transport through interaction with t-SNAREs homolog 1B	Q9UEU0	0.0823	0.1976	-0.21	0.9823	0.9874	0.00	0.0495	0.1241	-0.24	0.1854	0.3125	-0.13	0.2047	0.4064	-0.12	0.0999	0.2336	-0.32	0.3736	0.5340	-2.55	0.8708	1.0954	-0.33	0.0651	0.1571	2.06
5005	Vesicle-associated membrane protein 2	P63027	0.4108	0.4940	-3.31	0.5752	0.6916	-1.75	0.5661	0.6302	-1.19	0.5388	0.5964	0.89	0.2938	0.3963	-1.88	0.9510	0.9682	0.09	0.0066	0.0836	0.42	0.2652	0.5949	0.09	0.0142	0.1030	0.36
5006	Vesicle-associated membrane protein 3	Q15836	0.2131	0.3872	-0.15	0.6860	0.7708	0.05	0.0492	0.1235	0.30	0.1228	0.2265	0.21	0.1168	0.2800	0.22	0.4527	0.6750	0.09	0.5881	0.6370	0.09	0.7873	1.2302	-0.02	0.1056	0.1620	0.40
5007	Vesicle-associated membrane protein 8	Q9BV40	0.0043	0.0412	-0.50	0.6640	0.7525	0.07	0.9601	0.9678	0.00	0.7131	0.7514	0.03	0.2889	0.5191	-0.06	0.9395	1.1758	-0.01	0.3736	0.4321	-2.55	0.8708	0.9070	-0.33	0.0651	0.1124	2.06
5008	Vesicle-associated membrane protein-associated protein A	Q9P0L0	0.0161	0.0739	0.19	0.3532	0.7050	0.06	0.0128	0.0530	0.22	0.0172	0.0548	0.16	0.0657	0.1950	-0.10	0.0217	0.0949	0.19	0.1407	0.3215	-0.19	0.0010	0.0264	-0.52	0.0257	0.1368	-0.34
5009	Vesicle-associated membrane protein-associated protein B/C	O95292	0.6650	0.7244	0.03	0.3232	0.6853	0.20	0.0812	0.1748	0.21	0.0397	0.0979	0.27	0.9333	0.9492	0.01	0.1202	0.2648	0.17	0.0472	0.1740	0.41	0.0468	0.2019	0.22	0.6551	0.7105	0.02
5010	Vesicle-fusing ATPase	P46459	0.0645	0.1681	-0.11	0.8079	0.8616	0.04	0.0555	0.1345	0.11	0.7963	0.8246	0.01	0.1384	0.3098	-0.07	0.7982	1.0357	-0.01	0.0683	0.2057	0.27	0.9590	0.9710	0.00	0.3750	0.4468	-0.06

Supplemetary Table S2. Overview on all relatively quantified 5180 proteins statistical analysis

			MCF-7																		MDA-MB-231								
			Dai SC20 vs control			Gen SC20 vs control			SSE SC20 vs control			Dai IC20 vs control			Gen IC20 vs control			SSE IC20 vs control			Dai IC20 vs control			Gen IC20 vs control			SSE IC20 vs control		
	**Protein name**	**UniProt**	**p value**	**BH q value**	**log2FC**	**p value**	**BH q value**	**log2FC**	**p value**	**BH q value**	**log2FC**	**p value**	**BH q value**	**log2FC**	**p value**	**BH q value**	**log2FC**	**p value**	**BH q value**	**log2FC**	**p value**	**BH q value**	**log2FC**	**p value**	**BH q value**	**log2FC**	**p value**	**BH q value**	**log2FC**
5011	Vesicle-trafficking protein SEC22a	Q96IW7	0.0422	0.1301	-0.24	0.1275	0.6412	-0.19	0.5212	0.7421	0.08	0.1351	0.2437	-0.11	0.7517	0.8058	0.03	0.1632	0.3256	0.22	0.3736	0.5113	-2.55	0.8708	1.0542	-0.33	0.0651	0.1461	2.06
5012	Vesicle-trafficking protein SEC22b	O75396	0.0261	0.0977	0.12	0.2176	0.6607	0.21	0.0018	0.0240	0.26	0.0018	0.0189	0.26	0.1121	0.2729	0.07	0.0057	0.0614	0.34	0.6461	0.6908	0.05	0.9089	0.9325	0.01	0.3515	0.4229	-0.10
5013	Vesicular integral-membrane protein VIP36	Q12907	0.0005	0.0173	0.31	0.0994	0.6365	0.20	0.0011	0.0222	0.20	0.0001	0.0096	0.29	0.0054	0.0551	0.11	0.0430	0.1368	0.28	0.4795	0.5340	-0.04	0.0851	0.2889	-0.13	0.0567	0.2119	-0.16
5014	Vigilin	Q00341	0.9851	0.9889	0.00	0.2811	0.6652	0.21	0.0056	0.0363	0.27	0.0049	0.0282	0.29	0.1542	0.3336	0.08	0.0324	0.1160	0.20	0.0761	0.2194	0.39	0.8435	1.2870	-0.01	0.8982	0.9190	0.01
5015	Vimentin	P08670	0.2734	0.4665	-0.14	0.4273	0.7627	-0.11	0.6529	0.7044	-0.06	0.1040	0.1995	0.25	0.2655	0.4872	-0.14	0.4694	0.6917	0.20	0.0046	0.0720	0.87	0.0074	0.0710	0.48	0.0008	0.0351	2.04
5016	Vinculin	P18206	0.0011	0.0223	0.16	0.3667	0.7154	0.17	0.0059	0.0371	0.35	0.0001	0.0084	0.36	0.0029	0.0415	0.12	0.0001	0.0324	0.43	0.1086	0.2695	0.28	0.6611	1.1011	0.05	0.2776	0.3495	-0.09
5017	Vitamin D-binding protein	P02774	0.0354	0.1175	-0.26	0.0200	0.4731	0.14	0.2919	0.4722	0.12	0.0377	0.0941	0.11	0.0040	0.0469	0.35	0.2002	0.3768	-0.06	0.3736	0.5024	-2.55	0.8708	1.0379	-0.33	0.0651	0.1420	2.06
5018	Vitamin K epoxide reductase complex subunit 1	Q9BQB6	0.5798	0.6473	0.03	0.3155	0.6810	0.15	0.6821	0.7314	0.05	0.0504	0.1156	0.14	0.3082	0.4058	0.05	0.1996	0.3760	0.09	0.3506	0.6256	-0.09	0.2501	0.5730	-0.14	0.0231	0.1291	-0.31
5019	Vitronectin	P04004	0.4108	0.4938	-3.31	0.5752	0.6915	-1.75	0.5661	0.6301	-1.19	0.5388	0.5962	0.89	0.2938	0.3962	-1.88	0.9510	0.9680	0.09	0.3652	0.6456	-0.47	0.8428	1.2871	-0.09	0.6303	0.6875	-0.22
5020	Voltage-dependent anion-selective channel protein 1	P21796	0.0056	0.0450	0.38	0.2146	0.6609	0.19	0.0006	0.0192	0.51	0.0011	0.0163	0.21	0.0787	0.2164	0.08	0.0056	0.0617	0.47	0.0774	0.2218	0.08	0.0297	0.1556	0.15	0.0122	0.0950	-0.13
5021	Voltage-dependent anion-selective channel protein 2	P45880	0.0040	0.0398	0.18	0.7894	0.8484	-0.02	0.0092	0.0448	0.21	0.0054	0.0295	0.04	0.5143	0.6031	-0.01	0.0955	0.2277	0.23	0.3897	0.4456	0.10	0.3367	0.6943	0.09	0.0274	0.1416	-0.26
5022	Voltage-dependent anion-selective channel protein 3	Q9Y277	0.5644	0.6324	-0.03	0.1247	0.6421	-0.15	0.5118	0.7328	0.02	0.0332	0.0862	-0.12	0.0291	0.1242	-0.15	0.3965	0.6100	0.05	0.4023	0.4582	0.06	0.0580	0.2300	0.17	0.3125	0.3840	-0.14
5023	Voltage-dependent calcium channel subunit alpha-2/delta-3	Q8IZS8	0.0013	0.0242	0.19	0.3253	0.6858	0.18	0.0036	0.0314	0.24	0.0008	0.0147	0.25	0.0313	0.1297	0.09	0.0015	0.0505	0.25	0.4526	0.5075	-0.10	0.0449	0.1966	-0.35	0.0300	0.1476	-0.43
5024	Voltage-dependent L-type calcium channel subunit alpha-1C	Q13936	0.3291	0.5359	-0.06	0.5907	0.6959	0.05	0.8508	0.8809	0.01	0.3106	0.4788	-0.06	0.4969	0.5861	0.05	0.3484	0.5534	0.10	0.3736	0.4744	-2.55	0.8708	0.9864	-0.33	0.0651	0.1296	2.06
5025	Voltage-dependent L-type calcium channel subunit beta-1	Q02641	0.4108	0.4937	-3.31	0.5752	0.6913	-1.75	0.5661	0.6299	-1.19	0.5388	0.5961	0.89	0.2938	0.3961	-1.88	0.9510	0.9678	0.09	0.1629	0.3568	-0.20	0.0636	0.2421	-0.14	0.1772	0.2433	-0.05
5026	Voltage-dependent T-type calcium channel subunit alpha-1I	Q9P0X4	0.0060	0.0464	0.29	0.0618	0.5906	0.22	0.0597	0.1415	0.48	0.0030	0.0222	0.50	0.0222	0.1077	0.26	0.0007	0.0412	0.62	0.3736	0.6507	-2.55	0.8708	1.3007	-0.33	0.0651	0.2253	2.06
5027	von Willebrand factor A domain-containing protein 3B	Q502W6	0.0231	0.0909	0.39	0.5078	0.8298	0.11	0.0132	0.0536	0.52	0.0174	0.0553	0.45	0.0077	0.0635	0.53	0.0162	0.0845	0.45	0.0390	0.1638	0.19	0.0485	0.2061	-0.20	0.5079	0.5743	0.02
5028	VPS9 domain-containing protein 1	Q9Y2B5	0.0577	0.1581	0.34	0.0168	0.4604	0.54	0.0154	0.0584	0.55	0.0610	0.1335	0.34	0.0397	0.1461	0.40	0.1150	0.2580	0.24	0.0741	0.2160	0.30	0.0477	0.2039	-0.26	0.0049	0.0675	-0.27
5029	V-set and transmembrane domain-containing protein 4	Q8IW00	0.2393	0.4238	-0.15	0.4580	0.7900	-0.23	0.0367	0.1006	0.35	0.0815	0.1663	0.27	0.0139	0.0840	0.48	0.1157	0.2588	0.27	0.3736	0.5290	-2.55	0.8708	1.0864	-0.33	0.0651	0.1546	2.06
5030	V-type proton ATPase 116 kDa subunit a isoform 1	Q93050	0.0926	0.2139	1.06	0.2021	0.6597	0.23	0.0377	0.1022	0.10	0.0030	0.0223	0.50	0.2005	0.4015	-0.05	0.0675	0.1820	0.68	0.3736	0.5503	-2.55	0.8708	1.1246	-0.33	0.0651	0.1653	2.06
5031	V-type proton ATPase 116 kDa subunit a isoform 3	Q13488	0.0630	0.1661	-0.15	0.0922	0.6309	-0.19	0.7176	0.7628	-0.03	0.5506	0.6016	-0.05	0.4077	0.5033	-0.05	0.1446	0.2999	-0.11	0.3736	0.5181	-2.55	0.8708	1.0666	-0.33	0.0651	0.1493	2.06
5032	V-type proton ATPase catalytic subunit A	P38606	0.7283	0.7787	-0.02	0.4768	0.8035	0.07	0.5110	0.7320	0.05	0.0429	0.1032	0.16	0.3057	0.4033	0.06	0.0171	0.0853	0.25	0.1800	0.3803	-0.03	0.0089	0.0774	0.10	0.0144	0.1033	0.10
5033	V-type proton ATPase subunit B brain isoform	P21281	0.6760	0.7336	0.02	0.1494	0.6603	0.17	0.4659	0.6831	-0.04	0.0612	0.1337	0.16	0.2720	0.4956	0.06	0.7083	0.9500	-0.02	0.3927	0.4486	0.04	0.0718	0.2601	-0.14	0.2097	0.2785	-0.06
5034	V-type proton ATPase subunit E 1	P36543	0.7416	0.7906	0.04	0.4673	0.7976	-0.25	0.5556	0.7795	-0.09	0.5025	0.7064	0.09	0.9165	0.9375	0.01	0.6534	0.8905	0.07	0.3874	0.4433	-0.11	0.0098	0.0814	-0.39	0.0504	0.1969	-0.19
5035	V-type proton ATPase subunit E 2	Q96A05	0.1198	0.2554	-0.23	0.4003	0.7440	0.12	0.8029	0.8394	-0.03	0.9190	0.9290	0.01	0.2496	0.4666	0.15	0.2766	0.4718	0.14	0.3736	0.4860	-2.55	0.8708	1.0078	-0.33	0.0651	0.1346	2.06
5036	V-type proton ATPase subunit G 1	O75348	0.0007	0.0193	-0.18	0.4397	0.7760	0.05	0.5637	0.7879	-0.01	0.0063	0.0317	0.11	0.0056	0.0556	0.14	0.0562	0.1621	0.10	0.2681	0.5109	0.14	0.0139	0.0981	-0.53	0.0401	0.1732	0.32
5037	V-type proton ATPase subunit H	Q9UI12	0.1422	0.2886	-0.17	0.6798	0.7658	-0.04	0.7384	0.7812	0.03	0.6350	0.6803	-0.05	0.6513	0.7189	0.04	0.0782	0.2000	-0.23	0.0267	0.1421	0.49	0.0059	0.0616	0.23	0.0116	0.0939	0.20
5038	WAP four-disulfide core domain protein 8	Q8IUA0	0.4108	0.4936	-3.31	0.5752	0.6911	-1.75	0.5661	0.6298	-1.19	0.5388	0.5960	0.89	0.2938	0.3960	-1.88	0.9510	0.9676	0.09	0.0087	0.0929	0.73	0.0000	0.0000	1.97	0.0001	0.0305	1.65
5039	WAP_ Kazal_ immunoglobulin_ Kunitz and NTR domain-containing protein 2	Q8TEU8	0.8161	0.8518	-0.04	0.2882	0.6686	-0.47	0.3674	0.5647	0.16	0.2002	0.3327	-0.25	0.4341	0.5281	-0.15	0.5138	0.7412	-0.11	0.3285	0.5966	-0.18	0.5460	0.9713	0.07	0.0124	0.0960	0.35
5040	WASH complex subunit 1	A8K0Z3	0.4108	0.4935	-3.31	0.5752	0.6910	-1.75	0.5661	0.6297	-1.19	0.5388	0.5959	0.89	0.2938	0.3959	-1.88	0.9510	0.9674	0.09	0.0820	0.2286	0.25	0.0498	0.2099	0.57	0.0157	0.1074	0.41
5041	WASH complex subunit 2A	Q641Q2	0.0189	0.0809	-0.18	0.0422	0.5707	-0.06	0.9188	0.9358	-0.01	0.8143	0.8398	-0.02	0.1644	0.3493	-0.03	0.1392	0.2926	-0.08	0.2368	0.4668	0.19	0.2507	0.5736	0.18	0.1162	0.1740	0.30
5042	WASH complex subunit 2C	Q9Y4E1	0.0223	0.0888	-0.53	0.3649	0.7133	-0.57	0.0880	0.1857	-0.33	0.0709	0.1491	-0.76	0.0601	0.1845	-0.38	0.0503	0.1506	-0.91	0.3736	0.5605	-2.55	0.8708	1.1428	-0.33	0.0651	0.1707	2.06
5043	WASH complex subunit 2D	Q5SRD0	0.4108	0.4934	-3.31	0.5752	0.6908	-1.75	0.5661	0.6295	-1.19	0.5388	0.5957	0.89	0.2938	0.3958	-1.88	0.9510	0.9672	0.09	0.4759	0.5304	-0.13	0.0887	0.2951	0.12	0.0085	0.0823	0.41
5044	WASH complex subunit 4	Q2M389	0.4108	0.4933	-3.31	0.5752	0.6907	-1.75	0.5661	0.6294	-1.19	0.5388	0.5956	0.89	0.2938	0.3957	-1.88	0.9510	0.9671	0.09	0.3367	0.6075	0.26	0.0002	0.0150	2.71	0.0172	0.1121	0.75
5045	WASH complex subunit 5	Q12768	0.8558	0.8857	0.02	0.2338	0.6614	0.13	0.0125	0.0524	0.41	0.0242	0.0691	0.35	0.2264	0.4356	0.12	0.0806	0.2042	0.30	0.3736	0.5435	-2.55	0.8708	1.1124	-0.33	0.0651	0.1618	2.06
5046	WD repeat and FYVE domain-containing protein 2	Q96P53	0.0881	0.2066	-0.14	0.3208	0.6841	0.12	0.1139	0.2265	-0.15	0.0194	0.0592	0.23	0.0167	0.0923	0.28	0.0133	0.0767	0.25	0.3736	0.6063	-2.55	0.8708	1.2238	-0.33	0.0651	0.1966	2.06
5047	WD repeat and HMG-box DNA-binding protein 1	O75717	0.4108	0.4932	-3.31	0.5752	0.6905	-1.75	0.5661	0.6293	-1.19	0.5388	0.5955	0.89	0.2938	0.3956	-1.88	0.9510	0.9669	0.09	0.2483	0.4843	0.08	0.5302	0.9533	0.05	0.7660	0.8075	-0.02
5048	WD repeat and SOCS box-containing protein 2	Q9NYS7	0.2096	0.3822	0.10	0.1646	0.6610	0.20	0.1447	0.2714	0.22	0.0438	0.1047	0.21	0.6089	0.6831	-0.04	0.7858	1.0240	0.02	0.3736	0.4398	-2.55	0.8708	0.9217	-0.33	0.0651	0.1154	2.06
5049	WD repeat-containing protein 1	O75083	0.9522	0.9643	0.00	0.3500	0.7046	0.08	0.0829	0.1774	0.10	0.1909	0.3201	0.06	0.1261	0.2932	0.08	0.0624	0.1737	0.12	0.5428	0.5939	0.06	0.7328	1.1759	-0.02	0.2571	0.3287	-0.04
5050	WD repeat-containing protein 19	Q8NEZ3	0.4108	0.4930	-3.31	0.5752	0.6903	-1.75	0.5661	0.6291	-1.19	0.5388	0.5953	0.89	0.2938	0.3955	-1.88	0.9510	0.9667	0.09	0.0007	0.0394	0.53	0.0123	0.0926	0.27	0.0087	0.0835	0.32
5051	WD repeat-containing protein 44	Q5JSH3	0.0900	0.2100	0.15	0.3414	0.6995	0.09	0.5757	0.6333	-0.04	0.4556	0.6525	-0.06	0.7748	0.8238	-0.02	0.2905	0.4875	0.08	0.2826	0.5319	-0.17	0.9605	0.9719	0.01	0.1982	0.2667	0.18
5052	WD repeat-containing protein 46	O15213	0.4108	0.4929	-3.31	0.5752	0.6902	-1.75	0.5661	0.6290	-1.19	0.5388	0.5952	0.89	0.2938	0.3954	-1.88	0.9510	0.9665	0.09	0.0437	0.1682	-9.14	0.3355	0.6929	-2.02	0.1629	0.2276	1.64
5053	WD repeat-containing protein 5	P61964	0.3864	0.6031	-0.15	0.6579	0.7477	-0.05	0.8628	0.8907	0.02	0.4184	0.6102	0.09	0.7548	0.8078	0.03	0.5175	0.7452	0.10	0.3736	0.4590	-2.55	0.8708	0.9577	-0.33	0.0651	0.1232	2.06
5054	WD repeat-containing protein 55	Q9H6Y2	0.4108	0.4928	-3.31	0.5752	0.6900	-1.75	0.5661	0.6289	-1.19	0.5388	0.5951	0.89	0.2938	0.3953	-1.88	0.9510	0.9663	0.09	0.7198	0.7597	0.03	0.6405	1.0786	0.03	0.2877	0.3596	0.10
5055	WD repeat-containing protein 61	Q9GZS3	0.0280	0.1016	-0.77	0.5206	0.8411	-0.36	0.6418	0.6945	-0.08	0.1222	0.2260	-0.60	0.5381	0.6226	-0.13	0.0154	0.0826	-0.87	0.4756	0.5302	-0.05	0.7084	1.1521	-0.02	0.3021	0.3733	0.08
5056	WD repeat-containing protein 62	O43379	0.8084	0.8456	0.01	0.0193	0.4650	-0.20	0.0125	0.0526	-0.13	0.0005	0.0122	-0.29	0.0105	0.0734	-0.26	0.4861	0.7111	-0.06	0.3736	0.4614	-2.55	0.8708	0.9622	-0.33	0.0651	0.1242	2.06
5057	WD repeat-containing protein 70	Q9NW82	0.0439	0.1331	0.14	0.2262	0.6594	0.34	0.0047	0.0335	0.30	0.8095	0.8363	0.02	0.0513	0.1675	-0.12	0.0604	0.1702	0.13	0.0194	0.1292	-0.53	0.0018	0.0349	-1.11	0.7510	0.7946	-0.05
5058	WD repeat-containing protein 81	Q562E7	0.2010	0.3717	-0.07	0.0301	0.5062	0.14	0.1216	0.2391	0.08	0.0500	0.1150	0.12	0.1115	0.2722	0.08	0.3246	0.5269	0.04	0.0348	0.1546	0.29	0.1800	0.4674	0.11	0.6702	0.7237	0.02
5059	WD repeat-containing protein 87	Q6ZQQ6	0.0182	0.0790	0.19	0.1951	0.6684	0.23	0.0062	0.0381	0.26	0.0119	0.0438	0.28	0.6239	0.6955	-0.02	0.0164	0.0847	0.21	0.5322	0.5844	-0.03	0.0659	0.2475	-0.11	0.1683	0.2335	-0.15
5060	Wilms tumor protein 1-interacting protein	A6NIX2	0.4108	0.4927	-3.31	0.5752	0.6899	-1.75	0.5661	0.6287	-1.19	0.5388	0.5950	0.89	0.2938	0.3952	-1.88	0.9510	0.9661	0.09	0.0031	0.0604	0.45	0.0358	0.1725	0.26	0.0409	0.1757	0.53
5061	Wiskott-Aldrich syndrome protein	P42768	0.0369	0.1207	-0.09	0.3218	0.6837	0.21	0.0234	0.0752	0.20	0.0094	0.0390	0.20	0.0005	0.0179	0.17	0.1908	0.3643	0.02	0.3736	0.5046	-2.55	0.8708	1.0420	-0.33	0.0651	0.1430	2.06
5062	Wiskott-Aldrich syndrome protein family member 2	Q9Y6W5	0.0036	0.0380	-0.40	0.2146	0.6613	-0.18	0.0286	0.0857	-0.30	0.0024	0.0208	-0.41	0.0006	0.0198	-0.69	0.0003	0.0432	-0.59	0.8698	0.8924	0.02	0.0580	0.2297	-0.22	0.7859	0.8246	-0.02
5063	WW domain-binding protein 11	Q9Y2W2	0.4108	0.4926	-3.31	0.5752	0.6897	-1.75	0.5661	0.6286	-1.19	0.5388	0.5948	0.89	0.2938	0.3951	-1.88	0.9510	0.9659	0.09	0.0136	0.1101	0.51	0.0595	0.2331	0.32	0.0038	0.0602	0.95
5064	WW domain-containing oxidoreductase	Q9NZC7	0.4108	0.4925	-3.31	0.5752	0.6895	-1.75	0.5661	0.6285	-1.19	0.5388	0.5947	0.89	0.2938	0.3950	-1.88	0.9510	0.9657	0.09	0.2346	0.4635	0.16	0.0384	0.1784	-0.23	0.0208	0.1236	-0.31
5065	X antigen family member 2	Q96GT9	0.0544	0.1526	-0.20	0.0658	0.5948	-0.19	0.2122	0.3668	-0.10	0.0197	0.0597	-0.28	0.0601	0.1846	-0.20	0.0214	0.0943	-0.30	0.3736	0.5922	-2.55	0.8708	1.1990	-0.33	0.0651	0.1883	2.06
5066	Xaa-Pro aminopeptidase 1	Q9NQW7	0.0021	0.0311	0.24	0.3733	0.7207	0.15	0.0135	0.0544	0.18	0.0014	0.0169	0.21	0.1257	0.2929	0.05	0.0041	0.0600	0.29	0.5229	0.5758	0.05	0.5919	1.0241	-0.05	0.0484	0.1918	-0.27
5067	Xaa-Pro aminopeptidase 3	Q9NQH7	0.2946	0.4924	0.08	0.2036	0.6600	0.19	0.0721	0.1604	0.17	0.0186	0.0577	0.28	0.3020	0.4002	0.08	0.0793	0.2018	0.17	0.3736	0.5442	-2.55	0.8708	1.1138	-0.33	0.0651	0.1622	2.06
5068	Xaa-Pro dipeptidase	P12955	0.4108	0.4924	-3.31	0.5752	0.6894	-1.75	0.5661	0.6283	-1.19	0.5388	0.5946	0.89	0.2938	0.3949	-1.88	0.9510	0.9655	0.09	0.3812	0.4370	0.04	0.0001	0.0106	0.44	0.1458	0.2079	-0.19
5069	Xin actin-binding repeat-containing protein 2	A4UGR9	0.0099	0.0578	0.23	0.2010	0.6615	0.20	0.0031	0.0302	0.35	0.0066	0.0327	0.30	0.0599	0.1845	0.13	0.0118	0.0739	0.34	0.0258	0.1402	0.41	0.0808	0.2794	0.22	0.1954	0.2634	-0.13
5070	X-linked interleukin-1 receptor accessory protein-like 2	Q9NP60	0.4108	0.4922	-3.31	0.5752	0.6892	-1.75	0.5661	0.6282	-1.19	0.5388	0.5945	0.89	0.2938	0.3948	-1.88	0.9510	0.9653	0.09	0.3611	0.6397	0.16	0.6041	1.0372	0.08	0.0578	0.2140	0.54
5071	X-ray repair cross-complementing protein 5	P13010	0.0023	0.0326	0.24	0.1070	0.6313	0.29	0.0012	0.0230	0.29	0.0003	0.0113	0.35	0.4528	0.5458	-0.02	0.0130	0.0765	0.25	0.0332	0.1525	0.44	0.9307	0.9496	-0.01	0.1246	0.1840	0.13
5072	X-ray repair cross-complementing protein 6	P12956	0.0053	0.0441	0.19	0.1935	0.6682	0.26	0.0033	0.0307	0.27	0.0027	0.0220	0.23	0.1743	0.3633	-0.05	0.0289	0.1089	0.13	0.0610	0.1952	0.30	0.2019	0.5004	0.12	0.2945	0.3659	0.09
5073	Xyloside xylosyltransferase 1	Q8NBI6	0.2663	0.4581	0.12	0.0305	0.5096	0.18	0.0252	0.0789	0.19	0.3356	0.5093	0.05	0.0138	0.0839	0.49	0.0384	0.1275	0.17	0.3736	0.5709	-2.55	0.8708	1.1614	-0.33	0.0651	0.1763	2.06
5074	Xylosyltransferase 1	Q86Y38	0.0562	0.1551	-0.31	0.0986	0.6353	-0.15	0.0019	0.0247	-0.81	0.0072	0.0345	-0.81	0.0595	0.1838	-0.21	0.2152	0.3963	-0.26	0.8721	0.8944	0.01	0.0035	0.0481	0.53	0.1583	0.2222	-0.30
5075	Y-box-binding protein 2	Q9Y2T7	0.7201	0.7715	-0.02	0.9551	0.9703	0.00	0.2365	0.3979	0.09	0.0114	0.0428	0.26	0.0735	0.2074	0.15	0.0210	0.0938	0.23	0.3736	0.5927	-2.55	0.8708	1.2000	-0.33	0.0651	0.1886	2.06
5076	Y-box-binding protein 3	P16989	0.4025	0.6227	0.03	0.7244	0.7987	0.05	0.0148	0.0573	0.23	0.0279	0.0762	0.09	0.0642	0.1921	-0.06	0.4815	0.7062	0.02	0.0109	0.1023	0.56	0.0482	0.2053	0.23	0.0005	0.0285	0.30
5077	YEATS domain-containing protein 2	Q9ULM3	0.4108	0.4921	-3.31	0.5752	0.6891	-1.75	0.5661	0.6281	-1.19	0.5388	0.5943	0.89	0.2938	0.3947	-1.88	0.9510	0.9652	0.09	0.0009	0.0402	-1.30	0.0008	0.0257	-1.34	0.0229	0.1281	-0.57
5078	YjeF N-terminal domain-containing protein 3	A6XGL0	0.0028	0.0352	0.33	0.1171	0.6325	0.23	0.0015	0.0229	0.46	0.0043	0.0268	0.20	0.4091	0.5046	0.02	0.0131	0.0768	0.13	0.0397	0.1641	0.28	0.5288	0.9524	-0.06	0.0143	0.1027	-0.26
5079	YTH domain-containing family protein 1	Q9BYJ9	0.2033	0.3749	0.14	0.0227	0.4761	0.16	0.0236	0.0756	0.19	0.1099	0.2085	0.12	0.1736	0.3622	0.06	0.0636	0.1759	0.10	0.0103	0.1020	-0.52	0.0050	0.0569	-0.45	0.0128	0.0987	-0.49
5080	YTH domain-containing family protein 2	Q9Y5A9	0.8534	0.8834	0.01	0.5748	0.8862	0.07	0.8307	0.8635	0.01	0.7492	0.7839	-0.01	0.1112	0.2717	-0.05	0.0719	0.1892	-0.08	0.0601	0.1940	0.43	0.7727	1.2166	0.04	0.8190	0.8536	0.03
5081	ZBED6 C-terminal-like protein	Q96FA7	0.0246	0.0946	0.24	0.1902	0.6698	-0.27	0.0113	0.0493	0.57	0.0002	0.0093	0.94	0.0216	0.1061	0.30	0.0003	0.0361	1.59	0.3736	0.6534	-2.55	0.8708	1.3052	-0.33	0.0651	0.2271	2.06
5082	Zinc finger and BTB domain-containing protein 18	Q99592	0.0744	0.1849	-0.20	0.8560	0.8967	-0.02	0.0465	0.1192	0.28	0.7306	0.7675	-0.03	0.1296	0.2974	-0.15	0.2150	0.3963	-0.13	0.0018	0.0524	1.06	0.0019	0.0361	0.86	0.0172	0.1118	0.66
5083	Zinc finger and BTB domain-containing protein 47	Q9UFB7	0.0666	0.1710	0.39	0.0242	0.4840	0.55	0.0402	0.1070	0.51	0.0104	0.0409	0.66	0.0057	0.0558	0.89	0.1118	0.2527	0.47	0.3736	0.5306	-2.55	0.8708	1.0893	-0.33	0.0651	0.1554	2.06
5084	Zinc finger and SCAN domain-containing protein 23	Q3MJ62	0.0005	0.0172	0.33	0.0457	0.5636	0.40	0.0026	0.0282	0.30	0.0017	0.0184	0.25	0.0000	0.0000	0.65	0.0013	0.0514	0.40	0.3736	0.6477	-2.55	0.8708	1.2954	-0.33	0.0651	0.2232	2.06
5085	Zinc finger and SCAN domain-containing protein 30	Q86W11	0.1003	0.2254	-9.16	0.0925	0.6288	-7.61	0.0389	0.1045	-7.05	0.0052	0.0290	-4.97	0.1130	0.2742	0.95	0.0269	0.1050	-5.77	0.3736	0.5838	-2.55	0.8708	1.1842	-0.33	0.0651	0.1835	2.06
5086	Zinc finger C2HC domain-containing protein 1B	Q5TFG8	0.0004	0.0155	-0.83	0.1086	0.6300	-0.68	0.0015	0.0231	-0.44	0.0294	0.0793	-1.06	0.0750	0.2103	-0.34	0.0479	0.1458	-0.56	0.3736	0.5622	-2.55	0.8708	1.1460	-0.33	0.0651	0.1716	2.06
5087	Zinc finger CCCH domain-containing protein 13	Q5T200	0.4108	0.4920	-3.31	0.5752	0.6889	-1.75	0.5661	0.6279	-1.19	0.5388	0.5942	0.89	0.2938	0.3946	-1.88	0.9510	0.9650	0.09	0.0782	0.2220	0.40	0.0908	0.3000	-1.10	0.0611	0.2212	0.35
5088	Zinc finger CCCH domain-containing protein 15	Q8WU90	0.3268	0.5328	-0.06	0.4700	0.7995	0.09	0.0556	0.1347	-0.15	0.8147	0.8400	0.00	0.0918	0.2388	-0.12	0.3316	0.5351	-0.16	0.3529	0.6288	0.10	0.5381	0.9642	0.04	0.2999	0.3711	0.08
5089	Zinc finger CCCH domain-containing protein 18	Q86VM9	0.0129	0.0662	-0.26	0.4690	0.7989	-0.06	0.0470	0.1201	-0.17	0.0265	0.0734	-0.23	0.1725	0.3612	-0.09	0.0364	0.1236	-0.23	0.0942	0.2467	0.34	0.7236	1.1691	-0.02	0.9481	0.9592	0.01

Supplemetary Table S2. Overview on all relatively quantified 5180 proteins statistical analysis

			MCF-7																		MDA-MB-231								
			Dai SC20 vs control			Gen SC20 vs control			SSE SC20 vs control			Dai IC20 vs control			Gen IC20 vs control			SSE IC20 vs control			Dai IC20 vs control			Gen IC20 vs control			SSE IC20 vs control		
	**Protein name**	**UniProt**	**p value**	**BH q value**	**log2FC**	**p value**	**BH q value**	**log2FC**	**p value**	**BH q value**	**log2FC**	**p value**	**BH q value**	**log2FC**	**p value**	**BH q value**	**log2FC**	**p value**	**BH q value**	**log2FC**	**p value**	**BH q value**	**log2FC**	**p value**	**BH q value**	**log2FC**	**p value**	**BH q value**	**log2FC**
5090	Zinc finger CCCH domain-containing protein 4	Q9UPT8	0.0112	0.0607	-0.29	0.6911	0.7739	-0.04	0.2083	0.3618	-0.09	0.0178	0.0562	-0.27	0.0087	0.0669	-0.41	0.0113	0.0731	-0.40	0.3736	0.6103	-2.55	0.8708	1.2308	-0.33	0.0651	0.1991	2.06
5091	Zinc finger CCCH domain-containing protein 6	P61129	0.0145	0.0697	-0.40	0.0951	0.6308	-0.57	0.8125	0.8480	-0.07	0.0961	0.1883	-0.64	0.0906	0.2371	-0.67	0.6080	0.8453	-0.34	0.7132	0.7540	-0.07	0.4531	0.8569	-0.15	0.4381	0.5087	0.31
5092	Zinc finger CCCH domain-containing protein 7A	Q8IWR0	0.4652	0.5414	0.20	0.9983	0.9985	0.00	0.0014	0.0220	2.69	0.3466	0.5222	-0.27	0.4524	0.5457	-0.20	0.3309	0.5341	-0.26	0.3736	0.4764	-2.55	0.8708	0.9901	-0.33	0.0651	0.1305	2.06
5093	Zinc finger CCCH domain-containing protein 7B	Q9UGR2	0.0093	0.0568	-0.10	0.7046	0.7826	0.06	0.4950	0.7166	0.04	0.0051	0.0287	-0.12	0.0023	0.0375	-0.16	0.0742	0.1937	0.30	0.1059	0.2662	0.11	0.0109	0.0854	0.15	0.0082	0.0812	0.22
5094	Zinc finger CCCH domain-containing protein 8	Q8N5P1	0.4108	0.4919	-3.31	0.5752	0.6888	-1.75	0.5661	0.6278	-1.19	0.5388	0.5941	0.89	0.2938	0.3945	-1.88	0.9510	0.9648	0.09	0.7159	0.7563	0.76	0.3414	0.6998	3.02	0.1531	0.2162	3.95
5095	Zinc finger CCCH-type antiviral protein 1	Q7Z2W4	0.0027	0.0347	-0.12	0.6440	0.7375	-0.03	0.1913	0.3383	0.05	0.4247	0.6167	-0.01	0.0998	0.2528	-0.05	0.1273	0.2763	-0.04	0.0303	0.1474	0.17	0.7016	1.1439	0.01	0.0140	0.1023	0.21
5096	Zinc finger CCCH-type antiviral protein 1-like	Q96H79	0.4108	0.4918	-3.31	0.5752	0.6886	-1.75	0.5661	0.6277	-1.19	0.5388	0.5940	0.89	0.2938	0.3944	-1.88	0.9510	0.9646	0.09	0.5529	0.6041	0.08	0.0310	0.1588	0.24	0.0418	0.1775	0.32
5097	Zinc finger CCHC domain-containing protein 7	Q8N3Z6	0.4108	0.4917	-3.31	0.5752	0.6884	-1.75	0.5661	0.6275	-1.19	0.5388	0.5938	0.89	0.2938	0.3943	-1.88	0.9510	0.9644	0.09	0.3657	0.6463	0.23	0.0694	0.2542	0.26	0.0034	0.0579	0.45
5098	Zinc finger CCHC domain-containing protein 8	Q6NZY4	0.1832	0.3476	0.10	0.5720	0.8850	-0.10	0.5413	0.7632	0.04	0.0150	0.0503	-0.28	0.0125	0.0797	-0.34	0.6074	0.8447	0.03	0.3736	0.4519	-2.55	0.8708	0.9445	-0.33	0.0651	0.1203	2.06
5099	Zinc finger FYVE domain-containing protein 16	Q7Z3T8	0.0041	0.0400	0.17	0.4349	0.7707	0.05	0.8195	0.8538	0.01	0.0986	0.1920	-0.05	0.0061	0.0569	0.13	0.2461	0.4343	0.14	0.3736	0.4912	-2.55	0.8708	1.0173	-0.33	0.0651	0.1369	2.06
5100	Zinc finger FYVE domain-containing protein 21	Q9BQ24	0.2139	0.3881	0.07	0.3002	0.6732	-0.04	0.0608	0.1430	0.10	0.1936	0.3236	-0.06	0.0698	0.2012	0.11	0.0064	0.0623	0.26	0.3736	0.6237	-2.55	0.8708	1.2540	-0.33	0.0651	0.2074	2.06
5101	Zinc finger FYVE domain-containing protein 9	O95405	0.0075	0.0509	0.31	0.0013	0.2322	0.37	0.9651	0.9719	0.00	0.1006	0.1949	-0.09	0.0074	0.0625	0.30	0.0636	0.1760	-0.11	0.3736	0.5536	-2.55	0.8708	1.1305	-0.33	0.0651	0.1670	2.06
5102	Zinc finger HIT domain-containing protein 2	Q9UHR6	0.4108	0.4916	-3.31	0.5752	0.6883	-1.75	0.5661	0.6274	-1.19	0.5388	0.5937	0.89	0.2938	0.3942	-1.88	0.9510	0.9642	0.09	0.0022	0.0553	6.27	0.0009	0.0265	7.13	0.0053	0.0706	7.38
5103	Zinc finger homeobox protein 3	Q15911	0.4108	0.4914	-3.31	0.5752	0.6881	-1.75	0.5661	0.6273	-1.19	0.5388	0.5936	0.89	0.2938	0.3941	-1.88	0.9510	0.9640	0.09	0.0026	0.0561	-0.47	0.0092	0.0788	-0.53	0.0006	0.0302	-0.72
5104	Zinc finger imprinted 3	Q96PE6	0.4108	0.4913	-3.31	0.5752	0.6880	-1.75	0.5661	0.6271	-1.19	0.5388	0.5934	0.89	0.2938	0.3940	-1.88	0.9510	0.9638	0.09	0.1168	0.2843	0.30	0.3142	0.6627	-0.17	0.1623	0.2268	-0.25
5105	Zinc finger matrin-type protein 2	Q96NC0	0.4108	0.4912	-3.31	0.5752	0.6878	-1.75	0.5661	0.6270	-1.19	0.5388	0.5933	0.89	0.2938	0.3939	-1.88	0.9510	0.9637	0.09	0.1677	0.3623	0.24	0.0675	0.2501	-0.37	0.5074	0.5739	-0.09
5106	Zinc finger MYM-type protein 2	Q9UBW7	0.0867	0.2048	-0.30	0.1169	0.6341	-0.26	0.2331	0.3936	0.17	0.1821	0.3080	-0.20	0.7068	0.7677	0.05	0.2292	0.4124	0.18	0.3736	0.4952	-2.55	0.8708	1.0247	-0.33	0.0651	0.1387	2.06
5107	Zinc finger MYND domain-containing protein 15	Q9H091	0.0169	0.0759	-1.14	0.0358	0.5391	-0.67	0.0103	0.0474	-1.04	0.0116	0.0432	-1.23	0.5243	0.6113	-0.15	0.1976	0.3732	-0.70	0.3736	0.5028	-2.55	0.8708	1.0386	-0.33	0.0651	0.1422	2.06
5108	Zinc finger protein 114	Q8NC26	0.0091	0.0567	-0.33	0.2218	0.6584	-0.20	0.0633	0.1466	-0.17	0.0405	0.0994	-0.20	0.0188	0.0979	-0.33	0.0054	0.0620	-0.56	0.3736	0.6283	-2.55	0.8708	1.2621	-0.33	0.0651	0.2104	2.06
5109	Zinc finger protein 121	P58317	0.1115	0.2425	0.17	0.0445	0.5664	0.31	0.1320	0.2539	0.15	0.0468	0.1100	0.23	0.0139	0.0841	0.35	0.2861	0.4824	-0.13	0.3736	0.4842	-2.55	0.8708	1.0044	-0.33	0.0651	0.1338	2.06
5110	Zinc finger protein 136	P52737	0.0029	0.0360	0.36	0.1688	0.6614	0.29	0.0035	0.0314	0.45	0.0002	0.0105	0.49	0.0012	0.0278	0.30	0.0009	0.0471	0.48	0.3736	0.6501	-2.55	0.8708	1.2996	-0.33	0.0651	0.2248	2.06
5111	Zinc finger protein 174	Q15697	0.4108	0.4911	-3.31	0.5752	0.6876	-1.75	0.5661	0.6268	-1.19	0.5388	0.5932	0.89	0.2938	0.3938	-1.88	0.9510	0.9635	0.09	0.0166	0.1198	0.45	0.0444	0.1952	0.26	0.0129	0.0988	0.59
5112	Zinc finger protein 185	O15231	0.6740	0.7318	0.01	0.0406	0.5669	0.12	0.0042	0.0325	0.25	0.0730	0.1526	0.12	0.0533	0.1714	0.10	0.6654	0.9037	-0.02	0.0035	0.0650	0.41	0.0098	0.0817	0.30	0.5818	0.6441	0.03
5113	Zinc finger protein 2	Q9BSG1	0.4108	0.4910	-3.31	0.5752	0.6875	-1.75	0.5661	0.6267	-1.19	0.5388	0.5931	0.89	0.2938	0.3937	-1.88	0.9510	0.9633	0.09	0.0133	0.1090	1.81	0.1110	0.3404	0.65	0.0063	0.0748	2.53
5114	Zinc finger protein 248	Q8NDW4	0.4108	0.4909	-3.31	0.5752	0.6873	-1.75	0.5661	0.6266	-1.19	0.5388	0.5929	0.89	0.2938	0.3936	-1.88	0.9510	0.9631	0.09	0.1497	0.3357	-0.13	0.0012	0.0283	-0.57	0.0024	0.0543	-0.85
5115	Zinc finger protein 263	O14978	0.4108	0.4908	-3.31	0.5752	0.6872	-1.75	0.5661	0.6264	-1.19	0.5388	0.5928	0.89	0.2938	0.3935	-1.88	0.9510	0.9629	0.09	0.0188	0.1271	-0.21	0.6244	1.0612	-0.03	0.0947	0.1491	-0.09
5116	Zinc finger protein 276	Q8N554	0.8281	0.8620	-0.03	0.1111	0.6324	-0.40	0.6413	0.6941	-0.07	0.3804	0.5653	-0.13	0.1641	0.3488	-0.24	0.2803	0.4762	0.16	0.3736	0.4853	-2.55	0.8708	1.0064	-0.33	0.0651	0.1343	2.06
5117	Zinc finger protein 318	Q5VUA4	0.0034	0.0375	-6.12	0.2891	0.6691	-2.27	0.1553	0.2873	-2.87	0.0337	0.0872	-2.87	0.8744	0.9044	0.05	0.1191	0.2634	-2.75	0.3736	0.5275	-2.55	0.8708	1.0835	-0.33	0.0651	0.1538	2.06
5118	Zinc finger protein 324B	Q6AW86	0.4108	0.4906	-3.31	0.5752	0.6870	-1.75	0.5661	0.6263	-1.19	0.5388	0.5927	0.89	0.2938	0.3934	-1.88	0.9510	0.9627	0.09	0.5989	0.6474	-0.14	0.1095	0.3374	0.46	0.0758	0.1258	0.59
5119	Zinc finger protein 330	Q9Y3S2	0.0238	0.0924	-0.21	0.5167	0.8390	-0.14	0.0070	0.0395	0.24	0.0743	0.1549	0.10	0.0379	0.1422	0.24	0.0675	0.1821	-0.11	0.3736	0.5504	-2.55	0.8708	1.1249	-0.33	0.0651	0.1654	2.06
5120	Zinc finger protein 354C	Q86Y25	0.0109	0.0598	-0.54	0.4924	0.8152	-0.09	0.5316	0.7528	0.05	0.8529	0.8730	-0.01	0.0691	0.2001	-0.19	0.0224	0.0959	-0.39	0.3736	0.5898	-2.55	0.8708	1.1949	-0.33	0.0651	0.1869	2.06
5121	Zinc finger protein 382	Q96SR6	0.0002	0.0118	0.27	0.0745	0.6126	0.12	0.1792	0.3225	-0.07	0.1564	0.2741	-0.12	0.0073	0.0622	0.22	0.9715	0.9770	0.00	0.2773	0.5240	-0.10	0.1066	0.3310	-0.16	0.4358	0.5067	-0.08
5122	Zinc finger protein 383	Q8NA42	0.4108	0.4905	-3.31	0.5752	0.6868	-1.75	0.5661	0.6262	-1.19	0.5388	0.5926	0.89	0.2938	0.3933	-1.88	0.9510	0.9625	0.09	0.2026	0.4155	-0.27	0.6601	1.1009	-0.05	0.2521	0.3234	0.14
5123	Zinc finger protein 394	Q53GI3	0.4108	0.4904	-3.31	0.5752	0.6867	-1.75	0.5661	0.6260	-1.19	0.5388	0.5924	0.89	0.2938	0.3932	-1.88	0.9510	0.9623	0.09	0.3620	0.6409	-1.89	0.0591	0.2321	-6.09	0.6542	0.7100	0.79
5124	Zinc finger protein 397	Q8NF99	0.0086	0.0547	-2.38	0.2348	0.6614	-1.29	0.0734	0.1620	-0.77	0.0081	0.0363	-1.63	0.4527	0.5459	-0.24	0.0075	0.0650	-1.83	0.3736	0.6203	-2.55	0.8708	1.2481	-0.33	0.0651	0.2052	2.06
5125	Zinc finger protein 40	P15822	0.0405	0.1268	-0.17	0.0240	0.4875	0.59	0.0038	0.0323	-0.43	0.0006	0.0128	-0.67	0.0008	0.0228	-0.47	0.0020	0.0534	1.81	0.0624	0.1967	-0.85	0.0815	0.2805	-0.73	0.1276	0.1873	-0.58
5126	Zinc finger protein 42 homolog	Q96MM3	0.4108	0.4903	-3.31	0.5752	0.6865	-1.75	0.5661	0.6259	-1.19	0.5388	0.5923	0.89	0.2938	0.3930	-1.88	0.9510	0.9621	0.09	0.0165	0.1195	-0.22	0.6873	1.1292	-0.02	0.5627	0.6266	-0.04
5127	Zinc finger protein 428	Q96B54	0.0396	0.1254	-0.29	0.2736	0.6641	-0.14	0.4571	0.6723	-0.07	0.0819	0.1668	-0.54	0.3104	0.4079	-0.12	0.5602	0.7957	-0.17	0.0160	0.1179	0.39	0.0355	0.1715	0.23	0.5301	0.5964	0.11
5128	Zinc finger protein 454	Q8N9F8	0.9643	0.9737	0.01	0.5158	0.8389	-0.08	0.6740	0.7243	0.05	0.1035	0.1989	0.27	0.2561	0.4760	0.27	0.2127	0.3941	-0.16	0.3736	0.5001	-2.55	0.8708	1.0336	-0.33	0.0651	0.1409	2.06
5129	Zinc finger protein 468	Q5VIY5	0.4108	0.4902	-3.31	0.5752	0.6864	-1.75	0.5661	0.6258	-1.19	0.5388	0.5922	0.89	0.2938	0.3929	-1.88	0.9510	0.9620	0.09	0.0019	0.0518	0.31	0.0006	0.0243	0.71	0.0043	0.0649	0.42
5130	Zinc finger protein 469	Q96JG9	0.1738	0.3352	0.07	0.2022	0.6596	0.26	0.0252	0.0789	0.29	0.0028	0.0217	0.39	0.4884	0.5783	0.03	0.0170	0.0853	0.24	0.0240	0.1371	0.42	0.0029	0.0448	0.34	0.0068	0.0769	0.38
5131	Zinc finger protein 484	Q5JVG2	0.0461	0.1371	-0.48	0.2047	0.6594	-0.64	0.2510	0.4178	-0.26	0.1526	0.2689	-0.55	0.1249	0.2917	-0.58	0.0171	0.0854	-0.89	0.3736	0.5982	-2.55	0.8708	1.2096	-0.33	0.0651	0.1918	2.06
5132	Zinc finger protein 496	Q96IT1	0.0474	0.1398	-0.24	0.7132	0.7901	0.06	0.5319	0.7530	0.05	0.7638	0.7964	0.02	0.5009	0.5904	-0.05	0.2528	0.4430	-0.09	0.3736	0.4901	-2.55	0.8708	1.0152	-0.33	0.0651	0.1364	2.06
5133	Zinc finger protein 511	Q8NB15	0.4108	0.4901	-3.31	0.5752	0.6862	-1.75	0.5661	0.6256	-1.19	0.5388	0.5921	0.89	0.2938	0.3928	-1.88	0.9510	0.9618	0.09	0.0277	0.1442	0.31	0.0181	0.1150	0.22	0.0411	0.1761	0.26
5134	Zinc finger protein 518A	Q6AHZ1	0.0371	0.1211	-0.34	0.0299	0.5045	-0.35	0.1678	0.3066	-0.13	0.0441	0.1053	-0.24	0.0392	0.1445	-0.24	0.0954	0.2278	-0.20	0.3736	0.5359	-2.55	0.8708	1.0988	-0.33	0.0651	0.1580	2.06
5135	Zinc finger protein 541	Q9H0D2	0.5861	0.6532	0.07	0.1159	0.6360	-0.23	0.8173	0.8520	0.04	0.8963	0.9109	-0.01	0.1709	0.3591	-0.19	0.5744	0.8090	0.06	0.3736	0.4542	-2.55	0.8708	0.9486	-0.33	0.0651	0.1212	2.06
5136	Zinc finger protein 552	Q9H707	0.4108	0.4900	-3.31	0.5752	0.6861	-1.75	0.5661	0.6255	-1.19	0.5388	0.5919	0.89	0.2938	0.3927	-1.88	0.9510	0.9616	0.09	0.6279	0.6741	0.11	0.0450	0.1967	-0.32	0.0003	0.0229	-0.57
5137	Zinc finger protein 57	Q68EA5	0.0131	0.0666	0.20	0.5721	0.8846	-0.07	0.0203	0.0685	0.14	0.3027	0.4682	-0.04	0.0161	0.0906	-0.16	0.2408	0.4267	0.05	0.3736	0.4919	-2.55	0.8708	1.0187	-0.33	0.0651	0.1372	2.06
5138	Zinc finger protein 57 homolog	Q9NU63	0.3073	0.5079	0.06	0.9797	0.9862	0.00	0.1440	0.2703	-0.09	0.0418	0.1017	-0.17	0.0066	0.0590	-0.26	0.7222	0.9624	-0.03	0.3736	0.4442	-2.55	0.8708	0.9299	-0.33	0.0651	0.1171	2.06
5139	Zinc finger protein 578	Q96N58	0.4108	0.4899	-3.31	0.5752	0.6859	-1.75	0.5661	0.6254	-1.19	0.5388	0.5918	0.89	0.2938	0.3926	-1.88	0.9510	0.9614	0.09	0.5033	0.5567	0.07	0.0644	0.2442	0.18	0.2154	0.2842	0.10
5140	Zinc finger protein 616	Q08AN1	0.0962	0.2188	0.08	0.4035	0.7478	0.13	0.0414	0.1094	0.33	0.0161	0.0527	0.05	0.0037	0.0454	0.13	0.0046	0.0603	0.08	0.3736	0.6314	-2.55	0.8708	1.2674	-0.33	0.0651	0.2124	2.06
5141	Zinc finger protein 620	Q6ZNG0	0.0107	0.0596	0.19	0.0056	0.3868	0.42	0.0314	0.0906	0.38	0.0059	0.0308	0.39	0.0104	0.0731	0.29	0.5510	0.7852	0.03	0.3736	0.4569	-2.55	0.8708	0.9536	-0.33	0.0651	0.1223	2.06
5142	Zinc finger protein 648	Q5T619	0.0467	0.1383	-0.63	0.5678	0.8822	-0.11	0.0712	0.1590	-0.55	0.0322	0.0845	-0.86	0.7709	0.8213	-0.05	0.0279	0.1067	-0.93	0.3736	0.5822	-2.55	0.8708	1.1814	-0.33	0.0651	0.1826	2.06
5143	Zinc finger protein 674	Q2M3X9	0.6328	0.6949	0.02	0.3078	0.6776	0.15	0.4137	0.6201	0.08	0.0289	0.0783	0.20	0.4142	0.5089	0.04	0.1036	0.2390	0.10	0.3736	0.5325	-2.55	0.8708	1.0927	-0.33	0.0651	0.1563	2.06
5144	Zinc finger protein 678	Q5SXM1	0.4775	0.5528	1.56	0.2202	0.6597	0.96	0.5082	0.7298	-0.77	0.2173	0.3553	3.07	0.5972	0.6734	0.34	0.0055	0.0613	4.40	0.3736	0.6275	-2.55	0.8708	1.2607	-0.33	0.0651	0.2098	2.06
5145	Zinc finger protein 705A	Q6ZN79	0.4108	0.4897	-3.31	0.5752	0.6857	-1.75	0.5661	0.6252	-1.19	0.5388	0.5917	0.89	0.2938	0.3925	-1.88	0.9510	0.9612	0.09	0.0035	0.0641	0.99	0.0014	0.0305	1.12	0.0113	0.0934	0.64
5146	Zinc finger protein 763	Q0D2J5	0.0860	0.2036	0.14	0.9073	0.9355	-0.01	0.0913	0.1902	0.12	0.0642	0.1389	0.14	0.0765	0.2127	-0.18	0.0058	0.0618	0.23	0.3736	0.6265	-2.55	0.8708	1.2589	-0.33	0.0651	0.2092	2.06
5147	Zinc finger protein 781	Q8N8C0	0.7001	0.7541	-0.09	0.1325	0.6487	0.51	0.3698	0.5676	0.24	0.8713	0.8895	0.04	0.1322	0.3006	0.51	0.4570	0.6793	0.20	0.3736	0.4632	-2.55	0.8708	0.9655	-0.33	0.0651	0.1249	2.06
5148	Zinc finger protein 789	Q5FWF6	0.4108	0.4896	-3.31	0.5752	0.6856	-1.75	0.5661	0.6251	-1.19	0.5388	0.5916	0.89	0.2938	0.3924	-1.88	0.9510	0.9610	0.09	0.0484	0.1759	1.21	0.0059	0.0615	2.11	0.0032	0.0568	2.83
5149	Zinc finger protein 791	Q3KP31	0.5421	0.6123	-0.09	0.2154	0.6602	0.21	0.9344	0.9491	-0.02	0.6540	0.6973	0.07	0.4686	0.5599	0.11	0.0683	0.1831	-0.44	0.3736	0.5498	-2.55	0.8708	1.1238	-0.33	0.0651	0.1651	2.06
5150	Zinc finger protein 8	P17098	0.4108	0.4895	-3.31	0.5752	0.6854	-1.75	0.5661	0.6250	-1.19	0.5388	0.5914	0.89	0.2938	0.3923	-1.88	0.9510	0.9608	0.09	0.0929	0.2445	0.21	0.9382	0.9546	0.01	0.1866	0.2540	-0.14
5151	Zinc finger protein 816	Q0VGE8	0.0218	0.0877	-0.70	0.1327	0.6473	-1.07	0.5664	0.6242	0.05	0.0049	0.0280	-0.60	0.2016	0.4023	0.24	0.1648	0.3281	-0.75	0.3736	0.5106	-2.55	0.8708	1.0529	-0.33	0.0651	0.1458	2.06
5152	Zinc finger protein 830	Q96NB3	0.0117	0.0624	0.28	0.1630	0.6627	0.07	0.0144	0.0565	0.19	0.0559	0.1251	-0.25	0.3571	0.4550	-0.11	0.0996	0.2332	0.34	0.3736	0.5343	-2.55	0.8708	1.0959	-0.33	0.0651	0.1572	2.06
5153	Zinc finger protein 837	Q96EG3	0.1230	0.2604	-0.39	0.5422	0.8571	-0.08	0.0388	0.1043	-0.54	0.0059	0.0308	-0.21	0.3215	0.4185	-0.07	0.0422	0.1354	0.10	0.3736	0.5684	-2.55	0.8708	1.1569	-0.33	0.0651	0.1749	2.06
5154	Zinc finger protein 891	A8MT65	0.3170	0.5196	-0.83	0.1527	0.6592	3.61	0.0636	0.1469	2.05	0.2773	0.4351	1.88	0.7422	0.7981	-0.34	0.7875	1.0260	-0.21	0.3736	0.4397	-2.55	0.8708	0.9215	-0.33	0.0651	0.1154	2.06
5155	Zinc finger protein Aiolos	Q9UKT9	0.3069	0.5079	0.12	0.2068	0.6588	-0.52	0.0323	0.0922	-0.26	0.0106	0.0412	-0.55	0.0281	0.1215	-0.47	0.4505	0.6731	-0.05	0.3736	0.4641	-2.55	0.8708	0.9671	-0.33	0.0651	0.1253	2.06
5156	Zinc finger protein basonuclin-2	Q6ZN30	0.4108	0.4894	-3.31	0.5752	0.6853	-1.75	0.5661	0.6248	-1.19	0.5388	0.5913	0.89	0.2938	0.3922	-1.88	0.9510	0.9606	0.09	0.0037	0.0652	0.93	0.0059	0.0620	0.81	0.0591	0.2177	0.50
5157	Zinc finger protein castor homolog 1	Q86V15	0.0276	0.1005	0.15	0.0842	0.6222	0.19	0.1054	0.2126	0.08	0.0009	0.0145	0.28	0.0331	0.1329	0.11	0.0097	0.0704	0.15	0.3736	0.6151	-2.55	0.8708	1.2392	-0.33	0.0651	0.2020	2.06
5158	Zinc finger protein DZIP1	Q86YF9	0.4108	0.4893	-3.31	0.5752	0.6851	-1.75	0.5661	0.6247	-1.19	0.5388	0.5912	0.89	0.2938	0.3921	-1.88	0.9510	0.9605	0.09	0.0071	0.0859	0.85	0.0045	0.0541	0.75	0.0178	0.1143	0.60
5159	Zinc finger protein Eos	Q9H2S9	0.5726	0.6401	0.14	0.6282	0.7251	0.17	0.0471	0.1202	0.46	0.6332	0.6792	-0.05	0.5026	0.5918	0.12	0.1471	0.3032	-0.36	0.3736	0.5173	-2.55	0.8708	1.0651	-0.33	0.0651	0.1489	2.06
5160	Zinc finger protein GLI1	P08151	0.3372	0.5447	-0.06	0.0369	0.5508	0.23	0.2272	0.3864	-0.02	0.0338	0.0872	0.04	0.0987	0.2509	0.12	0.2535	0.4438	-0.16	0.3736	0.4898	-2.55	0.8708	1.0148	-0.33	0.0651	0.1363	2.06
5161	Zinc finger protein Helios	Q9UKS7	0.0002	0.0116	-4.03	0.1950	0.6694	-2.27	0.0030	0.0300	-3.49	0.0029	0.0222	-4.23	0.1203	0.2848	-0.58	0.0040	0.0595	-3.58	0.2493	0.4855	0.11	0.0312	0.1594	0.17	0.1030	0.1591	0.11
5162	Zinc finger protein neuro-d4	Q92782	0.6485	0.7096	0.03	0.4865	0.8103	-0.06	0.4527	0.6669	0.04	0.2854	0.4456	-0.08	0.4551	0.5477	-0.05	0.6167	0.8530	-0.02	0.3736	0.4509	-2.55	0.8708	0.9425	-0.33	0.0651	0.1198	2.06
5163	Zinc finger protein Rlf	Q13129	0.0016	0.0268	0.30	0.1563	0.6620	0.23	0.0016	0.0236	0.45	0.0006	0.0132	0.50	0.0013	0.0285	0.41	0.0106	0.0708	0.43	0.1098	0.2717	0.31	0.0398	0.1820	0.22	0.3865	0.4578	0.05
5164	Zinc finger protein ubi-d4	Q92785	0.4108	0.4892	-3.31	0.5752	0.6850	-1.75	0.5661	0.6246	-1.19	0.5388	0.5911	0.89	0.2938	0.3920	-1.88	0.9510	0.9603	0.09	0.7371	0.7756	-0.05	0.4457	0.8472	-0.30	0.3379	0.4088	-0.21
5165	Zinc finger protein with KRAB and SCAN domains 1	P17029	0.2147	0.3894	-0.65	0.2951	0.6716	-2.09	0.0130	0.0534	-3.44	0.0509	0.1164	-2.36	0.3024	0.4004	-0.40	0.3193	0.5219	-1.18	0.3736	0.4787	-2.55	0.8708	0.9942	-0.33	0.0651	0.1314	2.06
5166	Zinc finger protein with KRAB and SCAN domains 3	Q9BRR0	0.5660	0.6338	0.05	0.5572	0.8712	0.07	0.2033	0.3557	-0.12	0.8492	0.8700	0.04	0.0482	0.1613	-0.44	0.2936	0.4909	-0.32	0.0090	0.0940	-0.24	0.3995	0.7851	0.05	0.0578	0.2142	0.13
5167	Zinc finger protein with KRAB and SCAN domains 4	Q969J2	0.4108	0.4891	-3.31	0.5752	0.6848	-1.75	0.5661	0.6244	-1.19	0.5388	0.5909	0.89	0.2938	0.3919	-1.88	0.9510	0.9601	0.09	0.0175	0.1225	0.26	0.0332	0.1647	-0.17	0.0088	0.0841	-0.14
5168	Zinc finger protein ZPR1	O75312	0.2255	0.4042	-0.09	0.7954	0.8515	0.06	0.7224	0.7671	0.02	0.1568	0.2746	0.12	0.0468	0.1592	0.20	0.3197	0.5222	-0.07	0.1284	0.3022	-0.20	0.2290	0.5421	-0.14	0.0893	0.1424	-0.29
5169	Zinc finger protein-like 1	O95159	0.7224	0.7730	0.01	0.1371	0.6509	0.06	0.0121	0.0515	0.16	0.0160	0.0525	0.13	0.1204	0.2849	0.07	0.3716	0.5796	0.08	0.0124	0.1053	0.20	0.1227	0.3642	0.08	0.0844	0.1364	0.19
5170	Zinc finger Ran-binding domain-containing protein 2	O95218	0.1371	0.2818	-0.11	0.9051	0.9338	0.01	0.3525	0.5469	-0.06	0.3818	0.5665	-0.06	0.8105	0.8525	-0.01	0.6493	0.8858	-0.11	0.2878	0.5396	0.11	0.4736	0.8847	-0.06	0.2179	0.2870	-0.09
5171	Zinc finger SWIM domain-containing protein 1	Q9BR11	0.3348	0.5414	-0.04	0.4850	0.8091	-0.35	0.0224	0.0732	0.15	0.0264	0.0733	0.12	0.9235	0.9426	0.00	0.1472	0.3032	0.11	0.2042	0.4176	-0.45	0.6051	1.0379	-0.06	0.0052	0.0694	0.36
5172	Zinc finger SWIM domain-containing protein 4	Q9H7M6	0.0007	0.0192	1.13	0.2480	0.6588	-0.28	0.2154	0.3702	-0.13	0.0962	0.1884	-0.30	0.1774	0.3679	-0.15	0.6767	0.9152	0.77	0.3736	0.4461	-2.55	0.8708	0.9335	-0.33	0.0651	0.1179	2.06
5173	Zinc finger SWIM domain-containing protein 5	Q9P217	0.8488	0.8795	-0.01	0.1005	0.6333	-0.06	0.0003	0.0216	-0.18	0.0605	0.1325	-0.19	0.1082	0.2666	-0.09	0.6597	0.8974	-0.05	0.0324	0.1511	0.29	0.2341	0.5495	0.12	0.6697	0.7235	-0.14

Supplemetary Table S2. Overview on all relatively quantified 5180 proteins statistical analysis

			MCF-7																			MDA-MB-231							
			Dai SC20 vs control			Gen SC20 vs control			SSE SC20 vs control			Dai IC20 vs control			Gen IC20 vs control			SSE IC20 vs control			Dai IC20 vs control			Gen IC20 vs control			SSE IC20 vs control		
	**Protein name**	**UniProt**	**p value**	**BH q value**	**log2FC**	**p value**	**BH q value**	**log2FC**	**p value**	**BH q value**	**log2FC**	**p value**	**BH q value**	**log2FC**	**p value**	**BH q value**	**log2FC**	**p value**	**BH q value**	**log2FC**	**p value**	**BH q value**	**log2FC**	**p value**	**BH q value**	**log2FC**	**p value**	**BH q value**	**log2FC**
5174	Zinc finger SWIM domain-containing protein 6	Q9HCJ5	0.0168	0.0757	0.40	0.0307	0.5097	0.35	0.0731	0.1618	-0.24	0.8410	0.8635	-0.02	0.0335	0.1334	0.39	0.7985	1.0359	0.02	0.3736	0.4388	-2.55	0.8708	0.9198	-0.33	0.0651	0.1150	2.06
5175	Zinc finger transcription factor Trps1	Q9UHF7	0.4108	0.4890	-3.31	0.5752	0.6846	-1.75	0.5661	0.6243	-1.19	0.5388	0.5908	0.89	0.2938	0.3918	-1.88	0.9510	0.9599	0.09	0.1596	0.3525	-0.16	0.1865	0.4766	0.20	0.8250	0.8585	-0.10
5176	Zinc finger-containing ubiquitin peptidase 1	Q96AP4	0.0115	0.0616	0.16	0.5325	0.8498	0.08	0.2652	0.4361	0.07	0.0049	0.0280	0.21	0.1632	0.3473	0.06	0.0155	0.0829	0.22	0.1476	0.3326	2.31	0.3235	0.6765	1.73	0.1084	0.1651	2.90
5177	Zinc phosphodiesterase ELAC protein 2	Q9BQ52	0.0683	0.1737	0.24	0.2648	0.6639	0.10	0.7355	0.7786	0.03	0.3201	0.4911	-0.11	0.2690	0.4906	0.11	0.1955	0.3707	-0.12	0.0560	0.1876	0.17	0.6944	1.1368	-0.02	0.1354	0.1961	-0.07
5178	Zona pellucida sperm-binding protein 1	P60852	0.4108	0.4888	-3.31	0.5752	0.6845	-1.75	0.5661	0.6242	-1.19	0.5388	0.5907	0.89	0.2938	0.3917	-1.88	0.9510	0.9597	0.09	0.0009	0.0398	0.97	0.1118	0.3417	0.22	0.0063	0.0747	0.66
5179	Zygote arrest protein 1	Q86SH2	0.0311	0.1090	0.40	0.2298	0.6609	0.13	0.1527	0.2836	0.17	0.4823	0.6832	0.07	0.5269	0.6133	-0.07	0.2252	0.4083	0.16	0.3736	0.4967	-2.55	0.8708	1.0275	-0.33	0.0651	0.1394	2.06
5180	Zyxin	Q15942	0.0914	0.2119	-0.07	0.8058	0.8599	0.03	0.8946	0.9162	-0.01	0.0919	0.1819	0.11	0.0121	0.0785	0.13	0.0411	0.1335	0.09	0.7409	0.7785	-0.04	0.3661	0.7356	-0.09	0.2423	0.3126	-0.10
