# Supplementary figures and images for: From Proteomics to Personalized Medicine: The Importance of Isoflavone Dose and Estrogen Receptor Status in Breast Cancer Cells
Source: J Pers Med. 2020 Dec 19;10(4):292. doi: 10.3390/jpm10040292 (PMC7766658; doi:10.3390/jpm10040292)

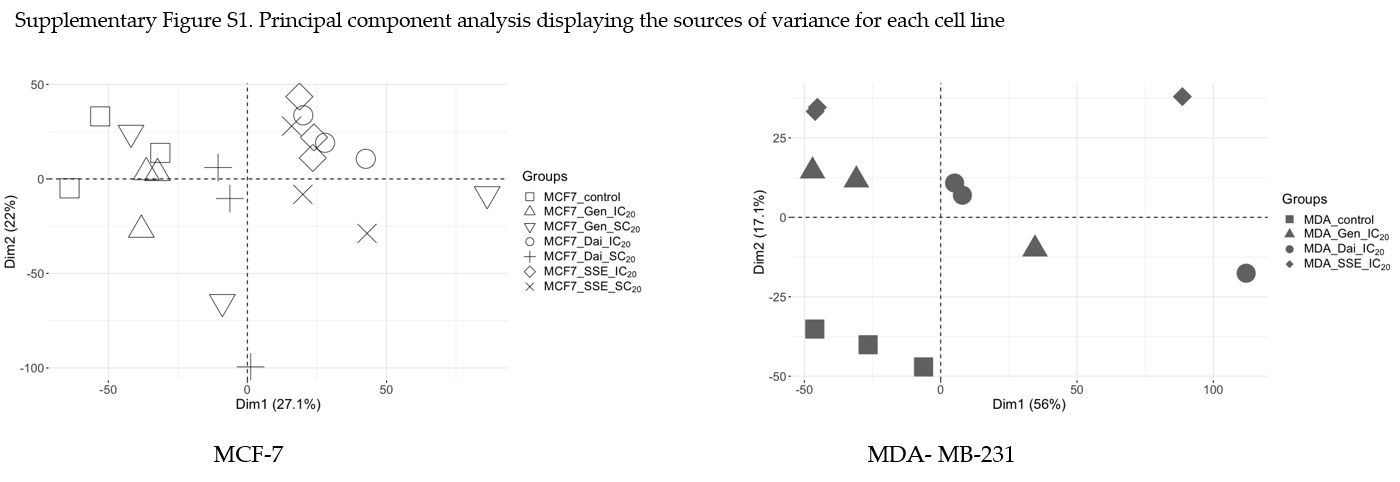

Supplement: Supplementary file 1 [file jpm-10-00292-s001.zip › Supplementary Figure S1. Principal component analysis displaying the sources of variance for each cell line.tif]
